# RNA-Seq reveals large quantitative differences between the transcriptomes of outbreak and non-outbreak locusts

**DOI:** 10.1038/s41598-018-27565-0

**Published:** 2018-06-15

**Authors:** M. Bakkali, R. Martín-Blázquez

**Affiliations:** Departamento de Genética, Facultad de Ciencias, Universidad de Granada, Fuentenueva S/N, Granada, 18071 Spain

## Abstract

Outbreaks of locust populations repeatedly devastate economies and ecosystems in large parts of the world. The consequent behavioural shift from solitarious to gregarious and the concomitant changes in the locusts’ biology are of relevant scientific interest. Yet, research on the main locust species has not benefitted from recent advances in genomics. In this first RNA-Seq study on *Schistocerca gregaria*, we report two transcriptomes, including many novel genes, as well as differential gene expression results. In line with the large biological differences between solitarious and gregarious locusts, almost half of the transcripts are differentially expressed between their central nervous systems. Most of these transcripts are over-expressed in the gregarious locusts, suggesting positive correlations between the levels of activity at the population, individual, tissue and gene expression levels. We group these differentially expressed transcripts by gene function and highlight those that are most likely to be associated with locusts’ phase change either in a species-specific or general manner. Finally, we discuss our findings in the context of population-level and physiological events leading to gregariousness.

## Introduction

Phase polyphenism of locusts is one of the most notorious cases of phenotypic plasticity due to its association with extreme increase in population size (outbreak) and the consequent devastating effects on the ecosystem and economy of the affected area. It is a reversible density-dependent change from the usual solitarious phase (in which the locusts lead isolated, cryptic and sedentary lives) to the outbreak-associated gregarious phase (in which the locusts are more active, voracious and swarm)^1–4^. The morphology and colour are different between the solitarious and gregarious locusts^5–7^. With regard to behavior; the gregarious locusts move and eat more, aggregate and migrate^2,4,6,8–11^. The differences between solitarious and gregarious locusts also include significant changes at the metabolic^12,13^, physiological^14–18^, reproductive^4,19,20^ and developmental^21–23^ levels. For instance, gregarious locusts have a more active immune response whereas solitarious locusts have longer lifespan and even an extra molt^24^. All these differences are due to changes in gene expression that are triggered by changes in environmental *stimuli* that result from the increase in population density. The enhanced mechano- and, to a lesser degree, visual and odorant *stimuli* in a crowded population are the triggers of locusts gregariousness^4,25–28^.

Several molecular factors were associated with the phase change in individual locust species. However, no molecule has so far been found to be unequivocally involved in all the cases of locusts’ phase change. For instance, juvenile hormone (JH) induces solitarious traits in gregarious *Schistocerca gregaria*^15,16^, and JH binding proteins (JHBP) are over-expressed in solitarious *Locusta migratoria*^29^. Corazonin^30^ induces gregarious colour and morphology^31–34^ but not behaviour^18^ in *S*. *gregaria*. Serotonin, a neuropeptide related to aggressive behavior, learning and circadian rhythms in insects^35,36^, was proposed as a trigger of gregariousness in *S*. *gregaria*^18,37^. However, it seems to have the opposite effect in *L*. *migratoria*^38^. The involvement of the catecholamine pathway is more consistent as some of its genes are over-expressed in gregarious *L*. *migratoria* and altering their expression changes both colour and behaviour^12^. The catecholamine pathway is responsible for synthesis of L-DOPA, a dopamine and melanin precursor present in the gregarious egg pod foam^22,39^, which was found to contribute to phase maintenance by maternal effect^21,23^.

While the search for molecules key to the development of the gregarious phase is advancing, the characterization of the cascade of molecular events that accompany the shift from the solitarious to the gregarious state requires the identification of genes that affect or are affected by the locust’s phase change. This is now possible thanks to the high-throughput sequencing technologies. Since locust phase polyphenism is due to changes in gene expression rather than mutation of the genome, RNA-Seq is an appropriate method to analyze it^40^.

Almost all studies focusing on the molecular aspects of the phase change in locusts use either *L*. *migratoria* or *S*. *gregaria*^12,13,16,18,33,41^. While Illumina-based RNA-Seq has already been applied to *L*. *migratoria*^29,42–44^, the only transcriptomic study available to date on the main pest locust species, *S*. *gregaria*, is Sanger-sequencing based^45^. That work was by no doubt gigantesque and, as such, provided a large set of sequencing data. Still, it did not provide any quantitative information on differential gene expression between phases. One might think that such information could be inferred from the studies on *L*. *migratoria*. However, apart from a non-quantitative study^43^, there is only one RNA-Seq report on the CNS of *L*. *migratoria*^44^. In addition, the molecular aspects of the phase change differ between species (see examples above).

In this first RNA-Seq study on the main pest locust species, we assembled, annotated, and performed differential gene expression analysis of CNS-enriched libraries from solitarious and gregarious locusts. We chose to analyze the CNS due to its obvious importance for a phenomenon so tightly linked to perception of *stimuli* and to changes in behaviour. In addition to generating new transcriptomic resources for locust research, we also established a list of genes whose expression is significantly different between phases. Furthermore, we validated some differentially-expressed genes from our RNA-Seq data using quantitative PCR. Based on our results as well as results from previous published reports, we highlight genes that seem genuinely important for the phenomenon either in a species-specific or general manner. Finally, we leveraged our expert knowledge, both on gene functions and locusts’ phase change, to infer and propose a “big picture” of the likely association between quantitative changes in gene expression and the cascade of population- and physiology-level events that accompany the change of phase.

## Results

### Quantity and quality of the sequencing reads

The sequencing library of the solitarious CNS-enriched tissues yielded around 25% more reads than the library of the gregarious tissues (Table S1 and SRA files SRR6315672 and SRR63156723 of the Bioproject PRJNA381887). About 90% of the nucleotides in both libraries satisfy the Q30 quality threshold, especially within the first 75 positions of the reads (Figure S1). The percentage of unidentified nucleotides (N) is less than 0.005%. In addition, the similar G+C content of both sequencing libraries suggest little contamination (the 1% difference could be expected given the differential gene expression).

### Transcript assembly and annotation

We considered all the assembled contigs that were shorter than 75 bp and/or were assembled from less than 4 reads as potential artifacts. Their removal left 117309 valid contigs (Table S2), around 65% of which produced significant BLAST results (51% in our *insects’ proteins database*, 5% in the *NCBI nr* database and 9% in the *NCBI nt* database). Around 30% (34696 contigs) produced no significant BLAST hit against any sequence in any of the three databases. These are sequences with no known annotation to which we will henceforth refer as non-annotated. The remaining 5% of the contigs were considered as potential contaminants as they showed significant BLAST similarities to either non-animal proteins (168 contigs) or non-arthropod nucleotides (6049 contigs). This low number of contigs (5%) further confirms the little contamination of the sequenced RNAs. The 76396 contigs that produced significant and acceptable BLAST results in our local database of insect proteins, the *NCBI nr* and *NCBI nt* databases can be found in Tables S3a, S4a and S5a, respectively. They correspond to 17620 unigenes (Tables S3b, S4b and S5b, respectively), since like all RNA-Seq assemblies, they contain partial sequences and isoforms of the same genes. The size-, coverage- and BLAST-filtered contigs that we retained as reference transcriptome for *S*. *gregaria*’s CNS are in Table S6.

**Figure 1:**
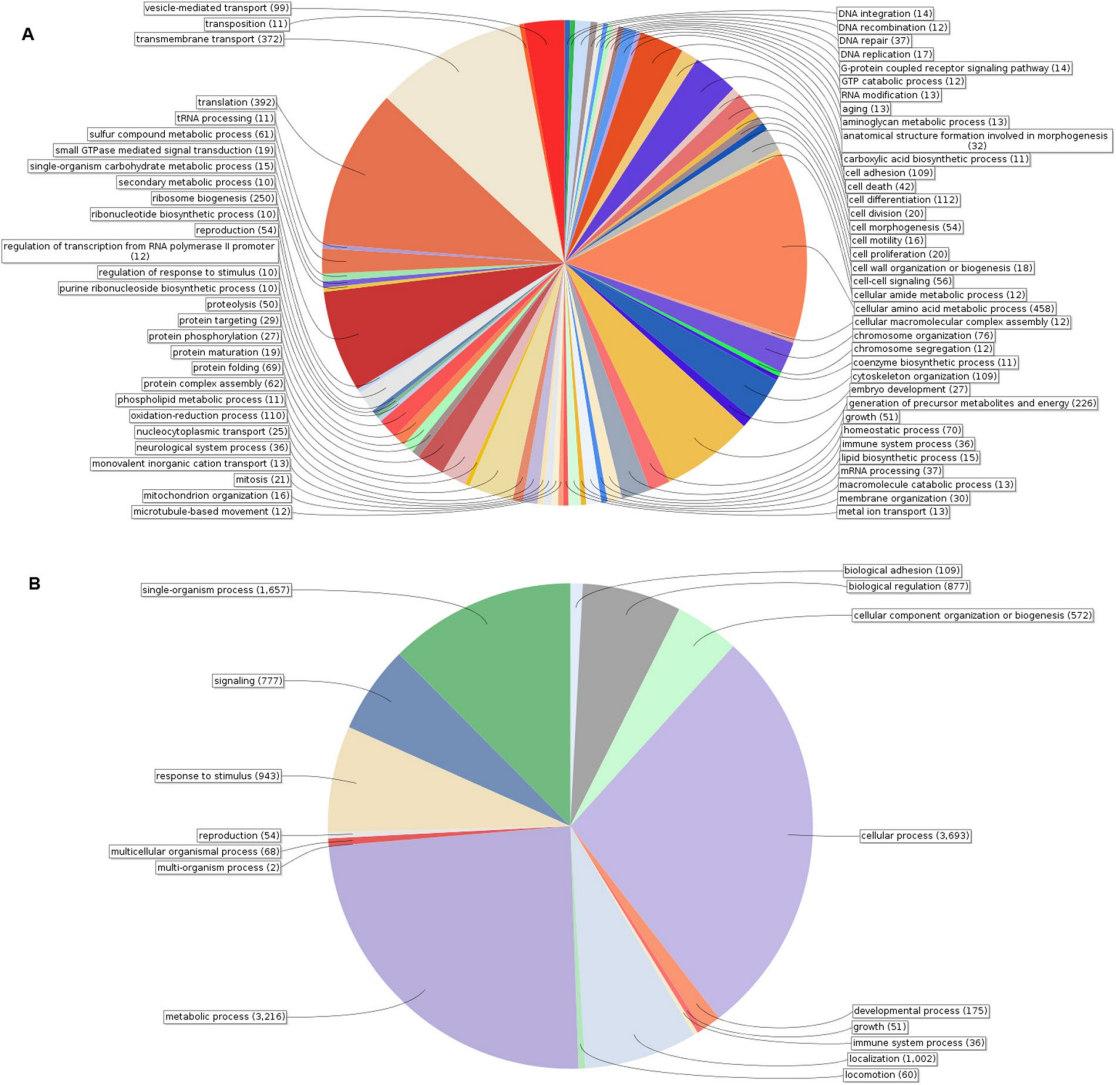
Distribution of the number of sequences in each of the GO terms to which belong the functionally annotated part of the reference transcriptome of the CNS-enriched tissues of the gregarious (**A**) and solitarious (**B**) *S*. *gregaria* adults (see also Tables S7 and S20).

Categorization by Gene Ontology (GO) shows that we covered a large portion of the biological processes (1043 processes distributed among 16 GO levels) with the usual predominance of ribosome biogenesis, homeostatic, synthesis, metabolic (including nucleotide, DNA, RNA and protein synthesis and modification), energy, structure-related, cell differentiation and cell organization processes (Fig. 1a,b and Table S7). Relevant to this work, we recovered many genes involved in processes related to biological regulation, signaling and cell communication (including G-protein coupled receptor signaling pathway), response to stress, response to *stimuli* and neurological system process. The species distribution of the BLAST results (Figure S2) confirms that we sequenced mainly locust RNAs and the noticeable presence of CNS-linked molecules proves that the material was indeed enriched with the CNS.

### Comparative analysis of the reference transcriptome generated using different methodologies

We compared the sequences of the NGS-based reference transcriptome that we report here to the 34672 ESTs from *S*. *gregaria*’s CNS that were Sanger-sequenced by Badisco *et al*.^45^. We assembled those ESTs into 4714 contigs and 8365 singlets of between 200 and 4954 bases (sequences in Table S8). This is similar to the 4785 unigenes and 7924 singlets reported in Badisco *et al*.^45^. Both transcriptomes show similar G+C content (39.53% and 40.31% respectively). The 1296 *vs*. 827 N50 values of the NGS- and Sanger-based transcriptomes, respectively, suggest that our assembly of the NGS reads (75 minimum and 35064 maximum contig sizes) produced results that are at least as good, in terms of contig size, as the assembly of the Sanger ESTs, with the differences in contig size homogeneity that one would expect given the quantities and sizes of the raw results (reads) of the two sequencing technologies.

7977 of the sequences that we assembled from the Sanger ESTs gave significant BLAST results against sequences in the *NCBI nr* or *nt* databases (i.e., were BLAST positive), while 5102 did not (Table S9). 7719 of these ESTs (5780 BLAST positive and 1939 BLAST negative) were NGS sequenced and assembled into 29666 contigs (Table S10), 23497 of which were BLAST positive, while 6169 were not (Table S11). The remaining 81098 NGS contigs were new sequences that had no match to any assembled Sanger EST. 52572 of these contigs have significant BLAST similarities to 13057 unigenes either in the *NCBI nr* or *nt* databases while 28526 do not (Table S12). Of the 5360 assembled Sanger ESTs that our NGS sequencing missed, about half (2556) were BLAST positive while 2804 were not (Table S13). Table 1 offers a simplified summary of the degrees of overlap and complementation between the CNS transcriptomes of *S*. *gregaria* produced by the Sanger ESTs in Badisco *et al*.^45^ and by our NGS assembly. For its part, Table 2 lists genes which we can confirm as associated with the locusts’ phase change as they appear so both in our and other works.

**Table 1:** Comparison between the numbers of sequences obtained for the CNS transcriptome of *S*. *gregaria* either in this NGS-based work or in the Sanger-based sequencing by Badisco *et al*.^45^ or in both works.

BLAST result	Transcriptome		
	NGS assembly	Sanger ESTs	Both
Significant	13057 (52572)	2556	5780 (23497)
Not significant	28526	2804	1939 (6169)

The table reflects the degrees of complementation and overlap between the two works. The numbers outside the parentheses are of unigenes, in case of significant BLAST results, and of assembled contigs in case of no significant BLAST results. The numbers within parenthesis are of the NGS-assembled contigs that correspond to the unigenes (in case of significant BLAST result) and to the assembled ESTs (in case of no significant BLAST results).

**Table 2:** Genes with confirmed association with locusts’ phase change.

Confidence	Gene description	Accession	Phase	Confirmation	Tested species	Contig
Agreement between our data and data published elsewhere	hexamerin 5 precursor	NP_001164204	Solitarious	Microarrays in Guo *et al*. 2011 and our RNAseq	*L*. *migratoria* and *S*. *gregaria*	104268
	PREDICTED: protein takeout-like	XP_001950683	Solitarious			37604
	PREDICTED: similar to glutathione-s-transferase theta, gst	XP_975048	Solitarious	Microarrays in Badisco *et al*. 2011b and our RNAseq	*S*. *gregaria*	103559
	PREDICTED: arylphorin subunit alpha	XP_001600430	Solitarious			85614
	PREDICTED: similar to adenylate cyclase	XP_975639	Gregarious	qPCR in Kang *et al*. 2004 and our RNAseq	*L*. *migratoria* and *S*. *gregaria*	83877
	PREDICTED: similar to Annexin IX CG5730-PC	XP_967931	Gregarious			85392
	adipokinetic hormone receptor	NP_001161243	Gregarious	RNAseq in Chen *et al*. 2010 and our RNAseq	*L*. *migratoria* and *S*. *gregaria*	83922
	PREDICTED: similar to calcium/calmodulin-dependent serine protein kinase membrane-associated guanylate kinase (cask)	XP_972920	Gregarious			86801
	PREDICTED: diuretic hormone receptor-like isoform 2	XP_003427176	Gregarious			92428
	gamma-aminobutyric-acid receptor alpha-2 subunit precursor, putative	EEB18262	Gregarious			102894
	glutamate receptor Gr1	ABD36124	Gregarious			103430
	PREDICTED: protein Malvolio-like isoform 2	XP_003424930	Gregarious			37276
	tyramine/octopamine receptor	NP_001164311	Gregarious			47154
	synaptic vesicle protein, putative	EEB16248	Gregarious			43963
	PREDICTED: ATP-citrate synthase-like isoform 2	XP_003425261	Gregarious	Microarrays in Guo *et al*. 2011 and our RNAseq		85903
	CSP precursor	AAO16783	Gregarious			108631
	pacifastin-related serine protease inhibitor precursor	CAD11970	Gregarious			77531
	chemosensory protein 2	AAC25400	Gregarious			108608
	*Locusta migratoria* clone LmigCSP3 hypothetical protein mRNA, complete cds	GU722578	Gregarious			Sg_CNS_NA4Plus8496
	chemosensory protein 4	ABH88177	Gregarious			88200
	Fasciclin-1 precursor, putative	EEB13818	Gregarious	qPCR in Badisco *et al*. 2011a and our RNAseq	*S*. *gregaria*	52795
	PREDICTED: slit homolog 2 protein	XP_001603014	Gregarious			42904
	cytochrome P450, putative	EEB11101	Gregarious			91157
	PREDICTED: 10 kDa heat shock protein, mitochondrial-like	XP_001599992	Gregarious	Microarrays in Badisco *et al*. 2011b and our RNAseq		77390
	PREDICTED: RNA-binding protein Musashi homolog Rbp6-like	XP_001606007	Gregarious			41415
	PREDICTED: unc-112-related protein-like isoform 1	XP_392367	Gregarious	RNAseq in Wang et al. 2014 and our RNAseq	*L*. *migratoria* and *S*. *gregaria*	47840
	PREDICTED: basement membrane-specific heparan sulfate proteoglycan core protein-like	XP_393220	Gregarious			86324
	PREDICTED: similar to Switch-associated protein 70 (SWAP-70)	XP_974449	Gregarious			43957
	PREDICTED: E3 ubiquitin-protein ligase hyd-like isoform 1	XP_001605335	Gregarious			93157
	PREDICTED: similar to mitogen-activated protein-binding protein-interacting protein	XP_967919	Gregarious			31687
	PREDICTED: glycyl-tRNA synthetase-like	XP_001606827	Gregarious			103685
	PREDICTED: polyphosphoinositide phosphatase	XP_394455	Gregarious			35564
	PREDICTED: dynein heavy chain, cytoplasmic-like	XP_001951535	Gregarious			93074
	PREDICTED: serine/threonine-protein kinase mTOR isoform 1	XP_625130	Gregarious			42592
	PREDICTED: niemann-Pick C1 protein-like isoform 2	XP_624752	Gregarious			32806
	PREDICTED: vacuolar protein sorting-associated protein 16 homolog	XP_392642	Gregarious			48556
Agreement between our RNA-Seq and qPCR results, when using the same as well as when using different samples, and no known disagreement with previously published works	CSP12	ABH88185	Gregarious	our RNAseq and qPCRs	*S*. *gregaria*	88095
	Yellow h	ABB81847	Gregarious			49891
	—	—	Gregarious			Sg_CNS_NA4Plus202
Agreement between our RNA-Seq and qPCR results, when using the same as well as when using different samples, but disagreement with previously published works	asparagine synthetase, putative	EEB18196	Gregarious		*S*. *gregaria*	85647
	Larval cuticle protein 9	P82384	Gregarious			29324
	*Locusta migratoria* heat shock protein 20.6 mRNA, complete cds	DQ355964	Gregarious			Sg_CNS_NA4Plus277
	Glia maturation factor beta, putative	EEB15105	Gregarious			103230
	Phosphoenolpyruvate carboxykinase	P20007	Gregarious			33808

Accession: a sample accession number of a homologous sequence; Phase: the locust phase where the gene is over-expressed; Confirmation: the methods and laboratories that report results on the gene similar to ours; Contig: code of the contig in our reference transcriptome that correspond to the respective gene (see Tables S3–S5).

### Differential gene expression analysis between locust phases

Among the 51448 unigenes and non-annotated contigs of the NGS-based reference transcriptome, about 48% (24770) show differential expression between phases in *S*. *gregaria* (Table S14). Just above 10% of these (2537) are significantly over-expressed in the solitarious sample (Table S15), while about 90% (22233) are significantly over-expressed in the gregarious sample (Table S16).

As a first overall assessment of our results, we gathered 86 genes present in our reference transcriptome and mentioned in the literature as differentially expressed between locust phases. We used qPCR-, microarrays- and NGS-based studies that were available by 2014 either on *S*. *gregaria* or *L*. *migratoria* (see^29,42,44–47^). 28 of these genes appear over-expressed in opposite phases in our results and in the literature, 22 show no significant differential expression in our data (9 of them showing more expression in the same phase as in the literature and 13 showing more expression in the opposite phase) and 36 were confirmed by our results as significantly over-expressed either in the solitarious (4 genes) or gregarious phase (32 genes). Our data therefore support the published results in 42% of the cases, could not do so in 26% of the cases, and oppose the published reports in 32% of the cases. 56% of the cases are in agreement if we do not consider the statistical significance of the differential expression in our RNA-Seq results (Table S17). Interestingly, our data show similar consistency with the published results on *L*. *migratoria* (29 consistent *vs*. 22 opposed, 56% consistency) as they do with the results on *S*. *gregaria* (7 consistent *vs*. 6 opposed, 53% consistency). Our results exhibit stronger agreement with the Illumina-based RNA-Seq on *L*. *migratoria*^42^ (Table S17), which implies higher consistency of the results between studies that use the same technology.

**Figure 2:**
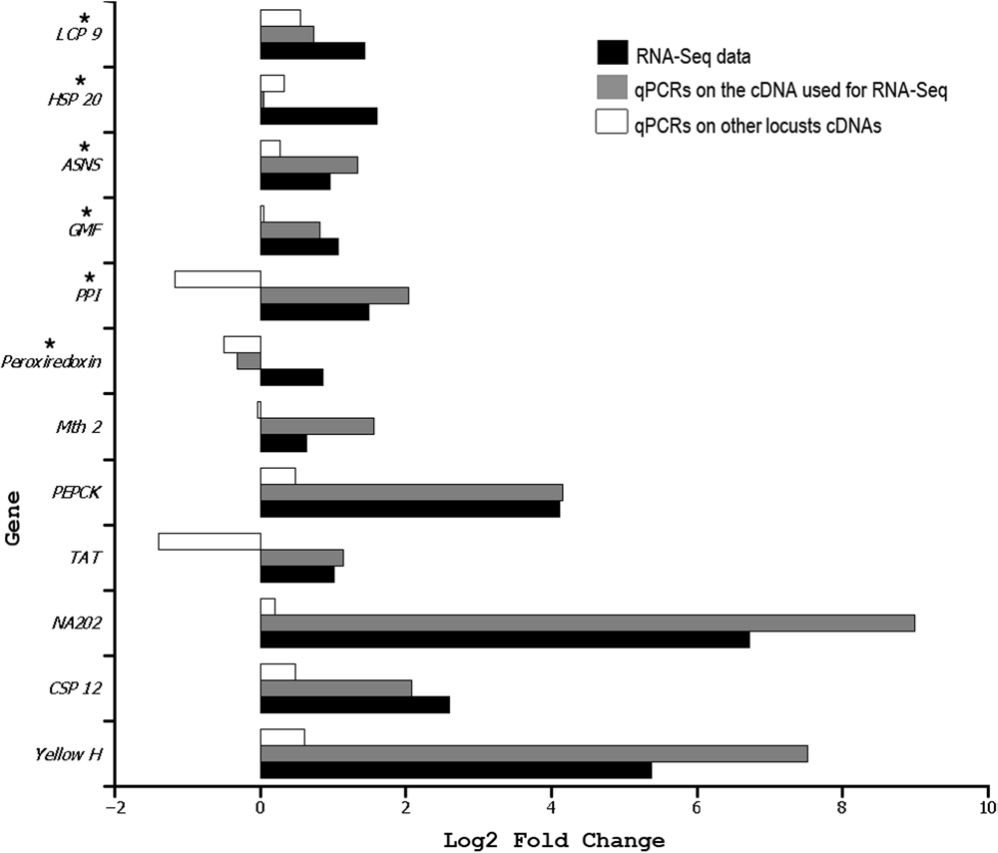
Direction and magnitude of the differential expression of the 12 genes tested by RNA-Seq (black bars), qPCRs using the cDNAs used RNA-Seq (grey bars) and qPCRs using cDNAs obtained from different parts (heads only) of other *S*. *gregaria* locusts that were at a different developmental stage (nymphs). *LCP9*: larval cuticular protein 9, *HSP20*: heat shock protein 20, *ASNS*: asparagine synthase, *GMF*: glia maturation factor, *PPI*: cis-trans peptidyl-prolyl isomerase, *Mth2*: methuselah 2-like, *PEPCK*: phosphoenolpyruvate carboxykinase, *TAT*: tyrosine aminotransferase-like, *NA202*: a transcript with no known annotation, *CSP12*: Chemosensory protein 12. The 2-based logarithm of the fold change was calculated as the ratio between the expression level in gregarious and the expression level in solitarious locusts (positive values reflect more expression in the gregarious cDNAs and negative values are due to more expression in the solitarious ones). The reference genes for qPCRs were as in^79^. The asterisks signal the genes that we choose for qPCR testing based on disagreement of our RNA-Seq data with those previously reported in the literature.

To further evaluate our RNA-Seq results, we used qPCR to validate phase-specific expression differences for 12 genes. 11 of the 12 qPCRs that used the sequenced mRNAs as starting materials were in agreement with the RNA-Seq results (Fig. 2 and Table S18). When we extracted fresh RNAs from other animals (nymphs this time) and performed validation qPCR, 8 of the 12 tested genes were in agreement with the RNA-Seq data (Fig. 2 and Table S18). Furthermore, the qPCRs that used the sequenced mRNA supported our RNA-Seq data on five of the six genes chosen based on disagreement with the literature (Fig. 2) and only one of these five genes showed opposite result to our RNA-Seq data when the qPCRs used other cDNAs. Overall, eight of the 12 tested genes showed consistent direction of higher expression in the two sets of qPCRs and RNA-Seq data (Fig. 2).

It is noteworthy that our RNA-Seq results show similar sets of most expressed genes in the solitarious and gregarious libraries (Table S19). In numbers, about 90% (4618) of the 10% most expressed genes in each of the two locust phases (5145) are the same. The remaining 10% (527 genes) are either absent or do not belong to the 10% most expressed genes in either library. Furthermore, paired t-test shows that, as a whole, the expression levels of these 90% shared genes are statistically similar between the two libraries (t = 1.882, p = 0.06). On the other hand, only less than 7% (349) of the 10% least expressed genes (5145) are shared between both libraries and their expression levels are significantly different (t = 8.971, p < 0.0001). Even when we consider only sequences that have at least 10 reads in at least one library (40573 cases), only about 15% (618) of the 10% least expressed sequences (4057) are shared between libraries and their expression levels also are significantly different between libraries (t = −8.184, p < 0.0001). Accordingly, the correspondence between the rankings of the genes in each library based on their expression level weakens as the expression level goes down (Figure S3).

The set of annotated contigs that are over-expressed in the solitarious sample is dominated by translation, catabolism and metabolic processes (Fig. 1B). Whereas, in addition to these processes, the set of annotated contigs whose expression is significantly higher in the gregarious sample shows a notorious presence of processes relating to protein modification, ion transport, energy as well as behaviour, response to different *stimuli* and stress (Fig. 1A). Tables S14 and S15 show the full list of genes and their annotation and relative expression levels. 26 of the 114 detected GO terms contain significantly different numbers of differentially expressed genes (Table S20). 14 of these 26 GO terms are significantly over-represented in the set of genes that are over-expressed in the solitarious locusts. They mainly correspond to growth, metabolism and biosynthesis processes. The remaining 12 GO terms are significantly over-represented in the set of genes that are over-expressed in the gregarious locusts and correspond mainly to molecule modification, response to *stimuli* and cell communication and signaling.

Highlighting all the differentially expressed genes is not possible given their large number. On the other hand, the comparatively reduced set of sequences that are over-expressed in the solitarious phase contains sequences with little information; as inferred from their annotations. Several proteases and the genes encoding for vitellogenin, vitellogenin receptor, yellow-y, yellow-12, hexamerin, and hemocyanin are among the sequences that are over-expressed in the solitarious phase.

The large number of sequences that show significant over-expression in the gregarious sample reflects the rather complex nature of locust gregariousness. Still, to have a glimpse of the general picture, we opted for a meticulous bibliographical search of the functions and implications of each of the thousands of annotated genes that are significantly over-expressed in the gregarious phase. Table S21 groups some of the most significant ones into functional categories (the 19 sheets of the file) and subcategories (the columns of each sheet of the file) and, when necessary, the table clarifies why we consider the gene important for locust gregariousness. Among the annotated genes that are significantly over-expressed in the gregarious phase, in addition to genes whose differential expression is more likely due to the presence of other tissues or to general processes, we can identify genes known to be directly involved in dealing with *stimuli* (including perception, transmission of the signals and response), genes directly related to the neural cells (including their function and plasticity) and genes involved in behavior. Interestingly, the list of genes that are over-expressed in the gregarious CNS includes genes related to each of the steps of gene expression.

As to the 34696 non-annotated sequences of the reference transcriptome, 2059 are significantly over-expressed in the solitarious phase (Tables S15 and S16). 380 have over 10 reads and are absent from the gregarious library, 1380 have over 10 reads and over 1 fold increase of expression in the solitarious phase, and the remaining 299 have either less than 10 reads or show less than 1 fold increase of expression in the solitarious phase. From the gregarious side, 11061 non-annotated sequences are significantly over-expressed (Tables S14 and S16). Only 28 have over 10 reads and are absent from the solitarious library, 5940 have over 10 reads and over a fold increase of expression in the gregarious library, and the remaining 5093 have either less than 10 reads or less than 1 fold increase of expression in the gregarious phase.

## Discussion

We determine, for the first time, the changes in gene expression levels that differentiate solitarious and gregarious *S*. *gregaria* locusts and confirm 59% of the ESTs Sanger-sequenced by Badisco *et al*.^45^. A third of the assembled sequences had no known homologs as of 2013. While some of these non-annotated sequences might be artifacts, many are genuinely novel (given the high number of reads mapped to them) and some are significantly associated either with the solitarious or the gregarious phase.

qPCRs using the sequenced cDNAs confirmed the RNA-Seq results for 11 of 12 genes. Thus, the sequencing did not significantly alter the composition of the cDNAs and the *in silico* quantification of gene expression was accurate. The RNA-Seq results for eight of these genes were confirmed even when the qPCRs contained other cDNAs. Furthermore, the qPCRs confirmed our RNA-Seq results for a non-annotated sequence as well as for four out of six genes whose results were not congruent with the data reported elsewhere. This incongruence does not necessarily mean error in the published reports as it may be due to differences at any level from species-specificity to inter-individual variation. qPCRs and comparison of our RNA-Seq data with those of previous studies allowed us to identify genes differentially expressed between locust phases either in a species-specific or general manner. They must be genuinely associated with the phase change and their functional testing might highlight some as potentially useful for the fight against locusts.

The colossal number of differentially expressed genes between locust phases should not be surprising as the differences between the solitarious and gregarious states are due to changes in gene expression, not in the genome *per se*^40^, and they affect almost every aspect of the insect’s biology; including the external morphometry^2,7,8,32^, size of internal organs^48^, colour^5,6,49,50^, behaviour^4,11,51–54^, diet^49,50,55^, metabolism^12,13^, physiology^14–18^, reproduction^4,19,20^ and development^21–23^. Locust population outbreak is not a simple increase in population size; it involves a phase polyphenism as adaptation to recurrent drastic changes in living conditions. Life in a crowd exposes to increased chemical, mechanical and visual *stimuli*, and leads to more stress, infections, competition and movement. The resulting hormonal and metabolic changes cause a change in the colour of the locust, which serves for warning or camouflage. The unavoidable exhaustion of food leads to more movement, changes in the metabolism, physiology, fat content and body size, among others, and requires developmental and structural changes (e.g., gregarious locusts develop wings earlier so they can migrate in search of resources). The increased exposition to *stimuli* leads to restructuring of the nervous ramifications as part of larger CNS changes (e.g., gregarious locusts have larger brains^48^). These modifications require changes in the expression of genes involved in a plethora of functions; including gene expression itself. The large number of differentially expressed genes between locust phases is therefore logical. In fact, there are even more pronounced cases; such as the over 85% of 5500 genes found to be differentially expressed between nurse and forager honey bees^56^.

The fact that the most expressed genes are common to both phases and have generally similar expression levels while the least expressed genes are not, suggests importance of the changes in expression of low expression genes and stability of the expression of housekeeping genes between physiological states. Most of the differentially expressed genes are over-expressed in the gregarious phase. Gregarious locusts are more active and exposed to a more stimulating and challenging environment than solitarious locusts; they are expected to have higher levels of sensorial functions, metabolism, detoxification, DNA and cell damage, apoptosis, neuronal activity, immune responses and gene expression. Hence, the genes over-expressed in solitarious locusts mainly belong to the metabolic, catabolic and translation processes expected to be over-represented in large organisms that live in less challenging conditions, whereas the genes over-expressed in gregarious locusts show over-representation of processes related to the response to *stimuli*, stress and behavior; processes expected to be over-represented when facing infections, high levels of interaction, competition and a need for processing the inputs that originate from such conditions.

**Figure 3:**
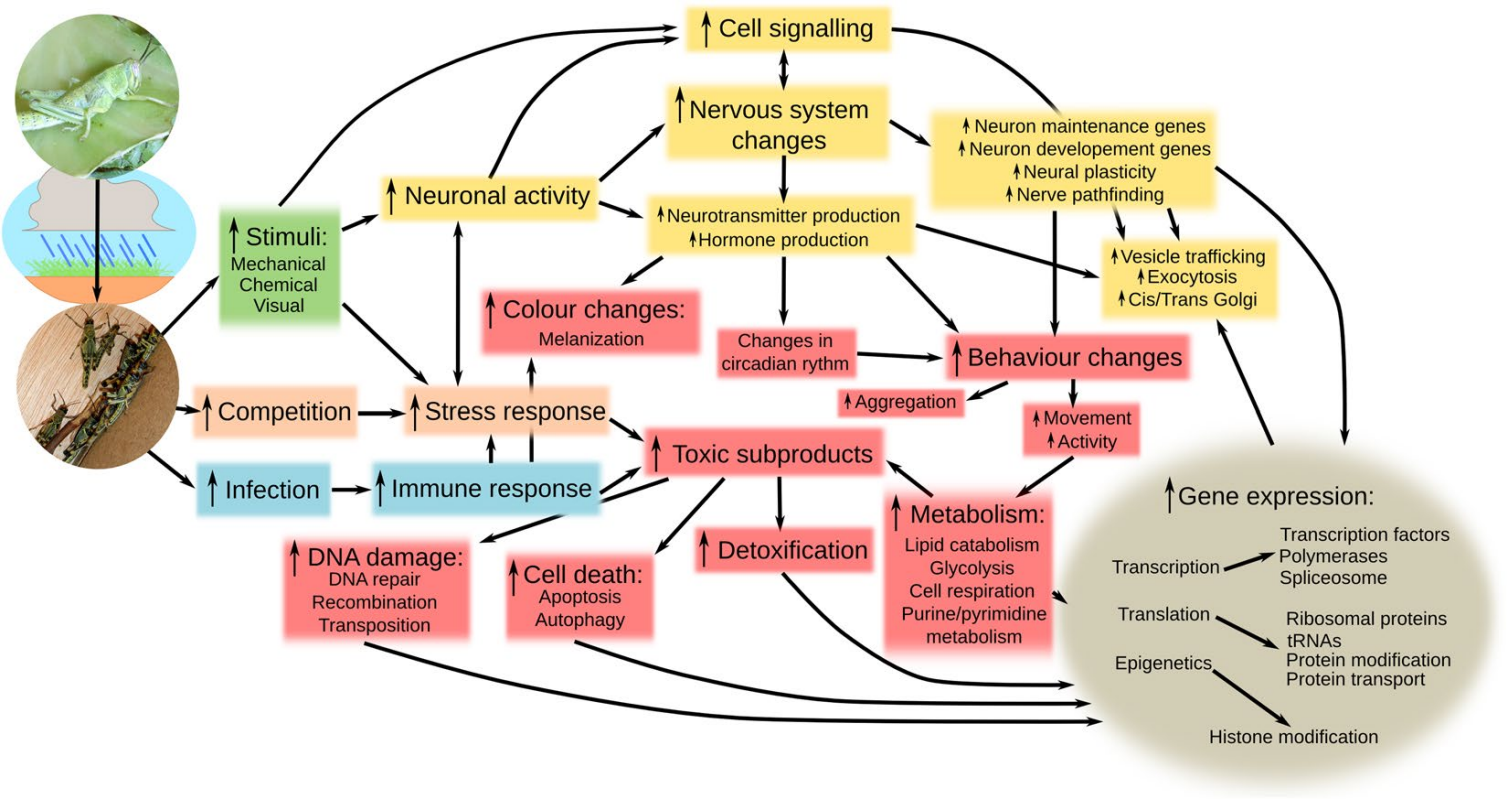
Resumed schematic representation of our interpretation (hypothesis) on how the changes in gene expression and the population- and physiology-level events likely match and interrelate between each other in the cascade of happenings that leads to gregariousness in *S*. *gregaria*. Supplementary Tables and Figures can also be downloaded from: http://www.ugr.es/local/mbakkali/CNS_sup.zip.

The differentially expressed genes could be categorized into functional groups (Table S21). We establish links between these groups and we hypothesize on how the cascade of events leading to gregariousness potentially affect or are affected by the genes in question (Figs 3 and S4). However, we want to stress that while the population and physiology-level events listed in Figs 3 and S4 and the categorization of genes in Table S21 are based on scientific knowledge, the association between the gene-, population- and physiology-level events that led to both figures is based on our interpretation intending to generate a hypothesis for debate and for inspiring future studies.

A locust population outbreak is caused by an extraordinary survival rate of eggs and hoppers due to favourable environmental conditions. The consequent crowdedness leads to increased visual, olfactive and mechanical stimulation and a likely increase in activity of genes related to those functions (for instance see^57^) and of genes involved in remodeling neural structures in order to handle and enhance perception of *stimuli*. Crowdedness also results in higher contagion rate and, thus, over-expression of immune response genes. The depletion of resources and competition in large populations are stressful and require a more active behaviour. This is very likely linked to over-expression of stress-related genes and changes in expression of genes related to neurons and behaviour. A more active behaviour is associated with changes in the muscles, fat-bodies and metabolism. The latter increases the toxic metabolites inside the cells and could be the cause of the higher activity of detoxification, DNA repair and cell death genes. Both the hormonal, immune and detoxification functions are known to promote the production of melanin, which changes the animal’s colour. All these would need changes in gene expression regulation, which would explain the increased expression of genes related to gene expression. Following the same reasoning, we could fit the differentially expressed genes into a coherent and logical cascade of population, physiology and gene expression events that appears concordant with the known changes that locusts undergo when they shift from solitarious to gregarious.

In conclusion, we reveal for the first time the positive correlation between the increment in population size, behavioural activity and gene expression. We show how the gregarious phase affects every aspect of the biology of *S*. *gregaria* locusts through differential expression of a large number of genes. The genes whose expression changes are involved in every aspect of the normal functioning and reactions of the cells of a living being. Whether such magnitude of changes is a locust-specific adaptation to recurrent exposure to the episodic selective pressures that the stressful conditions of life in a crowded population cause, or if it is rather a generalized characteristic that differentiates crowded from non-crowded animal populations is an interesting question for future research. The fact that stress affects the expression of many genes in humans (*e*.*g*., see^58,59^) would suggest that the psychological effects of life in crowdedness, increased *stimuli* and other social interactions might generally affect the expression of potentially as many genes as the ones that we show affected in gregarious locusts. Of course, functional testing is needed in order to confirm our interpretations.

## Material and Methods

### Locust colony and tissue collection

*Schistocerca gregaria* was chosen for this work as it is the main pest locust. Laboratory colonies of solitarious and gregarious locusts belonged to the same population. They were started during summer 2009 using four egg-pods kindly provided by Profs. Arnold de Loof and Jozef Vander Broek (University of Leuven, Belgium). The original stock, established in 1985 at Aquazoo Löbbecke Museum (Düsseldorf, Germany), was of Nigerian origin. After two rounds of collective crossings, the hatchlings of each egg pod were divided into two sets, one maintained as gregarious and the other solitarized for three generations.

Rearing conditions are described in^60^. Briefly, gregarious animals were kept together in 60 × 60 × 60 cm wooden cages, whereas solitarious animals were kept isolated in 15 × 15 × 30 cm individual wooden cages. Since we wanted to detect the differences between the solitarious and gregarious states of the locusts, we ensured that the animals used were fully solitarious and fully gregarious locusts so: (i) the solitarious locusts were reared in individual cages in complete mechanical, visual and olfactive isolation from other locusts and the gregarious locusts were reared in crowded conditions. (ii) Both locusts were reared at these different conditions for three generations in order to erase any homogenizing maternal effect. (iii) The solitarious and gregarious locusts showed the typical differences in nymph colour (see Fig. 3 in^40^) and adult size and activity (see^60^). For complete mechanical, olfaction and visual isolation of the solitarious animals, the small cages contained only one individual, except for a short period when each cage contained a male and a female for mating. The cages were placed so that locusts could not see each other and a stream of air from outside the building (2 liters per minute and cage) prevented the animals from smelling each other. 60w and 15w lamps where used for illuminating the large and small cages, respectively. The temperature, humidity and photoperiod were 31 °C, 60% and 14:10 hours light:dark, respectively. Animals were fed organic cabbage and a mixture of ground maize, barley and wheat seeds.

We collected tissues from a pool of five adults for each locust phase. The pooled samples contained three males and two females (the size of adult females is about double that of adult males—see^60^). Cold anesthesia was used in order to avoid the effect of chemicals on the CNS. We also opted for quick separation of the central part (0.5 cm) of the ventral side of the thorax and abdomen and the head of each cold anesthetized locust followed by immediate immersion in liquid nitrogen. The whole operation lasted for about two minutes and no animal was awake.

### RNA extraction

The tissues belonging to each locust phase were pooled in separate 50 ml tube containing 30 ml of RNAzol^®^ RT (Molecular Research Center Inc.) and immediately homogeneized using an Omni-TH mechanical homogeneizer and its hard-tissue tip. Extraction of the total RNAs was carried out for each homogenate in 10 separate tubes (replicates) following RNAzol^®^ RT product manual. The total RNAs were then stored in RNAse-free buffer at −20 °C until extraction of the mRNAs using GenElute mRNA Miniprep Kit (Sigma Aldrich) as recommended by the manufacturer. The 10 mRNA replicates of each locust phase were then quantified using Quawel Q3000 spectrophotometer and pooled into one sample in equi-molar conditions. Part of each of the two resulting pooled mRNA samples was stored at −20 °C in RNAse-free buffer and another part (500 ng) was added to two volumes of ethanol and 10% sodium acetate 3 M pH 5.2 and shipped to Macrogen Inc. for sequencing. Once at Macrogen, and after quality check (see Figure S5A,B), using Aglient 2100 Bioanalyzer RNA 6000 chip (Agilent Technologies), a 400 base pairs library was constructed for each pooled mRNA sample using the TruSeq RNA Library Prep Kit (Illumina, Inc. Reference number 15026495, revision B, February 2012). The resulting DNAs were then quality checked using Aglient 2100 Bioanalyzer 1000 Assay (see Figure S5C,D) and sequenced in an Illumina HiSeq. 2000 system. A 101- base Paired End sequencing approach was applied following Macrogen Inc.’s application of the Illumina multiplex sequencing protocol (we aimed for 4 to 5 Giga Bases outcome per sample; over three times the sequencing depth estimated by Wang *et al*.^61^ as adequate for detecting all the annotated genes in a transcriptome).

### Transcriptome assembly annotation and analysis of the expression levels

After initial quality assessment using FastQC^62^, assembly of the solitarious, gregarious and joint sets of sequencing reads was carried out using ABySS v1.2.0^63^ and Trans-ABySS v 1.2.5^64^. Instead of arbitrarily choosing an assembly k-mer or using the median length of the assembled sequences as a less subjective selection criterion, each set of sequencing reads was assembled using all the odd-numbered k-mers from 95% the size of the read, 95, to the smallest k-mer that would not produce excessive mis-assembly (the probability of having a 19-bases sequence in a set of equi-based sequences is 3.638 10^−12^, and the probability of having it again by chance among the 8.2 10^9^ sliding 19-base windows of a 100 million reads library and its reverse complement is less than 6%). We used only odd numbered k-mers for sequence assembly in order to prevent reverse complement matches and inversion of the de Bruijn graph in case of palindromic k-mers^65,66^ and the ABySS options were: OVERLAP_OPTIONS = ‘–no-scaffold’ SIMPLEGRAPH_OPTIONS = ‘–no-scaffold’ MERGEPATHS_OPTIONS = ‘–greedy’ n = 1 c = 1 E = 0 e = 0 q = 3. The −0 option of Trans-ABySS v 1.2.5 joined all the 39 assemblies (one per k-mer) of each set of sequencing reads into a non-redundant fasta file. The resulting three fasta files (one per set of sequencing reads) were then joined and their redundancy removed at 95% identity threshold using UCLUST^67^. CAP3^68^ was then used for further assembly and redundancy removal using 16 bases minimum overlap and 95% identity threshold. Illumina sequencing reads typically start losing quality at 75% of their length^69^, so any contig that is shorter than 75 bases is most likely produced by less than one read. We eliminated those contigs and kept only contigs that had significant BLAST hits (e-value 10^−6^) in the *NCBI nr* or *nt* databases or were produced by at least 4 sequencing reads (*i*.*e*., they came from at least 2 molecules).

We downloaded, assembled using CAP3 with its defaults options, and annotated the ESTs Sanger sequenced by Badisco *et al*.^45^ (Accession numbers: JG662739.1 to JG697409.1). Local BLAST databases were separately built using our reference transcriptome and the transcriptome assembled from these ESTs. Reciprocal BLASTn searches were then carried out in order to compare and assess the completeness of each transcriptome using 10^−6^ as E-value threshold.

A local database was built using the protein sequences of Acyrthosiphon pisum, Anopheles gambiae, Apis mellifera, Bombyx mori, Drosophila melanogaster, Nasonia vitripennis, Pediulus humanus and Tribolium castaneum. BLASTx searches against that database allowed an initial annotation of our reference transcriptome. In continuation, BLAST2GO^70,71^ was used to complete the annotation against first the NCBI nr then the NCBI nt databases. The E-value cut-off in all the BLAST searches used in this work was 10^−6^ and sequences that were significantly similar to non-animal proteins or non-arthropodan nucleotides were considered as contaminants. BLAST2GO was also used with the default 10^−3^ E-value cut-off for functional annotation against the KEGG database and establishment of the GO terms and enzymatic maps. The sequences that we assembled from Badisco *et al*.’s ESTs were annotated in a similar way.

Following similar steps as in^72^, the sequencing reads from both the solitarious and the gregarious material were separately aligned to the assembled reference transcriptome using BWA’s *sampe* and default options^73^. After processing the resulting SAM-formatted files, using *xa2multi*.*pl*^73^, removing entries of unmapped fragments and compressing the files into BAM-format, using SAMtools^74^, the *intersection-nonempty* option of the *htseq-count* script, HTSeq^75,76^, was used for summarizing the counts of fragments that mapped to each contig of the assembled reference transcriptome as in^72^. The solitarious and gregarious counts of each contig were compared using contingency Chi-squared test in a costume-prepared MS Excel worksheet. The test takes into account the library size; as it compares the distribution of the 2 × 2 contingency table of the read count of a transcript and the rest of the read counts in a library and the read counts of the same transcript and the rest of the read counts in the other library. Correction for multiple testing was completed using the False Discovery Rate method^77^ and, after normalization by each library and contig’s size using the FPKM approach^78^, the 2-based logarithm of the ratio between the gregarious and the solitarious FPKMs was considered as fold change and its absolute value was used for sorting the contigs whose expression levels were significantly different between libraries.

The distribution of the biological processes and functions of the differentially expressed genes was compared based on graph representations of the relative abundance of transcripts belonging to each GO term in the sample as well as on contingency Chi-squared testing for significant difference in such abundance between libraries. We did not test for GO enrichment compared to the genomic representation as no reference genome is available yet for the studied species. To compare the distribution of the expression data, the genes in each library were organized by their level of expression in that library (using the *Linux Bash’s sort* command) and the top and bottom 10% were extracted (using the *Linux Bash*’s *head* command). The genes that belong to these selections and were present in both libraries were extracted (using the *Linux Bash*’s *fgrep* command) and an overall comparison of their expression levels between libraries was performed using paired t-test in *Statistica*.

### Quantitative PCR

We used qPCRs to validate the RNA-Seq results that we obtained for 14 genes. Eight genes (troponin C, larval cuticle protein 9 and asparagine synthetase from^29^, heat shock protein 20 and peptidyl-prolyl cis-trans rhodopsin-specific isozyme from^47^ and RNA helicase, glia maturation factor and peroxiredoxin from^46^) were selected because our RNA-Seq results were not congruent with results reported (Table S18). The remaining six genes (chemosensory protein 12, G-protein coupled receptor Mth2-like, tyrosine aminotransferase-like, phosphoenolpyruvate carboxykinase, yellow-h and Sg_CNS_NA202, the latter being a sequence with no known annotation) were selected based on their potential relevance and differential expression. qPCRs were carried out for each gene in triplicate using both the sequenced mRNAs as well as RNAs from a different material (heads only) that belonged to other locusts at a different developmental stage (four gregarious and four solitarious 4^th^ instar S. gregaria nymphs). The primers for troponin C and RNA helicase did not work. As reference genes we used actin and tubulin as in^79^. cDNA preparation and qPCR were as described in^80,81^.

## Electronic supplementary material

Figures S1 to S5

Table S6

Table S8

Tables S1 and S2

Table S3a

Table S3b

Table S4a

Table S4b

Table S5a

Table S5b

Table S7

Table S9

Table S10

Table S11

Table S12

Table S13

Table S14

Table S15

Table S16

Table S17

Table S18

Table S19

Table S20

Table S21
