# Supplementary material for: RNA-Seq reveals large quantitative differences between the transcriptomes of outbreak and non-outbreak locusts
Source: Sci Rep. 2018 Jun 15;8:9207. doi: 10.1038/s41598-018-27565-0 (PMC6003920; doi:10.1038/s41598-018-27565-0)
Supplement: Supplementary file 1 — Figures S1 to S5 [file 41598_2018_27565_MOESM1_ESM.pdf]

Active genes in active populations: The colossal gene expression differences between outbreak and non-outbreak locusts

M. Bakkali* & R. Martín-Blázquez

Departamento de Genética, Facultad de Ciencias, Universidad de Granada, Fuentenueva S/N, Granada, 18071, Spain

mbakkali@ugr.es

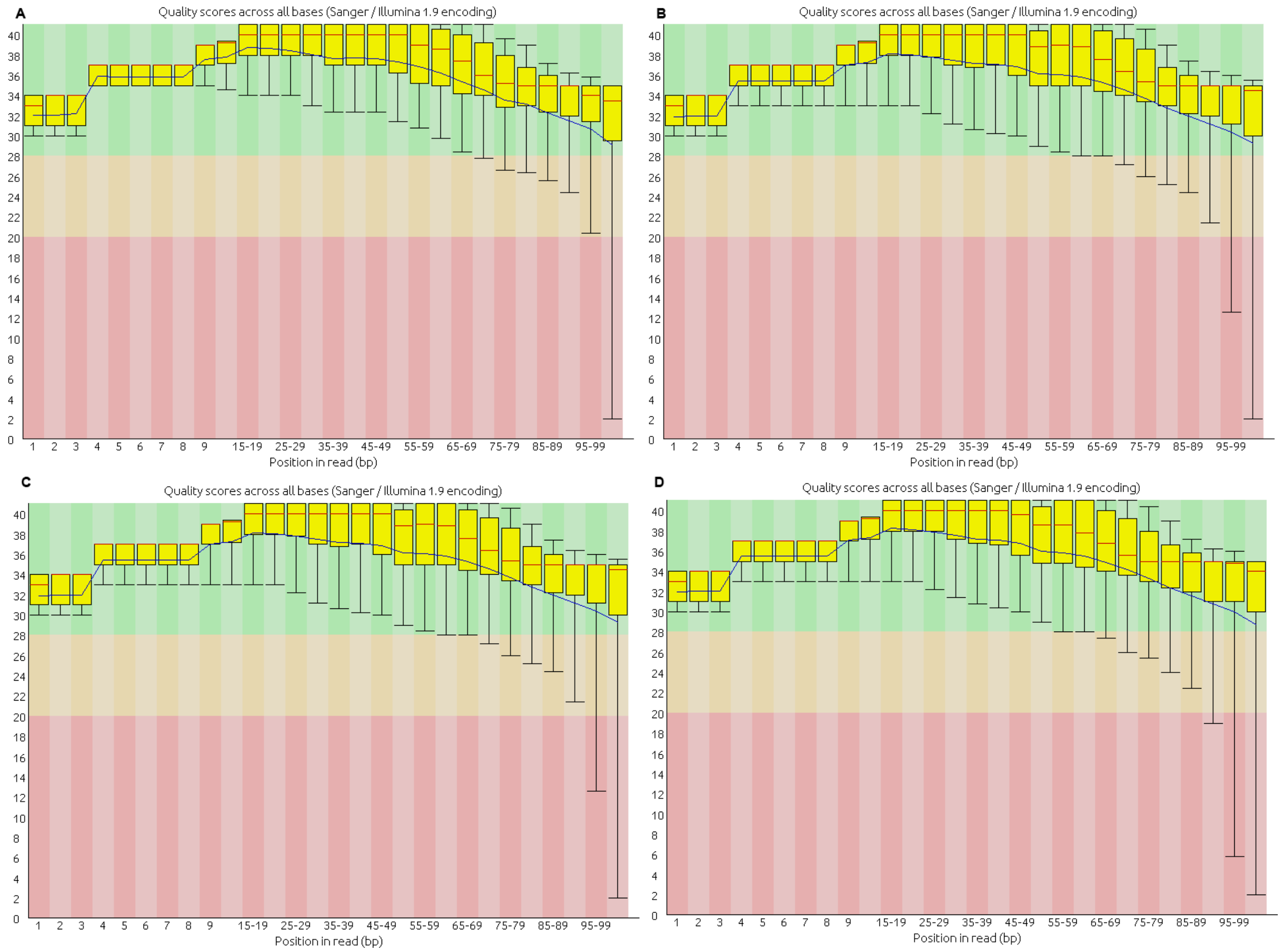

Figure S1. Distribution of the mean sequencing quality values along the 101 positions of the Illumina Hiseq2000 Paired End forward (**a** and **c**) and reverse (**b** and **d**) sequencing reads of the CNS-enriched tissue from solitarious (**a** and **b**) and gregarious (**c** and **d**) *S. gregaria* locusts. The positions of the sequencing reads are in the x-axis and the y-axis shows the quality values. The Q30 value is defined as less than 1 in 1000 probability of error.

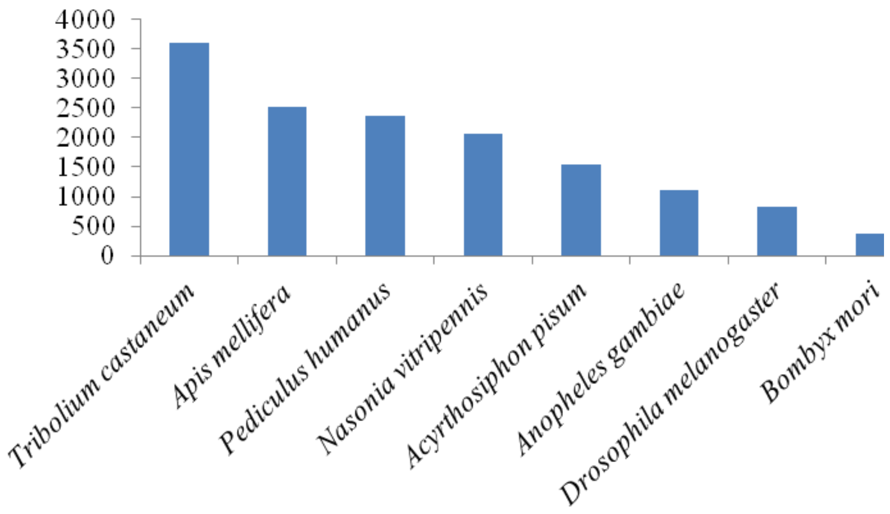

a

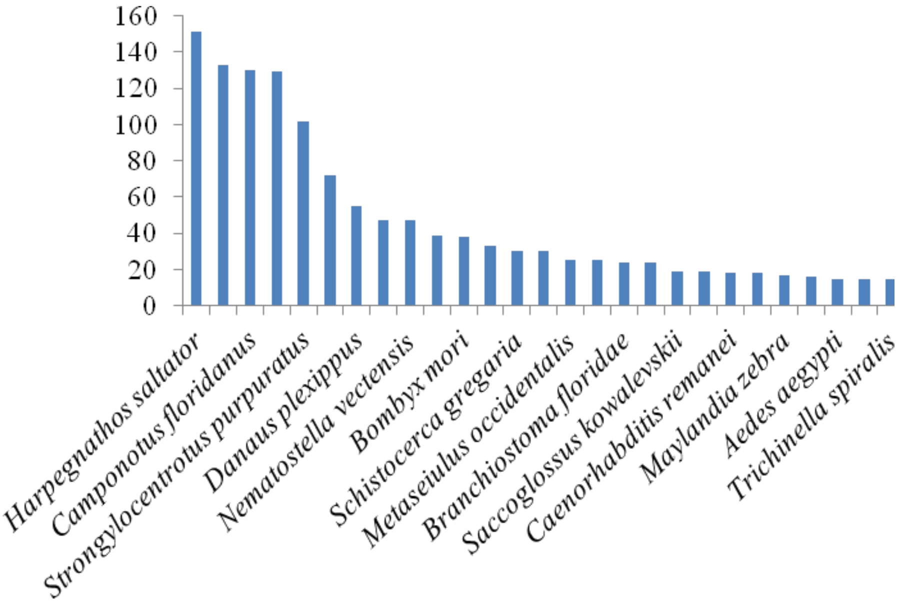

b

Figure S2. Distribution of the species against which the sequences of the reference transcriptome of *S. gregaria*'s CNS-enriched tissues gave a significant top BLASTx result when annotated using our local insect proteins database (**a**) then using the *NCBI nr* database (**b**).

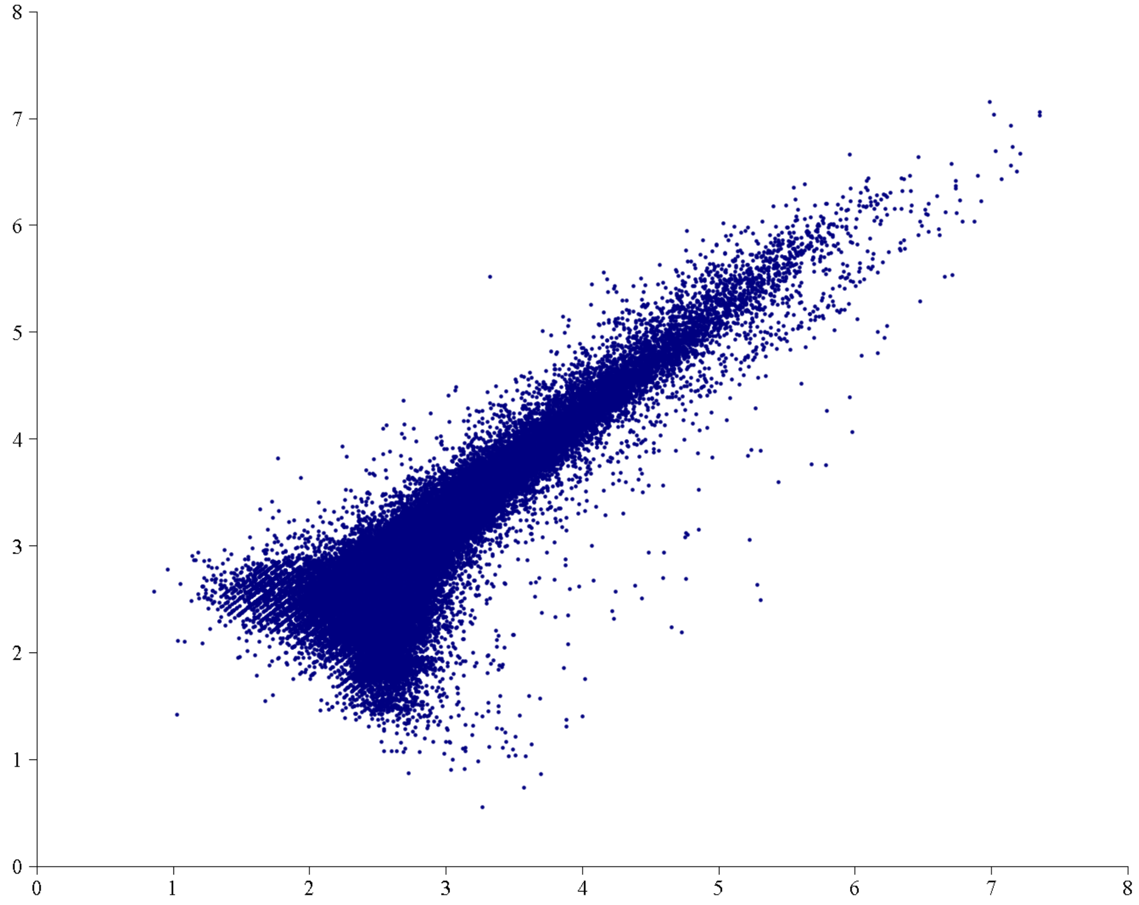

Figure S3. Correlation of the rankings by expression of each sequence in the solitarious and gregarious transcriptomes of the CNS-enriched *S. gregaria*'s tissues. The rankings were calculated in each library as the positions of the sequence in that library after sorting the sequences by expression level in the same library (the least expressed gene being first and the most expressed last). The two rankings of each sequence (one for each library) were log-transformed for better representation in the figure. The narrower is the cloud of dots around the 45 degree axis the more similar are the rankings of each of a set of sequences are in the two libraries.

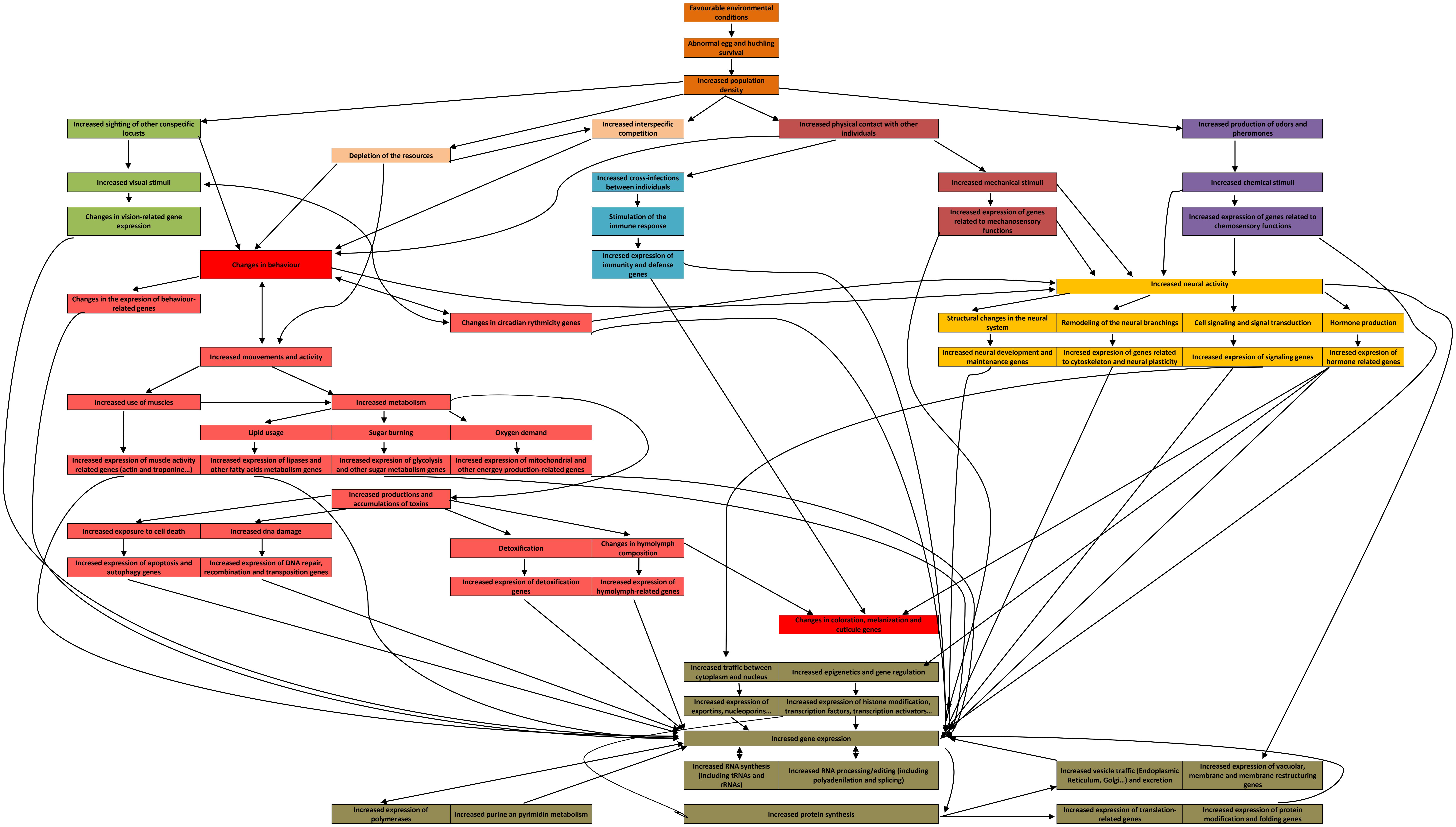

Figure S4. Detailed schematic representation of how we think genes and population- and physiology-level events possibly match and interrelate between each other in a cascade of happenings that leads to gregariousness in *S. gregaria*.

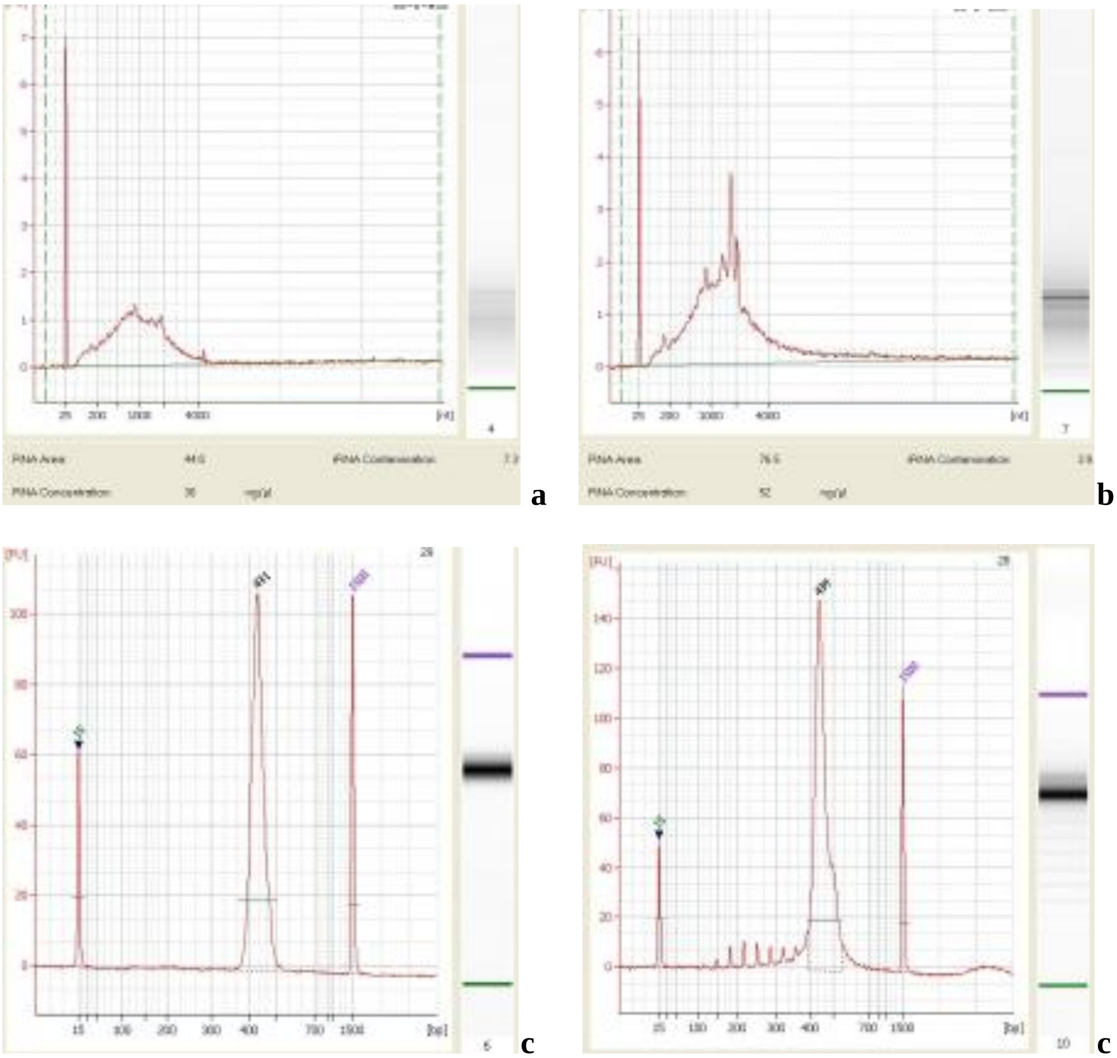

Figure S5. Bioanalyzer results of the mRNAs (**a**,**b**) and cDNAs (**c**,**d**) used as libraries for Illumina Hiseq 2000 Paired End RNA-Seq of the CNS transcriptomes from solitarious (**a**,**c**) and gregarious (**b**,**d**) *S. gregaria* locusts.
