# Supplementary material for: RNA-Seq reveals large quantitative differences between the transcriptomes of outbreak and non-outbreak locusts
Source: Sci Rep. 2018 Jun 15;8:9207. doi: 10.1038/s41598-018-27565-0 (PMC6003920; doi:10.1038/s41598-018-27565-0)
Supplement: Supplementary file 3 — Table S8 [file 41598_2018_27565_MOESM3_ESM.doc]

>Singlet867|zinc-dependent alcohol dehydrogenase

cttcgactcagctgcagtcgggttaccggatcaatctgaggcactccctgctttcctagttacgccgaggcttcagatcctttgacagattttaacatca

cgtgacatagaagttgccacagaaagatcgcgaagtgtacctgcggtcttcgacttgggtttataagaaacatcgaagtcttctgctgcttagtcagctg

ctgatatactcatcacttgcatatgcgagagaacgaaaagtgagatctggaacatcagcaccgttttggtgtgccgtatttgattgtgagagagcatatc

cagaatgaacaagctgtgtcggcaagtgagcatcgagagcccggggccgagtattaaggactgcgtcttcagcttcgaagttcccgtccccgatgtgccg

cctatgggtgcgcgaatcagggtagtgtgcgccggagcctgctaccggcggcgctcgccgtccgtgtcctcgatgtcgtcggtgtcgtcgcaggagtcga

catcgcaggagccgacgcagagcccggcgcactacggcgtgcgcgacggcgcgctcttcccgggctacgaggtggccgggatcgtcgagtcgctgggcga

gcgcgcagaccccgccacagccggccccggggggctctccgtcggagaccacgtcgtgctctacccctacgagggcgttcccaacggatatgctgagtac

attactgtgcctgagctgaaatatttggtgaagattcctgagagcataccattaagtgtcgctgcaatgcttcctacnnnagcacttttagcaatgaata

ctgtgacatcagcacaccagtatgttgaaagtatatt

>Contig1299|zinc-containing alcohol dehydrogenase

ctgtcggcgagaagtgagagtgtagtgcgtgatacagtagcagtgcagagatgccggcagacgttatggatgcgctgcagttcgatccgaagacattgtc

actgagtttaaaacgcgtgcccgtcccgaaagtgagtcacgataccgatgtcctcatcaaagtagcgtttgccggtttttgtgggagcgatttgcacatg

ctgaagggccaacatccttgcattgacacaccattcactctaggacatgaatttagtgggacagtagttgatgtaggaaaagcagtacaccatgtgaaag

ctggagacagagttgcagtggaccccaacagaggctgtggtgcttgtcagttctgttctgttggagactatactcactgtgtacttccaaagaaatattc

aggagtgggatgctttcgtgatggtggttgggcaaattactgtgtgatacctgctgtacaagttcataaattaactgatgacataagtctacagcaaggt

acactggtggaaccactgtcatgcatttgccatggatgggatcgcatacaacctatcagagtggcttcacgcattttagtaacgggtgcaggcattattg

gaactctctggacagcaatgctacatgctagaggttaccaaaatgtgactgtgagtgagatggtagagcacagaagaaatgtagttgctaaattaggtac

aggatgtgaagtcatcagccctgctgaactaaaacagcgtttgcagaaagatccatcatacaaatttgatcttattgttgactgcagtggtgccatacct

gccttnnnagcagcattannantgcttaatgatggtggaaagctgctcctttttggatgccccctatgga

>Singlet4828|zinc transporter zip9-b-like

ggcccnnncatatgactaaattgaaacacatcagcagtcatgaannnctgtttatgttgtaatttttttatcccaggtatgcagtattgtaagacaaaat

atcgtaaatttctttaggttatttaatgatgaacaaacaacatagttttttattttgtaatgaaatcagtttgtgaagtagtatcttcgtgtgttttgca

aggacattaggtttattacaataatctacatgtctttaatttgagtaagaggtggcacatcccgtgcaaccctggcactatcagtgcaaatactgttcaa

cattggttgtcattttgtggaaagtattgagtgcacatgcagtgtgacatgcgcacaaatcagcacaactatccttaaaacgtaccacagtgtatatgaa

tggttgtcggtttgtaagacacagggtgagcagtagtgaactgtcctgtatttttttcatttcaacggtgtgtgtgtgtaaaatatgtcattgtgttatt

gtatattttgagttttaagcagaatgatacaaaactctgcaatcactattatgaaagggggctatactgatgtatataagggtgccagctctgaactttg

cttatatagtgtttttgtgtcagtcctttgtcacagttgtgtactgaaaaaaatgtgtgctagagctattttaaatggtttttaaatacatctgtgttcc

tttgtgagtatttgacaggttaccatgtgtttatattctttctttannncattgttagttggtataatttggcatatcaataatttaatttattgattat

ttttctgtt

>Singlet3831|zinc transporter zip8-like

gcaccagacttatatgggtctacactgtattatccatctctcagttctgtatgtgaaaggattatgaataatgtctcagctaacccacggaaaccaacag

gattgccacaacctgagtcaggcccaactactgtgcaaatttgggtgtatggaaatttgtgtgtcttgtgcataagcatctgttctctatttggatcatt

gctggcaccacttcagaaaaccaaactctacaatactgcacttacaattcttattggccttgctgttggatcactttcaggaagttcagctttccatctg

ataccacaggcttttgaagttatgggatcagaggatcatccccagtacttacacattgcattaacagtgtggttgggaatatgggtattcttccaggttg

aagtaatactcaaaattatagaaaagcacaagaacattccacatcactcacataaccatgcatctgagggaactatgccatcagaaatgacagctttcat

caatcacaaaggagaaatttttccagcacaaccagaactgcaattaaaagctattaccaataatgtacaccaggaggcaactgatggacatgagatttca

gcttggaaaaatggctatcagccttctttagatgttcctcattctgtgaaagctaagaaacaaacaattgcaccagtggcctatatggttgtttttggag

acgccattcgcaattttattgatggagtatctattggagctgcattcactggcagcatctggacaggcattagtctcagcattgctgtcttctgtgagga

actacctca

>Singlet2251|zinc transporter zip1-like

ctgtcgaataacgtgaacaaaaatgtgtcccaaaaatgaatttttatttatgtgtgacttggatgctgtcgacttgtatgctgtcgtcttttcaatgtat

gatagataatacactgagcgatacaaatctatatcaaacggccgttattactctttaagctaccaagagcgcactatgagatatgtaatcttttttgttt

cccggccagttgaccagtgcaaacagagtagagtgcaagaagtctcacagcaattgtgagactatttcacatgttatttttaatagagaagagaagactg

ccttttatataaatctaaatacacttcaataaataaagaatatcctgaagaaaagaaaacatggaaatcaatgaagcaaagggacttgtacttggtactc

tatttgtcctaacatttctttttagtatgttaccacttttacttattaaaaagtttcgtggaaccatagatccaagcaaacgacacaggtatgctgtggt

cataagccacttgagttgttttgcagctggcgttttcatggctacagctcttctacatttgtttccagaagtgaatgaaactcttaaaagggccctgaat

ttcctacaaatcaaaaccgactttcctgttgccgagtttgctgtatctgttggattctttgtgatactgatcattgaacaaattgtgttggactataaag

aaagaacgttaacacaggacatccttacacagccggcagaagcggaaggtgaacagtggctgtcacaacgtcgtaatagcatagaaggcatcagtgatca

ggtgcattctaatgagcatgacgttc

>Singlet2971|zinc transporter zip1-like

ctgtcgatgcnnnnngtttgatgactatgaatcattcgtgtaaagtgagatgtggtagtctggtttgttttcattgtaggagcagtctcacgaaacgaat

taaactgtccatatgtgaggttcgtacaatacttacaagggtatatatttcaataatcaccgctgttgcgaagatgatataagtagaacaataaggtttc

ccggccagttgaccagtgcaaacagagtagagtgcaagaagtctcacagcaattgtgagactatttcacatgttatttttaatagagaagagaagactgc

cttttatataaatgtgagatacataccctcattgcattcttattgaaatacgggttatttattagtcatggattgccttatactcagtgtaatcatcatt

ttaaggctattatgtcatttccttaactaatttattgcaattaatttcattttcagctaaatacacttcaataaataaagaatatcctgaagaaaagaaa

acatggaaatcaatgaagcaaagggacttgtacttggtactctatttgtcctaacatttctttttagtatgttaccacttttacttattaaaaagtttcg

tggaaccatagatccaagcaaacgacacaggtatgctgtggttataagccacttgagttgttttgcagctggtgttttcatggctacagctcttctacat

ttgtttccagaagtgaatgaaactcttaaaagggccctgaatttcctacaaatcnnaaccgactttcctgttgccgagtttgctgtatctattggattct

ttgtgatactgatcattga

>Singlet6103|zinc transporter zip1-like

gtttgatgactatgaatcattcgtgtaaactgngatgtggtagtctggtttgttttcattgtaggagcagtctcacgaaacgaattgaactgtccatatg

tgaggtttcccggccagttgaccagtgcaaacagagtagagtgcaagaagtctcacagcaattgtgagactatttcacatgttatttttaatagagaaga

gaagactgccttttatataaatctaaatacacttcaataaataaagaatatcctgaagaaaagaaaacatggaaatcaatgaagcaaagggacttgtact

tggtactctatttgtcctaacatttctttttagtatgttaccacttttacttattaaaaagtttcgtggaaccatagatccaagcaaacgacacaggtat

gctgtggtcataagccacttgagttgttttgcagctggcgttttcatggctacagctcttctacatttgtttccagaagtgaatgaaactcttaaaaggg

ccctgaatttcctacaaatcaaaatcgactttcctgtt

>Contig1159|zinc transporter zip1

atataaggtttcccggccagttgaccagtgcaaacagagtagagtgcaagaagtctcacagcaattgtgagactatttcacatgttatttttaatagaga

agagaagactgccttttatataaatctaaatacacttcaataaataaagaatatcctgaagaaaagaaaacatggaaatcaatgaagcaaagggacttgt

acttggtactctatttgtcctaacatttctttttagtatgttaccacttttacttattaaaaagtttcgtggaaccatagatccaagcaaacgacacagg

tatgctgtggttataagccacttgagttgttttgcagctggtgttttcatggctacagctcttctacatttgtttccagaagtgaatgaaactcttaaaa

gggccctgaatttcctacaaatcaaaaccgactttcctgttgccgagtttgctgtatctattggattctttgtgatactgatcattgaacaaatagtgtt

ggactataaagaaagaacgttaacacaggacatccttacacagccggcagaagcggaaggtgaacagtggctgtcacaacgtcgtaatagcatagaaggc

atcagtgatcaggtgcattctaatgagcatgacgttccagcagaggagccagctgcacactcagtattccgttcagtactactattggcagccttgtcag

tgcattcactgttggaaggtgtagcaataggactacagcctgatgtaatgagtgtactgcagatatttattgctgtggtgctccataaagttattatagc

cttcagcttgggactcaatcttgtacagagcagactgaaacttcatgcaatcataagatccaatattacattttgtgt

>Contig4679|zinc transporter 7-like

tgggcatggattttggcatctgacgtttactgaggaaacggggaaatagtgttcaccgttagagactatgaaaaaccagcgaaatgggccgtgtaaatga

aaacagaagccaaaattcgtttcctgcttcgtgaagtggaatgaagaaccacatttggaaacttaagttacaagtacaacatgttacctgtaacacacaa

ggatgcttccagatctctgggatcccgactgaaagagaaggtcagcggatgggcaaggctgatattttcagaccgtaactcaagaaatctttttctcttt

ttattgctaaatctttcatttgcattcgtggaattagcttacggaatatggaccaacagtttaggtctcatatcggattcgtttcacatgtttttcgact

gcacaggccttctagcaggtcttgctgcttcagtaattacgaggtggcgagcaaatgataaattttcctatggttatgtaagagcagaagttttggctgg

ctttgtgaatggcttgtttttgctcttcattgcattctttattatgtcagaagctgttgagagagcaattgaaccgccagaagtgaagcatgaaaggcta

tttgtagtatctgttctgggacttgttgtcaatctggttgggatttatgcattccagcatggtcatgggcattctcatggaggcagtagtggccatggac

attcccatggcaatcatggtcatatacattctcattctcatggaagtcattcacatagtcatgaccctgagttgagtggcagcaattctcaaataatgaa

aggagtatttttgcatattttagctgatactttagggagtgttggagtaattatatcggccatcctgatgcagatgtttggatggatgatagctgatcct

atatgttcaatgttcatagccattctaattgcattaagtgttttggcactgataaaggataatgtgttaatattaatgctgagacagccagtagctttgg

atcatgtacttcctcagtgttatcagaaagtcagtcaattggctggagtttacagtgtgcaagaaccacatttttggacgttgtgcagtgatgtttatgt

aggtgctttgaaactggaggtttctaaggcagcagatccaaaatatattgtgagccacacacacatgatatttgcatctgttggtgtcaggcagttgtat

gtacaattggactatgcttctatgtgattcagacaatataagaattgtgtaggtaccatgtggttgtttcgttaatggttgtactgttggttatgttcca

gttggaaggcagttgaagagccttgcaactgacacaaattttatttgttgacagtattattgttgtgtaggtatattttattctttaaaaatgatcccat

aatttaatgcctacacttgcatttaaaatcactgattacctatttgtataaacagnatgttttgttctgataatatta

>Singlet3777|zinc transporter 7-like

ggatnnnggcatctgacgtttactgaggaaacggggaaatagtgttcaccgttagagactatgaaaaaccagcgaaatgggccgtgtaaatgaaaacaga

agccaaaattcgtttcctgcttcgtgaagtgcagacactgtctacatgttgtctccattggagttcattgtcaaacaaagtgctcaaaaaatgtatacag

gaatgaagaaccacatttggaaacttaagttacaagtacaacatgttacctgtaacacacaaggatgcttccagatctctgggatcccgactgaaagaga

aggtcagcggatgggcaaggctgatattttcagaccgtaactcaagaaatctttttctctttttattgctaaatctttcatttgcattcgtggaattagc

ttacggaatatggaccaacagtttaggtctcatatcggattcgtttcacatgtttttcgactgcacaggccttctagcaggtcttgctgcttcagtaatt

acgaggtggcgagcaaatgataaattttcctatggttatgtaagagcagaagttttggctggctttgtgaatggcttgtttttgctcttcattgcattct

ttattatgtcagaagctgttgagagagcaattgaaccgccagaagtgaagcatgaaaggctatttgtagtatctgttctgggacttgttgtcaatctggt

tgggatttatgcattccagcatggtcatgggcattctcatggaggcagtagtggccatggacattcccatggcaatcatggtcatatacattctcattct

catggaagtcattcacatagtcatga

>Singlet130|zinc transporter 6-like

agtagggatgtagattatacgaattacacttgtcagttaaaaaatttggaagatgtcaattgagaagaaagaaaaagtgaaccttgtggcttgttggtct

ttggttgctacagtgttaattgatggtatgaatgaatatgtgcatcacatagggattaaaatatttctcttacctttataatataataaagtaatgtaaa

attccagatcagtgtgaaataaggagtaaaagtactattcatttgggtgagtagaagtctgtgctccaatcctttatatgtgtgtgtttatcatctgtga

cttatcaaagaaatatactgtcctttttaatattaaaaaatggaaattaaaattattttcatatt

>Contig586|zinc transporter 1-like

gggccgcgggagatgtgccgcgacacgctgggttccatattggtaataatctgtgcagtcattgtgtacttcacagatgtgtatgtagccaagtttgtgg

atcctgtcttgtctatcatctcggcagtgtcccttctagtgctaagttacccatacatgaaggagtcgggactgatccttcttcagacaataccagatac

gatcaacatagactcattcagagtagagctcatacgagagttccccgacatcataaatgtgcatgacctgcatgtgtggagactgacaccaagcaaggtg

ttttgcacagcacatataatttttcttaatccaaaggattatgcgaagaaatctcaaaaaataattgacttctttgaagaacaagggataactcaagtga

cgatccaaccagaattttttgagactgacaatgacaccaacattatcgcactgcccagcattgaggagaaaagctgcctggtacagtgtcgtggccaggg

ctgcattgagagacactgctgcctcaagttggtgcctggcatctctgtggaccccacctgcaaacagctgccccagaagggtgccccagacaaggagtgc

attaagatctcctcacaaagtgtcgcggagagtacaacacttccctctgatgacatttcagagaaaacagaaagtgccgaagagcataacgcttgtcagt

cagctgggaggcaaacaccagatgaagaagaacaagtttctgcagcaaatgaaacccaagtgccaactatagatgaatcttctctcacttcagaagtggt

taaagacttatctcaaaatagtgacaa

>Contig3544|zinc knuckle protein

gaannnnnngcccaggagagagtgacactgcattgacgccagcccactttctaaatggtgggaaattagtaacaattccatgtgggccagagccagcaac

tagaaacgaccttgccaaggagctccgactcagacaaaaggtcaatgacgacatctggcgcaggtggaagacagaatacctcctgctgctgagactatat

cacgaggtgaaggtataccctttgcaaaggaaaccgagaattggagaagttgttctgctctaagaagacagcaaaccacggcacttgtggaagagggacg

tggtagaagaagtgcggcatggcagagacaataaaatacggtgcttcatcctcagccagccagacggtatgaagatctgtcgaccggtccagctggtcat

ccccctcgagatcgaccagggtggggaggatgtcggggaatgaagacgtgtacgccatctgttggtgctctgttaagtccgtacattgtgctgccctctg

cagcggatatatttcaacggtaactgtaaaatggacgtgtgtgtgagcagaagttttactgtatccttgatttcactagtgtgtagcaacaactgtgttt

tactctagtcaactatttgctctgctccttttaagtgtcctctatttcgtgttattgaggtgttcattttagctctgcgccccttggtgttccctccctc

tgcgtgcagaggttacgcttttattgtataggcctttataaatatgtctgtttggaacaggggactgatgacctcgacgtttagcccccatcaaacccca

aaaccaaccgaccttcatctgccccctggggccttttcgtgtcgactatgaccggctgtgtatctgccatgnntccctcggccgcccgtaggaaggcggc

gtggt

>Singlet3569|zinc iron regulated transporter-related protein dzip1 protein

tttnnttttttgtacgtagagaacggagacaagttctggacctgnntttcgttggaagcgcagtggcgaagtcgtaaacgtaatgtgaaacgtgttgttt

gtgcgtaattttgtttcacttgtttagaggaagactgatcagtcttaagtttttcacctgactggaatgtgtcgtcagttttcttcaatatttcgcaact

ctggtgtaaagttgcagtttcataatgggaacgagattgacctgtagtgatttctgcagtcggtatagtcattcttaacaagaacagtaattcaccagta

ttcaagatctgacaggttgctggaaacttccgtcctacatatttcattactgtggataaagcagtcacaacatggaagtaatcatttttcagtttgtttg

tttggtagttctttttgtcattatgactgtaagtgggtcaatacctatagtcattatatggaggcgcaagaatgctcatttaagtgatatcgctggcaca

ttaatatctgtaagcaactgtgttgctggtggtgtttttatggggatgtgttttttaggactatttccgtttgtcaaggaaaagttt

>Singlet4425|zinc finger rna-binding

ctgtcgacnnntnnnnataaagaccgaaccaaatattaaagaagattcaaagaaggaagatggcagagatggaaatctattctcatttcacagagataaa

gatgacagccaagttccaagggttttgaaaggtgttatgagagtaggtgtacttgcaaaaggacttttgttgcatggtgatacagcagtaaatctagttg

tcctttgtggggaaaagccaacacgcactcttctaaacaaagttgctgagtgtctccccaagcaactaaaggtggtgtccccagaggatacttataagat

acaacggaaagttgaagaagctgccatagttgtgatgggtgtaacagaaccacacatcactgttactatcacgctaacttcccctgttatgcgtgaacag

ttactgtgtcctccagaaacgagtggtgactcggtgtctgtgagtcaggcccaggtgatgaaggatccaccagacgtgctcgacaagcagaaatgtttgg

atgccctggcagctttgaggcatgccaagtggttccaggctagggcaactggtttacaaagttgtgttatggttattcgcattcttcgtgatctgtgcca

aagggtccctacgtgggcgcccctacatagctgggcaatggaattgttagttgaaaaggtgataagttcagcaggacaaccactaagtcctggagatgca

ctacgccgtattatggaggctttggcatccggtattctgcttccgggtggtcctgggctgatggacccttgtg

>Singlet3174|zinc finger ran-binding domain-containing protein 2-like

tttgtttgctaaattgcctagtttggctgtatacagtgtttttaataagtgtttacaatcacacgtactaatagcctgattgtggaaagtcaccagtttc

gctcgtggacgttttattgacgtgaagctttgtggcgtctaccggcaagaagattnnncaaggtgctgacatgaatggtgtgagagctggatactgggtg

tgcccagatccaagctgtggcaacttgaacttcgcacgtcgaacagcttgcaacagatgtgaaagagagagagatggtgggccagggggaggaagcagca

tcaggagacggaggggtgctgcacctgaaataggacgagctgctgctgagaagagtcgtggcctatttaatgctgatgactggcagtgtggcaaatgtgg

aaatgtaaattgggctcgccgcactgcttgcaacatgtgttctgctccacgtgtagctgatgaagctgaagaacgcactggtcttggaggcggttacaat

gaccgtgatgtagttgagtatgtagagcgtgacagcgattcatatggttatgatgaatttgg

>Contig3442|zinc finger ran-binding domain-containing protein 2

gggcannnnntggaagtttgtggtgattgtttgggatcggaagagtggggatttctttgatatacaactaaatttagcaacacaaattaaaatcttaaaa

ttttctattccgtcgtaacgatttgatacaaagtatggacacggcatcgggtaacagtcgatttaaaatgagcgaaggtgattggatatgcagtgatcct

cagtgtggcaatattaactttgcgaggcgggacacctgtaatcggtgcgataaggaaaaagtggaaagcttgaaaaagaagaagttaggccatgaaattg

gtaaagctgctgcagagaagagccgtgggcttttcagtgcagatgattggcagtgtaataagtgtgggaatgtcaactgggcccgccgacagtcgtgtaa

catgtgcaatgctccaaagtatggtgaggttgaagaacgtactggttatgggggaggttacaatgaccgtggcgttgtggaatatgtacgccgttcggac

tcagatgatgaatatgatgagtttggaagaaaaaagaagaaaaagaatggagagtcgattccagaaaaaccacgcagagtagcaaaactggatgtagacc

gtgatgaagaagatgaagaagaggatgatgaggaagaggatgatgatgatgcggatctgtccaaatatgacttagttggttggggtgatgacagtgatgc

tgattctgnnnaacaaaaaaaggacaaagaaaatcgtccaaaaacgaggcggggtcattcaa

>Contig4222|zinc finger protein zpr1-like

gttttnnntatgtaaaacttgtgtgttgtttacgcgtgtaatttggttaacgtttgcaagctgagtatgtcgaaggacattgataaaaggccgatattta

gagacctagctgcagatgacccagatcccgaaacaacggaaattgaaagcctttgtatgaattgtggtgaaaatggcattacaaggttattgcttacaaa

aattccattttataaggatgtcgtgttaatgtccttcgattgtgaaaagtgtggattccagaacaatgaaattcagtcgggtggtaaagttgcagaagct

ggtgtaaaaattgtgttgaaaatcagtactcagagagatttaaatcggcaagttgtgaagtcagattatacttcggtagaaattccagaagtcgaatttg

aaattccagctcagtcacagaaaggagaaataacaactgtcgaaggtataattgacagaagtataactgggttgaaacaagatcaaccaaagaggtgcga

agaaaatccagaagctgcagaaaaaatagagcagttcatatcaaagttgaaagatttaaaggagattaaaatgcctttttctatgatttttgttgacata

agtgggaatatgttcatagaaaatcctaatgcaccacacagggatactggtgttactgttacatatttcaatcgaacaaaagaacaagaccattccttgg

gcatctacgagcatactgagatcgaagagagtagtggtgatgaaccaggaattttgaaaccgctaggagaaggagaacacactttggaggatctgcaagg

ggaagttctacagtttcnnnnnnactgtcctgactgtaactgtccttgccagacaaatatgaagttgac

>Singlet804|zinc finger protein with krab and scan domains 5-like isoform 3

gtannccttactgcacttttaaagcgtgtatctgtatttattacttttattgtgaagtagttccacattttgaaatgtgattttatagacgttttatttt

accttttttcatttgttgtgaaatattggttgtgaaattttacctgcttagttttttatggagtttttttgttgaagtgtgccattttccatagtattgt

aggcctagctgatgtaataaatatttttgccatagggatgtttaccagattctcacctgtgcagaggataagagagataaatactcacataattttttta

ggtcagttgtaacttttgaggaattttttccaggaaaagcattaatccagctggcaaagcagcttggtgagaacaagtcccttcatttgagattatgaga

aagtattattttttagtaaatgttagtgacagtattcaggcaggtacatttaaggtagtatgttagtaagaaatgtaatgtaataagagaacatgtaaga

agttttgaatgtatagagtgtaagcaggacacacataatcatcacaactggtacaaaacaaagcttttaagttagttgtattaaccgtgtacaatcctgc

cagatacgtgccaatttctcctctcgtgcttactaggcatgagcataacctcccgcatgcaatgcagtaatatgtgcatctgctttgacaccaaaagaaa

accagtcacaaatatgtatactgataataatgatggaagctgtattggggtaaaactattgactctgattgtagatcaatcatgaattttattatgttga

aacatgaaattccttgagatagtacaaaacaccatgactgatgtaactgt

>Singlet4671|zinc finger protein transcription factor lame

gtacagacttaaaattttcgtcttggaatatccataatactgattcaagaagaacttccttttcttttagaaatgatagcataaacttgaacagtataat

taggcattcctctacaagtcttctgggatcatcaacaatacaggagcacagcaatacgggtgcaatgggcagctatggtcacttatcagcaaggagcact

tgtagcccaccaagcctgagtttcatttccaacagcaacaataataatttgccagtaaacgggcactcacatagttcagaggcaatgaccttagtagaaa

ataagccattcaacacatgtgaagccctatcttcaaatcaggttgttcttcctaaatacaagggtgaaattctttcaaatttactgaataaacagcaact

accttcctttcatcaaatacagctgtcagctgaccattcaaaggataactatcattgccattatccaaagaatggaaatttgcctgattgcctcagaccc

cctcatgatcctttccttgggacactggaaagtgagaatacatcgttacaaagaattacttctgaacttgctgaagatgatgagacaacagctgaaactc

tccttgaatgtcgttggattgactgctatgctgtttttatggatcaggagtcactagtcaaccatattgaaaagagtcatgtagaaatgaggaaaggtga

agatttctcatgtttctggcaaggatgttcaaggcggtcaagaccttttaatgcaagatacaagttgctgatacatatgagagtccactct

>Singlet1555|zinc finger protein loc100301947

cggggatgttgttgttcttgcatatcaaagctggaaatgtgccataaatttgttgtatcgtgtttagatgctaattctaagctgtcacaattcttcgttt

gccacggacatctgatgatgagaacactggcacttcactgatgttgggagaacacattggaccatttgagactgtcgttatcgagaaagagagtagtttg

gctgacagatttaatgaagaggtggatgtacatcccaatataagtgagtccagtctgttaaaaaatggaactgacatgtgcaaaaatcagactaattttg

tttcaagcagagtgctgaatgataataaaaatgacaatatcagaggaagtaatgtaatgaaagtagcttctccatatgaagaacatacgtttcttataga

aggaaaacaggaaatgaataaacagttggtgattgccaatgtgcgttcttacattaactctggcaaaaataaaataaataagaagagaaatcttcaaaga

aagaaatacaagaaatcaaaatttgaagttgcattgatgaagttaaaaaaggtatatagctcatcacttgcagtttctccatctaagggacagaggcttg

atgcaaaagacgtattttattggaatcgcagtttgcagaaacgacaatggacaagtaacagcatcaggtgtgatgggtgtaaccagctgtttatgaattt

gaacaggctacgttcacacagagtagtagttcatagtcgtttgcattttaagtgtcagatgtgtagcaaactctttgg

>Singlet5858|zinc finger protein loc100301947

ttgttcaagcgatcattagttgtacatgagcgtatacatactgaaaacaaatcttttaaatgcaatgtatgtgataaatgttttaactctgtaacaagac

tgaagcagcatatcattacacattcagatgactggccatttcgatgtacattttgttccaaagaatttagacgtagagaagctttgaaaaatcatgtagc

aaaacacacagatgaatgtaaattatgtaatcacaaacctgatgtaaaaggaagaaaagaaggtagtggcatactagaagaagttgctgatagtaaaatg

gaggaaccaggttcttcctcagataacactattatctttggtgaatttggttcatcagatattgttattgaaagccatgcagattccacacctttgataa

atgacagtaatgttgcttgtgaatatgtcatttgataagaatttgaaggaaactttgtctctcaagatcaatacagaattgtaccgtaaaattgtatgac

agagttagacatccattgtattaatttgcctgcttcagttcagaagccaaacccattttgcctgtaatagaactgtaaattttattgtaattatcttttc

tatgtgttacttgtgtataatatattttgtaatgaaatcttaaaaaaaaaaaaaaaaaatg

>Singlet7834|zinc finger protein dpf3

agggatatgtgtaaatctggccttctacaaggccaggcattcgttcacgggagctcataaacaaatttgagtgattctgggcaacgcctgtttgagaatc

aagaaatggcattcgcagtcttctttcgatacacaagcgtgtattataagttgccgaattctcaattgtttctctgtaagtggcatcattgaagaagctt

tcaattttacttaaaatggatggattaacaacttcaatttcacatttttccattccctcgttcacagccataacgatttgcctactactaactacacaaa

ttcacactaatgtttcgagctcactcacaagcacacctgacaacaaaaaaaaaaaaaaaaaaataaaaaaaaaaaaaaaaaaaaaaaaaaaaaaaaaaaa

aaaaaaaaaggaaaaaaaaaaaaaaaaaaaagggcccc

>Singlet3583|zinc finger protein dhhc domain containing

gaggtgatccaggagtcctttcaagcactcaggaagaaaaatatagaaccatcattgaactggcagaacgtgggggttttgacccacaaggtttctgcag

ctcgtgccttgttagaagaccggtccgatccaagcactgttctgtttgtaatcgatgtgttgctaagtttgaccatcactgcccgtgggttggaaattgt

ataggagccagaaatcatcgttatttcatgggatacttaataatgcttctcacaatgtgtgtatttttgctgtatggttgtgtacagttttggcacataa

tgtgtcctgttacaagtgacagtatctgggaagctgttctcaatatggcaaaatgcgatgcatgggttgcctgggttgctggcaatgctcttctacatgc

agcatgggtagcaacgctctttgcttgtcagatgtatcagataatgtgtttgggaatgacaacaaatgaacgtatgaatagtggccgttacaaacatttt

cacacaacaggacgattgggtcaggaaacaagaagtccatttcacagaggactttgtcagnacctcattgactttgctgaattccgttgttttggcttat

ttaagccagacagtactgattggttgacacggtatgatattaaagactctgttg

>Singlet4340|zinc finger protein dhhc domain containing

tgaatgagactataggtgttttgaggttgttggtaggatggtggtcgtgttatgacatactttgcaataaacatgacgtccagttgtcgtgttattttgc

gattcccatcactctcacttttcctatagttatctttgtaaataattaataataatgatgtccggcgaagttgaccccacttgtaatcccgttcactttg

tgacacaggatgcttgcaagccagggcctacgtacagaggtgatcaaagcgaagctcatacgatttttcaagagcctgttacagctgtgacggagcaaga

ttgcagtggatttgatatagttaaagcaacacagtacggttcgttggcgcgctgccacgagcttattgaaggcggctatgatgttaatcagccagacagt

gaaactgtcacattactccactgggcggccatcaacaaccgaagagatatcatacggtattacataagcaaaggagctgttgtagatgcaattggaggtg

aacttgctgcgactcctctacactgggccacaaggcagggccacctcggtacagtgacattgcttatgcagtacggagcagacccctcattacgtgatgg

agaaggatgttcctgtattcatctggcagctcagtttggtcacacagcaattgttgcatattttatagcaaaaggtg

>Singlet6474|zinc finger protein ccch domain-containing

agaaaaagaacgtgaaaaggaacgagaacgtgaaagggaaagagagaaggaaaaagagaaggagaaggagagggagcgagagagagaacgtgaaaaggag

agggaaagagagaaagaaagagaacgagaaaaagaaaaggaacgtgaaaaggagcgagaaagggaaaaaagacggaatgaaaacaaccaccattcatctt

ctggatcagaatcaccaccactatcaaagatttcaaattcccacagtggaaatggtcatactaaaagggaaccttcatcctctccagaaccacctccacc

aggagaggaaccaaacattgagcttccaaaaatgccagtgtcaagcctgcaggcgaagctgatgagccttgcgggtggaccccggccgaagacaagatcc

aggtcagcctctaggtcgaggtcacgctcacataccagctcaccatccagatcacgatcgcactccagccattcgcggtcgaagtcaaggtcacgttcag

tggacaacagcagcaaaaggctgccaccacagaagtcagaatctcggtctcggtcacgatcgcgatcccgttcccgctcgcgctcacgtggcagttcatc

ggcctctgacagaggtggtgattttggtgaaggatctgatggtcgtgcatctcgttcctctagcaaacattctagaagttcagtttcaagtcgtagcttg

tcttcatcttcggaccagtcagacaaaaatgcagggggcaaagaagaggacttcgaaatacataaaaaggaggacagcattcag

>Singlet1170|zinc finger protein 91

tagtctcagtttaattaaacactagtaaaataaacttacattatatgaactaaagcattgtttaattaaataataataaaataatcttgaattatatcaa

tctgttgccacatacaagtgttaccccacagtagtgacccactcaaagcattaaacagtccagttggatcagatcccagtgtgctgcagctcatgatcta

gatcattttcttgcatggatcataaatttttttctcatgtagctctctgtttattttatattacagactctcacatggtcgtatgacaacagaacatatc

actgtgttagctgaagcaatgatgaaggaataggtgtggacttgaaaatgtaaaaaaacagtggtatggtaaaagtcaatgttatttgtggttggcgcac

tttacacacaggtgcacccactcaagggtttataaatgtattgtaaattaattcgatatgaagtttagtgcaatttttgaatgaatcttctatgcattaa

atcaattaattaaagttgaaactaataataaattaagattctgcttcagattcttatatttatagaagttcctttcatagcaggtggtttttcatgcacc

aaatacctgaacaaaggttctgaaaaaactgctactggtaaccaggcaccaccgcaacccaaactattacgatgcccaatttgccgccgtgaatttcgat

accgtaacttgtgcagacacttacagcaacacagaaaattgcgaccatgtagatgctgtggccgcagtcttcctggcaatatagctcttcatgaagccta

ttgctctc

>Contig1589|zinc finger protein 862-like isoform x1

atattaacatcactggcgcttttaggtgctaataccttttttcaccaacaattctgccttcgtcttgccacaggaaagttttacagcggtttctgaatct

gtgaatgtacatgatgtcaatttattgctacaatccaaacttgaataacttaggttgtgtttgactgtgtgatacaaaagtcctaactcacaagccatga

caacgttggtttcagatttcttagttcacaaaatatttggaaactttcgtggtttgactacttgctttctcctttaactgatgtcatttgtactcagcat

gttgctcgagatcaaactgtccaccatgagaaacgctgaattccttcctgcacaatgtacaaaaagcatgtttatcagaagttaaacagtgcctaaccca

cgcgaaagtatcatcccatttctttagtacacttgactgtactgctgttgcttgctgcatttcttcactggtgaactcattacaattcttgaacaaaagt

aaattaagaaaactagactatccactgtatggattcgagaaattatcacagcaacgaagtttattagtttacagacatctgtagttgcacacatcagaat

tggaaataagtatatagacaacattattgatcgtgcgattgagtgattgtcagaagtaccaatttaaacgtagacaagcctaaaatctgtatctgaacag

taataataacaaatataaccaaattaatgtgaatttttgttatatgtatattaaggggaaggattgttgggatctgcgatagtttagt

>Singlet695|zinc finger protein 850-like

gtgnnnatgtttgaatgttgtttacgttttactatctgatgttgataggattgtcaaacatctcagtggattgaatcgacagttatgaaaagaatgtgtg

tggaatcagcttgggcataaagtccattccagtgccagtattgaagatatcaaggaccagttacagcagttgtgcatcagcttggctcaggagaggatat

aacggctttgacacctttccagaccagttcatcggtacatttagggcagagggggtgcagtgtcatacagatcagggaggtcaagcagaacaaaccaggg

cacagcaaatggtatatgtgaccctggacctcaagtaaatgaactaggtgatagaaataccccacaaattaaaagagaagagatgttcatctcacaggat

tgtgagagtaacctctctggtgaagataatgcctctggatcaagtgtgccagaagaagctgagtgtcggactttggtttgtggagactgtgggaaaaatt

tttcacaaagacaccttcttactagacacaagagagaagtacactgtccaaatcataaccttgtatgtaaggaatgtggcaaaaaaagtagcagtagtgg

tggactgtatcatcacatgaaatctcatttaccaccagcttacgaatgtgcagaatgtggtagaaaatttgataggaaaaagacacttgcatcacattgg

cacagactacatggtgacagtacccaaattcaaaaagaagttcatgtgagacctaaatcgcctaaatgcaatactggtactgatcnnntgcttttaaaat

gttgtgattgcgataagacttatatgtcagcaacaggcctaagtnnncaccgtgctactgttcacttacaaaaa

>Contig2266|zinc finger protein 845-like

tgaacgntctcataagtgtactatttgtaagcagagatttgagacatcagataaactctttatacatgagtttacaaagcatagcatagaaaagttgcca

gacaacaaaaatgttagaagatacagaaagagagagctacataaatgtgaagattgtggcctagttttttgcgggctgaaaccttatcgaaaacatgtaa

aagaacatcacattcccgcagaaaatatatgtgaattttgcggtgcccattataaaattaaatcccagctagaaattcataggcgactccatacaaatga

gaagccatacacatgtactatttgtcaagaatcatttcatttcaaaaaggcattgagacgtcacaaacgtactcatattgaacccaagtctttaacgtgt

agtatctgcaataaaacatttgccaatagaagtgcaatgtggcgacatgaaaaaatacactctgacagcagaaacgttttatgctacctttgtggcaaag

tgttgtcaaacccccaaagtctaagagttcacatgagaacacactcaagtgatagaccttgttcatgcccaacttgtggtaagagcttcaaagacaatgt

ttctgttaacaaacatctgataattcattcaactgaaaagaacttccattgtgatatttgtggtcgagcattctacagcaaggctttggtaaaacagcat

aagttaagccattctggagtaaaaccgcatacttgtgagacctgtggtgctgcatttaatagactgggaaatctaaaccagcacaaaaagaagcacttat

atgactttggtaatgttcaggatgtaagc

>Singlet3096|zinc finger protein 845-like

gaattgcctaccaaaaagtctttcaaactctgtttaaactgtgctttatcttaaatcaattttttaattgttgctgtcaatttattgaaaatgtgtgttc

ctgaatactggacccctttttggacgaaggttcataaaaatgatggtttaccacaccatgtgtgttgcagttgtgtttttaaattgagtaagtgtcacca

gacagtggaagcctttttagcagctgacaagaagctacgggttctgtttgaatctcagagcaaatcaactcaaaatctaacatacattaaactagaagag

gtgcgtcaaatggtgacacagaaattgcaacatgtgccgaaatcagcaaaatccatctaagagccttttatcagatcaagtgggtcttcctccggatctc

tcagaatttcttactgtaaagaaaactactacagggaaacagtctcctgcatctgtaaatgagtgttcagatatcgcggagaagaacgatgttatctatg

ttttcaccgattccggcaatcagagtgatgcaaatgttggtaatgattcttgtgacagctctataacatcatcacaccaaacatcattgttttctggttg

tgaaacatctaaatcggaacctaatatttctgactacaaagcagctggcagttttggact

>Singlet5609|zinc finger protein 76-like

tcagaagcacttacgtactcatacgggggaacgcccattcacatgtccagttgctggttgtggacgctcctttactacatctaatatacgaaaagtgcat

gtgagaacacatactggtgaacggccttatgtttgtaatgttgaaggatgtacaagagcgtttgcatctgcaacaaattttaagaaccatctcaggatac

actctggagagaagccgtatgtttgtagtattaaggattgtggtaaacgtttcactgaatattctagtctgtataaacaccacatggtccacactcagct

gaaaccatatttatgtggagtttgtggaagacattacagacaagcctctactcttacaatgcacaagagaacagcttggtattgtagaagttgacgatgg

caccgaaatattttttggtgacagagtcctcgagtttgcaaacaacgaatctgtcacaaaagggaaaaaacttaaaacttttccaggagtgaaaaaagat

ggaagcacattggtggccttacaaacatcagaagatccaaatgctgtagatgcagtccagggcacaacaagtcgattagtttttgtcactgatgctgtga

atttggcagacctagaacagtttggcgtgcaagcaaaagaagcagttcctgaggaaacatgtcacattcaaatggtggaagatgaatcctccaacagtat

ttcaacataatgctagttgctgagcttagccatggaggcctatcttaagttatttaacctttatgtgacactgtatttatacactgactgttatgcatct

gctgttgtctatggttttgatgcct

>Singlet4802|zinc finger protein 622-like isoform 1

gaagagtggttgtgtaagaagtgtggaaannnnnntcaattttttatttaatgtttttttttttaaattgggtgtgaagcatagtctaaattattatttt

ctccgtttatcagcgccaaaacgtttgacatgagtggaaaatacacatgtatcacttgtcgtgttgcgtttaccagtgcggaaatacaacgaatgcacta

taagactgactggcatcgatataatttgaaaagaaaagttgcagagttgtcgcctgtgacagctgaggaatttcagcgaagagtactgctgcagagggaa

gaagataatgatttaaataagaactgttcttttcattgcgattcttgcagaaagctttttaatactaagaacgcctatgaaaatcatttgaattcgaaaa

agcatcaagaaagtttactaactaatagtttcagcagttcaggaaccccagaaaagaagaaacctgaaaatgatgtagattcagaagattccgagatcga

agaggttgattcagacgaatgggacgacgactctgaagataacccaatactgaataataattgcttattttgttcacatcatagtagaaacttaatgaaa

aacttgaagcatatgtctgttgctcactcgttctttataccagatgctgaatacgtggtcgatttgaaaaggtctcctaatgtatctgggtgaaaa

>Singlet3197|zinc finger protein 62-like

agagcgtgctgctatcctgagaacaaccacaggacaagcaagtgaaataaagtgtgaaatatgtggcaaggtgctgtcaacaaaaatatcattgcgttac

cacatgatgacacattcaggcgaaaagccctatgaatgcgatttctgccataagaagttcactatgaagacaactttggcttatcaccgacgcactcaca

ctggtgagaaaccatatcagtgccgtttctgtgatgatgcattccagtcgaagcaaggacttgttattcacgagcggctgcacactggagagaagccatt

cccttgtgaacattgtccaatggctttccgttcccgagttaatcttaatcagcacctggttgtccattcagaagaaaggccctttacttgcccatattgc

aacagaggttttcgtcgccgtgacacccttgatgttcatgtgcgcacacacacaggggaacgaccttatggttgtcaagtgtgtggcaggcgttttaagc

agaagggtgactgcaataagcatcagaaatcacattttaaggactctgacgttccaatagatgtgccagtgaaaagtgggagaggagctcacagtagtcg

tcagatcaagcaaaaatgataattccaggtgcagctgcaagtactgtttctcagcaatctatacaagaggatacagcaccagctgaaagctatgcttgtg

ctttatgtggagtagtctttgataacaagtttgaaacagaacagcactttgaatcattccattctgttggg

>Singlet5840|zinc finger protein 62-like

cccagtattttgtcaatcaaaatggattattttaaagatgcaattttagatttatgtagtttttgtgtgttctgttattaatcagaatagattttaagtt

gcaatgagtacaatagtaatttttcagcaattcctgttttaaggaattatgtacaattgtaaatacaatgcattgtttttaatggagttctagaaaactg

tgcacaaactgtcgagtatattttggcccgaggcaatggacctgaatgaccacttactacattttttgtttattatacagttgatttgtaaaaagataca

atttaatgtttatatactgaataccttgatgtcatagtacaatcaaaaccgttcaactttgtgatgtgacagattttaattgtatgtagtttatagaaac

aggaatggatgtcaagtcttaggcttggcaagcatatcagaaatatctctttctgtttcaaaagaagaaagctctggtcattgatgtgtcatgcaagaga

gtaaatggatgaaagagcatatttttggattataaaagttctccttgatttgtgcagaattcagtggttgccttaagcactcttcactttcttactctgt

atgaatatgtatgtacatattttatatcattgttcaacattttgtcaggaaaaaattannnaacaaagttcacttaattaaaaaaaaaaaaaaaaa

>Singlet425|zinc finger protein 511-like

tgccatgaagttgtttcagaattcccatgtggtgtccctggctgtgatgcaaagtttgagtctcttctacaatttgatgtgcattacagcacagctcatc

ggtatatatgtgtggtatgtcggaagacgttgccatcaccacacttactcgatcttcatgtatcagaaagtcacgattcctttttcagtgctcaagcgga

gaggaaaccaatggaaatggagaagacgttggaagtactgtcgcagatcaaagcatgtcattgatattcgaaagttccgttctcatctcctggagaagca

gcgtgcaatacatttactaggccgtgtatgtgtgacattgttggtgaagaacattgccgtggcatgaaatggagaagacgttggaagtactgtcgcagat

caaagcatgtcattgatattcgaaagttccgttctcatctcctggagaagcagcgtgcaatacatttactaggccgtgtatgtgtgacattgttggtgaa

gaacattgccgtggcattgtttcagaatcctgaattggagtccctggatctggcacaaagtctgagctgtagctacaatgtgatgtgctttgtagcacaa

ttcataattctatccaagtggtgtacagaaagccattaccttctttacacctgcttgatctccatgtatcagaaattcacaactgccttttcaatgctca

gatggcttatccattatgggaattcatctaaggcttgtgaaggcgtatnnnnagtctgccctttcatttccaactgttatgaaaaaggaactgaattcaa

atgtggtcatatttttcattc

>Singlet3650|zinc finger protein 511-like

ccnnnnnngtcgcgtctgccaagctatacgagttacttaatacttttatgtgggcgcggagacgcaggaaatggagaagacattcgaagcactttcacag

gtcgcagtgggtcgacgatctccggcggatccgttcttcttaccgggagatgcagtatgcaaaacatataacaggcttggtgtgtttgatattgatgatg

aagacctgtgccatgaagttgtttcagaattcccatgtggtgtccctggctgtgatgcaaagtttgagtctcttctacaatttgatgtgcattacagcac

agctcatcggtatatatgtgtggtatgtcggaagacattgccatcaccacacttactcgatcttcatgtatcagaaagtcacgattcctttttcagtgct

caagcggagaggaaaccaatggaaatggagaagacgttggaagtactgtcgcagatcgaagcatgtcattgatattcgaaagttccgttctcatctcctg

gagaagcagcgtgcaatacatttactaggccgtgtatgtgtgacattgttggtgaagaacattgccgtggcattgtttcagaatcctgaattggagtccc

tggatctggcacaaagtctgagctgtagctacaatgtgatgtgctttgtagcacaattcataattctatccaagtggtgtacagaaagccattaccttct

ttacacctgcttgatctccatgtatcagaaattcacanctgccttttcaatgctcaggtaaagagaaaaccaatgatggcttatccattatgggaa

>Singlet3730|zinc finger protein 511-like

agatgcagtatgcaaaacatataacaggcttggtgtgtttgatattgatgatgaagacctgtgccatgaagttgtttcagaattcccatgtggtgtccct

ggctgtgatgcaaagtttgagtctcttctacaatttgatgtgcattacagcacagctcatcggtatatatgtgtggtatgtcggaagacgttgccatcac

cacacttactcgatcttcatgtatcagaaagtcacgattcctttttcagtgctcaagcggagaggaaaccaatgtttcgctgctttgtagaacagtgcag

cacgttatgcaacaatcctcaggagcgacgcagtcattgtatttcacagcacaatttccctcatgatttccggtttgatagtaccaaaaaacagaagcac

acatcagctgcaggagctctgatggagacagatattcatcctacaaatagatcacatctaaaaccaaagactttcagttttgggcgaggagcaaggaatt

ccaagaactggtttaaccaagggagaggtagaatgaaaagtactgatattgacacagtgatgaaagatctacaggacagtttaccagatcaaacataacc

tatgaataaaatttaaattttc

>Contig3790|zinc finger protein 500 isoform x10

ggcccttcacatgccaacagtgtgggaagacctttgcacagtcatcgcacctgtcacatcaccgccgtactcatacaggagagaggccatttgtgtgtaa

ggaatgtgggaagtcatacatgtactcatcacatcttgtagtacatcgacggtctcacactggtgaaaaaccatttatttgtcgagactgtggtaaggca

tttgcagaatcatcaaaattggcacaacattcacgtactcacactggagaacgtccatactcctgctcagaatgtggcaagacctactctcattcttcac

atcttgctgcccaccgccgcacacatggtggcgtggaagacgaaagggcatttgcttgcccttgctgccctcgagcttttgctgcagctacaaaattggc

tgcatgccagatcacaccttcagcagcagcagcaacagcagcagcatggccacagtacaactacattgtcatcttgcaaggtgtgtggaaaagctgttac

aagaagcgctgattctcctgcaagtgatatcccccctgtctgccaagaatgtgaggagaaagcattttcacattgccttgctttgtcatggcatgctcat

atgcagacacagaatggagaacgccttaagttgactgtaacagcaacacaaccagcactgacactgccacgtccagcaactgcaacacgctgagaatgtt

ataatcagacgaatatcttgtatgtctaatctatgatgtttttggatatgattactacaaactatcaggcaatttcagaacccctccaagtgcatttttt

aacaatctgttacttaccataattgattatttcttattaagaatggtgggggataaggttattgaccacctaatttttgtgaatcatttgtaataaaaag

tgt

>Singlet7565|zinc finger protein 395-like

gttcctattgagctgtaagctgaaagtcagaaatgtgcataatttttatgtcagttcaaactgtctaattgtaaacaaatgcacaggagctggtgagctt

tctgtcagggcatggaccttgcaagttaactgactgtgttaaagaatgagtgcacagtacttcacaaaagagaagtgcagtattgttgtgaagtgtaata

ttgcaaacttatctttcattttaggactccgaagttagcgttaatggaatgaggactacatctcgtccactttagataccaacttaaattttaaactatg

tgaaaaccttagtagaagcccatttcaaatttaactgctgctttacttctttctagctttgatgaaaaacatgtcactgtaccaggaactcacttctgct

tgatgtaaaatcttatccatttgatgcaatatattctttattattgactgatatgattctaaaggtatgtgagtaatggggattgtgaacgctattgagt

cacaaaaatgtcttaacagtaaagagtagtcagttgtgtgctttttattgcaatgcga

>Contig1936|zinc finger protein 294 (ring finger protein 160)

aaatgtttaaagacttactggaagaatggaagctggtttatgtaggagaaatttacaagaccttcacagatattgtcaaactcttagcagatgctggtaa

agaaacagttgtgaccttagatcgacagatattactccaagaaattgggaagtctcttatgtgtgctgatgttgggcagtatctccaaggaagtcagcat

gaagcatttatagaaagctgtgaaaaattaatttactcaccactaaatgtgctccaactaactggttttcaaatgctgaaaaagttggtgccattctttt

ctgtgaaagattcaagcacggggcaagtgactggcgaggagatgcccactccgggcctcagcctcctgaccagtttgctggacagaggtgaagccgcgac

cggggctctgcttactgagctgcgctcatgcatcgccacagctaagataccaatatgcagctaaactcagtgaaaatggtagtctgaggtgtctgctgca

gaacctgttttgcctaatgccagcagaagttctcaaggtgtctgatataaagccaaaaccaccaaaggcctttatgtttnntgcggaaccgtcacttcag

ttttcagaaccatgtgatgggcagtatttacagcatctggcatgttgggtgtacttttctactttaaggcnnctgcctgctatggtgcgccagtggtggg

g

>Singlet3359|zinc finger protein 294 (ring finger protein 160)

gtgtacgttagtttttatttacaaatttggatatgcgaaatctttgttggtgtcagaaaacattattttcagtagtagagtgaaattagattcagctatg

ggaggtaaacataaacaagcccaaaggacgaagaataatgttcggccatcaagcagtggtagaagtgctgcactgctagcaaataccacgccaaatttcc

ccggtttcgatgctgtcaaagaatgtggctacattccagcactatctggatttacagcaccaagccatgaagatgtggaccccaacattgacgtaaactt

tcagttagtactgaaaaaaatgagcaagaaagattcaacgacaagactcaaagctttacaggagttcacacagttggtatcagcaggagattcagctgaa

gcggtcaaagtcttgctcccatgctgggctcgtctgttttgccagctggtgcaagaccctgaacaccgtgtgagggaagctgcacacgtagcacagcgtg

ccattgttcgggccgcccgccgcaacttggctccgcaccttcggcagctcgccggtccgtggttcaccgcacagtatgacactcatccccctgctgccag

tgctgctgcacttgctctccaggatgcttttccaccaaagaagatgattgaagcaataatattttgccaacaagaaatattaaattacataatcgataat

gtcactgtgcagactcctcagaccctgagtaacccaaagacatcatctgctgaagatatggaatccaaatatcagnnngtacttgtgagctccttacaag

gatatgcattatttctacagaagatgccaccagagcatcttgaggctgtaaatgatctcaataaaaaactt

>Singlet2094|zinc finger protein 277-like

tgcatgccattccagacacattcttcacctaactcattagtggaaaaattgtttgggtttcatttgtgtctgtgccttgggagtttttttaacatgtttt

tttaaatattttttatactttttgtaatagtgatgaaatatgttccaagtatcacatactttctaactgcagtacatagaaaccttgtaaaaaaaatcat

tgttatataatataacaagcatccctttgtaatgtatttagatataaggccattcgctatcttatggagttgatttcacaaattttaatttttcaacgaa

gtatgaagttaatacaacaagacgtgtttggaattcacatggtataaattatttaaatgtttcagagttattttagtttcatgtactttaccattctttt

ttgtatattttaataagctgctagcattccatcctctgtgaaactgtatcaaggaaagaaagaaaaagaaacaatggattttagacatatcctttcgtct

tggaacatgatctttcagtctgcatgtgggaccatatgggcacatggcaactgaaaggaaaatcttatcctttggcggaaacagatgaactggattaaga

ctttcgaaagcttttctttttttgtaggggtgtttgatattgaaagtgttgtatccagtattttgaagactatgagaaaggtttttacaacataaaaata

ctttaggattaatggagatcatgcaccaataagcagaagttctgagttgtcagtnnncacacgcaaaaggaagagaacttgtagctttgctttcnnnnnt

accctttgacaacaggagataacaggacacacacacaccc

>Contig1128|zinc finger protein 271-like isoform 2

ctgtcggagcaccgtccctttgtctgtgatatttgtgggaagggcttccacttccactggtacctcagctcacacaagaaggcccaccttgatagtaaaa

cttacacttgtaggacttgtggcaaatcgttcaacaagaaaaactgtttactctcccacacgaggtgccacgctgaacagggtttatgatattacgctta

aaagcagtacaaagtgttgttgttttatgtcttatattttgtacaaataattagccatatttcagcggagtttgggtgcaaagtttgtaactgtaatgtg

ttttacatttacattttgaagtctgttgatgcttcagcctaaagagataatgttgacagtttcgtttgtttatatttacagttaattgtgtttggtttgc

tttgttagaagaatttatttggtctcagtaaccccattttgtgtgggaaataattttaaagttacatacagtattattttcaatatgtaaataaattctt

tcgacttatttttgtgattacacctcttatctagtttgttgttgactgtatgaaaccatttcaaatatggttctaagctatattttgttgtaattataaa

agagtggaagacatttttttaaatgtttatatttaagcagatgttaatttatcataggtcaatttctttcttcttcatcttgtggcctaaattttcactc

attggatagtagtgatctgcagtttgttgaaacaaaaagcatgtaaaggtaacactgttaattttgaaccagccacagtttgactgtcagtttgcggttt

atattcatatggcatagtgtttagacctatttgtgcggtgatacatttaattttgtaaagcatactgtcacattccagacctctaggtagataataggca

acaggattaaattacattaaaatcttttgaggtgtacagacacatcatcacattaaatactaaaataacacacgtttcagccattttacagtgtcctcct

gcaggacatagtctagtctagtgtg

>Contig271|zinc finger protein 271-like

aaggggagctgttgctgatttttaagaaatttacgaatggaagtttcgaacaagtgctcttgatttaaattcataacattcgcttcggatatggaatcta

caggagaggcagaggcagtttcggcatacgatgaaattgacatcaaagaggaaccaatatcaactgaacctgaactgcaaccgtttgaacagatcttcat

taaagaagagaatattgagtgtctactagaggatgaaaccacttctccacatgacgcaacagacatgaaaaaagaatccgtgctccaaaatgacgtggac

acgacaatctatggcagtgaaccagaaaggggaagagacgaaaactttgtggcttaagctgagaggcagcgacgacagctgaaatatatgaaaagcggan

nnnnngcagagaacatcaagccacggggatatcaaaaccaagtgctatttcttgaaccctttgtgtcagctggtccacgtggtagtaatataagtgatgg

cagcgaccacagctcacaagttataactaaaacattagcgag

>Contig1338|zinc finger protein 271-like

ctgtcgcgaggaatcaagtggtactttcgaagacgcctaaccatgttaaaataaaaaataaaaagccatttcagactttgtgctatgtcttaggtatact

acggtatatcttttttgtttaattaattagatattgattccgtgttaccgtccatgttcgttattacatatgcctttttgtctcatgatcttgtgtaagt

tcatgtgtagactaccacgttaggatcaataatgccttcattaacttttatcgttaacacagtcatataagttaagaagtcactaaacaggtaggcgtta

ttgtgtagtctccggacgtagttacggaaacatttattctgaagtgtcgctttgcgctttcttacatcgttttcaggatatgaattagtgtttgttgaca

tgaatattccatataaagaccgctgtgttaaactgctgtgaagtaatcagtgttgtgatgttgtattaccttctcttatgacaagattttgggagctcag

agcagtgtaccaacagccgcactgaagttgggggaaaaaaaaaatgctgcgcattaatggatgtgatggaatgtgtaagctgtattaagcaggaagagga

tgatggaactcttaggccattttatactgaccaacagtgtacccagatgttggtagcaattaaggaagagccgacatataattacctggaaggttgggag

gaactggagcttgtttctgtaaaagaggaagtaaatccagaaataccaattccttttagagaaattgaatctgatatatctgaagatggagtactgctga

agaaaagtgtcgatggctatatggttgtcaatgcaaaggagggtggcgagacgtgcccatcagttaatggtgccaaagtcttaaatgatgattctaacag

attacttcaaaaaaagaatgagcacaaaactgttgaagccactccaaaagccgtcagaaagcgacgcaggcacaaatcacattacagcagacatctttcg

gacaggcctttccagtgtaaaatttgccagaaatgtttccgaaagcccgaacatttacgctgccacgaattgttccattcagatgaacggcagtttttgt

gtgacacttgtggcaaaggtttttttaccggcggcgatcttaagaggcacggagtagtccatacgggagagaaaccttactcctgtcccatgtgttcaaa

gactttcacgcagttgtgcaacatgcagtcacatctcagaaaaacgatgccagataaaagggtatggttgtgatctgtgcaacaggatgtttgagaatca

ggaggctctccagaaacacagggcaaatcacggtacggtgccgtaccgccgcaacaagcggaagaaaccgttctgccaggacttgccgccattcatttgt

gacatgtgctccatgtgtttttctaacaaacgaaatttnntatcccacatcaaagagcacatgggagatctcangnncacgatcacttgtgagatttgtg

gtaagagctgcggcacccggagggaacgcttgatcgtgcacatgc

>Contig2036|zinc finger protein 271-like

ctgtcggctcnnnnngcaggaaaggctcaatttgcatgtgattactgtaaaaaaactctctcttcgagtgcagaacttgcaaggcatcttgtctcccatg

caggaagcaaaccgtacccatgcaaaatttgtggaaaatcattttcactgagtcaccatctaacaaggcactttcgaagtcatgcaagggatgaaatttt

cacttgcaatgaatgtcaaaagacttatgcacgcaaagagtctttagaaatccatttgcgaggccatactggtgaacgaccttatgtttgccgcctttgc

cagaaaggatactattccagcacacaacttagtcgccatatgaagatacatgctggtgtaaaaaattttacgtgtgaccactgtggaagttcgttcatta

gaaaggaccagatgaaaaagcacataaagagtcatatgtactgaatttcctgtaaggggacatgatgtcgtaaaataaaatgtaaattatgtgcaagcaa

aaacaacttaagtagcccaattgtgagcttctgctgttatagttaattagtatattatgcttttccatgttgctgtttacacattttatatctgttacat

gattatgtattttcatgttatttattatgggtgtctgttgttaataaagatgggaatccatctgcaaaaaaaaaaaaaaaaaaatcggt

>Contig4247|zinc finger protein 271-like

atagccggtaaccaataccgatcaatggataatgttgaaacaccattggccatcctacccgaaaatcgataatggtcgtaggtgatcgattttcattatt

aggcggcgctggttcgacagctaacagtttaaataaaaagcattaccatggctattgcagcggcagataactttcagctgaagtggcatagttacgggac

acatcttcattcatccgtagccactttgctgcggtcagaattattttccgacgtgtgtttagcaacagtggacggacgacagatatcggcgcatcggttc

gtattatcagcatgcagttcgtatcttcagcagctactcaaagctgtgtacccacctacagtcaatttccccattgtaattgttttaccctcagaaatcc

cttaccgtattttgaagattttggttcagtatatgtacagcggagaggcaacagtgagctatggacagctggatggaatcctgagagctgcacatatttt

aggtataaaaggactgtgccatgaaaggactaatacagtaaatcaggtggtacaggacagaaatggtaatgtccaaccctcagtacagagattatttcct

cttcagatacagactgaggcaacagggaagtgtcagaggaaaacatcctcaggttcaagatttcaggattcctcctcgactgctacaactgacccgaatg

catgtaacgaaaaggaaacagcaagtgtagatacagcttcaaatggaaaagaggacaagaatccagagaaaaagcaagatgaagtaactccttgtgctgt

tagcagcaagagtcctaccgaagcagaccttctgactgacagttcttctgagcacccgcctaataatttgccattgaatcctgtccagttgatagtaaaa

caggaacctatagagtgggtagatacagaactgtctgaaccaatggaagaagaaatagcaagtcatatgcatccagaagtgacagttaagtctgaggaag

atgacttccatgatagtgaaggaagggctgtacatgtacaggaaccctctgccttgtatacacccctcacatgtgatctttgccatgaaacatttagtct

tccaggtgactgggtacgccatatagaaaatttccatgaaaaacatgatggtagac

>Singlet3138|zinc finger protein 271-like

cggccttgggggagcattcgcagttgcacactggtgaacgtccattcacatgtggcaattgtaataagacattctcgcaaagtagtcaactcaaacggca

cattcggttgcacactggtgaacgtcactatatctgtgacgtctgtaacaagacattcacacagagttctcacttgaagcggcatgctcatttgcatatt

ggtgaacgtgtgtacacctgtggtatttgcaacaaaacattctcagaaagtagtcacctgaagaagcattcagagctgcacattggcaactgagtgatgt

tattgtgccataagaagttgaagagtagaaacgacttgaaaggcatgtgaaatctcacatccagtacaactcatgtatgtgtttgttctggtggttcatc

ctgggtaacaattaagtttttattttcatctgtgaaaagcagtcagatagtcgatattatgtacataccaaactaagttttaaaatcttgtaagtaccga

gtagatacgctttcacttgcaaaataataaattcgtgaaaactaataaaattattgtgctccaatcactgtggtagtgttccactgatttcactgctgtg

cttatctttggcatgcatgaaagtgtcact

>Singlet4300|zinc finger protein 271-like

aagttccacatcagaatacatacaggagaacgccctcacctgtgccaaacatgtggcaaaggattcataagaaaatcaaaattggatgatcacattcggc

ggcatttgggagagaaacgatacagttgtgatctttgtcccaaatgctatgccagtagttgggaccttaagttgcatcggaagaaatgtcatccagaggt

tgagtcttcaaatccccctcttgaagcttcagcatctactgaagctccttcagaactgacaccagttgctgtcattgatgatccagatgagccatcacca

ttacctgttccactctctgttgaaactgttgtaccacagcagcaatcagcatctagtaccccacctgtgctagtacagcttgatggaggacatgcaggca

>Singlet5801|zinc finger protein 271-like

caatggatcagaaaaacattatctggataaaaacagagataacagatgaggtgtcaaccttgccagactccacagtccagacttacccatccaccataaa

tgtaaaagaagaattgcaggagtgtgtgaatcaagagttatgtcctcctgaatttgaacatcatgtaattaaggaggaggatctcgaagatccattggta

aggtcagggtccactgagtctgtcggggagtatctggaactaaatttggaagcagaagaaactgaggactttgtcttggcatcagagaatgatcgtcctg

atgagtgtgatccattgagtgtaacagatggtgaggagccagcctcaaaaagatgtgcaccacacagccaatggatgattaagacgactactgagagtgg

tagcattgcatttgaagaaacgcatctgcagggaatgactggtgataaggtggactttgctgcagagaaatcatcacttttgcagaacagtgcattatcc

aatagaagtagtaactgcttaaaggaaaaagaaattcaagacttcacatgcagttcatgcagcgaaactttttcatcaaaatacaatctcataatgcatg

tgttcattcacattgatgatgttcagccgcctgcacatatctgtatgaggtgtggtgatgtatttctc

>Singlet5949|zinc finger protein 271-like

acggccggggagtcgtaattttacaacaaacgtgaaacgtcaaacacactcattgaaaatgtaatcaagttttgacttaatgtttgtgaaagagaagtga

catgctaaaagaaataaccttaactgaccctcagaacattcacgctgtagataatatggcatcagggatatgcagaagttctgtttgtggtgcaactctc

atcaaacaagagccagttgagactgacccacttgcatccccagagcaagagatcgagcacatatctgttaagcaagaaactcctgttgatgccaacccac

ttgcatcccccaagcaagagatggagtatgtatctgctaaggaagaaactccagagtgggatgtgcctgatgaccacaatagtacgcatgatccacaaag

catcatcaaagaggagtgttcatcttcagaagactttctcagccctagtgctcccagtattgaggacgacgacgaggaagacgaggatgcaatgagtgag

gacgattttcagccatcggagtcggtgcaacagccggtagtggaatgcatgcctggagccaatgcactggtagacactgtggtactgactggctgggctg

gaatgcgttgtcatccaggcactgtaatacgtccccgagtcgaaatggatagagggtcgactggccgaatgagaggatgtcaagtaccagcgactgcaac

actcacaggcaggaatcatgtcaatgggggctcaacagactcagaataaacaggggtaattcaacaatatgaatacagaacaggcccg

>Singlet7153|zinc finger protein 271-like

tgggggaaagagaaccataggaaaacagacgggtttgcaatatttgtcacctgtgtagtagtaacgtaagagggcgttgtgtgctgttacccacagaatt

atagttgttggaaacagctgtctactgtttcacgtttcagttctctatacaagcgaacataattagggaagtagtttatgaactgtggttaccacaaatt

ggttgttatgaaatgaagtgtttgaaatcaaacatcaatgtaaaacagtgtgactgtgataagtgagtgtagcaataaacaagatctctggtcatggaag

aatctgaatccttagtggttgatgtggaccctcgtataattaatttttcagaagatgtatcatcctgcagcattggtgatggggctaaaaatatttttcc

atgtaagcgatgtgacagatcatttcctctagagcagttactcattattcatgaaagcaatcatgacagagaaaggaatttccgttgtgatatttgtaac

agcagtttttactgtaaaggtgacttaagtaaacatcatgctgtacacattaaagacaagccattttcatgcattatatgcaaaaagtgtttttctcgga

aaaatctgcttaggagacatgaaaatattcataaggaagagaaaaaattttcttgcaagaaatgtactaaacaatttttcttgnnngagcaactcaaatt

gcatcaaaaggtgcacaggatatctaaaaaattttcctgtaagatttgtagaaaagcattatcatcaa

>Singlet2976|zinc finger protein 235-like

gttnnntgacgcggttggcctgagccggcacgtggccgaccgcggctgcgaggatcgtggtgggggtgagggcggggccgagggcggggccgagggcggg

gcccgggacacggggccccggcgggcggagctgccgcccgtcagccagatccggccgttcgcctgccgggcgtgcggcgccaccttcgccaggctgggcc

acctcaaggtccacctcaccatccacgccgacgaccgtccacacgcctgcgacgtgtgtgggaagcgcttccgccagtcggtgcacctgacgatccacaa

gcggctgcactcggacgagcggccgtacggctgcgaggtgtgcggcaaggcgttccggcagtcggtgcacctccagatccacctgcggcagcacaccggc

taccggcccttcagctgcgacgtgtgcggccggaccttcacggaccgcaccggcctcaacaggcacaaggcggtgcactcgcgggaccacccgttcgcct

gcgccgcctgcggcaggtcgttccgcgacgagggcggcctgcgcgaccaccgcgcccacaacgcctgcgccgccggccccttctcgtgcgacaagtgcgg

caggtcgttcgcggacccgttcgcgctcaagcggcacctgcgggtgcacgagagcaagaagcggaagccgtgcgacgtgtgcggcgacggcggcgccggg

ggccgcgcctgccgccacgacannccgtacgtgtgcaaggtgtgcggcagggacttcgcgcgcg

>Singlet1025|zinc finger protein 235-like

gtgttgtatgaaatgtttataaatcgatatttcgtcgagtaaatagaatattagagtatttaataattggaatctaagtaaataaatttcaggtctctca

gtaaactttatttacggaggatcccgctaacaccaggagaatgaatttcaaggattcagtatgcttattttctaatttttcgtgaattgaggggaactag

agagaagatctgtgaaaatgtgtgaaacgaaccaaacttctgtgcagccacagctatataactgtgaatcaactttagaaaatgctagctgtcaaaaatc

gacaaaagaaaagcctcgtccaaaaccacagacgtgtaaagtgtgtgggaaagtactcgcttcagcatcctcatattatgttcatatgaagctacactct

ggcgataaaccatttcactgtaagttatgtgaagcaagtttcagtcgcaaaccatatttggaagcccatgttagaactcacacgggggaacgtccatttc

agtgcaatgtatgtcttaaatggtttagccagaaaagcagtctcaacacacacaagcgggcacacacaggggagagaccctattcttgtgatatgtgcgg

aaaaaaatttgctgtcaagagctatttgacagcacataaatggagtcatgttgtagatatgcctttagtttgtggaaaatgtaatgtaacatttacctca

aaacaggattacgctttgcacgagagattacacgcaacaggcttgaactttgagtgtcatgtctgtggccgtacatttgctaaagacagttacctgattc

gtcatgtgaatcgcgttcatcatag

>Contig2407|zinc finger protein 184-like

aannccntggaaatgtgagtactgcaataaatcctttcttcatnnngacacatggaagtgtcataccagaaggcatagaggtgagagaccgtttcagtgt

cattactgtgctagaggcttcacggaacaatgggcactgaagaaacatttacgtctccacacaggtgagaaaccatatacgtgcaatatttgtgggaaag

cttttgctgactgctcaaatctcacaaaacataagaaggtacatagggatcctaaagcagacaatgcttccactagctctgcttcagttgaccgaacagt

ttggaatataatacgtaatcacttgacaaaggaagatggacaggaaggtggcagtgaagtaacaaccacagaagcacaagggaacagcgttgagcaaatc

atatatgttacgtaccaggatcctgacgatccaaatgaagcaagaacactccattttgtagaaaatgttgaggctcctccagaacagccatctactgaag

ctatacttcaggagactacttcaaatactgcctctgttgaaaataagatagaactgccaccagaagcaacataccagatagaatcagcccctcatgcact

ccaagttatggatgaagatggtaatcctatccaatttacaatgcaggatggaagagcattgcagattactacagttgatggtcaggcaatccatgttact

acaccagatggacaaagcattcctgtgcagttagcaacatctggtactcaagttttctcgggggagatagcatcccaacttgatagtatttcagaagcaa

cttctgagcagtcagaggcaaagcaagttgttgtcactgcacctcagggtgagcaggtcagtctgaatgaacatgttccagatgtcgaaggtgaaaatat

tatgggtgagcaaactattgaatttactacccctgatggccagaagctgagattggttgcctccttggggccatttaccagtgaattttaataatttcat

gtacatgtcacttagattgtgtgagttgaacaaattgtcattttgagtggatcatgcttttaattgaagtatttccagctgtaattaaaatcatg

>Contig2603|zinc finger protein 135-like

gttcagtaccaatacagaagatatgactnnngataaggaatgctcagttgccacccagtgtgaatgtagtacgagggaaaaggaattagatgtatatagc

tgtaatttctgccaacaaaacttttcttcaaaatacacactcataatgcatgtgttcatgcacattgatggaatgcaaccaccttcctctgtttgtaagt

ggtgtggtgaggtatttcacactaatgttggcttgaaaaagcatatgagaatgagtgagaattgtcaagatttaactgctgacactcatgaaaaatatga

acatagtgatgagcatcaaaccactacctcgttggatagcgaggcaaaagtttctgtcacagaacacaatgagcagtcttcatgtaagggaacttggaaa

gattcaaaagtgccctctaatgacatatgtaacacacatatgacaaatgatagagagaaagcaagtaattatggaactttgtctatagctgttaatgtca

gtgcccaggctgatctacttactgcaaacagaaccgacagatgtggtatttgtggcaaattgtttgctaggtcaggtgatctcgagagacatgtttctat

ccatactgggaaaagacttcacaaatgtgatatttgtgagaagtggtttgcccagtcacgttatctaaaggctcacacattaattcacactggaaagaaa

cctcacaaatgtgagatttgtgggaaatcttttactatattaggcaatcttaacaaacatttattaattcacactggaa

>Singlet4390|zinc finger protein 135-like

gatgtcaacatggaggaaaagcaagtgtgttactctgcttcaaagagtgtggtggataacttcctcttcatgagccgcacaggtgtgaaagaagaatcca

aggaagctacagatgaagaggttgtcacttttgaagagattcccatccccgattggaatgtgcatgttaagcaggaggtggatgagcacttggcatctga

gaatgattatcttgatgagtgtgaccctttgaagattgaggatgagatggaggtacctgcagaagggtgtgcaccaggcagtacacggttaaaatctgct

gtcggcaaggttggaagaactttgttacaagaaaattgtctgcatggagtgactattcgcaaagtttaatacatactggtgagcggcaacgcaaatgtac

tgcttatggtaaatcctttacccaattcagtaacctgaggaggcacaactcaatacataccggcgagagtccacacaaatgtgccacgtgtggaaaatcg

tttactaggtctgctggacttaagaagcctatgttctacattcaggcgagaagccacannngtgtgacctctgtggcaaaacttttagtatgtctgatgg

actgagggaacacaaattaatacatactggtgaaaaa

>Singlet3677|zinc finger protein 135- partial

caacttatctgcatgcagactccagtaccnnnnctgtctctgttgtgacagaaagcataataaacaatgttaaagaaaagttgactgatcaaggagacaa

tagcttatcttctggtgtaccactgaaggcatccagcggtgaaaaggagaaaatgagagataataaaatctcctgtggtgtatcaacaaatgtcacgttc

acatgtagcacttgtttcaaacagttcttatcaaaatcggcccttatcagacatgtctttattcacttgcctgatgcaccaaagccaggagatatatgtg

aactatgtggtgaagtgtttcaatcagcagaatttcttcaaagacatgttgtgtcattccatgttggaaaatctgttaggaatggtgcattgattggaca

ggatagtgaaatacctgtaacaaatagtccttgtgaagattcttttgaaccaccaaggaaaaaacagcgctgtgtggatacaactggcaatattctcatc

tgtactctttgtgacaagagtttttttgcgtttagacttactccggaagcactacatggtacacacgggtgagaaaccatatcgttgtgaggtatgtggc

aagacatttgtccagagaggtttgcttggtaggcatcagtttgtacatagtgctgtgcagccatttgcatgcaactcgtgccctgaacgat

>Singlet5415|zinc finger protein 106-like protein

tattctttcacccattgtcctggatgaggaagacggatcacaatcagatatgtcaggtggtggcactccagctcatgatgctgatgcagtcaatgagatt

cgaaggatgcacttaattttggttgggcactcaggtgccatcctagagcttgtgaccgttggtcggcaagcagttgcagcctctgaggatggttttgtat

actgctactctttgaagacaggacgattgaaaagagtggtctcctgtggaagtcagcccgttacgtgcctctgtgttgtagagacagaagagggtcagct

tatctttgcaggcagcctagatgggcacatgcgatgctttaccttcaagtctggaacccatatcttcgaggcaatagatgttggtgctccaatacagtgc

atagaccaaaattggggtaatgtttacatggggaacagagatggaacaattgcagtattcaatattgaaacacgtcagcttttgaatccgaggctatcat

gtactgatgaagccatcctttcattgaagactgccaaggagggtcccagacaggtccttttagtatcctgtcgtaatcagcctctcactataagggatgc

aaaaacaggtctcttaatgagaactgtgagtctcaatatgagtggatcagtgtactgtacacttctagactcatcccttatttactgtggcacaagtaat

ggaaaaatatgtgtatacgagttcacgagtggcgcagaagtttatcgattgactgcaggtgttggcgtagtagtctgtttacgactctttaggaacctgc

tctttgctggctgttatgatggtttcatcta

>Singlet1167|zinc finger protein

aagggggtaaagagacttgtaacatttgtcacaggaatttttggaatgtggcagcacgagatcgccacttcaggacgcacaatacatcattcggaaaggt

ggcatgcacagaatgcaatgattttaaatgccgtttcatgcgagacttaagaagccatctagaatgtcagcatggaattaagattgaaagagaagaaatg

gagtttgattccatggaacagttccttcagtggaaagaaaagatggaaggtgaaaaaagagtttcattcataaagcaacgtggtggcacactaaacacgc

agaagtctaaaatttattatttatgccaccgttcaggaaatttcaaaacagtgggtttggggcaaaggttggctggtccctcggtcaaaattggtcgcac

atgtcctgcctctttgatagcgacgaagatgccatcaggcaaagtcagtgtccagttctatgccacacacacaggccacagcttctctatgggttctctg

catctcactcctaccgacagagcaatggttgctgacttaattgcaagtggtctatcagatgctgaaattttaaagaaaataagaggatagtgcacatgaa

agcagcttaaaaagggtacacatgttaaaaaggaaagacatttataatatcaaaaaacaatataaattggacatcgcttcagataagaatgt

>Contig3250|zinc finger protein

ccgttgctgtcgccgttgctgtcgcacaattttatgtaacctgtggtgttcgggaagtagttttgatgtgtgttgttcgtgtgacgtacttggcattttt

atattctctcctatctagtatgtgcgatgtcctctgcattgctacgtaaaagccttcgtttgtttgaagacgatttgacagaaaaaggtgttgtgaatct

ggaagaaatccgtaaagaactgcagctgaaaaagaacttcacagaagaaaacttgaaacgtttgcaaattttacgttatggacaaatagaccaagccatt

gcaaataagatccttgagcgagcaagtaaatgcactaaaggagataagaatctgaaaactgaagacacaactgtttttacagaggaagattttaagaact

ttgaagaggaatacattgatacgtagatcattgtgtataacagccgtttagaatcgctaaagaaatattgtcatcgtattattgtagttacagtactgac

tgttttctaatgagatgttttcagaaagcaaaagtatggcttttaactatttgtcatcttggaagtctataggcgtgtctgcttaactgtcgaacaatgc

gtgacgctttacttggaaaaatattcattttgttcagtagtttattttactttctttacactgtttgggttatagctctaccctttgttgatgatggaca

tcctcttaggcttctgtttccatctgttcattatgctcttttgatccctgctttcatgggacttggttttattggagggctaatagtttttactgtatat

gcaattcaccagccatgaaaaagtggcttgtaattggtgtctcaggtgtaacgtgtggtgggaagacgacagttgcacgttctttgcatg

>Singlet3519|zinc finger protein

gtatgtgcgatgtcctctgcattgctacgtaaaagccttcgtttgtttgaagacgatttgacagaaaaaggaaagaagaaacgaaaaacagatcaaagaa

acaatgaacacacagaacagaaaggaataacaagatggaaaactaataagaaaaagaatccaaaacaaaaaggtgttgtgaatctggaagaaatccgtaa

agaactgcagctgaaaaagaacttcacagaagaaaacttgaaacgtttgcaaattttacgttatggacaaatagaccaagccattgcaaataanntcctt

gagcgagcaagtaaatgcactaaaggagataagaatctgaaaactgaagacacaactgtttttacnnnggaagattttaagaactttgaagaggaatacn

ntgatacgtagatcattgtgtataacag

>Contig1250|zinc finger mym-type protein 1-like

ctgtcgctcnntnnnnttagtgatatagacgattccatccctgtcttgacagtgaattgttcagcctcccacgcttccaagctgtccggcgtacatgtgt

tttatgtggaagtaagtgttcatttctaccagataccacacatttaataagctctcagtgtttcttaccccaacatcaatagtagtatttcgtgcttgta

gtaatttgcttctgtactgagttgatatcaacaactttagctaaataaatggcatgatgaaacatttgaatgatctcacataattcaaaacactcgttgc

cacagaatgtgtttcagctaacaaatttaacttattgtgattttaaagatcaattgcagtcttatccaagtaagctgcaattggtattgcagcgtcgatg

cgagcactccagttattatcagaaatgggatataaataacttccaactaagagttccagaatttcgcgtagttgtgaattactactgaatggttgtaaag

aagatgaattaccaaaaaaaagtatctgctttatgacaatcctccactagctgcatctactccgtgtacggcagtgaggagaacacagagaacattttgc

aagagtacttcactgaacaaaatgtgactaaactcctgtataagacttcgtaatatttcttatcttattataaccttgtccggggcactcactaataaga

attgagtgcttcacgagtggcgattgaattaaatgtgcaatagcaactgtcgctttctaattaaaatctaaaaaattccaaaaaccgatcattattttcg

tatttatcagttgctttcttgaa

>Singlet8159|zinc finger mym-type protein 1-like

tgggggatcaaacttcgccaaaaattatacaaggcctaagaattttccattatttggattaaattatttatcgaaaaaccctctgaatgccttattattt

tcaaccgaatagaaagttacgcccatcaatcactgaagcacattgttccatcgtaaacttcctttaaaaattagcttttgttcttccttgtcagtagtta

atccagccttaaacgtgatttcagcttcagttcaatgagtaaacttttttgtggttagggctattttaatgcattttcaaagcttcacttaaatatttcc

agtttttaaaaccctcagaggtggtcaaattagcagttgactttaagtcaaacagtcgacaacaaaagcaaaagacactgccatttgataccgaataact

agccatcgtcgtctgacagttccttcatatgatagatttcttgtgtaggctaatttgttgaagaaaaatgactcccattttcatctttttgaaaatttag

atatacttgtgaggctccacatagtataatgtgatctatatctgcggcattaagtactttgcgcatgtgtctacatcagaagcattctattttttattcc

atgatcgtcgaaatttcatcaaccacacaagtcatatattaattttgatttgcagaaatctcagagggttccttcaaggatttact

>Singlet3265|zinc finger mym-type protein 1-like

gttttcttgtgtaatgagttgaagagaaattatgcccactttcatcttttggaaagttatttagacatacttgtgagggtccacatattataatgcgatc

tatatctctagcattaagtacttttggccacgtacctacatcagaaacattgtattcttttattgcaagaacgttgatactttcatcgaccacagatgtc

atgtgttgtttttgacttggagaaatatcagaggattcatgcaaagatgtactgtgctggtcaatttgatcacctccaataggtgattgtatattttttg

tgtataattgttcacagtctaaagaaatatttgctgatacatggacttttgattcaatatctgaagtatttgccttagaatttcctgtaaggtacttata

aatattcatatgcattttagtttcttcgagaacttttttttcagcttttcttttccgattttctgaaccagaaagctttttttaatcatttccttctctt

ctggcacgatttaataatttgacaaattattgcttggacactgctctatttcgcgaccacaatattcgatctataacaaataaagaaattcacctatcag

tagacgtaactagaaaaaaacctgaactgaaagataaaaatgtttttcgcacttaccagaaaattaaaatgttgacacagtcacgacactcgtttcactc

acaattattcagccacgaaaacatgaccgctgcctcagactcttactgacaattactgacatgcgggtgcaaatcatagccctgccaacatatgcagtct

aacaaacattgtgtggcactgt

>Contig262|zinc finger matrin-type protein 2-like

catggcgtcgaaagagagagcagaagatcatcgaagaaagtgggaccgcgatgaatatgaacgtttggctgcagaaagaatacaggaagaattggaagca

caagaaaaagcgaagcaaaaacagccacctgttaaacgagacttattgcggcaacgtgactaccgagttgatttagaatcaaaattaggaaagagtgtag

tcataacaaaaaacactccgtcgtcgcaaacaggagggtactactgtaatgtgtgtgattgtgttgtgaaagattccattaacttcctagaccacataaa

tggaaagaaacatcaaagaaatttgggaatgactatgagagttgaaagatcttctttggatcaggtgaaaaaacgttttgaacttaataagcgaaagctg

gaagagaagaaaaaggactatgatatcgaacagaaaatgaaagaactccgagaagaggaggagaaactgagagaatatcgcaaggagaaacgcaaagaaa

aacgacgaaaattagatgaagacacagacacaacaccaagttcagaattagcagctataatgggattctcaggatttgggtctagcaagaagtgaatctg

gaatgtaataaatatcagcgacaaaccaaatggtcttttattgaactgtgctcttcgttactccacacacaataggaagtgttgccagtaaaatcatgag

actaattttattacaagaactgttagatataacttagtaatttaacagaatttgccattgcaaacataagctattggttctgtggataatgggcagttcg

tgaatgtgtattccaatttttgtaatttttccttagtgatctgtgtaactaaaatgcatctttcactgtcacgggactcataataaagcacaataaaaag

aatggaaaaaaggcttgtttgctatgaagtacacagagaaaatagcattgtatatttgaagctatgtatgtaacagatatctgaattccttcttt

>Contig1854|zinc finger homeobox protein 3 (zinc finger homeodomain protein 3) (zfh-3) (alpha-fetoprotein enhancer-binding protein) (at motif-binding factor) (at-binding transcription factor 1)

cgaagtaggactgaaaaagcgcgtcgttcncctgtggtttcaaaatacacgcgcccgggagcggaagggacagttccgagctcacgcccaggtcatcaac

aagcgctgcccgttctgtcctgccctcttcaaagtgaagtccgcgctcgagtcgcatctggtcacgaagcacgctgaccagtgcaccagaggggagatca

acatcgatgcgctaccagacgaagaactgagcatggagtcgacgccgtcattcacgtcgtcgcaaatgggagacgctagcaaaatggggtcatcgtacgg

cacggccacgccgacgcagaatctcatgccgccgctttttccaccattccacacggatatggagaactctctgaagaagtactacgaagagtctatgaag

cggtacctcagtgagctgcaagctcaccacacggcgcagcaaaacggctccattactggcgtcaaagaaggcggcattccgacagatctgagtatgaaaa

tcaaacaagagccggtgacgtccggcggcgagggtggcggcgagtgtccactagacctgagcaaaccggtcgatctcagcaggccgatgaagatctctat

ggaccacaacacatcgctgactgaggtcggtcctctgacggatctcagcgagcgcagcatatgcttcgaggacgattccatgtcggagacgacagagaat

atggacggttacgagagcagtccaacttctccggcgtcgagcacgcagagcggtcaccagaggcatatgaacgcttgcagcacccccgtgggcagcggaa

ccggcggcggcagcacgggcagcgggccagggagtgggggcaagcgcttccggacccagatgagcagcctgcaggtgaaggcgatgaagtccctcttcag

cgactacaagacgccgacgatggccgaatgcgaaatgctcggccgggagatcggcctaccaaagagagtggtgcaggtgtggttccagaacgcgcgggcg

aaggagaagaagaacaaactggcgctgcagaaggccctggtcggcgacggcaaccctccggcgcccacgacgaccccg

>Contig2683|zinc finger homeobox protein 3 (zinc finger homeodomain protein 3) (zfh-3) (alpha-fetoprotein enhancer-binding protein) (at motif-binding factor) (at-binding transcription factor 1)

ctcccnnttcccctgcagcaacaccagcacgagcgtgagcaacatgctgccgccgaagctgaccccgcctaacttcgcgtccccgggccagctccccacg

tcgccgagcattctgcccctggcgccggcgtccagaagcgcgagtccgtgccgatcgtacacgagcaactcgtcggacggcttcgggcacgtgtccgcgg

cgggtggcggcggcaacggcaacaccccgaccggaggctcgtccggcaagagggccaaccggacgcgcttcaccgactaccagataaaagtactgcaaga

gttctttgagaacaatgcctatcctaaagatgatgatctggaatatttatccaaactgttgaatctcagcccgagagtaattgtagtttggttccagaac

gctcgacagaaggccagaaaagtttacgagaaccagccaccagtggaagcgccaccaggtgtcgatgatggcggtgccaacagattccaaagaactcctg

ggctcaactaccagtgtaagaaatgtctgctcgttttccagcgctactatgagctgatacgccatcagaagacgcactgctttaaagaggaagacgctaa

gagatctgctcaggcccaggcggctgctgctcaaattgctgccgtgctgtcgtcagaagattccaattctagcaccatagctgatacgactcagcaccag

caaccacctgtgataccacagatacagcaacaacaacaggtgcagcttcaccaccaacagcagcagcagcaacaacagcagcaacaacaatccccacaac

agcaacagcagcagcaaccagtacaacagccaccgttacaacctcagattcagtccctgttgcctgcaataccngctccattgtccaccaatccccaaac

accgtcaaggactatcacgcc

>Contig913|zinc finger hit domain-containing protein 3

agggggaagtaagtgtttgtatatagctgtcgccagaaccgtgtgtgaagtgcaaggactggaagatgacaattgtttgcaatgtatgtgagcttggaaa

gtcgaagtataaatgtccggaatgtctgatttcttattgttctcttgactgctggaagactcacaaatcttcaacaacatgtaagcctgtagtatacaga

caggtacagcgtcgtcttaatttggtaagtgcagcggaagaaaatgctgaatccacgtcagaagatgctgttgccaaggagaaactacaagcgttagcgg

aatcggatgaactcaaagatgttctgaaaaatcatgaattaagagagatgttaatagcaattgataagtctaaatttccttcacaagcaatacaggcagc

aatgatggaacctatgtttgtggaatttgctgacatttgcctgaaaattgttgagccaccagcagaatgaaaagcctatttgtatactcagaatgctttt

tttccatgtaacatttgtgtgtgctgcattaagttctttatatgaaaatattgttgtgtatgtgaaaaattatttatttaaggttgttgtgatgtaatta

tcttgccacaaacatcattttataaatttgttatacattaaaaatgcttcgttttagtagcagaacaaatgtattgactgtggcataatatttatgttgc

tgttgcttaaatttctaatgaataaacacattttctagctaatagtatcaagaatgcagaataagacgacaagaacttacttgttgtttctggtgcaaat

aagccagtctttagtcatagggcaccatacatgtgaccactgtcccactccaaataatttttgttatccatatatgtaagtggaaaatatataaatg

>Singlet5665|zinc finger hit domain-containing protein 3

taaaagtttcgttgatgacattcgtccacaaagtctgtattacgttgaaactacagaaagatacactaccgctagtaaaatggtattttatcacacacag

aagcataaaataacgactgtgcacagcggcagggcagagcaggattaataaatatattttgttctcttgactgctggaagactcacaaatcttcaacaac

atgtaagcctgtagtatacagacaggtacagcgtcgtcttaatttggtaagtgcagcggaagaaaatgctgaatccacgtcagaagatgctgttgccaag

gagaaactacaagcgttagcggaatcggatgaactcaaagatgttctgaaaaatcatgaattaagagagatgttaatagcaattgataagtctaaatttc

cttcacaagcaatacaggcagcaatgatggaacctatgtttgtggaatttgctgacatttgcctgaaaattgt

>Contig4110|zinc finger hit domain-containing protein 2-like

ctgttannnggaaagacgaatagaaatgttattttgacaatgtagttgtgtattccattggaattgtattgtacattagagatttaaataaaagttgaga

cacccaatataatgccacttcaaccttaatgtcatttgtggaagtccttgaatacgagcgcagttcggcactacgttgttgattgtttatgggccgccaa

gttgtagctagtcctgcaatgttaacggggtttgcttcgaaactgtaatttaattatagttcgtcatggaggaaatcgatcaaaattcatgtcagatatg

taatcgctgtgcagggaagtacatgtgtccgcggtgcaatatttcgttttgctctgtccagtgttatcagtcagatatacatagtgtgtgctctgaaaca

ttttataaggaaagtgttctatgcgaacttaaactggaaaatgatgatcctgaagggcaaagaaaaatgctggaaatactttctaggatgcgtaaggaag

aacaggatgaactggactcggatgatgaacgtgatctcagtgaacgacttgaaggtgtggacttagatgatgctgattctgtgtggaagagtctcacaga

ttacgagcgacaagaatttgaagctcttgtaagagaaggtgatattacagatgtcttgccactctgggaaccttggtggacttacagaaaagagagagct

cttgtgcaggaactagatgaagcatgttgtactgaagaggagccatacataaaaacttgccctccgctaaagactggcatccctcatttttcacatttat

cgaaacatcctccagctcctgatgtgaaatggaacattataaacgttctggcggcatattgttatgtagc

>Contig1264|zinc finger hit domain-containing protein 1-like

ctgtcgcatgaccttagcattaagacttgggatgctttacgtgtgtattatgaagagctttggattgtttgtaagcagccagccggtgatgtgtgacgtg

tgttaaacagtgaactgtggaggatatatatattacattatttcaacatggtgaatgttatgaaaggtgtgttagtcgaatgtgaccaggcaatgaagca

gttccttttgcatcttgatgaaacactggcattagggaagaagtttattatccaggatttggatgaaacacatctttttatatcggctgatattgttgag

gttcttcaagctcgtgttgatgatctaatggaccagataagtttccccttggctgacagagggttgtaagaattgaacaattgtgattgctttcattaca

tttcaccatggcatctgcaagagaatcaggacggctgaaagatgccaatcagaaaagggtactagatgaagctgcacgtcacaggagggccagaaaagcc

cttgaagctcttgaacaagataatttccatgaagatccacatgctgaccttgtgatgagcaaaaaagtcccaaaatttcaagaaactctcgaatctcgtg

gtggaagaagaaagaagaccaaaagtgctgaatattacaagctaaggtttcgcaaaacatttgcccagttggttgaggaagatcgaaatattaacccaca

accaccaaattatatgtcggctcaggcaccaccatcaaggttcccagaaagacacttttgtgctgtgtgtggatttcccagcaattatacctgtattcca

tgtggcgcaagatattgcagtattcgatgtttaggtacacatcttgacactcgctgtctgaaatggacagct

>Contig1792|zinc finger fyve domain-containing protein 9-like

gctcctgactcaatacaggaacttcgttctgcattgatgaatatgaaagatttcagtatcagctgtggacccacaggagtagaacaaccagatgaagtgg

tttacatcaactgggtgcaggatgataaaaatttcaacattgngggtgaaaagtctaattgatggacgtccacttgatggtattccttctattagagtgc

ataatggcactgattatatgggtagctcaagatttattcattggacggaagtgttcattcttcagtctgaagaagtgtcagaacagggaagtgatcccat

agatatcagtcgcttaacagaatcaatagcaagagctacctgcctggctgtgattaatcttctagatttattagcacaggcaggtctaacgaagatagca

gttcgagcaatgatacacccagaaaatgtgggctatgaagctggaagccaaggagaaaaacttcctccaatttatatgaatagtcttgacaatgagctga

tccccattcttcacaaagctgcatcaatgtctcaggacagccctgctgttgttttggaactgatttttcatataatgttgcagtgatataagtactgatg

caaatttgtaacaaatatgtgcagtgtaatgcggtaatcatgtattctccacttgctgttacaaacagtaaggatgtgtttgtgttatttgaagaactgt

gttattactcacaattaatattaaatggtatatgacttgtataaataatngtcctcacttagttcaaagtgtatctctttctca

>Singlet3169|zinc finger fyve domain-containing protein 26-like

cacgacccccaagagatccacctctgcttatggaaatcatcagtatcctggaagaagtgtctctgggccactctaataactcctcagaatctagcatcag

acagtttcctgtggatgacaactctgtcttacatgctcttgcatcagtggatgacatttcatctggcaggtatatgctgtcagcacctgcctcaaataca

gttttcattggccccaaaattggtcacaaattctatgaggagagtattttttaccttaaacagtatggcagccattctggtattatttcattctacctaa

ggcatgatgatgaaactgctgctctgcgttacatacaggagcagaaagttgatcctgagatatttctccacatcatttacattcctagcctgcagaaagg

aactgtttcttctttgcatggtagaatggaatggatggatagttctttgttgcagtggaagccttacctgtgtcatatttgtcatcatcttgaaaagcaa

ggttatttaaatgtcctgtatcaactacagatatttatgaaggattatgtgcgtgctgcaatgacatgtattcgtttttatcacgctggtgccaggagct

atactgacttggcaaataatgtaaattatttggagcaagcaaataaacatcttcaaaatagcctgtcagccttacagaacactaatgtcagtacagttgc

taaatctcctccaaagagaagactttcaaatattcaaccaccagaggaaaaagtgggacttcagcttgattcacgagagcttctgcgtcacataaaaacg

atagagaggcaactggaaataacnngattcttgcacaagtgtgagtgtgat

>Contig4104|zinc finger fyve domain-containing protein 21 isoform x2

tgggtgatatgaaggcgaggctaattgacgtgttatcagtgtgggaattatgaaaaattgtacacaagaatggagaagacagcaccaaaaaaactcgtta

agtctaagtcgggattacgattagtaagccttgacggcaacttaagtccgtttactctgcaagaaccttcatggatacccgacagcgaggcagcacattg

ttcaaactgcaaactcaagttcgatatgttacgcagaaaggtactcatattgcagatactaattaagcatatccgtcatattgccgttgttaatatatat

gcattttcatttgggaactgacagctgttacctgtacatgcacatatttccgagaaatactgggcggaaagtttttttccactacatgtgctgattaagt

actccaccattagtagtgtaattttgtcacaataacaacgaaatgaaagtatcaaaactgcggttgcagcatcactgccgacgctgtggtaacatcttct

gtaactcatgctgtgctaccaagttgggtcttccacgtatgggcttcgttgatccagttcgtctgtgcaagccctgtgctgatgtaactgatcgcgagaa

cgatctcttcaacaatcagctgaagataccgacaaatggggcagctttcttgattgaagatctgggtcaaaatgccagtacatctggttacgtaaggtgc

aagctatctcgcgaccacaggtccatgtggtgtgaagattgtttacgacaatggtgagnnnnatgagccctatcggcagctaaagttatcttgtggtca

>Singlet349|zinc finger fyve domain-containing protein 21 isoform 2

gtaagccttgacggcaactcatgtccgtttactctgcaagaaccttcatggatacccgacagcgaggcagcacattgttcaaactgcaaactcaagttcg

atatgttacacagaaagcatcactgccgacgctgtggtaacatcttctgtaactcatgctgtgctaccaagttgggtcttccacgtatgggcttcgttga

tccagttcgtctgtgcaagccctgtgctgatgtaactgatcgcgagaacgatttcttcaacaatcagctgaagatactgacaaatggggcagctttcttg

attgaagatctgggtcaaaatgccagtacatctggttacgtaaggtgcaagctatctcgcgaccacaggtaccttctctttgagggacatgaaacagaca

aaccagaaccactgaagctctctgaaataaaatcactctgtgttacccgttatcaagattcaggtccatgtggtgtgaagattgtttacgacaatggtga

gaaggatgagccctatcggcagctaaagttatcttgtggtcaagaaaccgatcaaacagcatctggtctgtgggtctcggccattcatgaggccttcaag

ctgatttgtcggcgcaccacgggggagaacagataattgaatcagaaaaagaaaaatatttttagaagatgaatttacatgaagacagattttatgttct

atcttgcattgtgtggcaaatgtttagatcaggcagt

>Contig2226|zinc finger cchc domain-containing protein 8 homolog

gctccgttcgtgtgaagttgaggggctgtgataggaggaaacggttttctatgttttgtttatgtgtgtagtttcatgattgtatttgtatgagtataag

tgacagcattgattccgattttaatggaagttgctctgatgaatgcgttgccacggaaacctccctgaaacagtctccaacgatggttattaatgttgac

gaggattctgcaaatagatgtgatgcattgaaacctatgtgtcccttagtcgatttaacagacgcaagtgatcgatccagctgctcaagtagtaatatga

agggagatgccgtgaggaaaaaagaacccatattcagtataaggtttagggatcaagggacggcaaggaaataccagaagaaaattaaggactttttgaa

agatcttatttcaaagcaactaacagattcggaagatggtggctcatcggacttagagttagatatttgggaagaatcacttgaagaagcagacgatgat

gaccaactcgatgtgtcacatctctttacggttgatactcatccatccatgaaggatgacttagacattcctgcatataatcagaaatttaacgaagttc

ttcagaaacaagaagtaaagtcagatgagaagaatacaccaaatgcatcattgcaaacatgtttcaactgtctgggaaatcactgtttgagagattgtcc

agaacctcgtcgccctgctacaattgcaagaaataggagagatttcatgaacaaacatggtaacactccaaaagtcagccgttatcatatggatga

>Singlet3435|zinc finger cchc domain-containing protein 7

ctgtcgacagtctgttcctttgtcatggacatcagatatgataagattctattgtcattcatggggtggtcacaagtttaatcatgaagaactacagaag

caaatgccaggcaattctagcaattggaaggttctggagattgattttttaggaccattgcccaaaaatggcccattcaaacgtgctcgttgtttaaact

gtaaccaacttggccatattcttcagcagtgtccagaccccattagggagatttgttgctgtatgtgtggtgcaaggggacatactaaacacacttgccc

caacaaaatttgtttacagtgtggacggccagccaccaaatacagcaaaggctgtaagaaatgctgctttgctacacaacagtgcactggatgtggccaa

catggacactatatgtggatgtgtccagaggcatggaggatataccattctactacatctcctcatgaaaattctgttagttccaaatatccattgaaga

gaccaaatgaacagttctgttgcaactgtgcaagaagaggccatacgtcacttaattgcaagtttccaaacagtgagacttttgatacgtatgatgcaca

atcaagatctctgaactttaatgtatttccctacaaaaaccataaaaataatggccatcacaataaattttctgttccaaatatgaacaaaaaattgaag

aaaaataaaacaaataacaatgtaacaattgtaacagattctgaatcattcaatcacaggagaaagaagttaacaaatactgaaagaaaaagaataaaaa

agaaacaaaannnggtgttgcagaacaaaatggcaaatacatagcctcatat

>Singlet7732|zinc finger cchc domain-containing protein 4

atggnnnnnngcatggtatgatgcggaagggtagtgcagtgtattgctgattctgaagacgaaacacgaagaaaatggggagaggtaatgaaaatcagaa

cccggttttggatgtcattatggatgatttgccaaatcaccctcagtgcccacatggtccaatgctgctatttgcacaaaagaacaggaagttctatgct

tgttccgcatgcagagatagaaagttgtgtccgtatttcattcagcatgggactgaagttagtgcggcacatcttttatcttgcgctgctgaacgagaaa

aattggaacctgcaagcaatattgatattttgcgtcagaaagttgttaaattgctaaagaagaaaaagaaagtggattactgtcattcttgtgaatgttt

tgtacttggtactgaggaacagggtcaccgtcatcatgaagttacggccaacataacctttcaccattttacacacccatctgaggtgggtcatcaacag

aaattgtatcttcaccataaagctactgaaaccttcagagagggacaaaaaagaggctcagtacctgttttcctccagtgcagtatcagtaatattgaat

ttagcacagaaaaataatttgaggaaactgctctgtattgggtcaccacgggtacacgagtatgtaaacagttctgaataccggaacatgcacagtctac

ttcttgacatagacactagatatcattgcttttacagtcgagataaattctgctggtacaattcatttaacaactacttctttgactgttcttgcttgga

agnagtactgaaacctttcttactaacagacgccaatgatggcatttta

>Contig2161|zinc finger cchc domain-containing protein 24-like

ctgtcgcagccctttattcatgcagcttactaaactgtgatgtagaagtaannntaatatgtttgtgtgagctgattatttatgttttacctttatagat

cattgtcagtagcctttatttaatctggctgattcatactctgttttactttgtaatttaaataataggcaaaagaagatcctcacatgaatttatgttt

atgtatgtgataatgtttttcaaaagtaaaattgaactatgttaccattatgatctttacacagtgtgctttaagctgtgcattatgtcaccttagcata

aaagcaaggcattctgaagtcaggtaaagcaaccaaaaaaatttgctttatgaggagcaaacaattactgggcagattcgtaaataaataaaggtaattt

aaagtcagattcatactgatgaggctgtaaaaacaaaacatatgtaagataatgtgactgctcaaaagccaaaagaaaagtttggaaatgaatcactgca

gtaacaggcgaagtacgtcaaaattaagaaatgtgtattttaaacttctgatgtattttgttctcataacttcagtgtttcattatagagtgtgactata

acaattatctgaggtgaaattagcaaaagcacattctactataaattattacatttggttttggtaagcacttcaacatgtaatgtagacaaataaatgc

aacaacccaagaaataccatagatatga

>Contig137|zinc finger ccch domain-containing protein 15 homolog

tagttacacttgctcgtaattgtggatgttggtgtttacaaatctttaatgcaatgccacctaaagccaaacctgcccaacctagtaaaaagacggtcga

aaagaagaaggagaaagttatagaagataaaacatttggtctaaaaaataaaaaaggtgcaaaacagcaaaagtttatccaacaagtgcagcatcaagtt

aaaaatgctggtgctgcgaagaagaatgatgaactcaagaaacaagaaaaagaaaagaaactcaaggaacagaaagaaatgaacatgttgtttaaaccag

tttcaacacagaaagttgagaaaggtatagatcccaagtcggtgttgtgtgcattttttaagcaaggccagtgtacaaagggagataaatgtaaattttc

ccatgatttatcattagaacgtaaagcagagaagcgtagtatgtatgtagacatgcgtgatgatgaagatacaatggagaattgggatgaagaaaaactg

aaagaggttgttgaaaaaaagcacggtgaacaagaaaggaaaatgcctccaactgatattatatgtaagtacttcatagatgccctggagaagagcaaat

atggatggttttggcagtgtccaaatggcacaggctgcatttatagacatgcactgccaccaggttttgtattgaagaaagataaaaagaaagaagacaa

aaaagacgagatttcattagaagatctgatagaacgagagagagcagcacttggtgcaactttaacaaaaaatactttacannnctttactacttggaag

aaacgtaaaact

>Singlet1957|zinc finger bed domain-containing protein 5 isoform x1

tgggnnttcaatttgagatgtcttcaacaaatgtgtattggtatcagggttgactacattccctaatttgttacaataactcttgttggctttcatatgt

gaatgccttgaaaatggcgtatgaactcaaaaatgaactttactcttattttcttaatgagggacatttgtgttctgagaagttagtagacagtgacttt

gttttgaagctggcacaactttgtgacatctctgatgaactcaatttcttgaatgtaccatttaaagataaagagaccaacatcctgtaactgccaaaca

aaatttaactttcagagagaagctactgttttggaagaaaaatttggatgataaagatatacattaggttttttcaggtcttcacacagttttagaagac

aatcactttctgtcaagcagttgtagatcatttaattgaattcagtgatttctttctgaaatattcttcaggagaagttgatcaacataattggatcgaa

gattctttcagtacataacttccataaatacttaccactgacaaacaagagcagtttattaatatttcttcaaaagctgcattgaaactgaagtttgttt

cctcatcactgtttgcgttttggagcttggtgaagcaggagtatctgcaagtaggtaatgaggctctacaacacttacacaacatcctttttatgcgagg

taaatttttcagcatttttgatcatcaacttgaaatatagattgcatatgaatatgaagaaacag

>Singlet5387|zinc finger bed domain-containing protein 4-like

atcgtcctagtcgtgttggtttccagtgtccttggacttttagttctttttcccagtacctcaatactttctctagaactaggtagaacaatggtatgga

taaaatgtcgccttagtgtaattcagttttgatcatgaagggatcagagatttcacccatgaatttagctctcgatcgtgtgtcagccagtgtccgcttg

atcagccgtagtgtttttaggtcgaatcactgttcttctaagatgttgaggaaggagtgtgtcacagaatcgtgttgcttcttgaaagctatgaaaatgc

aaaggactggagcattcctgttggtcttctatgttagacttgttttcagactgaatgtttgttctgtgcatcaacgaccagggcgaaacnnngcatggta

ttcaccagttctagctattcttgtgctctctaggaaataagagtattttgtagttaactcttagcagctgtcagacatacagcaatggcaatctgaagtc

ttgcaatttcggccagctcacaagaaaggagcccaccatcgaatgttccataggcagtaagccaagtatagtacacgcatttgaatttagttgattttaa

acgggtgtgcaaggttctggtgaaccaagtgtcactaggaagaggggatcatttaccttctgtcatt

>Singlet89|zinc finger bed domain-containing protein 1-like

tgggactgatgaaggcttcaataagtaataactctgaagtgtgggaactttctgtacgcgagaaggacgacgttggtggactcaatttagtgtcttgcag

cttgcgtcggaaggaattcacgcattttggaaacacttaaaattgagagaaacacttacagtgtattcatcacatacaactagaacagagaaactgttca

agtcatgaggacaaatgtggacagctctgacgagggacaagagataacaaaaggcgtgagagtgcctcaagaatacatttccggtaatttgagtgaaaca

acaactcgtgatcaaaaacttttactctgcaagatatctcagcagttaaaactctttgcagtcagtgtggaaacaagccttaaataaatgaacgatgttg

attctgcagtgacaaaaatgcttgtaagagatcttcaaccattgtccgttattgaaaatgaagagttccgtgagttggtttacaaaccgaactcaaaaca

caaaatgcctccccggaaaaagctcgctgaagtattgctgccacagttctatgaatattgcaagaacaatctgttgtcaatgatgaatgaagcgaatttt

gtggctataaaggcagacacacaaatttctgattgcactaaatcatataaggcaacaacatcccatttcatatattaagatgttgtgtgctcgtatcaca

tttcaaccaaaaatgttgtatgttggtaaacttatttacgctgctgaattcaacattcaatgagtnnggtattcagaacaaaatcgtaactg

>Singlet6037|zinc finger and btb domain-containing protein 34-like isoform 1

aagggggggccacttcggacagcttgtctgctgttgaactgtcccagatccttgcgaaaccgggaccgtcgggagaaagccagagccaagattctctgca

gggtctgcaaaatatcagttaccagcagggaaactcatcacctggagcaaaaatgttcagctgtgatatttgtggagcggtatacagacatgctgcaaat

ctaacatcacgcaagaaaatacattctggagagacattatgtgcgctttgtaacaaagtactcagtcgcaaatcgaacttccgaagacatatgaaactgg

tgcatggttgtcctggatttgatagccctaacagtggtgttgggagattcctgcaactctaaaatgagggcttctcggcaatgtccaatatgctggaagg

ttttggctgtatctacatccctaacacgtcacattcacactgcacatgcggatcctggagctgagcctgaaccatgcacagtctgtggaaaattgttcaa

aaataagtacaatatgtgggtccatcgcagcaaggtgcatcgtctgtccaaagtgtggcaagaatcgtattcacatgactcatgctaattgacttatgag

atgtacacttacaattctcaaatgctgaatctgggtcagaatatagttattaactacttagtttcccactgtctgtgattgggacaacagtctgtgatac

aacaggagaaaaaattgtaaa

>Singlet6261|zinc finger 1-like

ctgtcggannnnnnaatgtagcaaggtcagatgatgtacggtcagttgcagtgctggcaacaaccagagacagagtactccaaaacgaacaatggtacca

aattcggactaccaacaaactgtttcagatgaaaatccacattccattgttcatgttgaagaaaccacttcagcatttagtcgtgcagagagcttcccta

cttcagcaggccatgcacctcgtcgagtatttgaagctctagatgaggtaaaagatacatcgtttgaccatgatgaaaataaatataagaggcgatctgc

aatagactggttctttagatggtggagaactgcttctggtcctgccctacgaaacaagaagccaggccctctgtatcggcgctacttgatggctgttata

cttttcttaataggcttgtttactcttataatgatattttcttggcttgggcgcatagcaacagaaaatgatccaagttttgatgtgcatttaaacccta

atgtgcgaaataaagacactgttcccatccacaatcgcaatgaagatgctaagtaggagaagaatgaacgaatagaaaattttatatatctggatgacaa

gtaacaagtagttttttgactgcttggtgaagttggaatcattatttgatattttacatgtactgcgtaaatagtcctcataagaaatggatttttaaaa

tttggtcaataataccagtgatatgataggatacatgatattcaaatgcagttgttactacatgaaaaataagactgcttcattttatattgttgtagta

c

>Contig256|zinc finger

aaggggaaaaagagataatgaaatgctcacagtgcaattacagcactaccaggccatatctcttggaaagacattcgaaggtgcatagttcagatttttc

gaagaatcaccttgtttatgaatgctcagagtgtccttacaaagctagtcgaaaagaacattnnngatcgacataccagtaatgtccatggctgccatcg

tcctttcctgtgtcatcattgtggtaaagcatttaaaagaccggatgcattgaagcaacattcagcggtgcatctgaatcatcctcctttcacatgtcct

acatgcgataaaggatgtcgatctcgagcacatctcaaacaacatttgtctgtgcactcagctcgaagactgtttctgtgtgagctctgtggagcttcat

tcaagactcgtgctgttcagcgaaaacatattctcacaattcatcatcatccaaaagcttattcatgcttaaattgttcccgaagcttcaacacaaagta

tgcattaagacgccacatgaaacaacatttggagagaaagcataaatcacatggaacatctttagaaataaatgcacgtactactcctaagggtgataaa

aactggctccacagtgcatttaattcagcatgaagtcacaactactgagaaaaacgttgtgaaacaaccttttggaaatgcagcagtgataataaataat

cctagcactggacagactgaaggtgaaaaggtggcagtgcacctgcggaaccatggttcagaaagtgtgcaaatcattcctgcacatgcattcattcaac

aaaccaatgagacagcaacagctttattatatttgacaggaagcttttcatcatactaaaatcaaagcactgtgtgtacattttcaagtggtataaattt

aaattttcgtcgctttagaaaggagacagcagattctgttgaactttatggtacatacaatcaggcattttaaatgtaccaaaagaaatttgtttgatct

gtggtacaattttannnnngctatgttagtccaatgaactttattttctttgctcagnnctaagtgaagtgatgatgtatacatgtagtttacaaatagt

tatttattaaactttacat

>Contig2237|zinc finger

tcataggaaaatagtaaattatttaagatgggtgaatctgtgaaagagctgctacaatttttgtgcagactttgtgcgcggaccgataagtcgtgtgttg

atgttttcagcaaggatggcagtagacatttcattcaaaagaagattcggctttgccttccagttattgtagaggagcaagatgaaatgccaaggcagat

atgcttgagctgtcttaataaattggaaagtacatatgaattttataccacctgcatttatgctcaatcgacaatccagagacttctgagagctgaatct

gtacaagatagtgatcatgttaatgaacttggcaataagtcaagccaacaaaccagggctgacaccaaaaccacgtggagctgttgtaacggcaacagtc

agtcctttcactccaactttgcggtctgcatcagagccacaaagacagttgcctgaaggccctccaccagacgtacaggtagagcctgtaagaccatcat

ctcagtcaccgtctgaagcggggcctggaagatcttcaacctcttcaccatctccaccatcagtaggaaaggaagagtgcccttactgccatattgagtt

tcctgttgatattctgatccaccacaagacacttcactacagagaaaggttctatacatgcatcgattgtgacaagaattttgttactgagaagggctta

gaatcacatcgatgtcagaaagaaagcaagggataagttaataagactgaatttttaag

>Singlet472|zinc finger

gtaaacaatggcgtctggttgtttgttttacgtgatgttttagtttgtgtgggcggcttgttgatgttgaggcgtcagttcttagcaataataataataa

tgaaatggcttgacgatcactgtgtttcgaactaataggaaaagtttttaatacgccctcccttacgagtgccctatatattttatggtgattgcttgtt

acctattttaccagcgatagctatgtcacagcagtctgaagccaaaaaacatgtttgcgttctttgtaatacaaagctcgatacaaaggaagagctgcag

gaacattttcggaaacacgcaaataaagaaattgataacagaggccgtccttgtaagcagctacagccagctgcaaagacgacaaaacagccagttccaa

atcgtgctagcgcgacaaagccatctggtaaatcagatgaaataatttgtgatgtgtgtgggcaagaattcgaaaatgtcaccattgccattcagcataa

gttccggaaacatcctgaaagtgcagctaagcacttctgtccatattgtggtatgcagtttcctcttaaaataaatcgtgacaaacatctttccgaccat

ccagataagaagcctgagaagatgtttccgtgtgaagattgtggcgttgttttctacaatgaagaagctcagtcctaccacgtgaagtcaacgcacaaga

gaattgtagctttctttcaaccagtggctactcctcctccaagtaag

>Singlet3817|zinc finger

agtgccctatgataattgttgaatatttatttaaatgtactaaagaaaattgagactatatcgtatcagagaaatttaagacgcaagtaacaatttttgc

acactttttttaaatatgtttggcaccatttcaacactacggcattgcaacaaaccgtaacataaattgtactattaagaccgctcactactgtgccaca

cgagaagagacgaaaactctcttgttaactctaagcattataatgaaattaaacacacaaatatctcgggtacaaattcatcggaggcggcgtcagcagg

aggcaaatatcagcagcagaattttacagctacgactttgaactgtctgaagagagacctcggtaaagaaattctggtcagtacagctaatcaccatgca

gacaacaatagtcgaatgccacagatctgtatttccagatgaagatgcaacaacagatcagtttcatcagctctgcagattttgtgcaaagaagaacagc

tgtatgatgcccatattcagaggacaaggacttactgatgacttaccatcaaaaataaaagcatatgtgccagttgaggtttctgaggatgacaccttac

ctttacaaatctgcagcccttgtgttcagtgccttacaatgtggcatgaatttatcactggctgtgtaaaagcagatgaaaacttaagagtcacttatca

agaaatactgaaagctaaagctggtagagatggttctaagtctgtttcagaagagagagataatcaacaaattacagaggccaggactcctg

>Contig1046|zinc finger

tcctcaagttgagttggaaactgattttgtaaaagatttagatgcaacagaaatttcttattgctgtcctctttgttgtcaaggaagtgtcagtgctcag

aatattgtcaaagaccttgaaacctgtactcctatggaaagttcaacctattctgatatggaagatgaagacgacgatgatgaagaagaagaagatgatg

atgttgattatatagatgtacatataatagatgacataagtgatgaatcaaatcttgtatatccttcacttgataactttgctttatctaaaattgatga

tgatagtaacacaacagatgaaaattattctgattggtggaatatggaaaatgctttagaggatgctgatattattgtggatacgttaaacccacaagca

ggtgaagtgtatcatagcttcgaatctcatgacaaccaagaaactcttataaaaaactttgataatgtaaaggatgtagacacagaggttaactacactt

gtataatctgcaataatacatttccaagtgttgatctttgcttgaaccatgcaaaggagcacagtgagactgacaagcatccatgtagcttgtgcagtac

attttttaaaactgaagctgaccttgtcaaccattttaaggatcaccagatagaggaagttaaactgaaatcaagaggtaagcgtttggtgtgccctact

tgcaataggagatttaatggggaaaaaacatacctccaacattcatgtattactcctgaaacaaaacagtttcattgcaagcaatgtacaaagtcgtata

attcagaggaaaggctagcatttcatatgaaattccatgagggtgccagagctaacttttgtgaaaagtgtgcaagggaatttgataatgaatgtgcttt

atactatcacactcgtatggttcataatggtgagagaccatttgcatgtccaatttgtcataaaagattgtactccaactcaagattagcagcacatatg

agagttcacactggtgaaagaccttttgcatgtgaattttgtgggcgtaaattttatgacagggaaactctaaaaggccactatgtgacacacatgtctg

taaaaccgtatcagtgtgactactgtggtatgtgttgtggacgcttaagtatattaaaacagcacattcaggcacatcattctgactgtagcacatccaa

acgtttaccctctcttatacattatcattgtaagacttgtaacaaaactttcactagttcatctgatgtcctgaatcatcgttcatctcactggggcaca

aaatctacttctgaaagtgcacaggagcatgcttgtgaatactgtggaaagtctttttcagtacttcgtgcattaggaaggcatcgaaaacgagaacatc

cagatgaaaaaccttatgtatgcagtgtctgcaaggaagcatctcatacactttatgaagctcgagttcatagaaaaatacatacagatagtgaattgaa

actgaaaaccaaggaaacttcagataattctgttatgtttgtatgtgaagcctgtggcaatatttttgttgacaaacgaacttttacaaaacacctcagg

gaacacaagccccatgcatcttataattgcaaagtttgtggaaagcaatttgcagataatcagagactcacagttcacatgagacttcacacaggagaaa

aaccctatgcatgtactgtgtgcaacaagaggtttacacagacatctgccctatacacacatgctctgctccacacaggtgagaaaccacatcagtgtga

cttatgtggtaaagcttttcgtataaaagcagatcgtgataatcaccgtcgtacccacacgggagaaaaaccatacaaatgtgaattctgcaacaaacag

tttcggactggacaagtgtactatcagcatcgcatgattcacacaggagaacgtcgatttccttgtgatatctgcggaaaagcctttaaacgttcccata

ccctcgttgttcataaaaggatacacactggtgaaaaacctaatatatgtgatatatgtggaaaaggtttccgacagcgtagtgacatgagaaaacatag

atcaatacatactccagctgaaccatcatgatgattgttagtgtctttcaagttaagatgtggaattcttatgcagaatagtttttgattttctgaatca

ctgaatttgttgcagcattcacaaatacc

>Singlet1437|zinc finger

agcacaccatccacctccaccttcccagccacctccagcagcgtctctgccccaaggccaccaacagcagacttcccagatgcaccagcaacagcagtca

catcagcaggccccacagcaatcacaccaacctcaccaccagcagcaacagcagacacaaacacttcagcagcatgttccacaacatcagcagcagcaac

caatacatgcggaacaaatgtgtcactcacaccaaacacacccacaacaaccacagggccagcagcagcagcagcagcagacacaacagtcacctcatca

agctcaacagcagcaacaagcacaacaactgcaacagcaattaccacagcaaccattacatcagcagtcacaacaaggacatcagccgcagccgcagcag

cagcagcagcagcagtcatcatcccagccaaagcaacagcagcagcagataactggcagtgaaaatcagagcaggccagcagagggtcagcatgcaagtc

gcagcacgtcatcctgctctcgtgaatctagtactcaagtagatactagctctgatcactcagacaaagatgagaaggcaactgtttcatctgcatctac

taaagaagaacgcatcttgttaccagggtttccaattcaagatcttaaaggccacacaatggatattcgcactcctgatggaaatattgtaaagataagt

gctcctattaatggtgaacaggacctatccaagacacttggagttgaaatggtccaaaacatgtacaaggtgaatgtagaggaattcaaccagttgttag

cgtaccatgaagtatttggcaaactacaaagtgagatggttgcaaacagtgcaca

>Singlet5349|zinc finger

agagtaaaacagttgtttggaacaacttagaacaacaagttcgtttgcgtgttgtggtgtgctggtatttttatttttttatatagtgatgtagggctta

tgttacactaaagtgtgacattggcagtgaaacgtttgtacaatttaaacctgcagcttatgaattaaaatgatcagaagaagaagggagagagtgcgag

ataagactagtcaacatattgaccattcgagagtcatggggtaaggcagcttattagagacactcaattctgcttcaaatgtgaggattgtggtaagaac

tacactgcaaagcaaagtctgacaagacatctgcgctatgagtgtggaatggaacctcagttttcttgcccattttgtccaatgaaatataggcagaagc

attatttaaagactcacgttgtcagagcacacagcaactggactgcacacacaaaataaacacactggaaaattagcagatgtgttaattgaaatacaag

agtcatttaaacatgtaactttatttaatacttcctgtaaaatgtggtatcttttttctaaaacagtaaaatatatacagagaagtcagctaagtttata

aaatgttcttcataaagatatctgaaaattgaaaataaagtgtgaattttgtannnntcttaaaagataattatgataccgtgttgattttactctctga

atctatggttgtctgtgtctcaacatatgtatct

>Singlet8092|zinc finger

ctgtcgtgactgtatgttatcctgtcggctgagtacattaatcatcatcgtaaagcgaacatttcataacaaaatatttccattaatataaacgtcacat

aattcccagaatgaaaccgccgttgccgaagccagaatttttctgaagataatgtattattgagataaacgaggaactagtacttttttgtgatcctttc

cctttctgggttcatttcgtaaattcaatgggtcacagctcagaatcaggcgaaacatccgccacagagacaggagacaatgtggattacactgatgcta

aagggtatgacaccagcgcatttgtgacggtagtagtatcagactctgaagatgactcaagtcctacaaaaaaacagcacatttgccactatcctgggtg

caacaaagaattcagtaggccatggcgtctggctgcccacctgtctacgcatactggggagcttccatacaaatgcagttttgatggttgtgacaaagca

tacactactaacagccatctcaggaggcacatagcgaacacccatcagcgcaaagaactagaaattttgttcagatgcccattcccaggatgtgaggcta

agatgaaaagccaatggaacataaacaagcatttcaagtcaaaacatgaaccaaaagcttttaaatgcactgtgtgtgtggagagtttccggaagcacag

tcagcttaaagcacatatgtatgagcacactggtgtgccacctattcaatgcacgatatgtaaggcaggatttctnnnnattggggatctcaacaggcac

atgcgatgtcataaacaacacacatgttccaatggtgaatgtaggcaggtttttgaaacatg

>Singlet2108|zinc binding

acggccgagggggcatagatgctgttcagntnnttgatgatgtgcctatcccatctgtaaagaaacctgatgaacttttgatacaagtgaaggcagcttc

tgtagatcctgttgatattcttatatgttctggctatggaagagcgctgcgtagactactccacaggtataatagtaatattagtccatactggatgtac

agctgttggagtgataatactgcagcttgcacgtctgtggggtgttcacgtaactgtgacttgtccacacagggcaacaccagttgcgcaagctttaggt

gcagactgtattgtttctgaccagcagccagatgttgagaagcagcttttagaacatgataggtttgattttgtcttcaatacagttggacatgcagctg

atgaactgtgcctaaaattttgcaaagatgatggccttgttataacaactgtagcaagcccattagctgctgacaactatgggctcatttttcgattgtt

gtacagcctctgggttcgcttaaaatgcttttttagggtctgttttggggctaatgcttggagtttaatggatgaatctcctgtatcactgaatgaatta

ggaaaattagtagaagatagaagtctgcagccagttgtggacaaaatattttt

>Singlet600|zgc:174162 protein

cagtgctctctatctccgccggtagggcctcttccacatctcagatgagatcccccggcaagcaggcagagtgaaactggccttcttccccaacctttct

gctatttccttaaggggttccatcacccgcctaacgttggagctcccaatcactaattaacccctcccccctgtgtgcctgctcggaccttgctgaagga

gcggccacatgtccactcacaggcagagcaggcgatgccacacggccagcctctacattgatctcccacctcgtgcgccacgaacactgctgaacccgtc

actccccttggggagagggtggcccaactgcgccctgtacccgcgaagatgtcttgacagcagggacagtgggtgaagcatgtaacacctggggtgtacc

gtgcaacacacaagactccccattgccgctacactccgaggcagcagcctgaagacggctggccacggccatcaacgcgttcagctgttcgcgaacagtg

gccagctcttcctgcgtccatacacagcagtcacacatcctatccatcctaagaaatcaatttactgtagagagttaatcaacttttaactagactgcta

attcactaaaggcggctgatagttgattaaactgtggttactacacacttcttgtagaaaacaatgaaaatagcactacctgtctcttgactatattcaa

aacaaacactagcactatggctgactaaagggactctccctgactgtgnnnnnnacaaaacacgaaatctatggaacattattactagcactcgacaatt

aaagttcctaaaagcaaaaatacatg

>Contig4531|zebrafish dna sequence from clone dkey-197a14 in linkage group complete sequence

cacacacacannncacacacacacacacacacacacacacacacacacagtagcaaatttatgcttgagctcatttcagaagacggatctgcatgtgcaa

accatattgaattacttggtatctgcttcttcagaagccagaaaaaatagataaagggctgtggaataatgacagaagttgcaaagacaaggatataccc

tatggaagttcttgcatttgtcacatgatggggttgactgactggcttattaagttcttttgctaaatttggcatgcatatgcatgtttttgttgacaga

tgatgaagcagctttcaatttgaacacccagtatctctgaacttctttctttcattggcattgcctcacagacatgtttatcaaattgtttctgatgtat

tataatattttaaagtaactgtaatgaaagtaaaatcgactgtagattacaggtgttcatattttgattgagtttcattgtttcagcaagctgtaatctg

atgttggattatagattcttgtatgttttcctctgcatttcagtttaaattgtgttataattctgttacagttaatatttctcacatagtttatatggaa

tgtactaatgtataccagctgtggactgttttagatgtaaatgtgacaattaagaaaaacattgaaactttggggtgattacaacagtgtgcaagactga

aagtctaaactggaaccttcacttttgcaggtaatgctgtaaggtaagagcacacccatgaaagccaaggttctggtcggacttccagtctcacacactg

ttttcatctactgtgaagtttccaaaataacacacacttcactgaagagtgaaagattaattccagatggaaatccttgtcccttacctcgtagttttcc

atttactgtaagtacaattttttgtaattggaagtttttctgaggccaaatactttgaccactctgcagttaaatccacatttttttatgttctattacc

tactgacagttttcatgaggaatgtgattattgaattatccactgatacaaagaaacaatgtatgcaagttccagatagtgacaacaaacttcatgtgct

attgcatgtttttaaaggacagactctgtggttgtgagatgataaaaatgttgtttagtaacagttgaacatagaattgtaatactttttcctttgataa

ttttctccccatgtgatgtaaaatgacctagtacagtgaatttaaaataaaaaagataattcaaaaaaaaaaaaaaaaaaaaaaaaatcg

>Contig4281|zebrafish dna sequence from clone dkey-183j10 in linkage group complete sequence

gtataaaagcactgcaccagtgcagcctgagacaaataataagtgctgatgaaattacatttgtattgtattatatgtatttatatataaatatatatat

atgtgtgtgcgtgtatatgtctattaaaggcgtaagtgaaatactactgggcttatatacctggcaggcaggaaacagatgttgcagtcacaaagaaatc

tgtttcataatgtgctataaaaatttcagatattaatttcagcatttattatttagtattcagatagggtataaccatttgactgctatatcagaatgtt

ctcttttagctcatatatttttgtaataagtgactaatttatgctgcaatacagtgaatattgtgttaatcctctgtcataaggaacagaatataactac

atacacatgtacacaaatgctcttacagtgctgctactgaaactgccatgactataaaattacaacacactcttaaagtatttcagttagaatttgttga

acatgtgtgcattagattaaccaagagttctgcaatactttcatgtgagtgcattggtgtatcatccgcaatgaatgctattacagaaattatgtttgct

ttagcttgagggagaactttgcagcgtttcagtcatcacttaagtagcaacagaacttaatccaaatgaataaacttatattttttgttaatatcattac

aaaatagttattactttgtccaaagtgaaatgctctgcgtctatcacatccattatctggcattggctactacagaattattatttccagtaatgctaat

aatctcaagaacatgaatgtgaaaactaaaataccattacaaaattacttaagcatggaggaaagtagaaatagttttaatgttttaactctcaacataa

catttaaaattaatagaatcagttgttggtgcctctctttactgacttttcttgtgaggtagtttaaaacccctagctaacagcattttcatcttctaaa

atatatttctgaagtcaccttacagtaaagcttacagttcatatttataatttgtgggccacaatagttattactgattgactgccatctttgcataaag

tattatagctgcaacccctgct

>Contig2064|zebrafish dna sequence from clone ch211-220m1 in linkage group complete sequence

ctgtcggtgtnctttgtagtgtagataccaccgacagttggcacccccaaaacaagtccagtaaaatcaacactatcaacaaacagcttatatcaaacat

ttaattattaatatgacatattcacagtaaatgtaaacttaatattgaggacacttataagaaggaacacatattgttcataaaagatatatgaaatact

gcaactgaaagaaacttttcattgtttggcagagtacttaaaacttttagttattgtcagtatttaatttattttcacaactacaactggaaactactat

tacagggtatatgtaattaatatgcatatttacatgattgcaaaaaaacgctacacatcatgtgcccttgtatcaacatcctatcaaaacaaaaaagaaa

ttatttagagatttccagagttcactagaggagagtctgtaacacgtctatttgtgaattagatggtatggttgaacaagacagaagtgtcattggtaaa

agacatctgtgctaaataatctctaaacctttattatttgcatatcatcatcatcatcatcatcactatcatctccatgtaagactttgaatctccacac

agtatacaagattttttgtgattgcatgccctcttcacttcagtcataatgtattcatagccaaaacaaaatgaaaactggagagaggctgaatttataa

acaataaatgaaaattacaaggtcagcagggatactaagttacactaacttaacaagggtggaagagatggcctctgtggaccaattaagtcagatccag

agggactaactacatttacagtgaataaatgtaggacaggattgttaatgctcgaacacatttctgcttgtaatgataatgagtactataactattgtct

tctgttagctttttaactctccactaatttcatcttatcatacttttaacaccatatgtggtaatctgacccacccgtattaaaatttctctctcttttt

ggccaaagcagtaccttgttgctgtcagcatgcaaagaagaaatattcattacaagagcttggtagtaattatataaaataatgtcatcctcagcaagct

aatggattcaacacctttttatcatcattttattttgtgtcagtactatcttgattcagcccaccccaaaagtaaatttttcagtttgtcatatttaaag

aaggatcttgcagttcacactccacaaaatattcactagaaatgatgttagattctacagtactgctatctctttgtagcttgtgaagtgtatattcctg

gttaaatttactgtaaaattttctatttttttcttatacccccagttttctgccagtcaggcttgtcannnttctctctctaantctctctctctctctc

tctctctctctctatctatctatctatct

>Singlet5500|yth domain-containing protein 1-like

attaacttttgctactttggttcaggagccatttgacattaaggaataaatgttcctgctaaccaaaggtatctgtttcatctttctcttgccagctgta

cgtcgtacagtattttgtggaacagacacatttgtagccacattggtttctagacatcaccgtagtataaaatcggcactcacccaaagtgtgaagtaac

atttcagtacatttaatattgtccccagcaaaatattattcattgctgccttattttttctgatttgtcatttgtttcaaaggaagagagagtgcactaa

ttgtagtaacgtgctgcttctacccagaaaattttatagatgttacattaagcatttgaacaagttaataatccaaaattgctttgacatttggtgcttt

ttgcagtgcaatgtggcttcactttgtacatgacgagtggcttaaatttcctatagtggcctgttgtgtgcccatggatagctgttaattgtcacgtgct

ttatctacgtatttagcttgctgtgtgtttgcaaaacacaatacagctaagaatagcacttgtacattttaatttttttaaaaaaaacattaattatgtt

tgccttacttgtattcacagttactagttttgcttattagtaatgagctaatattagaggatgggtgtataatgatattcctgaatgtgaaagtaaggtt

cattatctatgagttccattttcacattaatgaagtagttctttattcagtcagtggctctttgaattgtgatgtgtttaatatagtgaacacagtgaat

gtattttaagactttttttttt

>Contig2670|yth domain

tctgaggattatgagtagtagctcttgaatggatctaactccaaatttccagatacctgtggttaaagcatatatgaattccatatttgctttagacctt

ttccaatttgctggtagttgattattgtgttgattctttgtcccttttcgctccttggtataagtggctgccattgaaaaattagttatgttatgcaaaa

ataaatttctgtttgctgtagctcattataaatgcgtgctaagtgtgtgctgtatcaaaatgtgctgaaattgtcacaggtgaagttttaagtaaccctt

taatttctgcgcttttctgcttcgtactattcatgtgcccttcgcataattacaaccatgttgtgtatttgtgaactatataatatatgcatatatattt

aaaagtaattgttttcatggttggttggaaactttttaacagtaatataatgatgcagtaacgttgaaggaattatctgggagtaatttctttttatatg

ggaaggagaattacttgtggttatggtctatataaagaagaataatgagctcattgatagatgaaagccaatctttaaaaagttgctgtgaataaaatta

cagtggaagtacatactgaattattttctcatctgttttattaactgtttgtgttacgtatatttcttttcttgcatctaaggtagatcatttggtccca

cttgtctggatcataaaaagttaaaactgaagtgactgttgtactgctggaacaaaacagctgaaaaagtgcaaggtggaacttccatgtgttgatgtgg

agtgatttctgtggcaattcgtgcgttggatgaggacatagtgcagtgaatgaaaacgtttttgtgtgctggattatttaagcagtccgcgtatttaatt

cctttctttatagaacagtgacacagtgacagataggaccttttgtgagatttatgaagttagtgaaggcagaagattgtgtcatagtgaaggaacttaa

aattgattgtaactgaaaacagcttagtgcatcattttctttcacagcagcacactttctcaaattaacttgctttaatagccagaaatgtcatgtggac

tttatgaccctaaaactggaaaaagtgatgcgatgagaacattgaatgtcctttttaagcatttttcaaggagaaacagtctgctagaaatgaaaatata

gaacagtttgacaaatgactgctattagaaatctatttaaaaatatatacttgtaaaaattattacaagaacttgaagaatgttactgttgaaccatgtg

cgtagtgctgtttcctcatttaattatgtgcgtacttttttactcaagttatttttcccctccctgccaccaaatataaaatgtggactttaattaaact

taatttttatactttttgttgtatgttgtgggattgagccagaaatgtagtgaatatgatgatgctgttgaatgcttttggactcttgaaatatataaga

ctgcaataaattttaaactttgtttgaatcgataaacaataggttgtcagaaagagaatttaatatcagtgttggatttgctaacaatctcattttgaag

cgtatgtgggctttcagaagtctgttaaatgagaatgtaaactgaaaaactggacataaaatgttagaagcttcaagtgactttaaaaaaggctattgtt

tatgagcattgaactgaggtgtgtgattgatagtgtgtgtggtgtgtttccccaaccagattttatctcctcttgttaccattgatagcattaaaaatgc

aaatacatcaatagatgcatgcttgtttgatgatttccaaaaaattgtgatatttctacattgcagctgctgatttgaatgtaacagtaatcttggaact

gttaatatgtttgtaatcagattatgcattattttttaaatttaattttaaaataatgaattattatnnanacaagagaggagatattgtattttcatga

ttgaaattgc

>Contig4043|yrdc domain-containing mitochondrial-like

acgannngtaccatgtgatacgtttggttacgttgatcgtgtgatagtgacacttgcacatttgtggatgtgtttacgtggaagacacacgaattatgtg

tgtacatcgataaatgtccatgcctaaagcaatgattaggataactgtttttggaacgacaatttgtctccggcactgtaacagcaaaatccattacgaa

acggtggagttacttcgtaaaatggctctgcaatccattaaccgtgattataaagctcttggcagtgttcgtaagtcttttgtggacaatggcctttata

atagagtgacgataaaccataaaaaattggaagcctatgctgagcaagcagtccgtttattaatgcaagactgtgtgatagcggtacctactgacacagt

ttacggtctagcaggtgctgttcagagtgaagatgctgttgaaaaactgtacaaaataaaaggtcgggatgcagcgaaaccaattgcgatctgtgttagt

tctatcagcgaaatggaaaagtgggcagacacaagcattcttcctcctgccctcttacagtgtattcttccaggaccttttacaatcgtcctcaaaagat

caaaaactttaaatgctaaatttaatcctggaatcccgaacgtcggtatacgcattccacaagacagatttatagttgaagttgttaaaaagttcggaga

acctattgctcttactagtgcaaatagcagtggagaacagagtgcaattgaagtagaagaattctctcacttgtggcaatatatagatgcagtttttgat

gccggcaaaattccacaagaaatccgcactggctctacagttgt

>Contig2812|ylp motif containing 1

ggacagggacagggactgggagagggagagggaccgggaccgggacagagagagggaccgcggcagagaccgggaccgggaccgggacgattacgcaaag

cgtgatcgggatatgcggtgggagcgcgataggccaccacgagatgccgatgacaggagcaaagacgacagagtaaaagagagaatgtcacggtgggaag

aatctgaaagaaacagattcagtaatagcagtgatcgttggggaccaaagggaggcagtcgtggatatgacagtcgagacagaatgccactgaactcttt

cgacaatgacagcctggcccagaatgcccttgcagatgcggcgccccctcctccgccgccccctcccatcagcaacaactcagcaattggcgacggcatc

ccagccactatggatggaccaccgccgcctccctcacttgctccttcgactcaggaaccagtcgtacgttctgtcatggtggaagatttgctgcaaagtc

ctgggagactgcaacgccccacgagaatcgtcgtcatacttagaggtcctcccgggtcggggaagacattcgtcgcaaaactcatcaaggataaagaagt

tgaaatgggtggttcagctcctagaattctctgccttgatgattactttatggtcgaatctgagaaagaagataaagatccagaaacagggcggaagatt

attaccaaggtgatggtgtacgagtatgaagcaggcatggataagcattaccatggttcattgttgaaagcattcaagaaaacagtgaatgatggatact

ttccctttataattgttgactgcatcaatgataaagtaaagcaatttgaagaaatgtggagctatgcaaagcagaagggttttcaggtatacatctgtga

aatggatatggatgcttcagtatgctcgaagaggaacattcacaatcgttcagagaaagacatcagtgagatcatcgaaaactgggagcctactccaaag

cactaccttcgccttgatgttcgctcattgctacaatcggtcgctatcacggaggtggaaatggaggatactgcagctgatgtggaaaaggacgactcaa

aagctgtaactgaaacacaagacagtatagctgacaaggaaccagagggagatgaaactgagaaggatggtgatgaggagttggagaacagcactacgta

cacgagcaagtgggagcgcatggagccttctggtgataaactgaatcggcttgacgggcttg

>Singlet6184|yjef n-terminal domain-containing protein gf19489-like

tacgaaaatgtggttgtagaatttttattgttgttccttggaggggtaatgaatgtgaaagtagtgtcattggtgtcccgtatcagtttacgttttgcga

attccattcactcaagaagtatggtgaagtttattggccaagatgaagcaatcagcatcgatcaagaactgtttaatgactataaatttagcgttgacca

gttaatggaactggcgggattaagttgtgccgtttcaatnnngaagtgttatccgttagaacgaatccagacgaataccgtcttagtttgttgtggtccc

ggaaacaacggcggtgatggtcttgtttgtg

>Singlet3587|yippee protein, putative

aannnnnttgcnnnaggtctgggagaactggtaccaagggaggccttcacaagagctcttactttatactaatctttaaaaagatgcatcacagactgac

aagacaacacaccaaagaaaaaaaggtttcccttttatttctgtcacctcttatgtacacaatctgaaagtcattctcaaaatacagcattcatatttct

atagttaatatcacattaaacataagaacactcattctaattaaatatgtgccatctgataccttatggcagttatatggaagaatattaaattctttaa

tataagtaataaccacttgaaagttatgattcaacagatttcctgtggctgtatgatccacatgtttaataataaaatgtacaaaaaatctcaacacaac

attgcacaatcctaaattacaaaatattcttaaaaacaatttcttttcaacaaagactgttttacctcacacacatttgaaaatgcagcgaggatggaca

ttttctacacacatcacaagaaattaccttcaactcgtgtgaaggtcagtttttgtgtcacttctacatgcaaattaagggtaaatttaaaaaagtaatg

ataattgtattgtaaaccagttggtcattgttttagtgttcatccaagttcagctgctgcagtgtaaaatggaagaaaattgacaattttaaaaaatgag

acactaagaaatcgctcactctcacaccaaaaactgtgacaga

>Singlet4362|yellow-c precursor

ctgtcgggacgctcagggcgagcgcaccgtcttcttccacgcgctgtccagcctgcgcgagttctcggtgcccgccagcgtgctgcgcaaccgcacggcg

gcgctcgccaccgggccggactcctactaccactaccgggacctcggcttcaagggcaacgacacgcaggccaccgcttcctacctcgacgtcgacaccg

gcgtgctcttcttcacgcagctgaaccgcaacggcgtggcgtgctggaacacgcagaaacccctggagccggccaacgtgcacttgctggtcagcgacgg

cgagcgcttctccttccccaacgacctcaaggtggacgccgagggcaccctgtgggtgctcaccgaccgcctgcccgtctacgtgtacaagggcggcctc

gaccctaacacggtcaactaccgcatcttctccgcggccgtcaaagacctcatcaagggcaccgtctgcgaggcgtagttggtcgtcgaggatcgacgat

gacgccgcagaactgtccagcgccgcacctattcagctcccctcctcgctcttttgttatgctaacaactccgtgcaggcgtatagtcagcttaggacat

gaacttttcatcagtgatgtttttttattcaacactctcgtctccgctcgtcaaaacctcttagtcgccgaagtctaaaactttttttttcccttgtgga

gcaatgattagtcaaaagagcacttggatctcgccttattaaaggaccagacgctttatcacaccggatacattctatcaaagcatcagtcttgttgcat

caaatt

>Singlet5461|yeats domain-containing protein 2

agggatgccttagtggcactaagaagaagaaaacagtttgatatattcagtaactgtggacttggagttgatgatactagactagtggacaagtaaaatt

catttgtatagcattctgaaaaatctgtgataactgtttgtgttaaaaaaaaagttacaagaatgttgtgttgtgttgttaaaaaagaagttacaagaat

gttgtgttgtgtggagctctttccagtgttaaaagaccgattatatgaattattgtactgcgtgttagatttacattgtgtgtgtgtgtgtg

>Singlet5229|y+l amino acid transporter 2-like

ggacaaagtcctgggaccgatgtcgtggagcatgcccctgctggttgcgctgtctgcattcgggggcctcagcgtccacatcatgacgtcctccaggatg

tgctttgtcggtgcccggtacggacacttcccggccatgctgtctcacatcaatgtccagcgcctcaccccgaccccttctctagtcttcctgaatctac

tttcgctcatcatgctgtgcacgagtgatgttcacgtacttataacgtactcgagcttcgtcgagtcgttttttcatcatgttatcggttgctggaatac

tgtggttgcgctggaagaggcccgaaatggacagaccaattaaggtggcgttatggatacccataacttttgtgctgatatgcgctttcctagtcttcgt

gccattctacgagcgcccatacgaggtgggtatgggcgtgttgataacagtgacgggtgtgccagtgtactacatgggggtggtctggcaggacaagcct

gcttggttcctcagtggcctcgacaaactgacctgcaatgtccagaaattattcctgtctgccaaagaagagaaagaagactaattccatgctgcagaaa

accaaggaattatagtgttaaattataagtagtttaaaaattgagcttggcaagtactttttttgtattgcttgatttatgctggagattgacagccagg

tggtaggtagtgtttgtcagacaatgtatcagacggaagttaagaagtgctatattgcagtgtgtta

>Singlet6690|y+l amino acid transporter 2-like

ccgttgctgtcggttaaaatactccatgtgtacagaattcttcctggtagttttattttatttgtattttctaattgtatgtaaaatgtgattttaagaa

tgcttttaaaagaaacaagtggtgatgtttttatgtttacttagcaacagagaacaatgaatatgtgaaacacaaaatagaggattgtattttttaactt

ggaagtccatttgctttatgaggagcagtgttaaagtttgcatcatcttcattgtaattgcatattccccgaaattactgtatgaactttaagtctgcca

tccctagatggcagtgaaacttgcatttccttttgcatatacacaattggctcaaggtttgtactggtgcatggtgcaactgtaacagttttttttaatt

tttttattatttaaatacacagttccatccaaacattctccagtaacaaacttcagtcattactatgaaagtacttgtatgtcaatgctttcacactact

actcatagctcagacaatactaaattgtaagtcacacacccacaaacctgcatattataatgtaactgaagattgcatatgaagaaatgcccatttgtta

ttagcattgtgagggtatcttaagtgtaatgttttgacatgctttgtgtgtattttgttttgtggttggttacaaatgttaatggttcctgagtgtaatt

taatgccaacaaaattctgaattttgggatacaccaataaattttttatttataactgcaaacaaatcttttattgttggcctagtgcctcaagctgaat

ggaatttactgttcacatt

>Contig3514|xanthine dehydrogenase oxidase-like

gcacagcctgtgcattagctgcatatcttcaaaacaagccagttagattcataatgacacttgaagcaaacatggaatcaatagggaaaaggttagactg

tgctatggattatgaggttggcgtagatgatgatggagtcataacttatttggaagcaaagatgtatcataactgtggtgctcataggaatgatagttct

gcaggtgttgcacttgactcattcaagaactgttacgataacagcacttggaatgtaactgcttacaatgtcatcacagatgttgcacctaacacatggt

gcagagcgccaggtactactgaaggtacagcatttattgaaaacataatggagcatattgcaaagactcttgagatggacccaattgattttaaaatgaa

aaaccttgtgaagggtgatacattaattgctgatatggtcagttactggaagactgaaacaaattacttggacagactagcagctgtcaagaaattcaat

atggaaaatcgttggaggaagagaggaatttcagttgtcccaatgacgtacgggtttcagtattttggaaatttctatgcccatgtgtcaatttacgctc

gtgatggtacagtttctgtggctcatggtggaattgaatgtgggcaaggagtaaacacaaaggttgcccaagtgtgtgctcacgcattaggaatacctct

agaatttgtgagtgtaaaaccatcaaatgtcctgacagctcctaacaacattgtgacaggtggaagtgttgcaagtgaatcatgtgcatatgcaacattg

cgtgcttgtgaagaactaatgcaacgactggcacctgtaaaagccaaactcgaaaacccaacttggaaggaccttattgctgcagcacattcagcaaata

tagatctctgttgcagtttcatgatgtatgctggtatggcagacacaataactccatataaagtttatggtgttactgtggcagaagcagaaattgatgt

actgacaggacaacatcagttactacgtgtagatgtactggaagatgctggtgagagtttgagccctgaggttgacataggtcaagtggaaggagccttt

gttatgggcctaggctattggctggaagaattcatggtttatgatgacagtactgg

>Contig2751|xaa-pro dipeptidase

gacggacaagtgcgcggttccggcgcggtgtacatgatgggcgaccacacgctggccgtccccatggagctgttcgcggtaaaccggcgccgcctgtgcg

accgcctgcggaagaacacgagggtgcccgaggacgcggccgtggtgctgcagggcggcgacgagatcccccactacgccagcgacaccgtccatctctt

ccaacaggaggcctacttccaatggacgttcggcgtcgagatgcccggctactacggcgtcgtccaggtgtcatcagggaagagcatcctgttcatgccg

cgcctcccggaagagtactccatctggatgggacacctcttcactccggaagaaatcaaggccaagtacgccgtggacgaggtgttctacgtagacgaga

ttgtgaaggtgctatcgagcatgcagccttccctgctactaacactgagtggtgtgaattctgacagcggtcttaccaatcaagctgcaatgtttgatgg

tattagtgagttcaatgtgaatgacactcttctatacccagaaatttctgagtgccgagtgataaaaacaccaatggagatagaagtaattcgttacgcc

aacaagatttcatctgaagctcatatacagatcatgaaaaagattcgtcctggtatgtatgaatatcagtgtgaagccatattcttggaatactgttatt

atgtgggtggctgccgtcatgtggcttacacatgcatctgtggatctggaaataatggtgctgtgttacattatggacatgcttcggcacccaatagcaa

aaggatcaatgatggagatatgtgcttgtttgatatgggtggttcctactgtggctatgcatcagacatcacttgctcattccccgctaatgggaagttc

acaagagatcaacgccacatctacaatgcagtgttgaaagccaatgacgctgtcatgcaagctgctagacccggtgtgagctggactgatatgcacaggc

tggctaatcgtattttgctggaagaattgcgtgacaaagggatattaaagggtgatgttgatgaaatgctcaaggctggtattgc

>Contig180|ww domain-containing oxidoreductase isoform 1

tgggggaagtttatgttacgagtatggctgctgttctgccagattctgatagcgaagacgaattaccgccgggatgggaagaaagaaccacggttgacgg

cagtgtttactatgtgaaccatgcaacaaaaggaacacaatggacacacccaaggacaggaaagaagaaagttgtgtcaggagatcttccatttggatgg

gaaaaatgtgtgtctgaagacggaaaaatattttatgttgatcacgtaaatcgtcgtacaacatatactgacccaagactggcatttgcaacagaagaga

aggaacatccacatgactttcgtcaacgatatgatggttcaactactgctttacagattttacatggcagagatttgacaggaaaagttgctgtaataac

gggtgctaatacaggaataggctatgaaacagcaagatcattagcattccatggatgcatcgtagtatttgcttgtcgcagtcagtcgagggctgaggca

gctatagcaaaaattcaagctgaaagaccaaatgctcgatgtaatttcattgagttagatctttgttcactggcaagtgtgaagatgtttgtcgtcaatt

ttagacacatataccagtcattggactatctcattcttaatgcagggatttttgggttaccattctcattgacgggggatggatatgaaacattgttcca

agtaaaccacttgagtcacttttatctcacactgctgcttctgtcatcgctgcattcatcatctaggattgttgttgtgtcttcagaatcccacaggtct

tcggatttgtgtctcaacaatatttcacaaagtaaattatctcccagttcagggagtcattattggtctttgatggcttataatcattcaaaactctgta

acattctgtttgccaatgaactggcccgcagatgcttagataagggagtatgtgtgaattctttgcatcctggaaacttagtgagctcagatttacctcg

aaactggtggttttatcgacttctgtttaccttagtcagaccatttacaaaatcattgcaacaagcagcaacaacaactgtgtactgtgcaacagctcca

gatctagattcagtctcaggaatgtactttaacaactgttgtcggtgtgaacccagtgaagctgctcaagattacgatatggctcaagaactatggcgaa

tttccatgaaaatggtgggagacacaatgggaaggtctgcaatgattctaccaatgcgtaagagcccagtctttgaacaggcttcttctgatgatgaaac

aaacgtgggccagaatttataaacatgtgtttcataatatgcaggctgtgatagacttaaacttgtataagcatagtcaattttttatatacttttggta

ttaattgtttgcttattatgtggtggtttttctaaatcttaaccatgacttcttgtttaagagtagattaaatcatagctcacttagtccagatttaaat

gaattgtacattttatattatcatgtctggaggagttatgtgaaatggatttaagtgtttattacagaagcactactttatataattgatgtaaaatata

ttacttttattgtgtacaaatcaaaaaaaaaaaaaaaaaaa

>Contig3166|ww domain-containing adapter protein with coiled-coil

gctanngnngctgcaagctcgacaccttctcattcacagggccctgtactgttagctgcagtcctacctaggttgtcgtcacaaccacctgcagtagtac

ctccaccacaggccccaaccccaacctgcaatgttcctggcccgcctagcaacctcgttagtctaccgcgaatactgtcgcagattgcaggtggcaaggc

tcttgaacagtcagatgtcttgcagcagaaaaccttgcagagcttgcagaatgtgctgcttctcacaaatcagcgtagtgctacagagccactgaaagtg

gacactagtggccctgccagcatgggagagggacctcccactccgacacactcggagagccaggactgtgtggatgctagaaaaatggcaagtccgccca

gtaacctgagctccttgcagaatttgggcacagtaggcagttgcagttctttacatgcactgcgtccacaagggacccatttgacaccaagtttgttgaa

ccattatcgtgatgacttggtcaatcatgtacgtggctggcctgctgatgctttggaaaagcaggctcaaaaattaagtgaagaaggttatacaatgggt

agcctgcagtgtacacgagtatcggcagaactgaaaactgcaaggtctattgttcgacttactgaaatccaagcgacgctgcaagagcaaaggatattgt

tcttgcgtcaacaaatcgagactttggaacaattgaaatctcagaattcctttatgtctgatgattcgtagtgcagtatgtggaagtgtgtgtgtgtgtg

tgtgtgtgatatcaccgcacaataaggaagtggttgctcttgcagggaaactttctccattattgttcctgctggctacaggtgcattcttgctctgtgt

tgctatgttggattggcagattttgagtgaaccagcatttatcttgtatctcatttataaaatggctgtcatgtaattttcccttttttttattgtagat

ggagactaagtggacttcttttatgta

>Contig3679|ww domain-binding protein 2-like

cacattctcagtgaggaagttgtgattgtagctttcggctgggagcaaatagaaatttttgaaatgtcgctaaatacagcacacgccaacggaggcgtgt

tgattcattccggagaatgcatacttctttttagtgatcacgtaacaatggaattccatggacaggatcagccagagtttaaaggcaccaaagtaggaag

gttatatctgacaacacatcgcatgatattcaatgctaaagattcgagagacaaaatgcagtcatttagctttccatttgtaacactgagtgatgttgag

ctggaacaacccatgtttggtgcaaattatataaaagggaaggtgagagcacagcctaatggaaattgggttggtgaggcaaagttcaaacttctcttta

agtctggtggtgctattgaatatggaacagcaatgttgaaagcagcacagatggcatctcgcaatggaccgccaccagattcaccaccaccctattcagc

acctatggggccatggtatgctgctcctccacctgcctacacaccacctcccactggctactatggttgggtacctccaacaaatgtgtttcctgatgca

ccaccagcaaactcagtattcatgactgatatgccacccccatatcctggtattactggttatgctgggtatgctacagctcctccatctgaagcaactg

gtttcactcagccgcgttctgctgctgatgccaaagcagcagaagcagctcagagtgcctattatgatccaaatcgaccacagtgtgcgtacgtgccacc

tcctgcgtattatgagcctccgccaccatatcattcaacaac

>Singlet4881|wings apart-like protein

gtttttnnnnataacaccaaaagtgccgtggaagcactagtaacattcttctaccagcaagaacaacaagcacgtgttgaagagggtaagactgatgcca

ttttggatggaaagaaagatgctgaagtccaggatgaagctgcacaggcacccaaaacacaagaagagtttattgaagaaactgtagcaaaattgttaca

gaaagctggtcgtaatatggagcatactctcattggtgcttacgtaggtctgctgttgggatatcttgttatagacaataaggactatgaacatctgatt

cgtcagtacctgcctgagggaaatttcaagacaatagtcggtgtgctgcaaaaggtttatgacttcatgaatctcacagcttctgctgtaggcagcagta

gaggaatagctcaaacccgccgtataattaacttcctggtacagtgtgatggcccaagcaccagtgatcaggacaccctatcatctcattttagcttgtc

tgacaacatttcgtgataggacccaagcatgtgggattcgttattattgcttgtgcaaactgaaatatttgaagtggtttcaaggttaattttaaacata

tgaaaatacgatagtgtaagaagagctattatctgaaatgtacggtttatgttttgtgtatagttacttttaatttattttccatttttaagattaagct

gcagcaatttcatatttatataaaaagctgtgaagtaatgtgatacaattcgtgttatgattgtgtgtgttgtgttatctgtaaattttatctgagaatt

tgatcagcaccattttattcctctagttagcttacagtaattgttgcataatatttaagctgaccaaaat

>Singlet428|wilms tumor protein homolog

gtnannnnnnataagtattgtaacgttgccaaatgtgagaagtgttaccagtcgtcgtgtgagttgcgtgagacgtttgtcgtggccagtcagcactctt

gtgtgtgcgtgcgtactgtgcgttgcaacccacgtccgatagtgcgtagttccgcggtatgcgtagcgtctgtagagctctactgtgaggagcgcggaaa

aggagatagaaacgctggaaaacgaaattttcagaaacttttaaaaaaaataaggagagagagagagtatggtgactttctaagcaggagataaaattaa

aaaaaaaatcagggttcctaccaagagaggcgctacacgggaataatgctcaaaaaagctttacacgagacgaagcgtattttttgtcgtgtgacggtag

agtagtggagcaggtgcgtggcggtgcgtttgggggcgaggtgagccccactttgtgatgtggtgtggtgtgtgatgtaatgtgatgtgaagtaatgagg

tgtga

>Contig1319|wilms tumor protein 1-interacting protein

ctgtcgccgttgttgtcgcgtcggcaacgtcggttggcgagggcggcgcggccatgctgatgcagcagtcgtacgaggcggagcagcgccgcccgcacgc

gctcgtgctgcccctgcagagctgtatatgtcacacatgtggtgaaaaagtaactggagcaggccaagcatgccaagccatgggcaacctgtaccacaca

aactgtttcatttgttgctcatgtggtcgtgctcttcgagggaaagcattctacaatgtgcacggacgtgtttattgtgaagaagattacttgtactcgg

gcttccaacagacagctgaaaaatgtgctatctgtggtcatttaataatggagatgatcctgcaggctatgggaaaatcgtatcatcctggatgcttcag

atgctgcatctgcaatgagtgtttggatggtgttccatttacagtggatgttgataacaagatatattgtgttaatgactaccacaggatgtttgctcca

aagtgtgcggcatgtgggaaaggaataacgcctgttgagggtacagaagagacagtaagggttgtgtctatggataaggatttccatgttgattgttaca

tatgcgaggattgtggtatgcagcttacagatgagccagataaacgttgttacccactggatggtcacttgatgtgcaagtcgtgtcatattcagcgctt

aagtcaccaaccaaggcaacttcagggagttccagcaagttatcagtatctgggttaaagtacccattttatggaatgttgattttgcatccaatttcct

tcagtctgatgcagaagaaaagcacagctgcccagatatttcattgtacgatagctgcatggcttaagtataagtgttacactttttgtgaaaatattct

gttaaaatatgtttagtgtaaaacttggacagaacctgagtctagagtgctctaaaaaatcaattttgtaccacttttttttttttaaattatgtaacgt

gtgtgtgtgtgtgtgttcgttcaactcctagtgtcagaaattgtattgcacaagggaggattagtgaagttcctccagtaagtcataccattgggtgacc

tacagctcactctttcagactacaataaattatgcattaaactactactacagatatacctataatcattacttatcttagtacattcagatagcccata

ctgatgcataaaaggccctatgattagtcatttatgtaacattccttatccaattatctacaatacatgtgcatagatttttttttagaccatcatataa

ataaacggatttgt

>Singlet7797|wilms tumor protein 1-interacting protein

ccgnnnnngtcggaggcgtcggcaacgtcggttggcgagggcggcgcggccatgctgatgcagcagtcgtacgaggcggagcagcgccgcccgcacgcgc

tcgtgctgcccctgcagagctgtatatgtcacacatgtggtgaaaaagtaactggagcaggccaagcatgccaagccatgggcaacctgtaccacacaaa

ctgtttcatttgttgctcatgtggtcgtgctcttcgagggaaagcattctacaatgtgcacggacgtgtttattgtgaagaagattacttgtactcgggc

ttccaacagacagctgaaaaatgtgctatctgtggtcatttaataatggagatgatcctgcaggctatgggaaaatcgtatcatcctggatgcttcagat

gctgcatctgcaatgagtgtttggatggtgttccatttacagtggatgttgataacaagatatattgtgttaatgactaccacaggatgtttgctccaaa

gtgtgcggcatgtgggaaaggaataacgcctgttgagggtacagaagagacagtaagggttgtgtccatggataaggatttccatgttgattgttacata

tgcgaggattgtggtatgcagcttacagatgagccagataaacgttgttacccactggatggtcacttgatgtgcaagtcgtgtcatattcagcgcttaa

gtcaccaaccaaggcaacttcaggttgtgcagcaattcagattgacaattttgtattcatatctccaactttcatgtgaggctttcacattatttgcttc

tattacagggagttccagcaagttatcagtatctgggttaaagtacccattttatggaatgttgattttgcatc

>Contig2558|williams-beuren syndrome chromosomal region 16 protein homolog

atttgctgtagatgccaaggatggatacaaagtattcggctgtgggataaatacagattcacagatagggtaccatgcaccacgtagggatcatcccctg

gggcttgtattagcacctgcccctgtggagttgccagtgcacagtcgagtgaagaaggttgctgctgggcgtgcacatcttattgtattatcagagagag

aaggagcttttactcttggcaataatgcttatggacagtgtggcaggaggataattgaagatgagaagtactctgggagccagacagtccattgtattcc

ttcccttgatggagctgtgattgaaaaggtcttttgtggacaagatcacagtatctttctcactaaaaatggagatctatatgcgtgtggttggggagct

gatggccaaactggattaggcacttatgacacagtttatacgcctacaaagttgaaaggtgatgttgaaggagagaatatcataaaagtttcgtgtgcag

ctgactgtgtgttggcacttaatgctaagggtgaagtttttggttggggaaacaatgaatatggacagcttctgttgccaactgacttgcaacaactgaa

tgaaccaagacacatcaagcttgctggtgtgggcaaagtcctggatattgcttctggaggatctttctgtcttgtagtaaatgatgcaggtgatgttttt

gtctggggttatggcattcttggagtgggtccctcagttgaccatgctgccaactcacgtcgagtcactctcagtcacgaatctcgagcctaaaacggga

gcatcgcagaaccaatctacacacgcattcacagccctcagtaacactgtcatatgaagaacgcatacagaagctgggtctttcataaccactcaatatg

ttatcaacattgtttcaattccaactacagtctcaatataggatcaaatctgcaatacaggacaatattttttgtgaaagctctcgttcgtcattaaaga

tatta

>Singlet6829|wd40 repeat-containing protein smu1

ctgtcgagcgnnnnnggttttgtggctgacatcaataatggccattgggatacagtgcttaaagcaattcagtcacttaaacttccagacaagaagctca

ttgatctctatgagcaggttgtgttagaactgattgaactacgtgaactgggtgcagctcgatcacttcttagacagacagatccaatgattatgatgaa

gcagcaggaaccggagagatatattcatctagaaaatttgttagcaaggtcatattttgatcccagggaggcgtatcctgatggcagcagcaaggagaag

cgtcgtgcggcaatagcacaagccctgtcaggcgaggtgtctgtcgtgccgtcgtcacgtctgctcgctttgcttggtcagtcactgaaatggcagcagc

atcaaggcttattgccaccaggaacaacaattgatctgttccgtggtaaggccgctatacgtgaccaggaagaagagaaatatcctacacaactatcaaa

gcagataaaatttggacaaaagtcacacgtagagtgtgctcgattttcacctgatggtcagtacctcgtaacaggatctgtggatggtttcattgaggtc

tggaatttcaccacaggaaaaataagaaaagatcttaaataccaagcacaggataatttcatgatgatggaagaagcagttctgtgtatgtcattcagcc

gtgattcagaaatgttagtatcaggatcccaacagggcaaaatcaaggtatggcgtgtgcagactggacaatgtcttcggcgattcgagaaagctcattc

aaagggggtt

>Contig682|wd-repeat protein

ctgtaaaagttcatgatcttcgaagcaagacctgtgttgctgaattgaaagatgattcagaaggtccatcaatgttaaaaccaatatcaagctttgattt

ctcatctgatgagaactttggaagtgcaggaacagagttgtttgatggagatgcgtttgtgctgttctgggatattcgtacatctaaattacttggtggt

tactgggagtcacatacagatgatataactcaggtccgatttcatccagaagaaaaaaacaaattagcaacaggctctgttgatggtattataaatgtat

ttgatatcagtaaacagtgtgaagatgatgcccttgcatactgtctcaacacagagtcatctattgacaaactgcactggtttagaggcatggatagcac

ctttaatatatcatgtataactcatacagaagaactgcagctgtgggaaattgatggtgcttcacctttcgcgcactttacgagagaagatattgcaggt

attttgaaggatgacactgttgacaacacatatttggtcaactca

>Contig3344|wd repeat-containing protein mio-b-like

actatattgtatcagaaagaattacagttaactgttctccacgatttgacctagtttggattcatggtggaagatctctgaaaaaacttaatgaaaattt

tgcagtttatggattatgcggtgatatttccacaacaatgaagaaaagagctatgaatgaatatgggctgaaggtactctttggcggttggagttcgcca

gcagatacctttacgtcacagctagtactgcatggagcttccaacaccttgtggactgcttaggggaatgttggagtaggcaatgtcaggagtcatcaat

gaggatgttattcgacaatggatgtatgaattcttttgtaaaatatatgcagattgttttagtgtgaatgaaatctatactcgtttcagacttatgtatc

caactagtaataaacgtgaattgttatctgtaatagtgaacgttaatttcagtattaaagattggagaagaattaataagtgatatatttagaaggaaac

ctatgccagtaatttcatggtaggcactcgcatatcttctcctgtgtgtgaaatatgaatctgccctgaagaattttgattcgaagagtaacagagtaga

cgagacatgttgagaagaattaggcatttaaaagagaagaaacaatttattgaagaatacttcatttatgattattgttacacaagattgaagaattttc

actgctttcatgtnnaatgtgtgttatgaagtaatggtcgcacacggcatcacgtatgtgagcactgtcatacag

>Singlet3465|wd repeat-containing protein cg11141-like

aaggggaagtctttagtcgacaggcnntcagtgatacttgccataatggatataattggaaacgacttgatttatcacaaatagataatgttcggttcac

tcatgtgtcttgtgggcgagatgtcatctgggcgtgtgatgcacaaggagatatttatatggcatttggaattcctcaatctgtttcaagttccacgttc

tctcctgcatggataccaacggatgaaagaactcataccaagattcattttgtcaaagtatacgttggacctgtagcagacatggtgtgggcaattgatg

tcaggaggaaagtatatattagaaaagacatactaccaaattttcctcttggcagtggttgggtgctggtctctggaattgatgctgttgcactgagtat

cagtgggactgctgtttgggcattggatccaggaggcaatgtgtacaggcggtatggcatatcaccttccaattatgttggtgattactggaaaaggata

cctggagctgtgagtgctcttacagcttctgtgaatgatgatctgtgggccattgagaagaaggggtctcttttgaggcatcgaccaaagactgtaaaac

tgtcattacagccacaagaatcaaagagt

>Singlet661|wd repeat-containing protein 91-like isoform 2

ctgtcgaaaatgctgtactgaaattgtacctcgtcaatgctgtcactaataacaagcctgagaaagttacggacttttttgcaaaaatgactcctgaact

cagtagtcagtctgaatggaaagaatggtttatgttgccattcattaaatgtccagaggaaaaccccacctttgctgttcattttacacgccagtggcag

gatactctctttgtatccttacacaatttccttgcaatgatttttcagtgtatgccccttcctacacttgcaaactatgaagaggatgctgctagaatgc

gtcgtttgcaggaagaaaatgacatgctgagacataaacttgcacagatcgataacacaaaagccaccccatcagatggtccatcactgatagaaggagt

acagcctacagaactcatggacgatttttacatcattgcacaagaaacactgaactcaggagaggggcaaagtaaaactcttaagaatcttattcgtaat

attggaagtggtttgcctgcaagtccaattctgaacagaaagagttctcaggcaggtactgtttctcgcaggatgagcagttcaccaagtgtggaggaac

cacagaagcgtactgtaagtaaacagcagcagcaagttcatcgacctggtggtatgacgcgaagtactgtgtcagctgggaagcttgctcccacagatgc

aaagcggcgaacatcttcagaatctccgtcatcgtcatcagttgatcgaagaagtgctcagaaatcggttccattggatgaagacaatgacactccacgt

tttctgctgctgataaagagggaatatgggcagcatgctggatcagtgtgccactgc

>Contig759|wd repeat-containing protein 74

ggccggggaaggtagaatgtaaatattttgatttgaacacgtgcttcagttcgtgttaattgtttggcggtgttttacagttcgtgtgtaactgtgctgt

tacagatgtagtcgagaggttgtccaccatgtcgtacaagcgagattcgaatatatttgccggttgtcttttaggaacttttaaaggactgcagtttcat

ggagatgaaagtgaatggaaagtatttgcaaaaaacttgcagaaagttgggtccctgcaaaaagataatggaatcacacatttgagctggggtgatgacg

atgaagtggacattctaatagggctgaagaacaagactgtaaagacatacgacactgattacaaggctttcacaagttctgttaatgttgaatatgggga

aggtcagatttgtggtctaagcaaatatgatagatgtctcttaactgctgttgaatcaggacatataagagtatggagatttaaggaaaaagaaggtgat

atgtttaattctggatgcccaattcagaaaatgaaacacagtaaagtaaataaaaatatagtggcatttggcagcaaaaatcatgcagatttgaaactgc

ttgatttaaatacaaagcagtatactttcatatcaaaaaatgtaagaaagaatgagcttgagctgcaagttccaatttggccatctgatcttgtttttct

accagatggtgaccgcagtgtggctgtttgtacaaaatatggacaggtacgtctttatgacacaaaagggccagcaaaacgaccagtactcagtgttgat

attccactgggaagtcttccctctaagtcctctat

>Singlet2120|wd repeat-containing protein 7 isoform 1

tgataaaatgcagaatgaaatggctgatttgcttgttgaggttatggacatagtgcttcattgcttagatccaggccatctaaagacaaaccgcctgaat

gacatattccctgcagtatgtcgcttcaatcaagttagccactgtccaaatacacggcgtattgcagttggcgcaaaaggtggacaagtagctttgtatg

aactacgttccaataaatgtcagatgattccagctcatggagcagcagttactgccaacacattctcaccagatggaaaatacttagccacctacagttg

cagtgagaacaggctttcattttggcagacttcaacaggtatttttggactgggaaattcacagacccgatgtgtgaaatcatattcgacagcaccaata

ccagatgtttcgcgcctgaacccaatgcgattagctaaactagtgtggataaataatcgcacagtgtccctgatgcttgcagatggatctgaaacaagat

tcaatgtctaaaaatttgtttactcagtatattttatgaatggaaaagaatattgggttctttatgaaaagatcttttataatggagtttgtg

>Singlet8357|wd repeat-containing protein 7 isoform 1

gttcccgtttcttttggtgtactctccaaaggaggtcacatgagtttactgctaccaacatggcagacaagtgtagatgatgcagaactaatggctgaag

tagaagtatcaccaattgctttagatttaccagaagatgctcttcgtcttgaaaggctgacacgcttgttcacatcacgtatacattgggaactgagtac

aacactcactactaatcatcttcttgccatcattgctattgctaacaccctgatgtccatgaacaatgcaaccttcgtacctgaacaggaacgtaaccga

aaattgcacagacagagtacaagaatgtcactgagctggaacagtaagatagaggaagaaaaagaagaacagttcactgcccagcaggctcagatcaaac

agggctggagtcttttggctactctgcactgtgtgcttctgccagataaagtagtggctaacggttctcgtgcattcaagaggccacaggtggaaatgat

ggctaggaggtggcagcatcagtgtcttgagatacgtgaagcagcacaggctctgctcttggcagaactgggacgacttggtccgaagggccgaaaggca

cttgcagatagctgggcaacatatttacctatctacacacaaggtcctgatggccatggagtgttacaacctggtcactcacaacaacaggcgccagcca

ctccacagccttcaggttctgccagtggcgacagtcaacagcagcaacgacagggtcatgacgacgagatgga

>Singlet7713|wd repeat-containing protein 59-like

ggaggattcagaaaacaaagtttattcccgacatctttcccagccaaactcctttcttgagtcatcaaatatatttggcagttttcaagatgcttacata

ccatttcctcggacatctgggggaaaattttgtggagttgatatgttggtgtgttttggtcgtgctccttcttctcgaagattatcgacacgttcagaaa

gctttactcctagatctttgtcagctcttggtggaagccttaccagttttaatcttggtgcctcacctggtaccacatcacagtattcaagaatttatcc

atctgtaaaccagggaaactctgtggatactcctcttccagtgtcatcatattatttcccagaaagaacaaaacagacaagagtaagaagtagaaatgtg

catggtggaagtaggggttttagtagctcatcaagaggcagcaaaaagtcaaccagagctgttattacagtttatgatgcatcagccctattttttgtcc

atagggagcttggtgaaaaatatatacttggttctcatgatgtgccagctgtatgccaacacaattcttccgttgctgcatctgtaggacggagagattt

ggtgcagacctggtccttagcaggtctagctgcaactccagcaatggtgggaccttcagaacaagaagacgatgttccctggtcacatcatccatttggg

aggcaattggtggaatccttaattgctcattatgctaaacagtcagatattcagacagcagctatgttgtgctgtatatttagtacc

>Contig3389|wd repeat-containing protein 5

agaaagcttaacagaatttttcaagagtttggagtctttaaaatgacataattgttcctgttcctgcaaggcgtcaacactactgagagggttacgctgt

agtttccttcgcaagtgcatatagtggactaggtgtgttgagcagacagcccctaatttgattattatgaattccagacattacatgatattgatacatt

taacatagctgacactttttgaaaaccgttacaatggtaccagtaagctcggggcaaaatatttcacaaactggagtcaacaatacgactactccaggaa

ctcctaataatccaacaggaggatctgctaaagcaagttctggagtaaaacctaactatgcactaaagttcactcttgctggtcatacaaaggcagtatc

tgctgtaaaattcagcccaaatggtgaatggcttgcaagttcatctgctgataaattaatcaaaatctggggagcatatgatggaaagtttgaaaagaca

atttcaggacataaattgggtatatcagatgtatcgtggtctagtgatagcaggttattagtaagtgcttctgatgataaaacattaaaaatttgggaac

tgagttcaggaaagtgtttgaaaacgctaaaaggtcatagtaattatgttttttgctgtaacttcaatcctcagtctaacctaattgtttctggctcatt

tgatgagagtgttcgcatctgggatgtaagaactggaaagtgtttaaagacattgcctgctcattcagatccagtgagtgctgttcattttaacagnnat

ggttccctaattgtgtccagcagctatgatggcctctgcagaatatgggatactgcat

>Singlet6045|wd repeat-containing protein 48-like

ctgtcgctggaggtggccagagtcagggtggcaatgagtacttttctgtgccaggacatacaccagttatcttcagtgaagtaggtggtcgaacgcttta

tcgtttgttagtacgtgatgctggaggggaaactgaaggagttttactcaatgagacagtaccgccatgggtgaatgatattgttgttgagaaaaacttg

ccaaaatttattaaaataccattctaccttctgccccatgccacatcaggagtaaaaagtctaaaaaaggaccgattaatagccaatgatttcatacaag

tgagaaaagttgctgaacatgtgtatgaaaaggtgttgggtgctggttctgaatctggctcagtagctggtcctagttctcccagtggaacagatccaga

aagacaggaaacaggatctatagctgaagaaagggttgaacttctgtgtaatgatcaggtcctagattcaaatatggatcttcgaacagtgagacatttc

atctggaaatcatccactgaccttatgctacattatagaccactgaaatagtaccagggttgccccgctgtgttgtggttaatgcgcaagcggaatagtt

agatgaacataagctgacagattatgaatgtggaactggccagtacataaggaatactgttattgttagaaagcagttattcatattacttattgactgg

ttacattctgcaatgagcaaactttccacaattacagtgctcaaaattgcaattagttacatgtgagttgctgtaagggaagttctaagtgaatttatac

acccatcagccagttgttcctg

>Singlet1311|wd repeat-containing protein 43-like

ttataaaaacatttgcaggccattcaacagntcttttaaatttattatatgttgctccagtgactgaacattcggaaaactatttcctgagctcagccaa

aggtgatcggtatgtcaatgcatggtcattgaatccttccaataggaacaaaacgccagtggcttcttttgtaatggaggacattgctgtcagtgtgaca

gttttcagcagctcagaaactcaacagattactttgggtactgttacgagaagcggtgtgcttcatttgtacaggcatcagctgaacggaaaatgtcaga

aacctcttaaaccaaaagttactgtacaaatagcctcggatactggacaaaataaggaaactgtaacaccaataccaataattggtgctcatttgtgtga

tgaaagtacaatacttattgcacatggaaatatggtattccttacatttgaaaaaatagttccaaattgttatgagaagctcctatgcctagttaggaaa

gaccctcgacaaccagctgttacaaaggaggaatcatcattgaaggtgaaaattccggaaacagaagaaaatgttcagtacatgcaggcacccgcaacaa

caccagcaaaaaggagccgtagagttcagtctgaagtgccaatggaggagagactagagaacttacaactgaaagaaccagaacaaaacagggcagctcc

tcgtgntgatagcttagctcacctgcttgtccaggcccctgcacagcaaagncaagaaaatgttgc

>Singlet8269|wd repeat-containing protein 43-like

cacgccttttaaaaacgtggtgtggtttaatatggtgcaatgtgataaagtgccagttacctgtgattttaaatgtgttttagtaagaaaaccgagaact

gcgttcttacagttgacgtatataagcaggttttaagttttttttacaacataacttgaaacgccgggtggtcttggtttgtaccatgtggtgaggttgt

gtgtacaatcaacatggcgtgttcgggtagtgcagagttttcgaacgacgggagatatttatgtgcttgcggacaagacggaaagctaaagatatgggag

acagagacaggcctcttgaaacaagaatatacacccaatctacatctcgtctcaccttgcacgtgtctgacatggataagtttacagcggccttcggtat

cttcaaagaagaagcgccgttcgaagtcttcattagaatccccagaaaaaaatgtagacgtaatcgcaatgggaacaactgcaggtaatgtgctactcta

tagcattgcagtgggagatgtggaagcacagttacagggtggccacagcggctctgttacatgcttatcttggtcttcgggctctcatttgtatagtggc

ggtgcagatgctcatgtcacagagtgggcaatcaacgatggttctgtgaaaagcaagtggaaagcaggaactgaacagttgactagtatcttagttttac

cagatggaaactcactcctggcagcaagtcgaactattagttggtggaatttggatgacaaacaagttataaaaacattnnnangccattcaacagatgt

tttaaatttattatatgttgctccagtgact

>Contig640|wd repeat-containing protein 24-like

tgtaatgtttcacgtgatgagtcnntcttgagtgtcgacatggtagatggagatcagcgtactgaaacaccaacagttgtaacagtgccacaagccagtg

gtgatgatgaaactgagactgatgaaactcctgacctgccaaggagggctgctttctcggctctcacaccatctcaaggagactttttctttggtgaggg

tgagctggcattattagattttgaacagttgactagcaataccatggttgggcagcaagattggacactaccaaatgaagcattccaggtccggcatgaa

attcaagatcgctcacctccaccggaacaatttcctaatcactctccaacagaaaatttagatggtgtccttggacctctgctaccagaagaatcatctg

gacaccctgttctgagtgtccccaatgtgcctcggccacagccattggatccatcagccattgttgtggatgcacttcgacatcatgctgagctgggtga

tgtccagacagcagtctgcgtcctagctgtcttaggtgacaaaattcgtcgtcagttgtccagcatgttgacaacagttgagcaagaaacttggcttcta

ggttacctggactctttatcacggaggcgacagtggatagcatcagcannnttgactcaactgtcttg

>Contig140|wd repeat-containing protein 13-like

agcatgtgttgttactggaagtgaagactcctgtgtgtacttctttgacattgaacgcgatgggaaggcttgtgtcaacaagttgcaaggacatgcttct

cctgtactgggtgttagcttcaactatgatgagtccctattagcaacaagtgattatcaaggtcttgttataatatgggagagagaaaaacgacccggaa

gtgagaaatgtcggggaaatcctagctgatatactatagctcactatttaacaaataatttatgaaatgcaatattttatttacaacatctatgttcaga

ttctgcagtgtcttatcatttttgttgtgtattgttactagtttatttactaatttatttggaaaaaatgttgattgttactatttattacgagaatcct

attggagtttttgatgttaatgttcttaagaactttggtgggcacactgtaattacatgcttgttacatgaggacaataaaacacattgtattctctgaa

aaaaaaaaaaaaaaaaaaaaaaagaaaaaaaaaaaaaaaaaaag

>Singlet7359|wd repeat-containing protein 13-like

gtggcccatcaggtgtttgctttggatgcgaagtataatgctcatcgagcgcctaatcatccaaactttagaactttgtacattcgcagacgaagccagc

ttctgagagagtatgccaaaggagaggatacagtgttgcgaaagcagtatatcaaaataagaagccaactcctgcaacagaggtatggcctatcatttga

tcagagcagtctccgtagcaggagtgcgaggagtagcagtcggatcagcgaatctcctgagagctcaagactacaacaggaaagtattatgagtgagcaa

ggaatagtggtacctacaaaacaggcagaggcatcacgagctattgtgggtggcacaacaatcgctgagaactatgcttttgttggagtgcatcatatat

ttgatcagcacacagctgctgtcacagtattgaaatttgcaaataatgatcgttctcatttatgttgtgcatcttttgatggaactgtttccatatgcaa

tgtgacagcaactcctccatgtgttgaagttattttgagaggtcataagaaagctgttacagattgtgattggtctgtttcaaatgatctaatggtaagc

tgttcactggatgggacactgtgcttatggcatgtggcaacgcagaaatgtttaagagttgttaaagaccagatgggttcagaaatgctttcctgcctct

ttcagccagcaaataataatatggttgttgctggtaattcacgaggtatgatggaggtactgaatgtatcaacaggcatctatccaagaggtggtagcag

caagcta

>Singlet1626|wd repeat domain phosphoinositide-interacting protein 3-like

ataannntggctgcagtagagtagtggttacagcactactggggaggcatgtagataaaattaggttaaattttctttatcttggtaccgcacacgataa

caagagacatgaatttaggaaccaccaatccatacaataatgggctcctctatgcgggtttcaatcaggaccaaggatgctttgcatgcggaatggaaaa

tggttttcgagtgtataactgtgatccactaaaagaaaaagaacgacaagatttttctgaaggtggtcttggttatgttgaaatgctgttccggtgcaat

tatttggcgcttgtcggaggtggaccccggccgctctaccctccgaacagagtgatgatatgggatgatttgaagaagtcacctgtcattgcgatggaat

tcaatgcacctgtccgaggagtacgtctcaggagagatagaattgtcgttatactggaaggtgtaatcaaagtctacacattcacagcaaatccacaaca

gctacacgtgtttgaaactaatccaaatcctaagggtctctgtgtgctttgtcccaatagcaataactctctacttgcctttcctggtcggcattcagga

caggtccagttagttgatttggctaacactgagagagctccactggacatagcagcccatgaagcacctctcagttgtattgctctcaatctc

>Contig2025|wd repeat domain phosphoinositide-interacting protein 3

gtgnnnacagcactacnnnnggaggcatgtagataaaattaggttaaattttctttatcttggtaccgcacacgataacaagagacatgaatttaggaac

caccaatccatacaataatgggctcctctatgcgggtttcaatcaggaccaaggtgagaattgtcatgcgcgtggtgacaccgcaatgtgtacagtgtac

aacgtgcgcagttttgcttacagacgttcgacatttcaggatgctttgcatgcggaatggaaaatggttttcgagtgtataactgtgatccactaaaaga

aaaagaacgacaagatttttctgaaggtggtcttggttatgttgaaatgctgttccggtgcaattatttggcgcttgtcggaggtggaccccggccgctc

taccctccgaacagagtgatgatatgggatgatttgaagaagtcacctgtcattgcgatggaattcaatgcacctgtccgaggagtacgtctcaggagag

atagaattgtcgttatactggaaggtgtaatcaaagtctacacattcacagcaaatccacaacagctacacgtgtttgaaactaatccaaatcctaaggg

tctctgtgtgctttgtcccaatagcaataactctctacttgcctttcctggtcggcattcaggacaggtccagttagttgatttggctaacactgagaga

gctccactggacatagcagcccatgaagcacctctcagttgtattgctctcaatctccagggcacacgtcttgccacagcatctgagaagggaacattaa

tccgtgtgttcgatacatctactgggttgatgataaatgaacttcgtagaggggcacacactgctaacatttactgtataaacttcaaccatgattccac

atgtctttgtgtggcaagtgatcatggtacagtacatatatttgcagttgaagatcagaaactgaataagcagtccagcttggcatctgctacattttta

ccaaagtacttcagttcaagctggagtttctgcaaattccaagttccaggtggtcctcaatgtatgtgtgcatttggttcagacaacaactcagtcatag

ttgtgtgtgcagatggcagttactacaagttcctgttcaacaacaagggtgaatgctctagagatgtatatgcacaatatttagaaatgacagatgataa

attgtaacataagtaccatgcgcacttcatcaatattgtgaaaagtc

>Contig918|wd repeat domain phosphoinositide-interacting protein 2-like

agggtagtcgatatggagtgatcacaccaaggtcgttaattttacgcaatatttatgcaaaattttgctaagaacgtggatgttatgacgcaaggcttaa

aagctggcatatcaggactaatgaacctcgcaagccaaggaagtgatcctaatagcggggttttctttgttaattttaaccaagattgcacgtctctagc

tgttggaacaaaaactggttatcgtctattttccctcaattcggttgatcacttagagcagatatatgaaaatgatgctgaagacatttgcattgttgaa

cggctcttcagtagtagcctggttgcggttgtcagcttgtcgtcccccaggaagttgaaagtgtgccatttcaagaagggaactgaaatctgcaactaca

gttactcaaataccattcttgcagtgaagttaaaccgctctcgacttgtggtatgccttgaagaatctctgtatatacataatattcgtgatatgaaggt

actacacacaatacgagatactccacctaacccttctgggctatgcacgctctctgttaacagtgactgctgttatttggcatatccaggttcgaataca

attggagaggttcaaatttttgataccatcaatttgcatgcaaagaccatgattccagctcatgacagcccactagcagcattagcatttagtacgtcag

gtaccaaagtggcaactgcatccgagaaagggactgtcattagagtttttttagtgaacgatgggacaaagctctatgaatttcggcgtggagtgaaacg

ctgcgtcagcatatcaagtcttgctttttcaatagattccaacttcctctgctgttccagcaatactgaaactgttcatgtgttcaagctggaagaacaa

cgagaagaattgcgacgtgtgccatcagaaaatcaaggctggatgggttacctgtcaaaggctgtgtccgcatctgccaattatctaccgtcgcaagtaa

caaccgtgttcagccagggccgagcatttgccaccgttcatcttcctttccagggactcagaaatgtttgtgccatcacagttatacaaaaagtcctgag

gttgcttgttgcttctgcagatggttatttgtacgtttacaatctggacccaaatgaaggtggtgactgtactctattcaagcaacacagactggatggg

aaactggagcagccagcagaacaggcagctggtgcaacttgtataagccctgagaatgctcactctgcttccaaacctgttactaatgcgtctgcaccac

cagaatctgaaaaattccatgagatggcagctgcaacagaatcacccccaaaaggtggttttcgatttgataatgaaagtgaatttccaccagtcactca

aaaaacagaatagtgattgttctgtttatgatgcggcacatcgaccaagacctcttacagagtgtagaggcattttcattgtactgtttactatgtgtgt

gacacagttattgcaaggtgcataagttcttttcctaattcgtgatgtatgaattatgtaaataactggtgcatacgtacaagaataaacacaatgaatg

ttactgatctgagaagaagctgcaaaagcttgttgtgcacatgcatttgagatgtgtgtatcctcatagctacattatgcagagtggtagaattgatatg

ttattgactgaattcagtttacagaattataatcacattgttggcaatggtggtatcttgaatagcagttgttttacatattttagaattttgtatttga

taattccatcatattcaaatgagacagaattttgattaccactgacatttatagccaacacctgcttgtgtgtaaaatattttaatgtttgacactgaac

atttacattaatgatgatggagaacctgttctctttttgtgataaagaattttgcttgtgcttccatctttgatgttatgcctctctaatttcttttagc

agtggtatgattgagcttgaagaaaaaagttgcacttctttctatgaaaattgagtaaaaaagtaagattgtgtaatttcagctca

>Contig1659|wd repeat domain phosphoinositide-interacting protein 2-like

ctgtcggcaaccnnnnngatacacacaaactatttttaaaccaaaagtgcatacatttcaatgatttgtgtgttttgatattgcaaaaatgtacattgtg

gcgctgcacttgtacactccaccacagtattttcccacatctgataccatgttatgtgctgatgtgctttgtatctctcactgttaataaattaaagaac

tttcttattggaaggtcttacctgtaccttttttatgttgtccagtttctcactattacatgttttccatttgtttccttatggattttgacaagaaata

caatttcagctgtgagcttctgcatgtatctgaaaattttatattgttgtacatcattccaaatttttccatttatttaactaaaagacagtgtttgtac

tgataacttacaaatgataccattgttttaaaataattcaattattttgttgacagtatgaacaagtaattacttgctgtgattgtagtgttgccattat

taaagctcagtgtcatttttatgtgtgtgtgtatgtgtgtgcacttggtttacatttcacaattttatgaagtctgcacaattctgtacccatactagct

aaagcaaaattatactaacttgtttaattgtgacagtgattggtgggcatatcctctttgatctctgatgcatttaaaaaaaaaattctgttgtatcaac

agtctgttatatacatttcattcctggtaatattatactgatcttgcttaaagtatttttttttcttaaaatgtatgtca

>Contig4098|wd repeat and fyve domain-containing protein 3-like

ctatnnngtatgaggcaacagttccctctactgtttttctgtttttgcttgatctacacctgtacctccctcattttactcacttttctcatatcatatc

ttagatcaaatattttactcattggcaaaggcattcacttaatcattacactcctttgcaatttctgtctctcccaggtcgcaaatagcaggcatggatg

atgtagtggtgtgacattctctctctctctccctctctctctctgtctctctcgcacacaccatgactctcccattccctttttagtcagctcagttttc

gtaaaagagagaaagaaagtcaaaggttggatcgttttctggactatggaattgatctgctatatggaatagtactggttatgatgattgttgagaacat

agacattagtttagtaggaattgcagaattttgacaagaattaataatttataacattttgaaacagaagaataaactgctaagaatgcctaaagtatgt

tttacacagtgtacctaactgtacccaagtctgaagtgaattaaaaacaaaagactggaaattgttgaatggcaattaagtttaacagagctttggagca

gtttcgaaacagtgcagatttaattaaccaagtgtgcagcaaatttttccatttcaagatttattactgtgagaaggtcattgcaatgtacaaggcttta

tttctgtggttacaagagtaagataaacaaatttagaagataaaataagaaggcaactgaaatggataacacagaaactgctctagtgttttgttttgtc

tttgtagcactactattctgtcacctctgtgtgttgtgaccactgtaatgataatttaaatatgtaacaaagtaattgtaaattagccctctgacactga

gactaaaagaatgaagtagaagtgatatttggtacaacattaatagaacagaatttttgcattccatttttgtattaaacagacctgtgtacacaggaat

tgcataaatgaaggcatctcctccttgacatagactgtttatgtttagtacctaattaaaacaactagggggccatgtctttattagtaatttatttttt

tatggttagaaaatgtatgtttgtatagtaatgtgcattgtttttctttcattgctgtgtacataatgttgatataacaatgtgtgtgataagcattgca

tagaattcttctagtcgagtgcaaataataggcaaacaataaacaaaaaagtattacttgttaaaatttcacatgggtgttaacag

>Contig4706|wd repeat and fyve domain-containing protein 3-like

tgccttgtacacttaatactaaattattatttagtatttttgttgtaaagcccaaaaaataatatatatgagttctttctatattacaggcatttattat

acttaataaacctgtgaagacagtcaccacaccaacaaataaatgactgtgcaatataagagatgtaacgtaactgcagaactgtacgtatattcacaat

acaagtgggcaagcttggcacatacctacagtattttactaaactaggaagaagtcaaagcacttgggtagtacagaaataaaaatgttttttcattgta

atgctgcatttgtatattgccagacttgtatcaaaacatgactctttttgtgtgttctacataatacaagttgtacaatttttatacgcaagtgaaaaat

tcataaccctaatctccgagaaatgaaaaggctgaaaaatctgctcagctgctgttatctgttcctctaagctattattttctttattttggccatattt

taattggggtttgtgaaaaggacattctgtttgaccttgccgagtcattctacttcttacttctgaattgtgaaagtgtaagtaccattgagattgcctt

gtttcctcctggctgctatttgacttgaataaaaagttatgatcatacttccgaagaataaattgctagtttaagatcaacatcaattgtccaaagtatc

atatttgtcatttataaaatgacactcttcttctactaactacctgtgtcttctttctttttctcattgcaaaccattattttttattttaaaaatctaa

acatttacagcaccatggttttctcaagttccaatactaatattagattaccaaatacataggccttatggtatcaatagaactcctctcagaagtgttg

gcatatatatcaactatttaaatggcatcttgtccaagtagtggtgatgatgattgtggattattttgtgcattgtgatactttctgctatattgtggag

ctttaacggccaaaaaattgaatcattaaataaactggtcattctgactgctgcccacatttgtaagttcgcccttccttgcaatttgtctgtacacttt

tggtgtccacgtaactgcaaatttttgtat

>Contig2180|wd repeat and fyve domain-containing protein 2-like

gnnnttttacctgtatcttcgaatagtgccagatttagtgtaactaaaaagcctaccttacttcacaaactcgaaggttgtaacgacgatgtaaatgctg

ccataatcattccaggcgaggatggcgtcattagtgtatgtgacgaccggactgtgcgaatttggctgaagagagatagtggccagtactggcccagtgt

ttgccattacatgccagctggggccacagcactattttactgtgtggaaacaaggcagctttttgtagggattgaaagtggctcagtatccgagtttact

gtggcatcagacttcaaccgtctgactgctgtacggacttacccagctcatcaggctagggtaacatccatactatttgcattgaattgtgaatgggtgc

taagtgttggtagagataagctgttccagtaccattgctcagaaacaggccgccttcttggcagttaccagagtgaagcatggtgtacggcattacaatt

tgatgcaccttcaaagcatgcttttgttggggattactcaggacagataacaatgctgaaattggaaaatactggtgttcaagttataactgctctgaaa

ggtcatacaggaagtatacgtacactagcttgggactctgaaaaacagctgcttttttctggaagctttgatcaaagtgtgattgtctgggatattggtg

gtcagcaaggaacagcatatgaactacaaggtcaccataacaaggtgacagcactgtgctatgctagtct

>Singlet5828|wd repeat and fyve domain-containing

aagggatgagagtgaagagttagatctgactgactcgctgactttgagggatttcacaaagccgatgggagctcaaagtccagagagattggaacagttc

aaaaaaaggtataaggaatgggacgatcctcatggtgagactccaccttatcattatggcactcattattcatcagccatgattgtgtgctcttatcttg

tgagaatggaaccttttactcagcatttccttcgtttgcagggaggacattttgatttggctgatcgcatgttccactcagtacgagaagcatggttatc

agcgtcgaaacataatatggcggatgttaaagaactgatacctgaatttttctatctcccagagttcctttgtaattcaaataacttcgatttaggttgt

aagcagaatggtgttcagttgggtgatgtagtgttgccaccttgggccaaggaagatccgagagagttcatacgggtgcaccggctggcacttgagtgtg

actacgtgtcgcagcatctgcacgagtggattgacctgatatttggatgcaagcagcaaggacaggctgctgttgaggctgttaatgtgttccaccatct

cttctatgaaggaaatgttgacatttacaatattgatgatcctctgaagaagaatgctactattggtttcatcaacaactttggtcagattccaaaacag

ctcttcaagaagccgcacccagctaaaaaaataagtcatcgtacatcagtgatagatccaggcccaataacacc

>Singlet6883|wd and tetratricopeptide repeats protein 1-like

gtttgttgtagcaggttctgatgatggatnnntcttcatatgggatagacgaacaacaaatatcattagaatcctgcgtggggatgactcaattgtgaat

tgtttgcagcctcatccaacctactgtcttttggctaccagtggaattgatccggttgtcaggttgtgggctcctaagcctgaggggtatacagggtgga

acactttgtatgaagagctacatcccctgtcggcagcaatggctctaacctggatgggcctccgttaaaactgagcttggatgacaggatggttccaaaa

atgatagagaggtgttaaacctatatgatgctgcatctgcaaaccaaaaacgaatgaatgcagatccatttgaagtaatgcttataaatatgggttatcg

tatccaggcgatgcggggtggaaattcagagtctagtaatgagggagatcatcccacaattaattgccggccaagttaacagtagtagattgcatgtggc

agtgttatgattgaatgttccctcaaaccaaagtattagttgttgggaactgttggaaaactgaacttatactaaattgatagaattgtattgtacatta

tgcacatatttttaatatttgtgagatatttaattgcacggatagtgtaatgatataaagtgtgtatttgtatagacttttg

>Singlet850|von willebrand factor a domain-containing protein 8

agaatnnntcaattgcacagggatacaacagtacaaacattgaccctgcagccaacagtgagagacggtgtcgtggtatatgaagactctccattagtac

aagctgtaaagagtggtcatgtattggttgtcgatgaagcagacaaggctcccacgcatgtcacttgcattctgaagacactagtagaatctggtgaaat

gattttatcagatggccgccgtattgtgccacatggtgatccaagagtacagtctgctggtggctcggtcatcgctctgcacccagacttcaggatgatt

gtactggctaatcgcccagggtttcccttccttggaaatgatttcttcggagccttaggtgacttattcagttgtcatgcagtggacaatcccagccccc

agtcagaaagggcgcttttgaagcagtatggtccagatgtcccagatgctgttattaatcgtctggtaaaggcatttggggaactgcgctccatggcaga

ccaaggcctagtgcagtatccgtactctacgagagaagttgtcaacattgtgaagcatctgcaggttttcccacacgagggcttggtaagtgtagttcgc

aacgtcttcgactttgacagctacagtccagaggcacaggagactgttatacagatactacagaagcacggaattcccatgggtgccagcccatcagaca

tccgtctagcaagagagtaagcaattcactcagaaggtggctgcatcaaacaaactgagagaaagtgtctgtacttcacaagnnagtttgaaaacccgtt

gcccattctcatatttcctaccacctactaagtgttaataaaatattagc

>Contig2981|voltage-gated calcium channel beta subunit

gttgtagacaggtatgtatccagaagttaattttcagcattagataagatgtttttgttaaaggcacctaaaacttgatccatttctttccagctaacat

agactaaattacatttgcagtgcatcataatacattccttttgcaatgcaatttgatagtgcaccagtgcagttgttttcagatattttttatgttgcct

ggatacataccttctactactaatgacagaatatatactcaaaatttaaccttttctcttgacatcgaattcaaaatgctgatgtttataacaaagttat

tattaaaaaatcgttcagcatgtctcagtaaatgcagaatttcttccatttttggtagtggaaactttcagatttttaggtttccatggaacatgaggat

catacagcactctgtaagatactggtgcctaggtaatggctgagatacaggtattaatttgtttacttctcatcactgttttttttttctgtggtacaga

gaagtgaacatctttaccttcttattttaaggaatgtgatgttctgtcactattcagtatctgggaagaattagttttcctctcaagacttaaacctaaa

tttcacctaccatattgaaagtgtagctcattcattgtactaggaattttctaatttcattaagatctattttcactacacatctgctggaaaagactgt

atacttttattaaaatttctaataattaaaatgtaagtgcttttttgtctaattcatgcttcagtgtataagtacatgctgctaatagtaaga

>Contig2496|voltage-dependent anion-selective channel isoform 1

ctttccctaagttcacggtggtgctctgcctgtggatagaagttttctgattgtgcaactgaattatttataatagtcataatggcaccaccatcctact

ccgatttgggcaaagacgctcgcaatgtgtttggaaagggttatcatttcggcttaattaagcttgattgtaaaacaaagtcagcctctggggttgaatt

ttcaatgggaggtttttccaatcaagatacaggaaaactgttcggctcattggaaactaagtacggaatcaaagagtatggactggtattttctgagaaa

tggaacacagataatacattgggcatggaagtgagtgttgacaattaccttgccaaaggactgaaaatttcattagactcaacatttgcaccacaaactg

ggagcaagtcaggctgcctaaaaactgaatttaagaacgatatgtgtgcagttaacatggatgttgatttgattttggcaggtcctaatgttaaagtagc

aactgttcttggttataatggatggctagtaggttaccagactgcctttgacagtcagaaatccaaactaactaaaaacaatatttcccttggatactcg

gctagtgatttcattctccataccaatgtgaatgatgggcaggaatttggtggatcgatttatcagaaagtgaactcccagctagaagcgggagcccaac

tagcatggtttgcaggcacaaatgaaaccaattttggaattggctgcaaatattctattgacagagaatcctctattagagcaaaagttaataatgccag

ccagatagggataggttactcacagaaaatccgtgaaggtattacggttagtttgtcagcgctaattgatggcaagaatttcaaccagggtggacacaag

ataggtgtagctctcgagatggaggcctagaagtgtcacaaagtacagtgataacatggtgaagcttgaagaaaagtgttattggtagaagtatgccggt

gatggaatggactgcacatggaattgtgcccagactttgttcacattttaccagcattatttatttttacagctagcagcacaaaattgtgcttcgtgat

cctttgaataattatacagcatggtgaaaccaaacttgaggatgttaatattttagatgtcactattggagacacctatgtatttactgtattgttggct

gaattaaaacacaattgtatannnactactgtacagtaaaagtgctgaaattttcttcaagcctcacatttgtaaattacaaaatgaaaccattttt

>Singlet6729|vitellogenin receptor

ctgtcgggcacccgtgtgatggggagtgtcgggcgaccccggcaggcccactctgttactgcacggctggctacgagctcgggcaggacggaaagagctg

ccacgacgtggacgagtgccgggttcggcccccagtctgctcgcaggtttgcaacaacagccccggtagcttctcctgtagctgtgttgctggcttcatc

ctccgtctggataaggtgtcctgcaaggcaacagggcccaccatggagtacgtgttggtcacgggagggggccagattaggaaggtgtcgcagtctctgg

ccagtgtggacgtgttgcacaagcaccagttcctgagggtcacaggtctggatgtcgatgccagggagtacgccgtctgctggagcactcgggatacggc

aacaatttactgccagtcactgaagacccacaacaggacatatgtggaaggggtaggtaacccaacagaggtggcaatagactggatcacaaagaacatc

tactatgtgaatgatgtgcctgttggccagagtgacatacaggtgtgccacctgacggagaagctctgtgctgtcgtggtgactgctgaacatggcagta

ggattggcaacattgcagtagatccacttgctggatatctgttttggactgaaatgagcactttgatagaaaatggagtggccggcagcattaggagggc

agaccttgccggaggcagcaggacagttctggtgcagtcgggcctcggtttgctgagtggcctggctctggacacggtgcggcgcaccatctactggaca

gacgcctccttccaggttgtagagtcagcgacatatgac

>Contig3108|vitellogenin

caaccaacgaaggatccagttatcaacatcgtatctgacttgagggaacaacttgagggtcaaatagcaggaaacgttatggcccatccgtctataattg

cctttgacaacaacacaattaactcatgggtggaagctatcaggaacaatgtcaacaagctacagaatgggctgccttttaactgcactagtattgtaag

cgcagttgatgtcaggatctttttccccaacgctctgggattcccaagttccatcgtatacgagactccagttctctactcagttggtggagaactgcgc

ctgaagacttcaccgaaactcaacagtgctcctaagggccacctggcctggccgaccgtatggaatatcactgcagatgtgagggctgtatactcaagaa

gaagctcgggatccattttattcactgtcctccctctcgacaagagttattctgccggatatgttaagaaccagcaatatcaagtaccagtaagattcct

cgtcaacgttgatctcgaagacaatagcacatatgtgaaactacagcccatcaacaagaaccacaggtaccagctgtcacacatgagcagcgttccttac

acaacgatctaccgcatgtcccccgtggaaagtgctgctcttacggcgccagaaactgaaatagttcacactcgcaagccgcgctcgtggaaggctcagt

acggaagatcaactggtatggtgtattccgtagagtacaactcggaaaaggattacgatgacatgtacgagagataccaaaaatatgcaaaccgcgatgc

cctttctgctatcttgttcttcaatgcggagcagcaggtctactacagcaacatcagcctttattatgagccagaaaagtccatctccaaagccgtggaa

ctcagtgtccactattacgacgaactctcttcgtcctcatcacgccgtcgacctgagcaatctgaagagtcacaggagtctgaagaccagcccgagtcca

gggtagcgtcggaggagatgtctgacgaaccagagagcgctgagtcaagccaagagaagtactacagatccaaacgaggagcaatgttctggggaagagc

caagcacaggagagaagcctccggtcaaaacagagccgcactgagtaacgtgaatcccaacgaatcacataaggaactggtacagagattgaaggaacaa

ggacttgcgaagagtgtcaccacgaaggcgctgttggttggagtgaattttgtagaaggcaaaggcgcttcttcgctgctggcaatcgtctggagctcca

gtccagtgtccagcaaatcgcaactgctcgctttcgcgtcagttaaaggagcacaggctagcacaccttaccaggtttgtgtggaggcacaatccgtgtt

ccccaacgttccattcatgaacttggagaaagctctgaagaccaatccaaactcgtctgtatctgtcagactagattctggagaaggctcgtgcttgtca

ggaatgtcaattcaggccaatcttgatattggaagaagccaaagcatgtaccaatacctgcagaagtctagactcgtggcgcaatgcagatctcaaatga

aggaggataactacgtcctgcctgcatgtcgtaacgccacaattaaggctagtgtgctcaacaattacgacctagaggtcgagtatcagaatattccaga

agcactgaagcattcgatttacagagcttactccaacatggattacgctcatttcctttatggatcacaaaacgtcgtaaatggtaccggtgctcctgga

aaactgtatgccaaccttatagtcgcacccaacatgcgctcgctcaacttctccttctcatctgcatacttggacgccgcctacgacaacgttcgcctcg

tgatgcccgtagcaaaagccatcgtcagccatccactcttgcccagagcagaacgcgtcgctaactactacaccaactggcagtataacgcaacctgcag

cgtggattcttcatacattaagacttacgacaatctaacatacccgttcgagtcgaagagcccctgctggagaatcctgctcgccacagctcgtcagcgc

gacgtgaaaccatccccacttgtcccgtcacctaacgtcacagtgatggttcgggaaatctcagacaagagggaagtcagagtgctggtcgatgacaacg

ttgtgtcactagcataccagtcgggcaagtacatgctgagagccaacagcaaggccgctaccgtctctcagtcagaggtgacgcaactgaacgacaagga

aggagagccgctggtgctggcctacgccctcccagactctgtcaggatggaaatacaggatctcatcatctactttgaccagcagcgtgtactgttacaa

ccgagcgacatttacaggcaagcagtgcgcggtctgtgcggtaccttcgacggcaaacgacagacggacttcaaacttccggccaactgcattgtgcgaa

acgtcacggcgttcatcgaagcctacacgtatggtgacaaatgcgcagctcctcgcagagcacagccgaagcaatgctatgaggagaagatcattcctgg

aaatcaaactactcccgagccactaactgacgcaacatgcgtctcactccagaacagggttgtgctgcggggtcagaaagtctgcataagccgaaagcgc

ctgccgatgtgcaaatgggaatgcaaggcagtagacaccgtcgacaaggtggttccattccgctgctactccagaactgagactgttgaaactgtcgccg

ctcaagctcaacggggacgccatgttgacctctacagcataccaattgaaggtgacgataccaatatcgaggtttcggtagcaactgcctgcacacgtct

ggaggagtcagaagaaagcgacagtgacgaaaattaacagcttcatgttaaacgctaacgaaaaagcactattcgaccaaaacaatatgtactaaaactg

tgcaaataaaatctctttagacgataaaaaaaaaaaaaaaaaatgc

>Contig4158|vitamin k-dependent gamma-carboxylase

agtctgaanncaaaaaatgtgtaagataggtatcacaataaatgtgaagtatggcatataagatgggctttcaaaccaaatttcaaatgtgtttaattta

atatcacatcgtgacacaaagcgtaattttgtagatttactattgcctttgtattaaatcttaggcacgcagatgaaaagaggcacatcttcaatggtat

gtgctgctattttgcagagtccacatttgtatcatgttggaagactttaggttatgaagttttttttttttccttttttttctctctccacctcagccct

ctgggctctcacaagcagcattttactgtctctcacccctactctactattcctccccctccctgccccagcctcctccttgcccccactacccagattg

cttcccccgtaacatgctgccactcacaatctggtctcggcagccagagactgtggtcgtgcatatgtgagtgagtgtgtgtgggtgctgtgtgttgtct

aattctgatgagagtctttttggccaaaagcttatttgtttgactgtctttttgttgtgcagctgtacaaatcggcatctctgctgtatgttgagtagta

gttgatccttttcaaaataccatcctggtttttcctttttgtgtgtctggcagccctgttctcattgctgtttcctcagaaattaacacataagcatttc

attgtttttattttatatatttctaagatatgctatcaatttgcatgttctgaatttaaaatgtaagcatggttaatgaaatttccacgttgttgcactc

aaaagatattttgacttacattattcaaaaactgtgcattaaccctctgt

>Singlet2559|vitamin k-dependent gamma-carboxylase

atttnnnntttgtaaaacctaaaaattgggtagaaattccaagtacctgtggcaagagcaagccattatgcttattttccccttggcagaacatcaggtg

ttgaagtgcggatgtgtggaggccaaatagttagttaatatggataggagtagtccatttagtgatgcagttacaactgtgaagaaaacatcaggtcacc

aaatttgtgatgtgtttttgggaaaactgtttgatgcaggggaaaaacaaccaatacaatgatgtgtatacatattaaagtaccacgtatacaatatctg

cacctatacccattacacactgtatataaatttcacactacaaattatcacatgcatgtttatgtgtgtaaaaatacatgtaaagggtgactgaaaactg

catgttggtgatgatttctaaaagcaaagaatatttgtaactggacagagaactttgacagtggccaagccaccaaatatgcttgtcataatagtactat

gaccaaagtctagatgttttctacagaagtaagctaccctagtcatttatacactgtaacttggaatccaaactgtgaggaactcctggcaaactacact

tgcaaaataagtgtggctactgatgaaaggcacacagaaacttcttagcatcagaatttcaacaagaaacctaatctctctccatgtgttacatgaaaaa

tactgtgttgttacagtncgggactatntggtgctaacatcactttcaa

>Singlet4573|vitamin k-dependent gamma-carboxylase

tcttnnntnctgcttgctacatgtacacatatacgaattacacaaaacaattgcttgaagaagctgaccaatcagaaaatattgagatgccaaacccaaa

taatgatttcttttcagacttaaaatcccagataaaaacttttttgacagccttaggtatgatttttaatgctattctgagtttactgttctctgtccca

atgcctagacgtgttaaagtagcataaaatttgttgtggacaagatacatctttcacttttttctaatatgtgactacattattttggaattgacttatt

tacagaaaacgagaaaagtgccagaaaattattattcaggtactaattagaaataatgaaaactccagatataagtaagaaaaagaaaaatcctttacca

atagaaattatcggagattccatgttagtcaataactgtagtgtacgtctcatactctgcagaattctctagttttcgcttcagaaaaaaagtatgccct

gtgttagctatttttcattccccaatttttggttctatattaatataatttcacacctgtttatcttttatggtgttttcagttcgtttctgatcatctt

agtttattttcaatctaaactcataaataaggatgtggtaaacattgtctagttagtatagtaggcacatgtgagaaaataagtgggagatagtgacata

taaattacatatttgatttatcatttcagtgtcatctgatgaaaatggtaaaatctgtaatggaagggtgtaacaaaaattaagaaatgtaactcttatc

catcatagtgtaaaaatagcaagaaaatatctgtgttaattcagtattgt

>Contig472|vitamin k epoxide reductase complex subunit 1-like protein 1-like

agggggaattcgtgagtcgcatgtcgctcgttgtttgtgcagcaggaggaaatctgttattaagagctctgtcggacgctgtgtttgtgtttggtgtgag

attctaataattttcgttatgggtttgaacggtgctataattgcttcctgtcttttggggatgggattgtcgtattatgcgtatgttgttgaaacatcga

aagaagctgatgccaactaccaacccgtgtgtgacattagtgaacacatgagctgctcaaaagcgtttatgtcagaattcggaaaagggtttggcattgt

gcatcgtacgctgggaaaagattccgccctacatgtttccaatggccttattgggatgtttttctacagtgtttttgccgctctgagtttcacgagcagt

gtagtagcaagcaggattcagttgtttatggctatcctatctaacttcggctctgtctaccttgcatatgttctgtacttcgtactctatgatttctgtg

tgatatgtgttacaacatatattgttaatcttgttaatttagttttagtaatactgaagttacgaaaagttcctagttctactcagaagaaaactacgtc

caaaaaaaaaaagaattaaaatgagttatttgtacagttacaagctcattgtgctactgaaataccattccttgaacaggaatgaagagtgatctcattc

agggtatattttaaggccagtaacatggttaagtcaacaagtaaaatggggaaaggtgctacatgtactttactgcccaaactattaaatgacaaataaa

gta

>Contig3205|visgun cg16707-pc

tgaaaagatttttttgtttagtcatcattatcttatcatgatttgttttgtctcagaagttcctttaggccagttggagtacttaaatttgattctcatc

tggcagtgcagtggtcagttgtgacagcattgtataataaataagttgttctggaaagatctttgcagttagtgtggttagacttttctcgatgattacc

attctgaaagggccagtcatgaatgattatatatatatatatatattcattattctggtcgaaattctttgttcctttgttacatgtaatctattatggg

gtacttcatttgaagacatttaggcactgcaaacagattcagaacttcagtgtttggctggtttcatggcttttgtcttctcatagttttacaacattct

tatacatattctactgaagggcaaaaaagaaactgatatttttgctgcagaatttgttgtctaaatcatatgcaaagatattggataacatttcatcata

atgaattgtaaagccctctgacacatggataaatggtgcattttcagataagcttacttcagttcatagtacattggcaaggacttccttgtactactta

ctcattgcttgtgcttggggttaattttccttggttattaagtgacctttgcatttactttctgtcatatgcaattacacacaaatgcagagttcaacat

gtagagactacatgcattgttaaaatgaaattacttgggtgggaaggccttcataacagtgccttaagtttcaatgcataatttcaattgaacagtaaaa

aatagtgtgaaggaaaaagcaccatatatcaaagcttcaatgaacagatactcagtatcataggtaaagtgaagatgtttcatttgtactagcagacagt

tttattttgtggcacttaataaaatgggtcaaaggcagtctactgttttaggttgcattagtaaaatgtgaaaagttaaaatttcacaaatattgtttgc

tggttttcagtctgtccagatgtatttcatttgtaatgcaaagtcaattaggaaaactgtcatctcggggatatgaaattagcaatagaagcataaaatg

ttaaagtaacttggtgattattcaattggtatcaatggaattagcttggaaaaataaagtgctcttaatgtaattcacttacatattaagtgataatgtg

aatacttctggaacagaaatggtcatttaaagaagtgttgaaagctattgttgccttcctgtgtatttttaaattaataggggcttcaaacagaattgtc

atttcagtggtttacgatcttgcacacacatttggttagtgtgttaagtgtattcctaaagtttcttaaatatgcaaattgattgtgtgtgtgtgtgtgt

gtgtgtgtgtgtgtgtgtgtgtgtgtgtgt

>Contig1741|viral iap-associated factor homolog

ctgtcgccgnnnctttcgggtggcctacttgaatctactggtggaagtgaaccnnagcgttagaagattaaattctgactgtcaggggatgtactgatag

cttttttacttcaggaacggactgttgttaaatcttacaactgcaataaaatgcaggttcgtaggtacttggttgttctctttttcagataagggacacg

aggatccagtgcccttatatggtgcgtttgcccgtaatatgaaaatcgttttcagttagcgacgaatgtgcctctcattattgtaaacctatagctcctg

accgtagaactgtgtgtgattattgttctaataatgtgtcgagaacgccgtcgattaacatcagacgtggagagaggtgtggttatgcgaaacctcacca

ttgagtaatgaataaataatccctttatcgtaaatgacattaatttcaagaaactgtaaggaatagtttgcactacgaaaggatccaaatgaagatacgg

agtggaatgcagctttgcgaagacatggtattcttcctgcaaaggaaaaggaaattactgaagaggaagtactaaatattgttgaagcaactatagaaga

gaagcagtccaaaggaacaaaannattagaacagtgtactcttgatgaactggatgaattagaagatgaagaagatgagagagtattattggagtatcgc

agaaagcgaattgctgagatgcaa

>Contig1823|vesicular integral-membrane protein vip36-like

aagatctttgtcattgtcactgctaggtccagtacagtgcgcatttgatatactgaacttgcttatttttttttactttctttttatataattttgattg

tgtgttgcgacacttattattgtgggcttttgacatattttgcggtagcagtaaaaaaagaaaaaagtgtttcaccccgtgtagtcttctgcatcttgtg

ttttagaactgcgttgtgtagaagtattctgctgtatttttataaagactaattcttttcacatgtcattttactggttttaatgtgtatctgccgctga

agctgaataaaagtttagtgaataccatgagtatagaaagtgcttcgaaatacgtgttaacttttagttttttactgctattcagctttggtacaacggc

agaatggaacacacaggactatatgaagagagaacattcgcttataaaaccttatcaaggttctggaatgactataccttactgggatttttcgggcaat

actatggtgacgaataactatgtcaggctaacaccagatcagcagagcaagcagggattattatggaacactgtgccttgtcatgttcgtaactgggagc

tgcaaattcagttcaaagtgcatggaaaggggaaggagctgtatggagatggtttagcaatctggtatgccaaacatcgtatgaccccaggaccagtgtt

tggcaacatggattaccacgaaggcttagctgtaatactggacacttacagcaatcacaatggccctcacaatcatcagcacccgtatatttctgcaatg

gtaaacaatggctccctgcattatgaccatgatagggatggtaca

>Singlet3196|vesicular glutamate transporter 2-like

ttattgcttcttaatttcagctcaaattttatttgacagactcatttatggttttttctgacattataattatgtttggtgatctcatttaatgtttttg

acgcacccccatgggctaacatggccagctatgtatgccattgtaggccactggatacctgctgttgagaggagtcgcttcatgtcttctttccaagngt

tcagctttggcattggattaacatatcccctatgcagattcatcattgcccactttggatggagagcnctgttttacacaactggaactattnnngctct

gtggtgtatactctggtggctgcttgc

>Singlet7117|vesicular glutamate transporter -like

ccggcagtctgtgggggcgcgccgcgctcctgctcgcagccaccaagtacctgcgacacgtgtacggcttcgccctcacctatgaccgcgtgctgtcggt

gctgccacacgtgggacacgtggcgacagcggtgctgctggccggcctggtggacaaggcgcgcgcgcagtcgctcgtctccaccaccacggcgcggcgt

ctcatcgtctacattgcgcacctgccggccagcgcgttgctgttcgtgctgggctacagtggctgcgaccccgccgtgccagcagccctgtacacggccg

ccgtcgtcgtcacgggagccacccccgctggcgccaccgccagttgcatggacctggcgcccaacttcgcaggcgtggtgctggcggtcagccagacggt

gggcgccgcgtcctcactggctgccgcatacgtcgtcagcgaggggcttcacgggtcgctacctggctcttggcggatggtgtttggcgtgtcagcgctg

gtgctcacattcacgggcatcatcttcatggcgctgggcagcggcagcgtgcagccatggaacttcgtagcgagcgacatgcagcaccggcagcaggcgc

aggcgtcgccctctgtgccggccacgccgtcccctcgtcagcagcagccgcgggaacagcaacagcagcagcagcaacaacaacaacagcaacagcagca

gcagcagcagcaacaacagcaacagccgcagcagcagcagcagcagcaacacaccgagtcagcgccgcagccgct

>Contig143|vesicle-trafficking protein sec22b-b-like

gaggagtgagcgtggatatgtttgtgtgcgagtgaatgtgtggttccgagtgacgtgtatatgttgtgcctattcaggtgaaggtagatttttaatgatg

cattgtgtttaatagcagcagtactccatacattctgtttgtaacgtcaccacacctctactgtagacaaaatggttttgatgacgatgatagcaaggct

gtcagatggtctgccgctgtcagcatcaatgcaagaagatgaacagtttgggagaagcattcttgagtaccagaaccaagcgaaattattattccgcaag

ttaaacagccatagtttggagcgttgcacgatcgaaacaggaccatatgttttccactatcttatcgaaaatgatgtttgttacttggtattaactgagc

gtaatttctcaaaaagactggctttctcgtacttagaagacctagcgcaagaatttcatgctcagtatgggaagagagttaatgttgtgcaaaggcctta

cactttcattgaatttgatacatatatgcaaaaggctaagaaaatgtttactgatgcgagaacacgccgtaaccttgctgcacttaacacagagttacag

gatgttcaaagaattatggtgcaaaatatagatgatgttctgcacaggggtgcagtattgtctgatttggatacaaaagcaatgaacttgcagctacatt

cccaaaaatataagaaagacgccactcatttaaatgcaaaatcnntgtatgtgaaagcttttg

>Contig1929|vesicle-fusing atpase 1-like isoform 2

gatgccaataatctatacatagtaaacgatgtaaaagtcgtactatgtttgtgataattttgtgtgtgtgcttgtgtgaatgtctggtattcagacagat

tgaattttttaaaatgcattgtttattagtatgcaaaagattataacttctggaaatggtgtaaagattacctgaaaagaacattttcctgaagaattac

tcagtgacccactactctgtgtcgtatttaaattagtttgtatttgtgcgtatgctactaaagctgttggtaaaatgtgttgaatcaaaatgacattaca

ttttaattttgttaaaataatttgcatttcttctgatttaaaattcacaagaggtgtatcaaacacctggtcgttagtgatgtgtaaccaaaaaagaagg

tgctaaaagattgttttatttgacattagtcacagaaatctcacatgaatgttgttcagagtacactcgtgtaagacagaatcagcatttgttacaaaat

gtgcctttttaaaagtttgacttgaaatgctctcataacatttgtgtaagtcagtgtagtaaaacattctcacaaaagtgtttaagaaaaattcaaaatg

tttacttaaaatttacaatgacaagtacatttgatttccaggaagatactaaggttaacaattttgtattttgtcagcttcctcccttctacttattatt

agttaactggaaatcactttgtcattgagagaggtaaattctgcatcagggtaggaatcatttgacaattgcattcagcttgtgtgctcatgtactcaat

attttattaatagattcaattctcttactatgaagcttttaacttcatcattgtcaaggtgaaatttcttcactcattacttcattgttttttgcatatt

tattaatcatttgtattccttagcgagtcttccagcaccagaacttaaatttcatcttcctctatacattttgtggcttgatgtgagggataagaggtta

tctgtgataaagttctgggttcatgtatttatcaaaatcactataccaattcctttgtcttcactgtgtgtatttatttgccataggtaatgcaaaactt

ttatctgtactctctccatgaacttgaataattgtgtagaaatttgtgttactttccaaataaagaagttgaaataagaatttatgctatcttaccagtg

ttgtttaagttatcaagaaagacctcacatgaaattattttgagcatgttttgtcatttttcattgctgcactctgtaatttagatactttgcttgaatt

cctatggtgagtaaaatggaggaataaaagaaaattaaaaaaataaattaacactgcaaatgttgtgcaaatccatgcttatgaaagatcaggctgttct

atattcatttgagacaaatgtttttgagtcattgcatgctgttagttgtgtgtgctattgtgttttactgggagatagagtaaactggcacatagtcccc

aatttcattttcagagtaaaaatcttcattaaatagtaaattgtttgttgaagtttattgtaatcattaacaacactgttggctcttgacataatatata

aaatgccaataaatgtaaataattgttacatctttgttaaaactgtttttcaggtgtataaataatttttgtatgtgtcttgttggttactgtgttgatt

tttgtatttctcatattcatgttgtcactgtgtctgaagagaaagtgactgtgccatttattcaaaataatatatcactcaatgtacagttaaatgttaa

tagcgtccagtaatgctaaaaagctcattttatgatgt

>Contig2394|vesicle-associated membrane protein synaptobrevin-binding

cttnnnnnnnntctgatggatgcgaatccatgaaagaaacattattttgctaattttagatctgaagcaaatttatttttgctgtttgcactgggtctag

cagttggagtgacgtcatttcaggcagccatatttccgtcatgacgcatgcgtattggttagaaaactagaatcgccagtaccgggattagaattgaagc

catttctttacgcatacagtattttactgtaatttcggagtacactcatagtcttaaattatggctaatccaggttcaaagccggaacaggtgttaataa

tagaaccggaacatgaactaaaattccaaggacccttcacgtcgccggttacttcttatatgaagctaacaaatcctaccactaaaaaggtgtgtttcaa

aatcaaaacgacagctccaaagaaatactgtgtgcggccaaattctggtgttctggacgcaaaggcatcaattgatgtagcagtgactctacagcctttt

gaatttgatccgaatgagaagaataaacacaagttcatggtacagacactgattgctccagaggaagaattcaatttggattcagtgtggaaagatgcca

acccagattcacttatggattcgaagctgaagtgtgtatttgagatacctgtagacccagcaacagcttcaagagagaataacgttgatgcgtcaccagc

catccatgaagagaaatcaaagaggattggtgacggagcaaaatcttctcctaagcctaatgctgctgtagatggagaacttatgaaagcagcagctgaa

gtgaaacgtttgagggaggaagaaagtaacttaagacaagaaaacctccaactgaaggaggaaatcttgagacttcgtcgtgcaatttctaatgttgatc

tttcaccacaaatggcctcctctactactgcaatggcacctgcaccacttccttttatgtatttagcagctgctattgccatgggacttatgggaataat

tctggggaagtttttgctttgaaggactgaacaaaatggcagcacctaccaactgcagtactgtgagtcagcctgttgtttgcagcttccttaccctctt

tctctccagttcagaatcagaaattgtcttttgtgtgtgccacattcgattgtaaaaacttatttaaagccattttctcatgtcagtttctttgtgtccc

aacttatgtgattattggagagagaggaagaagggtttaaggaaaatttgtttttattctatggtgccacattttggtggtgtgcaatttcctgcgctaa

cttgagtaggcgactcacattgaatggcttaccaatgattgtacataacatgttgtatatggataatttagattgaaatcaattgagttgtagcactgtt

caaagccaaattagaagaaataaatgaagaaagctgcaccttttgattttaggtatcttgttaaaacagtttggctgtgtgagttgtggtagactcattt

acctggataattacctgaagctatttgataacttggaatgctatagttggtaactgttacatttatgttgcagaagaaattgcatcacccagcgaattat

cagcattttttatgatggattttttggtaaagtccccgtatatatatattgacggtttcccagaaactttaaaacttcttgattttattaacttttgtat

tgtgcatgaattaatttgcgtccttgtaattttgctggaaagagttacgttggtgattgattgattggtcttctatacatatatgtagcttttaatggtc

ttatcatacttaaatacgctgaatatccactagccgaaacgagtcatataggtgcatacaggtgcatttacggcattgttattattaatatgacaaagtt

tttcaggaattttatgaattccacttttcggtctacctgagtaaagggtgtgaggggaaggggtgtgcgtgtgtatatatacctgatcttttgagtcctg

attgaactgacgttaactcattgtggtagttcctttgacttttatgagtagtaaaatgctgagacacttctttatgcattgccttattcataaggaaata

gttgttctgtcaagagattcagcatgtactgttggaaaaacagttctctataaaatcactatcaaaatggctcttaggaggttgttgcaaattattttta

attgtgcttaaagtggtcagatctgtggtttctttaaatgtataccacagtttctccagtgaagaccatggtgctgttatgatgaacggactgttatcac

agcctttgctctctacgattgaacttcacttcctgttttatttgtacagattgattgtatgcttgtttgtttttaataatttaatgacaggaataactta

tag

>Singlet4279|vesicle-associated membrane protein 726-like

ccgcaaccgaaatctccacctctctatcctagcttgagggttcggggatcgaatcgatggggcagcaatcgttgatctacagcttcgtggcgcgcggcac

ggtggtcctcgcagagtacacggaattcaccggaaacttcaccagcatagcctcccagtgcctccagaaacttccggccaccaacaacaagttcacctac

aactgcgacggccacaccttcaactacctcgtcgataatggcttcacatattgtgtagtcgcagttgaggcggttggtcgacaagttcctattgccttcc

ttgagcggatcaaggaggattttactggtagatatggtgggggaaaagctgcaacagcagttgcaaatagcctgaacaaggagtttgggtccaaactgaa

ggagcacatgcaatactgtgtggatcatcccgaagagatcagcaagcttgcaaaagtgaaagctcaggtttccgaagtcaagggagttatgatggaaaat

atcgagaaggttcttgatcgtggggagaagattgagcttctagtggataaaacagagaacctccgctcacaggcacaagatttcaggcagcaggggaccc

agatgaggaggaagatgtggttgcagaacatgaaggtgaagctgatagttttgggaatcttaatcgcattgattctcatcatcgttctatccgtgtgcaa

tggcttcaaatgctgatgtgtccatagactcatactgcggactggatgacgttcgttcctcgcgctgatttctggtcgacgtataattatttttggcaca

tcctactttctgctatattttgcatcgtaagaccgtttgtatagcttgtattcgtgaattctttctat

>Contig390|vesicle-associated membrane protein 4 isoform 1

gnnttnnnttcttgcatgtaaattaaacgattgtgttgttcgtagttcctgcgaaaaataagcattaagaaatgaaaattgtggcaatattttgcttgtc

gtctttgaaggtataacttgaaaatatttttctactgtgccagtttcagatatgatgttgcagcagttcttgttttgacaagaaagatggtacgcgttgg

tgttacatgaacatataatgccgcccaagtttaagaaaactgtgtctcaagaagatttagtggccgccaaagatgaggaaagagcttcactgttggaaca

acaaagtggaagtgaggaagatgaaggtttctttcttaattctccatcaacaaaacgtgaaaagaaaaccttagaaaacgaacgtattaagcaagtgcat

ggtcagatcaccgaagtaattgaaacattaagagataatgtgaaaatcctagacagaggcaaacgtcttgaagaactacaggacacatcagagagattga

cagctgcgagtatagatttcagggaagcttctaaccgaatgaaaagacgtgcctgggctcagcagatgagaacgcgagccgtattaattgcagtttgcat

catactattggtgggtttgatagttccagtcatcgttcattattcccgagcctgaagacatccctgtgttcgaatatttgaagataatgtctttgaactt

aaataggatgtgagtgtcctgaagtgttctgcattttgaatagttggagtgtagttttaaccattccagtgtatattaca

>Singlet4320|vesicle-associated membrane protein 4 isoform 1

tggggcaggatagtgctttgcttcttgcatataaattaaacgattgtgttgttcgtagttcctgcgaaaaataagcattaagaaatgaaaattgtggcaa

tattttgcttgtcgtctttgaaggtataacttgaaaatatttttctactgtgccagtttcagatatgatgttgcagcagttcttgttttgacaagaaaga

tggtgcgcgttggtgttacatgaacatataatgccgcccaagtttaagaaaactgtgcctcaagaagatttagtggccgccaaagatgaggaaagagctt

cactgttggaacaacaaagtggaagtgaggaagatgaaggtttctttcttaattctccatcaacaaaacgtgaaaagaaaaccttagaaaacgaacgtat

taagcaagtgcatggtcagatcaccgaagtaattgaaacattaagagataatgtgaaaatcctagacagaggcaaacatcttgaagaactacaggacaca

tcagagagattgacagctgcgagtatggatttcagggaagcttctaaccgaatgaaaagacgtgcctgggctcagcagatgagaacgcgagccgtattaa

ttgcagtttgcatcatactattggtgggtttgatagttccagtcatcgttcattattcccgagcctgaagacatccctgtgttcgaatatttgaagataa

tgtcttt

>Singlet3554|vesicle-associated membrane protein 4

attgtgttgttcgtagttcctgcgaaaaataagcattaagaaatgaaaattgtggcaatattttgcttgtcgtctttgaaggtataacttgaaaatattt

ttctactgtgccagtttcagatatgatgttgcagcagttcttgttttgacaagaaagatggtacgcgttggtgttacatgaacatataatgccgcccaag

tttaagaaaactgtgtctcaagaagatttagtggccgccaaagatgaggaaagagcttcactgttggaacaacaaagtggaagtgaggaagatgaaggtt

tctttcttaagtaagttatttataacgaaagtaaacattcttgaactgttcagcaagtgacaaataatgggagaattcgctaatctgtttcctagtgaag

gctttagtaatgcctttctccatcaacaaaacgtgaaaagaaaaccttagaaaacgaacgtattaagcaagtgcatggtcagatcaccgaagtaattgaa

acattaagagataatgtgaaaatcctagacagaggcaaacgtcttgaagaactacaggacacatcagagagattgacagctgcgagtatagatttcaggg

aagcttctaaccgaatgaaaagacgtgcctgggctcagcagatgagaacgcgagccgtattaattgcagtttgcatcatactattggtgggtttgatagt

tccagtcatcgttcattattcccgagcctgaagacatccctgtgttcgaatatttgaagataatgtctttgaacttaaataggatgtgagtgtcctgaag

tgttctgcattttgaatagttggagtgtagttttaaccattccagtgtatattacag

>Singlet6936|vesicle transport protein sft2b-like

tttaannaatgagtaaaaagaagtgattagttagacactatttatgtnnnagtaaaatttcatcagtagcccatttcatgactgttgctgattatatgta

tcgtttctgtgaaaaagagagaatgttgtattggcatgttttagcatgtctgtgtgccactgtagctaattccttctgtacagtaaaggcattaacttct

cttaggattttccttgtgctgatgtttgcagccagtattggtacaattgtcattaactaagcagtgttggtcataccatgtcagcacaattgaatatgtg

tgttggtactggtttttggtacaatttgatatcaaatatagaaatccaatgtactgtataggactgttgtttatgtttctactagctgatgtatttaaat

agtgaatccatgtggcaaccagtcattgtctcagagcagtgtaaaggtataatttgaagctctgacatttttgatagctcatttatctgagaaagataaa

agaaaagataaccacaaactatacaatggaagtgttaagtagtaatcgggtttacaaatggattttttccattcgtgagagtgatcccataggtgccaca

aaaattttgtgtatgctgaagtacagcagacaaatcattttaaatttttcttctacacagtgcatagttgtgtattatcctaatattgtttatacttagc

cagtcaaattcacagattttttccatctgtgtgaacaaatgtttcttctgactatcttcttgttttgcttctcaaaatatcatgccacacacagtatgca

>Singlet6970|vesicle transport protein sec20-like

agggggatgcgaaaacttagttgtcatatgtttggcatgcgttagacaggtggtaaattagtaaacaaaattcaggatgtacagctgatagtaattgaag

taccttatcttttatcattattttgtgactgtgaattcgggaaaaatgttcaacaagacacacaaaccgccagatgagagctatgtaatagacttaattc

gtcaggaaattgtgactgaaaatctgaaagtgaaagcaattattcaaaatatttatgcttgcaaaggacccagggaagtgttggatgaactgaacagcga

aggtcgtagtaagattgccatcctcaggaaacaaataacaaaattggagacattggctaaggaaagtgacaatgaaaagactaagagagagattctgtca

gatgttgccaattataaggatgaactaacaagtactcttgcagcatttcgtaaagcaaacattacctgcctgcttagtatagaaaagagcccacgggaag

agctccttggggattcttcagaggaagcaagtttgagacacaggcaaagaaaagataaagaaagtttggtaaaactgtcgacagatgtaacagaacaact

tctttctatcagccgtgcattggctgaaacaacaaaacatagtgcagagacgttggatacattagccacatcatcatctgtggtacacagtacacatgaa

gaacttcacacgttgggtagtgttataaaccagtctggaaagctactatctaaatatggtc

>Contig181|vesicle transport protein got1b-like

aaggggatataagtgatgaatagtgtttatgatatttgtgtgataaatgggtctatcaacactagccgctgtgaataaatacgggtgatgcacaactata

gcttgcgtgctgttatccgaaattcggagtttgcgagacgccgacaatgcgcggatgacgtggaggtgtagacgccgtctggatttgtgagaagtgtttt

cactgcagaatacacgtatagaaatagaaagaaaatgttcgaaatcacggatgttcaaaaaattggtgttggtttggccggtttcgggatagcatttcta

ttccttggcgtgctgctcctatttgacaagggtctgtcagcccttggaaatatacttttcatctcaggattagcatgtgtaattggattagaacggacat

tcagattcttcttccagagacacaaagttcgagcgtctgttgccttctttgcaggcattcttgttgttttatttggttggccattagtgggtatgctgat

tgagacatatggatttgtgttgttgttcagtggcttctttcctgttgcaatcaatttcctacgaagggtaccagttcttggaacagtattaaatctacct

ggaattagagggattattgacaagctggcaggagattcctcaaggacaatggtttgacagcaagattaactttgcagtgagcaacttgcaatgtgcagca

ctttgaaagtgtgggaagttttgtgtgataatgtgtgaaggtttttgtgtgtactttacgaaaaattgatgcacgatgtcgtcattgaataacggtgaag

cagtggtgtaacacgatgtgaagccgttcacaatgacacttgaaaatgcttgccttcctgagtgttcttgggagtgttaattgtgacattccattggagt

gttggagaacatttctcactattgacacatattcatgtttctatcatgtaaatatgtgcaatcctgcttgagattttcgtggtggcggtggagtcactgc

tgctacatattattgctaagtggtgttatcaggtacttacttgttatatgcacagtcataagctcaacatttttatgatctgtgatatacacattgatga

gtggatata

>Singlet6075|vesicle protein sorting-associated

tggggttgttttacacggtagcaaaacaaatgtagagcgaaaaacgtgttttggatgggaaaaagagtgttgtgagatatacgacggtttttttgttact

agagcgtaatctacgacttttgcaaccaacaaaatgctgacattacgagaaaaacagattggtgctttaaagcagatgctaaacttgaaccagcctcatt

cgaaaacccagtctgcggaacccgtgtttaaaatcctcatctatgatagatgtggtcaggacataatttctccgctggtgtccataaaggagctaagaga

gctgggagtaactcttcatgtgcagcttcattcggacagagatccaataccagatgttccggcagtttacttctgtcttcccactgaagaaaatttaggc

cgtatcagccaagacttccaaaataatttgtatgatgcttatcacttaaattttatatcgccaatttcaagacagaaacttgaagatttagcatcagctg

cactgcaagcaaactgtgtctctcagatacaaaaggtgtatgatcagtatctgaatttcatttccttggaagatgatatgttcattctaaagcaccagaa

tagtgattctgtatcatactatgctataaataggggtgagataaaggacaccgaaatggaaagtataatggatagtgtagttgacagcttgttttcagtc

tttgcaacattgggagctgttccaattattcgcagtcctcgtggaaatgctgcagagatggttgctgagaagttagacaagaaacttaga

>Singlet6117|vesicle docking protein p115

gactannanntcctgtgggctgttagaaaacttgtgcaatattctgatggcaagtggtgttccagcagatattcttactgaaacaatcaatacagttggg

gaagtcatcaggggtaatatgagtaaccaggaatattttgccaatgttctggcaccttcagctcctccaaggcctgcaatagttgtcctcctgatgtcca

tggtgaatgagaaacaaccattcatacttcgttgttccgtgctgtattgcttccagtgcttcctatttaaaaatgaagttggtcaagcacaactggttca

aactctcctcccttctacatctgagatgagcaccctaacaactggtcagctactttgtggagggatgttcagtgctgatccactctcaaactggttcagt

gctgtggctcttgctcatgcattagttgaaaatcctgcacaaaaagagcagcttttgagggtaatgcttgcaactagcatcggcaaccctcctgtgtcct

tactaaaccagtgcacttcattgctacagcagtgtggcaaagtgcagtcgaaacttggaattttgatgttgctcagtacatggctagcccattgttctct

ggcagttaaacagtttgtgagcattgccacgagtattccctacctcacagcacaggttggatcaaatgaacatgatgaaaatgaagaactggtacagggg

atctgtgctttcttaatgggcatatgtgtagtattcaatgatgacactgtcagcagcttttccaaggaaaatctctgccagctgattgagnaacgcattg

gacttgaaacatttcttgataagctaagtagcgtctccaaacatgaactctatagcaaagctgcaaaacatccacagttgcg

>Singlet2847|ves g 1 allergen

gccgcgctcgccagccgcggtcccagcgtgacacacacaccgaaccaagtgctgtgcgtgctcgtcgtgacagttgctaggacgcctcagagcacttcgc

ctgcaaacaagtagtgcagtgtgtgcttcgtcaaacgcaatttcttcctcgagtctccagcccacgtcgaagtaacttccagataggtggcgatggaggc

caccgccttgtgaaaagccggcacggagcagtactatgccagcgcgatggcggcgtcgcgacgactcgagagtctgctacaggcggcggtctggctcgtc

gtcctgacaggatgctgcgtgtgggcgcagcgggaacagctgctggagcgcaaccgcacctccgcgaagaagccgcagtcagttcgcgacctgttccaca

cgccctcctgcgtggacccgcccgtgacttgcccgcaccacagaatacagttctacctctacaccatgtccactcatgacaacccgaagctgctggacgt

gac

>Singlet2711|very-long-chain -3-hydroxyacyl-

ggtgctaaaggaccacacacctactcattttccttggatttccactctcctattgataaagagggtagtaaccataagatatctgacagacaagtggatt

tcactttgaaaaaagaaaatgatagctggtggccgcgacttacagcaaaacctcagaaaccagcatggttaaagattgattttgatcgttggaagtcaga

agatgatgtcgatgatgacaccccaagagatatcagaggagattatccagatctttatgacaaattgcaaaaagaagaaataggatacagaaaagaagac

atgaagaaggtttatttggccttgtataacttggttcaatttgttggtttcacatatatcctgacagtgatgggaattcgatattatcgtgatggaccaa

attccatggaaggaacttaccaagctgttggaagtgcaatgaaattctgtcagtttatgcagtttatggaaattatgcatccactatttggttacaccaa

aggaggaatattagcaccaatgctgcaggtttcaggaagaggatttgtgttgtttgccttgattgatgctgaagaacgaatgcaagagaaacctgttatt

ttttacttattctttatctggagtgccatngnnnnngtaagatatccatattacatttctcaagtgtacaaannnnacttaccagttttaacatggccgc

ggtacacaatgtggatcccgctgtatcca

>Contig2900|very low-density lipoprotein receptor-like

ctgttatactaatattggcagctatagtggctccggtagtttaccggcattatcttcacagaaatgtgaccagtatgaacttcgacaatccagtatacag

gaaaactactgaagatcagttctcactggaaaaaaatcaatatcaaccacaaaggatctatcctgcaacagtgggtgaagaggctcatgagcccctgact

agtcctgggacaaatgattatgtataactgtgctttgaagttagttcagtaacggactactatccaggcatgttctcctgccattgtggacgagtgtgtt

gaatggtgcatggtggtgtcaaggctagaaataaagttgttgggattgcagcagcattgtctgtggttggcaggattaagttaagcaactgtgcattctg

aaatgaatgtggcacaccatgtgcaagcaagtgatagctcgcatttctagaattttttagttgagcaagaggatttgagtggattgtgaataatgggaca

gtgccaaatttctgtgatctgtttactataaagcatgtagtgtgaataagtgtatatacaggaagatggaaacttttagaggtgcatactggacatgaac

ataaaatgtatgttgtatccaaaactaattattttcaggctacctttttttgttactgtgtgtatcatctaagtatccattttccagactcctgtaaata

tttaattttattatcagcctgtatattttgaaatgtgtataaatcatattcctgtgtaaatactttcatactgtgtaaatggtgtagaaatctttcatta

cagaaagtgtcacattcggtttcgataatagtgtgagagaatgtgtgagaatgtctttaaactaagctgtatttacgtactgcatggatgactgatgtat

ttaattgcctgtaaaggatttgtacaaaaatataactttgtcattaagaaacgagaacgaaaatattttaaaaagagctatttaaatgttttgtaaatac

agtgatcataatttcagatttgtgtcctgcaattaattctattgagcaaatggaggtaagcatttataaaaacatcagttctatgtagaataaatgtgaa

attctgccaaagttttaacaaattgtgtgttaagtttattcgttaaaaagttgactacattcattgttc

>Singlet4599|venom serine carboxypeptidase-like

ctgtcgcatgcggaggtacttgtaaagcgtcttaacaagagtaacagtgcgtacgtcggtggtggtactatggcacggatccaggaagttgtttatggcg

tctgtttcgttttagttagtttttgtgcatttcatgtagactctcttttcaatgtttatccgcgaattcaacctctcccggttgagggggatccaggaga

accactttttctcaccccgctcattgaagaaggaagaataaaggaggcacagcggcttgctttagtaggtgtaggcattaacgaaggctgcaccgaaaat

tgtcatagagtcaagagttattctggttatttaacggtcaacaaaacgttcaactccaatcttttcttctggttcttccctgctgagaccaattctgcaa

ctgcgccagtgatcctgtggttacaaggagggccaggagcttcatcactttttggtctctttactgaaaatggaccattttcgatcactcgatggaactt

cctgaggagaaggaaatattcttggactaaaactcattctgtcatatactttgacaatccagttgggacaggattcagctttactgagaatgatgctgga

tatgccaggaatgaaacagctgttggaaaagatctttataatgctctagttcagttcttcacactttttccagaacttcagaagaatgatttctatgtca

ctggtgaatcatatggtgggaagtatgtccctgctgtatcatatacaattcacacacagaatccaactgcaaaactacaaattaatttcaaaggtatggc

tattggtaatggtctgtcagatcctgagcatatgctgaaatatggtgactacctctatc

>Singlet5666|venom protease

gatttcaacgacatcttcgagtgccgactgcgtctggcaagaggacaacaatggttttgcagagggctgctttgactctcctgctggctgtacttcttgc

tgcgaaatcagaaggtgcgaactgcaagcagaacgaggtgtgcatcaacatccggcggtgcgacgagctgctggcgctgctctcgcggaggaatgagccg

ggcgtgacgcagcggctgcgtgactctctgtgcggctacgagggttcagacccgcgcgtctgctgcccgcgccaggaggcgcagctgcagggccgcaccg

ccatcccggtgcccggcgagccggcgcccggccgctgcggctacagcaacaccacgcacgtccgcatcataggcggcaagaaggccgagctcggggcgtg

gccgtggctgactgcgctgatctaccgcggcagcaacggaccgcgcgtgctgtgtggcggcgcgctcatcacggaccagcacgtgctcaccgccgcccac

tgcgtctacaacagatccgacctatataaagttcgaattggagatcttaatcttctcgcagaggacgatggtgctacgcccatagaatc

>Singlet2165|venom dipeptidyl peptidase 4-like

aaggggaaatatcttttaatttccagcaaccattcaaagctattcagacatacctacctggcccagtatgaaatcatcaacatagagaagaaaacaaaaa

tgcctctggtaaatagtgagggactattagacaacaaggtgttgcagcttgttgtgtgggcacccactggaaatgcacttgtgtatgtttaccacaataa

catatattaccgtccaacagctgaacttgctagagagtttaagcttaccaccacaggtagctttggttcaatttatcatggtacaccagactgggtatat

gaagaggaagttcttgcatcaaataaagcactctggttttctccagatgggaagaaattggcttatgcaacatttaatgatagtttaacacgtgttatga

caattccatatt

>Contig1491|venom carboxylesterase-6-like

ctgtcgctnnngnngcggcgcactgcaggtctcgccgacgaggaccgccagcccttctaacgtcctggtgtggcaggaagttgactggagtattgtatct

tacatcactgaatttttttgtagtaagagagttattctaggaacaagaacgaacacacactgtattgtcaccagtgatttatttattttggaaacaatcc

ggcgttgatatgcacctcagacaagcgcttcctctaactgtattatttatggtttctgcggcaccgatctccatattcgcacaggagtacgtcacggtcg

atatcgatgctggtacgctgagaggacagatcagaacaacttacaccaataaaaccatgtatagctttgaaggcataccttatgcagaatcgcccgtgga

taatcttcggttccagcctcctgtgaccaagggagaatggagcggtgttcgagacgcactccagcctggatcggcgtgtccacagctagtcaacggtgca

ccatccggcagcgaagactgtctctaccttaatgtccacagcccgtcgttaacttcgtccaatgggactctaaacgctgtaatagtttggatacatggcg

gatgttttctgaggggaagaagtgacacctggacaccccacttctttgtggataacgacgtcgtattcgtcagcgtaaactaccgcctcggattactggg

tttcttgagcactggcgacgacgtcgttcccggaaacatgggtcttaaggaccaaacagaggcgctccgctgggtgcagaggaacatcgaggtcttcggt

ggcgacccggagcgagtcaccctgctcggccacagtgctggagcggcatccgctcactatcaccttctgtctccactaagcaacggtctttataggagcg

cgatatccatgagcgggtcggtgctttgcccctgggccttctccaagaatgccactgacagggccctacggtttgcacagtacttgggataccctgccaa

gaattcatctgatcttgtagatttcctcaagaccgttgatgccaatatcctcgtgaaggacgttcacaaagctctttccgacgaggacgcactgagtctg

accacctgtgtgtgggtgcccagtgttgaaccagagcacgagtcagcattcctggtggaggatccgcgcttaatggtgcannnnngtcgatacaatttcg

tcccgtacgtaactggatcgactgatct

>Contig3379|venom carboxylesterase-6-like

ccgnnnntgtcgtgttgtgcgagagagcgtggcgacatggcttcnnttacagcgaccgtccgccagggagctctgcggggaaaggtcgccaccactcaca

ctggcaagacttattacagcttccagggcatcccttacgccaagccgccggttggcccgctcaggttcaagccacctgagcctgcagagccatggaccgg

agtgagagacgccaccaaggaaggcaatgtggcccctcagttcagtgataccacacagcagtacatgggggacgaagactgcctcttcctcaacgtctac

actcctcagatgccgagtgatagcagcagctccctgacgccggtgatggtgtggatccatggcggcggttacacggtcggctctggcaacaccgacatgt

acggccctgactacctgctcgagcacggtgtcgtcgtggtcacgctcaactaccgtctcggcgtcctgggtttcatgagcacgggtgattcggttgtcac

cggcaacatgggactcaaagatcaagttatggccctccgctgggtcaaggaaaatatttctgtcttcggtggagacaccgacaacatcacgatattcggc

gagagcgctggcagcagggcctgtcatctgcacgtcctatcaccaatggccaaaggcctgtttcaccgcgccatctgccagagctcggtggcgtcacgag

gcagcctagacgcgccggtggcggagcgtacgttccggctggcgcaccacctggggctgaagcagggcgcctcctcagaggagctgctggccttcatgaa

ggaggtccccgccag

>Singlet3271|veli cg7662-pa

agggggaagagttgggtatcagactccacatcgtttattacacactagtgacactatacaaaaagaagtagtggcaatggcagctgttggcgagcaatta

acgctagcgagagatgtgaagagagctattgagcttcttgaaaaactggaaaagagtggagaagtgccagctacaaaacttgctgcactacataaggtac

tccagagtgattttctcaatgctgtcagggaagtctatgagcatgtttatgagacagtagatattcaagggtcacaggatatacgggcatctgcaacagc

taaggcaactgtagcagcttttgctgctagtgaaggacatgcccatcctagagtagtagaattacctaaaacagaagaagggcttggctttaatgttatg

ggtggaaaagaacaaaactctcctatatacatatcccgcataattcctggaggtgtagcagatcgtcatggaggcctgaagagaggagatcagctacttt

ctgtgaatggtgttagtgtggagggtgag

>Singlet3507|veli cg7662-pa

gtgtagccattattcagtttatacatattcaanntattttcgaggcagccattcctcagtattactgttttgcacaatgaatgcatggtaacttctttat

gttatctgtgcttctgttggcagtgtatagttatttaatttgtaattgatacttgtaagcagaaagtcatttaatactgatgcaagacctaattctttgc

tgcctcatattttccagaagataggttatgccacttacgctgaaaatgaaccatatcatgatttcagtatatatatatactaagataccatcttagtata

atgctcaccagccacttgactatcttcttcttctgtgcgaatgcacaaacagtgcctaaactcttacgggaatcggcagcacgctgcgagtaatgagtat

aatggccaggggcactacaaatgtagtgcaggacaatgcattgagaatgtgggtttgacgggaggcgtgccagagataaatccctgcagtcgcgtactat

cctctgtgtcctcggtggctcagatggatagagcgtcttccatgtaagcaggagatcccgggttcgagtcccagtcggggcacagattttatctgtccct

gttgacatatgtaagcagctaagggtggtcattgcattgtaatttcatgatttcagtattttttaaatgtgttcagttatacatttttagtaggagcata

tgacgacgacgatgatgatgatgatgatgatgatg

>Singlet5980|vasa-like protein

ggaggtacttcaaccatgcatcaagcccaacaagtagcgaggggctgccatattcttgtagcaaccccaggtaggcttatggattttttgaacagaggtc

gtgtgaacttccagtctgtccgctttgttgtacttgatgaagcagatcgtatgctagatatgggtttcctaccagatgttgagaagatgctggaacaccc

tacaatggttcctacgggagaacgtcaaacagttatggtgtccgctacattccctgaagaaattcagagactagcaacaaaattcctgtccaattactta

tttctggctgtcgggattgttggtggtgcttgtagcgatgtggaacagattttttacaaagtatctaaatttgacaagagagcaaaacttacagaaatac

tgagggaagaaggtggcaagaaggtacttgtttttgtggaaacaaaaaggattgcagattttttggcagcatttttatgtgaggcagaatttccaacaac

aagtattcatggtgatcgccttcaaagtcaaagagaagaagcgctttatgattttaagactggacgaatgggaattcttgtagccactgctgttgcagcc

agaggtttagatataaagaatgtggctcatgttataaactatgatctacccaaaagcattgacgaatatgttcatcgtattggccgaacannnagagtag

gaaatagaggaagagcgacaagtttctatgatccagatgtggacgcaccaatagctaggga

>Singlet7863|vam6 vps39-like isoform 1

ccgttgctgtcggcaatcttcatcacagtgttttagcactaacagatagaagggaaaaataatgtaaaacaaccctatatttgactgttagaagaaaata

agatgcaaaggataaagtaaaaggatactgttatgaacacttgtataaattttcacagttaatattttagggttgatgtctcactgtgtttttaagactt

attctgttggtttcagtcccctaaagtgtgtagaatttataacataagctttgtcgaaaacattcattgtaggttttgtttagtcttaggttggttgatt

tctgggaggggctttgttttatcttacataaatgtgactgctatcatataggctagttactttaattctgagactatctcatgacttttcaggaggtgga

cagttctccataacctaacttttactgttcagaactgggatatgtggcatgatgctgatattttgtgcagtgtgaacgaaattgctatttgagggctgtt

gtcattatgtaatatgacatccattgtgaacaacctgaaagttacaaaccatgtctccctttgggcaaagcctgtatggcacgaggaacactgagcagtg

gtggcacatctgaaattctggaactatgtgccaaatatcacgaatggcaaccttgcagtctggcttacctgtgagtgttgtggcacgtttgttgaacatt

gataccttataggtccaggaatatgtaccatcgatatgatactgagtcacaggtgttattgatttgcatgtaggcattgtattattaagatgaaggtgac

cagtttttgtctggcgagctttttatttaatgtgctatctgaaatatctccttacaaaaacttc

>Singlet7995|vam6 vps39-like isoform 1

tgggggaggtttcagtttaaataannnttggggtcaggaactcaatttattaagatctcaccatgtgtcacatcagctgtagctgggaaatgctgagata

atgtgcatttcacagaacacatcagagggtttagtgggcaacattaacaaactcggctgtaaacagccacatgagaccaacaaaagttcagtctggttgg

agtccatgctgtgagtacacccaaattggacaattcgaagcacctggttgtcgaaatattgcacataagctggaaacaacaacttgactgaacacccaga

agcacaatattaactaacaccaactgtcaatataatggaaagggatgccaacagctgaaacgtcacgattctgtagaataatgtctttatattgttggga

cagtggatgacatgactccttagttctttgcctagatctgatgttaacctattttcactgcacttatgtatgtgaaatctccattatttcattcacactt

gcacagtaaatatgtattgccataactagtttagactatgtatgtcaacttaaaatcggcccaaaaccaaaggaagtctatggtaaacatgtatcattta

gatttttactgttttataatggaaactgatgtgtaattttgtgcttttatgcagtattggaaaaactagtattgtttgttactatacatgtaactttcct

gaaacagcactaagtagcagtaataggaggttctcttttatgagtat

>Singlet7448|vam6 vps39-like

gtannccnncaccttggcaaggatcacataaatctgatcttcgattttgccggctgggtgctggagagaagtcccgaggaaggcctgcggatattcacgg

atgatttgcatgaagttgagcagctacctcgtcccaaggtgctggattttctgctcaggaatcatagtgaccttgtagtgccatatctggagcatgtgat

tcatgtgtggcaagaaaagaactgcatcttccacaatgctctggtgcatcaatacagggagcacagtttgcaacttctacaaaaccctgagacttttgag

gccggacgtgctttacgtgtgaagctgcaagactttctcgaggtgtctgaacactatacgcccgagactgtgctcattcacttcccgtatgacaatctct

ttgaggaacgtgctacagttctcggaaaactgggtcgtcacgaacaggccctgtcaatctatgtcagtgttcttggtgatgtggagcaggcaatgcggta

ttgtgataaggtgtataggcagcaggcagagggtgccgatgaggtgtacgcgatactgatgcgcatgctggtgaatccgccagagagctggcttgtcggt

gtcccgtctccgccgaccgtccagccagacctggaggctgccctcgcactactggagcagcatgccagcaagattcatcctgtcaaagccctgacggcgc

ttccagatcgagtccctctgatccgcatcaagcacttcctcgagtcgagtctgcagcacaaactgagtgagcgcagaacgacaca

>Singlet2520|valacyclovir hydrolase, putative

gtaaaagtgttttgcgtaagtagaccacaaatttaggcattaatagaatttttgtgtgatttctctgtaactgaaatgatattaatggttcaagaaaaac

tacaagggagatatcatcagttatcatgtctgtgacaaaattgattaattctagctttgcagttgatgttttcagaacaaagtatttttgttttcgatcc

aatatatctagttatgtagaatcaagagttgcataaacatgatcatgaagttgaagctgactgacataggtgaatctgaatcaaatgtggtacattattt

accaactaaagtaaactctcaaactgttgagagctgttttgacattgtacatactgaatcttgatgttacattagtacacacaattgtaataaagtcctt

ttttcccttctaaagctagtgagcagaataaattccttcttccacctgagtaagtgtagtgtactttgtactatggtttaaatgacaaatttgctgtctc

tttggtagcaaaagtagtatcctattaaacatctttaaaaggattttcacataatggcatatatcagatcaaagaatgaataacggtgccaccatgtgac

ggactacgtacaggcctgatctatgcgttgtacccaatggtctgtgctacaaagacctgcaaggttattcggtgttacgcttttaacatatgatgtgagc

acatacttgctttgatttattttactatgtatgatgaggacagtgtattttttggccatttctctaatcgtggtacccaaaacccacctaccaacactat

actggacaat

>Contig4156|vacuole membrane

gatgaaaagcttttttgatcgtgtactggaatattaaatttaaaacaggaagtcatcctgaaagcttgggcagagaatttgatacctggatgcataaaat

ttgagaaatgtagaagagatctgtttaaatttaattttgaattgccctctacctcattgaaaaatgtaattgcattttgtcttattcagtatgaggatca

ttttgtgactttgtccattactgaatgtttgagtcctatgtctgtgttgttaatagaaatatttattttcctcattccattgtgatatgnnttaatggta

tgtttatttaggtattgttacaaatgctgtgtttgaagcacttcagttattgttaaactgaaacctgaggcttgtgcacattaaatttcacattgaaatg

aaccacattactgttcttttttatttttttgccttttatgtcagggaacctcttgcagcttttatatacatatatgtacattttacaatgaccaaagttt

ttataatgttccatatttctacactaacatttttaaattcaaggctgaaagaaaaatttctgatctaagagcacttcagtgtaaaaagtctttcagacaa

actgtttcttaatatatgacaaagaattgtgcaatattttatactataaactattgaccagaattctcagaagtgcatccaattttcctcatttcaaaga

gttgtgtgatttctgtaaaatctgtacagggaagccttaaagttacctgtttcttttctacaatgtatttggaataaaaaatacttccgcctatcagttt

tcactgtagccataaccagtaattgttacgaagcaatttaaatttccttatggcatacagcaatgaaatatctgtcatcttaaagtctgtgccgatcaat

ttttatttttttatattactgttgtggctttggagattataccatactaattgtaaatatgccatagctgaatgagagtttaaaaaaccctgtgaactct

ctctctttctgcctagtggtagcaacgcacaatcttcacttgtttctcataaatagaaatggttcattgtattttttatagtttgagtcaat

>Singlet2451|vacuole membrane

ctgtcggtttttaaaaattagctgtatttcaatgtaagaacatacagcagtgaaaacatactttaaatatagtgatcccaggagtgtttgactccccctt

tcttttgttttgtgttcttctgtaccaatgtgatctaagcatgatattcaaactgcacttatatatagttcatgtataagacacatcctggaaaaagcga

gttacacattattaaaagtgatctcacacactgagtgatacatgtatatagaaacatacaactttacatgtatgcttgactcttttgtaaagttttacca

ctttatggtaaatttttaaaaagacattcgtctagaattggaatgccaaaggggaattgaaatgtgttgtttcttaaagataatgtatcataaattcatc

acttcatgtggaacatatgtacagtttttgtaatgttttgtacatgtgtgagggtatgtagcattgctacagataaataaatgcaatgttttattgc

>Singlet4586|vacuole membrane

atttttggctggtgacattgccagattggctgtaatacttgtttccttaatgtactggttttccttaaatgttgtactgtatactgccatacttgtaacc

tgttgcgcaattattattccagtttcattgagcctatttattgaagttggtaattccattcactatatgctgctcatttatgtcatagtaatgggaagtt

gtaaatgttaagtgacacaaattcgtgtaaataacactttccttcttgtgtagtctgctattaacattgatagaccagatagaaaaaggtgataatttgt

gttgatattgtcttaaaaccagtcaggccttccagataagctttgaatctaaattttttggagctattaaatttattttggttctgtggtgataatgtaa

gcaaggaatgagtgtagaaaaataatgtaaattatgatgagttacttagtcctactcccaaagataaatattacatacaaaacagagaaaatacaaatta

tggaatcaatgtatcatattgtttgatctgtagtttcaactgaatttgcacctctgttagatttcaccacatttgtttcttacgnctttctgtagcattt

taagacagaaacttgctcttcagnattcaaagatgttcccagtatgtttaaagaatacacttctttgtctttcctgatttt

>Contig734|vacuolar-sorting protein snf8

aggggaagtgcgaagtgtcaacactcaaccctcaacagtcaattccttcatagttcattccgaaagttgtgttgtgtagattgaggtgtgtttccgtaga

gacgtgcgatacccaagttaagcgtgaaagaaatatgtttatcttatgaacattaatatccagtggtttggtatcaaattaaaaattacgaaatacgtct

aaactgtctacgtaaaagaagttagctgataatgaggcggcgggcaggagttggtgctatacaaaagcaaagactggaacaagagaaatacaaagacaag

ggcactgaactgcaggaaaatcagttggagcaaatgacgaaacagcttgaagttttccgagcaaatcttgaagaattcgcatccaaacataaaaaagaaa

tcaagaaaaacgcccagttcagaagacagtttcaggaaatgtgtgcatcaatcggcgtcgaccctttagcgtccggcaaaggcttctggtcggttctcgg

aattggcgacttttactatgaactaagtgtacaaatagtggaagtctgtttagcaaccaactataaaaacggcggccttattagtttagacgaactaaga

caccgtttgatacaagctagaggcaagaataaacagcatcaggatattacagtagacgacctgctaagcgcagcaaaaaaacttagaatttttggagacg

gattttctgttgtaccaattggtaaaggacagtacttagttcagtcagtaccaggtgaactcagcatggatcatacagccgttctgcaacaagctgctaa

tagtgggaaagcaaatgtctctgtatccgacttacaggatcagttacggtgggagagnaaccgtgctcagaaagcactcgattatatggtcaaagaaggt

cttgcttggattgacacgcnagatgttaatgatgtattatattggtttcc

>Contig223|vacuolar protein-sorting-associated protein 36

tggtgggagtggtgttgtgaatgtgttgacagaaagtgaaattaggtatcttggagacagtagatgatgtttgacatgtttggggtttgataaataaata

acatagtctcgcgagacatcacgaacaaatgtcaacaatgagccaaaatcagcttatttcacgatatggattccaaataaaataagttaccgttcagtat

ttatcgcgttggctcagggtattaaaataatggatagatttgagtacgctgagccgctgctctcgaccggtgagtcgtacgtaacacgggaaaaagctgt

caagctgtatgacggtgatacaaagacatcattcgaaggaggtgaacttgttctcactactcatcgcttgttgtggggaagaccaggagatattcctcgt

ggcagaacatgcctatccttgccgcttcgttatgttgtatttgcagaagaggagtcaccaagttcatttgcattcacgaggagcaaaaaaatagtcctac

accttacggaaccaattccagggaagatccccggtcccgtcggtaaaagcagctacaattatgtgaagctgtcatttaaagaagggctggaacatgattt

cttccgtttactcagtgatacagttcagaagaaagcttgggaaactcttttgcctattccaccggcttcacaggcacaccaatcaaatattaaactacgg

actggtattgttggaattgagagaagtataagtgaaaagcagaaagctactgatgaaagtatcagtgttgccttccaagatttgaacaagttaatggtta

tggcaaaggacatggtgaatttatcgaagaatatttcacagaaaattagagaaaaacaaggagatatcacagaagatgaaactgtcaggttcaagtctta

tttacttagcctgggcatagatgatcctgttacaagaaatgatttccgcagtgaaagccagtactttcagcaacttgctaaacagttatctgaaattttg

gtggagccaattacggaagttggagggatgatgtcactagcagatgtatactgccgagtaaatcgtgctagaggcctagagcttttgtctcctgaagatc

tcatgcatgcttgcaatatgttggaaaaattaaatctaccgatcagactgcgcacttttgacagtggtgtgaaagtgttgcagcttcagtcacatgatga

tgaaagcgttgttgagaggactctcagtgagttggaagctcaaggttctcttacatcagaagaattagctcaaacattgggaatatctgtcttgttagct

agggagcgactgctcaccactgagaaatatgggaaagcatgtagagatgagagcatagaaggcctgcggtttttcccaaaccttttccttcagcgtgata

catagtccaatattgtaattttggaactggaagttgctgttcaataaagtcttatgtttattattgttacttcttgaagtctcagaaattgtgctgtgta

ttcctctcatagttgttctcatctaccagcgataacataataaggcaagaattatattgtggacttttccacagttttgaaaatgttgcaatttcaaaca

tgtatattatgacttgatgtggataagagatatacataataggaattttatacaggtaaagtaattcannctgtgctatttatactttaaagagaaagca

aaattacaaatctcaagtgagaaacttttatgtaaatcacattgatga

>Contig497|vacuolar protein-sorting-associated protein 25

gggtacacatgtgttgtgttgtcattcacaagtgttgaacgcgtcgaccaggatccgttggtgaatcattctcgaagtgtagaagtgaaagatagatggg

cgacgttcggcggtgaaattcatacgggtgctatagacaactgaacataatatttttgagggttgctgagatgtcaattgaaaatcccaatattgagttt

ccatggcagtttcacttcccaccattcttcacattgcaaccacatgcagagacaagagcaaagcagatagcagcatggaggtcacttgtactcgaatggt

acggagcgatgcggcaaagtaaagtagacgtgcgtgaagcagggcggggtccaccatttgccaatgccaccatcaaccgacggtttcccgaagagggcat

aacagctgtaatggaagaactggctcgcactggtcatgcggaacgtctcgacaagcagggctatacctgggctgtgcattgtggccgcactatagatgag

tgggcagactgccttctgtcttgggcacgtgataacggcatgagtggtacagtatgtacattatacgaactaacacatggagatgatactgtcagttgtg

atttccatggtttggatgaagacacactggtccgctcagttcttcgcctgcagcaacgtggtaatgctgaatttatggattttgatgataacagaggagt

gaaatttttctgattcatttttttagggagatattttctgattangcaaggttttattattttttgcagattttaatttctaattttgaaagaaagtttg

tttcgcnnnnnntgttaatacatttctgtaacagatgagctttaatgtggcgatgattgctt

>Contig69|vacuolar protein sorting-associated protein 8 homolog

gaaagtgcttgtgaatttgttaatccgctattagtttacgtttcccagtgtaactgactcgctgctnnnagnntctgttaacattcaaaggacggtattt

gattatggatccacttgtgtcagatctttatgccatccacgagggtattaaggtggagccggaatcgaaggagaatacggcccaggactcatcaaacaca

acacagtcagacagttctggccatccaccagtcgttccatcatccgctgcccaaccaacagcacctcaacaacctgcaacagccgtctcaccaaatcatt

caagtgtggacaaccagaattcatcacagaacgccacacagccagtggcagcagtgacaaaaggaactggaagctcagatgaaaaagctccagattcatc

agacaatggtaataaacctcgtgccggttggcctaagnnnnagaagcggaaaaagagccagcgtgatcaaactgcacctcgacaacccctgacaggttat

gtgcggtttttaaatgatcgccgtgagaaggtgcgagcagaaaatccaaacctcccttttccagaaataacaaaactgctagcagtggaatggagccagc

tcccaccagcccaaaaacagagctatctagatgctgcagaacaagatcgtgaacgctatgtcagagaactaaatgcctataaacaaacagaagcttatcg

tctatttacacagaagcaagtggagaagaaacagcgtcaagataaacaagtagagcaagttgtccaagaggatacagagaaggatgcagatttcaatgga

cttgacatccccatattcacagaggagttccttgaccataataaagctcgtgaggctgaactgcgacagttacgaaagtctaacacagattatgaacaac

agaatgctattttgcaaaagcatattgaaaacatgaaagcagctgtcgagaagctggaggctgagacagtgcaacaacagagtagtaataatgcactcca

gcaacatctggaacatctgaggacaaacctctcggcagcattttcatcactccctctcccaggaactaatgagctgcccacagttgctactgttgacggc

tatataacaagacttaatgcagttcttatggaccagtcagctccagaccatgacggacttgcagtccgtgtgcgagagttggtcaacagtttagagtttc

caggatgacc

>Contig709|vacuolar protein sorting-associated protein 8 homolog

aggggaggtatgttcaaaatattctggaacattgtctacaaattttttctttacttacttgttacttattgtgcattgtctccctcaaaacactcttctc

cacaattgatacaccactcccaatggtgttttcacttttggaagcagtcttggtatgcctcttgctggatcgtatgaagcaccatctgtgaattttcttc

gatctcatcttttgttgcaaatcttcatccattcaacaccgttttcaagttgggaaataaaaaaagagcccacagggccaggtctggagggtacagagga

tgaggcagcacagtcttttgtgcaatagttacacatcaacaggaatgaatgtttggatgcattgttgtgatacaagagccatgaattgtctcgtgacatt

caggtcatttccttctcatattttctcacaggcgttgcaacacatcccgacagtgccatggataaacactttctccctgtggcatgaattcatgatgaac

taatccttcaaagttaaagaaaggtatcagcatgggatgaacattttatctcacctgatgagctttttttggtcttggagaatcttccccagcccattgt

gaagactgaaccttggtctcaacttcataaccatagatccacatctaatcaccagttatgactcttatggaacgtctcattctcatttgtgtgatccaaa

agctcttcatagattgcacagtgaaggtctttctggtcttgactgatgagccgtgggatgaacttgatggcaacacaatggattccaagacactgtgtca

ggattttatgacgtgatccaactgaagtgccatatccttctgcaatctctcgtacagtcactctttgagtggcctgcacaattttgttgatgttcctgac

atgaacgttgtcagtagatgtcgaagggcacactgaacgagggtcttctttaacttccatccagccatttttaaaccatgtgaaccatttgtaacactga

gtatggcttaagcactcaccactgtaaacttcatccattatttggtgagtctctgtaaaggttttcttgagtttcacacacacacacaaaaaaaaaaaaa

aaaaaaa

>Contig1512|vacuolar protein sorting-associated protein 8 homolog

ctgtcgtatccannnagctttggcatttgacctgacgaacttttattggcccctgagaatctttcctgacccattgtaaagattgaaccttggtccaaca

tcataatctcagacccacatatcattgccagttgtgattttcttaaggaacacctcattctcatttgtacgatccaacagcctttcacagattgtgaagc

gaatgtcttccagctgttgactcgtgagccacgggatgaatgtggcggcaatacgatgcattctaagatgctgtgtcaagatttcacgatatgatccaat

agaaatgttacattcttctccattctctcggacaccgacggtgtcagtggacgttgaagggtgtcccgaatgagggtcgtgtttaacttccgatcgacaa

ttttttaaatggcgtgaaccgttcgtaacgtaaagtacaggttaagcactaattactgtaggcgtcctgcatcatttggtgtgtctctataaagtctcga

gctgtaattagttttggaaaaacattattcaagttttactgtctcaataaatgtttgtttttaagcgtaaagacctgtcattttaaagtcacatgtgaag

gcgtacagacattgacttttcttagtttattcacgagcgcatcagcaacgagaatttgggagaggggatgacaaatgaacatatggaataccagataatg

gcttgcagagtattcatgtttataaaaaggttctaaaaactgaaaaacataattaacgttgaacatatta

>Singlet738|vacuolar protein sorting-associated protein 8 homolog

tgggccagtatttgcgctagtgtgctaggtaacgcttcaaacctctcctcagcgtctcatgaagtgacggcacactgtccagcagattgggacgttgaat

ttggcgggtcacctcgtgaaaggcgtaggttgtgcactggaaaggggttcaccctctcccttccctcgtcaccatcatacaacagaaacatgacacagtg

agcaccaatccacaatcacacatgcagaacaacttgcgaggtctattcaaaaaattccggatcgtccgtaattttgcgcgaattatgtgttggagcgaaa

tgcggttggcatccctgcacacgtcggtgttttatgtgtaactgtctgaagtttcactgttgtgtagttatagttcagtgctgtattgagtagatcgttg

tatcgcactgtttgcgaattccgagatggcagattctgaagagcagcgcgtctgcattaaattttgcgtgaaactcgcgaaaacctttacagagacacac

aaaagggtgcgggaagcctacggtgatgagtgcttaagctgtactcactgttaataatgggttctcacgaattaaaaatagccggacggaagt

>Singlet1743|vacuolar protein sorting-associated protein 8 homolog

ttcgtcagaagcaacgtgctgtcataggattcctgtcctgtgaaaatgagacagtggaaaacatccacaagaggctgaaaaaggtgtatggagatgctac

tgtcgatcgcagtacagttagtctgtgggcaagcaggttatgtaatgtaagcgggcacggcaatattgaggaatgtcctcgcagcggcagacctcgttct

gcacacactccagacaatctgcagagagttaacgaaatggtgactgctgacagaagcatcacagtgaacgaattgtcactctacgttgggataggggaag

gagctgtttgcagaatactgaaagtgttggcgttagaaaagtttgtgccacgtgggttcccacccaggatgttgacag

>Singlet4115|vacuolar protein sorting-associated protein 8 homolog

gcagaacggcgagaagctcagtacattaagaacaaaattcattgtggcaatgacgcatacttgcggaaattctatttgaccaggaacggagtataatttt

ttaggcgtcgtgtgaatagtgaccaaaatttaacgcgcaaaatttttcagcaaagtctgtattacgtcaatgacacacgaaatttacatattacagtgcg

ttttttcatctctcctgtaaagcaattaaaatggagtcacgttctgatgcccgcacagcgatatgaagatttactggaccaaatacgaggtggaatccaa

aattttcgggactggtactgccatctggaaagcaggagtagtagatctttgcaccgctaggaggcgagagctgcatatctcatgagtcagtgtgcggagt

ggcactcagctgggaggacgtgttgcgtgtccacagtgatttccgtaatactctgtttggcgtgtggcgattttacgatggatccgcgaacagaacagtg

cctctgtagcaaattctgtgcgaatctcgggtaaagtgctacggagccccttgcaatgtttcaacaagtgtttatgggacagagcatgagccgtaagcgt

gtgtttgagt

>Singlet5734|vacuolar protein sorting-associated protein 8 homolog

ggccgggctcttctatgcggccgtcggcnnnntgtctcgccgcttctttagagcacttttctgtagttctcgcggtaagtagcactccgtttactacaat

ggtttctaagttagcaaagttgggtagtacgtggtgtgtgagaaaagcaatgagactgattttttatctaccaaagattttgtttcttcaaacaacaata

ttgtccctccaaagtagtttccttcggcagctacgtaatatgtcggtcacattcttttcctgagtgcaaaagtgacgtccttttaagacacttttcaatt

tcgggaaaagaaaaaggtctcaatgatacagatcaggtgaatagtggagctgtggacaaacaggaatggcttttgaggtgaaaaattccgtgatggaaat

agccatttgacaagggtttttgggcaccattttggcatgtctcatgtgcaaatctttgcttaaaatttgatgtgcgctataagtttttaacgggtcaccc

atcatccttgttaaatgtcgctctgatctcccaagagcacgcacacgttcgacattttcgacggcttttgaagttgaaggtccacctgagagaggttcat

attcaacgtgttctcgacccctcaaatatgat

>Singlet6450|vacuolar protein sorting-associated protein 8 homolog

aaaaactttcgcttcacactcaaagtgaaacatggaaattttaatgtaacggactgagaagcaataactgaagtatgggatatacaagtttagtgtatca

attgaaccaactttaattacgtgcataaatagtcaatcttgcacctctattttttcactaatgtttattagtgtttattacaataaccgtaagcatatat

gatgccactatttctcatttattaaaaggtacattcatttggtatgtaaactttgtcacatttaattatttttgtaactacaagtaaaagtaggaaccct

atgttaatatgtacaatatacagttatttcaccaatgttagtaatggaactgaaaaactactataggtttaattaaaacctaattaactctgcttgccag

gaaatgtaaatgttataaaagtgaaactaaaattatgcatatttcttatttttgagcagaaaatctgtatttacgttttgcaaagtatgccgtggtgtgt

gccaatgtcaatataattattttttgtatttgtaattaaaattctatta

>Singlet6842|vacuolar protein sorting-associated protein 8 homolog

tcccttgatgccaaagttcgggccttcaccgctgtacgttttcacggagccgtcgcaaaacgtcacagtagtacgtggaattcactgtttgtttgtgtgg

gatgaattctttgtgcacaattccctcgatatcaaagaaaactatgatcgtgctcttcactttactcctcatctgtctcgcttttttgggtctcggagag

cccaggctcttgcactgggacgattgttgatttgtctctcgggtcataaccgcgaatccagctctcgtcgctgttgataacccgtggcaagatggtttga

tcatcaaatgcagtctgacgaagttccgtgcgcaattcgacatgctgtgccttctggtcggcagtcgagatctttggcacaaattttgtggggacacaat

acgtgcccaattcatcagtaaacattcgttgacatgtcccgtaaccaatacccactacatacgcacggtatggaatggttcgacgtaaatccgcacgaac

cgattattgaagtttgacaacaatgtctggcgctgtgcagctaacgggccttccagtgttagcatcatctttgacttctgtacggctggnnctgaactga

gcatgccactcnnacacactcgtatggctcgtgctctgtcccccaaacacttgttgaatcccccaaacacttgtcgaatcattgcaag

>Singlet6859|vacuolar protein sorting-associated protein 8 homolog

gcaaacaataaaagtattctacatcatcttttttaaatttggtaaactgagataacctcagctcatgaggtcacttattaaatatgaggtctgttcaaaa

aagttttgtgaattttgagatgacaaggttaaaggagcaatgcatctgcattacattttgagtgaatcttaagaaaacctttacagagacacaccaaatg

attgaggaaccctatggtgatgagtgattaagctgaacttggttttatgaatgattcacatggtttaaagatgtccagacagaagttaaaggtgaccctt

tttcagattgcgcttcaacatctactgatgacaatcatctcagaaactttaataaatctgcgcatgccaattccagactgactgtctgagagattaaaga

agactgtaacatttcagttagaccatgccatgaaatcctgacacaacatcttggagtgcatggtattgttgccaagtctatcccgtggctcacaaatcaa

gaccagacagaccttcacctcacaatttgtgaagagcttttggatcatgcaaatcagaacaaggttttccttaagagaatcataactggtgttatgttgt

tgagaccaaggttcaatcttcacaatgggttgagaaaggttctccaagaccaaaaaaggttgtcagatcaggccaaaactcaaagccatgctgataattt

tctttgactttgaaggattagttcaccataaattcatcccaca

>Singlet8247|vacuolar protein sorting-associated protein 8 homolog

ccgggaagatgtgttgcgtacccacnnttatttccgtaatactctgtgtttggtgtgtcaattttaagatgaattcatgaacagaacagcacatatgtat

caaattctgtgcgaaaaaccgggaaaagtgctacggggactcttgcaatggttcaacaagtgtttgggggacagagtatgaaccataagcgtgtgtttga

gtggcatgcttagtttggggccggccgtacagatgttgaagatgatgctcacactggaaggcctgttagccacacaatgccagacattgtcgcaaaactc

caacaattggttcgcacggatcaacgttgaaccattcaagaccttgcagatgaagtgggtactggttatgggaaatgtcaacgaatgttgactgatgaag

taggcacgcatcgtgtcaccacaaaatttgtgccaaggatcttgattgcctatcagaaggcactgcgtgttgaactatgcatcgatcttcgtcagaccgc

atctgatgatccaaccttcttgtcatgagttaacaccggcgacaagagctggatatacagttatgacccagagacaaagcaacatggaagagcccggtct

ctccaggacccaaaaaagcgagacaggtg

>Singlet6481|vacuolar protein sorting-associated protein 54

gaacaccatcgtgtcagcaacaagctgcatgacctcatgacgtcagtgtgtgattatgctcatgaaagggtggctcaacttgttcaggcacaagacaggg

tcttggaacgacaatcatcctgttggctggcagataaagcaacagctagtgaagtgtgtgaactttctcgggtgatagacaagtttgctgtagcctgcga

gaagatctgcggccgttccagcacagctttacgatcagcatttaaaatacaggctagcaaatttattcaacgttttcatcaagatcgtaaaacaaagctt

agcttaattttggataatgaacgatggaaacaagctgatgtaccagcagaatttcaagatcttgttgaccagattgctacatatggacgtttctcaccac

ctaaaaaagatgatggagatggcagtgtcaggaaaccagaagcttttcttatttatggggaacagaagtttgctgtcgttgggacagcattgctgctaat

aaagatggtggcagagtactgtgagtgtgcagaggaactgcctgtaacagtagctctcatgtgtcgcaatcttgctgagctactgaagctgtttaactct

cggtgttgccaactggtactcggtgctggtgcgctgcactcagcgggcctgaagacaatcactagcaccaacttggctttggcgtctcgtgctctccaac

tgctgcttgcactcctgcctgctgtgcgtgtacactttgatgatttgctgcagcagcaacaacaacagcagcagcagctgacgcaacatgtcatgcnnnt

ccgtggagccgctactggcgtggggattctcgatacagt

>Contig1063|vacuolar protein sorting-associated protein 53 homolog

ctgtcggcagnnnnnngtgatgctttttctggtcctaatgccaaacattttgcacctactaagcaactagcagaagcttgtcgagttgtgtctgttctgg

atccaaaagtgaagcgagacctcttgaaatggttcattggtttgcaactggccgagtattgtcatctctttcaggaaaaccaagatactgcttggttaga

caaaatagacagacggtatgcgtggctaaagagacatttactcgattttgaagacaagtttggatcaatgtttcctccagaatgggaagtatcagagcgc

atcacagtagaattctgcaatattaccagagatgagctttcaaagttgatgcataaacgcaaaacagaaattgatgtgaagctgcttttatttgtaatac

aaaaaacagcaaattttgaaagtcttcttgcaagaaggtttactggtatcacattagaggagactgataggccaactgagagaagattgtcggaggcagc

tccttcaacaaacccatttgatgaaccttctgctccaggtaacccgtttgaggaagagacagaagacacaaaatccagagacagtgcaccagttaagcca

aaactttcacctttccagggcatcattggacgctgttttgaaccatatctgtacatttatattgaaagtatagacaggaatttggctgaacttatcgaga

ggttcattcaagaaacaaaggagcaacagtctgatgggataaatgatggcactggaaatgctgtgatgtcatcatgtgctgatttgtttgtcttctacaa

aaagtgcatggtacagtgcacacaactcagtactggagaggccatgttaggactcacagccacttttcagaagtatctacgagaatatgcagtaaagctg

ttacagaacaaccttccaaagctagctgctccatcatcaggaatgtcaaacttgacgagggacctgagagacctatcatcagctgggctgtctactgcag

gacttattcaaaacttccaaagccttctgaaagaaggagaagtaacgagatacaccccggaggaacaggcaagggtctgcagtatacttacaacagcaga

atattgcctggagacaactcagcaattagaagagaagctcaaagaaaaagttgaccctggattggctgagaagattaacctgtcccaggaacaggatgta

ttccacaatgtgatctccaactgcatccaactcctggtacaggacttggaatctgcttgtgaaccngcactgactgccatgagcaagatatcgtg

>Contig194|vacuolar protein sorting-associated protein 52 homolog

gtttgagtacatattccggttgaatatcaacagtgttcaggactgtgacccaatgaaattcagtgccaaagaaatgggtccacattatataacaaggcga

tatgctgaattcagtgcagcaattgttggcttgagtgagacctatccaagtgaacttgtaaaccggctgctagcagaattgcaggagcaagtagaatgtt

ttattttacgaatggcagcaatttttcctcaacggaaagaacagttaatttttcttataaacaactatgacatggttcttcaagttctgatggagagaac

aaaggataattctaaggaagcagaaacctttcgagaacagctaaataccagaagtgctgaatacgtggaagaaatactgtcaccacattttggaggaata

attcaatttgtgaaagaaggagaagtgcttctagaaaagggacaagcagaggatttgaagaaacaagaaagtaaatctctcgtcctggttcaatcatttt

ccaacaactggaaacggtcactggaggaattaagccaggaagtgttgacatccttcccaagcttcatcacaggatcctcattgctgcagctggcacttac

acagctagtacaatactaccatcgtttccacaagcttcttacacctaacgctcgttctcagttgactaacatccatcacattatggttgagattaaaaaa

tacaaaacaaatttttgaattccttgtataataaatcactatttatttagaattaaatgttcatttcaaaaaaaaaaaaaaaaaa

>Singlet6833|vacuolar protein sorting-associated protein 52 homolog

caaannnatgtcattaccagggttagtgtaaaagttgagtccaacatttatcgtcagttcttgtggggttacgaagaagtggagtgtaagatctttcaac

aatgaaggacatattttctgtttttattttctgttccgtgtgctcacgtagatagctgtcaaatgaaatgaatgaagtatattgagaaataggcctattt

gacaatagtttgatcaagatggttgaagctggactgatgtttgaagataatttggatgaccatcttgaacaaaatctccaggcagatgttgttcaagaag

tgctcaaaactggaactgacctccggcagtattcaaaacagattgaaaggaagttgaaagacgctgaaaacaaatcaattcaggattatataaaagagag

tcagaacattgctagtcttcataatcaaatatcagcatgtgataatatattagagcgcatggaatcaatgctgttgagcttccagtcagatttgggaagc

atcagcagtgagatattgttcctacagaggaaatcagttgcaatgagccagcagcttcataatcgtcaggctgtgagggcaccactaagtcagttcatag

atgagatggctgtttctgaaacattaataaaaggaatccttgatagtcctgtaacagaagaggattttctaacccagttaactgtactaaatcacaaaat

aaattttgtgaaggagcaaagtataaaagatgtgaaatcatgtcaagatgtgaaggaagtactcgaaaaattaaaaattaaggcagtaacaaaaatacgg

gcatatctgtt

>Singlet1293|vacuolar protein sorting-associated protein 41 homolog

ggnnngtgtttacttcgtagtggacagtagatctacgaccaaaacaatggaagcggataatggcgacatgcctagcgatgatgacatgaatagttcatca

tcagatgaaattgaaccaagactgaagtatgtgaggatgactaatgatgtactgaaaatattaaataaggatgctgcaagttgcatggctgttcatccaa

aatttgtatgccttggcacccattggggaaccgttcatcttctggaccatcaaggcaacaatattaaaagtaaagccttgcgttcacatacagtatcagt

gaaccagataagcattgatcaaaatggggattttgttgcaagctgttcagatgatggcaggatttttattagtgggctctgctcaagtgagcataatcaa

gaggtggtgattggaagactggtcagatgtgtagcaattgatccatattattcaaaaccaggttcaggacgaaggtttattactggtgatgaacgattag

tatttcatgaaaaaacattcctgtctcgtatcaggacaacagtcttgtatgaagctgaaggaacagtgcaaaatataaagtggaatggccaatttgttgc

ctgggctagtaatataggggtcagagtgtatgacatgtatggacaatgttcacttggcttgattaagtggacaaggaacccagaagcactgcccgaatgc

tatcgctgtaatctgtgttggaagactgcaactacacttctggttggatgggttgacacagtgcgtgtatgtatcatcaggaaaagaactcctatagaaa

tggttgca

>Contig1609|vacuolar protein sorting-associated protein 41 homolog

tgaatgttgggagagcgtatcttgatcacctgctgtcagttcaaaggtatcaagaagcagcagagctttgtgttaaaatactcgggagagacaagaggct

ctgggaagaagaagtgtttaaatttgttcggctgcatcaactaagagctgtgagtccatacttgccaaggggagaaccttctctggaccctcacatatat

gaaatggttttgtatgagtacttgaaaatggacccaaagggctttctgaaaacagtgaaggaatggtcaccaacactttacaatgttcctgctgttgtga

atgctgtcctggaacatctgctggtaaatgatacagataagacactactgttggaagcattggccatcttatattcccacgagcacaaacacgataaggc

tctagctatgtaccttaagctgcatcataaggatgccttccagttgatccaggaacacaatctgtacagttcaatctatgacacaatagaagaattgatg

gatctagattctgaacaggctattaatatgtttttggagaaagatagagttcctgttgatgttgttgtaaaggccttggaaaacaaccatcgttatctgt

atttgtatttagatgctctcgataaaagagatacaaaagaagtaagtcggaagtaccatggcttgcttgtcagattatatgcagattttgctagagagaa

acttcttcctttccttcgacgtagtgaccagtaccctattgtggaatctcttcaggtttgtgaagaaaggtccttctatccagagctcgttttcatccta

ggacgaattggaagacctaaacaggctcttaacataattatgcaacagctgaatgatatagatcgtgcaattaatttctgtaaagaacacaatgacaatg

aactatgggaagatttgatccagtattctcttgaaaaaccagaattcataacattcctcctacagaaaattggaacttacattgatccaagaaaactggt

ac

>Singlet5246|vacuolar protein sorting-associated protein 37b-like

cagctttttttatgcaacttgaagtcagtaccttcaaatatgtcaatattttttatatttattactgattaggtttgtttcattatattcagtatcagtt

tgattggatttagttcatgatgtacataagattgtctcaattatcctcaacaactgttccttgatgttcccttctataacttgcacaaaaaattttaaaa

agttatttttcttcacaactttttctgtgtttcaatgtcctcactgtaaaagtgcaaaaggcagacataactagttgcatacttggtattgtattttatt

ttcataattgaaaatgacaaatccaaaaaatgtcgaaagctgcccaatttctgtgaaatagctacagttgctggtatggtttccgcacagaaaaaaatac

acaagattgttggcattccaaattggtctttacaagaccagatgcagcagattgtaaggaatcagataaagaagtagtgatttctgttcaccttgtacgc

cactagtatacttctgcccttcaaaataaactgttctgtaattttgcgttcactctttgcaaaatttccaaaatctgtgtcatatattgcacaactgaac

atatttcctccccttattcttcttcttcttcttcttcttcttcttcttcttcttcttcttcttcttcttcttatttttaagcaatgtgatagcaagcaag

actacaaaaacacttcatgctagaaaatcaaactaagttacaccataaatgacgaatatgatgctaattataatattcccatttatgacttattgac

>Contig956|vacuolar protein sorting-associated protein 33b-like

caaatatgaaaacaaagtatgagaatgtcaatgcgttacagctttgatgttttttacgtgcaacaatgcttaggtccttcgaaagaatcctataataatg

ctgacgtgttgagaatcgaatgctatgtaagcttttagtgccgaaggttaccatggattcccggttgcagtgtttgaatcaaatctcccaaaggaaactc

actgaaattttggttaaaatacctgggaaaaaagacctgataatcgacccacgtttgatgaaacctcttgagcaaattacaggagtctcagtattgaggt

ctcatggagtacacaaaattcataagctggaaaaaagtggtttccgatatgtcagtaatcattgggtttatctcgtatattctgaccttataactacaaa

gtatatttgtgatcagataaatgcagatttacagtgcaaacccacaaattcttattacattatatttgttccatgtgaacttgttgctctcttgaatctt

ttggaagaggaaggtgtgtttggtctagtgactgtatacaggtttatgtgggaaatgatttctctagatagtagtgtactgtcatttgaattaccaaatt

tgtttcgtatgctgtttgtggatggagataaatcatttcttcctgcagtggcccactctctttggtcactgcagatactctttgggaagatacccatgac

tgtaatgtgtggaaaatttgcatcacaagtgtgtaaaatattgaatattctattgaaggagttggggaatcccaccaaacaagaggcagatattgactgc

ttagttatagtagatagggattttgattatgcatctgtgttgcttacagcagttacatacgctgggctactggatgaagtttttgg

>Contig1009|vacuolar protein sorting-associated protein 33a-like

ctgtcgctttgatcggcgtagaggtgaacgttggaaacttggagcgtatggtgaactgtcatgtgattgtaaacaatgtgaaaaaaatggtatatcacgt

tatcctcatgaaaggttacccgcttagaccttacgtgtgttaggagacagaagttagtttacgtagtaactcaaagcttaatgtctaccatggcttcaca

tttggccgggggtagggtgaatgttgcccaggttcaagaattagcccgaaaacagttggtccaattattggaaaagtgtcagggacgaaaggtaatagta

tgggatgaatccttagcaggtcctgtaggacttatagcgacatacagtttactaaaagaacacgatgttcctgaaatgttcccattacgagctggtaagc

tgccttcaactacacaaaaggacattaagaatatcatctttataacaagaccacatctacatttgatggacaaagttgcagacaatgtttatggtgaaga

acatagcggtggtgacagaaaggaatttcacttgttttttgttcctcagaaaagcttactatgtgagaaatggttaaaaaatcgtggtgtttttggcaat

ttcacttggattgaagaatttccatgcgacctcttcccttttgacaacgatttgatgtctatggagttggagtctgcattcaaggagtaccaagttgaac

atgaccctacagtactgtaccgttgtgcgagggcactgatgacactgcagcaactctttggccctataccacgtgtttcagccaagggtacagcagcttg

ccaggtgtgggacacacttctaaggttacgtcgagaacagatgccatctgcttcacgactgaggcggcaacagcaacatgcatcttcacagattgatcat

ttgctgctcatcgaccgttcaatagatctgctttctccacttgcgacacaacttacatatgagggattgattgatgagatatttgggataaccaactgca

ctgtgcaactgccagctgaaaaatttgctaagcctgatgatggtcccggacttgttggagaaaagaaaaatattattcttaattctgctgaagaattgtt

tgcagaaatcagggataaaaatttcaatgctgtgggtccagcactgagtcgtnnngccaaacaaatctcatctcaattggaagagtgccaaagtgaccgt

tctgtgc

>Contig870|vacuolar protein sorting-associated protein 29-like

gggaggcaaagattatacaagccatctgtggcggtagaaattactgattacttgtcaaggggcgtaatatggaagtggaaatattttccgtgtagtgtgt

tgtgtgtattaatgctgtcacgataattcctggttacataatcaacaatgcttgtgcttgttctcggagatttgcacattcctcacagatgtagcagcct

gccatcaaagtttaaaaaacttctggtacctggcagaatacagcatatactttgcaccggaaacttgtgcacgaaggaatcatatgactacttaaaaacc

ctcgcaagtgatgtgcatgttgtcagaggggattttgatgagaatttgaactacccagagcaaaaggttgtcacagttggccagttccgtattggattgt

cccacggccaccaagttgtaccatggggagatccagaatccttagcactaatacaacgacagttagatgtggacattcttatatcggggcatactcataa

gtttgaagcatatgaacatgaaaacaagttttatattaatccaggttctgcaacaggagcttataaccctcttgacacatcaattattccttccttcgtt

cttatggacatacagagttcaacagtggttacatatgtgtaccaacttgttggagatgaagttaaggtagagagaattgaatacaagaagagctagagac

aatagtttgtgcaaatagagaactacattctgaaaagaattgtttctttggaaacaatttacttgttcgagtctgtggtcgctgcacctataaggaagac

attttatacctgtcaatattcttgtccagatattaaagctgtatactttatatgaaagtatgtaacaaggagtatggtaacaactgataactcttgtgtg

ttacatgtctgtgactgatacttcattccggatgttcactacaagcatttgtcaaggaagacttgggcattcagtttggaataagttctgaaccagttaa

tctggaagaggactgtaacagtagcatggattaaattatgtaaatacaataaattctgaacatttgtgcatttattgctggagtgatgacagtgtaatgg

atgtttcagatatttttgacataagttaatgaggacccacacagttactatcaagaacagatacagtctataattgtttcaaactccagtccaggttaaa

catacatgtatttataagtaaaaaaactagaataagtcattttcagtatttattactgattttgggtctctagttttaac

>Contig699|vacuolar protein sorting-associated protein 28 homolog

ggggacggatgtaaacgtcaagtgtaactatgacagtgttatgattcgaatccttggaagtgtggataattatttgaaattgtgtaatcttttaaatgcg

ttagaatgtctgtgatgtcgggaatgccagcatctccagattctggaagaccagagctgtatgaagaagttaagctgttccgaaatgcaagagagagaga

gaagtatgataatatggctgacttgtatgccgtggtaaatactttgcaacatctggaaaaagcctatatacgtgactgtgtaacgccaaaagaatacaca

gcagcttgttcaaagttgcttgttcagtataaagcagcttttaagcaagtgcaaggggatgagtttcctactattgaatcatttgtacggaaatatcgtc

ttgattgtcctgctgctttggaaaggattaaggaagatcggccaatcacaatcaaggatgacaagggcaacacaagcaagtgcatagctgatattgtttc

gctctttatcacgatcatggacaaactaagattagaaataaaggccatggatgagcttcatccagacctccgtgatctgatggatacaatgaatcgctta

agtctattgggatcagattttgagggcaaacaaaaagtggcaagttggcttacaactttgtcgagtatgcaagcatcggatgaactgacagatactcagg

tgcggcaacttttgtttgacttggaatcctcttacacagcctttaacagaattctacatcagtcttagtgggctgtaattcacttgtacatagagcgcag

>Contig4481|vacuolar protein sorting-associated protein 26-like

gggtgttgtggctgatgtagttatgtacttgcaaagttttacgaagcggtagttgtctcaattcccaagcttttcatttcccgcttactgaattcaacgc

aggaagcacaatgagcttctttggatttgggcagagtgctgagattgacattgttttagatggcacagagaaccgtaaaatggcggatatcaagtcggag

gatgggaaaaaagagagacattatcttttttatgatggagagactgtatctgggaaggtgaatgtaaccctgaaaaagtatggctcaaaactggaacacc

aaggaataaaaatagaattcattggtcagattgaactctattatgacagaggcaatcatcatgaattcacatctctagtcaaagaactagctcgtcctgg

tgaacttatgcaaaatatgtcctatgcttttgagttccttaatgttgaaaagccttttgagtcgtacactggttcaaatgtacgacttcggtattttttg

agagttactattgtgagaaggctttcagacataattaaggaaatggatattgttgttcacaccttatcttgctacccagaaatgaacaacagcataaaaa

tggaagttggcatagaagactgcttacatatagaatttgagtacaataaatcaaagtatcatttaaaagacgttatagtgggaaagatatactttttgct

tgtgcgcataaaaatcaaacatatggagattgcaataatcaaaagggaaaccacaggatctggaccaaacacattcacggaaaatgaaactatagcaaaa

tatgaaataatggatggagctccagtcagaggagagagcattccaataagagtatttcttgccggctatgatcttactcctacaatgagagatattaaca

aaaaattttcagtgcggtattatttgaatctcgtgcttatggatgaagaagatcgccgttatttcaagcaacaggaaataacgttatggcgaaagggtga

aaaaactcgaaaaaatcttcaaacatcctcaaggacatcaccccaactgcctcacgcaattccaggaactcaggaacacacctccaaccaaccaattgaa

gatatgaaacgagaagatgaagacaagggtacagtgtcaggtgctgagaatgatttgagaaggaaagtggctgttgatggtgacaaggctgatgactgac

tgcacaaaagccagattgtgtaaagtactatgcagatatgttcacatgctcccattcctgatgcacatggtcatgtgaaatatgtaattgtgcttaagaa

cttcgagtatttactactgggagtgctgttctcattactcagtgactctgtacatagcacacatttgttgtaactatattcagtgtgcagtatgtatcaa

>Contig3761|vacuolar protein sorting-associated protein 16 homolog

acttcncatagtatggatatgattctggagtgtacttagtgcctgagatggatggtgttcgtgtgctctctctgttctctcatgaaatgattcagaaagt

gccaaatgtggtgcaagaaatttttcgaatcaatagtaatgatcctggatcttatttatttgaagcttcgaagcagttccagaaaggaagtcatcgagca

gatgaatatataagaattgttaaagacaagctagaggaagcagttaaaaattgtattgaggctgcaggctatgaatttgacacggacacacagaaaatgc

tgattcgggcagcacaatttggcaaaggctttgttcccagtatgaactcggattcatatgtgaggatgtgtcgactgctacgtgttcttaatgctgtgcg

aaatcgacacgttggaataccattgacgattacgcaattggagcatttgactacacaggtattgctggacagattggtgctgagacaccactactgcctg

gccatccacatcgccaaatatttgcgtctgccagagacagagggatcaagtcgtattcttgcgcattgggcctgctataaagtaaagcagacacagctgg

atcgtgaacaggtggctcgtgaaatcgcagagaagttgggggatacgcctggtgtatcatacagtgatattgctattaaggctgcagactgtggtcgtac

acaacttgcaataaagttaatggattatgagcctcgtgcaaatcttcaagttccgctcctgttgaggttgggtgaagtcaaaccagcattagtaaaagcc

atagaaagtggtgatacagaccttgtgttcactgtgctgctccatctccgtgagaatatgccttt

>Contig2202|vacuolar protein sorting-associated protein 13d

aaaccctnnaagccttgtgaacgatgtgtctgaggggatgtctgctcttatttttgagggcaatgttactgcacttctgaggaatgttacacatggcctt

tccaattcagctgctaaagttactggatctctgtctgatggcctaggtagagtaattctggatgacaaacatgaagagaccagacagcggatacgcaaag

tcagaaacagcagtagtggtgaacatttggtagccggactcaaaggcctaggttttggcctacttggaggtgttaccagtgttttcaagcagacatatga

aggagctgcaaatgatggcatgcagggcttcatttctggacttggtaaaggacttgtaggaacagtaactaaacctgttgttggagttttggatctagct

tctgaagctgcaagtgctgtaagagattccagccgaagctcaatacgaataaatcgccaaaaagtccgtgaaactcgttgtgtcataggtacgagtggtc

tgttgccagcatatgactcaaaacaaagcacaggattgcgatatctttatgatatcaatgatagaaattattcagaactgtttattgcatatgagtgtct

acgaagtggatcagaagatcttcgtatcttggtgtccagtgagataatacgtgtatttacttgtggaccagctgcatccagtcacaatatagttctggaa

ataaatttaggtgacttgtttttctgcaaagcagtgcaagaaatgtcactaagcagtggcagccccatgtattatatcgaactgacagtcagagctgaag

ctgccagtgaaaggcttgtggtgctgcaggaagcagtcaagaagccacgtgttcgctgtgatgatgaaattacagctaaacaggtatcacatcacataaa

ttatgcaaagggattatttgaagaacggcggaacacacttgtatctctagnnnatgatattttaaaagactgaatgtatagtaca

>Singlet6188|vacuolar protein sorting-associated protein 13c-like

gtctgaatcaggtagtttcttcacacactttggttaaacattgtaagcatctggttcacaatgtttaaccaaagtgtctgaatcaggtagtttcttcaca

caccaaagtttaatattttggtactgtttgttttgttactgagagtatgtatgtagtcatatttttgagagtgcttagctttttaaaacaatgtttgcac

aagtcacccggaaaaaagactagaatatatctttattttttaagtgttctttacactttttgatactgagcctttttacagcattctagaaatctgtagt

acttaatttatagcatccactctcatgtagtcgtcctaagtttaattgcactgtctatgtggaggaatgtgtgtcaacgtttgtttactttttggatgac

agggctttctgtggaacattgcataaagaacacaaattaaatgcttttgttgtattcagtatggttgcaatgaaggatattaaagaacagttcaagaaca

aaaaaaaaaaaaaaaaatgc

>Singlet6502|vacuolar protein sorting-associated protein 13a

gaaaatgataaaacccattgcacaagaagttagcgatagctaactgagcaagagatgggacttcagaagctgtgatcacaacaaacatccgccatgttcg

agggagtggtggcgacagttctcaaccgttatctcggcaaatacattctagatctcgacatcgaaagcctaaatattggaattttcagcggaaatgtaca

acttgtcgatctcagaatcaagcctgaagctctgtacgagcttgacctgccaatcgaggtgaaagtcggcgtcattggaaaaataaacttaagcattcca

tggggtggcctttacacacagtccgtagtagctactattgaggatgtttatatcatcgctggacctgtacttgacagagagtatgatccagaaaaagaga

aacggcttatgcgagcagcaaaacgaaagaaactggaagaccttgaagcggaaagtttattagggtcaggtccagaggattctaagactttcactgaaag

tcttctcaccacgataataaacaacttgcagatcttttttcgaaatatacatattcgctatgaagacactatcacaaatcgagatcacccatttttttgt

ggtttctgtgttcagaatgtctccctcgaaacaaccaacagcaagtggaaaccggcaatcactgctcagaacgctacatcgttctaccagcttattagac

tggaatctttctcggtctattggaatcctaaaactacggcgagcggacttgtgtcacaaatgctaatgcaggatattgcccaatacaactggag

>Singlet8332|vacuolar protein sorting-associated protein 11 homolog

aggagaatgtaaacactttgttgttgtgggttgaaattcgtgtttgacagatgtcataaatgaagttatttcgtgttctgagaggcttacgtggctaatt

aatgtgtgagatgtgtaaccataaataagaaatactaaataagtaaattaatacaagatggcattcctagagtggagacgattcaacttttttgagttga

ttcaagatgttgacaatgggaaaatagctgagacactaaaggacagaaaaattactgcatctactagtggccatgggcatcttgtatttggagactctga

aggaaatattcatttagtaagccgcttgtttcatgtgacttctttccgagcacatgaactgactgttaattttgcagagcagctacgacattctgctata

cttgttacagttgggagcgatgagcctggaattaatccagtgatcaaagtatggaatttggacaaaacagacaagcagggccatccaacatgtgtgcgaa

tttctcgtgcaataccaaacaacaagccagaaaatgttactgcactatgtgttcatgaaaatttgaatttgatggctgttggcttcaatgatggatctgt

aatactttacagaggtgacataacaagagagcgtacaaataaaacaaaagtactgaaggatggaaatgcaacagtaacaggcttggcttttcgaactaca

tcaaaattaagtcatctgtttgttgttacaacgaattcctta

>Contig1653|vacuolar protein sorting-associated

ctgtcggtgnnnnnnaggcttccatatgcaagtgttggtggcataacctgttgctgagatataatctcccagataataattagggaatctcaatgatgta

gagtttgttagaagtatattcatggcataatacctgccagaagttacacactttgataatctactttatgttacctgacttattcacttataggtacata

acaatgcagtatcaagaatttatttaatatggaatgtaacgggtctggaagaatgtgtattaaaaactcaaacttgagtttagctctgctgtgacattaa

catgggaaggaatctatatcattcataagttaaaaggaaaaatatgatatgtgcttggatgtacctttaagctttgtgaaataactctcctgttgctata

ctgtgctggtgtattctgatatatcactactgatagccaaactattgaaatatttctactcattttgcattaaaaaacataatttattactcccttcata

cagaaacatggaattttcatcagaataaaatcaatgaaggagaagttcttgttgattctgtggttaaaatataccatatttcatttctcctctcaacttt

tttattctgtaacgtcagttctgtaacttcactaacgcaatcagaagtttaaatattatgattgatacagatccttagagaataaataccatcatttaat

tgactttcagtaatatattacacagtacacgtatggtgaaatagaaatatgtgtttaattagtggctcttgctgtgcaaatgttgtaagtgttgttgaaa

tttacatgaaaataatgttacagtaattacaacattaagaactactaatgtcatgatgtgatactcttcaataatgtatttttgtatacagatagatata

tactaaaatatatgaaccaagttcaaatacttcacaggaaactgatttgaatataaaactcatgcagcctaataattctgtttgatatagaaataggaag

caagaaaagtgaagttcagtaacaacatacaacattaatattggtgctcaagcattttaaaactctgctacacagagttcaaaaaggtgaaagtggaatt

tttgtgggcattaataggaattttaaaatgaagaagaatgaaataacattatttttgtattttcatcttaatcaagacattaaaatcctaataacaaaca

ttttcatacaaatattgattctgagtgttcgaccttgtgaagaaaggaagtaccagtataatgtatttggaggaaaccactatctgttgctggcattact

tgaaagagtttccatataaaactcagggaaaagacttgtgattacagtttctatgttatttgacactatgcttgactgaggaaattactctatttcacct

tctgtactgataaaaatcctattgctgtgtttttagttcatttactgataaatatatatttgagatacattttctgaaaaataatacaatcattttcgaa

atccaaaaatagtgaaaaccatttggtgtctgatacatatatcagatgagaattaaaggtcaagataaaacatatacatttcttattgtgctattcttct

cacttttgtaaactgataattatattcaaaatgaaatttcagccttagtgtttgagttgtgtcac

>Singlet4439|vacuolar protein sorting 52

aggggatctggatgccgaaatcagtctctcgaattatccagaacgtacttcaaaacagtcgtgaacaactgtgtcccggtgacacgatttggaacatgaa

gtccatgaatgggtgcaaatggtctggaggtaatcgaacgtaaccatgtaaacacagcccacacgagattgcacagtgtcttgttcacaacttagctcca

tggcttcgtggggtctgcaccacactcgaacactaccatcagctcttacaaactaaaattaggactcatctgacgaggccagggttttccagtggtctag

agtccaaccgatacggacacgagcctaggagaggcgctgcagcagatgtcatactgttagcagaggcactcgcatcggtcatcagttgccacagccatta

acgccacatttcgccgcgctgtcctaacggacacgttggtcgtacgacccacttcgatttctgccgttatttcacagagtattgattgtctgttagcgtt

gtcagtggtgagaagtaatgtctgtaatttggtgtactcgacacactcttgacactgtggctctcggaatactaaatttcctaacgatttccgggatgga

atttcccctgcgtatagctccaatgcgtccagagtctgttaattccagtcctgcagccataatcacgtcggaaacattttctcatgaatcacctttag

>Singlet4503|vacuolar protein sorting 52

cacaaacaatattccatttcagaaatcatttgggaattcagtagtctgagatccaaagtgtcaagagtgcacctagaataccaaatttcagcattacctc

tcaccgtggacaatgcagtggccaacggccttaacttaacaaccgagagcagaggtgtttgtgtgtagtagtcagtgctaacagacaagcaacactgcat

gaaataaccacagaaatcaatgtggaacatgcgacaaacatatccattaggacagtgcagtgaaatttggtgttaattacctatggcagcaggcaatcaa

tataagtgcctttgctgacaacatgacatgacctgcagcacctatcctgagcttgtgaccatatcagttggaccctagatgactgaaaaaccatggcctg

gtcagatgattcatggcttcagttggtaaaagctgacggcagggtttcagtgtggtgcagactacaggatcctatggacccacgttgtcaacaaggcact

gtgcaagctggtgtggctccagtgtagtgtgagctgtgtttacatggaatggtctcaatactctgatccagctgaactgatcattgactggaaatggtta

tgttcagctatttggagacaatttgcagccattcaaggacttcctgttcacaaacaagaatggagttgttactgggccacagttgttcacagttggtttg

aagaacatactggacaattcgactgaatgatttagccgtccagattgcctaatatgaatcccaccaaacattaatgggacaaaatcaacaggtcagttca

tgcataaaatcctgcacaggaaacacttt

>Singlet5127|vacuolar protein sorting 52

ctcgttttgaaatgctgaatggcgcgttctgaccacccctaacaaactccgtgccgtcaagaacacgacggctgtgtggcggtcatccatgcgagccaac

cgcagggactcagtcgttctttgtcggctccgcattggccacacccgactcacgcacagttatttactgtgtcgtgaggatccccctctttgtcgttgtg

gggtttccttgacggtggtccatgttctgtcggagtgcgcccttttaaccgtgctcaggcagacttttgcaatgcctgacacggtctctgctcttttatc

agatgactctgccatggttttgtgttttattcgggcagggggtttttatcctttaatctgagttttttagtgttgattctggcttttagcctctgatttt

aaacttgagtttttaaaatgttcttggtggttggcttttcctctttttctctacggtcggccaaccaccgtcacactgtgtgttttaatttgttttgtct

ggtctctgtcagagtatttcatgtcctgtttcgtctgctgtctttcctgttgttcgttttttattctctttgggtggttttagtttttatggaacaaggg

accgatgaccatagtagtctggttcctttaatcccacaaaccaaccaaccaacagacaggagcgcctgtgatggtgtactcaacgacgaacctgtgtgca

cgaatggcataacgtcatttttcgtatgcatccattttctgtttacagcatcatgatagtcgcatccacgtttggcgacatcgcgatgaacacacattgg

aagcgtttattcgtcatcgcgatactggcgtatcacatggcctgat

>Singlet527|vacuolar protein sorting 13d

caagaggcggaagctgggggttctagtgaccttttatcagagaaaactcagcgagaagtgtcccgtctgctgatgctacaatttactgtggaacaccttt

caattgaggtccagtccagaggccgtagcatagctgagctgcaggtctcaggagtgcgggcatcattcacgaagcgtccatttgataccagcatgtcgct

cagtgttcatagtctactgttagctgatgccttacagacatttggcccagactttgagctgcttgttgcaagtcataagcatgttggaatggatagtgta

agcggtagcctgcgggacagtgaacctacatctccaacttcgccaggttcacctgatcccacagctcctcgttcattcaaagcgacatcacccatcgcgc

taactcaggcattatctagcctccagttagaccctctagcttcattacgagctgcttcacctacctatggtgtaccatcatccccacctcatatcacttc

tccaccactacctgcaaggccgccatccatcactatggagcctctggatgcagaagcactcattactgttgaaattctgcttgttagtgccagttgccca

tccaatgaaggcagtggagaagcacttcaagttgcttctatccagtttaataacctggatataatagctaaccaagagaccatagttgaacttctgggat

tcatgcaaactatctttccccaatttaatggagggaatggaagagctcgaataggaattccagctccttctcctgaacccatagaggaaagtgaaggacc

aaactgggaaccaacacgaacagaggtgacctttgac

>Contig3919|vacuolar h

ggcacgtgaagggtcagggaaagcagacgggaatcatgtcgtacaacaaagttataagtcctaatcaggcgaataaagaacatgttttggcagtttcaag

agattttatatcccagcctcgcctcacttataaaactgtatctggtgtcaatggaccactggttatacttgacgaagtgaagtttccgaagtttgctgaa

atagtgcagttgaaactagcagatgggaccatcaggtcagggcaggttttggaagtcagtggatccaaagctgttgtgcaggtctttgaaggcacatcgg

gtatagatgccaaaaatacactatgtgaattcacaggagatattctgcgcacaccagtatcggaagatatgttgggaagagtattcaatgggagtgggaa

acccattgataaaggaccacctattctggctgaggattaccttgatattcagggtcagcctattaatccatggtcccgtatataccctgaagaaatgatc

cagacaggaatatctgccattgatgtgatgaattccattgcccgtgggcaaaagatcccaatcttctctgctgctggcttgccacacaatgaaattgctg

ctcagatctgtcgtcaagctgggcttgttaagctcccagggaagtcagtcctggacgatcatgaggacaactttgctattgtatttgcagctatgggtgt

taacatggagacagctcgtttcttcaaacaagacttcgaggaaaatggttctatggaaaatgtgtgtctgttcttgaacttggccaatgatccaacaatt

gaacgtatcatcacaccccggcttgcattgacagctgctgaatttctagcatatcagtgtgagaaacacgtattggttattctcactgatatgagttcat

atgctgaggctttgcgagaggtttcagctgctagggaggaagtaccggggcgaagaggtttccctggttacatgtacactgatcttgccaccatatacga

aagagctgggagagtggaaggtcgaagtggttccattacacagatacccattctaactatgcctaatgatgatattacacatcctatcccagatctgaca

ggatacatcacagaagggcaaatatatgtagatcgtcagttgcacaacagacaaatatatcctccagtcaatgtgttgccatcactcagtcgtctcatga

agtcggctattggcgaaaacatgactcgcaaggaccatgctgatgtatcgaaccaactgtatgcctgttatgcaattgggaaagatgtgcaagcgatgaa

ggctgttgttggtgaggaagctcttacccctgatgaccttttgtaccttgaattcctcacaaaatttgagaaaaatttcatatctcaaggtaactatgaa

aatcgtactgtatttgagtcattggatattggttggcaactgctacgaattttcccgaaggagatgttgaagaggatcccagcagcaatacttgcggaat

tctatccacgtgattcacgacacccacaaactaaatagatataaggtgtggaattcatcttgtcatcactgttttaaaatatcttagggtggttgtgtag

agggagtagggaacattgaactttgctgtaagacggaactttggtccactaatttcatttgtaagtggccaggagtaaagatcattctgtacaatgagat

gtcaagaatagaaatgccagttacatttttccttaaaaatgtagtaagtctcagcattgttgtgtgcatttggaaaaatgtgatattttattgcattgtt

gtttatagctattaattactcgtgctgaaaaattgacaatactatactccttcagatccttgggatgttagtacctgccatcatagtttgatggatgagt

ttccattgcctagtttgatctccatcataaatcttcatgttaagcattccattgcatttacatgtggcttactgaacaaatgtatataaatgttaatatg

taataaacatcagtatttatgtgttgtaaaaatggtaaaagata

>Contig4455|vacuolar h

ggatctcgttgcaaagctgaagcgggggcgtgctaaatttcggctgctaaatcttgtttggctgtcattgatcggtatttaggacatagcgagacatgac

ggaatactggctaatatctgctcctggagacaaaacttgccagcagacatgggaaactttgaataatctgaccagtaaacagaacaatgtatctgtaaac

tacaaattccatattccagacctgaaggttgggacattggatcagctggttggtttatctgatgatttaggaaaactagacagttacgttgaacaagtga

ccaggaaagtagcagcctatcttggtgaagtactggaagaccaaagagacaaactccatgaaaaccttctggcaaataatagtgacctacctggatacat

cacacgtttccagtgggatatggcaaagtatcccatcaaacaatccttgcggaacatagccgacatcatcagtaaacaagtaggtcagatagatgctgat

ttgaaaacaaaatctactgcttataataatttgaaaggcaacctacagaatctggagaagaaacagactggaagtctactgacacgaaatcttgctgatt

tagtgaagaaagagcacttcatattggattctgaataccttactaccctactggtaattgtaccaaaaataatgttcaatgaatggaatcagaactatga

gaagattacagatatgattgtacctcgatcaagccagttaatttaccaggactcagattttggcttgtacacagtgactttgttcaagaaagttgtggat

gaatttaaactccatgctcgggagnnnaagtttgtcgtaagnnattttacgtacaatgaggaagaactattngctgggaaaaatgagatgacaaaattgg

ttac

>Singlet1367|vacuolar h

agnnnnnncacctgacttgttgtggaaactgatggaggcgtagccgcgttgagagagatttgaagtgcgttttgtttgaggaacctaaaggtaaagcaac

aacatggggtcgttatttagaagcgaagaaatgactctatgtcaattgtttcttcaaagtgaagcagcctatgcatgtgtttctgaattgggagaactag

gattggtccagtttcgtgatttgaatcctgatgttaatgcatttcaaaggaaatttgtcaatgaagtccgaagatgtgacgagatggagcgaaagttgcg

ctacttggagaaagagattcggaaggatggcattccgatgttggacacgggggagagtccagaggctcctcagccaagggagatgatagacttggaggca

acatttgaaaaactggagaatgagctgagtgaagtgaatcagaatgcagaggctctaaagcggaacttccttgaactcacagaattgaagcacatcttga

ggaagacacaagtgttcttcgatgaggctgatcagagctaccaacccacctaccttgcgtacccgcagatggctgacccgagtcgcgaggaagagcaggt

cacacttctgggggaagaagggcttcgggccggcggacaagctctcaaactcgggtttgtagccggagntctcctcagagaacgcattccagcttttgag

cggatgctgtggagggcatgccgtggaaatgtgtttcttcggcaagctgagatagaaacaccacttgaagacccatctaatggggatgcagtctacaaat

c

>Singlet2970|vacuolar fusion protein mon1 homolog a-like

ctgccctnnaagtgctttgctgctaagtaatggcgtcaagtgtaaatgaagtggaaagtaaacagttgctttccgaaacgaacgaagatcctgatgtcgt

cgctgactttgaaccaggggcatgcaaagaaagcatgctcgtcacaacagattcttttgaagaactggaacaggaaatggactctagttttgatgacaga

cagatgaaggaaagtactactagtactattagcgaaattcaagagggactgcatgacacaactgttccggaagattcagcatctgatgatgacaagctta

aaaatgatgtggaggaattagcctcacaactaagtgaagcaagtttgacttcaaatgaagagggtacaacagccgctaccccagttggttctgtg

>Contig1672|vacuolar fusion protein ccz1 homolog

ttactgcattgtgaagcaggtgcaggaggtcgtgttaaagattgcggaaatgcagtgtgtacaaattttgagacagaatttatttattatgaagtagttg

tgggagtatgtttttctgacgttaatcaagagttgtgtcgaaaaatggaataatgacaactaaatctgaaattaacctccaaaatttctatgtttacaat

tcttcgtatgggcagaaggaaggggaggaactcaaaaatatattgtattattacccgccgcaaacagacgttgatactcagatgaaaaatgttggcctta

gtgaagcaataattaagtttaccgatactttcagtccagacctaccatgtgactcactacatacacagaagacgaagcaactttactatgaaccagagag

cggtttttggatggtgatgactgtcaacattccttgcgtctcaaagacgaaagatgatacagagtatttggaatatcagagtgatgatgtccaagacact

gtctatttggctgttttaaaacagtcatatcagatgtttcgcttgttcaagaactcattcacagccattcttgatagtaaggatggagatgttggatact

tgaaacagaaactggaaaatttcttttcaagatatctcctcacactgaagctcaaccattgtgacatactggatgtatttcaaggaattcagttccttcc

attagataaacaaacattccttcagagtgcagtgttttgttaacctcattg

>Singlet2299|vacuolar atpase assembly integral membrane protein vma21-like

ctgcannnnnagtactgcacatttcgttggtgtttttcataattaaagcatattcagagactgaacctcctaagcctgttaccaaacgggattaaaggta

tcttcattgtgtggatacaatgaggaagcataggtgcccatcagatgattggtgatacgatgatagatactttgcaagttataaaatggaagtgagtgag

catgtgtcatgctgaactacatcaattttcatcaacacatttgaagtgttgtgggtcagtaaaaactgtcatgaaacagttgtgagtagccactattgga

acttttagagcaagggttttatgccttaaaaaggaaggagataatgaatataaagagtgattataaagatagtgattattggaacatcctgagcaactga

ttggaccttcataattttcttataataccgcagtttcattttaacaactactctgtggcatcgtatacatttatttaacttttgtttgagaatactgtgg

cttttatactggcaaatagtggttccttacttcatacttttgcttaatttgtagcgacatggctgatgacaatctccatttactgcaggtacatagtgtt

ttcagttactggcactgttctttgtgctgcattgtgctccacaaccctgggtcccacacgtaggtcttagaaaaggctatcaaatatgacagttctcaaa

tgttaattgttcagtttagtagttccatatttaggatctgtgaaaattcatttatgtggtgcattaatgctggatgaggacttgctcagaataactatac

tcataacattctaatatcaacatacaaaccagtcttccatttagta

>Contig829|vacuolar atp synthase subunit f

tgggggacttgggggcgtgatttaagtgttgtacggtttacgtgaacagcttgtgtaaaatggcattacattcagcctacaaaggaaaactcgtctcagt

cattggagatgaggacacttgcgttggctttcttctcggaggagttggagaagtaaataaaagcagacatcctaacttcatggtggtagacaaaaacacc

agtgttggtgaaatagaggaatgcttcaagagatttgtgaagagagatgatattgacatcatactgattaaccagaatattgccgagatgattcgacacg

taattgacagccactcgcagcccgttccagctgttctcgagataccatccaaggatcatccttacgatgccaccaaagactcgatattgcgaagggcgag

gggaatgttcaacccagatgacttcaagtaaaatcattgcattgcagatgccagatggaataatttacattttaccttatgtaattaaatgtatctgcct

tgtattgcttaaaattaattaagcatatactatatttaaggctcaaatgaaaatggtgttggataaatagctaattagaatatcaaacacattccagtct

tgtagtgtgactgttgtacagtcctgtgtaaatacattatttattgtgggatcgacaaaaagaaacactgtttaaattgaaaagcttgggacctgtgttg

tgataagggttgtaactctcctacccacagtattatagtatgatttggaaataagttgattgtatcatgtgtcacaatatggaattctataggtagcaga

cagtaacaattcacaaaatatgttaatatcttgttaacacttcttgaatgctttgttgaacatgatttaaagttgaatggttcatttatgtagaaatata

aagctacagttcttatactttgtttactgaagctaagcagttatttttacattattgttattattattattatgatcattgatatttgttggacatccta

tgcagcctacataatattatgtaaatcgtacgtttgatgattg

>Contig1160|vacuolar atp synthase subunit

ctgtcggaccgatagggcgttcgaagtgtgagagttttgttgcaaatttggttggaattgtggaacagtgagtcagaatgatgttcaatttgtatgtggt

tgtgctgtgtatttcctttgttggaacgaactccgaatacatacctgtattattgtggcagcccgatgaagttaatggtaaagttgctggatctccacct

tctctttcaaaagttagtgtcgaacaattttcggactatttttcaaagaagattggtgaacggaaacatattgtggtatttctagaagaaaacttaagta

tggaagattttggaggtttgaatttggatgacactttcagcagcttgaaaaatataaccagttcatctcatgttaattttctgccatctgtccaatctcc

ttacaaagccttgaaaactttgggactgaaaatgaaagcagtgaatattaaggactttgattacacatttccaaaagcagaaaaaagtatcttagtgttt

aaactgggcgacgctcggtcagaagaagatcgcccagacttgttaaagcgtcatgataatataattggtgaagtatacaacagtttggttaacaaatatg

atgatgtacttgctctatacacagcccatcattcatcgtggatagagccatatttaaaacccaatgttcgcagggtgaggcagttgttagaaaccagcaa

tgaactcgaaactaacggtagcttctggaatcggaataacacgcttgtgtacaccaaacgcccacctgtctttcgtcatggaacagacgaaattaatatt

accgatgtgatatcgataaccacttcaacaatagcaggcaatgtcatgttaagtgttagaataaagagtttaagttctgtctccatgagacttactttta

gtaaatctagcggatactggtccttcgataaaattgaagtgaagaacgacacgcaaaattatacccttttgccatcagctcccataactgcaccactcca

tttctcgtaccactgttcttccaacgttattttcacatcgaaagacaatgaagcaagtgtaacattttatgggctccaagtggagccctatgtttcttct

aatgatacatttaatgatgcatatgactgtgtcccctttttttctgctccaatatggtctggtattttcattactgtacttttggctatagtcatgatat

gggccctaacaatgataatggatatcaaaaccatggatcagtttgacaatccaaaggggaagacaatcaccattaatgccactgattaattgacacgagg

aaaattgtaacagctatttcagatttttttggcttgttactgtgcaggtgacattgaagagcagtcaaaacagtaaagtttttggacaagtagaagttga

aggtgacatgtaagctcacattaggtagtcacattgcacaaagaatgaattttattttgtaagtttatgaaacatctaatttttggtgtaatggttgttg

atttactgtaatataattgagtgtagataatgtatgcaagtctttcattggaactgtatgtggggtataatgttaatagctaataatattgtaaaatgtt

cattgttatatccatgtgaatgcccattcttcaccagtattataatgtaggataatttttttcataaagtgaaaacattccctatgtgtcggcatttgca

tcatgaagatttactatgactgttataaaagttggttacagctgaaccatatttaatattttggtgtgttttcagtatgttgtga

>Contig4289|vacuolar atp synthase catalytic subunit a

tgtcagtggagattgatggtgtgactgagtctatcatgaagagaacagcgctggtcgcaaacacctctaacatgcctgtcgctgcccgagaagcatctat

ctacacaggcattacactgtcagaatatttcagagacatgggttacaacgtatctatgatggctgactcaacatcccgttgggctgaagctcttcgagaa

atctcaggtcgattggctgaaatgcctgctgacagtggttaccctgcctacttaggtgcacgacttgcaagtttctatgagcgtgcaggtcgtgtgaagt

gcttaggtaacccagaccgagagggctctgtgagtattgtaggagctgtgtcgccaccaggtggtgacttctcagatcccgtaacaactgccacactggg

tattgtccaggtgttctggggtctcgacaagaaacttgcccagcgaaagcacttcccgtccatcaactggctcatctcgtacagtaaatacatgcgtgct

ctggatgacttctatgacaagaacttccctgagtttgtgccattgcgtacaaaggtgaaagagattctgcaagaggaagaagatctgtctgaaattgtac

aactggttggtaaagcatccttggcagaaactgacaagattacacttgaggtggccaaactattgaaggatgacttcctgcagcagaacagctactcacc

atatgatcgtttctgcccattctacaagacagtaggcatgctgaaaaacatgattgctttctatgacatgtcacggcatgcagttgaatctactgctcag

agtgagaacaagatcacttggaatgttatcagagattctatgggcaacattctgtatcagctttcgtctatgaaattcaaggacccagttaaggatggag

aagcgaagatcaaggcagactttgagcaacttcacgaagacattcagcagtcctttagaaacctggaggattaaaatggtagctgcctgcaattaactcg

gtgcagttgtctccttcggtagcctctctaggactgccaagtggcatggttgctcgacatctaagcattcctttgccccataaggactaaagcagatgga

atttcagtttcagactggtttcattggtgctaagattatgttgtgcccatttctgcttctcacattccagcagagaagaatttacttccatattcttccg

ttttccttcttcctgcttttaagtatcagtatggaggtaacttaaattataaatcccatttaactgtgtgtgctgcagtcctgtaaatttatttgcccct

ctaaatccctcctgtgtgtaacatgagattgccatctgtatgtatgtacacaccatactgcagtatttgaaatcagttagaagatgaatcacatcactta

ctcattgtttcacatagcatgcgtgcaaactgccatcaatttcctatttatttgaatagttctcacatttgcagtgttgcagattcttttaattattatc

ccattgagactgagtcaatcctgcagttgtaactcactatttacacacacatttaagtgttaaaaaaagaaagaaaaaaataactgttgtatattgaaag

tacaaggaacaaagttgcatttaaaattgttaatgtattttatatttctctgtagacacaaagttaattcagtttgcttaatggagatatgtgtaaacct

tacatagcagtttgtgcacaaagtgtgtatatgt

>Singlet5798|vacuolar amino acid

ctgtcgtagctttgtaaacatgtttacttcgcactttgtagtgtttatgcaatgtgatctgtgtgcatggagatcgtaggcagtacaagggagtctgaca

caattttacagaaacagatcttgatttgatatgcaatatgcacattaataagtgcaaacaaccagttctgcagtaaaatgagggagtacgtgaggtatca

agctactgcatcaagggatcccatttggacgagagtatcagacgaccctcaacgcgaacagttgtcagatggtaaccgaaaccgcaagaatacaccctca

gaggatagatatggctcaacaggtgtatcagatggtgcaaagaaaggccttacggtgctttcaactgcgatttttattgctggggaaatggctggcagtg

gagtcttagcattgccaagagctgttgtggattcaggatggattgggcctatacttgtcgtaatattctgcattaattctgcatatggtggtgctcgcct

tggagattgttgggcaattttagaagaaaggtatccagaatacagaacacctgtcaggaatccctatgctactattgcttacagagctgttggccggaag

acaagcttgctggtgtcaggatgcatacagttcaccctttttggtgctggtacagtatacttgttacttgcatcacaaatggtgcaggaacttctggaag

attttgtaacaagagtaaacttctgtgtctggttcctggttatttgcatcatactaactccagcaatgtggttggaatcgccaaaagattttagaataca

attggaaacggtgtttattgcggagcatcatgatgctgataatgctttttatt

>Singlet2854|vAMP-7, putative

aagtgtaaatatacaaactatacataacagagcgacctctgcctgttatctcacaacagattagtatctgtccccttggggtctgtgtgagttactattt

ctttttaacctcttccaactttctcctctctcctcctcaaggaacatgctgatagttctgaaagcaagaatactgtgtgtacttttatacaagtatgtca

acattatttaatttttttgcttcctggtcagcagttttacttcagaaaaacttttaattttctcagctggaatttccttgtgataaaattaatctttaat

gtatgattccaattttccaattgaatatattcctcactgtttgttcctgtgcaagatattttaatatcagttttataagcaaacattccatttgtgaaac

aaaggtgacatatattgcttgtagcttatgtaatgtgttttaatattattcttgcatgtgtacatggctaagtgggggtgcaatatccaacgaatctgaa

aaggtttcgtgatttcatgttttatttctgaattttacatagtatttattgcctgttgaaaaacaggactggaatacagaaagttacatttgaaatcatt

ctaacagccatgaaggcacttcactgggataaaactacaaaatggaaaacaaattaagttttggacatttttaaataaagagtaatttttatgtaatttt

aaagtgaagaataaaactacaggccattttagcctttgtagtttgagactgatttagaatgatgaatttctttataaattgctcatgctttctgaatgtt

cacctgtttttacatacgcattaaactatgctatgtg

>Singlet2080|v-type proton atpase subunit h isoform 1

tttttatacatttaaatgccttggatgtgctcattactattcaggtgattgggatttaaggaaagaaatttgaatgtggtgtttagattatggtgtattc

agtattaatgctcagaaatctaggctaaagagcatattgcagtgtctgaatttgtgttgaaccttatttacattccattggaaatgtaaagtattgtatt

ataacagctattacacagattttttaaattatatattgtataattttaactaaagttacaaataaatatgtaaataaagcatgtagatattgaaatataa

aaataaatgttgacataaataaatctcacttgccacaggaaacaaaatattttgtaatgagtggttctatgtagtagagaaatgtttttttatggtgtaa

tc

>Contig3477|v-type proton atpase subunit g-like

ggaaaatggcaagccagacacagggaattcnncagctcctagcagctgagaaaaagagctgcagagaaggtttcagaagcaaggaaacgtaaagcacgca

ggttgaaacaggccaaggaagaagcacaggaagaaattgagaaatacaggcaagaaagagagaggcagttcaaagagttcgaggcaaagcacatgggttc

gcgggaggacagagctgcacagattgaagcagaaacaaaagtaaagattgcagaaatggaaaaatctgtaaatcaacgaaaggaaaagttaatagaaagg

attttggaattggtgtacgatatcaagccagaactccataagaacttcagagctgcaaagtaggctctgtatttgatggaaataagtttcaaaattggta

tataaaattccagtgaatgtaataaattgcataaaatttttgccatctgttatttaaaaaagtattataaacatttattcgttaaagctcagatctgtag

ttgaaaagttggtggtgggataaccaaacagaaaacaaagaaaataaatatttgttcattattaatgtacctgtgcatttctccaccacataagagggga

atttttcccatttgggtcacatgcagtaataaatcaactgtcacacaatttttgtgatgtgtttttaattgctgatggcagataaattcagtattaattt

gaatagcagtatcagagtttaaggaacattattctaaaagtaatagtgattatttgaatacgctccatgtaattttgttcagtaataatgcaattcttta

atcaaatcctgtggcacttgtttcattgcttttcatatttatttcttataagattttactataatatgtggtgcatggccagttacaatcttataaaaaa

aaaaaaaaaaaatgc

>Contig1513|v-type proton atpase subunit d

ctgtcgatcancttacagttgattggaagtggagcaagtgggaagggccgagtgttatagaagtgcagtgtaaatatatatatatatatatataaaatca

attgttattttctttacaaattcgaaatgattgggtgtatgttcaatattgatgctggttacttggaaggcctgtgtagagggttcaaatgtggaatttt

gaaacaggcagattacttgaacttagtgcaatgtgaaactcttgaagatctgaagttgcatcttcagggcacagattatggaagcttcttagcaaatgaa

ccaagtccactccaagtgtctgtcatcgatgataagcttagggaaaaattggttatcgaatttcagcacatgagacatcatgcagttgaaccattgtcca

cttttctggactatattacgtacagctacatgatagataacatcattcttcttatcactggaaccttacaccagcgaccaatatcagaattgatcccaaa

gtgtcacccactcgggagtttcgaacagatggaggctattcatgttgctgcaacacctgcagaactatacagtgcagtcttggttgacacaccactagct

ccgttctttgtggactgcatcagtgaacaagatcttgatgaaatgaatatagaaataatacgaaacaccctgtacaaggcatacttagaagcattctact

ctttctgcaaagaacttggtggtacaacagctgacactatgtgtgaaattcttgcttttgaagcagatagacgagcaattattattacaataaattcatt

tggcactgaattaacaaaggatgatcgtgccaaactgtatcctcgttgtgggaagctacatcctgatggtctagctgctttggcacgtgcagatgactat

gaacaagtaaaagctgtagctgaatactatgctgaatacagtgctttatttgaaggtgcagggaacaacccaggagaaaaaacactggaggacaaattct

ttgaacatgaggtacgattgaatgtgaatgcatttttacaacagttccactttggtgtgttctattcgtacttgaaactgaaggaacaggaatgtcgtaa

cattgtatggattgcagaatgtgttgcccagaaacacagggcaaaaattgataactacattccaatattctgagacaatttccactactgtaatatattt

aaaagttatgtgctgaagaccagtgatatgtcttcacttagtgtgtaaaaacaaaacctgattcttcgttgcatgtgtcactctttttatttggtaactc

attaaattatgcaataaaaaagcgtgcataaagtacaccatgaatttgtaaatagaattgtcatattgtttcccacatcctttcacagtaaagtattatg

ggctggggtatataagggcaataggtgtaacaatggtttggtggaagtatagtgtgtacaggataatgattgtataaaagtattaaaattgaaagaatat

acattcttcaaatgttgtagtggttgtattttttgccttcaataataatcatacactgtcatttagaggagctatttcagtgtcttaaattagaacaact

tccatgtacatattttggcatgtgttcagtattaatatatattgatttatgccatagaagacatgtttttaagcacagtatgtaaagctgacattgttaa

aaaatactaattgttgtattaaatattcattgtgaccccaaattttgtacatataggatagtggtttacgtagcaaaaatgtgactgcagaaaattttat

tgtgatgtaatattttgtttcccagtccgtgttgtacattcctgttattacagacgcatgtgtcacatacctgtacatcatgtaatatttgtgtgtgtgt

gtgtgttaaagccacagattgtacaaactatgtattattcaagaaatacagttttcttcacaaatcaaaaaaaaaaaaaaaaaatgcgt

>Contig2039|v-type proton atpase subunit c-like

gcnnntttgaaaacaaaatctactgcttataataatttgaaaggcaacctacagaatctggagaagaaacagactggaagtctactgacacgaaatcttg

ctgatttagtgaagaaagagcacttcatattggattctgaataccttactaccctactggttattgtaccaaaaataatgttcaatgaatggaatcagaa

ctatgagaagattacagatatgattgtacctcgatcaagccagttaatttaccaggattcagattttggcttgtacacagtgactttgttcaagaaagtt

gtggatgaatttaaactccatgctcgggagagaaagtttgtcgtaagagattttacgtacaatgaggaagaactattagctgggaaaaatgagatgacaa

aattggttacagacaaaaagaaacaatttggcccattagtacgctggctcaaagtcaacttcagtgaatgtttctgtgcatggatacatgttaaagcact

cagagtgtttgtagaatctgttctaaggtatggactgccagtgaactttcaagccatgctgctacatccacacaaaaaaagtaccaagaggttacgagac

ctcttaaaccagttgtatgggcatttagacagcagtgcttcacaaggcctgggggcacatgacagtgtggagataccaggacttgggtttggccatgctg

agtattacccatatgtttattacaaaattaatgttgatatggttgatactaaagtgtaaaaagcttttctgtaatattgccaactcattacggatagtat

tatgtacagcaaataagggaagttggtatgtgttcattttttttttaaatgtaaaagcttctccagaacattccacattgtatcagacattatttctgtg

tagatggcacagtgctgccagtattcttgtagtagtgacattcagtcttccactggaatgttagtatttaaaataaattgaatgttagatgagagagaat

catctgaaatgcagcaagctgaaactgtgtacattgtctgtagcacaacttcaagtttttacttccaggaagcttttcactttaaacctaaaaaagaaat

gctctccatgcatctgccttgcctttgagatatcaaacgttaattatttgttattggaatattttataatcaaataacagtagtactttaacttcccaaa

ggaaaggaactggattccaaattgcattttattaatggtaatctgtggaatctcattcaatactcctgcttcatataatatcctcacccttctcatctct

cttaattacagttttatcgtatgtttatatttattgcatagttcactagattttattgaagaccataccacattgccaataaaaactaaaacatgcttga

acttgtcatatatgatcactcagttttatttactaactacaaaaaagtgtctcccatagtcagcagtatgcagagcattctttttattggaagtgtgtat

ataaaaataacttgacagttatgagggattagtgttacaactcagtgtggagtgctggaggaagttatatggtagggca

>Singlet1424|v-type proton atpase 21 kda proteolipid subunit-like

ctgtcggctgacaagttaagggtgttggttaggtgagaattccaaccgggaggtggctatttttttccccgaaaatgaggtacgggctgggatatacttt

tttagcgacgctttcgtcggcggtgactataatagtgctgtattatgttcttactgggaaaggagagaggattagttttggctggttcctaacggagact

agcccatacatgtgggcaacagttggtgttggattggctgtttctctgtctgtggttggagctgcactaggcattcatacaacaggagtcagtattattg

gtgggggtgtaaaagcaccccgaataaaaacaaagaacttgatatcagtcattttctgtgaagcagttgcaatctatggtctcattactgcaattgttct

ttctggactgatagagagtttcagttgggatgccgttcaaagtaatattgatctgaagagccagaattggctttctggctacataatgtttggtgctgga

ttaagtgttggtttggttaaccttttctgtggaattgcagttggaattgtgggatctggtgcggccctagcggatgctgcgaactcagcattatttgtca

agatcttgatcgtagaaatttttggcagtgctattggactttttggactcattgttggaatttatatgactgccaaagtaaagatgggggacaagcagta

aacaagtgatccatttcagcagacaagaaatatggctgtttttggattgtacagagacttcatgtataaattaggggtgctggtgtacagaagtgtgaat

gtgctttgtatatttgtaaaaaataaagtgggtgtag

>Contig534|utp11-like u3 small nucleolar ribonucleo

tgggggatagcgtattatctttggtaagccgattgtatgtttgtcaacacagacggaacacatgttccaggacagaagtagaagtgggctagtttctata

cttgtgaacaaaaagttaatttttcgcgatgtcctcatggaagaagacttcaaagacaaaccagaaaacacaccgagaaagatctcaactagaaagcagg

gcacacctgggaatcctagagaaaaagaaagattataaagagagagcaaaaaattatgggaaaaagaaaaagacactcaaaatattgaaaaaacgtgctc

tagatagaaatccagatgagttttattttcatatgatcaattctcgtgttatggatggtgagcatcacgaagttgataaaccagatgaacatacaccaga

gcagatcaaattgatgcaaactcaggatttaaggtacgttaccacaaaacgaacaatggagtcaaagaagatagataaacttcagtcacaacttcacttg

atcgatgtagcagatcaaacggaaaatacacacatattctttgtcgatacagaagaagaggccaaaaaatttgatgttgccgctaggttagacacacatc

cctccttgctcggtcggcgaacaaaccgacctagactgtcagttttgaagaatacattgttgccagaagtagatgaaaatattttgga

>Singlet5908|uridine-cytidine kinase-like 1-like isoform 1

ctgcaactgtgcaagtgtttgatccacctagctcagcgagttcggagagtgatgatggtgaaattgatacagtggatcaggtgttcctgccagacgatgc

agaggaggaagaagagagaggttgcgattactaccctgtaccgaacacaccacctccacagtcacccaggccaccctcgacaggctcccagaagtcgcca

cgttcaaagcggcaaagaactacttcactgtctcagtcgtcaaagaaaacggcatctgaatcaatacttcgcagtcataggcgcactatttacactgcag

gccggccaccgtggtacaactcagctggtcagcaagtagaaccctttgtgattggtatttgtggtgggactgcttcaggaaaaacaactgtggcaacaaa

aattatagagtcattagatatttcttgggtcacactacttagcatggattctttttataaagttttaaatgagaaacagcatgaacaagcagccagaaat

gaatataattttgaccatcctgatgcgtttgactttgagctattgataaacacactgcaacgattaaaagtgggaaagaaggtggaagttcctatttata

atttcgtaacacattcgagagagaatagaacgaaaacaatgtatggtgcaaatgtgatcatatttgaaggcattttaacattctacaatgcagaagtttt

gaagttgctcgacatgaaaatttttgtggatactgatgctgatgttcggttagcgcgacgattacgccgtgatatttcacaaaggggccgtgaccttgaa

ggtgttcttaagcagtactgcaaccttgtgcagcctgcattcttccattacatagcacctt

>Singlet2218|uridine phosphorylase, putative

ctgtcgccgttgctgtcgagctgactgcccgctccctatttccgtcggtgcgcggtgctgcaggcgcacacacccccgtctgttcccaagcgcgacagga

tgcggccgtccctgctctaggagaccgcgtcgcgcctacaccagttaccccgtcgcctcgacagcgcgcaccccgtctgcacacggagtttccgtcacct

ttccccgtttgacaccttttccgccgattctcagccatgtcgaagccggagaagctgatcatcatgtcgtccgacgaggaggaagtggacgagtatcctg

atggatcggtccgccttagaaacccaaacattgaacttatggaccaggacattctgtaccatattgctcttggtagcgaatcgcatgacctggtctcgat

gtttggggacgtgaagtttgtgtgtatgggaggaacgccaaagcgcatggagcagtttgcgttctacatcatgaacgagatcgggcacaagctgccgaca

ggcacgaccctgcaggacatcagccagtattcgtaccggtactccatgtacaaagtggggcccgtgcttgctgtcagtcacggcatgggcatcccttcag

tcggcatcctgctgcacgagatgataaagctgatgtaccacgctaaagtgcgagacccagtatttttccgcattggcacctgtggcggcatcggtgtcga

gggtggcacggtcatcatatcggaagacgcagtcgatggcatgctcagacctgttttggaactgcctgttctagnnannntgatacagcgaccagcaaga

ctggacaagaaactggtgtcccacctcaagagcatggctgaccctgatgacccgtatgacacaatcacag

>Singlet2477|uridine diphosphate glucose pyrophosphatase-like

tgggggatggaattannagggaacatgcaactactactccgtcgcttatacaacaaaatagaactgtactctgccgaaatactgtaacatttattcgtca

tcggttcgttgcacaaaatcgttcgtccgtttcccatcacattgtcgctattatgctgtttactttgatgatattgcggattctgatgtaaacagcccag

ttgataccgataagtatcctgtagaaaagggctttacaattgaactctgtgctggcattgtggacaagcaaaaaagcctggaagagattgcaaaagatga

attgctagaggaatgtggctatgaagtaccactttcagttctgcgcaagatcagtactcacagatctgggataggtgttactggagacagacaaacactt

ttctatgctgaggttactgatgaaatgcagaaaggcccaggtggtggaatcatagaagaaggtgaattcattgaagttattgaaatgacagtggatgaag

taaagcagtatttggccagtggagatgtcagaagtcctggtggttttttgtttgcagtaacatggtatcttcataaccgcttgcctgcatcacataaaaa

agatgcagtataacatttatcagtgaggttttgtgaccttgagtgaataataggtcaagtataacctttcaaatgccatgatttgtacaagcaatggatt

tgagnnttttatgacagtgccgtatttctgctgnnaaatgtgaaaggcaaactttgcctggttataaatctattaagcagat

>Singlet7094|uridine cytidine kinase i

gattactcttgtgtaaataaattgtaatccattgagtgcactatatttgtgtgattcattatgaactgtttataagctatctagtttgtgcttttggatg

ttattgacactagggaaagaagaaaaaaatcagaaacagaaaatatttggtcctgagaagtaaaaattgcatacctctaaattcacttagcaattactga

aaaaaaattaatcatacagattctgctacactgtctgtttctctaagttataagtatttgtgtagggaggaaaaaaaatacaaagctataaaagttgtta

ttttgctgcaataacagcctgtatcatcagtatctcatggttttcacaatttaaatggtgctcagaatgctgcagagattttttagaaacttaaggatcc

ttcagctgttatatcagtgctgaagaaacaaatcagagctgcagttcttgtcaccataacttaaacaaatatttgtgagtattcagtattttttattgag

gtttcgacattccacttgtgagactgagttttgatttggatgtacttaaaatgtggctttgtattttgaataaacaagtaagagcaaaaaaaaaaaaaaa

aaatgcgt

>Contig1538|uracil-dna degrading isoform b

ctgtcgccgnnnnnggacacttcggcaagaaacactgcgtcttgttagcgttttatccaaccaggggactttaccaacacaacaccagctatgcctaaag

taaaatctgagaaaagaaacagcccctcaaagagcccactggaaggggaaaagaaatcaggatggggcagtggtggtcagttatttggctcagagacaaa

tgaaaacacttttcaaggagcagggtttaaggacaaagacaaggcactggagacattaaagctacttgatggtcgtgacatctcgtatcagttccagata

ataaattccatgtatcatcgagcaaaggttattctaaaacgtacaaaagatgaggaaaaagttcacaatcttacagaagcaattgaagtttttgagaact

ggattaatgattataaagcacacaatagatcaagagagaacttcagctaccttccactggaaaccgttgactcctataaaccactagcacaacgttatgg

tgtttttgtggaaggtgaaaagtcattccttcacgtgtacaaagaagcagaaggcgatcacaaaaaactaagacaactgaaggtcgacaaagatgatgaa

aatagcattacctgggacatacaccgcaacaagaatctgaaggaaatttcaacacgcataaagactgaacatcttcctttatttgagactgatttagagt

acagaggactgccaagcaaagaacatgttgaaatgattcaatggggttatagtcctgaggtcaccaaaattaaaaagctgataccacaaatagcagagaa

gctgaatggagcaacaaaagaaaacatggttattgatagtaatggtgacagtaacagcaacagcagcaatggcagcagtgtcaatg

>Singlet451|uracil phosphoribosyltransferase homolog

gatgagatcagaagggctatatgtcccttataccggtgaaccagtttaaatataacaaacattttataatgagaagcattagttgaaactgagttgtgat

aacttaagtttgtacctgctgttgctaatggtattctagtaacttagtaagaattaaacagaaattcatagttgtggtgatcttttacatgaagaaatta

aaactagtcaggcagattttgtcagtgtcaggatgtgtagtaattactgcagggtactgatgttgttaactgcaatttactagtggactgctaaattata

gtgaaatgtcttttggtataagaactctgcgatgattatggaaatatgaatctcttgattcagtttaaatactagtcgtgtgtccaaaaattttacttcc

tattttgcctctttttgtgtaaaaatcattgtacaggcatattcagttttaattctaacttagtatttattggttaattttgttcctgaaagagctaatt

cttaatgttaaatttgctgcttgaaattctgatatcgccattgttacatgtacacagttttttagaattctcttaatttgcagatgactagaatttcttt

actttattaattgtacttatatgtaattctagttcaacatgtgataaattatgtttgatagccattgttcctgaagcagttgattattaatgttaaaatc

acagtttgaaatttcagagccttccattgtaatgcatt

>Singlet1232|uracil phosphoribosyltransferase homolog

gagtcagttgttttagtttcctatgaaaatagcacaacatatttttacagtgtgtaagtttccaaagaataatgaatcgttttttatttagtatttccat

agagcgttaattttctgtttttagacctgtctctccaaattaatggtacttattagatgtgtgtgccctgcaccacatttttccagtattgattgtatac

ttacataacacagtaattaactattatgttaaacatatttttaatgtcaaaacatgagcttaaaactgttcttagttagtacacatgcttattagcagta

gaagaagctaccaagatacagtgaaaagcaaacctaaggttctatgtttttatatttgttgcttttttcattaccagtctccattcaaaattgagctgaa

atcacaccagtggagacatgagatgaatagtttcatcaaaacttgaaacaagaaaatcaagtattgaatgaaactataagtt

>Singlet8339|uracil phosphoribosyltransferase homolog

attgtggatgtacgacagtgtggatcannnnnttttttcggtatttacgtaggcgcttacaagagcctgtaaagtgctgctctcatcgtaatcagattac

aagaaaacaatattatccgtcgcaatattatgggaagtgccggtgaattaagtggtttaatcagtagattacctgaacataaaaatgaaataaaagaaga

ttacggtccaaatttgaagatactgccgaacaatgatcaagtgaaggaattgcagacaatactgcgagacaggttaaaaacgagtggtttttggaaataa

tgtttacggctactttaagctgtagaataatttataattttacacgtaccttttttccagaaatacctccagaagtgatttcaaattttatgcagatcgc

ttagtaagttcatctagcgcgcaaggctaatgaagacacaattttcgtagtaccatggataggccgcgtgttcaatagtatgcgtttttcagatccggct

cgtaattgaagaaagcctgaatcagttaccattcacga

>Contig3236|upf0760 protein c2orf29-like

cgctagatgttttatgtaaatggcgtgttttgtgtagtgtggcgggaggctgtcatattgtactaatgaacttagtacatctacatacatcaacatcaca

ttccgcccccagtttctaagaacaacagatttattgaaaagagaaaaaaaatgtctctgtcagttaaagaattgtcatctttactaagcattcttagcga

ggataatgtcgataaccagacactggaaacactatctgcccaaatgcatcagtgcttttctaaacaggacttctacaaggtgggatgtgcaattatattg

ctactgcagcaagcagatcttttacccaaaccgacgcaacgaatggctgctatttgtcttctacatgaactttatagaggagaagggatgtcaacaaatc

cttttgctgcagtgtttgtgcacttgttgcatcccccagatgaagtcagcaaactcacaactaggaagcttgaatatgctggacagttaccgcgacttct

acaagtagagaagaacttcttgatgcatctgattacgaatcccacaaaagaggtattgaaaaagactccaaaccaaatattaaacagtgatctggcaaat

acacatcctaatgtagatatcactggcattcagctggcacttgctgagcggcagtctgagttgcctcttacttgcagaagtgggattcctgtaattctcc

ctgatggtgataataggtcagagccaagaatgggtctgatgagtatgtattctgagcagaacattgcaaaaaagacagctgaagctctcttgacaggcaa

caatccagttgttgatcagtactacaagccagaattccttcgtttagcaccaccattgcatagttgtgaagatgagctggtgtggctaaatgtcactggt

gtttcggagcttaaagcagcatatgatgtcacaatgtgtgtttcaaacagtgaaggagtagaggcaaagcgtctaatggcaaaagcattcaagggtgctt

taacgttacaacagcagcaacatttactgactgaactagataatgatccaaaacttgtataccatataggacttacaccaagtaagcttccagatctggt

cgaaagtaatccattaatagctatagaagttctactaaagctgatgcaatcaagccaaatcacagaatatttcagtgtccttgttaatatggaaatgtca

ttgcattcgatggaggttgtgaataggttgacaaccactgttgatcttccaacagaattcgtacatctgtacatctcaaactgcatatcaacatgtgaaa

ccattaaagatcgttacatgcaaaatcgtctggtccgccttgtttgtgta

>Contig2165|upf0694 transmembrane protein c14orf109 homolog

ccnnnnctgtcgaaatttgtgatataagtaaattgttttgcaagcctgattaaaggcagaaagaagttactgattttaatagcatttattaacagtttat

atccctctgagcttgtaaaagacaatgaaacctgaaacagcttactgaatgaaaagcaagtgtaattattcaaagcatagtaactactactgtgcaagaa

acatttaaccaaacatttgtgatgtgtgtttaacctattgtatcttcattatttaacagcaagtaaatactgggcagtgcgtggaaagtctcaaatatta

agaattcaggctttgctaacgcatgtaggaccactgttcattgtacacaaacattgaataactgctattttacaatgtgaacattcactgaaagtataat

ttttgaaataagtattttctgttgtaattactacatcagtatttaatgatgaataaaattacaaagcattaaggagctattgaagaaggtgctaataaga

caaagcagttcctgttgattcatttaaattatgggtagtaatcttattgttatgatcttgtatcagaagattgtattgtatgaacactttcagatggtga

agtccctgcccacatttattttaaagttgttttgactggtgtattgtgagccatgtcttcaccttgtgtagaaacaaaagaatagaatttctttgtcccc

ttgtgctctgcagagaataaaattggcatgtgaataaataacacagtagagataaaaaattgcattattttagaacatttttttgcccttgccataattg

gtcattgtttcaaactaattatcagtattatcaattgttttgtgagaaacaattattaaaatttcggtaatttgtggaacttctgttgtgcataagaaag

ttgtaataaccacactgactgtttctaacattagtcatgggtgtatcagccatgcacaagatgtgaaattccgtaaacagtgcaactatagatcattcaa

tttttctatcacttcaagtgaaaaatgtctaaatttccagattactattatactttaacacagaggagtgtaaatgctttatccatgtgttaatttgtaa

ttacagtatggctcttacttggaagttgcttttacccccacctcctctcctgttttcattccttcatggtttgatatgcacatctttcatccagttgagg

gaataaaatcagtttgataatttgacagtagaaactttcaaagaggatactttctgccacaggtatgttacagtgttcatacaccaccatgttatttgtc

acatcagttctgtggccatcagaaggtatgtcacactataaaccattcagctagtcaaattagagactggtgtggcattcacatagcagtcacagttaca

gatggcacacaattgtatcactgtttttactgtatattgtgtatgtgcagtatctgttaatcctcgtaaatgttaactgtttggcacacgagcgtttatg

tacaaccgaggaaatttgtg

>Singlet6328|upf0667 protein c1orf55 homolog

agggatataaatgaagagaaaagattaaaaaactggatagctcagcaagcagagagggaacaagaagcagcagaaagacggcagaagaaacttgaacgac

tttgtttggaaccaaaacatgaatttaaggacataaaatatgaagaagaacgatctgtgctaacagaaaaagtttgtgatgctgttgagcaaggattcaa

ggcatcaagtagtggaaatggcatgaagagaaaacaggagagtaaggaaatgagctcaaagccaaagaaaaagaaaacatgcttgtggattgatgctgat

aatgaacttgacactgaggatagtgatgattgcctcagtgacgagaatgatggcagtacatcatcaaaaagtggttctcaaaacagttccagtcagttac

atgatgctacactacatttaccagagacagtggcaagaaatgacaaaaggataacacaagctgtgtaatattattgactcagtctctttgtaaggaacca

agtgattgtactgtatgtttttgtaaaataaaatgtgtaaaaataattattatacaaatatattaacaattgaattgtgtatgaataataaaataacagt

attg

>Singlet5301|upf0636 protein c4orf41 homolog

gccttggggaaaaacaaggtgtccaagctctgcagtatcgggaaaagaatactgtcaatcattcgatgctaattattggacttctgagcagtgcaatatc

acagtttaaaacatatagatgcccaaaaatgagaagacaactggtggtccagatggctgatgaatactacagttcacaagattatggaaaagctctgacc

ttactctcacacatgctatgggattatcggagtgagaaatggtggcttttgctcacaaacattcttactcgtgcactgaattgtgcctatctttcggcta

atgttcaggactacatcacactttcccttgaagcactggctactacaactcagcttcccctgccagaaaaaataaggatacacaacaatctagaaaagct

tctgaagaagttacctcctgatcctgagcctaacgtaacacctgaagaggggcttcgagcaaaagatctttggagcaaagcatcgagagcagaacttctt

accgtcactgtagaaatgaacagtattacatcctgcattgaaagcagagctcgcttcacaaaatccaagtatcaagccgatgaaaatgttgttattgaag

tatttatcaggtgaccaaaatctcatactttcatttataaacagtgtgangtcacagcctatagtctgaactttgacttgtttttaccttattgaaaata

aatatctaatttggtgagaaaatgtgccatatcactaaatacatttattttcgtttaatcattg

>Contig987|upf0598 protein c8orf82 homolog

ctgtcggtnnnctgtaaacataaacataaaagtgtagtgagtacaacacatatacaaatgtaatatgtttgtgactttcacggttgaatttgtttccaag

aaatatttaattttaaatctgcatacgctgctctttattcattaattcaagaaatgtgttgtctgctacgcgacttacgaacactaaaccaaattctgta

tgtcaacgtttctactaaaggaaatagtgtattcaagaactggacgagatgcgtttcttatgtgcaagggcagtcaccagaaccgaatataagagaatat

ttttactacgttgaccaccaaggaatgctgttcctggatgactccagaatgaagaatttcacttcctgtttcaaagacaaaaagttcctggaattctttt

ttaaacgcctgaggatgaatacaacagggcgctacacagaagactttccttacttgtcactttgtggtcgagaaagaaattacgttagatgtgatgatta

tcctattgtattcactcatgtaatgaaagacgcgcagatgaataatgtcttaagttatggccatgccggaaatttactaacagttaagtttgagcctgag

aaagttataatgctgccagagactgggcgtgtttaccatcccgcaccggaaagagtcggtggtattggcttaattagatccaagttagcaattgaattaa

gtaagttttttatatttgaaaatggtgagcaacaggaaccaacacatttatcatgggatggtaaggtatatgagttaataacggattggtacaaagaagc

aaaacagcaggaacttgagtgctcaaattaaa

>Contig4707|upf0586 protein c9orf41 homolog

cattgaaaagtgtgtatcgtgattggaccgttgagggagctctcgagcgagaccaatgctataaaccaatacttgaagctattgacaaccatttctccac

tgaaaagatttcgtcaggagattaaaatattagtcccgggagctgggttgggtcgtctgatgtttgaagtagcatgccgaggttacagctgccagggtaa

tgaagtctcatatttcatgttgttatcatcatattttctattaaacagatttatcatgaaaatgcttactgggactgtgtagcaacttgtttctttattg

actgctcaagtaatgttgtagattgcatagagaagatatacagcattctcaaaccagagtaaacgaaatgaacaaaaatttcacaagagttacacatggt

catttcacactgaccaccattactttggtacacttaactttttcatacacgtataaaactaaacatattgtgaaagattttcatcaaaaattggaagata

ataacatgttgccatacctccatttatttgattgcatagatgagtaagtaacatctgtggtggcatatgggtgaacttgggcccattattgtatcactat

tcagatgcaactacaaaacagcccctggtggaacctccatttgatatagttctgagcattattaagaaatttggattcgatgtgcagactgtaagatata

atgtggagagtacttacgcacacaacacagaatctttgttgcagtataaatacaagagtatattttttgtctgcactaaacctgtaccagtacatcagat

ggaagcacacactgtggttgaagaaaatgtggaaaatgctgctggaacaagttcttcccaaagtgaaaatccttcatgcacaaacccgacagcttcatct

gaatcacacactaagaagagaaagtcgaaaccaaattctgaagaaacacaagataattcagattcacctacgactgatgtacaaagtgataaatagcaac

atgattctgacgtaaatacaactgactgaaaagaaaaaggaaatagaatgtaaaccctggggcaacattaatcctcacattccttgaatcattgtatttc

tgaagctaccactaaacagaagtttccttcacatca

>Contig776|upf0585 protein cg18661-like

ggcagacgaagttgacagcgcagtgtggtaggtcgtaggactgagacaagggattttgacttgagttgtgtgagcaaggtttcggcctgttgtggagcta

tagacagttaatatttgtaaaggaagtctcctgaagagggtcagattataccaccaccctgccaatcaggtgaaaagttctttgcagttgacacagaagt

tgaagatccatacgacagacagaaatggaccgcatgccatgtgttggagataaattgcacagccctgcagctgaacgcaacaaagaaccaattctcgagg

tgttaatgcagtacatttttaacaacaaggcaaaggaagaagctctgacagtactggaaatagcctcgggaacaggccaacacattgtgtattttgccca

atattttccagatgtgcagtttcaaccatctgaatttgaagaaagctacataagaagtattggagcatacatttctgagagtggtgtccgcaatgtgcgt

gacccaattgtaatcgatatacgtactccatatagattctgggcctttggagcaatcacagaaaattctatgtcttttgttatcaacatcaatatgatcc

acacaactgaaagtgaatgcactgaacacctattcaagaatgtacagaaagttctgaaacctggcggtctattattcctgtatgggccttttgcatttaa

tggagttataactccagagtctaatgttagctttaaccagagattaagacaagagaatcctgcgtggggcctaagagacgtagaagatctcaatgtannn

nnatttaaatatggtttggcactaatagcagcacacgatatgccagctaataatcacatgcttgtgt

>Singlet2107|upf0577 protein kiaa1324-like homolog

ccnnnnntgtcgccttgttttgtctacacaatcagtgacactgggtgatgagctcgttggtgttactagaagtaacacatttatgaatgtggatttcatt

tatgagttttctgatgttggttttcaggatgggttgcatttttattattcaacaccacttgctactcgctcttgcccccaaggacggaagacagtaatta

ccttactgtgtgcaccagatgagaaaggaagtggaacaataactttaccaaagaaatgtcctgatggaacatgtgatggctgtaatttcagtttcttgtg

gtcaagtgctgcagcttgcccagtgtgcacagaaaaagattataggatggtaaaatcagagtgcataaatggtgtacagtcagttcattttctgccacct

tcagactgcttgatgccattggacatgccacttacaagaagagtcacatgtactgcacaaattcctctgcagttacaggttgtgattgcagtaactatag

ctctcgctttgtttctatgtggtcttatgatttacttctggaagaagacacagaatctggaatataagtatatgaaactaaaacagtcatcaggtggcag

agatggtgacggagacaatgaacttgccccagcagaaagctgtgcactggatgatggcga

>Singlet4921|upf0568 protein c14orf166 homolog

ctgtcgattgcattgacggcgagtggtgtgtgaatgttgaattttgaagtgtgttattataaactgtttattgatagccttagccttagccgatcacctt

cggcatgatgtttaagagaaagcttgcagcccttgagtatccagtcaaggattttaacgtcaatgatgaaagagagtgtcggagcctgatcctgtggttg

gaggatcagaaaataagacattacaaaatagaagacagaaagggactgagagatgtggactctccagagtggaacaattccttctgtcaatacttgcatg

atttagcatgtccagttcagagtgaaaaaatgacagaacaacttgaatggctgctgagcttctctgttagagttgaatatgctgacaatgttgacaagta

caagaatcaaactgcagaaaagattaaaaagccagaactcagtgctccaaaagtcatatcttcaaatcccctggataacttggattttgaaagtccggac

tttaaaaagggtgtgaatgccttagcacaattgctgaatgttactgctcatccggaccaccttataacactaaaagctatcagtaaagttgtttgtcagc

gactgtctgcagatgcattggaaaatccacagagtgtaatagtaaagggaaagccattcccatttcaagaagctgatcttggttttgacttaggagatta

cgtactgaaccaggctgcaaaaattttgagattactatatatacatgacgtaagagatcttcaaaccaaaattaatgaatgtattgttgctgtacagagt

attactgctaatccaaagactgacaccaaacttggaaaag

>Contig2105|upf0563 protein c17orf95 homolog

gatggggtttcagagcaagtgaaaaagtttgttttcaaatcagttaacaatagagcgagtgaaaacgagatagagacgctagaaatactgataccagagc

aacttcaagcaggttacagtttctacacgtggccatcagcaccggtacttgcgtggttcttgtgggaacatcgacgtgaactgccaggaaaacatatact

ggaacttggttcgggcaccgcgcttccagggattgtcgctgcaaagtgtggtgctgctgtaacattaacggataatgcttgtctaccgaagagcttgcaa

cacgtgaaacggtgttgtgaaataaatggactccaaccaaaccaggcaagagtcattggtctgacttggggacttttcctatcaagtatatttagccttg

gtcaggtagatctcattttgggatcagattgtttctatgaaccaggtgtttttgaagacattatcgtaagcgttgcgttcttattggaaaagaatccaca

cgcaaaatttttatgcacttatcaggaacgcagtgctgactggtgcatagaacatttgttgcacaagtggagactgcagtgcgaacatatccccttaaac

aacttgggcacagaatcaggaatagacactagtgaattaatgcaagatcacactatccatctcttagaaataacaagagcttgatttgtataataggcag

ttaataatggcagcacgagctgcatataaaatatttgttggaaacctaccttggactattggacatcgtgaactgaggatgtatttttctgagtttggtc

ctgttgcctcagcagtcgttgtttttgacaaaaacactggcttatcaagaggctatggttttgttgtgcttggtaataaatcaggttttaacaaggtcac

caataaacagactcatgttttagaaggaaatcaaataacagttcaagttgcatcacactgacaacaataatatttggtgattatagtcatttgtagtcag

ttaccagtttttgtagtatttgtaacagtatggatgatactgatgagcttgtagaaaaatttacaaaagcagcaaagtatgctaaagagatttgtgcgga

atttgacagtaatgaacttctggaactgtatggttactataaacaagctacagaaggaccttgccaaacatcgaagcccagttggtttgacttaacagct

aaacagaaatgggaatcatggagacgcctaaaggatatggaccgtgagactgctatgataaaatatgttaaaaataatatctgaagtggatcctgtttgg

gaggaacgatttacagaa

>Singlet2168|upf0563 protein c17orf95 homolog

gcagtaagctgcatgacaaatactgatgagtacttaccagacacagagaaaacagtatttgactgggtgaaagaaggtaatgttcagaaggtcattgatg

cctctaggagcttcagttcacttgaaatgattaataagcaagatgaaggtgggatggcattacttcattgggctgctgacagaggaaatatggaaatggt

agactgtctagttgagaaactgaaagctgatgtgaatttaaaagatgctgatggccaaactgctttgcactatgctgcagcctgtggacatgttaacgtt

actaaatttttagttcaacatggtggagaccccaatattgctgatcttgatgggacactcccaaaagatatcgctgctgacaatgaaatattgaaggctc

tgacaatagcaaactgatattgtttcatgtaggcctatgagtatttgacatcatgattattcttgcattgataatgaagttaattgatcttttcccttca

tttgcttagctgataaatccttgaagtataatatgttgtaaataagtgctccatcatttaaaacaagacagtaaatttgaaataagtcattaaattctat

ttgagtttctattaaaaaaaaaaaaaaaaaaaaaaaaaaaaaaaaaaaaaaaaaaaaaaaaaaaaaaaaaaa

>Singlet2422|upf0551 protein c8orf38 mitochondrial-like

aggggcagttggggagatagggggaaccgggtgtgcagaggcggcgaactcaaaagctcaaatgaagtgcttcggtagtttacgcagaacttttggttat

tcggtgccacgcagcgaagtatgtaaaaagtcattaatgaaatatagtacttccaaagaaacttcttcagggtattgtgtgaatatggttaaaaacttcg

actacgagaatttcttgtgtacattactcctccctaacgatattcggacttctgcttttgcaatccgtgcatttaacatcgagacagcaagagttcagga

tagcgtgagtgatccacgtattggtcaaatgaggctgaaattctgggaagaaactattgataacatttacaatggacatgtgccaaagcaaccagttgct

agtgaactttatcgtgctatcaagaggcatactttatctaaaaggcacctgaaacggctcatattggcacgctccaaccatttttctacttcatttctga

gtctcgatgaaatggagtcatatgcagaagaatcaacatctccagtatactatatgcttctggaagcactgngcattaaaaacattcatgccgatcatgc

tgctagtcactta

>Contig373|upf0547 protein c16orf87 homolog

ggtgtcatgaatataatcgattagtgggaaaacatggaaacaccgtttgttcgctttatgcatgacaagagtcactatttagccacttgaggcaagtgac

tcacgtggacgatgtttattagtatgtaaacacgtaaaatgaaaggaatgctgtaattgatcattcacacattcagtgtatttaacaacttgaagcagat

aaggttgacatgaagtaaacgatgcatgggtggagtaaactataattcatgctctcatccttctgaagacctatgtacgtaaacaaagtgacacaatgag

gaaagttccaaaacacaagatgatagccaagagctgtcccaagtgctccatgcagttacctattgcatcaaagacatgtgcatgtggacatatatttgtt

ggaaggcgatcaaatgctgcacaagagggtgatggcacaagcattacccgtcgccgtacagaaagggtaaaacgagagaagcctaattactacgatgcat

tggagtacgacaaggagacaaggaagcagcaacaacgaaagcgcagtatgcgtacttctgctgaccagtcacagagcaaaagtgatgaacagattggtgc

agatgacgatgttgtgcatctcaaaaggaagcggaaacgcaacttcagaacagaacaggatagtgatgatagggacaattgtttacaaataatttcacca

gagaatggcttcaaatgttctgttattctctctgaaattaaccgaaaaatgggtgtaacatcatggagagtataattcctgggtttacattttgtgatgt

aataggaattacagtaatcttgtagacaggggaatgattcacagttttgcttcccttgtgtc

>Singlet2539|upf0536 protein c12orf66-like protein

cttgaaccaagtgtcaatgttgtgtttattgttatggatgtgtagatgtgcgtgtgtgtgatattgtttccgcgatgacagaccaccaggaagagttctt

aaatgacttttttaataatgtgtcgcagcttaattttgaaaaggccagggagctagcggaaaaagaacgagagatttcacgacttgggctaacaggacca

tggggaatgctgctaacacatttgtctcaggtagcacttgctgaacgttcctatgttgatttagggttttttgtaacgaaaaataaaggcttcctacgca

aagataactccctcaaatcaatgtatgacggtttacgtacagatctgagccgtttggaagagctgacaagatcaagtgggcctgataaaactgtgaatac

tgttgcaaatcagctgacacaatttttaacagctcgcattgaactaatagacttatatgaaaaaatgcaagtaatgggaactggaaagcaaatgcagtat

gaagagcttgtctcccatattgaatgtattgtggaaaaacattctttgtcatttccacatatggctctaacttccatcaaagctgctctcagtttggagt

gtgaaattctcgaccatttgttgcgtgcccaagttgaaatgcaaatgtggaggtttcttcagtccctaatgctacttcatggtgctaatacccgattatt

ggcatgggaaaataaactccaaaacagagagtcaaggaaactaggatttctgaaggcaaatcaacttcctgcagtgttccaatggtgtttaaaactgaaa

gctgcttttgtatccaaattcactttatatttttttactaca

>Singlet809|upf0534 protein c4orf43 homolog

gaatgaaatgtgagtcacgtgnntttgtatcgcccgtgcgtgatttaagtgcagcgaaaatgcctaaatcaattcagaatgatttaaagaagctcacaaa

agttatccatccaagaagccgaaaagcccagcagcttacaaagaagataagcaggatctcagcacgtgagaagattaagctaatacatcaagtaaggcag

aatttgctgggggagaagttacaatggttcaaagacaaccttgatgcaaatgctgccctgtgtacagcggaaatgcttatggaacttattgaacggtacc

ttcacagatttgatgatgagttagaacaaatagaacttaaaagaaccattggtggacatagatcacggcagcatgccagccgtgaggatataattcgtat

gaccatactgaaagatagagaggattatgaaactggaggtatagaactacctgatattcttgcaaccagccagttggcattgttacgtcagtggcaagga

gagttacgcctcctacaaaagttcagacttagactgttttcaaaacgtttcttgtcctccatcataggaaaa

>Contig507|upf0533 protein c5orf44 homolog

aagggggaatgtgaatgtgtcaagagctgcatctgttgacatggtgttgatgatgcagtttattcggaattgagtctgtagtgcgtcgctaagcctgttg

caaagtaaacgcttttatgaaagtttcctagaggcacatatatactatgatttcagtcgtatacagattacctgcgtacaaaattctaatctgaaaatga

agaaaaacttacgttgtcatttgggcatcttataagaaactaatttatttcagtgtttggcaacgagtgatgttcctaacaccattggatcgtggaaaga

tggaaggcaaggaaagaaacgagcacattcttgctttgaaagttatgcgacttacaagaccaactttatcaagtccattaatcgtgacaagtgatgcgaa

agatctgccaggaaacctgttcaacaatgatctgaagcatgacattacttctgtatcaagtgttgagacactagctgctggacagtttctcctgttgcca

caaagttttggaaatatttatcttggcgaaaccttctcaagctatatttgtgtccacaacgacagtaaccagatagcaagtgatgtttctgtgaaggtgg

acctgcagacaaactcccaacgtatacctctatcgggtggccactcagaagctgtcgctaaagagcttcatccgcaagagactgttgatgatgtgattca

ccatgaagtaaaggaggttggtacacacatactactttgtgaagtgagctacaactcacccatgaagaatcatttatcatttcgcaagttttttaaattt

gaagtattcaaacctttggatgtaaaaacaaagttttataatgcagagtctgacgaagtttacctagaagcccaagtacagaatattacttcaggtccca

tatgtttggagaaagtatctttggaatcttcacatcttttcaatgtgacagccctaaactcaactccagctggagaatctgtttttggccgagtgaacgt

tcttcagccacaagcaagccgacagtttctttattgccttgccccacagccgtcactgacttctgatttgagactacttgctggtgctacaaatatagga

aaacttgatatagtgtggagatctaaccttggggaacgaggaaggttacagacaagtcagttgcagaggatggcaccagattatggtgacatacgtttgt

ctgtgcaagaactcccaaatattgtggttttagaagaagccttcacgattgtgtgcaggataatcaacacatgtgaaagatcaatggacctcgtgttggg

actagagtcatcagcagcatctggtcttgtttggagtggagtgtctggcaggcagatgggtaaattggaacctggggcttcagtatatttctctctctgt

ttagttcctacaacaccaggtttacagaacatatcaggaattcgcctcacagacatttttctaaaaaggacatacgattatgatgagctggcacaa

>Contig1152|upf0532 protein cg3570-like

ctgtcgctcagcannnaaatgtttggttatgtttacggaggacggaaactgtttgttgcgtagaataaaccgcggagtctaacgttcagacatggtatga

ttaaaaggacacatcatcatttagtagtgttatgtacaagatacgataatcgcgccaaaaaaactgaatttgtggcttgtcaaatgaatgttcttcccgt

gcttgaaactaggaaagtactgttaaatcatcttgcagcgaaagtatggcctttatgtcattctaaatcattaggcagagaaattgcatctcgtatagct

ttcagcttaacagataacgaaattttgcaccttctcgaaaatgaaagacatcttgaagaagccgttggaaggtcactgacagccgtctgtccagaagaca

agaaacacgtactcggagaaaaactgtttgctgctgttgcaagtgtagaaactgaactatgtgcacaagtcacagggatgcttctagagctagatttcaa

aaccgtagagtttctccttaatgagccagatgccttgtcagcagctgtaaagaaagcaaggaatgaatatttgctgtacactcagagcacaggaccagtt

aatgatgataataaaagtgatgaaataggagaagccttatatgatctggtaatcaaggactacccagatgaagagctggcagcacgcttaacagggatga

tacttgagcttgatgttaaatatgtgagacaacttgttagaaatcctgatgatttaagagagaagctcttggtggcacataaagctttggaaaatgcaga

agaaagtataactgcttagggtgcacaatggcatcaccagaacatcaggaactggctctttttattaaatcagtacatcattctctcagaaggcaatgca

aaaaattaggtcatgaacaagcatggaagtaccactgtaatcagaagaatattttaaaaaaatatgcatcagttatgcatacactagctaccaaattttg

ggagcagtcaaatgacccacttgtgaatggttccagtaacaggatccaatgggttgttacgatgtgcatgaactattttctgaatggtggcagggtggaa

gaacttatcaaagaggttaaaaaacaaagttattgtacagaaaatacatcaaaatcatcattaatatgcgaaaactctattttcaatgtttcacctgaaa

aaagcatggagaaactgaagttattggatgtaggaagctgttataacccatttagtcagttctcagtgtttcaagttgtccctattgatctggcaccggc

cacccctgatgtatggcaatgtgacttcttgaatgcaaagatttctgattacggtcacaactctctaacagttcaacaaatcttaactcaaggagaaata

ccagcaaatttttttgatgttgtcgttttctccttattcttagaatacataccttgtccacaggagagatataaatgttgtcagaaagcatacacagcac

ttcgacctgaaggcctgctctttattattacaccagattcaaggcatgcaacagcaaatgcacctataatgaaacattggagaattgttttgggcaaaat

ggggtttcatagaatatattatgaaaaactgacccatttgcactgtatggcctatagaaaaagcattgatcacaatattccactgaagtgggctc

>Contig2115|upf0518 protein cg3558

ctgtcgggnnnnnnttcctcattgttgcgatagtaaatgaaaacataggcacagttgttttcgctgacagggcgtagtgtagtgatgattgttcatcagc

tttgtgagattagttgctaacttgcgtaatacactgtgttattgtacctgtttcgccttatcagtgtcgtagaaatgctgcagttttttatagcaatgaa

gttactgcattgtttccattttcaacggacaagatgtttaatattagacagacagtttgaagtcttgtattaatctgcaaagagaagaaattatccaaat

gttgtatattgttatggccgactggaaatgtgagcgacaggagcgtgccgaacattttttctgtaaatgtatcgcctgcaaaaggtggaaacatcactgt

agatagccacagaaatatgagctggcttcgcagtagccctctgcgggctagctttagcaagcggagacagtccaattctcctcctaaggactgtgatcct

gttgcatgctatgatactttctgtaagcattggcagcaggttgctgatatcattgaacgtacacagccaccatcttcatgcccatctcaggatgatgtgg

tcagtgttgtcaatcatttggcccagatggcaacattattaatgcttgagatgcagtccccgtctcctccagtttctagttgcggagnnngnntatcatc

tctctcttgcctggaatacctactgtcacagaatttattgaataaactttacaccctggagctt

>Singlet3685|upf0510 protein inm02-like

ccgttgctgtcggtgttttgaaaatgttatgtgatcagaagatggaattatttggtgctttgttctttttggccaaatgtttagctattacatatgggtt

tcagtcaggggtagactacgatggagtattaaatgtgaaactgcgccatgctcttgatcacgctcctagcccagagttcaccgatcgtggtgcaataacg

atacagagcatacgaactggagctgtttcagcacaacagatttccctttctgcatttgacagacagaaattgaagcacctggcagaaaaagacggcttgt

acaggcttaatgcaaccgttcggactgtggatggaaaggaaatttcatttttgacatttgtgaaagcttgctctctggtggagtcggggctctcagatat

actaacagtctcactggaccatacaggaatgcctgttgctgtgacagcaagtactatatcagatgggtgccatggagacctagaaattgttgaagatcta

cgcaactttaacaccagtgtatttgtcaagcacatggaacagggacctgtgcctgatactgctacctacatacagaaattggagaaagagaaagaagcaa

gagaaagaggtgaaacacaagacaaccgctctttcctagcaaaatattggatgtacatagtcccagtggtgatctttgtgctgctatctggtgctgctaa

ccctgaaggtgctgctggc

>Singlet4610|upf0493 protein aael009648-like

cttgggctgcttcgagtgtcagcggcgcatatctcggaggcggtgcacttccacccgcagacgctcaccaagcggcagacattcgtccgtgtctgtgtca

agctgctgaccaactgtgccggccggcacaaggccctcctcttgcgcagagaaaaggacttccgatctgccgtcgggaacatcctggaggacgtagagtg

tgtcgtcagtgccagcgtgccatccccgcagcaagtagcagagtgcggcttcctgctgtcggaggtagtgccgctggccgcacagccccagtcgctggca

ccgctcgccctgagtgaactctgcagctggctggcgagccgcgacggccgcagctgcgtgctgcgttcgctgctcaaggtagccgcgccgacgctggcgt

cgagcccgcaggccctgggtgccgtgctcgaggcggcgcttgaggcgttcttccgcatgcccgagccggaggaccaccagggcggactagggaccgagac

ggacccgcgcccgacgtggcaatgcgcggtgtcgatgctgtcgccgcccgcactgacaccagctctggaagaggcactgacctcgggcggccacgtgctg

acgctgcacgccttgctgctcaagaagctgccctcctgccgcgacatccgcgaggaggccgccgtgctcgccaagctcaccgactggatgggcgccgtca

agcctggggagagtgtggaggcaaagctggcactgctgtgggggcgctgcctgatgctggcacagcggcagtgcgaattctcggaggacacggnnngtgc

catctcctgccttcggacgcttgtttcgcgtctgct

>Singlet1612|upf0472 protein c16orf72 homolog

ccggtgctgtcggtagaagtgacgaaagaaacagacagaatacatggaaggtgatgtgattcatctgtgccatcagctggaagcattccaaagcaagcct

ttacaagaaacaggggagtctgtgtcgtgtatttgattttgagttccgtaatacaatgaacgaagacagagatcgcgaagatgacccgctgttagatatg

ttcattaccaattgggaggagcagtgtatacaacatgttgaaagtgaacccgactacgaaggacagcttcagagggagagggaacaaactcaccagaaac

tgtgggtgacttttcaaaacagcgcaactgcaatagcccaactttatagagaacgtcagcaaggcctttcgttatggatacctttccaaaccgccgctgg

tacggtgacaaatttgtacaaagattcaacggaaggaataaggcggactagtgaacttggaattcagtgcggttatcagagacgaaacaaggaattgttg

agctgggttcggaaaatgcgacacaaaatacgacgggaggatctactttcttatctggctggaaagccaactccacctcgaccacatcataggtcttccc

cacgtcccagaactctgatcgagcggcatggttctccagctggtttccagatggatgcacaacaccacgaattccatatcagcagcaatcattgtaacag

tgtctattctgatgacaatatgcgtacatttagggaagcaataactttcgctaatccagcaactgcagcatcacgccggcagcgttcatcagatcttggt

tcatttataacaaatgaatttgcccgtcacaagcgaccggctcccatcagttctcctttgtctg

>Contig3320|upf0468 protein c16orf80 homolog

gatagatttgttgttgtttacatgaaatttgtgtacattatttaataaacactatctttaaaaatacattccagagtggcttcttgtcaattttatatag

tatcgggagtaaaccgctacagatatgggacaaaaaagtcaggaatggtcatataaagagaataactgataatgatatccaatctttagttttggaaata

gtgggttctaacgtaagcacaacgtatataacgtgcccagcagacccgaaaaaaacccttggtattaagttgccgttcctggtcatgataataaagaatt

tgaagaaatattttacatttgaagttcagattctggatgacaaaaatgttcgcagacgatttcgtgcaagtaactaccaatccactactcgtgttaagcc

gtttatttgcacaatgcctatgcggctggacgatggatggaaccaaatacagtttaacttagcagattttactcgcagagcttatgggacaaattatatt

gaaactttgcgagtacagattcatgcaaattgtagaataagaagagtttatttctcagacaggttatattctgaggatgaactgccagcagaattcaaat

tatttcttccaatacaaaataaagcaaaggcatagtgttgcaaacatgggttgacagaaatggatgaatggttttgtagacatgtgtcattgatacatag

atgttcttaatggatcctcagataatcattcttattcaagatcataattcatcattacataagtgatgtatgtcattctcacagtatttctctgtctcgt

gtactgtataacagtaacaactctcacaaacacttgtcgaaaagtaacagt

>Singlet5002|upf0454 protein c12orf49 homolog

ctgtcgaacaannncatttcataatgtaggcgctattaagtttatcttgctattagaggtgaggaatatatgatcgaacatgtggacagcatttttaatg

cgtataataagaaaaagaattgtgctaggattgattttcgccgtctcttttacttactgcgtggtaagcttcgtgaaagagaatagctcccagaataatg

aaattgaatccttgcctgtgagaagatttcagcaaccatttctgtggcactctccaaaagatgattcaaatgggacagatgtcaaagttacaacttgcag

gaattctgtgcaagggaaggtgcttatagttgatgacagaggctatatatgttcacggactgatgttttatcaactggatgttgcaacacagatatagat

actacaaagcaatattcatgtgaaacctgcaaagacaatggatgctgtagtatttatgaatattgtatatcatgctgcttacacccagataaggcagtgc

cacaatacgtccgaaaaattgtaacataacccatagacacatgtcatattaacaaatgtaaatccagctgatgatggaggtttaaacatttgaaacgtgt

catggagaaaaatgttttgcagaatgtcttgggaaaagcatcagaaactttcaatgtgctattcgcctctgtcacagaccattttgagctttgtctagcg

aaatgtcgaactagctctcagagtgtgcagcacgagaattcttaccgtgatccacgtgcgaaacactgctacggtgagacacctgctgctccagcaggac

cagctggtgaaggtggcactgcaccatcatgagactgtcagagcagtgtgagtgcatctctttaatatgtgtgtgggctcagtgaatga

>Contig3638|upf0369 protein c6orf57 homolog

ccgttgctgtcggcgctcagggcgaatgtttttgtgtttcagcagacgaacgtttttgttgtagcagctggcttacggatcaacagtattctgcagagaa

gtgcctcgacaaaacctgagaaggagccagcatgttcaaagacagagagaatggcaaagaagctgagggagaaaactccaataggaaaactagatgaact

ggacgctggaaaacacccttatcaagaaaaggagcctcttcccccatggccagataatactaaccctcacactggggaagttggaggacctcgtgggcct

gaacctacacgttatggggactgggaaagaaaaggacgtgtgacagatttttgagtgctgaagtactgttcagttctaaataattggacacatcagcaag

ttcattgtgtgaatgaatgcttggctttatgtacatgtagataaaaataagggacattgagtaagtcattgttaaataaaatattaaaacctgttgattt

gttaattgtgtgttatagtattaatatgtaaatatatttatgtgaatctagacaaatatgcatatacaatttttccatttctgtattatgccctactgga

atgactgaaaccaaagacgcctcagtccagccatactttcatcaattcaaaatctattacctcttaaagcagtcagtgggctgctgctacagatcacatt

atatttttgtgacattcacactacttttgtgtaacactgactcttgatgtagactggctcttattatataagcatgttgtaaatgtgtccattcaaatca

atgaagattatcatggttattgtagatgatggtggtactacactatgatccttagtttgtaaagttatattcacactgaagtttgcaacatgattcatac

agttttcccgcaaacaactgggtgatcgatgtttgcaacatgtttcaaacacggaaacagctatcggtgaatgtcagatgaaactgtttccaaccagctg

tcacatattgccactgtatggctttaatgtttctggttgttacttttgattctgtgtgatgcatttgtggttgcactgattattattattattgaaagat

attggatgaggcagctagagttggaattaaaattaatgtggggaatgtaagcagtatagttttctcatcaatagactagatgatgtgagtactctacaag

gaatgaagatgcagatgatgtcaatatgccatgagtgatgtttgccaccagtgtggacataaaccttaaagcagaaacaatgttgtgaacagtgttagaa

atgtgtttgcaaacaaaaacatgttgcaaactttggggtgaatgttgcttaagactgtttaacagtgagtgtattctacaaaatatggttgctaggacag

tgtacaatctgtgacatacaagattctattacttttttctgtgaatctgctgtttttcctgcacccactggcttttcctctcctcgcacttctgcgtata

taatactatctgaagaacccggaattgcccaggtatttgttatgcccatttttttttttaattggaaatggaaataaaatgaactgtgtttgtcgtgtgt

tgttcctgtacactgggtgcagctgctgcatatgtct

>Contig4529|upf0364 protein c6orf211 homolog

gcaaatctccnncatcatacatgcgaaatatcgcgttttatgattttttgcttctattgttgacactctatacaataactcgtatggttgccaacctcag

aacgtacgaagaaattatccctgaaccaaagaatagcgaccagtaccaaaaaattacgctgctgaagttgcgtcatgtttgtttacaattgtgtcgtctg

caagtgtgaaagtctatattgttttccctcctgagttcattagtattgttggcacgtgttatttgagtttcgtatttgtatgccaaacagagtaggcatt

agaactagacttgcgcttcggaagcaaagctgcagatgtacggggtatcattaatttcgtttttttctagacgcgtgtgatttgataacaagaacgttac

ataaagatcgaaatgcgtgcatatgcaaagacttttctcaggtttaggtcgctggactgatgcagttgttcgagtggctttttaactttttgcatatttg

tattttatttgcaatgcgtgcgaatgaagattgcattgatgtggaaactccaccaggtgtgccactcagcgctcagtatatgaggagttttgcgtatgca

acagtgaaggatcggctacccgttatattgacccaggtcattgatcatttagtgcgagacaaggaagaaatcattaaagaacatggagaggtagcaagag

aagagctaaaaaccgtcattggtgcattttcacagttgaagaatgaaattcagacaaacaaaccactgacaccacttaaatcaaatagctctgacattaa

aatctggaattcagtcctggaagaagaaacaaaaaataacaaacacaagtggttcaatagtgactggctattttcagaatgctatatgtacaggaaagtg

agagaaatatttgaattaagtcagtctcttactgcctttgatccttttcgtaaatcgaaggaatctggactccataattcaatccattcaattgtgattt

tatcagatcatatgaaaaaacagttggaagaagttgaccagctgaagaggagtgacgtaagacaagcattgcagcgaattgtttatctgaacctctgggg

taaccgctttgacctatctctgtcaaaaggtgttcaaattctcataacagaaaatcctcttacatatgctgaaaacttnnnagatgatatcttagtagat

gagtctgataaaatatggggaagttttatctg

>Contig3625|upf0361 protein c3orf37 homolog

gaaagcggtaattttcgcagttgaaagttgatttgttgacatcgaaaatattgtgttatgtgaaaatttgtacactggtcatatacgtcagaaatgtgtg

gaagaacggcttgtacacttgacccagaatgtatatgcaaggcaactacttatcgcaggaaggactcgaacacttattgtcttccagagtggaaacacat

ggatgacaagtaccatgtctacagaccatcaacaaacattgcaccaacggaagtgttacctgctattatatcaggacatcagctgggcaaaacatccgat

agaataattgtcccaatgatctggggaatgataccaccatggcacaagggtgactacagaacacatggtctgacaactcataacatccgtgatgataatc

tcttcctctcaaaattgtacctcccagtctttaaacagagtaatcgttgtgtagttctttgtgatggtttttatgaatggaagaaagaagagggaggccg

tggtaaacagccatattttatctacatgccacagtcactcgggattgatatatatgacagaaaaacatggaatcttgagtggactgcagaaggaggctgg

aaaggaccaaggcttttggccctagcaggactttacaatatctgggtgtctcctgagggtgataaagtatacagttgttctgtcttcacgaccacaccaa

atgatgatttcaaatacatccatcacaggatcccagttgttttggagaacgatgaagccattgagatgtggctcgatgtagcgggaaaggcgccaattga

tgctgtggcattgttgaaagccccaaagttgcttgcttatcatcctgtatcaaatattgtgaataattcacgtaataaagatgacgagtgcatgaagcct

attgatcct

>Contig3525|upf0160 protein mitochondrial-like

caaannnnnatctgctgtatacgacaaggtatatgaattcttcattcaagaaatagatggcattgataatggagtacctatgttcaacggcgaaccaagt

tacagaatttgcaccaacttatcagctcgtgttgcaaggttaaatccattttggaatggtgtaagtgacagtgacatagatagatttaacgaagcctatt

gtcttgttggaaaagaatttgaagaccacataaaatattgggcccacgtttggtggcctgctcatgatattgttcgtaaggcagtaaataaccgttatga

gacacatccttctggtgagataatgttactgtcaaatggtggttgcccttggaaggatcatctctttgagctggaagaaaagttacacattgagccaaca

ataaaatatgtgctgtttcaagatcaagtagggaaatggagagtccagtgtgtacccattgcaccacaaagcttcgtgtgtcgcaagttcttatgtgaag

cttggtgtggtttgagagatgaagaattgagtgtaaaagctgggattgcaggttgtatttttgttcatgcaacaggttttattggaggaaataaaacata

cactggtgttcttgaaatggctgctaagtcactaacagcttagcgttttcacaatgtttaaaatgtctgtgcagtgataacattaaagtactatgtacca

acccagtcattacctttgtatgtatgacaacatatgacattcatcaattttgtgttgtaaagattttttttccagtggatgctacatgggtgggcataaa

caacctgaccagctcgtgtgtgtgtgtgtgtgtgtgtgtgtgtgtgtgtgtgtgtgtgtgtgtgt

>Singlet3857|upf0160 protein mitochondrial-like

gtgttaacgctgattgcgggaagtgttcaaataaatacttgagtaatgggaagatgacatttctcaaggacagtgaatgttgccatcagcactgtttggt

tccatccagattaagcgcttctgtgtagcatactgttgtcattttagatctcaccttcacataccactacacatttgtcagaaaggtagagctggtacac

tttctgtttctaatacatctgggaaaatgtgttctgtgtctagaccaagaatttgcactcatgatggaattttccactgcgacgaagctcttgcttgcta

tatgttgaagagattgccggaatataatgatgcaaaaattgtcagaactagagaggaagctattataaaagaatgtgatgttgttgttgatgttggtg

>Contig2384|upf0061 protein azo1574-like

cacannngtaaagtgtgtacatctaggaaaaggaagtaaagatcttttgacagaggttacaaaattgtttctgactggctgtaggatttatctatttatt

tattaagccagtccagtgatgtcctatttccgaatattagcaatacatggtagtgtgttaaagtattccaacagtaagttgaaacctgagcagtaatttc

taacaggattttgccctatgccagataatggaaagtggagacataagtgatgaggtgtcatggataattgagaggttggaggaatagagacccacacttt

cttccaaggggcagctgcctcagatgtcacttatcaggcagtctacctgctttgagcattctgattgctcttttgacatgacatgatataacatctccac

acatcatagatgcagtatatcatagtcaggcaagtgacattgcatgctgtgccattaataatcacacaataaagaaagtgtaggataagaatggggaaac

gtagcttctgaggtttttctactccagggatgatacaggaggaaaagactattttacattttgatcatccatagattaccaagagactgatacttaatta

ccacatgacacataagtgcccaaatattatatattaatattaaacataatataaatatataatattaaatatattactttaaatatttagttcccttaaa

tagtgaaaatctatcacttagaggagacatttcatcattgtgactttactaagtacattaggcttcaggtaagtcagtattttttcctccgatattctgg

tttaggctgctccaactctagtgctcacctgcaggcagtgcaacaggcagtcagccagcagagttgtagtgtatgttccatactactctggcctctcacc

catgaacacacgtgcaataggctacctgatggctagcttatactagctacgcctacctgcaaatgagctgttctgcctgtatttgttgaattattggtat

actgacataacatgttaatatgatcatttgcatacttgtttgctgtagcttataaattctgcaaggtataagtttgtaggaatacttcagctattgaact

acaacttctctgccttgtagtggctatttgcagtggaacctctactattactgttgtcagttcttctgatgtgttctccatgatggtacttgctgaatta

ttttaggctaactctaacaagtacactcatgttc

>Contig4320|upf0061 protein azo1574-like

caaatttattatagtcgcttagctgcaagtggaaaatatcgaatcagttatttgttatggcagcagttgacattcaaaatacagacactcacgaaaatag

gtgtggcgcctttaatgaagtttttcaatttacatgatgtatcccatttgtaattaatgaagattcttttgtgttcgacgattttgtgtgttgttgccac

ttcgataacctgtgacacttgtgataagtggccgaagaattacacgcacttagtgatggatataatcgagcgtgatacttgtcccaggctgaaaagaagc

ctaagtgaatggcagttttcttgtagcaaattgaaacagttgcccattgatgaaataaaagaaaactatgtccgtgggaatgtagaaaatgcggtgtttt

cagaagtgtcaccggtgccattaaaaacaaagctaaggcttgtgtcgatctcgcgagatgcattaacagacattcttgacatggatccggaaataagtga

cacagaggaatttattcagtttatagctgggaataaagtgctggatagttcaacgccgttagctcacagatacggcggataccagtttggactctgggct

taccaactaggagatggaagagcagtatcgcttggagaatacgttaacagcaaaggacaacattgggaattgcaactannnggctcaggaaggacaccat

actcgcggtttggtgatggacgagcagtgcttcgttcgtcagtcagnnagtttctttgctctgaagccatgtattatttagggattccaacttctagggc

tgccg

>Singlet7775|upf0061 protein azo1574-like

attaaagttcattgctttccaagtgaaaaacattgtcaatattttagttgagtatagataaatttttcttttgataaacactatgtgattggagacttga

cttttaaacatgtcactgaagagactgggcctagttccatgttgtgacgggtttagtttattttagagactagtaaatatttataatttaggacactaag

gtaaagtgtgtacatctaggaaaaggaagtaaagatcttttgacagaggttacaaaattgtttctgactgactgtaggatatatttatttatttatttat

ttatttattaagccagtccagtgatgtcctatttccgaatattagcaatacatggtagtgtgttaaagtattccaacagtaagttgaaacctgagcagta

atttctaacaggattttgccctatgtcagataatggaaagtggagacataagtgatgaggtgtcatagataattgagaggttggaggaatagagacccac

actttcttccaaggggcagctgtctcagatgtcacttatcaggcagtctacctgctttgagcattctgattgctcttttgacataacatgatataacatc

tccacacatcataggtgcagtatatcatagtcaggcaagtgacattgcatgctgtgccattaataatcacacaataaagaaagtgtaggataagaatggg

gaaacgtagcttctgaggtttttctactccagggatgatacaggaggaaaagactattttacattttgatcatccatagattannnagagactgatactt

aattaccacatgacacataagtgcccaaatattatatattaatattaaacatagtataaatatataatattnnntatattactttaaatatttagttccc

ttaa

>Contig2383|upf0047 protein yjbq-like

ggatcgaatcaaaacagatggcagctctagtgcaagtatgtgggaggtttactgacaagcaaggtttcgatacctgttttaatgaaacgtaaacgataaa

aatgtgttcttgagtgaaatgagctgattgcccactgatggaatagttatgaatctaataccttgcaacgcgtgctgcttgaactttgatctgcaatagt

agtgggagttggtgggttgctgcgtcagtgtaatggttagagcatgcaagaagtcggtggaagtatgatgaagaatctgaaatgaatctaaaagagagct

ggaacatgctgtcgacttcatagtattcgtgattgaagttacagcggcaagtgtatgtagtagtgtgctcaacatggcttcatcgaacagaggaatagga

ttgcagataggttcggcttggtttcagagaaaaattaatctcaggccacaacatagaggtgtacatctagtgacggaagaaattttaaggcaaatgccag

aaatatgtcaattctcagttggactgtttcatgtacagattcttcatacatcagctagcttagctcttaatgaaagttgggaccctgatgtaagggatga

catggaaatgatgctgaataaaattgtgcctgagggtttgccttatagacattcctgtgaagggcccgacgacatgccagctcatgtaaaagcttgcttc

cttggctcttcacttacaataccaataacggatggtaaattaaatcttggtacatggcagggtatctggctgtgtgaacaccgagaccacgcaggctctc

gcaaagtggtaattacagttaatggttgcctcagagactcgagtcgaagcccattgagccccgtttcaccgatagcatctacaagtagttaagggttcag

ggagaagtatatgatgtgtgggccgtagatttaacattccatactacagaagtgattgataataatttcatacatcttcatttataatgaagagtggtgt

gatgggaagtttaggtgagacaacgatgtttcttagttgcaaaacattgttaacagggaagcaagaagaaagttgataatggcacaagtgcaattctctg

atatgttgtgtaaacatatgaaaaaggcagtttttgtgtgctcaacttgctgcaacatttcagtgatcctacgaatgtcacccagttatccctcagggta

cgagtgtgtaaaaacactacactcacttcgccgatgaagttgtgaagttgagagtatttagttaaggaaacagagttcattccttgttgtgcaaggaaca

cacagcagatggtagtttggaaaggaaaaagatttggcctttcactgccacagcacaagaagtatactttattttgtgaaacacaaataaccacgtacgt

gctgtacctgggctttatgatatgacatgtacagttatatagaaataaacaaactgatcacttatacatacattagtatccttccacatatttgcaagaa

aaaacacacaaatgtaaaaaaaaaaggtaatccctaaatgttttctacacttgcattatggtttcctgagtattgcatcatacatgaaaattttaattaa

cattccatttatgattactg

>Contig157|unnamed protein product

ggccaagggggtgatttaatatgctttttgtaattcagattatggcaacttttctccagaaatgacagccctgcagttatgctgtatacactttaggtgc

ttctgactgaacaaaagtgcattattgtatcagttttactcataatttgcacctttcttagtaggcacctcagtattcacagttaataagtgtatgtaat

atttcagctaatagacatcaaaaacttaaggtcttgaaatgactttaatgaaatttaatctacatagttttaagtttttattttgacaagctgtttaatg

agcacatttggtaaaacagcagccaaagacatgttgcaaacaaattgaaaaattgtggggtatattttgaagaaatatgtagtaattagttttcagttca

actgctttcacaggttcttaaatatttgtttcagatcttatgagaaagttgagtttaataacatatgagtataaaactgaatgcccctgattcaaaagaa

tatcattagatgtatgcaaaaattcggaagtgtgttgttaagaatgtccttaaacagcagatgaatgcagagaggagtgtaatagtgacattttatgccc

tatcttctgcatttaaagctttctagtgttcagcaattacgtattttgtagcatttaggattgacagctgtgtgttcttttacaatttcacttcagtctg

atagtaactgatgacattgttatggaatatatttgtgatgaatttgttacatatctttcatgccaacatatttttgtaaataaatactacataactaaaa

ctgttttagttttccaccatattattgcacttggtactgagggcaatttggtctgcttatcatgagtcaatgtgtaatcctcttgatagtatgtgcacaa

ttgaaagcattgatagtgtgatgcattttccttattcgcaaatgtacttttttgctgtaatgtcaccatctttc

>Contig1774|unnamed protein product

ctgtcgcataacttgcttctgtgtcgtgaccaggaacttgctgtccctggctcatatgtgcccaatcagccaatagtacgcattgcttacataaacagtt

ccttacaagtcatcacatcaaagcagcgacctcgtaaactatgcatcagaggcagtaatggaaaagattatatgttcctactgaaaggtcatgaagattt

aagacaggatgaacgtgtaatgcagcttttcggtcttgttaacacgctgttgctgaatgatcctgatacattccgcaggaatcttacaatacagaggtat

gctgtgattccattatcaacaaacagtggactgattggctgggtgcctcactgtgatacacttcacacacttataagggattatagagagaaaaagaaaa

tattattaaacattgaacacagaattatgcttcggatggcaccagattatgaccacctaacactaatgcaaaaagtggaagtgtttgaacacgctttaga

gcacacacaaggagatgacctatcacgacttctctggcttaaatcaccgtcatcagaagtgtggtttgatagacgtacaaattatactcgctcccttgct

gtcatgtcaatggttggttacatcctgggcttaggagacaggcatccttcaaatttgatgttagatcggctgagtgggaaaattttgcatatagattttg

gagattgttttgaagtagcaatgacaagagaaaagttcccagagaagattccatttaggcttactcgcatgctgattaatgcaatggaggttacaggaat

agaagggacttatcgccgcacatgtgagtccgttatgtctgtgcttcatcgcaacaaggacagtctaatggcagttctagaagcatttgtgtatgatcca

ttgctgaactggagactcattgataatgccacacccaaagcaaaacgctcaannncccaaactgatttgtctgtttcatcatcatcaca

>Contig2164|unnamed protein product

gtgtcccatctgcattgtcggtattcaatgtaataagggtcgggttctctttaagcttgtctggaaccatgaagacattacaataaaatcctttttataa

tatttctttggctcttgtcccgttcagagaagaaaattcgtatacctgtatctttaaaaaaagtatgatgtttgaaaaatctagttctgccctgtctgtt

ttgttatttttgatgttttgatcttgctcttaacttaacatcagaatatttagtgtctttttcctgcattatcagtggatagaacttgttttatatgttc

cactgggacataataacaaaaagaaaaaaaaattcagaatagcacagttaaatagttttatttcgataatagtgtctttattgcttcaatgaaaacaaag

atagtggtgtgtttttaacagcagcagcccctacctgccagaattctatgagggtatgtcagtatggcaaaaaaatgagggagaatgttttaattccatc

gtgggtcattctatttgatatttctttttatcagcaagaaacaaattaatattaaaaagtagcatttgtctcgcataagaagactcgctaagatgaactg

ttggaattcgtggtgtactcatgtacttctaaaagtttccatgggaacccatgaaattttatttttcata

>Contig2780|unnamed protein product

canncnnntagaacttagaactacctaaacctaactaacctaaggaaagcacacaacacccagccatgacgaggcagagaaaatccctgaccccgccggg

aatcgaacccgggtacccgggcgtaggaagcgagaacgttactgcacgaccacgagatgcgggctcaattccagcaaaaccattttcattgaagatgaat

gtgatttgtgtccctaaattaaactggaatcttctgtgattggtagtacataaaaagacataagggcactgcacgttgcatgggtatgtttccagatcgg

caaccacgaccagcaccgcgtggccagccggtacagtccagagatggtagacgggatgaacatgctggtgacgctcctgccgggaaccgccgtcacctac

aacggagaggagatcggcatggaggacaactacaacatgacgtgcgaggtggcccacgaccctcaaggctgccacgataacgtcactctcgggaactcca

gagacccggagaggacgcccttccagtgggataacactgcaaacgctggcttcagcaacaccactgacacgacttggctgcccgtcaacgagaactacga

gactctgaacgccgcggcccaggaggcgggcgaccgcagccacctcaagacgtaccgccagctggtgcagctgcgcgccgacccagccgtccagaccggc

gacacaaaggtcaacagctcgggcaacgtgcttgcgttctccaggtcaatgaacgacagggcatctgtagttgtgctggtgaacgtgggaactgaggagg

aaaccgtggacttggtagacacgctgcctatcgtcaccgaggacgacgttctggaggtgtacgccagaagccttgagtcccaaaaggaagaaggggaggt

gctggagacgagggaagtgacgctgtcggggcaggaggcggtggtgctgcgccgcaagcaagagcagaatgcagccgcctccagaccgctgtctgcagcc

gcggttctgctcacaatcgcggctctgctgtgggctggtggggtcaaccgctaggcaggcgcctaccggtttgctgagacacacacctcgcagtatttcg

ttcctacttccacaccagtcattacattacttcatgtgcataaagttaccttttatatcttgcgtaaatgagtaaattattgtgtcaagactcgatcaat

gtaaagtacttgtaataaattattataaagcaaaaaaaaaaaaaaaaaaaaaaaaaaaaaatgc

>Contig3431|unnamed protein product

ctggtgacctgctgacctcggcatatctttgtccttcctcaaatcagcgttaaacaccatttaatccaactatggcaacgagaatcagttatctgtcgcg

tctatgtcgcagggcagcggttgcgagggagcatctgcacgagattggtgtgcaggattggaacagggtcctcatcggttgtgtcagaatggcgtcatca

tcttcgcacagagtcgagagagatacatttggagagcttcaagtacctgctgataaatactatggagcacagactttgcgttctgtcatgaactttccaa

ttggaggtgaaacggagagaatgccaaaacctgtgattgtcgctatggggattttgaagaaggcagcagccattgttaataaagagtatggcctggaccc

aaagatcgcagatgcgattgcgaaggcagcagacgaagtgatcagtggggatttgtatgaccaacacttcccccttgttatatggcaaacagggtctggc

acacaaagtaacatgaacacaaatgaggtgatcagcaacagagccatagagctattaggtggtgttctcgggtcgaagaaccctgttcacccaaatgatc

acgtaaacaagagtcagagctccaacgatacattccctacagcaatgcacattgctgttgcaactgaaataaacaagtcactcattcctgcccttgaggt

cctgcacaaggctttggataacaaggcaaaggagtttgagggcataataaagataggacgcactcacttgatggacgctgtacctctgacactgggacag

gagttcagtggatacgtgacacagttggccaactcgatagatcgcatcaaggcgacgctgccgaggctgtaccagctggctctgggaggcacggcagttg

gaactggactcaacacacgcatcggtttcgctgagaagtgtgcagcgaagatcagtgagctgacatgccttccatttgtgacggcaccgaacaagttcga

agccttagctgcacatgatgccttggtggaggtctcgggtgctctgaacgtggttgcctgtagcattatgaaaattgcaaatgacatcaggtttctggca

tcaggtccgcgctgcggtcttggtgaaatcagcttgccagagaatgaacctggcagcagtattatgccaggaaaggtcaaccctactcagtgtgaagcaa

tcacgatggttgcagctcaggtgatgggtaaccatgtggcagtgactgtgggtggctcaaacggccactttgagctgaacgtattcaagccgatgatggt

tgccaatgtcctgcgctccattcggctgctggctgactcttcttgtgcatttacaaacaactgtgttgttggcatcaaagcgaataaagaaaggattgcc

aaactgatgaacgaatctcttatgcttgtcactgctcacaatcctcatattggctatgataaggctgccatg

>Contig3792|unnamed protein product

gcattcaataaatagccggctgtgccgtagtaccacttggtcagaagcgatctcgccgaataaatatttgtgctggtctggatcaaaaatggcactaact

ttcaacattactcgtgtattaaccgggcgagtgtctggtatctccaggtggtcgtacgttgcttcgaaaaaatatgcaacaagtggtgcatacacacaca

ttctaactgagacagctggagagaaaaaaaatgttggtatcataaaattaaaccgacccaaagctttgaatgccctgtgcaatgatttgatgcatgaagt

tggaagtgctcttgatgcatttgagaaagatgacaatattggtgctgttatcattactggaagtgagaaagcatttgcagcaggtgctgatattaaggaa

atgaaggattacacatattcagatacagtgaagggcaacttcttgggtcactggactagggtgtctcactgtaaaaaacctgtgattgccgcagtgcatg

gatatgcattgggaggtggctgtgaacttgccatgatgtgtgacatcatatatgctggtgagaaagcaaaatttgggcagcctgaaatagctattggaac

tattcctggtgctggtggtactcaaagacttccacgaatggttggaaaatcgaaggcaatggaaatatgtcttaccgggaaccaagtcactgcccaagag

gctgaaaaaatggggttggtcagtaaagtgtttcctactgatcagttgctacctgaagccatcaagttagcagagaaaatatcatctcactcaccattga

tagtggctctgtgcaaagaggctgtcaacagagcttacgaaactaccctc

>Contig3847|unnamed protein product

ccgttgctgtcggttaggacctgagcaacgttttgtagttttgtgtcataaagtaattttgccaccattccgaatatcatggctgctattcgtaagaaac

tagtaattgtaggtgatggtgcatgtggtaaaacttgccttttgatagtgtttagcaaggatcagtttccagaagtctatgtgcccacagtatttgaaaa

ctacgtagccgatattgaagttgacggcaaacaagtcgaattggctttgtgggatacagctggtcaagaggattatgataggcttcggcctttgtcatat

ccagataccgatgttattcttatgtgcttctccatcgattcgcctgattccttggaaaatataccagaaaagtggactccagaagtaaaacacttctgcc

caaacgttcctatcatcttggttggaaacaagaaagatctgagaagtgatccacacactataaaggagcttggtaaaatgaagcaggaacctgtaaaacc

agcagaaggaagagccatggcagaaaaaataaatgcatttgcctatcttgaatgttctgcaaaaagcaaggaaggagtacgtgaagtttttgaaacagca

acaagagccgcccttcaagtcaagaagaagaaatccggcaggtgcaggctgttttaagctgcgaaaaccttgattatagtgagttcattttcagtatttg

tattcaaagttttagtaacaggtttgccgaaaattaaataaattgaaactttaaatatttagtgcttgttagaatatatgctgttagatttcaagaactt

aatgggaactcgatgtgcaatcacagtgcgtgtgtttagtgttcgtttcaaacactagataattttgaggatccattctaggcagtgttatggaataggc

atatccgaatgttttgttgcatttgtaatgtaatattccaatcgttgcaaatgatattgactgtagtaagatacttccatagtatcataaatcaacagaa

agttgcccccccccccccctcttgtcttatgatgagtgaccattggtgttgtgtgtatttgtattaatttggggaacgaaggacaaatggatgtatggac

aggtacatgtaagaaagaattgttaagctttaagcagttcatttattgcttcatacatttagtaatcagtcagcaagaagtcttgttagccttctggtca

ttcagctgtaagtatgatagtgtatgttagcattccagcttgcagcagttgacatgtttgcatccttataggataatgatttgttgaatgttagtgcttc

agttctgagctatatgtacctgcaagcagtaagctataaaagaaatcatgaggttttgttgaaatcatcccccccccccctctctctctctctctctctc

tctctctctctctctctctctctctctctctctctctctctctgactgcaatataca

>Contig3915|unnamed protein product

ctgaaacgtttatactgaaacgcagaaaaattgataaggattctgaatcactcttcatgcggctagtgaaatacaccaatgaacagccatggctatgggc

tgtgtatgtagtagtgattggacttcccatagttctggtgattgtgttttgttgcggttcctcaagcaaggaacgtgatgaacataggcgtactgctgaa

gcaaagaagacagatgccatatctcctgatgataacatagaagaaatacaggaactgaaaccaagaaacactgttccaacttcgaagagtgatctggagg

ttccagaagaggaagaggaagaagaagaagataaatcagcagcagcagcagaaccagaaccagaagtggcaacttctggagttcaggatgaaggggagaa

aactaaaggatctgaagactctaaacctgagcatgcatcagtttcggaacctgaagtggaaataattgaagaagaggaagaagaaaataaagatgctgat

ggggaaacagctgcaggagatgcacctcggtctccaaagaaaagacgcccccggaaggagtaaattgtcctaaaacaatgcagctgatctttgcattacc

tcaaagtgcagcttcagtttactgtgctttgtgactgaaagagaaactctgaacatagagtgctaattttgattgtgtttgttttacattgttgaaaata

ttgtgcagatgtttatagttttcatgtattactgtatgtgttcatgactgtcagtgtgaatcaaatgtgggtaacaaactcaattatttttnnnnnatgc

agtnnnnntattttcgttacactgcttcagttctggtcattttaactgtttcatttatgttttt

>Contig4682|unnamed protein product

ggtgttaaatttgagacacagtttggatgcatttgcagagatatatgctcagaatcttggcgtgaggaagattatacggttaaacgttagataaataaac

catgaggcttaaactatatcactcttcttacgtctacatttgtgcacttgtttttgtgtgcctgtggacacagtgcagatttgaagacatagatgatgac

gatgctgtggtgaccacggaagaaatagtaccggaagaggcagttgtaaatgaagaaataaattatgagagtcctaaaccatctggaaatgtttattttg

ctgaacattttgatgatgtgaatcgattcaaacagagatgggttagttctgaagcaaagaaagaaggcattgacgagaacatagcaaagtatgatggcag

gtgggaagttgaacctcctgtaaaagatggtttgaaaggagacttgggattggtactgaaaagtaaagcaaagcatgcagcaatttcagcacggctggat

aagccatttagctttgtagataagccactcattgttcagtacgaagtagtcctgcaggaaggacaagaatgcggtggcgcgtatctcaagctgctttcag

caggaaaggccactgctgatttgcgccaattccacgataaaacgccatacaccattatgtttgggccagacaagtgtggtaatgatcataagttacactt

tatcttccgacacaaaaatccagttaatggaactttatcagaaaagcattgtaagaaacctaaagaacgcctggaagagcctttcaaagacaaacagccc

catctctacaccctagtggtacggcctgacaactcgtttaccattaccttagacaacaaggtgattaatgaaggaagtttattagaggacttcagtcctc

ctgttaatcccccaaatgagattgatgatccaaatgataaaaaacctgacgactgggatgagaaagagaagataccagacccagacgctcgaaaacctga

tgactgggatgagaatgcacctgctatgattgttgatagcactgcaacaaaaccagaagggtggctagaagatgaaccagagatgatacctgacccaaat

gcagagaaacctgatgattgggacacagacatggatggagattgggaggctccattaattcctaatcctgtatgtgagaaagctccaggttgtgggccat

ggaaaccacctatggtagctaatcctgcatacaaagggaaatggagagcacctcttattgataatccaaattataagggcaaatggaaacctcgccgcat

tccaaatcctgacttctttgaggacaagtcgcctttcaaaatgacaacaatttcagctgtggggtttgaactttggtcaatgtcaaacatgattttgttt

gataatatcattgtaactgatgatcctgctgttgctgnnnnntgggcggctgaaacgtttatactg

>Contig4708|unnamed protein product

cagggcgaaggttacgtttggcagtagacgtcgatcagtctgacggggtgtttgtgtgtgtgtgcagtgaaggtgagaaggttaagttaataattgcgag

tgtaatatcagaaatcaacagtcgtcagctatgaagatctggacatcggaacacacattcaatcatccgtgggaaactgtagcccaggcagcatggagga

aatatccaaatccaatgaacccagccgttattggtacagatgtgattgaaaggaaagtggttgatgggatactgcacacacatcggctggttagctccaa

gtgggggtttcctaagtgggctcaatcgatatttgggaatccaaatgtgtgttatgccagtgaatattctcaagttaatccaagtgcaagacatatgaca

ctgaaatcaagaaatctcacattctgccgtcatatagctgtggatgagactgtaatgtatgtaccacatccacaagatccaaataaaacattactgaaac

atgaagctgttgtcactgtgcaaggagttccacttaattcctatgtggaagatgttttaacgagcagaatttcccttaatgctgggaaggggagacaagc

aattgaatgggttattggtaaaattgatgcagaagtaaaggaaattgcttcaaatgctgtgaagagcacagatgacttgctggcacagactaaaaagtca

tttgatgaaattacaagctctgcacgaaaatctatggatgatatttcatctgttgctaagaaatcactagatgatctacacaacttaacaagtgcaccta

atcgaacaaatcaaaatataccaaagttgtagcattgatattaaaatttagttcatttaacaactttgtgtagtaatcttttgtaatc

>Singlet274|unnamed protein product

ttagaaaatttagcagcgaaaatactgaaagnnnntcaacagccagatagtcaagagctgaaaatattgttgcttcagctcgcagaagtggcaaatacac

ctggcgacataaattgtcttattggtccggttgtcaagcttctgccatctcacaacactagcgtcaagtttacaatctacggaattttaccgagttgtaa

atacagtcctgaatgactgccgtgatcccaatccagaagtgaagcgtgtagctgtatctacattgtgcagcttgccaccacttttacaagaatatgtggg

acctgttgttgatgcagctctgaaggacaggcatcccaaagtacgcaatgctgctgtgttgggttgtagtaaaatcttcaaagaaatgcccaatttgctt

caggatattggaatgatagacaggctgtatgagacaattcgtgacgaggacccagttgtgtccacaaactgcctgctggttttggacactgtgctggcat

cagaaggtggtgtggtggtcagccgaagcatggcgaggtatttgctacatcgccttccaatgctgcctcatccacagcttgctacagttcttcagtttct

ggcaaaacacaaacctgccactgaagatgagctattcttcattcttagtgaggtggatccatacctgggagtaatgacgagtccccctgtggtggtgagc

tgcgcaagacttttcttgcatttgac

>Singlet897|unnamed protein product

atgaaaggcttgaacgtgaactgttcggcacgggaaacacaggcattaatttcaacaagtacgaagacattcccgtggaggcgacgggtgaagatgtccc

acctcacatcaacagttttgaagaggtgcagatgactgaaattatccaaaacaacattgcccttgcacggtatgacaagccaacaccagtgcataaatat

gctatacctataattatggcaggaagagacctgatggcatgtgcacagacaggttctgggaagacagctgcttttatggttcccatcctaaatcgcatgt

atgaatgtggtccgcctcagatgactatgaataacaggtcaat

>Singlet2148|unnamed protein product

ccgttgctgtcgcgacagcaacggtaaatattgctatgactctttgtaagttaaagtattcatgtattgtgataaatatcagcaatgaagcattatgtga

ttacttaccttgttgctgtgttttcttcacattaattggctgaatgttcttatgtgtgcaaatctaagtctggttatagttcattgtaatactcactaaa

gagcaactagagtgcagagtccctaatcacaaaaatctacaaattgtaattgagatataatgggataattattgtggtaattcataattatagtttcaca

gaactattttgagagtaaaagtttataagtaatacatagaaaagttcactttgtgatcaccagtctagcctttcattgttctaatgtgaaaggtaacggt

ttttggtttgagtaattttcttctggttatgttgcttataagtattttatcagttgaggttaataaaagaatgtccaaaatatgtcatcgatgttgtagg

ccttactgtcattgactgttgagtactttctgaaaaagcatttgtcaaattctcatcagcttttatcagtttgtatgtaatgtcttagtgtatattatga

atcttccagatgtttgtgagttaatgtttgttacctttatgattagaaacagtatcaggtaacatttctttttaagcaattgtttccatgctatgtgaaa

atcttactgtgtgtgtgtgtgtgtgtgtgtgtgtgtgtgtgt

>Singlet3767|unnamed protein product

gacaattatacagacacggtgatgatgactgaggcgtacggcactgtggagcaggttgtgggttactatggcaacgccacaaatcctggcgcgcacttct

ccttcaacttcttgctcatcaccgacctcaacagcgcatccaccgcagaagacttcgccaacgtcatcggaaactggatcgacgtgatcaacacacggga

actctggtccaactgggtgatcggcaaccacgaccagcaccgcgtggccagccggtacagtccagagatggtagacgggatgaacatgctggtgacgctc

ctgccgggaaccgccgtcacctacaacggagaggagatcggcatggaggacaactacaacatgacgtgcgaggtggcccacgaccctcaaggctgccacg

ataacgtcactctcgggaactccagagacccggagaggacgcccttccagtgggataacactgcaaacgctggcttcagcaacaccactgacacgacttg

gctgcccgtcaacgagaactacgagactctgaacgccgcggcccaggaggcgggcgaccgcagccacctcaagacgtaccgccagctggtgcagctgcgc

gccgacccagccgtccagaccggcgacacaaaggtcaacagctcgggcaacgtgcttgcgttctccaggtcaatgaacgacagggcatctgtagttgtgc

tggtgaacgtgggaactgaggaggaaaccgtggacttggtagacacgctgcctatcgtcaccgaggacgacgttctggaggtgtacgccagaagccttga

gtccca

>Singlet5478|unnamed protein product

tacggccgggcgacataaattgtcttattggtccggttgtcaagcttctnnnntctcacaacactagcgtcaagtttacaatctacggaattttaccgag

aatatgtaaagcacaacctgatgctgctgttttagttgtaaatacagtcctgaatgactgccgtgatcccaatccagaagtgaagcgtgtagctgtatct

acattgtgcagcttgccaccacttttacaagaatatgtgggacctgttgttgatgcagctctgaaggacaggcatcccaaagtacgcaatgctgctgtgt

tgggttgtagtaaaatcttcaaagaaatgcccaatttgcttcaggatattggaatgatagacaggtaattactactcatattgataaattcatgaagaat

ggcaataacttagttaaggtatatttcttatgcacattaaattctaagttagcagaaatattgaacctgttgatactaaccttctgctgttgcccattaa

ttcataaaaactgcaggcattaactaatcatcaacttctaagatgtgtaaaacacacaccataacaagtattctcaccatatcagtgcagggcacaccaa

ctaatgaccaaactgccacctcccagtctaccctagaccttggagtatgttacagataaatcgatctccataatctacatttggaaagatgatatagaag

cagaataaatgctgtcagttttgacagatgttctgttctcctcaccataatgccacagctacatcagctgtggcagcaaacatctttttaggcattgaat

aaatgatactattctcatatttgtgacagtgattcagtttgcacagactgatttgtgatgctgcgaactcgatt

>Singlet5948|unnamed protein product

aaggggaaattgtgaaaaacaagacacaatggtttcaaaaatacgtcctaaaagagatatatatacctgcagaaagatttatctgtattgggaaagaaat

tgcaaagagcttgtgaagtgtggcgctgaagttgttgcactttcactcaacaaggaaccattagaacaattggcccgtgaagtaaatgttactacaattg

ctgtagatctaagtgattgggataaaacaagagaagctgtgaaatcagctagacctatagatctcttggtcaataatgcaggagtagtgagcctgaatcc

ttttctagacattaaaccagaagaatttgacagtacatttgctgtcaatgtgaagcccataattaatgtcagccaggtcgttgttgaaggtatggtaaag

agaaagactggagggagcattgtaaacatctcttcgcaagcttcacaggctgctctcctaaatcatacagtttactgtgcctcaaaagcagcagttgatc

agattacgaaagtaatggctgtggaattaggaccacataacattcgagtgaatactgtaaatcccacagtagttttaacagaaatgggtagatttgcctg

gagagat

>Singlet6296|unnamed protein product

atgaattgtgagaaatagatgtttcttgtagtgagagatttgtgtgaatgttgttgttgcttgattaatttgttttaacttatgctgacagactgattta

actggctggtccagttttttttataagttagttgtagtctgataaagagaaatgacattatcaaaagaattactcttaaaatttgggacctatattttgc

catagttcatttacaatggattattaacaaccattacatacagttactcattgattgtgtaatggagtggagtaataagttgcactgatcactgtgtgat

ctgactgcatactaagcttattgtgatgtcttgctgtgattcactcaaaatttttacgaacagtggtattgcacctacttgtctgttaagtttaaatagt

gagcaactgttctgtgcaaagatgatacagcagatgtatgctctgttcattgcaggcgtttactggtgttaatttgctattgcagctgacatgcacagaa

aagtaaatatgcactgcagttaaaatgtgaaattttcttacagcacacgttttgtgtttgcttgttgcatcaataagaaatactgtgaacttggtgcagt

agagacatcataacaggcctttatgannngaaaaaagtgtacttttacactttcgatagtcttanntattgctatgactctttgtaagttaaagtattca

tg

>Singlet7548|unnamed protein product

gtcatattctatctggagcatgcattgaacctgtagcccttgatgagcttatacctgactggaaggaaaagggagcacctcttaatacacctgttaagga

agacaaatttgcataccttacagagaaagggaggataccagtccccatcttcagctggatgccaatgtacaatcatggcaactacgttgtcaggctgggt

catgttgttcgttggcttggggagcaggctgagcaacttggtgttgaaatgtatcctgggtatgcagcttcagaactgctgttccatgaagatggcagcc

ttaagggtgttgctacaaatgatgtaggaatagcaaaagatggttcacctaaggaaacttttgagcgtggtatggaactgcatgcaaagtgcacactctt

tgcagaaggttgccatgggcatttatcaaagcaactttttaataaatacaagctacgtgaaaattgtgaagcacagacatacggcattggattgaaggaa

gtatgggaaattgtaccagagaaacataaaccaggcactgttgaacatactattggatggccattggatcggcatacgtatggtggctcattcctgtatc

accttaatgaaccatctcctttggtcgcagttggttttgttattggcttagactatacaaatccatacataagtccctttagggaatttcagaggtttaa

acaacatccatctgtcagacccacatttgaaggaggaaaaaggatagcctatggtgcaagagctttgaatgaaggtggatttcaagcaataccaaagcta

acatttcctggtggatgccttattggttgcgctgct

>Singlet8014|unnamed protein product

tcaagcttctgccatctcacaacactagcgtcaagtttacaatctacggaattttaccgagaatatgtaaagcacaacctgatgctgctgttttagttgt

aaatacagtcctgaatgactgccgtgatcccaatccagaagtgaagcgtgtagctgtatctacattgtgcagcttgccaccacttttacaagaatatgtg

ggacctgttgttgatgcagctctgaaggacaggcatcccaaagtacgcaatgctgctgtgttgggttgtagtaaaatcttcaaagaaatgcccaatttgc

ttcaggatattggaatgatagacaggtaattactactcatattgataaattcatgaagaatggcaataacttagttaaggtatatttcttatgcacatta

aattctaagttagcagaaatattgaacctgttgatactaaccttctgctgttgcccattaattcataaaaactgcaggcattaactaatcatcaacttct

aagatgtgtaaaacacacaccataacaagtattctcaccatatcagtgcagggcacaccaactaatgaccaaactgccacctcccagtctaccctagacc

ttggagtatgttacagataaatcgatctccataatctacatttggaaagatgatatagaagcngaataaatgctgtcagttttgacagatgttttgttct

cctca

>Singlet8039|unnamed protein product

caannnnnnattggctggtgccctttctggtaactgctagtcactgcagtctctctacagtgaagtcatttgatttaagaacacttaaagctagtcaagc

agattatgatcatatgttaacaagaaaagtgtaatggtctgtgtgatgtgtaactggaaaattaaaaccatagctactgtaaatttatatgtatatgtga

ttgtatataattatgcatgtaatatattcttcagactgccagacatttttgagactgtatgaagtattgagtgtaagaactttcattccattgttatgta

aattatattcagatatattttgttgaatgctgtaataatgtttttttgtggatcaagtgaactgtaaaacagagctcaaaactctattttttgtagatta

agtgctccttcaaggtttaaacattcatctcaaattaataaaagaacaaatcttttgaaaaatcttatcatgacagacagatgtgatgcacattcacttt

attgcttgtttttgtctacttactgatgactgcaaaaagaaattgcagatatttatgaattgtaagttataacgtgtgggcctttttcacattaaggtcc

atcacagtacagtccaaagaagcaactaaacactatgaattgctgtaaaggtagcattcagagtgaaacttgctttcagtaattaatactattgtgtggt

tgacctaataggtttgtcatgaacagccagtgatgaatcatgtcattacattgctgtcatctgccactgttgaagtnnnatatcatgagtgaatggaggt

acttggagagagggataatgtgctcttctttgtgaaatg

>Contig221|unkown protein

tggggggttttgtaaacattgccgatgnnnnccaggtggtgtgacaaagttgtgggaggatatgtcagagaagaagtcacattcagtgaaagttccgagg

acgtataaagtagcggctaaaatattaaaggaagttattgctggtggaaacttgaagacgcttgtttacaaccaacgcttttatatttgattaaatttat

catggaaggcaaggaacccactgaaggtgagggtgtctcattcagcccacctgtggagaatacatcatttgctgattctgcaagagtgctggcctctttt

gttatttatagcctcgcaatgttcacattgccattcattgcttacactggtacaaagtatacactgaggactcagtttcatattgatggatttcagaaca

cagtgtggtcagttcttgcagctgttatagtaacaaacattatcatattgatgtatgcatgtaagggatacagtgaggcagaagaggatgaaagtaaagc

tgctgcaagtaaaagtgatttgaaccagaaaaagaaagattaaaagtgctgttattgttttttaagttatcagttcactgtactaagtaagatgctgtat

taactgcaatgctgatagtgttgccttggtaaatcaagtgaaatagccagacataattgttggattgtgttgccagtaattttttatgcatactttgttc

atgctgcatttgtattgtctcagctttatttataactctgaagaagtactttaagcttcatgctaataaccctgggagattttacagatacgtg

>Singlet4747|unknown

gaaaaatgttatacatcacaaaaggctgccnnnnggctctgcttttgcccagagttctgggatggcataggaccattgttgaactagcttcagagagtaa

caaacactggaatctgcatcacttacacaatctgataattatgatatctgctaacacaatggctaagttagaggaaaatagtaaggtagttcttggaaaa

actaaggaacttctgcttaggaagcaaactaatgatagctctgcacaaaacagtttatatgtgattaaaaggaagtgaactagaaatctcacaattagaa

taaaaagaccaacctgtgaaaagaaaagcagtatgaagttacttcagaattaacatcgtcaaaaagctgaacttccatgcacacaaagaaatctcctgcc

agccagagagacattaaaatcctaaataaaagagcaaaagctaaagaagttttcagctgccactaagtgcaataagaatgtaccacaacacactcttact

tgctatcctggggtatgggtcaactgtttgggctcatagagccactaaagtaaggatagcacaagctctacagcaagcacagaggaatgtactcttgagc

atgacaggcgtgtgtcgaacaacactgttgctggtggaggtactgggcttgctagctctagatgtagcattatgaaggagaggctcaaaaagtgtgataa

agtgatggggagagtgggcac

>Singlet7757|unknown

aggggggatgaatttcacaactttgtacatcatatagagggagtaggaaacaaatgttcaaatgtgataaataaaaactataaccattgcagataggtaa

tttcagttcccttgaaaacaatcagagtatcatcagtcataaaatacggtaaaagcatttgggctcatagattgtatctagcaaaggtatcctcattagt

gagaagagttcaacaaggagtgatactaagaataatgggtgcctcctgcactaccccgaatttcaccccaaatgatgcttcaatagtaattgtaggtata

tggccattggacttggaaatggaaaaaagaggactgctatactggttaaagaaagaccatcaaatgaaagtacgaagggactgagaccaatgcgaaaaag

ttaataaaagattacagttgttgcaaaatgaatggaacacatcagacacaggcaggggaatataccagtctcttgctaaaatcagggagggactgaaaat

gnnatttcttaacccgttgaagnnattgatacattaactgactgctcatggcctgtatgtcacttttctttacataaatcaatagacagatgaatgatng

cagtgtgngcatgccagaatacacattgtgtgacttgatcatggaactgtgaactcttaaagntgtaacagtgaatagtagaactgcttgttatactgta

acactctgt

>Contig434|unknown

agggtggcaaataagagggaaattatgagaaggggagaagaattatggcaggggacttgggaaacggaggaaaccggacgtagaacttttcagttcattc

cggacgtgagggaacgcttgcaaatgaagtattttgagccaacaaggggattggtccactttctcactggacatggaccctaccggacatacctatgtca

gtttgggaagaaggcaacgcccgcgtgtgactgtggcgttccggaaggtactcctgaacatgtgatctacgagtgtcctatcttcaatgatgttgcaacc

acactccgagacagactaccaaaccaagacacataccaacttctaagacgcgaagatacctttcaactgcttaacactctggcaaacgaggtatcacgta

aggtgttgatggaatatctgagggacatgaatgaacataaactcagaaatatacaccgattaacataaactcagaaatatacaccgatttcgtctggatc

cctgttcccataccgcctgtgcgcggactggccgactttccatcatagttgggatccgccgcgtgcaggaatagggggagtaggggtcaactctaccaaa

atggaaagcaccagatttgacttaggataagcttagtttgta

>Contig1576|unknown

ctgtcgattnnatatagtccacaatttnnntttttcgttcaggcacaattttttgccaataatttctaaagaatatgtgcagtgcaccacagtcatccca

acgtcagtttttcatcactatgtatcacttaacagtgtttattccgtcgctattgacctcaatctctattatcattattactacaaatcagttacaagcc

gtatgctttctttgctgctgatttaacaacttgtctctttttgcaatagccattcctactagcaattttaaaaaatttcaggtcttttcaacattttctg

aaatctgattctaggctattctggtagtctaattcttctgctacaatctatgaatttgtcttctttccaatacaattaacagtattaccttttatctttg

ctgccgtggcaagtccttcaaatagctaatacgactgtaaatttttcgagattcgccctcctctacgttcactgctgcaaaactattgtattttcctttc

aagatcccgatcatttattcgtttgtttttttttttgttagagtcaaggcttcaattgcataatttaaaatgataattaaaaaatagttgcagattaata

tttctattatttctagagatttttctactctttaggacatttttcaagct

>Singlet1926|unknown

agggggggggggatcatcagtcttctgactggtttgatcaagcccgccacgagttcctctcctgtgctaaccttttcacctcaaagtagcgcttgcaata

tacgtcctcgattatttgccggatgtattccgatatctgtctacaaacaattactcagtggtgtcacaacaacaaactcaggatagccgaaaacaaaaca

acatacactttactgaaaggatcactccaaaggaatccttcggttaaaattggggacacaaacatcaaacgagcacgcattacgcgctatcttggggtac

acatagacgaaaaattgaatttccacgaacacataaggctaacaacagacaaagcagaaaacatactccacaaactggtgaggctaaattcaaaacagta

cagacttcctctatcagttatacgtacataccactgtgcgctcttcgaatcagtactcagctttgcagctagcacgtgggcacatagactggatgcggtc

agcaacaaagcatccgtcagacgagggcagagaacttaggctatctggagccttcggtaccacctcagcggatgcactatgtgtagtgctcggaatatac

ccaa

>Singlet5026|unknown

acagtaaagagctacagctagatcctgcaaaagagtttcttcaatacccgtacccttttggaatggatccagtttggcagctggctgaaaataaaatcat

cttcatgaactcttacaagatgaaaatttcaatcattcttggtgtgctgcacatgctgttcggagttcttctgagtctttggaatcacttgtatttcaaa

aattccattaacatcatatgcgagttcgtcccacagataatttttttgatcttcttgttcctctacatgtgtatcttgatgtttgtcaaatgggtgaact

atggaccgacatttggtttcacagcaggtcctgcgtgtgctccatcaattttaatcactttcatcaatatggtattgttcaaaggtagtgttcctccaaa

aggttgcgatgagttcatgtatagtggacagaaaggcttgcagaggttctttgttgttcttgctctactgtgtgttccttggatgcttcttgcaaaaccc

attgttttgataatgaggcaccgcaaagcccaccaactgctttcaagccatcccgtacctgcagagaatggcatggatgcggaggtgggatcgatgtcag

ggacagcacacaaggacagtacagatggggctcccgcgccgcagagcagtgaagaccatgacct

>Singlet7235|unknown

acatggtccctatcttcacaggatannnagccaagaaacacctgaatgtgtctgtgatgcccctgtagggacgcccgagcatgttgtattggaatgcccg

gccttcgagccagttgccttggaggcaaggcaacaattacagaatataccgatatttgaaatactaagaaatccagaaacataccaaacattacaagttc

tggttgatgaagtttctaagtattcaaggttggtatttgaatagacctagagttaactagggaaaagccgtactcctcagacaatgtttgaggagtattg

tgcagcaaatagtggcaaaacaacagaagaaaccagcctgggaaggaatggaggcgtcctcatcttctggttaccgttgatgggggttgctgcccgccat

ccaggtcaaatacgaattgaattgaaggatcgcaaagagggcaagtaggaagacgttgggggggctcccgggtctatggaaacatagacgctgtggatgc

ccaggggtggttcctttggcaaggttgccttctagcgaatccacaacatatacataaatatatacaaataaatcaaaccagaccaaagagataattgcaa

atagttacagaagttgatgtttatctactccatgcttagttagttttattatcctattgtaaactaatagcgcagaggcgcaagttttgtaatcaatctt

gtagaagtagnnnttgtagttgtattgtagacttgtataa

>Singlet1719|uncharacterized transposon-derived protein

acaaaatatggtctgacacgaagatgctatgacttttgtcacatcaatataagtaagagcagtggaaatgaactgttggctctaaagtaaatgagtggtt

gataacaaacacttatttcgttggtttatgtggcggtgtgtttttaattgttactgtactggcacttttgactgcccaagagcatcaaacgctaagttat

gtatttgttgttgctatggacggatactgaagtacgtcatgacaaaaaagtggaatcagaaatttggacgacgacagaagcagatatgaacaggcagagg

gcatttgaaaggaaaatccttcgaaatttttatggtccagtgagagcggcaggtggttgtagagtaagttaaaacgatggaatacaacagtttgtacaat

cgaaagctatagtgaaatttgttaaatcgaaaagaatacgctgcttagggcacctggagaggattccagatgacaggatgcccgaacgattaatgaaaag

cagatggtatttcacgagaagaaagggactaccaattctcgagggtaggacaatgtgttcactcatcttaccaagagggagacccgaggatggaaggcaa

aagcagagaggaggatgattgaggaagattgttgaggagaccaaggcccatcaaaggctgtagtgcgaaacaaaaagaagaagagaagcaaaaaaaaaaa

aaaaaaaaaggt

>Singlet5290|uncharacterized transposon-derived protein

ctgtcgaatggatgtggtaatgttttcataactggtactgatctagtggactgctgcaaatgtgagtggaatggagcagtggttattaatgcactggact

tatactcaagaagtgcagggttcaaatctcaatttgaccatccagatttaggttttccatcttcttcttcttcagtagcactacagcccttggtgagcct

tggcttcttcaacaatcttccttcacgcttcttggttatttgctgctcttttccacctccgaactcgcatactggtgatattcattattacctcgtcgat

ccatctccttgtaggtctccctttttgcatagctgaatgaatcacacatttcatcatttcctttcgaattccatcccctgacattctctccaagtgtcct

agctatcgtattcgctgtaatttcacaaattttactatgtccctgccttgtattaactcttataattcggcattgtatcgtattctccagccttgtcctc

ccttattggcccatcgattttgcataatattttccgctcagtggttcttagtacatttttatcgtgttctgtcaacgtccatacctctgaccgaaatgtg

ataactgggtggactagggaattgtatattagccattttgcttttcttgtaacaaggctgtttttgaaaagttttccatggtttccccgaactgttgaag

acaaatactgggatgttttctttgaaaatgacagagccaattccttcctcacccttgtcccatccaatctcgtgctttgtatataacaatctcgtcttga

acaggatattaaacccaattttccttctgttataatatgtttctgttttctctctgaaagagttgactagtagctgtgac

>Singlet7257|uncharacterized protein LOC100578262

gtncnataagagtgacaggagttctgtgcatcgatctgggaatgcgagtgcgaagagaggttccagatccatcccatcactnntttgacatagaggttcc

aatccatgtgcagcggttagctgaacaaaaggatgttgacggagaaaattccccagcacgtcgatctacacccatgccagaatctgaaaatgaagatgga

acagactcacctacctcatcttcaaaactggagtcaaggccttctagaagcaaggaaaataggtgggtttctgataactcaatgccatcagggcataaca

aagaatttaatgccgagaaaacaccagagagaaagaatgactctgatgatgcaacatgatgtacaaaggccgactttccgtttctatcaagatactttaa

gttctgatgaaggggaacatcgaaagggagatgataagcataggaagagagaacacaagagcaaaagagaaagtcatcataaacgctcaaagctgtctcc

tgacgct

>Contig1089|uncharacterized protein LOC100322888

ctgtcgcaaatattttgacagtgatagtgagccgtgttgtgcttatcttcataaaaatgagggtagattgagatacggtgaattacatgcgttcgttgac

agaaacgttatgaatttcgttgcaggcaaccatgaagatctcagcacaattccgtgtgaattgtagatatctcatttctaacaaacaaattcgacctagg

tagttcctattgcttaatccgtttccaacaccgagtttcatttagcagcgaagaggaaccaaacaaacataaaaatttggcatagtctgtattgctgtaa

catgagcaggaaacgcatccatgattatgtgtatattataggttataattaattctgattgttgaagaagcaaatgaggacgaagactttcgtattattc

ctgagctgtattgtgatcacaagtgtttttttgatagcattcggaagccaaagaccttctattcaaacaatagtgagtgaaacgcacaaacaattgaata

acttgaagaattttaaggaaaaccttgaaaatgcagagtctatgcgacttgttgctgatgaaaaatatttgaatcttttgggatttactgaaagcccacg

tctatacccatcagaaatatggaaaaacacatctttaccaattgtcgtaacatatgttttaggaggacaggaacagcaaggcataggtttcattcgtaac

atagtacactttttgcccaaccacactatacttgtgtacaatttgggtcttggatcttatggtttgcaaacgatccaaggacactgcaatagcagccgct

gttcagtcattacttttgactacagtgtgtatccatctcatgt

>Contig4534|uncharacterized protein LOC100302406

atggaaggcatgcagctgcaggtgcattgaaagccagagatgacttcaagatgtatttcaactccataagtactgttccatggcaagaagaggctgttga

tgctgggaggtggtgatgacagtagagctttggacaacagtacacatttatctgtcattaagttttatgtattttatatatgttatgtaagaattttttt

tttttttgtatatcgtcaaatatttctgtcctgcaatttgtagtcaacatctatgtacagaagttagtgtactttcctgtacataatacgcataataaat

ataattttgtgagcacaagaaaaaaaaaaaaaaaaaatcggt

>Singlet2938|uncharacterized protein LOC100302406

ctgtcgcnnnnntatagcatcatggtctgtcagcctatttaggacttgactgactgtcaagctactgtgcctagacttaccaacaaacacattatgaatt

agagtactggagtggtcagttaccctagtgggaaattcaattacttgcattagattgtaacagctcattaattgctccagctccattctgtcataacttt

cattcaggaaatttacattaaagtcgcctagtattatgactgaaatgttttttctgtacaatttgtcacagattgcaatttcagttaacgtatttttgcc

acactacaacacatttttcagttttcactcacagagaaattaaatggaagaatggatgaaagttctatgtcagttaagccacttgcacacgggacgaggc

agctaatgttgatgtgcacgcaaacgggatacaccacgtcacgaggcacagcacagctgttgttgaatttatttagcgcacgagttttatgcccagcaca

cgtttcacaaagtgaatatggatcaaactcaatttgctagtgtggaagatgagcagagaaaacaccacagaagtttatggacatggcagtggattcggca

gcgcgataatggtagaagaacaattcattactgccctaatgtaacttattttctcacttagattacaagacgcagtcttttcagaacttcaccagaatgg

atgaagatgtgttttcgaagttactgagtataattggcagtgacatttcccaccaagacaccaacttgagacaattgataaaaactgttattgactgaca

gtc

>Singlet3122|uncharacterized protein LOC100163446

ccgtngctgtcgccgttgctgtcgggagataagcaggtggtttggagagctggagtcaacattctataagcatccaaaatcaccacatccagacgttttt

agagtttccaatgtagccaatgaaggctgtgtaggggttggtcgaactggctggcacattgatggttctttccagccagctccatttgcctattctttat

accatatagtgtcagtgccaaagaaaggcaacacagtctttgcacctttgacagaactgattgaaggccttagtactgaaaagagagcagagtgggagag

attgtggatggtcagtgacagacgcagtggtccaatacatcctcttatctacactcatccagtcaccaagaagaaggttctttgttttcatcttggtatg

acaaataattatctgtgggacaaaggaactcctaatgagcgttttgctacagaggaagaatttgatcgcattacgagagacatacataatgaatttgttc

gggatggtggaaaaatacaatattctcacaagtgggaggcaggagatttcatcatttctgacaatcttgctgttggtcatgaagccagccccgaaacaca

gcttccgcacagcgaagttggactacgtgtcatgcatcgtacaactgtcaaaggaacnnnacctccacaaaagtgaacagctttatttatggtacattct

agaaattatgttaaagattcactgagatgttttaaagttgttagtaaatgaagaaag

>Singlet885|uncharacterized protein LOC100162992

ccgttgctgtcgggcgctgcaaccggcgcctggcatcccggctgcttctgtggcgcaaggaagtcgcttgcgcttggagcaacactatgcacagggagtt

ggagcagctggctggtgcgcggccgtcgtggcgcaaggtgactgcggagggcctggatgtggactacacgcggctgctgacacctgcgctggctgatgcc

gtgctgcacgagctggaggcgggactcacctacttcacaggagacctggcgcgcgtgcgcgtcttcggccgctggcatcccatcccgcgacagcaggtgg

cgttcggagacgcgggcctgagctacaagttctccgggcagacgctgcccgccagcccctggccgccgcttctgctgggcgtgcgcgatgccctcagcgg

cctcaccggcgtgcgcttcaacttcgtcctggtcaacaggtaccgagacggcaacgaccacatgggcgagcaccgggacgacgaggcggacctggacgcc

ggggcgcccatcgcgtcgctgtcgctgggcgccgagcggccgttcgtcttcaagcaccgcgacgcgcgccgccgcgggccgcagcacagggacgtcccac

cagtgaaactgctgctggagcacggcagcctgctgatgatgaatccgccaaccaaccagctgtggttccacgcgctgccgcggcgcaggtcctgccagca

gccgcgcataaacctcaccttccggaagatgctgccacaaccacagcctgcacagcgcaagnnntcagctcccaccactcgctgaccgatgtgcttctat

ttcctgttactctgctgtcaccgaacctctcgacactgaagcgcaactgctagtttatgtattta

>Singlet7904|uncharacterized protein LOC100162417

ccgttnntgtcgccgttgctgtcgaaaatttaaaatattctagttttcaattcactaaatgtgatacattctgtggtatcaaataatggcacaaagtagt

tgcaatattcgttctgggacgaagactgggccctcagtagaagaatttcagcgaaaattacatcgatggtttgaggaacaaggactgttatcagatcttc

gagcacacatgcgacaattgattattgctgccctaaaagggactcaactggctccacaagctggccatagtatgcatcagaacacatctcctaaaataca

agcaattaatttacttgtagcagagtttttgcttctgcaagagtgtcattacacactttctgtcttcacaagtgaagtgccacttttgagaaatcttcca

gaattttctccatcacttaaagatgcagctaaaaacaaactagataccaagaagagtggtgaagtacatgctagtgcctctagactccaggagcgccgtg

tgtgggatatactagaagcactaggttttgctcctgattcaagtgcaggaaaagcagtattcaaatcatatcaagaaaataatggtgaaccacttcttac

atgtctcatacgttcagtatccaaagttgttagacatgagagaaagaatgagaaacctctcacagaaattgaggagaatatttcagttcctgatagtgac

tcagttttgctgattactgaaagttctatcacagagaatgacaaaaatgataaaaaactgtcagttgtacacgatatcctccagaaactcaatgtgaa

>Contig3615|uncharacterized protein LOC100123423 precursor

cgcccnnntctgcagcacagcacggcagcgctcctccaccacaagcacagggcagcccggctcctaggctcagctcagcacggcacacggaccagaaaat

tgcatcagctgctcactatggaagcgatatttctgctgttatctggactgctgcttctagtacctgttcagtctgcgcccagcaacaacatccctccata

tgtcaaacaatgccggatgggtgacccgaagatcatagactgcttcatcgaggccttgcaccacacgaggccgtatttggcgaaaggtatcccggagata

gagatgccatccgtggagccattccgcatggacgagctgtcgctgtcgctgacgacgggccccaacggctaccgcgtcaccctgcgtgacgtcgacatct

atggcgccagcaacttctccgtcgacaggctcaggctgggcaagaacggcgcgcccttcgagagccgcatccgcatcccccagctgcgcatcaacgcgcg

ctacaccagcagcggcgtcctcatcatcctgcccgcctccggcaacggcaccttccagtccacgctcggcgacatcgtggcggtggtgcgcggcaaggtg

agcacgccgacgcgcgacggccggcagtacctgcacgtcgacacgctcgacgtcgacctcagcatcaagacggtgcgcatggccgtcaagaaggtcttca

acaacaaccgcattctcacggaggcgaccaacctgttcctgcgcgagaacggccacgaggtgctgaacgcgatgatgccgcagctgcgcaccaagctggc

cggcgtcttcatgagcatcgccaaccagctgctcaagcacgtgcccacagacgtcttcctgctgccggcggancacgcagtcgccaagcgccgcgccgcc

cgctg

>Contig1216|uncharacterized peptidase c1-like protein

ctgtcggtgcctcctgggccatctcgacggcggacgtcgcctcggaccgcttccacatcatgtccaagggcaccgagcccgtcaccctgtccgccggaca

cctgctcgagtgcaacagccgcggccaacgcggctgccagggaggcaacctcgaccgagcctggctcttcatgcgcaagttcggtgtggtggatgagcca

tgttacccatacacggcacagagtggcacccctggcaagtgccgtctgggtcgccgtgcaaacttgctgactgctcgctgcacaccaccaaggactctgt

acgggccacccaggactgagctgtaccgcactggaccagcctaccggcttggctctgaggaggacatcatgcatgagattatggagtctggacctgtaca

ggccactatgaaggtgtaccatgacttcttcatgtaccgtggtggaatctaccgacactcaatgcttgacacaactcagagaactggttatcactctgtg

cgtattgtgggctggggagaagctcaaggaaccaaatactggattgtggctaattcctggggtgaccaatggggtgaaaatggatatttccggattcgca

ggggaacaaatgagtgtgaaattgaaaattttgttgtagcagcatgggcagacacacaaacccctgtcatggataatatgatgagcaaccacgacaatcg

cctcagcaatgttgtatactgaggctaaggaatgcaaccactagatacaaactcagcaactcagtcagtcaaaggttgttttcacatgttagtggggctc

tttttcagtggtgagacatgtagattcttaatccggaattcaaaaaacaatggctgcatataattgtctttctttttggatttctgtccttttttcagta

cataaaatcctgaaacatgtgacaccagttctttgcactttaacttgattgtttcaccatgctggagtaatggctgtgctgaaattcagaatactgctga

acatttcttgacagaaatcgtgcacttgaaaactgttgttcatgtacatggtactaaggaagaagaggtttgttaatgctggatggactgctaaaaactt

atcctttcactgttcatcgcatcaatttgttattattccaaaaaattaacagttgttcctgcttaaaggaattactgtatggcttctactataaaaagtg

ttttatcctttgtattaggcattaataaaacacagatatttaagtaaagagttcaacaaatccaaaacaacaaactgcactttctaataagaaagcaatt

agcaatcagtcaagtacttgtgtgtgtttcatgcgtcacttagagatactgtactcactacagtattgactttgctttaagtggactgctttataaaaat

tattaggtttaattttgattataaactgtcgtgatattaaaaatcatgaaagtttatgaatataaatagtccaaaggcattcagtaacataataatggca

acatgtgacaggaagatgttggttgatgtaccatgaatcttctttgttgcattaaacaaaactataatctttgcattccttatgaaaggtgcagcagaag

ctttttacatggaaaccaaagttctgagaagtcaagtagaaaaataaatgaatataatgttggttactgaaagaattaaaatatttctgtgaaatataaa

tttcacctgtaagatacaaatgaattgttcttttgactaaggccatattgtattaattaatttattgatgctagtgtaagataaagagtgtaatatttta

aataatgtcaggctttattataattgaattttgcattgtgcctaacacttatacatatagtgttcgagggattttaacccataagatactgccaaaacaa

ttcacacaatgatttcatgacagaaagatgatatttctgcttgaaattaactaccactatacttttgtgcatgtgtgtaattgaatgacgcagtcatgag

gtggacaatctgctagatatgtaagtggaagtaatgataaccaagttgtggtctaaatatgtgaaatgtgattgaaagaagtgttattaagtgagaaaaa

attgtatctatattgttctgtctgaatatgggtagtgtagtagtggttatcagaaataattcatgaattccagaaatttgcagagtagtgtgaatatttc

ttattcttgatgcaaatgtaatgtgataccgtctatgtacagtaggtgcacaatgtgagtgaatgtactttttttataacataactgtaaatattgttta

acatttgattatttaagttctatttactgttcactgccaatatagttatactataattatacactgagtcttaccttgaataaaatttttatttccacaa

aaaaaaaaaaaaaaanngcgag

>Singlet2690|uncharacterized oxidoreductase dhs-27-like

ccgagaccgcgccgcaggctggacgcgcgcactgccgccctgctgcccacgtgcctccggggacgcactagcaggtcgagagcggattcgcgatgtctga

ggaagggggcagcgcagagggctccggcagcagctgcggcgaggaggaggaagacgaaagcgaggagtgccccgtcaccagcgcctggatcgccgaagtc

atcgcgtcgcacaaggggcaccagcaggtggaggtggtggacatgtgcatccggccgggctgcgacgcggcggacgcggtgctgagcgacatcgtggcgg

tgcgcgccacctaccgccaccaggcggaccccgcggaccgctccgccgcgctcatcgtcaagctgttgcccagggagccgttcagcagatacttcgtcac

cgaggcgcagttcgacttgcgtgaaatcaaattctacacccaggtggttccggagctggaggcgttcgcgcggcggcaggcgggcagcggcgcggacgcg

gtgcggctgcccatccccgagtgctaccacgcgcgctacgtccctccggaggccgagacggagcccgggcccgacggcgagagcgtgctcgtgctggagg

acctgcgcgcgcgcggcttcggcggcgccgacttctctcgcggcctctcgctgcgccaggcgcacgccgcactcgccgccgtcgccaggctgcacgccct

ctcgctggcgctcaaggtgaagggtgacggggagggcagcggaccggtgtcgctgccacagcggtacccgttcctgttccagacggcgcgcgccacagac

tcctaccagcagctggtggagcgcggcctgccgcagctcgcttccnnnctcgagcgccgcccgggcctcgaacccgtactggcggcgctgctcgcgctgc

ggc

>Singlet5114|uncharacterized mfs-type transporter c19orf28 homolog

gcagtnnncacaccatttgtaggatatcagtcggatagagatgacaacttttggctttgcaagtacggaagacgcaaaacatggcatttgtttggtgagt

gttttaattttttaatttttttcatcaaaatatatgaaattaatagaaaaatagaatgtacttaaactgacagatagaaaatacccaaaagaaataattt

gttttgtgcatcttttaagcttgtaaacaatgtgtggtctaacacttcttaccatcaacaattgtgtaataaccattacagaactttaattgcatatagg

tgaagcaacattatccagaacctagacaaacttaaacattaaattatgcttggcacatatgacaggaacatatcaacagcaattgatcatttacagggtg

taatgcatcatcatgatatccatgattgctacccaaatacaatttctgcactgtcttaagcatatgatgaccatactcattgtgttacaattttatttaa

gatgtttctacacttcaaatgtaggatgtttcatgagaggcatgaattggaggtgcctatgtcacatcgaactgaatgaggaatatagagtgcagcttgc

tggattttgtgtgatctgtgatggagagatctgtctggtgtaaatttgcaagctacaatacatacacttttaactagttttgtaactccttagcttgtat

tataggtagtctgaaaactctggatctccaatatttaatgcagggatgcttgctttatacctcagcaggccagaaaaaaaaaaa

>Singlet7876|uncharacterized methyltransferase wbscr22-like

gtttnnntttgttaggtactccgactcgaaggaacatggaaaagcagtagaatagtgatttgctcatttgttggaagaattttattgaagttcaagaatg

tcaagaccagaacaccaagcacctcctgaggtgttttacaatgagcaggaggcaaagaaatacacccaaaactccagaatgattgatattcaagtgcaga

tgtccgaacgtgcaatagagcttttagcactaccagatgacaatccatgcttgcttctggacctgggctgtggatcgggattaagtggtagtgtgcttga

agatgcagggcacatatggattggtgttgatatagcccaggccatgttagaaattgcactagagagggaagtggaaggtgatttgattcttggtgacctc

ggccatggagtaccattccgagctggctcgtttgatggggctgtcagtattagtgctctacagtggttatgcaatgctgataaaagaagtcacaatcctg

ttaaacgactgtataagtttttcagtacattatatgcatgtctgaaccgaacagcaagggctgttcttcagttttaccctgagaacagtgatcaaattga

gctagttacaacacaagccatgaaggcaggattctttggaggacttgttgtggactacccaaacagcacaaaggcaaaaaaattcttcttagttctgatg

acaggagcagcagtaccattaccgaaagcccttggtactgatgaagatgccaacggagtgtcatacaccagtaaaagggaacaaatgaaga

>Contig4537|uncharacterized family 31 glucosidase kiaa1161-like

gtttgtnnngttacgtttttttcgttatctgtcataacttgaatattagaaatttcataaagcgaaagtactcaatggacagcaaatatgaagcactgcc

aacagtgccttctgcagctgttttccacgacaatggaagcctatttaggcataaaaacgctggggccgttagaaagcagaaattgttgaaatggattggc

attctctttattgtgctgacagtctttggatgcgcattttatcggattgttgcagaaaccaagcattcatctaatttactacaacaaaatgtcttccatc

ttttgtctacaagaaattctgcaaaaatctatatgtgggatgtctctggtgcaaatgtaacactgctcctgcagaatcaagtgactttctctgaagatgt

tggaaacccagtttcatgtgacaacagttcttttgatctgtgtctgaaatgggaaaaagaaggcagcaagcttccaaatgcaaaattaactttaaggcag

ttccagataagtgagggtgttgaatgccataaattaaatgtgtctgctgcttggaatgcagatataacatcttgtgtgtccataacaggagagcatctgt

atggtggtccagttttgaaacaatctcagtactggcccagtgagaaaaatgaaatgcagaatgtgccacgtgccactggacagggcacaattaatggagt

tgcagaatcattctggataacatctggaggaaaagttttttatgttccacgtgatgttccattgtttatctcccaagacagtcgccaactttgttttcga

agcaaacttgcatcaccctacctccgagatactgaaaccactgttaatatgaaactttattactgccagagcaatgatgcacagaagctgcacagggcaa

cagttaaacgtttcttggggcttccatcaggcattccagacaaagaaatgatacaaaacccagtatggtctacttgggcaagatacaagagagaaataaa

tgccacagttgtaacagactttgctgctcagatagcagagtatggctttaacaggagccatttagaagttgatgatcggtgggaaagttgctatggtgat

tttaaatttaatgaaggattcccagatcctggacagtttattcaagacctcaacaataaaggttttagagttaccttatgggctcatccatttgtcagta

ctgtatgttcagcatattctgaagcaaagtcaaggaactatcttgtgaaaaatctggatcaaagttgggacagaactgtatggtggaatggaattgctgg

tcttgtagatttcacaaatcgtaatgcaacatcttggtggaatgggaaactaacagctttaagagacaactatggaattgatggttttaaatttgatgcc

ggtgaaacttcttacatgcctggatctccagaagttggacaccttcagtctcctataagactgcagccaaatattttcacttcacagtatgtggaaaatg

cagcgtcacttagtagaaatgaattgatgactgaattcagagtaatgtacatgaatcaaggtctgccagtgttcttgcgaatgtcagataaagaatctac

ctggggttatgaaaatggcttgaaatctttaataccaaccttattatcactcaatattgttgggtattcatttgttctgcctgatatgattggaggaaat

ggatatagtactaaaccaagcaaagaacttttcatacgctggttgcaggcctctaccttcatgccttctcttcagttttcatacactccttgggattacg

atgacgagacagtagaaattgcaaagaagtttactagccttcatgcagagtatggaaatatgatcattgatttagcagaacagaaggttacgaatggaac

acctattaatttgccaatctggtggcttaatcctgctgacacaactgcacttggaatagattcagaatatctattgggtgagaaattgcttgtggcacca

atactagaagaaggagcaacaagcagagacatatatttacctgttggaacgtgggaacagaaagtgagaagtgatgcaacaactgttattactggccctg

catggatcagaggttatagagttgaacttgatgagttagcgtattttgtacgcgtgtcatgaataagttcatacagttgcaaaaatatttaaattcgtat

gcaatgtattaatcgttcatggagagttatgagccatagtagtgcaaacagccacatgaaatgtgtaacacttattgtcaggcattcatgaagttgtatg

ttctgaagataagtaagaagagataaatatcttttctctaaagtgaattctctggttcagttatatgaacctgtgatgtcgtttagcctgtctgtagcac

ataattttggtcatgttgtctctctttaacgtaacttttactttagtttcctatttttaattttcagagtataattttataaattgctgattctcatcaa

aagttgaagtgtttgtcatgcattgtgttaaatatttcatggtgttacactttcagtgctctcttatatgcttttattttgtgagttgaatcttgtatgc

atatttatacaccgtgcaatgcaaaggctgtgaaacttggtgttatactgtatacatagttattatgttactaagtttgtatactgatattaatgtctga

attattaaacctgcctttcaagtttgaaaaaaaaaaaaaaaaaatgc

>Contig309|uncharacterized conserved protein

aggggagtgtaaattgtttggactgggatgtgtgtatagttcaaatattgtggtgtgattatactggcagccctcgtattttgatcgtgtcgatcactgt

agtagtggtgaagtatatcagctggaaagcaagtttctaatgtgtacatagctgccaacacagaatctgtctggagctatctcatcaaggtttttgtgtg

aagatgagcactcaacggggaaatacacagagaaccaggccacaggcacaccaaaatacaaaagcatttaaaaatgacctacatgatacctcttctcaga

caaagcttataaataatattgatctgtctggtgtgtgtgaaagatgcaagagcatcttggaatggaaaattaaatacaaaaaatacaagcccctaaaggc

tccaaaaaaatgtgtgaaatgtgaacagaaaagtgtaaaaaaggcctaccatacagtctgtttcccttgtgccaaattactgaatgtatgcccaaaatgt

ggtaaaaatgagaaagttattgaaaaactcatttctattgaagaacaactgaaaaaggatgctgaactccagagacaaataaagctgctgtcattgcgaa

gacgacgtacactgctacgttacattgctagccttaccaagggtacaaattcaggctttgctgtgggagaagatgaaatgaggaaactgcaggaagaagt

tacaaaaaaactggaatcactgaaaataaacaaagatgatgttttnggatgatttattagatgatcttactgatgaaagtgatgatgattgattactctg

tctcaaagattttacnnnngaatataacttatttcagtaatatccttcatatattcccaaaactgtagagtacataatgtgttttc

>Singlet8185|uncharacterized conserved protein

gcagccctcgtattttgatcgtgttgatcactgtagtagtggtgaagtatatcagctggnnnnnnnagtttctaatgtgtacatagctgccaacacagaa

tctgtctggagctatctcatcaaggtttttgtgtgaagatgnnnactcaacggggaaatannnagagaaccaggccacaggnnnaccaaaatacaaaagc

atttaaaaatgacctacatgatacctcttctcagacaaagcttataaataatattgatctgtctggtgtgtgtgaaatnnnnnngagcatcttggnnnng

ggaaaattaaatacaaaaaatacaagcccctaaaggctccaaaaaaatgtgtgaaatgtgaacagaaaagtgtaaaaaaggcctaccatacag

>Contig138|uncharacterized aminotransferase sso0104-like

gatggacagcgcgaaagnannngacgatccgcatctcttgcatcttttcgatggagggcctactaacgtttattctaaggatcacgataatttgtccgtt

ggtgctccaggcccagatctcctgaggctttgtgtgaaaatcatgcagaacgcgacactacacagaatggaaagtgagaaagaaaatccgtatcttttcc

aatatggtattccttgtggactctgggaatttagagaagatctttctcattttctatcagaactttatggtggaaatgtgaacaggaaagatcttgtgct

aacatgtggggcaacacatggtcttcagctgattctggggacgttcctgtctccaaatgccgttatatttgttgaggaggctacatacatgattgcattg

tcagctttcaaacagtatcctttgatgaaaatagtcccagttcctttgaacaacgatggtgttgatacagaggcatttaagaaaattgctcttgaagaaa

aaagtaaagggaaatggatgacagatgaacaaagaccattctgggctgtgttgtacactatccctctatttcacaatcctactggcataactatgtcgga

aggctgcagtcaatccttaatacatatggcacgagaactggacatacttattgcctgtgatgatgtatataacttactatattatggcagtaaagagtat

cctcctaaaagattgtttgcttatgatactgaaatggatggttatgagggaggccatgttatatcaaactgcagtgtttctaatattatggctcca

>Singlet7650|uncharacterized aarf domain-containing protein kinase 2-like

ttgttgtttacattggtaaaggttatgttggaaaagtacgatgtttaacttagtacagaccttctgtgtaattcttccgcgtcaccgtacaacaggaaag

ctatggctagatgtagggtcgcgctttcgttggattttcctgagtgattcgaaaaatattaggtctagaaacgggtgtataattgggtatcgaagaaagc

cgaatatttatcagaatagcagggcactttgcactttatgaaggttttgtgcaacagttgcctttccagttgtgttagcgaaatccacaatgatggaaaa

tcagatgttcaagcaattgcccggtgctgtcatcaacag

>Contig2427|unc93a protein

gtctcgnnngcgccaacgcgtccagccacgtgccgctgctgcactctgcgcgccgacatggaccgcgccgtctcgctctacacgttccaaccgccgccag

aggaggagagcggcacggagatgagcgacatgcggcgcgactctgcggctgagcggctgcagaagctgagcggtccagaaccggaggaggatgagcccgc

tgctccccttgtctgctccagtctgccaaccctggttctggagaagggccaacaacgaccaaaagacctcgaagcgcaagaggaaagcctactatcaaac

aaccacgcactagaccaagggtcgaagaagccctcgctgacgggcgactgcggcgggaaaccggggccgctgagtgctgccgaccgccggcgggtgctgc

gcaacctggtggcgctgagcgcggcctacatgatgcacttcaccgccttcgtgggcgccacgaacctgcagagctccatcaacaaggcggacgggctggg

caccgtgtcgctcgccgccgtctacgcggcgctcgtcttctccaccgtcttcctctcggtcacggccatacagtggctgggatgcaagagcgcgatgctg

gcgtcgttcgtgctgtacgtgccgtacatgtgcgcgcagttctcggcgtcgtacgcgaccgtgctgccgggcgcgctgctcgtgggcgtgggcggcggtc

ccttctggtgcacgcagtgcgcctacacctgcgcgctcgctgacgtctacgccagcgccacgcgcctcaagccgcaggtcgttgtcgtccgcttcttcgg

catcttctacaccgtctacgagtacggacaaatcttcggcaacctcgtctcctccgt

>Singlet547|unc-112-related protein

gtgaaagatctgccactcattgaagcaaaaatgacttacatcaaagcttggcagtcacttcctgaatatggaatctcattgtttattgtgaaatttatgg

gccacaagaaagaggaattgcttggagttgcttacaacaggttaatgagaatggagatcaacactggagatcacatcaaaacttggagatacaatacaat

gaaggcatggaatgttaactgggaagttaaacacatgatggtccagtttgaagaggaaaacattattttcagttgtctctcagctgactgcaaagttatt

catgaattcattggaggatatatatttttgtctatgagatccaaggaagcaaatcagacactcaatgaagaaatgttccacaaattgactggtgggtggg

tctgagtcaccaactagaacatttttttacgtaaatgcaagacacaagtaaacatgcaacaaggtgccttctacaactgtgaacatatatatgatcaaag

ttgaaatatgacatttaatatgagctttggcaacattatttttaaaagagaagtatttttaatgtttgaaaattttgaagtttaaataaagtttactgta

acaaagagagagagagagagagagagagagagagagagagagagagtggtgcttgtgttgcaacaacctcataataaaaaataaaatgaacagtttcctt

nnnaatgtgttatatccagtgataggtgaaattgactccataggacagttgttttcagtgtttgtcacattcagtgtt

>Contig462|ufm1-specific protease 1-like

ggaatatctttgatatacgaaacatagcaagtaaacaaagtaccaccgcttacgatttgatgttctgtgaacccgaacatgaatcaatttgaaggacaga

atatagttaacgcccattaatggatgtcaagcgtcttatctaggaagatgcctccaaaagactatcgcgacagtctgcttagtgatgtacacgtgggttt

gaaagacgttacagattgtgggaccgtcaattatgttaaaggaacatacgactattaccattacggatgtgatggattttcagataagggttggggctgt

ggatacagaactctccagacattatgctcctgggtgaaggaaagtctgaaaggaaagggctcaccaccaccagtaccacatattcgagaaatacagagag

tccttgttagtatagaggacaaacctacacatttcataggatcgagggactggattgggtgctttgaagcctgccttgtcctagatgtactctacagtgt

atcctgcaaagtgatacacatcccaagtggtaaacaactgtgcaagcatattcctgagctagtggaacacttctctcaactagggtcaccagtgatgatg

ggtggtgacaaagattgctccgcaaagtgtattgttggagtacatacagatgttgaaagaaatgaagcgtcgcttctagtagtggacccacacttttggg

gcgttgctgcagatcgttaccaactacagagagaaggctgggtgtgttgnnnncagatttcagnnntcctggatagctccttctacaacctgtgcctgcc

acaactttcttcagaattggagccttcagtttgatccataactgcagtcagaacagcacctcacattcagtagccataattttatcataatctttgtttt

caaatgtgtcattgcacttcatgtctt

>Contig3263|udp-n-acetylglucosamine--peptide n-acetylglucosaminyltransferase 110 kda subunit-like

gatannnatcgtttaccgtcagttcatcctcatcattcaatgttgtatccattatctcatgaattccgtaaggctatagcagccaggcatgccaatctct

gtcttgaaaagattcacgtcttacacaaaccaccttacaagtaccctaaagagttaaacgctaggttgcgcattggttatgtttcttctgattttggtaa

ccatccaacctcacatctcatgcagtctattcctggattacataacaaatctaaagtagaagtctactgctatgctttgagtcctgatgatgggacaact

ttcagagcaaaaatagcaagagaagctgaacattttgttgacttgtcacaggttccttgcaatgggaaagcagctgacagaataaattctgatggaatac

acattttagtgaacatgaatggttataccaaaggtgctcgtaatgaaatatttgcactccgaccagcgccagtgcaggtaatgtggctggggtatccagg

aacaagtggagcatcatttatggattacctcataacagatgtagttacatcacctcttgaattggctagccagtacagtgagaaattagcatacatgcca

cacacatatttcattggtgatcacaagcaaatgtttccacatctcaaagagagagttatccttacagacaggtcaactgggaagaaggaagtgccggata

acgtagcagttatcaatgctacagatctgtcacctattatggaaaacacttctgtgaaagaaatacgtgaggtggtttcttcagatagcaaacatagttc

gcggcctgttgaaatttctttaaaggtagctgagctaccaactaccacaccaattgagacaatgattgcatcagggcagatccaaacatctgtcaatggt

gttgttgttcagaatgggttggccacaaatcagactaataataaagctgcaacaggagaagaagttcctcagagtattgtgataacaactcgacaacagt

atggcttgccagatgaagcagttgtttattgtaattttaaccaattatacaaaattgaccctctcactctccaaatgtgggtctatattttgaaacatgt

gccaaactcagttttatggctattgcgatttcctgctgtgggggagccaaatttgcaggctgcagcccaacaattgggtttgccaccaggccgtattctg

ttcagtaatgtagcagcaaaggaagagcatgtgagacgtggccagctagctgatgtctgtcttgatactcctctttgtaatggccacacgacaagtatgg

acgtcctttggactggtactcctgttgttacattgccaggtgaaacgctggcttcaagagtagctgcatctcagttggctacattaggatgtccagagct

tgtggcaagcacacgccaagaataccaggacatagctgttcgtcttggtacagatagagaatacttgaaagccactcgtgctaaagtatggcgagcacga

actgagagtccattgtttgactgcttacagtatgctcagggtatggaaaaactgttccagaagatgtgggagcgttatcagcgaggtgataagcctgacc

acataactgacgtcaaataggtgcactatttgtactgagattcttccctttctttagcctgtaccaatcagtacattagccgccatacgataggcggtag

tatcctgatctatttatgtcatgaagctgtgactgcattagttcttggccctgatttcacataaggcaccaccataccgtagcctctctttgataactgt

gccagatgaacattaccatctgtgttaattatgcaaagttgggcatcagatgcatgattgtttactgtgagtggtggttgcctctaggtatttttccggg

ttgaaaatgaacttctgcgttcccagaagtattatcggctgcatccagttgttgcttgcagagatcagttgtgttgctttaagatgtatttccgttgtgt

cgaacattaaaaagtatttggactgtgctgtgtgactgaaactgttgacaccacgtgtcttcagagatagcatgcacaatatttttaagtgtctgtatgt

tccgttttcagtgattgtgatatgttatatatgttatgtgatatttcctatgcccgttgtggagtaaaatgacg

>Contig1471|udp-glucuronosyltransferase 2a3

gaaccgctgatgaaggcgctggctgcgagaggacacgatttgcatgtcctcagctacttcccgcagaagcagccgctagccaactacacggaccacatcc

tggaggggccgatgctggcgccagccacgggcggtgtgtcgctgcgcgcgcgcgcctcggagaaccccgtgcagcagcagctacatttggcctacatggg

gctcagcacctgcgaggccgtgctcagccacaacaccacgcagcacctgctgcgctcagacctgcacttcgacctgctcatcacggagctcttcaacacg

gactgcttcctcggcttcgtacacaggttcaaggctccattcattgcactgagttcatgtgctcttatgccatggtctaatggaaggatgggtaatcctg

acaatccagcttatataccaaatctttttgctgcccttggtagcaaaatgtcattctgggaaagaacatacaatactggtctttatttgttgcatcaaat

aattcgtaaactgctagttgacataccatcacagaaagtggcaagtcgtttctttggtnctgacttgcctccattgccag

>Contig1450|udp-glucuronosyltransferase 1-8-like

ccgttgctgtcgccgttgctgtcgccgggccgccggcagctggaacagcgcacagcacggccggacctccacatgctcccatctcgcacgcattcgacgt

ccacgaaaagcatcgttagcagctgagtgcagtaacgctgttgatgttgacacgtcgccatgaatgcctgcgtcctgctgacggtgttgtttatgctggg

agcctcggaggcggccaggatcttgtccgtcgtgccgttcccggccgtcagccaccagagacccctcaggacgatgagtctcgagctggccaggaggggc

caccaggtcaccttcatcaccaccaacccgtccaaggaaaacatcaccaacttcacggaaattgacatatctgccgcatatagctatctgaaaacatcca

ctgattggatggctacagtccattggacagaaattgatatgctgtttttttggaagcaaatagggagagcagcttgtgagagcgaactcagtactccgga

gatgcagcacttcataaagtcagctcccaaattcgacctgattattctggaacgggtgatgacgccgtgctactacggtttagtccacaaactgggctcc

ccgcctctcatcgggtacgtctccttaggcgccactaccgccacccactgggcagatgggaatccgagtaaccccgcttacctcctagactttca

>Contig3409|udp-glucuronosyltransferase 1-8-like

gggnnnnnnacaagcagagggcggccacgctgcagtcggccgccggcgtctagcggcgatttagaggcgagatgagttcctgcccgtggtctgcgctgct

gctgttgctgctggcagtgggcggctgctggtcggcggacatcttgttcatcgcgccgacgccatccgtcagccacgcgctgcccatcaacgccgtgatc

ggcgctctgctggccagggggcaccgcgtcacgcacatcacgccggaccccatgcagctccaggacacaaagtacaaggcgattgacctatcagcgtcct

acccgttcctgcagcagctaaacaaaacagtgctggccgacacgtggcccatgaaactggccgaggcgtaccacgagctgggagtcttctgctgcaccga

tgagctcaaaaaccaggctctacaggactggttaaagtctgaacaccggttcgatctcgtaatcatcgagaggcttccgtatcagtgctactacgggctg

atccacaaggtggggtcccctccgatggttggcttcttgacactaccggctatggtgccgacctactacgccataggaaaccccataaatccagcctatc

tgacagatgtctttattgggtacaccgaccacatgaatttctggcagaggctgtacaacacttacttcgtcctaagattcctggttttctggcagaacac

agtactgccaactcaagaagcaattatgaggcaacactttggtccagaggcaccttcagtctacgaagtggaacggaactacagtttgctgcttttagcc

gttcattccagtgggcattacccaagaccaaacgtacccaacgttattgaagtgacgggattacatgtaca

>Contig4695|udp-glucuronosyltransferase 1-8-like

gaggccgnnnactcaacgtgcagcggcgagctggctgtccagaggtccaacactctctagaacagcgtggtaccaactctccggcatggggagcccccgc

ctgctgcagctgctgctgcccgccctactggctgcggggtgcgcggacggcgccaggatcctggccgcggtgccgttccccagcgtcagccaccagtacc

ccctgcggatgatcgtccttgagctggtcaagcgcggccacgaggtcaccttcatcaccacggaccccatcaagaaaaacatcgcaaactacacggagat

tgacatatctcctgcgtacaggtacctgaggaagaccttcgattggatggaaatggcgaaacatgcttgggacccagtcgagatgttatggggcatggag

gacatggcaggtcccatctgtgaggatggcctcaagagtccggaaatgctcaagttcctaaagtcaggcaagcagcagtacgacctggtgatcgtggagc

ggctgctgacgccgtgcttctacgcgctgcagcacctaaccggctcgccgccgctggtgggcttcgtgtcgctgggcgcctacccctacacccactggag

ccacggcaaccctgacaacccggcctacctgcccagctgggtgctgcccttctccgaccacatgaccttctggcagcgcctctacagcacctacttctgg

ctctacctcaactacgtctggttctacgtcgtactgccgcggcacgacagactccaaaagcagtactttgggcc

>Contig1643|udp-glucuronosyltransferase 1-10-like

tggaaacgctagctaccgagaaaacatgaagcggttctcagcggtataccgtgagcaccaggagaggtccctggacactgccgtgtggtggatcgagtac

gtgatacgtcacaagggagctcaccacctgcgcagcgcagccctagacctgagctggtggcagctgcttctgctcgacgtcatcgcattcttcgttgcga

cggcgtcaatcgcgatctatttgctctacaggatagcctgttattgcaaaagtcttgtggctgttaatctgaagcaaaagactgcgtgaagctaattttg

gaacgaatctgtaggcgtttcatcgttttcgactatagctagggccgagtgttaggccacatagagggttgtgttgcaatgtgaacctcagcaagtgact

tctgaatttcacaaggcactgttataaaaaggtattactcctgctatgtagataacagggacataaccccctttgtatttattaattgaatgactgtatt

ttgtataaagtaaacatttttgattcgtaaaaaaaaaaaaaaaaaatcg

>Singlet6053|udp-glucuronosyltransferase 1-10-like

ctgtcggaacacgcagaaatgggaggtgccttttcaggaagggtacttctggtgctcaccatgctggctggcggctgcctggcctacaacatactggtgt

tggctccagcaccgtcctacagccaccagcagccgttccggtacctcacaaaagctctgctgcgcaggggccaccgggtcacattcttcacagcaaaccc

actcaacatatctaacgagaatctaacagaaattgatttgtctgtggggtacaaagtatacaagagcattgaccaagtgaaattggctcaacagccgcca

acgcagagtctctcattgcttcgagatcccccgccaaaatttatttgctgtttaaggaaccggcagttctggacttcatgaggaggaaccacagttttga

tctcgtaatcatggagtggtttccgtaccatgctttctacgggctgatccacaaggttggatcgcctcccatggtaggcgtcatcactctgacgccattc

gcccccatctactacgctatgggaaacccgatgaacccggcgtacatgccggacgtgtggatcggctactcccaccacatgagcttctgggagcgcgcgt

acaacgcgttcttctacctctggatccactacgtgtggttctacgacttgatggccggccaggaggccatcatgagggacgtgtttgggccggacccacc

gtcagtgtacgagacggagcgcnnctacagcctcctcatcgtcaacaaccacttctgtctcgagtatccacgcccgcacctgccaaacatcatcg

>Singlet1344|udp-glucuronosyltransferase 1-1

gtggctgcgcctctcgccttccaccagccggttaccccaagccgcactcgctcgcggaccaggccagcgtcctgtaagcatgaaggccggtggcccactg

tgggccgcgctgctgccgctgctgctgctgcagaacgcgccggtcgccagcgccgctaacatcctggccccagtctggttcgtctcgcccagccacttcg

tcatgctgggccggctcttctcggagctggccgccaggggccacaacgtcaccgtgctgagccacttcccgcggcagtcgccccagcccaactacaccga

catcagcatcgctggcagtattccctcttacgccgacaagatcactatggaccttgtggagatggtgtccagcccgtttgggctcctgtacaactggcac

ttctgtatggacgcctgccgcaccgtctaccaacacccagaagtggagaagctggttcactcgcaagaccactacgacctggtgatcgcggagatcttct

acgtcgactgcatggccgttttcgcgcacaagttccgcgctcctcttgttggcatcgttaccagcatggcctttccaaccgcctacgacaggatgggaaa

cccagaccatccggcctacatggactcgtacttttctccccactcggcgcccttcaccttctggcagagggtgaaaaactcgttcctgcacgtcgtggcc

cacttctgcgactggtggttctcagnncgagggatggaccagttgctgggcgnnntgttcggcgatgcggtgcccccgatcagggacatagtgcgcaaca

cgagcctgctgctcgtcaacagccactggagttacagcaaccccacgcccacg

>Contig1456|udp-glucose:glycoprotein glucosyltransferase

ctgtcgggnnnnnntgcaatcgagtggaaatatccgtgtgggtctcctattaaatccagaaactgatgccactgaacagcatttgaatatgttagctatg

actgctctgcgagtactgaaacctccacaagcaacaaagctaatgtcttcaatacttactgatgaacaatcagcacaagccatggctgctggccagaagg

acataagtgatataaatgtagcaggaattgatgtgcctgaactggtagcaactatgaagaaagatagagccatttatcaaagagagttagctctccacaa

tgatttctgcaagactgtactgaagattgccagtggtgccagagcagttatagcaaatggccaaatggttggtccatttgaaacagatgaagactttata

tcagatgatttctctctgttagaaagatacagcatgaacagctatggtgaaaaaattatggaagtcttgaaggaaaatgttctcaccgaagaggaagatc

ttattgagaatagtgatgtgctgatgcaagctgttggcttgctggtatcaagacaacaaacaagaagtcgatttgacatacctgtcaagagagagctcta

tagtgttctaaaactacctcctcgttctgaatctgaaccagtctttgaaattgctgtgnnngttgatccagtgtctcgcggagcccaaaaagtgggccca

atcctctccatcctctatgaagtcctgaactgcaatatta

>Singlet3055|udp-glucose:glycoprotein glucosyltransferase

cttannttaacgtttcttacgcattatgatgctagtgtcctgaagcatacgaagacaccagtcaaattctggtttctgaaaaattatctctcgccaacat

ttaaggattttcttcctcacatggcacgtgaatatggatttgaatatgaattggtacaatacaaatggccaaggtggttacatcaacaaacggagaaaca

gcgcatcatttggggctataaaattctctttcttgatgtacttttcccactggatgtaaagaagattatatttgtggatgctgatcaggttgttcgagca

gatatgcgtgagctacaacaacttgatcttggaggagctccatatggctatacaccattttgtgagagtagacgcgaaatggatggtttcagattctgga

agcagggttattggcgtaaccacttgcaagggcgccggtatcatattagtgcactctatgttgtagatctgcgtagattccgtcgaatagcagctggtga

tcggctcaggggtcagtatcaggctcttagccaagatcccaactccctctctaacctggatcaggatctaccaaataatatgatccatcaggttgccata

aaatctttgcctcaggaatggctgtggtgtgaaacttggtgtgatgatgactccaagaaatatgctaaaacaattgatctatgtaacaacccactgacaa

aggaagcaaaattgactgcagccaatagaatcatttcagaatggaaagagtacgatgaagaaatt

>Contig3302|udp-glucose 6-dehydrogenase-like

acagaaagcaggtcaattttcctgtaacttgtgccgtatgtgaacacttgttagcatttaaagacttattttctgtataaaacatataacttcgagatgt

atgttgttcattgtatattaatcatctacatcttttacacttttgtataggtgagttctgttgttttataattcttgttttctgtcttttaatgatcact

gttgcaacttgtggagataatattcatttagttttataaagatctagtttttgcctgcatagtagcgcacataggtagttttatgtttattgttaatttc

attttgtagtttttgtggtcaatgtaactttcatgaaggacactacaggtgtcgtcgtcataaccttcatagcacttttgtttcagtaagtgactgttgg

tgacagggcactgatggagacagcaagctactagcccctcagccccgtcctctccattacatttatcaggtgccctgtcataatcctcctgtgatttcca

tcatatagactaattttttctatcccctctgccattgctttccctctccctcaatcttcacctccactttctggtataaggcaagaggaagcagaaagca

caagttaaaaatgtttatatgcacttcctcctttcatattaatccattatctacgttaccattgagagccaggaatgtggaatttaatgatttactttca

ttctgtgctttcaaggtggtggtaatttctatgggaaattgcctttgccatacagaaactagagcaagataccatcagtattatcagatatatagcataa

cattttaaaggaaagtattctgtgtgtacttcaatataaattctatttacacctaatagtttcacaatatattttagctgtttattgttccatctctttt

tcctatagtgaaattaaatttcattatgtagaacaacaaatataaatattgactgctgtcagttgatactgtgattgaagtatcatctcatttaaaattg

attcctgcttgaagtatttgcagtattttgaggactgtcgttgtcagggtacttgtttatgggacatttaattaaaaatttccccactgatcaacttctt

aaataacaaactagttttatcctctgacctactgcaatattctttgaagaaggcaacccttaagagagtattacgttaaaaagaaatgatgtacagaagt

aagatcttcattgtgaaaacaagttattcatttgttcccagcttcattgtacatgtgttgaaagcatttaatcgtataataaatgtactacagcaagaca

aatatgcatttacaaaaaatattatagaatgaaaagtatttaattatatgtgtgatttttctaattgttaaacttgctttttgtagacttcttgatgtgt

tcttttgatgcagcaacaaataaatgccagtgatcattatgtacacatgaatctgtaatatgtactctttagttgagtgagtctttagaggatgtaacta

tcatattaatttgtgtatgctaaaagctgtcatgttgttcaggaaaatattttgtatgtggtatttcaataatgaaaatatcaagatagaaacaaccaca

gcaaaaacgaggggaagtaactagtcacctgcagataaatgacagtacttacgagtgggtgtgtggttacctctccccatatttcatttgtgctagactc

atagattgatcttgcttgcctgatgttgggaagattggggagtgaatgacagcatcagcaaataaaataatgcaaacttcagatatcaaaagctaaagtt

tctgcacaagtctttataacagaaataaaaaagagaatggatccataattatatgataaagaatgtgaaatgcatctcagtttattgttactactgacta

cttgaagttgatggaaaaaaatatttttaatatctccgatagagctggcagtcattcacaaccctctgttcatccagcattcacatctttcttctcagnn

attctttgtatgtgatggttctgtacttcaagttaatgcaccaatctgaattttatt

>Contig3459|udp- c:polypeptide n-

agnnnttctgttgtagcagaactgtcaaaatgtttcgataatgcggtgtgtatgctttgttatataagtggtatgaatggtgttgtttgttagcggcact

tccgtaagactgccaacgtttacgcaatgatgtcaagtatacagtaccatgcctttgtgctaactagtgaacacgtgaactggaatgcatgcctttgttg

aagaatccttattggcactcaaatttgagaggtaatgaaaattaaagtgcttcaacaactgtcatgtctcttcgctacaactcattcattgtaggcgtgc

tggtggcgtcggtaacatgggcggtcagtttgtttctttactggagaattgctggcatagggttgcaagttgaatctccgtcaagtcgcatgccagttga

tccagatagcaagagtcatgatgcagggagttacaaaaatagtgacaagctcttgtcttaccttcagcagaaggcattcaaaccactatcagtgttggac

cagctgggcatggtacggtcagttgcagaaagacaagccagggatgagggttacaggaagcatgccttcaacacacttgttagtgaaagaattggtccac

ttcggaaaatcccagatactaggcatcctctgtgtgcaagagaaacatttgacacaaaagatctgactgccagtgtagtgatttgcttctacaatgaaca

tcccgcgacactatttcgaactgttcattctgtcctgaaccgttcatcatcagacacactgtttgaggtagttctggttgatgactgcagtgatcaggat

ggcttgcatgatgaagtgagaaactatgtctcttcacatttccctgctaatgtgatgctattgcggacaccaagaagagagggacttatccgagctagaa

tgtttggagcatcagccagcaagggcaaggtgctggtgttcttggacagccatgtggaagtgaatgaaggttggttgtctccattactgacaacaatcgc

agactcaccaactgctattgcagttccaataatagacattattggtgctgacacatttgaatacaaatcctcacccctagtccgcggtggtttcaactgg

ggactacacttcaagtgggataacttgcctcccgggacactcacagaaccaatggacttcattaaccaataaagtc

>Contig3060|ubx domain-containing protein 6-like

gagtcccnnnngtttattgtgaatattaaatacagacaattacatatgtgatgacattcgtagatgactattttagctatttctcttttttgaacgtgat

acagcatcatgttggccaattgtttaatgaaattttcagtgcatttaacataacctccactacacctttgacggctaatttgtcgtcgagctacctccaa

gacaatagaagagctgaagaagagtgtgtcactgttgaaattgcccaaggagcattcgccaagagtttgagttggcaaaataaagatggctgataagatt

aagcaatttttccaaaagaaaaaagtagatgcaaagttcaagagagctgggaagggatacaaactaacagatgaacaaccaaggtcaaatacagtgacac

cgaagagcagtgtgccaacgacatcacgatctgccctttcagcggaagcaagccaagcagctgctgctgcaatggctagactaggtggacagaagcaaga

cagtgcaacagccttcacttcattagctgcaatccaggcacaagtacgcaaagaactagaggcagaaaagaaagcagcatctgcagcagcaaaagaagaa

gaagaagaagaagtagttatagctcagcagaagctatcatcggaagttgaagcttcaccgcacttggcggtgaatggagtgtatttcaaatgtcctctta

taagtgatgaaatactttcaaaagaagagtggaccgtaaaaattaaagaattcttatactctcagttagaagaagaacgtggattaacatcttgtcttat

cattcactcctgcaataagaacagagaaaaaatagctcagtgtgtggagacactttgtaagtatttagagaacatagtgcaaaacccaacggaagataag

tacaaaaagattcgaatgtcaaacagaatatttcaggaacgtgttgcacccaatgaaggtattcaagatttcctaatggcagctggatttgaaattaaaa

aggaagaatttcaaggtagtgaagaagactaccttgtattttctgaacagaatgttgaaagcttagaatatttatcaacattatgtgatgccctgagatc

tgcagaaccaatttcgctggaattagatcgtaatttgcaagtgctgcttccttcacaagcaacccataggacagatttgccaccagttttttatacaatg

acancagaggaactgaagaaagagcaacagt

>Contig2169|ubx domain-containing protein 1-like

ccgnnnnnngtgtttgatataccgtgagtggaaagaagtggacattgtttattaaaaaagaaatattaaaaaatcggattgcaatggcaacccaggacat

tgaaacgttaattgatatgggattcccgaaagaaaaagcggtacgagctttggaggttaccggaaatcaaggtgtggagccggcaatggaatggttgctt

gctcatgcagatgaatcgattccattttcttcttctgaagccaccaccgatgcaatggagacaacacagccttctgtgatagatacagcacctattggtg

atagtactgaaaattctgtggagccttccaaaagcaatgaaagtgccagcagctctgaaacagccaagtctttaaagtgtaatgactgtgggaagctttt

ttctacccagcttgaaatagagtaccatgctgcaaagtctggacatagtagtttctcagaatcaacagaagagaagaaaccattgacagaggaagagaaa

aaagaacaacttaagaagctagaagaaaaaatgagacagaagcggctagagcgagaagaaaaggagaagcaagaagctttagaaagagagaggttgagaa

ttcgctcagggaaagaaatgactgcagcaagaaagaagcttgaggaggaagaaatgaaaaagctggtggaacagcggaagcgtgagaaattagaggagaa

aatggctcggcagagagtcaaggatcagatagaacaggacaagattgcaaggcgcacaaaatttgcaatgggcccagcgccagaagctggagctacttct

cccaccacatcacctgtgcctacgccagcatctca

>Contig3616|ubx domain-containing protein 1

tgtggtgtaagctaatgggttggaagggcgtgtgttttgtattttgttatgggagtcgctaacgtttattgtgaatattaaatacagacaattacatatg

tgatgacattcgtagatgactattttagctatttctcttttttgaacgtgatacagcatcatgttggccaattgtttaatgaaatttttagtgcatttaa

cataacctccactacacctttgacggctaatttgtcgtcgaggtatgcaacggatattctcgacagtctgattaactggctagcatttgatgaatttcag

ataaaattgttaaatggtttcttgttcatacttgttgcaaatttggtactcattttcttgtcttggagggtgtacggagaacaaatatcagagcgatttt

tgaaaccagctacctccaagacaatagaagagctgaagaagagtgtgtcactgttgaaattgcccaaggagcattcgccaagagtttgagttggcaaaat

aaagatggctgataagattaagcaatttttccaaaagaaaaaagtagatgcaaagttcaagagagctgggaagggatacaaactaacagatgaacaacca

aggtcaaatacagtgacaccgaagagcagtgtgccaacgacatcacgatctgccctttcagcggaagcaagccaagcagctgctgctgcaatggctagac

taggtggacagaagcaagacagtgcaacagccttcacttcattagctgcaatccaggcacaagtacgcaaagaactagaggcagaaaannnngcagcatc

tgcagcagcaaaagaagaagaagaagaagaagtagttatagctc

>Contig3483|ubiquitin-specific protease, putative

tcgtcatccactgacactacaattttttgcgatgaaatgcatgtttgaatatctagtttccttaatgttacgtgctaatggatactgagaaagtaatact

gatatctggactaggattagtgatagttgttggggctttcatcctttttggacctaccacacgtcctagacggaggagaggccaaatatgtggtcttgtg

aacctaggaaacacatgttttattaattcattattgcaagccttggcctcttgtccagccattgtggaatggttgtcacgatatgccaataagaagaata

atttcattgaagctttgcatacagcccttctagttgtgaatgggcagcatccaaacatatctgaagatccgtacacagcggaaaatgttattaaatgtct

cttcagcaagggctggacaatttcacctgaggagcatgatgtacatgaacttctgaacgtgataatgacaacaattgaggaagagatccatcattctaat

accacgaaagtgtctacacttgccgatctgcttgattttgaagcagaacaagaggaaagcaatcaaagtgtatctagcttgccggaaagtgaaggtagcg

gcgattttgagttgacttcagacagtgataaagaaaattttgccactgtaaacagttatttggatgcatcttcagggcaaggacttacttgtctgacaga

gaatactgacttgactgttgagaacaagtctcnnngtaatcacatagttcaaggtgggaatgantgctcaagtattgctggttctatgac

>Singlet7647|ubiquitin-specific protease, putative

aggggaacatgtacatcaccatccacattgtatggacaagagaagtcccagtgactggaagaataaagacagtcaaagtaggcagtcattgtcagccagt

ggcattgttaggtgtatacagtcaggcttaaaaaatgcccacctacctggtgtaaaaggatctgtgtgggcagtaccaccttgtgaaggtctgctagctg

gcaagactacatgcacaagatgcaaatacaagtcacctgtgaaatatgacaagtttggctgcatatctttgacactgccccccaacgaaaccgtatttcg

taataaaacgacattgtatcaactgttggagaagttcatgtctccagagctaatcaatggtgtaagctgtgagaactgtgaaaaagtatttggtgattca

agatcttcaggatctagcatctttaaaacttgtaacctaggaaagcttcctccatgccttatttttcatatccaacgcactacgtggcagagtaatggaa

tgacaaaaaggcatgattacatagaattcccagaaattttatccatgcactcatatatctacaatcaaacagagaagaaaaagaatgatcttacttctat

tatgggaagtgaaggttctggcagaaaagcacctgttgtaattgacaggttcaaacatttgtacagactgtctgctgtaatagtacac

>Singlet925|ubiquitin-protein ligase e3 component n-recognin-

tgcagagaagtggggacttgaaaacctaattgaagagttgtgtggtagtgcaagagttgaggcgcacaaagatttgctacagtggacactaggaaagtat

aggcaagttgctgcagctcgcaaacttggtcacgtacaacctgcacctcaacctcagcatcaagagggggtaggtcaggcagaagattctgcaaatgagg

aaaaggaatggagagcaaaaatggcagctcagaagagggcaaaaataatggcccagatgacagcaatgcaaaagaactttatgaaggaaaatgccaagtt

atttgaatcagctcttgcagagagctcttcacaaaaagaaaatgtggaaatgttcatggatgtttcagaacagtcagaatgtcagcccattgccctagga

ccgaaacagacaacacgggcaacaacggaccgcacacatacttgcattttatgtcaggaagatcagacagtttctgctggtggacaagcacttgttgttt

ctgcttttgttcaaaaatcaacagtactctgtactcacaggggactgtctgaagaatatctggaaaaaatgtccgagcctttatttctctcagcttctat

tgggccagctccatttactggcacttgtggtcacgtaatgcacagtaattgctgggaaaaatactttgagaatgtcctggccaaagagtcgcgacgaccg

tatcggctccggcagccagcaagctttgacattgagaaacaggaatttctgtgcccactttgcgagtgtctcagtaatacaccgtgcccacttgttcctc

ctgttggatctctaatatc

>Contig3329|ubiquitin-protein ligase

ctgagtatgagacaaaggaaaagttaaatgatcnncttatgaaggcaataaagtattccaaaggatttggaatgttgtaacccacttcgctttcctccta

aaatttaggaaacaatttactttgaagttcccatcatgtctaaagattgtggaatttgttaataaactcctgtgaaaatgaaaatttttggtgcaattta

gcagtggatgaaatatggtgcaaaatgagctgctcataagtgttacataagtgtgtaataacaatttgttctatgttaccactccctcttgcctctgggc

tgttagtccagtgatgaatgtaaactgatttgttaaatatcagtgacagtttcctggtggtgtttagtaaaaaaattattaatactactgacagtgtcta

aatatggaggtatctgcaaaatactactgtaattaactttgtacaaagtgctaagaatattgtcatcttcagtgaagcttttatgaacatggggaggaag

aatgaggtgcctgtttagtgtgtttgtatgaatgaaattcacagtgtatgattcattttgtgtgtgatgactaattaatttgtctcttgtacattttcca

aaattttgaggcagctattctgtttccctccagaaaagtgcaaaaaaaaaaaggaatgacttcatatccttacaaatttgcttcatatctggtgcacata

cacacacaaactaatctattacctgtcatgtttacacataaagtttccaatttt

>Singlet351|ubiquitin-protein ligase

gccaaggggttttttgctacgtttctccacagcaactcacagttaaagaactgttgcttaaattcattccaaagagaatcatcacatttcgtctgtgtcc

atccactaagttcacctttctaggaaacaacaaccagtatggtcaaccttcattcattgtggatgatggatgtcagccaaaagttgaactcgcagcaaag

gaccggaatgtcattgctgcaacatttactcatttcctgctgaagaatattggaggatcagagactttcaaagataaacaggactttttctaccatgaag

ttcggaagtaccaccagaaacattatcatgaaaaactgcccatgaaaatctgccgtgaaaagcttctggagtcgtcaatgaaagcaacaaaaaacttctc

tgtgagtgactggtgtcgaaactttgagataacctttcaaggagaacaaggtctggactggggtggtgtacgacgagagtggtttgaacttgtgtgtgca

gcattgtttgatgcacgcaatggtctcttcatgagcttttcagatggtcagcaagcacttgtacatcctaatgttcaaaggcctcctcacctgaagttga

agcactttgagtttgctggtcgaatagttggcaagcgcctttacgaatcatcacttggaggttcctacagacagttagtacgggca

>Singlet3179|ubiquitin-protein ligase

aagggatagaacaacttgaaaaaaggcaaacacaagatgacgctgtgcttaatgttgtgaatcgttattggaatcagcttaatgaagacatccgtgttct

gttgcagcggtttgatgcagaaacggctgatgaatcagaaaatcggaatgagaatgaggccacaacttccttcttgatgcagttatcgacatgggacaaa

gaagaattggatgagaagctagcaaatcgtgttcaagtttctaagagggcagttgcgaaagtgatccaggcctttgatcgactcatgcaaccaatgaaaa

aatcacattggctcttaaaggcgaacttgatggaggttattggaatcttctcccatcttgtgatcagtgtatgcttgctttttttcatttttagtttcta

tactttaaatgctttatttttgatcactgttaatattatttactgcacataacagtagtaaaattgtgtaatagcttatgagtttgtaaaacatgtgcac

aactctgtagtttgtcaaggaagtagtgctggataaattaagttggaatcagtaatatgtcacttggtgcacataatgattttgattgtaacaaatagaa

tagataaataggtgcaaaaacatctttttttaagtgcttcaatattaaatacagagcaagaagctggagacaagtgtgtgtttgtggactcgagatagtc

aaagttttaatatcattagatgaataaatttcgttatatttatcttcagcagaagatgctttcttagcagttacgataatctctgtata

>Singlet4515|ubiquitin-protein ligase

tgggggaagagtgcactgtgcatccccagaaatatcttatcctttctttagaacataaaatgtctgacagctaataaagaaaatctgagaacactactaa

aattgtataatctaggcaactcctctcacactgatgaaactttaattaaaaagaatgttattttactagtagtttacattatttggaagactaagttcat

ttttatctgtttgctctaggaggatcagagactttcaaagataaacaggactttttctaccatgaagttcggaagtaccaccagaaacattatcatgaaa

aactgcccatgaaaatctgccgtgaaaagcttctggagtcgtcaatgaaagcaacaaaaaacttctctgtgagtgactggtgtcgaaactttgagataac

ctttcaaggagaacaaggtctggactggggtggtgtacgacgagagtggtttgaacttgtgtgtgcagcattgtttgatgcacgcaatggtctcttcatg

agcttttcagatggtcagcaagcacttgtacatcctaatgttcaaaggcctcctcacctgaagttgaagcactttgagtttgctggtcgaatagttggca

agtgcctttacgaatcatcacttggaggttcctacagacagttagtacgggcacggtttacgaggtcatttttggcacaactcattggtcttcgtgtgca

ctacaagtactttgaacaagatgatccggacctctatcttagtaaagtgaagtacattctgg

>Contig435|ubiquitin-protein e3

aagcaacaactttgtgtgacagatactattttattattgtggtcaaaatttaggaaaaatatgaggaactagtggaaaaattcatcacgttgaaggatag

aaacaagtagctattgtgataaaaggtggaaatttctgtttctttgtataatgaaaacaaatatttcaattcaaatatctttacataacttgtatatact

tacattctatttttaagctatcatgattttatgaaatacatttgagcaaaaattttgggtattcggagttgtacagaattcatttcaaagaaggcaattt

ttcttacataatgacaagttttgatgtattgattttgttcatgttgctcagattcattttctgatgaattaagaagacattatcattctgcattcagtgc

agacttatgtatcaaaaactgttttagccatttgtgccaagttgcctatttaatttagaatgtaggaaattcaacaggggaaagttctaatcccttacac

atgactgttgacattaatgactttagtaaatgaagtgctttgtctgttgtcagcatacattttccttcttctttccccacactgcactgtagaaacatcc

tctggagcttgagtgttataatcaagttaagtattgttagatgcatatttgcaatcttttatgttccatattttgcattgtatcttgaaaaataaataat

aaatcagtgcgaaaaaaaaaaaaaaaaaaa

>Singlet8232|ubiquitin-protein e3

gttgctgtcgccgttgctgtcgcatctacatnntctagttcagttcgtcagcatgagccttcattgaatccgtggaatctatctagccgtggcagtaaat

caaagcataagtcaaggaggcatgacaggaaaaagcggcgaagaaaggagcgatatacttcttcctcatcttcagaagattttgtatttcgtcataccaa

acggaggtctgaaaatgccgtaatttattcagactctgactaaggccttaagagccaagtgcgttttccttttattgtaaataatatatatactggtgtt

cagttcaaagtatttcagtaagttaagcactaaatgtgtaaatgttactgaagttgtgctttttttttaacgtagtgcagtgaacataataatgtgacag

attgcaatgcaggaagactacaagagccgtacaacagtgcttgtagtgtttgtaaatacagtgctaactgcaatannngaagttcgtcataaatcatcca

gatgtgcccttatttattttttgccctgttttcttttccacttggtcgtgtgnnnacattacgggttgctgctggttagagtaaagaccatttaaaggtg

cttt

>Contig350|ubiquitin-like-conjugating enzyme atg3-like

aagggggatgtaatacggcttgtagatgtagacagaagggaaataaagtactagcgttgatacacagccgcaaagatgcagaatgttattaatactgtta

agggaacagcgcttggtgtcgcagaatattggactcccgtactaaaggaatctaaattccgtgagacgggagtgctgacgcctgaggaatttgtagcagc

tggtgatcacctggtccatcactgcccaacatggcagtgggcagttggtgatgagagccgtataaagtcatatcttccccatgacaagcagttcctacta

acacggcatgtcccatgttctcggagatgcaaacagatggagtactgtgaggaacaagaagaaataattgaggctgatgaccctgatggtggatgggttg

atacacatcatcatgatcttggtgctttgggtttagatgagaaagtttcggaaatgacgttagagtcgaagacagagtctgctgctttagaactaacagc

cagtggaaaaacatcaaaacccatttctactccaagtggcaatagcaatggtagtgctgatggtggtggtgatggcgacgatgatgacgacgacgacgaa

gaaccagttgatatggaggaatttgaagagagtggcttactggatgaggaggatcagaatactgtaattgcttccaataacactacgaaagaaatggaag

cttctgctggtgaaattattcacacaagaacttacgacttgcacatcacatatgataaatactaccagacaccaagattgtggctttctggttatgatga

gaaacgcaagcctttaacagttgaacagatgtatgaagatgttagccaggatcatgctaagaagacagtgacaatggaaacacatccacatatgcctgga

ccaccaatggcatcagtacatccatgcaagcatgcggaagtgatgaaaaaaaataatacaaactgtaactgaaggaggtggagagttgggggttcacatg

tacttaataatattcctgaaatttgtccaggctgtgataccaacaattgaatatgactacactcagaattttgcaatgtgatgggtatgatttttatctt

ccatttaatggaaaacactggaaagcattccatcttttcttctcttccatacagtgttctgtgaagctattgaaagagatttgaaatacaagaatactat

acagttttgccttctattttaattattggtttctctgtattcaggagccttctgcagaaaatattcacatttcctttgagtatatgcaagcagtaagata

tttgcatgtgtattctcttgctgtacaagttttagactgcctttttcatggaaaaagaaaggtcattaacagtttgtgcacaaaataaatttgttccttc

attaaaatgtaagaaaaaaaaaaaa

>Contig105|ubiquitin-like protein smt3

ggtgagctccttggaacgaagcgaagtgttaggcgttaaagtgcgcgctcgtattttttttaaaatacgcctttggcaaattcttgtggtcttggctgat

ttgtattagcgtaatatcagccatactttgtattgagtaaataagctgtcttacctatacgttgggtttgttgtggagaagcaagttttagtgaatttcg

tccgggtagtgctttagtaaaaatgtcagaagataaaaaggacgtgaaaggctcagagtctgagcatataaacttgaaagtattgggtcaagacaatgct

attgtgcaatttaagataaagaagcacactcctttaagaaaattgatgaatgcatattgtgatagagtggggattgcaatggctacaatgcgcttccgat

ttgatgggcagccaatcaatgaaacagatactcctctttcattggaaatggaggaaggtgataccatagaagtgtatcaacagcaaacaggtggaagagc

atagaaagaaactgtgtttattgaaatggctttgtgttatgcaccgagagaagtggaactcgagtgaatatgtgaaatacaagcacactttttctattaa

atgtgacaacttcaatagtgaatgtaagtgttgggtaatcatgtgaagtattcctaaatatcactttgaatttggggagcccatttgtgattgagtggaa

gtaaaatttgttctgttgttttggactgctaaacttgtttttcttgtgtttttttttttcatttttttttttaattcctcaaagtctgcattgtactaga

agagaacttaacaattccaatggccttgcatttttctgttcattccaacaaggaattgagtgttctccagtctatattttgttatgtagaagtgttcaaa

ctaaggattccacattgtttaaatgtacttacatgataatggcatgaacattt

>Singlet3499|ubiquitin-like protein fubi-like

agggaaaactgcgtcaacagaaattgcttgtgttatgcttggctgctctaatgctagctagaagggattattctcgcggttgctcgcctaatcacatatt

tccatttgtttatgctaataaaattcggatatagcgcgatagtacctctcatcgtcgatttgtcaatattgtacagacatttggtcgtagaaagggacct

aattctaactcttaaatttctagaatgggaagtgcaatatgatttgcatgtaaaaatgcttggattgatgataatcttaagtgtgagggagttatgttac

caaacattggcatcttgtaatgtacagatttttgtgtatcaataaacaaattatttgatgataaaaaaaaaaaaaaaaaaaaaaaaaaaaaaaaaaaaaa

aaaaaaaaaaaaaaaaaaaaaaaaaaaaaaaaaaaaaaaaaaaaaa

>Singlet1054|ubiquitin-like protein 7-like

aaggggagattcatacttcatagattggaagatgacagaaaatgtagcagtttaatgtttgtatatgagaggtttagattgaatatccaaattgaggtgc

tacaacgatagttctcgtactgaatacaacagcaatttctagaaatgtcaagaatagtgtttggtatcagactttcacccaatacatatcagtgcgttaa

aattgaagggattgatttggagtgtactgtggaccacttgaaacaagaagcttcaagagtgacaaatttaccgaaaagctttctagaacttgtttactgt

gggaatatccttgaagatgaagccacccttcgagcttccggacttaaatctgatagtacagtccatgttctgaagaagagagaaaaagaagcacctgttc

catcaagacccctgtctgaaatggacattcaagagttggtgatggcgttcaagacatttcttaagcatccaagttaccgtagtgccctacataggctgaa

tcgagcagaagtagtagagaatatcatacaggccacacctggattgctggatgatcctatagcaatttccattctacaggaaccagagttgcttgtgcat

cctgagaatgctgacactgttaggagaattgcagaagctcatccggcacttgcagaagcagctgnnnatattgcagctgctgt

>Contig3273|ubiquitin-like protein 3-like

agccactgcttcttgcccgtgtgcccaagaaaagtgtaaaattatttggtttcgtataaaaaaatgcaagtttcacatccgtagaagaatggatagataa

cgtggttcgatattattctcgtcacttcgctaaactgtgcgtcattatggcgtaataagaagtttagtgttgtgtgttaatgttgctcattttcgtctgc

tgccaccagctgcttggtgctacgaaaaaaatcaactccctgggaaattagccatccacttctacgcttattggtttaaacgacgcaatttctaagatga

gctccagaaacataccagcggacaagatcaacttgcggctcatactggtcagcggcaagacgaaggagttcctgttcagcccgagcgactcggctggcga

catcgcgcagaccgtcttcgacaactggccggaagattgggcagaagaagcagtggccaaggcagaaattcttagactaatataccagggacggttcctg

cacagtaacgtgacacttggagctctaggtctgccgtttggcaagacgacagtaatgcatctcgttcccagggagaatctaccagaacctaattcacaag

atcaacgccagaagagtaagggtggtggaagcagttgctgttcggcatcgtgttgcatactctaatgtattgcaagataacattgtgatgactttaccaa

gaattggtgcattgacagtttaggacagaaatgcctacgtacatagtcaggcacctcaagtattactattgacttaaacaagcaaagcatctccccctga

cagaagataaagatcttgtgagatttcatattgttgtgatattcaagtagcataacagaaaattttaaaaatgaagacgagagtttttttagtgggagga

tatgtgatagtggagactgattgcccatcgcagtgaaatgtacagtgaaagaaattaagtttctgtgttactttatttttgtattaaaattctaatatta

>Contig1861|ubiquitin-like modifier-activating enzyme 1-like

tattccacaaaaccgtgatcgtgaagctgttgctcgaattgctgcgaaggtgcaagtaccagttttcgttccaaaacaaggtgttaaaatatcagtaact

gattcacaggatcagatgtctaatggaactggtgctgttgatcatgatcgcataatacaattgcagaaggagttgcctggtatcagtgagctgggtaatc

ttcagataaagccattggaatttgaaaaggacgatgacaccaatatgcatatggactttattgttgctgcttctaatctgcgtgctgaaaattacggtat

tccaatagcagatcgtcacaagagtaagcttattgctggtaagattattcctgctattgctacaactacatcagtagttggtggccttgcgtgtcttgag

ctatacaaattagcacagggctttaaaaatattgatgtcttcaagaatggttttattaacttggccttaccattcatcagcttttctgagccaatttcag

ctccaaaaaataagtattatgacacagaatggacattatgggaccggtttgaagtagtgggagaactcactctcaaagagttccttgattacttcagaac

taaatataatctggaaatcacaatgttgtcccaaggtgtttgtatgctttactcatttttcatgtctagatcaaagtgtgcagaacgcatgggacttcca

atgtctgaagttgtaatgaaagtctcaaaaaagaaaattgagccacacgttaaagctctgatatttgaattgtgttgtaatgatgatagtggtaatgatg

ttgaagttccctacgttcgttacacactcccaaattaagttattgtattatttggttctaaattcaaccagatattttctcatgttttcaatgtttaaat

ctaatttgagcttacagtgtggctgtgaattttatattttcacattcagtaatttttgttcttctgtgtttagaatttacagtatttttaataataatgc

cacaaagttccaatattttttatagaagtagggtgaaatttatatctgtggaaagttattgaggcagttcatttagattgtgcagaataatttacaggaa

gtttacttcatgcatttaacacacctgtgct

>Contig3360|ubiquitin-like modifier-activating enzyme 1-like

ccgnngctgtcgccgttgctgtcggtgtctttgggagcagttgaaggtaacaaagattattacagtgttatgaactatgcaattgtcttttaacgaaaag

acagtgttgatttgtgatcgaaacattggagtatacgccttcatagtgaaaactttgtgtgtgcccgatgtctagttctcgtgtgctcgatagttccgta

gatcctgcagccaagaagagaaaagttgctacaggagactcagtagtagcgtcctcttgctcctcgcgctcagcagaaatggagcaaccgggatcatcca

actgtgcccccgagatcgacgaaggactttattcaaggcagttgtatgtacttggtcatgatgccatgcgacgcatggcgtcctcgaatgttttaatatc

tggtcttggcggcttgggagtcgaagtggccaaaaatgtgattcttggtggtgtcagagctgtcacgctacatgatgaagtttcttgcacaatgtcagat

ttgtgctcacagttttacttatctgaagcttcattaggaaaaaatagggcagaagccagtctcaaacaactgtctgaattgaataattacgttcgtactc

aggcgtataccggtccactgactgaggattttttgaaacagttcagagttattgttttaacaaatagtaaacttgacgagcagctgagaatcgcggaaac

cgcacgtgccttcaatatcgcacttataattgcagacaataaaggactattttcacaggtgttctgtgactttggtgatgaatttactgtcgtggacaca

aatggcgaggcgcccttaactgcgatgatagccagcgtatccaatgacattgagggagttgttacatgtctggacgatactcgtcacggg

>Contig3990|ubiquitin-conjugating enzyme m

caacnnngaccatacgggcctatactcgatatagtttttgtttttcgacaagaacatgttgagcaagtgaataaatccttgtcagaaattatagtatttg

aatgacagtgtttttgaggattttcgtgtgaatgctgtgaaactacgaaaaatttataccacctttccgaagtgacaatgattaagttgttttctttgaa

acagcagaaaaaggacggagaagcagcccccaaatcagggactcaaaagaaagcttccgccgcgcaattaagaataactaaagatataaatgaacttaat

cttccaaaaacctgcaataccgagttcccagatccggatgacttactcacgttcaagcttattatatgccccgatgagggcttctatcggggaggaaaat

ttgtatttagctttaaagtaggtcctaactatcctcatgagccacctaaagtgaagtgtgaaactccagtatatcatccgaacattgacttggaagggaa

tgtttgccttaacattttacgagaagattggaagccagtgttaactattaattctattgtttatggactacagtatttgtttttagagcctaatccagaa

gacccacttaacaaagaggctgctgaaatgctgcagaacaacagacgtatttttgaacagaatgtgcagaaagcaatgagaggtggttatgttggttctg

tatactttgaaaggtgtttgaaatgaaaaactgtgaatggaacacacaacctttttttttctgaagaaagatgtgacctatggtctagcaacatgggttt

gattaaagtattatttaagccagcacattataatgcttcggccagacaggtcacttgcttgagaaagtttttatttaaaccagctttcagtacgaactct

gaagtttt

>Contig1462|ubiquitin-conjugating enzyme e2i

ctgtcggtannnnttcattccaactgatggtcttctacattttgttagaattttatgtattatttatcatttgtcaaaactttggaactgtttcgtctga

ataattctgtaagctagtgtttattccgccaaagcattagattcaacgaattataagtttacggcaaatagcgaggcttttgcttccggttattggaata

ttgggtaataaggaagatggcagcgaccaggaggttgcagaaagagctcggcgacatccgatcgtcgggcatgaaatctttccgagaaatccaagtggat

gactctaatatattaacatggcaaggtcttatagtgccgaatacccattcaaaccaccaaaaataaatttcaagaccaaaatatatcatccgaacattga

tgagaagggccaagtctgtctgccgatcatcagcgctgaaaactggaaaccagcaacgaaaactgatcaagtgatccaggcgttggtagcacttgtgaat

gacccagaacctgaacatccacttcgtgctgacctggctgaagagtatctcaaagaccgtaaaaaatttgtgaaaaatgctgaagagttcactaagaagc

atagtgagaagcgaccgtccgattaaagtctgtgaaatgatgcagttcctgcctggactttgggaggagaggttttgtgatacacnncttgaacatttgt

ttgtgattgtaattctggtgtatgtacagtgtgcactgaaagtgtagta

>Singlet7910|ubiquitin-conjugating enzyme e2i

tgggggtaataaggaagatggcagcgaccaggaggttgcagaaagagctcggcgacatccgatcgtcgggcatgaaatctttccgagaaatccaagtgga

tgactctaatatattaacatggcaaggtcttatagtgccggataatgccccttataataaaggtgcgtttaggattgaaataaattttcctgcagaatac

ccattcaaaccaccaaaaataaatttcaagaccaaaatatatcatccgaacattgatgagaagggccaagtctgtctgccgatcatcagcgctgaaaact

ggaaaccagcaacgaaaactgatcaagtgatccaggcgttggtagcacttgtgaatgacccagaacctgaacatccacttcgtgctgacctggctgaaga

gtatctcaaagaccgtaaaaaatttgtgaaaaatgctgaagagttcactaagaagcatagtgagaagcgaccgtccgattaaagtctgtgaaatgatgca

gttcctgcctggactttgggaggagaggttttgtgatacacaacttgaacatttgtttgtgattgtaattctggtgtatgtacagtgtgcactgaaagtg

tagtaaacactcctcccccctctgtgacagtgttagcaccatctacactcaccaggtgaagcggtgcatgggtgcttgtcaggtgttgccaacactggtg

tgcatgagtgcataaagaaacgcccttggtactatttgtcactttgataaacaccagcccagatacacatgattttgagactactatat

>Contig1074|ubiquitin-conjugating enzyme e2-32 kda

ctgtcgggcnnnnnttggcaggttagggattttctctgagctcttgctcagctgctattgttcgtgtgcgacaaagttatcttttcggagttattttacg

tgacgcgttcgtgtgctgacgcgtcgcgccgaaaagtaattggcgcaagacgagctccaagtactgaggagagtttgagaaagtttctgtgactacgtaa

cagaaagcacgagagaacaaaagctggcagccattgctgtggggatctgctcagtcgcagtgtagaaactggagttttggggactggcgaaatatccccc

ctttaaaattttgtggtgaacataattcgtgacaaaattggactgtgttttacaaatttaaaatggcgcagcaacccacaagcagtgcagttagagccct

tgcgcttgaatacaagagcttacaggaagaaccagtcgaaggcttcagggtgaaacttgttagagaagaaaatatgttcgaatgggaagttgcgatattt

ggacccccagagactctttatcagggtggatacttcaaggcacacatgaagtttccacctgactatccatattcaccacccagcattcgttttcttacaa

aagtatggcatccgaatgtgtatgagaatggtgatctgtgtatttcaatcttgcatcctccagtccatgatcctcagagtggagaacttccttgtgagat

gtggaaccccacacagaatgtcaggaccatcctgctgtcggtaatttctctactgaatgagcccaacacattcagtcctgccaatgttgatgcgtcagta

atgttcaggcgctggagagattcaaaaggcaaagacaaggagtacgagaatatcatcaggaaacaagtgttggctgcccgacttgaagcagagaaagatg

gggttgtagtgcctacgacattagaggactattgtatcaagacacaggtgtcaaagccagccaccgaacctgtgctcgatatgacagacttttatgatga

tgactacgatcttgacgacgatgatgacgacgatgatgaagaggatggtgatgcggacggtgatggtgatgcagatgacagtggaaatggcgagtcgtga

tgacattcgtcttggcgtgtgtttgctgttgacatttgtatccagtcaatggaaattgctggtgtcacgaaacggcatggcaggggagagtgcttggagg

gggttgacaggtcacaatttcatgtccatgattgtatctctgtaatgcataatgctagtagtagtatttgtgcttactgtttgagaagtccttaagaaaa

agagcaaagaaaatggaaagaaacagttgtatgtcattacccatgttagtctgtagttaaaatgctaaaagcatgcagaaatcagaggtgtgtagtataa

atgtgaactgcattgcacttcacattgcaagcacatgtgcattacagggtaatctactacacttnnggagaatttgtgcattgttggaaac

>Contig3091|ubiquitin-conjugating enzyme e2-24

aannnnnttgatcggcaagccgatcctgttgactgatgtcaggggcaggatctagtggaacagggcggggccgtggcacagcctcattgactgacaataa

gactgataataaagatactaagccaaatccaaagatgtcgaaagcgcttagtacttctgcaaaaaggattcagaaagaattagcggaaatcacactggat

ccaccaccgaactgcagtgctggtccaaaaggtgataatttgtatgaatgggtgtcaaccatattggggccaccaggatcagtctacgaagggggcgtat

tttttctggatattcatttttctccagaatacccattcaaacctccaaaggtgacatttcgcactcgtatttaccactgtaatattaatagccagggggt

tatctgcttagatatattgaaggacaactggtcacctgctcttacaatatcaaaagtattactatccatttgttcactgcttactgactgcaaccctgct

gaccctcttgttggcagcatagcaacacagtatttgcagaacagagaagaacatgatcgtattgcaaggctttggaccaaacgttatgcaacgtgatgtg

tcaacaaaaaaaaataatgctcttccaatgactttttgtaaagacatagtgataaatgacaaacagcccttattcagtgcagcatttgtttcattggcaa

aaatataattgtgtgtatgccacagtgtatgggtgtttttctcactgtttggaactcctgtgaaaatgagatgttttgaacatagataatcatttattca

taatgtagtatcaaaacaaagatagggaaaaggatattaaatgtaatgtgcggcttcagaatagatagctttctgtggattgtttcct

>Contig719|ubiquitin-conjugating enzyme e2-17 kda-like

agggggacagagtagtgtggttgttgtgaacccgtaaacaaatgacatgaaatactgtcagtttatgagttcacgttctgaggaaacggtgtaggaatta

tttgtaattgatagacttctgttgtaggtactagatatgtctacacctgcaaggcgaaggttgatgagggattttaaaaggttacaggaagatcctccca

ctggtgtaagtggagctccaactgacaataatatattgatctggaatgctgttatatttgggccacacgacacaccatttgaggatggcacgtttaagtt

aactatagaatttactgaagaatatcccaataaaccgccaacagtaaaatttgtgtccaaaatgtttcatccgaatgtgtatgcagatggtggcatatgt

ttggacatactccagaatcgttggagtcccacatatgacgtttcagccatacttacttccatccagtcactattaaatgagccaaatccaaactctcctg

caaattctgttgctgcacaactatatcaagagaacaaacgggaatatgagaagagagtttctgctgtagtagaacagtcttggattaattaccaagaaag

cagagaagaaactgtggatgacaatggccaaccttagagagtaacagaagacattgtatgtgggtgtctccagtgtctacaaactatataaaaattatat

gtataatgatgtagtgagaatgaagtagctcttttgttgaataacaaaaataaatttcacttttgtttttatatgcagaggtatttttgtttcaaataga

gagaactgctctctaccgctgtctttcaaaaaaaaa

>Contig2152|ubiquitin-conjugating enzyme e2-17

ctgtcgggtatcactcttgaagttctatttacttaatgatgtaaatatagactggcaccattaatgctacaacattttcctatctagtttggaaatgtta

ccacacacatgtagattttggctttgaattctgaggagcagtgaggctagatacaaaatgaaatttggtacaaagtttgacgtagtggatgtaaaacgtg

tatctttgtctagtatttgaagtgtgatataatggtgcaaacatcctaaaatatatattcagggtatttaaacattggctcaattcttctttactcttgc

ttaaaattttgaacttcaaactatcaacaaaaatttccaccaggtagtgtttttttgatagatgtaatgtaatgttgagtcataaaattgattcctttgt

agcttttagaaaagactgtttcgataaataatttgtttgtaattcagatcagtttttgtgatccacgtattttatttactgaactgagaacaataactcg

gacattgctcatatgccgtggctgtttaatgcctgatggtgttcgttgctaaaatacagatttttactgctaatgcaatctttgtggataaaatttgttt

gttaaggtcatatttggacctgtcagacacattgtgtaatttaagtaaattccattttagggcactgcatcatcacagcagtacatcggcagttgcatat

ggtgatttttgggaagagnnntactggtgttgagtgagggttctttaccagtcagatgcata

>Singlet7915|ubiquitin-conjugating enzyme e2-17

gacagttgccgcaatctcgataaaattgctgcagagtttcatcaaatttggggttatttttatgccttttcgtcattgcgtgaccatataaacgaaaaat

ggcaaagccacaaaatattaaagttgttgctgttactgctgtcagttcattaggttcggattgggaccctattacaaatacagattggcagaaggagacg

ccgtcagcacaatgtcttctgaggattaaaagggacatgatgtcaatctacaatgaaccaccgcccggtctgtttgtcgttcccgacgagaaggacatca

ctgtgattcacgcgctcatcacgggttcatttgacacaccctacgagggtggcttcttctacttcctcgtgcgatgccctccagactaccctatacggcn

nncacgtgtcaagctcatgacgactggaggaggcactgtcaagtttaacccaaacctgtacaagnatgggaagatctgcctca

>Singlet3639|ubiquitin-conjugating enzyme e2 s-like

agcaactgtgtatcttcaaatgatgtccgcaatgagttcaatgtcaaatgtggagaatttatcaccacagataataagacgtgttgctaaggagattcat

gatttagctaatgaccctccagaaggaataagagttaatattaatgatgaagatgtaactgacatccaagcatatattgaaggtccagctggtacaccat

atgctggtggtgtgttccgtgtgaagcttgctcttgggaaagatttcccacaagcaccgccaaaggctttctttctcacaaagatatttcatccaaatgt

tgcgaaaaatggagaaatatgtgtgaatacattgaagaaggactggaagccagaccttggaataaagcacatattattgacagtgaaatgcttgctgatt

gtgccaaatgcagaatctgcacttaatgaagaagcagggaaattgctgcttgaacaatacgaatgttactcccagagagcaaaaatgatgactgaaattc

atgctttgtcatcgaagatgcctaaacctggtctggaagttggtgcctcttccggcgatggaccaatggcaaagaaacatgcaggagacaaaaaagtggc

tgaaaaaaagaagctgctgaaagataagaagagaactttgaaaaggttgtgaaattattttgcaagatgtatgatctcaccacttcatccggaaatcaga

aatgaaagtggattgcaattttgtacgtgtgttgtcaacaaaggattgtgtactgacactaaaagctctagactgtcatacttctatggcaaagtaatca

aacagtcctgttaaaatgatttctcgtgtgcaagtgtcatcaccatcaccccaccccaaaataaaaac

>Contig517|ubiquitin-conjugating enzyme e2 q2-like

aagggggaactcgataaaggtcgccattttctgggctagtggtgaatggaagggtagtaattttgtgttttcaaagtcgaaatcaatagtgttgctgatt

tatttatgcaggccgtgtacaaatggcttgtctgataactctgaaacaagaaataaagactttagaagccgttttcccaaagaatcatgagaggtttcag

atagtgtcagctagtgtggacgaactgaattgtcgattcgtcggaaagaatggaaagaagtacgacatacacgcaaacataaccgaaacttatccttcta

ccccaccagtgtggtttgccgagtccgaagaaacgagtgtgactaatgcagttcaaatactgagcaacacctcgggaatggataatcacgttctgaatca

agtcagcattttattgagagaactatgcaggttgcactgtgtacctgaaccaccagatattgcactgctgaaattgcctggtccccagggctcctatcgt

cttcctgatcgacatgatgatgacagggaaatggatgatgaagatgaagatgaagacgatgatgatggtgatgatctgcatcttgacatggatgaaggag

aaagtgttgctaaaagcaaggtggaggagatggacacagaacatctggcaacacttgagagacttagacagaaccagcggcaagattaccttcggggttc

tgtttcaggatctgttcaggcaacagacaggctaatgaaggaactgagagatgtgtatcgctcagacagctttaagaaagggatgtacagcattgaactc

gtcaatgatagcctttatgagtggaatattagactgatgagtgttgatcctgattcaccactgcataatgatctggttctcttgaaagaaaaagaaggga

aagacagcattctactcaatatgacttttaaggaggcgtatccttttgaaccaccatttgttcgcgtggtgcacccagttatttctggagggtatgtctt

ggtgggaggtgccatttgcatggagttactgacaaaacaaggttggagttctgcatatactgtggaggctgtaataatgcagatcgctgcaacgctagtc

aaaggaaaggcccgcatccaatttggagccgccaaggtctgtaaccagagccaatatagtctcgcaagagcacagcagtcct

>Contig2913|ubiquitin-conjugating enzyme e2 o

tgtctcctacaacaccaactacgttccgtcagctgcatgagacagaattaacaaatgacttgaaagtcccgctgcctgattttcctctgattcctgcctc

gaaaggattctgtctcacactacgcaaaactttggtcacattccgtgaggttcttgcatcggtgggtgtcctgacgcccgccagttagtacatgcttcat

ccacattgcctacatttagcaacaagttgcctcagcgctcactataggcactctgcggtaggccatgctgccatccctcccaaatatttatttattcttt

ctgcccagtcactggcactaccgtataatgaagaatggacacggacttaactgttgttttgttggtgattatagtattggtgacgtattgaaacaatctt

ttaccttcatctttatttaagagtaactccagtgtcaaaaaacttaactgcagcataggaatttccttaaaatcaaactacacctcttctacatcgtatg

tttggagaaaagactataaacatgtagaatttgctagtattttttgttcatactttaattgtaaaatttagatcggtcataataaataaaaatcagtaaa

gagaatattgtacagtacatccactccttaggtatgagcagtcactttttcacacaactgtgatgcaacgaaatgctgtgctgctactgttgctgctgat

tgtaagttttgacatcatgtcaatatgagtataggcattgctcctgacctttgtttcttctgttaataaattttctgttatctcagggagttctgataat

tttgtctggctcttgtgatcgtgtgtaatgtaaggacattgaaaagtggtgctgaaaaatatatcatcatgtagataaatatttaaaaatgaactgttgt

atatttaatgtatgtatatctgctgtgta

>Singlet4924|ubiquitin-conjugating enzyme e2 h-like isoform 1

ggggcagtgcagcaaagncnnngaatctgtcatactgtcaaagtgtgttggtaatggtggcgaagcacatctgacgttaaggagtatgcgacgtgagttc

catatacgtcaacgatcaggagcagaagtgtccaagaatctggtggttaaatcacgcagcacatcgcgccgaaaggaacatcgctaaataatgtcttcac

ccagtgcaggcaaaaagcgaatcgacacagacattattaaacttatagaaagtaaattggaggtaacgttcctgtcaggtctgaatgaattttgtgttaa

atttaatgggccgaaaggaacaccgtacgaaggtggaatatggaatgtacgtgttcacttacctgaacagtatccttttaagtcgccttcaatcggtttc

atgaataaaatttatcatccaaatgtagatgaatcgtccgggacagtctgtctggatgtaattaatcaggcatggacagctctttatgacttgtcaaata

tattcgagtcatttttgccccagttgctcgcgtatcccaacccaacagatcctttgaattctgatgcagctgccttgtatttacacaaaccagatgatta

taagaagaaagtgcaagnntatgttcagaaatatgctgcaaaacatgtatttaaagatcaggacagtaatgatgattcatctgattctgaattatccatg

actgacaacacagaggacgaactg

>Contig4015|ubiquitin-conjugating enzyme e2 d3 isoform x1

tcaagcggaaaggaagcactatcattgctgagttgttgaacatatgaaagaggagaaatgtcatatcgttttggagcagtggttatcaatatataaatca

tattaattgacgttgttggtgtgcgttgttttcagtggttaatcggtgatttttgtcttacttctgtgaaagtgcaggattcagcttgactagctgtgta

tcatcgcgttagaattagaaagcagccatcttacttacccaatcaaaggtctttggctagcggtttcattgtctggtttcaaaccagaaatatataatac

acagcccgtcaaatggcgttaaaacgaattaataaggaactgcaagacctgggtagagatcctccagcacaatgctcagcaggacctgtgggcgatgatt

tgttccactggcaggccacaataatgggaccaccagacagcccgtaccagggaggtgtattcttcctaacaattcattttcctacagattatccatttaa

acctccaaaggttgcatttacgacaagaatatatcaccctaatataaatagcaatggcagtatctgtttggatattttgagatcgcagtggtccccagcg

cttactatatcaaaagttttactgtccatatgctctcttttgtgtgatcctaatccagatgatccactagtgccagagatagctaggatatacaaaacag

atagggaaaaatataatgaactggcaagagagtggacgcggaaatatgctatgtgatgctctcaacaccagcagcggcccggctcaccaaaatcagctgt

cggttaatttccaaaaaacaatagaacatgaaacaacaaaagatttcaagcaacttaagggacttcctgacactggtgttttgattctgaccgttgctag

catttcagcagaaagctccacagcttttactttttgagaaataagaaagggaaatgcaattggtcctactgaataattttcacttaaacttctgtggttt

taatgaagaagaatgcctgatttattttgtggtgttagacttagctatgattttcttctttttaaaatgtgatatgttgttgaaatatttcgagagactt

gatcagtcaaggcaggaaaggcttgggaattgtatgttggaacttactttatgcccggtcacaggaagttcctctgtccttctgtcaccttttggtatgc

ccttattgtgtatgcaagtcagatatagttagtaaaaatattttgctgccattatcttgtatccagtttacttgatcattgctgtctcatggttccatct

taatttacttatcatagcattgccctcccattctaatcctaacttaaggtgggagctactagtgctgtgatcttcaaagaacaagaaaaataacaatgtg

tggtgcagaatgcgaagaaggcaccaggttttgattacttttaacatcaaatgtggagcccatattaatttttctcattgtacattttctccatactcca

tctaaagtggacatgtatgaaacttatgatcttgttaataaacattgaacattaattagttaaattatttatcagggatagattttgagaccagtactat

ttattacactttaaaaccttttttctgatatgttttgatgatgcatcctgcttagtattggtttttagtcacttatt

>Contig4026|ubiquitin-conjugating enzyme e2 c

atatcttattggctgtgagtagttttaaaaactgtggtgccacatttatattggttttgaaagcaacgtactttaagggatggcacaagagaccaatcgt

cttgtagatgacagtaggacacttgcgaaagcaaatgaagaatcgcaaacaatctcaagagataatcatgcagttagtaagaggctacagaaagaattaa

tgtctctaatgatgagtcctgataaaactgtgtcggcattccctgatggtgaaaacttgttcaagtggattgggactataacaggtcctgtgggaacggt

gtatgaaggtctggtgtacaaattaagcctggaatttcctcatagctacccatattcagccccactagtacgatttgctaccccatgttatcatccgaat

gtcgatcaggcaggaaatatctgtttggatattttgaaggaaaaatggtcagctctatatgatgtgcgcacaattctactctccattcagtctctgcttg

gtgaaccaaacaacgaatctccattgaatgtccaggcagcagccttgtggtcaaataaagttgaatacaaacgtcatctgcatagcgaataccaaaaaca

acgaaagggacattgagaatgataggggattaatatgtcataggaagaggatgttgctgatgggaactgaatgtgcaacgtgacacgtgtgaagcacaag

gacaatttcactgattgatattgtgatatttattttaaactgttcttgctttatcttttatattcatttatttatacatattttaataattttgtatgtt

gtggtttagagcatactagtacatccttcccattttactg

>Singlet625|ubiquitin-conjugating bir-domain enzyme

taaagttatacgacctcgtcttgtgggctccaccataaactctgttgcaacattggatctgctgaaattgtccaactggaatccagcccgggatatgaaa

tcagttttggaagaaatcaaggactatcttgataagaacagcagactcaatattaaaacaaaacgaaatgacagacttaggttcccccagggagcttatt

tagacattgaaaatcatttaatgcgcctaacatttgtaagtgaagttccattagctaatacaaaggctgctgatagttctggttggactgcaaaagttcc

atgtactagtgaaagtgaaattccagttgttgcaggaaagaagaaagattacaaaaagacgtggtttccacgtggtactggatacagttcatctacacag

tctggttgggatgtgaaagcataccttgcagctcaaaaggaaaaggatcatcagatagaactagttctcagaggaattctacgtgagattgaaaaagttt

gtgggatatcgaaaggtagatcttcattcctagtgtacaacagtgtttcatttccaacatctagctcatttgcaaagtttgagacttcaggcactgaagt

tggaggttctagtgcagctgttggaagtgcaattgcgactaatgaaacaccgtgcggagatattgaaatgcctttagagaatttgagtgagagtgaaaat

gttgaattatcagaagtctcgtcagatgacgaaaagtgggctattgatggtgt

>Singlet5601|ubiquitin-conjugating bir-domain enzyme

agcacannttcgctaatgaattttgtcgcatgagccaagctgttaaagaagcgcttcagaattgtgggctgctagacaaatcagcatcaaccagtgaaga

gggtgaaacttcagaaggaagacgcattggtataaacacccttgatgttgcacgggcatacaaggatgccttgaaagacctgcagtttcttagttgtgac

attgacgtggaaggcccaagagcacatttgttcagcagccaatttaaaaaggctctgcctccatcctcacagcaagtgatgagaattgctcaagaagtgg

gtgctttatcaacatctctaccccttgatatagggtcagctatttttgttcgcactgatgatgcaaggttcactatgctgaaagcactgataacaggtcc

ggagggtactccttacagtggtggctgctttatatttgacatattcctgcctatgcagtatcctcatgttccaccaatggtgaagttatgtaccacaggg

ggtggcactgtacgcttcaatccaaatctttatgcttgtggtcgtgtgtgtttatcactgcttggcacttggcatggcttacaaggagaagcatggcttc

caacatcaacattgcttcaggtgttagtttctatccaaagtcttatccttgtcccagaaccatttttcaatgaacctggatttgaaagtatgataaacac

acctaaaggtaaaactgagagtgcaaattataattatggtttaatggtcaacacaatcaagtatgcaatgctggaacagataagacatcctgcacatggt

tttga

>Contig3631|ubiquitin-associated domain-containing protein 2

gtactgttgtgtattgtgttggagatcatgagacgtaattaccttgtgaaattgttgtgtgctgtctaacttatatgcgtggtgtaatgtttaggtcgat

cgtcagaaaatattcgcgaaagaaattgtctgaataatattacgaaatcaacaatggctgcaattctttcaccatacagcacaacaggattttacaaggc

gccagtatgcaaaggtttgatgggcaccatgtttctgacgtgcaccgcaatgaatgtgcccctgttggcacatatgcggaagtatctcgtatgcaagttg

cccgatatatttgtagagggtgaagtatggagattactcacatcccgtgttacatttctggagacaaaggatttggtctgtggtgcccttctgatttatt

acttcaggatatttgagaggcgatatggttcacataagtttgcttcatatcttttggcaacctgtacaatagcaacaattttagaaacttcaacaataat

gattctccaatatttaaatatggatgtccatggaggtggttaccttccaccaggaccgtatggcctgatatttccattgtttgtgagcttcttctttgat

gtccctcgtgttgcacagactcatattcttggaatacctattactggaaaaactctgacgtatctacttggattacaactttgttcctcatcatttgcaa

acggaatttctgcagtatgtggaatagctgcaggtttaatgtaccgttacaatgtggtatatttgagaaaatggttgaaaataccaaaatgtgtggcaca

aattagtaacaaatcttttggctggatacttcgttctggagcaccacaggagggagcaatcggagcaacactggaaatccaaagagctcagcagatggag

attttggagcagcagatgcttctcaacagagcaagggagttcaggagacagcagggtcagggctttgctgaaaccttggttggacctgacaactattggc

cacaggaaaatgggacattatttggaggttttctcagaaataggcgaccaaatgctgtacctgaagagaacaatgttcagccgtcagaagaacaagtcca

aacactagttgagatgggatttgaacgaacaagagttctgagtgctttgcgaagcgcgaacaatgacatcaacagcgcaacaaatattttactgcatgaa

tcg

>Contig1138|ubiquitin-associated domain-containing protein 1

ctgtcggcgagcttcatgtgaaaacaagaaattaatatgtataaattgctttcatgctgccttgggtgcaagagaaaatttcaggaggtcgtcaaaggct

gatgagcatcttcagcaggcgtctcgatagtgacatacggctttcttcacaaaataaagaaactatgtttgtctcagagggtaatgctttgtgcggtgaa

atgattgaggttactgtcattagccctgaaggcgataaactaacagtagatgcaacagctgattgcacagtagataaattgaagacaatggtagtcagtc

gcttttacaattcaacagatttatcaaaggtagcccaaaattataaactggtgtcggtgtcaggaagaaaaccattaaacgataacaatgctattagtca

agaggatattaagaccaatgatcaggtactattaatgaagcggcggcgaccaccggtaaaggagcagatttctgtggacaacttaaaagctcctactgtt

gaggagattcaagaagcaacaagtaaactggatacaaagaacacaacaaaggagcagcctcagcttggctgttcactagactttgaaagtgatatccgca

aagtattaatatcactggtggaggcatctgcaagaattttgacaccaagcccagagtcagaagaggtatttcagataatcagagagagacttgaaaataa

aggcgttgaccatattgttaagcaactaacagacatgggatttccacaggttcttgtggttgaagctcttcacctgaacagcatgaatccgagactggca

ctagattggctactgaaacattccactgtgaggcctgggggattatgtgatgatacgt

>Singlet7121|ubiquitin-activating enzyme e1

aaannnnntgagacagacatggatctcattgagaagtcaaatctcactcgccagttcttgtttcgaccacatgatgtccagcgcccgaagtctataactg

ctgctagagttgttaagcagatgaatccatatttaaatattgaggctcatgaaaatcgtgttggtccagagtctgagaaagtgtacgatgatagtttctt

tgaaggccttgatggtgttgcaaatgctttgataatgttgatgcccgcatatatatggatcgtcgatgtgtatattatcgcaaaccacttctggaatcgg

ggacgttaggaacaaagggcaatactcaagtagttgttccatttttgacagaatcgtacagttcgtcacaagatccaccggagaaaagcattccaatatg

taccttaaagaatttccccaatgcaattgagcacacactacagtgggcaagagattcatttgaaggtctgtttcaccaagcagctgaaaatgctgctcag

tacctctctgacccttcattcatagaccgaacaatgaagcttccaggagtgcagccattggaaattctagagtcacttaagaaagctttagttgatcagc

gaccattaagttttgatgattgtgttgcatgggcccggtgctattggcaagagcaatataatgaccaaattcgccagcttttg

>Contig703|ubiquitin-60s ribosomal protein l40-like isoform 2

aaggggccgttcggatttgttctttcttttggatggtccaggaattataattcaagatgcaaattttcgtgaagacccttactgggaagaccattacttt

ggaggtagaagcttcagacacaattgaaaatgtgaaggcgaagattcaagataaggaaggtattcctccagatcagcagagattgatatttgctggcaaa

cagttggaagatgggaggacactctcagattacaacattcaaaaggaatccactttacacttggtgcttcgtctccgtggaggtgtaattgagcctacac

tccgtattttggctcagaagtacaattgcgacaaaatgatttgccgaaagtgctatgcccggctacatccaagagctactaactgccgcaagaagaaatg

tggacataccaacaacattcgccccaagaagaagatcaggaactaatctcaacatgctacatgtaattgctatgtcttgaacagaccacttgcaaaattt

gcagctcatgtataagaaatcaatgaataaaactttcttccacttttaaaaaaaaaaaaaaaaaaaaaaaaaaaaaaaaacaaaaaaaaaaaaaaaaaaa

aaaaaa

>Contig3542|ubiquitin-60s ribosomal protein l40-like isoform 1

ctttcttttgtgaagaccctgaccggtaaaacaattacgttggaagtggaggcatcagacacaattgaaaatgtgaaggcaaagattcaagataaggaag

gcatccctccggaccagcagcgtctgatctttgctggtaaacagctggaagatggtcgcacactatctgattacaacattcagaaagaatccacactgca

tcttgtgctgaggctacgaggtggtatgcaaatttttgtcaaaactctgactggtaaaactattacattggaggtagaggcatcagatacaattgaaaat

gtgaaggcaaagattcaagacaaagaaggtattccaccagatcagcaacgtctgatttttgcaggcaaacagctggaagatggaagaacattgtcagact

acaatatccaaaaggaatctacactacatctagtgctgaggcttcgaggaggtatgcaaatttttgtgaagaccctgactggtaaaacaattactttgga

agtggaggcatcagacacaattgaaaatgtgaaggcaaagattcaagataaagaaggcatccctccagaccagcagcgtctgatcttcgctggtaagcaa

ctggaagatgggcgaaccctgtctgactataacatccagaaggaatctactctgcatcttgtgttgcggctgcgtggtggtatgcagatttttgtgaaga

ctcttactggcaagaccattacgttggaggtggaagcttctgacaccatagagaatgtgaaagctaagatccaagataaggaaggcatccccccagatca

gcagcgacttatcttcgcaggcaaacaattggaagatggacgcacactttcagactacaatattcaaaaagaatccactcttcacttggtacttcgtttg

agaggtggatattgataaatggaaagtgttctcaaatttaaagttgactgcaagaaatgttttatgc

>Singlet7951|ubiquitin-40s ribosomal protein s27a-like isoform 2

ccattgcanngcagtatggtagagatttcgtgttgaacgtagttaacgtgtggatataagaaatcaaaatgcagatatttgtgaagactttgacggggaa

gactatcaccttagaagtagaagcttcggatacaatcgaaaatgttaaagccaaaatccaggataaagaaggaattcccccagatcagcaaagactaata

tttgctggcaagcagttggaagatggccgaacattgtctgattataacatccagaaagaatctactttacaccttgtcttaaggctccgtggtggtgcta

agaagcgaaagaagaagaattactcaacacccaaaaaaattaagcacaagaagaagaaggttaagctttcagtcttgaagttctataaggttgatgaaaa

tgggaagattcatcgcctccgccgtgaatgcccatcagaacagtgtggtgctggagtattcatggctgccatggaagataggcattactgtggaaagtgt

gggcacacactagtgttcagcaaacctgatgaagcttgaacatgttgctgtatggtttcttggcattgtaatattactgacaaataaaaaataaacaaaa

aaaaaaaaaaaaaatgcgt

>Singlet6332|ubiquitin, partial

ctgtcggctttnnncacaccatttaagcgaagttgggactgtcattgtcatacgactttgaggtgtggcgtagctggttgaaattttgactgtagttcgc

ttttgacagtcgttctgtgtttgtgatcccacaaagcagatatgcagatctttgtaaaaacattaacagggaagacaataacacttgaagttgaaccttc

agatacaatagaaaatgttaaagcaaagattcaagacaaagaaggcattccacctgatcagcaacgtttgatatttgctggtaaacagctggaagatggc

cgcactttgtcagattacaacatacaaaaagaatcaacacttcacttggttctccgtctcaggggtgggatgcaaatatttgtcaaaactttgactggca

aaacaataacactggaagttgagccttcggacacaatagaaaatgtaaaggcaaaaattcaggataaagaaggcattccaccagaccagcagaggctaat

ttttgctggaaaacagttggaagatggacgtacactgtcagattacaatattcaaaaagaatctacattgcatctagtgttacgtcttagaggaggcatg

cagatttttgtaaaaactctgactgggaaaacaataacgttggaagttgaaccatcagacacaattgaaaacgtaaaagcaaaaatccaagacaaggaag

ggattccaccagaccaacagagattaatttttgctgggaaacagcttgaagatggaagaactctctcagattacaacatacaaaaagaatccacattgca

tttggtgttgcgtcttagaggtggcatgcagatatttgtaaaaactctgactgggaaacaataacattggaagtggagccatcagacacaattgaaa

>Contig1358|ubiquitin thioesterase trabid-like

ctgtcggcttccgcggagaaacaattggatttgcaaggtttgaaggtgtttacttgccacttctttgggatcccagtttctgtatacgttcgccaatagc

ccttggatacaccaggggccacttctcagcacttgtacctgtggaacagtacagtccgctgggagctggtgccggagccaataacctcagggcagatgaa

gttaccttcttgcctctcacagatcgtgaccggaaactccttcctgttcattttctcacacaaacagaggtgggtcatgaagatgcaatcttgcgacagt

ggatggatgtctgccatacagaaggaggaattatggtggcacagcagcatctccataaacggccacttttagtagctcaaatgttagaagaatggctgaa

ccattatcgaagattagtacaggtagcaaatgcaccattcgttcgtccaactccaatacaggattattcaagtgacggtggtgaaactgatgatgagtag

gtagatgtgaagatctgctttgtaaaagagtatgtaatggatccaagtaagtgcacagaagatagaactagcggaaagtgctagtgaatgaaaatgggac

actgcgttagccatttgaagattttgttaacagtgtgcgattaaatttttaaaatatttattagaggacttttagtgactgtatttgcagctgagtttga

agatgaaacgttttctctctatctgttcaaaacttaaataaaagatacagtgttgatccaattccagtaataatacttataaactgtgacttactatgga

acacttgctaatatatttttcttccaaagaattattgtgctgtagaattatacttgaaagtctattatttgttacttcacaaacagtaatttttgcctcc

aaaaatgttgtgatgttgacacaagagtcaatcttctgtaataattgtacactagaaacactagattgtcattcattttcacaagataacctaatttttt

tatctccccttttttatattctctgcaggtcatcaagcacagttacttataaatgcgtagatttattaactttgcatgtacaaagctgtgagatatttgt

tttgtcagagctggcaatagtcagacttaagatatttttcttcgttttgttgtaattaaactgttgattgttttaaaacaaaaaaaaaaaaaaaaaatcg

>Singlet7615|ubiquitin thioesterase otubain-like

tctatgggggggtgatgcgaattattgaannntgatgatttattgaagcgtactatgcctgagtaattgtttcactattttaattagtgtacgatggagg

acggtaatgatttgcataataataaattcgatccaagtgccaaccaagatgagctgattttgcagcaacaaagaagaatagaaaaagaaatttcagagac

aacgcctcttgttggtgatatccaaagtatttcttcgttggaaagcgaatattcggccgacgacatatataggcaaaaagtactggatttagctaaaaaa

tataagagtattcggcgaacgcgacctgacggaaattgcttttttcgtgcctttagttacgcctatcttgaacgtctgctcgggaaccgcgaggaatatg

aaagatttcgtgattgggcactcaaaagcaaggataatttagtggctctgggttttccagaattcacagttgaagattttcacgatacttttatggaagt

attggatcgtgttggagaaggcagtgatgtattttcacagaaagatttacataaactatttaatgaacaaggctattcagattatattgtagtgtatctc

cgcctaataacttctggacagcttcagaaggattcagaattttaccaacattttatagagggagatcgttcaattgctgatttttgtcatcaggaagtgg

aacctatgtataaggagagtgaccacatccatatcatagccc

>Singlet5232|ubiquitin thioesterase otu1-like

tannnnnnnagttactgtcagcaatttgttacaacgaataattgaaaaagaagaagtttatgatacatcgttttcttcatattgtatttctaggcaggca

gacaaacactcgtatcaaaatattctgtttgtttcagatctgttattggcactcttgaaagtctttcatggcgcagtataactagcagatgatacggctg

tgattgtttacaaatgaatatatatgtcctggctgacgtacttttgttgattatgtgttaaatttttaaagtgcaagtcttctcattgcagaacttttaa

tgtggtgcactataatcacatacgtatagataccaggcctatttttaagttgtagtccagaactgccttttaatatgtcttcacttgtcttgaaattaaa

aacgaagtcgggtcagaatgtagttgaaggattaacagcagcaagcactgtacgtgatctaaagacaaagctctgttcgttgacgaatataggagaaatg

aaccttcatgtgctgagtggctatcctccgaaagcactagacttatcagacaacgaagcgaacttaagaaacgtcggaatatcgtctggtgatgttttaa

tagttgaaaataaacctggtgcaaaaccgaaagtgatttcaagaaacgatgtaattcctgcaactagactggataacgacagagtgagacctcacgtaac

ggatgcaattagtgactttccagggattcttatgcggaaaatagttcctgccgacaactcttgtttgtttacgagcattggctttgtccttgga

>Singlet5482|ubiquitin conjugating-like enzyme family protein

aagnccnnnaccttccttccttagatccgtacgatctgcgaggtccatccatggcttccaaacggatcttgaaggagctcaaagatctccagaaggaccc

cccgacctcttgcagcgcagggcctgttgctgaggacatgttccattggcaagcaacgataatgggtcccccagatagtccttatgcagggggtgtcttt

ctagttaccatccatttccctcctgattatccattcaaaccacctaaggttgcctttaggactaaggtatttcatcccaatatcaacagcaatggaagta

tctgccttgacattttgaaagagcagtggagccctgctctcaccatatctaaggtattgctatccatctgttccctgttgacggacccaaacccagatga

tcccttggtgccggaaattgctcatatgtacaagacagaccggtcaaagtacgagacaaccgcaaggagctggacccagaagtatgctatgggctagcat

gctttgtcgcgagggcgtgtaggaacatcggtttgtattccttgttatgtataaaagaagcgtggtttcttctacttttgcaatagacattgaagaatag

ttctacatgttccctcgcaagtcatgacgatgtttcaggtggtttaatggatatttttcgtaacaactatgaaatatcagcagttaaatgttcaaaaaaa

aaaaaaaaaaatcgtg

>Singlet7288|ubiquitin conjugating enzyme-like protein

aattcaaagaagtcattaaaagcgaagaggtnnnaaaatgtgcgataaaggtggaactagtgaatgacaattacactgaactgaaaggagaaattgcagg

tcctccagaaacaccatatgaaggtggaacctttgttttggaaataaaagtaccagaaacttatccatttaatcccccaaaggtacggtttataacaaaa

atctggcacccaaatatatcgtctgtaacaggtgcaatatgtttggacatattaaaagatcagtggctgctgcaatgacactgagaacagttctgctgtc

ccttcaggcacttctggctgcagctgagccagatgatcctcaagatgctgttgtagcaaagcagttcaaagaaaatcaggacatgtttagacaaacagct

cgacattggacattcatttatgctggaggtcctcacaggaattcagattttgacttgaaaatacgacggcttacggatatgggaatagaagatcatcaag

cacgagtagccttatcgtcatacaactgggatctggagagagccacagaacagctcttcagttagttaacggtgtcatctgtgaaatgacgagaattaac

tgatgtgaaatagttatattcatattgtcacctctttggaatccatccttaagtgcagttatgcaagtcatgttatgaaacggaacattgtcacatggca

gtcaagtagtttactaatgtatgtatactgtgaaaatggatggagggtatgtgaaatgtaaatacgaatataataccttacatgtatgcaagttgtcatt

tttaagttttctttttg

>Contig1337|ubiquitin conjugating enzyme j2

ctgtcgggaaccctgcatggagtgtgtccacaattctcaccggcctccttagttttatgattgagaagagtcctaccctggggagtatagagtcgtctga

ccatgagaagcgggtcctggctgcacagagcctagagttcaatctgaaggacaaaatttttcgtgagctgttcccagaccttgttgaaaagataaagtcc

gagctggagcagaggcggaaagtggaggaggagagccgaagaccggcagcggcggacgcggccgtggcgggggcgggaggcggcgcatcgtcggggctgc

ccggggcgggggtggctgcggggcgcgggggcgggctgggccaacagcagccgcagggcgcgctgcatcacgccattgccaatatctgtgtcatcattgg

tttcgcggccttcgcctacaccgtcaagtacgtgctgcggactattgcggcggaataactgaggtatcagtgggacgagcatccgcagcctgtatttgga

gtttcttctcggacaatctacagttgctgtcattatttaagatgcgtgtgcagttatgtgtaacagataaatacttaaatccgttggccacaggtgacta

aaacagtttcagtgatcgctacaaacaagacggcaagcctcctcgagtgggagagtaaataagaagtatgtacatggtgtcaatcagcagcagttaattg

agggagatatcttctattgtattcaccgtgacaagcattaatttgttgttctacattcttagatgtagtaattgttcacagaggagattctaatatacta

aaatcaactaaattacagaaaaaattactaagaaagttttaattttccagtaactgatgccttttttgccccattcatcattctggaattgaattccatc

gtatatttgtggttaaaaggctttgtgtcgcagtaaaatatttttaaaaaagctttgctgataaagttaactgtgtaatccattacttctacagctgtgt

gtggaatttgcatgaagattgaagcgatgtgtatattttccattttgtgattgatgtcattgtaaatgctattgatactatttgcaacattgcaatgaca

cattggtgtctacaatagaaagcattccannnnacacaacaaagttgataaaattaagagatgacatgctgctgatcattt

>Contig3963|ubiquitin conjugating enzyme e2

ggaagaattcggaattttattcctacggttgtaagaagctgtggcaagttgtgattaggtagtattttgtcttgtacgcagtacaaggcgtaactcttgt

atttttaagaagtttacgtcaacatgacagcgttacctaggagaattatcaaggaaactcaacgcttgatgcaagagcctgttcctggaataagtgctgt

gccggacgacagtaacgcacgatactttcatgtaattgtcaccggtcctgaagattcaccgttcgaaggaggattatttaagttggagctttttctaccg

gaggattatcctatgtctgcgccaaaggttcgatttattacgaaaatataccaccctaacattgacagacttggccgcatttgcttggacattctaaaag

ataagtggagcccggcccttcaaattagaacggtgcttttgtctattcaagcccttcttagtgctccaaatccagatgacccgttggctaacgatgttgc

tgaactgtggaaagtaaatgaagccgaagccatccgtaacgcgaaagagtggacaagaagatatgcaatggacaattgatatgcatcgattccaaggaaa

ggttaagagcgttgtatgggtttattttttttatgaaaactatttcactgaatctgtatcaccatgttatattttaagtaaggttttttgccaagctagg

ttgtcgctggtaggaaaaatacactaaacttgtcataccttttgtaaatgtgaaatttcctatgaaacccatacgttgctaattataatcatgatttacg

cgtgagagtatagtataaagtaaaacagaaagagcggtgatttcagatgttattccatgaatctgtccatttatattcattatgtactctcatttttgtg

tttattatctttcgaaatacttcacacttacagatcatgtgcatttagctactgttgctgtaaatattggagagtaccttcagcttaagtcctctgacat

ttcgcccttgcttcagtttgtcaccagataccaatttagtgaatgcggcttgatgtactaccagtgtaataatgtccatattaaatgtaaagtctacctt

gtttatggcaaattttatcagagcagacattagtatgatattgataattttgctgcataaagtatgtattatttgtgatttctgtttttgtggtattgat

gcagccttttctcatggacagctttaaattatgtctgacttggtgtaacaccaagagtagtggtaagtaagacacctttatataaaatttttgtcaggcg

ttgtgaattaaaacattatttccttcgtaacaagtgccggtcaaactacgaatgaatgaatgttccagtgtgaatacttacctacttgatctctcttatc

tggcttctaggcaagcaaggtgcatacacacattaacagaaacaacttacaggagatctttcatttccagtttgtttttaagaaactgtactgatgtatt

ttattcgttgaaagtatctgtgtaattgtattgtatgtgactagagatttatgtggattaatctgttttaccagcaatatgtgcctgttggccactgagc

agtttcagtggttgaaaaattagcaatcttttggttttaaagactttgtagaatgtcttgaccttgtgttgatataacttatttctggagctg

>Singlet1796|ubiquitin conjugating enzyme 7 interacting protein

gacccaggtgattggaatatttgctggctttggaatcctgttgcttgtggcttcaccattgctgctgctggctgcaccgtgtattgtgtgctgcaaatgt

cgcatctgcagtggtggcagtaagcttgacagtgaagatgagttgcctgatgaatctgcatcttgagcaactagcatattgactcttttaaagaagtgct

gattctttagtgttgtgacaggataaaatgccagtgagtgtatccttaagcacttagtgaaggactgactgtttttcaactgaaaatgtgcattagggat

aattccctgctgtcgtgaggactgaattagtgccagcttttgtgggtgactgaactaatgcatactattttgtaaatggaaaaaatcactataatgttca

tctttttcttggagttatttggtatttgtaaggatatccatctgtgttgatggtcactgaagttaataaagagttactatcacttttttgcttcattaat

gaagtatgggtgattgttacattcaattatatttcactgtttattttcaggatttttatagcatatcatcaagagggttatagaagctgtcactttgaga

tgtgcctatgaaaataaataaagacagaacagactgatacccatttttcagttctcatttatgattatcatccttgaggaagatattgcaagagacaagt

tgtatggtttaggtgatatgttcctcttaactgtttgtttcctccaaaatcactttatacacatctactcactacaaccattaacacacaattacgtga

>Contig3069|ubiquitin carboxyl-terminal hydrolase isozyme l5

gccgaacaggcgaacagtgagtgttccgatgtctgtgtcatggtgaggtgaattaaattcacttaaaaatggctgacggtgcgggaaattggtgtcttat

tgaaagtgatccgggagtgtttacggagctaataaaggaatttggtgttaaaggggtccaagtagaagaattgtggagtctagatgcagaacagtttgag

aatttgaagcccattcatggtttaatcttcttgtttaaatgggtacaagatgatgagccttcaggctctgttgtgcaggacagtaggctggataaaatat

tttttgcccgtcaggttatcaacaatgcctgtgccacacaggcaatcctgagtattcttttcaactgcatacatgctgatattagtttaggcagtacttt

aacagaattcagagatttctgtcagtcgtttgatgcaaatatgaaaggtttagctttaacgaattcacagacaatccgaacagtacacaactcattcgca

cggcaaactctatttgaatatgaatcaaaacttgcgtccaagaatgatgaggtctatcattttgtgggatatgtgccaatagaaggccgtctgtatgaac

tagatggcttgaaggaaggtcccatagatctgggacctgtagcatctgatgctgactggctagacgtagtgcgacctataattgaaaaaagaattaaaaa

gtacagtgaaggagagattcattttaatcttatggcaattgtatcagacagaaggatgatatatgagaggaaaattcaagatatacagaaacaggt

>Contig565|ubiquitin carboxyl-terminal hydrolase isozyme l3

cttnaagtacggccgagggggcagtgagaggacgctgttcttaagttgtttgtcaccggctcggagtcagtattgaacaggttctcctagaagttgcagc

gtttgttctgtgtcctggtgtcgtttgttgtgagctgtcattaataatggcgtgggttcccttagaatcaaatcctgatgtgatgaataagtatttgcag

aaactgggcatgccttcaaaatggcagatggttgatgtacttgggttggatccagagttgctaccctctgtgccacgtcctgctcttgccattattctac

tgtttcctacatcaccaaagtatgaagaacataagactcagcaggaggaagaagttaaggagaagggacaaactgtttctgaaaatgtattttatttgaa

acaagttgttgaaaatgcatgtggtacaattgctctcatccacagtgttgcaaacaacactgataagatccagttgggtgatggccatctgaaacaattc

ttggaagatgctcaagcccttaatcctgatgaacgtggtgaactcttgcagaaggcagatggaataattaacacacacaagcagctagctttagaaggcc

agactgaggcaccagactccaacacacctgtgaattaccacttcgtggcatttgtccacaaggatgggagtctctatgaattagatggaaggaagtcatt

tccaataaatcatggaccaacaactgaggactcattcttggaggatgctgccaaagtgtgccagcaatacatggcaagagatcctgatgagctccatttc

acagtagtggcacttactgctgcagaataagtgttttgcaggcttctctaacacttgcactaatagtgcagtgatgtgatcatttttgtcttaagtgttc

tatttttatttttgtgcaagtatctgtctcaatatttgtctttcgaagttaattttatttgctgctgtgtttaaggcagttccaaataaaagtgtatagg

ttttcaactttgttgactagcttaaaaatgcttcatgctagttgtgctcagattattataaaatgttacaccaaagattttgaaacaataaatgtgatgt

gaaacataatgatctacagccaacaataactacttaattgctactttattaaaattatagtatttcataacatatcatatatatatattctctggaactt

gttggcttgactgatttttcgtagttaactagtttagctttgtttggacatgaaaaatattttgattacaataagtattttaatgatactcttatactat

at

>Singlet5501|ubiquitin carboxyl-terminal hydrolase isozyme l3

ggtncccttaaaagttgcagcgtttgttctgtgtcctggtgtnnnntgttgtgagctgtcattaataatggcgtgggttcccttagaatcaaatcctgat

gtgatgaataagtatttgcagaaactgggcatgccttcaaaatggcagatggttgatgtacttgggttggatccagagttgctaccctctgtgccacgtc

ctgctcttgccattattctactgtttcctacatcaccaaagtatgaagaacataagactcagcaggaggaagaagttaaggagaagggacaaactgtttc

tgaaaatgtattttatttgaaacaagttgttgaaaatgcatgtggtacaattgctctcatccacagtgttgcaaacaacactgataagatccatttgggt

gatggccatctgaaacaattcttggaagatgctcaagcccttaatcctgatgaacgtggtgaactcttgcagnnnncagatggaataattaacacacaca

nnncagcttgctttaaannnccagactgaggcaccagactccaacacacctgtgaattaccacttcgtggcatttgtcca

>Singlet7251|ubiquitin carboxyl-terminal hydrolase calypso-like

ctgtcgagagagagtgaggtgtcacagtgtttacggccttcttcttaacccccccccccaggtgggaggatgccatatgctgggtttcatacggatacgg

ttccctataatcactcgagacagatgtctggccaatcctcgccccccccccccccccccaccatcttctatctgaatgtgggttccgcgtctactgacct

cactttgaggaggaggacgacgctaaattctcactgtcccacatgtgcccttaactcatggttgtacctggcagatgctgccagcgtaaaaagttctcac

atttttcaatggtagctgtgtagcaacttgaaacataaagatgtatacgaacatgaattggtatgactgtacagagacttaccctgtgcccaacccaggc

agtactcacatagtaactacaagtttctggtaagtagccaactgagtttctctgttagtaaattaagctctatagccattacctccttagcgtacaccac

cattttaaatgttgtattatcggcagtttcattactttgcatcctgtaataaatgctgtagttgtgcgcactcagacaatagcaaaattacatgtacgtt

tataaactgactttagtctaattacagcaggaaataaggcatttgtgaatagagagagatctgtcaaatcctagaacaatattagatgtgctaccttcgt

gtgtaaatgcagttttttagttacagatcacagaagccaagaatttaggaaatctcacagacacacagtactgaaatgtttgatacagtttgaagctctg

ataatgttagttttgagcaactcattc

>Singlet141|ubiquitin carboxyl-terminal hydrolase 8-like

ctgtcgaaagaannngagaagttgaggaattaaatctggcaaaagacaagaagctaacagatcttgaaaaggaagtaaatgaaagaattaatcttacaga

aacagaaatgaaaaagatcacccaacaaagggaacaaaaagaacaggaaagaaaagtcgctgaaaagaaacaaagagaactcaaagaacaagagaagttg

aggatagaacaggtagcgaaaagaaaagaaaaggaacatgaaacacaagagaaggaaaaaccagttcctggaaaaggtgtttattacacccacctcagac

atgatggtccttcaacttctagcccaatgaaacgatcccattcatctccagacttagctaagctggatgaacctgatcggagcaccaaaacaccccaagt

tgacaggacaagcaaacctggaattgtttctcgaccaaatgagatgttctacacccctcaccagaagaaattgtactctgtttatggtgttgtggagcga

ggattaaccggattgaaaaatcttggcaatacgtgctacatgaactcaattttacagtgcctcagtaatacttctcaactcaaagaattttttgtgcagg

ggacgtatcgtaactatgtaaacaaacagtctgatacaagaggctgcattgtggaggcagtagctgttgttataatgagtttgtggagtggtcaatacag

atactttgtgtgtgatgacctgaaggcagtggttggacgtttccgagagcagtttcgaacatctcaacaacaagattcaaatgaattcctcactgtactt

atggattggcttcatga

>Contig1926|ubiquitin carboxyl-terminal hydrolase 7

tcctggaaaccctctccggtgcacctatnncggaacattaaaagaattgctagtttattgcaagcctaagacgcctaagaaaatattttatcagcagctt

agtatacgaattaatgaacttgaaaacaagaaacaatttaagtgtatatgggtaggcccaaaattgaaagaagaaaaggagctaatactgtacccaaata

aaaatggtacagtatctgacctcttggaagaagcaagaaagcaggttgaactgtccgaaaatgggtctggaaaacttaggatatttgaagtaaactgcaa

caaaattatttctggcccaaaggaagatggtccactagaaacactaaactcttctgctagtaaaacattcaggatagaggagataccacgagatgaactg

cacctagcggatgatgaaatgctaatgccagttgcccatttttataaggacattttctctacatttggcattccattcttccttaaagtgaagcatgggg

agccattttcaaagattaaagatagattactgaagaaattacgaattcaagaaaaagaatttgagaagtttaaatttgcactagttgtgatggggaggca

aacatttattgaacagccagactactgcataaatttgcaggatttcaggtgtcatccaaaccaaggaaacactgcaccaatgccttggcttgggctagag

catgtaaacaaagc

>Singlet2986|ubiquitin carboxyl-terminal hydrolase 7

tggggaacaccattttgtttattttcgtgtgaaattggtgaattgtgatctgtgcaacataagtttttatatgtttaagatttagtgattataaaattac

gtgtattatatatggttcggtcgtttcccctggtaacagggggtgaagaaatccgcagacattgggcgcattgcaagtaaacacgttaaagtgacggtgt

ccggtaaaaactcagtcggttctgggaaagatcgtacagttagtcttaatttaaaaaaaccaaaaattcagatcacgacagttgaaaatatgaatcacgt

aaatgacaaggaaaatcaaaataatgacgtagaggagatggacacacaagaaggtggagtagacggtggcggagagacccaaatgaacggtgagacacaa

gatcaaaccgtgtcacaggacgcggaaatggaagaagatgaagcccgttcggaagccaccttcaggtatactgtaacaaacttcagcaaactgaaagaca

cacaattgtcccctccaacctacgtgagaaatctacagatgaggtccacgagatgctgacggagaaatctgcacgaaagttggatattgataaagtgaca

cttaatgaagcagccttccgttggatgttgaatgaagaccagatggccactgaaaaactgtcagaaggcatccgaaaatttgcagaagattccaggaaac

ttgagaagatgcttactgaatacttggctgctaaaggaaaatgatgtgtttaatg

>Singlet6855|ubiquitin carboxyl-terminal hydrolase 5

cttacgacatcaacacgaagaggtctatcagaaattgtgcgatggctgctgtggatttaactcgcttcttgcctgatataaggattccatctaacggaga

taagatatacaaagacgagtgcgtgttctcctttgatactccagagactgaaaccgggttatatgtctgcttgaaaacatttttgggcctaggggcagat

tttgtcgagcagcattatcgaaaaacagggaatgctgttttcctgcaccttacaagactgaagaaagagattgctactgaacagcagggagatggaccag

agaaaaaaattacaaggcttgcaattggtgttgaagggggatttgatcctgatgccggaaagaagaaatttgaatatgaagattcatattccattgtggt

gtttccatcacgcgataccataaagtggcctaattctgatctaccggaaaaggtgcaggaatgtgtcaaagctattctagctgcagagtcagccacaaaa

ctagctgaactggaggcattaacaggcacatgggatggtgaagcaagaataatatcaaagcatgcaaataatcttcttcaattaaataatggcaaaaaga

ttcctccaagtggatggaagtgtgagaaatgtgatctcactcagaatctgtggctnnntctgacagatggagctgttctttgtgggagannnttttttga

tggctctggaggaaatgaccatgctgttgaacattatcgtgaaacaaactatcccctggcagtgaagc

>Contig2069|ubiquitin carboxyl-terminal hydrolase 46-like

accnnctnaantgttaccatattttttattgggaacagtgatttcctcaagtaatgcagcacccaaactgatttacagttgagtttgttacacgactcac

actcccttctcgtcagcgcctgtgtacagacgcttgtgtggacaaaattcaggatgtcgtagatggcgtttcagaagttacgataaaacatggcagaatt

gggcgaaagtccaacatagtacgctacaatgtgattactgtagtgcccataatgggtgcaaacatttcgcagctagaacgagatattggatcagatcagt

tcccaccgaatgaacactacttcggattggttaattttggcaatacttgctacagcaattctgttttacaagcattgtatttctgcaagccatttaggga

caaagttttagaatacaaagcaaagaacaaaaggaccaaggaaacccttctgacatgtctagctgatttattctacagtatcgccactcagaagaaaaaa

gtgggttctattgcaccaaagaaatttattgctaggttgagaaaagaaaaagaagagtttgacaactacatgcaacaagatgctcatgaatttctcaatt

tcctcataaatcacataaatgaaattattttagagcgtcaccagagtagtaacaagccaaagacaggaggaaatggagagacaccttcatctcagcagga

accaacttgggttcatgaaattttccagggtattcttacaagtgaaacaagatgtttaaactgtgaaacagtcagcagtaaagatgaagacttttttgac

cttcaggttgatgtggatcagaacaccagtataactcattgtcttaagtgttttagtaacactgaaacgttatgtagtgataacaaattcaaatgtgata

attgcagtagctatcaagaagctcagaaacgaatgcgtgttaagaagttaccaatgatattggctcttcatctcaagagattcaaatatatggagcaata

taacaggcacataaaggtttctcacagagttgtgttccctcttgaactacggttatttaatacgtcagatgatgctgtgaatcctgatcgtctgtatgat

ctggttgctgttgttattcactgtggaagtggtccaaacagaggccattacattagcattgttaaaagccatggattctggttactgtttgatgatgaca

tggttgataaaattgatgcatccacaattgaagacttttatggtctaacatcagacattcagaagtcttccgaaactggttacattttgttttacc

>Singlet1283|ubiquitin carboxyl-terminal hydrolase 38-like

ctgtcggtncnnntnnnagatacattcaccgatctacagttgtcttttcccccnnnnggaagaggacaacaaatatgatgtgttagatgtgcagaatttg

ctggattactttgtttctgcagagaaactgagtggagataattgctataggtgtgataagtgcagtgcattgactgagggacagcggtctgttaatattt

tgcaagcaccgtctcatcttattctgacaataaaacactttcattatgatagtaccacaatgcaacgaacaaagttacttcacaagatgcattataatga

aagattatttgtgcctgttgtagctgctggacagcaacagaaacactgtgagacatataatttgtatgcagcagttgtacattctggaaggagtgtggat

tcaggacactattacacgttctcacgagaccaaagtggttcctggtttgtatttaatgatgagtcaatatatcctagttctcttgatgagctaacaaacc

ttaaagcagcagatactccctacatattgttttatgcgttacctagtgaaaatctccaactggaagtaggctcacacccactagaaaatctaaaacccca

cctgttagaggtagttctcaaagacaatgctttattcatagaacaaaagaaagaagaaaggcgaagattgtctatgggaactat

>Singlet7461|ubiquitin carboxyl-terminal hydrolase 38-like

tggtggggtgcttgaaaggaaggcacagtcattagcagactgtagtcaagatggcactgactcatagaaaacagagattctaagcagcacattgtcagga

tcagcagacagtggaatccattctgtgtgtggtgatgaagcaggtgttgcaggaacattaacagaggttcctagcttacatctaagccttgttcatcaaa

catttggtggaaaaatagtcacttgtaatgagtgtttgacttgtcattcacagtccgtccaaacagatacattcaccgatctacagttgtcttttccccc

acaggaagaggacaacaaatatgatgtgttagatgtgcagaatttgctggattactttgtttctgcagagaaactgagtggagataattgctataggtgt

gataagtgcagtgcattgactgagggacagcggtctgttaatattttgcaagcaccgtc

>Contig4364|ubiquitin carboxyl-terminal hydrolase 36-like

aaaaggctcnncatgttgggtgctaaattgaataagcatgttgaatttcctcagcggttggatctcaccaggtacctctgtccacagtcacagtaccgtg

gaggacagcctttgacttacagattgacatcacttgttctccacatgggctcatctgtaaactgtggccactacacagctgttgcccagacatccactgg

tcactggtatcaatttgatgacagctcagtaagacctatatcactaaattcagttcttgatatgaatgcatacataataatctatgaaatggataaatct

gaagcaaatatacctcagacacagcctgcagtgtctacaaaacctgcaacagttacttctgtgacatcacaaaacactcttacaaatggaagtacaggct

acaagacaaacttacaacggctcgttagccctgcaatgattcctacaagtgtaaaggcagcatctcagactaatggcctgttaaataaccacaagccagc

agcccctgttacaagtaaactgtcccctgttaaaagccctgtgaaagtcacaaaaacggaagcccctattgccataaataatgtatctagtccagctcag

tcatccttgaaagaaagggtacactcgtcaccactgccacagagcttggttccatacgatcccggtgactccaccggtacagattccgagccggagggag

atggagcagccagagactccagctccggtggtcctggtgatgacagttcggtccccagccccccctccaacagcccgacaccacccccgccccctgtcac

aaccgcgacttccacccccacacccgctcccagccccagacgtacgttacaaatcccccgcgcaggttggcaagtcacggagcagggcatctgcacgaag

ccccctccaccccctgccacatctgctcccgtagcagctgcaaccacaacgtggggtgtgtcatctctttcctctgccnnngactctactccggggccac

cgcagcagccccagcggcctgtatctgtgccttctcagtcagatcggcactgtaatggcagtcggaacaacgatgacagcccgnnngtacatcgagacga

agccagatccatagttgg

>Singlet720|ubiquitin carboxyl-terminal hydrolase 36-like

caagcaacagtggaaaaggaccaagtcagatgtgggatggcgctgccagaacagaagtcactggcaaactgaagcacttgtcacaccaaggctatgcggg

agcacaagtgactagttggggtggtgaacggagctatgtggacattgaactggagaaagagcgtgataaggaacgcaagaggtctttagaaaatttgtat

gatgaagagctcgacagaggaagggcaaagaagatgaaattcaacaataacagcagctcgcagcacaattcccgacccaagtacaattcgttccagcagt

atcacaatgagaagattcgttggaataactctggtaacatgttcaggccacaggtctattaccgcccacaccattcgcatcctcatcccccaccatacca

tcacaagggattatattacaagaaccgtggtggccattcctttcacaaattcaatggtagtgggagattcggtggttcaaaacaagggtctcgttggcag

cgctgacatctctcctgtagaggagagatgtaccctcgagcagatatctcaatggatgtaaatatttttctttttcttcagtaccttttatttttactca

gttgtcaggcagtcacattgtaaaaataaaaggtactgaaaaaaaaaaaaatgcgt

>Singlet3270|ubiquitin carboxyl-terminal hydrolase 31-like isoform 1

aggggaaacagttaatgctattttttaatgtacactccttcactaagaaatattgtgggtatatcatccttcacaaacttctgttgacatgtttgtgttg

aagtgaaaaatttttgtgtaagcgttgtgaaatgggtttctgtacacgcaccataaagtagaattttgcctagtaaacaggaggaaacagctatgaccag

cgatttattagtactgcctttccatgaggataccttaaaaaaggaagtaaaaaaaaagataaaaaaatcaattcttggtgtaacgcattagatgtgaaat

ttactaataatggatgttaattaaacaaaataaaaatgttggtacacagaaatcaattcaacactgcaatgattagtccattggtggtattgtctttgag

atttgacaacaatttttatgttgttgtgtaacttgttgtcagaaaacatcatgtggatactgattctcatcatcttggtgtcatttgttaacactacatt

tgtgatatgatgtgtgttgttaatttactgtgttagtgtaatat

>Singlet5184|ubiquitin carboxyl-terminal hydrolase 31-like isoform 1

ctgtcgtgtataccaaaaggagttgaatacagataatccacttggaatgaggggagaaattgcagttgcatttggagatttgataaaaacaatctggagt

gggcataatgcttacacagtaccacataacttcaaacaccaggtaggaaaatttgctccacaattctcaggatatcaacagcatgactcccaagaattgt

taacctttttattggatggattgcatgaggatctaaatcgaattagacagaagccatacattgagctacgcgatgctgatgaacgtccagataaagaagt

tgcagaagagtcgtggaaaaattatcgtagacgaaatgattccattattgttgatgtgttccacgggctgttaaaatctactgttgtctgcccagaatgt

tcgaaggtttcaataacattcgatccattttgctatttgtctctgcctctacctatcagaagagaacgggtaattgaggttacatttgtgccacatgata

ctacaagcaagatatgtaaataccgattaaacatgatacggaagggtactatacaaacattgttaactgcattcagtagtatgactagaattaaaacaga

acacttaattgtggctgaaattcagagccaccatttccataagttttacactgtcaatgatcctttggatagcattgatgaaaaggatgaaatatttata

tatgaagtaccagataggaacaattccaattcgataatgattccagtctgtatgtgggaatataacccaaatcaaacatttaattttagtcagctatttg

gaattcctataattgtgacattaccaaaggatcagntttctcatgctgtgttatatgatg

>Contig1708|ubiquitin carboxyl-terminal hydrolase 3-like

ctgtcgggttctctgacttggttcatgaggaagtgtcatttgcgaaaggcaaggactgactgcagatgtaggctcttgttaaccgcaggatggagtgtcc

acatctgttatccagtgtgaaatttcatgtatctgcatatgaaagtaactcagatggtataagagattggcaatgtgcagtatgctcatcttcagaaagt

ccgtggctgtgcttatattgcggaacgataaattgcggtcgctatattaatgggcatgcgaagcatcattcagaacagcatagtgatcatacactcagta

tggactgtgagaccctagcagttttttgctacacatgtgatgaatttataacgaatgatacgcagtcagatgtcctacagggaattcgtcgattgtttca

caaattagatgcaaagactaatgaagaaaatgaatgtcgtacagcagaaaatgaacggaagccttggaagtctcggtccctcacgcccagagacagtcaa

gaaaacgtccggattttgcggcctaggtcgcgcaaaaggtattccacatcagatgttaatggtgaagtgaagtacaaaaaacagcatatagaaaaagttg

acaaaccaaaaaataataatgccaaattaaggagggagaagaaagttgttggtataagaaacttaggtaatacctgtttcatgaatgcagtactacagtc

tttaagcaacattcaagaattttgttgctacttcaagcagttgccatcactagaaaaaacaaagcaaaatggcagacgcgtttatcagtctagaacactt

agagaaatgaatgatgcaataatggca

>Singlet5795|ubiquitin carboxyl-terminal hydrolase 3-like

gttaaccnnnggatggagtgtccacatctgttatccagtgtgaaatttcatgtatctgcatatgaaagtaactcagatggtataagagattggcaatgtg

caggtattccacatcagatgttaatggtgaagtgaagtacaaaaaacagcatatagaaaaagttgacaaaccaaaaaataataatgccaaattaaggagg

gagaagaaagttgttggtataagaaacttaggtaatacctgtttcatgaatgcagtactacagtctttaagcaacattcaagaattttgttgctacttca

agcagttgccatcactagaaaaaacaaagcaaaatggcagacgcgtttatcagtctagaacacttagagaaatgaatgatgcaataatggcagaggagtt

gaggaaagttcttgtaagtttaacacaaggtggcactaagggtgccatttcgcctgagtccctgtttcttgtaatatggaaggtggtacctcgtttcagg

ggttaccaacaacaagatgcacacgagtttctaagatacatgctagatcgcttgcacacggaactgctgcaccttttgcccgacttttctctgaaggaga

gcccttatatctccctacagaaagggcgtacatcaatcgtcaccagtgtgtttgggggaacactgcagagtgaagtacggtgtcttaactgcagtacaga

atcaaagaaacatgacccttttctggacctttctcttgacataccagacaaatttcagctcagcagaaagtataaagaccatgagggtggcattcctcca

tgtaacatatcag

>Contig3791|ubiquitin carboxyl-terminal hydrolase 22-like

gaaggtgtcatgtgattttgcattaaaaaatatacccagatatgagaaactttgaaacactaacgtacctggtgcagaactgccatttctctgtgtttgt

atttgtgtgtgtgtgtgtgtgtgtgtgtgtgtgagagagagagagagagagagagagagagagagagagagagagagaacatacaaaagcatttgtatgt

accattgtattaaaatgagaaataagtatctttaaaaaaatctgtaaatttgtggtttatgtacttccatatttgtgtaatttaacataaatcagaacat

ttatttattgtatgtaatggagtagtgaaggtataccctaccttgataaagtggatgtgtggtataattttatgcaaggggttttgggagcaggcagaga

ggagagtcaaggtgccaagtgtgtaccagtgcccatataacataaatggatgtcctcagtgttttaggtggaggaaaaaaaaaaacactttctggctcag

gtattacattaactctttcatgtactttgtttcgggggcagggagttttcttcctgggtttattataatatctttattgcactcatttgttaacatttga

tacagaaaattatcaggtacttcctttatttagcaataatagtccttaactgtgtccctttaatatgaacagaattttagtgtcat

>Contig2857|ubiquitin carboxyl-terminal hydrolase 14-like isoform 1

ccnnnnnngtcggaattttgtgttgtggtgtgtgtttagaaatgaaatactgaccccgtaagcattgtcacttcagtgtctcttgtttgtgcagcgaggg

tgtgaaaatattcgattatcgtaactgcttgctacactcatttttgtaatgccaacttacaaagtgaaagtgaagtggggtaaggagacatacactgatg

ttgaagtcaacacagatgaagatcctctggtgttcaaagcacagatatttgccttaacaggcgtccagcctgagcgacaaaaggtaatggtgaaaggcac

aacattaaaagatggtgattggggaaatacaaaattaagagatggtgcaactatattgttgatgggcagcaaagaagaggatgttccagtagaacctaca

gaaaagccattgtttgtagaggatatgaatgaaagtgaactagcatctgcactcgacttgcctgcaggtctgactaatcttggcaatacttgttacatga

atgcaacagtgcagtgcctcaagactgtccctgagctacgagaggcactgagggacttcaagggaggtgtgacattatctggggaaatgattcctgcaca

gtcaattactgcagctcttagagatttgtatgagtcaatggagaagggagccactattcctcctatactattgttggaagtgttacacatggcttttcct

cggtttgctgagaaaagtgaacatgggggctttactcaacaagatgctaatgagtgttggacggaaatggtgagaatgttgcagcagaagctaccaccaa

agacagctgaaggccagacttctactttcaagtcactggtagaccagtactttggtggcacctttgatgttgaattgaagtgtac

>Contig2626|ubiquitin carboxyl-terminal

gcnnttttaatatattttattatgcctgtagtattgtttttagtgccatttccatacacatttcatattgtttctttttttataggaaaatgtctggaag

aaatttttatatgatcattatttacctttccacataaaatttagtcctcaggttcttgctgtttcttctgtaagacaaacaactcattatgatctttaat

caatagcattattcccagcagaatgtagctgtattttccattaaccatgtagttaaagcaacactagtcagtaaatgttattatgaacatattaattcac

aaaaactacattatttctgtaccttgtgagccctttcagaagtctgaagaaataagttgtgtgttattcttttaattatgtatggaaaaatattaattta

acaattgacatgtgcaagtcatttagantgtctcatacaagtttataaatgtgtgttgtatataaaattgttacacatttcactgttatgtaaatgataa

tgataaaaaaaaataataataatagttatagtagtactgctaatgttctgccttgatattgcacacctgtgttagatattgatgagagattattaataca

gaagataaaatgaagataatatgtgcggataaaaataccacggagcaattcagcctaattgttgatttttgcgaaatatatatgaattccagagattaca

ctattggtgagaatttaaagaaaattttttgttaatgtaaagaatacgtttattgcatctttatacataatttgtaaatatacagagatattattgtttt

atcatatatcagtgtgtgagc

>Contig3318|ubiquitin activating enzyme 1 isoform 1

aatattttgtgatattgtcttcctagaataggcctatgtaactgcaatgctgaaatattaattttggtttaatgtttcgatttgttttactatcttcagt

cgaaataatgcttaggttatacattttggttgtaatttgttggaaggcttttgatctctacatgcttcacttcaaaattgtggtgctcatgtcttaagac

atcctattcatatttatttttctgttaagattatcacatatttttaaatacttcaaattctgaagtgtagtacaaatgtgaaagtctgaaacagttctga

aagatccattaccacttgtatacattgttgaccacccacaattgctgtattatgtggtgttgttgctggagcaattttatttatctaaaggtttaaatag

ctagtcaagtcttgcaatgtttctcattcctaggatactgtactcagtgctgtatttaaaatgctcatttttaacaaaacatttgatatcttagtacttg

tgaaactacaaagttgaatggcacaatttttcactaatgcatttttgaagcttgctaatgtgtgtacatcaacataatcttcagaagtgataaaaaatac

aaaagttctcatgtaatatagtttattacactccacactggtggtttgtatgtgtgcagttgtaaagtactgcctctattttgttaatgattgaaaatac

taagtgctttgcaaatgatgtgtgcagatattttttgtgtagagtccagctgtttcatggccttcagtttaatgcctacattgaaatgaaaattcttgaa

aatcttaagcactgtgtgtaatgagaaccttggtatataagcttagatactggataaatagcttcattggagcaagcaaatgatcagtacagttgaaatc

>Singlet7339|ubiquinone biosynthesis protein mitochondrial-like isoform 1

ctggcatttataaaatgacggagctctacatgatacaagataaatcatatggtttccaggaaacatggcaatttctgcgtagacgaattgatgatgctga

gaggttacatacgtatctgcaccagtcagaagaagcgtcaaccatagctcgagaaactgctactgctgcttttatcacagcacggaacatacttggatta

aataaagcttttcggtgatgtaaaaataagtagtgctaatttatctttgccatgcaaagttacattgttagcaagtgcatttcatcctctatgctgaaaa

caaagttgtgttaataaaatacttaggctatggaaaaaaaaaaaaaaaaaatgc

>Singlet2158|ubiquinone biosynthesis protein coq4 mitochondrial-like

gtctgcataattcgtataatgcatttcagcaaagtacaggaagatttgaggcagatgctctaataaacataagccatcaacagaactttgaagctgacta

tgcctcttcacacattcccacttcaacttttcaaaaaatagcactgttctttggttcatctgctgttgccctcatggatccttcaaggggtgacatgatt

gctactttgggtgaaacaggaggagagggaccaattaagtatatgcagcagaagatgctacagaatggtgaaggaaaacaaatcttaatggaaaagcctc

gaattaattctggaactgttgaccttcaaaagttgagggaactaccagaaggaagtctgggaagaatctacactgacttccttgataagaataaagttgg

tcctgactctcgtttaatggttcagttcattgatgatgtggaattggcatatgttattcagaggtaccgagaggtgcatgatctagttcatgcaatccta

ggaatgccaacaaacatgcttggtgaagtgacagtgaaatggatagaagcaatacaaaacaaattnnntatgtgtgtggggggagctgtttttggagcaa

>Singlet6186|ubiquinone biosynthesis protein

gaagatttaattcgtaaacaccgtgctaggccgactgtcatgctgccactgtggaatattgcaggatttgcacttggtgcaggtagtgctctcttaggtc

ctaaagctgcaatggcctgcacagtagctgtagaatctgtaattgttgaacattatgacagccaactgcgtgagcttgttggaaggccgnnnnnagctgt

ggatcaagaactgctggagactattcgacagtttcgnnntgacgagcaagaccatcacgatacaggccttg

>Contig376|ubiquinol-cytochrome c rieske iron-sulfur polypeptide 1

ccaagggggaggtgaaggtgggattggcgttgaaggagtgtaagacgtaatactaagttaacacaagcatgatgaatatcgtatctcgtgcgggaaattt

agcaccatacctaaaagcaacatcgcaggttgttgcacctcaattaaaggtaccagctacgccggttgcaatttcatctgaaaagattgtgaccccgagt

ctgccgaagcaacgcacaagttattctttaagccagactttacccaagggtaacttgagggtgtgttctggcccaacagttactactcaagtgagatgtg

cgcacacggatattcaggttccagatttcagtgcatacagaagacattcagtagcagatgcaacagtgaagaatacggacccggaagcccgaaaatcttt

tacgtacctagtagcgggaactggtgccattggaggagcatatgctgcgaaggcaatagtaacccagtttgtctcaagtatgagtgcatctgcagacgtg

ctagctttggcgaaaattgaagtgaaactgagtgacatcccagaaggaaagagtgttacattcaaatggcgagggaagccactgttcatcaggcacagaa

cagcagaagaaattcagcaagaacagagtgtaaatgtaagcacactaagagaccctcagcatgataatgaaagagtgcagaaaccagaatggctggttgt

acttggagtgtgcacccaccttggctgtgtacccattgccaatgcaggtgactttggtggatactactgtccctgtcacggttctcactatgatgcatca

ggtcgtatcagaaaaggcccagcacctctcaatttagaagttccaacatatgtgtttcaagatgaaggaaccttagttgttggctgattttactcataac

tactagcatgtactagttatgtgaagtaaatcatatgttcccaagataaaaaaaaaaaaaaaaaaaaa

>Contig3367|ubiquilin-1-like isoform x1

gtnnncntaaaaccctgcactacagggccagatgcggcagatgctannnaactttctacagcagttgcaaaatcctgaaatccataatctgatgtcaaat

ccccaggcattaaatgcattgcttcaaatacagcagggagtagaacagttacgtaatattgcgcctggttttgttaacagtatgggcatgactgcacctg

caacattgggaggtactactgctgccacgacgacttcttctagtgcaactaccagttcacctgcagcagcaacaaacacttcatcagcaacaactgctac

atcaacacctacaagcaccacgaccaccaccagctctattgcaggagatactcagggagcaaaccaacaggatgtcttctcgcagttcatggctagaatg

ttgtccagcatgtctacacagtctggagtcggtaaccagccaccagaagaaagatacagggcacagctggagcagttggcagccatgggatttgtcaata

gggaagctaatctccaagctcttattgcaacatttggggacataaatgctgcagttgagaggctactgcaaagtcgtcagatgtcacaaagctaacaata

ctacagaggtaattccatattccctgagtagtacttgcccaggtttgtttgggtttccactgtggggttactacattaagagagatattttcaagaggac

agcgaaataataaagatatttggacagcgcagcattgaaacttgtggatactattcactattgacacaaaaatttgtcataatttgaactttacaggaat

aataatgatgataataataataaaaaaaaggaaaccaagaaaccagatcacccaacacttaaattttagaagttcatataagcatgcaacaatatggcat

actatagcttcttaagattgtgtgtgtgtgcatctgtgtatttatataaataatatatatatatattacctgtttctctctagtatgttaagccttttct

tcagtgttatgttaaatatgtagttggaaagttcttttcctacttttccggatcttgaaaaaaccattgtaactggcttcaggcctaatactgtaatctt

gttactaatcatcgtgtaatgtaattttctgagtaatgaaaacacgtaaacccaagaacagtgctagcaatattgccaaaggtctgttttgtttgtgtat

ataattaggtaagtgtgtggatgaatatgggagtccacaaatgaaagactggtacagtgtaacagttatattcaatactgaatagcatccaagttttgaa
[truncated: 11,406,983 more chars]
