# Supplementary material for: RNA-Seq reveals large quantitative differences between the transcriptomes of outbreak and non-outbreak locusts
Source: Sci Rep. 2018 Jun 15;8:9207. doi: 10.1038/s41598-018-27565-0 (PMC6003920; doi:10.1038/s41598-018-27565-0)
Supplement: Supplementary file 2 — Table S6 [file 41598_2018_27565_MOESM2_ESM.doc]

>54694|-alpha-glucan branching

aaaaagaacttctgctctttcaaatgcaatgactttatcatcttcatgtttccagctgacataaccctgtaaccaaatacattgcaaataatctttgtga

agccacccaaacctttcttcagctgcattcattgccacatcaaaattgttcaggaatttgtactttagaagctcatcatcaacgaggtgccactgccggc

gtgcataatggtaagagtcattattcccagcacgagggaagtccaaccattcagggtgtccgaactcgttaccaataaaattcaaataagcttctccacc

aagtccatgtgttattagcctgatcattttgtgcaaggctagtcctctgtcaatgacaggtgtgggctctgaaagtactgacatgtgtgtatacatttct

ttgtccattagccaaaatgcaagtgttttatcacctacaagagcctggtc

>83020|-anhydro-d-fructose reductase-like isoform 2

tttgggattggtattgcacccaactgaaactgatttataactggcttaatggtagctgcttcaattaatctgctgagctgcttactattgaaattggaga

caccaatgctttttgctagacctaagtttacacactcttccatttgtttc

>86614|-atpase isoform j

ttccaattttttaatcaattagatcctttacagtaattcgtattcacttaaacctgcaaacaacgttttcgtgatcgggaaacggaagaaagacttaaaa

caaagatcgtctatgcacggacagcaggagtcaggttgtagggggataatgttgtgtttatatttccattgtgtgacaagataatgtgtggggtggatgt

aacaggtgaaatgagagtatttcgcatggggatgtttattcacgaatttgactgattagtgtcatgggaatttgtacgcggagatattttctactgacaa

tatgcattttacaattggtttcaacagttgaacgtcaagttttcgacttcttgggttacatgtgggcaccaattcttgcaaatttttttcatataatatt

tgtgatatttggattttttggtgcttttcagtacagatcaaagtatcttataacgtatactgtttggaatatagtgtggattggctggaatgtgttcatc

atctgcttctaccttgacgttggcatattggacaaagatagtgatgtgttaaatcttggaactggcagtgttagctggtgggaggtaaatggagctggtt

gcaaacctgtttatcccacaaatcttactgctgaggatactgatgtatttcgacctctaagaccagaatatgttcctggttgtttccttgactatcagta

tgtagaaatcttgcatgcttcggtgcagtgtttattagcggttcttggcacagttggtggcatatttgtttgccatattctctatgaagatgatgatagt

tcccaagttaacagttcctatggaaagaaagcaaataatggaaaccgaaactctctttattcaattgaatttagttctccagataataatgcagatgaca

gtgtagaatatgatgaaacatgtcaaaatggtaactcacacttatcagctccacaactttctccacgtcccatgaccccacgcagagtgaaacgccgtag

tgtaatttcgcgtggttccaccggccgtaactcacagcaagataaacgaggtagcgtgaggcatggaaacacagtgcgctcatcagttagatcttcagga

cggcggccgaagctgcaacatcagaaccctgtgactcgtttaatagagcagcagcagagtttgcagcaacaacaggctctaggagactcgtccacatcaa

atgattctcatcgattgaacatgttcgaccctgcatcctctcaagcagacttgttaaacagctcacagtggcggaagactgctggtcacacaaacccctt

gtatcagcagagttctatgcagtcgttaaatgaggaagaagattcatttaacagaccaccttcagcaagatcgagttactcaaactatcatggaacacgt

cccatcagttgttatgtaggttcaagtggaatgaattcttattatgtgccaggacaagcaacagctcaagtacctgtcataaatacacaccgccgtagca

atcggtcaataccaaaaagtagcagaccgagtactgaatttttgagttgtggtccaccagcgtatcagttgcaaggtgccatagactctgaaactgttat

ataaatttttcttttagcacaaaaagtgtcgagcaagccttcactgccagaaacaaatgtgcctcttgtaactggatgttgtgaaaagttaaaattttat

ttctctatggaactgtgtactgcaattattcacacaaagtgctcttgtatgtgattgatttgtatatgctgtgtgtacagttcgatgaatgtttggctca

tcatacatatattttggagcattatttgaagatttgtggtttatttaaaataactgaatcacatataacataacagtgctgagtaatattttgtgccttt

ctagtcagctattgaatgtagtgttttgtacgtgagcataccatcatatgttatatgtgatggctaaccctcattaggctgttgctggtaaattctagtt

ttcacgcgtctctggaaaactccttcacgtaaaatgttacacctgggaatcattttaaaaggtatcaatgtggaactgttaaatatgtaatttgctttgg

aagcaatgaattcaaaaacttacttttaatacatttcccacacattgttattgaa

>86615|-atpase isoform j

cgcattgtcttctccagagttcagtatctcagtcgtcgggtggggggaatgctgactcagttttaggtagacagaggggaggacatgaagacaaacatgg

agcatagttgaatgtttctggtcctggagtcaaacagactggagttagcacaaggggcacagcgccaccttcataattcagaagtcatcaccaggtgtct

ttagtgtgtgtggtgacacaaattggatgtaaatcaaagaacatcgtataatatggagtgattgagtaaggtggtgcagctattaaggtactgaccccca

tatttgggaggatggcttttattctgtccggccgtactgatttgggttttgtttggttttcctaaatcattttacttaaattccaggatggttccttcaa

ggctaggcctatcacctgtcatattcatggggtacatttatgaattctgtgtgcatttatattttgtgttactgtgtttcattttactaacacaacgtgt

gatccttagatgaataaaaataaacagttcaacgtatctgattcacaaaatgacctgcaccatgtgatataagatgtgtaaaataacaaatttatgatta

cttaataaactttgtagggtgatgagctgaagtggtttgaaagaagttttggaaagatagataaaaacctctgagggactgatgcagttacagtaacagt

ttccacaaatacgttttatttctgatatttgtactactaatacaaaaactgatcaaattttccatttaactttttacaatcacaataatagttttcaatt

atattttgaaaaatcagtcttaatatgatcaaggcgattgtaaaattttcccaattctagaaatttgttatatttgtctgtgttatgtaaagtgatgtta

taataaacatatgcatcatccaaagttgtcagaaagtttcaaagctggttatgaaaataaatctttacagccttattactacttactatttattaagttg

ctttcttctcaccagaaccacctatcagtgtttcaggctgtcaaaatgtataaaaattgcttcagatgtaagaaatgcattttatgaacttttaaaaagt

attaggccttttttgttaagaagataatgtggattctaaattgatactgtgacagatgagcagaaacatttgatgaatgaaaaaagcagaaattgggccc

aacactaactgacagtccttgcagcaccatctccagtaacgatataaaaatcccgggagcagagacttggtttgaagtgcagagacatccgttcacaagg

aagtggaaatctttaacagtcaaagatatagcctgtaatatcagtgttaaggataaactgaaagctattctctcattgtttgttgagggtgcagtaatac

gttaaaaataaaatacccagaagaaaaattaaaaacaaacatcaaaaaactgtggtttgtcatgttctgtcacatttattagtacgtagtcagattataa

tggatcactgtgggttgaatccaacttgcagaacgtacaagtgagctgcttcttggatttggggaatatgacggatggaagcatttaataaaaatttgta

aacaacagctagaaaagaacggtccaactatatattcctgaaaattatgttgcaagtgtggcactactggccaagttgaatttgacactgccaatgaaca

gaagttactcaaacctggtgctttatggcatatttacagcttcaataacaatgtgtgggaaatgtattaaaagtaagtttttgaattcattgcttccaaa

gcaaattacatatttaacagttccacattgataccttttaaaatgattcccaggtgtaacattttacgtgaaggagttttccagagacgcgtgaaaacta

gaatttaccagcaacagcctaatgagggttagccatcacatataacatatgatggtatgctcacgtacaaaacactacattcaatagctgactagaaagg

cacaaaatattactcagcactgttatgttatatgtgattcagttattttaaataaaccacaaatcttcaaataatgctccaaaatatatgtatgatgagc

caaacattcatcgaactgtacacacagcatatacaaatcaatcacatacaagagcactttgtgtgaataattgcagtacacagttccatagagaaataaa

attttaacttttcacaacatccagttacaagaggcacatttgtttctggcagtgaaggcttgctcgacactttttgtgctaaaagaaaaatttatataac

agtttcagagtctatggcaccttgcaactgatacgctggtggaccacaactcaaaaattcagtactcggtctgctactttttggtattgaccgattgcta

cggcggtgtgtatttatgacaggtacttgagctgttgcttgtcctggcacataataagaattcattccacttgaacctacataacaactgatgggacgtg

ttccatgatagtttgagtaactcgatcttgctgaaggtggtctgttaaatgaatcttcttcctcatttaacgactgcatagaactctgctgatacaaggg

gtttgtgtgaccagcagtcttccgccactgtgagctgtttaacaagtctgcttgagaggatgcagggtcgaacatgttcaatcgatgagaatcatttgat

gtggacgagtctcctagagcctgttgttgctgcaaactctgctgctgctctattaaacgagtcacagggttctgatgttgcagcttcggccgccgtcctg

aagatctaactgatgagcgcactgtgtttccatgcctcacgctacctcgtttatcttgctgtgagttacggccggtggaaccacgcgaaattacactacg

gcgtttcactctgcgtggggtcatgggacgtggagaaagttgtggagctgataagtgtgagttaccattttgacatgtttcatcatattctacactgtca

tctgcattattatctggagaactaaattcaattgaataaagagagtttcggtttccattatttgctttctttccataggaactgttaacttgggaactat

catcatcttcatagagaatatggcaaacaaatatgccaccaactgtgccaagaaccgctaataaacactgcaccgaagcatgcaagatttctacatactg

atagtcaaggaaacaaccaggaacatattctggtcttagaggtcgaaatacatcagtatcctcagcagtaagatttgtgggataaacaggtttgcaacca

gctccatttacctcccaccagctaacactgccagttccaagatttaacacatca

>109534|-carotene 9 -oxygenase-like

tcagcaatattgatttgaggtgtctcacatccaagattgcacagaagttctgctttcaaattaccatgtggaaacatattagtgaaaccagaacttctgt

gcaaacttggatgtgagacacctcaaatcaatattgctgactgtttgggaaagcccg

>109531|-carotene 9 -oxygenase-like

gggaaagccctacagatatttctatgcaatatcagctgatgttgacatagaaaacccaggctgtctaattaagactgatgcagagacacgtcagacacaa

atctggtttgagaaaaatgtttttccaagtgaaccgatatttgttcgggatccaaatgggacagaggaagatgatggtgtactactatctgctatcattt

ggggaaaaggagaggagaataaagcgggcttacttgtattagatgccaaatcctggaaggaagttgggcgagcagtttttgaaacgccaggacctgtgcc

caaaagtctgcatggctggtttactaatacagtgacatgaagtcttatttgtacctgttatgttttctacaaataaacattttttttaatgttgtaatat

ttgagcttttttcccttcaaagccaacattgtaatgcattgtccggtcacattgtgtgaccacttgtcaaaagcctgaatagtcaatgatgttctggaag

gcaccaccaaagatgtagtcatgccagctccagtgttgtggcaagctgtgctaggttaaacgtgtgcagcctgcttgatgtagagccacactctcttgac

cgagttgaaatccggg

>109530|-carotene 9 -oxygenase-like

attcagatttatgttagcagttaataaacatagttgttaaagtatctacatatgtaaatcctatacatagttgcagtgcactgacttttttaacatctgt

atcacacagtggcattttagatcagagaggccaggcaatttaccttgttcaatagaaaagtaaaacacacaacaatgttttcacttatgcttggaatgta

taggtaacacagaatatcatgacaatttagtgaaagactcagctatcaattgtaaataagaagttttaagatgtttagtagcattttacacattcctgtc

acattaatgtcaccaccatccatatttgatgtcaatgttcaaaaaccactcacagatggcagcactagcagtaaagagcatataaagcatgtttggagga

catagaaaacaatgctgttattgtcataatgtggaaacagagtgatttatctgacattcaaaagggcatcattagctttcaggcaaagggtgaaagtatt

tttgaatcatctaactttgtaatctgttcacatgctgccatggttagagtatactgtgcatggaaaaattgtgctatccaaaactggcaatggggcaact

gggcaactgggcaatcttgtcattttaaaaaacacagtgatctacacaagtacacatttatccttgggaactatctccacccttacatgcagtctgtttt

tgtttgtcacacagcttgcaatgtatgtgtgtggttcagagaggatcaggatgagttatctatactcccctggccaccatatgccccagatttcaactcg

gtcaagagagtgtggctctacatcaagcaggctgcacacgtttaacctagcacagcttgccacaacactggagctggcatgactacatctttggtggtgc

cttccagaacatcattgactattcaggcttttgacaagtggtcacacaatgtgaccggacaatgcattacaatgttggctttgaagggaaaaaagctcaa

atattacaacattaaaaaaaatgtttatttgtagaaaacataacaggtacaaataagacttcatgtcactgtattagtaaaccagccatgcagacttttg

ggcacaggtcctggcgtttcaaaaactgctcgcccaacttccttccaggatttggcatctaatacaagtaagcccgctttattctcctctccttttcccc

aaatgatagcagatagtagtacaccatcatcttcctctgtcccatttggatcccgaacaaatatcggttcacttggaaaaacatttttctcaaaccagat

ttgtgtctgacgtgtctctgcatcagtcttaattagctgtaagtaatataataaaacatctgtcagtaaaattcatgcacctgaaagactatgtcatatg

caaaaatgaattaaaatagttttatctataaagtaatgtgtaaaaacttcaatttctgatgttatgatatctgttggatgaaatgtgtgtagacctgaaa

gccaactcttctgaatgatattaaaaatgtggctttcctcgatgacattaaaaatgtgcatacatacttgatatgtttctgcccactcttacaaaataag

taagttgtcacaaagatattttagactgcaaagatgacctctggaggcagtacag

>15521|-dihydro-8-oxoguanine triphosphatase-like

gtcacaatgttcaaatcatgtcgcagaattgtgctatgaccttcaaacagaaagtaacctttgatcttatgcccttgaagtattagtggtaaccagtgtc

gatcatcagcccacatcttatcaaatggtatctcgtcaattgaataccactttggcaacatttcatctgattctgagacatttcctgaccattcatgggt

tttaaacacatgtacctctagaattgtcatgtcacccacaaattcaaaatctattactcctacatgttccaaatctagtgctgtcaatccactttcttct

tgcaattctcttgcagcagcacactgtattgtttcgccaggttcaactttccctccaaagccattccacttcccttttccaaatcctctcttctttaatc

ctaacagaatttctctgtctctctgtactattaccaaagtaagcagcttacgaaacatcagctgaattccagcgttggatgccccgcgtagccggaagat

ggtcatgctgctgctgttcgaagtttacgttgtgctgcacctgttcaggactgtagattttacatgattatcccacttctgacaccacttctacaaggag

gttatttgtgttgttgcaccggatgataattacacgaatattttgttaggatttgtaaacagtgggtccttgaggtacggcaatacgcgaacaccgag

>35464|-like 2

gtgaagaagaggactgtgaaatgacagatgatgctctgacagttttgacaagaattgcactggagacatcattgcgatacgcgattcagctcatcactac

tgccagccttgttgctcgtcgtcggaagagcactgaggttggcattgaagatgtgaaaaaagtgtactccctcttctttgatgaaaatagatctgcacag

ttcctaaaggaatatcaggatgaatttatgttttatgagatggaagaagaaaaaatggatgtctcctcagcataaccagagaataacccaccagtatgct

tgaagctgtaaattgtttgttgatgtgaactgcaggacacatgaacatcgacgtttcgaacgcaca

>40140|-like mitochondrial

aggcaacagaaagtgtaccaaaggaagaaattaaagacacaaatccccatctgaagagtttatatgaaggtcttaaaatgacagaagttcagttgccttc

cccaacacatcagcaacctcaagtaagactttacagaagccctgga

>40138|-like mitochondrial

gcagcctgtaaaaggaagaaggcgtatcaaaaattgctacatactagaactcaggtagacacagaaagtgtaccaaaggaagaaattaaagacacaaatc

cacatctgaagagtttatatgaaggtcttaaaatgacagaagttcagtta

>40141|-like mitochondrial

accaaaggaagaaattaaagacacaaatcctcatctgaagagtttatatgaaggcaacagaaagtgtaccaaaggaagaaattaaagacacaaatcctca

tctgaagagattatatgaaggtcttaaaatgacagaagttcagttacataaggtttttaagaga

>42676|-specific partial

gcatcgagaagagttctcaccgcttcgacgtgcctgttccatgccgcagtgtgcagtgcagtcctcccgtagatgtcctttccgtctatgtctgcccctg

cctccagcagagcccttagctcttccactcgcccttgcttggctgcttcgatcaacttcatgcatttctcatgtcctgaaaggctccggaatggagagac

atcgctgacgtcggctggggcggcgccatcgtcatctgggaggggcgtctggtcttcgtcgttgggccaatgagcggtgcttacctctgtggaggctggc

gtgctttttactgcgggtggctcagacagcagctgactcactctgatgaggtcttctgtgtgagtgcgcattacgtcggcccagccctgtgaggccatca

cgtcaaagatatgagccctgacgaaggcgagggcagcgtccctgaggtcagtacaggagtacctgacagcgaggacggccacggcggccgcagtctcaac

agagagtggcagggcagcctgcagggcgcaatctgccgacgagtcgtactggggagccgcgctgggcacgcgtggggcctgcagagtgcggtgggcggcc

agcagtcggcgcagctcccggccgtccacgtccagaagggtggcccgcgggctgccgaggtccggcgcgccggggcggagcgcggaggccccg

>47921|-trehalose-phosphate synthase

cataaacatcaggcatgtttgataatcgttccaagacactcttggaacgaggaggcagaattgctaagtctgggtgaggtgcaataggagccaggg

>47919|-trehalose-phosphate synthase

attctttcacatctgttcttgatggaactgatatctgaagaagtgtaagcttctcaatatgtactggatatttttccagtacatattgagaaggttacac

ttcttcagatatcaggtccatc

>47928|-trehalose-phosphate synthase

aactttataagagaaggatttgttttagtaaaaagtttgtatgagtatgtgcctttttacttcacaggtgtgcagagaaggagcatgggtggaaaataaa

ggggctctgttaacattccactatcg

>47920|-trehalose-phosphate synthase

tgcttcaaacctgcaatgtagcgagtgatatattctaatgtaacaggatcttcctttacaacatttcttccagatataatagcaacataaacttcaggca

tgtttgataatcgttccaagacactcttggtctctggaggcagaatt

>47926|-trehalose-phosphate synthase

caagtttgactcatattataatggttgctgtaatgggactttttggcctctttttcactcgatgccagacagggctgttttccttgctgaacattggaag

gtaatgcgaaagtaatattttatttttgttgtcagtgatgacagtttcattctgatatggtggactgctttatttatttactttcatcagttagatcatc

tccaataactgtaaatgtaatggtattgtggtaattctgctgtattattgccaatgactttcttactgttgtatttacatactgttgtatttacatctgt

tctaagagtttatgtatctggttttcagactgatattgtgtcaagaccattagccttaacacttacgcatttgcatttgtttacagaattattacagtgt

gaatgaactatttgctgagaaaacaataaaaggtttaaaaagtttactagaggagcagccaaatgcaacacctcttgtttggatacacgattatcatctt

atgttagcagcaaattgggtcaggcagat

>47925|-trehalose-phosphate synthase

gtattacctacaaaccagaaatttgttttgggagtagatcgtcttgattacacaaaaggccttgtgcacaggctgaaagcatttgagttactactggaaa

aatatccagtacatattgagaaggttacacttcttcagatatcagttccatcaagaacagatgtgaaagaatatcaagatctaaaagaagagatggacca

aatggtggggcgcataaatggtcgtttcacaacttacaactggtctccgattcgatacatttatggccaagtggggcaagatgagctggctgccttctat

cgagatgctgctgtcgctcttgttacacctcttcgagatggtatggttgactttcaattcccttgttgatctcagatgaaatcagactgccagaagcagc

tgaatattttaatttcactgacttggtatgcatattgggaatgatgctcagtgcacattctggaagttgtatccattgcttttgtatcattaaatgccaa

gatatagttttctggtgttgtgtttttcatgtatatgacagcataatct

>47918|-trehalose-phosphate synthase

gtattacctacaaaccagaaatttgttttgggagtagatcgtcttgattacacaaaaggccttgtgcacaggctgaaagcatttgagttactactggaaa

aatatccagtacatattgagaaggttacacttcttcagatatcagttccatcaagaacagatgtgaaagaatatcaagatctaaaagaagagatggacca

aatggtggggcgcataaatggtcgtttcacaacttacaactggtctccgattcgatacatttatggccaagtggggcaagatgagctggctgccttctat

cgagatgctgctgtcgctcttgttacacctcttcgagatggaatgaaccttgtcgccaaggagtttgttgcttgtcagattaatgagcctccaggagttc

taatagtttcaccatttgctggagcaggtgagatgatgcatgaggccctgatctgtaatccatatgaggtacacatggctgctgaaataattcacaggtt

agcagcgtgatgttcaatactactacttctaaaatattaggtgtttgttttttttatttgttaatatgtaacaacaagttcaatgacggttgcagatgca

ttattcattatgtagaaagtgcatgtacaaattgttataaggagtaaaacagtactgtacttttgatttactgtatctagttatgttcagtatggtagta

acaacatggaacttccccaattgcacaagttaaggaacagatggattgtttcaaaccaaagggttgttaaccagatgtcccacacaccaaaagtgagcct

gct

>47914|-trehalose-phosphate synthase

gtcaccctgaaagtagctgccattccctttagagcctccatggcatcttcatcagtaacatcatcaccagcgtaaattatacggattcgttcactccaat

caactccaaaggcagttcgcagaatataaagagatgctcgtcctttgttccactgtaccggaggctttgtttcgattgcgcagtgggcagatccagcttt

gaatcctgcctgctcaatcagagtctgagcttgtgcaaccatttcaggccgcaagtgcataggtgtctcgcgatagtggaatgttaacagagccccttta

ttttccacccatgctccttctctgcacacctgctcctgaagtgcttttaacaaatcagtcaccttgtcttcaaattctattggcattggatgaacaaatt

tgcttccatcaggatgcaaaatttcaagaccatggttaccagcatatgttatgccctcaatgcccacctaaaagacaaaagcaaattcagatttcctgtc

aatctcacaatcatgagcagccttataaaaacaaaaactgcaatgaaaattaatatgaccttataattctatctacatttattccccagtagtctctctc

tctgagaaaaaaggccgcacataataactctcagtgtgtatctaagttgctgcacccaaacatactattgcaacagacagaaacgaaattcacagattgc

aaccctttaattggagtgagtggtggaaaacagggagggagggagaatgtgaaacaatggggaagtcatgcaatcatgatgtccaaaagtctgggtctgt

aactctgaacttcaaacaaaacgtgaatatcctttacacattttgtgccaccctgtgcacaaaagaactgtgacatgtttacataagaagtggtacagta

gaacctcgctaatccaacctcagatggtcagactccttgctcagaccgactatcccatttccagtgtctgtttaaacagccttcacacaactccactcag

tggcactgctggcagaccgagcaacaggtcaccacatataaacaatttgcaagtgatgtcagcatagaagcagtggtagtgacagaatcaaagctgctca

ctacaatagttgcggccattgccagtgaagccacatgaaggccggagcaaagaagagaagcacttctcagtcgtgtcatcagaagcagccctcacatgca

caagccactgctacagtcaggtctagacttgttgaggaatcgtcatctgcataccagtcagtaagcagtcaacagcagataggtgcggtactttgatcaa

ctttttcctagcacaatcataa

>47916|-trehalose-phosphate synthase

ttaatcttgcaggaagtttcagatcagctgatactacactgcagagtgaaatattcgttccagacccatagtgttctttttaatctaatgagttatttcc

gtcacacaaaatttttggtgagtagaacaatgactgcaataggcatataacttttgcagatttcaagttttgtgctcatatacttctatctttttcattc

atgtttatatatttatactacttctttttcattggtctcctctgcccttgaaagaaattgatctttctttttttatgttattgattttcctattctgtat

actgaatctgtctccttataaaagagcgggtttataaaatgaggatgttaataattcagtgtagtctctctctccactgtcactgactctgactcttcct

ttttaaagataatggcttttgttcttaaccagcattttactttaagttttgaattacatatgtaacatgtgtatctctttaatattaatccgatctggtt

ttattcctatttggtattttattttaattgttaaagttgttgcaatgagtgtaaaagtgttacttgaagtgaataatgaatttttatggcagggcattaa

caatgcctgaagatgaacggacactacgaatgaactaccttcgtcggagagaaaagcttcatgatgtaaactattggatgaaatcatttttcaaggccat

gggctcactgagggaagatggtgatgatggtttacccacaacaatgcagccagtgactatggatgactttgatgaatatttgtccaagtaacaacacaga

gtattcattgtgaagtaattaagagctagtttaaaatgttgggatccatttctgatcatggcctctctcaggtacctgggcaaccaccacaagctggccc

tgctgctggattatgacggaaccctggctcctattgcacctcacccagacttagcaattctgcctccagagaccaagagtgtcttggaacgattatcaaa

catgcctgatgtttatgttgctattatatctggaagaaatgttgtaaatgtgaaacaaatggtgggcattgagggcataacatatgctggtaaccatggt

cttgaaattttgcatcctgatggaagcaaatttgttcatccaatgccaatagaatttgaagacaaggtgactgatttgttaaaagcacttcaggagcagg

tgtgcagagaaggagcatgggtggaaaataaaggggctctgttaacattccactatcgcgagacacctatgcacttgcggtctgaaatggttgcacaagc

tcagactctgattgagcaggcaggattcaaagctagatctgcccactgcgcaatcgaaacaaagcctccagtacagtggaacaaaggacgagcatctctt

tatattctgcgaactgcctttggagttgattggagtgaacgaatccgtataatttacgctggtgatgatgttactgatgaagatgccatggaggctctaa

agggaatggcagctactttcagggtgacgtcatcccacatagtgaagactgctgccgaacggcgacttccaaacaccgattcagtcctcacgatgctcaa

gtggttagagcgccacttgagt

>47917|-trehalose-phosphate synthase

cacacacacaaatcaatagtggccttccctgagaatcaaattctccaactgcataattagaaaagaaaacacattttaggggttacaaacatgtttttat

taaatcattatcttactggcttgttttacattcatttcatagcatgaaaggaaagtagaaaataagatacactagataaaaaaaacattagaatggcatg

actaattcatatctcactatttcctacataaaatcatgcattacagagtatttgagcacagaccatctattgcattttgaagaacttaatacaggatgga

tggtaatcagaaaatctgcatacactttagggcttagagtaaggatacatattagaaatgggaagcttgtaaagagtaaaattgggatacacatgaagta

tgaagcagattagaagggtcagtacacagatcctttcatgttagctgtgatgatgatgatgatgatgatgatgatgactctgttgaaagaatacttatca

ttatgtacctcccagtgcttttgcatgcattagtacataggtgcaaaggacagacaatggaactatatcacttaaactcatgtgataagaaaacttcttc

agtatttttccatgcttactttttttatatttgatgtatgtatctgatctacttcttatttttaaagtaataaatgtaacagacagcgagaacgcactga

aaaacacagcattcagcacctacagctgaaatgtaggactgaaaagttattatcaagggcatatgagcacatctgagactgcattattttaatgtgccga

tagaggtctgtctatgcaatataaaatcattttcattttgtttataggctccaaacacacagtcatgaaaatttctgataattttgacacttctcttctc

tttgcattttatcagcatctgattgtttatgtaggctgctgcatattgtcttttccctagctttatcattttcatgaagtttggcaacttctttcagctg

gcactctacctaaaatcaataccctcactaactagtttgtcaaccaatctgtattagtgcagtaagctgtaaccaacacaaaatagactttggaatggca

gatagatatactttcaaaaccatgtgaacaggtgcatcacagataaactaaaacaatgttgtcagcttgtgttctactcctgtatgacaatgcttcctat

cacagcagatatgtttgacctcagttgtctgtctgaaactgtgtcttccaaatcttgtaaccaccactttactttcaaactggttttaactacaggtaat

aatgataatgacatatttgacactgaaatatggttaccggtctatgacaaaatattttacttacttaaaattgcaatatttgaaagggaagtgggggggg

ggcagatactgcagaagagggtggggttcattacaatgtggctttctatctgtaagaatggtagaaattatccagtaacattactggaactaggccacag

cattaactttctgcacatttctcttaaatcaaggaaatttgtatgtgatatgattatatgcatgccattcataatttgtcttaccttctcaatatgtact

ggatatttttccagtagtaactcaaatgctttcagcctgtgcacaaggccttttgtgtaatcaagacgatctactcccaaaacaaatttctggtttgtag

gtaatac

>47922|-trehalose-phosphate synthase

aaaaaaagccacatacacaagtgatgaacttccctacatagcagacagacaggcagagatggactgaccacctaattagagactgctggtgattgaggtt

ctggtgacacggaatgtggtgtatatgacatttcacttgtaacacttaatcgagatcccttacttttggaagggcgcctgtaggggccaccatttgcatc

ctgtcgaggctctctcccactcaagtggcgctctaaccacttgagcatcgtgaggactgaatcggtgtttggaagtcgccgttcggcagcagtcttcact

atgtgggatgacgtcaccctgaaagtagctgccattccctttagagcctccatggcatcttcatcagtaacatcatcaccagcgtaaattatacggattc

gttcactccaatcaactccaaaggcagttcgcagaatataaagagatgctcgtcctttgttccactgtactggaggctttgtttcgattgcgcagtgggc

agatctagctttgaatcctgcctgctcaatcagagtctgagcttgtgcaaccatttcagaccgcaagtgcataggtgtctcgcgatagtggaatgttaac

agagcccctttattttccacccatgctccttctctgcacacctgctcctgaagtgcttttaacaaatcagtcaccttgtcttcaaattctattggcattg

gatgaacaaatttgcttccatcaggatgcaaaatttcaagaccatggttaccagcatatgttatgccctcaatgcccaccatttgtttcacatttacaac

atttcttccagatataatagcaacataaacatcaggcatgtttgataatcgttccaagacactcttggtctctggaggcagaattgctaagtctgggtga

ggtgcaataggagccagggttccgtcataatccagcagcagggccagcttgtggtggttgcccaggtacttggacaaatattcatcaaagtcatccatag

tcactggctgcattgttgtgggtaaaccatcatcaccatcttccctcagtgagcccatggccttgaaaaatgatttcatccaatagtttacatcatgaag

cttttctctccgacgaaggtagttcattcgtagtgtccgttcatcttcaggcattgttaatgccctgccataaaaattcattattcacttcaagtaacac

ttttacactcattgcaacaactttaacaattaaaataaaataccaaataggaataaaaccagatcggattaatattaaagagatacacatgttacatatg

taattcaaaacttaaagtaaaatgctggttaagaacaaaagccattatctttaaaaaggaagagtcagagtcagtgacagtggagagagagactacactg

aattattaacatcctcattttataaacccgctcttttataaggagacagattcagtatacagaataggaaaatcaataacataaaaaaagaaagatcaat

ttctttcaagggcagaggagaccaatgaaaaagaagtagtataaatatataaacatgaatgaaaaagatagaagtatatgagcacaaaacttgaaatctg

caaaagttatatgcctattgcagtcattgttctactcaccaaaaattttgtgtgacggaaataactcattagattaaaaagaacactatgggtctggaac

gaatatttcactctgcagtgtagtatcagctgatctgaaacttcctgcaagattaa

>47910|-trehalose-phosphate synthase

cgaattccgaaaataagacgacaacaacaacaacaacaaaaacaacagttggttggggcgctaaaatactacaacaaatctgctccttttgctggagaaa

actgtctgataagttcttgcaggaaatttcgtttgtaatctttatagccttcacacctctaggaagctccgcccacaatttcctatgcttcagtgcggcg

cgggcagaacttgtataaatgaaggcggttgctggtagcagctagtaaaattatctccactggtttttgtgtgataggaacgtgacctcttcagaaaggc

agagtccgggtcacggcgtatcaagcagctatttgcagtctgcgtgatagtgcgtcgaactttttgtagcctgcgaagaaaactttcttattccttcact

caggactcattatggttgacgatggtttctccgacacggtgggtgacccggtaacttcgaaatgcatgatagttgtatccaaccggttaccctttgttct

taagaggaacgagaaaaccggtgcattggaacgaaaagcaagtgcaggaggtttggttactgcagtagcgccagtagtggtccagggtcatggtttgtgg

gttggatggccaggtattcatttagaagatccagatgagcctataccagaatccaatccaaatgataaaactccaactgccggcctgttgtccaaaaagg

tagttcctgtgtgtgttgaaccaggcaagtttgactcatattataatggttgctgtaatgggactttttggcctctttttcactcgatgccagacagggc

tgttttccttgctgaacattggaagaattattacagtgtgaatgaactatttgctgagaaaacaataaaaggtttaaaaagtttactagaggagcagcca

aatgcaacacctcttgtttggatacacgattatcatcttatgttagcagcaaattgggtcaggcagattgcagaagaatcaaatttaaggttgaagcttg

gattcttcctacacataccatttcccccgtgggacatttttcgactgtgtccgtggtctgatgaaattctgcaaggtatacttggtaatgacatggtggg

attccacattgaggattactgtctcaatttcatcgactgctgccaaaggagactgggttgccgtgtagaccgaaaaggactgcttgttgaacatggtggc

cgcacagtgctcgttaggcccttgcctatcggtattccgtatgaccggtttgtcaagatggccgaagttgcacctaaagtattacctacaaaccagaaat

ttgttttgggagtagatcgtcttgattacacaaaaggccttgtgcacaggctgaaagcatttgagttactactggaaaaatatccagtacatattgagaa

ggttacacttcttcagatatcagttccatcaagaacagatgtgaaagaatatcaagatctaaaagaagagatggaccaaatggtggggcgcataaatggt

cgtttcacaacttacaactggtctccgattcgatacatttatggccaagtggggcaagatgagctggctgccttctatcgagatgctgctgtcgctcttg

ttacacctcttcgagatggaatgaaccttgtcgccaaggagtttgttgcttgtcagattaatgagcctccaggagttctaatagtttcaccatttgctgg

agcaggtgagatgatgcatgaggccctgatctgtaatccatatgaggtacacatggctgctgaaataattcacagggcattaacaatgcctgaagatgaa

cggacactacgaatgaactaccttcgtc

>47909|-trehalose-phosphate synthase

gcaattcagaaaagctggtgttggtaggaaggatccatatggcacaggcaataatattgtcattattccatcctgaattttccattgttcaataggtagg

acatctacacctagcacagctgatgccaagttattcatactcagtgaatttattttgatctaattttgaatggctgtcaattgatatgagtgtaaacatt

acacgctgaggagggtgcaaaatgataacttcataggtctcaattccccactgtcttcttctcattggtgcagggatacaaggttgggctgtgagcagtc

actgatgaatctagtagacaatgatatagggatgtcaacagtatgacatactgaagactgggttggtccatgaagcatgcatgggcaacctaatggtaag

gtgactgctatcgataagcaggaaatccagcttcgtgtcccagactggcaccaataggtaggatatctgtacttaatgcagctgatgttaagtcattccc

attcagcaaatttattttgacacaaataaaagagttgttggagtgctgcactgatgtgtcaagctagcatccactttcggcacttcctagttctttgaga

gatggctagatgcacacttccacaaactttgtcataaccaaatgtttcatgaattacatgaggcacagctccattctgaattttcacatcagcaacagac

tccatattgtgatctgacagagctggcaagcgatacactcacagttgaaactgtcacagcagtaatgacaacacagacatgttcaagccaaagtaagact

ttaatgtatcactatttagcactgatatgttgcaaaatataagacactgctttctgcaatgttatccattctacaacaaatttctgaagcaggctcactt

ttggtgtgtgggacatctggttaacaaccctttggtttgaaacaatccatctgttccttaacttgtgcaattggggaagttccatgttgttactaccata

ctgaacataactagatacagtaaatcaaaagtacagtactgttttactccttataacaatttgtacatgcactttctacataatgaataatgcatctgca

accgtcattgaacttgttgttacatattaacaaataaaaaaaacaaacacctaatattttagaagtagtagtattgaacatcacgctgctaacctgtgaa

ttatttcagcagccatgtgtacctcatatggattacagatcagggcctcatgcatcatctcacctgctccagcaaatggtgaaactattagaactcctgg

aggctcattaatctgacaagcaacaaactccttggcgacaaggttcattccatctcgaagaggtgtaacaagagcgacagcagcatctcgatagaaggca

gccagctcatcttgccccacttggccataaatgtatcgaatcggagaccagttgtaagttgtgaaacgaccatttatgcgccccaccatttggtccatct

cttcttttagatcttgatattctttcacatctgttcttgatggaactgatatctgaagaagtgtaaccttctcaatatgtactggatatttttccagtag

taactcaaatgctttcagcctgtgcacaaggccttttgtgtaatcaagacgatctactcccaaaacaaatttctggtttgtaggtaatacctagaacaca

caaaaaacctgtaaaaattttagtcacttggtacaacatttcactgctacaactgttcatatgtatcagcaatgcaataatccctcaaagcaagtaacaa

atcagaagttaggaaaaacattctcttccataaattaaggaatctatggaacttacaagaaaatcttgtattgtcatggcaccaaatagactaaactgca

gtggggaaacacaggaaatatgtggcaaacttatgttatacaagtttgatgcacagtagcaggagccacaaaaaacag

>47915|-trehalose-phosphate synthase

tgaagcaaaatgatggttctggtgccagattggaacacacactccaaccttacctgtaatgctgacccccaccctcccccttttccattgtacaatattc

cctacaacctcgaactgatgctacaagagatgtagaaatcgtgacacactttgtgacgaatgtggcagccttctgtctaccacagatttgtgtatgggta

gttccatccaggtgtgccagctgtcccacgctgttttttgtggctcctgctactgtgcatcaaacttgtataacataagtttgccacatatttcctgtgt

ttccccactgcagtttagtctatttggtgccatgacaatacaagattttcttgtaagttccatagattccttaatttatggaagagaatgtttttcctaa

cttctgatttgttacttgctttgagggattattgcattgctgatacatatgaacagttgtagcagtgaaatgttgtaccaagtgactaaaatttttacag

gttttttgtgtgttctaggtattacctacaaaccagaaatttgttttgggagtagatcgtcttgattacacaaaaggccttgtgcacaggctgaaagcat

ttgagttactactggaaaaatatccagtacatattgagaaggtaagacaaattatgaatggcatgcatataatcatatcacatacaaatttccttgattt

aagagaaatgtgcagaaagttaatgctgtggcctagttccagtaatgttactggataatttctaccattcttacagatagaaagccacattgtaatgaac

cccaccctcttctgcagtatctgccccccccccacttccctttcaaatattgcaattttaagtaagtaaaatattttgtcatagaccggtaaccatattt

cagtgtcaaatatgtcattatcattattacctgtagttaaaaccagtttgaaagtaaagtggtggttacaagatttggaagacacagtttcagacagaca

actgaggtcaaacatatctgctgtgataggaagcattgtcatacaggagtagaacacaagctgacaacattgttttagtttatctgtgatgcacctgttc

acatggttttgaaagtatatctatctgccattccaaagtctattttgtgttggttacagcttactgcactaatacagattggttgacaaactagttagtg

agggtattgattttaggtagagtgccagctgaaagaagttgccaaacttcatgaaaatgataaagctagggaaaagacaatatgcagcagcctacataaa

caatcagatgctgataaaatgcaaagagaagagaagtgtcaaaattatcagaaattttcatgactgtgtgtttggagcctataaacaaaatgaaaatgat

tttatattgcatagacagacctctatcggcacattaaaataatgcagtctcagatgtgctcatatgcccttgataataacttttcagtcctacatttcag

ctgtaggtgctgaatgctgtgtttttcagtgcgttctcgctgtctgttacatttattactttaaaaataagaagtagatcagatacatacatcaaatata

aaaaaagtaagcatggaaaaatactgaagaagttttcttatcacatgagtttaagtgatatagttccattgtctgtcctttgcacctatgtactaatgca

tgcaaaagcactgggaggtacataatgataagtattctttcaacagagtcatcatcatcatcatcatcatcatcatcacagctaacatgaaaggatctgt

gtactgacccttctaatctgcttcatacttcatgtgtatcccaattttgctctttacaagcttcccatttctaatatgtatccttactctaagccctaaa

gtgtatgcagattttctgattaccatccatcctgtattaagttcttcaaaatgcaatagatggtctgtgctcaaatactctgtaatgcatgattttatgt

aggaaatagtgagatatgaattagtcatgccattctaatgttttttttatctagtgtatcttattttctactttcctttcatgctatgaaatgaatgtaa

aacaagccagtaagataatgatttaataaaaa

>47911|-trehalose-phosphate synthase

catcacaaaatcccttggatcataaggtttatcacgaatcaaaatttatcaaagaaaagtttgtaaagctgtacaagagactgcttacaatttttaacac

ttaagaaattagcacctgaattcaacatagccacatttctcttgaagacaatctatgcgtagcacatcctgaaattctaaaacattgggaaagagcacaa

cacaatatttggcaattggcaattcagtctatacaaaacaactggcattttagacacggctacaaaaacaacatggtagatttttcttgatgaattcact

aaggagacatactgttgaatacaggtgctacattcattaaaagatgacccaaaacaaatgctagaacaaatctcttaccaacacttggatcactttaaaa

agaattgacttagctgggctgatagttaaatggtccaccacggaaaaagagacttagtgtccacagtaaacaaaatattgtgtggaagaagtcagcaaac

ctgcaccaaaaaagtggaagttgatttattatgacataaatgtaagggctagtgttttgtaggataaaaaaaggattatttttgccaattgcctctcaaa

agctaaaacaataaaagatgaataccacacatgtattatggcccagacaggtgaaaaaacacaatgcaagagttctgtaatgcaaatgaaaagaaaaaat

aatcaggatattgtgacactgatgagcaagacggtttgaaggacaaattactgggatattcaccagatctggtgcccttgccttccatttaattcaatat

ccaaggagatacgcagctggaaaacaaactgagccatacgtcagtactttgcagaccttccagtgtcaaacttcagagatggtatctactaattgaaaaa

aaattgataaagtacattgagcttaaagtgcatggaataaaaaagaagtacattacaattaaaaacaaatattctgctgaagggctaagaactgccttca

tagtatgtgagcagttaaattgcgtgagttgataatacatccacatttaacaatgacaaacggagcgaatacattaatgagatgacatcactgacagtct

caactgtttcttaatactaaatgttagcaacagagtcttgtaataattagagctgatatttatgctgtcccccccccccccctctctccaaccaaacatt

taagtgaatttaacagtttgacctctataggagagatttttaatagtaaaacaataattaccagaaaagaagtcactttttagagaacatacctcacctg

agtgaatcacaagcaagaaggaagtaagataaatgatactattaaaattatgctaaacagaaacatgcagccaatggaataaagaaaagaaaaatatttt

tgaagcagtataaaatacaaatgttatgttcctgcaccaatagacatcaacaagcctatgacccaagtcagtcaccagttggtggtaaataagaccactc

acagtcagtacaatagagttaggtttatgtttgtagataatcttttacatctgacatgctgatgtgcagtgaccttcatggtagtgggaagtgtgaagca

atttgtaacagtaatggataatatgttttaaaaattcaatgacaggatagcccaataaaaaccagtcatctaataaaatgattttttagcaatcttggtt

gtgacgttttctacacttaccatatgctgcacactgccccgagtgaccatgtcaggaatatataacacgttttatcacttacgtagagtcagtttcttca

aagcactacgttttcttacattgaagaggaatatgactggactcaacatacacagatgagtcaggcagagattagtgatacagttgtgtatgattatcaa

taaagttgaagcaaatcattctgcgtcagtctctctcatataatttctcagaaacaatttgttatacttaccaagtataccttgcagaatttcatcagac

cacggacacagtcgaaaaatgtcccacgggggaaatggtatgtgtaggaagaatccaagcttcaaccttaaatttgattcttctgcaatctgaaatcaac

atatccagattgacaaaagtgtaacaagtttcacacatgaagggatgccataaacgtaagaacaaattccacaatgcaaaaatgattgttaaatgaggca

gttagctaaaagcagtgaaacagcaaaataattcactattctctaacgcaatagttcactgaaacttacaagttgtcaaaagtatgattcatgaccc

>47912|-trehalose-phosphate synthase

ctttcttattccttcactcaggactcattatggtatgcggtattggcgatattacatctacacaaaagagtgctgcgtttgcattagaaaactgttttcg

tccttggcaatgacagcttcgttcaattgttttgtgtttaaggttgacgatggtttctccgacacggtgggtgacccggtaacttcgaaatgcatgatag

ttgtatccaaccggttaccctttgttcttaagaggaacgagaaaaccggtgcattggaacgaaaagcaagtgcaggaggtttggttactgcagtagcgcc

agtagtggtccagggtcatggtttgtgggttggatggccaggtattcatttagaagatccagatgagcctataccagaatccaatccaaatgataaaact

ccaactgccggcctgttgtccaaaaaggtagttcctgtgtgtgttgaaccaggcaagtttgactcatattataatggttgctgtaatgggactttttggc

ctctttttcactcgatgccagacagggctgttttccttgctgaacattggaagaattattacagtgtgaatgaactatttgctgagaaaacaataaaagg

tttaaaaagtttactagaggagcagccaaatgcaacacctcttgtttggatacacgattatcatcttatgttagcagcaaattgggtcaggcagattgca

gaagaatcaaatttaaggttgaagcttggattcttcctacacataccatttcccccgtgggacatttttcgactgtgtccgtggtctgatgaaattctgc

aaggtatacttggtaatgacatggtgggattccacattgaggattactgtctcaatttcatcgactgctgccaaaggagactgggttgccgtgtagaccg

aaaaggactgcttgttgaacatggtggccgcacagtgctcgttaggcccttgcctatcggtattccgtatgaccggtttgtcaagatggccgaagttgca

cctaaagtattacctacaaaccagaaatttgttttgggagtagatcgtcttgattacacaaaaggccttgtgcacaggctgaaagcatttgagttactac

tggaaaaatatccagtacatattgagaaggttacacttcttcagatatcagttccatcaagaacagatgtgaaagaatatcaagatctaaaagaagagat

ggaccaaatggtggggcgcataaatggtcgtttcacaacttacaactggtctccgattcgatacatttatggccaagtggggcaagatgagctggctgcc

ttctatcgagatgctgctgtcgctcttgttacacctcttcgagatggaatgaaccttgtcgccaaggagtttgttgcttgtcagattaatgagcctccag

gagttctaatagtttcaccatttgctggagcaggtgagatgatgcatgaggccctgatctgtaatccatatgaggtacacatggctgctgaaataattca

cagggcattaacaatgcctgaagatgaacggacactacgaatgaactaccttcgtcggagagaaaagcttcatgatgtaaactattggatgaaatcattt

ttcaaggccatgggctcactgagggaagatggtgatgatggtttacccacaacaatgcagccagtgactatggatgactttgatgaatatttgtccaagt

acctgggcaaccaccacaagctggccctgctgctggattatgacggaaccctggctcctattgcacctcacccagacttagcaattctgcctccagagac

caagagtgtcttggaacgattatcaaacatgcctgatgtttatgttgctattatatctggaagaaatgttgtaaatgtgaaacaaatggtgggcattgag

ggcataacatatgctggtaaccatggtcttgaaattttgcatcctgatggaagcaaatttgttcatccaatgccaatagaatttgaagacaaggtgactg

atttgttaaaagcacttcaggagcaggtgtgcagagaaggagcatgggtggaaaataaaggggctctgttaacattccactatcgcgagacacctatgca

cttgcggcctgaaatggttgcacaagctcagactctgattgagcaggcaggattcaaagctggatctgcccactgcgcaatcgaaacaaagcctccggta

cagtggaacaaaggacgagcatctctttatattctgcgaactgcctttggagttgattggagtgaacgaatccgtataatttacgctggtgatgatgtta

ctgatgaagatgccatggaggctctaaagggaatggcagctactttcagggtgacatcatcccacatagtgaagactgctgccgaacggcgacttccaaa

caccgattcagtcctcacgatgctcaagtggttagagcgccacttgagtgggagagagcctcgacaggatgcaaatggtggcccctacaggcgcccttcc

aaaagtaagggatctcgattaagtgttacaagtgaaatgtcatatacaccacattccgtgtcaccagaacctcaatcaccagcagtctctaattaggtgg

tcagtccatctctgcctgtctgtctgctatgtagggaagttcatcacttgtgtatgtggctttttttctcgagtttgaatatggggttaccagtagcgta

tgttcccatatttgtctatttgtctctaattacgatttattgagaaagacatagttagaggatagagagattgttcccagtgtaacatgttgtaagataa

caaatgtctacaactgtacctacagaagtttttatgcattttggtgcacaaatatgtgacaatgtgtaagagtatctggtaagaaatgatacaagtggtt

tgctaattgtttacaaaaatatttttcatacataattcttcaaacagtgtgtataaaaggaaagcaaaagatgtcatgagaatgccttctgtgtctttta

ttcttacggaatacttatttagtgttcttttcgtacttggattctgtcctctgtattgtgttcttgacgttgcaactaaatggaatcagatttgtagaga

ttttgcctgaatgtttacattttattttgtggaaagatttatcttttgtattttacagatgagattaaagctgtaatttgttgaattcagtgatatatgt

ttttaggccatttttgtatgtatattggaaaaagaatgtttttaacaagagatttaatgttatacagtgtcttcctttgcaacaactactccagtcactg

tttttgttgtttcttcctctttatacccataatcttgtatgttgcatgtatggaaattaagattttcatttctatgtgtattttgtgtgctgctcttcat

cactttctcagtgtaatgcaatcagaaattttgttgttgcattagagagaaaatacctgtaagatgccagcagttcaattttaatctcatgtagaaagat

tgtttcagatatttaaattggagatgacagagtgatgttagggcagatgtggggcagtatgtagccgctaatttgtccaatgttcagtgctttgcattaa

gggctgacacatttgtaaatagtcagagaaactatcttaaaaatatggtttctgtattctatctttttgtgtttttcttttttagtctttgtatttcaac

agccacatgtgtgatatattgtctgtggtatgttattaaaagtataatgtaattatggaaagggatgtcacatacattgtaaaatatgggacacattgag

ttgttaagtttatcttttttgtagtagaaataattgttggagtaagatctatgtaattatttttattccaaccttaaatcaactttcagctttaaaatct

gaagctttaataaatatctcaatatcagtaacagagaaacttatcgataagaaagtcattgaagcatcaaaatgcatacgcatttccaatttgaaactaa

acagttttattgtagtagatattatatgtgacaaagatagtgagcagaggtcagtaaagaagcttagttgtttaattcgtgatattcacattctgtggtc

cattgggtatctatataggacagaggtacatatttttatatacattgggtggtatttaagtgtgccaaaagtgactgtcaccagtttcacttagtgagga

cccagcatttctgtgtcactgtggtaagtggtgaaaacacagaagtggagccaatgtgttatatttattcagatgaattttacagtagaaaatatctaat

catacttgtcatcttttatggtgtttcaccctaaaatccaatatttttttccaattgctgttgtgtctaccatttacctgattaatgattaatgattaat

gcagagttaaattgccaccactcacataactataaatacggactctacacaagcctccattcctcacagcctgaaatttttctattcaatttatgaatta

aaatgccttttatcaaacagatagtgttacacagaactccgtcagtcaccctacacatgataattacgacagcttgaggtttctgcctcacggaggtgca

gagagtattatatctaataaatgatgtgaagctgaactatgttgaagaccaaaatggtctgaaacaggaatccaattgtatcagcctgtcataaattgca

agtttaaaattacataatattctcagctaagagtaatgtggccatttagtcaactg

>51317|0908303Coxidase II,cytochrome

gctggtaaagctgttcagattgtttcaattaaatgtccatgaagtatattacggttagtagtcgaaatccatcattttctaggtcttgttctggtgttat

ataagtgtcaaattctacgtctatgaagtctgaatattcatatcttcagtatcattgtcgtcctatggtcgtattattattaattacagtaattgtaggt

tatgccctaagttatatattatttattgcctatactaacggtaatatacttcatggacatttaattgaaacaatctgaacagctttaccagcaattacat

taatttttattgcccttccatcattacgactattatatttgcttgatgattcagtagatgcaataattacaattaaaaccattggacgacaatgatactg

aagatatgaatattcagacttcatagacg

>51626|0908303Doxidase III,cytochrome

taaccctatacaaattccatgacttaatacagctattcgtttagcagcaggagtaacagtaacaggagcacatcatagtggaagggaatctaat

>51625|0908303Doxidase III,cytochrome

attagattccattaaactatgatgtgttcatgttactgttccgcctgatgctagaagaatagctgtattaagaaatggaatttgtatagggtta

>51616|0908303Doxidase III,cytochrome

ctatacaaattccattacttaatacagctgttcttttagcctcaagggtaactgtaacattagcacaccacagccttatggaatcttgcccagag

>51623|0908303Doxidase III,cytochrome

tagagcttgagtatgattatattccattaaactattatgttctcatgttacttttactcctgatgctaaaagaatagctttattaagtaatggaatttg

>51634|0908303Doxidase III,cytochrome

tcctaataacactgtgaagaatagtccttgtagagcttgagtatggttagattccattaagctatggtgtgctcttgttcctgttactcctgatgctaa

>51622|0908303Doxidase III,cytochrome

ccattacttaatacagctattctattagcatcaggagttactgtaacatgagcccatcatagtcttatagaatctaaccatacacaagcattacaaggac

>51617|0908303Doxidase III,cytochrome

ttgctccaattgctcctgttaatggtcaaggtctattgtctactaagtggaatgggtggtttgagtgagttatacagttattatacatcttttcagcaac

atgagataagtcaatttgctgaccatttccacgatttctt

>51615|0908303Doxidase III,cytochrome

aaccacccattccacttagtagactatagaccttgaccattaacaggagcaattggagcaatagtactagtatcaggactagcataatgatttcacctat

ttaatattaacttattcataatcggatttggaattaccctactaaccataattcaatgatgacgagatgcagtacgagaaggaacatatcaaggattaca

tacaggatttgtatcaattggattacgatggggaataattttatttatcgcatc

>51881|0908303aatpase 6

aatttcataggactattcccttatatctttactaaaacaggacacctagcactaacattcacaatcgccttacctatatgattaagatttatattatttc

gatgaatcagtcacactaaccatatatttatatactttgttccccaaagtacaccaagtacaccgagg

>51880|0908303aatpase 6

gtgtaccttgtcgtacaaggtatgtaaatatatgcttagtatggttaattcatccaaataatataaaaattagccatataggtagggcaattgcaaatgt

taatcaattttgtcaactatctggaaaccaaaaagagaaaagacatctaacccaaaaatattttgagccagcagcaaattaatgacttacgatata

>52197|0908303fprotein urf2

ttaaatattttattgtccaggcaatagcatcgacaatactattattttcaattttgttgagtcaaataaagtatccaataggatgggaaacagaatttat

cccctcaataataatcagatctaaattattattaaaaattggagctgcaacttttcatttctgatttccagaagtcataggcgcttcaagatgaaataac

tgtttaacattaataacatgacaaaaaattgccccaataacagtactatcatattgtattcaactaagaacctttatttgaaccattttcattttaagaa

ttgttattg

>52449|1,4-dihydroxy-2-naphthoate octaprenyltransferase, putative

tctctctctctctctctctctcacacacacacacacacacacacacacacacacacacacacacacacagtgacaggtaataagctcacagacacatttg

tgttgatacagtaaataagttcaagcaccattcagctatgaagctgtaactacacaggagtgaacacactgagtctttgttttggcttgaacatcccagc

acaaaaattcaaagaaatattaacaattaaaaacaatataggctctaaatcagaacaaaaaactgaaaataacagatatatgtgatgttaaaattctttt

gaagtgtagcatttcttgcttgtttagtctgtacagtactaactaaatctgtgaaagaagattttagtgccacttcatttactgccaaaacagagacatg

ttcacagaatgctaagcatggtatagactattatttgctacactgcagctacgttatttcaacaaaaattgaatttattttagcttatactgcttgtagt

taaatggctgcagatcataaaatcattgaatcctcttttaagtactcaaaccacaatataaaaaatgaacaatgacctacacatattgacaatgtttcaa

aatagaacatctaaatatttaaatattagtcttgataacttaccacatacagtcacaacaatgattacattagcacatattcatacaatgaaacatctct

caaagcagctgctcttattattgatatttaggcatatgaggtaatcctgtattgaaaccaagtcctgcataacgcacttacacctatcttggtccttatt

gtcaaacacatcacgaatgcaagtaggtatggtcattaactatgagatagacaagctaccacaacagagagagatatgaagagttaacatttcttcgtta

ctgactcagtgttcatggttttcctctggaatccattcagttaactctgcattctgagtatatcattaacttacaccacactttcacaactattgtcaca

acttaaatagcctaacacttcagtacaaatcttatcagccaaagaacacagcttcctacacgtataacatgaatttctcttcaagcacttgaagagggct

ttcaataaaattttgtgattcagtagcttgtgctattaaaccttccacatttatttctacaggcttgttactgcaaggatattctttgtttagtaacaat

acttgcttcaacatccattttgacaagcattttagataaaattgtaccactgccagtgacctaatactggtaatgctgaagaccattacagcgacagtta

aaaaattgagtaaagcaaataagtaacattttacaaattatgtttccaaaataaccagaaaggtaactgacagctaactatttaatgctgttctaaaatg

ccataggcctgaatagcaggaaatatgtataaagtttacaagatattacttatactgtcttttccattcatttttgacgtaacttcagttacatatttcc

cgaagtaatttaagatgttttaagccaaatctctcattataagtaaatcatttgatcagttagtaaccaaattaatcacttcataaataaaacaaaattt

tgaggtgctgataaagattcaataattgcacccagaatgtacataaccaatacacctctttagtgtgattgttgtagcactacactgaaaattatcagtg

tagaaatggcatatgatttgcatctactaggctgcaggcaagaacatacaaaaaaccaaaatataaatttaattttgcagtttttctaggtactgtctga

atagtctccaggcagcgaaaatgtttttctatgcgaaatgctgcaggtaatgtcgtaagtggtagcaggaaccacatagagtatctaagacctagagcca

caaataaaatatatggggtaaaaaggagaaatgcatacaaaatgtgtgaagctgtttgtccaatcaatatagcaagtgttacaattccagcacggctgtc

actttccagatctcttgtattgttgctgtgtaagatagcttctgtgtttaacgctaatggaattgcataataaatcgttccccagtcgacatgccctgtc

tgggacatgaaggaaaacaacactgatataggtccaaatataatgagtattataacgtcacctaatgcgatgtacttcaatcctattccaccagtataca

gaaatgaactagaaagtccgccaaaatacaccaaagccaagtgttccatttttgccggtgaaactgctgccaagcaaataaacccaaggcagcctgctgc

atacaacattgcaccaagtgtaacaacttcatccttagacaaaatatggtctacaaggatacgatcatcactctttcgtccatctatacccttcacgtaa

tcgaagtacgtgttcacaacattcccagcaccatgcactgtcacgactgtaaacaacgtcagtaataatataacccaactgaagtatccacacttgtatg

atagtgcacatcccaataaggtaggcatcaaagacccgcttaaggaccaaggccttagtgccaaaacatatgatgacagtttcataaacttcggtttggc

agtaactgtgtggtttgcctcttgatggcgaatattgttggtttcttctgtccccatatcttcaaaaatgatgtacgttcatttaataatatcgacaatg

gttttacagaattctgcctctatccgcgctcaacataaccattggataaggttagaattcgctttcgggaaaccaatacatgacagtatgccaataaaat

gataaattgctcactacctcagaagctctattttagagcgaaataaagcttagcagaaataggtatttagtgtttcctgctattccatgacactcggcat

ttgccttcaatttacagaacacattggtgacgtgtttgtttgtaacctacaacagctaacaacaatctgtgtttgatcatgaaaagaaatacgtttacgt

agtgctttaatgacctctacaattgtttataaaactgcctcgattttattttaggtaaaaatcgcctaaatattgcaacactatctttgacatatcaagc

atctatgacgctaaagtgaac

>52706|1-acyl-sn-glycerol-3-phosphate acyltransferase alpha-like isoform 2

cagcggtagtagtaaattcaaatacaagtgtacacagttgtcatgcatacatttaacacgcctaaccaaagaaatctcccaacactggagtccctcagaa

tattaatacacttaaatgagattatttgaaagtaacattcacaaaacacagagaagttatttaaatatgtgcaacttgcaagatgcagtacaatggtcac

tttacatcggtataacttaagtttaacatcctataaacagtgtgcgctcctttctacacacacacacacacacacacacaacttcagtctctttgcagct

tctctccacatctacatttatatactactctatgccctctccacaaatcaatgtaatggttacatcaattaatgaaattaaactgtaaaacacctatcac

acacaaagcccttaataacatagtaatttcagcactgtgagctgagttgcctacaaggaaggaagcacaaacattaatatacattcagatatatacaaaa

ggtcatgaagtagagctgcaggtttgctatgtttagcacaataatttaattccatcacatagtattgacttaaatcatcagttttttaactgaagttgtg

aatgtcaatactttcccactaatgttgttctgtcatacataaacattaaaattgtggttacagatgagatcatgaagatatgcttttgctgttactgtcc

aaccaactccattaaagttcaaaagcactctctctttattacatgtccttgttttttatattctgcagcagtagttctgttgatgaggcatggacatcca

acattagctcacgagttctctccacaagtgatcccacatcttctttcttcagtcctgtagtgtctattggtggaaggatggttatgacagcttttcctgg

attaaaaattttctcctttgactcaaggaaataatagcgtgtcacaacaactggctgaacaggtgtctgagaagcaatggcaacatggaaagcacccttc

ttgaagggcagcaatgtaggtccattgtttcttgtaccttcaggaaacatgcacagtttcatctttccattttttatggtgtctgcagtgctgtttattg

ctgcctgtgcttgttcaacattcagtctattgatgtagatggttccccacaaccaggcagcaagtccaaaaggccacaggtaaaaaacttctttctttga

aataacagtgcatcgtttcagctttggccaaagctcagcaagtacaagtaaatccagacaactttggtggtttattacaacgacacacccagagtcagta

acaacattgtccagtcctcgaagatgaaatttgatgccaaggatttttgatatttgccttgcaccccatgctggtaacagtgcgttgcgccagtccctcg

gccggaagagcatgagaggaatgaagacagagccgaagaagagggagagcagcaagaagacggtgaacttgatgtagtagcggcacgcgttgctgacgtg

>52705|1-acyl-sn-glycerol-3-phosphate acyltransferase alpha-like isoform 2

gctcgctacgggacggtcgtggaggcgacgttgctgctgcctgcctggtccccgtcgccacgcataccctgggtttcccgagcgcaaccctcactgaggc

cgtgtgcgacgcttcctgtgttccatcccgctgttggattttccaaagtaatcttccacacctggcagtacatcaggatcctttaggacgtgtagcaaga

aaaacgaaggatattcctccgtaatcagatagctgaaagcgtcttctacgaaggagcgatgacgctggccggggacgtggcgctggctgcggccgcggtg

ctcatactgctgctgctgtggcacgtcagcaacgcgtgccgctactacatcaagttcaccgtcttcttgctgctctccctcttcttcggctctgtcttca

ttcctctcatgctcttccggccgagggactggcgcaacgcactgttaccagcatggggtgcaaggcaaatatcaaaaatccttggcatcaaatttcatct

tcgaggactggacaatgttgttactgactctgggtgtgtcgttgtaataaaccaccaaagttgtctggatttacttgtacttgctgagctttggccaaag

ctgaaacgatgcactgttatttcaaagaaagaagttttttacctgtggccttttggacttgctgcctggttgtggggaaccatctacatcaatagactga

atgttgaacaagcacaggcagcaataaacagcactgcagacaccataaaaaatggaaagatgaaactgtgcatgtttcctgaaggtacaagaaacaatgg

acctacattgctgcccttcaagaagggtgctttccatgttgccattgcttctcagacacctgttcagccagttgttgtgacacgctattatttccttgag

tcaaaggagaaaatttttaatccaggaaaagctgtcataaccatccttccaccaatagacactacaggactgaagaaagaagatgtgggatcacttgtgg

agagaactcgtgagctaatgttggatgtccatgcctcatcaacagaactactgctgcagaatataaaaaacaaggacatgtaataaagagagagtgcttt

tgaactttaatggagttggttggacagtaacagcaaaagcatatcttcatgatctcatctgtaaccacaattttaatgtttatgtatgacagaacaacat

tagtgggaaagtattgacattcacaacttcagttaaaaaactgatgatttaagtcaatactatgtgatggaattaaattattgtgctaaacatagcaaac

ctgcagctctacttcatgaccttttgtatatatctgaatgtatattaatgtttgtgcttccttccttgtaggcaactcagctcacagtgctgaaattact

atgttattaagggctttgtgtgtgataggtgttttacagtttaatttcattaattgatgtaaccattacattgatttgtggagagggcatagagtagtat

ataaatgtagatgtggagagaagctgcaaagagactgaagttgtgtgtgtgtgtgtgtgtgtgtgtgtgtgtgtgtgtgtgtgtgcagagagagagagag

agagagagagagagagagagagagattccagccaaactcaacaagtatttataaatgcctgaaatcagggtaataaaaatactttcatgcatgtgaatgt

ttgtatccagaaaatagggtaataaaatacttccatgcgtgtgaatgtttatattaagaaaatagggtaataaaatactttcatgcatgtgaatgtttat

aataagaaataatgcatttagagctctgcatgatcagtaacctttccattcttattctaatgtaataaaatggagagactgtcatacattgcagtggtaa

acattataaaccgtaggttctacatactggactgatctagaaaaatccagaaagtgaaaatacttattttattatgaaacaagctcttttgtgtgatatg

ggtgtagagaacagatgtttgtgttgtctcagcacattctgcagtaaaatattcaccacacagttgaatgtgttgtttgtgctgcgttacgtttgattac

tgtgtgataatcaatacatggagagcagttcaatcacttaattaaatattcattcacacaaataataaaattttctgcacaaatatgaggaaatgtaatg

ataatatgaaaatttaattcagataatattttattaaatttttcacatttcattgatgtcgcatgttccctgaacacaatgctgaatttatgttcatgga

tacattcacaacagcataaaagtgagctacagtacatttgtttttataacttcttggcagaaataataagttcagagggggaaataaattgttaagtatg

actgcaggactgttaataaacactgattagccaacttatgtaatcaaatacaagtcttgaacaaataaaaacagtaaaacttttaatgaaaactttttgg

ttgtgaaatatgtattcattatttcaagtcttctccacacagtgtgtcattcaatttttattatctattgttttctttagcattcatataaaatgctact

gcaatgctttttaaattatgtgaagaaatgttattgactacagagccacagtaatacatagatgtatttttctatttcttaagactgaatgttaaaagac

aaatattatattttgcaaaggccttgaaatgttgagaggaaaatgttcaataagaagttagatatgtaacattgtaagcaaaattaaaatattaagagta

gtctgcagtatgtaactaaatttacatatgttagttaatatgctattgtttaccgtagaactgttcagaataaccagataatatctaatcagtagtatgt

aaacattcatttaaatttcttcatcttttatgtctagttatatctgaagttaccacatttgtttcattgtgcttccagctcaccaactgaacattctttt

gattgtctaaa

>52911|1-acyl-sn-glycerol-3-phosphate acyltransferase alpha-like isoform 3

gttctctccacaagtgatcccacatcttctttcttcagtcctgtagtgtctattggtggaaggatggttatgacagcttttcctggattaaaaattttct

cctttgactcaaggaaataatagcgtgtcacaacaactggctgaacaggtgtctgagaagcaatggcaacatggaaagcacccttcttgaagggcagcaa

tgtaggtccattgtttcttgtaccttcaggaaacatgcacagtttcatctttccattttttatggtgtctgcagtgctgaagaaagaagatgtgggatca

cttgtggagagaactcgtgagctaatgttggatgtccatgcctcatcaacagaactactgctgcagaatataaaaaacaaggacatgtaataaagagaga

gtgcttttgaactttaatggagttggttggacagtaacagcaaaagcatatcttcatgatctcatctgtaaccacaattttaatgtttatgtatgacaga

acaacattagtgggaaagtattgacattcacaacttcagttaaaaaactgatgatttaagtcaatactatgtgatggaattaaattattgtgctaaacat

agcaaacctgcagctctacttcatgaccttttgtatatatctga

>52912|1-acyl-sn-glycerol-3-phosphate acyltransferase alpha-like isoform 3

ttccaccaatagacactacaggactgaagaaagaagatactgactctgggtgtgtcgttgtaataaaccaccaaagttgtctggatttacttgtacttgc

tgagctttggccaaagctgaaacgatgcactgttatttcaaagaaagaagttttttacctgtggccttttggacttgctgcctggttgtggggaaccatc

tacatcaatagactgaatgttgaacaagcacaggcagcaataaacagcactgcagacaccataaaaaatggaaagatgaaactgtgcatgtttcctgaag

gtacaagaaacaatggacctacattgctgcccttcaagaagggtgctttccatgttgccattgcttctcagacacctgttcagccagttgttgtgacacg

ctattatttccttgagtcaaaggagaaaatttttaatccaggaaaagctgtcataaccatccttccaccaatagacactacaggactgaagaaagaagat

actgactctgggtgtgtcgttgtaataaaccaccaaagttgtctggatttacttgtacttgctgagctttggccaaagctgaaacgatgcactgttattt

caaagaaagaagttttttacctgtggccttttggacttgctgcctggttgtggggaaccatctacatcaatagactgaatgttgaacaagcacaggcagc

aataaacagcactgcagacaccataaaaaatggaaagatgaaactgtgcatgtttcctgaaggtacaagaaacaatggacctacattgctgcccttcaag

aagggtgctttccatgttgccattgcttctcagacacctgttcagccagttgttgtgacacgctattatttccttgagtcaaaggagaaaatttttaatc

caggaaaagctgtcataaccatccttccaccaatagacactacaggactgaagaaagaagatgtgggatcacttgtggagagaactcgtgagctaatgtt

ggatgtccatgcctcatcaacagaactactgctgcagaatataaaaaacaaggacatgtaataaagagagagtgcttttgaactttaatggagttggttg

gacagtaacagcaaaagcatatcttcatgatctcatctgtaaccacaattttaatgtttatgtatgacagaacaacattagtgggaaagtattgacattc

acaacttcagttaaaaaactgatgatttaagtcaatactatgtgatggaattaaattattgtgctaaacatagcaaacctgcagctctacttcatgacct

tttgtatatatctgaatgtatattaatgtttgtgcttccttccttgtaggcaactcagctcacagtgctgaaattactatgttattaagggctttgtgtg

tgataggtgttttacagtttaatttcattaattgatgtaaccattacattgatttgtggagagggcatagagtagtatataaatgtagatgtggagagaa

gctgcaaagagactgaagttgtgtgtgtgtgtgtgtgtgtgtg

>53431|1-acylglycerophosphocholine o-acyltransferase 1-like

aattgctgacactgatctgtacatcctctccgtctgtgctgaattaacccttccatagaaactttgctttgacatcaatgatgttgtaatgtctcttaca

ggtgctcatattcccagaaggaacatgtacaaacagatcatgtctaatcacattcaaaccaggagctttctacccaggagtacctgtgcaacctgtctgt

ctacgttatccaaacaaacttgacacagttacttggacc

>53430|1-acylglycerophosphocholine o-acyltransferase 1-like

gtctcgccctttgcaacaatactcggagcacccagatatacaactggcagtgcatcaaagtaggaggaatgcggcgctaatgccaggacgggcgcttcct

tagccgtcgcctgttttcccttcaccgtcacccagtggaatcctccacacaggaagagacccctcatcacacagcagatcacagcacgcgcattgcgccg

ccagccggtcatgggcctggagcgcaggtcgtcctcggagagccccagcagccccacgcacgccagcagccacgccagcaccagcagcaccatgatggcc

gccacgcggaacggcagcaccgccaccgtcagcacgcccgtcttaattttgtcgtacgttgttgacagttcgaggcggtgtacaaatggatttaacagat

cttctgcattcggcagatgttctgccgcatgtagctttccattcatcatggtgagatgaagacgccaacaataacaaataatcacgattagtccattact

tcacttaacgcgcggacaccacaatcacac

>53696|1-associated eps domain-containing

ttggtatctgcagacagccctcccagcccatcaccatcagcaggtggtgacaccgattcaccactacggggaagtaacaaggatgttagtaagtggacaa

agtttgtggactctcccacgagttctgtatcatctccaggtccaaagccagtgaattttgatttccataaatcagctgttgaacaggatcctaaaattct

gcatcctgtagctttgagagtcacaccggagtcacagggtgtcagtgtggttgcggcagttgctagtgcggcagttgaagaagaaccttgcagatcacca

cgaaaaccatctgaaccacaagccattccatatgaacaatttggtggaagtgcagactcaggcagccccaagaaagtactctctactgctgtgacgcaca

cagatgcagcaccagcagaaattcgacccattcagaggccacaagcaaagaaaccagcagctccgggacctggagcaataccccctccaccacagccagc

tgtcatcccaacctctgaagaagtcaacagcatacctggcgcccagtctgggccaaccagtcttccatcatctcagatagggcccaaaaaggagcctcca

cctcctcctccaaccagtcttccatcatctcagatagggcccaaaaaggagcctccccctcctcctccacctagaccttatcgtacgcatgcaaggagtt

catctttggacctcaacaggctgggtaaatctggtggcctgttaggtattcctccagttgtacctcctcgtgtatccccaagcacgacatcaccaaacaa

aaagctgatcgggcagcatagtgaaggcgatgtgctgtcactagtgtcagaccaaactggatttgcggattttggcagatttgggcaggaggaagaagag

actgcagaaatgcagatgccaagaaaacatggggcatttgaagtttaccggaaacctactgcccgtgcatctgatgctggagttggtgtccttcctgaac

aggatagcccgcaggatgacagtcagcaacaaaggcaccagcatgagccatcaacttctaggccatcgaacaccctgcgtcaacttcaagaacaaaacac

ac

>53695|1-associated eps domain-containing

aagtgggagagatgccatgttaatcagagtgcaggcagccctgacttaatacagctcagtgacactgatagaagtctgaatcaggtggctgctgggagaa

tggcttgcaatagtgatgtgattgggaggcattccccaactcataatgggcgtgctctgtcactatcaagtggacagaagagtcgatcgccagaagcatc

aagcacagccagtgactctcctacccccactaacagtgtacaggagcgtaactgggcagcagctgcacagtggcatggaatagtatgtgaagaacaacga

cagttacttgggactgaagaggagtcatcggaccgccatagcagtgatgatgatggtgacccatcagaagtgtggacaatcactgaagagcaacgagaat

actacactgctcaattcctttcactgcagccagatcctgcaggattagttgctggttctgttgcacgtcttttctttgaaaaatcaaggttacctgttca

tgagctgagaaaaatatggcaactcgctgatgtaacaaaggatggtgcgctctctattgaggaattcaacacagcaatgcaccttgttgtactccggcgt

aataacatagatctgccagactgtttaccaccttctttggctccaccactcccggcctcggtgacccagcctccagcacccccacaagcacctgtacaag

ttaatgcagttccaccccctgcagtggtacccgtacccgagccgacgacaactttggtatctgcagacagccctcccagcccatcaccatcagcaggtgg

tgacaccgattcaccactacggggaagtaacaaggatgttagtaagtggacaaagtttgtggactctcccacgagttctgtatcatctccaggtccaaag

ccagtgaattttgatttccataaatcagctgttgaacaggatcctaaaattctgcatcctgtagctttgagagtcacaccggagtcacagggtgtcagtg

tggttgcggcagttgctagtgcggcagttgaagaagaaccttgcagatcaccacgaaaaccatctgaaccacaagccattccatatgaacaatttggtgg

aagtgcagactcaggcagccccaagaaagtactctctactgctgtgacgcacacagatgcagcaccagcagaaattcgacccattcagaggccacaagca

aagaaaccagcagctccgggacctggagcaataccccctccaccacagccagctgtcatcccaacctctgaagaagtcaacagcatacctggcgcccagt

ctgggccaaccagtcttccatcatctcagatagggcccaaaaaggagcctccacctcctcctccacctagaccttatcgtacgcatgcaaggagttcatc

tttggacctcaacaggctgggtaaatctggtggcctgttaggtattcctccagttgtacctcctcgtgtatccccaagcacgacatcaccaaacaaaaag

ctgatcgggcagcatagtgaaggcgatgtgctgtcactagtgtcagaccaaactggatttgcggattttggcagatttgggcaggaggaagaagagactg

cagaaatgcagatgccaagaaaacatggggcatttgaagtttaccggaaacctactgcccgtgcatctgatgctggagttggtgtccttcctgaacagga

tagcccgcaggatgacagtcagcaacaaaggcaccagcatgagccatcaacttctaggccatcgaacaccctgcgtcaacttcaagaacaaaacacactg

ctgcttagagtgtgccaggaactgaaccaagaattagcagaagttcaagaagaaagggcctctttggaaatgcagttggagcatctcagaagtcacctaa

cagaatgaaagcccttaaattaaagagtatattgtgtactggaatttctcaaagtactgtcttgtacttttctggatacaaggcaattttcagttataga

attctttattgcatggataagagacagttgtggagtctaaaaaatatatatgtttattatgctattttgtaataatctatgaagagaattaaggttgaaa

ctgctagatagtttttgacatagatgtatgttaaaatgtgcagtttattttatatgttgacatgtatgtcagaaagaagggaagaaatctgttagataat

tttaagttccaggcagttactaatatagtaaaaagacctccttaaaatgtgttgtttactgagaaacagtcaatggcattgactgctaaatgtgctttgt

atgtagtcaagagatcataaaaaacctatgctggacactccagattggcttgttttgcagagtatatgcagttactatggtgttctaaaataatgcttca

aggtgccacaagtattattgtgttagtgctgtacattttgtacattggtgctcttgttcaattgagttgttttgcctgagaagtttggaagttgtggcca

gaggtgtattttttatcttgggagtttttcctagacagattacctctcttgtgctcaatatatgccttttatattgttattgttgacctttgtaattgaa

catgtagttattaatatatgagagcatgtaaaagcaaagattccaaattgtgaatgtaatttttaaatttactatttttttaattcattgtaatgataat

ggggatgttattcaacattatttttgtttgtttgtatgtgaagattgaagtatacatgtttgtctttaatatactgacagttttggttagttactgttgc

tgaaacatttatgtgaagttaaagaggcagctaatccaaaaaagttgtagaagccattatatttgcctgctgaccttgttgacagaaaattgtcataaat

tgcccattcatatggcagcatctccattatttactgtctgtgtagagatggaagtgctctgattgaaattttatagcaaaattcttagtgcatgtgtatt

gatagcaggttgaagtatggaagagttccagaccagatctagttgcatgagtgtattgtccagtgagtgacgtgggaaaacttttttgtgccataaaata

agataatttggaagtaaatgaaactgggaatgtacagtcagtgtcacaaaaacattggatggagcttcatgtaatgctcacctttggttacacacattca

gaattagagatacttggtactgtcgcagtacttgttgtgttaagacttttatgtgctgatgagttcatgaatatttgtatgatctgattttcttttcttt

ttctgttttgtatcctaatgagagtgaagctttctgtgcacttgaaaggatggtagtctacattgtgtgtgctaatttaaaacaaaacaagaaaatccca

ataattatgaactagagtagataattaaaaagactatacaaaggaatattgtcaactgtgcgatattgtaggaaaaaaactgaattgaatcttcatattt

tgcaaccaaagatttgctttcaggccaatggatagcagagattaggacatgattaaaaataggatcccagaatctatagaaaatatactatgaggctcct

taaccacccttcccaatcctcaattaaaggtgttgcatcaaaagcttgtataggtagtgtattaagattgaagtattttgctgtttttaacatcatagat

ctgaattacatgagtaattcagttaagcatatgggtaaattgcaaaattccaatttccattgtcaatctcatttattttcatctctctggctatttgttc

tgtgattaaagagccttgtagcagttcatattttgtgatcgaaagtccatactgctaactcatgaattacctcgaagcaagagtttttttttccaaaagt

tatgtattcacataattctgtttttggtaactgtaaccagtataattccagcaagttgtatattggttatttttttcacttaacgtggttatagctgtac

aaagccaattcacacagagtttattgatccatgccgtaccctttcttatcagtatgttaccattatttttatacaaaagaaaaggaaaggaaagaaacaa

gcaccaatttttgttccttcttcaacaacttaatattcaaattctgtttgttttgttattgtaattgtttgtaattaacaaggtcatttctttttctaac

agaatctgcaattcagactaatacacagaaaacattgtttccatctctctaatccctaatcccagcaatcaatttaaaagaacttggggatgtgttccat

tg

>54344|1-o-acylceramide synthase

tctgaaattattgttgcaatctgtttgctattagttcattgtttggttgtttatggtgtttacttctttttgaggttggtgtataaaccaggaggatttc

ctagtgcataccctgacttcttatatggtgatggtgatggaacagtaaataaacgcagtttggagggctgtctccattggcaaggcaagcagaaacaaaa

agtataccatcagacttttccaggtgtggatcatatgaatgtgttaagagacattcgtgtgcttggctatctgaaaagtttactcaataaattatagttt

ctgtatcatagtgtagtgttttgaatttttttattctgttgtaagtagagacatgagtaaatagtattttgcagtataaaagtacacctgagatttcctg

ctgtacaaaaactttgattgttgcaaagagaacagggtttccatttttatccacatgtgaacttagaaaaaaatctcattaaaatattcaaactattgta

cattagaaagtgagtcggaagaaaaattaatttgttaatgattgatgaaacagtaatttttgataacttgctattagatataaaaaaatatatagatata

ctagaaaaggagacatggaataaatacagcaatgagggcttaccgtatctccaaagaaataaaaactgatctggctagaaactgcttggtgattgtgaac

aaggtcatcaacatttttttacagcctagaagaactgttggctagaaagtactgatagatgaaatgacggaaatgtaaaaatattattgggattttaaat

atacagggtgatcaaaaagtcagtataaatttgaaaactgaataaatcacagaataatgtagaaagagaggtacaaattaacacacatgctgtatatgcg

gatggatatgtgtgtgtgtgtgtgtgtgtgtgtgtgtgtgtgtgtgtgtgt

>54688|1-phosphatidylinositol- -bisphosphate

cttcatgtaagcaaataagaatattaaccgaagtgttggagtaaatgaaggaagaagaggaagagagtggttttcatagacctaaggagggcattccaaa

tgacgatctgcacttcagtgaaaaatggttccatggaaaactggcgagtggcagaacagaagcagagacgttactaaaagattacagtcatttgggagat

ggcactttcttagttcgggaaagtgaaacctttgttggtgactactctctctctttctggcgacagggaaaggtcaatcactgtcacattaggtcaaagc

aggaaaataatcaaaca

>54692|1-phosphatidylinositol- -bisphosphate

cagtatgttattctggtctgtgcaaagtacaaataatttttttttcttttgaaaaaaactttactgacatgcaaaatgtaaaaaaagcatttctccacaa

aatttcaggcatgaactcaaccccaacatgtatttagttcacttttaataaaatcactgtcttacagaagtcaccagaaaaattgttatagcatgtcaca

taaattctgattctctaagtttacatttttattcatgttacaccatttcttaaatatcttcctaaatacagaacacataatatagtattattgtattacc

caaaacttacttgtgctatgtctttctttgtgcaacaaaaattaatatatgtaagattaacaaaatcagaaccataacaaatggtaacagtcttttcttc

taaagtgtcttgggatcccattgtaacaatctgcttcagcttagagtctttaggaattttagcatattttccagttctcgcatctctaatagaagtaatg

tccagcatgtccatttcctcatcccacttgatgaatttttcaccatcttgcatacactgtggcacagatacaggtttcaaaaggctgcattgtgatgtag

attttgtaccagccatacttctgtcaacagcacaccacaaactttggcctacggatgcactgtaggcagatttttacgctcattactccttcccaacagc

cacatcctagtgtcaaatcataggtaatgctgg

>54691|1-phosphatidylinositol- -bisphosphate

ctcgctccctctcctacgtatttctggacacgataaccattactgcttccatgagttttatatattttatccacaaatttacagtttctgattattatta

ttatattgtaatcttttgtgaaattactttaaattctaagtagcgtttattacaggtcatatctggccagtttctggccgacaagaaagttactacttat

gtagtggtggaaatgtatggtctgccaacagatacaatcagggatgagttcagaacgtccaaggttacaggaaatggcctgaatccagtatacaatgcag

aaccattcctgttcaaaaaggttgtgctgcctgagcttgcgatgttgaggtttgctgtgtacagtgagaaagggaagcttctggggcagcgcattttgcc

tctggatgggctgcaggctggctaccggcacatctccctccggacagaggccaacttccccatgtcactgcccatgctgttctgctacatagaactcaaa

atatatgtgcctgagcactttgaagatttaatgaatgccctgtcagacccatgtgctttcttgtctgctcgagagaagatggtggagaatatgaaggcaa

caggtgccgaacagatggaaatgttgactcctgaaactgtggtaggtgatggaagtgaaggaggagctggcgacagtggtagcgatgatggcagcagagg

attgggcagcagcagtggcggcagcaggaaatctgggaagtcttccgaccgaaggtccagtggccctgcaacacttgaggaacactatgaggagatggag

tttgatgcaatcac

>54693|1-phosphatidylinositol- -bisphosphate

cagcttttaagacttcagattaccctgtgattctgtcatttgagaatcattgtaatcctagacaacaagcaaaaattgcccagtattgcagggaaatctt

tggtgaaaatctcttggatgcaccgttggaatctcacaagctggagcctggctcagagctgccacccccttctctactgagacgcaagataataatcaag

aacaagaagaaacaccaccaccatcaccataaaaaaaatcaaaactcatcaaatacatctgatggtagcagtgcagttgcagctacccagggaaatggtg

atgttccacatgtgcatgcccctcctcttcagtcacgccaaggaagcaaagacagcacacaagatgatgatgacgacgatgatgagagcagcagtgctga

tgaaatagaagatgatgttgcagaagaaacaaagggtttacatgataagattcctgctgagaaagcagctgctcaagagactgaggctggtgctgaaata

tcagcattggtcaattacgttcaaccagtacacttctcgtcattcgaaaattctgaaaaaaagaatagaccctatgaaatgtcatcttttgatgagaagc

aggctacaacacttttgaaggagagtccagtagaatttgttaactacaataagcatcagttgagtcgtgtctatccttctggtacacgttttgattcttc

caacttcatgccacaagtgttctggaatgcaggatgtcaactagttgcccttaattatcaaactctagaccttgcaatgcaacttaacttgggaattttc

gagtacaatcaccgttgtggctatctgctaaagccagaatttatgcgaaggcaagaccggagactggatccttttgcagagtcaacagtagatggcatta

tagctggtacagtttcagtacatgtaatctcagcacagttcctcactgaccgtcgggttggtgt

>54689|1-phosphatidylinositol- -bisphosphate

ctggtttccttcaaactatgtggaagaatgggattctcaagagaacagagatgataattcttcagattcactactccttggcagtcttcagaagggcagc

cttgatgtgatgggtgctattgttgaagtacatcgaaatgaccgcccaggactggagtgggtactacgtatacaaaatccaaatatgtgctcagtatttg

aagtagctgtacctaggatagaacttgctgttgaatggaggaatgccattaaggaagtggcacaaaatgccagtgtacgggaaaatcagcacaaggaaat

ggagaagcaattaagaatagccaaagaaatgtctgacttgatagtttattgtcgatctgtagcatttaatatggaaaaattcaggagaggcttcaatcat

tgtgaaatgtcatcatttcctgaaactaaagctgaaaaactgatatgtcagcaagagacaaaattcttcttgaaatatcatcagcaccagttcagtagaa

tttatccaaaaggtcaaagaattgattcctccaactacatccctgttccaatgtggaatgctggcagtcagatggtagctcttaactatcagacaccaga

taaagcaatgcagctgaatcaaggaaaatttcgtcagaatgggttctgtggatatttgctacggcctgatttcatgttccttgaagactttgacccatat

gataagaaaactttagttggagtagatccactgaatatttctgtacgtgtaatttgtgcacgccatctcagcaaatggggtaaaggaagggcaagaggga

ctgttagtccttttgttgaaattgaagtgattggtgcggactttgataacaaaagcaggaacactcaaccagtttctgacaatggttttaacccagtttg

gaatgaaacttttgactttgaggtttccaatcctcattttgccctgctgaggtttgttgtccaggatgaggacatgtttggagactcaaatttcattgga

caagcaacctacccagtaagtatgattttcagtttgatattgtgatcaaaagggctagaatcttattctctttatgttcttctgtttgccatttgaaaga

aatgagaaacaaactccggctgtagaacttttgtatgttaagcatttaaaggaggggcaagtcactcaggtcctacactgagagggacatgcttggtgtg

agggtaagagcagacaaaaatggaggggg

>54687|1-phosphatidylinositol- -bisphosphate

caaggaaacagtggctggtggcgtggagactatggaggaaagaaacaacactggtttccttcaaactatgtggaagaatgggattctcaagagaacagag

atgataattcttcagattcactactccttggcagtcttcagaagggcagccttgatgtgatgggtgctattgttgaagtacatcgaaatgaccgcccagg

actggagtgggtactacgtatacaaaatccaaatatgtgctcagtatttgaagtagctgtacctaggatagaacttgctgttgaatggaggaatgccatt

aaggaagtggcacaaaatgccagtgtacgggaaaatcagcacaaggaaatggagaagcaattaagaatagccaaagaaatgtctgacttgatagtttatt

gtcgatctgtagcatttaatatggaaaaattcaggagaggcttcaatcattgtgaaatgtcatcatttcctgaaactaaagctgaaaaactgatatgtca

gcaagagacaaaattcttcttgaaatatcatcagcaccagttcagtagaatttatccaaaaggtcaaagaattgattcctccaactacatccctgttcca

atgtggaatgctggcagtcagatggtagctcttaactatcagacaccagataaagcaatgcagctgaatcaaggaaaatttcgtcagaatgggttctgtg

gatatttgctacggcctgatttcatgttccttgaagactttgacccatatgataagaaaactttagttggagtagatccactgaatatttctgtacgtgt

aatttgtgcacgccatctcagcaaatggggtaaaggaagggcaagagggactgttagtccttttgttgaaattgaagtgattggtgcggactttgataac

aaaagcaggaacactcaaccagtttctgacaatggttttaacccagtttggaatgaaacttttgactttgaggtttccaatcctcattttgccctgctga

ggtttgttgtccaggatgaggacatgtttggagactcaaatttcattggacaagcaacctacccagtgctctgccttcgaacaggatacagaagtgttcc

actgaaaaatggctatagtgaagatcttgagctagcatcattgctggttcatatcagtataacaaatccaatggaaagcagggaagtgcttcaaagtact

gccttttcttgtgtaactgccggaggagacagtgaagactgaaatgccaagttgatgtggattttctgcaatgagaaggtgaacaaatgtgactttttac

aacttgagtgtgaaatacttggtttgaaaaccaaggtgcatttaatgaacaaaag

>54690|1-phosphatidylinositol- -bisphosphate

gaattctataaaatggaaagtggcagagaaactgtcacactgaaagacctaaaggcattccttcctcgtgtcaactgcaaaattccaacaaataaacttc

gggatgtctttcaggaagtcgatacaaggaaacgaatggaactaggatttgatgattttgctactctttaccaaaagcttatgtttgataacaatatgta

tagtgaaagttttagtaagttattctctgattactctgaaaatggtaaaacagtaacattgcaagaatttcaaagttttcttagtcaagagcagagagac

ccaatgggagatgatgaaagagaagtctcttcctttatgaggaattatttgcaagattcacagcgaaatgtccaggagccatacttcacaatctttgagt

ttatggattttcttttctcgaagcaaaatgaaatttgggatgaacgatatgataaagtctaccaagacatgacgaaaccgttgtcacactattggattgc

ctcatcacacaatacatatttaacaggtgaccaattctcaagtgaatcatccactgaagcttatgttcgctgtttacgaatgggttgccgatgcatagaa

ctagactgctgggatggtcctgatggtatgccattcatctaccatgggcatactttaacgacaaggataaaatttctggatgttgttaaaactatcaagg

aacatgcatttgttacatcagaatacccactaatattgtccatagaggacaactgctctgtaccacagcagcgcaagatggcaaaagtaatgatggaagt

gtttggggaaatgctactcgtgcagctttatgagaagaatgaaagtcagcttccatcaccacaccagctacggcgtaaaattataattaagcacaaaaaa

ctcccagatggtgtcgatgaggatgcttttatgagcaaaaatgatgatgcaagggacgtggatctcagaaacacagttaaaaatggcatcttgtatctgg

aagaccctgtggacaaggaatggaataaacacttctttgtattaacaaaaaataaattattttacaccgactcctttcatgatgagcaagaaggtgaagg

agaggaagaagaggaagagagtggttttcatagacctaaggagggcattccaaatgacgatctgcacttcagtgaaaaatggttccatggaaaactggcg

agtggcagaacagaagcagagacgttactaaaagattacagtcatttgggagatggcactttcttagttcgggaaagtgaaacctttgttggtgactact

ctctctctttctggcgacagggaaaggtcaatcactgtcacattaggtcaaagcaggaaaataatcaaacaaagtactacctcattgacacaaattgttt

tgatagcctgtacagcctgatcactcattatcaatcacatccacttcgtagtcaggagtttctcatcaagctgcagaaacctgttcctcaaccaaataaa

catgaaggaaaggaatggtattatcctcaggcaacaaaactaaaagcagaagaactactgaaaagggtccccgctgatggagcatttctcgtgagaccaa

gtgaatctgatactgatgcatatgctatatcattcagggctgagaaaaagataaaacactgtagaataaaactggaaggacggctgtacacaattggaac

atttcagtttgaaagcctagttgagcttgtgagctattacgagagacatc

>54686|1-phosphatidylinositol- -bisphosphate

cacacactcacacacacacacactcacacacacagtgaattcaagcacttctcactaacaaaaaacaagtctaatcatgtaatgcacgaagagaaacaca

taatatttcaattaagcactttttcttaggaaaaatggcatcaactcttacagaataaaatatgatcaaataataactgcacacattaagtatggcaaat

gcacacttagaaataaaaaacagcatgagaagaaacattgttactaacttcattataatgaaatacatattatgaatcaggaagagattctcatctaaca

aactagaccatgaacctaaagcactttatcagttaatatttccatgaacatgttattcccctgccttctttcacattaataatcaccttacagttcgtat

cattacttggcacatcttgtgcacacaaacactctagtgcttcaaccattagatcatgcattttttaaaccgcatataacataactgtcatattcttcct

cctctagggttttccctctatgtttattccattactataataatcagatcatttaaaaattaagccactcattagtgaatgcaatttattataagttatt

ggcctgacaaaaaatactgttgcgtgtatgatacagacacatattatttaaaactaactattgacatgtggctcttctaaaagcttaaggaaccagttac

attttgcacacaagcacacattattgtacaataaagataaaaacatatcctgcatttgaaaaatattaaacaagcacatacctttatgattaaaaagaag

gctctccattggaccttagtggcatttagtatcatcctttttgtaacagaatcttccatattcttctcattgcagaaaatccacatcaacttggcatttc

agtcttcactgtctcctccggcagttacacaagaaaaggcagtactttgaagcacttccctgctttccattggatttgttatactgatatgaaccagcaa

tgatgctagctcaagatcttcactatagccatttttcagtggaacacttctgtatcctgttcgaaggcagagcactgggtaggttgcttgtccaatgaaa

tttgagtctccaaacatgtcctcatcctggacaacaaacctcagcagggcaaaatgaggattggaaacctcaaagtcaaaagtttcattccaaactgggt

taaaaccattgtcagaaactggttgagtgttcctgcttttgttatcaaagtccgcaccaatcacttcaatttcaacaaaaggactaacagtccctcttgc

ccttcctttaccccatttgctgagatggcgtgcacaaattacacgtacagaaatattcagtggatctactccaactaaagttttcttatcatatgggtca

aagtcttcaaggaacatgaaatcaggccgtagcaaatatccacagaacccattctgacgaaattttccttgattcagctgcattgctttatctggtagct

cttaactatcagacaccagataaagcaatgcagctgaatcaaggaaaatttcgtcagaatgggttctgtggatatttgctacggcctgatttcatgttcc

ttgaagactttgacccatatgataagaaaactttagttggagtagatccactgaatatttctgtacgtgtaatttgtgcacgccatctcagcaaatgggg

taaaggaagggcaagagggactgttagtccttttgttgaaattgaagtgattggtgcggactttgataacaaaagcagg

>55141|1-phosphatidylinositol- -bisphosphate phosphodiesterase

tcttcctcttcttcctcatcttcttcaatgacaaactgtccctgccagaacagttccaactcctgcttttccacatcaggatttagcttcttgtttttga

tcagaattttctttttgagggcatttggtgaaggcagcggtagtccaggttccagcggaaaatctgggagtggttccttgagcagcagatcacccaggat

gtcattgcagtatctggccagtttatactgttgagccttgctgcagtggttctcaagagacaggatgacggggtactccgatgtcacaaacgcagtgtcc

cgtattgcatgtatgacatccttgaacaaaatatctgtacatgctgctctgccatgtgttattattggttcttcatcatccctcgttccatcccaacagt

ctaattcaatgcacctgcaaccagcaagcagcacttggcggtacatttccacagaggatttgccacgaaactggaagccggtcaggtatgtgttgtggga

gctgttaatgtagtaatgcgacaatggctggtccatgtccatgtacaagtccagtcgatccaagtagacaggtgcattctcatctgacatcaagtactgg

atcaagccatcttttgataatttgttttccagccttgtccgctcatctggctcatatgatgatattatcttctccacacgccttttgtcatataggggat

acaggatctcattcagtctttgatcacgttgcttctcattgagaaatgtaatcaactgttcaatactgatcttgcagcttttgccttgagtgattgattg

gaaaagttcttcaatgtcagttcttggacagatcttgtggtacagtgcataaaacttgttgaatgtgaaatctgctgcatcaattgaagcatgttttctg

tggggcaggcccaactcagatagacatttgtagaccagcttctctgtttgtccagatgcaaaagttttggccacaactctaacaggaactttgcccttcg

gattcacgagcaaccgaagacgcatccagtgtttctttaagcacatcattgggcatatgttgtctgctttcacattgtgtgtgattttcctcagcccatc

taaccagaccttggcgatgtcagtatctggacatattacatgctggtagtttatgttgacataatctgtaccactgcatatagttagtgacttttcttcc

acatcatcaccatgttttccacgtaattgtgacagcagtttctgatcctttggaaggccaccagctctaatgtcactaacttgggacagttctatgacgt

ttccatggtgg

>55140|1-phosphatidylinositol- -bisphosphate phosphodiesterase

atttttcataaatttacatttttattattgaaagaaatacaaatgtacaaaatagaaccttacaaaacttttacatattctttattttgggaaattctac

ataaatatcattacaattttttttacaaaatgaggagttttatatcagtacaaaacatacaaaagcctgcagaataacaagagtatgaactgcagtagct

gcccagaaaatcagacatgtgtacaaagaaataattatagtacacttcaaaatgtatataaaaatttccaagcgcacaacagcattttgaaatacccata

aggctctctatgtctactagctggtacactcagtgtaagcagaaatgactgtcatcagctttagaaagcagcatctgcattcaacacatacaacttaatg

tgctagtacaactatatcttatatttaccaaatgcaatatatttcactgcacgttgctctcactgtgtgcaatagttgaattcagtatttgcgtttctac

gtggcctctttaatggacacatgtacaaaaagtgaagcaggtgaaacataattcagctcataatccattaagtaaacatgcctgtatccttccgttacgc

tgttaaacgggagtgcataacttccaagaaatgcatcttttcctgtactattcatatccttcacaatgaactgcaccatagcaagctcaggcattcttac

cacaaaatacattctacagtcccaccttggattgaaaccattgtctcttacaggatctgttttttgtttctgaatatcaaggggatgtcccagtattttt

actttgacatagggatcaactatttctccttctgagtcttggccaggcttaggaatgtgctgcccacttatgacagtcagtgtgaggtacttgggtttgc

ttccactgtaaaccatttctggatcatatccaacatccttcctgagatacataggtttcaggacgtatccacaagcaccattctgtcggaatttggcttc

attgtgaaacattggctttcctgcactctgatagttaagggcaacaatttgacacccaacattccaaaatggaattggtttgtaattggatgacattgct

cgtctaccagaaggatatatgcggctaagctgaataagattgtgtttcacaaattcgggaccacttttctgaattaatttcatggcttttctttcattaa

atgatgacatctgatagcatttaccttgagttaaagtcttctcaaagccatgaaagtgaacagcttggcaaatatttacaatgttggataatgtgggaga

aaccactgttccctgagctccttttgtctccatcttattattacagtcttcttcggagtcctcatcatcatcatccatatcatcatcctcactataagaa

tcatcttcatcacttctatcagaaggttttaactttttagctttcacaattattctgtttttcagatcttcaggagatggcaggaactgcattccttctt

catccactggttccatatataagctctctcctaaaacatccactaaaatttgtaccagaacatcctgttgtttttcactacagtgattttcaagagataa

aataagagggtattctgaggaatgaaatgcatatggttttatggcatctgtgatcacatctctaaataaaatctttgaagtcagagtgtatccatggtaa

ataactggttcaccatcagcaccatcccaacaatccaattcgacacagcgacaatcttttttcagagcgctgatgtatccctctacactgctgtcccctc

ttagctgccctcctgtgagatatgtgttatgggatgatgcaatgtagtagtgattcaacggttgagacatatcttgataaacgactttgtgatctggcct

gaatatttcaaattcttctgacatcagaaggtttgtaaaaccaataaatgacagtttttgattatttctcatttctgactgttcaaaagcctcaatccat

gataaacattgctcctccgttacattgttcaaatgttgttctttcttcataaagttttgcagctctttaacgcccatttcttcaggatttttagcatacc

taataaataatggcttcagttcttctcttgacatgaggctaaagaagaaaccaacaaactcctctgaatctaaaacttgttctccatctcgtaagttctt

gttagtattggcaatctcaaattttttctttacttctgatttcaccatcttcacattgagctgctttaaaagtctcagacattcttcaaagttcaagcta

ccattttcatcttcatctgcctcttgaaattgttgttttaaccaccgttcgaagcgctgtccctgctggaggcccttgatgactgtgatgatgtggcgca

gccccacgacccaggtctgagccagctctggactggtggccacgaggtccaggctcttcttgtttctgccgtgtatcaccgagaaacagcacgcttcgtc

gatgaatggtggccagccagtgttgttcttcccgaacttctcctccaccttcttggcgattttattgaaagtgtccgtcttccagccgtgcctcacgtcc

agaatatcgtccacttcaatctcgtcaggagcgctggagcagagcactttacgcgagacctcatatctgacggtcccctcgtctgtgtccagcacgtact

tccgccggtaccacttcgagtaggaccgcactttccacagcaccgttcctttccgcagaacactcagggtctcttccagtggcttgctgtgcctgtcgat

ggcatccctgaactctggcgactgcatctgcaggctgtacgtgaccttggtctccatggcgctgacgaggcgtgcgttccgcgcgctgccacagccggcc

cgcgactacggcgac

>55462|1-phosphatidylinositol- -bisphosphate phosphodiesterase classes i and ii isoform 1

atcccatatttgacgaggaagctttcgtctttaaaaaggttgttctcccagaattggcgagtctacgtatagctgcatatgaagaatcaggcagactaat

aggacaccgtgtgctgcctgttgttggcctgtgcccagggtaccgtcacattgccctgcgcaa

>55463|1-phosphatidylinositol- -bisphosphate phosphodiesterase classes i and ii isoform 1

ttgcgcagggcaatgtgacggtaccctgggcacaggccaacaacaggcagcacacggtgtcctattagtctgcctgattcttcatatgcagctatacgta

gactcgccaattctgggagaacaacctgtacaaaaggaacatctaatgttaatgttctcacactgtctgaaaa

>56148|1-phosphatidylinositol- -bisphosphate phosphodiesterase classes i and ii-like isoform 2

atgtaattatgtgttcaagtactgttttgtttttttatgctgctgtaaaaaaaaaaagaaaaaaaaaagctaggtttaaatctaaagaagcttatcatga

gatgtgttgtgtttgtgtcacttaatgcctgcaatgtaaatacatagatggcacattgcattgacacatgaaagaaacttcagatgtaatgtgcaggaca

tcttaaatattataaaatatacaagaaataggaataattctatttgtatctgcttggcttctaaaattgtattaaagacatttaatatctattcagaggt

tgtctccataaacacaaaagaaatgttagttaaaatccaagaacaatatattccattttcaacaataggtagtgctgtgaatgactatcaaaaggcttaa

aatctaaatatcaaacagaggctcccatggaaaactcagttatagataaaatgagatggtataatattgactgttatagcactagtgatgtaataaaaat

gcacaaatgtcaagcttaaaataataaaaagagagtggagggttctattaatagggaaacaatgttttagtatgaagcatttcagtagtaccatactttg

tatgaaattaataagatggtggatacaacaaatcacacaacacaccgaaagaacaaaatgccagttctttctattagagcactgtagagcaaaaagaagt

taaagtaatgatagtgtacaagttggcacatatctaagtagataacaaacagaaacagatcaagcacaatggattgcacattacaggcattctatcaaac

tcaagcaaaagctctaaagagaagactttgcatgaactctatgagatcaaaagcaaaataataagatattatattgcacagctcttaaaacctctttcac

aagggaaagaactgactacggacattgaaatacagtaagggatggaggaggaggaggaggaggaggaggagcaggaggaggagcattaaaatttaaatct

gtgttttttaaattgctgactataaattcaaaatctcacaaagtgttcaattatgccagtaatctaaaaagaagtacataattatttcttagcagcattc

aactaaatgtggcataccacaagaaacaatgaaggcattgtgttaaataataagtgacaagggaaccctgtgtcattgtaatactagacacacacagtga

tgagtggctaagcggtgcaccgggctcatgtactcttcattttccttataatagcatgatctttcaactttataagaaaacaactacaatcaacactaga

aagtagagtgtaattactgtaacacattgtgttaatatgttgcaacagacttaatttaagacccattcgctttctggtcatttaatataaatacaatatg

aatgggatctatttttttgagacgacatgcttatttttttgaaacagcgttgcatgcttctagcagtctagttgctatcgtctttatgtactgtaaaaac

catcacaatgagtattttatcatcttatagctaaatgatgctttggtataatatgtacaatgatgtttaatgtcactaatcttagctaatgacaatacat

attaagtcgtagctataaacaatgcttacagagtatttaatgactggtcatctgtgctgcttagcagcagctagaaattttaaagtgtgagccactaata

caagttatttctttacttttacaataggaatgggtatcctttgtagcagctactgattgtcctaacttttaccgcagattcttaaactttaagtatacaa

tgatagttaagatcttgcctccgcaacccttcaaaagggatgttacatcaccgagattcatggctccatctccatttgtcaatgttttttcaatagtgct

gatgtgacctgcattttcaatataatactgagctgctgctattgctgttactgctactgccaggagtgggttctcatctacactccccagcatgcagtat

ggcaagacaccacatcaaatctttggttttcagcacacacaaacttgttagacaattcattgggcaacatcaaagttagcctttgcagcctttagttctt

cttcaattttagcaaaacgtccctcatattctttctttagtgctgtttgtgccttttccttttcagcttgaaatttctgccggacctgctcatgggcagt

ctccagtcggcagcgtctgcgctcatataactgtgtcaaacgcatacgctctgccacacccctgtctactgttgctgcatcaacctcacgtttcacacgt

accatttcatcacggtcacggtgaactttagcaagagctttcacctcatcacgtcgtgcactttgtaaacgcctcataacttctgctgtctcacgatcta

gcaaggcccttaacagtttcatttgtgatgcctgagatgctgccatcaacttttccactgctgagtaaaccagttcatggtacctttcatgtagctcacg

ctcctgatgtatgtgatcccgaaacatagcaagaagtctctctgagtgacttcgaggtagccctgtactacctgcatgacgtgttgtttccccttcactc

tctgacagttctgatgattcatggtgatgttggtgttgcatatcagttatatttttgcttgacagcctcttgactagcttgtgactcatggagaacttgg

attttgacttctctgaatcttgggaacttttatgtatttgaatacgggctctctctttctcatgcttttttcgtagtgattccagtttcttttccaattc

tagctttttctcctttacagctttgttctccattaatttctccagtgactctgctacaagctcttcttctccactatcagacgggcagttgttgactggt

ggtggaccagttttggtagtgttctccgttctttcctgagtcttttgtggtagagtaccatcaacaggtgcaccatcagctgctccatccacagggaccc

cagagaaagaacctgacgagtcagttgaaacaaacgcatgtgccagcgatggccgtgggcttcgatcaggacccataagagtcagttcagcactcaccat

ctttactgccattgctggcacacaagttgaagggctgcgctgctgtcgtgcagtaggtgccgtttcctcttgatcctcaacaatatcggtgtgatcagtg

aggatggccaactgctgttctcgcttttccaactcagactgatatttgattggatttgctaaagcctctgcaaagtccgacattgtgtcaggaacataat

ctttgacaaccac

>56363|1-phosphatidylinositol- -bisphosphate phosphodiesterase epsilon-1

gttcctgcgcttgagcgtgctggatgcgaggacgagccacgtgctggcgcagcgcgtgctgccgctcaagtgcctgcggcccggctaccggcacgtgcgc

ctgcgctcgccgcagaaccagccgctgcagctggccacgctgttcgtgtactcgcgcgccgaggaggagagcctcgactacgacggcg

>56361|1-phosphatidylinositol- -bisphosphate phosphodiesterase epsilon-1

gtgcccgtcagcgcctgcgcccaggacgtgctcgcccaggcgctcgtcaaggcgcgacgaatggaggatccggcccgcttcgtgctggtggaagagctgg

agtggagcagcggcagcggcagttccgtcacccgccaacagagggttctgacagacgacgaaaacgtctacagcacgcaggcacactggcagaccatcgg

>56610|1-phosphatidylinositol- -bisphosphate phosphodiesterase epsilon-1-like

aacgcccagaagatgaccgggttgaaattggacgagtcgatgcgcatgcccgccgggtaggtgcgcatcagctgcgtctcgg

>56608|1-phosphatidylinositol- -bisphosphate phosphodiesterase epsilon-1-like

cgggcctgtgctgggtggtgcgcggcctcaagcggcagatgcagctgacggaccggcgcatgctctggctcaaggagcagtacctgcagctctacttcga

cgagggcctctgccaggggcccatgacagccgacgccattcgggtgttcggaggccgggactgggccatgacgggggcggtagccggcatgtcgcccc

>56609|1-phosphatidylinositol- -bisphosphate phosphodiesterase epsilon-1-like

ctttttcgttcgttcagcttaagagcgaggaaggacttgcgagacttgtttgaccaactggctataacgtgcagaagtatgtccgacagttcccttaacg

acggcagcctcaagaggtctcctgaacacctaatgaaacctcctcagaaaataggactgctcacaagaaacagctccttggacctttacgagtaccgaag

ctctgggacgctacaaaagaagaaaatcttcgacgcaatagcaacggccagtattgtaaccaactgtgcgggaatggacacctcgaaatcacaagtaata

actctgcagacgttttcaaaatttttagaaacccgccaaatggagcagaggacggaagacgaaataaaagcattaatacagaggcacgaaccagatcctc

tgttgcgaagtcagaactgcttttcttttgagggctttgcgaggtatatgatggacaaggacaactacgcctttatcagtgaaagaatgggcccagaaga

aagtgaaatggaccaaccgttgtcgcattactacattgcatcttcccacaatacatacctcacaggccatcaactgaaaggcgaatcttctgtcgagcta

tatagtcaggtgctactaacaggatgcag

>57364|1-phosphatidylinositol- -bisphosphate phosphodiesterase gamma-1-like

aattctataaaatggaaagtggcagagaaactgtcacactgaaagacctaaaggcattccttcctcgtgtcaactgcaaaattccaacaaataaacttcg

ggatgtctttcaggaagtcgatacaaggaaacgaatggaactaggatttgatgattttgctactctttaccaaaagcttatgtttgataacaatgtaaga

caatttaggcctgtaaaatcaatatttgtttgccacagcttcaacacagaacaagtgattaag

>57363|1-phosphatidylinositol- -bisphosphate phosphodiesterase gamma-1-like

atttattttttgttaatacaaagaagtgtttattccattccttgtccacagggtcttccagatacaagatgccatttttaactgtgtttctgagatccac

gtcccttgcatcatcatttttgctcataaaagcatcctcatcgacaccatctgggagttttttgtgcttaattataattttacgccgtagctggtgtggt

gatggaagctgactttcattcttctcataaagctgcacgagtagcatttccccaaacacttccatcattacttttgccatcttgcgctgctgtggtacag

agcagttgtcctctatggacaatattagtgggtattctgatgtaacaaatgcatgttccttgatagttttaacaacatccagaaattttatccttgtcgt

taaagtatgcccatggtagatgaatggcataccatcaggaccatcccagcagtctagttctatgcatcggcaacccattcgtaaacagcgaacataagct

tcagtggatgattcacttgagaattggtcacctgttaaatatgtattgtgtgatgaggcaatccaatagtgtgacaacggtttcgtcatgtcttggtaga

ctttatcatatcgttcatcccaaatttcattttgcttcgagaaaagaaaatccataaactcaaagattgtgaagtatggtgatcttattgtatcgttaac

caagtagtcaaggcctcttatccacaaagtgcactccgattctgaaaaagctgctattgatagtgttttcagtctgaattccaagccataatatataaca

aaacattttaaattctccattcgctttgcttcctccggccatttttcaaaatcctttgagttttttcctatgcgaacctcttttacttccctcaagtcca

cagctacttcaaatgtccttatatttgttgtacgggcccagactaattgccgagtttcccgtcgaagcattagtgttttcctttctggtcgcttgcgctg

gaaaaactttgttaccaccgttcctcgttcaagctgactaattatctgctcggtttccgtgatcatgctgcctaagggagtaccatttggcatacccaac

aaagccatgattatttgtaaaatatcacgctactacttggcaacacgtttcgtgatggtaccattaaaagcattaaaatcgtgtctcacaacacttgagg

aaaccgcgaaggaaactaatcaccaagtcttcagcgtcctacgaaaaaatatccatttttcagttcttttgtactcagtctgaaccgcagtagcatgttc

tgagataaccacattccgtatgttttcctggttcctgacagtcagtcagcacatgtcaacacctttaca

>58723|1-phosphatidylinositol- -bisphosphate phosphodiesterase-like

ctggaacagtagtcccatactgaagcaatctgttccaggatattaatagttagcacacagatatctgaaagcaaaacttactgtttctttaaacacgtca

tggggcaaacattattcgctttcacgttgtgcgtgatttttctgatcccatctaaccaggccttggcggtgtcggcgtcggggcagacgacgtgctggta

gttga

>60690|1-phosphatidylinositol- -bisphosphate phosphodiesterase-like isoform 2

ccaagggcaaagttcctgttagagttgtggccaaaacttttgcatctggacaaacagagaagctggtctacaaatgtctatctgagttgggcctgcccca

cagaaaacatgcttcaattgatgcagcagatttcacattcaacaagttttatgcactgtaccacaagatctgtccaagaactgacattgaagaacttttc

caatcaatcactcaaggcaaaagctgcaagatcagtattgaacagttgattacatttctcaatgagaagcaacgtgatcaaagactgaatgagatcctgt

atcccctatatgacaaaaggcgtgtggagaagataatatcatcatatgagccagatgagcggacaaggctggaaaacaaattatcaaaagatggcttgat

ccagtacttgatgtcagatgagaatgcacctgtctacttggatcgactggacttgtacatggacatggaccagccattgtcgcattactacattaacagc

tcccacaacacatacctgaccggcttccagtttcgtggcaaatcctctgtggaaatgtaccgccaagtgctgcttgctggttgcaggtgcattgaattag

actgttgggatggaacgagggatgatgaagaaccaataataacacatggcagagcagcatgtacagatattttgttcaaggatgtcatacatgcaatacg

ggacactgcgtttgtgacatcggagtaccccgtcatcctgtctcttgagaaccactgcagcaaggctcaacagtataaactggccagatactgcaatgac

atcctgggtgatctgctgctcaaggaaccactcccagattttccgctggaacctggactaccgctgccttcaccaaatgccctcaaaaagaaaattctga

tcaaaaacaagaagctaaatcctgatgtggaaaagcaggagttggaactgttctggcagggacagtttgtcattgaagaagatgaggaagaagaggaaga

agacacatctgccatcagtgaagctaaactggatgttcctgggtatgatgctacacaggagaatggagacatccccagccccgtgaagtatccaccatca

acaaataagattcatccatggctctcatccatggtgaactacactcaaccaatcaaattccagggattttatgttgctgaggagaacaacatacactacc

atatgtcatctttcgaggaaagagatggccttcagtacgtggagtctcaggctatcgagtttgttgattacaacaaacgccagctgagccgcatctatcc

taagggactccgtgtcaattcatcgaactacatgccacaggacttttggaatgcaggctgccagatggtgtcactgaacttccagactcctgacacaccc

atgcagctgaatgagtcaaagtttgaatacaatgcacgttgtggctacctactgaagccagattttatgcggagagctgaccggagctttgatccatttg

ctgaatctcccgtcgatggcgtgattgccgcgcagtgccttgtgaaagtcatatctggccagtttctggccgacaagaaagttacta

>60689|1-phosphatidylinositol- -bisphosphate phosphodiesterase-like isoform 2

gtaatacacccaacatgatgagcggtgtgaagaagctgagtggtgtacaatgagaaacgactgttttgcttttcacagcataaagttaactttcaagtgt

ggagtccctaatatgtttctcttaagtaattccattagttattacatttttgtggtattgtatcttcactcattagggcaaccaaaaacgtaagaaaact

ataaaaagtcagaaacaaatgaggtgtgtgtgacaggtctctatctgtatatccaaatgtagtgtgaagaagccctaacaagtgctacaatcagaaatac

tccatgtcatgcacttcacagtgatttgcagagagcaaatctagatgtagacagaccaaagagcaaataaaaattgctgcttgtacatgcttactttcac

aaggcactgcgcggcaatcacgccatcgacgggagattcagcaaatggatcaaagctccggtcagctctccgcataaaatctggcttcagtaggtagcca

caacgtgcattgtattcaaactttgactcattcagctgcatgggtgtgtcaggagtctggaagttcagtgacaccatctggcagcctgcattccaaaagt

cctgtggcatgtagttcgatgaattgacacggagtcccttaggatagatgcggctcagctggcgtttgttgtaatcaacaaactcgatagcctgagactc

cacgtactgaaggccatctctttcctcgaaagatgacatatggtagtgtatgttgttctcctcagcaacataaaatccctggaatttgattggttgagtg

tagttcaccatggatgagagccatggatgaatcttatttgttgatggtggatacttcacggggctggggatgtctccattctcctgtgtagcatcatacc

caggaacatccagtttagcttcactgatggcagatgtgtcttcttcctcttcttcctcatcttcttcaatgacaaactgtccctgccagaacagttccaa

ctcctgcttttccacatcaggatttagcttcttgtttttgatcagaattttctttttgagggcatttggtgaaggcagcggtagtccaggttccagcgga

aaatctgggagtggttccttgagcagcagatcacccaggatgtcattgcagtatctggccagtttatactgttgagccttgctgcagtggttctcaagag

acaggatgacggggtactccgatgtcacaaacgcagtgtcccgtattgcatgtatgacatccttgaacaaaatatctgtacatgctgctctgccatgtgt

tattattggttcttcatcatccctcgttccatcccaacagtctaattcaatgcacctgcaaccagcaagcagcacttggcggtacatttccacagaggat

ttgccacgaaactggaagccggtcaggtatgtgttgtgggagctgttaatgtagtaatgcgacaatggctggtccatgtccatgtacaagtccagtcgat

ccaagtagacaggtgcattctcatctgacatcaagtactggatcaagccatcttttgataatttgttttccagccttgtccgctcatctggctcatatga

tgatattatcttctccacacgccttttgtcatataggggatacaggatctcattcagtctttgatcacgttgcttctcattgagaaatgtaatcaactgt

tcaatactgatcttgcagcttttgccttgagtgattgattggaaaagttcttcaatgtcagttcttggacagatcttgtggtacagtgcataaaacttgt

tgaatgtgaaatctgctgcatcaattgaagcatgttttctgtggggcaggc

>71921|1-phosphatidylinositol-3-phosphate 5-kinase

gcagtatttaaaatacattatttacataaagtcatatgtttttatgctattaatactgatgaatggtgttttattccattttggaatgaatcatgcaacg

tacgcattgttatttcataactctcacatgcaacaatgtttttccttattacgagaaaaataactgttatagagaatgcacaatttacaagcataacaat

taatgaaaatcatgcagatagttactttacttgacttattaacatttttgattttttaacaaaacaaatactctcactgttagctgtatttcatattgac

ttagttaatgtgcaaggtagaaacaattttctatagatgactgattacatataattgttgaaacagaacaattaaactaagcatcaatgtatggtttaaa

caaacatgactagaaaaccataattaaagcaaaccagaaaattcacaccagctgtgtggacatctttatgaacaaaatgtaggcaatcagctttcacact

cctgttatcaattgttagttctagcaaccacaacatcattaacattccagtcccttacccagaccagtccactgatctggcacagctaggaaataacgat

gcattgcacttgtaaagcgagcacggtatagttcaggtgaaacaacagttggaagctttccttggccacccaaaatgcctgatgatttcaccatagtctc

cagcttcttgtcccaagtgaatgtacggatgtaatctataataccaacaacaagttctctcttttcttgatccagtccaactagtagagagtaatccata

actgactgagaagacaggaactctgtgtcattatttatagcttgcattaagactttttttgaatggaggtgtatgtacaatggagaatcacatgccattt

tcaacaagttttcatcaagcaacacaatttcaccttcttggtctgtacttggatttacaagacgattacgcacagatcctttcaaatcaaatttgtgtgt

gactaagcgtgcatgaaagagattctccatcacaagcaagtttgaacgcaaggctgcatttgtaaccgagtttcggaaaacaacacggtacactccaaga

atttttccaagtaatgtaggctggcctgaactaaggcatcgagacatgtatgcaaaatagttaggtgcaaattcaagaaacacttgcatttcaagccgag

acatctctttcaatataaatcgatcatctctagttttacaaaagcttgagcctgattttccaccacgagctgcccactgtacacaccgagacaaagagcg

aatgtaagcttcttctcctgctggcagtacttcagccctaagagttgcaaactgttccacaaaatatactctacagtagaagttacagcttgcatcttca

aactgcacttcaatgtgctgtgaaggctgtgatttagcagccttcgagttaggggatgcttcttcttgctgttccaccgagtcccccggaactgccacag

tggtatactgaacaccatccaaaacaccacctttattggggccactagtagatgcaccacgcaagaatgaaagaactccagaacgtcgtatttcagatgt

ctgctgaccactggtaacagtatctcgctcactaactgcaccagtagatttcctcttgtgaacaggacttgacatgacctgatcgttattcatttcatca

tgaaatcgtcgataatcctgcgaacttaatgcatatgcaataatagaagaaggttcagtttcatacacaacaattggtacagctccatctgttggtggca

gatgatgctcctggggactcagtggactctgtataggagctgaagatgtggcagaaggcaagagttgtgacaatatagttttaacactcttctgtgatga

ttttccatcatgactgtcttcaagtgattcagaatgcatttttatttggacttttggagatgatccttctaacacatccacagttccaacaactgtatct

ggaggcaaattgtagttatcttcagccagatcttcatcaataatatcagagcccccttgcatacttggagacgagagacattcaacttcttggccttctg

agcaaggactggtggatccttcacatgtctctgtatcattaatatcctgtggcactgcctcttcaatgtcaagagaaagactacttgaactgatgcctcc

actcttccgttgacgtcttgtggcagccagcagctcagtaagacgcgcattccaggctgttacaacatcccaaatgtgtcgtttcagctgcacgacacaa

tcatcaatgtgccacatcaattgatggacatcttgttcagccatttttcctgccaacttctttgcctcaagagtaggtgatgtcagtttcagctgaattt

cttcaattcttgatttatacaaagcctgctctttcatcaatatttgtttcaaagcactaacatctgcatcaactgccagtgagcaaagacgttctaatat

actactgtaaacatcataacccttaagggctaatttttttatctcttcaatgacattgctctgctgttggggattgtaactcatttctataataggaggc

ggcagacagatttcccatagtgtgataccagtgtacttgaaagatgctacaatttttccaagtccaaagtacagataatgatcatgatgtaatgaatgct

ggcagctgtgatgtcgccttgtatacacaccaccatgaaacctaagttccaggaatttcgcaaatgacagtgaccatgcattgtgtgtcattggcactac

aggagaagtagtgttgcatagattacaccagctccacataagaatctcgttgtttgttgcaggaagtggctgatccagttcctttagcaacacatgtaca

cagccagcatcatgaacaaatcgtcgtacatgactcaacattggagtgtcacaagtgtcagaaggacataagtaggatggtcggaagcagtaacgttcca

ggaaattccccagtgaaatatcattgcgaccataaaactccatgttcacaatccaaggattcacacagaatgcgggtgcattatttgactcatatgcata

gctacagaacaacactgatatgtgttggtgattggctggatggagggcatctataacttcctgttcctgctgct

>71922|1-phosphatidylinositol-3-phosphate 5-kinase

gtgatgttggggagttgttgagtggctgaggcaggcatacgggtctatcatatgttaatggaagacgccccccacatgctctaaaatgagcgagcagagc

ttgtacttcacggctaccagcagggctcgtcagtttcgctttcacaaatggatgttgtggctttaccttggctgtcttcactgcgtgattatattcgtgg

ttaactgaacagtttgctgataatgagtttctatttgcatttctatctgagctacaaaactgctctgagaaaaatatttcttcagcaaaaaatcgacgaa

gagtgcagtttcttcctgcttctgtctcaaggtagggcactgcaaactttatatatggtgaacctgacagaattgtatcatctagagctttcctaaaccg

gttacaaagcggaagctctgaaactgagagagagggtccattctgactgtcagtactaaaaacatcatcttcaaggttgaggtatgaatgtagtggatca

ctaaaatcactgatagattccacattcatcttcctgtcatctgaaggtgatttttctttatttgaaggatgagaacatgtgcacgtggagatattttttg

tgtccagtaactcttttctaacactaaaatcctgcacattttcattctctgtttttttgtagggttctcccatttctacagaaatattttctatagtttc

acaaacacatttagtaagcacagtttcttgatggagcatctcacaaatttctttcatgttttcattggaatagtactgttgttgagcagaggagacagaa

ttttgtttattttgtttcccattttctttagggtatgcacacgtagaactcaaaatttcttctcgcactgacttaatgggtgcagctccagaataactgt

tctcctccataaacgtatcttttggtagtggtggtggacaagcaaattcatccatcaagaaggctctttccaaacaccagttgtaacgtacataagtcat

aaaactagtcactcttttaagtttggcaagttcttgttctgaagcaccgcgcagcaagactgtacatccaaggtgtggtaaaggacatccttcaaaaacc

ataagtgtttttatggcacctgattccatgtggaatgtacgcaagtagaatgaatggcaagttccaagttgaggacgaccaatgtgtgcgtcaacagaag

acacaatgtctgccttggtgcagcgtgccactctattcaaaacacttgaacgtacattcagtgctagggttattccttgtgcaagcaacaaatcctgtgc

cagacgagacactccacgccctacaagcaaaatatctggttgcaaggccacaatacgtgcaactgcattacgcagatattcgtgctcctgcatcatgaca

ggttcaagagacagaagtcgcccttcaatgcgttgatagactattgagcatcccaaaagtagtattctggggtttgttaatcgagcagccatggacctat

gagccacatttttagtacacacaattccattaactatacagctttcttgtcgattccctccaggtaccttcttgaactggacgtattgtcttatatccat

attgtcaatatcgttcttagtatcaggttttaccatgtctacaattcgcgaaatgagaggaagtataatatcagtccaagattgggacaagccttctgaa

ttcagcaactgcctcagaagactagattcaaggtggctgtaagcattctcaagtctcgagagtgtttccttctccatatcttcttgctgttgaccagagg

cctgtggctgctgaattgattcttcatggggagacgcctgttgtagccaaagtgtgccagacaggaactcctttgtcagtccagcacatacatcttgtga

gcaagcacttctgctagttttctcttgtttaatgctatgctctccatcattctcctttgggcgtgataatgtgactatattctcttccacattcatgtcc

agataaaatgatgaatttgatgatggaaggcgtggagattctagttcagaaatctgcatttctggttctgagtcggtagtagtagctgagtcttgctgag

ggatctgctttacccaaagtggctcctgactgtcaacatgcactgtgtcaccaatctcttcttcatttattttttgttgttggtatttttcttcttgctt

cagatgacggtatagagcatagccatcaataaaatattgttctgatacacattccagaaagttgccttcaattaaagcttggccgattgctgttgcttgt

gacctcgtgcctgctctctctctcgcaagaagccagtcaacaagttctgagccaagaaagcaacctctgtaattgcgaagcctatagcgatgtgtgtgca

gccccagacctgaaactggatcacgcagttcttctgccaaagagcaaagtgaggccgattcctgtaatgcacgtactcgttcctccatgttcaagtagcc

tgagccctgagcccttccaagggcaaagcgatcttcttgaaagccaacagatgttttcctacgtgcagtttgggcatcttgtgcatctgctgcaataaca

ctgctgctacctagactaccctgaactgctgtctgtacaacagaagaattcccgaacttcacctgtaggtcttcttgcaccagtcgcagatcagctgata

gatcagctgctgtgtctgctgactgaagatacgaaagcacaactttgcaacaatatgtgcacactcgaagatcaccagtacatcccattatctttccagg

aatttcttgattgcaacaccgagaacaaaatatctgtccacaaacacggcaatgatgttttctacgaaaagtggtgaatttttcaccacagtcataacat

tccttactgactgaatcaggcatccagtattgtttgaggtcagtatccttatatgactgcaatcctgtacttttgagggcaagaagattacttattctcc

tcaggacatttggtaagcttcttccatctgttacatcaatggtaaatggtgaattttgctccgggtttggattttcctgccaatcaggagaatgagaagc

tgacacttcatctgtagattgagattctgtagtctgaacactgctagttccttcagatgttgcttcacttcgactgaatttaaatagtttggataggaat

acactaactccaggttgacctgtttctgggctgagaggtgcaaattctgtaagtttcgacggtgactctaagtttttactcattgttgccgagttacgca

gaagaaccagtc

>73619|1-phosphatidylinositol-3-phosphate 5-kinase-like

gcattggacataaagctatacactcttaaaaatataatgaacttatcagcataccagtacatcccattatctttccaggaatttcttgattgcaacaccg

agaacaaaatatctgtccacaaacacggcaatgatgttttctacgaaaagtggtgaatttttcaccacagtcataacattccttactgactgaatcaggc

atccagtattgtttgaggtcagtatccttatatgactgcaatcctgtacttttgagggcaagaagattacttattctcctcaggacatttggtaagcttc

ttccatctgttacatcaatggtaaatggtgaattttgctccgggtttggattttcctgccaatcaggagaatgagaagctgacacttcatctgtagattg

agattctgtagtctgaacactgctagttccttcagatgttgcttcacttcgactgaatttaaatagtttggataggaatacactaactccaggttgacct

gtttctgggctgagaggtgcaaattctgtaagtttcgacggtgactctaagtttttactcattgttgccgagttacgcagaagaaccagtcacgtttatc

agtattcgcttcatgaaaaaatcgcgggccgct

>77180|10 kda heat shock

tgcagtaggtactgggagatgaagaagcttgcacaggatagagtgttggttcagagggcagaggcaataacgaagacgaaagggggaattgtaatccccg

aaaaggcgcaggagaaagtgcagaaggc

>77387|10 kda heat shock mitochondrial-like

aaaattacttttgttcccccatattctggtagcaagaaacaataaaggagaacacatccccccaaaaaaaaaagtgggcgaccaagtcttgctaccagaa

tatggg

>77390|10 kda heat shock mitochondrial-like

aatttcttcgcagcagctgcagcagccattttctacaatgtgcacctctaagttacaacaacaccgccttttcggggattacaattcctcctttcgtctt

cggtattgcctctgccctctgaaccaacactctatcaaacataggaagcaatttcttcgcagcagctgc

>77389|10 kda heat shock mitochondrial-like

tggaaacgaagctgctggaaaagccggtgttgttgtaacttagaggtgcacattgtagaaaatgaaaggagaacacattcccccaacaataaaagttggc

gaccaagtcttgctaccagaacatgggggaacaaaagtaattttggatgaaaatagagagtaccatctcttcaaggagtcagacat

>77383|10 kda heat shock mitochondrial-like

ccatattctggtagcaacacttggtcgccaacttttatttttggtggaatgttttctcctttattgtttcttgcgccaggtcctacagctactacagtag

ccttctgcactttctcctgcgccttttcggggattacaattcctcctttctatacaccacagcgtcatcaccacacagtcgcagaaagctgctcacccta

tcttccag

>77388|10 kda heat shock mitochondrial-like

ttttctacaatgtgcacctctaagttacaacaacaccggcttttccagcagcttcgtttccaccacgcgataacgaagacgaaaggaggaattgtaatcc

ccgaaaaggcgcaggagaaagtgcagaaggcgactgtagtagctgtaggccctggcgcaagaaacaataaaggagaacacattcccccaacaataaaagt

tggcgaccaagtcttgct

>77385|10 kda heat shock mitochondrial-like

cccgaaaaggcgcaggagaaagtgcagaaggcgactgtagtagctgtaggacctggcgcaggagaaagtgcagaaggcgactgtagtagctgtaggacct

ggcgcaagaaacaataaaggagaacacattcccccaacaataaaagttggcgaccaagtcttgctcccagaatatgggggaacaaaagtaattttggatg

aaaatatagagtaccatctcttcaaggagtcagacattttggctaaactagacggctgacgtattaagttt

>77386|10 kda heat shock mitochondrial-like

tagcaagacttggtcgccaacttttattgttgggggaatgtgttctcctttattgtttcttgcgccaggtcctacagctactacagtcgccttctgcact

ttctcctgcgccttttcggggattacaattcctcctttcgtcttcgttattgcctctgccctctgaaccaacactctatcaaacataggaagcaatttct

tcgcagcagctgcagcagccattttctacaatgtgcacctctaagttacaacaacaccggcttttccagcagcttcgtttccaccacgcgataaccccaa

gttcaagaccgctgcagatcccagttgttgtaacttagag

>77384|10 kda heat shock mitochondrial-like

gccggtgttgttgtaacttagaggtgcacattgtagaaaatggctgctgcagctgctgcgaagaaattgcttcctatgtttgatagagtgttggttcaga

gggcagaggcaataacgaagacgaaaggaggaattgtaatccccgaaaaggcgcaggagaaagtgcagaaggcgactgcagtagctgtaggacctggcgc

aagaaacaataaaggagaacacattcccccaacaataaaagttggcgaccaagtcttgctaccagaatatgggggaacaaaagtaattttggatgaaaat

agagagtaccatctcttcaaggagtcagacattttggctaaactagacggctgacgtattaagtttgtgaagctaaaatcagatgtgcaaatgtagtaaa

tcttagaactgtgcgatctgaaccatggatttgtgttggaagtgaatgttggactacaggaactgttagtgcagtgtgcacatgttaaatctgacagccc

tgaaactgagctaactttgttattgagttaattttgccaaacgttagataactataattgtgttatataaagtataaactgtgttcatctgttttacagt

aagttttcaataaaatccttctggaaagata

>Sg_CNS_NA4Plus6328|10 kda secreted protein

cttcctcgactggaacaacaatcaggacgacagagatttcctgatgacagtcagaaagtgcaacagggccatccattttcagggctagttgcttcggcag

gagagttgttacacactccttagcggactccgacttccatggccaccgtcctgctgtcttaagcaaccaa

>77703|10-formyltetrahydrofolate dehydrogenase-like

ctttcacagtggtatcagcttcttttccatagtttgaagcgggtatcatttttccagcaacagatattcgttgtacattgagcagacctccatctaggcc

acgcagtagtaacccaccagaatgaacaacaccacatcctgccatgccatcaatagtcacttcattgccactaggttctttaccatgccataaactcgaa

ccaaataacttcccctctcgaccatcaataacagtccatgctccaggtgagctatccatcccacgaataaaattatgtatattttgtaccggttgatccc

atttcacctgttgaagttctttcttactaagggatggctcgtaagaagctccttcttcagactgcttgatgctcggagctgtgccatttgccaccatgtt

aacagcttctgccatagctttaattccctcaggatacaagaatttgttatagagggagtcaactgtatcatttggctcaacatcacaagctctttgtagg

agtattggaccagtgtctagcccatcatcagcccagaagacagagaagccagcaactttgtcaccagatatcaatgtccagtttattgcactaactcctc

tgtgctttggtaataacgatgggtgatagcaaatgctcttatgatctggatgattaataacatccataggaataaactgtgagcagaatggcaacacatt

aagatcagcatctacacttttatatttattcactatctctgccaatggctgaccacctttcctccatgctttaactttgaatactggtgtcttatcagct

gcagcagtagatgctaaaggatcttccctgttccctttgtctggaattgtgaatactccaacaattttgtgtccatctttcttcaaaagtctgtagactt

cagctgcaaatgtactctgtccaatcacagcaatttttaatttgttgaaagtgctctgactactagaaaactgtctacttagtatgaagaacgctcgtgt

gcctaacatgttgactacttgctactgctactgcacactaaacggccttggttcccgcaactaaacatcgggcaaaacgtgaagtcgtgtttccaagtcg

ataagcaatgttctggcaaaacggcagacttagactaaaagagcaaattacattccgtcaagattatacaaggagactggtttattacatcaatc

>78446|1101404borf 2

cagacggtaaaaacttgtgatagtatgctgtttttcccaagaaggcctgcagttccttaacagacgtagggtgaggaagggcatcgattgcagctacagt

ttgctgaagcggacgaataccatcccgagatagtt

>78443|1101404borf 2

gtttttgcgtaacagctgatgcacaggataagctaccgccgctgcgaatggaatgaatttgtgataataagcaatcttacctagaaacgcctgaagttct

tttaccgtagacggccggggtagagcgttaatggccgcaacgtgctgacgtagaggacgtataccctcacgggacaagt

>78440|1101404borf 2

tcttcttggtgtagcaattttaatggccagtagtgtacatcaatttcgggttagtcctcacaccatccttaccaagtacaggagcaaagtaatttacttc

ttccaatgcaaaatgacactcctcagtactcagtattaaaaatgcagcccttaacctctcgagcacttctgcagttgctgcctatattcttgcatttctc

ttataaaaacaattatgtcattgaaatactctaaacattgacaaggctttactcactttgattaatgctggaacattgctggtgcattctttaaatcaaa

tggcagtcctctatactggtaagccccaaggtactaaatatgcagtgtttgatctgcctctggagccacctccaactggcaataccctctccttccatca

accactggcagcagcgatgttggctctcttgacaccactgtatagagtggtggagagcaatattttattagacctcaattacacatt

>78439|1101404borf 2

ccgaaatattgtgcccgttggacactatagacctgcagtacacccgcagatattttgattattagtggcaatgttagaaattctggaagaggaagattgc

tgtgcaaaaggtgtacaggatgaacaagcatggggtgtcactaagaacacactgcatggaaggttaaggatttgaggggaacagagagatcacaaatgaa

gaactgttgttagagttccaagatttgttttttccatgaaggcccttgcctgcaacatgtgatgcaacacaggattcctaggggaatgaagtgctggtgt

actggaagatgtacagaatatcatgagccctgcagtcaattttaggagactttatcacttagcagttagctgactgtagtagtagcccattgagggcagg

aattataattgtacctgaaaagtgtgtggttggttcaaggaattattgattctgctgtgattaccaccatttgaacaataagatgactgcagatgcttac

ccatttgcaaacatcatggaaacaattgataaacttggacagtgccagtatttcttgatgatggatttgcagagagggtaccatcagttggaggtgattc

cagaagacagaccaaagactgcattttcattaccttggggaaattataagtatagaagaatgctgtttggtttgaaaattcatcagcaacatttcagcat

tggttagatggtgtgttgaaggggttaaatccttgtcaatgtttagtgaatttcgataatttctttctcaaagagctgcaagaacataggcagtgactga

gagaagaacaggaggttacagtctgcacatttaatttggatatgtaattagtacaggtggtgtgagtactgatccaagattgatacaggtagtacaggat

tctccagtaccagggtctattctgggtgtcacaaattattataggaaattcgtgagagggtctacaggtatagtgtgactgcttatacaacagttaagaa

agggtatcagaattctgtggtctgaagagcatcagaaagtatttgatagattaatggaagtattgacactgagtccaatgttggcattcctgaattttga

gaagaaatttatattattatgtgataggttgacccatgctctggggcaagttttgagtcagcagttcaatggagaagaacattcaattgcttttgcattg

tgatgactgaatagacctgaaagaaattattctactacagagaaagagatgcttagctgtacttccagttttatttttacatgagaaagttgtaaaaaaa

gtgatggatcatgcggctttgaagtggttacttagtttgaaggatacttccagtagactggtgagatgagcactaaaactcagtgaatttggatatgaat

aaatccacaaactggggaagaaacactggaatgtggacacgctatgaaggaaggagggtgcaccagcactaggttgagacctcgctgagtggcaagcaga

gcagagtgctgaaacagaatgtaagcagttcagtaagcagcagcatttcaacatgtgtgatgggttgttatgtaaggagacaacactgagaccaccagtg

gcagcatcagtgaagctgagggataatgtgctaagagagatgaatgatcacattttgtttggccatggagggtgcagagcaacaaattggctggtgaccg

agacgttctggtgggaaagaagaaaaggtgatgtgagtcagtatatcgagcattgtgtgaattgagctcaacatccagatatgaatggaaaatgagtacc

gtggcagagattgctccaagtaatgaaaccatttaggtttattggggtggatgtgttaggaccatttagctgaacaccagcaaggaatcattttgtgttc

actattacagaccatttctctgattatatcaaaatggtgccaatactgaaccatcagacagtcacagtcaagggagccagccacccaatgaccattgctg

gcttcccatgccagagtggagtatgtctgcctcagactgatgttggaggaatgtgtaactgaaaagtaataaagtgcttttcttgaccatcaatatatga

ctggacacaccggtccaccactcagcc

>78718|1110268agene f1

ttacaaacccgacaacatccccttcgtcgccatctccggtttcatgggagacaatatgattgagaagtcaagcaacatgccatggtacaaggg

>78712|1110268agene f1

tccaccacatttgtagatcaaatgtccggtcgtggtcgatttgccggaatctacgtgaccaatgacaaagatgggtaaggaaaagattcacattaacatc

ggcgtcattggtcacgtagattccggcaaatcgaccacgaccggacatttgatctacaaatgtggtgg

>78715|1110268agene f1

gactggagttcagacgtgtgctcttccgatctctattccaccacatttgtagatcaaatgtccggtcgtcgtcattggtcacgtagattccggcaaatct

acgtgaccaatgacgacgatgttaatgtgaatcttttccttccgcatctttgtagtttacagggagttattcagtctaatacctccagat

>78714|1110268agene f1

caccacatttgtagatcaaatgtccggtcgtggtcgatttgccggaatctacgtgaccaatgacgacgatgttactgtgaatcttttccttacccatctt

tgtagtttacagggagttcttcagtctaataactccctgtaaactacaaagatgggtaaggaaaagattcacattaacatcgtcgtcattggtcacgtag

attc

>78711|1110268agene f1

gcccatctcctgggcttccttttcgaatttttcgatggttctcttgtctattccaccacatttgtagatcaaatgtggtggaatagacaagagaaccatc

gaaaaattcgatggttctcttgtctattccaccacatttgtagatcaaatgtccggtcgtggtcgatttgccggaatccacgtgaccaatgacgacgatg

ttaatgtgaatcttttccttacccatctttgtagtttacagggagttcttcagtctaataactttactgtgatatccacccgatacgcagaacactgcag

acagtcctactcacctccagatgccacggcagaaagagaacgacagagccaatagacatgcgcacttgaagtggcacaaaaaattatt

>78713|1110268agene f1

gcttatccaacacccaggcatacttgaacgaacccttgcccatctacaataaattgcatagtgaacattttacaagttacaccaatatcgacgaatatca

ctaatgtcgctgtaagcaatattttgcacttacctcctgggcttccttttcgaatttttcgatggttctcttgtctattccaccacatttgtagatcaaa

tgtccggtcgtggtcgattcacattaacatctacgtgaccaatgacgacgatgttaatgtgaatcttttccttacccatctttgtagtttacagggagtt

cttcagtctaataactagctagagagaacactttctcacacaaatcaaaataattacgactaagaagaaagcatcttcactcttgctcaattcaatacct

aacgaaactactaagatagtcaaatcctaacatggcagacatctttgtaac

>78972|1212243bubiquitin s5

caccagatcaacaacgcctcatctttgccggaaagcaacttgaggaaggacgtacacttgccgactacaacattcaaaagga

>78968|1212243bubiquitin s5

gacaatgttcggccatcttccaactgattgccagcaaatatcaatctctgcttatcttgaggaatgccttccttatcttgaatctt

>79268|1212243dubiquitin s2

aaagtagattatttttggatgttatattcagtcaatgttcggccatcttccaagtccttgccagcaaatattagtctttgctgatctgggggaattcctt

ctttatcctggattctgtgtcttactagctacattgtcaactatagaaagtagctttaatgcttttgttatcaaatgtaaggaactactcccagctatgc

ctcagcatttc

>80185|1212243hubiquitin s7

aaagatgagtctctgttgatctggtgggatcccttctttatcctgaatttttgcatttacattttcaatggtatctgatggctccacttctagagttatg

ttttgccagtaagagtttttacaaaaatttgcataccaccacgcagacgcaa

>80377|130 kda phosphatidylinositol -biphosphate-dependent arf1 gtpase-activating

tgactgcaataaaggcttctcgaagaactacagctcagtgttacattcacaagttccttattgtgcatgtcgacacaaaggttgttaaaggttttaatgt

tttggtttcttgtgacattttattttacacaataaacccttccctcgcccattcagattatggaagtagcaaaaattctgaatctgcaagaaactttcaa

aacttgcattcttaattgaaggaaatgcatgcttcaaatgatagaagaagagcacgcatagtacttctggtcacttgtaataataaggccatcacacatg

agaatgggaggaagtaacaatcagtaatgtatcaaaactctgaaactggggtatcataacatattaaatgataaacctgattgtaatgctcttgttatga

cattaattaggatcataattaaaatgtgagggtaaaactgtaacatgataacacaacattgtttatcacttctctcaactttaattgctctctcaaaaat

tctatgaagcatagatcttgtaaaagatgacagtgagtttgaacattgtcgtttgtgtattagttcctgcccctaatgcccatggaagcctgaagaagag

agctgctccattacctccagtcaccaacatctatggaacattgcccaatagtcatagccgaacgccatcagacccaattgtggccaacagccatggttat

cacacgctcaatcaccataagaggtcaccatcgagtgactcaggtgctgtgagcattacaaatagaacagccttaatgttagctggtgcaaagcaggttc

tgcctcctggtgagccaccatcactgaagtcgcttaatgcggttctggataaaagcggtggtgtgcagcgaccacgggggcctccgccaccagcaccaag

cccggtttccagtaccgggaggctagccaatggtcggtccactgagagcatatcgtccgttgcatcagatgttgagactgcaagctctggaattggcctg

cacaatccaattcctcctcctcgaaag

>80646|1303225ahobo transposable element

cacctgattatgcgaatcagtccacatatcaactgccgcagcacatgttttattaataataacttccgcaataacagaaggcattatgctgtttcgtatt

gcatcagctcgttctttgacatgtctgcttatagtagagggatgtggcatgacatctgccacgataacttctccataatgaccacctagatgtattaatt

cttgg

>80647|1303225ahobo transposable element

tgctcagacacatttgaaccatttagtaacgactgcaataacagaaggcattatgtttcgcattgcatcagcttggtctttgacatgtcttcttatagta

gagggatgtggcatgacatctgccacgttaacttctccataatgggcacctagatgtattaattcttggcctagttctctgaaaccaccagaaacagcat

taaaaggtctcatatctttagcacacaag

>80640|1303225ahobo transposable element

gttgcaatgtgtgctaaagatatgagaccttttaatgctgtttcacataaagatttcagagaactaggccaagaattaatacatctaggtgcgcattatg

gacaacctaacgtggcagatgtcatgccacatccctctactataagcagacatgtcagagaacaagctgatgcaacgcgaaacagcataatggctttggt

tgttgtggaagttattaataaagcaagtgctgcgacatttgatatgtggactgatttgcataatcagctgcactatttgacacttacaacacattacatt

aacacaggttggtctctagtgtacaatgtttatttt

>81720|14-3-3 protein zeta-like

gaatgttgtgggtgcccggcgtacgtcatggagagttatatcgttaattgaacagaagaccgaaggttcagaaag

>81719|14-3-3 protein zeta-like

agggagattattacaggtaccttgcagaagttgccacaggagaaaacaggaatagtaagtaatactttcttgaggaatatgaaagacataaaattattgg

agcagtagttaaggcacatttataagattttatattttggaggagtctttcttggtccttttatggttaattgatttctgcaaagtttagtcttcacaat

tttctgtagtgataaaaggttagtttaaaagactggaatacaaggtgaacagacatgatgcattgtcacagttcaatttcatttgtaaacatgttgagag

ttctcactgcatgttgagaatttgtaactatcatatactgtaaaaaaaaaaaaattgcaccagctttgagttggacttttaggtagtaataaagggacat

atttgtaagggaacaaaattaagatttaggcaccacctgctttcctttgaaaacattttaagcatatgcagtttgtacagttcttggaaaatcaccttta

cagctaaggtatagttctgaggaaactgtttcataagcctattttcattttgctccaatcatttctcaacattactttaaacaaatgttgtcatagttcc

accagttaatatatgctttttcattgtaattctgtaatgtctaacttctcccagataattgagcattaccacctgcgtagtagccacagtgaataagact

taaaactggacaagtgtttgtcacatacttgtcaacatttttacaaattttatttccagcagaaccaagaagttttgatgactttcacaatggaattgtc

agaaattgacataacaaattaaatattcttttaaatttgatatggattacttccctttgataagtggaaaactatttcttgcagctgttgtggatgactc

tcaaaaggcataccaggatgcctttgaaatcagcaaggccaaaatgcagcctacccatccaatcaggctggggctggcactcaacttttccgtgttttac

tatgaaatcttaaattcgccagacaaagcgtgtcagttggcgaaacaggttggtttctttgactttaggtgacggactttcttctaggttgaaggcagtg

ctgatggcagactggttgcttagtttcatgttacaaaactacttaaatgtgtgagaaaagttaacaaaattgctgaccgacacagttcagggaaaataaa

ctgcatctaatcgcacatttaagtaagtttaatttcccgtgtggtatcttcgaaaagtaatggtcaaatgttttcttttgcctttttattagcggtagtg

gatgattcgcagaaagcgtatcaagaagcatttgatattgcaaagtcaaaaatgcagccaacccatccaatacgtttgggcttagcactaaatttctccg

tattctattatgaaattatcaattctccagcgagagcgtgtcatcttgcaaaacaggtaggcccaagactgggttacggaggttcccatttgtgttaaga

gttgctgatacctcttaactaatgtaatcagcatgccctatcatgactccaacgctaacatattgtgtgttatgaaataattgtctggcccttccatgat

ttgcatgttatctgaattactccttgagttgtggagtttactaaaatgtatttgatagtggaatgaaaatataatggatgttagtgaacaaaatattgtg

tagacaaaaccttaaagtagcaaaccagtctgcacagcagtgttgcactaatgttttgaatcaatttacataaaattactggtgttaaacattgtctaat

gttacaaactttctatagttcaataacaaataatttttcatatagtgtaattggatagatgaactgctctagtggtgacaagaaaatgcaaatagttttt

gtattagtataacctttctgctgttgctgcttgatgcatagttaatctatgcagttgctttataaacgtcacgttgtcactaatgtgtcctgtttaatat

ttaccttcatcaaaaatagttatgccgcttcatgtggccaactaaatactaacagtttccaacaaattggc

>82286|140u_drome ame: full=rpii140-upstream gene protein

ctcttgcttaggtttgggaatgtgtaccctagtcagctgatacgggtaatgttgccgaagtcgcgcgtggttacgttcctgggcggtctgttaccgacaa

cgataacatctttagacagtgttgacaaagagaaacatgactatttatcactttacaagtcttcgaacgctgaaaatggatgggatagagtacggatgat

gttcagcacagatgaatttggaaacatcagtccagaggtatttagtgttctgcagactgcattctttggttcctttctaggaggttgttatggaggcttc

ctttttttcacgaaaagcatatgtggaattcattgaacgcaatcaggccacatcatttacaagtcacttagaagcaaagaggcagttacagaaccatgta

acagttaattttgcaaaaggtgcactaaaatgggcttggcgtgttggattttttagcagttcatatgtgttgctttccacaatgttccaaacataccggg

gagagtcacgcctagttgattatgtgggtgctggtggaattacaggagcttgctacaggttacatcttggtgttcgtggttttctggttggaggattact

gggttgcgttttaggagttatggctggtggatcgtcaatgctgttattgacggcagctggtacttcagtggaagaagtaaggttctggcagtacaaatgg

aagacatcgagagatatgatgaagaaagagcaggcaggtgctgccagaggttacgatgatcctctgataaaatggcatgacgaagttcacacaaaggact

caaaccagtcgactcagaaaagtgagaaacagtgactgtaatgcggagtaacatgactgtgatagttgtgaattgttgatacgctttacattttatgtga

tgtttttataatgcttgctctgtcaagagaataaagtaaaacctgtgtaggattataatggtgtaaccaatcaaatctcctctgtaaaacaccctgttac

aatgtgaagttcatttcttctggaattcagcagccacagaaatagtatagcacacatttaaagattgaaatagttctctataacggtaaataaatagaaa

attgcttgaaacaaaagaaattacaaaaatttttggttcactgttattttgaaataaattgacttgttacttatcta

>82496|1433z_bommo ame: full=14-3-3 protein zeta

agagcgctttcgatgaggctatctcacagctcgaatcggtcgatgaagactcgtacagggactccactttaatcatgcaactcttgcgtgataacctcac

tctatggaccacagactctgagacaacaagaaacaaggaagctgatggggaggatgatggacttcactgactttaagtcctattagttcccacgcatcga

tggattgtcgccgttgcccgccgtcatcagcaattgatgcgtgggcaaaagcagtgaac

>83019|15 kda selenoprotein

tgacattttttatgtaaatcacagcctccataaaacaaacattacttatctttttatttaacatgtaaatacttgtatgtgtgtcttgtgtagctcacaa

attttacaggtccatctcagattgcaaatcacaggccacagccagacttccaaagctgtttatacacagaattatctccttactacctgtgccctatctt

acaagctcttactttggcaattgtacagtaacataaaacttttgcatgtaaaaaacagcacagcaagacacatcaaaccatattactttccaagtagtct

ctcttttcctcttctacagattccaaatgtgtcttcaaaaattcttccactgaatcggtattccacttatcaattgccagtacctctttaacgtttccat

ctttatccattagttttattatgggatctaagcctcgaacatactttatctgcaaattcggaaattttgaaggtctctcacttttgacaaaagctgcaat

ctgaggatatgcaccaaacttacaagtgcagacttctaaccgtgcttgggcataacgttttacttcgtatgtgttctcatccttctggcagcactgtgtg

cagtgttctttgataagatccagatcaaaagatttcagctggtcacacgacgaacacagcaaattggctttgttgaagcccaacgcccagcaatcctcag

tcgtgtattccgccaaagaacagacaaccaaatttgtgaagagataaaaagcaaatgctcctgaaataaactgcatattgaaacaatgtaacactccttc

cctatcaccactctttcatccacgttatacagacactgacggacgtcacaagcatctaaagctagcgaaatgagggatgctcaactacctgacctaccga

tag

>83471|15-hydroxyprostaglandin dehydrogenase

ctgccgtttgtgatacccctcctgctgtatctcgttgccaagcattctcatattcaggtgacagcagcagtttccttacatctttcacaaggtttgtgtc

agtggcaaaggagctgatacagt

>83474|15-hydroxyprostaglandin dehydrogenase

aggaagtgttcaaaacagcgaaaagcaccttcaagcgcctggacatcgtcatcaacaatgctggtatcatgcgggacagcatctgggaaaagcaaatcga

cattaatatgaaaggggtggtgcacgggatgctgctggcgttcaagcacatgg

>83469|15-hydroxyprostaglandin dehydrogenase

gggcgtgaggatggtggcgctgtgcccggggcccacggacacgccgctggcgagaaacgtccacctctactgcctacacgagtacgccaaggagccgctg

ctcgacgtcatggccgccttgggcaccctctgcacgcctgacttcattgctgaaggcttgatgactgccgtccgggagggcaagtcagggtccgtctgga

tggccgacaacagcaagatcaaggagattgtctacgagaggctcgttcaataaggactgttctacatgccgttttgtacacatctcattatcgtgaataa

agt

>83470|15-hydroxyprostaglandin dehydrogenase

atacagcagctcccaataaatggggaggacgaatatgatgtgcctcagttaccttggtgtacaaaaattgtgctattcaaaaactctgtacttttcacag

catcacaccatgtttatgtaacagtacagcacagtcagcaactgtttgaataaggaactgtcaaccaattacacagaggttgcagtggtgagtaattgaa

attgattaacggtgcacgtattttaaatgtggcaccataccctccctaacccctactagttagtctagtctttaaaattgtggcacaggctcattttgtc

tacttcgaaaacgagaaaaatgttttgctggcgctctttgtatttaactcactcgcgaaaaggacggattttaaatttgacataagtaaaaaatgattat

gataaggtagcttctacactactaggctcattttatcacaagcagaaccgtaaatattgaagtatttatttgttaagcatgcaaaaacgtcgagcgatcg

caatgctcaaaatgtagcaaaccttcgtattgggggtaatccgtgacatcacactgacaaaacagcactttgtctttgccatatttttcttccaaccgtt

gaacaagctgctcccctgcctcagcatttatatcgcatatagaaaccttcaccccatgtttcagcaactcttcgcagtaagcgcgacctattcctgcagc

tcctcctgttactagagctactttccctttcaaatccatgtcaatattctaccaactacagagacactaatatatgtacatattttgacagagtaccggt

gtttcatctgacagaactcgtcaaagcctttc

>83467|15-hydroxyprostaglandin dehydrogenase

cgggtgatagtgattgcaacatgaacacttcattttgtttctcaaagttagtaatacaaaaagtcttacataagtttattcaacagctctcccaatattt

cagattcaaaaaagttatcctctcggaagcgttttataaagaatgtctgctttttccaagcaccatcaaagtaaacgttgtccttcacagtttgtgaaga

ttattcgacttttttagtttgttgttaaggggtttatgattatttcgtacgattgtttcgtatgatgaatgaagaagcagcgcacttcttatggaacagg

gcaccagcatatacaagcagtgcgtccggagcacggcagctacaccaggcgcgggaagtccacctgacgaacgactccttcgttggccaaccacactgag

ccagagcgaccctcccggatgactcgagtcagcgcttccgcgatgaactcaggcctgttgatgtgagtggcagacaggagttgggctagaggttgcttga

tgtactcgtgcatcaggtgctctggagagtccagcagcaatggtgactcagaagggcccggacacagcgccaccacagacacgcccgtcttatccgcgta

cgtggcatctccgtaggcgcgggtgagggcggcgacggcggccttggtggagctgtagactggcgccaggaaactcggctccaggcctgcggtcgaggag

acgtttaccaccagccctcccctgccgccgctgtctctgcccatgtactggaaggccagcagcacgctgccgatggtgcccttcaggttgatgtcgacct

cctgccgccacacgctctcccgcaggatgcccgcgctgttcacgactatgtccacccggccgaacgtggacaccgcagtccggaagacctcttccagtga

ctcggagtccgccacgtcggctttgacgaagatggcgcttcccctcccgaactgctcctgcagcccttcagcggccttcgtaccggccgcctcgttgata

tccgtaagcaccaccttggctcctgcgccgagcagcactctgcagaaggctcccccaagcactcctccagctccagtcaccagggccacttttcctgtgg

gatccatcgtggttcgtctgaaacgctacgacttgcggagtgttttaaaagtccgtctcccttccgtgtgcccgtatcagcgcgcgctcgctgcagctgt

tgttgacacacttacttcaaacactaacttacaggaccaaatcacaatgcaattcacaggaatattataa

>83475|15-hydroxyprostaglandin dehydrogenase

gctctctctctctcaacaggatgaccccacctcctcctcctccactgcaataattttatactccattttatgacccttccattttttgaccaatttgctt

tattacttactttagacatgtctgacaggtgaatcggccttgggaagtacagccaagctttcactttaatggcgtagaaagtgacttaataggttacaat

ttactgggatttgtgtataaggtagaattatcttaaatgtagctcttaataatattaatatccttttctgcaaaattcggtacttttgtaaacaatgcag

ataattaacagctgaaccattacactacacaaatttggactaaaaaaattatcatcagaagttacttctagcttatttattgatttgttttaataaaaga

ggacagcagcagtactaacggtcttaacacaaagtggcctcttacagaagaaaatatcagttacttattcgaggtaaattacatgtttaagcactatccc

tttggctcactgaataatcggtaagtctttccttcccaaaatggtccactgaatcaacaaaattccatgtttacccttatatgcttctagctcccatcac

atgtgcacagcaagttggtagtatgtaatcatgtcctctcttaaccgatattctgatcaattctagaaaaaaggtgaacagtcattctgttctttcttaa

gtgtaacatacagtgcataaatatcaacaaatatacaaatgtttaggccaaattacaaaatgtacatacgataaatgtgacttgaatgaaaagccacaaa

aagcccataaatctgcaaagctccggaagaaaattgtgcataatatttgtagtgtgttagatgcacttcaacaggctacttgtgcaattaaacttgtcat

aacagtaaaaccactgtttactgggtagtaaaatgtatctcccgtgctggctggttcttctccaccatccaaacagaaccactggctgccttctgcaaca

actgtatcagctcctttgccacatgttctgccgtttgtgatacccctcctgctgtatctcgttgccaagcattctcatattcaggtgacagcagctgttt

ccttacatctttcacaaggtttgtgtcagtggcaccagggcagacagcaagtacgcgtacaccagttatcttctcatggtaagggtccccaaatgaacgt

gtgaaaccaacaattgcatgtttagtggcagtgtaaatgggaatgcttacatatggctttacaacaacatttgagccagtattaacaacagttcctccat

tgccaccacggtcttttcccataaaacgcagagccagtaaggtaccacgaatgactccattcaagttgacatctacttccagctcccaaaatctgtcatt

cattattccagcattgtttacaacaatgtcaaggcagccaaattcagaaattgttgtctgaaaagattcttcgtattgggggtaatccgtgacatcacac

tgacaaaacagcactttgtctttgccatatttttcttccaaccgttgaacaagctgctcccctgcctcagcatttatatcgcatatagaaaccttc

>83472|15-hydroxyprostaglandin dehydrogenase

gaaaggctttgacgagttctgtcagatgaaacaccggtactctgtcaaaatatgtacatatattagtgtctctgtagttggtagaatattgacatggatt

tgaaagggaaagtagctctagtaacaggaggagctgcaggaataggtcgcgcttactgcgaagagttgctgaaacatggggtgaaggtttctatatgcga

tataaatgctgaggcaggggagcagcttgttcaacggttggaagaaaaatatggcaaagacaaagtgctgttttgtcagtgtgatgtcgcggattacccc

caatacgaagaatcttttcagacaacaatttctgaatttggctgccttgacattgttgtaaacaatgctggaataatgaatgacagattttgggagctgg

aagtagatgtcaacttgaatggagtcattcgtggtaccttactggctctgcgttttatgggaaaagaccgtggtggcaatggaggaactgttgttaatac

tggctcaaatgttgttgtaaagccatatgtaagcattcccatttacactgccactaaacatgcaattgttggtttcacacgttcatttggggacccttac

catgagaagataactggtgtacgcgtacttgctgtctgccctggtgccactgacacaaaccttgtgaaagatgtaaggaaacagctgctgtcacctgaat

atgagaatgcttggcaacgagatacagcaggaggggtatcacaaacggcagaacatgtggcaaaggagctgatacagttgttgcagaaggcagccagtgg

ttctgtttggatggtggagaagaaccagccagcacgggagatacattttactacccagtaaacagtggttttactgttatgacaagtttaattgcacaag

tagcctgttgaagtgcatctaacacactacaaatattatgcacaattttcttccggagctttgcagatttatgggctttttgtggcttttcattcaagtc

acatttatcgtatgtacattttgtaatttggcctaaacatttgtatatttgttgatatttatgcactgtatgttacacttaagaaagaacagaatgactg

ttcaccttttttctagaattgatcagaatatcggttaagagaggacatgattacatactaccaacttgctgtgcacatgtgatgggagctagaagcatat

aagggtaaacatggaattttgttgattcagtggaccattttgggaaggaaagacttaccgattattcagtgagccaaagggatagtgcttaaacatgtaa

tttacctcgaataagtaactgatattttcttctgtaagaggccactttgtgttaagaccgttagtactgctgctgtcctcttttattaaaacaaatcaat

aaataagctagaagtaacttctgatgataatttttttagtccaaatttgtgtagtgtaatggttcagctgttaattatctgcattgtttacaaaagtacc

gaattttgcagaaaaggatattaatattattaagagctacatttaagataattctaccttatacacaaatcccagtaaattgtaacctattaagtcactt

tctacgccattaaagtgaaagcttggctgtacttcccaaggccgattcacctgtcagacatgtctaaagtaagtaataaagcaaattggtcaaaaaatgg

aagggtcataaaatggag

>83473|15-hydroxyprostaglandin dehydrogenase

caacccaattttattgttttgtaattaacacttgcaaatttttgtcacagttccagtaatgaatgaaaggtcaaatattacatttcagctgaagcatact

tttgttgaaatccaccctgagaatgcagcatctttcaacacctcatgtggcattttcaacgcctaaacaacaacagtggtttttatttttaagtcaacaa

aacatatattaaatattgaagtactagttgtataacatatctgtggttgcaaatgcaaaaaaatacacataaatatcattgttctttcatgcaaacatca

cagaatcaaaaacatttacatttagtccttaaataatcactacttaagaccataatttcacaatcgaacttgccctgtaaaacagcttaatcatcatcac

attcagaaaatttgtccagtgtaacttttcttgcaacctccaatttaattctttgtttattttccagatttgattttcttcggccccactcgtttttctc

gcttaccaaagattttcttctttcttctgccagttttggcttttgcaaatgcctttggaaaaggaatgatgtcttcacctaggtctcatttacttgtgct

tgattgcagtcctttgtttttaatgttgttggcctaagactacacaccaacactgctggattttgtttgcttgtgctaggtattcagttgtgcagtttac

ttccggaggcaagaaacctactaatacatccatggtgtatgttcagtcttatatgaaagtaactgaatgagtatcgtatccctaatataaaatatatatc

tgtctgatgtttccttttattaacagctgttgctcttgggtttcaggtttccataagggaagcagataacatctctgactgagtcaaatttgttattttg

tttgtgtccttaatactatgtatgctagaaccccttagttttgcattttgtatgcaagatatatttgtcagtgaatagttaatatttctaacttttaata

atgtaagtgtaacatttaaattcctcatctttgaaaacttttataccatgagtgaagctggtgcttgattactgtcacccagtgtaatattacctgcatt

catgtttctcaccctatcaaatgcactacactagacatcccaaatagatctacagcagttttaagagacatcccatattttactgctttcatggcttttg

tgatttattcctcactcacttctgatttaatacaagtctctctataatttcataagatattacccaaatcaatgggaaagcaacttactttggacgtatt

attccacatagttatatattactgcccaaataaattattttgatgggctgcaggtacactttgagagtgttgaaatctaggcctacaggtttattaaaat

cttccccaagtgcagccatcaagattaatgcagcgttgcacttactttacaaaaagtgtttcaagtagtgtaaggaatttcaaatacagcagctcccaat

aaatggggaggacgaatatgatgtgcctcagttaccttggtgtacaaaaattgtgctattcaaaaactctgtacttttcacagcatcacaccatgtttat

gtaacagtacagcacagtcagcaactgtttgaataaggaactgtcaaccaattacacagaggttgcagtggtgagtaattgaaattgattaacggtgcac

gtattttaaatgtggcaccataccctccctaacccctactagttagtctagtctttaaaattgtggcacaggctcattttgtctacttcgaaaacgagaa

aaatgttttgctggcgctctttgtatttaactcactcgcgaaaaggacggattttaaatttgacataagtaaaaaatgattatgataaggtagcttctac

actactaggctcattttatcacaagcagaaccgtaaatattgaagtatttatttgttaagcatgcaaaaacgtcgagcgatcgcaatgctcaaaatgtag

caaaccttcgtattgggggtaatccgtgacatcacactgacaaaacagcactttgtctttgccatatttttcttccaaccgttgaacaagctgctcccct

gcctcagcatttatatcgcatatagaaaccttc

>83750|150 kda oxygen-regulated protein

gtggtctttagtctttggttggcgtaatagaaacgctccgcataatggtacagaagcgtgtgtaaagcaaagtgaaatgtgtttcgattaatagtacttg

cgaaaattagttttagtgtttttcgtcgcagtggttcttcgtttgcttgagtaattaaagatctagttccagtgatgcggctgctggcggccatttccgt

tgtgctgtcacttctgcatgcagtgcagcatgctgacggcatagctgtgatgagtgtggatttgggctccgaatggatgaagattgctattgtatcgcct

ggtgtaccgatggaaattgttctgaacaaagaatcaaagaggaaaacacctgcaaccatcgctttccgggacggagagcgatcgtttggtgaggatgcac

tcgtggttggagtgcggttcccccagaacagttacagctacctgctcgatttgctggggaagagtgtggacaatcctttggtacaactcttccagaacag

gtttccctattacaaaatagaagcagaccctgacaggggcacggtagtcttcaggcatgacagcgagtcgacgtacagcgtcgaagagctgctggcgcag

atgctgcagtcggcgcggcagatggcgcaggcatcagctgggcagccagtgaaggaggcggtgctgacggtgcccggcttcttcaaccaggcagagcgcc

gggcgctgctgcaggcagctgagatggctggcctcaaggtgctgcagcttatcaatgactataccgctgttgcattgaattacggcatattcaggcggaa

agacttcaacgagacagctcagtacataatgttctatgacatgggagccagcagcaccacagcaacagtcgtcagctaccagatagtaaagaccaaggac

cgcggcttcgtcgagactaacccacagctcgccgtactgggagttggatacgaccgaacattgggcggcttggagatgcagctccgcttgcgcgactact

tggcgaaaaagttcaacgagcagggaaagacgaagagcgacgtgttcggcagcccccgtgccctggcgaagcttttcaaggaggctggtcgattgaagaa

tgtgctgagtgccaacgccgagcactacgcacaggtggagggtcttttggacgatgtggatttcaaactgctggtgactcgcgacaatttcgaagagctg

tgtgcagatctgtttgataggatacaaaagccagtagaacaagctctcaaggcatctgggctcgacatgaatgtcatcagtcaggtgatccttgtggggg

ctgggacacgcgtgccgaaggtgcaggagcggctggcagcagcggtgggcctcgagctggggaaaagcatcaacacggacgaagcggcggctctgggagc

cgtctacaaggcagccgacctcagcactggcttcaaggtcaagaagttcctcaccaaggacgccgtcctcttccccatccaggtggtgtttgaacgagag

ttagaaaatggtgctggaacaaaacagattcgtcgaaccctctttggcctgatgaatccctatccgcagaagaaagtactcacattcaataagcatatga

acgacttttcgttctatgtaggctatggagaccttgatagtcttcctgaacatgagatacaagctcttggttcgctcaacctgagtcgtgtggaccttac

tggggtgagcgaggcactgtccaagcacaaggactcgcacggagtggaatcgaagggtataaaggcgcacttctcgatggacgagagcggcgtgctgcag

ctgtcgggagtggagctcgccctggagaagacggtctcccctgaggagcaggaggctgaggccgccgacgctgaggggacactatcgaagattggcagca

ccatcagcaagctcttcacctcttctgacgagaagaaagatgagcaaaagcaagaaaaggatgagaaagcagaagagaagcaaagtgaagacagttcggg

gaaggaaggtcaagagggaggagacgctgccagacaagaaggtgaagggaaagagcaaaagtctgatgatgagaaatcggctggagagaaatccgaaacg

gaggagaaaagtgctaaagagggtgctgaaaaggcagatgcgaaaaatgtaacgaagccaacggaaaagaagcctaaaataattttgctgaaagagccaa

tcaatgcagcagaagaaaagagcggtgtgccagctcttaatagcgagcagttccagtcatcaatcaagaaattggagtccctgaacgaatttgacgaaca

gaggaagcgccgtgagagtgctctcaatgccctggagagttttgtttttgatgcacaaaataagttgtatgcagatgagtacagagcagctgcaacggag

gaggagctggagaaaatcaagcaaacctgttcagaggtgtcggactggctgtacgaagatggctcggatgcagatgctcaggcttacgaggacaaattgt

cggagctaaagcagctgacacggcccttgttccgccgtgtctgggaacatcaagagcgaccgactgcactcagttctcttggtgaaataatagaaatgag

ctcaaagtttctagatggcgcaaagaacggttcagaaatatttacatctgtggaactcgaaacactggagaaggtcatcaaggaaacaaaggagtggaag

caaaagatggaggaggaccaagctcagctgaagttgtctgatacgcctaagcttacagtcaaatccataacagacaaaatctctctcctggaccgggagg

tcaaatacttgatcaataaagcaaagatatggaagcctaagaagaaagatgacacaaaagagaaagccgatgacacatcatcttcaggaagcaaagacaa

aaatgaaactaccacagacaaaaaaactgaatcggatcaaccagtccccgactccacctctgatgacggagaagagaggagtcccgacatggaccccgag

gctgttccgctcccggaggacgtgacgggtggcgctgaggagggtggaccggcggtgccggagccggatttgccggcttcagcggaccctctgtccagcg

acaagaaagcggatgtcgtgaaagaaaaggacggggaaagccacacagaactgtaacgtttgaagactgtagagttccttacctccactgatttgtggaa

ggtgtgatttaattccggaagattaaaacacttcgttgtcgcatttagtggccccggtgtggggagagataaagggacctaaactgtacagagttaattg

taaggtgcgagtgagtgcgtgtgtgagatctatctgttgcacaaaccgtgtgtttgtttttctttggagtcctcccctgctgttgttctcacccctatgt

ttactctgtataaacatttgtcacaattatttatattatttataaaagacatttttgtcagcatccagttgtttaaaattgggtcatgtagttatacaat

gatatttaaacattgtcacattagaagtgtgtggattgtctgcaagtttttttgttttgtgcaatcttgagcacatttcaggtaactgtgggagagatgt

cttttttttccagtgtcacagggtcactcgcctgtgcatttatgataaatttttcatgttgtttgtcttcttgagaagtagcatcctgtaaatattcatc

tcgtgtgttgttagttgtcattggcgtgaaacaaattcacttgtaaaatctgttgaacaaaaattacgcgtataaccaattttggcagtgcacactgcac

ctcgtgtgttcccttacccgtttttgcgaaagtattcgtaccacctaattaaaaagcaaaaaaaattgtttacctaccagttatgtggcaaggaaaatgt

gtaaccgaaaattgtgcgttgtggtttattttgagaattgcagacaaaacagatgtaattgttatatttattcagataaagtgtcaacattttttgtatt

gcacttaatttgagtaagtgtttaactgtttgaatatctgtaagatctttaccgtgaataccttatttgtacatcttttgatttaaatttctgtgtgtgt

tgtacttttccttcagaaaacataaatgtgggatctcaatga

>84062|16 kda thioredoxion

tcgttgaaacactgagagctgtgcacgaagaagcaatgaagtttgagctgcctatccaggtaatttacatatcagcagataacaacgaaactgaaatgtt

gcaacacttctcagagagccatggtcattggtatgctgtgaagtacgatgacgacgtaagaccgctcttgcgaatgaaatacggcatcacatcaattcca

gtcttactggtagtacgtcctgatggatatgttgtgagcaagaatggcagacggcaattggaa

>85214|1802231avitellin-degrading protease

gataaatttttagtactagaaatagtgcaaaaaagtttttcttagttcaccaacctttgaaattgcaaattatattacatttaaattttgtaaaatattt

tttggacaataataattcagttgtgtttttgatgtttgtatgtaaagtaaagactttcagtaatcactccttactctgacctttcagatttcctttgcat

aggaaaaatgtgtaacgaatgtaatagacgtatatactatggtactctaactttaagtactctggaaatttggaacgttgtctttaatagaacaatattc

agatttaaaaaatcacaacacattctttcagattaacatttaaataatactatcattttcactatgatacataagaagacaatattgcagaagaattaaa

tagttgttagcaaatatcacttatacagttaaaaatgacactaatgacatacaaaacactgataaataatacaaaaatcagtccacattcaaacacattg

tactttataaaaaatatactttaaaaaagaaaccattgtaacagtcacagatgaagtataaaaacagctcatccacattacataatgaaaaatgaagact

atgtgttcattgaaatgacaattctggcaataatgattcttttttacaaactcgttgtagttgacaaagtcattctgctccacttccacgcaccatttga

tgagatgtgatacttagttttatttacacatttggtttcagtgaaatatagtttatcttaagtaacaacatatttattttagttgtatgtgtatagaaca

gtcactgagtcagccttattagtcattcagacatttgtgttattgagtatccagttactgaatgcagaaactcgagtgtacacatgtggatattcacttc

tcgcacaaccaattgcccatgaaacgagtccaaccagcttccgttttgctaccaaaccaccgccattgtcaccctggcagctgtct

>Sg_CNS_NA4Plus10392|1chain 60s ribosomal subunit in complex with initiation factor this file contains 26s rrna and proteins of molecule

cagagccaatccttatcccgaagttacggatccaatttgccgacctgcccaacacatacatcggggacgttgtcgtctaccacaagaccattgacgagat

caatcgaccagctgtctagtcatgtattccccagccactgccaattcctagtgccaaataaaagttcgttgtcgtctaccacaagaccattgacgagatc

aatcgaccagctgtctagtcatgtattc

>85444|2 -phosphodiesterase 12-like

tttccatttcaaatcagcaactagtgctaagtgatctgaaggaaagacaacatttggtaatgcagtgtgctgttttagttcctcttcagtaggtaaagga

acaacctgtgtcacctgtaagttatctgtctggaagaatatataatctaaacatccagcgaatccttcagtgtagtttgtgaaatctggtgtcccacatg

cacttgcaatgttgaatttgtgcgataaacaaacacctttcacagtctctgctacattgcttgaccagtcgacagcatcttccgtaaccatctgttgcgt

cataagttgatagactccacattctggtgtgctgttgaagtcgccacacaagagaagtgtcgcctctttgtctggaagctacaacaataatttaatctac

agcatttttggaccagattcacagttttgaaacattattttgtgtgacagacaatgaggcatattaggactgtacattaaatttctttgctctacatata

tttgggtaacattattctatacagagtacgcgaaagaacttgcccccttctaaacagccgtgtaccgcaagtctctagaggaacg

>85677|2-acylglycerol o-acyltransferase 1-like

gcatctgaaatgtatttgtgcttgtacttctcaaaaaagtttacaagttcttcagagaatttattgtggagttcagtgatttgttcctctgtcggatctt

caactttcttcacttcaattggtgagccaacgacagtagtaacaggattcctacgaggtacaataccaaaggagtactgaaacaaaccacgaccaattgg

aagcacaggtgccattccagtaatcttgcggcaaaagtgctggaatttcagaagtcttgtaccttctagttgatcatagagatcggtctccccaaatgag

ataacaggaacgagtggacaaccatgtttcagtgctagcctgatgaaaccacgtcgtcgcttcagaattactcgatacgtgcgaggtttgcatttcagtg

cttctgatgcaccacccacaattaacacagcagcatttcctcctcctggtttgtccataagatacccaatgctttcagcagaagcagaacatccacctaa

ggataagcagagctctctgaagaaaggaaccttgaaatgttgttccagtgtaagaggataaacagtaattcctgggaaaagtttgtcccaccccatataa

tctgtggagaagcagccaaaaacccctgcactcagcacaccatgtggaaatgcacagaataggtaattgcgatttggatccaatggttcaatacattcaa

ggcgtaatggaaagaagtccctaaagtagcgccaccacagccatcgccgtgcccattgtatcctgcgcccaccacgacggcagacctcccagtcataggc

acaccaggcagcataggcaagtgcaatgtagcgcaggcgtgtatacaggactaagtagaccaacaacagccatgacattaagccgccaaatggcagaatt

acaatccacattagagctgccaatgtctgcaggcgccgctccagtggcacgaacaatggtgcgaactttattcccagcagctccatcctgcagcaatgaa

aagggatatggaatcacaaaaatgatctttcactggctcacgtttactgattctccttgaaatggggctgactaaaccagaattacttgtagatacagat

aggtttaatgaaacattaggtacatgcatatatttctttctaatgcttgccttcttgtgatattggcattttcatatggtcctgtgtagataattttatt

ctggatgttaagagtttttgcttttttttaaattatttgtcatctttgtcaaagttagtaacgtgagaaatataaacaagtactgcctatgtcattgttg

taagcagtataaattgcacaatataaataggtaattagctctttagaaaaaatatcaatgtttgtatcagaaagattagatttgtaaaagattgtaaccc

aattttacatggaaatatcacattccatcacaaaaagtctcaaactatttgagacaattaattgcaattaaatttacaacacagaaaaatgttgtaat

>85678|2-acylglycerol o-acyltransferase 1-like

gcatctgaaatgtatttgtgcttgtacttctcaaaaaagtttacaagttcttcagagaatttattgtggagttcagtgatttgttcctctgtcggatctt

caactttcttcacttcaattggtgagccaacgacagtagtaacaggattcctacgaggtacaataccaaaggagtactgaaacaaaccacgaccaattgg

aagcacaggtgccattccagtaatcttgcggcaaaagtgctggaatttcagaagtcttgtaccttctagttgatcatagagatcggtctccccaaatgag

ataacaggaacgagtggacaaccatgtttcagtgctagcctgatgaaaccacgtcgtcgcttcagaattactcgatacgtgcgaggtttgcatttcagtg

cttctgatgcaccacccacaattaacacagcagcatttcctcctcctggtttgtccataagatacccaatgctttcagcagaagcagaacatccacctaa

ggataagcagagctctctgaagaaaggaaccttgaaatgttgttccagtgtaagaggataaacagtaattcctgggaaaagtttgtcccaccccatataa

tctgtggagaagcagccaaaaacccctgcactcagcacaccatgtggaaatgcacagaataggtaattgcgatttggatccaatggttcaatacattcaa

ggcgtaatggaaagaagtccctaaagtagcgccaccacagccatcgccgtgcccattgtatcctgcgcccaccacgacggcagacctcccagtcataggc

acaccaggcagcataggcaagtgcaatgtagcgcaggcgtgtatacaggactaagtagaccaacaacagccatgacattaagccgccaaatggcagaatt

acaatccacattagagctgccaatgtctgcaggcgccgctccagtggcacgaacaatggtgcgaactttattcccagcagctccatcctggttttttttc

ttttgagaaatgttcgtacttaagtcgtcagtcttctcaaaccagaggcatgagtgatgctccctgacagcactacaatatcgtcaatcgccttctcaat

aaatttctgtattggaaacccagagataaggccgcaggaggcagaggcagaggcagaagcagagggagagggagagggagaagggaaggaagaagaggaa

atatcagcgaaagaaacggagcaaagagagaaagggaggtgacaagctctccaccgcgcccccgtaagattttgtggtaagagagccaagtagatagctt

tgcatgtgtaataacaaaaatcttgcctttttacaagtatttcttctcctgtttatcgaaacagtgaaataagctgatatgccgctttgttacctgaaaa

atgtattacatagcacgtaatttttatataatttacgcaattataaaatacattttaattactggtcatggacgctgaatagaatacgaaaagaaccaga

agttctgagttcaaaaaa

>85676|2-acylglycerol o-acyltransferase 1-like

cgacttataatcggatgaagcccgcgctccataattgtctggtgtcatattttgtaaagagaatatgattacttctacaatatcatttcagtggtacagg

gtggcgcttgaaaaatcggccacgattactagactgctcattgttgtccaccaatcgttatctgtggtttcgaggttgtttgcattagcttatttgttat

ttcgtcactgcctaattattacttgtttatctattctgctcgtagcggtcaacaaatcgtgtgcagttggagagcagtctgtactcggggccggtttttc

gtgcgccaccttatattatttcactgtgatcagccacataatgctacagttggctaaataagaggttttgcagaatcacgaaaattgtttcagagttgtg

ttatgagtaaaaactctgtactccagggggaagcatgtgctccctcttggcgcctttcatcttcagtcacctatgctactaattttcaattccaaaaaag

agctatcaccagtgaagtagttcttaaggatgaacgagtttgcctatctctacgagtcacgtcttgaccttggaccgttcactgtctctattttgcttct

ttttcacagttcagtacaacttctttctgttctaatgcttgatctgtcttctgtttttgacaggctatctacttggctctcttaccacaaaatcttacgg

gggtgcggtggagagcttgtcacctccctttctctctttgctccgtttctttcgctgatatttcctcttcttccttcccttctccttctccctctgcctc

tgcttctgcctctgcctctgcctcctgcggccttatctctgggtttccaatacagaaatttattgagaaggcgattgacgatattgtagtgctgtcaggg

agcatcactcatgcctctggtttgagaagactgacgacttaagtacgaacatttctcaaaagaaaaaaaaccaggatggagctgctgggaataaagttcg

caccattgttcgtgccactggagcggcgcctgcagacattggcagctctaatgtggattgtaattctgccatttggcggcttaatgtcatggctgttgtt

ggtctacttagtcctgtatacacgcctgcgctacattgcacttgcctatgctgcctggtgtgcctatgactgggaggtctgccgtcgtggtgggcgcagg

atacaatgggcacggcgatggctgtggtggcgctactttagggacttctttccattacgccttgaatgtattgaaccattggatccaaatcgcaattacc

tattctgtgcatttccacatggtgtgctgagtgcaggggtttttggctgcttctccacagattatatggggtgggacaaacttttcccaggaattactgt

ttatcctcttacactggaacaacatttcaaggttcctttcttcagagagctctgcttatccttaggtggatgttctgcttctgctgaaagcattgggtat

cttatggacaaaccaggaggaggaaatgctgctgtgttaattgtgggtggtgcatcagaagcactgaaatgcaaacctcgcacgtatcgagtaattctga

agcgacgacgtggtttcatcaggctagcactgaaacatggttgtccactcgttcctgttatctcatttggggagaccgatctctatgatcaactagaagg

tacaagacttctgaaattccagcacttttgccgcaagattactggaatggcacctgtgcttccaattggtcgtggtttgtttcagtactcctttggtatt

gtacctcgtaggaatcctgttactactgtcgttggctcaccaattgaagtgaagaaagttgaagatccgacagaggaacaaatcactgaactccacaata

aattctctgaagaacttgtaaacttttttgagaagtacaagcacaaatacatttcagatgcagacaatgtacatttagaaattgaagaataactacctta

gttgtcagcagagcatatgactctgttttatacatcacttccaattgtctgtcgtttacagtattggataatatgcatttcatttttcttgtaatatgaa

tgcattcacattcctgcattcagttgataggaaaaggaccactgttactcttgtttaaatatgccagaaattgtacaaattattgtaagtcattttgctc

aatgaaattattttgtgtgtgtgtgtgtgtgtgtgtgtgtgtgtgtgtgtgtgtgtgtgtgtgtgtgtgtgtgtgtgtgtttttttttgttctcttgtgc

gggcgcgcgctctgtatgataaatatacaaattttacttaacttttgcttagatacaaatctgctgcaatgctgaagtataacactgttaaatcttattt

ttttagattaaaatttattgaagattaagtattacatataaaacagtaaacacattcaatgaaccataaattgatactggtattttatttcaaacttctg

tattgtggcagatttgtaaggagtaaacttttccaagacagtaatggctgtgattagttgtaagaatagagacataac

>86120|2-acylglycerol o-acyltransferase 2 (monoacylglycerol o-acyltransferase 2) (acyl :monoacylglycerol acyltransferase 2) (diacylglycerol acyltransferase 2-like protein 5) (diacylglycerol o-acyltransferase candidate 5) (hd

gagtaacagtggtccttttcctatcaactgaatgcaggaatgtgaatgcattcatattacaagaaaaatgaaatgcatattatccaatactgtaaacgac

agacaattggaagtgatgtataaaacagagtcatatgctctgctgacaactaaggtagttattcttcaatttctaaatgtacattgtctgcatctgaaat

gtatttgtgcttgtacttctcaaaaaagtttacaagttcttcagagaatttattgtggagttcagtgatttgttcctctgtcggatcttcaactttcttc

acttcaattggtgagccaactgtaaaaagaacatgttaatgtgttagttggtttgttataaacaaatttatttccatttaaattcactcacttaaggtca

caaaagtgaatatttctactatgtattgataaattcctcctgccataataatgaactacaatactgacttcacaaaatcttatttatgcacttcagtgct

atcctagaaatacttttttcgtttcagtcagttccagcaacaaactgtacagttcaggccattatgtacagcattttttgttcaaactgtcagtgtaaaa

atggaacaactgctgtcttgactctccacatccttgatcaggatacccaaccgaaggttttgtctgtctggtttcatcatcagtcttttcccttgcaaca

aaagtgacaattataaccccctcatttatgtcaatgaattatttaatgtacaaacatgtgtgtagccagtttgatatttttggcacgttgagttaaaaat

attttgttacctaattgcctttaccattcaatgaaaggtacctacatttcccatagaacatgtta

>86400|2-amino-3-ketobutyrate coenzyme a

tcccataattatcttggcttagctactcatcctgatgttatatgtgcagctaaagctgctcttgatgaatttggtgctggacttgcttcagttcgtttta

tatctggaactcattgtttacac

>86611|2-amino-3-ketobutyrate coenzyme a mitochondrial-like

gcctctacacaacatttgttctgcaaaaatagttgcaagtttagaatcatcctatctgccctgtcatgttaggtgatggtaaacttgcaactatttttgc

agaacaaatgttgtgtagaggcatt

>86613|2-amino-3-ketobutyrate coenzyme a mitochondrial-like

ccacaggataactgaagccaatcacataaatgcctctacacaacatttgtatgtgctgcagatatctgcacccgaatcctggctttatctttgggcacca

caggataactgaagccaatcacataaatgcctctaca

>86610|2-amino-3-ketobutyrate coenzyme a mitochondrial-like

aaagatgcagttctttctgatgaactgaaccatgcttccatcattgattgtatacgtctctgcaaggcacagaagttcagatacaaacacagagatatgg

caggtactcgggtgctatatgtgtaatcaaccagtttgtaacaagtgaaatttattgaat

>86835|2-deoxyglucose-6-phosphate phosphatase, putative

gtgataagtagagccaaatttaattccacgagctgtaaccgtgtcgatatattgacacgcaacatgcaggacttgctcgttaatacattgatccgtagca

ttctgatatttgttccgcaggtcagttgcgttgcgggcattgccactggttatttacggaaagctaccaaggccgcaagaatctatggggttccaaccag

taacacattgtattttcgatttggatggacttctacttgatactgaatcaacatatttgaaattatatacggctattgccgcgaaatataataaggaata

cccatggtctgttcgcatgaaactaatgggtacgttggaaagagattcggcaaaaattatggttgaagatcttggtttgcccataacagtggatgaattt

ttgtcacaacaaaatgctggtcttcagaatgttcttccagaagtcgaccttctgccaggagctgagaggcttatacgtcatctgcataaatccaaaatac

caattgctgttgcaacaagcagtggagaagatagttacaatcttaaaataactcgtcacaaagcaattatgagcatgttccatcacatagtgtgtgggag

ttcagacacagaagttaaaaaaggaaagccggcgccagacatatatcttatctgtgcttcaagatttgctgagaaaccaaagccacaaaagtgcctcgtt

tttgaagattcgccaaatggagtgaaagctgcacgtgctgctggaatgcaggttgtaatggttccagatgaacgagctactgatattgagacgaaggaag

ctacaattgttcttcgaagcttagaagattttaaaccagaactgtttggtattgctccattttaaaatacatggtaaattagtctcctgagactacgcga

tatggtgcttttatgcagttgtaattttggaatttataaaaataattatactaaatggagagttgtagctctagctctaaataaaatgctgctgcta

>87119|2-deoxyribose-5-phosphate aldolase homolog

gtcatcaccagacaatgtagtcaggtcaatgcaagtgactgccctgagaagccatgctgcttcatggtcacctttgagtgacctccgagaaagaatgtca

tcagcttgacgcattatagcagaagtattcacaattgcttcattgatccacttaacatctaaatgacaaccaagatttggggcatctccaataattgaga

aaggtagaagaaacgcgtttctttcctcccatcctgatcttaagttatctattctactgtgccccaacgaatccatatcgcgtgcacggcaaacgtttga

catagaatgagtgtcgattct

>87661|2-hydroxyphytanoyl- lyase

ctttgtgttggaaatttcctttagtctatcctcttcttttaatgtttcctgccattcaggatcacatttaaaaccacctattacaagggagagttcggtt

aaaaaggttccagcatcaccacatacagccaatgttggtttccaaaataaatctgaattcttataaagttgttcacggctcctgttgacagcaatgattt

tagctgtgcgaggcagtgctcgaccgtacttcaaacgaaaatcacagactgtgcccacaagaagaatgaggtcagccctgcgaagtgcccctgaacgatt

atgacggagctgtagtgggctttctttgccaagtaggcctcgtgccattccaccaaggaagcatggcactcccagcctctcaacagctttgcgaacggat

tcagcagatactggtggcaatgtcacctggctgccaagtaccagcaatggttttcgggaacaagacagcagctcaactgctttctgaatctcagaatctt

gtggaagaggaatgtttagttttagtggtgtaacttcacgaggttcccacgcaccagcaaatatgttgtccaagtaattatttagataccagttgatagc

ctttgtcaatatattccctggttttgtacctgatgcaacttctttcttaacaagtttgtatggatatagcacatccacaggaagttccacaaaaacagga

cctggggttcctgactgtgcctcttttaatgcctgccttagagttggagcaatgtcacgaactgctgaaactgcagcacagaatttgcaaagtggggtaa

acaatgacatctgatcaatatcctgcaaagcccctcgtccttttaacagtcctgcagaagctcctcctaacagaagaactggagattcagccagctgagc

attcttaatagcagtaacagtatttgttaagcccggtccagcagttacagcaacaactccaactgtctgtgatagtctcgcaactgcatctgcagcgaag

acagctgatgcctcatgtcttgtatccactattctgatgcccagtttctcagcaccaacaagtataggtgaaatgtggcctcccaccagagtaaaaatgt

atttaactccatgtgctttgaggacttcagcaacaatctcgcctccatgatgatcagagtctgtgtctacttctaagaacgctatgttttcatagttacc

ttgtgcaaccagatatatatgatattaaaccagcttgcaaaaaaaccgacaaatatgcccacaaaagcacaaagaataaacaatataattaacgaaactg

agtgttcttcaatcagttcttcttctaacccgctgccaaacgaggtcatgttttccctttctcaagtacgtaaaatctgcttaaacagtgctgaaaaaat

gcaggtcatctttattattttaagtgataattaaag

>88323|2-hydroxyphytanoyl-coa lyase

ttatcacttaaaataataaagatgacctgcattttttcagcactgtttaagcagattttacgtacttgagaaagggaaaacatgacctcgtttggcagcg

ggttagaagaagaactgattgaagaacactcagtttcgttaattatattgtttattctttgtgcttttgtgggcatatttgtcggtttttttgcaagctg

gtttaatatcatatatatctggttgcacaaggtagacacagactctgatcatcatggaggcgagattgttgctgaagtcctcaaagcacatggagttaaa

tacatttttactctggtgggaggccacatttcacctatacttgttggtgctgagaaactgggcatcagaatagtggatacaagacatgaggcatcagctg

tcttcgctgcagatgcagttgcgagactatcacagacagttggagttgttgctgtaactgctggaccgggcttaacaaatactgttactgctattaagaa

tgctcagctggctgaatctccagttcttctgttaggaggagcttctgcaggactgttaaaaggacgaggggctttgcaggatattgatcagatgtcattg

tttaccccactttgcaaattctgtgctgcagtttcagcagttcgtgacattgctccaactctaaggcaggcattaaaagaggcacagtcaggaaccccag

gtcctgtttttgtggaacttcctgtggatgtgctatatccatacaaacttgttaagaaagaagttgcatcaggtacaaaaccagggaatatattgacaaa

ggctatcaactggtatctaaataattacttggacaacatatttgctggtgcgtgggaacctcgtgaagttacaccactaaaactaaacattcctcttcca

caagattctgagattcagaaagcagttgagctgctgtcttgttcccgaaaaccattgctggtacttggcagccaggtgacattgccaccagtatctgctg

aatccgttcgcaaagctgttgagaggctgggagtgccatgcttccttggtggaatggcacgaggcctacttggcaaagaaagcccactacagctccgtca

taatcgttcaggggcacttcgcagggctgacctcattcttcttgtgggcacagtctgtgattttcgtttgaagtacggtcgagcactgcctcgcacagct

aaaatcattgctgtcaacaggagccgtgaacaactttataagaattcagatttattttggaaaccaacattggctgtatgtggtgatgctggaacctttt

taaccgaactctcccttgtaataggtggttttaaatgtgatcctgaatggcaggaaacattaaaagaagaggatagactaaaggaaatttccaacacaaa

gatggctgaggaacgtacaggagaatatgttaatccattaaagatattgaaagaagtagaaaatattttaccagaaaactctattttggtagtagatggt

ggagattttgtgggctcagcagcatatattttgcgacctaggggaccactgtgttggttggatcctggcgcatttggtactttgggagttggtggaggtt

ttgcgttaggtgcaaagctggcaaaacctgatgcacaagtgtggctgatatatggagatggttcttgtgggtacagcattgccgagatagatacaatggt

gcgtcataagcttcctgtggtagctcttgtgggcaatgatgctggttggacacagattgccagagaacaagtgcctctctttggcagtaatgttgcctgt

aaactagtgcacaccaactatgaacaagttgcagagggttatggtgggaagggaattctgatatcaagctcatctgataatttaatttcctctcttaaaa

aggcagtagatctgcacagcaaggaccacacagttttgataaatgttctaattggtaatacaaagtttagagagggaagtatttcagtgtgatgatttag

tgagtaaatgcactttaaactggagccttatttcatggacactgtacaaggagaaatcataaacatcacacaaaggctctgataattaagtaaaggtagc

actgagaaatgttcaaaaggtggtttggatattaagttcttcagctatctctgtttaaaaattgagatagtttgtattaaatggcattttatggtgatac

ggatcattttggtagctttagtgacttaaaatggtgaaatagtgctgtgactgaaaattgtattacttgatgtactagtttataatttttaatcagtatt

gaagctgggacagttttcactgcagctatattgcaagtgtattgtaatatttttttcatacctgtggatgcttgactgattctccattattcctcaaaaa

tgcattcccagaagaaaggcagggttatttttatgaggtcgccccatgtgttgcccaaaccttacagttaaagggtctgacttataattcatatgtgaag

agcatcatgtcagtcttgccatataaacataatactatttatttatgatgtttgtgttgagtattgtggaaatagcagttctgatactgattttgtccca

tctgcactatgttattttgtttgtttcatatacaagaggtgttctgatgttattgtagtcacttgacaaggtcacagtggattgtcaaaggaaaatgaaa

ataagtaactggcagttgacagatttttgatttttcagatatgatgggcacagatgattatatctaaattattcagttaactagttgatcactgagaatt

ataaggagcattgtttgtaaatttagtaatgggctgctctacaaggaagctatttaagttagtgtaaactggatcctatgactgccactggctgggtgtt

catgtttaagtgaattttactgttttgacatttctcatggaaaataatgttattcagtcctttaggcagattcacagtaaattatgtaagatctctagag

gacagagaatattcaaaaattgctcttattaaaaaaaatttggcttttaattggtaaatcatgtgagagtaagtaatttactctgagagtagtacttcat

ttctctgttcatatcagcactttaagacacacttgttcagctccttgttgtagtgtagccttaaggtagttgtacattaaaggaaagttattgtttatct

gttgttttgttggtaccattgatgtgtgattactgaaaaacagtattttggctatgaaatcctgtgatattagcaaatataaccacatggacataatatt

ggtttgcagctggacatatggacatattgaaaaacacaatgtatgatatgaagcttcagaagtcactttacaaataaattgtacctacagatgtggaatg

aaaataaatttatacctgcttcataactttctaccacattgatttccttccttctgtcctgctgttagtcttttgtttattgaaatgctattgtgtattg

tctggattacattaccctgtgagggtattgcagccagtcttcctccaaagtttagatacccaacaaagttgtcttccatggaggagttaaaattgtctaa

ccattaagacattttcttacacactctctaattacactgaatctgaaccaatttttgctaatgtagtggtcttactagaactgttggaatgatttgtcat

agcgcagtagtctacagcagttacctttgtttgcatctgaacaaaattgtaaaagtataattaattgtgtggaagaatcagtaaaatgtatttcacttgc

a

>88324|2-hydroxyphytanoyl-coa lyase

ttatcacttaaaataataaagatgacctgcattttttcagcactgtttaagcagattttacgtacttgagaaagggaaaacatgacctcgtttggcagcg

ggttagaagaagaactgattgaagaacactcagtttcgttaattatattgtttattctttgtgcttttgtgggcatatttgtcggtttttttgcaagctg

gtttaatatcatatatatctggttgcacaaggtagacacagactctgatcatcatggaggcgagattgttgctgaagtcctcaaagcacatggagttaaa

tacatttttactctggtgggaggccacatttcacctatacttgttggtgctgagaaactgggcatcagaatagtggatacaagacatgaggcatcagctg

tcttcgctgcagatgcagttgcgagactatcacagacagttggagttgttgctgtaactgctggaccgggcttaacaaatactgttactgctattaagaa

tgctcagctggctgaatctccagttcttctgttaggaggagcttctgcaggactgttaaaaggacgaggggctttgcaggatattgatcagatgtcattg

tttaccccactttgcaaattctgtgctgcagtttcagcagttcgtgacattgctccaactctaaggcaggcattaaaagaggcacagtcaggaaccccag

gtcctgtttttgtggaacttcctgtggatgtgctatatccatacaaacttgttaagaaagaagttgcatcaggtacaaaaccagggaatatattgacaaa

ggctatcaactggtatctaaataattacttggacaacatatttgctggtgcgtgggaacctcgtgaagttacaccactaaaactaaacattcctcttcca

caagattctgagattcagaaagcagttgagctgctgtcttgttcccgaaaaccattgctggtacttggcagccaggtgacattgccaccagtatctgctg

aatccgttcgcaaagctgttgagaggctgggagtgccatgcttccttggtggaatggcacgaggcctacttggcaaagaaagcccactacagctccgtca

taatcgttcaggggcacttcgcagggctgacctcattcttcttgtgggcacagtctgtgattttcgtttgaagtacggtcgagcactgcctcgcacagct

aaaatcattgctgtcaacaggagccgtgaacaactttataagaattcagatttattttggaaaccaacattggctgtatgtggtgatgctggaacctttt

taaccgaactctcccttgtaataggtggttttaaatgtgatcctgaatggcaggaaacattaaaagaagaggatagactaaaggaaatttccaacacaaa

gatggctgaggaacgtacaggagaatatgttaatccattaaagatattgaaagaagtagaaaatattttaccagaaaactctattttggtagtagatggt

ggagattttgtgggctcagcagcatatattttgcgacctaggggaccactgtgttggttggatcctggcgcatttggtactttgggagttggtggaggtt

ttgcgttaggtgcaaagctggcaaaacctgatgcacaagtgtggctgatatatggagatggttcttgtgggtacagcattgccgagatagatacaatggt

gcgtcataagcttcctgtggtagctcttgtgggcaatgatgctggttggacacagattgccagagaacaagtgcctctctttggcagtaatgttgcctgt

aaactagtgcacaccaactatgaacaagttgcagagggttatggtgggaagggaattctgatatcaagctcatctgataatttaatttcctctcttaaaa

aggcagtagatctgcacagcaaggaccacacagttttgataaatgttctaattggtaatacaaagtttagagagggaagtatttcagtgtgatgatttag

tgagtaaatgcactttaaactggagccttatttcatggacactgtacaaggagaaatcataaacatcacacaaaggctctgataattaagtaaaggtagc

actgagaaatgttcaaaaggtggtttggatattaagttcttcagctatctctgtttaaaaattgagatagtttgtattaaatggcattttatggtgatac

ggatcattttggtagctttagtgacttaaaatggtgaaatagtgctgtgactgaaaattgtattacttgatgtactagtttataatttttaatcagtatt

gaagctgggacagttttcactgcagctatattgcaagtgtattgtaatatttttttcatacctgtggatgcttgactgattctccattattcctcaaaaa

tgcattcccagaagaaaggcagggttatttttatgaggtcgccccatgtgttgcccaaaccttacagttaaagggtctgacttataattcatatgtgaag

agcatcatgtcagtcttgccatataaacataatactatttatttatgatgtttgtgttgagtattgtggaaatagcagttctgatactgattttgtccca

tctgcactatgttattttgtttgtttcatatacaagaggtgttctgatgttattgtagtcacttgacaaggtcacagtggattgtcaaaggaaaatgaaa

ataagtaactggcagttgacagatttttgatttttcagatatgatgggcacagatgattatatctaaattattcagttaactagttgatcactgagaatt

ataaggagcattgtttgtaaatttagtaatgggctgctctacaaggaagctatttaagttagtgtaaactggatcctatgactgccactggctgggtgtt

catgtttaagtgaattttactgttttgacatttctcatggaaaataatgttattcagtcctttaggcagattcacagtaaattatgtaagatctctagag

gacagagaatattcaaaaattgctcttattaaaaaaaatttggcttttaattggtaaatcatgtgagagtaagtaatttactctgagagtagtacttcat

ttctctgttcatatcagcactttaagacacacttgttcagctccttgttgtagtgtagccttaaggtagttgtacattaaaggaaagttattgtttatct

gttgttttgttggtaccattgatgtgtgattactgaaaaacagtattttggctatgaaatcctgtgatattagcaaatataaccacatggacataatatt

ggtttgcagctggacatatggacatattgaaaaacacaatgtatgatatgaacagagaaatgaagtactactctcagagtaaattacttactctcacatg

atttaccaattaaaagccaaattttttttaataagagcaatttttgaatattctctgtcctctagagatcttacataatttactgtgaatctgcctaaag

gactgaataacattattttccatgagaaatgtcaaaacagtaaaattcacttaaacatgaacacccagccagtggcagtcataggatccagtttacacta

acttaaatagcttccttgtagagcagcccattactaaatttacaaacaatgctccttataattctcagtgatcaactagttaactgaataatttagatat

aatcatctgtgcccatcatatctgaaaaatcaaaaatctgtcaactgccagttacttattttcattttcctttgacaatccactgtgaccttgtcaagtg

actacaataacatcagaacacctcttgtatatgaaacaaacaaaataacatagtgcagatgggacaaaatcagtatcagaactgctatttccacaatact

caacacaaacatcataaataaatagtattatgtttatatggcaagactgacatgatgctcttcacatatgaattataagtcagaccctttaactgtaagg

tttgggcaacacatggggcgacctcataaaaataaccctgcctttcttctgggaatgcatttttgaggaataatggagaatcagtcaagcatccacaggt

atgaaaaaaatattacaatacacttgcaatatagctgcagtgaaaactgtcccagcttcaatactgattaaaaattataaactagtacatcaagtaatac

aattttcagtcacagcactatttcaccattttaagtcactaaagctaccaaaatgatccgtatcaccataaaatgccatttaatacaaactatctcaatt

tttaaacagagatagctgaagaacttaatatccaaaccaccttttgaacatttctcagtgctacctttacttaattatcagagcctttgtgtgatgttta

tgatttctccttgtacagtgtccatgaaataaggctccagtttaaagtgcatttactcactaaatcatcacactgaaatacttccctctctaaactttgt

attaccaattagaacatttatcaaaactgtgtggtccttgctgtgcagatctactgcctttttaagagaggaaattaaattatcagatgagcttgatatc

agaattcccttcccaccataaccctctgcaacttgttcatagttggtgtgcactagtttacaggcaacattactgccaaagagaggcacttgttctctgg

caatctgtgtccaaccagcatcattgcccacaagagctaccacaggaagcttatgacgcaccattgtatctatctcggcaatgctgtacccacaagaacc

atctccatatatcagccacacttgtgcatcaggttttgccagctttgcacctaacgcaaaacctccaccaactcccaaagtaccaaatgcgccaggatcc

aaccaacacagtggtcccctaggtcgcaaaatatatgctgctgagcccacaaaatctccaccatctactaccaaaatagagttttctggtaaaatatttt

ctacttctttcaatatctttaatggattaacatattctcctgtacgttcctcagccatctttgtgttggaaatttcctttagtctatcctcttcttttaa

tgtttcctgccattcaggatcacatttaaaaccacctattacaagggagagttcggttaaaaaggttccagcatcaccacatacagccaatgttggtttc

caaaataaatctgaattcttataaagttgttcacggctcctgttgacagcaatgattttagctgtgcgaggcagtgctcgaccgtacttcaaacgaaaat

cacagactgtgcccacaagaagaatgaggtcagccctgcgaagtgcccctgaacgattatgacggagctgtagtgggctttctttgccaagtaggcctcg

tgccattccaccaaggaagcatggcactcccagcctctcaacagctttgcgaacggattcagcagatactggtggcaatgtcacctggctgccaagtacc

agcaatggttttcgggaacaagacagcagctcaactgctttctgaatctcagaatcttgtggaagaggaatgtttagttttagtggtgtaacttcacgag

gttcccacgcaccagcaaatatgttgtccaagtaattatttagataccagttgatagcctttgtcaatatattccctggttttgtacctgatgcaacttc

tttcttaacaagtttgtatggatatagcacatccacaggaagttccacaaaaacaggacctggggttcctgactgtgcctcttttaatgcctgccttaga

gttggagcaatgtcacgaactgctgaaactgcagcacagaatttgcaaagtggggtaaacaatgacatctgatcaatatcctgcaaagcccctcgtcctt

ttaacagtcctgcagaagctcctcctaacagaagaactggagattcagccagctgagcattcttaatagcagtaacagtatttgttaagcccggtccagc

agttacagcaacaactccaactgtctgtgatagtctcgcaactgcatctgcagcgaagacagctgatgcctcatgtcttgtatccactattctgatgccc

agtttctcagcaccaacaagtataggtgaaatgtggcctcccaccagagtaaaaatgtatttaactccatgtgctttgaggacttcagcaacaatctcgc

ctccatgatgatcagagtctgtgtctaccttgtgcaaccagatatatatgatattaaaccagcttgcaaaaaaaccgacaaatatgcccacaaaagcaca

aagaataaacaatataattaacgaaactgagtgttcttcaatcagttcttcttctaacccgctgccaaacgaggtcatgttttccctttctcaagtacgt

aaaatctgcttaaacagtgctgaaaaaatgcaggtcatctttattattttaagtgataa

>88575|2-methoxy-6-polyprenyl- -benzoquinol mitochondrial-like

tgattctgctcaagtgttatttcatcttttagcacacacatgcttacaagttttcacgtcataggttgatggctgatacttaaaactcacagtgcgcatt

aacatttcctgtcaagaattggaatcactatgttttccttagcatctttaacaaagacaggtttaatgtagattcctgtttacaaatactgtgaggatgt

gcacctgaaaaatcaatgaaattaaattttcctctttcagggcatatgaccagtactccttccaagtaataccagtaatggggcaactcatagctggcca

atggaaaccatatcaataccttgttgaaagcatccggcagttcccctctcaggtaagctcaacagatatgcaaaacaataccaattacacaatatacata

tagtggttgtgtgtacaagtttattgtgtactttttatcatttgtgtcagtagttttcaaaagtttttgaaccagatgtggaaatttggtaagacttaaa

tttaagatgtagacatcagttttattacatatcttatgatgatttttttttctgtcttctgcacaggatgcgttcaagaccattattgaaaatgctggct

tctcttgtgttacatacgagaatttgacttttggggtggttgcaatacattctggattcaaaacctaaatgtgtaattaaaagaaagtaataaaggtata

aaaggttgtttgaaaatgactttttgaaacttgtgataagtaatttaatgtaatttgcatctatatcttttataatacacattatagatgtggccattac

tccacaatttaggagtcactttagaga

>90944|2-oxo-4-hydroxy-4-carboxy-5-ureidoimidazoline decarboxylase-like

tttttatttgaagggaaatattggtttttatttcatgtaagttgaataacaagttattatatacacgacacaaaagttcagtcttcgactaaatctttta

ttcttaaattgcatatcttctttacttcagaaatacctgtttccagttcatcttccttagaattctgtaacctccttctcaacccttcaagaattgtgtc

tgctttattttcccgtgcacaaattacaaatgggaatccaaattttgatttatagctgaggctcagctcatgtatgccactgatggttgccttggagttt

gtctgcagaccagccatgtgctgctctctgttggactcctctgtcagctgctccttgctgcccaggtctgggtggctgcgcagaacctcctccttgtctc

tgggcgatctggagccacgggttggtgtggcgcggctgctgcgctgcccgcaggccgccttatcgtgtggacagccgccaagccgtggggtcagtcggcg

gcaaggacaca

>90941|2-oxo-4-hydroxy-4-carboxy-5-ureidoimidazoline decarboxylase-like

ggtttttatttcatgtaagttgaataacaagttattatatacacgacacaaaagttcagtcttcgactaaatcttttattcttaaattgcatatcttctt

tacttcagaaatacctgtttccagttcatcttccttagaattctgtaacctccttctcaacccttcaagaattgtgtctgctttattttcccgtgcacaa

attacaaatgggaatccaaattttgatttatagctgaggctcagctcatgtatgccactgatggttgccttggagtttgtctgcagaccagccatgtgct

gctctctgttggactcctctgtcagctgctccttgctgcccaggtctgggtggctgcgcagaacctcctccttgtctacaggggaaatgttcgtatagtc

tctgggcgatctggagccacgggttggtgtggcgcggctgctgcgctgcccgcaggccgccttatcgtgtggacagccgccaagccgtggggtcagtcgg

cggcaaggacacacacac

>90940|2-oxo-4-hydroxy-4-carboxy-5-ureidoimidazoline decarboxylase-like

gggaatccaaattttgatttatagctgaggctcagctcatgtatgccactgatggttgccttggagtttgtctgcagaccagccatgtgctgctctctgt

tggactcctctgtcagctgctccttgctgcccaggtctgggtggctgcgcagaacctcctccttgtctacaggggaaatgttcgtatagtctgctgttgt

caagcagtccaggtagtcaccagcagcagcggccacatggtcggcactttggtagggcctgccagagcagacggcctcggcagcagcaggccagcgctcc

accacgttgagcagcagtgctgcgcactctccatcagtcatggcattcaagtcacgcacacgtactgttggcgtcttctttgtgtgtgggcacagccatg

ccaatgccatctccaacacctgataaactaatcaaaatatccatgggtgtactgccggtctacagtgtccaacgggcacaatatttcggcgatcatacat

gtcgccatgatcaggtgaactgacggactgagctcccgtgaacatgccagcacg

>90945|2-oxo-4-hydroxy-4-carboxy-5-ureidoimidazoline decarboxylase-like

cacgataaggcggcctgcgggcagcgcagcagccgcgccacaccaacccgtggctccagatcgcccagagtttatcaggtgttggagatggcattggcat

ggctgtgcccacacacaaagaagacgccaacagtacgtgtgcgtgacttgaatgccatgactgatggagagtgcgcagcactgctgctcaacgtggtgga

gcgctggcctgctgctgccgaggccgtctgctctggcaggccctaccaaagtgccgaccatgtggccgctgctgctggtgactacctggactgcttgaca

acagcagactatacgaacatttcccctgtagacaaggaggaggttctgcgcagccacccagacctgggcagcaaggagcagctgacagaggagtccaaca

gagagcagcacatggctggtctgcagacaaactccaaggcaaccatcagtggcatacatgagctgagcctcagctataaatcaaaatttggattcccatt

tgtaatttgtgcacgggaaaataaagcagacacaattcttgaagggttgagaaggaggttacagaattctaaggaagatgaactggaaacaggtatttct

gaagtaaagaagatatgcaatttaagaataaaagatttagtcgaagactgaacttttgtgtcgtgtatataataacttgttattcaacttacatgaaata

aaaaccaatatttcccttcaaataaaaataaaggtgtatcagc

>91140|2-oxoglutarate dehydrogenase

taatggttgggtgaacatgggctcatcaatttcattgtggccattgcgtcggtagcagacctatgatagcaaagaaaaatcacatacatctaataaacac

tacgacaaaagcaggaaattaataa

>91141|2-oxoglutarate dehydrogenase

tactttatgtgtgtctcttttctcgcattttctaaagcatcttcgcatattttttcatatttgtcacgaacatcctggaaataaattttatcatgtttca

cataaataccacgataatcttgtactatcttttttttttgtctgtttactctttaaagccaatggttgtcatctgttgaatctttatccagg

>91142|2-oxoglutarate dehydrogenase

aaggttcatggacatacgttcaaccacgattccacacggcaataaatggaaccagggatattgtaacgcctgaggtgcagcacggtgggttgtttagcag

gttctttggcagacagtctgaacctgctgaaactgagagctcgaaacttcaccctccctcttctgcaatcagacaaattagttatgcgggtcgaccaaca

gctgcatctccagcgaccggtagcaaagtccatcaccttagagaacttgctgctctccttgatgaggcaatggccatttgagcagtgtctttctagctca

tggttaaagttcaaaacttgtcatgtcacattcatacatgacagtggctagaaggcttgtattccctgtgcacattttcccaacagagcctgaggatttc

caccagatgctttaatagcacagagcactatctcggtaggcaagaacaactgtcttagttgtagcggtgtgcagttttcagggcatttttgcccaataat

aaacagctatgttctgtgttggaacatggtgtgcgctgactttccggcagcttagtattcagtacaataatgttcatgtgaaagccattaaattttcatc

gcagagtggttgtgtcacttaaaatagaaactgatatgaagattgtatttgttatccttttgtatatgtgagaatttaattttcttatggactttacatt

ttgtgcaaaaataatgtatgctgagaaataggtcatcaaatatttgttttaattgtctataaatcagtattttagaacatatgtggaacatttagctttt

acttacatcatagtgacactgctgctttctgatgtttttacatatcaatatcagcaatgcaaatatcaacctttgggattgagggtctgcttgtaatgat

agcaagatattttttttt

>91939|2-oxoglutarate dehydrogenase, putative

ggatcccctgggaatctcacgtgcagacctggatgacaaatctcctccagaattgctgtacagccactacagctttgag

>91940|2-oxoglutarate dehydrogenase, putative

gagagatcctgagaagacttgaaaatgcctactgtcgtcacattggggtagaattcatgttcataaattccctggaacagtgaattcatgttcataaatt

ccctggaacagtgcaactggatacgcc

>91937|2-oxoglutarate dehydrogenase, putative

gttcaggctcctatcttccatgtaaatggtgatgatcctgaagctgttatctttgctactcaattagcacatgatttccgtcacgaattccgtaaagatg

tagtcatcgatttgttctgttaccgtcgtcgcggccataacgaagcagatgagccatctg

>91941|2-oxoglutarate dehydrogenase, putative

ggtcagggcgtgaacatggaaaccttccagatgtcacaaactcgtcggtatactgttggtggtacagttcatattattgtgaacaaccaagttggtttca

caacttctgatccacgtgatgcgcgttcgacagaatactgtactgacgttgctaaaatgattcaggc

>91938|2-oxoglutarate dehydrogenase, putative

agtcaccaaattgtgcttcccaaataatcaagctcttaggaattgtagttgcgtaaccatattcgaacgcaagaactgcttcttcagataaaagagagtc

ataaatcgcaaaacgcggttggttctctttcacatggcaaagaggaatgtaagttgaaccatcaacttggttatgcaatttcgcatgacggtgtgagaag

gtaccacgc

>92392|2-oxoglutarate mitochondrial-like isoform 1

atgacattttcttgccaatgtgaatgagtgtgtcttctttgacacctgtaggagatactctcattggatctttgccctcaaagaacccag

>92394|2-oxoglutarate mitochondrial-like isoform 1

gttgcgttcaagaagtgctacaagctcactgtcggttaccagcatccgaaattacagtgcatctgttgcaccagaaccatttctcaatggaacttcatcg

gtatatgtggaagaaatgtacaatgcttggtt

>92390|2-oxoglutarate mitochondrial-like isoform 1

aatttattagttttagttataagattgatattttgagtgttctgtttgtggaactaatcttcaaacgacctgctttcatacagaggagaccgacatggac

cgtgttttcaagctgccctcaactacattcattggtggtaaagagaaa

>92389|2-oxoglutarate mitochondrial-like isoform 1

ggcattcacgctcatccccgtactgcactgatgttgccaaagtggtgaatgcacctatcttccatgtcaactcagatgaccctgaagctgtcatacatgt

gtctaaaattgctgcagagtggagagcaacattccacaaagatgttgttattgatattgtgtgctaccgac

>92391|2-oxoglutarate mitochondrial-like isoform 1

aaattctgaggagacttgaaaacacttactgccaccacataggtgtggaattcatgttcataaattccttggagcagtgcaactggatacgtcaaaaatt

ggaaacacctggagtgatggaaataacaaatgatgaaaagaggcttatacttgcaagactgacacgagcaactggacttgaagcatttctggcaagaaaa

tggtcaagtgaaaaacgatttgg

>92393|2-oxoglutarate mitochondrial-like isoform 1

cgagtcttcccctgaacaacaggatccacagcctcaagatgggatggattagctaccacagctagtcgcatatttttgttagtcacacggttcaaacgtt

caatgtaagtacccaaatgatatttgacatcacctgacccctcatcttcagattccagagcagcaaactgggtaaaaatatgttccagtggcttccgaca

aacattggccaaaacattcaggcgacctctgtgagacatgcccattacaacagattctataccaaactctgttgagtgatcaata

>92388|2-oxoglutarate mitochondrial-like isoform 1

gccacaacctcactgtcagttaccatgctacaaatggtagctggtggtgagtagcggtgaggttcgtgcggagataacacctaggaaaattaaggcaacc

caagatgcatcgtgcaagatcagtattaacaaatctgttaccacttacaccattaattccacacgctgcaggacctgaacgatttgcagcatggttattg

cgatcaggaagtgccacaacctcactgtcagttaccagtactcgaaattacagcgcatctgtagcagcagaaccttttctcaatggcagttcgtcggcat

atgtggaagaaatgtacaatgcttggttggctgacccaaaaagtgttcatgtgtcatgggacacatttttccgaagcagctcagcgggtgctgcaccagg

ccaagcataccagcctccgccttcactagctcctccagggaagaaccaggttcccataacgagtcttgctccttatttgggagcatcagtgcctgctctt

gatggacaagttaatgaaaagataattgatgaccacttggctgtacaggcgatcatccgttcgtaccaggcgcgaggccacttggtggcagacctggatc

ctctggggataatgtatgcagatcttcacagcaagtataaagatcgccagggcgctccacccgagattgttgtccggcagtacatgctggaggagaccga

catggaccgtgttttcaagctgccctcaactacattcattggtggtaaagagaaagctctttctttacgagagatcctgagaagacttgaaaatgcctac

tgtcgtcacattggtgtagaattcatgttcataaattccctggaacagtgcaactggatacgccaaaaactggaaagccctggtgtgatggaaataacga

atgatgaaaagaggcttattcttgcaagactcactcgagcaactggatttgaagcatttctggcaagaaaatggtcaagtgaaaaaaggtttggtttgga

gggttgtgaaatcctgataccagccatgaagcagattattgatagctcaacagagtttggagtggaatctattgtgatgggtatgccacacagaggtcgt

ttgaatgttttggccaatgtttgtcggaagcctctggaacagatttttacccaatttgctgccctggaagctgcagatgatgggtcaggtgatgtcaaat

accatttgggtacttacattgaacgtttaaaccgtgtgactaacaaaaacattcgactagctgtggtagccaacccttcgcatctagaggctgtagaccc

tgttgttcaggggaagacacgtgctgaacaattctaccgtggtgatggagaaggaaagaaggttatgtccatgctgctacatggtgatgctgcattctgt

ggtcaaggcatagtgtatgagacctttcacttgtcagatctgcctgattacactacacatggaaccattcatattgtagtcaacaatca

>92812|2-oxoglutarate mitochondrial-like isoform 4

aaaaaaaatgtagaggtgatacgagccttctaaccattggcatgtatgatatgaaatgccaataactgaaatggaaacatgaactaggacaactgttcaa

attgacattgcctcatcaaggaaagcagcaagttccctaaggtgaaggactctactacccgttgctgaagatgcagaaggaggccgcccagcataaacaa

tattcctggttctatttattgctg

>92813|2-oxoglutarate mitochondrial-like isoform 4

ccgccttcactagctcctccagggaagaaccaggttcccataacgagtcttgctccttatttgggagcatcagtgcctgctcttgatggacaagttaatg

aaaagataattgatgaccacttggctgtacaggcgatcatccgttcgtaccaggtatgccctagactcacgttacagctgctgtgagagatttgcacatg

tgttattttattgctgtgtcagttcttttggttattgccataggaaaggagaaatgtattgatacacagtaatgattgctgtgtgttgtggtgctatgtg

taatccagtgcctgctcccttaactggcatttggtcagttaagtagtgtaatgcatagccacatctgtaaatatgtattgtattattacatgtaatagca

gaaacacaaatactgtaaataaaataatttgtgaaaggcttgatcagaacaaatactaatgcgtagctgaatgagtgactgtgtgaaagctcttttttct

tcctcctgcagatgaattacaattgtagcccttgtgtaattacaaaaattaatgaattaataaaactaatgctggcctcaaataatttgcaaatatgaag

ttatgacatcgttgttgttttccacatttataactacttttttgtagattttattgaagtgtttaccttcactataatttcagaaaatgctattttgaca

ttctttgctatgattaagtatgacactgccctgattagcacttatgcacaaggtactgagcgagatggtgcagtgattagcacactggactttcatttgg

ggggacaacagttcacacccgtctccagtcatcctgatttaggttttccatgatttccctaaattgtttcataaaaatgccaggatggttcctttgaaag

ggcacgatttccttccaatctttccctaatcagagttggtgctctgtctctaatgacctcattgtcaacgggatgttaaacactgatatccttcccctcc

ttatgcacaaggtgccatttaatttctgggggttgtgggatggggcaagcacagctcgggcatgggcagtcagtgttaccatattctgactgagcaggag

ttgggctatatgaagtgctgtggaaataattaaaagggttgctcaatagaaagagtggagactgtgcagaaatgaaagtgtctttattaggattgtgaat

tttgtttaaatttcaaaatgatggtaatttgtaataagattccataggacctggagatcatccatcatcttcagtctttgtttgaggatcagtgcacatg

ttgtatgtactgttttaagtgcaggaaaaaacagcaagtaattctgcctcatgggattacttgctccttatcatgctttacctggtagtaagttgctttt

gtctattcagctgaatttgttgctgcacattgaggtacattcttaatatgttcattaaattaagtatcagagtacaggtggaacactgttgctgtctatt

ctttctaacaaaaaaagtgctatttctaatgtagagggaaggataatatgtgcttgctggaag

>93300|2-oxoisovalerate dehydrogenase subunit mitochondrial-like

gaacttgtttctgctctgttagtagatgtttgtaatatttcaaacttttgtattgtatttattacaggaagagtgttttctacacctggaagcaccggta

caaaggggtacaggctttgatacaccctttccatgtatcttcgagcctttttac

>101525|2021248btroponin c

tgttgtgagtaatacagctgtacaccggggcaccgtgttcaccgcagaagaggtagctaagaggctcagatggcgtccaaaaccaagtgttgtgaaggag

gcggcagcgacgcgggggggccgtgccactggcaggcgggtcgggtcttgaatcggcttgacgtgattggcgctgcccgcagcgcctccagcacccccta

ctctccggtcatcacctccatgaactcatcaaagtcgacggtcccggagccgtccgagtcgatctcctcgatcatcat

>101524|2021248btroponin c

cgataaaatattttattgacaattactttgtattacatagttggtggtcacagcactgctttctcagaatgtgttgccacatagatatttcatagataca

gctcgatacatttatgttgttgttgcacactcacagagtacacttaactgctatgtgtaaacaaagatgaacaaaattttcgtgtggcaacgagtgtggt

ttttaccgttatcaccaaaactggaacgcttgttgtgaagtgcggttgcgcactcggttccgacgctctcgccgacgagggtggcggcgcgggatgagaa

agcggagaggcgaagcccatccgggaatgtagagacagccgtgggagctgagcgggggcggggggaggcgggcggcggcgcggcgcgggggcggcgccta

ctcgcccgtcatcacctccatgaactcatcaaagtcgacggtcccggagccgtccgagtcgatctcctcgatcatcatatcgagctcctcctcggtgatc

ttgtcgtccagctctcgcaggatctccctcaggactccagtcgtgatgtacccgttaccctccttgtcgt

>102027|2104214acys protease

cttccaagatgtcacattaaataaagtcaaatcacatgtaccactgtcagtttctcaaacaagagggtaggttgcagaacttgcaattccacagttgtta

tcacggtttcattccaattttattctaaaggagtttactatgagccgcagtgcagttctgagaaccttgaccatgga

>102266|2108277aribosomal protein l11

taacaagaccaacgaaggtgttggaacagcccacaggccaacaacctgttttttcgaagtcgaggtacactgtaaggtcgttttgtattagacgtaatga

>102267|2108277aribosomal protein l11

tacaatgtacagcaatcttctcattacgtctaataccaaaagaccttacagtgtacctcgccttagaaaaaacaggccaacaacctgttttttctaaggc

gaggtacactg

>102265|2108277aribosomal protein l11

atcttctcattacgtctaataccaaacgaccttaaagtgtacctcgccttagaaaaaacaggttgttggaacagctcacaggccaacaccctgttttttc

taaggcgaggtacactgtaaggtcgtttggtattagacgtaaaaagaagattgctgttcattgtacagtgagaggtgccg

>103323|2109232ad- sp receptor-associated protein

tgcgactctgggagctgccctattcagtttttatatcataatggatacccagaaaattgtcggcgggaagcatcgtaaataccagtttagtattgatgat

tacgccttcgccgctttgacactctacctcgatatcatcaaccttttcatgtacattctccag

>103648|2111232aactin-related protein

cttgactaatcttagcttttacttataaaattgcagtttcacgaaatgcatagagcactccagttgtatatttggtctcgttgtcgacgtggctgtattt

taataacaaaagcaaaaaaaattctatgctacaattgaaactgtttgttctagcaatattccaacagtactatttgaaaactaaatgatatctacttaca

gatattatttagttcattcagaatctatttatggttcagtaagtgtgagaattttaattagagtccatctgtttgaatcaaataatatttgtagtatgtt

gcagaattcttctttaagcagtatttctggcttctaagtgttggattattccccttaactgatgaacaattcttttgtttcagcctgaattttaccaggt

ttgtcacacaaaagcagcttatgaagaatatgggccaagtatatgtcgccacaatccagtttttggaactatgacataaataatgcacaaacatttttaa

cagagataattatttgaaaagcagcagtacgataaaggtgaatttattattggaacagtgaactcgagttattcttaattgacagccaaatccagtttaa

ttatgtggattgcttattcattgtgaacttgaagatttattttggcctacttgatactgtctgttcctg

>103840|2122243ae63-1 gene

tctcgatctacatcagccatagccaacatttcattcaggtctccttctgttactgtctctcctatcatatccatggctgacttgagctcatccttagtga

tgtagccattgcagtctctgtcaaacaccctgaaggcagcaatcaagtccttggtgacatcgtcatctccctcagtgtcctgtgtcaaggcctggatacg

tgccacccactggaggaactcagcctcattgatgagcccattttcggaccggccggcgtccttgaccagctgctcgatgatgtcgtcgctgacggggatg

cccaggttggtcagcatgaactgcagctcgctggccgtcacacgcccgtcgcggttccggtccagcagcccgaacgcagtccgcaagtcttccggcatgg

cgagctactagtgtgtcgagtgggaggcggtgtctgcgtcggaggcggcagctgcggcggcgtcggcgggcgggcgtcgactgacggtgatccggccggc

gtc

>103841|2122243ae63-1 gene

acatcagccatagccaacatttcattcaggtctccttctgttactgtctctcctatcatatccatggctgacttgagctcatccttagtgatgtagccat

tgcagtctctgtcaaacaccctgaaggcagcaatcaagtccttggtgacatcgtcatctccctcagtgtcctgtgtcaaggcctggatacgtgccaccca

ctggaggaactcagcctcattgatgagcccattttcggaccggccggcgtccttgaccagctgctcgatgatgtcgtcgctgacggggatgcccaggttg

gtcagcatgaactgcagctcgctggccgtcacacgcccgtcgcggttccggtccagcagcccgaacgcagtccgcaagtctttaagctcttcctctgtaa

actctctttttggaagcgaatcttgccgtggcaagctccgcttcctgtgggccatggcgctcagactctgacggcgctcgggccgccgaatacgacaaag

tgccgctgctgaacaagttctcgttggcgtaaatcctgtcccggtcaaccttatccacagacgaaggcggcagcaggcgctggtcttaaccacgcaactc

gctgtaagtctcggaacgcacaagaccggctcactgctcgtggaagctcgacacacgactgcccggccggcgcttcgctgagcgcgtcggcctgcaagcg

gcggactgaggcggcatcgcg

>103839|2122243ae63-1 gene

gcatgttgaatttatttatctcagaatctgtacaaaatattttgacagtatgtaactatcagcaactacatcattcatttaacttgaaaaaaataacagc

ttttgaatattattttacatcattattaccagataactggagttacttgtacagtattataaacaattgcaacaacataataggcacattacaaaatata

tacaatatccttaacaaaaataagtgtgcatttatgtaaacaatgtcagcactaccattacaactggctgtcatcacttttacattcatttcaataacaa

aaatttagctttgcaaaggtaacagctgtatttcacagtgatgttagaatatgatgctactcacacaggttttgtctttctttacaagaggagccgtgca

aactcttcatagttgatcctcccatctcgatctacatcagccatagccaacatttcattcaggtctccttctgttactgtctctcctatcatatccatgg

ctgacttgagctcatccttagtgatgtagccattgcagtctctgtcaaacaccctgaaggcagcaatcaagtccttggtgacatcgtcatctccctcagt

gtcctgtgtcaaggcctggatacgtgccacccactggaggaactcagcctcattgatgagcccattttcggaccggccggcgtccttgaccagctgctcg

atgatgtcgtcgctgacggggatgcccaggttggtcagcatgaactgcagctcgctggccgtcacacgcccgtcgcggttccggtccagcagcccgaacg

cagtccgcaagtctttaagctcttcctctgtaaactctctttttggaagcgaatcttgccgtggcaagctccgcttcctgtgggccatggcgctcagact

ctgacggcgcttgcacagtgagtcccacgctcggcctatcgccgccatggccgcgccgctgtctcgctctcgctcgccgccgactgccgccagcccacag

ctctcgggccgccgaatacgacaaagtgccgctgctgaacaagttctcgttggcgtaaatcctgtcccggtcaaccttatccacagacgaaggcggcagc

aggcgctggtcttaaccacgcaactcgctgtaagtctcggaacgcacaagaccggctcactgctcgtggaagctcgacacacgactgcccggccggcgct

tcgctgagcgcgtcggcctgcaag

>104052|2209312aprotein kinase pk92b

cagtcttactcaacaagccatataaaaatatagttttaactttatagaaaataaatagtacattaagagtgttgttccatccttgatttaagtctttaaa

ctttattactacaggtaccaatatattcaggtgtgtttatgaagcactcatgattaagtagttcttttgctgtaggccgcagttctggttttatctgcag

acacttgtgtgcaacatttttaagtaacatgccaagagtatgaggaatgaatggaggttccttgcttgaggctattttgtacataagagcaaggtggttg

gacatctgattttctttccatggtggttttgtggtaaccatttcaataatgcaacaaccaacactccaaatatcacaggagtgttcgtaattttctcccc

ttaggacttctggagccataaatgcaatagttccaagaatttggccttgcaattctcctgacaccactgtctgagtttctaaatgcactgctgccccaaa

atcacctatgcataaatttctgcctgtgctatctattaagagatttgcacctttcaggtcacggtgcaaaatatggtttttatgcagatagttcaggcct

tgaagagtttgttttgtatagttgatgataacagactcactgaatggaccatagctatccaggagcgtggcaatagatccaccagccatccactcaacaa

atatgttgaaatggccattctgttttgtagcaccaagaatatgtagtacattgggatgatgtaattttgacaacataactatttccttccatatttctga

ttcaactctttcctgctcttgttgtgaattacgtaagaaggacacctgttttacggccattatgcccccagacactgtatctctagcttgaaaacaggtg

ctatatgctcccgtgccaagagctgggcctctcttccagttaataccttctatgtaagaacgtgtgtttaagtgattccaggttacctcagatactaatc

tgcagtagaaatcaggtgctgtgccttcggtttctacagagtactgtacatcacttccatcaccagctgtccttgtactaaacacagaagtacaaggctt

atccttatttgcttccacatgaccaaagtcctgacaggaaatattgagagtacagctcatattttgtgctggcgacaacagagactcactttcacacgta

tgagtaaaagtttgtacgtctgatccctgactagaatcagctatcatgctaaagtgttcctcattaaacgtttctgttaagttttgagaaactttatctt

tacttgtcacattttcctctttcttgtttattaaaaccaaatttgatgcttttgaaagagaattcatccatttatttgtgggtgaaaatgatgaaacagt

atttgattctgaaaaatgcttcaaagacttgcattttttcaagtttttaagttcaaggtcttgcatcttagttgcaaccatactggttaattgtttagat

tcacttagaggaactgaatttactgctcctgttctatttctcatgttgctctgagccacattcctcaagtgaatttgagttacagattttgcacttccat

tctcacaggaagcttgactgtgaaattgagctacatccaacagatgttttttcaatgttacttgcaagcttatatccaggggtgctaacatttctaagac

atgattcagtacaccattttctttgatacttaagctcgaagacaacaaaaaaacatcttttgctatttttttcactgttctatgaggactgcgtaatgct

tttactgaaaattctataacagaaatcagtcttctaaatttattaagtctgtcaccatcatcagtcctttgccctagatgaaactcatcagggaacaggt

gaaacattgttttcaacatcagcagccttccacctaaccactgccaggagacactgtctacagtgaatggctcaagaatgcaactgagaacaacttcaag

acccgttaatctttgaatgctagtgtttctcatcttgaatggatttcctatttctctctcagatatttctgccagagcttcagcactcaactggctcgca

cgccgatttcgatcagcacacttcttgagcattgttgttattactgggtagattagatcaacaaattccttcagctgagttgcagttgaacagcaaacac

ttctgatcagagtacaaaagctgtgcacacaagtagaataaactttgaaaataggatctcccacaagcatagctaaaatctttgcacaacagcgaataac

tttttgttgccattcttcattactaagctgaacttgtcgatgaagttcatcttgcaagagctgcaagccacgctcc

>108526|24-dehydrocholesterol reductase

cagtaatcgcattttaattgttcaataaaaaagtctttacaataaacttttgagcatctgtggagcatgctagatcctggcactcttgttgcatttttca

tacacttctggaaatgcatctttgcaatttaattgatcgcggaccttccagtagagtgtgtgatcaaacatgacttcaaactccttcttcgtcatgtaac

tgtctgcgtacagcatctgatacccttttacattccgtacatgttcttctaagcgccttgttgtgtcgacaggatggaaattcttaactttaggtacccc

atatactcctatatcgacatataactcatctttatcactaaacgaatgaaccatgcctggctcgttggttaatttgaacggacacagccacagtgggtag

acctcaacctctttgtgaaacacttcgattgacttcttcagttcttgcatcgggacgagcatgtcctgtataatgtgattattctcatacagcttcttaa

ctgtttctccctgtgtcaatttcagtagagaaaccttgggcggaaccatccaaccaaaaagccaacggaagagtatattgtttccgaaaggtactatgtc

ctgaatctcccagaagattgatcttgtgtgtcggtggtaatagtctcttagcggaatgtattctgtcccttcgcccttcttcaggaaagatttcacatgc

atgaagaaccatggcttgtgccatttacctatttcgtttagctttccaggttcataatcgtccaccatgttgccaaccatgatgacgccctcgttcaagc

tgaactgcagaccctcgatgaacatgtttccttccttcctggctgctgcctctgagaaaactttcaccgtttcgtctagagatttgacgggtttataatc

gagtttcacatactttttggacggaatgattctgaattcaactgcagtaaggaaacccagagttccataagaccaagggactgcgtaaaacaggtctgcg

ttgttctccttatcgcagacaacaacactgccgtcagccaagacaagctcataggacacgcagatgtgctggatgaggccgtgcttgtgcgaagagctct

ccacaccagtgcccatcacaaggcccccaactgtgaggtcgtcgagttccgggacaacagggatggtccagccggaggagagcagaagtgccgtcagctg

ccccatgctcaccattggctccaccagcaccgtctgcttggtttcatcgacgcgcacgacgtccatgaggttgacgctgacgcagtacaggcggctcttg

taggcgggtctgcggaagctgatggtctgccagccgggccgggccgtgcacatctgtgtcggccgcccatcagaaatccactgccgcacctgtcgctgta

cgtgtttcactttgtcgccatgcttattcggagcgctgttcagcttgaagatgatgtaactcctcacgtagtagaacaagtcgtacaccagagacacagg

cagcagaacaaagcacacgagaacccatctgtaatgcacgataagatactccagcggtgacaaagatgctgtcatggtaggaggcgcggcgtgaatttct

gat

>Sg_CNS_NA4Plus35982|26-kda lectin

ctctgacataaaagcagtgcctttatttctaatgtaagcaaacctcaacaataacctcatatttactctatctctgatggctatggatcatacaataaaa

aagttgattgaaatttacatgttattcttcagcaaaataatgattctagtcgaaaaattattaacaatgacttacagtctattagatgtaaacagttaac

atttacatgtcattttggacagagtaattcatgtcacatttaaagacattgacacggaaacatgatggattttaaacaaaaacatgtgccagacatgact

ccaagaccaagtatttgtctggttgtattcaaaccaatactgtcatacaagatgtatcattttttttgtttacagttttatttaatatacaggaaatgag

aatcaggatggctttaatagaatttgactcctacaattgcctaatgtgttctcagagcatttaatgcaccacatcattcatttcatttatattactgaat

tgtattttgaactttgaagtcactggagaataatcccatgcagccaaagtacaaaaatacaaagcagccaatgtgcacccaaagttatctcaaacataag

tgatctgcagaattcatttcttctatagttcaatgatataacaggtattgccatataacttcatgacattctccgaataaataaattcacagaatataca

aatatgtggcataacatacagccattgttataaaatagacgttttatactttttttattatacatcttaaaataaattttgataactattactactaatt

ttcataatgcacttatctctaactgctaagcaaaagaggtcaccatcatgtcaaagtattttactgtcatttatatctaggttctctataagataacttt

taaatgacattttaaattttagatgtgctaataaattaacaggtgagccattgtcaactacaggctaccatcgttggtggtctgtgagcagtcctgacaa

cctgggctatagcagcataagtgcaggtgaagactgtggttctgttcatcgcaatggtggcttgaatgacatttcatgctacatgaaacatgcttttttc

tgtgagcaggagttgctggaataatttttcacggttaatttatgtaaaaaaaagatctatctcacattatcactggtgttggactatttctaagaacaca

aagtaatgatttattttcttttttaaaaaattctgatcagcagattaatcagagtgctttattcatgttatgcagttcaaatttcaaattgcaaactgtg

agctgaagcatcatatctactaatgggaatcattatcacaacagcttacttcaaagcaatttaaccaataaaacttaattctgccaatttaaaaagaatg

ctgtttggtctgataagaaagtagacaagccacatctctcagcctttgggggcaatgaaggtggaatttagttgcatagacaaggtttctaaaagtcagc

agcaccactgtggttgggaatgtacaaaattcagacagacatttttcctggaaccaatatggaaggataggcaaaagcatcaatttatttctggatataa

gtcctgaaagtaaaatgttagaaagggcaatggagagagaaaggaaatagtcaacgaggagtttttgacagtgaaattaatttgtaatagtcactcacag

aacaagtaaatagctgaatttgactttcagtaacaaatgtgatgatcagtgcactgaaatactgggagacttggttgtgactaagcacaaatcccacaaa

tgttattcactgacatatacaccacaaagaagttagccactgcttttattcatactatcagaataagaaacaaaagttactcttaattatccttctacct

ctcatcattaaccatgcttgtgaaatgaattttctattgtgatatttatctttcaaaaaaacaaaaaataatatttctgtcacttagacatatgagggta

attttctgccaccttatgtaaatgcttactcattgacttctaatatattatatttgctacagattattttttatactgaatttaaaaaatgtggaggcag

atctaagttgttatgactggtaattgcattaatagttaaccatttatgaaccttatgttgatagtttttatgtctacactattgtattttataaatgtag

agctttgtagagaatgcttaacaagttttgtaactattatttgtgttttacaaatccaaggtatacaatccctaataataacaataggctcttttctata

tctatgtaatggctga

>108882|26s protease regulatory

gaggcagggatgtatgctatacgtgcccggcgcaagtctatatcagagaaagacttgatcgatgcaatcaacaaagttatcacaggctacaagaag

>108883|26s protease regulatory

gagaaagattttctataggctttaaataaatttataaaatcatattcaaaattctcatcaactccaatatatattacatacaattaaatttattttcag

>108880|26s protease regulatory

gtccgtccttccaagtctggcaagccaaactccacttttctgtccaatcttcctggtcgcatgagtgctggatccagagtgtctggtctgttcaatctac

ctggtcgcatgagtgctggatccagagtgtctggtctgttcg

>108884|26s protease regulatory

aactccacttttctgtccaatctacctggtcgcatgagtgctggatccagagtgtctggtctgttcgtggccattagaacctaaaatatactatgtctta

gtgtgaaactaaaaaaggaaaactagtaatattatatatataaccaaccacttatgcctagaaaat

>1957|26s protease regulatory subunit 10b-like

cattgacattctgaagattcactcgaagggcatgactaagaaaggagagactgactttgaggctctggtgaagcttacagacaattttaatggcgcg

>1956|26s protease regulatory subunit 10b-like

tctttggatttctctatctgctgatgttccttctgagaaccttctaccaccaatagcatcaatttcatccataaagatgatacatggctgatgatcacga

gcataattaaacatttctctaatcagacgagcactttctccaatatatttatccacaatagcactagagacaaccttcaaaaaattggcatctaactgac

ttgctactgcacgtgcaagaagagtttttcctgtgcctggaggtccataaagaagacaacctttaggtggggtgatgccaactctctggaacagctctgg

atttaacagtggtagttcaattacctctcttagttctcttatttgctcactgagaccaccaattgcagaatatgtaacttctcctggatcttcatgtgac

atgttgtacactaatggatcaacttctctgggcaagtaacgcataattgtaagtgttgtcatgtccagtgctactcgcgtacctgactttagacgagctt

tgtccagctgccttctgcagcccactacatatcgagggccatttgttgctttcactataaatttttcctcggttagctgcttaagaacttcaccaacaat

ctgtccaaccctctgtaatgctttcaagtcgttttctgacttgtcatattgcttagtcagttccttcagctgttctcgcactgtaaaattaaataaaaac

tttgttaataatatggtttgataccgttcaacatcgtggtaggtaagtaagggtcggtgaatcagcagtatcctttaaccttac

>9634|26s protease regulatory subunit 7

tccagctaatcattttcaagtactgttttgtgtgtcacaatgttttattgacagggattttatcacctgtttcactgttgtcatagcaagttagaaagag

gcacaatgtagctgctgtaacttgaacagtgaagtttattaaaatttgcaatcaacagcagccattcaaatttgatatcactgttatcatttgtttttat

agggagcgagaatggttcgagaactgtttgaaatggcacgcagcaaaaaggcttgtcttatattttttgatgaaattgatgctattggaggagcacgttt

tgatgatggtgccggtggtgataatgaagtccagagaactatgttggaacttataaatcagttggatggttttgatcctaggggtaacattaaagtaagt

acagtgtaatgtcttgggcagctttttggactataataaagtgagagtaaacagagaggggtcacaaatgatgcaaggtaggtaggagagttctgcttcc

caaatacagaattgtagttagtgactgcagtagtgaagaatatttggtgttagactaccagaagcagagttttcttcttcagttttgagataacaacatt

ttattgtagatttttattcattctaattcctaaattatgttcctttattgcactgcatcattacactctgtgagtgagtgagtgagtgagtgagtgagtg

agtttgttttaaggagggggtggggggggtgtacattgtaatccaaatggaaa

>13434|26s proteasome non-atpase regulatory

gtatgagttttaatttaataatgtttcagtagatgaatgtgacaaattaatttgactttgtgcaacttaatgtaacaaagcttactgtacaaatgactat

atttattgtcttgttggtgacaaggaatgggcataaatttggacaatgtttgtaatattccagttatggctaggcaaaagattttagcaaacaattaaaa

atgtctagtttatttttatatgtttgtgaaagggcaatgatttcactaatatagtacccacatacttccaacccaatcaaggaattacacacaaaataaa

taatagtgaagagcttacaatttatgttttgaacatgggcacagaacaaactgatttcaccaattatgggtgaacaaataaaatatttgccaaaatgcta

actatgatgccaactcctaagtttcatctcatggatgccaccatacaggaaagtttcaattataacctaatagtttaccaaagttttgtaacacatacag

aaagcaataagtacatgtattcatgtgatgcaaggtgacaattttagagaacaatccaatgaatttttaaaggtttaccctgtgaggttttgacagttta

ccctgaactcaaaaagttagcttatgatggtgtttacagtaagatggtaccttatggaataattcaatccctgttattagtaacctgctcttcctaaaat

gtttacattgtaagaatttgtaaaatgtattctcatgaagaaggaattttagtgtttactatgaggccttctggaaaataactagttcaatatacaaatt

tactaactatctttgtcagcaacagttaattttctgagaaacttatcacaagaaagattatgacatgtattggagcagtattttagtatacaggtggagt

gacagaggagaaaatcattaaaactctatgaaaaatgttacagatggatgatttatcacacatttccacttctcattcatacacacatttcagcaaatta

aatcaaaactgttataaaacaattaacactgctgttacaaagtaagaaattcatgagcacgtgtttccaagagtgtttccattgcactaacatcattgca

ccaaacatcgaggcgttgaaccatggaggagatctgttgcttatcaagtacacggggctggacccatgtcatgtggacggtggctgcaacctggtctatg

gcacctcggaccaatccttgggcaagggccttcattactagaagttccacttctccaataggaaggcgagtttctcgtgctatttcttcgaaagtgagtt

gtctgtccgtagcagctcgcttaaaagtcatctccataagacagagcaatgagatcttctggcgtaggcgtaactgttcagctgccaggtcagggacagc

agaccactggggcttcatacgttcaaactgtgcaatgtcacctgcattgaaagcagacaacaattctgctagccatgccttatctgtatctcttaaggtc

tcaagtacaggatgtgccaacagctctcccagattgtacactccttcaccgagaagagcagctagtccaagaaaaaatgcatgttgcacctgttgttccc

tagataatttagacaactcaatacagcccaagtaacgtaaagctgtgcgataataatctgcatgtttcccttgcaatctgtacagctggctagccaatag

gtaaaaacgtccgtgtactgttgttatgccatcttcttcatcgagcatcttttcaatttcatctatcaatttcttggtgccatcttgatcatttagtttg

tcaagtagtatctgacctgctaacactttacacaacgcaacagcttcattgttactttttactttggcttcgattttctccaaaaatgaaatcgcttctc

ttttatcttcgaactgtaccacaacatgggcaacgatttctaccagtgacagtggatttatcttattctcaaacacctgtatgaagttgttgtacagctg

aattaggctgtccttcttttgcaaggagtcatgagtgacaaaagattccagcagaagagttatctgatgccataatcttctgttgtataaatcctcaagc

tgagcccaaactgacgcgacttctttactagattcttctcgttgtttggttgataagtaattactaacatctttcacagctacagtcgccatgtttctaa

caatcacgttgcatattttgaactgtgtaagcactggatatcgacatcaacacatatcgacaatttgag

>14418|26s proteasome non-atpase regulatory subunit 1- partial

agagaaaattcgtcgacgattaagtcgagtttcttcagtgcaaagagtttgtgttcgtttgtaggcgtgtaaactgtaagcagcagtaatgaatataaca

tcagcagcggggattatttccctgttagacgaacccatgccggaactcaaggttttcgcactgaagaaactcgacttaatcgtcgacgaattttggccag

aaatatcagaagcaatagagaaaattgaaattcttcacgaggacaggggcttcaaccaacatgagctagcagctctggtggcaagcaaggtgtactatca

tctgggatcatttgaagattccttgacatacgccttaggagctggagacttatttgacgttaatggccatagtgaatacgtggatacaactattgcaaaa

tgtattgatttctacacacagcagcgtacagcaaatgctgaagatgc

>16379|26s proteasome non-atpase regulatory subunit 10-like

aatctggcttcagtctcacgattctcttcacatgctaagtgcctgagggtatgaaatgagaagaattaatgctggagtacaaattctactcacagcaagg

aaataaacagttgcacaaaatcttacaatggtgtggttccacatgaatcaattggattgatattcaattcagatccctgatcaatcaagagctgtactac

tgcagtatttcctcttgaagcagctctgtggagaggtgtagcaccacgtttgtcagcaatgtttatatctgcatggttggctatcagaatctgagcaata

tctttccaattcttagagcaggcatattgcagggacgagtgcccaccatcacttgtggcatttacatcagcaccatttgctattaatgttctaacaattg

tttcttttccagcagatgctgctagtatcaaaggcgtcattccagtctcatctgctggatcgacaggagacttgttttccaataaataattaacgatcaa

ctcatgtccgcctaacgctgcccagtgaatcagcaatcttccatcctgtcagggaaaacaagtaccaacccatctttttgggtgataattttactatctt

tcaatattttttctttcagcgtcagtatgttccctgcgtacgcagattcaaacaccgttcccgacgccattttgctcaaagatatattgcttgattgcta

at

>16377|26s proteasome non-atpase regulatory subunit 10-like

gatacaaggaaatacataaaagttttatataagttccacatagcaccaaaaatgcacctaaaaagtacttttcagtaaaaaaatattgtagaaaactggt

gtccgttagtaatcgagaattattcagtgtgttttgaatttttagtaaaaggagagcccccgtttgaacttatagtagtctataagcacttatggtgtaa

tgatactcgagtcgttgtatgcactatgtaaatatgggtgcaaaccaaggctgtgggataaacttttctgttttatttattttataagtaacaaagatca

catggaaggctttttttcttttactccagagtcgtttggactatatttagcttgatcctaggatgtctatgagctgctggtggttattccgctcggctag

gtccagcggcgtgtccccattgtcattcctgacttctctgtcggcccctgcctccagcagcgcagtcgccacgtgtgggtggccacgccgtgccgcatcg

tgcagtggcgtgttccctgtgtgatccctggcgttgtggtcggcagaagatgctgccagcagccgcaccccgggtgcgtggtcattccatgcagcctgat

gcagaggcgtgttctgccagcggtccctggcgttgacatcagctccatcctccagcagacgcctcgccccctccaagtgtcccctggctgctgcccagtg

cagtgagttctgctggttctcgtccaccgcctccacgtccgcccccactgtcagcaacgtttgcaccttgctcactgacccctccttagctgccgcaatc

agcatcttgcccttctcttctccagaaagatccc

>16376|26s proteasome non-atpase regulatory subunit 10-like

acacgctggaggccagcagtggccaagtcaccattccagacgtggaaggcgaagtgctgaggcagatgctgacctatatgtatacgctccaggcccccaa

gctgcccagcatggcccccaaactgctggcagtcgcagacaaatacggcttgtcagccctgaaatctgtgtgtgagcagcaggtgattgcacagctggcc

atcgacaacgcagcggccgcagctgtgctcgctgtgagatactcatgtcagaacctcactgcggccgccatcgctttcgtgaaggcccacacgcacaagg

tactggccacacagtgttgggcagatgcaatgcggaaccagcttgaagatgtgattgaagttactaggctcctcgctgagccatcagcagaaaccggtac

agcctgaagagtgcgacctcctgaccgccacatatatgcactcacccacccactgcctctatgcacagcatgctggccactgcacgggacatccccacct

ccggctctcagttccacagggaggacagccagactcgtgccaccactgcgctacctacagttcccagctatgctcctccgccagatgatgccgtcgtctc

tcgcatgaggagtcttcctccagaagagaaaggaagaaagctgatcgagtcagctcgggtgggtgcagtgcaagagctgcgcgcactggtggcagcaggg

gcggacttgaatgcttgggacgaggagaagaggactgccctgcagtttgcaggagtgggtggccacgtagaggcagtgaggtctctactggagggtggtg

ccgaggtaggcatcaggaatataaggcagaacacgcctcttcatgcagttgcagatactggccatgcggctgtggttaggctactgacatcatattctgc

agaccccaacgccaggggtaatttggggaggacaccgctgcactgggcagcataccgcggtcattttaaggcagcagctgcattgttggaggcagg

>16378|26s proteasome non-atpase regulatory subunit 10-like

tgaaagggttaagagaaccgtgttttaatttgcatgcttgaaataatttgtgtgacagttattactgaaaattagcatattaattgctaaacattttgca

agaaaacatttattacgggtttattaaaaatacactaagactgtaaataaattgtgaattccaatctataaacaagaaatcactatccacattataaata

aagcagtgaaggtcatatcatctgcagtttctattagtctttccatcagtttaacaactgtcttttcttcacaaatggccatgcaatcatgtctccttaa

cattcatttcccttatatggtggcaaaatatatcactgttcagatgctttacgtaactggcgagctagtgatggctgtgcaagatccaatggagttttct

tccccttgttctgtatgtccagtcttgcaccatgtgcaacaagcaatctggcttcagtctcacgattctcttcacatgctaagtgcaatggtgtgtttcc

acatgaatcaattggattgatattcaattcagatccctgatcaatcaagagctgtactactgcagtatttcctcttgaagcagctctgtggagaggtgta

gcaccacgtttgtcagcaatgtttatatctgcatggttggctatcagaatctgagcaatatctttccaattcttagagcaggcatattgcagggacgagt

gcccaccatcacttgtggcatttacatcagcaccatttgctattaatgttctaacaattgtttcttttccagcagatgctgctagtatcaaaggcgtcat

tccagtctcatctgctggatcgacaggagacttattttccaataaataattaacgatcaactcatgtccgcctaacgctgcccagtgaatcagcaatctt

ccatccccatctttttgggtgataattttactatctttcaatattttttctttcagcgtcagtatgttccctgcgtacgcagattcaaacaccgttcccg

acgccattttgctcaaagatatattgcttgattgctaat

>19289|26s proteasome non-atpase regulatory subunit 13

gtggtacagttcgaagataaaagagaagcgatttcatttttggagaaaatcgaagccaaagtaaaaagtaacaatgaagctgttgcgttgtgtaaagtgt

tagcaggtcagatactacttgacaaactaaatgatcaagatggcaccaagaaattgatagatgaaattgaaaagatgctcgatgaagaagatggcataac

aacagtacacggacgtttttacctattggctagccagctgtacagattgcaagggaaacatgcagattattatcgcacagctttacgttacttgggctgt

attgagttgtctaaattatctagggaacaacaggtgcaacatgcattttttcttggactagctgctcttctcggtgaaggagtgtacaatctgggagagc

tgttggcacatcctgtacttgagaccttaagagatacagataaggcatggctagcagaattgttgtctgctttcaatgcaggtgacattgcacagtttga

acgtatgaagccccagtggtctgctgtccctgacctggcagctgaacagttacgcctacgccagaagatctcattgctctgtcttatggagatgactttt

aagcgagctgctacggacagacaactcactttcgaagaaatagcacgagaaactcgccttcctattggagaagtggaacttctagtaatgaaggcccttg

cccaaggattggtccgaggtgccatagaccaggttgcagccaccgtccacatgacatgggtccagccccgtgtacttgataagcaacaggttctgtcaat

attataattgcacagctctgtaatagtagagagagattggagaggaaacatatccagtaagcacagtcagttatgagtgaggtgaggccagtcctcccta

cagcactcgccttctcacatgcaaccgctagtgtctgcaatttgtaccagtcacttattttagcataaacagtttcaatatagcactcaaaaactagaga

gacaaggcaccaagaaaaataagcccatcattcttagctgtgac

>19288|26s proteasome non-atpase regulatory subunit 13

cgatttcatttttggagaaaatcgaagccaaagtaaaaagtaacaatgaagctgttgcgttgtgtaaagtgttagcaggtcagatactacttgacaaact

aaatgatcaagatggcaccaagaaattgatagatgaaattgaaaagatgctcgatgaagaagatggcataacaacagtacacggacgtttttacctattg

gctagccagctgtacagattgcaagggaaacatgcagattattatcgcacagctttacgttacttgggctgtattgagttgtctaaattatctagggaac

aacaggtgcaacatgcattttttcttggactagctgctcttctcggtgaaggagtgtacaatctgggagagctgttggcacatcctgtacttgagacctt

aagagatacagataaggcatggctagcagaattgttgtctgctttcaatgcaggtgacattgcacagtttgaacgtatgaagccccagtggtctgctgtc

cctgacctggcagctgaacagttacgcctacgccagaagatctcattgctctgtcttatggagatgacttttaagcgagctgctacggacagacaactca

ctttcgaagaaatagcacgagaaactcgccttcctattggagaagtggaacttctagtaatgaaggcccttgcccaaggattggtccgaggtgccataga

ccaggttgcagccaccgtccacatgacatgggtccagccccgtgtacttgataagcaacagatctcctccatggttcaacgcctcgatgtttggtgcaat

gatgttagtgcaatggaaacactcttggaaacacgtgctcatgaatttcttactttgtaacagcagtgttaattgttttataacagttttgatttaattt

gctgaaatgtgtgtatgaatgagaagtggaaatgtgtgataaatcatccatctgtaacatttttcatagagttttaatgattttctcctctgtcactcca

cctgtatactaaaatactgctccaatacatgtcataatctttcttgtgataagtttctcagaaaattaactgttgctgacaaagatagttagtaaatttg

tatattgaactagttattttccagaaggcctcatagtaaacactaaaattccttcttcatgagaatacattttacaaattcttacaatgtaaacatttta

ggaagagcaggttactaataacagggattgaattattccataaggtaccatcttactgtaaacaccatcataagctaactttttgagttcagggtaaact

gtcaaaacctcacagggtaaacctttaaaaattcattggattgttctctaaaattgtcaccttgcatcacatgaatacatgtacttattgctttctgtat

gtgttacaaaactttggtaaactattaggttataattgaaactttcctgtatggtggcatccatgagatgaaacttaggagttggcatcatagttagcat

tttggcaaatattttatttgttcacccataattggtgaaatcagtttgttctgtgcccatgttcaaaacataaattgtaagctcttcactattatttatt

ttgtgtgtaattccttgattgggttggaagtatgtgggtactatattagtgaaatcattgccctttcacaaacatataaaaataaactagacatttttaa

ttgtttgctaaaatcttttgcctagccataactggaatattacaaacattgtccaaatttatgcccattccttgtcaccaacaagacaataaatatagtc

atttgtacagtaagctttgttacattaagttgcacaaagtcaaattaatttgtcacattcatctactgaaacattattaaattaaaactcataccataaa

aaatggcactttgtattgatccaaattgattcttttcgcaatttggatgtgttatgtgtctttcattttgatgtgattataatctatacacacatagtgg

catcatgacaacactataggtaataattacaaaagtgcactcatgtaatagaggctgattctgaggtgaggccaccgcacaataaaatactttaggaaag

aaggattttttgattccccatttttttactctgattctcaggtgaggccaccacacaataaaatgctttaggaaataaggattctttgattccccttctt

tcttcctttttttctcccccccccccc

>24106|26s proteasome non-atpase regulatory subunit 14 isoform 1

caccagacacagtgaaatcacattctggctccttttgtattgtaatatgacaaaatatacatctatgtataattacagatgataatccatcgatacaatc

ctctccaacaatgtgttttacacaaagggttaggaataatcaatacattattaaaaacaacactcttccgtttattatctgccattgctggacacgtgtt

acacaatttcttttaactatgcaaattagaacatttgcataagatgaaagcagagccatcagtttgtgagtccactgttttccatagtgcgttatcaaag

gacaaaactctttaaccacatattaataaattatattcacttatttgttcatcaacttatactgcaatctgatggtggaattgcagtttagaataacact

gttcacttaaacacaacagtatcaagcatggctcctagacattgcacaatattagttgtcattaaaatgtcccctttttcttccaaatgtcgttttggat

cttgctttcccacattcttaattgccaactgttcaggcgtcattttctcttcatcttctaatgccttgttgtagtt

>25192|26s proteasome non-atpase regulatory subunit 4

attcagcataatgaactgacactgatatgttttaaaataatatcataaatgcaaacagtaaacacagaaacaaaaattctagacaagaagatatatgtga

agattacatcagacaattattactttattgaagttcacacaaatcgtactcctttcttcacaacaacactattaaatgctgtgaggcaaataaaactaaa

aattttacatttattaaggaagatggggcacatacaaaagagaactcattcaatgaaggtcaaaatctttaattgcaacataaatatatctgtttcacct

tcagacagttgaatagcaatttcttattaacattgcttaaaaactaaaatttacaccaattttgtgtattgaattaggacaagaaagctgtccagcaact

gcaatgaacagctggggttaagtatattcagtgttaatgctgcattaattttttgtcttagtattaaaaattttccagtccactggtggcctcataacca

tccattcttcagtatcattgaaactgaacacactgatagacaaatttggaaacctcagaatttataacttacagatcagtattttgttgctttatttctt

ccccttatcatcatccttacgatctttttttgtcactttctttcttgtcttttaaactaccaactgctttgcgtatagcctcactttgtggatcaactcc

aggtaaattttctagaacactctgcaaaaattcaggatcattcatcacttcagaataatcatcatctccctcaacttccattggagtttccgttttctct

tctttcacagagctgcttccttcctctttcattggtacttcttcaggctgagcatcttgcatggacatttgcattgctagagcaatttgttcctcttctg

tcattttggagaaatctgggattcctgcctggctgctctgttcttcggcaccaccctccagagacattgccaatgctctttccaggagtgcctcttcact

ttgttcttctttaattgtatctgctggctgtggagcaccatctggcgctgaagctgcttgagctcgacgagcctcttcctcttggcgctgtcgttgttct

tccatggatacacgcaaggccaaagcaagttccggatcttcatttggatctacaccaaattcaaatcctgaagcaccaaggccagcacctcccataccat

cctctccctgaataactggcgatgaaagcagagcatcagacaaatgtgggccaggaggcactgtgaccaaatggctacttccaccatctcttccatttaa

cgcatttataaatgctgtgaggagttcattgtttgaaaccttgggattaacttcttctccgaagctgacaatatcaacattgactttttccttcttgagc

ctttttgccagacggatgagatcacttccttttgcttgcacaggacttcccacaaatgctacaatgcgcattttatggttttttccttgtcgatgtttca

gtgctaaatgggcaatacggatgccagtaaggaatttaatatttccttgaggctggacttgatgcaactttgacagtattctcccaacatcagaagtgag

tgttgcaagaacttcaacatttgctaaagtaagcagaccaacattgttctctggatttgatctgatttttgaatggcatacaaggttcactgcatcctgc

tgagcttgtagccgagtaggaacaaaatcaccatttctcatgtagtcactgttatcaacacatatcatagtactttctaaaaccattttttccactctac

tcttcggtaaccaccaatttttcacaccaatcacaatactttacagtcactgggtacaaaatgcaaac

>27093|26s proteasome non-atpase regulatory subunit 4-like

aataagttggctgtgtcatactgtgcgtctagacggcttggtgcaaaatctccattccgtgaccaatctgagttatccacaca

>27683|26s proteasome non-atpase regulatory subunit 5 (26s proteasome subunit s5b) (26s protease subunit s5 basic)

gccagtaagccttgctcacagatcactggcaacaggctctcgaattccactgctttcagaaggactttcaaaacaatggatctaacttcgttcattggat

gattctgcgctctttgtaagcacggcaaacaacgagtgaacacaatttctggattcatatctggtaaaattttatccaaaattgcacaagataacttgat

ttcttcctcgtccgatgtattcagacagtcaaacaagcgtggcaactgtatttccacttcaacacgttgcagtgtttctttcctgacgcttccaactaag

tttaatatctccttcagtgtgtgtcgtcgattgacatcaatcgacaagatgccgaaaagacggcatatatcatcaattttttcctccatattaaatgtca

acgtccgtgcagcttatctcagagcacacaaacaacactaccggcttctcaataaacaaaaccagaaacgccactcattcttgtatatccaaag

>28957|26s proteasome non-atpase regulatory subunit 6-like

gactgttgatgaagtgctgttgtctagactaaagcagcttaatatggaaaaacttaaacaacttgatgatgaaattgaagatgcagagaagaatttaggg

gaaatggaagttcagggagcattcatgaaaaaatcagaatacctatgcactattggagcaaaggatgaagcagtgaaaaattttcaaatcacgtatgata

aaactgtatctcttggccagcggttgg

>29269|26s proteasome non-atpase regulatory subunit 7-like

aaaggagagacagtggtttctgtggattcatggttggcgtaacgcattcgaaatgccgagtcaagaaatagtaacctcaaaagtagtagtacacccatta

gtgttactaagtgttgtggaccattttaatcgaatgggaaaaattggaaaccaggagagagtggtgggtgtgttattaggatcatggagagcgaaaggag

ttctcgacgtctccaatagctttgcagttccatttgatgaagatgaaaaggacaaatccgtctggttccttgatcatgattacctggagaacatgtacgg

catgttcaaaaaagttaatgctagggagaagattgttgggtggtaccacactggacccaagctacatagaaatgatgttgcaatcaatgaactcattcgg

cgttactgcccaaattctgtccttgtgataattgatgcaaagccaaaagatctgggtctcccaacagaagcatatcgtgctattgaagaggttcatgatg

atggttctccaacatccaaaacatttgagcatgttccaagtgaaataggtgcagaggaagctgaagaagttggtgtggaacatttgttaagagacattaa

ggataccactgtgggcacactttcacaacgaattactaatcagctccttggactaaagggactacactctcagttgcgagagatacgtaattacctaacc

caggttgttcaggggactctcccaattaatcaccagatagtctaccagttacaagacattttcaatttgcttccagacataagccacggaaattttgtga

attctttatatgtgaagaccaatgatcagatgttagttgtatatcttgcagctctaatacgttcaatcatagcacttcacaatcttatcaacaataagct

aaccaacagagatgcagaaaagaaggaaggtaaaaaagatgatgtgaaagacaaggataagaaagatgagaagagcaaggaggatagcaaaacggaaagt

aaaaagtgatgtttgtagacttagtgttgatgaatattttgtttgtcaaccactttgaattgtataggacaagtgttctcatgtgttaacatgcaagact

acaaactcgccaatacttaaaactgagtgacatttcagtagtgtcgcataactgctattagcatcgtgtgtgttgtaacactatgaagagattaaaagtc

caacattttgctatttattttataactaaatgttttggatgttctggcagattttagttgatgaaccttggtgagtgaagctaacatacctttgttaact

ttttaatgtttgatatgactaaaatataacatcattcactatgtatttttgcaggctgatggttcccagtaaatgatctctgaaaaagtttgttcatttt

gtgtctattctggacctctgtaaattgtattactgtttgcactgttaaaaataatgacatttaataaagaagatctatgaatttgtacaagttaaattaa

aactgtatgtaatatttcattagtattctcaaacctgaatcct

>29478|26s proteasome non-atpase regulatory subunit 8

ctgtaatccaaatagtaacatttcagttgtgccatatatctctcaaatgagggtatatctttcatagcaatgctccactgagcaccaatctccagaatgt

ctcttgcaagtaacagctcttgctgtgaagcagtcgagttggaagttggcaagaacattaaatgtgtaaggccaacctttaatttcgttaaaagttctcc

acatttgttcaggttcggtggtttcttgttccattctgttttcagttgctggtacaatgacaccacttctttcattgccgccatgacgcttaccttatta

tgatgtttggcaaaagatctgtgctcccttcacaaagatacagctgtgtgcgcgcatagcattgagtataccacagtctc

>29479|26s proteasome non-atpase regulatory subunit 8

ctttgtgaagggagcacagatcttttgccaaacatcataataaggtaagcgtcatggcggcaatgaaagaagtggtgtcattgtaccagcaactgaaaac

agaatggaacaagaaaccaccgaacctgaacaaatgtggagaacttttaacgaaattaaaggttggccttacacatttaatgttcttgccaacttccaac

tctactgcttcacagcaagagctgttacttgcaagagacattctggagattggtgctcagtggagcattgctatgaaagatataccctcatttgagagat

atatggcacaactgaaatgttactatttggattacagggaccagcttccagaatctgcatacaagtaccaacttttgggactgaatctgctgtccctact

atctcagaacagggtggctgagtttcacacagagttggagctgttgcctgctgatcagatccagaccaacgtctatatccgccatccgctttctcttgag

cagtaccttatggaaggctcctataacaagatcttcctggcaaaaggcaacgttccagcagaaaattataatttttttattgacattctgctggacacaa

ttagaaatgaaatagcaacttgcttggagaagtcctacgaaaagatatctgttcaagaagcagcgagaatgctgaacatggcgagtcttgctgccatgaa

gaaatacggagaaaagaattggactcttaatccagataattactactactttggtactgaggagaagaagagtgaagaccctcttccatcaatagaactt

tcagagcaagtgatagattatgcaagggaacttgaaatgattgtatgagtgaacaaacttttgtggaaaattaaagctttatttaattttactcatctgt

tgaaaacttttgtaaataacaaagtaaaaagtttaatagtcattttgcattgtcttaatgaattgaatccaacaatcacttgttgacagtaag

>30147|27 kda hemolymph

tccatactgaggtgatagcagttgctgctccagctgtcttttcattcacccactttgcacagggagtcatctttatgatgaatctgaacatgctttcaat

tatattggcaggtgtcggctctgcacatttttcttccattacattcacagtacatgtctgcatattttgcattgccacacactccttttcacccaggacc

aactgcggaagagattctatgttagacttttcctgaggaaccaatttaccaagagttgaattcatacattcctgtatttcagttgaagttttattcaggc

aatcttgaccaccttctgcaatgaatactgcaagaaatataaaataagcatcaacatttaccgacaaatgaggcatacctaagaaaatcaggttgtgcat

aattcattcttacatgcaatacgatcaccctgcttgtggcaaataaaatccagaattgcatttgttatgtttaatgtagttcgaacactttctttctct

>30440|28s ribosomal protein mitochondrial

gggtgatgttagaagcagcacgttttgtaccaacgaacaaaactttgttctttttgcttgctaattggttagcgaagttcaaagcatcatttaacgcagg

aacagtgtgctcaaggttgatgatgtgaattttgttacgcgcgccaaaaatgtattgacgcatttttgggttccagaaacgagtttggtgaccaaagtgc

gcacctgcttgaagaaggtcgcgcatgcttacgttgtaatctgccattttctttaccttaaaagttgggttaggcctccg

>30435|28s ribosomal protein mitochondrial

cctcaggcgttactccacttcacagaaacttaagctgagtttaagatttggaaaaattttacacatggataaatttgtatcagtctttggtcaaataaag

taatgttttattagctacaaatgtatttacagacttatagtgtcaatgtaaatttcaattgatgttgcagcacggaaagcaattaccaaaaagtacaatt

tcccccccccccctctctctcttccatctatccccatcggtaatgtgcgtatgctctgttagcttcacagagtttatgtagttcttgcttccttctaaca

acttttccctgtaacaaaaaacacagactgtaatgaatcttcatgacactaaaacatttttatcatgggtacaagaaatatcactgccacaatgaaatct

ttagatttggaactttaattgaagcaaatgcaatgtgctcactccttgattagctaatggtgatcacaatgtaatccaaaataatatgtttctgcatttc

acacattattattcacaattcagttgcacagtatccagtttctttcttcctgaatggattctgtgtagtaagaaagctcagtaatgaaatgtatcagcag

ctgctattgttctcatctgactttaatacataattgttattt

>30439|28s ribosomal protein mitochondrial

gctaattgcattcaaatttgtttcgctgtcacttttccatggaaggagaacccgccgcctcacccgttctttttaagcaacgggggtgctgcacttctgt

ctgagttgttagaagatgtggaataaaaggtgataaatttgccagtaaaattctgtttatttgttttattacagaggctgcaactgcattttccatttta

ccctcatccagattatttctgcaatccagttcttcctccctccttctgcatttgtactcgaataccagtgcgtcctggacatcattcttaattttgttga

acaaatcatctgataaagtgccattattttcaaaactaaaaggcagttcttcaacagaagaaaattgtgttctcgtaatatatcggaaaaatttgaacgt

atcgtacagtaaatgaccctctgttagcagtaatgacctccacccataatatcttggcaagttcaacgcgaagagcttctcttccacggtttccaaagac

ctgagcttttcgtgccactctctttccttacgttcgctaatggctcgccgtgataggtctaaaataggtggatattttggtctttctgtatactcagctt

catttgcactaacactgaaattatgtttaacactattaagaattgctgcaacgcactgtggtcttaactttatcaaatgaacgactttctctcgcaacgc

gcataccacagccatcatttgtcaccaacctcactgtttacaaatacgaaagtgcg

>30436|28s ribosomal protein mitochondrial

atcgcgttcgtccttgtgaagcttcggtatatctttacatacgaagtggttcgtcggtataatctggctgctttgaaagaccatggaggtgtaaacaaaa

acaaaccatggccggcagctgtagactgttgtttaaatcatttacgcacaggctgttgcaagcaaacttacgagaatcgcatttttgtagattgagcatt

gccagacgtttctctgcggaagcgagaactgatgctgatagagatccagcaccgttcttctttaacaaagatgtgcaaaatttgcttaagaaattgacaa

gggttgatttacataaagttttcagagcgagaaaagaaggaaaggatctaaaagaaccagaatacaaatttatgtcaacggaagaagtaaacaaggcagt

agaggaagcatggcagcgagctgaagaaagattgcagatgccacctttcctttcaccaactccagacgtccgtcgagagatttgtaaggatccagaacta

caaggctattgcgattcaaaattcctgtttacagatatcacatttggtgtttgcaatgaggacagattgattgttgctcgagacccagatggtactttac

gacatgctgacagagatgaacgtttccgttgcaatcaaatctactttcctcttcctggtcgcaaattaaggacacctcatatgtttgaagaagaacacct

taagccgttgctcgatactggagagtatgaattcatactagatcgagcatgtgtgcagtttgatccagactctccagatttttgtagagttcgagatcaa

acatttgaccatgtggagaaatcacgtcatttctcaaagttaagatcaactcgtcattttggcccattagccttctaccttgcgtggcacagaaatattg

attctcttctcttagacattgtgtctgatggcaggttagatgaagccacagctttgattaatctgtttcataaactgaatccaagtttggagtctgcaat

agctgctgctgaatatgaaggagatgaccttggcttgataaggctatatatctcatcaagtgactgcagacggcgagggcaattggaattggcaatacag

actttgcttgagctccagaaacaacaagaactagtggcagaaggaattcaacatgcacatggcactggcagctagagcagtatgtgtaaatgttgtgatc

tggacacaaatgtgtgtgaaattttgtactaaaacactggttgtttgttggctgaagaatgttgtactgctgtgtggaaagaataataagcagaagattt

caaaaatgaaataaaccattcctccccactcctctctccctctccctctccctctccccccccctcttcggtttatttggcttacagtagaggatgctgc

tctttgttacattaaaaattctatctgtttcaccaacaactgtcagtagtacattcatttgttatattttttattttttgatgcaaaaatgtatttaaat

taataatgttaattggaaaaagtgagctgcagtactgtcaaaaactgttatgtatgggaacatgtgaaaggtggtctctatgagtgggttacattttctg

taactctataataataatctgaggaaagtaatgagatatggatgaaatgaatgcagtaatgtttgacatgtgtaaaacatctgttcttctaaggccaaaa

gaataagttgttgtcacagacaatgcagaaataaaatgtgtagtaacttag

>30438|28s ribosomal protein mitochondrial

tggcaactttattcacaatattcagtgcttacaaaataacaaatttgtagatcacaaatctactttctacttttcttgtaatcattttctgacaagggat

aataagtcttcttccacttagttttcttgtcctcctctggccgcaaactttttggaatgtactctggtacatgttcatcatactgacgtctgaatggatt

gatacctagctcaaatgggcgtctgcgagcatccataggtctagttttatggtccaccttgtatatcttctcccaatgatataattcatatggcaacctc

attcgtggtctacaagaatacatatgtttgaacctagtttcaagccattctcttcgttctggatcatcaataagtgcaaccaaatgtttgccctctttca

gatatggtcgtaggttcactcccactctctcttgtggtgcagtagtgtacaacccaataatcatcttcagcagcttgtcattaaaacctacaacttctgt

gtctttcacttcttcaaacagttttaagggggctgtaccccagcagaaattgcattcctcattccttaatgcatactcagaatgtaccaaggttgtgttc

aactgataagtataaagtgaccacagttgaccatttgttacaacagtttgagtgacaaatggataagtcacatcattgtatgtagagaaacctttgtaac

aggcttgtgccagaagccatgcataggaagccaagattgcttgactgtgtaaagcttcctcctcatcgtaagtgctacagaattctcgtctgtgatacaa

gtgtcctcgtgtatggaaagaagttagaccaaattcacactcatctcctggccaaaaacctggtatagtagtaccattccgatgggtgaagccataacct

aagactctaggatcaaactgaaaatgttctatagatgagactccttctggttgcagtctctcagtattgatgactggcaatggaagttcatgcctcagtt

gtagcactggtgagccaaaatactgtaccggcctatcagctggcctttccacatcttccttagtaagaaatttcatgccttctcttcctttacgttgtcg

tctacttggttccattcctccaacgaaccaaaatgcttctacacgaggttcaaagtcaacctgagttgttagaagatgtggaataaaaggtgataaattt

gccagtaaaattctgtttatttgttttattacagaggctgcaactgcattttccattttaccctcatccagattatttctgcaatccagttcttcctccc

tccttctgcatttgtactcgaataccagtgcgtcctggacatcattcttaattttgttgaacaaatcatctgataaagtgccattattttcaaaactaaa

aggcagttcttcaacagaagaaaattgtgttctcgtaatatatcggaaaaatttgaacgtatcgtacagtaaatgaccctctgttagcagtaatgacctc

cacccataatatcttggcaagttcaacgcgaagagcttctcttccacggtttccaaagacctgagcttttcgtgccactctctttccttacgttcgctaa

tggctcgccgtgataggtctaaaataggtggatattttggtctttctgtatactcagcttcatttgcactaacactgaaattatgtttaacactattaag

aattgctgcaacgcactgtggtcttaactttatcaaatgaacgactttctctcgcaacgcgcataccacagccatcatttgtcaccaacctcactgttta

caaatacgaaagtgcg

>30437|28s ribosomal protein mitochondrial

aacaacatgctgtgttctgtttgctaaaaactcttcaatccagccacacagctggtctgatattccataggctcttactttgtttatcaggcgacagtgc

gaaactgtatcgaacgccttccggaagtgaagaaaaatagcatctacttgggagcctgtatctaatattttatgggtctcatgaacaaatagagcaagtt

gggtctcacatgattgctgtttccggaatccatgttgattcctacagagtagattctgagtttcaaaaaacgacatgatactcgagcaaaaaacatgttc

taaaattctacaacaaattgcaagatgttcatctgtgattgattgtatactgtaattttcatattggttttgaaaaattgaatacgcaatagtcctgaat

aggtagtttactttttgttttgattagattagattagattagattaatacttgttccatagatcatgaatacggcactttgtaatgatgtggaacgtgtc

aggttaatgaaagatgtctgtacaagatattacattacacaaaatattgcatgacactaatgcttaagttagtttttttccctcccttaatttatatcta

aaaattcagccaatgagtagaaggagttgtcatttagcaattattttaatttatttttgagtgttggttgcctatctgtcaggcttttgatgctgtttgg

taggtgaccaaagacttttgtgggagcataatttacccccttctgtgccaaagtcagtcaatgctgttaagtgatgcctactgccaaatgctaagggatg

catatgtagtttgctatgtgacagttgtttgttatgacccgctctactaaccctctcatttattacattcattttagaaacaccttgaatatagactgtg

agattagaggattattttttccaatgaaaacctttttttagaagactaacagtggtttcaaaaccacatccagataattttaataatttgtaggagaaag

gcgtatattatacatttttttgtaattcccagtttactattttatgtttgtgtacagtttattaattacctgactggaatgcactctaaacttctcacag

gaatccaaagcctgagtggaataattttcatatactgtttcacagtgtgtatatcaaagcccatttgtaactgactttaattattagttacatttctcat

ataaatgcaagtgatcagggatatgtttaaagatttccatcatcctcaaaaatcctattaaagatcagctatatgtactcttaactgcagatgtaacata

atgatatactacaccacagttaaattgaaaaaagtttatttgagattaatgcttttattataaatgaaaatgtaatcttgtttcagacagaacttcctgt

ggctactatagagactcaaatgtttcaggttgactttgaacctcgtgtagaagcattttggttcgttggaggaatggaaccaagtagacgacaacgtaaa

ggaagagaaggcatgaaatttcttactaaggaagatgtggaaaggccagctgataggccggtacagtattttggctcaccagtgctacaactgaggcatg

aacttccattgccagtcatcaatactgagagactgcaaccagaaggagtctcatctatagaacattttcagtttgatcctagagtcttaggttatggctt

cacccatcggaatggtactactataccaggtttttggccaggagatgagtgtgaatttggtctaacttctttccatacacgaggacacttgtatcacaga

cgagaattctgtagcacttacgatgaggaggaagctttacacagtcaagcaatcttggcttcctatgcatggcttctggcacaagcctgttacaaaggtt

tctctacatacaatgatgtgacttatccatttgtcactcaaactgttgtaacaaatggtcaactgtggtcactttatacttatcagttgaacacaacctt

ggtacattctgagtatgcattaaggaatgaggaatgcaatttctgctggggtacagcccccttaaaactgtttgaagaagtgaaagacacagaagttgta

ggttttaatgacaagctgctgaagatgattattgggttgtacactactgcaccacaagagagagtgggagtgaacctacgaccatatctgaaagagggca

aacatttggttgcacttattgatgatccagaacgaagagaatggcttgaaactaggttcaaacatatgtattcttgtagaccacgaatgaggttgccata

tgaattatatcattgggagaagatatacaaggtggaccataaaactagacctatggatgctcgcagacgcccatttgagctaggtatcaatccattcaga

cgtcagtatgatgaacatgtaccagagtacattccaaaaagtttgcggccagaggaggacaagaaaactaagtggaagaagacttattatcccttgtcag

aaaatgattacaagaaaagtagaaagtagatttgtgatctacaaatttgttattttgtaagcactgaatattgtgaataaagttgcca

>31069|28s ribosomal protein mitochondrial-like

gtggcatcctcggttcctctgtcactttttattaataaaatacaatacctacagtgcaacaacaaaaaattacacactgctccaacatatcacttttcaa

tattactatacaatgtttggctcttagtaacaccaaagagtctatctatagagacagttggcttttcactttcttttgcaactactgagacgttatcaac

accttggttacatacaagtggcatcctcggttcctctgtcactttttctcccctggctaggcaatcacggattatcagctcttttagtagaggtggaaaa

ggagctgttcttgggagcagtctcgccggtttctcactggattctgcattctgggcatgggtatatgcatattcctcgtgtttatggataagcctgtagt

ccgctttgtatgtgacgctacttattttcaccaagtacggatacttcctgcccctgaatatcttttccgcccagacgcaaaccttgcgagtttcctgcag

tttggaataagaacacagtttcaatatctcacagcataccatctgacgggcatacttcattaaattacctcattcggcatcgcttcaacttttcgtatga

caaagtaacatggttcgtcgtatctttcgaacatacttctcaccacaattctgcccacaccaaaatttttcaggttgcctaatatttcccacaggggttt

ccccctaaaatcggttgtcctgcctatatactttatgggcattgtaatatttttcactagc

>31071|28s ribosomal protein mitochondrial-like

aaaaaaaaaaaactaattatctggatcttaggaaaatttagaggagtctcctttggtacctgacgacccagtaacccgaaataatctattcaaataacaa

gtgagtgaactgagagggcaaataatgggcgtaacaagcgtattacgtgcgatgatgacagtgaaaccacatgttccactaatcaaattccgcaaggggg

gtaaggttcaagagaccagcacaatgctttcaccagcccaagggccaacccccactgcatctaaagggaaggtacaggcaagacctgttattgaagatta

tcagctgcctgcaaagtaccggaggaggccaattgatgagaaagaaattgaatatatcaatagaggtggtcctgaatgaggtttgggcttacaaaaaaca

gcagaatcaccagtatttcatcaaggatgtgaaatagtgaattagttgtatcctgtacatattgtatataacaatatcaaataatatttttcctctcttc

atatgatgcatcatctgttgaaataagattgtgtgtattgactaggaaacatgtcgctgctgatgagaccaatataagtatgcagtgatgatgtaagtgt

tgaatttgggaacctgttggttacagtatcgttgcgggcagctgtttttcagttaacatgtgtccttttgcctttatctacttgccctcatatacacgtt

tggtcactgaaaaaactcaggccaaaaggtaactggaatactggggagaaatgttcttgccagagagattcccctgctctccattcatctgcatacaatg

ttatcaatattttgagtggttcagtgtatactgttttaattccacaataaataatcagactggctcttccacaaaagggttgcttcttctccatagatag

agcagaaaaaatttagtggtttttaatgaaacaagatataataatagggatgcctatcattctttaatgccaaaatatttgcagtcatttcctgttaatg

aaaagaaaagattgttgaacaggaactaatgttttgagaattgtatttacccaagaagagttaaataggaaaacaccacaagaaattgcactggcacaaa

atgtcattaataacaaaatgaatgtggctgtaaatgagaggtctattatgaagaaattgatttgaaaactaaatcaaaaacaaagcatttcaaaaaagta

gctatttgagcgagagaaacatatggttaagaagtgttgcagaaagtcgctttcaggatccagaggaacacatttgtaggtacgaagttacatagaatgg

tgtgtgtataggtttctaacaagagacagattgatttaggaacctaataattgcacaggggatgaaacaaattagaaaatgccaagatatctcagactgg

ggctatgtatgtttcttcattgttgtttgtcaggcacacttccatgtaaaacaggtttgctctttgcatttgtggttattgtaatttatgtttacatttg

attcttctgaaattgctttatgaattctggctctttcaaatgtcataaccaatttattttcccgtccttcccaactactaggcctactagctgtgcacct

cttcgttattgactgaccgctatttttgacttccacaatgtattacttcataatgtagaagaacctttaaaggttcaagaaatacattagtgcaaagaaa

tagctgttgcgaactgttgcagtatgcaacacttatttcatacagtaattattgataatctgcccataacattataattattgccattatacttaaaata

gctgcagctagcctgtcttacttaatattgagcaaagaaactactgagcatgaagctgcatttacagttacattgttcagttgtcagcatactcttaaca

ttaatgttgtttattataattgtgtgtatacaagaagaatggaggcttatttaaccaggttcagtttattacagtactaacacagatttataatctcaga

aatttggcctctaatttatagggcaagtggaaaataaagtttatcttg

>31070|28s ribosomal protein mitochondrial-like

tccacttgccctataaattagaggccaaatttctgagattataaatctgtgttagtactgtaataaactgaacctggttaaataagcctccattcttctt

gtatacacacaattataataaacaacattaatgttaagagtatgctgacaactgaacaatgtaactgtaaatgcagcttcatgctcagtagtttctttgc

tcaatattaagtaagacaggctagctgcagctattttaagtataatggcaataattataatgttatgggcagattatcaataattactgtatgaaataag

tgttgcatactgcaacagttcgcaacagctatttctttgcactaatgtatttcttgaacctttaaaggttcttctacattatgaagtaatacattgtgga

agtcaaaaatagcggtcagtcaataacgaagaggtgcacagctagtaggcctagtagttgggaaggacgggaaaataaattggttatgacatttgaaaga

gccagaattcataaagcaatttcagaagaatcaaatgtaaacataaattacaataaccacaaatgcaaagagcaaacctgttttacatggaagtgtgcct

gacaaacaacaatgaagaaacatacatagccccagtctgagatatcttggcattttctaatttgtttcatcccctgtgcaattattaggttcctaaatca

atctgtctcttgttagaaacctatacacacaccattctatgtaacttcgtacctacaaatgtgttcctctggatcctgaaagcgactttctgcaacactt

cttaaccatatgtttctctcgctcaaatagctactttttttaaatgctttgtttttgatttagttttcaaatcaatttcttcataatagacctctcattt

acagccacattcattttgttattaatgacattttgtgccagtgcaatttcttgtggtgttttcctatttaactcttcttgggtaaatacaattctcaaaa

cattagttcctgttcaacaatcttttctttacattaacaggaaattactgcaaatattttggcattaaagaatgataggcatccctattattatatcttg

tttcattaaaaaccactaaattttttctgctctatctatggagaagaagcaacccttttgtggaagagccagtctgattatttattgtggaattaaaaca

gtatacactgaaccactcaaaatattgataacattgtatgcagatgaatggagagcaggggaatctctctggcaagaacatttctccccagtattccagt

taccttttggcctgagttttttcagtgaccaaacgtgtatatgagggcaagtagataaaggcaaaaggacacatgttaactgaaaaacagctgcccgcaa

cgatactgtaaccaacaggttcccaaattcaacacttacatcatcactgcatacttatattggtctcatcagcagcgacatgtttcctagtcaatacaca

caatcttatttcaacagatgatgcatcatatgaagagaggaaaaatattatttgatattgttatatacaatatgtacaggatacaactaattcactattt

cacatccttgatgaaatactggtgattctgctgttttttgtaagcccaaacctcattcaggaccacctctattgatatattcaatttctttctcatcaat

tggcctcctccggtactttgcaggcagctgataatcttcaataacaggtcttgcctgtaccttccctttagatgcagtgggggttggcccttgggctggt

gaaagcattgtgctggtctcttgaaccttaccccccttgcggaatttgattagtggaacatgtggtttcactgtcatcatcgcacgtaatacgcttgtta

cgcccattatttgccctctcagttcactcacttgttatttgaatagattatttcgggttactgggtcgtcaggtaccaaaggagactcctctaaattttc

ctaagatccagataattagttttttttttttttactgccctatacacaaatcgcgcaagcacgtgttgccaaacatatatttcgcaagacaaccaattta

tttaatcaaggcttaaaacttgcaacgatacatccactacggtaaagagcagacggcgtcaaattac

>31958|3 -5 exoribonuclease csl4 homolog

ttgattgaatcccacagaagttctgtatcctcactgttttggggacttgtacatttttgttttaaaaatgtaaataaacagtgctgtacaatagtttaag

gaagtctgagaaaaggttgatatgtaaaaatggttatctgttaaacttgtttcttaaataagcctgttttctctctgtttaattaattatagtgttctat

ttatgtttgatatgttagtgtaagactattttattcctgcattgcatgtttgttattctatagagttgagcataacagttttccttgattaattactcct

gcagctaccaatgacagacattcactcgtatcaactgtcaactgctgaaaatgaacttggagttgtgatagctcatagcgaagctggtattgagatggta

cctatcagctggacgcagatgcagtgcccaaaaacttttgtaaaggaatggagaaaagtagcaaaagttgttccagaagatacaatagtagactgagaca

ttacaaaatgttttc

>32811|3-hydroxy-3-methylglutaryl coenzyme a synthase

aaaaaaaaaaaaaaaaacatttaaatgaatgagaattacttcatttgcaactgacataccatgtgtaaggactaatgtcacaaatgaattcaaatgatca

ataactgtaagttcttcatcagaatttcaccatcatctaaacactgttaagcatgttaatattacacttattgatatcacatgcttgatgataaatatta

gcatgtgactgtttatatacttaccacagaagattcataaatgctttggcaggtgatactggttgaacactaaatgctgctgaagaattgttgctcttga

atttagatttttatggccaagtatttacattacttaacaacttccttgggcacaaaagaaatagtcaatccatttcatcttcatgattaacacaaaatca

tgtagagcataagcaacacttctattggtttctctctcaatgagctagttcaagcgaacacaaatcaagggatggtacacttcttcgagtaaacagtgaa

ctgattgagaaatatttgatatttaaaactgcaatgtttctaaaaaaattactactttaaacaaacattaggcagcctttttgtacactcgacgatgcag

ttcatctattgactccaaataccaggttcctggaaagagcgtatctatgggaccttcaggagtgtatggtgccttatgatggtttttttcacgaagttcc

attatagaagaaaaatcgcttggagacacacatgttctgcttttcagttgagtgctgatgtgaccaagattagtaataatgtgtctcaaagatgatcctt

ctgaaatatcttctgccaccttcacagaatacatggaggaagccaacccggagccataagaaaaaagaccgattctttttcctgcaagatgttcagcact

tttacttataagtaatgataccagacatccatataaagatggggtgtacatattgcccacttctctcgctaacagtagagagggaagagtcttttcttca

aaggcatttttgctgcaagtcatgaattccttttctatgtctctgtcaaaatacgtgttttcaagatctacgccttgaaatttctgaagccctggatact

ttgcaagaatatcttgaggagaacgtataaaatcattcaacattaaacgtgccagtgatttttgcactaatttacaataaggggaatggaagatcattgc

atcgaagtattccaagttaatcttgttaccattaagatgtttttctgctttgctgcagtacagctgatagcagttgtccagtgcacttaaatagcactta

actgacaatttaccatcaactactggatattcagaagagagattgggcttgtaaaaatcataagcatgtttcataaatgttgcccgagcacttgtttcta

tgacaagaggagcattagcaccaaccagcatagcaacggctcccgccccacctgttggccgagcattacctttcgcgtacacagcaatatcaccacatac

gacaagggcataacgaccatcccaagcactggattcgacccatgaaactgcgttgaacaatgcagacgtgccaccataacatgcatttgtagtgtccaca

ccctcgacatcaaaattaccactttcctcgaaaagctgcatcagcactgtcttcacacttttcgatttgtctataatagtttctgtgcctacctccaacc

ttccaatctccttatatgaaatattgtttctttgcatcagtttcgacactactgtgaggcacagtgaatttatatcttcgcgatctgagcaaaaacccat

ttttgattgtcccaggcctatagtgtattttcctgctgaaactccatcgaacgcttccagctccgattggtcaacgtactgggatggaaaaatttgttca

atagcaacaacaccaacattggatggtctggcactcattttcgctgatttgatgacgaaaaataacgtattggttcggcagtgcactgcagtatcgcgac

actcggtggaaagaactggaggaatgtcaacaagcactcgcacacttcacgacaccatccaggcgaggagaatctcgcctgtgtcgcctctcacggtact

tcaagtacttggcctactaaaaatacacgcataactcctgtaccgttactgtgcacgttgtcaagatgccatcacaatgtcaaagattaa

>32810|3-hydroxy-3-methylglutaryl coenzyme a synthase

ggggggggggaagatgtgtgacagcctgaatacagatagtacttacagtaaatggcaaatagctgcattataatgttgcttaaacaaagcataaaactgt

gcagagatctctcttatgaaatataaattttgtaatacatgaccaattcaatgactggaaacactatcgtttacagatatttagggtagagaaccaccca

ctatcagagaagggtaagaaaaagaaaaaaagtctacaagaaatacaaattcttgacagaaagtatctagtggcaccagaacacagaaaaatcaagtgaa

gtaaagatgggtgaacaacccatcaaagataaaggtaagcaaatgtcagttattcataaatgtgaggaaagttttaaatatggcttgagcagagaactaa

ttatgataaaactttccctataaactctacccacctttcctcctgccaattctttgcttagattacttttagctgctgaggtgaggtgaaaccaattcac

tgctgcacatagacaatccacacttctttagaaatggaaattcgtccatcgaaatgttactatcaagcaaacaatttactcactaatggataacatctaa

ttcactgttttctctgccctttaagaaatgtaatcaaagtaatcatttctgtgtgtgctgtgacattactagagcattaaaagaaggttgcttcataatt

cattgcagctcatggaaatttttgacacatgaaagataacattcattaaaaaagcaaaaaaagtttatcctactacctaaggcaacaatatggaaatgac

aaattgcttctcttagacaagcacaatgaaaaagacctccaaatatttaagctttcagacaaacatctatttccaatgtaggaaacacacacattcactc

ttgcacttgtttgaatgtgcctgtttgttcccctttggaagacagggtgttttgtcccaaagcttaaatatttttcgttttgcctgcctgtgactcaaca

tctcctccatgcggtgaacagcaatctgttattccatactggaatttccattgtttgtttatgacatatgacattaataccagaagttcaaaaaagatct

ggctaatagagatggctaaaaccacaattattcctgtactttctaaatactcggtattgaaaaagaatgcaaaatagtgatgaataacaaataactgatg

gggtcagtggaagaagtgctccttcaaaagagtttattttgacatatggcataatatatgtctggtcagattcaacaatttatctaccagatagtatttt

atagtgatacacaaggaactactttccagaataaatatttactctgaagtggagcgtgcgctgaattgaaacctcctgccagattaaaactgtgcgccaa

accaggtctcgaagctgggatgtttgccttctgtgggcataggctctattgacagctttctcagcatgatttaaaacccacacacctcaacttctgtcag

tacctcatctcctaacttccgaacttcaagcattcctggaagaaaggatattgcagaaatatggtttagctgcagacctgtctcaaaatatatcctttat

tccagatattcaggtaaacacctatgaagtctggaaggtagaaggtgaggcactgacatgtggagttgtgagggtgggatgacagttgggctctgatagt

tcggccagtggagcacttgcctgcaaaacacaaggtcccaggttcgagtccaggtccaacatacagttttaatctaccaggaagtttcctgtgaaagcac

aacttatcagcatatgacaacttcataaacacaacaaataaagttattcgtttaatccttactatatcgatgttagcactttcttccgcagaaccctcca

gttatattacaggaggtgacacacagtattcacccaaaaataaatctaaaaaaaaaaataaaaaaatagatttgaacatttaaaaggtcaccagctgcaa

tcacattttccatattcaaatatgtgcatctatcctacctttccttttgcaatccctattgtaaagatgaaatgagcaattaaccacagacacacagata

aaatctttatggtacatgtgaagtgataaggtcgaggcagctttatatggaatgtagtacaatataaacgtgattacatgaataatgaccacttctactg

cagtgtccattgatgaagatgctctgttaagcacacaaagtcaattgtgacatcaagataaccctgatctcaatctcactctttggtattatatgtcatt

attagtacaaactgaaaatgagatgtgcaggtacagaaacaaaacattaaagagagataataagataatgccactcttatgtcatactttaaaattctgt

gtttgttgctagcacataagcaacacatcaggaacaatgaagatcaaccaaattggattgctgcacgggtgactgacaggaaccaggaacaccacagaag

attcataaatgctttggcaggtgatactggttgaacactaaatgctgctgaagaattgttgctcttgaatttagatttttatggccaagtatttacatta

cttaacaacttccttgggcacaaaagaaatagtcaatccatttcatcttcatgataaacacaaaatcatgtagagcataagcaacacttctattggtttc

tctctcaatgagctagttcaagcgaacacaaatcaagggatggtacacttcttcgagtaaacagtgaactgattgagaaatatttgatatttaaaactgc

aatgtttctaaaaaaattactactttaaacaaacattaggcagcctttttgtacactcgacgatgcagttcatctattgactccaaataccaggttcctg

gaaagagcgtatctatgggaccttcaggagtgtatggtgccttatgatggtttttttcacgaagttccattatagaagaaaaatcgcttggagacacaca

tgttctgcttttcagttgagtgctgatgtgaccaagattagtaataatgtgtctcaaagatgatccttctgaaatatcttctgccaccttcacagaatac

atggaggaagccaacccggagccataagaaaaaagaccgattctttttcctgcaagatgttcagcacttttacttataagtaatgataccagacatccat

ataaagatggggtgtacatattgcccacttctctcgctaacagtagagagggaagagtcttttcttcaaaggcatttttgctgcaagtcatgaattcctt

ttctatgtctctgtcaaaatacgtgttttcaagatctacgccttgaaatttctgaagccctggatactttgcaagaatatcttgaggagaacgtataaaa

tcattcaacattaaacgtgccagtgatttttgcactaatttacaataaggggaatggaagatcattgcatcgaagtattccaagttaatcttgttaccat

taagatgtttttctgctttgctgcagtacagctgatagcagttgtccagtgcacttaaatagcacttaactgacaatttaccatcaactactggatattc

agaagagagattgggcttgtaaaaatcataagcatgtttcataaatgttgcccgagcacttgtttctatgacaagaggagcattagcaccaaccagcata

gcaacggctcccgccccacctgttggccgagcattacctttcgcgtacacagcaatatcaccacatacgacaagggcataacgaccatcccaagcactgg

attcgacccatgaaactgcgttgaacaatgcagacgtgccaccataacatgcatttgtagtgtccacaccctcgacatcaaaattaccactttcctcgaa

aagctgcatcagcactgtcttcacacttttcgatttgtctataatagtttctgtgcctacctccaaccttccaatctccttatatgaaatattgtttctt

tgcatcagtttcgacactactgtgaggcacagtgaatttatatcttcgcgatctgagcaaaaacccatttttgattgtcccaggcctatagtgtattttc

ctgctgaaactccatcgaacgcttccagctccgattggtcaacgtactgggatggaaaaatttgttcaatagcaacaacaccaacattggatggtctggc

actcattttcgctgatttgatgacgaaaaataacgtattggttcggcagtgcactgcagtatcgcgacactcggtggaaagaactggaggaatgtcaaca

agcactcgcacacttcacgacaccatccaggcgaggagaatctcgcctgtgtcgcctctcacggtacttcaagtacttggcctactaaaaatacacgcat

aactcctgtaccgttactgtgcacgttgtcaagatgccatcacaatgtcaaagattaa

>33102|3-hydroxy-3-methylglutaryl- reductase

gattagtgctagccaccaagcatccctctgttgtagccatgggaatatgcacttgttgaccatccagtaacagaggaccagcaatacctactggtactgg

cagatagccaacaacattttcacagcatgcaccaagcacctttgaataatcgtaaccagtatatggtagatccgcaagtgcaccaggtttgtgagcatca

aacagtttcctccgaatgccaacacctctctcagggtccttaacaactgcttccagttggtaagaaggcaccacactagcacgtacaagctgaagcattt

cttcatctgttaaagctgttgctccctccttggacctatatatatccacacactgttctaaacttcgacaaatttgtggttcctctttatcttcatcaag

agatgtcttattcactgttgtttctgtaactgcaatatcttgttgtgcctgttgacatgttggagatacttcagttacaggtttaagctcttgtggtggt

ggttgctgctgctgctgttcctgatgatctgtctgaactgatttgtgctcagtattaattgcaatgagttcttcacgagatgggacctccacccaaccgg

cttcaagcattggacgcatacgatcgaaagcatggccatgactgtaactcctgtgcctacgttccctgtcactggcccggtcagataccagcatttgctc

ccgaatatcctcctcactctcaaagaaaacgaactttacagcaagtgctaacagcaggataagaatgacaatgtggtcagcactcacagtcagccaccta

agcaggtactcgcggatagaggatgctgtgaagtcatcagtatggttgtagacagcagtagaggagacagaagtagtgggggaaaccaccaatggcaggg

atgttcctgggactcccacagaaggcacactgcctgtatcgtcgccgccaagtccccaccgactatgtgcatgaaccagcacaagacctgcagacatgat

caccttgactctctgcaccactggatttggtttttcctcctggagcacatggcgcactatagctgagcgttcctgccatgcaggcctgccgtgcttacca

gctcgtgataactctaagatgagagaaaggcatgctggatagaatgtcatgaagacaatgtaattaacaatgacagacatgcaagcaaaacagcatagga

cctccaatctgcgaacacctgagagagtcccaacaccaatgacaagcgcctcaacaagtgtgtccagtgtgagggcaggtcccagagttgccatcccccg

agcaatattacgtcgcacttcttctgtgttgctagcactaagtgctaactgtgccagagcacttgctttagatagatcgatcagcaacagaaagaaaaat

aacgcatctttgagatcagatatatcactgtgaagaacattgatgacacttgtgctgaatacgaaactggagaagacagtaaagagtccggcaataccga

gaatgtatttggaaccgagcttgtggagcctgcagaactggtagtagcagtacagaacggcgacgcagcggatcaccgtcatcatgatcatgtcggcggc

tgcgctgtactcctctgcctgagaggcggcggccggcgcggcgacgggcagcctggaggcgcgctgctccaccgtcaacatgctgatggccagcgtcagc

gtcgccacgatcacctcccacgggtggctggcgcacagctcgccgtgcgcgctgaacactcgcgatgccgccatctcgcactagctgtcgctatacaact

gccatgtactaataatatatttcgaattaaacatcgccgcaaaatttcaaaacacactacataaacaacgttccgcacaaacacgccgcag

>33519|3-hydroxy-3-methylglutaryl-coenzyme a

ttttctttctgttgctgatcgatctatctaaagcaagtgctctggcacagttagcacttagtgctagcaacacagaagaagtgcgacgtaatattgctcg

ggggatggcaactctgggacctgccctcacactggacacacttgttgaggcgcttgtcattggtgttgggactctctcaggtgttcgcagattggaggtc

ctatgctgttttgcttgcatgtctgtcattgttaattacattgtcttcatgacattctatccagcatgcctttctctcatcttagagttatcacgagctg

gtaagcacggcaggcctgcatggcaggaacgctcagctatagtgcgccatgtgctccaggaggaaaaaccaaatccagtggtgcagagagtcaaggtgat

catgtctgcaggtcttgtgctggttcatgcacatagtcggtggggacttggcggcgacgatacaggcagtgtgccttctgtgggagtcccaggaacatcc

ctgccattggtggtttcccccactacttctgtctcctctactgctgtctacaaccatactgatgacttcacagcatcctctatccgcgagtacctgctta

ggtggctgactgtgagtgctgaccacattgtcattcttatcctgctgttagcacttgctgtaaagttcgttttctttgagagtgaggaggatattcggga

gcaaatgctggtatctgaccgggccagtgacagggaacgtaggcacaggagttacagtcatggccatgctttcgatcgtatgcgtccaatgcttgaagcc

ggttgggtggaggtcccatctcgtgaagaactcattgcaattaatactgagcacaaatcagttcagacagatcatcaggaacagcagcagcagcaaccac

caccacaagagcttaaacctgtaactgaagtatctccaacatgtcaacaggcacaacaagatattgcagttacagaaacaacagtgaataagacatctct

tgatgaagataaagaggaaccacaaatttgtcgaagtttagaacagtgtgtggatatatataggtccaaggagggagcaacagctttaacagatgaagaa

atgcttcagcttgtacgtgctagtgtggtgccttcttaccaactggaagcagttgttaaggaccctgagagaggtgttggcattcggaggaaactgtttg

atgctcacaaacctggtgcacttgcggatctaccatatactggttacgattattcaaaggtgcttggtgcatgctgtgaaaatgttgttggctatctgcc

agtaccagtaggtattgctggtcctctgttactggatggtcaacaagtgcatattcccatggctacaacagagggatgcttggtggctagcactaatcgt

ggctgtcgtgcattactttcctgtggtgtcacatcccgtgttgttgctgatggtatgacacgagggccagttgttcgtttcccatctgcagcaagagcca

gtgaggctatggtatggatgcaaacaccagaaaactttgagggcatcaaagctagctttgatggcaccagtcgatttgcacgtctaattaagttgcatgt

acgcattgctggcaggcatttgtttgtgagatttgtggctaccacaggcgatgccatgggcatgaacatgttgtctaagggcactgaagttgctttgggt

tatgttcagaaaacattcccagatatggaactacttagtctcagtggaaatttctgtactgacaagaaaccggcagctgtcaactggattgaaggccgag

gaaagtctgtagtttgtgaagctgttgttcctggagaagtagtgtcaaaagtgcttaaaacaactgtgcaagctcttgttgatgtaaatgttagtaagaa

catgattggctctgctgtggctggaagtgtgggtggatttaatgcacatgcagcaaatatagtaactgccatctttatagcaacaggccaggaccctgca

cagaatgttagtagcagcaactgcatgacattaatggaaccatggggagagggaggcaaggatcttcatgtgtcatgtacgatgccatcccttgaaattg

gaaccgtagggggtggcacagttttgcctgcacagggtgcctgtctttccatgttgggtgttaaaggtgcaaatgaggagtctccaggagaaaatgctaa

aatgttggcaaggatagtgtgtggcacagtattagctggagaactctctctcatgtccgctctggctgcagggcacctggtacggtcccacctgaggcat

aacagagcttctgttacttcaagaccgagtgaagaaaagtcgtgaaacgtgtcttagatgaagctgcatgcaactgcatgtaggtcactgacaaagaaga

ctgccacagtgactgtaatcagtgacattccatgcatcaggatgactggcaaatatccacgagaacatgattttctgtcatggaagaaaaagtggtttga

aactaggcagtgtatattctgagagaacagcccccaaaaccactgccagtcactgaagtttaccaaataaattaaacagaaatgttttaaggtgtcttgt

cttactgactgaatggagtactagctcattgaacagcatcacacagttcaaattaactaagtgatcatgctcaataatttagagtaactgttcaattaaa

tcaaaatgtgatgagttagatgcatttgaataagaattctgagcttcaaacttgtttatacaatcacattttttatgtaatcagactgactataattatt

tggatatatttatagatgtaaaataatcgtaataatttattaactgaaatgagtctgcagaaaagctggctttatgtgacagtacagtacttaaaaactg

cttttgaaactgagtttttgtagctgcaaaggaggagagcctttacaagaaatctgtgtacaaccttaagaaacattacatcttatttgcagtcctcgtg

tcttcttaaatcttactctatcttaacaatgaatgtgcttgagcgtttagtgtactcataccttttgagttaaatagagcctcaaatgtaattgagaaac

attatattatatgtaaatacatgtacaaatgcctacctgtcatcagaatagattcttgattaatattttcgtgacttgtttattattgacacatacatat

cttggtggtttcttgaccgacgcagactgaatgttcatttattgtgacattttaggcattttttaaatttcactataatgtgcttgtcagatttaggtta

caactgatgtgtgattgtctttgcagtattccttaatgttagtcacttgtaaccagtatttgtacaaaagcaactgcaatgaaaagatctgtaatcatgc

agttgcagtttatccaccacaaacttaatttatagtgctcaatgactgtgaaaagtcaatttgtgccagtgaatgaaactgtaaaaatattagctgttca

tagccatgtcactgttggaaaacgtaacagtattttctatattttttaattaagtaatgtataatgttagtcacagccataagttatacagttgtttcat

ttattgcaaaaaatcctttagagtgtttatattgatcatgtgaaggcaacagcaacattgcaataatttcttaagtcatgacatttttccagcagaaaga

gagcagaacattattttttacaacctaagcagagaaagctcccgtaacttattatgaataaataaaaagaacattattttttacaacctaagcagagaaa

gctcc

>33786|3-hydroxyacyl- dehydratase 2-like

ggagttgcaagaagaacaccacaaactgcaaatactcgtgaaaagacttggaatgttgttaccactacactggatggtacaagcttcaaggctgcatgaa

gtatctctagggcagctgcattctggaagatgcatataggaagtttaacaacttcccacaatataacacttttcgagggctgcaaatgatgtcttatagt

aaggaagagaatatacgaccatcccaagaactggacaacgttatatgcgaaaagatacgctgtgcctagagttccgggccctttacgcacgttcgtttgt

gatttcttggccatttcgctgagatattcaaataccacccacttgctgctcagtcaacaaacaggtatgcaagaacccaagtgggaaacttgtttaaaag

ctgactggcaatattgtaatatgctgccaacacaaaactgtatattcaaccatcgaaatactagacccaacacaaacaataaacaggtacagggtaatga

gtgctaactggtatttattctttaaagcggttttctattataactgtaacacacagcaatgtacgaattcaaatccagtcgccttttatagactccgtaa

gtctgggcgtcataaacaaatattgtacgatgcaagcggctttgcgcacgatgaaaattctctgcataaattagccctacatctattgcgagattgaaag

tgtcagaatgaaacccataaaaatgctatgctttaaagccattattacaacggaaaccaaaacagtatctacagga

>35463|3-hydroxyisobutyrate dehydrogenase, putative

aaatatttgtctgggccaccaaccatgaaagttaactttccttcttttgctgcaaccacgcctccagatactggggcatcaaactgcaaacagcctcttt

ttgtagcttcctcagacagttctacagataaatctctgtcaatggtgctgcagtctatcagaaatgtgtctctgctgacagatctgaataagatgttaaa

atatacaagcattagtaagaatctaatgatcaacatgaactgaagaaaaagtgtcttccattgctagcctaagcttttgcagtcctttgtccaatcctac

atgactgctgtttcccttcatcctatatcccagtaggttattttattcatgcccatggatcaatgagaccaagttatggaacaagacaatacagatacag

gccacagaatgttgattaggagataaaagcaactgatttgcaatactgtatacatatgattgctacagtatagtgaaaggctctaaattactgtgaagaa

ctcttatgcagaataaaacaactgtgctacaaggaagtctttcaactacgatttacaattctgaaccttatacatttttaacttttgatgataacctatt

ggaacaaaatttacagaatatggtgatagttcttgtagactagttagaaacattttatccatatgccaatgttttc

>35462|3-hydroxyisobutyrate dehydrogenase, putative

ggagaaagggactaattgcagacataattccaggatagaaattgtaaaacacatggtctcaaataatatttaaggaagtattcctggtgtatacaaaatc

actttgctactaaaaactgtgctgataactgtgctaacataaaacacatacctccattttcaatacaaagtcgattttgacatttcttaatgtaatacat

ttatacagccatttgtatagaaaactatgagtgacatattagacaactgcagatttatttcgcttcacttagaaacttatatataacagaaaaatctttc

tttccatatccttgtgcaactaaaatgcgataaatctgatgtgctaatgaacctaagggagtaggcacttgattttttgttgatgcttcttgtgcaatac

caagatcctttgccataagatgtgtgccaaagccaccctcatagttattggcacttggtacattgctgattgttcctggtacaggattatattgttctga

tgaccagcaacgtccagagcttgtgttgaagatttccataaggacatttggtttcaggcccagccttatgccaagattcattgcttctgcagtaccaatc

atacttattgccagcaacatattattacacattttaactgtttgtcctgaacccacaggaccacaatggacagcacgtgaacccatgtgcaacagaagat

ctttaacagtttcaaaatatttgtctgggccaccaaccatgaaagttaactttccttcttttgctgcaaccacgcctccagatactggggcatcaaactg

caaacagcctctttttgtagcttcctcagacagttctacagataaatctctgtcaatggtgctgcagtctatcagaaatgtgtctctgctgacagattta

aagattcctctctctccagtatatgtatctcgcacactagcactgtggggcagcatagtgacgatgcagtccgactgtgctgctacttcctcagggcagc

tgcaggctgtggcaccttcttgtgcaatggtatttacaacttctggttgtacatcaaagactgacaattttttcccctttttcagaagatttcgtgccat

tggtccacccatgttcccaagtccaataaagccaattttgtttatgcatttcagtgtgtttgagatcattttcgagcttcagagaacaattcaaagtttg

taccgtagcaacttagaagaatacttaaaatttattttggtatcaaggcaaaaatctggcagctagaggacgctacagttcacgcactccaaatgcttcc

ccacacgtgaaagctacggaaattacgtaaacaaagttacggaaggttagtaagcgttcttatctggc

>36130|3-hydroxyisobutyrate mitochondrial-like

caaacacactgaaatgcataaacaaaattggctttattggacttgggaacatgggtggaccaatggcacgaaatcttctgaaaaaggctgggcctgaaac

caaatgtccttatggaaatcttcaacacaagctctggacgttgctggtcatcagaacaatataatcctgtaccaggaacaatcagcaatgtaccaagtgc

caataactatgagggtggctttggcacacatcttatggcaaaggatcttggtattgcacaagaagcatcaacaaaaaatcaagtgcctactcccttaggt

tcattagcacat

>36129|3-hydroxyisobutyrate mitochondrial-like

cagttaaaatgtgtaataatatgttgctggcaataagtatgattggtactgcagaagcaatgaatcttggcataaggctgggcctgaaaccaaatgtcct

tatggaaatcttcaacacaagctctggacgttgctggtcatcagaacaatataatcctgtaccaggaacaatcagcaatgtaccaagtgccaataactat

gagggtggctttggcacacatcttatggcaaaggatcttggtattgcacaagaagcatcaacaaaaaatcaagtgcctactcccttaggttcattagcac

atcagatttatcgcattttagttgcacaaggatatggaaagaaagatttttctgttatatataagtttctaagtgaagcgaaataaatctgcagttgtct

aatatgtcactcatagttttctatacaaatggctgtataaatgtattacattaagaaatgtcaaaatcgactttgtattgaaaatggaggtatgtgtttt

atgttagcacagttatcagcacagtttttagtagcaaagtgattttgtatacaccaggaatacttccttaaatattatttgagaccatgtgttttacaat

ttctatcctggaattatgtctgcaattagtccctttctcctacgcagttgtaatgtgtactaaggcttcagcttctttttcaacattttctgaaatacaa

gactttctgtccccatccattaactagtcaacagcc

>36745|3-hydroxykynurenine transaminase

aagtacaggctcaaagaaatatctatgtatctgtgtgtgatgtgcagccatagatcactatgccttctcctaggggaatgttacatagatctgtagaggt

gacattcttaatgtctgcacagatgcttacactagtgtaattagtgatgactgaatcttataaagcaaagctacataaataaattgtacttctctcacca

gtgaacatctttaactgcaaaaaattcaggacacaaagcagcctgctcattctgctgaggtgaaacacagcctaccaactctatgatactttcaggaggc

tgttctctgtcagtctctcttgctgttatgagtacaggtcagttagaaaaagggcattcactttatggagaccatagtcatcatcattaacagcaaacct

cactacagagtaatcacatttcttgagctatttaaaactaacacattaatttttcattttaaaataaagtctcttttcaaaaccatgttttatttttgcc

atattttatccaatttttatcacattatacacaaagtgaagtcatctgagttgttgtttttgtaagtctttcttgatttatataagtcagtttataatga

caatattaatacactttgtgggaatatacattgtattttaagtatttgcttctaacattacagtataaacaagtgcaatatcagaactgtttttctttgc

gtgcatgtttataatttgatgtgtatcagaatatacgattatcaggggttggatcagcttttagttaaagatttaagaaagatagatattttgatgatgt

gggactgttaagagagtgacttaaaggtacatataatatgaaataagacatttgatctcaggcagataagtacttgggaacctgatactttgcatgttcc

tttctgaaatgtaatttagtacactcttttgctgtgcatacttggtaaatatttactcttgtttttgtttcaggtatcactatactttgtctatgaatct

tctttttgcactgcgtgaagctctagctgaaattgtagaagaaggcctggaaaaccgatggaaaaggcatcatgaaactgctgagagagttcgtgcaggt

gttcgtaagcttggtcttgaattgtttccacatgagggagaaacccctcttaactgtgtgacaacagtccgtacaaagccaggaattgattggaggaaac

taagacattatgcaatggatgtttacaaaatggatttgcctggaggatttggcaaaacagcaaaacagg

>36996|3-ketoacyl- mitochondrial-like

ccctatgcccattatgcttgggtccaccccaacaaccgaataaccggcaagtcgtgctaatggcttataaccatattccttacaagcctcttca

>36995|3-ketoacyl- mitochondrial-like

attacaaatttcagggtcatgaagtcttttctgaatagtaaccttatatagaagtggaacatggaaaaattgaatgtgaatggaggagcgattgcactgg

gacatcctcttgctgcatcagggtcacgtattacgagccatcttgtacacga

>36997|3-ketoacyl- mitochondrial-like

gctccaaacctgacattacgcacaaaatatggtgtctgactcatattttcagctcctccagttaagaccacctttgattcacccacaagtatgctctgag

caccactgacaatggattgaaaaccagatccacagagccgatttatgctcatggcatgcctgtctattgggacaccactgtataaagcagcatgtcgagc

tatcagtattccatctgctgatgaaaccttttgtcagagatgccatggcgtagatgttctcttggcaaggcagaggtaattgcagccctgtattgggtgg

caggtgtgctaatactgcaagtctgcccctgagttcagagagccacaaaccagctgctcaccagataacgaaagcgactgtaacgagtacgataagacaa

aaaagaatactccattcgcgggagacctttgaccgccactgggcggtacgcgggacggcagttcgtc

>36994|3-ketoacyl- mitochondrial-like

gttctgtaaaaaaatataatgtaactcaacactgaatgaagctatgctctaagaacgacacataagaaatttctgaatttaggtgtcataatgggagtta

tactgaaaattacttgtctttttgaatttagagcacatttttgtaactggttttatagtaaatagtttattttagtagcaagaatacttgtctattccat

tagcacagagcatataccttacagaagcaagaaagttttctcttaataagaaataaccacacatatgagagcagtgtaatccagaataactgttaaagtt

ctctctacaacatttgaacaaaattttgacaattatatacctcaagaaatgtacattttttcacattggaacttaagaaagtgcagtaaagatgatatag

tagtttatttcttactataaacgtatgaaatgaagtaatactggatgtgcaaagtgcaattttaacacaacatttgcactaggaagttaattattattat

gaattatcatgtcagtcttctcataaaaaacattcattagtgtatgctttcaatcaagagagcaattccttggccgccaccaatacaagcagatcctatt

ccatattttgctttcctcctcctgagctcatgtacaatatgtgtggtgattctagcccctgatgctgcaagtggatgacctagagcaatagcacctccgt

tgacattaagtttctcaatatctagtttcagttcttcctggcaggccattacctgggcaccaaatgcttcattgatatcaaccaggtcaatatccgctaa

agattttccagaaaccttcaacagattttgtattgcaggtgcaggaccaatacccattatgctgggatcaactccagcaacagagtaaccaactaggcgg

gcaagaggttggtaattgtattccttgcaagcttcttcactggcaattatcacagctccagcaccgtcacatatacccgatgcagaacctgcagtaacaa

ctccatcttttttaaaaactggaggaagagctgctagtccttcagctgtggagaatggtctaggatgctcatcaacattcacaatttcttcacctttcct

tgtttttatggtaactggagctaattcttccttaaagtaacctttttcattggcattcttccaaagcatctgtgatcgaagagcaaacttatcaacattc

tccctggttagtttatactttacagctaagttctccgctgtcatagccatatttaaattacaatatgtgtctgtgagaccagcccagaaggagtcttcaa

attcaattttggttccaagagg

>37700|3-phosphoinositide-dependent protein kinase 1-like

tcgctcattgatcggaaaggcgggagtccagccaccatgtggcatatgatgcaaccgagggcccagagatccgaactgtaggaggccgacttgtcagtca

acagctcaggtgagacatactgcgctgtgcccacaaagctgttgcggcgacggaagttagggttatccttgttttgatctgtgaaacaaaaaatagagta

aacagagatgaaaatacaaatagaacaagattatcagggacagtaaatgattttgtaataaaaggacttaaatgattttgtaataagtgatctaatatgt

atgaaaggtgtttgcaattagtttaatgtatataatgatgagaaaattctgaatacaaaaa

>38164|30s ribosomal protein s10-like

ataaccatcagtttcaagtgttttgatttgcgtaatgcggtttggatcgacttcgatgactgtaacaggtacagaaacaccagcatctgtaaagatgcga

gtcataccacatttgcgaccgactaaaccaatagccatgtgcataaacctctaaaaaagcggcctatttaactaatcgttaattaaccgaaagccttaac

ccaaagcgatctgaacatcaacaccagctgcaagatctaacttcttcaatgcatcaacagttttatctgtaggttgaacgatgtcgatcaaacgtttgta

ggtgcggatttcgtactggtcacgcgcatctttgttaacgtgcggtgaagtaagaacgttgaagcgttcgatgcgag

>38165|30s ribosomal protein s10-like

gaacatcaacaccagctgcaagatctaacttcttcaatgcatcaacagttttatctgtaggttgaacgatgtcgatcaaacgtttgtaggtgcggatttc

gtactggtcacgcgcatctttgttaacgtgcggtgaagtaagaacgttgaagcgttcgatgcgagtaggcatcggaattggaccacacacttgtgcgccg

gtacgcttagcggtttctacgatctcttgagcagattgatcaatcagacgatgatcaaaagacttaagacggatccgaattctctggttagacataccag

taaactccaagccaaatatataaagaatcacctttgacgcatctcacctatctggtaagtgtcaaaggcagtgataatcaccaaggtcgtgatcgatgcc

acgactgtaatgcacatttactactttcttcgcagtaaatgcc

>38374|30s ribosomal protein s9-like

aacagtgatgtaaaggtcatatttttcagtagcttctaaaagctctaaaggttgacgaacaaccatacgagcagtttcacgaccgaaatattgctctaaa

gtacggttgttaataacgagtttacctgtaccagctgacaagaaaacacgtgcagttgcggtcttacggcgacctgtaccataattagtagccatgtgct

gtatcccttagatgtccaaaacttgtggctgttgagcagcgtgtggatgctcgctaccagcgtacactttcatcttcttgatcattgcataaccaagagg

accttttggcaacatgcctttaacagcacgttcaaagatttcttcaggtttgtgagcaactaatttttcgaaattagtttcttttaaaccgcctgggaat

tcagtgtggcgatagtatttcttgtcaagcgctttgttaccagtcacttgaacttgttcagcattgataacgatgatgtagtcaccagtgtcaacgtgag

gagtataagaagttttgtgcttaccgcgtaaacgacgtgcaatttcagtcgctaaacgaccaagagttttacctgtagcatcaacaacgaac

>40137|33 kda inner dynein arm light

cctgagagacgccagggagagggagcctgatcatcggcaggggcagaaaaacatccaagatggccgccgagtgagatcacaggcatgggaaggaaagtct

gcatctggcattctgtcacctacacagcctgcgggaagagcaaagtcagctgtagcacagagcgctcaaagtgttaaggaaacagaagaaatacttaatg

ccatactacctcctcgggaatgtgaggagaatggccagctttggagacagcaggcagaacaaagtaaaggtgatcttgaagagttggttcagacactgaa

atcagaaaaaagagagttggaaaagcaagttattgaactcaggcaaaaggcagaacaagtagaacgaaggtcaaaggaaatgcaacaggctgaagagaag

aaacacactgaagaagttgaatttctgagaaagacaaatcaacagctaaaggctcaattggaatcagccattgcgccaaagagaaaacgataatctttta

aaagctgataaattttatttttaatacaaagcttttgttgttttgtacattaaatgacaaatacttggtggtaaaaacttttttatatggagaaactgtt

acttcagctccaatttctgtacatgtattcatgccaaagcaagttctttcaagagaagagatcgtaataaaaagaaaaatgaacatttttttttaaaaaa

agtaatgagaatacaaggattattattaatttccatctcacctacagcagtgtacgtcattgtcctgcaggtttacaatgattgcctttgtagcattctg

ttaggcaactggttgtagtgaactttctcctgaaaatgatttgtcttaagaaaagacataaataaactggc

>40578|34b cg9354-pa

atatggtggagtcttgtgtcataaatgtgtaaaggaaagcacgaataattctttcctttacccatttatgacacaagactccaccatatgcccttttcac

ag

>40573|34b cg9354-pa

gtgccgcaggaaaaagactgtgaaaagggcatatggtggagtcttgtgtcataaatgtgtaaaggaaaaagactgtgaaaagggcatatggtggagtctt

gtgtcataaatgtgtaaaggagagaattattcgtgctttcctcattgaagaacagaagattgtcgttgaggtgctgaaggcacgtcaatc

>41203|39s ribosomal protein l13-like

gcgtacactttcatcttcttgatcattgcataaccaagaggaccttttggcaacatgcctttaacagcacgttcaaagatttcttcaggtttgtgagcaa

ctaatttttcgaaattagtttcttttaaaccgcctgggaattcagtgtggcgatagtatttcttgtcaagcgctttgttaccagtcacttgaacttgttc

agcattgataacgatgatgtagtcaccagtgtcaacgtgaggagtataagaagttttgtgcttaccgcgtaaacgacgtgcaatttcagtcgctaaacga

ccaagagttttacctgtagcatcaacaacgaaccaatcgtgttgtacttcagcaggctttgcgctgagagttttcattcaaacactacctattatagttg

gtttgagctagacatcgc

>41427|39s ribosomal protein mitochondrial

tcccgatatttcttttcctggttccatagcacccctttgtatcccagcagcttgttttaggaaaaatgtagcaggttgcttatgcataataagctgataa

ctcctatcagcattaactg

>41425|39s ribosomal protein mitochondrial

gatcaaatttgactgaaatctttttaacaggtttcagagtcacagagcgtaactgtttcgaaatcgcgctgataagtccactagatcttgtgaacgttcc

ggaattgtagaacaagcacataacac

>41428|39s ribosomal protein mitochondrial

ggcttatgcataataagctgataactcctatcagcattaactgtaattctacgtggaagaggtattccctcttttaaatcctttgttctttcattgaaat

ccttgcaaaaagctgctatttcatacaaatgctttagtgttatttttcccgatatttcttttcct

>41921|39s ribosomal protein mitochondrial-like

gcgttctttcagcagaacaaaccataacttatgcagatcggtgttgttctttattcgcagttcctctttcctccatgctcttccgactcccaccccttcc

acttgcaactttcctccttgctaatcttc

>41912|39s ribosomal protein mitochondrial-like

ctctctctgtcatgcaatttaagcacagagtccgcttccttctctttagcaggtaatggtaatgaatgttcataggggaatggttcttccgggtgccaac

atactacagacgaaccactatcagtgaaaacaatttttggtccagaaaaagtccttgcattgtttgcatgaaagcgtatttgctgaactctcggtgaaca

cactgctcgcaagcaaccgcatacattagaaagcatcgttaacacataaaaaacactgacttccacacgtaatgacaaatagtacaaccttgctaaagag

aaggaagcggactctgtgcttaaattgcatgacagagagagagttatggctcagtttaaaagtaaacatcccgaag

>41914|39s ribosomal protein mitochondrial-like

gttcatcatgtggaaacagacagtggcagcctgtacaggtttcagggagtggcactgcccagaggactcatgctgaatcactatgcatatggtatcctgg

agtctcgtgcaagcaagttgaacactgtgcatctcccagacagttacagagcagaaccagctaagacggcacctgtcatggcctcaactgtggcatgact

gtagccgagaactaggatctccctatcaggaggattattctcccaaaaatgacagcagtgaatccacctcgaggctttggtctcacaatgtatttatatt

ttttattgatgtacaataataaatagattttctttaataaatgtggttttcaccattttcagcaatatattaaatcagaatatcac

>41918|39s ribosomal protein mitochondrial-like

ctcctattgaccctagatgtatgctgcttgattgtcaaaaaaccatgcactatcagggatttacttttggtagtaggataagaggtcatggtccaagaaa

tgcttctgttcctgatgtctcagcctgaactgcgctggacatcctcagaggtgctcctccactgagtcttgctggctgactggtcagatgtccaagagtg

acataaataaccacaccagttactgggtcacgcctccacttccctggttctggcacgtagtggacaggctccggctttctaaccttccattcatcccaaa

attttctatacgcttctggaagtcgtgcacccactcgtcctggatcaaatggcccaggcctcttaaaatataagagtttaagaccgtttgctagcctaga

ttccatttcaacttgtaaccgctttcctcactttgtgcatccacacatacgtacaatcc

>41923|39s ribosomal protein mitochondrial-like

gtttgactttgttactctgttatctttgcgaaagttctgtgtttttatgttctccgagcgatttgtttacatttcttcgaagcccatactgtgttatgat

tactaaagtatggcagctataagaaaactgctgtctgtgcatcatattctccgaaacggcgacctactagcagcaacccagcagagatcagtgaagcgtt

gggtggcacctacgttgcgggaattgaaaaagaggaaagacagacttggtccagaaccaatacaaaagcgatcctcctttttggaatggaactatcatgc

tgaaatctttgcatttggcaagcgcctgaaggaagattttgacaggacgttactgcagcaagcgtttacaacacgctcttatattgttcaagaggaaatg

aagcaggagcagctaggtattgaagatcccaagctagcacttactgacaaccaagaattggcaaaagaaggcatgaagtttatgaaaaactacatagaga

tttatctgagagaatctttgcctaaatttccagaagaaggtgttgtagccatttgcaagttcctgatggcagatagcatgttggcttctatctcaaagct

tattggaacgaaagatcttattctttctgctgaatttcctgtggaagaatcaacattagtgcaagtgttctgtgctgttgttggagcacttttgaagagc

tcaggcagtgagaagacgggcttgtttgtacgtgattttgtggtggctcaacttgctggccgagatgtgagtgacatatggtctccagaacagccacttc

tgaccctagaaaacattcttgaacaacaaggaagagctgcaccagagccacgactcattggagtggccggtccaaataccatactggctgcatacagagt

tgctgtttatagtgaccgtcaattccttggtgctggatttggagaatcagtagatacagcagttgagcaggctgcactggatgcactgaaacgattattc

tctgtcacagaaaacatgaagccattctcgtacaaaataaatcctgatgtagaaatgtacaaagcaaactctaatgccagtctggaagactggggtgtga

aacatccacaaaatatagtaaactgttaactttgtattgctcatccaaaaatttagagctgtatgtgtacaaagttttattaaaatttttgatctcaaat

aaaattgctactgtatttcttcagattagattaacatttggcatgtgatagccgtcatccagcag

>41920|39s ribosomal protein mitochondrial-like

gactccttaccctctcccttaaaacccacatcctttcgtctttccctctccttccctctttcctgaagaagcaaccgttggttgcgaaagcttgaatttg

gtgtgtctgtttgtgtgtctaccgacctgccagcacttttctttgcacgctgaaatgccaaactgccggcctgcaaacaacacctgtgaggaacgatctg

atggagttttttgacaagaaggaaaattggggtgaaaatcaagtgagagtcggaagagcatggaggaaagaggaactgcgaataaagagcaacaccgatc

tgcataagttatggtttgttctgctgaaagaacgcaacatgctacttacaatggaacatgaatctaaggagcaaacaaaactctttccaaatccggaaag

gatagacaaggtgaaagagagtatggcaaatctggaagaggtggtgagagagaggaatagagcatactttgaactggaaactggagaagatggggagagg

ccttggaagatgcaaaccaatcccctcgggctgcgtttcttctaccgcaagagggagtaccccatcccgtggttcatgaaccggcgctggcaggagacgc

actacatccacgtgcaccgcggccaggcagtgcgcaagttcctgctccgctaccgtgagaggctgtggctcgccaagaggaagcagaggacgagggattt

caaccacgtggctcatctgttcaagcgcttcccggatatggatgtcgaagctgtgaggaagcactaccctgacgtggatatcgagagggcgcgactgtgc

aagcgggcccgtgggcaccacaccaagaatctgtcgtagagtcccacgcctgtactgcagcgctcccagcacgggtatcagtcgtagttgggaattctca

gaccggcaggcatggttactcaaacaaactgcaatctttcgtaccgggtgacagccatacgctcgtgttcacctgtgcagcgagagtgacaggttgtcag

atgttcgatactgcgaggtcgactgacattcggcagagtgacattaattagttttgtttgcctgctgcacttggagctacaaaatgtatcagattgcaat

acgagtcacaatatctctaaaacctgtgggtgtctactgttcatgatagtagtaaatgattttgtgatatcttctgtttgcagccattattacattctaa

cagtttaatggaaagaacactgtttttgttaagcactaattatgtttataatataataaataaatttgcagcg

>41916|39s ribosomal protein mitochondrial-like

atcaataaaaaatataaatacattgtgagaccaaagcctcgaggtggattcactgctgtcatttttgggagaataatcctcctgatagggagatcctagt

tctcggctacagtcatgccacagttgaggccatgacaggtgccgtcttagctggttctgctctgtaactgtctgggagatgcacagtgttcaacttgctt

gcacgagactccaggataccatatgcatagtgattcagcatgagtcctctgggcagtgccactccctgaaacctgtacaggctgccactgtctgtttcca

catgatgaactgcagttacagtacaccgaccaataaagccagtattgcccactaggggacccctgcttatatccacatgctctcccattctgtacactgt

aatatccccatttggatcactgcttgcaatatctggtatctgttgcagtttgtgagggttgtccgcaaacatttctagagcgatatctgctgggacagag

aggcgctcaatacggtatgcatgttttgccaattccaccatctgaccagacagggatcgtaactcatcctggtttggctgccagtcactaagacttaggt

ccacatcatacacaaaactgccactttttatgtttggtggaggaaaactgtgtagacgaacagtgatgtcttctttgaaggcattctcaattaccgcacc

aagtgccatggaacacgtgcgccaaaatgcccgatttacatgtcgaggatcttcatcttggaaatggagtaactgaagactgcaggaacgtggtaatggg

cgatgcatgtcccacaattcactgtcatccagcaaagccaaagcagaacgctgcatcagcatttcactcatatgttcagcacagttaaatggagttgata

ttcctttgttcatgacaagggttgcatcagcaggagttccaatatactgaacttcaattttctcaatacgcccaacagctgatttctgccttttcttctc

atcactaaatagcttgcttctcctcttccttgttccagcagtgtccacagttgtgtaagccaatccacgaacaaccgttcctgataatttgatactgcaa

catagattctttccttgatacacaatcttcgcagcagagggtagaatcattcttacaacactaactttacgttactgtcttctactttattagtaaactg

cactatatacttgtcgacttaacctccggcaagttgtaaacaataaacaacgccattcacatgtcaacaatgtcaatgagttttattatcgtaggttata

acaagcaaagcgacgaatacggcgttgactgaaa

>41917|39s ribosomal protein mitochondrial-like

accacttcccgaaacaaaggggtgaaaatacatgaattaataaactgaaaagtgtatttcttactttgtttcatacagatatttaatggcatatcaaaac

atcaaccttgccacaaaacaactcattttagtttggcggtgtctttggtccatagctcattcagttaacacatagcttatatataaggaaagaaagaaaa

ataatatgaatctcaatatggcatatatgccttcagaaatcaattttgtgtgttaccacactgtgattatggacttctgctttggacttgttttgacagc

agttgacactcttgcttgttatcagcaatagtaatttgcaaatctatttcgttggctatatgatttctgttaccatataacattaagttgttggcaagtc

ttgaaaattaatagattttaaaaaacagaattaccagttatgtgatagcatattctttttatgggaaaaatgtggaatgtaaaaaccagacctaggattg

tttgagttgcagatcaatagctattaatctgggacattaactttatgcagacctccctcaaaagcagatgcactgagttttgtgttgttgtgttgcccaa

gttggttattgcttgtgtagttgcactacacttcaagtaaaagtcagccaacatgctactggttacatcaattttctctcataaaaaaaaatattttttt

atgaaacaggtaatttatttagtcataaaacttttatatataagggtttcttcttacgctgatggtttttcatccttagtagcaaaaggatttattcttg

acagccatgaacctccactttcctctgggacaattttatcagtgactagagtttcttctagtagttcagcaagcagttcagccctgaactgatgctttag

gggctttggctcagacatagcctcatctgactgtttgagttttctgcaggcttcacgataggtcagaccataccactcaatttcctcttcagtgtacgat

ttcaaataatgaccataatcttcatgtatctgtttctgcttttcagagttatcagtgtgataatctttcttcagcaaagctgttagaagctgtcgcttaa

ttcgtactgctagctctgactttagatcacatgcaggggtcttcaaaatatagtggtcaaatccacaattcttgtggatcaagtttattgtcctttcggt

gacaacaacagacatatatttatctaagatttcactgtacacaactgatcgttttaaatttggagtccagaagtatggatttcttctgacagttcgtttc

ttctttgtgaaaccttggataacaccttctccaccccaaagtcccttatgactttcagctggatattttacaggaattggtacattttgtattggagtta

ccacaccagttactgggtcacgcctccacttccctggttctggcacgtagtggacaggctccggctttctaaccttccattcatcccaaaattttctata

cgcttctggaagtcgtgcacccactcgtcctggatcaaatggcccaggcctcttaaaatataagagtttaagaccgtttgctagcctagattccatttca

acttgtaaccgctttcctcactttgtgcatccacacatacgtacaatccgaacgcaccttccgctcattcctacgaccctactactgatcaagtgtttac

acccgatttcaattgtttcaccgactgaggtacacgttgagtaacatcaaagttaagctgtgattcgagctgtctgcagtcgaggaggaaagaaaagagt

taagatttgaaatacgtgagccagtaaaataatgtttggaggatatttaatcgttgggaacacagatgaacttcttatattggccgcccctgcgtagaaa

attctttaataaggacaccgtggtatcattgcagcactaacttctgttaacagagagtaacatatcttcgtatcgagttaacgaatatttgatatcacat

tcgataatacgaaccgaaacgaacgacgcttagaagggtatgtgtcacttctcggagcgaagtgttctatttaaacttcctaaaagcccagaaaaatcag

tgaggtgacaatgtggtggagcgagagttctgatccaccaggcgaccagacgcaggcgcctgcacaaaatatggaagtaaaagaacttcaagaccagttc

gaacagcagcagacgctaatcgcccaactgaaggagatgttgaggaaaaacgagcaacagcttgtcacaaaagagaaggaagttgaggaatatgcaacca

aactctctggcattaagtcacgagtttccaaaaggggacgtacaaaagcagaaaaaactgaagctacagtttctgaaagtgtgccggatgctgttcagca

agatgtggcaactgcagatgccaaagacgaggaattgaaatcaactggaaaaagaaaagttcagagaggcaacctgattcttttaagaaaacaattagaa

gagaacagaataaaatgtgagcagcgaaacaaggaagttacaaaaaataagcgtggtattgaggagatggttgaacacttacgatcacagttggaagaga

gagaacaaactattcaagaactgaaaggagaatttaacactaggttattagcagctgcttccttgacacacacagttcctactgcagaggtcactcaatc

gtttgaaataccagaaggtctgtcaccagaacatcagattcaagaactgcactcacagatatctctgagagacagtagaattatagaacttaatggtaaa

ataattgaactggaaaatacaataattgatctacaagagaacttgaaggagaaggattctgtaattgaagctcgcacaaaggcaataacattaatgtcag

aagacttgtcacgcaaaagcaaggcaactttagataccttagatgaaacgcgcgaggagatgaggacaatgcagcaaaattttgtggcccttgaagcaaa

catgaaaagagagaaagaagacttgaataaacaactggaagcaaaaaacaagaggttacaacagatggaagacagaaacaagatgctggaatcaacaaga

tttgagctgagcactcggaatgcagagctccaagagaaagttgttcttttgcaatctgaattacagaaacagtctgaagaaaatcatctaactgataatg

taagagcattagaagaggctcttgaggaagcaaacaatgagattagtagcctgaaatctgttattcatgaaaacaagattcgacaaagtaatgaagatat

cgagctattgcatcaaagaattgctgagcttgaagaagataaaggatcagaagatctgtggaagcagaaggtaatagagctagaagacagagtgaaacag

caaaacctcgatttggattcccatttgcatgcactttctcttattgagtctgagaaactggatttaatacaagcactacaagagaagtcacatgcaacaa

acgcacttgaagccaaagtagcagacctgcaacaaatcatatcagaaactgagaactgtaaggtcacagctgaactgaaggcagtggaacttgaggagca

agttgacatgttgttaaaacaaaaggatgaccttgccagtgaactagaaaatatgaaaacagctttagttcagtttcataatcagcctcttaaaaatgat

gagcaattctcaaaccagtttcatgataaacatgaaaagtgggatgtacaggaactctcagaaaaagaggagaaggattcttccaatttggaaatatcat

tgaaacctcttgaaagtgaacttgaacatgcaaggaaaacaattgcagaacaaagagaaataatatcagagttaaatgagaaactcagacaaagagaaga

agaaatggaattacaactggaaattatttcaaaattggaaaatttaggggatcaggtacaagaaagagaagaaagtaatgaggatggtaataaaatttct

gaaattgtgaaagaacttggagagatggcaggagacttaaatgaatggaagaagcgatgctctgaagtagaggataggttggagcaactgggaaatgaaa

aagttcaactagaaagacatttagaagaagtgataaatgaaaatcaggtcatatctacacaacttagatttcagaaagaaatttctgaggagctagaagc

aaagctaagtgagcagtatcaaattcttgccacacgagatgaagctattaataaactgaaagatacggtaaaggagcaacaaaaagctctggaaagtaaa

gatgttgctttgttggatgtttctcagaaactacaagaactagaacaagaagtagataagcaacatcaggaactcgcagctgaacttatgtgtagaacac

aggaattagaaaaagtcaattctgaatatttggaactgaaacttgcttttaatgacaaagaacaagaactgaatagtttgaacatactaatgcaaaattt

atcagtacaagttgatgatgttattaatagaattggcttgggcagcattgatgtgaacaacagcatttgtggaaaactggaggttgctatgtcagtaatt

ggagaaaagtgtgtcggtttgatttcagaaaatcaaatgcttattggcactattgaacaaaacaacactgctttaaaagaaatgagtgaaaaattgtcat

acatggaaaagattgctgctgagcttgagggaaattttgaaattcagaaaaagaaagatgatgaactttctaatgtcaaagaagatcttgcaaggaaatt

ggatgagctgagtaataaagatagaactatcagtgaacttgaacagaaaattacagagcaaatttcaaaaaacaaaaagcttgcaaaatgcctgaaagcc

aaagcagttgttgtccgaaattatgaagagaaagaaaaattatgggaagcagcaaatcagtcaaaggagcaagaggctgctgttggaaaatctcttcagg

aaaatattagtgctcagcaagaaagtgaaatactttcgctgcataaacagttacaggaaaaggatgatgacctgaggaaatatagtcacaaatgcagtga

acttgaaaatgaaatttctaatcttactcagacatgtgcagaattacagcagcaacttaagctggtatctgaggagaggaattatacaacctgtatcatg

caggaaatgagtgcagaaatgaaggaaagagaagaaaagatcaatagtcttatgaaagacatcaataaattgcaagaagagcttactgctaaagatatac

tcaaaaatgaattaacggaagaaaataaaaatatattgatgaaacgggtagagcttgaaaatgaattggtagagctccatg

>42367|4-coumarate-- ligase 3-like

aatagccatcctcgtcgtagtacgcgacatcgcccgtgtggagccaaccgtcactgtcaattgtttcttgggtcgctttctcgttgttgaagtatcccat

catgaccgtgatgcccctggacaggcccaccagcaagtatgacagctg

>42366|4-coumarate-- ligase 3-like

acacaatattgcattgaggctggtttatacatagctttatttataccaaaacatacacacatgcatatatacaacattgtaacactgacagaaagactac

cagtgataagtgataacaaaaataatttttacatttatgtgttgattacttacacaccatatgtacataaacatatacaaataaacagttctattcattt

aattagaaaacatacatagtaaattaaaaatagctaccgaattaactgtaaaaaaaggaatgccttcaacaacataataatacagtaacatcaaacataa

gaacaagtaatggggaaagaaactaatttattacacaaagatcatctgcaataagtactggcctggaaacttactgattttcaacaggtcttgtcatcgt

acactgtgaaatataaagtatatgactgaatgaagtcaacaaaatttgtcttctcaaatatcacatttcataatttgtatcaggggatgcattcagaatg

taatatttcttataattctgaacagcagagtgttctgtatggagaacagacagtattattttcttgttacagtggtaaacgaaaatataagttgaaatta

aaatttgttccactttttttcttgtcaaacagggtcacagttgtgctgaaataaataacaaccttccattcaaatatactggcaggcaaaaataataatt

acaccagtattacatgaatgtctgtaacttctattcctctctgcttgtttataacataaatactcaacgaatagcctagggatgtgttcattttgggtga

aacatatttttgactgatatttcagtataacttacattgcctgcaaatgcaccagaacacacatgtagcaccaggttaactccaaacaacagccttgtct

gcagcatcaaactgtgtaaatcaacagttcatatcttagttcatttgatttataaacaatacaacagattctcacagtgaaaactggaacagaatgtaac

agccacatttctcttatgttaatttatactagtccatcttcctttttattcttccacgagccatccctcagctgagcacgcagtactttaccagacggat

tgcgtggaatttcactgataaactctacaccaccagcgaggcgtttataacgggcgactcggggagttacgtgttcttcaacagcctcggccgtcaactt

ctgctcagatttgagtacgatgaatgcctttggcacctctccactaatctcatctggcataccgaccactgcaacgtctgctacttcaggcaactccatg

atgatgctctcgagttctgttggtgacacctggtttcctttgactttgatgagctccttcgtgcggtctacaatgtagaaatagccatcctcgtcgtagt

acgcgacatcgcccgtgtggagccacccgtcactgtcaattgtttctcgggtcgctttctcgttgttgaagtatcccatcatgacctgtggaccctttat

gaggagctcgccagtttctccaggaccctggtcagtgccatccttcagcgagacaatgcgagccttcgtaccaggggccagttggccacagctgtcatac

ttgctggtgggcctgtccaggggcatcacggtcaccacaggagacgattcagtcatcccaaagccttgaccgacgaagatgttgtggccggccttgtcga

ggaacttctggatgaggctctccttggccggagccgcaccactcattattatcttcaggctcgctaggtgctccggtttcaccatcgggtgtgttgccag

gaacagcagtaatgatggaacgacaaacatcattgttggcttgtaatcctctagggccttgacatagctggcaggagtaaacttgggcagggtgacattg

tggatgccgagtgaaacagtgaggttgaggatgatgttgaagccgtagatgtggtagaagggcaggatgctcagcaccacctcttggtagctggctgttg

tgggctggaagaagagcagcctggagtggttcatcatctgcaggttggcgaccaggttgtggtgggacaggcagacgcccttgggcaggccggtggtgcc

gctggagtagggcagcactgcgagctggcggggcttccccggcggcggcagcgccacgggggaggcccgcgacagcaggcggctccagttgtgcgcgccg

ggcgcgccgccgtccaccacgatggtgccgcggaagcgcgggcccagctggtggcgcacctcctccaggggcaccagccgcgagaacgtcgtcaccaggc

agcgcacgccgctgtcccggaactgccggcacacctcctcggcggtgtaggcggggttggcgaaggtgacgacgagcccggcctccagcgcgccgtggac

ggcgaccaggaactcggggctgttgggcagcacgatgcccagcacctcgcccggctgcatgcccagctccgacagcagagcccaggccagccgccggcac

tgctcgcgcacctccccgtacgtcaggctgcgccccgtctccgcgcacgtgatggctttcttgtcgggccacctgtggtagtgctgccagaggtactgcg

tgacggtgagatccggcacctgcacctcgcccagcggggactggatcaccctggagggggcggcggctgacgaagacgcgaggcagcgacttccttggcg

gcgggccgacgcccacacgcagcggcccacgacgctccacatgtcacgcacagcaccgcaccgcacagcacacggacaggacactgctctgcgaccagct

gcggccggcgttcgcggcggactggtctgcgagatacgcgcctcgcgcaaggtcgccggctctgtcggcgccccctccactgcggtccacagctgactgg

cgcgcctcgccagctgatgcactaaaaat

>42670|4-hydroxybenzoate mitochondrial-like

ttgtcgcaatgtagccagtgttttctgaagtgctgttacgtatactatgctgcagaatagtggcatcgagtagaagtgcacataacttagttctctcctg

tatttgtgtgtgattgaatgtgttggacatttcaatacgtaaaccggtagaaactgttcatgctctattaactgtatgggccttctaccttttgcaaaat

aatcggcaagtttcaataggatggctgtcacagtcttctctgttttgcgcaagcgcttatctccaatgcatatatctgcgagacgacttatttcttggac

tgtgctaacgaagaatgaacaactacatcggcacacaaggttgtcatccgagcaggtgactttgagaatagtgtgtggaagatcttggaagttgcgctgc

atgaacctggttgctcagtataataccactgctccgaagagctactgttataaagatgacacgacctccgttgttaggaaagaatcaactgaaaattcac

cagataaaaataatttattgccagctctcttgccatatgcccagttaatgcgacttgaccgtccgataggatcatggcttttgtattggccatgtgcttg

gagtatagctatggcagcctctcctggttcctggcctgatccagtcctactgggactgtttggcattggtgccattttgatgcgtggtgcaggctgtact

gttaatgacatgtgggatcgtgatatagattcaaaagtacttttataagagcatttattgcaaaaatgtccaggtgactcgaacgaaaactcgtccattg

gtgactggcgctctgtctcagtttgatgctcttgtctttctgggaggtcaactggcactggctttactcgtgcttctgcagctcaacacgtacagcattg

ttctgggtgcctcatctttgggtttggttgccatgtatccattggcaaaacgtatcacacactggccacagcttgtgctgggagcaacttttaactgggg

tgccttgttgggttggagtgcagtgcacggcacatgttcctggccagttgtcttgcctttgtcctgtgctggtatatgctggactatcatatatgatag

>42874|4-hydroxyphenylpyruvate dioxygenase

agaatatgcaagatcgaccaactctgtttcttgaagtgattcagagacataatcacaatgtaagttcaactaatatgtgccacataatatatttatttta

ggtttttttatttattaaatatgtcatacatattttcagggatttggtgcaggcaattttaaggccctctttgaagccattgaaatagaccaagct

>43092|4-hydroxyphenylpyruvate dioxygenase, putative

aagaatatgtagattattatggaggagctggagttcagctcattgcgctgagcacacaagatattatcagttctataaaatatttaaaacaaag

>43090|4-hydroxyphenylpyruvate dioxygenase, putative

tctctttacaattgtgtcaagatcttcaactgtaaatgcaacatctttgactccatctccgtgtttgaccaagtgcttgcccattgcttcattatttggg

tcctattttgggaaaaaaaaagaaaaagaaaaattttttttttttttttttttttt

>43096|4-hydroxyphenylpyruvate dioxygenase, putative

ctgattccgtaaaccacgcttgtagcctgtgaatattgccttagcgtaaagtcgaccatgacgacttacaccgacaagggcgacaagccagatggaggac

gatttgttcaccttgatcacatcactttctgggttggaaatgcaaaacaggctgccagttactactgtaccc

>43097|4-hydroxyphenylpyruvate dioxygenase, putative

cccagaaagtgatgtgatcaaagtgaacaaatcgtcctccatctggcttgtcccccttgtcggtgtaagtcgtcatgatcggcgttgctcggctggctgc

agagcgtcggtgtagcgctggaggggtcagcatagccgcagctagagcgagtgaaagcggggcccggcggcgcattgtggtagcg

>43093|4-hydroxyphenylpyruvate dioxygenase, putative

agcctgttttgcatttccaacccagaaagtgatgtgatcaaagtgaacaaatcgtcctccatctggcttgcgcgggcgcgggtacacgccgttgccgttg

tcgaggcgcacgtccggcttgctgtcgcgccgctgacagccgccagcgacgcgtggcgaccgctgcagcccgttggccacggcgcgcccca

>43091|4-hydroxyphenylpyruvate dioxygenase, putative

ccatatgtttgtactgttgcaaatcgaacaattccatcatcatctctttcttcccaaatatctttaacaataactgcaccacgttctttggctcacttta

caattgtgtcaagattttcaactgtaaatgcaacatctttcactccatctccttagctgagtgctcaggttggcagaacgctgtgcgagaggtccgggtt

tgattactggtcaggttggggattttctttacttggggactgagtgttttgttgtcttcttcgtcatttcatcctcatcgacatgcaagtcactgaagtg

ttactaagggaacggtccatccacctgtgggccctagccacatgca

>43483|40s ribosomal protein

acaccgcgcacagttagtgcagcgaacaggattcccctggcctcgcccctgtttcgatcttccactatttcttct

>43498|40s ribosomal protein

agggtccggttcgaatgcccacgaagatagtgaggactactactcggaagaccccctggggtgaagggtcaaaga

>43505|40s ribosomal protein

cctgttcgaatgccccctaagatattgaggattcctgctcgcaagcccccctgtggtgaagggtccaagacttgg

>43410|40s ribosomal protein

cgaaagttgaaccttcactggaagagcatttccaggcatctagattgcttgcttgtatttctagccgaccttttcag

>43502|40s ribosomal protein

gtcctgttcgaattcccactaagatattgaggattactccttgtaagtccccctgtggtgaagggtcaaagacttgg

>43497|40s ribosomal protein

gtcctgttcgaatgcccactaagatattgaagattactcctcgtaagtcaccctgtggtgaagggtcaaagacttgg

>43348|40s ribosomal protein

ttcaaaaatgtacgcaatatgttgcgccatacggagaaggggggagccagatgattgtatagcaaggcttgcaaaaaa

>43493|40s ribosomal protein

gtcctgttcgaatgcccactaagatattgagaattactcctcgtaagcccccctgtggtgaaaggtcaaagacttggg

>43501|40s ribosomal protein

gtcctgttcgactgcccactaagatattgaggattactgctagtaagcccccctgtggtgaagggtcaaagacttggg

>43500|40s ribosomal protein

gtcctgttcgaatgcccactaagatatggaggattacaactcgtaaaaccccctgtagtaaagggtcaaagacttggg

>43494|40s ribosomal protein

gtcctgttcgaatgcccactaagatattgcggattactcctcgtaagcccccatgtggtgaagggtcaaagacttggg

>43421|40s ribosomal protein

ccatggccattatcacattctcggcatgacctctatcctttgccatactttctcggatgataataccaaatgtttcgga

>43495|40s ribosomal protein

agggtcctgttccaatgccccctaagatattggggatttcttctcgtaaggccccctggggtgaagggtcaaagacttgg

>43370|40s ribosomal protein

ttgaagttgacccagaaacgaaagacaggtggaaaatgttggaattcaacaacatcaccggacttcaacttactcagcca

>43369|40s ribosomal protein

tgttgaagatgttggacttcaacaccatcacgggacttcagcttcctcagccagcaacacaaggatttggcagacgcccgt

>43359|40s ribosomal protein

cgtgaaaggcgtgataattctgttccagaggtgtctgcttcggcgcaggatattattgaagttgaccccgaaacgaaagacg

>43492|40s ribosomal protein

gaagggtcctgttcgaatgcccactaagatattgagcattctttctcctaagtctccctgtggtgaagggtcaaagacttgg

>43392|40s ribosomal protein

gataacttcttaaatatgttatgcctacatttgtaaggtaacaataaaaacgcccccaagcaaactgctgtgttacatatcct

>43360|40s ribosomal protein

catgttgaatatgttgcacttcaacaacatcacgggccttcaacttccccagccagcaacccaaggatttggcagacgcccgt

>43351|40s ribosomal protein

tggcgccatacggagaatgggggagtctgatgattgtataaaaaggaatgcaaaaaaggatggcattctcgcaaagaactctt

>43380|40s ribosomal protein

cccatctcggcagtgcgggtacgtggtaataactattagtggaaaaataatggataatgaagaagcgaggagaaaacatcttg

>43471|40s ribosomal protein

tgatgcatggccacaacaatggaaagaaattaatggcggtgcgcattgtaaagcctgcatttgagatcataccccttcttacg

>43375|40s ribosomal protein

catgttgaagatgttggacctcaacaacatcacgggacgtcaactgactcagccagcaacacaaagatttggcagacgcccgtg

>43518|40s ribosomal protein

cgtgtatagtgccaatcatcatcatatggtgcaagttctttaatccttgcgcttttcaccaattctacccaatctggaaccttc

>43545|40s ribosomal protein

aagagtacaacttattaagaaaggaaagaagaaaacagcatctgttcctcgacatggctgcttgaactacattgaggagaacgat

>43365|40s ribosomal protein

agacatgttgaagatgttggacttcaacaacatcacgggcctgcaacttactcagccagcaacacaaggctttggcagacgcccg

>43374|40s ribosomal protein

acaaaacccgcaattttatttcttagtggcttgctggggattttctcaatttccccgcatatccgtttgtttgtgtgaaagtcaag

>43491|40s ribosomal protein

tgaagggtcctgttcgaatgcccacaaagatattgaggattattactcgtaagcccccctgtggtgaggggtcaaagacttggttc

>43401|40s ribosomal protein

gggtgcatcgcaattttcagcgagaacacacagcagtgccgcgcgctggtctagcgcttttgccgcgtcgggaagaccatgcgctag

>43443|40s ribosomal protein

cccattacgttttgtggatattgcaacaccatgtaacaacaagagctcacattcaattgggctgatgttgtggctgcttgcaagcca

>43391|40s ribosomal protein

cagctgggagatgaagataacttcttaaatatgttattccttgatttgtaaggtgccaaaaaaaatgtatccaagcaaactgctgtg

>43469|40s ribosomal protein

gattctctcccgtaaaaaggtgtatgatctcaactgcatgctttccaatgcccccagccattaacttctttacattgttgcggccatg

>43353|40s ribosomal protein

aaaaatgtacgcaatatgtggcgccatacggagaatgggggaatcttattattgtatagcaaggattgaaaaaaaggatggcattctcg

>43373|40s ribosomal protein

cgaaagacatgttgaagatgttggacttcatcaacatctcgggattccaacttactcagccagcagcacaaggatttggcagacacccg

>43362|40s ribosomal protein

cagaaacgaaagacatgttgaagatgttggactccaacaacatcgcgggacttcaacctcctcagccagcaacacaaggatttggcagac

>43356|40s ribosomal protein

cagaaacgaaagacatgttgaagatgttggacttcatcaacatcgcgggactttaactttcacagccagcaacacaaggatttggcagac

>43366|40s ribosomal protein

acgaaagacatgttgaagatgttggtcttcaacaacatcacggggcttcaccttactcagccagcaacccaaggatttggcagacgcccg

>43407|40s ribosomal protein

cgccctggtcagtgtgggcttgcagatggatacatcctggaaggtaacaaactagaattctacgtaagaaaaacaaaagctaaaaagggc

>43346|40s ribosomal protein

atgacggacacttcaaaaatgtacgcaatatgtggcgccattcgtagaatgggggagtctgaggattggatagcaaggcttgcaaaacag

>43413|40s ribosomal protein

tatttctagccgccctggtcagtgcgggcgtgcagatggatacatcatggaagggaaagaactagaattttacgtaaggaaaacaaaagctaa

>43481|40s ribosomal protein

ttgtctttcggtacacaccgcgcacagttagtgcagcgaacaggatgcaaatggcctcggcaatgtttcgatcttccactacttcttgtcttac

>43525|40s ribosomal protein

atctatttcaggtctccagttggagttagttcaataacaaaaatatttggaggtcgccatagaaacggagtctgcccatcacatttttgccagg

>43352|40s ribosomal protein

ggacgtaggacggacacttcaaaaatgtacgcaatatgtggcgccagacagagaaggggggagtctgatgattggatagcaaggcgggcaaaaaa

>43403|40s ribosomal protein

ctgtgtgttctcgctgaaaattgcgatgagccaatgtataagaaactggttcaatccctatgaaatgaacaccagattcctcttatcatagtgtac

>43372|40s ribosomal protein

acccagaaacgaaagacatgttgaagatgttggacttcaacaccgtcacgggtcttcaacttactcagccagcaacccaaggatttggcagacgccc

>43400|40s ribosomal protein

gcatgggttcatcgcaattttcagcgagaacgcacagcagtgcctgtctcttgtctagttcttttgccgcttcgtgaagaccatgcgctagcgttcc

>43357|40s ribosomal protein

ttgaagttgacccagaaacgaaagacatgttgaagatgtaggacttcaccaacatctcggttctttaacttactcagccagcaacacaaggatttggc

>43463|40s ribosomal protein

tatctaaactgttagagctccacggtgatggcaagggtggcagtaatgagccaggtgctaaagttggcaggcatgagggttacgagccacctgttcag

>43538|40s ribosomal protein

tacaggcaatgtgtccatacgagggcgtattttaactggtgttgtgcacaagatgaaaatgcaacgtactattgttatcaggcgggtctacctgcaga

>43543|40s ribosomal protein

agtgtgtaagagtacaacgtattaagaatgcaaagaagataacagcatttgttactcgagatggttgcttgacctacattgaggagaacgatgaagtg

>43341|40s ribosomal protein

tgtggatccagctacaggacgtatgacgcaccctataaagttgttcgtaaaaggggccgccatacggagaatgggggagtctgatgattgcaagccttg

>43399|40s ribosomal protein

acatgggttcatcgcaattttcagcgtgaacacacagcagtgcctgtcccttgtctagcgctttttccccttcgtgaagtccatgcgctagcgttccac

>43479|40s ribosomal protein

ttttttatggctttgtctttcggtacaccccgcgcacagttagtgccgcgaacaggattcacatcgcctcgcccatgtttcgatcttccactatttcct

>43345|40s ribosomal protein

tgtggatccagctacaggacgtatgacggaccctacaaaaatgttcgcaaaaggtggcgccatacggagaatgggggagtctgatgattgcaagccttg

>43452|40s ribosomal protein

aatgcgcaggattttattttgtgacacaaatctcacttgtgcactcccatgtgaatccctgagaataacacttatttgagaaggagtaagacccttacg

>43338|40s ribosomal protein

tgcggttgctggcggaacatttgcgaggtcgatataagtcgacaaactctactgcttcgttttccataactccggttagttgaatcagttatatatcttc

>43350|40s ribosomal protein

atgtagatccagctacaggacgtatgacggacactacaacattgtttgcaaaaggtggcgccatacggagaatgggggagtctgatgattgcaagccttg

>43358|40s ribosomal protein

ttgacccagaaacgaaagacatgttgaagatttcggacttcaacaatatcacgggacgtcaacttacgcagccagcaacacaaggatttggcagacgccc

>43361|40s ribosomal protein

tgacccagaaacgaaagacatgttgaagatgttggacttcagcaacctcacgggacctcctcttactcagccagcaacacaaggatttggcagacgcccg

>43482|40s ribosomal protein

tctttcggtacacaccgcgcacagttagtgcagcgaacaggattcacatgtcctcgaccatgtttcgatcttccctgtacttttctccgtcattttcttt

g

>43458|40s ribosomal protein

tatctaaactgttagagcaccacggcgatggcaagagaggcagtgatgagcctggtgccaaaattgacaggcctgagggttacgagccacctgttcagga

g

>43546|40s ribosomal protein

agagtacaacttattaagaatgtaaataagataacagcatttattcctcgagatggtttcttgaactacattgaggataacgatgaagtgttggtagctg

g

>43367|40s ribosomal protein

ttattgaagttgacccagaaacgaaagtcatgttgaagatgtttgccttcaccaccatcacgggacttcaacttactcagccagcaacacaaggatttgg

ca

>43426|40s ribosomal protein

gccattagcacatgctcggcatgacctccatccttggccatactttctcggatgagaattccaaatgtttcggactcccatctgcgtttatctcactatc

aac

>43415|40s ribosomal protein

ttgaacctgcactggaagagcagttccaggcatctagagtgcttgcttgtatttctagccttcctggtcattgtgggcgtgcagatggatacatcctgga

tgggaaa

>43448|40s ribosomal protein

ctggaggcaaagctaagaagaagaagtggtcaaaaggaaaagttcgtgataagctgaaaaaccaggtgctctttgacaaggcaacatatgagaaactgtt

gaaagaaggtacttctt

>43423|40s ribosomal protein

gctcggcatgacctcgatccttggccatactttctcggatcagaataccaaatgtttcggaatcccatcttcgtttatcacacacctatttgggaataag

cactacacagacacacac

>43389|40s ribosomal protein

tttggatatccgcctcaatcgtcgaattcaacaagcactacatatcctcttgacttcaatgactgcatggcttttatgacgtgaagattaggtatggttt

gtagttcgagagcaggatgtttc

>43344|40s ribosomal protein

aaaaaaggatggcattctcgcaaagaacttttgatgcctgacgtatgacggacacttcaaaaatgtacgcaatatgtgtcgccatacggagaatggggga

gtctgatgattgtatagcaaggc

>43490|40s ribosomal protein

atttggtgatgttgttttattagtttctactacctgttcgaatgcccactaagatattgagggttactcctcgtaagcccccctgtggtgaagggtcaaa

ggcttgggacagatttcaaatgag

>43521|40s ribosomal protein

cggcatctctgtgatgacattattggcgcagttatgcgttctgccacctccaaatatttttgttactgcaccaactccaactggcgacctgaaatagacg

ggcctagcgagagcagcacaacgtgta

>43478|40s ribosomal protein

ccaattaggacattcccaagggacatgcgacaaggaggcatgtgtaccgaaagacaaagccataaaaaagttggttagccgtaatattgttgaatctgct

gccgtaagagctatcacagaagctagtgtttacac

>43412|40s ribosomal protein

agttctttcccttccaggatgtatccatctgcacccccacactgaccaggtcggctagaaatacacgctgaaaaaaaagttttttcagttttcactacat

ttgaaacaaaattaaatgccaaatcatttatctatt

>43480|40s ribosomal protein

gtgtaaacactagcttctgtgatagctcttacggcagcagattcaacaatattacggctaaccaacttttttatggctttgtctttcggtacacaccctc

acttgttggcctccttgtcgcatgtcccttgggaatg

>43425|40s ribosomal protein

tggccattagcacatgctcggcatgacctcgatccttggccatactttctcggatgagaataccaaatttttcggaatcccatcttcgtttatcacacta

tcaacacgtcgcgcgcgctcacacacacacacacaca

>43477|40s ribosomal protein

gtgtaaacactagcttctgtgatagctcttacggcagcagattcaacaatattacggctaaccaacttttttatggctttgtctttcggtacacaccgag

aacagttagtgcagcgaacaggattcacatggcctcgac

>43385|40s ribosomal protein

tcttccagtaagattaactacaatcttcccagcgcgatggtcatccactatttcaaattctccaatgtaacctacaaggtaagtttcgaatgaacatttg

atatgcatcacttcattaagcagttaactaaatcaagagtccgtaattcac

>43383|40s ribosomal protein

gacgaataagctgcgcccatgtcggcagttcgggtacgtggtactaacttttagtggaggaataatggatcatgaagaagcgaggagaaaacatcttgga

gggaagaaattgggtttctttttttattgacacaagtcacagttcattaaaaa

>43378|40s ribosomal protein

tttcgatggttctcttgtctattccaccacatttatttaatcttccagtaagataaactacaatcttcccagcgcgatggtcatccactatttcaaattc

tccaatgtaaccatgcttcatcatgacagtcaagctcctcctacacctactccttcctgcgcg

>43342|40s ribosomal protein

caaaaatgtacgcaatatgtggcgccatacggagaatgggggagtctgatgattgtatagcaaggcatcaaaagttctttgcgagaatgccatccttttt

tggaagccttgctatacaatcatcagactcccccattctccgtatggcgccacatattgcgtacatttt

>43424|40s ribosomal protein

tatcagaccatggccattagcacatgctcggcatgacctcgatccttggccatactttctcggatgaggataccaaatgtttcggaatcccatcttcgtt

tgtcacactaataacacgtcgcacgctgtagaaaccacagaacacaacacagcccacacgcaaaaaggag

>43539|40s ribosomal protein

atattggtcttggtttcaagaccccgcgagaggcaattgagggtacgtacatagacaagaagtgcccaattacaggcaatgtgtccatacgagggcgtat

ttaaactggtgttgtgcagaagatgaaaatgcaacgtactattgttatcaggcgggactacctgcactaca

>43519|40s ribosomal protein

ctggaaccttcatttttccagtctttttttaagaacgccgaaaacgctcgctccaaggactgtaatgcttttcttgcaacactaccggccccccgggaaa

aatgtgatgggcagactccgtttctctggcgaacctgagcagcaatgcgatcaagatcccgcctgccctgctca

>43336|40s ribosomal protein

tcagacatcaaaaggtgttggcgagaatgccatccttttttgcaagccttgctatacaatcatcagactcccccattctccgtatggcgccacatattgc

gtacatttttgaagtgtccgtcatacgtggcgccatccttttttgcaagccttgctatacaatcatcagactcc

>43386|40s ribosomal protein

gattaagacctgccttttacctctcttcccagcgcgatggtcatccactatttcaaattctccaatgtaaccatgcttcatcatgacagtcaagaactta

actatgactttggagcaaggcctgattaagacctgccttttacctctcttctcagcattattgattgacttgaga

>43377|40s ribosomal protein

cattatttgtaagctttattgatacataagcaaaagcaagtaacaaaagttcacgggcgtctgccaaatccttgtgatgctggctgagtaagttgaagtc

ccgtgatgtctttcgtttctgggtcaacttcaataatatcctgctccaaagcagacacctctggaacataattatcacgcc

>43532|40s ribosomal protein

ttcctgtggcgtttctcaaacctattatatttacgaatgtagtgcaggtagtcccgcctgataacaatagtacgttgcattttcatcttctgcacaacac

cagttaaaattcgccctcgtatggacacattgcctgtaaatgggcacttcttgtctatgtacgtaccctcaattccctcaatagtac

>43419|40s ribosomal protein

ctgcctgtaatgaacatacccgatgattcccactgaaattgtacaggcaaatgctaaagtgacttttgtgcagcgtgcgacgtgttgatagtgtgataaa

cgaagatgggattccgaaatatttggtattctcatccgagaaagtatggccaaggatcgaggtcatgccgagcatgtgctaatggcca

>43416|40s ribosomal protein

cataaattgagcccgtacttccgtatcagaccatggccattagcccttgctcggcatgtcctcgatccttggccatactttcttggatgagaattccaaa

tgtttcggaatcccatctttgtttatcacactaataacacgtcgcacgctgtagaaaccacagaacacaacactcagccatcacgagg

>43449|40s ribosomal protein

aggtacttctttcaacagtttctcatatgttgccttgtcaaagagcacctggttgttcagcttatcacgaacttttccttttgaccacttctatttacaa

accttcttcttagctttgcctccagatcctccttccttttttttctgggttttctgaggttgctttgaagaacccccttttgaatcctttttgg

>43537|40s ribosomal protein

cagacatattcctgtggcgtttctcaaacctattatatttacgaatgtagtgcaggtagtcccgcctgataacaatagcacgttgcattttcatcttctg

cacaacaccagttaaaataataggtttgagaaacgccacaggaatatgtctgtccacttatcaccttgcttcagggatgtggagattggcgatg

>43427|40s ribosomal protein

ttgagcccgtacttccgtatcaggccatggccattagcacatgctcggcatgccctcgatccttggccatactttctcggatgagaataccaaatgtttc

ggaatcccatcttcgtttatcccactatcaacacgtcgcacgctgcacaaaccacagaacacaacacagcccacacgcaaaaaggagtttgccaaaga

>43529|40s ribosomal protein

aatgggcacttcttgtctatgtacgtaccctcaattgcctctcgcggggtcttggtttcaagcccccgcgagaggcaattgagggtacgtccatagacaa

gaagtgcccatttacaggcaatgtgtccatacgagggcgtattttaactggtgtagtgcagaagatgaaaatgcaacgtacgattgggatcaggcggg

>43418|40s ribosomal protein

accatggccattagcacatgctcggcatgacctcgatccttggccatactttcttggatgagaataccaatgtttcggaatcccatcttcgtttatcaca

ctaataacacgtcgcacgctgtagaaaccacagaacatcaactgggagtactacggcgcatacttcaggaacctcatctccctcttcggccacattgtcg

a

>43484|40s ribosomal protein

tacggatcacgaacttttttatggctttgtctttcggtacacaccgcgcacagttagtgcagcgaacaggattcacatggtcgaggccatgtgaatcctg

ttcgctgcactaactgtgcgcgttgtgtaccgaaagacaaagccataaaaaagttcgtgatccgtaatattgttgaagctgctgccgtaagagatatcac

aga

>43402|40s ribosomal protein

gtggttatccactttgataagaaaaatctggtgttcattacatagggcttgaaccagtttcttatacatgggttcatcgcaattttcagcgagaacacac

agcagtgcctgtcgcgatgtgagtgaaaaataactcgttccacatcattacgatttatcgtgcataattatccatggaatacgaaaactagtgcacgatt

agtac

>43476|40s ribosomal protein

gcagcttcaacaatattacggatcacgaacttttttatggctttgtctttcggtacacaccgcgcacagttagtgcagcgaacaggattcacatggcctc

gaccatgtttcgatcttccactatttcttctcttgcccgtcattttcttcgacagcaaattttacacgcgcactatacaacagcgaagaatatcaaagac

caaac

>43534|40s ribosomal protein

cggttaaggttgaggtgcggctgcttttggaatgccttctctgtttgatccgccatggcctgataacaatagtacgttgcattttcatcttctgcacatc

accagttaaaatacgccctcgtatggacacattgcctgtaaatgggcacttcttgtctatgtacgtaccctcaattgcctctcgcgagacatattcctgt

ggcgtttctcaaacctattatattta

>43404|40s ribosomal protein

tttgttcttactagttcattgttggaagcattatatacaacatcaatgatacgcgtttttcgcgtacaaccttcagatccccaggggaactttgcctggg

gatctgaaggttgtacgcgaaaaacgcgtattattgatgttgtgtataatgcttccaacaatgaattagtaagaacaaaaacattggtgaagaatgccat

tttagggattgatgctgtgccatttagacagtgg

>43379|40s ribosomal protein

tttaatcttccagtaagattaactacaatcttcccagcgcgatggtcatccactatttcaaattctccaatgtaaccatgcttcatcatgacagtcaaga

acttcactatgactttggagcaaggcctgattaagacctgccttttacctctcttctcagcattattgattgacttgagagcatcactcaaaacattcat

gcgcaccattgctcctacacacacacatacacacaca

>43533|40s ribosomal protein

taaaacaccaagagaggcaattgagggtacgtacatagacaagaagtgcccatttacaggcaatgtgtccatactagggcgtattttaactggtgttgtg

cagaagatgaaaatgcaacgtactattgttatcaggcgggactacctgccctacattcgtaaatataataggtttgagaaacgccacgtactattgttat

caggcgggactacctgcactacattcgtaaatataataggtttgagaaacg

>43406|40s ribosomal protein

tcttccagtgcaggttcaactttcgctaaacgctgtcgagctttgtatttctttacgtagaattctagttctttcccttccaggatgtatccatcggcac

gcccacactgaccaggtcggctagaaatacacgcaagcactctagatgcctggaactgctcttccaggatgtatccatctgcacgcccactgaccaggtc

ggctagaaatacacgcaagcactctagatgcctggaactgctcttccagtgcaggtt

>43408|40s ribosomal protein

caaaaatcatttaacatatcacttagatatgcatcaatgttaaaatttcatcagtgtgaacttaaaaataaatttaaaacatcaatgcaaaatccactta

ccaagcactctagatgcctggtactgctcttccagtgcaggttcaactttcgctaaacgctgtcgagctttgcctggggatctgaaggttgtacgcgaaa

aacgcggatcattgatgttgtgtataatgcttccaacaatgaattagtaagaacaaaaacattgg

>43530|40s ribosomal protein

attttgctagtcctgatactagtactattgctccaattgctcctgttaatggtcaaggtctatagtctactaggtggaagtggttggttgcgtcgggagt

gaacgaagttttcttcctttcttcaacgaatatggcggatcaagcaattgagggtacgtacatagacaagaagtgcccatttacaggcaatgtgtccata

tgagggcgtattttaagtggtgtggtacaaaagaattaaaggcaccgtactattgttatcaagcggg

>43515|40s ribosomal protein

tccaatatacctagagatgttaacactcgccagaagttcacatactgatggtaaaaatactgacagatgcaaataataatatgaatactaaaatatgtta

gtctactctgctagaagtgccagtacctccaaatatttttgttactgcaccaactccaactggcgacctgaaatagatgtgcctagcgagagcagcacaa

cgtgtatagtaccacttcatcaacaaaacctcctgctggccttgcgccggcggctcctggtactgaccactc

>43514|40s ribosomal protein

tcagcgtcacggatggcatcttcgtatttcactttccaaccaactcacaacaggtcctccaaatatttttgttactgcaccaactccaactggcgacctg

aaatagatgtgcctagcgagagcagcacaacgtgtatagtaccaatcatcatcatatggtgcaagttccttaaaccttgcaccatatgatgatgattggt

actatacacgttgtgctgctctcgctaggcgcatctatttcaggtcgccagttagagttggtgcaataacaaaaatatttggag

>43405|40s ribosomal protein

ccactgtctaaatggttcagcatcaatctctacaatggcaatcttccccaatgtttttgttcttactaattcattgttggaagcattgttcagaacagca

atgatacgcgtttttcgcgtacaaccttcagcaaatacaaaacttggtctaaggcgtatccacactgtccgcaccaggggcggtaacaagaaatatcggg

ctctgaggttggaccaggggcactttgcctggggctctgaaggttgtactcgaaaaacgcgtatcattgatgttgtttataattcttc

>43474|40s ribosomal protein

gattcacatggcctcgaccatgtttcgatcttccactatttcttctcttacacgtcatttgctgtcgaagaaaatgacgtgtaagagaagaaatagtgga

agatcgaaacatggtcgaggccatgtgaatcctgttcgctgcactaactgtgcgcggtgtgtaccgaaagacaaagccataaaaaagttcgtgcagcgaa

caggattcacatggcctcgaccatgtttcgatcttccactatttcttctcttacacgtcattttcttccccaccaaactttacacccgca

>43473|40s ribosomal protein

atctcttacggcagcagcttcaacaatttatggctttgtctttcggccactatttcttctcttacacgtcattttcttcgacagcaaattttacacgcgc

actatacaacagcgaagaatatgacgtgtaagagaagaaatagtggaagatcgaaacatggtcgaggccatgtgaatcctgttcggatcacgaacttttt

tatggctttgtctttcggtacacaccgcgcacaattagtgcagcgaacaggattcacatggcctcgaccatgtttcgatcttccactatttcttct

>43382|40s ribosomal protein

tattaaccagactttcatcagtatactagcggtgcaatattgagatagaaacttagaagaagaagcccagaatttttccacctgtgtgtcgtctcttggc

ttcatcatgatccataatgccatatgtagtggtcaaaacgacatgtccgaattgtcttgatggtaggaggtcagatgtaatttgttccagctttgacaag

ggaacatcatatcgtggtgagatgacagcgcacttgttcagtcgtcccaaaagatttactacgatcttgcctccgcgcttgtcatcaatgatctcaaatt

ctcctatgtaacc

>43487|40s ribosomal protein

ttgaaacttccatctgaataaccaacatggtcttcttccatttcaatgccagcaatttttcttcttttcttatcctcagctacagtaccaatccatcgaa

taacaccataatgaggcttcaatttaactctcacttccacaactgatccaacttctagatcagttggtactacagactgctcagagtcatcaaggaaagc

agttacatcatttgtaggactaaatcttcttggcaagatatggtcaggaaaataagctgataacctcttatgtgttttgttgacaatctcattcattaac

ttctgaaatgcatggtctaaaggttgaaag

>43354|40s ribosomal protein

aatcaaactacaggaagaagaacgtgaaaggcgtgataattatgttccagaggtgtctgctttggagcaggatattattgaagttgacccagaaacgaaa

gacatgttgacgatgttggacttcaataacatcacggtacttcaacttactcagtcagcatcacaaggatttggcagacgcctgtgaacttttgttactt

gcttttgcttatgtatcaataaagcttacaaataacaaaagttcacgggcgtctgccaaatccttgtgttgctggctgagtaagttgaagacacgtgatg

ttgttgaagtccaagctcttcaacatgtctttcatttctgggtcaa

>43542|40s ribosomal protein

gttctagttgagagaatgggtaaacctcgcggtcttcgtactgctcgcaagcatgtcaatcacaggcgagaacagaggtgggcagataacgattacaaga

aagctcatcttggtactagatggaaggccaacccatttggtggtgcgtcccacgcgaaaggaattgtcttggaaaaagtgggtgtggaagctaagcagcc

caactctgccattcgaaagtgtgtaagagtacaacttgttaagaatggaaagaagataacagcatttgttcctcgagattgttgcttgaactacattgag

gagaacgatgaagtgttggtagctggttttggccgtaaaggtcatgctgttg

>43355|40s ribosomal protein

cgtcggccaaatccttgtgttgctggctgagtaagttgaagtcccgtgatgttgtttaagtccaacatctttaacatgtctttcgtttctgggtcaactt

caataatatcctgctccaaagcagacacctctggaacataattatcacgcctttcacgttcttcttcctgtagtttgattgaaatacctctgacttggct

atgtctcaatcgcttcatgagatgtgtcacaaatcccgcaattttatttcttagtggcttgctggggattatcgcaatttgctcgcatatccgtttgttt

gtgtgaaagtcaagcgtgagcttagtgtaatatttctcaattataacacgagctgctttcttcacagttttcgtcctcacacgacccatgttgcgaagct

ttcc

>43486|40s ribosomal protein

cctcccatctttctgacaactattcagtggcacaaacatagcttttcctggtctacagtgaaagtatctgcggccattgaaacttccatctgaataacca

acatggtcttcttccatttcaatgccagcaatttttcttcttttcttatcctcagctacagtaccaatccatcgaataacaccataatgaggcttcaatt

taactctcacttccacaactgatccaacttctagatcagttggtactacagactgctcagagtcatcaaggaaagcagttacatcatttgtaggactaaa

tcttcttggcaagatatggtcaggaaaatcatgagtttaaaagcagagcagtaacagagtttcacatgccaacttcacttctaattccactttccagctt

atattgtggtaacagtcactgtcactagcaaggtgtg

>43335|40s ribosomal protein

ttttattttagtacaaatttgtagacaccaacgtcaggcatcaaaagttctttgcgagaatgccatccttttttgcgagccttgctatacaatcatcaga

ctcccccattctccgtatggcgccacatattgcgtacatttttgtagtgtccaacgtcaggcatcaaaagttctttgcgagaatgccatccttttttgca

agccttgctatacaatcatcagactcccccattctccgtatggcgccacatattgcgtacatttttgaagtgtccgtcatacgtcctgtagctggatcta

catcagcaatattaatctgtatagacgcatgatcctttgcgtgaatgatgcggttgctggcggagcatttgcgagggcgatacaagtcgacaacttctcc

tgcgtcgttttccataactacggttagttgaatcagttatatatcttccacaaaccaacagccaaa

>43531|40s ribosomal protein

ccagatgttaagctctatcatttgggcttttgccacttgctcactatttttaacattatggtgctgatacagtaaaaagtaaaaggtttgttttgcaact

ggaaactaaaccatatgagagtccatcctatttgggtagcttaaacaattcagcaaagattgaggtgttggaaaacagtggttgctggttgcatgcagtt

agtcctaagcttaacaatattaagataagaacatatactaagttgctgaggcatttggaaaaaaaattgcatctgcattgctttcatacagaaacttgta

ctttctccacaggcaattgagggtacgtacatagacaagaagtgcccatttacaggccatgtgtccatacgtgggcgtattttaactggtgttgtgcaga

agatgaaaatgcaatgttctatttttatcaggcgggactacctgctccacattcgtaaatataataggtttgagaaacgcc

>43475|40s ribosomal protein

aacttttttatggctttgtctttcggtacacaccgcgcacagttagtgcagcgaacaggattcacatggcctcgaccatgttccgatcttccactatttc

ttctcttacacgtcattttctcacttgttggcctccttgtcgcatgtcccttgggaatgtcctaattggtggtgttcggatgcgcctgtctgcctttgaa

cgattgcgcacaacctttgagtggatggcacaggaaacacagtagtgcagctttgcatacagttttggtagctgatatgctgtgtaaacactagcttctg

tgatatctcttacggcagcagcttcaacaatattacggatcacgaacttttttatggctttgtctttcggtacacaccgcgcacagttagtgcagcgaac

aggattcacatggcctcgaccatgtttcgatcttcctctatttcttctcttacacgtcattttcttcgacagcaaattttacacgcgcactatacaacag

cgaagaatatcaaag

>43343|40s ribosomal protein

tagtacaaatttgtagacaccaacgtcaggcatcaaaagttcctgtaaataacaaacagcacagtattagttaatgcaacaaactcaaattaacacattt

aggtaattaatgggcttaaatcaacacttactttgcgagaatgccatccttttttgcaagccttgctatacatcatcagactcccccattctccgtatgg

cgccacatattgcgtacatttttgtagacaccaacgtcaggcatcaaaagttctttgcgagaatgccatgcttttttgcaagccttgctatacaatcatc

agactccccctataaagaaagtaaattattactttgaaaactgaaaaagcaggatgaaaattagatatggaccaaaccaaaattgaaaagctaagatcaa

ttacaaacagcacatcagttgccacctgtagatatggtgttaaactaggaaacattttcaacaagtaaccagttttttggcacaacagaaaaccttgtga

aagtacttacgatggttacatgttttattggaaagatcatttgatggaaatacttcttcatgaatataatcaaatggtggtacacattttctcatcttac

agatacaggcaaggtttgagtaagccttgtctcttaaaggtaataaagtaagcaacacaatattctttaatttataaagacatataattaaagtgtgata

ttcatactggttcattcttcaaccaatt

>43337|40s ribosomal protein

atacacgtgctcattccagacgacacattgcataacaatcatatcacaaactattcaggatatagaaacaatgtttttacgaagtatttcatatgcagca

gtgaggggtcagcagctcaggatgcatacaggcgtccacagtgctgtaggaaaacccaagtggaatgtgtacaaatgtccacagaagctgagtaatggta

tggtaggaattttatacatgtctacagcagtgaatggattacgtacatctgacgctgagactctcattcacttacacaccatggcagcggcagataaggc

aaaagtcagtaatcttttcacctcccttcctcaactattaccttcttgcaatggttctcatttgaaaaaaattagaaagttattcggcaccaaccagttg

aaagctaataaagacctgaaggacaggaacaataataagacacatattatttaggataaagtttttattttagtacaaatttgtagacaccaacgtcagg

catcaaaagttctttgcgagaatgccatccttttttgcaagccttgctatacaatcatcagactcccccattctccgtatggcgccacatattgcgtaca

tttttgaagtgtccgtcatacgtcctgtagctggatccacatcagcaatattaatctgtatagacgcatgatcctttgcgtgaatgatgcggttgctggc

ggaacatttgcgagggcgatacaagtcgacaatttctcctgcgtcgttttccataactacggttagttgaatcagttatatatctgaaaagaactttccg

tatcactatttttcaaactaaatgccaaaagccaccaacaaatcaccacaattgcaaccgaaactaatcatacaacacataccttccactacccctatta

ctac

>43485|40s ribosomal protein

aggggcatccagggtcatcataattcgtgctacttagatgctactcttttcagtatgtttacttttaccagcgcctttgacagtctgttgttcagacctc

caacagagaatgatatcaaacagtatgatgaagtgcagcgagttttgagggaagaaattgttaatcccctgcggcagaatctctatgtaagagccgacag

agtaatgaagttgcggactttgcttgagaatttaagttcagttacagggttaacaagtgaagagaaagatccagaagaatttttgacttctctggttgcc

cagatattaaaagcagaaccatttttaaagttaagttctggtcaggaagcataccactaccaactgtttgttgagaaggatgagcaacttgttctgccct

ctgttcagcagcttttcgaccagagctttttaaccagtgatataaaacttaaagagacatcgttgttgaggaggactggctcaaaataaaaggggataaa

cttcagatggttaaagaagtgacctgactgtggtgttcaaacatcctgtctctcagatcaatcaaagtaattttctctcaactttgtttaacttatgctt

cttactaagatatgaagagcctccagggtgtggtgcttggggtatcactgaaacaatgtgctacatttaatattttgctaggatctgtaataatttccaa

caaatgtatttgattatttaatataaatttgttttgtgtttttatgtttgtgctttctttttccactgtgctataatggtttcattatcgaatgatactc

agtttactttgttaattggcattggaaattatctgaccattttgttgaatgcccaaaatccaacattgatacctggcaatcttgatttctttaggtaatt

cgtacagtgtgataagatattgtaacctctacaggttgtaaatgttaaattttaatagattttgag

>43420|40s ribosomal protein

atcagaccatggccattagcacatgctcggcatgacctcgatccttggccatactttctcggatgagaatactaaatgtttcggaatcccatcttcgttt

atcacactatcaacacgtcgcacgctgcacaaaccacaaaagcccaacacacacactcactcattaaccacaccaagcaccatattcaatgatacacttg

ttactaaaccacacttgcactatatgccagaacggatagtttattcgacatcctcccactcgtcatcactgtaaacatccggctcaagctgctcttccat

atcgcccgcttgtgcccatggaagccatgtagatctccaagggctattgtctaacctatccatctgctctcgaaaagcctgatcctgccatgcaagattt

aatctagccgcttggtttcctatcctgagatcaaaataagcttggggtaactggttgtaagccaaattacagcactgacccctggtgatgcccctgcccc

acaagtgggtggctaacccacctacatcgagatgttccccagccaggaacatcatgtattggcaagagccccatatcctcaaagccttgttgaacatacg

acgagctcgatcagttgcatcgaggtggtcaggcccaatgcgtatgatatcatgatgattggcatctggctcacaagccacgtccaagcccagattcgct

actccgagctgaagttgatctaagatgtatttgtagatcccatagaaatcgatggggttacggtgtgggacagcttcctcaatgtggtgcagacactcgg

agatgattagagcatcaaacatgagtagtatctctgagtatgtcaggcgtcttcccacagctacagctggccggtggggctgagcaacatgtcgaagctc

tcgtgctctcctggtgccccccagtgtgaaatgacgcatgaagagttgatgatataggagctggctcctttcagctagagcattcttgatactagacaga

agtgaacacaaatggagaccttgctgggcagaaagcgagcaatggggatccaccatattgaagacattaataggaggcagactacaccttgagaagtagg

cccagacattccgcaatggagtggaatgaggtgtcctcccggtctcgtggaattgtcgaagctgggctgtgacctcttggtatactctggcaactaagtt

gcgtgcctctacatgtagctgaggcacaagaatccctgtgcgaagtagcgtccagtctcccgaaaaaatcgcattgccccgagctccccctatgatcacg

aatatttctaaggaatgtgatcgtgaatgtgagcaacgatggagatggaagtgcgtgaagaaggtggcacaaaagtgtatccaccgtgtcatgtctggat

ctgcaagtaagaacgtcttgattattgccagaccctgaggatgtagggtctctcgtgccaaccgtgttacattccacaatagtttcatatgatgagcaag

acctttcttccagttgatgtcggcatcgcaagtcagcagagtcacttgaccacacgcacgggctgtattcatgatcgattgacacacctcccacattgtt

aggtctccaggataaaatccctccctgcggattctctctgagtgcggtctcccccacacactacaaatctccgcgggaggcccatgcacgccggcatctc

taacaccatcaacctgttctgggatgtcaagcaatgagttatacacaactgttgcctcagggcacatggtagcacagtatactgccacggctccgattcc

gtcagcagtgcacacaatcacagatcttgtccccaaggactgacagagtcgaaatgcccctgttaggtgacacatcacagcatacttcgtcactgccgtg

ttcaagtgcccaccgactctggcaggctcaagg

>43767|40s ribosomal protein s10-like

gaagataacttcttaaatatgttattcgttcatttgtaaggtttctataaaatggtgtccaagcaaactgctgtattacatatcctcttgacttcaa

>43995|40s ribosomal protein s10-like isoform 1

gaaggcataacatatttaagaagtcatctacatctcccagctgaggttgtcccatcaactctaaaacgccaagcacggcaggaaactgct

>43996|40s ribosomal protein s10-like isoform 1

atggaacaatctcagctgggagaggacgataactacttaaatatgttatgcattcatttgtaagggaccaataaaaatgtctccaagcaaac

>43990|40s ribosomal protein s10-like isoform 1

cggcctcggtcgagcagtttcctgccgtgcttggcgttttagagttgagggaacaatctcagctgggagatgaagataacgtcttaaagatgttaggcct

gcatttgtaaggtaccaagaaaaatgtctccaagcaaactgctg

>43989|40s ribosomal protein s10-like isoform 1

tccgatctcccgcctgtacgcagcacggtcttcagctggccgggaagcttcagagcgaggggcacttggcctcggtcgagcagtttcctgccgtgcttgg

cgttttagagttgatggaacattctcagctgggagatgaatataaattcttaaatatgttatgccttcatttgtaaggtaccaataaaaatgtctccgaa

acatcctgctctcgaactacaaaccatacctaatcttcaggtcataaaagccatgcagtcattgaagtcaagaggatatgtaacacagcagtttgcttgg

agacatttttattggtaccttacaaatgaaggcataacatatttcagaagttatcttcatctcccagctgagattgttccatcacctctaaatcgccaag

cacggcaggaaactgctcgaccgaggcgttttagaggatatgtaacacagcagtttgcttggagacat

>44904|40s ribosomal protein s11

caagatgaagatgcagaggaccattgtcatccgccgagactatctgcactacatccgcaagtacaaccgcttcgagaagcgccacaag

>44909|40s ribosomal protein s11

ttgtgcagaagatgaaaatgcaacgttctattgttatcaggcggtactacctgcaaaacattcgtaaatataatagacttgagaaacgcca

>44906|40s ribosomal protein s11

catttacaggcaatttgtccatatgagggtgtattttaactggttttgttcagaagatgaaaatgcaacgtactattgttatcaggcgggac

>44907|40s ribosomal protein s11

cgtaaatataatagatttgagaaacgccacaggaatatgtctgcattccccaacagttaccacatcgccaatctccacatccctgaagcaaggtgat

>44908|40s ribosomal protein s11

agtgcccatttacaggcaatgtgcgtcccctcaattgcctctcttggtgttttaaaccccaaaccaatgttacgagtgtaacggagagacttccgtttct

tggcacctaag

>44903|40s ribosomal protein s11

ggcaattgatggtacgtacatagacaagaagtgcctctcttggtgttttaaaacccaaaccaatgttaagagtgtaacggagagacttccgtttcttggc

atctaagccttttttacggttaagaaatattgttggctgtttttgaaacgagcgctcactcatgcaccagactcctccattgctatgaacaccagctgaa

ctctaagtaagttcctgacatttaacatttattaaaatgatatgcattttctgaacattctgtaagaaagtatgcatgggca

>44910|40s ribosomal protein s11

tcccggctgataacaatagtacgttgcattttaattttctgcacaacacgagttaaaatacgccctcgtatggacacattgcctgtaaatgggcacttct

tgtctatgtacgtaccctcaattgcctctcttggtgttttaaaacccaaaccaatgttacgagtgtaacggagagacttccgtttcttggcacctaagcc

ttttttacggttaagaaatattgttggctgtttttgaaacgagcgctccctttgatccgccatattcgttgaagaaaggaagaaaacttcgttcactccc

gacgcaaccaaccacttccaccaaa

>45502|40s ribosomal protein s11-like

ctgttggtgaatgcagacctcttagtaaaactgttcgcttaaatgttctaacagagtcgaaagaaacaagagcaatgaagagtttcaagaa

>45495|40s ribosomal protein s11-like

tttgatccgccatattcgttgaagaaaggaagaaaacttcttgtctatgtacgtaccctcaattgcctctcttggtggtttaaaacccaaaccaatgtta

cgagtgtaacggagagactt

>45493|40s ribosomal protein s11-like

gttcgcttcaatgttctaaaaggtctgcattcaccaacagttaccacatcgccaatctccacatccctgaagcaaggcaatgtgtccatacgagggcgta

ttttaactggtgttgtgcagaagctgcaaatgcaacgtcctattgttatcaggcgggagtaccggc

>45499|40s ribosomal protein s11-like

acgttgcattttcatcttctgcacaacaccagttaaaataccccctcgtatggacacattgcctgtaaatgggcacttcttgtctatgtacgtaccctca

atggcctctcttggtgttttaaaacccaaaccaatgttacgagtgtaacggagagacttccgtttcttggc

>45497|40s ribosomal protein s11-like

cctcagccttttttgcggttaagaaatattggtgggtgtttttgaaacgagcgctctgtttgatccgccatattcgttgaagaaaggaagaaaacttcgt

tcactcgtaacattggtttgggttttaaaacaccaagagaggcaattgagggtacgtacattgcctgtaaatgggcacttcttg

>45501|40s ribosomal protein s11-like

gaatatgtctgtccacttatcaccttgcttcagggatgtggagattggcgatgtggtaactgttggagaatgcagacctcttagtaaaactgttcgcttc

aatgttctaaaagtgtcgaaaggaacaagtgcagaagatgaaaatgcaacgtactattgttatcaggcgggactacctgcactacattcgtaaa

>45494|40s ribosomal protein s11-like

ttgagaaacgccacaggaatatgtctgtccacttatcaccttgcttcaggtaattgatgtatatttgtctgttaacagtaccttaaagctatgatgataa

cattgacggtcttctgaaaaactgattaaaatttcttcatctgagaaatgtctttcaacttattaacttcttgttactttacatcgtactacattaggat

tttttctttacagggatgtggagattggcgatgtggtaactgttggtgaatgcagacctcttagtaaaactgttcgcttcaatgttctaaaagtgtcgaa

aggaacaagtgcaaagaagagtttcaagaaattctaactggtgttgtgcagaagatgaaaatgcaacgtactattgttatcaggcgggac

>45492|40s ribosomal protein s11-like

aaaactgttcgcttcaatgttctaagaggtctgcattcaccaacagttacgtacatagacaagaagtgcccatttacaggcaatgtgtccatacgagggc

gtattttaactggtgttgtgcagaaggtgaaaatgcaacgtactattgttatcaggcgggactacctgcactacattcgtaaatataataggtttgagaa

acgccacagggatatgtctgtccacttatcaccttgcttcagggatgtggagattggcgatgtggtaactgttggtgaatgcagacctcttagtaaaact

gttcgcttcaatgttctaaaagtgtcgaaaggaacaagtgcaaagaagagtttcaagaaattctaactgtatggatttcaatcaaaataaaaacatataa

attaggatgtttcttatcttaatttctacatgaaaaccaactatgtaaatctgtattgaaatataattccttattacttgtggcgtcattcagaaataaa

acgggccacatttcttaaggagtagtagttaaatggactgatctgtcttagtgattcctttaatgaaagaattgacaacaggatagagtgaaatgcaaat

gaataatttttattatgatttcatgagtagcccaaattctttgaagttctgttttatcaaattaaaaaaaaggttggctctgaagtaaaagatcagattt

ctctgactttaatatctagt

>45777|40s ribosomal protein s12

cctttcaaagtttcccagaactgtctattttgcaaagcccagtccattcacccagcttcttgttgttatccactttgataagagg

>45775|40s ribosomal protein s12

ttcaagccatatgtaatgaacaccagattcctcttataaaagtggataaaatcaagaagctgggtgaatgggctgggctttgcaaaatagacagttct

>45773|40s ribosomal protein s12

acaacaagaagttgggtgaatgggctgggctttgcaaaatagacagttctgggaaagcgcagcccattcacccaacttcttgttgttatccactagacaa

gcgacaggcactgctgtgtgttctcgctgaaaattgcgatgaaccc

>46049|40s ribosomal protein s12-like

ttcattacatagggcttgaaccagtttcgtatacatggcggcatcgcaagtttcagcgagaacacacagcagggccggtcgcttgt

>46045|40s ribosomal protein s12-like

cttcttgttgttatccactttgataagaggaatgtggggtttattacatagtgcttgaaccagtttcttatacataggttcatcgcaattttcagcgaga

acacacagcagtgcctgtcgct

>46047|40s ribosomal protein s12-like

gtttcttatacatgggttcatcgcaattttcagcgagaacacacagcagtgcctgtcgcttgtctagcgcttttgccgctccgtgaagaccatgcgctag

cgttccactagttaacgcagttttcagaacttcttgaagagcagtgtttatgtccattcctccggcaacgggtgccggtatcaacgtccgacatcacgtg

ttaatgagacgatctggataatgaatattgaactcttcacagcgtcaacttccac

>46041|40s ribosomal protein s12-like

cttcttgttgttatccactttgataagaggaatctggtgttcattacatagggcttgaacaccagattcctcttatcaaagtggataacaacaagaagct

gggtgaatgggctgggctttgcaaaatagacagttctgggaaagcgcgaaaagttgtaggatgctcctgtgttgttgtgaaggattacggtgaagagcct

ctagtcgatagaataatgtaggtaagggaagtcggcaaattggatccgtaacttcgggataaggattggctctgaggatcggggcgtgtcgggcttggtc

agggtggtcagaatgcagtacaacctgtggacctggacaacaagttcgcaagagagag

>46800|40s ribosomal protein s13

acctgtattacctgattaagaaagctgtaacaatcagaaatcatttggaaagaaacaggaaggacaaggagtcaaagtttcgcgtgattcttgtggaga

>47057|40s ribosomal protein s13- partial

attcttgactccacaagaatccaccggcttgctcggtacttcaagaagaatagtcttccaccaaactggaaatacgaatct

>47587|40s ribosomal protein s15

atcaagccagagatgattggtcattatcttggtgagttttcaattacctacaagccagtcaagcacggtaaagtcggag

>47588|40s ribosomal protein s15

gcctgaaatgattggacactaccttggtgaattcagtgtcacatacaaacaagttaaacatggacgtcctggaattcaccaaggtagtgtccaatcattt

caggcttcacc

>47584|40s ribosomal protein s15

actgatctagatccaccccacggaaagtaaatttccggaaggtcctattcttcttctgggtttcctctacctccgccatgatattgctaagtcctgtctt

tccaccaactcctgtctttccaccaactcctgtcttt

>47582|40s ribosomal protein s15

ctaagtcctcaaagccacatcttcgtaatatgataattgtgcctgagatggtaggaagtattgttggagtttacaatggaaagcccttcaaccaagtgga

ggtgaagcctgaaatgattggacactaccatgtttaactggtttgtatgtgac

>47586|40s ribosomal protein s15

cccaacacaaccggacgcccgatcctccacgcgacactcctgaaagcttttcttctttacttgaggggaatgaaacgagaagagtgggtggcaccgatac

cgggacgaccgtgcttgacgggcttgtaggagatggagaattctccaaggtagtgg

>47592|40s ribosomal protein s15

aactgatctagatccaccccacggaaagtaaatttccggaaggtcctcttcttcttctgggtttcccctacctccgccatgatattgctaagtcctcaaa

gccacactacagcacacacaggaactcgaagggtaacttcctaccatctcaggcacaattatcatattacgaagatgtgtcttgacaatttcaggctttt

c

>47575|40s ribosomal protein s15

aaccaagtggaggtgcagcctgaaatgattggtcactgccttcgtgaattcagtgacacataccaaccagttaaacatggacgtcctggaattggtgcca

ctcacagttcaagattcattcctcataagcttcccccattattcagaattttacaagtatcagtgaacatttttctatgaatttttgatcctaatctgtt

aaaatgaag

>47574|40s ribosomal protein s15

ttgttggagtttacgatggaaacgccttcaaccaagaggaggtgaagcctgaaatgattggacactaccttggtgaattcagtgtcacatacaaatcagt

taaacatggacgtcctgaattcagtgtcacatacaaaccagttaaacatggacggcctggaattggtgccactcacagttcaagattcattcctcttaag

taaaaaaaattgtaaat

>47578|40s ribosomal protein s15

aataagcttcgtaaagccaagaaagaagcaccaccaaatgaaaagcctgaaattgtcaagacacatcttcgtaaagccaagaaagaagcaccaccaaatg

aaaagcctgaaattgtcaagacatatcttcgtaatatgataattgtgcctgagatggtaggaagttttgttggattttacaatggaaagcccttcaacaa

gggggaggtgaagcctgg

>47598|40s ribosomal protein s15

taaagccaagaaataagcacccccaaatgaaaggcctgaaattgtcaagtcacatcttcgtaatatgataattgtgcctgagatggtaggaagtattgtt

ggagtttacaatgtaaagaccttcaaccaagtggaggtgaagcctgaaatgattggacactaccttggtgaattcaggcttcacctccacttggttgaag

gtctttccattgtaaactccaacaatacttcctacc

>47597|40s ribosomal protein s15

aactgatctagatccaccccacggaaagtaaatttccggaaggtcctcttcttcttctgggtttccgaagcttattgacgagagctaaaggtttgcgctt

cagacctcttgaaaacctacgccgagctctagcatggacaagctccataagctgatccatagtcatgtccaataactaatattatagatgccccaccacc

tcttcctcactacctcaaatcctgggtacgcccttgctgattttgaac

>47576|40s ribosomal protein s15

ggccccaattccaggaagtacatgtttaaatggtttgtatgtgaaactgaattaaacaaggtagtgtccaatcatttcaggcttcacctacacttggttg

aaggtctttccattgtaaactccaacaatacttcctaccatctcaggcacaattatcatattaagaagatgtgtcttgacaatttcaggcttttcatttg

gtggtgaagcctgaaatgattggacactgccttggtgaattcagtgtctcatacaaaccaggtaaacatgggcgtcctggaattgg

>47577|40s ribosomal protein s15

attcagtgtcacatacaaaccagttaaacatggacgtcctggtttgtatgtgagtctgaattcccaaaggtagtgtccaatcatttcaggcttcacctcc

acttggttgaaggtctttccattgtaaactccaacaatacttcctaccatctcaggcacaattatcatattacgaagatgtgtcttgaaaatttcaggct

tttcatttggtggtgcttctttcttggctttgaggacttagcaatatcatggcggaggtagaggaaacccagaagaagaagaggaccttccggaaattta

ctttccgtgggg

>47599|40s ribosomal protein s15

acctttagctctcgtcaataagcttcgtaaagccaagaaagaagcaccaccaaatgaaaagcctgaaattgtcaagccacatcttcgtaatatgataatt

gtgcctgagatggtaggaagtattgttggagtttacaatggaaagcccttcaaccaagtggaggtgaagcctgaaatgattggccactgccttggtgaat

tcagtgtcacatacaaaccagttaaacatggacgtcctggaattggtgccactcacagttcaagattcattcctcttaagtaaaaaaaattgtaaataaa

aaggaataaagtatgcttttgagtttttatttgttatgttaccaaacagaactttcc

>47600|40s ribosomal protein s15

ggctttgaggacttagcaatatcatggcggaggtagaggaaacccagaagaagaagaggaccttccggaaatttactttccgtggggtggatctagatca

gttattggacatgactatggatcagcttatggagcttttccatgctagagctcggcgtaggttttcaagaggtctgaagcgcaaacctttagctctcgcc

aataagcttcgtaaagccaagaaagaagcaccaccaaatgaaaagcctgaaatcgtcaagacacatcttcgtaatatgataattgtgcctgagatggtag

gaagtattgttggagtttacaatggaaagaccttcaaccaagtggaggtgaagcctgaaatgattggacactaccttggtgaattcagtgtcacatacaa

accagttaaacatggacgtcctggaattggtgccactcacagttcaagattcattcctcttaagtaaaaaaattgtaaataaaaaggaataaagtatgct

tttgag

>47904|40s ribosomal protein s15a-like

tcaagaacttcactatgactttggagcaaggcctgagtaagaccaacctttttcctctcttctcagcattattgattgacttgagagcat

>48456|40s ribosomal protein s15aa-like isoform 3

attgagaagtggacgaataatctgctcccatctcggcagttcgggtacgtggtactaactacacgtgaacatgtctacacacaagaagaagacgaggaag

ctcagaggtcatgtgagtcacggacatggacgaataggaaagcacaggaaacatcctggtggaag

>49376|40s ribosomal protein s16

ctggttcctgaagtttatactgaagcacacgaggcttttccatctccagtggcctgccatttgctctcaagtttc

>49367|40s ribosomal protein s16

atggccgcctgcgtttacacgaactctgatctcaactgctaaaaaacgctccttccctaacagcagtactggttcctga

>49366|40s ribosomal protein s16

gaaaggagcgtttttccggagttgatatcagagttcgtgtcaacggaggtggccatgttgcacagagttctgtcatactgg

>49379|40s ribosomal protein s16

aacttcttgggctcgcagcggcgcgggtcggcgacgaggagcgtccggtcgtagcggccgaagatctccttcacctccttcttggccgcctcgtcga

>49380|40s ribosomal protein s16

atttcctcttttacaatatgcaacagcagttgcacttttctttctagcgaacccctgcacggaaggaatcggctcctttgctggcttgtgcattctggtg

gtcaccgcgtaataaggaagcg

>49378|40s ribosomal protein s16

tgttaggagaggagcgtttttccggcgttgatatcggagttcgtgtgcttcaatataaacttcaggaaccagtcctgctgttaggaaaggagcgtttttc

cggagttgatatcagagttcgtgt

>49369|40s ribosomal protein s16

tctgtgcaacatggccacctccgtttacacgaactctgatatcaactccggaaaaacgctcctgtgacagtgaaaacaacaactttcctaacagcagtac

tggttcctgaagtttatattgaagcacacgaggctctac

>49371|40s ribosomal protein s16

aatagcataaatctgtgcaacatggccacctccgtttacacgaactctgatatcaactccggaaaaacgctcctgtgactttcctaacagcagtactggt

tcctgaagtttatattgaagcacacgaggctctaccatctccagtggcctgcca

>49372|40s ribosomal protein s16

agacacttccttaccacgcggtaaccaccagaatgcagaagactgatatccatttcatgttgcagccagcaaaggagccgattccttccgtgcaggtgtt

cggtagaaagaaaagtgcaactgctgttgcatattgtaaaagaggaaatggaaact

>49370|40s ribosomal protein s16

gccagttgttgttcagcagcgaatactgcacctccacaccttggacagccttggccagctggagccgattccttccgtgcaggtgttcggtagaaagaaa

agtgcaactgctgtggcatattgtaaaagaggaaatggaaacttgagagtaaatggcaggccactggagatggtagagcct

>49365|40s ribosomal protein s16

gtaatcgctgacagcacgaaggatgtcacgcgactagttagcaggctagagtctcgttcgttatcggaattaaccagacacttccttaccacgcggtaac

caccagaatgcagaagccagcaaaggagcgtttttccggagttgatatcagagttcgtgtaaacggaggtggccatgttgcacagatttatgctattcgt

c

>49362|40s ribosomal protein s16

cacttttctttctaccgaacacctgcacggaaggaatcggctcctttgctggcttctgcattggtggttaccgcgtggtaaggaagtgtctggtccaaaa

aaaaagtgcaactgctgttgcatattgtaaaagaggaaatggaaacttgagagtaaatggcaggcaactggagatggaaaagcctcgtgtgcttcaatat

aaacttaaggaac

>49363|40s ribosomal protein s16

aaaagtgcaactgctgttaaatattgaaaaagaggaaatgaaaacttgagagtaaatggcaggccactggagatggtagagcctcgtgtgcttcaatata

aacttcaagaaccagtactgctgttaggaaaggagcgtttttccggagttgatatcagtgttcggtagaaagaaaagtgcaactgctgttgcatattgta

aaagaggaaatggaaacttgaga

>49361|40s ribosomal protein s16

aatcgctgacagcacgaaggatgtcacgcgactagttagcaggctagagtctcgttcgttatcggaattaaccagacacttccttaccacgcggtaacca

ccagaatgcagaagccagcaaaggagccgattccttccgtgcaggtgttctgtagaaagaaaagtgcaactgctgttgcatattgtaaaagaggaaatgg

aaacttgagagtaaatggcaggccactggagaggggaaagccccgtgtgctttaatataaactgcaggaaccagaactgctgtta

>49364|40s ribosomal protein s16

cgtgttacaaaatatttgatggatacgatataatttggcttgtgttccattaatacataaaataactgcataaaaattggaataatttccaaggtgaaat

aaatgagaccctgttttcaaagaatgtttattaataagagaatatacactttaacggtatgatttctggtatcttgcacgagcacctggtccaccgaact

tcttgggctcgcaacgcctcgggtcagctaccaagagagttctgtcatactggatcaaaatatcctttatctctttcttagacgcctcgtctacatattt

ctggtagtaagaaacaagagcttttgaaatggcttgacgaatagcataaatctgtgcaacatggccacctccgtttacacgaactctgatatcacctccg

gaaaaacgctcctttcctaacagcagtactggttcctgaagtttatattgaagcacacgaggctctcccatctccagtggcctgccatttactctcaagt

ttccatttcctcttttacaatatgcaacagcagttgcacttttctttctaccgaacacctgcacggaaggaatcggctcctttgctggcttctgcattct

ggtggttaccgcgtggttaggaagtgtctggtccaaagaacacctgcagtcactcaactcacaaaatagaccaccact

>49652|40s ribosomal protein s16-like

gttcctgaagtttatattgaagaacacgaggctctacccgctccagtggcatgccatttactctcaagtttccagttcc

>49650|40s ribosomal protein s16-like

acgctcctttcctaacagcagtactggttcctgaagtttatactgaagcccccgcgtctctaccatctccggtggcctgccatttac

>49659|40s ribosomal protein s16-like

acactctttccctccacgacgctcttacgatctaacggtataatttctggtatcttgcacgagcccctggtccaccgaacttcttgggctcgcaacgcct

cgggt

>49651|40s ribosomal protein s16-like

tgctcgcaagcatgtaaatcacaggcgagaacagaggtggccatgttgcacagatttatgctattcgtctagccatttcaaaagctcttgtttcttacta

ccagaaatat

>49648|40s ribosomal protein s16-like

cagcagttgcacttttctttctaccagaaatatgtagacgaggcgtctgagaaagagataaaggatattttgatccggtatgacagaactctcttggtag

ctgacccgaggcgttgcgagcccagagggagtcttcccccgcaacgtcgacattctgtacgtggaag

>49657|40s ribosomal protein s16-like

aaaagaggaaatggaaacttgagagtaaatggcaggccactggagatggtagagcctcgtgttcttcaatataaacttcaggaaccagtactgctgttag

gaaaggagcgtttacacgaactctgatatcaactccggaaaaacgctcctttcctaacagcagtactggt

>49653|40s ribosomal protein s16-like

ttactaccagaaatatgtagacgaggcgtctaagaaagagataaaggatattttgatccagtatgacagaactctcttgggctcgcaacgcctcgggtca

gctaccaagagggttctgtcatactggatcaaaatatcctttatctctttcttagacgcctcgtctacatatttctggtagtaagaaacaag

>49646|40s ribosomal protein s16-like

ttcatgacgatcacgtttcccgaaggaagtagcatttttcagcgagataatgcgtcgtgccataaggcctggagtgtgattgagtggttcgaagaacgca

gtggccatgttgcacagatttatgctattcgtcaagacatttcaaaagctcttgtttcttactaccagaaatatgtagacgaggcgtctaagaaggagat

aaaggatattttgatcgagtatgcaagaactctcttggtagctgccccgag

>49647|40s ribosomal protein s16-like

gcctcgggtcagctaccaagagagttctgtcatactggatcaaaatatcctttatctctttcttagacgcctcgggtcagctaccaagagagttctgtca

tactggattaaaatatcctttatctctttcttagacgcctcgtctacatatttctggtagtaagaaacaagagcttttgaaatgacttgacgaatagcat

aaatctgtgcaacatggcgtctaagaaagagataaaggatattttgatccagtatgacagaactctct

>49656|40s ribosomal protein s16-like

aatattgaaggacccccgtcaaaaccccaagtatacttgtgtattggagtgattaatgtatcctgaaacttggggtcattttttaatttctctgtagctg

tgaagtctaaccacagatttcgtaagtttcctgtggacttaaatcttggaagagctggctggagcacagaggatgtatttctcatatcactggtgagcac

ctgtcactcaactgacaaaataatgtaccttcagtcactaatgaagtaacatcacattcttcattttctgagctgctaaattcaatgtcaaacacaggat

tatagtaatccttttttgaaagcaatgcctaaacagcaataaaggagggagtgaaaaaatttctaggcctcatggtgaaaaagatgtcaattgcttgcct

atcttttgaacaatttgttttaaatgcgcacagggcaccgctaaatcagcaaacaatatttgtgttacaaaatatttgatggatacgatataatttggct

tgtgttccattaatacataaaataactgcataaaaattggaataatttccaaggtgaaataaatgagaccctgttttcaaagaatgtttattaataagag

aatatacactttaacggtatgatttctggtatcttgcacgagcacctggtccaccgaacttcttgggctcgcaacgcctcgggtcagctaccaagagagt

tctgtcatactggatcaaaatatcctttatctctttcttagacgcctcgtctacatatttctggtagtaagaaacaagagcttttgaaatggcttgacga

atagcataaatctgtgcaacatggccacctccgtttacacgaactct

>50573|40s ribosomal protein s16-like isoform 1

atatttttatccagtatgacagaactctcttggtagcttacccgatgcttttcgagcccaagaagttcggtggaccaggtgcccg

>50572|40s ribosomal protein s16-like isoform 1

gcgtctaagaaagagataaaggatattttgatccagtatgacagaattttcttggtagctgacccgagggggtgcgaccccaataagttccctggcccag

g

>50567|40s ribosomal protein s16-like isoform 1

agaaagagataaaggatattttgatccagtatgacagaactctcttggaagctgacccgaggcgttgcgacaccccgaagttcggtggcccgggtgctcg

tgcaagatacca

>50568|40s ribosomal protein s16-like isoform 1

gtctaagaaagagataaaggatattttgatccagtatgacagaactatcttggtagctgacccgaggcgttgcgagcccaagaagttcggtagatcggaa

gagcacacgtctgaactccagtcacgccaat

>50563|40s ribosomal protein s16-like isoform 1

tctaagaaagagataaaggatattctgcaccagtatgacagaactctcttggtagctgacccgaggcgttgcgagcccaagaagttcggtggaccagctg

ctcgtgcaagataccagaaatcataccgttaaagtgtatattctcttattaataaactatggaaaacccagtatatctgtacattattttggaaataaat

acggaaatctcaaggaaaaaaattaagcgatatt

>50565|40s ribosomal protein s16-like isoform 1

ccttaatgtattgtaatgatctttctcacaaatatgcactctttttggtcactatctgatgatcaaaataatcaaaaccagttactgaaatgaacaacaa

atttgtgaccaaaaagactatttcacagtttaaccaatatagtatgatgtaagttttttcacagtggggtaagcaacacagtatatgacaggcacaagtc

ccaatatagccagtgaaagataatgggaaacacagtttgtacatgttagaatttctctaagacaatggaaagtcattcatacatactgcaaaataaatgt

attttacatcaactacactatgtccataatgaaaaatagtgtcaactataacagcaaaaattgttgagcacaacaaagcatgtcctctgaatgacactga

acattcaggagtgcatgaatgctacaatttccaaacaggtacagcaggtgccctggcagctcagttttacacagtgtgaaccatctctaatgataaacat

gcctgagtggcaagcattttttccccagctaaactcaatttatgtgtatgcttttaactaaaactgatagctggccaactgattgaagcagagaatgcac

aaagctggaagaacaccacttgctttcaagacctggtttgtgctacacacactgcaaacaaaattttacacgccgcacaagaggctatagataaaaaaaa

attgtctacaaaattaaggaaaattgggtttaactatacatttgcactgcacctgcaccatagttggcaccacagtgacaaacataaatgaatgacttca

aggacatcacacagccagacacacagtactgtgccttccactgacccaaaactgccatcatttgcagtttcattggtgtcaagtgagaacttattggagg

gcaaggtggaggtatgtatgttttctgattaaacttagttctgcctcagcactagtgaaggctgtgtgctggttactggcctttaagtgtctgcaaccaa

cctgcctgcatgccacacacaggacatatagctggagttatgatctgggacatgattttgtacgacagcaggagcattctcatggttatcccacaaaccc

cgactacaagtcaatatgatgattcaacctgttgtgctggcattcgtgaacagcacactagggggtgttttccaacaggataatgctctcccactgctgt

tgcaagccaacatgctctgcagtgtttacatgttgcattggcctgctggatcaccagatctgtctcaaatctggcacataagatacatcagaaaactcca

gcttcatccacaaacagaatcacccccataactgattttttcaaattatgttccaaattgttttattaatgagaaatataataaatatcacacacagata

aagacagcttttcaagatcaaaataataagttacaaaatattgaaggacacccgtcaaaaccccaagtatacttgtgtattggagtgattaatgtatcct

gaaacttggggtcattttttaatttctctgtagctgtgaagtctaaccacagatttcgtaagtttcctgtggacttaaatcttggaagagctggctggag

cacagaggatgtatttctcatatcactggtgagcacctgtcactcaactgacaaaataatgtaccttcagtcactaatgaagtaacatcacattcttcat

tttctgagctgctaaattcaatgtcaaacacaggattatagtaatccttttttgaaagcaatgcctaaacagcaataaaggagggagtgaaaaaatttct

aggcctcatggtgaaaaagatgtcaattgcttgcctatcttttgaacaatttgttttaaatgcgcacagggcaccgctaaatcagcaaacaatatttgtg

ttacaaaatatttgatggatacgatataatttggcttgtgttccattaatacataaaataactgcataaaaattggaataatttccaaggtgaaataaat

gagaccctgttttcaaagaatgtttattaataagagaatatacactttaacggtatgatttctggtatcttgcacgggcacctggtccaccgaacttctt

gggctcgcaacgcctcgggtcagctaccaagagag

>51086|40s ribosomal protein s17

agaaatattacactaagctaacgcttgcctttcccacaaccaaacggatatgcgaggaaattgggataatccccagca

>51084|40s ribosomal protein s17

ccgcaattttatttcttagttgcttgctggggattatcacaatttccacgcatatccgtttgtatgtgtgaaagtcaag

>51087|40s ribosomal protein s17

tgctttggagcaggatattattgaagctgaccaagaaacgaaagccatgttcaagatgttgggctttaacaacatcacgggacttcaactttctcagg

>51088|40s ribosomal protein s17

gacacgtctcataaagcgtttgatacatagccaagtcagaggtctttcaatcacactacaggaagaagaacgtgaaaggcgtgataattatgttccagag

gtgt

>51085|40s ribosomal protein s17

cctacccgacgctcttccgatctcgcgtttcacgttcttcttcctgtagtttgattgagatacctctgacttggctatgtctcaatcgcttcatgagatg

tgtcacaaatctgattgggaccagccgaattgacacagacaaagtactctactattgtacaatcaaggcctcacaaaaattcgcttccaattgtactcta

taaacttggaccacagtaagtgctctacaggtaattc

>51212|40s ribosomal protein s17-like

catcggacaggtctgccacagcgacagagagaaaggccagcagcaacatgggtcgtgtgaggacgaaaactgtgaagaaagctgctcgtgttataattga

gaaatattacactaagctaacgcttgacttt

>51213|40s ribosomal protein s17-like

gcgttagcttagtgtaatatttctcaattataacacgagctgctttcttcacggttttcgtcctcacacgacccatgttgcgaagctttccggaaagcca

ctaaaaaataaaattgcgggatttgtgacacatctcatgaagcgattgagacatagccaagtcagaggtatttcttagtgg

>51211|40s ribosomal protein s17-like

acactttcaaaaagatcgctgcacagtagtttgcttcagatttgcgatgtataccgcctcacttagacacagactctgaaaaagatcacgacgcacttgt

gggacctctcaagtcttcctccactactcggcaatatttgcatacacaaacaaacggatatgcgaggaaattgcgataatccccagcaagccactaaaat

aaaattgcgggatttgtgacacatctcatgaagcgattgagacatagcc

>51247|40s ribosomal protein s18

ataaaattccagactgttttcttaataggcagaatgacattgtcgatgaaaaattttctcagttaacctcaagttac

>51244|40s ribosomal protein s18

aaaaggatattgttgatggcaagtcctctcaagttatctccaacgtacttgataccaagtaccgtgaagatctcgaacgtttgaagaagattcgtg

>51240|40s ribosomal protein s18

aaaatgtctctcgtgataccagagaaattccaacacattcttcgtgtcattgcgaacataactttcctctttccgtaaatattcgtgcccatgacacgaa

gaatgtgttggaatttctctggtatcacg

>51248|40s ribosomal protein s18

cctcctcttcagtgcattcaccggctctcttgtccaagtaaatattagccttctttaaaactatattagcataacgtcgaccgtctccattacttggggc

agttgcccccctccgacataaacactcccatcacagcacaagacattaaa

>51245|40s ribosomal protein s18

caccattaccaacatcaagtttcaccttcatcatctacattttcaaatatttcctgttctcaacatcagcgcaactcccaccccttccacttgcaacttt

cctctttccgtcaatattcgtgcccatgacacgaagaatgtgttggaatttctctggtatcacg

>51239|40s ribosomal protein s18

gtcatgggcacgaatattgacggaaagaggaaagttatgttcgcaatgacagcaattaaaggcgtcggtcgacgttatgctaatatagttttaaagaagg

ctaatattcgtgcccatgacacgaagaatgtgttggaatttctctggtatcacgagagacattt

>51238|40s ribosomal protein s18

cgtcaatattcgtgcccatgacacgaagaatgtgttggaatttctctggtatcacgagagacatttggaaacgctatcctccacctaacgtggcctgcta

gaaaatcgattgatacgggaaaagaaacttacaggcaatcgtccactcattctgacgtccatggggc

>51241|40s ribosomal protein s18

tccgtcaatattcgtgcccatgacacgaagaatgtgttggaatttctctggtatcacgagagactgagaagaacaataaccatattaaaacactgtagaa

atacatatttcttccacaatctctagaaaacactactctaaaggcagatgggtaaatttgcaacagatt

>51246|40s ribosomal protein s18

tgcgaacataactttcctctttccgtcaatattcgtgcccatgacacgaagaatgtgttggaatttctctcgtgataccagagaaattccaacacattct

tcgtgtcttgggcacgaatattgacggaaagaggaaagttatgttcgcaatgacagcaattaaaggagtcggtcgacg

>51237|40s ribosomal protein s18

tgactattatgagcaatcctcgccagtataaaattccagactggtttcttaataggcagaaggacattgtcgatggaacatattctcagttaacctcaag

ttacctggacagcaagctccgagaagatcttgagaggctgaagaaaatcagggcccagtagtgcctcattccacgttgg

>51233|40s ribosomal protein s18

aaatgtatgttcttgcactgaccatctaggtgggaatatgacaaaatcagcaatcctcgccagtataaaattccagactggtttcttataggcagaagga

cattgtcgatggaaaatattctcagttatcctcaagttacctggacaccaagcgcccaaaagatcttgagaggctgaagaaaatcag

>51242|40s ribosomal protein s18

ctttccgtcaatattcgtgcccatgacacgaagaatgtgttggaatttctctggtatcacgagagacattttctagcaggccacgttaggtggaggatag

cgtttccaaaatgtctctcgtgatgccagagaaattccaacacattcttcgtgtcatgggcacgaatattgacggaaagaggaaagttatgttcgca

>51235|40s ribosomal protein s18

ataaaataccagactggtttcttaataggcagaaagacattttcaatggaaaatattctcagttaacctcaagttacctggacagcaagctccgagaaga

tcttgagaggctgaagaaaatcagggtccatcgtggaatgaggcactactggggcttgagagtgcctcattccacgatgggccctgattttcttcagcct

ctcaagatcttctcggagcttgctgtccaggtaacttgag

>51234|40s ribosomal protein s18

atgtgttggaatttctctggtatcacgagagacattttggaaacgctatcctccacctaacgtgtccttctttaaaactatattagcataacgtcgaccg

actcctttaattgctgtcattgcgaacataactttcctctttccgtcaatattcgtgcccatgacacgaagaatgtgttggaatttctctggtatcacga

gagacattttgtttgaaatgtgggaatttatatacttttattttatgtccctcctcattct

>51232|40s ribosomal protein s18

ctttgcgatttgatccgatcgtattgacatgctgcattactatcacattcacaactaacttctttggcgttcattttctagcaggccacgttaggtggag

gatagcgtttccaaaatgtctctcgcgataccagagaaattccaacacattcttcgtgtcataggcacgaatattgacggaaagaggaaagttatgttcg

caatgacagcaattaaaggagtcggtcgacgttatgctaatataggtttaaagaaggctaatattgacttggacaagagagccggtgaatgcactgaaga

ggaggtggagaagattgtgactattatgagcaatcctcgccagtatgaaattccagactggtttcttaataggcagaaggacattgtcgttggaaagtat

tctcagttaacctcaagttacctggacagcaagctccgagaagatcttgagaggcctaagtaaatcag

>51236|40s ribosomal protein s18

tagtgtaaatacaagaacaaatgtaccatcaaaatgctgaaagcatgtacattaaatgcatccacaccatggctcaatgtccttgcaatgctggtatgat

cactgttatatgatttaaagaatgagaaaacttaaatatttcagcatcaaatacacccacattatccaatatcttagctctgagtcaaccaaccacaaca

tgaacttgcattaaaatgattttcaaaataaacatttgcaacaaaaacctttaaccgagcaatatggaattataatcacaccaacatattttgaatttct

tgtttatttgaaaaagtcaatgtacagacaccatgaaagaatagtttacttcttcttagacacaccgactgtccttcctctgcggccagttgttttcgtg

tgctgtccgcgcactctcaagccccagtagtgcctcattccacgatgggccctgattttcttcagcctctcaagatcttctcggagcttgctgtccaggt

aacttgaggttaactgagaatattttccatcgacaatgtccttctgcctattaagaaaccagtctggaattttatactggcgaggattgctcataatagt

cacaatcttctcggagcttgctgtccaggtaacttgaggttaacttagaatattttccatcgacaatgtccttctgcctattaagaaaccagtctggaat

tttatactggcgaggattgctcataatagtcacaatcttctccacctcctcttcagtgcattcaccggctctcttgtccaagtcaatattagccttcttt

aaaactatattagcataacgtcgaccgactcctttaattgctgtcattgcgaacataactttcctctttccgtcaatattcgtgcccatgacacgaagaa

tgtgtttgaatttctctggtatcacgagagacattttggaaacgctatcctcttcttcttcattcgctcacctcgccttcttc

>51269|40s ribosomal protein s18-like

gtgactattatgagcaataatcgccagtataaaattccagcctggtttcttaatatgcagaaggccattgtcgatggaaaatattctcagtt

>51265|40s ribosomal protein s18-like

acagtctttggaaaggagtcggtcgacgttatgctaatatagttttaaacaaggctaatattgacttggacaagagagccggtgaatgcactgaagag

>51274|40s ribosomal protein s18-like

gccagtataaaattccagactggtttcttaataggcagaaggacatcatcaatggaaaatattctcagttaacctcaagttccctggccagcaagctccg

ag

>51267|40s ribosomal protein s18-like

aagttgagcgccttacagcaataatagctgctccccagaatttcaagatcccatcatggtttttgaacagacaacgagacttcaaagatggccaagataa

gcatgtagccgcaaatgcagttgattcatctctcagagatgatcttgagcgac

>51294|40s ribosomal protein s19a-like

aaatagatgtgcctagcgagagcagcacaacgtgtatagtcccaatcatcataatatggtgcaagttccttaaaccttgcacttttcaccaaatccaccc

attccacttagtagactatagaccttgaccattaacaggag

>51298|40s ribosomal protein s19a-like

ttccagtcttttttaagaacgccgaaaacgctttgacgaacttctgttggtcgacatccttcagcgtcgcggatggcatcttcgtatttcactttccaac

caactcacaacagggcaatcaaacggaagaaggacctggccacagattttaa

>51291|40s ribosomal protein s19a-like

tggagcaactgaagctgattgagcgaactggtgatggtggacgccgactaactgagcagggcagccgggatcttgatcgcattgctgctcaggttcgcca

ggggggccggtagtgttgcaagaaaagcattacagtccttggagcaacttaagctgattgagcgaactggagatggtggtagcccactaactgagca

>51296|40s ribosomal protein s19a-like

acttctgttggtcgacatccttcagcgtcacggatggcatcttcgtatttcactttccaaccaaatctacccattctggaaccttcatttttccagtctt

ttttaagaacgccgaaaacgctttgacgaacttctgttggtcgacatccttcagcgtcgcggatggcatcttcgtatttcactttccaaccaactcacaa

cagggcaatcaaacggaagaaggattaccttcctcgactggaacaacaatcaggacgacagagatttcctgatgacagtcagcaagtgcaac

>51290|40s ribosomal protein s19a-like

ttcgtcaaagcgttttcggcgttcttaaaaaagactggaaaaatgaaggttccagaatgggtagatttggtgaaaagtgcaaggattaaggaacttgcac

catatgatgatgattggtactatacacgttgtgctgctctcgctaggcacatctacttcaggtcgccagttggagttggtgcagtaacaaaaagatttgg

aggtcgccagagaaacggagtctgcccatcacatttttgccggggggccggtagtgttgcaagaaaagcattacagtccttggagcaactgaagctgatt

gagcgaactggtgatggtggacgccgactaactgagcagggcaggcgggatcttgatcgcattgctgctcaggttcgccagaaaaaaacactggaataaa

cgatccttttgtatgttgtatctaaaaataaaatctatattgtaaagaaaagcattacagtccttggagcaactgaagctgattgagcgaactggtgatg

gtggacgccgactaactgagcagg

>51292|40s ribosomal protein s19a-like

ccccggcaaaaatgtgatgggcagactccgtttctctggcgacctcttttcaatgaaggctgtggtatgagtgaaatttttgtgttattcgtatctttgc

tactgctgcaattctgatattttgtatgtgctgagttccaaggaatgaatatatttgttcctgcattgtacccaattaagagtgtgacaagtgtcccttc

ttgaaatcttgattgtggtgcaagacatctccaaatatttttgttactgcaccaactccaactggcgacctgaaatagatgtgcctagcgagagcagcac

aacgtgtatagtaccaatcatcatcatatggtgcaagttccttaaaccttgcacttttcaccaaatctacccattctggaaccttcatttttccagtctt

ttttaagaacgccgaaaacgctttgacgaacttctgttggtcgacatccttcagcgtcacggatggcatcttcgtatttcactttccaaccaacccacaa

catgcttcctatcacgtcaaaaatc

>51339|40s ribosomal protein s2-like

accctaggaaactttgccaaggcaacatatgctgctattccaaaaacatacgcaaacctgaccccacatttgaggaaagaggagcccctcctgaag

>51405|40s ribosomal protein s20

gaagggtcaaagacttgggtcagatttcaaatgaggcttcacaagagactaattgatctcttttgtccttctgaagttgtgaag

>51411|40s ribosomal protein s20

gattcacaagagagtaattgatctctatagtccttcgtaagttgttaagctgattacatcaaccagcatagcgcctggtgtggaag

>51412|40s ribosomal protein s20

tctctttagttcttcggaagttgtgaagcagaaaacatcaatcagtatagagcttggtgttgaagttgaagttactatagctgatgaagg

>51408|40s ribosomal protein s20

tgtggtgaagggtcaaagacttgggacagatttcaaatgaggattcacaagaaagtaattgatctctttagtccttcggatgatgtaaagcagattacat

caatcagca

>51407|40s ribosomal protein s20

gtggtgaagggtcaaagacttgggacagatttcaaatgaggattcagaagagagtaattgatctctttagtccttcggaagttgtgaagcagacaccaca

ctggacgcagcggtcgtaccacaccacactggatacagcagtcgtcccacg

>51403|40s ribosomal protein s20

tttttttttttttttttttttttttttttttttttttttttttttttttttttttttttttttttttttttttttactttcaaattctttattaaagttt

cacatttgaacaggtacaattaatcttttccttcatcagctatagtaacttcaacttccacaccaggctctatgctgattgctgtaatctgcttcacaac

ttccgaaggactaaatagatcaattactctcttgtgaatcctcatttgaaatctgtcccaagtctttgacccttcaccacagggggtcttacg

>51432|40s ribosomal protein s20-like

tgtggagggtcctgttcgaatgcacactaagatattgattattactactcgtaagaccccctgtggtgaagggtcaa

>51431|40s ribosomal protein s20-like

gcttctgcttcttagccccaaatattaggtcactgttcgaatgcccacagggggtcaaagacttgggacagatttcaaatgaggattcacaagagagtaa

ttgatctctttagtccttcggaagttgtgaagcagattacatcaatcagcttcacaacttccgaaggactaaagagatcaattactctcttgtgaatcct

catttgaa

>51429|40s ribosomal protein s20-like

tcaaagacgtgggacagatttcaaatgaggattcacaatagagtaattgatctctttagtccttcggaagttgtgaagcagattgtatcctttatcaagc

ttaattcatatatatgatataaggttgcatgttaagattgtgatttatcttgctatgatgtgatatcttaggacaccttatgagattcatgattacatta

gaacctctgagcaagatgtgacacttgaatattagtagttagtgatacaggcgtcataattgagcagaaataatcacttgttgatactacaacacgcaca

aacactctaagctttcaaagccactggccctctctggcataagttaaggggaaggaagagaggtgaaggaaaatgacaggagaggactattaaaagggat

ataattcagaaaagtcacccggagcccttggtcagaggagactattggatggcatgagaaggaaatgttgcttcctggtctacccttctcatcctgtctg

ttagcacacagggctcctgggtgccttgttttgaactgtggcactttttaaaaccttgagcccttttttcttcattcctgttacttacccttcatcttgt

ctgccaggagtcactcgctcaaagagcttgtaaaggttgcttttttgtgttctgctgctgcttgctgagtaggtttctcgagacttcataatgtatttat

actgcagatctgggttatatcttctgttcagcctttagattaccattagtggtaattagtgacatgcaacagcttttattatggcatttgtgattgtgaa

ttcacaaagttggaattgagtactatgtctgatgagaaatgttgtttaagctaaaaaaaaaaaaaaaattacaaggagtctataaatgactgaaaaactg

gtaggcctatatgagggaattgcacactttcttgtggaactgttggggagcttatagaaagagaatgtttgtactgcaccacatgttaactggtgaggga

agtgccctgtgtaactgcagtgtgggcatcagtctgatacttggctgtctcattgttcctaattgtgtggtaaaaagtgggtctgtgttcagttatggtt

gcagaactctccttaaaagtgatatcactattgtatgcagattgtatgttgattatataacatgcactgaggcaatctgaaggacattgtttgaattcag

ttcttgtggcggtgtatgaatgcagacagcaaacacttattgtgtgcatagactgactttcccagtgttagcactataagtaattgcaaattcgttgttc

atgttattacctttcaatctaacgtagatgttgggtgatgtcacagtgcagcatgttaaaacggagtttaaaagttgtggtgtaacaaaacagaattttc

atatctggacagttacctcggttaagcttaaaacatgaagcggtgtggaatgttggtggcaattcatatccacatcaataaattttagcctgtttcatcc

tgctaaaattttcagatctttcataaagttcagttcacagttgcattgcctcttaaaaacatgtggatttccagtttagtttaatcctgtccttcccacc

tttgatggcctggctagttcttaaaattttttcagattgtttgactggggaagctgtggtttgtggggggtaaccttttcatctgttttgtctgctgatt

gctgtgagacaatgcttctagattttgtatttaatttttatgtaatgtaataatttgtattataaattccttaatggttttgtagacatcaatcagcata

gagcctggtgtggaagttgaagttactatagctgatgaaggaaaagattaattgtac

>51457|40s ribosomal protein s21-like

gcgtgaatgatgcggttgctggcggaacatttgcgagggcgatacaagccgacaaaatctcccccctcgttttccacaactacggttagtttaatcac

>51455|40s ribosomal protein s21-like

tgcgtgaatgatgcggttgctggcggaacatttccgcgggcgatacaagtcgacaaacgctcctgcgtcgtggtccataactacggttagttgaatcagt

t

>51456|40s ribosomal protein s21-like

atacaagtcgacaaatgttccgccagcaaccgcatcattcacgcaaaggatcatgcggttgctggcggaacatttgcgagggcgatacaagtcgacaaat

tctcctgcgtcgctttccataactacggttagttgaatcagttatatatctgaaaagaacttccacaaa

>51454|40s ribosomal protein s21-like

agtttcgtttgcaattgtggtgatttgttggtggcttttggcatttagtttgaaaaatagtgatacggaaagttcttttcagatatataactgattcaac

taaccgtagttatggaaaacgaggcaggagagtttgtcgacttgtatcgccctcgcaaatgttccgccagcaaccgcatcattcacgcaaa

>51453|40s ribosomal protein s21-like

ggacacttcaaaaatgtacgcaatatgtggcgccatacggagaatgggggagtctgatgattgtatagacgcatgatcctttgcgtgaatgatgcggttg

ctggctgaacatttgcgaggtcgatactagtcgacaaattctcctgcgtcgttttccataactacggttagttgaatcagttatatatcttccacaaacc

aacagccaaagaacaacgtaatt

>51480|40s ribosomal protein s23-like

taacagcatttgttcctcgagatggttgcttgaactgctttgaggagaacgataaagtgttggtagctggttttggc

>51473|40s ribosomal protein s23-like

cgactttttccaagacaattcctttcgcgcgggacgcacccccaaatgggttggccttccctctagtagcaagatgagctttcttgtaatcgtta

>51475|40s ribosomal protein s23-like

agacgtgtgctcttccgatctccgactttttccaagacaattcctttcgcgtgggaagcaccaccaaatgggttggccttccatctagtaccaagatgag

c

>51482|40s ribosomal protein s23-like

cgaaaggaattgtctttgaaaaagtcgttgtggaatctaagcagcccagctctgccattcgaaattttgtaagagtacatcttattaagaatggaaagaa

gataacagcatttg

>51481|40s ribosomal protein s23-like

gaggtttacccattctctcaactagaacagacccacaactccggaggcaaggaactgaaagctcatcttggtactagatggaaggccaacccatttggtg

gtgcgtcccacgcaattcctttc

>51479|40s ribosomal protein s23-like

ttaataagttgtactcttacacgctttcgaatggcggagttgggctccttagcttccgcacccgctttttccaagacaattcctttcgcgtgggacgcac

cacctaatgggttggccttccatctagtaccaagatgagctttcttataat

>51478|40s ribosomal protein s23-like

tttccaagacaattcctttcgcgtgggacgcaccaccaaatgctgttatcttctttccattcttaataagttgtactcttacagactttcgaatggcaga

gttgggctgcttagcttccacaccaactctttccaacacaattcctttcgcgtg

>51613|40s ribosomal protein s25 isoform 2

tcaaaaaggtctgataaagcaggttataaagctccatgcccaggtcttgtacaaaaggacaaccaaaggagatgacccaccagcataaacctga

>51655|40s ribosomal protein s25-like

gtcaccgagcgattgaaactgaatggcagtctatctcgtagaattatcagagacatggaaaagaaaaaactcatacaacctgtctcaaaacaccacgcgc

aaatgatttacacacgtatcaaagtcgaaaaaacagaagctgttgcataaaaaatgtcaaccataatatacc

>51654|40s ribosomal protein s25-like

cttttaacctttcactaacaacagaaggtgtgatgagtttgtaagcatgtacttctttcaacagtttctcatatgttgccttatgctctctctgaaactc

tgtacaacctctggtttagtcagtttattcatgtcccttctccctctctcccccctttctctctctctccccccctctcc

>51653|40s ribosomal protein s25-like

gttagtgaaaggttaaaagtacgaggatctttgactaggcgctcgtttgaagacctgcgtcaaaaaggtctgataaagcaggttataaagcatgcccagg

tcatctacacaaggacaaccaaaggagatgaccctccagcataaacctgattcatttaagtgtacattcaagtgataataaaaactcatcacaccttctg

ttgttagtgaaaggttaaaagtacgaggatctttggc

>51676|40s ribosomal protein s26

gaacttttttatggctctgtctttcggtacacacggcgcgcagttagtgcagcgaacaggattcacgtggcctcgacc

>51675|40s ribosomal protein s26

acaatattacggatcgggaacttttttatggctttgtcttccggtacaccccgcgcccagttagtgcagcgaacaggat

>51682|40s ribosomal protein s26

ggggggatggcgttgtcgttcgggacacaccgcgcacagtgagggcagcgaacaggattcacagggcctcgaccatgttt

>51677|40s ribosomal protein s26

aatattaaggatcacgaacttttttatggctttgtcttccggttcactccgcgcacagttagtacagctaacaggattcccatggcctcgaccatgtttc

g

>51680|40s ribosomal protein s26

cttatatgaacagtggccaacacgattttttttttttgtgtgtgtgtgtgtgtgtgtggagtgtgccgaaagacaaagccataaaaaagttcgtgattcg

taatattgttgaagctgctgccgtaagagatatcacagaa

>51679|40s ribosomal protein s26

tttatggctttgtctttcggtacacaccgcgcacagttagtgcagcgaacaggattcacatggcctcgaccatgtttctatcttccactatttcttctct

tacacgtcattttcttcgacagcaaattttacacgctaacgaaaaagcactattcgac

>51707|40s ribosomal protein s26-like

caaaggttgtgcgcaatcgttccaaggcagagggcggcatacgaacaccaccaattaggacattcccaagggacatgcgac

>51705|40s ribosomal protein s26-like

tgcgcaatcgttcaaaggcagacaggcgcatccgaaccgcaccaattgggacattcccaagggacatgcgacaaggaggccacgtgga

>51709|40s ribosomal protein s26-like

gcagtcgttcaaaggcagaccggcgcatccgaacaccaccaatgaggacactcccaaggggcatgcgacaaggaggccaacaagtgaggaagg

>51719|40s ribosomal protein s26-like

atccgctcaaaggttgtgcgcaatcgttcaaaggcagacaggcgcaaccgaacacctccaattagaacattcccaagggacatgcgacaagaag

>51717|40s ribosomal protein s26-like

atcacgaacttttttatggctttgtctttcggtacacaccgcgcacagctactgcagcgaacaggattcacatggccccgcccatcgttcgtccttcc

>51708|40s ribosomal protein s26-like

agttcagacgtgtgctcttccgatctccctcacttgttggcctccttgtcgcatgtcccttgggaatgtcctaattggtggtgttcggatgtgcctgtct

gcctttgaacgattgcgcacaacctttgagtggatggcacag

>51711|40s ribosomal protein s26-like

agctgcactactgtgtttcctgtgccatccactcaaaggttgtgcgcaatcgttcaaaggcagacaggcgcatccgaacaccgccaattaggacattccc

aagggacatgcgacaaggaggccaacaagtgaggaggtgatgtgtatgaagttcaattaaaataaaatggccaacaagtgaggaag

>51762|40s ribosomal protein s28-like

cgaataatttggcggttttgttccccgataaattctacttccccttgcgtacactgcccttgcgagccagttcttcccaaa

>51761|40s ribosomal protein s28-like

cgttccgaataatttgtcggttttgttcgccgataaattctactttcccttgctttcactgcccttgcgagccagttcttcccaaaactttca

>51765|40s ribosomal protein s28-like

caccttcgcgaacggggcctttgacgtaccgaataatttctcggttttgttccccgataacttcttctttctcttgcgtaccctgcccttgcgagcc

>51763|40s ribosomal protein s28-like

gcgaacggggcctttgacgttccgaataatttgttggttttgtgcaccgataaattctacttatacttgcgtacacagcccttgcgagccagttcttc

>51764|40s ribosomal protein s28-like

agaagcaagaagattaagataactttttggctgccctgctctgaaatatctgcagtggccattcttctgttgaagttcaatgtgcagttacacaagtgaa

agtagaatttatcggtgaacaaaaccgacaaattattcggaacgtcaaaggccc

>51802|40s ribosomal protein s29-like

ttagcacatgctcggcatgacctcgatggtagcagccacaacgggaatgcagttagtcaagcttgttgaagccaatgtcatttgcatattccctgaagca

ctgccggcataaattgagcccgtacttcc

>51799|40s ribosomal protein s29-like

gtatgttggactggtaggtgaatactttgagcttgcagcaggtgagtattgatagtgtgataaacgaagatgggattccgaaacatttggtcttctcatc

caagaaagtatggccaaggatcgaggtcatgccgagcatgtgct

>51800|40s ribosomal protein s29-like

gaggtttagtaaaattttaataagaaagttgttggttctttttcaggctataggaacaggatgaaagtgaccttttgatacaaaagtattcatgaaaaca

tttttcatttaatactctgaaccacctgaaaatgtctcgatggtagcagccacaccgggaatgcagttagtcaagctttttgaagccaatgtcatttgca

tattccctgaagcactgccggcataaattgagcccgtacttccgtatcagaccatggccattagcacatgctcggcatgacctcgctccttggccatact

ttctcggatgagaataccaaatgtttcggattcccatcttcgtttatcacactatcaacacgtcgcacgctgcacaaaccacagaacacaacacagccca

cacgcaaaaag

>51789|40s ribosomal protein s29-like

aagagaattgcagctcacacatctgtctctgacaattcttttgtatttctattattgtgtcaatggaatttctcactatcttatcaaaatcccaatataa

caaatacatttacaaagacaaggtaggtacactgtaatactaagagttgacacctttagtctggatcaattccatgtctgatgtgggatttaaagcaata

aaacaacacatttagaggtttagtaaaattttaataagaaagttgttggttctttttcaggctataggaacaggatgaaagtgatcttttgatacaaaag

tattcatgaaaacatttttcatttaatactctgaaccacctgaaaatgtctcgatggtagcagccacaacgggaatgcagttagtcaagctttttgaagc

caatgtcatttgcatattccctgaagcactgccggcataaattgagcccgtacttccgtatcagaccatggccattagcacatgctcggcatgacctcga

tccttggccatactttctcggaagagaaagttggtgactgaaatttcgtaaaaaggtctcccgcgacgaaaaacgtctatgctgtaatgacttccatccc

aactcgtgta

>51878|40s ribosomal protein s3a-like

aaggcaatgcatgaaatcctgagatgataaccatctagagttttgacatcacaagtcgcttcaatcagcgaatgccatttgcgcaccagtgagcacaaat

>51876|40s ribosomal protein s3a-like

tgcaattcgtgcccgaatggtgaagataatccaagatgaagctaataagtgtttgttgaaagatttggtccggaagtttattcctaaagctgtagagaat

caaattaccaagtcttgccgacgaattttccctctagagcacgtatgtttacgcaaggtcaaagttgtcaagaagccaaaaagcgat

>51981|40s ribosomal protein s4-like

ccaccaagtttgtccaacatacatgccttgggcgcgtttaggcgcttgaaatgtttcttgggcccgcgagccattgct

>51984|40s ribosomal protein s4-like

gtgctgggccttggcgcatagaccacaccaagtttgtccaacacccaggccttcggcgcatttagacgcttccaatgt

>51980|40s ribosomal protein s4-like

ccaccaagtttgcccaacagccatgccttcggctcattcaggcccttcaaatgtttcttgggcccgcgagccattgctgc

>51978|40s ribosomal protein s4-like

tttgtcagcacatacttgagtctgtttccaaggaagattacccaaggccatgactctctaagcttgtgagggccagtgctgg

>52119|40s ribosomal protein s7

tcaaatcactattcattaccagttcgatgagtgcttgtgaaattgatttttctaaagcgtccggctctccgccgccgcttttaatgatcttggcacccg

>52121|40s ribosomal protein s7

tttttgccgttgagttcaacttctttcgcctttgtgatatacaattcgcgaagttgcgccttcaaatcacaattcatttccagttcgatgagtgcttgtg

aaattgaaggcgcaacttcgcgaattgtatatcacaaaggcgaaagaagttgaactcaacggcaaaaagtcaa

>52125|40s ribosomal protein s7

tttttgccgttgagttcaacttctttcgcctttgtgatatacaattcgcgaagttgcgccttcaaatcacaattcatttccagttcgatgagtgcttgtg

aaattgaagttttgaaagcgtccggctcaccgccgccgcttttaatgatcttggcacccgataacatttcaaggcaaaataat

>52126|40s ribosomal protein s7

ggaagtctttgcgatctcggacggcaagatgttatcgggtgccaagatcattaaaagcggcggcggtgtgccggacgctttcgaaacttcaatttcacaa

gcactcatcgaactggaaatgagtagtgatttgaaggcgcaacttcgcgaattgtatatcacaaaggcgaaagaagttgaactcaacggca

>52122|40s ribosomal protein s7

tattcatttccagttcgatgagtgcttgtgagattgaagttccgaaggcgtcgggctccccgccgccgcttttaatgatcttggcacccgataacatctt

gccgtccgagatcgcaaagacttccacctactacacaaagacataaagttcttccgaaaacagttgtctgtgtattgctgctctaaattttt

>52155|40s ribosomal protein s7-like

ttttgtacggctttttcgagttggcttaggcagaattctcctctgagcgataaagacgacatgcttcccactgaactttttctccaatt

>52192|40s ribosomal protein s7-like isoform 2

cggctccttggtcgtttttgcttattctttgtgagagttttacttgttggcttaggtaatattttcctttctccaat

>52196|40s ribosomal protein s7-like isoform 2

acaggaaagaatgtgctcttcattggagaaaagaaaattttgcctaggccaaccagaaaaactctcacaaaaaataagcaaaagcgaccaa

>52193|40s ribosomal protein s7-like isoform 2

cgcggctccttggtcgcttttgtttattctttgtgagagtttttctgggtggcttaggtaatatttttctttctccaatgaagaccacattctttcctgt

gacttgtttctccaactcccgctcgaggcgtgtctcaatcccagggaaagctttcagttgtggaattggt

>52219|40s ribosomal protein s8-like

tcttccttccaagtggcagagcataatggcattcgtcccactgtctaaatgctacagcatcaatctctacaatgg

>52221|40s ribosomal protein s8-like

tcttcatcctctggagtcaatttagctcccttctaccttccaaggggcagagcataatgggatttgtacccctgtccaaatggtacagcatca

>52216|40s ribosomal protein s8-like

gtgcggacagtgtggatacgccttggaccaagctttgtatttgctgcaggtctgcccaactcaaacttccgcttcatcttcacttgcttcctcttcctct

t

>52217|40s ribosomal protein s8-like

cgatatttcttgttgccgcccctggtgcggacagtgtggatacgcctgggaccaagttttgtatttgctgcaggcctgcccaactcaaacttcctctctg

cctgtcctagccaat

>52218|40s ribosomal protein s8-like

tgttgtgtataatgcttccaacaatgaattagtaagaacaaatacaaaacttggtccaaggcgtatccacactgtccgcaccatgggcggtaacaagaaa

tatcgtgctttgaggttggaccaggg

>52214|40s ribosomal protein s8-like

ttgcgaggtgaggcgcacctagagcgtacacgttgggacccgaaagatggtgaactttgcctggggatctgaaggttgtacgcgaaaaacgcgtatcctt

gatgttgtgtataatgcttccaacaatgaattagtaagaacaaaaacattggtgaagaatgccattgtagtgattgctgctgtaccatttagacagtggt

acgaatcccattatgctctgccacttggaaggacaaagaaagggaaaaggacaaggataaagtgagagagaaggaaaaagagcga

>52213|40s ribosomal protein s8-like

ctggtgcggacagtgtggatacgccttggaccaagttttgtatttgctgcaggtctgcccaagtcaaacttcctcttcttccggagcggctttcttttcc

ctggttggaagcattatacacaacatcaatgatacgcgtttttcgcgtacaaccttcagatccccaggcaaagttcccctggtccaacctccaaggcgta

tccacactgtccgcaccaggggcggtaacaagaaacaccgtgctttcaggttggaccagggggactctgcctggggatctgaaggttgtacgcgaaaaac

gcgtatcattgatgttgtgtataatgcttccaaccatgaattagtaagaccaaaaacatctgttaagaatgccattgtagtgattgatgctgtaccaatt

agacagcggtacaaaagacattatgctctgccactttgaaggaag

>Sg_CNS_NA4Plus27820|43kda collectin precursor

tattttgatcgatcggtagcagttgcgatgtatcgcccgcgctcggcagcagtggcgcggtgtggtggcgccactgcggcgcctcagaagagcggcacct

cgcagacgccgtagaggcggcgcgttttgcacgacagctcggcccagtgcccgtcgtcgcgcagcgcccagcagttcttgtccgtggcgttcctccagtg

ttgcggcgctggcttgaagaagcggcgttccgacacagaagtaccgtcactgtgcgtggggtctccctcgcggccgtagacgtcacgcaagccgatccag

gcgtactgtgcgcccgcctgccgcagcagcgacgcggcatattcgttgagcgccggcgacgtcagcgccgccaggcggccgccctgcaactcgcacagcc

ggtcggcgcccgcgtagtcggcctgctgtgacaccaccttcatgcaggagccggagttgggctcgtgggagaagccgtcaccgcgcacgtgcggcctggc

ggcgaagtggtggtagtcgtggtgctcgtaggcgaccttggtgggcgcgcagcgcgccagcaggcggcacttgtgggtggccgcgtggtagttggcggcg

tcgcacgagtgcggccgcagccggcacagctgcaggcagcgctccgcggagtcggcgcgcaggctcgtacccgggccggcgacgcgcgcgtgggccacca

gccgcggggtcgccactctagggtagacaggctctccgtcgtcgaggtcggccatgagctgctcgcggcgctcggcgaggctccacgccttgtcggcgga

cggcttgcgcagctgcggcggcgccgccgcgtccccgtcctccacggccaggtccggc

>52344|45 kda calcium-binding

tcatctagaactcatcatggaaagatcgagctgtatcgaccatcttgctgttgaggaagggtcctgcatgagccagtacttccattagactcacagcacc

atctgcatctgtgtcagcaagctgaagcaactgcgaagcttcctgacgtgcgtgtcgtggatttcgtgggtccatgtacatctgaaagtgaaagatatta

cgcacaactcaaagtcatgccactcaagttaactctttatcacccattgtgatgtgtgtgcccatgatatgctatcagggaactagtggacatcgctgca

ttaatattgctatgtatatactttccactggtaaaggaaaccttgctaaaagtttgcagaattcttacagttacagactgcgcctgcaaacagtgctatc

tttgagtttcattgttaaccctatgctctgtattgtctcatcaaattagttcatgcctggaaataggcaacccaatgaacagatttgcaaactgggctaa

tgtcacaccttcttggacaattcctgtcaatgatttgcacatgcttattaatgtttgatgcatcacaaatagtgcctgtattgagcac

>52365|5 -3 exoribonuclease 1 isoform 1

ctacaatggcgtgtgctcgtggtcgtggttctacccacaccactacgccccctacgtctcggacatccgtgacttctcgtcgggcctgcggctcgagtat

gacatggggaaacccttccttcccttccagcagctgctcgctgttctgcccacactcagcaagaaccttctgcctgagccttaccagcatctcatggaag

acaaagactctccactcatctcgttctacccgacagacttcaagacagacctaaatggaaaaaagcagcagtgggaagctgtggtcctgataccgttcat

aaatgaggaggaattgctgaaagcgatggctccctacgagtctcagctgacagaggaggagcggaagaggaacactcctggcccgatgctagtgtacaag

tacacagacgacgatcttggtgtatacgaagcaccagaatatttcccaccactggagacaaaccacgcaaaagttatcgaagtgtggcgagatgagctga

atgtggagaagaaagacctcatcaagggactctggcctggtgctaagctggacgtttactaccctggctttcctaccatgaaacacatcccacacaaggc

aaaattggagaagcgtgaggtccatgtatttgaccagccctctagaggtgaaaatatgattctatccatcgagaaccaaggcaaaccagatcccgaggaa

gttgcgacgcacctcctcggcaagaacatctttgtggcgtggcctcatttggtggaagccagggtgtacgctgtttcgaaccacgagttcaacctgtcgt

tggctgcatcaggccagcttaacaaggagagacagagtgccgaaggcagggatgcgtggtcgcgccaggtgcaggccatcaagagacagtacatgaccaa

gaggggcgtggaaatcggctctacgtcagttttggttcacgcagcgactctttgtggcataaaacacatcatcagtaacagcgaggtggctcttcacaaa

cagtggagctccacacctgtagcatatgctctccagacgacggtcaaggacatcaagacaaaagacaaggttgccgatggcccgaagtaccgcaccgttg

atgatctcttcccgcgtggttgcgtgtgctttgtgatggctccaccgcactatggtgcgatgggagaggtgaaggagagtataatggacaacaggaccct

gcgcctgaagattactgtgtcggtgccggtagagcccgacctctcgcagctgttcagaacgtacaagaacatacaggccaactatatgactgtgggacag

gcagctcagaggctgagcatctcaccacacctgttgtcacgcatcacaggcaccgtctacgtcatcaagcaggctggggcgtcctcgtccgccaagctga

acctcggcctcaatctcaagttcaacaagaggaacgaagaggttcccggctacacacggaaagaaggtacgacctggttgtacagtcaacgtgcagtgga

tgttgtggcagcgtatgtgagggagtttccggatttgtttcagtacctcacgtcaaatgttggcaatgactgctttgtcgctagtgatgtctttccgggg

aaagactggaactatgtttcacaaataaatacgtggatccgcaaccagcactgttcgtctgttgaacggaggccctgtggatcacagtatttggaaccgg

aagttggtgaaatgctcatggagatcgtggatgcccaccaggcaaagaagttggcaagcaagaatgttggcatgcatgtgaaaccgaggcttctgtacag

gcacgaacttcaggacgggaacctcgcgcctgatacgacggccacgtacagtctactggaccgagtcataaatgtgcgggagggctacacggtgcccctc

ggcctcagggggaccatcataggtatacggcagatgggagacaaggaggcagatgtgatgcacgacgtgatgttcgacgaggcgttcccaggtggactgt

cgctgctgtgttcggagaagcgcagctactcgctgccggcagcggccctcgtcaacctgagtcacggggagcgccagcagcgccgtcgccagctgcagcc

cctcagcaagcccaccgccgtcgtgcaaccctccggaggaccaaacctgccagtggcaccacccatacacacgttcacacccccctggaagaagagtgca

gcccccgtgtcagtgtctcccctacggcagcagccgaaccagcagcagggttcggcgtttgtcagctggacggccggcaggggcgtcacatccccgcaac

gcccggcacacagccaagcccaggtccagcctccgcaccagttcccgcctgcggtggcgttcgtgcagggacctcctctggtgagtgctgcaagcaaaaa

cccaccccaagcagctgttctccaggagaatacggcaggtgccgagttccgtgctctgtggaacaacttgcagaaactaccagccaacaacagtatggtg

cctaagaaaccggatgggaaactggtgcctaaatctgcagaggtggaggaccaggggacagagctgttgaagaaagtgctcagaatcgtgccagatagcc

ctgacggtgcggcagccaaagagaaacgcaagacgggtgctgcatctgtggctgctgagacacagacttcgccagccaagagcatccagaaaccttcaac

agagagcagctgcatgaaacttctgcacatgttccaggccagaggacttggcatcccccgctatgcatacattccccaggaagacgggacagtgcaggct

aacgttcagatgccggacaacactacctaccacggcgagtgcgctgctaacagggaacaggcatcggagagtgcagcccgagtggcacttgccgagttgg

aagctgctggcggtgctctgcagtttggagcatctggccagagctggatgcagttgcaccaaaaacagcagccgtttggcagtgggagtggagtgcacca

cggcaaaggcgcaggggtaggtgcactgccggcaccgccccagcagtggttccagaatggcagccccagtgcccggttcaatcagcagccgcacggacag

tggaggtcacacacagcagctgacacgtcagaccggagtcgtctgagggaccatcaggggcaccctccgaaaggatctcggggcagcccggggtggcagc

accagcatcaacatcagcagcagcatcaacagcagcacctgcaacatcatggacgtcatggccagggattccaaccaccatttgggcagggttcacaaca

gcagcagtacaaccgccgctccggtctggctgaggccggacacgcccagtacggcccgggtgctgccggaggcctcccgccgcgcctgcaaaacctcaat

caccaccagccacaccagcagctcttccagaacgtggcccctccgccaggggcactgccccctcgttttctcgggcagcaggcgcagccattcgtgccgc

tgcaggcggccgtcaagtcccgcgccgtcaccggtcgcggttcgcagggcgaggagggacgtcacagagggcaggagagtgagcgggtgcagaactcgac

aacttccgtcgagcagtcacctgtgaataagtcgagtgagggcaatgtgagaactcccgacaaagttgtctccactgccagcaagggctctggtgggccg

gcagcacgcaagacccgcaaagtgcgtatagcggcacgcttcacttacggcctggacgttgatgatgagaagcagccagctctgccaaacgaggaagaga

aaaaggaggagacgaagcaggcagcgggcagcgactccggggctgttgagggccccaccaccgagcagaactagaggcattttaatagtggtcggcattg

gtcgtaggctgtgaactgttactgtaactacgcttcaaagtgaaagaaaaaaaaa

>52364|5 -3 exoribonuclease 1 isoform 1

gaaatacagcatttggaaactgtgtgtcgtcacaaatgcagagagagatgtaaagctgcagaatgcagcttacatcagcagttttcatattaaccaaatc

gttttttatgggcctattagaaaatacactgatcaaagctacaaccataaacacaaacatgcaaactaaaaatcagcaattcaaaagtcattgcacatac

aacatccaatctgataatttgatcagtcatcagatttgccaagagtacattaaaatgcaatcaactgcagtttttcagtacaaattcaaaaagaactttt

ttttttctcatgcatatgacctcactacatataaatggtaggtttatgatagttcacgaagcgtttttttttctttcactttgaagcgtagttacagtaa

cagttcacagcctacgaccaatgccgaccactattaaaatgcctctagttctgctcggtggtggggccctcaacagcccctgagtcgctgcccgctgcct

gcttcgtcgtctcctttttctcttcctcgtttggcagagctggctgcttctcatcatcaacgtccaggccgtaagtgaagcgtgccgctatacgcacttt

gcgggtcttgcgtgctgccggcccaccagagcccttgctggcagtggagacaactttgtcgggagttctcacattgccctcactcgacttattcacaggt

gactgctcgacggaagttgtcgagttctgcacccgctcactctcctgccctctgtgacgtccctcctcgccctgcgaaccgcgaccggtgacggcgcggg

acttgacggccgcctgcagcggcacgaatggctgcgcctgctgcccgagaaaacgagggggcagtgcccctggcggaggggccacgttctggaagagctg

ctggtgtggctggtggtgattgaggttttgcaggcgcggcgggaggcctccggcagcacccgggccgtactgggcgtgtccggcctcagccagaccggag

cggcggttgtactgctgctgttgtgaaccctgcccaaatggtggttggaatccctggccatgacgtccatgatgttgcaggtgctgctgttgatgctgct

gctgatgttgatgctggtgctgccaccccgggctgccccgagatcctttcggagggtgcccctgatggtccctcagacgactccggtctgacgtgtcagc

tgctgtgtgtgacctccactgtccgtgcggctgctgattgaaccgggcactggggctgccattctggaaccactgctggggcggtgccggcagtgcacct

acccctgcgcctttgccgtggtgcactccactcccactgccaaactgctgctgtttttggtgcaactgcatccagctctggccagatgctccaaactgca

gagcaccgccagcagcttccaactcggcaagtgccactcgggctgcactctccgatgcctgttccctgttagcagcgcactcgccttggtaggtagtgtt

gtccggcatctgaacgttagcctgcactgtcccgtcttcctggggaatgtatgcatagcgggggatgccaagtcctctggcctggaacatgtgcagaagt

ttcatgcagctgctctctgttgaaggtttctggatgctcttggctggcgaagtctgtgtctcagcagccacagatgcagcacccgtcttgcgtttctctt

tggctgccgcaccgtcagggctatctggcacgattctgagcactttcttcaacagctctgtcccctggtcctccacctctgcagatttaggcaccagttt

cccatccggtttcttaggcaccatactgttgttggctggtagtttctgcaagttgttccacagagcacggaactcggcacctgccgtattctcctggaga

acagctgcttggggtgggtttttgcttgcagcactcaccagaggaggtccctgcacgaacgccaccgcaggcgggaactggtgcggaggctggacctggg

cttggctgtgtgccgggcgttgcggggatgtgacgcccctgccggccgtccagctgacaaacgccgaaccctgctgctggttcggctgctgccgtagggg

agacactgacacgggggctgcactcttcttccaggggggtgtgaacgtgtgtatgggtggtgccactggcaggtttggtcctccggagggttgcacgacg

gcggtgggcttgctgaggggctgcagctggcgacggcgctgctggcgctccccgtgactcaggttgacgagggccgctgccggcagcgagtagctgcgct

tctccgaacacagcagcgacagtccacctgggaacgcctcgtcgaacatcacgtcgtgcatcacatctgcctccttgtctcccatctgccgtatacctat

gatggtccccctgaggccgaggggcaccgtgtagccctcccgcacatttatgactcggtccagtagactgtacgtggccgtcgtatcaggcgcgaggttc

ccgtcctgaagttcgtgcctgtacagaagcctcggtttcacatgcatgccaacattcttgcttgccaacttctttgcctggtgggcatccacgatctcca

tgagcatttcaccaacttccggttccaaatactgtgatccacagggcctccgttcaacagacgaacagtgctggttgcggatccacgtatttatttgtga

aacatagttccagtctttccccggaaagacatcactagcgacaaagcagtcattgccaacatttgacgtgaggtactgaaacaaatccggaaactccctc

acatacgctgccacaacatccactgcacgttgactgtacaaccaggtcgtaccttctttccgtgtgtagccgggaacctcttcgttcctcttgttgaact

tgagattgaggccgaggttcagcttggcggacgaggacgccccagcctgcttgatgacgtagacggtgcctgtgatgcgtgacaacaggtgtggtgagat

gctcagcctctgagctgcctgtcccacagtcatatagttggcctgtatgttcttgtacgttctgaacagctgcgagaggtcgggctctaccggcaccgac

acagtaatcttcaggcgcagggtcctgttgtccattatactctccttcacctctcccatcgcaccatagtgcggtggagccatcacaaagcacacgcaac

cacgcgggaagagatcatcaacggtgcggtacttcgggccatcggcaaccttgtcttttgtcttgatgtccttgaccgtcgtctggagagcatatgctac

aggtgtggagctccactgtttgtgaagagccacctcgctgttactgatgatgtgttttatgccacaaagagtcgctgcgtgaaccaaaactgacgtagag

ccgatttccacgcccctcttggtcatgtactgtctcttgatggcctgcacctggcgcgaccacgcatccctgccttcggcactctgtctctccttgttaa

gctggcctgatgcagccaacgacaggttgaactcgtggttcgaaacagcgtacaccctggcttccaccaaatgaggccacgccacaaagatgttcttgcc

gaggaggtgcgtcgcaacttcctcgggatctggtttgccttggttctcgatggatagaatcatattttcacctctagagggctggtcaaatacatggacc

tcacgcttctccaattttgccttgtgtgggatgtgtttcatggtaggaaagccagggtagtaaacgtccagcttagcaccaggccagagtcccttgatga

ggtctttcttctccacattcagctcatctcgccacacttcgataacttttgcgtggtttgtctccagtggtgggaaatattctggtgcttcgtatacacc

aagatcgtcgtctgtgtacttgtacactagcatcgggccaggagtgttcctcttccgctcctcctctgtcagctgagactcgtagggagccatcgctttc

agcaattcctcctcatttatgaacggtatcaggaccacagcttcccactgctgcttttttccatttaggtctgtcttgaagtctgtcgggtagaacgaga

tgagtggagagtctttgtcttccatgagatgctggtaaggctcaggcagaaggttcttgctgagtgtgggcagaacagcgagcagctgctggaagggaag

gaagggtttccccatgtcatactcgagccgcaggcccgacgagaagtcacggatgtccgagacgtagggggcgtagtggtgtgggtagaaccacgaccac

gagcacacgccattgtagtagtagtgcaggttccactggatggcacgcacgtagccctccgcctgcgaccgcatcacctccggtgtgactgcctcgtatt

ccagtttgttaatatagtagtccctcttgtggaggtcgaactcctgcagaaatatcttctcctcttcatcgctccctgcagttgacaattcctcatcctc

caagcagaactctggctcctcactgtccgagagtccatctgcgagcatgtcttcagtgcggctgatcaggtcactgaggtcaggatccaacgctttccta

gggctgtcatccactgcatcttcagtttgatctggcctgtaacttttcctttcatttccaggccgcctaccagtcttcatctcaaattcccgcaagtcag

cgtatgtatcccgaaactggtcgacgtcaaatgtcgctaacctctgcatgaactgctcaaatctctttaggttcaggtcacctgcttcattaatatatcc

tccgagctgtggcagcacatcgatgtaagccttgtacagcattggcaatgctccgctggtgatgtgcatgtctggaagcggagggatgaag

>52390|5 -3 exoribonuclease 1-like

ccgatctttaagcagggaaatagaaaagagtgcaaaaactatcaggggataacactgatgagtcattgtgcaaagatttttgagaaaattttggaagcac

ggattcgggcaaaattagaaggaagaatgagagaagagcaacatggcttcagaacaggaaggtcca

>52386|5 -3 exoribonuclease 1-like

cagccaaccagcatatattccgaaaacatcgatcatgtgaaacccaacacacagttttctcgcatgacatactgaaagctttggatcaaggcagtcaggt

agacatattacttaccgatttctgaaaagcatttgactcagtgccacccctacacttactgttaaaagttcgat

>52392|5 -3 exoribonuclease 1-like

gagcctctatctaatattttctgggtacaagtaaagagagttgggtctcacacgatcgctgtttccggaatccatgttgattcctacagagtatggtttc

cagaaatgacatgatacgcgagcagaaaaaatgttctaaaattctacaacagatagatgtcagagatataggcctatagttt

>52403|5 -3 exoribonuclease 1-like isoform 2

ggtaaggctcaggcagaaggttcttgctgagtgtgggcagaacagcgagcagctgctggaagggaaggaagggtttccccatgtcatactcgagccgcag

gcccgacgagaagtcacggatgtccgagacgtagggggcgtagtggtgtgggtagaaccacgaccacgagcacacgccattgtagtagtagtgcaggttc

cactggatggcacgcacgtagccctccgcctgcgaccgcatcacctccggtgtgactgcctcgtattccagtttgttaatatagtagtccctcttgtgga

ggtcgaactcctgcagaaatatcttctcctcttcatcgctccctgcagttgacaattcctcatcctccaagcagaactctggctcctcactgtccgagag

tccatctgcgagcatgtcttcagtgcggctgatcaggtcactgaggtcaggatccaacgctttcctagggctgtcatccactgcatcttcagtttgatct

ggcctgtaacttttcctttcatttccaggccgcctaccagtcttcatctcaaattcccgcaagtcagcgtatgtatcccgaaactggtcgacgtcaaatg

tcgctaacctctgcatgaactgctcaaatctctttaggttcaggtcacctgcttcattaatatatcctccgagctgtggcagcacatcgatgtaagcctt

gtacagcattggcaatgctccgctggtgatatgcatgtctggaagcggagggatgaagtcgttgccaactaggaatcccatcagaacccaatcatcaatt

attttctccaggtcgaatgggaatggcagcttctccttaagttccgagaattcgtgctccagatattctcgcataagcgaaaggtgaagcaagtagaaag

tcgtctcttcgggtgccacaatcctcttctgttggttccttcgcccaaatttcacctcttcgcggaggagggagaagtgtggctcgtggctgcagagccc

cagcatgatgaggtcagcgtccagcccgtacaggcagtgccgcgtgttggggtcgtggtccggccgtgaccgcgcgtacctgatgtagtccattatcttg

tgctccccttcacctggggtctcgtgaccagacaatatgacagtgacgttctgccacagcctgtcagtggacaccttgtaggtaacaaagtacttgagtt

gctggtgtaggcgatccatgaagcacgtaccaggggtgatgcagttcgagtcaaaccttgcctccgaaggcagctgctcacccctctcacgggcattgcg

ctccagaatttctgcctctttggctgaacgaaatcttcgtccacgctgctggttcatctttgctctaggggctacaccatccacagctaggaagaatagt

ttctttggtttgatcatacgaaacaatacctcgatataatggaatatatctttaaatattttctcttctgtaatccgaaaatgtggatcaaagtcatttg

ggtgtgagcatgtgtgaatgattccattcatgtcaaggtataagttgtcaaaatctggtatctggtattccttcacaatttcgcttagacaagggtatct

ttcactaatccatctaaaaaacttagggacacccatttttttctactaaaatttaaaacgctacaccaccacaatatcagctgcttatacaactgttt

>52417|5 -amp-activated protein kinase catalytic subunit alpha-2 isoform 2

cgaagaaagcatggctgaagcacaaggtcacgtgactccagtccgacaattaatgtagcatcgctgtcgaatgatcatttatttacatcaatggtgcagt

tgtatttgtaaactgatgttaaaatcatgggagagaaaggacaacaatcaacccagcagcctatcgttaagataggacattacactttaggacagacatt

aggagttgggaccttcggtaaagtgaaaattggtgaacatcagcttacaaatcacaaagttgctgttaaaatattaaacaggcaaaaaatcaaaagtctt

gatgttgttggaaagattagaagagaaatacaaaatttaaaactctttcgtcatcctcatataataaaactgtatcaagtgataagcacaccaacagata

tttttatgataatggaatatgtttctggaggtgaactttttgactacattgttaaaaatggaaagctcaaggagtatgaagcacgccgtttcttccaaca

gataatatctggtgttgactactgtcacagacatatgattgttcatcgagatttgaaaccagagaatctactccttgatcacaacttgcatgtgaaaatt

gctgactttggcctgtcaaacatgatgatggacggagaattcctgaggacaagctgcggttctccaaactatgctgctccagaagtcatatctgggaagt

tgtatgctggccctgaagttgatatttggtcctgcggtgtgattctttatgcgttattgtgtggtaccctgccctttgatgatgaacatgttccaacact

gttccggaaaataaaatcgggggtattccctatacctgaatacttaaataagcaggttgttagtctgctttgccatatgcttcaggtggaccctatgaag

agagctacaattgaagacataaagaaacatgagtggttccagaaagagttaccagcatacttatttccatctccagtagaacaagatgcatctgttattg

atactgatgcagttaatgaagtttgtgagaaatttggtgtgaaggaacatgaagttcacaatgccttgctgagtggtaatcctcatgatcagcttgctat

tgcgtaccatttaataattgacaataaaagaattgccgatgaagctgcaaaggcagagctcaaagatttttatgttgcaagcagtcctccacctgtttca

tttagcccaaatgagctgaatcctagtcctattcgaccacatcctgaaagaatagcgcctgccattcgtgataaagctattgctacaagcaatagtgggg

caactgacagacgctctagaggaactccagtcaaaagagctaagtggcatttgggtattagatcacagagcaaacccaatgacattatgaatgaagtcta

cagagcaatgaaagccctaaactatgattggaaagttattaatccttaccacgtacgtgttcgacacaagaatgaactaactggaaaatatgttaagatg

tcactgcaactatatcaagttgattataaaagttatcttttggacttcaaaagccttagcagtgaagctagtgaagatagtgaaagcatgatcttatcat

caggagaagaaccaaaaccagctgtaactgcacctgtagagtcaagtgaacagcagcagaagcaaactggtcatcatacaatggagttttttgagatgtg

tgcagccttgatcactcagcttgctcgctgacatctgtcgtaatagtcactgatccatacagtggctatatattcagttgcagaagatatgatactaaag

tggtttgccactgttcttctatggctgtatatgaggagaccaattgtccaaagatcaagcgacaagtttagttgtaatgttgtgatttcagcgtaaaaca

ttcaaagaaatttaaaagttaccttcaaagtcacatatataggagccttttttgttagtattatagtaattgaaaggttgttatttatagctattgttta

ggattttaagtgcagaaacggatgactggtatggtattgccagtatttttgtgctcataccctaagctacaggcattaatctaaacagttgactggtaga

gaaatgcagcattttacaaatatgctcttgtacaagaaactgttgaagaaaactaagtgcaacttttaagaagcgacaagaaatacattttgccctatta

aggttatatatgaccacttaatgtgtaatgttagcacttccagtttatctgagttcacgaacaattctctgttataaaaagttcattttattcttacgta

ccaccttctgtttcacatgggtgccatgcattctgatacagattgtttgctccatgtgaagtctctgtcattgcacagctggtattagaacacctttggt

aatcttttgacagttgtagtgcccggaccaacctcaccatttgtcagtgtctctgaattgaatcttgcgagagggtaaatggtgagggatggatagaggc

acaaaggcacattggaatgaataaaagcaaaagcttggatcatcctcgctataacattcacaagaagaatttttccatttgtatctttctgcaacacaat

ttcagctaactgttcatccacacattctgatttcatcagttgtatgttttcatcccactacagggtgtaggagcacttgtaagaagaaggtggaaacctt

tcataaattgatagattggatccttccaacatgtctgtcatcaattaaacatggacaaagtgaaactaatgtggcttgtaagttaccttgtcagtaacag

agacatcttgcccacacttctgaaaccagctctatcctcagaatctgatgagccacaaaagagaacacacaatggttgtccttttactgccaagtgcttg

aacaggttgcacaatttttcatagccttctagcctaatgtgtaaccatgaactagccaaaattctcagcgtgaagattaggtgactgtcttaatgtcatt

gtcgctgtctaagctgttatagtgaacatgtagtctaaccctttcttatagaattgtgaaagattgcagcaatttgttcaaatgtagattcacatgaata

ttatattgtgtcatgttcagagagcaagctaaaaaccttttcctcccttttaacattgtaagctgttgatagatatctccttacatcttggtgcgaaagt

agtacctctagtttccaacagatagcagaaaaatgtggtcttatgtagcactctacagctagcaaagctcaggttaccatcgttctttatgttaccctat

agagtgagtattgacttatctcctggcattaaggattaaataataaagggtgtttcaaaattttttattcgatttcacaagactatcttgtatgtgaatg

aagatacgaatttatactcgtgtattagattatgacgtttttgttttcagaagaaccctctgattttaaaaggggatatcttttacatgcatacaacctt

gttgtactttttacaactatacttaagatcaaaggtttcatttaccttcaacaaatgttcagtgtcgcttccactggacacatgacaaacaaccagatga

tagtcaaagtcatctcacacttttacaagcactcactactgtttctatgccctgttacagttcaccaaaagttgttggaaggggaggcacatagacagag

ttccatagtttaaaaaataaaaataaaataaactcactagttatactcagcaagcctggagaccaatggtgcaatgtacaccccatgcggtagatgcact

gtcacagtagtcatgaaaatgctgctgcacaggtgtaagagaaatgcacctgttgaaaaaagagaatgcaaaatgcccttttgtcaccacattattacca

ttttgacttgacgcaccttgggtaatgtccaaccaagcaaacaagctagctgcagcagctagaccactaccaacagctgcatgaactgacagcttacagc

tggagccaagataaatttgatacttaaattaaaatttacttgagtttatatcttcgttcatgtagaatatatagtcttgtgaaattggatgaatcttatg

gaaacatgctgtatattgactatagaacaatattatttatgttgtggtcgtcaggagtcagaaagccattaacattaattgtttttaaatctttcagttt

taatgtttcagtcaatgggtgaaactgtacaacaagaaaagtggaaaaactttggaagcctgaataaaagttcccttcatccaccacatatttaattcat

actaaagaagaaaaaaattaagttttcttttgaaactttttggaattttttaacatcttgaagaatttaaagtctggttcttcatcaaatcagtgtccgt

ttttcttctctaagtatcaaaaatgtagattcaatttcatgttgtacttaagcatatacaaaactaccttcatagattccaagcaaaacttatgaggtag

gggttccatttttgtcagttccattattttgtaagtactggtgatgtaatgcttttatgtgaaatgttcttctctcatttggtgtaccaactgctttgtt

taatttgcctcagtttgcattcaggataagtgattctcctttaatgctgccctcgcttcatcaccatgttgtgtaaatctgttactgttcattttgatga

tttggattaaattctacttttcactcaatttagtagtcatgtcacatactactattcttttttcccatccttataggacatcattgctttcggctctatt

tggtatgtggggaccacatcatatattgatgagacacagtgttaatcatctttcttctgtctattgtacacttattatcactttgttcctatatgctgca

agtgttccactgctttaagctgcccagtctcttcttacatgcctgcttccactgataatctttctgcctcctcctttctgtacatgttgtggaagccaga

agtgtgtatgtgtacagtccttatcagatgtgctttggcttatgaagatggttcaccagtatatttccttctgatgttattttgactgagttttataatg

gtgctgcttctaaaaacctcagaactttttgctccagacattagtcattgcaagatgatactcgataagtcatcactggtgttactgtgtgacatgtcaa

gtttactttctgtagctgccaacaaatataatgtgataaaattgtccctaagaccgtaaacgtatcgaaactgtacatagcacctgtgcaatgtgagtgt

ataatttgtcatgtgataaacattatttttaccaatgatgataattttaaaaggtttcttctgtgcagtcactgcatgtcttatgaagacaatgtgtgtg

atagctcttcaaatgcctaaaaagtgacagaaactttcaggtctgtattttctgcatagtaataatgataaaactaaactgataacatgttatattattt

attgtataattagttaattcttataacagatacttactttttttcatcatctacaaaaaagtattataatgtgcaccaaaagaaagcaatagaaagtgtg

tgtgtaaaagagagagagagagagagagagagagagagagagagagagagagagagagaatttttaatcagtgaatctgaactgttcatgatgtgaaact

aaactgtaaataattattatgaaacagtgtgatgaacaatggacatattttatacctttgatgagttattaattactcaagaggacttggaatgttcaac

tatgttagtttataatagtaaaactactgaagaaataaccatttgtcagaaagaatgctaacaaggaagaaaattggttgtgggggagttatggaggtga

ggaatactgaaaagggg

>52435|5 -amp-activated protein kinase subunit beta-1 isoform 1

agcttttgtggtatttaaattctctatctttaaacaaataaatcagaaaaattaaaaagtaatcataggtcagaagatggcagatctttcgcatactcag

ctttacaaggctacgatttcaagtcagctgtttgatacgcctgtgtgtttgttacctgctgtaagtagtgtacggcgtaatgcgcgcaagtggttctagc

gttaaaatctggccgtgagccgtattaatgggtgcttcttaagtgttgacaatgacataaaatattttgacgtttcatctgtgaaaacgtcgaagacaat

cagaaggtcttgacatcttcgaaaggacataaacagagacatctctttacaacatgggaaatgctggaagcagtgcgagggagaggcacaagggaggaga

gcttgcccctcctccttctcctggaaaggatagtcaagcatttgtctttgacaagaaacctaacacaaaacttgtcttccagtcctccaatgaagaagaa

gaaccctattatactaaggaaaagccaccatcagagcaggaagaacttggagctccacggcaacgatctgcaacagtttctgaaggcagcaccaaggttg

atgacaaaacattgccaacagtattcaagtgggaaggtggtggaaagcaggtttttatcagtggtactttctctaattggaaggctcttcccatggtcaa

aagccatggtgattttgttacaataattgatctccctgaaggagaacatcagtacaagttcctcgtggatggagaatggaaacatgatcctggagtgcgt

attatagataacactatgggatccaagaacaatttggtgactgtcaagaaatcagattttgaagtctttcaagctttggcaatggacagtgaaagtgttg

gtcaccatcaacaaagcgagtactcgcaggaaattccagcaaacaagccatgggaaaaagtatcagggccaccaatattgcctccacacttgcttcaggt

gatactgaataaggacacgccactctcgtgtgagccaacattgcttcccgaaccaaatcatgtaatgctcaaccacctgtacgctttgtcaatcaaagac

ggtgttatggtgctgagtgcaacacaccgatatcgcaagaagtatgtcacaactttgctgtacaagcctatatagcctacgcatcgaccacacactggtc

aagttcgtccacgactggatcgtgcagatactcggtgaatcaccgcatgatgcataataaaagtatgaaatactaaagtgtattgtacaaaacaaaaagt

ggccacctgtacccacatgaattacttaatggtttatgtgacagatgtattaaatttttattttagtcagattgacaaatgtttgggaagttagcccaac

tacgttgtgatgaattgcagttttgaaatgttacccaattgtgcctgttttaagaagaagttacctcttattttctgactgataatataatggtatcaga

aaatatttaagaaatgacacataaatttacatattattgccttcttggcttatagcattattgacaggctccaactttgtcagacgatagatgttgtttt

tcttattgataactatgacctcatttgtttcataatcttaaaataaaacataaattgtaaactaacaacactgtcatatgccttattgtaacttacacta

ttatcagacagtataagtcacctaaactgcagaagtatcctagttatttttttagagatagcaagatatagagattttgttgcatagtatatgtataaat

aagatgtattatagtccattgttaaagtgccgtacaagttccagaagttactagaaacatcaatagctcactgaacagactgtagaatggaggcaaacaa

taaacttaagaatgaatgttatgctatttttgacagagtgtaagttgaaaccacaataagtgtaaatataagaaaaactagaaaaataatcgggtgtgta

aaaagattatatataaacatttattgttactgactgttgtgtgtatttttgtttcttggaaaaatgaagcctggaataacatcatgacaacaatattttg

caacattaagtctatttgagaaaatgggatgtccaaatagaatctagtttatttggtaagatagtgtcgatacttaaatcacgaaaataagacaatgttt

ctaataagcctccaggtgccaactgatggtgattttgtactggagaagtatgttgttggcctcatctcacagctgtaatctaatcagtaattgaacaaag

cacttaaatatttgaagtcttctagaattttgtaacaaatgacacacacacacacacacacacacacagagagagagagagagagagagagagagagaga

gag

>52448|5 -amp-activated protein kinase subunit beta-2

caaagcttgaaagacttcaaaatctgatttcttgacagtcaccaaattgttcttggatcccatagtgttatctataatacgcactccaggatcatgtttc

cattctccatccacgaggaacttgtactgatgttctccttcagggagatcaattattgtaacaaaatcaccatggcttttgaccatgggaagagccttcc

aattagagaaagtaccactgataaaaacctgctttccaccaccttcccacttgaatactgttggcaatgttttgtcatcaaccttggtgctgccttcaga

aactgttgcagatcgttgccgtggagctccaagttcttcctgctctgatggtggcttttccttagtataatagggttcttcttcttcattggaggactgg

aagacaagttttgtgttaggtttcttgtcaaagacaaatgcttgactatcctttccaggagaaggaggaggggcaagctctcctcccttgtgcctctccc

tcgcactgcttccagcatttcccatgttgtaaagagatgtctctgtttatgtcctttcgaagatgtcaagaccttctgattgtcttcgacgttttcacag

atgaaacgtcaaaatattttatgtcattgtcaacacttaagaagcaccctattatactaaggaaaagccaccatcagagcaggaagaacttggagctcca

cggcaacgatctgcaacagtttctgaaggcagcaccaaggttgatgacaaaacattgccaacagtattcaagtgggaaggtggtggaaagcaggttttta

tcagtggtactttctctaattggaaggctcttcccatggtcaaaagccatggtgattttgttacaataattgatctccctgaaggagaacatcagtacaa

gttcctcgtggatggagaatggaaacatgatcctggagtgcgtattatagataacactatgggatccaagaacaatttggtgactgtcaagaaatcagat

tttgaagtctttcaagctttg

>52460|5 nucleotidase

cacaggcaagccgccgacaggggacgcagcagagggcccctacccgacgatcgtgacgcaggacgacggccacaaagtggcggtggtgcaggcgggctcc

ttcggcaagtacttgggacacttggtcgccacgttcgacgcgaacggcgagctgctcagcgcgacgggcaacccggagctgctggacgagcacgtgccgc

aagacgaaaccatcctggaggaactgattccgtggaggaacgtggtggaagagactggttcgcgagtcttgggccgcacgaacgtgctgctcaacgcgtc

caacttcgtgtgtcggggttacgagtgcaacatcggcaacttccttacggacgccatggtggacgagcacgtggtgattggacaacagggtaggtggaca

cgcgctgcggtcgctcttatgaacggaggcaacatcaggacgtccatcgacgaactcaggaacggtggagacgtgacgtatagcgacctgctgaccgcat

gtccgttcagagacggcatagacacggtcaccatcctgggctccacccttcgagaagtgctggaagcgtctgtggagaagagccagatctacactgtaga

acaaggcggagttaacttgaccgtccactacgctggaggaaatttcttacaacagtcagggctgcgcgtggtgtacgacgtgcgccagccagagggtaac

cgcgtgaacagtgtgctggtacgctgcgctgagtgccgggtgccgcggtacgagccgctggtgccggacgcgtggtacacggtcgccatcaccacctaca

tggcgcagaacagggacaccttccgcatgctgcgcgacagcggcgtcaaccgcactgccggtcgccttgacacggacgtgttcattgactacatgaagaa

gaacaaccccatcacgcatgggattgatgggcgcaccgtggtcctctactgacatcacacacgagtgttgcggtcaacagaccacacgggacctgttgcc

ggattcgtgctagtgaaggcgactgctagtgccatgatgcgcaagaacaaatgacaaccgagagactttgtttaatgcttcactttagttcctaagtacc

tacgcaatgataacagatatttattttatatcatttgtttcatgaacgataattgtaattctggttacgcaggtaaaaggttcgatggatcgcagtcgta

agtgtgagacactttatggtttgtaatttggttgatatgagaaatatattttggggttgcgtttggttttcctctagacctacggaaacacac

>52474|5-3 exoribonuclease 1

gtacagaagcctcggtttcacatgcatgccaacattcttgcttgccaacttctttgcctggtgggcatccacgatctccatgagcatttcaccaacttcc

ggttccaaatactgtgatccacagggcctccgttcaacagacgaacagtgctggttgcggatccacgtatttatttgtgaaacatagttccagtctttcc

ccggaaagacatcactagcgacaaagcagtcatttccaacatttgacgtgaggtactgaaacaaatccggaaactccctcacaccacttacactaacaca

cacacgg

>52489|5-aminoimidazole-4-carboxamide ribonucleotide formyltransferase imp cyclohydrolase

ctctgctgggggctacccgataaagggtatccacaatgcgtttggttgtatgctgagctcagatcaacaagtgtccagtttcgtcgtataggtgttcaag

tgattcatttgaaatggcagcaggtggaaatttagctcttttgagtgtctccgacaagacgggcctggtctccctggcggaaaagctgcactccctgggt

ctgactctggtagcatccggtggaacggccaaggcactgcgtgacgccaagctcgcagtcaaggatgtggcagaaattaccggcgcacccgaaatgctgg

gtgggagggtgaagactttgcacccggcagtgcacggaggtatcctagcacgtttaactgattctgatcaagctgatctgaagaagcagggctacaactt

gataagagtggttgtgtgtaacctgtacccatttgtcaagactgttgcaagatctgatgtcacagttcctgatgctgtggaaaacattgacattggtggg

gtaacgctgttacgtgcagctgccaagaaccatgatcgtgttactgtgctctgtgatccttcagactatgattcggtagttgccgaaattcagaagtcgc

ctggcaaggatactgaacttgcaacgaggcagcgcctggcactgaaggcattcacacacacagcgcagtatgacgatgccatctctgactacttccggcg

gcagttcagcagtgggcagggccagctgacactgcgctatggcaccaacccccaccagaagccggcacagctgttcacgacgcggggtgcactgccactg

cgtgttgccaacggttcccccggctacatcaacttgtgcgacgcactaaacggctggcagctggtgcgtgaactggacagggcgcttgggctgccggcgg

ctacttcgttcaagcacgtcagccctgcaggggcggcagtgggtgtcccactcaccaaagatgaggcgcggctgtgcatggtggaggacctactggagca

gcttacgcccctggccactgcctatgcccgtgcccgtggagcagacaggatgtcgtccttcggagactttgtggccctatccaggacgtgtgatgctgtc

actgccaagataatctctcgagaggtttctgacggtatcattgcccctggctacacatcagaggcactcgagattttgagtaagaagaaaggtggtgcct

attgtgtcttggaaatggatccgaactacgagccggacctcatcgaacggcgcactttgtttggactgacattggagcagcggcggaacgatgcagtcat

cgacgagtccctcttccaggggcgcacggtgtcgcagaagaaggagctgccagcagaggcagtgcgggacctgatagtcgccacgatagcactcaagtat

acacagagtaactccgtgtgctatgccaagaacggccaggtgatcggtgtgggtgcaggccaacagtcgcgcatccactgcacccggctggcaggggaca

aggctgataactggtggctacgacagcacccacgagttattggcatgaagttcaagaaaggtgtgaagcgtgccgagatatcaaatgccattgacaacta

tgtgggtggcagtgttggtaaagacatggagtatgatacctgggcatcagtttacgaagaggttcccccattcctcacacagcaggaacgtcaggagtgg

atcaagcagttgaagtcagttgctctcagctcagatgctttcttccccttccgggacaatgtcgatcgcgcctaccagagtggagtggaatacattgtca

gcccagcaggatctgcaaatgatgctgaagtcatcaaagcatgtgatgagcgcaaaatcactcttgtacacaccaacttgcgtttgttccaccactaaac

tgctgttatctgtactttataatcaaacggagtatcagcatgcaaacagtagacacatgcaaaagttagtctcgcacgcttgatagttgtaatatgatcc

actgtcacaactatcttttccccgtgaattgtcatctcattgtttttaaatgtggtcactaaagcagtttgttccttctgcttgttagttttttaccaca

tgaaatgcacacttctgcagaaatcttctagcacaggctacgagcttaggtccttcaatgtaatgccgtaaaaaccagacttccgtgcacaaacagtaag

gtccaacacacaacttgactgtgtgtcaacagctttccgtctattgctacacattgaccataacaggccttatggatcctatagagggacattgtaggtg

cccatctgccttggaactatccatacacttatattaggcatgatggcccacaatgctattagtttcggcaccttaccacatgcaaactttaggtgttctg

taccagtaaggatgcaatatttagaattattgcatccatactgttaacagagcacctgaagtttgaatacggtaggtattcaacactactagcatagtag

gccattgggcctaatattggtgtattggtagttctaatgtctgcagaaggcagatgggtacctacagtgaaggacgtaagctcgtagcttgcgctaaagg

ctttctgcgttaagtgcaatttcatgtggtaaaaactaacaagcaaaagaaacaaaccacttcagttattgcacttacaacataaatagtgaaatgacaa

ttcaggacaaaaaggcgtagaacacttgctgaaattttgactgtc

>52502|5-aminolevulinic acid synthase

tttatcttgagtgtttatatatttgttccattttgaacacacatgcagcataattagtttcttttcacacttataatgaaactgccatatccagagaaaa

attacaattatcaacgactagaatttattacaaaaataatgattcttgtgcacacaaaatattgacctgaatttgtaaatactgtatactgaactctcat

tagcgttcagtaggaatgatgacacagagcattaaaagaattacatattataaattaaagtctcttaatgctgcccatacattaagacatcaacatttat

agtctgcaagccacctaacagtgtgtggcagagagagaatttaaatatctctcacacatacaaactgagaatcacaaaatacaaggtacataacagatga

caaatacgaagttgacagtttaactagtttggttgtgaatttacttcaaatctcagtaccttctcagtacctaagactcttgaacatgattgtatatagt

gagataaaaataggtttttttcatttgcttcagtactacaaaatggaaaaaactaaaaagaaaaatctgtttcttaaaatcatctcaaatctgaatcaag

taactaattacttaaagactgtttattaaaatgaagactgacagaaaacagattacaaaaccaaaaacaaaacagtagaacatgcaggtggtaaattaaa

aatcacattcaacattgacatgaatcacaatcaggctacagaaaaagtcatccatgtgtcttttcacaaatgtgttgtgcagcagtcagaccaaacttgt

gttctttttgtacatcatgttttcaaatgtatgccactgtttttcaagactaaaacataaaattctcctgtcagcctgattatgatttttttaacactga

ggagtaagtatatctaaataacatttctttgatacaacaatggtaagtgcttttcacaatcaacagtgtaaatgcaacattcactacagaacatgaaact

agttcagtgctcataatgcaggacaaagaggagttcaaaatgaccaaaaatatcttgcacatctgattatactaaaatccaagttactgaacttgcagag

cctgtgtgcctgcaagccacataatttcttttacatgttcattgtttccttgacataaggtattaaatattttcatttttaaacaaaattttttccaaca

tcttgtcaaatacttctatctgccagttaaatggcaaagtcacagaaatattcacattttctttaaactaatcagtactattcaccaccaactagcacaa

aaaattgtataattaatactaatgttgcattaagaatgtagaaaagaggaaggactattaagatttaatgtctcctcaataacaaagtcatgtgattata

aatcattatcctctccacagtttgcacagcatgacattacaattaatcagtggtttaatttgtgaacagttacatgttcaaaatttttatcccatattga

cttgggcctatgtcaatgaaacacatcagcacataatatatttgagtgagtggtctccattagaaaatggctatgtaagtctaactgcaaacacatgcgg

cagtgaaaggcaaaggaatgtgcaacatacactcaatccaacacgtggagatcacctgaaatgaaattttgtgctcctcatataggaacaaaatatgatc

acgttaataaagtgtcattccaaattaatggctgcatgctgttattgatccttatttaatgttcaaacaaagaaaaactccaaaatatagcttacactac

acaaaatgttgtgctagttccacaaaatatactcccaataaatatattcaaaataatgttcctgtgtattttatttataaatcccagtgatactttcttt

ctatagtaatgctgataattttaatcttcctacattggagctttttcttcaagcatttaatttcagagaaggatatagttaaattattactttattgttt

taggatatattggatgcagtatcaccagaaactgaccagtgatagtgaacaatttctaacacattaaaagtttcacattgaattgcaagttaaaatttgg

acctttttttccaccaaacttgcaatttctcccctctctcctgcatagacacactcagggtttgtagggaaaagtattctttttaaattacttgttactt

ttttaaaaaatgcaaaatttcaaatttcttgtaaaaataaacaaaggatcagaaagaaatccatgctttgcatctagaaatgtagcattacatcataatt

aataccgtcatcaaatacatttccacacaaacatggaggaatatgatcatcatctgtaaaagtagagtaattaaaacctgagagctcacaataacatata

aatactgtaataattttctaaaactgttataaaaataaatttgagacacaataaatcagatcactcgatctacagagagcaacctattaagcaaattatt

tgtttttggacttttggtcataagcatcttattccttgaaacgaccagcgttgagatgcaaagatgctgttaaaattctggaacaattaaaatgtcccac

tttgttcagactctggcagcactgatgtttgtcatagtgaaagcctggcaatcttatgagttgtgtagaggaagaccaatagtgagccaaacatctcgca

tgtctgccacaagacgattcatcatttcaggagtatgatggggtgttggcgcaagtcgtaatttctcttgccctttgggaacagtagggtaattgatact

ctgcacatagtggccaaacctttgtatcagctcatcagatagttgactgcagctaattggatcaccaacctttattgggataatatgagacggtgtgggt

tctactggaaaaccagcagctatcaatctctcacgcaaatactgaacattctgttggtgcttagcacgcaatgctcgaccttcaggtgaacgaagaatct

gcactgcagcataacttccagataacaccgtaggtggcaaagatgttgtgaagatgaaacctgctgcatagctccttatcatatccacaagatttgctga

accagcaatgtagcctccaacatttccaaatgctttccccaatgttcctgaaataatatccattttatccagctgcccatctcgctctccaataccagca

ccattatgtccatacagacctacagcatggacttcatctacaaatgtaattgctccatatttgtgtgctacattacagatctcttctaatgggcaaactg

ctccagtcattgaatgaacagtctcaaatgcaacaatttttgggacagaaacaggtaacgttgataaaagttcctccaaatgctttacatcattgtgacg

aaaaatatgtttgggcacatggctgttacgaattccatgaatcattgaagcatgatttcctgcatcagaaaatatgtgacaaccaggcaaagctttggca

agagtaaacagggtagaatcgttagctacaaagcaagaagagaacaggagtgcagcttctttttggtgtaatgatgctaattcagcttccaacttctcat

gaaacatagaattcccagaaatgttacgagttcctccagctccagctccatgggtctctaaggcttgcctcacagcctccttaacttttggatgacatga

catcccgagataatcattagaacaccaaactgtaataggcctttcaccccaactatattcatatgcagcaggaaatgcggatgccaatctgttaactttc

ttgaatatacggtaagaatgatctttcttttttcgcattatctgttcatgaaagaattcctcatactggaagatgtcatcaccagttttgctctttttca

aaggaatttcattttgttcagtaatatctttttcttctaacttagaaatttcttctcttatctgctcaactttttctgctgatggagacagctcaataac

atcatcttgcatttctttgctagcctcttttaccaaaggtgtaacctctgctagaaaaggacactttttcacggcatctgctgcttcagtatttgaatca

actgatgccacagtagtgacaaggcgtgagatcacaggacagaattttccataggttttaataagattggctccataattgcgaacaaaatttggagaca

aatatgacataaaaggacacggcatatttgtgcctttatgaatgtgtagtatatttgaagaagttgtgttaaaagtctgtaaaaatctcagtgactggcg

tgacagcatggtggtggcggcggcccaagcttctgcgcggaatgctgctt

>52552|50-kda dystrophin-associated glycoprotein

tcacgggctgactatcacaaagtgcagccagtattgtcaaggacaatgtggtcagcatgtgtggctgatatgagggacatcacagataagttcagcgaag

tgaatgagacaacgccaatctaaacaacggcagttacaatgggcacaggcatttagcagcaagtgcagtggataatggaacacaattatgtagaaaattt

tgaaccttaaatagctagactacttcaataatcaacaaaaaggcaataacacggaagaaacaaaaatgtaagtttttataactattttagatatcaacaa

agctttcagcttcaaaatttgaaaaataactattatttttgtgtacaatatagaaaaagatagattgatacttaccgtaaagatgacgcattaagttgca

gacagtcgcaattaaaaggcacttacacaaagccttcagacacagccttcatcagcaaaagagaaatacatgacattcattcacacaagtgagcatgtct

catgcacacatgactgccaactccacattctggcctgagctactgatgctggcggtcacgtgtgtgtgttaggtgaatgaatgatgtttgttgtgctttt

gctgatgaagaaaatgtccaaaagccctgcgcaaatgtctttcaattgcgtctgtctttagttttacgtgtcatctttatggtaggtagcaatctatctt

ttcctacattgttgacattactacctggagtcccattgttttattatttttggctcattacttgcatgatgaatatgtacatcattcaaatatatggttt

gctgcaaccactgcacaagataattctgaattgcaggaacaaattaatactcttttgaatgatgaagctagtaatactgcctcacatcacacatcaggaa

atcacttttcttgttgctcagcacacactggtagcaataatcctgttttccacagatttaacaataccacattttgtatttgtccggaagtcagaaatcc

acatgatctgatgtgctctgtggaaatcctgaatatggtggaggattgggcctgtagcaggaattttcacccacagtcctaggaatggttgtttttagta

atgaattgcttgggctggaattctgaactttataagaagcaacttctgatggaacagtgcaggcatcacgtcggtttgacaaagagcgcaatgtatttgt

tgctcgatggacagcagtgtactgaaccatttgcactgcaggcagctgcttgttccgtttgcttctcaataattgttcaaatatatcaaacagtgactca

aagtattcatctgattcatcaaacatcccttcgtggtgaaagcaaagtattaaggacaatatgataacaaggcatatcataatcagcattggcactaaaa

ttgttgcaacaaactcattaacataacttctttgtggaacttctgcctttgagggtgcggaccatattgtatctgactttataagattattgtaatggtc

cactacacctacttcatgatgtgccagatgtctcaaagatgacaactcttcagtaattaccaatctgaatgcacaccaatctaatgcaaagcctgcatct

ctgaagaatctgtcaactgttgtacgtttaaaatctcttgggcaggacagtaatttccacagtggtttcacttcttcttgcagttcaattagtgcagcag

aaaattcaacttggcttcccagcttcaaaactactccttccctttcatttgggctcaaaggtaatctggctcccagctgcacagctgattcaaggaatgt

tacatataaatcagcatggctctgtggccacagattatttcgaaatacgtctaagagtctgttcattctaagttcatcaaacatatcctcaacattcaag

ttatcaatcttcagctggacttcatattttgctggatctggtttttcttcaactttcaacaagactgtctcacactgtgtttcatatgttcctttgttta

gtccaacaatttccaactccaaatcattctgtttcattggtggtacaccatacaaatatcctttgccatttgatctgctgtgtgtataatggatccactc

tggaagatctggggcattta

>52576|50s ribosomal protein

acagatacgatttcaagaccttgtactggtacgcgttcatcacctaagtgaccagccatttttttgcctttgaacacgcgtccaggagtttggttttgac

ctgtagaacctaaaacacggtgagaaacagagttaccgtgagtagcatcttgggtacggaaattccaacgtttaacaccaccttggaaacctttaccttt

tgattgaccagttacgtcaacaatttgaccaactgtgaacaaatcaacgccaatagtaccgccaacttcacggccttcaagctcagcttcagtaacacga

aactctttaactaaacgaccagcagcaacacccgctttagcgaagtgacctttttgagcgttagttacgcgagactcgcgacgttca

>52604|50s ribosomal protein l4-like

ggtaatttgtaagaatagctgcgtggtgttgggccgaatacgacaccgcctccgcgccattgcggtgaacggattgaaccttgacgggcacgacctgtac

ctttttgac

>52603|50s ribosomal protein l4-like

ataagttctcatctacagcatctgtaacgatcaaagcacgagctgcatttaaatcgttaagttttgcaagcaattctttagtttttggagcagcaacagc

gaactcttcaactaaaacaaggcgatcttggcgaacaagttcagctaagatacattgcattgcaccgcgatacattttacggtttactttttgagaccaa

tcttgtggacgagcagcaaaagttttaccaccaccaacccagattgggctacgaatagaacccgcacgagcgcgaccagtacctttttgacggaatggtt

ttttaccaccgccagaaacgtctgcacgtgatttgtgagcacgagtaccttg

>52631|52 kda repressor of the inhibitor of the protein kinase-like

acagtagaaagaaggttttcagcagtgcgacgtacaaagacatggatgaggtcgacaatggactacgatcgacttaatggcttagctctgttgaacactc

>52665|52 kda repressor of the inhibitor of the protein kinase-like

aaacatatgatggtgcagcagtcatgagtggttcgtcacgtggggtgcaggctcttgttaaggaagagtttcccaacgcttcatacattcgttgttatgc

c

>52702|52 kda repressor of the inhibitor of the protein kinase-like

taaaaaaaagcttcttatcatattagccacactcccggtgtcaacggcgtgccctgagagatgtttctcgactatgaaaaggctaaaaacttacataagg

a

>52675|52 kda repressor of the inhibitor of the protein kinase-like

catctattgctacagtagaaagaagtctttcaacattgcggcgtaaaaagacatggttgaggtcgacaatggaggaggatagacttaatggcttagctct

gttgaac

>52635|52 kda repressor of the inhibitor of the protein kinase-like

agaaagaagtttttcaacacagtggcatacaaagacatgactaaggtcgacaatgaaggaggatcgactcaatggctgagctccgttgaacactcaccct

gacattgagtgtcc

>52700|52 kda repressor of the inhibitor of the protein kinase-like

tagaaagaagtctttcaacattgcggcgtaaaaagacatggttgaggtcgacaatggaggaggatagacttaatggcttagctctgttgaacactcaccc

tgacattgattgccctattgacgatg

>52672|52 kda repressor of the inhibitor of the protein kinase-like

tgagtgttcaacagagctcagccattaagtcgagcctccttcactgtcgaccttagcaatatctctgtgcgccgcagtgttgagaaacttctttctactg

tattagtagatgaaggtggacagggaaatatttgtacactgtgtacaacgcag

>52686|52 kda repressor of the inhibitor of the protein kinase-like

gtgactattgcacttagcacaaactgagtaagatttttgcttttatacctgcatctactgctagaatagaaagatctttttcaacactgcgaggtacaaa

gacatggcagaggtcggcagtgaaggaagatcgacttaatggcttagctctgttgaacactcac

>52673|52 kda repressor of the inhibitor of the protein kinase-like

tacaaaatatttgcttgcctaaccgcctctactgctacagcagaaagaagtttttcaacattggggcggagaaagacatggctgaggtcgacaatggagg

aagatcgacttatggcttagctcaaaacaggccgcatagatttcatcatttaaggaagagaaccaca

>52669|52 kda repressor of the inhibitor of the protein kinase-like

ctggatttctaaatatgaagtcgttgatgactggtcggatacccaatccatccaaaatatctcggtgggcatgaagaacagccaagttgttcaatcactt

ctgtcccattgttgattggagatacgacttcagatgtctaagggcactaaatgaccgttctgctgttgct

>52639|52 kda repressor of the inhibitor of the protein kinase-like

agcagacgagaagaccttgtgaagaaagacccagtttacctgtataataatattaggttttgtcagctacatttcgaagaaaaccagttcatgaacgcag

acaataacaaactcgtgaggaatgcagtacccacactgtttgacatcccaaataagccacctcaattgacga

>52646|52 kda repressor of the inhibitor of the protein kinase-like

ttaaaagcaaaatattcctctgtgggggcttagggatgatgggagtctgtcaggaaatgaaaatgattgtgaaagtatagtgagtaatgagggaaacttt

agagctcttctaagatttagaattggagctggagacttgcaactaaaacatcactttgagaccactgaaatga

>52678|52 kda repressor of the inhibitor of the protein kinase-like

tcgattgctgggagatcctaggattacattgtcaccatgaaatggaagccttctcgatgccgaaaattttccagcagctaccatcgatttaagtacattt

ctccagtaaacaacttctttttgatgttgagataataagtctgtatttattccaccaaccactttcttcataccagat

>52637|52 kda repressor of the inhibitor of the protein kinase-like

tgtcagctgagggggtttatctggaatgtcaaacaatgtgggtactgcattccaaacgagtttgttattgtctgcgttcatgaaccggttttgttcgaaa

tgtagcgaacaaaagctaatattattatacaggtaaaatgggtcttttttcataaggtcttctcgtctgctattaaccag

>52697|52 kda repressor of the inhibitor of the protein kinase-like

gtggtgtacaaagacatggcttaggtcgacaatgaaggaggctcgacataatggattagctctgttgaacactcaccctaacattgattgctgtattgac

gatttaattaatcaatttgccaagaagaataggcacatacaattcatcatttaaggaagagaaccacaattgtaactttttt

>52658|52 kda repressor of the inhibitor of the protein kinase-like

acaagactatgtcatcttacgatgtaaaaaagttacagttgtggttctcttgcttaaatgatgaattctatgtgcctatgcttcctggcagattggttaa

ttacattgtcaagaggacaatcaatgtcagggtgagtgttcaacagagctaagccattaagtcgatcctccttcattgtcgac

>52670|52 kda repressor of the inhibitor of the protein kinase-like

ggtgagtgttcaacagagctaagccattaagtcgatcctcctccattgtcgacgtcagccgtgtctttgtatgcttcaatgttgaaaaacttctttctac

tgtagcaatagactgtagtctgtatttataatgctatgtatcgaaggtagaaaaacagtagaatattcgcaacttttattgaa

>52661|52 kda repressor of the inhibitor of the protein kinase-like

ttggcgacgtcatgaatctgctgaaataagcggctgctatcccatgaggacgtgtgaagcagttaggtgtttccgcaatctgtcgcaggtcaaacagttc

cttagaagtaccacaggtgaagataggctgtgtactcttgccatgctttccattgaaaaggagacgattcacaggtctacagatttcaac

>52691|52 kda repressor of the inhibitor of the protein kinase-like

tcttccttaaatgatgaattccatgcacctattcttcctggcaaactggttaaatacataatcaataggacaatccacgtcagggtgagtgttcagcaga

gctgagccattaagttgatcctcctccatcgctgacctcagccatttgttagttatctatgagccagacagaaacgtataaaatacgatgatgcggac

>52677|52 kda repressor of the inhibitor of the protein kinase-like

aagcaaacgagcataaacgccctttatgatcgtgtttctgttatacaagcgttgtctgtctgtcctactttgcacacggtgtacaaaataattgcgtttc

tacctgcatccattgctacagtaaaaagtttttcaacattgcggcgtacaaaatcttggctgaggtcgacagtggaggaggatcgacttaatggcttagc

>52634|52 kda repressor of the inhibitor of the protein kinase-like

acttgaacttgacataaatataaaaaaattctgttacacaattcactggttatttccatcttctgctcaatttgtcatgctcattcgtctctgtttcttt

ttactgaatatgtcaatgatattttcaatgtccatatctgtttctttatggatatttgcaagagccaatccatttagtctcacctgccccattgtgttac

acaaataagtctttatttgtg

>52638|52 kda repressor of the inhibitor of the protein kinase-like

gtattaatgattaatgtttcgttttaggagcagaaaatggctagctaatagcagacgagaagaccttatgaagagagacccagtttacctgtataataat

attagtttttgttcgctacatttcgaacaaaaccagttcatgaacgcagacaataataaactcgtgtggaatgcagtacccacactgtttgacattccaa

ataagccacttcagctgacgatga

>52674|52 kda repressor of the inhibitor of the protein kinase-like

tgaacaaaatatttgcttgtctaccttcatctattgctacagtagaaagaaggttttcaacgttgcgctgtacaaaggcatggctgaggtcaacaatgga

ggaggatcgacttaatggctcaataggtgaacagaattcatcatttaaggaagagaaccataattgtaacttttttatgtcataagatggcattgtcttg

tatacgtgtataaataaaactttcttaaacttttca

>52648|52 kda repressor of the inhibitor of the protein kinase-like

tttcagtgtagagaaagatcttttggctgttgcagttgagacaggaagacccgaaggatcaacattaaagatgtaatcaaggggaaaatgtctgtagtac

aaaactgcaaacactcaactgcagagtttggtagaagtgttttctttgcatcttctcttttccattttgtcacccacaacttgacttctgccctcaaatt

agagatgcagtcactttcaactgttccaaacggtttataatattttctagccactgtttcggtatcact

>52692|52 kda repressor of the inhibitor of the protein kinase-like

tggatgtttttcctagatctgactatcctacttttcacacgctgtacaaaatatttgcttgtctacctgcgccttgcattgctgaagtagaaagaaatta

ggagcttacaaagacatggctgaggtcgacaatggatgacgatcgacttaatggctttgctctgctgaaccctcaccctaacatcgattgtcctgttgac

gatgtaattatccaattttccaggaggaataggctgatagaattcttcatttaaggaagagaaccacaattgta

>52657|52 kda repressor of the inhibitor of the protein kinase-like

tttaaatgaattgtcatcaaggcgagcccattcagatgttgctctctagttgttgtattccttagctatgtcttgatgctcttaagagtggagaaactgc

actcatttgtggctgtagaaactgaaacacactaagatatatctgacagttgcaggcagaaaaagtttcctttgcattccctgaaacaactactccagtc

acctcacatgttgatgaaagtgtgtcaagtcagccattgccattgttaaatcggtgagtaatggacctgaatcataaaatgaatgtagttttgtaactcc

atcaatg

>52649|52 kda repressor of the inhibitor of the protein kinase-like

cttgtaagattttgctgaccggatatgttatgctcacaatgccactcagtgtaaacagagtggggataaactcgcaattagagacagctcaaaagagaat

attggctttggcagccattgttctatccctccaacattgtatttctttaagaaatcagctgcgaattgaataacagcatcatgttgcatagcctatctca

tttgacaatgtgatttcagctagtgttttaggtgattcttcaattatgtgaaccacttgctagacgctgtaaagaacaatactacttcttcaatagtacc

aagtgag

>52652|52 kda repressor of the inhibitor of the protein kinase-like

gtatacaagataatgacatattatgatataaaaagttacaattgtggttctcttccttaaatgatgaatgcgatgcgcctattcttcctggcaaattggt

taattacatcgtcaataggacagttaatgaacagggtgagtgttcaacagagctaagccattaagtcgatcctccttgattgtcgacctgagccatgtct

ttgtacgccgcaatgttgaaaaacttcttgctgcactcatttttcaatgttgcttctgagttactggtttttcgccagctatcttgctggtctttgctca

aacaggaaggtgccaaatgtgtgaacgtgacacccggtactgtaatacttgttgaag

>52687|52 kda repressor of the inhibitor of the protein kinase-like

taaagatatcatgagctgtctttacttttaccgtagtaaaaaagtaatcaagtcacatgcaacgcttaagaaagttttatttaaactgattatacacata

tacatgacaatgccatcttatcgtataaaaaattacaattgtggctttatttgttaaatgatgaattctatgtacctgttcttcctggaaaattggttaa

ttacatcgtcaatagggcaatcaatgtcagggtgagtgttcaacagagctaagtcactaagttgatcctccttcattgtcgatttcagccatgtctttgt

atagaaaaacttctatctactgtagcaatagaacaaaatgaaaaccaatacagactaccataattatgatacaaggcaatgtaaagatctccacttacaa

gctgtaacaaggagccaaagtcagaagcatgtatgtaaacaaggcatcaaactttgcagtgc

>52640|52 kda repressor of the inhibitor of the protein kinase-like

gaaaattcaacattatgtatgtatttaattctctattttattaaggtttttaatattgctctaaatgccatgattaggactagtgtctttagaaagatgc

aaatgtcaaccgtctgacgattactgaataggaagtcatccacgactggtcagatatcaaattcatcttggtgggcatgaagaacagacaagttgttcaa

ttgcttctgtcccgttgctgattggagatgtgacttcagacatctaagggcactaaatgaccattctgctgttgctgtcgtgattggaactacttggaaa

accttaatgcacttcactaacatttctccaattgcaggttcttgtgtaatgtagtttcttacatcacacatgtttttttaagaccagttgtttttattag

caatattggctaacatattcaagtgtaagtgcagtctctcaatgtctaggtcatttttaaagaactcagttgatgtttccaaatttgtttcacctgtgtt

tactaaaagcaagcattcttgg

>52679|52 kda repressor of the inhibitor of the protein kinase-like

cgtggaaaaagcccttttctccgaagaaatgtatcctaatgatttagagactgacttagttcaagaatgcatacattttgattgccactttttttctgat

ttttttttgttaaaaccagattctggataattgaaaagtctatcttagttcctgcgaaaacagaatttgcaaaatgtttatcctaacttggatacaatag

tttgcatggctctatgtatactagtgacaaattgttcgggtgagagatgtctttcttgctttaaacaggtgacaaattatgctcttttgtgcatacagtc

gcaactaatgtacaagattccatacgatgatataattgatgatcacgcaaaaagaaaatcgcacaaaaaggtactgtagaactttcataacttaattgaa

tttcattatgatcagctagatagttaccagataattgaatacactgtttttaaactgtaagtaagtgcaataatgcccggccggctagccgcatggtcta

acacgcagcttcccgagcaggaaggcgtttaggtccctggtatgaatccgcccggtggattagtgtcg

>52693|52 kda repressor of the inhibitor of the protein kinase-like

gaaaaattgtactcaagcaagagggagacataatttgcgggatacaagctttcaccaagcgcaaagcatatgtttccacatacaacaactgactcttgca

accggcaacaatgcaatatgtccttgtctcgacctacgcgaatcgccatcgttcgtggatcgggctgtagctgcccatacatttagttacttgaagtcgc

agacctgtgttcttcgtccactttagaatcttcttcgccaaatagtaagtccatatttgcactgtgctactgtcatgatgtgcctgagaagagtttggta

cactggcggatacagaaaaacctcaaagaagagatgctaaagatatcatgagctgtctttacttttaccgtagtaaaaaagtaatcaagtcacatgcaac

gcttaagaaagttttatttaaactgattatacacatatacatgacaatgccatcttatcgtataaaaaattacaattgtggctttatttgttaaatgatg

aattctatgtacctgttcttcctggaaaattggttaattacatcgtcaatagggcaatcaatgtcagggtgagtgttcaacagagctaagtcactaagtt

gatcctccttcattgtcgatttcagccatgtctttgtatagaaaaacttctatctactgtagcaatagaacaaaatgaaaaccaatacagactaccataa

ttatgatacaaggcaatgtaaagatctccacttacaagctgtaacaagga

>52663|52 kda repressor of the inhibitor of the protein kinase-like

gaggaatgaagttttgtaactgcatcagtgatatctatgtctcaattttttgtggtataagatgaataaagctgttaaaaagtgtgtaatgtatcaagaa

ctgatccttcagtgaattaatgaagacatccagaaagacactttgtactcacagtaaccctcaacatcattaccagtatcctgattcttatgacgtgctt

gctcggtataaacacacggctgcttggtttcaactccactggctgaagctaattctttgactgctaagaaaatccacctgaaagtatcattcacattttc

tctcattgccagaagttcccttttgaggtccctcgcaacgttaatcacctctgcaaaatggatatctcagcactgaagttgacaacagatggactcatcc

agtttgaacgtttcagaaagtacaaccaagcatgtgacaaaatctaccttgtttgttgctggtaacagaatcatggcagcatgagatgtggtactggcaa

atttttgtattttccccagagctgaaacaacacatccatatattttcttgaagtcttgtgcaatgtgaagacactcaatccatctagtgagtgtagtaaa

ttccaaactgctgactcgaataccggttaatgattcaggggcatcatttaatatttgcctatgttttggttgtccaaagatattaagcacaatttccagt

gttcttagtgaattccttgtaggtgtaatgttgcaagtgttgctaactacaaacttgaaattatgggctccacaatgagaatacataactataggataat

cttgttgtatgaatgcatgagccccacaggcttttctt

>52680|52 kda repressor of the inhibitor of the protein kinase-like

ataatgaatgggaaagggggctaatccatttagacttctctggaaggataaaacacagttatcaactcaattcaaacagaagcatgcattatttcttttg

aaaatttgtaaatactaataaattgtaatctaatgattgtcatacgacttaatgtgaggcaaaaaaaaaattcaaaactttcctgtcctcttgtttttcg

cagttatgtcttgaatggaggtgagttctatgctgtggctgtagttgcaagaagtatggccaatttcttcaggttgttgatatttggatatgtgtctttg

ttgtataattacagtgcttccattgcttatgaaggtctgtaatctcctttcagagatttctttctccataagagaaattcagtaagaacatcttgaacgc

tgtggtctataccttcaatgtagaattcaattaaggccttgaagtcccttcgttttcctctgtttaaggacacagtactgactgtaactgggaacaatga

agaaaatccagacagcactttcttatgttctgtaaacattaaatttgtcataaaacaatttaaaaatggaataaaatccgctcctcggtagtactatcca

ggatcctgcatcaagaatttcagtctgttcttttgtattttaacctcccaggaaaccaagatggaaatgttagctgttaaaaaattctggttggtctctt

aaaaaaagtacttaaattttttgcagcatcagagctcagattttccagtacttttgtactgtttcatctgcttccatagctttcctcaagcacagtactg

gtttctgaagtcacttttccactcttcaagtgttttttttcctaacaggtctctcttatggagcattgcccttatttttaatacatctaaggccacgcga

atcacactgtttgtgatttatttaaatgcaaacccctgaaccttaatattaaatggatgatacaaaagttacatttattttcgtgtgaggttagcgtgat

taacacagttatttaataacacttcagtcttccaaaatctaaataaagaaacttacctaagtatgacttcaggtgaactggtcaaacgacacaattttag

tcatgctaagaactggaagttctttcttgctatcagcgtgacggagcgttttaaaacttccacagatatattcatcgctcatccttcatgtaaacaaaca

cacactcagtggtcagtaaccctaggacagagccactgggtgtcattacgaattaggtctagcttatgtattacatatatcattgttgcatcaagatcaa

tgaaacactgagacgctctgaagtctaaagcgttaaaagacttcaagtttttggtaaccactacgttttttagctgtgtttcttttatttcaagttttag

ctatctaagatgctatatacagatatcgacaaaattcggtattagttggaaatttgtggtaaggactatgggaccaa

>50718|52 kda repressor of the inhibitor of the protein kinase-like

ttagcccagcaaaacctcactttccatagtcattttgaagacactccataagaaaacaaaaaaatttcttagaattggcacagttgatggtgaaatatga

tctagtattgaaagagcattatttaaaacttgaagagttcactggtagctccaagagggtaccttcatatttgtccaaacggatataaaatgaatatatt

tttattttgtgtgaacatattatagaaaaagttactgcagactcaaaaaaggctcactattatgtaataattttgatagtattcctggtgacaacaaaac

tgaccaaatgagtcaaataattagatctgtcccaatagaagacaacgtggtgaaagtttgagaatctttc

>52740|6-phosphofructo 2-kinase fructose -bisphosphatase short form

tttaatatatttttcccatgtggaagtttctttctattttatttacaattctatattcacatttttttggctttgctaactcacaaccaaactgtcacct

aacctaacctaaatgttgtgcagtcctatgattcagtctgccaccataaccaatgtttcatatttcatttataactatttacccataacaagcacaaaag

tagtttacacttcactaacaaaatcaaaatatgaggcaaacataaaaaaagcagcaattcttatttcccggcataggcgcatcaacaatgtgatctataa

tgtgcagaggaatgtatggttgctccttcataaattttagccacaaaagctatacaattaatatgagtaaaggaagactccctgatttaggtggctgaac

cctacaggatatcctagctaacaatgccacatgaatttcaactgggaatttcattacacgcttcttaattgatcatcctcctccctccctctctctctct

ctctcaaaaaactcttcacctacgatattttatttcacccttcacttctgcgatttccgtaccagttaacttttacatttttcaggcagtacattgatca

ttgccaatttattttcaaaataagtggtgatgtatgtttttttaaacatggcaaaaaaatggttatttccataaattaaatatcaaatgaagagatcata

taacatatcatctaaacttaatgagaaagatagatagttctgcctaagaactctctggaggagagattattattaatattcaatgaccataatgcaatgg

aaattgcaaatgaaaatagtaatactaatgcttaccttcggtttaggacgatgtgtgtcaactgcatcaataggaagctttatgtgctcaactttgcatc

catatgccactggtgttagcttaatgattgtatgtagaggaacttgcaggtacggtagctcatctgttgaaatgagattactgatttaatcattcacaaa

atatggtttgctggacaaaacaataacagaatcctttaacactttaagataaaaaattacagcgaatgatgaaaaattcaagtttcttaaagagatgaca

cctattaaactaaagatttctgcatgccattttttcctgactgaacttttggagtgaatctttctctctgcagtacagtatgcaatggtttgaaacttcc

tgtcatattacaattgtatgccagggcagggtttaaagccaaaacaatgcaattttcagacagtgcacttatcaacaaagctatccttgcatgacacgat

atgcccacacagcttcaatattattaataatctctttcctactttgcacagttcacagaagatctttgcatacatagttagaccctctttactttttaac

gtagtacattttgtaccaagtttcactacatgttgtatcattcacttgaattgatctgtgatctttaaacaaaatttttcatgtccactgcattggaatt

ttgaaatataatcacccagaataaatgaagagatcccctgttataaatttaggggctaaaggcatgtgacagcaatgactcacatcttcactaggacagc

taaagcaactacctttgactttacaagaatgagacatattctagctggcatgttcatcagtcagttttgagctttattcccaagtttgaaggctgacatt

atttttacaggggctctattaaatcctcgtgaaattgtgtgatcatttatacagtttctgactgtggaatttctatcaaatattttatttatttattgaa

ttcttccctttagggcagaatatcaagcttgtcttttctttctgtattttgttcagctatcttgcactgctattgttttgtatggtaagcacaatcttta

ttttattaccttctaacatttttgtgttagcaatcctatgtgatgatattagattagctacatgctatgagatggcagaaaacgttgaaagtgtggtaag

aaaacaagaaggaaggacaatacccacagccaatacgtcctagagtgtcaaaggataccagcagcagtatagcattctaactgttgagatattcatgtta

gataaaatatctttaaacaaaattattacatgcaaagctttgaagtgaattaaatctcctgttttgttggctggttgctttaaggttttggtcactttca

aaaaacatatcaatatttcacttacatcgctacctacatcaaactgaggctgcaaatgataatgcagcagctagaaactaaattaaatactatagtatag

gaacaacataagctgaaacactttgattacatctacaatagataaagaaaactgcattattcaaaatttgataaaaccaagaacacacttaaaatgaagt

atgtgttgtgagttactcaaagatgtcagtttcttaaagagaatttcagcaaaattaccataattgttacatgaatatgctgaaaagcaaatttctttcc

atgctgagaacatacctgctgacttgtcaaggaaataagcaagcaaacagcgtaacacagctt

>52758|6-phosphofructo-2-kinase fructose- -biphosphatase

catccatatgccactggtgttagcttaatgattgtatggaattggaacgtcagggcaatgtcttggttgttggtcatcaagctgtgttacgctgtttgct

tgcttatttc

>52760|6-phosphofructo-2-kinase fructose- -biphosphatase

tttgctgcccgtgaccaaaacaaatttagttatcgctacccgaggggagaatcttatgaagaaatagctgagaaataccctgaagactttgctgcccggg

accaaaacaaatttagttatcgctacccgaggggagaatcttatgaagatctggttgcacg

>52757|6-phosphofructo-2-kinase fructose- -biphosphatase

gccgtgcaaccagatcttcataagattctcccctcgggtagcgataactaaatttgttatggtcacgggcagcaaagtcttcagggtatttctcagctat

ttcttctattacatctagcctaatttcgacatttagtaagccgctgaactcctttctgactctcttcatcactttcgtctgtttcatttatccttgccca

aaatcaggctctctattccat

>52755|6-phosphofructo-2-kinase fructose- -biphosphatase

tatttgagatgtgctgtcacagaagaatgttgaaaattaggtggaaagctcttaatgaaatagatgctggaatttgtgaagagatgacatatgaagaaat

agctgagaaataccctgaagactttgctgcccgtgaccaaaacaaatttagttatcgctacccgagagtgagcagaatttagaaggacgaattggtggag

actctgagctttcctatagagggcaccagtatg

>52756|6-phosphofructo-2-kinase fructose- -biphosphatase

tcccctgttataaatttaggggctaaaggcatgtgacagcaatgactcacatcttcactaggacagctaaagcaactacctttgactttacaagaatgag

acatattctagctggcatgttcatcagtcagttttgagctttattcccaagtttgaaggctgacattatttttacaggggctctattaaatcctcgtgaa

attgtgtgatcatttatacagtttctgactgtggaatttctatcaaatattttatttatttattgaattcttccctttagggcagaatatcaagcttgtc

ttttctttctgtattttgttcagctatcttgcactgctattgttttgtatggtaagcacaatctttattttattaccttctaacatttttgtgttagcaa

tcctatgtgatgatattagattagctacatgctatgagatggcagaaaacgttgaaagtgtggtaagaaaacaagaaggaaggacaatacccacagccaa

tacgtcctagagtgtcaaaggataccagcagcagtatagcattctaactgttgagatattcatgttagataaaatatctttaaacaaaattattacatgc

aaagctttgaagtgaattaaatctcctgttttgttggctggttgctttaaggttttggtcactttcaaaaaacatatcaatatttcacttacatcgctac

ctacatcaaactgaggctgcaaatgataatgcagcagctagaaactaaattaaatactatagtataggaacaacataagctgaaacactttgattacatc

tacaatagataaagaaaactgcattattcaaaatttgataaaaccaagaacacacttaaaatgaagtatgtgttgtgagttactcaaagatgtcagtttc

ttaaagagaatttcagcaaaattaccataattgttacatgaatatgctgaaaagcaaatttctttccatgctgagaacatacctgctgacttgtcaagga

aataagcaagcaaacagcgtaacacagcttgatgactaacaaccaagacattgccctgacgttccaattccatgatgaccggttccagccgtgcaaccag

atcttcataagattctcccctcgggtagcgataactaaatttgttttggtcacgggcagcaaagtcttcagggtatttctcatctatttcttcatatgtc

atctcttcacaaattcccttcggtttaggacgatgtgtgtcaactgcatcaataggaagctttatgtgctcaactttgcatc

>52779|6-phosphofructo-2-kinase fructose- -biphosphatase 2-like

gaaataagcaagcaaacagcgtaacacagcttgatgactaccaaccaagacattgccccgacgttccaattccatgatgaccggttccagccgtgcaaat

tttggacgtattgtgccctctgtgatggtcaact

>52782|6-phosphofructo-2-kinase fructose- -biphosphatase 2-like

ctcgcttgaacctgtcctctagcgtcccaggaatcttcggtttaggacgatgtgtgtcaactgcatcactaggaagctttatgtgctcaactttgcatcc

atatgaagctttatgtgctcaactttgcatccatatgccactggtgttagcttaatgattgtatgtagagga

>52777|6-phosphofructo-2-kinase fructose- -biphosphatase 2-like

gtactgtcatacattagtgtatttattcacaaaagatcacaaagaccaagtataagtagattatttgagtcaactgttctaattcattcccaatactaac

attgtcaaaattggagattacagaaagctatcaaattaataagatcatttaacatttcactggtgttccaatgatgacatcctgagattaccgctccact

tccttcggtttaggacgatgtgtgtcaactgcatcaataggaagctttatgtgctcaactttgcatccatatgccactggtgttagcttaatgattgtat

gtagaggaacttgcaggtacggtagctcatctgctgacttgtcaaggaaataagcaagcaaacagcgtaacacagcttgatgactaacaaccaagacatt

gccctgacgttccaattccatgatgaccggttccagccgtgcaaccagatcttcataagattctcccctcgggtagcgataactaaatttgttttggtca

cgggcagcaaagtcttcagggtatttctcagctatttcttcatatgtcatctcttcacaaattccctgcggaacaggtgacttaagtttatcattttcct

ttacaattgtttaaaaaaaatgcaatgaaatataaaagattttttttcgttagtcaacaagctggagaaaaaactgacttggccacttacatcatctgtc

actataattccactacacaattaaaaagacttgaataaggttctgaagtttattactccacttaagctacacatgcccaccttttagcttcttccaaaga

tgtacaaagctaag

>52778|6-phosphofructo-2-kinase fructose- -biphosphatase 2-like

atatgaagaaatagctgagaaataccctgaagactttgctgcccgtgaccaaaacaaatttagttatcgctacccgaggggagaatcttatgaagatctg

gttgcacggctggaaccggtcatcatggaattggaacgtcagggcaatgtcttggttgttagtcatcaagctgtgttacgctgtttgcttgcttatttcc

ttgacaagtcagcagatgagctaccgtacctgcaagttcctctacatacaatcattaagctaacaccagtggcatatggatgcaaagttgagcacataaa

gcttcctattgatgcagttgacacacatcgtcctaaaccgaagattcctgggacgctagaggacaggttcaagcgagcaaattttggacgtattgtgccc

tctgtgatggtcaactctgacgcatcttgatgcttgatggaagaactagctccagaaatggcttcacatttggaccagtattgtaactgactctacaaaa

tataaatgtgatttcataacaagtaattaaaagtgtgttaacaggtataacacagtcattggtattctgattccagatgtttaattacagataagtaaac

actgagagacttttatacccttatctattgataaaggttgaaactgatggtctcttgttgatttttctaggattgtcagcatgtgtacctaaatgttgat

tttatttgcaagatttacaaagttcaaccccttcctaattactaaaagattagttcaatttatatgtgatatgctttaaattttgtcatttgcttgtatt

aaaaaaactgtatttaacagttacagtattatttgattgattgctctatgcaatacatataactcagtacaaggtctgacaatcctgatagttgttagtt

ttaaagatattggttccatatttgaacaattactctgtctcatccttttagaaaatcaaatcactttgtgactacagtaatgatacgatttgtagatgtt

aatgtatctttgtttttatataacaaatttaaaatatctttgtgaagtatgtacgaatattttgtcataatttttttattaggatgaataatagcctcat

taatgtatggttcacaagtatgagtgtgtgtacatgttacagtgtatgtggccaatttaaggcacacctatgtattttgtgcagtgtaagacaaaagtaa

attggtttgctgggtgctactcaagtaagtggattatattgtgttcagtcatttggacgagtaaagaatgttcatgacatgcccatgtgctgaatgattc

tgcagtagcgggactactacagtaaagaaaaataattaattttcgttcttaaataacttaatgactggtggaagacactgtgaaatcaacactgtcacag

tcaacagcattaactgtttgtatgactgtggcaattaccaactagattactgacaggtcacttcagttgtaagctttcttctctgttatccagcatgcag

tatgtctgcataggttttgtaacatagagttactgccagaaagcctaaaaatactgtcacagtggcaactgtttgaactctgtgaagaaaggtagatttt

tcttgtttctgtaactttgcc

>52823|6-phosphofructo-2-kinase fructose- -biphosphatase-like

taacaattaatgccactggccaactgaagcacctcgtcaaataaatcagtacagtgaaatatttgaaagtacttacattgacattccttgaaagaatgcc

attgtcatcacaaacacattcaataaccaagcacctatagcccatacgtttctcaaaatggtcatgtactttctgccgc

>52822|6-phosphofructo-2-kinase fructose- -biphosphatase-like

atacatcagaactttagcctcaagatgacctgtgaccttgtgggtagcaattgactctcctgaaattggagcagggcacatttaattaaaatcctttatg

catgaaacttttatgtgagttatctaatcaattgtaagtttcagtaagcataataaccaactcaccatcattgatgtacttgatgaaacggtattgcggc

tcatgttttgagctcataggttcatactgttcaatatagtgttgtattttatgattgaaatcatccatagccttttcagaattcatatctttataatctg

ggctattctgtaatgtttcattgacattccttgaaagaatgccattgtcatcacaaacacattcaataaacaagcacctatagcccatacgtttctcaaa

atggtcatgtactttctgccgctgataatgagctatgtgtgtcccatccaggatc

>52821|6-phosphofructo-2-kinase fructose- -biphosphatase-like

gttgcaatacactattgtaaaaaagtgtttatgttactactgtatcacactattgtagattttttccaaagaagtaaaaagcaattacagtaagctacat

ttgagtttcagaaattagtaaaacttccactgtttgtcttacatctctttcttaaaaattggtaagatgcaaagttttcatacgtctttctttttttttt

cagatcctggatgggacacacatagctcattatcagcggcagaaagtacatgaccattttgagaaacgtatgggctataggtgcttgtttattgaatgtg

tttgtgatgacaatggcattctttcaaggaatgtcaatgaaacattacagaatagcccagattataaagatatgaattctgaaaaggctatggatgattt

caatcataaaatacaacactatattgaacagtatgaacctatgagctcaaaacatgagccgcaataccgtttcatcaagtacatcaatgatggagagtca

attgctacccacaaggtcacaggtcatcttgaggctaaagttctgatgtatctttcaaatttcaggccagctccaaaaacattttacttttcaagg

>52842|6-phosphofructo-2-kinase fructose- -bisphosphatase short

atgtcatctcttcacaaattccagcatctatttcattatgagctttccaatatacctgcaccacaggaaaggtggaaagctcttaatgaaatagatgctg

gaatttgttaagagatgacatatgaagaaatagctgagaaataccctgaagactttgctgcccgtgaccagaatttagaaggacgaattggtggagactc

tgagctttcctatagagggcac

>52867|6-phosphofructo-2-kinase fructose- -bisphosphatase short form

gtggcaaactccgcctctcgcgttagtgattggcgaattgcgggtggtgcgacttgtgtcaagctaaacatgaggttataatggatctgagtgggtcagt

ttgcgtgtgcgattattttaaagagaaaggggtaaggttataccctgaaagttattccatgtcaccagaaactagaaaaagcataccgccaccaacggag

acaatacagacgaaacctttccctgtcagaggagagcgtgccaactatgtcaacagtcctcatgtcattgctatggttgggctgcctgccagaggcaaaa

catacatctccaaaaagctttcacgatacctgaactggattggcatcaacactaaagttttcaatttaggagaataccgccgtcatgcaacaacagccta

caagagtcatgaattctttagaccagacaataaagaggcaatggccatacgcacacaatgtgcaattgacgcattgcaagatgtgtgctcctggctggag

tgtggtggagaagtggcggtatttgatgcaacaaattccacagctgaccgtcgaagaatgatacatgaaatagttgtaaataagatgggtttcaaattat

tctttgtggaatctgtgtgtgatgatcctcagataatagagcaaaatataatggaagtgaaagtaagcagcccagactatcagaacatgaataaggaaat

ggcattgaaagattttctgaaacgtatagaacattaccaggaaagatatcagcccttggatgagaaatatgaatccaaccttagtttcatgaaaatatac

aatacaggtgaaaaggtggtagtacataaacatgagggacatatacagagtcgaattgtttactacctaatgaacatacatataacacctcgtactattt

atctcacaaggcatggtgagagtgagcagaatttagaaggacgaattggtggagactctgagctttcctatagagggcaccagtatggagctgcactgtc

tgcatacattcagcagcaagatatacctggtcttcgtgtttggacttcttggcttaagagaaccatacagacagttgccaatatacctgcaccacaggaa

aggtggaaagctcttaatgaaatagatgctg

>52887|6-phosphofructokinase, putative

gaatgaatgtattaggtcacatgcaacaaggaggatatccaacaccgttcgaccgtaatatgggaacaaagatgggagcaaaaacagttgaatggatgtt

ccacaaactaaaagaatcttccaaaggtgatggcactgtatatactgcaacaccagattctgctacactgcttggagtattgaggagacggtatacattc

agtccactggaagaactaaagaaaggcacaaattttgattaccgtataccaaagaatcagtggtggctaaaattacgtcctctgttaagaattttgtcaa

aacatgagagtgcttatgaagaagaaggcttgtacatgaccatagaacaagaagaagaagatgacaaaacagtagcaaaataagacaaatggtaattctt

gaagctcttatcaccaaacaagtagtctatatacctttattgtttttctccctttgattatgttcaatgagaacaaaggtctcttttatgtaagttaata

tatgttc

>52910|6-phosphofructokinase-like isoform 1

aatccatgcatctggctgaaccaataatggtcccacccttatgtataatggacgaaactgtcgcccagccagcttcctctat

>52907|6-phosphofructokinase-like isoform 1

aatttctgtactactgcacgtactgcagcattcatcccttgagaatcgccgccactcgtgagtactgctaacccttttccaacatgt

>52908|6-phosphofructokinase-like isoform 1

ataccaaaacgtactactgcacgtactgcagcattcatcccttgagaatcgccgccactcgtgagtactgctaacccttttccaacatgtgaacctctct

gcaagaagcgtacaggaatttctcccgaaaactggccggctacgtcacttccttctctaagcgtagggacatctc

>52906|6-phosphofructokinase-like isoform 1

gtcgcgggggtaagtgaaggtgaccttggcggggtttggaagtcgtctgttgtgtagtgtcaacaaattaagtttcaaacatggcagatcaagatccaga

tacaagtcaaagacgtttcatagagagaggttcacacaaaggcaaaggcattgcagttcttaccagtggaggagactctcagggaatgaatgctgctgtt

cgtgctgttgtaagatttggtatatatttgggttgcaaagtattcttcatcagggaaggttaccaaggaatggttgatggtggagatcacatagaagagg

ctggatggtctagtgtttcctctattattcataagggaggaactgtaattggctctgcaaggtgtaaggactttagagaacgtgaaggccgcttaaaggc

tgcaaaaaaccttgtgaccagagggattacaaatcttgttgtgattggtggagatggttcactcacaggggcaaatctctttcgacaagaatggccctct

cttcttgatgaactactgcaaaagggtgaaatcacaaaagaaagcagagaaaagtacacatatcttcatattgctggtttggtgggttccatagacaatg

atttctgtggtactgacatgaccattggtacggattctgcccttcatcgaattattgaggctgttgatgccatagtctcaacagctttctcacatcagcg

cacatttattatggaggtcatgggacgacattgtggatatttggcgctggtgactgccctgacaagtgaagctgattttgtgtttattcctgaatggcca

ccagagcatgattggccaactaaactctgcaagaagttagcacaggcaagaggagacttctcatttgtgcatcttgtcttcaccgcgttctttctttcca

tcagctacattactttcctacttaaatcacactgttgaatcagactcaggcagaaaaaaaaagaaaatgtgaaaacccctttttacaaactattggtggg

gcttgcaggccaacgattaaacattattattgttgcggaaggagctatagaccgtgatggacaaccgattacttcagagaaagtgaaacaggttgttgta

gaaaacttgcagcaagacacccgtataactgtacttggacatgtgcagcgaggtggcaatccatctgcatttgaccgagtattgggctgccgtatgggag

ctgaggcagttctggctttgatggaagcaacaccaggttccgaaggagttcttatctctctagctggtaatcaggctgttcggttgccattaatggaatg

tgtagaaaggacaaaagctgttgcaaaagctatggctgacaaacaatgggataaagcagttcagcttagaggcaggagttttgaaagaaatctgtacatc

tacaaaatgctgacaagactaaaacctcctactccagttggtgatgctgtacag

>52909|6-phosphofructokinase-like isoform 1

gaaaagtggggagaagtatagtggaatgcattcactacatcagttaaaagagtataaataatcaacaatatataaaataaataagtcaacaactgagagt

agtgcattcctaccagaggataacacaagtactttaaaaaaataatcgtctatacaacaccacaccttcatcataaaattcttttcaagattaaaataac

aattttagatataaaggattctctatacaggatacatttttggtattttagtatacaacgacactggagaaattaattacaatatcaacacaatataaga

acttgtaacttctacaacagcactcaatttaaccatttggcaagaaaaaaaacacagtattatctttcacattgtgtagtataattgtgctatagatgaa

caagccttctatattatgttttttggtatgttacaaaaggcaattataattacagatctatgtgaaatcaatgtaccagaaacaaatcagttgttaaaaa

tacaataaagcagcaggacaaataaaagaaaaaaatgtatgtcttttcacatactggttaccatcccagatcattctacattagaaactcttaacataaa

aagtgtattcccccaaaatatgtttcgatcacattgttttgtctgtaacacaatttttgtagttctagaaacatttgtcttattttgctactgttttgtc

atcttcttctgcttgttctatggtcatgtacaagccttcttcttcataagcactctcatgttttgacaaaattcttaacagaggacgtaattttagccac

cactgattctttggtatacggtaatcaaaatttgtgcctttctttagttcttccagtggactgaatgtataccgtctcctcaatactccaagcagtgtag

cagaatctggtgttgcagtatatacagtgccatcacctttggaagattcttttagtttgtggaacatccattcaactgtttttgctcccatctttgttcc

catattacggtcgaacggtgttggatatcctccttgttgcatgtgacctaatacattcattcgagcactaaaaagcccctttccttcttcagagtacaaa

cgataaatgaagtctgttgtgtagttttcattgcatttttcatttctaagaataaggcctctctggacaccttcagccattttggatgccatgtggtaca

catcttgctgtagatcttttatcgaaaatttctcttcgaatatgtatgccgcatcagcgcctccagcaagagctgctagtgtagcgaggtatccacagta

gcctcccatagtttctatgacaaacactcttctctttgtgccctgggcagactgccggattctgtcacaaatctctgtaatttcattaagtgctgtatca

caacccaaggaaaattctgtgcctggaacattattacttattgttgaaggaataacagcaatcggtatgcagaactctggttgtgttggcctgctctctg

caagttgcagcgcaccttggtaagcctcaaaaccgccaataatgagaaggccttggatgttgaattccttgaagcgagctgcaatttgctttattctatt

tccaggcagagtacgttttgtccccagcatggcaccacccaaagaaacccaaccagtcacatctgaccatgccattttactaatattaccagccactaga

ccttcaataccatcatgaattccaagaactgtcacaccgcggtaaaggcaatttctgacaaatgatctcacagcagcattcattccacaagctggtgctc

caatgtgcataactgccaaagtgaaggattcctgtacagcatcaccaactggagtaggaggttttagtcttgtcagcattttgtagatgtacagatttct

ttcaaaactcctgcctctaagctgaactgctttatcccattgtttgtcagccatagcttttgcaacagcttttgtcctttctacacattccattaatggc

aaccgaacagcctgattaccagctagagagataagaactccttcggaacctggtgttgcttccatcaaagccagaactgcctcagctcccatacggcagc

ccaatactcggtcaaatgcagatggattgccacctcgctgcacatgtccaagtacagttatacgggtgtcttgctgcaagttttctacaacaacctgttt

cactttctctgaagtaatcggttgtccatcacggtctatagctccttccgcaacaataataatgtttaatcgttggcctgcaagccgctcctgtgctaac

ttcttgcagagtttagttggccaatcatgctctggtggccattcaggaataaacacaaaatcagcttcacttgtcagggcagtcaccagcgccaaatatc

cacaatgtcgtcccatgacctccataataaatgtgcgctgatgtgagaaagctgttgagactatggcatcaacagcctcaataattcgatgaagggcaga

atccgtaccaatggtcatgtcagtaccacagaaatcattgtctatggaacccaccaaaccagcaatatgaagatatgtgtacttttctctgctttctttt

gtgatttcacccttttgcagtagttcatcaagaagagagggccattcttgtcgaaagagatttgcccctgtgagtgaaccatctccaccaatcacaacaa

gatttgtaatccctctggtcacaaggttttttgcagcctttaagcggccttcacgttctctaaagtccttacaccttgcagagccaattacagttcctcc

cttatgaataatagaggaaacactagaccatccagcctcttctatgtgatctccaccatcaaccattccttggtaaccttccctgatgaagaatactttg

caacccaaatatataccaaatcttacaacagcacgaacagcagcattcattccctgagagtctcctccactggtaagaactgcaatgcctttgcctttgt

gtgaacctctctctatgaaacgtctttgacttgtatctggatcttgatctgccatgtttgaaacttaatttgttgacactacacaacagacgacttccaa

accccgccaaggtcaccttcacttacccccgcg

>52925|6-phosphofructokinase-like isoform 2

ggaaaagtggggagaagtatagtggaatgcattcactacatcagttaaaagagtataaataatcaacaatatataaaataaataagtcaacaactgagag

tagtgcattcctaccagaggataacacaagtactttaaaaaaataatcgtctatacaacaccacaccttcatcataaaattcttttcaagattaaaataa

caattttagatataaaggattctctatacaggatacatttttggtattttagtatacaacgacactggagaaattaattacaatatcaacacaatataag

aacttgtaacttctacaacagcactcaatttaaccatttggcaagaaaaaaaacacagtattatctttcacattgtgtagtataattgtgctatagatga

acaagccttctatattatgttttttggtatgttacaaaaggcaattataattacagatctatgtgaaatcaatgtaccagaaacaaatcagttgttaaaa

atacaataaagcagcaggacaaataaaagaaaaaaatgtatgtcttttcacatactggttaccatcccagatcattctacattagaaactcttaacataa

aaagtgtattcccccaaaatatgtttcgatcacattgttttgtctgtaacacaatttttgtagttctagaaactgaaaacgaaagaacacagttattagt

tctccagttagcacctgcacactttgtcaaagcctagtttctaacattccatacaacacacaacggcagtataacagaagcaaagagggcaaacacattt

gtcttattttgctactgttttgtcatcttcttctgcttgttctatggtcatgtacaagccttcttcttcataagcactctcatgttttgacaaaattctt

aacagaggacgtaattttagccaccactgattctttggtatacggtaatcaaaatttgtgcctttctttagttcttccagtggactgaatgtataccgtc

tcctcaatactccaagcagtgtagcagaatctggtgttgcagtatatacagtgccatcacctttggaagattcttttagtttgtggaacatccattcaac

tgtttttgctcccatctttgttcccatattacggtcgaacggtgttggatatcctccttgttgcatgtgacctaatacattcattcgagcactaaaaagc

ccctttccttcttcagagtacaaacgataaatgaagtctgttgtgtagttttcattgcatttttcatttctaagaataaggcctctctggacaccttcag

ccattttggatgccatgtggtacacatcttgctgtagatcttttatcgaaaatttctcttcgaatatgtatgccgcatcagcgcctccagcaagagctgc

tagtgtagcgaggtatccacagtagcctcccatagtttctatgacaaacactcttctctttgtgccctgggcagactgccggattctgtcacaaatctct

gtaatttcattaagtgctgtatcacaacccaaggaaaattctgtgcctggaacattattacttattgttgaaggaataacagcaatcggtatgcagaact

ctggttgtgttggcctgctctctgcaagttgcagcgcaccttggtaagcctcaaaaccgccaataatgagaaggccttggatgttgaattccttgaagcg

agctgcaatttgctttattctatttccaggcagagtacgttttgtccccagcatggcaccacccaaagaaacccaaccagtcacatctgaccatgccatt

ttactaatattaccagccactagaccttcaataccatcatgaattccaagaactgtcacaccgcggtaaaggcaatttctgacaaatgatctcacagcag

cattcattccacaagctggtgctccaatgtgcataactgccaaagtgaaggattcc

>52937|6-phosphofructokinase-like isoform 3

caatatataaaataaataagtcaacaactgagagtagtgcattcctaccagaggataacacaagtactttaaaaaaataatcgtctatacaacaccacac

cttcatcataaaattcttttcaagattaaaataacaattttagatataaaggattctctatacaggatacatttttggtattttagtatacaacgacact

ggagaaattaattacaatatcaacacaatataagaacttgtaacttctacaacagcactcaatttaaccatttggcaagaaaaaaaacacagtattatct

ttcacattgtgtagtataattgtgctatagatgaacaagccttctatattatgttttttggtatgttacaaaaggcaattataattacagatctatgtga

aatcaatgtaccagaaacaaatcagttgttaaaaatacaataaagcagcaggacaaataaaagaaaaaaatgtatgtcttttcacatactggttaccatc

ccagatcattctacattagaaactcttaacataaaaagtgtattcccccaaaatatgtttcgatcacattgttttgtctgtaacacaatttttgtagttc

tagaaactgaaaacgaaagaacacagttattagttctccagttagcacctgcacactttgtcaaagcctagtttctaacattccatgtatctcgcctcgt

tgttttgtgataagcgcttcgggcattcccatttgtcttattttgctactgttttgtcatcttcttctgcttgttctatggtcatgtacaagccttcttc

ttcataagcactctcatgttttgacaaaattcttaacagaggacgtaattttagccaccactgattctttggtatacggtaatcaaaatttgtgcctttc

tttagttcttccagtggactgaatgtataccgtctcctcaatactccaagcagtgtagcagaatctggtgttgcagtatatacagtgccatcacctttgg

aagattcttttagtttgtggaacatccattcaactgtttttgctcccatctttgttcccatattacggtcgaacggtgttggatatcctccttgttgcat

gtgacctaatacattcattcgagcactaaaaagcccctttccttcttcagagtacaaacgataaatgaagtctgttgtgtagttttcattgcatttttca

tttctaagaataaggcctctctggacaccttcagccattttggatgccatgtggtacacatcttgctgtagatcttttatcgaaaatttctcttcgaata

tgtatgccgcatcagcgcctccagcaagagctgctagtgtagcgaggtatccacagtagcctcccatagtttctatgacaaacactcttctctttgtgcc

ctgggcagactgccggattctgtcacaaatctctgtaatttcattaagtgctgtatcacaacccaaggaaaattctgtgcctggaacattattacttatt

gttgaaggaataacagcaatcggtatgcagaactctggttgtgttggcctgctctctgcaagttgcagcgcaccttggtaagcctcaaaaccgccaataa

tgagaaggccttggatgttgaattccttgaagcgagctgcaatttgctttattctatttccaggcagagtacgttttgtccccagcatggcaccacccaa

agaaacccaaccagtcacatctgaccatgccattttactaatattaccagccactagaccttcaataccatcatgaattccaagaactgtcacaccgcgg

taaaggcaatttctgacaaatgatctcacagcagcattcattccacaagctggtgctccaatgtgcataactgccaaagtgaaggattccatgccgagaa

cagaatcttgctgtacagcatcaccaactggagtaggaggttttagtcttgtcagcattttgtagatgtacagatttctttcaaaactcctgcctctaag

ctgaactgctttatcccattgtttgtcagccatagcttttgcaacagcttttgtcctttctacacattccattaatggcaaccgaacagcctgattacca

gctagagagataagaactccttcggaacctggtgttgcttccatcaaagccagaactgcctcagctcccatacggcagcccaatactcggtcaaatgcag

atggattgccacctcgctgcacatgtccaagtacagttatacgggtgtcttgctgcaagttttctacaacaacctgtttcactttctctgaagtaatcgg

ttgtccatcacggtctatagctccttccgcaacaataataatgtttaatcgttggcctgcaagccgctcctgtgctaacttcttgcagagtttagttggc

caatcatgctctggtggccattcaggaataaacacaaaatcagcttcacttgtcagggcagtcaccagcgccaaatatccacaatgtcgtcccatgacct

ccataataaatgtgcgctgatgtgagaaagctgttgagactatggcatcaacagcctcaataattcgatgaagggcagaatccgtaccaatggtcatgtc

agtaccacagaaatcattgtctatggaacccaccaaaccagcaatatgaagatatgtgtacttttctctgctttcttttgtgatttcacccttttgcagt

agttcatcaagaagagagggccattcttgtcgaaagagatttgcccctgtgagtgaaccatctccaccaatcacaacaagatttgtaatccctctggtca

caaggttttttgcagcctttaagcggccttcacgttctctaaagtccttacaccttgcagagccaattacagttcctcccttatgaataatagaggaaac

actagaccatccagcctcttctatgtgatctccaccatcaaccattccttggtaaccttccctgatgaagaatactttgcaacccaaatatataccaaat

cttacaacagcacgaacagcagcattcattccctgagagtctcctccactggtaagaactgcaatgcctttgcctttgtgtgaacctctctctatgaaac

gtctttgacttgtatctggatcttgatctgccatgtttgaaacttaatttgttgacactacacaacagacgacttccaaaccccgccaaggtcaccttca

cttacccccgcgac

>52959|6-phosphogluconate decarboxylating-like

ccttgataaagcaggccaaaaaggaacgggtaaatggacaagccaaagcgctcttgacttaggcgtgccgcttccgattatcactgaatctgtttttgc

>52958|6-phosphogluconate decarboxylating-like

gcccacgaccttggtgcccttggcctcgttggccaggaaggcgtccaccttctccgtggtgcggttgtaggcgcacaccgtgaatccgtggtcgttcatg

ttgaggatcaggttctggcccatcaccgccagcccgatcagcgcgatgtccgccttttcactcattgttttccgtgcgaaggtagttgtctggtgataca

ggtgctggaacaagcggaatttcgagacaagcttgggcgtccacgtctcggcggcgcagtgagagtgcaactacggcgggtggcgatattaacgacttat

atcaggcgcacagaatcactcgcctattgcataacggcactctaacacgtcggcaacgtcacacgtgtgctggaacaagcggaatttcgagacaag

>53016|60 kda ss-a ro

taggggaaaacttttttcaaccaatgcctcctccttagacagaggaacagaggagaggctacatcacggtgaggaaggaacagtttaatttcctggaatt

atttaaacaagctacagaaagaagtgtacctaaatggcccactaattttaagtccagcactttattgcctattcatccagtttcgtcgtatcagaaacgt

gggaacgcattatttatgctatatgctgtattaatatgattattatgagaactgtgtcattatagttttagtttgccctacataaatgttcatcagtaaa

gtacatcacacaatagtctttccttgcctgtgttacacacaccaagttctattccctctttcccttccactgtgtgagaactcttccaatacgactgctg

ttttttaactatcttccaatgcaaggatgtctgaagttaatgtaaggtcagccggtacactgactatacaaagtgttacattaaaaaactctgcagtttg

atattccgagatgtttttacctcttctcttcttaaaccaattgtttacacttcttccactctctctttcttgtaaaatgtttactgatgcttctccccat

acatggccttcttattgtctcttcattccacatcttataaatttattttgaaccattcctcctacaaatccacatttaaattttttcaagactgtctctg

actttcacaatagacatgttcaaacgtcatagagagagatgaatgattttaatgttgtctgaaaatgtagtatatttgttctatattatgaaatttcaga

tttattttggccttttccttcaattcgtttacagttttgaagttgaaaaacaaaagtgacaatttttcacatctcaacaaaaatgttgggtttctgcagc

ttcccaacttttatttacaagttgtcttagctttataattcagaaaatattctgttcaattttatactgtcagtagctacatcctgacctacaattgcta

catgtctgagttttttctcagttcattcttcagaattacatttcatagctgctattacttcttcagtaattctaacttttctgaaatcaaattaattctc

tagcatttcttcaacttcccagtctagtgcaaatcatttggttaatttaaccacacatagaaaacaaagtaaaaaacgtttagctgaataagaatgacaa

aaatcattcttactgctgttgttgatcactaacttgtctctttcttaaacctagaaagcagtatagacatatgaatttagcgagcaatatgtttatgata

atcgtgtttgcttaaaatctctcaaacactacttcataaacctggatttgtgttacacgattaaactaaatacataacgtgcagcagttagaaaaaatat

taaacatcaacaattaatgtaataaataaaataacaagccataccaaaagctgttatcaatatagtaaacaattcaagtgaagaatgacagtgctcataa

gtgaatccaatcaaaatacacatttcttctgagaaaaagagaaagagagagagagagagagagagagagagagagagagagagtagaagttaaactttat

ttgtatcacaaactgtcactctaatcttgcatcagatttcactcgcaataaacaaaactaaaatgctcctcttgaaaatgcttcaattatagtgggcacc

ttttcatcaaatcctttgatctccaacataccaggatctgctgggtcaatacaattcaatccacacatatctagtccaaccgcaatatactttgtttgag

gaagattcatttcagttctatactgatttaagcatgttggtaaagaggcagaacttgtttcattgttgttatttgttataacaacaaacacatcaactgc

ctttttgtttttctgtgcccacattacaggttgtgccatattcactttagcagcagaacaacgcgctctcattgttgaaattgtttcattgaaagacagt

gatttatccaatggcacttcctccatatctgatgctgaatatataagaacagtagcatctgtttccgttttgtaaatacaatatgttatcaggagtgcag

ccatacaacatgtcaattcacgacactgccaacactttgtcagtatcatatcaaatccattgttgactgctattatatacctcaagccagttggtttaag

aagactgactgatgtattcaaaagtgcattagctgcatctacgacagcagggttgactggtggtgggggcttaactggtcttgatctgtcaataaatctc

aacctagatgacgattcatacttttttaagtagagcaagatttctgcaggctgtactttctcctctataatggcctgttcctcctttaaacgatccacaa

taagtggaacaatcactgactttcgtcgaagtaaccgatatcttgacaaacgtcgcagaaccttcaatatggtcaccacagacatctttggtattaacac

atgccaaatttctttcgatctatagaggcctgatggcacacattcaaatgaaaatgaatgtgctgatataagtaatgacgctgtagtggaatttgtgcta

tgtttaagattctcaacatttttcaggtactcaattattgattgtgcctctggcttatcattaaataatttctgtgctgtctcaataccatttatcacat

aactgatgacagcattttgggctggatttttagaacatgtatgagccaatttcagaatatccttgtgtgtccagctatgcattcctacaaaacgagtaac

attttcagccattttctgtggttcttgatgtaaataccagttattaactgcacggcgtagccctcttccccagcctgttgttggtttcgacaaatcagtt

atgtacttcacaaaagcaaaaaatgactttggtgtcttatacactgtttcaatagcagcgtaggctgctcggcggacttctaatgactgactctgcttca

tgcataacaccaatgcaaacaagatggcttctggttcagcggagtaaccaccattgtaagcctcaattatgtgttttactacatcacttcctttcccttc

agaaattagagaatcaactgatgtcagttctactgcttcatagtttggagaggcaatatttattttccatgcaccagcttcataacatggaagttcagat

ccatagtgtaagaaccttttcagtcgtgtttccgctgatactgccattttctctgtgagattagttactatcatccatcaaagtgctaataagtgtacat

ccatcaaggaccatccgcttagtataaggtttgtgacacacgtaatgcctggtttctatgcctcctcagcagcagcttcattctaatttttactgacagc

aatgcttcgaactgtcacttaaaatatacaaaattgtcgcactgtattattcggatacactcgataaaaaaaaagtttttagtaagtacagcaaaaagaa

agaaaagccctatgaacatcataacaaagagcttagtaacaacactatttgcggaagataattttttgtttatttaccatatgattcaaacacgctacat

gcacacgcacactcacacacactgccttaaaccaataaaccttcaacagcaagaacctccagcacgcacgaaacgggaaacagcgatattcttt

>53030|60 kda ss-a ro ribonucleo

tttgtgaagtacataactgatttgtcgaaaccaacaacaggctggggaagagggctacgccgtgcagttaataactggtatttacatcaagaaccacaga

aaatggctgaaaatgttactcgttttgtaggaatgcatagctggacacacaaggatattctgaaattggctcatacatgttctaaaaatccagcccaaaa

tgctgtcatcagttatgtgataaatggtattgagacagcacagaaattatttaatgataagccagaggcacaatcaataattgagtacctgaaaaatgtt

gagaatcttaaacatagcacaaattccactacagcgtcattacttatatcagcacattcattttcatttgaatgtgtgccatcaggcctctatagatcga

aagaaatttggcatgtgttaataccaaagatgtctgtggtgaccatattgaaggttctgcgacgtttgtcaagatatcggttacttcgacgaaagtcagt

gattgttccacttattgtggatcgtttaaaggaggaacaggccattatagaggagaaagtacagcctgcagaaatcttgctctacttaaaaaagtatgaa

tcgtcatctaggttgagatttattgacagatcaagaccagttaagcccccaccaccagtcaaccctgctgtcgtagatgcagctaatgcacttttgaata

catcagtcagtcttcttaaaccaactggcttgaggtatataatagcagtcaacaatggatttgatatgatactgacaaagtgttggcagtgtcgtgaatt

gacatgttgtatggctgcactcctgataacatattgtatttacaaaacggaaacagatgctactgttcttatatattcagcatcagatatggaggaagtg

ccattggataaatcactgtctttcaatgaaacaatttcaacaatgagagcgcgttgttctgctgctaaagtgaatatggcacaacctgtaatgtgggcac

agaaaaacaaaaaggcagttgatgtgtttgttgttataacaaataacaacaatgaaacaagttctgcctctttaccaacatgcttaaatcagtatagaac

tgaaatgaatcttcctcaaacaaagtatattgcggttggactagatatgtgtggattgaattgtattgacccagcagatcctggtatgttggagatcaaa

ggatttgatgaaaaggtgcccactataattgaagcattttcaagaggagcattttagttttgtttattgcgagtgaaatctgatgcaagattagagtgac

agtttgtgatacaaataaagtttaacttctactctctctctctctctctctctctctctctctctctctctttctctttttctcagaagaaatgtgtatt

ttgattggattcacttatgagcactgtcattcttcacttgaattgtttactatattgataacagcttttggtatggcttgttattttatttattacatta

attgttgatgtttaatattttttctaactgctgcacgttatgtatttagtttaatcgtgtaacacaaatccaggtttatgaagtagtgtttgagagattt

taagcaaacacgattatcataaacatattgctcgctaaattcatatgtctatactgctttctaggtttaagaaagagacaagttagtgatcaacaacagc

agtaagaatgatttttgtcattcttattcagctaaacgttttttactttgttttctatgtgtggttaaattaaccaaatgatttgcactagactgggaag

ttgaagaaatgctagagaattaatttgatttcagaaaagttagaattactgaagaagtaatagcagctatgaaatgtaattctgaagaatgaactgagaa

aaaactcagacatgtagcaattgtaggtcaggatgtagctactgacagtataaaattgaacagaatattttctgaattataaagctaagacaacttgtaa

ataaaagttgggaagctgcagaaacccaacatttttgttgagatgtgaaaaattgtcacttttgtttttcaacttcaaaactgtaaacgaattgaaggaa

aaggccaaaataaatctgaaatttcataatatagaacaaatatactacattttcagacaacattaaaatcattcatctctctctatgacgtttgaacatg

tctattgtgaaagtcagagacagtcttgaaaaaatttaaatgtggatttgtaggaggaatggttcaaaataaatttataagatgtggaatgaagagacaa

taagaaggccatgtatggggagaagcatcagtaaacattttacaagaaagagagagtggtagaagtgtaaacaattggtttaagaagagaagaggtaaaa

acatctcggaatatcaaactgcagagttttttaatgtaacactttgtatagtcagtgtaccggctgaccttacattaacttcagacatccttgcattgga

agatagttaaaaaacagcagtcgtattggaagagttctcacacagtggaagggaaagagggaatagaacttggtgtgtgtaacacaggcaaggaaagact

attgtgtgatgtactttactgatgaacatttatgtagggcaaactaaaactataatgacacagttctcataataatcatattaatacagcatatagcata

aataatgcgttcccacgtttctgatacgacgaaactggatgaataggcaataaagtgctggacttaaaattagtgggccatttaggtacacttctttctg

tagcttgtttaaataattccaggaaattaaactgttccttcctcaccgtgatgtagcctctcctctgttcctctgtctaaggaggaggcattggttgaaa

aaagttttcccctaaatgattttatgagatgattccacaggtattttgtttctttcagatatcttcagtgtgtg

>53097|60s acidic ribosomal protein p1

caatcctggccagtacggatcaacatccactgctgctgccttcagaatagtctggattttctcgcccttaaaagaaaaattatattataaccaatgtcac

tgagaatcaaacaacgttacgcagttagaaattac

>53122|60s acidic ribosomal protein p1-like

agcaaaggagatagtctatcatgtctaacaagtctgaattagcctgcatctactctgccttgagaatagtctggattttctcgccagttactgcgatctc

atcgtcaacgaggatcaaggcagagtagatgcaggctaattcagacttgttagacatgatagactatctccttt

>53121|60s acidic ribosomal protein p1-like

cgaggaggtcgcagtagctggcgagaaaatccagactagtctgaaggcagcagcagtggatgttggtccgtactggccaggattgtttgcaaaggcactg

gaaggaattaaatctttcacattaattccttccagtgcctttgcaaacaatcctggccggtacggatcaacatccactgctgctgccttcagaatagtct

ggattttctcgccagttactgcgacctcatcctcgttgacgatgaggatcaaggcagagtagatgcaggctaattcaga

>53139|60s acidic ribosomal protein p2

acctaacatctgaggtcatcagtgctcggagccatttcaaccatttgaaagattttggtttgtcggcatcgaagctgatagtgagagactgaagaaagtt

atcattgaattgaatgggaagaacattgaaagtttgatagcagcaggtcgtgagaagttggcctcgatgccatttggtggtgccgcagctccgccacctc

cagccgcagc

>53138|60s acidic ribosomal protein p2

tttcaatgttcttcccattcaattcactgataactttcttcagtctctcactatcagcttcgattgggacagaaaatccaacttctgcagatattgaaaa

ggttctcagttctgtcggcatcgaagctgatagtgagagactgaagaaagttatcagtgaattgaatgggaagaacattgaaagtctgatagcagcaggt

cgtgagaagttggcatcgattccagttggtggtgctgctgctcctgctgctgcggctggaggtggtggagctgctgcagctgctgctccagagaaggagg

agaagaaacctgaaaagaaagaagagtctgaatctgatgatgatgacatgggctttggat

>53140|60s acidic ribosomal protein p2

ctgatatcctgttcctctgcatgcaacttaccctcatacgcatgacgccatagttagtgcgaggaagtggatacacgtttcgtgtcgaaaggacttaaaa

aatgcgttacgtggccgcttacctgcttgccgcccttggtgggaaagaaaatccaacttctgcagatattgaaaagattctcagttctgtcggcattgaa

gctgatagtgagagactgaagaaagttatcagtgaattgaatgggaagaacattgaaagtctgatagcagcaggggaaagtctatgcatgcgatactccg

ctgatattcctgcacgatattggcggtgtttaccctgggtatgggaagaatggatttgtccctgcagcttatacatctcgcggactgttaaaagacgata

a

>53142|60s acidic ribosomal protein p2

tatcagtgaattgaatgggaagaacattgaaagtctgatagcagcaggtcgtgagaagttggcatcgatgccagttggtggtgctgctgctccagagaag

gaggagaagaaacctgaaaagaaagaagagtctgaatctgatgatgatgacatgggctttggattatttgactaagttcggaaactgaacagggaaagaa

aatccaacttctgcagatattgaaaagattctcagttctgtcggcatcgaagctgatagtgagagactgaagaaagttatcagtgaattgaatgggaaga

acattgaaagtctgatagcagcaggtcgtgggaagttggcatcgatgccagttggtggtgctgctgctactgctgctgctactactgttatgacagctaa

cttcagaatgcacactggagttcaatgcgttggtatcttctatgtgacgattttgttctatcccagcaacagacgaatgatcacaatacagtgactgtat

atcattcaaatcattactttgatctggacattcagaatatgggggcaaaagtttactatttaaatttgttgaca

>53284|60s ribosomal protein

gaaacaagttcctttgaatcgctgtaagcattttgaacttggtcctgacaaaaagaggaagggccaaatgttcca

>53270|60s ribosomal protein

gcttcgtatttgtgaaccatatgcgacttggggaatacgaagcctgaagagtgtgcgtgagtttatatacaagagagg

>53232|60s ribosomal protein

tgtaatctgcatttgcctctgcatttttcaataactgatgcaagaacgctccagatttcttaggccagcggccctgag

>53177|60s ribosomal protein

acctattccgcagtgggtcaggatgcgaacagataatcccatccggtacaatgcaaagaggcaccactggcggaggacaaagctcg

>53289|60s ribosomal protein

gagaatggattgcccagaatgcaaattcaggaaacaagttccttggaaacgctgcaatcattttgaacttggtggtgacaaaaagag

>53229|60s ribosomal protein

gacatcatggcaaaaaggaatatgaggcttgaaattagaaaggcacagcgagagcaggcaatcagggctgctaaagagcagaaaaaag

>53209|60s ribosomal protein

gtgggatgtactgttgaagggaacccaccacacgatttaattgataagataaatgctgctgattcggaagttccagacgattgaaatgag

>53212|60s ribosomal protein

cacagtggaaatttgtcatttgatgaaagtgtaaatattgccaaccaaatgagggccagagccttttcgagaaaacttgaaggaacggtg

>53304|60s ribosomal protein

ttctgtggcgactgttgaaaaagtacagggaagcaaagaaaattgacaggcacctttaccaccagctgtacatgaaggcaaaaggtgaaagag

>53243|60s ribosomal protein

atgcaaaccagtgatgaagcatcgggagctgcgctgtctttccgctgctgcccgcaagtctcgtggccttggcaagtgtcatctctacagccag

>53174|60s ribosomal protein

ttccgatctccaggcttcttcataaaacgcactactcttgcgagcgagttaggtggcctatttatttttgacatgaacaacctccgtaaaaaata

>53210|60s ribosomal protein

taaaacacagtggaaatttgtcatttgatgaaattgtaaaaattgcaaaacgaatgagggaccgaaccatgtcgagaaaacttgaaggaacggtg

>53218|60s ribosomal protein

tcaaattccttaacctcggggctgtaacctgggaatcttttggttcagggaggtatattcaaaccaccgtaaacagctcccttcatagcaccaaac

>53253|60s ribosomal protein

tcatttgaaacttaattgttgctttcacttcatcaattgttgccatcactgcttcaaggtctgaaagaagaccaggaaacttgcaaaccttatttaag

>53271|60s ribosomal protein

cacaaatacgaagcatatgtattgtcgccttgttcaggttcaggaagaggccaggggggatctgtttcagccggaacagctgcagaacttttcgcacctt

>53222|60s ribosomal protein

agaagaatggtggtaagcgtattttgttaatcaagaagaggaggtttttctacccaacagcagacaggatcaagatgcgaccaagcaaaaaactgttcaa

g

>53225|60s ribosomal protein

atttcacagagctgttgtcggtgcatctcttactgccatcatgacaaaaaggactacgaagccaaaaattaggaaggcacagcgagagcaggctatcagg

g

>53275|60s ribosomal protein

tttttgactgcaaccttcgattttcaaaagatcggtgttctcgttctgattccgtaacccacgtttgtagcctgtgaatatcgccttagcgtaaagtcga

c

>53311|60s ribosomal protein

tttttttaaaaactttcctttttgccttcatgtacagctggtggtaaaggtccctatcaattttctttgcttccctgtactttttcaacagtcgccttag

aa

>53219|60s ribosomal protein

cgatctgcatgtggcagattacatcgaaaacacatctttggcctgcatgtaatctgccacatgcgggagctggcggaagaagatgatgaggcgtacaaga

gagatc

>53255|60s ribosomal protein

aggaaattggcctgaagaattatgatccgaagaaggccaaacgattttcgggcccagggaagttaaagcatatcccaaggcccaaaatgcaagtttgcat

cctagtt

>53239|60s ribosomal protein

tttcctgggataacgatcaatgcctgcgacgagagcatgtccgaatggtttacctgatgagccatcatcaaaatttttcaagacaattgctttctttccc

cctcgccttctttctctt

>53306|60s ribosomal protein

gcaaagaaaattgataggcacctttaccaccagctgtacatgaatgcaaaaggtaatgttttcaagaacaagcgtgtgctggtggtaaaggtgcctatca

attttctttgcttccctgtac

>53301|60s ribosomal protein

cagcggatgagagttctaaggcgactgttgaaaaagtaccgggaagcaaagaaaattgataggcacctttaccaccagcagctgacaagatactgattga

gcgcaatattcttgtaatccct

>53274|60s ribosomal protein

acccattgccttcgaaggaagatttgttttgaactttgctctcactgctctagaacatccatgtggcctcgtcactttgccccacacagctccagaacat

ccatgtggcctcgtcactttgccccacacagccctaac

>53215|60s ribosomal protein

ctcatcatcttcttccgccagctcccgcatgtaatctgccacatgcaggccaaaaatgtgttttcgatggatctcagcatcaaatacccgtgccaaccca

gatcgaaagaagaaagaaaaagatgctgcagctattaccaa

>53201|60s ribosomal protein

gtaaaacacagtggaaatttgtcattttatgaaattttaaatattaccaaacaaatgagggagagatccatgtcgagaaaacttgaaggaacgctgaaag

aaatccatgggacctgccactctgtgcgatgtcctgttgacg

>53234|60s ribosomal protein

gcgccatgcctgaggagaagaacctaccgtgcccacggaagaataaacccatccatgagctctccctgccacattgaggtggttctcactgaaaagaaga

aagtgtcaaagaagaaattggctaggcagaaagaaaagatgat

>53252|60s ribosomal protein

gtgcgagagcacggcacctatacactcgtacagagtatccctggaaaccttcaacttcactgtgcccgaaaatcgtttgtccttctgcggatcatatttc

ttcaggccaatttggatctccacagtttcaacaaatttcctctgctttt

>53217|60s ribosomal protein

gtggctgtatgcagaacaaagaatcatatcaccttcaattcttgaatacgccacgaattgccagagtatggagttaaagttgggctgaccaactatgcag

cggcttactgcaccggcctacttctagctagaagattgctaaagaaacttggtttg

>53261|60s ribosomal protein

ggaacataccactgaagtacgacagggagatgtggtcaaaaactgtggaagcaatgaagcacgacagggagatgtggtcaaaaactgtggaagcaataaa

gcgtgtagacgagatcaggcagaagcgagaggcccaccatatcatgcagcggctgcgg

>53178|60s ribosomal protein

atataaaaaaaaacacatccaacacacacacacacacacacacacacacacacacacaatggacaccacagcatgaactgacaaaattacagtttgagct

ttgccctccgccagtggcgcctctttgcattgtaccgtatggtattatctgttcgcatcctg

>53220|60s ribosomal protein

aaataattacaattggtagaattagtgcaatttctacatcaaaaattaaaaagattacatgcgggagctggcggaagaagatgatgaggcgtacaagaga

caggtttcccaatatatcaagctgggcattgatccagatagtattgagggaatatataaaac

>53233|60s ribosomal protein

tgaacacgaaggttagagcctcgtgctttacaagactttgtagcattgtcaggttcgtgtgaattccgccccatctccaactatgcctattctccacgac

ggagctcttcttcctcactaagaacttaagttaagaaataaactagattatcgtatattacaat

>53236|60s ribosomal protein

gtggggtacaactcagggccgctggcctaagaaatctgcagagttcttgcttcagttattgaaaaatggagagtcaaatgcagattacaagggcttggat

gtggatcgcctggtcatagaacatattcaagtcaaccgtgcgccatgcctgaggagaagaacctaccgtgc

>53282|60s ribosomal protein

ggacatggacgaataggaaagcacaggaaacatcctggtggaagaggtaatgctggtggtatgcatcaccctcgcatcaattttgacaagtatcaccctg

gctacttgtcaaaattgatgcgatggtgatgcataccaccagcattacctcttccaccaggatgtttcctgt

>53206|60s ribosomal protein

tttctcgacatggatctgtccctcatttgtttggcaatatttacaatttcatcaaatgacaaatttccactgactcatttcaatcgtctggaacttccaa

atcaccaacatttatctgatcaattaaatcatgtggtggtttcccttcaacagtacatcccacagactggcaagtcccaa

>53279|60s ribosomal protein

cagggtgatacttgtcaaaattgatgcgatgttgatgcatgccaccagcatttcctcttccaccaggatgtttcctgtgctttcctgactgtaatacatc

actccaatcaacaccacctccgaaaatgctagccaatgatcaaaaatatgcgacaagaaattacctattcgtccatgtccgtg

>53223|60s ribosomal protein

acagtccgatcttcatcttctctcacaggaaggtgacatggacagttttatacaggcggaagcacaagaaaggtcaggaagaagaacagaccatttagtg

ttccgtttcctgtgagagaagatgaagatcggactgtgtgcctacagtggggataaaatataccctggccatgggaaaaccatgg

>53208|60s ribosomal protein

gattgaaatgagtcagtgatggaaatttgtcatttgatgaaattgtaaatattggcaaacaaatgagggacagatccatgtcgagaaaacttgaaggaac

ggtgaaagaaatccttggggcttgccagtctgtgggatgtactgttgaagggatggtctgttgagcgtaaggaagggaaggccgag

>53213|60s ribosomal protein

tcactgttatcttcaagcctttccagtcagctgttgctttggcaatgtcatcaccaacctttttaggtgaatggcagctggagggactggcgacacactg

atcccatgtcctacaaagataagatcaatcctcatcctgcctcgtacgcagctgtctgccaagcgaaactgctaatctttcattggtg

>53307|60s ribosomal protein

ggcaaaaggtaatgttttcaagaacaagcgtgtgctgatggagttcatccacaagaagaaggcggagaaggctaggccaaagatgttgaaggatcaagct

gaggccagaaggcaaaaggtgaaagggcgtcactgtggatttggaaaaaggaaaggaacagctaatgctcgtatgccacagaagaccctgtggat

>53257|60s ribosomal protein

cattttttaaccagcttcttattcttgttcaacttcttaagagcttcaacatccatatagggaacattattggcctgaagaattatgatccgcagaagga

caaacgattttcgggcacagcgaagttgaagcatatcccaaggcccaaaatgcaagtttgcatcctaggtgatcagcagcactgtgatgaagcaa

>53200|60s ribosomal protein

atataaaacacagtggaaatttgtcatttcatgcaattctaaatattgccaaccaaatgagggaccgatccatgtcgagaaaacttgaaggaccggtaaa

agaactctttgggacttgccagtctgtgggatgttctgttgaagggaaaccacgacataatttaattgatcagaatgcctccaaaacttgatcca

>53296|60s ribosomal protein

gtacagggaagcaaagaaaattgatatgcccctttacctccagctgtacatggaggcaaaaggtaatgttttcaagaacaagcgtgtgctgatggatttc

atccacaagaagaaggcggagaaggctaggacaaagacgtttacgtgcctctttcaccttttgccttctggcctcagcttgatccttcaacatcttt

>53298|60s ribosomal protein

cttcttcttgtggaggatctccatcagcacacgcttgttcttgaaatctttaccttttgccttcatgtacagctggtggtaaaggtgcctatcaattttc

tttgcttccctgtactttttcaacagtcgcctctgatcctctactttctgctccatttttctcctttcttccatatcccacagcctcccgaaaatgtg

>53235|60s ribosomal protein

tgagtattgaagtaatctatatgcatcgttaaaatatatttcatttgattttactgcaccgtaaattaattgctgcactagttggagatggggcggtatt

cacacgaacctgacaatgctacaaagtcttgtaaagcacgtggctctaaccttcgtgttcattttaagaatacaagagagactgcaatggcactgaggc

>53180|60s ribosomal protein

aggatgcgaacagataataccatacggtacaatgcaaagaggcgccactggcggaggacaaagctcaaactgtaattttgtcagttcatgctgtggtgtc

cattgtgtggatgtgtatgacatttgtgtttaaataaaacagtaaacacaaaaaaaaaaaatttaaaagtaaaaaaaacacaaaaaaatagaaaaaaacg

>53305|60s ribosomal protein

gcgtgtgctgatggagttcatccacaagaagaaggcggagaaggctaggacaaagatgttgaaggatcaagctgaggccagaaggctaggacaaagatgt

tgaaggatcaagctgaggccagaaggcaaaaggtaatgttttcaagaacaagcgtgtgctgatggagttcatccacaagaagaaggcggagaaggctagg

acaaaga

>53186|60s ribosomal protein

gaactgtactgcaggaagttgggacattacagtccccgcctcatcaggtgcagatgtttcacacttagctctctggtgtgtgaacacatctgacatggac

cccccaacaacctgttttttctaaggcgaggtacactgtaaggtcgtttggtattagtcgtaattagaagattgctgtgcatggtacagtgagaggtgcc

aaggcagaag

>53310|60s ribosomal protein

gtgctgatggagttcatccacaagaagaaggcggagaaggctaggacaaagatgttgaaggatcaagctgaggccagaaggcggagaaggctaggacaaa

gatgttgaaggatcaagctgaggccagaaggcctcagcttgatccttcaacatctttgtcctagccttctccgccttcttcttgtggatgaactccatca

gcacacgcttg

>53203|60s ribosomal protein

gcatttatctgatcaattaaatcatgtggtggtttcccttcaacagtacatcccacagactgtaacagaaacaaagcaataacaacacagactggcaagt

cccaaggatttctttcaccgttccttcaagttttctcgacatggatctgtccctcatttgttttgctatatttacaatttcatcatatgataaattacaa

tcgtctggaacatc

>53263|60s ribosomal protein

ttcttggctttggtacttcttgggaagactactagcttgttcaaataattcttcagacgatggatgttcacggctaagccttcttcattcttattcctcc

gacgcatatccacggagataccaatcgttcgcgcagtagctggtgtcaatccacatgccttgagcgcttcaaggctaaagccacgtccagctcggacttt

gtaattgtattttt

>53297|60s ribosomal protein

gactgttgaaaaagtaaagggaagcaaagaaaattgctaggcacatttaccaccagctgtacatgaaggcaaaaggtaatgatttcaagaaccagcgtgt

gctgatggagttcatccacaagaagaaggcggagaaggctaggacaaagatgttggaggatcaagctgaggccagaaagcaaaaggtgaaaaaggcacgt

aaacgtcgtgagggg

>53226|60s ribosomal protein

tacaagtaagatatatatcgaatcgcatacaacaaagtcaacatatttagccgatgttataaaagaaagccacttaataaaagtgtggttcgtagatttt

tataccccgtagagagcttaaaacccagaaatttcacagagctattgttggtgcatctctttctgagatcatggcaaaaaggaatatgaagcctgagaat

aggaaagctcagcgagag

>53202|60s ribosomal protein

acaaatgagggacagatccatgtcgagaaaacttgaaggagcggtgaaagaattccttgggacttgccagtctgtgggatttactgttgaagggaaacca

ccacatgatttaattgatcagataaatgctggtgatttggaagttccagacgatggaaatttgtcatttgatgaaattgtaaatattgccaaacaaatga

gggacagatccatgtcgagaaaa

>53237|60s ribosomal protein

tgaacacgaaggttagagcctcgtgctttacaagactttgtagcattgtcgggttcgtgtgaataccgccccatctccaactatgcctattctcccgact

gcacatagctgccctaccctctttccacctcatccctgtgtgctccccaacagcaagtcactgtcccccacccctatcctgctgtccctccccactccag

cctcctccttacctccacccagtc

>53231|60s ribosomal protein

tggtcatagaacatattcaagtcaaccgtgcgccatgcctgaggagaagaacctaccgtgtccacggaagaataaatccatacatgagctctccctgcca

cattgaggtggttctcgctgcaaaggaggaagttgttgcgaaagcaactgaggaagagcccacaaagatgaaagtgtcaaagaagaaattggctaggcag

aaagaaaagatgatgagagattaagaagttgtga

>53224|60s ribosomal protein

gtgacatggacagtgttatacaggcggaagcacaagaatcccaggaaggtgacatggacagtgttatacaggcggaagcacaagaaaggtcaggaagaag

aacagaccaaaaagcgaacccgtcgcacccagaaatttcacagagctattgttggtgcatctcttactgccatcatggcaaaaaggaatatgaagcctga

aattaggaaggcacagcgagagcaggctatcagggctgctaaagag

>53299|60s ribosomal protein

accttttgccttctggcctcagcttgatccttcaacatctttgtcctagccttctccgccttcttcttgtcgatgaactccatcagcacacgcttgttct

tgaaaacgttacctttaccaccagctgtacatgaaggcaaaaggtaatgttttcaagaacaagcgtgtgctgatggagttcatccacaagaagaaggcgg

agaaggctaggaacagctaatgctcgtatgccacagaagaccctgtggattc

>53300|60s ribosomal protein

tgttttcaagaacaagcgtgtgctgatgagagttctaaggcgactgtttaaaaagtacagggaagcaaagaaaattgataggcacctttaccaccagctg

tacatgaaagcaaagaaaattgataggcacctttaccaccagctgttcatgaaggcaaaaggtaatgttttcaagaacaagcgtgtgctgatggagttca

tccacaagaagaaggcggagaaggctaggacaaagatgttgaaaacgttaccctttgcc

>53173|60s ribosomal protein

tctgtcatcatttgtgaccgttccaactacaacagcaatcaggttttctcggccaggcttcttcataaaacgcactactcttgcgagcgagataggtggc

ctatttatttttgacatgaacaacctccgtaaaattatcttattgaacttcgcttccgtacgtcgagcgagatatcggtacaacttaaccagaagcctaa

gttatacatcttgcgacttcgtttcagtgcgccgaaccttgcggtcccccttgtgattaa

>53187|60s ribosomal protein

cacattgacttgggaataaaatatgatcccagtattggaacagctcacaggccaacaacctgttttttctaagtcgaggtacactgtaaggtcgtttggt

attagacgtaatgagaagattgctgtacattgtacagtgagaggtgccaagtcaatgtgttcttgaattccaaaaccaaagttaccagagtcagagaagt

tgtcttttcgtaattcatattctcgaaccttcaaccctcgttcaaggatttcttctgcctt

>53248|60s ribosomal protein

acggccagctcagcactctcacacgagcacgacaggaaatgcagaggaaatttggatctccacagtttcaacaaatttcctctgcttttccttcgcgtgg

gagagcacggcacctatacactcgtacagagtatccctggaaaccttcgaactcatttccccagcacaacggccagctcagcactctcacacgagcacga

caggaaataaaacgttatttatgccccaataagtgaaactttcaaggtaactgaagcgtaacttataa

>53247|60s ribosomal protein

aacttgcattttgggccttgggatatgcttcaacttcactgtgcccgaaaatcgtttgtccttctgcggatcataattcttcaggccaatttggatctcc

acagtttcaacaaatttcctctggttttccttcgcgtgcgagagcacggggcctatacgctcgtacagagtatccctggaaaccttcgaactcattttcc

cagcacaacggccagctcagcactctcacacgaacacgacaggaaatggtcaaacctcaaatggaagatc

>53214|60s ribosomal protein

gaggttaaggaaaatgatgctgagatccatagaaaacacatctttggcctgcatggggcagattacatgcgggagctggcggaagaaggtgattaggcgt

acaagagacagttttcccaatatatcaagctgggcattgatatattgggaaaactgtctcttgtacgcctcatcatgttcttccgccaggtcccgcatgt

aatctgccacatgcaggccaaagatgtgttttcgatggatctcagctattaccaagaagaggtggggccgtgccaagctgtcccttgctg

>53246|60s ribosomal protein

atgcttcaacttcactgtgcccgaaaatcgtttgtccttctgcggaacattattggcttttgcttcatcacagtgctgctgagcacctaggatgcaaact

tgcattttgggccttgggatatgcttccacttcactgtgcccgaaaatcttttgtccttctgcggatcataattcttcaggccaatttggatctccacag

tttcaacaaatttcctctgcttttccttcgtgtgagagtgctgagctggccgttgtgctgggaaaatgagttcgaaggccaatttggatctc

>53293|60s ribosomal protein

ccgggaaatacggtaccagatatggtgcctcacttaggaagatggttaagaagatggaagtgtatttggaatgctgcgtaatttccatcttcttaaccgt

cttcctaagtgaggcaccatatctggtaccgtatttcccggtgattccaaccttcttcgtgcgcttagccatttcgcttgtatgatcacgctacctcact

gtagctgcagctgttgtgctgtacacccacgctccagccactgtcctcttgcaacgcttgcacgaccagataccaacacaactcctcttcattgc

>53241|60s ribosomal protein

agggtgctacttacggaaagcctaaaagccatggagttaaccagctgaagcccacacgtaacttgcagtccattgctgaggaacgagttggaaggcgttg

tggtggtctgcgagtgctttactcttattgggttgcacaggattccacatacaagtactatgaagtaatattggtggatccggctcacaaggcaattcgc

cgagatgctaagataaactggatatgcaaacctgtgatgaagcatcgggagctgcgatgacccttgccaaggccacgagacttgcggccagcagagg

>53262|60s ribosomal protein

ctgtcgtacttcagtggtatgttccttcgtttctcaaattcaaaagctggatctacagcaagttcttttcctgctgtttttcggaaagctttggtccacc

ttactttacgtggattcttcttcttcttgaaagccgcatgacatttcgatcggcagaatttgaagatcttacaatcatttctaacgaactgtattccatg

tcccggatagatacgggaagaacaaaaataacacgtttctatacgcatggtttacaataacattacaccacttcacgccgagcaccgtgaaagacagtag

aagatcgaattcggcatttg

>53244|60s ribosomal protein

ctccttcgatgagtttctgcgtgagtgatgtatttaagcggcttctctattttacatgtgtccaataaaatgggtactatggggagttgcaataaaattt

tcaatgatggtatttgcatgtattatcgtgatcttgagtgacgagaatacaaactgagagctccattaatacattttactgtttagtgttaagtacagtt

atgataattacattaaatttacaggttgaagcatatcccaaggcccaaaatgcaagtttgcatcctaggtgatcagcaggactgtgatgaagcaaaagtt

aataatggtcccaatatggatgttgaagcaag

>53295|60s ribosomal protein

tgttgatgaactccctcagcacacggttgttcttgaaaactttaccttttgccttcatgtacagctggtggtaaaggtgcctatcaattttctttgcttc

cctgtactttttccacagtcgccttagaactctcatccgctggatccacagggtagcaaagaaaattgataggcacctttaccaccagctgtacatgaag

gcaaaaggtaacgttttcaagaacaagcgtgtgctgatggggttcatccacaagaagaaggcggagaaggctaggacaaagatgttgaaggaggaaaggg

cgtcactgtggatttggaaaaaggaaaggaacagctaatgctcgtatgccacagaaga

>53249|60s ribosomal protein

ctctgcttttccttcgcgtgcgagagcacggcacctatacactcatacagagtatccctggaaaccttcgaactcatttttccagcacaacggccagctc

agcactctcacacgaaggaaaggcagaggaaatttgttgaaactgtggagatccaaattggcctgaagaattatgatccgcagaaggacaaacgattttc

gggcacagtgaagttgaagcatatcccaaggcccaaaatgcaagtttgcatcctaggtgatcagcagcactgtgatgaagcaaagccaataatgttccct

atatggatgttgaagctcttaagacgttgaacaagaataagaagctggttaaaaatgcatcatacttctttg

>53204|60s ribosomal protein

ccacaaccttggtctatcacgaagaagggagttactatgtgtgaatggatatgctggtaataaaaattctgatgtatgtctgatgcagtgtttggtacct

tttttgcaccatctttagcagttttgtactatttcccctttgcagagaggatgtattctgtaataccacctggcaaattttctatgttatatacaccaca

tgttacattagtaattcagtccaatgcaccactttcattacaatttacacagagtaaattcatgttatccaatctgtaggttggcacttaagaatttatc

aaagtttgcttggaacgaatatgggtacaaaattaaaaggacaggcaaatgaaagaaaaatgcaattatagcagaaaatgtaattttattccatcactga

ctcatttcaatcgtttggaacttccaaatctcccgcatttatctgatcaattaaatcatgtggtggtttcccttcaacagtacatcccacagactggcaa

gtcccaaggatttctttcaccgt

>53230|60s ribosomal protein

caatttcttctttgacactttctttctgcctagccaatttcttctttgacactttcttctttgtggtctcttcctcagttgctttcgcaacaacttcctc

cttttcagtgagaaccacctcaatgtggcagggagagctcatgtatggatttattcttccgtgggcacggtaggttcttctcctcaggcatggcgcacgg

ttgacttgaatatgttctatgaccaggcgatcaacatccaagcccttgtaatctgcatttgactctgcatttttcaataactgaagcaagaactttgcag

atttcttaggccagcggccctgagttgtaccccgctgctttgcctgagcacaacgaccaactccaccattgaatctgcgaaacggaacacattctttctt

ctcaacaacattcttcaaatattagacagctctcctcagtggcattcgcctcagtgccattgcagtctctcttgtattctagaaaaattaaaatcaaact

tgtcacgtacatgcattcgacagttaagtggtcga

>53245|60s ribosomal protein

aagggaccgtttgatatggaatgggtggcagctgtcataatggaggtactgttgctcgtgtgagagtgctgagctggccgttgtgctgggaaaatgagct

cgaaggtttccagggatactctgtacgagtgtataggtgccgtgctctcgcacgcgaaggaaaagcagaggaaatttgttgaaactgtggagatccaaat

tggcctgaagaattatgatccgcagaaggacaaacgattttcgggcacagtgaagtatgtgatcacctgactcgagatacctttatttctactacagagt

gtttccaaagcactgaatagcttaacaaaattcataactttggggaatagtgtgagctctatttcatttcgacgcggttgtttgtgaaataaagggatct

catgctacgtcagggagctgggcagatcctggcggacctgcacccagggtcttaaaataattggggaggtagtttcctaccgttattttattcgttgtcc

tgtatggcggtaagtcaacaaaagtgcagtaggctagtttaattcgtaaacgatgcgagaagtgtgtatgtaaccacgtggtttgtctatgcaggtactg

agattactttggcagtgttttgttgctgaaggaacttaacgttaggttttcttttcgcagaattgcattgctacttaaatttaggttattgttgcaaaaa

ctctgccatgggttgatactaggtggtagtcgggaaagtgtagatccactttcttcatttatggcatagggtaactgtagacttgtgtgtattgcttgga

gtgtaaggctaagccgttatgaagttaattagtgttaaaaatgtagtaaaatagttcagcattttatgctaagcacgaaggccccatattaagactaatg

gtaatttgattcgtgcttagatttttttttttttggaaatgtaggaaggaatcttacattttgagttataggaagcacaagaaaatataatttcctacaa

gatctttttaactgtaaatgatatttgacaatttttctcaagtggtttgataactgggagacacatgtagcaaacagtgtgcacatgtgggcttgggaat

gaactgatatgctaaaaactatttgctgagtgtgactgcctgtgcaaattgtaagatattgcgttaatttcgttgcattggacatggtgcagcccattct

gcccaatgtcttcacactaaatggaatgaatggtgctgacgagtggaattgtggttgtgaggaaagtctgaatatagtattaggttctggtagtggcatt

acattccatggtttaagtgaaatccataatgaatcttacttatgtaggttccatacgctgaaattagtcaaaaagtcagatactgaactattttaataga

atgttgctaaactgttgaaggcactcttataaaagttttttgtacagtcatgtttttcctccttcaggcagtaaaccatgtatatatagtatttgttttt

ctgaagttgtgtaattactgattcataatagggtaaaatatcagaacacactgagtttttttggaaacaagccaagtcaatgaggataggttaacccttc

aatagctgttaacttgtcctgttctgaaatgcagttttgagtagcctttgggtgttaaattgctgttgatgtaacctggggctacaggccatcccctgag

agctagtcatatctgtgcttggggatgtgagctgttcacaaatattataccgtggattttgtgctgcttgttagcttcagttctgcttgctaagaatgta

aaagatctgacagcttgtgtgaaaggtacattaagtgtaccagtactgtcagatttataaaacattttagttctttctaaatgtagaacagctcagatgt

ggaaatatgaatagcaacaatgactgcttatttgatgctgatatgaataactctttcacaaatgtcagcaggtgtaagcagagtattgtcacaagaagag

atgtaattgacagttcgtatgaatctgatagtgaaacacttagtcgataatgaaagaatggtgtagtaacaaatttgaaagaatgtttctatgtgcttag

tcacacagttcacattcagtactaaagtcaattggggattggtccaagtagtaagctcctcttagttgtttccttagtatattttatgctgacatgtgta

aaatggtggtatggaggtggtgatttgtatgtgaggtaggtgtttatgacttgtggctatgaagggatgttgaattactgatgtgtattcgtaatgtgta

actattggactttaacaattacagaaactatgggaagaaaagccactttcaaatctctgggatatgtaacaaatattaatgcagacaactttcataggca

aactaattttttctctgcaataaactggcatgaaacattgtgacgaagcatcctagaatctcacagctcagcatgtacagcaccagctggaggattaact

ttttgttagcagatgtcagtggcctatcaagattgaaagctgccatagtgagagtccagttgatatcagttggtttgtgtgaatgatattcgaaatgctc

ttggttttagatgtccattgtggattgatgtgtgcacatgcttgtgcttcagtatgaaatgttggaccctgttaaaattactaattgggcactcaaggca

gattgctgcagtagtaaaattatttcagttcagatagtaactgttgtgcaacatactactcaatgtatactgacattggtttcagtgttttcttaaatgt

gcagaattttttggttcttaactttggatatgatgcttgctttcatacactgtgatcctacctcttaatgtctgacctccttttacatgtagtgcagcaa

ctcaatgtggcatcaactcaagttgttgtatgttccctgcagaaatattgaaccatggttgagaaaaactttccgtgcaggattttgtggatgaactgac

cttcagattgtcccataaatgtttgatgagattgtttggtaattttggtgagcagatcattcacttgaagtgtccagaatgtcatccaaccaattttcaa

cagttgtggtatggtatatggcagattgtcagccttaaaaactccaaagttcagaaaacatgaagttgtgaatgggtgcagatggtctcaaagtagatta

tgcagttggttggtagtctgtgttgttgggtatcattgatttgtattttcacttggtcattttaatattaggtaatacaattgttttttgtggattctag

ttccatggactcagaattaggtgacattgtccataaatttaaggcttgcaggttgtactggttggatttcaaagtttaatgaaatcataagttagaaatt

gaatgttttggtccagtaagttgttggtaattttattttgaactttgttcaatgtttatagtttctgtgtgagtttaggcctctctattttacatgtgtc

caataaaatgggtactatggggagttgcaataaaattttcaatgatggtatttgcatgtattatcgtgatcttgagtgacgagaatacaaactgagagct

ccattaatacattttactgtttagtgttaagtacagttatgataattacattaaatttacaggttgaagcatatcccaaggcccaaaatgcaagtttgca

tcctaggtgatcagcagcactgtgatgaagcacaagccattactcttccctagatggatgttgaagctcttaagcagttgaac

>53349|60s ribosomal protein l10a isoform 1

tttcacttcatcaatttttgcccacattgcttcttggtttgaaaggagaccaggaaatttgcctgccttatttaa

>53352|60s ribosomal protein l10a isoform 1

aacagaaaggcacagcttctttttcatttgaaacttaattgttgctttcactccaaaaatttttgccatcattgcttcttggtgtgaaag

>53348|60s ribosomal protein l10a isoform 1

cttaattgttgctttcacttcatcaatttttgccctgattgcttcttggtttgaaacaagtccgggaaatttgcctgtcttatttaagcc

>53350|60s ribosomal protein l10a isoform 1

gcactggcagaatgtcagatcttttcatgtgacgtcaactatgggacctccaccgagactgtattaaccctgtttcatttccatgaacaaataaatac

>53353|60s ribosomal protein l10a isoform 1

aggcacagcacctttttcatttgaaacttaattgttgctttctcatcatcaaggtttgccctcagtgcttcttggtgtcaaaacacaccagcaaatttca

c

>53351|60s ribosomal protein l10a isoform 1

atctttacatgtgaagtcaactatgggaccaccacagagactgtattaaacctgtttcatttgaaacttaattgttgctttcacttcatcaattttcgcc

atcattgcttcttggtgtgaaagaagaccaggaaatttgcctgccttatttaagcctgg

>53355|60s ribosomal protein l10a isoform 1

tttggcctcagagtctctgatcaagcagattccacgaatcctcggcccagatctaaataaggcaggaaagttcccttccctgctcacacacaacgaaaac

atggtggccaaagtggatgaggtgaagttcacaatcaagttccaaatgaagaaggtgttatat

>53371|60s ribosomal protein l10a-like isoform 2

gtttcatttgaaacttaattgtggcttgcacttcatcaatttttgccatcattgcttcttggtgtgaaagaagacctggaaatttgcctgccttatttaa

gcctgggccaaccaaccttggaagctgttta

>53375|60s ribosomal protein l10a-like isoform 2

aggaaatttgcctgccttatttaagcctggtccaagcaatcttggaatctgtttaatgagtgcttcacttcatcaatttttgccatcattgcttcttggt

gtggaagaagaccaggaaatttgcctgccttatttaagcctggtccaagcaatcttggaatctgtttaatgagt

>53372|60s ribosomal protein l10a-like isoform 2

ggcacctatacactcgtacagagtatccctggaaaccttcgaactcattttcccagcacaacggccagctcagcactctcacacgagcacgacaggaaaa

gcagaggaaatttgttgaaactgtggagatccaaattggcctgaagaattatgatccgcagaaggacaaacgattttcgggcacagtgaagttgaagcat

atcccaaggcccaaaatgcaagtttgcatcctaggtgatcagcagcactgtgatgaagcaaaagccaataatgttccctatatggatgttgaagcatatc

cca

>53391|60s ribosomal protein l10a-like isoform 4

ttttgccatcattgcttcttggtgtgaaataagaccaggaaagtggcctgccatatttaaacctggtccatgcactcttgcaatctg

>53423|60s ribosomal protein l11-like

atttatggtttggacttctatgttgtggctggacgaccagagttcaatgtagcactcaggagaaggaaaacttgtgtggttggtttccagcaca

>53428|60s ribosomal protein l11-like

tcttgttaatctgtcaccagattcaccgacgcaaatatttagccacaatttcctgatgcgaacttcgcgcatcggattcttggtcttgcctttcgctccc

tgtattttacccctgccaccttcagaatt

>53427|60s ribosomal protein l11-like

ggacttctatgttgtgcttggacggccagggttcaatgtagcacacaggagaaggaaaactggtgtgggtggtttccagcacaggcttacaaaagaagat

gcaatgaagtggtttccagcacaggcttacaaaagaa

>53425|60s ribosomal protein l11-like

ctgtagtccaatacgagaattacgtgatcgacgcaaagcaactcgtccaccagggcgggctagatgtacgaagaaattcaaaccgtaaataccgactgct

ggatcatatttgatccccagatcaatatgctcgtgaattccaaagccaaagttgccagtatcagaaaagcaacgcttccttaactcatattccttcacac

gtagacctttctcaaggatctcagcagccttttcacctcgaacggtcacatgacaagcaatcttttcatttcttcgaataccgaaagatcttatagtcaa

acgtgctttcgataaaactggtt

>53426|60s ribosomal protein l11-like

ccaagtgattgatggcgtgaaaactcgcgttcacaaatcaactggtgaatcagtggcggtagcgaagtaataggttgaaaaggcaatggccagacttaaa

gcacgttacaacgacgaacttaaagcgaagttacaagaagaacttagcattaagaatgtgatggaaattcctcgcatcacaaaaatcactcttaacatgg

gtgtaggcgcagcagctactgataagaaattattagacggcgctgtagctgacatgcaattaattgctggtcaaaaaccagtagttactcttgcacgtaa

atctatcgctggtttcaaaatccgtgatggttggccaattggttgtaaagttactttacgtggcgaccaaatgtacgaattcttggaccgtttgatctct

atcgcaatccctcgtatccgtgacttccgtggtttctcagcgaaatctttcgacggtcgtggtaactactcaatgggtttgaaagagcaaatcgttttcc

ctgaaatcgatttcgacaagattgatcgtattcgtggtatggatattactattactacgactgctcgcaccgatgacgaaggccgtgcgcttatgcgtgc

attcggcttcccgttcaaataagaggtcgatatggctaagaaaggtatgattaatcgcgaattgaaacgcgaaaaaactgttgctaaatacgctgcaaaa

cgtgctgaattaaaagctactattgcaaatgtaaatgcgagcgacgaagaacgtttcgaagcgatgttaaagttacaagcattgccacgtaatgcatctc

cggttcgtcttcgtaaccgttgtggtttaactggtcgtcctcatggttacttccgtaagttcggtttaagccgtaacaaattacgtgacacagtaatgca

aggtgatgtaccaggcgttgttaaggcaagctggtaaggagcgactaaatgagtatgcaagataccgttgccgacatgctaacacgtgttcgtaacgcac

aaatggctaagaaacaaactgtttctatgccgtcttctaagttgaaagttgctattgcaaacgtacttcaacaagaaggttatatttcaaacgtagaagt

tgctcaagaagagacaaaatctactttgactattactt

>53452|60s ribosomal protein l12-like

ccctcggcctgtaaaacacagtggaaatttgtcatttgatgaaattctaaatattgccaaacaaatgagggacagatccatgtcgagaaaact

>53450|60s ribosomal protein l12-like

ttgaaggaacggtgaaagaaatccttgggacttgccagtctgtgggatgtactgatgaagggaaaccaccacatgatttaattgatcagataaatgctgg

tgatttcccttcaacagtacatcccacagactggcaagtcccaaggatttctttcac

>53448|60s ribosomal protein l12-like

agattcaaaacaggcaagtatcatgtgaagttattcccacagctactgctctcattctgaaagctctcaaggagcccgtacgtgaccggaagaaagtcaa

gaacattaaacactctggcaacttggacttcaatacaatagttgatattgctcgtcagatgcgcg

>53449|60s ribosomal protein l12-like

ttggcaatatttacaatttcatcaaatgacaaatttccactgtgttttactgttttctccagaaatattaatccattgatgccacccatctgctatgatc

cttgatatcaagactgctgacaactattcttctgcttctttctgtctcgagtgggctccttcaaagcctttata

>53476|60s ribosomal protein l13a isoform 2

agaagaacgaggttggtggtgcctggagccatgagagttaggtgcttgaagccaggacgaaagtactgccatttgggca

>53482|60s ribosomal protein l13a isoform 2

tttgtggtgcctggagccctgagggttatgagcttgacaccaggacgaaagaactgccatttgggcaaactttctcatgaagtg

>53480|60s ribosomal protein l13a isoform 2

agaagaaggaggtttgtggtgcctggagtcatgagggttatgtgcttgaacccaggacgaaagtgctgccatttgggcagactttctcatgaagtggga

>53475|60s ribosomal protein l13a isoform 2

aagaaagaggtttgtggtgcgtggagccatgagagttatgtgcttgaagccaggtcgaaagtactgccatttgggcagactttctcatgaagtgggatg

>53473|60s ribosomal protein l13a isoform 2

gaaagaggtttgtggtgcatggagccataagggttatgtgcttgaaccctggacgaaagtactgccagttaggcagactttctcatgaagtgggatggaa

gttcaagcagg

>53483|60s ribosomal protein l13a isoform 2

catgagaaagtctgcccaaatggcagtactttcgtccttgtttcaagcacataactctcatggctccaggcaccacaaacctctctctctctgtccgtct

ctccggctctctctctctctctctctctc

>53470|60s ribosomal protein l13a isoform 2

agaggtatgttgccacataagacagagaggagtaaaatggcgctgaagaggcttatagcttatgaaggttgtcccccaccttatgacagaagaaagaggt

ttgtggtgcttggagctatgagggttatgtgcgtgaaaccag

>53474|60s ribosomal protein l13a isoform 2

ctggattaacattgcaccttttgcgaaggaatgagaggtacttccactttgccctgaagaaatttcccgaaatgttgatctgttctcatctaacgacagt

tcctttatttccttgtagaacagtcttagcaactatatctgtacg

>53469|60s ribosomal protein l13a isoform 2

tcccacttcatgagaaagactgcccaaatggcagaactttcgtcctggtttcaagcacataactctcatggctccaggccccccaatcctctttcttctg

tcataaggtggtggacagccttcgttggctttgagcctcttcagcgccattttccc

>53468|60s ribosomal protein l13a isoform 2

gggaaaatggcgctgaagaggcttaaagcttacgaaggttgtccaccaccttatgacagacgaaagaggtttgtggtgcctggagccatgagagttatgt

acttccatcccacttcatgagaaagtctgcccaaatggcagtgctttcgtcctggtttcaaacccataactctcatggctccaggcaccacaaacctctt

ctcttcatccaattccagttccaggtctcgcagcct

>53541|60s ribosomal protein l13a-like

agacagagaggggaaaaatggcgctgaagaggcttaaaccttacgagggttgtccaccaccttatgacagaagaaag

>53546|60s ribosomal protein l13a-like

ccctgaagaaattgcccgaaatgttgagctgttcacatctcactacaattacttagtttccttgtagaacagtcttag

>53543|60s ribosomal protein l13a-like

tctgttcacatctcacgacaattactttgtttccctgtagaaccctcttcccaactttaacagcaagccgtcccaggacatggcctctcc

>53538|60s ribosomal protein l13a-like

cgctgaagaagcttaaagcttatgaagggtgtcctccaccttatgacagaagaaagaggtttgtggtacctggagccatgaaggttatgtgcttga

>53536|60s ribosomal protein l13a-like

ctgggtttagtgtggaggcaatcttgatcgacgggaggggccctctccttggacggcttgcagctatagttgctaagcctgttctacaaggaaacagaat

agttgtcgtgagatgtgaacagatcaacatttcggga

>53544|60s ribosomal protein l13a-like

cgctgcttgaacaaatgcatagttttctagttttcaactggccttaacagtaagtttacaaacctctaacagttctccacagtatcttgcttggtgctct

aaaattgtatggtcctcgtgctggattaacattgcaccttttgcgaaggaat

>53540|60s ribosomal protein l13a-like

acagtcttagcaactatagctgcaagccgtcccaggagatggcctctcccgtcgatcaagattgccttctcctgggacggcttgcagctatagttgctaa

gactgttctacaaggaaacaaagtaattgtcgtgagatgtacttcaactttgccctgaag

>53539|60s ribosomal protein l13a-like

tcttaacaacctgcttgttcttccatcccacttcatgagaaagtactgctatttgggcagactttctcatgaagtgggatggaagtacaagcaggttgtt

aggacccttcctctttgtttcaagggtcctaacaacctgcttgtacttccatcccactttctcatgaagtgggatggaag

>53537|60s ribosomal protein l13a-like

tgtttcgttgtagaacagtctttgcaactatagctgcaatccgtcccaggagatggcctctcccgtcgatcaagattgctttctcactaaacccagtcat

ctttcatactccacaagaaaagccacatttggaaagatggtgcatatcccagtcactggcgtgagactgttctcattcccatacctaag

>53535|60s ribosomal protein l13a-like

ctttgccctgaagaaatttcccgaaatgttgatctgttcacatctcacgacaattactttgttttcttgtggagtatgaaagatgactgggtttagtgag

aaggcaatcttgatcgacgggagaggccatctcctggggcggcttgcagctatagttgctaagactgttctacaagtaaacaaagtaattgtcgtgagat

gtgatcagatcaacatttcgggaaatttcttcagggcaaaggaatgagaggta

>53534|60s ribosomal protein l13a-like

atgaaatgtattatgtgataaaagaaattattcagatagttaatggagatgaaaatttagtagtcatgggagactagtatgcgactgcaggaaaataaga

gaaggcaatcttgatcgacgggagaggccatctcctgggacggcttgcagctatagtcgctaagactgttctacaaggaaacaaagtaattgtcgtgaga

tgtgaacagatcaactttgccctgaagaaatttcccgaaatgttgagctgttcacatctcacgacaattactttgtt

>53532|60s ribosomal protein l13a-like

atttggaaggaagtggcttccttttcttgtggagtatgaaagatgactgggtttagtgagaaggcaatattgatcgacgggagaggacatctcctgggac

ggcttgcagctatagttgctaagactgttctacaaggaaacaaagtaattgtcgtgagatgtgaacagtcttagcaactatagctgcaagccgtcccagg

agatggcctctcccgtcgctcaagattgccttctcactaaacccagtcatctttcatactctctgctgttcctcttttggtgacagttctgttatgttta

caaataaatcttgcttatcacttctcc

>53561|60s ribosomal protein l14

caattacgtccacttcggaacatagttttcctttatgtggccccacggagatgtaagccaccctccccgtttccacaaacctcttgaaa

>53562|60s ribosomal protein l14

tcaattacgtccactacggaacatagttttactttatgtggcccaacggagatgtaggccactgtccccgtttccacaaaccttttgaaaggcatc

>53566|60s ribosomal protein l14

ggaacatagttttcctttatgtggcccatcggagatgtaagccactctccacgtttccacaaacctcttgaaaggcacttctaccggaagaagaaacagc

g

>53568|60s ribosomal protein l14

tcaattacgtccactacggaacatagttttcctttatgtggcccatcggagatggaagccactctccccgtttccacaaacctcttgaaaggcatggctt

gatggaaaa

>53567|60s ribosomal protein l14

atcaattaaagccttattttgatcaattacgtccactacggaacatagttttcctttaggtggcccatcggagatgtaagccactctccccgtttccatc

aagccatgccttcctca

>53575|60s ribosomal protein l14

acaggtccatcaattaaagccttattttgatcaattacgtccactacggaacatagttttcctttacgtggcccatcggagatgtaagccactctccccg

tttccacaaacctcttgaaaggcggaagagaca

>53571|60s ribosomal protein l14

cttattttgatcaattccgtccactgcggagcatagttttcctttatgtggcccttcggagatgtaagccactctccccgtttccacaaccctcttgaaa

ggcatggcttgatggaaaagttgatgaaaaatggaaggaaagtttgtg

>53573|60s ribosomal protein l14

gtaaaacttgttatataggaagttatgtgaaaatataaaacattttcgttaaaatttgtcctttcaagaggtttgtggaaacggggagagtggcttacat

ctcggatgggccacataaaggaaaactatgttccgtagtggacgtaattg

>53574|60s ribosomal protein l14

ttcttctctttcttcgcatcctttgatttcttcttaccgtacagggttcctgcacgagttgcaatgacagacttcgatcgcttcaagttacgaaaagcaa

aacaacttagaaacaaggtccgtagcttagcgtattacaagctcaagaagaaggc

>53569|60s ribosomal protein l14

cgaagaaattggaagccaagcaaaagagagttgcaatgacagacttcgatcgcttcaagttacgctaagctacggaccttgtttctaagttgttttgctt

tacgcaacttgaagcgatcgaagtctgtcattgcaactctcttttgcttggcttccaatttcttcgccc

>53560|60s ribosomal protein l14

tccatttgcagtttcccgcaagttgtatttctcagaattcaggtacaaatatttcctgaagtttataaagcattgttttaattttcaatggtccatactt

acgtagtagacccaagtgttagcacagaatcaactaaattagagaactaatattcttattaagaaaaaaattataaaaattaaaatgtaagatgtgattt

tttcccttgtaatatacataatatgtggaattaaataggtctattaccttagccatcatgtcatgcatatctggtaaaaatttagtctccttcaaatata

taacatttgataaaatggtaactcaatttgaggaagcattttggaaaaatatacgcatcgagttctttgtaatttttctaataaccctaaacaggttcaa

aaacaattaaaataccaattcgtttagaaagtgtgctacattacccagtaactacaaaaacctgcaaaacatatgcattgttaatagtgtctgaaagaca

ttggtgatttttacataatattgagccagaaaaataccgtgtatatttcaagtgcaataagtatgacaaacttgcatgctctgctggttcaatttaagct

gtttcaaattttgcataatgtacatttctttgcatgatctcaccaacaaacacgtcaaaagagttgcaggaattgtgccctacaatcaggaattttaatt

cagctaagtaattcgtgggcactgtatgctgcagctgtacactcacagcacatgtttgtaatatgtcacagcattcgtcttatatagttattgttataac

attcatttcactcgactgcagagaatagcttagttgtggtgacatactaatctgtaatgcagctcgagctgcaggtttgttttgaaggtgactgtttcat

tgcaagttgtcttcttcacattgcttttccactcaaaactaaaaacatgaaatccacttcaatatggaacttgctctactaacaaacttccaaattaaag

gcatatattcactgtaatagtgtaaaataatcacacagaggggaaaaaaaagagtacctgcagatctcattggccaaatgaacaaactactttgatgctc

tgctaactgcacgaaattttacctttatacacaccaaacttgtggcacccacacacaaaatgaaaaagtcacctgaagatttcatggcttagacaatttg

gtgacaaaccacataacactctcctgccaaatttactttatgcataaagcaaactgccctcaccacacacacacttttcacaaataaaaagtataacaag

tacataacttataaattttatttttctcagactttatacattatttcttgggtgctggcttggatttgggtttcttctctttcttcgcatcctttgattt

cttcttaccgtacagggttcctgcacgagttgccttcttcttgagcttgtaatacgctaagctacggaccttgtttctaagttgttttgctttacgtaac

ttgaagcgatcgaagtctgtcattgcaactctcttttgcttggcttccaatttcttcgcccacaaactttccttccatttttcatcaacttttccatcaa

gccatgccttcctcacaaccctactgcgagcagtaaatggaaacttgaccctaaacttagttaggtgtagctgtgtcaatcttatctcaactcgaggcac

accagttacaggtccatcaattaaagccttattttgatcaattacgtccactacggaacatagttttcttttatgtggcccatcggagatgtaagccact

ctccccgtttccacaaacctcttgaaaggcatccctaccggaagagacaagatttaaatgaaagactctcctcttcaaccgagtccagtgttcctgggtc

aaagatgagacatccaaag

>53597|60s ribosomal protein l14-like

ccctgtcattgcaactctgttttgcttggcgtccaatttactcccccacaaactttccttccttttcttatcaacttttccatcaagccatgccttcctc

actaccctactgcgagcagtaaatggaaacttgaccctaaacttagttagg

>53593|60s ribosomal protein l14-like

tggcttgatggaaaagttgatgaaaaatggaaggaaagtttgtgggcgaagaaattggaagccaagcaaaagagagttccaatgacagacttcgatcgct

tcaagttacgtaattgatcaaaataaggctttaattgatggacctgtaactggtgtgcctcgaggtgagataagattgacacagc

>53596|60s ribosomal protein l14-like

ttgcttatgcttgtactgcttccaatttcttcgcccacaaactttccttccatttttcatcaacttttccatcaagccatgccttcctcacaacccttct

gcgagcagtaaatggaaacttgaccctaaacttagttaggtgtagctgtgtcaatcttatctcagactttatacattatttcttg

>53592|60s ribosomal protein l14-like

tgatcaattacgtccactacggaacatagttttcctttatgtggcccatcggagatgtaagccactctccccgtttccacaaactttccttccatttttc

atcaacttttccataaagccatgccttcctcacaaccctactgcgagcagtaaatggaaacttgaccctaaacttagttaggtgtagctgtgtcaatct

>53591|60s ribosomal protein l14-like

gaaggcatggcttgatggaaaagttgatgaaaaatggaaggaaagtttgtgggcgaagaaattggaagccaagcaaaaaagagttgcaatgacagacttc

gatcgcttcaagttacgtaaagcaaaacaacttagaaacaaggtccgtagcttgaagcgatcgaagtctgtcattgcaactctcttttgcttggcttcca

atttcttcgcccacaacctttccttccatttttcatcacttttcccatcaagccatgccttcctcacacccctactgcgagcagta

>53612|60s ribosomal protein l15

atatgcaaaccagtgatgaagcatcaggagctgcgaggacttacctctgcgggccgcaagtctcgtggtcttggcaagggtcatcgctacagcca

>53609|60s ribosomal protein l15

cgaccagataaagctcgaagattagggtacagagccaaacaaggatttgtaccctaatcttcgagctttatctggtcgtgaggggctaggagccctatgc

attttcgtcagctgtctgtattgccaacatcttat

>53611|60s ribosomal protein l15

cctcgtgaaactctgccagctcctgtaacgccgcgacattcacgatgtttcatatgaggagcgcagatccaattgatcttcgggtcattacgaatggcgt

tgtgcatgtaatccaccaatatgacctcaaagtacttataaatggagtcttggccgacccaatatgag

>53646|60s ribosomal protein l17 isoform 3

gcatttttcaataactgaagcaagaactctgcagatttcttaggccagcggccctgagttgtgccccgctgctttgcctgaggagaagaacctaccgtgc

ccacggaagaataa

>53671|60s ribosomal protein l17-like

cctgaggagaagaccctaccgtgcccccggaagaataaatacatacatgagctccccctgccacatcgaggtggttctcactgaaaaggag

>53668|60s ribosomal protein l17-like

aggaggaagttgttgcgaaagcaactgaggaagagcccacaaagaagaaagtgtcaaagaagaacctaccgtgcccacggaagaataaatccatacatga

gccctccctgccacattgaggtggttctcactgaaaaggaggaagttgttgcgaaagcaactgagga

>53667|60s ribosomal protein l17-like

aatttcttctttgacactttcttctttgtgggctcttcctcagttgctttcgcaacaacttcctccttcagtgagaaccacctcaatgtggcagggagag

ctcttgtatggatttattcttccgtgggcacggtaggttcttctcctcaggcatggcgcacggttgacttga

>53670|60s ribosomal protein l17-like

cattgaggtggttctcactgaaaaggaggaagttgttgcgaaagcaactgaggaagagcccacaaagaagaacctaccgtgcccacggaagaataaatcc

atacatgagctccccctgccacattgaggtggttctcactgaaaaggaggaagttgttgcgaaagcaactgaggaagagcccaca

>53669|60s ribosomal protein l17-like

tagccaatttcttctttgacactttcttctttgtgggctcttcctcagttgctttcgcaacaacttcccccttttcagtgagaaccacctcaatgtggca

gggagagctcatgtatggatttattcttccgtgggcacggtaggttcttctcaacaacattcttcaaatatttgacagctctcctcagtggcattcgcct

cagtgccattgc

>53691|60s ribosomal protein l18a

gtatcaatcaagcagattcctgagaagtccccgattaatataaagaactttggtatctggttacgctctgactcccg

>53693|60s ribosomal protein l18a

atagtggagtccccttcggtttttctgatggtaacttccggcctataacctcaaattccttcaactctcctttggctttcattttcgcctatatacgcgc

acgtgtattcccaatccaatggtaacttccggcctataacctc

>53689|60s ribosomal protein l18a

gccagatcggggggcatagcgtaaccagataccgaagttctttattttaattggggacttctcaggaatctgcttgattgatactatttccccagtagcc

ttcttgaacttctttagttgccgcaggaaataccagaagactactggggaaatagtatcaatcaagcagataccaaagttctttatcttaattggggact

tctcag

>53688|60s ribosomal protein l18a

tcaccttctgtttttcagatggtaacttccggcctataacctcaaattccttcaactctcctttggattgggaatacacgtgcgcgtatttaggggaaaa

tgaaagccaaaggagagttgaaggaatttgaggttataggccggaagttcccatctgaaaaacagaaggtgactccactataacctcaaattccttcaac

tctcctttggctttcattttcgcctaaatacgcgcacgtgtattcccaatccaat

>53686|60s ribosomal protein l18a

caaaatcccgcttctggtatttcctgcggcaactaaagaagttcaagaagactactggggaaattgtaccaatcaagcagattcctgagaagtccccaat

taagataaagacctttggtatctggttacgctatgactcccgatctggcacccacaacatgtaccgtgagtacagggacctgagcgtctctggtgcagtc

actcagtgctatcgggatatgggtgctcgccacagagcgagagcacattccattcagataatcaaggtggagcaggtaaaggcttcaaactgccggcgac

ctcaggtgaagcagttccacgactcgaagatccgcttccccctgcccaagaggatccagcagcgccacctcatgaacaagttttcagtccgcaaacccag

gtccttctatatgtaaacatattgtggacatttgttgtgggtttctgagttttggatgtgaaacaataaagt

>53687|60s ribosomal protein l18a

ctgagcgtctcaggtccctgtactcacgggacatgttgtgggttataggccggaagttaccatctgaaaaacagaaggtgactccactatacaaaatgag

aatatttgcaccagattccattgtcgcaaaatcccgcttctggcatttcctgcggcaactaaagaagttcaagaagactactggggaaattgtatcaatc

aggcagattcctgagaagtccccaattaagatcaagaattttggtatctggttacgctatgactcccgatctggcacccacaacatgtaccaaagttctt

tatcttaattggggacttctcaggaatctgcttgattgatactatttccccagtagtcttcttgaacttctttagttgccgcaggaaataccagaagcgg

gattttgcgacaatggaatctggtgcaaatattctcattttgtatagtggagtctccttctgtttttctgatggtaacttccggcctataacctcaaatt

ccttcaactctcctttggctttcattttcgcctatatacgcgcacgtgtattcccaatccaatggcaactccggaaggaccctgtctttggacgcaa

>53716|60s ribosomal protein l18a-like

gcctcaaactgccggcgacctcaggtgaagcagttccacggcacaaagatccgcttcgcactgcccaagaggaacaagcagcg

>53717|60s ribosomal protein l18a-like

aggctacaaactgccggcgacctcaggtgaagcagttccacgtctcaaagttcctcttccctctgcccaagaggatccagcagcgc

>53715|60s ribosomal protein l18a-like

caaggtggagcaggtaaaggcttcaaactgccggcgacctcaggtgaagcagttccacaactcaaagatccgcttcccactgcccaagaggatccagcag

tgggaagcggatctttgagtcgtggaactgcttcacctgaggtcgccggcagtt

>53714|60s ribosomal protein l18a-like

gtggcgctgctggatcctcttgggcagtgggaagcggatcttcgagtcgtggaactgcttcacctggggtcgccggcagtttgaagcctttacctgctcc

acctgaggtcgccggcagtttgaagcctttacctgctccaccttgattatctgaatggaatgtgc

>53713|60s ribosomal protein l18a-like

tggagcaggtaaaggcttcaaactgccggcgacctcaggtgaagcagttccacgactcaaagatccgccttgattatctgaatggaatgtgctctcgctc

tgtggcgagcacccgtatcccgatagcactgagtgactgcaccagagacgctcaggtccctgtactcacggtacatgttgtgg

>53711|60s ribosomal protein l18a-like

gtgagtacagggacctgagcgtctctggtgcagtcactcagtgctatcgggatatgggtgctcgccacagagcgagagcacattccattcagataatcaa

ggtggagcaggtaaaggcttcaaactgccggcgacctcaggtgaagcagttccacgactcaaagatccgcttcccactgcccaagaggatccagcagcgc

cacctcatgaacaagttttcagtccgcaaacccaggaccttctatatgtaaacatattgtggacatttgttgtgggtttctgagttttggatgtgaaaca

ataaagttatactgtaagaataaatggtgtaatttttactaccttttaatgaagtgaaatgtgcatattgtgtgagagattcagtgagctactgtgatat

tagcccccctatcaaaatcttggttgtgaacacactgatctgcagcacaatgcaaactctagattatattgtaaatagataattcagttgagttggggaa

gccaacaaatacgaat

>53793|60s ribosomal protein l19

tttcattaggatccacccaccccttttttttcccacatggcatcacagaggcggcaagcctcttctggagttttagg

>53800|60s ribosomal protein l19

ttcattaggacccaaccaccccttttttttcccccatcggaccacagaggcagcaagcctcttctggagttttaggga

>53802|60s ribosomal protein l19

ttcgttgttttcattaggatccaaccacgccttttttttcccccaccgcatcacagaggcagcaagcctcttctggag

>53806|60s ribosomal protein l19

ttgatttcattaggagccaaccacacctttttttttccacaccgcatcacaggggcgccaagcctcttctggagttttaggg

>53804|60s ribosomal protein l19

cgttgattccattagaatccaaccaccccttttttttaccccaacgcaccacagaggcagcaagcctcttctggagttttaggg

>53807|60s ribosomal protein l19

cttgaattggtattggcaatttcgttgatttcattaggaaccaaccacaccttttttttcccacatcgcatcccagaggcggcaagcctct

>53785|60s ribosomal protein l19

atttgcaatttcgttgatttcattaggatccacccacacattttttttgccacatcgcatcacagaggcggaaagcctcttctggagttttag

>53789|60s ribosomal protein l19

tttgcaatttcgttgatttcattaggatccccccccaccttttttttaccacatcgcctcacggaggcagcaagccccttctggagttttaggga

>53784|60s ribosomal protein l19

atttgcaatttcgttgatttcattaggacccaaccactccttttttttgccacttcgcctcacagaggcggcaagcctcttctggagttttagggaactc

a

>53795|60s ribosomal protein l19

ttgcaatttcgttgatttcattagggcccaaccacaccttttttttacctcaccgcatcacagaggtggcaagcctcttctggagttttagggaactcat

g

>53797|60s ribosomal protein l19

tttcgttgatttcattaggatccaaccactccttttttttgccccatcgcatcccggaggcggcaagcctcttctggagttttagggaactcatggtcac

tgag

>53781|60s ribosomal protein l19

gggaactcatggtgactgagttccgaccaccatcaaacagccgctgttcctttcctttttccaaatccacagtgacgctctttcctcctcgcttctgtat

tcttacgaacacgcgcacg

>53794|60s ribosomal protein l19

ttcgttgatttcattaggatccaccccccctttttttttcccacatcgcaccaaagaggcggcaagcctcttctggagttttagggaactcatggtcact

gatttccgaccaccatcaaacaggtcctttttccaaatccacagtgacgccctttcctcctcg

>53779|60s ribosomal protein l19

aaagacgattcaccgatattggaatgccgggttccagctcgtgattggcttgtgcgaaactggggaaagtcaaaaaagagaaatgtgcttttggaaccga

gctgacgaggaaacggggagaaagggcgtcactgtggatttggaaaaaggaaaggaacagctaatgctcggaatccacagaaggccctgtggatccagcg

gatgag

>53778|60s ribosomal protein l19

gcttctttatgattaacccatctttattcaacttccttatatgctgtcttgaattggtatttgcaatttcgttgatttcattaggatccaaccccccctt

ttttttcccacctcgcatcacaggggcggcaagcctcttctggagttttagggaactcatggtcactgagttccgaccaccatcaagacccataccaact

ccaacacgacagccaacacaaggacagccatcctcatgcca

>53836|60s ribosomal protein l19-like

tccacagggtcttctgtggcatacgagcattagctgtgctaaggcaactcttgaaaaagtccagggaaccaaagaaaattgataggcacctttaccaccg

ggtgtacatgaaggctaaaggtaacgtt

>53839|60s ribosomal protein l19-like

tgcaaataccaattcaagacaaaatataaggaagttgattaaagatggtttaatcaacttccttatattttgtcttgaattggtatttgcaattgcgttg

atttcattaggatccaaccacaccttttttttaccacatcgcatcacagag

>53840|60s ribosomal protein l19-like

ctctgtgatgcgatgtggtaaaaaaaaggtgtggttggatcctaatgaaatcaacgaaattgcaaatagcaattcaagacaaaatataaggaagttgatt

aaagatggtttaatcataaagaagccttctccgccttcttcttgtggatgaactccatcagcacacgcttgttcttgaaaacattacctt

>53838|60s ribosomal protein l19-like

tgtcatcgtcatcatcaccatcatcgcccgctgctctcctcttcctggtcctcgcttctgtattcttacgaacacgcgcacgtgagtgaacagcaacagg

cttctttatgattaaaccatctttaatcaacttccttatattttgtcttgaattggtatttgcaatttcgttgatttcattaggatccaaccacaccttt

tttttaccacatcgcatcacagaggcagcaagcctcttctggagttttagggaactcatggtcactgagttccgaccaccatcaaacagagatcaaaaac

tgcac

>53835|60s ribosomal protein l19-like

atgttattctagggggcacaggggcacaatctttcactgaacattgtcatttaagacttgtactcacttcaacatctttgtcctagccttctccgccttc

ttcttgtggatgaactccatcagcacacgcttgttcttgaaaacgttaccttttgccttctggcctcagcttgatccttcaacatctttgtcctagcctt

ctccgccttcttcttgtggatgaactccatcagcacacgcttgttcttgaaaacattaccttttgccttcatgtacagctggtggtaaaggtgcctatca

attttctttgcttccctgttctttttcaacagtcgccttagaactctcatccgctggatccacagggtcttctgtggcatacgagcattagctgttcctt

tcctttttccaaatccacagtgacgccctttcctcctcgcttctgtattcttacgaacacgcgcacgtgagtgaacagcaaccggcttctttatgattaa

accatctttaatcataaagaagcctgttgctgttcactcacgtgcgcgtgttcgtaagaatacagaagcgaggaggaaagggcgtcagtgtggttggatc

ctaatgaaatcaacgaaattgcaaa

>53837|60s ribosomal protein l19-like

taatgccaaatgtgtattatctataatatattcttcgactcactaatttcacaattaaaaaaccacacacacacgcgcacttcataacaaacctgcaata

tttaataaagctaaggtgtcatcaacttaaaacacccttttgtggcccattgtaagcacaatcaatctgaaggacattgatctgtcctggtaatcaaaat

acatagcacatactgtaacctttcactggttatgcaaaactgacatgctgtagtttccacacctcatgaggaaaaattcactggcttgcttcacttttat

tttaggctgcctggccatcacaaattatctcgttactgaaaagtgccatcttcatattaagtgctacttacggtctgctttgatttctgtacgtgtgtgt

agctcaaatgacaacaggtaaatatcgcctctagttcttgtttcatcattagggttaaggtcccaacatcaactcaattgaaatcaaagtgtttttagat

aaacttgagaattttaaagagccaagtgacttgcacaataccccatatacgatttacaatttgttgcaaccataattgggtttacatcctgcagcatatc

aaataccaccaattagataaaaatttaccaaacatgttagtcaatatatgttggtgtcattgtttttcaacttcaatgtgtaagagccaattttttacaa

gaattctttgtagccgtggctagtagtttggtcatttatttttctcaaaaagccacaatttacaacagaaccgaaagaaaataaagaaaatgcatcattt

cttggcagctgcagttgctgcatcatcttcacgttggtacgactgtagcagctcctgcttctttttgctgattctttcctcacgacgtttacgtgcctct

ttcaccttttgccttctggcctcagcttgatccttcaacatctttgtcctagccttctccgccttcttcttgtggatgaactccatcagcacacgcttgt

tcttgaaaacattaccttttgccttcatgtacagctggtggtaaaggtgcctatcaattttctttgcttccctgtactttttcaacagtcgccttagaac

tctcatccgctggatccacagggtcttctgtggcatacgagcattagctgttcctttcctttttccaaatccacagtgacgccctttcctcctcgcttct

gtattcttacgaacacgcgcacgtgagtgaacagcaacaggcttctttatgattaaaccatctttaatcaacttccttatattttgtcttgaattggtat

ttgcaatttcgttgatttcattaggatccaaccacaccttttttttaccacatcgcgtcacagaggcagcaagcctcttctggagttttagggaactcat

ggtcactgagttccctaaaactccagaagaggcttgctgcctctgtgatgcgatgtggtaaaaaaa

>53879|60s ribosomal protein l22-like

cgttcctgaaggtacttctcgaaatttgcgtcatccattatattgtcctcaacagggtgggcgcagcccacggtgaa

>53882|60s ribosomal protein l22-like

ccttaatgcgttcctgaaggtacttctcgaaatttgggggggccatgatattgtcctcaacagggtgggcgcagtccac

>53884|60s ribosomal protein l22-like

aaaattattggtttttccgtcgaccttagtgcgttcctgaatgtacttcacgaaatttgcgccatccatgatattgtcct

>53883|60s ribosomal protein l22-like

gaccttaatgcgttcctgaaggttcttctcgaaatttgcgtcgtccatgatattgtcctcaacagggtgggcgcaggccgcg

>53885|60s ribosomal protein l22-like

cgttcctgaaggtacttctcgaaatttgagacatccattatattctccgcaacagggtgggggcagtccacggtgaacttaag

>53888|60s ribosomal protein l22-like

cgttgaccttagtgcggtccagaagggacttcttgaaatttcggacatccatgatattgacctcaacagggtgggcgcagtccacggtgaacttaagagt

aaccttct

>53887|60s ribosomal protein l22-like

gtggactgcgcccaccctgttgaggacaatatcatggatgtcgctaatttcgagaagtaccttcaggaacgcattaaggtcaacacgtttgcataatggc

tgcagtgtctgctaagaaaccc

>53881|60s ribosomal protein l22-like

caagtacctcttcgaaaagggtacatcagaattaatggaaactttcgctttgtttctctccagaaacacattgttcccaaaattattggtttttccgtgg

actgcgcccaccctgttgaggacaatatc

>53878|60s ribosomal protein l22-like

tttcgctttgtttctctccagagacacattgttcccaaaattattggtttttccgttgaccttaatgccttcctgaaggtacttctcgaaatttgcgaca

tccatgaagacgaggaagaagaagataacgattaaatgtatgtgtaaatttttgtaagatcaatgttgtgagt

>53872|60s ribosomal protein l22-like

gactggagttcagacgtgtgctcttccgatctcgttgaccttaatgcgttcctgaaggtacttcccgaaatttgcgacatccatgatattgtcctcaaca

gggtgggcgcagtccacggtgaacttaatagctacactacctatgttctgatttgcaaacgatctatttctccgcagggacaacagggagactcgaaacc

gctatagtatcaaaaattgattctgtggcttgtgacggc

>53873|60s ribosomal protein l22-like

ttacaaaaaattaaaatgcgttgaaacacaaatcacaatcaaccaccacgtgcccgacatccatgatattgtcctcaacagggtgggcgcagtccacggt

gaacttaagagtaaccttcttcttctttatgcctttacctcgaatatgttgttttttggacgaggtaaaggcataaagaagaagaaggttactcttaagt

tcaccgtggactgcgcccaccctattgaggacaatatcatggatgtcgcaaatttcgggaagcaccttcaggaacgcattagttcggaagagcacacgtc

tgaactccagtcacgccaata

>53874|60s ribosomal protein l22-like

ttattcttcgtaagaacagcgtcaacacgtttgcataatggctgcagtgtctgctaagaaaccctcgtccaaaaaacaacatattcgaggtaaaggcata

aagaagaagaaggttactcttaagttcaccgtggactgcgcccaccctgttgaggacaatatcatggatgtcgcaaatttcgagaagtaccttcaggaac

gcattaaggtcaacggaaaaaccaataattttgggaacaatgtgtctctggacagaaacaaagcgaaagtttccattaattctgatgtacccttttcgaa

gaggtacttgaagtacctcacca

>53871|60s ribosomal protein l22-like

gatactttattccactttaacgccagggaaaaccgtggcactcacaacattgatcttacaaaaatttacacatacatttaatcgttatcttcttcttcct

cgtcttcttggctgtttatctggaagtacctcaattcgtaagtgtctttgccgttggcgacaactctcagccaatccctcaaattatttttcttcaagta

ctttttggtgaggtacttcaagtacctcttcgaaaagggtacatcagaattaatggaaactttcgctttgtttctctccagagacacattgttcccaaaa

ttattggtttttccgttgaccttactgcgttcctgaaggtacttctcgaaatttgcgacatccatgatattgtcctcaacagggtgggcgcagtccacgg

tgaacttaagagtaaccttcttcttctttatgccttcccctcgccc

>53907|60s ribosomal protein l23-like

ttaggcagcgaaaaccgttccgaaggaaggagggggtcctcatatactttgaagataatgctggggtaatagtaaac

>53908|60s ribosomal protein l23-like

caggtgatgatgaaatctccaactccagccgcaggtaagcggttcaaacgacccttatagccagccacagcgatgacaaaaagagatttc

>53904|60s ribosomal protein l23-like

attatcttcaaaggatatgaagaccccatcctcccttcggaacggttttccctccccaatgacaactgctggcatcaccttcttcctcagctctg

>53906|60s ribosomal protein l23-like

ttgtcattaggcagcgaaaaccgttcagaaggaaggttggggtcttcatattctttgaagagaatgctggtgtaatagtaaacaacaaaggtgaaatg

>53905|60s ribosomal protein l23-like

aacaatcatgtcacccgaaccggccgctgttaaagggtcgtctcacccgtttacccgcggccggttcaggtgacatgattgttgctacagtgaagaaggg

aaaaccagag

>53902|60s ribosomal protein l23-like

aaagtttgtgcacggtgggttccaagaatgttaaccgatcagaataaagaggcaaggacaacaatagcctcccaacacttgcagcgcttccgtttggagg

gaaggatggggtcttcatatactttgaagataatgctggagtaatagtaaacagcaaaggtgaaatgaaaggcagtgcgatcactgggcctgttgcaaag

gagggtgccgatttatggcccaggattgcatcaaatgcaagcagtattgcatag

>53944|60s ribosomal protein l23a-like

ctccttggaacagacttcctagggtattttggatttctgggtggacagaatgtcttcggtctaccgaagtgcaccgatgttctgatctttctaaaacaat

atccatgttcacccttgagaaccttcctctgaatcctgagtgccttctgcactggtcgtactatcttgcctcctttctgactttttggcgcccatccctg

at

>53945|60s ribosomal protein l23a-like

taacaagattggaattatataaactggtttgtccttggaacagacttcctagggtattttggatttctgggtggacagaatgtcttcggtgtacggaagt

gcaccgatgttctgatctttctagaacgagatccatgttcccccttgagaaccttcctctgaatcctgagtgccttctgcactggtcgtactatcttgcc

tcctttctgacttttttggcgcaacatctctcttgggcttttcaacaaccttcttcactgctggcttgccagctacagctttgcctgctgccttaccaac

agcctttactgcaggctttgctcccggcttggcggcagccttaccggcagctggcttagctgccggttttgcaccaggtttggcagcagccttcccagct

gcaggcttagcggcagacttgggcttggcagctcccttggcctgtgctgcacccgcagctggcttcgccttgccagcagctcccgccgcaggtggaacgg

gtgcctttgct

>53971|60s ribosomal protein l24

aaatttcaaagagctattgttggtgcatctcttacagacatcaaggaaaaaaagaatatgaagcctgaaattaggaaagcacag

>53975|60s ribosomal protein l24

taagaaaagtaaacgtctttccatctgtcgtgcccacggttttcccatggcccgggtatacgttatccccactgtaggcccacagtccgat

>53973|60s ribosomal protein l24

aaagtaaacgtctttccatctgtcttgaccatggttttcccatggccagggtatatttcatacccactgtaggcacacagtcctgctatcttcagagaaa

acaatacactaaaaacg

>53980|60s ribosomal protein l24

gttcttcttcctgacctttcttgtacttccgcatgtataacactgtccatgtcaccttcctggaattccttttcatcaaatgtgaagattctcttttcgc

attaagaaaactaaacgtctt

>53979|60s ribosomal protein l24

tgtcaaagcagcaaagaagcaggctgcacttcctaaggcgaaacctgtccatgtcaccttcctgggattccttttcatcaaatgtgaagatgcacatttc

gcattaagaaaagtaaacgtctttccatctgtcttgacca

>53976|60s ribosomal protein l24

tgttcttcttcctgtcctttcttgtgcttccgcctgtataacactgtccatgtccccttcctgggattccttttcatcaaatgtgaagattcgcatttcg

cattaagaaaagtaaacgtcaatcgagccctttacggatacttcgacgcctgctattaagct

>53969|60s ribosomal protein l24

tcaaatgtgaagattcacattgcgcattaagaaaagtaaacgtctttccatctgtcttgaccatggttttcccatggccagggtatattttataccctct

gtaggcacacagtccgatcttcatcttctctcacaggaaacggaacactaaatggcagatccaaac

>54006|60s ribosomal protein l24-like

ctttcttgtgcttccgcctgtataaccctgtccatgtctccttcctgggtttccttttcatcaaatgtgaagattca

>54010|60s ribosomal protein l24-like

ctttcttgtgcttccgcctgtataaccctgtccatgtccccttcctgggatcccttttcaccaaatgtgaagattcac

>54004|60s ribosomal protein l24-like

tcacagagctcttgttggtgcatctcttactgacatcaaggaaaaaaggaatatgaagcccgaaattcggaaggcacag

>54005|60s ribosomal protein l24-like

tttcacagcgctattgttggtgcatctcttactgacatcatgtcaaaaatgaatatgaaacctgaaattaggaaagcacagcgag

>54008|60s ribosomal protein l24-like

gttcgctttttggtctgttcttcttcctgacctttcttgtgcttccctaatttcaggcttcatattcctttttgccaggatgtcagtaagagatgcacca

acaatagctctgtgaaatttctgg

>53998|60s ribosomal protein l24-like

gacgggttcgcttttttgtctgttcttcttcctgtcctttcttgttcttccgcctgtataacactgtccatgtcaccttcctggggttccttttcatcaa

atgtgaaaaattacatttcgcattttt

>54001|60s ribosomal protein l24-like

ctgtgccttcgtaatttcaggcttcatattccttttggccatgatgtccgtaagagatgcaccaacaatagctctgtgaaatttctgggtgcgacgggtt

tgctttttggtctgttcttcttcctaatttcaggcttcatattcctt

>53999|60s ribosomal protein l24-like

gctaaagagcagaaaaaagctgtcaaagcagcaaagaagcaggctgcacttcctaaggcgaaacctgtgccgaagcaggctgcacttcctaaggcgaaac

ctgtgccgaagcacaagaaaggtcaggaagaagaacagaccaaaaagcgaacccgtcgcacccagaaatttcccagagctattgttggttcatctcttac

tga

>53997|60s ribosomal protein l24-like

gaaaatttaataactgagtactgtcctaaaatcacatattgtcctcagactttctgttgctcgtgtttcactctgtatctttttaggcggaagcacaaga

aaggtcaggaagaagaacagaccataaagcgaacccgtcgcacccagaaatttcacagagctattgttggtccatctcttactgacagcatggcaaaaag

gaacatgaagccggaaattaggaaagcacagg

>53995|60s ribosomal protein l24-like

ggcgttgcagcgacatgtcgtttgtgcggtggatgcggtttgggttggcgacgggttcgctttttggtctgttcttcttcctgacctttcttgtttttcc

gcttgtataacactgtccatgtcaccttcctgggattccttttcatcaaatgtgaagattcccatttcgcattaagaaaagtaaacgtctgtctctctgt

ctctctgtctgtctctctgtctgtctctctgtctctctgtct

>54000|60s ribosomal protein l24-like

cagtccgatcttcatcttctctcacaggaaacggaacactaaatgcgatcgctttttggtctgttcttcttcctgacctttcttgtgcttccgcctgtat

cactctgtccatgtccccttcctgggattccttttcatcaaatgtgaatcttcacatttgatgaaaaggaatcccaggaaggtgacatggacagtgctat

acaggcggaagcacaagaaaggtcaggaagaagaacagaccaaaaagcgaacccgtcgcacccagaaatttcacagagctattgttagtgcatctcttac

taacatcatggcaaaaaggaatattaagcctaaaattagcaaagcacaacgag

>53996|60s ribosomal protein l24-like

aaaagtaaacgtcttcccatctgtcttgaccatggacagtgttatacaggcggcagcacaagaaaggtcaggcagaagaacagaccaaaaagcgaacctg

tcgcacccagaaatttcacagagctattgttggtgcatctcttactgacatcctggcaaaaaggaatatgaagcctgaaattaggaaggtacagcgagag

caggctatcggggctgctaaagagcagaaaaaagctgtcaaagcagcaaagaagcaggctgcacttcctaaggcgaaacctgtgccgaagcagaaggcat

caaaaataacacagaaaagtgcaccacgagtgggtggtaaacgttgaaacctgttatgtaatttttgaatgtataaatt

>54002|60s ribosomal protein l24-like

accatggtcaagacagatggaaagacgtttacttttcttaatgcgaaatgtgaatcttcacatttgttgaaaaggaatcccgggaaggtgtcatggacag

tgttatacaggcggaagcacaagaaaggtcaggaagaagaacagaccaaaaatcgaacccgtcgcacccagaaatttcacagagctattgtttgtgcatc

tcttactgacatcatggcaaaaaggaatttgaagcctgaaattaggaaagcacagcgagagcaggctatcagggaacagtccagtttgggtgaaaaattg

gcaatatattgctcttttgtagcattgggctatggtccctttccctcacatttctacagacattgtgaacttcaaattagattgaaaataaattgatt

>54026|60s ribosomal protein l26 isoform 1

ccaattcgtaaagacgatgaagcacaggcagcgagaggccattacaaaggacaacaggagggaaaaatagtccaagtttataggaaaaagtttgt

>54027|60s ribosomal protein l26 isoform 1

tctttcgataagggcgcagacatcaaacgtcttctgatatgggatggtcctgtaaagtgcctcttacgattcttcctacgcgacgaggacaccatcttgt

ttagcttcatggtccccctagtactagagcctccagccgaaaggcctta

>54064|60s ribosomal protein l27-like isoform 2

ctttattttaggtcttttgtgaatcttagcttcccccaccctcttgtgttctttcctgggataacgatccatgcctgggacgagag

>54060|60s ribosomal protein l27-like isoform 2

agatctaaaataaagccatttgttaaggttctgaactccaatcatcttccccccacacgatacacactagaattgaaacttaagacatctgttaagg

>54058|60s ribosomal protein l27-like isoform 2

acaaaagatctaaaataaagccatgtgttaaggttctgaactagaatcatctaatgcctacaagatccacagtaaaattgagtcttaagacatctgttaa

g

>54054|60s ribosomal protein l27-like isoform 2

cgttatcccaggtaagttcacaagaggatggggaaagctaagattcacaaatggctttattttagttcttttgtgaatcttagctttccccagcctcttt

tgtactttcctttgataacgatcaatgcctgcgacgag

>54061|60s ribosomal protein l27-like isoform 2

ttagatcttttgtgaatcttagctttccccatcctcttgtgtactttcctgggataacgatcaatgcctgcgccgagagcatgtccgaatggtttatctg

atgagccatcatcaaaatttttcaagacatctgttaaggtcctta

>54055|60s ribosomal protein l27-like isoform 2

cacaaaagatctaaaataaagccatttgttaatcttagctttccccatcctcttgtgtactttcctgggataacgatcaatgcctgcgagagagcatgtc

cgaatggtttatctgatgagccatcatcaaaatttctcttcttcagaaacgctttccttgccattaccagtcacatttt

>54063|60s ribosomal protein l27-like isoform 2

gagccatcatcaaaatttttcaagacaattgctttctttcctgcgtaacgtccacttaggacgattacgatttttcccgatttcattattttcctcatgg

ttaatagatggtttccgattccctattcctaataaccatgaggaaaataatgaaatcgggtaaaatcgtaatcgtcctaagtggacgttacgcaggaaag

aaagca

>54107|60s ribosomal protein l27a

ttgtcaaggttgattgtgggacacctcttcgttttgcgtcttaggtggtagttgcgcatcccaagcttaccgaag

>54108|60s ribosomal protein l27a

ctgaagccgcaatgcgaaggggtgtcccacaatcaacctggacaaagtatggacccttgtcacggaacagacaaggca

>54092|60s ribosomal protein l27a

cctctcccaccaggatgtttcctgttctttcctattcgtgcatgtccgtgactcacatgtcctctcagcttcctcgtc

>54094|60s ribosomal protein l27a

gtagccagggtgatacttgtcaaaattgatgcgatggtcatgcatagaacaagcattacctcttccaccaggatgttt

>54105|60s ribosomal protein l27a

tgtgctttcctattcggccatgtccgtgactcacatgaccgctcagctccctcgtcttcttcttgtgtgtagacatgttaacg

>54111|60s ribosomal protein l27a

gtttcttgtgctttcctattcgtccatgtccgtgactcacaaggcctctcatcttccttgtcttcttcttgtgtgtagacatgttcacgtgtct

>54104|60s ribosomal protein l27a

aatacgaagtggtgtcccacaatcaaccatgacaaattatggtcccttgtcgcgtaacaggcaaggcaaaagtataagaacaattctgaggggag

>54106|60s ribosomal protein l27a

tgtttcctgtgctttcctattcgtccatgtccgtgtctcacatgccctttcagcttcctcgtcttcttcttctgtgtagacatgttcacgtgtctct

>54097|60s ribosomal protein l27a

cttcggtaagctgggtgatacttgtcaaaattgatgcgatggagatgcataccaccagcattacctcttccaccaggatgtttcctttgcatcctgcaag

agacacgtgaacatgtctcttgcaggatgtttcctg

>54101|60s ribosomal protein l27a

cttccaccaggatgtttcctgtgctttcctattcggccatgtccgttactcgcatgacctctcagcttcctcgtcttcttcttgtgtgtaggcatgttca

cggacatggacgaataggaaagcacaggaaacatcctggcaggggcaca

>54099|60s ribosomal protein l27a

caaattcttctccaaacaggctgaggataaataaaagaaatcggtggctgttgtgttcttactgcgcaatacgaagtggtgtcccacaatcaaccttgac

aaattatggtcccgtgtctcggaacagccaaggcaaaagtataagaacaatactcagggg

>54096|60s ribosomal protein l27a

ccgaagtagccagggtgatacttgtcaaaattgatgcgatggtgatgcttaccaccagcattacctcttccaccaggatgtttcctgtggaagaggtaat

gctggtggaagaggtaatgctggtggtatgcatcagcattacctcttccaccaggatgtttc

>54113|60s ribosomal protein l27a

gtccgtgactcacatgacctctcagtattgttcttatacttttgccttgtctgttccgtgacaagggtacataatttgtcaaggttgattgtgggacacc

acttcgtattgcgtcttaagtggtggttgcgcataccaagcttaccgaagtagcctgtgatcgtgaaagccaaattcttatcctcagcctgtttg

>54100|60s ribosomal protein l27a

tggtagttgcgcataccaagcttaccgaagtagccagggtgatacttgtcaaaattgatgcgatggtggtatgcatcaccatcgcatcaattttgacaag

tatccccccggctacttcggtaagcttggtatgcgcaactaccacttaagacgcaatacgaagttgtgtcccacaatcatccctgacaaattatggaccc

ttgtcgcggaacagacaaggc

>54098|60s ribosomal protein l27a

ttcttataattttgccttgtctgttccgtgacaagggttcataagttgtcaaggttgattgtgggacaccactttgtattgcgtcttaagtggtagttgc

gcataccaagctgaaagaaaatgagcattgaaaccacactctacaaccaaaaaatatacagcataatttgaaaaatacagtactattccaatgttataat

aatgttgagtgcaactattttaacaaactgatgtgagaaaattaaaacaattaacagtcattaagtggaaattacatcatcatttaagactgattatgcc

tttcagcattcagtctgga

>54144|60s ribosomal protein l27a-like isoform 2

acgaagtggtgtccctcaatcagccttgacaaattatgaacccttgtcacggaacagtcaaggcaaaagtataagaa

>54146|60s ribosomal protein l27a-like isoform 2

tacgaagtggtgtcccacaatccaccttggcaaattatggtgccttgtcacggaacagacaaggcaaaagtataagatcaat

>54148|60s ribosomal protein l27a-like isoform 2

cgaaacagcctgtgatcgtgaaagccaaattcttctccaaacaggctgaggataaaataaaagaaatcggcggctgttgtgttcttactgcgtaatgcag

atgtggtgtaagaaagttttttcttaataaatatatcaaccttgacaaattatggacccttgtcacggaacagacaaggcaaaagtataagaacaatact

gagg

>54147|60s ribosomal protein l27a-like isoform 2

tcctgtgctttcctattcgtccatgtccgtgactcacatgacctctcagcttcctcgtcttcttcttgtgtgtagacacgtgaacatgtctacacacaag

aagaagacgaggaagctgagaggtcatgtgagtctcggacatggacgaataggaaagcacaggaaacatcctggtggaagaggtaatgctggtggtatgc

atcaccatcgcat

>54145|60s ribosomal protein l27a-like isoform 2

tgggtaagggtcgcctaccgaaacagcctgtgatcgtgaaagccaaattcttctccaaccaggctgaggataaaataaaagaaatcggtggctgttgtgt

tcttactgcgtaatgcagatgtggtgtaagaaagttttttcttaataaatatatcaaccatctctggtaaaaaaaaaaaaaaaaaaaaaaaaaaaaaaaa

aaaaaaaaaaaaaa

>54185|60s ribosomal protein l28

tgttgatgttccttttcttcaggagatacgcattgttgttccggagcagcatccatgataaatgagaagacattggacggcacctcaccaccac

>54190|60s ribosomal protein l28

gctccgtactgaatggtttgttgatgttccttttcttcaggagatcggctttgttgttccggatgatcctccttggtaaatgagacgacattttacttca

c

>54192|60s ribosomal protein l28

aatggtttgttgatgttccttttcttcaggagatacgcattgttgttccggatggtcatccatgataaatgagaagacagattacttctcctcttcaact

tgtacaggg

>54188|60s ribosomal protein l28

tttgttgatgttccttttcttcaggagatacgcattgttgttccggatgatcatccttgataaatgagaagacattttactccacctcactcaggttttc

aactctacttcaaaccatacaccac

>54193|60s ribosomal protein l28

ggaggactaatctttggttggtgtatgagtgtggtggtgaggtgaagtaaaatgtcttctcatttataatggatgatcatccggaacaacaatgcgtatc

tcctgaagaaaaggaacatcaacaaaccattcag

>54183|60s ribosomal protein l28

cccactgaaccgatatgaactgacagtaattaggttgtttgtctctgtactgaatggtttgttgatgttccttttcttcaggagatacgcattgttgttc

cgtatgatcatccatgataaatgagaagacattttacttcacctcaccaccacactcccggca

>54309|60s ribosomal protein l28-like

tgctttcattgtcctttttgtaactgattttgcaggtttgtgcgaggcttgaaatttcttgcaaacaacagtgaagcccttgctacctggaacagcaact

actccaactctcttccgg

>54302|60s ribosomal protein l28-like

tttaaatttctcgcacacaacagtcaagccattgccatctggagcagcaacccctccaccactcttccggtgaatcaacccactgaaccgatatgaacag

acattaattaggttgtttgggtttgtactaaatggtttgttgatgttccttttctt

>54303|60s ribosomal protein l28-like

ttcattgtcctttttgtaactgattttgcaggtttgtgcgaggcttcaaatttcttgcaaacaacagtgaagcccttgctatctggggcaaaaactactc

caccactctttcggtgaatcaacccgctgaaccgatatgaactgacatttattaggttgtttgggtccgtactgaaaggtttgttc

>54304|60s ribosomal protein l28-like

cagcaactactccaacactcttccgtttaatcaacccacttaaccgatatgaacttacattaattaggttgtttggctccgtactgaatgatttgttgat

gttccttttcttcaggagatacgcattgttgttccggatcatcatccatgataaatgagaagacattttacttcacctcaccaccacactccctaacgga

aa

>54343|60s ribosomal protein l29

gggtgtggatgcaaagtttttgaaaaatcagcgttttgcaaagaaccctaatttgaaaccaaaacagcaggtggagagggccgaagaccgcaaagcaaaa

c

>54341|60s ribosomal protein l29

ttgagttctacaaacttgtcagcactccaacgaacagaacactaaccatggggtgtggatgcaaagtttttgaaaaatcagcgttttgcaaagaaacata

atttgaaaccaaaacagcaactgaagagggcagaagaacgcaaagcaaaacgagaagctgctcagcagaagaacgcaa

>54340|60s ribosomal protein l29

cgccattttcagtcacaaaataaattcctccaacagccttcgctccgtcctagtattgaacaacgtgttacaaaataaattcctccaacagccttcgctc

cgtcctagtattgaacattgggtgtggatgcaaagtttttgaaaaatcagcgttttgcaaagaaacataatttgaaaccaaaacagcaactgaagtgggc

agaagaacgcaaagcaaaacagcaactgaagagggcgg

>54435|60s ribosomal protein l3

gaaaagaccattacaccaatgggaggtttcccacattatggcaaagtcaacaagtactacgttatgataaagggctgctgtatggg

>54444|60s ribosomal protein l3

agataaacttgaaattcattgatacttcatcaaagtttagccatggacggttccaagctgcttctgacaatacctcatttatgggcttg

>54445|60s ribosomal protein l3

tagccatgaccttttgtaaccccaatcacatctatcatctaatcaggagcaaacccgtgctgaacaagtataggtttttccaggtgttcacgtggccatt

t

>54441|60s ribosomal protein l3

aactctcttctttggccccatacagcagcactttttcataacgaagtaattgttgacttccccataatgcgggaaacctcccattggagtaagggtcttt

t

>54439|60s ribosomal protein l3

ccactggtatgtgtggggattttaactttataaataaacaatgcatcttgaaattcattgatacttcatcaaagtttggccatggacgattccaaactgc

tgctgacaaaacatcatttatgggcttgcttaaaaaaga

>54432|60s ribosomal protein l3

acctcctcttcctcctcctcagagtagacttcctcatcatctgacatggttgcaagaggttaaaagagactgaccgcgaagaatccgagggccgtggaat

gtggagtttcaatgtacccaacaacaccaacaatgatcattggaggtgtctccaaaattgtcacagcttc

>54442|60s ribosomal protein l3

gggaggtttcccacattatggcgaagtcaacaatgacttcgttatgataatgggctgctgtatggggccaaagaagagagttataactctgcgaaaggta

aggcggtgataaaatcgtcaagaatagtttgtaattagaattaacatgatgattttacagtagagccaaaagga

>54464|60s ribosomal protein l3-like

aatgcagtaagaggaacaggtttcgttggagcagcttttggaaacgcgggcactttaccacgatgcctctgtgatcgcttgggcggagaaaatcc

>54465|60s ribosomal protein l3-like

ctcttccttggacccataatagtgcccttcagcagcaagaaatcattgtcaacactgccataatgcgggaagccacccattggggtgatagctttgtttg

taa

>54470|60s ribosomal protein l3-like

cctcttcgtgtgcacaaggagcgactttcgcagagttataactctcttctttggccccataccgcagccctttatcataacgaagtcattgttgacttcg

ttatgataaagggctgctgtatggggccaaagaagagagttataactctgcgaa

>54473|60s ribosomal protein l3-like

acacctcacattagtgtaattgtcttctctgttcaatgtatgcactgtttttcatcacctgaaatattttagcaagtcaattatccacccttataatcta

acaacaaatacgtctagttcatcaccatagcaaaatacaaaacctttccatctttcacagcaattccctgaccaatgcgacattttctcactgatttgct

ctatggtggcaaccaagtttggatacaacgaatgagtatcagaatggcaagcaccgttacataaacccttgcctgttttatatggcagcttctctgtgtg

ctaatgcaatgtgacatctcggtgtgctccgatcaccttgccatgtctcaatttttttggacgtcacaatcatctgacttatcaacccaaatcatattac

cacttcttggaagctttttccttgacttgtaccattcaaaaacatcttgggatagtagcagataagacagaatgctggtattgggaaaccagattggcaa

agaagtagccatcacgccattacaagtggttatgattattcttcacccacttgaaccaggcctatcggcgtctctcatgacgtgacccgtaccagcttta

tagctaagaagtggaatagcgtgaaacgctttcgtttcacttaaccgcgaagcctcagtgatctcttctttggataaaaccccatggaactttggcgacg

ggcgctaaccattcgacgagacatctatccaacacgaacgccgaacaaat

>54467|60s ribosomal protein l3-like

gaaaaaaatacattttaaactggctctggagagagaagtgacactgagttgcagggaactttgcccactacttgtaattgtttcaactatttaacagttg

agttaaattatcagtttaatgacaatattttgtgaagtgatacacaccattggtatatatttgataaatgtaacagattaataacaaatcatgtcaatca

ttttatttcaatcaacatattatgcaaaagctgcatcttccttgatgaggtcttttttaagcaagtccataaatgaagttgtcatcagcagcttggaact

gtccaccaccgaacatggatgaagcctcaatgaattataagttgatctttccgaagccttctttgtgtgcgctagtagtgacttttgtagttataaccct

catatttgatctcttacacaaatattttatgataaagtcatcacagacattaccataaagtgagacacctcacattagtgtaattgtcttctctgttcaa

attatgcactgtttttcatcacctttccatctttcacagcaattccctgaccaatgcgacattttctcactgatttgctctatggtggcaaccaagtttg

gatacaacgaatgagtatcagaatggcaagcaccagtgcaagcacccttacataaacccttgcctattttacatggcagcttctttgtgtgctaatgcaa

tgtgacatctcggtgtgctccgataaccttgccatgtctcaatttttttggaggtcacaaccatctgacttataaacccaaatcatattaccacttcttg

gaaacttcttccttgactcgtaccagttcaaaaacatcttgggatagtagcagataagacagactgctggtattgggaaaccagattcgcaaagaagtag

ccatctcgtcattacaagcgggtatgattcttcttcacccacttgatccaagcctatcggcgtctctcatgacgtgactcgtaccagctttttagctata

aagtggaatagcgtgaaacgctttcgtttcacttcaccgcgaagcctcagtgatctcttctttggataaaaccccatggaactttggcgacgggcgctaa

ccattcgacgagacatctatccaacacgaacgccgaacaaat

>54570|60s ribosomal protein l31 isoform 2

taagaagagagcaccgagagttattaaggaacttcgacaactggatgttaagcacctggacacgcctgactcacgaattgactcaagagttaataagcag

>54565|60s ribosomal protein l31 isoform 2

gaattttcgaagttccttaatggctctcggtgctctcttcatggcgttggttttcagaagagagcaccgagagccattaaggaacttcgaaaatgcgctg

ttaagcaaatgggcacgcctgatgtccgaattgac

>54561|60s ribosomal protein l31 isoform 2

gacaggacgaaggagactgtggccgagagacaagaggtaatgatagagacacacaaagagatgatgacaatgggcacgcctgatgtccgaattgacacaa

gattgaataagcaggtgtggtccaagggcataaggaatgttccattccgcaggcgggttagactgtcgagacggcggaatgatgatgaagattcaccaaa

caaaagtaactaaagtgt

>54562|60s ribosomal protein l31 isoform 2

gaagaagaaatcggcgatcaacgaggtagtcactcgggaatacacagttaatttgcacaaacggcttcatggcggtggttttaagaagagagcaccgaga

gccattaaggaacttcgaaaattcgctgttaagcaaatgtgcacgcctgatgtccgaattgacacaagattgaataagcaggtgtggtccaagggcataa

ggaatgttccattccgcatgcgggttagactgtcgagacggcggaatgatgatgaagattcaccaaacaaattgtacactttagttacttttgtacctgt

tccatccttcaagggactacaaacagaaaatgttgacgccagtcaagaataatttggttggaagttataaaatatttgtaca

>54563|60s ribosomal protein l31 isoform 2

agaaagaaatccgttaatagtgttgccatcgatttattttttggactaataactcaacatggtgaagccgaaaggtgagaagaagaaatcggcgatcaac

gaggtagtcactcgggaatacacagttaatttgcacaaacggcttcatggcgttggttttaagaagagagcaccgagagccattaaggaacttcgaaaat

tcgctgttaagcaaatgggcacgcctgatgtccgaattgacacaagattgaataagcaggtgtggtccaagggcataaggaatgttccattccgcatgcg

ggttagactgtcgagacggcggaatgatgatgaagattcaccaaacaaattgtacactttagttacttttgtacctgttccatccttcaagggactacaa

acagaaaatgttgacgccagtcaagaataatttggttggaagttataaaatatttgtacattaaaataactctgaaactccagtgtttcatttcaaaaaa

caccccacagcaaaccttaaaacagcttcatttcatattaagtatcatttatcattgtagtgttctatggctcattgggtatgttgtcaatgcggtatgt

aaaatttacttgagaaataggttgatgaatattattagttttttcagttttgttaatttggtggaaggctgttactaacaaaatggattgtacttacaag

tagtttgattttgttaaagtgaagtagacgcacataattggacatattaaacttcagaatagaataagtgatggtcatgaagtgactcgtatgttgtcga

tttgtcaactcaaggagccagtagtagaattgtaggtaatctgagagtatcaaggaaaaaggtggtgtttcatcatgaaattgttgtctttctaattaag

agctttggtttctttgacaactttgttggtgtcgtacagggggttatctggtagtcctgtggtcatgtagttgctctgtgcataatataaataaattata

tgaag

>54586|60s ribosomal protein l31-like

gttgcccgagaatacactatcaaccttcatagacgtatcaatggaatacccttcaaaaagcgtgctccacgcgccatgcgcgaaatccgtcgtt

>54614|60s ribosomal protein l34 isoform 1

aataattctttcctttacacatttatgacacaagactccaccatctgcccttttcacagtctttttcctgcggcacattcttgatctttacacatttatg

acacaagactccaccatatgcccttttca

>54617|60s ribosomal protein l34 isoform 1

caacaggaggcgtgttgtccgtactcctggtggaaagctggtataccaatatttgaaaaaacctcagaagatcccaaggtgtggacaatgcaaggaccag

ctgaggggcatagttccagctaggccaatggaaagatcaagaatgtgccgcaggaaaaagac

>54613|60s ribosomal protein l34 isoform 1

ctccagatgattaacgggtcttcaggacctcaacgacaatcttctgttcttcaatgaggaacgcacgaataattctttcctttacacatttatgacacaa

gactccaccatatacccttttcacagtctttttcctgcggcacattcttgatctttccattggcctagctggaactatgcccttttcacagtctttttcc

tgcggcacattcttgatctttccattggcctagctggaa

>54641|60s ribosomal protein l34-like isoform 2

aggttttttcaaatgttggtataccagctttccaccaggagtacggccaccgcgcctcctgttgcttttcgtgttgtccgacaggggtcgtcg

>54646|60s ribosomal protein l34-like isoform 2

catagatgtgtaaaggaaagaattattcgtgcttttctcattgaaaaacagaagaacgtcgttgaggtgctgaagacacgtcaatcatctggag

>54640|60s ribosomal protein l34-like isoform 2

ataaatgtgtaaaggaaagaattatccgtgcgttcctcatggaagaacagaagatcggcgttgaggtgctgaaggcacgtcaatcatctggagatg

>54644|60s ribosomal protein l34-like isoform 2

cttggtatcttcttaggttttttcaaatattggtataccaactttccaccaggagtaaagacaacacacctcctgttgcttttcgtgttgtacgacagg

>54639|60s ribosomal protein l34-like isoform 2

atgtgtaaaggaaagaattattcgtgctttcctcattgaagaccagaagatcgtttttgaggtgctgaaggctcgtcaatcatctggacatgcaaagaaa

g

>54642|60s ribosomal protein l34-like isoform 2

tccaccatatgcccttttcacagtctttttcctgcggcacattcttgatctttccattggtaattatttatcagctttctttgcatctccagatgattga

cgtgccttcagcacctgaacgacaatcttctgttcttcaatgaggaaagcacgaataattatttcctttatacatttatgacacaaga

>54637|60s ribosomal protein l34-like isoform 2

atcaagaatgtgccgctcagcttgtccttgcattgtccacaccttggggtcttcttaggttttttcaaatattggtataccagctttccaccaggagtag

ggacaacacgcctcctgttgcttttcgtgttgtacgacaggcgtcgtcgatacagccccacttctccacactattcaccaacaagtccttcttattctcc

aac

>54638|60s ribosomal protein l34-like isoform 2

tgtccttgcattgtccacaccttgggatcttcttaggttttttcaaatattggtataccagcttgtccttgcattgtccacaccttgggatcttcttagg

ttttttcaaatattggtataccagctttccaccaggagtacggacaacgcgcctcctgttgcttttcgtgttgtacgacagtcgtcgtcgatacaccaga

cgctgaaccatttttatcaccccctaaacctccaccggaagaagaagaactgatcaaagagtaatctctaatacagtcaccaggggtaccaacagtgact

tgtgttcgaaaagaaagatccagtctgaaat

>54636|60s ribosomal protein l34-like isoform 2

aactaataagaaaaaacctaaaaaatacaagaaagcttttgtgtgcaaaatgtcatttctgaataacctttcacccttctcatatttaagacaaaattca

ctatcgtcactaagttgaaggaactagtctgatcaatgagaagatcaactctccacacagaattgtaagtcacgaaagaatacaggtatcattacaaaaa

aaaaaatcagtttaataacttttatagtttattaaatatgtccattgataattatttatcagctttctttgcatctccagatgattgacgtgccttcagc

acctcaacgacaatcttctgttcttcaatgaggaaagcacgaataattctttcctttacacatttatgacacaagactccaccatatgcccttttcacag

tctttttcctgcggcacattcttgatctttccattggcctagctggaactatgcccctcagcttgtccttgcattgtccacaccttgggatcttcttagg

ttttttcaaatattggtataccagctttccaccaggagtacggacactatattgcattgtgaaaatgtaagaatgcaatttgtggagaaacacatttgca

tcatcctcagctccaaaggataacaacacgcctcctgttgcttttcgtgttgtacgacaggcgtcgtcgatacaccagacgctgaa

>54775|60s ribosomal protein l35a isoform 2

atgtttataaggcaaagaacaagacaccagtgcctgatagatcaccacagaagacgaaagttccggctgtgtggggcacagtgacgaggcg

>54774|60s ribosomal protein l35a isoform 2

tgactgcaaccttcgattttcaaaagagcggtgtgctcgtgctgataccgtaacccacgcttgtagcgtgtgaatatcgtcttagcgtaaagtcgaccat

ggcgtttcttaggacgcttctt

>54805|60s ribosomal protein l35a-like

atgtttataaggcaaagaacaagaccccagtgcctggtcgatcaacaaagaacccgaaagttagggctgtgtgggggaaagtgtcgaggccaca

>54799|60s ribosomal protein l35a-like

cgttggtaaacgctgtgtttatgtttataaggcaaaggacaagcctccagtgcctgatagatcaacaaagaagacgaaagttagggctgtgttgggct

>54803|60s ribosomal protein l35a-like

agcagacagctggttctacgttggtaaacgctgtgtttatgtttatcaggcaaagatcaagaccctagtccctcgtagataaacaaagaacacgaaagtt

a

>54809|60s ribosomal protein l35a-like

ttgctctcactgctccagaacatccatgtggcctcgtcacttagccccacacagccctaactttcgtcttctttgttgatcttcccgtttgctctctgtc

gctccaagctgag

>54795|60s ribosomal protein l35a-like

aactataaactatgtggtacagattcatacggtatttaacaagacaccagtgcctggtagagcaacaaagaagacgaaagttagggctgtttggggcaaa

gtgaccaggccacatggat

>54800|60s ribosomal protein l35a-like

gcagtcaaaaagcagacagctggttctacgttggtaaacgctgtgtttatgtttataaggataatttcttttttaaggcaaagaacaagacaccagtgcc

tggtagatcaacaaagaagacgaaagttagggc

>54808|60s ribosomal protein l35a-like

tttcaaaagagcggtgtttatgtttataaggcaaagaacaggacaccagtgcctggtagatcaacaaagaagacgaaagttagggctgggtggggcaaag

tgacgaggccacatggatgttctggagcagtgagagcaaagtgacgaggccacatggatgttc

>54807|60s ribosomal protein l35a-like

taaaccacgcttgtagcctgtgaatattgccttagcgtaaagtcgaccatggcgtttcttaggcaaagaacaagacaccagtgcctggtagatcaacaaa

gaagacgaaagttaggcctgtgtggggcaaagtgacgaggccacatggatgttctggagcagtgagagcaaagttcaaaaca

>54793|60s ribosomal protein l35a-like

ttctcgtgctgattctacgttagtagccgctgtgtttatgtttataaggcaaagaacaatacaccagtgcctggtagatcaacaaagaagacgaaagtta

gggctgtgtggggcaaagtgacgaggccacatggatgttctggagcagtgagagcaaagaacaagacaccagtgcctggtagatcaacaaagaagacgaa

agttagggctgtgtggggcaaagtgacgaggccac

>54794|60s ribosomal protein l35a-like

tgatgctatgtaactcactgtaaaatggaaagctgttggaaatattgcctaatgtttgtaattatttttgtttttcttcttttgcagatgctgtatccct

cacggatttgaatgtttatatttaatgttgtgactcaataaagacaccagtgcctggtagatcaacaaagaagacgaaagttagggctgtgtggggcaaa

gtgacgaggccacatggatgttctggagcagcaggtagaaaaacccaaactgaagaagcgtcctaaga

>54806|60s ribosomal protein l35a-like

ctcttttgaaaatcgaaggttgcagtcaaaaagcagacagctggttctacgttggtaaacgctgtgtttatgtttataaggcaaagtgacgaggccacat

ggatgttctggagcagtgagagcaaagaacaagacaccagtgcctggtagatcaacaaagaagacgaaagttagggctgtgtggggcaaagtgacgaggc

cacatggatgttctggagcagtgagagcaaagttcaaaacaaatcttccttcgaaggcaatgggtcatcgcataagaattatgctgtatccctcacggat

ttgaatgtttatatttaatgttgtgactcaataaagacactttcgtgtaatttgtg

>54798|60s ribosomal protein l35a-like

cacaacattaaatataaacattcaaatccgtgagggatacagcataattcttatgcgatgacccattgccttcgaaggaagatttgttttgaactttgct

ctcactgctccagaacatccatgtggcctcgtcactttgccccacacagccctaactttcgtcttctttgttgatctaccaggcactggtgtcttgttct

ttgccttataaacataaacacagcgtttaccaacgtagaaccagctgtctgctttttgactgcaaccttcgattttcaaaagagcggtgttctcgtgctg

attccgtaaaccacgcttgtagcctgtgaatattgccttagcgtaaagtcggccatggcgtttcttaggacgcttcttcagtttgggtttttctacctcc

tctttctccttagcaggcttaactttcttgtcctttggtttctccgcactcttcgtctttgctggcttctctacatcagccattt

>54830|60s ribosomal protein l37

acatcgtctgcaaagcgtatgagtcttgttacgccttttaccaaaactggatgttcccttcgtcatttggcgaccacgcgtataaccatgtgcaattgga

tttcactcattttcaatccaagacagtagcggtactgcaagagcatgcttacatatgcaccactcaaacggaagaactag

>54909|60s ribosomal protein l37a-like

cctaggtgaggccccatatctggtaccgtatttcccggggattccaaccttcttcgtgcgcttagccatttcgcttg

>54910|60s ribosomal protein l37a-like

cactggtgtagtagttgcgagaagtttcgtacgagaacctgagtgtgggatggcatccagcagcgagtgtgttggaagcgtgatcatacaagcgaaatgg

ctaagcgcacgaagaaggttagaatcaccgggaaatacggtaccagatatggtgcctcacttaggaagatggttaagaagatggaaattacgc

>54908|60s ribosomal protein l37a-like

gcaatgaaaaggtgcaagtgtatttggaatgctgcgtaatttccatcttctttaccatcttcctaagtgaggcaccatatctggtaccgtatttcgcggt

gattccaaccttcttcgtgcgcttagccatttcgcttgtatgatcacgcttccaacaccggaaacagaaaggttaggggcacgaagaaggttggaatcac

cgggaaatacggtaccagatatgttgcctcacttaggaagatggttaagaagatggaaattacacaactc

>54938|60s ribosomal protein l38

gtacgcccttgtcattacagacaaagaaaaggctgagaagctgatacagtcgttacccccacgtctccaggtgaaagaagcaaagtgaat

>54937|60s ribosomal protein l38

tttttgtacccccttgtcattacagacaaaaaaaaggcggagaagctaaaacagtcgttccccccaggtccccaggtgaaagaagtaaag

>54934|60s ribosomal protein l38

tcagatgctcgaggtttttgtacacccttgtcattacatacaaataaaatgctgagaagctgaaacagtcgttaccgccaggtctccatgtgaaagaagt

aaagtgaatgtatatcttaaaataataaaatatttaaactcagatgctcgaggtttttgtacccccttgtcattacagacaaagaaaaggctgagacgct

gaaacagtcgttaccaccaggtccccaggtgaaagaagtaaa

>54935|60s ribosomal protein l38

aggtttttgtacacccttgtcattacagacaaagaaaaggctgagaagctgaaacagtcgttaccaccaggtctccaggtgaaagaagaatccagaaaat

acgaagttcaaggtcagatgctcgaggtttttgtacacccttgtcattacagacaaagaaaatgccacgagaaataaaggaaatcaaggagttccttttg

aaagcgaggaggaaagatgcaaaatctgtgaaaataaagaagaa

>55047|60s ribosomal protein l38-like

ctcgaggtttttgtacgcccttgtcattacagacaaagaaaaggctgataagcggaaacagtcgttcccaccaggtctccaggtgaaagaagtaaagtga

ag

>55053|60s ribosomal protein l38-like

gctcgaggtttttgtacacccttgtcattacagacaaagaaaaggctgcgaagatgaaacagtcgttaccaccaggtctccaggtgaaagattcaaaatc

tgtgaaaataaagaagaatccagaaaatacgaagttcaagg

>55052|60s ribosomal protein l38-like

cttcgtattttctggattcttctttattttcacagattttgcatctttcctcctttcacctggagacctggtggtaacgactgtttcagcttctcagcct

ttttctttgtctgtaatgacaagggtgtacaaaaacctcgagcatctgaccttg

>55046|60s ribosomal protein l38-like

tctgagtttaaatattttattattttaagatatacattcactttacttctttcaactggagacctggtggtaacgactgtttcagcttctcagccttttc

tctgtctgtaatgacaagggtgtacaaaaacctcgagcatctgaccttgaacttcg

>55048|60s ribosomal protein l38-like

aggaaagatgcaaattttgtgaaaataaagaagaagccagaaattacgaagttcaaggtcagatgctctaggtttttgtacacccttgtcattacagaca

aagaaaaggctgtgaagctgaaacagtcgttaccaccaggtctccaggtgaaagaagaatccagaaaatacgaagttca

>55050|60s ribosomal protein l38-like

aaaaaaaagaaacgagacagctatatttatatcacatcagcaattttccatattgttaaatgactaatatttagtgcttgaggatcaaaacaaactttaa

tgactttgcccaaaatggatggtcatgagaatttactcacctggtggtaacgactgtttcagcttctcagccttttctttgtctgtaatgacaagggtgt

acaa

>55044|60s ribosomal protein l38-like

ttcttctttattttcacagattttgcatctttcctccacgctttcaaaaggaactccttgatttcctttatttctcgtggcattttcgtcccagactaca

ggaccagctgcgaaaccccgattcttcacctactcaacccagcccaaccacgccgtcgacacacgaacttccaaagaaaaggctgagaagctgaaacagt

cgttaccacctggagacctggtggtaacgactgtttcagcttctcagccttttttttgtctgtaatgacaagg

>55139|60s ribosomal protein l4-like isoform 1

atcaacaccaggaatgttgcggaaggcttttgtaaggccctgatcttttcagtagattatcaatgggcctcgtctctgaatacggcggcggttcctcatt

ttccgtagattatcaatgggcctcgtctctgaatacggcggcggttcc

>55185|60s ribosomal protein l44-like

cttgtactgagttactttgtgctgcttatgatttttgcctttcttgcagaacgtcctacgttcattaggaacgttaaccatg

>55183|60s ribosomal protein l44-like

ttctgagaatggagtgcacagaatgcaaatacaggaaacaagtcactttgaaacgctgcaataatttagaacttggtggtgagaaaaagag

>55179|60s ribosomal protein l44-like

gttctgagaatggagtgcacagaatgcaaattcaggaagcaagtccttttgaatcgctgcaaacattttgaacttggtggtgacaaaaaga

>55186|60s ribosomal protein l44-like

ctttagacttcttgtactgagttcctttgtgccgcttgtgctttttgcattccttgcagaacgtcctacgttgtttccgaacgttaaccatg

>55182|60s ribosomal protein l44-like

cttcttgttctgagttactttgtgctgcttatgctttttgcatttcctgcagaaagtcctacgttgtttcggaacgttaaccatgtgccctgcaataaaa

g

>55178|60s ribosomal protein l44-like

tctgtcgtaacggcgtcgtccttgtgacgcttgcctttctttagacttcttgtactgagttactttgtgctgtttatgctttttgcatttcttgcagaac

gtcctacgttgtttctgaacgttaaccatgttgcctgcaatcaaagatctacacagacagagttgagagtaccgtgcagtcg

>55205|60s ribosomal protein l5

aaactgtctcttgtacgcctcatcatcttcttccgccagctcccgcatgtaatccgcacaactactggagctcgcgtgtttggtgctatgaagggagcta

tggacggtggtttgaatatacctcacagcaccaaaagattcccaggttacagcc

>55235|60s ribosomal protein l5-like

ttcttgaatacgccacttgacaggtaatatctctgttggaaaaacgaacgatcaggagatatttcggcgtgttgtacttgttcttcacctgagttgtaag

gcgaagcctagcgcggttcccatcttccaccgcttcaacgttgtattcctctccagtaacttctgtgcagccga

>55257|60s ribosomal protein l6-like

gttatactgaaagctctaccctctggctttgtggccgttgtcggaccctatgagatcaatcgggtacctttaaggagagtacatcagaagttcatcatcc

cgacctccactgttgtcgatatctccaaggctgatacttcaaaggtggatgatgcgctctttaagaagcaaccggtcgagggtagcaaggagaaac

>55276|60s ribosomal protein l7

ctggtggttggcgcacgaagacaacccataatgttgaatgtggtaactttggaaatagggaagacaaaataaatg

>55280|60s ribosomal protein l7

tgggggttggcgcaagaagacaaaccattatgttgaaggtggtgacgggggaaatagggaagacaaaataaatgaacggctccgcagaatggcg

>55279|60s ribosomal protein l7

ggtggttggcgcaagaagacaaaccattatgttgaaggtggtgactttggaaatagggaaacttgatcttttgaaaaactctccccgtttcctgaatctc

agaaaacaatttttcgcaggctccataaaatgtccgccgcatgcgtgaacattttacaccatggcgcgcttgcacaatcg

>55273|60s ribosomal protein l7

agaagaagttcaaagaaggcagcacatttcatattatcacacaatatgcgaggaacatcacaaatgacacaggatttgggatggacattaataaaacaaa

ggcatttttcattgcaacagaatcttctcacgaaatttcaatcagcaactttctcctccaaatgagaaaatattttgttatctaccaggaacttaaatgt

gatttgcagagtatccatgtggatgtaggtttgaaaagtaagtactgcaaacttcacgtttagttacatgtttattaaagtctcttaattacaccattct

gcgtagcagttcatttattttgtcttccctatttccaaagtcaccaccttcaacataatggtttgtcttcttgcgccaaagtcaccaccttcaacataat

ggtttgtcttcttgcgccaaccaccagttggtgtattgagc

>55278|60s ribosomal protein l7

tttacaagcgaggatttgtacgtgtaggcaaattgggagctcgtcaacgagtgcgtgtacaacacaatgaactaatatcggagcatctcggacagtatgg

aattcatggagtagaagatatggtgcacgagattgctacggctggaccacatttcaaacaagtttccaatttcttatggactttcaaacttaaagcacct

cgacatggctttgtttgcaagcgtcatggatttgccgagtttagaaagggtgattggggaaaccgggagacaaagatcaatgagcttgtcaatcgcatga

tctaataagatggctgcatacaacaatattgaaacgttcttcttccgctaaataaaactactccaacattcaatgacacccggggagacatacatacaaa

cacgtgcgtagagagacgcgccctgagcatgacacgctattttaagacgttttagagttctt

>55307|60s ribosomal protein l7-like

catttaagctcaatacaccaactggtggttggcggaaaaaagcaaacccttatgttgaaggtggtgactttggaaatagggaag

>55308|60s ribosomal protein l7-like

cctctggccatttaagttcaatacaccgactggtggttggcgcaagaagtcaaaccattatgttgagggtggtgactttggaaatagggaag

>55306|60s ribosomal protein l7-like

ggccatttaagcgcaatactccaactggtggttggctcaagaagccaagccattatgttgaaggtggtgactttggaaatagggaagccaaaata

>55303|60s ribosomal protein l7-like

cgcaagaagacaaaccattatgttgaaggtggtgactttggaaatagggaagacaaaataaatgaactgctccgcagaatggtgactttggaaataggga

agacaaaataaatgaactgctccgcagaatggttggcgcaagaagacaaaccattatgttgaaggtggtgactttggaaatagggaagacaaaataaatg

aactgctccgcag

>55300|60s ribosomal protein l7-like

tctggccatttaagctcaataccccagctggtggtgggcgcaagatgacaatccattatgttgaaggtggtgactttggaaatagggaagacaaaataaa

tgaactgctccgcagaatgttgtaattaagagactttaataaacatgtaactaaacgtgaagtttgcagtacttacttttagttacatgtttattaaagt

ctcttaattacaccattctgcggagcagttcat

>55400|60s ribosomal protein l8

ccaagttgcagacaattggcccttctggcattgtcccaacaggcatcacatatccaatttgcaagtttgctttctt

>55398|60s ribosomal protein l8

ttcctgtactgtggaaagaaagcaatcttgcaaattggagatgtgatgcatgttggtacaatgccagaagggacaaatgtctgcaacttggagg

>55399|60s ribosomal protein l8

ttatgatatgcacgtcaagctttaagaattggattatcaatccttcctcctccagcaaccatgccttccatggctagattgtttgaaggtataactttct

t

>55485|60s ribosomal protein l9

ggccccaggtgttactgtgacaacctcggaaaagaaaaaagataaattgatagttgaagaaaaccgcattgaagatgtttcccgatcagctccccttatc

c

>55487|60s ribosomal protein l9

ccccaggtgttactgtgacaaacgcggcaaagcaaaaagatgaatgtagagtggaaggaaacagcattgaagatggttcccgatcagctgcccttatcca

g

>55488|60s ribosomal protein l9

agaagtttcagtataaaatgcgacttgtctacaatcacttccccattaatattaacctcgtggatgatggtcgtgtagtcgagttacgaaacttccttgg

ggagaagagaactcgtattgtcaaaatgctcccaggagtcgtctgtactaaaggaacccttaaagatgagctagtcttgagtggc

>56016|7-dehydrocholesterol reductase, putative

ctgagtaatgccttttcagctctcttcgatttgtagctgttatcaaatatatcaattgcaacttttagtcatatttatgtctcactctttataactgaga

aatcctagtagaattcgattttttctgttaccacatcttattataacacattaactttcaatttttgttacgttagtataaagaaaaacaataacacaca

tgcggttttctgggccgctttattcgtcacaaattcagttcgtacaaaacgggtatctcgttattaaagtgaagtgaggcagaggacagatgccaatcag

agatgctatatgtaatgtagttcctaagtgtgtagttaatagtaagtctcaaatttatggtccgctttgtacatttcacactgcgttattgactatccgc

gtaaattttatacacaagtggtagtataaaatgcaatggtagctgatcttgttcgtgaaaatacgaaatttatgtcaaaattccaaatcttctttttttc

aaaatacagtaaaatattccgattttcatttatagaccttatttccgctttttggtacagtctgtttttccgtcacagagcaccaataaacggaagcagg

atcgtgagctatgttcggctctttctaccgttaaccttcttcctgtagcttgctgcaaggtagggacctagacacttttcgtatgtaatttttctagatc

agttttccatttttctgggggcgtttttctgtagcttgctgtggccgagaaaagtagtcacttaatgtgcatagtttttctgaagcagttttccattttt

gctggcggacatcatttgacacagattgggcagcttagtcatcatcttcgtcttcctcctccagctcgacctcctctaaagtggcctccagctggtgcat

gacgctggcggcggtggccgcagcggcgaagctcagaaaatccacggcagcaggcggtagggcaccagacgacagtaggcttgccagctgctcccgtatt

tctgctgacacttctcctcgtccctgtagacccgatggaccaggagcagggtcaggaagatcacgtacaggaacggcagaattcccaggcccatcgccgg

cagcgagtgttgcagccgccgccgcctcctcgcttcacacgtgctgctgcttca

>56038|71 kda protein

aaaattaagcaatatatttaggtcaaacatttctgcagctgagggggggaaaaaaaacctggtcaggggcaacaaatgcttccaatcaagacagtgacta

atgccacagcaatacaaagttgattaaaacatcaaatgccttgtttttcaaaattttttagcatatagacaagactttatttcattcaaaattaaaatat

cagttgcatcacagtcaaaattaatacacctttcacataagatttacatttcacatgtaaaatttttttaaataaaatattgtcagtatttttctactga

tgatacaaaactagattatctgacttaactacaacaataataatctttacaaaatggaattgtagcattaagaatgcccattgctttgtttaatcaaagc

aattctctttcctttgtactgaatgaaacgatttaaatttgtacacacagtagtaacagtgatgtagtgttcacccaaagacttcttctttgagcgcacc

ttatgctttagacctggcactaatcctttcagtctgtctactaatgatatttcagcagctgctacatctgtttcatcctcagcaaaaacatccatgattg

tatccaatgtttctgctgtgacccagagttcagtttctttgctagctgtatcaagtaaaaaatttccaacaaccttcaaagtcgaactgtctcccttcat

cattcctaatattccactgatctttaagaggtttgcccgagcacttggatcggagcactgctgtgtccattcaagcaataccttcaaatccgactgggtc

atatccttaaaacaactgggtgcaagtggtgcagatttttgaaggattgctcgcatagcagctgttacagcttccatcaatgtagcgtcattggaatctg

atctgagtgttgcaatgtctacccacattttgtaaagacctttttcaccttcaaaatctgatacatcaaggcaggttagaaggttatttaaacataaaag

tgcgcggcacctcagcactttggatctgttgtaaatattcaaagtttcagtgttttcttgcagtaatttacatacattttcaggtgggaaagcaattttc

atccagactttttgcaccaactgatggtgcactactgcttcatgaacctctgcagataaagtcacaacagatgcttcgtaagatggaggttcatcatctg

ggatactcgagtcactcaaaatattatcacttgtttcagaagagtcaacttccattgtactatcttcatcatcatcaccagagcaaatatttgtcaaaat

ttccagacatgtctgctgcccctcaaacaatgtctgtgtatctgctataagtttctcctgtttcttcagtttttcagtgtcttctatcactttatcatca

tctttactctgagcaagtgaggttgccaaggtgtccagcacttggcagtgatctacttccaacacagatcccagtacagcaagaatccgagctgtagata

tctttgctctgatgtttaaagccacaccacagataagcactctaaacaagagaaattctgtctcttttccttctagttccagcaagctgtgccaagttgt

ttccatattttgtagaaccttaattgcaattggattgttttctgttattgtgtgcaaacattgtgcaacagcaacagcagtttcaattccatacttttga

acatctaaacatctggcaagtattttaatattgtcttcatttaggtacattaaagcggttgtgttattttcacacaggttaagcaagagatgtactgcct

gaatgaaggtatctgtatactctgatacaattttcttgtcgccacactcctgccaatcatctgcatacttctgaattaaggcactaagtgatgtcattat

atcttgctgaactagtaagtcacacaagtcagggtctccacaagcactaagattccttaatgcacctgctgcagcatgtcttacaactgggtcattgtca

agcagcaatggagctgctatccttattattcgattttgaacaaccatttctggatgtttatcaaataccgatgcaatagagctcaggccaaatattttat

cttctgtgcttccctgtagctgttcaataacggctttaatagcattttttcctctgtatacattagccaagctcttagcactgagttcggcttcttcatt

ttgaatgtctttcacgcttggcagtcctgtagggttcttattcttccgtttacgtttgttttgtttcacttttcccattggggtaaataggtagacaagg

ataataaatctaaaattcaatcactaattatatttccactgtttctttaaaccgcttgcacactcacttgtgccacagaaacacacagaagaaagcatgg

atcagagatataatggacgtgtacaaacacagcgcag

>56113|72 kda inositol polyphosphate 5-phosphatase-like

ctagcgctgctgaatggagcaggagatgtgaaggtccaagtgtttcctgtaaactcacttcccactcaaacctctccgaataactctcttgagtgccaat

tgcaacaacatctgggacgtgttctaaagttgctgggagcatgaagtcattaagttcatgtggaggaggctgtccattcatattccatgttcccacaaag

atccgcagttcacggtggggcagtgtgcgttccagttccactgatccaagaagtgaattggcggcaatacggccatgtaaaaagttcc

>56114|72 kda inositol polyphosphate 5-phosphatase-like

gatgaagtcttgcagtgggtatcagagcaagaattccccttgcccttaccacatagtttacacagtgatcagttgaaagccattattgcagaaggaagtg

tcttccgagattttgaagaaggtcctataacattcccaccaacgtataagtatgatccgggaacgcagatgtttgacagttcgcacaaacagcgcacacc

agcatacacagatcgcatcttatacaaatgtcgaagtggtcgtggcgttctgcagtgcttgctgtattcatcagctccatccatctgcacatcagatcac

aagccagtgtggggcctctacagcagtgctgtgcgacctgggattgacactattcctttggcagcagg

>56130|72 kda inositol polyphosphate 5-phosphatase-like isoform 1

aaacagcgcacaccagcatacacagatcgcatcttatacaaatgtcgaagtggtcgtggcgttctgcagtgcttgctgtattcatcagctccatccatct

gcacatcagatcacaagccagtgtggggcctctacagcagtgctgtgcgacctgggattgacactattcctttggcagcaggaagaagcagcagacatga

aggtcacactagcgatagca

>56147|7up2_drome ame: full=steroid receptor seven- isoform a ame: full=nuclear receptor subfamily 2 group f member isoform a

aatatctgcaggtttccatcgtttgcaggttgcaaaagctttttcaagagaagcgttcggcgcaaccttacgtactcgtgtcgcggaagccggaactgtc

ccatcgaccaacaccaccgcaaccagtgccaatattgccgcctcaagaagtgtctcaagatgggcatgaggagagaagccgtgcagcgggggcgtgtg

>56145|7up2_drome ame: full=steroid receptor seven- isoform a ame: full=nuclear receptor subfamily 2 group f member isoform a

gcattggccgtagcgcgacgtggggtagggctcggcgcgcagcagcagcgagatgtagctggacaggtaggagtggccgttgaagcccgccgccgccgct

gccgccgccgccgcgtcgccgttggtcagcgccagctggcccggcagccccgggggctgcgtgggcggcacacgcccccgctgcacggcttctctcctca

tgcccatcttgagacacttcttgaggcggcaatattggcactggttgcggtggtgttggtcgatgggacagttccggcttccgcgacacgagtacgtaag

gttgcgccgaacgcttctcttgaaaaagcttttgcaaccttcgcacgtgaactggccgtagtgcttgccgctggacttgtcgccgcagacgacgcactcg

atgttctggcccttgtccgaggagttggacccggtgccgctctgcgaggagccgacgacgatctgggatccgccaccgccgcccccagagctggccgacg

cggagccgggcgtcgagctgagcaccaactcgtcgggggcgggcgggggcggcgcgggcgccggggcggcg

>56161|8-oxoguanine dna

atagttagccaatttatgtcatatgaagtgaatagagatgtgaaaactaacatcaaaacacatgctctattccttttagaatgttgtataatgtacacaa

aaagcactgcatttctcacatttatctgtataaaataagtacagctggtggattaaaactgttgcacaagaagactgcagattataagatgaatgataaa

aatgtgtctcatatggaaaataggaaagtaacaacacaaagcagacctacatggaagttcacagtcatttaatgacaacatcaattagctgtacatttct

ctgaatattcgtcatctggtgatcagcttggagtgtctccgcaaaagctggcaaagggatgataaaccatttaaaaagactgaaaactaaatgtctgcaa

tttattctcacaacactttttttcagatgaaaagctactttgcagcagttaggagtcgagaagtaaatttggtgagcctgaacacggatggttatgtaac

atggcaaaaagagccaactaattatacttctccaaatgtatcatgtatttataaaagaaacaacgtaacactgtatgacagacctttacaaattgttatc

agatcactgcaataaatctgtgaagttgataattaagtggttacaaatttgtaaatttacttgaagattccattacgtctaacagaacaacacaataatt

acaaagtctgtcataccacagcaggacaataagataatttaaaacagtaatactatagtcatacttttacttcatagtctctcattatttacagtaattg

taatacacacacagtgaacacaaagagtctacacatgctgctataaaacaattacataatcatagtgagcataatgcttcaaaatgatttgtgtgtatga

aatctgttgtagctgacaacaaacaccttccatttatctgagaaatttcaaacatgaaaaaatattataaaaactgaacaatgacttttttagtttatgt

aaataagttaaaattttatgtcacaaattggtgggactcataacagcaagccacagaatgtcaaaagtaaagtgcatcctaccttggggaactagcaatt

gaagatcatcatctggcttactctcaatagcagatttacttctttctctagcacattagacagatctcccaaatgactgttacatatgaagggcttattt

caacagtggcacaagtccatttttgttcatcctgagatagagtcctaaagttaaatgagtgctgaaaaatctgcagttgagatatcagaaatattcaagt

ataatacatgaatatttctggtatctcaactgaagatttttcagagctcagttaactttaggactctgtatctcagaatgaacaagaatggacttgcacc

actattgaaataagctcttcatatataaccaaattgtcattcactttaaaatattaatatgttcccttcaagagcagaccgtaaaagtttctaacattct

ttcgcccaccactatcattaaactcagaaggtgtaacctgctacatctttacagttttttttatcagcttctttctgttacttgacatggggacagcact

caatagtttgccactttcatttatttagtccgcttcttttttgtttttgtttctcctgtaccattttcttcaggagtttgctcatttccaatattcttca

aatcagcacaaaacaataccgtctgtgcccatccagcatctgggccatgaatgtcctgaaagaatttcataatgtccaaatacaaagtattagagagggt

ttttgcatttcgcagacttttcatataatgctgttttgcaacctgatgtatgtgtgtatccactggtactgcatttaagtgacccaatgacataaggcat

atacaatctgctaccttgaagccaacgcctggcagccttacaagttctttccttgcagcttcatacggcagcagcttaagcttatttatccactcgtatc

cacccaactcttggatcatacatgctgatttgtgaataaagccagctctatacccaaatcctgcttttgccagtgcaccatggacagaaggtgccacaag

ctgctctacatcaggaaaatcaaaatacttcacaccatcaataaccccaatttcctccccatacaatgaacacaatttctctatcatggaggtaattctt

ttaatgtgattgtttgatgaacaaatgaatgtcagtattgtctctagtggttcctgacacagaagtcttacaccataacatttgctagctgctttctgaa

atataggatcacgttgaccccaacggttatagcactcttctaaattagtgtccagtctaaggtatttacagagcagcattccattcagttcatctgctgt

atcacatatttgaagtttcttttcttcttcactaagagtcactgagtttgtacttaaatgagcaaggaatttttttagagacaaatcttcatgcagctca

ctaagcaaaacagaacacttcttagaaggtgcaacttgtttagcaactttgtatagtatgaagttatctgtttgttttaacatccaaaatctgctgcaga

agattccccaccattcattttcgttaatcttcttccacctgaaagattgtccgccacctaacgtgtactgcagattcaaatctcttctcttgcagaatag

tcggtaccatggctcttcaaggaggtttatcataattattttctaaaggagaatcaaaacaatgacataaacaatacacaaagcaacaagataacacgct

tcaattccgcgcgaattcgtc

>56181|80 kda mcm3-associated

gttcagtacagatttacatacaaatcccatcgcctcgagcaagtttttttagtgaggtactgccccatctccaatttatcttctcacacagcactggaag

gagttctgccacacgaggaactattttctgtcgagcgtagaactccttggcagtactccttactgctaatttattgaagttgttggctttttcaatatca

ctgcatctcttccctgttctttaacttctctggaagtccctttctagtgatggttgctctcttgttgttagcaagtaggcagcaggataaaaagaaactc

aaagcagtagtcaccccactggtttactagttcaacaacacaggattacatattttgtccttcggcacataggtctatgtgccacttaatggttactgct

tcccaaatgatcacacaggttctcccagcaacaattttgtgacgtctgccgcagccagcttccggtcgactgctttcaggcggaccggcggacgctgcgg

aggtgcggcacgtgtaatgcggcgaggcaggcgggcagtggcggcaggagtggcaggcggcgcagcgcccgcacgtagttccccgagtagcaggccaggc

tgaactccagcgcgctcttcactgtgtcacacctgtacgccacaggcagctgcagagcccgctgcagcgcatctgcacttcccggcgccagcacaaggta

gagtgcctcggtgtctgcccgaccgtcgcagccagtcgcactcccaccgctctcccgctccagccggtcatagagaaccagcagccgctgcaagcactcc

agcaagtgctgcctgtggatcactgcgtcaaagcgcgatgctggctcttcgcacaacct

>41817|AAEL017254-PA

gcctcacacccaaaagcttttaagtttggtagatttggtttcttccctatcctcgctccataggggtttttactttttattctctggtgggtgaaacctt

tcatttgtcatcacaccttgctgttttcttatattccacaccccctgtttaatgtctgatcatatgaaaacaaacacctgtgtacagtgtatgttctgca

acttgctctctacttaacattattctgtcatgatcatctttccttgtattgcacaaggactgtgaaatgttgtttgcctaagcaccaagtaacactctat

gctgttgatccaacttctgcccaaaaaacatttaggtaatccagtatcagttaacaaacttctttccttttcaacaatagtcctttgctctctatgcaac

ttcattctgagatgggctgtgtctgacagtggtttgatgtctaatttctagttccttcaaatatttcatgaatttctggttggtatactctgtcccatca

gatctgatgatttttaatcttgtttcacatttctctttctaccatattgtggaaattgtgaaaaatatcactcacctgatctttataacttaaaaagtaa

acttagttacacttgaacagttgtctataaaggtaggaaaatgatgacttcatccatatgcatgaactgctagatcaccttggttcttgttttattaggc

ttagagggcaacatagtcaatgtagtctttttccattgtatatctgtttcacaaggttccttgcatttactgccgtcttccattcctattgccatatcct

gtataattgtcatcccttttcagtgtaggttttcaaactccctgtgccataagaaaccttttgttgagtacacagaatggtatacatcttcaaatacact

tttagtaatatctaacttgataatttatctcccatttgcaccacagctctaatttttttttttatattgattaattatttttgctccatttctagcaaag

attactctgttgtccttcttgattgagtatttcacttgcacataaaaagttagatgtgacgtcttttacatagtaaacatcatgtactataatactttct

ggttcttcatttacaataacattaaaatcagctctcctggtaacttggaaccgtctactgtagatatacgccataaacagttgttttatttctgataatc

tgtactgatggtacaaaatttaatatccattctggctgtctgttacccatttttctaaaagagtaataaatggagactgccttacattgattatcagttc

tcctgtctagactatttgtatcgtacctttttccctttgtactgacttacctgaggtgtgtgtgatccatgtcctgacacttgtaacacttgactggttt

cttcttgggaagttctatagctcttttcctctgtgaagtatggcagtctgctgtactttccacattctgcaggatcgtcacttttatgcggtctcctgta

acagtacgcctgagctgtctaaccccataatcatgggtgcacattttctaggtagtccttctagcagtaattgacattctcatcccagttcttgcacttt

tccagttgagtggttataaattcacagagaattcctacttttcttgtaaggttttcatcttcaaatgcactctgtaacttgtcccaaactacttttgtca

tcacacatttctgtatgtatacatagttcactgtatcagtcaataatctcaacttcgactttgctcttaagaccttattcgtgtagatttgctcagtgga

cttccatttaccattcactacatcccagaaatcatccatatcttaagtatggttctacagcaaacttccatgtaatatagttcttgcaacctacaaggca

ctctatttgcgatgtttgtgccatgctcatttttcacagttatccttattggtaatatgcacagtattaaattatgcaaatcctaaacaaccgagcttgc

>Sg_CNS_NA4Plus26849|AAEL017254-PA

tggcagtctgctgtactttccacattctgcaggatcgtcacttttatgcggtctcctgtaacagtacgcctgagctgtctaaccccataatcatgggtgc

acattttctaggtagtccttctagcagtaattgacattctcatcccagttcttgcacttttccagttgagtggttataaattcacagagaattcctactt

ttcttgtaaggttttcatcttcaaatgcactctgtaacttgtcccaaactacttttgtcatcacacatttctgtatgtatacatagttcactgtatcagt

caataatctcaacttcgactttgctcttaagaccttattcgtgtagatttgctcagtggacttccatttaccattcactacatcccagaaatcatccata

tcttaagtatggttctacagcaaacttccatgtaatatagttcttgcaacctacaaggcactctatttgcgatgtttgtgccatgctcatttttcacagt

tatccttattggtaatatgcacagtattaaattatgcaaatcctaaacaaccgagcttgccactccagttttcactatgtgcttccttattcatgactat

tccaaagacatttcttttctatatgttacatcagaatacttattctgaatgaattcactagtgtttggggccacaacctgttagaactagtgcattgaaa

aagtaattatgaacactaaacagtaagaattaggccagttatattttaatattcatgactgcatagaatagctcataactgtacatgtagtacactttaa

t

>54996|AAEL017254-PA

ctcgcgtaataatcctacttttctcgtaagaccaccatcctcgaatgcacttcgtaaattttcccagacttctttcgctgtagcagctttttctacgtga

acataattcaccgaatcaatcagtaatatcaattttgattttgctttcctatccttactt

>56209|ABC transporter, putative

agagcacgcccagcatgatgccggtcatcatgtggtgcatgaggatgcagaacagcaggttgtagttgtccaggacgcgcgcgccgtcgtcgccg

>56202|ABC transporter, putative

ttataaatacactgaccatctgacatgatgagcaagtcatcaaacacttgaaaaaacccagacctcggctgatgcacaacccatatcacagttcggc

>56199|ABC transporter, putative

tgacattgttccatttatggcatcagttggtcttaactgcccaacacattataatccaccagattttgttattgaggtatcaagtgttgactatggag

>56204|ABC transporter, putative

gtatgtcacggtacactgaccatctttcccgcgtcacattaccgagtgacaactggagaagtactggccaaatagcaacacatgcaacagcatagtgaaa

tccttgtctgtcaccaagtactagctgagagagtgcagaaataatttgaagagaaaagcatag

>56200|ABC transporter, putative

cccgcagctggccggagttgaaggtccgccggcccacgatgcagctcagcagcgtcgtcttcccgcagccgctggcgcccaacaggccgtagatggatcc

gacggctacagtcatattgaggttatccaacaccagatggctctgagagccgcgaccgtattgcttgcaggctccccgcaccaccaccgcgttctgcatc

ttggagccttctgtagaaatgacttgcgacgacattcctttgccctcagagagcgggtgattcaccagataagtctaccagttggagctgtcatctaccg

ttttcccacgttaagtgaccgctggaacaaacgaaccgaacttcttgtagccgaaatccttacattatttacctcctccccttgtggg

>56208|ABC transporter, putative

cgcgttactggtcatgcatgctgtatgacgaacctttttcatgtagtgcccatacgatagcgccaccatagtgttcagagccatttgaaccatttgaaca

atcctgctcgctccacggtcagacacgcgacggagagagcaagtgccactagctccccggttggttattaaatttgggtatggataacttctaacatcgg

tcatttttgactgtgtgctggcactagtcatttacggtgtttttgttgtcattctgcttatgtcgtattgtggttctcggttttggtagcccacatgtca

aagttttcagcttccctgatcaggactctcttatttcatttgtgccttgaaaatggcggtgtgtgtcacatgtcgaaataacggcgttagtagaagatgt

cgctttgctgcattcctgtaagtagtgaaaaaatgtgattacggaaggcgctactcacaggtgaggatggtgagcatcatggtggtcatcatgtggtgca

tgaggatgccgaacagcaggttgtagttgtccaggacgcgcgcgccgtcgtcgccggcctcgatgaagagcacgcccagcatgatgccggtcaggatgtt

aaccagcagccgcatgtgcgtcagcgtctgaaacggacgatcagcactggtagaggcccagcattcccagagctgaacgaaatgaccgtacggcat

>56203|ABC transporter, putative

gtgctcttatgttccagacatcagtcagctacaggtgagtttccaagaattagtattgttattgtactcatataattacacagttcatatctgttgaaac

taaaaattttgtacccactgtgactccagatgtaattctgaattttatttaaactaacataacttcattccaagctgattagttatgagcactttcagca

gcactcaagttcagtttctggtgaacacgtaagttgaaacattcacagtatgatctctccaggagtttgtgtggagaatggtaagtggtcctgctttgct

cctggacattcaaaagacaactgtggaagcaaaagcgaataaacaaaaagttttggtgaattaatatatggttatgtttcaaaaaacatgcattatatga

gtttgaatagctataatatcattcacaatgagggtgaggcagctgactcactgctggatgtatgaaactaatgaaccagcttatgcactaaatggtactt

cattttcctttcaaaggtgaaacatgttcataggcatggtaatatcactagtttggatgttttacatgccgtttgcataatatcatttcattctacattg

cttacaatatcaggcataaaaccaagagaaaagcacaacaaatcactgtagacagataggagtacaatatattgctgcttacagaaaaataaaattattg

taatttttttaataaaaacttttgaagaacacaagtttcttaaaaattatttctgttttccagttcctttagaagcatctgtgtttctacgagagcacct

taacaactggtactgcctcacatcttactactttgcaaaactgctttcagatattccattacaggtactgtgtcccacttgttttgtactgatagcatac

ttcatgacgggacaaccatatgaccaccagagaatctatatgttccttttggtttgcatgttgatgacaatgattgctcaaactcttggactagttgcag

gtgcaatttgcaatatacagcttggaacatttatactggctgcagtaacaattccaatgttccttttctctggttt

>56206|ABC transporter, putative

gtgtgtgtgtgtgtgtgtgtgtgagtgtgtgtggcggcggatggagagggggagggtgggttcgcgcgtgagtgtacgacatatttactaaacgagttca

cgtacagccggccggaggggccgtccggttctaggcgctgcagtctggaaccgcgagaccactacggtcgcaggttcgaatcctgcctcggccatggatg

tgtgtgatgtccttaggttaattaggtttaactagttctaagctctaggggactaatgacctgagaaattgagtcccatagtgctcagagccatttgagt

tcacatacagctattacaaacttgcttgcgttgcaacattacgggatttaaaactttgtttttattttgattgtaatcgtcttcgccgccatgccgacta

aagtagtaattattagcgattatataactgtgtcgtttagcacacttcccgttatgcatcgtcagatctcacctgtagccagagaggatgtcgaggagcg

tggatttgccagcaccggatggccccatgatggcaatcagctgctttggaacgaagcggccacatacttcgtgtaggatctttttcttgcctgaaataga

aaaaagaaggtcagatagaactgtgtcaaagtgcaaaagaaaccacattcctacggcaaatttcacatacagcctgatgagctccagttgtatctaagtc

gaaaacaaataaacctggatatagctatcgagaaactcgatactgacatgtatacaccattaaaatgggcacaagatatcggtaaaaagctcagccagtc

caaaaacccaagcgttactgattggttattctaggctcgttagctcgaaatttcgggaatccctaccacttcaatcgtaaaagaagcaagtatcagcttc

actccctcagcaagagtctaggagtaataataatagatgaaaatctaaattcgactgagcacgtaactacattatgcaagagggcatcagtatctctcca

tggcctacaaaaatataaaaagctcgaccctgttaacctgaaaaaagaaacttttagaaacacttatactttcaataactgattactacgatgttaccct

gcaaggtctttctatgtaaggctcacggcgcctggaaattgttatgaacgcgtttgttcgttatatctgtgattcttgacactatgatcatattatacca

tcatacatatagctatcctgtctatgtatacacaaacgcagagatttccatactctctgtcttctctactgttttatcaatgaatactgtccctcacatt

tctcctccatcttaacactcttatccgaacaacatgccagaaacaccccatcagtgcaagaatctttctgttgcaccccatagttcagccacgttcaaga

agtccttcccagaagcagaaactcgaatctggaataatctcccacattatattagacaactgaataaattatttactactacaacaaaaataactaccat

tgtcgctgtactcattcattactcctggacatccttcttttcatcttcctcttttcccagtattcttagctggtgcagtctccttaaatttcttttccca

gaactcgctatatcagaaaacatctattttgtaaacctataaattctttttatttcttccactagtattgttattgttgtcatagttattattgttagcg

gcagcagcagcagtagtagaattagtgccagttttaactttactatttcacagttaatattatttcctctcactgtctctgtagactctcaaatcatttt

aataatatttatcacatcaaaattgttttttcttaagtaactattatgtaaggaaggtactatatgtttgaatccccggtctggtgtaagaaatcgcttc

attgccctaatcggatccggttaaataaataaataaataaaataaatagttctcagaacagggaacaactcgggctattgttcaaagttgtcgaatttca

aagatttttcttgacatctcgtaggcaaagtcaagaaattaaacacacatgattttatacactacattacg

>56205|ABC transporter, putative

taatggcataatgtcctgcaaactagcttctttgtttaagagcgtttgcgcactattttacagcttgtttattttagaaaatcaggaatgatttgtgctg

cagtcagtctcacaatatcttcatataaatgttgagaaaactggaaaaacagaacttaacaggacatactacacaaattgagtagcgaaattatttgtca

tgattcattgaactttaagtagtcttggcaggctctttttaaattcaactttgtgcagaactcagacacattaaagagacgttgtgtaaatgctcatatg

cctattttcagtaaagtttccacatttaacaaatcacgccaactgtttgcgattaaaaccctcaatatgcgatcatagagtcacgtttgcttgtggctgt

agtgtattcctgtaattcctagaacacaactgaaaatcgtagatgcaagaatttctcgtgttcttgcttttctaacggcaatgtcaaaacacagtgaatt

ttttgtaatgctcttaaatatagttgcattgttttgagtgtgtaaatgtgtaacatcattttattttgacaattgtgtcgaactagataagctcattagt

cagttttaaactcgaaacgcattgctttggtgtttcttgctactttcaggagcggaaaagggacgtactcacacacctcgtcgtttattgtctttagaac

tcactggctctatttagcagagaagtattctgatttaaacatttctatcagtctattctgatggatgctccatcacaaatatacagataaattctcaggt

acatttaaaatctgaccggagtaggtagtaatgtagctgttcgacatatacaaaatatatcgagttgtacattcttccgctagcatacacttacgaatta

caagagaaacttaattttatggattatgagagaaagttaattgactatgcatcactgatctcagtaaaaaaaaatatattatttttgcgttttcgtttat

agaagtcctcagttcctaagagcatcttctgcgagcaaaaggatgtaataaatagtagaagaccaaaataaaatggtttaaaatagtattgtagagatcg

aattcttattgttgaagaggtgacgaagtgataacagtcttaggccacatgtttgaaaacaggctttcttatgtgagattgtccgaaacgcaagatttca

tttttggtattgtttcacgtatggcagctaaagaaatttaagggttaaaatttgactcagatgggtcgcaacacgaacgcgcttgagcagtagttattca

ttgttggctttaaggcaacgtgtgtcatctttgttggtgccgaacaccgttaacagcgttgcttcgtaggcggctgaggttggttttcgaatgtgtcgcg

gcgtcgttggcggcgtgaggcgcatgcgcagttctcttcaccggacggcgtagagcttccagcgcagcagaaagtacgcggcgaccctgagggccagcaa

gatcccgcagagtgcgacgacgtcgacccagaacggcgtgtggcccatggcgatggtatccaggaagacactggggatgcggtagtggcagtagaaaacg

gagcagtccagcttgggccggtccatgccgtagatggcagtcacgtagccctcgagcccatagcgcaggtagctgatgtatgagccccagtacaagtatg

agggcagatctcgcaacgtcacgccgaagcccgcgaacatcatcatgggcaccgacagcgtcggtcccaggaacgtgccattcactacgctgaagacggc

gccgatcatgagaccaaagctttgcgcgacgaagacgacgagcatgctgatgacgaagaacattccgaatcgctcccacacaagcggctgcgagctcatg

aggtataccagcaccgagaagatggcacagcacaggactgctacaggaatgtcgacgatggttgtggacacgtaatacgacttgagtgagtaccaccgat

taaaatgttctttcttcagaatattcatttccattggaaaggtgaggatggtgagcatcatggtggtcatcatgtggtgcatgaggatgccgaacagcag

gttgtagttgtccaggacgcgcgcgccgtcgtcgccggcctcgatgaagagcacgcccagcatgatgccggtcaggatgttaaccagcagccgcatgtgc

gtcagcgtttgatctcgtttcactttgataaagcctcgtcgaagcagcaccttcagttgattcaactgcgaagtctcctggagatttcgggagaccttct

ggaagccgagagatttcgatctcgacctgcgcggtggtttctggtaggccaaggcctcaggttccgcgaaccactgcagagtgcggccgttctcggtacc

ttccaccaacctgtcgatcttgtcctctccgtagtcaccgcacgccagttcgatcacgtagtcggcggggttgtggtacatggggcagggcagctgcacg

gccgagaggtagggcaccaggttgtctgcggaaccctggtagaggcactcgccgcgcgccagcacgtacacgtggtcgaacagctggaagagcgacgcgc

tcggctggtggatcgtgcacacgattgtccggccctgccgcgccagcagcttcaggaggtacaccacctgggcgcacgtggagctgtcgaggccggtggt

tggctcatcgaggaacatgacgagcgggttgcagatgagctccagcgcgatggacatccgcttcttctggccgcccgacagccgccccgccagcgtcttg

cgagcctcccataggcccagggtgcgcagcgtctcctccacgatctgcgccttctcgctgaggtcggtgtcctcgcccagcttgaggtccgcggcgacga

gcatgttctcgtgcacggtgagcagctcctgcagccggtcatcctgcgtgatgtagcaggatagcctgcggaattcgtccagctgccgctccacgccgtt

caccagcaccgagcccgtcacgccagtcagcctgtagccagagaggatgtcgaggagcgtggatttgccagcaccggatggccccatgatggcaatcagc

tgctttggaacgaagcggccacatacttcgtgtaggatctttttcttgcctttgcgaaagccgagcgacgcggtgtagccgacgttgtcgaagcgtatgt

cgacgggcggcttggtgggcaggcgcgtgaagacctcgtttggcaggcggctggtgtccctcttgagctccctcttgcgggccggcaccacctgcgtcgc

ctcctgcggctgcggctcgctgctgatgacggacagctccatggcggcggcggcgcggcgact

>56201|ABC transporter, putative

gagctgctcatcctggacgagcccaccgtcggcctggaccccctgctccggcagagtatttgggatcacctggtggagataacagccgccaagaaagtga

ccataattataacaacccactacatcgatgaggcacgccaggctcacagaatagggctgatgcgccagggccgactgttggcagaggcatcacccgacac

gctgctcacccgccatggctgcgccacactggaggacgtcttcctgctgctcagcatgcagcaggagacatcacgggaagtccggcaactggaaaatgat

gtaaatgacacaaccagcatcgacacctataacatcgatggctccaaggaaacattggcattaggcagtacaactgaacttttacaagaaccagtcaaga

acaaggagcctcctactaaggcattgcgttcatcaattttgagaatgaaggctctcatatataagaatcttttacttgttcttagaaatcctgggtgggt

gctgtttattgtggtatttccagtcttgcagatttcactcttcttcttagctgtggggagagatccaaaaggtttaacagtggttgtagtgaatgaggaa

cttggtaatttaacaagctgtgaaaaaatggaaccttggacagctcatatgaacgatgattatatttgtgaattatcgcatatcagctgtctgtttgtac

agagaattgacaacagtcttgcaacaaaaaaatattaccgggaccttgacagtgctttgcagctagtgagagatgggtcagctattgcagtgttatattt

ttccccaactttcagcaaagacttggaaacaaggcagtatgtgggaagatcagcatcagatgagattctcaacagcagttcagtgtctgtctacatggac

atgtctgatcggataaaaggtgttcacttccaagcagagctctattctgtttatcaagaagttgccaaagatattgctggtagatgtgaaatcagttaca

gacatgtcacgctgcccatgcatttcaacaagcctgtacatggttccaaagatctaacatatgcaggatttcttgctcctgggattatgtactcgatgat

ttatttccttgctacaattctgacgtgcactgtcctcatcgcagaacagatagaaggagtctgggatcgaaacctggtgtcaggtgtgacaatgttggag

gtcctgctgtgtcatctgctgtgctactctttctggatggctcttcagttgtttgaggttctgctcatgacattcctagtgtttgatctcaattgtgatg

gagactatgcaacagtgtcagctctagcattcctccaaggtctttgtggaatatgttatggtttggtgataacagttgtctgctcagacctcacaatggc

aaactatgtgtctatgggaagctatttcccctatctgaacctcagtggagtgctgtggcccaaggaagcaatgccgactgctttgcgctggtttagcatg

tgcctgcctggtacactacccacagagtctgcacgctacgttctggagcgaggctggagcttcacgcatcctgcagttctctctggtttcctcatcacca

ttgcatggatcttcggccagtgtgctttctgtgttggattcatgacaatgaagaggaagctgtatttgcagtgatgttctctcaatgccaccagtttatg

atctgaatcagcagtactttcaaagtactcttcatccgggtcttatggttgtgctcctctccacaacactcacttaaactggagtgggactttgccaggc

tctgagtactgcactgttctgtaccctctgaagtgtaacataactggtgtggttgctacttttgattcatcaggtttttatgacagatgtgtaagactta

aagcgtctgactcatagatgttgcactttatgatggattcaattatgtatacacatattgtaagagacaccagaaccatcagtcttgacacctgtgcaat

tacttcaagaggttcagaccagcactgaatgtgcagtgaagcacttactgtgatactctttacttttttgtgattggaagaacaggtccccatgccaggt

gccagtttgtctgggatttttgtatagaaacatgtgaaaaactttattgttcaaaatgttgttggacatgaaactcacaatgccagtattaagtaagcca

tgtgctatcatttaatgaaaaaagtttatattacacattttttgccacagcaaagcagaaactattgccgtgtgtcatcaagctttccctctactgcaga

tacatactgtctaatagatgaaaactgtgtaccaaagtgggactcaaacaggacccttgactctcgtgactgacagttctgaaaaagctgctacagagtg

tttgagctccatccaccaaatggtattaagctgccagaaaagttatcggagtgcattgtattgtgctaagtgtatttttagtgacacatcattttttatg

tgaatgcaacatgcacaaaatttgttcgctatcactttaatcctgcatccatatggtgaaacatgcccatgaaatactaagtatccttaattcatttttt

tgccactcattgtccattctgtatcacaaaatgatgctttaatgtttaatctgccaaaggaatctgtggtctagttacagcaagtgttaagttagaatta

ctgcacagtgcattagcataacaatgccacaatatatttgcaaatgtcgcaggcgacgtgtgtccttctgtgtggctgtttgtatgtgttggctgtgtga

tttttgtgttcttgtattttgaatatcatatgtttaaatttttttgttcagtcaaaagactttatttatgtctagaacttgtgcttgctgttatatttca

cttctttacaagcttgcagaacatagacagatggttacattaatagaaatgtttacattaatggaaatgtgaattaagtattttctttgtatattcaaaa

tatgagaagttactgccagagtgaaaaccagaactgcattctttttttttttttttgttcattttgcatcttacatttttcaagtccttgaaggcatctc

gctttacacaagaaatgtcaactgatacagcagtttttcatcatttgaatttatgtttataaagtttcaggctttggagaaaatactctatgattgacaa

aattaagttaccttctttgcactgtcagaatatttgaaaattaaaattctacagaaatgcaagctttattggtgctctgatgtgatgaagtattctcaga

ctgtcactgccatttaaagtactacctgtggtactaatatgagcaggaagttgaaaagtcttcatcagatcaaattttaagttattcagaaaatgttttt

acaaattacagatttctaaacagtagatgaagttaagatgttacacttgtttaatatatcagatgagtgtcttatttatgattaaataaatattttaaaa

caaa

>56224|ACYPI000022

gcattctggaagatgcatataggaagtttaacaacttcccacaatataacacttttcgagggctgcaaatgatgtcttatagtaaggaagagaatatacg

accatcccaagaactggacaacgttatatgcgaaaagatacgctgtgcctagagttccgggccctttacgcacgttcgtttgtgatttcttggccatttc

gctgagatattcaaataccacccacttgctgctcagtcaacaaacaggtatgcaagaacccaagtgggaaacttgtttaaaagctgactggcaatattgt

aatatgctgccaacacaaaactgtatattcaaccatcgaaatactagacccaacacaaacaataaacaggtacagggtaatgagtgctaactggtattta

ttctttaaagcggttttctattataactgtaacacacagcaatgtacgaattcaaatccagtcgccttttatagactccgtaagtctgggcgtcataaac

aaatattgtacgatgcaagcggctttgcgcacgatgaaaattctctgcataaattagccctacatctattgcgagattgaaagtgtcagaatgaaaccca

taaaaatgctatgctttaaagccattattacaacggaaaccaaaacagtatctacaggaaatgtcaacagaattgtacatttacgacagtgtgaatgatt

g

>56273|ACYPI000068

tatttcttgactgggcgcagctcttcgtttagctcgtaaacgagctgaacgcctgtaggaatatttagttcaagaacctctgcctctgtcatcccgtcca

ggtgtttcacaaggcctcggagagaattaccatgagccgcaatcagaactctcttgccctcaaggatggacggggcaatgtgatcatgccagaaggggag

gactctggc

>56359|ACYPI000205

tgggttcaactcatccaagcagtcatcatagtgtaagccatatttattgaaaccggaaagattgtacgcccattttcgtacgtcacccccgctgtgtccc

aggagaagcaccccatcctggag

>56360|ACYPI000205

tgggttcaactcatccaagcagtcatcatagtgtaaggcatatttattgaaacctgaaagattgtacgcccattttcgtacggcacccagaaacccaaat

ccaagccagcacccaagaaataatgtataaagtctgagaaa

>56357|ACYPI000205

tgttgtgtatgaatttgttgttgctgctgttgtggttgctgctgtgttgttgatgggtgccgtacgaaaatgggcgtacaatctttcaggtttcaataaa

tatcgcttacactatgatgactgcttggatgagttgaacccagatgtggctgaagcactg

>56394|ACYPI000585

gatgcggatgccattttgagttgtaagacagaaaactgaagaaaatcaagaagaacttagagtacaatatgaaaaggagcaagatttgtacaccaacaag

gcgctgctgagcaaggagagcaaggacaaacttagtgtcaattttatgtatgaacc

>56438|ACYPI001113

gaaaaattaatgcgtgatgcaaagatatatcaaatttatgaaggtacagcacaaattctgcgcctgattgtttccaggaatatttttgagaaggcagatg

tttttgccttctcaaaaatattcctggaaacaatcaggcgctgaatttg

>56439|ACYPI001113

ctccttttacttttgaagcaaactgatgaaacttgcatctttgctgcctttacttacttgatgatttgcaatagtagttccaaatgtcttcctctcgaga

gaggattttgttgcctcatccaatgctcgttgtgctaagaccactgcaccagcagcaaccgg

>56470|ACYPI001237

actcacagctttacttctgccagtatccgtctcctaccttccaaactttacagaagctctcgattacctactaattcattaatggcagtttctagatatt

caccaatgaaaaatgtctccagaagccaaggactaagagattcccactacgagctagtattcagccaggtatacaaacagcacgtcacaggtcggtcaac

actttgggggacggtgtcggcccagtacgcggtcactcaggtggctctgaaccgcctatcgatccgcgacctcttgcgcttcctctgactttgagtgcac

aggcagtttttgaggaggatccaggtgagcgtggtggtgaggcagaaggtgcaggcgatgaaggcgagcacgtcgagcagcaggtactggtaccagctga

gccgcagcgaggcaggccgcatgtgcggcgcgccgcggtgccgcatcacgtactccgtcc

>56532|ACYPI001292

gtttaactggcaatcaagttactgcaaaggaagccgaaagaatgggtcttgtgagtaaagtgtttcctgctagccagcttatgagtgaagccatcaaatt

agcagagaaaatctcttcatattcaccactgattataactctgtgcaaagaggcagtaaacacagccctagaaactactcttcaagatggccttcaattt

gaaaagtgcctagcttatgcaacatttgcaactgatgaccgcaaggaaggggtgacagcctttgttgaaaaaagagcaccaaatttcaaaaatagttaat

tttggtaaaaagaaatataatgatgggattttacaagtcaggtgagtcattgttttactaataagacagactaaataaataactatagcatctgtggcat

gtttacagagaaatgcatagttatttacagcatgtcacttgctatttcaaatttaatttcttggcatgatacgtgaagttattcggttcacttgttgttt

ttacaccatttgttattcatgaaaaagtatcaatgtcaagatacgataacagaatttttacaaattattattaatatttgttgctgaacagtcatgttta

tcatcttttcttgcttttatagcatctggatactaagctggatttgctacacataatgcaatttgaaataaa

>56579|ACYPI001339

acaattactttgaagatgtttttgtggaatttaaagataagtagggtgaaatagaggaaatgaatgtatgtgacaacctg

>56582|ACYPI001339

tgaaattgcagaaaccactgcgagtgcactctcccatttcatcctgacaacaacaagcttcacggaaatctgtgacagggaccgtag

>56581|ACYPI001339

tgttgtgtattctggaacaccgatcgccatggcgacatgcccctgtcttgaagtagaatgaacaattaactttgtccttttctgcccaaaaataactatg

caaatctcctaggacaccaaagt

>56577|ACYPI001339

agagttgagccctgtcacagatttccgtgaagcttgttgtcgtcagcatgaaattgcagaaaccactgcgagtgcactctcccatgtcatactgacgaca

acaagcttcacggaaatctgtgacagggctca

>56580|ACYPI001339

atctatacacgaaactacaatattggcaaagcagccggtgaagcagctaacaaaatattgttattcctgtaaataattaagacaactatacccaacacaa

acagaatgtcatctttcactttgtattctgtggcaaagtaaaactttcaatgtgtgttcacactaattttgtttttgctggtgttcatttgcagatttta

cgtaattttcacagtatgtgatgccttaatatggattttccacacaactgtttttgtcaaccaaatccaacaattactaacttaaatttgtgccaatcca

tggtagaaaatctttttgtgacaaaagattaccatcttttgatttattgtggctgtttaacaatccacaatggaaaatatttttagaagttaaaagctga

tgatgctctaaacatatgagaattacattagtgtattttgcttcaatatcaaactctctttactacctaaagatctgtcagccatttataacacaattat

acattttaacctaaattattgttcatgccaggagctcagatgaatcaggttctcacttattttattaaaacttacttaacatacttagtgcactagatga

acatacacaaagtcttaatgaacctaatattcacattggaaatgcacgacacttaacactacacttagtaacggccagaacggccctctcgatccttctc

tgaccgaccccggtcacctcccttcttcctacggtccctgtcacgatctctatcacggcctcgtgatcttgatcttgaacgcctatatccagcaatcctt

tctccatcacggtcacggcttcggctgtgtgaacgggatcttctgcccctcctgcgtctactatacaaataacgacgaagctctcttgatattggtttca

agtgcatgaaattgcagaaaccactgcgagtgcactctcccatttcatactgacgacaacaagcttcacggaaatctgtgacagggctcaactctgcata

cacagg

>56666|ACYPI001601

cttgcattctttccattaaggaagcccgttaggttgatctggattttcttgggatctggatgctttagctccaactggttgaaaacaaattccacaacaa

catcatcttctattcctaggagacttgttatcttctgagctatccagggttttattacttccagctttactttggacatatcaaccttccgactcaagct

atcgccaaacttcatctgtttgagaagtttcttctctttatcactaaagcggttatcctgctccgcggacgtaccccgaaaaaagcctgcatcactcatt

ttgagaagcttaaactttgcaaaacttaattcactaccatgctactaaagatg

>57111|ACYPI001706

gacaaacattatctctccctgtcatgaaagataataaactgcacactgaacaatcttcaataaaacaagatttatgccagcacatgttatttgtcctgaa

accttcattcacctaacaagttattaaaatattatatactttaagcaaaacaaacatgttttaggaaaataagattttaacaaatttgctttatgatagc

tccatgcagctcgctcattcctcgccgagaacatatccacggccccagttgtggcgcccccctggaggatcagccactggccgccggctctgtggtggga

caccaaaaccgtgcaccgatccttgtcggtttgggaaccacctgtacaaaattgatggtgttgttaagaggcttggacctcccatttcctggggatattt

aaacatgaggaagaagtacaggtgtccaactaggatacccaccaactcattaagtcctcctccagaaataataagatgaaaggcaaagaggacccatggt

aaatatgtagctctgaagcgagttccaaaccaaaagctgactatgacgtctttgtttagctggcaccaaacatacagaacactgaggaccatggaaacca

taaggagattaatatcagccatgagtgctacaataacaatacatatccagttgaaaagaagtaagaacagataatctgcagggcgaccatcaaaaatacc

agtttccaaaagtaatgaatacttgtataagaagtataaatttattaagaaatggaatcctgttgaagcagtaatggggtagtagaagacagctgtgact

ggcctccatatctggaatcggtggatgaaaggttcgtaaagtaaggcgagataaatgcctctcagcaatccaaagcgtcccagaagagagaacgccgttg

tcagtgtcatccaccatctcgtgaagactggtaatgaatagaaccagttacttaaatcactcattcttcttcgcagccaggtacccgtcttcaaaataat

taaatgccttcaagtaatcccattggcgttggaccctccaaacttattttattcagctctcatcaccgtggcgtttatttaggaataagtagacaaagaa

tactcatgtcatgaaacttccatatctggactgattattggagtgcacactgtcacacgaattatttgtagacacgtgtcatattatacactacacagta

cacacaactttaaccaacccaactcaaacagcttagcagtgcatgactagattctttctc

>57218|ACYPI002076

actttcttgtaatgtcctggagctataggtagatggcaaagaccgtaccctctcacaacatcatttccaaacaagtcaagtccataaacactaataacta

attgtggccagccaaatggattcgtgcttttaaaagtaatttctagcggaaaattccagatacatgtttgtctttcatctttgcttcgttttgataactg

tgaaataccttcttcaattcctaaggttcagcaaatttccggccagtttcaaagacagttggttactagtgacatttgcagcaaagaaaccaaaaccttt

ttacacgaaccttagattttcgtttgcaaag

>57275|ACYPI002216

cactttcagatacgaatacactgaacacaatcttaccattagcttttcaaaagaaacaatgctctccttttcattaatatcacctctgtctagatattca

ttctcataactcttgagtctagcagtggcatcctctatcacatcatctggtaatttggcaaccttagcaacttcaactccaaagctcctgttgcaaaatc

ctggctcaattttgtatagcattgtaagtttaccatccacaatcattg

>57274|ACYPI002216

ttttagtaattaaatacaaaaattaatgtacatgtttaagtgcactatgttacacaattaaatcaaattataaaatgacactctagcaaataaaagcaga

acataaaacaatactgtcaacatcattagaaactgccttcacaaagagcttgaatgtaatgtgtggttgaatggctgatgtaaaagacacaattaacagc

aatgagtaacggagaggtaggttggctgaaaattacaaacacaggcctaaacaacatgtgatttacttacaaaatacacatttcaacggttaatactgaa

ctatagagtgactataaaacggtacggcctcattttatttcagtcttaaagtcaatatttaaaaaaagaaagggacaaaaatgggataatgaaacataaa

taagggcaaagagtctctctctctctctctctctctctctctctctttctctctctctctctcctgccttgcatgacctcacataattatgcaagtgaca

cttgtgattcagcacaattcaattcttgtggtttttcagtattgagcatcttatggtctgatgcataacacaataattattttgtaaccttttcttttat

acctgaacctaatactaataatctcatcacaaatcatgcaataaaaattatcacaacacgccagaagatttccatctctggtcaaaattttgtcaaagca

gttaaatgaaaaactttaaattaaattgtgttaaaaaactgcatgtcttgatgaaatgcaatcacagctgtctaagaatggagcacagtgatgccttgtt

gaatgaagtgccataactgcttgtaagcatttaaactgtgttgcagcttcagtttgttagagcaggctggtgcctttccagttatattacagctttgtaa

cacattttatagaagtcaggtacttatctacaggaactttcaaagaaaaaaaaaagtgttaaattcattcaaaatcatataataaagacacagccctttc

cctttcttctatgaccttccgaaattttgcatttggtgacagatcttcttttgcaatagttttcaatttgttcagaagtttctccaacacatctgggtgt

tccattagcttttcaaaagaaacaatgctctccttttcattaatatcacctctgtctagatattcattctcataactcttgagtctagcagtggcatcct

ctatcacatcatctggtaatttggcaaccttagcaacttcaactccaaagctcctgttgcaaaatcctggctcaattttgtatagcattgtaagtttacc

atccacaatcattgcagttgcatgtagattgtgaacatttggtagttcatttgccagctcagttatttcatcaaaatgagtagcaaataatgtgaaacac

tttgttttctctgctaagtactttgaaattgcccacgcgatgccacaaccttcgtaagtagatgtaccccttccaagttcatcaatgagaacaagagatt

ttcttgtggcacttctaatgatgctcgatgtttccatcatctccttcataaatgttgacattcctttactcagatcgtcgcttgcacctactcttgcaaa

tatttcatccacaactgatattt

>57362|ACYPI002360

gtttattattgtaacatccataaataaaatactatataagtcattacaagacatataaacacttaaagtagtacattggctcatttcctcctttgccaaa

cgagccatcaagataatgaaagcttgatatcagtaataacgtatggcatgatttagatctcttatgtactctgttacagttcgaagcttgtcatatgaat

ccacatggtttgtcacaaatttctgtataccatattgtgcaaattttgtttgccatatttcactcttgtcaaaacttttcagtgctctaacaattgtttc

cttaagttcaccatccatacgagagttgacgacaatgggatatggtggtaatggtcctatgctgtccaatacaagcacatcctttccctgatcctgcaag

tatcttttattgtaagcaagtgtatttgaatcaacagctgctgcctctgcttgtttagtcaacaccatctgaatggattggagatgagatccagattgca

atgtatttccaaaaaatgtagagttttcgccatgttcttttagtgttttcaatactattttgtatccacttaatgattcgtcagtgctgtatgcccatgt

agaaccccgaagatccaacagctccttcacatgtttcttgccatcaatgtgaatgatgatgtcagaataataacctgctacatcatccttgttttttctg

tgcctgaacactggtgtaactggcaacagctctgcatatttattcttgttgtctagtagctccaagaatgataacacactcatgaatcctatgtctactg

tgtcgtctgtgaaggggtcaactcttcccgggagggggccagctcttctggactcatactggagagtggcttccagcccagtgtagcgctccaggtacgt

catgatcagctcgtacatttccaccgggtgactggggcacatgtaagtcacaagccgcagcttctgcttcttctggttgctcatgttgatcgcttagctt

gggacgccgaacgcgagcgcacttgccggcagcagacgctcaactgtcaaaacagttccagcgctacacgctctgccggcgtgctcgcgggcgctgttgc

cgcgttatcgaccgacgctgtgctcg

>57473|ACYPI002694

tccagaattgatttctcgccattggatgcaaggccaccagcagtgatcatcacatggcgagaaatcaattctggacatcatccctacgaagattcactaa

cgtagccccattttcatggggtctcgtgaagatgttgaggaactgcttgaaatc

>57491|ACYPI002825

ccattgtaagaatatttaatttctgtgaacaacaggggaagagtaatacttcacaaattattcaacaagctttggcctttgtgtgatcgtcgaaaattcc

tattttgctctccacagctctggaaggcaatttctggagcagacctctgcttagagtagcggtctcggcagtcagcgcgagcctcatccaatgagcgttg

tgggcaatgtgtcgtacggacagcaagctccgacaaagctgctgtcaatcacacagaagtagggcaggaagtcgtcgaggctctgagtgaagttgggcag

cacgcagttggccaggtggcggcacagcccgcgtcctccaccaggggtcgcgcactccaccagctgcgactccgtcttgttgagggccaattcgtaggtc

tctgctgtgccattcacttcgagtgtgacgtgtgaggagttcgagacaccgcctagccaggtgatctcggatatgtgggcctcctgcggttgtccggcag

aagccgcagcaccaggccgaaaacttgtggttgagtatggactggaagactcgattaattcctcaaaccggtctgccaacaacaatactggcgctcctga

tgcgaagtagtcttcgcggtttccaccagcgcgttccaggtccacagccgatgtgtcgttaggtttcaccacgaccagtgacgtcacaggagtcacgaag

gagtacttgagagcgatgtccttggctctgtccttgatcgtctggttgggtgtggcggc

>57557|ACYPI002894

aacgagtcgtctaaatggcggcctagttcggtcgcgataacaccagggtgcaggctgtatgtcgttacgccgctaccttccagtcgctttgccagttctt

tggcaaacaagatattagcgagcttgctacgtccgtaggcgcccactgcactgtacgatttctcccacatcagatcgtcgaaattgatagatccattgcg

atgagctagggaagagacgacgacgat

>57613|ACYPI003541

catccagccacgatgtcacaacagtggtgataaagaattgtgatccgttggtgtttcttccggagtttgccatagaaagcagaccaggcttgttgtgagt

cagagagaagttctcatcctcaaactttttaccatagatagattcaccgcctgttcc

>57614|ACYPI003541

tgccattatggtttgtaaaatcacctccttgaagcatgaagtttggaataacgcggtggaactttgatcctttgtatgttcctactttgtccttgacctc

accagagcaaagagcacggaaattctcggctgtcttcggtaccacgtcattgaaaaggttgaagttaat

>58137|ACYPI003740

cttgttacaaaatgtgagaaggtgtttttgtactctagtaaatgtttcttgtttcaggcatgcggtttggtctggtgcaaacgaaggtcggccttataaa

catactgtcgaggtacagcgttgaacccagcgagaagagcgttcgccacctgtccctggaaccgagggctttcctgatgacacccaagggcgggatacag

ctaaggctcagcaagaggcttgcagcgtagcgtccagccacaagacaacacttcctctgtaaaagactgtcacccagagattcccaaacctgtacatagt

gtatataatactgtattatttagttactgaatgctgactgattgtttgtaataaagagattgacatgaa

>58266|ACYPI003905

ccgccaatattaccagggcgccgatgtgttttggtgacaccatgagatgctgggccacctttgaatccccagcgtttaaccacaccttgaaagtctcgat

caatggttttaccacgaacatctacaacatccccaactcgaaaatgggaggcatataacggggttccaggctgaattactgcttctggtgacaccatgaa

ccgtcctagtgtcttcttaggcataactccagagtctttgaataatccacaatattcctttgtgaacatttgtggatctgcgctctctgctccaactagt

aaacatccatagcgatttggatatttctgtcttacagcacgcttccgagggggatcaaattcttcgggtggaatataccgaataacgtgattgtcgacca

cctgcaatagcgttgtatgtattttggagccatctttcagccacattgggtaacaaccaattttccttgcaataacacctgttctcctcgtcttaggagt

ccattcaccacgttccaaaggtgcatttttgagtgggctttgttgacaggtaagtactcctccaataacagctggtggtccatatttatcttggactact

tccttgataaatgctgagttttcttgagtcaaattttcattataattacttctttccttcttgacaaaccacgtcgggtatcttaagcgtggacggcatt

tgagcctctttcctctgctctgcgtaataatgctatcgaaactcggtttcaaaaagcagatattagtttgtatagacgcaaacactttccacaccgttcc

ggcagccatctttgttgttatgccagtactcaatgcattggaacgagcgttgcatcagtgttttacctgttatgaacaatgtttacatcagaacagcgtt

atgaatccaccaggagcagtatctggaactgagctctatggtaatgtgatcaaatgttgatgtactcaagagctacaattatatacagtggttaatatac

tgtaagcttgtcaagggcctcatcgatcttttttctgtaaagaaaaaaaggacattgtggagaagtgtgggtgatgcatgtatacggtgttcctgtcaga

tttcaaaacagtcccctgacattgaatgtatcattgagaaatttgttaagtttgacgattggtctgtgatttaagagtgaataattgtacacaagcatca

ctatcatcaataaacttaacgttattgctgcatgacgtacgttcttcctatatgtcatgttatttaagtgaagtccctgttcggtgtcgaaactagcgtt

tgattgtgtctataatgtagttacaggtaccctgcgctgtgcatgatttccggtattccttttagcgaaacacaaattttgggtaacgaagccacattcg

tccaccggaatgttctcgcgcaatctgtttgtttgattcatgactcaccaaaaacggcaatatctc

>58720|ACYPI003921

ctttaatgaaagaagttatctatacaaaatagcaattttctgcacaccaagaacaatcaaaagctactgctcttacatgcagttataaaattctaataac

tttattttcaagaaagtgatcaataaaaagagagtattacatgagtattaatgttatgaatgcccccaaaccagtttatgaagcatttaaaagctagaag

atttactgaaatgttatttttgtatctacagatttcaactatcacaaatagcagcttctggtggattaattcattacgtttagtattctgactaatatga

atattttgaacaataattatactgttattaactgtaaagtttcagaggagaaagtgttctacaaggtacctgtgactactttaatattggcaccattata

accaaattgttaatagaaacactgaaattaatgacaactgcattcacaatattgaagcactacaacagttctgttttacagatagaaagtgttttgcgtt

ggaagatgaactgggatgtctggtaatatattgagaatagagtatttccctttgtgaacataagaatttatatgtactatgtgcaacacgactatttgta

agcgacaaatgaactatttcagcatagtatatcatatttcataaagctgcctgaaaagagagcatataatagtcaaaaacttcaataaagtcattacagg

attccgataacaccgcaagcctccctggtgccagcatgaccagttgtaaggctgtcttcaaaaccaccctttcctaagtcatctttgtccgaat

>43899|ACYPI003921

gtgatcaataaaaagagagtattacatgagtattaatgttatgaatgcccccaaaccagtttatgaagcatttaaaagctagaagatttactgaaatgtt

atttttgtatctacagatttcaactatcacaaatagcagcttctggtggattaattcattacgtttagtattctgactaatatgaatattttgaacaata

attatactgttattaactgtaaagtttcagaggagaaagtgttctacaaggtacctgtgactactttaatattggcaccattataaccaaattgttaata

gaaacactgaaattaatgacaactgcattgacaatattgaagcactacaacagttctgttttacagatagaaagtgttttgtgttggaagatgaactggg

atgtctggtaatatattgagaatagagtatttccctttgtgaacataagaatttatatgtactatgtgcaacacgactatttgtaagcaacaaatgaact

atttcagcatagtatatcatatttcataaagctgcctgaaaagagagcatataatagtcaaaaacttcaataaagtcattacaggattccgataacaccg

caagcctccctggtgccagcatgaccagttgtaaggctgtcttcaaaaccaccctttcctaagtcatctttgtccgaatgaacaacaacagaacgtccaa

gaatgttgttgcggcctgtcaaagagatcactctgtctcgaatggaaatattgaggctggtgaaaatggagtagccaatatttccattcgagacagagtg

atctctttga

>58985|ACYPI004051

ggggccccagaaaatccgataagaggaacttttccttctaacttgtgacgcgtcagtgtgattgcttctgcaacatagccaagtttctcatttacattaa

ctggcacttcaagtttggtaatgtcttcgggttcaaccagaggttccaaaacggggcccacactaggtttcatttcaacatgcactcctaaagcttgagg

aattaccaagatatcagaaaatattatgcatgcatcaagatcaaatctctgtataggttgaatagacacttctgctgctagagctggggtttgacacatc

gtgaaaaagtcatacttttggcgaagatcacgaaattctgggagatatctgcctgcctgtctcattatccatactggaatcttgtccacttcctcaccac

gagctgctcttaaaagcctgtcattctttaaagctggaaagctcattttgatattggaaagtcacacctgtgttgacagcaagaagtctataggatttca

gcacaatttatcaaacagaaatttattcattgaaatcctcgacgccgtttgaagctttattttacagaaacagttctgcgctatcagaaaaaataactaa

gtactgttgttaactgagcaggaattatacaaccacgtcttagaacatacggaaagttcgtactactgatgctacatcatcacaaacaacaaaaacataa

cctcaacaaaaacataacctcttacgcggagcatcaatcctcgtttagacacagcaaaaagcttaagaaactatgtacgcaaatttcggcg

>59646|ACYPI004224

aaaacatcaattttactgtctgggacgtcgggggccaagacaagatccgtcctctctggcgccactactatttaaat

>59645|ACYPI004224

gtttggcaagaaggaagtccgcatcctcatggtcggacttgatgcggcaggaaagacgaccattttatacaagctaaagcttggggaggttgtcaccaca

attcccaccctaggtttcaatgttgagactgttgagtataagaacatatcatttaccgtttgggatatcggtggacaggatagaattcgt

>59832|ACYPI004271

atattcctgttatagccctgtgtaattctgattccccaatacgttttgtggatattgaaataccatgtaacaacaagagcccacattccattgggc

>60419|ACYPI004549

caacttcactgaaatatgtaaaacattacatcggcaaccaaagcatttgctagatttacaattagcagagtaaggaaccagtggg

>60417|ACYPI004549

gaaatatgtaaaacgtacggagcctatattctcatacataacttactttattttgtaatgttaaatattaattttggtatatttttacagattacatcgg

caaccaaaacatttactagatttcctattagcagagttaggaaccagtgggtctgtagatggaaacaatcagctcaggaggaaaggacgcttccagcaga

aacagattgaaaaggtatgaaggag

>60584|ACYPI004704

gtcacatccttatcacttcagaagcagtcttgaagtctcttatttaggcctacaaccactgttttatatgtcacattttgtttcttctttcttgaaacac

tttgctttgcatgtttcgcttgcttcctcagcttctcggtccttttctgtagtgcctcataattctgtttcaatttttgggggctaggcaaactataaga

atgatctgtatgatcattctgattcactctttcacctggttcatttgataaggaagctgattcttcactgacaggctgtctccttggttgtctctatact

atagttgttttctgcagacagtcaagaaacataaatattatcggtactgcgtttgccagaagtcgccttttctcgtttgtactatacatgtatctatctt

caaagtgaaaggtgcatacgtagctatattgagttggctgccacttatctcgcctcatattggctacccaacgacttaacagctctttctttttcagagg

aaattgaaatatgatattcgaattgttttatcacttctattagcgcaattgtacactgcacatacactgggcatgattcgcgataggtcaataagacttt

taaaacaaattacaaccggaaaacagttattttctaaagccagtactatacaacaaatgtaaatatactgacatatagacgtctcaaaaaat

>60747|ACYPI005374

atttgtttcttgagaattgtcgttcacggtgaacaaattgtagggaatgcatcagataagcaaagttaaatgtaaaatgtttcactttttctcaggtgga

tagggagagatggaagaaaggaaaagtgcaccgtattacaatattactagttcgaaagtttgtaggagtttttaatgtcacccaaatcagttatttcact

agcagtaagctcagctgtaatctttcctaaggaaattatagcgaacgtatagtctattcagatgcagtatcagctagctattgggctgtggctccgctat

tggacaacgtgacgtagcgttaatgcgcaggaaaaaccaatcaggaatttcagcatttcgagagccgatgtcgtgtgtctttccaaatcacgaggctaat

ttgtgtaaattgacatctaatcactgctagtccattactccctgaccgagcttctagggaacaataagcaacaagtggttgagaaaacataaccaacgaa

acgttgaagaccagttgaaatgtcaaaagaagtgaaacgtgtatttctcacgggtgcatctggattacttggtcgatcagtttaccaaaaattttcagag

cataactggacagtatttggtacagcgttttcaagagttcagccacatttgcacaaaattgacctaagagacgagaatgctgttcagacagtgttgtcag

aattcaaaccaaatgtggtcattcatactgctgcccaaagataccctgacaaggttgatgctgatcctgaagctgcagtggatatcaatgttcgtgcaac

agagagtttagctgcactttgtggtaattgcataagctacagcatttccttgaaatgggtatatttaacagtttcactgcagtgtatgtaatcttctgaa

ttgaagaattgacaaatttaatgagaaaacaaatttactgtgaaacttagtatctttcaggttcacatgagaaggatctaat

>60744|ACYPI005374

ggcatcaggaatttatcaaccctatttagaaatgcagggcgtttgtctagagtccaaaatgattttaacgtgacactctcaaaaacggtaacaactgttg

atccactgctacacccagcaatccaagcagaagtgggacgaagcagagtcaacagctaaaaaagatttctacgtcatcgcagagttcagccacatttgca

caaaattgacctaagagacgagaatgctgttcagacagtgttgtcagaattcaaaccaaatgtggtcattcatactgctgcccaaagataccctgacaag

gttgatgctgatcctgaagctgcagtggatatcaatgttcgtgcaacagagagtttagctgcactttgtgccaaagaaagagtgcctctgatttacatca

gcacagattatgtatttgatggtaaaaatcctccctattcagaagaaagtgtccccagccctctaaatctttatgggaaaacaaaactgcagggagagga

agcttgcatggcagcttcatcagggaacattgttttacgtatcccagtcctttatggccctgtaatgcatctggaagagagtgcagtaacagttcttcta

gaagcactgctgaatacttctaaagaaaggcaggtgtcggattacgagcgtcgccgaccatctcatgtagacgatattgctgaaatctgcctgggacttg

ctgataggaaactgcagggtgaaccgattcatggaatatatcactggtgtggcaaagagatatttacaaagtataaaatgctgcaagagatggcagatat

cttccatttgtccagttcacacatttctcctgctcctgcacctcttctcagttcctctgccacccgaccttatgacacaactctaaacactagtagactt

acggaactcaatattcagtgtcacacaccttttaggcagggcataaaagcagctcttgaaccatggataagacaaaaccaaaattaagtgaaattagaat

tgtacatataaaattttagttacatatagtttgtctgagtattaaaaaggcaaacaaaaggaaatactcaatatattgtgtgtgtgtgtgtgtgtgtgtg

tgtgtgtgtgtgtgtgtgtgtgagagagagagacagagagggagggagagagtgcgcatggaggtgaaacgcctagagcctgtgtattttgagcgact

>60745|ACYPI005374

tatccatggttcaagagctgcttttatgccctgcctaaaaggtgtgtgacactgaatattgagtatttccttttgtttgcctttttaatactcagacaaa

ctatatgtaactaaaattttatatgtacaattctaatttcacttaattttggttttgtcttatccatggttcaagagctgcttttatgccctgcctaaaa

ggtgtgtgacactgaatattgagtatttccttttgtttgcctttttaatactcagacaaactatatgtaactaaaattttatatgtacaattctaatttc

acttaattttggttttgtcttatccatggttcaagagctgcttttatgccctgcctaaaaggtgtgtgacactgaatattgagttccgtaagtctactag

tgtttagagttgtgtcataaggtcgggtggcagaggaactgagaagaggtgcaggagcaggagaaatgtgtgaactggacaaatggaagatatctgccat

ctcttgcagcattttatactttgtaaatatctctttgccacaccagtgatatattccatgaatcggttcacccttcagtttcctatcagcaagtcccagg

cagatttcagcaatatcgtctacatgagatggtcggcgacgctcgtaatccgacacctgcctttctttagaagtattcagcagtgcttctagaagaactg

ttactgcactctcttccagatgcattacagggccataaaggactgggatacgtaaaacaatgttccctgatgaagctgccatgcaagcttcctctccctg

cagttttgttttcccataaagatttagagggctggggacactttcttctgaatagggaggatttttaccatcaaatacataatctgtgctgatgtaaatc

agaggcactctttctttggcacaaagtgcagctaaactctctgttgcacgaacattgatatccactgcagcttcaggatcagcatcaaccttgtcagggt

atctttgggcagcagtatgaatgaccacatttggtttgaattctgacaacactgtctgaacagcattctcgtctcttaggtcaattttgtgcaaatgtgg

ctgaactcttgaaaacgctgtaccaaatactgtccagttatgctctgaaaatttttggtaaactgatcgaccaagtaatccagatgcacccgtgagaaat

acacgtttcacttcttttgacatttcaactggtcttcaacgtttcgttggttatgttttctcaaccacttgttgcttattgttccctagaagctcggtca

gggagtaatggactagcagtgattagatgtcaatttacacaaattagcctcgtgatttggaaagacacacgacatcggctctcgaaatgctgaaattcct

gattggtttttcctgcgcattaacgctacgtcacgttgtccaatagcggagccacagcccaatagctagctgatactgcatctgaatagactatacgttc

gctataatttccttaggaaagattacagctgagcttactgctagtgaaataactgatttgggtgacattaaaaactcctacaaactttcgaactagtaat

attgtaatacggtgcacttttcctttcttccatctctccctatccacctgagaaaaagtgaaacattttacatttaactttgcttatctgatgcattccc

tacaatttgttcaccgtgaacgacaattctcaagaaacaaat

>60803|ACYPI005467

actgttgggcgtgtgacggcaacgttgctgtagtctcacgctagttgggctggacctggtgcgacgacgacgagacttctgtaccgaactaagaccaaga

ctgaacagttcaacgctgctgtttcatctcgcatcccactgctcaagagccgaaaatcgaaactttctgccaaagttgtaccagctgaggaggcccctac

tgacgtctcacctgattctggtatcaatgaacttcagggtttgagtcctgaggaacaagagagacagaaacaggaatggcagcaggagcttgtaaaggtt

gaagaggaaattcagactctccgccatgtcttggcatcaaaaataaaacattctcaagaacttaaacgtaaacttgggataagtgtgtggaaggaatttc

aagatgatatgtcacagagcattcgaaatgtgaaggaatcaaatatttatcagaatgttgaagacaaactagggaagttcagcagggctgttacagaagc

gccattgtatcagaagactgaatctgtgattaaaactacagctgaaaaaa

>61073|ACYPI005806

gttctggagagacagaggatacattcattgccgacttggttgttggtctgggtaccggtcagatcaagaccggtgctccttgtcgatcggagcgccttgc

taagtacaatcaattaatgagaattgaggaggagttgggcagcaaagccaagtatgttggcgccagcttcagaaatcctcagcattgaagtgttttttta

gtgagtttgatagaattaaacatggaacggag

>61935|ACYPI005885

gaagatgaaaagtttgatctagagtatgcagttaaaaagaaaaactttgaaatttccgatctgaacagccaagtaaatgagctccgag

>71294|ACYPI006276

cgcccagcacggccccggcggcaaccctggcgccggtcctgcccccggccaggacgcagcgcgcgcgccaccgccacccgccatcctgctgcaccgccag

gcactcaccacggacggcgccttcaactttgcgttccgcgctgagaacggtctgcagcagggcgagagcattgcgcccgacggcagccgccgtggggcct

actcttacgtcgaccctaacggccggaccatcagtgtgcgttacacagccggcaaggacggtttcaaggtgctggaaggcgagccatcacctggtgctgc

tgcaccacctcagcagttgcaggctcgaccacagcctcagctggcataccagcagcagccacaaccacaactggcctatcagccacagccacaactgcag

cctcaaccacagctggcataccagccccagccacagccacaggcacagcttgcataccgtcagccacaacagccagcactctaccagacagcagctgcaa

caccaaggcctccaccgtctccagcagcttaccaaccagctgtacg

>71504|ACYPI006288

atggaattgctgattatgataaattcttgtaaaatagcgtcttctgctcgagtgtctgctgttattccatgtttcccatatgcaag

>71502|ACYPI006288

aaagaatccctgtatttgagatgcatggagatccattgtaataaggtgatccgcaccagccacagataacatatttgccaccaagcctcggat

>71920|ACYPI006308

aaaaaatgacaccaattgcagtaagacgtaacgaacgaaacactggttatgtaacttgtggatacgtgcaaatagaagaacatgtctcctgcaaatgtgg

ttgtacagtaatgcctggtgactgcaatgacttacagaaatacagtgcaagtgactgtcagtgtaactgcattaatggcaggaagcctgaagactgtgtt

aagccaaaggacatgtggaatatggataaatgtgaatgtgaatgaataaacaatgaagagtctttaaactgacagctcttagtttacagaaaacataaat

ttctttacgcgactaagtttgtctgtctactctacaactttttgttttatcttcttgtaaaatattgagtacttagtaacaaagtaaataataatccttg

aggcataagctgtgttaagcaataaattaaaacatatgttttaaatactgagcatattatggagattttatatcacataataaactatattattctgaat

aattttatgtacaatgaaaatcaatttaatgaccaattctaaagtcatgaaatatatttttcctcctgag

>72113|ACYPI006410

gatggccgaacattgtctgattataccatccagaaagaatctacgttactccttgtctaaaggctccgtggttgtgctaagaaacgaaagaagaagaatt

>72351|ACYPI006530

accagacaaacaatagaggtgtaccccctccactctctcagggaaggggggccgcagtgttagaccaaaaagacaagggagggctctcctcaggcccctt

ccggccagccgcagtaagacggcagcgggggtgttctccgcaggcacaggcgctgccgcccccccttcgtgcccctgccgaggctaacgactagcgacga

ctcgttctgaccctctgcatctggacgtacccctaggtctccttcttcgtcttgaactcctcgcccaggattgtagcaatctctccttgcatgaatgccc

acggcacattggagttagccagtgggcacttttcaccactcgggcagtatacctcagcacctgcaccttgcctcttgatggactctctgctgcacgggaa

acagaacttgtgctgtggaacagaaggacactgcacgaagtgcgtgtcttccagcctctcctgacacagcgtacacttcagtgtggtggcagagctgccg

ccggtgcccgccgcactgccgccggtccccgccggcgtctgcccgggtcccgaggctgtggtggaatttccgtcgtgcccggtagcagctccgccactac

ccgtgccggcagccgcaccgtccgcttcgctggacgtcgtcactgtggtcgagctcacagtccggcttccagaactacggcgactccccgtggaacctga

ggagttgggagaatgctgggagcccctggaagcagagcgcggtgggcctggagggctgcctcctgcagagcgcggagatccgggtggcaagttgtctgcc

actgacatgagagcagccatgggggactgtccaggggcggcggctccagcgccactgcccgctgtggcagccgcagactccggaggcgtgttggtgcgcg

gacccagcggagagcccgctccacccgcactcacgctcatcgccacacctgggtatcctcacaatgcaatttctatgtggaaaatttcgacagctgcgaa

cggcccgacgagaagactgagttcgacagtactgcgataatccactgcgatggacgcaaagaacagctaaacgaag

>72352|ACYPI006530

tgtgtgtgtgtgtgtgtgtgtgtgtgtgtgtgtgtgtgtgtgtgtgtgtgtgtgtttgtttttgttcttcacaagctgttagtgaattagatggctatgg

tttagaagctagaagtcatatttatcacaccatttatatacttcaactcaagctgtattacagcaaacatatttatttgtacaaggcaaaaggaacatct

aagttaattcatcatttcagtaacaattataattaaaaatagtccatctgagctatcactgaccaaaaaaaactttatctttttacaaagttacttgtta

tacaatagagatagttttgctgcaaaactacctttcattagagacactattagtactaagttttgctagtaaaatttgtcttactacacaagggttttaa

tcaaattctgattaattgttaaaacacaccagcgagttttggagttcctttataggacattgcagtccaggcattgactagggtaagaaggttatctact

acacaataaagtgtggctgatgactgcaaaagctacaggaagttaaaaacaaaacaaaaaatcattgctgcagagaaaaagcaaaatattccttataatg

tggcagcaatggtctaccatattgtgagaaaacttatcatataagcagccagtgactgcctgcgatcaaacagcaaaaaacgttattaagctgccagact

cattttctgatgcttctggtccactgtactgcagcaaagaaagagaatattgggcttacacagctgtagaactcgagataacctctggatctattccctt

catcaactagctcatttgaccaaccaaagattgtggaattgcctcaaacaactaatctctttatttcaacatttgaaacatattgatgtacactggctat

ttaagtctggtgtgaaagatacctacgttttgcatgtgataattacttttagtctacagtacttcactgtacagtctggcaatgactattttttattgtg

aaaaggttatacaaaacaaaataatgtgataatatgtattatgtaaaaaaccgaacctctccacataaaatgtgcagcacatattagtaacaacagaaaa

caggagtttcttatccattcatacacatacatcactgaagacaattttaaaagcatttgaaaactcatctataaaattaattatctgcagccattatcag

aactgcataaaattgccatcctttcctatacgtaaatattttagaacattcttttattaacagcagtaaagttattctgattctataggtaacatgatca

tcacaataacaggcctacaagcggtgaagagacaagagtactgaagccataaatggagacataacataatataaaaaatcatttgaaatgggcagcctca

catgtcaaaagaacatcaacagcacatcacaatccacatatgatacacaatcgacaaaagtaaaacaaggtatgtagctgagacttcacaaagatcagac

attaaacaaggcttgacacacatgcattcaaccacagtcaattcaacaaaattactctcaaatgctacaacatccacacacaaaaaaaaccacaaaaata

ttgtgcaaccctgttttgaacactttcactctttatttcagtccttcaaatccaaaagagtagctgaactaatacatttgtccagcacatattagtttgt

atacaaaaagatgaatacacacctaatttgtaaacaagttaaaaaatcactcctaaacttagtaagtagcttagtgctaaaaactaagagtcaaaaagag

tttatcactgtttgtacctaggaatcatttcgggaaaaaacaaactttccagtcccttttcatgaaatgttacagttaaaatttaactgtgtaagatcta

caagttagccttaattttaaaaaagtctacaacagttcaatgtaacactatataaagggaaattaatatatactatgtacagttcatttatttacaataa

atgatatatttatatacactgtaacgactccccatgatgtgtacttttgtgcacagaaaaactccgtaatgcggctagcgcaattacgcagctcgatata

aagatcaataaaagtggaacagattctgtgaggggtaaaacagtcccctctacctggtactgcacgagtagtgaatccgtggcatgtctcttgcataaat

gcatcacataagtgtaatgtgtaatacacaaagcactgtaaactgcgtatttacattcagaaaaaggtatgagttgttattcttattattatgaatatta

atatgtttatttatgactgatttttatactatatacataatatttgtttgttgtccctactagatacaagcgctgctaatacagtcagtccgactaaaaa

caattctgacaaaagtctcagaggacgtaattctcaccgtgcaaattaaatcaaaaactgctatcaaaactgaacagcagtacaatttcaggaaaattct

taataccttcccaagaagactccaaatacacccttccgcaccgtggaaggttattctatatacacaaaatggtcactgagaacagctaaatggcaagaca

gtttgacaaatggtctcagagaatatatcaccaacattgtttccatcagcagatttacttgtttgttgcttttagaaaaggtctcagcttcggggttaag

tatgatacactgccattctgtgacagcccattatttctccagtgaccagacaaacaatagaggtgtaccccctccactctctcagggaaggggggccgca

gtgttagaccaaaaagacaagggagggctctcctcaggccccttccggccagccgcagtaagacggcagcgggggtgttctccgcaggcacaggcgctgc

cgcccccccttcgtgcccctgccgaggctaacgactagcgacgactcgttctgaccctctgcatctggacgtacccctaggtctccttcttcgtcttgaa

ctcctcgcccaggattgtagcaatctctccttgcatgaatgcccacggcacattggagttagccagtgggcacttttcaccactcgggcagtatacctca

gcacctgcaccttgcctcttgatggactctctgctgcacgggaaacagaacttgtgctgtggaacagaaggacactgcacgaagtgcgtgtcttccagcc

tctcctgacacagcgtacacttcagtgtggtggcagagctgccgccggtgcccgccgcactgccgccggtccccgccggcgtctgcccgggtcccgaggc

tgtggtggaatttccgtcgtgcccggtagcagctccgccactacccgtgccggcagccgcaccgtccgcttcgctggacgtcgtcactgtggtcgagctc

acagtccggcttccagaactacggcgactccccgtggaacctgaggagttgggagaatgctgggagcccctggaagcagagcgcggtgggcctggagggc

tgcctcctgcagagcgcggagatccgggtggcaagttgtctgccactgacatgagagcagccatgggggactgtccaggggcggcggctccagcgccact

gcccgctgtggcagccgcagactccggaggcgtgttggtgcgcggacccagcggagagcccgctccacccgcactcacgctcatcgccacacctgggtat

ccgttgctcttgaaggcggtggccgtgtcgaaggaaggcgccctcatggggtgcttgtccttgaaggacctctcgaggaagggcggcgtgctgatggaga

cggcgggcagcgac

>72523|ACYPI006609

ccgcattcgattataacggtagattccatccataatcaccctcttcgcggcatcagcatcttccataccttgtgtctcaactaccttacattcaagcttc

ttatagtcccgaaaaaatcgctcaatctcgcgtaaagtgtgagggggtaattcattcaccgcactgtaatgagcgtaagctggatcgtcaagatgaacgc

atattatcttctcatcttgatcaccctgatctatcatacccataactccgataggtttgaccttcatgatgcagcctggataaacaggctcttgcatgag

taccaatacatcaagtgggtctttatcttcccccaatgttcgtggaataaagccgtaattagcaggataaacgacggatgaatagagtactctatctact

ctcatcaatcctgactttttgtctaattcatactttactttggagcctttgggaatctctatgaccacgttgatctcatgagggaccgccaaaccaatgt

caacgtcatgccacggattcaccatgctccttttcgaatgacttttggcaacctagaccaaaaaaag

>72892|ACYPI006711

ttgcaatttgtcctggatgattcagatcaataacctgtgctgggaaatcagcaacacccttgggtgggttgttcttag

>72895|ACYPI006711

gtataaccatttgcaatttgtcctgaatgattcagaacaataccctgtcctgtgcagtcagccgaacccttgggtgtgttgtcctta

>72889|ACYPI006711

tgctaggtaccaagtttacgatagcagcatcaccagacttgtttgatttaggattcgcctcagttggcttgtcagtacgacggtcacactactct

>72893|ACYPI006711

gcactggtgtataaccatttgtaatttgtcctggatgatttagatcaataacctgtgctgtgaagttagcagcacccttgggtgagttgttcttag

>72891|ACYPI006711

atcctgtaaactctgtgccaggtgtgtgtgtgtgtgtgtgtgtagaaatgtggcaggagttccctccattgggtagatttgctgtgcgtgacatgaggca

gacggttgctgctggtgtaattaagaatgtgacattcaaggatgtcaccccaggacaaattgcaaatggttatacaccagtgcttga

>72896|ACYPI006711

tgcgttgaagctttctctgattatccacctctcggaagatttgctgtccgtgatatgcggaacaccgtcgctgtaggtgtcatcaagtccgtggagaaga

aggagtacaccaccagcaagggtggcaagaagtagatagctttttagtgatttgattttacatctgatctcaaacccttagtagctattgagacttttta

gaattgacc

>73407|ACYPI007196

gttctatcacacggcgtatgggagacaagacgtgagaactgataacgactttattgtccaatctgagtgcgtgttggcgcagttccgaagtgaacaaaat

caatcccacgacgtgtgcatttggtccatgacagttaagcagaaagctaattttttttcgctgcaccacaagcgctagaacacccaacccattttcggaa

tatacaaactgacagagaaagtgtgctcccacacaaaatgacaaagattcaagtaataggtctactaattatcagttttgctgcaatgtgcaccacaaat

aactcaaaggaacatgactgggatgtacttatatttactcagcactggccagtaactgtctgtctgcagtggaaagaaaaggaagagcagcacacttgca

gttttccaacagtaaagaacatctggaccattcatgggatatggtgagtataacaacattctgtacataaaattttagcacttgaaacaaaatcaaac

>73406|ACYPI007196

gaacacccaacccattttcggaatatacaaactgacagagaaagtgtgctcccacacaaaatgacaaagattcaagtaataggtctactaattatcagtt

ttgctgcaatgtgcaccacaaataactcaaaggaacatgactgggatgtacttatatttactcagcactggccagtaactgtctgtctgcagtggaaaga

aaaggaagagcagcacacttgcagttttccaacagtaaagaacatctggaccattcatgggatatggtgagtataacaacattctgtacataaaatttta

gcacttgaaacaaaatcaaaccatacctatatattgtctcactgtacaatatacaatacataacaggtagaataccctagtttttgaaataaaaacaaaa

tatgattcacaaaaatatttcatttatgttcaaatacatataatctaggtctttatgcatgctacaaaacagtaagttatttagatggtgtcccccactc

ataatgcaattgttataccttctttggaaatgtcccataattttatgcattatttccaggaaccagtattacacgaagtattttggaatatactacagac

ccctgcttatagctcttttaaggaccaaaagcaatacaacagcagtctttttttcccccctacgattctgctttgagcaaacacaattctcttctttttc

attcatacattttgatagtgttgcgccattattagtgggctatttgttttcataacagcacatactgatctgtggcaactgcagtacagcttatattttg

gtacagttggccatctgtgatttccacattatcatgctggtatggcaataggtaatacttatcacaacataatttggaaattgcttaaactgaacagata

catctagatggataaaaagaatgacacactattgtataactagtaaatgaaaagagggagtagctatatatcgtagcccctacaggtaagcaaatattgt

gtctattgggtccttgaatactgtgcctcaagtttgcatatataaactcatatacttgttgtactattggactgtgctttatttaccgaccagcaggtca

cagcggatttgttaatggatctgccctctactttagataacattcaaatgaacatggttagggttttaaagttttgtgaattttgcaacattttcgtttc

aagattttagggtgacgattttcatgtactaacagaacgacagctcacgcacatttattgtaagtggtcagccagtgacattttcctgtgcatttctttt

agctcctttgtcattttgcttgttttttaatctcatgtacagcaccttttactctttctccgattgtgtggtcactttgagtggtgggagtgagtgagtg

catagttgtcattttataggtttcctcttcactgtatgacagcattttcaaaattttactacgtgcatttctttcaatgtgcataagggtgttgatagag

actgataaaatagcttcacagatttaggtgcaagggcaaaaagtatgtggtttattggctaaagtgtacttatggtatttggcagttcttgttgcatgct

gttgttggcaatctgttgatgttcagtgttagtttgcagaaaccttatttctttggagagagaatgtgaatttattatactggttttagatgctggtgat

agatctagatatgctgacaaactgccctataacatagccaaggcctaggttgaattcaaatgttataatttgcttgtgagatggtaaagtcaacaaagct

taacacaaaaatatgatcaccataataagattgatgtgcagtgtagccacatgttgtatctgattttaccaaaattctaacctagtagaacattattaca

gctaacttcatttatgaaccacagaaaattatgaacaagcattctgtttggcttttgacataactgcacctttaccagtttactcgtccatgatggtcaa

atgctgacaataaaacatagtaagaaaaattctgcattaaaaataaataaataaataaaataaaccccacacatatgactctgtcacagcatttacagat

tacttaattctgaagatatttacagaggagacatcactgatgaaaatttaactctttcttgatatatccttttagaacttttaagtccc

>73442|ACYPI007377

tggcagtcagttgcgctcaacttattcatatcattttattttttcagcatctcggagaaatcgaatggctttaactaatgcagaccgcgcagagatcatt

gctaaatttgctcgcgctgaaaacgacactggttcaccagaagtacaagtagctttattgactgctcaaatcaatgatttacaaggtcacttcaaagctc

acaaacacgaccaccacagccgtcgcggtttgatccgtatggttaaccaacgtcgtaaattgcttgactacttaaatggtaaagaccacgagcgttacac

tgctttgatcggtgctttaggtctacgtcgttaattcgattttgctttgttttcggtttatcgcatgttgctatgctttgtttctgaaaagaaagtttcg

tctaataaaccattaggcgatttata

>73459|ACYPI007380

gttttgctgacgctcagttcagcagcgtgtgcgttctcgtgtacactgtgcgcgctacgtctgcagaaggaatccgtttccgtctgccagcagaaaatat

cagagtgcgtcggcggttatacggtgctgggaactcacgccagcaaagtgtgtgggagcttcggtcgtaacgggtattgtgcgtgcctggtgattcgcat

gaacggtagaaacaccaactgccttttgcagccgaaatcggaggtgtttcgtctttgtcttacagtccggagcgtgtgaaacattccagttgttgcctgc

gaacttggtcagaaagggcgatatccctgtaggacatctgtgagggcgtgtggcgtgttttcgcaatgctgggcgactgacttcgcgcggcgcggcctgg

ccatggagctgttccggcggggcgccatctttgcgacgttcttcggctcgtgcctggcgatcgcgctgctggtggccgccctgggcaccaagttctgggt

ggtggcgcgcgcgcgccgcatccccaacccgcaagagtcggatggccgcgtccacttcgggctcttcgacggcaagaaggagctcaacgtcgcctacgga

tggcgcacctacgagatcgacatcgtgtcggagatgcgtccgccgcggcagctgctgacgtggggcctgtggctggccacggtagcttcggtgggctgcg

gcctgctgttcgccgcgctcgccgccctcttcgccgtcgtcaacacggcggccacgcccccagggcccatcaccgccgtgccggggctctacctctggaa

ctccgtcgccatgttgtttgacctggcagctatcagtctgtggactgtccaatactatcagaaacttcaggacaatgttatgagtatcgaagatctggac

aacatgtggacgtcagctggcatggcacatctaggttattctttctggtttgttgttggaggtgccatagtacatgctcttaatatggtggcaatctacg

tgggcactcgccgctcaaggcataaggatgttccccagcctatgatggaaatgaagaacagtggtgcaataatgttgtactgaattcgttttgttattct

tcagaaatttt

>76226|ACYPI007511

agaatttcgagaaaaacagacatggttttgaccagttgtaccgccagttgtctgatgaaatgtgggaacaagctattgatttgataa

>76228|ACYPI007511

tggtaaaatattttatcaaatcaatagattgttcccacacttcatcaaacaacttacggtacaacttttcaaaaccatgtctgtttttctcgaa

>76225|ACYPI007511

acttggttacaacttgcgttctttccactaactacagatctcccaacttgcggtacaacttttcaaaaccatgtctgtttttctcgaaagtcttgaaata

cgaagacagcaaaagatattggtaactgtgattgaaat

>76221|ACYPI007511

cgcaagttgtctgattgaaatgtacgttcatgtagcgcaagttgtctgatgaaatgtagtaacatattttatcaaatcaatagattgttcccacatttca

tcagacaccctgctgtacaacttttcaaaaccatgtctgtttttctcgaaattcttgaagtacgaaatcagcaaaagaaattgataactgtgaacggaat

gtacgtccatgtagcgtgaaag

>76503|ACYPI007765

gcttcccagcaaagataagcctctgttggtcaggtggtattccttccttatcctgaatctttgccttaccattctcaattgtatctgaagcttcaacatc

cagagttatagtcttgccggtcaaggtcttcacaaagatctgcatacctcctctgagcctcagcactagatgtagggtagattcctt

>77114|ACYPI007905

catacgagatattggcgtgactggagttcagacgtgtgctcttccgatctggggcctggttttctggttcatcttgactcagatgggcccacagctcctt

gcttagttcaaaaacagcttttgggtctttgacaagcaattcacctaagacacattgcaaactttaatgtacttaggtgaattgcttgtcaaag

>77130|ACYPI008176

cattttctgttgtagtgtgtgtcccaaattgaatccaaggtcctttgtttgtatttgagggcgacgatacatagatgttgcttgctttcaacagaccagt

ccgcaggagtgctcctccgatagcttgtgccatattcccagctcccacgaagccaacctttcccacgccttcaagtgtcttgcctgaatccatattctct

tgatatgaaaataacctatttaaggaaagggagactgtcgccgcaacgaaacaccaacaataacaagaagctagatcagcggggctccgcagacagtatc

atcagaaaacaatatgactcccacaattcccgttcacaatatgcaccgtttcaacaaataacaatgatggcactgtcaccccctcttcccccccggtaaa

acggtacacgaaactgtcatagcaaccacatgtcaattaaatatacagcggtctgtttgccatcccctctacgactctgattggctatctctggtgacgt

ttacttagcatgtaaacacgtcaatgccaccgatcggtggcccaccgataaggaaagatggcggcgtgtttacaagatcttattaatccgcaagaccatg

gtccaagtgctaatcgcgcggtaaataacgagtatttgatgaatatcacaaatcaatgcttctacgcaaatcacatcccatgttcattaagggtagagca

cactggttattgttatcactgtgactgtttcatcacacttgtcaagcctcttgatacatacgtagatgcacgcacagaacaccaaaaaatcggaagtgca

agacccgtgttaaatccccgaaaactggcaagcttatttgtcaaatgctcccttccccacccacggatttgtcaaacaagccctt

>77162|ACYPI008316

gccagattggcacataaagagcgttcagcaccatttgatgtaatgagaacggcacaggtacctgtaggttctttatctgtatactggtatttcacatcta

ctccctcagctcgggcctttgtttccataatcgtgctgaatttgtctttgccaacacaacccatgaaaacggtaacattaggttcgcaaagaatccacgt

aggtgaggctccatgcttcgaaattcagtcaaaggcagaggtcaaactacaagataactaaggttatcagtcttagtttctactaacatttcaaacaagc

tctgcgagtgcgggaaccatatcgcccacacaaagcgtgttttcgttcgtctgttgccaacacctgcacatggaactacagccacaaatatgtaaataaa

cgtgcattttcttggatattcagaggaatactgccatttacacactaatcacagcacacgtagactaatgaatcttttatcttactggctcatgatccgt

atggtcccgtcgccaagctaaagttcttaatgatcctaattttagcacccacgcatgtgaaaacagagacggcgctttacatcagatctcgactgcgatt

ttgctt

>77179|ACYPI008716

gcattcttccaatctgattgggattcaagtctgacaaatttcttccacaatactctaaatatgcaggaacctgtgtttgaatatgatttcccacctccat

atcatccgccacaaaaatggtttcctctgaaacaagctttcaatttatacatggatagatatcgtgatcagaagcagattgcaaaagaatacttgctgat

gaaattaagcaaaacacatccatttgagaaacctccaccaccactgaaatatccaaatgcacatcgttatgaagagggaacaccttcatggttgaagacg

gaaattaggaaaaacaggcttggttggggccgaattaatgatttttaatcatattattataaacatatttttgtgaataaacttgtcactgagaaaaact

agttataatttataagtcagatgttttacggtatcaaaatttgttaaattttgaatgtgatgttctgtacagaaattttgtttggtagttgtactgtatt

tacccaattataagacgaggttccttcccaagttctctatttgaaaattacacagtgtctacattcgcaatttaaactaatgttgctgagatcacagttt

ttatgttttgtaattatttaagtaaagctttcatataccaaagtagcattttgtgtcagtttagcacaagaagctgtacttaaaatgacgtgtttttgag

ggaactgtgagacagtgtctcactgaaactacatacttttgtattacacaagtataaatatgaaaaccttcg

>77278|ACYPI009439

atactctactaggatgccatgcaacaatgcaaccaaccttacccaaccccttgtgagttttacgagacagcttctttgtg

>77283|ACYPI009439

tttctgttctgtggtggtaccccttttggccagctctggctactttgaatgatactctactaggatgccctgcgccaatccaagcaaccttacgcaaac

>77281|ACYPI009439

tgttgatcattgtacgatgatggtaccccatttgaccgtgtctggcaacctggaactgaattctagctggatgccaagctccgacgcaagcaacttttcg

>77353|ACYPI009769

cagctgctcttccggctctccagtctttgccacctttagatggaccgtcaacagtcagttggttggcggtcatagcatggacagtggtcatgagtccttc

cacaataccaaacttctcgtggatgaccttggcaaggggagcaaggcagttg

>77354|ACYPI009769

tattaacggtttcgggcgcattggacgcctagttctacgtgcctctctggaagctgatgacgtgaaggttgttgccatcaatgaccctttcttagacccc

gagtacatggcttatttgttcaaatatgattccacacatggtatatacaagggagatgtggcagcatcaggagacagtctcattgtaaatggccaaacta

taaaagtttccaatgagaaggatcccaaatccattccgtggggtgcaaacagtggggtatatgtagttgaaagtactggtgtattcaccacaatagagaa

agctagtcttcacctggaaggaggcgctaagaaggtcataatctctgctcccagtgcggatgcaccaatgtttgtgtgtggcgtcaatttggatatgtac

gatagcaatatgaaagtagtgtcaaatgcttcttgcactacaaattgccttgctcctttagcaaaggttgtccatgacaattttgaaattatagaaggtc

tgatgacaacagttcatgccaccacagcaacacagaagacagttgatggaccatcgggaaagttatggcgtgatggtcggggagctggtcagaatatcat

cccagctgctactggagcagccaaagctgtcactaaagttattccatcattagttggttgcctgactggaatggcttttcgtgtccctgtacctgatgtg

tcagtagttgatctcacatgcaggataggcaaaggtgcaacttacgatgaaataaaacagaaagtgaaagaagctgctgagggtcccctctgtggaatat

tgtcctacactgaagatgaagttgtttcgtcagattttatagggaatgcaaactcatctatttttgatgcaaaagctggcatgtcactcaacagcaactt

cgtcaaacttatttcctggtatgataatgaatgtggttactcccatagagtgtgtgatttgattcgtcatatgcacaaagttgggtaactatgaaaagtg

tgctgattgcggcaatttattattatatgctttgtctttttgcgctagatgtgttgaggtattatgattattgtttttctgtatgctgtcaatgaaacaa

gaaatattaaataaaaattattgtaggtaaaaacgtaattatccagtaatacattcattttctgttaatatgcatacattattaagaatctgagg

>77382|ACYPI009963

acacacacacacacacacacacacacacacacactcctatttactgcaacattgaatgtgctgacggaggtattatacgtttatctgatacagtttatgg

aagacttaaattgacaatacatttttcaccttaaatactgccataaaattagcataggataatttgtacaacttctttggtgctaatactacaaaaaact

gaacaaactatattttttttccttgcagagcaatcttactactctgcatgcacagtgattgctttaatgttccatcagacagagaaaaataatgtgtcca

agtacattatttaaaactatatacaaagcaccaacaacattttggatcattatcaagatctactgctaactaccatcttttccccagtctcctttgcaat

gtcaggctttgtcattctgccatgatacacagtaccatcaacatccaacgaaaaattgtagtcaacttcattgtctagggccttctctatggcttcatca

atattttcaggagtgatgtagtgtttagatctttccttttctttggaaacaatgtcttctactttctccaagtattctttttcttgctgctcatgtttta

tgatagcatcaagaattgcttctttctcagccagctcctgttttgctagtcgaactgcccgcagttttgctacttctacattccaggcatcattttcctt

catacataaaacatgctgctctctctctcttcttagctcctcctgagcttgctcactagccgatgctgatttttctagttcttctttcaggaaagctcga

atactcttaatgtaagtcttgtaattgttatgcagtcgcaatagttctgaattttcttcttcgggaagccgttttctttctggcactcgaaacattttgc

tttttgccataggtaaatgtctaggtttcctccgtgccatacgtatactctcgtactgaaccgataaattccgaggtgctgcagatctacacacactaac

tattgcattcattgtattgcacggcacaacaatactttaatcgccccagtataataatgggcagctaagttctgtaagtccacacgacgcctctctctcg

tgcgcagtcgcaaacgcacagatatatatgcgcatgctttg

>77538|ACYPI22936

caggcagcaatggtcacacaagtgttggtaaataactggattttgaagtttggtgtaccggagacaataattactgaccaagggaccaccttcatgtcgg

atttattgaaggagctgtgtaaatcgattaacataaagaatctga

>77535|ACYPI22936

caaaattctgcagatacgtgcaccataaaaacactaacaatcattcggcaagtattggcacaatcaagcaatggcacctgtcccttaagctacactcgta

tcatgagacaaactggatgacaactctcctaatagttctccttgatctgatgaacatgtgcaaaatagccataaagtcactgttagcagaacttgtttat

ggtgaagcatta

>77534|ACYPI22936

aggcaggagcaactccatcttgatggtgctgtattcgttgcctggaggaggctctaggacatcagctggcttgagcctgtctatggagacagttgttgtg

gccatgtttagacgtatggagaccatcttgtctttatgttgcaaaaccaagtgtggggtcaaatactgagggtggagtggtggctggtacgcttccatgt

gaagcaccatgtatacctgaagcaggacgtgtttttatgctgtgaggccacagtggatgaatgtatgatgctcagcatg

>77565|ACYPI24120

cttatttaccttcaaaagtaactgtgaaaagatgtgctggacattgtatgtcgggcatgacatgtattccaagaaggaaaaaaatgacaccaattgcagt

aagacgtaacgaacgaaacactggttatgtaacttgtggatacgtgcaaatagaagaacatgtctcctgcaaatgtggttgtacagtaatgcctggtgac

tgcaatgacttacagaaatacagtgcaagtgactgtcagtgtaactgcattaatggcaggaagcctgaagactgtgttaagccaaaggacatgtggaata

tggataaatgtgaatgtgaatgaataaacaatgaagagtctttaaactgacagctcttagtttacagaaaacataaatttctttacgcgactaagtttgt

ctgtctactctacaactttttgttttatcttcttgtaaaatattgagtacttagtaacaaagtaaataataatccttgaggcataagctgtgttaagcaa

taaattaaaacatatgttttaa

>77587|ACYPI28835

tactgcatttcacacgagtttgttattgtctgcgttcacgaactgtttttgttcgaaatgtagcgaacaaaacctaatattattatacaggtaaaccggg

tctttcttcatagggtcttgtcgtctgctattaactagccattttttgctcctaaa

>77588|ACYPI28835

gtaatagtttcgctcgatctgcacattctggcagttcacacacttttgtaggacacgaacagctgcacttaatctaaccacttcatcgccaaagcaggtc

tgtgttaacgattcacacgaatctggcattgatacatggagagtcgaactggctgcttctgtgtcgtaggactgttttacagctttagactgactgtcga

atatttgtggcagtttcctcttcgtcagttgagatggcttatttggaatgtcaaacagtgagggtactgcattccacacaagtctgttattgtctgcgtt

catgaactggttttgtttgaaatgtagcgggcaaaatctaatattattgtacaggtagattgagtctttcttaataaggtggtctcgtctgctattaact

agccg

>77658|ADA2A-2

ggggcttttctgattctgctgtcttctgcagtttaattggaatgccaaccaaacatccaccttcaatcaggggcttttctgattctatctccactcacaa

cacagaagtgtctcagagtacacatttatgaccctgcaaatcaaaacagccacgtatcatgcttgctgaaacacaccctgctgaataaggaaatcgtaca

gcttccgagttttgttgacatctatctttataagctgccttgcttgggctagtttcaaggaaccaccacagcgacgactttcattttccaatatgcgccg

aaagttaaggtatgcctgaggaaggattctggtaacagagcaaagctctcgctctgattgtg

>77657|ADA2A-2

tggaattgtaacatatcactcacacttcccagtgaaaataccacatttatttgtcatcactcagatgtgtcacatgtattaaataacagtgcaacaaatg

aacaaatactggaagaccagcagctatctagcggggaaatacactatcagtcgtataaacaagaaaaaaatatgtcttcagaagagttcttggaagcttg

tggacaccgcttggctgctgcggaaattttgccagagtatttgtcacaatacatgcacacagtacgtggtacagggaataagcgactacccgttccttgt

ggcaaaatacagaaaaagccaggcaagaaaccactgcacaggctacgacgccctcctcctagctcaatacccggatactcagctgcacgatcagagtttg

atatagattacgacaatcatgcagaattaatgacagtttgtgtagctcatgttgactggtcagacgatgacacagatgctgacttgcagcgtgcactaac

actggcagctgtcactgcatacaataggcgcctgtcaaaacgacttgatgctcacaagttgataagaggccttggtttagtttcatcatcaggtggatta

actttggggccaggaaggttagcaccatttgcacggctgctagcaccactgccagccgcgtatcacatcctgctgacatcactgtcacacagtgtcgacc

tccgccgacgtataacgtgcctgtcccagttacgatcagaagccggcctgtggcgcatatcccagtgtgcactctaccggcgacttgctcgcaacagaga

gcttgccattcgagaaagacgtgcagataggcaaaggataaatctcacttcgggcccttctacagtccgtctggatgggatacctgcagcgccacgctct

cgagctgttgcagctccactagatgtttctgccctgcccggtgcaaatcgcctgtcacaatcagagcgagagctttgctctgttaccagaatccttcctc

aggcataccttaactttcggcgcatattggaaaatgaaagtcgtcgctgtggtggttccttgaaactagctcaagcaaggcagcttataaagatagatgt

caacaaaactcggaagctgtacgatttccttattcagcagggtgtgtttcagcaagcatgatacgtggctgttttgatttgcagggtcataaatgtgtac

tctgagacacttctgtgttgtgagtggagataggttgtggaaaacttagcaacaaatgacactttttcgcagtttgcagtgacttgagattatatagtat

ttttgagctgtttctgcaaaccaccaagtaatttgcattgagaagccattataattactttgggtgtgaacagcagaatcagaaaagcccctgattgaag

gtggatgtttggttgggcattccaattaaactgcagaagacagcagccagttgttgcaactaaaaattcctgttcattatttgttaaaattagcttcatt

ccctgtctaaaagtacaaaatattttgtgtctcttaattgtttttgtactgaacaaaactgtgtcacaattatttttcaatattgtgaataaatgtgctt

ttt

>77678|ADP,ATP carrier protein, putative

gatgcgagattcccgccaaagttcctccaaaacggtgtctccttatcaactcctccaaggaaaatttgcttatacttgt

>77995|AF160895_1GM13640p

cttcactgctgattttaatgagtagcaccagatttgtacaagagactttaaaggattaaaaaaacatagtcatatgtacatcagatcagcaggcattaca

ccttaatattttacaacacacaacatatagtacataatagttatagtccagttctccgatgatcaacccaagtgaatggagagataaattgaaagagaac

cagaggttactacacaaaagacaaagacagcaaaaacattgattatttacattctgttataaaaggtgcttctattcatcacaacattatacagtataat

actctttctgaaaacctttatcaccatctctggttacacagacaggaaggaagctattagccactactgcacacagtatgaatctgtatgacttttagtt

attttgaggcacacaaaaatatgtttatgaaatgatgggagcacaatcactttcattaggctctgtttttatgtttgcttcaatttttgttaactccagt

gaagtcatatctatatcatctccccagatgtcttcttttggctttgtctttgaagattgttttagtgacgcaccattttctgatttgaaatgttttccag

caataagcttctgactctcttcaaaatatttattgggatgattaacaccactgtcaatgggtgcactgtgagtgatttcgaaatacttggcacatgcaat

ttgaaagtgaccacgagatacataatctgccatttcttgaatacctgctgcaggtattccataacttaggagtagctgtttgagggatgataaatcatta

tgtttaaaagggcagccatgagtgtcaccaggtccaactgatgacattatgattttggcacagctgtaaggtgtgtagttcacccttttcccttcttttc

cataattgtggcgtatgttgtaggcatactgtttgtcaaacttatctgcatccattgcttttacaaattcttctctccagaatttcatagcattttccag

agataaaccaataccttttaagaaaagaccatactgcatacggccaccatatttcaaa

>78326|AF160941_1BcDNA.LD19727

agctgattcacgcacagctacatttgatgctatacagtctggattgaaatgctgcggggcatctggtccaggtgattgggctaatagtaaattcaatatg

cgtgacaaaaatatagtggaattgacaattaagaaggctgtcaatgtttacaacattcctgctagctgctgcattgatggaattacaaaaaaggaatgtg

aagaagggcgcacagtggtattaactgctgccataaacagacaaatttattctgagggctgtgtcgacaaactgattgaactccttcagcagtatgcaaa

tattttgatgggtgttggcctggcacttgccacagttgaagttctgggtcttatattctcactcatactgtgctgtgctgttcgttcagaggacagatac

aaagcttaaattttttcctctacagctatgttaattgtgtgcattgagaaagaagttgttttatatctcatttaacaaataaaatagctgtagaggaaaa

aatttaagctttgtatctgtcctctgaacgaacagcacagcacagtatgagtgagaatataagacccagaacttcaactgtggcaagtgccaggccaaca

cccatcaaaatatttgcatactgctgaaggagttcaatcagtttgtcgacacagccctcagaataaatttgtctgtttatggcagcagttaataccactg

tgcgcccttcttcacattccttttttgtaattccatcaatgcagcagctagcaggaatgttgtaaacattgacagccttcttaattgtcaattccactat

atttttgtcacgcatattgaatttactattagcccaatcacctggaccagatgccccgcagcatttcaatccagactgtatagcatcaaatgtagctgtg

cgtgaatcagct

>78325|AF160941_1BcDNA.LD19727

atgaattgcaaatataatttcaaagttgctgaacttccaacaagcactcatcgtacacagaaatatagtttcctgtgctttgtatgtcaatgaactttaa

aattatatttgcaattcatcacacacaaaatgttgtactaacaaattatggcattacaaaattgtgctgtattttttcacaacggcttccacttcatcta

caattttttgtctctcaatgacttgctactgaacaattgatactgatacgtataaattaaatgactaacatcagcatattaagctaccttttttttttca

tctcttccaactaactcagagaatagggccgaaaactgaattacagataacctgtgacattgactgctagcaaattccttgcttgagtgctcttaagttt

tgtgacaaattttaaagcctgaaaaagtttaaaacttaaaatttctagttacacatttactgctagatactttaaattcttaaatatagtacatcactac

aacagattttgattctaaccaaacaactcaactttatctagtgttacaagcagtgagacctcttcctgtaagtgccacaattttaagacattttgcttca

accaataagtcatttcaaaaataataaaaatagacttatatcaactatgctacagtgcattagttgttaaaaacagtgtgaaattaaaatgcaagttact

gtgcattaatgactgacagatttttcttttctagtgcaaatatgttatgaaactttactaatccacagatctttgcaggaagaagactgcaggccacaaa

tatcaagctgctaggaggaaaaaggttcagacattaaccaaagacaaaattctggcttcccttaggcaaaatgtggtcttccaatatttttgaatgtctc

acatgacttttgtcccgtgtgtgtgtttgtgtgtgtgtgtgtgtgtgtgtgtgtgtgtgtgtgtgtgaagtagttgacagacaacatgtaacagctaagt

actactgttctgggaacaaaaagaacattcccaacccagctatcaactttaaacttgtataaggtgaacttgcctattaatctatgacaatacttcatca

caacatacaaataaattagattgtcatgacttattcaagcaaataacaaaaaatctgaacatttgcacatataaaaagtttatgaaattctataaacaat

gatttaaaaatcttacagcagaagtgagaagtaattataaatgattcaatgctacctccagatctaacaaataagacaacatgccaaaaaacaggcaggc

tgagagattcaaacttcttgcacagtataagtttcataaaaatattctgtataaatgtaccatgaagtatgtaagcgcattatggtcctttatgttaatt

gtgtgcattgagaaagaagttgttttatatctcatttaacaaataaaatagctgtagaggaaaaaatttaagctttgtatctgtcctctgaacgaacagc

acagcacagtatgagtgagaatataagacccagaacttcaactgtggcaagtgccaggccaacacccatcaaaatatttgcatactgctgaaggagttca

atcagtttgtcgacacagccctcagaataaatttgtctgtttatggcagcagttaataccactgtgcgcccttcttcacattccttttttgtaattccat

caatgcagcagctagcaggaatgttgtaaacattgacagccttcttaattgtcaattccactatatttttgtcacgcatattgaatttactattagccca

atcacctggaccagatgccccgcagcatttcaatccagactgtatagcatcaaatgtagctgtgcgtgaatcagctcctgatgttccataatcagcttgt

acggttgcagtaacactctgactgactagttcctttaagcggtcactattgctataagcccatgccccagcagcaatttccgcaaccaatattatgagaa

ggaaacagaagaacgatacaagcatacaaggagactccctgtatgctccacaacagcccaaaaatgcaactataaatattagtgcaccagctgcaagtag

aatgtataagcctgtataataatttccatcatcttcagttatgctcaggtatgatgtcttgtcagtgagcagtaaaatagtaaaaaccaaaactgtcagt

cccaccaaccagaatacgaagttaaagataaacaaaaggaacttcaaaacggcgtaacatcccgttaacgccatttctgaatgttatgcgtggactgcct

acacaaaacacaaccacaaaattgtctcctttaactgttctaccaaacagtaaccctctgcatcaattctgataactacgcccccacttcagatctctgc

gtttcgcacacctctgttcacagttcctatttaggaattgcctatactacattcgtaggaactgt

>78365|AF181629_1BcDNA.GH04637

catatgcccaggaagtgacgtgtgtttatctatgcagcgtgtgcagtaaacatcgccacatctccaacaatggtactttctgctaaaatgatcaaatgga

gttgaacacacacattcccttatattccgcagtgtcttccagtccagagcaactgaatttaattcttctgctaactgatcaactgtgagaacttctttac

ttttagcagtattgggaactacgcatagagagagagaaaacacaaccatagctaaaatacaccat

>78364|AF181629_1BcDNA.GH04637

tgggaattgtgaaaaggacaggttggatctgaggctgagtgagagaacatattctctttggggctatatggcaaatcatatgaacgagtacattaatcca

ctctactgttcagaagggacatctgatatgctttctccaaatttggcaccacaaaatatacggttttggagaggaatgtactgtcgctttgaaagtggtg

tccacccacgtgaaccattgggtgatttgttgttggccacttgtgatcatagtgcttctcttgaggatcacataaaacagcttaacaagaggatcaactc

attgaaagaccagttggcagaaatgaaagtaagaaatgtgagaactgtacctgatgggagtgatgttcttgacaacaaatacctctatgaaaattgtgga

ggattggaacctactaaggagagtacagagaaacttgctattaacaatgttcccaatactgctaaaagtaaagaagttctcacagttgatcagttagcag

aagaattaaattcagttgctctggactggaagacactgcggaatataagggaatgtgtgtgttcaactccatttgatcattttagcagaaagtaccattg

ttggagatgtggcgatgtttactgcacacgctgcatagataaacacacgtcacttcctgggcatatgtcacaacgtgctgtacctgtttgcaagccttgt

taccgtctgctgcgtcgttcctcatctgtagagtcaccatgatgcggtgaccctgttacaagggcatcatgtgaccacagtccacctgacgatgctgctt

atgctccatgcctgggactgccagtccctgcttccttgctggcagcaattctcagtgctcagcactcagccagcctcttctagtgtgtgaactgcaaatc

ggatttgctcatcatcgtttcgtggatagtgatatgactctgctattctttgaatgaacaaagattagctcaggcagacctgatacttctgcttgtgtta

acagtgtaatgaagattgagactacaattatatgattcccagtatgacattaatgttgcagattgtgatcaggtctgtttagttcgtattgcaactataa

aggtaaagttatgatgatttataaatggatacaaaagacttgtgcaggtttgttaatgagagaaccttaactgttccaaagtcctgagtttacatgattt

atagctgtctgcatattctttttataggtgctctcaaaaaaacatttcagcattgatcagacattatgctttgtttatgttaatcacaccatgttgcctt

gcaaataaagtgtaataattgattattgcttttagattacagatgtacagattaaaaaagaggaaaatatggtaggaaaaccaattaatcatgtggaata

tatcaaaatgaattatttgtatagtgtataatattatcttcaggaaggaaaaccatcagcctctccagtgtttgtttgatgagcggaactatggaatgct

tctagtgtcagtgactgatatgccaatgaatggattgctattctagccatcctgtgatgcactctttctttttttgactgactgttttttccagtcttca

taagtgaattactagggttgtgtttaaacatttcgtgctattatgaagtggagtattgacagcaagattttgtaataatttctgagtgtctgaaactctg

ttggagaaaaagcaattccgatttctaacttcatattgcgttttattttttatgaatttttaaaacactgggttagattaatctgccatcatgtccttgc

tgtgatatttattgctgttgcttttaattatgagctctaaagatacatgatgaatgaattttacttgctattttattcaaaatttgtagaggtttacatt

ggacttcagaatgaaatgactgaatcagccatgtataatgttcacaaatatatttattgaccttagttttgttatctgagaataatgtgaaaataaatat

agttagtgggtcac

>78385|AF199369_1unknown

ccgccaaacatggtgtctgtttctgggtgaaatctgtgatattcatactgaatatgaagattctctggcagctccacctttttcaatgtatcaaatggaa

tagtcacatctacgggtgtttcatagaaatttattacatcatcaagtgactctattgtatgcaagagtgaatttggcactgcgtgttggaattcttggta

gcattttgccaacaagctgaacttaaattctgaatctgctattggattgctgtctttcaagatttgtttcttatcttgccatactgacatacagatatcg

tcaatttttttcttaatgtcaccttctgggatgtactgtttctgtggccgcacgaacctaaagataaagtcattcgatagacatcatcaaactgctctat

ctgtaacgagatggtaaatattcatcgtgtgtcaatgagtaccctcgtgaagcaagagattcccctgtcgattcaattgttctagctctaggcccctctt

tcttcttatttttggtggccagaagctttttgctagctgtaaacatacacatgacatttccaaagctatatttaatttcgtcccctttcctcagcccatg

cctcgcgagcgctgccatgatacagttacatgtataccatgtgaccataacaagccaatagaaatcacagagtctctgcgcttgttgtttcccatccgcc

attt

>78384|AF199369_1unknown

gactgtaaaatggcggatgggaaacaacaagcgcagagactctgtgatttctattggcttgttatggtcacatggtatacatgtaactgtatcatggcag

cgctcgcgaggcatgggctgaggaaaggggacgaaattaaatatagctttggaaatgtcatgtgtatgtttacagctagcaaaaagcttctggccaccaa

aaataagaagaaagaggggcctagagctagaacaattgaatcgacaggggaatctcttgcttcacgagggttcgtgcggccacagaaacagtacatccca

gaaggtgacattaagaaaaaaattgacgatatctgtatgtcagtatggcaagataagaaacaaatcttgaaagacagcaatccaatagcagattcagaat

ttaagttcagcttgttggcaaaatgctaccaagaattccaacacgcagtgccaaattcactcttgcatacaatagagtcacttgatgatgtaataaattt

ctatgaaacacctgtagatgtgactattccatttgatacattgaaaaaggtggagctgccagagaatcttcatattcagtatgaatatcacagatttcac

ccagaaacagacaccatgtttggcggtgtaagcgcatttccattaagttcaactatagttactgggctcagatataagaagaaatacaaaggtcacattg

ctaaaccaaagtggatgtgattattggatgaaatatgactttttttttagtaatgagcattaaatagtaaaactgcattattggtcaaactgccaaggga

tgcaataaattattgtttgtaaatattttatactgttttgtgatgtgtattaacatttggagatattggggtaagggggcatctgtaattacgtgaggca

atttcggcattttttcaattccacatccccaccttgtgagatttggatcggaccctcccatcctcctccccccacccctacagcctcatgtgagattttt

cagaatacatatttttagtgtaaatatgttaatctgtgcttaattgcattggatttatttaaagaacataatttgcataattatttttatctcagactag

ttaaggctagtatttaagacacaatgtagactagaatcaaagtgaggaaataaaacaataaaatgcttatgtaaaaggaactacaaaaattcattaaaca

atgttcatttcacttcagtccaaggcgatacatgtggtcttgagaatgactactggaaccaacatgtccatgtaattttcaataaaatcatctgctcctg

>78404|AF212322_1SP71

atcggcctggaatgtttggtggagctggtggcacaggtggtggcagtggtggtggtggtgctacagctgagcttgataagcactctttttctgcataaag

ggcaccactctgaggctggaatgcaccaatatctatagtggaacccaatgacccactcagcaaacaatgattgttgtttatacctgtctgtcccaagaaa

gtataagatcgacacacaaattccctttccacatcacatgcttgctcacagcttttgtcgctgaaagaggacagtgatttatctgtgtatatgagctgtt

gtcctgatttgtggtatgtgtaggcacagtgtgtgagacgtggtgcacattgattctccaaataatcagagtcaccattcactatgaatgcttgtggctg

agtataacgatcttcacaacttagtcggcattctttccttagataattatatgtgattgatctgcacacaaatgatctctcttctagacatctttct

>78481|AF221110_1SCYLLA

gcttaattaaaagtataagtaatacattcgaatgtgaaaacactactctgctgaaaatctgaggggtgaggagtcgacttattcactcttcattttgttg

taaaataatcacaccaagatgtcagaagatttttaaaatattacctattgattttgttaaaaattgtagaaacgacagcatgtaacatcttaagttgtgt

ctagttttgagctaattctatagttcttctagaagacgaccgttcggtcgagtcgagttgcagaacaataaaactaacgtttttaacagctcggtgtggc

tattttacaacaaaatgtcttttatgcaagctctaccgaacaaagaattgttttttttttttctatgcaatctgccgaaagcgaagctgaatggtactca

cttgaggatgtgtggcagggcgactttccacgaggtctcctgtcgcagggtgacgaacacttcgaacgtggagacggtgtcgggcgcacacttgaatgtg

gcgagccgcagccgctgctggctttggtctgccccggcctgcttgcccgtctccaggtccacgtgcagccggcagccccggatgccgt

>78602|AF295693_1polyprotein

gcatgttaaaggatgcaggattacaaaaatgttactgggtagaaacagtatcacttgcagtgtatctaaataatagattgcttactaaaattgttccaaa

taagactccatatgaagttttgactggaaaagaacctaatctagatcatctatgaatgtctgcttgtaaggtg

>78601|AF295693_1polyprotein

atttgagatttttgcatcccactttcctcttaatggctttggaatctgaaccatggcttcacatccaaagacttttaagtgctttagatccggtttcttc

ccactctatacttcataaaggtgtcttgtgtcttacagctttggtaggtgatctgataaatacactgctgttaacacagcctctgcccaaaaacacttag

gtagttcagcatcgcttaacatacttctaaccttttctacaatggttctgttggctcgttctgcaacttcattctgagaaggactgtagtgaatggtagt

ctgatgcctaataccgtctgtttacatattctgatccattgtctgatctaatgattttaattttcttcccagtctgtctattgaccat

>78629|AF387850_1integrase

ctcctttctattttcaaagaatactacatctcttcttgtaactatccttttactcaatggatccattaatcagtagccttttgaattctcacaatagcct

ataaaaatatactttacagctttcgggtcctattttcctcttagctgttttggaacgtgt

>85246|AF391290_4unknown

caacttcgagtttgaagagtttagttttgcatggtgtttggtgagtcgtgtgtgggagtgtttgacgtttaacataagattcttacattgtatagtgctg

tacacaagagccaggctgtaggttacttacaaagcaaaactatgatccgaagggctccaacacgtattgagctgaagctcgaagacttacaagaatttga

agctgcgcgacgggagatggaagttcgacgccaggagcaaggtgcctcctcgtcgtcttcagtggatgctaccacaccacttggctccagcccacctggt

ggactttgggcaccggccgcacctaatgccaaaaggacataccgaacgacgagggttgtcggcttcctgccacagccgcactttacataatactgacaaa

ttgtgatgtttggatctagttccactgttgagccttttcacttcatcacaagtttaacatcagataactctttggaagagctctgtatcatatgtaaatg

tttccatatatccagtacaccacaatgttactaatctttaaataaaatttctatattgtgattattgtattgttcagcaaaatacagcagttttagagtg

ttgtttgtgcttgtcaccctgtcagaatgtc

>78681|AF444782_1Toll9

aggtaatgcattttcagtagttacgttgatgggagaggaggtgcgtggagcaacggctttgttcatcggcagataaattaagtggaagggataggttctg

gctggaatgtgcttgtggaaatatcatttgtttttttgactttcgtgctgaatactgtctgtctgcggctgtgatgtgactgcaaggttgtgaccacaac

cgctgcagtccagatggctctggcagtgaggtgctgtcagcagcggctgacgtcagagcaggtgaagcagctggcccccagcgccctcttgagtcggcgg

aacgcctgctcctcgtactggacgtcgcccgtctggtgccactccacgtaagtgcgggtatccatgaggaagcgcaggtgccgcggcagcgccgaacgtt

ccagcggttccagctccagcaggatgatgaactcgcgctgcgacgggtccagcgtacggtggttcgccaagtccatctcccacaggcaccaccggctgga

caggaagctcttcgagaggacgacgatggtgtgccgactgcggtccatggagctggagatattctggacaatgacggtgcccaactggaagtctcgctca

tgaaggcagatgcgatagcgctgtggtccgttctccagcagtggaaccagcgtgcctaccacccaagggcggtcgctgtagaggatgaacgcgtc

>78682|AF444782_1Toll9

ccgggccccagtagaggaggcgcggccggtgctcgccctggctctgggtctggcggccttggccgcgctggcgctggccgccgcggcgctctgccaccgc

taccgcttcgagctggcctacagcgcgcacctgctccggctgcgcctgcgcgctcgggccacagccggaactggtggaggcagtgctggcgccgccttcc

tctacgatgctttcgtcttgtacagtggaggtgaccggcagtgggtggttgagacgctggtgccgctgttggagaaaggaccgcagcgctaccgcctctg

cctgcacgagcgagacttccagctgggcaccgtcatcgtgcagaacatctccagctccatggaccgcagccggcacaccatcgtcgttctctcagacaac

ttcgtcagcagcaagtggtgcatgtgggagatggacctggcgcaccaccgcacgctgaacccgtcgcagcgcgagttcatcatcctgctggaactggagc

cgctggaacggtccgcgctgccgcggcacctacgcttcttgatggacacccgctcgtgcgtacactggcaccaggcagccgacagcgaccaccaccagca

ggcactgcgctctctgaagcgggccctggggccaggcctcttcccccgtactgagaccactggctgctgaccacgaggggcctccacctccaacgaatcc

caaccgtcttactgcgcttcaccaaattgtccttatacggggtgaacatt

>78680|AF444782_1Toll9

tttggtattaagtaaaagagcaatttatttctgttcaaaatatctttcttagcgttcgcaatcaatattttattttctagaacgcatgttatatttcgtt

gaacagaacactaggtcgcatatgtgcacttgcttgcctgatataatgaattctatcattccacaccattcctctgaacgcttctaacatacttatcatt

tacactacgtttataggtgctaatgttctcgttttctgcgcccaaaaaagaattcatcatcaaatgcaaacacgagattttcatgcatcgtcgcatctat

tcgtggcaaggtaattaagagcaatgtttgtactaatgtcgtggagtcataaaactgactactaccacaggcatgtaaatcagaggaacggtccagatgt

ttcgaatccggacccatatcgtaatcagtatgaaccacacagaatggagtgctattctgaaaacacgcaaacccactcaatttcaaaggtacagtacgtc

cgttgcaaagtagcatatagatgcgactgttccttggccgaagtcacacagtgaaggcagtggcgtccaccatccgtagctgctgctgctcgttgtcagg

agtgtggtcgtcgacgtctgcaccgtcagtgtcagtgtcagcagtgggagatcccgccggcgcggtgaggacggctcgcagcctcctccacatggagtgc

tgggcggatggctgcgacggccacgtcaggtaggtgcgtgtgtccatcaggaaacgcacgtgtcgctccagctggcgccgctccagccgctccagctcca

acagcaccaagtggtgcaccttctgtgacgccaggtccagctcccagcggcaccaccggctgtcgatgaaagcttgagacaggacaatgactgttgagcg

gctggttttcatgcaggtgacaatgttgtcagcaatgtaggatcccagcgggaagtccctctcgtgcagacagacacgcagcgccacttcatccggggaa

tcccccgcattctccaggtgcggtacaagttggtccacaacccactcccggtccgcactgctgtagcagacgaaagcgtcgtatttgtagctgcctgaat

tacgcctctcggcagccactctctcacgtcgcatctccatcaggtgcatcatgtaagttatctcatacctgcggcagtagcagacagcacagacgacggc

gaccacaaggacc

>78679|AF444782_1Toll9

gacgcgttcatcctctacagcgaccgcccttgggtggtaggcacgctggttccactgctggagaacggaccacagcgctatcgcatctgccttcatgagc

gagacttccagttgggcaccgtcattgtccagaatatctccagctccatggaccgcagtcggcacaccatcgtcgtcctctcgaagagcttcctgtccag

ccggtggtgcctgtgggagatggacttggcgaaccaccgtacgctggacccgtcgcagcgcgagttcatcatcctgctggagctggaaccgctggaacgt

tcggcgctgccgcggcacctgcggttcctcatggacacccgtacctacctggagtggcaccaggcgggcgacgtccagtacgaggagcaggcactccgcc

gactcaagagggcgctgggagccagctgcttcacctgctctgacgtcagccgctgctgacagcatgtgtgccaggggtcacctcactgccagagccatct

ggactgcagcggttgtggtcacaatcaaacttgcagtcagatcagagccgcagacagacagtattcagcacccacgtcaaataccaaataatgtctgcag

tacaccagtgcctttcaacatccctatagccactatacctcctaaactaaatcaccactcgatgaacatgtgtgctaggtatcgtcgcacaagccataga

aaaaggaatgttgatctgtcaggcatgaaacacatctgttgtctttttgcaaagataacattctccaacagcgcaggtactttgacaggcagagatagct

gttcattttgtccagttcactgaatggccatgagagtttgccaccaacgatttctgcctactaaataaaacgcaatgccttgcttatgtgttggcaccag

gaattgcacctgcccatacatatagctactatggcgatttattatcccattctgcgtgaatcccgccccctcgcaaagtgaaaccaatgtgcgaactgtt

catctttaacattcattaagtggtgagcgagttgctgagttcgggaagagaataagatactaacattacctactggaaagatatggattgaggttatccc

caagagaaaataaaagcattgaagcattatgttgtgtgaattagaataatctcattactacaatcattgttatttctattatagtttttacaaaactact

aatctgtgataatgcttcatgttcataatgtattgcttaaaatgtaattttcacttgccttctgaaataaatcttttta

>78735|AF465602_1PKG-II

ttgtagagtatggcgagctcaccgaacactttgcccgccgcgagcgtcgagaggtacttgttctcacgcgacacctccacgcggccctcctccatgacgt

agacgatgctgcccacgtcgccttccttgatgatgaggctgccagccgcatactccacggggtacatgcagtccacgatctcgcggatctgcgccatctc

caggttcttcatgaagtcgttgtccaggatagcagccttgatcagttcccgcgatctgtcatccttgggcacggtggggaaatcggcgatggcagagggc

gactgcggctccgcagagataccctgcgccctctgcgtcttgcgcaggacgccgacgggcccgtggtggcggccgccgcgcagactcagcacgctctgga

acttgtcgaggtgggagcgcagcgacgcgatctcctggtcgcgcgcgcgcagctcggcctcgagggcgcggatgcgctcgtcgcgggcggcgatctgcgc

gcgcgcctcctccatctccagctgctgcgtctgaggggcggccgacgacgaggcccccgcgccggagccgcgcgaagcgaagcacagactttgcaagcac

acacgcatcggcggagctgtactcgcgccgacgaac

>78752|AF492763_1polyprotein

gtgcctcacgtcaatgtgttttgttcgagcgtgatgcactccattgtgactcagtgcaatggcacctctcttatcacaggatacacttactggtttttca

ctatgcgtaacacacaattcacgaagcaatccttgaagttataccgcttcttgtgctgctgctgatagagcca

>78753|AF492763_1polyprotein

tacgtactccacagtaatttgtaccatttctattttttcccttatgaagtggagtcttgtatctaaatgtttggtttgagtcttatagctgcttttctta

gacaggtcaattgctgctttgttgtcacatagttgtttcacagggtctttttttggggtttggtgatatttcattgcgtagcctttgaagccatagagcc

tcataacataacgag

>78756|AF492763_1polyprotein

ttctctttgagtttctttttgaagttgttcttctcttgtgggttattactgaaaattaaaatgttgtctatataaattgcaatagttaatggtatttcca

ttacccatctgataatcactttgatcagctgtacttcccataaaattcaagtttaagagtgcttcattcagcttaatattccagtcgtgattgtcttgtt

tcagtccataaatagccttctgaagccttgtgagacatttgtctgcaacagttcttgtatgaaaataaatctctgagattttgacacaaatttcctcttt

caaatcaccttgtagaagtgctttaacaatgtctatgtgctctatgtctacatcatatttagtagctaagccacacactacatgtcattatttatgtgga

taattcatgaatccatgtaactggtgaccccattaagagtttcccatacctttcggtgaacggcataagtatcgagcagcagagatacgcacagagtaat

gtgccatgacagctagctattccatgccttttgtaatgaaattagcaggctccagattgaagtgaaggctttggagcttgacattctgataaatatggac

atgaaatctgtccaggacaatgaagaccatgttaattggacaacacatatgcattgcaacatggtggataccctcggtgtgtcacag

>78754|AF492763_1polyprotein

tataaattgcaatagttaatggtatttccattacccatctgataatcactttgatcagctgtacttcccataaaattcaagtttaagagtgcttcattca

gcttaatattccagtcgtgattgtcttgtttcagtccataaatagccttctgaagccttgtgagacatttgtctgcaacagttcttgtatgaaaataaat

ctctgagattttgacacaaatttcctctttcaaatcaccttgtagaagtgctttaacaatgtctatgtgctctatgtctacatcatatttagtagctaag

ccacacactacatgtcattatttatgtggataattcatgaatccatgtaactggtgaccccattaagagtttcccatacctttcggtgaacggcataagt

atcgagcagcagagatacgcacagagtaatgtgccatgacagctagctattccatgccttttgtaatgaaattagcaggctccagattgaagtgaaggct

ttggagcttgacattctgataaatatggacatgaaatctgtccaggacaatgaagaccatgttaattggacaacacatatgcattgcaacatggtggata

ctctcggtgtgtcacagaagagtgttaagcaagattgaaagtatctgcaggctcagggaaaacatcctctgtcacagcttaacagtcaactcagcag

>78757|AF492763_1polyprotein

tactaccttctcaatacaaccaactgcacctatgtcataaaatccatacctgcttatcactgtacagaaactaaattgcttcaagaagaaatgatagcat

gtgcagtgaattaaacaattgctgctgtgttttgtagccatcaatgaatttacatgccagccaccagaagttctctttgttgggcatcggcagttgtttt

gtttagacatgctccatataagtcatggaagtacacaatgagaacttgtttgcttatttgatagatatcatgcttaacattaatttcaagttcttccttt

cttcaacatatatgtaccaataaatgtttgttgttctattggatattggctccaattagtcatttgagagattataaagtcaacttgggcaaataaaatt

atgtatgctgtattcccaggaacatatatattatcaaacactatttattcagtgatatatttgtaaaatagaccaaagatgaataaacaagaaattataa

gtactatacaagctagcagtaaagatgaagcatatttttcttaacagcctcacaagttcttcagagcttaattcatgaaatattatcacagaacacgttt

attggtttttcactatgtataacacacagttcatggagcactccttggaggtatattacttcttgcactgatgttgatagggccatgccctcagattctg

tagttcatacaggaactgtttgttgccatcttgggttccaggaaactgctgccccagttaacttggaaatgcagccagttactgatcattgttgatcgag

atctgatgtgtaattggcatcacagaagtcttcaattttgtttgttcctgtttttaagaatgttagttctatgtttgactgcttgtcagtgaatttttag

gtaattgaggttgatgaactcccacaacataaacaatgtctggccttgcaccttgag

>78781|AF506020_1polyubiquitin

gtggtatgcaaatttttgtaaaaacttttactggaaaagcaataaccctagaactggacccatcacataccattccaaatttaaa

>78786|AF506020_1polyubiquitin

aatcgtgtctgatgcttctactacgagtgtaattgttttaccagtgagagtctcaacaaaaatttgcatgccaccacgcagcctcag

>78791|AF506020_1polyubiquitin

cagtcagaaggaatcgactcggcatttggtgcggagattgcgggggggcatgcagatttttgtgaaaacccggacgggcaaaacaattacattggaag

>78780|AF506020_1polyubiquitin

tcttcacttagtactaagactgaggggaggcatgcagatcttcgtgaagacgctgactggcaagacaactgaggagaatcctaaatcaatcaaatctggt

g

>78783|AF506020_1polyubiquitin

gtggtggtatgcaaatttttgtcaggtcatgaacaggtaagacaataacattggaaaaggagccatcagatactatagaaaacgtgaaggccaagatcca

agacaaagaag

>78796|AF506020_1polyubiquitin

aggtaatagcgccaaatactagaagaacgtcctggattataacatacaaaaagaatcaactttgcatctggtgttgcgtttgcgtgatggtatgcaaatt

tttgtaaaaactcttactggcaaaaca

>78789|AF506020_1polyubiquitin

cctgttaatgtcctgacaaaaatttgcataccaccacgcaatctgagcaccaagggcaatgttgattctttctggatgttgtaatctgagagggtacgac

atccagaaagaatcaacattgcacttggtgctcagattgcgtggtggtatgc

>78779|AF506020_1polyubiquitin

cttgcacctgtcaccgcacatacagctcttgtatgaataccttcaactggtagacacttacagtcctgagtgagatcaacattagtaagtgaatcagcaa

tgtgctgaggctacgaggtggtatgcaaatttttgtcaaaactctgagtggtaagactattacattggaggtagaggcatca

>78817|AF506021_1polyubiquitin

gtatgcaaatttttgtcaggacattaacaggtaagacaataacagtggaattggagccatcagatcctattgaaaacttgaatgccaag

>78814|AF506021_1polyubiquitin

caatgtcctgccatcttccaagtgtttcccagcgaaaatcagtcgctgctggtcaggaggaatcccttctttgtcttgccctccgtgtcgttcaacctca

g

>78815|AF506021_1polyubiquitin

caactgcttaccagcaaaaatcagacgttgctggtctggtggcataccttctttgtcctgaatctttgctttcacattttcaatatgttgtattacagtg

taacagcttct

>78822|AF506021_1polyubiquitin

cttgccagcaaatattagtctttgctgatctgggggaattccttctttatgctggattttggctttaacattttcgattgtatcagaagtctatcacctt

agaagtagaagcttcggatacaatcgaaaa

>78816|AF506021_1polyubiquitin

tgttgtaatctgagagggtacgaccatcttctagttgtttccctgcaaggatgagtctctgttgatctggtgggatcccttctttatcctgaattcttgc

ctttacattttcaatggtatctgatggctccacttctagagtta

>78823|AF506021_1polyubiquitin

gcagaaggaactacacccagctatgcctcagcatttcattacatttgataacaaaagcattagtgctactttctatagttgacaatgtagctagtaagac

acagaatccaggataaagaaggaattcccccagatcagcaaagactaatatttgctggcaagcagt

>78844|AF506023_1polyubiquitin

tctgacaccatagagaatgttaaagctaagatccaagataaggaagccatccccccagaccagcagcgacttatcttcgcaggcaaacaatta

>78894|AGAP000010-PA

tgtgaaatgtaattggtttcaggtgtttactgatggtattaccaacaaactggtggggtgcttctatagtgactgtcctgacgatgtggttcttgtaaga

gtatatggccagaaaacagatctcctaattgacagacatgcagaaacacggaatattcaggtaacgggaactgctgttctgaaactgtagtccgaattat

ccatttaatgttctgg

>78895|AGAP000010-PA

ggcacctaggtactcctggaccacaatcaagacgatgcatgttcgccatcatacgtgccaccaagggaaaaacagatatatgatgacaagtgtccactgt

aaggacatcccccgacacatactcatatgccagaccattatcaaatgtggcatacaattgcggggcatagcctgcagcatgaagtagctgtaggcacaag

cacgagccacctgtttaatcaacccagtcactgtacgtttttgtttatcactaagagcatggtctaattaattttgtttaattgtcatttagtagcaaat

aaatagagtccctattgccacaagcaagcaataatgtgtataaaataatgtattctgtacactaataaggtcaaagaagacacttcacgaggcagaaaat

aatgtctata

>78898|AGAP000010-PA

cacagtagcagtttacgacgagtcttctgttttcttatgaccgggaactgaaagtgtgcagaaatgaaatctgccctaattctatattttatgttgcaac

ttcgaacgaagaggagtggtggttattgggggtcgtgctgcagtatataagctgcgatcggcgcaagagccccaccagttacactaagttgccggtgcgt

gaagggacagctcgagatgtccttcgaacaaaattccctggtaatccccatcacgattgacgaggatcatctcatcgatggagcattctccgtgctgcaa

catattcgttcatcatggagcagagacaaaacaaagttcaaggtgtttactgatggtattaccaacaaactggtggggtgcttctatagtgactgtcctg

acgatgtggttcttgtaagagtatatggccagaaaacagatctcctaattgacagacatgcagaaacacggaatattcagctacttcatgctgcaggcta

tgccccgcaattgtatgccacatttgataatggtctggcatatgagtatgtgtcgggggatgtccttacagtggacacttgtcatcatatatctgttttt

cccttggtggcacgtatgatggcgaacatgcatcgtcttgattgtggtccaggagtacctaggtgccctgccttatggtctaaggtgcggcagttcatgg

acatcatgccaacaaagtttgaagatcctgcaaagcaaactag

>78897|AGAP000010-PA

ggcacctaggtactcctggaccacaatcaagacgatgcatgttcgccatcatacgtgccaccaagggaaaaacagatatatgatgacaagtgtccactgt

aaggacatcccccgacacatactcatatgccagaccattatcaaatgtggcatacaattgcggggcatagcctgcagcatgaagtagctgaatattccgt

gtttctgcatgtctgtcaattaggagatctgttttctggccatatactcttacaagaaccacatcgtcaggacagtcactatagaagcaccccaccagtt

tgttggtaataccatcagtaaacaccttgaactttgttttgtctctgctccatgatgaacgaatatgttgcagcacggagaatgctccatcgatgagatg

atcctcgtcaatcgtgatggggattaccagggaattttgttcgaaggacatctcgagctgtcccttcacgcaccggcaacttagtgtaactggtggggct

cttgcgccgatcgcagcttatatactgcagcacgacccccaataaccaccactcctcttcgttcgaagttgcaacataaaatatagaattagggcagatt

tcatttctgcacactttcagttcccggtcataagaaaacagaagactcgtcgtaaactgctactgtgaaattaaacactttcaacattatgtgaaatcaa

acgctgaattacatttaataagatctataatttcaagcgtttcattctcacacagagataaacacacgaacgttctagatacgccactatacgtcaaaaa

ttctctctcgatactaaaacagctgaaccatcaacccggcagtttcaccgtattgacttgacaagcggttgaag

>78896|AGAP000010-PA

ccggtgcagggatcctcagtagctcacggatgctggtagtgtgacgtgacttttgttgcgctgatgggttcgcaaaacaacgcgctccgtttgccgctga

ccatccaagaggataaccttcctggtggctgcgcggctgtgcttagacacatcaagccgcactggaacgaagaaaaaaccaaattcaaggtgtttactga

tggtattaccaacaaactggtggggtgcttctatagtgactgtcctgacgatgtggttcttgtaagagtatatggccagaaaacagatctcctaattgac

agacatgcagaaacacggaatattcagctacttcatgctgcaggctatgccccgcaattgtatgccacatttgataatggtctggcatatgagtatgtgt

cgggggatgtccttacagtggacacttgtcatcatatatctgtttttcccttggtggcacgtatgatggcgaacatgcatcgtcttgattgtggtccagg

agtacctaggtgccctgccttatggtctaaggtgcggcagttcatggacatcatgccaacaaagtttgaagatcctgcaaagcaaactagatttgaagag

ctcattcctccaaagaagaaattggaagaagaatttgaaaatttaaagttacagttgacacgtctgaatagtccaattgtgttttgtcacaatgatttat

tgcttggaaatgtcatctataatgctgaaaagaactctgttaccttcatagattatgagtattccagctacaactatcaagccttcgatattggaaacca

ttttgcagaatttgcaggagtgtcagatgtggactttggccgatatccaagtgaggacttccagcgtcagtggattagtgtgtatctggaagcatatgaa

gatcgtgagcctacaaatgatgaagttgagcagctgcttattcatgtcaataaatttgctcttgcagcacatttcttatgggctgtatggtcccttatac

aggctgagcattcctctattgattttgactttcttggatattcctcaataaagttgaaagaatattttggcaagaaagaaaagttcctgaacatggagtt

tcctaattcatgataactgtgaattacttgcacaggtgtgcagctatggttgtttcacttatagaagtcagaagatacaacaacaaaaaaaactctaaac

actgggggcaatatttttttaatacttcatcttaacatgtgataactggtgtgatgatgaataaaggacattctattgagagcattctctctctctctct

ctctctctcactcactcactcactcacacacacacacacacacacacacacacacacatacacactgtgaaatgtaagtaaagcctgttatcactgcttg

gtgcctgtgcatgtttgcaaagaaattagagaaagagagaggag

>78917|AGAP000011-PA

ctgtagtgttcatcagacagtatagcgtcgcacgagggaaagcgctcgcaaagtgaatgcgtgttagtaagctacggagcgcgcgtctgtttagaactta

gtgtaaacgcaataactgtggaccagctgacttaaaagaatatgaatgtgatacagaggtgacagtgcgttttaaacacttattatgttccaaagacgta

aagagtattaccaagacaaactgttcgttgtgccgaggcagccaattaacaacattccgtcttcgttttgttgatagtgttaaagtgatacttgtgtaaa

cgggattgaaatggcacggcttacacatttttcgcagctattgttactacgtaagtttctttccagaaatctcatccacagccaggtgaaatcagttcgg

aggttgtctttaaaagtgcatcatactttcaagttttcagacttgtctgtgaggttggcatcacctgatgaactgcattctaaaccagatgttagtgaac

ttcagtttggcaaacattttactgatcatatgctgaaggttgcataccatgcatcattgggtggatggcagaaaccagaaattacacctatggagaatct

cgtattacatccagctgctaaagttttgcattatgccatcgaggtctttgaaggtatgaaggcatatcgtggagttgatgataaaattagattattcaga

ccagatctcaacatgcgtcgtatgaatggctcagcagctcgcacaagtttaccaacatttgatggtgatgaattgatcagatgtttgtgtcgtctgattc

ttatagaccaagagtgggttccacactctgaggcatcaagtttatacatccgtccaacaattataggcattgatcctacgctgggagttgccacttcaac

atctgcacttttgtatgccatcatgtgcccagtgggtagctattttggtcctgacagtaaagcagtttcattactggctgatcctaaatacactcgtgct

tggccaggtggtgttggtgataagaaagtaggatccaattatggtcccacactgcaagttcagtgttgttacttttccaggcagaggcagcatctcgtgg

tatgcagcaggttctgtggttgtatggggaagaagattatgtaacagaagtaggaactatgaatgtgtttatacttcgcttgaatgaaaatggagaaaag

gaacttgtaactccaccactagatggtcttatactgcccggtgtcacacggtactccattatagacttgtgtaagcagtggccggagtttaaagtacttg

aggaaagattcactatgggtgacctgattaagcttctcagtgaaaacagattgttggaaatgttcggtgccggaactgcgtgtgtcgttagtccgatagg

ctcaattctttacaaagatgtgttgcacaaaattccaactgttgaatgggaagaccctctgtatgctcgtttacttaaaactctactggacattcagtat

ggaagagtagaccatccttggggtgtttgtattgatcaatactgctgatagtggccattgccagtatgatttactggtgtgtttgttttctaaagactta

cgaataacttataaaaaccactgtacaaatttctaatggtgtgtgcagcttttgcagtgtgaaaagactgactgtgttttgtaagaaagtgttgtaagtt

gtgtgttgaactctatctcagtgcttaggttttcagtttaattgtaatcacgtgagaaagtgtggctgaatgtagatggtatttttagggcttatttttg

tattgtgtgagaaatgcctcctgttgtgtttaacaacaaagtgttgtatcttggtctgtaaatttggagagacacttgggtgtttcctaagtaatgaaag

aaagtctgttgtggcagggagcttgtaagagtctatgttgtattttttatttttgtgtcattgctttgtatgttcagatgatcatgttatttatttattt

tgaacatattttaatgttactgctgtgaaaacaatgtagatgtaaagttttactaccttgaatatcacatagtgattgcatgttttgaataagacattgc

tatagatgcatgtagcatacataaagtaagaaaagaaaatgtgtatgtatggtgatgctgttattttggcctcttatgaagcgtgtgtagtagaataaca

ctgaatgaaggtatgtgaattacaatgaagccctttgagacatattgcagtgcatccaattaacatgtcttaattgcaccaaatggaaaggaaaaaggaa

ggaagaaaattcttccatcctttgacataatcataatattcatattgttcaggttaatatcaagtttccttatgttttcataagaagatgtaacgatcgt

tccattttattgttttatttcagtgagagcttatacaagctaattttgtccattggacagagtggaaaccatgtgacatttatttattttaccataaaat

ttaaaaatgttcaccatgagtacaacttgatcgaatctatagagactgaataactacttcaaaaaattgcttatcttctggccaaactagttgcttggga

tatgaattattttagtttatatctgtatgccagatgacaaggaaatgattttgaattactccattcatggtacaaaatgtatctcagtggtttttactgt

gtgctatgatttgaaggcacatgacaaagttcaagtttagtgtgttgtgctaatgcatagaatgcagggtatattatatgtacagtattttgtgtatatt

agggtacttgaaattagatacatccagacatttattaattttccttgaaacaaagcgtaacagtttacgattttttttcagcagatttgcagtagagaat

gcagagtcatttgtcattaggaaagtacagcaatcctgcaaatatagttcagtaacacattttgtgtagtatacggttttgcagtttcttctctaatacg

atacccagtttgttcctatgtgtgatctaaagagaggaaagaggacttttttttataaatttacacaatgagtgcttttaactcaacatgtgggctcaga

atagataattttaaatttagttacctatgtaccagtgcaatcaataattttcttaaaagcaactatacttttgcaagaaattctgttttttagtttacaa

agtgcaagtagtttattttagccaatgtccaactgaacaattttactataacagtactgcaagaaggtatttatgttgaaattaattttgctgttattac

tttccataacatacaaattcaggaattacagtgttacagaatagttttgtgtgttttagcattcaccatttataaaatggattgataaggatgtgccatt

ttagatagcttattttatttttgtattgtgttgtttcttcctgaatttcccacctgatttgtagaacatgtgcagatgctcagaagcaggtataaaataa

aatatggtgcattaatgtttttcatggtggctgtaggtatattttgttaaaaaccaaactcgtgctcataatgttatcagtactgtgtcatacatgcggt

ttttatgaagcatataagcaatctaatctcgcactgtgtgtgtgtgtgtgtgtgtgtgtgtgtgtgtgtgtgttttttcaggtaaaaacatgtgcttggt

tggtcttacataaaacactaaaactgtaatcattggcttctttgtaatgcaagaaaatgagatggttagtcgctcacttcagtctttttttgtgtttctt

tattccctctgttttcataaatgctagattatttaagaaaattcatttgtgctgttctgttactgttaaaatagatttactattttccaaagtgtattaa

actaccttcacatttgttagtagtacgtaaacttgttattataataaaaacattagaaatgaatgccaacaaaattaaatgattcctcaaaagaagagac

tgtttagttaaagcaaatttattcttctgtgttgtcagaaaaaactgtgttctcagggactgtcgacaatataactgtgataccttcctgctgacatatt

taatcactatcatgtcagcttccactattttgagaagatatttattttttgtaatatgcattcagagagttgctgaattgctctctttttatcatgagaa

ccatgactgtagagtaaaacagttgcagtacatgaagaatgactaaattatactacttatatttttgtaatagtattgttgtacatttttgattccacta

ctgttgataaaatacgctcaactg

>78945|AGAP000017-PA

cgggcgggcagtggtccagcagctccagcatgtgcttgatgttctgcgcgtcctggatcacgaagttgagctccatgtcgaactcgccgccgaccagcag

atgaagcacggtgtttacaacttctctgttagacacctggccaacttctattaagccgatgaggacggcgaacttcaagctgtcgttcatagccatccta

actacttcttcaggtcttttgatgtcactgaatggcggactctgaaggtcctcc

>79073|AGAP000026-PA

attatcttagaaatgaaacatcaaatcattgaaaaatatgagcttggtgtgaacattgctgatttagcaagcacatataatttatctacatcaactattt

gcaatgtcctcaagaacaaggacaa

>79090|AGAP000026-PA

cccaagagtgctttaatgtagagcttcttaaagttagaaataatacgctggtccacaggttggagtaacggagcggtgttgggaggcagaaattggatct

cgatgaactgaaattcttcaaggaggtggtctt

>79086|AGAP000026-PA

ctttaagaagctctacactaaagcactctttgaacattgctttgagctgactgaagctaccaatctctctctctctcggttttggaaatatcatttcaac

atggttccctgcatcaaaatgatcaaaaaggtgtgggaagg

>79029|AGAP000026-PA

cagtcaactaaagacagtgctcaaagagcactttagtgtagagtttcttaaaattagaaataatcggctggtccataggatggagtaatggagtggtgtt

gggtggcaggaattggatcttgatgaattggtcttgtagag

>79084|AGAP000026-PA

gtgttgcctcgcaattgctttccatttatccatacaagcagcaaccttcccacatcacccagaatacgaaactgttgtttagatactcttgtcactccct

ttgatgcatatattttcttaatcttatccttgttcttgaggagagtgcaaatagttg

>79051|AGAP000026-PA

cgtggagtttctaaatggtaaacaagaagctctttaattttcaaatcgccgcttgcattgtcacagaatagcagtgtgagacggcctttcattggcttgg

gactggcaatgtattctcctctgctattattgaggcacgcttcgacatcttttcccagaat

>79083|AGAP000026-PA

tctcttaaatctctcaaaccaccgacagcttacattaaacatttcttcagctgctgatgatcctggcatcttcttaatgaggtcagcaaaaatcagtctc

gccttctcacaaatgatgttctgtttaatagtgttgccttgtaattgtttctcatgtatccat

>79042|AGAP000026-PA

gaagctctatatcaaagcactctttgagtgtagacttgagttgactgaagtgaccaatctctctctcacacagttttggaaatatcatttcaacatcgtt

ccctgcatcaaaatgatcaaaaaggtgtgggaagggattaccaggagaaccctcatttctgttt

>79087|AGAP000026-PA

cgtaaggaccagcaaattatttccaagtttaagaagttctacactgaagcactctttgggcattgctttgagttgactgaagccaccaatctcactttca

gagggatttggaaatatcacttcaacatcattgcctacatcaagatgattgaaaaggcatgggatgggg

>79040|AGAP000026-PA

ctccagcctatggaccagcagattatttctaactttaagaagcgctacactaaagcaatcttcaagcattgctttgaattgactgaagctaccaatctca

ctctcagagaattttggaaatatcatttcaacattgttgcctgtgtcaagatgattgaaaaggaatgggaag

>79032|AGAP000026-PA

tcatcaagatccaatttctacgtcccaacaccattctgttactccagcctatggattagctgattacttctaactttaagaagctctacactaaggcatt

ctttgagcactgctttgcgttgactggtgctaccaatctcactctcatagagttttggaaatttcacttcaac

>79017|AGAP000026-PA

tgaatgaccttctcacactactattctgtgcaaatgcaagtggtgatttgaaaatgaagctgctgcatgtttatcattcagaaactccgaaagccttcaa

gaagtgtgaaatccagaagagcaggttaaatgtaatgtggagatccaagaacaaggcttcagtgatatgtgacctttttttgt

>79071|AGAP000026-PA

ggcatcttttccagaatagacccatctcgtcacaattaaaaacctgttgtggcagacaaccctcagactctatgtccatcttgaagttgctgatgaagtt

ctctgctgcctttgttttggagctggctgctttgccatgcctcacagtgctgtggatgccggtccttctcttcaacttcctgaaccacccg

>79045|AGAP000026-PA

aaaagtatttaagggaagccatgggtggttcaagaagcttaagagaagaaccagcatccacagtgtagtgaggcacggcaaagcagccagctccaacaca

cgggcagcagagaacttcgtcagcaacttcaaggtgctcatagattctgagtgttatctgccgcaacaagtttttaattgtaatgagacggatctattct

ggaaaaggatgc

>79044|AGAP000026-PA

ccgaaacggttgtttagatattcttgtcactccctttgaagcatctgtctccttaatcttgtccttgttcttgaggatagtgcaagtagttgatgtagac

cgattgtatgtgaatgctaaatcagtaacgctcacaccatgttcacgtttttcaattatttttgtgtttcatttctaaggtaattttctttcccttatga

tcatcttcttgtggcttta

>79027|AGAP000026-PA

agtttcatattctggatgatgtttaaaggttgctctgtatatggataaatgaaaagcaattgcaaggtaacactgttaatgagaacattatttgtgagaa

ggcaagaatgattttcactggtgtctttaagaagatgccaagatcatcagtggtcgaagaaggggttaagggaaaccttgggtggttcgagaagtttaag

aggataaccagcatccacagcattgtgag

>79012|AGAP000026-PA

ctcaccttctcataaatgatattcttgttaatagtgtcaccttgcaatttcttttcatttatccatataaggagcaaccttttcacatcatccagaatat

gaaaccattatttagatattcttgtcactccctgtgaagaatctagattcttaatctcctccttgttcttgaggatagtgcagataattgatgtaaaccg

attgtatgtgcatgctaaatcagcagtgctcaca

>79074|AGAP000026-PA

caaggtttcccttaaccccttcttcgaccactgatgatcttggcatcttcttaaagacaccagtgaaaatcattcttgccttctcacaaaagatgttctc

attagcagtgtcactttgcatttgcttttcatttatccatataaggagcagtctttcaacattatccagaatatgaaactgttgtgtagatactcttgcc

acttcctttgaaccatcttccttcttaatcttgtcc

>79024|AGAP000026-PA

ggggtgaccattgttgatttagcgcgcacattcaatcagtctgcatcaactttttgctctatcctcaagaagaagaagaagaagattaaggaaacagatg

cttcaaagggaatgacaagagtatctaaacaatggttttgtattctgaacgatgtcgaaagattgcttcttatgtggataaaggaaaagcaattgcaagg

tgattctattaatgagaacatcatttttgaaaaggcgag

>79063|AGAP000026-PA

aaaaaaaaaaaaaaaaaatcttgtatcatccaagccttgttgttggacctccacatcacatttcatccactcttctggactttacatttcttgaaggctc

atggagtttttgaatggtaaacaagcagaggtttagttttcaaattgccacttgcattggttcagaatagcagtatgagatggtctttcattggcttgtg

acaaggcaatgcatccttctctgctgttataaaggtacgtttcagcatctttttccagaataa

>79072|AGAP000026-PA

actacaggctccacaggtactgactcaaatgcctcaacgagttacattagactatgcactctggccaaagcttctccaagtgggagtgagttctcttggt

aagctcttcccaagccttttcgatcacatcgatgcaggcagtgatgttgaagtgttatttccaaaactctctgagagtgagattggcagcttcaatcaac

tcaaagcattgctcgaagagcgctttaggttatagcttcttaaagttagaaataatctgctggtccat

>79022|AGAP000026-PA

agccagggtcatcagtggccgaagaagtgttagagggtagttgtgggtggtttgacaagtttaagagaagaaccggcatccacagtgttgtgtggcatgg

tgacgcaataagcttcaacacaaaagtagcagagaactttataagcaacttcaagatgctcatagattctgagggttatctgcagcaacatgcttgtaat

tgtgatgagatgggtctattgtggaaaaagatttcaaagcttaccttcataaaaacagaggagaatgtattttctagtca

>79060|AGAP000026-PA

caacttcaagatgctcatagattctgagggttatctgccacaacaggttttcaattgtgatgatataggtgtgttctggaaaaaaaagtcaaggcatctc

tgtacaataacagaggagaatgcatcgcccagtcactagccaatgaaagattgtctcacactgctattctatgctaaagcaaacgatgattttaaaatat

tgaaccatttcttgtttaccactcagaaactccatgagccttcaagaagggtaaagtccagaagagcaggttaagtaggatgtgcaggtccaacaacaag

gcttaggttaaaca

>79075|AGAP000026-PA

ctgactcaaatgcctcagagttacattcgataacacactctgcccaaagcttcttccaagaagaagtgagacttcttctcttgataacccccttcccttg

ccttttcgatcatcttaatgcaggcaacgatgttgaagcgatatttccaaaactccctgagagtgagattagtagcttcagtcaactaaaagcaatgctc

aaagaatgctttagtgcaaagcttcttgaagttataaataatctgctgatccataagctggagtaacagggtggtgttgggaggcagaaactgaatcttg

atgaactgaaattcttc

>79028|AGAP000026-PA

gtaaccttcttcaagctcatcaatattattgttatccacttctaatcccatgcccctggccaaagacaattttgttgactacaggctctacatttattga

ttcaaatgcctcagagtcacattcaacaattcactccaaacagaagtaagagtcttcttggtgaccgcttcccatgccttttaaatcatattgacacagg

caacgatgttgatgtaacatttccaaaactccctgagatagagattggtagcttcagtcaactcaaagcactgctcggagagtgctttagtgcagagctt

ctgaaagttagaaataat

>79039|AGAP000026-PA

actgctgcttcgccgtgcctcaaaacactgaggatgccggttcttctcataaacttcttgaaccgtccatggctttccttaaacacttctttggctgttg

aggatcctggcgtcttcttaacaaggtcgccgaaaatcgttctcactttctcacaaatgacgttcttgttgatagtgtcaccttgcaattgattttcatt

tatccatgtaacgagcaacctttcgatattgtccagaatacaaaaccattgtttagatactcttgtcattccctttgaagcatctgtttccttaatcttc

ttcttcttcttcttgaggatagagcaaaaagttgatgcagactgattgaatgtgcgcactaaatcaaca

>79056|AGAP000026-PA

ttcttgaaggctcatggagtttctgagtggtaaacaagaaatggttcaatattttaaaatcatcgtttgctttagcatagaatagcagtgtgagacaatc

tttcattggctagtgactgggcgatgcattctcctctgttattgtacagagatgccttgacttttttttccagaacacacctatatcatcacaattgaaa

acctgttgtggcagataaccctcagaatctatgagcatcttgaagatactgatgaagttctctgctgcttttgtgtcggagctgtcagcttcaagcagcc

tcataatgctgtggataccagttcttcccttaaacttctcaaaccatccatggcttcccttaaaaacttcttcagccactgatgatcttagttttg

>79068|AGAP000026-PA

accagaagattactgctttactgttgccgcatcctactactgctcctctgtagaactcctctccacaacttcctgctgtgagacacactgcaactccata

agctcttcagtgactatttcttggctgtgatattccacaagctcatcgatatctttgttgtccacttctagtcccaagttcttggccaaagacacaatct

cgttgactacccgtttcacgggttctgacataaatgcctcagagtcacattcaacaatgcactccacccaaaccttcttccgagcagaagtgagagttct

ctaggtaaccccttcctatgccttttcaatcatcttgacgcaggcaacgatgttaaagtgatatttccaaaactctgagagtgagattggtagcttcagt

caacttaaag

>79059|AGAP000026-PA

catcttctagaaataatctgctagtctgtaggctgggataacagtggtgttgggagacagaaattatattttgatgaattgaaatttgaacagcgatgtc

cataacaagcaagacatggagtggcagattcatctcaagcaaatattgttttttccgaaggatcaaacacttcattgatccaatcaccaaaaaaaaacat

gtgtcacccaagccgtgttgttggacctccatatcacatttaatctgctcctctggactttacacttcttgaaggctcttggagtttctgaatggtaaac

aagcaactgtttgcttttcaaattgccactcacattgagacagaatagcagttgagaatgtttttcattggcttgtgactggggaatgtagacccatttt

gtcacaattaaaaacctgttgtgacagataatcctcagaatcttcaagcatcttaaagttgcagatgaagttctctgctgtctttgtgtcgaagctggc

>79085|AGAP000026-PA

ggggagaggggttgagcacatgccagacacccgcactccgcgtggagttcacatttttcccaacagaatgcacctcgtcccatatcgctactgcaagcca

gtccctagatacagcgctgggtgtgtgacatgacaatcgggttacgtatatgggccttgcatcccatagcgactgttgcatcgggccacatatatgggcc

tttcatcccaaaagggttatgaaactctacactaaagcactcttatgcactgctttgaattggctgaagctaccaatttcattctcagagagttttggac

atatcacttcaacatcgttgctgccgtcaagacgatcgaaaaggcgtgggaaagggttaccaacagaactctaacttctgcttgggagacgctttggccg

cagtgcattatcgaatgtggctctgagagatttgagtcagtacctgtggagcccatggtcaacgacattatgtctttggcccagagcatgggaccagaaa

tgcctaacaatgatatcatcgagcttgtca

>79026|AGAP000026-PA

agccaataaagacaatctcgcactgttatactgtgccaatgcaagcagagatttgaaaactaaaccgctgtttgtttacattcaacaccacaaaccttca

agaagtgtaatgtccagaagagcaggttatatgtggtgtggaggtcctacaacaaggctcgggggacatgtgatcttttttgtgattgaatcaatgaagt

atttggtccttcagtgaaaaaatatttgttacagatgaatatgtcttgcttgttatcgacaatgctcctgtctatttctccaggcctacaagaccaccac

cttgccaaatttaattcatcaagatccaatttctgccttccaaaaccactccattactccagtctatcgaccagcagactgtttattacttcaagaagct

ctacactaaagcactctttgagcattgctttgagttgactgaagctagcaatctcactctgtttgttttggaaatatcatttcaacgtcattgcctgcat

gaagatgactgaaaaggtgtgtaaggggttgccaagagaaatccctattctgcttggaagaagctttggttgg

>79067|AGAP000026-PA

ttgtcatttcttggctatgattttccacaaacacatcagtatcattgttatccacttctgttccatgctcttgaccaaaggcacaatttcattgactaca

gactccacgggtactgattcatatgtctcagtcacattcaacagcacactctggccagagcttcttctaagcagatgtgagttttcttgtaaccccttcc

acatctttttgatcatcttgatgcagaaaatgatgttgacgtgatatttccaaaactctcttagtgtgagattggtagcttcagtcagctcaaagcaatg

ctcaaaaaatgatttaatgtagagctccttaaagttagaattaatctgccagtccatagtctggagcagcagagtagtgttgggaggcagaaattggatc

ttgatggattgagattcttcaaggaggtggtcttgtaggtctggagaatgggcagaagcattgcccataagaagcaagccatggagtggcaccttcatct

caagcaaatgttttttcactgatagaccaaacacttcattgacccaatcacaaaaaagatgaagtgtcactcaatccttgttgttg

>79031|AGAP000026-PA

ccagaagataaagctgcaagaattttaccataagagttagaaaataaccttagatatgaaatgtaaaatcactgaaaaatgcgaatgtggagtgagtgtt

gctggtttagcatgcacgtacagttagtctacatcaactatttgcacagtccttaagaacagggacaagactaaggccacagatgtttcaaagggagtga

caagagtttctaaacaatagtttcattttctggacaatgttgaaaagttgctccttatatggacatacggaaagcaattgcaatgagacacttttactga

gaacatcactgtgaaaaggcaagagtgatttctgacagtctcgttaagaaggcgacaggataatcagcagccgaagaactgtttaagagaagccttatgt

ggttcgagaagtttaagagaagaaccaacatccacagcattgtcaggcatggcagagcagccacctccgagaaaaaggcagcagaaagcttcgtcagcaa

cttcaagatgctcgtagattctgagggttatctgctggaatagggtttttaattgtgatgagattggtccagtctgaaaaaagatgctgaagtttatctt

tataacagcagagg

>79009|AGAP000026-PA

tttcatttatccatataaggagcagtcttttgtcatcgtcctgaatatgaaactgttgtttagatacttttgtcactccgtttgaaacatgtctctccat

cttggccttgaggatagtttaaatagtttatgtagaccaattgtatgtgcttgatgcatcagcaatgctcacaccatgttcaagtttttcaatgattttg

tgtttaatttccaaggtaattttctttctcttatggtcgtcgtcttgtgactgtctttggagacatttctatgaaggtaattaaaatttgcacgcacaaa

aaaacactgtatgaaccccagtttagaaaaacgtgtcatcacaagctgcactaagatgataccagagagagcactaaacccagattgcagttctgaagac

attgttcatatgccattgccaacaatgaaaggtgttcgttctgctgaacattttcccagccatacaaacagttggtaatatcacactaaactgtgaaatc

actgtcactcattactcaaagcatcgttcatcatgtgaggcgttttttctctcttttcttcttttttttataatttgttttggtcattatgcgaattgtg

attttgagagatgctcattaagtgaggttccattgtat

>79048|AGAP000026-PA

tttcgtacttcggtgaaaaaatatttgcttgagatgaatctgctacttcttgtcttccttatggacaacgtttctgcccattctctaggcctacaagacc

acctccttgaagaatttcagttcatcaagatccaatttctgtctcccaatactatagtgttactccagtataaggaccagcaaattatttctaactttaa

gaagctctacactaaagcattctttgagcattgcttttagttgactgaagctactaatctcactctcagggagttttggaaatatcgcttcaacatcgtt

gcctgcattaagatgatcgaaaaggcaagggaagggggttatcaagagaagaagtctcacttcttcttggaagaagctttgggcagagtgtgttatcgaa

tgtaactctgaggcatttgagtcagtacctgtggagtctgtggtcaatgagattgtgtctttgaccaagagcatgggactagaaattaataacactgata

tcgatgagcttgtggaagatcacagccaagaaatgatcacagaagaacttgtggaattgcagtgtgtttcacagaaggaagctgtggaaagtagttcgga

ggaggaggaggaggaggaggaggagatggtggtaacagcaaagcagcaatcttctggcacaataagagaaatgctgaaagtgtggcaatcagttgcaatg

tacattgaaacctcaataaagcaatgctatgtgtgctactagtttatctgatga

>79010|AGAP000026-PA

catgggtggtttaagaagattaagagaagaatcggcattcactgcattgtgaggtacggcgaagccgccagctccaacacaaaggcagccgagaacttaa

atcagcaacttcaagatgttcatggattctgagggttatctgccacaacacgtttttgattgtgacaagacgagtctgtgctggaaaagaatgccgaagc

gtacctttataacagcagaggagaaggtataacctggccataagccaagtcacaagccaataaagacaatctcgcactgttatactgtgccaatgcaagc

agagatttgaaaactaaaccgctgtttgtttacattcaacaccacaaaccttcaagaagtgtaatgtccagaagagcaggttatatgtggtgtggaggtc

ctacaacaaggctcgggggacatgtgatcttttttgtgattgaatcaatgaagtatttggtccttcagtgaaaaaatatttgttacagatgaatatgtct

tgcttgttatcgacaatgctcctgtctatttctccaggcctacaagaccaccaccttgccaaatttaattcatcaagatccaatttctgccttccaaaac

cactccattactccagtctatcgaccagcagactgtttattacttcaagaagctctacactaaagcactctttgagcattgcttcgagttgactgaagct

accaatctcactctcagagagttttggaattatcacctcaacattgttgcctacatcaagatgattgaataggcatgggatgggg

>79054|AGAP000026-PA

ctcttggctaaagacacagtctcattgactacaggctacacaggtactgactcaaatgcctcagagctgcattcgacaattcactccagccaaagcttcg

ttgaatcagaactgagagttctcttggtaaccgcttctcacacctttttgatcgtcttgatgcaggcagtgatgctgaagtgatatttacaaaactctct

gagagtgagattgatagcttcagtcaattcaaagagtgctttaatatagagttccttaaagttagaaataatctattgtccataggatagagtaatggag

tggtgttgggaggcagaaagtgatcctgatgaattgaaattcttcaaggaggtggtcttgtaggcctggaaatgggcaggagcattgtccataacaagca

agacatgaagtggcagattcatctcagcaaatattttttcactgaaggaccaaacactacattggtccaatcacaaaaaaaaccacatgtcagccaaacc

ttgttgttggaccaccacatcacatttgacctgctcttctggactttacatttcttgagggctctgaatgttaaacaagcagcagtttaattttcagatc

accactggcattggcacaaaacaggagtgtgagatggactttcattggcttgtgacagggcaaagtatcctcctctactgttataaaggaacacttcagc

atctttttccagaatagacccatcttgtcacaattaaaaacctgttgtagcacataaccctcagaacctatgaacaaacagctgatca

>79011|AGAP000026-PA

attatttctaactttcagaagctctgcactaaagcactctccgagcagtgctttgagttgactgaagctaccaatctctatctcagggagttttggaaat

gttacatcaacatcgttgcctgtgtcaatatgatttaaaaggcatgggaagcggtcaccaagaagactcttacttctgtttggagtgaattgttgaatgt

gactctgaggcatttgaatcaataaatgtagagcctgtagtcaacaaaattgtctactggtccacaggctgcagtaacgaaggtggacttataggcctac

agaatgggcaggaatgttgtccataacaagcaagacatgggagtagcagattcatctcgagcaaatattttttcaccgtaggaccaaacacttcattgat

ccaatcacaaaaaaagatcatgtgtcacccaagccttgacgttgggccactacatcacatttaacctgttcttctggatttcacacttcttgaaggctca

tggtgttcctgaataaaaaagcaacagagcagcagttcaatcttcagatcgccacatgtgttgacacagaatatcagtgtgagacggtcattcattgggt

tgtgatcgggcaatgcattctcctccgccgttataaagatacgcttctgcatttttttcatgagagactcatctcgtcacagctaaaaacctgttgcgac

agatgactcccagaatctacaagcatcttgaagtcgctgatgaagttttctgctgcctttgtgtcgtagctggcagcctcctgtgccttaaaatgctgga

gatgccagttcttcccttaaacttctcgaaccacccacggcttcccttaaacacttcttcatcctgctatgatcctggcgttgtcttaaagaggtcag

>79033|AGAP000026-PA

ttggtcatttgtttctactgatgcttcggcaacttggtgacaatgtgacacaccactcttgtcaaataaatttgtagcatgtgtagtcacttctttactg

ggattatgattttcaatgtacaatgcaactgattcccatgctttcagtgtttctctctttgtaccagaagattgctgctttccagttaccacctcctcca

ccttctcctcttcctctgaagaaccctctctacaacttcctgctgacaggtactgacctaaatgccccagagtcacattcaacaatgatctttggccaaa

gcttctttcaagcagaaataagtcttttggtaaccccttctcacgccttttcgatcatcttgatgctcaaaggatgttttagtgtagagtttattaaagt

tagaaataatctgctagtccataggctgggctaatggagtggtgttgggaagcagaaattggatcttgatgaattgaaattcttcaaggaggtggtcttg

taagcctggagaatgggtaggagcactgtccataataagcaagacatagagtggcagattcatctcgagcaaatattttttcaccaaaggatcaacactt

cattgatgcaattacaaaaaagattacatgccatccaagccttgttgtgggacttttacatcactttgtacctgctcttctggactttatacttcttgag

gctggtggagcttctgaatggtaaacaagcagtggtttgactttcaaatcaccacttgcattggcacagaatagctgtgtgagacggtctttcattggcc

catgattgggcaatgcattctcctcttctgttataaaggtacacttcagcaccttttccagaatagacccatttcatcacaattacaaacctgttgtgga

agataactgtcagaatctacaagcttcttgaagttgctgatgaagttctctgttgcctttgtgtcagagctggctacatcatcatgcctcactatgctgt

ggatgccggttcttctcttgagcttctcaaaccacccatggcttcccttaaacacttcttcggctgctgatgatcctggtgtcttcttaacaggctcggt

ga

>79089|AGAP000026-PA

aatacaccaagatacatgaagtcctgtatgtacatttcaacatgaaatgaaagtactgaatttcatactaatctttcctaattgcatcaaccaattgatc

agattgaaaacaatgacttttttgacttgaattctatcagcttccaaatatactccttgtgtcaactagaaggtcagaacttgatttagtatagccctag

gttaatactatattagaccataagaacgttgtttaaattgctgcccaagaaaaaggtcatgacacattatttttattgtcacataaatattcagtttcta

tagtgtgattaaattttcaaatgatacaataagtatcaacattttcttacatatgcagcaatcactatccatagttgttagagcaaactgaacctcccct

cttctgctttaatccctttacatttaaagcagaaagaataacaacaatgcatcatcatcttaaatattcttggtggctgcctttttatagtgtttctata

cagtggaacctctcacagtgagcataattcgttccagaatctttctcgtagtgcaaaacactcattaagtgaaacaatttattccatataaattaatgta

aaatacaaaaagttttattttactcatgccatttattcaaagacaattacacatacagttcaatgtattttattattcaaaaaacataactatttctttt

ttgctaggaagttagctacagtcatttgtttctaccaatgcttcaatacttggtgaaaatgcgatacagcattatcatcaaatttgtagcacaagtagct

actgatttattgtggtgatgattttcaatgtacaatgcaaccaattcccatgctttcaatgtttctcttgttgcaccaaaagattgctgctctgctgtgc

tgcttcctcctccactgaaatgctcttcccaacttcctgctgtgaaacacactgcaactctataagctcttcaatagtcatttcttatctgtgatgttcc

actagctcataaatattgttgttatccacgtctaattccatgctcttggtcaaaggcacaatctcagtgactacaggctccacaggtgcttactcaaatt

cttcatagtcacatttgacaatgtactccaaccaaagcttcttccaagcagaatagggatttctcttggcaaccccttacacaccttttcagtcatcttc

atgcaggcaatgacgttgaaatgatatttccaaaacaaacagagtgagattgctagcttcagtcaactcaaagcaatgctcaaagagtgctttagtgtag

agctttctaaagttataaataatctgctggcccacatgctgtagtaacggagtaatgttgggaggcagaaattggatttgacgaattgaatttattcaag

gagctggttttgtagacctggagaatgggcaggaatgttgccagtaacaagcaagacaatggagtggcagatccatttcaagcaaatattttttcaccga

aggaccaaatgcttcattgagcccatcacaaacaagatcacatgtcactcaagcctttg

>79013|AGAP000026-PA

gttctgcaagatgtccaaaattccaagaaaaataggagtaatctgtagggaaagatgagtaatacagtgaaaccttgcatagtgagcatgattcatttca

caatcttactcatagtgtgaaatactcatcaagtaaaaaactttatcccgtataaattaatgtaaaatatgataatgaagcccatgtagaaaaaatacta

caagttttaccttactaatgacacatatctaatgcttttttactaagaagctatctacaatcatatgtttctgccaacacttcaacacttggcagaaatg

caacacatcattatcatcaaataaatttgttgtgcacatagccactgctttactggggtgatgattttcaatgtacaatacaaccaattcccgtgctttc

agaatttctcttattgtgccagaagattgctgctgtgctgttatcacttcctcctctgaagaactcctctccacaacttcctactgtgaaacacactgca

catcttaattctttggtggccatttctttgcattgatcttccacaagctcatcattattatttttatccatttttagtcccgtgctcttggtggagtaat

aatctcactgattttagactccacaggtactgactcaaatgtctattcgataatgcactctggccttagctttttccaagcagaagtgagaatcctcttg

ctatcaccttcccatgccttttcaatcatcttgatgcaggcaataatgttgaagtgacattcccaaaactctctgagagtgagtttggtagcttcagtca

actcaaagtaatcctcagagggtactttaatgtagagttctttgaagttagaaataatctttagtccattggctggagtaatggagagtggtgctgggag

gcagaaactggatcttgattaattgaaactcttcacagagatggtcttgtaggcctggagaatgggcaggagtgttgtccataacaagcaagacatggag

tgccagattcatcacaagcaaatattttttcagtaaatgaccaaacacttctttgattcaatcacaaaaagatcaaatgtcacacaagccttgttgttgg

tcatccagatcacaattaacctgctgttctggaatttacacttcttgaaggcacatggagtttttgaatggtaaacaagcagcggttaattttcagactg

ccattttcattggcacaaaatagcagtgtgagatggtatttcaatggcttttgatcaggcaatgcattctcctctgctattataaagatacacttcagca

tctttttacagaacagacccatctcatgacaattaaaaacctgttatggcagataaccctcagaatatttaagcatcttgaagttaccaatgaagttctc

cactgcctttgtgttggagctggctgcttcaccatgactcacaatgctgtggatgctgattcttctcttaaacttcttgaactatccacagcttccctta

cacattttttcagctgcctatgatccttgcacattcttaatgtggtcagcaaaaatcattgtcattttctgacaaataatggtcctgttaatagtgttgc

cttgcaaatgcttttcatttatccatataaagaggaacctttcaacataattcagatactcttgtcacttgttttgaagcatctatctccttaatcttgt

ccttgttcttgaggatagtgcaaatagttaatgtaaatcaattgtatgtgcatgctaaatcagcaatgttcataccacattccatacttttcagtgattt

acgtttcatttctaagtcattttctttctcttgtggccatctgcttgtggttcatcttcagagacatttctatgaaggttgttaaaatttgcacacaaaa

acaatgtgtgaacataaatttagaaaaatgtctgatcacaagctgcactaagatggtaacagaaagaaccctaaactcagactgcagtaggaaataaaga

cattgctcatatgccactgctgacaatgaaaagtgttcatattgattaatgtttcccccactgtctgcaaatggctgatgacatcacactaaatcatcat

cactcatcacacaaaacattgctcattaagtgagtcattttttacaatttgttttgcttgttaagctcattagatgaggttccactgtatacaatacact

caacaaccaaaaaggaatgataaaattggaagaccaagaattatgtgattggattaaaaatgagtaaggcaggaatgcaatctttcacccttacagttca

atctatgtatcaaagaagcactgacagatatgaaagaaaggtgcatgagtgggattcaaattcaatgtctagagatatcagtgaaaagattctggtgaca

ttgttctcctcggtgaaggaaaataagaattacaagatctgttgaatgaaatgaagagtctaatgagtacagaatatggacagacagtaagaaagactga

agtgatgtgaagtagcagaaatgagaatagtgagaaacttaatgtaaaaagtagttatcgcaaagaagaccaagataatgcattctgctaccttcaaagc

aaaataatccatagtaggtgaaacaattagtacataataaacccaatagtacaggcagagagggcattcttggccaaaagaagactactagtgtcaaaca

taggctttatctgaggaagaaatttctgaggatgtacatttggagcacagtgttggatggtagtgaatcatggatggtaagaaaattggaacagatgaga

atcaaagcatctaagatgtcatgctacagaataaggttgaaaagtaggtggagcgataaggtaaggaatgaggaggttctcctcagaattggcaaagaaa

ggatcatgtggaaaacactgaggagaag

>79108|AGAP000032-PA

gaagaaaatagctgtatcagcatcatatgcaccaaccactatgtcaggatactgattgccatcaaggtcaattccgccagaaagtgaaaatccaaatgtt

gttatctgactatagatactttctccaaatataacttgtgaagcttctttcataatgccagtagctgatccatgatatatgtacacagcaccaccacctt

tcaggccatcgtaaggagctccaacagcaatgtctccatatccatctttattaagatctccaactgatgctaaagaaaggccaaatcgtgatttagaatt

tacaccgtcacgccaatgatgagtggcaaatctgttcgttccctttttttggtagattacgtagatgcggccagtttcatattttccttcattattttga

aggtgggtgtagaggggtgcaccaacaacgagatcatcagttttgtcaccatccatatctgatacacaaagtgcatacccaaagtatgcacccatctgtt

caccagtgaactccccaactttttgcagga

>79144|AGAP000047-PA

ggcatccacggctccgtcgccggcggctactcctacgtcgacggcaacggcgtcgtccagagcgcgcgctacacggccgacgccgtgaacggcttccgcg

tgtccgccaccaacctgcccgtggccccc

>79166|AGAP000054-PA

ctgtctcaccaccagatcatcaacgggaaagtgatgatgatggctgggaggaacgagaaaagtctagagcgcactctgtcaaatttacagaagacctgga

cactgaactgaataaagagacgccatttgtacgccagaatactcctcatccaaaggaattgaaagcaaaggcccataaactttttgccaaaggaaagttg

ccagatggaaaaatgcctcatgagaatgaagacggaaaacatccaggagataagcagacaatggaaagtgaattgcaacagaaagatcaaaatgccattg

cagagaataatgttgcatcagatcaagaggaaagcaaggaggtatttgtttttatctagaattaagtatgattgcag

>79184|AGAP000065-PA

actcatcagatcgtatcaaagtccgagtgtgggatgaagataatgatctcaaatccaaactgaggcagaaacttacacgagagtcagatgattttcttg

>79183|AGAP000065-PA

attctgtacagcgaaccccttgtcttgcaatgccccaaagcagaccctcacattcgtagcagtaagttggtgatgttgctgtccacagaacaaagttgtg

gggtgttgtgctggatattgggtagatgagggcttgcagtcttctt

>79206|AGAP000080-PD

tatcagcatctggaaaagacattcctgcaaaagaaaggaggaaccactgtgatacattatctttaaaagtcctcaaatgctatcaagatctacaacacat

ttgactgttctaatgaatgtattttttttaagtgtatatttttgcttttatcttgtataagcaataaatgtgtgtgtgtgtgtggaaaaattgtttcaaa

cttgagaaaccactacttaaaacacaggcctgaaaggcatcagtgccccatttgctttgcaactactacaagagcagataatttacgtcgtcatatacga

atgaaacattctgatgagtcatgcagtaatatggtatagacaagtaactgatcagcactacaaaagtaataggaaacagcattgctcattaaacaatggc

tacttcattgcattgcattcgaggtgtgtgtgtgtgtgtgtgtgtgtgtgtgtgtgtgtgtttcatcag

>79252|AGAP000090-PA

caatatgggcaaacaagctccaccaacttgaattctaccacgaatacctgttgaatccattcgactagcaatgttaacagcatctccccaaatatcataa

tacagtttctttgttcctatgacaccagctgttacatcaccaaaattataacccactcgcaaaactataggaagcacttttatggcagcatcaggtctga

a

>79290|AGAP000096-PA

gtttgctatttatttctatgaaatacctgtaatatttatagtatttatatattcttactctgaaatgaagcctttttaagaatattatgtacaatacatt

tatatacagtaactacacaaaataagttccgctactacttctgaaaatttgttctttactttcagagaggcattactcttgtgttggtttgtcacgtgtt

tcttctgcaaaccatcgttgcacagcttcatctttcaacttgtggtacatccttataatcggaagatcttttctctcttcctttggcacaaattcctcca

cagattgaagcctaagccatccccaatggataccaatcaaactaggtatcattacaatggctaccaagttccggttgacaaagcttcttaatttggggtt

agaaaacattcctacttgcctctatcatttcggatccctatttattcccacgtaaatatgtacattggtagctatgtacttgtacagtgttgcagttttt

tgaagtgtgcctatcggtaaataaattccgtctactttttgatcacaatactcctgac

>79356|AGAP000130-PA

ccgcgctgcccagctgtagcagcacgcgcgtccctggcttgcggcgccacttgcggtcgagtacagctgctgctccgagtccaacagtgcccagcaggga

cgcgaagacgccggccagggagatcagctctagcacaccggcgtggtcgccccactgcaggtcggcgtctgtcaccagctgcgcgaagtgtgtcaggtgc

cggcagcggcacacgtcgatcacctggccacgacggtacagtgtacagctgccgaagtcccaggcgttcgtcgtcaagttccagatgccgcatcgccgcc

gcacgccacgtactcgaggcttcagaaagacatccacgtactttgcgggtaacgaagagttccaagtctccacctgtgcgccgatgacgtcgctgttaac

gtggagcccctcatcgaggaaactggtgttaagcaagtcgtgccgaagaacggtgatggcaatagctgcggcagacacgcctgtgtcctcaggcagcacc

acccccacggaggcgggccgcgccagcatccacgccaccgagtagtcctcctggccatatcgcaggagttcgatattctctccgtcagtggagaccagcg

cgccgacgatgccggacgcgatgaccggtgtggagtgctcgtgcttcactttcacctcctgtcctttggctgtggtgttggccatgagtattgtcttggc

aaacagctcgaagccagttagcgccagggacacattattttgtgtcagattgttcgagaagtttataatggtgtccaggttcgtgagcatctggtccggg

tccagcgttctgttcttgttcttcagagcgccgtcctcgatggtttttggctcaagttgttccagcgcctcctcaactctatcacgaatcgtgttggtgg

cttcagttcttgtcgtttggctgtccaaccatgaggtagttgtttcctccgtaggtggcagcgttcttggagtcatcatcgttttgcatatgccaggtaa

tccttctgcatgctctatagctgtctttgtagatgcatcttctgtttctgtcttgcaacctctgaactcgtaagggttttcttcggcaaaatcaatccac

cggcgacagccttcgcatgtgaacgtaaagctgtccacgactcttagtgttgggtagggttgacgactattttcattgcaagagaagtagcggaacagct

tggggatttcgtcattggttattacgtatgtgtgattttctcctaagaagtctggttcggtgtggctggtgaagctgcagtgtctacattgatatgtgcg

attctgcttcaacgacatagccgtctcttgattgatcgtccggtcgctaagactctccgacgtgttgtggtgccagcagggcggggcattgtgtgtgacg

agccacacggcgtaccgctctcggcaggggttagcagacgcctcccagacggcgccaaagtacgcgtcgctgaggatgcggtagctgatgcggtccgcgt

tgcacacggtccaactggtgttgcgccccaagagggcctcgtagtcgggcgacgtggcgaccagcgtggggtcgctgtcgctcggcggctgagcgttgct

gaaggccgcgccacaggccagcaccgtttccggggcgggctggcggaaaacggccgagcaccagcgactcacctctggcagccgcggaaggacctgcg

>79371|AGAP000132-PA

ttaacaactgtaactcaaaacagcacctactaatatacatgtataactccacattccaagtactaagctaattcaaaactattttcttgtattccggaaa

gctgtcaatggccttctgtaaatattcacatgtcagcttcattttgagttcgttgttgtgtacataatcaaaggcagccttagctagtatcctcccaaca

ccctttccttggaagactgatgggacttcagtgtgtagtaagtcgacaacaccacaagcgtcctcattgtattgcaggactgctctttctttgcccaaag

taataaagaattccttgacgctcttgtcgtgactaacttcaagagcgtctgtggaaaattgcattattgcagttgtactcgcatttggtttaacagtttt

tgccaaaatccttgcagagcgtcgaagcatcgatatcgctaaggcttgcacttgtgtagtaaaacagacgaacttcgccgacaaacgtagaatgtttttg

tttacgtttgcgtcattgcgtgtcttcagtgccacagacaatctacacacatctctgtggtgggtgtggatatctcccaaaattccaagaattgtcggac

aagattcgtgaatctgtaccatcggcgaacataacaggaaaggagg

>79386|AGAP000136-PA

aaaaagcttttcctaactacattagagattcacaatcatggaacaaggcatgagcacagtcttcgcaaagactcaataactctcaatacagcgaaaaaat

tgagttacccagcccagaataaaaagtgtttacaataaggttccatggatattgtcatatatttaagaacaaactgaaacacattttttttataatttta

atgagtttttagaggtattcattaagcaaaatatacatcacattgcaaattatactgtaatattgcttactgcatatgtatcatgtaatgttttgtgtct

tgtgtacatattgtttgcagttttgtatgaaccaataagtatggtagacactcaatttcaacaactggactgaaaataatgataattaatacccatacag

aaaatttttcatttatacttttctttttctgcctctttctctccaagtaatgttgtattctttgacttgtcctactatacagtacatccattgttctgaa

tataatttaacaatttaacataaataaaagtcaaatcaagtccaaagctctaatatttcattctcttagaagtgcttcaatttttacatcaaaaattaag

tgcattgcaatgttttaatgattaaaaacagaaacaaaatccaggttcccatgtatataatataaaaatgagctgcagactcccaaacttctaaaggtga

cttgtagtgactttttctagttgtgatgatctactgtcacacagtttcaaatactcaagtgaactacatatttccaataacttgcacacttttttaaaaa

gctatatttaattttattttgtatgataacaaccttcagaaatatgtattcagtgttcatgagggtgtcataacagctaaatacagagaacactaaagac

ttttcaggtagaatgtaaaataccatgtacagaagacaaataacagaactgcgtagttcaataagcttataaaagctgcttactgtgttaacttaaatta

tggacggatgttcacaatgtctctcattacaaaatccattaaggctgagacagatcaagttcttaaattattacgacatttttctagaatatccttgcgt

atacggggaaaatctggttgcagcttaagcacttgctggcagacatcaatggcttctgcatacctttttgctttcatgtgacagaaagccagtttgctac

caagcacaggattagaccgcctactgaggacccatgctgtagaatagctcatggcagcatctcggtaccgctgttctgtttctgcagcttggccaagcaa

ttcatgcgcacgcacgcaaccacggttatgtcgcaagacacgagctaataattcagaagctccagaactagtgctaccttgtgcttcagcaagtgtcaac

caacactgttctaaatagtctgcctcctcaaaacgccaaggtgaacgtgctacacgtttcagttggttac

>79385|AGAP000136-PA

ctgcagaatctgttcgtgcgctggaggcactggcagctgatgtcgtgtcaggacaagctctgggacctctgatagccctggctgctgcacacaaggcctg

tgctgttcctgaccatgaagctttgtcaacagtggaggaacgcctggctgatgagaagaagaaagcaggagaaaatagtttttaccaggctgtgtttgca

catgtcttattgggacagtatgatgctgcacgtgaattgggtggctcactgctgcggatttcttctgcctcctctgatgcacttgcagtgtgtggatggg

ctgagctacttgctgcttcaggtgacaagaatctcgctgaaaaatattttcagcaagctttggatcatgagggtggacaacacacagaggcaacactggg

caaagcaaccctcctgcatttgcgtcgggagtctgaagcagctgcaactcttttggatcgtttggtgattcgagattctttgccagcacttattgaacgg

actcgttgtcagttagccctctatgattgggagcaatgcctggagtctgcagatcgtgccctcagtgctgatgctggatgcctacaggctcttgaacaca

agatggtggtggcattgtgtcgggatggtgactgtgaaggcacagctgcagggttacagcagtacatgtcagaacttgagcgccgtgagccgagaggagc

aaagctgatgttagaagctgcacaactgttttcaagatgttgtggacgtaacagaactgttctggcagagactcgacatcttgttgaaaaggctgcacaa

ctgcgacctggtattgatgctgcatgcgaacttggaagacagagttttctcagtggaaaactagcagaagcattacggcacttccgtgctgctacacgtg

tcgacaactcatcttcagcagccctcacaggtctcagtcgttgccagatggaggaaggaggtgcctcagctgagcaggctgcacgacgggtggaatttct

ccatgaaattcatccatctgcagaagtgttattcatgtcatctcaagcagctgcagccaatggtgattttaaaaatgcaataaaagagctagatgaggct

gttgaaacacattttcgaacagtacgtacaatgccctttgggacatcttacttgcgagctgtggatccagatttcctaatacaggtggctaaacagtaca

tttcactcagtccaccccatccaccagaagaagggcagcagccagcatctctacgtcaggctgtgacagtacttgaagcagcaacccgtgcatgccctgg

attgacagaagcgcaattgcttttagcccgtgtacaattcctgagtcatgatttgtcaagagcagcagagactttgcatcatattcttgatttggattca

acctctgctccagcttatcttcttcaagcacagatactcatacagcaaggggatttcagtcaagcaatgcaaagcctggagatggggctaagctacaatc

tgcatgtgcgtgaacagccattgtaccatcttcttatggctctgattcagaaacatcaaggcgaggtggatgagagcttgaaatcactgcaggctggtct

ccaactggattgttctcagatgtcactagcggatcaagtgtcactttcacttgaacttgtggacacacttattgtagcaggtgatctccagttagcaaac

aagcaattgtcagcagcagccgagtcctttggtgccacttctgagaggggtcggattgtcctgatgaaagccaaactacttctagcacaaggagatgtgg

cagcagcattacatgagttagaatctgtttcccctggacaatcctcatatagacaagcgcagtgtcaaatggctgagatacatctaaagcatcgtggtga

tcgtatggcttatgctgaatgttttcgtcgtttggtagaacatgcaccag

>79429|AGAP000149-PA

gaagaaatcggcaatgaagtggctcagtttctaaatctcagcattacagaatgccgtgctattcttatgcgttatcaggaagaggaagaacgagaaacaa

aacgcatcaaagctaaatatcgggagatgcgtagacgaataaggcaaagaatggatgatcttaaggtaaggttatgatattttgatttttctttcttggt

ttggactgggtggatagcagctgtgaggaagatctgcacatatactccactttgctgcaagtttattttttctgtgtaaattgttgttaaaaagtcagca

atatagtagaactaaatgaagaaatgtactgttaggtgtgcttggtttgaaataagtgctatctgcaagctgaacactgtgttagttttgagtggcttgt

aacttcagtagaaaatttgtgaaacctctctgcaacagaaagcaatgtcagcatatagtgtcatgcttccacagtgcaatagttttggctctggaagctg

ttctgtgtggtgtattactgattacaataagtttgtattagtgtttgaagtaacttgatgagatcagattatgttattttctgatagatctgcatgtatt

gaagtgttatgtaagtattctatgagatattgattaattacttgtaatgctttatgtaacctgcatttgaagctggatttatatgtaaggcagcattcta

ctcagttctgcatattaggagcaaatgattattaattaactaatgtaacataactaataccatctgagaagaaaaaaaagtaacattgctctgacagttt

tagttttatgggcacatgagtatttctgtaactgttttctttggttgcagtcatgtgtgaagaagtgggtaaaaggcatcattttcgtgtacagaaacat

tatttcactcccagatctgtgatgtaagcgaattaccttacatccttcatttacttacatttacttatgttgtactaccaaaaatgtttgatggacacaa

agcaattaggagattatttgccctttgcatgtagttaaatttctcatgatgtaagggaaggaggtgagagttggactcataaattattagatacacacac

tcctcagaaagtccgcagctcatggtctaggcgagtgttgctgcctctggatcacggggtctcaggtctgattcccggccgggttggttggttttccctg

cccagggactgggtgtttgtgttgtcctcatcatttcaattgggactttgtagttgagtggcctacaaaccaaacattgtcgttgttgtcatcatcatca

tcatcatcatcatcaacaccc

>79449|AGAP000162-PA

agctgtgggcaacactccaattattcgattgccacgcttagaagctaagttgcaattgaaatgcaaactttatattaaggccgagtttatgtctattgga

ggttctgtgaaagatcgcattgggaaggctatggtaatggccggcggggagca

>79470|AGAP000167-PA

tgtatacacgttgtgcaagtttactggatagacggcccactgtactaacagtatgagcaaccttgtcttctttagaggaaactggaactgaaaatagtgc

agtgttaaaatactgcttaccaacgcattttatattacttcaatgaaatatatttggaaagacgtttacagcttacctgcttgcttttcatcttcgccag

gggggaaatagtagtctaagtatttttccgctactgctgcagtgctgtcaacagttgtgacagcagcaaggccatactttgtgctcaacaactcatttgc

tttgttccagctgagctctttcaggctctttgctttctgcacaccagcatccttcactgttgacactttgctgcaggttggccgtaaagttgcagcaaca

tagttctttgttgtttcatatatctgttgcggctgctctttgataattggtaatctttcttccaccagcgaaagtcctttgcatagcgttccatcaacca

tttgaatgggttgttccagttt

>79468|AGAP000167-PA

cttgcaaaatttttaataaccataaaattacataaataattaagtattgttaaataatatgttcagtcttaacatgcaaattttatacaattttctacac

ttccttcacgaagaaacattttggctttctggtttctcttgttccgttttaacttcagctggtttaagatatgcatttatataatttgtaagttgtacaa

cagctatcaatgaagcaatatactcctgtacattttctctgttcagattctgaacttgtttcaccagtgtactgtatacacgttgtgcaagtttactgga

tagacggcccactgtactaacagtatgagcaaccttgtcttctttagaggaaactggaactgaaaatagtgcagtgttaaaatactgcttaccaacgcat

tttatattacttcaatgaaatatatttggaaagacgtttacagcttacctgcttgcttttcatcttcgccaggggggaaatagtagtctaagtatttttc

cgctactgctgcagtgctgtcaacagttgtgacagcagcaaggccatactttgtgctcaacaactcatttgctttgttccagctgagctctttcaggctc

tttgctttctgcacaccagcatccttcactgttgacactttgctgcaggttggccgtaaagttgcagcaacatagttctttgttgtttcatatatctgtt

gacactttgctgcaggttggccgtaaagttgcagcaacatagttctttgttgtttcatatatc

>79469|AGAP000167-PA

cgagcggcaaagtggaactagttgtgttcaacacttgctttgtgttgtggcttttcgttagtgttgtttgtggtaagctggtagtggttgaagtcgcatt

ttgattacagtacgtatccgtgccgttagtgttattgtactgtaatttacttttaaaggaaaggattacaatggccgaagcgcagaatatgtctgtagca

ccgagactggaatgcgtcgacagagttaggacgctccctgttgttgaaatggcgttgaatcagtcggctgatatgtataacagggttaaaggatctaata

gtgtctttaactggacattatctactgctgaagcaacagtgaataaggcaatggaacatgccgctcccttggcgaagaaactggaacaacccattcaaat

gtttgatggaacgctatgcaaaggtctttcgctggtggaagaaagattaccaattatcaaagagcagccgcaacagatatatgaaacaacaaagaactat

gttgctgcaactttacggccaacctgcagcaaagtgtcaacagtgaaggatgctggtgtgcagaaagcaaagagcctgaaagagctcagctggaacaaag

caaatgagttgttgagcacaaagtatggccttgctgctgtcacaactgttgacagcactgcagcagtagcggaaaaatacttagactactatttcccccc

tggcgaagatgaaaagcaagcagttccagtttcctctaaagaagacaaggttgctcatactgttagtacagtgggccgtctatccagtaaacttgcacaa

cgtgtatacagtacactggtgaaacaagttcagaatctgaacagagaaaatgtacaggagtatattgcttcattgatagctgttgtacaacttacaaatt

atataaatgcatatcttaaaccag

>79484|AGAP000173-PA

tagtaatgttacaaagtgagaattgtgctcatgctaccttaattcctgaaagcattctaagatctgttgaccaatctaccacagactcagaaaagccaac

agaagagaaacctacaaaagagcagcagcagcatgtttatgagacactgaaaaatacactcccaaaactgtttgttcagccaatggactatagtatttac

catccagagataatcttcgtaaacaacataagaggaacaataactcaagggattgtgccatatgtttgtcagatagctgctgtaagagtagttgggcacc

tcaaatttgcattcgttaaaatggatgtgttgaagataacactccacgttaaagaaaaaacggtaaaagttcgctggaggattagaggaatttcagggtc

gaaggtgtttttgtatttttggaagtacaaaatatggaatatggatggtgttatggaggagttccaagaaacgtggtatgatgggttttccatttattgt

attggtgaggatggaagagtaataaaacatattgttgataaggtaatgccagatgacgacatttgtcctgctaatattcagaatccatcacccttgacag

caaacagctgaacgtaactagtttcatatgagtaatcagctgcacgcagtcttcaagacaccagcattttgtgcattttgcgatgagttgttagacaaat

gtgtacatatgagctagctgtgatacaaattataaattgtcagtttctacatgctccattgtgtgttgttacagattatggttctataaatgtttgccac

actttcatgtcctcctcaaatggaatgaagtccaagcatttatcattgtcataatttgaatgcctgttgaacgaggcatgtgacacaacttatttattaa

acaaaatataaaagttttgtttcattattaatggcacagacagatgtcttgttcaaaatttgatattaagtttgtgaaaatatatggctcatttgcaagc

tttttgagttttgtttgtaatccttcctccttgtatctgctgtcattattcatcaatgtgatgttttttgtattgtaaacctcgaattatgaattgctta

agttccatgaaagatttttcattttgtagtacacaattttggaagtattccacatttgctctcttataaactatgttcagtgtcagttgtagctaaacta

aattcactcagtgtaaataagtaaa

>79540|AGAP000181-PA

aggagacagatttgaaataaaaaatatatacggcatttattgtaaatggcagtatacatataacacgtaaacaattcgtagacagaactgatgtccgtgt

tagtttcacagcacaaagtaatgtcatctgcagatcgtgttcactgggcagtgtaaataaaatagcacaaagtaatgtcatctgcagatcgtgttcactg

ggcagtgtaaataaaatagcacagtcactgtgtgacgtgcgcagcttgtgctctcgtgcacactgtctgtggaagtgatcagcatggagagtacgaatgg

aagatgccggcgtagttgaccaggaagaaggtgaccacggaagcgacggcagaccccatctgctgcgcggcgccgcaccagaacaggctgcgaccgccct

ccgtgcggaagatggacgctatcgccagcttcatgtacgaaatggctcccgtgaagatcacccacgaaataaccacgaggaactctccacccgcgtggcc

gaccaaaggcggtgacgggctcatcagcgcggtgacagcgatgtagatggtgaaggcggcgctgaccactgacagcgcggatattgtcctcactgacgtc

ttcggcaggaagaactgcaggaaacacgccagggggttcgccatggcggagaggctgaccgccaggtggtatgcgacgttcccgtacggcaggcacgagt

acgactggatgctggggaacgcgccgttcccgaaagccgccaccaacgacaccagcaccatcaggtagacgtaggtgcggcgcggaagccgagtcacttc

ttgcaagtccgaccgccggctagctgccggtccggcgctgccctctcc

>79539|AGAP000181-PA

gctgctcggagtctctgaaactgaaaacatgtctcaggactaaataagtttgtgtttacatactcgcgctacaagaagtgtttcaccgtttgaagatcat

ctctgtaaacaagtcttctggatgcagtcgtctccatgttgcctgaaatcgacgaaggcactccacttctgttgcctgctgtcccaagtacctttcaaca

caaaatcaacatgaagttccacatcaaaggactgtcagaccgggaagtagtggtggacgtcctggctgttctcttcgggatcggaacatggttggcgatc

aacgggatgtacgtccagttaccactgctcgtccagacgcagccagaagggtggaacctgccctcgttcatttccatcatcgtacaaattgccaacatcg

gacccatcctatatggcttgacacagaagttcctcccgggcaaactgcgggacagtttcattatttacggattactgataatggggaccatcgctctgtt

gctcatggctctgctgtaccaggagacgacgtacatcggaggcgaaaagcgcagtacctctctactggtgctgacgttcttcattgcactcgtcggctgc

accagctctgtgctcttcatgcccttcatgaggaactacagagagacgtacctcacttcttatttcgtcggcgagggcctcagtggtttcttgccgagtg

tgttcgcccttatacaaggagtcggaggcaacccgacttgtgtcaaccaaactgtcgtgaacgccaccaacccagacgagtcttatacggtagtcgcccc

gtacacgcctcctccgaacttctcgactaccgtttttatcttcatccttttcgcagtcctcgccattagcacaatttctttcgcgcttctaaataatttg

aaagtgtgcaagggtgagagagtgagatcgcaaagagagcagaaggcggaaaagtccgttaaagacggcagcggagagggcagcgccggaccggcagcta

gccggcggtcggacttgcaagaagtgactcggcttccgcgccgcacctacgtctacctgatggtgctggtgtcgttggtggcggctttcgggaacggcgc

gttccccagcatccagtcgtactcgtgcctgccgtacgggaacgtcgcataccacctggcggtcagcctctccgccatggcgaaccccctggcgtgtttc

ctgcagttcttcctgccgaagacgtcagtgaggacaatatccgcgctgtcagtggtcagcgccgccttcaccatctacatcgctgtcaccgcgctgatga

gcccgtcaccgcctttggtcggccacgcgggtggagagttcctcgtggttatttcgtgggtgatcttcacgggagccatttcgtacatgaagctggcgat

agcgtccatcttccgcacggagggcggtcgcagcctgttctggtgcggcgccgcgcagcagatggggtctgccgtcgcttccgtggtcaccttcttcctg

gtcaactacgccggcatcttccgttcgtactctccatgctgatcacttccacagacagtgtgcacgagagcacaagctgctcacgtcacacagtgactgt

gctattttatttacactgcccagtgaacacgatctgcagatgacattactttgtgctgttttatttacactgccctgtgttctgtctacgaattgtttac

gtgttatatgtatactgccatttacaataaatgccgtatatattttttatttcaaatctgtctcct

>79580|AGAP000189-PA

acacacacacacacacacacacacaaatcatttgatttgcactataaaaatgacgctatgactgggttaagtataaaatctaattgccatttcaaacaca

caatgcattctaaacaattagctccttgattattttgcacaaggagtaatcaaaataacacgttttgtttgttttgcattctgacgtaacaaattagatg

caaatacattaatttacaaacctttatgacactcataattacagttacatgtacatgatgatcaccattctactcatgtaaaacatgaagtagttcagta

aaactagatgcaaatacattaatttacaaacctttcatcgtaatgaaccttataattatagttacatgtacatgatattcaccattctactcacgaaaaa

catgaagtagttcattaattcacatactttcataagggcaaaaaacacacagttttctctaatggaaagtttaaaaattatcatgtcttgaggttaccat

taactttaaggaatactgacactgaaacaattttctaaggaacagcaaagtatcttcatatttcttacgctagcttggcatagaaaagaggcatctttca

cttcactcaggcaatctcctagtcttatgaaacacttacaataccaggttaattagcaacaagagtcacatacagttttgttcctgcttttctctgattc

aagtgttccattttaattactttcatagtaccaacatgttaaagaaacagtccaacacacagattacaacacactttgaataataatatgatgctaatca

ttatttctttacacaatgttgagacatgcactgagagagcaggatttgaatgcatgaaaaatgaattggaaacgaaaaataacaatgtttgtatgcttgt

aatactctgtgcaacatctttaagggaaactattattcagtttaaaaacgaaaaataacaatgtttgtgtgcttgtaatactctgtgcaacatctttaag

ggaaactattattcagtttaaagatgaaaaataacaatgtttgtatgcttgtaatactctgtgcaacatctttaagggaaactattattcagtttaaaga

agtatcatttttaccagcttatgagatccagttcatcaacaatatttgtaatgacatttgtatcaaatgtacaggacaagcactaactgatcatcactat

tatttctggtccatgtatcataaatagattttttcactgaatgcttaatggatatttagttacctatgatgactgacatacacccaaataggtttgttag

aaatcacaaatgcaactttctgcagcacgaatgtttagaaaaatgtctacatcagtatctcagatagatagggagaggtagagggaatgggaatgggaga

gggaatgggaatgggagaagagagggagaggagggaaggagagaggagaattttcagattctgagctttgtgttcacttactagttctaaatcatcttca

ccagctgagcaatcttttctgatccataaacagtctgaaacttcacaccttcacttcatgggacaacttttttctttctagatttcttctttttgtcaac

aatattctcctgcttcctgacaatgcatacacggcagtaccagtcttcattttcatctggaggaacttgtatgcccacacatacccagtgataccatgca

tcacagtcatcacagccgatcattggactgccatcatcttgaccaccacatgcagggcatatccatactttgttgccatcattatcctgcaaaggtccac

tcgcaatcgtcttcagggcaggggcaggttgtccactctctgcaggcttcagcccacattctaaggg

>79636|AGAP000194-PA

atctggtgactgaggacacccccctggcagggggtcggtatgtggcacctcgtggcagcactgtaatggtggcttattacctgttgcaccgccaacccga

gctgtttccagacccgcacaagttcgaccctggtcgattcctggagggtggcaacgcgacatccaggaaaccctacagctacgtgccttttggcgtcggc

cctcgcaaatgcgtgggcggccggta

>79638|AGAP000194-PA

acgaccaggagctgcaggaggaagtggacaccttcatgtttgagggccacgacacgacatctgttggcatgttgtgggttttggtactgctcggacatca

ccccggaatccaggagaaagtcttcgctgagctggatcatatatttcgagacgatcccaacagacgtccaactacacatgatctgaacaacatgaagtac

ctcgagatggtcataaaggaagccctcaggctatatcctagtgtgcctggtatcggtcgccgagcgacgaaggaagtggaaattggtggctacaaaattc

ctgagggttgcacgataaccctcaacatccggaacactcatcgcgaccccgaccactggccggacccggatcgcttcgacccggacaacttcctgccgga

gagggtgcagggcaggcaccccttcgcctacgtgcccttcagcgcggggccccgaaactgcataggccagaagttcgccatcctggaggagaaggcggtg

ctgtcgtacgtgctgcgaagtttccgcgtggagacggtggacaggctggaggatctcaaattgaatggcgagtttctcctgaggccaatgaacgccgtca

acttccggctgtggccgcggtagcgcaacgtatgtggtggcgcctccagctgtctcctgtaccattcgccgacggtgcttcacgttctcaccaagcaatc

aagtgacgtgaatttttaataccattttatatatttttataatatatttaataaatattactttctcggtgaa

>79635|AGAP000194-PA

ctgttgtaaagatagtagatatgcatgccatattttgtatattatcatcttttgttgttacacaacaacctcaattacttacaatctgataaggagaatg

cattcgagggaagcgtacaattacaaaaagtcaatatggctggccagttgacctatttctcattagaagacaaaactccagttttatcaatagacacatt

aaaaatagagaaaactattcataatgttcagaattatcagtttctccagcaaagggaaataagaggtggatctcagaagacttggaaggattggaaagtc

ctctgaattatacagaagtttctctttgtggcatgttactttgaatccacatgttatcaattcctatttcctaattcagattcaggggaaatgacaaacg

tgtatgtttcaaactgaattaaataaattatgaatgcataaatttttttagtgctggtgtatttcattgttaacatttgaccatagtgagatagaacaat

ttactattatatttacatatgactcattgtatttatttgggtctatgactgttgcactataaccttaaattatttttgagaactttactgtgtgaaataa

aacaaaatttggcatcaacaactcctgctgtctttgatctggtagattgagaatcatttctatgctggccaaatgttagcaattaagtctttttctacaa

tcacctctgaactggtagcaatgcactaccaactacaaagtaatatatctatataggcaatgaccaacaaatatcacacattttaaacaatgcacttatt

aactaataaccacaaaactgacttgtaaaaacctgagattttcttttaacattacaaaataaaaatcattctcaaaatttctctacgtctttcaacaatt

ttccaaaattgaaacaaattagcactgtataagtaaacaggcaaataattttaaaacacataaagaatatttttcattcaatttatatcaaagtatttct

tagagaaataccagcaacattatactaatggaagcacctgcttccagagctacctggtcctcagcttcaccttgattccattcatggggcgaagaatgag

ctctgcagtgactttcatattttcacgtctctctacagactcaatgacatagttccgcaatagtgaagagaggacagtcttctcttccagcagtgcgaat

ttttgccctatgcagctacggggtccaccgctgaagggcacgtaggcgaaggggtgcctgccctgcaccctctccggcaggaagttgtccgggtcgaagc

gctctgggtccggccagtatttggggtcgcggtgagtctcgtggacatgcaggaccaacatcacgccctctggcagcttgtagccaccattttctgattc

ctttgaattgatacgaccgataaaagggacgcttgggtacag

>79637|AGAP000194-PA

gttagcctcgctgccagcagcatccctcgacggttccacgagatgctggacgtcacgtcgctgcttttaacgatcctcgtggtcgcagtgtctgcgagta

tcatcgccaggaaatggcgatggaggcagctcgagaagcttcctgggaacgtagctctgcctttcatagggacttcctacgtagcgttcctcacgaaacg

cgaagatcgaatgcgcaaatttgacgaggtgcacaagaagttcgcacctatctttcgcacctggatgggtccttacccagcggtacagatacaaaagccg

gaacacatggagaaaataatgtccagcagcgagataatagacaagtcaaacttctacgccttcttgtatccctggctgggacagggtctccttacaagca

caggttccaagtggcacacgcaccgtaaaatgattacgcctgctttccactttcgcatattagacaacttcgtcgaaatatttgctgaaaacggagagat

tctagttgagaaacttaaaccaaaagctgatggaaaagactttgatatctacccgttcattacccgctgtgctctggacatcatatgtgaaacagcaatg

ggaacttcagtcaacgctcaaaatcagacacactctgcttacgtcaacgccgtttacaggataagtgaactcaccgtgaaaagaatgattaagccatggc

tgcactctgatttcattttcaaaaggacttcgacagggcaagagtattatgaccaactgagtatattgcacggattcacaaagaaggtgataagcgaaag

aaaatcagcccgtaaggcaaacggtgcccaaccgaagacaccacaagaggatgacctaggtaggaaacgacgtgtggcttttctggacatgctgttggac

gcttctgagaacggccaaaaacttaacgataaggagttgcaggaggaagtggacaccttcatgtttgagggtcacgacacgacgtctgttgccatgtcgt

gggttctggtactgcttggacaccatcctgacatacaggaaaaagtcttcgctgagcaggaaaacatttttcgagacgagcctaagagacgtccaaccac

gcatgacctgaacaacatgaagtacctcgaaatggtcatcaaggaagcccttaggctctatccgagtgttccagccgttagtcgccacctgacgaaggag

gttgatattggtggctacaaaattcctgagggctgcacggtgtcactcaacatccggcagacccaccgtgaccccgaccactggccgaacccggagcgct

tcgacccggacaacttcctgcctgagagggtgcagggcaggcacccttacgcctacgtgcccttcagcgctgggcctcgaaactgtataggccagaaatt

cgccatcctggaggagaaggcggtactgtcgtacgtgctgcgcaacttccgggtggagacggtggacaggctggaggacctccagctgatcggcgag

>79639|AGAP000194-PA

gtcgtgttgttagctgaaaggtgcgttgattacagtactacatctctggtgccttgtacgtgttgtgctgcagtcacttcttcgactcggctgccacgtc

taccgcggagtgagcctgaggaagacgcctttgaagggccgcaacaccagctcgcctatcagcttcaagtcctcaagcttttccaccgtctccacgcgga

agttgcgtaagatgtacgacaataccgtcttctcttcgaagatggcgaatttctgaccaatgcagttgcgcggtcctccgctgaagggcacgtaggcgaa

ggggtgcctgccctgcaccctctccggcaggaagttgtccgggtcgaagcgatccgggtccggccagtggtcggggtcgcgatgagtgttccggatgttg

agggttatcgtgcaaccctcaggaattttgtagccaccaatttccacttccttcgtcgctcggcgaccgataccaggcacactaggatatagcctgaggg

cttcctttatgaccatctcgaggtacttcatgttgttcagatcatgtgtagttggacgtctgttgggatcgtctcgaaatatatgatccagctcagcgaa

gactttctcctggattccggggtgatgtccgagcagtaccaaaacccacaacatgccaacagatgtcgtgtcgtggccctcaaacatgaaggtgtccact

tcctcctgcagctcctggtcgttgagcttctggccgtcctcagaagcttccagtagcatgtcgaggaaagcaacacgccgcttactgcctacgtcgtcct

cttgtgctgccttgacttggcctccattcgttttacgggctgctttcctttcacttatcaccttctttgtaaaaccatgtaagacatgcagctggttgta

gaactcttttcctgttgggctccttttaaagatgaactcagggtacaaccatggattaagcattctttctatggttagtgcgctgatcctgtaaatggca

ttaacatatgctgattgtgtctcatgctgagcgttgactgatgttcccattgctgtttcacatatgatgtccagcgcacagcttgtaataaatgggtaga

tatcaaacgttttcgcatcagctttaggtttaagtttctctactagaaccttcccattttctgcaaatatatcgacgaagttgtctaaaatgcggaagtg

gaaagcaggtgtaatcattttgcgatgcatatgccatttggaacctgtgctggtgaggagaccttcccccagccaagggtacaggaaggagtagactgcg

gccttgtctattaattcgctgctggacaatactttctccatgtgttccggtttttggatcattaccactgggaatggtccaatccacgctcggaagattg

gcccgagctttgcggccaactcatcaaatttgcgcactcgatcttcgcgcttaacgaagaatgctacgtaggtagacccaatgaagggcagaggtacgag

accaggaagcttctcgaactctctccagaacagtttcctggagatgatgcttgcagagactgcgatcacaaagaccgctatcagcaaagtcgttacgtcc

aacatttcgcgaaggaagttcctgctagcaaccttcgcgcgtac

>79634|AGAP000194-PA

ataatcgccccaccgttataggcaagtacagacatctcggcgctcagtctcgtccgtaggtcgcttccggcaacgttttgagacggttccgtgcgatgct

ggacttaacggcgctgctgctgacggccatcgtggtcgcagtgtcggcgagcatcatttccaggaaatggcgctggagccaggtcgagaaacttccaggc

catgtagctctgcccctcctagggacgacttacgtcgatttcttcacgaagcgtgaagaccgaattcggtcctttgacgaggtgtgcaaaaagtttgggc

ccatctttcggttctggttaggcccttacccagtggtacacatatcaaagccagaatacatggagaaagtactgtccagcagcgagataatagacaagtc

ccacttctacgccttcctgtatccgtggctaggacagggccttctcaccagcacaggttccaagtggcatatgcaccgtaaaatgataacccctgcattc

cacttccgcatattagacaacttcgtcgaaatattcgctgaaaatggagagattctagttgagaaacttaaacctaaggctgatggaaaagcgtttgata

tgtacccgttcattacccgctgtgccctggacatcatatgtgaaacagcaatggggacttcagtcaatgctcagaatcagacgcaatcttcttatgtcaa

cgccgttaccaggatcagtgaactaaccgtggaaagaatgattaatccatggatgcagtctgatttcattttcgaaaggacttcgtcagcaaaagagtac

tacgaccaactgaaaattttgcacggatttacaaagaaggtgatagctgagaggaaatcagcccgtaaaacgagtgaatcccaagtgaaggcaccgcagg

atgacgacctaggtagaaaacgacgtgtggcttttctggacttgctgctggacgcttctgaggatggtcagaaacttgacgaccaggagctgcaggagga

agtggacaccttcatgtttgagggtcacgacacgacgtcggttgccatgtcgtgggttctggttctgcttggacaccatcctgacatccaggagaaaatc

ttcgctgagcaggacgacatatttcaagacgatcctaagagacgtccaaccacacatgacctgaacagcatgaagtacctagagatggtcatcaaggaag

ctctgaggctctacccaagtgtgcctggcgtcagtcgccgagtgacgaaggagattgaagttggtggctacacagttcctgagggctgtacgatattcct

gaacatacgccagacacaccgcgacccagaccagtggccgaacccggagttctttgacccggacaacttcctgccggatagggtgcagggcaggcacccc

tacgcctacgtacccttcagcgctgggccacgaaactgtataggccagaagttcgccatcctggaggagaaggcggtgctgtcgtacgtgctgcgcaact

tccgcgtggagaccgtggacaggctggaggacctccagctgatcggtgaactcgtcctcaggccgatgaacggcgtcaacctgaggctgttgccgcggta

gtgcagagtctgtggcggcgcctctacctgtctcctgtgaagtccgccaacagcacttcatgtcttcatttttttcgtcctttgatctgactattttaac

aaccttgtacatatgtagaatgaaattttcactctacagtggagtgtgcgctgatatgaaacttcctggcagattaaaactgtgtgctggaccgagactc

gaactcaggacctttggcttccgcgagcaagtgctctacgaactgatctacccaggcaaagatcccgagttcgagtctctgtcgggcacacagttttagt

ctgccaggaagttgcgatcgtacgcgttgctttaagccgctacgtaaatgacaac

>79739|AGAP000198-PA

cttgcatattatgagatctgtgaaaacctgcagtctgatgactggaaagtggtacaaccaaatccaagtgctatgggaccttatgcgtataaaggcaacc

aatgggttagctatgatgatatggatatcattaagaagaaggcacagtatgtgaatgacaatggccttggaggaattatgttttgggctattgacaatga

tgatttcag

>79772|AGAP000210-PA

ccccattgtgcgcctggacccatctgatgctgattatgtagatgttgtacatactgatgcagcaccgttcatgagtggcggtgagtgtctgcaaaatatg

aagatggtggcatgtttgcattttttccttcataataaagaaatataatattttgcttaatgcttttaggccttggactgaaagaacctgttggtcacgt

ggacttttatccaaatggaggcattcgtcagcctggctgtactgatggagtctttgggcatataggaagacag

>79821|AGAP000252-PA

ttcaacaaactgcaaacccatcagagattcgcaaagcataaacaatccagagagcaccttcggtaatggaaaatactcccgaatggaatcttcatccaac

ccaaattccagtttctgctgcctcgtcttccaataaggtacatcccatatttgcatgggaccttcaaatccctgttctgctgcaaatgtttccaattgca

gaagttcttcctcttgaacagggagtgctttgtctaaaaggtgatttaacatcttttgaacattttccacacttcctgccatctttgtttccattgacaa

atcggcatacttgtcgtatcccaagagctttgctaaattcctcctctgtatacggatctcctggacagttgtgctgttcgacacacaatgagaaatcgtt

ggagaacagcacaactgtccaggagatccgtatacagaggaggaatttagcaaagctcttgggatacgacaagtatgccgatttgtcaatggaaacaaag

atggcaggaagtgtggaaaatgttcaaaagatgttaaatcaccttttagacaaagcactccctgttcaagaggaagaacttctgcaattggaaacatttg

cagcagaacagggatttgaaggtcccatgcaaatatgggatgtaccttattggaagacgaggcagcagaaactggaatttgggttggatgaagattccat

tcgggagtattttccattaccgaaggtgctctctggattgtttatgctttgcgaatctctgatgggtttgcagtttgttgaa

>79851|AGAP000255-PD

gtcggatgttgactcaaatgacgtcgcggtagcacacaagtgatgagtgcggcgaaaaagtgaaacgttacagtcagtttagaaataatattcgtgtgtt

gttcaccttcggcaacagcagcgtcgtaactgtgtgacaagacaacgatgacgagccgccggaaaaagcgccagcaggatggcttcatgaagaaggtgtc

ttcgctcttcaacctagacacggcgcacacgccgctgttgcccggagatagcaaacacgtgaacggcgatgcggagtgggagttcgaggagatggtgctg

gag

>79869|AGAP000264-PB

tcttgtatgctaacttccaactgctcatagatccgtaagcgagagtcattgacttctcttagggcagctgtctgtttagtgaggtactctatctcctgtg

cctgatcctctatgactccctggtgctgtttgaggcttgtctccaactccttgttcctctccaataaagtctttcctaactctgctgccaactgcagatc

ttccagcatgccctgctcatccgtctcgtcgtcgtcgtaatccatgttgtcgggcgcagccgctgccactggcgacggcgttacttcgcagagtctcgcg

cccaccacacacgcacctcacacgtcgagcacgcgcgtcggcggctccgattgccgacgcccgcagtagttctgctgaggcttgtctccaactccttgtt

c

>79902|AGAP000276-PA

cgtgatattaaatatgtacatttgcagaatggcagggtaacggggaccgattgaagtgtgtaactcgatggtatgccactcgggactggtactccgagtc

agggctggggctcccgtagggtggccaggaaggtgtggcaggcgctctcggctggcaggcactggccgaatgcctccagcagcagcggctccgcctggac

cgccttctggaagtcctggaaggaccccttgccgtcgcggtccgcgtccagcttgcgcagcgccagctccaccaggtccttcacgccctcgtccggatct

tcgtcgttcgggtgcttcaccaggcagttcctgagcaggtggaacatctc

>79983|AGAP000301-PA

aaattagtactggagttattcatccctcattttattgagatggccaactacagcaacaaatacagatcacatcagcaggctatattttacctcaaacatg

ctgaacggcttacaagactgcttgcagaacaaccagcagctaacaagacagatattgaaatgctgatgacacagatcaagtcagcacaacaggccac

>80026|AGAP000314-PA

aacacattaaagaaattattactggtttcaattttcaggtgaaactagcaaatcaggagtcagaattagtaagtctcttgaagcgcatccctctaggagc

ttgggaacttaaaattattcgggaacgtgcagagcaactaaaagacggaacattgcgatctcgtggggaagctctgctcccaatcatgcttgccagctat

ctttttgatgtgcttgtcatgccaggaggacgggaagtgaggtgtcagttgcagaatcagtcactgcggttgccaacagatgagactctgggttttgagg

ctgcagttgctgctcttggtttgaaagctaatgtgtcggaggctgaccacccactcctctgtgagggaactcgaagacagagaggtgaccttgcaattgc

acttctggtccattacaaggatgatccaaacaagatttccagaataatggacaagcttttagatagggaagttcatcagttggtaaaaactaccatccat

gcgtcttattattcaaacaatccacctaccaggagtcatttgagcaaggtttcgggtcgaagaaaagaagaacagagctcagcagaacctcctctagggg

cagtagtcaattctgttgggacttcatcagatggcttgcttccagatgttgcccctggctgttctggcagtcgtccacacagcgcaacaagtgcggagtt

ggagcaggcaatgtcgggactttgcattagtagtcctccaaaccccatgccaccacctcgtacaaaggagtcgcggtgagtaattgtttattatctatga

ataatcaccataaaataccc

>80045|AGAP000331-PA

tcactattgtacaaagtgttttatttcaggctgacaattttcagtcaatatattacatttttttgtaaaaaaatattcaatatcttggtacaggtcattg

taaaaatttacatgatgagtaattattcagtaattactctaaaaagcttgaattgtgataacattgctgctctgatatcacagaatttggtaacaagctg

aagatttaaatgcacacaaattaaatgtattacatattattttgcagttgtaattggccatttattctcaattattagtttgctatggatctcctggaaa

tcatgtcgtcctcactatgctttcagttgcttgtttttaattataggcacagattaattataatacaatttctgaaacaagaacaacttgatcttaaaca

actcccttaactttcgccagaatttttccaagatttctttaattatttatgtgttggcactaataatttaaacgtattacattttaaatagaaatgtcct

gcaaacaaattagtttccagtttttccatagcattgttgaattcctattcatttcttattcatcttttataataatatagagcataccacaccacaagag

agatttacaactgcaaagctgcatatggagccacatttgtgtgacaaaataaaatttgctatttatctagaaaaataacatccactttatcatttgtgct

cagtgacaaaaatggttatcacaactgtcagttcccaaaatccacaaatgtttgggaaacattctggaggagtaacaaatatatttatatagtactataa

ttccattttcttgtatgaagtttttgttaaaatcaaaaactttgcatacaaagaagatgaacatggacggtttcattacaatatttgtgtattttcacaa

atcttaattcaaaacactgcataggggaaagtgtgcaggagagcacctgtatccttatgaaataaataaggcagttgtttggaatttcaacagcatagtt

aaactttcatattttaacattaaaaacaaatcaaaaatttacacaacaaaaaatacttttagatcaacagtgatgaagtgtcataaatggtcccatgtaa

aggtctgaagtatgtacagaaatatagatttccacattttaggaagcagatatgtaaattcatacaggataaaattttgttcaaaattgttaccctgtct

taaattacatgtggaatacttttttatactaatggaagatttttacttagttgtttggcccattggaatgccatatttgtatgtatactttacatgcaac

atttatataaaaataactctgcactatttataatgatagttatcactctgtgccacttgtaagttgttaagaccttcaacattttcagattagggggaag

taatttaaaatacaggaaggaattacaggttatggccctgatatcaaagtgtcatagtccagtcagtctttctctgcttttatagagtattagctgcagc

ctcacataaaacgatgttctgatcatatttacatatgaatattcacaaatctgcttaaacttcaacaataaaacatatcaacaatatacactccttttca

cactcctgggatgcacaacagatttaaaaattgtaatactgcacaagctcttaacagagacaataggatcctctacagaaatatcttgtgacaaattcat

atgtacctgttatgaaaatatatcatagtatttctgcctttgtcttcattatgacttctgcatcaaagttaagaatatttacatttttaatgtcaattat

taacacacggcctaccagtgatgtcctttcacaatcaagtttgtaaatattcagttggtataataattgcaccacactggttgagatacgcagtatccaa

attctctcagagaacttgttgtgttgacagttcaatagatgtgtgcatatttcagcattctgatttgtcctcctctacaagcttttgagtgtctttgcca

gtatctgtagaggtgggatccagttctctgtgcctgatgtgaatctgaggtgatgatttctgaattatacttgaaggtgcgagaatagctgatgctggtt

caggcatcatatctacttctccattgcttccaatttttgatttagcaacatctgctggaacttcatatatccccccaccaccgcctccatttccagtttg

tcccccagttacatttccaagggcatcatacatcggattgctgaagtctctattttttgggccaacatcacgcatgttgtattcagcatctatcggttct

actcgtgtcccttgtgggccattagctgcaaaagctggtgagccaaattcaacattcgttccttgtctgaaggaaacactctgggaatttgttaatccac

cgaggccaggagcctttccaaatggatgcttccggaaaactataaacagtgccactgcagatgctagtacaagaagaataaccacaataggcacaacaat

tgttgcagtactaggtccagatccaccttctgttgcatgctgaagataggttccacagaatttgccagcaaattctttgggacattcacaaacaagatca

ctggaaccatcatctgcttcttggcatacgccaccattctggcacctgcatatccgtggttctggatgtggctcttcattaggtgcatcacactgcactt

cattgttgcctatgtgtgtctgcgtattgtctggacacatacaccggtgcccaccaggcacaataacacacaggtgggaacatgggctattatagcaggg

atcaatcagtgacatgttgtaacgaagtttgtggtacactttcactcctgatggactttcaatatcttttgcaactactactggaacaccacgtccaaat

ttgtcctgtctgacaagttctccattgtctctgttcacccagtacaggctgctttcaaaaacatccaatgatatgggatgacgcaaggactctcctttca

aaacatcaactctgttggacccatcttgtcgcatcctcacaatgtagttcagtttagtgtcggcccaatataacgtgtggtccatagcgtagtcaacaac

aaggcctgtcgggtgcctgataccatctgtgaccagtggcctgcgtttgctcccatccatccaagctgtctcaatctttggtgctgccccagcatcagcc

cagaacatgcggcctaactgaggatcgagagccactgaagttggtgattccagcccagttgtcacaagggagcggcggaagcgtccatcttgctttgcaa

ccattactctgcctctcggtttgttgcctgaacggtcagtctctgtccaatacagattttctgcaacccaatccactgcaatgccagttggctttgaatt

acctttcatattcagatcttgagcatatcccatttttacttctccatttctagcattaatcatatatgacctcttaattgttttatcatatgaatctgcc

caaaacacatattccattcttggatcatagtcaattgcttcaatcctcttctcattctgaattacattttgtgctcttcgttctttaggttcatatgctc

ttatctctggtccattgccaaaaagtaatgtaagatcaccgtctgccaccttgcatacaccactcagtctatcagagagaacaaacccatctcgacagga

gcaactgaaagttccatttagattggtacaagtctgtgagcagtgatgggtcccagattcacactcatctatatcaaggcatttctttttattttcagga

gatataatgtacccactgtagcaagcacagatatatccaccacctgttatattgatacagtgatgttcacagcctccccttgttgcttctgtgcatgatt

tcaaatggtgacaaccaagctcatcagaattgtcaccacagtcatctgcaaaatcacacaattttttcttgtcaacacagttcttatttgcacagtgata

ttctgaataatggttgcatggcttgatacgagaggcacacaatgtcatattattttcatcacttccatcaccacaattgtctgctccatcgcacacctga

tatcttggaatgcatttatgattatcacactggaatcttcttagggtatcacagttaaagtcattgcacaatgctggactttcatctgaattatcaccgc

agtcatctgttccatcacagaggtccccttggttcacacacagatgattggaacactcaaatttactctcgggacaatatctaccattaggatatcgtgg

tggacaatgcatttcatcactcatgtccagacaatctctgtctccatcacacttgaaatatgctgcaatgcagtggccactagcacactggaatgttcca

ttcctgcactgaaatcctgcacagtttacttcgtctgaattatctccacaatcatcatcatggtcacagcgccatcttgttggaatacatttgttatttg

cacaacggaactctgattctgaacattccctgtatgatccactacacactgattcatgttcatcactattatcaccacagtcattctcaaagtcacacag

ccatcgccttggaatgcatctgttgttcatacactggaagtcacctgtctcatggcatttcggacagttctctaccatttcatcactgttatctctgcag

tcatcttttccatcacagaataaccattttggaatgcaacgataatttgcacggccaggacaacgctgccacccatttgtacagttacgctgcctgcaca

tgtaagctggttcatcagaatcatcaccacaatcattgtcaaaatcacacatccacaatttagggatacaccggccattcttgcaagaaaattctgtttc

aggatcacactgtcggtttcggcatattgcaggatcttcatcactggcatccttacaatctgcatcaccatcacacttccaggcattgagaatacaccgt

ccagtggttttgcatttaaattcaagaccagggcatggtaaatcacattttttctcatctgaattgtctccacagtcatcaactccatcacatatagtgg

cagttgcagtacagtttccgttctcacactggaatgtccctgcacgacactgccggccagggcaatctgaaggttcatctgatccatccctgcagtcttt

ttccccatcgcacttccagaaccatgggatgcaactctcatctggtgcaccacaacgatgttgtcccactgtacagttggcaatgcaggtcttattatcg

gcaggagagaggaaaaactgattgggacatgcacaagtgtagccaacagtaccttcaccttctccataaccctctggggtttggtactgtcctgagcctg

gagctagcaggcataaatgtgagcaacctccattcccattaccacaaggattgtggtatgctagctgcctcagagggtggaacacatgaatatcatacgg

tctgtgcgtggtattgcgaattacttcc

>80070|AGAP000339-PA

gtgcaattgaatctgtagatcacgttgtgaaagatatgttcttctgcaccccgtgtttccataacgcgtaccaccatgtgtgcacataaaaacagtt

>80086|AGAP000344-PA

gcggaagctgctgctagggcgggtgttgtagccctccgacttgtagtagacgctggggtcctcgcggtactgaccgtcgtcgtagccgtccgggttgtcg

taagacggcgacgccgtgttctgcagcgtcggtggaggcacgttgatgcccgtacccgcgggctcgaatccccgcctggaggcgccgtactcgatctctc

gcagg

>80101|AGAP000348-PA

atgccctccacgaagccgatctggaagtcctttagcagtcggcgctccatgacgcgacccttcttgcccactgccgactcctgcatgtcgacgttgggcg

tggtcatgccgttcgtgcgcaggatgtcgatgaggtcgtagcgcagcgtcgagatgtcctgccggatctcgatgacgtcgtcctcggtgatgccgaagtc

gtcgcgcttgcgctgctcggccgtc

>80142|AGAP000369-PA

gcattcttaaattcagtgtttttccatgcatatatgagtggatttatgcacgagttggccatacccattgaaaatgctgctttgtaaaccacaaaggatg

tgtatgacttcgcaccagcaacacgacaacagatgactgtgaaatagggtagccaacagatggagaaagagcccaagactaaaagaacaacctggacaga

tttccaatctgactgagagttagtaacgttcctaagtcttctggcttgttctgaagcttccttccatattcgccagtaaagtaccaacattgctacaagt

accagaacaaatgctggtgtgatgatgcatatgatgtaggcttttggtagcacattgtcaaccaaacagtccttgtcctcactgtatttattccaatagc

tgggcattgttgatactatggcagaaagtatccagccaactccgactatcactaatgctttccttgctgtcatgtacgttgaatagtgtagtggatacac

aatggaaatatacctgtcaatagcgatcgctatgagattatatatcgaagaactggctggaagtatgactagtatgaatcgcaataaacaggtgttcttg

aacttgtttagttcatcgattattgagaatgccatatgatatggcaaaaatagacctactaacaagtcagatatcgccaagttcaggatgaactggtttg

atagtacacttgacagacgtcgacaccataaaattgcgcagatggttacaatgttccctgctaaaattggaatcatgagggctccgtcaatagttgccca

tatcactctctctggcagtccaaatagtatctcgtcttcagtgtctgttgaatagttccccgaaaaggtctcattgaagagtcctgacatgtcaatctca

cgcaaaagatgtccgttttcccgctcatatataacactaaaagatcactgggaaaatgcacagtttctgatataaaataagtgcttcttgtgtgaagatg

agtgggaaaacggacatcttttgcgtgagattgattcttagatcccacacatatggtttgcctcctcctaagccatagactcacatagcattttctttca

tcatatgtcttccatcatgaatcttacttttcaacatttcacattcataataatttcacttcattgatgttgtcactggatataagctattttcattgtc

tctgctgccgctaatgtgggatgttttgtttaatcagtg

>80141|AGAP000369-PA

attttttcagaactatatttacaaaaagaagattaaacaatcattgacaattaagtgaaacctccacatgaactgtcaatagtgtgcagttcggtatgtg

tagttatagtttctagtagatgatctgctcggtttggtgacttgcaatgaagtatatttctaaaagcattcttaaattcagtgtttttccatgcatatat

gagtggatttatgcacgagttggccatacccattgaaaatgctgctttgtaaaccacaaaggatgtgtatgacttcgcaccagcaacacgacaacagatg

actgtgaaatagggtagccaacagatggagaaagagcccaagactaaaagaacaacatattcacctggacagatttccaatctgactgagagttagtaac

gttcctaagtcttctggcttgttctgaagcttccttccatattcgccagtaaagtaccaacattgctacaagtaccagaacaaatgctggtgtgatgatg

catatgatgtaggcttttggtagcacattgtcaaccaaacagtccttgtcctcactgtatttattccaatagctgggcattgttgatactatggcagaaa

gtatccagccaactccgactatcactaatgctttccttgctgtcatgtacgttgaatagtgtagtggatacacaatggaaatatacctgtcaatagcgat

cgctatgagattatatatcgaagaactggctggaagtatgactagtatgaatcgcaataaacaggtgttcttgaacttgtttagttcatcgattattgag

aatgccatatgatatggcaaaaatagacctactaacaagtcagatatcgccaagttcaggatgaactggtttgatagtacacttgacagacgtcgacacc

ataaaattgcgcagatggttacaatgttccctgctaaaattggaatcatgagggctccgtcaatagttgcccatatcactctctctggcagtccaaatag

tatctcgtcttcagtgtctgttgaatagttccccgaaaaggtctcattgaagagtcctgacatgtcaatctcacgctcatatataacactaaaagatcac

tgggaaaatgcacagtttctgatataaaataagtgcttcttgtgtgaagatgagtgggaaaacggacatcttttgcgtgagattgattcttagatcccac

acatatggtttgcctcctcctaagccatagactcacatagcattttctttcatcatatgtcttccatcatgaatcttacttttcaacatttcacattcat

aataatttcacttcattgatgttgtcactggatataagctattttcattgtctctgctgccgctaatgtgggatgttttgtttaatcagtgcttagtaat

tctgtgtggataatatatgaagagaattgctgtttcgggtacagctactttgactctagctttgaaggcaatatctatcacttatcagtctggtgtaaat

tacataggcaaacaacataaacattcagaaagctgagcttggtgataagagttagaatatttaatggtgttttgcatcttgcactgaattttcattttct

ttcccatttttctgctaggcaagttgttaaaacttatacccccactattgaccagtgttatcagaaaaatacaaagcttcct

>80155|AGAP000379-PA

cgttaaaaagctggttggcattatctggtttgttggtattgttggaaaaatgtaaaaatgataacgcttgtgtgaatcagttgtgggacatcgttttgcg

aaatatcagtgtgtgtatcaatggattcgttgaccagtaatcatcgatccttgctttttttacaattcccataaggataacgagccgaaaccattttcta

agttctgg

>80156|AGAP000379-PA

gcaaattatagtgtacatgggcttgctatccttatgggaattgtaaaaaaaaaaagcaaggattgacgattattggtcaacgaatccgttgatacacaca

ccgatatttcgcaaaacgatgtcccgcaaacgattcagacaaatattatcattcttacataagagcaaagagtaaggctccacacattttgctgcatagg

aaaggactgcaaaaagaaaaagaaataaaaagcactgacgatattcgagggttaatacatgctggtggtaaggaggacagttacactttattgtacattg

gtatgaaagaacaatgcactaaagcacggaacctggg

>80166|AGAP000379-PA

ctttcttctttcttgatgctttgcatttcagaatttgtggtaaacctttgtccattctaatagtaccacagattctgattttgtctttcagtagtaatta

tgcagtagaggaactgttataacaatgatcctgatatatgtggtgccacatggcaagacatttgccaagaatggagagaacagattcttcagatttcttc

ccttcagcagtgcaaatatccaaattgcagatataacctatagcattctcgcacaccatcctaacaagtaactcatgctgcataattttagcggttcctc

tccatggacttgtcccctcatggcaagacaactgttggtttggtttgtaaagtatcctgaatttagtgaagagataatccagcacagggtgcactttgaa

gagttttcca

>80182|AGAP000383-PA

ctcgtattcgggaggacagtggctacatcgagtcttcttcggacgacccgaaatactgtatgttttccgtcgtttccctgcgggctagaagactccgttg

agaatgtcgcagccagtttctttccgcgttcgtgtcccaatccgacacaccgcgtatctcgtcgtggaaaggctactagtcaatcagatggtggctgaga

cgtacgacaatggtgccacgtatgacgtcatatacaccatcttcgtgtggtttgcattcttgcacactttcaccacacctttctacttctatgtctggaa

cgatggtccggtgacgaaggagca

>80245|AGAP000398-PA

cccgtctcgcacctctcctcacttcctgcgactgccatcccgggcggtgatgcggcatcttcgaggactccacgtagagcactttcgacagagagtttta

cggggcagcaattcactccaggaatggttcgcatccacagggccagtgccatgctgagggtgatgtgtgcggatggcagctggctggcctttgcaaaagt

caccatcatcgggaaaaagcccatgtcggcaattgacttctataatggtttcctgtcaaaagtgcctttggatgaatggatcctcacatgaccactggac

caaatggaattactattatttgttcagtttttctttgcaccaacttcaggcccctctgtgaaaatggcaagcacaaggataccaggtagtcaccattata

ctttcaggtctagtaatttagttgtaaaactcatacttaagaggtcacaggaacgaatctgttgg

>80270|AGAP000402-PA

caaaattacttttttgttgacactgggcatcacatgattcaccccattaacatatattttttgctgtctgcacctgtaccctacaaatacaaaactgaaa

gtgtcaacagaaacaccagagaaagatatgtagtgagatagtacacacacagatttgctgttgtttgatattgaaaacaagatagttatgttctaacatg

aaatataatccatgaataagtgttccagtcccctgtcatttctacatgacttgattgagaattgttaccatggatttattttcaggtacaaaagcaatag

caaaaacacgggtaacagttttatcgcttaatgattcaaaggaaacattcctcaaagtgacaagatctctctgctattcagctaaaaacagagaagtgaa

atcttgtcacatcagtcaagatgtcataaatgaaatgttaattgaagagaccggtttaccagaacccgagcttgcattgtattgtggagaagtatgttgc

acatacgggttcttaccatggcatatacgaatcacagagtttctgaggttacgaagccatcacaacatcacagtaagagaatttgtgagattgttacaaa

cctatggtaaatgtgaacagagatttggaaagtgattaataaagacagtgcatcaagtgaagattggggaaatccattccatttctgtgttctttatcat

tattattataattattacgaaaatgaacctgtttttttttttttttttttttttttttt

>80292|AGAP000422-PA

tctctgtaactactctcttcaaagacaagaaatcctgcactctagccatttctgctgctgggtcgattacaagctgctctccactgacaaaatgcaactg

tcgcaatgggaaatatcgtaaccatcgttccaagtgccgtgcgtaaataccaatcttcacaggtccccatgaagtatccaccactgttttaccttctctt

ccagtaaagttaaaaaaagccaactcctcaaactgtggcatatcaagtttcttgctgacagcttgtgtataatctgaaattgctcttgttactggatctc

gtactaccacaagcagttttgttatggggctcatctcatacactctctgtggagcctctcttgtaacaaagtagcttggtgttttttccatagtcacttg

accttctagagttggtggcatgcggtttctgtaccactcaaaaccctttgcataaaatttatcaaaaaagtgaacttctgaccccgctgctcttacatca

ggatgtaatcttatgaattctaaaagagctcttgttccacccttcttaacgccaattataagagcatcaggaaatttacgacttggttcaagtccctgat

gtttcaggaatcgatattttggagctccatcctgcagatctgtgtatatagcattccaagtaagaggtacaacacggactacactttgcctttcaatagc

agaagtaagttgtggttcatcacgatttggttcaacattttccttttgtagagtgccaaatgaaaaggcacgtactctacgtcgaagtgatctgttgata

ttcgacaagagacaggaattaaaggtataaaacaaatatatgacagttccaaataaaacaaacgtgaaaatcagcgatctgttattcatgtgagtcacaa

ggtccacacatgtttgtggtatgacttctgtcaatagacatcatcaccatgctagtaaacacttcaaaccttcgttccaacttcttagggtgacttgaca

gacctgaactataaataacaacaggacacaccttcaggttcactaaagaaggcatcttactactctcgacaaaacaatcgcaatgaataatcaaatcaga

gttaattaagagcgtctcagggtaacaatcttccatgacatccaatcattttgaagccggcaccagatgtgatgtaaacaaaat

>80310|AGAP000426-PA

ctccagattttacggatcgccgccctagtccttgctgcctttatccgtgcagttgagcttgtagatctggtagttgagggcagctgccagagtcatccac

aggaagtagggcacgaagaggtagccggcgaccgcattgacgccgtagaaggagtagccgcaggccgccacgttggcccacaggagcgtgatgtcggcca

ggctccacgggatgttgcgcgccccgaagaagatgggtccccacagccagttgagcaccagctgggacccgtacagcaccaggggcagcctcgccggccc

gtggaagccgccgccgtcgcggcacgcgaggtaggaggcgtagcccatggcggtgtagagcgcagtccagacgggcccgaacagccaggctggcggccgg

agcgcgggtttgcgcagccggcggtaccagtcgctgccgagggccctgttgatgcagacgccgcccaccgcaggcagcaggcaagcacacaacgcctcca

cctcgctcctcatgccgccatctttacatcgttcttctcatgaaa

>80313|AGAP000426-PA

caagtgctggctctaaaatgacagacaccctactacagcaagagaggaaagggttgcaagaaatgatatttccagggatggagtgtgaagtcaatctgcc

cacgtaatgaagccatctaagagaataatcaaaactgctgcaaaatattctaataataccagtattatttcttttcacattttgcacctgtcaaatatta

gctattcactgctctacatttgagcaaaagtgcagattttggtgacattctcgagtaaaaatgttgtaattaccttttttatgttacatgatgtgctcca

tcagaaaatgaacacctcaaactgctgacatgcatcaggtaagtttcttctgaatttgtaactacaccatgatatgagatagcttgtgagaacagtataa

taaaatttattgtgaaaatagcattattataaggacatatcctaccactacatgtacatttaaaatgagatttttttgtacaaacatcaaaatatttttg

taaattgctaacaacaagaataatagataagaaataaagaatacaaagagcttgtagcttaaaaattcctgtaagcaattctgagaattatttcttccct

ttactgctgaatgattaaatagaaaatgcaagagaacaaaagttttaagatatcactatcatttgtctgaggtgtgtggtatcttaggctcattaaaagt

ttactttctgttgtaagtatcagatagttttgagaaatattgttttgttacgttattagtagaagataaattggtaatacactaaatatttttgtggtgt

tacagttaaaaatcacgaacattactgttacaatcgttatacgaataatgcaaaactaatgcactaaatattttcagtggtgttacaattaagaatcatg

aacattactgttccaattggtataagaatattgcaaaacattttctgagaactggattctttggttatgaattggtacaagtgagaaggctagtgttttg

ttgtgcctgctaccagcctttaccatcaaataataatgtttactgtagtctccatcatctatgtccgaacacttcgtcggaggatttagaatgaattgca

aggtagtttaccagtctgttgtgggtaccaccaacaacttgcttttaggaaagaatgattcctgtccacactggggtaagtacacagcacaaagcattgc

tgtagtggcagcactgaccaaagcagctggcgccacgggactccagattttacggatcgccgccctagtccttgctgcctttatccgtgcagttgagctt

gtagatctggtagttgagggcagctgccagagtcatccacaggaagtagggcacgaagaggtagccggcgaccgcattgacgccgtagaaggagtagccg

caggccgccacgttggcccacaggagcgtgatgtcggccaggctccacgggatgttgcgcgccccgaagaagatgggtccccacagccagttgagcacca

gctgggacccgtacagcaccaggggcagcctcgccggccctgggaagccggcgccgtccctccagacgaggtacgaggcgtaccccatggcggcgtagag

ggcgctccagacgggcgcgtacgcccagcgaggcggcgtccaccggggcttcctcaggcggtcgtaccagtcggagcggaccagcgggcggaacaccgcc

gcctgcaatactggcaccgtgtaaggcacggcgcacgcgaccacggccgggcacgagacgagcatctcgctgcagaacacactgccggcggctggcgtcc

ctccgcgc

>80311|AGAP000426-PA

gcgcggagggacgccagccgccggcagtgtgttctgcagcgagatgctcgtctcgtgcccggccgtggtcgcgtgcgccgtgccttacacggtgccagta

ttgcaggcggcggtgttccgcccgctggtccgctccgactggtacgaccgcctgaggaagccccggtggacgccgcctcgctgggcgtacgcgcccgtct

ggagcgccctctacgccgccatggggtacgcctcgtacctcgtctggagggacggcgccggcttcccagggccggcgaggctgcccctggtgctgtacgg

gtcccagctggtgctcaactggctgtggggacccatcttcttcggggcgcgcaacatcccgtggagcctggccgacatcacgctcctgtgggccaacgtg

gcggcctgcggctactccttctacggcgtcaatgcggtcgccggctacctcttcgtgccctacttcctgtggatgactctggcagctgccctcaactacc

agatctacaagctcaactgcacggataaaggcagcaaggactagggcggcgatccgtaaaatctggagtcccgtggcgccagctgctttggtcaggtata

ctgtactgagaaaacgttaaaaaatatcgctctctgcttcctcctcccgtaactgttctcgctcaattcgatcgtatatactttcttaatatctggcaat

gtggtgctatatctgcaaacaacttttcacttattatgctgcgattttatgagccgagccgatcagagattaaattgatggagcacataagtgacccctt

ccttcacgtaccgatggtgaagatatatcgataccagtggtgaagatatttctttctgtagcactccaactacattctattgcatcgtttgacgttctcc

cggtaatctggtacgcgcacctattaaggagagtgtaacgtgcaattctacaataaagtccacagcaaatattctctctttgccactcgtatttcacact

tttttattgcaatttgtaatacagaacaggaagtaaaatcagagcctaaatattattttatagtttcctgttgcaaattggaatgacagacaagtaactt

ttcactaaccagtgagaatcagaccatcactcttcacagcacaattgaaagagaaagcagaaacaattaaggtaacttcaggtaggcccagctgttaatc

ttagtggtaattcgtgtaatacaatacagctggtacactccagcatacagtctatcccatggggctgttactaagctgagagtccttaagacagattgta

gagatcatttacagttccgtacgccacatctcgtagtgaccggtccgtcagtaatgcgcgggcagttacacctttctgtggacaggaaattgttagtaaa

tactgaatgagatatcctttaatcactccgcgtttatccttctcacatcagatgttctgaccttattctaaccctacatctgcttaaaaattcttactgt

gcatcataatgtggaggccttacaaatttaaggaatcagctatttttttgaacaacagatccgaaaacttttcattcgtgtacttgtagatcaacttgct

gaatatttgtgctgatcggggattatgtaaatttgtgctaaacagtgataacatactgaaagagttgcgacaggagggcagcagttcaaatcctcatcca

aactcccagatatacgcttcttgcggtttccctaaattactcgtgcctcttttttgcagtgctgccactacagcaatgctttgtgctgtgtacttacccc

agtgtggacaggaatcattctttcctaaaagcaagttgttggtggtacccacaacagactggtaaactaccttgcaattcattctaaatcctccgacgaa

gtgttcggacatagatgatggagactacagtaaacattattatttgatggtaaaggctggtagcaggcacaacaaaacactagccttctcacttgtacca

attcataaccaaagaatccagttctcagaaaatgttttgcaatattcttataccaattggaacagtaatgttcatgattcttaattgtaacaccactgaa

aatatttagtgcattagttttgcattattcgtataacgattgtaacagtaatgttcgtgatttttaactgtaacaccacaaaaatatttagtgtattacc

aatttatcttctactaataacgtaacaaaacaatatttctcaaaactatctgatacttacaacagaaagtaaacttttaatgagcctaagataccacaca

cctcagacaaatgatagtgatatcttaaaacttttgttctcttgcattttctatttaatcattcagcagtaaagggaagaaataattctcagaattgctt

acaggaatttttaagctacaagctctttgtattctttatttcttatctattattcttgttgttagcaatttacaaaaatattttgatgtttgtacaaaaa

aatctcattttaaatgtacatgtagtggtaggatatgtccttataataatgctattttcacaataaattttattatactgttctcacaagctatctcata

tcatg

>80376|AGAP000434-PA

gattgatgaggaccacgtaacccacccaagatgactccatgtaaaagtaagctaacgctgttaatgttgcccacagtattgttccagtttttacagcttt

aacccacatgtagtacgtcaacagcatacagaaaatggcaatgcctgtgtaacataaatctgactgaacaatatccataaggcactaccaataatgaaat

aaaaaacaatggtgtgcataagctgtta

>80395|AGAP000472-PA

gtcgtagatctctgtgccgcgagccgccatgtgtttcctgcgctcgtcgtctgctccagctccagtgtgggtctgcacctccttcacagtgggctccgtc

ggcagcacatcagggctgaaggcgtcgtcgccggggcagaccttgcagcagcggccgggctgcagcacggggtggtcgcagctgggcgccgggcactccg

tcttcacgttgcggcactgcgcacgcgccaccacgcgccgtttcttcttcacggcgacgcactcgcagcgg

>80510|AGAP000506-PA

acgtcatgaagaagaagacaagggtcttcttgtcctcatcacagacttacactcgaatcatcaataatgtgacgggagccgtgctgcctggatcactggt

ggcgcttattggaggcagcggcgctgggaaaagtaccctgctggcggccctcgcacatcgatcccctgcgggcacgatcgtcg

>80511|AGAP000506-PA

gaacagctcgtgctggtgcatgtagccgctgagccgccgcatgccgggacccaccggctggccgttgacgcggatgtcgccgtccaccagcgtacccgct

ggtgagcggtgagccagcgccgccataagcgtcgtcttaccggccccgctgcctccaagcatcgccaccagggtgccgggtcgcaccactccagtcactg

ggagtccagcaggaaggctgcgcggcgcccggacggacgcggcttcgccatggctggagtgtactgcgccg

>80543|AGAP000508-PA

aatatctttggtttgtttatatttaaatcagtatatctatagtaggagaagcattatgtgatgaagtaaagcgacgacatactgtgtttgtggttctcaa

tattatattcactggatacatttctgaattactgattcttagggaatgtttctttaagtttctgttaagtgcaacacctgtatttattactacccagtaa

tgttttcgtgtgtgaaacaaatgtcagcattgctgccaagcattgcttgcttccacactagctcagttgccagtgcctggagtggcaggcctggtggacc

tcgtaagtggctgagtcacaacaagaaaatatatccaccacaattacctgatgaagaaccaagacctgcttatgtttgtcatatgaaagccaacatcaaa

tacagtccatggaagatgtggtatatagcatgtttggtacgtggattgtcggtggaagaagcacttaaacaactcagatttgttaataagaaaggtgcaa

catacgtgagagataccattttggaagcacaaactttggcagtagagaagcacaacgtagaatacaagacaaatttatgggttgctgaatcagttgttgg

caaggggttggttgtaaaaggcatacggagacatgcaagagcaagacacggcttagtggagtactttcactgtcattactttgtacggctggaggaaggg

aagcctccagaacactattatccatatcaacgagaactctcaggtcaggagttactggacaagtggctgactaagatgcggagcaggaagatcaccagct

cattgtagtaggcagatcttcaccatatttgcagtatcagaagttgcattctgcttttcttacaccacagtcctgaaaaatattcttgtaaatataatat

ttataataaaagtatattgaaaa

>80564|AGAP000519-PA

aactgatatgttgtgagtccaacaacttctataaggccatcatcagttgctggttccacattacctcctgctcgattccagggatgagtgccaccaccat

aacttggaatgttgagaaacacaattgcatggaccttatgatccctcaacttaggagttatgtctttaccatcacattctaatgtgactaattctgcaag

tcctttccatttccgctcgaattgttactaccaagacaatacccatgcaggtagatggtgaagcctgcaagctcaacccttcaataatcaccatgacatt

attaaataaagccccaatgcttgccaaaagaagaggtggtaaagcaaatgttcaacagactgccttggaacagctcaagattagtgtgtaccgaattagt

atgtcagattatgaacaacaccattatgacaaggaactattgaaacaagcag

>80584|AGAP000521-PA

cgcctcacgccgccctacgccgccatgatgtggctctacgcgacggtgctgccgcgcctcgccgctggtccgctgtggcatgaggtagcgggacccgcca

gctaccactgctcgctaggctggtggttcaacctgctctacgtcaacaactaccagcgtat

>80583|AGAP000521-PA

atacagcaagttgtaccaccagcggcgggtacattgctcactgtctaccaccatctcctcagaccaaaggggtccgttgccaaggcgaggcaagaatgtg

gcatagaaccaaatcattacggcataggggggcgtcaggcgcaggtagcgctgcaggtagaaacggaagaggccgaagggctggccgtgggcgcgctccc

tcaggaagccgaagcccaccagcatgcc

>80638|AGAP000523-PA

ccaggatggcatagtagaaaagctgctgcttatgtgatgggtttcttgcatagccaatgattaaggagccagaaactgttccaattccgg

>80637|AGAP000523-PA

gctgcttaagtgatggatttcttgcatagccaatgattagggagccaaatactgttccaattccagcacctgaaacaaggaaagaatttctattcactta

ttagcacagcaaattatcaagaagctgcaaatagtactgtacagggatttattttaagtggagg

>80629|AGAP000523-PA

tatgcaagaaatccatcacttaagcagcagcttttctcccatggcctcagacagggcaaaccccaggatggcataggagaaaagctgctgcttaagtgat

ggatttcttgcataaccaatgattagggagccaaatactgttccaattccagcacctgcaccagccagtacacacag

>80634|AGAP000523-PA

cttccagaccacaactgcaacgagagacattgactctgctgcaaagttcattggggcaggagctgccactgtaggtgttgcagggtcaggtgctggaatt

ggaacagtatttggctccctaatcattggttatgcaagaaatccatcacttagcctgcttccagcagtgcgaaacttccagaccacaactgcaacgagag

acattgactctgctgcaaagttcattggggcag

>80631|AGAP000523-PA

gccatcctggggtttgagaaaagctgctgcttaagtgatggatttcttgcatcaccatcgattagcctgcttccagcagtgcgaaacttccagaccacaa

ctgcaacgagagaaattgactctgctgcaaagttcattggggcaggagctgccactgtaggtgttgcaggttcagaaattctttccttgtttcaggtgct

ggaattggaacagtatttggctccctaatcattgg

>80630|AGAP000523-PA

ataactgctgcttcaatgtaggatttctagcatatcccaagattaaactaccaaaaacaactccaataccaacacctgaacctgcaacacctacagtggc

agctcctgccccaatgaactttgcagcagagtcaatgtctctagttgcagttgtggtctggaagtttcgcactgctggaagcaggctaatgaactttgca

gcagagtcaatgtctctcgttgcagttgtggtctggaagtttcgcac

>80632|AGAP000523-PA

gcttaagtgatggatttcttgcataaccaatgattagggagccaaatactgttccaattccagctgctgcttaagtgatggatttcttgcatagccaatg

attagggatccaaatactgttccaattccagcacctgaacctgcaacacctacagtggcagctcctgccccaatgaactttgcagcagagtcaatgtctc

tcgttgcagttgtggtctggaagtttcgcactgctggaagcaggctaatcgatggtgacgccggggcattctgcaataaggggctgtggctgttaacagc

tatgctgctgataggtcggatgtaggatcgtgctccgctcacaagctaaaatagtaaaaatgtatcagtgattatgttaaaacgtctgcagtattacata

acttttgtacgctaataacttacagcag

>80663|AGAP000532-PB

atatataatcttttctcatgctcacagctttataaaatcatgttataaaccataaaacaaaactaccttctcatcgcccaaagctgagtcatagcccgca

gacacgatcaacagctcaggttgaaactccaaaaccactggtaatagtacatgatggaaaatagctaaataatcagcatca

>80681|AGAP000541-PA

gggacgaataatctgctcccatcgcggcagtgcgggtgcgtggtacgaacgactaggggaggaataatggatcaggaagaagcgaggagaaaacatcttg

g

>80678|AGAP000541-PA

gagaggaggggtgcgtgggaaaggaggggaacagggaaggggaaagacagatgcattggcagttcgggtacgtggtactaactactagtggaggaataat

ggatcatgaagaagcgaggagaaaacaacttggagggaagatattgggtttctttttttaatgaactgtgacttgtgtcaataaatttgttcccatctcg

gcagttcgggtacgtggtactaactactagtggaggaataatggatcatgaagaagcgag

>80721|AGAP000562-PB

acttggtcaaacagttgtttcccagtcgtcgtgtgctggatggagaactccagctccgcgtccatcgtcgtcacgcggacgttcatcacttttccgccgg

caaccatgtttttctagaagtcagacgtgcacacagaagacaagctcggccagaactgagcaagagctccacccgaacatcaaccgggtgcacaggcgcg

>80738|AGAP000575-PB

cacacgccatattcacttttagattatgtcggcatattggaaatgatgtactactgtcagagttacacatggtagatcttgcaggatcagaaagagcaaa

gagaacaggcacctgtggtgatgctttccaggaagctgtatgtatcaatagaggcctgctggagctgggaaacgtaattcagagcttgtcatcaggccgt

catggaaatcatgttccatatcgaaattctc

>80791|AGAP000598-PA

gaactgttggatacaagaattcgccctactgtgcaggaagatggtggagatattgtttttatggtgagaaaaattagcttcagttctgcctggatggtgg

atttgaggacggaattgtgaagctgaagatgcaaggatcatgcac

>80862|AGAP000630-PA

ggaggatgccttccttgatgcagtttcacaagaaagagatatggttggaatggatcaaggaccaggagagatgatggaaaggtataattctctcagtgac

acagaaaggaaaaggttggaacatgatgaagatcgacttctttccacaatgttgtataacctgg

>80861|AGAP000630-PA

gaagaattgcaaagaatacagagtgctgaacgttccaccacaaattctgagaaccaagctttcctgaaggatgttgtaaatcaagttttagacggggaag

gtgttggatggttaaaattaaatcgcctgaaaaagcttatggaggatgagagctacagaaactttgtagtgagcaagttgaatcgtactctggagag

>80897|AGAP000653-PA

ttaatagtcttgtggtagttttcactgatcatactggctccatttttctatgtgaactttgctgatttctggttggcagatcaacttaatagtcttgtgg

tagttttcactgatcatactggctccatttttctatgtgaactttgctgatttctggttggcagatcaacttaatagtcttgtggtagttttccctgatc

tgcagttcctgatttgtttctatgccactaataagaactggatggttgctgaagatagccgccaatgtactgacctccgtggtttcctacgaccaatggt

tgggtgtctcccagcttggtggcgatttgcacagtgcttgcgccgatacagggacaccaaagaggccttcccacatctagtaaatgcaggaaaatatgca

acatcattttttgtcatcctcttttcagcacttaatattgcaaatgaaagt

>80940|AGAP000654-PA

gtgctggatctccattagcagatgatgttacagtggctgcactgccatccctcgaattagatttcactcttagcttaaaaggaggaaaagtccatggatc

cgtagctcgagcagggaaggttaagggtcagacaccaaaggttgagaagcaagaaaagaaaaagaagaag

>80937|AGAP000654-PA

gtgctggatctccattagcagatgatgttacagtggctgcactgccatccctcgaattagatctccattagcagatgatgttacagtggctgcactgcca

tccctcgaattagatttcactgttagcttaaaaggaggaaaagtccatggatccttagctcggcgtttggttcatcccacagcg

>80936|AGAP000654-PA

tcttttatgggaacttttgaaagagaaaccaaagtttgttactatggtagagattgatgagcaggtgatgttacagtggctgcactgccatccctcgaat

tagatttcactgttagcttaaaaggaggaatagtccatggatccttatctcgagcagggaaggttaagggtcagacaccaaaggttgagaagcaagaaaa

gaaaaagaagaagactggccgagccaaaagaagaatccaatat

>80938|AGAP000654-PA

aaccttattaggaaacacaggaccatctatagcaataaacttaatctcattactaattattggacaaatttctattaattctagaatcagcagtagcaat

aattcttgaattattgctactgctgattctagaattaatagaagtatttgtccaataattagtaatgagattaagtttattgctcagcaagtttgtactg

tgcaggagctccagttgcagatgatgttctggtgtctgctctatcttgtcttgaattagacttcactgtgagcctcaagggaggaaaggttcatggatca

ctagc

>80958|AGAP000659-PA

aaaggacaacaattggctgaactgaaatgtatagcaaatgcaagaccccttcacgagttggagatactgtggatgaaagatggaattatgatagagaatg

caggcatatcatatgacttcagttcattgtggaacaatacgctatcattattgaacgctaatctgacttacacaggccagtactcatgccaagttagact

gcgtagtggtggttttccagtggtttcagcatctgcaaatgtcatggttcttgagaaaccatcatttgcaaccaatctgagaactgaaacttt

>80984|AGAP000663-PA

tttctgtcttatttacatgtggcagtcatttatttggacgataatgaagatacatgaattgcacttccacaggtacaattatcacagtcagtgactcagc

attgtagctgaagtacataaaaagttattgttaatagtattattttgttgatggtaacaataatgattgtcataactttgtggccaatgagcatctcaaa

ttctaaaatgcattacataattacttactttattaaatactttatggcacattatttaggaggaaatagacatttgatgcaaaatgatgacatttagaca

cacagccttctcttactctatacaactcaataacattttttgttatgcagaactgtgcgtgaatctcaacagctttggtattatgcaccaagtaaataac

aatttgtagtcacaatttttaaaatgtttgttgttaattacactgatatcagtgcattattcaagcattgcaaaaatatttggcaaagcactattatctg

cacatttcagaaaaattgcatttggattatatgtcacgctttctcaattaaagagaaggatgtaaacataaaaaatgttaaataattcagtccttacata

atactgttctttgttgtttatgttgtcagacatagctggatacactgttacattctttatggaatcaagtatgatatttggcttaccctatgcaatgtct

tcacaattttagtcgagtacctcttatgttaaatactcagttcagtattatctacaaacgattaatgctagctacaaatgattcagtttactttgcaact

gtatgtagagagatactattattaatattgatgctacttctctcaaaatgctaattgcacctcatctcaagcattttgtacttcatacctacattagcaa

ctatgtgctaaattgttcagaggacaagcatgctgtttttaaatttgcctacgatttttcatgtagcagcagtaatattgaattgtgagtgtacatcaca

tgtggaggaacatcattattttaacaactatattcaaagtactttggatacagatgtcaaatgtagtgttatccattaatatggccatagcatctagaaa

attttctttctctaataagtacaacctttaaccattatatgttacatgtacagcctgcaagaaaacaaatgccaatgccaggaatgatgatcctgtgaga

tcaccaatggcagtcagaaatggtatagcagaattatctgggtcacttttccatctccacataatatgcacaaacaagtgagccaaatataacagcagca

tgacttgtaacagactggcaaatatgtaagtgagagcaaatctgtagtcaatggtggtttcaccggcatggaagtaatcagctataaatacaaaaattga

ttgtcctatgacagccagaagtatcaacagccttgcagtggcagcgtgtggcaatcctttgaaaagagctgaccatggccaaacccataatttagtatgt

ggtggaataattcctttgagtgaagtcttatgtagcatggtggagatgcgactagcttgtacagacacaaggttgccaccaattccattaattattggtt

gaaacacaacaaaaccattgaacatttggtagacagaattcatgacaataccaccaaacccactgatgagaagagcaccaagtactggaacccatccaga

atacagaactggctttgtgtacttgttacggtatacaacgtatatccatactggcagcaacaataagtaaaatgcaactaccgtagcagatacccaggga

ctgacatcatgtacttcataaaaaattgatgcaaagttggcaagaagtgtaagggaaacaatatcccctatacttgcagctaaaggagtagctagattat

ctggattcattttccgcttatgagatatcagtatgacagcactcagtacaaaatctaaaacgaagcatgatgttgtagctgtagcaagagatgaggaagc

aagtaggagagcatgcgtgtactgaaatgccccttccatgattgcattcacagagacagcaaatattgctactactatagcagcaacaattgcctgaacc

tgtacaagagctatatttcctgtcaccatgagaaatgtctccctcctgctatccatgagccctaagtttgcttgggtagacaatcgagatgcgagacaca

tatccaaatttcctttaagtccagtcagggaaggcaccaggatcatcagtgaagatatttccacaaacacagcccatttctcaacagtgccgagcagcag

ccccgagccgatggtgccagcacctgccaggaagaagggcacagagacctgcaggatggtgtggtaccagcgctcctgtttctcctgtggagtctcatca

acagtggccactgtggtgatggtggcaatgctggcagtgctggccagtgagaagccacctggggtggtgggtgacttatcatctggcggcactttcttgc

tggcattcagtgagggcatcttttcaagaggactcttttggccactgagcttatccacatctaccaacacatgctccccgggcaccacttcgggcaggtc

tgggtaagccaccattttcacttgggctgcaggccgagtagatctggaagacgtagggagagagttggagtgaacgaagtccctcgacgtctcttggcgt

ccaaagctagacgtcggatcgccttagcttttccagcgccagtgctgacagctgcaggaatgcgcgagtcagagttgatcatcgactctccaaagtttaa

tttcaacaaatggcacctagaagcaggctagagagctgcaaaaggcagcaaacgaattgtttttacagcaaaatcagcagaaaaagt

>81002|AGAP000697-PA

gttaaaagccttgtagggcggtggcgcgcacttcacttgtttcacacgatcctcagtgctcggcacacgggcaccactcgctctgctcagcgctccgctc

ctcgcacgaccacaacatgagccggctgcccctcctcgtttgcctggcgggctgcgtagtctcgctggtatcgtcgctgcccctggcgccggaacagcag

gaagtggtgcaactgccgctggatatacaggaggcgttggccaagctgcagctggcggagcagtcgtacgaggaagagacgccgcgggacaagaggacca

tctgcgtgctgcgccagctcttccccgggctgtcccagataatcgataggaaaatccaaatgatcactcagtacttattccgagtaatcggtcggctggt

gctgagaggtggtctgctgggaggcggcaaccgcggtggcggaggctcctctggcgatgggggcagcaggcgcatcagcataacattgccaacat

>81206|AGAP000697-PB

caagtgttgtataattctttttataacaaaaatattcgtacaatgtaacacaaatgttatataaaaatattacagtttcaaatattaagatttttacaga

ttgctgtgtatgtgatgtctcttcaataagacattcaatctaaaccaattataaaatactcattgaatgcattttcacataaaatataataaatttcagt

tcatacaattacacagatactatacaaaacaaatctaatacaattgtcattgtttaaataatgtagcaatactgccattgccagtaactttcaatttctc

cactgttatatacagtaattcactcgagtatttcaatggtaactattgaaatggttgatagtttacatagactgtagataaaattaaagcacaagcaaac

atgcagaagaaagtgccactgactagctgaatatacctctatatgcttctgttagtcacatcagtatttagaggaatggtagcaggccagtggccattca

gtctctcacacaacacgtactgtgtgataataatgcaacagatctgaacccatgatcagctgacctaaacatatgctctgttctttgtatcatgaaatta

tgatgttcacgttgtcaaaggaagaagaacatgtgtccagtttgctgcagaattcttctataagatgcttgcgctttaattataacacttttcatgagga

tagagtcataaaggtgtgatccttgtaccaggaacgaacagtttctctttaaataatggaggtgatcaggcgggccttgaagttcactatcctctcactt

gtctgagccaggcgcaagatcaggtcctgcatgagattggctatgccacggttggcaatgataaacaaccgtgtgattggtccagggacacctgcttggt

atccatcatctcttacaacaccagcatctgctgaagctcctgcactggcacttgcagattctccacttggctctgtgttgccactgccagcgaccagccg

aatgatgtcaaagaggaagttcccagatccgccacctgagcctccagtgccagcagaagctccaaacccaaagttgatcaaccgtttgttgcggtcagcc

tcctcagaatctgcatctgctgcaggtgcaggtgcagcattgtcactgggtgcactgttgcctccacttgctgccagttcttggtcctcaccatactgga

cacgaacttcactctgctgggattctgccccagacagctgattctcttcaggatttaattccgtctgggacagcccggggaagagctggcgcagcacgcc

gatggtcctcttgtcccgcggcgtctcttcctcgtacgactgctccgccagctgcagcttggccaacgcctcctgtatatccagcggcagttgcaccact

tcctgctgttccggcgccaggggcagcgacgataccagcgagactacgcagcccgccaggcaaacgaggaggggcagccggctcatgttgtggtcgtgcg

aggagcggagcgctgagcagagcgagtggtgcccgtgtgccgagcactgaggatcgtgtgaaacaagtgaagtgcgcgccaccgccctacaaggctttta

ac

>81225|AGAP000711-PA

atcgtttcttccctggatccaaaaatattgtcaagaaattttttaagtattctgtgaatctgcaccaacaataattcatgttttctgaagtaattcttcc

taaggaaatcttcaaaagttgtgtgtatatacctacaattgatttggagctcaattccagggcatcaaatagctccatcgaaatctttttgtgagctagt

gtatctattacgtgaataacaagtgatggtggtccatttgcaatgaaatcatagacataagcttgcaaacctatatcccaaaatattgaaattagtaaat

ccttactgacagttgggatgcttccaatgagatgtccgcagttttcatctgccattaacgggtcctcatcaaacatgtcagaattcaataataaaaatat

tactatttttttcaaaccatcatgcaaattttctgatttagcatttaagaaatctttgtacttttcatgtattactgctgctaacttttcagtgatctct

ttggtcttcaggctatctccagtgatagatgcatcgcacagagaaccatatttattttgagcgctattaagcgctctttggatactgacaaaatcttcgt

tgaattccatatttccctatgattacataaattcccgcttcacactttcaaatatata

>81242|AGAP000715-PA

aggcattgtaggcgtgtccgtcatgctgcaggtgtccgagtcgccgacgactgagctgcggatcatcaccagcacattcggccacatccgcttcaaggcc

tcgtcgacgccgatacggccgctgacgctgcagcagctgcttccccggacggac

>81241|AGAP000715-PA

aatgccttgcgatttgtgctgtgcttctgtcatgttgtggtacaggtccgcgttaaacccgtacagctgaatctccccagggaaggagtagccgtggatg

cggtgctcggagccgtggctgttgttggtgccatagtggaagtacatctcctcaaactggtagcggtaggcgagcgggccgcc

>81362|AGAP000732-PA

gcccagtgcgacctccggctcgccacgcaggacaagggcgttctcaacgctatctgctatatcggcatgctgtgcagctcgcacctgtggggctacctgg

cggacactcgcggccgccgcaccgtgctgctgctcacgctggtggcggactcagcttgcaccgtcctctccagcttctcgcactccttctggctgctgct

catcttcaggttctggaacggcttcttgtgagtacctcctggttcccccatactagctacacctctgtattctatctag

>81598|AGAP000745-PA

gccaaacgtgaacgatccgtcatcgttcaccttggagtagttgcggatggtctgcacgggcggctgcctgcggggttcggtgctggactcttccctgttc

gctctcggtctcgtggtcatatttagtctcagatggagttt

>81641|AGAP000752-PA

gctgctgacagaggaaatatggaaatggtagactgtctagttgagaaactgaaagctgatgtgaatttaaaagatgctgatggccaaactgctttgcact

atgctgcagcctgtggacatgttaacgttactaaatttttagttcaacatggtggagaccccaatattgctgatcttgatgggacactcccaaaagatat

cgctgctgacaatgaaatattgaaggctctgacaatagcaaactgatattgtttcatgtaggcctatgagtatttgacatcatgattattcttgcattga

taatgaagttaattgatcttttcccttcatttgcttagctgataaatccttgaagtataatatgttgtaaataagtgctccatcatttaaaacaagacag

taaatttgaaataagtcattaaattctatttgagtttctattatattgtgtttggcctaatttacccctacatttgtatgataaagtgttgccacagttt

acttgatggtatgcattttcaatttttacatgaatcagtggggaaaagtgcatgtaaatgtttccatttatgaagtaatggctgtaatttagcgtagttt

tcataattgctgtaacaccggtgcaaaaaccacatgaaagttatctttgtagagaaacaattttaaatgttgaaataggatggtctgtaaaccattacag

tgataatagatttgatttttcactggctattacaaatgttttaaaagtaaatctggtacatgcattcacaccctaggaattaaattttataaaattgtta

tcaaaagaagggtatttatttttgtgtatattgttttgcaatttctagttaagtgaattggctgccacatgttctttacctactattttttatgtagtgt

gttggcgggagggagttataatcacttccagtggtatgtcattatattcagtatcggttcagcttaggcttgaacctgtttaccaacaagtctttcttct

aatgtaaattataaacagaggtttgctcatattagtttgtttcatgtatgtggcttatgcctggagtgttgcatatgtaaactgctgtcatattttctca

acattagatctgtgtacatttggaattgttgacagtttgttggtgtgtatgaggctggaacaaatatttgtcacatcagcatcattatttcaatggttgc

ttgaaagctgtttggtcagaaccaatcttatttaatggccaatcgaaataactgaataacccactctgacaagtctaaattgttatattttcactctatt

cattttttaaactttttttttctctacatacaagaaattaattttagccatatttccacacagcaaatttttgctctcactctttgatatgtaatgcatg

ttctactgttgttacaaaacaatcaaggtaagtcatttcaagtattctgttttgaagaggctggagataaatcagtataaaatatagtaacaataaagat

aatctctc

>81654|AGAP000759-PA

agtagctttgtccatatgtataatagctgaaacgccaaaaataagtgaaggcttatggctgaagcgttcgagttttggacagttgaaagtcgaacttagc

ggcagccacaaactttgatatgtttatttaccaaagacaatgttgatgttttggtgtattttgtgccggttgtgcgatgttgaggttataaattgcatgt

tggtaaaattattgtgaattcgattagttttgcaaagcactatggctgcagtcatttttagattgcgcactgccttgaaaagaatagaagaagtatttct

tcaaatgtttctgggtcagaacttcccccgtggtaattacgctctagcatacgctggaaatgtaattccaacgccgtcaacaagtgccttttcgttagaa

agtattgtgggtgacggatttttgtgggctgtgccacgaaataggcgcactatcgaaaggaggctaaaaagaaagtatggcaatcccgaataccatctca

agattctcaagccaaagaagcatttactaacatgtaacacatgtggcaattcttacgaagcaagaaatctgtgtccccattgctatgagaaagtgaagct

ggaaacggaagcgatgcaagaacaaattcaaaaagaactaggcttgaatcctgttgaacaagaagtggtagtcgtctatgagggtgagaagcaagaacag

tcggatgaattttggaaggggaagcgaatagtagagatgaagagggaacgcccgtcgtggttcagcaaaaatcttctgcagcgttccactgaggaaccga

catctgttccaggagacatgaagccgaaagagttggcgtgagcacactggcgactcgtgtattgttcttataccagatggtgacagtggatattgttgca

aacagcaatctaaataagtgtaaggtaatgcttgagattgctcacatggactgaatcccgactgttgcccacggagtgtagatagcattggtgtttgttg

tattttatgaagagcaatgtttgttgtaactgtcttttcacttgtacccaaaattgtatcaatgcttgaagtttgtactgaagcatagtaattgcataat

aactttggatagcataacttgtatagtgagaaatatgatttgatacagtactagcataaacagtgttcataataaacagtgtttaaatttattcccacta

atccatttgaactggcacctttatagaatttaaatttcagattgtccacatatactatttccaccattgttgccaattaatttacag

>81718|AGAP000785-PA

gttctgtttgggagtggaacagggagagaggatgctcgttgtggtaccaggccagggccgcttccactggacggcgctggtggtgtgtggcttctgcctg

atgacgctgctgttcgagtcgctggcggcagcctacgtcacgccggcagcccagtgcgacttcgagatgtcgtccttcgagaagggcttcctcagcggct

ccatctacataggcatgatcctgagctcccacctgtggggcttcgtggcggacacacggggtcgccggttggtgctgtcgctcacgctggctggcaactg

cctctgctccgtcgcagccgccttcatgcccagcctgcccctcctcatcttcatgaggctcctgaatggcgtctttgtgtgtggaccctccgccgtgacg

tacgcctacctgggggaattccactcggacaggacgcgcacgcagcacatcgtctacgtctgcctcttcttcgccatcggcagcatcgcccagcctgcgg

tggcgtgggtggtgatcccccagccgtgggacgtacccctgggaggaggcctgcggctgagttcgtggcgggccttcctgctgctgggcgctgcgttgag

cgcgctcaccctggtgttgctcaggaccctgatggtggagagccccaagttcctgctggtggctggccgccaccaggaggcgttggccgtgctgcagcac

atgttctccgccaacaactccgtgcccccagacacgttcccggtactggccctccagaaggacctacagtcttcctcagcaatggccgggaaccccaagt

cgccactgtacatcgcacgccacatgtggaggcagacgtacccgctgttcaagactcccttccttccctacacgttgctcgcctgcctcatccagtttgg

actgtatgccagctcgggaggcttcttcctgtggctgccggacctgatgacccgcg

>81785|AGAP000794-PA

atggaggacatagcttacgatcatcggctttgatcatgccgctaggcatttgattaagacattgcacaatgatccgaagactttg

>81784|AGAP000794-PA

cccgtgatagactgtggtgccctctgcagtgccccttaagttatggttgtagtttgccactttacctctgcagatgtcttctgctgtcagagaggaaata

ctaggagaaagttttatacattgaccacagcccactagtgcagaaggtataagcagcgtcagtaccagtcatgaaatcttaaattgtatgattaatgagt

acattttattggattaactataaagcattcatatgaataatgtacagcctctataatagatacagggccaagtatgagatgtcaccaacaattccataat

attcattacatgaattatgcatagcgatgttattaatttaataattattaatatctgagaaaaatattgttatacttcttataaacggagtctatttcag

atatcttctatgtcataaaaactatattttatttcttcatatttttcttttctttgattctgtctagcgatcaatctcaccaaaaactaaatccagtgta

cctaggatagccacaacatcagctaacataagtcttctgctaattttgtcaatggctgcaagatgagcaaaacctggtgctttgattttgcagcgatatg

gtttggaactgccatcagaaataatgtaaataccaaattctccttttggagcttccactgctgtgtaggtagcacctggtggcacatgatagccttgtgt

aaataacttgaagtgatggattaatgcctccattgatgtcttcatttctgcccgtgaaggaggagttattttcatgtcatctgtcttaacttcaccagca

ggcattttgtttagacactgctcaatgattctaacagactgtctcatttcctcaaaacgacataaatatctgtcatagcagtccccatttgttccaatag

gtacatcaaattcaacctcatcataagcatcatatggctgtgtcttgcgtaggtcccacttaatgccagatcctcttagcatcactccactgaagccata

attaagagcatcttcagctgaaactaccccaatatctttagttctttttatccatatgcgattagttgtaataacatcttcaacttcatccagacgctct

gaaaatttgctaacaaattcgtatatatcatccaaaagaccaaaagggatgtcctgatggactccaccaggtcgaatgtaagcagcatgcaatcttgccc

ctgatactctttcgtaaaattccatcatcttttctctctcttcaaagagccagaagagtggtgtcattgctccaacatcaagcatgtgggaagctgtggc

tacaatatgattcagaaggcgagttatctctgcaaaaagaactcttatgtatttagctctctttggtacttcaatattcaacagcttctccacagctaaa

gcgtaacattgctcattgcacattgttgaaacatagtctagtcgatcaaagtatggtaaagcctgcgtgtatgtcttgtattcaattaatttctcagttc

cacggtgtaataaaccaatatgaggatctgctcgcatcacagtctctccatccaactcgagcactagtcgaagtacgccatgtgctgcaggatgctgagg

accaaaattgagagccatattctgcactgtcttctccactggagctattttactattgtaaggtggcattttccatttagctgtctcttcatttggatac

ataacaggattttcaaactgtttcatgtagtctggatctgggaaccatttcttcacattcctgctctgcctgtataaatggttcgttgctggaacgccta

agagcattttcaccgctccgttcactggcaagccatttttacgtaacaacatttgtacgattccaaccattgcaaattggtctgtgggcgatggtaacaa

ctgactgataag

>81842|AGAP000795-PB

ggtggccggtgctggcgtgcgcctgcctggtgcagctgctggcccacgggctgcagctgtcgcacggtgtgctgctgcggcccatctcggacgtgttcgg

cgccacgccgctgctcgcagggtggctgggcgcgctgtcgacgggcgtggcgctgctgatgtcgccggtgacggtggcgct

>81858|AGAP000799-PA

cgcggctgctggcgggcatgtggtggttcttcacgctgatcgcgatcgcgtcgtacacggccaacctggccgccttcctcactatcagcaggatggagtc

gcccatcaacagcgtccaggacttggccaagcagaccaagatcaagtatggcaccttcggcaggggctccacggcagcgttcttcaagaattctaacgac

tcgttgtaccagcggatgtggatagtgatgaagcaggcacggccagatgtgtttaccgacgataaccacgagggagtggagcgagtgaagaaggggaagg

gcaactacgccttcttcatggagtccaccagcatcgagtaccagac

>81860|AGAP000799-PA

gaggtatattttggactaggcttcttctgttggatcctttcttcaacagctacttttcgtgacttccagacaaactcgcaaacagcaatcacacaagcaa

cacccatgccacccatcagcacaacaaaaacacctccaacattggccagtccaagctcgtttgctgtgctgctgctcttggaggtgtcatctcgacatgt

cccaccaccacgtttttctttccaccagcgtgttttcagtatgtgcaactttccctcttcttgcaacttcaaaatagctccgcttatagcagtgcgatat

ggagaatttggtggcatggcaattccgtagccttttgaatccaaaagtcctcctacttgggtcagctcac

>81859|AGAP000799-PA

cggttccacagcggccttcttcagaaactcgaatgattcgctgttctctcggatgtacacggtgatgaggcaggcgagacccgacgtcttcacagacggc

aatgcagagggcgtggagcgggtcaggaaggagaagggcaactacgccttcttcatggaatcgaccagcatcgagtacatgacggcgctcaactgcgacc

tgcgaaaagtcggcgggcaactcgactccaaaggctacggcatagcactgcagctagactcgccgttccggacggcggtgagcagcgccgtgctgcagct

gcaggagcgcggcaacctgagcgcgctcaagacgcgctggtggaaggcgcccgagggcaaggagtgcccctccgacgtggacacggtcgacagcgcggag

ctcggcatcctcaacgtgggcggcgtcttcctcgtgctcttcttcggcaccctggccgccttcttcgccgcagtcctcgagctgctgtggaactgccgca

ccatcgccgtcgaagagaagat

>81876|AGAP000801-PB

gttgccgcgctcctgcagctgcagcacggcgctgctcaccgccgtccggaacggcgagtctagctgcagtgctatgccgtagcctttggagtcgagttgc

ccgccgacttttcgcaggtcgcagttgagcgccgtcatgtactcgatgctggtcgattccatgaagaaggcgtagttgcccttctccttcctgacccgct

ccacgccctctgcattgccgtctgtgaagacgtcgggtctcgcctgcctcatcaccgtgtacatccgagagaacagcgaatcattcgagtttctgaagaa

ggccgctgtggaaccgctgccgtaagtgccgtacttgatcttggtttgcttcgccaggtcttctacgccattgatgggtgactccattctgctgttggtg

aggaacgccgccaggttggccgtgtaggaggcgatcattatgagcgtgaagaaccaccacatgccagctatcagccgcgtcgagacggccttgggtaaca

ggtcgcagccttgtcccatgagggagcccatggagaaccagaagatgttgatcatgttgaacgtgttctccagctccgttggttccgggtcgcaggggtg

>82200|AGAP000812-PA

taaagttcctgagctagttgtgcaacaattatatcagtctgtggttctgcatctgatgtcccatacaacatatttttaatgagtaccagattcttgctga

catcttcttgtgccttttcagcctttttatctcctcgttccagtgcattgacggcttcttttaacgccttaactacttctgcagggctcttctgagattt

accaaacaagggcattgtaaaatgttttggatcccaatatatcacactctaagccgcatgtgaagcagatttcctataatctgagagaggaagagaacat

tacgtcgtatcttttataagtcacagaagaaacatggggttttcaacgcctctgacagaatgggctacaaccatgacattaactgtatcacagacaatta

aatgcatcacagatgatttacatgtgaaacacgtgtttttgttatttttttccaatacaatgtggcactgaatcacgaaattgtttcaccattactcgct

ctctgcactttacctggagtttgttctcttacgctgcacacacatttaacactgattgtct

>82240|AGAP000830-PA

cgtcggtgagcaccgacaccaggtccttggatctgccgtgggtttcaagttgagaacacagtgtctcgacgtaccacgagccttttgttggattgcggaa

ggagtacagaccttcgaatgtcgagtatgaaacgatgatgtcggcgtaagaaggcactgggtaagtgagctcctcggcatagccactgtctggggcgtct

gtagtgaggtcgctcaccatcacgccggcatcagtatctgttcccttacacgcctgtatgaagaagagcttgggtttgccagcgagagtggggcacagcc

tggctgtgaacggctgccacagttcgctgacgttgtactcgcggtccctggcgtccaactgtccgttcctctcaccgtgcgtgagcactgccactgcgac

gcagtcacggttgctgtggttctgatcggccacccgtctcagctcatctcttatttggtagtggcagagatcgtcgaaacagagtactctgaagccaaga

cttgtgaacatatcacgaagagcgtctctgtcgacttttgtgccaagcctatcttttggtcggtagttcccatctgcatactgacgaacgtcaaatttct

cgtggctgatgatgagggctacacctcgctcccggttgcccatgaagtactcctcagcgtcgcgggagacaccagggtttggcagaacgatagaggaagt

tggtatcgactccgcctgtgtccgcctgcacagtcagcgacgtcttcgtctcctt

>82301|AGAP000844-PA

cggatgcagaaatagctggaagggcaccaatgctctggctaatccagataccaagcatatcaagttgtagtagccggtaatatattttctccccatgttc

caggttcatgaacagatgataaataaagctgccaatccatgggcagccagctcccaaaaggtgacaccaagataaaaagtatgagtccacttcattccat

gggataagatctggccatgacacgataatgtacaggattggtgcaccgtcgtatttgtcagaagatagaattgtagccccacaggcgacaaatgctaaag

acggg

>82332|AGAP000849-PA

ggcgtaccactgacggggtttctcattggctggtacctcggccataaagagcacttttatctctgaacatcgttagcctccctgtctctttcaggtccag

gtaccagccaatgagaaaccccgtcagtggtac

>82333|AGAP000849-PA

tctctgtctctttccggtcgaggtaccagccaatgagaaaccccgtcagtggtacgccagcccagacatactgcttagttatgccaaatattgccattcc

ttctcgttgaatccacaatagaatagcaccaccttatg

>82331|AGAP000849-PA

cgtaatttcaaccaagaagtaaaaatcaaggactttaaatgttttaacttacactctccctgcaggacaaccaataattatttcacttcatttcaacaaa

aacaacaattcaatacactgtatatatttgctaacaactacacagtacatactaattatggccacgatggcttttctccaggtttcagttctcggccata

aagagcacttttatctctgaacatcgttagcctctctgtctctttccggtcgaggtaccagccaatgagaaaccccgtcagtggtacgccagcccagaca

tactgcttagttatgccaaatattgccattccttctcgttgaatctttcgtatcaaacttcaattagttaaaagttcctgttgccgtagcttttctgcct

taagccttttccactgacgagtatggtacaaatccgcaacaatcgcagtagtaactaccgcaggaaaaaaaatcacaacaccgtatcttctcaaaaattt

tccagtggtatttaacatttcagaagccaacctacaaagtgtaccacaata

>82355|AGAP000851-PA

ccatgggaacctctttgttctcagtctcagattttcataaggtataaattctggtcggtggtgagggaaacaagagcctgttccataatcctcatacaaa

tgccctccctgaaggttatgaaga

>82354|AGAP000851-PA

aggtataaattctggtcggtggtgaggctcttcctgatgcttaagccctcccgagggcatttgtatgaggattatggaacaggctcttgtttccgtcacc

ccatggggacctctttgttctcagtctcagatggttataagggataaattctg

>82350|AGAP000851-PA

tcctttattgtaagctgtttattgctatatacatattaaacatccatccaatacaataatggttcactacacagcattctgaaataagtaaacaattcaa

tggtgggcgtcttcataaccttcagggagggcatttgtatgaggattatggaacaggctcttgtttccgtcaccccatgggaacctctttgttctcagtc

tcagatgttcataaggtatagattctggtcggtggtgaggctcttcctgatgcttaagccaagcatttaacatacacaaaccaaccgctggtaatgcaac

gaagaaagacaaatttttccacagcttt

>82351|AGAP000851-PA

tatggaacaggctcttgtttccgtcaccccatgggaacctctttgttctcagtctcagatgttcataaggtataaattctggtcggtggtgaggctcttc

ctgatgcttaagccaagcatttaacatacacaaaccaaccgctggtaatgcaacgaagaaagacagatttttccacagctttacgccaccccctccatgg

cccgcaacagctgaagggcctttcacagctgcgaatctcgctgcagacagcaacatctttcttctccaaatgagtctcttgaaacacgaaacacgaacaa

atccctaggacttcctgaatcttgactacctcatctctgatggctccatccacacctctgtccacatcaaac

>82419|AGAP000877-PA

attctgagcagtcatgtacatattgataaatctgtagaatacagactgttcaaaccatggcttggagatttcctgctcattagctctggtgataaatgga

g

>82415|AGAP000877-PA

agaccatgtattgtattcagatgctgcaactgttacttagccattccaaccaagcggaatattggttcaagccttaaacaaaccttgaatgttctctggt

gaataatatcacacatctt

>82416|AGAP000877-PA

ccaccgggaagacatttatcccaatccggaagttttcaatcctgtcagcggtcttctctgggatgaagttgtcaggattgaaaaattccggattgggata

aatgtcttcccggtggtggatcttgtaag

>82417|AGAP000877-PA

aaaaaatgcaaaataggcattattatggttacattccatttagtgctggaccaacacaactccttggtccagcactaaatggaatgtaaccataattatg

cctattttgcattttttctggtaagaagttgtctggattaaattcttctgg

>82414|AGAP000877-PA

taaattcttctggattaggatagatctctggattgcgatgtaccttgtatgttccaatcacaactccttggtccagcactaaatggaatgtaaccctaat

aatgcctattttgcattttttctggtaagaagttgtctggattaaattcttctggattag

>82413|AGAP000877-PA

ttgaacattctgtattctacagatttatcaatatgtacatgactgctcaaaataatctcaatctcccttggatccatcagaaatacgaacagattccatg

ttccaatccatcctcttactacaggtggatagtccttgcttatctccagtgctttctccattatctcatttgaactggtaataccaaatatcttgtggat

gttaccgatgaaaggcagggtaggaggacctgggagtttgttgcccagttttacaaaacgt

>82478|AGAP000885-PA

gccgcaggcggtaggcggcgcccggctccagcgggccgtccagctggaggaccaggaagtcgcggtccgcgtccagctgcacgcgcctcagcgccggcgc

gggcggcccctgcgacgtcagccgcaccgcctcctcgtccaccgtcaggttcttggcgtgcagccgcagctcccgcaccggcgccgtcgccgacagcgtg

ataacaacgtgccctttgaacatgaagccctcgctgtcatcgcccaggtaggtctctaagaagatgtcgtagtgcgagggcagcacctcatttgagaggc

ggaatgatgacgaagcattggtgccagacgcctggctgacggtggtgctgtgcagctttccacgctccggcccagacacctgggtcgcagccgtgctgtc

tttgtcatcaggccccgtcccagacgccgactgggactggagcagcagcaccagcaaccacagcgtgccaaccactgcagctcgccaagggggcggttgc

agcatcgacaggcccatcctgaggctggtgtggcgtcagcggggactcggtcagccggtagaccagctggttagaagtcggcaggcagctggcagcaagc

acccgcagccac

>82477|AGAP000885-PA

cgcacttcctcggggagacggtgttccgggacgggctgaccagctacctgcgcaagcaccgcctggacaacgccgagcagagtgacctgtgggcggcgct

gacggccgaggcccaccgccagggggcgctgcccaccggcgtcaccgtcgaggccgtcatggacacgtggaccctgcagaccggctacccgctgctcacc

gtctccagggactaccgtagcggcacactcagtatcacgcaggtcaggtacctgtcgtaccaaccgtcggacgttcggcgctcgaacgtctcgcaatgct

gggaggtgccggtgacgtactcgtcggctcgcaacccagacgaggtgcggagagagtggctgcactgcgaccggggccagccaaacgtgctgacgctgcc

gctggatgctgccgagggcgactgggtgctgctcaacgtccacgccaccggcctgtaccgggtgctgtacgactcgcgtggctgggtgctgctggctgag

gcgctgaagggggccgaccccagcaagtcgggcatcgaccggctgagccgtgcgcagctggtgggggacgcactcgcacttgcgtggagcggcgacctgc

cctacgagcacgcactctccctgctgacctacctgcagcacgagcccgactacctgccctggaaggccgcgttctctgggctgtccaacatctaccgcct

gatgcggcgcactgccagctttggcctgttccggatgttcgtgacgcagctgatggcggagccgatgaagacgtgggtggatctgcagcggccgctggcg

tccgacgacctggaagggttcatgcaccgcaggctcatggtgtcgtgggcgtgcaagctgggcgtggccgactgcgtgcagcaggccaccaagctgttcg

ccgactggatgcggcagccggaccctgaccgggagaacccggtggccccgggcctgcgg

>82494|AGAP000889-PA

gttggtgaatcggtcggaccgctgcaaaaggcacgcgtgtcgaccgaccgactggttgtaaaggaggtcatccagaacttctcctacgagctgttcgcca

cgacgttggatgacttggacgagaagaccatagagaaggtcatcatgaagaacaccggtgctaactatggttcatctattaattgaacgtagtggg

>82510|AGAP000893-PA

aactccaggcttgctgttcacaacaacaatggctggcccacttgattgtcgctatggagaagagacctatgctgatggtgcactcattgaaacagaagaa

ccttgccaacactgttattgcatgcgtggagacattgtttgtgctgtccaagaatgtggcacaccacttgagagagaagctggaaactgtactgctttgc

ctccacaacctggacagtgctgcccccaacagtatcagtgtgataatgtgacagttccatttgacattctgtcacaaggaatggaaacagctcaaccatc

aaagtcaactgactcaacaaaatctgatgaacaggtttcattgtcaactgaagctactgtggttcagcactctactgtggataaagaggaacatgtccag

cagcaaggaagtcttgatgaggcacaggaacatctgaatgaagttgagcaaggtttagaacaacatacgcactatgaggatgaagagattgaaaagggaa

cagaaatagagcacatcacaaatcctgtagaacatgagggacaaggcacagttgagcatttcacacaagctgtagaacacttaccagcagagggcagtgg

gcatattacattagagagtgacagttctgtgactgaagctgtcattgcctcaagtgagaaacaaagaacaacactggaaggtgcagaaactgctactgaa

agtctaactgaactcaatgttgcaagcatctctgaaggcattcaaactattgaacaggtaccacagcaagagtttacttcttctccaacagaaaatggca

atgaagatgtacatgatgaacagtctttaacagctgatgcagacaaccatccatcagttgcccaaacaacattatcaggagaatctgcaagttctagcag

cattggtgaacttccagaaggtcaagtgacaactgaacaaaatgcacctgatattgttactctgaaagatgaaataggctttactcacaagagcacttcc

acaactcagacagtgagcgatacagatgaggaaagtgaacctgtcacacatgaaatcatagcagcagaaagtgattccacagaaaaagtggaacaacaag

atctcacaccaccttcctcgcagtcctctccagccaatgaattcattacttcagcagctgaaattcaatcctccacagataagactgaagaatctgcttc

tgttgaaaatattcctgattctcttgaaccatcagaaggtgaggaagcaatatcacatgttacagacacacaaatacacgaagatgaacacactgttgca

actccagtaagaacgtcaattgtacctgaaacaggtggaatcacaataccagtacaggatgtcgaaggtgaaattgtaactgaaggtctagagccaactg

attttgagtcaggcgatagagtaactgtcactgaaagcacacaagagcatgtcacaaatgggccagtagtagagatctcacctgaaacagacaagattga

tgaagtatctcatgaacagtctgtgacagaggccaatgtagctgaagaagcaggccatacaccaaatgatgtactgagtgaagtacatgaagcagaacag

cagacatctggtaaaccacctcagctgccaatggaagagcatgctacaatttctgaaggattagaactgtcaacaggagtagaatccatccatcatacaa

caagtatatctgattccgcagattttgtctctgagttagttacaactaccagtcctaacaagcaagaagagggcagtttccaagcaacggaaacagtact

caatg

>82512|AGAP000893-PA

cagaatctcattgtgcacatttgtaacatgaagaacataaaagtttcagttcaatgaagtagaaaattttagttaaattttgtcactgagaatgtggagt

gtttttagaaaacacatccatggaaagctcacgtaattacatttttatatataatatataaatactaaaaatactaacactgtatctatatgaccaatga

cataaatatacagctttttgtccagatgttcaagaggcagaaatttaacagtcatagttcaagtagtaaaaaatttgaagtaaagggcacatcacctgca

aatttctaccagtcaaactacctggacatgtgttccaaaagctcacttattcaagatagaataaacaaaaagcgagaaaatggaagtgtctgtttgttat

acctatgcttacactgagataaatagtaaaaaaatatttaaatagtataaatcatggagattttgttaaatatcttcagaataagtggctataatgaaca

ccaaatattaaaaaacaggaagtgccacgtctctgaattttctcctccaacccttcaacttgtgtataaagtttttccttgaataatatttggttggtac

aatgtgtgtagcaaaagattttgtgtaaagactcaaaaagacacaaaatatgtagatgtaaaatgttcataaaaaggaagatcagtctacctgtatcagt

gtgtgaggacaatctttgctttgattatcctcaatagcatatcactcataaaaagaaaacatttcatgagtgttaccagtttttacacagcaacaactgt

cttaaatttgcactaaaaactgtgcaactcagaagactgagggctgcgtagggcagaggtaaagaggaaaggcacagtcttctcagatcctctcctagct

tgtcctactagttttggcacccaatttaatctcttttaaacaacaacattcttgaattttaaaacaatatatgcatgtgttgccacaagattcactgtcc

acaatagttttcttgtcacaacacagtcaatgcacaaatattaactttgcttccattatcaccaaaggaagcctcttcaattgagcacatctgtgagagt

ttctgtactcttcttcaccttcttctggatgctgctgccatgatcatttttcgtaacattggttctggactgcattccttagggtcacacttcatttgga

tgctgctgccatgatcatttttcgtaacattggttctggactgcattccttagggtcacacttcatttggccatctccaccacacctgcactccaaacag

ggacccgaggcagagtgcaccacttctccaacacgatatgcacttccctgaacttgacacccagtcctgtgggctgcacccttgtagtggtgcgctagga

aatgtggtggcaatgtggtagtagtggtggtggtagttgttgatacattcagaacagttgccatagagacagggcactcatatcgtggacaacaaaagcc

ttggattggctcttcgtgacaacctgggataggaggagggcagctctgttgaagacatatgatatcacttcggaagcagaaacagaaatcacaagggtca

tcacgaggtatttgttgagctgatacgtagacctttcccccatagcgacaggttcctggaccaaaggcagcttgttcttcatcatcataatctgtatcac

ctcctgttgggtactgtccttctccaaatggaaactgctcatattgatcttcagggtgttgacttggatgatggtgtgatcctgtagttaatggtggtct

agtagtaaaatgatgtgatggtcgtttcgttgcttccgttatgtgttcgattccttcaccaattgccaaagtatgatcttctgggaaatgttcgtaagca

ggatgtgatgttgaagaatgagcaggcctctgatctggatgaaatggtgaagacgcattagcttctagttcattttcctgtacagctgatgcctggctag

tatcagaatgatccaaagaagttggtgcaggctgtgctgtagtgtgcagtgga

>82511|AGAP000893-PA

ttgatgatattgctcaacaagaattggtttctcagcagcctgtacagcagccatctgacacagaagaaatagcagaacagcagacatttgcacccatagc

tgcttcacagcagcctgatacagtaatgcaggaggaggttacagacaggattcctgatgacaaacagccattgtcccctacagaaagtgatgctcagcat

gttgttgcttcacatcctcatgaatctgatgagggatcaggggccatggaaacagcagaaattcaaacattgtctcctatcacagtggaagaagcatcaa

gcactgtttcacaagaatcagcattacctggaagtgaagctgtttcagctgcagaaacagggcaagaagaaggtttggcatcagaagaagaaaaggaatc

tgttgctactcagcaaccaatacaggcagtatcccaacaagaatctgtgtctgatgataagacacctgacagcattactgaaaattatgtggaacaacag

acttcattaacaggggatgtcagttctcagcatgacattatttctcaggagcctgtagttgacaaacaacatgaacaaacaacatttgcagctgatagtc

attcagaacacacacctgctcccttacagcctgttacaggtgacaaaactcctgacattcttgagcatgagactactacatctttagcttctgatacaca

aacaacagttgtttctgtagagcccatatcttctgaagaagaggaactagataagctgcagactctgctcccaccaggagaagacagtcaacatgtttcc

ataccacaccagcatgcaccaactacagaagaaatgattttgcagacatcctcaccagtatttgaggacaaagagagttctgcaagcgtagtcactgtta

gtgatgatgacaagacacctggatttactgaggaaactagtaaacagacagtagcaccactagatcatgatgtgcaacactctatggtccctcaagaggg

tgcacctgtcacagaacctgcagaacaacagactagatttactgcacaggatgagatggtgcacattgagaacacacaaaagccgtcactggatggtcag

caggtcactgaggtttcaggactgtcagacgtagtaactaatttacctgtagacgcagaaactgagcaattcttgataggtgaggagggaatagaaaaag

taacagaagctgtggatgtccagactacttcacctactgattcagaaaacaaacaaactgatgttccatcacagcctgttactattgagcaagtggcaaa

tgcagtaagtagttcagatgaggaacacagcatcccctctgttatacctgatattgaaccaactcacatatctcaccctgaaagccaagttgggcatgaa

acaatcgtcacagatgtattggatgaacaaagtacagcaagccctgagcaacctgaaatatccgaaacacccacagtaccagttgcaggtacggctgatg

aaatacaagtaactaacatccctgaagaacatgtatcacatgctgctgaagcaaatgttgatgatgcaggagaaataaatgtagacatgcaaactacaac

ttctcttgtatcacagtctcctgtagagactgtttcaacagtacaagaattaggagaactccacccctcaacaccagaggaaacagaagtccagcaggtc

actacagctcaaataccagaaacctctagtgagaaacctgctgaactagaagaaccagaaatacaacatagttctgtgtcatctgagactggaattcagg

aagcaagtacatttggtgaacaggaagcatcagtcattgaagacacaggatcacaaaacaccatttctcctattgagtcaggggcacacacagtaaacgt

tgatcagccagtctctgcacctgatgtggaacaagagcctgcatctccttcagagcctgaaattcagcaagcagcaaacactgaacagtcagtactggca

gaaggtgatagttcctctgtggcagaaacaagtgtagaaactgccacttcagcagttcagcagcctactccctctaaggaagaacagcattcttcggcag

aatcattggaacaaactgctaggcccacagagggtcatgtccaggaggccactgctacagaaagcatttcagaacagctagctacatctgacaaggacac

atccattagtttcagcaaacctgctgaccaagaatctgtgcctgaaagtacagtcacatcagatgaacctcatttaattgagactgttgtatcaacaagt

attccggaaggcttggagtctcaatcaagcagttccaaggctccagatgtggatcatggtgtgcacaatacaactagtgatttcgcacaatctagtggtg

tgcaggaaagtggcactgtaacactggcaatcatacacccttcccaggctcctatagaatcagcagatcatcatatagaaatttctgaacagtcttccca

aggtacagttacagaggcaacaacagttcagcacgaacatacaacttcagctcctgttaagccacctgtccatgaagaggaagaggaacaagtagccaat

aatgtccatcctgtggaagttacagaaggaaatcagattcttcctgcctcttctgaacatccactgcacactacagcacagcctgcaccaacttctttgg

atcattctgatactagccaggcatcagctgtacaggaaaatgaactagaagctaatgcgtcttcaccatttcatccagatcagaggcctgctcattcttc

aacatcacatcctgcttacgaacatttcccagaagatcatactttggcaattggtgaaggaatcgaacacataacggaagcaacgaaacgaccatcacat

cattttactactagaccaccattaactacaggatcacaccatcatccaagtcaacaccctgaagatcaatatgagcagtttccatttggagaaggacagt

acccaacaggaggtgatacagattatgatgatgaagaacaagctgcctttggtccaggaacctgtcgctatgggggaaaggtctacgtatcagctcaaca

aatacctcgtgatgacccttgtgatttctgtttctgcttccgaagtgatatcatatgtcttcaacagagctgccctcctcctatcccaggttgtcacgaa

gagccaatccaaggcttttgttgtccacgatatgagtgccctgtctctatggcaactgttctgaatgtatcaacaactaccaccaccactactaccacat

tgccaccacatttcctagcgcaccactacaagggtgcagcccacaggactgggtgtcaagttcagggaagtgcatatcgtgttggagaagtggtgcactc

tgcctcgggtccctgtttggagtgcaggtgtggtggagatggccaaatgaagtgtgaccctaaggaatgcagtccagaaccaatgttacgaaaaatgatc

atggcagcagcatccagaagaaggtgaagaagagtacagaaactctcacagatgtgctcaattgaagaggcttcctttggtgataatggaagcaaagtta

atatttgtgcattgactgtgttgtgacaagaaaactattgtggacagtgaatcttgtggcaacacatgcatatattgttttaaaattcaagaatgttgtt

gtttaaaagagattaaattgggtgccaaaactagtaggacaagctaggagaggatctgagaagactgtgcctttcctctttacctctgccctacgcagcc

ctcagtcttctgagttgcacagtttttagtgcaaatttaagacagttgttgctgtgtaaaaactggtaacactcatgaaatgttttctttttatgagtga

tatgctattgaggataatcaaagcaaagattgtcctcacacactgatacaggtagactgatcttcctttttatgaacattttacatctacatattttgtg

tctttttgagtctttacacaaaatcttttgctacacacattgtaccaaccaaatattattcaaggaaaaactttatacacaagttgaagggttggaggag

aaaattcagagacgtggcacttcctgttttttaatatttggtgttcattatagccacttattctgaagatatttaacaaaatctccatgatttatactat

ttaaatatttttttactatttatctcagtgtaagcataggtataacaaacagacacttccattttctcgctttttgtttattctatcttgaataagtgag

cttttggaacacatgtccaggtagtttgactggtagaaatttgcaggtgatgtgccctttacttcaaattttttactacttgaactatgactgttaaatt

tctgcctcttgaacatctggacaaaaagctgtatatttatgtcattggtcatatagatacagtgttagtatttttagtatttatatattatatataaaaa

tgtaattacgtgagctttccatggatgtgttttctaaaaacactccacattctcagtgacaaaatttaactaaaattttctacttcattgaactgaaact

tttatgttcttcatgttacaaatgtgcacaatgagattctgag

>82539|AGAP000928-PA

tatacttccaggaagcaaagtacggaaaaactggatgtttaattttaattaggaacaactttctccatgtacagaaggatgtggaatggaaacatgtgta

atctcatcacatcacaatcacaataatggcattgtttataaactatcaatcctcaataattaaagaagtaatcaaattttatctcatggaaaattctgta

gcattatcactctgttaggagttacaacatctttgcatacagggcactgttcctgtgatttcagccactccaaaatacaggaccaacaaaacacatgacc

acatgttgttgctgatggactggctaattcttccaagcataatgaacaagtatgtcctttaattctttggatattagtagaatattgtgttttttctact

gaatgtgaaaactcatgtaaaagttttctatatccgtgatacgatgcaagtaataagtatataacagatgaaattaacactgctttgaatacagtatctg

tgctgtgatctttgatccagtggtgatgaagaacatatcgaattcctgttaaacgcttggacaatgctaagtatgtgccatttaagtaaaacaatgtagt

atgtatcttctcaaaaaatggtaaacacttttcagccaatagcatcagttttgatgctttctgacatgcttcctcatcagcatcacttgttgatggcagt

ttttctatgtacactcgtaatttcttcattaagcaagtaagtagtagtagtccttcacattgtgcagaaatcatcacaaatctttcaaatttgcttggaa

cttttctcagtgactggtcaaccattacaatgccagtgtactcctctcctagtgtttgaaaaccacttaatgtagttaaagtataatacaggtttcttga

accaactgaagtccatttactgtatttacgaaaccaatgactgcccaatgtccttagcaacacttctgccaactgtgatcgcattttttcagacaccagt

tcatctttctgacttgctcgcagtatttctgcccgtccagcagtagtgaaaaccatttcaaacactctaaaatgtgtttttgctgcagtagggcgtttta

acttaactataaaagcaaaagataaggtgtcacaacgctacaacgtctctctataaccttacaggacacaattacaaaaaaaaggacgggcaataattga

tattctgcgctctactggcgcaaattaatcaaaaactttctcattagattatttgcgaagaaagcgttaaatgacttagtactgaacatcagataacatt

gatacaatccattgctgtaccccatcc

>82540|AGAP000928-PA

ttttagagtggtgagtaggtcgacctgtttcattcgtttgcattgcagatatcgaagcgaagctgtgcatttgtctttaatcaagacaaccttattttac

atgcgattttgaatattgtgcacatcataggttttgttattatggttagctacctaaggtactgtttggcattcaaacaagaaaagatacgagataaaat

taattaaaacagacattcgtaacgttctttaaaaatgacaacgacccaagtatgtcttcgtgatctgtcgtaactcatgcgtaataaaaggtggcctgtt

tagaagtgtgtttgcatacaatattgcgtttaatggaagcgttgggtaccgtttcgtcaacactcaacgaaacccacagtttcaacctaacctagaccgt

gagtttgaaatggttttcactactgctggacgagcagaaatactgcgagcaagtcagaaagatgaactggtgtctgaaaaaatgcgatcacagttggcag

aagtgttgctaaggacattgggcagtcattggtttcgtaaatacagtaaatggacttcagttggttcaagaaacctgtattatactttaactacattaag

tggttttcaaacactaggagaggagtacactggcattgtaatggttgaccagtcactgagaaaagttccaagcaaatttgaaagatttgtgatgatttct

gcacaatgtgaaggactactactacttacttgcttaatgaagaaattacgagtgtacatagaaaaactgccatcaacaagtgatgctgatgaggaagcat

gtcagaaagcatcaaaactgatgctattggctgaaaagtgtttaccattttttgagaagatacatactacattgttttacttaaatggcacatacttagc

attgtccaagcgtttaacaggaattcgatatgttcttcatcaccactggatcaaagatcacagcacagatactgtattcaaagcagtgttaatttcatct

gttatatacttattacttgcatcgtatcacggatatagaaaacttttacatgagttttcacattcagtagaaaaaacacaatattctactaatatccaaa

gaattaaaggacatacttgttcattatgcttggaagaattagccagtccatcagcaacaacatgtggtcatgtgttttgttggtcctgtattttggagtg

gctgaaatcacaggaacagtgccctgtatgcaaagatgttgtaactcctaacagagtgataatgctacagaattttccatgagataaaatttgattactt

ctttaattattgaggattgatagtttataaacaatgccattattgtgattgtgatgtgatgagattacacatgtttccattccacatccttctgtacatg

gagaaagttgttcctaattaaaattaaacatccagtttttccgtactttgcttcctggaagtata

>82538|AGAP000928-PA

ttacggaaaaactggatgtttaattttaattaggaacaactttctccatgtacagaaggatgtggaatggaaacatgtgtaatctcatcacatcacaatc

acaataatggcattgtttataaactatcaatcctcaataattaaagaagtaatcaaattttatctcatggaaaattctgtagcattatcactctgttagg

agttacaacatctttgcatacagggcactgttcctgtgatttcagccactccaaaatacaggaccaacaaaacacatgaccacatgttgttgctgatgga

ctggctaattcttccaagcataatgaacaagtatgtcctttaattctttggatattagtagaatattgtgttttttctactgaatgtgaaaactcatgta

aaagttttctatatccgtgatacgatgcaagtaataagtatataacagatgaaattaacactgctttgaatacagtatctgtgctgtgatctttgatcca

gtggtgatgaagaacatatcgaattcctgttaaacgcttggacaatgctaagtatgtgccatttaagtaaaacaatgtagtatgtatcttctcaaaaaat

ggtaaacacttttcagccaatagcatcagttttgatgctttctgacatgcttcctcatcagcatcacttgttgatggcagtttttctatgtacactcgta

atttcttcattaagcaagtaagtagtagtagtccttcacattgtgcagaaatcatcacaaatctttcaaatttgcttggaacttttctcagtgactggtc

aaccattacaatgccagtgtactcctctcctagtgtttgaaaaccacttaatgtagttaaagtataatacaggtttcttgaaccaactgaagtccattta

ctgtatttacgaaaccaatgactgcccaatgtccttagcaacacttctgccaactgtgatcgcattttttcagacaccagttcatctttctgacttgctc

gcagtatttctgctcgtccagcagtagtgaaaaccatttcaaactcacggtctaggttaggttgaaactgtgggtttcgttgagtgttgacgaaacggta

cccaacgcttccattaaacgcaatattgtatgcaaacacacttctaaacaggccaccttttattacgcatgagttacgacagatcacgaagacatacttg

ggtcgttgtcatttttaaagaacgttacgaatgtctgttttaattaattttatctcgtatcttttcttgtttgaatgccaaacagtaccttaggtagcta

accataataacaaaacctatgatgtgcacaatattcaaaatcgcatgtaaaataaggttgtcttgattaaagacaaatgcacagcttcgcttcgatatct

gcaatgcaaacgaatgaaacaggtcgacctactcaccactctaaaatgtgtttttgctgcagtagggcgttttaacttaactataaaagcaaaagataag

gtgtcacaacgctacaacgtctctctataaccttacaggacacaattacaaaaaaaaggacgggcaataattgatattctgcgctctactggcgcaaatt

aatcaaaaactttctcattagattatttgcgaagaaagcgttaaatgacttagtactgaacatcagataacattgatacaatccattgctgtaccccatc

ccctagtcaccgg

>82556|AGAP000932-PA

cgcggctgcacgccagcgccgcccaggaggcgccatgccgccgagccggctgcacgcatgcgcacacgcgcagcctcgactctcccagcgctgccaactg

ctcgcagctgcgcctgcagctgcgagtctgccatgccatcaggtactggattctatcatttcccatgcatttcgatctggaatatgaactggcagacaca

ttaaaagaattccaggcagttctcactccaacttcagctgaacttgtagacctgtcccaagtgcaaacatatgactggatgcgtcatatgtcaatacgag

acagccattcagcacatctgcatcaacatggcaataaagagcacaacagaaaagtgtctttagtgttcagccatttggaaccattacagatagctgagca

cattacatacctggagcacaaggtcataagaaggataactttcatggatttcaaaaaatatgctgaaactggatctcttcgggagaccccaaaactggaa

cgctctgtagctttgtttaatggacttactcaatggatacagtgcatggtcctcagcagaacaacacctctacaaagagctgaagtcatggtgaagttca

ttgaggttgcaaagaaactattagaactgcaaaattttaattcattgatggctgttgttggaagtgtcagtcacagtgtattggctcgtttattaaagac

aatggcctgcatacccaccgaaagtaaaaagtcactgactgaaatgacagatctgttgtcttccacaaacaattttagcaattatagaaagttgctgcat

gaatgttgtggtttcaggataccaatcctaggaatacatctcaaagacatgatcgccttacatgtggcactgccagatactttggatggagaaatgataa

attttcgtaaaatggcacagctatacttgatatttaaggatttagaagaggttcagctgagcacagcacctatcaatgttaacatggacctggtcaacac

tttgagagttccactctcaatgcaggtgtgactcttcagcatggaatcagtgtgacaagaacattttgggagctatttggctgatcagtaagaaagttgt

cgcttgacttggcctacactgaagatgaaatatacgagttatcacttgcacgagaacccagaaattcttcttcaccccagtgttcacctactcagtctgt

gctctttgctgaatgggcatctaatccatgccctcctccagatgcacagacaattgaaaaacatgtcaatgcaatggttgaagctgttttcaagaactat

gataatgaccatgatggatacatttctcatgaagaatttgaagctgttgcagagaactttcctttcattgcatctttttgtgtattagatgctgaccatg

atggtatgattagcaaagatgaaatgaaaacatactttattcatgctaattaccatgcattgaaaaatgggttcaagcatgaatttcatgaaacaactta

ctttaagccaacattctgttcacactgtgcagggctgctgtggggacttatacgacaaggctataaatgtcgagactgtggaattaatgcacataagcac

tgtaaagatcttgtcgtaatggaatgccgcataaaatatccttgcttgcaaggtgaagttagctcacatgctgctacttcgaggaggatatttcggcgca

agaaaaagtctagtgcttcagaatctgagtcatctcctccaagtcctaaatgttcagtgtcatcttccatgacaggtgaccatgaatctagtcaagactg

tggtacaggaatggtatgctcagagcttggagggggttctcttccaccaagcccaaagccagctgttatgtggacaggaccacggcgcaaaattagtgat

tccctcttagtaccttcacacagtcgtagcagtgccagactcaggactcaaaacagctgcccagagagtagttgcgcacattgctacaaacctctggaca

gcatgcccaaccactcttcacaacatcactccagtacaggacatcatacttcacctgttcaccaagcttcagtaacaagcctcaagaactgctgtcatag

tgaacagttaaatgaggtcaatcaaaaacttgctgctgctgaggagg

>82581|AGAP000943-PE

ggaagacagatgttctcaagaatacattatccttagcagaaaacagcatcagtgtcaaatgaaatctgtacactgtacggttacagaaaaaagtatttcc

ttggtaaaggagggtaacatgaattagataactaatttttgagaaagcacagcatatataaatctgtctgaagtgcagatagttactgaatatacaatat

attcaaattgtaaaacaatataaagagcaaacaactgcagataaagagttagatactcaattgttcatgtatttgattagtagtagattacatggattta

aaattcatgttttcctttttttatcaaacagcaaaggactcaaattttagtaattccatgtaatgaaacaaaagttcttaaatacagttcacacaatgac

acaaaacatgtgatgttttatacacacaggaaaacaacttaaatactgtatattaaaattctacaaatataatgaactttattgctttccagcaaaataa

aaatttgtattattgaataaaacattgagaaaaactgaatatcaaattgtaggtgccacatttgactgtcagtaagacaatcctttcatattcacaaagt

aaaaaagctttgtgaactaattcacagtaaatgaaccaatttatcaaatacactaccatgtcactgcctcagaaattcaatagaactaacaattgctgta

aatgtcctctaatttaatggcaaaattattcaacttcagcaaaatggcatagtgcatctcgggatgtgtagaaaattcttaaatgcacagccgtaaatgc

atagaaaaataaaaattaagatgcatttgacggttcacttcatgtcctgcagcactggctgctacaccttaatttattcattctactaaataaatgcact

gatcaacttctgcttcactttcccatatactgcatctggtttaatcttcaagggatatttcagcaggaattgatgttgtaaatgtaggtgtttctagctc

ttcatgtttcttcattttcattgctccaggtttattggtttccactttatgctgcttgtagtaatttataatgtcttggtcagctgggcagctctgtgta

aatgcatccaactgatacatatggtacatatagcgccacagtgctgtcagttccattggaatttcaaaatctgtaaagtatttccctgcaacccttatat

gctgcagtcgaggcatgagttcacagtcaaagcagcacatcgtatcacctgtgaggaagcgagaacccttacgagccaaatggtcattgatcttgcgaag

atgtgataggagactgtgggtgcttgttgaatctttcttaattagcatcagcttcaatttactgtagaggttctctatcaatgtagctacttctttatcc

tgaacaaacaaattatggccaccaggaacattcttcatgatatgccgttcaatcttttcattttccaatacagccaaaccattatcaataaggattggtg

gtggtgttgcttcgaagttggtcctgaagtcaggtggaggtttctgcatgtcaactgttgtcaccttgagactaatggttttgagttcagccagcaggta

caagtccataaagtattcttggcaaaagaggcaagctcctttgcggcggccatcgatagttgatgccttgatgatcagttcaatctccggtacattcccg

ttctctggagccccgttggtatattcatcagccatcgtagcaacccgtgaatgacacttttaagatgacacctactcctcacaacacgactaaactcact

ttgccgcaatgtgtcaaagacgtt

>82612|AGAP000954-PD

acagaggtgaaaacagtatctttcatacatttcgtttatccactgcgtctgtgttttgtgttcatggtaacattggaactgtataaagttgtgatttacc

tgtagaaaatgatggatatgccagcatcaagcgatattatgataattggagggaactcacatccagagcttgcaaatttagtcgctagccgcctaggggt

taaacctgcaggatgtgcagtgtaccacaaaactaatcgagagaccatggtggagattggagactctgttcgaggcaaagacatctacattctgcagacg

ggaaccaaggatgtgaacaataacataatggaattgttgatcatggcgtatgcctgcaaaacatcctctgcaaagagcattgttggtgttattccttacc

ttccatacagcaagcagtgcaagatgagaaagagaggatgtattgtaagcattgttggtgttattccttaccttccatacagcaagcagtgcaagatgag

aaagagaggatgtattgtaagca

>82634|AGAP000973-PA

gagtagcagtgctcctggcggccgtcgggcgccctggcgcaggtgctggacagcgggtcgcagctgcggcccgtctccttgcagcgagggcccgccgtgt

tgggcgacacgtcccccaggttgctcgacgagaaagccgagatgaacgggcccttgccaggcatggccccgccgttgaactgcttctccaggagtatggc

ggcgtagccgacgttgtcgctggagacgagcgtgttggtgttgttcatggaggtggggtgcacggcgtaccagttgatggcgcccagaggacggcctgaa

ggcgtctcgatccgtagctgcaccatcgtcttgtccacgtcgtgctcgtacctgctacgttctgaggtggggttggcgaggtaggcggtggggctacggt

tgatgctgacgcccagcagctccccctgcgacaggaagaggcgcgccggctgcaccgacaggtgggcccgtctgatgctctgcacgatgccggagagcag

cgcgttgtaggtctgtcggacgaaaccgaggctgctgatgtcgaagaggaagtcctgcataaagcctccgggcccagagtgcgtgtgcgtcgcgctgatc

atcacgttgcgctccgtgtacgtgtcaccaaactccttctgcagcttagagatgacgtctctgcgaagtccgttgccgatcatggcggcgtcgatgctgg

tgaagaccaatctgctaccgtcatcgtcctggaagatgtaggctctggagaactgtcggaggtgtagaccggtgccccgctgttccagctgcgcgtagcc

catgaaagtcacttccaccggtggccctgtgatgtcagcgattccgattccgaccatataagcggctcctgacgtggacagcagtgctgccaacaccacg

agtgtgcgtccctgcatcatctcgactcttgcagtaaactggcggggacggcgagatagagaatggttataagtttgtagtcttccagtattaacccaca

aattggcggcgctctggtggttggtgctgaacgttgtggtgcctattgtcagcgccggacaagcagcgggctctcagaagccggaagcgctgctcagctg

ttgttgttgccgttagagctcccgcgcgcatgcgcaccgcaccaccagaccgcgcgagacgcactgggcgaggagtgggccgtgtcccgctgaatgcgcc

agccaccacgtcacgtgaccgccttacatcaggcgcgcagctgagtctgtgg

>82632|AGAP000973-PA

gagtagcagtgctcctggcggccgtcgggcgccctggcgcaggtgctggacagcgggtcgcagctgcggcccgtctccttgcagcgagggcccgccgtgt

tgggcgacacgtcccccaggttgctcgacgagaaagccgagatgaacgggcccttgccaggcatggccccgccgttgaactgcttctccaggagtatggc

ggcgtagccgacgttgtcgctggagacgagcgtgttggtgttgttcatggaggtggggtgcacggcgtaccagttgatggcgcccagaggacggcctgaa

ggcgtctcgatccgtagctgcaccatcgtcttgtccacgtcgtgctcgtacctgctacgttctgaggtggggttggcgaggtaggcggtggggctacggt

tgatgctgacgcccagcagctccccctgcgacaggaagaggcgcgccggctgcaccgacaggtgggcccgtctgatgctctgcacgatgccggagagcag

cgcgttgtaggtctgtcggacgaaaccgaggctgctgatgtcgaagaggaagtcctgcataaagcctccgggcccagagtgcgtgtgcgtcgcgctgatc

atcacgttgcgctccgtgtacgtgtcaccaaactccttctgcagcttagagatgacgtctctgcgaagtccgttgccgatcatggcggcgtcgatgctgg

tgaagaccaatctgctaccgtcatcgtcctggaagatgtaggctctggagaactgtcggaggtgtagaccggtgccccgctgttccagctgcgcgtagcc

catgaaagtcacttccaccggtggccctgtgatgtcagcgattccgattccgaccatataagcggctcctgacgtggacagcagtgctgccaacaccacg

agtgtgcgtccctgcatcatctcgactctgtaatcgggaggtgtcggtgctcagcagtaatccttcaattggaccgacacatcaccagagctatgcactc

tgacagcacaagaagtggcgggaatttacggcgtatttcacagaaatccgatcagaacagtaatacggcacacactgcgcacatgtgaagacggatagaa

tatgaaattccgcgaaacactcggcaccactaagccctcacgtacgctggcaaaacgacggatggccagagctgggtgaggtgggacacagacaaagctg

catcggcttctgctcgaactggttcacacagagagctgccgttgcatcaagtagctccctgtgtggctggaggcgtgctccggagcgcggacctgcagca

actgagccaccgcca

>82633|AGAP000973-PA

ctccggagcacgcctccagccacacagggagctacttgatgcaacggcagctctctgtgtgaaccagttcgagcagaagccgatgcagctttgtctgtgt

cccacctcacccagctctggccatccgtcgttttgccagcgtacgtgagggcttagtggtgccgagtgtttcgcggaatttcatattctatccgtcttca

catgtgcgcagtgtgtgccgtattactgttctgatcggatttctgtgaaatacgccgtaaattcccgccacttcttgtgctgtcagagtgcatagctctg

gtgatgtgtcggtccaattgaaggattactactgagcaccgacacctcccgattacagatctcgccgtccccgccagtttactgcaagagtcgagatgat

gcagggacgcacactcgtggtgttggcagcactgctgtccacgtcagctgcatcgtatatggtcggaatcggaatcgctgacgtcacagggccaccggtg

gaagtcactttcatgggctacgcgcagctggaacagcggggcaccggtctacacctccgacagttctccagagcctacatcttccaggacgatgacggta

gcagattggtcttcaccagcatcgacgccgccatgatcggcaacggacttcgcagagacgtcatctctaagctgcagaaggagtttggtgacacgtacac

ggagcgcaacgtgatgatcagcgcgacgcacacgcactctgggcccggaggctttatgcaggacttcctcttcgacatcagcagcctcggtttcgtccga

cagacctacaacgcgctgctctccggcatcgtgcagagcatcagacgggcccacctgtcggtgcagccggcgcgcctcttcctgtcgcagggggagctgc

tgggcgtcagcatcaaccgtagccccaccgcctacctcgccaaccccacctcagaacgtagcaggtacgagcacgacgtggacaagacgatggtgcagct

acggatcgagacgccttcaggccgtcctctgggcgccatcaactggtacgccgtgcaccccacctccatgaacaacaccaacacgctcgtctccagcgac

aacgtcggctacgccgccatactcctggagaagcagttcaacggcggggccatgcctggcaagggcccgttcatctcggctttctcgtcgagcaacctgg

gggacgtgtcgcccaacacggcgggccctcgctgcaaggagacgggccgcagctgcgacccgctgtccagcacctgcgccagggcgcccgacggccgcca

ggagcactgctactctcctgggcccggcaccgacatgttcgacagcacccgcatcgtcgccaagcgactcgccggcaaggcttctgagctgtggcgttct

tcgaatgccagggaggtgactggtcccatcgcgttcgctcaccaatgggtggacatgccgaagcagacagccgagtacgtcgaccccgagaccggaacgc

catcaactggtacgccgtgcacc

>82652|AGAP000977-PA

cttatctctgactaaatttatattaactacttctccatattctgaaacacttactgtgaaaatacacatattacatctccttctgtcaagtcataaggca

gaccac

>82672|AGAP000984-PA

cagatcagtgcacgtaacattcggaggtggttgtggcctccttcctggtcctcccactgttcgttgtcgctgactccataactgagggtgttgctgcttt

atatgtcgctccatatttgaacggagtgtaaatcgtgcagaacagtgttcacatgcaaa

>82745|AGAP001007-PA

ctatgctgtaaggggaaaaaaactgatcttttgacttgtgttccaaagttcagccgacaaagatgtgttctgtgtatatctgtgctctcaggggggaaaa

aactgcttgtagattatacactttgcagaactgccagtgcaagtgaatgtttacaaacaatgcaacaagatgttaccaggtttggttagtgtgagaataa

gcagattattttataaatcagcacggtcatttgtatgtgctaggattagagtccctgtaggctatggacagagacgaattaatgtagtaggcacattgcc

acaaatcgaaatacaggcgcacaggttcaagtatacgaaaaaacgagagagtgccgaagaagacgaagacgatgatattgacagtgtatcagaagtttta

gacccttcagataaaagcataagcactaagaagtttaatgtcacttcgctgcgtgttgacctgattataaaggctggaatgggaatggcacgaaacaagg

tagaaacagccttttatgaaagtcgaatacgagtaaatgggaagaagatacttaagaaaagtgtacatgtaaatgttggtgatgagattgatataatacg

tggaccaagttcttccaatccagattttctggttgtaactcggattgagttactgtcagcagctccaaaggatgattctgtggtcgtgcatctgaaacgc

cataagaatttagtagttgaaaattatgatgaaccgtggagaggttcattggcctgagcatcaatttgttcaatacagaaatgtttttgtaacattttac

taaatatgattgttagattttcactgttttcatcaaataaaatatgtttttttcctgacctttcttttataattccttattc

>82945|AGAP001008-PA

aagcagaagtcccatttcttgcaccgcccattggtccggcagccgatcgactcgtgctactcgaggtagaacaaccgtttgtggactccgagtactacgc

ctggagcgagaatctcggtgagggcgatgaatcccggggggcagtggcgggggtccaggaccgcgacgtgcagcagcgcagacaggcacagcgcccacag

gggctcgagcaggcgcctgtcgcacaggtgcgccacccgcagctgtacgagcgtcgtcacggcggcgaaggcgatggtgaggtccgggtggagccagaag

gcagcgccgctcgccaggccggccaggagcgcatgagcagaccggtcctcgccccgcagccggcacaaaccacagcgcaccgcctggtagagagcgacgt

aggcggcgaggaagagccccagcgagttgtcgccgcacagggcgctcaccagcagggccgccagcccgcgcccggggcctgcaggccgcctccactgcgc

cagcaggtggcgagccgcctgcagcgccacgcccgccgccaggtacttcgccgccagcgtgaggccgtggttccagcaggttccctggtgcgagcagccc

ttattgctctcgatgtcgcctgagcgccgagacggcctgaagaacctgcggcaaggacacatacacacaatcgctggagcatcgacaaccgagaatgaac

tgctcctatatacatctaccagtaaattctagtagatttactgtcgtgttagctccagacgctaaaccgtaaccttcttgatcattctgacgttaaatct

tgacaaagctagtaaccagatatcagtctttcttcttcatctacttccacttcactaccatatcattgcatacgcatgtgtagaggacaattaattaaca

ttcatatcgagttcaaactttgtatacttcaaactgaagcgaatgcagtgatgtgcttccttcagt

>82946|AGAP001008-PA

aagcagaagtcccatttcttgcaccgcccattggtccggcagccgatcgactcgtgctactcgaggtagaacaaccgtttgtggactccgagtactacgc

ctggagcgagaatctcggtgagggcgatgaatcccggggggcagtggcgggggtccaggaccgcgacgtgcagcagcgcagacaggcacagcgcccacag

gggctcgagcaggcgcctgtcgcacaggtgcgccacccgcagctgtacgagcgtcgtcacggcggcgaaggcgatggtgaggtccgggtggagccagaag

gcagcgccgctcgccaggccggccaggagcgcatgagcagaccggtcctcgccccgcagccggcacaaaccacagcgcaccgcctggtagagagcgacgt

aggcggcgaggaagagccccagcgagttgtcgccgcacagggcgctcaccagcagggccgccagcccgcgcccggggcctgcaggccgcctccactgcgc

cagcaggtggcgagccgcctgcagcgccacgcccgccgccaggtacttcgccgccagcgtgaggccgtggttccagcaggttccctggtgcgagcagccc

ttattgctctcgatgtcgcctgagcgccgagacggcctgaagaacccacgtgtagtagtaaaatgctgtaattggttttcgtctgtcatcttcaatgtat

accgacaagccagcgaggaaagctggccaaaataatgcggttgacccgtgatagccgccaagtaaatatctgaagaggcagtggaggacgaatatgccgg

tggtgaccgaggtgccgaagcagagggagcgcagcaaagcgcgcagcgccgacgccgggctcggccgccagccgccgaccgcgacctgcgccttaaggat

ttcgaaagcacggaggcatcggcataggcgggcagcatgaggcgcgatgagccgaacaggaagatggcgaagacgcgcagcgacgccctccagcagctgg

gcgtccacgggtggacgatgtggcggcagtcgtggctggccagcagctccagcaccggcttgctggcggccaccatggtgtgcggactgccctgctaggc

gcgcaccatgagctcggagcggcactgacgctacgtgacgagcgcgcagcgacggcgacgcgggcgccacgcttcggctgcggccgg

>82971|AGAP001015-PA

tcttaccactgcatttgtgcactaggttatgaaggacgtgactgcatcacaaacacagatgattgtgcttcattcccctgtcagaatggtg

>82970|AGAP001015-PA

cctgggacgtctggtcctaactgtgaaattaatgtcaatgagtgcttcagcaatccttgtcgtaacagggcaaattgtgttgatggaatcagcaaataca

catgtgaatgccttcctggttatgctgggcagcattgtgaaaccaatataaatgaatgtgcaagcaacccttgtgcaaatgggggtcgctgtatggattt

agttgacggattccgctg

>82984|AGAP001027-PB

ggggaccaactaaaattgagttatattttggcgttcttcgttaccatggttgaaaagagaatacttcttcactataattaatgagtattaacgttaattt

tcttatataaatctcgatcaacaaatcataaatagagagtagtactggtcagtgcaatcgtaaagagtcctcacaaaacaaaggtgaatgacattcgacg

tcatttcttccagcagtgtcagttctcaggtaagaattatgctccatgtgaagcaccaagttttgctgcttccggttcagtcgtgaaggagtacagtgag

caccagatgtggagaggttccaaaacaaagattaagcaatcgcgtatctcttcagcgacatgctctgattgtcctatcgctttcaagcagaacctgtatc

tcttctaaagtcttccccttcgtttcaggcagtaaaaggtacacgtagagcgaggcgaacaaggagcaagctgcgaacagaaagaagggcccgtagttgc

caacggcgatggaaatgtcctcaaagaacctcggcaccaggaaggacatggtccacgacacacaggcagctgcgggtaacgccacagacttgacgttctg

cgcgaagatttcccctgtgacggtgaacggtaggccgccgtatccagggcgactgacatgccag

>82996|AGAP001027-PC

aatattccacagcagagttctcttaactaccgattgctggcagcacagatccgacgggcccgcccttctcttcgccgagcatctgttgtatttcctcaag

agtcttccctttcgtctccggcaccacaaagtagacgtaaatcgttacgagtagcgcgcatcccgcgaacacgaagaagggaccggcgttgcccac

>82997|AGAP001027-PC

gtgctggtgatggagctggtggcgccgcgcctgcgcgggctcgcatcggtgctcggcttctgcaccatctgggggctggccgccctcatgagcaagctct

accccttcatgtccgacgccatgggcgaatacggctccttctggttcttcgcggccgtcagcatcgcgtgcaccgtcgtcggactggcggcgctgcctga

gaccaaaggccggcctctggacagcatcctcagggacctcaacggcggc

>83017|AGAP001049-PA

tgaatgtgtctaattgactttctaacagatcgaaaaaccgatctatagcaactcgattaaaccccattgctcgtgcgattgttgtggcctctggttttcg

tattgttaaggtaggatttcgtgcaagaaacccatttaaccaatctcttc

>83010|AGAP001049-PA

tgattatcaagtataattaatgttgggtaatcaattgatgttacagagaatctgatgaaatgaaataacaatgtctcataattctcaccagtcatccatc

cattgttacggtggtagaactctgatcctagaggagcaccagctttcattaaatcactcactctttttctatggaacacatataag

>83014|AGAP001049-PA

taacacttaaagaactttgtgaacttgattttcatatacctgaacgaaacaacatcaaacatccatttaatactgcagccaaatctgctggaagagattg

gttaaatgggtttcttgcatgaaatcctaccttaacaatacgaaaaccagaggccacatcaatcgcacgagcaatggggtttaatcgagttgctatagat

cggtttttcgatctgttagaaagtcaattagacacattcaagtttacgggagaca

>83045|AGAP001053-PF

aacgttgtcgtaggcggcgtccaagtatgcagatgagaaggagaagttgagcaagcggtgtggcaaaccaaagaatgttgatgaggccaatgaagctgaa

ctcgtgagcgtttgcaaaatgtactacgacagactaa

>83046|AGAP001053-PF

aatttataaatatttcaagatcgtacgcatcacttgcaattttcattagtatggcgtagtacattttgcaaacgctcacgagttcagcttcattggcttc

gtcaacattctttggtttgccacgctgcagccttcttctgcagcgtggcaa

>83092|AGAP001053-PH

gtttgccaagctgcagaagaaggctgcaaaattcaacttcagaatccagctcaaagttgtcaaaaaaaaagaattcaccctcgaggaagatgacaagg

>83091|AGAP001053-PH

gaacaagaaaggaaagcaggcgagagctggagaatgattgaggagcggtgtggcaaaccaaagaatgttgatgaatccaatgaagatccaggtaagcgtg

tgctcaaggataatcacgctagaatccagaag

>83090|AGAP001053-PH

aagcagatgaagaattgaagaaagaacaagaaaggaaaacacccgagaggaggagaatcattgaggagcggtgtggcaaaccaaagaatgtagatgaagc

caatgaagaaaaaaccagattggtccaaaaagggag

>83219|AGAP001065-PA

aggaagatcaagtgccatgattctttcatgaggctttaacaatgcagtgtacacatggaagttggcaggagatcc

>83220|AGAP001065-PA

gttgttgttacgtcagcaatttgaactgggtttggatatacaccagcagcaacaagaccagcaacgtgagccatatcaacaaaaaggtaagcaccaactt

tgtccgcgatgtcacggaaaggttgccaatccacaacacggctatcagcagagaaac

>83248|AGAP001073-PA

ctaaatatgccaaataattaataggtttacagaaatactttttatagttcggttgggcaacaacaatttattctttatacagtgctgtgtaaagagaggt

ccagcttaaaagatgtgagagccatattggtatttatataatctgaaatgtgatcttcttttctccagactctccccagcgcctgtcgctaggctgctgg

ggaattccggatgcagtaagtaaacagggtaagggtaacataccacccaggtttacatgtaacatctgcgggaaggtgttcaagcactctggatccctct

cgatgcaccgccggctccatcgtggtcacaccctgtgccctatttgccaaaaagtgtttagtaggacatactacctcaagatccatatgcacctcatcca

taatataacaacagactgaagctttgaactttagtgatgctctg

>83292|AGAP001076-PA

aaaaaagcaaaatagggattattatgggttcattccatttagggctggaccaaggagttgtgttgggcgcaaatacgccatgt

>83291|AGAP001076-PA

atcggatctgtatactgtgtataactccttgtacctacgattgttgccatcatgagaatagtaattgtataatccaccaccgggaagacatttatcccaa

tccggaagttttcaatcctgacaacttcatcccagagaagcccgctgacaggcattactacgccttcatcccgttcagcgctggccccaggagttgcgtt

ggtcgcaagtacgccatgctgaagctgaagatcatcctgtcgacgatcctgcgcaactaccgcgtgtactcagac

>83323|AGAP001076-PD

tcccatagcggtttcgagcaagactttcgatgtccacgactacatgagcgagtgtaccctcgaaatcttgctcgaaaccgctatgggagtgaccaaatcg

acacaagataagagcggatacgagtacgctatggctgtaatgaaaatgtacgagctcgccgacaagataccgggacctccgggactgccagtcctcggcc

acgcgctttcgttcatcggaaagaatgaggagatcttcgagcgcgtgtacacga

>83465|AGAP001134-PA

acaggcttgtgccagaagccatgcataggaagccaagattgcttgactgtgtaaagcttcctcctcatcgtaagtgctacagaattctcgtctgtgatac

aagtgtcctcgtgtatggaaagaagttagaccaaattcacactcatctcctggccaaaaaccatcaattgttcttcctgtggtccattcataaatggtac

cttagggagtttagttgtgtttgtagataactgcagatcttgcatacgtctctttcagcaataaatcatctgattgtctcatttaatggatttaaaagtt

ctgttagctacagggcatgtagcagtgttcgctggtatagtag

>83512|AGAP001139-PA

gatcagcataaaacttattattgttacaagtaataaattaaattaaaaacatccatttcataaaaataacaattttgtttgtttttgtttcagtagaatg

gaaatagacaaaatcttgaattttagcaaagtgccatgaaaataatgttgaacatattaattatatacagtggaccacacttttattttgcagctacaga

atttttgtttacaaatagtttctcttcaagctttctttttaggtttaacaaatgttcttgttctctttattcttctacgactaatctccaatttatgtag

ctctgatgaaacaatacagggcaactataccagaagaagagcagagggaaatattttcagaaattcagtcagagctacataaattggagattagtcgtag

aagaataaagagaacaagaacatttgttaaacctaaaaagaaagcttgaagagaaactatttgtaaacaaaaattctgtagctgcaaaataaaagtgtgg

tccactgtatataattaatatgttcaacattattttcatggcactttgctaaaattcaagattttgtctatttccattctactgaaacaaaaacaaacaa

aattgttatttttatgaaatggatgtttttaatttaatttattacttgtaacaataataagttttatgctgatc

>83538|AGAP001141-PA

ataatcaggatcacccattttaatgggctctactgcttgtaacctcactattttattaaccggatccataattcccagaacttctactttcacattccgt

gacttcccaccatgtgctgttattcccaggcttaccactcctatttcaatctttttgtttggaccacccattctgctaaacttctcagccactgcagctg

tgcaaatgcttctcagatgtgtgtaaaatcccttcacatagagattgtctactttaacccattgaataacattttgaatacttgtctgtacaacccagtg

atatatgagtttaacaagtacagtgggaggatgaggtgatccttcaaatattgaaccactgaaaacagaaacaaatctttgtgggcaacagtcactaatc

caaacatatccaccactgaatggaaacttacttacatcagagtatatacccaattttaaaggtattttctcaccatatggagttacatgtgtttggcagt

attgatcactacgaactaacccttcctgaatcatccattcagaaaactttgttggcgtgaacatcaacactttcttcacttttttgtctaattcagtacg

gtcttcagacagctgcactgcagtaacttcttttacccgtatgctgggacgagctgtcacaacaagtgatgggaatgtcttccctattaacttattgaaa

gtaatttcacgagtgtcagcaggaatctcacttgggctgtcatcacatgtaagatcaaccactggagcgccacttgcactattagatggggggatttgtg

ggttatttggaagtctgctgggatttttattagcgggtacaccaggtggtcttcctacagttactggttgtaccattaacgaacgagcaggaactgtaac

acgaggtgcaggcatcatcggccgaacatttcttactaaagcaacatgactgcggctcctgagtgtaggcgatgtttgtggaatacgtggagccctaaca

ggactttgacgtggtccatagtgtgcaacacggatggatggtgtatgttgtgtaactatacctcctgaagtcgtgttgactggcctggatgctgaagcat

tttcacttaatggtacacgcacaggtgctgggtatcttgat

>83556|AGAP001147-PA

ttttggtattgaaagagaattaaagcaaatgctttatttaaatgaaaacagaatttcagtttacatgataatacagagtcacactatgacttttaccatg

tggtacaaatattgctactgaaagtatagaagaagaattacaattctgtgtgttgcttagtttgaaattttctgagaatggcataatacaagtttgcaaa

agcataaacactgacacaaataatcataacaagcaccactgcaaaaactacactgaggaagctcccatcaataatagaatctcttgctgatctccgtttt

ttacttctcttctgagtgttattctccattagtctcttgacgatgttcagcctgctccgaagctgttctacggattctgagaagtcatcactgctgtctg

aaccttcaaaatccatgtctgtaacattactatgcaccgccacgcagttgatggtttgtttacagcgacactacgctgtaatttgtattaaggtaccgtg

acataaacaagtaaacgtgcgactcgcgcaccgctccttcagctcagcccgggttattgatccagcagag

>83642|AGAP001156-PA

tacaaagtttcaagtttataaccactgggcgtacactctccatccttctataaaaaaaggaagatttacaaaatctgaagatgctataattttaagagga

gtgaagaaatatggagcagactttgggcgaatttcaaggtatttgccaagtcgtacttctatacaagtacggaacagattcaacacatttctacaatatc

aggaaccatttaagccctggactgcagaagaagatgaaaagcttttagacttagtagataaactgggtaaagggaagtggagccagataagtaaaatgtt

tgataatcgaaacagaacacaaattcgacacaggtatggtgcaatacaaaagtggattaaaaagaatcctggaaaacgtttaaacatgaccactcgaaaa

gtggttgccaaccattgtggcccacaaaagatgttggaagttatagaaaacactaagacaacagaacttccaactggtgacttacatgaaggtgaactac

ctacaaagttagtgaaacgtgaacatatgaaaaaaccatatgtaaaaccacatgtacacaagagtaccatcgacaatgctgaaaatatagatcatcaact

gatttctttctttaaatctgcgagtggacactttgggaacaacaaacagattcgctacaacaagaatgaaaatgcccttttctcacaacaagtaaaaatg

cagcttactgttctgtgtggtaacttatcacagaaacccacaactgtctccaagcctgatagaaaaatgcttcagtttctgaagggagagaatgcctctg

acgtggtacaatgccggaaaagtagaacatcaaaggtaaccagatattctaataacacccttccgacagaaagcagtactaatggcagcacctcaaaaaa

tacactgtttagtattccaccaaattacagtactcttgcggcatttaaaacacttacctctttttgccaaaatttggactgtaatatatggtctgcagag

gacaaatgtgtttcgcaagaattgtcgaatgataaacatagccagacaagtaagaaaggaagtcagtcaaatgttcacttgaagaaagtgtcatctacaa

gttcaggaagtacatccccaactgatggtgtaagggattttcagtcaagtatttcaatttggaaagatcgactgttgtctttgtttttttggccaaaaat

aatgtctgaggtgcctccaccaactctggataacttaaccactgcaaagacaactgtaaagacaaagcattcaggtcacaatgaacccaagaaagtattg

agacttagacaaaaaaatgaggaagctttgcggaaaagtaaagattttgttacggacactgatgcaaatattcatctagaagagatagatgtaaaggtac

aatcaaagcagaggaaaatttcaaaaacctcagacttatcttactgtgaagttactggtgctgagaaagtagcttcgaattacaatgaacctaagaaaat

atcaagacctagactaaaaaaagagaaaacaaaagattttgttacagagactgatgtaaatatccatacagaagagaaaaatctaaagttagaaccaaat

aggaaaatttcagaaacc

>83669|AGAP001185-PA

tactcatcttcgccaaatatttcgactgcgatccactcaccaccaaggtggcgcgggccaaggaccagatgctgcctctgctggtggtggactcgctcag

cggcctccctggactgacggggctcttcgtggccggcatcttcagcgctgccctcagctcaatgtctacggc

>83670|AGAP001185-PA

catcaacaggtcggtctgccgctccgacagccggcgggacgggaagaaagttttgaagaagtcctccagcaccacggcagacgtggaattcagcgccgtc

gacatggaactgagcgccgcactgaagacgctggccacaaacaggcccggtagcccgggcagcccgcccaaggagtccaccaccagcagcgggagcagct

ggtccttggcgctcgccatcttggtggtgagtgggtcgcagtcgaagtacctggcgtacatgagcaggccacagtaggaacagatgagcaccaggcacat

ggaccccactgtgaagatgcacagagctctgttagccttgg

>83673|AGAP001185-PA

gaccgcccagcgactccgccaccatcagcggcagcagctgatccttggctcgcgccaccttggtggtgagaggatcgcagtcgaagtacctggcgaagat

taggaggccgctgtaggtgctcagcaggatgaggctagcagcacccgcagtgaatatccacaccgctatgttggccttggtgagcgtgggcagcgcgagg

taccgctggaccattatctggttgacgccggcgttgtagaggtactgcatgaggccgcccagcagcaggttgggcagcgccatccgcaccctggggtcca

catccagcagcggtggctcgatgcggcccgtttcgacgttgcggctccagacgacggccagtccgccaacgtcgacggtgcccttgatgacgaccaccag

caccgac

>83671|AGAP001185-PA

cggcatgctgaatctgggggtctcccagatggcgatccagaggttcgcctccatcgaaagtcttcccaaggctcgcctaacaatgtggatgttcctgatt

ggtgccatgagtctcaatttaatgagctgttacaccggtcttctcagcttcgccacgttcttcgactgtgatccgatcagtacaaaacaagcgactgcca

aggaccagatagtacctctgctggtaatgcacaccttgcaggggtggcctggcctctcaggagtcttcgtagcaggtatcttcagtgccgctctcagctc

gatgtcgacagcgctgaactcgatcgccgcggtggtgctggaggacttcgtgaagacgttcttcggcccagactgtctcacccagcgccaaacaaacata

cttctgaagaccactgtagtcgttttcggtgtcatctgcactgtgctcgtttttgttgttgagaaactcggcactgtcctacagctgtccatatcgctgg

gttccatgacaacgggccctgtcttgggcgtcttcagcgctggcatgttcctgccgtgggtgaacacaatcggtgccatcctcggagggatggtggggct

cggctacatggcgtgggtggttctggggacgcaggcgtacatggccagcggccacctcaccatccccggcaagcccatgtccatcgagggctgcagcgag

gagctcctgcgggccgtcaacgtcacggtagccgccgtggagactacggcagcgccggagctcgtcagcgaggtgccgtacatcttccgtgtgtcgtacc

tctggtatatgctgatgggcctggtggtgacgctggcatcggcgctggtgatcagcttcgtgacggggcggcgagaccctgcgacgctagacccccgcct

ggtcacgcccatgatgcgctggctgctgcccaaagacagcaagaaggctgcagcgctggcggagcgcgtcagggccacggtgcccacgtacgaaggctac

aaggctgtcaagatgcaaatgatattcacagacgaagaaaagacgaaggaggagctgtgatgcagcgacaactaagacacacagtgtgttaaagtgttgg

aggtctctctgatagcataagtctcatcaagcacgggactgttcaagtcatcagcaaaaccaataaacaacacattctcgtgggcctgcaagcggaatat

gtgcaacacgattctacgcccggaacattaaaattcggcgttcctagagttgttgtttctcagattgagaaccaattgactactgtacagaaagtttgat

ataatgtttttaatgcattatgtgaaataataatacctaaataaagcaatgttcatttctgtg

>83672|AGAP001185-PA

ccaaggcgcgcctgacaatgtggatgttcctggctggtgcgttgtgtctaactttgatgagctgctatactggtttgctcacttatgcagtgtaccatga

ctgtgatcctctcactaccaagcaagcgactgccaaggatcagttgcttccgctgctggtgatggaaaccctgcagaacttgcctggtatgccgggaatc

tttgttgcgggtgttttcagtgccgccctcagctccatgtcgactgcgctcaactctatctcggcggtggtgctggaggactttttcaagacgttctacg

gacaggacgcgctctccgagcgccagaccgacatcctgctcaagagcgtcgtggtgatcttcggcgtcctctgcacgtcgctcgtctttgtggtcgagaa

gctcggcacggtactgcagttgtctatgtcgctgtcttcgatgaccaacggtccagtgctgggtgtcttctctgccggcatgtttttgccctgggtcaac

acttggggtgctatcttcggaggactgacaggcttgatcttcatgggctgggtggtgttgggctcgcaggcggtcatggccgccggactcattaccatcc

ccagcaagccggtgaccatcgcgggctgcagcgccgacctcctgcgccacctcaacaccacagccatcagcgcagccgccaacagcaccatcacgacgga

tgctgcaagctctgttctgcccatctaccgagtgtcctacctgtggtacacaacgatgggcctcgcggtgacgctggtggctgcggtggtcatcagtctg

gtcacggggaagagggacctcgccaccgtggacaggagtctggtgacgccgctgatgcggtgggtgctgcccgagggccggagggcgtccgccaccaggg

tggaccgtgtccgtagcagcatctccacgtacgaaggctacaaggctgtcaagatgcaaatgatattcacagacgaagaaaagacgaaggaggagctgtg

atgcagcgacaactaagacacacagtgtgttaaagtgttggaggtctctctgatagcataagtctcatcaagcacgggactgttcaagtcatcagcaaaa

ccaataaacaacacattctcgtgggcctgcaagcggaatatgtgcaacacgattctacgcccggaacattaaaattcggcgtttctagagttggtgtttc

tcagattgagaaccaattgactactgtacagaaagtttgatataatgtttttaatgcattatgtgaaataataatacctaaataaagcaatgttcatttc

tgtggtggtaatgataaaaaaattatatatcacactgaaaaatatgtgacatgctgtgtgagcggattgcaagttctctcattacactgactatatattc

taatttatcgccttccataactgcataaagtcattgctttgtattttacccatcttccatttactgaaataatcaacactgagctgtacaaaagaaattt

tatttccttttccgctttcaggtacctactgcccttcattagaacgttataattatcgcaatgtttgttgtgtatctcattgctgatta

>83674|AGAP001185-PA

accaaggcacaccacacctccagctgctggttcctgggagacgcgccgttaactggctggtccaccagcagacacacgggattgttgttccatccgaaac

cttacaaccggcaagcggtggtatacagtgaattatagaagcattaatcgaccactgaagcccaaaagatggacacgccttcgatgatgaacggcaccga

agtaaacgaccgcttgctcttcggcgttgtagattatgtcgtcttcagcgccatgctcgtgggttgctctggcatcggcgtctactttggtttcttcaaa

attgccactactgccgagcagtatttgctgggaggccgccgaatggctattatccccgttgctgtctcgctggttgccagcttcgtctcgggcgtaactc

tgatcggcattccaactgagctgtacctccggggcacgcagtacgccttcatgaaggtgcccattctaattgctcccgtgttcgttggcttcacgtatct

gccagtcttccacgagctccgcatcacgtcgacctacgagtacttggaaatgcgatttggaaaaagcgccaggctgttgggttcattccttttcattgtg

agcattataacatggattcccattgtcatctacattccagcactcgcttttaaccaagtgactggcatcaacgtccatgtagtgactcccttagtctgct

tggtctgcatattctacacttgtgtgggcggactcaaggctgtcgtgtggacagacgtcatacagaccgtgtcgatgtttgcggccgtcatcctggttgt

tgtcaagggaactgttgacgtgggcgggctcggagtcgtctggcagaggaatgcggacaccggcaggatggaactacccgagccaggttttgatctcacc

actcgtcactcctggtttggcattctcgtgggcggtttcttcggcatgctgaatctgggggtctcccagatggcgatccagaggttcgcctccatcgaaa

gtcttcccaaggctcgcctaacaatgtggatgttcctgattggtgccatgagtctcaatttaatgagctgttacaccggtcttctcagcttcgccacgtt

cttcgactgtgatccgatcagtacaaaacaagcgactgccaaggaccagatagtacctctgctggtaatgcacaccttgcaggggtggcctggcctctca

ggagtcttcgtagcaggtatcttcagtgccgctctcagctcgatgtcgacagcgctgaactcgatcgccgcggtggtgctggaggacttcgtgaagacgt

tcttcggcccagactgtctcacccagcgccaaacaaacatacttctgaagaccactgtagtcgttttcggtgtcatctgcactgtgctcgtttttgttgt

tgagaaactcggcactgtcctacagctgtccatatcgctgggttccatgacaacgggccctgtcttgggcgtcttcagcgctggcatgttcctgccgtgg

gtgaacacaatcggtgccatcctcggagggatggtggggctcggctacatggcgtgggtggttctggggacgcaggcgtacatggccagcggccacctca

ccatccccggcaagcccatgtccatcgagggctgcagcgaggaactcctgcgggccgtcaacgtcacggtagccgccgtggagactacggcagcgccgga

gctcgtcagcgaggtgccgtacatcttccgtgtgtcgtacctctggtatatgctgatgggcctggtggtgacgctggcatcggcgctggtgatcagcttc

gtgacggggcggcgagaccctgcgacgctagacccccgcctggtcacgcccatgatgcgctggctgctgcccaaagacagcaagaaggctgcagcgctgg

cggagcgcgtcagggccacggtgcccacgtatgaaggctacaaggctgtgaagatgcatattatccttgctgacaatgaaaaggcgaaagaagccaaaga

agaaccgtgaagccgcctctacattctgtgcgtctccctcagaagcagagcgtaatgacgcaaaaggacggtcacctactgcagaagtgcagaaaactgt

gctagactgtctcataacgaataactgttaatttattacatttactggacccatttcacctcagattaatatgtatccaattcaatgtaataacatgagt

taatatgtatcctggcgctgtctctcaatgtgaggaaatatgcaactaggtttttacgctgttgagagaatgaaatgaaataaaccaataacatagttcg

accatgatagctgttatcttgacttcatatctattgtccaaacatgtcattcatgaagtacgagatggtgctggttctgcacataaagtttgcaatatgg

acatgtagtgaattcttcacaaattattttttttataccttgttatattaataatgtacaactaattac

>83697|AGAP001187-PA

gagatttgagtgggcctccagcgtagttgccggtcctcatgagcttctcaggtgctatcttgtactggctggcaaccagcttgtggacggcattgtcgtc

ctcgaggaacatcttcattcgaatgaagggctcacgccccttctgcgtcagcatatgccacggtttgggccgtgctaggaggtctgacacgctgccttgt

gatagccccagccaccaccgccacccatgcttgtgggtccaggtgcattggggctggcaggctatggtgctgcactcactgctgacaagaaggaccgcca

cgggcagccaccgcca

>83715|AGAP001212-PB

ggcctacagcctgggaacgatgtcgcggagcggagaccagtggggccacttctgaatgctggcgaagaacgcgtcgccggggcactccgtggccctgacc

tgtcggtggccgagcagcttgtagtcggcggcgatctggccgcgggcgacgccgcaccggatcagctgctggcccgccttcagcatcgccggcgtcggca

cgttacttgtgtagtcgccgatgaagctgatcccgatgctgcggctgttgtagttgggcgcgtgggcaccgactattccccagccgcgtccttcgtacac

tttgccgtcgccgccaaccacaaaattgtagccgatgtcgttccacttgtgctggaactggtgcatgtcctgcatggtacgcatggccttcttgcagtcc

tcggtggtggcgcagaagcccggcttgtacgtgtggtggatcacgacgaacggcaccggcgtcttcatggtctcgcgggccgtcggcggccgcgcccccc

actgcgcccgggtcaccatctcggggcactcgtccgggtgggcgatgtcggtgagttgaagcgccgttgccagaagcaaagacaacaggggcgacatgac

gaatggccggtggtgtccggtagcgcgcggaagtcaaacgagtactgacccacttccgctgcggaggccacgcttaaaacaaccgctgcggccgcctgct

tctg

>83714|AGAP001212-PB

gacaattagattatcttacgtgacacttactcaaaatcacatcacatactgaccaaatcactttacacaagggaaaacaatagaagtcttagaggctttt

ctttcgtggctaagggtaggatattagcccaccattcatcatttgaaagtgtgacagatcgaacatttgtttacgtaactgatctgcacgaatgaccagc

tatgctgtcactcgtcgtaccctgccgattgagttcctgatatactgctacattgtgcaggaacagtaaagcaaacttcttcgtaacaggcattcattta

atatttgtcatcgtttttgcgatggtacagtataaaaaaaaaaaaaaacatttcatttacttcgatgagcatattgtcctcgcgtccacgtcacgagcta

aaaaggcgctccgtacattacacaagattagtaagatgcgcagttgcacagacgcttaaggaaacgccggcggctggaaggcctacagcctgggaacgat

gtcgcggagcggagaccagtggggccacttctgaatgctggcgaagaacgcgtcgccggggcactccgtggccctgacctgtcggtggccgagcagcttg

tagtcggcggcgatctggccgcgggcgacgccgcaccggatcagctgctggcccgccttcagcatcgccggcgtcggcacgttacttgtgtagtcgccga

tgaagctgatcccgatgctgcggctgttgtagttgggcgcgtgggcaccgactattccccagccgcgtccttcgtacactttgccgtcgccgccaaccac

aaaattgtagccgatgtcgttccacttgtgctggaactggtgcatgtcctgcatggtacgcatggccttcttgcagtcctcggtggtggcgcagaagccc

ggcttgtacgtgtggtggatcacgacgaacggcaccggcgtcttcatggtctcgcgggccgtcggcggccgcgccccccactgcgcccgggtcaccatct

cggggcactcgtccgggttcgggtgcacggcggccgcgatcagaaactgcaccaccagcattgctgcccgcctgtcgctctgcacttgcctcctgctggc

cgcccctcgttccgacagctcagaccggctcaccttgccccgccgcctctttttaaactgtgagccgtctgcg

>83749|AGAP001234-PA

atgtcgtgtgcttttgtagtgtgttaagacacctgtcaacatagaaatgggttgagtaccgcatgtgttttatctgtgcgcgtatggtggcatgaaactt

gcacaacgttgtaaaacttgataactgctgatagagaatagtgttaatctttaaagagattcaagaacgtcgacgctaggcgaatacattctccaatttc

tgctactcatagcgacaaattcgcgattgtttcggcttcagcctggcgtataaacagtgtgatatgaaacactatatattagtgtaaacataatggtgaa

agaataccctcgttttactcgtgccgtcagggcttttgccagcttagatcgccctaacccaccagaaggctcgaccctcgaagaacgactgcaatgggac

gcccagaaagcaaaagagcgtttaagctgggcagacatccgtgcaaatctggagacattgaatggctcaaaaccaggtgttgtgaagcttttaaatcatc

tgcgttcgttggctttggacatgactggtggtgaccaaagcaaagaaattgtagacgaagctgctgctttcttgcttcacctcttcttaaatgaaaggat

agtaactcagaaacatctagctgtattgcgacaatcatttggatttgtcacaccgggacttgctgataaggcattcgagtgtgtgaagaagattcgccag

gaattgccggatgatctaccagactacctgtcgtatgatggcagtgggaccagtcgtaagcgaacccgacccgcttatatagtgcttccaaagttacctg

aactgaacatctacctcccagacacaagctcccttgcagatctcactccatctgttggggctaaagcacttcctgctggtttcagcatgggatatgaagc

tgtcaaggctctgcacagcagtgctattaatggggcagatacgagcccccagaccaatagaaagtttgcttcaaaacacgccaagtatggtgtgtcgtgg

ttggaagatcagataaagaagtatgaaggcaaggttgtctcagccttttcgacacaggaactgtgtgacacaattttaatgatgctgaagtctacacgta

gcaacgaggagcttcagaatgagctttttgacctactggggttcgacagctttgagctgatagaagcactgctccagcacaggaccaatattttgaatgg

tgacttaatgactgaagagagaaaacagactgcacctccacctgcatctatatcggaggtcaggaggccagcatatggctgcaacgtaacagtacagaca

gaaaaagaaatacagttgctgaaacaggtccgcaaagatgaaaaacgtttacataagcacaggggagctgatgatgacgatagtggtagtgaatttgatc

ctgtcgatctgagagcaaaaagggtagcagctcttgaagtgaaagctgtacctgtactaatgaaaccaagtacacagcgacgagagatatacccaaatgt

gtatgattctcgacaagaagcacagcaaacttcaggttttatctgtggagttaaaatggtattgccagttggggttgatcgtgttgataccaaaaaattt

gaagaagtcaacataccagttactgaaagtgctccactgaatatcgggaataagtttgtgcaaatctcttcacttgatgag

>83748|AGAP001234-PA

aggggcagcatagtcgaagatcccagccagcggtcacttgtagcacggactatatactgagatggcagtggctcagacagtgggattgtcatcacaagtt

cttgtggctcacgttttattacctgcttcttcgttataagaaatagttcccagtgatgcaagtaattattgtctggatcttcaatccatatatagaatgc

ttcttgagttctcccatgaactttgtcattccacctgaagtctgccacgacctgcaggcggatgcgaagcacagtgcgtgtgatgggctggaggtttgcc

accaggtcgaccatcgggaattcctctgcacagcgcttcacaatgtgtcccattttgctgtgcctcaaaagaatacctatatcttttgcatccatctcac

gtaatttttccacagttaatctcttctcttcaattttgtggataatgtcagatcccagcatacgaaactgtctcatgggagtctccaagctccattgttg

ttgctcaaacatctgagcaaggatcaggaatcgtgtggccataacagcattgtttttgcgcagctgaatttcaaacagggcacgcaaaatccggacagca

ttctgcattacatacgactggtctgagatgagggagaaagagttgaggcgcccatgagacaggaatgtctgcagcaatatgttcaccttcccgtagacat

tctctgtgcctcctgccacaggcagttggcaggtcatgtctaccagctggtccagctcctccatttcatcatctcggacctttaattgatcaaattcttg

ggccttcgaaatcattgcaagtgcatcagcttctgacataactggtgccattagctcgttgaatatttccaccgtatcatacttgatgtaaaaatgactt

gcagttcgcccaagatctgtagcactgagcgatcctgtgcgtggatcataacgtatcattttagctttatccagtttattggctgcagaagttataagat

tcagtctcccatgctccaatgtttgatcttcctttacatcttgataatttatcccataaacctgggggttcatacgcatgcgtacatacaagtacgtgta

actcagccaattaactgcttccgatacatttgagatggtgcccaacacaacctgaaaaagcaaacatgacctgggatgcagatatatacaaaatttaaaa

taattatcactgcaacaaatttacacttagaagaaaaacaacatagattttatcatgaaatttctgaccaatagataaatgtcttctacctgttccacaa

atgatccgattttattattcacaactgcatttctgcacattgcattgtaacacctgcagcattctgtgacagaggcaatgacaaagacttgctgaactgt

aactctctgcaagtctttttgtctcattttgatttttatttgcatcaaagatatccttcatattcttactcaataaggctgctcccttctctcagtttta

taacatgttatttattgttgattgaatcaactacttcccacattcccaatgttgacatgccaggttctcctttacaaggttccctttcattcagtaccct

tctgtatcacccatgcagccatatggccattggggtcatggtgccatacaaataaagaattctgactgtggacacttttatgcagtgtgaaaaccttaac

tttttttgcttcaaatagctttttctaccaaatatatttgtaatcttccttcaatcagataaataatggccagggactcctaaaacaaagactaatgtgt

gttcacaactttattcatcacctagattgtcatcaacttctttcttcctttttttgtgggaggggaagactaatttttttggtgccagaacagtagtata

tgtgcacgagacgaatatagtgatataactattctgaagggtcaatcagtaattactcagtgaaaatacattgaatatgaggacttttacgctcctgtta

aagccatttaaatggtacgccatccagacattcactccactttcaacagaaattagtactgttgaggcaagtgatgtgtgaggttaatgttacacattat

atgatgtcaacttctgggtgacaagcctttaaaaaaaaacaaccacattcaatgtattctaatgggaaaattaataacaaaatccaggttatatgttcac

ctaataattatgtattgtttctgttacatgtatgcttacagaaaattaatattaaaattcctacagaccatttccggttttcctttcagctgctagacag

ctcaaatttttctaataatatttgaaaactttccaattgggcgtatacaacgcgtagtagtagtagtagtagtag

>83778|AGAP001236-PA

cagttagagcactggccacatacagcacttcagtgctattacacaatccactggcaatccatcctgaaagagatgcactgtttgccagtatcattgctat

cctcctccctccagcatcagcagttaaacctgatatgcaacttcctagtgccagcataatgtaggttgtaataccaagccaagaagcatctattacatca

actgacaacctctcatacaaagttggagtcatccttgtgaaggagatcatcattccaagatcaaataacagtagg

>83777|AGAP001236-PA

cgacgctccacagacgagacaggtgcaactgcgagccgacccgacccgagcgtacgccacgctagtgctgcttccagagactcttagacgccgccaccca

tgagcaaggacgaggcggactgcgaagcgcctccggcgacgccgccgccgtcttcgcagcactgcggcggccggcgcgtctcctgggccaacacggccgc

cgtcgccaccctcagccccccgccgccggagcccgcatggcacatcagcgtcttcaggactgcccttcctcagattctggcgagctcggtgaagaacctt

ctgctcctcgacctggggatggtggtcgcctaccccaccatcttgatacccgccctgcagaacgaaccgtctgccggcgaactcagcctcaacgacgaac

agacgtcttggatcggcagcatcgcgtacatctgccagccggtgggggcggtggtggcaggcgcgctgctcgagacgctgggccgcaagcgctcgatgct

gctggtgaacgcgccgctgctcgccggctggctcgtcttctactgggcggcgcggcccgaggcgctgtacgccgcctgcgccgtgctggggctcagcgtc

ggcttcatggagtcctccgtggtcacctacgtgggcgagatcagccagccggagctacgcggcatgctcacctcgtacgcagaactgtgcgtgtctgtgg

gcttcttcgtggagtacctactgggcacggtcaccgactggagaaacgctgctctgatcagtgccctgctgcctatcatctccgtcatactcatcactca

ggtgccggagacaccgatctggctggtgtcgcgcggccgcaccaaggacgcggagaacgcgcttcgctggctgcgggggtgggtgcagccggcggacgtg

caggccgagctgaacgagctgtgcgcctactacaagcagacgg

>83776|AGAP001236-PA

tatttattgttcaatatataacttatgtacatatcaatcagacgtcgactagactacacgtcacacatgcatctaaaacactcgcagactaagttgacac

taggcgcgagagctgttgggagcctccgcatatcccgtttacagtctcccccatcagccaaagcgccgactgtcaacttagtttgcacgtataatacttg

aacaagagtcacaattgctctctcctgcacttacacacaccatgagtctttgtacttaacaatataagatggtcattgtcgtccataattattgtttcca

gtcttcacatctttgcagaactctccacattttcagcgtctgttccagaaactgtggctggtttgtgatgtccattacactggttgctcacctggatatc

ctcgtcacacaaaacacccttcgaattttcggagtctgtgtttacatttgatacgttgtattctttgtggtcttttctgtcgctcgaagcggactcgtct

gtttgcgagcactgcagcttttccgaggtactgtcgttttgcgatggacagctgttattcgaaaagagagaaatttcattactcgacgtctggtcaattg

ggcgtccgacagcgccgtttggttcatctcccgaatttggagcataaatcgtgtctccgtcgacgtttggcttgggccgagactcgccttcgggattgcc

ggagactaattcggtacgaatgggagccgcaggctgacaactgtctcgtttcttggcagtggttttctcgtcgtcgtcggagatgacggttttgccggtg

gcaacatctgtttgagagtttggggcgtcactcgcgtgagagccatcgtgcggcgagtcgccttccgatgtagcttcgctcctggtagggcgggagtcgc

acccgttggcgacggtgctgccagtctcggaagtggcagaagtgcgcggctttttaagggccggcggcggctgcggcgagccgccacaggcgtcctccgc

ggggcccgcgtagtggcgctcgatgtcctgcagggtgcggccctcagtctccgccatcaggaagtagacgaacacgaacccaacggcgctgatggcggcg

tacaggtagaaggcgccgtacagggtcagctgctcctccacgttcaggtacgtcttggtggatgtgaaggcccacacgtacgacagcgccgcggccaccc

ctgcagccagccctcgggttctgtaggggaagatctcgctgagcagcatccaggggatgggcacgatgccgagcgaccagctgaggtagagcagcacgaa

gagcagcagcggcgcccagctgcccacgggcagcgtctcacccggcggcagcgcggtgtgtgcctgcgcgtagacggccagtagcaggcagcaggcggtg

cccagagccagcgaggccagcgtcagaggccgcttgccggtccagtgcaccgccaccatgcacagcacgttgcccacgatgccggtcgcgcctgtcgcca

cggtggcccagtgcgagtcgatgggcatgccgaacttgtcgcagacctcgacggcgtaggggcggacggcggtggcggcggcccagttctggaagaagaa

gagcacgctgaccaccagcagcggcttcagcgtgccgcggcccagcagcgccaccaccttgcggcacgccgcagactcgcaccacgccgcgatctcgtcg

tcg

>83905|AGAP001250-PA

ggggctgccttaatgtcatcaacgccgaccagccaacccccctcttgggaatctgaagaaccagcacgtagcgtatattctccgggagcattggatccca

ggacgcattgcgctgcagtcagtgcccatcggcttctgatgatggacgccccgcataggtgagaacccatcctcattaagctgagcatccaagggtgttt

ctcgatgccta

>83906|AGAP001250-PA

ctgccgcaagccaccactattcccgtcatccctacagtcgttcagcgcgttacgtcggaagttaacgcaaacggcaacgggaaagagacatcagatgaag

tctgggacccacgcacagtgtcccagtcgcaggtcgggcgcccagaactcccgcatgcttcgtgcagacgtgcttgtatggctctccagctgtctaaacg

tcaccgtttgcttcgcaccgccccgtatcttgggcggagaggaaacatctatcgaggaattcccttatatcgcaggtctcgagaggtggggtgtgtttga

atgtgcagcgaccatcatcagagagtcttgggccatgactgctaaacactgtatcgagcggactgaaaccgacggcgaagtaacactgcgagttggaaca

tctgaccgcgaaagcgggggatatgtgattggcgtcgataaaatagtttcacatcgcatcgctgatatcgctgttttacacgtgcagc

>83992|AGAP001255-PA

ctatcatctctgtcccacctgcgatttccgggaaacagagacagagtctgcaatagttgtatatcgtctgtgttgtgatgataggatgttgaatcaaaac

atgtaggaaaaacgtctgttttgttgtggtcatcaaatgacacagtaatttattcacatagattacttttcaagaagtgaagatgcggtgaacgttattt

ggtccgtatattaaatttcgtggaaggctataatccagcgtaaaatgtttgctggggaagctgctgacctgagcaacgccggtttattgaagcaactgaa

aaagtttggtttcaattcttttaagagtaagctgcaacaagatgcaacgctaaagatcctcgaaaggaaacaagatgtgtatgtttcaatgccaacagga

tctggaaagtccttgtgtttccagttgcctgctgttatctacaagcacaaggttgcagtggtattttctccacttcttgcacttataaaggatcaagttg

accatcttcgacaaaaaggaattcaagcaagtgccataaattcaaatatgcgtacttcggaagtaaaagcagtcatgaaagacctgttgagtgaacatcc

caaaactcaacttctttatgtgacaccagagcaggcgaggacagagaattttaagaatttgatggaggatctttctaaaaagaaaaaggtgtcttacatt

gttgttgatgaggcccactgtgtgagtgaatggggtcatgattttcgtccagactatgttaaacttggacagttgcgtactagatatccagacataccat

ggattgcacttactgcaacagcaagtaaaaaggtagttgaagacattttcaagcaactcaaactccatgaaccagttgcaacttttaaaactccatgttt

tagagagaatttattctatgatgtcgtcttcaaaagtcaaataaaagatgatccatatgaaaatttaaaggattttgtgatgggctgcctgtacactgat

agtgcaccatctattaaggctgctaatggatgtggtattatttactgcagaacacgtgatgcaactgaaactgttgcttcagtactcacaaaaaggggta

tacggacactggcttaccacgcaggtctgaaggcagcagatagagttcaagttcaagaagactggatggctggaagagtgtctgttataagtgccacagt

tagttttggaatgggcgtggacaaggccacagtgaggtttgtggcccattggaatgttccacagaatatagcatcttactaccaggaatcaggcagagct

ggtcgtgatgggaaaacagcatggtgtcgaatttattacagctcagaagacaaaagaagcatagactttctcttgaggcgtgaaattcagactgctgcaa

atactaacaaaatgaaaagtgctacaaatgcttatgaagaatttgaaaaaatagtggagtattgtgagaaagtacggtgccgacacagtaatttttcgag

atattttggcgacagtgaaccacaatgcaataaaagatgtgatgtgtgtagaaataagaagtctgtagaaatgtcagtgcagcaatttttgaatgatgtt

ttccataaaacaagaatcagtcatgactataattcctacgatgatgacctatacgaaggagggcgccgtggacagaaaagatacctggatgactatcgaa

atgatgatgacagtgatgataatactagtaaaagaaagaatgatgatctgaaatttattaaagagcaattatctctgaggaaaccttcaaaatcatcttc

tactgacgatgaggtaaaaccagaacgtgtgagagttcgagcctcggattacacgtcttcaaaggtttctggcctcacaatatcggctcgtgaagcttat

ctggatcttttgactgaagcattatccaaaaatcattcgacatgcaaaattgtagatcctcctgaggttgaacttacaagatcggatattgaaacttgtg

caatagatatggaatatgatattttttccaattgcactgttgctagtctttatcgccattcagtatcaaaaatgatatcccagataaaaaaggaaactgc

tgcactgtcattacataaaactgttaaggcctttgctggcaacagacatactcccaaaagacaggatggacagaatacatttgacccagacaccagccca

acaacagcacttgcatcatttgttcaagcatcaaaattattatctgcttataaaaatgcaaaacgtattgcacatactgcgaactatacaacttcaagtt

ctactgcaagagatgacaaatctcgccaacagccaagtataaaatcatattttcaagatttttcaaagttaactatacaaaaagacatgccaaaagaata

ctctcacaggagtaacagttcaaatactgttgcagattcagacattagtatatcgttctctggatctggaaataaatgcaaggacagagactcaagctac

cctgattctgaacgtacagtcttttctaatgaaggatcactgtcctttgaatcaccagggaaagattcagaagaatctttagatgcagctgacaaaaagt

ctgtaccttcatctgac

>84013|AGAP001257-PA

ccaagtttcacagtagttggaaacatccattgtggactcattacatgtgatccatgatttaagctgtacacattactcataacaagcaacaaatctgatg

ttttcttcaaaggcaggttacttgacccccttcactaataaaacactcattaaatttcactattacatactgtcatacaagacacagaaattgagat

>84012|AGAP001257-PA

tgctgtagtctctaaccgcatcttctggtagcttttcaattctattccagtcgattgaagggccctcttcactgagaaatctttcaaaaagcttctcaaa

cccattgaactgcttgcttatgcctgctctcttggtttcaggggcagtgctgaggagcttctcaaattcactctgaagttgagtgagagcatctcgtttt

gtcatttccttgaactcacgagagcctgagggagctctctggtgaccacgcacacagtatgaagctccccacgtcgaagtcaaagcctctagacgtcgtt

actgcgactgaaagcatgcctgagtatcttcttgaaaattcttccttctcgttgtttctacgagagaccgcaaatgatctacagtagttcctggaggacc

gtgtgacgaagaaattgccgac

>84014|AGAP001257-PA

gaggcataaacaaaaacattctaccacctttcatttgtttttggtaaacgcttaattgtccatgcccattgatcaaatgcaaagccatgttaggaaattc

tgatcacaagacactttaacagttgatgatttaaatatgtctcttggttcacagcatagcagtagtttacaaagtcatagtggaaatatttcagcaactt

ctacctttcattcacggaacttgtagctcaccttaacgtattgttaaagaacattggatatatattggttttaatgcaaatattcatatctagttgtcta

tgcttacttgtgtattacagggataaatgtgcccagaagtcgtttcctgcctgtgaagaaaacatcagatttgttgcttgttatgagtaatctgtacagc

ttaaatcacggatcacttgtgatgagtccacagcggatgtttccaacaactccacttgtgaaacttggggaaaatcattttgcaa

>84011|AGAP001257-PA

tgactccattatggatcacaacaacaacagataaataggcttcactttacgttctctctacaacaaaattctaggaaactcttagttgtttttcaagcta

ttgtctttccttcttttctgtaaaagagaaaatacaaatctttgccttaaagtaaaatagatgcaaacaattttaataaaataatgcccttgtttttatt

ttggtcttcattcataaacaacaaaacaacaaatattcagaattatgtatatatcatgaaatactgtaccacaatatagcaattattttacagttactga

actaacagcagatttgtatgtgcttggaaagcatactaagacaacaatttaagtcacacacacacacacacacacacacacacacactttaatttacatg

catcatgcatacttcagccactttaaattctctctcattttaacaattattaattgagtctaatacatgtttctaggcacatatctccatctagtgatct

aaaattctcatatttcctgatacaattttgttttcaagtaaagctcctgatgggatatcaattctatcaccatggtttgcaatgatgatgacagtgccct

tcagagaaactcctcttccgaatgtaacatctccagagactgtcaaatggtccagttcaattaagtctggaatgttggcaaatcttgaaagaaattcttt

aacctttgcaaaatgattttccccaagtttcacaagtggagttgttggaaacatccgctgtggactcatcacaagtgatccgtgatttaagctgtacaga

ttactcataacaagcaacaaatctgatgttttcttcacaggcaggaaacgacttctgggcacatttatccctaaaccaccatcaaagcacttcatagctg

ctccaacagctgtttcaagctgtatcacatttaaaccattatctagagttttgtgatttactataatttccatatttaatgtcttttcctccacaactcg

gtcaatagcatcaagtttgatccaaagattgtttgtgttaaagaacttgaaagtcttaacagacttaaagtcatctacatgctcctttggcacctgagca

atttcaagtaaacgcagtttgtcttcatattggatcagtgtaccacccttaacatcagcttttgttttgtctgttacttccataacaaactctggagtct

tcttagttggatgaaggagaagattcagtatattgaggtctactgttgctccaaggttgtcaatatttgaaacaaaacagtactcccgaccctgttcaat

aaatttctttaagagacctgagtttttaaaactttcataaaagtctccatgtccaggtggataccacgcttcaatgtccatttctacgtcacattttttt

gcaactggtagtaaagagtccctgttaatccgagggaaacaactctgattgaatgtatagatttcaatttgtaaacctttgtattttctaataattttca

gagtatcttcatcagtgttgaaggaattcatcaaaactagaggaacattggccttgtatgttttgttgagatgctctatctgctgtacagttaggtcaag

aaatgtcaggtcacttcttactggaatcacagattttggtccatggcaacccattgaagtaccaagcccaccattcagtttgacgacaaccaatttaccc

agcatatcgtggacaacatcggttgatggctgagatagtgtgctgtagtctctaaccgcatcttctggtagcttttcaattctattccagtcgattgaag

ggccctcttcactgagaaatctttcaaaaagcttctcaaacccattgaactgcttgcttatgcctgctctcttggtttcaggggcagtgctgaggagctt

ctcaaattcactctgaagttgagtgagagcatctcgttttgtcatttccttgaactcacgagagcctgagggagctctctggtgaccacgcaccttgcag

agtatgacattatctgttactgttgatggcaatgtattaactgagttccatccatttgttgcctctgggtgtaagaccatggtgtgtcgtatgtcactgt

cattaaacagttgcaggtgcggtgcttgtttcacttatgaaaagtgaaacaattacaggtacaggtgtttcaactgttgcgaatgtctaaatttgtttat

aactagtcgcgtagcgcgaatggaagtaacgtgtggtcgtttcacaggggc

>84039|AGAP001257-PB

agcaatactctcgtccctggtcgatgaatttccgcaggagaccagagctctggaagccttcttagaaatctccgtgaccgggcgcataccacgctcaaga

t

>84040|AGAP001257-PB

gcttttcaattctattccagtcgattgaagggccctcttcactgagaaatctttcaaaaagcttctcaaacccattgaactgcttgcttatgcctgctct

cttggtttcaggggcagtgctgaggagcttctcaaattcactctgaagttgagtgagagcatctcgttttgtcatttccttgaactcacgagagcctgag

ggagctctctggtgaccacgcacctggggcggagacaacttgtcggtcatgctgctgctgtgacagacccactgacaatggccgttcgaactgctcgg

>84086|AGAP001261-PA

gtttggcatttccctgactttcctttttgaaattacacgtctgcacaaactcatcatcttgacttccattatgtataaagtttcctgtaggatttatccc

tgcagatatctgaattggttttacgacagactgttgctgttgtctttccctcgccaatcggtccaaaaagctttcttttgcaagttccactttcacgtgc

tgaccatgccattttctttttgacatgtattcaagacattcatttatatgactcctgtcagcctctaaattgacgtaagcaaacactgagtcatcagacc

gtcgtttaatttcagttgattcaacttttccatacttggagaacctcttcgaaagatcatcttcagttaccactgtgggtaagttacccacaaatattcg

ttgtacatttaacttcatttctctgtacacaagttgacacctacacagtaacaaatactgagcctgttactgtataacattaataacaagtgctcctcgc

ctatacacaaacaacacgttcccactgccgctaacacgcagtgtacacatctcagagatcacacttatcggagtgtgcttgtaccacaacaaccagtgtc

gccagatccaaaaaaataaaatcgaaggttttataaagccgggttcactcctgcaacgaaagtgtcgccacagctagtggcatactccagttacatgtgt

gaactcccagtttttagaggcgcaacttaagagacgcatctttcgtcacgtcacagaaagttcagcttgattgtaatttgttgagcca

>84119|AGAP001264-PA

gatcttccacagtcatcatatctgctgctgagtgatggaaccagaaataatggccatttgcattaattaaacttgacccaggcacacctgcatttgtcca

cagggctatatctgatc

>84118|AGAP001264-PA

agtacttaaacttcttaggccactgaatgctagcaagtatactactccacaagacggaggaccagacataaaactatggacagatgctggcgtacctggt

tcaagccttctcaatgcaaatga

>84307|AGAP001280-PA

caagctctgttaattcctgtagcgagataccggcagacgcggttcagctcgcgtcgggtgcgcaagcgcgttatcttcaagaacggcgactgcaacgtgg

tgcagggcaacgtggcgaagcgccggcgccgctacctg

>84346|AGAP001290-PA

catcacattcattctgcaccagccacccttaaaaattgttcacaaatatctagtagtcagaataatggaaatttaatgataaactattcaacaccatctt

tgtgggtggagaaataccgaccaacaagttcaaagcagattataggacaacaaggagaaaagagcgtagtcaggaaactaagtaaatggcttgaaaattg

gtacaaaaatcgcactggtgggaaaaagtttaaaccaagcccctgggcaaaagatgacgatgggagcttctacaaagctgctcttatttctggtccacca

ggaattgggaaaacaacagcagcccaccttgtttctaaggaactaggtttcgatattgtagagttcaatgcttcagacacaaggtcaaaaaagcttttac

atgatgaagttacagaacttttgtctagcaagagtctgtatggtcttttccatggaaaagatggccgtgtagcaccttctgctaaacatgtgttaataat

ggatgaagtagatggaatacctcctctatcttcatttccagccattccatctacttcatccatt

>84362|AGAP001294-PA

ttgcgtgtttctgtacctcactgagccagtgtctccatagtcttgacaagtagtgtgtgaagtcagccgagtgtacaaatccataccccctcatatcaaa

tggaaaaagagccacaatccatactggaaagaatgacaattttcccaacatgcaataggaataattacatcatgagatacccatagacattcaacataac

acagccccaaataaatagcattacttaaagtgttgcttttcaaaaagtaaaccagcaacatatttaatggcacttactatgaatgctctttattcctaaa

caatgcaagagaacactgtcacacatatttctgaacatgtaagtgataaaaacatgcagttgtcataacaaataaaaagacactatactttatatatctg

tgtcataaaacagattaagagattcttcattcaaagttaacatttgctgtggaggagacatacattttgatgcttcatgagttcctgaatcactttcttg

agtaaattttgacggtgaaactgcagtaccacaagtattaccaatatacacacctgacttatttcctaaatcaggattttctgacttacagcttgttaaa

atattttgtttatcattaggaagatacacagattcagcactagtgttaaatactactggaatattcagtgtaaatggatcattgttttcatgaaatactc

ctgggtttttggaagcttcagattgtgactgagatgatataactatatttaacatatctgatgatttagaggaaggacattctttaatttgggatatatt

gtgtgtgttttttagcttggtgctatacattttagtagcagtagtgttagcagtcttactttgatttacaaaattagtatctttttcatccttgcatatt

tcttcataaaaaatttcatcattacccatagttttattaactggactatatttcaatggataacttcttgtaacagtttcatcattacagtctttctgaa

cttgctgtagctttgtatcacataaaccaactatctgtggcatatgcaaagattctagacctgcatcctttgtctgtgatttttcaacttctcccgttat

ccaaagtatgggagaagaatctaccataacatcatgtgacaatgcagattgaatgtgactgttcgccagaaagatatagtcattattttgttgcttcttg

accagctgtctctctttgctccaagtgtggtgcttttctaataaactgcatccttctttaacatttgcagccaatacaccatttgtgacaatgtgttttt

cctgcaatggtttgacctcaacagcttgtcgcagtttctgcaccaagtcacttgactgcctcactactgttcctggtggctgggatggacatttgtgtgt

tctctggctgaccttgtaccgaaaattttttccacatgcagtacatttgtaaggcataacacctgtgtgaatacgacggtgtactaagtaagacactcgt

tgacggaagcatttgccattagtacatttgatggtttgttagtcacacggcacacaacacagttaggtgaagatcaaaaattactgtagtttgatcacat

gtttcacatgcaaatggcttctctcctgtgtgtattcgctgatggttatggagtgttgaaagttccttgaaggctctgtcacattcagtacaaacgtgtg

gcttctcatcactgtggtacagcaaatgtttatgatatgactgttggaatgtaaatgttttatcacatataccacatctgtatggcatttcacctgtatg

aagtcttttatgctttttaagaaaatattttgttgtgaatagcttgctacatacatcacaagcccattgttttccatcatgtgtcttcatatgctgtgtc

ag

>84500|AGAP001318-PA

ccttattgtcgaaaggaatggtgttataaaatcctacaaaacacatccttctcttgattattgcaaatctgccataatctcagcacatttcaatgccaat

agcaatacccattccacctccaatacagagagcggcgacacctcgcctgcctctggtcctttgaagagcatgaagtaaagtcactattatccgtgctcca

gaagcccccagtggatgccctagggctattgcaccaccactaacattaacttttgctggattaagacccaactgcttcaatactgctatggtttggcaag

caaaggcttcatttatctcaaacagatctacatcagatatgccccagtgtaccttctctaactagaatgaagaataaaacaacactttagacaaaagcag

tccccttttcacaacacagtgttctccattagtaatttactacaggaacttcaaacaaataaacaacatctgacaaaccaatactgatacaaaaatacag

agacaccctgtgtagataatggcatttctttgagctttag

>84519|AGAP001319-PA

tatcaggatttcaggagttttgaattatttattgaaatttaatatctacacataaacaataatatttcacacatacaccaccaacatcgccaatatatac

atatgtttttccaactaattacacaagatactacgtgataatactgtttttagtctgtcaatctgttacacccactcacaaagtaaaatgagttcacctt

cactagctcattcattcacatgcttcctcccaccactgaattgaaactattacggaacatgctcaacgaaaaattacaggggcttctggatggaaaccat

gttctttgcaattatgatcttcaaatctaaatgaagtccacaagtaatcaaccggatagtttgcaaggacatcaacactcggtacgcatatttttgaatg

tttacgtataatatcactccatatactaataccatgaatttcacaaccgaaataagatgcccaaggccgtgaagacgacacccctgaactgctgctgtcg

acagacacttgctgctcttcttccaacatattgtccacaattgcaactgtatctaaatccacgtattttgatggtacatatatatcctggatcaagttgt

ctcctacatataaacatctgggattttgttttcctacagctttggagaaaacttgtctcaattccttccagttgccctgactgtaaactttattcaattg

cagatcatttacagtcacagatacagtttccttaccatcttcagttgcgacaaattcccttgaggctgtaaaaaatccaggcttcctggcgaaacaaatg

actatgtcgaataattcgacccaatcttcgcctaaggaattccgcgccgcaaatgatgcaaaatcaacatttgagcccgttaataaaaacgttcttgctt

gtttcttcagacttttcaaccaagaggttacgttatcactgcatcggtaaatatattgttcaggagacgttttcatggcaggaaagtagccgccagtatc

cgtaccaaagtgttctctttgatacatgaaatataaaccgtctagcacatcaggccaaacattatacttatctagttctcgtcctttttcatctaatgta

tctattatgcgggcaaatgcaagggaggcaggcatatcaaagctatccagtacagttcttaatttgagtgacagcggaccattccatgcaccgagcatgt

cctcgtaaaattccccagccaatttccacattctgtcttgtccataatattcgactatttcattgttggtcaacactctggtaccgtggcatgcccgaag

aactttcccacccttgccaatttgtaaaagattacctttatcaaaatcaatcatcaaccctttcctaatgtaattgaagtcatcatcaacaggtttcaat

aagtgctttccggggtagcccttgctgtcgacaagatatttcgacaagacatcgtactccatttttatcatattctctacattatatttacacagagtat

tgtctaaatcaaaaccaatccaatcgtaatctgtgaacttgaaatagttgtgttccgtcattttagatgcgttcatacgtgctttagaaaatatatcaca

aatatgtgtgccaaaacgcgtgtagttactaactgaaaccacgtaaggcaatactgcttcctgtgtttgttgacttctgttcttgacagaatgaaaccga

caacctgctacaagtactgaaaacactcttctaatcttcaggcagacttcactattgaacataactattgttgatgaaaattattttcttcacgcatatg

tggatcaacatacatcgactgattaattagcattccacaactcataatataacaaacattaaccacattcactaactgcacaacgtctaacagcgct

>84584|AGAP001352-PA

ccatgagcaagagcctcttcaaccgcgtcttggaccacctctccggaagaactatcctggacgggctggcgcgcaagctgaccaacctgacagaccgccc

gatagacatctcccggctggcggagggctacagccagcgcatgcacaagccgcagaggctgctcggtctccccgcactcgatcccctccggatggacagc

atgccgctcaacttcgacagcgacatggccaaggtgagaggtgaggcctggaacgtgacggtgctcggcatctcgagcatgcgggctcgccagctgcgac

tgcggcctctgtcctccagggtggacttcgagctgctcttcccgtcggtctcggccactggcaactaccgcatgaacggcaccttcgccaactccatcca

cgccttcggcgaggggcccttcaacataactgttaaagacctgaatgccacgggtttcgtgatcgtcacgccagaaggagaccacctcaaggtgaaggat

gtcttcgtcgaagtgaatcttaccagcctacagatggaccttcaagggctgttgggcggcggtgagacgggccggctgctgagcgccgccatcggcgacg

ccgcgccgcggctgctgcaggagcagcgagaagacgccacgcg

>84605|AGAP001358-PA

gtgtacattagatatgcaggaccaattatgttatgataagataatcatgcaatcagtgttcacaaaaggagtcatgttatctgtagtgaaccaagtagct

ttccaaacagtttgtataattcaaaatctaagtactgcctctgtcctttacgttaatcaaggaactgatcggaaaaaattctccaaaacacactcggtaa

ctgttaattgatacagtggggcaccaataatttaaaaccttcgggtatcttcctaactacaacttatgtctagcggcttatggtgacaagtaaatcaaca

tttaacagcaaaagaggagtttctacaatagggataaaatgcatttataagcaagacaaatccattaagaatcaaagaactttcatctaatggattgccg

aaggataatgtttaattacttaaaaaaaaaaaaaaatgttgaggcaatgttaattaatgcaacattgaataatgtagagttggtaccttcagccacttgt

caacacactttgcgtggaactcgtgtgaacaaggaagaactctaagcatctgcctggcttcaaaatcacacatgcatacaacacatgacgtctggtcacc

atggtgtgtgtctgcgttgaac

>85213|AGAP001419-PC

catgacaggccttaataggggatatgcttttattacttttaccactagggaggcagcacagcaagcagtcaaggagctgaacgattacgaaattcggaaa

ggcaaaaagattggtgttacagtatcatataacaatcatcgcttattcgttgggaatatacccaagaatcgagatcgtgacgagttgtttgaagaatttt

caaaacacgcacctggtctgacagaagtgattatatacagctcacctgatgacaagaagaagaatcgtggcttttgttttttggaatatgaatcccacaa

agcagcatccttagctaagagacggctaggtactggccgtataaaggtgtggggatgcgatatcattgttgattgggcagatccccaagaagagcctgat

gaggctacaatgtcaaaggtaaagg

>85212|AGAP001419-PC

ttttaccactagggaggcagcacagcaagcagtcaaggagctgaacgattacgaaattcggaaaggcaaaaagattggtgttacagtatcatataacaat

catcgcttattcgttgggaatatacccaagaatcgagatcgtgacgagttgtttgaagaattttcaaaacacgcacctggtctgacagaagtgattatat

acagctcacctgatgacaagaagaagaatcgtggcttttgttttttggaatatgaatcccacaaagcagcatccttagctaagagacggctaggtactgg

ccgtataaaggtgtggggatgcgatatcattgttgattgggcagatccccaagaagagcctgatgaggctacaatgtcaaaggtaaaggtgctgtatgta

aggaatctcacgcaagactgttctgaagaaaaactgaaagaatcatttgaagcttatggaaaagtggaaagagtgaagaaaatcaaagactacgctttta

tccattttgaagaccgggaaaatgcaataaaggccatggaggaactgaatggaaaagaaatggggggttctgcaattgaagtttcattggcaaaaccccc

atcggataagaaaaagaaagaagaaattctacgggccagagaacgtcgcatgatgcagatgatgcaagtccgtggtggatgctcaccatcacacccgtcc

atgttacctccaggtgtgatgcccatgaggggccctggaggaccgagagggcaagggggggcagcagctgcatccttgcgtggtcctatgggccgtgggg

attacgattacgattacgagtattacggttattcggattatcgaggcggctacagtgatccgtattacgatgactattatcgttatgaggattactattt

cgattacccgccgccgccaccacctgccagagggaggggcagaccgcctcagccggctggtcgtgggcgtggagtcgtggcgcgtggccggg

>85238|AGAP001420-PA

aaaacttcactgaaacaactggtttttaattggcatctaattcaaatacaaatggtattccagttggaatattcaaa

>85251|AGAP001421-PA

gtgaagatcggtgaacctgtgccgttcagagaggacagctctactggggcggcatgtgatgaacagtccgcacagccagtgctatcacctcctgctctat

ctcagccacaggaggatgacactacgggatccacctgtggttccattctgcgaattgctgatcttagcccagaccagaaagagtgggaaataaaagctcg

tgttgtaaataagacagcaataaaacaatataataaaggagaagggaaacgattttctatggaattggttgataactctgggcacatacatgcgacagca

tttggtaaacagtgtgatgaattttatgatctcatagaggtgggtagagtgtatttcatcagtagaggtaagctgaagccaacaaacgaggaatttacca

acgtcaatcatgtttaccaaatgacgtttacatataagacgacagttagacgatgtgagaaagatgacatgagcatccctcacattgctttcaactttgt

acgtctgaacaaacttgctgacactgctctttcaaaagtcattgatgtcattggtgtagtaaaggatactggaagtgttgaggttgttacagtccgaaaa

agaaatgagcaagagaagaaacgtgaggttaccattgttgatgagagcgacaccacggtaaagaagcttctataacaagttaataagtgctcgttgtgcc

atgcttaactgcaatactaattattttctgttagtctgtaccatttgagctttgtatgtgactgttttgttgctggacttgtcagattttcagctctttg

tttcattagtattcacttttgttaaactttgtgaggtgtagttttgtcctaatacagactacctcaaattttgtgtcacacatgctgttaagagctactt

ctgtactatgaacgttatttatgtactctctcccaaaaaggttgaatatccttacagctaacctagtcctttaaatcatttataaatgcatgatggaaaa

atctgttaaagataagacacatttcgtgccgagcaacattaatcacacttttatgatattatgaaataagaaaattttcatttaattgggcactactctt

ctattgcacatgtccaaaaaaatttcttaaataaatatggtactacatcacttttagattcccagaagaaatttatctactatttacaactttctctaat

tccgtcaaatttggtttgttcactatggtaaattgcgcacacttttgcattaatatgtttgcctggtctcca

>85252|AGAP001421-PA

ctgggaatctaaaagtgatgtagtaccatatttatttaagaaatttttttggacatgtgcaatagaagagtagtgcccaattaaatgaaaattttcttat

ttcataatatcataaaagtgtgattaatgttgctcggcacgaaatgtgtcttatctttaacagatttttccatcatgcatttataaatgatttaaaggac

taggttagctgtaaggatattcaacctttttgggagagagtacataaataacgttcatagtacagaagtagctcttaacagcatgtgtgacacaaaattt

gaggtagtctgtattaggacaaaactacacctcacaaagtttaacaaaagtgaatactaatgaaacaaagagctgaaaatctgacaagtccagcaacaaa

acagtcacatacaaagctcaaatggtacagactaacagaaaataattagtattgcagttaagcatggcacaacgagcacttattaacttgttatagaagc

ttctttaccgtggtgtcgctctcatcaacaatggtaacctcacgtttcttctcttgctcatttctttttcggactgtaacaacctcaacacttccagtat

cctttactacaccaatgacatcaatgacttttgaaagagcagtgtcagcaagtttgttcagacgtacaaagttgaaagcaatgtgagggatgctcatgtc

atctttctcacatcgtctaactgtcgtcttatatgtaaacgtcatttggtaaacatgattgacgttggtaaattcctcgtttgttggcttcagcttacct

ctactgatgaaatacactctacccacctctatgagatcataaaattcatcacactgtttaccaaatgctgtcgcatgtatgtgcccagagttatcaacca

attccatagaaaatcgtttcccttctcctttattatattgttttattgctgtcttatttacaacacgagcttttatttcccactctttctggtctgggct

aagatcagcaattcgcagaatggaaccacaggtggatcccgtagtgtcatcctcctgtggctgagatagagcaggaggtgatagcactggctgtgcggac

tgttcatcacatgccgccccagtagagctgtcctctctgaacggcacaggttcaccgatcttcacacccacctccgtaccacttgccagtattgtcaggt

cgacaattacaatgacacgctctgttcccctttccccactgttaaacatccaacggttcaatcggaccactgagtattctgacaactctccagatgccag

cttgtctttcaactctgttgcaagcatcgcgctgttctggtggcagccatcagaaacctggaggtggtacctctcacttgattctgcaccatctattctc

ccgctgcccagtatctgcattactggaccttcaacagtcccaccacgcattatagtggcaagtgctccctctgtgagttgccacttcatgttgacggtaa

caattgtatgttgctaaaacatcatgcacacacagctgagaatcattaactaaagttcaattatgttaggttatctcccgtcccccaaaacgcagatcca

cgggaaagc

>85265|AGAP001423-PA

ttcaccatagcggagcgattgttttttctcaaatgtaacggtgaattgctcagggtctttctcaccggcctcgcgtgttaagtaatcagcgattaatgcg

tcgtatgccgcggcaaggcggaatactttggccgcaagctcgcgttttcttttaagcgaaacaccgccgtgttctttaatctcattgagcacggagctgt

aatcggccggatctgtgatgaccgtcacatcctgatggtttttcgatgcggcgcgcagcatgccgggaccgccgatatcaatgttttctatcgcttcatc

gtatgttacgtcttctt

>85290|AGAP001424-PA

aggaggcggtacaaaaatttacttttcaggcggaagttagccgaatgatgaaactcataattaattccctgtatcgcaataaggaaatatttttaagaga

gcttatttccaatgcttctgatgcacttgataaaattcgtcttttgtctctgacagatgattctgcc

>85423|AGAP001477-PA

tgaccaggatcgtgcacacgagcagcagcaagaaggtgacgcggtagtgcagcttgaagacgaggttgtcgatgctgacgagcttcggcttgatgttgac

attcttggcgatcgaggcgaacgtgttgatcatggtgtcggctggtgcggccgagctgcgcgcaccgcg

>85477|AGAP001500-PA

aagccataccatgcggtaagagtgctactcccctagcaatgaaactgcagctcacctgttttcacagtttgccacatgacgaacagagcgcttcttcttg

ccagctgcaagtgggacgggcaacgatgcacgaccagcaccggctgagtcaacaccagcatccaccacgtccacagccatacgcttaaatttgcgcttcc

tgcgtgtactcggatgtaccggcgagaagttctcgttgaaggagtccgactcgatgttgccgtggagcagcggcacgtgacgtgcctggag

>85510|AGAP001504-PA

cttgcccatcacgggccagtaccgcgacgccgagaccgacaagtactacaacaacaacgacggggccaagattctggactggtcccactttgactacgag

tacacgctgggccacaagatcgtcttcctgtgccgcgccaagggcaacccgcggccgaccatcacctggttcaaggacgggaccgagctctactcgcaca

actacttgcacgtgcacgagtggcgcatcggcaaggaccaggtgaagtccaaactggagatcgaccccgcgacacagatggacgcgggcgtgtacgagtg

ctctgcagacaacatgtactccgtagacaggcgcagcttcaagacagacttcagcatcgccttcgactagccgctgccggagcctcactacccgccagct

tccaatagactgagcactgcaatgcctcgaacaagttataacagattgcttagcgtacacgtcggtactgtacagtaagttttgctgaatgtaaagtact

acaaacttcaaaaaaattgtaaatataaaggatatataaaaagattaaaaagtgagtaacaaaacaactaatgccacagtccttaccaagcacagcctca

gaaataaaattt

>85511|AGAP001504-PA

atcagtatccaaggctatgttcgactttattgaacaggttacatcgttgctacttctttctccatttattaacagtttttacattttgatgtaatgttat

tgttgcatgtatcgcaatacataagaaaagttcctcttctgtgcctctccataaatactcaattatgaatactgtaagtgcagtgtatttctactacttt

ttgatgttgtaaccatatagcagcctaaaatttacagatacgaaacgtatatcaaaaaacaaaacaaaattctggcttgcgcgaggatctgcaatagaga

gcgtaaatcgcaggaccctttgacgtcgtcataagcgatttcggctgttcgttgcattggcgcactaacgtgtaagcaatttttttttattgcaggaatt

gctgtacacaagtgtcataagctgatgaatatatctctgtgtgattcttgcgaatgtgagaagagacagatttcgattaccctcaacgtgacgcatattt

tgcacaatggtgtaagtgggctgtttaggtttttatgttggtaacgccacatagcgctctgtatgaaaatcactggcagtgctgtctgcagtctttggct

ggttggcattgttggaatattcgctattttagtgttgggcagttgactgttaacagcgcgtagcgttgcgcagttggaggtgagccgccagcagtggtgg

atgtggggagagagatggcggagttttgagagcggacgatctggacgtgtgtccatcagagagagtaaacttgtaagactggatgtcatgaactgatata

taatgacttttgaacactattaaggtaaatacattgtttgttctttgtcaaaatctttcatttgctaactatgcctatcagtagttagtgccttcagtag

tttgaatcttttatttagctggcagtattggcgctcgctgtattgcagtagtttgagtgacgaagagttttgtgaggtaagtgcttcatgaaaggtatag

gttattgttagtcagggccattcttttgtagggatttttgaaagtcagattaagttgcgcaaaaaatatagtgtgtcagtttaagcagagtcatttataa

tttttctaaggggacgtttcaatggttacacttgttgacattgaaggacagcatttgccgtactgataagtcttacgtgaccgttgaaggaaaaaattac

actttgtacttgcggtatttgctctcgtggggtattaacactttttcagcgtagtgttgttgtttttttttttttttttagggaaaacaaatgctcaaca

gcttgtcgggaggctgtatggcgacaaaagctctacttccgggcaccgacccgtgctgcacggtggtttcggtacgtggggcgtggctagtcgaaggcga

tgctgaagtccgtcttgaagctgcgccggtcgatggagtacatgttgtcggccgaacactcgtacacgcccgcgtccatctgcgtcgcggggtcgatctc

cagcttcgactttatctggtccttgccgatggtccattcgtgaacatgcaggtaggtgtgcgcgtacagctcggtgccgtccttgaaccacgtgatggcg

ggcctggggttgcccttggcgacgcacatgaagacgatcttctggcccagcgtgtactcatagtcaaagtgggaccagttgatgatcttggcgccgttgt

tgttgttgtagtacttgtcggtctcggcgtcgcggtactggccggtgacggggaggccgatctgcgcgcgggacttggtccgcccccgcgtccgcccccg

ccgcccctccgccccc

>85541|AGAP001520-PA

ccacagcgtctcgccacagccaaccaccacgagagaaagcccgggaagcagtaacagctccaccgccgccatgtgctcgctggccgccatgcccgaggtg

ctggtgtcgcacagcccgccggactacttctaccgcagccgctacacgccgcccccgccgcccaagcccgcccccaccaggccgtgcctcgcctctccgc

cgcccagagccgccaagaagaaggtcgtcttcgccgatgacaagggctactcgctcacacaggtgcgagtgatgcgcgaaccgtcaaatgcgccgcccac

gtggagcttgcagttcctcgcgcaggtgatgcgcgacctggagcccgccgccgtcgcagccgccgccaacgaggccgcctccgtcccgagcgccgcttct

tcgccctccgcagtcaccgcctccgaacccgagcgctgggtgccggaattcgcgcagcccgcctccgactacctgcagttccgcgagcggctcgactcgg

gcaacgtctccttggagaatgtgatagtgcgcgacgccgagggcgtcttcacgggcacagtcaaggtgcgcaacctcgacttccacaaggaggtgaagct

gcgctggacggcggatggctggaagacgtacacggacgagccgtgctcctacgtggagaaccaggtggcgtgctcttccgcctacgtgctctacgacacc

ttctccttccgcgtgtctctcccacctgcgaatccgcgggacgaatcctccccttcctcctcctcctcctcctcgtcgtcgtcgccgtccacctcgtctc

ccgcctctgcgacgccagcttcatcgtccgattcttcctccgcatcagcttcttctcctggctcaaccacttgttcctccgcatcctctccttcccctgc

ctctaacgcttcttcccctgcaccctcttcttcccctgcgcccacgacatcctgttcttccagcgctagtgacactaaatcaacgaccaccaccaccgag

ccagctcgaccacgggagccttcgcggcgagtggagttctgcgtgtgtttccgctgcggcggccacgagtactgggacagcaacggcggagccaactacg

cgctggtgcggcgggcgcccgccgtcgcgacgcccgcccccgcagccgcc

>85556|AGAP001522-PA

cttttttagaaaaaatgtttgtctctttaatttacttatacaacagacttcactagatgtacaatatttagatcttacatacaaaaatattacactttgt

cacaatttaacacacactgcaccatttgcagctcatatgtgaatgctgcagacagtaaaaatcttattttactgcaagtttacatttcacatgttagcta

ccaagaaatggtgcataagtacctaaaaaagggaaacggtgtggtttcatcatccaacacagttgcaatcattatttttatgtggtactcctccaaagga

aaacttttggtcctgtaactgtttcaatacagttttttccattgcttcaaggaattcttccaatgacgttgtttgtttctgaatataatacgaatgtgct

aaaaatggaatttttctaagtgatcttccacttagaccttcacttttcctggctatgttaagcaggaacaaactgtttttagtgagatta

>85578|AGAP001534-PA

gtgcagcggtcaattgccagtcctctcttcacaaatggctgtttccctctctcaggaaaacccattacacgaatggtgtctatgataaatgcatcatcca

tacaagtcatgccctgagctcctactagatttgtgagtgttacttcaacaattgcctcatcaatttctctctgtgtggtcaccagaaaatatatccatgg

atcagatggcgaatccagtgaattcaataccctcatttttccctccgctgaacagttccaagtagtatcattactgcagcttagagcggccaccagtgtg

acattggtctgtctagtttcctctgcagtactagcagctatctgcataggtatcacatgaccctctcttagcaggagcacagtttctgcttttaagctct

gcattgttatagctgagccgtttccagaaattctcttcccatctccaagaagatggaaaaagtttgcttttggtagacgaacagatactgtgtaggtaga

tggcttaacaactggtactgctagtagggcagaccccacccagaactggtcactcctgcccatcgtctgtaggatagccacatcaggatcatgaaaccaa

gatggtcg

>85593|AGAP001539-PA

tcgcacgcctgtggttcgctatagtctcttttgtcagtgtaattatgtactgtcccttatagtagtttatgagattgagtatctgcaacgtggagatcac

atcttctttcttgatgctcgtcagctcacatatctcattgatagtgatgaggggtttctcattctcc

>85648|AGAP001549-PA

cgacatttttaatatgtttaaaactttgtcctcaagtggaagaaaataataacttttgtaaatatgagacactcagatatgtagctttatttcagattca

tatttactttattgtagagtgtcattctaaccttcttaacattggattgttaattaagcggcaattaaatagcaatgttttatacaaaagcctgtattcc

tttgaaatattttgatttgtatgaagactagtttcctatttaaaaggaacaggtgtagatggataaccacaatggaatttatgcaaaaatttaaaacaaa

aaatatggtaatttcctgttctccactcacattcttctgaactgtttgcacaatcacaatttcagtacttggatgttgtttcttagtatgacattgcaat

tttgatctccaacatatagaactaacaactgaaggtaatggacttcactatcaatctgcatttcccacttacgaacattccttcaccactcagcatttct

tctgcactgtacaatttttcttcataaaggcataataaactcatgacattcccttacaaacccataagaaactgtacattaggcagaaacaaaattttaa

caatgtagtctgaggtaaacagtcaaataaacatcacattaccataacagcaaaaattaagtaaatatctggaaggaaaaataaatatgtatatatttgt

cttcttacaaaataacaaaacaaataattaacagaataaatgctttagtgcattcagtgaaaacatcaaataaagtcaaccacttggatcttcacatctc

ttctgtgaatctttgattgtaatgtgtccaagaccagcaatatctaactcatgaggtaaaaccctgttctgttcagctcgctgggctaatcggtgatgat

tgtgtgacacagaccacaatggtggtaggtacttgggttgcattgtttcaagaaagtgttctcgccacattctttctaactgcaccaagccgcccatacc

atcttgtttaaaaaattcaaccactttctgtccatgtggctggtagtcttggttaagtacacaagtttcaacaacagatgcctcctccaacatttctgga

gtcagttccacattattttctcctctctcttgaaaatagtttaaaattttcttcttaatctcttctttgcgtgcaggcggaattttatctccatttctca

gcagagctactgctgcagaacgaacttctttcaatccaggaatattataggtcttcaagttctcttttgtccctattggggcatcacatttttttgcaag

tgttgttctaagggacaagtcatgccaattgctgagttgatggcactcccaacagagcaataaaacatcatgtgatgtgtgatctttcattacagctggg

aagtatttcctgtattcacgaggaataatattttttcgcaagtatgactctgtacgaccacaaacaacacaacagttgtcttttacttgggtgtagtaac

ttcccacttctcccacggctctgcctgaaggttcaaatcgcaaccttacagtgaatgggtcctcagtaatcaggtcaccaagattgttctgtatgtacca

tttggccttgctattatcacaggtgcaaagaatttctccatctggtgcttgtaagtaacagttatcatacagaggtcgacttcgagtataatgtgctcta

gatgactgcttaaatttcttgacctctttaacagcacaaaaagctccattaacaccagcatctttctccactgctgtaccgtgctttctactcttgtatt

tgatatctaagtaacgctgacacgattcagttatctttgaaacattatcagcatcaggtccactctgaaaccatcctgatggtctggcaatatcaatgaa

aatttcatatgcagctaaagcatcaagtgctgcatattcacattgttcttgagtaaggtgatcacgctcccaatcactgcaacgaattttccagtcttta

ttcaagtgaacacccagtatgttctctgccaggcctgcaaggccacctgatattgtaaggccctttgctgcaagcaaatgcctcaagtccaaacagcccc

gcactgacactccatagtctcgaaagaggtattttgcatcatcagtaggagctacgcctactttcaaaatgcggctgtcttcgagtatgacctttaagtc

gtctggaacacatttaattttgcaaagcctgaacagagcacaaaacccgtccgacgatgctaactgaagcagcgctactggatgtgaccttttctcgctt

gttacccactcacaatcaaacccaagaactttaatggtctggcatttctcttttatgctgtcaataaccggcgcacatctttctaaagagtccactacaa

tgattttctttttccattgctgactgtggagtatatttgtttttaatctccggtatcttaatctcctaaacgcaaaaattacgcttactcctacagtggc

acaaacaatgccagaaatcaaatatttattgcgatcagtcattctgacaaagagagatctcttcgcgtctcgtgcaaacaaattaatttagggtctggga

tttctttttcaatgcatgcccattggcgcacttaaccctttgataatagcattgctaccataaatatacatacatccctattattattattgtacatcag

aacccaaacaccaaacaataacgctgggctgtcacagattttcttttacccttatccacgtgtct

>85675|AGAP001550-PA

gaatatttggagccgcacttagttccatgtctggttgccttaacgcgctatctgggacaatatacgaggatttcctgctacctctgatgcctggcaagaa

gtcggacgtcgtggtgaaccttataatgcaaagcatcgtgctggttgtgggg

>85674|AGAP001550-PA

ccggcacccagcccagcgccgtgtaccacacatgcgacacagcgaacaccggccacacccagctgctgcggtaatcggcaggcacggcgacggagccgtt

gttggcgcggtcgaactcgtagaggcagccctcggtggagaatggcttggtggcgaagcggat

>85670|AGAP001550-PA

ccagcacgataccttgcattataaaattcaccaggagatccgtcttctttttgggcatcaggggtagcaggaagtcctcgtagatagtagcagaaagagc

gttgagagtgccggacatggaactgagggcagcgccgaatatcccggcgacgaagaggcccggcagtccgggg

>85671|AGAP001550-PA

tagctgggaccatctacgaggacttcctgcttcccctgatgcctggcaggaagtcggacgtcgtggtgaaccttataatgcagggcatcgtgctggttgt

gggcgccatcaccgttgcgttagtcttggtcgtcgagaaactgagcaccatacttgagctgggatacagtgtcagcggcgtcagcaatggagcactgctg

ggactcttctcgtttggca

>85693|AGAP001566-PB

catagtctgcatcagcacattcctctgcagcatccaaaatgatccaatgtagtgtatagagaagctttgtctcagcttgtcctaaattctgaagatttgt

gtccttcctgttgtggagcaatgatgcagcacagtgcatgacatgaggaaatgcaccctgtatcagtcgccatcttggtattgttcttattgcttctgtc

aggcttggagaaaggcctatttgtatgttttgtaccaacaccttctcaaaagacttgcatgcttctttcagctcgtgatggccaggagcgtgctggcaga

acatacaagaggcctcgtg

>85754|AGAP001587-PA

caaatgttaacaatactgtactatactgagtaatacttcgcaaacacaatgaacatttaaagaaacaggcaacctggatagcagcaagagcaaaatttgt

ccaacaaagagacagtatgactaaactggaaacagaggcatctctcaaataaccatctctgatgtgatgttaaaagaatataaaatgctatggtatagaa

gtatctgcctaattggacaatacttggggctcatgcaatgtcagagagaatacagttaatatgttatgaagaattcaacatttgaaacaaaaaagacatt

gtttatcaaacaattcagtgttaaaatctcatgttaatctaggactgttacacattaccagaacaataagaaaacaattcatgaaccttttcacttctta

agtggattggactgctgcagtttctttccatattttgaaaaagaaatttgggaaatcatataatacttgccatattgtagttttccattctccttagtaa

ctacttctagtatgtaattatacaagcagattttattcggatttgttgccaagtaatactgcaaactactttgtttccatattttcagtataaaactctg

tttaaatattcatatagcaattttatcacatcatttttcttatgagtgaataaaatctacacataaccaataaaatatgtatttacatagacgaataata

tttttgtcacttaaaaatgagaatattatttacaaatattccccaatacaggatatgaattactcctatcataaaatgattttcatgcttctattaaaac

agcattatgatttctgactatttccatttgtcttctgttctgtagaacagtgagattcattcctaaaatgcattttgtatataaatatatccaatgtgaa

aatctgggaagatgattaatatagtacatgacagcagaaatgtataacagtcttttagaatccctattgggacagcatacatgatattttaataaaagaa

ataaaaactactcttagattttcaaaattcatttaaatccatgtcagaataaaaaacaacttgcaataatgtaaaatatatatttactctttctctcagg

agaacagatttatgaaagagcctgcaaagttagatgagcttaatatgacacatcacgtaaaggatttgaaaaccaattctacgcagcagcgtgaatgtga

aggaaatacgtgtaaaattactgtaactgaaggagatgaatattataaactacgtaaataaatacattgtgtggggctgaaaaacaatctgaacatcagc

tttcaaattggaaatggaaataataactatcagtgatgtattcttttcttttttgtatatacctttctttaactctctttcagctaagagcactaatgtt

aaaatttattaatttttcttgttgttgtacagtatgtcaattagcatgtcttacattttaaaaaccaagcgactttttcactacccatcccaatatcatc

tgttttcgtacttaattactatattacatggttcttaataaatacatattttacacatatctaacactatatacaataccatttctatatcatagtgaac

actactgttcattatcatcttgctctaaaatatttttgaatgaaaatggggcaaaaaggtacccagcaccagaataaaacttgctcatgaattccaccca

gtggagccttagtgtgtgaagaaatgctgaaagaccttccatcaaaaccaaaatggcaacagtgaggaaagaccaagctaggaacataatgtagagaata

attgcaccggtatagccttctgctgtcagtcccatccgcaggaccattgtccacagcacttcagaaagttgagaatgtgcaagtgacagagcccatagtc

gcagatatgaagctgtgtgagagatagtgctgagaatatactctatagtatgtatggactggtaaatgaaaacttcagtcatatcatcttctccatggcc

atcatgcccatctgctggagtaactgccccaacctcctcattttcctgacgcagttcaatgccttgatttacatcaccattctcagatgctgacacctgc

tgtctgcttttcttacgagaacacataatgtatagtggtttcgccagtaacaaccatgggacacaaagaagtgcaacaaacaccataatttgctgcactg

ttccttgactctcaaagatatatatgttgcagcagtctttgccctcactattgcacaatggctctgcttgcttgaaaagcagcatattgataaatgtgat

aagcacatttggtgcacaatgctcacttgtcagcagctcgttctttgcactgtatacaacccacttcacaaacatgagagagaccaggtatccaaataac

agaacgaggaagagaacttgaggtagaaattccaagacaatattgattcgcttccgaaaatgcacatgattcactacactaaggcacactccaaatatca

tgtgaagaactccaaatattatagataacttcattttatatgagttcaggtacatgatcttgtttgcttcagccagctgccaagcaggatccattcctag

ggggtatatggaatcaccataatcatttttgggacttagctgaagttcatgatttgctttgagagttgattcatcataagacacattccaattgctacca

aaaatattaactgatttggagaagaaatcattgtagattaacccagtgtacattgaaaatgtacccattagaagtatgatgtaacgaccagcaaaaaaga

tgttccagatctcattagtactcttctgtttcatatgtttcttctctgttaaaaccatatacagtccaaatgcaaacatgataaggccatgtccagcatc

accaaacattatggcaaacaggaatggaaaagtgatgattgtgtataaagctgggttaagctcctggtaacttattgctccatatgcatcaattaggttc

tggaaacctgcagtgaatttatttgttctgttgaatgttggtggcgtttctgatgtttcaattgcattgaggaaagatggaattgaactgccacaggctt

ttgaaccatcttgaagtgctttcctcagctttgggaggtcactcacaggtacccagcactccgcaataaggcacttgttcgttacatccatattgaaact

attcagagtatggtagatagctttcattttgcgtaccattaccatccagttgtttaattctcgagccacacctactaacactcgttgcctgtggtcctgt

gtctggttcagaaccagtgtgagatcctgaaggcgtgtgcgaacaccagccagcatgtctgcacgctcacctgcgacacttggacaggggtacagagatg

catggaaaccagcacacaccttctttactcgagacttcagttcctcaccttggaaaaaagccacaaaaactgttttatatacagagttgcctgtatttgg

ttcatctagaggttgatccagcccaacttgacgcaaaaatacattgccacgtgatatcctccagagcattcgttcaaatgctgggacccgttctcttggc

acaactccagcaacaaaccctagtctgcccctggctgatgcacttgctccagcttgtgtggagctctcctcctggatgagagccctggtcagtgagtcag

aagctccaacaccctcctgttctgtgaagaagccctctgtcttctccagtacatgtcgcatctctgtcaactccaaaaagtttagctttaggtttactgc

attctgactcagttccattatttcattctctgtcttctccagatttgcctcaaggtcaatgatctccctggggttcggtgccttgggtatttcatcaatt

tctgggacagatactccatctttgacgatttcagcctcaatgtatcgtaatttgcgttccagttcgtcgcagcgacgcacctcattgacgaatttgcgct

gaaaggcgtttacgtcattgttgaggtcccggaattgtgcaatgcccagttcgccgagctcggagacggaagggtaggcggcttctggttggatgaagag

ctggcagagggccatctcctcgctgcggaacattgcccccatggctgcggattacgcttcagatgttatgacaacaaacagttgcaacaccaaggactgc

cgtcgagacctctctatcaccaccgagcttggatcaagaattaggtagagtgatgttgagtgacgcggccccgttcgttacattcgaaaccttaaggatg

agagtagcttccgcgtgatgcgtcaccgtgttgtactgtaatagagacagggggcagcggctctcttaatcacggtctgatgacaaatgcagtaacggag

caaaatataaacacaacagaacacacgcggcgtatgcacgtggccaaacgtctagcaccgtacccacatcacaactgag

>85827|AGAP001623-PA

tgtacaggctccgttgtcctgttgttaagtcaaatggtgagcgctggctgaatgtcacttctccagacactccttcagactgcatgataaccttctgtgt

gtaaggagttatttgcctaattcttgcacatgctaaaaaactgtctgggtgttttgaatcaaatattacaatgtacaggctccgttgtcctgttgttaag

tcaaatggtgagcgctggctgaatgtcacttctccagacactccttcagactgcatgataaccttctgtgtgtaaggagttatttgcctaattcttgcac

atgctaaaaaactgtctgggtgttttgaatcaaatattacaatgtacaggctccgttgtcctgttgttaagtcaaatggtacagatgttaactctgagtc

atggtaggtgtttataacattggagtctggactgcctgaaatctgcactttacccaatcgtttatcaatgtcaccaacagtttgtccttcttttgcagat

gcaggattaaacacaaactgaaggaagttgcagttgcttcttgtctttcctgcttctgcgtctaaaatatctgttgcatagatcttccagtggtgttttg

tatctttttctggacttcctttctctttagttacccagtaaagattggtgtgaattagtgcatctatggaatcagaatgtaggcttccaatccaccgcat

gaaaacagacccagcaactggagaatagaattttgcttcagccactttttcatctgtttgatctgaaacactaatcgtaccacaagcagcatggattcta

tagccctcttcaaagatcaacagagatcggccccataagcctttttcaccagttaaggatatcaaatcagtgggtatcagtaaatcactggtgtttttca

caggaaattcaagaagtccaacagtatcatctag

>85868|AGAP001630-PA

agacgtgtgctcttccgatctctttgagataggcgcgaatttcttcatggacttcttgagcttgttccttgctagctactttgcctgtgcctatggccca

cactggttcgtaagcaataacaacgctagaccaatctttgatgtacggaatcacagcgtcaagttgtctcttgcaaacattcatggtttctcccttctca

cgctcggcaagggtttcacca

>85869|AGAP001630-PA

tggtgaaacccttgccgagcgtgagaagggagaaaccatgaatgtttgcaagagacaacttgacgctgtgattccgtacatcaaagattggtctagcgtt

gttattgcttacgaaccagtgtgggccataggcacaggcaaagtagctagcaaggaacaagctcaagaagtccatgaagaaattcgcgcctatctcaaag

ccaaggtagacgcaggcgttgcagagtcaattagaattgtttatggaggatctgttaacgagaagaattgcgaggaattggcgaagatgccggacattga

tggcttcctagttggtggcgcctcattgaagcctgcttttactgcaatcatcaagtctatgatttcgcgtgcgtaaaaaaattcagtcgtgtatttgact

cggcc

>85870|AGAP001630-PA

aagaagtatatagttggatctcagaatatctctaagactggaaacggtgcatacactggtgaaatctcagtggacatcctcaaggacatgggcattgggt

ggactctgataggacactcggagcgccgtactttgtatcacgagtgtaattcaatcgtgggcgagaaggtgaaggtgtgtgataaagcgggtgtcaaatc

agtcatttgtattggtgaaacactggccgagagagaagcaggcaagactatggaggtctgcaagcaacaactagacgctgttataccctctgttaccaat

tgggcacatgttgtcatcgcctatgagccggtttgggccattggtactggtaaagtggcaaccaaagagcaggccgaggaggttcatcaggagatccgag

cacatctgaaagagaaagtatcaagcgatgtggccaactcagtccgtattgtgtatggcggttcagtaaatgaaaagaattgcgaagagttggctggcat

gcccaatattgacggcttccttgtaggcggagcatctcttaagcccacctttactaccatcatcgcaagcctgaccacgaagactaattagaacgagctc

tatgtacaatgtgtaaagttttgttattcaaaccaatattcattgtgactaaaagactgtgcatactctgctgagccagcggggcgtttaggaggag

>85918|AGAP001633-PA

atgaatgtgtaatcatcacctccataacgcttcatgaattttacactttgtttcagtttaactctgttgataatgaatgtgtaatcatcacctccataac

gcttcatcaattttacactttgtttcagttcagcattgttatggtaccatgtcagcgttggtgtggctattgcaattactctgcaatagaacttag

>86062|AGAP001636-PA

aatctggagtgcccagcactcggtgatcacttgcaacttttcctcgacggacagattttggtgttctgaatggtatgtgtgctgaattaagtggcgtgac

aggactcatgacagagtgctggtctggtgttccagaatttccagacttgtcgcttgaaagaggaagttcaaatcttgttgttttcgtaatttttcttaca

cttaaagatgatataggtgtagaaacatatattttttcttgattgtggtggtcttttatgtcactgtaagtcaatggtgtggaatttcttagaacccctt

tcactggactggcacttctgaatgatgctgattgcaaagattcagagattctggaaatatttactttctttgctgttctccctgagacatttaactgata

aatttcttcagagagtcctgtctgagagacagttcccactgaatttagactgttatgaaaattacgctttcttttcatccccctaactattggacgttta

aacgatgtcaagcgattcacagggggagtgccattttgatatgccaactgtgcaattatgtcattttctttatctt

>86199|AGAP001652-PA

ctctggcggcatcaggaacataccaatgttcttagacgtcacacggctcagcgtcagtgcgtcctggtaagagacgaatctgtcgtttttgccgacaaac

acggctatgggggtggagacgttctgcaggcggtactccggcggcgcctcgcgtccgtatcgccgcagattttcttcgcg

>86246|AGAP001660-PA

cactttggagaacatgtttgccatggaacttcataaataccaagatattgctgaagagattatcaacaatgcagtgaaggaat

>86247|AGAP001660-PA

ctcgttgctcttccaaatcagctcgctcacaacgtaccacaacactcaatagctggtcttccaaacccgagactgttacagtgtagttgatgacagcagc

tttggcataaacagcagggttgaatgcaggattggacagtt

>86248|AGAP001660-PA

aatacttgcatagactggttgttcccatggccacgtcaagctttggttgcagttgcaagtgtttatttaatggagaatgaaaaaataccacagaattatc

gtgagaacattgtagagcatgctgtgtatgtacatcaaacagttgtggaatacacgacagaattcctgcttactctccgtcgaaggaattatgtcactcc

caaacactatcttgacttcctcaatacataccttagattgcttgaggaaaaaaataacttcattcttgcacagtgtcagcgtctggaagtagggctagat

aaaataagaga

>86311|AGAP001672-PA

cataccattcatttggacattctggaggcagatttgttggtgtgagcaaacaatcaaggagatggcataacatctggatgtgtg

>86313|AGAP001672-PA

actgttacaagagatggattagaagaacagctcttagcggaggttgtaaaagctgaacgcccagatctagagaaactgaaggctgat

>86312|AGAP001672-PA

gaaatctgttctggatgctattaagggcaagaagacaactttcaactttatgggcgaagtcatagctttaataccaactgtagggatgttcattactatg

aacccagggtatgctggaagaactgagctacctgaaaacttgaaagcacttttcaggccatgtgcaatggtggtaccagattttgaactgatctgtgaaa

ttatgttagtggcagaaggtttccaagaagcacgtctcttggctcgtaagttcatcac

>86309|AGAP001672-PA

ggagaatagcagcctgcattgctgcgaaagtcattacttgtggatacaaatgagacatcacgccttcggtttttcatggatcttctaatggagaaaggcc

ttccagtgatgcttgttggaactgctgggtcaggaaagagtgttattgtggcagaaaagctgagtgcattgccagaagattacattgttacaaatgtacc

attcaacttttataccacatctgaaatgctgcaagctgtgttggagaaaccacttgagaagaaagctggacgcaattatgggcctccaggcagcaaactc

atggtgtattttgtagatgatatgaatatgcctgaagtggatacctatggcactgtgcaacctcacacattgattcgtcagtactttgactatagacact

ggtacgatcggatcaagctttctctgaaggacattcacaactgccagtttgtttcctgcatgaacccaacagcaggcagtttcactatcaatccacggct

tcagcgccacttcagtgtttttgctgtgagcactccctcacctgagtcactatttcatatttatcattctattcttagccaacatttggctaatcctata

ctgaagtttaatccagccattctgaagataactcaagagttggtgaatgctgctctttttctccatggcaagatgggagccatgtttctgccaacagctg

tgaaattccattatgttttcaacttgcgggatctgtcaaatatattccaggctatgctgttcagtatcgctgaatgtgtgcccactgcaagagatcttat

acgactgtggattcatg

>86420|AGAP001688-PA

aaaggaacagatacttatcgactctatcagtagactttatggcttaccttatctccataaggtggttgttgtgtggaattctccaaagccaccactggaa

gacatgcgctggcctgatattggtgttcctgttcatgtatgttgatgataaatctactactctttcttaggta

>86480|AGAP001711-PA

gtgccaatgttacgccggcccctctgaaaagttcagtccaaaacatggtctgagctgcacggtctgttatatctttgaattccatactttcttgctctgc

attcacatatttataattactgcgggaagtggggaaagcaggtcggttg

>86479|AGAP001711-PA

aatagtgatggcctgtgccgggcaaattgcctcgcagagtttacactctatacagcgttcttcacctgatggatacctgcgtagggcatgttcacctcgg

aacctcggactcagtggaccctcgtgcacctgcacaaactgcgcatctggcaccaag

>86477|AGAP001711-PA

ttccttgttgtacaacaattcctcgtgagtttttgtagagaattcaaaatttgggccctccactatggcatcaacaggacaagcttcctggcagaatcca

caatatatacatttcgtcatgtcaatatcatagcgtgttgttcggctacttccatcttctcgttcctctgcttctctacatagtcgccggtacgacttag

acatttg

>86478|AGAP001711-PA

gcgacttctgtattttttattgtgaatgctaagtggacacactagtagttataggtcagagtggaattgctataaataatggctgtcacaaacagcaatt

tatttcttcttcatcaacaactgatatacacacacgtcaacaagtgtaataacacaaatttcttaatttttgcctacttaatacaatacacattacaaat

agctgcttatcggtacaagtgatccgcctggatatttgccgcaatttcagattcccacttatcaccattgttcaacagtttttccttgttgtacaacaat

tcctcgtgagtttctgtagagaactcaaaatttgggccctgtttatcatctgcatttcttcttggtgtagcaattttaatggccagtagtgtatttttgc

aggggacttccaacaacttgttgagtctacgtcacatcgagctgctgtattatgctccactatggcatcaacaggacaagcttcctggcagaatccacaa

tatatacatttcgtcatgtcaatatcatagcgtgttgttcggcgacttccatcttctcgttcctctgcttcaatagtgatggcctgtgccgggcaaattg

cctcgcagagtttacacgctatacagcgttcttcacctgatggatacctgcgtagggcatgttcaccgcggaacctcggactcagtggacccttctcgaa

aggataatttattgtcgctggctccttaaatatatgtgccaatgttacgccggcccctctgaaaagttcagtccaaaacatagtctgagctgcacggtct

gttatatctttgaattccatactttcttcctctgcattcacatatttataattactgcgggatggggtgacacaggttatgccatatacacgtctcaaca

accgacctgctttccccacttcccacaactttaacgcagccatatttaccagagactactactcaatgcacacaagaaaccatagaccttag

>86501|AGAP001722-PA

gatccatgtggcagagcccaagagaagaataggtatggtagatcatccgcatgtgaagcccctgaaaaccccctgactgtgttgagaatgttgctgaagg

agaattggtagtagaacacaggttctgcagacttctgggacaccatcctcacagttttatctattccagaattgaacagcaggtctgaaaacatgttggc

cgtgacgacggagtcggccgccgtgatgtcccggctggtggagccgtagtagaagcggcgcacctcccgggcggcggcggctcgctcctcggggctgtcc

agcgggatgtcgggccccacgcgctccgcgaagtgggcgctcaggtccgccagggcgcggcccgtcgacagctgaccgctcgtcgcccccagcagcgtcc

acggcagcgacatcacgcgtgtgatgaagaggcactcaacactggtggagccgatgagaagcggcacctgcgagaagttgccgcgctcctgcaggatggc

aggagactcggtgaggaaggcgccctccacgtcgggctctatggctggcgtgaaggcgcacgtgaagacacgacctgcgtcctctggcgagagcgcggcg

gtctcgttgctggtgagcgccttggcgctgagcgcgcgcaggaagcgcagcatctcccgcgagccgcctccctgctcgcggtagcccagcgcctccgcca

gccgccggctgcggtccgccatgttgcgggcgctgcccgtcgcgatggacgcggcgccgctctgcgcgatcacgcgccggaacagcccagcggctagtgg

cgaatacataagcatgttggcagcaatggctcccgcgctctggccgaatacagtcacattttctgggtctccgccgaaagctgcgatgttgtggcgcacc

caacgcagggccgccaccatgtccttgagacccgcgttgccgggcgctacctcgtcacccgtgctcaggaaccccagaatgccgaggcggtagttgacgg

>86520|AGAP001724-PA

cgggggataggagacaaacatcgaatgacaatcataatttaggtcagacatcgcaatgagaaaattgactacatcacaaacttgcctgaaagtggaaaga

accgcgaggaaatactcaaacaagttaaagaagaccgtgtttcgattagcaaggaaaataccagcagttgccaggagaattgacagtgagatgagtaata

tgagaaagacattcgaagatgaggtcagacatcgcaatgagaaaattgactacatcacaaacttgcctgaaagtggaaagaaccgcgaggaaatactcaa

acaagttaaagcctatttggaactaggagactacgattggaaaaaaggatatgtttcaggaacagtgtactttcataatcctgaacttattgaattgctt

actgaagtttatggtcttgcatcctatacaaatccacttcaccctgatgttttccccggtgtctgcaaaatggaagcagaggtggttagaatagctgcaa

atctcttccatggctccccctctgcatgtggaactatgactacaggtggaactgaatctatattgatggcatgcaaagcatatcgtgactacgcaaaatt

tgagaggggtatcaaacatccagagattgttttgccaaaaacagcccatccagcatttgacaaggctgcattgtatttcggcatgagactcaaacatgtt

cctgttgatcctgtcaccaccaaagtcaatataagggcaatgaagaattctatcagtaaaaatacatgcatgctagttggatcagtgccaaacttcccct

atggaactatggatgacgttgaggctatagcagaactaggaaggaggtacaacatacctgtacatgtagatgcctgcttaggaggttttgtcattgtctt

catgcaaaaggcaggctacaaattacc

>86553|AGAP001736-PA

caaccacacagacgttgcagccctatggataattgaatgccttctggcaaggacatcaatatttcttgagtaaccgcctccaataacagtggcacatggt

attcccttttttataactgtatccattacgtagaagtctctttcatataatcctttgtctgtgagctgcagctttcccaattcatcatccttgtgcggat

ctactcctgcatcatacagaaccaggtctggtttaaatgaactcagcaggtaaggaaggctttcacaaagctgcatcataaattcagcatcactggtgcc

tgtatcaagttcgatgtctagatcactcttctgcttccgaaatggaaaattcttcttgcaatgcattgaaaatgtaaacacatcctttgtgttcctgaag

ataaaagctgtgccatctccctgatgtacatcgaggtctacaattagcactctggctatcctctgctgatccagaaaccattttgcagtgacagccaaat

catttatcaggcaaaagccagtgcctctgtcaggaaaagcatgatgagtacctcccgctgtgctacaagcgagaccacgctcaagagccagagctgcagc

cagaagtgtgccaccggtttcatatctcactcggcttgccagccctggactccaaatgaaaccagtgactctttgttcttcttcagtagttcttccatta

aagaatttgtccacataatctgctgtgtgaacacaacatgctatattgcttgatactttcttcggatttattacctgcttcttcaaagaaattataccat

cattcaagagtatttcaaaaactttatgaaactttggcataggaaatctgtgaccttttggtaaatcacacacatatccatggtgatgcacaactggaag

tcccacaacttctttagggatttctgctaattctgtaacatgtgttgttaaatgctttttcctgtacgaaaagatgcaggatggtccacaaatacaacat

tttaatcttagtcgggtcgagctgtttacactacagatagaaaacattttcaaagtactttcatcgcatccctaaccagtactcccagacctacaccaca

gtcta

>86589|AGAP001738-PA

agacgatgctgaggaatatggcagcgacgttgggaggttatgtgtaaactctggcctaagcaggtaaacttcgtgcacaggtattcgtgccgtgacttca

agatttctggttccaatttgttatgggcaggccagaaattttgctgcgcgaaagggaactcgcgaaaaagcaagaaaaaagaaagttaaagtggagatca

aaaaagttgcgtgggttccgccaaagctgagagaaaagaaagataaattgccacttatttctaaacgtctgaacgacagccacaagccagtttcctgtga

cgatgtgtggattggcaggtattatcagtggagggtatacgactttgttgaagctgtacagtgtcatcgagaaacccaccacccaacaatgtacaacgtt

ccagatgcaccactcttcgcctttattgaactggacatgtccacagacaagaaaacaaaatttgttgataactttaccagagttgctggtattccacatc

catttgaacatggcgaagacagatctgtcatagtttttgcaaagacacaagaagaacaaaagattgcccttgaagccggagcaacagtggctggtggaat

ggagctcatcacagatatacagaatggtaatttgtccatgcaagatttcaagtacgcagttgctcatccgagtatcctacctgagttagcatctgtcaga

ggactctttaggcggaaatttccaaacattaagaatggtacccttgggccagatatagctgagattgttaaaagatttgcacatgggatttgctatacag

cttcaagagatgaacgtgaactggattatggtacaatagaagcacagattggaattctgacaatggatccaaagcatttggaagaaaattttgctgcgct

tctacaagatgttcatgctgcacgaactcgcaaaaaagcaaattttattacaaggactcttcttaccagtcccccatctcgagaacggtttaagattgac

catggactatatgttgaggtcttcaggggtgtcagagaagaagaatcagaagctaaaacacaagatgagtttgaagaagctgatgtgaaacaaagcactg

ctacaggatgatgggtgtactgttgtttgtcagtaagataaaaaaattttttgtcttattgaaaacagtgtacaggatttctcgaagataattcgccctt

taattctgataccatttcagaatttctgaactggcccaaatttttctagtaattatgatttttgtaatttattttccccatttttttcaacatgttaact

aaattatatttgtcatagaaaataataagtttttgaaaacataatgtaattggatagataaaaaatatgatcactaagaggtaggagaaaaattatataa

caggtaattacatttctcatatcattttctaaatcttctcagaaaacagtggccctgttatttatgaacatggagaatggtgccaggatgtctgtcaaat

ggaaaagcattatcaaggcaaatgaagtacctcaatattgtctgactactgctggtcctattaaatattcctggggaagtaacacactttacaacagtac

tttcaaatgcaaacagaagaaagaaaaatctgtcagtacacaaaggaatcaagtaaatgagtaacctaatactaaagaaaagtaaagaatgatacctaaa

aatatattcttccaaatttccagcaatactttgcctgaggaacaccactttgtaaggactggcaataataaaaaaaattgatattcaattttatccagga

agaagtccaaatatacattcaaaaaaagttatggaaataacttttttacattctgtaagatgttaagatatcagtatttcttgttgtacaatatcatgat

agttctttgccctattgttacctaaacagacatgctcttcttttaacatcactcaataaatcaaggaaattgtcatcaaacatcaatttgctaatttgtg

aacctgcgtcactgatcatgggaattacgtgaatcgtcatgaagttttgtccttgaagtttttgatgagaatcagtt

>86666|AGAP001748-PA

cctgtgtctgatcttactcttgtcctttaaatgtgcaatcatctttgttttcccaggtagtgtccaaacaagacgtcctccataaggtgttgggtatttt

ttttggtggtcgcactcttattcttgtttcatgtacaagagacgcagcttcatcaattgtatccaccaacagcttga

>86665|AGAP001748-PA

gaaaaatctgaactaaagtttatgtcaatatgttttgcttgcaggtaacaaaccttctttgttttcttcactgcaacctctcttgttccccatgaaacaa

cattcaggttgatgagagagtacaaaataagaaggagatacatagatggaatagaaagcaagtaaattattccaggaacaatacaccagaattcttgagg

gtgaaggcaggcagcaatgaagaaagatccagacaaggcaatgagaaaaattgctgacggtgaaccgatgccgtcttcaccgagttgcagagctgtaccc

acgatgacatccatcataatcagtgcatagcatgtcgacaaaatctgggccacaagtagctgtaaacttgatttgcatgtaaaacatactaacatgaaga

gaagtatgggaataatattatattggaagcttgtccagt

>86664|AGAP001748-PA

ctacggaataagtccgtgttcgccttcttcatgttcaatgctttgtttgtgcttattgttttcttgctgcaactgaataaggatatgctacatattgact

ggccacttggagtcaagacaaacatcacctatatcgaagagacagcagaggttcacataaccaaagagtatctgcagcttgagcccattggtttggtatt

tgtgttcttctttgctttgattttggtgatacaatttgtggctatgttatttcaccgatttggaacactgtcgcatatacttgcatccacagaacttagg

tggtgctttaacagaaagaaggaagatctgtcacaggactctataatagacaaaaacttcatggaaatagcaaagaatatgcagagattacgtgacttag

atggtgattatgagaatgactcaggtgctcagaaacctgaacacaggcggactatccataacttggaaaggcagagaaagaagacaaggactattggaac

acttgatgttgctttcaaacagcgctttttcaatatgacagctgatgggaatgttccaggtactcccgttcttggaaggaagctaacaatgaggaaagaa

actattaaagctttggaggtccggcgaaattcagtaatggcagagaggcggaaatctcaaatgaaaacacttggtgctagtaatgaatatgggctcactg

gtaacaacaacatagtaccaccacctcgcagccacagagcttcttctgcaagcatgagtgtgaaggatatgtttgaagtcaatggtggtggtcaagtgaa

tcgtgcttatgatggcctttcagaagtgaacacagacccaagggttagttctgttagaatacaaagcaatggcaggaatggagtaagctggagagattca

gagggtcaacggatgtaaacagctgagatatgcatacaaaaaatcttgtggtgaagacttctgaataaacaatcattaggtggtcaaaacgcttattaca

ttagaatggaagctcataattttaagagattctaaatgagactgaatgtttcaataaatattgaagttccatgtgagaaacatgagtcactgtaatattt

tatttattaaaaaaatctgatgttgtagttaatttaaacaattacccatataatatcagaaattatgaaattattccctcaccaatgcaataaaactgtg

agattattcagcaaattagacatatctcaacaattacactgccagtaacgtatttttgtagtttcctcatgatatgctgatgatggtaatcaaagataac

aacactgaattatcattattaaaaaaaaggttcatcactgccatatttcatgttaatgaagcagtttcattcatatttattcctcaaatatctgctcagc

ttatgtcaatggcagcagcaaacaataaactgtttcaaagtaacaaaactattttcccttaaggtgtgtgtgtgtg

>86694|AGAP001751-PE

gttaaatgtactttttaaaaaagaaaaaaaagaaacaagttatattgcgaagtataaagatacatttagttcaaactcttcatccacccataaaccagtt

ttagataaaatgatgtgaagactgaatgagaataaaatcaaacatactaaaagcttccattccaatgaatgaccaatattctatgacagtctcttaaaat

acaagtcaatgcagcactgaaatttatcaaagacagctgtctatgatacaaggataataaagcagagaactgcacaatgcattatttaaaaaaacacaaa

atgccaaatctttactgattacccacaatcacaaaacattcatgaaaatctgttaatttcacaactccagcaaaccacaaataacaaccacgtatcacgt

agcaaagttattttgatgtataaacaccttcccaaaccatcgagcagaggatagttaggtgcatggatatcaaatgaaacaaaaagttcaggcatgtcct

caacagacaccatactacgatagtccagctttggagcaacaggcttctgcggtggtgctgatcgaggttcttgttgctggcggatgaaagtcccaactga

tgatcgccttgaaaacactttggaaaacattgatgagtgctgctgcgcatctacagcggaaggcgttggtagtcccaccagaggatctttatcatctttt

tcaccagttattccttccatccacgaaagtgggtatgacagccttttcagcatcttattctttctgctctcttgtttctctggatcatcacttttttgac

caccttcttcacctttatcactgcctgaatccccctcttgatttgaatcaagactgaaggacactgatgaagttttcttcctaccagattcattaacagg

aggagagtcttgggacgtgtgaacatgagttggacctgacacagttcgaggtggtacccattcagtctttggtgcaagaattatgtctgcatcggaaaac

accttcttactaggtaatacaataggtatttcctcctcctcatcttcctgctgttcctggactgaatgagttccaggtggttgtgcctgatctccaagtg

ctcctacttctcggatatctatgccagaatcatggcagctgtgtttctctagcaggccatcacatggagcacttactgctgtgtcatttctttctccatc

tgcttcaagttcatctttttccaaggagcgatctcgttcgctgctaggtacagcccagtgatgatcaatacaagagcgacgtcgctcttctgcagatctc

agtgcctttataatatcatcatcatcatagtcatcatcatctgcttcagtcgtctcttctacaccgtcctttgtctgagaaccagaggtgcaagattcaa

aagacacctgtctgctgtccgcatcagtcccattttctgctgtggttgccaggttagtaggtgtcacgagatcccgttcaaaggccttgggaaaggcatc

accatcttcacgtccagggcatgaacactcgctctcattgtcctctggggaagatcgagctgggccagcaagctctgggaaagtctcatccttcactaga

agagaatccttgcctggtgagttctgtcctttctcgtaggtctgaaaggccttcgaatggtatatttccggcttgggtgtgtctgcccttttgttgtcac

cagtatggtggcccactctcatgtgggcaatgtcatagtagatagctgcatttaccacatattccattggagcaattacaccatacatctctggaccatg

ctcaataactagggggtcagatacatttgggtcaaacattactgcattaggcgtcacgagcagcacaccaccaacaacaccctgcccatcagttatgtgc

cgcacattgatcttgaggaaacgtctttcagctcgctgctcctccagcgatgcaccactgttgcctgtagtatcagggtgggaggggtgcgttgcttcac

tggtcacaggtggaccagttggtttgggtgatacaggccgcaagctctccagaatatctgtctctgcagagggccgcgcggcgtcagcggcggcggctgg

cgtcagctgcccttctgggtgcgcaggcgccggcggctgcagcggcggctgctg

>86770|AGAP001775-PA

tgctctgtggaattctagccatggtactcgttgtaaattacattctagtcgttccgatggcccttattggagcactgttcgtgtatgtgtctagactttt

catgacgacagcgacaagcatcaagagacttgagggtatccgaaggagccccgtgttctctcatctgtctgacaccctcaatggactgactacaataaga

gcatcggatatccagg

>86771|AGAP001775-PA

ttctagcaaaatgtaccatcttttcaaatggtttctcccaggcatacatctttattacttggattccacatatgatttcattcatgagtcttatcctgtc

atcacttcttattgctgtctgcattctaaactttgttgtcaaagtactcatataactctgaacaggtaccgtttgtaagaagagtccagtaacaccaata

atagatgcccagcccacaa

>86772|AGAP001775-PA

cagtctgcagaggcggcgccaggacatggagggcgcagcggagaaggcggagcaggccccggggcaggcgccgaggcccacggtgggcttcagcgacgag

gccgtcgcctacgaggcgaccagcctcgagtccgagtcggtgatatcggaccagcgcgccatgggcacccccagccgcaggatgaacccgcaccccaggg

cctccgccagccccatctccatcctgctgctgtggtggctgaaagacctcttcatactcggttccaaaaaagacctcgaaacagacgacctgtacgatac

actcgaaggagatctgtcggaaaagctgggcaccagactagaaaacaactggaataaagagatagcaaaggccaaaaaggatgctacatacaaaccaagt

cttctcaaggcccttataaagacatttggtcaacagtatggcctcttggggatatttgttgcagtgaacattgtaattgtgaacacttccaggccaatac

ttctaggtctcctcttgcagtatttcacacctgatacaacagtcacg

>86791|AGAP001777-PA

gaacaagaaacagcataacccaaaccctaaagcgactgccaatccgatctccaagttgttcctctggtggaccaggaaactgctgtggaagggctaccgg

cgcgagctggaggaacacgacctgtacacgcctctggagaacgaccagtcccaagtgctgggcaaccgcctcgaaagagcatggaagaaggagatcgcga

aggcgaggcaagcagggaaggggaagaagccgagcctgctgagcgccatcttccgcgccttctggctgcagtacgctgtctacggcgtcatgttggccat

ccagtgcctcgtcatcaggatagcgcagcctctgctactgggagagctgatcaagtacttctctgaggggaacgtggacatcagacaggcgtattactac

gcgggcggcatgatcgccaccgtgtacttcctcatctgcttccagcaccactacaccagggg

>86812|AGAP001778-PA

caaaaagtacaagttgtcagacacggacagagccctagtaattttcaacagtagcttctttctgcttgaataccacactcacgcagataatctcactcta

cagcaggcttttctggtggtcgatgagccttccaggaaaggtaagtgttccaaccaacacccacaacttgtgtgacaggtacctggaagttcacaggaac

gaagccaaagtttatcaattggacagcaggccatactttatagtttgttaccaaaatattcccataatcagctcccaacttttctttaatttcagctaag

cttttccctgcacatatgttaaggacagaaagggtcagcaataaaaggaaaggtgccattatacactggtccaataaaactttttgagccacaacagcat

aatgacttcctttcacttttgtgtcaagaagtccaa

>86834|AGAP001780-PA

ctactgaaacaaaccaaaactttttgttattcattaattagtcaaatgtaaatttttttccagaacatgagtgtgcatcacccgaagactttaggattgg

ggctgctatcagaacgggttactcacatttcaagtgctgtaattagatgttctgttgcaacagagagtggaaaagtagcaacgtga

>86865|AGAP001793-PA

ttggaatgttgaagtacttcgtagtgtgacaagtaaggagtgagacgacgcaagatattcaacaggttgcacagaagtaagccaattgtgcaaaagagtc

cttgcttgaagtcaaaagacttgtgagctaagaagatccataattaggaagcgatttcttcaccacgttatgaagagatagtatattttaaaggaaaaca

tggagaggacagctggaatgtctgctcgtaatgcaccaaatgttggtcttagaacagaaggagcagcaaaagatgcgcggtccagtagcaagacctggac

attgtctgattttgaaattggaagagcactgggaaaaggaaagtttggaaatgtttatcttgctagggaaagaag

>86889|AGAP001794-PA

cacgggtaggaggtgaagtagtaacatttgtatctacttttgcttcattttctttgccctccgaagactcagtattgtcacccagagaaaaacgaacacg

ttttgggacagaagctattacttgagagtttgcattactgggtgcagcaccatcatgtgatgcctgagttttaagaatagatttggctggctgtggtggt

gggacagaagccggaggttttgtgatgctctcaggattctgctgctggtcaacattctcttcactattcccactttcttttgcatttgcatggccatcat

ccctactcccttccccttcaacttcagcatcagcatcagcgtcagcgtcagcatcatcatcatcatcgtcataatccgccaccgccatgtcgtcatctac

atcttcactaccatcttctatttcaacgagatcctttcgtagttgatgttcttgtacatactccttcactgcctgcaaatctaccattttgggttgcttt

tctggtgaagcgccagcgctatcaccgtacgatggtctctttatagcaactgctgtccttcgcacctcttcaaaactagctatttgttgcacttcttttt

tcacattgttcaaagaaccaactggtgtactcgtcaaggagtgagaagaggactgagaccctggtgttttcggcgtatctgcaggcctcacaaggggttt

tgtttcatacgtgatgtcatcaaaactaggatggtaagtctgcggtttcaaatccacatccggccaatcaaagcgcatgggacccgagacatcttcgtga

cgggcatttgctccactctttgcaagtagaaggggtgatgagaaaatgacaggagttgagtcttcaatgtgtggagttataactggcagagtactcctcc

tcttccaaatgggaagttttcgacctggcagtggttctggaagctgagagtcacctgtcttcacaactttcttcacaggcaaattccaggatgtatggga

tggcacatgaagtttcttgacggttgcaggtttaagtgacccctgaacagatgcagaattatactgagcatccagaagctttgtcagtactgacatgctc

atcacttttgctgctgaatacaactttgagtgtagctcatatttgaactctaggcggcctgtatacatgaagtttataattggaacgacgacctctggat

ccatgtcctctggcattcgtagtaaatggctgcctggaggattctctttctctaaaagttggaagtattctgtacatgcataaagcacaacacgatgcac

ctttatcctttctgcattcttgaataatagtataaggtcacagaattcatttttgctgaaaaagtggtgcaacctttgcaggaaaaatacgccccagtta

tcaaccttcacttgcttcaagcccgtttcagtcattttgaacacatttgaatattaatgaaaacaatccgaaaatatgtcaccttttcaacaaatgcttt

ctgcatttccaactactgtcacattctttgtccatctcactaactactttaagaaatgttaaggcacaacaatttattcttcacgagatttcagacataa

ttccttcgataccgaacattaacatttaggtaaacgaaaatataacattacgcaaggtcattccaccgctattgtcgctaactaaaacataatcacagtg

tgcggtatcatcactatcccttcgaccatcactcaattcactatcgtggattgccatgatgtgttcaactaattcaaatatacaaaccagaagacgaaag

gcacaaatgtccgtcccag

>86930|AGAP001797-PB

tcatcctcaagcctgtcgacctccttctgcaacttctgcactgacctttcagcaaactcagctctggctttagcactcccagctggtggccaggtgacgg

cggttatccatcgtatatcccactgggctacgaggaccttggacggcaaggcaactagagcgacagcgctcctgctgtcagatgtgatg

>86954|AGAP001797-PH

gtcattaattgttcataagattaattctacgaaggcagaatcaaaatcgtcaccaatttttttgtatttctccttctctatccccagctcatcctcaagc

ctgtcgacctcctccttcctttctgcttaatgtgaatctttctgttgctgaatgt

>87135|AGAP001826-PA

agtgatgcgaattcagtagaatagctgttgcatcatatggtggtatcagtttatcaatatctaatgaaggagatttccattcttggagagaatataacag

agtcggtattcaaagcaaagctggtgtgaaagttacctgtgaaacaagtattgaactttgtacatttgaaataaatggatcctattttggaaagacaaga

ggattacttggaacaattaataatgagc

>87134|AGAP001826-PA

tgaagaagtgatgcgaattcagtagaatagctgttgcatcatatggtggtatcagtttatcaatatctaatgaaggtctgtaagaagctattggttaatg

gaagacctacagaataccctgcagatgaaggagatttccatgcttggagagaatataacagagtcggtattcaaaacaaagctggtgtgaaagttacctg

tgaaacaagtattgaactttgtacatttgaaataaatggattctattttggaaagacaagaggattacttggaacaattaataatgagccatgggatgac

ttcaccaaaccagatggacaagtcgctaaacaagcaaatgaatttggaaatgcatggaaagtcaatactcagtgtgctgatgtggatggtgttgatcatc

atggacataacattaaaattgaagaatgtgaggaggtgttttcaaaggccagtcttttgagcccatgctctctcctcgtggatccagctccttatctgga

agcttgcaatcacatagcacatgaggcaactacaaaagaagaaaagg

>87153|AGAP001856-PA

ccctcagaactggagcgacgacggcagcactacttcccagatgcccggagacccctcggtacggggcactagtcttgtggctcccatcccaataaaaaaa

tggatgtaagcaacattacacattttaagctctgaatgatatagaagcaaaacacgtgcagacactttgaacatttgaacacgacacactgaaagcagcg

cggtgatggcacaaggtataatactgtccgggcagagtactgtaggcaattccacacgaggcgaaagagcagacacggacagatggtggtgagtggaaca

ctctgcagtcctgcacggtaaaggaaattcggtagtctggggtctgtcactgtctgtccgcttttcaggtacacggggaagtaggctcgacaatcgggaa

gcaattcccgcggtggaaaatgcgccgttcccattgcgggaccattttcctcttgcgggaaactgcggtgaattcccgtgctggtgccttcgggatgaca

cctgctgcagtgactcacacagaggggcatggcaaaggtggcaggagaggccgtactgcgggcaggacacgggcacctgcgacgcttctccgtttcgcaa

gtgaaccgtcggactctgtcaagaagggtcggctccagtagacgagcgcttccagatccagaagatccgcaggcagctcggggccgaaccccttcgtggg

acacgggtgttgcgcagtggcgcgttgctggaagaagccccctggggctacgactgagaggtgtccactacgcaggctgcgatacgggtgacggcatcgg

tgcgttttcctcgaagatcatcaccagcttgctgtactccaaactgcgccgcttcttgcgagtcctgtggagtgaggcaccggcttctgccccggcactg

tcgttgccggaccccgagctgctggaagtgagctcgccctcctcgaagcccagctccttgccgccggcgcgcgccatgtccaggtacagctggcccagct

gctcgttgaccagcatcggctgtgagatggcacgcaacttctgcaggtcatccttcagcacagcattcaactcgcgttcagacacctggtacctgaacat

caggtctggcgtcacctccacattctccagccatggtggatccttcaggatgggtcgccgcttcgatgttccctcagatgagccattatccattctctcc

caaagcatgtcctccgcccccgtggcggccccgtccgcatcgcggcagctgtcgccgtcagagcgcaggaaggagtaggtgtaggaggagcttgactgcc

cccgcccccgcagcccggaccccgagccgctgtcctccgcttccgtctcctcctcgcccgatgtgcggcagcatttctgtgagccgtctgacgcacccct

gttgagcacggaagctgcgacggaggccgcactggagccgggctccactttgacggaagtggcgcaggaggcgggccgcggcagaggcggcggcggctgg

tgctggtacggttggtggtgctgtcggtgctggttttggtgctgctgcgcgacggagctctgctggcaggccgactcgggcagcggcaccgacgccgacg

ccgacgccgacactccactcctggctgctcccgtcagctggtaggtggacgggtggtggtggtgctggtgcagcagcggctggtgcagcgccagcaggtg

tgacggggacggcgggggcggcggggccagcgggtacagcagcggcgccgcctggtacagcacgccggccgccacgtacggcagcgggcctccttgctgc

tgcaggaaggcggcaggggcgtgtggctgctgcggggggcgggaaccgctccccacaggagtggcgccggcggaggcgggtgtcggagccggcaggtagt

acagcggcaggatctgcggggccgcgtgggagcagccacccgtgcagaagttccctcgcgtgctgcagggctgtggtgagtgcagcggcgtcatcgccac

agagaacggcggccagaggctcagccccgcgtcctcggcggcggcaggcgtctgcgttgcgggtgg

>87152|AGAP001856-PA

acgaggtgtgccgagatagaacaggcactgtgctttcaaataattgataagcctggctgggcaatggatccacattcttcagtgcgaatgcacaaatacg

tccgacatcctgtgggatctctaaatagcaaacaggagtataatggacaggcactgtggatatgtggcgctgctgattctgcttattgtcccactgcaga

tcatattagtgatcccatttcaatttacatgtactttctaataagtctctgttaagtcacctatatggcattgacctcaaattctattgatattctgcag

tactttagccgttcactcatgaaagaaagccttattgtggtgggctacatctgaagatctcctgggttcttgtgtttatatatagtggaaaaagagggag

acacacattagcagggattatgtcgaggatggtgtcaggatgtgaggatgttttggtgggctagttgccatccctggagaatggggaaactaattttgaa

ttgggggagttgagtatttcatgtgatgccatagtggcagggaccaaagtccctcaagttgtgctgcagaatgttctcggcggctggctgccagatgtgt

ctagtcgggcagttgttctgtgctcattcatacggtcaatcagtgtagtggtggtcgtgcctttataaaggttgtgcagtgaacagagcatgtgtggttt

tacgtgtcctctgcccgtgcaattgtaggatttagtagtgtcgaggctcgagtaggtggtagaaggagggcgagtgggccaagttttatagtgatgacaa

ttgcacgggtaggaaccttgtggtagggctaatggtttgggaatgtgatagggtttacctaggctgttacgtatgatgccaggcatggtgtagggaggat

gttggagagacagtgtcccattttaggctgaacttggggtacaggttactactttctaagcaccactcacagattaggccttagacctgttccgactgac

ccttttctgcttaccaggagtgatctgtgatcgtggggcatgcaattggcatgtcactgagccctccagctgttactgccagtagacgaaccctcacact

gccgtcctttaatactctagcagtttctcattcggcctgacgaggccatttcagacctccctagctcagaaaatatctgagtagtagtggaactgaaccc

gagtccttccacaccgaaggcaacgacgcaaacaaattggctacagaggcgatacgtataaagcatggccatcaattaacagctgatgtttatactcagt

tatatcagtctgcatccttgaagtaattacttaagggtgccacagcatttgacatttaagtccgtatacatcacagggcacgacacgcgagcactcgtcc

gactgctacctgcggtgtcgacgcacttttacggactgactgcgctcgccagtggcaccgttaaaatggtgtacacgacagtgcctaactttcaatatcg

ttcattttcaatttttgaaaactacgggtacacagaatcgtattacggcagtgcgggaattacgagaacgtcacaggggccggccgcgcctcccattttg

ccgcacatgctcgctctcgccctcgccctcactcgaattgccgcgtttcgctgtgccaccaccaccgcagccgctgactgtctcttctgccgagaagctg

acgggagcagccaggagtggagtgtcggcgtcggcgtcggcgtcggtgccgctgcccgagtcggcctgccagcagagctccgtcgcgcagcagcaccaaa

accagcaccgacagcaccaccaaccgtaccagcaccagccgccgccgcctctgccgcggcccgcctcctgcgccacttccgtcaaagtggagcccggctc

cagtgcggcctccgtcgcagcttccgtgctcaacaggggtgcgtcagacggctcacagaaatgctgccgcacatcgggcgaggaggagacggaagcggag

gacagcggctcggggtccgggctgcgggggcgggggcagtcaagctcctcctacacctactccttcctgcgctctgacggcgacagctgccgcgatgcgg

acggggccgccacgggggcggaggacatgctttgggagagaatggataatggctcatctgagggaacatcgaagcggcgacccatcctgaaggatccacc

atggctggagaatgtggaggtgacgccagacctgatgttcaggtaccaggtgtctgaacgcgagttgaatgctgtgctgaaggatgacctgcagaagttg

cgtgccatctcacagccgatgctggtcaacgagcagctgggccagctgtacctggacatggcgcgcgccggcggcaaggagctgggcttcgaggagggcg

agctcacttccagcagctcggggtccggcaacgacagtgccggggcagaagccggtgcctcactccacaggactcgcaagaagcggcgcagtttggagta

cagcaagctggtgatgatcttcgaggaaaacgcaccgatgccgtcacccgtatcgcagcctgcgtagtggacacctctcagtcgtagccccagggggctt

cttccagcaacgcgccactgcgcaacacccgtgtcccacgaaggggttcggccccgagctgcctgcggatcttctggatctggaagcgctcgtctactgg

agccgacccttcttgacagagtccgacggttcacttgcgaaacggagaagcgtcgcaggtgcccgtgtcctgcccgcagtacggcctctcctgccacctt

tgccatgcccctctgtgtgagtcactgcagcaggtgtcatcccgaaggcaccagcacgggaattcaccgcagtttcccgcaagaggaaaatggtcccgca

atgggaacggcgcattttccaccgcgggaattgcttcccgattgtcgagcctacttccccgtgtacctgaaaagcggacagacagtgacagaccccagac

taccgaatttcctttaccgtgcaggactgcagagtgttccactcaccaccatctgtccgtgtctgctctttcgcctcgtgtggaattgcctacagtactc

tgcccggacagtattataccttgtgccatcaccgcgctgctttcagtgtgtcgtgttcaaatgttcaaagtgtctgcacgtgttttgcttctatatcatt

cagagcttaaaatgtgtaatgttgcttacatccatttttttattgggatgggagccacaagactagtgccccgtaccgaggggtctccgggcatctggga

agtagtgctgccgtcgtcgctccagttctgagggaccggtggaacgaacgacaacgggaacggagggcgagacccgttttccggatctcgccgggatgcg

aggtgcatgagcgtacctcaccggctgtactgtccgtagctccatcttcgggctcggcaaatacgacagcacacagacttaattaattctcagtgagact

gagtagtgttttatgaaaatgagcaaggatgtttaaggaaaagaagagaattgatatcttgtttaaatacaacagactgaagaagaaaaaaagctgaaat

cattttcattaaacgtatatattatcagaagctgtggaatgctagtcagttgccaacttgcaaattcaaagtcgagaaaatttcttcatttttttccctc

tgttttgctgtgcttcagcacttgcttcatttttgagcaagttcttcctaatccaccattgtggatcctacgataagagactatattttgtgcaaacttt

caaactctattgagacttatttattttacacttttcttactatagtccagtaaagggcttgtgaagcctgtactgactagcttttgtacatgtagttatg

taagttgcaagaaatactacacatttcttttgtgtgtgtctgtatatatacatatatttgtggatatatttgctaaataacttcgaaataaaaaag

>87209|AGAP001879-PA

cccgacattttgttttgcacgttgtccccatccttcatccacgtgacacagttttcaggtccaaattctaagaacttctggtacaaaatgcgacaacggt

caaattctcggagctggatttcgagatcgatgtatccccggaagagcttgtccttgggacacattccaagagccatgcccagtgttttcctcgccacctg

cagattcttgcacctaacttcaaactggg

>87247|AGAP001888-PA

tagccacgtatatcttcctggctccgccgccgtttgttatctcgagcgtttcgtagatgtcctctactgcggccatgtactcgtcgacagcgtgatattc

agcttcgtggaacaatttgtcacttcttcgcacgtggatccccacgatgggcctggtgaagttgagacggcgcagtgctgccctcacgagctggtcggag

cga

>87248|AGAP001888-PA

cgcctcattaaaaagaacactgctgcaaatatacttcagaatatgtattttttttccatctttctcccacaattttacaaaacttcacaaaaccaaaaac

acttttttttttcaatgaaaatccatttaaataaaggaacagagaaagattctaaactacactgaaatacagccacaaggtctcttcacaaaactacaga

agtcacaatgcaacctgtttcttcagctctctctttggcagtaataatgacagctttgtatatacactatttgcaccatttaaaccaccatccacgtaat

acaatctcctgaacaacaaaaagcacacagttgtatgtgcaaccttcatatacagttgaatacctcacataatgcacaatgatatagtggcataacaaat

agaaaactatcaacactccttttctgttattaggccgctctaaattctagctcacgctagtgcattgttagtgctaatactgtccgtaagtagttgcgta

tatgagtctttgggctaagtatgagtgtcagtttgttgaacaattacaactggttaataaaattgtaagcttagctaagaattattgcagaattcccaca

cagtgacataagcaatccctttttttctgcaggcaggcaagattaaaaacaatgtaatggaaatctagatggttgcaatgcaggccaagtttgttacaca

tcaggtttagaaactttcagcagttcataaagtatttttttgttcacttttaatttatgatcaggaccaaagctggctctgttgtctgcatatcttctgg

tcttacatcccctcttttatttgtttaaccatgccctttattcaaaactatataatgtgctctgtaacacacagtacaaagaagcaatgcctcacctaac

gatcatgtaaccatttttatccaaataaattgcgagcggtgcccatgcaagtgagtacttcaaagagaatgaaagtaatatgtgcagacagaaaattctg

aaatgggaattgtgtttttgtgttttattcatctcatgacgtacatttgcaatccatttggtttgcatacacgagacatgtcaaaaatttattctgtgtg

gtccagtacagcccagtcatcaataagcagctctgttgtattcaaacacactaattttattaaggagaatgaagacagtttattctcttactgtttactc

ttcaaccagcttcataggttgctttttttgtcatttgctagcaattttgaaattctaagcttcaataccacaagtatcaaatatataatatgatgataac

cagacagagatgcctgcacaatactatatgagtctgattacctagcaaaagtacagacaaaataaggcaattaatagtacttattctatgtaataaaata

tcttaacagatgacatttccaattcttcctacactatggagccaacaagataattttgacaagttccttagatgtgtttattgcagtttcagctatacga

taacatctgtacatatatgacacataagtgtttactccacagtactgtgtacagcgatttaaaaatcttgcaagtgtgccaacctgagaatacagccaaa

tatagaaacaaagaattaatatctcagatgcatgcccaaaagatgatggaatatagaaatttcattagatgtttgagtgttacaaatcaaagtcatttat

gcacattagtactgttcttagtgcaaacatttataactcagggaaacatcatagtacatgcacttgttcttgttcctgtcacatttacagtctggagatc

ttactacctgatggccctgaatttatgtgatctctccagtgacgaaattgtgttcaacctagtatcattaaattttatttgaatgaatctgagcaacata

gacagctgcaaatgctgtgcaggccaactagtttcactggaatacaaaaggcattatgcaacaatgaaagtattcagttttaattactacaggcctacag

catgtaccatggaacataataaaaactggtgaagaagagtaggcaaaatcactcctcaaaagcagaggcaagtgttgtacaaacagtaataattgtgttg

aaaccatcattatgtacagaagtactctcttaaatactttaaagacacattacatgggctgcttctactgtgtagaatattgtagctgatctgctttgaa

tttttttgtgtcgtatctttctatacatttttgctgaaatgtcattacaaaaatgttaattttaatgtaagaagtttctcaaagagaaattgcatggttt

tgtagaatctcagaatcttaaaagctctgtaagtaactttccactgtgttttcaatgttaagtgtttcaaacaaaacagttcccatattcaaaaagtgca

gcatagttataaacactgtgatctgaacttacacaatactagcctcactaaataatttagaaatctggtatgctgtgacttaaatctccccagaatattc

attttgttcatcataacaagtgtcatataatacaatacatctgctgtacataaatatacattttatatataacagggacactttacatatacatatacta

ttgaataaacagtaaaataaagctgattacacttaataccattgctgatacaaaaataaaatattttaattcctcgctaataaaattaccaactaacact

ttacacacacaagattaatttcaaatccatacctctctctgcccccacctcactccacccctgaacaaacaacatttcagtgaatattacctttagtact

tttctggatttgagtaactctttgatttttacagaatcatttatcatccccgccgccacacagtgccttatctacgcagaatgtaagatataaaatattg

cacatgaatacctcagtatataatctaaaagattaatttgtgtatatttgtaaagtttgtccaacacacccatttcaagtagtcattgcttatttcagaa

ataccagagccaagatttcacatccacctgttccatttcatagtgaatttgtcatggaaacaagatatggagcacctcctgcaccactatactttgtact

gttgaaactaatggagataatcatacatctcacattaacttacattccttacactgtgtcataaaacacgtataaagaaagtgtacatttaaagtcctac

ataaagcaccagtaccccaggaacaccttagtacatgtacaagcccgtatcttaaaactgtactaagcactgccaacaacagtcacttctcaattgtctt

tctcctacagcattatcaatacaaaaagaataatttacaatgacttggtgtgaatttcctccctctgacacattacattacaacattgcaatggttactt

gaatactgtcctgctgtgcagcctaccagcgtaaataaattaataaattgttggcttattgctatttccattgtacttacagttatacacaacttacatt

ggataacacccttatctgttgctcaaaagaattttttaaagacaagtttgtcatcacacagataactgtacaacataaagatgcacaattcctgtacact

gtacatttattactcttgaattctgaaaaagagcacacaacagagagacataaacacactgtacaataagcgtaatccatttgaatcccgcacctcagag

tgacagaacattgtccattcacagtaacgtgctgacacttcagaggaagaattaaacaatttgtcttgaagtgaccctcctttctttgctgctcctcttc

aagacaataacctttgtctccttagaggtacttcggggtatgttgggaagtccactacatctaacttctccttgaccttgaatgatgggtaaaggccaat

ctgcccagtccgaatggaccggcctttgctccagccatcccagtggttacccgcaacactgactgcatctcccggcctcagttccagctcacctcggcta

ccagctcgaggcttatgcgggatgacagcaacctgtgtgtgctggttctggcccccatagtaatatatgtcgtcgagtgatttaaagcggtccgatgcat

ccgcatgcaaagtctgcatcaactcataggctacccggcagacctgggatgaaaatgtacagactaggtagtcacagagtgacaagaagtggatgtcaat

gatgatgccattcagtgaagagtcagagtagcgtgtggcaactgcggcagtcctcgcaatggatggatctccgagaatttcatactggggatacttgctg

cgggcctcaccgatgaccttggggtcgtcagaggcgaggtacacacgccgctcgacggtcttgccttgcttcagctcgagcttcaagtagtagtcttcca

cgtagcccatgtattcatcaatgtggtggaaggcggcctctgtccccaccttgtccgtccgcctcacgtgcaccccaacaatgggcttgacgaagctgag

gcggtcagcggcctcgtgcaacatgcgagcagtaccctcctgcggacgcagcaggaacttgaggaactgccccacccaccagacaggggggtcaccatgc

aagcgggcgaggcgaggtgccaggtctcgggggatggccagcggtaggtagggcggccgtggggacaatgagtccacaatgggcagctccaccacttgtg

tgtgtgacttgccgggccagtgagacctggagctgccgccggcatcggtgcacgtctcggacacaggcaggaacacctcctcccagccgcccttgtggta

ccgccagcccttggacttgaggatcatggtccgctgggtgccatacgccac

>87261|AGAP001894-PA

atactgcagttattcaaattggatggaggcaaactaggaattttcaactttctggtccaagttctagatgcatttggccccacaggttcatacacaatag

tctccattttgtctgttattctctttgtttccttacgaggtgaatgggcagtgaaacacacattcttctttagagaacatgtaacagcacttattgtttc

ttcagttgcattatcaacctctactgtcacaggtatttcttgtccagagacaaatcctgtagctggaactttagcaactaatgttaaagggccggatctg

caccagcagcagcacagagttttctcagtgacttgtgatactggttccttgaccaaaggatctgtattcagatctagatgtgataacacagtgtagaaag

ctttgactttttcatcaaatgcccatggacggttcatttttgctttgattgtgtaacgaatgtggccgtatgtgccttcaaatgacgatggttaattttc

tgggagtttgacagaaaaattatacacatgctctcctggtggaagtatgaggtcctcagatggacctccaacaaagtaatatttagtttcaaagtagttt

tcatgtgctgaatatctaatatgcttggtatggcctcttcttctcactcgtctggtccaccttacatttgcttcacctctac

>87292|AGAP001899-PA

aatcatgactgaggttgtttatgaagctataattgatgctggtgtgaatcctctaacactccgaggctctcgtactggtgtttttgtgggactgtgctt

>87294|AGAP001899-PA

tggaaagattgaggaaaaagggtgtggcagtgcatgttgctgtggataatatgtgcacaaatgatggagcaaaaagcattataaaccaggcaaataaaat

gggcactgtcattgcaatatttcaccttgctgtagtgctaagagatggcatcatagaaaatcaatcagcggaaacattccgtgaagtatgggg

>87293|AGAP001899-PA

tcatacatataaaaaaaaaacacatccaacacacacacacacacacacacacttacaggtgggtggttgcctgtctatagtttttgttgtttttgttgtt

aagcaaattagtacctcttcacacattcataggaagtaattgagactataacatgtcactgcactcagatttctgtgcacaaatttgtcatatgccaaac

aggattgatttatgcccaaatacaaatccagaagttaattctggatgaagaaacaatacatacatgccatattgttttgttatagagcacagtaatgtct

gacataattaactaatgcactataatctatcaaaaattaaaatcagactcaccagaaatgctgcaggtggaataacaatattccctcctagcacattttc

agtgtaatcccttgtttcttcctggaccatactgaattcaatagatatttcaccagaaacagtcccctctagagatgaaataactggccattcttccgaa

tggtcccattgacaaattggagatataaatggtgtgcctgccgatactggtaatggtacaggtggatatatttctgctaccttaggatctagcccacaca

gatacaatttgccaattgctgaaagaaggtattccacgccttgcttttcttgtcgccttgtgag

>87365|AGAP001913-PA

catatagtgattaggaatagtcattgtctggaacgtaacagcttttgcgtttttgaagaaagagtctggtctccacatgtttttcagccagtcaacttcc

agtaacctgtattctgatgtcatgttctctggtaagcgtaagcgaaaatctttccatgtctgagcaaaaaatatgtcagccgcatacgtcattgcatttt

cgtcaatggaatccagacccatgactgtaacgtgaaaataaacgactgttgggcttccatccttcttcggtggtcgcattttatcgtacagtttcggatc

ttctgggagaatgtcgttaaatgtgaggcttgttgacatttccttgtaatccccagcagacacggcgcccagcagcagcgccagcatcagccaaggcagc

tgtctctcccgcccggccgccattttcctgtgcggctcggcggctgctgcggcggcggcggtggtggcgtccgcagcgcgcgctcgctg

>87377|AGAP001936-PA

gggggggggaagtatgagaaattttcttctgtgatacagatatctccttaatacactgtggaaatgcatggtcttagatgtaaatggcaagatgacacca

caaactattttcaaggaattaacatctttaaaattatcatgtgtaaatggaatgctttcaaggaaattatacttttaatcacataaatatgatgaaaata

aattagcatttggcatatctatatagaacaaatacattttttacacctatgaaacatcattcaccaatatgagcaatttggtgaaaaccatacagtttca

ccattcttgtacatatattttcacttcctatgtgtattactgactagaatagatatgacacaacaaacaaatcacattcaactcataaatccagtgtacg

gacattcaaaaacacaacaaaaaattgtacgtgaacacaggcgtttcaaaaagcactcacatcacaccaactgcaatatttcaatggactgtgagtgtaa

ttgtcatttctggttttcttttcagtcacacattcgattttgcttcaaaatttgtccactcaaatcagttccttagaatgctttaccaattctcatctca

tcattcagagacgaaacacattttaatggatcattgtttatcatattctaacggataacagttttagggtcccaatgagtctgcaaattcaaaatgtcaa

aatgtacatctactttgattgttgattttttttttaatggcaaagagaaagtgtttcttccctgaaaattcacatgaacccatgagaaattttccattca

gtgactttttttctgtttgaattaaatgcacagcagaggaagatcctaaatgcttcatcaaacagtaacttatttcaaatacactcacaattgcagttag

tatactgcacagaattattttttgcagttttctccattctacccactgcatccacaccgcacagtgggtagtaaaccaatgtctgcaagtatcacaaaga

actggtcccggcaataatttttatttattctttcctcatattaaaatataagagcccattcacgagcagaaacatcaactcccctcattcaaactttgac

tgccgcaggcacaaatacttcaactatgtgagcctgctgtatctgtctgttacattctatggcacttgctttggtttgttttctgttatatgtaggcatg

aacctacatcaatatatcttacctaaaggagagagagagagagagagagagagagagagagagagaaagacaagtcctccagttattttacaagaaaaca

gttacacaatctttggaaagccacattctctctcaagttggcaaaattagtgtgcagcagggcaaactctgcggctcaagaatatgcccgctgtgggaat

aaatgctacaacagtggcacaggaagacagacttttacaggaaacggatgtaggttcggagagaggacggggcgacataattcgacgttgactcctccgc

acctgtccgggtcggcgtattggcaagaagtatggcacagcacatatgctgcaaaatttgacagtggcttatgcaaaaaattccatcttcggtgcaagct

cgtattcgatctcctcgtttttgtacatttcgatgagattctgcagttctgtctggacatcctcagcttgctttttatgcttcacgttcagcttctcacg

ttccttatcctgcttcatcttagccattcgcctctcatccatgaatttcttcgtgttgttctgcttcttctctcgtagtctgcggtctttctccccttta

gtcttcagagtcttgtcattggcaacttccttcatcgtttccaccgagatctttgcctgacgagaattcatctccttcatttccctttcgtgtttcattt

ccaattgctttatttgcatcgcctgtacggtctccatgagtttcttcaggatctcctgctgatcctgaaggtgtgtcctcagcatctcccactcttcttt

tcggtgccgctccaccatctctgaccactgcgatgtctgctcggctacgacctttcgaacttgcggatctgatgcgagttcatttttattcttgcccttg

atgagcttctcgatagcggcacactgctgcttctgcacggcgagacgttccttgagctggcgcttccgcagtgcctccagctccttctgctgcttgcgtg

ccgtcttctggaagcccttctctgctcgcagagagtcaaccgtgatcggctcaaacttcaagtcttctttcttctcctccggtgccacgccgccgcctcc

gtcaccctgcttgccggacttgctgggtttcttcccaccgccacctcccccacctccgccaccgctttcgccgc

>87404|AGAP001966-PA

ctgctatcgcactcagccaagtgtctgggatcagtgtccactggatcactcctctggtttgcatcgtctgcattttctacactatgctgggcggcctgaa

ggcggtggtgtggacggacttcctgcagagcggagtcacgctggcaggcttcatcgcggtgcttatcctgg

>87425|AGAP001968-PA

gcaggaaacaaattttatttacttgtacattccaattcaagtgtaacagcaacccataaaagaaatttgcagcaaatgatttcatatgttttaaatgttt

gtgacagatacaacttatttcttcagggtgtggagtataagatacaatcatttccacgatcagcttgtgctgacatcaagcagtgattctcgtgtcattt

tgacaagtgttgcatcaatctcttctgaaccttttggtcacttgccagatgaggaagaagctatactttcgcgaggcacaaccaatgttctgggacttcc

aaatgattatatctcttatttggggatgagcctgggacctcataaggcagatgccatgaaccatcatgatgatgctgagcaaatgggtggaatgtcaatg

gatccttaattacaactcttccattatttttaatttcaaagaagcattgatgtgaatactgccagcgcctaaaaatactgcgaagctccttgtccttcag

caggaacatgtgtgtacgcattaggtctcgtgaaacaatttttgttccaagcccactgtgtagtgctgctagtagtacaagaggatcatcctgtgataga

ttgtcagctagaaacaactgagcacaattagaaagtgattccatctgtcttttgggccagtttttcatgtgtttgcgaccaagtacaagcacttttttcc

cttgccgccagaactgttccactacaagaaaaagcagcttggcatgactgctactagaagaagagccactagcaaatgccacattcaggccatctattac

tacatcccaagttctgttcttctttaacatgtcttgaaagtgaacaacctctttaggtgatgtcttaagaaatacatcatccgatagtaaaacccggtcc

aacattgccacctgtaactgcttatattcagatggtgtgggaagctgtggtgtcaactgcttattacatgtctggcaaatgccccttttgtttatagttg

taaaatttgcttgaaatcctctgtcagctttgagcttctcagacaaagcacacactacttcgtcagtaggcttttctccgtgggttgcaataaaatgtaa

cagttcttctggatcgctgtgctgcagccaagccaaataggcctcaggacctggtcgtctatcatcccgttcaaatatttcctccatgactgagtgggca

agttcattgtttccattacgaaatgcagcaacagccaaggaagtgtaaacagcagatttaggatggtgcgtcagcttaatcatatccagtagttcaaaag

catcttgccacctcgaagtacgtgaaagtgccattgcagcactttcagatgtagtagcatctaacagaggatactgttttctcaagttgtcatatcgtga

caacagttccttttcttcataacttcctccacaagcatgccaaagcttgaagaaagcacttaatgtcgcaaggtttggtccagaagctgtggattccagg

tatgtcagcaatgatagacccacttttagattactggttctgacacactgtgacattatatatgcagccagtgttgtagatttcagtctgcctttgctga

ccagtatttcttcgcataatgacgaccactcctccagtgtaaaaggctcatctctcctcactgcattttggataaggagctcctgtggactttgttgagt

tgttattcgtgatggattaaaattgctgatagtactttcttctctgcgtccctcaacataacaggcactactaatattaacgcactttctagcaccggaa

aagaaactgctaaaatctctcgtgaacagcttttttcggatgaagtgctgcaagaccaactttgctgaccataccattgatcgtttcgacataactggaa

acacactttaacaatatatttttgatatgatattccgttaactgtattgcttttgccacataaacaatgacatttggtactttaggacacaagtcctact

tctgaacacaccacctcttcgtttgtttacaaacaaacttcgcttttaaatcgtccaattacaacattgcaaagcacagtctactgttagactgtgttgc

gaattttcatttgtg

>87528|AGAP002019-PA

gggtcattcaataagtaatacctcacatttaaataaaaataaaaaaacagccattaataaacagagacatacgtccttgtcagtgattcacatttgatgt

ttgttctgggcaccggtaaattttctccaaatggcaaatacatccttgcagcgacattggataacacattgaagctttgggactatagcaaaggaaaatg

ttt

>87543|AGAP002030-PA

tcaggatcaaaacttaatcttttccatcaagaatagaaacattatgaaaattcattttatcaaaacatgattctgaatgtactaatttcatttttgtcca

gcccgaagaaaattgtttacaatgaacgaatgagaccaggacaggcacaaatgatctgtgctgcatttagtccaggtggtttgttcctagctgcaggaag

tgcagatcaccatgtaagaatctatttaatgactgcgggtgcagaagatggaccaacaagaatattggaagtagaag

>87544|AGAP002030-PA

ctcaaaaagatcactgcactcacaatttatctattgttgattacgcagttaggataagaggatgccctctgattgatcctgctggtgtgaacaggcagca

taacttagttaaggtgctccagggtcgtgaactctcaggtcctctgaaacgccaccaggctgtctcatccaagttgtatagcaagctgcagctttattgc

cggactttaggtcatctttcagctgtatattgccttctttttgatcgaactggaagatatgttattacggtatgtatcgagcagctcaaatgtgttagtg

ataagtaactttgtagagagattttctgtcataactgtttgttttgcatgtaggtgagagaatagccaacaggtaacag

>87559|AGAP002047-PA

gtccttgcagctttgaatatatttttgctttcattgtgcagtttaaattggacaaacatttcatccaaatccttttcaagagcttccctatatttttctg

aaaattcatcacctcccattttacgcttacttcggaattggtgtattgctttatctttaacacgattatgttcggcctcaagatgagctgaactgaggta

cggctttgtgccaccacacacttcttccatgtcatgcttgtaagtgtcttttgctactgccacggcagccaagttgttaccctcagctgttgcaacaagc

atggattttggttctggcaactcgtctcctttatatatttcaatgtatgatttgaaatactgtagtaattcttttgctttcactttctgaccagcaattt

cttttggcactaaattttcaggtgctagtaacattggaatcagcctctggagttgaaccttgaagtcattctcaatatctgaaagtttaccatcaaattc

aggatgtgtggcgactttcagtcctgggtgaggcataagaaagcatgaaatatctgaaaaacacgaccttatgtgttgtcgcagtgactgcaattctggg

tgttgtttctttgaaatctgcaatctcctgtctaatattttgcgtcctccagattctccgtactctgcctcataagggaaactccagtctctaacaagga

actgcagtttttggaaaggcttcgtaccactgtcctcaagggccagtctgccatattctgtgaacaactgcagatgctgtaaatcatcttcctgtatgtt

gtgcaacaaattgtatatttgcactgaactgaccattgtgctaagtgcaaatattgttgcacagtctctcactgtactttcactgtcaaaggcaccctgg

gtatccattaataatactgctacctgttctccagttgggagatccactatgaacacttcagaccacagcaggattccagtagtatctctctcggaaccac

cacgccatgagaaaccatccagtggcatttcatctgtccccatccagtcagctcttccctgtgccttcaggtaacgcaggaaaaaatcaagcagaaaaga

ttttcctttgcggaaggctccagccacagaaacaacaactacagtcttgtctttcacatgatcttgcagtagtatttcctgaagtgctgcttcatccagt

tcaaaagtatgatcatccttagctacaactatctgcacgggtcttgctcccatcttttcttttcttcccctgatgttactatctccggcagcagcttcat

gcgatctctcctttgtatttgaatgtttacgtcctttactgtcagccatactgttctaattccaacgtcatatccttcctcggaggcgggagtggtaatt

tgcgcgttgttcatacaaaattgagatattcagacatgatggaaaacatgtaaattctgaggttacctatatctaggtttcaagaacgtgtccttgaact

ttgcgtcttttgtttctttc

>87558|AGAP002047-PA

gtccttgcagctttgaatatatttttgctttcattgtgcagtttaaattggacaaacatttcatccaaatccttttcaagagcttccctatatttttctg

aaaattcatcacctcccattttacgcttacttcggaattggtgtattgctttatctttaacacgattatgttcggcctcaagatgagctgaactgaggta

cggctttgtgccaccacacacttcttccatgtcatgcttgtaagtgtcttttgctactgccacggcagccaagttgttaccctcagctgttgcaacaagc

atggattttggttctggcaactcgtctcctttatatatttcaatgtatgatttgaaatactgtagtaattcttttgctttcactttctgaccagcaattt

cttttggcactaaattttcaggtgctagtaacattggaatcagcctctggagttgaaccttgaagtcattctcaatatctgaaagtttaccatcaaattc

aggatgtgtggcgactttcagtcctgggtgaggcataagaaagcatgaaatatctgaaaaacacgaccttatgtgttgtcgcagtgactgcaattctggg

tgttgtttctttgaaatctgcaatctcctgtctaatattttgcgtcctccagattctccgtactctgcctcataagggaaactccagtctctaacaagga

actgcagtttttggaaaggcttcgtaccactgtcctcaagggccagtctgccatattctgtgaacaactgcagatgctgtaaatcatcttcctgtatgtt

gtgcaacaaattgtatatttgcactgaactgaccattgtgctaagtgcaaatattgttgcacagtctctcactgtactttcactgtcaaaggcaccctgg

gtatccattaataatactgctacctgttctccagttgggagatccactatgaacacttcagaccacagcaggattccagtagtatctctctcggaaccac

cacgccatgagaaaccatccagtggcatttcatctgtccccatccagtcagctcttccctgtgccttcaggtaacgcaggaaaaaatcaagcagaaaaga

ttttcctttgcggaaggctccagccacagaaacaacaactacagtcttgtctttcacatgatcttgcagtagtatttcctgaagtgctgcttcatccagt

tcaaaagtatgatcatccttagctacaactatctgcacgggtcttgctcccatcttttcttttcttcccctgatgttactatctccggcagcagctgatg

cttgctgagtagacgaccgctgggcaggtaatcgctgagtagacactttctcaactgctggatgtgcagcaggcactggttcttgctgggcaggcacagg

tacttgctgagtagtaacaggtgcttgctgagcatatattcgtggggcaggcacaggtacttgcttaacaggcacagatcctttctgagcagatacctgc

tgggtatccacaggaacttgttgagcagatactggctcggcaggcgcagatacttgctgggtagccataggagcttgctgagcaggtacttgctgggtat

ccacaggagcttgctgagcaggtacttgctgggtagccacagttccctgctgggcaggggtcacttgccgacccgaactctgccgtctggccggtcggcg

aggccctctgcgccccggtgtcggggattcgttactacgcctgtcacccgtcattgcttaacttgcaagcagcttcgcaacgctagccaccacttgcgct

gtgctggag

>87615|AGAP002074-PA

tcaaaataccataacgttggctggtatactagatctactcctacaccagatcttttagatttctgaactttggataactctttaccgtaaacagttcgct

>87614|AGAP002074-PA

aagccaagaagatcaaaataccataacgttggctggtatacttggtctactcttacaccagatcttctacatttctgaactttggataactcttttcggt

aaacagttcgcaacga

>87616|AGAP002074-PA

aatttgagtacttacttcatcgatctcgcatccatcctcgtcatccaagtttgacatagacccacaaggagtatcttgattgccaataaaatcaaacaaa

tctaagtaccacaattttgatgtgtagacattgtctgttgaagctccagatttcattgacatataatgtttcttcttttctttacgtacattacttctta

aattatttat

>87659|AGAP002078-PA

agcagttattattattgttgtactgctacaaattattagattgttgtaaaagacgtctccaggactataacgagtcataacgtctcaaaattgatgaaaa

tgcgtcctaaaataagctcccttcgttgtctcataccattgttatgtatactgtctgatgttttggattccggaagagaaaaaccggtacattaactttg

taatcatttatttgatggtttcaggagaattagtaacatccagtagcggacactaatgacagcatcagagaagcttgcttccgttcctgatgtggatatc

gacccagaaggaacatttaagtatgttctaatacgtgtctttggtccagagggtcctgatggcaaagaaccaagcaaattggtggtgcgaggatacaagc

gagctggcttccattcggacgtttacgatgagacgatggacagggtgaaaggccttggcgttgatacagagtgcactggtggtggtagaatacagcacga

ccccagtgccaaggtcatcaaag

>87660|AGAP002078-PA

gtcggtattattaaaatccactgctccggtatgtcttcatttattaaaatacaatctctgaaaagcagtttatttggaggcgtacgaaatccagtgctgt

gtttatctaggacactaatgacagcatcagagaagcttgcttccgttcctgatgtggatatcgacccagaaggaacatttaagtatgttctaatacgtgt

ctttggtccagagggtcctgatggcaaagaaccaagcaaattggtggtgcgaggatacaagcgagctggcttccattcggacgtttacgatgagacgatg

gacagggtgaaaggccttggcgttgatacagagtgcactggtggtggtagaatacagcacggccccagtgccaaggtcatcaaagtgtacggctactcac

aggggtttgggaaggcagaccacagcaagactgtagaaattctgaagactgcatatcctgattacaacattacttggagtgatgaaggatactgatctgc

tcaggttgtaatatttgacattgcatcttttgaaaatggagcaggatgttgtgccctgtttcctattggagtgtttctctctgatcagaactggaggtgg

aaaattcacttgaacttccattgatgtctttgaatcatgttgtttcattgtgattaataaaaaa

>87905|AGAP002117-PA

gaagaatttgataaagaaatttgacggcatcatgtattatctgagtagttcaacaggtatcttgcccgtaagagtagatctactttgtggtttctgctga

gcctgttacggaagcaggagcagtagcagtgggagtagagaacagtcagctttcaaaatactggttacgtggctgtgtaaccggtgtcggaagaacaata

ataatgattaaacagattcaaatacgctaagagcgcaagagatgtcgtctaacaaatcattgtatattggttttccttacctcgagtaacctcgtgatgg

agaagagggagcagagggcccgtcgcgacagacgaagagatggcaagcgtaatacttttcagaatgatcaaggaaaagaaacagtcaagccgcagcagtc

ttctgtcgaggaaaagaaaattgatgctcaaaatgtagaagtatcgaataaaaagaatacgtcagaagagaaaggcaagaccattgaaggtgatgtacaa

tacagatgcaaaaactttaaaaaggaaaagctgtgcaatattttatgtttgagattatttgtatatataattttcagatgatcctctgagatttgcaaac

ttgaaacgagaaacagatgaaattgctgcgctaaaagcgaaaaagccacttaagagacaaattttcagcaactggtcgaaatatgaagagccattgacaa

gtcaagacgaaagtcgagccgtcgactttgaaaccctcctccatgcaccacagtcaggaggtttcaggtttaaatctgagcagcaatgggatactggaag

ttacggacgctttggagaaattttcacaccagatgccgaattgcttgctggaggtttgacttcactaccattctacaagaggctcaacgtagataaaaca

ttgttttcagaggatgagatccaaaggatggagagatgtgcaaaggaaaattacagagcatatgagaaattgagtaagtccaaaccaaaaccaaaagtag

ctgccactgacttgttcaagattgatcaggcagagaagtacagtgttgtggatcaggtaaacgaacagactgtagaactgagtacaaacacaagcaaacc

tgtattttcagtagaagagtccccagagagaacaccagcagaacttacactctcggcacatcctgccagcagcaatgcgagaccaaatgaccacagcagg

gagattagggtacccactgcacatgttcctgaagggcctgtgcaagattttcacttcggaaagccaccaccaccatcatcctcactcggtgctgctgaaa

acttgcactcacagacacccgacagagagcgtacccctctcagagacagtggccgagatgtgacatcaccagagttccattttgagagagcaccaccacc

ttcatacagctcagggaatctgaagtctccatttcacagtagggagagcacgccggcaaaagaagctgcacctgctacagttgtcagaaagccaccaccg

tcctcattcggtgcagctgaaaacttgcactcacagacacccgacagaatgcatacacctccccgagacagtggccggtgtgcagtgtcaccagagttcc

atttcgagagatcaccaccaccttcgtatgcatctgcgaatatgaagtctccatttcagagcagggagagcacaccggcaaaagaaactgcacctgtgac

agtgtctcgaggctcgggcagcttgcaagctcctgtgtcttggtctgtctccgacggatgggtcacaggtcgagaaggtacacccgagcccacagctcca

cctccacctgtccaggagagggaaacagtgagagacgaccgtccgggtgctgcaaagttcacttctacagtcggcacgccatctgaggacgtttctgtca

gaggtgagacatcggtcggggaacacgatgacgatagtgccttgcgagagacctccccggaacggaaagctacgtacgaccaccaacgaatcacgccgtc

tgcctctcgggaatcgaaacacacactcgacaaaattccttcagaaatggaaacagttggaagggaagagattccgagggagccagttcttcagaggaat

ggtgcagatgtgcgggaagatctgacacgtcagaaatctcctcgtgaaagagacatagtttcagagcagaagttggaggaaaatgggcagatttgcacag

aaaatgacagttattcgaagaaaggaggaaagtcggatactaaaaaaccggagggccagcagagaggtggagggaggcacggtcggagaaagaaagggca

gaatgcatctgctcgaggtg

>87904|AGAP002117-PA

cgtcgattttacttcattcttcatttatattttagttacataaattacaatttatgttataaaatgggtgacacacaaaaatgattaatttaataataac

aataacagtagacaaattgttatgttatacaaagcttttatatcttgctcaaatctgatattgcaaattttattttcagcactgaattaaatacagaaag

tgctaagacaaataaataagcacagatacattcttccataaattacacataaggtaatggtgttacagtaacgtatatcataccatattagctgtaaacg

tatatacatagattttcatgagcagaagaccttagagacataaggtcgacaaaattaaatggctcaaaataaaacattttttttacagcatagttattat

atgaaaaggagctgaaagttaaggctgtaaataaagcaaagaaagcaacacgcgtagagttcattattactgcgttatgggcaggtgtgccgagtatctg

cccacgtcagtcgtccagcatcgagtcgagccaatcttcgaggtcctctttcttttctgcagctatcactggtggaggctttggcttttgtgactctttc

atttcatcttcctctatcgcagatcttgatactgtggcacccgcagccaactttgccgcttccacttcactgacccctcccaccttctggcctctcagtg

acatcagaaaattcagtccactgtcgtccacacggcttgccgtgttccgctctttcagtacgggctccactttcgtacttgctgctgtctttctctgggg

agattctgcttggctgtcagttgttgcaggcactttcacttgttgagcctttggttcctcaggtttgttggccaaaggctgtgtagctttggattcgggc

tgtccaacaacagccggagcagaattcggctgtggtgccggcagaggtgactgctgtcgatcccgctgtagcactgccggaggaggcggtcgctcaggtt

tctttttcgccccttcggctggacgcggttcctcgtgcctcgtctcctcctgcacgtcggctttcacggtggtgtggacactggcccccacctccggttc

tgccacatcaccagactccacccggatcggctccccacctcgagcagatgcattctgccctttctttctccgaccgtgcctccctccacctctctgctgg

ccctccggttttttagtatccgactttcctcctttcttcgaataactgtcattttctgtgcaaatctgcccattttcctccaacttctgctctgaaacta

tgtctctttcacgaggagatttctgacgtgtcagatcttcccgcacatctgcaccattcctctgaagaactggctccctcggaatctcttcccttccaac

tgtttccatttctgaaggaattttgtcgagtgtgtgtttcgattcccgagaggcagacggcgtgattcgttggtggtcgtacgtagctttccgttccggg

gaggtctctcgcaaggcactatcgtcatcgtgttccccgaccgatgtctcacctctgacagaaacgtcctcagatggcgtgccgactgtagaagtgaact

ttgcagcacccggacggtcgtctctcactgtttccctctcctggacaggtggaggtggagctgtgggctcgggtgtaccttctcgacctgtgacccatcc

gtcggagacagaccaagacacaggagcttgcaagctgcccgagcctcgagacactgtcacaggtgcagtttcttttgccggtgtgctctccctgctctga

aatggagacttcatattcgcagatgcatacgaaggtggtggtgatctctcgaaatggaactctggtgacactgcacaccggccactgtctcggggaggtg

tatgcattctgtcgggtgtctgtgagtgcaagttttcagctgcaccgaatgaggacggtggtggctttctgacaactgtagcaggtgcagcttcttttgc

cggcgtgctctccctactgtgaaatggagacttcagattccctgagctgtatgaaggtggtggtgctctctcaaaatggaactctggtgatgtcacatct

cggccactgtctctgagaggggtacgctctctgttgggtgtctgtgagtgcaagttttcagcagcaccgagtgaggatgatggtggtggtggctttccga

agtgaaaatcttgcacaggcccttcaggaacatgtgcagtgggtaccctaatctccctgctgtggtcatttggtctcgcattgctgctggcaggatgtgc

cgagagtgtaagttctgctggtgttctctctggggactcttctactgaaaatacaggtttgcttgtgtttgtactcagttctacagtctgttcgtttacc

tgatccacaacactgtacttctctgcctgatcaatcttgaacaagtcagtggcagctacttttggttttggtttggacttactcaatttctcatatgctc

tgtaattttcctttgcacatctctccatcctttggatctcatcctctgaaaacaatgttttatctacgttgagcctcttgtagaatggtagtgaagtcaa

acctccagcaagcaattcggcatctggtgtgaaaatttctccaaagcgtccgtaacttccagtatcccattgctgctcagatttaaacctgaaacctcct

gactgtggtgcatggaggagggtttcaaagtcgacggctcgactttcgtcttgacttgtcaatggctcttcatatttcgaccagttgctgaaaatttgtc

tcttaagtggctttttcgcttttagcgcagcaatttcatctgtttctcgtttcaagtttgcaaatctcagaggatcatcttcaatggtcttgcctttctc

ttctgacgtattctttttattcgatacttctacattttgagcatcaattttcttttcctcgacagaagactgctgcggcttgactgtttcttttccttga

tcattctgaaaagtattacgcttgccatctcttcgtctgtcgcgacgggccctctgctccctcttctccatcacgaggttactcgaggtaaggaaaacca

atatacaatgatttgttagacgacatctcttgcgctcttagcgtatttgaatctgtttaatcattattattgttcttccgacaccggttacacagccacg

taaccagtattttgaaagctgactgttctctactcccactgctactgctcctgcttccgtaac

>87933|AGAP002119-PA

cgaggaacttctcgccattcttgatgatgtaatcgtggttgtcatcgtcatagccgtcgttgtacagcttgcgctctttcttgtgcgaagagtcatcgcc

ctgcgtctgctgcgcccgtcgtttcttcttcgcgtagtacacctcgttgatgtgct

>87934|AGAP002119-PA

acatgaagtgcctcttcagtttcactatataatacttgttgtctgcatctgctttgttcatcatttccagcagctttacctctatctgtgcctgattgag

gaaaggcttcttatttttaataattttaatggcgacttgacactgctcctcgtggtcatacgctttgacaaccttaaataagagaaatgactatttgtag

cacatgaaaggctctttggtttcacatttgg

>87955|AGAP002120-PA

ttggctttcgtgtggaggaagaagatggtccagaacagagttcaaagaaactattaagcattccatttgtagaggatccacagcagaggttgcaatgggt

agctcatttagaatttagtcacaataaggaagtatgtgatctgacatgggacaaagtagatgtcacacttccccgaacagagaaac

>87971|AGAP002124-PA

gcggtgacgacgacggagagggcgagcgcgatgtagttgatgacgagcgccgaggcgcggtagctctcctggtgcaggcggcagtcgcgcagcaggttca

gcgacaggaagagcacgcccatcacgccctggaggacgatggagggggagatgagcccgaccatgagcgagtagaactcgtgcttgctgcccgcctgcag

cacgtacttcagctggctggcgttggcggtgagcagcgcgatgtccagcatgccctgcgcgatcgtcttcttcgtcgcatatcggttggcgtccagcgtt

ttcggcatgccgggttccggggcgatggccgctgtggacgacatggtggctgccgtggttgcgcagagccgcgccgac

>87984|AGAP002135-PB

ctagatgaacagaaattggaatattttattataaccaaaatgttaaagatacaaacaattacacaacgctatgtatgcgttcattttcattctgcctcag

tcattttcctttcatccacatgagtctttccttttctgtatgtattaccactactttgttcgcgtactcttgcctggaaaggacttacatgaccaaccgc

tgtactcgtagtttagtcagtcagccctctcttcattcatacttatcaactcttctcttcctttccatcccttctgcgagcttcctcagtaggtacgaga

ttccacatcgttgagagtcacattcacagatgattgttctgtcttattagtattagcttgaagtcgcctatggagtatgtgaacagcgacggttgctgtg

ctgttgccttcagatggagtgcaggtgtagtttccagcatccaccagctggatgttgttgatcaccaaggtgctcttcaaagctgcatctctctctacga

cggtggtgatgcgttcgttgttatggaggtggacggtctcatctccattatctcgcgtccactcgatgttgacctccgatgttggactggggtcgatgac

acacatcaggctgacggagtcaccatccacaactgacctggtacgctcacccaaaatctccggctcgacgacagtgaaatgtatcgtgtgtcggctgttt

gccacaccggggagataacagaagtagatgcccgaatccttctttagcactgaacttatcctcatagcccttttaccagactgtaagttcctcatgctgc

ggaagcggtgtaacgtcttcctgtagcttgtgtccgtcacgagttcatcccgactccaatgcaccaatgaactgccaatgttgggcagactacacgagag

gtaggcagtgctgccgagaaccgccgtcaccacggaagacgagttttctgcgctctgcccgccagaagagccgtcagacccgatgacagagagatatacc

gagtgttccttggtcggtatgccagggacaaagcaggcatagacgccggagtcattgaactgcacggacagtatctgcagagacctctcaccagttcgta

cgtcaattatattgcggaaacgcttgtcagtggtgttgtagcttccatgtgtcaaattgtccttccgcctccatgccacctgtgaaactttgtccttgaa

ggacagggggcaccggaggttcatagtgctgccggcgatagctgtcattacggaagagtagttgctgctggagacaccgacttccgtgttctgtcccaca

gaagagcagtccgcgaagctcagtgaccctgtgaccacgagggcagcaaaagcgagaaacgtgctggaaatgcgggccatggcggtgagttgcgagtgac

gcatcttgttg

>88020|AGAP002176-PA

ggacatccagcacctgacggtgctcacgtcgaagcgcaactcgctgttcgacgcgaagcggcgcttccactggacgatgaacggcgtgggcctcgagttc

aaccacctgttcggcttcggcgtgctggacgctggcgccatggtggcgctggccaagcagtggcgcaccgtgcccgccaggttccactgcgagggcgggg

ccgtcaacgaagcgcacgagatcccgtcg

>88051|AGAP002181-PA

tttttttagcatatgttggcgtcccattgaactatacgtaaaacgaatttaatcctcgtttatataacttaatagcgcccttcgattacaagtgagtggg

taagacagtgtcatcgataaaatattgcaggagtcagtgatgtaaaaactacgttttacgtaagactgtagtagtatacgtcagtttgtcagaggcaact

ccgtttcctaaacagtactacttattaaaatgttcatttccgtgacagtttgtggcgagacaccgtcttccctaaattaaaatgttgattttaaaactat

agtttaacgatcgtgcacggcaaggtctgagaggcccgcccccgcccctgcctccggcccctaggcagtttcggcgcctggccgcgacctcaggagtgtg

atgtaggtgcgcatgatgctctcgatgcgcacgcggtagtcggggcccagcttgcgcgcccactcgctgatgcgctgcttggacagcgactcgctgccct

ccatgctgggcacgttctgcgggctgatcatcatcgccggcatcagccagaagcccatcagcagcccccagagcgcgtggcgttgcacctccgaatgcag

atggtccagagtgatgtggtccgcggcgccggccgcgtcgtgctgcagctgctggtgataggcgcccaccaggtcctgcaggtgttcgctgaggacggca

ctctgcacggtcgtgaacagcaggtacatcacgtcggtagccgccgatgcgtaccgcagcacctgcatgtcgaggaagcgcaggtcctgcggccgccgct

gctcgtcgtacaggaagagcatgttgttggcgctgctgtcgccgtggcagacgacggcggtggcctcgtcgacgtcggcgagcagcagccgcagccggtc

gtacagctggtcgtgcagcgggcgcacggcggccacggcggcgcgcagcggctcgtcccactggcgttcgtcgctcagcgactccacggccatgaggaag

gcgttgtccaccgagtcgctcgtcagcaggcgcgccgccgtcgcgaacgtcaggtccggctgcagcatccgcctcagccgctccgccgtctccgggctgt

ccctcttcaggaccaacgttgtcgcgtggaactgggccagtttcctgatcgccagctcggcgtgcggcaggtcgaggccgaggcggcggtcggccatgcg

gaagccgagcggccgcaggtcctgcagcacgacgacgccggcgggcgaggcgaacaggcagcgcgggaagagcgcggcagcggtggcgcccagccgagga

accacctcgccgtagccgcacgcctcgcggctgaacgcctgcgcgcagcggaacagccgacgccgcttcgcgtccgccggccacagcttcaccaccagct

gctcctcgaacgcctcttgtcgtcccgttttgccacgcactacggccctgtacacagtgctcatgtagttgtccccgggcccgctgcccggagcgacatc

gactttgtccacgacgagtccgtcgtcccggaggtggctccgcagcacaccctggaggaagggttcgtcgattgcaggaagcggctggtccacctcctcc

tcctgcccttccccgggcacttgttgctgcggcagctgctgttccgcatcggccatgctacggactggggaaggctgtgccacccacgttcgctcgctcg

cttgctcgccgactgcagagtcggcacctacctgccccctgtcaccggtgtcagccttttataaacacgagttcaccgccagcagtgcagcctgcgactc

ggacgttagccgctaggagcgatgcgagggacggcgctgccgctgtgac

>88065|AGAP002194-PA

ccttttttcacttgaccattttcttgccagaaatgcttcaaatccagttgctcgagtgagtcttgcaattgtaagtataagtctccgtcttcgcaggtga

cgtggcccttgtattctgggacgtagggcctgagcacgtcattcatgagcacctggaagcacagctcctctttggggcaaagcttcttcaggatcgtgcc

ttgctcgggaccggctttgaagtttccttgatggccagcaagctgtacccaggcatagcgctgtttcttgtacgtttggaagaacggagtccactgaacg

acgttccgtagttttctccaaccggaaggctttgttccctgggcatcggcagcagtcgaatccgtcagcggcgtgtctgaatcccactccgagctgaggt

cgtcactggaggcttcaccgtagtcgtgtgggcagggcacgcccagcagagactgctggtacatggtgtaaccgggcagcccacccccgatatagactgt

gttctgctcctctgtctcccagggtggaggcggatcatctgggggtggcggctctacagtcggtggagggggcgacggtttgg

>88066|AGAP002194-PA

agtctccgtcttcgcaggtgacgtggcccttgtattctgggacgtagggcctgagcacgtcattcatgagcacctggaagcacagctcctctttggggca

aagcttcttcaggatcgtgccttgctcgggaccggctttgaagtttccttgatggccagcaagctgtacccaggcatagcgctgtttcttgtacgtttgg

aagaacggagtccactgaacgacgttccgtagttttctccaaccggaaggcaccttgacgataatcttgccgagcgtcatcttcttgcggaaagacgcga

cggccgagaaagaatctgaagaagcgtcagtcgacgagtggaacatctcgaggagcatgttagcggtagacagatcggcgcacacgagagagaactgcga

gacgtagtcactcacacgctgggtaacacttaagatcgcacacgagagctgttctcactggagcggcagctagcagcgaaagacgcccggccacacgcag

cggatactctttaagacgcggccaggctacccccccaccaccacaccgccccgcgcacgtcacatcgccgcgcaagcgcagagggacttcctgcgcgcag

tgacgcttgcggctgatgtagccg

>88080|AGAP002194-PB

gtttggaagaacggagtccactgaacgacgttccgtagttttctccaaccggaaggctgcgaagttccatcggcctttgctagtgggattttgcgtcgca

cattctcagatgcggaaccgac

>88081|AGAP002194-PB

cgattgccgtgccgctgggcaggatcagtgtcttggcaaaatctatgagccacacgtttgcgttgtgcttgtcatgcacaaacaacagggaacttccgat

cacctcatgcgaagagaagaattcagaaacttccaaggtggccctgattgctttcaacctctgaatgtattttggctgtaacaaaaaataaaacctataa

gtaacagggtttcatacaatgtaggaatgtttcattatgcaaaacatttattatccctatgtatgcaaatgtgttacgacaaacataagttgaacacatt

caattaatgtgagaattgaacagactttattcttcttcactgttacagttcactacggaaccagtttttcaattaaattaacagcctggtcaaagaacaa

caattcattttaggccagttacaataacttaaggatgggaagtaaaccttcagtaccttaatccattcaaacctgcccactgttgggaaccaaggatggt

aagtcagccttccccattacacattcctagtccttggtgagtttgttggttggtcttggtcatcacgtatttcatctcttatgatctttgcagactgaca

acaagctggcaatatttcagtctctgcatctcccaccaaattacaggcagggtacgcatttgaggtgttcaagacaggagacagttgtggtatcacttta

tgccgttaactttaatttttgcaattcgcttcataaagagagggatgccaggacactgaagtgtaacatctgcatctgttcggaagacacttgtttgact

cagatggtgtcaatattgaccatcagtgacaacagtacttcactagctttcagtctttaactaacagtgattttctccccccatctttgattttaggtga

tcgtttagagttgcgtctcccccc

>88184|AGAP002215-PA

tgcctgtgatttttctcccagcatttttcccagcagactacgcagaatgaagtttatacacttgcgagagtaaacagcatcaacatgtgatgaagctgct

ttagggtttgcaaccaaatcaagaatatgtgcaaggagagtaggcaagtttcgctccaaccacacactgcccattgtctgaacaaagaccacatatgcat

gtgtcacaccaacacgcacttctctgttcactcctgaacttcctttaatcatttcaccagtgcctttcagaaaaccactgccaccacgaagaaagcccgt

cataagaactccaagtgcttcttccagtgacagtggtttaataccct

>88234|AGAP002223-PA

acgacattaccaatcttttctacacgctcatcagcaactcgctcggcaacaccaatggcagacaatctcctcggttgtgtgcagataatttcgacaagat

ggcgaccacttcctttgtgccgaagagtcaaccactcatccaacaggaactggggaacctgtgtgctcttcccacatcccgtttcaccactgatcacgac

gacctggtgtttggcaacagcagcaagtatttcctcctttttgctccatgccggaagcgatttccttccctctaggaacttaacgtactgcgactgtttc

tgcttttctacaaatcgccgagaaatgcgctcatcttccctcaatctggctgcaaggtctctgttctgacgatctgttttctgatatccatccccattaa

tcagaccattttcatttactgacattccttgctcactggggttcatccctgtatcaaatgtttcctgttttggaaacaagggctgcaatgggtctaagaa

gaattcgtaacttcctgctagcatctctatcatatcactgctctgatcgaccagcacctgggccagactgtagatgtagggagcctggaactgcgcaagt

tccttggacaactcacacagtttgccactcagccggagagaagcagtcaatggaaagcgtgtcgatgtcgtgctgagacacacgagggcgggctccagag

ggtaccgagatccttcctgaaagcgcacctccaattggaagaggtgtttctcctcttcgttctcaggagaaggcttctctggcacagcaacatcatggga

aaatctgcacttattttgaaatctgcagaagcctttttgaaaatagctgcaaggtttcgctgatgaattcttggtccttctgcttccacctccatgggtt

gtgttggagccccctccaccttcatcttcatcagaaggtggctttttcttggcatagagcctatgtaagtagtcgagttgcagttccaacacccacacac

gattcacaacacgttccttgaagctcccctcgtatattgattcaagcgaagacttttcctcggcacgctgcgtcgctatgtactccgcatcctccggcga

tagtggatcgcagtttaattcactctttcctgttgcactggacggcaaggcaatgttggaggagaaggtaatcggcatctgaaaatactgcgacagcaat

agctccagagccaagcctacatcccccttgctgatctccagggcttctacacagtggttcttctcaaagccatacccctcgagtcttcccagagcaaaaa

tgttggcgatagtttgtgcatcttgcttttgctttggtctggcggagtagtcgacgccaccttgaactctgagttggcctcgatccatccaatactggca

actcagccttccccctttgtcccggtatgatgactggtcacgcagcgtgaagttcggaccccgcagcttcctgagtgcctccaatactatactctcagtc

tcactatcgagatacagtgtctgcatttccaccttaagctgtgtcgatttggggggaactccaacagtagaactgctggaaaatcgctctggttttgtac

ttggacgatcatcatattcgtcatactgacccgcttgcgtgtcatcagtagcatttttgtcggtcatcttaattgaaaatatatttgttattaatacatt

tattaacgtaagtatctttcatgcatgccggcaacagacgtgttctcaatacccaacaataccactagtgcaccacactgatatcttctgtccacttctg

tcttctgtttcagaacaatacactttcgtatgtcgggg

>88274|AGAP002226-PA

aaattgctgttttgtaacttgtctgtcagttttcttgccagagtgctgggatggggcagctcttgtgatgatgacagcaatctgcctcaacttctggagc

gatat

>88272|AGAP002226-PA

caccgttggtatgcaacatcccatgctcgccccacttattgcaatgtctgcagagaagctttgtcaggcgtcatgtcgcatggtctcagctgtgaggtgt

gtaaattcaaagttcataaaagatgtgcagtcaaggccatcagtaactgcaagtggaccactctctccagtgttgggaaggacattatcgaagatatgga

tggaaatctcacaatgccacaccagtggatggaaggaaatctgcccgtttcagcaaaatgtgcagtttgtgaacgaacatgtggttcagtattaagattg

c

>88273|AGAP002226-PA

ctcctgtctttcttagtacaatttgagttactttactgaaaccgaggtacttaccttagtgtcttttgcgtagtaaagtttgcggcccttgagcttgaag

tagcgcctccgccaccgctggaaggaccacgtctgcttcatcaggaagccttctttcgttgccgcggatgacttgatctctttgttcgttgatagcctgc

gatggaacgatttagctggttcggtttctccatcactttctgacgattcttcgcctgtttgctcctgaccaactctttgcgttgcacagacgcccctccg

accgccagacgccatacttctttcg

>88292|AGAP002252-PA

caataatgacggagatgccaatgtttatgtttacaaagttggccatgcgatggattgttatgtgttgtgtttgagtgtacaagctgtttcacagtcatag

ttaataatggatgataaaataaactcagcatttatttatgctgaatcattgccacaatgtatatagcagtaactggacatttgtatgtatgtcccgatga

aggatgttaacgttgttaagtcaagctcgattctggacactgaaagatgaaatagttattaaagacacagacttcggtgctttcaggtatttagaaaaaa

aggacgtgtctgattcgccctacaatgaagggaagcgttcatttgcggaacaaagaggaatcacatgcgtggttcgaaataaaagaccaaacaagttaag

agagtcaattaaagatttagaggaacatttgattcagcataaagtgatctattgtcactggagggacaacagcctcttgcaactgattttcagctctggg

cttattgctaaaatatgtataaacatttttactggtgacatactaaaaatttcatttgacaagtatcttgttggaaaacttctttctgagtatattgctg

atgctattttcacaaaaacgcacttaattttttcatacaatgataaccaggtaacacttgtttactttgtgaaaccactaacaaactgcaatgtatcaga

aaaaattagtcgtttggaaccaaaagtacaaaatctggagttggctggaccatctggccggcgcttggagagaaaattgtcatcaaatgttaatggagac

atgatacttatttggtggcggtgcactcgtgatgaagtttatccgtggagtcccatcgttaaagatcaggacagagcaaatgttcatgtgtatatggtca

ctgggacaaaattcgagctgctgtgctactatagaacagaatttgatccagtcagtgtatctttcagctgtatacagccaaatgtcattcattcagtaga

gcagaaggtgtcaagaaaaggagaagtgacagtggagagctgcacgtacgagataagtaaatcacgtttacagcgaacagcagttacttctgtccctcta

cagactcaagtttgctgccacagcttcagtccggatgaagagaaacttttgcttggctgtattgatggctccctagtgctgtttgatgagggaagaggag

ttacacatacagtaaaggcagcatttatatccacaacagtatcttggcaccctgatggcatgttagttgtagttgccaatgaaaggtgccagctacagtg

tttcgacacagcactgggctgtatcaggaatcaattggtgagtgaagatattacaccatcgagtttgttggacctcggatcctactttaggactcagcca

acccttctgcacatggaatggaataagaagaccgatgtcagccaatatgcagaaaacttttgccagacagatgggttccttttacttcagttcgaaaggg

gaccactagctgttgttcagttggttgggggtggtggccttattggagatcttcatggaagtggtctcactgcagacactatggttgcaaagtacctgat

gttcaatcaggttgaccgggcgatcaatttgttgctaagtctcaactgggattcgtatggtcagcagtgtatttcttgtctcaaccgtatcattaattac

ctcttccggcaaccactcacaccagaaagagaagtgcagcttcaaacagctttaggcagctttcttttgcctccacggcccctttgccatgctacggagg

aggagtttggaagcactgtacgtgatttggcaagacgtttctttcaccacctgctgcgccatcggctacttgataaggctttcagactggccattgactt

ggatgatggtgatctgttttctgatatatggcactgtgccaaaggaatgggtgagaccgttatagctgatgctgcccgcgaacgtgcacgtctgatattt

agccgctgtagcagccggtcgagctcacactcagattcatctcagtgttctcgttcctgttgttctggatgctctggttgtgatagccgtgatacaacga

gtgacgactcaccggaaccaccacctcagccaccacctcctcttccagtaccaagacagcaagggagttctgtgagaacaccgggagagaaaccaaaagt

gaagttctctgacactgtcactcacattcttgtgcctgattcaagaccatcattgacagaagaggaggatttttgcctgaatgtgaaaccatctggcagt

gctttacaattgcagaaagaccactccaagagacagcatgtttacattccagacacgcagcaggaacttgctgacagtctgccactgtgccttggcaaca

aagactacttgaaagatttttcaaccagtgcagatatatctgaggaagaggacaatggaaatggcaccataaaagttgtccattttggtgttgtataaga

aaaaaaaaccccctcttgtttggattttcaggccaacagttcaagtgccttaccagtgtgcagttgtagccaagtggctttatgatgtgccttactcatc

tgtgtgccaaagtaaatgtttcatttattcagtattttgcacaatatatttatagaaatgatttttatttatcccttctcactgcattttccaaataatg

tgaatatgctgttcttggtgatattatcaatgctctaggtactgtggataaccactcacttctcttgttgtggtattaattcactaaatgtacatctagt

atacacctgacaagtactgtgtttattctgtgcggagtatacctttacaaacggactgatagatctaatgtaggagccttatagcccagggatgaacaag

ttatttgtttttgcatattactgatgcatatctgttgtgagatgttataactctggtacttaccaatgccaacatttattgatgtgaatcccagaggtcc

aattttaacctccacatatctactgaaaataactcgtgtcaactgtgaataatttctatctgaactgcagagtttggagtactttaaagaaaatatgtct

ctagtgcactttgattaatcgttatactcgttgaaaatacttccctcattttatccctactgtatggagcaaatattgcttctctttcctactgttttta

actgaaagcattaaaaaatgctgtctaaatttaatatttatacttggacagaagctattaaccctttcgtgttgctcattcactgtggacgagccctggc

ccactgcacagcgcatttttcgcgctccccgcctttactctgccgtgctttcatactcatagtgttattgcagttattggactgtatactagatatttgt

ttgggaaaatatttattcaaatcacacgtttttgatattcattttgaataaaaacagttttcttggagttttcagataaatggcataattttttcttacg

ttctataagatctgtgttttgattcagctctggtggggtgggtgctttattatttgaaacataaattgaatagtgtttttgttttcactacttcaataat

atttatttataattggtttgcattacattccagtaggattacattatgagaatatcattgcactgcgttacatttcaagagtcttttgtgtatggtaaat

catacagcagggaaaggcgcatagtgtcgtattgcattccttacaacacatttcagtctgcttccgcttcttgtgcactctgcagacaagcaagttctgt

agctgctccaatcgtcaggtaggtgaggccctgctcccagtctcaagggacatgctgatagttttcctctgctggttcttcttttttctactttatattt

ttccaggattccctgtatgactaccatttgaaagttggcatagctaatgttcgttacaagtttcttatataagaagtaagcattgaaaatggacatgtgc

acgatgtgaataaacagtttgtgatatcatttagtggattttcttatgctttctaaggtctttagtactgtgccacttttattctcagatcccattgatt

taatgtagtctaatactactgaatgtttcaattgcttctagcgtgttggacgatccatcttcaatgttt

>88321|AGAP002256-PA

ttgaactttgaattgtaatgtgtgtctgtactgtagtctgttgttcatgtcggaatatggttgtataaacagactatatttcactgcccgaacgctttgg

ctgatatcaaaatattgttgttaatattgacacgcaatagaagaagcttttctgtgtagtcaactgttagcactttgtctgtacacggtgaagttgatga

aatataacctctctgtcacataatgagtgaagatcaaagaacagtgttttgtggtaatatatcagaaaaagtaacggaagaaatattatttgaattgttt

cttcagtttatgtcaatagcactatccgactgttcagaaacaatcatcattttgaagagtgggatatagaggagatacatttttcattagatgaacttca

cagttgaagctagatagtgtaatggaaatcattattgtattatttttatatctagctgttatacactcaaaatctttgtaagatgtcaagagttatcaag

catgaactgtgtcttatcagtgtatcaaatttgttgaagattgttgcatattttttccagtataattcctcctccaggtgtcagagcaactttgaatttt

gattaggagaatttgtttttattcttatattatctttataaatgccg

>88322|AGAP002256-PA

gttcagtttagcaaaacaaacaaaaaaggaaaatcatgaataccgtgaaagagaatcatcagatatgagtagagaaataaaatctgctgcagcattaaaa

actatcagttcttgcatatggcttcctcaggcacttctttatatagttgcatcatcatatgacggaaatgtttcaataggtttgtcgtacttttcttcac

tacaaaaattttctcactcaggtgatccagtgtgataatcatagttgagtaaaacagaaaggctggcgcatttaaatgaaaggagcagaaaccaaaacca

agcagatactgcgttgacatcatattacatgatacgtcacttcaaaaatatacatatatctgaatattacccgctctaacccacaatctttacttacctg

aagaaacaattcaaataatatttcttccgttactttttctgatatattaccacaaaacactgttctttgatcttcactcattatgtgacagagaggttat

atttcatcaacttcaccgtgtacagacaaagtgctaacagttgactacacagaaaagcttcttctattgcgtgtcaatattaacaacaatattttgatat

cagccaaagcgttcgggcagtgaaatatagtctgtttatacaaccatattccgacatgaacaacagactacagtacagacacacattacaattcaaagtt

caa

>88472|AGAP002272-PA

tcaatgagaattttcacggtatttagtttcgaatctcggccctgtgcacgacattgttccgactttcggagagaaccctcttctgctgctaaatgaagtg

gcgtacagtttttgttatcagttacattaacttcggctcctttagccaaaagatatttgactacttcggcatgccggtagattgctgcgaaatgcaatgc

tgttgtctgctcgcaagtcctcgcattaacttcagcaccattggcgataagaatttttatagtatccaaacagcctgtaacagctgcactatgcaatgat

gtccactttcggtcgtcagtagcattgacgtcagctttattctcgatcaggatgttcgttatatcaacggaatcgtacattgcagcaaggttcaatggtg

ttattccag

>88475|AGAP002272-PA

catgtgtatgatggtttctgtcatttagtttgtgtgattatagttaagtctaagaatctcaatgttttgtgcaggggcctttctgaagaagagaagggca

ggaggctgatccaggcagctgagcagggttcagtggagaagctccaactgctgctcaaagcgggtgcagatgtgggggcggtggaggtgtgcaataggac

agccctccactgtgcagcagagtgcgggcacgtggaagtggtgaggtgcttggtcgacaatggggcagaggtggatgccaggatcacggtggcgatgtac

acgccgctgcacttggctgccacatttgaccgcactgatgtggtgaggctgctggcggcttccggtgctgacctcaatgccagagaccaccaggggatga

cgcctctgcactgggcggtagactatggccacgtggaggtggtggctgctcttctagaggcaggagctgacattgaggccagggatgagaatggggacac

cgccctagactgtgctacacgggacaatcaactggagctcatacagatactattacaaaggtagcagtgcaacgaacatatgtggcacaaaacaaacctt

gctatacatccttccgttttttgataacgaattaaaaatattaacgtg

>88474|AGAP002272-PA

cacgttaatatttttaattcgttatcaaaaaacggaaggatgtatagcaaggtttgttttgtgccacatatgttcgttgcactgctacctttgtaatagt

atctgtatgagctccagttgattgtcccgtgtagcacagtctagggcggtgtccccattctcatccctggcctcaatgtcagctcctgcctctagaagag

cagccaccacctccacgtggccatagtctaccgcccagtgcagaggcgtcatcccctggtggtctctggcattgaggtcagcaccggaagccgccagcag

cctcaccacatcagtgcggtcaaatgtggcagccaagtgcagcggcgtgtacatcgccaccgtgatcctggcatccacctctgccccattgtcgaccaag

cacctcaccacttccacgtgcccgcactctgctgcacagtggagggctgtcctattgcacacctccaccgcccccacatctgcacccgctttgagcagca

gttggagcttctccactgaaccctgctcagctgcctggatcagcctcctgcccttctcttcttcagaaaggccccgcatgtgagagatgggagcattatc

aatctgagaagtgtgacagggaggcaactgcagtgcagctgtgacaggagtctggccgtgactgctatgggactgacagtgagttccgggggtgcgtcct

ccctttgtagtggctggtgcactgcacagagagacactgtgtg

>88473|AGAP002272-PA

gttcaatatctgtcgtaacaatgtttgtaaacttgttgtttgtattcgaaaatctgtgtttgagacttgagtgacagctgccattggaagttggtagtta

agtgccatttttatctgaagaatcgtcctgatccttgtgactgcacagaataatagcacaaaggcaccgtgacgatatgaagtacttgtagtgcttcata

ttgtagttaaagttccgcaattgattttgtaaaattcaggtttcattattggcgcagaagaaataacacgattaaaatcaaagtaggaactgcaaatcag

tgtaaagggccactatgattcagctggatgcaccggatcccttgagaacacctttgctgtcagttgctgctcgtaccggcaatatcaatgctgtcagtaa

actgctcaaagatggacgaagggcagataatgttgataaccgtggctggacagcactgcatgaagcagctgcagcaggacatgttgagtgtgctgtactg

ttgctgagtcatgatggagacattaaggatttaagaacttatgaaggagaaacaccacttttcttgtctgttaatgcagaatctaaacttgatgtaatga

aaattcttttgaatcactcagcaaatgtgaatcttggcaacaatgaagagtttacaccattacataaagcatgtgaacttgattcattagaatgtgccca

gctgcttttagagtctggagcagacatcaatgctgcagatgctgacgacaatcggcctattcatgaagccatcaacaagaggtctgtagaaatggtgaat

ttgttgctgaggtatgaagcggacctgaaagtaaaaaattcagatggactcacacctctcatgcgtgctgcctacattggctttcatgagatcattaaaa

aattggataagcctgaactacacattgtaaatgagagagcatcgtcaggacgcacagcactgatgatggctactcaagcaggctgtttaaaaacgaccag

aatccttctagagatgggtgcagatgccaacatcgtgagtggtgatacgacagcactgcatatggctgcacatggtgatgatccaagacttgttgaatgc

cttatacctgtgacagatttgaaaatagtggcagcatgctcaagtattagtccgataatgttggcagttgatagcatgcaagtgaaatcgattgaactac

tgttggaatggatgaaagacaattcatatatgctcaagaagtgcattgaatgcgaaagtgagacgctaggactagagtacagagcgacgggtgctgtctc

atacctactctaccacaagtctgagctagcggaagcattgcctgtgctgaaactgcttcttaaatatgggcttccagtaaatgccagctacacatttgaa

gctccaccattagtagcaattgctacttcatcagcacctaaagaagtaaagagactgtttgttcagattcttttagaccatggtgctgatgctgaccatc

ctgttccttggcgtcagcgtacacctgtgccagcagccttgtattatgcatgtcttattaacccagatgaaaagtggttgatcaggttgctgatgtctgc

atgcagaaaatgtgaccctgatacattgctgtcagcaatctatgatgcagaactagaagaaggagaccaacgttcttcaaagcctgcacttcaaaatatg

ttggatgtagttcttgaacttggtttctcaaaaagtgttatcatgtcacagatttggaggaaatgtagaagatcaggtctcactggagaaagtcagcaaa

acttgcaagaggaaacatcagaaaactacttgagtgttccggaagtattagcgccaagttcactttcaaagctctgccgtattgtaattcgtagacatta

ctgtctgtcacggctgaagaaatataatttacctaatgtcataaatgatttcctgaattatgtgttctaatgttgggccctcttacgaaaatatcattta

tatgatgtgaaaaaaactcattatgatagtgtattatgttacacaaaatatgctccatattgtgatagtgaaaactggtgttcaaaatcttagacagtgt

atcattacagttaccttaatgctatatttccatacttcttttatgcagatttgaaaataggacttcttataatcatgagatatgacaagttgtatagcaa

cactattttattgtatgaaataaatatctgtatatcctttatatcaccatcataaggttacaaaaattcataaattattatatattatatcatatcatgt

catgtcacgtcttataaatatggcaatgacgtggcatgatatataagagagttattaactgaaaccagtagtgataatataggacttatagacaactccc

taacatgcattttatgaaatacatagcagctgttggcagtcaccttcaaaaagtgtttaactattttgttactaaaaaagttactcttaaccctgaaatt

ttaattcagcgagtatatttaatcaaaaataatcttaaactacatattttgtgcatatatctgttttgaatcatcaagttattaaatgaaaagttaagag

gctaatagttcttttcatatggcagaggaagattctgtgaactgctaacattatgtgaaatgatcggcacaattaaaaaaataaatagaa

>88489|AGAP002272-PC

agggtgttttgcatgactgatgataaagaggacaaaacattagagcatcaggagcattttactgaagttgcaaagagtagagatgttgaggtgctcgaag

gtaaaccacagtacattgaatttgcagggaacttggttcctgttacaaagtcaggtgaccagttacaactcggattcagagcatttagagagaatcgtct

tccatttactgtacgtgtcaaggacccacatgctgatacagtgggccgcatacttttcatgagagaaccgaaggttgcaaaaggtgaaccaccccagcag

ccagtctgcatattaaatattgtactccctgaaacgatattacctgaatcaggatttgacctcttgaaagaagagttaggaccatcaagaaatgca

>88488|AGAP002272-PC

ctaacgtgtgtacttgatctctcattcctgttgctcctgcgcttctgcggccaaggtggcaacgaacgcccgcgcacgcgacgtcgaagacgctccgatg

ttcaagaagaagttcccgtcgctgtccagcctggggtcggatggcaatactgccttcctgagggcggctcgcgccggtaacctcgagaaagtcctcgaac

atctcaaaaacaacatcgacatcaatacctccaactcgaacggtctcaacgccctacacttggcctccaaagacggacacgtcgctgtcgtcactgaact

tctgaaacgaggtgcttcagttgacgccgcaaccaagaagggcaatactgcgctgcacattgcttcacttgctggtcaagaggaagttgtgaaactgctt

gtaacaaatggtgctgcagtcaatgtgcagtcgcagaatggattcactccgctctacatggcagctcaagaaaatcacgacaacgtcgtccggtatctgc

tcgctaatggcgccaaccagagtttggcgacagaggatgggttcacaccactggctgtggcaatgcaacagggtcacgacaaggttgttgcagtattact

tgaaaatgacacaagaggcaaagtgcgtctgcctgctttgcacattgctgccaaaaaggatgactgcaaagcagcagccttgttgctacagaatgatcat

aatccagatgtgacctcaaagagtggattcactcctctccatattgcagctcactatggaaatgaaaatatagcaaatcttcttcttagtagaggtgctg

atgttaattactctgccaagcacaacataacaccacttcatgtagcagccaaatggggcaagaccaatatggtatccttacttttggggaaaggtgctaa

cattgaatcaaaaacacgagatggactgacaccactgcactgtgctgcccgttcaggccatgaacaggtagttgacatgatgctagagcgaggggcacca

ataagttcaaagaccaagaatggactggcacctttgcacatggcatcacaaggagaccatgttgatgcagcaagaattctcttgtaccaccgtgcaccag

ttgatgaagtgactgtagactaccttactgccctgcatgttgcagcccactgtggtcacgtgcgagtagctaagctactattggatcgcaaagctgaccc

aaatgccagagcactcaatggattcactcctctccacattgcttgcaagaaaaatcgcataaaggtggttgagctgctcctgaaacatggtgcatccatt

gaagcaacaactgagtcaggactaacaccacttcatgtagcaagcttcatgggttgcatgaacattgtgatctatctcttgcaacatgatgcaaatcctg

atgttccaacagttcgtgggaagacacccttgcatctggcagcaagagccaatcagacagatatcatccgtattcttttaaggaatggagcacaagtcga

tgcccgggcaagagagcaacagacacctctgcatattgcatctcgacttggaaatgtggacattgtgatgttattactgcagcatggagcagcagttgac

tctactacaaaggacatgtatacagctctgca

>88490|AGAP002272-PC

caaaagtactcgccctaattgttaatacatttatcccactgtgagacagaatgatcagtgccttcactgtctatggaagcatgactgcacccaggcgtgc

acttcgtcgttcaaaacaaatcgacgtccacaaatatctttctccaaatttgtaaattacgtgaggagagattgggtctatatggaggatgcgtttggga

tttccaagaatactgctctctagcacactctaaacaattgtgggaacttgtgggcgtgtccattaacagttatggcgattaattttgaaataatgaactg

cttacttccttttttccatctgtttcgttttaatctgactgctccttatataattattctcctctagtacaaatgagttgagtatgactgatactgacta

caaaaaatagtgctgtattgtgtgatgctgttactaaactttaagaaagctgtctactagtgcctgtaaaaaatatataaaataagatatgaacaaatca

aatgaccaaattgttatcagttttgtacaattaaaatgaatgtatgtccatttccatgtatcaccataattttatcatagtacaactgtgtatacacatg

ccatgtgagcaaatcaattgtcataactgcagcacatctgatggatgatttagctgtgacagttgtatatttttgtgtccttgagacttccatgttctgc

ccattgtttgatcagaagttcatattaaggacagtgaccagtctacctagaaagctttaacgataccatagacagcaaaagaaagcagtgaggtgaagaa

acagtctttgtcttttttgctacatagagcatatatagcataattaacattttatattgagtagtacactgcccagagcgaacacagatacataactata

tcagacatagaaaaatacatactgttacaacagaacattgattaagaggaaaattttccacacacatatatggaatcacatgatggctgttatgcacgca

catttatactacatcgtgcaggatcattcattaaaagctcttcttcaaacgcttcacagtcacgattatgcattatttagtaagacagcaaccccaatat

gcaggcatgttgtgttacgagaagtaacagtctaccaatacatatgtcatggacagtgttcactctatatttcacagcccaacaaaacttcatctaaaaa

cactcattttacacttccacctttctgctcaaaatgtttgaaacattcgtaatatcagtttttaccaacgactttttgtagtgaaagtagttgcagttca

gctgcggagattatagaagtgaacacaagcgcctgtgattttgaccagtatgacactttgtacctctggagcggacatcatcgcccacctcgaccacttc

ggcaccctgcaatcgtgccacacagaccacgttcccctcctcctgcgccacttgctgggctgtctttcctgctttgttctgcacccctacaggcgcaccc

gctcggagtagcacggtgatgacatcagcgtgacctttaccagctgcccggtgcagaggggtgtcaccatttacggtctgagctcgcacgtccgcaccat

tgtccagcagaaaggcggcacactcggcattgccgttcagtgcagcaaagtgcagcgccgtgtagccgtccgagtcctgtgcacggatgtccgacccggc

agccaccagcagggcggtgcactccacgtccccgttggctgcttcgcagtgcagcggcgtgccgccctccccatccagcg

>88508|AGAP002276-PA

ggctgttctaatctccttgctgtactgtcaaggaaaacaaaagcaatgcaatcttgtcacgttttgcctacatttcaaagattcgtttaattcggagata

taactgtcaggtggtactttaacaagattcattgaacattaaagcagcggagacttgtctttctcagccagtgagcgcaaggcatctgcttgaaagatat

atcgtcacatccagatatacattagttttactttggttctgttttgtaactgagggtaatgtgaatgcaacatgagttctccaaaaaaaggaaatgcaag

acatttatccaaaacaactgaactgtctggtcctgtacagaagagaactcagattgaaaatcccgacagcccaaagaaagagaaggacagtatacataca

cagataaaaaattcattgaagtcaccagacaatatttctgatatagagcataaccacaaattgcccattaaaacagaaaaatcatgtcttcagacatctt

cctgcattgtgtcaaaggaaacatcacaccagaacgaacctgattcatctatttcatcttgtgaacatgtggaaagtactcagtctgagctctcaggcat

aagctatacaaagaaaacaataagacaaaaaaaacaatatagccaagaatccctcaaagaacatactatcaaaaatctctcaaaaggaaaagagggaaat

actggccagttgaacagtcacttaccttatgacaccaaaaatgctgaagagaataactgtaaagttcaaatgacaacagaaatgtatgaaacatctgagt

cattgccaataaacaccactgctgatccctccactggtagttctgtagacaagaaggagagaaggaagatagtgaagaaacttgccaaagatgaaaaaag

aaagcaacgtgagcttgaatacaaaaagaatcttcagtacccaaagggtcaaaggattcaggttattgatcacaacacattccacaaaattttgaatgat

tctggatctttgcaaagtgaaaataagagatcattgctcaattctgaagaatttccagcattaaatgtattgaaacgtaatgacagatttcctgcaatga

cttgccaaaattcttcaatgcagtctgaaagtgaaaaatcaagggtgcaagataaggctactaacaacatgggaaagtgtacgaaaatatcagagactca

ccctgttgttaatgaagaaatgataaacaaggtgggaaagaaattttgtgttccaagtaaactaaatcccaaaggttccctgaagaaaataggaaagtta

ccaaagaaacgcgatcccatacaaattgacattctacagtgtataaagatggtaaaatcgaaagatgctgtggtagaaccaaggaaaaaagtgactgctt

tggagaggaagaatgataattcattgcttggtggcaatgaattggattcgtcaaatcctcaacgtagtcgggggaaaaagagagaaaaaccaaaaaagaa

gaggccaacaagactgaaacttgttattcttcatgaacgtgagctgaaaaatcagctgaagaagttacaggaatcagatttacttcaagcagagaaactg

ttttttggcacagaaaaaacgataaatttactaacaacagagggcgaaaagttaaaacaagtggaacgtattaccaatgtgtcagacaatgaatcaaaaa

aattattaggaacagggagcataaagctgggacatgtggaacctgctgctaatgtgccagacagtggaattggtaaaacagtgccaggatcaatgtgttt

accaacagcagggggtctaaagttagaacagttagaacatcatagtaagatgccagacaatgaagccgttgtaacagttccaggaacaaggaatttacaa

acagggtgcaaaaaattagaaaagctggaatgtgttactaatatgtcagacagtgaagttgttgaaactgtgccagggttcatgaatctgttatcagaag

agggcataaaattagaaaagatagaaccagttactgatgtgccagacaatggaattattgaagcaatgtcgggagaaacagatatgtcaaaagaaggcag

atataagtgtgaggcaccatcctctgatagcaggactgcagcagcaagtacgaatcagttttcgaatgctgaagatgacagtcagacctacacgctaagc

agtctacacataaacaacaatcagccatatgcaaatgaaagaggaagagttcctgatcttatgccatctttattgtactctgcaacagatattctgaaga

aagaaaagcaagagttcattagtttagtgaaggaaagtagttgcattgtaaatttgaagcaaggccagcattctgcagccactgctggtgatgacattaa

acctacccacatttcatcaaatgccataaaatgtgaaattatacttagctcaccaaatgagcattgtacaggtagtgtggaaaatgtcagttttatggat

tcaaatacatctaacaacctacatcagatcaaatgcataaagtttcccaaaggtgaagattgtgatagtccctcacaccatgaaaaaacaatacagaaga

ctgaaattcacagcagaaaattcaggccgtactgtgaccacttcataacacctgagattaacagttctgtcaccactttgcttcaagatgtagttaaatt

tcatgatcgccaatttgaaagggatcctgtaaaggctaatgctagacgcaggcatgtcagtggtctccgtgaggttctcaaatacctgaatcttaaaagg

ttgaagctgatcataatagcaccagatatggaactcattgagggcaaagggggaatatgtgatattgtggaacagcttaaggaaggtgcacggaaacagg

agataccatatgtgtttgccctacaaaggaggtcacttgggaaaatcacattcaaaaaagtccctgtgagttgcattggtatcattaactatgatggaac

tgagaaaaattttcattctttggtggcagcattgcaggttgcaagactgcaataccaagaagcattaacagatgtatgtttcaaggatacacagtcagag

gaagtggatataaacaaacaagtgattgaagctgcactttcaagattaacagaaaatagttcaacatcatgtttgtaacagaggcacttatgtgcggaaa

cagatcaggaaagggaggaataagtatttatattctgtattgggtgtacaagtttatattacggcattgaaataatggaacataatattattttttgtat

ataagatatgtgtgccatactgtattaaattcctatcttataattaaattactgtgcggataccag

>88527|AGAP002279-PA

aggcattcctgtatatctcgacctccttctgtcactcagaccatcaagtgattgaagaaaaattatacccggcaaaagcagacccagaaaagattatgaa

tattgtttcctcgatggatgacgatcttttagggcttataacaccaaagttgttggggaacctaccaaacacgtacgccttgacgaaaaacatgacagag

gagctggtggcgaggtattccgatcggctgccgatcgtcattgccaggccgtccattgtgacaccggtctggagggagccgctagaaggctgggcaccga

atatgaatggaccagtcggcttcttcgtagctggtgggaaaggcgtgttgagggctatgctggcatacctcaacttcaaaacggacatcgtacccgtcga

ttgcacggcgaacgccatcatcgcagtggcgtggcattttggagagacgaggcaagcgagcccctatgtttgcaacataactaatggggatcagaaccgc

atcacctggggagagctcatgaagaatctgatgaggataaatacaacagaggttcctctgagaccaattctgtggtatcctcagccaattgtgacgactt

cagtcgtgtggtacacaatctgccacttcttcttcgaattcttgcctgcatatattctcgacttcgtcatccgtctttctggaaggaagccatttttggt

gcacgtaaacaatcgtttacattcggccaagatcatgatgggatattacgtgcttcatgaatggacatttatcaacaaaaatattgtgtctatgtatgaa

tttatggctcctgaggatcaagaaaccttttattataatgttaaagaagtcaatcagtatgaatattgtaaaaagtcacttattggagcgaagcggtaca

ttctgaatgagagtgtggatgatatacccggtgatttgagatacatgagaaggatgtactatttgcacgttatagtgcggacacttttctatggcctgtt

actctggctactgtattcgatgtcagagccagcactggagatggttgaaactgcaatggaatatttgacttctgtgttttatggagctcctattattaga

gcactcactgacggtgttcctgtttcttctactgtgttgccagacgatgtggttgatgtgaaaaacagctgaatactctgttgccagttttgttcgaata

actaacctcaatattacactagtaactgtttccaataaaaattcgtcattccaaaatctgtataaaggagaaactttttttcacaaaaagaagactacac

atttcataagcacataatgttgataaaaaatcgtaattggtatggaacaatgaaatattcaagttaaaaacatggtttaatactttgaagataccctaaa

actctgcctgtcctgttactttaattcaatctacactggtttcaaaattattgaatttctgaacatcatgttgtatttgttttcataatatatcagtatt

aacaacattaggaatgtgtaaaccattagatttttctccatatgggagaattttgaaaaaaaaaataccgctgaaaatgaagccaagtggggtatgtatg

tctgatttaaattactaaacaaattaattttgaataaaatcagtaacatgtaaaaaaaaaaaaa

>88526|AGAP002279-PA

gtcgcaaccggaactgctcggaatgctaacgagaggtggagatcgatgttttgacgagaactgacatcactgccgtgaagcctgtcgtacgccgctcgtt

gctgcgtcgccctcgccgaacgaagaaatatctccaatggttgccgtggcaagacaggatgtgcgttcgggtccccaagtgccaaagtggtatgccggcc

ggtcggtactggtgacaggggccaccggcttcctgggaatggtcaccgtggagaagctgctgcggtcctgccctgacattggcactatctacatgctggt

gaggcccaagcgtggacgggacccgcaagagaggttgggagaatatgtgtccaatatgctgttctcgaagcttcgagaagagcggcccgacttcgcacgc

catctgtctgtgatctccggcgatgtcagctcgccggacttctggctggcagagcaggacatggcggccttgcggcgtgacgtcagcgtcgtcttccact

gcgccgccagcgtccgcttcgacgacagcctgcgcaaggccgtcaacaccaacgtggtgggcaccaagcgcttgctgcagctggccgagcagctgcccaa

cctgcaggtctgaggaatgtatggaacggagtaacatgttcttagagtattcctgtacatctcgacctccttctgccactcagaccatcaagtcgttgaa

gaaaaattgtacccagcaaaagcagacccagaaaagattatgaatattgtttcgtcaatggacgacgaccttttagagataataacaccaaagttgctgg

ggaacctaccaaacacgtacgccttcacgaagaacctgacagaggagctggtggcgaggtactctgaccggctgccgatcgtcattgccaggccgtccat

tgtggtgtcgacctggaaggaaccgctagaaggctggacagcgaatatgaatggaccactcggcttcctcgtagctgctgggaaaggcgtgctgagggtt

gtcatggcagacctcaacatcgcatcagacctcatacctgtcgactgcacggttaacgccatcatcatagtggcgtggcatgttgcagtaacgaggcagc

cgaatccgtatgtttgcaacataactaacggtcgtcagaactacgtttcctggggaggcgtcatgaagaatctggtgaggataaatacgacggaggttcc

tctacgaccagttctgtggtatagtcagccaattgtgacgacttcagtcgtgtggtacacaatctgccacttcttcctcgaagtcttgcctgcacatatt

gtcgacttcttcatccgtctttctggaagaaaaccattcctggtgcacataaagaatcgtttacattcggccaagatcatgttgcgatattacatggtac

acgaatggacatttatcaacaaaaatattatgtctctgcatgcatctatggctcctgaggatcaagaagtcttttattttaatcttcatgaagttgatca

gtatgactattgtaaaaaggggcttattggagcgaagctgtaccttctcaatgagaacatcgataatatacccagtgatttgagatacatgaggaggatg

tacttgttgcacgttatagtgcggatacttttctgtggcctgctactctggctactctactcgtggtcagaaccagcactggagatggttgaaacagcaa

tggaatatttgtcttctctgttacatggagctccagttattagagcactcacagaccaagttcctgtctcttctactgtgttgccaggcgatgaggctaa

agtgacaaacagctgacactctgttgtcagttttgttcaaataaccagcttagattttacactagtaactggttccaataagatttcctcattctcaaat

catttatgaaggag

>88525|AGAP002279-PA

caaaattttgaaaccagtgtagattcaattaaagtaagaggacaggcagattttttgggtaatttcaaagttttcaaccatgtttttaacttgaatattc

cattgttccataccaattaccattttttatcagtgctgtatgcttatgaaatttgcactattctttttgtgaaaaaagtttctccttcataaaggatttt

ggaatcaggaatttttattggagtcagtcattagtgtaatattgaggctagttatttgaacaaaactgacaaaaaagtattcagctgtttgtcacatcaa

ccacatcgtctggcaacacagtagaagaaacaggaacatcgtctgcgagtgctctaataataggagctccataaaacacagaagtcaaatattcaattgc

agtttcaaccatctccattgctggctctgacatcgagtacagtagccagagtaagagaccatagaaaagtgtccgcactacaacgtgcaaatagtacatc

cttctcatgtatctcacatcaccgggtatatcatcgatgctctcattcagaatgtacagcttcgttccaataagtgactttttacagtagtcatactgat

caacttctttaagattaaaataaaagatttctttatcctcaggagccattgattcatacacagacagaacatttttgttgataaatgtccactcatgtag

cacgtaataccccaacatgatcttggccgaatataaacgattgtttatgtgcaccaagaagggtttccttccagaaagacggatgaagaagtcgaaaata

tgtgcaggcaagacttcgaggaagaagtggcagattgtgtaccacacgactgaagtcgtcacaattggttgacaataccacagaactggtctcagaggaa

tctctgtcgaatttatcttcatcagatgcttcataatgtctccccaggtgatgcggttctgatctccaacggttacgttgcaaacatatggactcggctg

cctcgtctctgcaaaatgccacgctgcgatgatgatggcgtttgccgtgcaatcgacgggtatggtgtcggctgtgtagttgaggttcgccagcatagct

ctcagcacaccttttccaccagctacgaaggagccgactggtccattgatattcgctgcccacccttctagcggctccttccagactggtcccacaatgg

acggcctggcaatgacgatcggcagccggtcggagtacctcgccaccagctcctctgtcaggttcttcgtcaaggcgtacgtgtttggtaggttccccag

catctttggtgttataagctctaaaagatcgtcatccatcgaggaaacaatattcataaccttttcggggtctgcttttgctggatacagtctttcttcg

atgacttgatggtctgagtggcagaaggaggtcgaaatgtacacaaatacctgcaggttgggcagctgctccgccagctgcagcaagcgcttggtgccca

ccacgttggtgttgacggcattgcgcaggctgtcgtcgaagcggacgctggcggcgcagtggaagacgacgctgacgtcacgccgcagggccgcaatgtc

ctgctcttccagctggaaatccggggagctgatatcgccgccgatcacggacaggtggcgtgagaagtcaggctgctgttctcgaacctttgagaatatc

acattggacacataggcttccagcctctcctgagggtcctgtccacgcttgggccgcaccaacatgtagatagtgccaatctcagggcaggagcgcagca

gcttctccacgacggttaatcccaggaagccggtggcccccgtcacgagcactgaccggccggcataccactgtggcacgttctgtgaggggaacgcggc

tgctttctttctacggcaaccattggagatttttcttcgttctgtgagggcaaagcggcggcgtgcggcgtacggcgggcttggcgacggtggcgtcggt

actcgtcggaacgtcgacctgctgcgc

>88528|AGAP002279-PA

agtaatttaaatcagacatacataccccacttggcttcattttcagcggtattttttttttcaaaattctcccatatggagaaaaatctaatggtttaca

cattcctaatgttgttaatactgatatattatgaaaacaaatacaacatgatgttcagaaattcaataattttgaaaccagtgtagattgaattaaagta

acaggacaggcagagttttagggtatcttcaaagtattaaaccatgtttttaacttgaatatttcattgttccataccaattacgattttttatcaacat

tatgtgcttatgaaatgtgtagtcttctttttgtgaaaaaagtttctcctttatacagattttggaatgacgaatttttattggaaacagttactagtgt

aatattgaggttagttattcgaacaaaactggcaacagagtattcagctgtttttcacatcaaccacatcgtctggcaacacagtagaagaaacaggaac

accgtcagtgagtgctctaataataggagctccataaaacacagaagtcaaatattccattgcagtttcaaccatctccagtgctggctctgacatcgaa

tacagtagccagagtaacaggccatagaaaagtgtccgcactataacgtgcaaatagtacatccttctcatgtatctcaaatcaccgggtatatcatcca

cactctcattcagaatgtaccgcttcgctccaataagtgactttttacaatattcatactgattgacttctttaacattataataaaaggtttcttgatc

ctcaggagccataaattcatacatagacacaatatttttgttgataaatgtccattcatgaagcacgtaatatcccatcatgatcttggccgaatgtaaa

cgattgtttacgtgcaccaaaaatggcttccttccagaaagacggatgacgaagtcgagaatatatgcaggcaagaattcgaagaagaagtggcagattg

tgtaccacacgactgaagtcgtcacaattggctgaggataccacagaattggtctcagaggaacctctgttgtatttatcctcatcagattcttcatgag

ctctccccaggtgatgcggttctgatccccattagttatgttgcaaacataggggctcgcttgcctcgtctctccaaaatgccacgccactgcgatgatg

gcgttcgccgtgcaatcgacgggtacgatgtccgttttgaagttgaggtatgccagcatagccctcaacacgcctttcccaccagctacgaagaagccga

ctggtccattcatattcggtgcccagccttctagcggctccctccagaccggtgtcacaatggacggcctggcaatgacgatcggcagccgatcggaata

cctcgccaccagctcctctgtcatgtttttcgtcaaggcgtacgtgtttggtaggttccccaacaactttggtgttataagccctaaaagatcgtcatcc

atcgaggaaacaatattcataatcttttctgggtctgcttttgccgggtataatttttcttcaatcacttgatggtctgagtgacagaaggaggtcgaga

tatacaggaatgcctgcaggttcggcagctgctgggccagctgcagcatgcgcttggtgcccaccacgttggtgttgacagccttgcgtaggctgtcgtc

gaagcggacgctggcggcgcagtggaagacgacgctgacgtcccgctgcagggccgccatgtcttgctcttccagctggaagtctggggagctgacatcg

ccggcgatcacgaacaggtggcgcgcgaagtcaggccgctctcctcgaaccttggagaacatcacattggacgcataggcttccagcctctcctgagggt

gttgtccacgcttggaccgcatcagcatgtagatagtgccaatctcagggcaggaccgcagcagcttctccacgatggctatccccaggaagccggtggc

ccccgtcaccagcactgaccggccggcataccactgtggcacctggggaccggaacgcacatcctctcttcccacaggaaccattgcagattttgtttc

>88524|AGAP002279-PA

gagtaatttcaaatttttaaatgcgtattttgaacttaaatattctattgttccagaccacatacgatttttattagcgctgatacagatgaatatgtac

tcttctattttccacaataaaacaatgatacagtaacaagatgacagacatgtaaggaattctgccaaaaatattgttgttgcttgtacttgttagcagt

gattatgcaatacatacgatgaacgactggatgtctacagttttctttttagagacttgcttttgttaataaagtttctccttcataaatgatttgagaa

tgaggaaatcttattggaaccagttactagtgtaaaatctaagctggttatttgaacaaaactgacaacagagtgtcagctgtttgtcactttagcctca

tcgcctggcaacacagtagaagagacaggaacttggtctgtgagtgctctaataactggagctccatgtaacagagaagacaaatattccattgctgttt

caaccatctccagtgctggttctgaccacgagtagagtagccagagtagcaggccacagaaaagtatccgcactataacgtgcaacaagtacatcctcct

catgtatctcaaatcactgggtatattatcgatgttctcattgagaaggtacagcttcgctccaataagcccctttttacaatagtcatactgatcaact

tcatgaagattaaaataaaagacttcttgatcctcaggagccatagatgcatgcagagacataatatttttgttgataaatgtccattcgtgtaccatgt

aatatcgcaacatgatcttggccgaatgtaaacgattctttatgtgcaccaggaatggttttcttccagaaagacggatgaagaagtcgacaatatgtgc

aggcaagacttcgaggaagaagtggcagattgtgtaccacacgactgaagtcgtcacaattggctgactataccacagaactggtcgtagaggaacctcc

gtcgtatttatcctcaccagattcttcatgacgcctccccaggaaacgtagttctgacgaccgttagttatgttgcaaacatacggattcggctgcctcg

ttactgcaacatgccacgccactatgatgatggcgttaaccgtgcagtcgacaggtatgaggtctgatgcgatgttgaggtctgccatgacaaccctcag

cacgcctttcccagcagctacgaggaagccgagtggtccattcatattcgctgtccagccttctagcggttccttccaggtcgacaccacaatggacggc

ctggcaatgacgatcggcagccggtcagagtacctcgccaccagctcctctgtcaggttcttcgtgaaggcgtacgtgtttggtaggttccccagcaact

ttggtgttattatctctaaaaggtcgtcgtccattgacgaaacaatattcataatcttttctgggtctgcttttgctgggtacaatttttcttcaacgac

ttgatggtctgagtggcagaaggaggtcgagatgtacaggaatacctgcaggttgggcagctgctcggccagctgcagcaagcgcttggtgcccaccacg

ttggtgttgacggccttgcgcaggctgtcgtcgaagcggacgctggcggcgcagtggaagacgacgctgacgtcacgccgcaaggccgccatgtcctgct

ctgccagccagaagtccggcgagctgacatcgccggagatcacagacagatggcgtgcgaagtcgggccgctcttctcgaagcttcgagaacagcatatt

ggacacatattctcccaacctctcttgcgggtcccgtccacgcttgggcctcaccagcatgtagatagtgccaatgtcagggcaggaccgcagcagcttc

tccacggtgaccattcccaggaagccggtggcccctgtcaccagtaccgaccggccggcataccactttggcacttggggacccgaacgcacatcctgtc

ttgccacggcaaccattggagatatttcttcgttcggcgagggcgacgcagcaacgagcggcgtacgacaggcttcacggcagtgatgtcagttctcgtc

aaaacatcgatctccacctctcgttagcattccgagcagttccggttgcgacactaaagccgaatgtat

>88541|AGAP002290-PA

agcaccatgaactaggaatgttgccacttgaatgccatttcctcaacaagtcagccctagttagtgtggtgggacaacagacccagcctcttaagcattt

tactttgaaaaggaaaccaaaatcagctgcactgcgtgctgagctcttacaaaagtctgctgacgcagcgagtaatcttaagaaaaacactgtgccaact

gtgcctgttcggtcacgagttatgcctcgaaaactgaatgacacaacaccattaaaaggcataccaagtcgaattccatctggcggtttccgttcgccat

cactaagtagtcctattatgtctcgccctcagcatcctcgcacaccagctggacgcaaagatggtggaataaaacttctagacataaacgaacagcctct

cggatatgctcaggcaaagaaacgaaagcgaatgcaagatatggaagatgcaaagaaggcttcagaagcagcagctgccctccagcaacaatcacagcaa

cagcaacaacagcagcaacagagcccaacaagtggaacaacaccaagcacacccgactacgctgttggattgactgcaatgaatcctcccacacctactc

ctgcccctgctactccatcatatagcccggctacccctgcatctcagcacacagtcacaactattataactatccctagggaaccaggaacaccagccag

ttcttctgcaagtgcacttccgcagtctactgaaacttcaccagcaggacagtctctgttggcaaactcccccaatacttcaacaccaagttacgttcct

caagcttctcctacaacattgcagaatccaagtacaccacaacctcaggcacaatccctcttgcagtctactctcagtgctacaaatgtacagccagcag

cagtggcagccactgcagtaactggagttgctgttcaacctgttgctattgctgctactgctgtgcctgcagcaactataacaaaacaagttatccaaat

acggccagcaccccctattactcagatcagattccagccagtgaatgcagctgctggtacaaataatcctggacagcagagaaaagggctttcgttgacg

agagagcagatgttagaggcccaagaaatgttccgtaccgctaacaaagtgacacgacctgagaaggctctgatacttggtttcatggcaggttcaagag

ataatccgtgtccccatcttggcaacatagttacaatcaaacttagtgaaagccaagaaaaggtcaaacagcctgatgacacgttccagttaatgactat

tgagacacacttccagatgaattacaatacaggggaatggaagcgcattaagaagttacgcaggccggaagacagtgctgtgacggcagctttggtagct

acggcaacgccagtgtcgtcttcagttccgcaaccatctgtacaacctgcttctgctactgcaacgtagacactgcctttgcatttcactgtgcatttgt

caccatgttatagctgtagtatgttaattttgtaaatgtatatatgagatgcttcctatggaacttatgtattaatgtatatttcataagacagtatgag

taaattgaattactcatatgctatactttcacagtgtattgtatactctggtgacccacgagtaaaacaaactcagaaaaatacaaacagcctgcaattt

tctttttttcagaaattatgcagactgtcttggaagctatcctgtgtacttatgtatgtatatttttgttggcacctattgtctgtgcgtatgtatgtga

gagtgtgaatgaatgagagagagagagagagagagagagagagagagagagaga

>88561|AGAP002291-PA

gccacaaagcgtcaactagattctcataatcacttctaggaatgcgtggtactagccacttgtagaaaagaactacagtttcattgttgaaaatgaaaat

ggggaaccagttggagtagcatttaactttgatgcacatgacgaaccacctgttgaaatacat

>88603|AGAP002317-PA

aggacggcgctgcggaactgcgccatgccccggatggcgggccagcgatgctcgcacacccagccgccagtgcactggccgtgcgcgtcccacgccacgg

tccgtatctctcctgaggggtccgaaggcgggccctcgtctgaatctgtgaagttgtagctggacatcacacggggaatgccatagggccatgccagcat

aaacccaactgccattgtgtatagttgtcgtgacttgtgggtgaggatggtagtgccccctccaccatggccac

>90506|AGAP002347-PA

tggcagcatagcattgaataggtgagtgataaatgaagccccatgtttcacagcagcttctccttcaccaagacttgccatagaatgtcctaaagaaacc

gtaatgcctctttccacaaatttctcgataacttcagcggctttttctagctctggagctacagtaacaattctcacattgtccaaagatccataagttt

ccagaactgaatcaaatcccttctcgaatgtcttaataaattgttctggatgggcacccttcttgagaggatttatgaagggtccttctacatgaacacc

taaaactgtagctccttttgggccaccagcttgtttcttcagcctcggtagtactgtctcgtttgtaatcagcgttgttgaggtgggcccttgttatata

catgagctggagatgtaacgacagtcggacaaaatgaagtaacaccatgggccagaatacctttcgccactactctaagtccctcctcaacatcttcaaa

gtcgcaagagaagtcaactccgaaaccaccattaatctgtagttcaatgaaaccaggtgaaataagggctcccttgcagtcaatttgaacgtctgcactg

atcttttcgtcaaaaaacattttttctggatttacaatttttccgtctctcacccagatatcttccctaattatttcatgatttctcaaaatactacagt

taacgaactgcaaaagctgtgactgggtcattgctcctaagaaattgagatttcaatagactactgtgacagcttatc

>90505|AGAP002347-PA

ctctacagaaagttgaccaagctgatgcattccctcttctaggcccagtgcagaaattgcatcagtaaccagcaccaaacaagagagaatcagatatttg

ttatatggataaccaagctctggctttataacctttaggatgagttcgatgtgctattctcagagcagcagggtgagtgtggatgccatccgaaataata

ccaaaataaacaacatgatcatctggaacagcatctgatgccaagagacccacaaggccaggatccctgtgatgaaatggcagcatagcattgaataggt

gagtgataaatgaagccccatgtttcacagcagcttctccttcaccaagacttgccatagaatgtcctaaagaaaccgtaatgcctctttccacaaattt

ctcgataacttcagcggctttttctagctctggagctacagtaacaattctcacattgtccaaagatccataagtttccagaactgaatcaaatcccttc

tcgaatgtcttaataaattgttctggatgggcacccttcttgagaggatttatgaagggtccttctacatgaacacctaaaactgtagctccttttgggc

caccagcttgtttcttcagcctcggtagtaccttgttatatacatgagctggagatgtaacgacagtcggacaaaatgaagtaacaccatgggccagaat

acctttcgccactactctaagtccctcctcaacatcttcaaagtcgcaagagaagtcaactccgaaaccaccattaatctgtagttcaatgaaaccaggt

gaaataagggctcccttgcagtcaatttgaacgtctgcactgatcttttcgtcaaaaaacattttttctggatttacaatttttccgtctctcacccaga

tatcttccctaattatttcatgatttctcaaaatactacagttaacgaactgcaaaagctgtgactgggtcattgctcctaagaaattgagatttcaata

gactactgtgacagcttatcaccttaatattctgtcacagagctttttttttttttaacatagtcgatgatgacgacgtcagcagttccttaatcagcgc

atgacctgaatataatgttgacaagaagtgtttgttttgcgatacgtcaacccatggctgtttt

>90571|AGAP002348-PA

agagttatagcacaggtgtcctgtgaagttcaacaacccaaaccaacccgtccagaggaaggtctgcttctagtttatgtggaattgagccctatggctg

cacctgattttgaggcaggacgaccatcagaccttgccgtgcagttgagccgggttttggagaaatgtattaaagacagcagggctgttgacttggagtc

tctgtgcatcaaaagtgaagaaaaagtgtgggcacttcgtgtggatataacagtattaaaccatgaaggaaatttggcagatgctgcttctgttgctgct

ctgacagcattgggtcactttcagcgcccagattttactgctgagggtggcaaaataatcatccatagtgcagaagagaaagacacagtaccaataacgc

ttcatcattacccagtatgtgtctcctttgctgtgttcaacaatggggagcatgtagtggcagatcctacggagctagaagaacgtgttgctgaagctca

tatagtgttcggtatgaattcataccgtgagctttgtggtgttcacttgggaggcagtgcacttgcagatcctgagatgattatacagtgtgcaattcaa

gctgccgtacgtgcatctcgagttgtagagataatacggagtgccttggaaagtgatgccaaagccaggactgaaggagcagacgttggctttgttaaaa

gcatagaaaaagatagtctactgtccttagtgcgcgaaaatgtacacatctgtcttgatgccactgagctaaggacatctgcagtgaatgctgttgacaa

actgaatgactttagagaagatatggatctggatttgaataatggtgaagtgggaaggactaaggaggaaaaagtaattttcttgggtaatggcacagct

gaactggtggaagtcaaagaagaaaaaagacaaaacaatgtaaagacagaggtcgaggaagtgggaacaggaggaccgagcaagtgggaagtgtccagca

gcagcagcagtattgaagaagttagcattgacagtgatcagtcacgtgagaaagatgattcaccagacattgaggtagtcaaggaagtgagctttgagga

gaggctgaaaatagttgatagcattgaactgagtggcgacagtgaagaagaagaaactagtgttctacacgcagatgacattaatgaagttatggaagtg

gaaaagaattcacggaaagcagcagtaaaggatgaagaagtagaagaaaaagaaacacctagcagaggatggtataaatcaaagtggtagcaaatgtatc

tttcctacaaaaacgtgtcatacaatgtacaaaatgtgaaattttttattgtaatgttgactgttttgtctgagatatgtttcatctttgttgttataaa

gatttttggcagatacaaacttttattgtgtattagccagccttacacattaatacttactaactgagagataattttgtagcatttaaaatactatgtt

gtcctcctcctgaatgtaaatg

>90591|AGAP002349-PA

atcagatgtgtgccacctatgatttagcaagaacaaccagaaggtgaccgttggccttattcttttgcttactgaatttagcaggcataaatagtctcgt

catcttcagagctaacaaccagggggagactgtaaggagagactttctgaaatcgcttcggaaggctcttgtcagaggtcaattctacacaggg

>90592|AGAP002349-PA

cttctctagggaatgggatgctatagagatcagagctttaattggtttcttatatctgtgtggatctttgagatgttctaggaagagtatattgaacttg

tttgataactcaaaggaaaacggaattgaatcatgttagttatgcataagtgaaaacagattcaggtttctgtcgagatgtcttagctttgatcatatcc

gtg

>90600|AGAP002349-PA

aaggtggcctgcatgtatatttttccgcttacttgactaggcaggaataaattcgttacgcatttttctattcaacacaggtaatatgaaagaaaaaacg

agaagacaattcttacatgcgttatctttggaactgatgagcgaaaacctgaaagaaagagctaaattgaagcatttaccaaaagatttgttggtatttt

tggaaaattacacagagagtgacgtagtttatccagctgtattaacccctacaaggggagaaatatgttatttgtgtggctcaaaaaagaatataagaaa

caatgtgagaatgcagtgaatgtggaagaaacgtttg

>90595|AGAP002349-PA

catcagtctgagtctgagcaagagggagaagtaccagatgaggcaactgagaacagtcctgaattttcatgtgatgagccatcccggagaagcagacgag

ttgatttttatactggtgtagatgacaatttggtcaaaacctccacctctcaatataggaaggactagagctaataacactgtatatgtttctcctggtc

ctaaaggaaccgtacgacagaaaattactatcatagactgcatgttcctttttttggacgataatatcatttgtctcataaccaaatacgctaacataaa

aatagaccagatcaaggataatttttcgagagagagagagactctcttttactcatgaaggccatggctgaaatctt

>90892|AGAP002350-PB

tgtttcaagctttacaatagtttcccacagttctgttgcttttgcacgcagcttatctaaactgagcccatcaagcctcttgatattgatgggctcagtt

tagataagcttcgtgcaaaagcaacagaactgtgggaaactattgtaaagcttgaaacagagaaatatgacctcgaagaaagaca

>90891|AGAP002350-PB

ctgcttcttttcctcaatttcacgctgtctcctctcttcctcttgcttcttcctagctttgtgcctaagctgctgcttctgcctctcctacagttctttc

aggtcatagtcctgcctcttctgtctgtcttcgaggtcatatttctctgtttcaagctttacaatagtttcccacagttctgttgcttttgcacgcagct

tatctaaactgagcccataaatatcaagaggcttgatgcgaatgctcagggagattctcttctcttccttcaacatgtcatctgcacaattggagaggaa

caagaccaaggaacagttggaggaagagaagagaatctccctgagcattc

>90918|AGAP002350-PC

actgtgggaaactattgtaaagcttggaacagagaaataaggccttgaagaaagacagaagaggcaggactatgacctgaaa

>90916|AGAP002350-PC

tcatatttctctgtttcaagctttacaatagtgtcccacagttctgttgcttttgcacgcagctcatctaaactgagcccccatcaacatcggatttctc

c

>90914|AGAP002350-PC

gaaatatgacctcgaagaaagacagaagaggcagaacaagaccaaggaacagttggaggaagagaagagaatctccctgagcattcgcatcaagcttctt

gatattgatgggctcagtttagataagctgcgtgcaa

>90962|AGAP002367-PA

ctggcgactgctccgatcgcgggcgctgccgtgctggcgactgctccgatcgcgggcgctgccgtgctggcgactgctccgctcgcgggcgctgccgtgc

tggcgactgctccgctcgcgggcgctgccgtgctggcgactgctccgctcgcgggcgctgccgtgctggcaactgctccgctcgcgggcgctgccgtgct

ggcgactgctccgctcgcgggcgctgccgtgctggcgactactccacctgcgggtgccaccatgctgttgcctactctgcctgcggataccgtcgtgcag

ccggcttctcctcttttggacgatgccgtgctgccgacttctcctcttttggacgatgccgtgctgccgactgctctggtcgtggcccctgctgtgctgc

cgactgctccagtcgcggcctctgcttcgcatccaactgatccactcgcgttcggtgccgactgatcagctcgcggacaccgccacgctgacgacttatc

ctctcgtaaacaccaccgcgctgctgacatgcgctcgcggacgcagccgaacttgcatctactcctgtcacatggcctgccaacagctccactcgcgggg

agctgtcatgcttctgtctgctcaacttcgtgtggtctactgtgcgtgtctggtcagctccttgggggccattgtcgtcgtgtctgcttatctccacagg

aagctgtatggctggcactggctcagtccacggttttttgggttcggtaggtgcttgtcaggccaacaactactctgctcagagagtggttgctggacaa

ctcggtcgccatatcatcagatcagcttgctgttggaatgttgccgtcagatattgtatctgccctgcaagcaggttaatgacctggtaagtcactttat

tttgggaaatgatcagtataatggttaatctgtattcacatttaataagagacccattagtgacattatcttttagtattgtgtgattttcctaggtctt

ggcttcattgcgtagtttgttgattttaatggcgccggtcaattagtccaacaattttttaaattttccttaaaaaattattttt

>90963|AGAP002367-PA

caatgaaatattcacagaagagccccaaatgtttgacaaggcatgtttgttgatgaatttttagttttgatagttcctaatgacatagctttaaccattt

gatcatctgataatgcactcccaaaacatgttgtggtaaatatatcattcatttttgacaacttgagcaactgctccatatgtctgaatcccgtgagttt

ggattccatctggggccctcaagcatgacattaggtagcccaacactgcatggcttaaatgaatcccagtgatagaagaatctttaatttcaatgctctt

tataataatctacatcattacattaaaatctctaactactgcccatgactgaaaaattatttttaaggaaaatttaaaaaactgttggactaattgactg

gtgccattaaaatcaacaaactatgcaatgaagccaagacctaggaaaatcacacaatactaaaagataatgtcactaatgggtctcttattaaatgtaa

atacagattaaccattaaactgatcattgccagaaataaagtgacttaccaggtcattaatctggggcagatacaatatctgacagtaacattccaacag

caagctgatctgttgatatggcgaccgagttgtccagcaaccactctctgagcagagtagttgttggcctgacaagcacctaccgaacccaaaaaaccgt

ggactgagccggtgccagccatacagcttcctgtggatataagcagacacgacgacaatggcccccaaggagctgaccagacacgcacagtagaccacac

gaagttgagcagacagaagcatgacagctccccgagagtggagctgttggcaggccatgtgatgggagtatacgcaagttcggcagtgtctacgagcgga

tctgtcagcagtggggcggtgactgcgagcggatcagtcagcagcgcggcggtgactgtgagcagatcagtcagcagcgcggcggtgactgtgagcagat

cagtcagcagcgcggcggtgactgtgagcagatcagtcagcagcgcggcggtgactgtgagcagatcagtcagcagcacggcggtgactgtgagcagatc

agtcagcagcgcggcggtgactgcgagcagatcagtcagcagcgcggcggtgactgcgagcagatcagtcagcagcgcggcggtgactgcgagcagatca

gtcagcagcgcggcggtgactgcgagcagatcagtcggcagcgcggcggtgtctatgaggataagtcgtcagcgtggcggtgtccacgagctgatcagtc

tacagcgcggcagtgtccgcgacgtgatcactagccggcgcggctgtgtccacgagctgatcagccggcaccgaggcgg

>90976|AGAP002369-PA

gtcacttagaggctggggctgttgcacaggcggtggttcactcgctgccaccctaaacgggttcgtgtccgtttgcctagccggagcctgatcttgtgac

gacctcgcaacgctcgctgtaggcctctggtaggcctgagttgacggttggtagccttgttgcacttggtaattctgtggttgatggtatccctgctgtt

gggcctggtaactttgttgctgatagccttgactctgatacgcttgagcctgatacccactttgtgactggaatggctgagtcggtgccgctggttggta

gctttgctggactggtgcggtgtggtacggttgcgctcctgctggttgttgtgactggaaagcttgagtctgatttacttgaggctggtaacttggctcc

tgagcatactgttgttgtggaggctgctcttgctggtagtatcctcctgtaactgtgggggcatacccatttgcctctaatgccttgtcgacattcgtct

cctgaataccttgcgattcatcctcttgaaagcgagaatattcgagatgatttttgaggtctgtagaaaccggtcgttggtcttgcatcgtgtaggtttg

atattgcttgtcgggcggatccc

>90997|AGAP002377-PA

tgcacatgtgcggcaaaacaaacacacacacaaacacgcacacacgcacgcgcacacacacgcgcacacacacacacacacacacacacacacacacaca

gattatatataattatttacaatataattacattgtgatgtgcagaaatactatgttaagaactgctttcaggtggtggtacccagccaccaaattcctt

tgacaattcttgtgctacagcaatacacagcctgtcttggtgtggagctgcgaccacctgtatgccaattgggagtccctcactgttaagtccaagtggt

acatgtgttgctggcataccaaggacattaaataccattgtatacaaaacaccagcagtctttatgtatgaatcattgtgatatatggctgctgttgggt

gggttgggtatataaataccccactgtctcctaaaatatccacaaatttcttccttaaagcatttctctgagcacagtaatcgttgtatttgtaacgtgg

tattaacacattcatttccttcaacaaatagaaagccagtgctgctggcgagtactttgaaataccaaatattgacttcagtatttccacaaatagatta

tggctctcctttggattgtcttcattttgcaataaatttggtattcctggcattccaaagaaaacagctgcactcatttctacagagtctgacatttctt

cgatattccccttctgaataggtatgtcaaatttcttgtgcaaaaaattaacagctttttgtaatgcttttctgatctcattgtctaaatgtaatataac

atatgagaatcctgcctcttccatataaatgactcttaaatccttcatgttaacttcctcatctagtctaagaccagatgctttctcaccagccattact

tttaacactggtttcaaatcttcagaataacgtgtaagtggccccacagttaggtagtgcttgaactcatcactagtagcacttggataatgtccttcaa

ttgatatataccctggagtaggtttgtggccaaaaattccattgtacatagcaggtatgcggatagagccagcaatatcagaagcaacacctataattga

tgctccagctcctagcagagcagcctctcccccagaagatccaccactggttctgagaagatcatatggattacatgtttttcctgtgatgagattattt

gtttcccaactaaggcatagttctggtgtggctgttactgctaatggaatacctccagcctttttcattagagcaaccacctgtccatctttgtctgcct

tcataccttttcttgggacagaaccaacacagcagctcattcctttgagtgtgcagctctccttgacggtgaagggcactccgtgcaacggcggcagctt

gtgcaggtcacctgcggcaccgcgctgggctaccaggcggtccgcctctcgcgcctcctccacagccgcctcaaagcgctcctccaccaacgcgttcaga

aaagggttgaccgcgcggatacgctccacgtacgcccgcaccacctcctcgctgctcacctgctgtgtgcggatgagctttgcgagctgtgaggcgctgt

ggaacagcaggcggctctgtatgggcggcaggcgcggggccggcctcagggaacgcaggtactgcaggggtcgcaacatcagccgcaacagcagctggag

caggccgatcagccgcaccccgatctccatgactgctttttctccgagtcgcgccgaccagccgttatctgtgacgtgtggccgcggcttctgctgctgc

tgctgctgctgccacggcg

>91023|AGAP002381-PA

ccagtaccatcctccataggaggactacgaggacattcagatgatgttcctgtgcagcgggactcttgctcacatgtggcgtactgtgcatcacgacact

tgactgcgaccggcatgtactggcagttttggcagcaaggtgaattcttgtcactgcaaactgctccctgattacgacggagcttgcaatttttatcaca

acaggggtcattatcttctgtgcccaggagaccagcatcacattcctcatctccttcaactcttaaatttccgcaaaatgattcctctggctcagagaaa

caacggccagatttagcttggaggactttacgaatggaacgtagactgcagggtgaaaatctcttgttattgacatcatatccactgacactgtatgtat

acatcagatatgatcccccttgacttgcactgggactgcattctggtatgtcaggatcatgctctgagccccagttatgtccaaattcatgagcagtaac

gagatctgcttctcttgtaatgactctttgaccataatggttacgacttgaactcaaacctgaattcagatagagtgtgtaaccattcttgaaatattct

ggtgtgcaaatgcctccaactgaattccttcttggtgaacccacatatgcgagccctaggatacctccttcaaattttaggtcagtgaaaagatgggcta

aacagaagtccttatggctgtattcacgactaaaaacctccaataaattccggacatcccatttctctctaaccatattgtaatgagcttctccaccacg

tactctggtaggttcgctatggacaacgatcttctttattacaaatcccatgcccttgaagccatcattttcttggcggtcatgccatgttgtgtcattg

taaatcttatgaactctatctatcaagctaatcaaataatttattgtagtctttgtattactgcctcccatttcctgataaaaacggtaatctgcaacta

gaagcaatggacatcttgtctttgtgggtgtgtattcgtattgctcagcctggcgtttgtatctgactactacttcttcttggtcctcatcttcttcata

atcgtcgtcgtcatggctatgtcct

>91045|AGAP002391-PA

gtgcccagttgctccacagatcaacgatgtaactaacaagtacgtcggaaacgaggactgcctctttctcaatgtttacacaccagagttaccatcggga

gcgagtgatgcgctgaagccggtgatggtgtggatccacggcgggggattcacgacggg

>91052|AGAP002391-PA

cggctcttccgtcggcagcggcggcgtgaagacgttgaggtagaggcagtcctcgtggcccaaggggatggagcccttgcgctgggggcacatgctgccg

aagaacagcgccttccggacccccttccacggggacttgggcactggcgcctggaatcggaggtccggaaggcgctgttcttcggcagcatgtg

>91048|AGAP002391-PA

cagtcgacaagaaacctagaacaccaaggcggtaattgaaagtgacaattacgacgccattctcaagcaagtactcaggtccgtggagatcagagtttcc

actgcccacagaatatcctcctccgtgaatccacaccataacaggaaccagttgtttatcgacatttgtcggaatctttggagtgtatacatttatgtat

aagcagtcttcatctcccgcgtactctttcgtgatgaagtcacgttgcggagctatgtgaccttctttcattgcattaa

>91041|AGAP002391-PA

acacccagagcaccatcaaacccaaaattataacaatataatgggctgcgactatactttgcatggtactttgctgtttgatacaacccgtgtgctatca

ttatgtcagtctgcaacatcaaaataaaactgccttatcttactggaattacttctcatgaaggcatgatggagcttaaaaatattctaaaaaagccaga

tttgctgaaagaaatagacaatgactttgaaagattggtgccggaagatatggaactgggagaaaaaagtggaagattgaagaaagcc

>91047|AGAP002391-PA

gtgacaagcacctttatattgctgcaagcgcaatagcgtcttgtacacgttcagtcgtccatcaacgtcgaagacgtacaggtaaatgggcttcgagggt

ggagttgtggacagtttcttcgctgctctgagcgtagggtacacgaaccagaggtcacccttcatctcagcgaattgctgcattgtctcttcgttcaact

tcttatcaccaaaataaaagcttctcatgctctttgccacgtttgttcgatcttgcggattgattttagcgttagtggggagtacgatttctaagtcctc

gttaaaagcatccaaggcctctttactgcgacgaaggtcggctatttgatacaagccctccatcgaatttactcccatgatgaccggtaccgagctgtgt

tggcccttagccaacacatcttcgaactcttcgctgatgaaggccccgccttctgcgtcggtaggttccagaaccggacggaatgggctcatgtctagcc

gcagtttttcctgaaaacgtaagaaaagaacaaggaatgcttactttaaatgttttgtcgtaaatgaaatcgttggagacacagcacgagatcgtgttgg

acggatctagggaaagaaactgtccccatggcgtttcgaagaaaccataatgcctcttatcttaaatgaatgagaaagttagaaaaaaaaccaaaatttg

tatactgggcggctgtttcgttcaaaatgccaactgactactacactacttcgtacgtgtaaaatgtgatgagagtttgaagaacagagtaacagtttat

ttttgtgaatactagtcacgtacctcacgtgtcacaaaagtttactgaagttttttgaacttcataaattaaaaacatagtaggtaatcctaatgatgat

aacatgaaacaccatagaacttgg

>91039|AGAP002391-PA

gctctggaggagcttctgttcattaccttatattgtcaccactggcaaaaggtatttttatgtttttagatttgttccagcgtgctattgcacaaagtgg

cacagcattaattcctggatcattcagcagattttcaaaagagagaactttccgtttgggagaagttctgggctgcaaaactaatgactcacaagttctt

ttgaattttttcctggaactccctgtgaagaaactgatagaagctcaaaatatggctttgtctgatgaggagaaacggagagatgtacttttcccatttg

tcccatgtgtggaaccaaagattgctggttacaacgcagtacttcctgatgaaccaaagaccttgataaaaaggtgttcattttctgatgttcctttcat

tgttggtctgaattcacatgagggaatagcctttgctaaagatgttattcagaatgtgaaagtaagggaagaaattgaaaatgacttcaaaaggttgata

ccgctggatatgaatattgataacagtagtgatttagcacatgaagttgccaaagaaatcaaaactttttattttggagaacattcattgtgtgaagaaa

ctattgaacagtttgtgaatctgaaaactgatttgcgttatgcatatgctgcacatgagacagtgagacttcattcgttccacagctcagctcatgtgtt

ttactacctattttcttatgaaggtgacctgaacagcgtaaagaagcaaatgagagcagaacatttaccaggtgcgactcatggagatgaactgaattac

ctgttcagtaagtcttcaaatggcagagatattcaagcaaaagatcagaagattatatcagaaatgacctgtatgtggaccaattttgcaaaaacaggga

atccaacgcctaacaagagtgaccaagtaacagtattgtggccacggtactcacatgaagatcagagttacatttgcatagattctgagctgtcaataaa

acatggactgtttagtgaacgtatggaattttggactcagctatacaaaaagtactgtcacacattaacataataaataatatagttgattatgct

>91037|AGAP002391-PA

gttcctcaaacggccgcttggctatgcacagcccgtcgctggtgatgtgcaggtaggactcgtccttctctgtgaaagccggccatgacacctccacctc

ggggtctgccagttggcccgttttcgcaaagttggtccagaggcgcgacagctggtccgtcagtttggcttcgaaggagccgggctctggaacgccacca

aaactttccgtacagacgatataggcatagccccctccgtgtacagcaccgtcaaacatcttctgcaaattgcgtatcagttctcccaacgggccctgtg

gaacacggtcactgatgtagtggcagacaaagaagtaggctggagccttggacttcaaggagtggagtttagccgcacggacggcgtggtacgtgaacag

caagtcgcccagaagtgtgcagagttcgaatttggtatcaggtccaatatcacggtctccaaagtagaagcggcgtatctgctcagccaaggcgtccttg

tctgagtcagggcagcgtaggttggttgggatgagcagatggtgattctcagcatagccagatagtttaatggaccattctgattgatctgtagcgaagg

ctgagccctcatgagtgccaactcccgtgataaagggcacagcttggaagtctccagagcgcagcagctgtgctggctcccttgggaagaaggccccacc

atcagcgtcagctggctccaccactgtgccagcaaatgggaacagcagacctcgggccttgtcgtcttcactgcggccaaacaaggcgttctcgacgagc

agccgggccggctgttgcctcaggaagtccaccagctgttgcgggtcgtccgtcttgaggcccaggtgtgcagccagggtgaaggatcgagcccgcatgc

ggtcggagcacacgccgtctcccagaaccacgccactgtgcgctatggctctgtgaaacaggcccttggccaacggcgacaagacgagaccgtggcagga

ccagcctccagcgctgccgccaaagatggtgacgctgtcggggtcgccgccaaagctggtgatgttgtccttgacccagcgcagtgccatcacctggtcc

ttgaagcccatgttccctggcaccagacggtcccctgcgttcaggaagccgaggactccgaggcggtagttgagggagacgaagacgacgccatagcgga

tgaggaaatcgggaccgccaaagtcttcgttgccagagcccagcatcagaccaccaccgtggatgtggaccatcactggcaggagaccactggagccctc

tgacggcagctg

>91043|AGAP002391-PA

ctctctctctctctctctctctctctctctctctctctctctctctcatttgttttataatccatgcaaaatgtgtaattttttttcaaattcagttact

ccaataaaatggtttttgtttgttttttacaatcctttaaaaatttggtttgaacttatcatttaaacagtgatccatatgaggtcattctgttttctga

agtactaaagcgatgatctatttgaataataatttatttctatgatttttgttttgttttttgtgcttgttttcaatgtgaaatgaatatgctttcataa

ccaaatgtagcacattcctttctatcttaatcaaatgaaacatttaaacagcgcccacccaggaaatgcaaacagaaagatggagagaaatttgagaaac

ttaaccaatcaaaagcaacagagccttcatcaattgtttttagatagtcattgtttagcagattgaagacgtgctggatagtatgtatcccaccagaaat

tgaaatattggctgttaatatttcttttgattttcaattcctgtccaatgtctgcaaatgctgggtcttctaatgtgtaggctggccagtgctctgtcac

aacatcatttagttcatatgttggatcaccagttttggcaaagtttgtccaaagagaagccataatggagcgaacagtatgttggtggctgtctttatta

atgtcttgcctgttatcagcaaacaaaaataagtagcaaagttcagcagtgtgagtagcacctggaaatctgcgatcaaccattctggaagtaagtgcta

ggtcgctttcaaaggaaaactggtaacgataaatctgaggtggattgggtgctgatgcgatgagtctagcagttatgtctgaccccatattgaagtgaag

gtctgcacacatcaaaacaagcctcctatggcgatcatctggtgatgtgttgccaaaataaaattgttttaatttatcacaaactgtttttcttgtatgt

aggtcttttataagaagactatcaccaaacactaactcaaaattgtcaatcatgaaatccattttttctgtatcctttagaattccagtaagtgcaagca

tggcaatcccttctccggaggttccacccacaataactggaactctgtgaaaacgtccttgagtcatcagtaccaatggttcttctgtcaagaaagcgcc

tgagtgttccttctcaacacatggaacaaatgccactgtcaaagctcccatttgttccttttcaggaacaactttggggaatgtttcaacaagcttgtga

gggtcgacccctctgaggaattgaacaagctcctcactgctgtcagtatgacagcccagaagctctcccagtttaaaacttttctcgcggattccatctg

ctgctgcccacatgcttaaggctgaaccactctgcattatagccttgtggaataatcctttggagagcggggacagtagatggtagtggactgaggcagc

gcccgcactctcgccaaagatggtgacgttgttcgggtcgccaccaa

>91050|AGAP002391-PA

gtttgtatttctttattacaaatttggcataagtgacatttcttctaacagtagcttcctttacaactgcaaatacgttaagagactttggattgattat

tggcatctgtgcacagtatgaagagagcgctgaaggtttcacggtgcgtgatatgaggccaatacctcttgttgtagtgtagaagtactgtgcagtctct

ctagccgccatagcgtttgtacaggtcgtcccacaacgctaccctttcggcaaacggcctccgttggacagcgaatccctgccggccaacgtacaggtag

gctgcgtcgtctgcggtgaaaggtggccatgatacgtccagttcaggatctgtaggctgcccagtcttggcgaagttggtccaaaggcgagtcatcttca

tggcgatttttccatcctcggaatccttctccggcgggtctccatacatggcgatgcgaaacaagaacgccaaatctgccccatggctggttgctttgaa

tttctcgttggatctggcagcctcacccatgagaggtccttgaggaacaagttctcggggtctgtcgaagttgtagaagtacactggctggggagactta

gtactgtggactttggccgccctgactgcagcataggtgaacaccgtgtctccaaacaggtcgtaaagctgcttgtctgtctcgggcccgatgccgcggt

cgccgaagtagaagcgacggatctcctcggccacggcgtccttctcggattcgctgcatctcaggtcgacgggaaccaacaagcggggattgcgcgccat

gttggacatcatatccgtctccacgagcgcaactgtggccgttactgacccttcgaagtcgcagacgccggcgatgaagggcaccgcctcgaaatctcca

ctgagcatgacgtccgtgggatggcgagtcatgaacgctcccccctcagcgtctggagcctccagcgtgcccgtgaagttgaagatccagcagcgagcct

tgtcctcttctgtgcgacccaacatggcgctctcctgtagaagtctagcaggctgcttgctgaggaactccacaagctccttggagtcctgcgtcttcag

gcccaggtgttctcccagcaaaaatgacctcgcacgcacatccttcgagaagagtccatcgcctgtcgctactccactctgggcgatggctctgcggaat

aggcccttcgccatcggggagagaactagcgcatggcaggaccagcctcctgcgctgcagccaaagatggtgatgttgtcggggtctccgccaaagctgg

aaatgttgtcccgcacccaccgcagcgccattacctggtccttgaagcccatgttgccaggaaccacattgtcgcctgtgctcaggaatccgagcacccc

caaccggtagttaaaggagaccatgatgacgccgtgctgtatgagcaggtccggcccgaaccagtcttcattgcccgagaaggcagacagtccgcctccg

tgcaccatgaacatgactggcaggccgcgggcgccttccgaaggcaacttaggagtgtag

>91044|AGAP002391-PA

cgatcagctgatagaagcatgcgaaaattatgccccacagctgatcgctgcagttactaaattaaagtagttagaacgttttatttgcttggataaattt

ttgttgacgtctcttagtaaccatatcatttttgtaaaaggaaggttaattataacagagtcaatgaagcattaatttgtttgtattcacaactaagctg

aaagcgaaatctcgctgcgtttgagccgcggcttgttttgggattttgtaagttgttttgcgccttagcggagaggtggtgttttcgaacggctgtgttg

acgcattataactatcttcggtataatgattgttacgaactagagccacaaaataacatatcctgtacgatcttctaaatttttttataaaactgactga

aaatttcgttcgtttctcgaaatttatctttaagaacagttttttgcgttcgtacaagtttccagtatgaatgaatttgtgacagtcaaaacgtctgaag

gtatattaagaggaaaatcggcgaaaacttcatcggggatcatgtattacagcttccaaggcattccttatgcgaaaccccccataggaccgctgcgttt

caagccaccacagcctttgagtcaatgggatggaattaaggatgcactggaagaaggagacatttgttcacaaataaatatgaagggccagtacaaagga

agtgaagattgcctgtatctgaatgtatattctccaaagagtacatcaaatggtaatggttcaaaggcagtgatggtatggattcatggaggtggattta

caatgggatcgggaaactctgatcgttttggaccagagttcttacttgaaaaagatgttgtggttgtcacaatcaacttcagattgggagcactaggttt

ccttagtacacaagattccatcatacctggtaacttaggtctgagggaccaaattgctgctctgcagtggattaaaaggaacattaattacttcagtggt

aatccagacaatgttacagtatttggtgaaagctctggaggagcttctgttcattaccttatattgtcaccactggcaaaagatttgttccagcgtgcta

ttgcacaaagtggcacagcattaattcctggatcattcagcagattttcaaaagagagaactttccgtttgggagaagttctgggctgcaaaactaatga

ctcacaagttcttttgaattttttcctggaactccctgtgaagaaactgatagaagctcaaaatatggctttgtctgatgaggagaaacggagagatgta

cttttcccatttgtcccatgtgtggaaccaaagattgctggttacaacgcagtacttcctgatgaaccaaagaccttgataaaaaggtgttcattttctg

atgttcctttcattgttggtctgaattcacatgagggaatagcctttgctaaagatgttattcagaatgtgaaagtaagggaagaaattgaaaatgactt

caaaaggttgataccgctggatatgaatattgataacagtagtgatttagcacatgaagttgccaaagaaatcaaaactttttattttggagaacattca

ttgtgtgaagaaactattgaacagtttgtgaat

>91051|AGAP002391-PA

cgctgtctccgtagcagcagcggcgacagagcgctgttcccgacgcttggctgttccttcgcaggcgggatgcacgtggagctgacgacacggcaggggg

cgctgcgcgggaaggtcgtgctgtctgtcagggggcagacctacttcagcttccagggcattccttacgccaagccgccactcggcgaactgagattcga

gccaccacagccctgtgatccatggaccggtatcagagatgctaccagagagggaaatatggcccctcagtttagtcttatggcaaacaaatatctgggt

gacgaagactgcctttacctcaacgtatacacgccaaagctgccgacgaagagtagaagcgacatgctgccggtgatggtgtggatccacggcgggggct

actcgcgcggctccggcaacactgacgcctatggccccgactacctgctgcagcacggagtcctcctcgtcaccttcaactaccgcctcggtgcactggg

attcatgacgacaggagattccgtggtgccaggtaatatgggcctcaaagacatggtcatggccttgcgatgggtccgtgagaacatcgccaactttggt

ggcgatccccacaacgtcaccatatttggagagagcgccggcagtagggcgtgccacctcatgatgctgtcgcccttggcgaaagggctgttccagcgcg

tagtgtgccagagcacagtgtctaccagcggcagcatgcggacaccggtggccgagaggagccgacggctcgcgcgccacctggggctgccggagggcgc

ttctgcacgcgagctggtcgccttcctcagggacgtgcccgccaggatgctcgtcgagcaggcgccctacgcgcgctccaaagaggagacgatcctgcag

gatgcgtttcccttctggccgacagtggagcctgaggactcagagggaggatccttcattacgcaggacccagaggacatcatggccagcggaaactaca

atgcagttcctgccatcatcggtgtcaactctcaggagggctacatctacctcaaagatcttctaggtgacgtggcatccattcgcgacctcgacaacaa

ctttcatctgctagtcccaaaaaccatcgcaccgacagaagagcaacgttcatcagtggcgcgcctcatcagaagattctactttgcgaacaagcgcctg

gacgccagcacattgcggcagtactcacaggtgcgtggagacgcgacgtacatctaccagtgctatagggccgccaagagacaggccgagaagttccctg

ctcaacctgtatacttctatttcttcgacgttgatacacggctcaactggttcaaaagactgtacggggcgcaggattttccaggtgccagccacgccga

cgacattggctacttgttccgtgtcaattcgtttgcttctcacgtcgatcccgactctgtcgagggcagaattattgccaggatgactaaactgtggacc

aactttgcgaagacagggaaccccacacccgttcacggagaccctctgctgagtgtcacgtggccagcagtcagcactcgtgatcccagccacttgtaca

tcagcaaccacgggctcagcattgagaaaaatctgctcaaaccgcggatggacttctgggacggactgtatagcaagaaaaacgggctaatctcccatct

gtagtttttgagacgtttcaaatcctgaaacacttacttggatttctgccaacacagaaagaagccttacgtccattccaagaaagactgcatgtaacac

tttttcagagaatataatttcactgtacagatatgtattaactctaagtgtatgttactatagccactctgattacaaagatgaacacgggagtaactat

tgtaataccttcttcctctagtaatcatatctacagaaaaaaaatgttggtattttagttgcgtcaatggtaaattaaagtcagttatgaaggga

>91042|AGAP002391-PA

ttttattttctcacatataaactttcatatattagtaatatgtttgcacataacaaactatgtaagaaatatacatatttacatagcttttaagagtcat

tttatatgactctttttacataaataaatgttcagattgtgagtcattcacatgatcccaaatcatcttacattcctcagtctttttagttggtgaacta

atgtctttacattgtttagtaaatattacagtcttgcattcttacagaggtatttcattaccatatcagtcataagcacatattatcacaaattcagctt

gttgtagagatcttcccaaaatgacatacgttcttgcaacaagttgcttcctacttccaatcctgtatggctgatgttgacgtattctggattatttgca

ttaaataaaggccatttaacagtcagcagggggtcatcctcaggagttgggttcccagtcttagcaaaatttgtccacagttttgtcatcctcactatag

cagtttcttctgcagaatggggtttgacttctgttggcatggcagtatttttgaagaggtaggcagtttcatcagagtgacatgcaccgcgtttatctgg

aacgccaaagagtttcctagcatagttgagacctgtatctgcagaaaagtgatacaggtgaactggacatgacttctgcactgaatgtagagttgcagct

cgcacaacaggataattaaactgtttgtcacccagcagattaagcaatatatcaagtgtggactctgataccctttcatttttgaagtagaaagttctta

tagcttcagatacaggtgtaacatcctttattttgtcagctatgtcagtgggtacaaggtacttgaaattcttatccaggaatgacagtacttcagtttt

tgttaataagtctttcaaataaagtatgccttcctgttcatttatgccaagcagataggggactggtaaatagtcattatttttaagaatttccagtgga

tgcctcttaatgaatgcatcactctcaggtactgactcttccacagtgggcaagaatggaaaatatgaaaaccgtgctttgtccttttccgagaatcctt

tgacctgtttctcagttagagtctttgcagggaccttcatcatatagtccagtaggtcagctgacttgtttgtggagaggccaaggtactttgctagacg

aaatgccctttctggaacatctgtgttacaaactgtgtccgcaatggccactccactttgtgatactgcacgatgcagtaaacctttgccttttggtgaa

agcataagatactcagatgacattcctccagcacttcctccaaacactgtcacattatctggattacctccaaaaagtgcaatgttgtctttgacccacc

gtagtgccattacttggtcctttagccccatattaccagaaacaacagagtcaccagtgctaaggaaaccaagaacaccaagacgataatttaatgtgac

caatacaacttcgtgttcaaggagatactctggaccataaaaatctgcatttccagagcccataatgtaacctcctccatgtagccacaccatgacaggt

ttcagatcccctgagttctcttctatcgttgaagtgtagacattaaggaaaagacagtcttcatttcctttgcaagatccagtcagtaagttaatctgtg

gtgccacctcaccaaaagttgtggcatctctgacaccattccatgattcaggtggctctggtggctggaacctgagcttcccgactggcggcttcgcgta

gggtatacgcaggaagctgcggaacactttgccagctggcgtcagctgcgtcttgccgcgcagagagccctgcggtatgcgcagtgtcacgtgcgcgcca

gccttggcgctggcctgccgcacgcccgacatgcagcaccacgcacatcggccgagcgtcttgcctcccccccacatggccaacgccggttaattgccca

ctcttaagcgttatcgctactgcaggcacacaaacctactgccgttgctgctctcactccag

>91038|AGAP002391-PA

cccatgattaatatattaagtttattttttcacatttacataaaaatgaagtaatatctgcagtttaacagctcacaaatattacatttattgtgccaaa

taatgtcttttacaaagttcacagttttgatgctgatgtactccatttgtatagatcatcccaaaactccatgcgttctttgttaagactatgccttaag

ctgagatccttttcgatgttcaggtaacatgggtttgtcaggctatatggtgtccacttgacctttacatcagcatccaggtgtggtgtagggtcaccag

tctttgcaaaattggtccacaacttcaccatccttcgtatagttgacatttctatagagtcatgttctacagtgatatcaacaattgacatatggaataa

gtatccaatatcatcgatatgacaaacaccaggaagatgagatgcaccgacaaaacgtttgaagacacccagaacactatcaaacccaaaactataataa

tacaatgggctgtgactgtacttcacatggtaatttgctgtttggtacactccgtgtgcgaagtttatatcagtctgcaaatttacataatccattatgc

tatcttttgtgagtgctttaccatcaaaataaaactgtcttatcatatcactggctttcttcaatctttcattttctctcacttccatatcttccggcac

gaatctttcaaagtctgcgtctatttctttcagcaactctggcttttttaaaatatttctaagctccattaagccttcatgagaagtaactccagttagg

taaggcacatcacagaattttcctgattttacaatattttgtgggttatcaggtaggaagacatcttgtaaactgccggagtccttttctggtattggta

aaaatggtatgctgtttcctctccgtttgtcatcaggagttactatttttgcagtgccttccactatctgttttggagtgcaagtcctgagaaaatcaat

tagcacttttggatcatttgttttacatcccagcacagcacccaaggcaaacgacctctccttcaaagtttttctgcttgcccaaggattaaaaatagaa

ccactctgtgcaattgctttgtgaaaaagtccttttgacattggagatatcaaatggtattgtacagaggcaccaccagcactttcaccaaatattgtaa

cattatttgggtcaccaccaaagtttatgatgttctgctgaatccatctgagggcagctacttggtccttaaggccattattgcctggtatagttacatc

ttccaaacttaaaaagccaagagctccaaggcggtaattcaatgtgactacaataacatcagcagcaactaaataatctggaccataaagatcagtgttt

cctgatccaaatccaaatccacctccatgaaaccataccataacagctttcagtgattcattttctgaaggaatctttggagtgtagacattaaggaaaa

gacaatcctcttcacccctatacttctgccgcacccagtctgcatgcggggcaactgcgccttcttctagagcatctcttatgcctgtccaagactccgg

tggctgtggtggctgaaatctaagagggccgaccggtggctttgcataacgaataccctgaaagctgcaatatttccctccatactttgcttcaactact

tttcctttcaaaatgccttgggcaactttaactgtcactgtttccacaccagccattcttgtactatacagccttagcaccttcttgtttgttgtaggtg

cgaaaaagcagcgacgcgaaaaaagtgaaaggctggtttcacttattgttacataagttctcaagatctgtttaagaagtatcattttgtatgcatgtgg

tgttctgagcgtttagctgataaattcaatacatttttacacctcttggcgcattactgcaaacatcttttacaggaaaatgcgcatgcaaaatgcgaca

tacgtatcagttctctgtgtagaaatgaaggtgttgtctgtaggtcacaaacctcgccaccagattc

>91036|AGAP002391-PA

taaatatttgtgagcataaagaactaggatatcatacaacacattttcatctcgatgtcataaattgtaatatcttaattttcaaatttgtgtcttttac

acaaaaagctgtaattgtaaactatggtcacaaacatactgcataaattgtaaacattttaaaactagatcaactgttataaaatcataagctgatggcg

atgatgtattgtgcatttctgtcttttgtgcaaaaagatatgatgatgtaaactatgatcacatgctgccatatgaatagtaaacatcctattgcttttt

tgtggtcttaataagagtatccatgaatttacctttattgaggtttaatcacattatgaattgtaaaagaaaattaagtacagttatgtgaaagtaataa

tttgcttgcaggtttctctgtatgtaaactcttgtacatttgtaataattgtactgttgatattcatcacattgtcacatctgatcatcttgtaatactg

acttgtcctataccagtttttaactatgtacaaaactgatgatgaggttcaataatacaatacagtacagtacaatacatatgtttttcttctaatgttc

ctggcttcatattttaaacatccattggtgctcattttttggggcattagtgtactatatacaatctttccactgtgaccttcttgtaattgtctagctg

ttcagtggaatgtgtatttcagttcctgcattaggtaatgatatgaattttaatcttgtttcaaagaactttgatcaaacataatgctacacaatgaatt

atttttaataaatactatgctaatcgattagaattaatataaaacaataaatatcaacattttgaaatttattttggaaaagtatcacaactactgcagt

tctatataccagaattttcttgagaggaaataatgtagatgtccataaacagtgactgaaaatcaaaatgtgagtgtttcttgaacaattccaacaaatt

tcctgtagcagaatgttagcagtctgtgtgaaggtttcactaaaagtaccacacccacaatacagtgaaagggtaaaaatggacgtagcacaaatcatca

tctctgatttagaagatgttgggaaaaacagattaactttcacttctgcaccaaaagttttattagacgcaatactgtcttcaaatgcatgctttagctt

gggttttcagttccacaacttttcatgaagatgggaatatccctctccagaagccaagacgttttggttccagttccctctgtatggatgatttcacatt

tatatccaaaaactcgtgttctttaagtgtatactttggccagataatgttgaggtcatcataaggctcttcagttggtacaccagatttcatgaaattg

gtccacagtttaactatgattttccgaactttatgttcatcagattgaggactgaggtttagaggtgtgtctcccatagtaaataagtaaccaccatctt

ctccatgtgaggcaccagctacaccattatatgtgaagacagaaataggtgataaagttccttcatagctgaaacggtagaagtaaatagggaattctgc

tttagttgccatggatgaaactgtactgttcattccctctagaaagtacatatcacttaaaagatccacaagttcttcaaaggtttcagtggtcataggt

tttccaccaaagtaaaattctcttattttgtttgctgcagtctctctctctgctttgctgtgtaaacgaagatctggaccaataacgtctataaaattat

cattgagacgcttcactttctctggatcatctgccagaccagtccatttcatcagaaatttcccttctctgctggcaacaccagtcaggagaggcacatt

tatgtagttgcctttgtgcaatatatcttctggatgctctgtaaggaaggcaccctcaacatttggctccaccgatggtacaaaaacgaacataaacagc

cgttccttgtcctcatcactcagagcatgtttcattgcttgtggaaacttttctgctgggatagttctcagaaacttaactaattcttttgaatctgttg

ttgtaaggccaaagtgttctgcaaggtggtgagatcgttccttggctgtttctgagaatgaccaaggatttaatggtgagccacccatagctacaacacg

gtgaaataaaccttttgataggggtgacatcatatgcatgaaagcagatgaagctccagcactaacaccagacaaaattacatttctgggatcaccgcca

aaattaatgatgttctcctttacccaacgcaaggctgcaacctgatccttgaggcccatgtttccaggtaccacagagtctccagtggtcaggaaaccga

ggacaccgagccggtagttgacggtgacgagtatgacgccactgtcaaccaagaagtcaggaccatgtgcagccgccgtgccagagccttcgacgaatcc

acccccatgtagccacaccatcactggcagctttgttgtgctgtcatcatctccacttggcacctgcggtgtgtacacgttgaggtagaggcagtcctcg

ctgccaattagctggcgcacgatcaggttgtactggatgcaggccgcgccctctgacagcgcgtctctcacacctgaccagctctctgccggctgtgggg

gctcgaagcgaagctcccctacaggagggcgggcgtagggcaggccctggaagctgacgtaggagccgccttgcacggtggcggccctacggccgcgcac

caagcccccggacacccgcacagtcacgaactcctccggcgcagacatcgttcccatgagggcagcaaacgccagcaggacgtacgcagcgagtgtcatg

gcggaaggcgagcg

>91091|AGAP002412-PA

tcgacccaccaggcactaaaagacctccagctatgaggatatcaaacaatgcttccccataacgtctgtagtccagtttagacccggcagtatccagaaa

tttggaaactgcttctaagtcagtcccagctttgttaagcccttgtagtatagcatctctaaacccagctggatcatatttctctttttcatctcttttc

cgtgtcttgattcgctgccctgacagtactggcttttctgtttttttgactcatacaataactggaaaattacttgttaaacttgcagcaccaaacccct

cgtgaggcctttgatgttcagagagtattgagcaacgtgtgacgatagaggcggaactaaatactagtcaaattaactgataatttgcaaagatcggaga

tcagcaaagaagtacttgaggaaggaatgcgaaatggcggtgacgagtggcttgagttcggggatcagtcgactacgatttctgcagaaatgtatcatcc

ctggaacatattcatgtggcattaaatgtactttattgaattcacaacatgtgaggtgttataaacactggaatccaaagtttaagaggcttaggggcct

taaggttcagaaaattgaattaccaaattttgaagaagacactgatgaactgtctgaggaagaagtacggagtagacttaaggaaagaggtcttctccca

ccaaggccatgggtagaaagaccatttttcataagttgcacaggtggtgcatttgaaccttatgtgccaccagaaggtgatgggaaaatgtccataattt

ctgttccaggagcaaagcagaaatttgaaatggttgaaaagaaaagcaagtcaatgatggccattcgtaagatccgctcctttgaagaagagttcaacac

tcaggtcttcacagatgccgcacaagaaatttacataaatgctcacaactgcatggcaagcaaggattttgagaagttacaacagtttgttactgaacgt

gcctatccacaaattaagcataattcagaagataaaacaatacactggaaatttgttaaatcattagaagcaccaagagttgttcatgctcgttgtacag

acatcatctcaaaggaaaatgtatttgcacaggtgacagtgagatttcatacacagcagatcctggcagtatatgatcgtttcggccgcctgatgcatgg

aagtgaaattttagcaaaagatgtattggagtatgttgtcttcgaaaaacatatggctaatcagtatggaatttggcgaattcatgataaaatcatacca

gattggatgcccccaagagaaccaatggaaaaaacttacgtaaagactgaagagccaccagttgaaacccaagtgcagactgttcctgaaaacaaggcac

aagaaacagtttcaaatgtatgatctttgtttattgtccaattgtattttctgtacttatgctgtgttttataatctgtctgtgaatatttgaaggaaaa

aaaaaagaatttgtgtaagtgaataaacaaatttatttggaagttt

>91252|AGAP002421-PA

aatgacaccataatgacctgcacatctaatgacccatacgggaagagatggtgtctttagtcgagaaccaggttttctagattcgtcatcactccctgta

gcttcatcccaaagaagacctacttctgtccgagccagcactccaaattgtggtaatgcataatggtcttcatctcctacataaatgaccccattg

>91482|AGAP002439-PA

cactcttctctcacacacaaccttttattgcagctcttaacaaaaatatttagacacaatggacaggaaaactcattttctacaatatacagacaatcca

ctggtgtcttacatgtttagaatgcaccggccaataaacacactgcacactacgagccatcctgacaaagtgtccgttcgggaatattacagataaattc

cgacactgacattcgagaatcgacgtccccggcagttccggatcgagacacacaaatatacttcaccgtcaacatccgcttacccctcctccgagacagc

tccctgtagagtttaccggtggactttgcccgttcgccaaccttgagccgtcagtgcggacacggacggcgcggactcaggcgcccaccgacggcgcggc

gtcggtcagcgtctcggcgccctcgccgagcagctcctgctgcgcttccagctgcctctccttgacggcgggccgcaccacggtgaagtagcggaaggtg

ttcatgccgaggaggccacagccggcgacggtgagcgcgacgcagctcagcacgaaccccttgtgcagcttgttcagcagctcattcgcagccttcttcc

gcaacatgtccgctctgcgacctttcttttacaaattagtctgcaactacaagtcaacagatgcccacgaagccatgtatcgctactcgctagtcagctg

ttt

>91790|AGAP002470-PA

ttaaagttgaattattgaaggctgaatctggcgcaactattacatttgatgatgtattaatggttgtaaacggtgacaacattcaaatcggtgctccagt

tgtagctggcgctaaagtaactgcagaagtgattggtcatggtcgtcacgacaaaatccgcatcatcaaaatgcgtcgtcgtaaacactaccgtaaacaa

caaggtcaccgtcaatggttcactgagttgaaaattactggtatttcaggctaatcacgaggagtaagagacatggcaactaaaaaagccggtggatcga

cgaagaacggtcgtgattcaaacccaaaaatgttaggtgttaaagtttacggtggtcaaactgtaactgctggtaacatcatcgttcgtcaacgtggtac

tgaattccacgctggtgcaaatgttggtatgggccgtgaccatactttatttgctactgctgacggcgtagtaaaattcgaagtgaaaggtcaatttggc

cgtcgctacgtaaaagttgaaactgtttaagttttaa

>91865|AGAP002483-PA

atgccttgacgaactgctgtactgcgacagctatcctgtcgaagacacagttttaagagtccttgatcggaagttgagttcaggaggcaatgctgcgaat

aactgcaaagttttggcggaattgggtgtcaaaagtgatttgtttggaacgcttaacgttaactctacaactgggagggcgacgctggtggtgccgtggg

cggaagaaggagcggctgcccgaggtcctgagggcgtgttcttctcgcccgccttccctccggagcgcgtcgtggacacgttgggggcgggggacacgtt

ctgtgcgggtcttatgctggccctggccaggggtcggccgctggagcagagcctgaagttcgcctgccaggtcgctggtaccaaagtcggcgtgcgtggc

ttccgaggtctcggggatctgctcaggcagcgaggcgttcggctcgagtagccgccagcttcgcagaatacacggttctgctgcatcagcatctccttcg

tccatatggcttcttgtgttacaaaataaacttcactgactctatatttactgtatgtatctcatttccaatagagtag

>91936|AGAP002494-PA

tcccggcgtctccgctgctgactcgcgccaccagcagagagctgtgggcgcccgctggcgtcaggtccgtccgcacgctgatgcctcccctcgaggttcc

gtagttcagagtgacgtcaccgtgcctccaggt

>91935|AGAP002494-PA

cccgcggcggcataagcgtgaagacggacatgggtcctgggatggctaccagccggctgtacatcgccaacgcgaaccgccgcgactccggcaactacac

gtgcgccctggcggccgtcgccagcaccaccgtcttcgtctacgtcatcagtggcgagacgcctgcggctatgcaacacggcggggcatccgggtggtgg

tcacgtggccccggcgtggccggcgccctgtggcgg

>91969|AGAP002498-PA

ttacattagcttttacatacacaatacttactctattttagggtttagataaatactcaaaagagtataaaaaatatcatttcgatacttctgttccttt

ccttgtgcattattctcacaatacagttctgcagctttaaaatcctttaattggttcaccaaaatatcaagagctttctcgtgctcttcaatctttccat

acaatactgcgcattcctcatgcaggttgctgttaagcattttccccaacaagagctgcgtgcgacataggttggaagtctgaagcaatgtctgaagttt

cagtctaatagttttaatagtttccggtgaagttccatctttttggagctttgcaatgttttctaaatacaaaatagctaactgagtgtgaaacttttca

acctgaaaaataaagagtatactagttaaagggacaaaatatgac

>92000|AGAP002505-PA

gctggagtcggtgcgcgagcactcgtagctggaggcggccagctgcgcctcgcggtgcatctgctcggtcttcagctgctgctgcttggccagctcccgc

tgctcgcgctcccgccgttcgttgcgctctgcggagaagccttctacaaggatggcgaccagcaggttgaagagcacgtagttgccgaacgtcataagag

ccacgaagtacagggctgcccagtgcgtcgtcttctccatgccgttgaacaacaccacgttccaatcttcctgcgtcaggatctgaaacaccgtcacaat

tgcccagagcaaggaatcgaaattctttctgtcgcattcatctgtctctccgttgtcggccttattgcag

>92020|AGAP002513-PA

catgatctttccttgtgtcacagtttagttttgtgagaggaagccggagtggaggtgtggttaagttctgaagttttaaagaccaggtgcgtgaggggcg

cacctgtgatcgcaccatgccacgaaacaagaggcgtatcgagctcgtcacagaagatgagcgccagttcagcaagcgtgcaaggagtcatgtcagttct

agaagaattgcaagagacaattcagcatttactcaaagacgatgtgtgcagttgtttagggaatacactgcgcctactgaaccagacacattgggacctg

aaggaatggaaaaattctgtgctgacattggtgtggagccagaaaatgtagtaatgctggtattagcatggaagatgaatgcaaggcaaatgggattttt

tactcttaatgaatggctgagaggttttgtggatctacagtgtgatacaattccaaaaattcaggcaaagcttgactacctaaaatctcttctgaatgac

tacaatacatttaaaagcatctatagatatgcatatgattttgctcgggacaaggagcaacgcagcatggacattgagactgcaaaggccatgttgaagt

tgttgctgggaaaacattggtctctcttcagtcagttcaactctttcctagaaagctcaaaatacaaagttattaataaagaccaatggtgcaatatttt

tgaattctcgcgtactatagtgaatgatctgtctaattacgatgttgacggtgcttggccggtgatgctggacgagttcgttgagtggcttacttctgag

agacaggctcagccttacagaagctgaaagtaagctgtgtaggaatttccattgctgttggaaacttctttgctttcaccttcataccctgcactgctga

ttggttatgtttaaaccaaacacacacactaaacaatgtaacagttttgttaagcctcatgtaaaagttacagatcaacgtttacaatttacactcatgt

atttgtaaagtaacagagatatatattgttgctctaaggtccgattcataaatacatgtaaagtacctaaatcaatacattatttttaaatattgctaat

tacatggtgctcatcacgtacaattgtttatttggcgtggagaaagaatttgagatgttgaatatgtgtagtgaaaattatcgagtcagacaatgtatgc

acacgctctgtgacaagaaattgacaaaattctatttcactctgttctgtctgatgatactgcttgtttttccccactcatactgcagctaattatgtaa

tatacaatacatttgtgtgaagaactcagttcctagccactgctcagctcaaagtcaagtaagttttgttatcaaacattcatgtgaatagtgggaaaaa

gtggtgaaaaatgaccttggtatcaatatatttttaaaacatttcatgtcactacagctagtttggctgtttaatagagcattaacaaatgaggtggaga

attatggaacaatgtaaggagaagatttgaccaaagaagataatatgaactatttaagtatgtaggagtatttaaagtgtgatttattaaattggttgaa

actttgtgtgtccgaaactgtagtatgactttgtgtgtgtgtgtgtgtgtgtgtgtgtgtg

>92041|AGAP002519-PA

gccacatcttgcacaccacgatgccgaacacccaccgctggatcagcgagtacgccacgttgaacggcatcaccagcaccgccaccgtgagatcggcgac

ggctagcgagacgatgaagaagttctgtacgatgcgcagcggcttgtaggtgaagacgctgag

>92099|AGAP002521-PA

accagcaacctttaatagagacaacccacatacagttaacagttccattctttctaatggacaagaatactgttttacacaatatgaactgctcagacaa

tccataaagctagaaaaatacttctgtaacattctttcttcaagaatttgcctttaaagctattgaatcattaagtgcaaggctttgttaaacatggtaa

gtacacattataattaatacttcagtatatcttatcacaggaagccgttttcctcaacacaaacaatgttttaaaaaatactggttacaaatactttcat

aacaaggaatttctcgcattttgaaaaatatatcccatcactctacaaaaatatattgttagaaatttcaatagttcagagaactattttctgaataagt

ttatggtgtaccatatttatcgtgggcacatgcatcctcttcctccatcaactggaagggtgacaccagtgatgaaagatgcatcatctgaagccaggaa

agcgatagtaccagcaacttcctcgacattcccaggtctccccagagcatgtgttgtcttacacttctcaaggaacttttcataatctgcatcagacatg

ccaccacgtttgtgcacctcagtaacaataacacctgggttcacagcgttcactctgattccacgatcagcaagctctagtgcaaggcaatgtgtaaact

ggtccaaagctgctttggacattgcatatgccaagatacctggaaaagatctgataccagctatgctggaaacattgatgatagtgccttttgttttctc

aagatgaggaactgctagctgagtaagcacgataactgaccgtacattcacattcattgtgttatcatactgctctaaagtcatgttgtatattgatcct

ggggttaactgtccagcattattcaccagtacatccaactgtccaaagtgaccaatagtctcatctattatgcgcttacaatcatccacactggtcacat

ctccgaccacacataacacctcgtcttgaggcagaccagcttcggtacactgcgttgctgttttcttcaaattagtctcgttacgaccacaaagaacgag

gcggcaacccaattttgccattctaatagcagcaccagctccaatgccggaactagctcctgtaattacaactactttcgacttcgatgacatgactctg

tcttccttccacttcctcacctgctaatcactccctgcctcccggcgaggtgttctgtgtggtcgcatctccgac

>92120|AGAP002524-PA

gtttgtagtttagtaatcgatcaagaagggtgttgttgatgtagctgaaatgtgtgaaaagggaaagaacaaaggaactcctttgctgcgtaatggtgtc

cgttgcataggtgttgaatcaatggatgaagattcagaagcttctgactggcaaggcttttgagaaattaatgtaataaagctccctccttaaaaatgtg

ctattggtaagattatataaatgtttgcctgtattaatgaactgatgtaaatatttcacacatctatttttgtgatatcccctgtgggaatatttttcac

agtgct

>92121|AGAP002524-PA

ccagttttcaaattagttccacattcttgtttaatgagtcaaaaccggctgttgctaactgccatcatttatttcttaaaacatactttttggtggattt

tatggcaaattatcatgatttctattagtatatgtaagtcttaactatgtttcagcacactacatatgaatctaatacacaccattaattgattatggcc

tatttttaaactctgccaataatatggaataatactgcacactttctataacctgaaagctacatcctgcacacacaaactttaatgcactggcttctgg

ccatgtatctcccacagacttgccataacacagctacatgtgaactcgagcatgtcagtgattattaagatcttataaattaacaagaaaaccttcttac

ttaaatgtatggtaacaaaaacaaaggacaacactttgactccaaaaaaacttggaaatctgtaacaagtgaaactaaatttgtatttcactttaaaata

aatacaaattgaatagcactgtgaaaaatattcccacaggggatatcacaaaaatagatgtgtgaaatatttacatcagttcattaatacaggcaaacat

ttatataatcttaccaatagcacatttttaaggagggagctttattacattaatttctcaaaagccttgccagtcagaagcttctgaatcttcatccatt

gattcaacacctatgcaacggacaccattacgcagcaaaggagttcctttgttctttcctagcactctagccaacaggtgaatggtaaatcctttctggc

gggtaggctctgtaatgcgaatctcttcctttagcagtttctccttataagctattaggtgtaccgcaatatgcacagattcactctcaacaactgcggc

aataaggtaacaatttttatttggaatatccgagaagcatgacgcatctaaggactcggccggcctgaagtcgcaaacactacatggcacatcaaa

>92143|AGAP002526-PA

acatcaaatcacggtaatggcgaagccgtataaaccatttcacaaatagaatcgtctccacaaaacaactgagtacagaagatatctgattaaacgtggt

tgctacatacactggttggaaccacaaacttaagtgttgctacttttcttctgttcatcaaatatcccatttagactttttcttcaggaaaacttctgaa

ccttctttatcaacggcggtgtaataacccatgccttcgtattttgatctcatgtaagcgatgtatccgacgcatattcccatacacattaatcctatgc

ccattacgactttgttctgtaacaaaaagtttcatttcatacatgttccagcacatatctacttatggaaaataaagcaactgcaaacgccaactatggg

tagtatatgtttatgaagaaattatttcgtggctagctttgactttgaaacataagaacgaaattttaattttaacacgccactttttatagcttttatg

gtgctaagatgcaataatcctatttttcaagttgctatttttatatttgacatagcgaagttacataaggggggcacagttattacgtgtatcttacagg

tttggcatataattcgaagttggttacacgaaaaatggcagatgttcttaatgactttatgccatcacccggagctcgttcttgttttgcagcttcttct

tttccacccattatttacaaacagaacgctcttcagttacaacacactcatctttgctacgagaatattcgccaaagctaac

>92171|AGAP002532-PA

agcgaaatgtgcagctgttgggtgcacaagactccatttaagaaactgaagagtcatgaattccattgcaagaaattcagccactggatatggactttca

atcaatgcattcatatcagatacttttggtccatttatatcccgctcctcaaattttgctgctaacaagacagatactaatgttagtaagttcagacttt

ccagggcgatactatggttatccataaaaatgtcaagaatatatatagccaaatgaactgttgtactactaagtttcaatccttcagctatgttgcagat

ccatttaactaaatgacttctttactgtatctgaaaaaaagtaaataacagcaatatcagaaaatgaaaggaggcc

>92190|AGAP002544-PB

ctgtggtgaagaggacgatggccttgaggcaggagtattcggcggagtcgacgtgcagcgccttgagcttctcgacctgctcctggaagatccgtatgtg

gtccatgaaggcgacgacgcggtcggccgccatgggggaggcgtgcaggccggcggcggccagcagcggagccacgtgcagcggcatcgagcactgcgac

gcgttcaggacgaacagctcgctccacaccagccgcagcagcgccacctggtccgtcacctggagatcggggaagaagggtatgttccgcgcccactcta

cggcggagaagaggagtcgggcggcgagttcgcagatattgtcgatgcccatgatgttgttgggctgcatgcattggccgtagcgcgacgtggggtaggg

ctcggcgcgcagcagcagcgagatgtagctggacaggtaggagtggccgttgaagcccgccgccgccgctgccgccgccgccgcgtcgccgttggtcagc

gccagctggcccggcagccccgggggctgcgtgggcggcacacgcccccgctgcacggcttctctcctcatgcccatcttgagacac

>92277|AGAP002564-PB

tgatggtgaacatgacttggagagaacacagaaggtaactgaaccagtcttggctgctctatacaaggcacttaac

>92276|AGAP002564-PB

tctcaactccaatcgagtcaaacctcttcttaattgtgccagtggattcgtcagcagcgagaatacccttgccaggtgccgctactttgtgagcagtctc

gctcagctccttagcgatgtcttgcggaaggttggaaggcatagcgaggtagtatacgcgttagacttcgc

>92275|AGAP002564-PB

accgttcataaagtctaagtagatactacctcgctatgccatccaaccttccgcaagacattgctaaggagctgagcgagactgctcacaaggtagcggc

acctggcaagggtattctcgctgctgacgaatccactggcacaattaagaagaggtttgactcgattggagttgaga

>92299|AGAP002567-PA

gtttatccgaagtttgacagtagtgactagtgacgtgagatgtaggggtgttgtgttgttgctcggtccgcatgatagagcctagcgtagaattccgtcc

attttattaagactaggaagtgcctattaagtcgtcattgaaggatgctaattatgtaaagtagaatcatagaaagaaatgtcatcaacgaaaactgaaa

cggaatgtattgagtcttctgcaaatgtgatgtatcaagccctcagcgaactctgcaagaacaatgcagcgtttttccagcatgatgatactgaaagtgg

acaacgcctgtatgctggtttccttgccgttatggaccacgttgaagttgtctgcccattagttgaaaaagtaaagaaagtggctcctcactatgatttg

gatgtgcggacacctggtaacggatatcgcagtttcgtggtgatggttgactcctgtgttacccattgtatgaaactgaataaagagatttgctcatctc

gtgactctattctttttcggaagagtgcttacgtccggtgagtcttagattatgttaaagataaacatgtggaacaaaatttgtataaatgggag

>92315|AGAP002575-PB

atgagttatgggaaatatccaaatgaatcagctcatttaggtccgctaactcatctggaagcctgctcatctgattccttgccaagtttagatctgctcg

cttttctcggatttggtgaattttcaagccatctgtttcgaaacagtggttcgca

>92316|AGAP002575-PB

agcccacattcaccgaggcaaatggaaaaccgccttaaaaaccatccacagactggccagcacattgggcctcgacaccaatccactgggtggattcacg

ctggggaccggcacgccctcccgcccagaaagcaatgcattagactgcacagctaactgggtgagttttagtctcccttacatgaagatcagcacagaac

cattaagaaacatgaaaatgaaagcagttatgtaacatttatgtgttctctaggaataactctgcatgttgatgttacattacagtcaagcttaatccct

gttactcagtaactacagtctacttttcatagattattctctttattcaccagatttggcctagccattttgtgtctgttgaagttttgtaaaatggaat

acaccttacactaagtacagcatatgaacttgtgcacaaaatcacaaccaaagtatgctttgatgtttgtcacatttgagtgggaaagcattttcagtgt

tgtggatgtctgatgacaaaaatgacttagttttgtaattagtatttagtagccctatcttctgtctgctgccacagttagcctgtattctcaatgctgt

gatgaatatattattacttagaatattggtttagcttttaaattttcataattttatgcccatcatatcattacatactgcctttgcagataatgtattt

tacatgcatcctaatgtgctttaagtttggttgttggttatttcagtttgtttaatgactgaagcactgtttgaactactgataaagacaactaaaaact

aagtatgcattacaagtggtgtagtgtccaaagaatttaattttaatgagtttttattattgacagatctaaacttggcaaggaatcagatgagcaggct

tccagatgagttggcggacctaaatgagctgattcatttggatatttcccataactcattcacaacgctaccccatgctgtgtacaaaatgccaaagctg

gtgaaattgagtgcgcacaacaactacattattggtaagttacactccacgctgcaaaataaaatcagattttccttggcacaccagaatcatttcttga

attttgtcaatgaacattcttacacagtactgtacttattagtcacagttttaaaatgtgaaacaatcaacattttagtaaaaatggtgcactaaaattg

tgtttaattgttatttttctagtagtatagggtgcattcaaatacattctttttgatgtgtattgctgacagccgatccttacaaaatgccaggtgcaag

ctagcctattctctcaatgccacaattaccaactgcttttgtaaatattaaaggaataataatttcattgtggtttttaatgctcacatgatactgatgc

tgttgctaccaagtagtgtgttaccaactacaaagtaacccccccc

>92338|AGAP002578-PA

acaatggcaaacaatacaagcagaccaatctgcagcagtggtgccattgcttttattatggacttcagcaccacctgcagacttggtattcttgagacca

>92339|AGAP002578-PA

cattgtcatttttgccataattgggctcgaattctattcaggggccttacataaatcctgttacagtttagaagatttcgatgttatagttaaagaggga

gatttagaaactccttgtaatgcagacagcgagttgactgcaccaagaggaagttatgtttgtgatgctaatatttctacttgttatgaaaaat

>92354|AGAP002578-PB

attccagtgcattactatggaaggatggacagccattctttattggacaaatgatgcactaggtagttatttcaactgggtatattttgtgccattaatc

gtcctgggttccttcttcatgctcaatttagttcttggtgttcttagtggtgaattttcaaat

>92355|AGAP002578-PB

cttgtaaggataagacgatactggctcaaaaactggaaaaaacggaggcctatttccttggtattttttgtgtagaagcgtccctcaaaatattagcgct

aggttttgtactccacccgaaatcatatctccgcaatatttggaacatgatggattttgttgtagtagttacagggtcgatgaccatatttgcggagtcc

aatgtggatgtggatttgcggatgcttcgatcattccgtgtgttgaggccactgaaactggtctcaagaataccaagttcataacactcatccctttcaa

taat

>92410|AGAP002587-PA

gggtccacggtattacacacaataagccaaggacggtaagaacccagatacttgaactaaagctgccagtcaaatcctttagtttacctagaagaggccc

gaaggcgacgttgaggagcccagtgaagacactctgcagtccgaaggcagcggggaagcgatccgggct

>92413|AGAP002587-PA

tctgtttgctcgtctgtttgtagccagttaaggtaactactggttttgtttctgtgcactgtcttggacgcaggcagatgtctgtatttcttatcgcctt

tggaaaacaagtcagctctttgtctcttctgctgcgacaggtttcttcttcctcagtgcagttattatcagttccagcgtccacggtattacgcagagca

tcatttgcacattcagagcgtggatacatatagggtaactatccgtaacgtcacggatgagcccgatgagtgggccagtggacattcctatgatgccgct

gacggccatgtagagtccgtaggcagaggggaagcgctctatgctgcagtactcggcgaagacgagcgagaaggtgatgtggatccagctgcggaagaag

cccagcacacccgtgatcaccgccagcatcgtgaagccgctcacgtagat

>92412|AGAP002587-PA

tccgaccagcagctggtggccaacgggaagatcgtggcggagcagctatccgggaagcaccatcaagcgccgcggcaggaggaccgcgagaacagccggc

ggctgctgctggacccaggccagggcgccgacggggacagcgacggtgacgacgacgtcgacgccccaacggaccgggtggcggaggacgcagaggcggc

agcaggggacggcggtgcgcgcctgcgcagaacggcctcccaggtgtccgtggtgagtgtcggcgccgtggccgtcatggtgcccaccgcggacgccagc

ggccacccgatgtcgccgcgccgcttctcctgtaggcgcctgtggcggtcggtgcacgacttcctggacctgggcctgctgcgcgacccggtgtacacga

acctcaacctgggcgtgtcgctggcgttctactcggacttcacgttctcgacgctgctgccgctgttcctggcgtcggcgg

>92411|AGAP002587-PA

tcgcggtaaagctgcccgtcacatctctaactttaccgatggcaggtccaaaggccacggtcattacgccgcccataacggtctggatcccgagcgcggc

ggggaagcgctcctggctgcacgagtcggccaccacgagaggcgccagcacgaacatgaagcacttacagaagcccagcagcgcacacagcaccgcgatg

gccatgaagtcggtgaagaagatgtacagtgctcggatgaccagagtcgccgctgagctcagtaggaagacgagtctgttgtcgggcacgcagcaggctc

ccagcacagccacactgaagcggcccacgaggtcggcgatccccatcacggatatgcagagcgcggagtcctcgcgggagaactcgttgtggaacaggta

gagcggcagcagcgtgcagaacaccatgtcaacgaacatggagagcgccgtgccgaccaggatgttagcgcagcgcaggtcttgcagcagtctcaggtcg

aacagatccaccacggtcagccaccagcttttggacgag

>92416|AGAP002587-PA

gtacaccatcacgccggccgacagcaccgtctgcgtcgcgcccatcaccggcactcgccgctcgctgaagtacgtgttgaagctcgtgtacgacgcgggc

agcatcagccccaggcccactcctggtagtatggcgaaggtgatgatcatgtagaagacgctgttggcgaaaaccgtgaggaggttgccgacgaagaaga

gtgtggcgccagtgagtgccacctgccgacaggaatacgtcttgagggcgtagttcgccagcagtcctgtgaaggaatgcagcgccatgttgacgctggt

gatgagcgttactccccgggtgccctcgccgaggctctccaggaagtcgccgaagatgagaccgaagcacatctgagggcccagggccacaatgagcgtg

gcggcgagcccggcggcgacggcccacccccagccaccctccggcgccgcgccggcggtccccttgctgctgctgtcggcgcccgctgccatctgcccag

tactgctgccgctgccgctgccgctgccgccttccgagcgagtccgccccgccgactcaccgccgccgcgtccgaccgccacggatgtagactgcaggcc

agcccccactgtacgcttgaagccgatcaatctgttttccgctgttgtagcaatcagatggcttgctcgtgaaggtattgagacagtcaatgtttgtccg

tctcggtggatgcgcagtcggcgctgtttattaccaagcgaaaaggcctgcgtgcgatttccgtccgggtcggggattttcttcgctcggggagtgggtg

ttctgttgtccttacgttcgtgtcgatagaaatgacgttccacttgtgagatcgctgtataggcacacccttcactgactcgttggcgaaacctccagct

acgttttatgtgctgttctcacttttattctcttgaaactgatgcaaggcagagcggtcacttggcttcttcggtatttcgagaggtgttccttgtcgtg

cgtcgtctatggtggtgatcgacgagcgacggctgcctccgacagagtgatcccgtgggagcgctgctgaggacactatgccaccagc

>92414|AGAP002587-PA

acggcttctcagtttggactgtttgtacaatttgtgagtaagtctacaggatacttgaaccaccaaagtgactcttgtgttagtatatagtatattattg

aaataaaaaacatttatgtacatcgaattctattcttcagtgacacttgatcttcagcagtatgagtaacagaagtacgtcacataatgaaagctagtga

aaggtgttcggttcactcgtctgtttgctcgtctgtttgtagccagttaaggtaactactgcactgaaacatatcacgcactagttagtcatactacgaa

aagttcttccacgctacaactaatatattatatggttagtaccctatgagggctattatggcacatttatttctttcagtaatgctacaagcctttactg

acaattgcggctcagaaagttgagcatcctattcttagacacactgttataaaaattctagtttattaaacacaagacagagttctttgtactaaatatg

gtaatatatgaacacagcaaataattcgtgtgcggaactcaaacgagtccgccattgaatctctttaggagctataaatagattaattatggcagtcttg

tagctgcttcaactaaactttcaaatggcgctttcatacttctcaactataaatcagattctttagcgactacaatacttatttcagtctattctgttgt

gaaaataaactttgaaacatggatcctcaacgtaaagagaccctcagtctaattatatgccactcattgtggcaatacagacacaaggcttaagtcctgt

ctgtggaacatcagacctggttttgtttatgtgcactgtcttggacgcaggcagatgtctgtatttcttatcgcctttggaaaacaagtcagctctttgt

ctcttcttctgcgacaggtttcttcttcctcagtgcagttattatcagttccagcgtccacggtattacgcagagcatcatttgcacattcagagcgtgg

atacatatagggtaactatccgtaacgtcacggatgagcccgatgagtgggccagtggacattcctatgatgccgctgacggccatgtagagtccgtagg

cagaggggaagcgctctatgctgcagtactcggcgaagacgagcgagaaggtgatgtggatccagctgcggaagaagcccagcacacccgtgatcaccgc

cagcatcgtgaagccgctcacgtagatcatcactgtgcgggcgatgacgatgcaggcggagccgatgaggaagacggcgcggctggagaggcggtagcgc

gggcaggcgccctgcagcgagaaggcgacgcggcccagcacgtcgcaagcgg

>92409|AGAP002587-PA

gcttcaaataatagtttaattaaaaacttttcagtcaaataaatataacagtttcacaattcagttacatttgtatgaaaaaatgcacatactgcattca

aaataactaaattctaaacagcttctcagtttggactgtttgtacaatttgtgagtaagtctacaggatacttgaaccaccaaagtgactcttgtgttag

tatatagtatattattgaaataaaaaacatttatgtacatcgaattctattcttcagtgacacttgatcttcagcagtatgagtaacagaagtacgtcac

ataatgaaagctagtgaaaggtgttcggttcactcgtctgtttgctcgtctgtttgtagccagttaaggtaactactgcactgaaacatatcacgcacta

gttagtcatactacgaaaagttcttccacgctacaactaatatattatatggttagtaccctatgagggctattatggcacatttatttctttcagtaat

gctacaagcctttactgacaattgcggctcagaaagttgagcatcctattcttagacacactgttataaaaattctagtttattaaacacaagacagagt

tctttgtactaaatatggtaatatatgaacacagcaaataattcgtgtgcggaactcaaacgagtccgccattgaatctctttaggagctataaatagat

taattatggcagtcttgtagctgcttcaactaaactttcaaatggcgctttcatacttctcaactataaatcagattctttagcgactacaatacttatt

tcagtctattctgttgtgaaaataaactttgaaacatggatcctcaacgtaaagagaccctcagtctaattatatgccactcattgtggcaatacagaca

caaggcttaagtcctgtctgtggaacatcagacctggttttgtttctgtgcactgtcttggacgcaggcagatgtctgtatttcttatcgcctttggaaa

acaagtcagctctttgtctcttcttctgcgacaggtttcttcttcctcagtgcagttattatcagttccagcgtccacggtattacgcagagcatcattt

gcacattcagagcgtggatacatatagggtaactatccgtaacgtcacggatgagcccgatgagtgggccagtggacattcctatgatgccgctgacggc

catgtagagtccgtaggcagaggggaagcgctctatgctgcagtactcggcgaagacgagcgagaaggtgatgtggatccagctgcggaagaagcccagc

acacccgtgatcaccgccagcatcgtgaagccgctcacgtagatcatcactgtgcgggcgatgacgatgcaggcggagccgatgaggaag

>92415|AGAP002587-PA

gtctgcaagtcggagtaccgactttcgctcgggctttgcaagccagaggaccgatatctcaaagtctttgtcagtggatcacataaatgaaaggaatgca

gaggaacaaaacctgaacggagacaagtttaatatgtctctaaaccatttaacaacgcgaggacgtagcctgaacgataaggaaatgacgtcgaccgaaa

ggaaggaacatcacagagacgggggcgaaacgcagcagaagcgaccttcccaccacagtgctgggacactcaactctcagatacagccgtctcgaccagc

gagctctctaaaagatagcccatcaccaagagctgcagcaggcaggaacaccagaagatggtggtcagcagttgtggacctctttgacttgacattgatg

caagacttctccatagctaattctctggttggtatatctctgatattcttcgtcgacatgatgtttatgacggtgattccactgtacttgttcaacaccg

gactttctcgtaacgacgctgccttagccatgtctgtgatgactttctctgacatggcaggacggcttagtatggcaatcatcggcgcctgtggggtcac

gaagaacaggctgctgttccttctgggttcaacagcgtgcgtgtccttccgaacaatgatcatattcttcgtggagttcacgccaattgttgtactgtca

gtgctgatgggattctccaggagctacgtcactagcaccatgcctctagtgattggagagacgtgcagcaaggaacgctttcccgccgcctatggcctac

accaagtcatgtctggcatcctttacatcaccattggacctctaataggtttgctgaaagatatgaccaacagttttgtggctagtatcgctactttaga

cgtgattgcacttctttgcattgtatcctggattttggaaataattgtgacttacaaaaggaagcgagataagctggcacctaacatgtagaaggccatt

aacgacatttgtttacatggactaatggacaaataatgtgccagatgctgagtgtagcaattctggcagtgatacttgccaccattgcagtattattcaa

gcaaatctagtgtaagatattaattgtaaatgaaattcattttctgtttactgaaataccatatgtacaaacattcattttaccatttgtatttcattag

tgtataagtacatttttgagagagtgttacccatacttcatttataattaaggcacaaagataaactacaattgattggtccttgtaagattttctgcat

gaccttaacatgacaaattacgacacacagctttagaattagtcatagtctgttttgttttattccacacatttgtacaaaatttgtatatttgacagat

tttgtctcagcggagtggaaaagccaggaggtagccaaaataaaaatgtgatcataaattatttttttattttcaaaaattgtaaatattg

>92408|AGAP002587-PA

tggggtttactcagttggcaatgggactaggaattttcgggttcccgatgttggtgcaacatttagtggacagttttggcttcagaatcgcgcagtgcat

tatctgcgcgatcaatatgagcattattcctgtcttcgtaatcctgaggccactgcagaagaggagcccaggcaagagcgctgacaagcgctcgtctgca

agtcggagtaccgactttcgctcgggctttgcaagccagaggaccgatatctcaaagtctttgtcagtggatcacataaatgaaaggaatgcagaggaac

aaaacctgaacggagacaagtttaatatgtctctaaaccatttaacaacgcgaggacgtagcctgaacgataaggaaatgacgtcgaccgaaaggaagga

acatcacagagacgggggcgaaacgcagcagaagcgaccttcccaccacagtgctgggacactcaactctcagatacagccgtctcgaccagcgagctct

ctaaaagatagcccatcaccaagagctgcagcaggcaggaacaccagaagatggtggtcagcagttgtggacctctttgacttgacattgatgcaagact

tctccatagctaattctctggttggtatatctctgatattcttcgtcgacatgatgtttatgacggtgattccactgtacttgttcaacaccggactttc

tcgtaacgacgctgccttagccatgtctgtgatgactttctctgacatggcaggacggcttagtatggcaatcatcggcgcctgtggggtcacgaagaac

aggctgctgttccttctgggttcaacagcgtgcgtgtccttccgaacaatgatcatattcttcgtggagttcacgccaattgttgtactgtcagtgctga

tgggattctccaggagctacgtcactagcaccatgcctctagtgattggagagacgtgcagcaaggaacgctttcccgccgcctatggcctacaccaagt

catgtctggcatcctttacatcaccattggacctctaataggtttgctgaaagatatgaccaacagttttgtggctagtatcgctactttagacgtgatt

gcacttctttgcattgtatcctggattttggaaataattgtgacttacaaaaggaagcgagataagctggcacctaacatgtagaaggccattaacgaca

tttgtttacatggactaatggacaaataatgtgccagatgctgagtgtagcaattctggcagtgatacttgccaccattgcagtattattcaagcaaatc

tagtgtaagatattaattgtaaatgaaattcattttctgtttactgaaataccatatgtacaaacattcattttaccatttgtatttcattagtgtataa

gtacatttttgagagagtgttacccatacttcatttataattaaggcacaaagataaactacaattgattggtccttgtaagattttctgcatgacctta

acatgacaaattacgacacacagctttagaattagtcatagtctgttttgttttattccacacatttgtacaaaatttgtatatttgacagattttgtct

cagcggagtggaaaagccaggaggtagccaaaataaaaatgtgatcataaattatttttttattttcaaaaattgtaaatattggcacctaggtattttt

agttttcttattttattttgatgttaatgtaattatttttaacttttatggaaataaaagtgtac

>92468|AGAP002606-PA

ttttgactattaaactttttactttgttatttacaaaagttttcaacagatgagtaaaattaaataaagctttaattttccacaaaagtttgttcactca

tacaatcatttcaagttcccttgcataatctatcacttgctctgaaagttctattgatggaagagggtcttcactcttcttctcctcagtaccaaagtag

tagtaattatctggattaagagtccaattcttcttttctccgtatttcttcatggcagcaagactcgccatgttcagcattctcgctgcttcttgaacag

atatcttttcgtaggacttctccaagcaagttgctatttcatttctaattgtgtccagcagaatgtcaataaaaaaa

>92487|AGAP002630-PA

gaaacaattctcatttattcctcttcatctggtggtatgcctagctcttcgtctgtccattttgatggatcaggttatggaaattcgcctgtaatatgct

ctggttcatgccaaaggtgccacaggatccaccaccacatgtaggctccgactgctt

>92488|AGAP002630-PA

ggtatgcctagctcttcgtctgtccattttgatggatcaggatatggaaattcgcctgtaatatgctctggttcatgccaaaggtgccacagggtccacc

accacatgtaggctccgactgcttctgctaagaatattgtaatttttgcatgtggttttggggctgatcggtatacatattcgccaccgccatttagagg

tggtttcagcataaacgtacgcctctgtatactcttcgaagttgacgctaatccccgacacagcaacaaacccctagacgaaaacattttcacctcctcc

ttctacgaggtcattaacaacagacacccaggtcttcgggggaccaaaatatcaaagatcttcgacgctggagactgtccgtatttctacaacgattcac

tgcagttcgtcacaagactgcgcgatcaatccaacaagaaactgaaa

>92515|AGAP002638-PA

tctgtccactcatttcctgcctcatcatctggaggaagatctgatggttgaccagtaggaatttcagcttccttgttggagaggctgatgttgtcaccaa

attctccactaatttctgagttgaggtcaaatgagacaggctcttctggcggaggtactgttgccatgatctcctgcatgaaacttgatcttcttctctt

cccccggttttgtatcttgctcagttttaggaagacatcttccaaagagtcacaaccatggttacgaagcaagtcttctggagattcctctgccaagaat

tttccagcacgcatcaaaccaatcatgccagcttgacgtgtctcgtcgatgtagtgagtggtgatgatgactgtcctgttgccttctttggtgatgctca

ccaggtgatcccaaatgctctgcctgaggacggggtccacgccgactgtgggctcgtccaggatgagcagctcggggtcgtgcaccagcgctgctgccag

ggacacgcgccgctgctggccgccgctgagttccttgacgaacctgctggtgttgggcagcttcagcagttcgattaagaagtcgaacttggcatcgacc

tccttggctgacatgcctgcgagccatccgaagtatgtcagcgtctcccttatgccgaactcaccgtacagggctatctcctgtggcatgtagccgatgc

gcgggcccgggacgccggagcctcgggagccgggccggccgcccagcacccagatctcgccgccgtcgaggcgccgccggcccacgatgcagctcagcag

cgtcgtcttgccgcacccgctcgcgccgagcaggccgtagatggtgcccttggcgacggtcatgttgaggttggtgagcacgtggt

>92516|AGAP002638-PA

agtaactttattttcaatatactatacaatgacaaatgtacaacaaaatatcatctcaaattgtatgtttttggttgttcagaaacagagaaacaaaact

gaataaacatgaaaattaatcttaattttatatgtattgacacacaatgcattataaaagcatcaaaaagtagtaacacatttatgaacacaagttcatt

attaagaatgtattcagctccatattaattgtctatctctctctgtcacacacaaacactctatctcaactaaacatgaaaatgtgcagacagatgtatg

gtggcacgcccacaacacacacacacgcacacacacacacacacacacacacacacacacacacacacttttgtgtaactcactgttttggcaagactta

caacaaagtcaatacagatataaattcagtccccagagatgattaatgaatttgttacatacatagtcagttgatttattagcattattttttttatgga

cgtaaaaatgtctctcagatattggaaaatctgcttggcaagagaatttcatcctgattatatatctcacccacagtctatgctttgtgacagcatgacc

tggtagcatgactactggaaaaagtcatgtaatttaactttgtgtcatcagtgaacagtaatgtggctatttacagtcttagatagagaagaatagtgtt

cttcccagtcaattactgtatgcatgtaaatacgcacaaaacatacacataaaacagtaaatatatatatatatatatcacttatataataagtgttcaa

caaaaacatacatgtaacagaacccttacagtttcaccaattcttacaccatggaacagtgctgcctaaagattctatcagaattctttctgaatgttca

ttctttgttaagcaacagctccatgggaaacagaacaattctgcttcacagattttgtttgatagcttcaccagatgtgaaatacacagaagtgaatgct

gtcttcttacagcagttgctgtaatatttgtgtatgagttttaatttccactcatacattatcctttcttgaacttcaacactagtacactcacagtcag

gtagacaataatccaaatgaacgtagagacgaatccaaagtagacagttggctgatgaaactcccatcccctggcaagaacagttcgcaaggattctgtt

gactgagtcagtggcattatgaaacttatgtacctcaggatcggatgcatgccttcaattggccatataataccacagagcataacaattggtaaaaagc

ttcccatagcaagatatgtagcagtcctttctgttgcacacacacaggatacaaagaaaccaaaacacattccacagaatcctgtcagcattgtaagtgc

aatcacaatacccatgttgcccacacatgttatgccaaaaacagggaatgaaaatacaagcacaagagctgtttggccacacataaccatgaactgtgta

gctacatgtgaaaataatatctcggtcactgtaatacctgcaaccaaacccctttccagaagaccatcatttctctcaattaacattgcaccagatgtca

atgccacagacaggaagaaaatgatcgttaagaggactccaggagcagcaaagtctctgaagtttggaattattgcaccataaacaggcttctcaaactt

caatggcgtttgcgcaagatgtggattatagtcacaggcagaaagtatatcttgtgaaaaatccattaaggagtataccatatccctcctaagcatatta

ccaatttgctgatctgacatatccatccatacatttacatcactgccttctaattcataccattcagcctcccgattttcattaatccttttaacgaggc

tatgagagaaattagaagcaaactcaagaagtccccatgcatgtccattctggacagcataaagtccagcatcataatcttcatagggatccagtattac

tcctcgtttcgaaagatgctgcagataccggcaacttaatagttcatagctgcaaccttcatgttttgggcatggatccatagtattattgagctcatga

ttaacaatagataaatgtaatcccactgggtcacgtccaattgtaagacaatagagagcagtttctacaacaggcaaaccaaataagaatgccataaacc

catagtttcgccacatccacagtacattcttccaaagaagagccttcaggtgatgcaattgcgcgaccttgaacatatctgtccactcatttcctgcctc

atcatctggaggaagatctgatggttgaccagtaggaatttcagcttccttgttggagaggctgatgttgtcaccaaattctccactaatttctgagttg

aggtcaaatgagacaggctcttctggcggaggtactgttgccatgatctcctgcatgaaacttgatcttcttctcttcccccggttttgtatcttgctca

gttttaggaagacatcttccaaagagtcacaaccatggttacgaagcaagtcttctggagattcctctgccaagaattttccagcacgcatcaaaccaat

catgccagcttgacgtgtctcgtcgatgtagtgagtggtgatgatgactgtcctgttgccttctttggtgatgctcaccaggtgatcccaaatgctctgc

ctgaggacggggtccacgccgactgtgggctcgtccaggatgagcagctcggggtcgtgcaccagcgctgctgccagggacacgcgccgctgctggccgc

cgctgagttccttgacgaacctgctggtgttgggcagcttcagcagttcgattaagaagtcgaacttggcatcgacctccttggctgacatgcctgcgag

ccatccgaagtatgtcagcgtctcccttatgccgaactcaccgtacagggctatctcctgtggcatgtagccgatgcgcgggcccgggacgccggagcct

cgggagccgggccggccgcccagcacccagatctcgccgccgtcgaggcgccgccggcccacgatgcagctcagcagcgtcgtcttgccgcacccgctcg

cgccgagcaggccgtagatggtgcccttggggacggtcat

>92575|AGAP002652-PA

ctctcacaacaaggcagtttctatgatattaaccaagcacacaaagactctgcatgaagtaaaatgtacaatatctggctttcagttctcttctactgat

attacatcaaatgtgagaccttctttgtgcagctcttttattaaggatgtctttgaaaatgcagtagctgtagtaatcacacctcccctagctggcattt

tttcacgttctttaataattgtcactgctgaaaggacaagagcagtacaagtggcaccatacgctgggtttctacctttaatctgtacagttactttctt

atttattggcattacattcttgggtgttggttctggtaatctttccttccatccttcagcaaaaagcgtaaatctgaagagagtatttttcaagatttct

tctgcgggatcttcatgagttacataaccagatgtaaacattcctggatgcttaagaagaaatttaactccaagtctgcactgtgccagaagacccacca

acaaagctctgaagatgaagccaattgtggttaacaatgatggaagcacaaagtacatgttaaattgcacaggacgtttcatttcatttttatacaaatg

tctctgggaactctcagcaactgcaatatctgtccctggaaatggtaaacaccagccctcaacgtcagatcttctatgaggcagcaaccgttgttttagt

tttggagtaagtcgtggtaatctatcagggaacaatttccgttgcagtgactggatttcaccataatgggatgttacatgaatagcagattcccaggtgc

caaagtgtatggcgggtccctttcgatccttgacatcaacgtcgatctcaagatatgcttccacagagtttatgtcaccttcaaacttattttgcaggaa

cactactcccatgtctgttggaatgcagtcgaaaccacaggcattaataatgaagacacctttctcttctgccaatgtgttgtagctgagcataagtttt

tggatatactgaatttcaccagtaacatcaacttgatgagcaccactctctatacatgcttttactactggctcaccataaaatctatagggaccaacac

agttcacaacaacacgagctcttgaggccatatgaagtagtgagtcaaaattctgcaggtctgcctctataattggtatgtgtgacaaatcttcccctgt

tttcttactgatatcatctaaaatctgctttaacttcttagagctcctaccagctattccccatgtaaaaccatcatttactttcgataatcttaaagct

tcttcagtagtatattttccagtaaagccactggcaccaaatataacaaggtccagtttttctccttcaactgatcacattttatgaagcttgcttcacc

ggaattcggtggaatatgtgttcgcctggcacccgctgactttcgcttactactgaagaagctttaagattatcgaaagtaaatgatggtttaacttctt

agagctcctaccag

>92573|AGAP002652-PA

cctattagcaaattgggtgcatcatctgatgtagttactgacacctgtattcagtcattggtattttacccacaggtgcaactcattacctcaggatatt

ttttccatgtttcgatcatccataggatctgtagctgtgctcatgctatttactgtgctctgtgacatgcagtgtggagttgtggcttttgttccatatt

ttcaagaggtgattctgaacaacatgacttaactgatgtacacattgtgtacattgatggtctttatgctgaggaagcccagtttacactagcccacaac

atctggtgacaaatcatgtttttgtgagctaaaaacatttttgggggaaatgaagcacaaaactacattcatttcttggataactgttttagaaatgaaa

aattcaatttctaaagtcttgcattttctacacagtacaatctttcttactcaaaatgaaataaactttatagtaaaaactttacacatcatatagccta

acacagcaggttcttgttattggccacagttatgacaatgctttgaaattatgtaatctgacagcatgtgctttggaaatttcatatttcatagcgaaga

agatagtcaagggagaaatattttcatgtactgttaaaaggaaaaacttctctatctggaaagccacaagaataagtttagttttatttcagtagaacac

tacacattttatatggcatttgaagacgttatctcagaaagggaactacagtgatacataatatgttactctttacattaaatttttcataaaatacaag

ctagaagatctattaaatgttaaactgaagcagacagatggatcacatcattaagtgtatagtaccaactttggctatgcagatgagatccatcgtcacc

ctcccccattacacaacttcagtaaaaatgtctgaaaattaaaattttgttgacaaaatgcaccaacagtacaaagttaaaaaggttggaaatagtaata

tcagagtggcccatataagaaggaagaaaaatttttcaaaatgttagagactgtccacctctcttatgctaaaaaggagggaaaacatattcaacatact

atccccccctcccctccccctctttgtgcctttccattctgaccagtttgggtttgtaggtttctattttttttagaattgtgaaagtcaaacatttttt

ggatgttgcatacattgaaagcataggaaagatggcaacaaacttccccacaatgatttgattgccactgtgtgcagtttcacacatcatcaaacaatta

tgatgcaagttttttctaagtcaacagttttcgacctttttcttcatttttgtgctttacaagcactgaatgattgacacattatgacacattatgccac

attcattagttcacaaccatcaacacattaatttggtcagattttgttacaactctcagtttggagggtcaagactcacagttgcttattttcaccattt

ctcacaaatcactttagagattcgttactctccctcataggtctacactgcatccctttcagttttttcacacttttgagtagtatatggttgtggaatc

tgagactacagtctcaagaaaattgttgtaacattactttctatgtgctatatgctgagtgattaactgattaattggttgcttttggatacggaagttg

actgtttctatgaatcatgtctgtgcatacaaattcaacaacaactagtgcaaatcataagagaggtgttcacatcactaatagactagaagcttcaatg

gacaaccaaatttctacagcctctcacaacaaggcagtttctatgatattaaccaagcacacaaagactctgcatgaagtaaaatgtacaatatctggct

ttcagttctcttctactgatattacatcaaatgtgagaccttctttgtgcagctcttttattaaggatgtctttgaaaatgcagtagctgtagtaatcac

acctcccctagctggcattttttcacgttctttaataattgtcactgctgaaaggacaagagcagtacaagtggcaccatacgctgggtttctaccttta

atccaacaggaaagcgccggtagataggcacaatgaataaaacacacacacacacacacacacacacacacacacacacacacacacac

>92574|AGAP002652-PA

ctatccccccctcccctccccctctttgtgcctttccattctgaccagtttgggtttgtaggtttctattttttttagaattgtgaaagtcaaacatttt

ttggatgttgcatacattgaaagcataggaaagatggcaacaaacttccccacaatgatttgattgccactgtgtgcagtttcacacatcatcaaacaat

tatgatgcaagttttttctaagtcaacagttttcgacctttttcttcatttttgtgctttacaagcactgaatgattgacacattatgacacattatgcc

acattcattagttcacaaccatcaacacattaatttggtcagattttgttacaactctcagtttggagggtcaagactcacagttgcttattttcaccat

ttctcacaaatcactttagagattcgttactctccctcataggtctacactgcatccctttcagttttttcacacttttgagtagtatatggttgtggaa

tctgagactacagtctcaagaaaattgttgtaacattactttctatgtgctatatgctgagtgattaactgattaattggttgcttttggatacggaagt

tgactgtttctatgaatcatgtctgtgcatacaaattcaacaacaactagtgcaaatcataagagaggtgttcacatcactaatagactagaagcttcaa

tggacaaccaaatttctacagcctctcacaacaaggcagtttctatgatattaaccaagcacacaaagactctgcatgaagtaaaatgtacaatatctgg

ctttcagttctcttctactgatattacatcaaatgtgagaccttctttgtgcagctcttttattaaggatgtctttgaaaatgcagtagctgtagtaatc

acacctcccctagctggcattttttcacgttctttaataattgtcactgctgaaaggacaagagcagtacaagtggcaccatacgctgggtttctacctt

taatctgtacagttactttcttatttattggcattacattcttgggtgttggttctggtaatctttccttccatccttcagcaaaaagcgtaaatctgaa

gagagtatttttcaagatttcttctgcgggatcttcatgagttacataaccagatgtaaacattcctggatgcttaagaagaaatttaactccaagtctg

cactgtgccagaagacccaccaacaaagctctgaagatgaagccaattgtggttaacaatgatggaagcacaaagtacatgttaaattgcacaggacgtt

tcatttcatttttatacaaatgtctctgggaactctcagcaactgcaatatctgtccctggaaatggtaaacaccagccctcaacgtcagatcttctatg

aggcagcaaccgttgttttagttttggagtaagtcgtggtaatctatcagggaacaatttccgttgcagtgactggatttcaccataatgggatgttaca

tgaatagcagattcccaggtgccaaagtgtatggcgggtccctttcgatccttgacatcaacgtcgatctcaagatatgcttccacagagtttatgtcac

cttcaaacttattttgcaggaacactactcccatgtctgttggaatgcagtcgaaaccacaggcattaataatgaagacacctttctcttctgccaatgt

gttgtagctgagcataagtttttggatatactgaatttcaccagtaacatcaacttgatgagcaccactctctatacatgcttttactactggctcacca

taaaatctatagggaccaacacagttcacaacaacacgagctcttgaggccatatgaagtagtgagtcaaaattctgcaggtctgcctctataattggta

tgtgtgacaaatcttcccctgttttcttactgatatcatctaaaatctgctttaacttcttagagctcctaccagctattccccatgtaaaaccatcatt

tactttcgataatcttaaagcttcttcagtagtatattttccagtaaagccactggcaccaaatataacaaggtccagtttttctccttcaactgatcct

gacattttatgaagcttgcttcaccggaattcggtggaatgtgtgttcgcctggcacccgctgactttcgcttactactgatcctgcttgtcattatccg

acatcgtacctgaac

>92603|AGAP002686-PA

caggcaaaaaatcaaaagtcttgatgttgttggaaagattagaagagaaatacaaaatttaaaactctttcgtcatcctcatataataaaactgtatcaa

gtgataagcacaccaacagatatttttatgataatggaatatgtttctggaggtgaactttttgactacattgttaaaaatggaaaggtattacgctttt

acattattattctccaacatattgtcttgctgtttgctataattgat

>92625|AGAP002688-PB

ttgctgccgtaacctcctttcttcttcaagacttttttcctgttcagccaatttttgttcagcttcacttaagtgcttttgacagtctgctaaaactgcc

tgcagttgctctttatcatctaggtagcactgacattgcatcttaatatcagaaagcgatttctgcaagacctgcacttctgttgacagttgttgttttt

cttgtttaagactttcaaatttgtattccaaatcagaaagtgctgactttgcattattgcgagttaccttgcactcctcctgcaagtgcatcaactgttc

ctccaacctagtgatttcacactggccacgtaacacttgttcctgcattgtcgctacttggctctctagtgtatccttctctttcctgacattgtccaac

atttcttcaacatctgatttgtcttccaactcacgaggtgtctctaactcctcaatcttcgctcgtgcattttctaacagtgtggtgagtctctgcaatt

caatgagacgtgacgagctttcctgtcgaacctctgctaattcgcgctccaacgtgtgccgttctgcttgcacggcctctaatgttgcctctagtttacg

tgctgactctcttgactgagctgcctcctcctgggcaacatttgtagcttggcgtagctctgccacttgagtagttaattcctgctgtttaactaagagg

caatctcgcttctcctgggcaattttgaggaggtctaataatctgtcatcatggacaacttcaccgggtccaccccgcagcagcattccctgcaaagctt

cagcttgactccggctgtcttccaacttttctgactgctggcacagatattccacaagaatgtgcttgtcttcaaccagacgttcattctcagcctgcag

ctcctgaagctgagattgcaagtcagaaagctcctgaagagttgcctgcagctcttcattggtgctatagtgagtctcttccatttgttggatacgatcc

tgtaaacatgcaacagacagttggctggcactggaagctgactgcctgtcccatgatggaatagatgtttcaggtattgttgcatctgctgtatcctcca

ctggtagttgtaggtcactggcaaccatagatgcaggtgcagaactatgctgtcgagctccaaggagtagttgttgcttctcggagtctgagagaggtga

gtgtgcaacactcaggagctttgcttgcaaggacacgttttctgctctcaattgttccattgtggaatgctgttcctggagcctctccctgagagaatgg

acctcgtgctcggcgtcgagcagaaccgcgaggcgcgcctccaagcgcgccttctcgctgaccacctgttggtactgcgcggccagctgctgctgctgtt

gttgtagctgttgctgttgttgtagctgttgctgttgttgttgctgctgctgcagggccagagagccaccctcgacgtccaccatgctgggcc

>92648|AGAP002691-PA

tgcagaagtactgcacccagccgtgctggcccaggaacgtcgacggcagtcccagcggcaggcccagcttgaacgggaagctgtggatgcccgcggacag

catcacggcgccgcgctggcccagagcaggtgtctcatcttccagttccatgagcacgcgccccgacaggaactgcccggggaagtagagtaggttcgtg

ttgtcgaacaggatgaggaacttcagcagcttccgcgacatggcggggccgcgctcttacccacaccgcaaggcaaag

>92665|AGAP002704-PA

cacaaaccctgcaacatcgttgatgtaaaacatggctggtcttgaaagatatccaatattgccagacgatagctcacgtacatgggaaagaatatatgaa

agtgctgaagcaaataatgagtttcgaaatttggcacatgatatttcgcagcccacggaaaggtttaagccggcgcgtcctacctcatttcccgatgatg

atgttgcggtacggaaatatattggtgctggagtggggttagccagtcttataacagaaaatttacttagtcacccatttgttgtcctgcgtcgtcaatg

ccaggtccacaacaactctgttcggtatcacctcctgcctgtcacactgctgcctgtgatggtccatttacaccaacggcaaggtttcacaacgctatgg

aaggggctaggcagtgccttgcttgtcagaggaatgaccttggcagttgaggacataatcagtaaagttacaccatggcctaaggaaataacatggaata

gttcactgaaatctattggccaacacatcttactgaaatgtgtcagcctagctgtggtgacaccattctactctgcgagcctcgtggagactgtccagag

tgatatcgccagtgaaaagcctggtgtgcttgatgtcttccgtgaaggcttttgccgtctgttgagttggagtgcccctcagaaaggtcgcatgctgcca

gtgtgggcccttgttgttcctacagttgcatatggtctgttcagatattttttcaccttgattgtacgtggaactactagcaggctgctgcacatctccc

acaaatacgaacagagaaaacagggagcattgtcacgggaagtgactactcaagctgcctctcaagacattgaactgcatgctgcactttttgcagtatt

tgcagcagatgttatcttcttccctctggaaacaatactacacaggttgcacctacaaggcactcgaactattattgacaatctagatacgggcttggag

gtgatgccaatactaacaaattatgaaggtgctgctgactgctatcgtgcgaccctggaacaagaaggcacaatcggtctttacaagggctttggtgcct

tgttactgcagtatgctgcacaccttgccgttgtgcgtctgacgaagatcacactcacaaggctctctgttttattccctcgtcagcccccaagacagtc

cccacctacagaaaatcctgtcacaacagggcgaggttcatctcacagttatgcggggtcctttagacaatgattaataaaagaaatagttaaagatttt

gtactgcagcaagttgtactgtttgtactttgattattgtacatacaaaagtattttgtaaatagctgttacaactatcattgtatttgttgcacatttc

aatattattttcaggattcctcatcaagttttggtatccttagaacatggccctcccatgttgactaagacaacacttgtgtgtgaccattgtgtgcagc

agaaaaatgatgatcgtgtaaaaatgcaaagtcttgcactgatgaaatttaatgaaatattgttgggcttccagtcacatgtagttgataaacctgcata

atcttacagcatcattgtcatcaattactgtaaaatagttgtgattgactgtcgtgtcattgacagcaatgtctttgtgtaaagttgctacctgcatatc

acaacctatagcaatatctgtataaggaaataccaaaaactacataagagctggtagctatcactttgacaatagttagtgtgtatggcctatggccata

aagaaagaatttaatgcaactggaagcccaagataattatatgaaaatgatttggaaaaaaaaaaaacacatgcaatgcttggggggtgggggg

>92684|AGAP002706-PA

gggtattctacgtggatctgaagaagtttatggacaagaagttgtcgttgaaactaaatggcggacgtcttgttacggggattctacgtggatttgaccc

gttcatgaatttggtcgttgacgagactattgaagaatgtaaggatgg

>92731|AGAP002741-PA

caagtgaatgacactttcgcttattctgatacttgcggtacggaattcgtcttttaaatttggaaaatttcttctgattaattttaaaagataattttga

gttatctttcagattctttgtgtcatttgttttaatttcatgtctccatttggaaatttcatttagtttttcctcgactacatgcctttcttgactatga

tctactgtcatgttttttccacagtcgggagactgaggtacgtttttattacatagcacagttttaatgtgatatcgaactattctccacggttctatcg

aagaccaaaacaatcttgtacatgaaaacccatttggtataatagcctctggagtatcagaaacttcactaacaaattctcctatattttcaactgaaag

tgaaccaatcattaacctaacctttttaggttcagcaaacttcttctttttacggtctagttccacataaattgggcggcatatttcaaaatctgattca

ttgtgcagaactttaccagtggcaccttttgcatgcgatgggcagaaaacatttttgtcatccataaaagtgcaattcactttcctcgcacaggggaaat

ggaaggtttctggacaatttcgtgtgcaacatcctacacttgcacctctttttccacaatgatcacatcgtatctgacggcctcgagacattgcactgtg

aacattctgtagcgatccatcaatttcctcaaacacttctgctgaccacagagcacagtttgtgtgtgcccattcattctgtcc

>92769|AGAP002761-PA

tttgagccatcaacaatccgtgcaatcacctcttttacatcaaacctcttcttgaggttgcatccaccaacgccatatacatcatcaggaggaaacaag

>92811|AGAP002777-PA

gcctattcggggatgaccatgaagacgtcaatgatgaaatgaaacggctacgggaacttcgtgccaacgtgaaaccaaaaaaaggtgaaggtaaaaga

>92809|AGAP002777-PA

agttgactattatccacgacatccggagacacatttgttaatgttgaaacttagggagtacggcctattcagggatgaacatgaagacttcaatgatgaa

atgaaaagactaagggaacttcgtggcaaagtgaaaccaaaaaaaaggtgaaggtaaaagagcgttaaagaggaagtaagctgtgttggttctcacagtg

tagtaaatatttgtacactacagaagatgtaaatttaaattcataaaataaaacaaattttgtgttagaattacctgttgatattctgtttgacctgtgt

actttcagcaatcttgctggatgttccggctctgccggttctaaaatatgcatatctcaagacgactatttaacagagtctctattttacattcaacttt

tcaccacatgctaatacagtgtttgttgtgaattaccttatgctaaatatatattatatgtccgcatactgagatggttgttttagtgtattgaagtcgc

cacagctgttctcccatttgttgctcttataaattatatttgtttgtggttgcccaatcttcctctaattaaactgctgggaagaggtgtactgcttaca

aaattggttgtatttacattagacgtgctgcagactacattttagttatgtcacaatagtgtaggagtggtaggcacattgaaatgtttctgctgttaaa

tatcaaactgcatagatacaagtgtcctacaatatttacagtactattaagtggcatacatgattgtaatacaaatg

>92912|AGAP002802-PA

cctgccatagtcaactggatgcgcgacggccacatcgtggagacaggcgaccgctacgtggtcgaacaggacggcctcaccatcttgaatgtgaccgaga

tggacgatggcacttacacgtgccgcgccatcgtcatagcgactggggagatggcactccgccccatacgtgtcgaggtaagagaccttgctttgtatcg

ac

>92927|AGAP002818-PA

gacggagcacgtgtacgccgaggggctgcacaactgctcgtgggcgtcctgccgcgagggctgcacgtccgcggcgccgc

>92930|AGAP002818-PA

gtactgtgcaacaaccatctctggataggtacggctgtagtaacaaggaaatggttgtttcccaacttcaccatactgcttggcaaaaaccgtacagtta

accttgggtgggtatccgcagccttctgcgttcacgaagaagcgcgtgttgaccacgtcccattcggcgggcgggtctgcg

>92929|AGAP002818-PA

aaccataatactgttaaaactgagatccacaataatactgcttgggaggacacatttgtacagttgtgtgtcaataggctccttcatcaaagaagtcatc

gtcttcctccatatcttcatctttgggtgtgtatttctcacggtagggcttgtgacacagtctacggcaaccagggcagtaccagtatgcaagcacgcca

acagacaccccaaacaatagtgttggcactacaag

>92967|AGAP002825-PA

aattgtaacttttttatatcataagatggtattgtcttgtatatgtgtaaaatcagtttaaataaaacttccttaaactttgcatgtcacttgattattt

tttattacggcataagtaaatgttgctcaagatatctttagtgccccctccatgaggtttttctgtatctgccactggtacaatggggtacaaagctaga

ttatgtgctaaaggatagtcctagaaacacggacttgattaaggaaaatatttttcacctgttattaggtatgaatcaattgataatgttttagttttgg

ttgaagtgtatgatatggacatgacaaaattttgtatgaaaactgcctttttcagttgttatttgcaagacgatattttcatcaaacagccaaaaggttg

gcctattaaatatagtggtagcaatcttaaacctgtatttcagatctgggttgtgtcactatataaaccagtacatctgttaattcgacatctcacaggc

aaaaattgtttccagatctacaatcgacgtgtagattggtgcggcatctgatgcaggtgatggaataatatggcttgtgtattcttgcagaagctgagtg

acagtctggaacaaccaaatcaactctctgtggacaaccagagtacactgcaactgcctgtccatagtgtaatcagataccatcacaaacagatctgctg

gaagtccatcagtgttgccttttggaataagcatgtggtctggccatccacaaccacaatagttgaattcagcttgatttgctggatcaggattggttaa

gttacggaatgtacgttcgaatggtatggtaacagaagactggtttgaacttctttgaaatacattacggcctggtctcactgaaaagattcaaaacata

tgtatgtattgaaattctttggatccatataaagaaagttgcatttatactcgacatatcaccaattccagattactatctctgtcagtactgaacaaat

atctgagttttgttcaatgtttagtctctttaaaacttgctcactatgttccttccatatcagtttagtctacatttgaaaaatttaaaaaattgttaac

atgataaataattatttttctttctgaaggatgctagtctcatccttaaaatcagtactgaaagataccagatatgtacaagctatgcttagcataaaag

gactggaagaaataagaacatttcactgttggccctaccactagatgctgctttggcactgtttccaagacataaaataaagtggctagtattttaatta

aaattggggtttcaatgtgagtgtgagaactagaaacatattaa

>92983|AGAP002826-PA

gacttcgaatctgcaatagcgcaaacgggtttcggcagattcaactacatgctgatgggtgcgggattgttctccacgatcgtgtgcctgatggacacaa

ccaccatgtcttacattctgcctctggcacagtgtgacctgggcctcacggacctcagcaaggg

>92982|AGAP002826-PA

catgccctcgctgccactcctcatcttcgtcaggttcctcaatggagtctgcatctgcgggccctcggccgtgacgtacgcctacctgggcgagttccac

gcctacaagacgcgcacccagagcatggtctacgcctgtatcttcatcgctttcgccatcatagcgcagccgggcctcgcctggctggtgattccgca

>92984|AGAP002826-PA

gtcagtagatcgtccaccgccataatgaacacaaaggctaagaaggacaccgaagttcctgtagacctgaaggatgctgcagactttgaaacagctgcct

ccttaacagggtttggcttatacaattacctaatacttgtcacatctctggtgtcttccctgtcacatcagtttggtgcttcttcatcgtcatatctcct

accagcagcacaatgtgatcttgaaatgaagctgtctgacaagggtttgctgaactctatttcatttgcagggatggttgcaacatcatttctgtggggc

ttcttgtcagacatgtatggcagaagaaagcttctagtcatagcttacctgttggatggaacagtaaattttatagggagcttcacccagagcttttcac

ttttagtagccatgaaatttttgagtggtgcaataatatgtggaccaggttcaatacagaaaaccttcatatcagaatttcattgtgtgaagtataggac

tctaataatgatgctgtcaggatttttgtttgctgttgcaaacatcatcatcccagttgttgcatggctcataattccaaacccatggtcagtgacattc

tttggtggatacattacatataattcttggagactctttgtggctgttggctcttttgcaagctttctg

>93002|AGAP002828-PA

ttttgggatccaaattttaattcttggagagcctctaagcgacgcttcaatgtttttgttcggaggaagaatgccacgaacagcactattaccattgttc

caagtactgctgaaaccacaattaatattatggttagaatgccttctatgtttgtagcatctttgctgcctacttcctggtaactcttaaggttaaggtt

cagctctatccaaaaccttgactgcagctggatgaaggtgtcttgctcataaaattgccgtgtgattacatctctctcaactggatgtttcgctacagca

tcaacgaaatgagcaactacgtcagttgaatctgctagtgccagtccacttgaaggatcaacactttgcttggtgctctcaatgccacagtcatactgga

atacatcactgaaaatgtcttccatcatatctcttgtgctctcaacatatgtcctgttgttgaggaactggaatgtgactcgatgaatatttgatattac

atgcacctgcacttcagcatctgcataatgaccaaattcgtcatctggactaaatgaatccctagcacgtactttaaatgaaaagaatccatccagtgtc

tcatcttgtggttcaaaattgagaactatatcaccattttcttctaccaatagtgcattatctttaatactggttagactgttagtggatgcactaatgt

caccatacagacgataagttaccacgtcattctgatctggatcagtggccttaacagtcatgatgactgtaccaactgcaaagtcaatagtaaatcctcc

agcaatcaaatcattttcaaattctggaggcatgtcatttacatctgttacttgtattatgcatttcagtactgaatcattgttctcaagagcaggatcc

tcttctggttttttttcactattagttgctagtacatatatggtgtggcttgtagcattttcataatctaattcttgaccattcagtttcaattcacgag

aggtgttattcagctgaaacaaatcttcatctccaccaacaatataatagtatatagcctgttggtcaccttcatcaagattttcattgttgggatctgt

tgcctctggtattattctgatctcattaataccgctgctattctctggaaaacttgttgtccactgtggatttgagaaaacagggggctgttctgtaata

gttatgatcttgatgtgcaatgttatatttcttgattgcactggaactccaccatccattgctgtcagttgtaactcaaagcttgtgtttttggggaagt

cagaaagtggctgtaggagtaagagttggaatttatttttatccttactgtcacctattgtaaagatatcttcagccccacctgtgacagtaactgtcac

ctgtccatttccagttgatggatcatcctcatctgtgacttcaaaatctggtaatggttgattttgccagtcataaagtcggcttccaacacgttgtgtt

tctcttaagcgaatggtgtctccatcttctgggtagacaaaggaaggtttatggttatttacatctttaacacaaaaggtgtaggtgatattgccagtca

gaggtggtgacccaagatcatctgccttgaataacacctcatactcgccccacaagtctaagaggtcaacagcagctgacactactccagagggcccatc

aacagtgaaaagagctggttcttcccctgtacttatatctgtgatggatatgatggagtaggatacagttccattttccccggcatcagtatctaatgct

tttacttctccaatattttgacccttcaacacctcctcagtgatttcacccatgtccgatacgaaacatccatccaccatgacttcatcaggtgataaga

attctggtgcgttatcgttgatgtcttctagggtcacgattattgttgtcgagttggagttggagtctccatcaccaggcactccgtcgatgtagttgtc

ccgcgccacgatgttgatggtgaaggtc

>93069|AGAP002852-PA

cggggctgcgtgtgtacacgaggtctgcgttcgtcgcccgccatggcgcctgctgccgccaaagtgtttctcttcgcgttcgtcgcctgcctgctgagcg

ccgccgtctcagctgtggcttactacgactgtggttccacatacgacctcttgggtgttgacttcgcaggttgtgaaaacccgccgtgtcctctggtggc

tggggcaacgatcaacgtcacgtccacgttctacgccaagtccgcctctaacagcgtgctgaatgacgtgtacttcgtgatgaacacagtgcagctgccg

gccacggtgacttcgtgcacctgttcgggcgtggagggcacgtgcgtgggccaagaaggctgcgccatcgcccagggcgcgacaatggattaccacgcgc

aggtgctcgtcggcagggggttgccgccggtggaaggtagcctggtttgggagatgtacaatgaagtgcaacacaagatcatatgctacaaggtcgaagt

ccagattttttggccgtaggagatgtcagccgatccgtgaacaagactgctgaattaacgtcaacttgcattgttgcaagaaagacaaaagaacactaat

gttttaagattgctcccttgataaataaattaatcttgcattgcattcctgtttatagcctcattgtaagtgttttgtgttgaatgttcgtttcctattt

atttattgcaagttttttttgtgtgtaacacatttttattgtatatattctgatacacaaccatttacatgtaaataaagtattagtgatatgttaagag

aatataattcttaatgtagtgcaactactacattttccactc

>93119|AGAP002859-PA

ggagatcacggtccgcaaaaagtccggcgagacgcagatcctggtggtgcgcgtgtggaacgagaccgtcgccaacctgacgctgatggccctcggctcc

>93117|AGAP002859-PA

aaagaacaattcatcgaggcaatcacggtcagtccgggggatgatggtggaggaggtgaaggtgatgaagatgatgaaaaactgccatcatgtggagatt

acattatgcatttcctcactctcttctggaagctattatttgcctttgttccaccaacagatgtctatgacgggtg

>93118|AGAP002859-PA

cttgacaagctgccactttgcttgcaaatgtgtctgttggtggaacaaaggcaaataatagcttccagaagagagtgaggaaatgcataatgtaatctcc

acatgatggcagtttttcatcatcttcatcaccttcacctcctccaccatcatcccccggactgaccgtgattgcctcgatgaattgttctttccaggag

gacgtgccgattaacaaagaagcgtttgccctctgcaccaatttgtcgacggtgttcttgaactccttgctctccttgatgcgtatgagggtgcgggtga

cctcgccgagcttggggcggcccagcagcgccaccttctcctcgggcgtgag

>93137|AGAP002865-PA

cattattctccaccacacctaactgaaataattaattctatgacttgggaaagggccacgaatttgcattggcatgcgtttcggattgatgcaggttaag

ttggcactgatacagctgctgtcaaaatttgactttctgtccgttggagacaagcag

>93165|AGAP002866-PA

gttcaggatcgcccggttcatgcacgcctttgttccgactgtcgccagggcactcagagttggtgccggaaccgaagaagtgtccgcattcttccgaaac

atggtcagagagacagtggagtacagagagaagtacaacgtaacgcgaaacgacttcatgcacctgctggtacagctgaagaacaagggcg

>93164|AGAP002866-PA

agccgagctgccctccatgcttgttgagtgcttcctgcagttcctggcgcagtcgttgctgcacgtctgggtggagcgccaactcgtacagcacgaacga

catggccgtggagctggtgtgcacgccgtccgtcatgaaagtcattacttgtgccgctatgtccagatccgtgaagaagattttgtcggcgagcacctgc

cctgcgccgtccaggtatcccttggtcttgagctccaccagcatctgcaggta

>93181|AGAP002867-PA

gaaatgttatttactttaggtgaatggcctattacatatttgtcttgttcccagcaggaggaaataaatctctgacgatcaaagaattggcagcacaggc

gtgggtgttcttcctcgctgggttcgaaacatcctccaccaccgtaagcttctgcctgtacgagctctctaaacacccgga

>93202|AGAP002883-PA

gcgtatatgagctcgccaagtatcaagtagactggcatgttatgcctttagtcgctcactgtcaagcagaatggagacattgctgtcgagatcagagatt

cctataatcgacctcgctcacctaggtagcgtagagaaaccaactcgtagcaacttgaagaaggttgccgctcagctgcatcgatcactggcacaaaaag

gaattgctttccttgttaatcatggtattccaaacaatagggtgagttaaactctatt

>93248|AGAP002889-PA

tatgtagagttgcatcctgaagatttcccgactccaaatcgaaagaaatttgcggaggttttaaaccggtggaac

>93247|AGAP002889-PA

tggctgaaaaggatgctgttttaagccactatgtagagttgcatactgaagatttcccatctccagagccaaacaaatttgcggaggttttcatccggtg

>93251|AGAP002889-PA

attatctggctgaaaaggatgctgttttaagacactatgtagagttgcatcctgaagaattcccaactccagaacgaaagaaatttgcggagggaagagc

cgcttcttagcaaaatatgggctcctttgacatacggaggcatagcgt

>93243|AGAP002889-PA

gaaaaggatgctgttttaagacactatgtagagttgcttccttaagatttcccaactccagaacgaaagaaatttgcggaggttttcaaccggtggaacc

ctgttcgctgatactttgttcacatccatgtgaaattgagttacgttgtaagaatagtagtatgttgtattatatacacatctatggttaatcatcgaga

aatgtacatataaataaaaatttgttattcgtg

>93329|AGAP002909-PB

gtagtgcttagtgcaacgccggcatgacgttccccgccagctcgctcttcgtagtagcgctgcttcttgcaggaggcagcgaggcgcagaaatcgcgcga

ccaggaggacccgtgcaaagtgaagtcgcgcgtcgtgggcgacgtgacgtactgcgaccgctactgggagtgcgtcaacggccagccggaactctacgac

tgccccaacgggctggtgttcgcgggcaagcaccgcggcgtcaccgagggctgcgactacccatggcgttccaactattgtgacggcaagcagctagcca

acccacccatctccacggagcactgcgactggctgtacggaatcttcgggcacgagacgtcttgcacgcgttactggacgtgctggaacggcacctccac

cgagcagctctgcatcggcggccttctctacaatgaggacacgcattcctgcgactggcccgacaacgtagacggctgccagaagcacccgctgtgtaac

gaggacgcgaacggcaacgtgccgctgggcaagtcgtgcaaccggtactggcagtgccagggcggctacccgcggctgcagcgctgcccggccatgctgg

tgttcgaccggcggtcgctgcgctgcgtggtgcctcccacggaggactgcgacgtgcccacgacgcaggcgccgccgcccgaggagctggacgaccgcaa

cgtgccggggcccgcacgcggccgcgagcagccgctcgacctgccgcccggggccctgcccatcccgctgcggcctcggccgcgcaactaggccgcacgc

cgctctaatttatcgtgctctagctgtagctcacctctcctatctaatggactctcttctagtgtataagagaacgggcgtgaatggcgcgccgtgtgct

gttgtcctatccgggcaatagcgtcgtttacttcttccacactttcacatttgagtggccgcgtgtgaacgcaaaacccgattttaaatacgtttcttca

ttaatctgccaacgaaaggactctagcgcgtattccgttaactctcggccacatgatttgcagctagtttacctgaagatatctttaaacgattatgtgg

agaaaaaaaaa

>93411|AGAP002921-PA

tacaaatacgaccttcaaggctgaaaggttttaagacaaaacaaacaaaacttggtagagatttgattagagaagtgtgcgggcatgccccatat

>93415|AGAP002921-PA

tctcttttcatatggggcatgcccgcacgcttctctcataaaatctctaacaaattttgtgtgttttgtctggatccctttcagccttgaaggtagt

>93414|AGAP002921-PA

attcttttagtcagaaattgaaacaatggaaaatccaggatggcatgccccatatgaaaagagagcgatggaattgctgaaaggtattcagacaaaacac

acaaaatttgttagagatttgattagagaagtgtgcgggcatgccccatatgaaaagagagcgatggaattgctgaa

>93412|AGAP002921-PA

tcctctctcttcttcttggctctcagatgagaaccaagtctcttcttagagaatttaagtgctcgcttcatagaggaagcggtaccaatcttgatgagct

caataacacgcctctcatatggagcaaagccggccacctcacggacaatttcgcgaaccaacttttgacgagtagtctggagaccaactgggagcttc

>93703|AGAP002939-PA

gcgtttcatcagaaaatggtcgaagacggctcttgagcgacttatagacaccgaaatacaccatattgaaaatcccatgactaattattgtagcagacat

tcccctgttgaggccttccacaccatagcccttgttgctgacgatcttctttaggacatcccacgtgctgggcgccttctccacaggtgacctgtctgcc

atcagggtcactttcacacgctcgaatggattcatcacaatcgcttcggtcacaccgacacacaaccctgttgcaaaatacgtcaatggttttgctgttt

cagccccaaacaagaggtgctgctcatagttttcaaagagaagaaaattcattgctctcctcggagtctcaatgagtatgggagggatgactcccttcca

cagcgagaggaagctctcctggcggtagacagtccgaacacagttgacaactcctgtgtagtagtactgggctacgcccttgggtgtggatccggctacc

ggtgagtgctgcagctgcaaacacgtcttcaccacgtcaagggggtgcatagcgcatacttccaaaaatgctgtaatgccacctgcagctatcaccattg

cagtgttgtccgacatcttcagctacagacgtcccttccactgtgacacgctgaaatgaggctctcttcaactggtcacatgcaatatctgaactcagac

gtttcagcagaccatcaatgtgttgcttccagtttaacctctgatcaatggacacacctaaaaattttgaaaattctaccttagctacagacttctgttc

a

>93801|AGAP002952-PB

gtgtactccgtcgagatgaggcgccggttgtgtcgtactagaacaaaacatgattaaagagcaagaagtcggccacggctaacgggtcccgcagtggcgc

ccacatctcaaatgaaaaactgagacccatttgtatctttacccctgtttgccatggacagcaggaatggcttatcatgtttaagctcgaaattttcatc

tgcaaatgttcctccataaatagattcgccaccggtaccatttcctactgtaaaatcacctccctgaatcatgaaatctttcactactctgtgaaagata

attcccttgtaatgtaaaggtttgccggtagtttttccttcacctttttctccggtgcaaagggctctgaagttctcgcatgttattgggcatgtatcag

agaataactcgaaaacaattctgcccatgggcaacccccccacttcgatatcgaagaaacagcggggcctgtattgcttcgtcaccgtcattttcacagc

ttgcacttcgccttaaatgcaatctactactacttattttgagccgtatgtaaagccaaaaaattctctgacacgctacgatatttgttcccgaaaacaa

acactttccctaccccaacactattaacttcacaccaaagataaccagtaactagcattgac

>93830|AGAP002961-PA

tcctttgctctttgctttgcttcctttcatagcccccttctttgcaccctttataacacttgaaaagtgtcctgattgttttgcttttttccttttatta

agaaatttgcgagacaaaggaagataagcataaggatctggacgaccctttctcttcatgtctccttgagctttctttgcacgatattctgatcccaagg

tcactgaaggagctgtttttcctttgcctttatgcacagccttcttcacgggtcgatgaatgccagtacctccagctgatgaaaagtgaaaactacattg

gaccattttacaaaatatagcaagagagaatatgagtaaaagaacttgaactccaagtgaatgcataaaaactatgtctatgtactctatccaaaatgta

aattaaagctgtcactcaattttagaaagagaccacatagggatttataagtcatgcttttcctctacaaaactgtaattacttgttattccaacatatt

cagtcttcactagacggtgaagcaaaa

>101226|AGAP002996-PA

tacttctgataaaataacaaagaagtgatgacatttttcacatttcacgaatcttgttgaggctgttgggagcacaaacaaaactctttcattatctggc

tcttttattcatctacagtgttgtaaatatggatgttaactactgaaaatctaaataatgaaacataaaagtataatattaaatcgattttcttattaat

tccatatctgggctaaataatgaagacgagtagcaagataatgaaagagttttgtttg

>101382|AGAP003018-PA

cttcccttctttcatccaatcctcaccccccaccccaattgtaatggctcaagtttcatctctattttcatacttgggtacgaaattcacccccagtgga

taagtttgtgtgtagtaatagttatgctaataacggaagttgtaataggaaaataggcagaatttgatgcaagacatgtattggttgaaactcaagtttt

tataccgttattaaaaattaagatttgatcaaaacttggtttcactgctttgtcatttgttgctttgtatcgtgaaatcatattaattttattttcaccc

agtattataaatgaaggacatttaaaatgaattaaaattgtgctgacagtgctgaatatcaccctaatcgacctcccgggactaacaaaggtacctgtgg

gagatcagccagcagatattgagaatcaaatcaaagacatgttgctgcagttcatcacaaaggagacttgtttgatc

>101397|AGAP003050-PA

gctttatgttctcgcacgtgaccagccgcctgctcaagcctcacccacgcctccaggaggtcgccgccaccatcgacatgagcgacatcgaggccgactc

cgtcgtcatgttccagcacaggtactgggcgccgctgacgctgctgctgggggtggcgctgccgctgtggctgccgctgctggtgtgggaggacgtgccg

ctgcccgccaccgtcatcacggtgatgctgcggctcgccatcctctggcacgtcgccggcctcatcaacagcggcgagcagatctggtcactctccgtca

agaacaagtactcggtggactctaacctggtgttcttcctgaaccgcacgcactggccgcagtaccactacctgctgccgtgggactaccgcacgggcga

gttcggctcgtacgacgacgactgcgtgacgacggcgatccgcgtgtgggcggcgctgggctgggcgtggggactgcgcaccgtgggccgcgacgccgtg

cgccgcgcgctcgccgcctccgacgacgtcgtctccgcgctcgaggaggagggccgccgcgtcgccaagcagacgctcttcctgcagtaggccgggccgc

ctcaactctgtccaggctgacaaaggatgtcatcaacgaggtagagctgcagaattcactggaaacagcgaactcgactactggttatcgtccaagtgta

tcgaggcaatggtctgtttgttggagaccatcaacaactttagaatctctgcttttggcccccatgccctaactttttttttgtgtaaaacagtattgcc

atctctctcctcctgattacgttataataaaaatattttggcctagaattttagaattgcttttgctttttaat

>101568|AGAP003108-PA

taccatccttcatcatcatatgtgacattatgaagaaccagtgtctgaggatcatcttctggcttcaccgtgttactagactctgtttcattaaagtcca

tgtaatagcccttataccaggcaacatgaggatgaagatcagacaggactttgcagttaaatgaagctgtg

>101588|AGAP003109-PA

ggtaacgacggaagcggagatggtgatttgtgatgtaaacatgagaaagtctgtctcttcatatatttatagagaggatgaagaattcgatactgcagat

gatgtgctgaagtacgtgtatgacaatacatgtgcatggtgctgtgatatctttgatgaagttgaacctacattacggtgtgctgagtgtgatgcacacc

tatgtgcaacatgtttcagtcaaggtcaacagccaggacaacaccgtaactatcatcgctacactgttatacctcccaccaaattaagttctgcagtagg

cgatgaggccagcggatggactatatcagaggaattgacactgctctcggcactggagaagtatgggtatggtgactgggaagctgtttcagaggctgta

gcacgtaccccagaggagtgtcgacgacggtatgactctcgttatgtctcatctgctggaccgttttcgtggcttcatgaagtctccaactcaggaaggc

acatctgtcgtcatacagtgaagaggagcagtgatcagcggaaatcagaaaagcccctgattgaaggtggatgtttggttgggcattccaattaaactgc

agaagacagcagccagttgttgcaactaaaaattcctgttcattatttgttaaaattagcttcattccctgtctaaaagtacaaaatattttgtgtctct

taattgttttggtactgaacaaaactgtgtcacaattatttttcaatattgtgaataaatgtgcttttttagtgagtgg

>101606|AGAP003123-PA

ctgcgggccgcgctcccactcgcccaccagctccgaccactcgccggccgacagctgccgcgacggcgtcgtcagcagcgcgtacgccacgtccaggccc

atcggcgcccacctgcacgtctggtaatcaacgatgcagacttcttccgggacctgcacgccgttactactgcacgaatatcggaacagcaggttgctcg

cccaaatatctccatgggtgagcaccacgaagtcgccggtgctttgggtgatgaaagatttcatgcgttccaccactgacggcgcgaattctaacagttt

ctggaggtatacgccgttgttctgatatgcttttctcaccatctgcacagcgtctctagcagcaaattctaacacaggagcatacagctgaagaaatttg

tcagtgtaaatcacttcccgtatgttctgctgcataagagaatatgttgctggatccagggtgcgaacagcgagtgaagctgcctgaaagcggcccaact

ggtggacgaggagtcgcagctggtccagtcccaggccgcggtagcggcagggcacagcgaagccgcgtgcagacaaatcctccagcacgatgaccgcgtc

gtcggccgccaggcacctgggagcggcagtgaagacgccgagcctggcgaaggcggg

>101655|AGAP003146-PA

cgggagtcaacataatagcatacttattttattacacactttgtactacatataatatcagatacagttcagtaatgaaagcagaatgatactgttactt

tgagtgaactaaaaactttcaccagtctgcttattgtaataaaactttgtattggatagttgcagtaattaacttgttcagtatcacctctttcaacaat

aacttctttggtttgtgtgtcactaattcatatttgttttgactcttgtgagagttgtagaaggctgagctgaactctgaggttgacacatgcctttctc

tcatccatgatgcattttatgaggcttgccttcttttgctgcagtaataaattctctcgctgcaagtcagtaatgcgagactctatttctaaatcttctg

catcactaccatctgtatcagaggcagaactgctcccatctgaactagttatttctaggctcctgtagccacatttagatttaattgcggcaatctcagc

ctggagtcgcttcacttctgccttctctcctgctacgcggccctggaggtcagcctggagatttaacagttcacggtattccaaacgcaataatgtttct

tcatggtgagattcactggagtcctccacttctggggaatgatgttctgtttggttatgaggccttttctctgcattcttcccattatctgctacttttg

gttccttgacatcattatcagtttctacatcttcagcagctgcttgtgattttgaaacatttgctgtatgatcttgattctgtactacattttgatcctg

ccgaccatcaatgtcatcaatagtcacagatgttggtggtgaagttttcactgtgcgtgtttctgtttcttgttttacaactgcagttgtgctaacaact

gtatcatcttctgaacatgaatgaaacttctgtaatgataagtcaatatggaattcgtcctctgccggtttctcctccaagctcttctggccatgattca

cttcctgtgatctttgaacagtcctcagttttctcttgagttgtgtgattagcctttgcacctcccagagttgttcctcatttctctttgatacataacc

agcattcatttgttgatgaatgtggttaagtaatgactcctgctttgcaagttcaactgatattgcaggcactgtgtctggaaggcttgggctcccggca

ctcagcggcggcacgtatttgacaatactgatgtcagggaagagtgttcgacagtgacacagcaatgttgtaagcagtctgtggctcatttgcaaaactg

ggctcaaagttattgctatgttttgagcattcattttgttgtacttttcatgtattgttacattatcaaggtgtaccatcatccagcccaaaagatgcaa

attacattttgggagctggctaatgagtccttgcagttcagcttcacgaattgtaacttccttctctgcagcagcctcttcaaatcgagacagaagatca

tttgtcaaaatagtttctggtagttccctgaaaaattgtttgagaaggcttgtaacaactggaggctcatagtcagacagttggacacattctccttgat

tatataatctcctaagatactgaactttggacttaattccagagagcttgtatatagagtccatggtaagaccattttcttgtacgtaatcaatgcattc

tcggatcaccaatggaatatacgctccatcatgacaatgacttctttctgtagccacgtgtagtgggacaccaaagacaggttgctcctcaccaaagatt

gaaccatcttccaaatgttttgattttttgcgttcctttaatttcagtttcatttttgtttccttttccttctttttttctttttcagagtctctttcct

tttctttgacttccttttctctcgatttttcccgtttctctttcttgctcggaaatttaaatgatttagacttcttagacttggatgggctttttatctc

aggatcctcttcaggtgaactttctccttccagtgctgcatatcctctgtctttttttgagtctttcttatctttccttttaccaatcagtaagtctttt

tttgaagggcgctcatgtgcttcatcgctgaagtcactttcattactcttctttcctgattcagacgcgtaaagtcctggaaaatctttttcaacatctg

ggctttcaaattccattcttcactatctcctgtattaatgtactgtcacatgtctcttgccgcccccaatcagctggtcatgagcactagatgtaaac

>101732|AGAP003167-PA

ctccttcttctacttgaacagtaaagcccttcttgacaagagtagcagccacagctggagttaaggccactctcttctcattctgccatatctctttcgg

aacccctattttgagttgcttgtatggagtgccacgaactggaggtgcaggtgcagcaactttttccagcagatgcccggtgctggagaagagacgcttc

cagagatggcaggggggagcctgagtaatgccacaacacagtggtagcattgccaggcccctggggcctggatggtttaacgctctcagcactccctggc

gcatctcttactcctgcacgtacacgaggtccaactcgcagcacttcggcaaggaactgagagccac

>101801|AGAP003177-PA

actcacgcaggcttgtgaaactgtcttttatgtgatcacggcgtttcctctcaagtgcattgtggtgcgcccgcttctccgccattgacgtaagtaacaa

cacttccgtccacctcccacaaacactccaatccctcacgttgcttttaccctttccactggacaacaagcg

>101821|AGAP003179-PA

gtagaatgtgggtcaggaatttgtaggtatatttataatgagattattcacaatttaactgtgtttatacatcagtattttacgtttactgattctgttt

tatgttattttctaagcacagacagtttttccaaacaataatgtcggttgacagctcttgtaagtttgaaatactaattatttgcacacttatttcaggg

ttgacctctcagataaacatggatgtttctaaggaagcggaactgaggcatttaattcgtagggaacagcgtcgaaatcgaaggagacgaaggagaaatg

aagttggttcgtgtgtaatttgtcaagaaaatgtactaaggagtgttagcagtgttttatcttgtggacactactttcacacacactgcatttttgcatg

gattaatgctaatacctcttgtccaacatgcagagcaccagttgaatgttacaatatatgaacatgaacagtgatgtggtcttaagatactggagaagaa

tatttctttctttgttgtgtatgtaccagagacaaatgttaccta

>101847|AGAP003182-PA

gtagacttacattaatgtaccatactggtcatattgatcacaacttcaccctctctttctcctagtgtaatacttgactgaatacaccactgagtaattt

gagctggtcccaagacaaagaatttgaaacatgtaaaatgttatccttctaatctcagtcagtcaaaattttaaagcaatgactattttcatctacttta

gtatgttgttcactttgtttacagccttttgcagaaatcatggagatgacagatgttcaggagggaataattgttcgatgcattcagcgattgaatgata

cattgcgtgatgtcaaagatgcagcaacaatgattggagacacagttttgtgtcagaaaatggaagaagcagcaaatgctataaagcgtgatatagtgtt

cgctgcgagcttgtacactagtggaatgtcagtgttctaaagactgtaaattttgtacactatttattttattgttaattatattaacaaaacaaagaaa

taaatttattcaatg

>101889|AGAP003193-PA

aaagtaaaaaatgctgtggacaaagcaaatggatatatatttggagcgggggagcaacgtaatgtgcaagcgctgcttgcatgtgctgtgggagctgatt

atgaaacgggtgaagatatggacagatttacggagggtgcagtaggtgatgatgaggatgacgacgatcttatgatgaggggtgtatgggcataaactgt

gtgtgtttcactaacagatcacacaattagccatcattgatagcagtcagtttgttacttatgtttgccaaataggtcatcatgaacaagatagcagcat

ccaatgagtaattgtgtaaaatctctgtccatgacttcattttacacagaaaa

>101926|AGAP003198-PA

gagccactctactttatgggaaaaataatgttcttgttcttcctaaagaccggactgaacctctgcctggatatttgtcattacatcaaactgcaagttc

tcttaccataaaatggacccctaaccagttgatgaatgggtattctgaatcagacatttctgataaaagcatgtattgggagtatgctatgaa

>101927|AGAP003198-PA

cattcatagcatactcccaatacatgtttgaaaaacagatgaacaacaggtaacaaaatcttccactgtgtcaaactgaagaaaaccaacagtcaaaaga

gtctttaagtctatgacagacagaaagacttccaggtgcatgtcgacaggtgcttgtgcaaatcagactccgagtgccaagacagtgctaaatacaaatg

tgatattgttgatgttcgacgaaaactagtttgtcttcgagatgactctgttgatacaggcagactgccacgtcttttatcagaaatgtctgattcagaa

tacccattcatcaactggttaggggtccattttatggtaagagaacttgcagtttgatgtaatgacaaatatccaggcagaggttcag

>101987|AGAP003217-PA

gctctttggtgtgtactgtgggaagttttgtttgtttacaaccgaagaaaaacattctcacaaacatgccgacttatgaaatgccattattaattaaaac

acttagtcggccagaaacatttgttgtgctgaaacgtgcaagtcaaactatatttgataaaggtggtgtcatacgaaagattgaaaatcttgggacacgc

cagttacctttcaaaattagaaagcatggtcttgttgagaaaaaagccagttactttttgattgaatttgacacttcaccaaaaacaatagactacttga

ttgatgaatttcacagagatgtagatgtcctgcgttccaggatttacaggtcacatgaaaaagaagagtttgagtgcacatttgaagaggagtgcctgcc

tgcagcatacaggaaagatgttcagaagttgttagaaatagcaaaaaaagatgcaaaaccaaagttcaacccaaatacatctctggattactatccattc

aacaagtagtcatgacagttggtgactggctgcccttcctgatcccacaactctcctgggtatggaatgggtgtacctccatctgtctgcttctagagga

agaaacatgagaatttagcagcagttctgtgatatatttccactgttttgtcattggacatcaacttttaagaaaaagaaaaaaaatgtaattcccaaaa

atattagtggtttggacaatgctcacattgtaaagtgttagctgtagtggattaaccacatttagaagtcatcaactgataatttttgtaaaaaaaactg

atgtgctttcaggcaatgaaattacatattgtccagtatgttattttattttattttcatttgttatttaggtataatggtaattagaacttgagtttgt

ttggcagagccaccatgacttgaaagtcatgtaatttatgtaacatgcagaaatagtgagaaatttaaaattaagtgtacgctcaaaatgttccaaagaa

aaactgtcatacttagagcaggaaatacaagaaaagctctcattaaaacttgtttattaattgttggagtctacttaaatttaatgtgcactgtttggtt

gtaattacctaaatacttctggaaacagtttacattacttaatttgttgcatcgaactgatgaaatttcaataataaattgtacaagtgacttaggagtt

tgtatgatgaaacatgttgagtgatactgtttccaattattcagaaatttatatgaatatcactgcttgtgaacaagatattaaattaatcaagaagagt

cagctgaaaataatgtttagtgtaagtgcgagaggtggtcatgccaccatactgtaagttaatttgttttttgtttcagtaaatatttgaatgttcatag

catatcttttctgaacatgttaggatgaaggttcagaggaatagttatgagattcagattttagttggggcaggagttttatttgctttttgagtcttac

ctgatagacagtcctgcaatgcaaaagccggcatctgagtttctcggaaccttgtcagatagagttgcttactttcagtctcgtacctaatgtgttggga

ttttctaatcttcataaagtggactgtaggggagttacacaggacatacttaagttgtgcctactacatcatttggggttacggaatttctcttgacttt

ctatttgctttagatagtctcccaggccaggtaccatgctacaagaagtcatggcaccaggcagctctttcagagaagtactactggatggatgaccaat

atgagtgtttgcattctcctccagatgttgtgtttgatggaaaaggtggactaggcaggattaattaaagataaaaatgtcatttgtcaatcttgtttcc

actacatgtaataagtttatccaaaaatattaaaaagatacaaggggaaaccaattaaaagtgatcatagaagtttacatcaatggtacacacctgacct

caaaaaattaagaacacatgtcattaatctttataacagatcaaagaacagtgaagaagatgtacttcagtactttcaactaaggaagaggtacagatca

gaaattataaaacagagacaaatggaaaatgataattgtatgcagcaatcacaaaacaaatgcaaggctgcttggaacattatcaaatcatcttggtaat

actaacagaactgcttatgatatgaccagtatcacacccaatgaaatgaataacttctttattaattctggtaacaaagaacacccagctcttaataaac

atctcagggggtgtgggggaagatattcatattgcacaacaaacctgcaatatctacatacctggattccctgcctgtggacaatactgtgcccattact

actccatccaacacccccagcattcatatgttcatttctttgtttaacctttgcagagagtcaacagcctttcttccatacactggtgtcctgttaggat

cgactgtattaaggaaaatgataaagtgaactcatattctgctttagattgattgtgggcaatgtaaaaactgatttgggcttctgcaagtcagaatttt

>102004|AGAP003220-PA

aattccttggatgtgcttgaatacagaaagaatgagagatcacatactggatttgccaagcgtgacatctgaaaatcaattagacgagtatcttttatat

gttgctcatcagaaccatcataaaggaataaaaaatttggaccccatgtatctccattcagaataacagagtgaggcttgtcaggcttaacaaaatcaat

ggctttatcgtaaactgcactttcacagaattctctcatcttttgttcatatacactgtccggatattctaaggaaactgcctccatagaataatttagt

acatttttcatcattccttggtaccattctcgttgtgcttcactgtagagcacttcctccaagttggcagcagcggttctgaattcttccagttcctggt

ctttcatagcaaatgaaagagcatggaaattagccatgcatttcagaacttctgcacaatgttcgaagttatatgtgtcttgtccttttgctggctggaa

accaaacacactcaggtcttccaaaacaatgaagtcatgatcaccatctgtaaatgccttatagcaccttggaatttgtgtaaagggattttttggcttt

ttcctcttctggaatgccatcaacttctcaaatacctctttgtagaagtttac

>102099|AGAP003230-PA

atcctcttcacatgcagatccaatattgatacatatctttaaaataatgtaaatctaataaagtactgaattaaattgcttacagaaaattttacttggc

taaaactgtttttcatctaactgctcattccaagacacaatatgaacagaaaatttaaagaaaacattggcatccttaatttatttaatctttttggcag

aatagtcaaaaaaacccagtattcaattacaaattattccaaaatctgtagttttaaatgacatttgcaccatactggttattttgattttcagaaagta

atatttttaataagaccacgtgataacaagcaatcaatgcttttaaacataacacacaatattttacatggaatcaaacctcttaaacatctgtgatgac

atcagatcatcatacaaatggtttgcatcaaacaaagtgcaagaataacagcatgtacaggaagcttttccatcagatttaaaactgataaacattttac

aattttgttgaaactgcttttaaatctacaccactgacaatgtaatcttattattacataaatctcttgttttagaacataacttcaaaagtgtacgtga

aatgtgagctacatgctattttaattttcagcaaatcaggtgacactttcagtcatttcatgtaaaattcaaacagatattaagtgtgagaaccacactt

catgtataaaatcggcaaaggactcaggcaatatattcagtcttaaatgatgatttgtatattcttacatagctgcagttccaatgcctatcactgagac

tggaggactaagcaaatggaacaactatatacaagtgctaaagtaatacaacaagatatacatataaaaatagtaacaataccgttcgtcatcctttgta

tacaaggtatatcacatatttcattaggtgcagtcataggaaatttaaaacttgtacttgaagttaaccttcatcaaatactgaatgtttacttgcccaa

catcatatacaatgtattcattgtacaacaatgaaacattcttccgagcagttgggactcccttcccaacagggacgatgacaccatcaggtctgacatg

cacttcttttgggtttggctcagtgcggcccaaacccaatgtgctgtgtttgccaggaggaagtttctctatataatcagcctgcgttctttcgtacata

tttcccaaggcaacttcacacaatagcaacaagccaacagggtttgtttttgatgtgcagcaatagtttgctgactttgataccatgtcagcaaagtata

cacctttcccaaacatgtacccagtc

>102264|AGAP003292-PA

agaagagggccctgctgggatgaagcaccttgccgatagactactacgccttgccaggactgctctagggctgactggctctggaataccaaatgtcaca

ggctgcttcaaccctggcccctactggcattcctcagggagagcaacagcaatgctaccgtcatggggtggtggtgatggtgattgaaatatatacttgt

tttttgttttttttgtttttgtggaaatgtaaatagttggaaaaactgaggatttactacagataggtataatttatgctcaagtaagctgtagtttata

attcatttgtgtaaacaatatatacagacatatttcttctacaaaacaattacactattatacagagagtgtgaagatactaacaacattctgtgcactt

gtttctctaaacactccttttagcaactcagcggttttccctatgtcagctgtttctgctttatttttttttattttctgtagttctcattatggtaaaa

ataatgaagctgctgagacaccaaaattacatatttatagaaacaataaattaatctgatttgctaacaagtgtataaatttaccttgactgacaacagg

ctggaatttcaatacaattctttcaagagtggcttaaaactaaaggcagtcttaaagtttgagaaatatatattcatacaaaatatacggaaatttcatc

acaagtatgtaccaaaacgattaaaaatactgatttctaccaacagtctcttgtaaattttgaatgtttccctgtttctgctgtgaaatcccacaagatg

agtgaagttgattggtaaagacgggtcagtctgcgctggagagacccgagtggcagtgtgacaatgcaaggcattcatccaggtcacagcagtggcccga

actaaacaaaatgcagtgaaactttctcctggtccccaaaatcaatattgtataaaatgtacagtaccaaagtgtaaaagattccaaagcttcgtgacat

tgtgataaaatattacaactgaccacaagcctgaaatttaacactcttcgcacactctacctaaaaagcatcatttctttattgcaaaaaactgcaacaa

cattttgtttgcatctagacattcagggagactaggattacagtttccataattataatataaggacaactagctacgtcgaacaagtcacatctttggt

tatccatcgaggaagtaagtcattagctgaacatttggtaacattttcatgtgattcagctccagaatttaaaaattatagaatgattcttgaaaatgat

aggccagatttacattataatgcaaagcattcagtctcagttgaattcttgtcagaatcaaaaatgatgtgtttaaaaattcagactttacacagtgtaa

gatgaagcaatccattttccaccacagtattctggacaggaaaaccattgccatggcacgtgctggattatgttgatccctgtttcctttcctgagaccg

tatgctcttcacagggcagccgtgtaggtcctactggccaaggcgcatgttaccaccgacagcggtcacaactctctctcgaccaccgtccacagacgat

ccgggaggcgatccgttcattcgagccttgcggctgcgcttaaatttggcaacatcgtcactaaagacatctccagtccgtagggctgatatcagatcgt

caaactcccccttggctgctgaatcactctcacggcgccgcagacctatggcaatccggccatcagggccgggcacagtgccattgttcaggccattacg

gagtgaagaacctactcgagctaggatgctttcccgagagctcttgcgttctattgtgcgctttttcaattctgcttcctgtattgctctcctttcttct

tcctccctccgtcttcggagattctcattgtcctgatgagcttcattgaatgcattcaggaaggcatcaaagataccaaagaactcgtctggctgtactg

acgcagcattatcttctccaaatagccgaacagcccggtcaaaccgtgtcttcatatcctggaagagatcctcgagttccgagaagcggcaggtggcaga

ggacaggaactccctcatgacgggcaggaagcggtctcctggcaccacagatgcctgcgatctgtgaaactcgatctcccgctccacctccttcaggcct

cccctcaacgccgacatgtccttctcgagttcccccatactgacttttgcagcttcccttacatgagggaggtcttcatccagtttcagaatgtctttga

acttcttgtcgagcacctccacgaggtagtgcagcagggtagtaccgcgagctgcactggaccgtgtgtcggccagcctgttcagggaggccaggcggaa

accacgggcattgccacgtgcaccgcggttcatatagttgcccaatgccagcaccacttccagcagacgccgcaggcgccgtgaccgcgctacctcccgt

gaggcctccagcactgcccttgcgcgtggttccagctcgcacactgatgttgtgaagcgcttcttgtagtgcaggctccttagccgctgctcgtagtggc

caatctttgatatttcgtacaggaagcggtcagctcgtgccaggctgtcaatctcctctgcgtgctcttccagcagagcagcctcttctgctgatggtgt

gaacttaagcagctgctccaccatatcgattgggagctggtccctgctgtccatgcttagtatggctctagaaatttcttcgtctgacattttaagcttg

gacagtaagatggtgcaattctgggcacgtctaccatctattactgagatgacttttgtgcggttcttgcccagctttgatagatcttctacagagcctt

catttgtaatcccattcttctgataggcacagaaaagcttgtctatgctctcaaggtccattgccatgtacagcttgctgtcatccagttctgaccagat

agtgcctgtgactttggaatctggcagcttggaccagttgaacgacttaagtgggttggaaggctgtggcacattcttctctggccgaggtgtgagagag

ggtacaggtacgggcggaggtgcagttggaggagcctgaggcggtggtgggggaggaactgaggaggagagacactgtgtctgtg

>102935|AGAP003343-PA

accatgtcgaggtaggccgcctcgttgatgacgtcatagccgagctgcccgccatgcttgttcaccgcctcctgcagctcgcgacgcagccgctgttgga

tgtctgggtggagcgccagctcgtacagcaggtaggacatccctgtggagctagtgtggatgccgtcagccacgaacgtcatcacctgcgccgcgatgtc

caggtccgtaaagaatgacttgctggcgagctcctgccccgcgccgtccaggtatcccttcgtcttgaggtcgaccagcatctgcaggtagtcgttgcgg

tagacaccctcgtgctgccgcctcgccacagtccgggacaccacgtcacacaggaagttgtgcacctccagcggcagaatcctgaggtggaacaggcctc

ccaggaaagggaacatggtggccaggctgaaggagacggcactcagcgtctccggggcgagtatcttgcggagctcggccagcatgtcggacctggagtc

gtcgagggcgccccccctgatgcccatggcgcacgacgacaccacgtcggtcgtgtagcgcatgcacagctcccgtgcctcgagctctcctctactccct

tccttctgcagcacctgcaccaggtcgtcgcacacttccttcatcagcaggaacatcggcttcaggcggctcgctgtgaaggatggactcagctgcgagc

gtaactttctccacggttcacctctcataaagaaagggttcctcgccagaattgggtccagctcctcgttgatgtagatattgttgtcgtggaatgcggc

gaagtctcggatcatcacggcgcgtgccagctctggatccctcaccagcagcaccggctgccgtacccggtagaagccgcagaagggctggccgtccatc

tgccgataaatatcgtcagcgatctctgagaagtgcttccggccgagcatgagctctttgacgttcccaaaaggtacggagggctgcggaccgggcacgc

ccaggcgctgccagtggctgtagctccaggtgaggtacttccacagcagccacgccgccaccaccaccaggcagcccgtcatccaccagtcgaaggccat

cgcggtttaaagccggccgtgtctcggtagagaagcagcgacgcggcgtgggggtgcagcggtcggcgacaactcagcgccgctcacttggaacggcggc

tttt

>102948|AGAP003344-PA

ctacgagcttagttctgatccctttttgcacggatgatttgagctgttgcactcccgtcacgctttctttctcgtcgaatctgaaaatggagacatggaa

catttacacacacaatgaagaagagataattttgagcagtgatcgcaatgcataaactatgctaatgaatatagttatttctcagaaatagcagtag

>103232|AGAP003352-PA

gtgagcgacgcgcgcctgccggttcccggcgctgtgtgtactcgagccgtcacctgactggcggcgtgccaggaggaagcggtgctagccaccgactgta

cgccgctgcgaatggagagccaagtcgacatgcgggaggtgatcgtcgaccgagcgtccggctacaagccgtcaagagatccagagcctgtgggttgcat

tgaagtgttagccacagcattctcaattctgcttctcattctcacctttccagttgcaattttcatgagctacaaggtggtccaggagtacgaacgcgct

gttatcttccgcttagggagacttcgtaaggggggagcccgtggtccgggaattttttttgtcttaccatgcatagatacgtactgcaaagtagatctac

ggactgtttcatttgatgttcctccacaagaggcactcaccagggattcagtgactgtcacagtggatgctgtcgtctattatagagtaaatgatccttt

gaatgccatcgtctcagttaacaattacagccattctactcgcctgcttgctgcaacaacattgcgtaatgttctgggcacaagaaacctatctgagata

ctgtctgaacgagaagcaattgcccatacaatgcagtcagctctcgatgaggctacagatccatggggtgtgaaagtggaacgtgttgagataaaagatg

tacgactaccagttcaattgcagagagctatggcagctgaagcagaggctgcacgagaggctcgtgctaaagtaatagctgcagaaggagaaatgaaggc

ttcacgtgctctgaaagaagcttctgatgttatgtctgaaagtccagctgccttacagctccgttatctccagaccctgaataccatttcagctgagaag

aattctactattatatttccactgccaatggacattgtcactccttttctcactaatcttaaaaagtcttagtttgatctttaaacaaatttacatatat

tattaagtgatgtgtttattgtaatatgtttattctatgtgtgcacagcatttgtttgtatctcaggaatttacatctaattttatactttcttgtgctt

catcaagagtgtgtttcatacatacaaaggaatccacaaatttgtatcagagttcactttaaaagccaatggaatataatgttcaaaatattcatgaact

ttttttcgtacaactagaaaatgtagaaaaaatattgcctgaaatgttacattttgcattaacttagagtaatctatagaattcagctgtacatctcaag

aaatccattgcttcttattacccagatgttacgttaggtatgtgttgtacaatgagtatttaaggttagtatggaaaactgaactaaaaaggactgacat

aacacctcaatttctgatgattaaatcaaagtgtggtttgcaatgcatggtgtgacaataagaggtgaaaagatgagagtttcctacatacattctgcta

gaggcaaggaggagaggaggg

>103231|AGAP003352-PA

gattcttacaagcaaatatatttcataaactttaggctcgcacttcagaatcttacaagcaaatatatttcaataaccttaggcttgcacagagaactat

ttacacggcaaattcatattgcaacccataagttgtcaaacaatgtttagaacatactaaaaaagaaatggatacacactttagaaatttttcattattg

ttacagcaagtagtatgaattctggattaaattaacacaaattgaaattattctgcaaatcacaaaaaagtacaatatgaatcttggaaaagcaattaat

gcatgtccagtggcagttacaatgatagcagtacatcagtcgcaaatacacacagaaaccaatttccatctcccccttttctgtaaaacaaatatccact

aaatgggagaagtatcacaaaataccaatgacataaaatcctgctgtcaaaacaaacacagcagaaattttctggtagttactacttatcaatgtctcta

atatgaataacacttctgtgatattcacaggccataccaaaaaaacattacagagaaaaataaatgaaaaagtaatgaaatatactgatcatggtgagac

aaaatttagaatttaataattacactgacatatcactgtatcatagaatatattcgttcttttctttactttatttttatttaagattgcagagaatgtg

aactatagagtgtgggcaacttcacccattagttgtctcatttttaccatgctgtcagaaactaatctcatggatgtctgcattaattatctatatgatc

attttattttactctggtgttaaattgacgaaacaacacttaaatctgaaacactgacggatgtccactcattacgaatatttgtttcgaagatgtacaa

tctcatcatttagtattgcaggaaccatgactgtttgatggagttcagaatgaaatatgttcatcccattaatacacaaaactgtgctcatagactattt

tatatggataaacaggaaccaaataccacaggttctaaggtgttattgagacaacatacaagttgtactttcatacaaggatttattattattattatta

ttattattattattattattattattattatttataatgtgtgatatacactctcacagaaaagagcacaggaaaattagggtttagtgtcatatcaact

ctcataggaccaaactgggcaatgtgatggcattctatactttaggtaatgtgactagtgtaagtataaggattggattttcagggagaatatgaaaata

atacataaattcactttttactatgagcacaagctatagtaccaacacatttcacatatcatgtggggatactgtcaacagaatagtaaaatagtttcag

ctccatctatcactactgcattagttcctgacagctggtgttagtggtgagtggtgagtgtcttaattcccaccccccacccacccaaatcccctcctct

cctccttgcctctagcagaatgtatgtaggaaactctcatcttttcacctcttattgtcacaccatgcattgcaaaccacactttgatttaatcatcaga

aattgaggtgttatgtcagtcctttttagttcagttttccatactaaccttaaatactcattgtacaacacatacctaacgtaacatctgggtaataaga

agcaatggatttcttgagatgtacagctgaattctatagattactctaagttaatgcaaaatgtaacatttcaggcaatattttttctacattttctagt

tgtacgaaaaaaagttcatgaatattttgaacattatattccattggcttttaaagtgaactctgatacaaatttgtggattcctttgtatgtatgaaac

acactcttgatgaagcacaagaaagtataaaattagatgtaaattcctgagatacaaacaaatgctgtgcacacatagaataaacatattacaataaaca

catcacttaataatatatgtaaatttgtttaaagatcaaactaagactttttaagattagtgagaaaaggagtgacaatgtccattggcagtggaaatat

aatagtagaattcttctcagctgaaatggtattcagggtctggagataacggagctgtaaggcagctggactttcagacataacatcagaagcttctttc

agagcacgtgaagccttcatttctccttctgcagctattactttagcacgagcctctcgtgcagcctctgcttcagctgccatagctctctgcaattgaa

ctggtagtcgtacatcttttatctcaacacgttccactttcacaccccatggatctgtagcctcatcgagagctgactgcattgtatgggcaattgcttc

tcgttcagacagtatctcagataggtttcttgtgcccagaacattacgcaatgttgttgcagcaagcaggcgagtagaatggctgtaattgttaactgag

acgatggcattcaaaggatcatttactctataatagacgacagcatccactgtgacagtcactgaatccctggtgagtgcctcttgtggaggaacatcaa

atgaaacagtccgtagatctactttgcagtacgtatctatgcatggtaagacaaaaaaaattcccggaccacgggctccccccttacgaagtctccctaa

gcggaagataacagcgcgttcgtactcctggaccaccttgtagctcatgaaaattgcaactggaaaggtgagaatgagaagcagaattgagaatgctgtg

gctaacacttcaatgcaacccacaggctctggatcagccttgtatccagacagcgcctgactctccatttttccgacgtcccctctatcttcctccatcg

tgtctgaagtttgtttatccgtaatggttccaaatccagtagcagaggaaattccgttgtactggcactgcaagcgttgtgaacagtatatttatttgga

gaacgcggctagtatatatgggtttgaaacaccctgcttaactttcatgaaacatcagatcagatgccctgtcaaatgatctcttggctccgtagcccaa

cgaatgtgggtaattatgtcaacagaagcagctgcccacaactaacaacacgtaacttctgctacgttttattggaagtaatactaaaaattaaaataga

aagtgaacgcatatcgcacttatacagatgtttgccgtaataaaattgattagggcgtttcttatatttactaactaaagattagcatagatcttcc

>103304|AGAP003352-PC

tgctattatagaaatggatttcaggtgctgtcacgtgactcagtcacagtcaatgtagatgctgttgtattctaccgtgtcagcagtccaacaatggcca

ccaacaatgttgaagattacagccattctactcgcctgcttgctgcaacaacattgcgtaatgttctgggcacaagaaa

>103322|AGAP003372-PA

attaacttcaaataaaacacacttttccggcagttgccgggtgaagatagcgtgtggcgtgttttcgatattttaatgtgataatagcgtataaaatttt

tttgtagcggacgctgtgcagtcttcgtgttttactgcggatatttgtttgtgttcacgctccactttttgtagaggtgtggtttacacagctgtcaaac

atgaaattccaaccagtaacacattgtatattcgatatggatggaacacttttggacacggagaggatatacaccattgctacacaggagatatgcaaaa

aatatggaaagactttcacatgggaaatcaagcagcagcagatgggtatgacgggcaaagaagcggcggaacatataattaaagccttagatttaccaat

aacagtagaggagtatattacacaagcacaggctttataccaaaaacttatgccggagtgtaacgtattaccaggagtggataaactgatacatcatctg

catgaacgtggcataccaatagcagttgcaaccagcagtggagcagaaagtgtgaaaattaaaacaagtcatcataaaccactgatgagtttattccatc

acattgtgtgtgcaagtacagatcctgaagttgagcacggaaagccagctccagatatattccttgtctgtgcttcgagatttgatgacaatccggaccc

agaaaagtgtctcgtgtttgaagatgcaccaaatggagtaaaagctgcacgagctgctggcatgcaagttgttatgactccagatgagcaagttaatgat

gatatgaagcaagaggcaactattactctcaagtccttggaagagtttagacctgaatggtttggtcttccaccattttaagttatgtggtaaactaatt

gtggtttatagtcaagcaatttaagatgctaataaatctgtattttgtttgttaaaaaattatggtacaagaaagagagttcctcttgtagctataaact

attgttgatcttacataagcaacttttataaactgttgttgacattttttgaaaatgctttttagttatgtacttatgcttgttgtttagtgggttacaa

tatcacactattgtgtgctgattaatatgggatttgcttttggtctaatgaaactacttacttttttgcatgcatcctatttgtagctggcatgaaataa

atattatttttcccaa

>103458|AGAP003417-PA

tggtgcagttctgcatacagcagcagtggatcatcgagagcatagccgtccacgatccccgcgtctgcatcgtccctcttggtctggctgcccttggagg

gtccgccccaacgcccagacctcgaagagagcttcttcagtcttccagagagagtgctgatgtccggtgtggagcccttcttgagctgggcagctgcctt

ctccgagctgcgcaccaccacctgcgagctgaccacgatctcgacgtcgccgcccttgtcgcccggcggcaccgtgctgctccggtagtggtggtggtgc

tgctgctgctggcggtggcctgggcggcgctgtttcggagggccgaactgcccctcgtgggcgtggccagccgccgcgccccggttgaggtctgcccgac

cgccgacagtgacgtttgggagcgtcgggaaggacgtaattgccggctccgagtatttatctgggtcctttaggtgcagtgcgtggtcgatgatgtacga

gatgtcaactgctcctgggtacctcatgaggtaggtgaggcaagcagtgtagtcactggacagcaactggtgcctgatggc

>103480|AGAP003428-PA

tgggggtctttcttcatttaattctttatttacaagtatttagcgaacacacatgcagtcaagcagttaagatttatttcacacgtgtcagtcacttcac

attacttcacatatgtcagtcatcctcgttttctacatctagcagctgttcgcacagagccctgaggccaaccacgccataccggtcggcaacggccagc

agctgcgggcccagaccctgcagctgggagagcctaagagtatacatgtagcccacgagcctctgcagcaccgggccctctacgtcagggatggagagct

gatcgctgccgtcctctagcatgtcctgcagcatggcagcaaacacagggctcctagcttccagcacggccttgtgcaccaccagtcgcgtgttaccgga

cactagcgtcaccatgccgaaatcagcgtctagcagagagcccaggtccttacactcactcatacaaatcgtctttgctggtgacatgctggatgaacct

tcgaacaaatagcggtattcgcggtctcgctgctacggagcttctgcggtctgcgccacgaagcctgcag

>103488|AGAP003428-PA

accgaagtccccacagcgtctgaagccgaaggcgtccgcaggcgcagttagttctcaagggcaaacaagtgaaactgcgatggaaaagaacagcacatgg

accctgtcggccgacctgctggcgctgctgtcgtccggaaaagcttctgacatcaccatcatggcggacggcgtctggctgccggcccacagagcagtgt

tggcggcgcgtagccctgtcttcgccgccatgttccggcacgacatgctggaggcgcgcagcggctgggtgcacatcgcagacgtggcgggcccggggct

gcgccagctgggggcctacgtgtacagccagagggccccccagctgccggggatggcgccgcagctgctggcggccgcagacaagtacgggctgtcgggc

ctgaagtgtgcgtgcgaggagcaggtggcccggcagctcaccgtggagagcgctgcggccacggctgtcctcgcactgagccactgctgctccggcctga

agcgggctgccgtcgcgttcgtaaaggaccagttccaccgggtggctgtgacgcagggctgggcagacgc

>103487|AGAP003428-PA

agtcgagctgcgaatgaccgtataagaaatctaaacaactggagatgttgaaatgcgtagcatttgtgaaatttacacgtgcaacaggaaagttctacca

taagtaaactttattgagaagtgaacgactgttttcgtatgttatgaaatgtgagaaactttgttaggaactggaaccccacctattttgtaagagtagc

gtgtgaatgcaagtgtttacgtaccggtatgtaatatccagtgaaaaagttgggagacaaggcaagcgattttccagtcttcaggaagatcccagcgaca

gtgatggctgcgatatttccattggaaggtagaggtggtctatctggagacctgggagcgctgctggagtcgaaggaggctgctgacgtcacactggagg

tgtctgggtcacagctgttggtgcacagggccatcctaacagcccgcagtccagtgtttagagccaagctaagggacatcacaaaagagagcggcagcct

cactgtacagatctctgacatgaaggacgaagtcatgaagcagttgctctgtttcatgtacacagaccaggtgccagacctgtgtacttatgcagcacca

ct

>103491|AGAP003428-PA

agtcactcgcaaggcgtgacacagtgaggaggtagagcagtaggaagtgcacatcaagtatatatgcagcagaatcacctgcaatggaatccgttaccaa

ggttgaggaaactgcagctgtcgacctagaggccttactggacgctggggagggccccatggtgactctcgtggcaggagagacgcagctgaaggcacgg

agggccatcctagcagcgaggagcccagtcttcagcgcaatgttcagccacgacacgatagagtccagcagcaatgaggtcaccataacagacgtggagg

gtccggtgctaagagagctactggcctactgccacacactgaggactccacttccgtccagcatgactccccaagttttggcagcagcagacaaatacga

tttgtcgggattgaagacagagtgcgagcagcagctggcttcgctgctgactgtggagacagcggcagcaacagcagtgctcgctgtcaggcacacgtgc

cccagcctcataaaagccaccgtcgccttcataaaggccaacttccaggtgctggccactcagggttgggctgatgctgtgcacaaccagcccaaggatg

tggtcgaagtgtgtcggctactcgggcagccacctaaagatatcaggagcggttttggaaagaatgaaatcaggagactgactgaggcgtttgaggaggg

ggatgtagaggagctgctggaactcattgaggcaggggcggttgtgaggacgagggacagtgatgggatgaccgccctgcacttctctgcagaggtgggc

tacgtggagacagtgaggagtctgttggagagaggggcagaggttgacgccaggaacagcagtatggagacgcccctgcacttggctgcaatcggtggcc

actcatatattgtgcgactgctggtagcgtgttccgctgacgtcaacgcaaggaactgtcacaacagaacaccgctgcagtttgctgcgtggcatgggta

cgaagagacagcagcgtttctggtggaggcgggagccgacacggaagctagggatgatgatgggagaacctacctcgattgacaaccaggagatcatatg

gattccatcatataactaacagttcaataaatatctatgatataaaaaatgatacttcactacatcagtttccattatttttaaattaagaaatacaaaa

aatttcattcctac

>103477|AGAP003428-PA

cattatattattctttgatttgttttgtttttgttgttgtggtgttgcctgttgagctgtatcaaaccaatctcaggactgaagaccacaacaacaacat

gctaggtctttccgtggcgttaatattaatctcgttttgcggtccaactgcttcagttcttgctgggaatgatcagtagtgttagctacactgattgaga

aattaacaaaaaatcaaaaggcttattttaatgtattttgttgactattacataaaacattaggtcagacgcagtgaagcaccactcttaacgttgttct

tgaacatgggcagaggtagttaacgtttgtagcaggtggaattccctctgactactgccaaatgattggaagctgctgaacattatacccatcctccttg

ccaacaaagtcgagacgtttcccactgactatcccatgacttctgtcacagtgtggagggtacggtctaagggcaaagtgctgcagagtgactctgttga

ctgtacgcttccgaatctttgacagtggaaccagtccagagggataaggagaccgcagctgtggacctggccgcccttctggacgccggggacagcgccg

tggtgacgctggtggcaggcgacacgcgtctggtggcgcacagggccgtcctggctgccaggagccccgtgctcgccgacgtgttgcggaacagcacgct

ggaggccagcagcggccacgttgccatctcgggcgtggagggtccagtgctgcgccagctgctggcctacatgtacacacgccacgccccccagctgcac

agcatggcgccgcagctgctggccgccgccgacagatacggactgtcgtcgctgaaggcggagtgtgagcagctggtggccgcacaggtgtctggcgggg

ccgcagcggccgcagctgttctccaagtcaggcgctcctctgacagcctcagggaggccgtggttgacttcgtcaaggctcacctgctccacgtgatggg

agggactctgcacagccagcgtgaaggtcaggtggaagtgagccgtctgctgtctgttccatcagcaaaaaccaggtcgccggccactgcagacagccgc

acgaccggctccagtcagccgctcagcgaccacagctggagtcccgacgcagccacaccgaccacggcgacccaccacactgccccacctgacggcgttg

acatcgctttcctgcggacattatctgaagaggagaagggcaggaggctgatccaggcagttgttcagggctcagtggaagagctgcaggcactgctggc

ggctggagtgaacgtgggggcgagggacatgaacgagtgg

>103484|AGAP003428-PA

ctgtggttgctctgcagctgagaggtggaggcgactgagctctctgtagtggctggtgtgtgtagaatagaggcggtgccgccaccaggccagagtgtgg

agtgcagagtcgtgggcggcgcagctgtggccgaagtccagtcatgggtgctgggtggctctggggtgctcctggcgtctgcagtggctggcggtctttg

acgttgtgcagtggtgttactcggcgtctgcagtggggtgccagtgttggcttctcggtgagcgtcagcatatcgtaggagagagagggcgtggccatca

gttggagcagctgggcgctgagtggtgggtggtgcggctgtggcaggagtccggccgtggtcgctgtgtggctgagtggtgggggtgggagcggccccac

tgtcactgtctccaacagcgggcgcgctggtttccattggtgtctccaagagcaggcgatacacatcagccatgtcttgaggctgggagcgtactgcgtc

agcccagccccgtgtgaaaatcacctggaccaagtgagcccttatgaatgtaatggcctcctgcttgaggctgtcagaagagtgcctgaccgcgagagca

gccgtggccgccgctgtctcgacagacagctgtgctgccagctgcagctcgcagtccgccttcagggccgacacgccgtacttatcagctgcggccagca

gctgtgcagacaggccgggcagctgcggggcctggagtgtatacaggtaggccagcagctggcgcagcaccgggccctccacgtcctggaccgcgacctg

accgctgctggcctccagcgggtcctgccggaacatggcttcgaagacggggctcctggaagccaggacggccctgtgcgctgcgagacgagtgtcgccg

gccaccagggtcaccacggcgccctccccggcgtccagcagggcgctcagatccacagctggggtctccttaccctccgtttctggttccactgtggact

gtcctgggttttcagatcttaagcgtaaaacattgacttccagatcgggtcctgtaatagaatctgagtcactgcaatttgcagctatgactacggccac

caaaatgcagatgaaccaactgatgcttctgcggaacatgatgcaaagactaagatgtcggacccaagtgtgcataacttttgccacgaaatatttacat

cagatttgcacactgttgcacaaccagacacgtgtttctccggtccgcgccgtagcagcctactctatggcggtcggcggtcgcgagtcgctccttttaa

ccttgggctgtgcacggctcggcagggctgccacctcccac

>103485|AGAP003428-PA

tacgagtagaaattctgaaaatactttatttcattttctgtgatacaacagaaattaattacatatccatcttaatatttaagagatatggaagtagaac

aacatttatgtaatgtttagtaaatactgaatagttttctagtgaatgcagttgctacgtctacaggtaggagacctttgtgatcccttatatccttgtt

tgctccagccagaagcagtgcccttactgcttctacatcgtctgtggcagcagcccagtggagcggtgtctttccccaacgatccttaacatctgccttt

gccgatgccgtcaacagtgtccacatgatcttcacgtcaccccgatacgtagctgcatgcagaggtgtctgcagactcttgtccttcacattggcaggtg

ctcccgcgtccagaagacacttcacagtttctgcaccggtgtacgctgccacttgcgcgaaggtgttcgctctgccacgtctagccgattctgcacctgc

attcatcactgccaggtgcaagatggtgtacccattgttgtcccttgcgtcgactggtgcccccactcccagcaacctctgcaactcgtccactcgggca

gctttagctgccgctatcatcttctcagccaagtcctgtgtctgctccacagacgacgttctggttcttggcgtcgctgccgccaccagactacatattt

gagctgcagcctccgcattgccccgcaagacatttatccagccggcagaggtcatcacctcggggtgggtcgctatgaaggccacggctgctctctcgag

cctcggacaggagtgcagcacagcgaggagtgcggcagcagcagcattctcgacactcaggccctgcgctatctgttcctcgcacttttcttttagcaac

ggcagatcatatttatcggcggcagctatcatttcgcccagcatggtcgctagctgtggagctgtatcggtgtacatgaaacacagtagctgttttagca

catcttcctgcaagtctgttatctcaatttggcggctgcgggcttctttcatgttatttcgcaacatggcagcaaaaactgggctcctcgctgtcagtat

gggactgtgggccatcagctcagagtcaccgaccaccagcttcacgtccgcaccctcacccgattccagcagtgtgtgcaggtcagttacgatactgtgg

tcctgtgtgcttgttacagccattactatctctccttttaatactgaagccaagtaacaaacacagcgtaaaaagactagaaaccaataagcaacacccg

caagaaaatttacgaaacggaatcgcgtatcggctaaccctttgtgtcagcc

>103482|AGAP003428-PA

gaaattctgaaaatactttatttcattttctgtgatacaacagaaattaattacatatccatcttaatatttaagagatatggaagtagaacaacattta

tgtaatgtttagtaaatactgaatagttttctagtgaatgcagttgctacgtctacaggtaggagacctttgtgatcccttatatccttgtttgctccag

ccagaagcagtgcccttactgcttctacatcgtctgtggcagcagcccagtggagcggtgtctttccccaacgatccttaacatctgcctttgccgatgc

cgtcaacagtgtccacatgatcttcacgtcaccccgatacgtagctgcatgcagaggtgtctgcagactcttgtccttcacattggcaggtgctcccgcg

tccagaagacacttcacagtttctgcaccggtgtacgctgccacttgcgcgaaggtgttcgctctgccacgtctagccgattctgcacctgcattcatca

ctgccaggtgcaagatggtgtacccattgttgtcccttgcgtcgactggtgcccccactcccagcaacctctgcaactcgtccactcgggcagctttagc

tgccgctatcatcttctcagccaagtcctgtgtctgctccacagacgacgttctggttcttggcgtcgctgccgccaccagactacatatttgagctgca

gcctccgcattgccccgcaagacatttatccagccggcagaggtcatcacctcggggtgggtcgctatgaaggccacggctgctctctcgagcctcggac

aggagtgcagcacagcgaggagtgcggcagcagcagcattctcgacactcaggccctgcgctatctgttcctcgcacttttcttttagcaacggcagatc

atatttatcggcggcagctatcatttcgcccagcatggtcgctagctgtggagctgtatcggtgtacatgaaacacagtagctgttttagcacatcttcc

tgcaagtctgttatctcaatttggcggctgcgggcttctttcatgttatttcgcaacatggcagcaaaaactgggctcctcgctgtcagtatgggactgt

gggccatcagctcagagtcaccgaccaccagcttcacgtccgcaccctcacccgattccagcagtgtgtgcaggtcagttacgatactgtggtcctgtgt

gcttgttacagccattactatctctccgttatctggggggagcggagaatccagtttcagatcttttggaatatgtccatgtggcccacacatcgtacag

aaacaaagcagtagtatgttggaagcttttaatactgaagccaagtaacaaacacagcgtaaaaagactagaaacca

>103483|AGAP003428-PA

cgtggaagagttataagagttttattgcaaagcagattgctgggctggctcaggacagcatctccacgagccttctgttgttgttccccctggcgaaatg

tagggctgtgcgcccatcaacagttctgctccctctctcagcccctgatgcgagcagcaaagccgccacatctgtgtggtccctcgatgccgcccagaga

agtggtgtccacccagtgagatctgtggcgttggggtcggcggaggcctctagcagcatctgcacaacaagcgtgtggccgttctctgcagcaaagtgca

taggtgtgcgctggtcctcatccctggcgtccaccactgcaccggcgtccagaaggcacctcgccacctccacatgtccctcctgtgcggcacagtgcag

gggggttccctcccacccgcacccgccctctcttgcctcgacgttggcacccgcggcgagcagcaccctcacctcctccaccgcccctgcatcagctgcc

ctgaacagcctgtcatctctctcctctgcagagatgctccgcatctgagagacagtggcttcatcaagtggaggatatgtgggcagcacagccgtggcag

gagactggcaaggtgcaccagcagcagtagtggtggtggtggtggtgggcctcagcgatgtagcagctggtacactggtttgtggtggtggatcattaag

caattgacagacttgaatcaagtctttaggctgattgagcatcgcatctgcccacccctgagtggccatcacctcgaacggacaggtcttaatgaacgcc

agtgtggcgtgcctgaggtcactgcaggagtggcggactgcgaggacagctgcggccgccgcggtctcgacagtcagctgagcggccacctgccgctcgc

actctgccttcagcctcgacacgccgtacttgtctgctgcaaccagcaggtgctgggccatgccgggcagctgggggacctgcagggtgtacaggtacga

gaccaactcgcgcagcactgccccccctacgtcctcaatgttcaccttaccgctgctggcctccagcgtgtcatgcctgaacatagcagcgaacatgggg

ctcctgtctgccaggactgctctgtgcaccaccagccgcgtgtcgccggccaccaagatcaccatggcgcagtccccggcgtccagaagggcaccgatgc

ccacagccgttgtctccttagtgcccttaactgcttccattgtggacgattcttacctatctgttttgttgtagcggaggagagaacactcactgcacac

ttcccacgtcttctctgccagaattgaaccgtaaatacgcagcgcggaggtttgggccaagctaccgcacccagcgctgac

>103489|AGAP003428-PA

tctgcgaaatattttattacaaagattcctgtgatacaacaggaaaaaacagaaataaattacacatttattgtaacatttaagaggtacagaagtagag

agcttagttatgtcagatttagtacatcctgaacagctctctagtggacgaggtagcagtgtccgcaggtgtaatgtcttcattgtccttcaccttcttg

ctcgctccggcgagcaacagagcccttactgcttctacctgcttcgtggcagcagcccagtgcagcggcgttttcccccaacggtcccgaacatctactt

gtgccgacgacgtcagcagcacccacatcgctttcaagttaccccggtacgcggccgcgtgcagaggcgtctgcagactcgagtccttcgcatcgatgtc

agcgcccgcactcagcagacacttgactgtctccggattcgcgtccatcgctgccaggtgcagggcggtgcacccagtgccgtccctcgcgtcgacgggt

gctcctgcagccagcagccgccgcagttcacccacgtcaccagttctggctgccactgccagccactcgctcaagtcctctgtctgctccacagatgaag

tcctgtttttcggtgttgccgccgccaccagactgcaaatttgagctgcagcctctgcattgttccgcagcacttttatccagccatcagaggtcatcac

ctcagggtgcctcactatgaagtccacggtggcacttctgagctccagacacgagtgcagcacggcgaggagcgaggtggcggcagcgttgtccacactc

agtccctgcaccagctgctcctcgcacctctttttgagcaacggcaggtcatacttatcggcagcacctaacagttccgctgccattcttcccagtttcg

gaactgtgtcagtgtacatgaaacacagcaactgccacaacacatcttcccgcacgtctgttatctcaatttggcggctgttggcttctttcatgtcatt

ccgcaacatggcggcgaagacggggctcctcgctgctaatacgagactgtgcgccggcagctgcgaggcaccgaccaccagagtcacgtccgcaccctcg

cgagagtttagcagtgtgcgcatatcactcgcaaaactgccatccttagagtttctcgctgccattgcagtagctcggtagaatctgctgcgtattgtgt

ttacgtgcccttcacacgggcaccagtggcgaccgaccgttcggttgggcacaaactccgaagggatggagaattctatgaacgtagtagtatctgaaca

cctgccttcctgttccctcttcccgtcttccttccttacctctgcacccgactatctcctctctgatctcaccttcaccctccttctgtataacttctaa

ctgatgacaacctcataacctcca

>103486|AGAP003428-PA

gagcaggcgatacacatcagccatgtcttgaggctgggagcgtactgcgtcagcccagccccgtgtgaaaatcacctggaccaagtgagcccttatgaat

gtaatggcctcctgcttgaggctgtcagaagagtgcctgaccgcgagagcagccgtggccgccgctgtctcgacagacagctgtgctgccagctgcagct

cgcagtccgccttcagggccgacacgccgtacttatcagctgcggccagcagctgtgcagacaggccgggcagctgcggggcctggagtgtatacaggta

ggccagcagctggcgcagcaccgggccctccacgtcctggaccgcgacctgaccgctgctggcctccagcgggtcctgccggaacatggcttcgaagacg

gggctcctggaagccaggacggccctgtgcgctgcgagacgagtgtcgccggccaccagggtcaccacggcgccctccccggcgtccagcagggcgctca

gatccacagctggggtctccttaccctccgtttctggttccactgtggactgtcctgggttttcagatcttaagcgtaaaacattgacttccagatcggg

tcctgtaatagaatctgagtcactgcaatttgcagctatgactacggccaccaaaatgcagatgaaccaactgatgcttctgcggaacatgatgcaaaga

ctaagatgtcggacccaagtgtgcataacttttgccacgaaatatttacatcagatttgcacactgttgcacaaccagacacgtgtttctccggtccgcg

ccgtagcagcctactctatggccgtagtcatagctgcaaattgcagtgactcagattctattacaggacccgatctggaagtcaatgttttacgcttaag

atctgaaaacccaggacagtccacagtggaaccagaaacggagggtaaggagaccccagctgtggatctgagcgccctgctggacgccggggagggcgcc

gtggtgaccctggtggccggcgacactcgtctcgcagcgcacagggccgtcctggcttccaggagccccgtcttcgaagccatgttccggcaggacccgc

tggaggccagcagcggtcaggtcgcggtccaggacgtggagggcccggtgctgcgccagctgctggcctacctgtatacactccaggccccgcagctgcc

cggcctgtctgcacagctgctggccgcagctgataagtacggcgtgtcggccctgaaggcggactgcgagctgcagctggcagcacagctgtctgtcgag

acagcggcggccacggctgctctcgcggtcaggcactcttctgacagcctcaagcaggaggccattacattcataagggctcacttggtccaggtgattt

tcacacggggctgggctgacgcagtacgctcccagcctcaagacatggctgatgtgtatcgcctgctc

>103490|AGAP003428-PA

ccgccggccgccactacagcggcagaggtcggaggaggcgtggctggttgtttacctggcgtgataacttttacctggcaccatgtgttgcagaagcatt

ttgtgggtcatcttcatttttgtggcggtagtcacagattccagccacagtaacctggcctctaatacaggctccggttctgaactcgcagcttatagtt

taagaatggacaatggagtggaaccagtccagagggataaggagaccgcagctgtggacctggccgcccttctggacgccggggacagcgccgtggtgac

gctggtggcaggcgacacgcgtctggtggcgcacagggccgtcctggctgccaggagccccgtgctcgccgacgtgttgcggaacagcacgctggaggcc

agcagcggccacgttgccatctcgggcgtggagggtccagtgctgcgccagctgctggcctacatgtacacacgccacgccccccagctgcacagcatgg

cgccgcagctgctggccgccgccgacagatacggactgtcgtcgctgaaggcggagtgtgagcagctggtggccgcacaggtgtctggcggggccgcagc

ggccgcagctgttctccaagtcaggcgctcctctgacagcctcagggaggccgtggttgacttcgtcaaggctcacctgctccacgtgatgggagggact

ctgcacagccagcgtgaaggtcaggtggaagtgagccgtctgctgtctgttccatcagcaaaaaccaggtcgccggccactgcagacagccgcacgaccg

gctccagtcagccgctcagcgaccacagctggagtcccgacgcagccacaccgaccacggcgacccaccacactgccccacctgacggcgttgacatcgc

tttcctgcggacattatctgaagaggagaagggcaggaggctgatccaggcagttgttcagggctcagtggaagagctgcaggcactgctggcggctgga

gtgaacgtgggggcgagggacatgaacgagtggacccccctgcactgggcaggtatcaagggatacgtggaggtggcgaggcgtctgctggcggcaggtg

cggacgtgggggcgagggacaagttccagcagacgccgctgcacctggctgcgtggggcggcggcgcggctgtcgtcaggctgctggcggcgtcctccgc

cgaccccgacgccagggatgtggacgggtggacgccgttgcactctgcagcgttcgaagaccagacggaggcggcgacggcgctgttggaggcaggggcc

gacgcgggggccttagataacaatgggaaaaccccgctggacatcgccagacagtacaaccagcagcacgtgatagacgtgctgtgataaacctaagagt

cccacacgtgtctgtggactacaataaataaacatttttaacagtatttttggttgtttgtagaataaagcttacaaaaaatcctgcag

>103481|AGAP003428-PA

agctttatgttagcatctctacaagttgctggttgtttttacgttcagcaaagtatttgggagtgttaccatccttatcactcgccaacctgtcggctcc

cgcttccagcagtgcagctgtggtctctgtgtggccacgatctgctgcaaagtggagcggcatacacccacaacaattgctggcattggggtcagcactg

gacgccacgagaaactgcaccatagctgtatggccgttgtatgcagccttgtgcagaggtgtctcctgccatgtgtctctggaatccacctctgctccac

tctccaacaggcacttcaccacctccacgtgtcctttggcagctgcatagtgcagagcggtctgcctcagcacattcttgtccctggatcccacatctgc

ccccgcagccagcagttcctgcagctcctccactgtcccctcctgagccgctcgtatcaacctcttggctttctcttctgcagacaagttcctgaattgt

gccatctcaccgagcagccgactcacttcgactgcgtcatcgggctggtggagcactacatcggaccaaccctgcgtggccagcacttcgtggttggcct

ttatgaaagcgagcgcgtccgctatgaggctgggacaggagtgcctgacggcgagcactgctgtggccgctgcgctctccactgtcagccgagcggctag

ctgctccccgcacgcccgcttcaaccctgaaatgccgtatttgtctgcagccaccagaagttgtggggccacgctcggccgctgtggggcccgtagggtg

tagcaataggagaccagctggcgcaggaccgggccctcgacgtctgtcagggtggtgtggccgctgctgaccgccggcgagtcctggtggaccatggcgg

caaacactggactcctcgcttccaggacggccctgtgcgccgccaaccgcgtctcccccgcctccagggtcaccagggcgcactccccggcgtccagcag

ggggccgaggtccaacgccaccgtctcctcaggctccgcaacgggtcccattgaggatgattctagagttcgctcacactccactttcaacccagagacg

ccgtatttcattgcagctgccagaagttgtggggacacgcttgggcgctgtggggcccgtagggtgtagcaataggagaccaggtggcgcaggaccgggc

cttcgacgtctgtgagggtgatgtggccgctgctgaccgccggcgagtcctggtggaccagggcgcccaacactggactcctcgcttgcaggacggccct

gtgcgccgccaaccgcgtcttccccgccgccagggtcaccatggagcattccccggcgtccagcagggggccgatgtccaagcccgccgcctcctcagcc

tcggtagcgtcttccattgacgatgattctgctgtagacagtctcacacgcgcctgctctgccttagacacgaaacacagtgcattctgctcctcagttg

gctacctacgccacgtttcgcg

>103476|AGAP003428-PA

cgctggctgactgcgcagctggagagtcgctggcggtccgcgttctatcctcgcgggtcgagctggggcgtccgtttgtacaacagccggcgaactggcg

accatggcaggcgacaagaaagcccaggagacggcagccgataacctgggggccctgctggacgcggaggacggcgcggtggtgacgctggtggcgggcc

ggacgcggctggtggtccaccgggccgtcctggcagccaggagtcccgtgttcgaagccatgttccgccacgacacgctggaggccagcagcggcgtggt

gagcatcgcggacgtggatggcgcggcgctgaggcagctggtggcctacatgtacacgctgcgagcgcccgtcgtggccggcacggctgcccacctgcta

gcggccgccgacaagtacggcctgctggagctgaaggcccactgcgagcggcagctggccgcgcagctggccgtggagagcgcggcggccacggcggtgc

tcgccgtcagccactgctgccccaggctcgccgaagtggccgccgcctttgtcgaggccaactcccaggtcttggccacgcaggggtgggctgacgcggt

gcgcagccaccctaaagatgttgtcgaagtgagtcggctgattgctgcgacttcagtgagatccagtgtcccaaccactaataggaggaggctcgacccg

ggcacgcagccaaacagcgaccgcagtggaactcctgccacagctgcactgcctgccacttcccgtaacactcccccgtctaatgacgctgccatctctc

acatgtggagtctttctggagaggagaaagccaggaggctgaagcaggcagctaagtcaggatcagtgcaggagctgcgagcattgctgtctgtaggtgc

agatgtggatgcaggggacagatatgggtggacagccctgtactgtgcggcactagagggacacgtggaggctgtgaggagcctattggagggtggtgca

gacgtggttgccagagacaagtggcagagcacgcctctgcactgtgctgcttgctggggccatgcgactgtggtgtggcttcttctggcagcctcggctg

accctaacgtgagggatcagtggcgacaaactccgctgcactgggcagcaatgaatggccacgcagaggcagtagccgagctgctcgaggcgggagctga

caaggcggctagggatgaagaggggaacacccctgcggaccttgccaggctgttcgacaagcagcaagtcatcgagatgctgaagtaatgttaacataat

gttagtaaaacaaatacaacttgataaaggattttttgtaatttttaaagtaaagagtacaaacaattagtacatttgtcactcatttgccccacaccat

tacaaagtacatataactgctctagtaacttctgaacaagcacgggaacaagagatacccgtaaactaatgtaaccaaacttccttttgacgaaacaatt

aaaataattttcttgagcaaatattgtaaaagtcaacaataattctgaaatactcccttcattgccaataaacacc

>103478|AGAP003428-PA

tacgagtagaaattctgaaaatactttatttcattttctgtgatacaacagaaattaattacatatccatcttaatatttaagagatatggaagtagaac

aacatttatgtaatgtttagtaaatactgaatagttttctagtgaatgcagttgctacgtctacaggtaggagacctttgtgatcccttatatccttgtt

tgctccagccagaagcagtgcccttactgcttctacatcgtctgtggcagcagcccagtggagcggtgtctttccccaacgatccttaacatctgccttt

gccgatgccgtcaacagtgtccacatgatcttcacgtcaccccgatacgtagctgcatgcagaggtgtctgcagactcttgtccttcacattggcaggtg

ctcccgcgtccagaagacacttcacagtttctgcaccggtgtacgctgccacttgcgcgaaggtgttcgctctgccacgtctagccgattctgcacctgc

attcatcactgccaggtgcaagatggtgtacccattgttgtcccttgcgtcgactggtgcccccactcccagcaacctctgcaactcgtccactcgggca

gctttagctgccgctatcatcttctcagccaagtcctgtgtctgctccacagacgacgttctggttcttggcgtcgctgccgccaccagactacatattt

gagctgcagcctccgcattgccccgcaagacatttatccagccggcagaggtcatcacctcggggtgggtcgctatgaaggccacggctgctctctcgag

cctcggacaggagtgcagcacagcgaggagtgcggcagcagcagcattctcgacactcaggccctgcgctatctgttcctcgcacttttcttttagcaac

ggcagatcatatttatcggcggcagctatcatttcgcccagcatggtcgctagctgtggagctgtatcggtgtacatgaaacacagtagctgttttagca

catcttcctgcaagtctgttatctcaatttggcggctgcgggcttctttcatgttatttcgcaacatggcagcaaaaactgggctcctcgctgtcagtat

gggactgtgggccatcagctcagagtcaccgaccaccagcttcacgtccgcaccctcacccgattccagcagtgtgtgcaggtcagttacgatactgtgg

tcctgtgtgcttgttacagccattactatctctcctgaaacacacacacacaaagaaattttatttgaaaaggttaccaaacagtaaataagtgcattct

ttacctctcaacctatctattacattcttattttacttcccagcagtcatccttgtttagcaggattgtagtttgtgtgttggtcacagatacttgcctt

tgcatatttactcctttaactatgaaatgacatggtgactgcattattcactcactgtacagattcattcaattcactgttgcatcgcttgtatgaacta

gtttacacaattattcttcatatcacgaccagaactgcgctgcttctggataacacgagtcttccaggaaaaaactggaacactttacaataaaagactt

tactgcttcaggaaagggcaccagaagtcacctttggggcaacaatgataaatttgatgcgatacaagtggttaaaatgtataaaagattaaaaattcca

tactagaatatcaaaatactttcatgaaagttcaaacatgcacacacttgcaaaggtgtggatgaaacctctcaaaaactgcttgtaatagggaatgagg

ttaatgaagaaataatttacaaagggttgttgggtgaccagtgtggtaagacaagacgagtcagtcatactgacactaaaacattcagtccgtgaactta

gtccagacagctcacaatatttcacatgctttgatgaaatcacaggttatcagccaagttgatgtca

>103479|AGAP003428-PA

agttgattgtggaatttgcagctctctgctagctctgcgagtcgattttcctgggctgcgaacaaatgcttgctggatgcgtgctacattttcatcactc

gttctcggccatccagaacttttccctttgcacaaacacccattctctgtaaactgtttataccaacgtttaatacaccacctatcaggaggtttaacac

catacttcgttcgaaatgcacgctgaacaactgtcgtcgattcactcctgccgtactcaataacacaaaaagctttctgttgagcggtcgccatcttagc

atctactgacgctgacgcctagtcaacagcgcctcaagcgaacaaatgtacaactaaatgaaacttcatagctcccttaattcgccggcagatagtgctt

agctctgccttttgtcgttgcagaattttaaattcctaaagttgaggcattctttttgaatcaccctgtacattcaacaatgttttacacaatggtcata

cctaatgcaataagtaattttcctaaaaaaaattttatttctcttttttttaggatccataatatttaatttttcattgtttattgtaattttaagggat

tttggtttagtcttgcaactaggtaccgatttgtaatcaatactgaagttattgactggaattctttgagattctgaaggtgggagcggtaaaatgcagg

gagcataacgctatttacagtttgtacagaaaccagatggcagttataagagtcaatggacatgacctggaagcagtggttgggaagggagtgagacagg

gttgtagcctctccccaatgttactcaatctgtatattgaacaagcagtaaaggaaacaaaagaaaaatttggagtaggaaataaaatccacggagaaaa

aataaaaattttgaggttcgccgatgacattgtagttctgtcagagacagcaaaggacctagcagggcagctgaacggaatggacatggtcttaaaggag

gatataaggtgaacataaacgaaagcaaaacgaggataatggaatgtagtcgaattaagtttgtggaaagaagtgcgagatgggcgctgttgcaaacaca

tttcaccaaaaagcattaggcgtttagacacggcgacaaaacttctcaaagactaaagacccactcacgccgctgtgcacagagttaggggctgccttgt

ctgcgaccgctgcttgccgtagccctcgccactaagcgagaggctggctgtggctacaagcacatttccaggatcgatatatgcaatgttgagtgataag

acagaactattgacaggtcggtttgcaaatagtgccctacgttctatgcagtagcagtcctacaatttgacgttgacagaaacggactgagtaccagata

cgcttccctaagtaagtatattactgagaaatcgaagattctaatactagagaacaattctatcatcgcaaatgtcccgcagacgtgtttgtcccatatt

gggcagtgacttcgagagagtatgtagatatacaaatcgcgccgatatgtggtacagctttgcgatgtcgactgttgaaacaagacgaatggcacgttta

attagaggaattaaaaatgacaactgttgtgttcgtggtcattcaaaaacaaaatgggaagttgttttaatatataccaagaatgtgaaacagactacgg

tagaatttcgagatgtaagacgctggaggaatattattagcatgttgccaacagagtgcacgtgatccgtttgtcgaggcgaacacaaagccatgaagag

tataaggtttgcgcttgtagcccggttatttaaacacgatcggacagatattcgccatttggacgctttcatcactgcttcacctgtacgattctatgtg

tgtcacatttcgtttgcgaggtttgacatatgtttattctagattgtgttgacagttgaactctatcccctgcctcagttacaacttaagttctttgcac

agttgtattttattgacaaaaggtacacaccaaaatattacgttaatctaaattgattatattactgcttctggtcatttcatcaattgtttttcgacaa

gcaaaattttcaattttaaacaggtttatggtacgttgccattatatcagcaactgtaagataggttgtaggtatacttcgtagtaagcagaaatcctga

ttggcaactgagactgctgaaattgttttattgtctatcccctttatctgcatagcaattccatgaaacattacaagcgctatggggtttttttcttttt

gagtggagtagcactcatgtctcgtgtgatgtatcatctaattttgtgatatgtttgcttaagtttgtaacgtttccccatcagttccggcgttgatgca

gactttattaattagataacgtcggacgttgtttcagacaatgtacagggattcaaccaggtgacatctttgacagccaccattttaccattggcgcact

gaaaaaagaatgactgagttcttctgtcaaaatatgagtgcctgatgaagacatcacataactgcattcgcgtaagttatttgaacattatatgcaccaa

cagaaacttgagtatcacggagggagctggcatctgttggtaacgaatggcgaatttttcaattgctgttttgtagagctagctcaagaaatattttgca

gactgtttccagtagaaaggttacttacattagtttgcagtaatcgcctgtcatcactcttgttgtgttactagagcagttatatctctatgaaatgata

ggtgcaaatgaatgacagtagaattaatatttttgtattgcttattttaaactgtctcactctatggctgtagaactgaaagcttcatgttagcaactgt

accagtcgctgatggttgttcttcctggcatgctgacggggagtattcccttcatcgtccctagcctccgtattggctccaaactctagcagtacagttg

ccacctgtaggtggcctttgtatgccgcccagtgcagcggcgtccacccccataggttcttggcgttggcgtcagcagaggacctcatcagcagtcgcgc

cacagctgtgtggccagcagttgcagctgagtgcagaggtgtgttctgcctgacattcctggcagccgcctctgctccgccgcccagcagacacctcgca

gcttccaggcggccctcccacgctgcccagtgcagagcggtcctcctgtccccgtccatcgctcccacgtctgccccaactgctagcagtgccctcagct

ctttcatttttccctcctgagctgcctcgatgagcctcctgcctttctcctctcgagaaaggttccgcatccaacgtacagtggcatcgtcaggtggagg

ggtgtggcaggttgccacgtgtggtggggctgtgacagcagcctggccattgtcgtcgtggggacgagtgatgggtgctgagctgctgtctgtagtggct

gcggtactggtttgtgcaggtggctgtgcaatcagtcgagttaactctacaagatagtgtgggtggctgactgcagcgtctgcccagccctgcgtcatca

ccacctggagagtgtgggccttaatgaaggcgacggcggcctgcttcaggatggggcaggagtgcctcagcgcgagcagagccgtggcaaccgcgttctc

caccgacagctgtgcggccacctgctgctcgaacgcggctttcaggtggggcaagccgtagctgtcagctgcggccagcacctggggggccacgctgggc

agctggggggcctgcagggtgtacatgtaggacagcaggtggcgcagcaccgggccctccacgtctgggatgacgacccggccgccgctggcctccagcg

tgtcgtgctggaacatggcctggaacacgggacttctggcagccagcacggcccggtgcgccaccagcctcgtgtcacccgccaccagcgtcaccacggc

tccctcgccggcgtcgagcagggtggccaggtccgcagctgtgctcccctcaacctcctgagctcgttctgttgttgtagacatttctg

>103505|AGAP003434-PA

gcaagggtaaaaataaaatcagcaaagtgtccgaaagcaaagactggaactggaaagaacgtaaccatgaggcactgattgaaatatataattgcttaca

gttaccactgcaaaatctttgggatccagctgtctgcgaagaaccattttcagatattatcggaaatgcttgtttcaaatcactagaagatccaggagtt

acccaagtcaagatgaag

>103525|AGAP003475-PA

tccgatacagcttgcagtttctgtgctgactgttcttccctggcaggtccattatcacgtcctgacactctcagaagaaaggctgctggaaactgatgcc

cccttggagtacgtgatgagggttcattgttgctttggagcacatcagccacttcagctttatcttt

>103526|AGAP003475-PA

ggttcattgttgctttggagcacatcagccacttcagctttatctttaatgaattcaaagtcctgacactctcagaagaaaggctgctggaaactgatgc

ccccttggagtctgtgatgagggttcattgttgctttggagcacatcagccacttcagctttatctttaatgaattc

>103524|AGAP003475-PA

tctctctctctctctctctctctcgtccatatccgaacaacattgctatggttcattctgggacactttgacacttcccttgttgagagcccatcctggc

acaaagtaacaatgtggactgaataaaactgcggtattgactgtctaggcatgggtattgactgtctaggcatgattgaactacagacaacatgagctgt

atacttctttcctggtggaatgactggaattgatcagttgtccccctccatctaataggtgctgctcatgcatggttgtttacatctttgggcgggttta

gtgacatctctgaacagtcaaagggactatgtctgttatacaatatccacaatcaatgtctatcttaaggagttctgggaaccgaggcgatgcaaaactt

ttttttgatgtatgtataaaaaagtgataatgtctttttgtttccattttttcaccatcctctacttcatataaattaaaacagaggaataagcaagtga

taatattaaggcatacattttaccacaaatatgtaagtgaaatgaaacctttaacagaatatacactaaatttagtagacatgataatttcaacttgact

gacttaacaggacttgagtttatatatatatagcttttgcagacactatctgtctatttgaactgtgcgagaatatctcaagaatgaaagtaacactttg

actaatcagctatggaactttgagttttgtttgagcctattcagttatccatgttacaggacagccaacaaaaaatgtatatttgttagaaccctaaact

tttgctagtgtgttcttgtttcatacttactactactaaccgtacatatttaaaatgctagctgatagaagtgccagctcctcttgattccaaggatgga

cactattttttttttctttttgatatatatagataaagctgaagtggctgatgtgctccaaagcaacaatgaaccctcatcacggactccaagggggcat

cagtttccagcagcctttcttctgagagtgtcaggacttgataaaaatggaattgggtctgccaagccaatgaagtatacccatgtcgacattgcaggtt

cttctggttcactgccaaatgaaccgacaggatgtccagtagttggattctctacatattttcttcttcctcatgttaactaaagatattgtatttatat

ctcatgatctcagtatgtaacggtcgggtgcttaataatatatcaaagaactgtacttactcttttacttgttgttttatatacaaaatgtacttatgtt

gttgttatgccaatttaatgggctatttctgaataaaaagtatataaataaatgaataaataaataaataaatacaactgttgtattctacacaacaagc

tctagtttcctatcaaaattaagacacactcgctttaaggttgtaagcctgtatgtgatacagttttctatattgtgtatgcatttttaatttttgtaac

ctatcattacacattcaacaaaattagaaatgtttactacatttcagtgtagtaagttctcataattgtaataacaatatgactcaaattaccttgtgcc

ccttctcctactctccatctgaagttttgtgttgtatctaggataccattaaaatgtgcggcagattgtgctacttttctttgaacatttgtcagttata

tagagccattctgaacatatcaagaattgctaggtagttgaattctggtgcacttctaagaaatacatgc

>103554|AGAP003483-PA

gttgctttttcatctccaatatagcctttcattatagatgaaccccggcacaggatttctccttgtttatacgggcctagtgagcgcccggtgtctgtgt

caataccccg

>103556|AGAP003483-PA

cagtgggtgtcgctggctcgccattctccggagggccgactgcgctcggcaatgcagcaggtgcgcgtgtgtggggcaggaggcgccgctctgagcgcgg

agacgcaggcgtgggtggaggcgcgcacgggccggccgatgacgcagtggtacggccagacggaggccttcccgctgtgcaacgtgctgctcacagccca

cgcgccgccacctggctccgtgggtcggatcaacgccgggctacaagccaaggtattggacttgcagaccgcaaagtgtctcggtccaggggtcgacggt

gagctgtgcttcaaaggactggccgtaataccggagttcgtagacaaggagggctggatccacactggagacgtgggccactacgacgctgatggcaacc

tctatgtcgtcgacagagtaaaggaactcgtcaaataccgcggtgtccatgtgtcgccgtcagagatggaggcgtggctgctggcacacccgcaggtgtc

tgaggcggtggtggtgggcataccgcacgatgaagaccaggagcacccagccgcctttgtcgtgctgacccagggcgccaccgtcactcctgagcagctg

aagcaactggtcaaagagagagcatgcggcagcgaggagaagctgttgcatgggggcgtcttcctggtggagtccatccctcgcctcgccacggggaagg

tcagcagggggcagctgcggcgcgctctcct

>103555|AGAP003483-PA

ctattaaacattttgtaccgatttatcccactggagaaattcttcttgactgcttgtctcgtgattatttttaattaattttggcacacagtttttattg

agtaaggttagttcactttattacggaggttatgttacaccaggcggcgtggatgttcatgggatgccaagtgagttactgtgtaggctgctgacgtcag

accgttgcgaatccggagagccggcagctgctgtttctgggagggcagctacacctttgccattgttctctcctggagagctcgccgtaactgtcccttg

ctaaccttccccgtggccacacgagggatggactccagcatgaagactccaccatgtagaagcttctcctcgctgccgcacgccctctctttgaccagtt

gcttgagctcgtcaggagtgacggtggcgcctggggtcaggacgacaaaggcagcgggatgctcctggtcttcatcgtgcggtatgcccaacaccactgc

ctccgacacctgcgggtgtcccagcagccatgcctcaatctcagacggcgacacatggacaccacggtatttgacgagttcctttattctgtcaacgacg

tagaagtttccatcggtgtcgtagtgacccacgtctccagtgtggatccagccatcctcgtccacgaaatcgtctataatagtcggtcctttgaagcaca

gctctccatcgactcctggaccgagacactttccagtctgcaagtccaataccttgccctgcaccccggcatggaggcgacccacggagccaggcggcgg

ccggtgagcgctggcgagcacgttgcacaccggtaaagcctcggtctggccgtaccactgcgacagcggccggcccgtgcgcgcctccacccacgcctgc

gtctccgcgttgagagcggctcctccggtgaagcacagacgcacctgctgcatcgccggccgcagtcgggcttccgggcaatggcgagccagcgacaccc

actggttcggcggcatgaagatggcggtgggcttgcatctctcgatgtagtgcagtatagtctgggggttgaattccggcgtcacgatgcacttcattcc

ggtgtccaggccccagaacgtgaacagagttccgcttaaccagaagaagccggttgtgaacagcagggtgtcgtctctccctaaaggggcatcgctgcgc

gttagtatgagcatggctagggccatgttgcggcgaggtatgcggatgaccttgggctgtcctgtggtcccggacgtgaggagtgcgacggcgggggccg

tgttggtccccagcagcccctgggcgctgctcatcagcaggcgttgagcagcaggcactgcctcggcagtccccaacaccaggagagggcagccagtcgg

cctggcgtcggaaacggacttggtggcctgtggttcgcatagcactgctactggtttcagctcctgtagcgcttggcgcaggctctcgtgaggcagtgcc

gcgttgacgacggcgcagacggctcccgagcagagcgtggccagcagggctggggcgaagagcgccgacctgccgacgcagacggccacggtgtcgccct

cccgcaggccggggaccgccctctgcagcgccgacagctccgccgccgc

>103663|AGAP003501-PA

gacaacaatagaaagtgagcaatacagccacagcgtacaataaaggatgttctatttaaataatataaatatatttttgtcatctttgtttttactcatc

cctcaacaagaagacatcgttacgatcccctggttttgttaaaagggtggtgcaagaaacgcgtcatttgttcgaatatcttctcgtagacgagctccct

tgcatggattccccaaatgaaatccagatgcgaccactcgtccatatctacgcggaatagtccgatggggttcttcagctccttatatgctttcttcaca

tcctgctcgcgggccagacagtcgttgttcccgtagtacaaggcgacaggcgcagtggcggcggacagccggtagtcagggggggcgcggctgccgtagc

gctcgcggttcgccgcctgcccgaagtcgaactgccggaagtggcctccacttcggatctcctgggcgtagtggtcgagctgcctgacagacgctcctgc

cggtgagtgcgccaggtagacgggcagcagtgtctcgttgatttcctttccgttataaccagcgacgaggataatagcgttgttgcacacattttgcgcc

accgagcggtcgctacagaaaatgtcggttgccctcttcgtgatctcattggtgggctcgaattcgtctactcctaccatctccaacaagaccgtcaacg

gtccttcaaattctgaaaacagcttcagtaatggactggtgatgtggcgcatgtaggccaccggtgccatgaggaatgcagccctgatctttttgttgta

ctcaggcttctcggacgtcatgacgaagaacatggtggtgcccatggaatggcccatgtagaagaggtctcgatgtcccgttgtgtataagatgtgatcg

atcacagctgggatgtcgtacaccccctgctcgtgccagctgaatttccagaattttgaatgtttcgtgttcagacggacgtgatgcctagagtagacgt

taccgcgaccgttccccagccagacatcgaaaccggtgtcagccaagaggtacgcgagcgccttggcgggcccgttgacgacccagtcgtcagagctgga

gagcagcccgtgctgcaccagcaccggcggcatgtcgactggtcgctggagcggcgggatccggaacaggcgcagcacgtagccgtcctccgtgacggcg

tagtgctcctccacccggtagccgtgctgctgcagcatctgcgcagtgtccaggcgctctggttccttgggcggctgatggctcttctccaccgcctgcg

tgtgtttcttctccgctgcctgcggatgcttcttccccactgtgttcacaatcgatgtcagcgacaacagtcccatcaatgagaccgcctcgaacaattg

ccacctcatagccacaatctgtcggatggtcttcctgtggcgtactgccgatcctgctgagaagaccacaatccttgttgcaatggtcggcgactatgtc

agagatactgctgtatcctcaaatatcccctcttatatgattctcactattca

>103695|AGAP003513-PB

acatcagggacttcagctacgatggagaagggccagctgcgttcttctgggctggagacaccaagagcccgagcagctacggcttcaaggtgaacgacga

gaaaggaacaacgaacgtgctgccgcgctaccgcaagaaacacataacggtcaccctgccggacaacaagacgctcagggacatcaagtggttctccgtg

tggtgcgacgaattcgctgtgaacttcggcgacgtgaaaatccccaagaacttcgactatccgaaaccccagaagatcgatccgctggacggcgtgcacg

ccgtgtcatcggacaacatcgtcatcgtcgacgcccagacactgctcgtgccccacttctcatacgatggtgaagctccagacgccaagttctgggtggg

ccgcggcaacaagcccagctcgcaggggacgcgcgtgccggacgagaacgggcgcgaggagccgctgcggcgctacgaccgcaagacgctggtgctgacg

ctgcccggcgacctcaccgtgcacgagatcggccacttcggcgtctggtgcgaggcgttcgccgtcgacttcggccacgtgcgtgtgcccgccaacatga

acgtgccgccctcgctcaagatgctcggcgtctccgcccagaaaagaccgagatctcccgaaatatcccaaattcttcaacagtacgatcagcaacagct

tgcgccccacacgctcagcggcgtcgttgaccgtagcgcctcccacggtttcgaccagggccaacagactaacaccttctacaaccaacaacaac

>103710|AGAP003517-PA

aaacgacttttgcctttgtcaaatctgtagcagtgcactctataaacacattctttgtattaagtgttatttttgagtgatctccattaattataggagg

caaagataaaacaatgcctttgctgtcataaataactggatatactggactatctctgattattggcaaatattgtttcagttgtgcatgatttgaataa

agttccattatttcatgaccagtatattctttggtctgattaaggggcttgaatttgatgtctgctggtggcctagcatcataaatgaatggaccttcaa

cagaatctaggtcatgagtaccaattgccaccaaggaacgtttcctacatatgttttgatgaagtttgtcttgtaggtcaataaagctttcatatctgtt

tttattgaaagttatatttcttagtacagcagcaactacataaggccgtatcaatcctgtatcaggtttcatgataatcttctgaacactggatccttct

ggtttcactgctctatacctcggcaatggctttcttcccagaaaaacaagaagtcctgtgacaaggccctctaaacacaggagatcataacgattggctg

gtatgtcaatcctataaattacatcttctgatgctgctgtttcagtatgctccaatccttgttcctttgcctgcatctgcttctctgtggtcacttcatc

aagttccaagccaaatttaaaacacaaatcactaaactgttcatcagtgtatttctctccaagagcttcgaagagccagtcgcgtttgatgccgattgtt

ggcattttcgtccttcttcgaaaacaattttcttccactactgttaatgtttatatttacactgaccacatggctcagctgcacgtcacacactcacaca

ctcacacacacacactcacacacacacactcacacactcacactcac

>103730|AGAP003536-PA

ctgagaatcatttattagcacttctgcatttcgtgaatgatagaaaaacagtttctgcctcacttcaagcatgccacaaagaaggcatatatgattccaa

agctatcattaccataatctgattgctgttatacacaacagtatctttaacaagaatacttttcacacaaatccctctgcactgacaccattgtatataa

ctctctgggtaatctagtggcatctagattaacagttaacatttgaaggattatctccatcctcctcaccagcgtatcgctgtttattgccactgctttg

gtatagcaactgaagtataactcgccatgctggttttggctgacatcaacttattgtcttggagagggcagaattataataaagaagaggttcagatata

tataattactgttggaaaatgctaagcaatgaagactattttcaataagaatattctataaactacagaactgcaacagtgccattttctacatgttctt

tattcaggaatcaccactaatgcagttgctgttgcatagtgcagcgtatggttttcagttatatattatgttaaacatacatatccagacagtaaataaa

tgttttctaaaggacatcatatttactgctcactgtagaacttatttatttcacaatttctgcagtcaacagcaagtatgatgtccttttaaaaatacta

catgactgtgaactgctgtcatgagaagttaacaaatattttctgtttataatctgctagcttgtgccacagctttgtcaatgttttacagaatcattgt

gtattttacatgtgagggaatacagtgatctacttaatactgacaccacatttgcagcatagaactattatttagtgtaaggtatcaaattacaagaaac

tggtatcagtctagtactgaatataattctttcaaggccatcatggcacaggatctattaatatcagggagctataaaattatgagacaacatttcagtg

ctgccaataaacacacagcaaagtatgtacactatcctaacttttgaaacaatagtgccttcatcagggagggagggaaaaaggctcaaatggctctaag

cactatgggacttaacatttgaggtcatcagtcccctggaacttagaactacttaaacctaactaacataaggacatcacacacatccatggccgaggca

ggattcgaacctgtgaccatagcagcagcgcggttccagactgaagcgcggttccagtctgaagcgcctagaactgctcggccacagcggctggccatct

gggaggagacaggaatcggttggggaaggttaaaaaaaaggggagggggcaggttactcagaacccaggacccatcccttccctgagggaggaactatta

gttctgaaaactggaatagtaatgtacttttttacttatttatcagcagaactgaaattttgcctcataagggaaagttttggccagcaatccttcttca

ctagtaaaatttccttttgatacaattaatatcacataggtggctctttgggggtgtgattaacatggacatttagaagtacaatcccctgcaaattctc

tgtaccaattagcaactgttcttggtttttggagacttaatttcctttattttgtaaaatattatcatcagctatttaatatttactgctggataaagaa

acctccccccccccttccccccaaaaaaagaaaaatttgtggacatgagctatatgttttcatgtgtgatgtataatcctgcaatttgttttattcaatt

agaatggaaaaaaagttatgttcagtgaaaaatcacctgacatatcttgtagcccaagtttgtagacatgagagtgagtaacaagatatcaacctgagca

acagtgattgtgccagtggatgctgaatatatttcatacacttctttggcacaactttgcaatctaattttatttatttttattgtgattcagaattagc

caacatcaagcttttggagctgaacattttatcgtgttgtataagaacaactttcttcagcagcatgtgattttttccatgtagctcctaaagaaaaagt

ccctggttccagatgttcagaattctttgttatctgcatattactgcaatccaaactcctgctgtgcagtgaatgtagcgtatatttttcatatatccat

aatcacttcagcaatactgctcacaactgaagtatgaccccataataaagaagtgcacagtgtggatgagtgcatgtgcaatggcccattcctcaataca

tattcaagtgatcttgtcaagacaattgcactgtctcagctgttaacaaaaatgagcctgcagctcccaacaagttgcaatgctagcttctgctaggctt

ctttagcaccttaaagcttctcagagcacatcacttctgtatgttatacaaactgttagcacacataaactactcaatctataacctcattttgtttcaa

catataagaggaaacatcaactgcaacttcataactgccaaagaatacagcaccaccaagtgttatccaagtgattctgggcagaaatccagcaaataac

cctttaaatcctctttcagaatacacatttcttaatactcttgatattttcagtttcttgtagtcatctggaacctgaagttctttgtctgccagcataa

ttctggttttcgctacatcaagtggagtagttactgcagcagcaatgcaacctgcaaatgctccacagagtgccttttctgcagggttaagttcatcacc

ctttatttctctgagactcagcttaaaaaattcccagatagggaactgcacaacggagaatggaatctcccggacaactgtactagcaaagcctctgtag

aatcccataaagccttccttggcatagatgtggcgccacatggacaacaaggatgaattgtgcatagaagcttgttgtcgttgcttgaaaatttcagttg

gcactttaacaaggcaagagaccacttctgcaactgatgctgcacccatatgtaccaatggataataagcttcactggcatacggcctacaaattgattt

gactgtattgtaggtaccaaaaaacaatgaagctgttggtgcggaaccaatcattgttggtcccaaacctttatacacatgacgaaacccaccacttctc

caaaatccattagcactttgaagtcttgtcttcagagtatcgaggggaaataatgttatatctacagttaatccagccaagccacctgcaattaatgaaa

ttataagaggttgttccatttcgagatttggtactgtaccacagtatggg

>103800|AGAP003541-PA

ggagggaactcctggaacgattccacacatattggcttgctagtttccaagtttacgatagcagcatctccagatt

>103799|AGAP003541-PA

cctcatgtcacgcacagcaaatctacccaatggagggaactcctggaacgattccacacagattggcttgctaggtaccaagtttacgatagcagcatca

ccagggcacctctctgagaaactttacgttcgtgtgtgatccccattcaacatgacaac

>103838|AGAP003547-PA

tagaaagtttaaattgtcttttgttaaggaaaggcaaatagtatagcattttacactcagaatgtttttctacatctacaataacttttcaatcacttta

ttatttcactgccaagtaatactacattatgcaatgcatatttgtgttaatcttttagagttcatacttatttatcgatgcacttctaatagaaacataa

tctgttgcaccagcatttccattttaaaaatatgtagctcttcattttgtaggtggatacaaaagacaggtggtttccagctgtagccttatgcagcact

agtgtttgtctatctgcgcacgtgccatgagtgcaaccctctgggccctctgcacaacttctggacaacgcttcttgcgacccaggcggcaatcctggaa

gaagccttcatttcttgggaatccatggctttcatcactataagtgaccagtccgaggccccagatgcgtcctcctcggaactccccttcgtatctcatg

ccatcggcacgccaaaataccccgtgtccatggaaccaaccttgcatgaattctccttcatactttgcgccatctggaaatgtcataacaccaaggcctg

agcataagccattttgaaatcccccatcatatcgagtcccatctggcatcacaaggtggcccatgccatgtttttgtccacgttgactccagtctccaac

atagcgtgtcccatcttcatatcgataggcgccagtttttacaactcctggcagtactataagtcaagttattagttaaattatacttcaaacagaaata

agcttctaagaaaataacagtcttgcactataatgatctaatccttgcagttttgaggagatttgaatgaaaaataatacaacttagagaaatccataat

atctgattaactatatgatttttatcttaaaagtaacaccattttaaaaa

>103983|AGAP003621-PA

ctggcaactgattacatcatattcaagtttggtaacatatttacacgtttcaaagaaaagtatttattgaatagaaaatggttttattttaactaatatt

aacacatcaatatttgcacagtatccacatagtcaaaagtaatatctatagctgttccatgtcttcagtgcacattacataaaatcatcagaatcatttc

catcatcatatttatttgatgtcatattctcaggtttcattaagttactgtagctcctcagttcagcttcttctttctttcttctctcttcttccttctc

tctctcacgttgctcacgaagtaacctctttttgtcttcccgttctttacgatccctttgttcacgttctgcttgcaaatctggatgctcttcacgtttt

gttttgtttaatcgattcacaatttcattgtttcgttttgccactcgaatttttcgtacctctttatctcgatgaaatccaacttgaccaacttccatgc

tatctgtttttttcaagttggaccacattgtgtacactacatctacatcgttaagtttattgccctggatgctgttagctttcacaagttgtgctgcatc

ttctaaaactgaagttggtatatcatctagagtctgaccctcagtggtttcaaagcagtgctcatagatacatcatttccattacgtcacatagtatgtg

gaatatcttacagggtggagtcttaggtacacatgggcagatgaaaccttgtcaacatgaaaccacacatcttctggccatccccatttgatgaggtctt

cattttcgaatttgtctattcccataaacaatgtcactggtggtgacacgacgttactggtgaaataaaaaaccattgtgcttctgctctcctaacacaa

attgtcaacactgcgcctaaataataaacacagtacactccacacaacaaacattcacgtgctcaaaatcatattcaccaccgtgacacctatgacgcaa

ag

>104006|AGAP003623-PA

gcccaggtgacagctcttcagtgatctgacaggaaccagcacagccattggcataattatatatatgcatccaggtatctgactaaggcaacactgtcag

gagtgtgcatatgcagttctctttcagattgatgtaaatttatgaaggtcccattacttttaaatgtgtttgctataaaattgtaagttgctggtcagct

tttatttgagtttgtaaaattgaaaacttaaattttcatcatacccgtcatattctgtttgaagcatccatgagcagcaaatagccactctgcccttgtt

tcggcaaatatgacaacattttgttttggttgcaatccaagagctctcaaaccacagccaaaatgagaagagatggtttccacttcttcaaatgatttcc

atttataatctcccattataaactttttgaaaatgcgtccattgggttgtggctcatcttcttc

>104033|AGAP003623-PB

attctccaaaggccatgggagaagaaaagactttcaagtagagtaaaagcacaaactgttgccttagatgagacgtcaattacttaccgtagtcttcaga

ctccacagaagcatcacttgagaatggtgcgtgataaagtggacacaatggaaaaaatgttagtctacgtttcacgcctgtatcctacaaggcgctgtct

aggtaccagagaaattctggcagaagaagatgagccacaacccaatggacgcattttcaaaaagtttataatgggagattataaatggaaatcatttgaa

gaagtggaaaccatctcttctcattttggctgtggtttgagagctcttggattgcaaccaaaacaaaatgttgtcatatttgccgaaacaagggcagagt

ggctatttgctgctcatggatgcttcaaacagaatatgacggttgtcaccatatatgcaacactaggagaagatgcaattgcatcttctcctagtgttgc

atatatggtgacaaccgtcatattctgtttgaagcatccatgagcagcaaatagccactctgcccttgtttcggcaaatatgacaacattttgttttggt

tgcaatccaagagctctcaaaccacagccaaaatgagaagagatggtttccacttcttcaaatgatttccatttataatctcccattataaactttttga

aaatgcgtccattgggttgtggctcatcttcttctgccagaatttctctggtacctagacagcgccttgtaggatacaggcgtgaaacgtagactaacat

tttttccattgtgtccactttatcacgcaccattctcaagtgatgcttctgtggagtctgaagactacggtaagtaattgacgtctcatctaaggcaaca

gtttgtgcttttactctacttgaaagtcttttcttctcccatggcctttggagaat

>104051|AGAP003623-PC

caggtatctgactaaggcaacactgtcaggagtgtgcatatgcagttctctttcagattgatgtaaatttatgaaggtcccattacttttaaatgtgttt

gctataaaattgtaagttgctggtcagcttttatttgagtttgtaaaattgaaaacttaaattttcatcatacccgtcatattctgtttgaagcatccat

gagcagcaaatagccactctgcccttgtttcggcaaatatgacaacattttgttttggttgcaatccaagagctctcaaaccacagccaaaatgagaaga

gatggtttccacttcttcaaatgatttccatttataatctcccattataaactttttgaaaatgcgtccattgggttgtggctcatcttcttctgccaga

atttctctggtacctagacagcgccttgtaggatacaggcgtgaaacgtagactaacattttttccattgtgtccactttatcacgcaccattctcaagt

gatgcttctgtggagtctgaagactacggtaagtaattgacgtctcatctaaggcaacagtttgtgcttttactctacttgaaagtcttttcttctccca

tggcctttggagaatgaaatgaactggatatgttattatatcacagacaaatgcaattgctttaatggcacctattgtgccacttatcaaaatgctgtcc

atattctcattttcttcgaagtacatgtctgcaggcttcacttagcaactacgcggaggcaaggcacggcgtgagtagcgcttatcgggtgagcggcgac

ggcgacacaaacgactttgcgcgcaagcgtgtgcgcgttcgactgagcgaccacgcggcctg

>104085|AGAP003625-PA

cttcgcctcctgttgctcttgcttcttccctcaactaccccttcatttactacaccgccatcatcagatgcagcgtcttggtcttcagatgctgagtgga

gatcattggatggtaggttcctcatgtaattgaatgtactgttaaagggcaagccagtactcatccataacatcatgtctgatgtcccaagccgattccc

agaagtccagaagtcatatttgttataaccagcattcctcaaatactccattaaagaatctgccttttcacgtgattcaaatgatgctagttgcaaacca

agtgatcggcagtactgatatgctaggaaataattgagctccggtgagtatggattcatacgactgatgaaatattgcactccatccagctgtattgttg

tgattcgctgacctgaggcagtggccgcgaagacgatggcgagcagcaggaggtgcatggcgtcgcgtcggctggagctggtgtctgtcc

>104143|AGAP003636-PA

ctggtaggcgtgcacgatgttgggtacgtcgacgtccgggtcgccctcgacacgctgaacaagctggtggacgagagcgacccggacgccgacgtaccca

acatcgtgcacgcctaccagacggccgaacgca

>104147|AGAP003636-PA

ctacgacagtccagcacgccaactcaccgacacacgctccatcaaggagcgagtgcgcaaactctactacgacatgcacaagtaccagaccgtcgacttc

ggcaagaagcagcacgccaagtggctgaagttcgacaagttccg

>104142|AGAP003636-PA

acgccaagtggctgaagttcgacaagttccgcgcgcccatcatggacacgctggagcggctgagcggcatgcacaagtaccagaccgtcgacttcgtcaa

gaaccagcacgccaagtggctgaagttcgacaagttccgcgcgcccattatggacacgctggagcggctgagcggc

>104145|AGAP003636-PA

atgggcgcgcggaacttgtcgaacttcagccacttggcgtgctgctctgttaagacagacgacatacccgtaagagtaagagcaccgcaaccagccatca

ggaacaattcgcgtcatttctggttgcgcagagcggacttactcttgacgaagtcgacggtctggtacttgtgcatgtcgtagtagagcttgcgcactcg

ctccttgatggggtccttgtcgtcggcgctgcaggaggccgccgagaagacgaag

>104148|AGAP003636-PA

tccggcttccctgcgtacagaggttccggccacagacgcagctactgactgtccacggctgtctgcggtcttgctcttcagcaacatggccactgcagtg

cccctgccgcagccgctgctcgacccttcggagctgctcaggccggaacctctgtacgcagggaagccggtctcgcagttccgcgactacaccatcgacg

agaatgaccccgtcaaggagcgagtgcgcaggctctacctcgccatgcacacgcaccagacagtggacttcgtcaagagtcgtcacgagaagtggcgcaa

gttcgacaagttccgcg

>104187|AGAP003643-PA

tagcttcctgaaaaacctgtggaggttggttttcatgtgaaacttttatttttcagggcatgttttaagaagctgcctcatatcttgtgcttcaatacta

tgcgttatacattcaacatgtcaacaatgttgaaagagaaagtcaatacccatttttcctttccattgcgactagatatgtcag

>104211|AGAP003651-PB

tccatgtttattgttgacggggtttgagcttgacacaaacaatgtggttttttgttttctttattgaattacaatattaacatgctaacattttatttta

cacacaattaatgttaacagtaacacaaaagacatttagggacagatggccataacagtagctctcgtttctagaaatgtgaaagatgatgacacagaaa

ctgaactgtgactgtagcattagctgcctcaacatttcaaatctcctgagattctttagtaacagtatcttggctctaatttgtctggctgattaaacat

ttgtaatgtaaaataatttcagtataaaagaataggatgttgagcaagtccactaatttttaaagtattaacatttctgtacacatagcacaatgaatat

ccagcaggtaagaaattttgttcactagctgctttatactgttttacacttgttaatagctacacaaagattggctacatacgtctctgaaataaatgag

aaacttggcacaataatactgtattgacacgattatctcagccagacttaatgactagcacctgacaatgtatttgataaatacagagggtgttttctac

cagctgtttggaggcactcgggtcggactagtataccgctgtgaccactcttctcgaattcacttcggcaactacgtaatgacggtcacagctttcggca

ccatagtgacagtcgggccacgatctacctcgcagcgccgtacagagtctcgacgaccgtcaggatctggtcaaaatgtggccgcttctcgggcttgtac

tcccagcagctcagcatcaggcggtacatctcgtctggcgtcccatcgggcttcggcatccggtatccattgtcaattttctcgcgtgcctgtgaattac

tcataccattgtacggcactcctccacgagaaaatatttcccaaatgagaatgccataactccacacatcacacagtgacgtgtatttaccaaagtttag

ggcctctggtgctgtccacttgataggtatctgcttcattccatcagaaacaatgtactcctcttcttctcgagacatgccaaagtccgaaatcttgact

atgttgttctggcctatgagacagttcctggcagcgaggtcacggtggatgcagttgtgggactcgaggtagctcatgcccgcggcggtgtcgcggcaca

tccccagcagctgccgctgcgtcagaccgtcctcgtggctgcgcaggaacttgagcaacgagccacctggaacgagctccatcactatcatgatgggctg

cttctgcacgcagattccgatcagcctgacaatattcgggtggttatactgcttcaggatgcgcccctcttggaggaatttgcgtcggtgctcttcgggg

actgtcattcggcaggtttttatggcgacctccatcccagtttttcgcagtttacctctgtgcacgtccccgaagttaccccggccgatctgctccacga

ggatgacgtcatcgttgttgagctcccagcgctcgcggtagacggcgcgactcagcacggcgccggagcgcgccgtcaccggcagctggctctgctgctg

gtactggatcagctcctgcaccgtcgggaaggcccgaccctcgaacctgaagctgccgtcggcggtggtctgcacgatgaagtgcttgtggtggccggcc

cagcacacggagagcaccacctggctctcctcgtggcgcaccgtctcgcgcaccaggaagtcgccctcgtgcgtcagcaggcgcaccccctcgtcgcgcg

gcagcacgccgtgaaaccactcctcctcctgcagcggccggccgccgggcagcgcgctctgcaccagctgcgtgggcgtcgaaggcgcctctggcggtgt

gagcgcgccgccgtcgacgtctgcggcggtggcgacggcgccgttggtggcgggcggcggtcggaaggcggcctccacggagagcgagaggtcgcagatc

ggaagagcgtcgtgtaggga

>104236|AGAP003656-PA

aagaagtacaataatagaacaaaattgccataatactttattactggtcaaatatgtcaaaattatgtacagcaaaatatttctgtaaataaatgtaaaa

ttagcacatataacaaaacagacgttacagtaaatgctaacacagcagtttagttaatgcttttagactgctacatgccaaataccaggcaacaacagag

cattattcttacattggggcgtggctagtataaattgagattccatgtgccaccattatatcacatattttcaacttaagtagtatggcaattttgttct

attattgtacttcttatggaacataaatctagaaagccacacacatatcataatgattacagttacagatcattttaatgtacaataactgaaccagaga

caggtcacacatttaaacataaatctttatgacaacagtatgaataccagcttacacaaatgatttttcgatgggacaaaaggcacacacaactcgagca

cataagctaattggttcatttacttttctcctcatataccaatcaaatacacaaagctgaaccacaattacacaacactctaaaatgccatctttttttt

taaactgctatactttaatattacttcaaaactttctgcctttacacatacaattttctgaaccaaaggatgaaagaggggaagttacgaagctggaacg

actaagagctgttcatcctcatcacccgagaggtacgaaagcaaacttgttgacgaggtgacactattcctgctcggagattctgatgcagatgttatac

acgaatgagatggggaaaccgttcttgcaggagtttgaactacaggtatatttaaaatacaagtttcataaagagcgtgagggggcaagttttctgctac

ttgatttggcacaagtgctgcaggctggggaacaccgctaactgtattatttaacttattatttgtgttttcagctggtagtattgcctcaccctgttga

actaaaacagggtcatttgctcccgatactgcaccggaagactgagtgagaactggcaactggctgcttgtaagaccagcataatttggtgtcacagttt

gggactgcaaaccgagcgtgacattaggatcacagtgtggaggtactgtgaccataatattcctctgactcctcctcctatttgaatgtctgcgggacat

gcgcctgctgctcaaatgttgatgtctctgcatcatactgtgttcattgccaaacagcctactgtttggctgagtggctgcacactgtaaagggcaccca

ctgactgccacactgtacgctggtggtggttcccgattgaagaactcttcgtcgaggtgtggtactggtggctgggtagaaaagctcccactgttatggt

gaatacggtaactgtttgtaacagcaaggcgcagactgtacagacggcacatgcacccgacagcaatcaccaacaataaactgcacagcaaggatcccat

tatagctgctgctattacggagttcttgatacacccttgctcatcacttccatctttacagttttcttgtccatcacaccattcagaatgtggtatataa

tttccatcgtcacactgaaatcttctatagcaattttcctcatcagaattatccatacagttgtgtactccatcacaccgttcaacactggaataacatc

cgcgaccatcgctgcatttatgccttcctatacatgtgtcatcacagttagtttcatcagtgttgtcagcacaatcagattttgaatcacacacttgttc

aaatgtgtaacaaccctgtccagaacggcacggtcgttcatctgtagagcacttatgacggggacagttttcctcatctcggccagaccgacaatctagc

tctccattacacctttgtgtttcattgaaacagtcataactatctcccatacacggagctcctggttttccagaacaggaaaacatactgcacactgtta

agtcctgcacacatcttccatcagtgcaatgcatacaatcttcataataatcatatgtgttatatacgatcaaaaatcctctcccttctttatgaacagc

agatgaatgaaacttgattactgcttgtggttgccgcaatgtaaccatcacggaatgtgtcagtttgccgcacaatttttgttttgctcctcctcctgct

cttggaatagataaccaattgctcacacaacatggaggatgtttacagttagcatcatcttcgatgtcaagatctgttacccttatattaagaggatgct

gggctgaagttgttattacccatgtacaactggtgtaatttggatatggaaatggataatgtggacttgatattcttccttgtggagcactatagtgagt

gtgtccacacacatcactttgttgttcatagtaagttgcttctgctgaacatatagcagcaaaatgagataaaaatatgcatgccagaaaaatttccttg

tgctgtatcttctttgtgctattcatagtggagtattgctgcttcaaatctactgcatttgctgtgtcagattatgtttcttctgattgttaacatgatg

tcactggtgttcggccctgtacagtaaataacactggcatcacatcgagtcgcgcacacagcacagtgcac

>104275|AGAP003680-PA

ttgttctaattgtggaaacctgacaacgaaaggcaagatgagagcttttttacaaaagaatttcttacggatgtggcatctcttcactggggaaagaaag

a

>104276|AGAP003680-PA

tcttgcagatgaggagacactagatcagagtgaaatacatgtatggcttgacatgtccaatcagcagattggactgatgctaaacagagacttacagttt

gcttacagagactttgccaaagatctgttgacagcttgtggagaaaatcctaaactagctgatgttcccattcagttccaagaacctgtctatggcacaa

atgatccaagcttcactgattttgtagcacctggtgttatactcacgtaagtcttttctagcagcattaatatttgctgtacatatacagaatagttccc

cattaagaataaaatagcaagtgagtatatcaaaagtaatcttttattcaaattatgtgcatttatttaaatacagcaaaagcaatttacatccttcatt

caaaacttgtgaacattagtctactaccatagtcactgaaatggtcttaacaatggaaaacatca

>104300|AGAP003688-PA

gtgtccaaagacaaagctttgacaattactgcttcctttcctaaatactctgcctcttcatcagtaacctgctctaatcgactgcatagttctggtgctt

taatttttctttcaacattgaccataataatatttgtgtggacacttgctgggtcaatatggatatatttgcttccaaactctgatacaccattggcaat

gagagctgcattagcatggtcttgtgtaagtctgtcagaggcagtatccagtgccacaaggcccgctgctgctagcacacccacttgccgcattccccct

ccaagggctttccgtaggtaccttgctctggtcacaaaagtagtatcaccaagcagtagagaaccaactggagcaccgagacctttgctgagacagagtg

acacagagcggcagccgctggtcagcacagcagggtgcacacgcatctttgtcgctgcatttagcaaacgagcaccatccacatgcacaggtatgtcacg

cagggctgcctgcttccacaactcatcgagccattccatcggtagaactttcccaccgcataagttgtgagtattttcaatacagatgagagcagtgact

ggctcatggatgtcttcattccttattttcatctgcatttcatccaaatcaaaagttccatctggtttatttttaactgttgtcacttgaacccctgcaa

tatgtgctgctgatccctgctcatatttgtaaatatgagacttgtcaccagctatgagttcagttcctcttcgactgcaatgaaccataattgcaagtag

attggccatagtaccactggggacaaatagtccagcttctttcccaaataatgcagctgctcttcgttccaaagctttcacagtaggatcttcttcataa

acatcatctccaacttccgcattgtacattgcttctctcattgccttagtaggtactgttaatgtatcgctccgcaaatcaatcacatacatatcctgga

actttgatgctgccatctgaatatacagacccctgtggtgtaaattctgttgccggcagcgaaccaaaagtttcggcgcgtatctcgcaatacgtgtgat

catcgacacaccgacctctcgcgactctgggtcgtcacacgaggcaactgctgccaagaccgg

>104370|AGAP003709-PA

ctgcgctcgagctgcaagcaggtcttctgatggggatcctcgcataccgttatattattcatgtgtacgtaccgaggtgaatgccacgaaccccatgaag

tttgtcggtcaacctaggtaacccgacgaactattcggaatgtcgtgttgcgaaactacgaccgcgatatctccgcaggaagaagattgcctcaaggttc

gaaagtggtggtgttttttgttgtccagtatcttcacattcttagcaggactgctggtagtattgttgtggagagccttcgcgttcttgtgttgccgcaa

agaaccagaattttctccaaatgacccgaaacagaaagaacagaaagccgcccgacagggcaagcaggagttcgaagggacttttatgacagaagcgaaa

gactgggctggggagctgatttctggtcagactacaacggggagaattctggtggtgttggtgttcatacttagtattgcgtcacttatcatttacttca

ttgatgcgtccagtgaagtggtggaaagatgccaaaaatggagcaacaatattacacagcaaatagatttagcgttcaatatattttttatggtgtactt

ttttatacgatttatagctgcaagcgataaactgtggtttatgctggaaatgtattcctttgtggactactttacaataccaccttcttttgtctcaatt

tacttggacagaacgtggataggtttaagattcctaagggcattgcgcctaatgactgtaccagatattctacaatatcttaatatcctgaagacttcaa

gttccattcgccttgcacaacttgtgtcaatattcatttctgtatggctcacagctgctggaattattcacctgctggaaaactctggtgatccattgga

cttcaaaaatccaaatcatctctcatattggacatgcgtttattttcttattgttacaatgtctactgttggatatggtgatgtttactgtcagactgtt

cttggaagaacatttctcgtattttttttactcgtcggtttggccattttcgcaagttgtatccccgagattattgatctgattgggactagacccaagt

acggcggcaccttgaagaatgaacgcggcagaaggcatattgtagtgtgtggccacataacatacgagtcggtatcccactttcttaaagactttcttca

cgaagaccgagaagatgtggatgttgaagtagtcttcttgcacagaaaaccaccagaccttgagttggaaggcctgttcaagaggcatttcactactgtt

gaattcttccaaggttcaataatgaatccaattgatcttcaacgggttaaggtgcatgaagctgatgcgtgcttggtgctagctaataaatactgccagg

atcctgatgccgaggatgctgctaacatcatgcgagttatctccattaagaactactcggatgatatccgtgttatcatccagctaatgcagtatcacaa

caaggcctatctcttaaacattccatcgtgggactggaagcagggtgatgatgtgatatgtctcgcagaacttaaattggggtttattgctcaaagctgt

ttggcacctgggttttcaactatgatggccaatctgtttgccatgcgttcctttaaaacg

>104396|AGAP003709-PB

gtttatgctggaaatgtattcctttgtggactactttacaataccaccttcttttgtctcaatttacttggacagaacgtggataggtttaagattccta

agggcattgcgcctaatgactgtaccagatattctacaatatcttaatatcctgaagacttcaagttccattcgccttgcacaacttgtgtcaatattca

tttctgtatggctcacagctgctggaattattcacctgctggaaaactctggtgatccattggacttcaaaaatccaaatcatctctcatattggacatg

cgtttattttcttattgttacaatgtctactgttggatatggtgatgtttactgtcagactgttcttggaagaacatttctcgtattttttttactcgtc

ggtttggcggtgtttgccagttggatcccagagatcaccgagttggctggcaaccgctgcaaatacggagggcaatacaagcggag

>104395|AGAP003709-PB

ggctgtgtaagataaagctctgtgtcaggatcgtcatcatcatcttggtccaggaactgcacattgctgtcatttactagctctgttatcataggcacat

tggctccgtagaccgaccctctcctttgaagaacaataggactacctacaggcgtgagattgtcttgctcacttgctgcttggcttaagacaccaattgt

gtcgtcaaatgtcatagctttaatatttaatgatgctaagatggcttctttatcagccaatgttggatcatcattacttggtactttggctgacaggatg

acgcacatatcacataagttgacattcactgcacgcaggtctgcacggctcagtggcgaaccattcagaactgatatcttaggcaggttctgtaacatct

tccactcccttctaatgtaatctacagatcctactataaccacatgcttcaactcatgataatgaaaattggaagctcttaatggcatcacaaggtttcg

gagaccaataagtggtgagtctggatcagcaaagaggcagacaacaacatgtccattgagaacagtcattgcagcttggttgcgatccaaaatacattcc

tctaaattcttg

>104397|AGAP003709-PB

gggtttgtaattacatatcgttttgttgatgcatcacaagaagaacttgtgtcccgaaacctgtacagcccaatacaaagcatgccataagacttcagtg

cagctacaaacaggtcaccatactttccagtctcaccatactgagctagaggtccatcatacaaagatatctgacccactctgcacctatctctgtttga

caaactgtctgccgtactgtaacctcccctcaggcctgcaccttctgccaaaataagctctaactcaggagtagcaccaccagttattagtgaacggatg

agtgtcagtgcattctggttgaaatatgtagtagacataagagtgtcaaggacgctaacagcaaatgcagtaccacaggcaaaaggctgtgtaagataaa

gctctgtgtcaggatcgtcatcatcatcttggtccaggaactgcacattgctgtcatttactagctctgttatcataggcacattggctccgtagaccga

ccctctcctttgaagaacaataggactacctacaggcgtgagattgtcttgctcacttgctgcacacaatgttgaaaccagtgatgcagtctgcattttc

ttttgcatgaatggggcttggcttaagacaccaattgtgtcgtcaaatgtcatagctttaatatttaatgatgctaagatggcttctttatcagccaatg

ttggatcatcattacttggtactttggctgacaggatgacgcacatatcacataagttgacattcactgcacgcaggtctgcacggctcagtggcgaacc

attcagaactgatatcttaggcaggttctgtaacat

>108405|AGAP003715-PA

aatcatcacacagtcatacacgctatcttatctctacatactcactgttgcacagtatgcataactttgctcgacgtgtccctaaggtttttcaagaaac

taccagcacctccccgtaaactagagaaaaggccacctccagctgcctgttgttgtgcaacttgtccaacaggaggtggtgctggccttgacggaggggg

agccggcctgtgtggtg

>108404|AGAP003715-PA

ctgatgtttgacttgtaaagtcatacttactaattatttcatggaaacatgaatattttgcatcactacttggaattgtataatttccatttattatgcg

cagtttggccgaatcttcaaatgggtgtctcatataacataaggtgtacaaaatacatcctagtgcccagacatctgatgcctgtgtaataggatagttg

ttccaagtgtctaccatctctggagacctgtacatgggtgttgtataacgggccatctgcagaaaggaaataatttaatattaattcaaattaaataaac

aatcaaagacaag

>108442|AGAP003729-PA

gtgtccttccagtaggtgctgggggcttagctcggtgcatcctcgatttatagaatttcgcttctggattgtcttcaacatctaccgttcctgcagatgc

aaactgcggccctgcagtgtgcagtatacccttgtcatacaactttctcagtttcaaattaccgagaacttcatacgccgttgttatgtcacgaaatttt

tgagatgcttcttcgcttcctttatttttatcagggtgatataccattgataatttataatatgcattcttaatgtcactttgtgttgctttgggtgtaa

gacctaaagcatcgtagtgagatttgatacgttgtggactcgaagtaaaaaaccttttcggatgaagatgacctccgaactttgtcgcccgcaacaggta

cattgtgaggatgtcttcttattctgttgacattatcaaaattacacagtacttcgacatggaacttttacaaacactgtacccacatcttccgacattt

caaaacatcaactgcacagcttaaagatatttatttgtatgactgctgaatgctcaatcatacactctgat

>108498|AGAP003766-PA

gctggaagaacaattttatgtttacatatgtgaatgaaaatattacagatgttagacttgtagattttcagctcagtaaagtgcacagtggagctgcgga

cctgttgtacttcctgtacatgaacgccagcgaggaggtgcaccgcgaccacgtggacacactgctggccgactaccacggcacgctggtggcgctcctg

cgtcgccttgacatggcggccgaggcggacagctaccccctggaggctctcgttgaggacatgcagacgggaggcgcctgcgctgtcttcttctgcgctc

tcagctgcatgtcggtcacgtcgcacgagtacggcgccgagttcgagaagttgttccatcgtgcggactactcggggtctttcctggaagacgctttcag

aaatccctactctgtttcctatttgaaatacctcacaccaatatttgacagagagggtttcctatgagctaacttggcaacagtgtgctttagtgagttt

ccgtatcaaccacagcggtac

>108524|AGAP003767-PA

gccctccacgctcctcagcagcctgtgcacctctggcatcacctcacccagcatcatcgtctccatctgagtcgggtcgttgtccttcggcacgtcttta

gtctcccagttttcctgcaggcactttagtatgatgcttctcttttcacaggtggcggattctcctcgtctgacggtggctgtaaccctgaagatgttgc

ttgtgtggctcatgccctctttgcagccaggttggaggtcgatggtttccactctcagtttagtttcgtcgtgtccgttattcagcacagtctctatgaa

ctctttgttgatccaagaaggaaaggatgaaatttcagcttcagtgccaatcattttgccccagaagaaaatcccacaagaatctggatgatcttgttac

gatgcaggcaacagccaatggcaactcgccaggttgtgtgctgtctccccaccagtgca

>108651|AGAP003770-PA

cgtggtgttagtggaaatgaatatcggattttgcttcgaaagagaaggtagctcagggtccactgaaaagaagatacggatctaaagacaaaatcctgca

gaaaggaacattacctcatgttgatggc

>108650|AGAP003770-PA

cgctatcggtctcgtggtcatatttgcatgtgcttccaaaacaagcgtgtttcgaagttatgtggtgtgtgggaggatagtggatggtatacagtttatc

gaatgttaagtgatagtggcggacattatttgatctgaaattaaaatgaactggcttcagagaagattgattcaacctgtacaacaactctacccagctt

tgcagctgacagttgtcagatatagataccatgcagagaaggtagctcagggtccactgaaaagaagatacggatataaagacaaaatcctgcagaaagg

aacattacctcatgttgatggcaggaggcttccaatgcctgaatacaaaccaaaaaatgcctggagcgagagacgagcattatttggtcaaaatgattat

atagatattcttggaaatgagaaacttcatccaacaaggatcttgtacaatgttccgtcttggctccgtgggggtagtggaaatgaatatcggattttgc

ttcgaaagagaaaaatgttgcagcatggtatttttccaattgcaagaccaacaaagtggtatgaattggagaaaagaatacaatacttgtatagatttat

gaacagaaaaacaaaaacgtatgcttcaaatagttgatcttgtgctgtgtttcagattatgtatttgtgcgtaacagtctagcatgacagtattctgaga

catactgcattaaaaaaatgttaatacttacaagttaattatgatactgttgtaaacttcattttcctctgaaaaacaatgcatacagtagcagtatttg

ttgtgtattgggagaataattcttgtacattacgcctaaataaataatgtatgctgttgttagctgctgggtttttcatattgctgtacagacattttgt

gtcattttacatttgataattgtatttttccttgaatgccacacagtgattatcagaattttgcagctgtgaatgaaaaatttatgaattttggtttagc

cgaagtgaatgagttgcaatcttggttaaatctgtcctagctttttcactgaaatggtgaagactttagttaaagagtgtcctgctaaaaccataagtta

tttgagatacggcctacaaggttttcatcttgcagtgctttaactatcttcattgctttcttctggcactttttttaacagtgcgagtgtttttggacga

catattactgtaataaatgtgaatggaataatgtgcagtgcgaaaccagttgtgtgccaattacaggttttaatttgatcaataatgaagttgctacata

tagcttgacacag

>108669|AGAP003772-PA

cggcagtttcaactgcactctcaaaatgattcacgcatgaacctgaatgatgtgccattttttgaacatttataacaatgttttttaatttcttttgtta

atgatgttggaccactggcaaatactgcaacatttggcattctgtacatatttttccattgtttaaaaagactaccccactttggacgaccattctgaag

tcggtgtaacaaaaccttgtattcctggctaatttcaactgtgttacatgctgagctgtagttctttgtgacatgtagtattatgttcaaacggtcaggt

tggttgttcatccagaatctgtcatgcaatgaacacagtaagcttgcaaaccacagtagctgttccacatttttcagtaaccagacaaaatgtaaccgtt

ctggtaagtgaggaagtgctgtgcaatccaatagatgttgaaaaacgcttgcaaatggtgtcactccagaacctcctgcaatacagatggcaactggtgt

gtgaagaatgctttgcaatgaactgctaaatggtccatctaccagaaacctcactggttctggagatggccatctgcagagctcagttgtttgctggcag

tgcgagaaaggcaccaaaacacggccacagagttcacctgtccaatctcctttagcctgtatgagaatactgaagctggaacagccatgtaaattactta

cagatgtgatggagaatggatgccactcaaatgaagatatcacaggacactgaagcaggatatac

>108668|AGAP003772-PA

ttccacatttttcagtaaccagacaaaatgtaaccgttctggtaagtgaggaagtgctgtgcaatccaatagatgttgaaaaacgcttgcaaatggtgtc

actccagaacctcctgcaatacagatggcaactggtgtgtgaagaatgctttgcaatgaactgctaaatggtccatctaccagaaacctcactggttctg

gagatggccatctgcagagctcagttgtttgctggcagtgcgagaaaggcaccaaaacacggccacagagttcacctgtccaatctcctttagcctgtat

gagaatactgaagctggaacagccatgtaaattacttacagatgtgatggagaatggatgccactcaaatgaagatatcacaggacactgaagcaggata

tacagaccaggctggcatgtgaaggaacattgcagctgaagtctgatggctgcacctggtagtcttgtaacttgtaatacagttacagacctaacatttc

gtcgcattactctgtacgtggcatctacgctgtatatactcagtggtagtacaatccatagccaaccctgagactccattggaacaaaaattgcttcact

tttgcatatgatatgttttgaactttgcacttctgtgatgttgccagtgacatttatgaggcaaccaggaatatgttcctctggattttttaattccttc

agtacaccactgagagggtgacacaacagcaggagcaaaaaaggcaggaaaagatgatgtgtgaaccagaaggcattgtagtgtctcttgcgaacatatc

ccactgacatcactgcaaggatgcaaagcagcacaaccatagccactccagttattcctgccactgatgtgagtatcattgccaaaggggcctgtccttc

ataactggcccagttgattttgggaaaatcatgagaataatatctggagaaattcactgcatttgcaacatgagcagctgtatgtatagctgcagcaagt

agaattgtaatggcacaagttatatggatgaactttcctttctgcaaccagaaggaaagtgcagcatggccaaatgtagagtggagtgctgagctgaggc

tgcggcacatagggatcagtacaagacagcatgcaagattcaggacagctgcagttgcacgagataggcagagtcctcgccctaaaatgctccgaagata

tatatgttgtggtttgtttttatattgtgagtaggtcttccagaagattgtagcacttgcagaaagccagaacaccagcaaccagaaagacgcgcggcgc

agccagctccgacctctgcaccggcggcgagccatcgcagcactggctcgccgcagcagccgtgcacgctgccgggtg

>108759|AGAP003784-PA

taaaccattgcttgtctcttatgtgatgtttcaatgcctctaatccaaattcagattaaaaatacattagtactgtgtgtgtcatgagctcgtgataagg

ggatgttactgaaacagaaggacaaaggtgagcagcagagtgggatgtttttatctgttacaggtgacccgtgtgctcgtagacccacactcaaagaggg

ccacaggcgtggagtatgtgcgaaagggcaagacacgaagactcatggctgccaaagaggtcatcctcagcgctggaactgtcgcatcgccgcagatcct

cctcctgtctggaatcggtcctaaggagcatttgcagaaagttgggatacctgtcatccatcacttgcctggtgttggaagacgtctccagaatcatgtt

tcatacaagatgagggtccagctgaacaactggaagcaggaagcactgctgaacaatggaa

>108859|AGAP003790-PA

taccacttaattcactcttcagttcactgagcagatccttcccaaaaagtgtcttgaattcttcagaaatttctagtctctgaacaatgcttcgccttgc

cagtacatcaataattgcttgttcatctgttccaaagcctttcatagcagctcgcagagtttcagcatctgcctttgcatcgaaagggtctgctgggtag

atggttggtgtgcaatgaactggatagtactgcctcgagctcattgttgaatctgttggtagatcctggtagatgtgaacttggcgtagagcactgcccc

acacactcctgcacttgtcgaccgctg

>108879|AGAP003791-PA

gtcagtttacaaacaataattgtggtcaaaaaaattacacagtacgaagaaaggctttctttcacaagtaaataaaagaatcattaaaacacacatgata

aacaactaatttttgtctggaatatacacatatgtataatgtgctcaagaaccacttcctggtctcagtgtcaacatgcatcaagttttaaacatcacat

ggcatcaatagttgtgatgttagccattgccaatatctctcactccaatatttgatatctgaaagccgacagaagcacatgggtacacagtacttttcaa

gagcactgctgcaaaagtgccacctctctcagctcctaacatgatgcgatgattaatacggtgaccaaaactttctcccagtgatggaacaacttgtgag

gtgccatcagcaaagacttttgtagtcaactgatttgttacaacaaccgcaactttgaattttttagcacacagatgcaattcatcaaggatctgatgca

agagtcttgttctctggatgctgctcagttctccatataggagagggaatgtaatactatcaatgacaatcaattttacatttcttttttctaaaatgtc

actgagttgaaagactgcagccaacagctgtatgtggtttgttactgatatataagatatgccattttttattgcttccattgtaaaagttgaggcaggg

agtttaccttgttgtctggcaattaaagaacaatgactgatgcaagcatcagccatatcagagctgtgtctttcctgaacctggagccccacaaaattct

gtgatgctctgcagtggaatacctccatct

>108900|AGAP003806-PA

ctccccctctaacaccagctcttaattgctcaggtgggaactccccaacatatcattaattctcattgtaacaaacttcatcaattggctagatctcttc

atattatagtcttattgccagctttgtcatgaattctgcctatgttactgtttttgttacattgttactccactgatatatgaagattctggtgaataca

tttacagctataggaaaacagttgtggcagactgttaatattgcctgtcttaaattaccttaaccagagttatatggacagctgagaaagaaaacaccat

cttgataagggggtttcccattgtgttcatagatgttggtgtatgttgcagaaagcataatttttcttctcatcccgtatggttaaaatcctctgacctg

cggttctgggtgacattcccaaaatctaccccttttcctagacctctcccgtccttttccttcacctttcttccttccccttcaacccttctgcctgaag

aaagaaccactggctccgaaagcctaccaatcacaacagtcttttatgtgtgtgttctgctgccacttgatgagtagattttttatctacccaattaaat

ttttttatcaagaattgattgttttcattgttatagaaagcataatttaagttacaggttgctgctgctgattaaatgatctgtgaactcaataactagt

tgacagcaatgtgcagctgacaaccaggcaagaacaaggtagccagtaaagagtccaaaatctcactgatgcaggttgaaattacaaaaaagaaaaaggt

ttataagtaagttcaattctgacatgactttataacaatgtacgctgtgtcaacaggatactgctgttgctcttgaatgtaatgactgattctgagggct

agttagaaatgctaagtgacacctgactgcatttagtatgaggaaatactgtagtgttgattttaatttctgctaaaagttatttgtaggtttcactctt

gatgtttcactctgttgaactgcagtgatgtcctatggcagctgtttatctgaagtctcttttgagggtatgttctttccatggactgatggaccttctg

ttcgtctgcattttgtccttgaaagttttttcaagttaactgccaactgtgcagcaatttttgcattatttctcacaagagcaatatttgtgtcaagaga

tttccctgatgtcagaatgcgtacttgttccagcacatatggtgtgacatcttttcctttgataccattcttttctgcttcttgtagtgattgttgtata

gctttttctatatgatcaccagaaagtgcaaattcttctggtacaggaacagcaagtaacattcctgaattgagttgtgcctgcaatagactatgaagca

tacgggcagcttcttctgttgtttcagcattatatggtgaagaaaacccactgctggctgagtaaaatgctgggaaatccttattcttgccgtaagtaac

aacatttactccttgtgtttccaaatattctaatgttttctcaatgtcaagaatagatttcacaccactgcatacaatggccactggactacgaccaatt

tctaggaggtcagcactcacgtccattgtttcctcagcgcctctgtgaactccaccaatgccacctgtagcaaaaacgtgaataccaactccttttgcga

ctattaaggttcctgcaacagttgtaccaccattcaacttcttgctcataacatatggaaaatctcgacgtgatgttttgacagctttggaagaggccaa

ctctgcaatcatatctttgtttaatcctactttgatctggcctttcattattgcaacagtggcaggcacagctccctggttcctcactatatcctcaagt

tccaaagcagtttccatattttttggatatggcatgccatgagtgattatagttgattccaatgcaacaactggtaggtctctctcaaaagcatgcaaga

cctctgaagaaatgtctgctgcatttaatttactaataaactttttggaacacagaaatcttgagtttagcttcaaaagtttaccaagaacctgatacat

tgcagctatatggtaaaagtatccttcaataaccttaaaagatatgcaaattcctctgcaaaacttcgaacagatgctttcgagccgcagaatgacactc

gcgaaaac

>108928|AGAP003809-PA

acctggtacgaggaatccttgtatctcatcataaccagttgtgaatgggtcgtatatccatctgcatccaacaaaaaatcttctcatgaactcatcatct

ggttgcggtaaggaccgaaaacagtgagaccacagccccaattggttcaacttcgcacctgttattctataatcgctaacaagccaacttggtgcacaaa

atgccacagcaacacagacaagcgcaatcacaaaaacaccaattgcacacgagcctgatagcgtacgcctcatcttgacgttgtgagacacggtatgcat

ttttccgtttatttaacacttctacaaaataatggcttaatatatctacattcaggatcggcattcaatcaaccttccacactcacgccccaatgactgt

caaagataatctactctattaattatgttagtttatcatcacca

>108927|AGAP003809-PA

ttttaaaattataattctttgtacttgttgggacatattcataattacctttgataagatggctcatctgaaacgtaaagttagcggtcccttatctaac

ttaacactttatatgaatacacaaaaacaactagtatataatctttcatatatttcaccaaaatagaactaattaaaaatggacggaactgcctgagata

tttttcccaatgtgattacaaacttttaagccttagattcgtgctccatttcaaatcttgattgagattcctgtatgtgttttctcttcttgcgttgaat

gtttgcctctaccagaaataaggttcctgagacaaaagctgctacaacacctacaactgccagtccaaatgcccaaccaaggaaattattttcctgacca

ggcatccaatcattacgattaccaagggaagcaaacacaattacagctattccaccatttatacctgctgccattaacagtgctcctatggcaattatta

ataaaatataacgtgtctgctctggtccccaacacaggaaataaagtaacaccaaaaagaaagcagccaaaatacataggaaacagagtgtaaagaaaaa

ctgtgttgcgatcatgaaacctggtacgaggaatccttgtatctcatcataaccagttgtgaatgggtcgtatatccatctgcatccaacaaaaaatctt

ctcatgaactcatcatctggttgcggtaaggaccgaaaacagtgagaccacagccccaattggttcaacttcgcacctgttattctataatcgctaacaa

gccaacttggtgcacaaaatgccacagcaacacagacaagcgcaatcacaaaaacaccaattgcacacgagcctgatagcgtacgcctcatcttgacgtt

gtgagacacggtatgcattcaggatcggcattcaatcaaccttccacactcacgccccaatgactgtcaaagataatctactctattaattatgttagtt

tatcatcacca

>108929|AGAP003809-PA

gttgacatgcagggcgtcgtcagttgcatgcatgggactcgggcgcatacatgagacagttctgtacaaggaaattattgtaaactgtgagaaaagaagc

catcagcctttctgaatgatatttcgaggcttatgctgcaagttcacatttatggtttcacttattcgtaggctggctacaaattaagaaattcgtactc

tgcaggtagcaaaaataatcaccgtagaggaacgcgtcataggcataccgtgtctcacaacgtcaagatgaggcgtacgctatcaggctcgtgtgcaatt

ggtgtttttgtgattgcgcttgtctgtgttgctgtggcattttgtgcaccaagttggcttgttagcgattatagaataacaggtgcgaagttgaaccaat

tggggctgtggtctcactgttttcggtccttaccgcaaccagatgatgagttcatgagaagattttttgttggatgcagatggatatacgacccattcac

aactggttatgatgagatacaaggattcctcgtaccaggtttcatgatcgcaacacagtttttctttacactctgtttcctatgtattttggctgctttc

tttttggtgttactttatttcctgtgttggggaccagagcagacacgttatattttattaataattgccataggagcactgttaatggcagcaggtataa

atggtggaatagctgtaattgtgtttgcttcccttggtaatcgtaatgattggatgcctggtcaggaaaataatttccttggttgggcatttggactggc

agttgtaggtgttgtagcagcttttgtctcaggaaccttatttctggtagaggcaaacattcaacgcaagaagagaaaacacatacaggaatctcaatca

agatttgaaatggagcacgaatctaaggcttaaaagtttgtaatcacattgggaaaaatatctcaggcagttccgtccatttttaattagttctattttg

gtgaaatatatgaaagattatatactagttgtttttgtgtattcatataaagtgttaagttagataagggaccgctaactttacgtttcagatgagccat

cttatcaaaggtaattatgaatatgtcccaacaagtacaaagaattataattttaaaagtgatctgcattctctgtatgtttcatcaaccctgaagataa

tgattctcagtatcctgacagtattaactttataaacaaaatcgcatttgtaataaatggcactgaaagtttgctgctgtatggagaatttcatgaatga

tttgtatgataataggttctccatgtatcaggtgtacataaatttaaacttttgtggtttattgcttggtttgaaatttaatcggtctctgacgcacatt

agtgaatttatctcaaattatttctcgtctacaaggtgatataagaaatagaattttgtgtaaggatgatcctttcataacgtcaaacacctaatgtaat

aataattttgaagcctacttaaccgactggacttaataaatttttttatgtagaggcccatcagcttgtgaaatgggaatgtaatttgtggcactatgga

caattttaattttagtatgttgaactgaacagcagatatcaaaatttctcacattgcaaacattaaaggttttccccacatgcccagtcttgatatgctc

ttcaaaatgtcatgactgtatattcttttgtttgaaaataatgagtaagaattctcatgactatatatataggcatatattgctcagttaattttattca

ctggaatgtagacaatgaattatttttgtgtaatttaacattgtacagtatttacccctattaaacctttgtatttttacactttttaaatgttgtattg

tgaatgactgactgagtgtttggaaacgatgataagctgtagggaaattataggtgcctgataagccgtccactgtagagaactacgccaacttgtgata

tagttttgcaatcagtatttaatttttttaaatatttactgtgcacattgacaagtaaggtgcggagcattctaaaaaaactgaattagtgtggatgtaa

gtgagaggaaataagcttaattattacaaacaacaagagaaaaatttgtaaaagtgcaaacagctattgtgtctcttcttctacagttcttttgagattg

taactcatatatgactattgcatgcacaaatgagtgagttgtgtaactgatgttctcacaaaagttagctctgcttttgtattcattaaacatatatcag

tgaaatttgtaaccacagctacatgtataaaaaaaaatttacttttagcagaggacatactacaaaatgaatcggattttgtgtaccttgtttatgatct

agcagctgatcaaagcaatttctgtgtgatgtttcaatagttgccacaggcagaataatgacccatataatgagaactaaatataacaatgttatgaaaa

ggatagttgctactcaccatatggtggagatgctga

>108953|AGAP003812-PA

ccttaatgaataattgtaaacgcaatttgtttttagacagtctcttatcctttcatcatcttggttagattctgtgcaagtatattcattggattggggg

tatatttcattttcgctgtccacatcatttagccagcttcctctgaagtgatctttgtgcatttcaatgattttatggagtacacaaaatgtagtaacaa

gtttaggcacaaattgatagtttacgtctatgcatttggcaagcaatctccatcgagctttgagcataccaaaagcattctcaacaacaaatctggtgga

gctcaaataaacaccaaaaccttcttcttcaggtgatacagtccctgtatatggtttcatgagccatggcagaaggggatatgccgcatctccaagcagg

aattttggcacaacagtcccactgattgtgcatggtgcttctggcaccagcctgtgaatccttctgtactgatgcatcaaggacatttccagtatggatg

atattgat

>109023|AGAP003821-PA

atccctgacaaagctacatatcagttgtgtcatcataacccttttttttttaatttgttggcatcaccatctactaacaaaagtatgaacatatgcagca

gaataacacaacagcagccattaacattatggcactaaagccgttgactcgtatttaacatctctcttgaccccaatgaatcaagtttataccctctgta

agttccaacaataataaagttgttccgacgtacatacatacagcagagttggaacaatactcactttgttatgtgccgtactgtgatcattatgtacttc

tccacagccgatctgtaagcagcctcggtggacacaaagccacaggtttggtccacgagactcacaaagtgaacatttgctgatctccttcgctttctca

atatctgcatgtgtaacatcaacgctgttcaagtgtgggcacttcagtggtcgtccagacattgcacttcagtttttcgtagctcgcctagacacaattt

ttaaacgattaaagatggtaatcggttggtgctacagcagctatataccacatcactgcaatacacatctcactttcagatcagtcaatagttttttctc

ttcgctgtcatcatctcattactgttacgaagcacgcacgatttgaccacaaactaagtgagaccactagtcaatgcccatccacaaataac

>109051|AGAP003825-PA

tgaaatgttaatatcttaggaaggagcgtctgtactgtaacgttgaagttgcctgatcgtgttgtttgtgtttatggttagtagaaggtgttaaagcagt

gagtttattttgaaaaacattcgaatcatgggggagaaaaaactgccggaagaaaatctgtatgaattactgggtataaatatagaagcaactgcacaag

aggttaagaaagcataccgaaaaacagccctgaaatgtcacccagacaagaatccggacaatccaaaagccgcagaattgtttcataaattatcaaacgc

tttggagattcttactgacgagtgtgcaagggctgcatatgataaagtattaaaggcaaagcaagctgcgaaacttagaacacaacagctagactctaga

agaaagaaatttaaagaggaactagaagcaagagagagggcagcacaagaagcagcagcaaagaagtctgagtttttaattgacacaagaacagatgaag

aaaagcttcaggctgagattgataggttaaggaaagagggatcaaaagcagtggaagaagaaataagatatgttcagaaaataatagaagaagaaagaga

gcagagagcaaacaggagagaacaagaagcaaataatcaatatcgtcttcgtattaggtggaatgtaaataagtctgatgtgaataatggaggatattca

tatgacatgttacacaaattgttgtcaaagtatggaaatgtggtagcacttatattatctccaaagaagaaaggtcgagcacttgttgagtttgagagta

aagatgctgcaatcatggcacagaaatatgaaaagggccttacttcaaaccctctaattgtagagtggcttacgcagccacacaatgaagaggatgtcac

tacagtaccatcttcgcaattttctacaacagaaagcactgagagacatggactgtttccttcaatgaacactggaggtgctatgtcttctggttcttat

gctcactcctcaccaagtccaatgagtcacatgaaagtcactggctcagactttgaaagcttggtgctccgcaaaatgaaaaaagccgaagaaagaaaga

aacttgctgagaaaatgcaaaaagaagaactggataatgaggatgtatgatagaatgtcgtgcgtgaagtttacctcagaattccataaaatatgtacta

ctgtatgttttatggatcagagtggaaaaactgtaattagataaaaagttaatactcactaagtgtataaattgacagtagttataacagtgccaaaaca

taagaagtaaatatctgatggtctgcaccaatgattccttctttggaaactggaagaggaaaaaacagaaaacggacaatttttcctcttgtctcactcc

ccacgggtgttaaataccaatctttttaccactcatacattatttttacccttgatcttatgcttatcttgtggtgaaaggacatttggtcttaaaagct

gagaattctcatttagcatatcattgtggacaatgccttaattttgtcatttagtgggtataactattttatctatcttagctagtgtatatattttgta

atttgtaaagatgtgattctatatttcaatatttaatgctgaataaataatacttggt

>109441|AGAP003839-PA

cacacctgcctgatgattgcttgttacaagggtcacttccggatagccaagtttctcctcagtctgaacgccagtgtgaacaggaagagtgttaaaggca

acactgcccttcatgactgtgctgagtcaggcagcttggaaatcctcaagttacttattgcacatggcgctcagatggatgtagactcatatggtaattg

cacatattctgttaagatagtgttttacattaaaatgcttgacatatcatatttgaatggtgtttttgtattgcaattaacaaaatatattactaaaaac

cacttgacaatgaaccaatgaaaacaagtttttgagagggcccaatgggagctacagtattatgatctttggcttgtagtatctgttagaaagtcatcaa

tgaagtacgagttttggattgttaaattctttgattttagtttatttgagatataatcatcattaaaattcagaaacatatgacaacacagtattacagc

taaaagaatggttggttagaataaaggaagaagtataaagaagacaagttaaaggttaaataagaaatgacagtcattcagaacacagacaaatttcagt

tgggttgaaaggaaaacctaaaacaggtagaaaaaaagtttcagatattggttggttgtgagtacactagaac

>109458|AGAP003842-PA

tccaaatctgaaggagtgaatatagtttgaatgttttctgtataacagcagcacttcctcaaaatgatttatcacattttgagtagtccagtaagaaact

tcacacatgacaatattaagtatcactattaaaagtgacaataaagtaattttttatcattaatgcatagcttaccacattattaaagctcccagtaatt

taaacatagcaatatgcatctgaggtatcagacatcccacaattagcttatctgcagtaacatgttttgatatagtgttgctattaacaccaaaacccct

cacaaaaaggtttacgaacagttgtggcatcactcttttagcaacaggaagctcacgtactaacaattcctgaaggaagcatgagagattaatgaatctc

attgccaatctttgtattctgagaaaaggatcactttcttcaaatacaggtatgtgtaacttctctgtgccagtc

>109459|AGAP003842-PA

ccagtttgatctccacgctgatttttgtctacattatttttatcactgtctttttcttcttcttcttcttcattctcctcctcatctttgcctcctagtc

ttgtaacttcatgtttttcaggtaagaaattacttttaactgtctcatatttttctttggtttcattcttggcaacagcaagtaaacttgaaatacatga

atcctctgtagtttcaatatatctgtcctgtagttcatcctctgagtcatgtaattttagatgtagtggaatgttacagttattctgagtttctaactca

ctgtcattttggtcacaatattttgtgtgtgttacaatgctttggagttcctggacagttgtattgttgtgtgtcactagatccattggaacatcactgc

ttccatcattatcttggcacctagtaacatgatgtgaatgtggaacatcactactgcataaatcaaaaatatttttatcatgcaccgtctttgtctttat

aattctctcgtcagtattattatgaacagtacttgtatctcttccatgaagatgattctctgaagcattatgtatatgcttaggactgctagaaacttgc

agcagctcactatcttgctctgggctaagtttggatctgtcagtctccatgctttcactaaaaattattctattcttgtgactgttttctaattggatca

ctgcctgcatatcgtcaacagatagtgtgaaatgaagtggtctgctgagaggatgaactagtttgacaaagttgttcagcgactggctacagaaacttgc

aacctttacacaaggatctgaaagaccaacacgcataagctgaatggcgaactgtagtggtggtggccatgaacaatgtggccaaaaaacaagtgtgtga

agtaactgataaagttccaaacgacatttccagtcactatatggagcaaaaatctcaccacgtccaattttgagcagaattttcagcagttcctcttgaa

tcatcttatgcaatgttggtttaagaaatggtccagcactctgcacaatccattgcagtgttctcaatgctgcagcacatgtgccatgattggcagtttc

atcatatagctttgtacgtgaccaagaagattctgatgatgaagcagaactttcttgacctaacttcaactgtttacgttgttgtttagttagatgacga

cctctggcctttgctgccgaaagagttactgcagtatgacttattttcacatcgcccaacagtaagccaatgagctcatcaggtgaagatatttctacac

aacttccagcctttgctactttcaaccacagtgtgaaagtgttgtacgcttctttccgcaacagcccataaggcctctcagttccatgtggccagtgttt

gtcatacgatgtccacttcagtgtttgcaatacaaatttacagatgagagaaccatactccaacaagttctggccacagcacattattaaagctcccagt

aatttaaacatagcaatatgcatctgaggtatcagacatcccacaattagcttatctgcagtaacatgttttgatatagtgttgctattaacaccaaaac

ccctcacaaaaaggtttacgaacagttgtggcatcactcttttagcaacaggaagctcacgtactaacaattcctgaaggaagcatgagagattaatgaa

tctcattgccaatctttgtattctgagaaaaggatcactttcttcaaatacaggtatgtgtaacttctctgtgccagtcctctcactgctctgaaataac

tggacctcaggcactccttcaaacagttcatccaaagtctcctgaaggaatagaaccaatttcttctgtaaaacttcccaagagttcttgtgattgacgt

tcagaacaccaccacctccaacaagtggcaaaacagcaacacatctagccgccaactgcacagtttgtttatctgggttatcaatgaatttcataaggaa

ttgttcaatattactcttatgtggaccacatgaacctccatagttcctcactgcaaactccaaacagcgtaataaataagcagcattctctgtctgggag

tgaaagtttgatgaaacatgatgaacatgctccaagaattttgggattacagatccagaaaccggctttgttaattcctgtatcattcctgcgtactgaa

agagatttccaagtacttcataaaccagtaaacttatttccttttctctgggactgctacaaatcctgacgcattgttgcatccatgatacacagttatc

aatgaacacatcatctgaacactgtggaagaaatacgtttaacaaatgtaggcctttcactctgtccccatgagaattcaatttggtattgatgaaggca

acgactgactgaatttcgttcctccctttcagagaaaacgttttgtgttcgagaagtgaattcaaagaattacgaagataactgtatccattattaggtt

tgaagcaatcttctaagaaaaataatttcaaatgcatatccatcgtgtaatttttcaagtacagctacttcgtcggcaaactaaaacataacctataagc

aaacacgctgcaccgcaccataacaaatccccacctctcaccacaaatatcgaagatacttcacacagtctaacaagtaaccacagtcgcgacaattcat

ctcaattttgttgtctgctac

>109476|AGAP003843-PA

tggaaagacatacaaatgattactgtactttttatttgaaacgttatgccactgattatagttgtacaagactagaaatggaataattgagatggcttgt

aaaccaggtgcagacaccacagaatcatttatgactgacctgtaaggggtcacacaaaagaggaccaacaaagtgttactcaagctcttgcttaaataac

aatgtctccattcacagaagtgacagaacatcataattaacagcatttatgattcagagaatacacttgcatgttatatttaactgtaaccacagtcgtt

ttcttacaataccacaatttaattccatttgccagaaacaacattaaattctatataaacctaagaattcatgtgttgcaaaattttaacatgaaaacaa

acatgtttattttaaaactgggaagaatgaaacacaaactttatcacctgcgtttttccggttgttgtgttttggtgtttttgttttttctctctttttc

tctttttgtcttttttttgtgttttattttcaatcatacaaacacctagtttatagtatgtattttggtaaagttctttttgtctccttttcagttacct

tattagtcaactgagccctaagagtcatggagattctctttgcaatatgtttgtaacattatgcagttctgttcagttattttagctgtgttatcataat

ccatttagaacaatttattttaaacaatgagaacagcatcaagtgggtccagtagaataactaatgttgataaaaaaccattattttacataaaattgtt

gatccattgctacattaaagaataaatcacaaaaatgtgtgacccataactgcaaaatattaatccacaacaaatactgcttgtgtctgacagtggcaaa

atacctatgacgtcagttgagtgctgttcaaacacacaaaactgactaacagtaagtacaagtgtacagacagtttatactaaatcactgaacactcttt

aactaatatcagtttccaactttggatgccccttcagtgacattttcttcactgctatacatccatcgccagtacgccaccctcatacgatcccagatgt

tctcccacgagtccattgcaggacggacatcaaggagagcaaatgtataatcctttggattgacagacccaccatcataataatctataacatatcggac

gtcttttccacagcgatcaacaatccagtcatgtcggtcaaaaggaagctcatacccaagccagcttcgtatacgagcccgtggagagtaatctttggct

cgtccaccaaagctctttaactttggtttaccacattcatgagcatgcagagcttcccatttcaaaacttccagccaagcatgttcattattggcattgt

ggatacgaataatatcattcatatctttctccgagatatcctcttctctccatcgccatcctttgcgcagcatagcattccaaaacatctgggctgatgg

gtagacccaatattcattttcagagcctgctttaggtatagatgaagtctgcctctgtgttgaaagtgggaatggctgatctggtgctggatgctgattt

ggtgcaggcatcatgttcagtgggttgacagtgttatcgaaatcgccatgattcacggggcactcggatacagttgcctttgcctcttcttccttgtgca

tggggcagcctgccggcggattgccatacatccctgcagatttattgaaaccatctgactgtactgaaccctttgtgtcgtatggcccagttatttggga

agccgccacctgggcggctgttacagtgtttcccattcccaaattaacagggaccactgcagacgaccatgaaaactttacttcagcacaaagac

>109506|AGAP003849-PA

ctcgaagctggaggtctgcaacggttcgagcagcagccagaatacacggaggtcaacgcacgcgaggatgccatcctttcctgtaaaatcatcaacaaga

agggcacgtgttcctggcagaaggacaataagccagtgggcatctacgagaagaagtacgagtgggcaggcgcgcgcgagcagggcgactgcagcctgct

ggtccgcgctgccacg

>109524|AGAP003856-PA

gttgccttgttttgttgatttctgctgttaagataatgttgagatgaatacagttatttggatggatcgtggagccatctgatgagtgaaaaattgttac

gagtgtgtctattgccgttgcttattattctaaagtgagcaccagctgctgccttgttgttgctgtcagatcctgatatcccttgaatatttggaacgag

aatggttgccataagatcatgttggtctccttgtatatggaacagcaatgtcaaagttggaacctacatggttgcagcttatactgcatccttcagtgtt

gtcctaattacactgattgcatatatgatgagtggtggggaatcaacccagctatattcaccattatttgagacggatgtcagaggatctatgcttggtg

ttggagggtttttcattgcctaccttctgctgctgattgcattctctgtattcctcatttttggtgtggtggaaactaatcgaggaatgatgctgccatg

gatgatctgctttggaatagtaatattgtttcagctagtttttggcctgtggttgcttggtggatactacatatatgtaagaatattattgttcaatgac

tggattccgttatggcagctcttgttgattggctttggatggcatacaatgcatactgctggacctgtgtgtattctctgtaccagatatttaaggaaat

gcagtcaccaaatattgaactgctgtacccatgaactatagcagttgctctgatcatcagaaaagcaccatataaatctatgaatagattatatatagct

acgaccac

>11|AGAP003857-PA

caaccaacagcagagctttattagtctgtaaatgttacataatactgatacactcccatacatatagatgtgtgtaatatggaaacccacagaaccgttt

cagaaacatagcagcacagtgactcataacttggagaattaaaatcgttaacagagaatttcaaagtttactttaaggacaactgtaaaacaaattgcat

acttgatacataacagtatcaatacttatagtgatcaacgtatatgacatgctatactcagtaatagtacttattgacaaataataccaccagtttccta

cactccatgacacacaattttcattttctgtattcaaatatttttaaaaataacatcccttttctcacagaaaaagtttcacacaggcactgcaagacaa

atattttacatgcatctgttatcttttaaaataccatcatatgtgggtaaaatcccacacaggatattgaaataaaatgctgtaagttctatactgcccc

cacttttaagcatgtaactgatacactattttgatatgattaagtagtaaccagagagtcatgttaacactgaataacagtttaagtgctagttggatgc

catgtgctaaatgcagattttgcattgctaaaaagttaataacaacttatcagctgaaagtcatgtaactggaaatgattctttcaacagcaagtcttat

tgtaaacattttgaaacttttacttattgccataagagccttatcaatggatagtaaaatgctttcaaattgcacataaaatgcgatgttaaactgcgtg

aaagcagttatgtttcatgatagctgtgtcaaagatttaattacctatcctgataaaataatttgaggctctatagtcatacaatagttggctgtaaagt

catacaaaaagcactgtcaaaaatattttctggcaaatctacatatttagaataacaactcctgcgagtattattcaaattaaattccattgctctaaat

tttgtcacttccgactacaaactttcacccaacaacaacctaagaaatttcttgcaatgaactacgcggttcattggctgaacaatgtcgacaggtgggg

agttttctattacaagtctgtgtttgccatcaggcaaaatactgctgctccaaaacaactttccttcaaatgaaacaaaataagatatttcaaaatccag

tacatcaacagtaattttaattacatttctgatttaccagtgtttgactactttatctcttgacacaccaaatacccttaaccaggccccccaaacatat

actgacatgaaaaataacttgtccgaacattattatccacattttttacttatattgttctctgcaattttccaaaaggatggaccctaatcttagtttg

tgttcacttatctcacaactgagaagttttcaaatacccctcaccaacttcagtattcattcaatatttaaaaaaccatcagaacatcttggaaaaaact

gggagtagctaagcacgttttcacaaaggggaaattaattttttcccactcttgcattgccatagtcaaattattatgcatcttttctttgcacacatga

aggaactgatttccagcacctataaatctgataaggctgcaccatatcaatgacaaatgtttacaaaatatctgacaagcaataggggcttatttatcat

ctggcatgaatagtgagaaattgcacaatattagagtcactcatgcttataacattactaaatatgcactgaacattatattaacatacagttgcgcaat

tccacttaaataagtggcacttcctgtgaaacaattaagttccacctatttcaagcattttagtttaatacacaaaactctggggcatggcaaactaagt

aaaagatatttcatggatttaatatttcatttaattctaatcatcacagctatgacatataacatacaaattaacagtcctctgttagtagcattactat

tctaagacaaaaattggtgtgtcaatatgaaatctggaagctgttaatagctataatttagtgaaatcacaggccttaggcttatggggaacataatttc

cttagtcagtgttaaggtgcaatattccaatcacagttttaactataaacatgtaaacattacagccttacgtgtccaaaaagctttgctccacacatga

cttcacacgcttttcgtattctcgtcgattttccttgaaaagctgagctgccattgagtttgctggagagttcggattgggatcacttaatagtgattgg

attgatgtcagaatagcagacacatcatatgtcgggctccacttattctgcaagatgtccaaacagatgcctccatccgcataaacatttggatgaaaca

atttcgaaagaaatctcactgtgggcggcttgtttgggtactcttcggtaaattccaatgtcagcttgaatgttccatcttcgaaaggagtgtcgtgagg

cccaaaaatgacagcatgccaaagcataatgttattttcagtaggagccccactaataccagtaggcggatcctcttgaaggcgcttaaaatccctcatc

agacgtcgtctggcaggcgtagacatttctgatgggcttcagggagaatgcttgccctcggcagcaacaagcaggaaatcttgagcttaactatgtaagc

tgcgaatgtcagcaagctttaaataattagttcataacttcacaataacacgaaatatactagtaatgaataaaacactttctaacagaattttaaatac

tacaaaaattggcaattcatccagaatattgcacctctgttgcctgaatttcaattactgtgcagattctcagttacacaaatagaccataaccttctac

>35|AGAP003858-PA

atacctcaatataagaatgattcccataaggtacaagcctgtacaccttgaatgagagattcattttgcgtgccagagctgctagcagaagagcagtttg

tccccatgcagcattgatttcactccagtcaacagggacacttggcagtcgccccagccggaaattatttattgtaccaaaatgccctgaatgccatata

tggaatgtggcattgaaaacatttgttttcttcagtttgtccaactgtgcttgtacatatgttcgctgacattctaggctacatgcacatgaggagaaaa

ctttaggaattacaaacatttccaaaactcatttattagtttaaattatagttaacatgtacatcagtaattattgtataatatatacaggagagagagg

ggggagtagagagagaggggggccgggccgacacacacagagcggcagagcgccc

>58|AGAP003864-PA

gagtagtaggggagttgtcgcattgtttaagaggggatggaatgaggtacccgaagtaataggatcttcgtttatggcattccatcccctcttaaacaat

gcgacaactcccctactactctg

>56|AGAP003864-PA

aaattttacacgagtgcggagagaatggggggagggggtgaagttttgagtttgaggatggctgcagcggtgcagagtagtaggggagtggtcgcactgt

ttaagaggggatggaatgagatacccgaagtaataggatcttc

>60|AGAP003864-PA

aatgtgaagtgcctcagtatatgaactagtcctttcggattctggctgctctagggtcatcggatctgtatactgtgtataactccttgtacctacgatt

gtggccatcatgagaatagtaattgtataatccaacacctgctaatccaaatcca

>57|AGAP003864-PA

ggatctgtatactgtgtataactccttgtacctacgattgttgccatcatgagaatagtaattgtataatccaactcctgctaatccaaatccaatcaat

gccataaacgaagatcctattacttcgggtatctcattcccagattcatacccagca

>51|AGAP003864-PA

ctgctggccgccctggccgccctggccgtgctggtgtcctccgcggccgccgtggctgccctcgtgagaatagtaattgtataatccaacacctgctaat

ccaaatacaatcaatgccataaacgaagatcctattacttcgggtatctcattccatcccctcttaaacaaggcgacagctcccctactcctctgcaccg

ctgcagccatcctcaaactcaattcttcacccccgcaccggccccggccgtcatccgcacagtgcctgccgccaacatcatcatcggcccggag

>54|AGAP003864-PA

cagtatatgaactagtcctttcggattctggctgctcttgggtcatcggatctgtatactgtgtataactccttgtccctacgattgttgccatcatgag

aatagtaattgtataatccaactcctgctaatccaaatccaatcaatgccataaacgaagatcctattacttcgggtatctcattccatcccctcttaaa

caatgcgacacctcccctcctactctgcaccgctgcagccatcctcaaactcaattcttcacccccgcaaccggtcacttttctctacatttttt

>53|AGAP003864-PA

atgaactagtcctttcggattctggctgctcttgggtcatcggatctgtatactgtgtataactccttgtacctacgattgttgccatcatgagaatagt

aattgtataatccaacacctgctaatccaaatccaatcaatgccataaacgaagatcctattacttcgggtatctcattccatcccctcttaaacaatgc

gacaactcccctactactctgcaccgctgcagccatcctcaaactcaattcttcacccccgcaaccggtcactttggctacagaaaaagatgttagcaag

gttta

>52|AGAP003864-PA

taaattctcgagctctttttctctcgtaaattatttcttaatgttgaccaaacaaatccaagggggaagattcactgtatgaatgcagtgctgacaggac

aagaaaaagaggcccgcagatatttgaggaatggttgtcgcattgtttaagaggggatggaatgagatacccgaagtaataggatcttcgtttatggcat

tgatcggatttggattagcaggtgttggattatacaatcaatgccataaacgaagatcctattacttcgggtatctcattccatcccctcttaaacaatg

cgacaactcccctactactctggctcctacccttgcaatcgcccccagtgtaaaacctgtcctatgcaccctcccaccaccacctattccagtcctgtaa

cccggaaggtgtacacgatcaaagggagagccacgtgtgaaagca

>523|AGAP003892-PA

gcgtactgtggctccacacaccttgggcagtacatcatagcaagtgcactgagatgcccactggaaataccaagcagcatgcttgctacccaatacaccc

aatcattatgtatgaatactggaaactggcgaattctattctgaggttcgtacttgcacaccataaagaatggaataaataaaactctcagtaccacagg

aaaaattaaatgcttaggcttgggccacgtgaaaacagctgacaacaagcttccaagcatggcacaaacattgaatgttaagaaacatgtaacctcagtg

aagtacttctcactgatgataaaatctggatcactctgtttgatatctgtaggaacaaatataagattagtcatgatattataaaaaggtaatgttcact

atcaagttaggttcaatatagatctttaacagttctatctttttcaaatacaatatgattaaaacagaaatttacagatgctgtaattacatacttcaat

actacaatttgatacacttctccctgtcacaaaacaaaaacaaggatcagagaatgagtactgaaaccatgaaaatgataaaatagatcattccaccctc

ccctttcccccttcatgtcatgcacacagactagtgaaagtattt

>524|AGAP003892-PA

gatctattttatcattttcatggtttcagtactcattctctgatccttgtttttgttttgtgacagggagaagtgtatcaaattgtagtattgaagtatg

taattacagcatctgtaaatttctgttttaatcatattgtatttgaaaaagatagaactgttaaagatctatattgaacctaacttgatagtgaacatta

cctttttataatatcatgactaatcttatatttgttcctacagatatcaaacagagtgatccagattttatcatcagtgagaagtacttcactgaggtta

catgtttcttaacattcaatgtttgtgccatgcttggaagcttgttgtcagctgttttcacgtggcccaagcctaagcatttaatttttcctgtggtact

gagagttttatttattccattctttatggtgtgcaagtacgaacctcagaatagaattcgccagtttccagtattcatacataatgattgggtgtattgg

gtagcaagcatgctgcttggtatttccagtgggcatctcagtgcacttgctatgatgtactgcccaaggtgtgtggagccacagtacgcatcaacagctg

gcatgtttggtgcagctatgctgataactggaattttctctggtatcatttttacacttgccttaccagagtttgttgttggagttccttggaacttatg

aatatttgttactttaatactgtcaggaatttaaattgtgagacattattattttgaaataaaagtcaaactaatgtctgtcttactgtgtgattttctg

tgatgtgcttattttgattttataaatttttgttaattttatccaattttgtatgtccaaccaatcagggtatgatcttaatattaaaagaaag

>522|AGAP003892-PA

gtctgctgtacagcagttgtaaaggaaccaactagactgaagtctacacttcacctgaagaggtgggggaggaagcttgtggaaaagtagcgactgtagg

acgccactgtgctgataacccgcgaatatttcatcaattcatgtcttacttatcgtggtggaagtcgacctcggagcactaattacattcgtgcgtggta

ctttcattttgtgggaaaaactgaagatctttataaggacagaaatcgtacaagaaatgcttggatagaggtattcaagaattaaacaatgagtctgaag

agatgagcgaaaacgaaagacccgattatgcaggagcagaaggttatgaaaagcagccactgtttgaaagagcgggtagtgctgagcctgtacggttgac

tcctgcgtgggaagagacaaatctgcccaacgatgagcttaacttcaagggtgtcacaatggaaagagcagatcttgagatgaatccaccaatagacaga

ttaaagctggtatacctaacgtttgttctccatggagtggggacacttatgccatggaatatgttcatcaatgctaaatcttattttgtggactacaaat

taggaaaggcataccttggaactgaatctgtgtatgcttctaattttttggcatatgttggttttgctgcacaggttcctaatttgctattcaattggtt

aaacatctttatcacaatacgaggcaacttgacaacacgtattgtgtggagtatttttgtcgaagttcttgttttcattgtcacagtaatttttgccatg

tcagatacatcatcatggccaggaccatttttctttgtgacaatggccaccattgtgttgctgaatatggctggtggcatctaccagaacactgtttatg

gaatggcagctaaattaccttttaaatatacaggggctgttattttgggagctgtgcagcagtgggcatctggtcaaaccacactcaatgatcaactaat

aaatctgaacgagcgggggtggaaaacagcaaggagtagtcttgggccagtgccgaccacattgcaccctgcaccaggcttgatactctgaccatttctc

gtaaatgtaaaaaggctgccagtacaagtgtggctgcaggtaggccggccttcatttctccccagtctgctcatcgtgtgaagggctgtgtgaaaacgtt

gagcccccagaagacagcattaacaacgatacaacgatgaagaagagactgaaggaagagaccaagaacagatagaagaaagacgcttatattatgtgga

agatgaagcagccctgacattgatgatcgaacaaacagaatctgtgccaggatccgccatcttcgaagaacctcaaccagaactctctgagtgacaaaga

acatcagtggtgcaatttcatcaatcatcaacataatgtcattggcaatggcaccaaatgtgaggacagctgccatttattactttattacagccctctt

cattttgttggcatgttttgatacgtacttcgcccttcctttgaatagattttatcgttaccatgagcttctagctcaaaaagaacaacaaagcaaggag

tgtcaggctgttggcagagcatcaccacatgtgccctactttcagattttcaaacagtgtttcccacagcttttcaacgtgtttttt

>521|AGAP003892-PA

ccggcttgaaatgtatctttgcgattgcgtttaaatagatggcgatatacatctgtggttgtaatggcggaatgtgaattttttgtgtagcgtcagggga

gggtttgtagatgtttagttgattgtttgtttttagtaactgtgggtaacagtggcagctgtctgtagagtgtcttattaatgataggtatatgctgtgt

cgcaacatgtttaatactggtctggataaactaaacgcttgtacccacattagatcttaacctttacgtactgtcctttctgtaactctcagtagcaacg

tggttacattttagcagtgtgtttgtaacgtggttcagaattaaatgttgttttgtgttgtacgctgtgtgtgtatgaagaataaaagatcttgaaattc

cctgtattggaaacatggatccgtcgtacaatcaacgattggcgggaccagaaaatcagttccttctaaatgatgttgttccagggaatggaatgacagc

tagggggtcacggaagttacctacaggagcagaaggttatgaaaagcagccactgtttgaaagagcgggtagtgctgagcctgtacggttgactcctgcg

tgggaagagacaaatctgcccaacgatgagcttaacttcaagggtgtcacaatggaaagagcagatcttgagatgaatccaccaatagacagattaaagc

tggtatacctaacgtttgttctccatggagtggggacacttatgccatggaatatgttcatcaatgctaaatcttattttgtggactacaaattaggaaa

ggcataccttggaactgaatctgtgtatgcttctaattttttggcatatgttggttttgctgcacaggttcctaatttgctattcaattggttaaacatc

tttatcacaatacgaggcaacttgacaacacgtattgtgtggagtatttttgtcgaagttcttgttttcattgtcacagtaatttttgccatgtcagata

catcatcatggccaggaccatttttctttgtgacaatggccaccattgtgttgctgaatatggctggtggcatctaccagaacactgtttatggaatggc

agctaaattaccttttaaatatacaggggctgttattttgggagctaacatcagtggtgcaatttcatcaatcatcaacataatgtcattggcaatggca

ccaaatgtgaggacagctgccatttattactttattacagccctcttcattttgttggcatgttttgatacgtacttcgcccttcctttgaatagatttt

atcgttaccatgagcttctagctcaaaaagaacaacaaagcaaggagtgtcaggctgttggcagagcatcaccacatgtgccctactttcagattttcaa

acagtgtttcccacagcttttcaacgtgttttttgttttctttgtgacgttgtctgtttttccagctgttcattcagatatcaaacagagtgatccagat

tttatcatcagtgagaagtacttcactgaggttacatgtttcttaacattcaatgtttgtgccatgcttggaagcttgttgtcagctgttttcacgtggc

ccaagcctaagcatttaatttttcctgtggtactgagagttttatttattccattctttatggtgtgcaagtacgaacctcagaatagaattcgccagtt

tccagtattcatacataatgattgggtgtattgggtagcaagcatgctgcttggtatttccagtgggcatctcagtgcacttgctatgatgtactgccca

aggtgtgtggagccacagtacgcatcaacagctggcatgtttggtgcagctatgctgataactggaattttctctggtatcatttttacacttgccttac

cagagtttgttgttggagttccttggaacttatgaatatttgttactttaatactgtcaggaatttaaattgtgagacattattattttgaaataaaagt

caaactaatgtctgtcttactgtgtgattttctgtgatgtgcttattttgattttataaatttttgttaattttatccaattttgtatgtccaaccaatc

agggtatgatcttaatattaaaagaaagtgttacaaggcagtttttagtccactgtcacactgcagcactgactttcaaattgtgttcagtctgattaga

cacattggacttaaaaatattgtctttttgtgcattttctagttaataatttgggattagatttattgtcaattatgatttgttgtttaacaaatttacc

aaagttcaacatacaaagagaaaaacaatcatactttgtatataatcttcagtatgttgttcacatttttatattttttcatgacgtacaatgctctctc

tgatacgctttcgattcatatctatctctatctctgtgtctttctttcattttctctttcagccataacaaaatatataaatctttatcatattgtggtg

tagatatcattttattgaaactgcaacataaagaaaactaaaacaatattacgaacttcactttgtgcattgagtatatcacaagaatatttagacatac

aatgccaaacattatcaggcatgtatcatagtaaaatataaatgcagttataacataaagatgcagaaaacattattgttgtgaaacacaggaaatctgc

aatgtgaaaacaaatcttcagagatcccaagcctacatgatttcaggaaacgatatgatagcatatcaagatccgtttcattgcagttgatctgttgagt

tatttatcctgtgtaagatgacagtcaactggtggaatattattggcatactttatctaaaatgctatcagtaacaaacagttgattggtgagaatctat

aaaacagaaatatgttagtcttcaaaactttgaaaccttcacagcacttacgtgaattacttccataccatcatatactcctcaatgatagtcaaaagat

ttctaataaaaatggcaaatttctttgttttatagaaatgtttatgtcgaattttcgaaatatgtgaaatgtatgggatgtatattgttgggtatagcaa

gtgtcagctgattctgtcgaacagtagcagaccatatatgtggtgtagcttgcttcatgatgatgatggatgatgatgattcgcaagtatggctggtttt

catctttgtgttcaacactcaatccagttccactgtggcagattttttgttggccactgaggagccacagataggattctttgaagtcattacatcaatg

cacctctaaatatttcctgcctttcattctgttctgccagatacatcttaattctacagttatttaaaacttagtaagcatctgacagttggaagctttt

attttttgcagtacttcttgagataagtaattttcctgtctctagctggttttcattatcagtgaacctgactacatcactttcaataggtttataatat

taggttcatttgtaccccacatcaggttttgtgggcatcagaggtctatctaccaccatttgtattaaacacaaaactgattgcagattatctatttact

taccctcggaatacatttttcccacattgccacatttggcttaccttttattctaactgaaatatagttttcaacaagtttgcctttcacatttcttatt

aaaatcaccattctgtattagctgcatcatattgttgtctggtattggagtacctgacctaatatgtcgcaaagcaaagcagtgcaatccaatccatttc

aatgcaaagaccttttttatgtgtggggccattgctgcattatatttcttgataaaacgtattgtcctgcatcaggttccattttgccagtggcgtaaag

tgttatttcagataaaaaattgtctgattcagaaaatttgtttaagttgtgaacaggcacattcaacatgacataagtgagatataaactgaaaagtggg

gtatatttttgtacctaagctaagaagggtaggcaacatttaatggttattaatcctatgagcatcaataaaatcaaagtttcttcaatgttgcagtttt

gataagaaataatattttatgtaactaaaagcactgtattatcattgtaaacaattttattttgcaaacatgacaaatacccagtaagctgtcttccatg

ttttccgtcctaagatgctacatcagatgccttatctgcaataaaactgaggtggatatgggccaaaacatgaaaatgtttgaagttttcacagtcttgc

ttactgggtttctgttggttgcagaattttcttcacattgaattttccacttccatctgttctttttgtagtgatattctttttaacagtgaagatcatg

ttcaaatactcttaatttctgtgttatattttacatatctaatcatggctagctatgtattgactggaccaaagaacattttaaaactgtatagtaaatg

aatttattggtgctcaaatactttaagtaggttttatagagaggtcagcttagtattgttatatctacaagctgtagggcagtgctacagatcacagtaa

gagtagttcttttttttatgacattgtggaaacatgtttgaaagaactgagtgttttatataacatagtaatggggtagaaacttatagtacagatacct

gttgacaggtttggtgaattttacaaataaatctgtcttgttatggatttgaaatattgtatctgatttattttttatactagttgtgtttctacaatcc

tccctattgtgtaatatagtgtaataaatgttcataatcattcctgcttgccttattaccaacttaatgaccaagcaatgtcaatgataattcatttttg

tacatacctatcacatttgtatgcatgttcatttctgtaataagattttatccaagtataaaatcatccactgttaaattaaaaattaacaaaattatca

aagtgtgtttcagaaatatgaaattgtttgcttatctataaaat

>842|AGAP003894-PA

atttttggaagagaaaaggttgtcagagaaggttgttctcttgtttgataacacacatatgcattgtgatcagaggatttccgtatctgatgatggtccc

atcagaactaagtctttatcttctaacataactgtcattatacagccaatggaacaggacgtaattgcaccagtaaaatggcactactgggatggtctac

tgaaa

>834|AGAP003894-PA

ctacatttgctgaattagatgctgctcttttgcaatggtttagccaaaattgagcagaaggtgtcattgtttcagacattatgtgtactgaaaaagctaa

atgttttcatgaagctctagggttagagggagaatttaatgtctcatctggatggctaaccagatgcaaacaatatcatggaatttgcaaacttactgtg

cgaggagaacacctcag

>837|AGAP003894-PA

caacattgaaatgcattctttagacaaattttggttagcagctatttccaatacagaccacttttgtctgtaggacatctcttgctagtttttgactaaa

ccattgcaaaacagcagtacctaatctatcaaatggagatctcttcatgctttctcgttcaaatggtccagaactaaaatctcatttcctgacaaaatcc

tgtattttttgcttattctttgtaat

>844|AGAP003894-PA

actgttcaggacattaaaaagaataggcctaagcagaaaatataggattttgtgatgaaatgaaattctagtttggaccacctgcatgaaaaaaacttga

cgagatctacatttgatgtattagatgctgctcttttgcaatggtttaaccagaaacgaccagaaggtgtcattgtttcaggtgtgatgtgtgccaaaag

agctaaattttttcatgaaggtctagggttggagggagaattggatgcctacttagattcaaacagtgtcacaagattcacaaacttacaatgcaaggag

agcagcttagttcagatcttgtggtagatgattctctcttggaaaaggtcca

>840|AGAP003894-PA

ctcgttagtgctttgcagttgatagctagcaacagtgctatgagctgttgggcatcttcttgcaccatctcaactatagataacattatcttcacattgt

cccaagcattacagataccacggattgcatcacagctttcagaaattgatgatatctgaatctgaatcttctcagaaaagacgtttcataaaattcaacc

gagtttcacagccacaaacataccttggtccattggctgtgaaagtgatgcaataaaaggtggtaggtacttcacaaatacagttcgatcattcaaagca

agcaaaccttctgtacgatggcaaggtgcattgtccagcaaaagaactgctctttcagggaagcctttcccctcgaaatgtgtgctcacaacaggaatga

acacagaattcagtgtctcctttctaaagcctgtgagaaagttgtgtggtaacttgtccttagactagactgaaagtctttttccaatttttgagattaa

caagtggtttccatttgtattcatgttttaaatttgcacagcatataacagttcacttttctttgcttggcttatttctgatagcatacacttcagtcca

agatgctgtccgttccaagatacaaggagaaaactgttcagttagatgtgaccgcagaaatagcaaaaaagtatcagctgtttattttcctggtgctaag

tttcttaccctgatcagcactttctcaagttctgtagcagtgttttagagtgaaccagtcattgtgatgatgaaatattgacaagtgacatttcacagaa

ctcaaagaaattctggtcatatgtagttcagtccctagtgaatgactgcattttcgaatgttccttaaaaaaacaaaaccaggagaattgctccaattta

atccttgtgccactgaaaatatgaatgctataagtatgagtgtcagtggtgttgggaaaaagctgaaatcattaaaaccaaaaagctccaggcaccagtg

gaatccctgtcacattctaaactgaatctgtggctgagttatgccttcttctaactgtaatctgttgcagagcccttgaacaaatacctgtgcccaggcc

ttggaagaaggcacaggtcacacccatctacaagaagggtagtagaagtgatcca

>845|AGAP003894-PA

cctgaagtagttttctgtttctggtagttcttttagatctgagaacagttgtgtgaacagttgaaactagttatcaaatttttctgtatatttagtgatc

tagacaatttgcacaaaatttcttaaaaaatggtgaaaatacttaaagttggtatcttcatttcctaatgatgtcaggtactaactaggaactttacatt

tgcaacattttttcctgttttggtgtttgctgttcttaatacagaaaagtgagtttagtttccagcactactgtatcccacatgctattaaccactgacg

taccatctgcagtagatcaataattacacgtatgaattttgtctgaagtgggtgagaaatttcaaggaaaatacagtatactcctgattattgggggtaa

tatgggggagaagatgcttgaataattgaaaagcgtaaacaatcgagatatttttgttcaaaagtttatccgtgttctgtaagcctgtttatttcttaaa

agagccttgatgttgctctgcttctgagaggagttgacatatttttcacatggagttgcgaatatttcttggagcaataatgtcattgcactgaaaccct

tgtggtccatataatctaaaagagtgtcaatacattacagtgtggtacagtgactaacaaggttgctgttttcatcccctactttcatactcactgccct

cgtctttgtttcgagaatcctcttattgggatgtgaagggacattgtcaagcaatagaatagctttctgtggcaaccctagaaattgctgaatttgtact

gaatgtttgtagaaccagtttttgaaaatttcattacccatccatgctcctctcttattataatattgcacagggagatccttagctgtcaaatcactag

caatttcaatttgtggttcgcagaagtattagtggtaatgtaatgcttactttagacttatgttcaggagcacgaactttgctcttaaaaagcagggctt

ctggttggcagacatttccaatagagaccattttcatctgcgtaataaatttgctctgatgtgaaattcccttcttccacaaatttctggaactcatcct

ggagggaatcagctgctgcaatatttaaactaagcctctctccttgcacagtaagtttgtgaatgctgtgacattatttgaaactggttagtcatgcagc

tgagacactgaatttttgctctgatcctagagatttgtggaaaagggg

>865|AGAP003895-PA

cgttggcagaaggactgtagcactcgacgtaggagacggttgaagttccgtcgtagccgccgacggcgaatatcatgtcgtcgatgactgctatggcgtg

gttactgcgcggcgtgtacatctctgggattagcgcccactgaccggtagcagggtcgtatctctcgacgctccgcagtctcgaatatccgttgtttcca

c

>888|AGAP003900-PA

tagagttcgtcagctgtgttgtgagtgaataactgaatttattccaaacatgtcaaggaaagtagaaccacgtagcgtttcaccgttgttggagatgctt

agagagttccttctaggaagaccacataccaacgctctccgatttgcagatttcatcgcagctcgtactcaaccaccaccaaatcttccggaagggccag

ctcacaaattggctgcaaactattattactcaagagacccacgacgagaggtttgcccacctaccgtattggctggtggcagcaaaccagctattgcagc

gggcgcacaagcgttaacggctggcagtaaagttgctgcagtgggcagtggaagtaagaaaattccccgaccgggaaatatttggcactgggattaacag

tggtctgcaaatatttagtgtctgatatgcggtgctgccgaggagagcacttttataggcggccactgttgtactgcatacggacaaattgtacatatat

tcacataacaaaagaaatacgttttagacaggtttgcaaatgagagtgtaatttgagtgttaattcagcgtttcagatatgaatactaagcaactggaaa

aaataataaagtattgtttgtgttgatatattttctccttgttttgtttcataactattcgaagcagtccaaacatgttagcaagagggttgttaagtat

acttcaactcctgacgaaacactgagaatggtataatatgcctagtagaatctttattgaatgccttgcatactgtgcacagctttcctcggacacacta

gtgctggcgaaaatttagggacatttcaggatattactataaaagatgggtataatgagttcacaaattaaacaggcaacaatagatgtagatatgtttg

tgtaaaatgaataattttgcgaatttatgtgctgcacattttttttttcttcatgtaattatcatttcgtatcaatgatggacaaggtgattttttatag

tgttaatgttgaatatattttgagaaatgtcaatatctcaagtccttgatgcttgttcagatgtaaaagcatttggtactggtaatgagcttttagtgtg

aaataatttaaatactaaaagaaaatgtagagtcacac

>887|AGAP003900-PA

cttttgaacatgccgaccgaaagacttggtaggcaaggtattccggaaattttaggttttggactactaaaaggaattttaacgacaataaactgtgaat

gtatgcacttctgtttggagtggttcaactttagtggaaatcagagaacgattcggaaaaagttataggccaatttgttgttacttatcgatctttataa

agcgcgcctcatataaacagttgatcataaacttcgttactcgattgtcttcgtgtagcagactacaaacgctttccattagttaatatctttgggggag

gaggttaagtgctgtgtattgcgttctctatagagttcgtcagctgtgttgtgagtgaataactgaatttattccaaacatgtcaaggaaagtagaacca

cgtagcgtttcaccgttgttggagatgcttagagagttccttctaggaagaccacataccaacgctctccgatttgcagatttcatcgcagctcgtactc

aaccaccaccaaatcttccggaagggccagctcacaaattggctgcaaactattattactcaagagacccacgacgagaggtttgcccacctaccgtatt

ggctggtggcagcaaaccagctattgcagcaggcgcacaagcgttaacggctggcagtaaagttgctgcagtgggcagtggaagtaagaaaattccccga

ccgggaaatatttggcactgggattaacagtggtctgcaaatatttagtgtctgatatgcggtgctgccgaggagagcacttttataggcggccactgtt

gtactgcatacggacaaattgtacatatattcacataacaaaagaaatacgttttagacaggtttgcaaatgagagtgtaatttgagtgttaattcagcg

tttcagatatgaatactaagcaactggaaaaaataataaagtattgtttgtgttgatatattttctccttgttttgtttcataactattcgaagcagtcc

aaacatgttagcaagagggttgttaagtatacttcaactcctgacgaaacactgagaatggtataatatgcctagtagaatctttattgaatgccttgca

tactgtgcacagctttcctcggacacactagtgctggcgaaaatttagggacatttcaggatattactataaaagatgggtataatgagttcacaaatta

aagaatgatccaattattaatgcttcacttttaaaaaactgaattttaagttaactgaaggacttgcacacagttggtgcattttgcaaacactgaattt

ttaatacactgatcatggtaaaaatagcgtatttaaattttaacaagacatacttaagacatctgtgcaggcacaggcaacaatagatgtagatatgttt

gtgtaaaatgaataattttgcgaatttatgtgct

>924|AGAP003929-PA

cagtggtgcatgtgcattgtttgctatatacctgtgaagaaaactatgctgctgtctgtatccctctgatacacagcatctatttttgtaacagagtttg

ggaatctccagaacatatgccgaaaattcacaggatatcctggctgaattaaacctcgacctgacattctccacatcatctctcccttaaagaagaagag

ttcacctctaagtactgatactgcatcatagttaccaaggcaaggatcagatttgtcaag

>923|AGAP003929-PA

tactggtgggataatctggctgccttggctgtggtttatctgctgtgggagttacaggatgatgagtttcatcatctccttcagaactattttcatctgt

gcttggcacatctggaacgtggttgctatcctcatctctgtcctgagtttcactgtcactgtcatcagtaccactaggtacaaccccaggattaatttca

ttgtcatcttccagactacgagaaatgtataggtcatacatagcaagaatatcatcataaccaagctgaaatgattcctggaagcctttgtagtacggaa

acataatcgaggagggcacatcagagtgagccaatcccaggctatgaccaagctcatgtacagcaactgtgaagaaatccactccatcatcataaggtcc

ttctaattttactttccagttttcatcttcatcaaaatggacatctccaccatactgatccatttcatatggataataggcatgagccagtatactgcct

ggtccatcaaaaggaaatggatctccatgaggtcctcgaccaaaagcaatgataatatcagctgcaggatctgctatgagtgagaagtttagtcttgcat

atcctgcccatgtgctaaatgctcgctgaacttgttcaacaactgcttcttcaccaatcttaggagaccagttggctataaagtaggtaatatgtctttt

cttccagccatcagatcccacaatatatctcttgctccttgtcctgttggtatgcttgatgtcaggaactccacatcttggcgatttcattaactgaagt

gttgcattgtcaagaatgcctgtttggttaagagccccaaatctctgcacctgtttgatgacttcaaccatgtcttcatttttgtacagagactcagata

cttgtgagcttggatccagataaccatactgctt

>1308|AGAP003967-PA

ataatatctttaatatatttagcataaaaggtttgaattgcaccaatactactgtacttaacaagcggcaccagaaacttctcaatgctgcccagtcgtg

tgtttgaatcatacattctgttactaaacacggagaatgctgtttccaagcattgctgttgcttttctgatggcaaccttcccataagttttggtagcag

cttttctatgctcggagcacatatatgaccattatctccagctgagaatacaataatggctttcaggaaaatcttattccctgagatctctaaagcagaa

agcaatttatgaaagcaagtcacaaagctggtcaattccatacttccttcatgcagttcacttgttgaaagtggaaaatggtgagactgcagattcaaga

ggagatcccatagactttgatcttcagcattgctgacattaaccagtcctgggagcaagtccagaactctatttttctcatgcagagacagattttctgt

agt

>1307|AGAP003967-PA

ttgcaagtatccttaaagaactaagtgaaaatgaagaggacttcacagacagaatcaaacaagtatttgaagatatttttgaaaaaacagtagcatgtcc

accatccctcattggatccataattgaaatggctctcttaagtccacagtcaatcatacttgatgtcgagaaactatctgcagtttgcagggcagcaagt

ttgtttccaactggttgccttttgctagaaaattatattattaatgatgttcagagtcttgatccaataccaacaaaacgacagaaaggccctacgggtg

acattcattggttacgactagcagagttgtatacgggccttggagattggaactctgtccaaggaatatttcgaaacagtttggaaacatctgaaaactt

gcgaaaggctcttgtatgtcaagcagacatggaatggaaacctgcaagtttgcattatcgcaatgctttaaaggacacatcatgtgaagacctacaggac

ttctgtcgtagctcgtattacaagtgcctggctcatctctcctcgtgggataaaatcactgctgaaataaggcataatgagctaaacaataattggaatg

aggtttggtctgaaccatggtacacagagaaaatgctgcctgtcctgtttgcagcacagatacagcttaaattaaaaggatcagattgctctgaatttga

aagctgtgttgaagaatggctagctgatgaggaaaagaaatccactctagtgcaatccatgagtgaggagctctcaatattagacattacaaacaacaaa

tttattaatgctcgcatacacgcagaacagtacctttcactctttctgcaacaatgggcagaaataggtcctgcatcatatgccatcagaagcaaaagac

tgatggacctgcagagaataacagagataatgctctatctggacattgcaaacgtctccaatcttgaagctgatctcgcaaaactggaacagtttatgaa

gccatgggctacagatctaccaagtgcaacaagttcacttgaagactgggaacggaaagttttgtacaggtgttacttcttaaaacaaacagctgatagg

ttgtcatcagacagtgccactgatgatcattcctggttgaaaaccactgaagttgtttgtagaatgactgtaactgatttggcactcttacagagtgcct

ctgtccccgagtggatgtttcttggatgggtgagccagattttggcttctttggactcgcctgtggggattgccttacatgagcttgtaattcgtatggc

aaaagcctacccacgagctctaatttatgcttttaaagttagtgccaaa

>92649|AGAP003967-PA

atataatacaaaatttataatatttcataagatgaaaaatgcatgcagaaaaaattataaatatcccagccatttaaatatatatttaaaaaaaattggt

ttaaaaagaatgtattaaaactccacaggttatgacacaaatgacttcatatccatggaagccatctttcccaagtcttgcctaatatgtggggatctgt

tgcttgctcaatgagacacttaacctgatcatgtggtgataaaagttttctaccaaaagtatatctcagtgtcgtactcggaccccacagaactgttcga

aaatttgttggatctgagtgtccacttagtagctcttcttctgtaatcactgctggacttataccttcaaatttttttcttgctaactccactttctccc

ttgaaaatcgcttgggatcactttgcacatttcctggtgacttcctctttgcataaaacagccaatctaatgatggttcctgaacaaataccttcattgt

tgccaataataactgatggttattacgtagcaatcgcaaaacgtgtaccattgtctcttccagcactcctgaagtcttgtgcggttccattaagttaacg

atatgcggagttaacctgaatggcataagttctggcactgggaggaattctgtagctgttccaaatgcatacccaaagtcaacacctaggcactctccac

tgtgaggaaacaccaagcagttgccaaggtgtctgtcacctataccaagcaaccagtgaccaatacacatggttgcataactagaagcaaagcggtggcg

ttttgtaaagaagatttctgctgttgtgcttacattccgaatgacacgccacataatatcccaaggtacgctgtttacattctcattgtatattttaact

gtttcttcaggtgaatatttacggtaagcactttcatatttttctttgtccgtcaagttttttttacttgaacttgaagcagagagccagtctctatagc

gtcgtaaagcctcatccacagatgttttctcttctcctgtgtatgaagattctattaattcttccagagtttttgtattatccacccactgtatgaaacc

aagattgctgctaagtggtacaacctgataggtttttatgcgcagctgctgttctctgcacactgaatcctgtgttaacattttagtcataagactgaat

agttgttgaattctctgatcaaggcgcaagtcctcaccatgttttattatacagtgatgttcctttgcatcattgccaagtattgttagccttataggtt

tccgcactgaatgtaatacgatcacctctggatggaaatttgcaatcttaatgtgcaaatgtggaagagggcgtgattctccagtatactgtccaggtat

ttctaggtcattcccccacttatctccttgaaagttcttaagccatgggctgaactccagtagaaagtaactgcttttagttgaccttgacttgttgtgt

aattccatcctgcatttctggatgatttctagtgcttcttcaatattaccactattgacggtcgttaacactgcacccaagtgtatttcacctttctgaa

tctgagattttgaacttcttttgttttcatctggataatattcatcaatttcactgaaaatctcaccacataatgcactattgccttgtgaagtcatctc

ccttcgcgacacaaacaatctgttcaacatttcttgaaattcagttttgaactgatttaagtttgccactccagcttctaaaagctgagataaatttttg

agttcattgcccaacaatatatgtggcaaacacagccaagaaaatgcatttgtaaattcttccacaatggtgtcacttaacaggatggtctttaatctgt

ctacaatgattctggcagattcactggtttctgagatctcatcaaatgtgtactttttggcactaactttaaaagcataaattagagctcgtgggtaggc

ttttgccatacgaattacaagctcatgtaaggcaatccccacaggcgagtccaaagaagccaaaatctggctcacccatccaagaaacatccactcgggg

acagaggcactctgagttttaaattcatataacagttcagttggcaaacgatgtagctccagaagacatggaaatagctggcgagcttcagctgagccat

acttcatagccttcagtagtgaggttacaatggtgatgtcataattttgggtgtcaggaccatagtgactgcgacaatactgtgcaagttttagatgtaa

atctgcaagtctgtgctccatgcttctgtctcccacagtgctacaggaattcttccacaactgatgttcagctacatgaacagcttctgtggcagctatg

aaaccatgtttcttaattgctgtcaacagttcctctggctctactctaggagaacccacacatctcaaaagaatgtccatgtctgatgaactcagtgatc

gtagtttatcctgatttgttttaagtaccgacagaagatcttgtgaaaatctgcactgttcttcattaacagagataagttcagaaaggcagttgttatt

tttagacctcctctgaagtgatgataatgtgtcaagaacatcaaccataagtgaaaatttctttttgagctctgtgcttttacttgctttaagtgaggcg

agtttgagaatatgcaagccatgctgaatgttcattgcttcatcaccacaaatttcaaccttctccttcatatccatagcatatttttttgccacaaaat

aatttttctgtaagagtgccaaatcagttacagtcattctacaaacaacttcagtggttttcaaccaggaatgatcatcagtggcactgtctgatgacaa

cctatcagctgtttgttttaagaagtaacacctgtacaaaactttccgttcccagtcttcaagtgaacttgttgcacttggtagatctgtagcccatggc

ttcataaactgttccagttttgcgagatcagcttcaagattggagacgtttgcaatgtccagatagagcattatctctgttattctctgcaggtccatca

gtcttttgcttctgatggcatatgatgcaggacctatttctgcccattgttgcagaaagagtgaaaggtactgttctgcgtgtatgcgagcattaataaa

tttgttgtttgtaatgtctaatattgagagctcctcactcatggattgcactagagtggatttcttttcctcatcagctagccattcttcaacacagctt

tcaaattcagagcaatctgatccttttaatttaagctgtatctgtgctgcaaacaggacaggcagcattttctctgtgtaccatggttcagaccaaacct

cattccaattattgtttagctcattatgccttatttcagcagtgattttatcccacgaggagagatgagccaggcacttgtaatacgagctacgacagaa

gtcctgtaggtcttcacatgatgtgtcctttaaagcattgcgataatgcaaacttgcaggtttccattccatgtctgcttgacatacaagagcctttcgc

aagttttcagatgtttccaaactgtttcgaaatattccttggacagagttccaatctccaaggcccgtatacaactctgctagtcgtaaccaatgaatgt

cacccgtagggcctttctgtcgttttgttggtattggatcaagactctgaacatcattaataatataattttctagcaaaaggcaaccagttggaaacaa

acttgctgccctgcaaactgcagatagtttctcgacatcaagtatgattgactgtggacttaagagagccatttcaattatggatccaatgagggatggt

ggacatgctactgttttttcaaaaatatcttcaaatacttgtttgattctgtctgtgaagtcctcttcattttcacttagttctttaaggatacttgcaa

atatggctgtgagtaatcgctgagcaattactacatccatcttagctaaggcctgcaaaggcttaataaatgctgaaaattggatttcaatgtctggaag

gtctcctttccgatatttccgaaacagagatacagttgcttcctttttagacctttctgcttggagttcctttatcttcatggtgcgtcttacctctctt

tttgcaaagtaatagccaattttcttctcatctcttacaaatcttcgcttaagtttgtcagtacttacattttgttcactaatcccttgaagtgctgcag

aacaacttgattttgtattttgtggggcagtattacttctagttgaaaataacgaaccactggttaaagtgaaggctggattttgttgatagctctcagc

aaatgtatgtctctcagcgtttaatgttggtgaaaattccaggttgttttgagtagccctgataatggctgtagcatccatcagagattcatttgcttga

ctagctaatgtctcagcaaaaagaggaaccaaagaaatgttctgggctctccgagaaacagagatagggtactcctcaaaatgacattcttctagaggct

tgaggtatatgtttcggttataatccggtgatgttgtgcaagcttctaataaaacctgaagagcatatcccagaaattgtacttctgtttctgtgctgta

caggtgggtcaatatatatataagtctttctgaagcagaaactggtaactgcgaatctgaccgccagtacctcagaacaaattcctttatatctgggtct

gaatctgccaatccagaaaggagggcttttcgggcaccctcatgggcatcagtgtcactggaaaggatctgaaatgcaaggtgacgacatgaggcagcag

ttgaacgtttaactgcaataggacatactgcatccaaaagactctgcttttcagcatctttcatgtggcaaatgctgctgttgtctcggtctagcaacaa

gagtttctccagcactcttagtgctgcaagttgagcaccactatctggttggtcaacgaagtccagcagcccattactttttaggaatccatatgcatct

tcacctaatgtttcaattccagataacattatttccaaacttaatgtcttaaagcttccttgacatttctttagattaaaagccacatcttgcataaaag

ttttgtcaactacttgagggtaatgtttctgcacaccgtgtaaacacagaagatatttgtctcgacttttgtaacttagtttattaagaacagatctcac

attactcaggaatttattgtcaggttgtacactaatatcatcatcatcatcatgcatacgcttcaaaataattccgataagttcagaacatggtaagtat

acttctctactgttgacatccattttagtccaaaggatgtctagaacattattcactgtgttttcatcccatggattgattctgttgcagcagattgctc

caactaactgtataccaaattctgcatctttatctgtatcatacttcctttttaggtaatctaaaattaaatcatatggtacagtcaaacaactcttcca

tagctcaacaactgtctttattagttctaaattgtgcttcataatatcccgtctcctgtggaatgagtgttcaattaagaatctaaaaacttcactagtc

aattgcttttcataatcattagaatctggaatagctcttccactccatgatagtagtagtgctaaaacgtcagtgatgaaataatttaattcattaccaa

aagacccatcttttataagtaacaacaaggggtacaaccaatgatttgcaaatggcaagaagatattctcattgttcagtatcagtttcacaataaatat

tttcacatttctgtgcctatcagataggaggcatttcttcatattctccataaaagatggcaaatttcctttctcccctggttgtggaactgttatgaga

ttttgtttgtacatgttctgtagcagtgcacataagactggcatacacacatgctgattaacaacatccatttccaagtcgattacagtgccatccatct

gttcattgacattcccagggggaccctgaggacgcacaacactcagcgtgaagtcaaattgtgtcacatcttcagccagtgagctatggagaagaaactg

tgaatgtgatggaatataactgatgggttgtgcactgtccatggcattgcctgctccatcacatttctgtctgaggtttactaacattttccgtcttcgg

ggaacattttcccagtccacgggaaaggttatatttgtctcacagtctacaagattttcccaaatcttctgttggttctctgaaaacaggaatccagaaa

caaaagtttcttcaaattttgtgtttgtaatgacagctgctagaccattgtatgcagcacattgcatcttgcgaaacagttccagtacttcaggatcttc

cctagcatttgatggtttctcaatactccgaaatgctttcaatctcttaacaatttccctcatcagctcttttccttcaatgtcagcacctggaaaagct

gcatttgtgatggggcttgctttgtcttcaagtacttccttactgcacttgaaatgtaaaacatctaataactgggaagcaccaaattttgaaacaatgt

catgtttcgtgatgaatatgttggtgtcttccaccatgctagacaaatttttcatcttatctttaatatatt

>1461|AGAP003970-PA

tgacaatggcagtaacagtgacggatgaggatatacgcaatgttcaagacaagttatttgactgggaacgttctgaaaatcctatagcccccttgacaag

tgaccagatagatgctctccaagatttagaagaagaattcagtaaatttgaattttttggtgatttgtctatatctcaagaagatgacttacaccagtcg

gcgttaaatgttgtacgtcagtccgatggcgacgcgggaaagtgttttgttaatctactgcagagcaagtccaggatagaaactacacgacagtttctgt

cttggtattctgatgtagaggaggaacttctacacgaggaggatgtccagtacacacagttcatgaaacagctaactgagcgaaaagatgaatgcactgt

actcttgacacagatagaagctgcacttggagacctaactcatctcactggccagtaccagtctgtttcatcgagaacaaactcacttcatcaagtgagt

gagcaacttttggctgaccaggtgaaattgaatggactttatgaagaagtgaacaaaaggttgcagtatttcacatctgttgacagcctttccagccgac

tggcatcaccgacattgtcagtagcgagtgatgcttttgtagatattttaaatcgtctggatgagtgcatagagtatatgactgttcatattaaatggaa

ggaatctaacacatatcttgttaaatataagcactgtttatccagagcaattgcgctgatgcgttcatatgtgattgatgcattccaacatgccactcag

caagttttggctccaagagctgttggtggtaatcagacagaacagctagagagagatacttcatttgctttgtgttatggtaaatttcaagcatcagcac

ctaaagtcaagcaagtgataatccaaattgaggaaagagttgcaagaaatcaagaatatgaaagtttactcgcagaatgccaccatagctacttttcaca

gcgagaacagttgctcggaactggagtcgccaccgcagttagtgagctggcaggacagcatcgtggtgaccactgctccctcgtacgcagtgggtgtgct

ttcctcgtacatgtgtgtcacgacgagtaccagctgttctatcagttcttcactctccatacaccacaactcacgtgagttgtaatcagtattgtaatca

gtactgtgctatgtaaatatggtgaaaagttcttgcagaatgcaaataatgttggaaaccattattctttctttttgctgttgaagtatgttatgtggaa

agatacaatagttgttgatattgcagggaaacagcttgaaattagtaagaattaactctcattgtagaaatgtttcacatttttatactattttgttttt

aactgcacatgtaatttttcacgagcacacaagaaattataattatttctttcagaacataccttgag

>1489|AGAP003972-PA

ctccggggggaagtgcgacggcagcgccgccgtgctccagggcgacggctgcgacgtagtcagccagggcgggctcggccggtccgtcgtcgtcgtcgct

ggcatgcactggtagtgggcctctaagtattttagagtgcccgggcaaggatccccgaacatacttgtagcagcaacaatactgcagttctgctgctggc

tacatctg

>1512|AGAP003979-PA

tccaattgctcctgttaatggtcaaggtctatagtctactaagtggcatggttgtgattgagttctgttagtttacgatcgtaaaatgaggaatctttcg

gtttcccgtcatattgcattaaatattcctgaaagtaatgcgttttcacagtttactgtagatgtagctacaggtgacatcttaggatatagtgagaaga

cagtgtataaattgtctgctaagagcggtgagatagaatgttttctcagtttggaagaactcggtcatttgaccacatgttacgatatcataggcatgaa

cgtaaattctgtcagcgaagaagtttgcttggtgctagccagcggtgacatattaacagtagaaagttactccaaggaagtggaatgtgtcggaagtgtc

gacagtggggtatctgcagttcagtggagtcccgacgaagaaatgatagttcttgtcaccgggaaagaaagcattattactatgactggatcattcgatc

ctgttaatgaagtgga

>1526|AGAP003995-PA

ctcgtccgcaaacgcgctgacaatgttctcgtcgatgtctcgcttgtatcgggcccaggtcgaccagatcgggtggcggaccatgcgctcgtcggggacc

gccgagggtttccagaggaacttctgcaccgcctggaggtgcgcttgcttggcgtcttcg

>1642|AGAP003997-PA

cagatgtaatgtattgcttttagaaggcatcccgcaagtgtactactacgggacgtgcgatggccgctacaatgccatggtgatggaactgctgggcccc

agtctggaggatctcttcgttctttgtaaccgcaaattctcgctgaaatgtgttctcatgatagccaaacaggtgcttcataggatagcg

>1677|AGAP003997-PC

agggtggaggaaaaggtgtacctgcgtggcccgatgtgccaaagcagcaaagtaatgcaatgggtaaccttactccggcagacaggcatgggggatcagt

acaggttgtgagttcgaccaatggtgagctggccaatgatgatccaacagctggtcattccaatacacctatcactgcacaagcggaagttgaaatagtt

gatgaaacaaaatgttgctgcttcttcaaacggaagaagaagaagtctggccgacagaagtgactagcggtggtacagtgcagcccagaacgtgaggcag

tgacactgctacgtgccacctcccattcaaactcacgtgacagtatgctgaacggtagctgtccagctgtgccagcgggctctgcatgagccactgtgcc

ctggcactgctttgtcacacacgccattccatgagtttcaaactgtgttccctgtcaagtatgaaagcaaattgtgtgcttcagcaggaatggaaatagc

ggactgggaaggggaactgtagcagatgaaattttacccgaagtataggggattagtggccatttcta

>1676|AGAP003997-PC

agggtggaggaaaaggtgtacctgcgtggcccgatgtgccaaagcagcaaagtaatgcaatgggtaaccttactccggcagacaggcatgggggatcagt

acaggttgtgagttcgaccaatggtgagctggccaatgatgatccaacagctggtcattccaatacacctatcactgcacaagcggaagttgaaatagtt

gatgaaacaaaatgttgctgcttcttcaaacggaagaagaagaagtctggccgacagaagtgactagcggtggtacagtgcagcccagaacgtgaggcag

tgacactgctacgtgccacctcccattcaaactcacgtgacagtatgctgaacggtagctgtccagctgtgccagcgggctctgcatgagccactgtgcc

ctggcactgctttgtcacacacacacttggaaaacaaaataattactctttagactagtgactgtgagcactataaaatttgtggatccataccgttact

catcatttcgggaggacttaactggaagcaattttgctgtaatctgatgctccttgcacagtagttctgcctctgtaccgggtgaaggtatctctccttc

tgtttatgtcttaccccgcttacagagtgttcctactattctggagatgaattctagtatataattttgtttattacgggtaattctgaaactgacacag

tcc

>1955|AGAP004000-PA

aacgaaaaactgccactggatcagcaccaactagttctcgaacaaatgccatactgctgttttttagtacactgacaatatcttgcctcataagatccaa

gttcttctccctcatctctgctgcctggtatttaacttttcctgcataatgccttacaataaatgctgcttcaagctatatgtgtgggagtgatgaagag

agtgaaagtcc

>2118|AGAP004031-PA

ataatcagccacttggaatattggagcttccggatccttatttatagccacgattgtttttgaatccttcatacctgctaaatgctggatggcacctgaa

ctcacttctaaactttgagtttgtgttttggcttcagattataaaaataaacttact

>2117|AGAP004031-PA

tcaggtacaactttaaagaggtcagcaactaatccataatcagccacttggaatattgcagattccggatccttatttatagcgacgattgtttttgaat

ccttcataaacaatgtctcttaagacattgtggtcctaagtatttttttgttatgaggaa

>2119|AGAP004031-PA

acctgagctttacattgctgtaggtatttctggtgcgatccagcacttggctggtatgaaagattctaaagttattgttgctatcaacaaagacgaagaa

gcaccaatcaacagcgttgctgactactggttagttggtgatttgaacgctgtagtaccagagttggtatctaagctttaatctttattgattaaagtt

>2842|AGAP004066-PA

ggcatctgcatctccaccgacagcgggcccatctgcgagtgccgcaacgtcgacttcgagggcatcttttgcgagaaagacaaggcgccgtccgaggcga

cgttccggggcgccgagttcctctcgtacgacctgagccgcaccggcggcgagcccatcgtcagcaaccaggacgccatctcgctcgtcttcaagacgcg

ccagcccagcggactgctcttctacacaggggacaagggcgacttcatgaacgtggcgctgaaggacggcggcgtggtgctggccatgagcctgggcaac

gg

>2886|AGAP004068-PA

gttgtccctaaaatattttatttttctgtttaaaatacaagcttgaatataaaagttgttcacataataatgtctttttttgtttacttcatacacattt

ttatgcttcaattaagaacaagactgtttttgtctaaagtaaaagttattgaactacaatatgtttaccaacaccagtatatgtaatttgtttacactgt

ctgcaaagatgcaggtaagtctttgacatctttcctctattttcagttactgttaagaacccttctcatgtttcagtatttcaatagtgatttaaatgat

tattatttcagacattttacaatatattccgttacagattttatatatgagtcctacttctttgtagtaacagaaacaaacattgagtaaaatttacatg

gtttttgtaaacatctggctttaaatggctcttttctgtagttgtaagctacaataaactaatactctcaaatgacaaaattttgcagaaataggcttta

attaatgaaaaatttgcacaataagaaataatcaagacttaaaaaaactgattaagaatatatatgaatcatcatcatggttacgcatatttcatctcct

cataacattagcttttgtgattttcatttttatctgattccaatactttttgcataccgtgttagcttttaagtttactggcatgatcagaattggtatt

agttcttcacatccatgcactaagcacaattttgtgtaagtatattactcgttaacctccatatttaccatacatatttatctttctctgtgaatattaa

attaatcagataacttctttaaaattacagtaacattcactgtggttctaataaacatatactcatgaaatacatcacctcaaaactctttctaaaagta

gctgagatcatttaaaaaaataaaatgacttcgtaattcaaagttggcaatactttgaaatcattgtgagaattttgtcgaagctagttttcatggtcga

aataatcattatggcacacacatattaatttcacaatcacattcaagccttaagcgactattacacttgtctcaggtgtattttggtgagggtatagaac

aaggattttcattttattactcgtattgccacagttgctttcgatgaatatcaggtgtgatacagtaaattatttctgaaatcatactttcactctacat

atagcgaagaaaaaataattaagtaattgaccacattttagattaaactgcgataaagtacagagtgaggacagtggctactgaatagacaagaacattt

tttacataccgaagacaaagcaactatttggttggtgtgtgcttagattatggtcctaaatcacagtcatactctgactatggaacaagagtcatctagc

acatttattgtggctgcttagtaatggttgcacccctgccaaggtctagcctgcagtctaaggttcctaactacatatttaacatgtcaattatcacctg

tagatctgaacataatttaaccacagtatgaataactgcatgaatttttacaaaaagggcattatttcatataaatatgacatgttagaccagaaaatga

caagataatatgacagtttgaagagtctaagttcacagtcctttatattaaatgtagctacaaaactatggccctgtaatcaaacattttaatgaaaata

tggttttacattattggaggtaattattatgtaggagctatatcagtatgaatcttatactgatgtaattttgcagagtttgtatttgtcatttgtttta

gttaatggatttacctgtagatatttctgaattctgttgcttgagtgtgtgaaacattcagttttaatctaataagaactcctgcgacaattttgacacg

tctggtcaaaaaaaaaaaaaaaaaaaaaaaaaagtattttcgtgcacctaatcaaaatttttagccaattattaacaaaagaataaattatgttttgcag

atgtacttgttatagaattgcagccttttaatattttagtacagataaggttacatgagcttttaatgacatttatcgacgccgtgccccgaccttccct

gcactggttctagcagcaggtgcaggtttcttattatgctgcatgttggcacatcgggggatgtgccgttcagcagcagcctcattaaaccgccgctggc

agtgagggcactggatgtagtcagatgtgtcaagcggtggtggtgggggcaaatctgacagcttccctccagctgccaaatgtgccttcatctcctttgc

tgcacgaattgttgcaatgaattcctcatgccgttttctccagtctggcttttttgcttgagtttccttctttgctggcgggcccttgagcaacatcttc

ctgacgaaagtctccgcctcggtgcccttgac

>3882|AGAP004091-PA

tttttcttgtgtaaaacaactctgccaccaagagcgtactgctgcatcagtcctatgaaaaatgcctttacaatatggctaagatttgtagagccttcaa

ctttagcataaaggtctctaatgcccagaacttgacatattgtacgtatgcctcgatgacatacaagaccatacccttcaggtctcttgctaacaaagag

ttttgttttcccaaactgactaaagaaatcatggaacactgttcttccttcaaacagatcaacatgcataacctgctgaccagctctattcttggccttg

cgtaaagcagcccttggctccacagctttagcaagagcaaaacccgccag

>3883|AGAP004091-PA

attgaatatttagctttaatattttctttacaattacatagtaaaataatttattagttgtctccttctacatttttatctgatttgctgaatcttattg

gtttacattcaggatacttctctgtcaaaaagctgcgtacttccccatactcactcagcatttccaatctcaccttgtcatggtttctaactttctccat

tttcttcataactgtatcccagaagggtagattggtgtaaaatggaggccattttttcttgtgtaaaacaactctgccaccaagagcgtactgtgtcaaa

tcacggatttcatctgatggaatatcactagaagaacggcacacagttggtgaagcaacaacacgtggaaaggaagcattttcctccctcatttccacta

gatgtaatcctttttcttctgaaagttcatggtgactcttctgctgcatcagtcctatgaaaaatgcctttacaatatggctaagatttgtagagccttc

aactttagcataaaggtctctaatgcccagaacttgacatattgtacgtatgcctcgatgacatacaagaccatacccttcaggtctcttgctaacaaag

agttttgttttcccaaactgactaaagaaatcatggaacactgttcttccttcaaacagatcaacatgcataacctgctgaccagctctattcttggcct

tgcgtaaagcagcccttggctccacagctttagcaagagcaaaacccgccagtccattgccattaccagtcacaaccatcacactcattcttcgataacg

tccaaaattgcctttcatgtttattacagttttcatctctaagacccttgtgtcaaagccctcaaaagtatcttcacctatgtcatctggaggaccaatg

ctgcgtccaggcatctttgcaccagaccagccccgctcagtaggagctaggcgcagtcttcgaaagacacccatctcattgcgcattttaatgaggcgcg

cttcacgttctgtgtcttcaggaagttgctgctgttgcactaactctcgccctcgaacaactggaccagtcaagcctggccatactatgtttgcctttcc

aacacctataacttgacccttatttaagttcttcgctatcttctttcccactcccttaccccgacctcttttcttgcccgcattgctaacgctggttaca

cctttccacagttcttcagcaggcaatttattgaagaatgtcgccggccttatttgctgctgtaaaacgttgctgggccttatatttggcaaatcacatg

ccgactgtgttacagacgatatcaatttctgctggagtagtgccccagtctgttgcgctagtttaaaaatactgcggcagtttaacattttactaaacaa

cagatacacaatgacagttaactagtcgtaactctagctgtcatcataacactacttcagtacttttcatatttgtctccgaacttacaaacaataacac

actactgagtactgatactcaaac

>4043|AGAP004098-PA

ccctgcttcacgtgctcaaggatgaccatttgtcaactcatgtacccacactacatccacactccgaagagctcatcatgggaggtgtcaagttccagac

atttgatctaggaggccatgagactgctcgtcgtatatggaaagattattacgccaccgttgacggagtcatcttcatgatcgatgctgcagatcgttcg

cgttttg

>4516|AGAP004104-PA

ccttcgccatcatcgcagccatgaatgtggtggcagcgttttgcatgtcgttcctgccggaatctgcgctgcagcgtttgccagagacactacaggacgg

cgccgtctttggcagagggcaacgctactggagctggaagcctaagccaccatcagagtatgccacagaatcaagcggttccaattgtcaaaatttgatc

cctgtcaattgatggtgtatctaacctacactcactatagcg

>4517|AGAP004104-PA

tgacaatttacaatatgttcaatactaaagttaaagctttttttcccacttcagtagacgagttgttcttcagtttgaggtatgtaagacgaaatgggag

actggaccgtaactgagggcgcctattgtccctgaacgtatgacggcggggcgatgagtctccagctccagtagggacgccctctgccaaactcggcggc

gtcctgcagagactccggcagcctctgcagcgcagtttccggcaggaacgacaccgacagtgctcccagcaggtgcatcgctgacaagatggcgaacggc

accctacggtccactgagcctccctgaaagacaatgtacggaacaagagacatgc

>4518|AGAP004104-PA

cgagtggtggcttagcacacctgcagagcgcggaagtgcaccccacgtgcctccggcagattactgccagcctcgaatggtcgatggctgccatctttct

ctccctccttccgtacatagtcttcaaggctggatctgtagatgaacggctgccttttgccatcctgtcagcactcaatttactggcagccgtttccgtg

tcattcttgccagaatcagctctgcaaaggctgccggagaccctgaaggatgctgcagtctttggacgaggccgtcgttactggagctggaagcctaagc

cgcccccgatttctacaacagagcaacatgttatcacatgattctctcatggtatagtgtccacacctcttgtaatatctgtgtcataaaaccaataaaa

atgttgctagaaatgaattcaat

>4519|AGAP004104-PA

agtttacgccagataggcatcgcaattgaatacgcctttgtccaatgtattatgagtgttccaccttatattgctcacttgggaaaaacttatggaatgt

acatcgtattcggcgtactagcagctgtgaccctaattatgtctgttttgatgtcatttgtgccggagtcatttaatgagaatcttccggagacaaacca

agatatttcactctttggaaagaagcaaaagtactggagtcttccaagcagaaagtcaaaggaatcagaaaacacgacgttgtagaattttgatgcccag

gagatgatctctggaaagtgtttcgggaaactctgaacgaaaaatgattaaagaaattttgtgacgagacaaatgtgtacacaaaatgagagcatttcga

actggaaatgataatacgacaacgatttaagtggtataatggaaatgcgatcatatgaaacaattacatgcacaaaagaggtagttacagattgagaaca

ggtagaacacaaacctgactattccagcggtttatactgtagaccatgcaaaccatgttacatttattaaagtgtcacagttttgtctttggtttcttat

tatattttattattatttttctacattttcgcatgtatataccag

>4520|AGAP004104-PA

agctgcccgcctgctgcattgcccacctcactgcggcgcgctttggccgccgctggtccgccgtgggcagctactttctggccggggtcatctcagcggc

gatattcttcatgctggacgctaacatcagtggagtggccatcacgataatggcggcgatagctcagtttttcacgatgggcacaaacagcatggccaac

ctgcaaagtgtggaggtgcaccccacgtgtctgcggcagatcggcgcatgcgtcgagtggacagcggccggggtcatcatgtctgccacgccatacgtgg

ccctcatgagtgacagaaggatgtcctacgccattctcgcagctgtaaacttgggggctgcgtttgcagtatcgtttctcccggaatctgcactgcaaag

gttaccggagacactacaggatgctgcagtttttggtaaagaccagaagtactggagctggaaaccacgaccatctccagtgtataccatcgagcccatt

cagtaatcgtccgcagactgtattcctacgctgacacaaaagagtaaatgacagaagttacttgttaatgtgacggaagggcacatttacatgatttggt

agtgtgacatctcataaacacgaagtaacgcaagttattttaaagtttaatcattagttgaagatataacaacacatttcttgagcagatgctcgggaag

tattacagttagtggtagaataataattacaaataattgcaagtatttaatattagtcacgtacggtttattgtttgtaaataataacgtagtagtcaca

tgtggtacctttacagagtggatttcattaacagtgaatggatgggaccaaatatttttataatatgcctactgtaaatagttccctcaatacactttgc

gactatgtaacataatattctattacaaagtaatgtgttgtgaaaactatttgagtgcccatgcctgtcccatcagatgcttatacccgatgaaactgga

acagctttctattccattcagcgttatcggtgtagatactcgatgtgagcggttcggaatttctaactcccatt

>4986|AGAP004107-PA

agggtccagccaacgcactgcagcattgcgacgaagcctttagtctcttcgtcgctcacctccaagctgaacacgagaggcgcctggaaggtggcagcgt

tggtggcgctggcgagcagagtgactgcgaagaagagcgggtagatggacgccgtgc

>4985|AGAP004107-PA

tgctgcctttgccctcttcattgctgctgcatttgctgcagtgcctgaactgtcatggatgtgctcaggtgtgactatgtttgcacggttgtgcctcata

gtgcagagctatgtgagctatctgcaggccatggaggcatttcccacttgtgtgcgacagacgggtactgctgttggcaatgtagcagcaggtgcccttg

tcgttgttggaccatacatagtgtacctgggaaagacagacccatggctgccatttgcggtactgggtgcagtgacactggcaggggctctactggcatc

cttcatccctgagacactgcatgtacgccaacctgagacactgcaggaagctcaagtctttgggtctgaccagccatacttcagctgtataggcatgaag

cgcaggtgccacacttctggataatcattatcaaccgtgcagtgggctctcaatggaatcatacaacattttttcatatttattcttacctctcatgagc

aaatttattataggactaaacaaattactgctagaatgacagaaaataactatttcagcaaaacctgtcccccatcctcacaaggaatagatctaaagaa

aaggaaggaatatttagagttcagtatcttgtcgacaaagtagaaatgcagcacagttactgaacaacaatgaaaaacatgttaacaccaaca

>4984|AGAP004107-PA

gttggcttgttgccaaaggatatccagagggtgctcttaaagtgcttcagaaagtagcttcagccaatggcacggtattgccaaatgatacccacaccaa

gctaaaatacttgtctgaccaaagaacagaaaaaatgggtttgaagaccttatttgtgaaccgagtgttgtttagaaatacacttcttctcatttgggac

agggcagctgcaggatttactatgcttactctgctgctgagtgataagacttttggaggaaatccttttgttccttttattgggcaagcatctcttcata

taccagccataagtctgtcccattatgttggcaacaaatttggaagaagatgttcacatttatttatagtgctaactgctgctattgtatcctacctaac

tgtctgctcaatcacagtctcttcaccacagtgggtgataactaccagctctctgtttattcaattctgtaatcaagcgtcgctctcactcacaaactta

cagagcatggagatacatccaacatgtgttagacaaaccagtgtggccatggaatacatgctttcaaatgccattaattgtgtggctccatatgtcgcat

atgtgggaacagaaatggagctccgctacatgtttggtatacttggaactacaatggtttttacttcactttcaatatcatttctgccagagtctttgaa

tcagaagcttccagagacaatacaggatgctactgcatatggccagagacagaaatattggagactttcacagaagaaacattgtgaaatagaaactgct

aacttacattcacctgatcctgcaagatgacaattttcatacaagaattgatcttttacactgcgaaaaaattcagagtaacacttcacacttatgtttt

ccatcttatcattatttcttcactgaggatccatatgctactaactggatctttaattaaaaacaagacatagcttacaatatacagataaaacatcagt

gatgaaatcagcacatggaagttactatacgtaaaaaaaagaatttattgctgtcttgataacttctgtttaggtgatcaaagtattagaataatttact

gtttggattagtattcctagatttggagtgaa

>4987|AGAP004107-PA

cagtgacgtaattaaaacatatagaaaaacatatcttagaaatgatactttacgcatgagattgatatcttcatgtctgtatactgttggggaaatgcag

ggagaaaaattgtttgaggattattgagaatcgaagtcacaaatttgcacatagtttcctcataaagtagtattatctacaaacatatttgaaattgcag

ggtacattttacccagcttcacagaaacaaaggataattactgttgtacatcattttcttatttactaagaaaatcagaagacggctattgcaagcatat

tagaaagcctatcgtgatgaaaactatcaacgaaacaaatatccttcagacgtacctcaccgtatcaaaatgattatttcgcacattgttgtgaaacata

tacctagcaacaatgaataactctttcaaattttgcgtacgctatatgtctgtctgtgtattgccccactttatctattgtattactgttgcatgaagca

aaaggcatacagtggaccacgactttttcatattttcgtttagtgatggtactgttaactgtctcgtagccacactacggcagggagactgtccgcatct

gctatccaattagttttcttctggaagaataatatcagatcatttccagacaatcatttttcgaagttatatttgtacaatgtatctctttctgatgtgc

tgggccttcttctcggaaggctccagaacttttggtcacacccatatgtggaagcgtccttcagagtttccggaagtttttcattcagtgattcaggcag

gaatgacatcaaaatagatgtcgccagcgtcaccactgccaacacaccgaacatgtagtacaggccagacgttgctcccatgtaagcaaggtaaggaggc

gaacacatgacgcattgaacaactacgtattcgatggcgatggctatctgccgtatacatgttgggtgtatttcaatactctgcaggtttgttagcgaca

gcgaggagctggcacagaactgtagcaacagagacgcagtcgttaacatccactgaggaaatgaatttgcagtcaggaaaacaagtacgacacacacggc

ggcagccgtcagtaatatgaaacagtgtgttattcgtctcccgatcctgttaccaattatgtgaacagcatatcctgcaaagaacttcagaattccttga

gcaatgtaaacaatgaaagggttctctccaaaacttctgctgttcataagcaaggtgaaaataacgagggaactcgcagacctgctaatcacaagaagaa

ttgtgtttttgaaaaggttcctgttcgttaagatgtttattattcccatattgggagttctttgatgagacaaaaatttaagcttcattgtggtatcagg

aggtaacggctttccatttgttgccgccacacgttcgagaatcttcagcgcctcatcaggacgccctttgcttagtagccatctaggactctcgggt

>4988|AGAP004107-PA

cagcagttagcgaggagtttcacagaagtcatccggtcctccggtgtgcacgacgctttgtgcaaaacaccgcaagaaacaccacgctatggcagagtgc

aaagatacagctagacacaacatgattaacgaaaatgttagatttgaagatataatagaagatcttggaagtgatgggaagttccaaatacaaatgaaca

ttgtcttcaatgtcatcgcaatattcttttcaacaatgtcttcatacgccaagataattgccctgacagtacctgaacactggtgccatgttccaggaat

ggaaaacacaaatttaaccctggaaatgtggaaaaatcttacaataccaagacaagacgggaaatacagcagatgcctgatgaaggcgccagaccaagag

aacgaaacaatgtcctgtatctacggctgggactacgacaccacatggttctccatgacagccgcatccgaacagaactgggtgtgcgataaggaactgt

acccgaatactgtgtattccgtgagccacatcgtttccaccgtgtttgggatacttctgttttacattggagacagatttggacgcaggaaacagtttct

gctaggcataagtgccacggcgttgtcacagacgctgctccccgtatcagcgtccgtgtttcctctgtacattgccctttccgcagcctctgaaggtgca

cacttgcccatgtcagaagcaaccatggcgacaggagtagagttggccagtatcagtcaccgttcgtctgtaaattttttgtcctacgtatcttactgcc

ttggtgtgattgccatggcatcagtggcttggtttctcaaaaattggacatacttcactctggttgcgtctattccttgtttcattccactactgcttca

taagtatttgcccgagtcaccgcgatggctgataaataaagggcgcacgaaagaggctttcgaggtcgtggaacgtatagcgcgcacaaacggaaaagct

ttgtcccctgacatcaagaagaaaattgagtttctttctcagcaaatatcaaagaatatgggcattaaaaccatcataagcaacaagactctcctcaaaa

atactgttttacttgtactgagcaggtcagctgctggatttattgtattcactctactttttaccaacaaaagtttcggcgcgaatccttttgttgcata

cttcggacaaggatccctgcaactggctgcttcatatgcggctcacgctgtcggaaacaggtttgggagacggataacacactgtctcgtactactggct

gcaggttgcttgtgcatcattctcgtatgcttaaccgcagcatctgcagcccagtggatcataacgacactgtgcctggtactactgctttgctgtaaca

cttcacattcactcaccaacctgcagagcttggagttacatccaacgagtttacgccagataggcatcgcaattgaatacgcctttgtccaatgtattat

gagtgttccaccttatattgctcacttgggaaaaacttatggaatgtacatcgtattcggcgtactagcagctgtgaccctaattatgtctgttttgatg

tcatttgtgccggagtcatttaatgagaatcttccggagacaaaccaagatatttcactctttggaaagaagcaaaagtactggagtcttccaagcagaa

agtcaaaggaatcagaaaacacgacgttgtagaattttgatgcccaggagatgatctctggaaagtgtttcgggaaactctgaacgaaaaatgattaaag

aaattttgtgacgagacaaatgtgtacacaaaatgagagcatttcgaactggaaatgataatacgacaacgatttaagtggtataatggaaatgtgatca

tatgaaacaattacatgcacaaaagaggtagttacagattgagaacaggtagaacacaaacctgactattccagcggtttatactgtagaccatgcaaac

catgttacatttattaaagtgtcac

>5584|AGAP004107-PB

tgctgctgtgatatacatatttattataaaacgatgataacgaatgtaaatttagtataaatatagtgtatatgcaggtacaaatgaaagacagaagctg

tccagacgtagcaacacggttgaggtgacggggcgagaggtcacgttgcggtggcgttagaggggcggcggctgcggggagcgacgctggcgccgccggc

gtagggcgaggcaactgaagtagggctggtcgcgtccgaaggagcgcgcctcgtgcagcgtctcgggcaggcgcacgtgcagcgtctcgggcaggaacga

cgccagcacggcgcccagcgtcgtgatgccgcccaccaccaggaacggcaggcgcgcgtcccacgtgccgaggtgcacgatgtagggccccaggacgccg

aggatgcaggcggcgatgttgccaatggcggtgccggtctgccgcacgcacgtggggaacatctcgttggcctgcaggtaggcggtgtagttcaccagca

ccaggctcagccggatcaccaccgacagcaccgtcatcgccg

>5581|AGAP004107-PB

tagctcactaaggaacaatatttctaattcacaactttttttattcttgttagaggtcacaatgttagaaaacttattctacagagtattaaaataatac

aagagttgaaaaagtctggatttttgaaggagggctcagtatttgcggtctccgtaattaaccatactcacaaattgtatctagttttgttacaagtttt

acttttctgttttcacggcggtagtacttctgcggttctacaacagtaacagcaacttaaggtaggccgcgttggctatttccatcttctccgacgttac

cgttgttgacgtactctgctgtcaggtctggcgccgagagtttcctcacttttagtgcctttttcattctgtgtacgtctgccaaggtctctggcatttg

tcgtttccttgtctctttgaggaagaaggcgggaagtgagccgattgaagctagtgcagacattactaggaacggaatctgaacatctttgttttgaccc

tggaagacgaggtagggcgatgcgacgctggcagcggctcccagcatgctg

>5582|AGAP004107-PB

ggtcgcgagcaggaggcactggcagtgttgcgtgacatcgcgaccaccaacggctccactgtgcctgcatttgccttgcaggttatccagaccgtgggca

acggcaaggcggacaggacaggcttcctgagtatattctccagctggaacatctttaagaacaccacaatcctcatcatcgccaggtcggtgcacctgct

gaccacgtactcaccggtgatagcggtgagtcagctgagtgcgaaccctttcctgggaatagtggtgcagggtgcagcccagttcccctcctacttctta

gtgaactactgtgcagcaaggtttggccgccgttggtgtggcgtggccagtgcactgttggctgcagcgaccagtgccatcatattctgtctgctaaacg

tgaacatcacgggcacagccatcatggtggtgaccatgttgatgcagttcagcaccacagccaccaacagcatggcgaacctgcagagtgccgagctgca

tccaacgtgcctgcgtcagatcgcagtcagtgtcgaatggggcattggcgggatcgccctgtctgctctaccctacctagccttgatgagtgacaagcga

cttgcc

>5585|AGAP004107-PB

attgccagatgagactctcatcaaactgaagaatttgtctaaacaaacaacaggaaaacttagtttgaagactatatttttgaataaaattctattaaaa

aaatacattgcttctcatttggagccgggcagcttcaggacttattacacttactctgctgttgagtaccaaggcatttggaggaaatccttttgttccc

ttcattgggcaggcagctcttcatataccagccataagtatgtcacattacgctggcaacaaatttggaagacgatgttcacattcgtttacagtgctaa

ctgcagctattttgttataccttatcatctgcttaatcactgcatcttcaccacaatggatggtaactgctagtactctggcagtgcagttctgtgctca

agcatcaatctcattcacaaacttacagagcctggagatacatcctacatgtgtcagacaaacaagtgtggctatggaatatatgcttttgaatgctgtt

atttgtgtagctccatacattgcttttgtgggagcagaaattgaatctcggtacatgtttggaatacttggaaccattatgcttattacttcgcttctga

tatcatttctaccagaatccttgaacaagatgcttccagagactttagtggatgtttctacatttggccagtcgcagaagtactggattttttcccctag

aggagcgttaggaagcaaaaggccctagtttatatatcagtgcacctcaaactgtaagcttatttttatgtatcag

>6002|AGAP004113-PA

caatgaattacaaattcagatatttccctatttaacttatagctgatatctcactgttatttcttaacccaccagaatggcaaaaataccatgtacatgc

attgttgttagccttgaatatgaatgtatttatcatcccttaacatctttgccaatgagtcatgctgtgctgatcccaccatgtaattaaagcagattca

atttgtaggacagtagtttatgatgacatgaagttaatttgtaggcaaagtggaaattcatacttctatttcaaaactagtatcataataactatacttg

gcaatgttttgtttttgtcaccactttcaaagttaccagtattataacatgttctatggcatgagagaacaattacaaggtgttagtttttcagtaatga

tacattactggccaccaatgccatacgctcattattattattattattactactggaaatacaatacttgtttctatgcattagaatttgtttttctgat

gtatttcagatatttattaacatcgaagtcatcttcatagtcatcttctatcttcaccttggactttggcttccgcttttccaaacggagtttacgttct

tcatcactcagtatagacaaatccaatggaagctttatgattttccttggttcatgatatctgatgtttatagtggcaccatcgggcaaaacaacaagaa

caggataggtccgcggatatacctgtcggtgt

>6004|AGAP004113-PA

tttccctcttatttttggaactctaaataagtatttctgctgctgtaaccaatattttgacatgtgtgttgaatatatatacactataaagtaatctatt

atttattttcaggatgttttatttgactttcctgtttttaaatataatgcagaacactcagggctgagatttgttaatcttctcctagtattgccactag

aatactgtacttcaccatgttttttatagatcttacaccagcttttaattcttgccagaatagctattaaaacttacaaaactttgtactaattgtcttt

gttgttacttactagtcaatagaagaaaaggtttacttttcacttaattcacatagccctacagtgccagtctttactgaagctataaaatcattatgtt

tatggcagcttccattggatttgtctatactgagtgatgaagaacgtaaactccgtttggaaaagcggaagccaaagtccaaggtgaagatagaagatga

ctatgaagatgacttcgatgttaataaatatctgaaatacatcagaaaaacaaattctaatgcatagaaacaagtattgtatttccagtagtaataataa

taataataatgagcgtatggcattggtggccagtaatgtatcattactgaaaaactaacaccttgtaattgttctctcatgccatagaacatgttataat

actggtaactttgaaagtggtgacaaaaacaaaacattgccaagtatagttattatgatactagttttgaaatagaagtatgaatttccactttgcctac

aaattaacttcatgtcatcataaactactgtcctacaaattgaatctgctttaattacatggtgggatcagcacagcatgactcattggcaaagatgtta

agggatgataaatacattcatattcaaggctaacaacaatgcatgtacatggtatttttgccattctggtgggttaagaaataacagtgagatatc

>6003|AGAP004113-PA

taaacattggaatatgttggtttgtgatctttgagttaaaactttgttaaaagagtataatgaacttttattgtagttgacagtgctaattgtttcaagt

ttacaagatttcgttttgttccacgtggaagtaatgtcaggtttgctgcgaagagaattcagacgcttaccatgtctttatgagagtggaaagtgtgaaa

tgaaacgccatttaaatagtaatacggcaagtgtatgtaagatacaccgacaggtatatccgcggacctatcctgttcttgttgttttgcccgatggtgc

cactataaacatcagatatcatgaaccaaggaaaatcataaagatttgacaactgtgcagcaaaggtgcatttaaaccacaaagtgcagcaatctgttcg

atcacagaggtgcttgtctggctgagttagcttttgtcttccattggatttgtctatactgagtgatgaagaacgtaaactccgtttggaaaagcggaag

ccaaagtccaaggtgaagatagaagatgactatgaagatgacttcgatgttaataaatatctgaaatacatcagaaaaacaaattctaatgcatagaaac

aagtattgtatttccagtagtaataataataataataatgagcgtatggcattggtggccagtaatgtatcattactgaaaaactaacaccttgtaattg

ttctctcatgccatagaacatgttataatactggtaactttgaaagtggtgacaaaaacaaaacattgccaagtatagttattatgatactagttttgaa

atagaagtatgaatttccactttgcctacaaattaacttcatgtcatcataaactactgtcctacaaattgaatctgctttaattacatggtgggatcag

cacagcatgactcattggcaaagatgttaagggatgataaatacattcatattcaaggctaacaacaatgcatgtacatggtatttttgccattctggtg

ggttaagaaataacagtgagatatcggctataagttaaatagggg

>6001|AGAP004113-PA

tcattacttgattctgaacattcatatctaatgaactacctacgccattccgcaaatctctcatttggttttgtgatgtttgagttggaacctgcattac

catttggtcactttctcccataaaatacatatttctgtcacgatctgagtttccttgggtcatataacggtccccattcactgtatcagtataactggag

cctaaaaagtattgagaattattagatgttccataaatattattatctgtgttccctgtaccataatactgactctgattattctgttggaagtaatttg

gctgagtattttgccaatacgttgaccctgcagtgttagaactacagtggtatgaattaccactagtgttctgttggcctggtgcatgcgagtagtatgg

tgagctgaatgttgaagtctgactagaactattcgtctggtaatatgatacacctgggttatttgcataagacacagcagtatttccttgatcggggcag

ttattgtaggcagtcttgacaaagtttccaaatgatgcattagttgcattgggcccaaattctgtaactccatagtttccctgtgaaccataagaatgcg

taacattatgactagaattatggtactgcatatcgtgataggagccactatggtcatttacaaaggactggttctggtttgcatgccccatcatggcaaa

gttctgattatgaagctgctgctgctgacgctataaacattggaatatgttggtttgtgatctttgagttaaaactttgttaaaagagtataatgaactt

ttattgtagttgacagtgctaattgtttcaagtttacaagatttcgttttgttccacgtggaagtaatgtcaggtttgctgcgaagagaattcagacgct

taccatgtctttatgagagtggaaagtgtgaaatgaaacgccatttaaatagtaatacggcaagtgtatgtaagatacaccgacaggtatatccgcggac

ctatcctgttcttgttgttttgcccgatggtgccactataaacatcagatatcatgaaccaaggaaaatcataaagcagcaagtcatgtagtctcttcct

tctgtactgtagactgagagactcaataaaacttgtagtcttcttagacaatgttggcagagaccatcagttttgcatgttgatgaattctgcacataca

gactgaatgcattccagcagagcctacaacaagacacaactatgccttgagtcattaaattatgaaaaactgatctgcaccaaccttgttacaacatgtg

acaaacgtaaaaaccctgcctgtgtttgaattttactgatgtacactcaacctaggtggaattaaaatttgacaactgtgcagcaaaggtgcatttaaac

cacaaagtgcagcaatctgttcgatcacagaggtgcttgtctggctgagttagcttttgtcttccattggatttgtctatactgagtgatgaagaacgta

aactccgtttggaaaagcggaagccaaagtccaaggtgaagatagaagatgactatgaagatgacttcgatgttaataaatatctgaaatacatcagaaa

aacaaattctaatgcatagaaacaagtattgtatttccagtagtaataataataataataatgagcgtatggcattggtggccagtaatgtatcattact

gaaaaactaacaccttgtaattgttctctcatgccatagaacatgttataatactggtaactttgaaagtggtgacaaaaacaaaacattgccaagtata

gttattatgatactagttttgaaatagaagtatgaatttccactttgcctacaaattaacttcatgtcatcataaactactgtcctacaaattgaatctg

ctttaattacatggtgggatcagcacagcatgactcattggcaaagatgttaagggatgataaatacattcatattcaaggctaacaacaatgcatgtac

atggtatttttgccattctggtgggttaagaaataacagtgagatatcagctataagttaaatagggaaatatctgaatttgtaattcattg

>6618|AGAP004148-PA

tccgcaacggacaattaatgtaacaggaaaatttctagtgaaagtagcttttcgaggtgacactttcatgtgcagaaagtgccatgggtatctaaggagt

gctcgtagtgtactgcaaatataaccaacatgaaatagaaatcttcacgtgatgtaagtataacgacttttgcttgcacgtagactgaaactttaatgtt

gcatcggtttccctgttttaaacatttgtttattattaacaacaatagtgagtaattgttgctgctataggcgcaaacggagaaaaatttacttggcaaa

ggtaattgttgcaagggtctaatccgagttatcataccagaaagaggtggtttatatgttgattaatatcgagacaaagtgtaataaggagttaatgtcg

tagggatatttcacgtgtctgcatttttacaagacaagtaccagataaatttcggtggtgtgttgtcagtcagttcattacatacatatttcgttacatg

catatttcgaccagttttcacctacaagtaagatttatgaaaaacagtgtgtaagtcattaccttcggattacacagatctccaaagaatgtcgcatatt

ttaaagtagccacaatagtcatggacaatgagtatgtacgattaatagattagttcttgaaaaccgtttcgtccgtaaaaatatttcattgtgtggtaaa

ttgataattggaagcatatgatagctccagattaaatattttttctgcactgtttcactttggtgtggtatgaggccatgttaaaacacaaactcggata

tatgatctggtgcagtattaaacctaaaaatagctgtgatctggtgcagtattaaacctaaaaatagttctgctttctttccctatagcggaagaattag

tgacgtcttatcgcaagtaagtcaaacgctctcgcctttatctcttataaggcaaaatcattccagatataaattggaaattattaagatgcctaggaag

tgcgattacaaaatgcacggcgctttgaacgggatctgtctgtacgagcgatgtcacgacatccaaaatcctcacagtgcttacggatcagccttacgcc

gcaccgcagtcatgtgactttcgggatcggtttatgtaggtaacaaaaattaatttctggccaattgaattttcactcgatggatattcgttccttgcat

gtctaaagttcaatatgaaatttacaaaaaattaccgaattctcagcatcagctgtagctttgcaactgactaacaaaattttaaattgtagggtggttt

ttttcgttttcattcagaggagtcctgtgtctcagggcgctgcctgagcgaccgacagatcgtcttagtagtaggacaagtgtttcgtcacgacttgtta

atagcaaacagacacgtagcgctgtaaccagtccagtaacaggaacaaagaaacatatttttatttttaggcaaattaatattgcaaccaggacgtagga

agtgtaaactgtccgtgaagacaaccccggttgatttaaaaggaagcgtctgtccccggagacggcagagtgtgaaggtgaggacgggcgctggctaggg

ccgcatggagtcgagtatccagggcacgtaggcggcgacgcgcgtgtagacgccgggccagcccgcctggccgcactggcgcgggccgtacgacacgacg

cccgccacgtggtgccggttgacgccgtcgacggcgcccaccgccatcagcgggccaccgctgtcgcccgtgcacgagtccttgccctcctggccgccgg

cgcacagctgcgtcgcgtagctcaccgtgcgccgtccgctgtacacgcttttgcactcttcctccgtgactgcgggcactgcgaccttcagcttcactgg

gctgaacccacgtgactcggtcttgccccagccagcgactacgaggtcacgaccggcgtaagagagcttcccagtgctgcccatgggcaggcagataggt

cggatgtagtgtgtggtaggcgcgtcgcggtcgaggcgcaagagcgcgatgtcgttgaactggttggcgctgaagggctcgtagtcggggtgcggcagcg

actgctcgacgccgaagtcctgcggcgggtcagcgcacgtctcgccgcccgtgtactcctccacgcagtcgcggtccaggtccgtgttgtactcgcccag

ccgcacgctcaccaggcgataatcacggatttccctcccgttgacgcagtgggacgctgtgactacatatctgctgttgatgaggactccaccgcaaagg

aacagcagctgcccatctcttgcgcggtacctgagcaacgcgagccagggg

>6948|AGAP004174-PA

tagggcactgtagtgtacagttctcagtacaagaggcactgactagcaacagaaggccgagtaacagacactaataaaatgaacataacatccaacagtt

tttctcggttgtttgattaccaccagatacaaaccttggtgatgggacatattaaataattattttaaaataatttcttttattttgtacagtttgaaca

ttccatgttacttttctttgtcttctgtatttcacgaattatctccatctgggcatccattatggcttggagtattcggtgttttgctcttgtcttaata

caaaagtaattgtccatttttcgtaactcttctgccacaaatttccccagtgtgtcgtcttcagttgttcgatctaaatatgaactaactttctccaaca

ctgacaatgtggataagagcaatgtatcatcatcatctttcttttgtcgctttgaaggcttttcataatgtcgttgtgtttctgcatacagatgatgtgt

ttctggtggctctggagcatcttgtgtctcagtggtgacacactctgagggcatcacatcaatttcagttttcacttcaacgatttctgttgaaaactct

gagtgattgtcactgccactgtctgcagtgtcctctgtacttctggattccactatctgcaactgaggtccatctgaaagattataacaatccattaggt

cagaggagtgactggaatccatggatactgcctggggcttatatgaaaaaaatctttcgggaatatttgagtgtgatgatctgggtacaattcggttttt

cagaaactccatcagtggaaaccaccacacagtgggtgagtaaactactccggtttctttaccggttcgcatcttcttaagttcagcgccatagtgcgac

cgcaaggttgcaaatttgcttttgcagtcattggctgtaactccaggtagcacctccccaaccctttgtgcaacttttgcaagtttttcctttctattgg

ccttcaaatgatattgggggtgcctgtgatcgaagagcgagggttcctctttgtatgcttgaatgagaagctgaatggattttctgttccattccatatc

gataaacacaggagcccacacgtatttatatcagtttaatagaaagaaatgacctgccaaagcataaaacgtactcacaaggcgaatcatgaggatacct

cattatttgagcgcccaaaggaattctatttgaaacacattccacaactgcgcgttaacgcctacgcagctaataaaaaaagaagcaaaatacctatg

>6949|AGAP004174-PA

tttcactttttataacactatttattcacatagaaagcaccaaccagtattcgaaaacagcacaatgtttttttcattttgtgaagagcataattttaca

tttctgaatatttaaaataaagatctgacagaatatatgggcagctgttttcagatactaattcattaatagtaactgcatcagctgtagagagtctgag

gttactgttaatgttgtctgccaggttattaatatacaacatgacgggcaaaggtcccaacagtttcccggggtggacctgaagttacgtctacagctgt

ggttctcttcaaggcatcacaacacacaaacaataaatcactcttcagtacactaccacatacaaaataaaataggtaacaacactcaactggcaaatat

aagaacatatgatcttgcacagctctgatatcactgttaatgatattacaatactacccatacccaatcacactgttcttcagactgatcagatttacag

tgatttggtagcactcatattattactattgctgacagccatcattttgatcacagccaggtgtcacatgaacaatgaattgcaggattcacactgaatt

cattttctttcaattaaatagttacatttttagaaagtaaacacaatgcaatatacacaatgttttattcattatctaaagtatcataaacaatttttag

tattttttgcttacaggcattccttgcttctctgcattttatttgtctcatttctgttgccagatattttacaaaaacagttatgtcatcatcataactt

tcctgcagtctgtctacaattaattgtattgcactgtcataactgtcactgttgtttctttttcttgcagtagaggacctggatgtagcactctctgcag

aactccatgaggtttgtgccagtggcacttgttctagtattgaatcagattcaactagtcctatgtccttcacatcactttcttctgaaaattctatgac

attgctggtggaagattccacaaaatcactttcatgtgatgtgctttcatctgtctgaaaactgtcactacattccatagtctgcagaggggcagtgttc

ctaattcgtggcatgtaatgagcgtctagaaattcgagtttctgaaaataccacaagcctggccgatatatgtcatcttcgcaggttgcagtcctcatac

tctctttcctctttcgattttcaacgttgaacatgttcctcatattcaaaaatttcgtttggcattcctttggattgctcaaatgaggcctaacacattt

cactttaagaaatacacgacaaagcgcctccgcgcgacagcgtttgttgtaataatttttactttttacattgtaaagacattgttcttctttcatagcg

tttattagcactgacatttgttctcttgtccatgcggtagccatgttgctcagaac

>7215|AGAP004189-PA

tactattacgcagctgcgcagtaaatagaaataaagggtattaaacatactattacttgccctttgaaaagtatgtaacttcatactcagaaaaaagtaa

gactgaaacataatttgcgttttataattgccagaatcacagtaacactacggtgttcagcaactggataggactattcgacttaacatctgtcatttta

gtaccatttcaaatatgttttttctcagcagaatgctgtgatgctcattaggggcagatccaccaaacctgcgtaactgctgtacgtaacatgctgtatc

aaggctgaaaatgagataaccacattttacgcaaataggcagaagttaaagagcactcgtgcgtgtcgggagtaaggatatgtaggcaaatgaagtaggt

tatgtgtgcgtttgttatcggcttgtagtgtagcttataaacaactctcagacaacgggcagttgccaaagtaaaaggtgttaatgacacaaaggtgata

atatctgctcgtcctccagcagggttgcaagaatacgaaaggccaccatgcatatccagatggctgctcgcttcttgttggtgcgcagctctcccagcag

ggtcatttcagtgagccagagaaggcatctctctaccaaagctgcacagtctgttgaactgacttcagaaagatacccagatgtcagaagaggaaatttt

aataaactcaatccagggcatattagtttctttgagagcattctagggaaaaaccgtgtcttaactgatcgagctgatgtagagagttacaatgtggact

ggatgaagatggttagaggagctagtgaagttgtattaaaaccaaagacaactgaagaagtttccaaaattttgaagttctgcaatgaggagaacttggc

tgtttgtcctcaagggggcaacactggtcttgtaggaggcagtgttccagtttttgatgaagtcattctctctactacccttatgaatgaaataataagt

attgatgaagtttcaggtgtccttgtttgtcaagctggttgtatcttagaagttctggataattatttatctgaaagaggtcttatcatgcctcttgact

taggagccaaaggcagctgtaacattggaggaaatgtttctactaatgctggaggcataaggctcttacgttatggaagtttacatggtagcgtacttgg

tattgaagctgtgaaagctgatggcgaggtcattgattgcctgagcactcttaagaaagataatacaggttaccatttgaaacatttatttattggatct

gaaggaactttaggtgttgtgactaaggtagccatacattgcccaccagccccaaaagctatcaaccttgcttttctaggtttacaaacttttgaagatg

ttcttaaaacactccaacttgcaaaacactcgcttgctgaaattttgtcatcttgtgaaatgatggataacacaacaatggaggcagttacacagaatct

gaaacttaagtgtcctgtagaggaatatccattctacatgctgatagaaacttcaggcagtgactcaaaacatgatgaagagaaattatcaaaattcttg

gaggaggctatgtcaagcaacttggt

>7235|AGAP004192-PA

aagaatggcagagtagaaataattgcaaacgatcaaggtaatcgtattaccccttcatacgttgcattcaccccagacggcgaaagattcgttgtaagct

gattcttag

>7320|AGAP004218-PA

tttctgtagtaaaataatttattaaacactagattaatgggatcacagctcagacaaacttctcaaagaagttttcaaaatatttacaaaattcactctt

tacataaataatgaacacaacaagcataagtccaatgtcactatataaagtaaaagttttaggcagcttctggatatacagtgacacttaggaatattat

cacaaggaatctgcctttttttcatgcaaaattatgttggtgaaatgctgaagctattttatagctttgttggtaatgctcttattgccttcaaaatcca

caggaggattaactatatgatatttaatagtgctcctgttgttgttatgtttgcagttttgttgcactgccactgaatttgttactatttgttcacttat

ttcatttttttttccttcgcctccaatatatgcacatgaaccatgcagctgttggcacaagaaccatgattacaggaatcagaaagagaagatggacaga

cccatcaactccttgcccaagaacaggattattagaagtccgtggaagcactgtcaaatgaacttctctatagacaaaacctcttgagttagttaccaca

caagcataatcacgagaatccttttgttgagcagcaggaagattaagtttaccaatgtaagtatcttctgatatagctaattgcccactggagttaagtg

cttcatatatattgtctccatagcgtatagtttgtgaagctgagctgctgtcatatttacggacccaatggaactgtgcaaaaacattacttagtgcttt

gcaactgaaaataatttgtgagccttccagaactgttacattctctggaaacacctccaaaatttgtgggcattctgcagtttcaacaacttccaaaata

tatgaaacaattgtttttctgtcagcagttttgtttccataaggataaacacactggtacatcccactgtttgctactgagacattattcattaacagat

tgccacggcacaatttctttccacaaaggtgtagaggtttattaatgtccccttcctcaatatgctgtgggcatggatactggccatatctctcaccaca

cttctggaatatccacatagctttgtgcttcttgaatccactactaagaccatttttccaatgtggatggcatttcagctctacggtatcacccactttc

gccatcttccgctcaaatttaggatgcatctgcgactctggcagagcggcggacggcgaagagacgcagcagcagaggcaggcggtcgcggccgcgagcg

ctaggacgggcgccggcagcaggcgcatcctgcgcggcgcggcgcgacgctgtgggagcgccctctgagctgagccgtgctgccccggcaggccgctgg

>7340|AGAP004232-PA

tactcaacagtcttcttgtttgctatgtgcccacaaggattgaaggcgtactgcaaaggtcctctgtctacataaaaggctgcttcaatgcctagggaca

gtcttgctgttggcccatactctaaacaaactggacaagctgcataattcctcgtggtatcaacacaggtctgcacatgtccacatttgaaatacacgtg

aggtaagcattttttctgtgagtccatactgcctgacgaactcaatatcgtggaggaatctgagtctttgtattgcatgcacatccctgtgcatttgaat

attgactcctcagagagtgcctggcggaggttgtcttttgtctgaaacaaaacatttaccgcatcacgttaaatcattaaaaagcaaaacaataataaca

tcaatctatcacaaacttcaccaatagcagatttactattaacatcattgtaacggaattcttatattgttttgctttaactttattagttaaaacaaaa

atgcaaagaattgtgaactatacaaattttttt

>7341|AGAP004232-PA

tgcaaggccgctgttcggttgttagctgctgagtgcagacgtagaaactggaaacacatagtgagaatgaacagaagaggatcgtttatgtttcaggaac

cagtaactactgcactgaaaattcaacattcgagtataccagtagctgaaaaaaggcgaggtgacgaatgttatgcaccattgctaaaacatcccagggt

atctgaaccccatggctttataccgcaaataatcccttacggtaccttctacatagtagggtacaatggttttaaaccaccatttaatcgtggaagggtc

aaaagctattgccaaataattagaaaatattatggggatggtgtcaaatacgtgagaacttgcattgcaaaaactccacaggtgttgaaggcggcgctgg

attcaaaatcacataatgtgatttataaattttccgtctctgaaactgttcttgttcagtatgcaagagacagggaaacagacatgtttcagattggacg

ctcagctgagccaccaatcgattttgtagtcatggatgtaaatcgacatgctgggagggcaacaagtactgtatcgaggtatgcttgtcgtgtcctagca

gatcgaaagaattgcagagatgtccgaatcttcgcagcaggcttcaatgaggcttgcaacatattcttgggtgagaaggctataaagtggcatgaggagg

agtctgtggatggtctcaccaccaatggagtcctgttaaaccatccgcaaggagacttctggggaggagatgcaaaacctggttgttggagagaagtctc

ggttaatgggaaactttacacattaagaccaatcagggattcagacgtgagaggtgctcctgttcaacatgaaaccaacttactccaggatgggtctctt

attgacctgtgtggagcgactgtgatctggcggtcagctgatggccttaagaaatccattacaaaagacaacctccgccaggcactctctgaggagtcaa

tattcaaatgcacagggatgtgcatgcaatacaaagactcagattcctccacgatattgagttcgtcaggcagtatggactcacagaaaaaatgcttacc

tcacgtgtatttcaaatgtggacatgtgcagacctgtgttgataccacgaggaattatgcagcttgtccagtttgtttagagtatgggccaacagcaaga

ctgtccctaggcattgaagcagccttttatgtagacagaggacctttgcagtacgccttcaatccttgtgggcacatagcaaacaagaagactgttgagt

actggtccagtataaaattaccaaataggaaaagtagttttgaggcaaagtgccctttctgtgaggctacgttagcaaagcctgtgggttatgtgcagtt

gaagcagatgacctgagcatatgactaaaggctggagcatttgcagtttgcttctaagttttgtaatgtcccataagtatgatgtaagcgaatggtggta

cttgcccctggcaagtgacaatgtttccatgctatgtttggagaatcagcttctgagaagttcaataccaaccagttaatatattcaagctaatattggt

tcttgtctgtgacaagtgaaaactgatacacgttactttaagagacttggatgtacgtatgggtggggactggaaaaattcgatttattttacggacaat

ttcggttctactttttgccattttcagtgttatttcctcgatacatatttttcatagttcatcttgatttttgtcttatttaattgtttccagtactgcg

tttaaaatcaaaatacacctctcagttacattatatttgttagatattaaacatcaattattttcagtttttggacagaaaaagaaattttcccaacaaa

tattacagttcgttcattttgtcttatttcatggtaatttagtattttgctgagattgaaagtgtacatttagactatattgtatttattaatttaaaga

tctaatgtattacatcattgtatagaaaggtaaacatttatattacaccaattgatatctgcaaagaaaagttaacattaactgtacaccaagtatggtt

tttcatatgaaatcattgccattagttccaagttctcttctgagatatagtgctaccagtctgtttgtacttcattatcttgtgagaataactgctccaa

atctctgtttaaacatggaatcccaattgccttcagtgcaactgcagcaaattatgggttttaagagtagttccctgcagacatttgacaatgttcacag

ctcctgctgaaagctgttgttcagcttgatcctgaagagatttgcatccttttagaatttctatattgctatatgccaggtgtgcatgagagactgtttg

catgtcagcattaatatagttcatgattactgtcctaagattgaaacacaatcctaggaccgaaaacacacatacatggatcagtaactgcaaggatcat

cagctttgtagataaaatctgttgcatttaaatgtttctctcatttctgcctgaagaatatacctcttcaccttttacacaagtttgctggtgtactgac

aaattccacgtgcacaatggcaaagctattttacagctgtgttaacatgagtgtaattcatttgagggaggataatttgaagacttattcatcatatcat

ttgcatgtaccttaatgaacatggcctccaattgaacttacatcctcactagaaagttaattaccctaatcagttcctaaaagcactccatcttcaggcc

acgagtggcctaccaggaccatccgtctgccgtgtcatcctcagtggaggatgcggataggaggggcgtgtggtcagcacagcattggataattccagca

tatcatcactttcttccaccatgtcttcaaaatatactgccacacaagtggctgtggaaagccaagagtttcagctttcaacactggaagaggaaatgcc

tttggataccttttctcctcccttaaaaactgtagatgtaaatgttttctcttcctggtatttgagaatgtgctctttaagctaaatataactaagttca

gtttcaaaagttccttggaaaaccatctgccaacaaattcaactagtgtgcccaaaactgaaaatgatgcaaatggtctcctataattgtttttcaatga

attgactcattttttttatatacatagcattgtcacttaagataggtttatcatcatcatattgaatattatagtcttatcaacagtcttcactgtcaca

gcaaaacatgataacggcttttttcagtgctcttatctgctaaagctaaaacatcctcatcagctagttcttgcattatcccattttttggttgtttttg

tattgaaggtaccacagtttccctcgttgtcttgatatgaggaaagtcattagcaccatattcaaacgaacaccactgttagcattcaatttaaaaatat

ctaaattgaatctgtgctaacaaaatttgtactttttgaaatgtacaatggcaagctgaaaaatacaatttgattattttgctgtgaactaacagtaacc

acagcttatcttcgatgatgaatttgttagttttctgccgaaaatcttcattttccagtttgtcctttgaaccagatattttcagtgggttttctttttc

ttggtgacttcaaagctgctgctattgaagctggctgcttacctacacactggaattattgagtctattccctttgtgcttctcagtttgcatgtatttt

aatgcatatcttttctgtcccatgatattctgctattgaaatacttaaagaatacgtagatttatcaggagcatataggccatcggcttcgaacttccgt

gctcgtatgttggcagtaactgcatgctttggcatattgtacaacctcgaaaactatttctttgaagtagatcaaaataagcagtgagaaagcccaagaa

tatttggacactactgtactgttggacaaacataacagctatcctgagaagccaaagatcttgcagcaagatgaattttgaccgacaaagaaattcttca

aactgcttttgatagcgatgtctgcacagtccagtcactatgaaataaataatttttgttggctgttaatttcgaatttttcctgtccctaaatatgggt

tatgtgaccagaattgattattttttaaaagtattatgaaagtacaatatgttaacaaataatagactaatatctctctctctctctctctctctctctc

tctctctctgtgtgtgtgtgtgtgtgtgtgtgtgtgtgtg

>7443|AGAP004235-PA

aagaaatcaaaaaaaccagcactaattgcaaaggcaaatattattctgaatgtgaaaccttgggaccatgaacctgacataaaactgttggaagcggaa

>7442|AGAP004235-PA

tcagcttccaacaatttcatgtcagtttcatcgtcccaaggtttcacatccagaataaaatttgactttgcaattagtgctggtttttttgatttctttg

ctgcatacagtgagggagttagaacttttgtgttgagaagaaaacattgaaactcagga

>7441|AGAP004235-PA

tatccacactttggacataatcttcaattgcttctattgcttcctgaagtacatcaacagattctttgtcatcttccacaacacacgaaatctgaagttt

atgtattccataatcttcaattgcttctattgcttcctgaagtacatcaacagatactttgtcatcttcc

>7440|AGAP004235-PA

catcaaaacttgtacctcttgcttatggaatacataaacttcagatttcgtgcgttgtggaagatgacaaagtatctgttgatgtacttcgggaagcaat

agaagcagttgaagattatgtccaaagtgtggatattgctgctttcaataaggtttaaccccttcagaaaaagtgccaacacaagatcatccatgtgatt

ttaatgatatgagaatttgtaccagtttgatagatttcaatttatggctttcctt

>7592|AGAP004261-PA

atatccatggattgatgctgtcatgactcaattgagaacagaagggtggattcatcatctagcaagacatgctacggcttgtatgctcacacgtggggat

ctttatcagaattgggag

>7591|AGAP004261-PA

attctctacaaaaatgaatacaatgaaaacaattccactcagaaaatttgttggctaaatgaaaaaatgaaaaacaaaagcaaaaaggcagcatgcatag

ttttccgtggtttctctaacatcacaccagacgaatggcgatttattcctcccacacgaccaattcctttgcccatccagtaacaaggtgttttatcgtt

tctaataattccgccgctctcaacgataagttacagtcggatactcctccacttgttaatgccgacattagatgaacccgcgaaccaagggtccaggatg

aaaatacaccgaaatgtcgtcgcacctatgagtcccgcacgcagactagggttgtcatgtagacgaagtccccttctgaaccaatgcacagtgtgttcct

gagtggttttcgcacacatagttgatttatcccggacactgattcactggatgcgaagcgaagctcatcttggcattcttcacgaacggcgggcagttgt

cgacgctgcgtttgctctaacaaaagttgtgacgcgggtcagggacacagctgctcgctgccaaacgttgaacacttctcgaaacgtacgcggtgggcgc

ccagtggcgggcgaagtcagcacgctgcgtgca

>7590|AGAP004261-PA

ccgtgcataacacatgaccacagtttacttgttgtatctttacaaattaattaaattttttgtgacacatcaaaacactgcaacctagtgctctgatatc

catgtctacaatataaaaaaggaaagactgcttttatagacaaacaactaaactaaaaacaatatacgatggtccatgcattaatgtccagatatgttat

tacaaactgcctagttgtttgtcacaaagggaactgaaatcattcctgatagataagtgcatatactccctgaatgaatattaatattaaactaaattga

aatctttttatattctgttctgtaaaatctgaatatatacatataataattatggtgtatcagacaattttgtggtcttattaagtacactgacatctgt

attatgaatatataaaatgtgtgtattctctgtttgtacaaaacatgtaaaaaaattactatggagtttgcaaaagtcatcagttggtggctacatgcaa

aaaatacaataaataaataagctactgctcaacagttcctgtatacaatgaattcttttctaggcacattacattgtactctatgaggaattttccatta

tgattatgacaatttaattactaaatgttactttctttgcatattttaaaaatatgagatgattttcctgtgcataatatgatttaaataagaaaatatg

tagaaaataaattcatattatattggaaagacattcacagtaaatgctatgactactggtagaaaacacatacaataaaaggtaaatcataatgttgctt

tataaaagcaaaattgctacaattaacattgtgagaaatgaacatgagtaacaaatcaagacaaaaatcttgcatcactggaaatatttttaaaatagtt

ttagttaaaaaatgagaaatttgtgatatatttacactctatatacaatgactggaattcagtgaaatgtctcattttatactgattatttcccacatat

gtcctcacattcagtacattatcacagactataatttaacttcttaaaatgtgcacccaacacttcctgtaaggcctactaatgaaagacttctgtttca

taacacattagttttatgggatttcactggttactgccaatgtaaagaatcacatgtttctcctgcaaaactcccagtcacattttgtttcttgcggtgt

catctgctgttgagtttcaacatacatactgcaatcattgtttgcactcactatcctgttttctggtgtagttttgtcctctttgctgtcgtattgacag

cccttaactgaaggtgatcggattgtaggaactgttgccaaaagacctaaaaccttacacgcgcttgaagtaatatcttccttgcctgcaaaattgttga

aaaatttcatttaaaacagtggttgatctgctgccatgaaaattttgctggacctcgatatttgcttaactgttgatacacttgcttcattcgttctatg

tttattcgacttgcgtattggtgatccactataggcaaaggaaactccttcccaatgatgcacttcgctgttatctgtactgattcaggggctttccaag

gttcatggatataccttgttggaaagttcttcaaaatgggcaggtacttccttatgaagtccccatttggatctgccttgcgaccaaaccggacaggaca

ataacaatggaagaactgttgaaagaaggatgagcaagacagccacatccacattccagcattaacagaccagtcagcatcaagcagtagttcatcaaac

accttcatgccttcttcccatgaaatccacaaatctcctcgtgtaaggaaacacgccacagcatgcctagctaaataatgtatccaaccttcttcacgca

actgtatcattatagcatcaatccacgggtaacctgtttggcctgtggcccacttcgcaagtgcttctggattcctgtcccatggaatttgtacacatat

tgggtttccatacattttatcaaaatatggattttttgttgctgcacagtaaaaaaattcccgccataacagctgtccgtgtaaagaaagaggcgggcat

gccttttttatctttttgtaaagatcagtaagctggtaatagaaaagcctcgtagacaaacagccaaatcttaaatacggactgagacctgtctgactgg

caagcaatgactgtgatgtcattcttggtcttccaaaggatgccacccatgcctttctttcgaggtgcctttccagccttgcaagtgcctcagattctcc

tccctgccacacaggaggcatcaagccatcagtatcaaaacctaattcctctaaggtaggaacaccaaatcgttcgtcatggtcatcagaaatgggggta

aatttatctttcacgcatgctggtgtgatactgggttcagctggaggtggtgagtccatggaagccactatattttgaaactggtggtaagttaaaggtg

ctttccctccatttttttcaatgatcgtatcaagactgtacagtgtgtgtgaagctatggaaattactgagattcccatttctctgcacattgccattat

gttctgatcacgcacttttccaaatggttcaggatcttcctcaaaggtcaagtttgttgtcccccattccttaaacaattttggaagggcatcagctggc

tgccctctaatgacgaacagccgagaattgagcttccgcaagctagaatcgaggtcttcgagacactggagaagaaacctccacttgttaatgccgacat

tagatgaacccgcgaaccaagggtccaggatgaaaatacaccgaaatgtcgtcgcacctatgagtcccgcacgcagactagggttgtcatgtagacgaag

tccccttctgaaccaatgcacagtgtgttcctgagtggttttcgcacacatagttgatttatcccggacactgattcactggatgcgaagcgaagctcat

cttggcattcttcacgaacggcgggcagttgtcgacgctgcgtttgctctaacaaaagttgtgacgcgggtcagggacacagctgctcgctgccaaacgt

tgaacacttctcgaaacgtacgcggtgggcgcccag

>7806|AGAP004263-PA

ctcggacagcaagtacgacctgagccgcgcctacattaacctggaaggtctggtcggtggcgacaaggctctgagtgacaacacaaacaaaatccttaac

gaaaactggcaggacgtggcgaaggaaattgg

>7805|AGAP004263-PA

cgtcctgccagttttcgttaaggaatttgtttgtgttgtcactcagagccttgtcgccaccgaacagaacgtccaggttgatgtaggcgcggctcaggtc

gtacttgatgtccgagcgcacaggcttaatgtactcctttccgtcgtctctcttctccagctcccagtggaccgtgacggtgagg

>7853|AGAP004268-PA

tcatgacctatccagtgttcaacattgcgcttgcgtccagtgaaaaaattatcaaccacaataacttcatgacctatccagtgttcaacattgcgcttgc

gtccagtgaaaatattatcaaccacaataacttcatgaccagtcatcatcagttcatctaccaaatgtgacccaacaaatccagctccacctgtaataag

aattctttttctcagtttatagttcaagaaagtaacatctggatatctctgtggaatccttgcttctagcttcttaatctgagcttccaattccttgatt

ctctgctgagcttcaagaatatttttggcggcatgatactgttcatgggaaccaccgacatccactggtgacagatgcgtaacatatttgctatcagcgc

tgtgcaatctggagtatgcgtgctcatatgtgtatagttcttcactgtgtacccaatttttatagaaacacaaaagtataacaagcacaatgacacaaag

caataattttttcacatttctattaataatgttcatttcaaacaatgttcaccattgtgaccagtgacaactgcactcgaagagcag

>8203|AGAP004274-PA

ctttattatatagtaaaagtgtcagaggtggtacactatttttatttacacttgcatcagctgaggctgatctggaaggccgttgacataagttgaaaga

gctcatacatactcaaacagtgggcctcacaactatatcatttgcatcactgacttggtataataagcctcacactcaggtacaaaaatgtggaatttaa

agtcataccgcttcacaacatatcattccatttattcttctattttagtgaacagaacgatgtggtgatacagtgctccagacagaataaggagcctttt

aaaatttcagtacagcaattttcactcaggtttcccgatatacctaaattacggtttgtaacctctgagatgattccttcatccacctttgcaaaaactg

ttcaagtggcccaattcttgaacactgttacacctcacgtaattaaacgattaaaaaaaacaagagtgtaacctcttctttgtacaattaacagaaacag

tttacactctggtgagactgaaaatttttgtatttcaaacatcacaacagctatgatctaacaggaagggaaaaaactgaacagctatggtacataagta

aaagagcagctccatttgtattaataaaaagctttatgcacgagtaaaaaatcagaagatattagacacatcttcacggaaagtaaatcataaagttaca

cctcaaataaaataattttgtaattgacagcttgcagtagagaacacagcattgccagcgcgcagccagccatgcttgacaaggctatgcatgtacttat

aaaaacaggcaacgcaacaagcatgtggcacttgagacagtgccggcaggtagccctgtctcttgactcactgaatgtttccatcctttctgatgccctt

tatccaatcaatgatggactggtactgctacggtacttcaattttctcgcagagaccaaccatacctgtatacaggctattattgtgatggggtctccac

tcccaaaggtatttgaaagttaatgccagcttccccacacaggcccttccccctcagaggaatccatgtatccccatctgtctgtatctagtgtaatcac

atgatgaaatgtgacgccatttttcaaagtatttgttcatcatgtatctgaggtgggatgtagtccattatttacctaggaggatatggcaaacacctaa

aaaccatatccaggctagtcagcgtaccagctcccatcgtcagtctgtgaagcagattcgattcaatccgtgaagcggactcgattcagggcttgaacac

atccactgttccggaaatggtcactatggtggcaggttcactgactaaatcatcaaacataaagctattttagtcaaattaaaaaatcaattttgttgtc

caaattctgaaaaaagagcaataactgcaaagacagtttagtcaaattacagaatacaaatacgtaagttgcaacatcccacataacacgaagatatctg

gatctcaaagagacacaggcaaacaacttggccctaaatgaccaaaatgtttcagcttcattgacccacgtggatgtaactcaagctatgaccaagttac

agcagtttctaaacaaacttcaaaacaagaatctcttaaaagacattagattccatatacaaactgattatatgtattgaatgaacaaattcaaacgaag

cccaatgtggtacacagctacagaaactagagttctacatttcaccacaaaacaatattatttttgaaatgtacagaatctcatgtcagattctgagatc

atacactgaaaaagcaatttctcaccaggttaaatatttgttgagccgttgaatactaaagcaaatatgggactcagtaaaaatctcgaaatttggacgg

tgtgtaaaagtaatttactctaatgccactttattggcatttccatatacaataattattttttttttcatgttttaaagaaaaccacgtaagttcttgg

caatattcacaaatcactcctcgatctgtgtgcttctgattttttactggacactgcaggtaaaacattcttcgatttaggttctgatgtaatgttcttg

gatttagattctggtgaatcattcttggatttagatcctgatttttttgatttggaatgatgtttcttggatttctgacattcattacctgtgccatttg

ctggctgctgtgaggaaactacaccagtgtcagtatcactattgctgccactgctactggaggtgctactgctactactactactgccactgtcgctctc

ctctgagtcactactgtcactgtcgctactgctatccccactgctcttgtcactgccactacttgagctactgctactactgctgctgcttgtgctgctg

ctgctgctcctctcagtgctgctactactactgctgctgctactgttggaatcgttctcctccgacgaactttctgacgatttttcggtatcttgtttag

caggtttgtcctttttcttttttcttttctgaactttttcttctttcttctgagtattttcttctgaatttgtttcagcattttcagtcttttctttaag

tctttttcttaacactgctgttctcgatgatctgtggaggtacttgcgcttgcccttacattcgtatgtccagtggccatactcgaggcatttttgacac

ctcactccctggggataagctgcagaatctttccgctgctgaatttttttgtgcttcagtaattggtgacatttctgttataatggcaatcaatcaactc

ctcattctgaaacagcagccccaacacgaattaagggcggactttgaaaccaaatttctacgtttctctgcatgactacctctaccactaaaataaataa

acaaacaaaaagttaacaacagttccgacgccgcgctatccaatatggcgcctcaatgctt

>8607|AGAP004309-PA

gcaaagcagtcaacaagtaacctattgtgaagaaatactgacaaacaacacctgcaaacagccttttgcttggaccaaccatttccattgctgtctgaaa

gttcaccgaagattattgaaccaagtaaaacgcctaccatgaaaagtgcatcagcagttgctctcaaccacgcatttttgcaaaccaggtcaaactcagt

tacgacactgctt

>8609|AGAP004309-PA

ctagctggcgtgtttctgcaagcaaaacctaatcacagatgttggcttccttatgaagaaaacagtgccagctttaatttatcagaaagagaattaaacc

tgagtatcccaagggatgaaagtacaggaagctggtcatcatgttacagatttgatacaaacttttcaagtgaatattttgaaaacaatattccagcaaa

ttgcactgttggctgtgacaagtggatctatgatacaacagtttacaaaagcagtgtcgtaactgagtttgacctggtttgcaaaaatgcgtggttgaga

gcaactgctgatgcacttttcatggtaggcgttttacttggttcaataatcttcggtgaactttcagacaggtatgggcgtaagataatcttcttcatct

cacttgttttacaagttatttg

>8608|AGAP004309-PA

agtggagcaacttagaaaggagagcaacattcattttcttgcacaaagagtttctacattacaaccaattaaagtcattcacagcagaccctttcaaaac

acaagtggttgtaatgtatttgaatgtcaccacattagaccatacgtctttcgtcactttcataaacagttttacttgcaaataactatccttgcatcac

atattctgaaatatgtccaacagctcagatttctgaaaacactaaacactagtttgtacaaaaatattgctgcacacataaaaatatattcacacaaatt

tgtttggcagtgtaaaaatactgttgaatttgtacttattattttgatgctataacaataacagagccagttttcttcacaacatcttccagatcttctt

cattctcctttccaaagtcttctccctcttccaaggtgtcaggaagttttctatttaatgtttctgggagtaggagggccagaaagcctccaacagaaat

catagcaccaaataccaagaggggcaaaggttgccagaattcagcctgaaataaataataggatcatttctagatatttcagaatattataaatgtgatt

agcagtatctacttttaaacaccactgtgtccaagatcagcatcagtttcatgcctcattattctcgatcattgtaagagaaagatcatgagttactgct

aatcttacagatctttttctttttgtaatgtcttccttcatttgtcaaaataggatatataatgtgatctcccaggtttcctcaatattcacaatatttc

aaagactgacttagccatctttttcgggtgctgtgggatttattgtctgcttagattcc

>8611|AGAP004309-PA

cacaaataaaagttttattatgcagtagcttaataaaatcacataaactgttattaacaacattgaatacaaaatggaaatgtatatttaatttctcaat

atccactaaaaactggtaacaaattcacattcctttttttccctttcagctgtcaattttaattggagttcttgattcactcataacaacaccattattt

tcttctgctctgtggaagtcccgaccaaatctctctccctcctctatggtgtctggcagtttccttcccattgtttctgggagaactagagccatcaaac

ctcccatcagtgccaaagaaccaaatattagcaatggaaaaggttgccagtactcagctagaagattgatgtacggtgctgatatgcctcctactcgggc

acatgttgaactagccccaagtgcaacattgcgtattacagttggaaactgctcagtagaaaagacatagactgtgccataagatgctgtaatggccaat

tttccagtcatagccagcaggatcatcaaccaagacattcttgatggaactgcaattgtgagcaacagagcaagacctccagtgagcatgcagccacaca

gtatggttttgcgaccccatctgttcaatgtcaaaagtaaaaaggtatatgcaggaacttcaacggctcctgatatgaaaaaattgagatagtcattgcc

tcccaagttagatgtgttccaggacaggccatagtaagtgccactgttaacaaaccagttgaaaaaaatattcagagtctttcttcgcaagttgggatac

aaaaataaatcaaaaatagaaggcttcctttcaggctggcttccatcataatggtcgtttgcacaagacaaggc

>8613|AGAP004309-PA

ttggtttcttctctgacgatggtttctcattttcttcagacaacaggccatcaagaacatcagtaggaattttaactttgtttactttagcagcttttac

aattagctgttgtgcttgttctttccttccattggacaaaagccagcgcgtagactcaggaacaaaccaccagtaggacagaaagaatacagatggcagt

gtgaatgctaattgcaacattctccaatctggtatgaggtaagctaacaaagctgtcagtacatatccaaatgtgaagaaaaactgacatgccacaccag

caaaaagccgcctagatggtccaaccatttccattgcaataacatagccaattaagaaaactcctgaagcagtcatgccaacaaccagtctgaatatcat

gaagaaaatatactgtggtgaaacagataccagtattccaccaagaatctgtagcaccaaagcaataacaaatatcggttttcgtccatatctatcagaa

agaaaaccaaatccaaatgagcctatcatgtcaccaagcataaacattgagtctgctgtggcacgtagccaggcactgtcacatactaagtcccactcca

caatcgtgctgctcttgtaaatgctgtggtcgtagacccaggaatcgcatctcacactgcggttggcaggaatatttgagctgaaatattccgcactgaa

gttggcatcaagcctcctgcatgaggaccacgcttgttgctcactgtcccaagggtagctcatgtttattacgccttgaggtaactgatatgtagctgtt

tcattttcaaaaggcaactgacatctgtggtccactttcgcctgaaggaagacacctcccaacttatggagggcactcacaattgctgtcaggcacagca

aaaagtaaatacgccgctggtagcggccaaattctcccaagtatgggaagatgtcatcaaaagccatttttcaccgacttcttgctttccaatctgaatc

ttagctgcaagaggagagagtgcaaatccagtcagttcgtgacgcaacaatggagcgcggcgctctagcacggaagctggtgggccaggcgttgtgtgac

gtagccgacccgcgcgagcagagggcagtgtgcaa

>8615|AGAP004309-PA

caacatgctccacttggggggtatctctgtgggattcctctggatcccagggcatgttggtatctgtggaaacgaggcagccaatatagtggcaaaggca

gcagtctgtcttcctcagctcgctgttcacatggttccctttgctgacctacagagcattgtgtctcgtcacgttgctcttttatggcaacacattggtc

tgcacttcctgataataaattgcgggacataaaacttcctccctgtgcttgtagctcttccctgtgcttgtagctcttccttctggccttgtcatcggga

ggaggtaatttaactggactccagatacggtactgtgtatttagccatcgacatcttttaagtggcagtcctcacccgctttgtccccactgctctccat

tgttgacagtgagatgccttttactgcagtgcccctattttaattggctatgcacccatttacagctgtcactcgatatatcttccctttaagcagatta

cacacactcagcagattatgtcctatatcagtgattcgaagctcttttttggggaaaacaatctcctcttcaatagcagttttcttggatttccattcgt

ttttagtttccctatttcgctcctattgctgctgatttaaaattcggttttttacccttcactaagccacagaatgggtgcttatgactgtagcagtttt

gtgccctaaaactataaaaaaaatcactgcatggaatctaagcagacaataaatcccacagcacccgaaaaagatggctaagtcagtctttgaaatattg

tgaataaaatggaaattaaaccaggcagaacacctgaaaatacactgttaatcagtcagattgaggaaacctgggagatcacattatatatccaattttg

acaagtgaaggaagacattacaaagagaaaaagatctgtaagattagcagtaactcatgatctttctcttacaataattcagaataatgaggcatgaaac

tgatgctgatcgtggacacagtggtgtttaaaagtagatactgctaatcacatttataatattctgaaatatctagaaatgatcctattatttatttcag

gctgaattctggcaacctttgcccctcttggtatttggtgctatgatttctgttggaggctttctggccctcctactcccagaaacattaaatagaaaac

ttcctgacaccttggaagagggagaagactttggaaaggagaatgaagaagatctgg

>8612|AGAP004309-PA

ggtgctagctcaacatgtgctagagtgggaggcatacttgcaccatatgttaatcttttggctgaacactggcagccactacctttgctgatatttggtg

ctctatctttggcagcaggagttactgcacttttacttccagaaacaatgaatcagaaattaccagacactatagcagaaggagaaaattttggaaagta

agaacaattgatccagttttaatgcacctataagtgattgatgtgcaaaatttgtgaatttctttacatattgcagtccttttattttgcctgaatgtta

tctgtgtaaccatttctgtcataaattgaggcactctacaagtcacatactgaggtacactttcaacagttcttttcagccagtgcgaccttggcctact

ttgtaagtattcagatgatgttctaagagttcaagaaccattttgacatcacataacctttttgtatggcttcattgaactaggataaaaatgtgtgttt

ccaaatacttatcagaaattgaaagaaatgtatttttttctttttaacaaaatcttgaagtagtattcctcttcagtaatgttgctgtggtaacgagatg

gctgtactccccctgtcatacacacagtgctctccataataacagataatgacttgcaggggtgttattctcttaattgcaagtgctaagcaacaaaatt

cacatttcattatattttttaagtgatttgcagttcatgttgacagctgaaatttgtgtattttaactgaggttaactatgagttacaaaaacgtcagag

cagatttgtacatgatgaatctgaccattcccttgtcagtaacaggtcacatgtcacttgtccatttttttaagccttgtttttcaaccattgagaggtc

ttctttgtgaattcatttattgtctaagtctcagagctctaggtcagcatatctaaactgtcatctacatatttctttccttctttgatctgaaggccat

tgaatgttcccaaaaaccttgccatccttgtttgatatgaaataaaatttcatgtcttacattacactttatacttatgagctagtaaagataggtacac

tcattgacattttctataatcatattgtcagtgcaaatatttcctgctctctcccatgtgctggacataattttagttagatacttccacatagaattgc

atggtaatgtggctgactagaacatcataaacaacaataaaagtaaagcttttagattttctgtcctgtttctccctttttttgtggcaaaggagaaaat

gcgagacttgatatttttcattactttaattttccatcatttatactttcatgttg

>8606|AGAP004309-PA

cttggatttgatttcttgccaacgttatgttaggccattatcatttcacctgcaacacatttctcattgaattaattccattattgtgtgtgtgtttgtt

ttttttccctgtgtggttgggtcaagtacagttcagaatttgcagcagctgtctggatcaaatcatctcagattttccatcgctcataattttcaattat

cttcactatattttacatctgcagaacagtcattgaatgaatagttgtcatttacatataatgtgaggtgttgtttgatgttgtttctcatattccaatc

agttctttttcaaataacatgaaaagtccctataaaaacaagagttgatcacatttttcttaaataagaagtgtttctaaatggacaaaaatttctgtca

aagaactttataactaagttcagattagtgcaaaataacttcagacataatgtctgtaattgcctttgatattttttgctcaatagacaactgaattttg

ttaaggtcctgtggtgtaactatgaaggatggtatctgacataaatgttttcttgcttttcagtgggacttagtatgtgacagtgcctggctacgtgcca

cagcagactcaatgtttatgcttggtgacatgataggctcatttggatttggttttctttctgatagatatggacgaaaaccgatatttgttattgcttt

ggtgctacagattcttggtggaatactggtatctgtttcaccacagtatattttcttcatgatattcagactggttgttggcatgactgcttcaggagtt

ttcttaattggctatgttattgcaatggaaatggttggaccatctaggcggctttttgctggtgtggcatgtcagtttttcttcacatttggatatgtac

tgacagctttgttagcttacctcataccagattggcgaatgttgcaattagcattcacactgccatctgtattctttctgtcctactggtggtttgttcc

tgagtctacgcgctggcttttgtccaatggaaggaaagaacaagcacaacagctaattgtaaaagctgctaaagtaaacaaagttaaaattcctactgat

gttcttgatggcctgttgtctgaagaaaatgagaaaccatcgtcagagaagaaaccaatacctgagactaaagtaacaaaaccatcaattttggacatta

tgcgatatcccaatcttcgtaaacgatgtctcaacatattcttcaactggtttgtcaacaacagtgcctactatggtctttcatggaacacatctaacct

ggcaggaaaccagtacttaaatttctttatttctggtgctgtagaagttcctgcatatacttttttactgttcaccctcaataaatggggccgaaaatca

attctgtgttcttgtatgcttctgggaggagtatctctgctagctgctctggctgtacctggagagatgacatggttactggtgacatttgcaatgatag

gcaaaatggcagtaactgcttcatatggtacaatatatatatttacagctgaacagtttcccacagttgtacgaaatgttgcacttggtgcaagttctac

ttcagcacgagctggtggaatgactgcaccatttatcaacttactggctgaattctggcaacctttgcccctcttggtatttggtgctatgatttctgtt

ggaggctttctggccctcctactcccagaaacattaaatagaaaacttcctgacaccttggaagagggagaagactttggaaaggagaatgaagaagatc

tggaagatgttgtgaagaaaactggctctgttattgttatagcatcaaaataataagtacaaattcaacagtatttttacactgccaaacaaatttgtgt

gaatatatttttatgtgtgcagcaatatttttgtacaaactagtgtttagtgttttcagaaatctgagctgttggacatatttcagaatatgtgatgcaa

ggatagttatttgcaagtaaaactgtttatgaaagtgacgaaagacgtatggtctaatgtggtgacattcaaatacattacaaccacttgtgttttgaaa

gggtctgctgtgaatgactttaattggttgtaatgtagaaactctttgtgcaagaaaatgaatgttgctctcctttctaagttgctccactaccaatgat

agtatttataataaaattaactttatactgcttttgtatattcattgtatataattgccttatataaagaagacttgtaaattgttctgtgtatgtttgc

ctctatttatatattttttactcaggcttataaaaagtacagaatgctcatgtgtgatattagaaagtaattcaatgttgatccaatatcacaatgtgtg

atacatttgcattacaatg

>8610|AGAP004309-PA

acacacacacacacacacacacatctgcttcttagtcataagaaataaaatactgacaagcagttaacaaatgaggaaacataaaaagtaatgtgtaaag

ctactaatatttcagaacttgatagctctattgtccagttgaatactttctattaaacaagaaattttgagcaatgtgtaaataaaaataaaatgatatt

atgagtcaaaactactgtaatttatcagtttgcactttgaaatcattaagctgttgcaaatatgcattctcaagggcaatatggaataaggattgttctg

aaacgacatgaggcaatacagttagctgttggaaatttttctcacaaagtgcaagaacctacttgaagctatatgcctctttgctttcctatgccttctt

aagtggctcatcgagattgttaatagggcttctaaattatacatgatatctgaaccaggattagattctgtgtgcataggtttgacctaaaaggtaaggc

catttttaaagaaatgcaacataaacatgtatagtctttaatttcaccgacactggtgtgatatcctctgtcttctgttcttcactcttaacaacacaaa

caattaaagtaagggtttcattgatgtacagcatgtatgccatacactgcgtctcagatgctctcctttggaacttcatctatgtggtcaccaacccttg

caccagaaagaggcttttcttgttcttccatatcttcaattgtatctggtagttttcgatttagagtttctggcaatgtcagcgccataaatgctgcagt

gatagacactgtaccaaatattagaagcggtaatggtcgccatataatattcaaaacattgatgtatggagcagccatgccccccatgcgagcacatgtt

gaactagctcccaatgctacattgcgaaccacagttggaaactgctctgctgtgaagatatatattgtgccatatgatgcagtaatggctaattttccca

gcattgcaaatgtgaccacaagccaagatatgccttgtggtacagcagttgcagcaaatagtgacactccagcaattatcatagaggagcacagaatttt

ctttcgcccccatttgttaagagagaataaaaggaaagtatatgctggcacttccacaaaaccagctattaaaaaatttatgtaatcattgcctcccaga

tttgatgtattccaagaaagtccataatatgttgcattatttgcaaaccaaataaagaaaatatttagtgttctccttctaagtttactatgcttcatca

tatcaattatagaatatttctgagagttgttttctgactgaactgaaccttctcccattatggctttggtcagcacatcatctggaatagttactccatt

ttcttttgcagctttcaaaatatatttccgtgcctcttcctgttgaccatttgctattaaccatctaaatgattctggaataatccaccagtagcttagg

aatatgagagatggcactgtaattgctatttgtagtaatcgccactctggtataaggtatgcaaatagtgcagttataacataaccaaaggtgaagaata

actgacaagcaacaccagcaaataagcgttttcttgtaccaaccatttccattgcaattacgtatcctacaagaaatagccctgagacggtgcttccaac

aaacagtctgaatataataaaccatacatactgtggtgaaaatgaaaccagtaaccctgcaattacttgcatcacaacagctaccatgaagattggcttt

cttccatacctgtcagaaagcagaccaaaagtaaaggatccaataacatcaccaagcataaacatggaatcagacatggctggtaaccatccatatgtac

acactaagtcaaactccattattgtactgctcttataaatacttttgtcatatatccactgagtgcaggtcacactttcattagatggtctaccactgtg

aaaataatcttctgtgtaatttgcagccaacatctgacatgatgaaaaagcatattccctctcatcccacgggtatgacatatttagtatttctggaggt

agagtgtacgatgctgtttcattctcaaatggcagctgacaccgataatccacgctagcttgcaaaaaaactcctacaagtttatgtagagctcctgtaa

ctgctgtcaagcacagaagcacatagattctcttctgatagcggccaaagtgtccaagatatggaaacaggttttcaaaagccataactgtctattgtag

cttgcagataaatctttacggcgtcgatgaaacggctgatcgaacgagcccccaccgccacaaccaggaaacgcactccgacgacctgcatgacgccaag

aactcgctgagcgacttgacgcg

>8616|AGAP004309-PA

cattgtaatgcaaatgtatcacacattgtgatattggatcaacattgaattactttctaatatcacacatgagcattctgtactttttataagcctgagt

aaaaaatatataaatagaggcaaacatacacagaacaatttacaagtcttctttatataaggcaattatatacaatgaatatacaaaagcagtataaagt

taattttattataaatactatcattggtagtggagcaacttagaaaggagagcaacattcattttcttgcacaaagagtttctacattacaaccaattaa

agtcattcacagcagaccctttcaaaacacaagtggttgtaatgtatttgaatgtcaccacattagaccatacgtctttcgtcactttcataaacagttt

tacttgcaaataactatccttgcatcacatattctgaaatatgtccaacagctcagatttctgaaaacactaaacactagtttgtacaaaaatattgctg

cacacataaaaatatattcacacaaatttgtttggcagtgtaaaaatactgttgaatttgtacttattattttgatgctataacaataacagagccagtt

ttcttcacaacatcttccagatcttcttcattctcctttccaaagtcttctccctcttccaaggtgtcaggaagttttctatttaatgtttctgggagta

ggagggccagaaagcctccaacagaaatcatagcaccaaataccaagaggggcaaaggttgccagaattcagccagtaagttgataaatggtgcagtcat

tccaccagctcgtgctgaagtagaacttgcaccaagtgcaacatttcgtacaactgtgggaaactgttcagctgtaaatatatatattgtaccatatgaa

gcagttactgccattttgcctatcattgcaaatgtcaccagtaaccatgtcatctctccaggtacagccagagcagctagcagagatactcctcccagaa

gcatacaagaacacagaattgattttcggccccatttattgagggtgaacagtaaaaaagtatatgcaggaacttctacagcaccagaaataaagaaatt

taagtactggtttcctgccaggttagatgtgttccatgaaagaccatagtaggcactgttgttgacaaaccagttgaagaatatgttgagacatcgttta

cgaagattgggatatcgcataatgtccaaaattgatggttttgttactttagtctcaggtattggtttcttctctgacgatggtttctcattttcttcag

acaacaggccatcaagaacatcagtaggaattttaactttgtttactttagcagcttttacaattagctgttgtgcttgttctttccttccattggacaa

aagccagcgcgtagactcaggaacaaaccaccagtaggacagaaagaatacagatggcagtgtgaatgctaattgcaacattcgccaatctggtatgagg

taagctaacaaagctgtcagtacatatccaaatgtgaagaaaaactgacatgccacaccagcaaaaagccgcctagatggtccaaccatttccattgcaa

taacatagccaattaagaaaactcctgaagcagtcatgccaacaaccagtctgaatatcatgaagaaaatatactgtggtgaaacagataccagtattcc

accaagaatctgtagcaccaaagcaataacaaatatcggttttcgtccatatctatcagaaagaaaaccaaatccaaatgagcctatcatgtcaccaagc

ataaacattgagtctgctgtggcacgtagccaggcactgtcacatactaagtcccactccacaatcgtgctgctcttgtaaatgctgtggtcgtagaccc

aggaatcgcatctcacactgcggttggcaggaatatttgagctgaaatattccgcactgaagttggcatcaagcctcctgcatgaggaccacgcttgttg

ctcactgtcccaagggtagctcatgtttattacgccttgaggtaactgatatgtagctgtttcattttcaaaaggcaactgacatctgtggtccactttc

gcctgaaggaagacacctcccaacttatggagggcactcacaattgctgtcaggcacagcaaaaagtaaatacgccgctggtagcggccaaattctccca

agtatgggaagatgtcatcaaaagccatttttcaccgacttcttgctttccaatctgaatctcagacacggcaaggcgcgtacgaaacttcaagcgtaca

agtaggcagtaatttcgggcatcgagcgtagagacacgctgtcgtcctgcttcggtatcttatcagagcaggagcgaatactgtgccctgtgcagctcgc

tgggagacacctgtgagcttttcttcgccagataggcccctg

>8614|AGAP004309-PA

tcacaacatcttccagatcttcttcattctcctttccaaagtcttctccctcttccaaggtgtcaggaagttttctatttaatgtttctgggagtaggag

ggccagaaagcctccaacagaaatcatagcaccaaataccaagaggggcaaaggttgccagaattcagccagtaagttgataaatggtgcagtcattcca

ccagctcgtgctgaagtagaacttgcaccaagtgcaacatttcgtacaactgtgggaaactgttcagctgtaaatatatatattgtaccatatgaagcag

ttactgccattttgcctatcattgcaaatgtcaccagtaaccatgtcatctctccaggtacagccagagcagctagcagagatactcctcccagaagcat

acaagaacacagaattgattttcggccccatttattgagggtgaacagtaaaaaagtatatgcaggaacttctacagcaccagaaataaagaaatttaag

tactggtttcctgccaggttagatgtgttccatgaaagaccatagtaggcactgttgttgacaaaccagttgaagaatatgttgagacatcgtttacgaa

gattgggatatcgcataatgtccaaaattgatggttttgttactttagtctcaggtattggtttcttctctgacgatggtttctcattttcttcagacaa

caggccatcaagaacatcagtaggaattttaactttgtttactttagcagcttttacaattagctgttgtgcttgttctttccttccattggacaaaagc

cagcgcgtagactcaggaacaaaccaccagtaggacagaaagaatacagatggcagtgtgaatgctaattgcaacattcgccaatctggtatgaggtaag

ctaacaaagctgtcagtacatatccaaatgtgaagaaaaactgacatgccacaccagcaaaaagccgcctagatggtccaaccatttccattgcaataac

atagccaattaagaaaactcctgaagcagtcatgccaacaaccagtctgaatatcatgaagaaaatatactgtggtgaaacagataccagtattccacca

agaatctgtagcaccaaagcaataacaaatatcggttttcgtccatatctatcagaaagaaaaccaaatccaaatgagcctatcatgtcaccaagcataa

acattgagtctgctgtggcacgtagccaggcactgtcacatactaagtcccactccacaatcgtgctgctcttgtaaatgctgtggtcgtagacccagga

atcgcatctcacactgcggttggcaggaatatttgagctgaaatattccgcactgaagttggcatcaagcctcctgcatgaggaccacgcttgttgctca

ctgtcccaagggtagctcatgtttattacgccttgaggtaactgatatgtagctgtttcattttcaaaaggcaactgacatctgaatagaaacaacaacc

ctttatgtacaaatgcttatcaaagctatggaaaatatttgatgtttataattagcacatatggctaattggtgatggagttataagatgtaatctgtag

gtctgaagaagccaatctgttgatgacaaacaggtttaaaatttaaattgctaacatacttaattgttgcattattagttgcctctcacagtatgctaga

aaatattcctgttaactgaagacatatatattccagtaacaaaacacaagaagtgtggaggcctctgaaaacatctgtattgtgtacttattttgtatgt

cttatttttatgtgtaaagcatcacatctgaagaccaaaatcaatccttatttaactatggttagatcattcctgcgatactgagaagctatatgttata

ttggtatgaattagccttgaaaaaaaattacacatacttaaaacaaagagatgttaaaataatgtatttagacaagcatgggatgaagaaaaacaaaaca

taacattcaggcatagtgttgtcctcagcacttacaacacaaatttttattatacatgtccacctgtatgcaatacaagttaaatttgactgaaaaatga

aatataacttagaaacagactttgaatgttgtcctttgcctgacattctgaaagcattgtacagagttctaagaaagcagtgggaataaggtgaagtatt

ttgctaaaaagaacaggaagctggacaccagaaacttttgcctcagatctgaaatagtttccatgtactacatattaaattataaagtgaataaatgtga

acaacactgaaaccacagcaatttatagaaaacatactgaatctcagaattaatgacttaacttcaatttagattaataagggacactttttatctatat

aagcaacactgctgcagagaaaaagtcacatgcagagaagagaatcaaattaagtcactcatataaatttctattacaatggtgtattcttgtaacagtt

ttgaagtaatttctctgaaagcacacacaaagcatttattatggtatgtttagaaatgttatgaagacacctgctatcaaatgccattgcatgcacaatt

aaaagacgaatatgttacccccacacaaagcagcttggcaataattggtttggacatttgtgaagcagaataactcagaagaccgcagtaatcaacagtg

ccaatgcaacacaacacacatcacaatgagcaacataattaaacaaaatgcatctttctttaccaattttcactgtgagttagctgagtgtaaccagtag

tacataaaaatatcaaaattcctctcttctataaacagaaacaagcctgatgttttcctggattaagggcataagataattcattcctttgtaaatatta

gtgggtgctgttgtacaattaagaaatcacacactattaaaaataacagaacaaacatattaaataaaaacctttaaagaaaaatcacatataacagcct

gcaaacacaaaaccctaaattattcatagtagggtagaaatccagtggaatgctgcaaggctgaataaaaatcaaataccacatatgttcaacatgaagc

atgcacttccagttaaataattactgccatacagtaaatatcaactgtttcaggcactggaaaggtaatgcaggtaacttcttgtgatatgttttcccag

atagtagttctgaaaatgtggaagcatatacagtgctttgctccatattaataatcagaaattctcacagagaaaaattctgttctacaaatgatttttc

tggaaataaaacaaaaaagtaatgcagaacattgaactgctttgtgttaaataaatc

>8824|AGAP004323-PA

tggtgggctgcatcaaaccagtcagaagactgatgtctcaaaaaattaagttacaaagaaaaaataaatttacttcctcagctctaatgtgcattgttaa

ctcgttattcttgccattgtaattttagtgcacacatacaaggaagctattactcactaatcacttcaaattaatttggtacagctggaaggggaaaaca

agacgacaagtttaaaaatactgtatcacagttgttatggttttctaatacattatattaatggtttagattcagttgtcacaaatacttctctcttaat

accagtaagcagcagcataatttgtaagtgttatgatactttcataacaaatgtaagctaaagtctattatagcaattacatatcacagtatgcttattc

tgaaggcaccagacacctcactggaagacaaaacttaaaaaaaaaatgatatttactctctaatatatatctatgataaaatgaaggataggaatgtaaa

ataagtctaaatgaatgatgtactgggtagatatcttggatggcatggacccaagctatttgggaaaggtgtgtgaagaaaaggcagggcaaagagaact

actgtcagtaaatatgggtgtgaagaaaggtaaatggctgtggactggacatgtgatgtttaaggaaaataaatcacttccactattgcacaatataatc

cttataacctctgatggctttcagctgcttcttaagctgcctcacaaagtcagaaggctgtactaaaacagaaacctctttgccgacagcattcagacgg

tcacgaacttctccgtccttgaagaacaggtttaagggtcgaccatttatctccgtgagagtccacgtggtgagtgacagtccatcacacgttggtgctc

gaggagccaacccggcccgagctgctggagagttagccacaacatcacgtacttcagctgtattgccttcctgcaccacacctaaactctgtccttctgc

ctctcgatgaatggcaaacacgcgtccaaatggtacacgacgaataacgagctccacatagagtcccatggctcctctaattagcctgttggcatctgtt

gcactctgaacaggaactcctacaactgttaaaagctgatcaccaatatgcagggcattgtacaacattggatgttcacgctgcttccagccagctatcc

acacagaaccaaatatatcaacaagtgcaacagatcccacacagtccttacgacgtaactgcaatctaactccaccaggatgtgccatttcacggcgtaa

ctgaaagacttgttgctcccttagcgtcattcgactaacggcaacaccactattgccacttgttacactttctaatgaaacctgtctgtgataaggatgt

ggtgcaggctgtagttgcctctcaacatcggctcctctcgtggcagaagcagaaatatgatctgacctctctgggattctaccagtcgaagtgattacag

ctctttgtgatgctgatgatgcgccttgcaaagatgtttcacatctttctatcagtctacctgctgtcagtccagatcctccaactctcgatgtgctgcc

tgccaacgtttcctgtctttcagtttgattagtagaataaccgccaggtctcctcacaacacctgctcttggagaggtcgcaggcacgctgtcttgccta

tcagtcggtcttggaggcaaataggtggtactctgcccctcagcatcggaagccctgtgcaaatttgcagcctccatagcaggtggatttaatacagatc

ttaaaacctgtgtctctgaccctgaaacagctctgaatctgccatttctctgatatccatctgtaggaactactgtatcttgtaatgtactagctagaag

aggactattcaccaacagagttggactctcatcttccgattttagggttgtagctgtttctgcaggtgtgcgagccaaaaatacacattcgtaatgactg

tcactggaatctgaggcatctggcgccacattatttgagtgtactacttctgtctgtacatctgatagattctgtctagcatctgtcaaatttctaagtg

aggtatcacttacttcattctgttcaactggtccagcagcattgtcacattcagtctggttggcactcatctgtgttgcagctgtgctaaagtcaaagat

ggctgctgggtggtaacctgccgacagtggaacttggtcagagttgacacggacaactgtcacattatcatctgtatctgtgacggaattatgagcatct

cttgctggacttcgtagtgctactggagaacgagctaatgcattcaaagaacttgaatcaggaacagatgatgtacgacaaagaacatgacgagaccttg

agtaaactggtggctctgattgagtgatgtatagcgatgtggtgcatgcccgctgttgcacagtaacatcatcataattcaattgctgcacagaagaagt

tgtgtcttctgaagatgaagcagaactatttggaaaatcatgcgactgctctgtacttgaactttctcctccggtactggcaacttgttcaataccaggt

acagctgtggcagggcctgcaggtggtggcggaagtggcgagttgggatcacgtgtaggtgctaacggaagtcgaggccctgtctctgggtttttgctgt

atacattttcacgtggagacagtatgtgcatttcccgtagttttgagcttatgcattccacccattccatcattaggtcccaggaaggtgctgtaagacg

gacaacatcagaactcaaggtaattacaaactcatattcatcttcttggggacagatcgtaggtgaaacatgcaagcagttgctgagagagacaaaccag

tctggcttatgagtaacagctttgttcttacagttataaatttcaagtagtggctccgaatcatcgtgtacacaaaaaacaacccagacgcgttcacctt

tcttaggaaatgctcccattctcttctccactgtcagttttctcagcaaagcattcttgtgaatttcacggaagagtacatttgcaaagggtccagtttg

tgactcatcttgccttacactgccagctgtatcatcctgttgtgttcttccagtttgtggtggagcacttcttgccagatccatctcttactcaaacaat

gtgtcagcctacagatgtaaggcccggcagacacagcaactgcctttgtcatgagttagcctgatgaagaattcaaaatgctttgcataaactgtttaca

gatctcagcacctttaccataaggcatacggtccatgtaggagggtcctgacggtttaattggtgattctgtggctacagcatcagcttcgagtaacacc

acgcagccatattg

>9031|AGAP004333-PB

ctttcttctttttaatttaatttttattgaaccagtataaacattatcattagttgttgctgattgtacatggacaatattatgaatcttttatcctata

agttactgcacaaaaataatgtttataattgattattatcattatttcatgagatatttcacattactaaaaagtcaacaattttcacacttacaaacag

gtaaggtaggacacaagaaatctgtgcatgagtcatccttgacagcaacacattcttctccatcaccacatgtgaatccttcacatggattatcacattc

acatgttcggcatccatcatctccagttttaaacccatattcacacacttgcgcacagatattgagatcacaggatggagatgaactgcgccctgttgct

cttgttcgtgacactttttcacaattgactttagctccaggccccattgttccagatatcttgtctcctttggaatcaacacaccaacaaaccagtccat

tcctactacactgctttgcttcaaattctccagtactctctgaacattgtgggatatatccttttccttctttctcattcatggacagtagttcagcaag

caatttttgctgaaggcaaactgtcacatcctgtggctgactacagtgtttaccacaaacatccgtctcacaacatttctgcattgatgggcattccaag

tcataattacacttcattccacatgttatagaattatttgggtcattttggtcccaaattgggcaagagccaggtttttggagactgaaagatgctggac

aacacacaccataatcatgaccggtg

>9690|AGAP004358-PA

gcatcactccgcgtggaaggggaggtacggaacggagaattcagttgtctcattgctgtacacaaacgaggtgtgataggcaaaatgttctcaaacggaa

gtaaggatgacagccaacgcgtgaacgcaacgccggttgcgacccaacctccgaaagagaaacaggcgggaagggacaaaaatttaatgagtgatcggcg

tgaagtgggcgtaactttgagaaacactaaaccaaaaccaaaaaaagttaggagtgctattccaaagcacaggcaagagttgttaaagtggaatggttgg

ggttacaaggactctcagtttgtagttaaagatgggataatccatttcacgggaaaccggtaagttttcatttcatttttttatacttgtatgcgttgct

tgcgttttcaaagccaaacg

>9794|AGAP004362-PB

tgcaataatctttgcttggatacgatgagcattttgctttgaatggctgaagaacttaaatcacgtatcatatggtagttagactcccgctcatacttga

aggttagccggccatatgatacgtgatttaagttcttcagccatgcaaagaaagatactgtgacaccaccgg

>9814|AGAP004362-PE

atgcagtccagaaatcttcatctgcaattgcttgtgccccagcaaaaccaccacccccttggtttgcatttacgtgatcacgtagcgcaacaagatcaat

accatcagcattgaaaattgttccagtgtggtcttgaacgtgagtgatttttgcttttgattcaacaaataagaaagcagcttcactacctacattacca

aaaccttgaacagcaacttttgcgccttcaattggtaagttgatttttgcagcaacttcacgaccagtaacgaatacaccgcgcccagttgctttcacac

gacctaaagagccgcctaagtgaactggtttaccagttacaacacccgtcactgtgtgaccttggctggtagaataagtatccatcatccaacccataat

attggcatttgtaccaacgtctggagcaggaatgtctttttgaggtccgataatgtgaccaatttcagtcgtataacggcgagttaaacgctataacttt

cgag

>9835|AGAP004373-PB

ccgaagcacaaaaaagaatgtatcaaaaaattctgtgaacttggagaaataataccaccaacatgcgtggaccatccttaatgtcttgggattatttgaa

tagtcgacaggttgacaacggaaattatacaggcctgttaaccaacctgacatgcttatctcgtaaaaaagccatgcactgaatataacttggaaaaagt

tgtaagcaatcagtagattttttagctcgaatggcttacgattttccataagtcttgggccaagtacttttacaatatatacatatgtgagacagattgc

agcagtagggaatgggcttgacattaggaaccatttgctagtcctcggatctccttgtctgtccattaagtcctggaaatcgtctattaaactgaggatg

gcagccatggtctcgcgtcaaccttcccactttggaatattcactgattcactcccagatggttttccttggatgttgaatattcccgtgagtcctgcgc

cctggacgcggctactccgactgatgtctaacaaatcctcggagctgtagcacctcactcgtcaacgtcggcgtcgcag

>9861|AGAP004375-PA

ctgagatgtggtctttattgctaaaacaaacacattttttaaaaatcaacaaaattccaggaaataatcagcaagtaggtgatttggtagtgataatgta

taaagaagtaattcatgatagattatgggtgtgcattgatgaataaaacccagcaatataaacacagcatttcttgacagaaaatgtcataataagtcaa

ataatggttgttttataaatcatagggtgtgttcatttagagagcatgtgaataatgctgtgtgagagaatattagtattatcaacaataagattaagtt

cactgtaatgtcagagtcattaagaatgcaaatcaccagtaacaaactatgtatgagaaagttaataaagacacagaaaataattgcttcaagttgtttg

taccattgatcattcaatggtcattacagtagtttcagtttgtagtctgtattacctgctgtggtgaagagtggcatgtttgaagacgtggtgttacagt

gccctctggtacagcgaggcacagcttaccatgctggatagtatgtaggtgtgaattgtatgcccaaaggttttgtgatgtggacatttcactgcacaag

gcaagtgatacaggtgacttttcattttcaacttgtaagcaatgtgatccttgacgtaactggccaaatgcactgctgtcttcatttggaagtctgagtt

caggaaaaatattttccagataccactgaaatggcttgcactgcagttgttctttaagttttgatctttcttctaagcttccaatctcaacatcaacagc

atttggccttgcttcataaaagaatgttttgtattcttcaagccatacttctgctacacgtctgctgttcctg

>9888|AGAP004375-PB

ccgcctgccacgagatgcgcgccgcgcgcagacgcctgcgcctcgcctgcgccgccaccgccggggcgctgctgctgctgtacgcgctcgccgccttcag

tcagcgcgccacgcgcaaggtgcggacgggtgggtctgacggcgcgcagagccatctgtgggcagcgctggtgcaacagggcgcctacctgcaggcaggc

gcagccggcaacgagccgttctcgcgtcacgccttcagcgccgccgccagcagccgcctccgctgcgaccgccccttgcccgacaccaggcaccacagtt

gcagagagatgatatataccatagacagcgacaagacaacgagcgtagtgatttccttccacaacgaggcacgctccgccctcctaaggactgtcgtcag

cataatcaaccgcacacccgccgagctgctggaggaaatactgcttgtagacgacttcagtgacaacgaggaggacggtacgcttcttgaagccattcca

aaagttcgggtcataaggaatactgtcagagaaggcctaatcaggtcacgcaacattggagccagggaggcaagaggcaactatttagtttttctggaca

gccactgtgaggttaacactggatggctggagccacttctcaacaggctttcacagaatcctctgctagttgctagtcctgtcattgatgtgataaacca

agacacatttgaataccactcttcttcatctcaactgaaaggaggttttgattggagtcttcatttcaaatggttgcctctctcaaatgaagagaaagaa

agtaaaaaagatccatctgaacctttcacgagtccaaccattgctggtggattgtttttaatcagcaaagattggtttcaccaacttggggcttttgatc

caggcttggacatatggggtgctgaaagcctagaaatttctctcaagacatggatgtgtggaggaaggcttgaaatcattccctgtagcagagtgggaca

cgtgttccgaaaaaagcatccttattcattcccacgtggaaatgcacaaacatatctcaggaacagcagacgtgtagcagaagtatggcttgaagaatac

aaaacattcttttatgaagcaaggccaaatgctgttgatgttgagattggaagcttagaagaaagatcaaaacttaaagaacaactgcagtgcaagccat

ttcagtggtatctggaaaatatttttcctgaactcagacttccaaatgaagacagcagtgcatttggccagttacgtcaaggatcacattgcttacaagt

tgaaaatgaaaagtcacctgtatcacttgccttgtgcagtgaaatgtccacatcacaaaacctttgggcatacaattcacacctacatactatccagcat

ggtaagctgtgcctcgctgtaccagagggcactgtaacaccacgtcttcaaacatgccactcttcaccacagcaggtaatacagactacaaactgaaact

actgtaatgaccattgaatgatcaatggtacaaacaacttgaagcaattattttctgtgtctttattaactttctcatacatagtttgttactggtgatt

tgcattcttaatgactctgacattacagtgaacttaatcttattgttgataatactaatattctctcacacagcattattcacatgctctctaaatgaac

acaccctatgatttataaaacaacca

>10380|AGAP004414-PA

agaagagtgtgtgtgggtgtgtgtgtgtgtgtgtccaatgttgacaaacaccattggccaaaagctttaagtgtgaaaatttttttgctgtgcctatctg

cgactcagcatatccggcatattgttagtttatgatcgtgagaccggtaaaccaaaaggttatggattttgtgaatataaa

>11455|AGAP004431-PA

ctgggttctgttccaattgaagaactgtgtgtccaattcctttatgtatgaactgttcatatcagcatgaaacataccagtgtcattgacatcgttgaat

tcccacaccttgtcgatgatgattccaaaactataaaacggatccgtggaaaacgttaaagatccatcttcgccatcaatgaaacccctccagtcaatcg

tgagatttgaatccttgtctgtaagtgccagtaaaacagaataatgtgaagtattgcattgtgccaatgtgcaatgtggattcaattctgaagaaatcct

tctttccagtccgtctcccaacggcaggcatggtgcagctgttagtaaaacaatagtaagcaacttcaaaacaaaatgcaaactgtgaacagccatcttc

acggacgctttgttgtttgactgcaagttgcggccgtcgatgacgcaccttcacatgattcccatactccaccaaatagtagctgacgtcacattcatgc

taggccaaactacggactactaatcaatgacttctcccctaaagaaccttggccttcacgctcatgtatgaataaataaagtcttgcactgtatctcgtc

tccgctgtcaccgagctcaaagatgctccgaagatgcagacgcacaagaaatcttcttttactttacaaatacatttaatactggcaaatctatctaatc

gctacgagtggtggtaccactgaataaacaaaagtattcatcacagatttcatacgttctcatggaggttggttaataagttatgtgttctatggatcat

tt

>13136|AGAP004458-PA

attttgtgatacatcataacattcttttcccttagatatgcttctggtatctcactgtgtgtctagtattcagccgataaaatgccgaagttctttatca

gacacacactttggactgcatagaatggcataaggaatgttgtgaagggcaggaagtcaactaacagaaagaaaaactttgcttatattgacattctctg

cacaacacacatcctattccttacactaaaaatcatgtcaatgacttgccaacatcagcagcaataagcctgtaatgacaaaactttgcagcgagtcaac

aatcagcctagcagtactctcttcaccatatgacatgcttcctcttccgaagactcgcttgctgatgaaggggacgattcgctgatacaacgtgaagagc

atagatattgcatcagcggtatcctgtatttgccacagaagtcgacaaatttcacgtgttccactcggctgtcgaaatacactcctttgcagtgaggatt

gacacagtggtgtgcactgtccaggtaatgtacaagagttcttgggaggtcatatggagagtatgtgacgttagcaagcttcacggtgcgcgctgccagc

tccaacagtgttggagggtccagagacatgttcgtggcaaacctcaccacaagtgggttgtccctgagactcaattctgcaaggctgtttagacccacaa

tttctggtggaagaactgttaaacggttcttgtgtagcagaagtgacttcaggtacttaagatttgctatagctgctggcagactttccaacaagttatc

acacagcaccaaagcctgaaggtttttgagtttaccaacagatgcaggtacttcttgaagcttattgccacccatgtacaagtactgcaaactctgtaat

ttccagatatctttggggatgttggtgatgctgttagaaccaaggtacaagtacttcagtgccgtaagttccaaaatttgctgtgggaatgacgttagac

tattaccacttaaattcagttctttcaaactggtaagtgcaccaaacgatttagggaaggagtcattttccaacatattattccttgctatgagggaggt

gagcctgcattccgttaatgcttctggaaggtgggttatattattgtaactgatatcaagcatttttagatttgtaaacttcgaaacgctggccggcagt

gtatccaagtcattgttgtacagcaataacgtgtctacatcctcagtagcttttttgtccttcgctgcatactcttccaagttgtacatcaatgtgtccg

aatcgagtaacagcctcgagtagtccaacgtaacgtttctgtcagcatcactactgtcagacgtcgtgtactccggttgcactgcctgatacacactcat

ttttacttccctgcgtcaggacctggcgcggattctccgagacactcaccactactggactaaggcagtgtcaccactcagtacttataggtcaattaac

tgcaattcatttcaaggcagctgataacggaaatgatacagcaatatcctatgacgtcatctgctgataagttacggaattaggcagcacttttgcggca

aacactccagtacgaggatgcattaagccccccttcttttgcacttgagattctcaacctttctctagtaattgtcgcagcgtctctgatattttataca

cgtaacagtgtatgttccatccaacttttgctgtgtgtgacacaaacatttcaatttcaaaacaaaaccgcccacctactaagcgc

>13135|AGAP004458-PA

cgcattttatatctcaattcttatgaaattttataaaactacaaatataacacaaaattcacacaataagtccagctactgtacacacaaccacaatcag

aattagttatcagatcattatacaaaggcttgtatttcacacatttcattaaattacgacttttttcctctattgacagagtagtaataaaagtaatcca

gtcgattcttaccaaataatggtcaattacatttctgcagcaagtcagactgtccctattttagcaagaaaattacttcataattaaaatggtcccataa

tccaggggaatgcagaatgtatcaggcagtatgagtgttgttgagcaataaggtacatcattccatcaaatactaggtgacaactgcattatgagaatat

aatacaatgatactgaagtctgtgttcctatttaaaacaaaatattctaacaattctattttccacactgatttacaatactttgtttctgaagagagac

tacgatgtcagtatgaaaaattctctaaaaggtgcatagtgaccgtattttatgaacaacaacagtgtctactttcctactttgtgacagtatcttagta

gatcagtttggaaacaaacatggcatctatttcactgaagcaataaaatgaagtttctaaacagaatgcaatacactgatacatctcacattcaacatct

gctattttatgtaaaatggtaggagccaaacacagttagtattcaggtaaaggtgtcattctgtattgtagtacagttgcaacagaaaaaaaaaataata

aaataataattactgaattccacgaagtgcagctctttgaacagttacatgttaacaaatgtgatacacatcaagtttaaaaatttgaaaatttcctttt

caaatcagtttgctgcagagtatgaaatttttaatttccacgaattccaacgacaagtaaaaaatctatttataatggaggcaatttccaaggcatcaga

aaccattcacatatctgtcaagtgtgcctttataaaaggcacatcttgcagcaacaaacataattccagcacaatcaaggcattaagttgcccaattgca

aacgtatctctctctcctccccccccccccccctcttcctcttagtctctcacaagcttcaatcctcttcttcccttgttcactacagaaatactaactc

ctccatcggtgcatcactgcttcacctcccactgttacaatcagagaaatgtttattccattatactgaatgtttcacggggcacgcaccataggtgaca

ggctttcagagattttgtgatacatcataacattcttttcccttagatatgcttctggtatctcactgtgtgtctagtattcagccgataaaatgccgaa

gttctttatcagacacacactttggactgcatagaatggcataaggaatgttgtgaagggcaggaagtcaactaacagaaagaaaaactttgcttatatt

gacattctctgcacaacacacatcctattccttacactaaaaatcatgtcaatgacttgccaacatcagcagcaataagcctgtaatgacaaaactttgc

agcgagtcaacaatcagcctagcagtactctcttcaccatatgacatgcttcctcttccgaagactcgcttgctgatgaaggggacgattcgctgataca

acgtgaagagcatagatattgcatcagcggtatcctgtatttgccacagaagtcgacaaatttcacgtgttccactcggctgtcgaaatacactcctttg

cagtgaggattgacacagtggtgtgcactgtccaggtaatgtacaagagttcttgggaggtcatatggagagtatgtgacgttagcaagcttcacggtgc

gcgctgccagctccaacagtgttggagggtccagagacatgttcgtggcaaacctcaccacaagtgggttgtccctgagactcaattctgcaaggctgtt

tagacccacaatttctggtggaagaactgttaaacggttcttgtgtagcagaagtgacttcaggtacttaagatttgctatagctgctggcagactttcc

aacaagttatcacacagcaccaaagcctgaaggtttttgagtttaccaacagatgcaggtacttcttgaagcttattgccacccatgtacaagtactgca

aactctgtaatttccagatatctttggggatgttggtgatgctgttagaaccaaggtacaagtacttcagtgccgtaagttccaaaatttgctgtgggaa

tgacgttagactattaccacttaaattcagttctttcaaactggtaagtgcaccaaacgatttagggaaggagtcattttccaacatattattccttgct

atgagggaggtgagcctgcattccgttaatgcttctggaaggtgggttatattattgtaactgatatcaagcatttttagatttgtaaacttcgaaacgc

tggccggcagtgtatccaagtcattgttgtacagcaataacgtgtctacatcctcagtagcttttttgtccttcgctgcatactcttccaagttgtacat

caatgtgtccgaatcgagtaacagcctcgagtagtccaacgtaacgtttctgtcagcatcactactgtcagacgtcgtgtactccggttgcactgcctga

tacacactcatttttacttccctgcgtcaggacctggcgcggattctccgagacactcaccactactggactaaggcagtgtcaccactcagtacttata

ggtcaattaactgcaattcatttcaaggcagctgataacggaaatgatacagcaatatcctatgacgtcatctgctgataagttacggaattaggcagca

cttttgcggca

>13402|AGAP004494-PA

gtcagtgacgcggccagcgacgtgaccggttcgagtgttgtcatcgccagcctgggaggggaactgtctgctgctactgtcgactgagtttcgtgaaaac

tgctgtggctgttggaactactgcagctgtctgactgtggacttgttgctggtctcaggaacatcccaccgtcgcaaaagcagcatttgtcttcaacttg

ccaatcactgctgctgggttctatgtctgcttgcggtctacttctcaaaactgactcctgataatgtttccatttccttctgcccattaactcctccgct

acccgttccaaacctactacgaaaaccatattcttcgtccatctttgcagctcttttttgattgctcggcgctcctgaacacagcgagcataggagcact

ccgccatttttgccttcaccctacttgtgacgtcatgtctctcatgacgcaacacgttgcaccgcctattgccgcgtcttcgatgcgatctttagcagaa

cgttgggggggaacgactggagggaggggacgagcgcgacagatggagggagagaaagagagagggagggagattcgttcagcaaaggaagagggggagg

gggagtagtttcgcagcgctggcgctccccaccacagcccgaactcgcgagtccgtactcggcgctcgctcgtaaaatcgcacacacaactcgccccctc

taccaccttcctctctgcc

>13416|AGAP004501-PA

ttccatatttacaaggtttaaattacttaaatattaccattcactaaaaaagtcatgtaataaattataagttacaatataaaatctttatacacagcca

tctacaatttatgactagatagtaacaatgaagcagtgaaggcttcctgaagttaaaattcatgtgaagaagtgcactattttgtctgggaccatattct

ggtctgaagcctgtttcatctataattggaacatgccaaaacattcttgtattatctgagcagattggtaatctcactggggtcctacaagacttatcaa

ttttcctcctctttcttgacttcataacagatgaacaatcacataaacaaaaatatgctgaagtctcagttataactatggaaactttcataattgaaat

ctcattcaatatatagtgtagcctcatgaaatgatgtatgtatcacagcctcagaataactaaacttctaatctgcaggtctaaacttcaacatagcccc

atagacgaagacggcttataccaccatcaggtgcaatggtgacacgaacatgagtgaatggaccttttgatctcagctgatcctttgagaacaaatgttg

tttatgtggaaatagcttttgaggtggaagcagtgtcttccatggtacattgttattctcacacataacagtatcttccacttgatgatcaaaatatacc

ccttctatccttattgaatctgggtaattgcctttgaagtgagctgtgtcaacttcaaccatgccaatatttccaggatggcccatacgaaaaatggccc

actcagatcctggtacttgaagtattccttgagtatcctcttccagtacagcaggtcggtccaatcgcctagcagtttcccaaccatctcccatgttgat

acccggaccagggcgaattatgttccttgggtgaccaaagtgagcatctgaatacccaaggcagacaccgccatttttcatcaacaagagatctaccagc

ttgttg

>13430|AGAP004509-PA

gtggtcccggtggacgaggtggaagggctcgaacatgaggaggaaagaagaatggcggtggaccaggaggacgtactcctggtggaggccgaggtacagg

tggtggtgcagccaaaagtacagtagctgggttgggtggtggaggcataggagttgtggcttgtacagcaggcacagttgcaactgtagtgggaagcact

gtatcatcgctagcccctaaattaaagaaattgtctcgaagttcaactgggggcattggcagtgttccaggcaaaccaggtacaggttcaagcccctcag

aaccttcagcaccttctcgtccaccattagctccttggcggccttgtgatcttccccatttgatagtcaggcgacgtcctcctaggattaacttgttgaa

tgtccgttctgctgcagcttcagcagccgagcgtaaagtaaactgaacaaatgcacactgctgtcgaggaaccagtgtaatagaacggatctctccatat

tggtaaaagtggtcccataattctttctctgtgagcttacctccgcaatgcctaagctagaaccaccagaagataagactattacaactttgtacattgg

caacctgggagataagctcacagagaaagaattatgggaccacttttaccaatatggagagatccgttctattacactggttcctcgacagcagtgtgca

tttgttcagtttactttacgctcggctgctgaagctgcagcagaacggacattcaacaagttaatcctaggaggacgtcgcctgactatcaaatggggaa

gatcacaaggccgccaaggagctaatggtggacgagaaggtgctgaaggttctgaggggcttgaacctgtacctggtttgcctggaacactgccaatgcc

cccagttgaacttcgagacaatttctttaatttaggggctagcgatgatacagtgcttcccactacagttgcaactgtgcctgctgtacaagccacaact

cctatgcctccaccacccaacccagctactgtacttttggctgcaccaccacctgtacctcggcctccaccaggagtacgtcctcctggtccaccgccat

tcttctttcctcctcatgttcgagcccttccacctcgtccaccgggaccac

>13492|AGAP004523-PA

tgatttcagatattgtgacgacgttccgcagcacctcaagggcagagtctcgtacctcccagttgacatcatgtaagcgttgcctcacaactggtccaag

attatttattgaactgtccttcacagggaccactaaaagtgccagatttggaggcatgaaggacactaaaacttctcccataagcttcagggcctgaact

gcatccctgcaactgaggtttggattttggagtaaaattgatagaaatgtgaagatgcaaatggattctacactttgtgaccatacaatgtggaagtttg

tgattagcatcattaaagcctccaagactgttgagagtaactctggatactctattggaaagtcatacgaatcaatggtgtatcctggaagacactgcac

agatggtggcataaactcttttaagctatacattaaaacctggaacacaaccactgctttatcctggaaaaaatgaataattggtgaaatcatgaaagct

aagaaagaaaatattgccatcacaatgaaaataacaactgttagctgagtttgactgatctaggtaaatttctacattctgtacaatcatgcaaatcaaa

aaagagaaagtcataataaaacagaataaatataaaaacaaagtt

>14096|AGAP004546-PA

tttccctttgatactaacattgcataatctgtatgttttctgtacatatcagcgtatattttaattaaaattttctgattgacaggttctcgatgaagaa

gcggaagtgttcgttgttaaaatgtggcggttactgatatatgaaatagaagcaaagaaa

>14109|AGAP004549-PA

gaaatatcagggcagagcggagtaatagcggtggatccaaaagggaagttgctattcttccccgtgctagatgaactggctgtctactgttgggatacga

gcaagcctcacgacccaaagaacttccgcttgatcgttcaaaatgatgaacatctacagtatatcagcagcataaccatagacacgaaaaatgaaagatt

atatgtaacgagcgatcgattaacgtcctatctttcaaataccaccaactgctctgaacacaagttccagattctgtatattgacataaagaacatagaa

tgttagttctatgtgcaacagattttgttttgtgatttcgaaggaactcggtgcctcagcaagatccatcgaatgtaattaccactgtatgatatattga

aaagaatgaaatatagagcctgcag

>14110|AGAP004549-PA

tctgcgtcttaaaagtgagtacattaagttcgtcttaattttttgcagattctgtatttatttcttagcaatatttcatacagtggtaattacattaatt

gaaccttgctgattcagtaacttccttcacacattcacaaaatcaaatctgttggtacacaaaactaacattctatattctttatgtcaatgtacaaaat

atgaaagttgttttcagagtagttggtcgtattcgtcagaaatgatgtcattcgattgctctttatatataatccttgtgttttgggatctatgtcgatg

ctgctgacaaactgtagacgttcatcgtcttgagctatcatgcgaaagttctttgggtcgtgaggcttgctcgtgttccaacagtagattgccatttcat

ccagcacggggaagaatagcagctgtcccttcgggtccaccaccattactccactctgtccagataattcccctggcagatcaacctgcgtcttgatgtc

caacacatccccgtcgcttgcatctcggatgacctgcttgtccacaacgccctggatgttgctggcgaacgcctggaagtagagtttgccgtcgcagctc

tcgtcggctgtcactccagtgaggccagcaggtaggtagtaggctggacctcccgccttcgtgaagaagtagccgggcttggggtggaagtactggctct

gcaggattttggaggctccagtggtgaggtccgtcaccgacagcacgttgcctctggggtcggtggtgtaggccgtcaagctaccatctggacacgcatc

cacccacagattgttgtaccatgaatcgcagaatgggttctgaattactatggacttgatgatctcatctgttcgcaggtcgaagatgacaatttttggc

ggacacacatttttggaagtcgtacacaggtccaaagcaccgttgtcgaggacgtatagagtatcatcatccgcccatgttttcgtcgttctgatgacgt

ccaccaactgggagttgcagtcggtgatggagtcctcggtagtgtggtggatttccaggttggggtacggcttcaggagcggactccgctctccgttgta

ggagatggtgctcagagttaccgggatgtcaccccaggcaagaggcgtcgtgacaaacatgcggttacgctggtgattgtccaatgttaagtcaaatgaa

acgaccaatttagggtagtagcgtccagtgtccttcatttccttttccaacgtcccattgcctccgaagtcgtatttgagctctttggtggtcttgatgg

tgaccagcagcggctcctcggaggcgcccttgctcctcgtccctgggggcgccttgccggccctgtgggccgctctgactcgacctccgtgcctcgaggc

gtggcctggcctgtggagagcctcaccagccgccaccaccagcagcaccagcaacgcgagcgtcgacacgcgaggtcccatctcgtacctgacctcggtc

tacc

>14130|AGAP004550-PA

ccaatggtaaatatacatatattaatatatatttcagaagttaaacaaaaaataatttctcaatatacatgcagtcttacaatttcaaaaagggaatggt

atgtataaatcagcagtacaataatgcacaagcttgataaaatgctctctcatctgtcagaatcaaacacaaaacctttcttcctgagatcatctttggc

ttcttgaatttccatctgtaattctttcgctgcgtcctcacaattgttgaagttgtagaccctccacaatattacagctatggagtatagaccaaacagt

atcaaaacgattaatggaagtggtttgatgacaaacatccattcgtcaagtaatctcgagtggacttgacgagttacgagggcaatccatatgcttgcga

aaaggctcacgcctaaaagccactccataagcttagtcattttaacgtctgcaagaaatagtcttgttcgagaccggaatacacaacaaccaaatagctt

tataatttatcgttttccttttcaaacaatgatacattacactgaaacatagccagaaaatcgtcactgaagtcagcacaatggagccccagcagtaagg

aaatcgctacacatgacaacgtcaacatacagcccataacattccaaacacgacattcccatcaacacgttgtcaacataacacgttaaccaacgatgtt

tacagtactcaatattcaattcagagataccacac

>14152|AGAP004562-PA

gtcagcatcagcggcttccgccggccgtgccggtccgaccacgcgcccaggaacagcagcgccgccgcgggcagcgacgcctgcagcacggcgcgccacg

ccagcaccgccgtcaccagctgctgcaccgccgcctcctgctcctggtaggcggcgctctccttgagggcgagcgcgtcgcagacggtgtcgttgaaggc

gaggttgacggagca

>14149|AGAP004562-PA

tggacctaaatagaacatccatacttgtatgctaaatgcatacacaaagctggcaagaattttacttatgcagcttatgatgccaattaaggaatcatct

agttttagggtcctgctgaacaggctgatcaagataagggtcccaacaaagttaataaattcagcatatgctgaaaagtagcttgcatccacttcatccc

aaccaaaccgatttctcacaaa

>14150|AGAP004562-PA

ggggacgtgacgactccggaggaacggacgcttcgcatcggcgtggtcaacgtgtgcctgatgctcggggttccaatcggcatggcgctgtccggcgtcc

tgctcagggccatcggcttctacggcgtcttcctcacctccgcctgcatctacacctccagcatcacctacggattcttcttcatacacgacaagccccg

cgacacaccggctgccgccgatgagaagccggagaagcagcagcagcagcagcagacggcgccgcagaagagcccgccgctgcggcgttgcatggccggc

gtcagcgacctgctcgacgtgcgacacgtcgcgcgcaccttccgcgtcgccttccggcacggcaacaaggagcgccgcatgcgggtggccatgctcatgc

tctgctgctgcgtcatcatcgggcccatgcacggcgagatggctgtgctgtacctgttcacgaggtaccgcttcaactggaacgaagtcgacttcagcat

cttctccacctacagcatgatcaccaacctgataggaacaatggtgtcagtgggtgtgttcagccacgtaatgaagatggatgatgccctggtgggagtc

ctctcgtgcaccagcaagatcatctcaagcttcgtgtatgccttcagttacaccacatggatgatctacatggcaccaatcgcagaaattcttaacggca

catcattcatcgcaatgcgctccatcatgtccaaactggttccacctgatgagttgggccaggtgaactccctgtttggcgtgtgcgaggccctgatgcc

gctggtgtatgctccactgtacaccagcgtctacgctgccaccatggactccctgcctggggccttcttcctactgggaggcggtctgaccatgccagct

gttgcaatcttcctatggctctatgcatcccacaaaaaggatgctagaaagaaagatgaagaagaaaatgcagccgccaggacaacagcagttctcgaag

ctgctgccactgcgacaaagacggacccgttggccttccccatggtgaggaatggttctgtcaggttcagtgacaagtcaaaagcagaaagcaaagcagt

ggatgcatccacactggaccaaagagcagcagaagaaggactgactcataggaagatctttggtgtcgaaaatccggcgtacgagcaagactctgacaca

aaaccgggagaagctcgactatgaagaacattatctctagtggcaatgagagtgtaactcatcagtttggacttcacggtgcaccatgacacgccttctt

aattctgccttccttttattttttgcgctagtgggacctgacaaacgattacataattgtttgaaacaatggtgaaaaaatttgttggaagaaggcagat

ttctgacattagtaaaaatatgtaaaaacaatatgaatggcagtcgtaaaagattctcagtatcacaccttgattttacaagcctagtatgctgtgagtc

ttttacatgcagatgttg

>14151|AGAP004562-PA

ctccagcatcacctactgattcttcttcatacacgacaagccccgcgacacaccggctgccgccgatgagaagccggagaagcagcagcagcagcagcag

acggcgccgcagaagagcccgccgctgcggcgttgcatggccggcgtcagcgacctgctcgacgtgcgacacgtcgcgcgcaccttccgcgtcgccttcc

ggcacggcaacaaggagcgccgcatgcgggtggccatgctcatgctctgctgctgcgtcatcatcgggcccatgcacggcgagatggctgtgctgtacct

gttcacgaggtaccgcttcaactggaacgaagtcgacttcagcatcttctccacctacagcatgatcaccaacctgataggaacaatggtgtcagtgggt

gtgttcagccacgtaatgaagatggatgatgccctggtgggagtcctctcgtgcaccagcaagatcatctcaagcttcgtgtatgccttcagttacacca

catggatgatctacatggcaccaatcgcagaaattcttaacggcacatcattcatcgcaatgcgctccatcatgtccaaactggttccacctgatgagtt

gggccaggtgaactccctgtttggcgtgtgcgaggccctgatgccgctggtgtatgctccactgtacaccagcgtctacgctgccaccatggactccctg

cctggggccttcttcctactgggaggcggtctgaccatgccagctgttgcaatcttcctatggctctatgcatcccacaaaaaggatgctagaaagaaag

atgaagaagaaaatgcagccgccaggacaacagcagttctcgaagctgctgccactgcgacaaagacggacccgttggccttccccatggtgaggaatgg

ttctgtcaggttcagtgacaagtcaaaagcagaaagcaaagcagtggatgcatccacactggaccaaagagcagcagaagaaggactgactcataggaag

atctttggtgtcgaaaatccggcgtacgagcaagactctgacacaaaaccgggagaagctcgactatgaagaacattatctctagtggcaatgagagtgt

aactcatcagtttggacttcacggtgcaccatgacacgccttcttaattctgccttccttttattttttgcgctagtgggacctgacaaacgattacata

attgtttgaaacaatggtgaaaaaatttgttggaagaaggcagatttctgacattagtaaaaatatgtaaaaacaatatgaatggcagtcgtaaaagatt

ctcagtatcacaccttgattttacaagcctagtatgctgtgagtcttttacatgcagatgttgtgataaaagactaagttatgactttagtatagcaggc

agaaaaactcttccagagtatacctgtgtggaagataatctggtattccaacagctgtggtatgtgtttcatcagtatttcatactggatgccaggttgc

tagattgtttctaagtatttaacgcgatggtggtataggaatgataggaatgaggtaagttgtgtaaaatactagacacttatgtaagaatcagatagac

aacaaacaccctgataatgtgtacacaagtgcatttttctcactctttggaaagatttattataactgttgttatgccacagctagacatttcactctat

tatcttaaataatttattttgcttcaagttctaaaatctccttatgataccatgacttcattgctgattttctcctaaagcagccggtactcattaccat

acaataatggttaaatgtatggtattcaaagttattttatttttacacacattcctcaaacaaacacaatgctcacaattaatgtttgtcaaagcatata

tacaagtaatgataaatcaagagagcataacatgacaaaattagactgcttaccaatttatgtatacaaagtgaagctgtgactttagacttttaagtca

ttgcttagataatacccaagatttgccactgccatattgtttcaataaatctagctttcatgatatatggcacttattaaaatgcagttagggccaagga

tattctgttatcttaaccccccccccctctctctctctctctctctctctctctctct

>14383|AGAP004564-PA

cgcaggcctgctcccacaccgactccccgtactcgctctgcacgtagtgctgcacggactcgagcagcatcccatacatgatggagcggtgcgagaggag

cggagctggggagcactgcgaacgcgcggtttacacgtccgtccaggccggctgccg

>14398|AGAP004569-PA

ggaagtaatcggcatcgtgtcgtggggtcgcgggtgcgcgcgtcccaactacccgggcatctacacgcgagtggccaactacctgccatggctgcaccag

cgcctcgccggggactgcctctgcgcgcctccgtaggcgggccaactgtcgctgcacccacctgggacaaggagttctcctcgcacgtttgaagccacgt

ctcgcctctacaccaactaatttattctagca

>14524|AGAP004581-PA

tgctgctcgtcgacgtggcgccgctgtcgctcggcatcgagaccgcgggcggcgtcatgaccaagatcgtcgagcgcaacgcgcgcatcccctgcaagca

gaagcagaccttcaccacctactccgacaaccagcccgccgtcaccattcaggtgttcgagggcgagcgcgccatgaccaaggacaacaacctgctgggc

acgttcaacctgacgggcatcccgccggcgccgcggggagtgccaaagatcgaggtgaccttcgacctggacgcgaacggcatcctcaacgtgtcggcga

cggagagcggctcgggtcgcagcgagcgcatcaccatccagaacgacaagggccgcctgtccaaggccgagatcgagcgaatgctggccgacgcagagcg

cttccgcgccgaggacgagcggcagcgcgcgcgggttgaggcccgcaaccggctcgaagcgtacgcgctctcgctcaagcaggccgccgaggacgcgggc

agcaagctgagcgacgcggacaaggcgacggtgcgtgaccagagcgcggaggcgctcaagtggctggacagcaactcgctggcggagcaggaggagttcg

aggaccggtacaagcagctgtcggccgcctgctcgcccatcatggccaagctgcaccaggcgggcggcggcgccggcggcggcgcgggc

>14523|AGAP004581-PA

ggcacatttgacctcactggcattccaccagcaccgcgtggcgtgcctcaagtggaagttacatttgacttgaatgcagatggcattttaactgtgtccg

cccaagagaagagcacaggaaggtctcgtaacatcaccatccgcaatgacaagggtcggctttccaaggaggaaatcgatcggatggtagctgatgcaga

gcgcttcaaggaggaagaccagcggcaacgtgagagaattgaggcacgacatcgactagagggctatgccctgtctgtgaagcaggcactgtcggatgca

ggcagcaggctatctgagtcagacaagacagcagcaatggcacgttgtgaggaggaattactgtggctcgacaacaattcccaggcgagctgtggtgagt

gtgagcatcggctgcaggagctgcagcgggtatgcacacccataatgactaagctgcaccagggcactaaggatcccggcagtagctgcaggcaacaagc

aggtacaggagcggcacacgcaggtggaccaactattgaggaggttgactaactgaactgttaatataaaagtggttttatgtgtcactgtgtgtaatat

ttaaaaatgtggtgtacatagcataggagagtgaagcattcacatttccaagacgctttctttgtgtagtttttgtctgcattcttgttgactgatacac

atgctgttataagcatagtatttatgtaagactgaattcaattttaagttcctctgatcttcattatatttatttaacaatttgtagtttatgattgagt

cattgtttttatgattagttttgt

>15308|AGAP004603-PA

ctggtgtgcttggcaagagctatcctgcgaaacaacaacattcaagttttggaagaagcaactgctaatgttgacacag

>15309|AGAP004603-PA

ctgtttcgagcatttgctgtgcacgaaatgcacgtatagtggatagcccttgcagtaatgcattcgtatgagaaaaaactggactgcgagctggaag

>15310|AGAP004603-PA

ggtgctcgatgaagccaccgcaaacgtcgacccagagaccgacggccttatccaggctactatccgtaggaagttcaaacactgtacagtgctgaccata

gcccacagacttaac

>15429|AGAP004614-PA

cgggaaacagttcggaacaacagaagtattttttttcaaaacattaaacaagcaatggctgaggcacgggtgaggaagttagtgtcgaccacatttaaac

taaatggcttttccattctgagtgatgcagcatcattcctggtgtcacagctaatgccatttgaagaaagagaaagagaagaatggctggataaaattgt

agaacatattcaaaaacagtgtctggaaacaacgaatatcggaaaggacaacattgagctagcaatacatgagtgcatcaggagtggagcagaggaatct

gaaaccatactcacagttataaatgcatttgaagttcctcgctttgaatataaccaagaaagaaagaagttttttgtaaaaaacatcccaaaagattcag

caccaaagttgtttgattcagcagaagaaaaggcacagttattcagatacaggtatatattgctccatcaacgcacggctcgccatgaactatttacacc

ttcagctgttggatcaaatgagacacagaaattcactttaaggcctgtagactacttccttagctctagccgaaaagtggatgatgctgttgtaatgggc

atactgacacagctgaaagaaggaaagtattatctggaagatacaactggaactctgcagttagatttgtctgaaactgtatcctttactgattgctgtg

agacatgttaaatattgtaataaataatgc

>15500|AGAP004616-PA

tccagatccgattcagcagttattcctcgataaagttagagcttatgccaagaaaagccaggcagcaggcggaaaattagtggatatgactcctgaaata

gaac

>15499|AGAP004616-PA

tatcattacgacttgtcccccatcggtaccactctcaagggcagcaggaggaaaattagtggatatcactcctgaaatagaacgtgaatggaaagcagaa

ctggaaaaggtagcaaagcagtttggt

>15498|AGAP004616-PA

caactttaatatacgctattggagctggaattaccgcggctgctggcaccagacttgccaccaaactgctttgctaccttttccagttctgctttccatt

cacgtgctatttcaggagtcatatccactaattttcctcctgctgcctggcttctcttggcataat

>15519|AGAP004625-PA

ttcgtgtttagattacaaatattgttttaaggccaacaatgctattaaagcaaaaaatgtcacagcattgtaccaacaactgttatacaaagatattcag

tctgaaaatatttaaacttgtaattgagcttgaaaacacttactggaataagcagcatttatgttgttaactcttaacaactaccatatgggttctgcag

tgttgagacatattccagttattttacattaacagaaagtctgaaagtaaacagacttcactctaataatccatgtctcactactattaacatatagtgt

tgtttctgttctcttcgtacataattgctggttcctaaaagttttcagggtaattcactgcaggtagacaataacttttacaatctatgatataaaaaat

ttgcccaaaacaaaattattagaaagtgatgaaaatagttaacaataaataaataaaatttcataaaaataaactttagaatgctggaattatgaaactt

gtgaaatggatgatggagtttgcgatactggtttcagtaaacagcacaactctgtacttttttattctacacacattaaatttaatcagtatgttggcca

aaacgctgtgcagttctggggaaaatgtcattggcactgaaatttgagtaactaaactgctgaaggactattaacacctgaaatctgtacgtgtaggtat

tcaccatctacacatacaagggagatgacatatcaggtaagcattagatcacacacgtactcacattacatgaaacatacaattatgaggaaattgagtt

aaaaaagtgggcacctcaaactactttgaaagaatgcaagaattctataataaactgtcaataacaattcaatgcataaggtgcaggtgcacagaatttg

gcaatatttaacgacatatttatcacagaaatacattaaaatgaataataatgacattataaagactagggaaatctaaacaactttctctaaaatgaac

agtttggatttattagtaaggcttacaatttcaatcttgaggaacaaagcttttgcaataatgacatctacataaaaaagacagtaacagcaccaagaac

aaaatgttacaaaaaccatttacataagaatgttacaaactctttgattacaggccaatgaaaataatacatctggagtcatgcaacccccttgatagac

agttatctatttcttctttaattttcagtgtgtttgaaggctgttaaatgtttcatctagcaatgtcagttgtgtcacggatttaaaaaatgataatcac

gctatgcctgaagaaaatatactatattatggaaaatacacaaagatgggacagggggaggcacatttacaatgatccaattctcttactctaccactct

tcaggcttccaattaaataccaatcattggaagacttatgagactttggttacatgatatgaatggtagtgtatcttcttgttcctattttaaacatcca

cagtgagtgctagtattatgtatgtttcaaaactctagcatgaccaatatatgcaagatccacctgtatacaataggatacagaaagtaaaaattttctt

tttgtttactgagttctcaacatggaaaagttatataataggaaaatacacttatatggttgtcaagcaaataaatgttatacatagtcatatcattact

atcactaccacttcccatacaaagtctacgacctgtattaaacacttttcaaaacagtttaaaagttgtgtttcagaaggatattttccccataaagcca

ttcacgctagtaaatcaatcaataagccagagttcctccataagtaagcactttcttacacagcctcacaaagctgagagagttacatttattcacacat

acactgttttccacaaagctcatattcaggaataccgagtgatttaaagagctttattccatatgtattctgtttcacaacctcggcatgaatactggat

ggcagcttgcagtcagtcactgatttacacaaagatataaattagtgggatgacctgcagctagcatataacagctgacatggctgctctctgtcacttc

tttgtttgcactttgtgtcaatcagatgactgtaattgtgcacactgtacctgagaccaacagaaattattggattttgactatggctgtttgtttcatg

actccgattgcctctttcttacctgtgtatgttcagaattcattaccgattctaggcttcgtattacaggatcgcattgccctcgctcacgtgttgtttt

aaccttatttacgatgatcttgctcatcctaatcactgttattattgtatctgtgtttaagtgaatatatttaatgcagcacaataaatgagttaaacaa

tgactcgagagtggaaatatcaaaagaaagatatgctctgcagaagttttttttcctttttccttaagtaaataatcaatcaattgaagtcataacattg

agatgaaagaacatgaaaattaatatctgtataattacaaaaaggcttaatctctactgccactattaccattagcaataacagcagcagtagcataagc

aataattcttgctgtgtttctcaattccacgttattaatggcctccccttccacacaatatacagtgtaaaatttacatttcttctactcaattatgcaa

ctttacttgtatacttccatataagcatatcattacaattatgaacagtcttatctttcattatcatttattaagaatattttgaaaaagatagaattgc

tactcactgtaggtgacacgctgtggtgcagtcggtcatcatcatccctgtctgcaactcagcatgtcatatttatggtgagtaacaatctatccttttc

ataatattgttgatattccaacctggactttccattgtttgattagcataaattcactaatctgtctctaaacaccacagaatagcttctctaagtactg

tttctgaacatatgtatcttatgctccaccacaagtaaaatgtgttacagaaggtactcaataactatatatgacaatgtatggtaaacaacttactcaa

aatattaaaacatctttatgattgtgcaaagtagcacatagccatttcaagttactctaattgaatataccaacttatttggctgtaagacaacattctt

aatgcttttaccaattatgttctaaaaagaaacgatgactgtttcttttggaacagtttaatctgaagttacaattgcttaaaatcttctaactcatttc

ctaaagtactgataatacaggtaaactcaactcaaaaccacagatagtatcagtttcttttcaattaagtacttgccatattttacatatataatgattg

tatcttcaatgtacttttggaaaatcattcatgcgtatgttagcaattcagaagagtgtaaggtttgtcttccacttcaggtaggataggatgtctatga

ttagttatcaagtaactctaagaagatcagtgtgttgcataaatatacatggttaccataaatgtactgaaatttgtaaatgttcactgcaagaaagcca

ccatttgtaaacatatcagtgttttataagactttctgtgttatgagcaatacattctaaaaacagagataagcaaaaactattattcgaggtgcaagca

gaaagaaaatacacttatttctgctgattttaaaatcataagaatcacaactataatgaaattttaagcatttgtatataaactttgattaagaaagtgt

gggattgtacacgttcaatggtgacattttctggtggtgtacttgcccctgatttaatatgacactgaagcttaccttgttgctacccactgaaaaggat

atgtgtgctttggtatacttggtgatttttaacatatggaatgaagaatttgcttacattttgcattaagaatgatataaattgcaccaccacattggaa

atgttgaatgttgtttttgctggaacttctatgaataaaacaattagtaaaaatgtttataaatggccaggaacacactgacgacaacaaaaaaacaatg

taaaaagaaagaaaattattattgatgacaaagtttgcaaattatttggctaacataaagcaaaatgtttttgcaggtatcaggcatgaaatgtatgata

gcaaaatttactcagcagctcgtcacttctgacaaaaagcaattccaattgaacactgtttaggagatgatgaatgaagttcatcatgatccagaactgc

tcaaaagaatcattactggtgatgaaacatgtgtgtgtgtgtgtatgaagatgaaactcatcaaattcaactggatttgaaggttgtgcacactctgttc

ttcaatctcaatggtgtagagcactttgagttgattgtacagtcactaagtgccaggaagttctatgccactttttgcgaaactcgggtttttggtgaaa

ataaaaattccatattttttttatcaaactatgtataggttgaaggtcctgatacaaataaaacttcactgaatttctcaggctggtttcatattatttt

acattttaaaggtgaccaccttgaagtgaatatagcaagcaaatttttttttaaaattcaagatgataaaccgttgttatataaacagtcttcttagtgt

aatacttatttgttcacattcattcattcattctttcattataaatctttaaaataaaaaataaaactgtgaaaaatgaaaggtgtaaaatcttagtaaa

atcattcggaattgataacaaatgatatactgtaaaaaattacggtgtatctagtttacacacatcagaaataataaataaataaatatatgttttcttg

aaacttatttgctcttttacttagcaatttagaatgtaaagggtttaaaatactgaacaaatgtatactacaacaagttaaaaaaatcaagatacctcta

ttctgtatgcataaaatccaatacccagtgtgaatgaagatgtcaacacattctcataaaagtctgtacaatataatttctgatgggatcacaatggaac

tatagccccacatcactatgactgcaactggacatagttagcagggaacaagccatactgtcccctacaataccctctccaccaaccttcatctatcatt

tctattcctgtaatgatgtcatctggatcaaaggatatttcatcttcagcagctgcttgatagtcatacaatgctactgcagtatagccagtatcttcta

cttcatacaatgcagtattttctgattgcagattatcttctattgtaggtggtagcatctgttcatcagcaactgtgctctggtttgtgactgctggctc

taaaattagtgggttctgtgtagtcgtgactgttggtgtaggtgggctcactttgtcaggtggttctgcaatttcttcttcttcttcttcttgtccgtga

tgtactgagggagtcaccagctgtgttttcaaaatctctgcttctcttctaatatggtcttcagttttcactatttggttactaggactctggggctctc

cttcaatattttgctttggttcttcagttttcgccatgttttcgaacttctcacgtatgttggatggcctgagagatccaacatctggtttcactttttc

ataatttgttcctactttttctggttttgcattgaaatcatgtgctgatttatcaactcggtctgtctggagtccaaattttccaccaaatccctgttta

tgatccgtttgactttcatgttgatgaagattttcatgatgatcccaaccaactgctgacttatcttgcctgtcagcttggactccaaattttccaccaa

aaccaacagcataatctttttgacttgcatgcttttcagtcttctctacatgatcccagcccaaagcagacttgtcctgtctgtcagtttcaactccaaa

ttttccaccaaagcctttagcataatctttctgtgactcatgtttttcgatcttttctacataatcccaaccaactgcagacttatcctgtcgatctgcc

tgaattccaaatttcccaccaaatccagaactgtagtccctttgtgacgcatgtttttcaattttctctttatattcccaaccttctgcactcttgtcaa

tccgatccttctgcactccaaatttgcccccaaagcctgcactgtaatccttctgggacacatgcttctctacttttgcaacatagtcatgtccaacagc

acactgatccatcctgtctttctggactccaaactttcctccatagccaaatgctgctttgggtccatcttccaactgtctttgtttaaggactgcatct

gcctccgcagtttcttcacgtaattttttcatatctattgcaccaacagttcttcctgagccttcaactgttcgggatccccatcgctgttcctgttcag

taacatcgttaacaaaatctgggtctgtctcccagtcatcgtcatttccatcagctatctgtagattttgtccagcagtcgccttccacattgtgcaata

tttaaacttgaaccacacacctactgcttacatagatacacaacccacatgttgtct

>15546|AGAP004637-PA

caaccaagaggtggtgctcgacgaggtggccgagggcgagatggtcgagaacaagctcgtcatcccggacgacatgctgcagtacctcagcgaggtggcg

gacaactcgaagggctcccagagcccccggctccctgcggcagcggcggcggcggcggcaccgaccgccgccgacaccgtcggctaccggcagcagcag

>15564|AGAP004651-PA

tccgagccaggcagagttggtgacgaggtggtagggtgcgatgaaccacgtgatgagtgtggtagttgttgatacatagccaggttttcaattaagaccc

gttccaactcctttctagtgattaatttcaggtgctgtggtattgcacgcaaatgtggtaataaactcatgagaaagaaatagtcttcaccatactcttc

acacttttgattcttggaagtcatagcttctaggtatactagcttagttttctccagttgtattagattgtgaattggatctcctttctttcgctgaatt

ttttttgccaattttggcagtttttgtgcagtaatacatttgtctggagttaaacagtgggttctttcatcatcttgttctgaaaatgaatcctcttcca

gtggtgttccttcattatacgatgcatctcgtacatctgtctcgtcgaccattgagtgtgttgcatcggttctctggggctttggaacatttccaacaag

atgctttgaagcatgtgtgtcttttaaaaagtgtattttgtggaagtacttccgtttagaatcactgcccgatgatggtgaaccattatgcttgagaagg

tgagtcttgagcgcgttctcacgtgtgaagcacttggggcagtacatacaagggaagagtcccccctccttgtgcatcctttcgtgggtttccaggcggt

agcggctgtgaaacatcttgttgcactgcgagcactggtattcctgcttctcaccggggctccgcaccctcgctgtgacggcaggcacggccctgcggat

gtacgggcgttttgccctcctaactggagccattgtgtcctcaggtgaggcggaagaggacggcaccccggcagccggcatgtcaacggcgtgggcagag

tggaggtggcgcatgagcattacggagctcgagcacagctgaccgcagagcgcgcaggtgaggcggcgctgcagctggtgagacaacacgtggcgcgaaa

gcgatgcaccgtccggcagctgcaagccgcactccgggcatgacgccccctcgactgctgtatcaccgctttccacaacatcctctgcatcccattcctg

cttgcactcttgggtttctacttgcactagaggttccatcacattgtgagggaactcttgaacatc

>15576|AGAP004655-PA

cagtgttcgggcccagcagttcgccgctctccgggtgcaccaccttggcctcgacgttggtgcgcaccgtcccgacggagccctgcggcgggctatcgcc

gctgacgcagccgacggccagtagcacctcggtcatgccgtaccactggctgacgcgcacgcccagcgccccctccaccatgcgctgcgtctgcgcgtcc

atcgtggcgccgcccaccaccagcagctgcagcgacgacaggtcccgccggccggccggcgccttgctcagcgccaccgccgagttcggcgccaggaaca

tgaactccacctacaaacagccacaccacttgtactcttctatcgccttggctatcatatcctcgtcaaagcgaggcaacacaataaggcgacc

>15634|AGAP004707-PC

tctaaagaagcatcaaaagggtgtttgtggttcatggcagcagacatgtagggcacagtgtttgggggagcaggattgttacgacttcttccacgaagtc

ggctctcctttgtgacaggaacaccaccaagtaggtcaccatgagatgtatacgaaactcgtgatgcatgtgaggtatacgatgaatttcgtg

>15646|AGAP004721-PA

tttttgtgtctctatcggctgtactgagctgagacaccaatgcagaaagcacagaccctaattggagacatgggagttacatttaacaatgcgtttgtta

caactcctgtttgctgcccaagcagaacatcaattatcagtggactttaccagcacaatcatagaacagttaacaacacagaggaaggaaactgttttgg

agaaagatggagagaggaggttgagccttatgcatttccta

>15647|AGAP004721-PA

ccttctttttctgttctgttattaggtgaaatttattattacgtaaaaaattgtatttattttgcacataattctacaaatgtgcagcactttcattgtt

ttggtggactgttgcatgctgctcctgcacatgtggacatgtactgcagtgtactttcatattcactcactacatctgcactcagctggtatgctaagtt

gtttaattgatctgggtctgcattgatgtcatatgcttctttgaaagcctcatcatcatcaaactggcagtacaggatattttctgtagctgatatacgc

cgcagacagctataagtgttgttacgtgaatcctggcatttacaagaaccatcgacggagcaatacatgagcttattatccagcactagagggcaatcct

catcaacagtgagatcagatccctcacctacatattctattacaaattctctcccagttgtgttctggcctgctaaggattccttaaatgatttgccatc

cagttccaccagagatgaaagatttgcaatgtcaataacag

>15661|AGAP004742-PB

gacgaaacacagctcacagtagtgttcgtggagtggtaggggagttggtgtgctgctggtttcagcaaaacgctagcgattcggttcagcgcggtccgca

tcacgatcgctcttcggacctgccgttgaacatcgctacgcaaacacagcagccccttacggtccagattcagcgcgtcctgcatctctgtcgttcgccc

gaccggtcgttgaacatcgccacacacacatcacagcagcacaatgttgggagtctgcctggcacggaagatcctgtttcacccacagcagcggcaatgt

gcacactggaacattgccacacgcctctactcctcacacgtggagtacaggcccattcggagcgtcatgtgtgcaaacagaggcgaaatagcaataagag

ttttccgggcatgtactgaacttggcattcgatctgtggccatatactcagagcaggacaagatgcacatgcaccggcagaaggcggacgaatcgtacat

ggtgggcaagggcttgccacctgtccaggcctatctcaacatcccagagatcatccgtatcgctaaggagaacgatgtagacgccatccatcctggatac

ggattcctttcggagcgctcggacttcgcacaggcctgcatagatgccggcctccgcttcatcggcccatcacctaaagttgtgcagcagatgggagaca

aagtggcagctcgcaaggctgccatagaggcaggtgttgctgttgttcctggcacaccaggtcccataacaaagctggaggaagcaaaagaattctgtct

gaagcatggattgcccataattttcaaagctgcctatggaggtggaggccgtggcatgagggttgtccgcaaaatggaggaactggaagaatcgtttcaa

cgagcaacttcagaggccaaggcagcttttggaaatggggccctgttcatcgaaaagttcattgagcgaccgcgacacatcgaagtgcaactgctcggtg

accgcggtgggaacgtagtccacctgtacgaaagggactgctccgtccagcggcgccaccaaaaggtggtggagatcgcgcctgctcccaacctcgatcc

caaagttcgtgacaggatgaccactcaggcagtgaatttggcaaaacatgttggctatgagaatgctggtactgttgagtacctactggatgaacaaggc

aatttctacttcattgaggtgaatgccaggcttcaagtagagcacacggttacggaggagattactggaattgaccttgtccagtcgc

>16375|AGAP004767-PA

cattaacaaactgtcctcttgagttcagagcatctcatctagtgaagagccctgttcccttatccacaaagaaaagaaatttctttccaaatttccataa

taatcctctatcagaaacaagtataacattcatcctaagactctgctgccacatatgcataatgtactcttctgagacactttaaactattcatgaacct

caatgtgagtgttccattgtcactgaacaatcagtaatgtttacatcatcaaatttaaattaatgtggccattaattaacactttaaaaaatactggaac

tgccatcaactcaggttataatttgcagaattcactttttagtacaaatattttgtcattgcaagagacctttcttgctttgctacaagatctatgacta

cagtcacttaaaatatttgccagtaaacatctttatgtttgaataaaacacaaagatcactcttaccaacacttaataaaattttcctttgtcccctaaa

aattaatcacatgtaaatccacttttgtatgtttccctaatttttacagacgaagtatgttacagggtctacatgaagtctccctatggacaaattttgc

tatttatttacgagtcttgtgccaagtaatttttgacaaaaagaaattgtggtattttcttataatgaacagggatcacatgccaaataaggttaaaaca

cacacaccttaacaacaacaatgcctacaataaaataacaataagagatgaaaacatgaatggtaaaaaaagacctgatgaaaataaattacatgaaaag

ttgacagccaacttctctttaaaaaaaggaaaaaaaaatcattagattgtaaaagataagactatggcgaaggtggctacatcgtctggggatgggtaat

atccattagttagtacttgatttcccatcaagttcagttacatgacaggagaacattggaaactgcaagagcaacaaaacatttaacgcaatattgactg

ttcactctaacatgactcgatgaggaggtgggggcggctgataggcgtaaccattccaatagaagtcagaagattgtgtctgtgacttcagatcaatatt

cacttgtttgcagttgttaaatttcaatactgcgtgctagcacattgacattgtaacgtaactgtttccatgttaactttgccctgcgattacagccaca

tacagaggatatccacttgtcagtattaaattttaataatatagttccatctccaagtatttagccacacacagtacagtgttatccattaacaaaggca

gagtcagttagctcactaataaagctgtgggtatctcaaaacagctataaatatactctgtgtctgaccctcaccgagccttcttagagtcaacaactag

tatacccagcaagagtggaataaccacataaaactagtagtaaggaaatcagattcacttcaaaggctcagggaaatgtgatttctcaaaatggaaatgg

gcaatgtatttgtgccctatgctttattttagacagctggctacctcgagtaatgacataacagtgtacacataggaaattttggccacgaaacacacac

ccatcgttaggtgctctgtatgtccccaatgcgacatacctgtagttgcaatcccagtataattaagagactatcgataagaataattaccaacataaaa

taagttttagcattaaaaatccagtacatcgcaaaccaaaagaatggttgactgaatgagtgcgtgctctattacatgaccaaaatttcatagaaagtaa

aaatgctaatgtctaatggtaagaaattatcaatgaatattgagataaaacatgattaacaaaattcactttgtggaagttagatataacactttcgtgc

atatccgctcctgaataaaacttacctcccagtaccctcactattttttctcttcttcgaacatacagatttttgaactgattgtgttttactatggcct

tttgagttactcagtctactgctacttttcacatgtggtgatagttttattttatgagaagtggcacctgatagttctggcagcttcttgtgaacagaag

atgaattcaaaatctgtgttaaatgcgtcttttgtacacccgcctcactatggagtttttccgtttttgcttttgactgtatctttatttggtgataagc

actttgttctgtggcttttgactgtttcacaatttggtgagtacttgatttttgatgagcaaagtgagagttgctgctgtgaaaattatcttgttcaaca

gacattggcactgaaggcaaggaatgcatgcgagatgtttcattactttgtaatgattttcgcactgaacttttagcatggcacacatttaaatctgtat

gagtatcactgtgtgagctcatcacttttctccaagatccaccactacttgtggcattagaatacataacactttcactagtctccaaacttatctgtgt

atgttcctctgtttgagaataataactgtcagaaagactatgctgtatattcctaacatccagattctgcactgtgttttgactatcattactaggtaca

ggctgcgtcagttgaatattttgacagtcttcacagtcttctatctttggtactatggatgcaaaggcaatgcttgtatcaggtatcttcgctggtgtat

ggaaagcttctctggaagacacgagcgaagacgaagtgcatattccgcgttctgtatttgcgattgggtttgcttcgatgtcagttgcttgcgacgtttg

cgagtaatgagacccatttaatacctttaccacgtcagcaccggggaaattcatacctagcccattacaaagctcccggaattctcctagttcttcccat

gtaagtttcataataccttcgcagaggaacgtcagcaacagagtaacaatattataatcgaaatcaggtaaaaacaaggtagccatgtcatcggaattgt

tcatttcttcaataacagtctttagaaacgggctagctgaagccagtattaacttgtgggaatacacagtctttttgttcttgcatacaagtttcacatc

cacaaacgaatgttgttgcacaaaatcattaactgcgttcatggttaaaccatagtgtctggggcagtaaaattgacaagccatggcagaagttatctct

tcttcaataccaagcgcttcgccccctgccttctaatttcaatacaatttgccggtcggtgaacgcggcaaattgtattgaaattagaaggcagg

>16506|AGAP004777-PA

gtacaatcttaacagagtttttactaaaagtaagaatgtaagtaaggcatgtggaagtggaagcaagaagaaaaacagaaagttgtaacaaacaatttaa

taaatatacattttcaaaatgcagtcaataaaaaaacataaatataacaatctttgtcacaaggaaaaactatggctacattggggaatttctcaactct

tgaaataactttttgacaggggacatttcctctctttcattataacataccatcaaatctaatggctcaatatctgccaagttactgtgaggtttttgtc

acagacaatttactcacaggaaatcttccattaaaaaaaaaaaaaatagaaagacattgcaacataaaactacattatcatgatgttacacacaagcacc

ggtatggtattcatttacaggttattttttaagtacatgtttagaaaatgaccactataagatcacaatactgaatgtgaaggagtaatcactctccatt

cactgcctcaaagcatacaatgaacttaactagctgaaatgagacttgttacaaacccggcaaaggctgtaacacttaaaaatgaatgacaaacattatg

atgggtatgatgggttgtaatgactacgtaagtctggattacgctatgagttcacacaagatttgtctgttgaagaaacaagacagtatggctcctacag

cagtattatgctgaagatagtttaactggtctacatggaattaataaagagtattataaataacaataatattgaaataattgccccctccatgaatatg

tactcatataggacaaaacaatcacaccattaaaatgatggatatgttgcagttgttatattacacttcatcaggttcagatctgttgatatggcgcatg

cacaacaagctcacaaagttcatgttgaggaaagataaaaaacaaaaaaggggcaaatgtgaatgcacatatgttatcatgtcttagtccttacataaga

ccctcccaagtctgtatgtaaatgtatgtctggaattagtacagaccacaaatttatctctttctaatgatgcacaatactgtttccaaaaactgttctt

tagtacatctcatgcttttccagtgaaggcaggctcccaattattcataggttgcacaagttttccttacaagacaacaacatacaatcttaacaagatg

acatatctttcataaattttcctgtatccggaacacttttacacagggatctcagcatttttcattctgtcttctatatattcaattaaactttttggat

gaactttcaatctggccagttcattgtacttaaggctaacaactttaagaccaagttgcttaagatgcctctttcgcatcacttgtggccctgttaggtg

aacaccatctgaacaataatgttctggcacatggattagaagagcaacaaataagttcctattctgtcgcaaattaaactgcagcatagaagcaggcata

ttactaggatgtaacaatatatctatcacatacagatctaggacaggaagctggtttaatagaaccaacgtgctcaccctgcttggatgacctgtaatgt

ctacaattgtatcatatattgtgttgatcatgcgttttatgcgcccatcttgccagagactttttgcagatccatctttgggtagtaatggtccagaata

ttgcaagcattctaaagtcatcgcaatatcaaaaataactagattctgtctcaaaatttgtaaggacattcctgatcgggtagtgtgcaacctatcaagg

aaatagggattaaagatttttttaacaaaattaattgggtatttctctaaatacacacaactcaacattatatcaacaatatcctcaggcctaaactgca

caaacttactgtgcacagaattctcaaggacttgccaaaacttccagccattgggaggatcataatctaatatgccaaatggtgtaaacagtgattttat

taaaactggagaaagttctgatccaaactttataaaatactctccacaaccgtgcaaaataaaattatttcggaacctaaatttaacgcaataatccatt

acggcccccattacagctgcgttctttatctttaaactcttggctttaatgtatctttcaaggcctttctccaaattttcatctatatagttaacggcac

aaaatcctgtaatgatctgtaaaatttcatcttctgtcacaacatgtatgtttgtatttgtccatctggacaagatggttaaactgcgaattggttgaag

gctggtttcggcaaacactgataatatttctgcaactgatgcgcctgataaacgccaccaaactgctgctagccgcttttctaatattttaacgacaaaa

ttcctactgctatcgagatgtgctaatatgcgatatacttcaactatgttgttctccgttatatctttctctttttctaacaaaccagtccataacttgt

cggtttcacacaatgcatcttttttgctttttaacgatgcaaaaacattaataatttcacagatttgcaaaacatcgaagttcccgtcagaagcacacac

taaagctgcattgaagagcttgtcccgatactctgtagtattcttcaaaaattttacacgattcaataactttaaacttgctaacaaaacgtccgcctgt

ggggttgacacaataaactgaaaaatcttctcaaaagctttgtctctagtaaacacttccggggcctcgttgtataagctgacattacgaaactgctgat

tgttctctaattcaacaatcctcctgaatgcctgaagtgcaacaaatgaagttttttcatccaaaggtatggtttcaaaaagaacgtagaccccattttt

tgtggaacatcggctgaatgcatctataacttctttcgcatcttcctcataatgattatcttcgatcctcgatgctgtactttcagaagaaacatcttca

gatggcagaacctcagcgtctttcactttccgcactatcgtcggaagttcttgaatatccaaaccatcttgcactataatagttgtctgacctaactcac

tattctttttactgctatatcttaatacggaagctgtcaaacatctagcacacgaaaaattacttttgacaaaacttttgctagacaattgaagagctaa

tgtaatggcgcgagccattgcagcacgctcatttcgcgttcttttctctgtaaatcaactgttttcaaattacgctccaaaacttcatcaaaccaacaaa

taaacacaaacatacacagcacaactacgtaccacgcaaag

>16754|AGAP004793-PA

gaacacgatagacctcagcagggcggtgacttccgcttcgatgtagcggtgtccgtgctcgacattgacgtgggtgtggccgacctccccgaaccgtgag

caacaaagcagtcagtgcgccagcaggcagcgagggacacaggcgctacctaaccatgtctgtcgtacgagtaacaacgcgcgccttctccacggcgaga

tgtcttctcaaagcaagtgccgcagcccagccgaagatgtctgagaagctgacgacggagcaggtgctggagagggaggagaagtacggtgcccacaact

accacccgctgccagtcgcactctgccgaggcaagggtgtctacatgtgggacgtggagggctgccgctactacgacttcctgagcgcgtactccgcggt

gaaccagggccactgccacccccgcatcgtggaggcgctcaagctgcaggcggacgtgctgacgcttacgtcgcgcgccttccactcggacgtgctgggc

gagtacgagcagttcgccaccagcctcttcggctacgacaagctgctgcccatgaacactggtgtggaagctggagagacggcgtgtaagctggcccgca

aatggggctacatgaagaagaagatccccgagaaccaggcgcgcatcgtgttcgtggagggcaacttttggggccgcacgctgtccgccatctccagctc

cacggacccctccagctacggtggcttcggtcccttcatgcccggtttctccgtcatcccgtacaacgacctcaaggcgcttgaggaagctctgaaggac

agcaacgtgtgcgcctttatggtggagccgatacaaggcgaggccggcgtcgtggtgcccgcagagggctacctgaggggcgtccgggagctgtgctcgc

gccacaacgtgctgtggatcgccgacgaggtgcagacgggcctggcgcgcacgggtaagatgctggccgtcgaccacgagggagtgaagcccgacatcct

gtgcttagggaaggcgctctctggcggcgtttacccggtgtcggcagtgctagctaacgacgacgtgatgctgtgcataaagcctggagagcacggctcc

acctatggtggcaatcctttggcgtgccgtgtgggcattgctgctttgaaggtgttgctggaagaaaatctcgcggcaaatgcagagaaaatgggaaagc

ttttgcgtgacgaactctcgcaacttcccaaagacgttgtgtcgattgtgcgtggaaagggtctcctgaatgccattgtcatcaataaaaagttcgatgc

gtgggaagtgtgcctgaaactgaaggagaatggactgcttgccaaacccacacatggagacataatccgtttcgctcctccactggttatcaccgaggag

gaaatgcgtgattgcattaggataataaaggagactgtactgtcatttgcgaagagttagctgtagaaactttgtcctcttgacattcaagagatggatg

aatactacaacaaagataacttttgtgtgtattataatctttgtcaaaataaagtttattgctca

>16826|AGAP004802-PA

ctgttgcaaatatttcccaagaatatgcaagatcggccagctctgtttcttgaagtgattcagagacataatcccaat

>16820|AGAP004802-PA

agaccatgttgttggaaatcagccagccctacaaatggcatcagttgctaaatggtatgaaaagaatttgatgtttcatagattttgatctgttgatgat

tcccagatctacaaatggaatcagttgctaaatggtatgaaaagaat

>16827|AGAP004802-PA

ggagatggagtgaaagatgttgcatttacagttgaagatcttgacacaattgtaaagagatccaaagaacgtggtgcagttattgttaaagatatttggg

aagaaagtgatgatgatggagtgaaagatgttgcatttacagttgaagatcttgacacaattgtaaagagagcc

>16821|AGAP004802-PA

ttgtggaacgtggcaattacaagggtgctttcctgccaggcttcaggaaagttacatgtaaagagagccaaagaacgtggtgcagttattgttaaagata

tttgggaagaaagtgatgatgatggaattgttcgatttgcaacagtacaaacatatggggacacaattgtaaagagagccaaagaacgtggtgcagttat

tgttaaagatatttggg

>16824|AGAP004802-PA

gatgatgatggaattgttcgatttgcaacagtacaaacatatggggacacaacacacacttttgtggaacgtggcaattacaagggtgctttcctgccag

gcttcaggaaagttacaaataaagatggccttagtaaaatattgcctcctgggagacttgatttcatagaccatgttgttggaaatcagccagatctaca

aatggaatcagttgctaaatggtaagttatgtaaactctgtttgaaatactaatacatgaaaatcacaattttgctaatccacttattaattacaggtat

gaaaagaatttgatgtttcatagattttggtctgttgatgattcccagattcattcacagtattccgctttacgatcacttgtgatgacaaattgggaag

aaacaatcaagatgccaatcaatgagcctgctcctggaaagaagaagagccagattgaagaatatgtagattattatggaggagctggagttcagcacat

tgctctgaacacacaagatattatcagttctataacaaattt

>16822|AGAP004802-PA

attggaggaaagtgtagggcgtaaaaatcatagaggaagcccgagggatgaatccagtacgcagattcagaaggatgtaggtttcagtagttattcaaag

gcgaataggctaccacaggatagagtagcagtcttcagactgatgaccataacaacaacacatgggtcaaccactagaagtttccaagctatttgtgctt

tcagatgatattttcccaaacacttgataattcagagtgtgtaatcatcaaaaccacactataaatcaatcattcagaactccaaataggtaaaccagaa

gatgaaagatctagtgaattataactcagagagataataaattaaacttgcaatgaaaaatctgaatagacaacttagttcttacattactcttaggtat

tcaccagggtcaaaaaaatctgaggaaataatattataaattatgagagagaattgttggtcactatttacattcaggatgtgaagtatgtgtacagaca

ctcagagatgagtatgcaataggttggcaaagtttaaaattaagggcaggtcactcagatcccaggatgagaggtacccatccgtagtgagaacaagggt

aggcaaagatgaaacaggaggtgtaatagtttctctgaactattagttccaaaagctaggaagtgtatctcttttacttttacgtatgtctgctggcagt

actacacatcctccctgttaaagatgaatataggtcagcaattttaccccataacattaatttccttgatggaattttccttcaatacaactgaggaaac

aattttggatacagtgttcttagcttatgctctatgacaatcagtgaaaaatcataacttgtttcatttcaggaactcaaaattttaattgactatgatg

aaaatggttacctgttgcaaatatttaccaagaatatgcaagatcgaccaactctgtttcttgaagtgattcagagacataatcacaatggatttggtgc

aggcaattttaaggccctctttgaagccattgaaatagaacaagctgaaagaggaaatctctgatgttgtgatagcatatgcaggttcataatgtggaca

actggtgcacatattacactgatgcagtgttcatgttcatgatttgtatttagatttcattttctggagggacagtagttacctgtaga

>16823|AGAP004802-PA

ctcgtttttttctatttttttgtgtttttttgacttataaattaaaaatctttatttaataaatctaccagtattacaaaagatttatcatttcactttt

gaagtatggtgtttaaagatactttagtatgtggtatatacattacattgtgagggacaaggtaggctgtaatcaattttttctctacaggtaactactg

tccctccagaaaatgaaatctaaatacaaatcatgaacatgaacactgcatcagtgtaatatgtgcactagttgtccacattatgaacctgcatatgcta

tcacaacatcagagatttcctctttcagcttgttctatttcaatggcttcaaagagggccttaaaattgcctgcaccaaatccattgtgattatgtctct

gaatcacttcaagaaacagagttggtcgatcttgcatattcttggtaaatatttgcaacaggtaaccattttcatcatagtcaattaaaattttgagttc

ctgcaaagtgtcaagatcttccatgatatttacattatcattcttcagtctttctttcaacattttataataactatctggaactgataggaactccatc

cctctttgttttaaatttgttatagaactgataatatcttgtgtgttcagagcaatgtgctgaactccagctcctccataataatctacatattcttcaa

tctggctcttcttctttccaggagcaggctcattgattggcatcttgattgtttcttcccaatttgtcatcacaattgatcgtaaagcggaatactgtga

atgaatctgggaatcatcaacagaccaaaatctatgaaacatcaaattcttttcataccatttagcaactgattccatttgtagatctggctgatttcca

acaacatggtctatgaaatcaagtctcccaggaggcaatattttactaaggccatctttatttgtaactttcctgaagcctggcaggaaagcacccttgt

aattgccacgttccacaaaagtgtgtgttgtgtccccatatgtttgtactgttgcaaatcgaacaattccatcatcatcactttcttcccaaatatcttt

aacaataactgcaccacgttctttggctctctttacaattgtgtcaagatcttcaactgtaaatgcaacatctttcactccatctccgtgtttgaccaag

tgcttgcccatttcttcattattgggctcatatggtgaaacaaattggaacaggatcttattttgttttacaacatgggctgcaacttttctagatcctg

tttcaagccctttgtatgcaactggctcaaaccctagccgggtacagtagtaactggcagcctgttttgcatttccaacccagaaagtgatgtgatcaaa

gtgaacaaatcgtcctccatctggcttgtcgcccttgtcggtgtaagtcgtcatgatcggcgttgctcggctggctgcagagcgtcgtgttttg

>16895|AGAP004845-PA

aggtggctggtaacattccaacactccgcttccagtttccaagaaatgtgtttgattcagcatatcaaaatccagagaatgcttgcttctgtcatccaga

tgctgatttttgtcctccatcaggagtatttaatacttcagcatgtacttttggtgcacctatcttcttgtcattcccccatttttacctaggggatcca

agcctaataaatgccgtggatggtctaacacctaatgagacactgcatgactcatatgttgatatatatccagtaagttttaatgttttataaaccaaaa

atgtaaagaactctagtattttaaatagcacagagcattttccattcaaaaattatttgttactaaatacaagggtggtcattgtgtaaatacaatatca

tacctgtacacagtcaacacagatattaagttcaatagtaatatactaaatttttattgctgagtatatgcatcatgtttatgtgtcagcatcctctcca

ctttagacaaaattcac

>16894|AGAP004845-PA

cgtgtccatccatgtatatgttaagttcaatctcaacggtttcccagtcaaattaaacgcaaaaagtaaagaaagtttaaaaccagacggtcaactcccg

actaattaaaacagacagagtcgcattaaaaagtttactgaataaaaacgagcatcccttaggccagcacggaaatgtgtcagactgacgtggtaaccgc

ctcacctgtaagtgtagggcccgacctcgttgacacgcggccgcaagtccttaccctcatacagctgctgggggttgctgtaatttaagacgtagatctt

catgagtggctgcacaggcggcttctgccaccacatgaacgcctgcgtgttgttttccagcaccacgctgtcctgcagagtcctcttgtagacgtcgtgg

gcccacatgtagacggaaccacccaggctggccgccagcagaatactaaggagcagcgtgcgacagacgaggggtcggcccggaggagccctcttgcgca

cgcggccgtgggagtagttggctgccgcagcggcggagtcggctctcgctgccgacatgtctcgggtaacgcagcagagcagagcagtcggccgccgtac

gtaccgtcccgg

>16917|AGAP004845-PB

atgctgaagtattaaatactcctgatggaggacaaaaatcagcatctggatgacagaagcaagcattctctggattttgatatgctgaatcaaacacatt

tcttggaaactggaagcggagtgttggaatgttaccagccaccttgaaacaaatataaaaaaaaacattatcattgcttttgacagtactgatcatgagt

tagagcttctcttcaatacttttaatagtttacatgaaaaaatttcc

>17041|AGAP004877-PA

cagagagcaccctgtcactgattcgtgccaagcaccgctcctttgtctccacgagcacagtgcccggcagccaggtc

>17043|AGAP004877-PA

ctgcagagagcatcctgtcactgattcgtgccaagctccgcacctttgtccccacgagcacagtgcccggcagccagttcttcttggtgcgtgagcctca

gccccaccccaccactacagagagcatgtaggcagcac

>17042|AGAP004877-PA

cagatagcaacctgccactgattcgtgccaagcaccgcccctttgccgccacgagcacagtgcccggcagccaggtctccttggtgcgtgagactcagca

ccacaccaccgctacagagagcatgtaggcagcacaccagccacggagggcatgacacagcagagtgtgacca

>17256|AGAP004891-PA

cctatccaattattgaaatcaataacctcttcctctctgccacattttgtaaaatcacctgcatgtatgaaaatatctccatctggaatgtcaaatctga

tatgtggagtcaatgagtgtgtgtcactcatacacactattcgaaccttatcatctggtatcaaaggtttcggtggacccgtttgaactttaatcacttt

ctgattttttgacagttctttccatgcagcagttggattactcgacagttcatgaatagggatgctcataataatggttccgttatcgaatcccagaaat

aatttactaacggaaaacacactaatcttggtccatacttttatttccaagcagtgcgaccgactgattcacattaccaacaacgttagtattcaaacag

cccgtccatcgatgacgttaacagaacaatatgtcaatgctcagagtttaggcaacatttggtatatcgttagttcaactagaggatgctttctaaattt

ctgtaagtaaaagatttaatcgaatggctcgggaacgtattcataaacttttttgcattacaaggttattgttttgccatacacggtaaacacttgc

>17406|AGAP004900-PA

gtcagcggtggccaccgcgcacaccattgtgggcctcagcgccaacccgtacatctcgtcacactcgtcccacgacagcaccgtgctgttggtgaccacg

ggtccatctttgccgtccgccgtgtggcagaattcgatcagcatgcctgtagttcctgcggtcacttgatcggtcggtagggcgatgggcttcacagtgt

cggtgaataagaagggctggtacacacgcatcacagcgatgtcgtagcggcgactgaaggcgtcgtagtcgccgtgcagtacgtaagagctgacgaagtg

gacggcgctcaacctggcccccgcgccggcagccagcagcatgatgagccacgggctggtgtgcgccacgcagtgggccgcggtcagcgcccagtcactg

ctgatgatggagccgctgcacgccgtaaacggccgcagcacgaacacgtgccacgggaaaccgaccgtgtctccagcaccttcgcgcctcaagagtccat

tcaatgtcgaatggcgtttcctcagtaggcactctgagtgtaccatcccaccaacagcgatgatgagaagtatgccgtagatacgagccattgtagcttg

gagctccagcgttcgttctcccttctgctgcaggttggacgtg

>19286|AGAP004903-PA

gtcaacattcataccatatttaacatatcttgttgtatgtaatacactttttgttataaaacccgaaactggagataaagtcagaaaatttatattaggt

aatatacaagtctctgacaagtattgtgatgcatataatgtacataaattacgagaaatgaaaggggggagggtgccacccccctctacgtaactctcgt

caaatgtacaataaatatgtaaggctttgaaaggaatctcttatagcctaaaactcaatagctaagttgagaacaagttacatacttcgacatgaaacaa

attttttaactcagaataaatttctacatgtttaataaatatttttaaagctgaaatttaaaactgaaaccatccattaacaaagctgcataaattgaag

tgaaatatttcattttgctataggtggttatgccaagacgcgcactctctgatacactatgttcgacagttgaacatgatggcttacgcacatgtggtgt

ccaagacattagggtggttggcaatgcccattagctgccacaacaagacgttcagctgcactgtgccttcaataatttattagtgctaaacagcactaag

cagagaaaggcactaaactggtagacatggggaatttcccaaaattatacattaacacttgaagattacactcacattaactactttcaacatagtcact

caatttatttcagtttattctcaatacacactggtgtgaacttatcacgttttcttatcattttccaggtctccaagcttcaagtctagaggtttgtcat

atcccattatacgagcataatgcgatttgtcttcaaacagcctaggatcaacaaatagaggaatacctcttacgtggaaggaagagaacatgccaggaac

ttctggtacttcgacatcctctgggcgaaatattatctttttttccttcagaaaaaagttgtacactacagcagtataaacttccctatctggcgtgtat

ctaatctccgtcacatacttttttgttatccaatgcaataagaaaggcgttacgtatgtgaaacatcctataaatccgagcattgcaatggacagcggta

aatttccgaaagttgttgcatgttgaataaggtacggctgagcaaccagaccgcctgcactagtaaccaatgaaaataacttcactcctcgaatctgctg

tgccaatggtccatgatacaccttaacatcggtttgttgggtactttggttcctcgcagcaaaaacatttatcgttaatagtcgatggttcaacaactgt

ctctcgcagaaaatactttttcgcaaatttcttgacagcacacacgctactcgcaaagtctccatttctctactcctcctaagttctcgatgaccgagat

ataccattgaacaacgaatagttgga

>19331|AGAP004912-PB

ttgtcgatcaggacgaatttggcttataatctttctaatctgtggttcaaatcccatgtccagcatactccacgccgcctgccgcaccgcaaccaccgac

cacgccatgacggacgcactggactctgagcaggtccaaatgctcaagcgggccttctccatgttcg

>19438|AGAP004920-PA

caccatgacaatataatcagaaagttttatttaagttaaacacttagttccaagaaaatcagacgaaagaaaagatggaagttagagttattccacatta

ggcaaggcgtccggggcagctacaacaggcggccacaggtgtacccggcgtgtgagggaggagatgaaacagggaccctgcaccaagccgtccaaacacg

gctcggacgggtcggtggagcggttgtcctggcccacgatgcggttgacgtcggttagcagcgacacaacgtccttgtcgcccgcatgctctctcagctg

agtacacagcgtctggatgaaccacgagcctttagtgagatggcggaaggagtacatacctgcgaaatcacaaaatcaccgacggaagagtgaggcaggt

gcaagtaactgcgacatcacaaaatcaccgacttaagcatgaggcaggtgcgtgtcacgctacttaaacatattgtgcttgctatttcaacagacagaat

gaaattcacagagcaataaattgcacattttacatcatttaatgactgacaaagtacaaataatttcatatttatgcgaaataatatgtgatttccggcg

caaaagttttggaacgaagtgcagaagaggtgcagaaataaactaaaagccaggaaataatgaggaattagtactgtgtccttccaccgcgacgttgtaa

catttgtttcagctgttttcacatactttgtac

>19634|AGAP004934-PA

gatggtagcactccagtgcggaaataaattgttctgtcttcacttcctactgcccatacctggtcatttggtgcaacagatactagagccatgttgccaa

ccatctcaacccaaccagtcccagttgctgcctcttcactcacaccacagctttctcctctaatgcctttgcgaaaccaaacacgatgatctcttgttac

agcccaaacagcacttgtgccaacagacacttgagaaagtttcccttcctctggtggagccacttcaatccagaattcacctgtagtgttctctctagtg

acaccagctcttacaagagctctcccattccaaaggactgcccatacaaggccggtaggtccaacactaatctggttcacttcacatccagggggaacag

gtattgcagtccatcgttgtccttcaggagctgtagtgctcacaccggtgcgaaacatgactcgtccatgtgctgtttgggctgtaacacg

>19664|AGAP004944-PA

cttattattaaccttattaggaaacacaggaccatctatagcaataaacttaatctcattactaattattggactaatacttctattaattctagaatca

gcagtagcaataattcaagccagcattgaaatagattctctctatgaaggtattgacttctatacatctataacaagagcaaggtttg

>19754|AGAP004945-PA

gcttaatactaccattgctaactatgcaactcacatggggcccgtcatgccagtgaagactctacccgtgagatagtctcggctcacaggccggatccac

agcaccaccagtagcagtggcagtgcaaagctcatgttcagcagcaggcgcagaacacgattctctgcagaatacctgaaacaaaagaaatcaacctata

ttaggccactcaattcaaacagc

>19755|AGAP004945-PA

ttcagagagagagagagagagagagtgtgagtgagtgagtttcatgttagtctgctctgagtaagggtgctgtctgaaagctcagccacctttactgtct

cctgtagttatactttctgttgtaactgatactaaaactgtgctgtttgaattgagtggcctaatataggttgatttcttttgtttcaggtattctgcag

agaatcgtgttctgcgcctgctgctgaacatgagctttgcactgccactgctactggtggtgctgtggatccggcctgtgagccgagactatctcacggg

tagagtcttcactggcatgacgggccccatgtgagttgcataatgagtacacatgaggtgacttcttgcttgcctgttgagcacacg

>20387|AGAP004954-PA

cctatacccgtttgctggccgaaatttggatggagactgctctgcttctcatattccgatagaaatacagatgcaatattggaaggctgcgccttgatac

caaaaagagatgctgcttccgaataaaatgttggtggtggggttcgaaaattaaggagcgtaccaataacgtcgaaagtcactaaacggaaccgatgcat

tgcacgcccaacatacacttctacgaagaacagctgatcacagaattgttttttcagcatacaggtggcttttttttgttctgtgggctcagtgaccttg

gtcaaagagctcaacactaagttaacgacagtctgcttgacgccagtctctggaagaatgcgttgttggattgatgtaaaatcaaatatttgtcaatgct

tacaaagaagagttgtg

>21035|AGAP004955-PA

ctttgatttactgtggtgcgttgaattgtttgtttgtttatgtttttagtgttgttatgaaatgatgctgcgccgacttgtttttcgaagtagacgctta

tttgcacctgagcctgtagttaacttcgcaccttcatcatataaaatctgcagatatttctgtgaacaccggaataattctgttggagactcaaagaaac

aaaatgaagagactaagatttcgggggcttttgcgggcaaatatcgcatatttagggatgaggattcgcctataattctagatgttcaagaagagagact

tattagtgatggcgttgcccaagatattgtaacacacgaagacttcgacgaattctatggattatctcttgctcgaggagtgactggtgtttttgaagta

gaggaacttgtgagtatcctcaggagagagaatatgttagatatttttgttgtgtcagtaccccctgaaattaagtatgtgaattacatggtaatagtca

ctggaaaatcaccaaggcatatgtatgcaacggcagaatttataagaagagttttcaaaaagaaacgacacccacatgatagaataccagagatagaagg

taaagactcaaaggactggattgccatggatctaggtaatattgcattgcacattttttccaaaactgcaagactgacttatgacctggaatcattatgg

tctgttggagctgaatatgacagacttagtaacaagaaagatgatccagtggtggaattactcttgcagcattctgtatttttaggtgacataaagcctt

cagaaaccaaaggttaacctaaagcagaaagctgttttgtgcctttaattgtagttcactgagtaaagactgtgattgtcttttttctgtacattgctgt

agtgaacaataaaaagataaatttcctaggttccagcagcattgtcaaatagattgttacataatattttgagtgcgtgaattgtctttaggtgctgtga

gcagcgatctctgatataatcactgccagaaacagtaaccaattgaagtcaagacacaatcactttagttccagaagaagctgagcaaccatcaaattgt

ttatgaaaaatgaaaccaaaggacttaactgaatcaggaaatcatattttatcaagaattgtacattcaatctaaaatatttgtgtttaatttattgtgt

gttaatggattaggattgtttgcttctatgtatatattatataaatatatttttataacaatca

>21048|AGAP004983-PA

ttacattactcgcccaattcttgttattattctggtactatttttccctttggtattgggtcaccgttacactgctatactatttctcatttcttattaa

agttgccagtacagtgtggaagaagatcataatgctgtcacatttaggcacaaggctttctagattacagttaaaaacactatgtatcagacattgcatc

acagtaagaaccagctggaaaagtactggaaaactgcaaactgaatatatatgtgctgaaccacttttggaacatgcagtatatcgtgttgaccaacgcc

tgggtatgaaaacatgggcatcacctcacctcgaagaaaagattcgaaaacaagacaagttatcaagtgattacactttaatatatcgtgctccaatgat

caactatgttaaagtagctcagggactttgcacaggtcctccgttagttttttgtgggcttcttactgctaactatttctcagacttcttgtttgctgaa

gttaaggatagctattttggactgacccttgaccaaagtgatatgatgatcttcttggtgtcatattttctttttaatgtagttctttattcgtttatat

caagatatgcactaaggatatacagtaataaggaccatgtgtataaaacaatatttcagggtttgttgccctggaatacaaaagaactgagttttaagaa

aggagatgtagtgagaaaaccatcattatatttcctcccttggaaagattcccagtacagcatagggaagaaaaaagttattctgttagaacagtatttt

agaaggccagttgacctgcatgacatgtttaaaaattaacttatatatagaaagccttccacatggcaggtgtatggtacacaggcacacttgaagatca

gataatttcattagctatcactctgctttaagctttagcaagcatgtgcatgctgcccctcccccctatcctctcccccca

>21270|AGAP004994-PA

cgtaccgctgccagtggccggagtgtgagtggcggttcgcgcggtcggacgagctcacgcgccactaccgcaagcacacgggcgccaagccattcaagtg

cgccgtgtgcgaccgctcgttcgcgcgctccgaccacctcgcgctgcacatgaagcgccaccagcccaaggcgcccaagtgagcgaccgctgcccggccg

cgcgccaccgcgtctcggtgcgacccgaggaggacctcggcactgactgcgcgccacagtgtttcttacgttcgccgcgctccaagacttttctgttttc

caagaaacacagacaccgccccagtgctctcggtgaacctctcggtttcagctctcttcccccacgctttatacgagtacagtgacgtaggggttataat

aggagaacaaagggaccgtgaataagaaactggaaagtgtgcggtatatttaattagcaactgtttcccgtcgttcccgttgctgctcgatcaaactggc

gatgagaaaggtagcagttttcgtacatggcattaagtacttgatacaagcaagtagagcttttctgccattaaatataaggtgcgcaaacgatcacttg

ttgctgatggaaatatattagtctaggtcaccaacgtaattttcatttgtcatctcatcattgtgcgtttgtccgcagctcgtggtcttgcggtggcgtt

ctcgcctcccgcgcacgtggtcccggcttcgattcccggtggggtcagggacttttctcgcctcgagatgactggttgttgttgtcttcatcatcatcat

cat

>21507|AGAP005043-PB

cgctcttccgatctgagcggttcgccatcgactggggcacgtacatgtccagccggaacgacgtggtctacgtgaagctggacgtgcgcggctccagggg

gcagggcagtcgcgcgctcttccgcaggctcggcggcgtcgaggtgcaggaccagatcgccgtgctcaggtacctgctagacgagctcaagttcctagac

gagacgcgcgtcggcgtctgggggtgggggtacggcggctacgtgacggcgatgctgatgggctcccagcagagcatcttcaagtgcggcatcgccgtct

cgcccatcgccgactggctctactacaactcggcgttcacggagcgc

>21755|AGAP005046-PA

tggagagaaacaaggcgaaagtttccattaattctgatgtacactttgcgaagaggtactttaagtacctcaccaaaaagtacttgaagaaaaataattt

gagggattggctgagagttgtcgccaacggcaaagacacttccgaattgaggtacttccagataaacagccaagaagacgaggaagaagaagataaacag

ccaagaagacgagg

>21775|AGAP005052-PA

actttggcatataatatttgatacttaagagttaagaaaaattacaaaccaaaaacaaaatacaaggaacaaaacacaccttttccattgggaaatttgc

acatacatacacacacagaattgcaaagtattagtatttaaatcagttcaccctattacaactaataatttcactctacagcaagaccctctaaatatca

atcatgcatttttaaatcattaactttgtatggctcacgcttcaacaccacttcacaagtgatacacctacacattacaatttcaaattttaaaaacaaa

aaataagtcgaaaactaataacaggaatagaacttcttaaagacaacgagattatttccgtatttctaaaaatgaataacacttttacaaaagtttaaat

aatttgtaaactatgaacatacgagactcaacagattacctagtttaaaagaaaagagaagaaaaaaaaaatagctcacttggcgaacaggaaaaattca

tgatcatcatctaatttcatatcatacaactttaggaaaacagcatgctacaatcatagcttatagactggcattcaattcttccatatagactgaggaa

attagctaaaatatttttaatctcttaattaatactgaagacgaagtcttaccaggcagtgcatgaaaacacaaaatcggataaacaactaactacagtc

agaaggaacacaacattctgaggtacctctattactttttgaggtttcaaattcacattttctttggacacagaaagattaaccaactacaaattagggt

gtaaatcttttttgaaaactactacaatggcttccctgagtatctgcagtttttggaacacaacttaagaaattaaatgtcatttctggaaagtgcctgt

aaaaagttttgaaccctatattgtacctacaaatttgatatgtgctcaactacaaagagaaagttatttcattaaacaaaatacatatcaaggcacttat

ttttgataggataatacctaagctttctttatatatttatgactttttcctccaaaacgacttacatatgcagcaatattaaactttcgcttgcaacctt

cataattcatgtaccgtatttttccaaaaactggtctctctagccatccttgatcgtgtattccacacacagaccacatgcacccgacaaatccattagg

atctcctccatccaatgagtatctatcatttaaataaattgcgattcttaaagcttcttctggcgatggtgtccattctaatatcttctttgcccaatac

atacgcatgaagccgtgtatttttccatccttcactaactgaatttgggcagaattccacaaatcatcatgtgttaaagacttctccaattcttcacaag

tgtatatatatgacctcttgtcctttctgtgatcatttagggtcttcaatgcccacgcatttgttccctcaatactgtcgtacttgtcattgtaaaagca

aaaattatctgacagttctcgacggataattgcttcctcacagaaggcatctactgactctgtgtatttggatctgtatgccttcacttcaagaatagct

ctttgaacagagatatggccaaaatgaaaccagggagacaggttgctaagtgcatcaattgtcggatcattccgcttggaaccataattttttagaccct

ttgtgcaaaatttatgcaacattaaaagacccgcctcagtgcctggagttgcccaagtcacaggctgaacacttcgatcagcttctatgaatttttcagc

tgctaaccagttagcttcaatagcatgcttcttaggtgaatgaggatgtggcacaacgggtggatattgtgttaagtactcctgcagtttgctattaatt

ttatttcgtattgtcctggcagcatattccaatttatctgaggcgatccagcacggaacaatattgtgagcatcaacctgacataacggtacatcttttg

ggagacatttctttacatcatctacccacttacgaggctccctcaatggtgagaaatcagtgacaacagctcctaatttatgctgtgtcaaaaactgtgg

caaggtgctgaccggttcaccaaggagtaaatgaaattcaacagccagtttcttacattctgcttcaacctcttgcaatcctttcaagaggaaactgtaa

tgtcgatatgtagtatcaagaaatttgggaaccaaacagaaacaaacatgaagtgagagtgaagtttttaaagccaacttctgtgcatagagcaaggccc

agttatcttgaactctctggtctcttgacatccaatacacaacaccctctgcccattctggcacttcacttgcttttgataatatttgtactcgcttctt

gttgaatttaaattcatttattgatggagctgctttcactctgtcgttgcgaatcttttctataaattctgtccgttcatccgacgttgactttttcaat

tctgacttgtttactgattcatgtgttgttttcagtttctttgacggactgccagacgacattttcaccacgatttctgaaacggctgaatttgtacgaa

ttactaacaatgagcggacagaaaggttgtaattgtacatgccccttccacataatttcaacaataacaatgacgtatggccaagggcaatattgtttgc

acttaggatctctcatgcaagattattcccggtgcccatcaccttaagattatataaacaattccacgtgctatttgtttgttctgtttctgtcattcgc

agcgctaaattttatagagaaaaagtaatctgaatatcagagccacacttcccaagcatatgatttgtttctgtgttaaggtgttcctatctgcaaaaca

aaaccgaagtatgttttctttatataattaaatattaataacaaaccttacaaacacaaacccacttacgcaatttcacgcttcg

>21961|AGAP005079-PB

ccccagtgcacttcagcgttagttcagtattttaatcttgttaatatacacgtgcgcgcaaggctgactaggttttcaaatcgcattactgtaacaagat

ttgtcgtaagcgccgtgtcccagtctgggggtataaagcgtctcgtttgtttttgcgagcggggaaaggaagggttttagtaggctgcctgtagtattgc

gtcgtctgtaccacacccaacaggcagaaaacatcgagtgtgtactgtgcgctaatgtgtttatgcggaaagatttggtggttattattaatgttcatag

gagtgctacgtttgtgagaagccaagtgagggtttgtaaaacggctcggaaaacggctcgaaacagataatattggctgagccaaccctccgaagctaaa

acatggcgtgctgcctttcagaagaggcgaaggagcagaagcgaataaatcaagaaatcgaaaggcagcttcggagggataaacgtgatgcgagacggga

gctgaaacttttacttctaggtactggtgagtcggggaagtccacattcatcaagcagatgcgtatcatccacggctctgggtactctgatgaagataaa

cgaggtttcatcaaattggtttaccagaacatattcatggccatgcaaagtatgattagggccatggagttactaaagatacagtatgcgcagtcttcaa

atatagaaaaagcagaactcattcgaagtgtggactttgaaacagtaacaacgtttgaaagtccatatgttgaggccatcaaggatctgtgggcagatgc

aggaattcaggaatgttacgacaggcgtcgcgagtaccagctcacagactctgccaaatactacctgtcggagatagaccgtgtggcggctcccaactac

ctcccgacggagcaggacatcctgagagtgcgagtgcccaccaccggcatcatagagtatccgtttgatctggaggagattcgttttaggatggtagatg

taggaggacagcgttcggagagaaggaagtggatacactgctttgaaaatgttacttccattatcttcctggtagcattaagtgaatacgaccaaattct

gtttgaatctgaaaatgagaatcggatggaagaaagcaaagcattgtttaaaacgatcataacatatccctggttccaacactcctcagtcatcctgttc

ttgaacaagaaggatctccttgaagagaaaatcatgtattcacatctagtagactattttccagaatatgatgggccaaagcaagatcacattggtgcac

aggagtacattctgaagatgtacatggcacagaatcccgaccctgatcgcatgtgttattctcacttcactgtcgctacagacactgagaacattaaact

agtgttctgtgctgtcaaagacactataatgcaaagtgctctgaaggagttcaacttggcctaatgtaactaacgtgtgcatgcacatattttattttgc

tcctattattaacaccactgttgttgattgcatgttttcaaatcttcttttttctctgtgtcaaagaaatgtggaggcagaatttttttaaatgtatttg

tactgtatcgtgtatttcagtattgggatcactaagcagatattttaaaggttccacattgtcgtattttgagatgtttggaatatgtgggcaagtgaca

atttggataaattgatgtttttataccatttcagggaattattgtaaattcttttcttgtatattaatgaagctgcgaaaaattgaaggtggtgtatatg

agaaaagaattgaaatattgttcacattttatgcccttaaagaaagaaagataactggcaaatgggcaatttttaggtgcaactattcccttcagtatta

catctgcgtgtattgtattgggttattaaactgttctctctgcgaggctacaatgaatctctaaatatctttattataacaatgacaagctggtaatgct

gaaaataacagataactgttttacgaagttcttaaatattgttaaatacgtggggatgcatagacatcgttttgctgtttgcaggtatttcattaattca

tacaatatttgttatggtttctccacatgctaagagattttgttgccacattttgtgtttgatatgggtaattcccttaaaaatgtaaactcaggaacac

tatttgaatgaacctgagatcagtttttgtagatggcttgttctgtatttaaagatgtagaataatttagtgtgttaatattataattactaattttgta

aataacctattcttcagaatcaaataactaatgtttaaatacaattattttctgatatgagtttggaaatgcacaaatatagaaactgttatttgttgaa

cactgattatttttttgtatcactttacataaacagtgataactttgtctggaagaccaaataatgattgcatg

>21977|AGAP005079-PC

ccaatttgaagtggttttctgacttgaccgaccacaactttatcatatcatatcgtttcccttcacttcgcttgcacaactatcaaatgttaacagttat

catttacaccctttacagacaaaacactccaccacgatctggactgccatgggacacttgtgtggtaacttccaaggaaaaagaagtaaatgagtactga

tggtacaatattgactaacaaacaaaaaaccaaaaattttatttcacaatggagaataaagactaattaaacagccaaagcccataaattccaacaatat

agaagcaaacaaattaattatttttacgtcattttacactttcgaggatacaaaatactttcacacacaacgcatatcttcacaacaatgctttgagctt

taacttagtaactaaaacggctgttcaagtttaggcagcgtaccagtgtgatagcctatctttacttaatctgtctcttgttcctcctcctccctgtata

actttaattgttttgaaagcacatactatctggaagctgtttgccagaaatttgctaagaagaggaagacaacacagatacagaaaaaattcatgcacac

agaaagaggtcccagcagcccgtagagagaaaagaaccgcaaagccaacacagtttccattccacatgcagcagtagagaatggaaagccaccatcaggt

cacgagatgacggctgtacctaaaacgaatctcctccagatcaaacggatactctatgatgccggtggtgggcactcgcactctcaggatgtcctgctcc

gtcgggaggtagttgggagccgccacacggtctatctccgacaggtagtatttggcagagtctgtgagctggtactcgcgacgcctgtcgtaacattcct

gaattcctgcatctgcccacagatccttgatggcctcaacatatggactttcaaacgttgttactgttt

>22105|AGAP005095-PA

ccgtcaggcagctcgtacgacttctccagggacgtggaggcggccgcggtcgccatttcctgctcgaagtccagcgccacgtagcacagcttctccttga

tgtcacggacgatttcccgctcagcggtggtcgtgaagctgtaaccgcgctctgtcaggatcttcatcaggtagtcggtaaggtcgcgaccggccaagtc

cagacgcaggatggcgtggggaagagcgtaaccttcatagatggggacggtgtgggaaacgccgtctccggagtcgagcacgataccggtggtacgaccg

gaggcgtacagggacagcacggcctggatggcgacgtacatggcgggcgcgttgaaggtttcaaacatgatctgcgtcatcttctcgcggttagccttgg

ggttgaggggggcctcggtgagcaggatggggtgtccctctggggcgactcgcagctcgttgtagaaggtgtggtgccagatcttctccatgtcgtccca

gttggtgatgatgccgtgctcgatggggtacttgagggtgaggatacctctcttggactgggcctcgtctcctacgtagctgtccttctgccccatgccc

accatgactccctggtggcggggcctacccacgatggacgggaagacggctcgcggagcgtcgtcgcccgcgaagcccgccttgcacattccggagccat

tgtccacgaccagagcagcaacgtcgtcgtcgcacatgattgcggtgtaggtgtcgggtcctgcagcgcaatcatgtgcgacgacgacgttgctgctctg

gtcgtggacaatggctccggaatgtgca

>22609|AGAP005124-PB

cttatcaggcttcatccattcttccaaattcatcaatgtatttgttaggtcatattttagaaactctacttctgtgacaagggcctcttgtttgcatttt

cggagatctgacgctaaagctgcaatcattgcaccttcattctcttcatacattttgtacaattgttttaactgcttttcacgaaatgctgctggttttg

tttggccgctattaaaggcatctcgagcccgttgcacaatattggaataggccatgcttgaatctttcccgttcgccaccggacccatttccgatttccg

ttgtgcagtaaataaaacttagttctgagtatagccttccatcaactagcagccaccaacatgatttttaaacaggacacaaattcactggatgtcaaat

atcccgcataacctcatacatgcacatacacctcagcgcagtcaatcgccatattcaatgatacacttgttactaaaccacacttgca

>22612|AGAP005124-PB

cgactgttgatgaactttatcgcatcaaaagcactctccacatttattataggaaggattgggccaaatacttcttcttgcataacaggtgctgttggtt

tcacatctgtgagaatcgttgggctaatgaatctttctgaagcatctgtttcacctccaactgcaatgcttccattactcagataaccatgtaatctctg

gaattgcctatcagagataattctgcacagatcaggcgagtcctttggattatctccataccattcttttagtatttctttagctttagcaataaaattt

ctctccacttccttagaacatagcacatagtcaggtgctatacaagtttgaccagcattgatgcactttccccaaagaatacgttttgcagctatatcca

tatttacagtggaatcaatgtacacaggacttttaccaccaagttccaaggtggtaggagtcaaatgttcattggctgctgctctcacaattttaccaac

atgtgttgagccagtgtaaaagatatagtcaaaacgttctttcagcaattcggttgtttccggtactccaccctgaacaacatggtaacattcctgatct

aagtactttggaagtaactcagctatgagttccgaagatcgaacagccacttctgatggtttcagcacaacacagttacctgcagctatagcaccagcca

ttggcaacagagataactgcagtggataattccatgcaccaataattaaaactacaccataaggctccttaaaaattaatacgtcatccattaaattgac

aaatcctttcgctggcttatcaggcttcatccattcttccaaattcatcaatgtatttgttaggtcatattttagaaactctacttctgtgacaagggcc

tcttgtttgcattttcggagatctgacgctaaagctgcaatcattgcaccttcattctcttcatacattttgtacaattgttttaactgcttttcacgaa

atgctgctggttttgtttggccgctattaaaggcatctcgagcccgttgcacaatattggaataggccatgcttgaatctttcccgttcgccaccggacc

ttttcagggatgctagttgtcagtgtgactgctggcgctcggtacgtaactctgccggacgctaactggccgagtggccgaccaactgcgcccaccgcct

tgtcttatcagggttgccacgcgcgttgaaccggcgggataacagcgagcggaccgctaagcacggactgtgtgcgccgccagtagctgccgacaaagcc

cgctgcgcgcatgcgtcgggcgacacgtcctccag

>22610|AGAP005124-PB

cgactgttgatgaactttatcgcatcaaaagcactctccacatttattataggaaggattgggccaaatacttcttcttgcataacaggtgctgttggtt

tcacatctgtgagaatcgttgggctaatgaatctttctgaagcatctgtttcacctccaactgcaatgcttccattactcagataaccatgtaatctctg

gaattgcctatcagagataattctgcacagatcaggcgagtcctttggattatctccataccattcttttagtatttctttagctttagcaataaaattt

ctctccacttccttagaacatagcacatagtcaggtgctatacaagtttgaccagcattgatgcactttccccaaagaatacgttttgcagctatatcca

tatttacagtggaatcaatgtacacaggacttttaccaccaagttccaaggtggtaggagtcaaatgttcattggctgctgctctcacaattttaccaac

atgtgttgagccagtgtaaaagatatagtcaaaacgttctttcagcaattcggttgtttccggtactccaccctgaacaacatggtaacattcctgatct

aagtactttggaagtaactcagctatgagttccgaagatcgaacagccacttctgatggtttcagcacaacacagttacctgcagctatagcaccagcca

ttggcaacagagataactgcagtggataattccatgcaccaataattaaaactacaccataaggctccttaaaaattaatacgtcatccattaaattgac

aaatcctttcgctggcttatcaggcttcatccattcttccaaattcatcaatgtatttgttaggtcatattttagaaactctacttctgtgacaagggcc

tcttgtttgcattttcggagatctgacgctaaagctgcaatcattgcaccttcattctcttcatacattttgtacaattgttttaactgcttttcacgaa

atgctgctggttttgtttggccgctattaaaggcatctcgagcccgttgcacaatattggaataggccatgcttgaatctttcccgttcgccaccggacc

ttttcagggctgctagttgtcagtgtgactgctggcgctcggtacgtaactctgccggacgctaactggccgagtggccgaccaactgcgcccaccgcct

tgtcttatcagggttgccacgcgcgttgaaccggcgggataacagcgagcggaccgctaagcacggactgtgtgcgccgccagtagctgccgacaaagcc

cgctgcgcgcatgcgtcgggtgacacgtcctccagcgcccttgaagcgctcagctgacgctgatgagtttgttttgtgctgctcgcacgggcagccccgg

catctccgcgctgtgtgcggtgcgctccactccttccggcactgtcggagatcggatgagctg

>22611|AGAP005124-PB

ttccacaacacagcagtaactgacttacttatatattatttacatattataaaatatttacaatatatatatatacaattggaatcaatattattacaga

ataataccaatatcaaagattgtttacagatctaattcaagtacttcttttggcaaagtactgcttcaaaaagtgttgattgttgtgtatcactatactt

tgagatagtaaataaaatcaggtaaattgcaccaggaagagtgatcttccttaagaaccagtgagacacttgcaacgcatctgtagaaactggcaggttg

ctatgatatccattgtactaaatcccgctcttaatgcagcacctaataagtattactatttaaatgaaagagaactcttcctggatactgtatgtcacct

tctgaggtaaaagaatttaaaatctctcagttccctccttggcttgcctgcacaattccccagatggcaacagttgttaaaacacccaacacaaacatta

taaaatggattatatatttgaaacttattccacggcgtttcttcattataaaacttataagatttatttttttctctgagtaaggtgggtaccttatcga

attaagtttttccactaaaggattgaagttttttcccaagcaacttttcttgtgagtaaaagtgttgaaagtatggacaccatgatatgcccccattcca

ctagatccaactccaccaaaaggaagtgcttcaactgccaaatgcaatactgtgtcattgatgcagactgaaccactacttgattgctccagaaacagtt

tttgtacagttttgtcagttgaaaaaatataaagtgtcaaaggcttcggtcgattatttatgaatttgagagcctcaaaagcactgtccacatttattat

aggaagaattggtccaaaaatttcttcttgcataacaggttctgatggtttaacatcagtgaggattgttggactaataaatctttctgactgatcagtt

tcacctcctatagcaacagttccattactgaggaatgcttgtaacctctggaagtgtcgatcagtaacaattcgagcaagatcaggtgaattctttggat

tatctccataccattcccttagcacctctttagctattgcaataaatcgcttttctagatccttagtacacaacaaataatctggccctacacatgtttg

gcctgcattaatgcactttccccaaagaattcgttttactgcaatgtccatatttgctgtagaatccaagtaaactgggcttttacctcccagttctaaa

gttgttggtgtcaagtgttcattagctgctgctcccacaattttaccaacatgtgttgagccagtgtaaaaaatgtaatcaaaacgttctttcaaaagtt

cacttgtttcagccacgccaccctcaacaacatggaaacagtcccgatcaagatattttggaaccaattcagcaacaagctttgaagtgtgcggagacaa

ttctgatggtttgattatgagacagttacctgctgctatagcaccagcagcaggcaatagtgccaattgaaatgggtaattccaagcacccaaaatcaga

acaacaccataaggttcattataaatgacaacatcatccaacagataaacaagtcctttctcaggcttctctggtttcatccaatctctaaaatgaatca

gtacatttctaacatcatttttcataagttctacttcagtcagcatagtctcttgtttactcttcctcagatctgaagataaagcagcaacaatagcatc

ctcattctcctcatacaatttaaggagctgctttagttgtttttcacgaaaatctgcagatcttgtttttccgctattaaaggcatcacgagccctctct

acaacatttgagaataatgtgcctgattgtttcccattgggtactgacacgtcgccgtcgggctgtcgatcgccgacgagcacggcctccagctgcgcac

cccccctaggcccgtcttcagccgccgccatgctctcgccgtgcttcggtgtagtgcactgccgcgagcc

>24094|AGAP005134-PA

aatgatggcgaagaggagaaaaagaaattgtactggatttatgtggctattgggcaaaaaagatcaaccgttgctcagattgtgaagaggc

>24095|AGAP005134-PA

cctttataagcctgtcattaccgaatactacaactccaacattgtcgggttccaagttcagggccattcccttccaacccgaagcgcatcccacc

>24103|AGAP005134-PA

tgatggcgaagatgagaaaaagaaattgtactgcatttatgtggctcttggtcagaaaagatcatccgttgctcagattgggaagagtctgaccgattca

gg

>24096|AGAP005134-PA

agcagcggctaaacggatggagagtagagccatttcgactgttctgaaaaatatacaggccgacgagatggtggaattctctgcgggtttgaagggtatg

gccctgaacttggaaccagacaatgttgg

>24104|AGAP005134-PA

gcagactggcaaaacagccttggctattgacacaattataaacccgaaacgattcaatgatggcgaagatgagaaaaagaaattgtaccacataaatgca

gtacaatttctttttctcatcttcgccatca

>24100|AGAP005134-PA

tgcagttatttactgtggagtgagaggtcaccttgacaaactggacccctcactccacagtaaataactgcaacttgttcctctattgccattggaacat

attgtccctgtttcagaagctccgttaacctcacac

>24093|AGAP005134-PA

cccttcaaacccgaagagaattccaccatctcgtcggcctgtatatttttcacaccatagactcgagcaataccatctccaatactcaattattctccag

gcttacactgtattccatgtcattatattaataacagg

>24098|AGAP005134-PA

tctgagagtctcttcacaatctgagcaacggttggtcttttctgaccaatagccacataaatgcagtacaatttctatttctcatcttcgccatcattga

atcgtttctggtttataattctgtctataggctacagcctg

>24089|AGAP005134-PA

agaactgattattggagacaggcagactggcaaaacagccttggatattgacacaattaaaaaacaaaaaagattcaatgatggcgaagatgagaaaaaa

aaattgtactgcattgatgtggcttttggtcagaaaagatccaccgttgctcagattgtgaagag

>24091|AGAP005134-PA

cattaccgaatactacaacaccaacattgtctggttccaagttcagggccatacccttcaaacccgaagagaattccaccatctcgtcggcctgtatatt

tttcagaccatagactcgagcaataccatctccaatactcaagactctccccgtttcttctaaatcagcctttggagcagcaccaagtatcctctcctcc

aaaatt

>24105|AGAP005134-PA

caaactggttataaatcagttgataccatgattcctgtgggtcgtggtcagcgtgagttgattattggtgaccgtcaaactggtaaaacagcaatggcga

tcgatgcgattatcgctcagaaaaactctggcattaaatgtgtatacgttgcgattggtcaaaaacaatcaacaattgcaaacgttgtacgtaagctaga

agaaactggcgcta

>24101|AGAP005134-PA

gagaactgattattggagacaggcagactggcaaaacagccttggctattgacacaattataaaccagaaactattcaatgatggcgaagatgagaaaaa

gaaattgtactgcgtttataattgtgtcaatagccaaggctgttttgccagtctgcctgtctccaggaatcattcctcgtatctctgtgagggaacctat

gcagacaggaattaaagctgtagattctct

>24088|AGAP005134-PA

attgtgaagaggctgacagattcaggtgctattggttattcagtcattgtatctgctactgcatctgagcaacggttgatcttttctgaccaatagcctc

ataaatgcagtacaatttctttttctcatcttcgccatcattgaatcgtttctggtttataattgtgtcaatagccaaggctgttttgctacccagtctg

cctgtctccaataatcagttctcgctgacctcgtcc

>24092|AGAP005134-PA

gtctggttccaagttcagggccatacccttcaaacccgaagagaattccaccatctcgtcggcctgaatatttttcagaccatagactcgagcaatacca

tctcgtcggcctgtatatttttcagaccatagactcgagcaataccatctccaatactcaagactctccccgtttcttctaaatcagcctttggagcagc

accaagtatcctctccaatactcaagactctccccgtttcttctaaatcagcctttggagcagca

>24090|AGAP005134-PA

ccgtgaaccaaacttaactaaaactgaacttagtaagtttgagtcgctttaaagctttcaataccagctttgattgctgcttcgatgtctttgttgtagt

caccagtttcatcgatttgtttcatcaacggagcatattctgaacggaagtaagcaattaacgcagcatcaaagtctacgattttcttaacttctacgtc

agccatatagccttcgttagatgcataaacagaaacagcttggtcagcaattgagtaaggagcatattgtttttgcttcattaactcagttacacgttga

ccatgttcaagttgtttacgagttgcttcatcaaggtcagaagcaaactgagcaaacgcagccaattcacggtattgcgctaaagcggtacggataccac

cagacaattttttgatgatcttagtttgagcagaaccaccaacacgagatacagagatacccgcgttcacagcaggacggatacctgcattgaataatga

tgtttctaagaagatctgaccgtcagtaatcgaaattacgtttgttggtacgaatgcagatacgtcacctgcttgagtttcaataattggcaatgcagtt

aaagaaccagtttttcccgtttctgcgccattagtgaatttctcaacgtattcagcagatacacgtgaagcacgttcaagaagacgtgagtgaagataga

atacgta

>24301|AGAP005170-PA

agaaaatgattgtgcgacgattttgttatgttatgaaatttatactggagatgttcgttgttgggacgacgtgttttctgttgttaagaggcaacagcgt

tgtcggttgaggtgactttcgtgacggtgatcttctgtgacgtgataccgacccagaattcggagtagctcttcacgaatgtggccgtgaaaacgatttc

accgccgtcgtttggtgccttccagtcgatatccagcgtgctaagtggcgtcgtgtacgagatgtacgagtaggcgttcttgtgtccgggcgggcagtcg

gtcagggcgatttccttagtttcgccgctgggcagca

>24322|AGAP005176-PA

gctgattgctttaacaatcacttttatcattggctgcctgtccaataattttcaggcacaaaaatacaagcaagtctcaacaaataaattacaatgcatt

ttctgttaatgctaataatgctgttatggtaagacagcagacttgtcattcataattgctgcaagaagttgtttgtcctttagagcagcagcaacttctt

cctcatcccactgtaagcaaacctttgtgagccgcgcttctttacgagcaccttgtaaccggataataccacgtatttcaagcatttgacacaaactcct

gaactcagcctgatccacagctaaaatatttcttctactacaaacacgacggtacacttcatgtagctttgctaaagaaatgtccttttttggtccactt

ttcaacaatagtaacaatgtgcaaacaagaaccttttgttgtataggaaatgcactttcttcattaagggtttgtgatgtcccataaatgttattcaaaa

catccataactagtttagtatcaacacctccagtgcttttacgtggtgatcttggattggctgaattcaggagtttgtgtcaaatggttgaaatacgtgg

tattatccggttacaaggtgctcgtaaagaagcgcggctcacaaaggtttgcttacagtgggatgaggaagaagttgctgctgctctaaaggacaaacaa

cttcttgcagcaattatgaatgacaagtctgctgtcttaccataacagcattattagcattaacagaaaatgcattgtaatttatttgttgagacttgct

tgtatttttgtgcctgaaaattattggacaggcagccaatgataaaagtgattgttaaagcaatcagc

>24335|AGAP005188-PA

agatattcaaccacttgacacagctgaaatctcatgaaaattttatcagtatactttgtcatgaaactacacatttgtacatagaatattcttcattttc

caccagggagcaaagggttgaatgatgtcggtgacaatcataaagcaaagaagattgcctagagactgggttagggataaaaaggcacaaaagagaactg

ttgcttatacaagacaccctttattgttgtcgcttggtaagtagattttttttatctatactatattacactggatgatttgcatgtttgtgaaggttaa

acaaacaaaagcttggaaaatcagagcaacattttgctgataactactggccaatatgacaaggtatcaccagacctatgtgctacactgcacacataag

aactgacaaaaatttacaacaaagaagaaaacaaaacaatattatgcacatataaccgggagcagtgtgcaagagagcttaattcctgtactttaaaaca

gtttttacatatgaatatcacaagtagcaagcaaatcaatttccatgaagaacatactgcttcaattaagtacaactgtacccacatattccaatgttca

tgttaaaaataaggacaatgcatttttcagtcatgacaaggtgaatgactatgagaatgacaaggacagttgacaaactattacccgttttgtttcttta

aaaacagttggtgtcagcacatgcactatgttcttgtctagaatttatggaaacatctatccattttttgggacattgttcatgttgtgaaaaatatatg

atgtagaacatgagtagattttcttttcttcattgatgcttagtgctgacatttctgttatatgtcatgaaaaatttgaaggtcttattttcatattgaa

cctgaatatatatacaaaaactatgataccacacacagtgcattctttggatcatctttcatctatgccgctagtcaagataatttttttgttgttgttg

cagaaacaacagctattataaaatgtttcaccggtaatgaacttacagttctgacttgaaataaatacccattataggtcattggcatctcactgtctga

tgcccatcatcatctggaattatccatataaaaataaactgttcagagacaacagacagtaacacaataagctgcctcaagcttataatagtaggcttca

tatcttctcaatgcacagagcagcactcatcgctcatgaaactggtgcttcctgctgatcactcagcatcatcaccgcctcacttggtgcagcgtatctt

gggctttcagaatctccaatttcagggagtttgtctgactttcctcctagccagaactgaactattacatgcaaaacgaagcatatcagtgaacctatac

caaatataaggaaagtatctgctccaccatactgatctatcagtacacctccaattaaacttccgatggacactcctattccttcaaatgacgcaccaac

aagaccttgtactgttgcttctgttccaggtggtgaaacaatgcttgcgtatgaagtcatggctgagtaaaatattccaaatgtaagaccttggaaaagt

tcaattggcaagcaccaccatggatttgtaagaaaataatacagaataaagcggactccaaatgccagtaaaacaagagacattgcatgtatgtgaccaa

tcttgcgtaatatatgccctgagaggaagaaaaatggtagctctccaccaaaactctggactccatttattaaaccttgtagtgttttgacccattcata

tgtctcacaaccactactttttgccagatcttctatatgccaaaacaagaactgccacaccattgcagtgcacaggccaacagcaatagtccataccaag

aagacaacaattcggatttccgtgaaaagccttccaacatcacgaataatactagcagacagcttagtctgggtgtacttaaggcgtgaggagaccaata

tatccagtgccatcatcacaagcaatatataaaacacaactgtataatctttcttagtttgacccctgctaaactcatcaactaaaatgccagctaagat

tgaaaacgttccccaaccaacagctccccataaacgctgctgaccaaaatttgagggttgatcacccagcattccaaagcatatggcatctccaacactt

actacaactgccatgccgacccaactcaaaatcatgaggagaaaaaacagccaaaactggtaaagaccagcaacatctgcaatttcttcatttacagtta

tcatattattaatcacagtattgttacaatatgtgtcacatttcaaatgtctcacagcactgcacgagggatttaacacatcagtgttcataatttttac

tttatgcacagggaagtaaaaacaagaatacacagaagttgtgtgatttaagcttgccacagctgttatatggagagtgtcattaggaagtacaccactt

cttagcctttggctcttgcaatagtctgacacattccagtacatacagatatctgaaaatattgcagggtttgtcacactacacgccatctcacaatgaa

gagcacctgagctaccatcttctgtaatacgttctgcatcacactggtctgaagatttgttaccacacacattaatgaatgattccccttctccacagtc

aaatattgctacagattttgattcgacaggtacttctggtatgaactgtatagtaaaaaacgttactaaaacaagtacttcacagagaatgaacatcatt

ttctgacgtcgaaaatggtctgcaacagcaccaagtaatggcttggcaagcattcccataattggcagtatcgtgtagattaaaccaactactacactgg

agaatccaagctgtcgggcatacgttggtaagtaaggcaccactggagctgtaccggcattgaacagaaagtactgccctttgacagggagtaaatctgt

gttaatttttatcttcattgtcagttttcgcaaataatgtccttttatatttataaaaatcttttggaaatcaaccacacgcgcgtgctcgtcacgggtt

cacctttaaaataaaacacttaaccaccaatattgggcactaccacatattcatcgaataatataacgcaaacaagacaattaactaaaatatgagggtc

aaacaatatttaaaaacatatttcttcactgcgaacttaaagttcctcctacactcggtacagtggtttcatgcaatgtttgaaatcagtagcacaactt

caattgttt

>24377|AGAP005193-PA

gacatttcaaagtgttgtggcgctatcgcgggtgtaatgtatgtagtacatcgtgagtttgtgtatggaattttcatgtaactgaaatctttaaatgagt

aacaagtctttttaacgcaattgtatattattcattcccactgtggcgtgtgcaaaaacagtaaacagtctgggtgtttacaatgagattgaggttataa

caagtaaacatggaattgccagatgatacagtgtactgcaagtcattatggaccttgttatctggttctatttatgacatttctagtgatgctgttgatg

atgagcttgataaagcccagcctctattaaaaaatgggttgaattactataagagggcaaaagaaccatctgatgatctcctaaaatcaaaactctctgc

tccagtctttaagtttgttaccacattaggaagttttctggatcttgatgctgtgaaagcatggaatctgttgtcgatttatttacttcatgagttccat

gggtctgtgactgggttgcaacagtttgtcgaaaatgatgatcttactgagaacttaatatatgatatttggacattttacagaagtgaaagaatatttt

tgttgaaatgtattaactatattcttatccatcacaatgatgataaacatccatttaagaacaatttcagccggattattacctcttttgaagacctcta

cagatctttaattgatcagctaactgttctgacaaatgaagatggccctacaacaaaaaaatatggtccccttatggttgagaggtataaaaggatgtgg

gtttattcaaacctcagagagcagattgaagttcttcataatttaatattggttactgatgtattggaaactcgcaatgcagaagattttgcaggaatcc

tgagactgtgctctgaaaatggatttggagagcaaaaagaagcttggaaactcctcactgatgtcccagatgaaccttgcaaaattattggcttcttaca

gaatgtacttctcttacgtactttcaactacgaatcagcttgtaacaagaaactttatgaaaacacaaatgtgttttgcttaatagaagaccaggttaca

tctctgggacatgctgaagaacagtctccagtgatgttagtttggggactcgtaaatgcactttgtaatccagatgtgcatttgttagaatcttaccagc

tactttctgagctagctgtaagattaggtgtatttcagtacttgaaaaaatgcat

>24376|AGAP005193-PA

cttcacaacagtttaactaaaaatgctgttaataatatatgccatcaactgacaataattctcacttgaacattctttaaaattaatcagctgcgttctt

gcacgagtaggagaaatgtcttcatagagtgtcggcaactgcgactccttaataacttttgcagtctgtggttttatcaacttttcaaacattttttgta

actcagtgcttagtgactttcctggagtagacataactggaacatttgtggactgttgttcgttgcactgcagaaaggtacgatctccatctgatggcaa

ttgtgcatggtcacgatcttgacgaaaatagggtgtgtctgtcaaaactctgcgaaatggtgatggtgatcctggtgactttaatggtgatccaatattt

ctgccaggaaaattgtgaggtaagttcttttgcacattttcctcctctttagtctttggactctctgcatttaaagatcgacgtaaggtcctatttggat

cactaagactacgcttcagaggaaccattgctgctgcaatttgatcaacatcaaaacctcgagcaagacttctaggaacagattccacattacctgtagt

gtcagacttactaacagaagtttcccttgcaaatcgtttagtgtaatccagaaaaagtgtcatttccatgctcaagtttctgtggaacatctctctctta

atctgtgggagctctgctgaatgaaggagcccctgagtgataacaagttcaaggcacagctccaatagcacagtgcacttttgtttattaagtgttgaca

cccacattgtactcagtccagatgatactgtgctataccgcacagatgtgcttggagtggactgtcctgttcgactaccctttgacagtgcctgtgtgca

aatgtttatcattgtaatcaaagctccaaatgtcaaatttggaattggagaatcttcatctaacattggtgtgctgaaaatagtatcaacaataggctgc

cagtcaacatattcttccatttttccatttagtaatggtagcaatcctggtccaaatggtcttaaaatcaccaagcacatgactgccatttccagtaacc

tgttctgaacttctactatctctggagctgattcagtttcatcgcctgttaaagaagtaattttggtgactggttttgaccgtggctgtttcacaagcat

tttaagaagagaaggatgtatcaagagggcacctgtatagtagaaacaggcttttactccatctataatgtatttatgagaatgtgaatgtgagaacatc

cagttctcatggtaatttgacagcactcttataaaatttagaactttgattgctaagcgcaggacatcctgttttaatgtatgcctgacagtaagaattg

cacttgtaaggtactcttcatgaattgctgcaaacgatatggcatcctccaggaaatgatgaccttgtgatgtcagcatccatgtgagaacatctaaaga

tggcccgtagaccaacttccaaggtgtttcggcccagaaatcactgtccagctttcttggtggtaacagctcgagccacatggccgacttcatatccatg

tgcagaagagcaccagagaaatttttattcagcagcaacagcaacacatcgattaatgaaactgcaatattaaaatgacgagcaaccatgcctatttcac

gtatgtgagacagaattcttccatatgtctgaagcagtgttgggattaaacttgaacatctgtaattattaagccagatttgtcttccctgaaattgcat

caaaaatttgcgaagaagacataatgaaagaatgcagcttccagctttttcagtttctttagatgatgctacttctgaggtattaagccaattttcgatt

tcctttgagccatgtttcccttcttctgtcattgcttctttaataacaggcttctccaagcttgtcatttcaaaagtcacaatagaagatatagcatata

atgtactttcagatgcaaagtcatcttcttggagttgcttactacaccttccatacaactggatgcacaatgtcaatataagttctcgtacattttgtgt

catgactgaatatgaaacaactaggtactgaagcagattcattaactgcacttttgtaagttctttattgcagtggcatgtgcaatgccactcattaact

acaagcagatatgcttccgccaaaatagtaaacagccgtgcatcatcatttcccacctccagttgtaaaacactcattaaatcatgtgacaataactcct

tctgatatgcagacattgatatttctgcatgttttagaaccatggcaagaaattgcttccatgatgtgaggagctttatcttttcatccatatccaaact

aactcctgtttgcttctttttataaatattgttttcaatagcctttctacgagtctgtggcactaatttacttttactactttgtgagttgtcagtgtta

tctgtcattgctcttttcccacttctttctgcttcaggattgattccactagttgatatggtgtcctcaggctttcttttgccaggagaaactgttttgt

tgactgctgaagagcctggttgcgcatcatttgcacttcgtaatccatgttcttcacactcttcgatgttgccaacattatggtgtttctcagcagctat

tccaaatattgctttattcaaaccaatgttccattttggttctaggctctccacaagcaaagatgatatatacaatgaccaattttctaagtaggaagca

tcctccttaaaaaataatttcagtgattttgtaacctcactgcagttttgtcctttgaagcgatataattcaacggttaaaacattaaaaatctttggat

atgctttggagtattctcctggatcacaaaacaaaggacaacaaaaatcggcccaaaagttctctttcgatctaagatgatctataacagagaccatctc

tcccagccatattgtacaaatcaaatcaacaacaacattataaagacctgaattaattagcaatggctcctctttcagtctggaaagaactgaacaaaca

aaaactaaaattcctccagatggtttggtatcattcttagcttttttgccaaatgttgtgatatctggggatgcatagacatggaaaagggactcagtta

ttccaggctgagtatcacaacaagctatcaccaactctaaaagtgcttctttaagtgaatcactaacaatgtgtgattccaaatgtctcacaaacatctc

tctcacaatatcaccctcctttgccatacaagggaaaagatttcttggagattccatggcaaatctcttcaaaagctttacagctaatattggtatactt

gggttgaaacagtggtagatgtatcctgttaagttctgaattactttcaaactggtctcctttaaggggctattgtagatttccatttccaactgtgatg

taagtggttcatcctgaacaagatgtttcaataacattactcggttgagcaaagacaatgacagttgaactatgtagatggcatttgtaggtttgtggat

gtcccaatgatactggtgctccacttttccttgtattgctggatcacctgtggcgacaatctttaggagtgcaaatccacttggtgtatgtagaacacta

tgcaggataagctttctaaacaaatgaaggtaagtggtttctggaccatttgttacctctcccaccaaaagcaactcatggaatactttcatgcatgtaa

ctccaatggacagctgatcacaatcagacacataattccatgtttgatatcgagggaatacttcattcaaaatgaaaataagtcctggtaaaaccaattc

actgtaggcatatgtcttttcttctggtgtaacagttgcgttaggcttgataatcctatacaagaaatttaaataggatttcaataatgcataagtgcca

cttgggcattcttcattataaagccagtcgccaatggttctgacatacaggtaattgcccgatatgagtgtaagatttaatggggcattctctaatgttg

gaaatatctgtagctgcttcagaactgtataaactttcattcggtcttgtgatgcaagtgtggtcattatgtctaagcatgagctgaataccttagctga

agctcgcttaattcttttgaatctagtaatcattgtgacagtgtagttgacagcatttgtaagttcgggacataaatccactttagctaaaagcatgttg

ttgattatatgaagtcctgttagcacattctcttcagcacttgacaggtcaccatcatcacttgataacttctcttcaaccagggccttttgctcattat

aaacagcttcataaaaactgaaattctcaacatgccagctgattaacggaggtgcaatgaatgtacctcgagtgttttgaagaagaaaatttgagaattt

cattggctttctagtgccctgaagtctccaagtgtccgatgattcttgacaaatattcctgttcatatcttcttcacttacaatatcgccaaaccgttcc

atcctcactaactctgaatgtatcttccgagcactgtctgcactatcagaagataatgcctgaacaactctagcaaatggtaagaattgtccgatgggaa

atttgagagcaaggagctcacataaaagcctcaggccatgattttttcttgttttccaataagtatcaactattgttggatgatctagcacggagccaat

gagatcactcaatcccttcatgcaacatattgtattttcatcaaaattatttgcaattaccagcagaagtaaatatacagttcggtatgcaactgctgat

agtttggtaacatcctgaaatatttcactttcaat

>24415|AGAP005209-PA

ctgaatacttaaaaaaaaaacctagccgtcaccgtgcttcatcaagattactttgacacagatttcacattgttgttctcagtgtaaattatttacagct

taaatgtgatgttaaaacagatgacataaacatcttaaagtgtatattgtaacagttttaatgttttatttgttgttacaggtgttcattctatagcata

tgcgttcccccatgtccgtatagtcacatctgctttggataaagaaattaatgaaaaattttatgttgttcctggtattggcaattttggtgaccgttat

tttggaactgagccatcagaggaggccttctgatattgtgcatgaaattgttgcagtttgaaataaacagtatttctgtaacatcagtgacagatagtta

actgcaacaaaagaaaacgtgctctccagaattgttcagtggagcagtgtttaatgatacagggtttttataa

>24980|AGAP005223-PA

tacaatactgacacaaaggaaagtattaagtatataaaagagatgaacatccttatccaagcacagtctcttcatacacaagtactgttgacaacaacta

taagactgtgcatacacaatctcaaagccatgagttacttaaataaataaaatattttgaatgtcactaacagagtaagtttgtgcaatgagaacgttca

agttaaacacattttgtaaatgaatgtgcactatggaaatgaatttatgttgatggaaagaagtccatatgccaattgtgtaagttcacatcactgcctt

gtcactccatattagtgttgtgatcgtaaagatcctgcatttcatcttagagttttggagtaacatctatagaggccattttgtgggtttttacttggct

acatgtaccacttctcaatgtttcacttgggcaatgtctgtatgagttatctgctgtacattctcactccttgaggaagaacaccaagcagttaaaagac

agggcaattcctttgacgtgatagtgtgagcctttcagtatcaataccaagttcaacgagtttgttgtacacttgggctcggacttccactggaagttct

ttcttccttcccagcacccaaaccaaatctgcatgcataacagcgagatcggagcaagaccacagaattgcatagttgtcataatctgtatgtagaattg

tgtacaagccaacaccaggcatcatccttgcaactgctgaaggaagccttgtgttcactctgtagttgagagttgcagaaccttcatagagtgggttggc

tgagccaaggcttgtactcatgctgcctgttaatctgtttacagtcttaatagccactttcacatttccagaaggctttggtgagaagtagattgaagta

cagctgctgatgatctcatacaagtagaacgagcgctcaacttcgtaccacttcccttcaaacttcttgatgtcgaaatcccgcatgccgcggtgtgacg

ggcacctcccgaagccgagcctctgggccgcggcgcctggcgcgtccatcgacacgag

>25006|AGAP005234-PB

gacagatgacatgaaaagggagatatttcaagctgtattaatgctggaggtcatttcaatcctgataaggtgaaccatggtggtccaactgatgcaatcc

gtca

>25005|AGAP005234-PB

agcttgaaatatctcccttttcatgtatgtggaagccatgagaaccgggttctcatggcttccacatacatgaaaagggagatatttcaagctgtataaa

tgctggaggtcacttcaatcctgataaggtgaaccatggtggtc

>25023|AGAP005237-PA

cccatcgatcagccacccaaccgcctgtcaaagacccggtcatgccacccaccaggaagaccgaaacaatgacagaccagagtgtgttcagttcagattt

agacatctggattccgtaagtctctattactgactcgttgcagaactgtttgataatctgaaaaagataaagaaaaaattgtaacacaaaacctttaaat

ttcattttctcgttctcattgccaacccttagacttacgtgagctggagtattcataacgccaatgttgtacccaacaggaatgcaggaacccacagcag

ta

>25091|AGAP005245-PA

ggcgatgttttgtccgtgtaggacaaagggggcactgctgtgcagcttctttcccacactccacacgtaaatgcctccgaagggtatactcgcaagtgta

tctgcgaccacatctttcacagaaataatttccaggtggccgcgtgaggctgagccatagcttatcgaacttacgacggcttactggcgtctttggtcgg

tttccaaacctgtaaattcaacagagttcacacaagaatgcgggtctgtaaataaaacgtttttgattttgttgcgattcacgttactagtccgttgctg

tcttggtcattattaccgattcataaaatactataaaaatcaaacgattctatgcataatccggcaaatcaggaatacaattaaaaaaatcgactttttg

cactgttccgtctagcgcatcctcattcatcatgtttacaaacacaatgtcacgacaaggcacaattaaacaaatgaataaaaa

>25092|AGAP005245-PA

ctttgaactgccacttgaagatcacaaaagtccatgttgttctttactgttgcagtatggctcagcctctctcggccaccagggaagcatttctgtgaac

ggtgtggtcgcagatatacttgcgagtatacacttcggagacacttacgtgtggagtgtgggaaagaggctgcacagcagtgccccctttgtcctagacg

gacaaaacatcgccacagtttaatgcgacatctttttagctgtcatcctggtgcatttgcttaaagttgtgtgggggtgatttttttattttaagatcaa

acttttcttttccatgttcgttttttcaccataaatatgcagatcatttaagtcacttcaagcagtagtattaacatggtattcaaatatgatcagagat

gcccctctgtgcattttgttgagtttttgaagtgaacaataacacttatgagtttgaatagttacattttgattttgtgataccatattgttttatgatt

ttcatgtatacattatatgcgacagactaccagggtagctgctatcccataaaagattacatgtatagaaactggaaagaattatattaaaaactgagtt

caaaaacttggctgaaaatctttattaatataaattcactgtgagacatttcagagatggcattcctattatatttgtaagcattgagaatgtcacatag

cctacaatatgcatctggactagtgcgatgtacaaggtgtgggaagcacacttttacttacaaaa

>25090|AGAP005245-PA

gagagagagagagagagagagagagagagagagagagagagagagtagtactttgtcatgtcaccagtgtactctgtgtctttgtcacactccacacact

caaggtgttcagctgttctctctgttggcaacttgcagttctcgacaagagaacaactacttctgattctctcttaaacaatttaaatcatcaaaaaact

atggctactgcttgttgagaaaatatttcacatgtttacatcactcacaatgaacactacaatggctgaataccttaccttacctacactaaaacaaaag

ccaatgccaaaattctacatatttcctgaagcgtaaacttcagacagttgcaccagaacaacactaatggcccctgtgccatttagtacactggctttta

ctattaagcatcaccttagcagccaccttcttaagtacacatcactctagaatatgtgtatttgtgatgaatcaatacaaaaatctaaaacaagtctgat

ctttaataaaaactccatccctacgcagcattacacaaaatcaccaggatgacatctgaaaagatgtcgcattaaactgtggcgatgttttgtccgtgta

ggacaaagggggcactgctgtgcagcttctttcccacactccacacgtaaatgcctccgaagggtatactcgcaagtgtatctgcgaccacatctttcac

agaaataatttccaggtggccgcgtgaggctgagccatactgcaacagttaagaatgatgtgggcttatatgatctccttcaaatgaaagctttaaacac

aataacataagaaatataaatttctataat

>25139|AGAP005245-PB

tttttattcatttgtttaattgtgccttgtcgtgacattgtgtttgtaaacatgatgaatgaggatgcgctagacggaacagtgcaaaaagtcgattttt

ttaattgtattcctgatttgccggattatgcatagaatcgtttgatttttatagtattttatgaatcggtaataatgaccaagacagcaacggactagta

acgtgaatcgcaacaaaatcaaaaacgttttatttacagacccgcattcttgtgtgaactctgttgaatttacaggtttggaaaccgaccaaagacgcca

gtaagccgtcgtaagttcgataagcggatatttgcactgcacaggatatcatccttgggtataaatctcacagcaaatattctaggatcatctgcagcat

tattcagatgcaatgtctgtggtagagcatatcgacaccgtgaaaatctgacaagacatcagcgagtggagtgtggcaaagaagcacatatgcattgtag

gcactgccaatacaaaacaaaac

>25159|AGAP005245-PG

ctgctttatgtttgcatctgtacatgcataatggacaagctacctgtgcaggttttccacattcaagtctgatatgacgaataagacttgttctgtaacg

ataactcttatgacactggggacatataaat

>25161|AGAP005245-PG

agtctcattggaggtgttttgtatgctacaaaacttacaagttgcagagttctctgactaggcatatgaagtatgaatgtggaaaaccacagcggtttca

gtgcccttattgcaacaaaaaaaaccatcttaagagtaatttgaaaaagcatgtaagaag

>25158|AGAP005245-PG

aatactggcctcatgaaacactaaataaatgcagtgagaatattgtgtttgaccatccctttgggataaatgtgatacttcctgataatcggaaagcaga

ttacctttatacatgtgccaagtgctgtaagacgtacaagtttcagcgatctttgaataggcatcttacatatgaatgtgggaaactacctcagtttcag

tgtccatactgcaatgaaaagagacatcttaaatgta

>25157|AGAP005245-PG

gcacaataactcattgagataaacaaataaaacatcaaaaaaaatttctaatgcacagtattgtcaacacttatatgaatttatgggcaattagtagaaa

tataaatgctactcttaagatcttattaaaaaaaaaaagtaaagtcacattgtcacagcctgccgatgaaccactctgatgtgtcttcgaaaattggaca

tttgtttagctctgtacacacagaatgggcactgcagtgatggttctttgccacactcattttttaaatgtgctgtcagcgaatgtttccaccggtagtg

tctaccacagttgtaacaataaaaccctgtcgtagttcttttgttctggaatgccactcctgatctcgaaaattggaaatcagtaggtctataagttttc

tgcacatcacagtgcttagat

>25160|AGAP005245-PG

tgtgctttaattaagaaagaaaaatatacaaaagtaatgaagagataattacttatgtacaaacaggcatccacattctcagacatctataacccacaac

acacatgaagaaattggaaaacacagactctaacttcagaaacaggaacataaatttagagacactcatttagaggaaacacatcaaacatcactcatag

tgtcccacatagtttgcataaataacagtctaatgttccagagaaagcactacaaatattgctgttaaagattcatttcaaagcttgaaatgtatgttca

gacagcctgctgatgtacatctctgatatgccttcggatggaagacatttgttttgatttatacatgcagtatgggcaccgcagagatggttcttttcca

cactcattttttaaatgtgctgtaagtgaatttttccaccgatagcatctaccgcagttgtaacaataaaacccagcagctgtttgtctgttctggatcc

ccagtcctgatcttggaaactggaaatcagtagtcctatcttgagaatccctatgagcacc

>25156|AGAP005245-PG

cacatttttaataccacagtaaaccaaaagtttcaataatagacataggcaatatgacattacaatcaccttgtattaaacagacgatgtgcaatggagt

cataattgatttggttggtcacatgttaaagctttacaacaatgcaaaatagttcaaacataattggaatttgcttagtgacggaggttctaatacagtt

cttaaaggtctatttcactcacagtttcaagtcttctttgtcccaaccgaaaactatgtaacatttaattaccaagttcacgtgtatgaactgcaaatat

gtgtttcttgagatttcctttcagtttgcatttcttggaacaatagggacattcaaactgaggttctttcccacactggaaacgaacatggtttctgagt

gatgcttggtgacggtatgtgtgtccacactggaaacaggtaaatgggagtctgccaactatcccgctgaatgaagacaactcaggttgatcaataatgt

ctagaagactggatatacctgtaaatcagtacaacacttgaagcacacgtacagaaaggaaacgacactgcaattccaagtattaag

>25190|AGAP005245-PH

ggattctcaaggtgtttttctgtgcacgcgatgtggccactcatactgctcaaaaagcaatttatcaagacatcaacgatttgaatgtggtgtggttcgc

caatttgtttgcaccctctgtggaagaaagtttaagcgtagtcaccatctctcagtacatatcaagtcctatcacagagcttatcaggagacttccaaca

cctagatcaagatgacaacagcaattaagtccattttcatgcacccgctgtggccactcttattcatcaaaaggcaatctctccaggcg

>25189|AGAP005245-PH

gaagcggttggtgctcatagggattctcaagagtcatatcgaagagaaatgaaatttcgaggaaaagcactgctgccttcaaatatctacatgaagcttc

tcacaactggtgtatctacatctactgcatatcagcatcaaacagtagccagctttacttgttcacgctgtggtaattcctatgcgagacctcacagttt

gaacaggcacctgcgatttgagtgtggtgtcgagcctaagtttgagtgccctatttgccataagaagtcaaaacacaaacataatcttttgttgcacatg

cggac

>25186|AGAP005245-PH

ttctgcttttcttttgtactaagatatcattgaaaatgtttcctcatgtgcataataagattgtgcttgtgccgtgctttcttgtggcatacagtgcact

caaattggggttccactccacattcaaagcgctgatggcgccgcagattatcttgacgtgtgtacgtattgcagcaacggttacacatgaaagatcctct

cctatgtatcccctgcatacgatgatgttggccataaaactgttggtatgacctttcatgtttatgtggcaacacagtgtcccagccttgagaatcccta

tgagca

>25188|AGAP005245-PH

agggattctcaaggtgtgagcacacagttgctgaaagaagacataacattcttccaggcatcacaacagtggccacaaggaacaaaaggaagcaattttt

cttgcactcgctgtcacaacacttacactcaccgaaatactctattacgtcacatgcgatttgaatgtggtgtagagccacagtttgagtgtcccatatg

ctacaaaagagcaaggcacaagttccatctgatttcccatatgagaagacatacaagactataattttctagtgagactgtttttaattgtgtaaaatta

ttgtatgttttctggaaatgttatgaaatgaaacataattaaaaactgtcacatcagtatgtttcaaccagaataaac

>25185|AGAP005245-PH

gtcatttttaattagagtttattggtaaatagccagtaaaatatacaatagcattatatataaaaaaactggttcatctctcacaaaaaatttacttgct

gtaaattttcaatcctgttatgctttttcatgtgtttaatcaaagaaaatttgtgtttcatttttatgtggcatacaggacactcaaattgtggttctac

accacattcaaatcgaatgtgacgcatcaggttgtctcggcgagtataagcgttgtgacagcgggcacaagcaaagcttctggctttcacaactggggac

cactgggaaattcccctaaacagtacagaatctccatccagcagctgtggactcacaccttgagaatccctatgagcaccaaccgcttcctgcgcagcca

tgaagacttcatccgtcgatctgtcagttgcagttacatgccac

>25184|AGAP005245-PH

catcacatttgcaaactttcctcaatacaccatcattacagctaacagctctcatgagttttgttttagtagagataagtagacaccactagaaccagag

gtggttttgttatttaaattcaataactgtgtcatttttaattagagtttattggtaaatagccagtaaaatatacaatagcattatatataaaaaaact

ggttcatctctcacaaaaaatttacttgctgtaaattttcaatcctgttatgctttttcatgtgtttaatcaaagaaaatttgtgtttcatttttatgtg

gcatacaggacactcaaattgtggttctacaccacattcaaatcgaatgtgacgcatcaggttgtgtcggcgggtataggcattgtgacaacgtgcacaa

gtaaaacttctggcttttacatctggataccattgagaaattccttgaaagaatacagagtctccttccagcaactgtggattcacaccttgagaatccc

tatgagc

>25191|AGAP005245-PH

cactgagtgaccaaagtgtagtgtctgtctttaccttttgttctgcagctgtttcttgaatgcctttttgtaactccttttcttacctgtttctttcatt

tacttctgtttctttttcaaaatctcctatatttaatatatacttggaatcacaatttggcactaatagtttcaatttttattctgctgagtactagtag

ttttaccagctatccatacatctgctttcaacttacaacttttcttctaatcagtacctacatttgatttaatatctgcaccagctacatatttattttc

aaattcttactgctcattcagagagcagtaattttaacaacaaaaaacatatgaggtaagttatcattttaaaaattttgtagatataactaaaactttt

taacgattctgatgtgtccgcatgtgcaacaaaagattatgtttgtgttttgacttcttatggcaaatagggcactcaaacttaggctcgacaccacact

caaatcgcaggtgcctgttcaaactgtgaggtctcgcataggaattaccacagcgtgaacaagtaaagctggctactgtttgatgctgatatgcagtaga

tgtagatacaccagttgtgagaagcttcatgtagatatttgaaggcagcagtgcttttcctcgaaatttcatttctcttcgatatgactctgcagaaaat

gtaaaaattatttacttaatttataactggcatattgttcattactctatttctatcaacaagtgactacaatgtaaattacagtgatcactgtcgcaca

aagaaaatacattaacacaagtaggtttcataacaacacattcgtgacacatttaagtaataaaaagcactttacttatttacaacagtgaactgcttac

attatatccaaataggtaacattaagttagttataacttagtttatatggagctaatcaagacaatgaaagaaaagaaactatttacattgttcattgga

cttcatt

>25206|AGAP005254-PA

ggcggtgatagcagcacggatgacgtcgaccccgctggggtaccagcgctcgccctgccggagcagcagcagcaccgtgttgttgggtagcgtgcggaag

tactcgccgtcctccacctgcgtcccgtctgcttcaagcacgaggcgtaccggctcacctaccggcaggccgagcttctcccttcctctgacgatgagct

cctcgaagctgctgaccaccagtcctttccggatgtttctccagctgtcccatatcttgaagggcctcttgcctcgtgcttccgtaattaatatgcctgc

aagaaactggttcgttt

>25262|AGAP005262-PA

gtattgaaatagtgacatcagctgtataatttgacagtattaaaaacagagtatgacttacctcctgtgagtacgagaacaataagatcacatgccatct

tcatgagattacgtgcttcttcaccatcagcctctggtttgtgcttctccagaagtgttatgagcaattcacacagtcctgccactgccaaacaatgctt

tacttgttcattctctg

>25891|AGAP005313-PA

gtagtttcaaaagatacacagtttctgtctcaatctttaatatattgcatacaaaattatgtcaatttttttctagttatattctgaccagtaatttaac

atagctgtaaagtgaatataaaaaataatattgatgcaacatgaaagcgtgtgggcagggtacaattattttgatttgtctagtaagaattgtgcaagaa

atgcttgtaatttacatttatatacagaacaaaattcaggtccacatggcagaaacaatgctgacagttgcacacgcataatggaaaatcgtgtctcttt

acaattgtcctggattgcagtattactgtatgtgcagcaaactacagtacacttttgaaaagaaactagtgattatgggtttaacataatgcctattatc

tgtcttaaacacacaacttatttcttgcattattgatataatctgtagcataaattaatgaattccttcaagtggggaataggctttatactgatacaag

tctgaccctccatatccatacaaaagacgtcctcttccttgttcatcataatatgggtatcgatactttggcctctcaaaatactgaaaattcggtgccc

tcggttgcagagttgtttccaccagagtaaacagtgtcagagaaaggacagcaacagcacactactgtcacccccttctgcactatcgcctgtcattagt

atttgtcaaccgaatttcgtgtgctagttggtattatttcgtgagaccgtttaacttgtacaatgactttgtcttgactcttcactagcaaccagtcatt

catccaaacacacactaaagacgtcatttcttcgcgctggatgactgacggtaaaacctcgccaagtc

>25984|AGAP005317-PA

taataccacccatattgtttgagaagatgtaaataaccaagtaactgtaataattcagtatgtttgtttgaagtaatcccaaacaaatgtttaaaaaata

ttattttgtttttattagaagattagtagcataagggctacataacatgcaaagcatgtaatacacaaaaagttcattcagaaaactcattttgtaccaa

tcatcgggttggaatgggtccataatcactgctgggatgattctggaaatgcagaactatcctcataaggacagtagacactgaataaatcacagctgag

ataagaaagaaggcacttgggaaggtggcaatcgtcccactgtaaatctgactatatatggtagctgctaataaaggtgttaatgcctctgcagctgctg

tcattgagaaaattttcccaatatctttcccagggaccaccttagcaacaattgtgcggttaagaggtcctgagactccagaaaacaatcctacagcaca

gcctaagtacaggattccaacatttggtgctgcagcagatattaatgaccctaaa

>26844|AGAP005323-PB

gactgggagtgtgacgtgcgcgacctcagacttctcgaaggggaatacgacgccgtagtcaactgtgccggcctcggcgccgccaagctcgttaaggatg

acgacgaccccgtcttccccatccgcgggcaggtcgccagggtttatgcgccttggatcaagcatgtctttttggaagagttca

>26843|AGAP005323-PB

gtttgtgttacacacaacaagattgtgtgcttggaaagtttattttgacctttatactcttcgagtaagacagagacaaatgaattacaattaaaatgcc

ggtcatacgcctaacttggtaaggttcacaagtttgtaactaccctttttcgtgcatccaaacgaagttgtttctgaacacttccaaaatcttcactgtc

gcgagtatcgtataatgcaagaaactaattcattagacatcctccagagctgatattggcagctgtttttgtaatggtgaacaatttgaacttaatggca

cgcgaaactatcataattgtactgtagctatcattcttggaagtcgtgtgtaggtacttctttccattgcgtacaacgtatgctactgccgataaacaga

agaccccacaaataacacagcgtagtcctcatgtattcgttacgtaatgtttttaaccttgaatgtgataaggtttgcagtagccaagtggacaaacaga

taagattttaacaatttcccccggttttcatgatgagcctattcgcttgacaatatttcaggtaaccggatcaccatgtctaacatacgcattggtgtct

tgggagcaggcgtggtaggtgtgacaactgctttggaactacaaaaggaatatccttcagcttctctaactattattgctgacaaatttggtcaggaaac

tacaagtgatggtgctgctggcatgtttagaccagcctctagtttctgtggtcctactgaagatattagcaggcagtggatcaatgactcctactactac

tacgaagatatcagaaaatcagctgaagcagcacatgctggtgtaatccagatatcagggtacatactttcaagtataaacgaagatattgttcagaatc

actacctggaaaaacttttgcctctattcagaagagcaacagaagaagaactctctttgtacccaggaaactggaagtatggaaccttttacacaacgct

tgtgacggagtgtcgaaagttcttgccttgggggatgcacaggtttgaggaagctggtggacaagtcaaaaacaaacttcttagcagctttaatgatgtt

tctggtgactttgatattgttgttaactgccttggtttgggagcaaaatatttgtgctctgaccgtattatggtacccatcagaggccaggttctcaagg

tgaatgcaccatggataaaaacttttctccatggtgattttgatacttacataatacctggtatagaggctgtaacccttggtggttgtcgaaattacga

gagttatgatttgcgagtgaaccgttacgatacatcgtctattttagaacgatgcactgctcttgtcccaagtctcaagaaagcttctattatcagagaa

tgggttggactgagaccacatcgcagtccagttcgagtggaggctgaaaacataaatggcctgaaggttgttcataactacggtcatggtggttatggag

ttacatcagcacctggaacagcaaagcatgcggttcagcttgtgaaagaaatgcattctgcacgcagcaaattataagggcttgttattaagaggaggag

atgttgttcctgatcacatttacacatacttgccactccacaagacattgactgtaacacataacatcttaatactgtttagcttaagaaaaactcaaag

tttacaatgtacagaaataaaaaagttcttatgacgtgctgtctttatgttgctttgaattttttaccacctgttacatacaaataaatagcctggcttg

tactcatggaattgaaagttaacagatgcaaaagcagtatagttttaggaaactgttgtcataacttgagtaaatgtttgaatgtcatcgttgtaaagca

attcatgtctaattttcttctataagtgtaattttgtgacagtgcttaagtgtttaattgcacctatgtttttgtagcacattttaatgcacgtatgata

ctattttaatctaaaaagattggtttcaccgttgtttcttttatgtcacattccatagtaatgtaattttgtgttcctcattagtgcgtaattttgtcct

ttttttaaaataaaaattttggggaggtattagactcctgaatcacacacttacatgggggtgttatacaaaagtcagttgtttggaaagcacacattct

gaaaattatggaaaaataaatcatttaaatttttctgcaaacttttgtttgtgatgtgtaattgttaacataatttatcttgtccatagttttcttcaag

ggcattattccactccttttcgagtttggtcaacttttgcttttcatttaaatagtttgttagccaattctggtgtaacaattactaattacatcatgtt

gccttcatccttctaattgacctattcaatccagtataaatttcaccaggtcatggcaatacattgcttcctgaaatgtgggtgtatttcattaacaaat

taaagatcattttcactaaattccctttgaaaaatgaatgaattactaccagtttgttaaaatgcataattgccttagttgatacccaatttaattcatg

tgaagtgaagaatcatgtttaattagtgattttactaatttctactcttgatttgtatttcctatcaacataatagtaatttttatttattttgttcctt

tctgcacacttttgtttattttttagtaactgaagcagcagcctaactaattaaaacaacaattgtaacattttccatgaatggaagtatgtgtcactct

attagactcagcccggaatattacagtcaacgatctttcgctgacatatgatagaacagaatccattatacttgctactcttgcttgtgacaactactgt

ttgcccacaccaaatatattgtttgttttttgtgttttgtttctctcttcattctttaatatcatttttaaattcattaaaattatatttttatagctta

taatttgcattctaattacactaacaacattccaagctagtacagaaattccaaattcattatgaccattagtcaattgtgttctgtcccacagaaaaca

ggagagcttgttaaaacattcatttggtatcctacaatgagctctaataccttgatagagtagctacatggtacagctgctatatgacaatgcatcccag

cctgcctgattttgttttgaagttgtcgtcttcaacctttgaagctgcttctccataggtcagtgtggaaacctttcattcatcaaccctgtcacgttga

agagcattttctctgctgccactaaaaagctcattgaatccaccttagcactacttttcagggatcctatattttttactgaagcacataaaatcccaac

ttcgtcagtaaaggtctaatgttgtgctattgctcgtctcccatgacttaagtgaaggttaaacatttaggattcatgtctattatacttatgtaggttg

gtataagtaagcaagatacttgtttctttttaattgaaataaaaagttgataaactatgctgacaatctgattatatatagcttttaaaagctgtgcgca

gttgtatattagtagtgggcatcagaaaatgcattatggaagactttgtggatcttcgtcaaagattttattgagatcaatgatgaatttaggaaaaaca

cagccagcacattttacaggctattgtaattacaatgttaattctgtaactgaagttatgtccagttgttaatgaataacatctcaaacggtggtacgaa

atgagtatttgacacagctcagcatttagaggtgcaaataaaatttgtctcatcatatagtgtac

>27010|AGAP005325-PA

caaggatttatataaatggtctgtgtttaaagctatctgcatattggctgcacctgcatattggctgcacctggtagaagacccagtgttccataaatga

tggtaccaaggcctggtcacgtgtaaatttcacttggacggtaagatgtttaagttgtgagccaaacctac

>27200|AGAP005342-PA

cactttattcatattttattgttttatttgtttacagagatgaagtggttttcgttgtggcgaacctacattaaacaaacactattcttcccactatgat

agagcaattgtcactaatggctccttagtttcaaatagttttaatgccttgcaatttcctatggttaaatacttaatgcttttatgaagatatctttgtt

ataaatgaagggaaagacaacacaaaatttcgttctttgcttttgcatcctagatatctacaagcagcaccagaaattattgtaaaaaaaaaaagctatt

tagaagtgttatttcctaggttcaaatgtttgatagtcaagatagtaaacactattttctgattggttttgtatagcctgtttccagttcttagctaaat

aaattcttaattctaactctctactgattagtcaacgatcattggcaaaagcacattgtaacataagtgcctagcctctacgatagtctgagaagtttat

tcttcatttagccaacttaaatatgcagtaatattacagcatgccatctagtaaacaatgaacctatgagagctggcagctggagtctatgaatttggcg

gttctagttccttcccagttttgtctcccgttttattgtgtagttcagctgtaaaggggtgcctgacatttcagatgtcactttacaatgtatgacaagc

aaattacaggtattacactgcgaccaagcgtgttcagagtgaggagcaccctgtgggggcacagtatatgaagaaatggacgctgtgtatcgtgtctgta

tgtggtaagtgctgcaaaagggatcgccatatcccaatgccaaggagcgtaatcattaggccaatttaaaatgttcgaatggtgtgaacggtcaggttac

gtgaatgccgttcgtcaaaacgaaatttcacattacaatgttgcgtatctggaaaaatgtgaacctaagggaacaggaagagttgcaggcatggaagtgg

atcacaaaatgtgacaacagcttttttagttctgccagatgagtaggacgtagtaataaaataagtaaagccatcatcacccagaagtttgaacactaca

ctgattcagtaggcattgcattgtcgtattagaggcagtacgccattaccttcctccgatcatcaaactgactcatccagctgtttctaaggggaagccg

aactacaatgcatctcgctatctttcgcatcaacaaacgttgccagaggtgaaagaactgaatacgacagattaaaattttaggagggaccagggatcga

atgcttgacctttgtatttgcaggctggcactgcagcactgtgcttccaagtacttctgctataatataatcgttcctgtttgttctctatcagtgttac

aaccatcatcgtgagccgttgagaaagaagggatgttctagatggttctcactatgcaaaaaaaaaaaaaaaaaaaaaaaaaaaaatagcgcagccgtcg

aaggaaggggagtgcctggtccgtaagcgatgctcgcgtgaaccagtagattgcaaggatattgtacatgttggacatgtaactgatatggacatttcat

ttgctacagaagttcaatagccagctgctatggtacccatcacgatacagttccccaccaagtataagattcctttcaggaattccttgcagccactatc

ctttacatccttttctcaagaggtgcagtacctatagctacctggaagagcctccatggaggtttctaagtaatggaagacctcatctttgaatctgtta

gtcaggcgcattctaatagttcaactgaagttccagagcctagaaaactttggcatctggatgggagatcacttccttcgaaaatttctgaaaatgattg

aaaggaaggaaagcgactagttcgtcagatacagagcataatcttgggttgggtatgaaccgaaaaggaaatttgccgcgtttcaaagaaaacacatagg

cttccgcattaaccggtttgtaggtgttctggaaaaacgagttatggtcgggcaggaatctgtatctcgttcctctcgattgtaagttcaatatatttaa

ttcccaatcgtctcactctaatctgttctacagtgttactatgatccacatctgcatagcacgcgttagacgtgaaaaattccgaccccagaatgttagc

agtcatttaccgaagccttcgagaatattatcagcttgttgattgccagtaattcctaaatgacaagagacgacagcaggtttaccctgtggcttttccc

tagcctcgttggggcttaatgacgttctaaaatgctctctacagaatgagaataattaagaatgcaacgtttctgctcctttttgtctcaactgccaatt

gtgaagcgtattgccagaagattcacactctctgaaagtgccagaaaagaggacactggttcaggtaagtagtaatgaaatccgggaactctcgtctggt

tacgatgtccataaagacttccgcagcagctcgcatctttgcgacatccgccttgcagagctggtgctgctgtacgaagtccgttgtcagctgcttcact

agatgcttagtcaccattacgttcgtcttctccatgcagacctgatgtcgtatccactgccagacttgtgacctagagatttctgcggtggcagagtcct

cgacgcaacccctgtagaagaagtggcctcggcctttgaaccagtggaagacgaagagcacggcgacggcgatgttgtggcgcaggccctcctctgtcac

ccctccggctggcatctgcagcaggtcctcgcgagacaccaccagcgcatggtggatcaccgacaattggttgacgtcggcagcggcgcgctcccacagc

tgattcacgtagggaaccaaccgcaaatcatataccatgaagccgtctacaccatgctgaatctcctgcagtttcgcttgacaaactttgtccactacga

gcttctctttgctgtcgtcgccttcaactacgaacgccgccatgccccccgtggcgtgtgcgcctcttctgtggcaggtctgcaccacgagtcgcatgta

ggacaccaggaagtgtttctccatgttcacgtacttgttacggtcaggcaggacgaattccttgcgatcaccgaattttgctataattgaagcagcgtag

tcccatattccgcaattcagccctagagagtgctctctcaactcataaagaatttcttccatctcgaaagacgacagaatattttctattaagacacatg

ctttaatggtaccccgtggtattcgcagagcatcctgggcccatacgaatatttcgttccagagccgcgcttcgaaagcgccttcaacttttgacaggta

gaaaaatggtccactctgattcgtagcgaggatgtgaccgcagtgaaacatcaacagagcgaaatcgaaaacagctcccggcacctctttgccatctacc

agcaggttgcgctctgacatgttccaagccctgggtcgcagcatcagcacggggcactctgcaatgggaggtgtgccgtccaacgtaccgtgcacggcgc

ggtacaccgccaagaggccggtcagctggttgccccacgtgggacagtgcccgtcgtcgaagtccacctggatgccctgtacgtcggctttcagagcggc

cctgaagtggtcgacgtctgcagggctgacatcgcccaggtcgacgtgacggttggtcagcctgccgggcaccggcgctaccctccagtcggcggacgcg

gccctgtcgtctccgtggcggaatgctgggagccgccggccgtcggcgaactcgcacttactccgcgcccgccgccggtagagctcctctatgccatgct

gaaaagtgcgcgtcagatctgcaacgaaggagagggctgccgttgtcagcaatgtccggtgagcttcttccgcgcctggtggcgcaccttccacctccac

attccagagaggcttcgcactggcgtttttcttgcggacgccagcaatgaccctggagaggacgcgtaggcggcgtacagtagctgcgtctgccgccatg

gcggtcccacgaaaaactgcaaacagccgcggccgggctgataagccag

>27221|AGAP005366-PA

tggttgagatgttagatgttcacatattattttgttgatttttaccatttcagtaacacccgttgcggccctctgccactataggactttgaattgcagc

atgtaacatggtccgtccatactcttcaaaccactgtgaagtgtgtggcacagggttgatatgtccaaagtaaagctggaagtaataaaaccctggatag

ctcagaagataacaagtctcctaggaatagaagatgatgttgttgtggaatttgttttcaaccagttggagctaaagcatccagatcccaagaaaatcca

gatcaacctaacgggcttccttaatggaa

>27537|AGAP005378-PC

catttcattcaggtctccttctgttactgtctctcctatcatatccatggctgacttgagctcatccttagtgatgtagccattgcagtctctgtcaaac

accctgaaggcagcaatcaagtccttggtgacatcgtcatctccctcagtgtcctgtgtcaaggcctggatacgtgccacccactggaggaactcagcct

cattgatgagcccattttcggaccggccggcgtccttgaccagctgctcgatgatgtcgtcgctgacggggatgcccaggttggtcagcatgaactgcag

ctcgctggccgtcacacgcccgtcgcggttccggtccagcagcccgaacgcagtccgcaagtctcacggcttctagagatgtcccgtggctgttgtccca

cttgtgttcttcactgtcgctggcgagctctgacgcttccctttaagctcttcctctgtaaactctctttttggaagcgaatcttgccgtggcaagctcc

gcttcctgtgggccatggcgctcagactctgacggcgct

>27642|AGAP005423-PA

ggaagctggaggtttccccagtgtttgatgctgatttgttcgtgcggttacgcagcgggtttggtgtatgttgagaagaaggtagtagtgtcgtgataca

atgggttctagatatgatagagccatcacagttttttctcctgatggacatttgttgcaagtcgaatacgcgcaagaggcagttaggaaaggctctacag

ctgttggtgtacgtggaaaggatgttgtt

>27681|AGAP005431-PA

aatgagcacaccatggtgcgtagaacattactagccactggccatctttccgaacatccaaaaatccaatcctcctttgaacatgatttgttcccagtca

cagaatgagaaactcaaattagtgtcttgcctcacctggaaaaaattctgcatatacctatcactgagttccaaa

>27682|AGAP005431-PA

aatgagcacaccatggtgcgtagaacattactagccactggccatctttccgaacatccaaaaatccaatcctcctttgaacatgatttgttcccagtca

cagaatgagaaactcaaattagtgtcttgcctcaccttattttacatacaaagtcaaaccaaattgcacaaaaagattgtcatcacgttcagctcttatc

gaatacgataaacacaca

>27718|AGAP005447-PA

ggggactgtaagtatatttaagtttctttgatgagtagtatggttatagcacagagcacattgacagtgatccctcaaccatatataccaaaattgagct

ggctctgggaaataatcattaagttccactatcagaaactaaaaaatcagaagccagttttgtg

>27717|AGAP005447-PA

caacaagttggtcatttgatatccagcagttctgaggtttaatgtcactggggactgtaagtatatttaagtttctttgatgagtagtatggttatagca

cagatcacattgacagtgatccctcaaccatatataccaaaattgagctggctctgggaaataatcattaagttccagatgactctcttgtaattttagc

ttcccttttttgacatgcttgccattctgctgacgtaatgcagatccactatggaaatgaagccttggaacagacgttaaacgacttgcctttgagagat

tatatgatggtttctgcaggtgatggaggaagactgctgccaagcgcatcttcaaaactttcattgccctgatttgtgtatgatgaaacctcactcacgt

gtgcgatgtcgactgcccagcagacgcgcag

>27839|AGAP005490-PA

gatgctgcattgtctgttgattttggtctgaaacacacagcttgatagtcaaaaatgtgtatactttggaacacattctgctctatcagctgattcataa

ctgaatgtcggacttcaccaaaaaactttcccgtgtctctatgattttcagaaacgacgacatatccgttgttatccaggaggtagcattccaaaatgtc

tgacgcacatgttctgttcctagtgcagtttggtagaacgtctaaacaactgtatgttatattcctgaacaatgttgaaagagctgagtgctggaactga

aagccaacaactgcagctggggcactgagatggtcctttgagtggaatatggcatgactggctgtcaccaggcttccattcttctcacctgcatcaaatg

gtacagagtagacaaacaaagaacaacacacatcaattaaacaatcagaggaaaa

>27859|AGAP005501-PA

tcatccctaaggattccatctagaacggatcacaggtcgggtggattaggatgtcatgaacctgcaattcacagtgaagggtagatgccgtgcgtttatc

cgtgcatcatcactaaggattccatctagaacggatcacaggtcgggtggattaggatgtcatgaacctgcactctcggatgctgggacaaaatgtagat

gacagcattcgctatatcttctggttccagatatggtgctgtcttgtacatttcaggtgtcaagtgtttagttgtattgaagaactctgtcttcaccaat

ccgggagatacttgctataacgaaaaagaaaaatataaaattagaaagagaattgttccaaaacagaatacaaatagtaattttggctctgaatcgtata

acaaagttctcgtaggttgttttaaactatgttatttgagatacgtagcatacagaaataaatctatagtatattctccgcaataatatacagttctctc

tttatggtgagctttttcctcaataccaagtgtgaaggaatcagttaatttttcaggtttaaataggcgtccagccaagttcgag

>27860|AGAP005501-PA

agggttacggcaatatgtccaggcctagtgaagactgaaatcatggataacaccaaggaaattgataagaacaaagttttttcccagcttccctatctgg

agccagaacatatcgctaatgctgtggtgtatgtcctttcacagcctccaattgtgaacgtgccgcagcttgtaattcagcctgtggggcaaagaagaat

aagtgatgtataaagaattgaaaagctgtaaaatatatgctaaaaaataaattaaagaagaataagtgatgtataaagaattgaaaagctgtaaaatata

tgctaaaaataaattaaggaaaataagagacacagagttactgcattttgaatttgtagcagaacatttagaattcaaagatgctgtttccatggtgctg

aagtatcatagattttatacgtgaaatgtattaaatctaattatagtctcttattcaaagttgtatagagctgctcatatttgtctttttatcattatta

catcaaggagactggataacctctggtatagtgaattcacaatgcttgatataatgaataattattgatgatattgcaaatccaatttttcaattataaa

aaatgtcttttgtcaccaaacacattttgttttattgatacaaaacaacataagtgctggtactttatgtcagacttttccttataacaagaaatgttat

ctgcttgcacatttttgaggtattttcttatttggcactaatgtttttgatgagtctcacagtgttaaccagaactgtaagttttgtgaattcaataatt

gtaaagcaagtacagatactgtgcctacacaaatgaagaattaaat

>27978|AGAP005502-PA

cgacatgataccctgcctggagtctgaggacgtggcggaagccgttctctacatgctgtcgcagcaccctcgagtacaagttcatgacgtccttatctat

cctactggcacttcctagtgacaacgctgtgtatcgtgtcaaacaaatcagcgg

>27996|AGAP005507-PC

ctgcaaacacacaacttcttttaaatttattatagtgctaaaaaataacaacttcaaggaatatgctttacaatgtctactactcataaatgccagatat

ctgaattaaagcttcacatacatttaaatttcaatctagtcatcaggcatactttattaaattagtttcaacagtttttaaaattatttatatactccac

agatactttgtctcacttactttactgatagcaaaagattttctgtaatgcacgtgtaatggaaaacagtactacaatttttccattaaaatcttagcat

ttgtacatagtaagattccttgtctgttaattttggtttgaagttgtaacatattatggtcaacagtagtgcaatatatatgtacaggttatatgaaaga

gtgctattgtgctcacttcagtaactgctttttttttttaaataattcatgtaaacttcacactgaattctactcagtcagtcatctttaaaagaagtgg

tgtatgagaaacacattaaccttggactgctgacattcttccttgtttgtggactaggaagaggttcttaaagaggatgatttcaccattagtacacatc

caaagttatgtttggttggttgaagtatttgtatgttgaatacttatcattcttggtgaagtttattttacgtattaaaggctaaatgtcaacttcaatt

acctaaggtttaaatttttgagagtatgtcaccatcaaatctcaagcactgcctcttcaaattctatttacataggccaatcctaatctctacgacccca

caaacaaatcggaaaaaaagaaatcaaataaatagtataactggtaaatggtatcccaaacatagacctttgaagtctacttctttgtttttaaagaata

tatgttagtacattttacattccattcatttcaatacctatcatttacattttagcatggatagtattattttcatcatctattatcaataatctactca

gattgtaaatgaagtcatacatacaatctccagtggcaaacagttagagtggttatatgttccgaatatttgcataggcataaagacaacaaaaacattg

ttaaagtgtgctataacactaaattattctcactataaccaaaactgaatagaattgctagttatatatatttgccagtctgtattgtcttgtccatata

tatgattatcataatatttttcattgaatttttctctcaaaccatacaaaaaattcagaaatatttgaaaaaagtagtacagttctaatgatctggcaca

cctgcttcaaggcatgtggaaaaaatctttgtaatgatgtattagtatgaactaactgtggatttcatttgctcacttcacacatagctcagaaaagatg

atcataacattatttgtagtcacagcattctatatctttatcacaaagagcagactaagaccaaaatagaaaagctctaaattctgctgcatctgaatat

ctgcaaatgcagtaacaactaaaaatataatgctgaaattatgtgtaaacgtcatatcatatttacaataattaattcacattcacaacatcagtacttg

attgtgttgttcacagtaaggcaaatatcagaaaacattaacactgaagttcatttctttaaatggtatgctaatgcactaatgatactttcattcaaca

catggtatgaatattacagtatagtatagtccaactacccttaagtatgcaccataaatttaatgacaaaagttgtcataacaaactatacataactttt

tacatgtaataatatattatggttatcatatattcgttaacaagacaacaatacccgtgaactttcacaaaggtaaatctaggtgcttgcaccccggtgt

tcttgatagtatatgcagtatagggtaattcataaacataacagcacatattgtagtatcaaatacaagtcagacctttcaaaagaagagtaataagtta

aaatgaaagagaaatggctttctttgaatatgtaagatgaaatatgcttgtggataattattttcccttagcgagtttttggaggttcttgtggagcctg

aggaggtatctgtcccacattctgagtcattccaggaggtggtggtggtggtggaggtgctacaggagctgaatcaggcatcagtttaacaacaattata

gctaatataaccaatccaataactcccattataatcatcattttgagattttgcaaccaaaacttcctcttcagctttcctgcttgttgttcaaattgtg

aggctccttgctggagggcatctgctcgatcatcaagttctgatagtttctgatctcgttcaagtactttctcgacattagttttcatgatgtcaacaac

ctcgtcgacctgcgcctgtgtttgctggagcctcttctgcgcggctatctgctgcggggtcctgggcccgcccacgatggcgccatcctcccctggcgtc

aggccggcacctcctggcgctgcgggcgctccctcggcagacatggtttcgcgtagcactgagcgtaaaacggaaaatctctttcacacaatatatccag

cgctggtattgataatgtgcca

>28081|AGAP005533-PA

gcggacattgaaaatggacctgcttgttgggatattgcaaccatcgtgtagtgactgatcacgaacaaaagtgtctgcagaacatgcgagcgtagttcct

ttattgcagcagctaccaacattgctgtaagagcagtctgatgagttgccctggatctggcatcagggcatttatatgggac

>28082|AGAP005533-PA

atgggacaccttgtattggatttatcttgacactctgctctgtgaagcttggatgagagaaaagcctcttcatatctttcctatcattctccaagcttat

ggatgcaattaagtagcaattgacaacttcccagctctgtctcctatagaacggatcagtcgagctgctcttgagggcggtgaatgcagtttctataatc

ttctccacaggtaagtttatctgcctggaatgatcctggaagcagactgccatctcaggtccaagactgtcagttgtgctgtatgtcagtttctgaggct

ccaccatcatcttacggtttcctcctccaaattttccaagaactctaaat

>28180|AGAP005557-PA

gaaaaattagcatattttcttgtagattctgctaatttttcggaataaaaactgtttatcttcggtagttctttatcacagtagtggaaaaattg

>28178|AGAP005557-PA

gaaggataaacattttttgcataattattattcatacaactctgcagtaagttatataacaacaattattaactacagggatagacggcaagaacttctt

aagagcataatctttctttttccccccctagagatcattacatgtaataccacaccccccccacacacgcacacttgtgagattccataatgatctagaa

tgaacatccataaaactttacaacacatagatgcattatttgttgtcattttttttaaaatgaaaattattgatccaaatatgtctcgaataacctgtag

gctaggtacaaagcatgacttaaaatgattatacacaaatacctttagtgcaacatgcattcaaatagacaagatactagaaatttgtgtagtgtttacc

ctggaattagtcttattatttgtactagactatttgctgttgggcataacaaaaactatgcaaaatctgaaaaaaatattaaaagtcactgtctgtagat

caatataatactgtgagattaagtgatacagtacaaaactgtttatgtaatactcaataaaattttgcacatacccacaaaagtttctaaagtaaggata

gtaacttaatttgtactgatttaaatgttcagtttatcatcacagacaatggagcatacaaaaatctatgtaacaataaatatagcttctctatcttcct

ttcattccagaacctgcaacaagttggtctcctgaaccaagtctgatgctatacagcaccactgacaatgatcttttataatatctacacaaaagtacag

cattgaccaccttcaacttatattaggtactataaaatgtatccaagagttgtctgtggctcattaagaaagtgagatcatcaacatctatcactgccat

actgaattacccaagtttactctttctagcagtagccagatgcttcatatgagaagcaaagtatggtgtagtaaagagagaacttttggtaggaaaactc

agctatctggatcatccgttgattctccttccaaaaggaggcggtggtcttctcttttcttactagcatccaatttcttcttgcgccgattcacaacacc

attttcttcatccatcattttgattatttgtgtctgatctgatgaatccataggtgcaactgatatatccctcacagctctgaatttaccacagttgttc

aaatgctcattctcaagtcgaaagaagttccatacaaacctcctgataacttcaagtggtgacagaactgataccattagatctgaatgcacatatccca

tttctgttaaagacattgaaagggcccagccaaatcttaagataaagtcttccacaatcgcaaagtaatagaaccaagttgaagaatatacaatttcttc

tcgtaagtatttgttgtctcctgctttcttgtcaaacaatccccaatccatttttatatcccaagtatatgcataacaggagctaaggatatatgaaagc

acccacaaataaaaatatggattttctgaagtaaacgtgtatttacctgcagaacagacagatttcataagatgacgataagatcaactggatagaaaca

aatgaagat

>28179|AGAP005557-PA

caagaaatcacttgtcagaacagcatattatggaaatggcagcaatatttggagttgtttggacactgagtgtactgagtttcttgtacagctctgctct

tggcattccaccatatgcttgcccactcatgctcataatactgatggtactgttcacattcaacccaatgaagatttttcgtcatgaagcacggttctgg

gctattagggtgctgacacgaatcatactggctccatttttctatgtgaactttgctgatttctggttggcagatcaacttaatagtcttgtggtagttt

tcactgatctgcagttcctgatttgtttctatgccactaataagaactggatggttgctgaagatagccgccaatgtactgacctccgtggtttcctacg

accaatggttgggtgtctcccagcttggtggcgatttgcacagtgcttgcgccgatacagggacaccaaagaggccttcccacatctagtaaatgcagga

aaatatgcaacatcattttttgtcatcctcttttcagcacttaatattgcaaatgaaagtaaatacacgtttacttcagaaaatccatatttttatttgt

gggtgctttcatatatccttagctcctgttatgcatatacttgggatataaaaatggattggggattgtttgacaagaaagcaggagacaacaaatactt

acgagaagaaattgtatattcttcaacttggttctattactttgcgattgtggaagactttatcttaagatttggctgggccctttcaatgtctttaaca

gaaatgggatatgtgcattcagatctaatggtatcagttctgtcaccacttgaagttatcaggaggtttgtatggaacttctttcgacttgagaatgagc

atttgaacaactgtggtaaattcagagctgtgagggatatatcagttgcacctatggattcatcagatcagacacaaataatcaaaatgatggatgaaga

aaatggtgttgtgaatcggcgcaagaagaaattggatgctagtaagaaaagagaagaccaccgcctccttttggaaggagaatcaacggatgatccagat

agctgagttttcctaccaaaagttctctctttactacaccatactttgcttctcatatgaagcatctggctactgctagaaagagtaaacttgggtaatt

cagtatggcagtgatagatgttgatgatctcactttcttaatgagccacagacaactcttggatacattttatagtacctaatgtaagttgaaggtggtc

aatgctgtacttttgtgtagatattataaaagatcattgtcagtggtgctgtatagcatcagacttggttcaggagaccaccttgttgcaggttctggaa

tgaaaggaagatagagaagctatatttattgttacatagatttttgtatgctccattgtctgtgatgataaactgaacatttaaatcagtacaaattaag

ttactatccttactttagaaacttttgtgggtatgtgcaaaattttattgagtattacataaacagttttgtactgtatcacttaatctcacagtattat

attgatctacagacagtgacttttaatatttttttcagattttgcatagtttttgttatgcccaacagcaaatagtctagtacaaataataagactaatt

ccagggtaaacactacacaaatttctagtatcttgtctatttgaatgcatgttgcactaaaggtatttgtgtataatcattttaagtcatgctttgtacc

tagcctacaggttattcgagacatatttggatcaataattttcattttaaaaaaaatgacaacaaataatgcatctatgtgttgtaaagttttatggatg

ttcattctagatcattatggaatctcacaagtgtgtgtgtgtgtgtgttgtgtggtattacatgtaatgatc

>28299|AGAP005599-PA

cgagcagaaatacattacgtatgtgtggaaaaacagtgaggagacattgcgaaagtctcccagtctgaccacccttaatgcgtatctcatcaagaatgca

accactgaatgcgagaagaagtccatctggagaggtaggtaaaactttatactgag

>28332|AGAP005616-PA

ttatttctgcattcattaataaacaaaaaacattgtcagtattctcataacctatgttctgattttttttttaggctcaagctcctgaggacttatcagt

tcgacgtggtgattggatatatgcagacctcaataatcagacagtggatggttggctgtgggcatatgctccaaaaactcgcaagtacggtttcataccg

aaagcatatgctcgaccaccagctatgacaagtctatagaaagaatagtctacgaagaagttttactccagctgccattaaatttattttgtttgt

>28874|AGAP005621-PA

gtgggcaggtggaggaacttataatccaggctgagaacaaactgactctagcacgtaagatgctcgggtagaaaccgag

>28873|AGAP005621-PA

gtttgtcaaataatttctgactaagaaattagtatttgaaatgttaaacaagaccccgcttatagaaagtacacgcaagaaatagtcatggaacgagtac

gcatcgtaaagacaacttcaaatgttcaggaaattgagaagaaaataaactgc

>28872|AGAP005621-PA

ttctcagcctggattataagttcctccacctggccacagtttattttcttctcaatgtcctaaacatttggtgttgtctttacgatgcgagctcagcctg

gattataagttcctccacctgcccacagtttattttattctcaatgtcctgaacatttggtgttgtctttacgatgcgtgctcgt

>28875|AGAP005621-PA

gtcctgaacatttggtgttgtctttacgatgcgtgctcgttccatgactatttcttgcgtgttctttctataagcggcgtcttcaggcattttctgcaga

gccctaagtatcttaccataaaggactcctagtgtatgatgaggatttacggcaactgccaacccagtcaatcctgttgcctttttcag

>28870|AGAP005621-PA

atcaggggctcccacggtttccactggtctggtggtggttctgtcatcaggggcccccacggtttccaccccagcattttacgtgctatagtcagttcgt

tctcagcctggattataagttcctccacctgcccacagtttattttcttctcaatgtccttaacatttggtgttgtctttacgatgcgtgctcgttccat

gactatttcttgcgtgtactttctataagcggcgtcttcaggcattttctgcagacgccgcttatagaaagtacacgcaagaaatagtcatggaacgagc

acgcatcgta

>28871|AGAP005621-PA

gccgtaaatcctcatcatacactaggcgtcctttatggtaagatacttagggctctgcagaaaatgcctgaagacgccgcttatagaaagtacacgcaag

aaatagtcatggaacgagcacgcatcgtaaagacaacaccaaatgttcaggacattgagaagaaaataatctgtgggcaggtggaggaacttataatcca

ggctgagaacgaactgactctagcacgtaagatgctggggtggaaaccgtgggagcccctgatgacagaaccaccaccagaccagtggaagtggccacca

accaagtagtgtggtattcatcttgtgtagcaagtactttaatgttaataaaaggccataatattttcaaccaaaa

>28920|AGAP005624-PA

tctacactctttccctacacgacgctcttccgatctctagtacatgattctgatacacagcagtaacattaccacttgctgtgtatcagaatcatgtact

agccagatatgaaaacatacttcaagaaatgcctgctattgtaaaggaattaaaagcagagacggcgtacgctgtgaaaaaattccagtggacagaaaag

caagcaagagtagtatataaccgctcattgcggaagcagttaaaaaagttaattataagggataactgtcatccagccaaagcatcattactagtgtggg

ttcagattccaatgtggatttgtttgtcggcatctctgaggaatttggtgtacatgttaccacagcattcagtaggtgccgagataaattttgttcaact

tactaaggagggattgctctggttctcagatcttactttgcc

>28919|AGAP005624-PA

gtccatccttctattgttcattattagtcgtaatagcatttttcgcaagtgccaatgacagaatgccctctcacatcagtatttgacccaacagaacatg

tttagatcacatcggagaatggagatatcaaagattctagctcgaagaagcatttgtttatttattgtttacatgggaccctagctgttctagttgtatg

tttgtgaacttcgcagaaaagtatatgtcaacagcattgaacgtgaagtaataggtcaatagtgcttacagtgcaccattcaatgaaacttcctgtctca

cctatcgtggacaaaatgttttcacgatattcgaatttttgtgtttctaatgttaaaagcgctgcgttaagtcgggcatacagcgtttgtacaaacagat

cttattcaggccttgagttggactatttacacaacgagaaaaaatgtgggctcttcttggttagtatgggaaaaaacaaatgttgtcagcgaaggtccct

ctcctcacaattttcaccggaaatcgcgcctcatccgcccatggttttaaaatttctgtccgaaagcagcccagtgctttttgctcaagaaaacctagtc

tttcttcacgatctgacatgtctaccgtggtgggctacgatcatttcttcaactatcatactccgcacagcagtaacattcccacttgctgtgtatcaga

atcatgtactagccagatatgaaaacatacttcaagaaatgcctgctattgtaaaggaattaaaagcagagacggcgtacgctgtgaaaaaattccagtg

gacagaaaagcaagcaagagtagtatataaccgctcattgcggaagcagttaaaaaagttaattataagggataactgtcatccagccaaagcatcatta

ctagtgtgggttcagattccaatgtggatttgtttgtcggcatctctgaggaatttggtgtacatgttaccacagcattcagtaggtgccgagataaatt

ttgttcaacttactaaggagggattgctctggttctcagatcttactttgcc

>28987|AGAP005627-PD

tcacctccgaatcatctctatgcagcctggtggcgatttgggacaggtttaccgtcgtcttgtacatgtgagaaaggaatgcgcttctcaatttcattca

gagcatgtacaagacgacggtaaacctgtcccaaatcgccaccaggctgcatagagatgattcggaggtgttcttcctcattgcaccaaaccaagaaggt

cttgttgtcattgtggtaaatttaacctccaggtccgtggaacacgtggtgagcacacagaggctgagggtgg

>28988|AGAP005627-PD

tctgttgcacttccttgctcatgccagtcagagggtagaactgacccttgagctcaccctccttgaacaggaaatggtcatcaatgagcttctgttgcac

ttcctcattgcaccaaaccaagaaggtcttgttgtcattgtggtaaataccacgcccactgggccaaaatctgcatgcatgtcacgttcctgggaagaca

gattttcagattgagatttcccaccccagcaatcgaggtcgtcttacatgaactctgcccgttctcgtttctcgagaccttcacagt

>29029|AGAP005635-PB

ctgaagctggcctactgtacaatgggtgccctcctgttacaattgtaaggcccagccagccatgcattagtccaacttcgtttggagctgtctctgaagt

caggctaggcaggcagaaagagataagcccaggccttgtccgtgaatcttcagtgcagtcagatgtgacatctcatggaagccatggctcaagtgtagac

tcgttcttggagtcccgcaaacctgatcctgaagaagtgctgcttaatctaggatttggaggacctcctatagcagagaatgaagtgtcacgcattccac

gtcgcttccttcagccatcaaaagtgaaaggtgttgcaattgatgacttcctacgctatcagcaggatctgatagaaaattttgagacaggcttttgtgg

atacagagggctaactggctcgtcgcacacgccgccgtcggtgatcgtggcgaagatccgcgagaagctgcgcagctcgcactcgctgtgcgcgtcgctg

tcgcagtctgaggagg

>29052|AGAP005636-PA

aataatgcgatctcgtagctcggagattatgacgtagcacaaggcgaggctggaaaaggtgactttttagttaattgagtagcaggtaggcatttgggtg

gatcaaaagcacagatgcatctgctgcattggcacttgaaggagttgtagcatattatgattcgaaggacataccagggaaaaactcattctccttaccg

ttaagtggacttgttacaattgatgaagaggtattctgttc

>29095|AGAP005639-PA

cagctaaaaccagcttgtctttatacctgtctacttccttttgttgaccagcaaatgcaacaactgtgcgaacagcactgagtacttcttccaccacggc

tccagccctaccatatgaattctgctcttgagctgccagtgaactttgaaccttcgcaacaacagctgtagcaataacaattattggagcacagctgaaa

atcacaagagtcaatttccagccatgtaagaatgacatcacaactgaggcaacaaaggacatcagtaagtaaacgaacatgccaagtttctcaccaattc

catcctgaatcttatccaagtcccctgttattcgactggcaaacgatgctgcattagttgtgtcaaaccatgccatatcttgtcgcataactgaccgcag

aaaaagagccctgatcctttgaatttgtttctgtgcagcattgttgagacatataatgcacagtatacacataatgaactgtgctgctgttgttgaagtc

agcccaatgccaaaggctctcgcatcttctagtattgcatcttttgtgtcttcctcagtaccattaatgattaatcttccaccaccaaaccagctgagca

tgactgcttgggttatagtggtatttgacattgcacgatctactagcaaagaagtgtattcaccataaagtacaataaaagctggtaatcccatagctgc

aatgattccaaatgtgataccaaggagcacaaggaacttctctgcacatgttgcatatcgaaacaactgatggtagcttactggtttatactgctcttgt

tcatcttttggtgagaggaaagatagggagaaaatgcccttcttcttgttggatgataacgatgtctcatgtgttgtcacccgtaacttcctcatattct

cttgggtgtcctgatttttagtgcaggttttatgtcctttgcaagcttgcttgtggtggtgcacttcttgcgaacttcgcgtaactgcctctgcactttc

aatacgcatgcgacgcacttgtggaaacgagtagtctatctcggaaaaagaagataagcccactcgcaaatcctgttttccctgcagccctgaagggtcg

ccacggccagctgttgcgcctcgtttcccgggcccactaatgcgcgaagcacg

>29213|AGAP005644-PA

taattggtggagttgcactgtttgtcatactgctgataataatagttgtgatcgctgcatctgggccaaggcgagcaccacctgtttctcatcttataga

tggaatttatgtgcaggcagttacgggttgtggacctgttcatgggcttgtggaagaaggagcgcatgtttttcgtggtattccatatgcacgtcagcct

gttggtgaccgtcgatggcagccagctcaattattgcagcacctagcagattgttggaatggtaccttgcgtgcagtgaaggaagcacccccttgtctcc

aacgccttcccaatggaacctggtttggttcagaagattgccttcatttagatcttgtgactcctcgtgtcacatatgatggtcagctgcctgttatagt

acttgtaggagcagatacaatgctcggtccttctcctagtgctctgcgtacaccatcacctgagctatcacgtacccgtgaagtggtatttgttcgacca

ggatttcgtctgggcccaatcggtctgttagctgcccatccattgtctacttcatcacgtcgtcatacctcagggaattatgctctatcagatctaatag

ctgcattgacatggatccagcataatattcacaattttggtggcgatccaaaggcggtgactatacttgctcatcgtgctggagcaacaatggtgacagc

attagccacatccaaacaagcccaaaaactgtttgcacgtgcttggttgtctagtggctcagcaatctttcctggccagccactaagtgaatcagagcgt

gaatcaatgacatacttaagacggattcgagcttgtgaagacaacctcactgctgaatgtttgcaatctgtggatgcttcagaacttgttgagcttttgc

ctcctgaatgggaacctcgacaaccaggattgcccaatccagacaattccacccccgagattcaccagtggcttgtacttgatggacacattttacagag

acatgtctatgaagtcctgaacgagaacaatgtctctgtcaaaatggttataggaacaactgcccattctgagggaagtgatgagttgcatcagaggtac

aaagactggcaaataaacaatgagactgtgaagcaattggaacagagtgaagttggacgaaaaggtcttgcagaggaagcagtgagacgttatggaacca

gctggccaggaatagcatcattggtgtctgacgttcgcactgtgtgtccattgctgcatttggcaagacaaactgttccacccattcccttctatgttgt

gaagcaaccacgaggtgaaattgctgatgtagatgttgatgttgatgcaatattaggtcgctacattccaagaaataccgaacagaggcgctatgtgaca

gctatgcagcaagtcttctaccgctttgttgcccagggtgagctccgccgccgggaaggtggacaacaggtgctgctaattgagcaagatgctaatccta

gtaatgaatatcctgattgtgacatgtggctgagtaaagacattgttccaagatatggacgtgtagactgaaaatatttttgacagaacttgtgtgcttt

tgagaagacttcacacaggcagttcagtgaaagaaacatgtattgcatggtcagttgtatgaaataattacatcatcagcctgtgatatttgctagtccc

tctattcactttagtttgttcctcatttactttggaaataaatcaaactgctggctgtcagttggtgggtactaaaattttcgtcaacagctgcatattt

gagaccaatcagtctcagggactttttgatgtttgagcaaaaggaaattaatatcttgggtcttatagatgtatacagtttgtatggacaaaactatttt

attgaaaaactttgacttttataaagaaacataaattttccaatatttgagatacaataaaactttttctaccatgagaaccaatattagagttgtactg

acaacagttttcactaaaaagtatggcgtagcaagctgagaagaagttgaaatacattcctgagtcatagcaccaccagcatatgcaatagataagccag

taattaaatggttgtttttcacatcaaaaattaaatttcagaagactttactctctttattccatcacattatttagatgacaaactggtatgctttgac

agtgtttaaaaaaattaacttcaaacaaaagagtgtaagaatttgttttatgtggtcaattttgtctgctgatgatgtatcagagtgttgtacataagaa

tgtgttgaatatcgtgatcagagaatgttttgaataacaagcaaatgttaaatgcttcgcttgtacatatatatattacatatatttagaaagaaaagtt

gtcttgtaaatgtgaatactgtgctgtttcattttacagtaacatattttggtgtaatatgtcatgccaccttctaggaaactacttctgccttcagcta

aatgtgtgtgtatatatatataaatgtttgtgtataaaatagttcacctgacataaaacatagttatgaatgccagttgttgttgcatttacattttctt

gttgccccctcaagttaggtgctctgtgaaaaagcataagaagaagggtaaagaaaacaatggtctgttaagacatacatggacatgaataatttatgtc

attaaaatatgaggatgcagtgtatgtgtaaatattgtcatattaaaggaaagcatgaggaaaatttaatgtatggcaggaactgttattttcagcacta

tcatttcatattttgtgactcggacactgtttgggtttatatctgaactgcatactgaggtgtggaaaaagttttttaatctcatatgatgtatgaattt

atagtgtttcactgtacagtaagtgctttctctgatgcaattttgtcattggtgtacttataattgtcaatctattgatagcttttgatgtttccatttt

aacgtgaaatcttcagtgacatctagtttggtacgacagtaaatacaactcagatcacacagaaaacttagtttatttctctctccatcttaggagaggt

gatctttcactaatttgatatctgaaatcattaaggagcaggtggatcttttcttgtatgaacttcacaaaactaagaaaacatcaggttaccaatcact

ttataaatggtataatgttcaaaatatggaaactcagtttggttttaaaggtttcacctgtgtgagaaactgaaagaacaaagggatttgaatttaacaa

taatggaaattacaagacacaagaataaaaatgtgtaggggcaaccactacttttgatgagtaaca

>29267|AGAP005653-PA

cgcgctggagcggtctggctctgtggggctgtgcctcgtcgtgtgggccgtgtgcggcggcatctccctcgtaggtgcgctggggttcgcggagctgggg

acggtggtgccgcggtcgggcgcggaatacgcctacctgctggaggcgtacggcccgctgcaccgcttctggggcccggtgcccagcttcctgtgcgcct

ggatctacg

>29292|AGAP005656-PA

gctgtcataatactggtactgaaaacaattatatttttgtgttgttgcaatgttatcgagatttgaaaataatatcatatggttttggagcaaggttaag

gccttccaaagtagcatactttaggtcagtaccatctgcaggaacaagttgaaatttttgtaagattccagcaaacatttggaataaacagcattttgcc

aaatgctctcccaagcaccgacgtcgaccaagaccaaat

>29309|AGAP005657-PA

gttttgtgcagcgttcgtagcgtttacgaaactactgcatctgcttccactattcgtacaccttggctgtgagaagggaccacagggcacctcctacggt

cactgcacgaagattggtttacaccagagctgcgctgacggaatcaatgagaatgtgtcatgtcataccaacacgtggatatcgacgggtactcgaagac

accacgctacatggctgtagacttccagagaccactggggtgatcccgaagtgtatcgaccagagcgtttcattactgaagctggaaagtttagagacga

tgatgcgttcttaccatttggtcttggtcgtcgtcgatgtttgggagagcatttggcaaaatgttgtttatttcaaacgtttaccggaatcttgcaaaaa

tttcaacttcttccagctgatggtattaacctgaagtactctagtcttgagggcgttactcttgcaccaaaaccttatgacattattttcaaatctcggt

aatgttgcaacgccacagaaatataatgattgtcattattgtaattaaaaatgaaatctaataggtgaacatgtcttactgaaatgaaattgaaatgaca

aagttcagtaaaatgaaacttcattaacctatttccata

>29308|AGAP005657-PA

tgacggccagctgtggcaggaacagcgcgctttcgcaatgcgccacctgcgttcgttgggcttcaatggggcagccatgcaggctcaagtggaggaggag

gttcgcaccctgctggaagaactgagcgacagccgtgggaggccgacacagctcaagaacttgctgccgccctccgtgctcaacgtcattctccaattct

gcgctgggtaccgcttcccgcgcggcgaccagcgcctgcagacgctgctcaatctagtagagactcgttcgcgcgccttcgacatggctggaggcaaact

cagcgccatgccgtggttgcgcttcatccagcccgaggcaactggctacaacgcgatcatcaggttcaacgagatggcgcgcgcctattttttaaaaact

atcgaagaacataaggcaacatacacgccaggaaagaagagagatctcatagatgcatttctgtatgaaatggaaaaagcaaagcaggaccaagatgaga

acacaacatttacggataaccagcttcttatgctgtgctacgacatgtttgttgctggctcggaaacgacaggcaactctctccgatttgctttccttat

gatgttacgacatcctgacgtccaggaacgagtgcacaaagagataaaagaagcaattggcacccacaggctcccaacactagaggacaaagacagattg

gtttataccagagctgctctgacggagtcacagagaatgtgtcatgtcataccaatacggggaacaaggcgagtgctcgaagacaccacgctacacggct

atcgacttccagg

>29310|AGAP005657-PA

ctagcacgcagcgcgactcgcctgttccagcacagcgctagcctgtcaggttggcgagctgtgtgaaacgcgaaaccatgggttactgacgtcagcgaac

agctgatcgacaacaaaaacaagcttcacggcgcgggactcgctgttatgacactgtcactaaaattccaccaatttgcagagacaacgagtctacagtc

cccggcggcttcgatgccacagccctgtaattcatcgaaagctacgaatacatgtgttttgcatcgcgtcatatagggaagtgaaaaagcacagtaaaca

gtgaaagtgttattgttttgcggtggtatatccccggcgtcatcgatgccacatcccagtgcgtagtgaaccagcgtgtgatttacgactcaacatatag

gtagggaccgaaaccacagtaattagatatgaagtgctgcgtaaaaaaagtgttttccaaggtgactatttctttatggattactttccaagaaacttat

tgaagaccacaaggccacatatgttccagggaagaagagggatctcatagatgcatatttgtgtgaaattgcaaaagttgaagaagcccaaattaataac

agaaaatttatggaggatcagcttctcagtgtgttatacgacatgattgttgctggcttggaagaaacaagcaacactcttcgatttgcattccttatga

tgttacggcatccggatgtccagggacgagtgcacaatgagataaaagaagcagtcggtactgacagatttccaacattcgaggacaaagacaggttggt

ttacaccatggctgccctaatggagtcaatgagaatgtgttacgtgtttccaataaatggatttcggcggttactcgaagagaccacgctacacggctat

agactcccagtgaatacaattgtgctggtaaaccactggtcactgcttatggataaagaactctggggtgatcctgaagtttatcgaccagaacgtttca

ttagtgaaactggactacttagagaggatgacacattcttcccttttggtcttggtcgacgtcgatgtatgggggagcagtttgcaaaaagttttttatt

ccaaacttttgctggagtcctgcaaaaatttcaacttgttcctgctgatggtactgacctgaagcctactgcttttgggggatttaatcttgcaccaaaa

acttatgacatcattttcaaatctcgataac

>29348|AGAP005673-PA

cacgtgtgtcgcgtgaggtcactggaaactacaaacgtgggatgtgtttggatagtgagacgttaatagcgacgaaatgctgcaccgcgtagtttcaaaa

tcatcacatcgaaacataccgtcgcattaacgaaaagaaagtgacgattatatttcgaaagacttatgagttgcctcgcaagaaatattaagaactagga

tttgtaaaatgtctggcgaagacgacaatgaagaattggcttcctcggagcttggaacgattgaatactgggacaaaacgtattcaacagagattcagaa

ttttgaagaccatggtgacataggagaagtctggttcggagaagacagtgcattgagggttgtcaggtggttatgtgataatccagaagtgaaaagagat

gacaggattttggatgttggctgtggcaatggaatgttgctcattgagctggctcgtgaaaagttttgtgatctgacaggcattgattactcccagaatg

ccattgaactagccaatgctgttgcaagaaagcagaatattgatattaaatactatgtctgcgacatgttggcaaattacgatgagctaattgagggtgg

tacattgctgaaagagtatgacattgttttagacaaaggtacatatgatgcagttagtctacatccagtagatgctaagaaaaagagagaaaaatacatt

gaaaacatccacaggatactgtctcacactggaatgtttgttataacatcttgtaattggacacaaggtgaactaatttctcatttcaaagcaaaatttg

aactccttgatatcatcccaacaccaaaatttcaatttggtgggaaaactggcagcctgatcacatctctcgttcttcggaaatgctaaatattgtgcct

gtaaaaatgtttgcagtgaatttgatgcttttttcttcatgtttccattaaaatattacaaagacaagaatataaagcaatttcatgcatatgtaatggt

tgttataaaaattctttgttcaattatttcttcttagtgacattatttttaaacatttgcttgataataagatgttccttggttgatttgctgtttattt

taagtgtatgtttacttattgctgtaatgttacatgtgtttcgtgtatttgttgtttcacacaatttagtgaacaaaatattgttattcattgtaaatgc

aagttatcactgagtggacagcaaggtggggtagtactttttaagcagaattttttctggacacaaatatcttgcttttgtaggcagttcactctggtaa

tggaagacatctttcagaatttgagtcccataagattttctttctttaactgaatgtgaaagtcaaaagtgaaatgcttcataactttctctaactgaaa

gcaaaaagtgtatatagttatgctcttactcatctatttatatcttaatacatgcctttttcccttccagtcttgccctgtactctccctcccttcaccc

ccttcccatcccacctctctttcacacacacacacacacaccttattgcaactgtgaagaaaaggaatatttaactccatacttttagacattcatcttt

gtttctttttagcttgatcatattgttgccagtcttggagtaattttttacaccctagggcatagcgaatgcattaaagtgtcaaaaatatatgcacctc

catacacattagtgtgtgtttgcaaaagagttttcgtagttcactgcaatatttttttttatttagttcctctttattttgacaatttaatacatgcagt

ctcaaagtgaatagtagacttctaggagatccctgacacgacatatttaacacagtttatacagttgctgctaaaaatattgtgattacaaaatcaatct

ctcctgtttgcaagtagttttataggcaaattattttgctagtttgtacacatttagggttaaatttaatccagttgaaattaagtagataaactttgta

gtgagtatgtgacatttgtttggttatcaatctcaggagacatgttattgccttgcaataattaactcaggtatttactgtagaaatgtaaatcttgttg

tcagattgttgcagtgttgtgaatatactatttatttctgcagttttgcctgtagcttattctgcaccataaagtgtcttacataaatgcagactgaaaa

aggctgcacattatttatataaaatggctgttaacttattaccacatgagcctttttacagtattctactttttttatcatatgtagatttcactgaaac

tattttgtacatgtttgtgactatctgtaatgttctttttcataaatcacaaataaaatgcaccagcaaagcaaa

>29380|AGAP005701-PA

ttttatagtagtatgattttgggattttacctccttggttgattttgggtggacattttggatcaccatcaggatctgttagagttcctccataatatgc

tggcagcagatcagctgccagcatattatggaggaactctaacagatcctg

>29461|AGAP005729-PA

gacgattcgtcatcaggtactgtctgtctgtctgtctccacgaccaacaccagcggcttgctgctagcactgggatggcctgggcgaccgcgaccgagtg

acgcggcagctgtccgtgtcgagcgactccaagctgctggacgaggacattcgcgaggaggcgagagtcatcctgcggcccaagaagccgccgcggccca

agtccgaggtcttcctcaacaaggagggccagcgccgcaccaaacgctactccgccttcgggggtgattcgccttttggcaagtcagaggcgtacattaa

actggaacagctgggagagggatcatatgcgactgtcttcaaaggttacagcaacctgacgaaccaagttgtggccttaaaggagatcagattacaagag

caggaaggtgcccctttcacagccatacgagaggcatctctgcttaaggagctgaagcactgcaatatagtaaccttgcacgacattgtgcacacacggg

agacacttacatttgtttttgaatatgtgcacactgacctctctcagtatatggagaagcatagcggtgggctggaaccacgcaatgtgcgactgttcct

ttttcaactacttcgtggcctggcttactgccatcgccgtcgtgtcctgcaccgtgatgtcaaacctcaaaatctactcatcagtgagataggagagctc

aaattagccgactttggtcttgcccgtgcaaaatctgtgccctcacacacctactcccatgaagtagtcactctgtggtatcgcccaccagatgtgttgc

ttggcagtacagagtattccacctcattagatatgtggggtgttggatgtatatttgttgaaatgatcacaggcgtacctacgttcccaggagttcggga

tacgtatgatcagctggataaaattttcaaggtgcttggcacaccaacagaagagacgtgggaaggagttactcgcttgcctggctataaaccacacaag

ctggggttctatcgtggtcagaaacttggcttgacctttccaaggctctatgatataatggaaggcgagaatatggcatctgcactcttacagctcaatc

cagacaacaggataggtgcagaagatgcactgaggcatcgttactttgcttcacttcctcggaagctgtatgaattaccagatgaaaactccatctttag

tgtagagggagtctacctgtttcctgaaggacggtcttgttctgtgaagacctgaggttcgtgggaatttgccatatattcaccaccatcatgaaaaatg

tggctgatggttgtactgaagcttattgatggccataaaattgccttggtgtgtgtgagagagagacagataaaaaagtgacttgattggctgtaacttg

ttgacaccatcaatatttttgtttatttcggatggaacagctcagggacgattgttatgactgttgagattttgacgttatgaacattgcttgctcaaat

cacatttgcttacatctactgagagtacaataaaagtgttctcagcacatgtgtgatctgtctttaatgcagttttgtaatagccaaggcaatatgtacc

agacggaacatttttttctaaataggtacaatgttgtgtttcagaactgccctttatgattgcagtactttacaactcgaattttaatagtgaagatgaa

aatttccccaatgagtgaaaaaattagtagaatgaaactaaaaaaaagtacttttaatggtgaaatttaagcggagaggataaagtagcagtgcattagc

aaatctattctaatggctttcatcgaagaaaacattctcacctgttgccataaattttgcacggctctgcacgttcattccttccagagtgctcaattat

tattgttgatctacttattcaggctgtttagcaagatgatatgtattgatttgtaaataaaagttaatgtgaagacatctctctgcttcttacagtgtat

actttctgtgctttgttcaatagtctgaagtaatgtatgcacaaatgtgtcatgtactgctcatagtcattgtataaaaatttatgtggattatggcaaa

ggaatagtttgctgggaataagctgtttcgggaagacccgatcagtgtgttctagatctgaaatgtgatttctctattgacagcatgtaaaatgttcctg

tttcattctgtttacatatattttttagcaactgtatatgataaggggccaacagttatatgtaagtaaaataagaactggataaatttgttgaaccatc

aaattctgtctggtggtattttcaaataataacactgtaagcgcacatttgtgttgagatactgactgaaattgtcagtaaatatagcaaaacaaatgga

tttgcttggtaactggatacttctaatgttcatgaacagggtgtttcgattcctacgatattcttgtatggttcacttgcttttggccttgaagagtgta

tgtgctgttgttggtaatgttgctattttcttaaaattcgatttgcagtttctctattttaaagaaaagtcaatgaacgatatacactgcactgaactgt

cagattctatgtaatatattttagttgactatgtattcatgatgtgccaaataactctttaagtagcagtgtttctgcagtcagcagtctgtggcatttg

aactgtgatttttcagagcattagaattgattcacgacaatcccattttacttcccatgctctaaaaccaaaacggtggaatagtgaggcttaaaattac

tttcattagtttaatgaagtacaataaaaaaattagagtaggatgatgtgacatgttggtaattttaggtacaggaaatcattttcagccacccacttat

ttattaactgtgggagtttactagtatatttttataagcatttgaattagtgtaaattatgatcagcacacattctcccttgtaatgtgggagaaaccgt

gaagtacaagaaaaccttcttacagtttttcagattccagattttctcctaaaattaacagtaatattatattaacttacaaacatgggggggggggggg

caaaccaaaatgtttgtccctggttaagttcgaagttcttaatcacttacctgtatttatgacataaaacttacttttttttcaatactttgatgagaag

tattaggaccaacatgtcacctcaagtgggttggtcaaatttaggtatagagggtaacacagtactatgtttaggaactagggaaaaaattcagaagtgt

tttgaaagggagagcactattccatactgcaccaacccttgtcctaattttttacaaataaatcatttgagaaacaattatgttcagacaaagcagttat

acagatattaacagaggtgattagtagcacatacatcttctgcttgtatactgaaagaaagagcaacagactacaaacatttgtatgacttttcagcacc

tcttagtttacattacaaagagtaatttaatattatacatgataatatacatgtaaaatttaaaatgtaaatagttattggttgtttataccactaaaaa

tc

>29643|AGAP005844-PA

atcagagacgccgcaaatatttaccttaagtgtttggtttgattgtgttgtatatgtttgcgtgaaggtgttgacagacgtgtttgggatttttggttga

tttagaacagttttattatgttcacaaataagtgaaagtgcgatgaaattaatgtttgctgctgcaacctgtttaggagagtgaatagcccacagtgtgt

gcgactcaaaggataaaacccttaatcctgcttgccatccgtagctgataatctttactttgcgtatttttagtattttttgttcgaaacagaattgtaa

tcgatggagaactttatcggaatattatctcctaaattatcggcggaacttattgcaaagcgggcagctcacgtttcaattgtagatgatggtataaaac

tcttgtcagaggaagtgtttcgggctctgaagtgtgggaagcttgctattcaaaatttctcacagaatgagttgcaccctagtcctgataatgaatcagc

tgtaccttggttgtttcttgtggataccttaaatttttccttttggtatccagagggctatgccaagaaatgggaagtaacttggcaaggtaaaacatac

acaggatattttgccctttgtgcggctgtaaacagagcaatttcggtacgtcagtgagaatagactacacaacgaaaagtttattgctataagaagcaat

ggctacgttcctgcagccccaagtgaatttctgattatggatttgcaggaaggagtgcccatgactgatgcaaaattttgttccaatgtgaccctggatc

aactacg

>29741|AGAP005849-PA

tgcggatcagcggcggaaagtgtacctcgaggcggtacgtcttgttggcgggcggcgggatgccgttgtctgcgatgcactggtagacgcccatgtgcgc

ccgcgacacgcgggtcaggttcagcgtgtagccagacaccgacacggtctgccaggagcccagtgggatgggcgcgccatccagtttacgccactcgacg

tgcggtcgggggttgccggcggcgccgcagctgatgcgcacgttgtcgccctcgtgcaccgccgttgggtcctttctctccatcgatcctgaacctgtga

tggagggcgggatgagcagctcattgctgatggtgccgttgacgcgcaccttccacgtgttgatgcccggagtgtgtgtgtgtagcgacacgctggctac

tggaataggagcagcggccggactgacctgtgatggagggcgggatgagcagctcattgctgatggtgccgttgacgcgcaccttccacgtgttgatgcc

cggagtgtgtgtgtgtagcgacacgctggctactggaataggagcagcggccggactgacctgtgatggagggcgggatgagcagctcattgctgatggt

gccgttga

>29758|AGAP005853-PA

tgagtgtgaacggctggaactgatgggtggcgcactacaacgatatgaacaaatggtacgacagcgtggacagactcagttccacagcatattccccatt

atgacatcaatattagaagcaggactgaaacactgcacagagcaggcatgaatgtcatgtgtcaatgatgttatgtatttgtagacttaataatgagatt

gtgatatatacagttcagttaacagttgtaaaatatattgttgcacacaaccaataaatccatactgtgatagaatgaaaaaaaaaaaaaaaaaccaaa

>29794|AGAP005865-PA

cattcagaagtgggaatatcagccccttgggccgtttctagccaagaatttaggaacaactataagtccatgggttgtacctctactggcactggagcca

ttagagttacaacccatggacttatagttgttcctaaattctt

>29793|AGAP005865-PA

agaggataaaagtgtctgaattgtatcacgtgcttctttccatgctgctggtaacacagtcagatgctgaaatgcatcttccagcaattattggagatta

tgctgatttttattcatcgataaatcatgccacaaatgtgggcataatgttccg

>29795|AGAP005865-PA

tgtggcatgatttatcgatgaataaaaatcagtataatctccaataattggtggaagatgcatttcagcatctgactgtggcaccaatgctctgtgaaaa

aatttaacagtttgttaagagtaaataaatatgttattaaagcatatacattaatcaatgctgacggatttttttttaattgggtgtgaa

>29834|AGAP005886-PA

gtccctcacaattgctgtcgatcaggctcaccttatctgggtcccaattcctctgacggtcatcagcaccacaatcagttttcacacttggtatgacaaa

aggactgtcattgcaagacaatgatttacgactagttttacattcactttcacagcccaaaatgctattactacatagaaatctattacctattttgcaa

atttcagaattacataaacttttccaatgactgtccaagtaaccatgctttttactacgttcttgctcaactagctgtgtaaaacctgtacaactgcatg

aatccctacatagaaaagtacttttacttttaggacaattagttggaaatttatcacaatctgtagactttatattaccattactatctacagcactcac

aactggtttgtccacttcctgatgacttaaacttaaatgtgaagtccatgtttctccatgattattgtttttaagtcccactagttcttcattgtctccc

agtacaaatataacattttcagattttgtattagcactaccgatatgactatcaatgcctgaatcaggctcatcaatacagtcatatgaagtattattaa

tgtgagattcaaatgataccatgctgtcaaatttaccacttggtacaggagtctgagatggaaaaactgatctactagtatctgctgctgacatctgtcg

ttcacagctcactctttccagaagcctgctcgagttatctggttccgaatattccttctggagatggtacataacagcttctgttggaatgcgacagaga

cgattgactttttctgaaatcacttctggatgaagtgcattgttattgacactgtcctcaagaacttcaatcgttgttgtagtgggatgcagctgagaag

acactgaagcagcaccagcaagagaacttgtcatccggttgcaaaagctggtggaccgtttcataccacacaaactcactggactctgtgtggaattttc

tgtcaatgttctagaactttctgtgctcagtgccctgtggaactgaacatctggctcagggttactgtctgatgcaattacctgagaataagtaataact

ggagctggagaactatcactgtcacatttcttcactagattgctgacatcatggttatcccattcagctcgtgctggagaacacatattgcctgctgtca

tctcacatgaaaattttgctgcactctttaacttaaacaactccctcgccacacctttcttttgaactgcctcctctgattcagttacttttgaatcact

aatattgtcacccattgcaatgttaacacttttctcacttaactgataattggaaacattaaatggtgttgccaacatatagtcagcaggcttgtgaagt

acttttgatgacttatgtaagtcatctactgtccctttgagatgtaactcattggtagcttctgtgagagttgacacagagactgcactggatttcatgc

tggcaagtggcgaaccgtcaaagacacaatgtgcatcactgggacaaatttcagatggcaaggggatgttgctatgcttctctagagctccacagcgtat

gaagtatgtcaggcagctcactattttgcccacaagatcggtctgcttgctgcttgatgatgtcactatggtacgtgccactttagttggtgaagaaatt

gcaccactgaggtccccaagttgagcccacaaggcactgtacgggtgtgacttcgacagtgagttgagcaattgagaagagcaacaatttcttgaagcac

ttttacaagtgtttttatctggcattactgtggctacccagcccaagtgatgtgtcagtacagctgttatcaaggtactcacaaaaaaattgttgctctt

agtgtcaagagcagctatcaggtgacagaggtcttgaaggaagccataagcaagattagtatgcaagtgtgtaggagccattccaagacccatccaaaga

ggacatgatggacgaggagctgaaaaaagcccttccagccactggcacaagtcttcccaggcttccatcataagatgtacgaacatatccttgcggaggt

aagcacgatcaactgcaagctgcagcctctcaatcattgattccagaatagccatgtgctcaagcagaaatgtctccatgtccttttcttgtcctgatga

aagtgcaatggccactgccaaccccagtttagtgcgtcgctggttggtcccaacagaggatgaggtctcactgctagaggaatttcttgaactgaacagc

ccagagctagcactgcaggccacttcaagtgacgtactaacactacgaagccaacgtcttcgcaaacttgccaaactccctccagacacaccatcaacag

aaccagaactgttacgggaagcaatgcttcctgtatagcttggccaatatccaactgaactaacactgcttggcgactgaagagacgcatctccacagaa

accgctatcagcttcaaggctgctgtactgcccttttgtgtgcagatctgcatatggcacatccagtggtccacttccaggcttcccagcaattaaatca

gagctgctagacagcaggcctagagtaccatcactcactgaactaatcgagctactgtacgagtcttccaagctgtggtcactgatcttcttcgtacggt

ggcttgctcctggtgattgaaacactttagtgcacataagtctatcaggggactttaatgcgtgaactttaaatgctgtacccttgaacgacatcgcaac

tgatccaaatatcatttcacccattacagttacatcttgatttggacgcaaatactgatagccaaatccatttgtaatttccacgaatgtttcattcttt

ttctttttatcagatttttgctttgatccaaacttcgttttagcctgttgtgtttcaatggcaattttctgaactgtttcagaatcgaacaacaacttac

gtcctctccagtcacactctctgaagaggatgacacggacttgatccttactaaagctaaatggtttccagcttactgattcatttttgccaaacgaaac

ataaccttgtttctgatttttcttcctagacgagaaaattttgtttagtaaagccatttcaaaatacaattacactcatttcatcgctcttgtgtagcac

atcttcatggaaccttatttcagcgcagtgcaaaccagtgctatatttacgtgcgcaccagactgtaaataaacgtcacgtgtgctgccttcacgagtca

gtcatatgatt

>29850|AGAP005898-PA

acacacacacacacagagagagagagagagagagagagagagagagagagagagatcttactatgtaacacattatttataatgctgttgaaaatattaa

atgcaaacataaatgtaagcccaacacaaaacaaaagttaacagagccatgagatttttttattctgcttcatagctaaaaataataattaaaaacataa

catactgttaatagctactatacatcttcacatcgcaatgtcctcacacaacagatgaaatacacacaaaaattacaattagcaaacctctttcctatat

taaaaataaggatgtgtcactattttatgaacatcagtatcagatatttccccatcagttaacttagtctcctacactaacagttactaggcatcttgct

atgcccacaacttaagcagcacttaaatttatgctacaactataatgaaggtgaaataagacaaggaaacgtgttagccaactggaactacagacatatc

aagaaagtgttttatgagttaggacacagataacgaagaagcaatgcagtaactgaaagaaaccaatttcagatctcactatttaaacaccaaaaaattt

gaatgcactgcagtctcagatgtatatgataactcaccactaaatatttttacaatacataaataaagatacatgccttattaagtagataaattaacag

tcaaataaattttgtatcaaatgcactgatttaccatgaaactgccttccagatactaggcagataccattattttgtgtacaaatccactggcgcacat

gtgtgcacgcacgcgcgcccaaacacacacacacacacacacacacacacacacacacacacacacacactcacacttttaatacagtgttcaatcagta

ctaatgaacacacacttcttttgccacgtgacacggttactctctgaacaggcctgtcagagttaataccacattttgaagcaaatatcaaatgagtgtc

acacacattttcacttgttcattaacactgcactgattgcatgtaatggcatcattaatacaatcacatctcggtatatacagaggcacagttcactatt

tacacttcccaatttgtttattagtatttaccaacaatacgaaatcttttatttttgacggttgccatcgtgatgtgtccttactctcccgacagccaca

ctcctacacacttcaccaccacgtttagaaacgttagttcgtgaagattagtctggaatcaaaattcaggtatgttaagttattaatttgcattaggtgc

aaaccataccccactcggctgccacatgtggaagagaagcaacccaacaaccgggcgagaacgagaatattctagacattttcacaattggatgctagga

tgaggatagcgtcactcccattgatggggcgagggaggtagttcagacgggtgggcaactgtagagcctctcagcatgtccggagtacgacatagagctt

tatcgatcattatcggggaagggatgaagtaggagaagagaaggccctccagatgtttgcctcttgtggctgtggttgcagccttacctggcaaaactct

tgaaagctgcagattgaggattaatgcacaacgggactctactggtattcagttgttctgcaataaacttgtgcatttcctctttgacctgctgaacaga

agtcagcttcttctcccacagatcatcgaagctgtggggtgcgacaggctcgaagtccggcgtgtactgcctgaggcctcccggagttgtgtagcaacac

ttgcacatgcaggagtgatatctcaagcggccctcgtccaagtacgggtgagccagggcatcacccactgttattcgcttgtcagggtcaaaaaccagca

tctggcacagcaggtgcactgcctcgtgtgtggcctgtgaagagagagtgtatagggcagccatggagggcggcttaggcgcacgtcgcagcatgtgtgt

tcgtgccccgtcacaagcgtaccgcatgtcttccagcgacggtgtgccgagtagttctgttattaactccagctgttggactgggctctgcgcttgaaag

aggatgcgtcgcccaagaagctccccgaagatgcagccgacactccagacgtctacggcagcggagtagtgtcgcgcgcccatcagtatttcaggcgccc

ggtagtactgtgtcaccacttcctgagtcatgtgcttcgactggtctggctcctctacgcgtgccaacccgaagtcgcagatcttgaggacacaattgct

gttgacaagtaggttccccggttttatatcacggtgcaagatgcgtgcagaatgcaggtatttcagacctctgaggatctggtagaggaagactttgatg

tggtcggcggacaggtgctggggagatactattattttgtgtaggtcactctgcaggagctccgtgatgacatagatttcctggaagaagtccaggtgcg

gcggctggaggatgtccagcgcggacagcacgttctcgtgcttgaagaagcagagcatcttgagctcccggaagacgcgcttggacgagacgagcgactg

gaagacgttgggcagcttcttgagcgcgacgcgtcgcccgtccctggggtccgtcaccgcccaaacgactccgaaagctccataaccgattggtctatcc

ggttgcacatcctgtgtgtgagaatgtgcaggcgttgctggagtttggtgagaatgttgtgggtggtaataaggctgggcagctgctaatatggcttgtt

ggtgtcctccccctcctcccccgccaggcatgagcggcatcgacacggacatggacactctggagtgtctgctaggaccaactacagccatgaagttgct

atcgaacacgggtcagaaatatccgtcccgagaatggtcttttgtcggttgtcttcgtacgtcgatgaaatcctttaatcgctcgaataactccagaatt

aaattcttgaacgaatttttctgttcttggtgttgtcaactggcggagaaagagagaaaagattttcaatatcacagccacatctgaataatacgctgag

tcctcaacgtcgactccttgagtacaaagacgtacgattccacgtaaacggcacgaaacatgtgttgcagcagcctctttgcacgttgtctcattccttt

tcctgtcttactttttcaattcatttacatatatatcacttcactccatctgtcgttaatccacgaacattaatttcaccaatacgtgacaaaatattgc

tactcactactctattaattttaacacggtattcgtcgaagtcttgcccttgctttcatgtctgagtccttcattcatgcgaatcttctctcgtgttgtt

agcactttccagaacgaaaaatacacacattaatatgggtaacgtcgcaaaagttatagtttttgagaactttctacccatcacacacttgtcagtaatg

caccatcacaacactcacagcaacacgcaacctccccaagcactgcgtccctccagacaaagggaatcca

>29887|AGAP005925-PA

ctccgagctctgcgacgactactcgctcatcgacaacgagtacttcttcgaccgccaccccaagtccttcagctccatcctcaacttctaccgcacgggg

aagctgcacctcgtcgacgagatgtgcgtcctggcgttcagcgacgacctcgagtactggggcgtcgacgagctgtacctcgagtcgtgctgccagcaca

agtaccaccagcgcaaggagcacgtgcacgaggagatgcggaaggagg

>30029|AGAP005927-PA

cctactacctccggtgtgccctatgctgacatcggcttgtggattcaaaatgaaggccaggcttaaaagacgctgtaaggattgctattttgtcatgagg

caagacagactgtatgctaatatgatcgctacaacatgttttcaaacgaacttcaacacacttc

>30053|AGAP005940-PA

ctccatcataatagcgtggatactgaggctccacagaatcatcaacttcttcattttcctgttggtaatgctgtggatgccgatcatgcctctctttctt

cttggagctgccaggagataaccctcttgctttcttggcatgcttcctggaatctggttcgacatgtccccatgttgccctcccaaaagagtggctgtgc

ccataggtatcgaattctctttcttcttgttctatataccggtgcctggaatggctacttctgtgccttcttcttggaatctttgacttatgtaggattg

ctgttaggtcaattccttcatctatgggaaataatctgcacagcttcttcccaacatggggttcaatttcctgcccatctctgcttattctaacaggttt

atcatcattccatgggttgcacaaatggtttgtttctacgaacagaaggcttttc

>30081|AGAP005961-PA

agtattgctcactggttgaagctgaaggtggaataactcttcttgaagaaataattcatcatgaaaatccataccctcgcatcaaagaacttgcaacaat

ggtgataacacagtgtaggcaaaaaaaagaacatgagttaattgaaacgaattgtgcttgtgagaattaacttggcaacaaaaaagttaatcataaaatt

cagttgagtactgaaatttgttataaaattgtgaatatttgtataaattgtaaaatacctaaataaattgaagaatgtttttcatacatttgtaacttaa

gaacaggaaatttacaagagatttgtaagttcttaagttacaaatgtatgaaaaacattcttcaatttatttaggtattttacaatttatacaaatattc

acaattttataacaaatttcagtactcaactgaattttatgattaacttttttgttgccaagttaattctcacaagcacaattcgtttcaattaactcat

gttcttttttttgcctacactgtgttatcaccattgttgcaagttctttgatgcgagggtatggattttcatgatgaattatttcttcaagaagagttat

tccaccttcagcttcaaccagtgagcaatact

>30108|AGAP005968-PA

agagcaatattcttgctggaaactaaagagagaaaagtttcagaatatgcattgtgaggccagcattgggcacagacttgcacgagcagatatgtggaag

tgcccaagtaaactaacgaaggcagcagcttatccaaaatccatatgaatacataacgggcaagaataagtcagccggaaacagcgcagattttctattg

gccagattgacgtttgtgcctggatcaggtcatgagtcttcagtaataccaaccgtgaccatgatcagagcctccgtccccagcgccagctccgccacca

tatccatgatgcgagtggtgcacgacggggtggaagccggacttgtctgcgtggtacttgacggtgcgcacgctgccgtccgcctccttgagcgagtact

cgcccgtcaccacgtctccgtcgcggtgctccttctgggagtggtggtcccctgtgtgcccgtcagacaccccgtactcgaactcgtacttcgggtacgc

c

>30128|AGAP005978-PA

acaaacctcacatttgtgtggcttaacaggtgaatgtactactgtgtgttgcttcaggcaggaaatcgtacgaaaggttttactacatatgtcacactta

tgcgctttcacatccgaatggattagtgcgtgggtcttcacgccctcaactctagcaaacgatttgccacaaacctcacatttgtatgacttaatatcaa

aatgtacagctgcgtggtgcctcagattggaagtcgtacggaaggatttactacatacaccacatctgtatggcttaaccccagaatggatttgtgtatg

atgcttcagataggtggcagtaagaaacgatttgccacaaacatcacatttgtgaggctttactcgtacatcagaatgtactgttctgtggatcatcaat

tgggtagctgtacgaaaggatttactacacacaacgcatctgtgtcgcttcgcgccatgcacctctttatgcttcttgagacattcggcactagcatagc

atttagaacacacgtcacattggtacggtttcagctcacaatgcaaatacttgtgtcgcctcaaatattgagctctagcaaagaatttactacatatatc

acatttgtgcggtgttacttcagaatgattcactgagtggtactttagattggtagcagtactaaatgacttgccacacagctcacatttgtgtggcttc

acagcacagtgtactgctccgtg

>30127|AGAP005978-PA

tgtggtctctcgccagtatgaatcaactcgtgctttttgagactgctagactctgtgaacgatttaccacagatgtcacatttatgtggtttcagaccag

tgtgtgttaatgcatggtgcttgagatggtaaaagtaatggaaggctttaccacacacgtcacatttgtgtcgactctcgccagtatgtagtactgaatg

cttcttgaggctactagactgaatgaaagctttcccacagacaacacatttgtgtggtctctcgctagtatgtgttaatacatgtttcttgagagtacca

gactccgtgaaggattttccacagacaccacatttgtgtcgttttacgccagtatgtattaattcatgcttcttgaggtgaccagactgagtaaaggctt

ttccacagatttcgcatttgtgcgttttttcgccatgtgttaattcatgtgtcttcagacaatcaactcgtgtaaaagatttaccacaaaaatcacattt

gtgacgtctctcaccagtgtgtagtaagatatgtttcttgagagaaccagacgcagtgaaagacttcccacagacagaacatttatatggcctctcgaca

gtgtatgctaatatatttttattggcttgagcagatggagtgaacggctcctcacaaaactcaaatgtgtgtggtgtcttggcagtggacactacttcac

agaactggagactatcatattcatcagaaagagtgctttgttgatcacatttcccttgacttttcaccttctgcaccacagaatgcttgctgaggctgtc

atgggaggtgaatactttgccacaccacatacagatattctcagg

>30129|AGAP005978-PA

ctccacgctcctagaggagccatcaggaatcacaacttcaagagatgatgtgaaaatggatccagaactaaaagtagaagtgacttctagtgaatggaat

gtttatccacttgctgttgatgcactttccaactgtgatttccggagcattaagaaagaggttcctgaagagccattagcagtctcagagttcactagct

gtattaaagaggaaccagacctagatttagggatcgacacaactgacagtaatgttatctcagatgtccaatggaaactacgtgcttcttccttctgcag

tgcaagtaatatgagcatgatggggaataattttggtgacttcacctgtagtacatgcctacaaagattttcatcaaaatacagccttataatgcacgtg

ttcattcatattgatgctgtggatgtgccatcacatgtttgtagatgttgtggtgaagtgtttcacagtgataattgtctgaataagcacatgatgaaaa

aagaggaagataggactctaattgctcacccacttgctgacaccaataaatgtagtggtacgtgttcatgtgggcagtttacaaacattgatcatacatc

acagaggtgttatagtgaacccgataactactctctctgtcaaaacaactctaaattagtttctacatgcacggtacacacaaagccatacaaatgtggc

aaatgttgcaaatcatttgcttatgcttctcgtctccaagcccatgctgtagtgcattctggaataaagtcatacaaatgtgatgtttgtggcaaactgt

ttgcaagatgtgatcatcttaaaaatcacaaattagtgcattctaaagtaaagccatacaaatgtgatgtttgtggcaaactgtttgctagagttcataa

tctcaagaatcattccttagtgcattctggagtgaaatcatacaaatgtgatacttgtggcaaattgtttgcccgagttcataatctcaaagcccatgca

gtagtacatcttagggtgaaaccacataaatgtcatgtttgtgaaaaattgtttactagaattgataatctcaaaagccatatcctattgcattctaaag

tgaagtcacataaatgtactgtttgtggcaaaatgtttgctagagctgatcatctcaaatgccatgggcaagcactgcatcctggcacgaagccacataa

atgtgacgtgtgtggcgaatcttttgctaaccttcatgatatcaagtaccatttattagtacattctggggcataatcacgccagtgtaatgtgtatcat

aatttatttactggagttggtgtacttaaagtccatttagtagcgcattctcatatgaaggaaatcttttgcagggattttttgtctcaaaattcatatg

ctagttgcattcttaaattaagggacatatagcttcagataaaataatgaaagatgctcatactagttgtgtgcacacaaagtttgcacttagttaattg

tttgaacattattgcgagatttgccatgggagttttatttgtgtgtgtgaatctgtccttcttgtcacaataaatattcatattttttgtgacacatatg

ttttgtatgtgtaagcagaggaaagtgtatataaaaaagataatctctgttgctgtagttttgttccataacttgtttatagcattgatgtgcagctgtt

cgaagattctggcaagtatgcttaagattaatatttcagtccttttatttgtcttaaaagtatagaaaagcatgtgtgtagtgagagagttgtactggaa

tgtgtagttagttaaaaagtaccatataatgttaaaattcagttccaggactgacttttgccatgtccaatgtttcttgtaatacgtaaagtacataaat

accatttctgctataaactccatgattttctg

>30145|AGAP005980-PA

ctttcatgaatacacaatttaaaatctgatactaaacatatatcactgatgtgtcaaatgtttagttttgtccacttctcactaactagccacagccatt

tggctgttacatcctctttcccttgaggtccaacgtcttcttccttacagttactaaaatattttcctgaaacattctgtagagactcatccaatgcaac

ataaattgtcgtttgtgcccctttccttggacttttaataaacaatttcacaaatggtttcaaaaacacagctgagatggagctattatagaagctcata

tgccgagttatttctgtatcaacaattccagggtgcacagcatttactgtcacacctgtgccttccagtctctttgcgagttcctttgtaaaaagcacat

ttgccaacttgctctgattgtaagcttctccaggatcatactgtttctcactgtttagatcttctatgtttatttttcctcgtttgtgtgctatacttga

tatattaattatcctgcttggtgcagaagcctgccaacaaatttccgttgctttattgtatctagcaacaagttagtcaataaaaaatgacccatgtgat

tcactccaagctgtgtttcaattccatctttagttcttgaaagaggacacctcattactccagcattgttgattagtatatcaagtcttttgtgctcatt

tttaaatctttcacagaaatttcgaatagatgctaatgatgccaagtcacatgttctgcaatatacatatttattcttgctttccagaacgatttcatgt

cttgtcttttcacacttttgaatgtcacgacatgccatgacaacagtggctcctcggagtgcaagttctcgggctgtctcctttccaattcctgtattag

cgcccgtaattattactaccttgccatcagcacgtgttttattttcgtatttaggacctccagtatattctttgatgaacagtgaacctccaactgctgt

tcctatagcgctcagtatcaccattggctttgatggcttccagaccatgtctttgagaaaaactgactactaatttaaagtaaaactcggcttcgtcttc

acacactttcaatatctcacatggcaatacaggattgccttttgtttactatttacattgccggtatggcaaagatgcaatgtcaatggagctatatgct

aactcaaagatat

>30218|AGAP005988-PA

ttattatgtgtgaattccattcacatttgttgacatgcttgtatttacagacattcaggtacaagtaatcattaaaagtacacagtttatggcttgaact

gacagatttttctcgtttttgacaggatagcaagtgtcaatagatgtattatgaaatatttctaaaaaaggtttcttatccaaaaaagttatgtcaaatt

tctgtttctcattcaaggaagtagtaacaagtttgttcactggtacacacatacaaatcaaatgcagctctattacaagctaaccttcagaactgttaac

agaatgtttagtacattttccttttcagttaaactttttagcattatatatgcatttaccttgtacttgtgagcagataatcctctgagatttgatacat

gggagcttcttgtgacagctatcatttcactgataggaatacattgttttatataacttgtaccattttgcagctaaacttttgttcatgaacaccagtg

attaatttctgtcatacaaaatctaatatgtgtgacatggttctctcagcagttttaatctctacatattatgtaaatctctgaatacaaactgtactga

atttggaactttttagtcaagaaaccagttatttttaaatagctcatctaaaaaattggtagccagtaatatatgtaatgacactctgtgtaataattat

tttcagggataaaacttttagacctctgctacagtaatcctattcctataactcataagtgtttttaagatcataaaagtgtttggacattcatttaata

atcctcatcatatttattatgaacaaagttgacacagcaaagtctttgaagacttgttgctatagcttgtaaagtaggaggacacagtggcctatacttg

ggaacaattacgacacattaataatcgttccctctagtcaactcactctgcaaactgaagaaagcctgtaccctggttagaattctatttaaaaggtgat

ttaaaattctcaataaatatagaaaagggaaataggagtatggtttcttcacttctgtaatctcctaccacacaactaatcacattcaaacataacattt

ccccattctactgggaaatatattagttgggtattgggcaatggtgttggctctctgttaataatatgtgtgatttgatgaagaacatactatattatta

ttgcctgaaagcacgatgtattggtgtttgtatgtaaaatcaataccatgaatggcaggtaactgaaacaagttttcagtcagtggacatctatatgcat

gcaggggattcaaaaatatcgtatggtttacaataattgcaggtattacagtgaacacattttagttgtacacagttgtagctatgttactggtacatcc

agtgtgcacatttaatactactacttcacaattttaaaaaattaagtaagtgatcttgtgacagaatgatactctctgtatcccaatttcatagtcattt

tgtgaatgtcactccttatggtgtagaaatttcgacagtcagtgttgcacatttaatttaattctgctgccatcttgaataatctttgtccagcattgtt

tcatccattacttttgctcaaaaggaagtcataattgtatcttgctgcccctcagaacattgtagaattagttccagaatctaaataatatgaattttca

tcaaccaaaatacctgcaatacaagctgtcatgaaacaaactgcagatccagctataagtgctctgaatgctacttctgttattgctgcccttcggttag

gagccaaggcagataatgcaccaatcattataccaacagagctaggattagaaaacccacacagtgcatatgtggctattatttcagatctgggtgtgag

aagattctggtccagcatttcaccaagtttctgatatgccacaaattcatttacaagtgtctttatgcctatcagctgggacacatattcaacttgatca

gcttctacccccatgacgtatgtaaatggaataaatacttttgagagaatgaactggaaagtgacttctggatgaccaactaggcctccaagccaggaga

taaggccatccaagaaggccacacaggccacaaatgctatgatatttgcaatgataccaagtacaagatttgtagctgtcattgctccctgtgtggctgc

atctaaaacaccagttgcttcccctttgtcaactttgatgtttttaatactggtcttggattcttctgtttctgggtagaaaagcttagagtagcacaga

gcagcaggggctgacataacagatgctgttatcacatgggacgcttgaacaccgaagctaacatatgcagctaatactgttcctgcaactgttgcaaatc

cagatgccatgatagtatgaatttctgatttggtaagatctttaatatatggcttgatgagcagtggagactcagactgacctagaaatatgctagcggc

agcatttacggactcacagacagttgttcccatacttaattgcaacagccaacctaattttagaactatccattgcattactccaagatggtaaaaaatt

gataccatgaagctgaagaaatatataacagaaagaacctgaaaagcaaa

>30264|AGAP005989-PA

acaaaatctaatatgtgtgacatggttctctcagcagttttaatctctacatattatgtaaatctctgaatacaaactgtactgaatttggaacttttta

gtcaagaaaccagttatttttaaatagctcatctaaaaaattggtagccagtaatatatgtaatgacactctgtgtaataattattttcagggataaaac

ttttagacctctgctacagtaatcctattcctataactcataagtgtttttaagatcataaaagtgtttggacattcatttaataatcctcatcatattt

attatgaacaaagttgacacagcaaagtctttgaagacttgttgctatagcttgtaaagtaggaggacacagtggcctatacttgggaacaattacgaca

cattaataatcgttccctctagtcaactcactctgcaaactgaagaaagcctgtaccctggttagaattctatttaaaaggtgatttaaaattctcaata

aatatagaaaagggaaataggagtatggtttcttcacttctgtaatctcctaccacacaactaatcacattcaaacataacatttccccattctactggg

aaatatattagttgggtattgggcaatggtgttggctctctgttaataatatgtgtgatttgatgaagaacatactatattattattgcctgaaagcacg

atgtattggtgtttgtatgtaaaatcaataccatgaatggcaggtaactgaaacaagttttcagtcagtggacatctatatgcatgcaggggattcaaaa

atatcgtatggtttacaataattgcaggtattacagtgaacacattttagttgtacacagttgtagctatgttactggtacatccagtgtgcacatttaa

tactactacttcacaattttaaaaaattaagtaagtgatcttgtgacagaatgatactctctgtatcccaatttcatagtgattttgtgaatgtcactcc

ttatggtgtagaaatttcgacagtcagtgttgcacatttaatttaattctgctgccatcttgaataatctttgtccagcattgtttcatccattactttt

gctcaaaaggaagtcataattgtatcttgctgcccctcagaacattgtagaattagttccagaatctaaataatatgaattttcatcaaccaaaatacct

gcaatacaagctgtcatgaaacaaactgcagatccagctataagtgctctgaatgctacttctgttattgctgcccttcggttaggagccaaggcagata

atgcaccaatcattataccaacagagctaggattagaaaacccacacagtgcatatgtggctattatttcagatctgggtgtgagaagattctggtccag

catttcaccaagtttctgatatgccacaaattcatttacaagtgtctttatgcctatcagctgggacacatattcaacttgatcagcttctacccccatg

acgtatgtaaatggaataaatacttttgagagaatgaactggaaagtgacttctggatgaccaactaggcctccaagccaggagataaggccatccaaga

aggccacacaggccacaaatgctatgatatttgcaatgataccaagtacaagatttgtagctgtcattgctccctgtgtggctgcatctaaaacaccagt

tgcttcccctttgtcaactttgatgtttttaatactggtcttggattcttctgtttctgggtagaaaagcttagagtagcacagagcagcaggggctgac

ataacagatgctgttatcacatgggacgcttgaacaccgaagctaacatatgcagctaatactgttcctgcaactgttgcaaatccagatgccatgatag

tatgaatttctgatttggtaagatctttaatatatggcttgatgagcagtggagactcagactgacctagaaatatgctagcggcagcatttacggactc

acagacagttgttcccatacttaattgcaacagccaacctaattttagaactatccattgcattactccaagatggtaaaaaattgataccatgaagctg

aagaaatatataacagaaagaacctgaaaagcaaatactgctttctcaataacaaggaagtcaccataaacaaatattgaaccgtcataagaatattgaa

ggaaagtagtaactttatcacctatgcatttgaagatatttcttccaacatcccaacgcaaggtaatcaagccaaatacaaattgtaggtataagcccca

caacactgggtgccaatctatctttccaggatgtctggagaatatgaagccaagcactacatagacagcaaggcctgccagtgacattaatctgttgctg

tcattttttgtgtcaataaatacaaatattagcaaggctaccattggaacaatgtccactgtccacctgactgctcttctttgccactgcttagtgaaac

aaggaataagaggtgaatgttctccctcaaaatattttctgaaatgtttcttcagtatccttttaagaggatagtaaatggcaattgtatagccaatcac

aaaaattattatcaacattcctacaccagagcaccaatccatatcacatgaaccttcataacatgtcttcgtccgaaaatatgccagggcccatgcaaaa

tatccaataaatacaacatttaaacaaaaaagcagcacatttttgagcagacttttatttttctgatataatcgtgatgttagatcacagactgatccat

tgcattttgagcagcatccacagcttcttcctccctcatgttccttgttctccggtactgaatgtgagttgtcaagcctatgaccatcgcccatcaactc

tgcagtggtcccagtggttgccatgtccacaagctctagtgcagcctcggacggtactgctgagttggccaccacctccaagtttggcctggcgttggcg

tctactccacaattctgttcccccatctgcaggcggcgcgcgtgacgctgtcgcg

>30297|AGAP005990-PA

ttggaagcaaattcacatttaataaaatttaagtacaccacctcaaaaattataggaacataggaaatcttcccattattaaagctctgaactgaaacaa

gaatgctcaaagtaaatgcacatttctctttagtaatactgactactgttatggcagaccatgtaagtcatgtttatcgtagagattgttgctacgctta

tgtacctctcccatcacatccaacatttcaggtagaactaaacttgcagcttcaacttcctctttgcttccacaaaccttttgaagcaatccctcttttg

ttggcaacacatacattgcagcttttgctcctttacgaataaggaaagggtggaagttgcctaaagtttcatcataagcactctggcatactccagacgt

tttgtctttatcatttagctccttcactcgtgtcaggaacagtcttataaaatctaaaccacggtgaaggcgtaaaaacgttctgcttcctgacacgtat

ttggaattattcaaaagtccagatgatttttcatagagtatcatagtttttaaagaaacaaagttactgctctctttttctgctctaaattgctgtaata

tttcaattttattttctacatcacttgaaacaaaactgaacacagaacccaaaagatggaagaatttcaggagctccttgtaagcaacaatataactgtc

catttgaacgtcgtcatcttcaataagtgaatttttcaaggcatcgcggacaatcactaaattgaatttagaatcggcatcgctctttcctgcagctgcc

atcgtcaattcaaacacgaaaccgtaggcacggatacaatcagcacgataatagaaattttatctcagtacttcacatctcaaggctactgaatttgaga

taaaaagtaacctagccatgccgtagtttacaaacacaagctactgacaagtgacagctactgactccgacaacgcaaagataattaactcaacacctgc

agacatcctgcagaacactaccacatgttaaggcccg

>30317|AGAP005991-PA

cacgtaatcagtcaattggaggcgatttagggtaatgacctggcggtttactcctgatgttggtccatctatgatgacacgattcaagtcgacaatatcc

acaatgattgcgggtttatcaaagtccgctccatatcggatgacaactacccttccagcttgtacaaatcgcctgaaaagcgccatttctcccgccttta

g

>30333|AGAP005993-PA

ctttttattcatatcaaatttgtacaggctcgagtgttacagttcacaactgtactatttgcactgcaatatttaatataaattacttgctaccgtcgtg

taagatataaataaaaaagtgtctaatctactctagcctaaaatgttcaacgtgaaacagtgtgataaggaataaactacgtcaaagcagacaaacataa

cagttacgttatgaaaatggttaacattgcgttgctgtcctcatattccaacacgacacatccaacaccgacgtaattcgctagcagccgttttggacgt

cgtcttaaaagttattaggtgggagtatagctgcttgtagtcactccagtgacatcactatcctcgttagtgaaaaccaaatagggaaacagcatgtaag

aaggaatttatgtacaaatatgatcacttttgagaatggatgaacagatcctgggatgtgggaagtttcaggtatactaagtggatcgtttcgttttttt

tattcttttatgttccttgcaagtagttatgtagtgttctgcagtaatgcaagtggaaggtacttaaatgtggaaaatgtcctttataagactggaggct

gggtagaaaaatatgtaacaaaaaacggcgtgtaagattaaaaacttgttattaaatgtaaggatgtgaaaatgggagatgtgaaaattttagtaattgc

tgataacatatctcagttcctgatcaggtaaattgtacaatacaccgaagtataatcaaacaatgacaatgtcaattttgtaattattaaataacgaatt

gtgctagttatagaggaggaccacataaaaatgtcattcctactgtttcagaaagaacagaatatataaagacgagatccagtaggtaaacggaagagta

cttcccttacggttaatgctagcaactttactttccgagacattgagacgtctatggtttgaaacagatttgtaattttttatggaattggtgagtcttt

gaaaagaagtgaacagtcatatgacgctcgttaaagttagcgctatcacatttcgttaagaggcgtggaaagtcttgtaattatggataccgagagagct

gctccattacgcaatgtttatgtggcactggagatgaatcggcgttttcatatttaacacatccctcgcctctgtcttcgaacagtttcactatatggag

atttgacaagctaatcattgcactgctctttccaagaactgtcgaatatttcggaagaagtatatagttccattaacaaatatatcgatgaatcatcatc

tccattcataaaaatgttattaaccacatagaaaattctttcccttttgcatcttaccacagtctgttaacgtgtttcggtactaccaaccgtttataaa

ttattacttgaaaagatatgtctgagtgtatgggtatgagtcgatgctgccttaatgatttagggtgtcagtaaatgccttgtaaatacagagaaatgta

ctcctttttatgttagaaagagtgtcgtattaattagatggttttccgcgttaatggtgggggtctgaaggtgcgtaagaacgaagggaagggacggcag

cacgtttactcggacagagcttcgacctcgaggcggaaactctggagtagacgggcaccgcccggcgaccactcacgggctgtgcgacggacaacgcgtg

ggtgttgctctactggtgcgactctcgctctgtttgctacggggacggtgagatgattccgcggctccagcgcatccaaagctccaatcctattagtgcc

tggaaatatggcccttcttctcgtcgatcagtattactatatccgcgagtgaaataaaaaaattgctgtggcgcccgtatattaagtgaataaaggatta

tgactgctataagagctgtcacagacgttcccatttatttctagaatttgtaggaccaatgatcagaaaaccagcttgataatttttgagcgatgaattt

gcaagttgtattgtcatgatgcacagataccaacggccttttggcggaaagcagagaacacttagaattgacaggctacgttgcatctaaatttgagtta

ataacagagataggaggaaatatatataaaaaacaataggacagatcataagaagttcacgcaagaaggaactgagaggatttggattgcagtggtgcag

aacaatgttaaaaatcagataggcatatagactaagaaaataagaggtagaaaaaagaataatctaggttagaagtctagaagacagttaccaaagtgaa

ggacagctggtgcatccaagatagctaacgtgacgatggacgaaaattcttgaagaaaataaaaaccgaaatgcgtaatatttaatacatattctggtgg

atgtagcttataacaagatgtgagagatgcatagagctatcgtgaatatagtgaatatagtgaatatggtaactacatattccgagaagatgcagtaata

gattaagcgagtgaatcatgctgttaagaaaacaaaattatagctggtggctgaagcacgtaacgaatcagaagtagattatggctggaaaacgaaactc

tcttcagtcaaagagagagtatgccgtgaaatattgtcacagaatagacgatcgagtttctgaaactgtacatgtctgtcaagaaactgaggggatttgg

aatgcagtggtatggaacactgttaaaactcaagtaagcatatagattgagaaaataagaggtagaaaaaagaataatctacgttataagtctagaagac

ggttaccaaagtgaaggacagctggtgcatccaagatagctaacgtgacgatggacagaaattcttgaagaagataaaaaccggaatgcgaaatatttaa

tacctattccggtggatgtagcttgtaacaagattttagagatgtatagagctatccaagataaaaagagctggacgacgttaaacctttcgaaacactg

acttcaaaaaaggaaaatatttacatttatttcgttatgtacgaagagggacgcgaaaccgggaacagattctcttgttagtttatttgcacaacatgat

tctcatatcggacttgggcatctacaactgtcgttataagttttattaatgacagttccattagcacgaaaagtgcataattacgtttgggccttaggcc

attagtacgtgctcagcgacaacaggagttgtgttgtgacggagctcttgataaaggtctaggaatataaagtgtttgtaaagctatcattgtcatttaa

taaaactgattctcctgtgttatggtataacactgccaaaagctaaatgaattctgagaacgtagtttacgtattccaagtttgcgttttcatcagtatt

ccattatcgtctcgattgaaataatcgaatatttgtactgcattatatccgccttaggtgtaaactcagagaaaatttgaaggccacgtgaaaataaata

aataaatctgtagcagagcgcagtgtaagagaccgtggtatcttctatcatgggagcacctcaaatatttgcgaatgagcagaaggagggaacgtacaaa

aaacgatctcggaaactctcgaatactaacgtaacaactatgggcgttttgcctttgcgaaagcgggtataacagcagtaataaagaacaagtgtccggc

agacgagggccatatttccaggagagctactactggatcttgcaacgcgctcagaagcgggggtcgtagggagtcgcgtcgaagccgacgtatcggttgt

tcttgcggtggacgaagccggtgtccggggccgtgttgtagtagatgacgcgccggatgccgaacgggtcgatgtaaccgaagctgcccgcgcggcgatc

ctgcgacagcgtctcctcctcctggtactgacggggcaggtagtacctgaagccgttctgcg

>30363|AGAP006003-PA

taattatttgtactgttatatgcaatcaagaggccaatttatgaattaaatgccagacataaacgtggactcttttacagacagacgaagtggcttgtca

cgaacttttaactttcagaaaaattcatgcaaacgatatcgtctttcagtttgccacactgtcagaggcgatgacatttcaagaagattctaaggtttaa

atgtctcactgagacattattcacgaagctttctttgacgtatgatattagagcttgaggaactacatcatatttttggacacacattaggaaatatttg

aggataaatcggaagtgaggataatatttgacatcgtttccaaatgaattcattttgaaaccatagaaaatgataatctgtcagttctcgtgacatgatc

acaaggttgcccattcaaacggtgagtagagcctacgaatttatctcgtctgtaccttcgcctgatcacatctccttccaggatttgtatcagtctcaga

acatctgcagttgtttacacacactacctcaggagggcagaagatgaggtttcggcttgtggtacgttgtatgttggcaagaactgcgggtgtcctttag

cggccgatgagcgacagtttggcgttggggctgaggaggacttgcttctccagcttgggcggatccaccgtctcgtcgacggcctccagcttggactggt

agccgtccttgtcggcgacgtagttgacggagtacttctggccgctgggcgacttccataggtagctcccgaccgtcttgaggaatccgtccactattcc

accaacctcctgtcgctggctgccgtcactcgacttgtaactccagcgccagttgtcg

>30434|AGAP006027-PA

cagctgtccaaaaactggcggacgtaagttctggatgagtccacgatgcgaaaccccgtgatgttgatggcaccgtactctatgacttcg

>30433|AGAP006027-PA

ccatcccagcctccctccacgtccgggttctccgctccgtactggccgtccttcactatcctcagctcgtagttgatgccgagcttctgggcgatgaggt

ctgccaggtccttgcagtatccctcaaagcggtcgttgcccgtca

>30431|AGAP006027-PA

cgtgtcctgtttgtcgccatgcttgcattcagtacggtcataccaccagcgattcttcagttttgtcagctccccattctctattagcgacagtaccgcc

aagttaatctcatcccctgaagaagtcccaggtggatccgtggtagagcgtcccgtactcgacctccgtctgcgaggccaggtcctcagggctgttga

>30490|AGAP006040-PA

taaaaataagatataacacatttgtcttaaaaaaatactgtgaaaacataacaaaataaaaatttcaggactccctgtacaaggaatgtgaggaaattca

caattcactaaacacgaatgtaacaggttttaatgttacaacagtacttctttctcgttactttcacttcctccatgcaatagtttgctgcactgaagat

ggtcctctcattgcttatttcctttcctttctcttgcttgcctcctctttgccagatatatgatccagaaaaatcctatcaaatttgcgaaaagcactct

caacattggtggtactaatcttagattcagatactgcactactgttaaccaaatccaattggctctgacggtgggtccatagatgcgtagtagctgcccc

atggcttcattgtgtgattttccctaggaaatagaaatgaaatacaaagtaaaaacacacaatacattactagtgaagaataggaactatctttgggaga

aaagaactataggaagctgcatgctgctgctgaaaattcaaaatttagtctctgaaagttgcaaaaaagaggagg

>30489|AGAP006040-PA

attgattcttcttccatttaatgttgttacttcttgatttcaaataggatttggttgaacattgcacgagctgccattccaccccttctcctcctcctcc

tcttttttgcaactttcagagactaaattttgaattttcagcagcagcatgcagcttcctatagttcttttctcccaaagatagttcctattcttcacta

gtaatgtattgtgtgtttttactttgtatttcatttctatttcctagggaaaatcacacaatgaagccatggggcagctactacgcatctatggacccac

cgtcagagccaattggatttggttaacagtagtgcagtatctgaatctaagattagtaccaccaatgttgagagtgcttttcgcaaatttgataggattt

ttctggatcatatatctggcaaagaggaggcaagcaagagaaaggaaaggaaataagcaatgagaggaccatcttcagtgcagcaaactattgcatggag

gaagtgaaagtaacgagaaagaagtactgttgtaacattaaaacctgttacattcgtgtttagtgaattgtgaatttcctcacattccttg

>30569|AGAP006065-PA

tttgtttttggcgggaaaagttgtcaattggaattggaattacgcgaaaggcatcgaaaatcgctatggatatagccgagttcaccccggaagtgctgcc

agatttactacccatatactataaaagaatttttccgtacggccctttttatagatggttgagctacagtgatgtgacacctaattactttcaaaacaga

gaattttctttcacactgg

>30568|AGAP006065-PA

ttttataatctttgactctcttgttacgatcatcttctgttaataattccttcgtcttagcatcatatttctgaatttcatcaattaactgactgattgt

tggaacaagagagtcaaagattataaaaagacgagtatgtacaagggtgtcgtagtttttgaagaattcttacggaaactcgaaaaatcaggaaaagatg

atgttatcagcaggcatgatctcagcatggaattttaagaagccattggggtgaaatgatgcagttgctaaaagaacaaaagactgctgctcccacataa

cacatcagaacatgcttttctctgtgttataagatgtcc

>30567|AGAP006065-PA

cagatgatgtatatatacgttttttgtctttctcaaatcaagaggaattagaatgtgaaatgcaaaagaagaatccatttaagatagatttaggtgcagt

gtacaatttcaggcctaaagatcatcgctcagtttcactttttcaaccactggaaaaagaacttgtgtttgatattgatatgactgattatgatgaagtg

cgttcatgctgcagtggtgcagatgtctgcattaaatgctggcgatttatgagtgttgcttgtaaggtgttagatgctgcccttagagatgactttggtt

ttaaacacctattatgggttttctctggacgaagaggtgtccattgttgggtatgtgatgga

>30966|AGAP006068-PC

attcttcactgaagcagagtagggtacatttttagaactgtggtgtactccgtctaagatctgtacatgtgtgataatcatacattgcaaagtaacaagt

gaatgaagttttataatgaagaaaaatacaaaatattcagtgctcaagaaattgactgttctgaaaattcagcactccagaccagctggcatgctgaaag

aaaaggggaaacacaacagtaacatttctcattaggtagtctgcaaccagtacaatgagcatcatctatttggccttggaacacatttaacacatttgta

ctgtcctcatgcacttgcaggaactatgatttcctgaactctatgaacaatcatagacatcacttcagtcaccaaaaaaaataattaaccatacagaaaa

ggtaagttcatattaacaggatgtaatacatagtccttgaaagtatgagaagtacttttaaacacaacataaaaaaataataccaactagataactgctt

tcattgcaactacttcagtcacaatcttaaaaagcagccactgaacagtttctatataaaatttagcttcacagccatggtaaaagaaaaaaaaatcata

aaaattcttgtctgcaaaataatttatcttactgccattacaacatgacatttcattgtttcaggaacatatagatgctacagaatggaaccacatttga

gctacctggtaacactcattgacgcacaagaaatagtcatttcttggaaattttgtgaatcacacttttttatctagacagtaacagtaaagcttcagat

gagcatctgaaatgcacctactttgattggcataaatatttagcctgcttatttgtcctgtgagactgaaagatgctggatctttgtggaagtgtctgac

agttgcttcaaacagtccatcaccaggtgtagtctgaaacacaactacgtagtccacatgatccatttctgctttaatatcgagatcattgcttttccct

tcaactactcgtttcaacttcaaatcagcacaaagtttcacttttctctccttgtttgcagcatctaacatagactgcagctcagaaagtgcatattttg

cacaagcctgcatggtagtatctttggtagacaccgtcctgtactctgtgcatgcacaccagtgaggctgaatacctgcatccttacatttcctatcata

gggaatttccctgaagagactgttacacgttgggcattcgagacttgcatttttgacactcatttcactgtctgtccttcctgccctttcaagtacgtct

ctcagagtcgcatgaacatcaaaaggtgacgttagtctgttgctgtttgtgacaatattcttataggcatcagggtaactactcctaaaccaaggaggta

tccagaaatacaggaagggaagtctgtcctcaaaccaaccaacaactgtttctcgtatcttcccaaagcgcattccgtgatcactgaaaaaaagtacaag

tgtattgttcagaacaccactgtctgtcagctcttgaaggaggtacatgattttaatgtccatagcactaggggtgttgaggttatcgtgactaaaattg

ttcacccaaaagagtgcaaaatatgaggcattctgaaaaacagttgcgaaatctactgcatacctaagaatgtgttctgaaacagaaagagacccggcac

atgtagctgactgatcagtagatttagatttcagtttcttgtttgtggcaaggaaaaagggtcgaagatagtagtctgtaggttgatgcttgaagccacc

tttctggtagttgaatgtgctaataaatacttcatcttcagcgtaagcagttatgtatccttgttgatgaaaatccttccagataagagggatgtcatcc

attggtgtttcctttgtcttgcaacatactttcttgaccttttcatagctcagcccagtcagaagagctatcagatttggaaatgtgttgtctcccacct

tgaaaattgtagcctttttattggacccagttttgggtctgcattagttcgttcgtgtgcggcatggtgcggaagaggctgagccgcgacatggagtcta

tgccgagcagcaggacgctgatgcccggggctgcctgctcctgctgcgcgtccacgcgcctgcgcacagcttccg

>30987|AGAP006082-PA

ttcttggaaggaaaatgatgtgcactaattaaaaatcagttacgttacaaataccgcacatttgatatttattacatcattagtaggatacatgaattta

aaattcatgggaactggatacagaaaagtctcatacaaaataaaaataaataaacagtaagggttaactagactgtattcagaagcatgttctgtaaaaa

tagatgtgctgccagaatttgccgtgaaaaagttgtattgattactccattccttcagcttcctcaccttttactgagtttgaatttcagaggtccatct

ggtgcatacatgaacgttactttgtattccaatggttgatgatgatattccagtttatatgatcgaattagcttggcaagtagtatctgcatttccaaat

cagcaaatctgcgacccagacacatccgtggaccatggccatatggcagtgaagcaaatgggtgtacatgcagccctgagccaccatcattcgcttttag

ccatctctctggtaaaaactgctttgcgtttttcacatacttctccatgct

>31030|AGAP006084-PA

ctttgcaggagatgaacttgttataatggcaacctcagcagtcgtgccaagaccttttgaagtttccaatgtttccttttgttcaatttcattgcgcaac

ttctcattctcattgtcagttgtcattgccaaaagctggtcaatggttgcatctacagcaccttgatttgcccttaggactgcctctataacttcatcgt

ccatatctggaaacattgttctgaagtctgccatggcttggtgaaattccaattgtgtggttggctgttgaactgtggccatcgtgtaatcggcgagtat

gttaaacctacttttctcattaagttaatgtttcacattgccttaaaatatcttgtgaccatgttccataagagcaaaaactgcaaaataaaacgactgt

atctgttgtaaaccttagtaaggcaagactcaaaatactttattgtgtaatacaacagatgacagctaaccctgtatcacgctacggacgatcagtgttg

tgagaattaaggagacgcatttccgacatcagctgctgggcaattcaagtaaagagcaaattaagtttgctaagattttaatcctataaggtcagttatc

gaacgaacttcaaccgttggaagtaacagtcagctgtaaaaatgcaaacatatattcaaataaatgactcattttcaattttctttcaatgagacgtgga

attcctgcaaatatggcctcatttcggacattagttcaataaaattctactgaactatacaaattctagcgatgtatacacgaacagccattttttttaa

cgctgctcatca

>31044|AGAP006097-PB

tttcatttcagtgccaagtcgataaatccattattaacagttcgaggtaatccgtttgctacgcctgtggggcagagaatagagcaggcaattgatgcat

cattggcttcagagaattgggcacttaacatggaaatatg

>31097|AGAP006126-PB

gtaatgacaatgatctgtagccatacttgtgtatttttaccagtctttttaatactgccaattataaaataaagtaaaaaataataaaaatgtcgctaat

ctacaaaaattatattaaaatttcaaaaaagtgagaagataagctatagaaatacattgttacaacctaactgaggtagttatatttcaaaatgtggtta

caagtttgggtcctgttttggtgaatgggtaatagcaaggccatttgatggtaaactaacaactgggacagctgttctgtttgtattactcatctgggct

tacaccctaccttgggcgatagtgccacttcttgaaatatggagcagatttgcaccagagggctaccttactagctgcacatttgattacctaaccagct

caccaagcaatcaaatgtttgttctaacaatcttcgtgttttcatatgtaattcccatgagcctgatcatgtttttctactctcaaattgttagccatgt

tgtaagccatgagaaatctcttcgagaacaggctaagaagatgaatgtagaatctctacgcagcaacacagcacagaatcagacatctgctgaaataagg

atagccaaagctgctattggaatatgcttcctctttgttgcttcgtggactccatatgctgtaatggccctcattggtgcctttggaaataaagcactgc

tgactccaggtgttacaatgattccagcttgtacatgtaaagcagtagcctgtctggatccttacgtgtatgccatcagccatccacgatacagggttga

attgcaaaagcgattaccttggctctgtattaaagaaaatcaacaatccagtgacacaacttcaactgtcacagcaacatctactggtgcaactaccaca

ccaagttaagaatattctggcatgttacctcggatgatgacttctgcaatgtctatggtactggcagttcttgaatggaatgtgtgagacctgtagatat

atccatttttgttcttggatgttgtgagatattgcaatttttggtac

>31114|AGAP006131-PA

ctttggttgtacttataacattgcagaatgcagtatcatgtagatgtgtaatattcaatcatggcaacgagaaggcgcccattgcatcaaaatgcagggt

cgagacttaacttcaggcctcaggaaagttattcctttgaagcaaggggaaggaagcctctaccagagccttcgtcgatatgcaacgtcctactgatgat

ggtgcttcatgtttgcaggaagactttgtttgtagatacaagcattaaggtggctgtgtatggtggttgtttatttctcctttcactcatagccgacata

ttaccttttccacgctcgtacttttcgagttctgcgaatgtgtttaatgtttatttcgtgaaactcggttggggatggacgctcctcgtagcttttccat

ttctcattttgacaagttacacttattgttgtggtaagcgagagcttgtcctgaaacacttaatgagacttggtatatccacgtgctgttggtttttctg

ggttaatactttcagttacatagaatcaaattatggaaggtgcaatccaaagaaacacagctcgaaatcagtatgcctgggagcaggtggaagttggtat

gggtttgatttatcagggcatgcatttatattgatttatagcaatctagtgttgattgaagaagcgagagctataatcggatgggaaggaatacgcgact

tgatacggaatgaagaacacgcaagaagcaacagtgatgcgtctgtcagcaatagcccattgaggttgttgacagaagttgagtttctgcgtttgaaaga

gtcataccaaaaatttacgccctatatacgttccttatttattggaatgactacgttaagtgtcttgtgggatgttatgctggtgagcacaattctttat

tatcatataatgattgaaaagttcatcagcggtgctatagcaattcttatgtggtttgtgacctataggtatttatatactttaatgcgcaacttacctg

gtgaaggactgtttaaatacagagatttaaatgtaaacagaagtgttccattaaacaaaagatcatcaacaacaaatgtgaaaaatgatattcccaggtt

tatggggatgcctttatatgcagcaagaaatgtagaggaagaatgtcaacaaaatgaagatcagtcttcatctgtgaaaactcagctctgacacatgtag

gtgtccacttctgaatgacctgctgaacaattagttacctgcacaacttctcctacttgttagcatcggagggagagctctggaaaagtaaaaaattgtt

gatcattgattatttcagtgaagcaggcattaaatttaggacatctgtaaaaaaaaaaaaataataataataataaaattgaatgcactcttgtatcaga

gtgattaattttcaatgtggtacctcatttgcaatgcaattactgcttcacttttctgttagaaatcctgtgtgaaataggtattcagatacgtttatga

tgctgctaattaagattcgtgataacctgtggtaaatatttgtttatacagtatttataagcgatattcatcaagaattttttttattggtagaatgtat

aacccatcagtggtaattatcaacataagataccatggatttcgtttaagaagtggtatttccttgctaaagtatctgtccatatttttactatccttct

gttacctgtttgtaatgtttctgcttggtataggacagaagtaaccagcaaatgacatgttactctatatctctgtgttttggaagtctttttctcagag

ctctctgtttccacagattttttttggaaatgtagtctaatagtaatgtgtaactgaattttataatgtgacagtcactgaggagctatcagttcagttt

tgatactgaatggtactcatgtacaaaacttgtatcgttgtagtttatttacaaggatgatcatgtgtgttttgtaaaatctatgacatttttcccaaaa

ttttactgtagaagctataaccaaaagggagataacataaatacttcttttgaggttactattctcttggtctgtcttaaaggcacctaatttttttcat

gcaggaaaccaatttttgtaatggaatgttatttataattgtagaaatttgagatggcttttttaaagatgtttcccactgaaagtaacaattactttgt

catgatcgttttatttatttggaaataagagcacaaatatctctttttcagttgatgactgtttgacacaaaaagaatttgtgttgccattgctgtattc

agtccttaccttatgagttttgttgcactggtgttctttgatagatgatgaaacagcagcaaattgcgcccatttctttttatattgaaattgcactgcc

tcccaatctgactttctagttaaattttacatatggttagatgatttctgtgattgtctggtgttgaacacttcagtcaaaacagcctctcacaggcacc

agtttccttcctttgtagaatatacacagtatgtctgttgcaattatccttgcgtccaaagggttcattcgatttgtcctgattttttaaagaagactgg

cagcattgttgtattctgattaaaattattttgtttgacattccagtgagtcaagtggtagtggccatctgccatccaatagtagttgagtgcctagtgc

ttgttgtgttgcctgtaatgcaggtttcatatattgccttgtaaagtatgattggctgcacacaaccaacactgctctccacaactgcaattcgtcagtc

ccaaaattaagtttaattggaatatagccactgacacaaactttgcattatgacatcataataatccctaactggaaatggggtcaattctgtggggacc

acatatgccagtagcatgaatagacagcagtttgtatttgatggacttgtgcctcagagttaacacttttaatgaaaattcctttttataaaaattttgg

taatgtgtgcttttaagacttgggttatgtgatatctactcttgtacagtccttcagggaaaaagagataattttaagctgcctgaactgtatttccaag

ttttggatgagtatagtcatatttgcattgatgtaaatgacccgcgcatttggtatctttgaaatgctgaagttgctggaaatgatctgtccgtgttgat

ccatatcaccatcaaaatccagtgtgtaaaatactcatataaggagaattgtagtttatctcataagaccacatttgttggcttttcaacttccaactta

gttttacaatttaactgagatgtaagggtagtgtgagagaagtcgtgacaccagaacctacattccaaaaggtaataatgaaagaaaaaaatgtcactgt

agtcttctatttctgtgtgcacttgtaatgaccacatcattatgccacaggtcaaggtttgcatccagtactgatgggccttactgacctgatatgtggg

aactgtagccacacttcatttttctcatagctatccttatcgtaatctgaattactgtatactgtccataaatgttttctctaatgattcataaattctg

tgtttgtgtatgtatgttgacagaccctacttttgaaaattattttggcttgttataactttaagcatcagtgtagtttctcactgtgtgttcactcttc

agtttttgtaacattcaaatctgtctccgttgttttctagataatgtttacttaatgggagggggacgaataaattaactcagttgtatgcaaagactta

ggtttactgcaagattggtgttactattatcttattgttatgaaatac

>31130|AGAP006159-PA

cttgtttaccagtagcggcaagactgtgcccgtatgtcaaatttgttgtgtatgtcaaaatgtaattgcgttgtacagctgttatcaattaacaataacc

agaggttgcaggacagtgtgtcagctttccaaaatgaaaactatcgaggcgaaaatcgtggttttgggcgctcaaggtgttggtaaaactagtatggttg

tccgatatattggtaaaatgttcagccatcacatcagcccaacaattggagcatcgttcttcacatgcaaaataaatattgaagatacaagagtaaagct

gcaggtttgggacactgctggtcaagagagattcagatccatggcacccatgtactaccgcaatgcgaatgctgccctccttgtgtttgacatcacacaa

tatgactctttcttgtctgtcaaatcatgggtgaaagaattgcagagaaatgttgatgaaccaatggtgttgagtgttgttggaaacaaaattgatctgt

cagatgggaggaaggtatctaaagaagaggcctcagcatatgcagagtctataggtgcacggtactttgagtcatctgctttacaggatgaaggtattga

ggaagtatttttgaatacagcagtgggtctcatacgtctttgtgaagactcctcatgttccagcctcagagtgtatgactcctcagaaagaatgagccca

cggaccaatgtttttcatgatcctgcttctccaccttcaccaccaactgttactcaaccggtagttcatacagatgtgccttttgtaccagcacaagctg

aagtagaacgttctcctttctgttgttgaatggcttcagtcatgttgcattttattgtaatgaaaatgactgattctgcatggttaccctaacatttcac

gagtacgaaatgtta

>31143|AGAP006165-PB

gttgtgacacccataccattcctcctgaagcattccttaagagagtatcagcatatcggattggattggcttgttacaatgtttgatcgtaaactgaatg

gcattctggctgatgaaatgggtttgggcaaaacaatccaaacaatttctttgctcgctcatttagcatgcgagaaaggtaattggggtccacatctcat

tatagttcctacatctgtgatgttaaattgggaaatggaattcaagaaatggtgcccagctttcaaaatactcacatattatggatctcagaaggaaagg

aaacagaagcgtacaggatggacaaaacccaatgcgtttcatgtgtgtataacctcctataaactggtaattcaggaccaccagagttttagaaggaaga

agtggaaatatcttatattggatgaagctcaaaatataaagaactttaaatcccaaagatggcaacttctgttaaattttcagactcagaggcgccttct

cttgactgggaccccactgcagaataatttgatggagctttggtctcttatgcactttttaatgccaaatgttttccagtcacatcgggaatttaaggaa

tggttctctaaccctgtaacaggaatgatagaaggcaattcagagtataatgaaagtatcatcaagcgattacataaggtgctgaggccatttttgttga

ggcgtttgaagattgaagtggagaagcagttcatacaaacatagtccacaaatgtttagttatggagttacggctaatgtggggcccatttggattgtaa

aaagtatcttcattaatttt

>31162|AGAP006179-PB

agctggagttcgacgagttcgtgcagctggcggccaggttcctggtggtggaggacgccgaggcgatgcagcaggagctcagggatcaggctattgaaaa

taaacaggaagacgataagccagcaaagaaa

>31165|AGAP006179-PB

ccttgtcgtacagcctgaacgcctccctgagctcctgctgcatcgcctcggcgtcctcctcctcggtgatcttgtcgtccagctctcgcaggatctccct

caggaacctggccgccagctgcacgaactcgtcgaactccagctgcccagagccgtctgcgt

>31164|AGAP006179-PB

gcagacggctctgggcagctggagttcgacgagttcgtgcagctggcggccaggttcccggtggaggaggacgccgaggcgatgcagcaggagctcatcg

aggaggtcgacgcagacggctctgggcagctggaggtcgacgagttcgtgcagctggcggccagg

>45646|AGAP006179-PB

ctggcggccaggttcctggtggaggaggacgccgaggcgatgcagctggcggccaggttcctggtggaggaggacgccgaggccatgcagcaggagctca

gggaggcgttcaggctgtacgacaaggagggcaacggttacataacgacgcagaccctgcgggaggtccagctctcgcaggatctcc

>31161|AGAP006179-PB

cggccgccttctctagtcatcgccacccgtcattacctccatgaactcgtcaaagtctacggtgccggagccgtccgagtcgatctcctcgatcatcata

tcgagctcctcctcggtgatcttgtcgtccagctctcgcaggatctccctcagggtctgcgtcgttatgtaaccgttgccctccttgtcgtacagcctga

acgcctccctgagctcctgctgcatcgcctcggcgtcctcctccaccaggaacctggccgccagctgcacgaactcgtcgaactccagctgcccagagcc

gtct

>31206|AGAP006184-PA

gtgagacgggacaggaggggaagtgtcagggtaagctggtctagaatcaggcatgggaccagatgtgtacggaggctgtgttactgaactgctactagaa

ctgtagttgtaagtgcgatatgcagtgacagttgttggcgcactattgggctgtggagcataacgatcaggcttgtctgaagtgtctgcaggtggtgatg

tctggagcctgtggggaggtggtgaaggctctctgtacctgtctggtctaggagagggcactacttttgacacatttcttgtttcataatacacttcctg

tctctgtggtggaagggtagatgaagttttcgtttcataatagacatcgtgcctcacaggtggaagtgtagacgtttttgtgtcatagtaaacatcctgt

ctctgtggcggtaatgtggaagttttgtgttcataataaacatcttgcctcggaggaggaagagtgctggatttctctgtttcataatacacctcgtgtc

ttgacgttgatgatggtggaggagcaccaactccaggttctgcatcgtaataaacttcgtgccgcacagttgtggggtagggtgtcgaatccttcgtctc

ataatacacttctttctgaagacgacggttagaaggtgacttgtcacgatatctggcacttccatcagtctcgtaatagacttctcttcgagtgacagat

gtagctacaggagaaggtgagcgtgtgcgagtggaccgtgctggtggtgatggctcgtaagtgagttcacgctgtgcagaagttgaacgacgtggctgta

gctgtggtgcatttccaacactggtgtcagcactcgcactgcgagctgaactgtactgacgatactgcacggaccgagaaacatgctgctgttgccgagt

tggaggagctggtgtgcttgtgtccagaggttccaacaatccagggtctactcctgtgcgtgacacttcaactctattgactgtggtgcttgcagttcct

tgtggtgttccaaagccagctttctgtgcctggtgaagatcaacaagcaggctatcaagttcatttagattctcccttaacctcacatcaggtgtctggt

ctgaaggactgccagcactgctgcttagtgcatgtccaggctgaccatcagatgcactgtactgataagaaacagttttgtatgcaacacttctttgttc

tcctgcagcaggctgcaacagggggctgggctcccgctcagcgaccacagtcgtggggttggcaggagacaggtattgcacctgtggggagccggacacc

tgctggcgtacttgacgtatttcgcggtactcctggtgctgctggtgaccgccgctcggcccgttcatggcgctgcggcctcgtgacacactagtctgca

ggtcctccagcagtgcgtcgaggctgttgtccagggggtgcgggtgcagcagctggccgccgccctgcgtctgcgtctgcaccaccgtctgcgtctgccg

caccgccgccatcggtcctgtcccgctgatcccgtccgctgaacaaggcgccgtctatgtcaccgcctcctctgccgttgcagaactctgctgcggggtc

gctactggtcgctgtcgcgttgtttcgctcaaccccgttcacatccaccgaacgtgccaaatgccgggttaaaagtgagacgagacacggcacccctccc

ctgcccgccg

>31242|AGAP006220-PA

ctaacctgccaaactgctatgacacaagtacatggaaagtgacagctaatgttgtttttacagatataccatgcaacacctggtgtagagctccaggtac

tactgaaggtattgcatt

>31243|AGAP006220-PA

tgttacagttggtggtaatgtgactcttaatgaagctatggatctgtttcatgatcttgcaaacactcagaaaggttttaaatacctgaaaatgattgct

gagcacatagacttggtagctaatataccagtaagaaatatagggaccattgctggaaatttgtgtgttaaacaccagcacaatgaattcccatcagata

tctttatcattctggagactgtgggtgctagtctgtgtataattgataaagctgatgtggagactgttttgaaccctcatgagtttctcaagtttgatat

gactcataaaataatcaaaagcattagtttgataccatatgatgatgacacgtatattagaacatacaaggtaatgccaagatcacaaaatgcacatgct

tatgtaaatgctggttttcatatgaaagtagatcaggctaccgttggaaaactgaaggctaaacctgtaataatatttggaggaattagcccccattttg

tac

>31241|AGAP006220-PA

ctcttttggtggaaatatttgccgctgcactgggtatcgcccagtacttgatgcatttaagtctctttcctctgatgcttcaacagaacttaaaggcaaa

tatccagatattgaggatataactcaaaagatttgtgcaaaaaatgggaaaccatgtaacgaaaaatgtgccaatgacagtgacacatgcagtcaacaga

atgtgctctcagcactgcatttggaaacacagtcttcagaatggtttagagttgataaaattgatgatattttcaatattctagctacaaaatctgataa

aaaatacatgcttgtagcaggcaacacagctcatggtgtatatcgaaggaaacctgacattgaactattcatcgacattagtgatgttgaggagctcaag

aaattcaatattgattcagagataacaattggtggaaatatgtctctaactgaagttatggagctattttatgatatttcaaaatcaaacaaaaatttta

gctacaccaaaatgcttgcagatcacatagacctcgtggcaaatgttccaattagaaatgcaggaacagttgctggaaatctgtcaattaaacacatgta

ccaagagtttccatctgatatttttctcattctggaagctgcagatgctaggatcaatatagctgacaagaatggaggtcaaatttctgtcagtccagca

gagtatttgaaaactgacatgaaccacaaaattattaaaagtataacattgccgtcctatgatgacaattactatttcaagacatataagatcatgccaa

gagcacaaaatgctcatgcctatgtaaatgctggttttctattcaaattggacatgaataatgtgggaaaagttatttgcaaaccaagagttgtgtttgg

tggtatcagtcatgatctggtacatgctacggagttagaacagtatcttgaagatgaaaatttgtatgaattgagtacacttgaacaggctctcagaatt

ctggattctgaaatacagcctgtaaagctgccacctgaagcctctcctgagtaccgcaaaggtttggcagtagctttattctacaagtttgtgctgagca

tgtctcctgagaatgtgaaaccaaacctgagaagtgggggtgaaatcttgaagaggccactt

>31270|AGAP006221-PA

ttgttctttcctggtatgttaagtacaatggacaaccagtgggggttgttgtagcacaaaatcatagtctattttcaccaacttagcagccttgactgca

gtatcatgagattgagctacaacaacccccactggatgtccattgaacttaacattcccagaacaaaata

>31269|AGAP006221-PA

gcaatagatgtaacagcaaatgcacagtacaattcatctggtatggtgagcacatcatttacatattttgcttctccagaacattgagacagagcttcaa

gttttgataccggttttcctaatggccacaattccttctttgtgtcatattcctgttttccagaagaaag

>31505|AGAP006225-PA

tgaaggcatccagtattggtcggtatccagtgcaacggcatatattcccaccaaaagaattttcagtatactggagagaaaatcgaatagcaagt

>31506|AGAP006225-PA

gagctccattcctgtcaactatattgatcttagcatcagcagctataccaaaatgcttgcagaccacatagacttagttgcaaatgttccagttagaaat

gcaggaacaattgcaggaaatctgtcaattaaacacatgtatcaagagtttccatcagatatttttctcattctggaagctgctgatgctaagatcaata

tagttgacaggaatggagctcaatttagtgtcagcccagcagagtatttgaaaattaacatgaatcacaaaattattaaaaacataacatcaccaccca

>31507|AGAP006225-PA

ctaaattataccccatttttttattgccaattccttcaactgttgttattctacagttgtggcatgaaaatactggcatcaaacaagaattgaccgcaaa

tgattccacttttttgctgacaggatgtttctttgtaaccataacaatacatgttccacagcctccttcatggcacattgctttagttcctttgaggttg

gcataatttcttatgtaagtgttcaatgaggtacccacaggaactgtgttgtccacttgatacttctttccattgatgtgaaacactgcctccttctgaa

actcagtagaagcggaggcgcccattgtgcggccggtgtgctgtcggtatcagcaggtgtgcaac

>31504|AGAP006225-PA

tcgattttctctccagtatactgaaaatactgttgattgtttcaactttgtaccattctgttgaaggcagctctacatgtaatgatgaaggcactgtttg

ttgagtgcagaccccattttcaacagtgcaaagtttcttgcatggttttccattctttttgcaaactttttctgcaaagtcctctatatcaggaatcttt

tgcttgagctctgctgtagcatctgtacataaggatttgaaggcatccagtattggtcggtatccagtgcaacggcatatattaccaccaaaagaatttt

ccacatcctccattgttacatctctcccactctgcagcaaactgtacatgttcattaccatcccaggggaacagtacccacactgagtagcattgaatgc

agccagagttttctgcagtgtattgtaaccaatttttgcatttccaattccctcaattgttgttacagaacaattatggcatgaaaatactggcatcaga

caagagttgattgcaaaagattgctcctttttactaacaggatgtttctttgtaagcatgacaatgcatgctccacagccaccttcatgacacattgctt

ttgttcctttgaggtttgcatagtctcttatgtaagtattgagtgacgtatttggaggaattgaactatccacttcatactgctttccattgatggaaaa

tactgcttttatttctgatttcagttccggcatgttggtccgcaattttcggggcttgcagctcaactgacatacgaaagcgcgtgggatgcccgttatt

gaggcgcctggcgacggcagag

>31510|AGAP006225-PA

caacagattgcccctgatgaagactaaggtggtagctgtagaaatcttgacattttaccaagaacttacatgacaagaaatctgagaatggtttatacaa

gaatgtcatcacaagaaactttgttctcatattttataaagcttacatctctgctacatatcagcattcagttaacaccaattccatttgggctgggact

ttattgcactaaacaactcagttacagttaatgttcttgcaaaatactcatgtatcatctaaaatcaatggcttagggaagcatttccattgagaacttt

tcaatcatccatcctacagttccaagtttgctctagacactgttatattttttggacatgaaacattgatctgaaggtgatgaggagctcaataccacca

ttgtcaactgattcaactcctagatcgagatattctatgcaatggggtttaagaggctagcacaatgttatggaagtgtttataagtgactggcctttag

agtggaaatgtgaagtaggtttgtagggaactcaaatctctagtgaaaacttttctaatgcgcatatagaaatcaaatagctcttgctttttcagtgtgc

ttcatattatcatgaggagtgtccttaccacaaccattcaaccccctctccattgttgctatatgttactgaaacataaactcatgtttcaaaagcatat

tcatttaatgactattgttaaataattagactaaataagaaaatggaagcaccattaacttttgtaagtttttaaagttcaaactgcttgtagcttgtca

ggctggtaaagaaaactttttctggggtagcaggaggattcatctcaaaccacttggcagagtgacctgaatcttgtctagctgccagtaccgcttcacg

tattgcaaataaagcactaatgctcatgcagaggggtggttcaccagttgcttttgaacgaagaac

>31509|AGAP006225-PA

ccacaatcatcccaattggctgcccattgtattttacatttcctgagcaaaatatctcctcatcagtaaaactgaagaaatgttttgggtagaagttgtt

cttccctggaatatcttttgcgtcatagtatgccaccactccaggtacagccaaagcatcactcggatctgtgtgtcttatgtgaccatttgcaatagat

gtaacaacaaatgcacagtatagttcacctggtatagtgagcacatcatttacatattttgcttctccagaacactggaaaagagcttcaagttttgata

ctggttctcctagtggccacaattccccctttgtatcatattcatgttttcccgaagtaagtggcctcttcaagatttcacccccacttctcaggtttgg

tttcacattctcaggagatatgctcaggacaaacttgtagaataatgctactgccaaacctttgcggtactcaggcgaggcttcaggtggctgctttaca

ggctgtatttcagaatccagaattctgagagcctgttcaagtgtattcaattcatacaaattttcatcttcaaggtactgttctaactccttagcatgta

ccagttcttgattgataccaccaaacacaattcttggtttgcttataacttttcccacattatttatgtccagtttgaatagaaacccagcatttacata

ggcatgagcattttgtgctcttggcatgatcttgtatgtcttgaaatagtattcatcatcatagggtggtaatgttatgtttttaataattttgtgattc

atgttaattttcaaatactctgctgggctgacactaaattgagctccattcctgtcaactatattgatcttagcatcagcagcttccagaatgagaaaaa

tatctgatggaaactcttgatacatgtgtttaattgacagatttcctgcaattgttcctgcatttctaactggaacatttgcaactaagtctatgtggtc

tgcaagcattttggtatagctgaactttttgtttgattttgaaatatcataaaatagctccataacttcagtgagagacatatttccaccaactgttatc

tctgaatcaatggtatatttcttaagttcttgaacatcactaatgtcaatgaatatctcaatgtcaggttttcttcgatatacaccatgtgctgtattgc

ctgctaccagcatgtattttttctctggctttgcagctagtacattgaaaatgtcatcaatttgatcaactttaaaccactctgaacattgtgctttcaa

atgaagtgtagggagtattttctcttgatcacatgtgatactatcatttgcacagttttcattacatggttttccactttttgcacaaatcttttctgtt

atatcctcaatatctggatatttgcctttgaggtctggtgaagcgtcagatgaaagagacttaaatgcatctaatattgggcgatatccggtgcatcggc

agatatttccaccaaaagagttttcaacatctgccattgtaatgtcttttcccctctgcagcagactgtacatgttcataaccattccaggagaacagta

cccacactgtgtaccattgaaagcagcaagcgtcctttgcactctattataccccacttttttattaccaattccttcaattgttgttatcttgcagttg

tggcatgaaaatactggcatcaaacaagaattgactgcgaatgattccacttttttgctgacaggatgtgttcttgtaaccataacaatacatgatccac

agcctccttcatggcacattgctttagttcctttgtgattagcataatttcttatataagtgttaagtgaagtgtccacaggaactgtgttatccacttg

atacacctttccattgatgtgaaacactgccacttgctggaactcagtagaagcagtggcgcccatgatgagccggtgcgccgttggtatcagcgggtct

gcagctccacgcgctatgcagcaagc

>31508|AGAP006225-PA

gagagaccaaccagcgctcgtcagcggcactgtctgacgtcatgggtctcaccggcagcagagagtacgggaaggaggcagtgttctacataaatgggga

gaagtatactgctgacagcagcattccagctaccacatctcttaattctttcattcgggatcatgccagtcttaaaggaacaaagtatatgtgctatgag

ggaggctgtggtgcttgcattgtcatgatcactgctcttcaccctgccacaaagaaaaaacagtcttttgcagttaattcgtgcctgttcccagtattcg

catgccatggatgtgaaataacaactgttgaaggaataggcaatcagaagattgggcaccacaaagtacagaagactctagctgctttcagtggtactca

gtgtggctactgctctcctggaatggtgatgaatatgtatagcttgctacaagatggaaaagatgttaccatggcagacattgagaactcatttggcggt

aacatatgtcgatgcacaggatatcgtccaatccttgatgctttcaaaagtcttgctagtgatgctcctcctcatctcaaggagaaagcacaggatattg

aggatatgttgcaagacaaaatgtgtcttaagaatagggagtcctgctgcaagttgagaggaaaaggaagtgaacaatgtggactgcagctttccacaga

aatacctccactccatttacaatttcagacaaaagactggttcaaagttgatcaaatttctgaaatatttagtattttagcagaacgaccagataagaaa

tacatgcttgttggtggcaatacagcaacaggtgtctacagcagagatcctgacattgaagtatttattgatatcaacagtgtcaccgaactcaaaagcc

actctgtaggttcagaattgatcttaggtgctaacatttctctgactgaaacaatgaatttgttttatgatctcgcaagatcaaatagtcagttctccta

tacaaaagctattgcagatcatattgatttaattgcaaatgttcctgtgagaaatacaggaacattagctggtaatctgtcaataaaacacgcacatcca

gaattcccctctgatgtctttctctgccttgaaactgctggtgccctaattactatagttgaaaaatctggatcagagcaatgtgtcagtccagaaaatt

ttctgaaagtagatatgaaccagaaggttattaagaatataaaacttccatcttatgattcttcatactattttcggagctataagataatgccacgagc

tcagaacgctcatgcttatgtgaatgcaggtttcttggttcagctcgacgggacgaaagctggccacatagttggcagaccacgaatagtatttggagga

attaataaagacacaatacatgctacaaaaactgaagaatttctggaggataagaatgtttatgataaggatgttctacttcatgccctggaaatacttg

attctgaactgataccagactacagacccccagatgctactcctgaatatcgcaaacaactagcagaaggactattttataagtttgttctcagtatatc

tccagaagatgtatctgaaagattcaagagtggaggccagttactgaagagaccactttctagtgggaagcaagtatttgaggacaatcaacagtactgg

ccacttggaaaagcaatgcctaaattggaagctcttgctcaatgctctggtgaagcagaatatgtaaatgacattccaacaacttcaagagaactacatg

gtgcttttgttgtcactactgtgggaagtggtacaatacaaagcatagatccttcagaagccctgagtctacctggtgttgtagcttattatgatgccaa

agatataccaggaaacaatgtgtttacttcagcaacagtaggacttgttggagatgaagagatattctgctctgggacagtaaaattcaatggtcagatt

gttggtattatacttgctacatcacataatactgctactcaggcagctgcacttgtcaaaattgagtacacatctgtcaagaaacctatgattcatttga

gggatgctgcaaaatcaggagatcctaacagagtttttgtagctgctgaaatgaaaccaacagatacatcaaaaaaggagaatacaaagcatacaattaa

aggcagctttgatgttggaagtcagtaccatttctccatggaaacacaaactgcattatgtataccaacagaagatggaatcgatgtgtatgcaccgaca

cagtggccaaatctgacacaagttgcaatatcagaagccttgaaaattccacagcacagtataaatgtcaatgtcagaagggttggaggctgttatggat

gtaagataacaagaaactctcagacaactgcagctggagcccttgcagcatatcttcagaagagacctgttcgcatccatcttcccttacaaaggacaat

ggaatcaattggaaaaagatttgatgttgctatagattatgaagctggtgttgatgatcaaggcaacatcacatatgttgaagccaacacataccataac

aatggctcaggcagtaatgaccacacaccagatgattttctcattcagatggctagctgttacaacaacagtacatggaaagttacatcttacactgtga

aaactgatattccagctgcaacatggtgtagagcaccagattctcttgaaggaatagcatttgctgaacaaatgatggaacatattgccaaaacaattgg

gaggaatccagtagatattaaaatccagaatcttagtccaagttatgaagaagttggtaatatgataaaatattggaaaactaaaacagagtttgaggaa

aggaagaatcgtgtggagcaattcaataagaaaaaccggtggagaaaaagaggtatcgcaattgtgacaatgagatatctcctgaagttttatatgaatt

ttcatgctgttgtgacaatttatgctggtgatggcactgtttctgttgccattgggggagttgagtgtggccagggcatcaataccaaggttgcccaagt

tgctgcacgtgctttggggatttcagtggatcttgtaaacataaggccaaccactaactttacatctcccaataactttacaacaggaggaagtcaaact

agtgaatgttgtgcatatgcaacactgcatgcttgtgagatactcaagcaaagactggctcccatacgaagaaaaatgacaaacccaacatggcaagagc

ttattgcccaagctaatgcagaatttgtagatctctctgcaagtttcatgatgtatcaaggtatggaagagaagttggatcaatatcctatttatggagt

aacagtgggtgaagtagaagttgatatgttaacaggccagtatgaggtactccgtgtagatatcttggaagacgttggtataagcatgaatcctgatata

gatgtgggtcaaattgaaggtgcatttgttatgggacttggttattggctgacagagtacattgtttatgatgaagaaactggaaaagtgctgacaaata

gaacatggaactacaaaccacctggagctaaagacattccaattgaattcaacatagaactgaagaaaaaatcacccaatccacttggtgttcttcggtc

aaaagcggttggtgagcctcctctgtgtatgagtattgtgaccttctttgctttgcgtgaagctgttctctctgccaggaaagatgctggttccacagac

aactgggtagatattgcacctcctgcaacaccagagaagatattacttgctgctatgacggattacaaacagtttgatttgtaggagaatcttgtgtatg

cagatatatcattgcgcagacactgcgaaaataactattcttttgtataaccatattttgttgtttaatgtactgtttaagtttgtgaaactatagaaat

ttattgacttaatatgttttttctttgtatgtatgaagggaataataaatctatctcaaagtctgtcagatagcg

>31591|AGAP006232-PA

aaacactgatttgtctttatatagaagtataaatttaactttggtctcttgtggccatcggaagaggattctccatcatcttcaatctgcaaaatatttt

ctgagaagttgcaaaatgaaataagtgatatactagagcaacagtttaacatactgcacattaaattccattttatttacaaattatatcttgtacaaga

tacttgatacacagtttactaacagcccaactggcagaaactttactgcaagatgaaaaatttatttactctgtatacaatctcctaagtggaactgtgg

tcagacactggttaaaacaacttcaaagtatttaccaggttaaaggacggaacacaaataatcaaaaagatagtaaaaacactagctagtggaaccaaat

attactgagctcgattaaaggaaaacaaaaagatggaggaaactgggctaacaggtagttacagattgtcatttctcaatggggaacatggcaggaaaag

gagcaagcatcgagcatcggtgaaatgccagctatcaattcattaatctgacactgcacttcctcaaaatcgaacagatgattacaagacttaaatgcag

catatggaattcacaccaatgtgtgcaataaaatttattttcccccacctgtgatatctagaaccatacagaaacaacaaatgaagtctggatcaagata

cctttgattttatatgtactaatgactacctgcatgtcacaacacactgcttgaaatcgatactacacaaattcaaatattacaaaccattgcagtcaat

cacaacggaaataattaaatggaatgcccatgagctataacaaatatagcaacattacaaaatattaaaaaaaatagatttttttaaaaatctgcactgc

tggttctctgaaaacttttgttttacaaaatcactgagcaatgactaagcttatggcagtcatttaaagtactgataatgtgtcactataatatatggca

aattacagtgtttctgaattacaagatgccgcatttattactacatcgttatgagatgctattctctgattaactccatactgctatcacttccattgtt

atttatcatttcgggatcacttgagcctgatccacaagcctcatcactgacaccttcagtcttggattgcactcctgtaccaccttcctctttttgaggc

atctgccaagaaggaattgcagttgatactaatggggtcttttttggcacaggaaactgtgacccactaagaagcacactcttgagtgaggagaactctg

ccttgagatcttggatgtgcctacttgttgtatctgcattttgtactaggttgcttaaatgtccaatttcaatactagttttcttcacatcatcctgcac

actctttactttccctgtcaatgatttctctaagtccacaatagaatcttcaagagttttcttctttttcttcccaaagaaaaatgagtgtaagaatcgc

ttatagaacagatataatgcatagagagcacctgctattagagctatactgttgccaatatctctaaatctgctccagaatgttggtcgtggaagaacta

tctgttgggggggtattgctattgatgtctgagtgtctggcaatggagtgaagctacgctcaaaggcaatgctgatttcttcatctgtcagtcctttaga

ttttaggaatttctgcctgtatgttaaagggctctgttgtatcttctgattactcagaaaccttgtagcaatggaaatgagctcctctcggggtttcgta

gattcatcgttggagttaggtaaattggtattgtctggcacacgcatgattgcataacactggacaggtctttaaaatcaacacgtttcagcgtcagtag

tacgaagggcgcattatcaacaattcaaaacataaatgtcatctttatagtttcagagattaaaacgaacgaacgctactaaaagccgtcgcgtatcttc

gaaattaattgggacctgattctgcatattcagcacatttacttcagcttatacgtgcgacataatttcctgagtatgttgttttcagtttgtaatagaa

atgaagttaaaaaaagaaaagaaaaggaacataaactgcacaactgttactgttaacgacctaaaattttagaatctcagtttattgtccaatttctgta

aatgcag

>31656|AGAP006254-PC

ccttagcttcatcaagtcttccaagtgctttaagcagatttcccagatcacttctcacacaatataaatctggattgtactggagggcagtaacatatgc

ttgtactgcctgttccatgtcacctgcagccacaagagcagctgccaaattaatgtaaccatcaataaaatctggtttaagacgaacagcatgtctgtaa

ttctccagagcttcttgcaactgaccacgctctttgtacacattgccaagattgctgtatgcctcggccaataatggattctgtttaatagccaaagtac

tgaagtgggctgatttatcaagcctgcggcactggaaatgtatagatgacaggagcagtaatactccagtgttgtttgtctcctgtctccataattgcat

acagtgtctttcagcattttcatagtcgcctgcttggtactcccgatgcgccaattccagcaagcctggaaacagaacttcggatgagcccatttagcac

gatcgacggcgcacaaaactaaccgtaatacagctcatctagattacgtttct

>31655|AGAP006254-PC

tagttttgtgcgccgtcgatcgtgctaaatgggctcatccgaagttctgtttccagtgatggtgcagctcatcgtcgtccgccatttgcgcccgcgagga

agcgccggcgtccgccatctcggtgacgctcatcgccgtgcgcacgtttcctatcgctgtcacgagagaaatagccggcaacggggatctgagggtcacc

aagcacccaggcaccgactcaacgcctgcttgcgaagtcaaggcttgctggaattggcgcatcgggagtaccaagcaggcgactatgaaaatgctgaaag

acactgtatgcaattatggagacaggagacaaacaacactggagtattactgctcctgtcatctatacatttccagtgccgcaggcttgataaatcagcc

cacttcagtactttggctattaaacagaatccattattggccgaggcatacagcaatcttggcaatgtgtacaaagagcgtggtcagttgcaagaagctc

tggagaattacagacatgctgttcgtcttaaaccagattttattgatggttacattaatttggcagctgctcttgtggctgcaggtgacatggaacaggc

agtacaagcatatgttactgccctccagtacaatccagatttatattgtgtgagaagtgatctgggaaatctgcttaaagcacttggaagacttgatgaa

gctaag

>31657|AGAP006254-PC

tttctaatacaactttcaccagaaaattaaaaaaaaaaaaagaagttacaacaaggtttgagcatgacctattcccacatttcctacaaagactaatttc

catttacatgttggaatttattcactactgaattacgtgtgtgtgtgtgtgtgtgtgtgtaaagtcagaaatgaaaaagcaaactaatgtaatagcttca

atgtaaccaatgatgccaattcacagcaaatcttaatagtattctatgaaagcttcactactcaaacaggtatgtactttaataaactccatccaaccaa

acaaggaaagctgtatcagaaacagatgattttaaatgaattggaaaccccccgtctccagcactttggttccactcagcttttatgagtcaatgtgttt

gaatctttcaaaggctaattgcagtacattaaaaatttttaaaccactatcgcctgaaattggctgtaaaccatatgataagttaagacattatgcacac

atgcttaatctgatacattgcgcaactcaagaaaattgcctgaaaatatgaaacttatcaaatggttttgccaacttgcaagtaaatataaatttttcat

ctgggtccaggtaagatcatacatacaaatgaaattttcaacaactttttcctgtttgatatcgccaaggactggaaaatggaaggtcacagctgcgcga

gttacccgtcgagaattgccaagtataaaacagtaactgtaaacaaagtaacactttttgacgtaactcatgtcgtctccctcacgagtaactcttaaat

ggaagcaaaataattcaagaaatatcagcatctgttacaaaccacattgctgttatttcttgaaaactgtggcgctgcattttgtgagtctccatagttg

gtactggcaaatgcgctgtccaataccacttatgttaccagcctttactttagcatgctaatgagaaattagcatggtaaagtcactgggcattaagctg

caagtggaagcaggaggaaggaaggaatgcagatgtaatgtcaaattcatcattagtgctccaacaacaagaaaacaccacctggactcatagctggtga

atgtgacaatacaatcaataggaaaaagctgttttactcctatgtgcccaaaaatctggcaacgtgaggcactccattttctttttttggaacaaaagtt

actgcaacctccttttcttctaacgttgtgtgtgatgacacagtggataatcttttaccataaaactcagacaaaaagaaaaatcttaaccatgaatatt

tacgattcaatacacttggatgcatatctactccttcaccaggcaagccaacaaattttgtgaattacttttagcagcctttgaaaattcataccattgt

gttaatgactattggtaaagatagttgaataggattttggaaaggagagagctcactcagcttgctaaattttacacaaagatggttttccagcaagaat

tgtaacattcaatgtttaaaagaatacttaaatacagaggaagtactgtccccagtcatttcctctataattgctaagctgagtaaacttcatgtgataa

ggaaaggaatggaaaggtcaggtgtaagacaaaaccttaccttagcttcatcaagtcttccaagtgctttaagcagatttcccagatcacttctcacaca

atataaatctggattgtactggagggcagtaacatatgcttgtactgcctgttccatgtcacctgcagccacaagagcagctgccaaattaatgtaacca

tcaataaaatctggtttaagacgaacagcatgtctgtaattctccagagcttcttgcaactgaccacgctctttgtacacattgccaagattgctgtatg

cctcggccaataatggattctgtttaatagccaaagtactgaagtgggctgatttatcaagcctgcggcactggaaatgtatagatgacaggagcagtaa

tactccagtgttgtttgtctcctgtctccataattgcatacagtgtctttcagcattttcatagtcgcctgcttggtactcccgatgcgccaattccagc

aagctgacagtcgagagctgctgtatatcattcattttgagaatgacattggccccggtaatttgctgatgctgggcttgctgctgcacttgctgtgcct

gcatcgaagccgcccacgac

>31681|AGAP006256-PA

ccgtctggagccagtgggtgggctacagtcaggagaagctgatcttgttacttcggacattccccttgacagagaacatccagatatgttgcagagcgga

ggcatatatactttccttgtcaaggcaactgaacttataaatggtgaactgccaggtgatacagctacatcccag

>31682|AGAP006256-PA

aatttcaccacctggaccttcatctggatctgttgcagtgacagtaatgacattagcaccgagaccaacattttctggtaccactgctgagtacagtcgc

tttccaaattttggagcattatcattaacatctaagacctcaataacaacaggtgcactggctgctcctctgggactctgatccttataac

>31751|AGAP006340-PC

ttaggtgtagaaagataatattaaaatttcgaaaagagagctttttcaagcaaggtcaggaaaaggcacaaataagtcatatttgctattcaacaatttg

tactctagtcaacttcatttttcatggtaaagtttttcaaaatctgtatcgagaggcaagtacatctcccgtcattggagaaaggtgcacaacttttttt

tctttcttttagaatgtacattcacagaacaaaataggaaggaaataatggtcctttaagtacttattatgcacgtttgttcattaagaaatggaataag

cactaagtgaatatcagtgaaatttatttcatcattcactgaccaacgcctttttcttttttttataattctactttgtacctacactattctacagccg

ttcaacagtcttttggatattttgaaaataaataatcatgtcattaggcaagtaatgcatgaatggtcattttcctgagcactgtttctttacctcagga

agcagcatccaattttgatcatgcatcccttatgcaggataagatttggtgttttgagtattttataacagttgccactaatataaactatcaccaatta

gccataccaggcttgttcagtgtcatgaaatagatctggcaagatgaccctccttttgctgacgagaagaacacctgcaataaagattaataccatataa

ctaatctctctgtgaatcaataatgtctacatttctgagctgaatgttaaagaatgtttgcaaagttttctgactatgctgttagacaaaaatagcacac

acctgaaaatatgatctgttggcttaagtatataacacataagaaatagctatctgccacacattgatcaaattacagcaaaagaggccatacaacttct

attaacattttattactggtaaagatgaaaaatatttaaaacaagattaattacattctctattctttctgaaacaaattgaaagtggcttaaacaaaga

ttccaagactta

>31775|AGAP006366-PC

ggaaacatgaactaggacaactgttcaaattgacattgcctcatcaaggaaagcagcaagttccctaaggtgaaggactctactacccgttgctgaagat

gcagaaggaggccgcccagcataaacaatattcctggttctatttattgctgtgttgaatcttggctgaacatatgtccatggaccttgattcttgtgtt

cctcttgcgcccagcacaaaagtgcatttggatacttcgcacactcattcttcaccaagtcataggggaaagggcatatctgttcaatccttgtaatggc

ta

>31810|AGAP006385-PA

agccagctgcgaagtcggtcaggcagtgttctgagaacacaaagctggccacgccaatcaccacatagcccttggtggtcccaaacgtaaacggactgcc

gttgtcgccgttgcatatgcttgctccatatactcctccagcgcagaaagaggttctggcgttgtagtttggaaagtaggctttgcagttagcgttggta

tccacgaccagtggcatatacagcaacacttgggaacagcgtgacagaatatgtgtcctcccccatccggcgacgatgcccattctacccacaagatctt

ctttcatcatatcagttggtagcctcacggtcttgacgtaggctgaattgagaactgcggaagttttcagaaatacgacag

>31869|AGAP006396-PA

ctgggcgacagcctcaccgccggctacggcaacgtcgcctccaacctgctgcaggtcgctatcgagaataagggcatgtcctggtccattggaggcgagg

gcagctggcggcagttcctgacgctgcccaacatcctgaaggag

>31870|AGAP006396-PA

cctggtggcggcgctggactgcctgcgccagcagctgccgcgcacgctcgtcaacatcgttctcgtcccaaatttggaactgctgagaaacttcaagggc

cggacgtcagaatgcgctgcgctcgtcacactgctgtgtccctgcatcttccaatcgcgggagcagcaccgccttccggagttcgtcgaggtgatgcgtc

gctggcagcaagtcgaaatagacgtggcggcggatagcagatatgatgggctcagtgacttcgccgtggtgagccagccgttcgcgaagaacgccacgtt

tcctacggtgagccttggctacacggatttctcttacttttcgcctgactgtttccacttcatacaaaaagcgcatgccagagctgcgaacgccctttgg

aacaacatgctggagccggtgggctacaaatctgttgattgggaggagcccttcacgagattcctgtgcccgtccgaagagcggccgtacatttacaccg

gcggcaacagcgatctgtgaccacacgtcgcttagtcggtgtttgctaattcaagtcatgaaaaacggaatgtaagagcggtgccatacttctttagcct

catacgccaatgtgtttcatatgtggttcaaaccattaccgccttaggtcataagaaattgatgtgacacaaactttcatcatgtgttagaggagcaatg

tggcataactcagtccgttgagaaccctagttgcagtctgttttcatttcacttggatgtgctcatatgtagtagcgtcaatggagcacgtatttaagac

tgataccagttatgtagtgatgaacagtttatttcattctgtacaaatccttatattagagcataataacgattataataaagcaatatgcatgtcaatg

tgaac

>31928|AGAP006425-PA

attattttgtggattatgcgaggatacttttaacacacctgggatcaggatggcaaaggagagaacatatgggaccgcttaacgcacgaaaaacctaatg

ccatacaggatgggagcaacggtgatgttgcgtgtgattcgtaccaccgctacaaggaagatattaaagcactcaaggaactcgg

>31957|AGAP006427-PA

ccactgtaaggtctccttgaggtaaaagttccaagtcacgttgcagggcgcatgcgtgtatgacttgtttgtatcgcactttgtcataaggaagaccaaa

cagtatattctggcgcactgttgctgcaaagacccaaggctcttgggatgcatatgaaaactttcctgttgtatttagtgatccctcctttagaggtaac

tctcctaatattgcttgcagaagagaac

>31956|AGAP006427-PA

ggccagtacctctctctgtcataaggttcaccaaagagtatattttgtcgaatagtagaagcaaacagccagggtccttgtgatgcatatgaaactttac

cacttatagccagagttcctgaagtggctggtagctcacccagaatagtctgtagtaaggatgactttcctgcacctactggccctataatggcaaacag

cttccctggaacaactttgagagtcacattctttaaagtatcttcagcactgctttcccatttggctgtgacatctattaatttaatacaaggagtttct

ttctctgcatatcccttttcagttttcgtatgaacaatacttctcttatgaatattacctaggttcactggctttatttcaccattgttgagaaagttct

caatgcgcttgccagaaatgagaccttcagctagtgctgcaatagctctggggaatgaatcactcattgcttgtttcatgatattgtaatatgcagaaac

aacaaaaaccttgtcggcagttctgttttctccaatcatgacaaatattacaatgctgacaaatgttgacagtcgagtaacaaaaagacccattgacaga

gtaacagctcgaatgtactgcataacttttatagcattcatttccttctttcgtgcagcagcaactaaacttgtgaatgacttctcccatgcatacatct

tgataacctggattccggaaactatctcattcattatccgtaccctgttatctgtgcgaattgctgtcttgtacctgaatctggaagttagtctaccaag

ataaccttgtaaaggaataaacagcaaaacacaaataataccaccaaatgctgctactccaatttttgtccatatgaagtacgttatgatagctgtttct

atgggtccaatccacaagtaatgtccaagtataaacatcatatcaaacctattgacatcgttagccattagatttaccacttgtccgat

>32015|AGAP006427-PB

gcgcacacccggatcacctctcggtagcgccgccggtcgaaaggcagcccgaagaggatgttctgtcgcaccgtggccgcgaacacccacgggtcctgac

atgcgtacgacagcctgccgcgcgccttgcacgtcccctcgctcaccgtcagctctcccaggattgcttgcagcagggagctcttgccggcgccgacggg

cccggcgacgcccaccagctgtccacggtgcacgcgcaggtccacggcgtccagc

>32042|AGAP006434-PA

attctttttcgtcattgcatctctttgtatttgttccgacgatcactaccgtttcacgctgacgagcagccggcattcgcagcgtagtcgcacaccctca

gctttgcgttgtagtgaagaccagcaggacatggtatcagccaggccactccgttctcgtcgcacttgtagaagctgccgcagt

>32163|AGAP006454-PA

ggaatggatcccatgatagcactgatgatgatccattatccattcatataccaccagaaagtgaaggcagcaatcctaaggtggaatcaaatgtgtcaga

gtacactcaaagtgaaactcaaagccagcagtctcatcaaatgcacatctctccacaagcaggattgctgtggtgcctgcccgagctgccgtcctacctg

gacccagcaccagcaccccaaggccccccgctccgccctgcccccccgacatcgcgtcgtcgctccaatccacaagctggcgagaacttcagtcgtgcac

tcgacgcagtacgcttcggggggttgggcttctgcaaggcggcacgaatttatggggtcaacaaccgcaccttgtggctggagtaccggcgacgtggtta

tcctgtagcgatgccacggcctccgcgaggtccttctgcaggcatgcggccggtagctgttattccagcagctacaacacaagcccagcaacttgtatct

tcctttgctaattgtcctgatcctgcctcagttgtcgggtttggagtggcatcgcaaccttcccaacagatgagttgttggacttgatgaataattagat

tattgtttgatgagcatattatttaataattctaagtgatggaagatgtaaaattagttgaccttgtgcctaacgtaaatgacaattgctacagacctag

tctgccaaaagatagtcattgaagtccagttgcctcaagaaataattgaagtgcaatatgtgcaatttttcctcaaaactctggctgttttactttattt

ttaactgttttcattttcccctccagtattgtaccagtcaaaattaaaattatatgattttgataactgtttccctcctatctttgagaattattgaagt

acatatactctgcttattttttattatgcaggctgtattttacttcgtgactaaaacttgggtagcccactttttcctacctgaatctctgtgcagatct

aacgagacatactgtttgtcttccaagacacctgacaatggagggtataagaaaagagaatgaaagtaaggtaaacataatattttaaaaaatacggtaa

tgtatttatgaaagttatagtcatgaatccaggaattctggtaaatttgacagtaacatagaattgcatagtgaacattgcccagctggcagctggacca

tggtacaggagaagcagatggtaatctttatctttttggtccataagtcagtgacagacttagattaaagaaatgaaaaggaattgatgaatttcaggca

ttagtgtgaatttattaagttggtatttttgtgtgtcattagatagagtggaaataggatcgtttttagtagtttaatcattgtctataaggcaaccagt

caataaatagcattgaaacattttagaactgcataacgagatataattcaaatcaagacactgtccatacagatttttcctcattcacttactgcagttg

accccaaagtactttagtaatattttgccagcaaagctaagatttattaagttttctggccatgtgcccccccccc

>32209|AGAP006456-PA

aagttcatctgtataggtcttcacccttatacgtgaattgaatcgaagtgagaggaaatttgatgctgcacaacgtgcacaattgagtggttatggctgt

tatgtagcagaatgtcttccaaagtttgtgcagaaagtccagct

>32294|AGAP006478-PA

aagctctttgatgagaacaagaccctctctgggttcaacattcgtccccttttgtatcaacaaagtggagcaccatacattatttttttaatg

>32295|AGAP006478-PA

aggaaaaactgcttcgtgcttccggataacatcagcttgagaagcgcagttgccttgggtcttaactcgttgatggcacacctgtgtctgttcgaactag

gtggtctcagagaggggcaaactgtgttcatgcagtccatagcaggtggcgtcggcacatctgttatccagttagcccggactgttccaaatgtcacagt

cttagggacagcttcagag

>32341|AGAP006508-PB

attttttaacccaacaattttcttgtgttgtaaatactgatacagcaatttactaagacttagtgaagaaaaaaaaaagagtactgctgaaaacaattta

ctgacacagcagcagtaatacagtaaaataaatcctgacaggcacagacaaacggaatgtatataatttaacagaacatgcacaagacaaagtaacatga

tgagattcagagtatagtgtgtcaattaactgacacatacctcaaccatcagtaagtaatagctctagaaagtatagtctgggtctagaccattctttag

agtttcaatcaagatgtagttagtctcttgaatcatttatgaagaaaatgcaattttaagagtaatattagtctggcaattgtactggattcgctaaaga

tgaatgagcttctcaaattttaagttaattcccagaaaactcaatactgattaatcattaaacaacatttctgttacagaattgtatgtcttacacacac

gcccacacacacacacacacacacacacacacacacacacacacacacacagacaaaccaaaatatttgctaaggaaaacaccttttttggcaaagactt

tttaagaaaggtatttatccggacacaagaaaattacacatacacagaactcatacatcatatacaaataatgacaaatgtcttgtaaacattattaaaa

aagaactatcactttcacaaaatttgagaaatactcagaatcacagattaactgtaactgtagtttttcagttacaaccacaaataaattatgacggatc

tatcaacagtcatgaccatgtggtctttaggactcttgtaaattttaaataaacaattcctaaagcaagatggcatcacgtcatgttcactcctagtgcc

tgacgaactctttcataaacaacatagctaatactgacggcaggcgccactttcaaaaagttgggtgttattccacggtacagtcctaggaagccttcat

cttggattattgtccggattactcttgacatagttccattgtgggcatgagcagacccattgccagtggttataacttgtgcttgtaaccttgtcctaac

aagtgctagtggataactgcaaacctgcccacacgtacttgatatagtcccacaagttagaagtagtaggacacttggcgttgacccgtcattgtgcttg

cgaaggtatgtgttcttgagtgtctcatacacagcaaggtcgattcctgcatatggaataattcctagtaagttaggaacatagcctctgtagaagctcc

ttagtccttctttcttgtagatctttttggccgcatctgctattcctttatattggccagttttacgtaaagctaatcttgttttcagcacctctagtgg

gtatattgcagtctggcttatgccacctgcaagagatccagctacaaatctttcatagatcgaaagttcacgagtattatttcctcggattaatctttta

cactgttcataagccatgaacttaatagcagattctggagcaatcttaacaacatttatcccattgcctctccaaaagctccaaaaacctccttccttaa

agagatggtttaagcatgacctcatgcttgtttgttttgatccgtggacctggaggtaaactttcagtctatcaagaggggcagtacatgtccgtgaaac

tgctcctgccattcctccagctacaagatggcgccaccacatccctgtgttcatctccttctgcgagaagtcatctggtacgctcatatcctctccaata

tcgagatactttgagaggaggttgtgcacaacgagaatgatgtgctggcagatctcgcagtctggagcggccgtggtggtggcggccacggctccgtggt

ggacttgcatggtgccgcgagcactgcacgcttccgccgcgctgggcctactcgcgcttactgatagtgcggagctctgcccctggctccgcagtcggcg

gtctaatgccccgccactgttacgtaaaggtctcccggcaaagcgcctgtcctctacaacagctgacagtcggtgcagagctcactagccagccgatctg

cgct

>32381|AGAP006514-PA

gttctcttccctcatgatgtgcaggagtcaatttgtatagccgtgccaaaagcaggggatatttggtaactcgctgtactggtaccattaagaaagagtt

gagattcatgcgtctcaatacagcattttccatttgggagaccctaaggaaaatgcgaagaatttccttttctttctcaagattcgataacagcattgaa

gctgctccctgtctggtgcagtaggactcgaaggcgtgcagcatgggcgcagcctcgaggaagaggcggccgatgttgacggtcagcaggtcctcgtcgc

cctgctccagcgccacgtccagcgagtcgcgcagcttgtgtgccagcgcgcgcgagtgctccagcagctcctgcatgttgaggaagatggcggcgagctg

gtctggggtgagtagtccggcgacgagcattggtcggtagaactcgtcgagagcgatcttgaggtcgcgtgcgtatttctcctccgtgtccacgatctcg

gccagcagttctttgcgctcggcccgacgcttggcgcacttgcggcacaccatcggcgcgaacataacgccactgtcggtgacgtgcgtgtcgacgcgct

ggtcgcagtcttcgcactgcagctggtcctggtcgcccgcggccgcgccgcccccgccgcctccgcagcccgcgccgcccgactgccacctgctgcggct

gctcttgctcgtctcgctgccgacgccggagtcgttgcgctcgatctccg

>32563|AGAP006534-PB

agtcatactacgtgggagactactactactactactactactactaccacctctctctctctctctctctctctctctctctagcttgttctgggaacca

ttacaccatcaaaattggatgtgagaatttatgttaactgcgagattattgagaagacttcaacttcatccaattaagtacaattaacaattaatccagt

tgctcctcctccagttatggataggtgacaagttacagtatttttgtcaagtcctaaagtttcaccacaacaggtgaacaaagtgagcatagaaagtcag

aaggatattaagtgccccttgctcaatactactcacatcctcatattcgtaaatgatacttctgttttgtttcacttagtgtagtagaagttacaggttc

catcttggtaacaaagacagcagtatgtatttctccaatcattgtttccacatagcctctcatttctgtgatgtagtatgtaaaattagatttttgttgg

cagtttctacatttcctgtttctgcatccttgctatccagtagtttaaatcctttggcaataattcacagcagaaatcacacctataactactttgaagg

tgtctctgcaaatggaagaggaacactgacttatgtttactttgaaagttgcagattttgcactgataattttgcttatcctgctcagcatcagtctttt

tttcaggccctctggctataactaatgtttggttcttcttttttttcactcggagccaccgtcttactccataacagagcaccattagatgtcactgatt

tactccgtggcacaacttcattcgacgtttccttttcaggcaggccaaggcaaataacgtgcttctccgcaggaattctggagtttttgctgcaattttc

tatgctggcgagaacagcttcaggcatattattatcaccttggatgtaaccaagaccaagtgtccttcgcatacagtccagaactgattttgtgagcaat

ctatgctccacttcaattcggtccatgtagttcagcagacgataacagccatggcaaagtactccatcttcatcaacaacaagatccaattctgctccta

taactctacccagcttttgatgaagctggatttttgtggtggttgtaagagctgtcataggtgatcctgttgacccagctgctttgccgcacacaaagca

tttcaccccaggagcaatgacagctgcagacacctcaggttcttctacctttgtagcctcctgcctttgtggtggtggtggtggtgtt

>32603|AGAP006569-PA

ttgttactgcagatggagcatggcgaggagagaaattgttgcgccttaaagaaatatgcgataatgctatggaaaggagcaacaatttctctcctcgcca

tgctccatctgcagtaacaa

>32602|AGAP006569-PA

ttgcagcaccagaagttattcagtatgccccagcattgcctaaatcccgttccggaaagataatgaggagggtactaagaaaaattgcccaaaatgacag

agacattggtgatatatcaacaatgctttcatctgctaaagttg

>32604|AGAP006569-PA

ttccacttcatgtcctaatttcttgctcctttccatagcattatcgcatattcctttaaggtgcaacaatttctctcctcgccatgctccatctgcagta

acaagtaatgtgcttaaatcccacggtgtgaccaaaggtgacagagttgctatctat

>32655|AGAP006592-PA

ctttaacccagaatgtactacagaatggtacttgacctcacgaaatgtagcaaacaatttgccacaaacatcacatttgtgtgccttcacccccaaatgc

aataatgcatgggatttgagattactgactaaagcaaacgatttttcacacacaacacatttgtgtgccttcaccccagaatgcactaattcatgattct

tgagctgcccaagtctagaaaaacattta

>32693|AGAP006612-PA

taaggataggacaaaaacagctctgcatcaaaatttttccaccgagatttttggcgcacctcatcgaaggagggttatagctggattagaatagcaagat

ggccaaatccaagaatcacaccaaccacaaccagaaccgcaaagcccatagaaatggcataaaacgcgttaagaggcccctcaaaatagagagttccaag

ggacaatgccctaaatatttgagaaacttgagatactctcgaaaacagagggatgagtcaagttcggaagcgggttctgcttgatcattgttcatcttgt

ctgtgtaccattagaacattttctaggtgtgtcagttacgcctgatatgatacatccaccaaaattgctcttgtaaaacaaaagctgacgaaatgtttca

taattttctagtccccgatgcaggggcagtatagcgcgc

>32707|AGAP006619-PA

aacagttcataaaattgtttttaatctgaagagagatatgtacaataatatgcaataatttactgtataaattagtacattatatattaagagttctcta

cattttgtttgatcttttcctgaatgaggtttgcaacagctgctgatgggtcctttttaaaactggggtcgtgaagcagcctcttaaaactggcaacatc

ttgtaacatatttttctgcctccattttagttttggaactgatctcttcttaggtgcaggtctttttctttctttctttttatgacattcaatggccgga

agagcatcaactagaggctttaaatctcctattactgctcttttctgacgttgtcgttttttcttctcctccatcttcaacttttgaattgcatttattt

tgctaatgaaggtctcatgcctggcaagtctcctttctttctttgatgtaatacggtttttgaattccctcttgttaactgagactacacttcttgagtc

atcaaaacaaaggtccttcttcaagtcttcaaatttaatatccattccagcaaaaatgttattctcggataagtttggaaggggtgttaaagtatctgtg

ttctgtgggatagtttcatccttcagtgagacaatgaaatttccagcatcttttacttcaggctcctctcttgataaatgaaatttcttcctcaatcttt

tcactttgcccattttgaaggccttctcagttacaaatacgatgctatcaagatatctccagatgcgcttgatggcaacgttgtgactgtacttgtttac

cctacacctgaagttagtaaacaacctgtgaggatatcgatacctaaggccatcgatagcgagcgagg

>32724|AGAP006630-PA

cacgcacacacacccactcctctgagttgtttaggaatcagagcacaccgatcagcatagcacccaccgaaacaggtgggaaaggttaatgtatacaaga

gatatgagactaacaggaaatgcttataaaacaaacggactggaagcaataccagac

>32723|AGAP006630-PA

cctttccaggtgagaatagtgtttttacattccaggtgccacaattcactctacgtagatactgtcaataattgctgagacggaaggaatagtaaattaa

ggccagccttaccggcgtaacctaacaaacaggtgggaaaggttaatgtatacaagagatatgagactatcaggaaatgcttataaaacaaacggactgg

aagcaataccagacaaataagtcaacttttgatagggtagtttttagtaaatgtatatacctgtaaaatatttacatttgttataaaaaacaataaatta

catttgaaccataccagc

>32725|AGAP006630-PA

gtaagttcagtatagattattttatttaagtattatatgtgtacaaaattaaatattagttgttaagagttattcaggatatgaaagttaccagattaaa

gtatgtttgccaaagttttacctttggcatgaagtggaattaaagtgcaaggcacaaaactttgaattccagaattttttcatataatttcacacacgca

cacaaagtttaattgttaaatcagtaaattatgccattatagaaagaaaaacagtctgctctattattaaacatctctaaatttgcataatatagctgga

atatttttctcttctagccttaccggcgtaacctaacaaacaggtgggaaaggttaatgtatacaagagatatgagactatcaggaaatgcttataaaac

aaacggactggaagcaataccagacaaataagtcaacttttgatagggtagtttttagtaaatgtatatacctgtaaaatatttacatttgttataaaaa

acaataaattacattt

>32727|AGAP006630-PA

aatgtaatttattgttttttataacaaatgtaaatattttacaggtatatacatttactaaaaactaccctatcaaaagttgacttatttgtctggtatt

gcttccagtccgtttgttttataagcatttcctgatagtctcatatctcttgtatacattaacctttcccacctgtttgttaggttacgccggtaaggat

ttcccgatatgcctttctgaacatagtacatggcaactgtaggaacagtcatagcacccacaattatagcaaaagaaggtattatttcataccacatctc

cacaacactgcttatttacccaaccgagtcccaagccacaattctccctcacactagtggtgggctcacacaattccacaggcgactatcgattaacgta

tccctctttggtcgagctataacagtagcgcgaaacctggactatagaaatatctatagaataaatttccggatgcgcaaaacaagaattttatcatcgt

catctgcagctcgagtcacgtg

>32749|AGAP006638-PA

agaagttcgccaccaagaaggccttcgtcttcctcaacggcatcgtggggtgcattctgtctgccacgttcgcctactacaacggcaccatcaccaccct

agagaagcggttcaagatacccagtcgcaacataggtatcatcttcgtcgcgggcgacctcggcacagtgctggtgtcgggtgtggtgacgtactacggg

ggcagcgggcaccggccgcgctggatcacgctgggcatgtacctggtggtgggctcgtgcc

>32773|AGAP006645-PD

gtcaggatgcaagagtatcaggtcacttccacttaagttgaggtgcatcagacctggtaggtttgagaatgcattctcatgcactcgtcggaatgagctg

tcaatgatgcggagattcgtaacctgctgcagtcccatcctcttgaaggtgtcatggtcgagcactatgccgtgttccttggggacgccactcacgtgaa

gatggcgcacctcagcagcaaacacctgccttgctgggtctaacttggtgcagcgtgccaccatctcagagagcccagctcgccgtgtgcacacacagtc

ctctgggcatgacgacagtgggtctgccgccagtgccaaccctaccaacagcagcagatacaactgccgcatctctgacagcggacggtagacctgcaac

tggctcgggcacccaccgg

>32809|AGAP006656-PD

ggcgccactgtgggactggtcgagacttacctgtagtggagtggcagcggcagcagcagcagcagggagagtctactgctggcctcagtggtagccgttg

gtgaacgccgctgggatgagcggcccggcgatggccgggaagccggagggctgtgggctagcggcctgcacgtatccgtcgc

>32845|AGAP006662-PA

agagtgctgcagaagctaaacctatgtatttacccaggtgtgaagtgttcttaagcgtgacgttatggcagcagctatgatagattcagggaaacagtgt

aatattcagtcctttgcagtgcttgatttagaaacaacaggcttaccagcgtttgaatgcaacagaactagaataacagaattgagcattgtagccgtaa

ctagaaaacatattatcgagaccgggaagtcaaaagagaagcttcctagagtgctgcagaagctaaacctatgtatttacccaggtgtgaagtgttctta

agcgtgacgttatggcagcagctatgatagattcagggaaacagtgtaatattcagtcctttgcagtgcttgatttagaaacaacaggcttaccagcgtt

tgaatgcaacagaactagaataacagaattgagcattgtagccgtaactagaaaacatattatcgagaccgggaagtcaaaagagaagcttcctagagtg

ctgcagaagctaaacctatgtatttccccaaaaaaaagaatatcacccgagtcctctcaaatgtcaggactgtacaatgacacactagaggactgtagcc

catttgatgatgatgtgttcatgttattggatgcatttctcagacgactgcctcaacctgtgtgcttaattgcacacaatggttttaggtttgacttccc

aatacttcagactgaactatacagattgggaaagg

>32864|AGAP006670-PA

tgttctgcaaatgatgttgctcttcctctagagtttacacatgatttcaatgaaaccagcctttttggtgcagctccagagaatgttataactgctcttg

ggtcacagaatattactgccaattaccacaactactgcgtaactcaaaagaacttaacggaatttggtttgagtgggcagtggcatgtcctctctgtgaa

ccatgatataaatggtttggaatttatatcatctatccagtcaagactgtatcctttttatggtgtcatttt

>32863|AGAP006670-PA

ttttttttccagtcgctgagatgggcagagttcgctgcctcagccctcaaatataattctgttaatgcgaaagataagtccaaaggctaataatggagaa

gcaatagcacttttgggtgttgggtcacagaattttcttcatatgtgtttgtcaagtaggcttatttaagtgcagcgatcttagttactctagatgaaac

agttaagaattttgttcgattattgtcataatttatgtatgtaccgtaactgtataatgcatctctgaaaaactttagcttagctagcgtgaattgctcg

tcgagaatttgagatgcatcgagctgttcgatacacattgtgaaaccgcgttactaatttaaaagtgttattgtttggctgttcttatcaaaaggctcac

tcgaaagataattatgcgataagtattatctgtgcaagtacggaagacaaagtcgtactgaaactgttaattatgcatattgccgtatgtctgctgatac

tttttacagttataaaaggaataccactcagacggaatgacagaccgataatagggattttatctcaagaactatcgccaacagtttcaggaatatttgg

aaatcattctagctatatcgcagcgtcgtatgtcaagtatgtggaaggggctggaggtagagtagtacctatatgggtgaaccagactgagtcctattac

agagaagtaattagtagaatcaatggacttctgcttcctggtggtgcctcatattttacgaatactgatggctatgcagcagcaggaaaaatcttgtaca

atttaaccgttggtttaaatgaaaatggtgattattttccactttggagcacatgtttgggatttgaactgatgctctatttagcagctggtcaaagaga

acatcgttgttcctgttctgcaaatgatgttgctcttcctctagagtttacacatgattttgctttactacatacccacgaaattaatttagaactgatc

tgaattttctcacgaggagagaagacatttttccattcccctgagattttaagattgcaaactaagcaattttccagcacattaaaagtctactgatctt

cacttcaaacaacaaaaaagatttcaatgaaagcagcctttttggtgcagctccagagaatgttataactgctcttgggtcacagaatattactgccaat

taccacaactactgcgtaactcaaaagaacttaacggaatttggtttgagtgggcagtggcatgtcctctctgtgaaccatgatataaatggtttggaat

ttatatcatctatccagtcaagactgtatcctttttatggtgtcatttttcatccagaaaaaaatgcatatgaatggaaatcaaataaaaacatacccca

tagttcagatgcagtggttgcgatgcagtactttgcaaacttttttgtgaatgaagctcgtaagaactttcacagctttccatcagcggaatctgaagag

tctgcacttatttataaatatactcctacgtatacaggtgatcatcttatctatgaacagtgttattttttttattactaacagttacaggtgtactcgc

ctgacatttctgaaagaataatttgtcaataaatgagatgagatggagc

>32901|AGAP006684-PA

atccaaatctttttatttacaaacaaactttattaagcacatatttacaaaataaatactgtaaaatgagaaccattcacactctccgcattgtaatcaa

actacaagaaactggctgatgctgcgatagtaatctctgtgcaaaatggactgaagttcatcattattgagccactgaaaatcatttaacttattcttat

ttgttttgacgtcaccagaaaggtatcgagctttgaagaaaaacacctttgcgccaacagattcattgcgaactgctgttgggtacctatacttatagaa

tccacatggagcatttccaatgaactgaaccttcaaattatttccgcaactttccttgaggactcgctctgcagcctgtcgcattgtttcaccttcaacc

cgcacgccttgaggaaatgcccacacaaggctattaccaattttcatttgtcccaaaagcgttaaagttgttgataaacaacgttccagtgatttcttat

tgcctttacgatctgcatctgttaacctagatgcaggctgaaatttattcagttcttcttggcatgcatcttcaaagtcttgggcagtttgcttcaccac

ctgatctaagtctacatctttaatatcaccttttttcaacagctcagcttgtttcctgtctcgctcgtgacgtatttcatgatcgcttttcatgctgttc

tcgaactcaatagtttgcaagtactccgaaaagcgtttctctatgtccgtcatatcagctgtaataatcgggtctctctccaaacatacgccgctcagca

aatcccatctttcttttccggaagtactgcaacatctaatatttcctttacaagtaacgcgtgttcctacacagactcgcagtaaggaaaacacacgcga

gtttatcattttcatgtttacaaacttcaaacgtaaacatccgaacacccgccagaaacactggttataaatctcgaagatactcca

>33030|AGAP006701-PA

ggagaagtggccaaagacaacagcagtttcatctccactttcttcctcagcagctgattgtcacaatgcagagaaatcttcaactgaggaagaaagtgga

gatgaaactgctgttgatcaagaagaaagtacaaactgcagtggagaagtggccaaagacaacagtgttcagaataaaaaggtagaactgcagcagagct

tcccactgggattgactaatcccaagggtgggccgtacatctgtacagcgtgccacaaggaattccgggacaaggcgactctcagcaggcacttccgcag

acatacaggtgagaagccattcaaatgt

>33029|AGAP006701-PA

gcagactttgctctcaatcaatggtaagtaaaatgatgtgcttaacttcgaaaataacacacaaactgactcaacaaaatgcctcatatgcgatattgat

ttattgaaatgcttgcaatcagactaaaatccttttacagtaaaaattgctcttatttcactgtaagttcctgaatctgtcacagatgtttcttcttaca

tgtgttgcgagagtaaacctatgcttaaatttgcgaccacatgagaaacatgcatgtcttccttcttgtccacattctacgcgaagatggcgttggagag

aagtattcaggtgataatattttccgcagttattgcacacaaatcttgcctctaaaccaattctttcattagagtcctccacacaattttcagaccagtt

agctgcttccactggccatgtaatccctgcgtctgtgaacacctaagtgattaacaaggtgatgcttatgcttgaagcgacgactgcagatggagcaaca

aaggtttccctcttttccacattctaatctcatatgcctgcgcaatgagatcatccagcgatatactttgaagcaattagggcatgaatactgctgattc

cagttgctgtctggagcagcccagctttgtatgtgtggaacagttagcatctggcctacaaaattgattatcctgtattacaccatcacagaacacctgg

aggcattctcctactgtataatggatacataagcattctataagcctcaaat

>33084|AGAP006720-PA

aggtcattttgcaaagattgacgctgtacggcaaatttcgcttttgatcatacaagtaacagcccctctgtcgaacagataactccaggacttcaggtga

cgataccagctggaagtttttgaacgccgcgctcacgactctctttccttccagatagtggaaccatgtacctgtagctgctatctcatacggagaatga

acaaatatttttgtggcgatactcttcatttcgccggcaatgc

>33317|AGAP006727-PA

ttagacatcggagacagcatgtgcagatatacactggtggagccagcactatagccactgagcgctacattatctgggtcacctccaaaagaagcaatat

tctgctggacccacctgagaacactaaccagacctctgaagactgcttgtttctcaatgtgtacacaactaaattgcc

>33318|AGAP006727-PA

tacttggcgaagtttgtccagtatcgaagcatattcctgcgtactttatcctcgtctgattcaggatctaggttgtactcagtgtcggatctcacgaaaa

gtatcgtcaggtctgcagcgtgatttacaccccattgtgtggagttgatgtgttccccgtaaaaatcaaactcataatagaatacggggacgtcagagag

>33316|AGAP006727-PA

gtaacacacaacggtgaactgcctttcctcttcctgagaaacggcgtgaactacaatttggatccaaattccgaggaggaccgtgtacgaaggcatttcc

tgcatatttggaccaactttgtcaaacatgggattcccactccatggacggatcccgtgatatggcatccgtacaacgcgacgagccgcaactacctgct

gatgcaagccaacttcaccgtcgctcacgacagactcggcgaacgcatgggcttctggcatcagaacgtcccactgatgcccttccctggaaccttctaa

tatctgccgcagccgttctcagacatacagtcacgggccattccaccagcatggtgcaattttgctgacttcctaagtagtgcttctccaggctccgctg

taaattaataattccacaattaatatattattaaacactataaataacattttttagttttaggatcttaagtaaagtattttatgacagtgactgtgtc

catatgtaagtccactatgtaagctgctcagaattcaatgaaaacgattgttatcatgacagtattttggttttgttcgacgctgtttccaacttcgatt

tcaaaagctatttccaatacattcgcttgtgctcctttgtattatgattcctattgaaataaaagcacaccagctactctattcgtgtcattgttgtcca

aaataaaaaaatgatgaattaaaccatctacctgtttcagagaattgaaaaatcatcctgtgcttgccgatgtattacatgtataaatggttaacaattg

attataacttttttcattcggg

>33367|AGAP006746-PA

ccccggacctgctccagcacctggtggtggccgagtggcgcagtgacgagcgcaccctggtgcggcgcgagtcctcgaccgccggcaccaccaccaccac

caccaccaccaccaccggcagctgcgcgacgcagaggcggcagcgcgcccagttcccagtccagtgagccccagctgttgcttgttccgcagaaggcgca

tgacggcggcccaggcgctgcgccacaagtggctgcagggagtcgccgctgtcactcagctgtccacggctaaactcaagcgctacgtcatcaagaagcg

ctggatcaaggccgtcaacaccatcatcgcgctgcgcaggatgggcgccaagctggagcccgacgacgccggagccgcgtgaggcggccgcctcctcgcc

ccctggcgaccggactgccgcatcctcagcggtgtgcaattcactccggctgctgcagcgtctgtcacaagccgcccctcacctcgaccccctttaagcc

tcaactcttaatttccacggtgatggaagattttgggaagttcctcttagttctgtcgcacttgtctcgagcctgaatttcacgatcctgtaatttctgt

agtgatggaatatcagtggagctactcctcactttatctccctttaaagcatatgctaagcataaattctacagtcttgtagattctctaatgatagagc

attacatgaagctatttctcaccatgttgcccgtttaaacctatattaagcctgaatttcgatacttatgaaaaatggaagacagtctctccttgcctca

ccgcttttttatagcctacgttaagcctgaatttctagtattgtgcgaagggcgttcacgacggttcagtcccgcgtccggccatcctgatttaggtttt

ccgtgatttccctaaatcgctccaggcaaatgctgggatggctcctttgaaaggtcatgaccgacttccttccccatccttccctaatccgatgagaccg

atgacctcgctgtttggtctcttccctcaaacagcccaatcctttcgaagttagtaatacaacacaatttttttttctaaaagcaagttaattttcttct

atattccagtacaccatatttcccattcttttgacaaaagaaatctatttttaaactatctccactggatgtgaccgcattgtgctatcttacagccaca

cagggtagctgtgtggtctgaggtgccttgtcacggttcgcgcggctgctcccgtcggtcccataggacttaccaaaatttccatcttactctgaggccc

tatatgcacgcgttataccactcgactggttcacgtcggagccaacgtcttgttgcattagtaatctcaccatcattcatgcacggattccagtgcagtg

catccttctttgggccaaagtgatggaagtcagaaactgtgagatccgggctgtagggaggatgaggaaaaacagcccaatgaagctttttaaactcctg

ccaggtgcgcagacttgtatgaggccttccgctgtcatcgagaagcagaatttagtttgcatttttgtgatgatgaacacgctgaagtcgtttcttcaag

tcaacagccggccgacacgcgggagatcggatgggtttgcgctaccttttgcgatgatgacagacgcattgccctgcgactcactgtgcttttgtccact

gccaggtctccatagacattctgtatgcgcctaccgatatctgagatactgtggttttactccaaaagaaactaaatgaaagcttttgcttcgaatgtac

atccattgcaaaacctactttgaggtgtacgtatagtgctgccacctgtcggagcttcatataactatatgggctgaagctggaatattccacgatgttc

caaaacaaactccgcattttttctatcgaaactggacgagaaaaaagtgtgttgcgtcgctaattgaaagcccctggtagccctcccagcgacagaaaat

ggcggaggtccttcttcaaccaaacgcccttgcaaaacctgcgttaagcctcagcttacggatattttagattccatagcgtaggaaggcaatgatatat

ccagtgaagggcagggtaaggcacttgcactccagcccctcttccatccccttcttaagaatcgacactaactagttcctacagcagtttgttgtcaaat

ctcaaaactatttcgtcaatcacatgagtagctccctccatgactagctccagtcctgcgttcactgtaaatggatgacggcgaggagtccctcttcacc

ccatttcacttgtacagtcattattaagcctgaacttccggatcttgtagggtctctagtgatgaaatatggtgtggagtccctccctaccccatcaccc

ttgtaaagcaatctccagcgtgaatttccgtaattgtagattccaaaatgatagttgacggtgggtagttactccacactagaatgagtgtgcattaagt

tcgaatatcaagtgttggacgatgctgaggagtcactcctcattccattatctttgtgaatgctgtgttaggacagttctgtagattctgtagctatgga

agatagtgggaagttactcttagtctaactgccattgtaaacactgtgttaaacccaatctcgggatttcataggttccatggctgtggaagacggggga

gaggggggaatcactcctcacttcttcttcaccattataaaatcgctgttcagcctgaatttcgggatcctgtagcttgcataatgatggaagtgataga

taagtctgccaccaaggctttcattgcagaaaataaatactacctattctctttctatttctatatccaaacaactgtatttattgtgttccaatataac

tgctatcatttttggatccatgacactgcacctgtttcagtattcgtgtacctgaagatggatccttgtgatttcgaagtcggtagtctttttattggaa

tacgaaagagacacatttggatttggaattagaaagagaataaatagttgtttaagttatttcatatttgtaactactaccttcaatagttattcatcaa

ttacagacacggtgcctacacggaaatgaaaaataaatagcatttcttttccgatgtggaatgaatttaaggacgaatgtgtctacgatcatccagactg

caggatgataataaatcaggtgtgagaactcgccattaagtacgcaatgtgatgttatccagtttaaggaagcatataagatggcggatgatactcggta

aacaataataatctcgtagctgtgctcgaaattcagtttatcagcaatctgttggtctcaaggtttatatttgcagacatcaggagctgctctttttttt

tttttcagaagtccatttctgcctagaggaatctttgtgctatgttttctcgtttcttccttcttttactttttacatccgaggtagcgaaattcatcta

ctttttcatcagtcttctgtcatagtcttacattttgccttacggcatctccattcgatgtgtaaacaattgaattacctttctttgtgtgtattacagc

catccgcttgcacgaattccatttcattacgctcccgaatcaaataagagtcggtttcggcgaccactgctatctcgtcagcgaaccaaatcatactgat

tttctatcagtgagaaacaactatcacatgaaagaagttgtttattttcttcattaccccttcaacatatataataaatatcagtaatgacgatgactaa

tactgaggtttatacctcgcttggttttcgtttcttgaactattccatcaataatgactactccagtttgcctcagataaagagatggattctccttgta

tctccagaattgctatcacactgcctggtaatggaaaacattttattgtatttcactttatcaaaatcctctaaatctatgcatgcgacgtatgtttcac

tgtttttcctagctatgcttcttaactcccctatatctcctaaaatcctttattgttccctagtttggtatacattttaaattcctaagaaaattttaca

cagagaaaagattttctggaactggcaacatgtgaatgaccagcacctatagcgtcatccacaagtaactgcgaaaattattatttattatactcagcac

aagaccgcgccgtctcaaatgaccgtaactgtgctctccatcatttccactgtgcattttccccgacttgaccactccttcaaatgtacacaagcttcct

cagatccatttgacacactagttacattaagtaattgtcttttaagaaacatgctgtgttttttatgatgggttatcgtagtgttgagaataacatcttt

atatggatcatatgctcactttggatagtcagttgcgtctgtttaatggattatcatgcaaccagaaagacctacatttaaagtcatctctgttaatttt

atttgtcgatggacatagtggtagcagctgatgaggttcggtggtttagttagttgaaaaaacagcttgatagtgtatatttatatctactttaataata

gtcttcctttctgaagtatttgacattgaattatgggaggttttgtctaataaatattattaaagtgcagtagtttccacatttgcttgttttcatcatt

cttgtttgatgaaaagagcagtgccaacaaaaagagatttattaatatatcaaattgtcgaccagtagaaagtcgaactgatgcttttccttcagtatta

aaagctaccagtggtcctaatttgttttgctgtctatcagactaaggtagaatggaacacactagagtttgaagaagaagtacaaactaattttcctcag

ttcttgtgcatatatgctttagtgctgcacgattcagtacagtcaagatttggcaacacagatggaacgccaacacctcattgtgggaaccaagacacca

aataatttcagcaaggagcatctttcctttttcatagactctcacttatagctaaacgtgaactctgaaactatagcaaaaaacctgttgaagacttttt

gaaatacttttttgcaacacgccgaattcttgacaaacagttcaaattgtgacgggccagtgacagatgccatggtagttgatgtgatgaaactctaaaa

cggcatctactgtgtggcctaaataggcattgtatcagctcccttcattccacatattgatatttattcttaaataatttgagttagtcatataatttat

tttaactcattactttcattcaagctttagaaccaaagttgttattaattttaaaagtatatatacatattgttgtgtttaatataaatatacttaaatt

tcttattttcctttctgaatagtcgagttttagtgttctttatgtccatagtacgaatgctggaacaaaatgctgaccaaaagttaaagaacaaagctgt

ttgtgtaaataagtaattacctagtaacatgtgtttcattcacgtggcagttgtatagccacagcggttatgtgcatgtgcattggtaagtgtattcact

tcccacaatgagcacctcatagttaccataggtttaaacactgttttcaaaacattatttcataaatacagtcacaatctgcatataactttcattgtga

acttcatagatgtgttgtagaataaagctttgtattcgataaactggtcatggttttattcaaagtagacggctactgtgtttgtgactctaaatgaaat

aaatggtgaccactgatgtttatgattgtcgtatggtcagttgtaaattatgattactgaactactttgatcttttttttgggtgctacaatgtaagcag

tacgtttttctataatgtatgtatctgaacattgtgaaactctagtcgcaattgtagcctagtatttttcttccagtgcttagacgtacacagacagtag

aattttactaagcaggaaaaaattatattttttctgtgatattgaaatgcatctctatttttaaaaaaatattgatatttcttagaacaatataatttgc

gttggaataacatgtttagttttattattttaagttttacttttctgtaaatatgtgtacaataaagaaacttgg

>33407|AGAP006782-PA

ctggcaacattcctttggctgtatcgaagcagccgacgtacgctgccgggaagataatgattccttgcacggaaaccccgaagcctctatacagaccca

>33462|AGAP006799-PA

catctcttacaaaatattttcttttcatgaaatactgttgcaaattactttacaattactctttattacaatatatttacaacatttatgtctatacttg

ctttctatgtgagattcccattacaatggagcacaacatttttatacaaaactgtagccatagagtacattaaaaaaacacacacacaaaaaaaaatatc

tacacaggcactaatatgttcatatagttaagtgaacactttagtgaggctttggtttgtgtccatcaacataaaagtttcgggttgcttttggaagctg

aagaaccatgccatcatactcctttttcaaaattatctggcaacctagtcttgaattttccttaagaaagggagcaagatcaagcaggtcctcttcttgt

tcttcagcaggtggaagtttgtccaaataatctccaagcacataaatatggcaagttgtgcaagctaacgatgcttcacaagcaccttccatctcaattc

catatctatgagccaaatacaggacattgtcaccaactttccctcgtacttttatttcgtttccatctttatctatgaatgtcacattaactacttcttc

ctcagactgtggatctttccattcatattctccatgggaaacactgctggttatatgaaattttctctttgataaacgatctggtgttacaaattttcct

gctctacgtagacggagtaatgacgagaccattaaattagcaatacgacatgacattacgcacaactcaaattgtttgatgaaatcaacacatacaacaa

ccaaatcctacgccgccacaatactataccacgatactgtgaatcagagacatta

>33461|AGAP006799-PA

cgcaaatatctgcgcagatagatcgttgcgctctaacttgcctgaatacagtgcaaaatcgttgtcacatacgaagtgctgctgtgtctaatgtctctga

ttcacagtatcgtggtatagtattgtggcggcgtaggatttggttgttgtatgtgttgatttcatcaaacaatttgagttgtgcgtaatgtcatgtcgta

ttgctaatttaatggtctcgtcattactccgtctacgtagagcaggaaaatttgtaacaccagatcgtttatcaaagagaaaatttcatataaccagcag

tgtttcccatggagaatatgaatggaaagatccacagtctgaggaagaagttgttgcctgcattgatcaaaatacagtcctcacaaagtttataaagctg

gggaaaattaggaacatgcgaagctgtacaaaccataggactgatcttagaagagttaatgtgacattcatagataaagatggaaacgaaataaaagtac

gagggaaagttggtgacaatgtcctgtatttggctcatagatatggaattgagatggaaggtgcttgtgaagcatcgttagcttgcacaacttgccatat

ttatgtgcttggagattatttggacaaacttccacctgctgaagaacaagaagaggacctgcttgatcttgctccctttcttaaggaaaattcaagacta

ggttgccagataattttgaaaaaggagtatgatggcatggttcttcagcttccaaaagcaacccgaaacttttatgttgatggacacaaaccaaagcctc

actaaagtgttcacttaactatatgaacatattagtgcctgtgtagatattttttttttgtgtgtgtgtttttttaatgtactctatggctacagttttg

tataaaaatgttgtgctccattgtaatgggaatctcacatagaaagcaagtatagacataaatgttgtaaatatattgtaatgaagagtaattgtaaagt

aatttgcaacagtatttcatgaaaagaaaatattttttaatatgtaatatacatatatttacatctctt

>33487|AGAP006818-PA

tgcacagtcattttcatacaagccacttatagcctgttgagacagttagttgtgtaattgcatataaaaaaatcccaaccataaggagacatttgagaag

taaattttcatatgcaaactgtctttaggtacagtctgatcaattatagccacaaataaataaacacatgcataacgtgacagtatggaagtaaagttac

aggagtggagatggtgattgatgaatactacagaaatgacaagaacaatggcagagacaaacacataaaatatacaaatttacaaaggatcacaggtgca

caaggaaagagtataacttttcagaaatcagcgtctagtgtgaagacattttcttctttgctcgccattacaccacacttctggtattctcctactttct

tctcaaagaaattagtcttgccctccaaagaaatgtgctccataaagtcaaatgggttttctgaaaaatatacctttgaacagcctaactccacaagaag

tctgtcagcaacaaattcaatgtacttcttcataaggttacagttcattcctatcattgctacaggcaatgcctctgttaaaaattcttgttctatttga

acagcatccttaataatgttagttatttccacttctgtgggcttctgaacaacatgcttaaacatgaggcatgcaaagtcacaatgcaggccctcatctc

ttgaaataagttcattgctaaatgtgaggcctggcattaaacctcttttcttaagccagaaaatagctgcaaaactgccagaaaagaatatgccttccac

agcagcaaatgcaattacacgttctgcaaatgttgcagattcatcagcgatccacctgagtgcccaatcagctttcttctttacacatggtagcgtttca

attgcattgaaaagaaactctctttcagcagaatcttttatatacgtatcaatgagaagactatacatttcagaatgcacattttccatagcaatttgga

aaccatagaaacaccttgcttctgttacttgcacttcttggctgaatctttcaaccaggttttcattaacaatgccgtcactagcagcaaagaaagcaag

gacgtgggaaataaaatgtctttcttcaggtttaagctgctgccagtgaaccaagtccttcgataaatctacttcttcagccgtccagaacgatgcttct

gcttttttgtacatagcccaaatatcatgatactgaatcgggaagatgacaaatcttcttgggttctccctcaataagggttccagctgaatatcgaact

ttcttaaagatttcttgtctttctcaatgaaattctcgtcgccgaagttgatttgttgggctatattgtgccgcgtagtcacatccaaaactgcatttcg

gtgctttccgctcgaattcacattaggattaatctttagtggagacacagtaagtagcacattttctttatcaaccgcttgaaaaggcatgttaacaaag

tttacacttcgactactctacagcagaaacccaagccaaactgaccgagttagtgcat

>33518|AGAP006819-PA

ccttggactggctggaggatggctgtgcccctaaatcaccaattgttattatccttcctggtttaaccggagctagtcaagcagattatgtcaaaacact

tgttagagctgcaaacaagcttgggttgcgtgtagttgtgttcaataaccggggtcttggaggtatatcactgaaggtaggtttcagttgtaaccactga

tgtggcacacattcatatctcagttttaccctcaaatcagacagttgttaccaacaatgttaacaaagaaatattttattttataaaaagaattgttggt

gattggcattgaagagaataaatatttaagtgcaaggtgcttttatttatttatttatttaagtaaatggg

>33547|AGAP006825-PA

acggcactggctctagtgggtaacttagccaacgccacattgcacttccaatgacgaatcagccaacttaccggttctcctccttggggttggggatgta

cttgatgcactccctcttctggaagctggcctcgatccagctgcgagccgccgccttggccttggttcct

>33546|AGAP006825-PA

ctcttattgtgtttgttccaccttcaattactaaacataccactggtgtgctacagtgtgtacctggatgaagcttttgaatagatatatatttttcaag

ctttctcctgagtattatttcagcaccatattttccagttattccattatcaaccagaagaaaatatgcatggcggctat

>33548|AGAP006825-PA

gtcgaggcagcgacgacaggattggtggaagagccgtggagtccagccaagcatactcaagcctcgcctaccaatgcctatggaactattgagttccaag

gtggtcctcatccgagcaaagcacagtacgtccgcctcgcttacgacacaagacctgagctgatagtgcaattattcactcgtgaatggagtcttgagtt

gccaaagctgctgatcacggtacaaggtggtaaggctaactttgaactgcagccaaagctaaagaaagttctgagaaagggacttctaaaagcagcgaag

actactggtgcgtggatattcactggtggtacaaatactggtgtaacaagacaggttggtgatgcactgttgttggaaaggtctcaacgtagtggtcgtg

tcatcagcattggcattgccccatggggaattgttgaaaataaccatgaactcattgggcataacagggatgtaccatatcactctatttcttcacccag

gt

>33593|AGAP006858-PA

gctagggtgggttgcggcttgtgcagcaaaagttcgaactgaagctactcgtaaaatcggtttttttcaaagcgttgctagccattctctactattttac

tgccccagccgtcgactactactcgtaccccaagtacgcgtacgagtacggcgtgaacgacccgcacaccggcgacgtgaagcgtcagtgggaggagcgt

gatggcgacgtggtgcgcggcga

>33617|AGAP006872-PA

ggagatggcacatttggttgtttgggtcatggtgactggaataattgcttgcgaccaaagcttgttgagaatctgctaagtgttgatgtcatcatgctag

cttctgctgctcagcatgttgtgatcctcagtgcagatggtgatttattctcatggggaagaggtgatggtggaagactaggcattggaactgaggatga

tcgatgtgaaccacaacaagtgcctctgccaagggaaacagtagtcaggtctgtgcacactggtgttgattgtacagcaattattaccacagatggtttt

ctgcttagttgtggacgtaatactcatgacaagctgggattttctgtcaaggaaaatgatggcaaaatattattgctgaaaagagtgaaaagcattccag

ccaaggttcatgatatttcactgggtcccaatcatacagctgttctcatggataatggtgcggtaataacaatggggcgcaatgcagagggacagctagg

aagaggacattgcagagtaacagggccagctgctgtcaaggtgttggcagacagggtcattacctttgtcaaatgtggtccaacgtatactgtggtaggc

acaagtgagaatgctgtgtacttctggggaacaagattttttgcacctgctggcatggatggaaataagacagcagatgcctctgcagcatggcatacat

cacagagaggatctcattatttcacagcattgaatgcctccacagttactaacagtcaacagtcagaattgatacttgaaccccaagaaatacttgcact

ctatgcatctccaaatcacatagagaaaggacacttgcttactctctgtggcctcttcccactaagggtcagtgtgatggttttggtggagactacagca

ccacttggtcatgttctggcacaagatgcagatagtgataactctgctgatgaatctacatatcaaaacaacctacaacatcgtaattcattgtcttctg

ctgaatatgacagctttggaccgattccagattggataaaagttgaactggctcagtcagaaaatgcatggaaggggtatcctgaaaactgaagtgaatg

taagaaaaattgttttgatattaacacaatttaactgttcttattctgtaaattctgtaaactattcacttgtattttgaacctatttaattattacatg

aagtaattaatgacactgcacatcagattgcctaccacaattgatgtatcaataacaaaactgttgtatgatcacttgtgaaataaatg

>33638|AGAP006873-PA

acttgttgccggcgagaactttcaccacgtccggggatgcgttctcctgtatgtttcgcagccaatacgacaggtggttgaaggactccatgctggtgac

gtcatacatgagcaggatgcccatggcgccgcggtagtaggccgtcgtcagagtgcgaaaccgctcctggcccgccgtgtcccatatctgca

>33660|AGAP006879-PA

cccgtaaagcactccagccaaaaggaaactccatcggccaaactttatcagaggagatgccctcactggtggtggaagatccgccatgattgcacgaatt

gccttttctttttcctctatttctcgcaatgcagcttctttcttggaaagtgatctttttcttgatgccccgtaaagcactccagccaaaaggaaactta

atctagccttttcttcctttaacttagcgtcacg

>33704|AGAP006887-PA

aatggagggaaaagtttgttgtaaaatagatgtctttgtattgtctttaacaagaggatatcagaaagatattctttaataccatgctcacaccaaacta

tttcttctcttaaagcagtctggtacaaatatcagcaacaatccagacaggggatgagtacgtgcttataaaggaaaagtaatggagaaagacaaagtaa

aaatgtggatgagaataaattaatctgtgtagaaataacaagtcattcagtgtaattgtttctaacaaaatgccacattttaatttttgctttacttttc

cccttcttacatttcattctaaaattcttgctgccatgcccaaaagcaagggattcagtatatttttccatttgtgctactgacaaatatttctgttttg

ggactattcttttggcactcatctcctgttattagcaacatccactttgtaattactctgttttgcttaatgataagcacttcatctctccacaagctga

aagctggaaacttttattcatgcacaattgataaatcaagggctccaccaccaccaccagtcagtcaagtacatgtatattagggagattcaagagcatt

tccaaacacagacaccaacattaccaccaaaaaacatgtacaaaagattcttgcactcataagatatttcaaagccatagccttcaccaacaacagcatg

ccaggatggtccaaactttttatccattgcctccttaatcatgcgagcagccatactgttgttgagaaggcaataagctgtgactcaaagctcgagtgga

aagaaaagactcatcattcgttgttcgcagcatgcttctcacatgctgttacacaaagttccattgcttctcctctcatctcgtcaatcatatcgcagtg

ccttactaatggatacgtatggataattttctttgtttctgcttcttctttcttcccttctgactcgctcattgcgtcttcccacttctcacgcaatgtc

aattgtcaatatttacaaacagccgacacttgcgccaaac

>33720|AGAP006890-PB

taaaacaaaagtactttcaggtaagtacttttattttaacccactaatgatagtggaaaaatgagagtttcaactttctaaggccacttggaaatttggt

tacacgtatgaaaacaaaaccactttctcccaacattatcacaagagagaatccatccaactaacccaagtacatacaactatatctgcaatctgtttac

tcaaacaatataataatatttgttcttttacagttagaattccagtttcttagacatttaggttgaaccattatgcatgacactggaacgaccagcacta

tctttcaccacagaataaaattgtatatatataatttaggtccctacattgaaggaactaacagttttgtttcaatacgcagcatgtttcacatatctga

gacttcttttatggacttccacaagcacacacgttgatacacagcttgaagctaaagctctgtcaaccataccgtcacattcaattatttattgtggtgg

actcaaactgaatataataaatcttctaagaaaagagacaggcaggttcataacttatgatttgaggtcagccacgtaagtccttcatacaggccatccc

ctgtagtcgcacatgagggttggacataccaattacgatctcttattcgagtgagtcccagtttttcctggatttcatgaggtttcatagcatctggaag

gtcctgcttatttgcaaatattagtatgattgcatctctcatttccctgtcatttattatcctatgaagctcttgtcttgcttatttgcaaatattagta

tgattgcatctctcatttccctgtcatttattatcctatgaagctcttgtcttgcttcatcaattctgtctctatcagcacaatcaacaacgaatataag

accttgcgtgcccgtgtagtagtgtctccataatggacgtattttatcttgtcctccaacatcccaaacattgaacttaacatttttgtatgtcactgtt

tccacgttaaatccaacagtaggaattgttgtgactgactgacccaatttaagtttgtatagaatagttgtttttccagcagcatctaatcctaacatca

atatcctcatctccttattgccaaaaatttttgacaataacttccccatcttgctgtcagttctttcacagcac

>33803|AGAP006916-PA

ggattagcaggtttctatcccatccatccaaggacatacatgacagcctggaggaaccgccgcagtgctgcagaaaataaagaagaagttaaactagaaa

atagtagttagtatgaacacagtgatgtacattttacagcatatattttgaaaaatgtataatacaaacaattatagtctattaaataaaacagctcttt

atctacactttcgatgtatcacttatttcaccatgcacagtacacagatgcacctaaaacttctttatcttttgcaactgattatttcttctcaatgcca

ttttcctcagtcttctccaatactctttagtgttaggatccaattcctctaaagcaggtggcttgcctagacaaagagataaactaacattaatccagct

tttccacacattattgacttatccaatgaagaaacaatttttttttactgtaagatgactattctaaaacaatttgtgtcagaatgaaaagtgagtgttg

aaattctggataacaataatacagtaaaaccccctttttacacttttcagtggactgactcaaaaggagtgtaaaatgcagcaaagcagagaaaacacaa

ctgcaaatggataaatttctctgtcatatagttcaaaatatgaaattgatgtaatttaaattttgcctatttgaaaaaaaatattttattgtttttgttt

ccttctgac

>33816|AGAP006931-PA

ggggtgtcttcgacgggcttgggggcctcgagggcgggggcctcgggggcggcgggggcgacgggcaggttggaggcggcgacgcggaagccgttgacgg

cgtcggccgtgtagcgcacgctctgcagctcgcccttctcgtccacgtacgagtagccgccggcgacggagccgtcgaaggcgcgcgcctccgacttggc

ggagaggccgccgtggtagccgtagctgtactggcccagctcgtcctgcgcgtggtactgcgacgtcagcgcgacgggggcggcggcgtaggcgatgggg

gcgtggatggccgagtagggcagggcgccgtgcaggaagccgggcctcgccaccgccacggccaccagggcgctgagtacaaccaggagcttggccatgt

gtgaggtgatgtgagcggcgctgctgagaccgtcccagtactcgctgctgcg

>33871|AGAP006936-PA

gtagagagcttgtgccatccttatagctcccccaggaaagtatggcttgtagtactggccttccctgagaactaca

>33906|AGAP006943-PA

ccgcactttctacatagtagccatccaacactgtttcacaagagcctccttgggaaatgcaagcctgcttttctgattcagaactacagttattgtcttg

ggccatactgcatgccttgtaagttagtccatcaacaaaccacaaagcaaccgttgctggccagtttccacccaaatttgccactgtattaagcaaggtc

atgtatgttcctccaacagctggatcacttatacgtgcaaagaatgccatcacagcaacaaacatgctgtataaagccacctgatgtaatccataaacaa

taattaccataacatagtaatgtattggtataatgccatcatgaatgaagctaggtgtgatccacactaaaagtgcagcaataaggccacatagcaatcg

atagggaattgcagtcaggtaaacatccattggacgtggtccaacagtatacttgcttaacaccaatggcaaaattatctgcagtggaacaagtggtatg

gctattagtgccagcttctccttgggaacaccagcatccactagttttaagctagtcacagcatcacaggcagaaaatccaatcttgctagttaacagaa

caacaatggttgtacggactgcaggcaggcgtactatatcccacaacaacttgtacgtctctttgacacctaaatcagtgtcatgtggttcatttccttc

acgacatggaatctcatgtttgaacaggccaacaagtgttgttgtgactagaaacactagtccccagaagtaaagaaaacctgataatgttactaatcct

tgaggttctggctcagatctaaggtatgcattacagaactcagctgattctaatcccataaatacaacatatcctaagaaatatccagcagtctgtccaa

cactgttgcaagttgaggcatgtcctacattgttcctcttgagcatggtgagagcccatccatcaactgcaatatcttgtgtggccgctaagaagttgag

acaaaagaaaaatgctgtcaaggtcccagtactagggccatttgttcctgcagattctccttcaagccagtgttctacattagctgataagaacatcatg

aaaaaaccaatcaggtactgagttggtaccagccatgacttccgtcgaccaaagcgtgtggagaaaactgagtccacaattggggcccacaaaagcttca

ggctaaatggccaatgcgcaaaactaaattcagcctgttgtcgatagctcacgcctctgtttcggagatacatagggattgccgcagtaagaccaatagg

gattccttgtaacaagtacagaaagaacagaatagcgatatttttttcatctcctttgatgtcacttctcctttctggtaaatatccgtcttctatatca

gtcgactgattcctcacttccgtccctgttcgtttacgggtgctcatcttgttattcgctgagtaactttcagtatcgtcgtactccacctgtcaaatgt

tatcacatcatgcatccttgtcagagtgaaatttgcttattgtctgacacacacattagcaaatgccactgaaaacgtaaatcaccaacaacacattaca

acttctcttccactctcacgctcaaaccaaaacaaacttacttcatcattttacgtatcacatgtttttattcccagtcagctacttattaaatgtttgc

cagccaattagtagtattagagctgtgtgaatggcgatgtatgtttcatatgt

>33924|AGAP006949-PA

tgcaacaaatcgtgttgatatactagacaaagcattgttgcgacctggtagatttgacagacagatatttgttcctgctccagacattaaaggaagagca

tcaatatttaaagtacatttacaacctttaaaaactagtttagacaagactgatctggcaaggaaaatggcagccttaacaccaggttttactggtgctg

aggatgtatttaaatgcggtcactgggtttttcttaaagcgcttttgacatgtacatgtgcctcgttgtgccaacctctcactgctgtggacgaattact

tccatatatctacgaataaaaaagtttaaagtattttttggtaaaacatatgtgcctctttgtgtgctatcatttgtaaaaagccttgttcggatttctt

taattgttaataaattgacaattgttgagacggcataatttctgatgtga

>33949|AGAP006953-PA

gtggcatcctcggttcctctgtcactttttattaataaaatacaatacctacagtgcaacaacaaaaaattacacactgctccaacatatcacttttcaa

tattactatacaatgtttggctcttagtaacaccaaagagtctatctatagagacagttggcttttcactttcttttgcaactactgagacgttatcaac

accttggttacatacaagtggcatcctcggttcctctgtcactttttctcccctggctaggcaatcacggattatcagctcttttagtagaggtggaaaa

ggagctgttcttgggagcagtctcgccggtttctcactggattctgcattctgggcatgggtatatgcatattcctcgtgtttatggataagcctgtagt

ccgctttgtatgtgacgctacttattttcaccaagtacggatacttcctgcccctgaatatcttttccgcccagacgcaaaccttgcgagtttcctcatt

cggcatcgcttcaacttttcgtatgacaaagtaacatggttcgtcgtatctttcgaacatacttctcaccacaattctgcccacaccaaaatttttcagg

ttgcctaatatttcccacaggggtttccccctaaaatcggttgtcctgcctatatactttatgggcattgtaatatttttcactagcgcttacttgttta

tttgtaaacactagatgaaatccaaggaagtacggtggacatacgagccacttcactcacacgacctttaacaagtgagat

>33964|AGAP006957-PA

ataaacaccaaaaccttcttcttcaggtgatacagtccctgtatatggtttcatgagccatggcagaaggggatatgccgcatctccaagcaggaatttt

ggcacaacagtcccactgattgtgcatggtgcttctggcaccagcctgtgaatccttctgtactgatgcatcaaggacatttccagtatggatgatattg

atgttcctaaaactttgtaaaggcttataac

>33984|AGAP006966-PB

tggaccaatggaagaacactaattcatgggaaatatatgtacaacatacatgaactaaagcacaagataagatgctaaacaaaaaaatgcattcatcata

agaattttgtgcaacacataaaatatgaaaagtacaatggaaaaaataaaacaaaatcagcaaatgcaaattacagtctttaaattgcagaatgttattt

atgatataagtgaattgtcagtttttaaaagtattatcaaatgttacactatcttacaccatctctgtggaaacaatcttcaggaatattggcaacattt

acagtttttttttttttttttttttttgcgtttttagatggctttcgcttttagttgcactggttaatctctttggtgtcttcaacaacagactcttttt

aagactgaacacaacaatcttgcaggagttaaaatctcacaatcataaaaaatcaatacaaaaattttacaacacaaagacattattatgccaacaaagc

agcaaatgtaaaacagtttaaaaaattaataataaaaatagttaaaagtttgtcgtgccagtaaataaagctctattaatgtagtacacatcaacttcag

tgaattattaaaatttttacaaataatattaggtaagtataaaaaaaatcatttgcagacatgttttttcttctgttttccaagaagataatgtattaca

attgtttgcttctctgagctgtgtatacagtttattgtttatgtaaagaaatttaaaaatccaataaatttctgtagagaagtcacactagtaggttttt

aaaattaggccccagaactgatctccacaactgggtttatttagtttcacgtagaacttgtcggaattggtccagaaaaatttcagcatgttgggtctgg

aatatcagagctggtcgtatacgaatgctgtactctccgcatccaccagattgtattcctttctgttttaacctgttcaccaagtcatcacgcaactttg

ttgatgtgcagctgatagcaagaaatgtcccccggcctcttgctgcaaaaagcagagcaggaaattctttctggaaatcatataatccattgagaagttt

ctctcctgacttctggacaactgagagaagtgaatctctctttataactgaaagaactccttgaagcagcagtagtttaccagggtcacccatccatgta

ttaaatactctgtatggttgtgaaggcctaaactcttccttcatgtagtaccctccaagctgcatctttttgctgaaactcacaatatcaggtggggaat

ccagattgaaatgttcatggcaccacattttgcctgttggaccaccacctgtttgtacttcatccacgaaaaatgcacagccatactttttacaaattct

ctgcaactgctgaaagaattctggagaggcagcattgtctccaccttctgcttgtattggctcgactattactcctgctacattcttcccacgtttatta

tattcagtgatacgatcctcaacctcagccagacatcttctgtcctcagcttcattttctgacttgtgctcttctaaaggatatttgtactctggaaatg

atgcaattggccagtcaaatgctggcacatcaattttgtgaatatattttgaatgtgtggtagatagcgcaccaagggtgcgtccatgaaatgcaccttt

gaatgatagaatgctcagaggtggagagccaggtgattgatttatcatgcaagatgacctttcatcttcagtgaaatctatattttcacctctctgagtt

ttgcgataccagatgaagatgttcttgaatgcattttcatttgaacatgaaccacacatcattgtagtgacatttttcagtcccttgggggcaacatcaa

gaaggatatttttaac

>34015|AGAP006992-PA

tacacagtcggtgtatacagctctcgattgtggactgcattcctggctatcacactcctgtttttccagtgtcgaagactgtggttcgcattgaaattgc

agccatattttatgcttacaggctggggaattcccctggtgattgtaatatttttggtgctcttcgttaagcaagaggacatgtctgtggaaaagtgcaa

cccaaactttcagtttggaggggtacaagctataa

>34014|AGAP006992-PA

cgctgtgctacacaaaatgtatacagaagggattgatcatgaaaatgatgaaggtcatttatatgcctaatgaccctcccctgcaataaatcagtggcat

actgcacagcttcctgtcggtctcgtgccagaccagcttctatcaaccacgtcacaagtgcagatcccttgaacacttctcgatatgtgcggaagtgcca

tcttctatcttcagaaatatcacttctacatttctccaaatgatgcataacaaactgatcacacacatgtcgtgtctcaaaattcagttcttccc

>34046|AGAP006993-PA

atttccactgtgaaagaatataattaacatagcttgcttttacatgactgtatcattttaactcatatcaaaaaataatcttgtgcttcactttttaaat

caagttagctcttcagatactgaattttttacatttcacttggacaacagaaataaccttaacattatatgtgacacacatttctgcaaatccatttata

atactgacagtaatttgtaacatatagtgctttatagaataaatagtttgataaaaagaaagactgtagtagtgcttgagcattactcttgtggaatgcg

atctcgaaggtaagatagcagtgattcagtgccctgcaataatacgcactgacggggatgacgaaagcaatttccttccaacttcacagtccttaactgc

ctcaagttcccaagttgtggtggaacagatgatatgttattgtttgataaatcgagtactgcaagtcttcttagctgagagaggacttccatatcaattt

ctgccaaggtgttccctttcaaatccaaaatttccaaactttgtaaggttgcaataataggtggtatatattgaaacctgttgcaagcaagtacaagttc

acgaagcataacaagctctgaaattgattctgggagatctgttagtttattaaactgcagattaaggtactgcaagtatggaaagtctcctattgagact

ggcagactctccagtgaattacattcaagattaatttctgtggcaatcttattcaatttagacattccttcaggtaattcagtgagtctgtttcctgaca

aatcaacgactgttacagatgcaaaagcagcttcatcaaatacatatgggggaacatctgctagatttttctttgccaggttcagacaacgagttactct

caagttaaacctgttgggaaactccacttcttcaacctcatcagccctgccttcaggatgatttgcctcatctaacttcttacgcaaccactgcagtaag

taaggcgttccacataatacaacatccctacgtacttctgacaaagggttaccatctacatgcaagctgctcaaatgtggtagcagggacagtgtagcag

gcaaattttctagtctgtttcctctaaaatcgaatcttactacctgacacatcaaatgaatattttcaggtagcgaactcaagcagttgttatgcatgtc

aagcgttttaatcccaattaatccctcaacatcagtgacagaaaattcctctattgcattgtttcccataaacagttctttcaagcattcacatcccata

aaatttgggacttttgtgagtgagttctttgccaaattcaggaaatacaattttctgagctctcctaatggaggaaggtctgttatcttattgtcattca

gccaaaattgttttaacacaagaagattgacaaggtctggaggaatctcggtgagttgattatgagatagatccaaagatgttagatgtgttaaaaagcc

aattccaggaggaaggaatgtcagactgttgaagctgaggtccaaagtttgaagcatatttaagcatgcaatatcaaatggaatgctctcaaattcatta

cgtcctaaatttaaaaccagcaaactcttcaaatttgacatctcttcaggtacggtcttcagcttattgttgtcaagatacagttccatcaaattgcgca

gcatgcccatttccttggggagtgactgcagactgttgtacgaaagtttcaatatggtcaaggcatccaagtttgatatatcaggaggaattcccacaat

gcaattatgactcagattcagtgttgttaaaggtgagtattcccaccacttctctccttcatcctgtttctccatgtcaacatttaaatttttacattca

atgtcatctaggttctccaaagtccacactttttctggaacaaatgccaaaccattcttcgataaatccaactctccagttcttctggtttccttgatca

aatctgttgttaatactttatcacactcttccttccccagccttgaaaatagtgcagaatgtcgcattttcggttaccacgtttgtacgtatcggctcgg

atattactttccctgcacacagtaacacacaacgaagcttattacatcatattttctaataactccaaaacacgataatagcgcttaacaggtcaaccac

ttgttttgacaactatgacttctgttctgtttctgatcaatacttcac

>34062|AGAP006995-PA

atctgtaagaatttgaaatttaaatgcagctgctcacctcaaacagtatcccatctcctccaactactacaattgcacgccattggtaaatatctctaga

cctcacaaaatctctggcgtatccagcatgcttagtaacatgcagatcaaatggtatctctgcctctgcaaatacaggtacaactcgctgctgaaagaca

tctctagctcggc

>34097|AGAP006996-PA

ctgctttgctaaaaggaggtgctcccttgtctgtggcatggccttatttataaagaccactatgtgatttacaccaacctgctttgctaaaaggaggtgc

tcccttgtctgtggcatggc

>34096|AGAP006996-PA

ctccactggaagcatgaatggtttatccagttctcttatgggtgccttcatagtaccaggtttacacattatcatcccactgcgaagttcatccctcttc

acacctcttaccaatgcaccaagat

>34094|AGAP006996-PA

cttaattcgtcactatattatgcttatgcagttactttagcaacaacaccagcacctacagtacgaccaccttcacggatcgcaaaacgtagacctgggt

ccattgcgattgggtggattaattctactgacatttcaacgttgtcacctggcattaccatttcaacgccttctttcaactggatcgcaccagttacgtc

agttgtacggaagtagaactgtggacggtaaccatttaagaatggagtgtgacgaccaccttcttctttagaaagtacgtatacttctgcgtcgaattta

gtgtgcggcttgattgtacctggtttagcaagtacttgaccacgttgtacttcttcacgcttagtaccacgaagtaagataccacagttctcacctgcac

ggccttcgtcaagaagtttacggaacatttctacgccagttacagttgttttaactgtatctttaataccaacgatctctacttcttcaccaactttgat

gataccagcttcaacacggcctgttactactgtaccacgaccagaaattgagaatacgtcttcgattggcatcaagaatgctttgtcgattgcacgctct

ggctctgggatgtaagagtcaagtgctgctacaagagcaagaactgattcttcaccgtaaggaccagcttcaccgttaagcgctgcaagagctgaaccac

ggattactggagtgtcatcacctgggaagtcataagtagaaagaagttcacgtacttccatttctactaattcaagtaattcttcgtcatccacaaggtc

gcatttgtttaagaatacgatgatgtaaggtacacctacctgacgagaaagaaggagatgctcacgagtttgtggcattggaccgtcagttgcagcacat

acaaggatcgcgccgtccatctgagcagcaccagtgatcatgtttttaacgtagtcggcgtggcctgggcagtcaacgtgtgcgtagtgacgagttggag

aatcgtattctacgtgtgatgtattaattgtaataccacgttctttttcttcaggtgctgagtcgatttgtgagtaatctttc

>34095|AGAP006996-PA

cttccttgcgttaacaaacaaaacagaagaactaagagatctgtggcaaagtacctggaaatggcgtgggttacgacgagaattatttttagtccaattc

ttcgacagacagtcaagaaagttctatttgcagcagaagcacgtcagttgcattgccatgtcagtgtcaagaaacttttcttaccatcagcacagtttac

taggtcatacgctgagaagaaagtatttacacgtgacaaaccacattgtaatgtgggaactattggacatgttgatcatggaaaaactacactaacagca

gctattacaaaagttcttgctgacaagaaattggctcaagcgaaaggttatgcagaaatagataatgccccagaagaacgtgcgcgtggtatcaccatca

atgttgcgcatattgaataccagaccgacagtcgccattatagtcatacagattgtcctggtcatgctgactatatcaagaatatgatcacaggtacagc

acagatggatggagcaatactggtagttgctgcaactgatggagccatgccacagacaagggagcacctccttttagcaaagcagattggtgtaaatcac

atagtggtctttataaataaggttgatgctgcagacaaggagatggtagaattagttgaaatggaaatgagagagctgttgacggaaatgggatatgatg

gtgataacataccagtgataaagggttctgcactctgtgcacttgagggcagaaatcctgaaatgggtgccgagccaattttgcagcttctggatgaaat

tgataagcacatacctacacccataagagaactggataaaccattcatgcttccagtggagaatgtttactctatacctggccgtggcactgtagtcaca

ggacggttagaaagaggaacactcaagaaaggtgctgatgtagaattcataggccacaacaaaagtgtcaaaactactgttactggcattgaaatgtttc

atcaaatactggaagaagctcaagctggagacaatcttggtgcattggtaagaggtgtgaagagggatgaacttcgccgtgggatgataatgtgtaaacc

tggtactatgaaggctgaggatcatgtggaagctcaggtttacattctgagcaaagaggaaggaggaagacataagccatttatgtcatatattcaactg

cagatgttctcaaagacatgggacattgcatcacaaattgtaatacccgaaaaggaaatggtaatgccaggagaggacgcaaagttggagctgaaaatgt

tcaggccaatggcgttagaacaaggacagcggttcacactcagagatggaactgcaacactgggaacgggtgttatcacaaaagtactgagtcggctttc

agaaaaagaaaggatggcattaacaggaggaaagaaggcacgtgataagcttgcagaagaaagcaaataaataacacagacattaataaaattaatgtag

attcaaaatagactgattctactgaaacatacagtctcaagtggccattgtacatatgaattactacgtaattctccagtcactgtaaaatgttccagaa

agaattgcaaaagacagtgaatgttctgaagagtgcatatattgtgaattgttttgtgttttacctgttgcttcttttgtgaattgttcacgaatcatag

ttgtgtactgacagttttttacatgtaatgtttgtttaaaagtgcattagcatctctgattctgagctacacattaagattatatgagcctgggaaattc

gaagtcttgtgaggaatggtttagctaccatttctttcatgtattgtgttaacttatcacatcaggaaaaacttcaggattttgtgttttccattaaaac

agttgatgtattagctacacattgatgacaaactttcatctctgaataacttgcattattgtgaaaatatgtttctttgatcaagtaatgtgaactcata

ttattcttgtaatggaatttattactttctgtggtagttattgcacttacatttaagttgtgtcacagtaaatggaccttgtttcagcttttccaagtct

ataaatgttaaaactggcatgtgtattgtttgacagagtatataaaaatttccactttattatcaataagctgttttctgagctatctatctctgccctt

tcctactttgtctgggacttcgattatatttagtaatataattatatttgtccttgtgtgctaccatgtggct

>34158|AGAP007029-PA

cagtttgatgcttataaacaatcacttcagtataaactaccctcgaccgaacctgccaaacatgatagaactgactggactgcatgtggaagccaaccca

ccacctcttcccaaggacatacaggactggctggatggggcagaacacggcgccgtgtacttcagtctgggttccactctgcaggcgagtacgatgc

>34157|AGAP007029-PA

gtcaacgtagacggtaatctcaacgcattcgaagcggcctcgacaactcttttggatttggcgaaggagttccttgtggttgctgaaaaaatgtgtcacg

gatttcttagggacgaaaatgtacagaagttgcataagtcgggcaagaaatttgacgtcattgttctggagaacctttttacagaatgtttacacggata

cgctcatggactaaaagcgcctgtaattgtgttgtccagtttcgcggggacggactggatggccgacatggtcgggaatcc

>34155|AGAP007029-PA

agtgcgagggagagcagagagttgtaggtcttctggaagaggctcatacggctcgtgtaggccacgccgccctcgggcaggtaggacagcgggtacgggt

tcccgaccgcgtagttgatccacgtgttgccgccgccgaaggcgcacgcgagcacagtcggcgcccggtacttgtgcgagaaggcgatgaagcagtcgtg

gaagaacgtctccatgatgagcaggtcgtacgtccgctcctgggagtcgatcagctcgcgaacgaccgggtcgtccatcaggcgctggcagaagtccgtg

cccatgtggaacaggatcatgaactgtgtgaacgtcgagagcgacccataccccaggaagtcgaactctcgaagcgggccttcatgccggactgcgctca

gcgagtgtggtgcgtgtgcatgctgtgaggaggcga

>34156|AGAP007029-PA

gaaggcccgcttcgagacgtcggctgtggcgggggcggtgtgcgcggcggcgttgttgtgctgcgggtggctccccgccgccaggggtgccaacatcctg

gccgtgttcccgatggagggccgcagccactggatcgtgaaccgcgagctgctggtggagctggcgcaccgggggcacaacctgactgtggtgacgccct

tccaccagccgggcgacccctgggagctcgtcaagcccaccgtgcagctggcgggcagccgcaagttcgacttcctggggtacgggtcgctctcgacgtt

cacacagttcatgatcctgttccacatgggcacggacttctgccagcgcctgatggacgacccggtcgttcgcgagctgatcgactcccaggagcggacg

tacgacctgctcatcatggagacgttcttccacgactgcttcatcgccttctcgcacaagtaccgggcgccgactgtgctcgcgtgcgccttcggcggcg

gcaacacgtggatcaactacgcggtcgggaacccgtacccgctgtcctacctgcccgagggcggcgtggcctacacgagccgtatgagcctcttccagaa

gacctacaactctctgctctccctcgcactgaacctctaccgggacctggtctacttcccggccctagacgagatcgtgcgcaaggccttcggtgacccg

agcatgccccccctagcggacatggagcgcaacacgtccctgctactgctcaacaatcacttcagcttcgactacccgaggccgctggtgcccaatgctg

tgcaggtcgctgggatgcacatcaggcctcctaaggccctcccaaaggacctgcaggagttcatggacggcgccaccgagggtgtggtctacttcagcat

gggtagcaacatcaagagcgccgacctgtccgaggagctgcgggcggtcttcctgaagacgttcgccaggctgaagcagaaggtgctctggaagttcgag

gacgactcgctgccgaaccagccgcccaacctgagaatcagcaagtggtttccacagagcgacctgctcgtgcaccgtaacgtgcggctgttcgtgacgc

acggcggcatgatgagcctgcaggaggcgctgtacgccggcgtgccgctgctcggcttccccatcttcggcgaccaggaggcgaacctggtgcgggcgcg

cgacgccggcttcggcctgctgctgcacatccgcaacatcagcgagcagtcgctcggctgggccctggaccagctgctcggcgaccccaggtacgcggag

agggcgcggcggtactcgcggctgttccacg

>34159|AGAP007029-PA

gccggacctaaggagagaggacgcagctcgtaccagacacaccagctcacacggaacgaggaagcatgctgaaagcggttgcagtgtcgctgttggcggt

ggtgacagcctgctcggctgccagaatcttgatgatggcgccaaccccgtcgttcagccaccagatgccgttccacatcctggccaaggcgctgctccac

aggggccaccaagtcaccctcatgacgacggactcggcgaagatatctcacgaaaactatacggagattgacctatcagtgtcttataactacattcgaa

aagcgtatgattttttgaagacgtaccatcagccaccaacgcagtctgtaactatgatacgctacataacccatgctgtagccgacatggagttcaacga

ttcttcagtgcagaagtttataaggtctggcgaaacgtttgaccttgcgattctggagggactcccctaccattcattctacgggctgatacacaagatg

ggttcaccgcctgtggcgaagcttctgactctcatagctccgtcgtccgtctactacaacttcggaagccccatggagccggcatacatgccggacatgt

ggctcggcttctcagaccgcatggacttctggcagcggctgtacaacacgtacttctacctgaggctgatgtacatgtggttctaccaggtcatgccttt

acaagaagaagttatgaggaaatactttggtccagatcctccgtctgtctacgaagcagacaggaacgtcagtctgctcataactgccaaccatttcgtc

tttgaatatcctcgtccgcacctgcccaacatcatcgagataaccgggattcatgtcgccacaaaaccgaaaccgctgcccaaggacatcaagaagttcc

tggacgaggcggaagacggagtgatctacttcagcctaggctccaacgtgcgcagcaacgcgatgccagactggaagcgacaggcgttcatcgaagcatt

ccgggagctgccccagagggtcctctggaagtgggagaacgactccctgcctggtcaaccagacaacctcatggtcagcaagtggctgccgcagcaggac

atcctagcccatccaaacgtgcggcttttcatcatgcaaggcggcctgcagagtctgaacgaagccacgtaccacgccgtgccgctgctcgtcatcccgt

tcttctccgatcaggcgcacaacgccgccaaaatacagcaggcggggataggcgtacggctggagtacagtgacgtcaccaaggacaccttgctgagaga

catacgaactgccctgcaagatcccaagtacaaggaaaacatgaggactttgtcatcaatattccgcgagcacaaggcggactctgtggag

>34182|AGAP007032-PA

ccatacattaaaatcacaatcagtgcagctgcacatacacagacaaaacactactatcaaagatatcacagataattaaaagttacaataaacctatcag

tttcaactttacttaaaagttttatggtttcaaaaatcacacaaatgttattcaatacagtacgatagtatctctggattagtgtttcacagctacaaca

aagattttttacattcctaattataataaagggcaataaaacttaattagaagcaccatccacaactacataaaaaaataatgatatcaggaactttgtg

tttccgtaagcagtgtctaacaaatccttcatacaaaactgtgtttgtttacttgaacagttaagtagagttggtttgaaacttcagcagttcgtacaga

tgttaacacaccatgaactttaaaaagctacagacttttatatgataagggaacagaaaaaatgtatagctgcttcaagagctttgtccaacatggtccc

tcataaagaagtcatcttcagttctatgtgattctcaaataatggtgcattcatttttgtacttttctgtgcatcaacgatgctattaaaataaacttct

tacactatacataaaacaataatatatcaatctgccatactaatcttcttggataccaagaaatatagaaatatacataattttatggtagcactatttc

agcagcatgaaaaagaatttacttgcttgttatatgactgtgataaaagattacattttgtttctttaaaacagtatattatcttctactataacaaaaa

ttacgttcttttcaatatgaggtccctgaaaaataaatgaacttttgcaatggcactgaacactgccttatacaaaaaaaaaagccacactatcacataa

ttcttttaatcaaagactacttaagtaccatcggagtcaaggctcattggaggactatcatcacaagataactctgctgtctgatcagtcttcagaggca

catggaaattctttttgagcacacgacttcgtggggtcagagccaacggtgagagactgggaggttcgccatactgttccagcttgaacagcctacgctg

aactttttcactcaattctggtgtaacagctggtttacttagctgtgataattctgctgttgaaggttttgtttgtctcacattaagagatgcatttctc

actggagtcacaaaattctcatttaggaatgacaaaacaattccttcacaagtatatgaaaaattgtcaatatccatattaagattccttctcagtgagt

caagagctgcaatgaggtgcttttgacgaggatgttgcgaaaaaattgaaaattctgtggcatatgttgtgggtatccagtgaacagcagcacccacacg

actttgtagattactacaagccactaaagccttctgctgaagtatatcctcccaacctgaacccttaagaccattccagaaagaaatgcccacaatatcc

atgtctgcatcagctaggtagactgtctggtaatttccagagagaggagcgacatgtacaacaactccaacacagtccatttccccaaatggcgcatgaa

gattgccactagacacctgagacaatggtgttgcatatctggctgggaaatcttctggaaggtgactaggttgtggctgataacgagactggcggcacgc

agaaagttgcaagtctccattccttaatccattggcagtgatgctaaatatcttcaatacactaccctcctggaacagtgttaagagatcttctgaaggg

cgccaaatggaaagaactgctgacctagcagaacagtctttagcatccagacagcgaaccttcagtagcgctaatgacgaagtcccacttctttcttctt

ctattctcttgcgaactcttgattccagttcttgtagcaactgctgctggcgcatctgctgatagttagcaacagcattttgctgctcctcagtcaataa

agactgaagtgacgctggatcaggtgcgttgaaaagtaactggcaaaggtcttcagcgcttgtagcagaagctaactgttgtcgcgtaggagttgttacg

tgttgtttattcttccctttgcttttactttgttggctgcaggcactgaaccattctatctgactcaattgtgcacgttcctggctgtatagtttttcta

tatgctcttgccactgtcgctctcgttttgaagcacgaagtgcctcttgctttgctgaataaaat

>34211|AGAP007055-PA

gcattcacccatcacaaaagcatttggtggtgtacccacaggcagtacataagcataagaacaacatagacatgctggcatcataagatacattggatga

atttctactgccacagccatttttgcaaggactggtaaaacaatattacaaatggctgcattggatgcaaactgggttagaaatgttgtagcaat

>34233|AGAP007059-PA

gttgtggagatgaattacaggctgtttcctcgccctgcgcccagcctgcgttgttggcgccggtgccatctcagccgcactgcagctcgcccaccacctt

gagtacctccttgcccttaaggtccagagggctggcgcagatggcagggtcggccctgaccacaatgccgtgctttcggatcttggtgaagagcggctcc

agcgcgcagtcgcacgtccacggattgccctccagagtcagtcccttgagccagacgagcggctctataacggagacgtccatggtctgcagtaggttgt

gatccagctccaggtactggagacgggagttgttgcggaaggtgccgggctgaaccagggatagccggttgatgcgcagtcgcacctcctggaggttcgg

gttgcggctgaagaggtcagcgggctgaaccagggatagccggttgatgcgcagtcgcacctcctggaggttcgggttgcggctgaagaggtcagcgggc

agcctctcgatggcgttcccgatgacgctgagcacggagagcagcgggttgtcccagaaggtgtcgcggtcgacgctcgtcacccggttgtcgttcacga

tcagcgtcgagagccgcggcaggcgggtgaacatggtccggcggagagtggtgagtctgttgccgctgagcacgaggaaggccagctcgccctggtgatc

gaaggtgcccacctcgatgtcggccaggtggttgttgtccaggtagagggacgccaggcgcccgttgaagcggaaggtgcccggcgcgacgcgagccaac

gagttgttgcgcagctgcagggaccgtaggctcgtctgcttgacgaacaggtcgcggtgcagcgtggtgaggccgatttcgttgaggcggagctccgtca

gctctggcaggttgtccagggcacctgcagtcaagctggacaggttgttgcgagacagaagcagcgtctggagctgctgcagttcccggaaggcgccgcg

ctcccacacagagaggctgttgtccgccaggtccaggtactgcagagacggcatcgctcctgagatgtccgctggaacctccgacagactgttcttgccc

agcatcagggtcacgaggttggggttgcctccgaaggtctttggtggaagcaaggacaggtgattatcttcgaggatgacttccttaagactcttgaggc

cggtgaacgtcccaggctccagcgacctcaacatgttgctgcgtaagtccacagtctccagcaatggcgtcgacgcaaaagtcgaattgtctagctcagc

gatgaggttgttggcggcacgcagtaccctgagctgggccaggttgtcgaagagccgtggcggcagcgcggacagctgattccagccgacctggaggact

tccagcgcggggctgccgtcgagcaggtcctctggcaggtcggctagcgagttccagtccagagacaggacgcgcagtgccgtcaggttgaccagcgggc

ctccagacacgtcacggatggcgttgcgctccagccgtagctcagtcaccttgtctgcgggctggtccgagaggtctggcacggacgtcaggttgcgcga

gtcgcacagcatcgtgccctcctgcagcacgcaggggaacacagccagcgtcggtgccggaaggctcagcagcaggatggagaccacaaccaacggcggc

ggtcccaggtggcacctcatggttgcgtcgggttccgcggacgtcggccacaggcagtgcgacaccacaggcgcggtggagcgcggctcggcgggcgact

gaccactgtggggcgaggctgtcagctggcctgaggcgtggcgcggtgtgggccgcgggagagacgtccgtccctagcggcagctcaacagctgactggc

cgccgtcgcccggggcactggcgagagatcggccgcgagcgctcgccgcagcaaaacacggaaaccgcgggtgacctccaaccaggacagcagtggcggc

ggcggcgggaagacccggcccgttctcacagcgacgccgctgcacctgcggacgtgtccgagttacgaaacgcagccgcgccgcgcgcataatgccagcc

agctgggaacccggcggagagctcgctgtcggacacgcatggctgacgcgg

>34249|AGAP007069-PA

cccctctgggactccgtggtggggccggaggtgaacttctgccgccactcatggtgggtcaacatgctctacctcaacaactacgtgcacctcgaccagc

cgtgcatgaaccagacgtggtacctggcggtggacatgcagctcttcctggtggggctgctgctgctgaccgccgtgtggctgtggccgcgggccgcccc

cctc

>34269|AGAP007085-PA

agcaaagcaagcatatacacagttacgtaaattcggaaggactcttggaaagttgtacatcccgaactagttgcagcgtcgctgcaagattttgtccacg

ggtgcacatactccaagcaagtgtgaggcactgtttgaaatttactgaatgcaaccatcgctcacaactacgttatgatagtcatttgtgactccctctc

acgactagcctatagttgccagtcactgacctgtcatcacaacatgtaccttgttacaacagtcactgagataacaatatgcatatataaacagagacct

tgttactgttgcca

>34327|AGAP007101-PA

tacagtacataatttaaatggttgatataaaagctaacataccattcaaccattatccaattgtgtttatggaaatgtgaataatgttttataatttttc

ataaccttgcttgttcattttacagatatgtcagaaattcaaaataatataaacacttttgatgttttgtgaatagttaaggaattgaaagtatttgggt

aagtgtccacaataaaagtgcaataattttttgttcattttattcaacattatttaagaattatgtcctccagaatacaaggagagttcaatctttaaaa

agcagaaacctatgcctcactcttttctcaaaaccatgtattttatgctaactgaatgtatgatgtaaaaatttaaagacctgcaagaaattcagactta

tcactactgtactaagtataaaatagaaatgcaagtgttaacccatcttattggataacatgcagatactatgtagaatactgaaatgaatctgcaaaac

caaatttttttaggtataaaatctcacaggttttcagtaactcacgtgaaatggttatatggtgcaccacagtacacattattgtatacatttccatcac

tgtaactgaattaaatgtattccacatagattacaaattcaatcattaacataaataacaagtgtacaataccaatttctaacttttgttcataaactag

gtttgttccacaaataaaaatgaactaatacattaaaaatacacttcatacttaaattttgatctactaagtatgttaccttacatgaccttagtaatta

tacaatttttaatgtaacaataagtgcttttctacacacaagtggttgaagctgtgcattgactagttgctgagttaaaggctgctccttgagggcagtc

atataaatattggtagaaagtaccatttgcactgagggaacacatataatattgaaagcaatcaagtgggtttataaatcggcctacacttgtgcaggta

aaaggtgcaacagtagttgttagagttccagttggagcagttactgtaggtgataaagaggtgcttgatgtaccacatgtaacatttgtggaaagtgtac

aaagtcttctcaccgagtcaaaaaaagagccaccagggcagttatacaggtattggtaaaatccaccagtagatataggggagcacatataataattaga

gcaatttgttggatcagggaaccttccagttgaagtgcacacaaagggttgttctgttgtaggggaaatggttgttgttacacatggaactgtttccaga

cactctagggaacagtcagcactgcagtaagttcctgttgggcatacattgacaacaggagttgctggaggtgtgccacctatgcaggcaacacttgcag

tttcgtttacacagtaaaagccatcagcattacagacttgtccttccactgggcagtacaatgctacagcaaccaaagaaacaactgtccaaaacaggca

ctgcatattggaaaacaagctggtcctatttcacactacagctgcacacaaaacatctgatagactactgagtgaataaatagcccctctcatacttttt

ttgcaaattgagg

>34326|AGAP007101-PA

agctaaaatgcataaatcctcacagtgtctttcagttttagatgtaaggtagctgttgttaattaacaggaatgccgtctataccatattttattttctt

tgtattgatgttccatgcaatattacatcaaactgaaggacaaacttgtggccctaatgtggatattatttgtgtaaatgaaacggcattcgcatactgt

tcgaatggaagtgtagtctctgattttatacagtcatgtagccccttgaactgttatgatggctgtgtactcttctgcgcagagagtgaggcatactgtg

ttccggtagcaactgaaacgactactactgaaccgtcatcaaccacaacggaattaacaacagcaccgacaacaacgacgactgagactaccacagaact

aactacagaaggaaccagtacaacagaaacaacgacagaattaacaactacagcaatgccgacatcaacgacgacagatatgaccatggaactaactaca

gaagaaaccagtacaacagaaacaaccactacggaattaccaactgcaacaatgccgacatcaacgacgacagagaccagttcaacagaattaacaacaa

caacagcaacaaccaggcagccaacaaccgctatggcaacatcaactgctccaacagaagccccacctactgaagcgccatttacatgtgaatcagcagg

agagtttcaggatctcggaaattgtagcaatttttacctctgcataccactatcttcaggagactttgtatacataatgttttcatgcccagatggttct

cagtttgattctgtgactaagaaatgttctcaaaacgcaacatgtattactccatcatgtaatgactttggattctactgtctcagcacttcacaagtcc

agttatgttatgattcagagccaccaagtggggatgctttcacatgtcctgcaaacacatactgcagtatggactgtcagtacccatgctgggcagatat

accttgtacaaccacagcacctccctcaacatcaacaacagctttgcctacatcgacagtgacgataccaccgactactactgcagaaccgttcgtttgt

tcagagactggacggtttcctgatcctgctaactgcagtctttattatctctgctctcccaagtcttcaggtgggttctacacgatgcacttttcttgtc

ctccaccatcattgtataatcctaacacgcgacaatgttctgttagttttacatgttctcctgcacaattgcttgacaattcaagcacaacagtgtcgac

aattgtcacatcaccttcacagccgtttacctgtgaagaatctggacgttttgctgatccagaagactgcaaatcatactacctctgctcaccaaaatca

tctgggggattctaccagatacattaccagtgccctcccacatcagcttatgatcaaaacacaacccagtgcagcgcatcgtctagtattaactgttgag

ttgctcgtatgtgacacataatttttaaaaagattccttcttgtgtagctaaatacacgtttttgcattatactttacactaagaatattattgtacgaa

tcatcatattatgataagttctgttccgaaataaagagtgttatagaaagt

>34341|AGAP007104-PA

aagataatggcataccacctacggtagagctattctagtaaaccattgttgtgttcataataacctttgggctcataatggttttcactttttttccgaa

tgagcgacgtagcgagattcccagtaaacaaggcgccgttactttggcgtcgagctgcggccagtggtcagtactcaccatctcgagcgcccagcccatg

atgtgcgccagctcggtgttccgggacgccagctcctcgtggcgcgccagcagccggtaggacagcacgacggccagtccgcggttcttcttgccgcccg

tcatgttgtactgcagcacctgcaaccgaccacacacggcccttcacactccgtgcctgcgctggacaactggatgctgggcgcaaccagccggtcgccg

ggtctgatgtgggcatgttcggctaacaacacgtgacccctgagcagtagtcaccaagctttagtaatagaaaaaaattttaataaatcgagttttgttt

aatagcgcgt

>34357|AGAP007106-PA

cttccttaccttgccacaaacatgaaaatcaaactactgaattaattcacatgtttcacctcacttactctcttttgctgtgagtaacatgcttaatttt

tgaaacatttatacagaatatttcgcttacttgagcatgtatttacatatcttgtttttttcacacattcctggtagtttactgaataaactacttgcct

cggtccatactaccaaaaggaagtttcttactaactaaatgaatcctaataaataatacacgagtgtaatgataaacaatctcttagtgttaccagaatt

gtgtgcaggcatatgactatccgagattaaataagataaatatcaaaatgaaagacagactgtttctgagaagctgtattaacgaatcagatattagctc

cacaaaatttggtcagggaattattaacaaatgcaacatgatatgttttaaggtacattgggcaaatattaaacaaataaggaaaatcacttatgtatat

aaatatatatgtatatatgtacacaaattaactataaaaactacatagaaaatacctacaaacacaaaccaaatgcaattggccgatcagtctggaacag

aggtacacgatccttgcacagtggcgacggtcagaacaatatcacacaagtctcaaatgcagcacacatcaacgtgttttgtcattagccaacctaactg

ccagtcagaagttcattgaaatatcactgcttcatgaacagacaacattacacaatattcagtaaacttcaccagtcacttaaaatgttcacacctcagt

caagttcatccacatcatcccgtttcttaggcgacgaatcaacataaatttttaaagctctttcttttcgaggggagtaagaggatactgaacccagttt

attcacgcgggatccttccctattagctatttctttccgttcttcattagtcagctcctttacaggctcgg

>34376|AGAP007115-PA

tagttgaaaacatcgttagaaatagttttagttgttagtggagatggtgtagctactcgttggggaccatggccgaagtgaacgtaagtcccgatgaaga

agaacgtgtccatttcagaagaatagttgctgcctttaaatgttacagagaactgtatgaaacaagactaaggagagcagaaacaaatttgagaaaactt

tctgcaaggcaccaacagctcttacaaagttataaattgcatctgcgcagcgtcagggaatgtattgagaaaaatgctgaggtcctttcagcatttgttc

aagatgcttatgcactgtttgacaatgcggatgaatttgaaagaggtggacgtgatgagccacctacctggcaagacattgagaagctgcaggcaacatt

gaaaagtgtgtatcgtgattggaccgttgagggagctctcgagcgagaccaatgctataaaccaatacttgaagctattgacaaccatttctccactgaa

aag

>35505|AGAP007122-PA

agtgatagtgaccccgttatcataaaagcaccactccgatgatccaaaaggagacttcttttctctccccccatagcattcgggcgtatactaaatcttt

tagcattcaatatatggatcgtcttgaccttcagcaactggattatcttgctgaacttcttcataatcccgttctaaacatgctaagtcttcacgtgcct

cggacaattcaccttcttccattccttccccaacgaaccagtgaacgaatgctctctttgcatacatggcgtcaagcttgacagtgattctttcaaagac

agatgctaatgatgtagagtttgagatcatggctactgcacgaggcaccactgcaatacccgatccaggtacagcggttggtgcttgtgaatta

>35546|AGAP007123-PA

ctttatcagctagcaaaacggggagttgacacagtgctattagaacgctccaaagtcacctctggaacaacatggcattcagctggtcttgtttggagct

tgcgtcctaatgatgttgaaattcagctcttggcagcaactcgaaatttgcttgcaaaactggaaatagagactggatttaatcctggctggataaataa

tggtggactttacatagctcacaacaaggaaagactggaggaatatagacgtttagctacaactcagccggaatgatgtttggaggtggttgtggagaac

aaatggctcagtggattattaatggacggccagagctgcatatgttcagctacgacataagacgctttcgatctgaccaaac

>35545|AGAP007123-PA

cacagaatgatggcacagctggcaacttaattaacttcagtcaaatcacatacagtgctcttgaatggaggtgaagctgccttctgataatttttcagat

tcagaaattaagcaagaatatcgttaagatgtttcttcgtgcatctaagaaggcattcttcattccacaaggaagcaatgacttcagtaagctggtgaga

tgcatgcaaaatggtccaccaaaacaaaaggaaaaccagaaaccagaagaaccaaatattccctcagctgctgatgttgtcataataggtggaggctctg

ctggatgcaacctactttatcagctagcaaaacggggagttgacacagtgctattagaacgctccaaagtcacctctggaacaacatggcattcagctgg

tcttgtttggagcttgcgtcctaatgatgttgaaattcagctcttggcagcaactcgaaatttgcttgcaaaactggaaatagagactggatttaatcct

ggctggataaataatggtggactttacatagctcacaacaaggaaagactggaggaatatagacgtttagctacagtaggccacagctttggtattgaat

ccagtatactgtctgcagaagaaacaaaaaagatttttcctctgataaatgagaaggtagtacatggaagtctatactctcctggtgatgggactgttga

cccgtctatgctttgtacagcccttacacgaactgctgtacaagcaggtgctaagcttatagaaaattgtcctgtaactaaaatattgagagggaaaaat

aactttggatctaaaaaagtttctggagtcatcacaccatatgggacaatcagaactgactgcgtggtgaattgcagtggtgtttggtcacgagaaattg

catctcttgttaatctaccaattccactgctgccaatgaaacatgcttatgtagtgtcagagggaatagatggtgtgaagggactaccaaatgttcgaga

tcatgatggttctatatactttcgcatacaaggaagttctctctgtatgggtggttatgaatcaaaccctgtcatgttggacaaggtaaaagaagatttt

gaattttctttatatgagttggactggacagtattcagctctcatgtaagggaagctgttaaccttgtacctgtgtttgaaactgctggtattaagagta

ccgtatgtggcccagaaagctttactcctgatcataagccattactaggtgaggatccaaggttgctgggattttaccataactgtggctacaactcagc

cggaatgatgtttggaggtggttgtggagaacaaatggctcagtggattattaatggacggccagagctgcatatgttcagctacgacataagacgcttt

cgatctgaccaaactaaagatcaagaatggataagggaaagaagtcatgagagctatgtaatgaattacagtattgtatttccaaatgatcagcctttag

gtggtaggaacttccgaaaaggcccattgcatgatgaattgcttaatgcaggtgttgtttatgaggagaaacaaggatgggagagaccggggtacttttt

gcagagtggtatagctcctgttcagaagtatgattggtttggctcctatggatcatctagaaatccagagactccttatgaaagtctgttgcaaggggat

agaacatttggattctcaaagcaccatcatgcaattggagaggagtgccttgcatgtagggaagatgtagctctatttgacatgtcatacctgggtaagc

tttatctgagtggtcctgatgcccagctggctgctgattggttattttctgctgataccagaaaggaagtaggccagtcagtgttcacctgtctccttaa

tcctcatggtggagtggaggctgatgtcactgttagtgccattgaaactggaactggaggtcaagcagaccctattttgaagggtagagggttctacatt

attagtggaggagtctcagcataccatacatgggctcacatgaattcaattatcagcctgaagaatttcaaggtagctctcactgatcatacagacaaaa

tgggagtactttctgtacaaggaccaaacagtcgagaactgttgcagtgtattaccaacactgacctatcagactctgcatttccttactcaacaacaca

gctgattcggctggcaggacatctttgtcgtgcattccggattagctatgtaggtgaactaggctgggaaattcacataccatgggaatcttgtttacca

atttataaagctattacagaacaaggaaagaagtatggtctgagactggctggatttagagcactgaactcccttggctgtgaaactggttaccatttat

ggaattatgacttaaggtctgatgacctgcctattgaatgtggattagatgagacatgcagagatgaaggagaatatgttggtaaggctgcccttgaagt

ggagaagcgaaaaggtttaagaagacgattaacattttttcaactgcaagagcaaattcctgtgtggggacttgaaacagtatggagagatggccaagta

gtaggttaccttaggagaggagactatggatatgctctaggagcaagtattggacaaggctatgtgaggcatccagatggtcgcaaaattactacagatt

tccttctaaatggtttatatcagattgaaataatggggaatcgatacgatgctgttccatacataaaaagcccatttgacgcagaagggaaaagacttca

aggaatttatgatgagccattaccaataaggcaatagcattaagaatatttatgtacaaataaactgagaggaaggcaaaacacagtctcaaaatgtatt

atgtaaagtaaaatgtatggttgccttggttagttaagattagaagcactttgtggcaaaatggactgtacattaaaattatatgtttttgaaactttcc

agttgtcaacatagtatgtgaagtaaaagaaaaagaatgttcttggtgccagaacaggaactaacatatttttcatcaagtttttggtaactgtacagga

aaagttcagtgtctgccaatggtttattgtgcatttccctttaccattctttcagttaatagcaatattgagcttcatgtcattttatgttagtaacaca

gtggatataaatgtcaataatatagttcaaatccacataaattgaaatgaaatggactacagataatatttatgtaaatacagtaccaaaaatattataa

ccaattataaatagcaattagtgaaacttttgaaggctgtggaaataatgactcaggacactgcattatggatggtattggaaaatgaaaatgagggtgg

ttaaaagcatttcactactcagagactacattgccagagaagaaaatcaaaacagatagtcaatggggcatcttgttaaataaatgtaagtcaaacaata

atatatatgaagaaaaaaacaagcaatgatcaattaaacagtggaccagcatttaagcttatatgtagtaccagtgcatatatatatatatatatatata

gcgtggggtgagtcctgttgtacaagaaagcaggtagtggcataaagaacaacgtacattaaaggaaatatacataaatggacttccttaaattccagta

ttgtgatagtacagaatcgagcaatcaaatgatctgtcaaccagtaattttattgttatagtattccataatgtcaacctcaaacagggattattgttaa

tcaaatgcttgctttgaaacacttttatgagattccatgtagcactgcacacactcatcagatatgtggtatatatccatccgtcaggtccctagaagcc

atgtcatatgtcactttaactatcagccatttcctccacattgtttagatccaagtaggccctactgtttagctgatgattagcatgtgcattttgacaa

caatgaataactaaatcaaggcacattgatttagggcacagtattctgatgcaacaatcctaagctatacacagtggagatccatgcataacatcatttg

ataacatgaggacatttctgctgatgatgctcatctaatttctatgatattccagctcatgttttcttatgataagtacttgcatattggttctgtggca

tacttcttacactgtattaatgatgctctttagttttaattctattctcatgcaatgcaccattcatatatattttcttaccatacttcacatacttct

>35561|AGAP007126-PA

cttgtatcgtcatgctctcatggagaaggcagatcagctcctgacagacagttttattatgtgaactctaggccatgtgaaccagcacaggtaatgaaag

tagtgaatgatgtttatcatgtgttcaacaaaaagcagtacccatttgtttttatgaatataaaaatggagaaagataatgttgatgtgaatctaactcc

tgacaagaggcaagttttgctgaaccaagaaaaacttctattggcatctataaaggtcggctgctctgctctggatgacattgcattgcttgctgctgtg

gcatggg

>35560|AGAP007126-PA

attacttttattcctgaatctggtagttcatttttgaagaaaatgctgaaatttaccaagtgccgcatagttggacgtccatgtgggcagttccagggac

tgtccatctcacccatatggtcaacaattcttcgcatttctaccagactaagatctgttccaatcataacggctttgcgacatgctcgtgatgcaaacat

tgcccgtacacgagttggtcgacacattacatggggagcaccttccaacataaacagcaactcatcaatatcatccttcccaaatgtccagtttttgcta

atgggaattgaactcaatttcaccctctttgttggttcagctgtttcatcaataataaattcaaaaccatttctattgaaaatgtccatattttcaatga

gtaaacattccttaatataagtgagatctaacttctgtggcacaacaagtttctggttttgaagtgatacagttttttgcaacatttcaaaattgtaaat

ttcatctgatgcatgctgatcaatgatgaagagatcagaatccaaatgtgttataatgaaacctttattaaactgcccaactattttcatcttagcaaac

atgtcttttgtaatctctctcctaagctcttgttctgcaagagagttctgcgctggatcaatttcagctttgaatttaaagttacattctgcatctgctg

tagacttggacaaccgtttaaaagcttccatttcttcagtaatggaatttaaatcaacattccatctaataggttctgatacataagtaacttcagaagt

gacaaattcatggtcacctatttcatcttctgtaatgatcacttcttcatctatagagtttatcttacttgtaacatctatgggtctgggttcttctgca

gtaacattattactactaccaacaaatatttcagtttccacatcagacgttttatttatagttctactggtcacacaagttctttgctctaaataatcct

cactgattagattcacattttctgaacatgcctggattctatctgatacactgtttttagtttcagctgcagaggaaacagatgtagtacgactgaatgt

agatactatactgttcagattctgttcctcagattcttctccacattcaaaaggaattttaatagattcaggtgactcatcagaaaggacaggcacattt

ctgctgaatgagtacacattcctttcaaaactgttgtcaccatttccctttttggtgtgtgtttgtgccgctgtgtctactacttcagatacacctgttt

ttttcacactcatatgggtaaagtagttgttcagacaatgctgttttgatcggccatatttatcttggcgaataatctcaagggtccttttaatgcctct

ctttgatgacccaagagttcctggagatccaatggctcctcctggagaactaaagttacttgaaggagaaccaacaccactcactggggacccagaatca

ctgcttggagaacggcgccactgttgtatgatatctaaccctgatagaggtgattctgatgatggtggtgatgtaggagattgtgtaaaagatggtacgt

tattcaccttgagtgttgaaggaaccgactcatatagtttcagcaatgatccctttatagatgccaatagaagtttttcttggttcagcaaaacttgcct

cttgtcaggagttagattcacatcaacattatctttctccatttttatattcataaaaacaaatgggtactgctttttgttgaacacatgataaacatca

ttcactactttcattacctgtgctggttcacatggcctagagttcacataataaaactgtctgtcaggagctgatctgccttctccatgagagcatgacg

atacaaggccctccaatgtgaatgggtaatctgcatcagcaagtttttcagagaggcctaagctctgcagaatttctacacttggttttacttgagataa

cttcataagtccatgaacctgttttgaaccaaatatgcaggtaatattttcagatatagtgttgcttccctgagtactaacaacagtagtgcggctaccc

ttctttggctggtttgtacacgtgatccttacaccggtggagatgagacaataagcatgtagaatctgcaccattttgctgaattctttgcgcaagttgc

gctggaagtccttatgtctcacaggaaatgttacaaataagttcttgatgcagaccgttgtccctacctgacgagcacatggtgtttgttttgttatgtg

tccattatggtcaaattccagtcttgttgcacaactagcgctactgtgacgtgttaaaattgtcatgtcaccaagggcacacaaagagttcagtgcttct

ccgcgaaacccaaaggtttcaacaaaacttaattctgagtaatcccttagtttagaggtatggtgtttcagagtcaaagcctgaaaatctctttcaacga

cgcccgatccatcatcacagacttctattgtatcacttccatagtctttcaaaaatacgtctatattgcgtgcaccagcgtccaaactattttccacaag

ttctttaacagcaactgccaaatttaaaactacctgtgctgagcaaagcttgctcgaagattccttgtcaatttgatggataacactttgttttgtttct

tcacttccgcacgtaaaatttttcaaatctaatgtcgtttcggtttcggattctggatacacatccattctgatccaagactgtgaaagggaaaattttt

attttggataaactaccactttggctgacacacaaaataatgttttcgctttcccagtcatttttcataaatcttttgactcaattttgtttgcaacagc

acgaaaccatgtaaacaaacatttcgcgccatccagagttcacatacacaaccacctg

>35727|AGAP007127-PA

ccgctggaatcctccgcctcggcccagcgcgcagcgggacggcgaaccggcggcagccgcacttctttagtgtttatctgttttatcggagagataacgg

agcgctcaaacatgaccatgtccagtcggtggaaggccgtcgtgcaccggatacggaaaagcttggcggtgaagagtggcgaagaccggcccaagccgca

ggatctgccaaccaacggcgaacctctgccttctctggaagaaattcggagttggggaaagtcgttcgacagactcatgaaaagtgccgccggcagaaag

gtgttccgcgactttctacgctgtgaatacagcgaagaaaacatccttttctggcttgcttgcgaagatctgaaaaaggagtcgaaccccgatgtggtcg

aggagaaggccaggttcatctacgaggactacatctctatcctctcgcctaaagaggtgagcctagattcgcgggtgcgcgagatcgtcaaccgcaacat

ggtggacccgacgccgcacacgttcgacgaggcgcagctgcagatctacacgctgatgcaccgagactcgtacccgcgcttcgtcaactcgacgcagttc

aagaagctggcgcagctcaccagcaacggcaacgacgccagcggcgctgcgacgcccaccggcaaggacaaggagaaggagaagga

>35743|AGAP007129-PA

gaccacagaggtgaactcccccacagtagccagtgatcatccagagaacaccaccagtcattcaggcaaaagtattaaaaaaccagaagttaaagatgga

ccaatggcagtcagtaccaaagaaatgccaccacttgtgaatcgcattccattgtacacaaaaattgtagctattatcttgaaaacaacacaggctgaag

ttaaaaagacagcaattccagtggataacacaaatacaaaaaagagtactgacagttcatctgtggtttctacagacattgcccacaagaaagaagaaaa

tatatcacctccagcttccattactaagcaggaaactgttagtgttaagccaaaaagcaaattgttgaatgaagaaaaaccaagtaatgcaaaactgagc

tcaaaattaaaaaagaagaagccgaaaaacaattggtctaaaccaaacaggaactgctctgcaagacacgtccatacaaaaaattccgcagtcaaactta

agagacgcaaaaaagctaaacaaaataatgatagttatatttcaaaatttgttgaaaaacttttctgttacaggtgcaaattttgtccattcattagtct

gcagaaatctgcagtaagtttacatattgaaaaggaacatgcggccaatgtggaagcttcaagtggtcccatgcagttaaaatgtgtcatctgtcaacgc

atatatgaaggtgtggctcctctcaaagagcacttgctcagtgtccataaactgactgacgaaaaagccaaagaactcgcaactagttcactcttcagga

ataagaatttagtaataaatcacgttgcagagaaagagaccaccattgtagataagagtgttaaaaatgattcttcagagatcaataccaaagttgctga

gaataatgaaaaaccaaaagataaaacattcatggagaaaagtagaataagagtaaaaaatttggagagtgagaaggtttcattttcagaggtgtcacct

tcagtgaaagagaaggatattgcagaacaagaagtagcaagctttaatggtcatggaaatgactgtaccagctcacctattttagaccagactgctgcag

atttgacagatatggctcaacagaatgaaggcactcataaagttaattcctacaactataagcctgcgtggctgcagtgtcgtgttttggtgaaagagga

tggagccatagaagctgcagatgaaaaactaaaaattgctagaaaacgtggcagaccaaaaggatccagaaatcaattaacctctggtagtagcagaaag

aaagcttcaacaaattcaacaaatgagaaggagactggcttcagctgtaacatagacaactgtgcagtccgtatgcgctctctagaaaacattgagtatc

acagacgatgtcataatggtgcccagtattcttgtccagaatgcagccatgttcactgccattggaatggaatgagcacacacttatggcggacacatgt

aataaatatggag

>36241|AGAP007237-PA

cccgggcgtcttcaagccggagcagctgacgcagatccggcaagcgcgccggcacctccaagtgcgtcgacaacgacggccgctggcgcaagaacaacga

ggtctggaagcgcgacgactgcacggactgcgtctgcaaggagcaccagataacg

>36265|AGAP007237-PB

cgccacggtgcgaagaacgccttcgacagcggcacgtgcccttcggggatgggctggaaggtggcgttgaggcggtagagtaccgggttgatgagcgagt

gtccgaagcgcagcgcggcggtggcgaacacgttgctgatggtggcctcgaggttggggtcgtagccgcggtactccacagatcggaagagcacacgtct

gaactc

>36282|AGAP007242-PB

ttttcctgtaaagcgagggccaccgattcgtaggcgcaggggctggcatggtggacgtgttttgttagttaggcggtgtgggaaccctgtgcgttagtaa

agtgaccgacaaagttgaaagcttggtgtgtagggagttgctaaagtatttgtggctggtctttaatgcctgaagaatcactaattatgttgcacgagaa

aagaaccctttctcgcgacattaaaatgtacccactcaccaactacacctttggaactaaggagcctttatttgagaaggatccatcagtgcctgcaagg

tttcaaaggatgagggatgaatttgaaaagattggaatgcgaagaagtgttgaaggagttctacttgtacatgagcatggtttaccacatgttttgttat

tacaactggggacaacgttctttaaattaccaggaggagaattaaacccaggtgaagatgaagtggaaggtctgaagaggcttctaacagagccttgttt

gccgtacctaagaattacaagctagttgctgcacccttgtttgagttatatgacaatgcacaggggtatggaccaataatatcgtctttaccgcaagctt

tgtgcaggttcaatttcatatacatgtgaattaccttcacactggtattaatgctgttgaagattttttaccattggaattaaaatggggtctccgtcag

ctccacgcagcagcagaagtgtataaagacaaaataatctgtgctgtgtagcttttcaggtacaaaattgtgaaagtggtgacagtggacaagtgtttgg

taaacattaaataaagcagttttactgtgttgctttcatcagtgataaaagtaatttatagtgctgcagttggactcgagtgtaaaaataactgaaactt

gcaatagtctgtgcactttcccttctatgatacatagttttacatttatagtgtgaatggctgtaccaagaaaatattttaaggctcttgagattgtcat

tactgtgacattcagatgacaaatcatgtatatttcaccttccttaaaaactatatctttattgtattgtgtctcttgtatatgtgaagtgatgaacttg

tgagaaaggaggttttttacactcattcaataaaagctattaagttagaatccgtaggcagaattattaaccctagatggctgtcagcgtgaaaaatggc

tgaatgaagaaagtgttatgaaatttatagaaattgattaatggtgagtcagtcatgtgtcgtatgagattgtatgatgtcaaggtattctttctgtgct

aaggcccaagttgacagaaaatacagtcagttttgtgttccattatgtgacgctattgtaaattataaacattgggctgattcagcagtactgcaaactt

tcatttcatacacctttggtgtgtcatgttcctatttatgcggaaaataaatgtgat

>36305|AGAP007245-PA

gagagacgtaaacatattgtgttttatcttcagtaacgaaagacactgattttgccacctgttactgacagatttgaaactgaaagcttgtagcgctctc

aagataatgagattagagtaaatatatgtacttggatagcagagaagatggatatgggaaatacttctgaaccgcgttccattctttctgggatcagaag

tgcagaagactctataaagtatggtttttatagaatagaagctctggaaaggaacctcagaagcaggcatctttcatctgatgaaaggcagcgtattgaa

ggggagcttgaagaagtggaagaagaactaaaaaggaatcagcatgaattaaagcatcttcagaaagaaaataccaaatcatttgctatggctgcaattt

taatgttcctttgtttcttagtatttggtgtatacaaaatgttacagaataagtaatagataacacacattgttcaagaatagaattttcagttttaata

tttttattactgatctcaaatactacagtgtataaactaatatttcagaca

>36343|AGAP007246-PA

ctttaagtgagtgttcatagcaacatcgacaccagtaatagtaccatgcactagggtcccattcttgagctcgatggtaaccgtttcatggctaagtttc

atcaaaaacagagacccagtgcagttggagacactgagcatacgtggaaataata

>36342|AGAP007246-PA

ggcgcatattgcaatcaactgcaactgaagatagtcaaaacatttatgtaacatgaaacttaccagtaatagtaccatgcacttgggtcccattcttgag

ctcgatggtaaccgtttcatggctaagtttcatcagaatcctcactaattgcatgattgcggtgaggccacggctgcctgtcaaatatttatac

>36381|AGAP007257-PA

cgttgtagaagtgacatttgttgtttggtgtaaagattatggctatgaaaggtagtaaatgcgccgttccaggttgttcgaattataaattacaaaagga

taaatcattttatagatttcccaaggataaggagctatgtaagcagtgggtaattttaactcagaaacccggtttagctgaagtgttcgaaagccgaggg

ccagaatatgtttatcgaacgtacactgtatgctcggaccattttcaggaaaaagatttccggaacaagatatggttaagccaaggtctttggcgtggtg

caataccttcgcggttgttacccaaacctaaacaaactgtaccagacagtggaagaaatggggaagtggcgacagtgtcaagtgatacgaacagcgtaca

aaatgattcagccacaaatcaaatggcgaggggattctctgcagaacgggtgtcagcagaaatataccttgcagaaaatcagtattgttttcagagttgt

acggaagaacctcaccagccatgtgagagtataactgtgaaggtggaacaggagcctgaatatggagggaactcctgtgataagcacagaccacaggaac

ctgaatacatagaagttaagggtgagccgctcgaggaggaggaagctactgatgagggtgtcacagagcaggactctctgtgtttctgatgaacattctc

ctcagagctgaaatgtctacatttgtggtccgaatgtgaccgtaactgtaggctgggagggagctgcactctggaactgcagctgccggagtgtggatct

tcacaggtctctggaaaggagggggggacagcctggatagttgtgtgccagatgtgatgaagctgacagacctgagagaagaaagccactgcaaactgga

aagagtcatctccagcaaaggccacagtgaaaagcgtcttcttctatgaatttgatgtgtgtgcatgactatggtttttacacctccatatgtattttcc

attttttatttcctcatttggggtaatattcccacttttctatttcatatgttcaggtaccatcttagctaaaaataaatgcagaagttttactgtcttt

gcattgggttgccataatcagaggacgaaaatcgaggacaaaattcggcgattataaagatgtgcttgaaatgcagatagatatgttgtgcaagtgacca

ttggtttgcatttcacttgaatcttagacattactttacattggtaattatgaaaatgtggttgaaacttggaatcgcacaatgtcctttcatctttcta

cacatcagccaaatcttataattaccataattcaaccagtaattctgtcagcaaagaagttgtaattaacaactaaaattaatccaagcattaatggtgt

ctataaaggagtggtcaatgatgaagaaaattaattgcaattgacattaaataactttttaattaaag

>36382|AGAP007257-PA

cgttgtagaagtgacatttgttgtttggtgtaaagattatggctatgaaaggtagtaaatgcgccgttccaggttgttcgaattataaattacaaaagga

taaatcattttatagatttcccaaggataaggagctatgtaagcagtgggtaattttaactcagaaacccggtttagctgaagtgttcgaaagccgaggg

ccagaatatgtttatcgaacgtacactgtatgctcggaccattttcaggaaaaagatttccggaacaagatatggttaagccaaggtctttggcgtggtg

caataccttcgcggttgttacccaaacctaaacaaactgtaccagacagtggaagaaatggggaagtggcgacagtgtcaagtgatacgaacagcgtaca

aaatgattcagccacaaatcaaatggcgaggggattctctgcagaacgggtgtcagcagaaatataccttgcagaaaatcagtattgttttcagagttgt

acggaagaacctcaccagccatgtgagagtataactgtgaaggtggaacaggagcctgaatatggagggaactcctgtgataagcacagaccacaggaac

ctgaatacatagaagttaagggtgagccgctcgaggaggaggaagctactgatgagggtgtcacagagcaggactctctgtgtttctgatgaacattctc

ctcagagctgaaatgtctacatttgtggtccgaatgtgaccgtaactgtaggctgggagggagctgcactctggaactgcagctgccggagtgtggatct

tcacaggtctctggaaaggagggggggacagcctggatagttgtgtgccagatgtgatgaagctgacagacctgagagaagaaagccactgcaaactgga

aagagtcatctccagcaaaggccacagtgaaaagcgtcttcttctatgaatttgatgtgtgtgcatgactatggtttttacacctccatatgaaatagaa

aagtgggaatattaccccaaatgaggaaataaaaaatggaaaatacatatggaggtgtaaaaaccatagtcatgcacacacatcaaattcatagaagaag

acgcttttcactgtggcctttgctggagatgactctttccagtttgcagtggctttcttctctcaggtctgtcagcttcatcacatctggcacacaacta

tccaggctgtccccccctcctttccagagacctgtgaagatccacactccggcagctgcagttccagagtgcagctccctcccagcctacagttacggtc

acattcggaccacaaatgtagacatttcagctctgaggagaatgttcatcagaaacacagagagtcctgctctgtgacaccctcatcagtagcttcctcc

tcctcgagcggctcacccttaacttctatgtattcaggttcctgtggtctgtgcttatcacaggagttccctccatattcaggctcctgttccaccttca

cagttatactctcacatggctggtgaggttcttccgtacaactctgaaaacaatactgattttctgcaaggtatatttctgctgacacccgttctgcaga

gaatcccctcgccatttgatttgtggctgaatcattttgtacgctgttcgtatcacttgacactgtcgccacttccccatttcttccactgtctggtaca

gtttgtttaggtttgggtaacaaccgcgaaggtattgcaccacgccaaagaccttggcttaaccatatcttgttccggaaatctttttcctgaaaatggt

ccgagcatacagtgtacgttcgataaacatattctggccctcggctttcgaacacttcagctaaaccgggtttctgagttaaaattacccactgcttaca

tagctccttatccttgggaaatctataaaatgatttatccttttgtaatttataattcgaacaacctggaacggcgcatttactacctttcatagccata

atctttacaccaaacaacaaatgtcacttctacaacg

>36553|AGAP007288-PA

ggaggtgatcttgaacctggagaagggccgcacgaagcggcggtagcggatggtgcaggcgccctggacaacggcgcctcccttctgtcggatcttgcgg

tagagccccgtgcgctcgtagaagtcgacgcgggcgaagtccagctcgcgcaggtagcgggcgttgttcatgtggaatagcagcgtgtccacgtccgtcg

tgaggcacacgccataggtgtaggtctggtcgaggatggcgaccttcgacttgaagaacctggcgatgaggacgctgatggccatgcgcatgaagtagtg

gaactcgagcgtgaggtagagcgtgagca

>36572|AGAP007301-PA

tgttaatggagctatccgtttgtgtttatagttgcaacagctgatcctgataccatattatgtgattgtacgcagcaaaagaaacggagtatagcaaaaa

ttttgcgaaataaatctcatttcatttgataatgtaagctgtgctgctttccaaacgaaagcgacacaaaaaattagaaatccgtatgatctaagatgga

aaactataccaagggtaaaaatgttgaagaacctctggaaaaggaaaagattccgatccccaacttgccagaaaagttcatatggatgcaggtcacaggt

ggtagcaaaatgaggaaccttctcaactatgctctgaaagcatttaaagatgaacgttcaattgtttggagtggttctggaggaggaatgggtaaagcaa

ttagttgcgctgaaatcgtcaagagacggaataaagatttgcatcagataactaagatctgctatcgcaaggtggaagagtactgggaaccacaaatgga

aaatctggacacactggttgtaattcgagaaattcctacaatacatatacttctttcaaaagacccacttgataaaaatgaaccaggctatcaggcacca

ggaagtttcgacacattctggcgatccacagaagggtcttctgaacatgtgaggaaacagaatcagccaaaacgcaacaggggtcctcgggtatctaatg

aagaatttgctgcaatgggactacgatcagatcagaggaaaccaaagcatgcgggcagtggtaggataccaccaaaatctggcggagatcggcagaggag

aattggcacatctggtgggggctctgaaagtggtcaacaaacgtgaatgtgaaatggaatttaatttcctataaaaagttcctatacatgaactggacgt

gtattttgtctgcatatgttctggtaataaggtattgcacaaaaaatattgtcaccattgagtcataagatgtacttattacctattgaacattgctgcc

atatggaatttgtacaaaataaataattacacagaatcagccaaaacgcaacagggg

>36592|AGAP007305-PA

gtctgagggtctcgccgcgcagacagcacttgtcgtcgggctcgaggatgcgcagccgcacgaagcagatcatggcggccttctcgacgca

>36591|AGAP007305-PA

catcagcggcctgcaggcctacatcaggcaccacatcgagctcgccaaggcgttcgaggcgctcgtgcgcaaggactcgcgcttcgaggtctgcaacgag

gtcaagctgggcctggtttgcttccggctgcgcggctcgg

>36610|AGAP007308-PA

ccttgtgctcgtccttcaggtggtacaggtacgagcgcgggttctgcgtgaagtaggtgcagtggctgcacgagtagttgcgctttatcttacactcggg

caggttcgcgtcctcttccgcctcctccgccgtcgtcgcgtccggctcgccggcctccgcggagtcgccgctgtcgtggccgtcgcagtcctctttcgaa

gtggactcgc

>36611|AGAP007308-PA

atttgtatggaatgaatttggtcaagatatggagagtgaccagaatagtcagcttccatcagatgaaatgcaggaacaagaaaatatatcagttgataat

gaaactgagagctgtctacaacttaatggaggctgcaaaccatctgacaatgaagtaggccttgttgggtttgagaccgctcctggttatggggctgtaa

gtaacacaagtgatgatgggactccagcaagcaacagtgactcagctgcgactcctggtacatatactgttgttaaaagggtattcaagtgcccacactg

cactttctgggcatccacagcatctcgattccatgttcatattgttggacatctcaacaagaaaccatttgaatgctcactgtgtgcttatcgctcaaac

tggagatgggatattacaaaacatattagactgaaagtagtccgtgacaagaaccatgcagatgcgaaagtcttgatcacagatgaaacag

>36638|AGAP007327-PA

tggcattacaaaattaaatttgtttattctctagttgtaagtccaaggttaagtcagataatgcttcacatcaatgcttgtacaatgatcttctgttgtg

tgaacagacagtcaatggtctgaatgtaaacaaacctgcaagcagtgggtttgcagcaataatgttcatcttacgtgcaactgcacttttgtttgcgtta

ctagaattgactgattctgcagaacccggggacacgttgttgttattttcttccttgtcaacaaagtcggggaagaaagtatccaactcaaatgttgtgt

ctcccaattgcttgaacagatcatcatctacctcctcctcaaattctcctaccagtcctgagagatcctgctgtgacagctcacccaggccagtcccccc

tccttcctgctgatttccagagtccaagcattgtgacaggtccaccatcttgccgtcgttgtcagggcaccacagcaggtcgtcgtcaatgaaaggcatg

cttacagcagacttgctgactggccgcgccgccgcagccactaggttccaagagcgacctggctatatcacggtacttctgaaacttgatccaaaacatg

aacagcgctggttggtgacctgag

>36658|AGAP007332-PA

gttaggaaccactgagaggcaaacatgtgtgactctacacctttctcagtaaaatgctgccacaatttgggtaactgctcctccatcaacctgtccagtt

gatacaatctcaagtagaggacatcaaagccatccttgtacaaatctcgtaggccataattgtacattagtttaataaggacacagaaggcttgttcttc

>36679|AGAP007334-PA

ccacaaaatatgttggcatggtattatgatgtcttattcacaggactctacctaccattaggtgctctgcagtcagttgtggggcttgagttacatcgga

ggctacgtttgttttcattgtgcttgtgtgccttattgtacagcactgtcaactttggaatgtcgaagcatcataccttggccacactgccctgtcaaca

actacattttacactgaaagaatggattcaaaacaacaagttactgaacaaatcagcagattatcaccatgatttagctgaaagaatactgtctgaagga

tcaagggctagagatgtgacagcacacacaaccaaagcaaacaagaaagaagttgacataaagttaaaggaacgaataacagacatccagtttcacaaaa

aggaactggaaaaacaaaaagaacaatgctgcaaggaag

>36695|AGAP007339-PA

gctttgacgccttcgaccgtgagaagaatggcagcatctcgaccaatatggtggaggagatcctacgccttatggggcagcccttcaacaagaagatcct

ggccgagatgatagaggaagaacagcagaccaccgtatcagacccagcagtatcttcagctctgtctccagctccaaccccagccacagccacagcccca

gctccagcagcttatattccgctaccagatggaattcctgtaccgagcacagctgcccaaaatgttgacacctcccagccacctccactgcgaactgctg

atataactggtgtaacagctccagctggagttcctgggctgcctgcacaaattcctactgctgttcctccaccctctggacctccagtgcccattgtaac

accgtttgggattcctaatgtgcctccacccattagcatgacaatgggacatggtattatgcctagtgtaccaattggagtaccaccaccaggtatgcag

agtttgctgatgcct

>36712|AGAP007340-PA

cagtcgtggtcgggagtgtgaacgcagccgtattccagcagtcacgcgcggaccactctctctacgagcagaagaggtagaggcgccggcggcctgtcca

gcgatcgcacaaagctccgccggcaccaaccgcaacagcacctgcagcgtcacgtgctcctccagtgctgccaactagcacgccactaatcaagctctcc

tccagccctgagacatggcgcccttccaatctgagtggaggcagccgggagtacggtctgcagaagcaggtgagcgtgatgagcacaaacctgagcaaca

agctggatgagctgcaggcgagaggagaccgacaatttgaaacgtctgtagcactgtgcgagatccggacccagctacaagagctcacaaaatcagtaga

gtcgtgccagagtgaagtgagtgaggtgaagcgtgatatggtggccatcaagcacgagctggatactgtccagcaagtgaaagaagagattgaagaacta

cgtgaatttgttgatcgactgggcgagcagagtcatcgtcgtaagcttcgccttctagaacagtttctgccattcttcctgtcatatgcaatccttgctg

cagttt

>36791|AGAP007363-PA

tcttttatgctgatatccagtttgaatttttctgtaaggttcctttccagttttatccgaatacaaaggctcttacacagtcttcatcaacaacatgcaa

atcttccagggtggctggcttctcaggatctcgaattgtctttatgtgatccggtttcagtactgggacaacaatgctttctcgccattgcgatgagaac

tcacacttgctccagatgcagttaaagacagaaaggatatgaagctgacaatccaccaataggtgtttgattatttggttgtag

>36813|AGAP007374-PA

ccagaaactactcgcaaatgaagcaaatggtttcctcagagtgacatactagctcacaaaaatataaagttgtttatgactcacggaggaatgatgggtt

cgcaagaagctatccataat

>36814|AGAP007374-PA

caggggtttttgtggacggtcatggaagagtttggaatactttctcgcattctctacatatcttggattcagaagtagttcatttactgcccattgaaat

gatttttcagtgatgttattaagcagaagcataatgccatatccaagactttgcactttcaacaagtttggttcttgatcaccatatataggaactccaa

gaagtgggacagcaaagtgaagggcttcttgaacactcatcattcctccatgagtgataaagaacttcaaatttctgtgagcagcagcttaaaactttaa

tttctt

>36838|AGAP007377-PA

atttctcccatcagatcactacttctttcattgcttgtagcctggttgttgttacagtctttgttcctattcccttcaccagtgtcaatttcccatcgtt

gctgcacatgagtcctagaatgtgacttcaggctatgcggagtggagaatgcacgaccacaatcccactgttcacacgcatatggacgttccccactgtg

ggtgcgactgtgagttttaag

>36892|AGAP007392-PA

cgcggaaattttcgcacgtttttggcaccacatctgcgaagagttcaagaatcatacgtcctatttcctacgaaagattaaaagccatgacaaaaataca

tatatacacatcactatgtaatttggcatgttcaacactgagtaaatgtgttaccgttgtccc

>36909|AGAP007395-PA

ggacttgatattactccatattcagatgatatgaaaggttatttccctcaagcaccgtaccatatcagcgaagaggaaatacgaaaacgagttgatttga

ggaaggagtgcattttcactattgatccaaagactgcgaaagatctggatgatgctgtctcgtgtaaattactggaaaatgggaattttgaaataggagt

tcacatttcagatgttacacacttcttaacagcagagactcctttggatgacattgtttcaaagaaagctaccagtgtatatcttgttgatgcggttcat

cacatgctcccaaaagaactgtgcatgctatgctctctattacctggtgaagataaattgacatattctgtaatatgggaaatgactccaaatgctgaaa

tactgtctcagcgtttaacacgttctgtcataaattcctgctctcaacttgcttatgaacatgttcaggcaatgattgaaaacccagacaagcagtggac

aaaagaggacctgccaccattagctggaggctttgaacccaaagatatcctttctcgtgttctgtggctccataaattagcattaatattgcgaaagcgg

cgatttgatggaggagctctaaaaattgaccagactaaagtatttttctcattggatgagttaggtctaccagtggcatataactgttatgtccagaatg

attcacatagattaattgaagagtttatgctgctggc

>36934|AGAP007420-PA

ctcaatgcgaagctgtgaagtgggagacggggtgtaggcgctgtgaagacgagcgcaacatgtcgaaacgagggctgcctctggtccttgtggcctacct

acttggcgttcttaccaccagacatgctgccggctataaagtcgagaagtatcctctcctaatgccgaacgtctcgccgaaaacgccggaactttatctc

tgcacaccaataaaagtggatccagccaagaactactatattgttggatttgaaccaaatgctacaatggccacagcacatcacatgttgttgtatggat

gctcagagcctggatcacagaagcctgtttggaactgtggtgagatgacagggggaactgattctgcttgggaggctgctagtccatgttccaaaggctc

gcaaatcctgtacgcatgggctagagatgctccaaaacttgagcttccagaaggagttggatttaaagtgggtggcaaatcccctatacagtatcttgta

ctacaagtgcattatgcccatattgatgcatttaaagatggaagcactgatgattcaggtatattcctgcactacactgaaaggccaatgaataagctag

caggtgtgatcctgcttggcactggaggtgttataccaccacaaggtgtagaatatatggagacgtcttgtgttataaaggaaaagaagactatacatcc

cttcgcatatcggactcatacacacagcctaggccgtgttgtgtcagggtatcgagtacgtgaagattcatctggtagagaccactggaccctgctagga

aagcgtgatccactcacaccacagatgttctacccggttgtcaatacagaacccatacatcatggtgacagactggcagccaggtgtacaatgtacagca

accgtaatcgctggactcaaataggtgctacaaatgaggatgaaatgtgcaacttttacttgatgtattgggttgaaaatggatctcctctggaaaaaaa

gtactgcttcagctctggccccccactctacagttggcgtgaatcagacggtctcaacaatataccagatgatgctagccagttggagtaagctaattag

aaaagaatagagtattactaaacctgaaagtattagtatttggaaaatgataatgaatttgaagtaagcctttatctaccctgcacaatcagcgtcattg

tcagcagcataatattttttacaattttagaagggttgtctgcactgaaagtgaaaagtgtaaatgtgttacatttggaacttcataacaaaatagatga

atgcacgtactttcattcctctattgtagaaaatataaattaaccactttcattttcacattttctagtattttgcaaaacatttcaagtttttttttgc

tcaaaacctgcagtatgagatgctactgtttgaatatatatttaaagagcagctcagagtaatagtgaattcctgtgcataatttatggaaccacacatt

tagagatctgctacacatatttgcaattcatttaaaaataaaattgcttagatgcactacagatgtttgttatcctttaagaataacatttttaatctaa

cttgtcatatacaatgccctctgaaacaattacatgaaaatttattcctctatttattttaaaagtggccttgagcctaaatgatcagtgatattcacct

tctctcaatgtatcttttttttacataattttaggaaatagaaatcattgtaaatattattgaaagtatgcacagaactatctcagttaaattattagtt

ttcttggattttaattatgtgattatcagtatgaagcaaggttatgcgcagttctgtgcaggtgggtggggtgtgggggaggaagagtttgaatgtagtg

agcagtgaaaggtaggcaaaagtaaagaatgaagcaaaaaagaaagagtgtatacttggatgtttttatacctatgtaaaatgcaagaaagaatggaagg

agctgacaaaataaatataagttcaaaccagaatcactgcaaaaatgtgtttgaggaatattttcatcttacaaagatacactttttgaaggaagactct

ttagaggttcatgtttaatgtaagacattgagctggcaactacagaaaaaagaaatggagtgatatagttctagtaaatacattattatttcattaccag

ttctgtgacagctgttgtatagtgggattagcacacacctgcttcatgacaggagcaattggatgactacagtcaatgaatacttctcatcaccaattaa

aatgtattaacagatactgttagtcacttggtaaacaactgttatgctcccagaaattttaaaaatcttaacctgcgaaaggtatttgtaagttaactag

ttctgacagtgccttaaagttcaccatgggcaagcagttttattctcacttatcagagttggctttccttttgaatacaccacttcaacactttaaaggt

tacacctttctgtatcttccttgtagttgcataagtacatctattcacggaaatacataaaaaatccaaagaattttcattctgtatcatgagtaaggta

tgctgaatatcatctgttatgatgtcagatatactgcagtacatagtccatcgccagtaccaacggcctcacaaaagatttaattgcaacaatcatcaca

atgctgctgtcaacaaacaagttggaagggtggttttcttttcaccattatcactcagtttcagagaaagacattctttgcaagtaaatagcatccacat

ctcctgcagataactcatttttcaccaccctttcgctccaccctggtatttaatcttgtgaaccacatgggaggcatttttcgaaataatattcagtttt

aacccatactctgaagtatttgcattatatatgtggactctgccttagcttttgttcatcactttgtactccagtcccacagaccttattatctgtggta

gtgtaaacagcaattaatgtacccagatatatcatctgctgtttgtatcccactaactgatctgacacacaacagcacacaggatgtcatatacaatgaa

cagtcactgacccataaagaagaaagtgttatctggtcactgaacatgctgcattagcagtttagcaattcagagatatattttcacaagaactgttttc

acgcattggttcctttgaatttcttaagtgggtgttatttctagaatgtacctgacaatccagtagttaatcataaatgaatctgcacaatgtttaccaa

ctcccctcttcattgtgtcttcctataatctcttctctctccttgaatacttatattttctatttcttgatcatttttccatttccttatgcttccaact

tctctctcttttgtatcacctgcttgccttgttttccattctttactgataatgtggtcctcattatgtacaggttatcacattcatgttattgagctat

tttaacagggctaatttttcaaccatttccttatttctcattctccagatattaatcaaatgaaaataaacagtatttggatagatgaagggtgtctact

cacttgtgactgaaacatgagcagctgtagcagaaatcaaacaccagaaattatgactttttccaattaaggaacaccaagtttcaaaaacttgaaatac

agttgcattttctcatttatccgatttctagaccagcttctatcattcctcttcttgcaattgataagcagacactgcccttctgtctttcatagtattt

atatgttaaatatttcaattgtgatttacattaaaagaactataaatggaatgaaggtggcacttccatttctgagctgacatctctgatgcttgttttt

taagctttttaatttgtctggttaaggtaatgaaatactgtctgatgatacatgtagcatacttttatttttccaatgtatctgtattttctctgcatag

taataaggaaataataaaacaaattcaaaactatattttcattaaaatagttttatttctgtatcatgaatatctgtaaaaagaaaagcaagttggtgaa

ggatgttttaaaccatagtctgaaaaagcataaacggtgatcagtatttccatgaaactgcaacagttttaataattatctagtcatctgatcaatttga

tacatgattatgaatttaatccaaatcactactaataatctttttttttacttgtcaattggattaggacttatgtaaatatgttataaatttgttattc

agttttgtatgttttttatatataatgctatgaattgtcagtttgaatttcatattattttattgcagatattaaatcatatttatacttatcttagata

aaagccaactgataaccagagttactatttattaaaaggatgatgtctctattgaaattgatgattacattagtttaaatgatagcatattcactagata

aatattgttgtattgggaaagaaactttcattctcaccacaatgagcctttgtttcatggcaaggtagagaggtctgtgcatttgctttaccttaaaatt

atgtggtatgtgcactactttcatggaacttaattttgcattactgtactgggtgactgtgtatcctcaatacagaggtatttagaaatcccctacatat

ttccccatattcttggtgtctaccatttgaagtttctactaaaaacaattttgtggtgaagcatatagagaatacaaatacagaaagcacaagttccacc

tctgatactgataacctcttttgtacgtatacttcagtgaaatactgtgtaacttcttttctgtgaatatatagtattttgatcagatgttttacaatgg

ttttgtacaaatgtattgttttttgaaatggctgatgaggaatttatagtattgtcataaaaaatattttatgtgcctatagtgtgagatatttccccta

ctttcttttttcctgttatcatcttcaacttgtgaaaatgcacaagatattgtctcccactttatattacaatgtattgtgtctgcacatgttatgcaaa

tggaaattgcacagatgtgccatagcaaagctagtgctactttctggctatgaactactttctggctatgcatttatgatccataattcatctgtggatg

agtcatcatttgtctcgactttttgtttattacttttacaagatttttattattaaaagtccagtagtatttctgtaggttaattttggtcattggatca

taactagctggtttactgtgtaatattcagactacaaagagacatatgcaaatctgaaaatgaaatacattactatgggaacatgctgggcaaataataa

agtataaaaattgtacttgaaattgtaaccatattctttgattggtatcatcttcagtgtttaaaaaggaataaaaaaccaaagactgcattaagtctga

gatcactgtacccacatagctacagatagatatttttgtgaatttcacattcttgcatttgcaccaaattctcagcagccgtcattccaatgaacagtgt

ggctattgtgattcgcagtgttattttatgacaatggtaacaggtcatcttgaaagtaaatgtgaattctctaaaagctttattcagagcaagaatgaat

atgaaaagacattttaaacaaatctctgtttggtgaatgagaaatcttaaaagcaggtataatacacaaattatttgtcatcagtggttaatatgtgcca

gcagtgtaattaagtttttcttttccatatttcttattcccattttacctctatttcatttaaaatcctcgtaatcctcttcacacaaataatgctatat

ataatttttttctcaaattctggtttcagtaccaaaattacatcatttactattcatgtagttttatttttgtgtattcacgtaacttccttgatattcg

ttgtactttccattccttccccagaggacagtacaccatagaagtaacatttagcgtaattcgtactaaaccacgatgtaaaagaatatttgtattgtag

agatagatacaaataaatgctgtatcattaaataaatatggtagctacagatgaagcaaatagtattacactatgtaaggatttcttggaatacacttta

ttttattacaaattacagtttaaaatgtatgtgaacaactagtacaatgttgtactgtactgtgaactatcttaaaataacttttatggaataacactat

ggtgcaggaaatgtagtagacatatcacagatatcttcattgtatggtaattttaagcacagtgctgagaagttacatatttttatccaagaggaaagaa

atgacaaattcttagaaattattaggccagtatgcttaaagaatgaggtattcaaaaaatcttttcaagttacatttttatttggaaatagtgtattttt

gttggcagcaaaactgtatcagttcaaaaattaaagaacaatgatttattttgatacctgt

>36952|AGAP007477-PA

ttccttgctattttcttcaagtttgacatagtaaaaaattgttgctttgagatcaaaatactcttaatatgattaatacaaaacagcatagaaatataca

atgttacaatgataacacacatctcagagttactcttcatcccttgcaacttgacacaaacgtgctaacattccactaagttggagcagagaagccaccc

cctccactatcctcacatgaatagtaccaatttcctttataaatttaattcttaagctttctggcattggtatgttcttgcatacacggaatgtattcga

cacaatatcttctgctgcatatccaagcttccacaagtgtgctattatcttgtacgctttctccacatttccatccatgcagtgctgcagcatttcttta

ataagtaatggatgtggttcatcacataccttgaaaacattttcactgctgacatgtccaaatccattaaatgttgactgcaaattgttaaatgcttgac

gcatgtctccctgtgctgtgaacacaattgcctccagtccatcatttgtgtaggagaccccttccttctggcaaacctcaataactttggcaagaatctg

agcatcattaagtttgccaaatcgcaacacagcacaccgagactgtattggttctatgattttttcactgttgttacatgctaatgcaaatcgtgttgtt

ttactgtacaactccattgtacgcctcagagcctgctgagcaccttctgtcatactgtcagcttcatcaagtatcacaattttgtgcttgcctggtggta

atgtaaccttctgctgagcaaacatctttatcttatttctaacaacatccagacctctgtcatttgatgcattcagctcaagaacagctgagttcactgc

atctcctagtaaaactcttgcaaggcacaaaatagtggtggttttgccaacacctggtgggccagcaattatgatattcggacattccccagtttttgcg

aactgccctaagcgggctacaccctcttcatttcctacaatatcttcaaatttcattgggcggtacttctcaatccatggtaaccggtgggccgatttcc

ccttagaacttttattagcaggacctggcttcgatgtcgacgcttctatttctatatcaaggttttcttcgtcacctgccattccgatctcacgatacaa

gtatcacccgcaaccaaaaactctcccgcctaaatgttgactacatctgtttctactgacaaatgacggt

>36975|AGAP007483-PA

cctggatctgctcggcgcacaccacccccagcgagatggccagagcgcggcggcagccgcgggaccggaacaggtccagcacggagggctcgttcagttt

ggactcctcaacggctttcttcatctgacgcagttccgacatgatgtcgcggtccgggtcccgcagccagcgcagcgacttctccgctgcagcatccttg

>36974|AGAP007483-PA

ctcagctgctggttggcgcacaccaccatcccgatggcgaaggcgcggcggttggccttggtggcgaacaggtcgcgcagagacacgtgccgctcggcgt

cttccagccgcttcttttccaccgccgcctgcctcattgcgaactccgtctccagttcctcctcggtcttgccgaccctgaaccagcgcagagaggcctt

ggcttcgtcggggcggcccttgctgaagtagtaccagggcgtgtccggcaaccagaagaggcccgccaaggaggcggcggacagcaccagcgagaccacc

gccgtcatcgtgtaggacagcgccaggggcagcgcgtagctgcagaggaggccgattatgtaggcgatgacgaggaaggagcagagcgacccgcggaccg

cgtcctccgagatgtcacgcacgtaggcgccgcacacgacgaagccgcccgcgccgctcacgcccgccagcgcccgagacacgatcagcacggccacgtg

ctgacccaccaggataagcacccagctgaggagcatgagcgcggcggcgaggaaggcggacggcttgcagccgtagcgcgccacgcagagcccgaacagc

ggcgtgcccacggtgccagtcagcgtgctgacggcgcccacccacgagccctcctccaccgtcagcggcacccccagcggggaggcgtccgactgcagca

gcggcagcgccgacgacgtccacccgg

>37017|AGAP007510-PA

ttttacctccatggttacggtaattccttggccatgtgtaacctccttggactagtgtctagatcacagatattgttggcaaacagcaacaaaacagtgt

gctgtatcagctgtgtttatagcgcgttttgttggaagggtgcatgttgtgcaacgttttgatacggtgttgtgggattttagagagatgacgttgttgc

gcttcatacataaacgacgacgacgccctatatcgacacgagtctgattcacaaattttagtttcacgtagataaaatggaattaaacactctcattgct

gatggtgctgctgaaaatgacttgcagcaaatgaagggggatcgaattggcaacacaatgtacagtgagagatctgtcctaaagacattaataaagctgt

cacagataacagagttcacaagtgaatttgaagaagagctgtgccttctttgggatatgacagttgaggaagatgtggtaaactttcttatgaataataa

ctttttggatcttgctactcatattataatgaccattgaggataacaggctcatagagataatggtgggaattgttggcaatatgtgctgtgtcaaatgt

gtcaggagccatttagcaggaaaaagagcatctgttgaaacatttatgaagttgttgggcattagggatgcacctgtgctcatacaattaattagagtgt

ttgaattgatattgattgacattattgaccagccaccacaattatcttcaagaaataatatggaagccaataacgatgagggaaaagatgtttgcaaagg

agatggacaagtaacgcagccacctactgaaactactgattcagaaagcagtgactcatctttgtcacaagaagactgtcagaatgtatccagtaattgg

tgggaaatatatttatccaacactgaaatttggtttcataatattccagaaattcttagcaactgtaaaaatgatgaactcttgagtaagctgtggcatc

tggttgataatctgagtgatgttgcttgtgacagcagcatttatcttgatgaatatttcctaaacagagaactgcttcgttcacttgtggtctccttcaa

tgaatattttaaagaatgccatcccttacaagacatagatgaaacttatcagttaaggaggtccgcctcagattggattatggctgttaattgttttcat

aactatgaggaaggcacagacattgtgagaacgtgtattggagacctcttgccctgtattcaacatatcttgagtcgatttagtatgaattttaaaacta

gggcagaaagtaataaattgtttatatccgtaatatgtttcctggataaatctcttcctgtaagaaatttaccacatctctgtgaagacgaaatgaagaa

tttgattagtaggctagaagcatttctggaagaagatcacaaagcgaactctgatgaatgggaagatatttctgatgatggggaagcaattaataaagaa

aagaccactgaatgtttgcagcatttactcaacaaagccaggaacttgctctcaacagcttcacatcaaagtgcccagtcattgttaaatggagaaaata

aaccaagccactgagagagaggtgttctaacattatcaatgagtaatggagttcctatgaagagatgaagaaaaacaaaactggagaggtcattcttatc

ttctgcaagtcacgcagcctccttgtttacatgaactttccagctattaattaaacacagtgaattatgaaactgttgctgaaacagcaaagatccaata

gtactcatggtttcattttattttttgattgtccctctcacatctgtcatacttcagttgcagggaggtagggcagcgtaactgctttttaccaatatgt

gaaatattaattaatttcattcaaacttttcttgggccatggtaataatgccatctgtatgtggaccgtgagttgttgaaatagtgaaatgaatgcacac

ttatgtgagacatagagtggtaagaacaaaaaatagaagagagacaattactgcacaacatctcagtcatctaattaattattttttttgtgtgaagatc

ttagctagttgtgcacagtgttgcttatgagctatcaacatcataaaaaaaagaggaactctttgtgtgtgtgaaaacaactatatgattacagagtttt

gaatttacttttcatatgtgtacattaattaatataattcttttctaatattttttacatttctggaagtgtaaaactaacaaatattgagcagtgataa

ttctgtaatgaactgttgacattgtatatatgtactagattaaaaagagaaataggatgtttgaagcacttgtttcgaaagtaaataaaattgtcatcct

gctcaacttaacaattctgctgttgagatgtaataatttagtccagtgaagattgctaactcgaag

>37016|AGAP007510-PA

acacacacagacagaatcttcataaaagaagatttaatgagaagggtcagatttttcacaattcaaatctatacaaagccaaatagtacataaaaagaaa

actccatacctatagtaaatagatttttctttcttttcaacctccaggtgaagtacccattagcactaaataatagcagcaaaagcaaataatgcacatt

tataattattttaaatgtggagaaataaaagaaaacaccttgtcttcatattccatgcacaaaaccatcaatgatgccagttaatttaataaattcagtt

gcttgtcttgactaatgtacttcttgaacagtttcaaattcataagtattctcagtcatgctgcaattcacagaatttggtgtataatactaacagcagc

caaatgaatttattagctacgacacattttatacaaacattacacaatgaaacataatattttccatcacaaagtttcaaaggaaaggtatatgtaagtc

cctttccattagttattcactggattttgtcttagaaaaatcataaaatatgtttctatgaaactggtaagctgatcatcttcctaatgggtatttttta

aggttttcatttccttccattctcacagatgatagtatttcaagttacttcagtatttgtgtaattactggcataaaataaagacagttactgtacaaac

ctgtcttgactaatgtactccttgaacagtttcaaattcataaatattctgtcatgctgcaattcacagaatatggtgcataatactaatagcagccaaa

tgaatttattagcataaaatacagacagttaatgtacaaacttagtaattctttctctatacaactctgtcactctgaaagtgatcataaaaacatgaca

aatggtcttgactaattgtgaaaactagtagccaggcacaaaaatgcttgtatgatgtaagtaatctgcagtgcacaaataaatgataattcataaaaat

agttaattataatactatactagagatttatatgttcaactatcattcccactttgacaatagtactcagcatgccatgttctggaggcacatgtgaaat

aacctattctgcctgctgcatttcaatttcttctccttcaggatgttcccctgtaatattagagtttttttgttggcagactcttcgtataaagagtaac

ccaattattgctgctaccagaactgctatgccaacagctgcacccacacctgctgaagctactgaaggttttgctgaaaactgaatgatggtagtggttg

atatttcatcttcttcacgttttatctttactgtacaagtccattctccaagatccctcatttccacactcttgatactcaattcacagagaccagcttc

cataatgtgcacataggatgttgcatttccaagaggtccaaatacagttccatcaggtcgcttaaatgaacagtattccataacatacataggtgatacc

atctgacatcctattgttatactgtcaccaacatgtgacacaaccacttcatttactgcagttaaagggatttttgtcactttcacttgaatctgtgatg

cataaatcgtgttggttggcctcaatgtcttcatttgacatttccagattccagattcttcctcagttgcagcaaacagtgtccctccacagtcacctgc

atcaatactaaaaccatcatatttccacaaaccatcatatttccacatttctgtactgttgtaaatcccttcttttgagtcaagcacaattgttgaacca

tttggatgaaccagtgaacattcacttattggtgtctctacacggcatttcaatgtcagtggttgccccaagagtacaacagtgttaccaacatcccata

cttgaattttttcagctgcagcaacatctcccatatctgaagaaatggagataaaagaagagagaggttttcttctgtgagcagttaaaacaatgcactg

ccatctgccgtaatccttctcctgaggagaatcaatagttataccacaatgaccagaacaaagtccatctccaaaataactgtattggccattgccagtc

ccatcaaccacgctgaatgttgcctgatcatatagacgaataaagtagcaagatgctagtctctccttgcgtttcaaattgcaaaatagtgttaaacttc

cacctaaagaattatgagttgtccatccagaatcaagttcatcactgttagtacaagaagcatttttaacacctgcattaattttcttgtacttattagt

catgtaatatgaagtacaatgctggagatcaccaaagtttaaaacaggcttagtaccaatcaaaatcttgactgctgaagaagaactagcacttaatgat

attttcgaagcaccctcaaccttctcataaatatgtgatattgaagtaagtcgccagtaccctacatcttcagcagttacattattaatccagacaccac

aatctgtatgtatgttaccaccaaaatattttatcttttgatattcattttgatgatgagcaaactctgatataagtggataaataactgatgtgtttgg

tgttttaagaagacatgatctttggtttggattgctccttagcaattttagcaaaacatcacctccttccacaacatgcacagcttctgggaatattaca

gacaaacctccctgcaactgaagtatgacagatgtgagagggacaatcaaaaaataaaatgaaaccatgagtactattggatctttgctgtttcagcaac

agtttcataattcactgtgtttaattaatagctggaaagttcatgtaaacaaggaggctgcgtgacttgcagaagataagaatgacctctccagttttgt

ttttcttcatctcttcataggaactccattactcattgataatgttagaacacctctctctcagtggcttggtttattttctccatttaacaatgactgg

gcactttgatgtgaagctgttgagagcaagttcctggctttgttgagtaaatgctgcaaacattcagtggtcttttctttattaattgcttccccatcat

cagaaatatcttcccattcatcagagttcgctttgtgatcttcttccagaaatgcttctagcctactaatcaaattcttcatttcgtcttcacagagatg

tggtaaatttcttacaggaagagatttatccaggaaacatattacggatataaacaatttattactttctgccctagttttaaaattcatactaaatcga

ctcaagatatgttgaatacagggcaagaggtctccaatacacgttctcacaatgtctgtgccttcctcatagttatgaaaacaattaacagccataatcc

aatctgaggcggacctccttaactgataagtttcatctatgtcttgtaagggatggcattctttaaaatattcattgaaggagaccacaagtgaacgaag

cagttctctgtttaggaaatattcatcaagataaatgctgctgtcacaagcaacatcactcagattatcaaccagatgccacagcttactcaagagttca

tcatttttacagttgctaagaatttctggaatattatgaaaccaaatttcagtgttggataaatatatttcccaccaattactggatacattctgacagt

cttcttgtgacaaagatgagtcactgctttctgaatcagtagtttcagtaggtggctgcgttacttgtccatctcctttgcaaacatcttttccctcatc

gttattggcttccatattatttcttgaagataattgtggtggctggtcaataatgtcaatcaatatcaattcaaacactctaattaattgtatgagcaca

ggtgcatccctaatgcccaacaacttcataaatgtttcaacagatgctctttttcctgctaaatggctcctgacacatttgacacagcacatattgccaa

caattcccaccattatctctatgagcctgttatcctcaatggtcattataatatgagtagcaagatccaaaaagttattattcataagaaagtttaccac

atcttcctcaactgtcatatcccaaagaaggcacagctcttcttcaaattcacttgtgaactctgttatctgtgacagctttattaatgtctttaggaca

gatctctcactgtacattgtgttgccaattcgatcccccttcatttgctgcaagtcattttcagcagcaccatcagcaatgagagtgtttaattccattt

tatctacgtgaaactaaaatttgtgaatcagactcgtgtcgatatagggcgtcgtcgtcgtttatgtatgaagcgcaacaacgtcatctctctaaaatcc

cacaacaccgtatcaaaacgttgcacaacatgcacccttccaacaaaacgcgctataaacacagctgatacagcacactgttttgttgctgtttgccaac

aatatctgtgatctagacactagtccaaggaggttacacatggccaaggaattaccgtaaccatggaggtaaaa

>37036|AGAP007531-PA

cctgtattgttgcagctaattcagcgccaaagccacttgtcagtggagcttcatgtgcaataacctctcggcctgttttcgtcacagac

>37035|AGAP007531-PA

cctgtattgttgcagctaattcagcaccaaagccacatgtcagtggagcttcatgtgcaataaccacacggcctgttttcttcacagaatgagttcgaga

ctcgctctggcacacagttttaatctgccaggaagtttcatatcagtgcacactccgctgcagagtgaaaatctcattctagaaaattaataatctatgt

acctcaacagtcactgcagtaaaataacacaagacttttttaacttttcagtaaggtacatttttgcactgtatgcgcttcacagtaagagtttcttctc

tgtgtatattacaaacaggtttcttgcacgttacacagtttcacacatttttggatcttcgtttctcttgcaatcttgacatcattgtggcattggtaac

ttggaacattgggtctggatcactgctgtaggaattgcaataccacaagctttcagtgcatcttgaacatatttatgaagaccattgatattttagctct

ttctt

>37052|AGAP007542-PA

aataagctttcggccaacaagcccttgtcaaaaacacacacacacacacacacacacacacacacagtgtttttgacaaaggccttgttggccaaaagct

tatatgtgacagtctttttgttgtgcctatctatgactaagcactcccctatatggtgagtagcaactttcctcttcatgatattgttacattccatcct

ggatcttccactgtttggtatatacaaagttcaggtaagctttaatatttaatgtatggatcatctgattacaaaatagatcaatgaataatctgtaaaa

taagagaagggcaaccatttacctttagcagactgatgtgtggcgtacacacacacacataacagaaaatagtatgcacaggaattgatggtcttatatt

gctttcaaaattaatgtggtcatagactctcaatatgtaattcaaagctagaaagaaatgagctcatcagataggaagttagtactgtagtgatgaacat

ttattgaagaagggggagctgtaaatattgcagtaagtttattaccttgaaaaataaatgttagagaaacgaaacaaaaatctatgtctgctagaaccat

attacattcatatgttgctgtggtcacaatagtgtgcgttttgttaactgtgtggttgtgagtgtgcactcgctgccagaagaacaaccagagctcaaag

gtgagtgcatactgttttctgttacatggctatctgccacacatcagtttgctgtaggtgagcagttgccttcttcatattttatgtatcgttccatcca

agaatttccattcttgtcatcaataaataattacgatatatgttgtaaacatctacaattccacttatttgctgaaaatcaatatctttatattcgcaaa

gatgactttacataaattatgctagtacatgcatacccatacgcaagaaatcatacattttcaataattatcaacaggaagcgaagtaccaagcatattt

actatgagcttttctccatcagttaacaacacatcgtaaaatggaacttttaattttgtaaacattttagtttgaaaagtatattagcataaaaaattgc

agaaactgctctttttagtttgttgacaattaggcaattaacccgtatctgcaccagttcgaagcagaactaatgctttctctaggttttctatggctgt

tggtacatcgtcatagttcaaggcactacttgcccacttgcaatacttctgagcctttgtgatctgtgctggattcagtgaagctgaacttccattacct

gttggcagaggcttactgatttgcaaagttggatttaatggcaagccttctgttggtgtaagtggttgtggaactgtatatggagcagggatgtctgtca

ctttattcgacac

>37066|AGAP007544-PA

ttctgtttatcagtaacatcacagtcactactttttgcttcacttggtcgataaacttcaccaagctccccaggtgaaaatgttataacagaacaataac

catcagtgcttgatacaacgagaatttgaccgtcttctgaccatgatacatctgtaagtcttgtataatgtatattggaaatgagagcaaatggttcagc

ctgctgcgtatcataaagcatcacagaactctgtgttgcaactgctattactatcctgtacgggagtgcaaaaacagaatctccttcatgaagctgaaat

aacacagggcagcatctaactgcaatagtgtactggtcaggtgaaggcaagtataacactggcctgttaagtgagcaccttgtaaaaatgtaagtagcat

tgaaatgtttatcggtgtctggtatttcaacaaaaccagaaggaactatcaaaagttctccatcaggtgaaaagcacagcctccggaagaaagatttcaa

agtgtcatcatgaaaaagtcgaactgtctttccctcgagttcagatccgtctggagcaggcagaattgccttgtttatatggtacattactttctttgag

ttcaagttgtatatcctacatgatctgtcactgcacattgttgcaacaaatttattttttggatcccaagatactccttgaacaaatccagtatgttctt

tcagtaacttcagtgatttacctttttgtacatcccaaataattgctgaattatccactgatcctgaaattagtgaggctgaatccatcgaccaactgag

atcgtatatatcttcaaggtggcctctcagtgtttttaaagatatccattgctctctgttgccttcgttttcactggaagggagctctggtgcatcctga

tcagtcttctgcttccagattataattactgcctcgtcatctccagaggcgagaagttcaccagttggtgaaaatctgacaacatttaccgctctctggt

gccgctgtaagtcagatgcaaactctatctcagctaatccagattccttcttgtgtatgtaccaaatcacaacatgcgaatccgtgccgccggaggcaag

cctgtaaggtccctcgtcgttgttacgtcctggctgaatatcaatactaagaactggatctctattatgccacgatatttcggggattgtacacttcatt

ctgcatataagtatctgattattttacgttattgtcctttcgcgtaagtacaaaattatattacactaatgacatttcgcgagacacatgacatacaatg

aacgcgacattcacccctagcgtcttttttcgcgctaaaactcaaccagagatgtttacgcaaaaactaccaacttcattggattccaacaaatataggg

taaagtacccattttagcg

>37067|AGAP007544-PA

aaatactgttttatattttttgcgatatttttattacctatttctaaaaatttctgtcatcgtacgtggatgctccataagaagcattcgtttatacatt

tctcttattaaattattaacatcttcaatcaatgtctttagttacaaaatctgccattaataaaatatatttttaagaaatgttgaacactttatttatg

gctaagtaaactaagttctctcaacagctgatgtattttaagcacagcatatcaaggtgtgttatagttagaagagtcttaatactgaagctaaataaat

acatcatttgattcattcctttccatcaaatgaaataattcaattataggacagccagaaattttcaaaatgttaagatataataatggcacattctcac

actgtaagaaaagataaattacatttatatcttttcagctaaggacaacgcagtattgcagtgaatagtagtcatgattatatagtatagtcgcttctaa

ataatatattgtaataactattacaaaaggtatttatacaaggtagggaagtccaacataaaatagttgcataaaagtctgagaaaactatacctagcag

gtagctaaagactgacagacacacaaaggtagaagacaacagaatttccagagagtgtctctccagctgatgaaagcaaaaaggatatacaagaagagaa

agaaactgtcactttcatgaatttgtgtttctaccgtgtgtatgtcaattaactatctgctatatagatgccttttctatcttcaaactagtttaaccat

gataaatagaatggttttattaaatatacaatttctgcaccacacaaataactagagagtaaaaagcagagcattaacacacaatcagtttctgcatctt

atgttactgtataagcttcttccttatcttagggcttgacagagtgatgagctggacccgcctaggtgtacgtctgttctgatcattgcctggagttaaa

ttgcatggggtagtagcagggggtggtggagaatgagcctgaagtgcttctgttttcccatcttctagctgctctttcttcttgtgtgtgacactttttt

cagctgtaccatcaccacttttttcagttcgttcattgtccacaacacacattttctctgtcgaacttccagattcattttttacttcatgtattactgc

actctcaactgagctgcacgccctatcatttttactggaagcactttggtcagtgtcactttgtgaagcatctttattttctttctctgaaatggtgttt

gtttggttagtgtcaacctccatgcaactggattccacttccttaccttgaactatgccctgtctcaaagtactggattcctttgttctattacagagca

cactctcatcagcacttacattactctctccgtgtgtgtcacaaagaagttcagaattactggaactgttttcttttgacacagttttacactcagcctt

caatgtttttgaactatcacccgattgacactcgacactcttaccatcagcacttctatttgacgtcgctgtggtaccttgccttctttctgtaggctcc

tctattttacaactttcttttcctttctgaacacttgaaagcaaactactgttctcaccaatcttttcagttgcactattctgtttatcagtaacatcac

agtcactactttttgcttcacttggtcgataaacttcaccaagctccccaggtgaaaatgttataacagaacaataaccatcagtgcttgatacaacgag

aatttgaccgtcttctgaccatgatacatctgtaagtcttgtataatgtatattggaaatgagagcaaatggttcagcctgctgcgtatcataaagcatc

acagaactctgtgttgcaactgaaaagattggtgagaacagtagtttgctttcaagtgttcagaaaggaaaagaaagttgtaaaatagaggagcctacag

aaagaaggcaaggtaccacagcgacgtcaaatagaagtgctgatggtaagagtgtcgagtgtcaatcgggtgatagttcaaaaacattgaaggctgagtg

taaaactgtgtcaaaagaaaacagttccagtaattctgaacttctttgtgacacacacggagagagtaatgtaagtgctgatgagagtgtgctctgtaat

agaacaaaggaatccagtactttgagacagggcatagttcaaggtaaggaagtggaatccagttgcatggaggttgacactaaccaaacaaacaccattt

cagagaaagaaaataaagatgcttcacaaagtgacactgaccaaagtgcttccagtaaaaatgatagggcgtgcagctcagttgagagtgcagtaataca

tgaagtaaaaaatgaatctggaagttcgacagagaaaatgtgtgttgtggacaatgaacgaactgaaaaaagtggtgatggtacagctgaaaaaagtgtc

acacacaagaagaaagagcagctagaagatgggaaaacagaagcacttcaggctcattctccaccaccccctgctactaccccatgcaatttaactccag

gcaatgatcagaacagacgtacacctaggcgggtccagctcatcactctgtcaagccctaagataaggaagaagcttatacagtaacataagatgcagaa

actgattgtgtgttaatgctctgctttttactctctagttatttgtgtggtgcagaaattgtatatttaataaaaccattctatttatcatggttaaact

agtttgaagatagaaaaggcatctatatagcagatagttaattgacatacacacggtagaaacacaaattcatgaaagtgacagtttctttctcttcttg

tatatcctttttgctttcatcagctggagagacactctctggaaattctgttgtcttctacctttgtgtgtctgtcagtctttagctacctgctaggtat

agttttctcagacttttatgcaactattttatgttggacttccctaccttgtataaataccttttgtaatagttattacaatatattatttagaagcgac

tatactatataatcatgactactattcactgcaatactgcgttgtccttagctgaaaagatataaatgtaatttatcttttcttacagtgtgagaatgtg

ccattattatatcttaacattttgaaaatttctggctgtcctataattgaattatttcatttgatggaaaggaatgaatcaaatgatgtatttatttagc

ttcagtattaagactcttctaactataacacaccttgatatgctgtgcttaaaatacatcagctgttgagagaacttagtttacttagccataaataaag

tgttcaacatttcttaaaaatatattttattaatggcagattttgtaactaaagacattgattgaagatgttaataatttaataagagaaatgtataaac

gaatgcttcttatggagcatccacgtacgatgacagaaatttttagaaataggtaataaaaatatcgcaaaaaatataaaacagtattt

>37085|AGAP007546-PA

ctgaaatgtgatttgtgtgtgtgtgttttgttctgtaaccatggtgtacctgaattactgtgttctatttgtgtggattcttagtttaaattacgttaaa

gcaactgattacgactgcagccaaccccttctggagaatgctgtactaaaagctacttcatctcttcgtgaacgagggccagaaaatgcacgccttgatg

gttaaagtacttggtcatttgtgcagcagagttagcattgcagtgggaaagtaaatactaaatattgcacttagcaccacctacaaatgcagtgcgtcgg

ctgtgccaaatgttgtttggtgtgaagatcactggcgatatgtatcttccaggcattcctcttctttaacgtctcagtgatgactaccgtgatacatagc

tggaacacattgcttttagtaaacaatgacttttaacaaagtgatattgtgcaagttcgaagaagtagtttcataagcagggaaagctgagatgcatgaa

tttggtgctttcacatgttgaactgttaagcattactgcaacgtcatgcatagtgtttaagcacttacgataaattatatacaaataatttcgcttgatg

tgttgtagttaaatctttgttaagttttataaaataatgtaaacagtttagtggaaaatatgtacatcctgcattaccactcttgtgcattacaatatgt

ataagccgtcttgtacaagcttgagagttttgttgcacactgcactagtacgttttgcagttgagaaaataactatcgaatcacatttggaaatttgtgg

taagtatgtataccacacaaatgtgtgtgggtataatagtaaagtgaccaactgtttattcagtgctcagaacaatggtgcttgccttaagtattggtac

caagtaagagagaaaatacattctttttgtttttgtggtattgtggtagcactgtcagacttcaaaaaggcaaatacctttatatatttgccagaaatat

ttttggttgctattaaaggaatgacacaagcaaagtcaagtgcctcgtgcctgaaactgagtgaattttgtggcagtcggacagatcaactggagggtgc

agatttgaataaaatggaggaggcatcacttgcaagctgcgaggaagatagagtaacaaggaaatctgaagttgactctgctgctgtatctcttaacact

gcacatgttgatggtatagcagtgagcacagatgttcctttagaaaccaataataagcaggcagccagcacttctccacgttcggtgaggtgcccagagc

gagatcttgcagaacctgttataaccggagggcaagaacagcttgcaagtgcaaagatatctgcagtgaaggagatgagtgtgcgatggatgtttgacgt

cagttcgtggtgtccatctgaggcagagtgggtctcggcactgtcgagtgttcagccggaagagtgcactcgaatccgtcgatttgcttttcggagagat

gctcgtgcgtccttggcaggtcgcctcttagcacgattgtttgtgagtcgcgctctgtccatcccgtggagtgccgttcgcttggaacgggacagtcggg

gcaagccgactgttcaggaagggctgacagatgccaaagtcagtttcagtatctctcacgccgggaggtgggcggtgctagctggtgaagcaggatacgg

aagctcagtggccgatactgtcggtgttgacactgtagaagagaaacatcgtgatgctgcacacttgttgcgcatcatgaaacgaaatctctccacagtt

gaacaggtttcaattgagaaagctggcagagatggccctggcgtgttcttcaggttgtgggcccttaaggagagctacctgaaagcaacaggtgaaggta

ttggtgggggccctgaattatccgaattgagtttttcattttgcagcagtcatctacccgatacccccggatatgtggaggtgggaactgctctggaggt

gaagggctgtcctctacctagtgggtggcactttgaagagtctcgaatcgatccattgcatattgtcgcagtggctgtttctggttcggaagataactgc

tgtaaaatgaaatcggcacctccagtgtttcaagaaatatcttggaatgaatttcagggtgctttggagtctgtcactcatcatgaaccaacattctatc

tggattattttaataagccagaaagccctgcatacttgcagaacacttcaaggcgatgacttaggagactttgctgaagtgagagagcagagtacacacc

attttgttagcattagtagtacatatgtagtaaacatgctttcttgcacagtaaaatttcttaaatggctagttttagataccaagcataattttcaggt

tttatacagaaaggtaaaacataaggctactgacattactctgcagaatctgcacaaagtgttcttatataatcgaactgataaattgctgacaattagt

ggtgaggctaccactttgtcacagacattttacaaagttgtttatgcaggaaactttttgaaatgtttgaaaaaactgggaggggggttatgacagattg

aagtagtgaagacagatgtgagatatgtctggataacttatttggtaatgatttttatctcatttaaataaattaatatttaacttggcctcacaataga

ccattaccttcctttggttctattggttggactgtgttacagaaatcttatgtaccagcatatttgatttattaatgttcttaatggtgttttgatgctt

actttggccgtacttttacatggagtatgatgttgagtacactctgtttctacatgctgttgcattattttgtttgatatatggtgcaaacagtgaaaca

tttgataatcagaagtgcccattagctctcagtagaatttgtgtttttttaacatagcactttccatctgtcgataagtcacatttctaaggaaccatgt

gctacttacatttgtttcttaagctgtacttttgtttctcgtgtaaaaatgttacagcacacagtggaacagcttgttatatttggttcaacttcactta

tactgcctgtcaagtaatttttgtataattgtattcacattttacatttaataaaatgtatggtattggaatgctagatcaaattcagcttttaatatcc

tttttctgttacagcataatgttatgttccagtatattagtagcacataatgtgccctatagctttacattttgcattatgttgcatgacatagtctgcc

gttcattttattatgagcagaagtaaatggggaaatatttcttcaatattacgggactgtctaatccagtatatagcagtttgatgtgtagaaagttcag

atttgttttgtgtgtctaagtaaagaaaatactatagcagtgtgcgtgtgtctgttaaaaattagatgtatcatgagataacaaatttttactcgtacat

cctcttgtgtgtttgatcttttagtactctaataatagtatttctttagaataatattgtgttttattgtttattt

>37105|AGAP007549-PA

gttacaagttcatgaaatcagatcgaccccaattttggcagattctatatttaattcgcttcagtatttcatacagtggtagttacattcgtctgatctt

ccaggacaccaagttgtttcgtcttcaaaaaacaaaatctgtggtacgcccaactaacattctatatcctttatgtcaatatacaaaatttgatagttgt

cctcagaatagttggtggtatttgacagataggatgtaaatcgatcgctcattatatataatctttgtgttttggtgtctatttccatgctgctgacaaa

ctgtagatgttcatcatcttgaacgattaggtggaagttcttacggttgtgaggcttgcttgtgtcccagcagtagatggccaattcatccagaacaggg

aagaatagcagctgacccttcggatccactgtcgttactccactctgtcccggtaattctcctggcagaccaacctgcgtcttgatgtccaaaacatccc

cgtcgcatgcttctcggatgacctgcttgtcgacaacgccctggatgttgctggcaaacgcctggaaatagagtttcccgtcgcagttctcgtcagccgt

cgctccattgatgccgtccggtaggtagtaggctggacctcccgccttagtgaagaagaagccgggcttggggtggaagtactgactctggaggtgtttg

gaggctccagtagtgaggtccgttacagacagtacgttgcctctggtgtcggagctgtaagccgttaggcaaccatccgggcacgcgtccacccataaat

aggtgtacaatgcgttgcaggctgcgttctggataactgtggaactgatgatctcatctttccgcaggtcgaagataacaatttttggtggacacacgtt

tttgggactcgttgtcaggtccaaagcgccattgtcgagagcatatagtgtatcgtcatccgcccacgttttggtcgttctgctgacgtccaccagctgg

gtgttgcagtcggtgatggagtcctcggtggtgtggtggatttccaggttggggtagggcttcagtagcggactctgctctccgtcgtaggggatggtgc

tcagcgtcaccgggatgccgcggcggacgagcggggtcgtcacgaatatgcggttccgctggtgatcgtccaatgttaggtcgtatcccaaaacctgttt

tgggtcgtagcgtccggtggccttcat

>37104|AGAP007549-PA

tggtggcggctggtgaggctctccacaggccaggccacgcctcgaggcacggaggtcgagtcagagcggcccacagggccggcaaggcgcccccagggac

gaggagcaagggcgcctcccaggagccgctgctggtcaccatcaaggccgccaaagagctcgaattcgacttcggagacgatgggacgttggaaaaggaa

atgaaggccaccggacgctacgacccaaaacaggttttgggatacgacctaacattggacgatcaccagcggaaccgcatattcgtgacgaccccgctcg

tccgccgcggcatcccggtgacgctgagcaccatcccctacgacggagagcagagtccgctactgaagccctaccccaacctggaaatccaccacaccac

cgaggactccatcaccgactgcaacacccagctggtggacgtcagcagaacgaccaaaacgtgggcggatgacgatacactatatgctctcgacaatggc

gctttggacctgacaacgagtcccaaaaacgtgtgtccaccaaaaattgttatcttcgacctgcggaaagatgagatcatcagttccacagttatccaga

acgcagcctgcaacgcattgtacacctatttatgggtggacgcgtgcccggatggttgcctaacggcttacagctccgacaccagaggcaacgtactgtc

tgtaacggacctcactactggagcctccaaacacctccagagtcagtacttccaccccaagcccggcttcttcttcactaaggcgggaggtccagcctac

tacctaccggacggcatcaatggagcgacggctgacgagaactgcgacgggaggtccagcctactacctaccggacggcatcaatggagcgacggctgac

gagaactgcgacgggaaactctatttccaggcgtttgccagcaacatccagggcgttgtcgacaagcaggtcatccgagaagcatgcgacggggatgttt

tggacatcaagacgcaggttggtctgccaggagaattaccgggacagagtggagtaacgacagtggatccgaagggtcagctgctattcttccctgttct

ggatgaattggccatctactgctgggacacaagcaagcctcacaaccgtaagaacttccacctaatcgttcaagatgatgaacatctacagtttgtcagc

agcatggaaatagacaccaaaacacaaagattatatataatgagcgatcgatttacatcctatctgtcaaataccaccaactattctgaggacaactatc

aaattttgtatattgacataaaggatatagaatgttagttgggcgtaccacagattttgttttttgaagacgaaacaacttggtgtcctggaagatcaga

cgaatgtaactaccactgtatgaaatactgaagcgaattaaatatagaatctgcc

>37123|AGAP007553-PA

tggcggtggcggtggaggtgtcgctgaggcggcgccgggagccggctttggcgcgccgccgcctccgcagcgcccgctgctcgctgcggtccagcgtggg

gccctcggccccggaggacgaggaggagctctcctccgagtcgctggccgtcacgtcgccctcctccgcttcgtcggccggcaggagagccgcctggccg

tcggcggctgcggcgtcctcgtactccaccacctgcacgtcgtagcggaacttgacgcgcgacgcgttgtccgggaaggcggccgagtcccgccgccaga

agaggtgccccgaggcgcgccggtccagcttccacttggggtccatggcgggcagctggccactgccgcggtcgcccccctcgtcgtcgtcgctgttcga

cgccgggtcacgtggtccgcgcgccacggctcgcgaccgccgctccgtcagcgccgcggaaggcattttaatccagcgctcgcggccagcggacgcctag

cgttccacggtgggtatgatacggcgctatcggcgctcgcaactgctgacacgccgtgccgccgccgcagtctttgcacttcagactttctgctggcagc

gggcgaccaaacaagcgccgagctgagcccgcagtcggtgtgggag

>37148|AGAP007562-PA

gctggctgcagttgcttctcctgctctggtgactcctcacacaatccttggtgcagtatgaaaacaacaaagcaacactggtcataacagaagcttttcc

aaaagatgctggtatttatgtagtgacggccaagaatatagcaggtgaggcatcaagttcttgcaatgtatctgtgaaaggacgactaccaactgaaaca

tcagactctgagcttgcaagtgacatggaaccaactaagccatccatccaggtgcccctgaaagacattattgtatttgaaggaaaacgagcaagactgg

actgtgtgatagtgggacaaccagaaccagaggtgatatggtatcatgatgaccgaccagtaaaagaatcaacagatattcagctactcttccaaggtga

tcgctgttcattaatcattcaggaggcctatttggatgatgctggggagtataaagtagtggcaatcaactctgcaggagaagctaacagcaaatgcagt

ctttctgtcaaatctctggctgatacagaaccaaaaattgaaccaaagccaccagaggtgacagaagaagcttcaggcaaagctccaaaattcactaagc

tactcgcagatgtcatggtcagtgagacagaatctgca

>37175|AGAP007563-PA

gccggcatcccgcgatgtgcagttctttataagcaaacgatgaatacggccctcttgcgccaccactcggtggtccattccactgagctcatgattctcg

tggaatggaccaccgag

>37174|AGAP007563-PA

gctgtttctgatgtgatttcaacttccaatatagcactttccctttctttcacttcgaaatcttgtgtctgtcagttgttcaccatctttgtgcgatgtt

acatctgctgtttctgatgtgatttcaacttccaatatagcactttccctttctttcactt

>37171|AGAP007563-PA

cctgatgttgcagcctgtcccttgctatttgtggctttacagctgtaccctccagaatcctctgcataagcatgaataatatccagagccacatagccaa

aatcataagtgtatttgtagcgagcaccagtgggtaatggcttgccatttatgaaccattcgatattgagtgttgga

>37212|AGAP007563-PC

gtgaacttggatataaactctgtaattagtgaagattccggagtctatatgtgtaaagccataaataaagctgggga

>37209|AGAP007563-PC

acattccagttcaacgctgatgtcggcacggccccatttgtttgttgctcttcaagtataagtgccagaatcttcaattttagttcctaatatctc

>37210|AGAP007563-PC

cccagattcacagacagtcatcatgcatatgtgagctgcatttgtacgaaagtatgtgtgtatgttgtatatttcaggggaatgccttgtggctgaaagc

ttgcatgtttagtagtctttttgttgtgcccgtcagcaactcaacatctgtccttttcataatattgtcattgttccagcctggattttccattgtttga

ccctttatatgcatcttttcaagaccacaatttcctatatttttcagtgggtatttaaagtgtgaataaagtaagttaacatgtattcctgtttgcatta

agtatatatttcctgatataaagtgatagacacaactcaagtaattgaagaaaattatgctattaactgactgaaaaaaaatttttgtagctaaatctgg

cctctacttggatactcttgatgaaaaccgcttgaagaaaataaaagacctggaaacatttgagaggcctcaaaaaccagaagttgaacctgaaatgcaa

aagcctgtgttcattactccacttgtcaggtacacaaaattaacaccagtaacaactgtgcataatttgaagtattagaaatacttagaaattaattaaa

atgtgaaattttctattaatgcctggtacacagtatttacataacaatttttcctcccattttcagcttagaaaatctgaaggaaggtgaacatgctcat

ttggaatgtcgtgtagaacctataaatgatcctaatctcaagatcgaatggtttgtgaatggaattgccata

>37231|AGAP007571-PA

gctgtccagtgaagttacatcactttcgcttttttgtaaattatcgctgggcttgcttttaaaaaatttattttctacctcttccaacaattcatcaata

tcgtctgccatcgtaacaacaacgaaatgttgtatgtaaaccagtcgtggaaaattgtgtacaaaagaggaagccgagtgttaacacacgacttggttac

tatggaaacagttaactatgtttataaacagagtgtgtgtgagagagagagagagagaacaataagttcattttaagcattttggttagaactaaagtat

gttcgaagacggatgtgacgctgcggagctatcaaacgtttcagaaaacgtgctatacttcagtattgggcgattgtagcgtctcaacaaatgagactcc

agattgcgattacattttcgatacaattcgattttatatcgcaaatcgtagagtggatgtcaacacagactgctcaagcgaagtgaatggaaacataaac

aagaatggatgttctcagaccggagctcacatgggtgaacggaaaatgctatcgtgtgaatttcgttgatccatcagtaacaaggggtggaggggacgat

tttgagacgtatggtgcggacagttacagatatgaagttgtttacagattctgaatgtacagttgatgatccatgcgatgaatttgaaattgaaacaaca

gatggtggcaggtttaggacatcattccgagtaccaagggtgtactttccctatgtcattggctccaaaggagctactagaaaacgtctcgagactgaaa

caaaaacaaacatatgggtgccaaaacaaggaatggatggtgatattgtgataac

>37230|AGAP007571-PA

gctgtccagtgaagttacatcactttcgcttttttgtaaattatcgctgggcttgcttttaaaaaatttattttctacctcttccaacaattcatcaata

tcgtctgccatcgtaacaacaacgaaatgttgtatgtaaaccagtcgtggaaaattgtgtacaaaagaggaagccgagtgttaacacacgacttggttac

tatggaaacagttaactatgtttataaacagagtgtgtgtgagagagagagagagagaacaataagttcattttaagcattttggttagaactaaagtat

gttcgaagacggatgtgacgctgcggagctatcaaacgtttcagaaaacgtgctatacttcagtattgggcgattgtagcgtctcaacaaatgagactcc

agattgcgattacattttcgatacaattcgattttatatcgcaaatcgtagagtggatgtcaacacagactgctcaagcgaagtgaatggaaacataaac

aagaatggatgttctcagaccggagctcacatgggtgaacggaaaatgctatcgtgtgaatttcgttgatccatcagtaacaaggggtggaggggacgat

tttgagacgtatggtgcggacagttacagatatgaagttgtttacagattctgaatgtacagttgatgatccatgcgatgaatttgaaattgaaacaaca

gatggtggcaggtttaggacatcattccgagtaccaagggtgtactttccctatgtcattggctccaaaggagctactagaaaacgtctcgagactgaaa

caaaaacaaacatatgggtgccaaaacaaggaatggatggtgatattgtgataacaggaagtgacaaaaaaggcgttgcagcagccaggagaagaattaa

ccttattgttatgtcagcacgacagaagcagccatttacacatttcatctccattccacttacagggaattcaattatagaaggctttaccaagtttagg

gaagaagttttggaatcttgtagaaaccgcggcatagatgcatcaattttccaagtaccagagaagctacatttaactatagggacattagttcttgcag

ataatgaagaacggaaaaaagctggtgaagtgctaaaggaatgcaaagacattgttatagacccacttcttggaaagcgtcctttgaaattacgtatgac

tggagttgagtacatgaatgatgatccagcagaggtggatgttctgtatggtaaggttcgagttgaagggagttctgaatacctgctgcaggagcttgca

gatggtattgtagaatacttcgcaaacagaggtctcatgcaaaagcaatacgaacaagtcaaattgcatgcaactgttatgaacacattatttcgtgagg

acaaaggaggagtgtctgaaactgaaacatcaaaaaagaaacaaaaaccaagagaatcatttgatgcaacagcagtactgaagatatttgagaattttca

cttcggtgaacagctggtggaaacacttcatctttctcagagatattctactgcgaaggatggttattatcaatcctcggcctccattagtctgactgag

tgaagtatgctcactaaattgctaaattgttcctgaatgttatttctccttgaatgaattacacattacaattatcttccatttttgatgtttttatttt

tataagttgacaatttgactgtcaatgaaatgatatttgttattagttaagaatgctcctagatacaccccatacatttcaatacatgtaatgctgctct

ggcactaacttaagagatattaattagcagttacagaggaagctctttgtgatgcaaattttcacaaaatggattatgaacttgtgtgtgtatactggtt

ctaatgcttacattgttgatgggaatgtctccacatggaaa

>37258|AGAP007573-PA

gtactaagtgttgaaatcgtggaacgtgtctggctacgctccagctgtccactcaattcagcctgcattggtcgaacgctggccacaggttttggcacag

taccccctgttgcctcagtccggattgctcttatttgaagacctgtttgtaaatttgttgagctgctaccaccagatccctggtaatatgagtactgagg

tctgctataatttatgttggcctgtttttctaattcatagcttcgctttgccaatcgcttttcagcctgggaaagtttcttctcctttcgatcaaccaac

agtgattcatgctggaaaggctccttcgacagtacgttgctatgtttgtccaaaattttttgcatcactacatcaatatatttgtctttctctttgctaa

aatcttttacatctccatcttgttcattgtcccagcacaagttagtcacttctttaattgataaatgggcatcaggattacattcatcaactacacgatc

tgccataccttgtttgtttatctggcgatcgtatatttttttctcaagacagttatccataacaagacgataaacaaaacatggcttcttttgcccatat

ctgtacacacgacaaacagcctgtgtgtcgtgacatggattccacgatgcatcaaacactacaaccctattggctccaacaaggttgatacctaaggaac

cagctcttgtggaaaccagaaataagtgtatgtttggattcgaattatattcatttattagtttttctctttccaaagctgttgtgctaccatccagtct

gtagtagttccagttccttgcccaagtttcctgccgtccaggaagatttgttcgttgaaggaagtcttcaattaggtttaaagtaaataatgactgactg

aacactaaaatacgatcaccaagtgccattgattcttcaagaatgcaaaa

>37280|AGAP007574-PA

agcatcactctctgttttctcccattagggaaaccaagcaacgggtcaaatgaaggctctttcattgggtggggggtaccctgatgacct

>37284|AGAP007574-PA

ctctttccctacacgacgctcttccgatctctcccattagggaaaccaagcaacggatcaaatgaaggctctttcatgggctgaggagtaccctgaggat

ctgacacatacaaagcccacgcattt

>37282|AGAP007574-PA

ctcatctattaatcaagtaccaggcttgcagaggagttgtttggccatgggcttacaaatgtgcaaatgaaaagcatgcgtacatgacctgtgagtttga

ggatgaaggcttaacagtctcagtttcaaaaggatgcttgtctacgcatttggcagaagtaggtggagca

>37283|AGAP007574-PA

atgggagaaaacagagagcaatgactccagctaaagaagagctgatatcgacctaaattacaccagaaaatcgcgattgtcttcatttattaatcaggaa

ccaggcatgtaaaggaagttccactgtgggcttacaagtgtgcacataagaaacgtgcatacctgatctgcgaatacaaggactatgttttccacatgga

gaagtatcaacgtgagaggctgttgctgcaacaacggaggcctactgttgctaagcctcacaaaaataaagttatacattttcagtatctcacatgagtc

gcacttttattttgctccacaaatgcatacatgaaacgtcgattttttaaaaaaacaatctgcgaaaccgg

>37281|AGAP007574-PA

tcgtttggcattgaacgctatttaattgtgtagctgtcttatctcattctgcgatggatatattactttgcagtttagtcgtatgtagtgggaaatccag

gtactcctgtttgtggctgtgtaaagggagtgatgcttgccactgaagaagagatgatatcggccaagattccaccagaaaatcgtgattactgtgctca

tctattaatcaagtaccaggcttgcagagcagatgtttggccatgggcttacaaatgtgcacatgaaaagcatgcgtacatgacctgtgagtttgaggac

tatgttcttcgcatgaaagagtatgaacgagagaggcgactgctgcaacgacagaagcgcattgctgctaagtcccacaaagaggaattagaggaatgaa

gttattcatagtttggtgcctcacatgagtcgtatttgtattttgttgcatatgtgtatataaaacttgtttactttaaaagaaagtattttaaattttc

attctcc

>37277|AGAP007574-PA

tttaaatttctgctcaatgaggcaggaattcttttaccagctgaaatgtctcccaaatggctgtgtgtagcacatgggcaacgtggagtctgcccatagt

gtagcaaactaaattttatacaaaaaatgttcatacatttgaataatagtattgtatgtgtgcaaccataatggtaggttattacagttataagttaatt

acttttgttatagttttcaattatatctatggagaatgaaaatttaaaatactttcttttaaagtaaacaagttttatatacacatatgcaacaaaatac

aaatacgactcatgtgaggcaccaaactatgaataacttcattcctctaattcctctttgtgggacttagcagcaatgcgcttctgtcgttgcagcagtc

gcctctctcgttcatactctttcatgcgaagaacatagtcctcaaactcacaggtcatgtacgcatgcttttcatgtgcacatttgtaagcccatggcca

aacatctgctctgcaagcctggtacttgattaatagatgagcacagtaatcacgattttctggtggaatcttggccgatatcatctcttcttcagtggca

agcatcgctctctgttttctcccattagggaaaccaagcaacggatcaaatgaaggctctttcattggctgaggagtcccctggggatctg

>37278|AGAP007574-PA

catacctatgagctatgtgggccaaccagcttatggggaaaagaaaagaaagtgtaaacaagtgtttttgattcaacaaatatatctaaaagtctacatt

acacaggtttttaacattttatggcttaacaagtcaccattcatcttccaaaatattaaaaataaaccatacaccccggcctggccccctcccacataat

ctaacggatgggcgcaattgtctaatactaaatttgttaatgtcatagttaaaactgcgaaaggtcagtgacaaatttcaaccaggagcaaaagtttcat

atgactcaagtagaggataacaatgattataatgtgaaatctcacacaggactctgtctgtcacaattagaacttctcaaaatagcgcttatcgctacac

ttttagggtgaaaaccttgactacctttgccttcgtgttttggagtaacattgaagtaaataattgttttacggtcaacatatttgtcttaaaaccatat

ataattttataccaaaattagagagcccatacctccctttacacagccacaaacaggagtacctggatttcccactacatacgactaaactgcaaagtaa

tatatccatcgcagaatgagataagacagctacacaattaaatagcgttcaatgccaaacgaaaataatttccatttattttttattctatttgaaacaa

cccatcatcagatgtcatcactgttgttaaatgtacaattcaatgggagcttgagcgatacgttattacctctctgttttctcccattagggaaaccaag

caacggatcaaatgaaggctctttcattggctgaggagtaccctgaggatctgacacatacaaagcccacgcatttcccatggtgtcaacactaatgtta

ttacaatctttaatt

>37311|AGAP007585-PA

gtcacctctagaccaatgcttccattcaatgctatcttaatggggttgctatggttggaagcataggagaaacctaggtcttctaggctgctttttcgtc

ttgt

>37310|AGAP007585-PA

gattaagtagtatcgtatcctcataaaattcaggtaattctgctggtcgcacaccaacaagtgctactccatattctctttctgaccttgctttgattct

gaatttccacagctatcagaagatgtagtgtgatccttgtcatctgttgctgtagttccactttctgcacacagctgattctgctggttcccaagaataa

aggcagagttctcttcctttgaaatgctcatgtagtaacatgtgtcactcttcttcattatatgtctcgggccaggattaagtagtatcgta

>37351|AGAP007588-PA

ctttatgaaactgaaaaaaatttcagcctgacgctgataaacaatcacttcagtataaactaccctcgaccgaacctgccaaacatgatagaactgactg

gactgcatgtggaagccaacccaccacctctacctaaggacatacaagactggctggatggagcag

>37350|AGAP007588-PA

attgaggaaggactgtatctcctgcggtggcggcttcggctttcctttaatgtggagtccagtcatttgaaccacggtcggttgcatcggctttggatag

gaggaggagaagtgagtagttgcgagcaagagactaatattgtactgcgattcaaagatgctgggaacatcgtgtccgaaatgttc

>37382|AGAP007599-PA

gcaactccgaagtgtgtagagcaatccatcgcaatgtacgcagtgataccacagtctcgtacgtcgctaacttgcagcttcagatgatctttggtattgt

aatgtattggactattggatgatgataatgtgttttgggtgtgaacaaagtgttagggaggatgtgttgtgatgtagattgggtatttccagcattaagg

aaaccttctcgtctgtggaatgttgctagtacggtcacactttctgctgttggaatgtttagtaaattaattacagaatggctgaacaccacaaaggtct

acaaccgtgacatcttgattcacgcactagagaacagaccacccactgtaccactgataacagtttcaaatcaccattcctgctttgatgatccaggctt

atggggttgtttaagcttgaagcatttactgaatcgtgaaatcatgagatggtccttggcggcacacgacatatgcttcacaaatatgtttcattcgtac

tttttttatgttgggaaaatgtgtacctgtagttagagggaaaggtgtatatcagcaagcattagatttttgtctggagcagctgtcaaagggcaactgg

attcatgtgtttccagaaggacgtgttaatatggccaaagaatacatgagattgaaatggggtattgggcgtctgatatatgaatgcccaaaacttccac

ttgttattccgatgtgccatattggaatggataacatattgccaaacactccaccctacattttacgaatgggaaagaaagtgactttaaattttggtaa

acctattgatttcagaaatatgctgcatggtctcagagaaagtaatgtccctccacaggaggcaagaaaagttatcactgacaaaatagaaaacacactg

atgctgctgaaggcagaaactgaaagacttcatcttattaccctgcaatcttgacattaaactgtttgttgcattattcttcaaaatgttggaggttttg

gtgatgtgggcattaacttgctgagcatgagactgcaaagtctaatattgaaggaaaaagtcttacatttatatttttgctagtcattttttaaattatt

tttatagttctctgcaaattatttacaaaaggaacttctgaagactataaaagcacaattcttgttattgatgtttgtaaattccatccattgtaatata

taggcctacaggggactcaaagtattatgtgagttttagttgccacagtggttatgttgtgtgcactcatctcgtcctgttaagagctgtaagccctgag

taattttacagtattggtggtctaaaccttcatgctttaaaacaagaagagaaagttaaaagtaagttaaaattcacaaaaaggtttgttattcatttgt

gtgaaatagaacatgctgtagataagtgcagagtaagggaaatgctgcccccttcatcctttcccccccccccctctctctctttccccccccctctctc

tctcttcc

>37397|AGAP007606-PA

tttcacgatcagcaaggaaaagtgtttccttttcttcagctactgcaagtgcgtggggcaaactgaaagtatatgctgtcaagcctatgccatccaaaga

accttgcagtatattacggccccattccataagtttttgcccactggtatcgaatttaataattcggctgttacaataaccatcagaaacaaaaaagtct

ccattggacaatatagcaacatctgtaggcttgcaaaaatgtgtatcatcatttcctggcacaaattttgtgccaagaattaaagctggtttcttccatt

gattccttttgaacataaatacttgatgcagagctacgtctgttacccatacattattctcagaatccactgttaggccatgtggaagataaaatatgtt

gtttccccattcgtacagaacatgaccagtcgttgcgttgaaaacaatgactgctgactgctttattgggcctagagaaatttcactgaatgtgttgtca

agcaggaatgtctgtgaaccccatgtccgatctcctctatggaagacaacaatattgccatctgtgtcaatgctcacagcagaaagctgccccacgtgtt

tacttggatctggccagtcaggcactaatttgtatagactttttgctgttgaagaatcactcagtgatgagtgtgccaaggtgttctgagactctgatcc

ttgcagctgtgaatcaagtattttctgtgggtagggagaatgtcgccatggtgatcctctttcaggggaagtatgatggttcccaagttctgtataatca

ccgtactgttctttctctgcctgtgtatctgacagttcattctctacaccagagtcatactgtgaaaccactgcatccagggatgtcaacagtacagcag

taaatagaaatatgtaattcatcattcctgtctcctgcaatacagcttctccccacaaagattttgtggtatattgctaatcagaactacattttctacg

acgtggcattcgcgtttagaatccctggatttataaactcaattacgttagtgtaac

>37420|AGAP007621-PC

gtgctgtacttgagcagattgcgaaaacgcagataggaggaagagagtaatattacttcccttttcgttaagaaacaagaatgtcgtcgacaggaagaat

tacgcagcttgtgaggaacttcatgacttccactgcaaggagaggtggccatgcccatgatgaaggaggtgtgcctggaagaaatctaccgttcagcctg

tcaaacccatacaaattgaccttattgtatatattatccttcggttctggctttgcagcaccctttttggttgtaaggtatcaactactgaagaagtaga

aacatttcag

>37419|AGAP007621-PC

ggaaaaacccgtaaggcaggtttctatagactagcatgtagcgtcgtccatatacctgcgttaacatgttagaacttctgtgttttttcggcagtgtata

gtactccataattacagttttatgtgcatagggagcatacagacaactgccaaaacaagaatgtcgtcgacaggaagaattacgcagcttgtgaggaact

tcatgacttccactgcaaggagaggtggccatgcccatgatgaaggtggtgtgcctggaagaaatctaccgttcagcctgtcaaacccatacaaattgac

cttattgtatatattattcttcggttctggctttgcagcaccctttttggttgtaaggtatcaactactgaagaagtagtaacatttcagggatgctaat

tagttgcttacagtatgtttggta

>37443|AGAP007633-PE

gttgtgtgtatggggtatttgtcagcagccacatcaaagcccttctcccattcgaaaatagcatgatatcaccaggaagttttgttggtccactgggtgt

gcttaacagagctatggttatcatcatatttctctttattgtaattggattatttggctacatgaaatatggaaatgaagcacaaggaagcataacactt

aatctgccagctgatgaaataacactcagctcttagtcattcagcacaatggaccaagacttgcaggcagtggattaataaccagggacatcaaggaata

agcaacagtcttcagttcacagcatctttgtctgcaatcttgtgatgctagattctgcagcaatgtgacagatcagtaggcaaaagaattccttatcttg

tacaatctttgaagttattgtatttcatttatacgttggcaatatgaatcttttgcactcatttccatattcatgggcttcttaccataataggatgaaa

gaaatccttttttgtaagaccctgtattgtgacattactgtgtagtgtaataagtacactggtaatgaaaaatccaatgtcatctctgaagtctgtgtca

aaggttcagtgaaatgcctgatattgtaatctaaatgagagcaatttgaagacttaaaatattatgcag

>37445|AGAP007633-PE

gccatgaaatcatacacattaaaaagatatatttgacatattaaaaagccacatgcaattaaatcactccttccaacaacacactgacatatttatttca

atttacacacacagacacacacacacacacacacacacacacacacacacaaatatttatgtgcttagtacaccaccattctcatttattaaattaatcc

aggatgaaaaatcctgggaacttcattaaaactgcatatccaatgcagtttgcttgaatctttctgtgtccttttatttccaccaatactcaatgtttca

tcaaacatcacagttccaccaaataaacactgagtctttacagttccaccaaaacacattccatccttacagtctcattggataagtaccaaggactgta

atatagatcctggagatgcatgtaacttttgaaattacatcatgacagcatcttttaaatggttaaagtcagtcttcaggtttaacaacatctataattt

ctcagtacttgtatatagttcaatgatttcttccaggcttgtataggtaccaaacaccagtgccattaatccaaacacactgaccagcgcatttttccaa

agtcgccatctgaatggtcctagtccattatcccagtatgtgacgacttcaataaatgctggtattatgagaccaagcaaggagaagcatagagaaccaa

taagagaaatgaaaggacctattgtgggcactgcaaccgccagaacaactgatgcaataacaaggattgtcctcacagtatatgaaactaggataggtct

gttaggaaacttgtctttcacagcatttagggacacttcaagtggcacgacgaactgcaggccatacgtgcaataaactccgagggcaattagaattttt

acagactgagcagctatttcctccacgggtaagttgagtgtgatgctgcctttagtatcatcaccgtatttgagatatccaaggaacccaatcagtgtat

aaatcaaacaaattgcagtcatgccctggttaagaacaccacacaatccaagaaagtgcctaggagtcttcatgttgttctcaagtggcattacaacccc

gactgcttccatggcaaaaatgacaatgctgaagaactgaggcaagccataagggtggccaaagtgcacacgatcccaaatagatggaaggtcctggacc

agataatacactgtaatacccagaccagcacccatgaataaattggcaatcatggatattggtgcaagccatttcagattgggtacaaagccaagcagga

gaagtggtggcagtgttcctcctatgtaccagcgttggtctattgttgaatttgtgtagtgctccactacctgttgaatgttgttggccacaatgactgt

gtacacactgcaggtgccgaagtatgttgcaaacaaacctataatgatggtgagcctagctgtgcggctgaacttcttggcccactcaggaccattggcg

aaggcaacctcaccaacatctgggaagctcatggcactgactcgggtgcggacatacaactcatgtgcacacttgacaag

>37444|AGAP007633-PE

cgtcgtgccacttgaagtgtccctaaatgctgtgaaagacaagtttcctaacagacctatcctagtttcatatactgtgaggacaatccttgttattgca

tcagttgttctggcggttgcagtgcccacaataggtcctttcatttctcttattggttctctatgcttctccttgcttggtctcataataccagcattta

ttgaagtcgtcacatactgggataatggactaggaccattcagatggcgactttggaaaaatgcgctggtcagtgtgtttggattaatggcactggtgtt

tggtacctatacaagcctggaagaaatcattgaactatatacaagtactgagaaattatagatgttgttaaacctgaagactgactttaaccatttaaag

gatgctgtcatgatgtaatttcaaaagttacatgcatctccaggatctatattacaatccttggtacttatccaatgagactgtaaggatggaatgtgtt

tggtggaactgtaaagactcagtgtttatttggtggaactgtgatgtttgatgaaacattgagtattggtggaaataaaaggacacagaaagattcaagc

aaactgcattggatatgcagttttaatgaagttcccaggatttttcatcctggattaatttaataaatgagaatggtggtgtactaagcacataaatatt

tgtgtgtgtgtgtgtgtgtgtgtgtgtgtgtgtgtgtctgtgtgtgtgtgtgtgtgttacgctgtgtaaatggaattgagtgacaagttatttgcgataa

tttagttaaaggggaaggaatattgtgacagggaaagactagggctctatttcaatgatcatttgacaccagttactctatttgttcttttttttctgtg

aaactaatgcttccatgttcaataaacagacagatgaattgcagattttggtatcagacaatatatatataaataaaacttctatggcacatatattttt

caacattattgtatatgggcaataaatgaaaacttgcacgacttgaaatataatgccatattttgttacataactgctctttatcaagcttttatctagg

tattgacttatactgtttgcttgaagtttaattttgaacctttcagcatattcacggtaaagttatgcaatgtttttaatagcttttacttatgcacatc

gtagaatgggtatttaatgcagtttatctgcacaatatggccttataatccatggtgaatgacattagtaaaaaaaattctaaagaaactttataacctt

gtgccagatttgttgatgctaataactttgtagttataaacagttgaaagagatttgaatttagttaataaacagtgttgcaaatgaacaagctatgata

ttaagttctcaagtattactaatgtggcagtacagctttacggttgtatcttggaagtatttcacataacagtgtgaaagcaatgactgcatctgttcca

gtgaattttgtgaaatgtactgcaaaaattactgagtaacagactgaaatatggaaaaaatcttattttagtttaatgtgttataaaatataaagtaact

tattttttaaagatactggaaaatgctgttgagtctaaatttttccttgtgaggatgtcacggtgccctaccttttctctcctaccatttggtcatgata

tgagtcccttctttgttttaaaagaacctgctcagtagtttttaccagagaacatctgtatatatata

>37511|AGAP007643-PD

gtagactgaagactgtgtttgcacgtgtagatgtgtgaatgtttccttataataataatataccgatttgaccaaaatcgaagggtaaaggatcaggatt

tgggaaggactggaatggaagcaggtacgaagataggaaggatttcaatacagttacttggaacagggatttgacggttctcgcggagggccagatcagt

tatcacccccttcttgaggttcatccccatccccttgcgtgtccgaggtccacagagtgaggttgtccctcagcagctgcattatgagggtagagtcttt

gtagctgtcttcgtttagcgtatccagttcagctatggcatcgtcgaacgcctgttttgcaagatgacacgctctcgctggagaattgataatttcataa

tagaatacggagaaatttagtgctaagcccaaacgtattggatgggttggctgcatttttgactttgcaatatcaaatgcttcttga

>37531|AGAP007666-PA

cttagacgccaacaggtacgctacgagggcgcacgtcgctgtcagccggctcgtgtttcagaacgctccaggcatatttctgtgctccagggccattcgc

agctctttctttgaattgcttgtacggggatgtggaaccgtccgcagtcagccaactcccgccaagaggcggacatggtgaacaaagcgcggcggcagtt

gtcggccgcaaaggcccccgtggacaagctgcgcctactctgcctctccaggggcaccacgggaatcctcggcctcggcagaatatttcgtcgcatggat

gacgatggcagtaagacgctgaacctggaagaatttacagagggcatgaatgataccggtatggagctgtcaaaagaagaggtcgaagaactcttcaagc

gattcgacagggatggcaacggtagcatcaacatggacgagttccttatcgccatacggcctccaatgtcagcatctcgcctcaaagtaattgatgaggc

atttaagaaattggatcgcactggagatggtgtaatcactttggaagatctgaaaaatgtctacagtgtgagaaacaacccccgttatattagtggtgag

gagactgaggaacagattctcacaaagtttctcaacaactttgagaccgatgatggttctcgagatggcaaagtaaccaaggaagaattcattaattact

atgcaggaataagtgcatctattgacagtgatgcatattttgatttaatgatgcgtcagtcttttaaactgtgaactgaacgtttaacttcacaaaaatt

aaaccaatgtatgtttatgttatcccacaaagtaagtgtgtcaaactttcctctcatcaatttaaagagaaccaaatttattaaattttacatctactat

gtttaacagataaagaatttttcaataataaacattgtatccataataattaaacttcaaaagtttctcaacaatgtgtgatttgttgagtttacttcat

atcctgcatttactgaaaatacacaaatttttctttgtcttatttgaacgttttggtacttttatgcacaaaattgagatactttgatgttgttggcagt

tatgcggtgaaaagaaagatgaaaaatgttaaataaaagtttttctgtggcatggttaataagatgtacctcagggtagctacaatcatttctcaggttg

ccttgcaaattgtaaaacagccatttttgctgttatcatatcatctcactgcactgctctacaaaagaataagttgctaagaaaacggaataaaatttga

ttcacacacatcttcaaaatatgagttactacttaatctgtgtgcagtgtgccatgattaatagtggatatcttttgtagtaagctctgttacgtattta

tcacagttgttaaagtcatcagcacttgcttcattctctcatacctttggcaaaagtgactacgagctacatccattcttcagagttctcatgttaaata

tatgacagtgaatgggggtgacagtgaaaacttgcagaatggtgcaataaactgacaaagatgaaaacaagtttaaacaggcagaccatgagtggtaata

ctatcaagtgatacggttactcttaaaggtacatttaataaatctgtgaaccagttatcaggacaatgtttattaaaatactgctaattacaggatattg

taataatcataatttcaagattcttaatcacacacaattttggcattatgtgtggactgagatttatgacactgccttgctattttctattaagtctggt

atagagttatggatgatgacaaagtggaaataaatatgatcattcctgtgtcttaagattttactttgatggatgtgtaactttatatgaaactaatgta

cagagtttctgcaactgtagcagtaaaccaagagttactatttgttgatttcctactatgagtgttatcaaaatgttacttacactagcttaattaggtt

aactttgttgtcagctattttgtaactgaaaattgcaaatgaatagaaagtttctgataatatattaatgtcctatttattgtgcttgtttgttgtgctt

gttatatatgtgttgtaatttattacatgaaaataaaaatagttcagtaataaataggcatgttgaagtatgccaga

>37553|AGAP007670-PB

cgaatgtttttgagtgagcgtgaatgtgttgcgtgtagttgtaaacatttgtgtatagtgtaaatctagcgatcatggtgaaattaacgccggacttgat

ttatcaagctatgcagtacattaaccctgtaagagatcgcgaattggatttaagaggttacaagataccagtcatcgaaaacatgggagcaactttagat

cagtttgactgtatagatttttcagacaatgacatcagaaagttagatggatttccacttttgaaacgtctgaaatggctgttactaaataacaatagaa

ttgttcgtatatctgaaggtttggaaaagtccttaccaagtttggagacgcttgtattaaccggaaatcaaatacaggaactggcggacttggatccact

ggcaagcatcaaatcactaagaactctaagccttctgcataatccagtgactacgcgacagcattatcgtgcgtatgttattttcagaataccacattta

cgtatactggattttcgaaaaatcaaaatgaaagagcgagaagatgcagctgctctattcaaaagcaagaaaggcaaggagctgcagaaagagattgcca

aaaaagcaaaaaatgcattccagaattccgcggctgcaggatcttcagaggagcaaaagcctacaggacctacagcagaagagctgtggaagattagaga

agctatttcgaaggccagttcattagaggaagtggaaaggctcaacagactacttcaagctggacaaatacctgggagagcacatcctccaggagtttcg

caaatggacgaagagatggaagaagaggagtcgtcacacaaccgcccgatgatgaatgggaactgaagtgacattggttttgtgagttctgttgctgaaa

actatcaatatctttaagaaccgtttattcttatgatatttctgaatttgagtcgtctccctgtttaatatattaatccattttgtcggcagaaaacatt

tatgtaaattgtcttaaaatgtatctgaagggtatcacctttccttttattaaattctgattgaggaaatggatatctagagacttatattctaatgtga

aacattgacacacatgatttgtgctatttgtaagctcctattttcatttatattacattggaagtttaatttatgtaattatgtgaaatattcactacat

tctcttgaagtacaaacagagagatagctcctaataaagttttgttataaaatgcgactacttacaccgcttctgacttatttatgatgatattgggcag

attatatctcataatttatttgatttttgggtaaaagttaaacattggaccccccttgcatatttcaatatggtgtggatgcaaagattatgctttgcta

ccagtcagtttgttaccacaatcagattttctgcttcctcacaaagatgtggagcagcactacagaccactctgtgattagaatcaagatttcgagatgg

gactgatacataacttaaggtgatgttttgggatttaagttgtctttcccatttggcagcaagtaatgtgtcaagtgtagaattaaatgagtttcttaaa

tgttcactgtgtgtgcag

>37635|AGAP007675-PA

ttcttgcagatatgccatccatatacagtttaggatccttaggtgctgtgctgttaattgcaaacacatggactactggcatactgacaaacaactgttt

tgggcgagactcaactagttttacagctcttttgttccagccacaaccttcaagaaatagaccatgcacatatacaccctcctgtaaaataacgatacaa

aaaagagaaatgagatctagtataatggggg

>37634|AGAP007675-PA

gtagcttttgtaacatgtcaattgcctggcgagcaacagcagcttctcgtgtttcgcctccagagccaccagattccttgggctgtatacttaaaattgt

gtccagaatcattttagttgtattgctctgatatgtaatgtcagcatttgcatgaaggccatatacttgtgggggatcaatgggaggcatgtcatctatt

gcctgaaagtattcttctaggtttttgaacttcataatgccatagcctttgaaaaattcaaatgagtctgaaaacatgtcctccaaaaaccacactttag

caaatgtgttcagtaatcttttgtcaaaatcatctgtaactcggccaccatactgaacttcaccaatcatgtacctaacagta

>37633|AGAP007675-PA

ctcgtagtacccaatgatccggtggtttgtcactagggagccacaatggagtaatgaatgtcaaatctgttcgatttggctttttgtagatttgccatcc

atatacagtttaggatccttaggtgctgtgctgttaattgcaaacacatggactactggcatactgacaaacaactgttttgggcgagactcaactagtt

ttacagctcttttgttccagccacaaccttcaagaaatagaccatgcacatatacaccctcaactggtggaattcgaacttcttccaatgcaagctttgt

cacctcattgtgtaaggtaacaacgtccaatgcccagcccttatgtgctcttgctacctcctgtctcattgctgtcaggaaaccttggggattaaaaaat

cctgtcatccagaatgatacagggcgtttcacccatatccaggcagaaaactgagcatttctttctagaagctctgtgaaccagaagcctaacgttgaag

aagcccatgaacctcttttccatgtagttggtacacg

>37657|AGAP007682-PA

cagatcttcgtatacgaacgtggaactgagtatcacccatacatcctgaatttgagtcaggaaatgctaaatagcaaatattagatttttcttgctccga

gagatttatgtgccctgggtaaattatctgcaaataaatcataaaacaaaaccgttagtacaaaatagtcactactgtaaatatcaaaataaaaagat

>37658|AGAP007682-PA

tgggcctccttgctaaagactcatgtaactcttcggcacttcgatgcgcaaacttaacacagttgccagtcagactacctctaatgcttgtcccagttcc

aaatcgaatgtaacaacacatatacaatgaatccatcgcgagaatctgtcccagggcaacacagctgcggctggaattgtgcgtccagtgtcatattcct

ctagttgtggaacgtcattatcttttataaaattttcttcagccatgctgcacttttttataaaacacttttcatccactcatcgttcccgatctagatc

gcactcacacacactgtcacatacttcactcaccactaccacggcaacaccagcatatcgcttgtcgttataagtatgctgcgtgaaatacgcctactac

tgttatacgagcatcttccgcggtcaacagttactttcatcgctgcac

>37656|AGAP007682-PA

cttggccaatgaaactttattcttaaaattgcagcacacatcagagctatacactatgtagtcaatatctcagcacacattactattccgcgatcggatg

atgtcctggcggtagtcgacgtccgtccatgtacacaacgttgaatcggtaattaccatacagaatacatcagaaatgcttcctcgtctaccgctgatat

accgtttttatcctttgtactactttctggctaaagccccaaaggcaaatagaaaaatgaaaaacacacgctgattttgggggaacgaggtggacatgca

gttgcgttccatcggatgatccaacgacgtgtgtccaccgacttactgaaaaaatgcattggccacattgtgactcctgaaatatgtgcaactgttgtct

aactccactagaagcatgggaagtcggttcatccttacagcttaacaatgacagtatgtaggtacctccttgagagcggttcaagatgtctacgaaatgt

ggactaccatttaactgacactttagtatcttatacgcgtagatgcgctgacatttgtgaagaactttgtgcaccggtgctcgtcgcatgccgaagttcc

gactggcaacatggaccgacttcttgggggcctccttgctaaagactcatgtaactcttcggcacttcgatgcgcaaacttaacacagttgccagtcaga

ctacctctaatgcttgtcccagttccaaatcgaatgtaacaacacatatacaatgaatccatcgcgagaatctgtcccagggcaacacagctgcggctgg

aattgtgcgtccagtgtcatattcctctagttgtggaacgtcattatcttttataaaattttcttcagccatgctgcacttttttataaaacacttttca

tccactcatcgttcccgatctagatcgcactcacacacactgtcacatacttcactcaccactaccac

>37677|AGAP007688-PA

tcatatttgcaaacacccatatagtgagcttataacatttcctgatcccactgtctgcctctccatctcctttcgcttcttctgatatgcgaatacatac

agggaatgtcgctccttctcgggtacgtcctgtggctttctgcttcttgacatcctttggcatgacctcttcggggcaaggcactttgagagatggtata

aggctggaa

>37678|AGAP007688-PA

ctgcaggttatataactattatcagggtaattattgtacaaggtatttaattaaaaatactttttaagaaattgtaaaaaaatgtattgataggttgaca

agaacagtcacctttcaccaagtcatcttttagacttccaaaagacataaaaggccacttttgctagattgctatactgaaaaagagatcatcaacaatg

taaattaacatcaacatggatgcatctcatgcaaattaaggcttacaaaaatactaaaactcagtaacagtgtatatatattggtggatctgatatgtct

cttgtaaaattcataaatatttgtactagaatgtatccatcaatacagataatctgtagtaaatggaggtcataacaaactaacaagaactgtacattat

atacaatacttattaaagaaatcaacttgatgtagttgtatggtactttcattattcatgcaaaaacacattaagaaagtgatcacaatcaagttgtcac

acatcactttggtgaggtaataacaattaactgtacatcttgaatcagaaatgaatcatactatatcacatcacaatgtacataaaattggtatatcagt

ctagtatttattgtacacaaatttcctttctttgttttctactttttaacatgcataggatactctccaatcccttaaaaataatgttatgattaaaaaa

ttagtaatcaattataggaccataaaaacaacaactttcaagaaaaactatcttgttcatcatagtagtaatctgggtcccagtcatcatcatctgcatg

ctcagcttcaggtacattgtttccaccttcaacagctgcaatattacttgtgacatccactaaattctcttcaaacgtatgtaatactttgctaagtcgc

tccacagatacctctgttatattttgtacacagccaagggattcctgaatttttgctaacctttcattttctgtcactgaagtaataacatcctcttcag

ttccaaaattttcaacagttgtcaggctaccaagtgagtttgagcttccaactgttctatcatcagcattggaatcatcttcatgattggacaacaatgc

agatgatgattctgtaactggaaacctatttgacaatgaatcatttgcggaatcacatctgtattctgaataatatacaggaggatgtgcttcttgagga

tagcaagagacaatgtcttgaaaattgtatcctgtcatgtcaacttccttactgatccaaggatgttctacaagctgtttcacagtacaacgttctcttg

gattcttttccagcatccagtggagaagctccataagctcttctgaaaccgatgaaggtgggtgcaattctgctttcataatttcttcaacgtcaaaaaa

agggttttcaccaaatgtcataacatacagtgttacaccaagagaccacatttccagttctggaccttcatacttattgcctgcaagcacttctggacta

cagtattccactgttccataaaatgtggaaaataaacgtccttcgctcatgaatgttgctgatccaaaatcaattagtttggcatgaaacttttcattta

ttataatattctcatcttttatatcacggtgtagtatttgtaaagaatgaaggtagtcaacggctgaaactatctgcctgaagatgtaactattcagtgt

ctcatcaagatacggttttctgtcaatgaactcgaacagatccatccctgctccatgtttttccataaccaattggaaaaattttgtattttcaaagaca

tctaggactttaactatgtttggatggtttaatgtagtaagcaaactaatttcaagtggaacttttgatcccaggctaggatcttcaacccatgactgtt

gatggactttatgtttctgtatgaattttgtaataactaaaagcccatcttcattgcggaatgccatctttacatatccataagcgcccttaccaatttg

ttgcaatgttgtgtatcgttcaccatattcacctgacgtctgttcatcttcacactgactcaggactgacactgaacctgaacgtgttctcgaatgtgat

atattagtttgggcgcatgacctgattgctctattactagcctgtcccagtgagttgtccacagtactgttgaagcttgatgtcaaagtgaggttgccac

aagttctgcctccttcagctatttcttcaggatctctagaaatccaaacacagtacacaagacgaccgcaaaccagcaccactgctcttacacgatatac

aatatctaaatttgatccatccttatgcctgccatatccaaaataactcccatcaggaaattgctgtccttcaacttgcttcacaacttctgtatgagtg

atgccagcatctcttgagataactcttctcagaaacctctttcctgtagctggggtggatgtcagattatcacacggaatatttgccgggtcaaatattt

ttggacactgctggcacagatttgattctcctcctactgatctggaaacacccgataaatccgcatgtatctccccttctgcattacacctacaaacatt

ttcgatacttatcacattgaaattgttctcagagacggggagagcttcattacttgatacattaagttgtaagctattagctgatttttgtactgctgct

gaataacttatgtgggctggagtattacatgtgccctgacaggagtcaatcaaattagctcctttctgatgaatgcatccttcagttattactgctggca

gaatttcaatactatcttcatcatctgaggcagtctgaaaattgtcttcttcctccatgaaatccatatgtggtgacacaattggagttaactctccatt

atcactattgttttctgcatctgtcacagcattttgatcccttgcaggtaaacccagggtacatggaggcacaactttttcttttctcattgcttcatta

aggactaaatttgatccaatgtccgaaatagactcattaacagggaaaatgctgtggtcatccacagttacagtggatggtctggcttctccacatatgt

cattcactagtcccaaattaagcctatttaaggaatctatgacttctggcatatcagtgctactgttgccattgataatgtctttgttttcattaattga

aaaaatattatcacagcttgcaagattttttaattctagcttctccctattgctgcagtctttagaacagacagcggaacagtctgattcataaactgca

ctgctactgcatgatctatctggatccacattttcaacagcagaatcatagttgctgttgtcatcatcaccatctttcgcaccatccagatgttgcaaag

ggcctggatattccaaatcttcacaaaaatttggaatgatgttagttattttctgtccaattagctcagactgtgggtacccgaacatcatgtgagtaaa

atgatggttacagctctccactaccccatcttctgccaatacaactaagc

>37676|AGAP007688-PA

ctgcaggttatataactattatcagggtaattattgtacaaggtatttaattaaaaatactttttaagaaattgtaaaaaaatgtattgataggttgaca

agaacagtcacctttcaccaagtcatcttttagacttccaaaagacataaaaggccacttttgctagattgctatactgaaaaagagatcatcaacaatg

taaattaacatcaacatggatgcatctcatgcaaattaaggcttacaaaaatactaaaactcagtaacagtgtatatatattggtggatctgatatgtct

cttgtaaaattcataaatatttgtactagaatgtatccatcaatacagataatctgtagtaaatggaggtcataacaaactaacaagaactgtacattat

atacaatacttattaaagaaatcaacttgatgtagttgtatggtactttcattattcatgcaaaaacacattaagaaagtgatcacaatcaagttgtcac

acatcactttggtgaggtaataacaattaactgtacatcttgaatcagaaatgaatcatactatatcacatcacaatgtacataaaattggtatatcagt

ctagtatttattgtacacaaatttcctttctttgttttctactttttaacatgcataggatactctccaatcccttaaaaataatgttatgattaaaaaa

ttagtaatcaattataggaccataaaaacaacaactttcaagaaaaactatcttgttcatcatagtagtaatctgggtcccagtcatcatcatctgcatg

ctcagcttcaggtacattgtttccaccttcaacagctgcaatattacttgtgacatccactaaattctcttcaaacgtatgtaatactttgctaagtcgc

tccacagatacctctgttatattttgtacacagccaagggattcctgaatttttgctaacctttcattttctgtcactgaagtaataacatcctcttcag

ttccaaaattttcaacagttgtcaggctaccaagtgagtttgagcttccaactgttctatcatcagcattggaatcatcttcatgattggacaacaatgc

agatgatgattctgtaactggaaacctatttgacaatgaatcatttgcggaatcacatctgtattctgaataatatacaggaggatgtgcttcttgagga

tagcaagagacaatgtcttgaaaattgtatcctgtcatgtcaacttccttactgatccaaggatgttctacaagctgtttcacagtacaacgttctcttg

gattcttttccagcatccagtggagaagctccataagctcttctgaaaccgatgaaggtgggtgcaattctgctttcataatttcttcaacgtcaaaaaa

agggttttcaccaaatgtcataacatacagtgttacaccaagagaccacatttccagttctggaccttcatacttattgcctgcaagcacttctggacta

cagtattccactgttccataaaatgtggaaaataaacgtccttcgctcatgaatgttgctgatccaaaatcaattagtttggcatgaaacttttcattta

ttataatattctcatcttttatatcacggtgtagtatttgtaaagaatgaaggtagtcaacggctgaaactatctgcctgaagatgtaactattcagtgt

ctcatcaagatacggttttctgtcaatgaactcgaacagatccatccctgctccatgtttttccataaccaattggaaaaattttgtattttcaaagaca

tctaggactttaactatgtttggatggtttaatgtagtaagcaaactaatttcaagtggaacttttgatcccaggctaggatcttcaacccatgactgtt

gatggactttatgtttctgtatgaattttgtaataactaaaagcccatcttcattgcggaatgccatctttacatatccataagcgcccttaccaatttg

ttgcaatgttgtgtatcgttcaccatattcacctgacgtctgttcatcttcacactgactcaggactgacactgaacctgaacgtgttctcgaatgtgat

atattagtttgggcgcatgacctgattgctctattactagcctgtcccagtgagttgtccacagtactgttgaagcttgatgtcaaagtgaggttgccac

aagttctgcctccttcagctatttcttcaggatctctagaaatccaaacacagtacacaagacgaccgcaaaccagcaccactgctcttacacgatatac

aatatctaaatttgatccatccttatgcctgccatatccaaaataactcccatcaggaaattgctgtccttcaacttgcttcacaacttctgtatgagtg

atgccagcatctcttgagataactcttctcagaaacctctttcctgtagctggggtggatgtcagattatcacacggaatatttgccgggtcaaatattt

ttggacactgctggcacagatttgattctcctcctactgatctggaaacacccgataaatccgcatgtatctccccttctgcattacacctacaaacatt

ttcgatacttatcacattgaaattgttctcagagacggggagagcttcattacttgatacattaagttgtaagctattagctgatttttgtactgctgct

gaataacttatgtgggctggagtattacatgtgccctgacaggagtcaatcaaattagctcctttctgatgaatgcatccttcagttattactgctggca

gaatttcaatactatcttcatcatctgaggcagtctgaaaattgtcttcttcctccatgaaatccatatgtggtgacacaattggagttaactctccatt

atcactattgttttctgcatctgtcacagcattttgaagatagtattgaaattctgccagcagtaataactgaaggatgcattcatcagaaaggagctaa

tttgattgactcctgtcagggcacatgtaatactccagcccacataagttattcagcagcagtacaaaaatcagctaatagcttacaacttaatgtatca

agtaatgaagctctccccgtctctgagaacaatttcaatgtgataagtatcgaaaatgtttgtaggtgtaatgcagaaggggagatacatgcggatttat

cgggtgtttccagatcagtaggaggagaatcaaatctgtgccagcagtgtccaaaaatatttgacccggcaaatattccgtgtgataatctgacatccac

cccagctacaggaaagaggtttctgagaagagttatctcaagagatgctggcatcactcatacagaagttgtgaagcaagttgaaggacagcaatttcct

gatgggagttattttggatatggcaggcataaggatggatcaaatttagatattgtatatcgtgtaagagcagtggtgctggtttgcggtcgtcttgtgt

actgtgtttggatttctagagatcctgaagaaatagctgaaggaggcagaacttgtggcaacctcactttgacatcaagcttcaacagtactgtggacaa

ctcactgggacaggctagtaatagagcaatcaggtcatgcgcccaaactaatatatcacattcgagaacacgttcaggttcagtgtcagtcctgagtcag

tgtgaagatgaacagacgtcaggtgaatatggtgaacgatacacaacattgcaacaaattggtaagggcgcttatggatatgtaaagatggcattccgca

atgaagatgggcttttagttattacaaaattcatacagaaacataaagtccatcaacagtcatgggttgaagatcctagcctgggatcaaaagttccact

tgaaattagtttgcttactacattaaaccatccaaacatagttaaagtcctagatgtctttgaaaatacaaaatttttccaattggttatggaaaaacat

ggagcagggatggatctgttcgagttcattgacagaaaaccgtatcttgatgagacactgaatagttacatcttcaggcagatagtttcagccgttgact

accttcattctttacaaatactacaccgtgatataaaagatgagaatattataataaatgaaaagtttcatgccaaactaattgattttggatcagcaac

attcatgagcgaaggacgtttattttccacattttatggaacagtggaatactgtagtccagaagtgcttgcaggcaataagtatgaaggtccagaactg

gaaatgtggtctcttggtgtaacactgtatgttatgacatttggtgaaaaccctttttttgacgttgaagaaattatgaaagcagaattgcacccacctt

catcggtttcagaagagcttatggagcttctccactggatgctggaaaagaatccaagagaacgttgtactgtgaaacagcttgtagaacatccttggat

cagtaaggaagttgacatgacaggatacaattttcaagacattgtctcttgctatcctcaagaagcacatcctcctgtatattattcagaatacagatgt

gattccgcaaatgattcattgtcaaataggtttccagttacagaatcatcatctgcattgttgtccaatcatgaagatgattccaatgctgatgatagaa

cagttggaagctcaaactcacttggtagcctgacaactgttgaaaattttggaactgaagaggatgttattacttcagtgacagaaaatgaaaggttagc

aaaaattcaggaatcccttggctgtgtacaaaatataacagaggtatctgtggagcgacttagcaaagtattacatacgtttgaagagaatttagtggat

gtcacaagtaatattgcagctgttgaaggtggaaacaatgtacctgaagctgagcatgcagatgatgatgactgggacccagattactactatgatgaac

aagatagtttttcttgaaagttgttgtttttatggtcctataattgattactaattttttaatcataacattatttttaagggattggagagtatcctat

gcatgttaaaaagtagaaaacaaagaaaggaaatttgtgtacaataaatactagactgatataccaattttatgtacattgtgatgtgatatagtatgat

tcatttctgattcaagatgtacagttaattgttattacctcaccaaagtgatgtgtgacaacttgattgtgatcactttcttaatgtgtttttgcatgaa

taatgaaagtaccatacaactacatcaagttgatttctttaataagtattgtatataatgtacagttcttgttagtttgttatgacctccatttactaca

gattatctgtattgatggatacattctagtacaaatatttatgaattttacaagagacatatcagatccaccaatatatatacactgttactgagtttta

gtatttttgtaagccttaatttgcatgagatgcatccatgttgatgttaatttacattgttgatgatctctttttcagtatagcaatctagcaaaagtgg

ccttttatgtcttttggaagtctaaaagatgacttggtgaaaggtgactgttcttgtcaacctatcaatacatttttttacaatttcttaaaaagtattt

ttaattaaataccttgtacaataattaccctgataatagttatataacctgcag

>37699|AGAP007698-PA

ttgagaaagaatttaagaaggacttcacattttctgtatttaattactccttggttgatatttttactctgctgggctcatgatgaagtcttgaccactt

tgaaaaaagttattcaaaataatttagttatgcagtttactatttattgtcattcatgaaattcattacactatattaagaaatcttagtatgaaataca

aaaataatgtcctgtgtattataaaaaaatttaaggaaagaaggcactgcttattgacaacttttaaattttaacctttctgttagtgactaatactttc

cctgcacatcatactgcaactacaatacaagtgaaattttaactaagttaaaacctagaaacatccaagagtatttgcatttggtgatattttatcatgg

ttggaaagaagtatatatttccaatgtgatactattttaccattccagtctgcatatgagcttgcacattatcctcccagccacaactgatgacacatct

gacattgtttaattttgattaacacattttatgagattcaaaaaacaatgcactaccaggaaagcttgtcaagtgaagaaattattcctatgggacagta

atcaagatgaattcttcataaaaaaatacaaagtaattatatagaaatattgaaatataaataaagcatctaatttttataaaatgcaacttgactgtaa

aaataattaaaatattttaaatgcttagaaaaatgctgtcaatacttctgttaacttttctctactatgctgtgacttaaatataatgcaaaaaggcatt

ttaagaattctactacatgcatcagaaaatatctctatgtaattagttttatttttataaaagaggtactccaggcagtttcatgtgatagaaatgagat

gaaaaactcctcactgccatgcagcagtagtggtactagtacttctagttgcctcaccaccatgctatggcagcagactgttacattaatgatttgctgt

tttatattgctaaactaataaggtagaagcaacttcgaaactacatttttaatgaatgttttgtattttaattatacataacacaatacagaataatgtc

aaaaaaaggcattgcggttttcaatgtaactagtgaataattaggcattttattcttttctttaaaactgtacaaacatgttcagacttccccagaaagc

caattatggagaaaaaggtattacccatctagttgacagagaactgacaggttttaaatagtatgaataacttgataaatttacaggaggcataaaacaa

taggaacttcattattatttatgctattgagcagcagcaagtataatgaaacatgattacatttcaacatccaagttttaaacataattatttccaacac

tgcctttttaacaaatatcataaaactggatttattagcccaccatatgaactgccagctataagaagtattctgtattattacaatcatctgctttcaa

agcaagaatattcaaatatttatcacactttttcatattgctatcaacattttcaaattatatattacaaaattattgttgttgtaatgaaaatatcata

atttactctcattttaactaatttactactatacattaattacacaatgtcctgaacatttcatcagtaatccaaacaagggacagaaaacacacacacc

cgagcatgtaaggtagagtttgcgcttacgtttcctcgcaaagccagcagatgtcatctcacttcaagacttcccagtgcaaaatgttgtaaatacctac

caaaactaatactttatgaaactgattcctgcactgtaacattgtttacaaatgctctcccttaacagattttcaggcacaacagactttagggacgtgg

ggttaacgcaagcgagtcttaggtaactatttcaccaggcattcagctcaaagtgactgccaaaataataattaaaatgatggcgactactacgaggcag

attattaacaggatctttttcttagtaaaactggaatgcaatcgccgagccttggttaacgttttactggcgaaatccactttttctctaccttcgtcaa

cgtgatactcaatcctgtcaattgttgcatcctggctttccactaacattgccatgtccatgaacatgtcatgcagttccctaataga

>37755|AGAP007728-PA

ccaatgctcttgacagtagatacgagtaatacttagcaccgtagccaaccaggtgagaaaatcgtaactgccatgcctaagaaaagagaaaatcaagtaa

attagcagatacaccaggattgaaacaataaacacaaatgagaaaaggctaccactcatctttagctgactga

>37756|AGAP007728-PA

ccctaggatctgcagcaaaatattccatcaatacagaaggaacttcagcaaagtcagtactgcagcgagttccagtaacgtgctgatactttgtacgagc

aagcattgagtgcatggcatgtcccatctcatggaacaaattatcaaccatgctaggtgtcaacaagctggggcttgaccatcgtggagtaggtaagttc

agcatcagtacaacaacaggattctggtaagatccatcaggtaactcctttccacctcgaatagtgaaatggcagtcctgatttggttttcctggtcttt

cataaaagtcacaatatatatgtcccagaagtccttcatctttgtgtaccactatggaacaaattaaaaaaatttaaaaaatgtgaaagttttacacctg

taagggcagtcaatgtaaatctgtcaaaaacattttctggtgtacataaaagtgccgtaatgtaaaagacttatcattaaatttgagaaaatatagacaa

tatc

>37754|AGAP007728-PA

caatcgcaatatttataaatttacagttctgtacaatataacactttaagcttgacaggaagatgttctttttacattatttatatgctctctcttattg

tctatttcagtaataagactgtgtgcaaggttcactggtgtcacatctctttgcagaaactgggatacaagctcttttgctggctttcctcctccgtgtg

ccaagcattcacgcctatatttttcacctgcagatgaacttaaaggatttgcctcgaagtattcctgccatatccaggaggccaatgctcttgacagtag

atacgagtaatacttagcaccgtagccaaccaggtgagaaaatcgtaactgccatgctgtattggcaacacatgggattccatagtatttattttgaagc

tgtgctagaacatctgttgttgagccctgcaatgggtggtgactgtgatagcattggtcaagtgctgaataaaacacttgtaattgcatttctgatgcag

caaacaaatgctttgatgcacataaacgaatgagcatgtcttctggcattttctcttgagtctgaaaatgtctggcaaaagtctgtacaaccctaggatc

tgcagcaaaatattccatcaatacagaaggaacttcagcaaagtcagtactgcagcgagttccagtaacgtgctgatactttgtacgagcaagcattgag

tgcatggcatgtcccatctcatggaacaaattatcaaccatgctaggtgtcaacaagctggggcttgaccatcgtggagtaggtaagttcagcatcagta

caacaacaggattctggtaagatccatcaggtaactcctttccacctcgaatagtgaaatggcagtcctgatttggttttcctggtctttcataaaagtc

acaatatatatgtcccagaagtccttcatctttgtgtaccactgcaagcttgtagacatcaggagaccaaacttctcctggacgtacttcatcattcact

aagctaatgttataaagactattcgtcaatgtattcagaccctccatacatgctcccagggaaagatatggtgaaaattctgagcttccaacctggagcc

aatcacgtttcactttctgggtaaaatatggagtgtcccatggagctaaattttgttgtgacagaggatcttcttcacatttcattctcatcatagtatg

gaaatcagctgctgctctatcccacaactgttcagttaaaatgtccaaaaactcatttacaacactgggcatttcaacaatgctagctttaagggcccta

tggctgtatgttggaaatccacaaagtttggcaagttcatgtcgagccttcaacatttcttccagtaaatattcttggtgtccatctggataaagaaata

tcctgtatgctgcctcacgtgcagctgcatttggagagtcagcataaagaccagtcactagcacctggtcaccatcgatagtgaataaatgtctgatgtt

ctttggcagaacttctttcggaatagcacgggggcttacagcaccagccataaatctctggccgagttgcagaatatagtcattgagctccacaacacga

caccggtcactttctggaaggtgtataccacactgctcaaagtcaaagaggaacagttgtgctacatgttcatccactggcgtagtttcaaatttatcct

tgttttctgcaacatttcttagagctgaatacagttctcggtgggtgtttaacttttcaactattccactgatgctaacacatgcatcttctgctgcttg

tgagtatgctgcttttggatgagcaattctaatgaactcagccagatctgcgactttgcacaacgtatctgacagttcgtcaaaaacttcaaccatcttt

cgtgacctgtttggatcacatgactcagctattagtttgtctgtttccaaaacagcattctctttcatggaataaaacccgtcataggttcgtagttctg

gcagtccaaacagacccacatcatcttgtgatatgctgaagtttaattttcttgctggtctactgttgaaagctgtagcaaggggtgaccacgtgctgac

tttctgaacgccaactttattaaatgtcccattttttatcttaacagcagcgagtttccgaaaaactgaagtcatatttacagcactataattcagtttt

taaaatgagaattagtcagtttccatatgattctgcactgttattttttaacacgccaagcagcaggcgacagaagaagttaataatttgtttttgttac

attttataagttattatttgcaacgattttcgcacgtcaacatacatatttacattttgaacaatatgagggcactttgcctcaatacaaagatcttcag

cattagtgaagactatacagtgaaggactattctag

>37786|AGAP007732-PA

cggcgcttcacggcagctgaggcacctgccggcacccaccgctggcggccagaaaccgccgcggcgcctccggaagctgacatggacgccgccgcctcag

catcacagcccgccgctgccacctgcgaccaccctcgtagaccgagactggcaggttgtgtgagacagggatatgtgcttgcagtgatgagcttcctggc

catagccaatgcctatacaatgcgcatatgtctttctgttgccataacagagatggtgcttccacaccaaacactaaaaagttacaacagtaatatcaca

cacagtaatgccactgacatcagtggtcaagatctggatgaccttcgatgtcctggagattatccaacacatgaaccaaaaagaatgggtacattagctg

aatttgattgggatgaagaaacacaaggccttatattgagttctttcttctggggttacattctcacccatctgcctggaggaatgctggcagaaaaatt

tggtggcaagtactcacttggacttggcatcttgtgcactgctgtgttcacactgctcactcctttagcagccagatatggtggagccacatggctcatc

attgtcagagctgctgaaggtttaggagagggaccaacatttcctgcaatcaatgccttgttagcacattggatcccagcaagtgaaagaggaactttgg

gatcatttgtcttttctggttcattaataggaactgtagtaggaaattcattgagtggattgctgctgtatttgacacaaagctgggagagtgtgttcta

tgtctttggtggcattagtgtgctttggttcattgcatggcaatacctctgctacagttctccatcatctcatcctttcatttctgaagaggaagcaata

ttccttgacaagacaataggtggtcttgagaaagataaggaacggcatccaactccgtggaaagaaatggccatgtctgtccctctctggggtctcattt

gtgcacaaattggacatgactggggattttttacaatgattacggaccttccaaaatatatgagcagtgtcctcaaattcagtattgctcagaatggatt

cctgtcgtcacttccatacatcgctatgtggcttgtttcaattggtagtggacgagtggctgactggattataacacatggatatctttccaccacaaac

acacgaaaactgttcactactatagcttctgtaggacctgcagttggtattatagcagcatcatatgcaagctgtgatagggtaactgtggtatcattgt

ttacacttggtatggcattgatggggacattttatgctggtatgaaggtgaacggcttggaccttagtccgaattatgctggatctgtcatggctctagt

taatggaattggggcaatcacaggtgttattgtaccatatgtagttggcttattaactccaaatagcactgtgttgcaatggagaatggtgttctggata

actgtggctgtattattagcaacaaacttgatctttgctctgacagcttcaggggaaattcagccttggaatgaccattcagaagaaaagaaaccaaaag

aaaaaggtgacaacagcataggtctgaaaattattacaacacaaactggtaatcagatttcatgacaatgaactatagttattgggtgcttattgtgatt

ttctgcatggaggatgaaattagcaaggagaaaaggaagataaaggaaatgaagatatgcagacatcatacatatgtaaatacttcaaacactgccatct

gtaaatattcattgtgtatcataatttaaaaaatatttattgtaatataaagggcaagcgaaattttcttgagatgtatcctgtacagctaatattatca

gtgtagaaaatgagagagagaaagagagagggaatcagtatttaaagtgaagaactgcatggctaaaagaagcatgtgctatatttgctctcaccattaa

tgtttactctgcagaaaaagtctatttttccctattaatatcctttcagttgtacatattaaatagttatttcaacaatgccaaaatgtgttatttggat

tttctccagttctgttctgaaaacaaattattatttgacagatgatgtatgcagtaatatttcaaggacagatatttccaagtattgtgtgaataagata

cattaaaatcattattgaaaataaaagtgcataaaatgactaaagaaataatttctgtgttttgatttccaataatttatatacatttatcattcataac

accagacagttgagaccggagtggataagtatttcaagtaaaaaataaaattatagattgttgcat

>37824|AGAP007761-PA

gcaatcctgaatttatgatggaagggcagcctataattgcctgtcaagaaaatggtcggtggtctggttcagttccaaagtgtgtccaagcctgttctta

cccagggacaacaattagtggaagaatgtcagctgttaagttctactacaaaattggagagacaattacattcacttgtgatgcagatttagagctccgt

ggagcacctatgctacgatgtctacgaaatggcaagtggtccaatgctataccaacatgtgtttcgaagacaagttaatttactgtgaatgtagattata

cgaatatgaaatactataagttttgataaagatgtagaagaacaaaaattctttctgttgttgacatttttgatcagcaatggtcaacacagtgggacat

ctataaattatgaatatattttcatggtgatcaataaagaagaaatttgtttagaatttatgcattgttggaaaatgttgaacattgtaaagagaaggag

aaaacaaaagtagtaattaagtagacaggaggccttaaattgtgttctttcatacccttatctgcttagcttatagaagtaaaacatttttttacacatg

caagtaatgttattactttaaaaagtatatgtaatgtttatagaagactaatgaaccagttatttcaagtgtgagatagcttgaatgttatttttgtaaa

attattattgaaaatggaatgaatatggctggcttctaaactgactttctctttgaccaatactagcaaaattggaattttgacacacacacacacacag

ttggaattaaaaccacagaacatattgacagaattattttccacttttcttacc

>37843|AGAP007769-PA

ttgacttgaagcttttgtattgtgccttaataagtaacaaaactgacatatccacataagctatttctcagcttacaaaatagcttctttatgttaaaac

cttgtgtttgcttgtaccaaatacaacaacatgtttctgctctcatggaaactgcacaaacaaagtttgtattcagccttaattaataattcttacaaaa

acctcaaattgggcctcctgacccatactgtttatcccatgtcacatattgtatcaggtctgtctgtgaaacactagctcttaccctctttaaggcagtc

ttgaaatcctctacagtcacgggccgaacctggtctgacgtaatgttttcaatatgtaggaaattcattgacctaataggtccaagagaggcttcctgac

aaaggtttttcatgtctgctcctgagtatccctct

>37994|AGAP007781-PA

ctttggtggtgtgcgcgccaatggggagttcctcagctgcatcaggaatgccctgtcatacacaattcttgtgccagttcagtctgcgcattagaaaaca

gcctccacgtgtataatgcactgccttacgaaagcaaagaactcgaaaacatctttcgcatacaataagg

>38130|AGAP007788-PA

ctatactcctccgattggtttattgcactcaggaccttccagtattgaatcaattagtattaaaacacagttactatgcatttattttattctgtcttaa

tcagcatgaatcataatacagctagaaaacatttttatatgtccatacaccagattaattgctttcagctatacaatttctatgttacactgcagtccaa

gtcatcattgcaacaagtcactgatattacacgtaaaacaacatgaaaattggtaacagaaatacttaagatttctgtgaatcaaatttaattttcttca

taacatgtcacctctcataacataccttcatcagttgttgtgtgagtcttgaggacaggctcaggaattttatcaggcaccctcatactagcctcaacat

tcccactttcattatcagttcctacaggtggcccccaaattctcatcaagaaaggtttcagttgtgtcttactaagttctaaataggcagcctcaagatt

tccatgattggctgttagaacagttacaatatcctccctagtaaaatttccacgtgccatcagctctgcatacttcttctggcgctgctctacacactca

gtaacagctgcccacacatttcctcgatgtattcgtaatgcatcacgagcttcagcagctgatattgttccaatattattttcatgtctctcatgcccat

agttagtggccagagttactacagtatcaatcatattgcgccagttatcacgcaaccatgtaactggatttttctcaccacagtgttccatagccacttg

gagatcttctgtcgtaaagttatgcatctctgcttccctgagtaatttgacgagctccaggccttcgttcttcatggcttcagtgcgctgtttctggacg

agctctgtgaggcctgtgatcttgtctgcgtagccagcgcccagtggcttctccggcggttgtgacagcgctctgcctccgtgcagtccatttcgctgat

ccgcgaaatctgcctgcctcagtagcgtgctggcccctagtcccagaggaggctctgcgtacgaccgcctcctcatgtcccactgctggctagcgcggtg

caagtcgaactggctgcggtgcgtcgaggccatcggaggcagctgcggccggtaaaacgccgaactcccgtactccggg

>38131|AGAP007788-PA

cgcatttattttattctgtcttaatcagcatgaatcataatacagctagaaaacatttttatatgtccatacaccagattaattgctttcagctatacaa

tttctatgttacactgcagtccaagtcatcattgcaacaagtcactgatattacacgtaaaacaacatgaaaattggtaacagaaatacttaagatttct

gtgaatcaaatttaattttcttcataacatgtcacctctcataacataccttcatcagttgttgtgtgagtcttgaggacaggctcaggaattttatcag

gcaccctcatactagcctcaacattcccactttcattatcagttcctacaggtggcccccaaattctcatcaagaaaggtttcagttgtgtcttactaag

ttctaaataggcagcctcaagatttccatgattggctgttagaacagttacaatatcctccctagtaaaatttccacgtgccatcagctctgcatacttc

ttctggcgctgctctacacactcagtaacagctgcccacacatttcctcgatgtattcgtaatgcatcacgagcttcagcagctgatattgttccaatat

tattttcatgtctctcatgcccatagttagtggccagagttactacagtatcaatcatattgcgccagttatcacgcaaccatgtaactggatttttctc

accacagtgttccatagccacttggagatcttctgtcgtaaagttatgcatctctgcttccctgagtaatttgacgagctccaggccttcgttcttcatg

gcttcagtgcgctgtttctggacgagctctgtgaggcctgtgatcttgtctgcgtagccagcgcccagtggcttctccggcggttgtgacagcgctctgc

ctccgtgcagtccatttcgctgatccgcgaaatctgcctgcctcagtagcgtgctggcccctagtcccagaggaggctctgcgtacgaccgcctcctcat

gtcccactgctggctagcgcggtgcaagtcgaactggctgcggtgcgtcgaggccatcggaggcagctgcggccggtaaaacgccgaactcccgtactcc

gggtacagtggcaagtcatcgtccatatagcggccgctatagcgcagcgggggagacgactcccgggacacccctggaggcaggctctgagtggagatgc

tctgcggaggaggagaagttcccgtcgacgtcaccattttgccagacttggaagcggagacctccggggctggcgcagtcgccgtctgcacggaggaagt

tgggggcggcgccggaggtggcacagctctcgggggagaggtgcccactgccgttgacaccttggaaggcttctgcggaggcgcttccgtcatttccacg

tctgcttgctcgtcgggcctgggagaaacttcggcgtcgctagaaagtgcaggggggaggggagagactgaagacgtcgacacggacagcgaggaatggt

cgacgatgtcggaagaggtgttggtgcccgtcgtcactttttttcggttagtggaagtcgctgctgaggtggtagcagtctgcgtgtcagaaaaggaaga

cgggaaagatttagtttcagacacactgtcactctgttctccctggcgagattgtttatgcgacgtttccgacgtcttcggcacttcttccgatagcctc

tcttttcctttcttcttgtcgcggtctttctccttgccggtctcgacctgcgaggccctgcgcgtctctcgcgactctgcagctgtcgctcgcttcccgg

tctgcggcgaggaagacgaggaagagatgcgcaggtgtttgccgaatttggcggcgacggtggcggtgtcgtcgctgtcggagcggtcactgggccgcag

gtggcgcttggaggag

>38129|AGAP007788-PA

gttgctttagttctggcaggcacagaaaatttggctgtcttgttactttcttcttttcctgctgacatatgtacgggttttgttgcatctttcactatcc

tctgacaaccactagtggtttcttgcttactctctcctttcgttggttcctctggctttctcagatcagtgggagcaccttctatttcttgtttatccac

actattttccaaaatgttttcacttttaacaattttgttatgttgagtttgttctttttcctgtatctgtgcctcatgtcttgtgtgtgtaacagcattt

ttttcatccaggacttctgtttgagtatgtcctgatatatccttcaagtgtgcttcttgatcttgcatattttcatctttgtttagcgtagacattattt

ttggatctgaagctttttgctgctttgtaatgtcttccacctgctcaacagctatattcttgttacaagctgatacagcttcaagagcttcttcgtttac

tgaggaattgttatttatttcagttattgtatctccctgcatagattgtgcttcatttgaattttccatgctctgttgagtattttgtttttcaaaagaa

atgctattattttcagatgcattgttttctttcttaagtatgtcactttctttatcaaggtgtgaaaataagtcactctgttttggttctttcttttgct

gtgtaatggatgcagttagttgtaaatgatccttctcatcaccagatgttgtctcctgttttggttttatttttactgtgcttttgcttgtgtctttgac

tgtgataacattacttctgttagctgtattttttcctttttctgtaacttcatcatgtctcttgactttaccatcgacattagatactattttgccattc

acagcttgctttggttttgtttcattcatttgtctgctgttgttactggactttcctgtcctaacggagttgtcagacccagaattgtttgtactagatt

cagtgccggccagtggctgacttttcccatcttcactgcactgtctcaaatctgatttataaacattgtcattatcattctggtctacatttttagtttg

ttctactaatactttaacttcttctttctccaatacaaaaggcgactcaggtttcttaccaacgtcattcaatatggcaatattttctggaaacccacca

ctcgtttctgtttcttttggatatctatcatttgattttgttgtgatatggctcatatttacaccaactaaattttcagatgtttttctttgatccaata

catctgaccttgaagtaacttcttgctttttactgacattctcagcccttaaatgtgtattgtttgatatttgatgtttggtatgttgagatacattgtc

aagagccttattatttgattcttcctgggagtaatgtttgaacacgccagtttcatttttgttatccctctctatctgttgtgctcttatctcataatca

gatttttcctctccctctatctcttcctcatcctcatcctcatcctcatcttctttctcttcaaactcctccacatcaacctcctcctcttcttcgtctt

caaaaccatcacttgattcttcatcataatcagttgtgttctgtgtcatctgctgtactatagatgtcagttgctgtaaatccttctgaagcatgtccga

ttctgtgttattttgttcagcataccgagacattttccgtaccaatagtgacaactgctctagttcttctttctgcaattctgaacaaccatcctctgca

ttcacagacatctggttgataagtgaagaaagctgtttcatttctctttgtaatgtatctatccttgtcaaagcatttccttccagtggtggatatgttc

ctgatatatcactattgctagttccttgagtatcttcaacttcctgctcgtcataatcagaatccaaatacgcagcactcatatcaatgtctgatatttc

agcactattatttatatcttctgataaaggagatgacatgtcattaggattcctttcaatttgcagaccttttgttagctgaataaatgtgttgctcatg

ttatcctcctgagcagtgagtggaatgttatctacattgttatgaggttctaatcctatactgtcttggaatgttgaagttacttctatattctgctctc

tattcacatgattttgggtgggaatgttttgatctctcttacctgtaggggcatctttctcacgtacctgatttaggtcctcatccttatttgactttct

cttggttgatttatttatttctttgtgtgctacagcactcatcttatgaactgtatcatcatatgttgcatgaggctctgaatggactaaatcattgact

ggactcaccgctttactttcatcagtcatgtcctcagtatcaacttcagaccttgcatcaacaaagtcagttattgagacactgtcatcttcatcagttg

ttgtgtgagtcttgaggacaggctcaggaattttatcaggcaccctcatactagcctcaacattcccactttcattatcagttcctacaggtggccccca

aattctcatcaagaaaggtttcagttgtgtcttactaagttctaaataggcagcctcaagatttccatgattggctgttagaacagttacaatatcctcc

ctagtaaaatttccacgtgccatcagctctgcatacttcttctggcgctgctctacacactcagtaacagctgcccacacatttcctcgatgtattcgta

atgcatcacgagcttcagcagctgatattgttccaatattattttcatgtctctcatgcccatagttagtggccagagttactacagtatcaatcatatt

gcgccagttatcacgcaaccatgtaactggatttttctcaccacagtgttccatagccacttggagatcttctgtcgtaaagttatgcatctctgcttcc

ctgagtaatttgacgagctccaggccttcgttcttcatggcttcagtgcgctgtttctggacgagctctgtgaggcctgtgatcttgtctgcgtagccag

cgcccagtggcttctccggcggttgtgacagcgctctgcctccgtgcagtccatttcgctgatccgcgaaatctgcctgcctcagtagcgtgctggcccc

tagtcccagaggaggctctgcgtacgaccgcctcctcatgtcccactgctggctagcgcggtgcaagtcgaactggctgcggtgcgtcgaggccatcgga

ggcagctgcggccggtaaaacgccgaactcccgtactccgggtacagtggcaagtcatcgtccatatagcggccgctatagcgcagcgggggagacgact

cccgggacacccctggaggcaggctctgagtggagatgctctgcggaggaggagaagttcccgtcgacgtcaccattttgccagacttggaagcggagac

ctccggggctggcgcagtcgccgtctgcacggaggaagttgggggcggcgccggaggtggcacagctctcgggggagaggtgcccactgccgttgacacc

ttggaaggcttctgcggaggcgcttccgtcatttccacgtctgcttgctcgtcgggcctgggagaaacttcggcgtcgctagaaagtgcaggggggaggg

gagagactgaagacgtcgacacggacagcgaggaatggtcgacgatgtcggaagaggtgttggtgcccgtcgtcactttttttcggttagtggaagtcgc

tgctgaggtggtagcagtctgcgtgtcagaaaaggaagacgggaaagatttagtttcagacacactgtcactctgttctccctggcgagattgtttatgc

gacgtttccgacgtcttcggcacttcttccgatagcctctcttttcctttcttcttgtcgcggtctttctccttgccggtctcgacctgcgaggccctgc

gcgtctctcgcgactctgcagctgtcgctcgcttcccggtctgcggcgaggaagacgaggaagagatgcgcaggtgtttgccgaatttggcggcgacggt

ggcggtgtcgtcgctgtcggagcggtcactgggccgcaggtggcgcttggaggag

>38145|AGAP007792-PA

ggacaaagaagcctgcaggcaatgcctgtcgtggccagccacgcccttctgtcatgaagatgtgcaaaggaccaccttgtactcaaatgtcgaggtgcaa

agatcaatcaaagtactgccaaatcgtgaaaacaatgaaagcttgtgggatacaccaatactggaaccagtgttgtcactcatgtcggaaaaaaacctag

tttggtgactgtatacctctagcatcatttgttgatgaattgtgtgaattattgcctcagtgtggactaaagacactgtgtggtaatagagtcaaagagt

ttcagagacatctatatattaatatgtcagaaatgacatattattgaccctcacatatttttggtgacatggtactaagtaccagagtgaaatgtttcac

gcaaaaccctaagtgggggtgaaaactttgagtactattttcatgaagactgaaaatattaaaaataactatgtcctgccattttcatatttgttcacat

tatcaaactagaaagacttccattggatacttacaaattctatctaaaattatgagattatttactttaagacatttataattcagttttccaagatgtt

tggtcctgttattagtgaagattggggctagtttaatactcttactacattacttatttttttaaaaagttgttgtcttgctgtaatgagatatttaagt

acacataaagtacgatggtgttgttaccagtattcctacaagcacacattccatttgttatttgtaaataatgtagcataagttgtagtatacttgtaaa

tattgtatttattgaaatacattcatt

>38163|AGAP007807-PA

cctgagactgacaagattctctggaaacttatcaaaaggcactccagtaacatccgaataatcgaatgtcagacagtgcaaacctgacaatttgaggatg

ctgctcacaccgggctgcaactctgtaaaatacgtggtaccaaaatacatggtacccattcgcaaagtgtgaagtcgcggtagcccgacgaggcagggga

ataggcctccaccgagtctgctgcaacctgtcacatctagtatttcaagatgttcgagacgatcaatctccgagaaggcggcaggcgacagcttcgtagc

gtgagtgacggagagctgccgcaaagcgggacatccgtggctgatggcaattactgtgttatcttccacaccatatccgtgcggtagatccagcactttc

agttgtggcagtcctccattcgtaaaagccagagcagcagttccgggtggtattcgatcggggatggacatcctgagttcccgcaagttcttcaagcttc

cgacggtagtgaacgcctctctggccgagtcacggactatgtcgtactcgtaaagttccagatgttccaagctgtcggcatataccggcagcaacggtac

gacacgcctcgttccacccaccgaccactttattcgtaagcactttagggtgttcttcttggcattcagaaagtactttagattgtccgtatcgacagac

atacgtccgaagtcaatctcgaccagtcggggacacccgtcatccagaggtctcagcacacaaggacgcaggatgcaagtcgttcccacgtatctctctt

gcagactcagcacacggaggtgctgcaggcgcgacaccgcttccgaaaatttctcggtcttcagcagttcgtcaggtgccgtcagcacgcgtagcccaga

gcactgctcgaccaagttcttcaccgtcgaatagccgagcatctgggcggcgtgaaggcggagatctgttagtctccggcacttgtcgcagagcacgtcg

atgacgtggggactgacagcgcaccacatgtgtactttacgcaggtttcgggctctgcggaagatacgtatcgcctcccgctcggtcctgccgctcttac

agttctccgagttgcagtgctgcgagatcccgacgtggtattccttgtcagcccacaacttcaaatcccgcgacagctttctccaacgacggcacacttt

ccgtaccacgtccaccagctcagtgaaagacaagtgagagaagatcttgatcagtatctcctcaggcaagtcgtcaatgctcgcacgccaggctgcagca

ccttcagccatgtggcacagtccactgtgctcagactctgaagccattccactgtcgagcccgctgccgcaccacagtgaggcggaaggcaccgggtgag

gtgagggtctgccgagcggacgtctgtcagatacgcttgcaggcacgtttgttgacgtgtagcagccgacgggagaggaaccgtcttcagcctcttcttg

tggcgtaatccaatgggtgataagacattcacacgcgcacacaaagcaccatagtgacatgctacttttcaggcacaaactgtcactaatttcgctaaag

acacagtgctgggacgatgtgttatcgacaacacataaacagtgcaatcacacacgatgagactttacggtgcgatgaataaaatgtgtcagcagagact

tttaataaatgcgagcttaaaaatacgcgtataaaagcactaaataagtaacaatgttgagacatttatgttgtggttattcaaaccgggaacgatttta

taacagtatcacaaacttcagtcgtgtgaactgttaacgctctagtgaataaaacagtgacaatttacattcatgctagagggatacttatcacacaaca

caacacgtgcttatccgagcctttttcccaggacgctacagtgtgcagtgatatatataggactgattctgccgcgttttatgcatttcaggagcggaag

aagcttcgcgtggattggctctacg

>38183|AGAP007811-PA

ctttgctgacgctactgcatggttgatgttttcggcggaagacttaatctgatctggaatttgttgaacatatttgtctgttaggacgaaataattgttg

cgacttgcagccaatctgttgattttctagcgtgacttcatttattatatgatggatgacacgactgtagatgagtggatgaatttcttccgggatgcag

gcattccaccaaatgtatcagcctcatatgcatctagcttctgtcaaaacagaatccatttaaatatggttcttgagttaaatagggatcttttgaagga

attgggagttaatctaattggtgatgccattgcaatattaagacatgccaaatctttccatgaaaaggtaaaataactttattagaatgtagacatcagt

ttatcattagatgcaaaagcctg

>38184|AGAP007811-PA

tggtatcctcttcttcattctgcctgactgcaatttcagactgaacaggtgcaatacgtttctttggggccacagtcaccacttcattttgagcagccag

tgagagaggctgttttcccgattcttttgatgcgttgtgtttccttgcctttactgatgtctctgttccatctttttggccacgggatgcagacttttgt

ggtgatgattgtgcagccaaaattctttctcgtgctctcttgtcatgaactgtttttgcatgtctcaatattgcaatgacatctcctatcaacgtaatgc

ccatttcctttagtatttctctgttcaaatccaacagcatattcctctgtatgcgattatcattgaatgtaatagcataatctgcagctattgctgcagg

aattcctgcttcccggaagaacttaacccaaccttcagtacccctttcagccatgttgaaactgagattacaatagactgtgttttgtttgttgtctaca

caagtcaattccgttcaaaacagtcatgtagtttgcatattaagcttcctgtagccaagcatcttccgttatgcatttctaaacattcatgaataggatc

gaacatttcacttttcaggcactgtattttcaactgatgctgtt

>38182|AGAP007811-PA

ctttgctgacgctactgcatggttgatgttttcggcggaagacttaatctgatctggaatttgttgaacatatttgtctgttaggacgaaataattgttg

cgacttgcagccaatctgttgattttctagcgtgacttcatttattatatgatggatgacacgactgtagatgagtggatgaatttcttccgggatgcag

gcattccaccaaatgtatcagcctcatatgcatctagcttctgtcaaaacagaatccatttaaatatggttcttgagttaaatagggatcttttgaagga

attgggagttaatctaattggtgatgccattgcaatattaagacatgccaaatctttccatgaaaagaatgttcgtcagaagatacttgacataatgtca

tcagaatgtcaaaaagaacataaaaaaactgaagaatgtagtaagaaagtagtatatactacagatgtggcttcaacaaatgataggtggtttgatggtg

aggcagagacaacagaagaaatgagtactccatgcagtgcttcaagaaaccagaatagtcttcggacatcttattcagaatgttttctaatgagaggacc

tgttgaatcaagcagcacattgcccgtaagacaagcaactgtttcttcacgtgtttcaggaggtaattacatatgcagaagaaatcagggtataaagagg

ccaatgcagcagagtccggtctctacaatagtttctatatctcagcaacctgcaagaaggttaccatgtcaaacagaaaatactcacccaacagcttgtc

agtcaatacataggatagaatgcaacacttttgaggatacaccagatgaaatccttgccacagcaaatatgaagaggaagagaacttcagcgtgttgtgg

acctattgaggaagaacaatttgatccaaatgtttgtatgcagtcagtgtgcactaggactgccaatcaatcggtctgtgactctcaaagtactgcagaa

agagttcagtcagaggggttacaggttcaagagtcttctgaattagatattagtttaccaagacaggatgtgaaaaatgaaatgatagaaactgaagacc

ctgtagaaaaccattcaagtttttacccaagatgtcagctgcctttacaaaggaggcaggaagaatcaccaacacagacatgtactcaagataaatctca

ggagagaatgcaggtgttccagtcactaccaaacaaacaatcacaatctcaaacacagtttccatctcatgtggcatcatcttcacagccatcattttta

agaaggccaaattctcatatgcagtatcaacctgcattaaagttacatcatcttcacagtcatcatttttaagaaggccaagttctcatatgcagtatca

atctgcattaaagttacatcatgaatcccagtcatcattgtgtcttcagccattagtgcagcctaagacagcacatgcagtatcctctcaggcaaatgca

caaaagaatgggaaattacagaataaaaaagtgatgcattgtaattctgctcgttcctgctccacaacaaagctgattcttgcaactggcaaaaatataa

ataaaaaatttaaattactgcattctttaccaaggacaccatgtgtgaccccggctccacattcaaccgagtgcactcaaggaaatgaggtacagagacc

tataagctcatgggttgtttgtggatgtaacaacaagtctgttgtgaaaaagactgaagatgaggaaaagggggaactttcgcagactgtgttagaatct

gataatgcaacatgtgtattgcagaatggatctcagactaagttcattgatgctggtggtgacttgctgggagaagaaatgaatgccttgacagatacat

ctgcagtaagacaggatactcagcagttctctaaaccattggcacacacatctgatagctgtttatatgaaggttcttcctctcataccttcaataactc

tagcaatgacgctgaaaataattcgcaacatgagagagttcctggtcaaatattttctcgcttaggcgtgaaagttgaattggtgtcagtggatgacgac

gactgtaacacatcagttaaaaatgatactttaccaacttgcagatttggtaacaatgttaagtacaagataaataaaccaaaaaaacataaaaatttag

caagtaaatttgctaaaataatgtcagttaaacagttgcctagaaacaaattactcaagaggaaaattatgttcacagcatttcagaaaacctccacaat

tggcagacaaaacattgtaccag

>38204|AGAP007827-PA

gaatcgttatttgggcaaaggcgtcttgactgcagtcaagaatgtcaatgagatcattgcgcccgctttgatcggcaaatcggtgactgatcaaaaagaa

ctggatagcctcatggtagagaaacttgacggaacccagaatgagtggggctggtgcaagcagaaattgggcgccaatgccattttggccgtgtcaatgg

ctctctgtcgtgctggagcggcccataagaaggttcccctgtatcagtatattgccgaacttgctgggaagaagacagacaaatactggctaccagttcc

ggccttcaacgttatcaatggaggttctcatgctggcaataaattggcaatgcaggagttcatgattctccctgttggtgccacaacatttaaagaagct

ttgcagatgggtgccg

>38240|AGAP007852-PA

gaactcacatccagagcttgcaaatttagtcgctaggtatggccacaatctgtaacatgggtgctgaaattggtgcaacaacatcaattttcccatataa

tcaacgcatgaaagactatttgttagctacaaatagaaaggaaattgctgaagaagcagacaaatatgctaagacactatttacggcagaccctggagct

ccttatgatcagacaatagaactagatttggatacactagaaccacacatcaatggacctttcacaccagattt

>38298|AGAP007868-PA

ccctctttgataaagctcttctaatgctggtatttcaactggatcagtggcaactataattttaccacatttcttgtacggaatattctttttatcaaag

tatttgtatgacaaatagcaaccttgcacacacaattttgccttaagtgaccctggcttataatatataccagcgtgaacgacaccactgttgttaccac

tttggtgagcagcaagcttatcttctttctctaggactgccattttgaaaactggatacctcagcttaagttctcgagctgtggccattcccacaacgcc

gccaccaatgatgacgatatcatagattttatcatcgggacagctggcaattttaactgagtgccgacgatttaagctaggtggtctgtcgaagcttaat

gcaaataggcaccgtgaacgtgaagcaaactgcaagttacgcccacaacaaatggataccattgtccgaagccttcgcaaagccacactaatgttatagt

ttattcagtgcagcattccagggcgcgaaaagacgcgttaatttgtgttccgtgcacctttacatagctagtactcttatatttacatttagctgccggc

gttaacgcagtttacgcaatagctttggatcagaatcagacgaagatgtttcacaaacaacgctcgagttcagatttctgtttctacgaaaacgagctct

atgagataaagcataatcttcagagtgcttagtccatctgcagtgccacttacgtaacattatataatttgtaggagaaactatgatgccaaactgaatg

ttacattttatttcaaacagtggaaaaaaattttcaatatctacaagttgtcaatactgatctttctcatacaacgcattgacaaccgccttcgctttgg

tacgtcgctcctcgcatatattgcaacattccag

>38317|AGAP007871-PA

caaatgtgctagttactctaacacctaacagcaaggggcagatacatacagaatgcaaatattgtggcaatcacatggcacaaaaagggtgagcaataaa

ttcatcacaaaacaaaaaaaacgaattttgtaggtctacgtttagctcaaaactgacttttctttataagtctctccaaagatctgagagtttaggagcc

ttggagtttgaatgaacctaaatacttcacatttagtcattcccgatatacatgttattgtatttgtttattttgtccttgcaaatccaagaaatgattc

agcaatacagtctcttgtgtcacattagataaaaaaaaaaatgcttacagctgtcattgttttgtgcaggtacatatttacaaaaaataagtcacatgga

aaactaacaaaatgtttggtacaaacgtatcaaatactatggctaatggaggtgatgtgtttgtaagcacctctgtatgtgaaagatcttgcaaattact

gaactgatcacagatctgcttcacattaacagtaaaacatctttagcattgtgaattataatatattctccaatgactattatattttattcaaacatat

tcctcaaatgcactggctttttattcaagcaatcgtgataccacagcctaaccgaaaattgtttttgctatgattcagcataccactcagtgaaaagcag

aatggtagaggctgtaacttcttctccagcacagcaccaacagtccaattggagtcaatcatagctctgaagcacacatctgctttgggaagatctactt

ggtaaccaatgacagctaaagtttcttgcatacgcagatttgtatccatctccactccgaactgcagctggtcacttactttttcatagtaacatacgtg

gactccagcaccacccacaacaccactaactgtgaagtcatttccagtgtatcttccaacagctgaaactatggcaatttgacctcctggcacctttgct

ccatattgataccctagctcagctccaagtgccacattcttcgtaactgattgtaaatagtgaccaatgataagacccgattcattgatgatatctaggt

ttccgaaggtcaatgatgcagtgtagtcatctcccttgtaatcagttgttagctggactcctgtgaatactttactctgtatctgagctgcaaactttgt

ccttgttcttgttccaaattgatgaatgatatttgcatttagatttccttcaggatctatatcgccgagaagtacaggaaatgcttctgttggagacatc

tgtttagttcccacatatgttgcaccaaatcggtaacctgatggtgcaatggcactcaggtttattgtgtgagatacctgaaagtaattgctaagccctt

tgttcactatcagccttgcaccctcaaatacggcaggaaatacatctttgcacttcttgtggagatcttcaacagtgccagggttttctagacctttgat

gtcgctagtgcctgctgtttcgcctatctgatggatattgctgctactaatgtcttctgcttttggtgcagcaggcggaaaaccaactgttggcggcggc

ggtggtggtgtcggggctgccgtcgtctgcggcggcggaggtggcgtcgtgaatgtgcttgccggtcccggtgatgccatagagttacccattttcaatt

acgcggtgatatttatttacttacaacagtaacgccacaaccacagatcaattaaaactttaccccacacgcctactaacaattcacatttcacaccaca

aatccaaaattcac

>38331|AGAP007874-PA

atacgtgttgtggcagtaataatctttgctccagtatgtgcacacatttcactgatgacttacattgaaacatctccctgaatataaaattgtaaaacaa

tttaatccacaataacaaatttttcacacatcacagtatgcaccactgttctaggcattcatctactatgtttcttcttttttgcagacgttttttgttt

ctttgtactgtctgcagtctttttccttttcttttttttaaggttttttgacttcctagcttcttcctctgaagacgatgacgagtctgatgatgtatca

gatgaactgctttctgaggaatctgatgtgcttgaactgctatcagaggaactgagtgccttcatcaaagcaggtagcatggaaacttttggttgagatt

ttagatgttcccgcagctcatctgttaggccacccaaaccaatcgaggtgaagaaatttattgcaaacctcgtgttttttggattatcacgaggaaataa

tccatcaaaagctatctgcattgtgggatcttttactctctcattcaattttgttagtcccatatattcagaaagctcctgaaagaggattttaataaag

attctgcttgagcttgttgtgtcctcttcattcagctttataaaagaaagcacttcccatgaaatggcatcagtaaataacaaatgcgcaaagaactttg

cagtatttctcaatttatttgtatccaaacgatgtattgtgttataggtatcacggaagatttgttggaatggaggtatgtataccttgttgatctggca

gaatctttgtgcaagaagaccaaaaaacttttcatatgttcgctgctcagcacaacaatccagaaacatatggcagagttcaatttcctggccaggtttt

aattccattctcatcaacttatgtgcacattcctcaaaatctaagctggaatgaattgtaaggtaaattgccctcctaagggcaacaagattggtttcag

tgttgtccagaattgtttcttccttctctccctcattctcactgtcaccttctgaatcttcttcactctctgagccagaaccactaccagattcatcact

gctctcatccaacagttctttctttagagcattatatttttcttcatttgtctcatattctggatcaaatttgaacacatttaatatctcttctgcattt

tttgcttcatccagcgttatcagatgagtgaactggtcctcctcttctacaaggtccagttctggaatgactgatgcatggtctctaaattcatctttac

gaatttggaatataacttctatcatatattgtacccttttttctagatgtccttcatgtaaaatattacgaagcatctcaaagatggcaagaactccttt

gttggacacttctgttagcttcatgccgcattctttgagaaatgcaattgctacttcaacagaatcatcagttggtgtttcaatcaacaatgtaaggatt

tccagtgctaatatttcatgagccactctctggttaacaagatgtgcgatgaatgttacagctgaaatgcatgtgcttttatcgtttcttctgaagccac

gtctgaactgaataacaatccgccgcagcaacaactctccaatattgggaaacttgctattgataacagcaactaaagcagcataaacatgagtaaaagt

gggtgatgcagcttgggcctgtataatagaccgacacaacaatcctcggcccctaacaatattttctcgaaggagttcccgaacaattttaccgatattt

cctgtgttaactttgttgatgtggccatgaatagactttttaagagcttcccaagccaatctctgataagcagcactcgttttgtctgttatttgggctt

gcatcattctcagcttagctggagggatataggcacctccagtttttgatgtaatcatatcgagggattttttctgtggtgctggagctgccggtggttc

catcgtcacgtttgcactttcggtggctgtcgattcactagtgggttttgatctgttatccaacacagtactactgtctttcttgggactgcgatggtca

cgtctcctactactgtctctatagtcagtgtgtttactgccagagtttttgttatctttcctgtctgtataagacttgtctgaataggaatccctatttc

tatattcagacctctgaaatctgaaattttcgccgttatcatggaaactacgtggatctgaataatatctctcataaccgtctttatctgccgaatagtg

tctgtcggaattgcgtctttcacttcgttcatgactactaccatacctgtctctcatggtcctcgaatcttcgtagcgcttgcttctacctgaaccattc

tgctctgtcctgctgtactggtggttttttgtttcatcatcagcgaacgaagcactgcaatctctacttctgctattgcgttctttatccttatactttg

accctgaactattgcggcgtcttttgtactgatcttcccttgtatcctcttcatgtcttttctgtttccttttttcgctgtcttctttccggtaagtttc

ggaatcccttccgtctttgtccgacaaacgatacgtcgcagtttcattgcggctcattgtgtctgttgctttcactgacaagcgaactgtataatctgta

acaccataaccactggaggcctacaccatacacttgagattacaaacattcaatactacggggctctgacatagcatacgaaatattcgcatcagcacga

tgccacttataacacttcaaaacaacttctcaacaacaacacacacagaagcacaacaggacacaccactaacatcaacaacattgcatttgcaaggaac

ttcagacacagat

>38346|AGAP007877-PA

catttttatatgtatctgtatcatcaagtttattaacatttatcccagtcaatggcagttttccctcatatatgaaggcactcattcgctgactaacact

aagcatcagaagtgttgacggaaataacaccaagtagcggtcccgatgatcaggcagtatcgcaacagatcccatatgaagaatttctcccaaacaagac

agattttcaccttcccaacctctcacacctccagttaatacttccagttccaactccttctgacgtcgagttgtagatatgaaggatgctatatctttgt

acacactaacagaacgctgtgtatcccctctgtcaggatgactttcttccacatgtcgttccagttcttgtaacataccggaatacttttcaagacgtct

aaatggtttgctcaagcctgttgtcaagacaagaataccgggacttactgcaccatgagactccatgaatgaatttaactggtccctgtatttgtcaagg

atgcacacagccctcggatgggaggagcaataaacatgatgtacttgtttcagtctaggtgcagaggtgaggaaaattttccccacgcgttgttcttgtg

gtggacgctgactgcattcctccaacgctgccagaagctgctgatgtgtttcaacaacagtaactatgttgctgactaactggcgatattcatccctgga

gagtatgtccgacttctccaggggactgaggaagtttttcaacaaagtctgcagctctgctacatgggctttctcagattcaataaggtccttcaaaacc

agatttctgtatgctttctgttgcgctgcaatatctggagggagtttcactggtgagatcttcgtagttcctgaagcaccactctctaagggagtgtact

ccttcacataattggaaggaaaccagccagtcttctcccctagtgttccttcccaccatcctccttcctctctctgggtgactgttataatgtccccttt

cttgaagcaaagctcatcattgttcttgcctttgaaagagtagacagcctgcaccaaacacacactgctgtctgaagtcatggtggatgaaattcacaac

ataccagtggaacatggatcacctcagacaaaaactgtgttgactcttttcagtctccacagaaattcttccgcatatcttgccaaactagcatccctgg

ggctgaatactatagagaaatgtattacaaacagccaccactgtccaaactgaaactttcactcttcagtacagtacgaactctcattcaactttttggc

acattaaac

>38345|AGAP007877-PA

cctgtcagagcactcttatggggaggtggtaatggttgtggcttgctgggaggtggtgatgttggaggtggtgatgaaaccgatgattttgtaactttcc

gaatctgttgctttaaatgatccacccattgtgtctgatcatctcttgtctgacaaacagctactattcgctcaatcattggacctgaaatttcaaaagc

atttttatatgtatctgtatcatcaagtttattaacatttatcccagtcaatggcagttttccctcatatatgaaggcactcattcgctgactaacacta

agcatcagaagtgttgacggaaataacaccaagtagcggtcccgatgatcaggcagtatcgcaacagatcccatatgaagaatttctcccaaacaagaca

gattttcaccttcccaacctctcacacctccagttaatacttccagttccaactccttctgacgtcgagttgtagatatgaaggatgctatatctttgta

cacactaacagaacgctgtgtatcccctctgtcaggatgactttcttccacatgtcgttccagttcttgtaacataccggaatacttttcaagacgtcta

aatggtttgctcaagcctgttgtcaagacaagaataccgggacttactgcaccatgagactccatgaatgaatttaactggtccctgtatttgtcaagga

tgcacacagccctcggatgggaggagcaataaacatgatgtacttgtttcagtctaggtgcagaggtgaggaaaattttccccacgcgttgttcttgtgg

tggacgctgactgcattcctccaacgctgccagaagctgctgatgtgtttcaacaacagtaactatgttgctgactaactggcgatattcatccctggag

agtatgtccgacttctccaggggactgaggaagtttttcaacaaagtctgcagctctgctacatgggctttctcagattcaataaggtccttcaaaacca

gatttctgtatgctttctgttgcgctgcaatatctggagggagtttcactggtgagatcttcgtagttcctgaagcaccattctctaagggagtgtactc

cttcacataattggaaggaaaccagccagtcttctcccctagtgttccttcccaccatcctccttcctctctctgggtgactgttataatgtcccctttc

ttgaagcaaagctcatcattgttcttgcctttgaaagagtagacagcctgcaccaaacacacactgctgtctgaagtcatggtggatgaaattcacaaca

taccagtggaacatggatcacctcagacaaaaactgtgttgactcttgacttatttaaattggaaaattgtggtagtggcaatgcagctgcttctttcac

agttcttttcacagtgtttacagcattttcaattccatcacccttcctgaaaatacatataagatatacaatcatttgaagtattctaatttttggtgct

gtttatttgtcgcagctgatagcagtactgagtcactttttaactgtttctcttgctttaaactcctatttgaaaatacatgtctttcttcaccacggat

acaaacattatactgatatatcgataaatcaacgctatgcaatagatgtcatacaggag

>38483|AGAP007928-PA

tttttcaaaggcacttgaccggcccctggtggctggccaccatttcttgcatcggtaactcggcaagcgaccatgttgtatgccttctcttgtttccgac

gcttcctcttgttgcatattttaacagcacacactgacaatatgattgcaacaatgaagaacagtatccctccaacaacacctgctgtgattgccttgtg

ttttacacgagcaggcacagggtacttgacttcatcactactctcatagctacggaatgaattggccaggactctgaagtgatatgttcgtccaccaacc

aagttcttaacaaggtaagaagtttcctctggacgaatctgacctttgtttaaggttttccaagctgcatctgtgcggtacttaatagtatagtactgca

caagatgggccctttctaaaggtgcttgccatgttataagaaaaccattgcctacttccgtcactgtcacattgcgtggtggtccaggttttggccctgc

agaaggtggagaaaatggaaagtctgttcctgatatttgatttacacgtatagttggtatgccctgatcaagtgtcctgacagtgataactttgctaaac

attccatctccaagttcatttttgcccactacctggaattcatacgttgtgcccggagttaagctattgatcgtgacctgagtgctcccagagggtgtca

caggtattgtgttccaatctgagaagccagattctcgataccagatagtatactcctgcttgtacccagatcctccactgtatcctggaagccactgcaa

tgtgacagcaaactcacttgcattgcctgagacgttatatggagcatgtggctgcgtgccttcaacaacaagctgggtgactgtcacaattgtcgctact

tcatttgaagctacacactggtagaatccaaaatctgcacgccgcaggccctcaattgtaatgttgcccccctgcactcgtacacggttccgctgcagag

gtgatccatctcgccgctgccactggatcgtaggtttctgcgtgccttcagcttcttgtgcatcacaatgcatttcaacagtctctccaactttgcgctg

atatatgctctcaggttcaatagtaaatacaggaggcttacgcaccaagacttccataggaccagaagatccctgagtgccttgtgcattgtaaggagtg

cacgtgtatcgtccctggtggctttgatttacacgtgtgaacagtaaggaaccattattcatgattacaatatctttggtctggtatggttccaagaggc

gtttatctttggtccacgtcacgtactgcagtggtgggtttgccttaatgtagcactgaactacaccggccagtcgaaagggcaggtactgaactgtggg

tgtaaaggtcacctttgctggatattctacatttagaaatgctgaggcagtctgtggttctccgataccattggtcacctcacacatgaactgcccagag

tcatctgcagcaacgggatttataactaaagatccatcccgcttcacagtgacacgcgtctcgagggaagacacttctttaatgggaactccttctctat

accaccgtactgtcacattgcccggcaatgcctttgcttcacatgagaattggactttctcaccttccatcttagtttggtttgaaggagggaccataat

cacagccccaccagcaatgatcactcttgctgtgtgactgatttgtccttctccatttcttgcaatacatgtgtaatccccaatatcctcatgccggata

ttggaaattcgtaactcagtcccatcattaaagatgccaatagtggctgagggctccaccggattggcatctttgtaccaaagtatttccggtgtgggcg

tgccctcggcctggcaatttagaattattgcatcgcccaaatttacataaataatatcttctggtgtaacactgaaccttggaggtgcgtgtacatccaa

atggaaccatgtgccattcctgggctgattaggtggtctgttcagaaagaccactttacactcataccagccctggtctgactcacggatgttggtcagg

ttgagggaagcaagcccgtagggcgagtccggtgagacgcgtgacacgcgaccctcataaccttcaccactgtgggtcgggtagctgtcgtaccagatgt

agatggggatctcctgacccgtttcgcctaccttcttctcccactgcaacacgtagggcacgggatgatcgcccgggaactcaacgtggcagttgaacac

cacagattctcccaagatggcggtgatgtgcactgcatcttggaagcaaaggcctaagggcgcgagtaggagtaggctgaagtgcgatgcggcgagcgac

agcagcaaggtcacagcctggcgacaccacgtgaccactcggtccacgcgcatgcccatccctctgtttccatagtcactgttagagcgccggcgagacc

atcacccaacagtggagtgcgtcctcggtctctagcagcatggcgcgaggcgctgcggtgatttcatgccgcccctctcaccagcccatggttttctcag

tactgcaggttacaaggtgaagatcagcgtctgggaagctgcgccgctgactcagcactgagggcgccccctgtaaggatgatagtttgaagctgctatt

agtgcttcatggtctgctaaccttgtgttctccgctgggttttatctcatccagtggcagcgttggtacaggaacgctcacaacgatatccaacaatgca

cagtttaacgaaacacccgcaggcgtcgcacgtggtcaatcgaaagttagcgcagcacttggtcagcgaggaaacgcgctggtggcagcgggcgagactc

ggttcgctgggacgcgttcactgtctgtcgggaggaacagaacaaaacaggctccgcggggcaggttgccggaatggcgggaagaagggaagggacgagg

tgcggcaggcagagtccgcggctggaggctcaccggggc

>38513|AGAP007932-PA

cgttggaccgcgacctgtcgacggagatgccgggcacggggaagaggagcgccggcggcgagtcgcgtggcacgtagcggtagaactcgcgcccgtcctt

g

>38514|AGAP007932-PA

cacggtgctgcaccaaaacgcgcgcctcgagtgccactacgacctggagggcgagtcgctctactcggtcaagtggtacaaggacggccgcgagttctac

cgcttcgtgcccagggacacgccgccggcgcagatcttccagctgcccggagtcgtcgtcgacatgaacaactcgacggcgagccagg

>38603|AGAP007933-PA

tatggcttgaaccttgtcggctctggagttgctggtctgcacggtagccctgctctccagcacgggcgccttctggaggcggtcgctctcgacgctctcc

tcgttgctcctccagtagacggtcgcgatggtggccaggcacttgagcttcatgtccccccgccggaagtggcgcggccgcaccttgaactccaggccga

g

>40101|AGAP007934-PA

gtgcttctgtaatattcatgctgtattcaataaaatgccacagatttgcaaactgcttaacggactggaaagatcgatg

>40099|AGAP007934-PA

gtgaggcacataattcatatgttttttgttattcacacacttcatggtatacaactttcattaattttattgccacaaacatataatactatggagagtg

aaacagaattttttttttccactcagcagacagcttaaaccaaaggtgactagaagatctgtgttaaataaacctcccttcatgtagcttataaaattat

tttatggtacatagtatgttgtacagttaacaaatatcacaaataatgtcaaaaataaggtgcacagtgccaagtaaaatcatcagttttaaaaagcaat

gtcacaaaaatagggatgtagaaaatgacggtggctacaaacaagctaaaataaacttttccccgtttcttcctgtggaaaacaaccactgtggatcaca

atttaagttaataatactaacaagatttttaggcttaagtcaaatagcaataaaggaatacatattttctattacaatacattgcatgttacatattagt

ataatctttcttaaacaaaaatgtttgatgtagataatatgcaacaaaagaaccagcctgagaataagtcaactttgtcacaggaacatagttctccata

ttaatgcaatcgtgaaacatctgtgatcacagatcagaaaaaaagaggtggctccatttttgacatttctcttaactgtcatgtagatataaaatttcag

gctaccaactgtttatatttacacaaactctttaaaggattaatttattcacaaatgatcaagagatcagatccacagccacctaagtgaaaaatcctac

ctagcaaaactaattcagtctgaattctgattttttttctctccattacaaaggaacttggagcttaatcacaaacaaacactgataataaacagtttgc

atattttacttaaaaggcactggtatccgattttaacatccacataaaaactggtatccagctgtcccagctcatgttccagtaataaacaccagcaaat

gacttaaaaaaagcacaacattaacagtatgctacgtcctggaagcaatactactattctgttaaaaagaggtggaatctggacagcactaatctgtatt

aataaataattaactttgcagaaatgttatttgtacataactctgcttttaaactaatatagttgtgtaaaaatatttatttgaagacaaattttatgtt

aatgcactattatttgtgctactttaacaatgtgatgaaagtagtactctctcaacacagccactggcatcttctgtacatagtctcgggcagtgactcg

catgaccagagataacttgtacactgaggtcagaacattcactgtcttctcttcccaatcgcataacactcgacaactgtacaggaagctcttacacgtt

atacttccaagtacgtatactgctgttttcactgcattttcgaaacctcaaactttgttgtcatgatttcctcatctgaatccaagataggtaatgcagt

gtcaggcacagatgatgggatgttttcttcaacccatttcaggtcatcttcaacagaattagaagactttggcttaggagaattactcctgttgcctcgc

attttcaccccaaaagcatcattgacaaattgttcatcctcctcgtcgtcatcttctggtgcatctaagagtggtgtgaaagcatacctttggttgttgt

ttgttggtctccacagcaccattatgactaacaggataatagaaaataaaagatgccagtaagcatcatcaacccatagttctttccagtccgttaagca

gtttgtgaatctgtggtattttattgaatacagcatgaatattacagaagcacccacagcaaatataagtgtgttggtgaaatggtggtataatgaaagc

ttcaccaaattcctacgaagtctcaaagttctagttgtttgaacaagactagtaaatatccaccaacaaatgatagagtcgagaacagcaagtggaatgc

cagatataagaacttgattgctgcggtcattcttctggtgcataacacggagatagctttctattgatgctaagacaaaatatagaagtccagtccccac

aacacggtgcagcattggaccaagccgtggcttgacaatgccaaaacctaaactgacaatgattaccaacatgcgtgccaacattcgttttgcacaagaa

accagctcagccatcaaaacagcaccctttacagactgccccgttgaatttatgctttgatattctgcatagaatgtggccttttcaagcatacccagaa

gtattactccgccaatccaaaactgaattcttagaagatctcgccactgacagaaagaaacaataagccaagcaattccgtaaagaacatacaccacaca

catggctccataaaatgcaagaagtggccaatcagcagcagaaagataaccatgtggacctttcatatctatctgaatagaaacattgcaaggtttctct

ggtttttctacaacgacattgaggagatacataccatcccttggaattgctattataattttgtggtcatgttcaacactgtcctggttgtcaagcttca

gtgatgttgctttgggctctgtcttttgtgggggcttcttatctacattatcttgtttttctgctatagttccaccttcactgtcaggactcctcttttt

cctggttttacttgcagagccattaccatctccttgagttttctcctttgtattgccctgctgcaaagcttttgatactgctccttcagacacgaaggac

tgcctcgaattcatgcatctaaatatcttcctggtctctgaagttttgttgtcagctgggacctgagcttcaatgatttcatcatcgtattcagggctcc

ctcgctgaaaaatctcactccagcaatgcagttcatacattgttgcttttagcaccaaatcagtttctgatgagtcagaagagcaatcaactcttacagt

tgccactgatccattgaacaaactcttgggaatagactcatgatattcttcaatcatatcatacaagttcaaaacccattttccttgttctggaaaaccg

ttaataacttgcgtattaaacacaacgcaggcaacaaaaagagctaacaacgaagaaaaactcatatttctcttaaaaacttcactttctgtaacaattg

tgtccactttaaatatttcaaacgcaaccacagtttcactctttcccctctgacacgccgcttccacgaaaacctcc

>40120|AGAP007961-PA

ttttttttttttttactcacactttctggcacaaatttctccatttgcgaggtataacagttgaactggactggtttgttccattgcgcccaagaacttt

agcattggcatctagagctaagagggtgtaacgtgtggttgatgtctcaggaagatacagagccaaatccaca

>40135|AGAP007967-PA

ttaagatctaaccaggaccaatattatacagttctcttcaacatcaacacagaggtttattaggagtaaaagttcaaaagctaatgcaattttctgattg

ttaggtgtatctatgtgacgcctgtaaatcaccttcagatgggtggtagtttttctacatttttttctactttctgttcaggaatttccattactgccag

ttgcttaccctttcttaaatgtgaaagaacaaaaatactaaaccaacagcaccccagtatcaaacaggaataaagtaaagaataatatgaaaaacaacaa

caaattctactaggttaatgtaaattgttttactaacaactgtgtttaaaattaagacagacacttcaatacgcacataacaaattcatcttgagtacaa

acacttgttgcaacacctccgataatacaccaaggattaattccagtgtggccaccagtcacacaggtgcctgctcgcttagcacaattctcaaatcctt

tagcgagcaatggtaatatcacatctctctccttgtcactaaacttt

>40241|AGAP008003-PA

ccatactgcccctgtcttgtgccttgcaaaagcaagtgttgtactagataacaattacatagttagttcttcagaaagtggagagatgtgctgctgggat

ctagttgatggaaaatgcagggaggtggttaagctagcacatgtgcacacaactattcaggcataccatatgtcaggctgtgatgacgttcgtctattct

gcaatggatattattctgaaattttagtaatggatccatttagtttggaaattctctttactctgtcatcaagggttaatcctgactggataagtgccgt

tcatgttcttcgtccaaacaagagaaaagatgatgtagtactggcattgactacaactggaactgtgaaagtttggactttgttgggccaagaaactaaa

tcccaagaacctctatatgaaaatgagagcaaacagattcgctgtttgaatgccttgtctatgacgtgctgctcctataatcagcgcacagtattaattg

tgtgttccaaattctggcaggtaactttacatgcattttaagatatgtatttaaaattcatacttgagaaataaattgtctaaaaattctgttaattgca

acagtgttgtagtatttcttaacatttttgaattttgtgtccttgtggaacctgaggctgg

>40355|AGAP008007-PA

tgtaataaaacacccatctgaaatgtcgaagtttgtaatgtatagcgcatgctgaaacagttagttacaagctcaccacgtgataatgaatgcatccaga

g

>40356|AGAP008007-PA

accagaactacttgcaaaattttgtgatttagtgctgaaaaaaagctgcagaattcctgaagaagcagaactcgaggatacactcaatcaagttatgatt

gtcttcaagtacatagaagacaaagatgtttttc

>40357|AGAP008007-PA

aagatcttggaagaatgtaccagttggtatctcacattttatttggtctacaagacttacggagcctgtttgacatccatgttacaaacatgggccttgc

tgccatagatcaagttggagaagctgctctgaatgatccaaaaatttatgtggagattatactggaagtgtataagaaatacaattatttggttttgaca

tcttttaagaatgatgcgggatttgctgctgctcttgacaa

>40358|AGAP008007-PA

aactgtaccgaagaaattctgtgctttctcgggtatagtatctttctgtatcagccagaaatacatcttcaaatgcctccttgtagagaatcaagttctt

tcctttaccttctggatcctcttcatttgaacccaattcaacataacagttgataactccacttactaatcttgtgtttattgtttctccattacgttcc

ctttcaattaatttcaaaacagcatttgtcacctgtttgttcaactgtttaaacaaat

>40419|AGAP008041-PA

cccaacgtccaattgatcccctgcacaaggaacaaaattagtggttgccatgcaaccagccatctcagccagtcgagtagctgtccatagagaacatggg

gtctttcccatggattacagaacatcctctttaagtctaccttctccttacaatatggacaagtctgtttcttacccacaatgcaccatccacggatgca

aaattcatggaataaatgatcacaggatagtttgtaagtgttttcaattaatccttcctcattttcctttgcttgtatgtgattgccacacacagcacaa

acacctctttctagatgcctcgtagggatgccatctgctcggtaatactagagtccattgacaacaccagaatagtctcagcgtgaaagaaaaatctggt

aacgtgtcatgctagatatttgaatgtaacgcttgtatgtgtcaagacacacacaaggtgttcctatggatgaataggtacaagcacacgaaaagaagtc

cgtccctccaacagctggagaagtaag

>40417|AGAP008041-PA

agtcaagcgagaggcatgggtgtgtgtgtgattgtttttctgtaagttgcattgtttgtaaatttgacctacaaatatggatgaactgaacagaacactg

ctggacctgtcagaagatgatatgcgaaggttggagcacttgcggatgcatcagcagcataaaggccatgaagccatgcatgctgaaatgatccttatac

tggctgtaacactagttgtagcccaagtagcattggttgagtggaagaaacgtcattttcgttcctatcagtttattactttgctggcattgtggataat

tccacaggtcatgtcactacaaaatcgatggtggcgcttcattttcttttggctacttttttcctgtataacaggatttattgttcgaaaagctatgcaa

aaaccaatactgggaacaaccccaaggttggtgtataaatggttttactttctatacaaacttagctatgctctcggaataataggatatgccattatga

tggcaactttctttggcctgaatatggtttttgaagttaaacctcatgtgtggatggatgtaggattaatttttgtcttttatggcctctattatggagt

cttgggaagagatattgctgaaatatgtgctgacaaaatggcatcacatattgggtattaccgagcagatggcatccctacgaggcatctagaaagaggt

gtttgtgctgtgtgtggcaatcacatacaagcaaaggaaaatgaggaaggattaattgaaaacacttacaaactatcctgtgatcatttattccatgaat

tttgcatccgtggatggtgcattgtgggtaagaaacagacttgtccatattgtaaggagaaggtagacttaaagaggatgttctgtaatccatgggaaag

accccatgttctctatggacagctactcgactggctgagatggctggttgcatggcaaccactaattttgttccttgtgcaggggatcaattggacgttg

ggacttgagtgattgcatattttgcatgttgttttatttgtttcattacaaattgtccattgtaatagatttgttttgtgtatgatgtgatagtgctgtt

ttttggaagttctgtccaatttccatgatcttccaaggttgcatttccccatttattactttgtgcctaaggacatgtacgaatgtgtacaatgggaaat

tttgagcagtgtgctataaacatctattttttacataccagtatcaatgaaatctacaaatagtacttcatgagtgagaggggacgagagagagagagag

agagagagagagagagagagagagagagagaatgtctgtaattgttttagttctaatttgcccattttatgttcatcttttttgtacacattgtctttta

gtggttcagattccatgtgtatatgcaacatatgagatcctgaaagttttatgttgtttaccattaattattgacttctgattccattgtaaactattaa

atatagatgctttataagaacctgtaataaagaagtgtatgacagtctgaataatttggacgaattcagcattttcataagtggaatgcactttcgtcat

gtgggaaaataaagaaaaaaaattgaaattatataataaagtttctcctctttagtgggcagctacaagtataggagagaattgggaaagggaacagtca

aacaaatgtgaaaacatttttaaatgaaatgtgctttgaatcagttattacgatacttcgtttacactgacttaacaaatattgttgatgttaatctata

cttatctttatatacagactgctgaaataaaaagacttctagtcagattttcaaagatcacctttttatatatcttctgcacccatacattaaattaatt

gtaacactgctcagataccaaaaattttgtaactattttccttgtagttacatgaataataattcacaaaaataaacttaaaatttgtgcagaaggaaga

atatgaaactaccatcgaatacatgatacaaaaaaaagcaatgtatctctggtcataagaaaactggctgctgtaataaaaagaaataattccgataaat

tttcacaaatctggtcactaggtctccaaaatttttgtgaaaactggaagcttaagtatgtaaacacacatttaagaatataaaaacatgtttcaaacat

tagcgtaaaacttttgttccgctatgtactgcctctccccttatttattgttcgtctacacctcttccaaactgttgtttgtcccatcccccg

>40521|AGAP008059-PA

ctccttgctcgggtcgtacttgtccagcagcttcttcatctgctccgggtagtgctgcgacaagtgagcaaagaagctgcccaccttctccttctgtgtc

tctatgcacttggcgcagtccgtcttcacgatctcggccagcaccgacttgagcaactttccggcg

>40519|AGAP008059-PA

tctcgtctcggcactggccgtcattgtggtcaccgcggcggaagagaagtacaccaccaagcacgacaacgtcaacctggatgagatcctcgccaatgac

cggctgctcaacaagtacacacagtgcctgctggagaacgacgaaaacaactgtacggccgacgggaaggagctcaagagagt

>40516|AGAP008059-PA

gtcgatcagcgagttccacaagtgtggcttctcactgacgaggagcgtgatcgccttcttagcctgcgccttctgcttctcgttacactggccgcagtcc

gtctgcagggcgtccggtaagatacttttgagctctttgccctcgtcggtgcaagcgtcgttggagtctgagaggaggcacttatggtactccagaagca

cgcgttcattgtgcaccacctggtgcacatcgatatgctcgaaatggttgttgcagccactggccacggctagaagtgtcacaaacgagaccaagttctg

cattttgacacttcgatgcaactgttcagg

>40518|AGAP008059-PA

gcaacaatgatgccagaaggtcgtagtgatatccagataaacaacaaaattacatcgtgcagttatatattttcactactgaatatattttcactactga

agttcagctacagagtagtcgtttaagaagagagctttgccagttcagcagcgtgcctctgcctgtaactgccgtcagggtcgtatttgtcgatcagcga

gttccacaagtgtggcttctcactgacgaggagcgtgatcgccttcttagcctgcgccttctgcttctcgttacactggccgcagtccgtctgcagggcg

tccggtaagatacttttgagctctttgccctcgtcggtgcaagcgtcgttggagtctgagaggaggcacttatggtactccagaagcacgcgttcattgt

gcaccacctggtgcacatcgatatgctcgaaatggttgttgcagccactggccacggctagaagtgtcacaaacgagaccaagttctgcatt

>40520|AGAP008059-PA

gcacattccgaggaacaggctcacattaaaatactttctgctttccatatttgactgtacttgctgaggaaatatccagttggatcgttcttagcaatta

gttgatacatctctctcggccgatgcagggagacgtgtgccagcagtctggagacacccttattgtagtcctctgggcagtcagcgcagttgtcccttat

tttgttgtaaatcagtctcttgacgactgagcccttccgcgagcaaggggcgtcgccgtcggcgaggaagcagcgcagtgcggcgtcgacgcgctcgggc

gtggccagcagcgcgtccacttctgccaccacgttacgcctgttctgggtggccgtgggcggccgctggaggctcgagcagagcaccgcgggcaccagca

acagcataagaagcgccgtcacggacagcgtggagtgcgccatggttcccagggactgctgtcgctgtgctatggaaactgcgcgttacgattcgagctt

ctggcgcttacaagctg

>40571|AGAP008064-PA

ttcccttaaaaaacttcttcaaatgtttattataggatcttcacaaaagttcatgcataaatattcactattatgttgaaagtaccacaaatgcagttga

caacaagtaacaagggttaaaacagctctcaagttctttattgtatactcatttaaaaaatgacataagaatgctttctcccatgaactgaagacaaaaa

taattaacaagaatttctgcaataatatattaacatcagcaacatactgaaattggatgagcggtaaaacgataaaaaaaattgactaataagaatatca

cattagagcaacaagttataaatgttctatgattgctttggttaggtgctgaggtgttggtttctctgccgttgtccacacagttaaacctgcagtcttc

agagaggcctccgtagtgggcccaattgccacaatcttgagtgtagccaagtcaatccccagtttccgaaacacaggtaccgcaaaacgaactccagatg

ggctaaaaaaggcaacaatttctggaagtccttttttggttatagctctcaaattttgttcaatgtcaggatgcggacaagtgcgatatgatggcacagg

atccaaatggatgccaccaactgctaatgcaccacttaggcgatc

>40629|AGAP008073-PA

atcgtattccttcacacaatcttggaaaattttaatttcacttgagcacattgcctgatcaaactcattttgcttcaggcatgctagcattactgccatt

tcttgtacacagctagcctttgatgttttgtctcctttccctgacacacttcctttcagcttcaggggtaaaatctcttgaaagggaacaggttccattt

gtggcctacggcccggacctttaaataatgctgtagttaacttcatgcctggtgtctgacaacacttcttttgaaaaaataaatgtgttgtacagcaaac

tcagtttgtaaacatcgcgtggataaacaacagccaaagactttatagctcttgaagacgtaaactcatttagcagccccagcaataaacacgaccagta

cgacttgttacaatcggaacttcattcgtctgtcacggctcccaaaactggatttcgtaaacggattctccaatatcgtttctgggtaattatgtaaaca

cacagcagctcagtgccagccgtactgcgtggatcgcgtgctgagctgcaagagtgcgtacggtactgcttagagccatagtcacctacttgcggattac

gtcctcg

>40798|AGAP008077-PA

tgatcatccccgcaggaaagtgaaaacttataaggactggaagccagttgagacactcacctggaactcgactttgtacagcaccgggaacttgcgccgc

tgttcgcaccgtttggtgaagtgtgacaactccaca

>40840|AGAP008118-PA

gtggaagtgatacaacatttctgttttcaatacttgctagttgaagaaaaaaccagtcattgtatgtgaaaaaggcaataaactcatagtaattagtact

gatgattaacaaataaagcggagtgcatcagactgaaaacattgtttagtacgcaataagtttggcaaggaaaagtaaaataatcatgtgatattaatgc

tagcgcctttatttttgacagaaacaaattgagttgcgcttttaaattaataatgaaaatacaaacttttctgttttatgcttgcattgtccagaagctt

tggatgtggatttgtctggtgtctcatacacaaccccttccagaagttggtgaggcgtatgacacgcttttttgttacaacaaactgtgaagatagtctg

cgtcgattatgtgatttgttggataaaattggtttttcatggaagattgcaacaacaagagtggtgaccataacaactgttgacaagagaaagatgcagc

ttgttttcaaagcaaatgtgatggtgatggacggtaatacgcttttggatttcaggctttcaaaaggttgtggcctagagttcaagaaaacttttattaa

gttgaaagaatgccttgaggatataattattaaggggcccgttatgtggccaattgcaattgctacaaacacagttccttgaagtgtgatgtgtgtatgt

aaactgttcccccacttgtatccaagtgggaaaagtaaattatctgtaaatgtcagccatctgcaggtttttagaaactgtgataatttttgcaaattgt

ataactgctcagtgcatatgatttctgaattttataaaatatataatgttgtgtacagtattgtctgaatattttaagctttgcttactgcaattttgtt

aaccag

>40955|AGAP008133-PA

ttttttttttttttttttttttttttttttcagacttgaagcgggaatgaaatggccgaatactcggagtatatttgcagaacaaataccttaagcagtc

cggcataccttggttgtgaggataactcaaggcagccatgtggtaaaggggcaataaatgctgtacgacaattgattcttaaaagcccaaccataggtag

tacacgcacagacgatgaatttattttacgtttccttcgtgcacggaaatgtaatgtaccagatgcctttgagcttctgtgcaattattgcagctataga

caacaaaacagcagcttatttgagaaatttggacccacagacacagctctccagcaggctcttcgtgatgggtttcctggagtattacctcaaagagaca

ggagaggaagaagagttcttgtgatttttgctgctaattgggatccctcttcatatgatttgcttacaatatataaagccattctttactctctggaaaa

acttattgaattgccacgaaatcaagtaagtggctttatcttcattgtcgactggagtggattttcattcagtcagtcatccaagctgaatccccgtgtt

ttaaagctaatgataaatggcttacaagactgcttcccagcaagattcaaaggggttcattttataaaccaaccatggtatgtcgaggccgcactgacag

ttatccgtccatttctgaaagaaaagacaagagaaaaacagcatgcaagaagagagtggagtgtgggtcctcccacacacag

>40954|AGAP008133-PA

agttgacaaaggccttaatggcccaaaggtatacttgtgtgaatcctattgttgtgcctatcgtgactcagcatctctgttgtatggtgagtagcagctt

tccttctataatagtgttacattacaccctggtttttctattgtttgaatttccgttgttgcaataggctcaaatcactgatatgccaaattgaaaataa

gaatctgacagtagtatatttctagtgatgtcactttgtttcattgattctgttgcaatgcacactactgcccaattttaatcttgacattgggttacca

cagatttgagcctatttcacttctgaaggtgtcagtttaaactggtgaagtgaactaagtgaaggttttcttatgctgttgatgattgactttttatctt

tgttgaaatattctgcaatactgaactacagctatgaacaaaatcataagttaaaagattcactagacctttttttgacacattttgaaaaggcaacaat

ttaaaaacatttacaagtgcttaaggttagaatgaaagaataataaaaatttaagttaaggtcagttgttttttcaaaatatttaagtaaatttaatgtg

atacagcattgagtataaccagtcagaagcaaatggcaaggttatttagattactttgtcatttaataggtgtaagtgatacaaacaacagtgtaattaa

agcatcactcttataaacaataacaaaaatttgaagcacataactgggcataaaacataaaacacctatctacaatagcaaaagtgtctgtatacaaaga

tcagacttcagagaatctctatacaaagatacaacatcaaagaaatttttttaaagctagtccatcttagtactaattgcgatgttcacagttccaaaac

attcaagtgaaggattttacaaatgccttaccgagttctgtgtagaaatatgaatattgctgacaatcatataaatttgatggaattaatatacaattta

cagaaattatatacaaccttgttgggctggcagacaatggactgccacataaccattttttgttgattagcagcaacaggctgtttgttttctgtacttt

catacataacagatttagtttttttcttctctgtcgtatctctttaacattctaatcctctctgataccattaagatcagctactactgtctgggccttg

acctcagcctcccaatgaaaaatggtaacgagagataatcccacatttgtcccacaaccccatccttagtcatggatttttcaagtctctataatatttt

tcattaataacataatttaaatcatataactctagaaaccctaaatcatatctagaatattgcaatccatgaagtctagtacagtttttgtcaaagtatg

aaactcactgtcaacaaccttttaaattgttaaaatttattcacaaaataaatctgatcaactagacacacaactgaggaaacactaatttaaaatatat

gatagctgcactttagtgtattgaatactgaaactgtccttgccttaacattttatgccaaaacaaattcagatatttatcatataattttttccaaaac

acattttttaaaaacagtatgcttttatgaaatatatgttttcttataaatataaaaaaatacaatttgtacaagttgctcatttgtgcgaacagtatca

caaatccacatacacacttattaagactagttgtaatactgaaataccaacatttttaataaagtgagatcattccataacatcataatacaagccaaaa

ttgtcatctccaccaactaataaaaatcacatacacttgtggaatgttggcaagctttactgataaatcctctggcaccaactaccgagaattctccttc

cttttgaaaggaggagtcagctgaacagtattattgtctgtcctgccgtcctcagcctccaaaactttgtctgcccacgagagctgattgtacgagggtc

cttctcctccaagctctgctggcagtacatctcgtgctatacactcatgtagtgtactcatattgttgccgtgtagatatattttttctcttgtcttttc

tttcagaaatggacggataactgtcagtgcggcctcgacataccatggttggtttataaaatgaacccctttgaatcttgctgggaagcagtcttgtaag

ccatttatcattagctttaaaacacggggattcagcttggatgactgactgaatgaaaatccactccagtcgacaatgaagataaagccacttacttgat

ttcgtggcaattcaataagtttttccagagagtaaagaatggctttatatattgtaagcaaatcatatgaagagggatcccaattagcagcaaaaatcac

aagaactcttcttcctctcctgtctctttgaggtaatactccaggaaacccatcacgaagagcctgctggagagctgtgtctgtgggtccaaatttctca

aataagctgctgttttgttgtctatagctgcaataattgcacagaagctcaaaggcatctggtacattacatttccgtgcacgaaggaaacgtaaaataa

attcatcgtctgtgcgtgtactacctatggttgggcttttaagaatcaattgtcgtacagcatttattgcccctttaccacatggctgccttgagttatc

ctcacaaccaaggtatgccggactgcttaaggtatttgttctgcaaatatactccgagtattcggccatttcattcccgcttcaagtctgtcacacacct

ggaaccaatgaccacagatacacg

>41039|AGAP008137-PA

caaatttgtgtagtaagataatgaaagattttgtgttgttgttgattaatgcaaaaatttcacttgggctgggtggtgtccttattgttcttgcatcagt

agcagcgtctgtaggattttttggatacatacgtgtgccagttactatgattattattgaagttatgccttttcttattttggctgtaggtgtagataat

gtgtttattattgtgcagtcataccagagaagcaaacgttttgagaatgagagtcaagcagagcacattggacgcattatgtcacatgtaggaccatcca

ttttactttcaactttagctgaatgcttgtgcttttttcttggaacaacatcaggcatgccagcagttcgtgaatttgcaatgtattcaggttttgctct

tcttgttgattttgtccttcaggtttcttgctttgtcagtttactggcattggattctcgcagacaagaagacaaccgttttgatatctgttgttgtgtg

cagagctcacagtcagactccatttcatctgatggtgttctgtatgaattttttagaacaaaatatgttccattcttaatgaattcagctgtacgggctt

ttgtgtttgttgctttctttggatttctgtgtttcaatattgcttttattcccaatttggaagtgggtctggatcaagaattgtctatgccaagggattc

atatgtatcaaaatattttgagtttatgaaaaattatttgtccattggacctccagtgtattttgttgtccatggggacattgattactcacaagatttt

gtacaaaattccctctgtggcagtcagtattgttaccataactcgcttacaactaagatatttagtgcttcaaggcagccaaatatcacatacatagctc

gaccagcaacttcgtggatagatgattattttgattggtgtagtattgaaaactgttgtaaatattttcccagtaatggctccttctgtccaaatgacta

tactaaatgcagatattgtgatatcaagctagataaccattatcgtcctgaagcaggagattttaaaaagtatttaccatatttcctggaagacaatccc

acaacaacatgtgcgaaagctggtcatggctcgtattctagtgccgttaactacaagtttgataatggcactttgtcagagtttagagccagttatttta

tgagttatcatactgttctgaaaagttcgagagattattatgaggcttttggggctgcagtttctctgtcagcaaatatatcaaatgcaataaatgaggt

gttaagaaacaacagtgttgattcagaagtaattgtctttccatacagcgtcttttatgttttctatgagcagtacctaacgatgtggcatgatacactt

aaaatcttaggtgtttctgttgcaggtattttcattgtgacatttgttttaatgagctttgatatagcatcagctctaatattgatgttaacaataattg

caattgttgttgacattgctggaataatgtactggtggcacatctcactgaatgcaatttcacttattaacttggtgttgtcccttggaatatcagttga

attttgctcacatctcctacatttttatatccattcagatgagagcaatagagtgttgagagtctcagattcactgactaatattgggagctcagtattt

tctggaattacactaacaaaatttgttgacattgctggaataatgtactggtggcacatctcactgaatgcaatttcacttattaacttggtgttgtccc

ttggaatatcagttgaattttgctcacatctcctacatttttatatccattcagatgagagcaatagagtgttgagagtctcagattcactgactaatat

tgggagctcagtattttctggaattacactaacaaaatttgttggcataacagttttagcatttgcaaactcacagatatttcaggtgttttacttcaga

atgtatctcctcattattctttttggagctggacatggtttaattttcttacctgttttgctaagttatattggtcccttgagaactaaaagtgtaccca

ggaatgtgcggataaattcagaaattgtgggagacagcagaaccagcagctagttcactggcattttatgatgtttggttgtgattgatattgtcatgaa

tttatccagtgtactagaagagttagaaatattcataaatactctcttactaaactcattgttgtagacttctaaatgtacatcgaaggaaggaatgtga

aaag

>41038|AGAP008137-PA

gttctgctcgcccctggtccactcgttcgcagtttctgtggaggaaacgagggttgcccgtgtcacggatgctctggtcaacatgggcagctcagtcttc

tctggtatcacactcacgaagtttggagggatccttgtgctggcgctggccaagtctcagattttccaggtgttctacttccgcatgtacctggggatcg

tgctgttcggagcagcgcacgggctgatcttcctgccggtgctgctgagctacgttggaccgccgatgaacaggcagaagctggccaaccggtcgcggca

ggtgacgtgcggcggcacggtggtgcaggtgacggagcgcacagctcagagcgctgcaccgctgcagccgggccactgcatggagacggcaatgactgaa

cggacctagactaggctcactccactcgccgtcccgaaactgtcaagagttctaaacggagtgtcgacggaatctcgagtgtgctcttgtccctccacaa

ggagtgctttgacagtttggtgttacttcctaaaaattgagtttgtgctgtggttttggggaagtgacttgcagtattagttataatgttgctttgatgt

tagtaagtccagtacaaattctcagctcaaaagaatcaaaattccataaaaaatcccctttgttcaggtgcagaaatcttcaaatgaatcctggtaaaca

tgtgtaaatgtgcttaatatcaaattgttcatgcagtaataaaatgtattgtattgtggttattatttaatcgatgatgcacatttaactaaatgtatat

aaaaaaaagtgaactctgtatgtgaggaatgtgggaagtgatctgacgtaattcacaagtgaagagtgattatgatgatgatggtgatgatgatggattt

gcttaacacttgatcttcagtaaatccaatccatcattatctgcattcccaatgtggctattcagactatcatgtgaccacttggcaaaggtggtttgaa

attaactagataaaatatttattctgttggagtatttagaaccctgaatgctctgaacacccctgacatttagaacagtattacctaaatgttctcctca

aagttttttttaatcatgaacttccaacacaaaaataatccttcaaatcacccaagcacacttcccaacacctgccactggttacagattcaaactcaag

acgtgggccagccaattgcatctctagctttctcagccctctgtctttgtggtctcattctcttccatgttttcaggttaagaaattgtgttccatttta

ttataaaactgtcttgccagacagttccacagttgaaaaagacacttgaggttcatgagagacttgtccgcaataccagacatgactgatttgtaatttt

tgaaattttgttagtgcgcagaacaaaattttttaggctgtttattttgatgtttgtgggatgttatttagcagaatctgcttttactgaattttggtca

acagaatctctcttccaggtttaatgaagtgaagttgaagtaagttgtggtccttattacttactgctgctgtgtttaaaaatatttatttttatatgaa

ttagaagccagtattaagataaaacttgtaggatttagttttgttgttagtttgtggagattgtctgaaggagaagtgaagctataaagtctcccacttg

ttttgtttcagtgtccatcaaactcttcagtgttttgtgtgtaatgacagctacttatcaggagatgcacagagaaattttgttttatgtttttgcatat

ttgagggaccaataaattttgtaatgtgaataccttttctgctgtggtaaagtgtttgtggggggaggaggggagggtgttgctacttaatggataaaaa

ggttcgtggtggcctcaaagccacattcttaatacataaattatttggaagtatgcatctgtcaaatttggattgtatctcctttattttactcaaagct

tgtaattgggcacctttgtattagaattttgtatcatgaggaattgcacatgaatgaaaagtgaaccatatttaaactattgaaatattccttgaggcct

gatgtactttaattttgatgtttagacttcttgaaaggaatattatctgaatttttaatatgaaacgttttatggttgttcacaaggagtttttctggaa

atttagaaagtgatgcatttgtcaccactatttttgacgttgaaaagataacgtatacatttctcgattgggtttgtgtctgggctctttcaggcaacag

tttgtgggatgtataagtatgtatttgttatatatatttttaagactgtcttttgtaatacatatcaataaaatttgctattgcagatttttaatgtgtg

tatggtgtgtgccaaccaaggaaactaataattggttcctcaggtttacaggattttgtttttaagcttcagatgctaataaactgtttcagctatcact

tgatggggtattactacaatttgatacttccaaaagctatactccatttttaactttttatagtaccctcccccctatggtcacatccttataacttttg

acttcaatgtttacttttgtaacactgacctcagtcagatttcagtgtgctttttcttcagaatgacgattttataatcatgtgtattgatttagtgttt

acaatgtgaaattctgtgtacattttatagctttatttttgtattgttaatattgctgttcttaaaatattctgtaaactgatgtgtactgaagaatgga

gttgcaggttgtctctgaacttaaagtggcaatttacttaggtagttggtaactggtagagaacgtctgtctgtccatctatactttttgatgtggattg

ctgataagctgctggcaaagtctgcctcggcacacagcagcattttttgggcagatttgcacttgtggtgttgattggtagttgtgaaagtgtgaatgtt

tgcactctgcagtatttcctatatatttattgtgtggttcactgtaaggaagtagatttttatatgttgaaactgactgtgtagtagtagtaaaatattg

gaataaaaaattaattttcaaaaaaacacaaaaaaatagaaaaaaacgag

>41082|AGAP008141-PA

aatgaacttcagctgataagctctaaatttagtgatgatattgagaagatatgggattacactaacagtgttgaacaatacagaagcactggtggaacct

gcagtgacagcattctgcttcaaattagaacattgaaagaatgggtagagaaggaaaaactgcagttttctaaaatggagatgcctgtttcttcaaacta

aggcattgccactataaaccatcactgacataaattgttacaagtcacatcctgatatgacattcacagaggaaaagaacataagaacatatcaaatggg

gctttgtttctcatctttgtactagatagagatattcattatgtattagacaacattaacacattcaaggaggtatgtggtgtgatttgcttctctgaaa

attttatgttgttctgagtcattattaagactgttgtaagagatttttattctatggagattgtggagttaagtcctattgaataacattctatgaatgt

tctacattcagaaaaaattgttgtttagtgttccagtcccatctaaacataaagaaagagtccatattcatattaaac

>41081|AGAP008141-PA

ctaatgccacagaagaagaatccagacagccttgaactcattagagggaaatctggatgcatttttggacatttttctggttttatgatggtcctcaaag

gtctgccttcaacctataataaagaccttcaggaagacaaaagagctatgtttgaaacatataacaatatggaaatgattttaaagataactaaggggat

tgtggaaacaatggaggtaaataaagaagtctgcctgtcagctctaacaccaaaccttctagccactgacgttgcatattacctagtgcggaaaggggtt

cctttccgtgaagcacatcatatcgttggtgaagttgttgctacagctgaaaaacagaagaaacagataactgatctcaccgtaaatgaacttcagctga

taagctctaaatttagtgatgatattgagaagatatgggattacactaacagtgttgaacaatacagaagcactggtggaacctgcagtgacagcattct

gcttcaaattagaacattgaaagaatgggtagagaaggaaaaactgcagttttctaaaatggagatgcctgtttcttcaaactaaggcattgccactata

aaccatcactgacataaatttgtgagccataattataaaatacatcattaaaaaggatattgtattactatttttaaggtcccatgtacacttttctttt

gtgctaatctgaatgatatattatgtatttaataagtgaataaatgagaaatttagtaagtgaataaatgagaataaaaatacaatcaatt

>41131|AGAP008143-PA

gtcatttacgtcttgaatttcattatcacatttttctgtacttcccaaaggaagaacaaatcgtgtctctgcatcagtcttaattagacagcctgggttt

tttatgtcaacatcagctgatattgcatagaaatatctgtagggctttcccaaacagtcagcaa

>41129|AGAP008143-PA

caaaaactgctcgcccaacttccttccaggatttggcatctaatacaagtcaacatttaaaaaacgtttatttgtagaaaacataacaggtacaaataag

acttcatgtcactgtattagtaaaccagccatgcagacttttgggcacaggtcctggcgtttcaaaaactgctcgcccaacttccttccaggatttggca

tctaatacaagtaagcccgctttattctcctctccttttccccaaatgatagcagatagtagtacaccatcatcttcctcctggaaacattgacagataa

catggaattaaacattaaaactctcaaattgtgttttcattgcaactgaagaagtcatttttccactttccaagttttataaaagaaagaaataatttac

agcaaagatgtttggtacttcagccagcatgttttgtatactgctcattactttgtatgtgaatctataatcatataatactaataattttgtttctgag

ctggtgaagtttgactctttctaattagcataccatttgcttcctaattgaaacctacgataaggtgactaagacacaaaacttgactatgagctgtgat

tttgacagattaaacattagtattggggcagaagcttgtttcccagtaggaataaattaactttgtattactgtaattttattgctgtaacagactaaaa

tttaaatggcacatttagtcatgttgagaagcttgtgaagtgctagaaattgagtggagtgacttgcagggtgcccataattgaaataccaatttcaaaa

cgctgtagaatgaaaaccactgggccagaataacataaaatttgaacagcatgtattgatgataggggaaacatcatggaacaaaaaaagtttaacgaaa

atttgaccaacagatcggcactgtaagcatcagaatatgcacaccaacagacatgaagcccaaggctgttcctcagtctgtgtctgagacacccaacatg

atctctccttgaaggaagtgctttgctggttaaacacttttacaagaatggtgactgtgcaccagtagcccagcagaggttgtggatactcaagaatata

aaaaatggcatcagtccaatgtctgttaagggactcgagaaaatgattacaaactttgaaagtaaggttctttggaagtgcaatgtgacagagagactaa

agcagttgatccagcatctgttgaggatgtggccacagcactgcaggtgagatcaagtggtggtgtgcaaaaatgtggcacttgggaaattgtccgtaca

atggagacatgcctgtgggcacagtgcataaaatcttttgaaacatcccacattgctatccatacataatcacccacgttaaggagttgcttcctgctga

cgtgccagcaagacagacattcagtctggaattacttgttcacatggaagtggacattgaatggccatggaacattctgtggacagaagaagcccacttc

catctccaaggacatgtcagtacacagaactgcagaataagggcaacagaaaatctggacacacttctagcagtaccacttcactccacaaaggtgaccg

tgtggtgagatttgacagcattgcttatcattgggtcatatttttttggaggagatcagtaccatcattggtaaaaactatgagaatcttttttgcactg

tcattccaacccttcaacaacatggatgtgcatgtaagattatttttatgcaagatggcactcctccacacaatgcacagatagtgaagtggctgctgta

gaggcattttgaaaatcctcaaattaatctccaatattcccctacagcctggctgtctagggcacccaattttaattcacgtgacttctgtctgtctgtc

aggttatctgaaatatgttgcattcagtgcttaaattacaaaaacagctaaactggagacattcattgtacaacgcactctgaacatgacccctgagaca

ctgaaatctgttgtggaacatgttgtttcctgatttcaacttgtggcagaaaacagtagactgaatgttgaatatgtcttgtaccagtctcacaacaact

aaaaactgatgttattttgctttttatgcagtttttcaacctctgataataaaaaaccaatgtcattttaccttttatgctgtttttgggtccaggataa

ttataaactaatgtcattttacttcttaagaagcttttgg

>41130|AGAP008143-PA

cccggatttcaactcggtcaagagagtgtggctctacatcaagcaggctgcacacgtttaacctagcacagcttgccacaacactggagctggcatgact

acatctttggtggtgccttccagaacatcattgactattcaggcttttgacaagtggtcacacaatgtgaccggacaatgcattacaatgttggctttga

agggaaaaaagctcaaatattacaacatttaaaaaacgtttatttgtagaaaacataacaggtacaaataagacttcatgtcactgtattagtaaaccag

ccatgcagacttttgggcacaggtcctggcgtttcaaaaactgctcgcccaacttccttccaggatttggcatctaatacaagtaagcccgctttattct

cctctccttttccccaaatgatagcagatagtagtacaccatcatcttcctcctggaaacattgacagataacatggaattaaacattaaaactctcaaa

ttgtgttttcattgcaactgaagaagtcatttttccactttccaagttttataaaagaaagaaataatttacagcaaagatgtttggtacttcagccagc

atgttttgtatactgctcattactttgtatgtgaatctataatcatataatactaataattttgtttctgagctggtgaagtttgactctttctaattag

cataccatttgcttcctaattgaaacctacgataaggtgactaagacacaaaacttgactatgagctgtgattttgacagattaaacattagtattgggg

cagaagcttgtttcccagtaggaataaattaactttgtattactgtaattttattgctgtaacagactaaaatttaaatggcacatttagtcatgttgag

aagcttgtgaagtgctagaaattgagtggagtgacttgcagggtgcccataattgaaataccaatttcaaaacgctgtagaatgaaaaccactgggccag

aataacataaaatttgaacagcatgtattgatgataggggaaacatcatggaacaaaaaaagtttaacgaaaatttgaccaacagatcggcactgtaagc

atcagaatatgcacaccaacagacatgaagcccaaggctgttcctcagtctgtgtctgagacacccaacatgatctctccttgaaggaagtgctttgctg

gttaaacacttttacaagaatggtgactgtgcaccagtagcccagcagaggttgtggatactcaagaatataaaaaatggcatcagtccaatgtctgtta

agggactcgagaaaatgattacaaactttgaaagtaaggttctttggaagtgcaatgtgacagagagactaaagcagttgatccagcatctgttgaggat

gtggccacagcactgcaggtgagatcaagtggtggtgtgcaaaaatgtggcacttgggaaattgtccgtacaatggagacatgcctgtgggcacagtgca

taaaatcttttgaaacatcccacattgctatccatacataatcacccacgttaaggagttgcttcctgctgacgtgccagcaagacagacattcagtctg

gaattacttgttcacatggaagtggacattgaatggccatggaacattctgtggacagaagaagcccacttccatctccaaggacatgtcagtacacaga

actgcagaataagggcaacagaaaatctggacacacttctagcagtaccacttcactccacaaaggtgaccgtgtggtgagatttgacagcattgcttat

cattgggtcatatttttttggaggagatcagtaccatcattggtaaaaactatgagaatcttttttgcactgtcattccaacccttcaacaacatggatg

tgcatgtaagattatttttatgcaagatggcactcctccacacaatgcacagatagtgaagtggctgctgtagaggcattttgaaaatcctcaaattaat

ctccaatattcccctacagcctggctgtctagggcacccaattttaattcacgtgacttctgtctgtctgtcaggttatctgaaatatgttgcattcagt

gcttaaattacaaaaacagctaaactggagacattcattgtacaacgcactctgaacatgacccctgagacactgaaatctgttgtggaacatgttgttt

cctgatttcaacttgtggcagaaaacagtagactgaatgttgaatatgtcttgtaccagtctcacaacaactaaaaactgatgttattttgctttttatg

cagtttttcaacctctgataataaaaaaccaatgtcattttaccttttatgctgtttttg

>41181|AGAP008158-PA

agtaaggatccttgttcccaagttggtgcatgtatagtaaataaggagaagaaaatagtgggtattggatacaatggaatgccatctggttgcagtgatg

atcaatacccgtggaataaaacaagttctaataattggaaacaaaatatttttatg

>41180|AGAP008158-PA

caaatacagccaactgaaaaaagccacaccattacatcatgggtcaaagcagatgggtggaattgaatgcttccattgatcccaaatatgcaagaggcat

gttcccgtacagttcatttagtagccatataaatggatctttgtctttgcaaataacagaatagaaaaaaaaaaaattggtgacatttatttataagacc

tggtttaaattgttgtaacctcatattgtacttacttgttttgtgatgcagtttgtcatgccgaaatgaatgcaatattgaacaaaaattcagcagatgt

gaaagactgttctatgtacgttgctctgttcccatgcaatgaatgtgcaaagataataattcagtctggcattcgagaagtcatttatatgtctgacaag

aattcccataagctggaaaccattgcttccaaacgaatgtttgactcttct

>41179|AGAP008158-PA

agtaaggatccttgttcccaagttggtgcatgtatagtaaataaggagaagaaaatagtgggtattggatacaatggaatgccatctggttgcagtgatg

atcaatacccgtggaataaaacaagttctaataaattggaaacaaaatatttttatggtctgtatgcctttgactcattgttcagtcactctttcttatt

tttacatgtatgcccattagatagtaactttcatgaaaatctgatatgaaaatgtttacattggtgtgcaatttatatgtttcctctaccactgataata

gtttaatatgttgactactgtaaggccaccagtagtcacagcagagcctgattctcggcctggccagtggcaaaaggtacttcactgtgtgtgtgtgcag

cagtcaatttcaaattgtcataccataatttacccatgtttggaaaccaaaaaagaagtgcttttagacactcagttttagcatgtgttcttacttagta

cttttgtgaatacaaaaaatgacctacacttattttctttggaaag

>41178|AGAP008158-PA

ctctcgacctctccctgacagtgcaagtccctatctttaaccttcctcccatccaactccaaacatgtaagcttaaattataatgcgctcaccaaagttc

ccaactcaattgttaattttttgtgatgatcttccaagtctaacatgcttaaatctgctatccaattcgttaaattcattaactgtgcccacaccctatg

cagctgactgtcactaggttgggtttcctccaaaagacaaatagtgtcctcaaggcctttaaaagcctccaacaaaaattcaattgccgctcctgtctca

ccccccactgccaacgtgatgtcaactggctgaagaatggcagcgggcaaacaggctaccaattctggaacactgtcctcaatcgattggccccttacgg

tcctttctgagcaggctgatccccggacactgtctgactgtcattatgattcaccagtacttgacaccagaagagtcaaacattcgtttggaagcaatgg

tttccagcttatgggaattcttgtcagacatataaatgacttctcgaatgccagactgaattattatctttgcacattcattgcatgggaacagagcaac

gtacatagaacagtctttcacatctgctgaatttttgttcaatattgcattcatttcggcatgacaaacataaaaatattttgtttccaatttattagaa

cttgttttattccacgggtattgatcatcactgcaaccagatggcattccattgtatccaatacccactattttcttctccttatttactatacatgcac

caacttgggaacaaggatccttactacgttttgcagcaagaaaagcagtggccataaagtattcattccactccaaataatcctctctcttcgaatttat

atccctgttattgcaagatgtttccgccatgtgccttgttgttccttttacagacgggtttatttaaaaggtccctttgtggctttactaattttagg

>41224|AGAP008206-PA

caataaagaactcgaggatttcctttccaagaccactcaagagacaatggactaccgcaagaaaaacggcgttgtgcgagaagacagcatcgacctgttg

atgcgcgtccttgacgagtcgaagcgcatggtggacgagactggtcaggaattcaaattcgaccagagggacgttgtggcgcaggtgctgagcttcctat

cggcgggcttcgagacctccgcctcgaccatgatgttcacactatacgagctggccctgaaccaggagctgcagcagcgc

>41244|AGAP008210-PA

aaagttttgtgggaaagcagagcaatgcagcagatgtcacggacgcgtacaaacagctaaaagggcaccgttttggtggaatttatttcttcaaccgacc

cgctctagtgttagtggaaccagatctaatacgcagcataatagtgaaggatttctggacattccaagacagggg

>41326|AGAP008246-PA

aaaacacaaaccaggtacaacaaattctgcatatctcaatacaccactacaaatgcatactgacctgccatactacgaatacaaaccagggttgaatcta

ctccattgtctggtacagacagaaggacctggtggagaaaatctaataacagatggattttatgttggtaatcatatgaagaagcataatccagatttct

acaaacttctttccacagttcctgtagaatggctggatataggtgaagaggatgggattaaattccacacactgtatagggcaccagtgatttgtgaaga

catgtttggacaaatcataagaataaattacagccagccacagagagactctcactttaacgtgcctttggagactgctgtatcatggtacaaagcatat

catgccttcacaaaagcactttaccatccagataatgttgttatgttgaagaattcaccaggtgacatactgacatttgataatattcgtctgattcatg

cacgtgaaggttacactgacaatgttggccgtgaaagacatatcataggtgtatatcttgactgggatattgcctacgcacgcattagagtgcttcgtca

aaatgctggaatgctaaaaacatgagacagaatttggtggcaataaaaaaggaagcttaagcacatcatttaaccatgaaatgatgacagtgcaaggttt

aagtgataaattacaattcaaacaacaaaagaacttataaaaccacattagatattgtgatacatctcgttttctccattaaacaaagtactgtttgttc

catcataagcatatgtaagatatcatacactgtaaaaatattttagtatggaacttcaacattcttgttcagttatatttatctggataagaaatgtgat

tgatggaaaaacttagaaataagaacaaagttagcagtgaaatggaaagactcagtattattactagtcacacttttctcaaaaataaactgccttttct

atagaaagtgtaataaataacttaattgtacagaagttatctagccacagagatctacgaaaatatcttagaaaccttaaaacactatgagaataagaat

tcaggtgacctacacagtaagtgtaggtatcaacaagccatgtaccacaaagaaaagtagctagatatttggaatgaaataacctaaactggaaaaatgt

taaacaatttttcttacatatcctaataattactatatcaaaaaactaccaagactgattaatttgcagtatatacatatagggtgctaggggtacaggt

acagatattgcggtcattggtaccttaataaatacccac

>41360|AGAP008275-PA

ctcatgacaaaaaatgtgtgaccgacgaccagaaaagcctgttgaccacgtaacagtcgcagactgggatgctcgcattcaggagctgcgagagacagca

cgcaggaggaggaaggaggctcacgacttgcgacaagagagtatccagctgcgtgctgacacggctgtcacaacacactggagcctgctgcaaagttcac

aatctcttgtgtc

>41424|AGAP008312-PA

gccttcaatgtgcagctggcactcctaccgttgttttcgcagcggcatttgacatgttgttgttattatttggcttatgtaatttagaaaatgtacaact

tagtacgaatgtcggaaaccgcctcggtttctgttttaatcaatggaaagaatgaaaattgatattacctttgctgttattcattgcagcttggcagtaa

gtagcttattttgttgttctgtgtagctactgtcttatcgcaaatcaacgtgccttgggtagattcctagacagaagatcatgaggaaggtttgtaaata

tgtcacaagaaataaatgttaaccctccggtggcaagcgaggatcatgctgacaatgacaacgatcaagaaaatattgtttcaaacgaggttgatgacaa

cttagtcgttccaacgagcgtaagtggtgcagttgatgaacaaacttcgaatgaagacggcgaacctgtgccagttgatgaacaaactacgaatgaagac

gacaaccatgtgcaggatattgcccccggtgacgatgacgagcaggaacttacattcgaagaagttcctgtttcacctgttatgcaagagaaaattaaga

gaattgaagctgttttagagacatcacgtgatcctaaaagtctaaaacagtttgctttgtctgaaggcggtctcgtcactgatgaaattcgaagaaaagt

ttggccaatacttgtcggaattgatgcaccatcagatactgaacttcccagccaagctgaattagaaagtcatgcagaatatcagcaagtagtgctggat

gttaatagatctctcaaaagatttccaccaggtattccatatgaacagcgagtggctttgcaagaccagttaacaaggcttatactgagggtaataatca

gatatcctcatttgcggtactaccagggttatcatgatgttgcagtgacattccttcttgttgtgggagaatctgttgcatttaaaataatggagaagct

ctctactactcatctcaaagattgtatggcagcaactatggaaaaaacttcctatttgctcaattacatatatcctctaatccagaggttgcacccgtca

ctatgtgattatttggagaggtcagaagtaggaacaatgttttgcttgccatggtttctaacttggtatggccacaatttaaataaatatcaagatgtgg

ttcggctgtatgatttcttccttgcatcaccaccactaacaccattatatctggcagcagagattgtagtatatcggcaagatgagatatttgcagtgga

ctgtgaaatggccagtatccactcacttttgtcacagattccagatgatttaccatttgaagaactattaaaaaagacaagtgagctatatctacagtac

ccaccagaaagtattgaagcagaagtttctgagaggtcacgtagagagttgagaccaaggagatctgttgttccacagagacctttgccaccacaacagt

tcagtacactctggaggtggttggagtggagtggagtagttcatcaaggagcacctcgccgaaaattcacaatgcttgttgttccagttgttaccacatt

agttgtcatttacgcctattgtaaagcagctgaggcatacacttttgatctcgtcacatcaataagatagtaaaagtagaatcaaggacaactgaaacta

tatacagtgttagattcattacagattatttgttgggcattttagcagaatcatgccttttacccgtgcaatactccaacaatactgtttttcaatgtta

cagtcattgtacctgatggcatgatctgcatctgtgggttgctagaacattactgtattctgacagatggtatatatggtgctaaacatggagtaccatg

attgatgctagtgaaatgaattaacaggcacatttgtgtgctgaggctacattcgtatcaaacttgtcaacatatataaagagcaacttatttttcaata

attatagggtgtaaagaatatttgtgagatttgtgagaaatacagaaatgtctttccttgagagtgttgcatcagtttaatagaatgtgcgaatagtctg

cctgaaaaaggccccaatagttcagaaagaatgctaatatttttatatgattcctttagcaatatatatcactactgtgtttcttttgtgatcagggact

agtgtacagctatagttatgattgattcctcaaaaaatggtatgttctgcacatgttcaaccctgcaacagtggtttggacacagatctgaagaatgatg

aaactcttatcttcaaatgatggatactgcatttatctgcttctgtaaaacagcagaggactgaagaaataagcatatatcatgattgttacataatact

tgttgatatatatatatatacatgcagaccagtgcccatatttttcacaagtgagaaataacgtgttcacagtatgtggcttttgtcattacaatactgc

agatttgtactgtactcgtttccaagtaaaaagttgagccttcaataacagcaaacattagaaagtaatgacttaatgagtgactataatgcacattcaa

aaacgccatactactaaacaaatgaagcaaagttgtgattgacagtgatgtttggaaatacacattccttggtgtggtatctttgatcatgaaagatctg

tattactaccagttgcctcacattattaagaagaaaacttgcttatgttaattaagggtctaaaagttttccaccataatatccataataggtaccgtta

aaattaacagaattgtgaagactgattagttactgttttttttattgatattgctttaatcaagtgattttgcatagaaagaaattacagtctgcaatgg

cactaagccacatttatcaggtgctttgaatgtttctgaacgcgtatgtcagtttatgccaattgtgttatatttattcctatacaaatacttaattcgg

tgcaaggagtaagctttgagataaacaacatacttgtgaagactggctcaagttactatcctcttctaacttcatgcagtaatttctataccattacaag

tgtctgttcctgtaacttcagtgcacatcatcattagaaccattacttatgtatctgaaagtgatgaatttacttctagactatgaagtaaaccaaaata

atagtagtttagtgaatgaacatgtttttaaacaaatatggcttttaatcataggatgagtattttttcttatagtattcccatccacattataacttac

agtcctattttgaacaatgtatcaaatttggtcccgtggcacctatgtattgcattttaccttcagtttgatttgatatctcaatcaacagcagagtttt

gaatagtcagtacattatgctggaacttccagtagctgtcaacagatttctgaaagcagatgcatagagattgtttcaggcagaatgtatatctatgtag

agaatattgatttgtctctctgcccattcagtgattttaaatatacttttacatttcttttaaggccatttgcaaactaagtagtgc

>41571|AGAP008321-PA

gccaggtgcctcgctgagtttgtgagtgatgagtcaaaggagaagattgacaaactgttcaatacgtaaagcgtggactatgcgtatcttgtagtattcg

tggctggagtgtattgtgaaatagcaggttcaggatagactcgaggatcctgaaactcctgctcagccagtgtctacaagtggtttaaagcttgccaccg

acgtagcaaagaactgcgaatcttcaagaagcagagatgcacaagaactgtccaacatactgaaaagcgcacactttaagggctgaataatggacaggaa

gtaagcacatactgtggagcagtttcctatctgcaaagttttgactcacttgcattagaagcaaggatccaaagcactcatattatgggcaagatttgaa

gcttactgatattgtgattatatgctctgcaacgtagaatgtatgttaccaacacagactcagcatctgtgtgtgttcagccttaatgatgacctcataa

tatgaataatgctgcatggctcttttggaatctcatgatactgtagctgaaaaaagttttgaaaagcctgaagtggaatctcaacctcaaacttttgaat

ttccagcaagtgagatgacagccgatacaattcgtatggttggcctcaggaaaagtccaaaggaacccctgggtttaacagtccaggtggatgagtcagg

gaatttagtgattgcaaggatattagctggtggtatgattgacagacaaggcctactccatgttggagatgtgatattggaagtgaatggtgttcctgtt

tcaactcctgaagaactccagcatcagatttcccagtcagatgaatcagtgactctgaaaataggacctggttttgaggatatccattctcagctgaata

gtggccag

>41765|AGAP008379-PA

atttttctgcttggacagtgtgtattctcatgttagttcccatcaactttatctgcagagtaagttcggatgtcgttcaagagcttgggcagaagtacca

gcaagtgatgagacaatgccgaaggtagcagtgcgatttgagcacactgtcgaaccaaggagagacctccatccgcgctcctggggaaagaacaagctac

aaggtgaagcgcatgacctggaaatggaactgaacatgagttcactgattgggcttgctgattttattgaagatgaaattataccagag

>41782|AGAP008391-PA

gcagcaaggagctcgcgtcgactaccgtgagcctcctgatgatcctttcccccgcccctctctctgcgatgagcctctgcgtcttgccat

>41909|AGAP008431-PA

cgggtatcactaataaatttcaaacagccgacgaaccattcttcattgatatctttctccagtaaactctcaactattttttgggcttttgcattacatt

ctctctttttctgcaacgttgcttcaaggacctccttgctcatactcctaacatttctcggtagtcctgcacgcaatgcagctttacttttccctttccc

cgtggttcgcgttacgttgcttcaaggacctccttg

>41910|AGAP008431-PA

aaataaaataagaaaaaacataaataaatacatcgatccctgaaaaaatttgatgaacaaatggaatgcacgaggaaagtgaaaaccaaacttaaggatt

tgtgcccagcgagtacttcaacggataccgactgaaacatgtttgagaacgagtgtcatgtcttttacaatgcatttcgcagtatttatgttgtgacaga

gaggaagaagttgtgtactagtgttgttctgttgtaatccgtggaggaagagttagggatatttaaagtggagaagatggggagtatggggaccaacgta

acgcgaaccacggggaaagggaaaagtaaagctgcattgcgtgcaggactaccgagaaatgttaggagtatgagcaaggaggtccttgaagcaacgttgc

agaaaaagagagaatgtaatgcaaaagcccaaaaaatagttgagagtttactggagaaagatatcaatgaagaatggttcgtcggctgtttgaaatttat

tagtgatacccactttcaagatgtagttgaagaaagggcaatcactaaactttgtgggtaccctttgtgtaatggtattttgaagaacattccaaatcaa

caatataagatatgcacaacacagaataaagtttatgacttaacggaaagaaagaacttctgcagtaatcattgttacaaggcagcaaaatatctaaagg

atcagcttctgacaagtccactttggttgcgtgacaaagaagaaattccacagttcaagctgttgtcaatgagtaaatcaagcggcatggttggagatga

ggtacactttcaacaagaaagtcttgtggatgaaataacacaattgaaagatgacaactccaaaaatttaagtgctatattaacagaatctgaaataaat

gcagaattacaaaacactacaaatgaacgtgtatgtgtaatgaagacagaatttaatgaaattctctctgaaagtagtaaaaatctcaatttaccttcag

aaagcaatgtaaatgtatttaaggcaagtgtaaatgcgaatgagtgtgctagagttaatgaaattaaagaggaggatatattggaacagactgaaagagt

aacactgacaaaaaatgagtgtagtggtgttgaaattcaaagtggtttggattatcatagcctcggtgctaaaatggaaagtttgaaggagaaaaaaaca

gcttcagaagatagtaaagactctgtaaagaagaaaaacacaagtattcggaaaaaagagaaaaaggaaagtttagataaacattcaccaaccaaagaag

ctcctttaacaacactgctggttgagaagtgtgtacgagaatggtttacacttgactcaatgtactttctttttggtgaggagagaatgaaggaaatggt

tgcagacaaggacataactcattacttcaaacctactgagagtgattcttgggatccaggtatgcgagagcgttacattgctatatgcaagaaactcaat

ttattggaactggaagatattaattatgacaataaaattaccaaggagaataaggagctgagaccactaccagagtattccagtattaaggaagagggac

tgaaaatgcaattgaaggttagggcattttttgctggaaaaactgcttacgaggactc

>41908|AGAP008431-PA

ccctttgtgtaatggtattttgaagaacattccaaatcaacaatataagaagttgtgtactagtgttgttctgttgtaatccgtggaggaagagttaggg

atatttaaagtggagaagatggggagtatggggaccaacgtaacgcgaaccacggggaaagggaaaagtaaagctgcattgcgtgcaggactaccgagaa

atgttaggagtatgagcaaggaggtccttgaagcaacgttgcagaaaaagagagaatgtaatgcaaaagcccaaaaaatagttgagagtttactggagaa

agatatcaatgaagaatggttcgtcggctgtttgaaatttattagtgatacccactttcaagatgtagttgaagaaagggcaatcactaaactttgtggg

taccctttgtgtaatggtattttgaagaacattccaaatcaacaatataagaagttgtgtactagtgttgttctgttgtaatccgtggaggaagagttag

ggatatttaaagtggagaagatggggagtatggggaccaacgtaacgcgaaccacggggaaagggaaaagtaaagctgcattgcgtgcaggactaccgag

aaatgttaggagtatgagcaaggaggtccttgaagcaacgttgcagaaaaagagagaatgtaatgcaaaagcccaaaaaatagttgagagtttactggag

aaagatatcaatgaagaatggttcgtcggctgtttgaaatttattagtgatacccactttcaagatgtagttgaagaaagggcaatcactaaactttgtg

ggtaccctttgtgtaatggtattttgaagaacattccaaatcaacaatataagatatgcacaacacagaataaagtttatgacttaacggaaagaaagaa

cttctgcagtaatcattgttacaaggcagcaaaatatctaaaggatcagcttctgacaagtccactttggttgcgtgacaaagaagaaattccacagttc

aagctgttgtcaatgagtaaatcaagcggcatggttggagatgaggtacactttcaacaagaaagtcttgtggatgaaataacacaattgaaagatgaca

actccaaaaatttaagtgctatattaacagaatctgaaataaatgcagaattacaaaacactacaaatgaacgtgtatgtgtaatgaagacagaatttaa

tgaaattctctctgaaagtagtaaaaatctcaatttaccttcagaaagcaatgtaaatgtatttaaggcaagtgtaaatgcgaatgagtgtgctagagtt

aatgaaattaaagaggaggatatattggaacagactgaaagagtaacactgacaaaaaatgagtgtagtggtgttgaaattcaaagtggtttggattatc

atagcctcggtgctaaaatggaaagtttgaaggagaaaaaaacagcttcagaagatagtaaagactctgtaaagaagaaaaacacaagtattcggaaaaa

agagaaaaaggaaagtttagataaacattcaccaaccaaagaagctcctttaacaacactgctggttgagaagtgtgtacgagaatggtttacacttgac

tcaatgtactttctttttggtgaggagagaatgaaggaaatggttgcagacaaggacataactcattacttcaaacctactgagagtgattcttgggatc

caggtatgcgagagcgttacattgctatatgcaagaaactcaatttattggaactggaagatattaattatgacaataaaattaccaaggagaataagga

gctgagaccactaccagagtattccagtattaaggaagagggactgaaaatgcaattgaaggttagggcattttttgctggaaaaactgcttacgaggac

tcttcagttgaagatagttcacaagaaaatgagtccagtggaaattttatcccactggttgatctgcattcaaagaatgctcttcgcaggagaatagtgt

tggaccgcttcaacagaatcttacccgacctgttatcaacatttggaatatcatcccgtgacataagtgatgacctacgtagccttgtattcagcttttg

cctgtcggctcacaacataactttcaagccaacagaatggaacctgttgggtctaattgtgttaaaaatgctatcactcagggacaagcggatcaaggct

gtgttagaaactgaacagtcaaggaagtacatgactatgatgctgatggttttccaagaagatgctggatatctggagagacttgtggcctggttgatgg

atattgactatcttttacaacagcatagtggcagaacagggagttgaaattgttggaatacaactggtattaatattttgattgtgtgaaatgtcattag

aaataaagtgaactggaaaaatgcttcctttactatagcagctcagaagcaaaatttaatgaatgttgtctgttttcaccacctagtgttatcaagttca

cctatttcaatgtgctccatactgaagtagcactacaaaccagatgttacagataattttatatcttcacttttccgacaatttaactgaaaaatagaag

tacttgtaaaaaatatatagcaaaacaattttatgtaatacaggtgtgaaaccattccacagattatcactttaatttttgaacactacatatttttaca

tggcagattggggtaagtctgtaagctaagttatagagtgcagattcatttaatgatcatccaaatgtggaggatcttaatggaagatcccttcaagtac

cctgtcagagtgactccctttggaaaaagttggaaatattctactaccaaatgcatttgaacacacagtatatagtgatgctgatatttttttctcaggt

atgacctgacgagagaagaaatagtgttgcatgggaaagtatcgtataaattaacctctgcttttgagcagatacaacagttatttttcgcagtgcagag

ggacagattcacacagcaataaggcacatattaattttacaattatcattgcaagaaggcttaaaagagaggaaagattagaaaggctgtaacatgcgca

tgagcgaggatggaccttcaaaggcagttgaatatgaaaaaataatattcttgagagtgatatcagtttgcaatgaagtctgcaaaaagaaaaggttgca

gatgacttagagtatggaaatgacaggtggaaaatatcattttcaagattaatatattatggatcaggtagaaggtaaggtttttgataactctttaaat

ctgaagtattttcatccaccaaaaaaggatcacgtattaaatgcaaaaagacccatcaacaataatatgacgtatgtaagcaacacttatgccaaaatac

tacaaaacttgctatagaaacaattttatgaagatatcaatctcaaaacttgaagcttggaatattatcttaaaatgcataaattgatctcggaactgag

gtaagaatctatgaagctaaggatctatgaaatgtgcatgttgttgagagggagaggggttgttgagagagagagagagagagagagagagagggggggg

ggggggggcatgaaaaaaaaatctggacaaatgtgaataggattcacactatgagagctctgtacaagcttaattatgtgtactgatgatgatgatgatg

atgatgatgataattttgtgtgacttagtggcctggtgtgagtctttctactggacgccactccagtgacttgcctaccccagttatccaacttgggaaa

ggggacctacagtttaacattgaatctgaaacacatgttgtttttggtgactcctcacatcattgagagataaaaactgggttaaaaagaaaactgaata

ctctgtgatctgactgagacttgatccagtgacctctctgtttccaggcaagtgctttaccaccggatcgccaggcccaacatatatacagataattcat

gtgtagatttttcattgccagggaacagtagatgtgtgtacctgtcacatatttcaacatgatgcccatttctgagaaaaaggggactaaatagatgggc

agacagatggacaacaagtggtaaaaaaagttatatgattaattaaaaattaacaatttccagattttttcctttacttgtactataaaattttggtttt

tgttaaatttcatggttctaggtcaacaggaagcaccgcttcacagcactaagggactgtagaccttggtatgtgacataaatttcaacttaatacatct

acccattcctgagaaaaaggggtcttaacagtcagtcggtcaatcggacagatggacagacagcatagtgatgctacaggggttcctttttaactgtttg

aagcaccaaaccctaaaaaaagaatagttcaagtgaggtgcagaacaaccaagaattaaaaaatattgaccctaaactaactgaaaaattggcttcgtat

attcccaaaaggctgaaagtagttgtaaaagaaaaggactatatcatgatattagagttttgtgcattttactgtgcccagtggtaaaacatggtttaat

ttcacatgacatggatagtaaatatttgtaatttactttgtcttacttctacatagcatataataaatgtcagtgtaaagatggatgatattgaagttac

acaggctttcacatgttgtgtaaataaaaatacctcttcttaagttggaaatgtattcatctgaacagctttgttaaaatggttgtgcttaaacatcact

gctgttgtgtaacaataagcctgtaaaaacctcaaatgacttttactatatatttctggtactccattgtatggtcattattttattatggactattctg

tgttataaatacaaaacattacagctgttgtgtaacaataagcctgtaaaaacctcaatatgacttttactatgtatttctggtactccattgtatggtc

aatagtttattatggcctattctgtgttattaatacaacaaaaatgaacaaaaagagaaaatttgtgttaatacagaggtgttcaaatcattaggctcaa

gttccgtacaagagaaaagacaatactgtaatgtatagctgagataacttggaa

>42261|AGAP008484-PA

ctctgacgttgatgtgcgtggatggatcagctgtggtgaaaaacactgtgccaacaacacgacttcagtgagaaaatggagatacaggtgacaccggtcg

aggacgacagcggatgcgcggcgtcagagctgaaggaaggagaagatggctgctccgaggcgtggcgctggtggcgctcgctgggcagcccgcgcttcgt

gctggcgccaatggtggacgccagcgagctggcgtggcgcctgctgagccgcgaactgggcgcgcagctctgctactcgcccatgctgcacgccgccgtc

ttcgcgcgagatccaaagtaccgccgcgacgcgctcgcctcctgccccgaggaccggccgctcatcgtacagctgtgcggcaactgccccgagacgctgg

tgtcggcggcgctgctggcgcaggaccactgcgacgccatcgacgtcaacctgggctgcccgcaggccatcgcgcgccgcggccgctacggcgctttcct

ccaggacgactggccgctgctcgccg

>42286|AGAP008487-PA

tcacccagatcaatatcaactgtccattcatatggaggctcagtctctgcacagtctaagtcctgcagaagaacactacataattgatttcctgtcagag

ttggggtgttatcaattatgtacaccattatatcaatacttccctcaatgttgcctctgcagacttcctcagtctgtatgttgaatgtgatacaagcttc

caagagtaaagcttctaattcatcttttgatttcccactcttgtaatactcgagtactgcattcacaaatacatcacagagtacacaagtggtctcctgg

tcctttgggctcagatctgaaagatattctgactcaatcttatttgtcagggt

>42306|AGAP008522-PA

ttctacatggtttttgtaagtttttactaaattgcagcgccagttgcgagtattgtggatcatgtattcctaaaataataaaataaaaggtagtgcggaa

gaaacgtcgacaccatgagtgacggtactgatatttttctaattgtcttttttggaaaagaggtcttgtggcgcctatataaaataatccttagcaaacg

atgtgttgacttcaccgttcatttcagaaattgaagtgagtctcctagtagttatcctggatactaatccaagtcagcggattattagggaagatgttca

tgttttgacgcagtgtatggaatcagtagtcgcctttgcaaattcacatttgatgctcaaatcgaccaacaaactggctgtattggcgtgccacgcgaga

agcacgcagtttctgttcccaccagttgatggaaactctggtcagcaagtaccaacacttcgtcagttagatgggcagtatgagctctttacttatgttg

agaaaacattacggcagaatctgcaacagatggtggtccgtgatgttgaaaaaataaaagctggaaatgcagagctctctggagattccctcattgctgg

tgctattgcaatggcactttgctatatccatcgattggatcaagagaaagctacaggaacgaagatgaattctcgtatcttagttgtgactggtagtggt

gacagtgcctcgcaatatatgaacttcatgaatgtattctttacagctcagaaacagaatgttgtcttagatgtgtgcagtttggacaaggagcttggac

tccttcagcagggctgtgacatcactggtggactttatcttagagttcctcagctacaagggttgctgcaatacttactgtgggtgttccttcctgagcc

tccaatccgaggaaagctggtcctccctccacctgtcaaagtggattaccgtgcagcgtgtttctgccaccgggaactcatagatattggttttgtgtgt

tctgtttgtctctccatattctgcaaattcagtcccatttgcacaacatgccacactgtattcaagagtcctggtcccttgccattgaaaggcaagaaga

aaaagataaaactgacgtaaggggtatactcttgttcagagtttcagctaatataag

>42462|AGAP008556-PA

gtagctcttgctgatttcaggaaatgcttgactatagtcatggaaagaatcaatcagctttcggttagttttatgattgttatgtgagataggcgtagaa

gtcagagcctgtgtaatcacatgaactgcttcagtgctctgtttcagtttccactgcagttcttcattatgctgtgaaagcctttgtttctgcacagcaa

catgatgaaatgactcgttcagtttcctgttctcttgggatagctgcctttcgaagtcaactttgtgctgcagttgctcctggagatcctcaactcgcgc

ctgcagtgttgctatcctttgtagtgctcctggtagctctccagcatcttttgctagttcagcattctgccttcttacctctgctagctcttcatgtctc

aaatccagcactgtcctcaatgacctcacttcttcttccagatcatgacatcttcctgctgtgacctgcaactgggcgtcacggtcttggcgaaggccgc

tgacaaggccagctacctgctgctgcaggtgagctgtctccgcatcggctgcctccaggcgcacgcgcagctctgcggcacgctgctcactttcactgag

ctggcgtttgtgctccgcctctgcctgccgcagcctcattacaccatcctccagcaatgctgcctctcgctctgcgtggagacgctccaggcgtgaaatc

tcaccctcgtaccgacaccgcgtctgctccgtctgcagaaaatagaaatgtttttctaaccgatggtgccttgtatatgctgaaactgtctagtcaacta

tctagtatcaggaatagctgacaaagtccttaatttgttgctcaggtagtactgttaacgtcagacagac

>42461|AGAP008556-PA

tttctccaaatagtagcaactctttatttttaccttacaagatagtgacattctgtaacaagacatgatacaattccaatgtacatacaaacaactgcaa

tatcaattgtgctttgaaaacacaacataaatacagcaaaaaaattcacacaaaatatactatttcatcagccacacattgtactcaaaatcacaataaa

atgtaagtagcatatggttttctgatataaaactaacaaattaaaacagctgaaattttataaaagttgatccacaagtatcacatatacaaacacagaa

tgcggagacagttcagtagtacgaactagctctggctgagttagttcacaaagcacgtattccacacaagtagtaattctaggtttatcatatctgtaaa

ttatcagtattttaacagcatcaagttttagatttattagataagatgtattgtgtgaactgtcaccactgtgcactaatttacatctctcaaagcctat

taaacactcttacttacataatattttgtagctatgacgtatacaaatctaacagtcatcaaacagcctctgataaactttattacaagcagtagtaaat

ttacaaatagctcacatgtatgtagccaaaggaaagctgtgtaataactcaacacaagatcatcctcttacaaatattacctcaaaaaaagaggaaatga

tgttactttggtaatgataatttgatgagtatacagtaattgttctcaagttagagtcacactttaccccatttctcaattactcatcacatagttcagc

atttttttgctcaaagttcagtggcttggcaaatcatcatgttagtatcacgaaagttatgtataattcttccaattggtccaaaacgattaaacactga

gaatagcagtactgtttatgcataaaatatatggtgatcaaagcatttaaaaataaagcagatacaccaacagggatgctcttggaagaaaaacataaaa

tggatattcttataatgataacttcgataatttcagtgcatgttttgcttgtattagttcagcactgtgtaaaaatgcagtattgcatgatagaaacttt

taaaatatgacatcacattcagtgatgttcagtaactgatgagtgaatgttccaattttaatcccagtgctgcagtatttagcatctggtgttttattct

ccaataaatcaatgtttaatcttgatgatgttcggcagtttatgatccacccacaccccaaaatattatgaaatggaaatggcattagtcctcacactta

attgcaattaagacctcccccaagtcaagtcaaataatttattatgcatattaacaagctgaaagctctctcaatccaattgataaattctatatacatg

attgaaggtgggagcaccaaggttttgctggaagaactctcatgagaacctccaacaaccaaaacaaaatattaaggcacaagaaaatgcaagaactaaa

agtggtccattcatcctacagatcattactgcaactatcacgacatgatttcacattttgaagcttccatcacacaaaccagcactataaaaccagttaa

atacaattaacaataacttcatcaggtactgatttaaccaaattatcaatgaaaaaaattaaggagcccagtagcaaatctgagtcatctgtaaaaaatc

aagaggtgtatgcaccatgctttcaaaaaagctgataaattattaatgagaaaaattaagaaacagcacaataaagagtctgagtcgtgtgtataaaacc

gagagctgtatgcaccatactctcaacatacctgatatacaatattcaatgccattgtgttttgtcttaggtttaaaccaacacacacataaatgtaagg

cttaaaatgccagagtattaagtctcaagtatttcagaacttctaaaatactccagcttcaagcaagcacgtacaccagcaagacattctgcctcaggcc

tcattgtctgatggtaacagccccacattctcccaggactggggatcttcaccatcagatgatgtatcgcccccatctccatcattctcattgtccagtg

tctcttcagagatcattgcctctcctgccgcatccttgggagctcccatcagaggtttggcagcagcacaggcagcagccaaaatggagagatcactgct

accagaaaggtggggaatgggaacaaccctcatatctgcagtctctgtctccacttgccggtaaataattgtgccattcaagtgtctgtagttgtgttct

gcttctgaacagctgcttgtggaactgcaggtattgctgccaggggggctggcactgctgctgttgctgctgccacaagtgcgcaaactcaacaactgtc

catcaaggctggagggaccactatctgcgttggacctctttgcgacacttccatttgcattatggtgctggcgcatgtcgtcgtcgtcgtcttcgtcacc

gttaacagagcactcgtcaggatcacggctgcagcgcttcagtggtgtctttccaggggatactgtgtaattatcttctccataagaatgtgctacaggt

gacggtgttcctgaaactgaacgctgcattgtaacaggagcagaggtagcacacgttcctgatgaaccccagcaggatacaaagttgtccgaagatgaca

ctgaccacgcccttgttctcgatcgtccagcactggttcctgttgatcgtcgatttccaggaccagatgcaccacggcttcgtggaagggtctgagactg

ggcagtagatggtggagataagccagaagagccacggaaactgcctgcacggcgcactaggcgagacacttttgcttcaggagattcatccatgtccagt

acccatgacacagagtcactcttctcaacaacagccttcactttcggtgatgctgggggactagggtgctcgagatcatgatgtatcacaggtgtgttgc

tcaagtcacaacggcgctgcatcagcggcgacctgctactcaatttagtgtagctcttgctgatttcaggaaatgcttgactatagtcatggaaagaatc

aatcagctttcggttagttttatgattgttatgtgagataggcgtagaagtcagagcctgtgtaatcacatgaactgcttcagtgctctgtttcagtttc

cactgcagttcttcattatgctgtgaaagcctttgtttctgcacagcaacatgatgaaatgactcgttcagtttcctgttctcttgggatagctgccttt

cgaagtcaactttgtgctgcagttgctcctggagatcctcaactcgcgcctgcagtgttgctatcctttgtagtgctcctggtagctctccagcatcttt

tgctagttcagcattctgccttcttacctctgctagctcttcatgtctcaaatccagcactgtcctcaatgacctcacttcttcttccagatcatgacat

cttcctgctgtgacctgcaactgggcgtcacggtcttggcgaaggccgctgacaaggccagctacctgctgctgcaggtgagctgtctccgcatcggctg

cctccaggcgcacgcgcagctctgcggcacgctgctcactttcactgagctggcgtttgtgctccgcctctgcctgccgcagcctcattacaccatcctc

cagcaatgctgcctctcgctctgcgtggagacgctccaggcgtgaaatctcaccctcgtaccgacaccgcgtctgctccgtctgcaactgggcttcgtgg

tccctactgacgaggtcctctacctgccgttgcaatcgggctgtctctgcgtcagctgcctccaggcgctcgcgcaggtctttggcacgctgctcacttt

ctctgagctggcgtttgtgctctgcctctgcctgctgcagctttgacaagccatcctgcagcaatgctgcttctcgctccgaatggagacattccaggcg

cgaaatctcatcctcataccgacaccttttctcttccgtgtcattgacgaggtactggacgagcacggcgaaagcctcgaaaccggtggcgctgtggcgc

agctgctgcagctgggcgcgcgtgggcacggccggggcggcttgcagctgcggctgcaatttgccggccggctccttgagcggcgcgccgctgcccgcca

cgcggttgtgatccctgagaggctgcggtttggcgggggtggcgaccggcgccgagcgtcgggacgccggcttgcgcgccacgggcgtcttcatgtccgg

caccccgttcttctgctggttctgcctgcgcgccggctggtgtgacgcggatgagttgagggacccatcgctgagtgggctgttcgctcgagtcgtatct

gctcgctttttcgctgatcgtagcgaggagacgctcttgcaggagggcgtggccggcgaggcgtgttctccgctgacgtcactgacggagctgcgcaccg

aactgtggccggacagcggcagcgtctgcgagatgggcgacgcgcgcg

>42543|AGAP008584-PA

tccattaggtctaaaaatatatgctccttatgatctgttctcaaatacttcagtatttccaaggaaccctttgtagtagtagaaatcctgcttaacttg

>42544|AGAP008584-PA

tcgtaggctactccgtgatgatggtatgggagttgcagaagcattacaagaagaagcatttggcaagggtttagttgccagaggtaagcactggctgtta

gtgggcaacataacaaggagtgatggttgtagtctggcatgccaagaacgtaaagtggctgaga

>42578|AGAP008596-PA

gcagcactggaggcacattttctccacctgcagttatgataaattccttgaggcgtcctttaatataaacaaatcc

>42572|AGAP008596-PA

ctgcagtgatgataagttccttgagacgtcctgtaatataagcaaatccactttcaacaagggttccctaatctccagaatgtaaccaaccttcttcacc

g

>42569|AGAP008596-PA

aaggaattcaaaaaggtatagatagggcaaatgaaaaagcaatctccaatacacaaaggatacagaaatttgctattctggagaaagacttctcgctgca

aggagattgctttttcatttgccctatctatacctttttgaattccttcc

>42568|AGAP008596-PA

ggaacattgtttcatttgtaccacgggtgacttgtaatgttccaagtaccaaatcccaaataaaggtaatggaaggaattcaaaaaggtatagatagggc

aaatgaaaaagcaatctccaatgcacaaagtatacagaaatttgctattctggagaaagacttctcgctttcatggttggtcc

>42574|AGAP008596-PA

atagatagggcaaatgaaaaagcaatctccaatgcacaaaggatacagaaatttgctattctggagaaagacctctcgctgcaaggaggtgaactaggac

caaccatgaaagtgaaacgaagcctgtaaatacgtcacaatttaagctcttgtatataagtgttacatgtgtaaattatttccttag

>42570|AGAP008596-PA

tttgaagggtcctgatcagaaggtaatggaaggaattcaaaaaggtatagatagggcaaatgaaaaagcaatctccaatgcacaaaggatacagaaattt

gctattctggagaaagacttctcgctgcaaggaggtgaactaggaccaaccatgaaagtgaaacgaaatgtagttgcagaaaagtacgctgatgtcattg

agcgattctacaagggtacgtaaagcgttgtgtgctgcatggagaagagcaagtactcaagactgttcaaggacgggtcttcatgaaagatgaccgactg

ctgctgtgttactggtgatgcttttgcaagccagaggtacttttgtatgatatacattaactatttttgaaaagatcctctcttcaagaaatccgatata

tttcggtgtttatttttcgaagctgatgtatttcttttctcagttaattgttagtactctacattgctgtgcctttattatgttgtacttattcgtacct

gttctgtaac

>42575|AGAP008596-PA

cacctgtcctaatgcatccatggttggatataatcattcagcttgcataggggcccgaagatgacattaatacagtgtcgaaactcatcgtgtaattaat

atattttataaaaagtacagctgttggtgttccttatttctacaagtttaataccatcaacagtcccaagtccgattcaatgatggatcatccagagcat

agctaagtcggtgcagacatattttcaaagcgagtacctttagaagagtaggtttcctgttaacagttttctagagatcattatgacaataactatcctc

aaaatatctaactgaacttacctagttcacctccttgcagcgagaagtctttctccagaatagcaaatttctgtatcctttgtgcattggagattgcttt

ttcatttgccctatctatacctttttgaattccttccattacctagaaagaaaagaagaaattaaagaaaaatttaacgaattttgtgacaaagtcatac

gttgctgtaatgtaaacatccagtatctgtgaagggtgatttgcttgtgcacag

>42577|AGAP008596-PA

aagacccgtccttgaacagtcttgagtacttgctcttctccatgcagcacacaacgctttacgtacccttgtagaatcgctcaatgacatcagcgtactt

ttctgcaactacatttcgtttcactttcatggttggtcctagttcacctccttgcagcgagaagtctttctccagaatagcaaatttctgtatcctttgt

gcattggagattgctttttcatttgccctatctatacctttttgaattccttccattacctagaaagaaaagaagaaattaaagaaaaatttaacgaatt

ttgtgacaaagtcatacgttgctgtaatgtaaacatccagtatctgtgaagggtgatttgcttgtgcacagcccaatatttcgttcaatatggaggaacg

ctgaatcagtttgtctaagaagcaaagaggaaggatccatgatgtgattaaaaactggaagtttccactgtgacgctcacataaaatatcagtgctaaaa

tatcagtgcttcttttataatacgaacgttatttacatatctacatcttatccttttaaaacgtttgtctatatttaatactaaggatttataaataatc

tataaatcgtgcatctgtataatgacaaatttgggaccataatattgatctaataggcatttattgaactctgtaaatattttagaggatttcattgcgt

taatccacagttcttaacaacattcagtacttagttttcagtggctgagattttataatcggaagaaaaagtaagacaagtttgtattacaaaatctgca

aagcagggttactgattattgccactgcattttttacaatggaactgcaacgtaaacagataccaacattgttcagatgatctgttcaatagaatgaaca

gcccacttagtgtgacacatgacttacagataattaaatttaacatgaacgtaacgaaagatagaaaaaggggaacagcaaattgcttagttaagagggt

gaaagcacagcagtataagagaaagaattctcctacgggaagcaagataatacaagatgactgaaccaagaaagttattagaagtaaaacactgcaggtg

aatcctaaaaaagagtaatactgatgtcaatttttaaatgcaaattacttagaagttattgaaaatgaaatgtaaaacatgagcaacttaaagacgaaac

tggaactatctgaaatttgatattatagaaggttgtagatagacaaaagaacagaaattaaatattgcgggtggagaaataaatctataggacaccttta

gtggtacgaagagatttg

>42597|AGAP008606-PA

gttgccgttctcgaacatggcctcatatttcgggtcgagcatccagtagttgcctttgcgatagccactccgacagtcgcctctgcggttgccgccctcc

ggcggcactttgacgaagcacctgtggagcgagaggttgtagcgaatggagttctgccagcctttcttgtgctcgtagtagggaaagtgactcacgatgt

aggcgttgatctccgacagcgtcgccctcctgtgcggtgaattggttatggccgtcgcgatgagctccgcgtaggaaaatggcggcttcacgtgtgcaga

gtgccgctcctctcccgatccattttcgacgctgtccgtgacggttccccctttataagtatcggtggcgccgctaccactgtcagaattgccactggac

gtcaaggccggagcagtggtgtcggccgtcaccggtaccgcgtccggtgccgagacgaggtgtgtcgagtcgggggcgtcacgtccgacgccgccgacct

cggagatcgccaggccggtcgaagtcggggcagtggtactgtttcggtggaacagagacagtttcggcagttaaagatttgaggcattctacacgggtac

agaaggacattcaaaaggcagtacagggtgtgacagaataaacggcacaattcgagtgacgcagcacgcagtcgctaatgcacctatcctaaataagtac

acg

>42614|AGAP008617-PA

ttcttaattgccaactgttcaggcgtcattttctcttcatcttctaatgcctgaaaataattaaaaaataaattaacaacttcaatggaaaacttactgt

tagttattgaacagtaatttacacaggctaggcatattccctaag

>42634|AGAP008622-PA

ttctacaagctggtgaacagacaaggtggcgttgtaaggctcgacgacagtgtcagagacctttggggaaggaaaca

>42633|AGAP008622-PA

cttcagtgttgtgccatcacctaaggtctctgacacagttgtcgaaccatataatgcgaccctctccgtccatcagct

>42636|AGAP008622-PA

tgtgtagtgctcttttgcccagttattacctgttccgctttgtccgaacgcaaagttttctggcctgaaaagctggccaaatgccc

>42632|AGAP008622-PA

cttcacctaaggtttcagacacagttgttgagccttataacgcaacactgtcggttcaccagcttgtagaaaacacaggatctggaatgggaactctgct

tatttcaaaaatc

>42652|AGAP008626-PA

attgtgacattggcattctggactatatattcaatagatcgtgaactcataatgcctaaagcactggacgcctttttgcctctttggttaaatcatgcac

tgcacacaaatgttactgttgctgcatttactgagatgttaataacatttcactggtatcctgcttcacgacggaatactgtgtatggtattgcagcaat

tacggtggcctatgctgtatgcttttactctacatactactattatggaacatggctctatccaatatatggagttttggactggacccagcgtatttct

ttatcagctgtaacatttatagcatgcatactgatgtctttgtttggtgaatatctgaattcgcaaatatggggccatgaaatacagaagatgaaagcaa

aggatttgtcaaaacaaaataaacacagatagagtttcaaacacaattttactattgaaacatctttaaaaatgacacaaaattgtagaagaaattgacc

aattccatttctgatatttcaaatttaacatgtgattagattgttaattcttaaggtatccttgctgttattgttgtggattttattggatttaagaagc

aacttatgttatgaatgtacaaatgtggagttgtggaaaaagagatacaacagttttctttcatagacttgtttgtatagtgtttttattctatccaaca

aatattgcacatacaactgaaatagttagatattggacacatgtctttcaggacacactacttctccatac

>42653|AGAP008626-PA

tatacaaacaagtctatgaaagaaaactgttgtatctctttttccacaactccacatttgtacattcataacataagttgcttcttaaatccaataaaat

ccacaacaataacagcaaggataccttaagaattaacaatctaatcacatgttaaatttgaaatatcagaaatggaattggtcaatttcttctacaattt

tgtgtcatttttaaagatgtttcaatagtaaaattgtgtttgaaactctatctgtgtttattttgttttgacaaatcctttgctttcatcttctgtattt

catggccccatatttgcgaattcagatattcaccaaacaaagacatcagtatgcatgctataaatgttacagctgataaagaaatacgctgggtccagtc

caaaactccatatattggatagagccatgttccataatagtagtatgtagagtaaaagcatacagcataggccaccgtaattgctgcaataccatacaca

gtattccgtcgtgaagcaggataccagtgaaatgttattaacatctcagtaaatgcagcaacagtaacatttgtgtgcagtgcatgatttaaccaaagag

gcaaaaaggcgtccagtgctttaggcattatgagttcacgatctattgaatatatagtccagaatgccaatgtcacaataagtgataatggaaatacaaa

cgtcctgaaagtgtaattgttcactatccttacatagtgtttgacctgtttcaagtagctatttgactgcagctgtagtacatcttggacaacacaaaga

gcaaagtgaaatgtctgtatttcaaagttccagatagtcaaaaatcttccccagtatttctcaagaccatgagtccattcaagatctactggtacctttc

cttcacctaatttagcagcgaaataataaaacacaaaaacatgatgcaaaacaatacctaaatgaaaaatgaagctctttgtactcattttagttccagg

cattttagtctctgtgtgtgctagctctgtcagcacaaaagtgttgtaccttctttaaaatcactcatgcagatgagtgaaggtcaaagtcttgtgtatt

tctacttgcatgctgcag

>42651|AGAP008626-PA

ggctgacacagttatgtaagtattactactgcagcatgcaagcagaaatacacaagactttgactttcactcatctgcatgagtgattttaaagaaggta

caacagtttgcgctgacacagctagcacacacagagactaaaatgcctggaactaaaatgagtacaaagcacttaatttttcatttaggtattgttttgc

atcatgtttttgtgttatattatttcgctgctaaattaggtaaaggaaaggtaccagtagatcttgaatggactcatggtcttgagaaatactggagcag

atttttaactatctggaactttgaaatacagacatttcactttgctctttgtgttgcccaagatgtactagagctgcagtcaaatagctatttgcaacag

gtcaaacactatgtaaggaaagtgaacaattacactttcaggacgtttgtatttccattatcacttatcgtgacattggtattctggactatatattcaa

tagatcgtgaactcatattgcctaaagcactggatgcctttttgcctctttggttaaatcatgcactgcacacaaatattactgttgctgcatttactga

gatgttaataacatttcactggtatcctgcgtcacgacggactactgtgtatggtattgcagcaatttcggtgtcttatggtgtatgctttttctctaca

tactactatgatggaacatggctctatccaatatatggagttttgaactggacccagcgtatttgtttctcagctgtaatatttatagcatgcatactga

tgtctttgtttggtgaatatctgaattcgcaaatatggggccttgaaatacagaagatgaaagcaagggatttgtcaaaacaaaataaacacagatagag

ttccaaacacaattttattattgtgaacaactttaaaaatgaaacaaaattgtagcagtaattgaccagttccattggtgatatttcaaatttaatatgt

gactagtttgttaattcttaaggtacccttgctgttattgttgtggattttattcgatttaagaagcaacttattttatgaatgtacaaatgtggagtgg

tggaaataagagatgcaacagttttctttcgtagacttgtttgcatagtatttttattctatccaacaaatattgcacttacaactgaaatagttaaata

ttcaacacgtgtgtatcaggacatattacttctccatacaaaaagatggaaatatttacaagtcaagcacattttcattttacatttccttgtaaagtct

tctcagtgcatccttgtataagtgttcaaatgctattttaagtgatgctcaacatattatgtcttattcatttttcattaaagacacaga

>42725|AGAP008653-PA

ctatgcgcgttctcagcgatgttggcgtgtactgacccttgtaactattctactatttaaagttctttggtcgcaaatcactaatgtttttgtgaggagt

gaggaggtgcgaaaatgcagaagtatttggcacttgataaaattgcgagactttttgaaataataaaagtaaaggggggaatacgaggcactttatacaa

actatataggatggatgatgccatacttgtcttctcc

>42746|AGAP008671-PA

actggcaggtgttcgcgccggagtcccagtttcgaggctatgtcggcaggattgtagcgtcgcgcatttaagctgctggtcatctccagatgtttgaaaa

ggatgaggcacgggactccctcgtgcaccaacagatcagtgtcgctggatagaatgccgagacaatggtcttccctagcggcgtactcggccagctcatt

gtcgcaatcttcggttgctacacgcactttgcagtccagttcggtacaaaaaatgactcttgcagtgggcaccacggcctctggcgtcaaatagaatttc

ctcttagtctctttcgtaagacgtcttgcacgaaaatcggcaaagagttgcgatatctgcaacaatctatacattcttcggtttgcccaagcattcatct

tttcttctactggtatgccatcgaagaagaaaactaaccggatcccactgtcacgaaatctctgcacaaaagttctgcacgactcccggaattgcttgta

ctgaccgccgcaaaacttatctgccgtaccgtatatgtgcctggcgcaggcagcaccatcaacagcaatgactggctccacagtctttgtgcgcctccat

tcctgtgcgacgtctctgatgttcacttcttcgtaccattgttttccatcaatgaacgactgaaggccccagatacccatcgcacttcttccgttctagc

gctgtatactgttgcgaattttaagtcgccgatacctgtggttgatgtgaactggaatgcgggaataaatgcttcaatgcgttggttctatgctcttgtg

ctttgtgccacttctgttcgtcttcataaatactagttgtaaccaacactgcacacagaaatattcggacaacccaccactccgacacatttgtaaacac

tacgcaggcttctgcgctgcacatccatgcactcacg

>42745|AGAP008671-PA

tttcaataacattgtggatacataaagttctaaaaattatttcattttattaagttcatttctgtacattcatattattacaataagaatacaaaaacat

aaaatatagtccaacatttcatttcactcaacaatatatctgtatgttattacctctgtatttcataaatatatttttgctgactgtaacagttaaaatc

tggagttatagagagaggctaaaactatatcaaataaattaacagcatgtatctataaaatataaacatttgtttgattaaatttcttattttgattaac

tgatgtgtgagagaggggaaaaatgaggaaactgccaccagttccacatgcagaatgttgaaagtaacaactgttaccaccaaaacaactaaataaaata

aactttataacggatgttgcacatccattgcacatctgggtacagatacagtatttattgagtaattgatatacaagacagaaaacttacaagacatgag

tagtttggaaaactggaaactgtcacacttttagaatagagatgaagctttaaaattaggaataatggagaggagggttataaatgtttcaattagactt

tgacaaagcttaattactgaaaatattttatatgtaagtaatatattatttttattgtttaacatgtgggttgatcggtttctaaatcaagggtttttgg

attgattccattttattacccgcatgagctgttcaaatttcacttcatgctgaggtttaaacatgtcaagagtttttggatcttttccatttcgtgtggt

gacacagatattgtgaaacacaatcccatcaaagtaggcatctggtaaacagtcaatttctctgggaaaggcaatcccacaggtggcagcagcatcaagc

acatgcagtacacaacgcatgaacagtgatgccaggtagatggcccgttcatcaagagttttcacaatgtttgcaggctggccagtcgcagggaggccag

caacagcagcagcagtggcacagaaggcagcgacctcgtggtcgtgcaggacgcgtgcctcgtgcttaaggaacagcagcgccgcagcaggcaccaccag

gaactgtgggtcgagctcctccagaagagacggttttgcgtagggggccaccagccaggtgaaaaagcgccagcggcgctgagcgttgtcgctggtccac

agctgcagcagccctgggtgctccatgccggcgggtagccgcttctccacctccacctcctctttcctcacctttcccgtttcggtgagaagatactcgg

tcacgcggcacaggccacagtcgctctcccacagtagaactttgtacatcctggcccgcattggtcgcaggacgaggcccgtagttgggaagtcctgttt

ggtttctgacaagtcctccagcacttcaccgatccggaacacctggcgcttcatcacggacagcagcgtccccggaatttcgcccgagctgtgcttgtct

tcaacatgcgcgaccagattctcccagctggtcggttcgtaactgtagctgcgaatgcccgcctccaccatgggtagggcatccctccatttgccgtgga

agacaattttacacatgttcacgatatcgcatgggtcctcgtctttcatcatttctgccacttgctcgatgttggtagtcttttcaggatagcgcttctt

cagattgccgtgtgccatgaataggaggtctgccctgacaaagtcattaccgacgaaagttgcgaatgttggcatgtgctcttgccctatgcctaggtga

gttgcgatgtcaactggtcgaaactggtacgtcttcagcttaccatctttctcgagattcctgaaggttagcacactcgatacacccttcattgcaaaga

agtccgagtcgttggtgagcactccgatgcagtgtttagtgcttgatacatatttggcaatgtcgtgcttacagttacccttggacagatagactctgca

gccgagatgctcaaatatgactctggcggccgggatgactgcttccggtaacgttctcactgtgctgtcggttcgcacagctcctgaacgcagactgctg

aataggttgctgacaaacatcgtcttatctttctttttgtctgtccatgtatgtatctcatcttctactgggacgccgtgaaagaacaacacgagatgaa

tgccgttgtcacggaaccgttgcacaaaattcgcgcaatagtctcgaaatttcttgtactggcctccacgaagaaaatctagcggctcgtatatatgcca

catgcatgcagatccatcgacggcgatcacaggatcttctccgcttttcttc

>42765|AGAP008698-PA

tatataaatgtgtgcaaatggaaactgatttttggaatctgtctgagattgtcttggtaattttttgtccttctatccatccttccataccttactatca

gccaaaatacaagtctgaaaaataaattttacttccagatacgacaatacgaatggtgctatgccagttctaagcgtctcaagtttaatgcaatcctaac

cacgtatgaaatagttttgaaagacaaagtatttctgggaagcgtaagttgggctgctttactcgtagatgaggcccatagactgaagaatgatgattca

ctgctttataaagcccttatggagtttgatacaaatcatcgtttgcttataacggggacacctcttcagaatagcttgaaagagcta

>42804|AGAP008718-PA

actgaggagaaaaagaggaaagtgctacatattgtgattggaaagtcacacacatgaaaatagatgaccagggcaagatattaccgcctgtattaatggt

ggaaaatgaaggtgtcaatggaaggggtataggtcatatgacatatgcagcattgtgcagtttgctgttggtaaacacgtcgttttcacaatagtggcaa

gttttcattgttatacagcattttccagttgttcatgtgttacatgttgtagacatgttccgtggaaaatattgtgtagtggtgaacagtccaacattaa

ggagattcctttgtcttccgaggatgtggaggcactaaattgacatgaggaatgtggtgctgtattcaccttcaaaaggttggagattatatgtccagtc

tcatacttcatttggtagtgctcacaaagcagtgcagaagttagtgcagtgcagtttctagtatgcaggggttaatatgccggataaagaaagaggtcta

gtgtactgtacatggttttggtcatttgttacatccatgaaatcagagctggattgtgtttcattcactttaggggatacatgaacaatcaaaaatcaag

gatttgatctataaaaagaagccacacactctggcaaaactaaagattaacacaagggagattaataacattactgtcagtgtgctcctcagattgttct

tagacgtggtaaaaagtccttgaaaagtggaattcttaactggtcagccattttgagcatatcttatgaacaatggatgtgagatatataattgagctga

aaacaaaattcattctctaattgtattaaatgtctctttgtctcaattgtatgtcaaaataatcatttattactgtctgtaggaggaatgttactgtttc

aggtattgtggctgtcctgttttgtggaatctgtcaagcacattatacctacaacaatttgtccgtagactctcgaacaaggacaaagaagttgtttgaa

ctgctcaac

>42855|AGAP008721-PA

ctccgcttcacaatacaacagctggaaaatattattcatatgtttctttttcaaaattggccacttctcatggctgtgcctttaagcccacataccagct

tttccttaaacctcactgagttataatctggtaaaattcatatggtagtatctcagtaaatgcagagggcacagcactgcaaaattatgtgcactgacac

cagccacttttcattcaaactcctttgaatgcctcctcactctctttctttgatgttgagacacaaacctgtaagatattaccgtgtcggaaattattct

caatctcttgcagggaacgcctgtatgtctctggtaacaataggcccacaaaaatagttcctgcaagtgaagttgctgcaaagaacaaaaagacatctcg

tgatgtccattctccatcacctctgtcagttgtcagggctgcagccaatgaaggatacagtttgagtgctgcaaagtttgccaggtatgcgagacatgtg

gtg

>42873|AGAP008737-PA

tatgtattgtcatttgccattattatgctcaatactagattacataatccaagtgtgaaggaaacctacagttgaacagttcatttccatgaatagaggc

aatttttgtggagcttcaggttgccaggagaagc

>42913|AGAP008753-PA

caggtgcggcttagattcgagtgcggcttagattcgagcaaattctgtattggggcagtatttggttcttgggaggaataaggagaattttgttggttgg

atgaagacaacagctggtgcaaatgagaaacaggctaatgattgttaccgttgtctttgactggagtgatcagtttttgtgagctgctg

>42929|AGAP008757-PA

cctacaaatattccggactatccgaatatggtgccttttgaaaatgtgttcaagcaggaggcatatacgtgtttactcagc

>42931|AGAP008757-PA

aaaaggtagccaacacaagcattttacaaagctgagtaaacacgaatatggtgccttttggaaatgtgttcaagcaggaggcatatccgtgtttactcag

ctttgtaaaatgcttgtgttggctacctttt

>42930|AGAP008757-PA

aagcattttacaaagctgagtaaacacgtatatgcctcctgcttgaacacatttccaaaaggcaccatattcggatctccatcacatcccgtgccagaca

gttcgccttaggaaacatcgtagtgcttcactaccatgccagctggccagatat

>42950|AGAP008760-PA

gttcaatttcttcctcagttaatttcttttttttttgtttttaagatatcaaacataaattttatttttaaaataaaaactcaacacgagaactaacaat

ggctgtatcacactcttgcatacaaggcagtagtctgttgactcatcttacacagtattcatcaccttcgagcaaaatttttgtccattgccattccaga

cctctggatgttgtttatatttgatcgaatacggccttcaacagtctcttgggatgcagcaattgaaagttgtttccttataaaatctttatcaaaatct

ttcatctgctttttctcatattcttcctgtgccttatttctggctatgttcttgtcccgctcttgttccctccatttggcattatctaacatttctgccc

ttctgcgttcaatttcttcctcagttaatttctttttttccggcttcttccagctcctgtcagccttgtatttatctgtataggttgg

>42985|AGAP008802-PA

gcttcttccacttctttcccaacacaatcgttgaggaacttcacatctttctgcaaaagctcttttaagattggaaccactggtgctagtgtgtacttct

cctgtcttctgccgtctggacgcccgcagtgtgacatcaacaccaatgacttggctcccttgctgaacacggtttgaattgtagggatggttgctttaat

tcgggttagatccgtg

>43005|AGAP008806-PA

gtgttttattgtcaatgtggaatgtataacattttaacaaatggttgtggtgtttactactttgcaaatatattgcatgtgagcaatatttacaggtaaa

taaatatttcataactgtcatacacataaaaaatttaaaagattgcattgctacaatatttttcaaataacctccaactccacacagccttaaacacaca

ttttgaagactgaagaaccattatggcacacacaaattgaatggcactttttaaggcaaatatcaatggaatattatttccatatttcaccataagcagt

acataaaatttacatgtagattctcataaatgattatctcagttactgctgtgtttgtcccatctactgttgcacacattttcactaaaccatgtaaaat

aagaaacaattctcataataattaaagctttaatgccatcagtaccatcattttaatgaactgctttgtgtcttttcatttaacaaaggaacttgaggct

gaaggggatttgtgcaacaaacatgtgccaaaatgcagtttatattattaatacacagtatattgtgaacaatgatactgcgaacaatttcctcaacttt

tccatataaatattttttcactacacatttctttttacaagacggtatccataaaagtaattgtgagcattgtgaatatattaaacctacaacaatgtac

caactccattagctgaaaaatacttagtacaaaaaaagatcaaagttaaaagttttggatttatcacagctgttattgcccctactccactaatgtgatt

aaatatagtcaatgttcaacacttaacatgggaaagtaatgtatgcaataattttcaaacactgaggcaactatttttaacagatcctgaattcaataac

caggtgttaagaatgtatatgtaccatcaatatgtataatttcttgggaaaaattttcaacataaagtttaatttcccatttcatacaggcaataaattg

caaacactgattacaaatttacgtagtggctgctactctttttacattatcctgaattcagtaacttgatattaactgtcgatatgtgacatcaatatgt

ataattacctgggaaacttttcaacattcaaaatagaaacatttatacaaaactgtggtgaacattttactattcacagtaaaagtcttcagctacatga

tgattcaatgatactcaaaaacactctgaatatgttgataaaatactgacatcgcctttatgttatcaagagcagttagcattttctctgtcctctagct

attatttcagttcatatgttaagaaatgtttgtgcagccgttcattttcacaattacatctaggcaatttattgtgtaataatgaaataaatcctgtttc

ttccaatagtacaagtattactattagcattaacagaatatgaattaagttatcaataaatataagaaaatgaaatgtaatctacagtaaataaaaatta

taatttaaaaagcataaagctggtgtgaaaaacagtatatacacaagagcttgaaaactgcataatttgtacaaatggttcaaacttccagaaaatgaag

tcaaatatggaagacacaatttaaaatcgcagcaacctaatactacatgacaaataatatggcagacagtaatagtaaagggtgagcatgaaataaaatt

ttttaaaatatgttaactgtgaaacagcagaaaatttacagttaatacagatgacattatgcacagcctagacagtcaaattaaatcctatcacgtactt

gaaaaatattactgtttaaacttactttgttgtaatgaggctgagataaatcagcaacagatgtgagccaaagataaatcaaaattaactaaaggaaata

aaagcactcagtatgttaccttacaacatatgacaagttgctttgtagcaagaattctgagctgaacttgtagtttgaaaaatgtaaatgtgatttcaaa

atcatagtacttcacactgggtatggtgctattggaggtgctaagctcttgcctttctctgacctttgaacagacttagtcagctataggggtcctacag

tttaaagtggattccaaaccacagagcaacttggtatttttcatacttccaaatcactgcaggaggtgaaagaagcaaaggtaaaaaaacaccccaggac

tactgaaaggctttaatcctagactaatgaagttttagacaccaaaccacactggaaatacacaaaatgatatgtcaatttcattatattctgaatgttc

aaatggttctgagcactatgggactcaactgctgtggttatcagtcccctagaacttagaactacttaaacctaactaacctaaggacatcacacacatc

tatgcccgaggcaggattcgaacctgcgaccgtattctgaatgttactttctctttgtcaaaccacaaggaattaagatttgggtttaacgtccttgaca

atggcaataaaaagggatattctgagattgaagggataggcagaattacttaatttcacttctaacatcccctgccatgttacaatgctcgtactgtacc

aacgtctgataaagtaatttagcagtaagaggtattaatcttgtgcttattgacattttttttttaatcttacatctcaacacaaccaacatacgatatt

gcattcaacatagaaagaaaattttgtttccctacattaagacatcatcccacacaagttttaccttgtctttgtcaggatattaaattacattatttat

gcacaatacaaatctgtgtttcttaattcttgcagaatggtaatgagaccctatttctaaacctcaaaatgaagactaaatgcttttcctgaaagctaag

tgaagtagttatctctttcaaatcccaagtcctgggtgtatcaataaccaattgtattgcaaaaagtattaaagcttcaataataagagctataaatcta

aatgtacatataaataaatagtttaatcctaatttaatggtgaaaggacatagaaatcctgactgtgctttaatcatacaatcattaagtagatgcagga

acttgtgaggcactgcaaacagagacatgttttcacacaatgtttaaagagaaaatactattgttttatttgatattccatacctgattgaaaaatactg

tgtttccccttagacagttatgataaatcctggtatgatgtctgatattgttgccaacattttgttctatgttcttgtgtggaactattaaacttaatat

ttatgcaatacaaggacagatgtaaacttcgtgttaactcctttaattctgatttctatgtttatcatcccactgtaaatgttgtatgaattgactcaca

cagaaatatatttcttttttgtttttaccccaaatgcctatgaaagctaaaagttgcaccaaaagctaaattaacttaacagtctacttaaaatatataa

tctcaaatgtctttgtgaagcttaatattgcagctatagttaatcactatattttcttatgaaataggtcggtagatctctcagcaaaagcatgactacc

aatgaacaatatctttatcagaactccttgccgctgaaatgtatgactagaaaaacgatcaaaatcagatatatgaaatactctgaattctcaaaaatct

tataattacttaatttatcacccatttcaaatatctaaactggaataaatctacttctttttaataaataatatttgtacaaattcagtaccatatttca

atcgatgagaatgtcatcctatttaatcataaatattgtctaccactacattaaatgacaagttctagactgagccactatttatgatatacgactgcaa

cagggtgttattacagtcataactttgtgcagataatgtgtcaaaagttagcaaacacattaggaatggttatatcgtctgtgtgtgcccatcctttgtt

aagtaggctgctgccccttttgttgaactaacatcacccattccaagataacgaagttcctctagtagtagtttcaagtttgtatcaagatagcttccaa

tttttatcacagatcgggatttctttgtagtctgtgtgtgccacacaagaacctgagtgcacgagtttccttttggttcgatatcctccaattttaaatg

aagacttcgaagaaagtggtcctcttcatacccagctttgtatggcatggttgcatttggatgttccaataaaaactcaatattcacagcaaatccagcc

atgtctactggaaactttctcttggatggccatgaatcaaaaaatcctataactcttccctttcttaacacaggagaactaactgcatagtcaccaatga

gaccaactggaaacattgaaactcgcttcgtatcacgaatttcttcaaagagctctaaatcaaatgtattatcatcatctccaaaatatatcacaccatt

cttcacatgctttcttatccagctgagtgcagctcgtctattggctacacctcttggaacaatatctgctttgcgatacacctctggcattgggcttgca

atgtgggtaaaaggaataccaaattttttcaagagttttgttatcattgcactgcaaaccatgttatcatcagcaacaatccaatgcaggttaggtatat

gcatcaatgtttgccctaaccttgtaagttctgctgcttgctccctccttggatatgtgggtgtaataaaataaattgtgggtatattatccacattttt

attcctgctgtcctcaaatgacatgtggcacaggaatacatgttctcgatgatcataatgtctcccaaactccttgccttgagagctccatacaaaatat

aagactataattgtcatagcaatggtaaaaaggaacatgttcttaagtgggagcattgcttccagctttgttagacattatcaaccttcgacaaaaagtg

tatgccgctctatagctctttcgttacatatttctcatcacatgtcaacaaatacttctcctccctcctcctccagacttcccctcccatcacaaccgat

g

>43069|AGAP008812-PA

cgcagcgccagcgccaccgcccttccccgcccctgcaccgccagcgccacccccaccgccgcctccaggtgctggagccttgcctgccgttcgtattgcc

actgagattccagatacacgcacaggtggacgtatagggcagacaggcaacacctatgagccacctgctgccattcagaatgcaatgatgaccaaggata

agaagcccttcacttacacaccagggggcctcgacctgtcacagattcgaagccccaggatgcagagacgcataacacgaaatgctaatgcagaaggtgt

accagaacagccagtacaacaaaaaacatcaccactggcacatggtcagctcccaccatcagctttggctgctatgcaacctcagatgccagttgctgtc

tttccaacaggcaaccaacaaatgtcaccaccagttcccccacctcctcctccacagccacaggtacagacacaaccacaagtgcagaaggcaccagttt

ctccacctcactcacaaccacaagtgcagaaagttccagttccacctcctcagcctcagccagttaatcagaatcaagccacaacacagccaccacaatg

gtctcaaacacaggcaaaacctgcatcgccacaaagtgaacctggaagcatctacatcccacctgttgtacagccacaaggtggaagagctcaagtgggc

tccatttacatacctcccgtgcaacagcagcagcaagatcagcagaagactgccagtccattgtctcctgtttcactgaacaaagcaccaacaccatgga

tgagtcagcgcccccagcagcaacaagtgccaacttgggtcaaccgtgatgaaactcctgtgcagagacaaccctcagcagcagcagcaccaacacctac

acccacacccacacccacaccaagtggtacacggatcatacccatacaagtggagggacgggatgctccagttcaggcaaataagtcaaccccttcagca

acaaatcagcagacaaacacgagaatcataccaatccaaattgaaggtgaaccagcacccagccaatcaaagccatcagcacaacaacatgtgcaacaga

gacagccagtctatgttcacaataaatttaatccttcagtgtcaccaacgagcccaactccaagcggtcctggaaccagtgaaattgcggctgagcacaa

tcctaggcagttacaacagcagcagagctggggccctcctggtgcaaatggggctccaatccagtctcgatcattcagagtgctgcaaaaaataacagat

actgacaatactgatgcaggcaatgagaatagtcagcatatggagtatccttatggcaatcagtcaacacaaggcgtgccagtgcagcaactgagaaaac

ttcagctgagtgaggatgatcgtgcacttatgaacagattcaaagctcaggtggacgaggactcttttctgcacaatgaatcagatcccagatacagagg

gccagcaattccctcgcgtgcttttcgtatgcttcaaagcatgactgatggagcaagtggttcagactttgttcctcctgctccagccccatctcgagga

cctccacaaaggccagccagtgtacagccagctacagaatctcctcagccatatattccaccatctgagcaacaagttcaggagcctaagaaatatactg

gaggttgtattccttctcgttcatttaggatgctgcaggcaatgacagcacccgagaactgtggtcccgggcaatctgatatgtaagataggaatgacat

tggaatcgtgtctgaagtgaaacatggtagttatcaacatccactggaacaaattgttgcttgttagcatattttaagttatctaaggatgttgtactga

agtgtacttaccacaataatgcttttcacttgtttgactttttattatttcttgtaccataatgcttgaataaggatgttgtttaaactttacatatgaa

aagcttcttgtctgagcatcgttcaggggtttataactcatgagttatgtgtgaagcacttgctataatctgtgcactcaatgaaggaaaattttctgtg

taaatactttacagtttaccaactatgaggacagccaaacacagtcattataaacgtaattgtcagaacaaggacatcattctgactgctgtggatagtt

acatgatgaaataacataaagtgaaattctatgagaaatcacttatagagtgtattgtatttaatatactatccataagaaataaacaaatgctacataa

attttgagataaatttataagataactcaataaagactttgaattttgctttgatctgcagattgcagggaacagcatgttagacagtttgaacttgtat

aaagggcagatcagtgcaccaaaaaaaattgacatttactgtcacctcaaatttatgatactcatgtaaaaggagtggaaggaaaaactgtgattgctat

tagtgataaacataacactatcattttgttgtttctcctgtagtaaaatgctttgtaatgtagtccagagtgtgtgtatagccataatataagaagaatt

cagttccttactaacaaaaatgttgcaaagaaagaaagagattgctcagaacatagaaaacttttagagctatttcctttctttttcttaaaaaaagtgt

aacgaatgttcaactctgctaagaaattgtaaatttggatcagtccatgatatactacatctctgacactgagctgcagagaggcacagtgtgagaagaa

aaacctatccctcaatttggttttacattatatacatgtatatacgtttgtgtaaaatttttatttgctatctttttagtttcagaatgatatataatat

ttatgtcacaacatgttcttagataaaaatgcagaaaatgcagttttatctatttttactcagatgaaagtcttcctaacatcacatatttgtaagggat

gatatcctcagatactggtccctctgatgcttcaaaacttgtgagactcgtatgaggacattgaggagctcagacacttgtaatgacatgtcatatgatg

ttgtacaacagaaatttagtaatatacctttgtaaaatctgcaataaaatagcatcatagtccagtctcccataaaacattaggtttttagaaaactgtg

ttttctctttaatttttgctctactcccaattagcattagttattcattatacttatccacagtgatacttttccatgaaaatattatttttcaagtaaa

tttagcaacatttagttttattcagggtgaaagatatagacatatcagagaaggctgtgactgaatgcaatttagttgtcttaaaatctgttggagaaac

atgacaggcactattggtagtatattttatcagtgagtagtgttgcaacatctgctgtaaagttctgttacatattaaatccaaggaagtagatagtttt

ctccagttatttcatactgtgtaggcaggagtagatgaaaaatcaattttcttactggactattttatgccgttgagtattaacacaggaataaattatt

tgtaggacaatataatgacaataagcaggattacattgtcagagccttgaggtagggtttttgtattatgaaagttgacaaaatttgggtaatgtcaaat

atgaattatacacacatgaaactgcctcttttattgttaaagaacaaaacattgctctgtataatgtggattttaagtcacaat

>43089|AGAP008839-PA

caaggaagaaaggcagaatattctgaaagatcagtattggtttgaatgtaaatgcattccttgcattcaggactggccaactttcgtagagatggactca

actgttctgaggttcagatgtgacgccggtacaaactgcaacaatgttgtgtgtgttccaacatcaatagagcattgtttcgtacaatgtactgaatgca

aaggctacacaaacattcttaagggccttaaagcactacaggctacagatgatatgaactgtagagcacagcagttagcaaaaaatggaaaaattccaca

tgcgctgtcggagtacatcaaggtcttggatttattggcagaaactcttgcaccacctttcaaggattactgtatctgccaagagtctatcagatattgc

attatgcattttggaaacaagtaccacttttaacttttacttcactggaatactttttgacagtgaaccatctcatgtactgtgttctttaaataaatca

tgaataatctcataagactgttgacttggtgattctgtagtgcag

>43216|AGAP008870-PA

tgagtgctatgtgttttacttctgcagatgttagccagcctgacagcattccgtttaagttcccattcattccagatataattagggtcaatatatgtcg

gtatagggtgaagttccgtttgtactgattgatttttttttatgacagtcttgcttcttcttg

>43236|AGAP008879-PA

ggcccattaatggtgatattagcatactggctaccagaatcccagtgctattttctattagaccaagagctgcaatcaatcctgcaataattaacagaac

caaaaagtcaaatgtaagtaaagcatctgatttaactccctcaacaacttcagcaacagttatccgtgctttcacagatgccactaaacgattccatgtg

gaaatatcttctttgctttcttttgctcctgacggattgggtccattagatgatgagtcatctactgtccagtggagggaacacggtatgacacttacac

ttgaatgtcgtcttgatccaattccccattcacttagttcattcaaaatgtcatcacacttgtctccaggctccaaagtaaatgtagcctgtttgtagtt

tccgccctggcattcagaccaagcacaagtataaatgtgctttgcctctatcaaatcttccacaaccttctgcatgcgcagccgcatgggaggtggttcg

acttggcctgctgcagccacctgctcctcgtattcggcggacggcaccgtcaccaggaacaacacgccgttcgacatggctcttgcctcgcactatctgt

acgtgttgcacagcgcaccgcacatccggcactgccccaccgcc

>43237|AGAP008879-PA

tgtctccatttatttccatttgtaagtgaatggcttgatgatgcaagagatggtggaatacttgagtcctcggcaatgactggaagacatcttgaagact

catctgggcttcccactgtaattgtaacttttggactggaaggtgaagcatacgaaatgccatacaagtcttgaagtgatgcttgctgaacagtttccct

taatgctgtaggcgaccaagtacattggttgtacattgtactaaaatgtgattgccttcggatatctgcacccctgtgaaacagatttaaacctgcaagt

gctcgaaggtcagtggaagttcttctatgacctccagaaaccttagcaatatcatttaccaacttcctgcctagggcttttgcttcatcaccatagtttg

tcctgtcatactcctgtgctaacttcacatcatgagtccagaacttcttttgggctgcagaggtacttggtgcaactgccttcaacctgagtacaacaat

ggcagaagcaaaaatgcatacaatgtttacaaatgttaagcagaggcttattccacccataattccaagctcagttacaggatgatctgcatatgtatgg

ggcatcttcacttgaggcacacttccgtaacttgaatgtatgcatgttagtgcccacagaaggcccattaatggtgatattagcatactggctaccagaa

tcccagtgctattttctattagaccaagagctgcaatcaatcctgcaataattaacagaaccaaaaagtcaaatgtaagtaaagcatctgatttaactcc

ctcaacaacttcagcaacagttatccgtgctttcacagatgccactaaacgattccatgtggaaatatcttctttgctttcttttgctcctgacggattg

ggtccattagatgatgagtcatctactgtccagtggagggaacacggtatgacacttacacttgaatgtcgtcttgatccaattccccattcacttagtt

cattcaaaatgtcatcacacttgtctccaggctccaaagtaaatgtagcctgtttgtagtttccgccctggcattcagaccaagcacaagtataaatgtg

ctttgcctctatcaaatcttccacaaccttctgcatgcgcagccgcatgggaggtggttcgacttggcctgctgcagccacctgctcctcgtattcggcg

gacggcaccgtcaccaggaacaacacgccgttcgacatggctcttgc

>43261|AGAP008900-PA

ccgagatcttctcgccgcgcatccgcgaactggccacctcgctcgggctgacgctgctgtggggcgtgtgcttcctggtcgccaagctctacccgtccgt

ggccgctgcctggggcgaccacgtctgctactggttcttctgcgcgagctgcgtcgcctgcgggctcttcatcgcgctggtgatgcccgaaaccaagggc

cgctcgctcgaggacatccagaacgacctcgcggccggcaggctctgcggcaggagggccaagaccgaggaggccatcttcagctcgtccagatggctca

cgctcagtggccagaacctcagcgtcaacaatcagaagaaagagatcgtctggtgaccgtctccacccgtaaggggcgtgccatgacctcgcgccactgg

atccgaatcttgatccgtccatgtgatttggaaaggctctactatttgttatgttgtacctttcacttcgaaggcggagtcgatcgagctctccaagcta

gtctatcgtgtgtaagtctttcagtctccacactacagcaatctgcgccgaattgcagaggttcaattggttcaaatggatctgagcactatgggatcta

acatctgagaccatcagtcccctagaacttagaactacttaaacctaactaacttaaggtcatctcacacattcatgcccgaggcaggattcgaacttgc

gagtgtagtgctcagttgcagaggttta

>43262|AGAP008900-PA

ggtggaccgggctgggcgtcgcccactcctgatggccaccaacggcgggatggccgcctgcctgctcgctctgggcggctactccttcgcccaggaccgc

ggatcggacgtggcttctgtctggtggctgcctgtggccgccatgtcgctgctgctctttctcaacgccataggcgtcggcccactgttcttcgtgatcg

tcaacgagctgttcgctccagaggccgcaccagttggtgtctccatcggcctcggttgtcagatggtggtcgccaccttcgtcatgaagtcgtatgtcat

actcgtcgactggttgcagctctacggttgctactggttctacagcacttgttgtaccctcagtaacgtgtacatattctttttcctaccggagacaaag

aatcgtcccattagtgaaattcttcgagatctaaggagagagcggagaaggaggaatgaagacgcagaaaagtacgatcaggtgtctacgaaggagagtc

ataagcaagttgcatagtagtgtacaacatctacaaagtattagagcaaaaccagtgatttgcccgatagtgatttaacttggagggcagggctgaattc

aggttcctagatcatccctttgtacttttgttagctttcctttcttatttgtgcagaggccaaatgaactcacttctggcagaactaaagtgcgtatact

gtgtgaaatgattctgttacaaaagtcgaagtcaagtatgtattgtgcgctatttagagtataaggaattacaaaggaagattttaactctgtggcttta

gggcttttcttctatcgacagttaaatttacagttatataacgatttaagaaccgtatcgaaagtgtctaaacccgaaaccgaaaacttcgaaataccag

gtg

>43283|AGAP008906-PA

acccagtgttgtttgagagttggggtttacatacaaatctttgctccattcaaccatacatttcaaaatggagactaggcattccaaaccacgtattctc

attgacttttcttggtttggagtggctcctaattccaatgcttgccttccttgagctatctttgacaaatcattcaccagcctgaaaaatagcctcacct

taaaaagccaaaggaaatctacatacacatacatacaatacaattataaataagacgtacagaagcagcaccactcacacaggatacagattaaataaaa

tttcttttcagcaaagaagtactagtctcacataaactttatattctaaagtttctagcaaatctgttccaaaatattcggcagaattttcaatgcaatt

cctcaggctctttgtgatgataatcctgactgtacatttacaacacaggattcaaaagacaatgagaaacatttttgcttttcaacatttcatcacagca

gaataccaaatcaaacagtgtccagtgaagcagaaaatacaaaactaggggaaaatatgctacaaaactacgag

>43306|AGAP008908-PA

tttgttttaggagcccaatcaataccagtcacacgtaaatcatgctggttcaacacatccagtaacttccagtcattatctagacgtttataaatgtgaa

cctcatggttgttaggagacaaagccacttgagttctgtctctgttccaagcatgacacgttatgggatttactccaaagctgtgtctttccaccatttc

gtttggctcggtcttcaccttctcccgaaatttctttttcttgtaagtttcaaacacccatctcttcacgctagcccataaccatcaacttctacaagaa

cattcatgaacacatggttcatcca

>43305|AGAP008908-PA

ccagtgtaaaggcacaaacatgttattgcattttgatgattcgtttcaagagaagtatcatttgcctctactcgggcctgccgatctagagactgaaatt

tcttcattgcacttaagccccctgcttctttcttctgtgactgatcaagtttacaatttaaatatatctgtccagcatcatttactgtgtacaacattgg

gcaacaactgtgaccagctgcaactactgagtttcgaccaacccatgcacagctcagaaatggcaggaactcagtacgtaatttgctaactgtcatttgt

ttgtttgcatcagctacggatatggaagagtcatgccccacccaacaaactctgttcccatcgcctgagaaggcaacagctcgtacccatcctcctcctc

ctgatgaatttggaaactctgcaagtaattgtcccaaggtcatcttcattccccatggagaaggttgtggcatttcctctatgtctttgataaaggcaga

aaagattctgaccttaaaatctgttgatccagcaacaagcaacacgttgtttggatgccaatcaattgctgtgatagtggagcggatgggcttctttata

tgttttgaaacccaccaatcattttcactttcaaagtagcacacagaaatcagtcgtgctccggaaccaacagcaaacttgttttcaagtggtgaccact

tcacacatgtggctgctctattgatgcgcagcaagacaagtgttggtttccactttccatcttcaccctgagtccacacatatgcatttcgatccacagc

acaggtcacaatcctatttgttttaggagcccaatcaataccagtcacacgtaaatcatgctggttcaacacatccagtaacttccagtcattatctaga

cgtttataaatgtgaacctcatggttgttaggagacaaagccacttgagttctgtctctgttccaagcatgacacgttatgggatttactccaaagctgt

gtctttccaccatttcgtttggctcggtcttcaccttctcccgaaatttctttttcttgtaagtttcaaacacccatctcttcacgctagcccataacca

tcaacttaacgcaaccgctcaaagatgatttcatcacagtgcgtgtaatcataacatttatg

>43334|AGAP008910-PA

agaaaccacactcaaattatggtaaaatatttttcattagtgtgtaacacgacaactgtgcaatacaaatgaaaccacactaaggaaacaacattactgg

ggatataatttttgcctgtcttggtaattacagaaatgatgaaaatattttattgcagtattttctattctaaaccttaacaaaatataagtcattacat

tcacatcagcagaacatatgtacatttttagatacaaatgtcattcacatatctatttccgatacagtcctatcatttatctacttcactttcagatgaa

aaatctgttccaccaaacgatacaggaaaacagtcaagcgatgccagatcaatgacagcacatgtaaatttctttgagaactgaggtacgcaaataagtc

tgatcttctggttgtctgtaccttcataaatttttgtctgaaaacatggctgattaccagcaaaatatatatcaggataatcagatattatgaatggatc

ctcttcatagtatggataagttgaaagtgtgtctggacatgttggagcaaggtgagcccattccagagtttgctccagaatggtcagtggatcttccaga

cgactgtatcgtgttatatcatgtaccggctgccctgatgtacctaatatacgtcggcctccaagttcacattcatatgggtttggaacaccatgcaaac

tctggtacgatgaagcctgtggaagcatacaatagtgcattggttgttgtggtatcataacattggcagggtcaaattctccaggcatcaggtccacttc

aatactgcttgtgagttgaacaagaaagtcatcaaaaagtcgagttgcagcaactgcatcaacaacactcctgctcgtgcttgtaagatggtgcactggt

ggtggattgcgatctctgatgctattgccagcaataataactctcactgcagatgccattcgctgttgttctccagtgtctcccagaagaccaccaagcc

agtccacaaaaagttgaagagccagaagacattcatctgatgcagttgacaggcctaacccactcacaattaccagaaaccttttttcagctggagtgct

ggacaaaggcctttccactgatgaggctagccctggccaacaatagtcttccactaaaaatttcccatcctcactttcgtgtcctaaaacagcacacacc

actccggtaacaacataatgggggctgatactgccaatcaatctgatcctctgcagttcatcttccaatattagctcatcatcttcactcacataatttg

ttcgtgatggctgtgggacaagttgtagctcatcacttatttcccttagaatacttggttttaattcttgatgtttgaaaagcgttcctataacaacaca

cgactcctcttcatctggtttcaactggaacagtttacgtattggtacactgtctccccacttttgtcgcactggcttttctattatgcatcgcatctgc

acaagtctgacggcataaatatgagcaaactgcttcgagaagtctttcgccttataggtaaacctggcagataaatttttgtaatcgcattccttccgag

aataaacatcttcaatatggtcattaccctcatggggtgcagacaacagcttccccacatcatctaaattttttgatggctcaatgatcatcataacaat

tacaatacttcactcacagtaacaacaaatcaacaatcatctctcggcgccaaactcaaacttacggagtttagacgtatatcagaagatattacactct

actgtaagcaaaggccat

>43634|AGAP008940-PA

ggaattgattgttgtaatgattatagctagcaaatgatatcatagatccaaatgctactccaagagaattgaaattctgtgcaagagcatgtacccaaac

ctttgaatctgccaaaagatgccactgtggttgaaaaaagtatctaagacctcgatctgcaccatccagggagagtgcgcgtgctaagaaagctataatc

aatatataagggaaagtggcggtaaaatacatcacttttccagaggatttaacgctcttccatatggagaagtaaacaagaatccaggcaactagaagac

aagctgctagctcccatcttatgtctcccagctcatctatgccaccactcatttttaggaccttattttcataaaattcttcagtaggtgatttggaatt

gtttgattttgtgaagttaaacttcagatttgatgaagtccaacagtttcgagtattccatcgatgaccacattcgtgccatggaacttcagtcttaaat

gcagtaaaaaagtagtaaactgcataagcaataatgacattgtagtatgtggacataatgaacgatattacaacacttgccagtccagcgcctttaaaaa

gtggacagagttttcctagtgcaccgatgggccctctcctggtgaactgcccaacagctagttccatgtacagcattgggataccacatattattagtat

aataaaatatggcaccaagaagacccctcctccactcttgtagcagagatatggaaacctccagacatttccaagccccacagagtacccaatgcaggcc

aacacaaactgtgccttattagaccaatgctgccttcttgggatgtgaaggtattctctactagggtcaaaatagctgctactgcttgtagtcgaaactg

atgcctcatcatcagaaggcgtccgctgctgcacgatgttgagcaggtcggaaggcgaggtgggcacgcggcgcacgacgacgcggcggccgtcggtg

>43660|AGAP008942-PA

tcatccctgagtcttttttgctccagccacgttccttggtaatttctggtatagcaagggggacagaagtcctgcttgcataaagagctgaggtccccaa

aaataatgtgagcagccatctacgtttttcatttctcgaccacaattcatccctgagtcttttttgctccagccacgttcct

>43674|AGAP008954-PA

gaaaattgcacttaccaaatgatgatcctagctccaattgagttagtccctctggctatctgtacacaggtgccaacatttttggaagtcttggaagcaa

gcagggaaattttccactgggacgacacttataaggtcatttgtcatggcctgttggatgtctacaatatcttgatgaaggtttcgtttcagtcaccttt

tcacttatggaaacaagaaaaaatcacaaggagtgagttcaggtgaatatggcggatggtggatggcaatatcagaatgtctggccagatactcagtagc

aggtaaaacagtatggggtcgggtcttgtcatgcagttagaaccaacatactaggtaacataaaagcaacatttgttccattttagtatttcaaaacaaa

acaaaacaaaaaaaactatcctggaacctttctgacaaagggagtatatacccagagacggtactttaagatttcattctcttcctcctactattcctcc

tcatgctctgcaatattttcctgttcctcttgtgaaagaaaataagctcttctttctaccatatccattgtagatttctgttacaggaaatattatggag

aacgacatcctctgcttgggatttcactcatgaagtgtggtaagatatgtgtttatctcggagacttctcagcagcacgcaataaactggaagaagcaga

acatgttataaaaataactcatggtgtgaatcatattttatacaaatcagagcttgaaccactgttgctaacatgccacgttgaactaaagagttaggct

ttttttcctatttatataacaagctgtttccccccctccttttttttttttttttttttaaggaactgactatgtgaggtaaaataaaccatattattgc

tgtgagtggatggatgttgtgtttggttgttaacagttggatctattatttgatgtgggattgacacctgttgttgctggtgagagtaaaatgatagtct

gaagatttataaaaatgaggaaaggcatcttctcaattgcagctgattgatatgttatacacacatgcatttcactagcttttgagttcttttttttcca

aaaagaagta

>43735|AGAP008965-PA

aaaaaaactgctggcactgtcttgggacttgctatcgccagtgtcatctgcagacagccctcaggccctccgcgcggccaccagcagcgtgggcagcaga

gcggcagcagaggccgacggcgagaaggccgcgcctcccttgtcgtcggtgggcttccccgagtcgtccgcgccggcgatggcgcccgcttgcgggtcgt

ccccattggcaccgacgtcaacgtcgacttccactccgccagagggcacaccgagcagcactactgcttcgtaaccagccagctctatcgccgtcgacgg

cagcaactctccttccttgtggctggatttaacactggtgatgctgacatttacattttccgtggtaatggatgagaagccatcattcagattgacggtg

gtggtaccactgccgaagttcaccagaacaaaaagcgggttggccccctgtgtcgtcctggcgacaacaagggcgctccctagagcagcagtagatacat

ctccctgctggacgacggcttcctcgcgcatgcgcaccagctgctggtagacgtggtaatggctgcccgtgaaccccaactacctgacgctgaacctggc

ggcacagaaggcggccgagcgcagccattaccacgtctaccagcagctggtgcgcatgcgcgaggaagccgtcgtccagcagggagatgtatctactgct

gctctagggagcgcccttgttgtcgccaggacgacacagggggccaacccgctttttgttctggtgaacttcggcagtggtaccaccaccgtcaatctga

atgatggcttctcatccattaccacggaaaatgtaaatgtcagcatcaccagtgttaaatccagccacaaggaaggagagttgctgccgtcgacggcgat

agagctggctggttacgaagcagtagtgctgctcggtgtgccctctggcggagtggaagtcgacgttgacgtcggtgccaatggggacgacccgcaagcg

ggcgccatcgccggcgcggacgactcggggaagcccaccgacgacaagggaggcgcggccttctcgccgtcggcctctgctgccgctctgctgcccacgc

tgctggtggccgcgcggagggcctgagggctgtctgcagatgacactggcgatagcaagtcccaagacagtgccagcagttttttt

>43764|AGAP008967-PA

aatgaccactcgtccagtgtcttgagcactttaacgacgtcgggcgggaactgcataagcgaggtgctggtgacgcggcggtagatgcggtcgacgaaga

tgccggcgcggatggcgtgcgccaccgaccggaacttgggcttctcgtcggccttgcgtctcgtcgtggcgagctggcgcgtgaaggtggacgccagcca

ctcgcgcacctctggcggcaccgcgtccggctgcccctccgacagctcgtcgtcctcgtccgccagccg

>43804|AGAP008989-PA

cgaagaccggaacaacaacgccccctgtattcgtatcgccgtcctcggagatgtcggtgtgggcaagtccgctttgacggtgcgatttttgacgaggcgg

ttcatcggcgaatatcgttcagatactgatctcctctaccgtcagagcatcgtgattaacaacatcctgtttgatgtggaagtagtggacattacgggag

aaacgag

>43825|AGAP008998-PA

tcttccaggtacagcacatcagcaagtaatgttataacattattataaattatctcttactcactaattagccctgagttgacactgcacttacataatt

agcctgccatacccatgttcattgttattctttatctttgtgaaaagaatattcattgcaaatcaaaaaaccaaacatgactttcaacaatgtctatctc

actgtcaatttaaggctacctccactttggaaacatatgtctttagtgcatgaacccatcaatatggtaacagaagctgacacattcactttttaccctc

aacatgagcaacaatccagtgagcagtgtcagccactttgtggtatataccaggttggtgatcgacagcacatccaaacccagcacttgtgattccagca

agagtccagcgtccattgaccttcactatcagtgggcctcctgaatcacccaaacaagcatccatgtgcccatcactgtgaccagcacaaaacatttcag

agtacagttccaaatttatattcttatgttcatgccactttaaacattcttcatcactgagaattggtactgtagctttctgtaagatatttgttccagt

cttcccatatgttgtgtcagtttttccccatccagcaacaacacctgtgtgtcccataaatgcttcattatgcttaggaaggcatataggtaaaatgttt

tctcggtagaagactggccgtgataatctaagtagagccaagtcaaacctatcaggctgtgtaatcctgaattggaaatttgggtgaattattttcttta

tgactctgaatgtttcttctggaaggggttccatgtactgaccagtgttctgtgtgtcatattcacccagaaatacaattatatctttcagtcgtgccct

atgtatacagtgggctgctgttgcaacataccactgactgacaagcactcctccacactggtaacccgctatgcgaatgtgtgcttgccaaggaaattcc

ccaaagtaagcctcattgccaccaattattcgtttctggaaagtaagctgtggtgtcccacattctcgttttggagataaagaaactgatatctcgccat

tgtgattataagtatttcgttacacgtaaactgcaatggcgtctcctgtagtcaagtgaaggaagg

>43868|AGAP009001-PA

acgcgccgcaggagcccgagccttgggaggacatcatggccgacgtggagcgcgtcgtcatgccggggatgacgcactggcagagcccgcacatgcacgc

ctacttcccggcgctcaactcgttcccgtcgctgctgggagacatgcttgcagacgccatcaactgcatcggtttcacttgggcgtcaagccccg

>43888|AGAP009003-PA

gagccgccgctgctccgggacgaagcgtgctacgtcagctacggagcgccggcgcagctgctgccgccggtcgtcaagaaagtcctcaatgtccccacgg

aaatgatctgcaggtcggaatgcacacgcgctagagaaaatttcgagttctactgcgcatctctgagcttcgggatgacgcgtggtggacgtcctgctaa

ttgtcttctgagtgacattgaacaaagagaccttcgtcccgggattgacttcgttcacgaagaagataactggctgtttgcctggaactccagagatcca

ctttgccagcctcagtcccccataaacaaagatcccccaattgaacacaactacataccacacacgggcggctcggacacgtggagtcggttcacggtgt

cggggcagccgtgccggcgcgggacgttctgcagcctgaacgtggctggcggcttctggtcctgcgagctggagggcggagacgcgggcgcctgggacta

ttgctgtcgccagggacaccactgcggctactcagacggctaccactacccttggtgctacgttggaaccttcagtacaaatcagtggagaccatgcagt

gaacgctatttaccatatcccagccatggttccagacatgactacggaccaccacagtactggccagttgcatacttgcataaagaatcaccaccaaatg

ccacatcaagttacacatcattgccgtttgagtcaggttttcataactatgtggatgacagacagttgcatgctaaaaatgtagataaattcaggaatga

taattacagtagaaggccttataactcgaaggatcaaggacacgtacctcttaaaacacagtctgacatcacagctgcagtagcgtccataattaaaagc

aaaggtagttcttcaaactcctctcttggtgatacaaacattataccctctttcgatgtagttgaagatacagaggcaatgtctgatgtcggaacagtag

ttaaggataccggcagaatctcttatcagaaagaaagaatgagagaacctggaactatcatcaagcttcaaccacgctatgttcatgaaactgataacaa

caataatggaagacttgtcaggacctggcagtggcgaacagcagacattaaaactgcaccacagccgacaaagatagagctcagagaaccacttatgaat

gatacaactctcacacatatccgagacaccaaatagtagcgtctaaaaattatcaactccctacaggcaagaaaaaacataatcaaacaaaatttaataa

ctgatggtaattttttgcgcatcactgaacgacatccgttgtgttgctgtaggcacaatttatttgacatcaagttcaacatttatctgtattaaagtaa

taccttgaggtgtctggaaagtggactgtttctgtgtgattcactaattaatattttttcattgattgtgaatgttaaacaagtacagtcttttgtactg

tgtaaactggtagtatgaactttttgtgttaaacataaaatggaaaattgtggatacacaaagtgtgaatttgtcccagaaagtcattctacctgcaata

taagattattcgaagtactcaaagtgcagcattagattacacagaattatcagcttgtactattaatttgagaatgctaaagagtgtgttgttgaataat

atgacatttttgtatatgttcgtgcatttcaacaaacaaggaatgtcttcaaaataaaagaattttgtttttattttactgtgtatttatgaatatgtgt

actaaataagagatttttaataaaaaataatttattaatttgat

>43916|AGAP009004-PA

cgctgaattccaaatttgctgattttgaaactactttgtttagtttgttgtcaaaatctctagaaagatcagttgaatcgtccgaagcaaatgagaagct

gaaccaacacattgaaagatttttactgatgctttcaccatatttcttgttgaaaccggcacacaaagtgttggagtggcttatatataggttagtataa

aacttctgttggtgtgagtctcatctgaaatgttttatcagtggtagaaaaataggg

>43915|AGAP009004-PA

acaacttcttcttattctgaattaattcaagcaaagtaacaccccgcttccatgctgatgttttgagaatttccaaggatggagctgtttttataaccct

tttcttccttgtatttggtggtggtgtgcatattttcgcattatacatgttgtccagttcagcaacaatcagccgtgcatcaagagtaattcttttccac

aaatgtgcaatagcagatatcacatctggcgcatcactttcatatcccaaatccagaataattcctaaaatgtgctgctgtagatccggaacagattcaa

agaattgacttgaaatctgttccatagcgagtacagatgggcatataactttttcatcttcatcgaaaacatgtgttttctgatcaagaagcattgtttc

aaagaatacgaaacatgctggatgcttcaggcagtctactgtctcctcatcaagtcgtttcacaatgcctcttaatattgaggaagaataataatctagt

ttcttgtcactctttccagcattaatcataattctgttacccaagcaaacaagtttcatcagcatttctgatgatgtcccatttgaaagtatcttcaaga

gggcagctgccatgtacgttggattttcttcaattgtggcacacgacactaaggcctctcttatggaatccatgtgagttctattcttttctctcaccac

agttcgtgcttctggatcagttgacaacagcacatacatgatgactggaagttgttctgcatccaagattatctcttctttccgtatgagaatttcattc

actaagtgaatatagctactctcactgagagcaccaccaaatgactttccaagaatagagagacaatccagtgaagcaactcgaatgagtgataccggac

acgaaagtgctatcagtagagctggcactgttatttcatcgtagttcaacatccacccaatagatgattcagactgccgaagaagtgatgtcgcaagaca

cagggaacgtaactggagttcgtagctcacaatattacattcaatgtcatttgcttcagtcattgcatgaccaatccacatattacttaggaaattcatt

tgattgctaaactgtgggaagtattcctgaaagaattctctcagttgtattatgtatgcacactttgtacgctcttcctggggttgtaaactgcaacccg

caactaggacttcaaacattcttatcaaaaattttgctgctgtgacagtttcctcttcatctctaacgcctttcagatcccaccatatacttgttctaag

ttttggtgggactgtggtgtattttatcaatactgacacacagtgaagaaaagcttccaatggaagcttctgcatccttgcaactttaaagcatttgtga

atattgttgatgcccaattctttgccatctccagctagtgttacctttgagccttcaacatatgctacagcctcatcaagaatctttttactctcttttg

cagtaactttcttggttattgctgaggatttaaacaggagagcaagaaatttttcaacagtgtcggaattctcaggttctacaagttgtgctgcctcctg

caaatattcactcattccaggtatgctatggcaggtaatgaggcctctaaatgttttaacacagatttgtgtggtgtcacatttatcagggtcaggcata

cctctcctgatgtatgtcaataacatgttagttccagcaaaaaatgatgttaaaatgtccctagcaatctgggcttctgctttccctccaggaaagaggt

atggaaaaacagtaaccagaatgtgaccatctttctcaaaaaaattgttaacttgttcactgcaaataatgtttagaacacttgcagcaatttcagtcca

ttctcccctcagtatccaacacttctttgcaagctgtatgagattcttcagtaaataatcaggacccagtaacaatgtgagaacagatgagtcaaagctg

ctgaacacttttttcacgactcttgggctatcatctttcatccgctctgatagactgtcaactaggagttgctcatcaatggccttaattttcatgtatt

ttgaacccatatattctattgcctttatgcgaacatctgctgatgggtgatttatcttttcaaacagtgctgtatttccttgccttaatgctgcatcagg

cataaaatgaagcaactccttgcacttgtcagatgtattggagtttgtcatcagtttacaaacagctgcatcaaatttgcttggatactgcttttcgatc

ctactcagcagacttgaatatatttccaagatatttttatccacactatctgaatcattatttacttcacttttataaaaagtatcaataaaagattgaa

caatcttaaatacttcatcattttctgaatctagtggaacttctgtaaacagttcatctgcaaaatttttcagttcttctatgcactccggctgctgctc

catggtgcacagtatttgtgacaacaatggtatcaacagaggtgtaacaacacctccagctgttgctagttttcctaaaataactggaaaatctttccgc

tttacaatattcagtaatgctttccttgggaaagtactaagtgattcattctgaaactggtacagctgtaacaacaggaggactatttctccagttaaag

aagcatttggattctttgagacctt

>43968|AGAP009037-PA

aaaaaaaaaaaaagggtaagaaaaattacaattaatgacctgctgtcatacatatcaatatatatatatatatttattttgcattctggaagcaagattt

gtggctttttaaatgggatatataataactactctgtggattgcaataactggaagataatcaagtcttgctaatcactatactggcttcactcatagtt

ttgattttatggcaagtgctgtgctcaagataaagatattcttgataatttatacaggttatctgtaataatgtggtgtacataacacaatttaaactct

gtgttacaaactgttctacacaacatcaaaaaccaatgagattcaacaaacacattcattatgtaataaatgtgtatacatatatatatatggttcatta

tgtaactaaagtacaagtatattaataaatactagaacacaatatttacaactactaacagtcagatctatctgtgtatacatacatctccttcagtcag

gtaagctacaaccatgagactgtgctatccaagcgtatttacttcacagtcattttaatacaaggaacaacacattatatcacttagtattgctaatctt

cagacagaagacatttagtattatcaactgaatttgtacaataacgctatactacagccactgcccaaatgtcctgagtatttatgagtgacagttgtta

aacttcatacgtataagcagcaggtatgaaaaagttacaaactttaacagaaagatggagtaagagcatgggcacgtggaagggaaaggcctttttcaag

ttaatggaaagtggtcacaaaattatcatgatattctacagctgacagaccatggataattatgaatggtcacaagaaagagaaggattcaagataacaa

gaatgctctatcaaatccaagaagaaataatattacctaacagatcttccaagattatttattaattcaaagtaagatgagtagtatgaaaaattaaaga

acagaacaccatggagaattatttaatgttaagggtctcctactactacaacctactaaaattaataagggtgctggagctgtgaaaccatatcatacag

caaatgtgaagtgcacatgaaaaagtgactagggcatccctttgcttaaaactcacaataaatactttaaacataatatttagaatattacgcaacaaag

gtgtacgactaggtcaccttacatccttcagtacttatattttaacaagatttccacagcactatattacagggagtgacacttaacatactgtggatga

caataaatttgtcatacaaaatgtagacacttttgtgtaatcttacagtttattaatatgactctccatcaatcactcaaattgaggtatggcaatgggg

caaatgagagcttgcaaaagtcagttgcatcattaatgaaactgtttcattagatgcatggagaaaagttatgatatgcatacacaatatataaacaaaa

ttgttataagcagactattttcacctacaaatcaacaaggactttattgagacggacatgcttacacacaattgactataggtggggacccactatcatg

caaaattaaagcgttcctggaagaagtaatggagggggaacaaggggcatgccaaacttcaacaattactaattcaaacagcaaacatagtttttctttg

catctacaggattaaatgaaatgaaaagggatatatgtgaagtaaaggtttaaaacctgctgccaaacaaaattgaggcaaagaatactgtcaacatata

cttgctgagcaataaagtctgtattagtttagagagaattttcatacaccaaatcaaactgtggcacaattttctctctattgtatatggtaacatgaac

actgcaatactcttcatgtaactaaaatacaaaactatattagggagaatacttcctaatagaaatttccccaacacagaagtaaaattacagccatcag

catggaatctatttagtatatttctttcacacattttttccacttcagcaaatgttataagacagatgcgctgaatgttgagtgggtccacaacctgtag

acaaggtgcgcttgcaaagtcacacaggtgccacataattactggggaaaagccctgtagctcctttcaattcaccccgccaccaagagggttcctcttt

tgctacaactgttataacgtcatctttctcaaagcttagttcgtcatcatttagtgcagaatatggatataatgctttaactttttctgttgtaggctgt

gatacctcttgagcaggttgttgttgttgttgttgttgttgttgttgttgttgttgcaatgatgtatcctcctgctttacaggtgtccctttgttactac

ctcctagcaattttacataagaggcagggaaccagccaatttgtctttttttgccctttgcttgcaattcaccttcccaccaaccagttgttgtcttctt

tcgaatcattattaactggcctctctgcaggctgagttgttctgaacttgtagcctgatatggggcaataactgttgctatttcaggtttcttccctttt

ccctaaaaagttcaaatttcaagctatgata

>43988|AGAP009042-PA

ccccggccctccgccggcaccagccggtgaggcgctcgacgcccggctcatctcgctcgaccagaaggtgagcgacatcgacttgaagctgtcgggcctg

aagtcgcaactggattcgaacctgctgcctgccgacgacatcaacgcagaggccagcgagaagaagcccatcagcatgagcctgggcgacgtcgccaagg

tggtcaccgccgaggtggtgacgcgcgtctctcgcgaactggagg

>44020|AGAP009052-PA

atatcataatatagaagttaaaataagtgcaagatataaattagttatcatatgctcccaggaatgcattgcatcccggacagtagtgctcctttactct

gcagcatcctgtgcagtaagggacacatatacagggccagcaaaggaagacaaagagaagaactgctactatgtgtgtttttgtgttgcttctgtatcta

atctcagttgttacagtcttgtgacatgatggacactgcaatgttgttggctcaggtcccagcggcgtgctaatcactgctgctgcctgcggcactgcgg

cgtactgctgcggctgcgccatcctggaatgcggtgaaacgcgacggtaacgcccttcgcgaggaaagccgggccgctacttgtccaacctgcaggatgg

actggaggctgctgccatgtgcgaaccggcgtgacagctgcacggataactcgcgcgccggccgccaggggcgctcgtgcaggccccgctgtctcgccta

acaaccgggaacctgtgcaacggcaaaggcagcctgcggtcgcgcaaaatccg

>44019|AGAP009052-PA

gagtgaagaataaatatttttattactcccaaaaagtcagaaaaatgaataattacagataaaggctgagctcaattttcaacttttttcatataaaaaa

gtatgaaaagtacaggaaacaaatggaagttaaaatcatagcaactaagttagtgatgactggttgcattgtgtgaattacatatatcataatatagaag

ttaaaataagtgcaagatataaattagttatcatatgctcccaggaatgcattgcatcccggacagtagtgctcctttactctgcagcatcctgtgcagt

aagggacacatatacagggccagcaaaggaagacaaagagaagaactgctactatgtgtgtttttgtgttgcttctgtatctaatctcagttgttacagt

cttgtgacatgatggacactgcaatgttgttggctcaggtcccagcggcgtgctaatcactgctgctgcctgcggcactgcggcgtactgctgcggctgc

gccatcctggaatgcggtgaaacgcgacggtaacgcccttcgcgaggaaagccgggccgctacttgtccaacctgcaggatggactggaggctgctgcca

tgtgcgaaccggcgtgacagctgcacggataactcgcgcgccggccgccaggggcgctcgtgcaggccccgctgtctcgcctaacaaccgggaac

>44051|AGAP009070-PA

attttgttcttgtctgtgtacaccatgttattattcagtctttttgggttaagttagtgatccttcttaagagaaaagcaatgccaaatttgagttggct

gaaaatttattttcaacacatttgaagcagaaaagcatggcccaaaaattacagccatatgtgaaataaatgtctgtgtgatcaaatagttaacataaat

gaaaacaatgtcatatgtgttttattcacaaaaaaaaaaaatctgtaaaatcaacaaatgaaaggaaaaattgcagtttcataactcggtatactgtaca

ggccataggttatcaattgaactgtaaaagtgacagtatcattttttaagaaaaaatatatataaatttaggaatctgtacattacaactctatctgtag

catggaaatgcccaaatgcaacacccaaaatgacatacgtgagatacttgtagagaaggagcagggaaaaaatgtacatcctacactattgaaaatacgg

aaggagcttaaagtcaattcatcatatccaaaacgaaagcccaagttttggcacatccaacccatcttttgatggtaatgtcttaagacaaaatatcaca

catttttgtttcttagctttagccacttctgttaaattacaaactgccaacctaagaattttaatttatgaggacacagttgagaacaaaatagtataaa

aagatgcagatccagttttaagagaagaatgtgtcccatttagtaacagtgtgcaaaaaatgtttcacagtgcaaacatgacagcaacattaaagaagaa

ggaaaaacatctcaagatcaaatataaataacataaattggtaggtcataggtaattaaatctgtaagtcagacagtagttaacttactgttaataagga

agtttattatgtctatttaaactcagtttttaacaaacttagtggttccacagtgctataacagcacttaggtgatggcgtcgaggcaaaatattatctt

cagttatacagtactgtttgaaatgtattttgttagaatgtgtctgtcaccaagcggcagtggatgattagtaacatatccagtctcccttatttaaatg

aagatgctctcctgtaactaatctacctgttaccagcaatttgttccttaaccacatattctgaatttacatgacagacagacatatctggaacaaaaga

ttgcctggcaaaagaacttcctttcgtatcacttcaacaatacacacctcagacttccatgtgagaagtcctgtgtgtcaatatatgatattcagagtga

gttttgaatttacagttcactatgattatgtaagcgtatttacttgtaactgcagactcattaagtgtaagcgttactttacgtcctcacaacgcctcta

ccaatataccagcatcatgatggttcacacagttcaaaatgcaattcggttgctacttttgagaaatgaagtatgagtggctttatttcattactcagtg

accgcattttcttaaataaaaatgactatcacttcatgtcaagagtggatattattacacaagtaatggatagatgcacaagaaaatctgcttctgacta

tcatccactatatgtaaaacatcacgtctccctctcagatacaattcatacttgcatattccttttatcatgacttatacaacaaatattcctttagtgt

ctttggtagtggtaaagacgccactgatgccttcttgatgtactgtaatatggcatcgcgacatgcattctgcagtgtagacactccatataactgtaca

atcctaacttgctttgttgtgccataaacatcgattacagcatgtaacggtccttctttgtatggtatgtctattgtgcatggcccttgatcttctccat

taatgatgaaatgcatttcagcataattactctctcttcctaatccttgcgtaggtacataaacgacaccaattctgcttccaacatctgtgggcaatac

ctggtggtcagcacttggtggtgaagaacgaaataagctgcgaggaatgcaacctcttgaggttcgtatactatgttcatcacccagcacattatgtctt

tcataactcggcatggagccgtctggcattacgctgtactcataaatactattatgagactttgtaatagcaaaaatccaagaagttcccatgttggcta

aatcaggtagcgcatactgcggcagaccacccacagcagcagcagcagtctgtggatcaagctgagtgagccctagtcgcatatgaccactccagccgcg

ttcgttcttctctatctctaaaaggaagatttccccaggtagaagaggtttttcactaaaggcaagtgcattggcaaaacttgctttgcgataggccacg

gtgttgtcatcgaataaaacaatattgccaccatggtacgggtgaaacctagtaatcattttacttcctcctgtacacaactgaagtaaaactacacccg

aacatcagacagattttgcactatcacataaaaattcaactatgcataaatgttgttactgcctcctctacaccaagatgaaccacacgcagcattatat

gatagtggccggtacgttacgaagtacaaaaaaataaccaacacaatactgtcgaatcctttccgtgtggttttgaactagagaatatatctttaaagat

acatttacttctcgtagatttatacgagctttaaacaaaatacactgcgactgtttgaatacaccaactttctgatatcaaatacgtcacgaagatattt

ctgagctgaactattagaacta

>44068|AGAP009081-PA

agaaaatctgtaattctaagcttgctgtcctatacacaaataacaaaacattacactctaagtgatgataaatatcttaattgtagaattcaaaatcgtt

ggtacttaactgaaaaaaaaacaataaaaattcattatccataatgcacactgctatatttaacttttatctgatacatagttggaaggtatagaaattt

tactatataacgctcttcactgcttgacttgaaggacataatagtgatttgttctcaacctgcagctctgatgatttgtcgacagcataagaaagctgaa

gcagttctcacaacaagagatgctgatgaacagccactatgtcatggtggagagaaagtcactgcagaactgcgatacaaggatgccagcaacaggtaaa

ctgtctaatagacattgtacaaagaatgtacaagaagataaaggaacattctgtgtaatcatctccaggcagattcctgtgagacagtc

>44083|AGAP009090-PA

cacataaatggatgcttactaactttgactgttctgctatgtggttgaaggatgcaacagcacttagcaagagtttctgtgtagagcgcatttaccttga

acacacataaatggatgcttactaactttgactgttctgctatgtggttgaaggatgcaacag

>44876|AGAP009118-PA

ctgagcaatatgttttgaaagttttcggtagtaaggattctttgccccctctatcttgttacgataagcagaaagcaagcgttcatatggctctctgaca

atcaggaatgaaagtgcgtgtggaagtgtctcctgcaattgctgaacagatggtcgtggaaagcgagcacgtgcgaggctcattggtgttttgcgtgttc

gccttagaaatctctcgttgtagccagctaatatattgaaatgatacatccatgttgaactagcagccttgaagatactacaccagaccagatggtgatc

cttgtcaatgaaaaatttcaaaacatattgctcagcactatcggtacaagcaagatccgatgatagatgtgccagaaa

>44875|AGAP009118-PA

acaggcacaaatattaataacattaatttacttttgatttggggtgtaaagtgtacaaatattgtgaatagcatctgcaacagtaatatttacctaattt

cctgtaaatgattagtaatttagaaagcaagaagcaaagtgtctcttcttattaattccatgatttgatgtaatgcaatagacacataattatagttcag

attgcgtaaaagtgaagtacttcgttgcattgtaatcaaacatttcaaaatcagtaccatagattttgtgtaacttctccatttgtttttttgtcagttg

tgagtagtacttttccactagtgcagctgtagctttcccatcccttgccttgttgattgcagttcttggcttataacggtagtgggaaggtaacagcagt

ctttgtaggccagccttatggatgatatactcctcatctcgatctagtgtttccatttttgcaataactgtaaagtttacatggcatggagtacagaatg

aatgataaggtgcccaatgttcatcaaactttttgctgttgatgatgtaatcaacaaattccggaaatgtgggacctgaccttacactttctggcacatc

tatcatcggatcttgcttgtaccgatagtgctgagcaatatgttttgaaagttttcggtagtaaggattctttgccccctctatcttgttacgataagca

gaaagcaagcgttcatatggctctctgacaatcaggaatgaaagtgcgtgtggaagtgtctcctgcaattgctgaacagatggtcgtggaaagcgagcac

gtgcgaggctcattggtgttttgcgtgttcgccttagaaatctctcgttgtagccagctaatatattgaaatgatacatccatgttgaactagcagcctt

gaagatactacaccagaccagatggtgatccttgtcaatgaaaaattcccaggcatttggctgccctctggtgttcaatcctttgtcgacacatacttcc

catacacgttcacgtctcatctcatatatttcttccaaataccctggg

>44902|AGAP009128-PA

ggcatcccttggatactgtaaaggtgattctaaacacacaaacaacaaaaatacgtatggcagcaaaaaaaccagtcacaatgcacgaaagccaaataca

gcaatggtacatcttcagacgcagaaccagcgcaaacctgcctaccgaggagccttcgactgcttcagacacattttgcacagggagtcgtattgc

>44901|AGAP009128-PA

gtgtgactgacgaccaaataaaggcactcagcagccactgaggctgcaagctagctatccttcatcatcctgaaattcagcgatatataaatagcatgac

attctagctcagcatcatttacgagtaatataaaaatatttatcggaatgggaaaaatgggaaattatctactgcgaagctcgttaagagttcagttatt

tgtccttggtggatggaagcagttccgtgactgtactggaatacaatttgtgttgaaaaggcagcagtcagtcacattttggtatgatgaatttttttta

gttaaaccgttaccagatccaagcatttgttacttatatcagacggattgtgcactctcccagttagcattacaatcgctcgttcgtttctttgttgttt

catctgctatacctttgcctacacatagtcctacaagctttagagcacgactcgttgtaaatgctgatgcacactttgtctattttgcgtcgtcttcaaa

cagtccgcaggaggagaacgcagtgtgtgtttaagcccagcgccagttgatgtgtcaaacggtgaaactgatcaatcttacaaaaaagaagcaacagtcg

agtaatttttaaccctattctcgagcgtatttcacagttgggctggctccaggtaacattacatttcttgtaaaaaagttccacataaacagttactgtg

agccacagaccgagagtgcactacctgaaaagaatactacgtcaccgacagaaatcacgacataaagcacgaagtgtccaatctggtaacgacttaaata

aatgaaatcagaatcagaatatgcctgattgcggcctattcattgtaagaaggccataattttacactacgaatgaaaactctccgtcctcacatttgga

gacgtgcgtccctactattgattcagtcgttcaaggaacgacttttgtcgggccgtgtggcggctagctttccgcgctcgaggtgattgccgactccttg

gtccagccacaggtcgtctggtggcacgatatacccttggaacaagtccacctgcgtcgccaccatcgtacggttcgcagcatcccggctgatgtggcgc

acggcgtccggcttcctcaccaagccactcgccggactgcccattatctgaatgtcgtccgatttgggatctaactttggcaccattgtcgagatttctt

cgctggttgaagaggccatttcgctttgggaccggactatttcagactcacgccctttcgacagattgtcagcgaccatgctccctacttcgcccttttc

cgaaaccgccaactgccccgcctcgatctttcgcccagcatcgccttcaggaggggagaggaactctgcactgctcaaccacgagctctcggtgaagtca

gccgtgtgtaagaagagtctgtgaacgctctcgtactggcccctagctgcctcgctctgatcctccctgttgaccgccgggccttccagcttgaggaagc

tgcagcccgtggctcgcgccaggctacgcctccaaccgaagaggctgtccaggtggcctcggtgccaggtggcggcagggcgcggcggctgcagctgcag

caagctcgagtcgtgctctccgacggcgggcgggagctgccggtgtccgtggctgctggtacggcggcgctcctggtcccagcgcagtaccagcgtcacg

gtgaaaaagcacgctgcgttggtggggaaggcgcgcaccaccacgatgcccagaccacgcgtcaggaaggaccagccgtccgagcgcacgctctgccgca

gacagtccgccatacccgagtacctgcgggggcggccgtcgccgccgtccgcctgcaggcgcgacttgagcacgtccacggggtaggtgaacagccagga

ggcggcgcccgccgtgccacccgccgccagcaccagccacgtgggcgcgggggcgctgccgcgcgtcatcgcctcgtacgtcgcgaagtagatgcccatg

gcgggggtgtcccgcagcacggtggcggcgagcccgcggaagaggccgcgcgggccttcccggcgcagcgtgtcggcggcgcactgcagcggtgcgacgc

ccagcagctgtgcgcgcgtcttgagctgctccagcggtgcgcagatggccgactgcgccagcccggagagcgcgccggcggcgaactggccgcgcagcga

gtcggcgcccaggtagcgcgccgccagcccgtacacgccgaacgtcaccgcgttcaccgccgccacgcccgccagcggcgccgccatgccgcggtacagg

ccacccaccgactccctgtgcaaaatgtgtctgaagcagtcgaaggctcctcggtaggcaggtttgcgctggttctgcgtctgaagatgtacctttacag

tatccaagggatgccccactaatatcccagcgcaaccgccgaggcagccagcgacgaagtcgagcgccatgatgtcgcaggtggtggttcagtcgggttc

agatcaccagggcgtctcctctgaatatcgcttggctgccaagaagcgcggtctggcttcgcgaggctccgaggcagcggctaccagcgtcatgtctcgg

tgtcgggttgctgcgccggcggtccacccccggactgactcgcgcgctgccgctacccagcgcccggccttggtcgcggaccgctcgtcgcttcttatcg

>44926|AGAP009137-PA

aaccgacggctcaccgaattgcaagtcgactgagccacggccaaggtcacgcattattgtcatcgactgcgtcagcgattcttcgtatgtcttcacaaaa

tcatactgaccccttatgtagttgtaagctaccgataagtcaatttcggtatagttctcgtgggatatcttaagggagttcgtcgtcatgagggtaactt

ggtgtcctctctgcagcagcgccttggacaggagatggaacggcagctggtggctgatggatggggttggag

>44950|AGAP009143-PA

ggcgcacaccagcccacgctggccgaaggcaacgcagacctacatcctgttgatgggtacatgctccgtacatgtctccggcaacagtgtgctccgctcg

gcattgagttacgtctatgtcttcttctccacaccgcacattactcattaatatgttttcacca

>44945|AGAP009143-PA

ggcaatagacaagtgtcgtattctgaacaattgcttgcaactttatgggaatttctctacttgtcagtctgctgttgaaattgatgtgcaaaatatacaa

agttcccagacattatctcatccatccgtgttcagtatctctcatcatgaaacaagctgtcagggttgtagaattagtgaaactatacacaatatcaaaa

ggaaaattca

>44946|AGAP009143-PA

cttcagtttgttacaatagaacatgcgggtttgctagacttcaatggaccaatgctgaagccaggactgcaagtggatctatggcgccatgcaatggaga

gcatccgggtggagaatagtttgcaggatggccttggtgtcatctattctgatttgtattcaacaggagctgcacatactgtgaagaacagtgaatttcg

tgccaatcacagaaatggtatcagtattaggcagtttgggcttaaagtttcagggtgcaccttagagaagaatgatgcagcaggc

>44949|AGAP009143-PA

tacaaattgtgagttgctggtatggactgctctgcctgccctctgctgtcagagttggttgttgcttgttgtgagcaaaggcgttgtgacccacaaaacc

acgcagtgctgttgcggagccacgtgagtctgcactaactgacaggccacccacattgccaaggactgcattgttcttaaaatataggccttccatattt

tgcacatcaatttcaacagcagactgacaagtagagaaattcccataaagttgcaagcaattgttcagaatacgacacttgtctattgccagaagtggat

tgacttcaccagcactctggtaacgtattgctcctaatcggcagcctgccaaaactgatcctgttatgttgtgctgtgcatcacggtcaaaaccaactgc

tgaaactggcaatgtgataatttcagcaatgaatccatagtcaccaccaccgcctgctgataccatgtgcaaccctagaacagatcctctggtgcgatac

aaatgacgccaccttgagggctctgttgtagccaacaaagttgctaatggtgcagttccattaacatatccctcaaaaagagtcagagagtcgggccgga

ccacagatccattttgtaaattcagctccaaaaacc

>44947|AGAP009143-PA

tcagagttggttgttgcttgttgtgagcaaaggcgttgtgacccacaaaaccacgcagtgctgttgcggagccacgtgagtctgcactaactgacaggcc

acccacattgccaaggactgcattgttcttaaaatataggccttccatattttgcacatcaatttcaacagcagactgacaagtagagaaattcccataa

agttgcaagcaattgttcagaatacgacacttgtctattgccagaagtggattgacttcaccagcactctggtaacgtattgctcctaatcggcagcctg

ccaaaactgatcctgttatgttgtgctgtgcatcacggtcaaaaccaactgctgaaactggcaatgtgataatttcagcaatgaatccatagtcaccacc

accgcctgctgataccatgtgcaaccctagaacagatcctctggtgcgatacaaatgacgccaccttgagggctctgttgtagccaacaaagttgctaat

ggtgcagttccattaacatatccctcaaaaagagtcagagagtcgggccggaccacagatccattttgtaaattcagctccaaaaacctgaaaccaaacg

gttttgagtagtaaacactggaaaagaccttaacacaactgaca

>44967|AGAP009166-PA

gagagagagagagagagagagagagagagagagagagagagaaattacattacactccagcacagccatcttcattgttccaaatgagtccaatccattg

acatgaaatcgcccattatactgcgaatttgttgaaaactttgatttaacagctcttctgtcctgaacctgagctcaaaattatcttgaacaatagttgt

agcatctgagacagcaaatggcgacactctgcccgcaagatgtggtgatgactctcgggagactctcacttcatttggagttggggtttcctctcttgag

tgagttctgctaagatttccatctgcaaggttgcgatctggcatgaaactgccaatcttgaaatcagtcacagtcagttcaggaacttgaagctctttca

actcatccaagtacagacttggagatggtgaaacatgaccgacagaattctgtgtagcatccagtcccattacagacctccaaatgtcattttgtctctt

cagttgttcctctttcagttcaacactgactttttctattaattcaattgctctgttgatgggattgtcgagcatctcaagatctttttgaagaacagca

agtaatgttaactgttcagaaaatgccttgtctcttaacaaagcatctcttcgttttaaacagctgtagacaatcttgagcatttcaatacaatctgatt

tctgttgcctgctttctcttgaacgtcgacgaagctgatcataacacttcagtccactttctgatagcatttccatatcctgccatggtggatttggttc

ttcttgtaacttcagaagctttgatgaagttctttcactcctggtcttcaaatcctgcagcaatacatcatgtagtgtttccactcgtgattttatttgc

tcaaaacccctggaccaacgatgcattactgagccaataactctgcctgcagccttctcttcatccagcttgaggctactatgcacaatgtccatttgtg

cttcaaccctctgtaagcttgtcactgcatctttcaagttttctgtgaccatattccccagttcttcaacatgtttcatcttcgtttgaaatgctgcaac

aagtgtatgatataagtctgcttcttggcgtaagaaaaaacttgcatgtgcccatgcacggcggagatgaacgtattccagtttggtgcgtggctcctgc

aacatctgttgcactgtccatggaattcttggtccaacctgtggtaacgtcagctcaccctttctgaaaaccagcagcattggtgtcccatcctcaatac

acttctgattccagcactgcatggccccagcatctggctcaggtggtttgcctgatggtaaaagcaactcctgatcattcacagctaatgcagtatctcg

ttcaagccacacttgtaatgttgagagtgctgttgaactgtctaccacataactgcagtgcaaatacaatggcactacaaacaactgcacaatcttctta

ctgagaatttcgtcaagcatatcaaagatgataactttatcacctggtcctctaccacgtttctttggatcccattccaaagcaagttttaaccatcgtt

ccatatcatgtttcaaggctagtgagatatggttctgatcataaagttccttggaaaaaacaatacttccatcaggagcttgatatgcacagatgtcatc

agatgacttgtttcgtacatgagacatccatgcagctggagtcatgttaggtaagaagggtcttactcctgtaatcacttcatgagaaagaagacctaga

ctccagtagtccactgagctggtatagggcttgtttgtgaagagctcaggggccagatactgtagggtgccaacaaaagaagtacataagctgctttgtt

caagctctttggcataccccaagtcaatcagcttgtacacaatctttttttccttttgttgaagtacgatattctctggtttcaaatctcggtgtgtaat

cttcatggtgtgaaggtactgtattgcactcttcacatcagaaatgagagcacggacatcagcttcattcagcccacaacagttttcccttttatttaaa

acctgtctaaggtcaccttttgagcagaattccatacacaacaatggcagctcagagcgaagaatagaaagttcccatggaacttgaattgccttgacaa

cattgggatgtgaaagtcgctgcattatttccacttcttttgtccatctttctttatgttttggcgtcatgatactgtcagtacctgaacgacatttctt

caaggctattgtttcacccgtttgtttattcttccataatgttacaacaccaaacccaccgcttcccaacactcgctgcttcaaccagtccccaagttcc

gaaggatcttctgtttccattatggaaggccttaaacaataaccttcctcccactactgtcacctgtggctcaggaagcgcattcttcctcatccatcta

agatct

>45491|AGAP009241-PA

ttagagagaggaacagagaatgagtttgaaggcatagggctataacgtcacatcactgaaaatgactaattttttactcttaacagaaattgaaacataa

gtccataagctgcaggacagaacatgttagaacttgcaacgccaagtaaaaacacgacgacattcaatgacgttacagataacatacatcaatagttatc

acatgacgtcacctgatcacatcaccaataagacacttcagcacccttatcacatgatgtcatctgatgacgtcaccgacaagacatgtcaggaccctta

tcacatgacgtcatctgatgacgtcactgataagacacttcagcagtctttatcaataacgtcatcatatgacgtcattctgacatcactgataagagct

cgacggcccccagccgtgcctgtgcttgaacatacatacgctatctacttctgctataatcaaaaagattaaacagataaaattttgtaggaatttcttg

aatagtttctgagaaaaagatatacacatttttcttgaaaataaaattttgaaattccataaaaagtttaatatatagaatccaaagaacttaacaataa

atgtgttaatttcatgtcaagcatggtagaaaatttcattgtcgtgtcttcaaatttgttgggttttgtgcatcttctaaatagtttgtattttttatga

atgtcccccccccacccgttaacaccatataacaatgcagaccccgtagggaaacgaagaaaggaaaggaacaagaagaagattgtagatgtcgattcta

tgcactgtgcatcttgtttagaaagaaacctggtccactcacggacggtaccttttcctaacaacgttaaagttcactttacttttctccacccaagttt

gtagccagtgatgctgggctttgcttttccgacagtgcaagttgtaccttaacagtattacactgcagtatatacaggtttaccgatgatgagtaggcca

ataaaacgtcgcatatggaagagaagcaaaatgtcgcgatgctcagctgttccagtagcaacagcacgcaaaatgtgatactcctttttcgtttcgtgaa

cggttccagatatcgaaatgagctttccgagagacgataacacgcgaaggggcacgtcctttggtatgggataaagagtcttaactcttctagcgatcaa

gatactgaagtatgtacgagttttttaaatagaatgtttacttgctttagtggtattcgaaagcgcttgaagagatctaactcttcgcaaaagaatctac

gaaatatttttaaactgaaagggaagtacggccggccactgtggccgagcggttctaggcgcttcagttcggaaccacgctgctcctacggtcgcaggtt

cgaatcctgcctcggttatggatgtgtgtgatgtccttaggttaagtcaggtttaagtagttctaagtctaggtgactgacggcctcagatgttaagtcc

cacagtgctcagagccatttgaaccatttgaaaggcaagtccgccggaatcggctgttgcgttttaaacggggccaacgagatatacgggcctcgcaacg

gacttgtgacgtcacactagtcggcttgtccaccacacatcggaaacgcgcggttccgcgcaagagccaaaggaagcaatatgttattgagtaagtaccc

agtaaaggtcttaattttgtcgtgtgctatcgagagctcgtgttaatgtaaacgcggagtcaattattattaaagtcgcctaaaacagttagtcgctcca

agtcgtagacggctactggctctcgtaagacctgcagtgtcacgcggtgtgaagtacgcgccacggcctgccttctcgtgggcgtcagttgtaaaaacgc

tcaagccacgctacatcgctgtagaaaccctttcgactgcttattgtctgcactgcagaactaccaggaactgtgttgtacatcggcaggtcatttctgc

ttcaacattccttgcatgcagacatgtgcaccaatgaggagacgataggcattctttcgtaaggcgaataccaaagaaatcgtgtcgaaactgtccggtg

gtatgcggatttgtattctatttgccgctgtccgtcgcagtaggtacttacatccattattaggtcactagtgcctataggcagcttccacgtcagaagg

cgaggacgaagactgcaactgacatagcacatgaagctacgacgcttctcagtacgcacggtggcaggagacgtattgccaaggaatgtgggattagcca

gacaagggtgtcattcccatcctgcatcgacacgaattccccgttatcatctgttactacatcaggcaccgtgaggtagtgacttcgaacgttgtataga

atttcgtcagaccgacactgctgttgaaggaacatccgcaactgagcattacactgtcagaggttcaaaatagtcagtttgctgtgcagcgcctaggagg

cgactctacgtttctctaatgtgtgctctttatcgacaaggcaacttttgcgaacaacggtactgtaaatttaagtaacaagaactactcggcagcggaa

aatccacgctggcctgatgatgtacacaaatggccgtggagagtaaacgtagggcgctgcatcctaggaaattccattgtgggaccataattcatcctag

aagtgttaaatgggaatacttactcacacttccgtgctttctaccctttcttctagaagacgtagcaccggataagcgagtgtgtgtgattccaacacaa

cggctgcctagctcgcttttcgagtgttcatcgcaaacactgaatgaaaagtttcctactcgttggacaggacggaatgctacagtataatggccggcaa

ggtcccctgacttggctccgtttgctttatttctatggactgaaggatgcagtctgtcacgaagaccaaactccgccaggcgatacgcgcagtcaaatct

ccacagcgtgcagcggcatatcatccaatgttctttcatgcgttcatctctctttcgaacggcgactgcaaatgtgccttttagttaattgtaaacaact

ggagcacatgcttaagtgtgaaactacgtcttttttttttaagaaaagtatttggtttgcagagacacatcgtcagtcagtctggataaaatgtttactt

taaacaccgactacacgtgttcggtagtcggtgttttacctttaagaagcattcaggcggctccagggaaagacaggtcaagcattatttttgttatttt

caaatctaagaacgtttatacagttgtttttcctgtgaatagggttgtttataagtttgaaaaccgacgtttctgtttcgggttcagcaaacaaacagca

accgtgagggcttaaatgcacgcaagcctgacacagttgtagctgtttccaaaaaagctggaaacgtacaaacgttgtgaacagtttttctaagatccat

ctgaagctccacaaactagcacatggtgtacatccataaatttaaactttctctgactctttatgtatgagattttatcttaatagcttaggcctgtctt

gccatacgtaacggcgaaacctggcgtgagcgctctgtttggcggactctggcggcatttgctttccagttttagtacatttctaggattcttctctgaa

aggttacaacgtcaacactaacaagcactatatcactgtaatagactttgcaagagctactgaatgttgttatgaaaagcatataattccattaaagaaa

atatatttgcttcagtatttcgatcgatagaaaagctaagacccttcatcacatacatcgtttcgatgtctggaactgttcacacaataaaaggggtgtt

atgtcttacgtgactcgccctgtatatcttgttcattccatattacaggatttttcatggcaatgaaaatatatgtgtagcaataaaggtaactggaatg

ataaacaggatgaatcataatgaaattgttactatgtaacgagtgctcgatagcctacgaagttctgtcggtggggtgcagacacaccgtatattacagt

tcggcaactgcccatgaaacattctgccgtctacagaaacttcttactgacatcagaagaatgttgtgtgaaacatgatggtctgaacagttcagtaaag

ttccgtaatatgtggaacgaggggtggaggaccagctgttgagcatagacgccctgtgcagcttacgtagggctgcactacacgacgcagtaccacggtg

tctgctaaggtggtggtaacgcctgtgttacaaagtcataggaactaagaatgtttattcaaggcttttgaaaagtgtgcctcgagaaggaggtaattac

gagcaacttctctcgttgctacctcggcgtcaggcgtaccattatcccttcaacaggcttcaggatgagctctatggcgaccttggtgtcctctggggta

tccaaggtttgcacgtggtagttgcgcaggatctgcgacagcactgtcttctcttcaaggatggcgaaacgctgccctatacagttgcgaggcccggcac

tgaagggcacgtaagcgtaagggtgcctgccctgcaccctctccggcaggaagttgtccgggtcgaagcggtccgggtccggccagtggtcggggtcacg

gtgtgagtggtagatgtgcaggttcagcatggcgccctccggcacagcgtagccgcctatttcgatatctttggtgaccttccttccgataaacggcaca

ctgggatataggcgtagcgtctccttgatgaccatctcgaggtatttcatactgttaagatcttgcaacgttggaaggcgatctttgtcatgtccgaata

tatcgtagagttctgagtaaactttgtcctggacgttctgatgatgtcccagaagaagaagagcccaacacatggcaactgaagtcgtgtcatggccctc

aaacatgaatgtatcgacctcttcctgtatttcttggtcactaagtttctgtccattctcggaagcgtctagtaggaggtccaagaatgccaaacgtcgt

tttcgaccaatgtcgtccacgagtgcgtctttggtcacaccctctttcaacttccacgataactgcctttgtttaatgaccttcttcgtgaagctgtgta

taacgtccaagtgtttgtagtattctctcccggtactagttctctgaaagatgaaatcgctgtgaatccatggtcgcactatcctttttatggtgagagc

gctaatgttgtacactgctgacacatattctgatccggtttcattttgggcattgacagatgttcccatggccgtctcacaaatgatatccagggcacaa

cgcgtaatgtatggatagatgtcaaaggcatgcccgtccaatttgctcttcaatttctcgaccagtatttgtgaattctctccaaatatgtccacgaaat

tatctaggatcttgaagtgaaaagctggcgtaatcatctttcggtgcgagaaccacttctgaccagcacttgtcagcaggccttccccgagccaggggaa

caggaagcggtaattggccgccttgctgataagctcgctgctcgccaggattttctcgatatgctctggtttactgacgtttattgaggcactcggaccc

atccagccgcggaatatcggccagtattgtttcgtcaccttgtcgaagacacgaagtctgtcttgcctttcgacgaggaacgctatgtacgtagtcccta

tcagcgggaacgccagagggccgggaatcttgtcgacggctctgcagaaagtccacctccggtacagcgccagagcggccaggcctacaactgcggccgc

tgccacgaagagtattagctccatggggcagaagacgcaaatgccggtgaagttagaagtaccgcagaggagcggcgtgcactcggctcggcagagcgcc

ggaggtgcactggccggggaaagccatcagcgcagacgagatcgcagccatcagcggccagcgtgctgctgctagttccgttatgggccgcggcgcgccg

tagcgctagc

>45559|AGAP009246-PA

aatgcgcaaatttgacgaggtgatcaagaggttcggtccaatcttccgctcctggattggaccgtatccatcgataatgatccaaaaaccagaataccta

gagaaaataatgtccagcagcgagttaattgagaagtccaccttctactcattcctctacc

>45558|AGAP009246-PA

ctgtagctgtccgccttagttatcagttcactgctcgtcattatcttctcgagatgctctggtttgctaatgtggacttcaggaatgggaccaagccaag

atcgatgtataggccagtatttcctggttattttgtccacgtgagcgactgcatcttcccgagcgatgaagggcaccttgtaggtgttgcctagcagcgg

tatggccagctcccccggcagcttgtctatctcgctccagaggcgccacctcctggacatgtacgccgcggtgagcgccgccacgacagacaccaccagc

agcgccgtcacctccagcatgctcgagcttccagttccagcacactagccctcaaacacgtgctcacggcctgccgtcgcacgccggactgcgcgatgct

cctcggcggtcgccgtacgcaatgccgagctcagcgcagcccgctatctgtccg

>45589|AGAP009259-PA

gccaacaatcaatctttttgtctcatctgacctgataagtctcccctgaatcagggttcagggtaacttttcaaaactgtgtcaattttctaaacctcac

ctgtcctttcccttcatcccatcttcttccgctcaaccattctgccagaaggtgccagtggcaagtagattttttttatcagtccaattactttaaataa

atattttagtattagaggaaaaattaagctctcttactgttggcactttctcctaatcaatcacttaatgattaacattttaactctgatttgtcatacc

atttatcactatttttctgattaacataaatataatgcttagaaattaaatttattttaggaaatgtagtacttctccagtagcatattccccttctttg

ccttgttatgcatgattatacctcactccctgagcctgaaaagtatattatccactgtcacaaccctcttatgatcgttataaaatttagttgcttgctt

cgcaggattgggttgcagccataatagaattatacacaataccattcccaaaacatcacaaattgagacacattacatattactaacagaggtctctcaa

gctacaaaagatgtataagagagaaaatagaggcatcatgaaaacatgatatgtaatcagtgttctttctttttcttatgaacacagtatttgatagata

tgccgacaaggtacagcactgcagcaatgagatgcccaatgaaatatatggatttccaatagtgtaaggtcttgtccaagcgaagcagtagaaatgcagt

tgtcatgtatccaaattcttgcatcttgaagaaccacaggataatgtcccacacttttgcctcatttcctttggcatctttccggaaaagtcgaacatac

aggtcttcaacaggcaagtagaatggaactgtgcacatgcacaaatagtagcctgcatggacgccatgccagtaggctgacacagccatagttatcagag

tccgcagctttttaaatggtagtcttttgtagacatagacagccaaccaatactgtacacaagaattccagtgcttcattgcagcacgagtagttggaac

aaagtcacttaagaaaggatcaacattatggattgtttcaaaatcataggattctctctttccatttccagagttaacacacacatctatgtatgctttt

gttggtccatgacctcttcgagattcagctgaagcaggataggccccaaatccagccatagtacagacacattcagacagtaccatgccagtgtacagcc

tcatgcgaaatatgaagaacgtaggagtcaggtaccatatacggtagagaatagatctctctttgtaaaattcttctgactccatgtactgtgagaacac

caacataacaaaaagcataatgaaatatttccaatccatcataattaatttcttcttgctgttggagttctagcttatcgctggacttttctgacacgtt

cttactttcgga

>45606|AGAP009260-PA

gattatcatgagttgaagaccatcctcaaagctttaggcttcgaagtcacaaagcaacaggtcctagaaatgatgaagcagtatgatccctcaaatacgg

gatacatcctttacaacgattttgtcacattgatgaccgacaaaatgttgtcgcgggatcctatggaggagattgcgcttgcatttaagctcttcgatga

tgataataccgg

>45628|AGAP009265-PA

ctcatttctctctgttgaaatatttgatcaaaaaataggcccaggataaatgtcttacctgttagtatatacagacagactttgcctgaaagcggagact

ttgtgaagcagctctgaccttgcctccttcagtgttgtgtggtctgtgggggcaaatgtgatctgtgtcgagcctccaccaaggtccagtgctgccacag

taccatcacccccacttccacccagccg

>45689|AGAP009280-PA

cttcatgctgggcggtgttatgtatatatatagtaacacggaatgttgtgtggaagtatgcggtggagtttttgagtagcgaagaagaaatactcgattt

ggaagtaacacaactatctcggattgatctggagcccgcattcagcattgttgtgaattctattaatgagtttaatatcattaatgtgaagataaacgtg

tattggaataatttatcccgttagcccacaacatttcgaaacaccttcggcgtgacaatgtacactttacgtatcagatgatcagtgcgtgtttttcacc

gtattttacagaatttgtgagctattaggacactgtaatgatggacagcgtacaatacaaaaagcttcagaatgaccatgtatcaaatcttaatggaaca

actcgttttgaaaccatattacaagtggcacctatcccattctcaacattactttcgttgttagcggtaccgctattggtgtccgtgagatgcggaagcc

gcacagcatactttgttaagttttctgccgaattttgtgtattagttttgcctattactttaacactgacagtctttaatgatgcgttacatttggttat

tattttcttaatgttgctggtgtcattagcaacatatacacacacaagaaactgtcatcgcacagattatcttaactatttacaagaactttgggtatca

aaaatttcttctaagaatttgccctttgtaaccatgttcagagcaataacgaacttggtaactgtgctgtgtatattagccgtggatttcacaatttttc

cacgaaagtttgcaaaaacagaatattttggctattcggtaatggatgtaggtgtaggtttgttctgcatttcaaatgcaattgtttctcctgaagttag

acatgcaaataatctggcatataattccgcaaaatatattttaaagagtgtaattagctgtctaccactatttgtccttggagtagcaaggtttttcacg

ttgacaaacattgaatatcaggtacatgttacagaatatggtgttcactggaattttttttctaaccctcgcttttgtgaagattttttcatctgtgata

ctatatttaatcggctttgaatatgtgggattattgtcagtattagtgtcattttttcatcagtgttttctgaatgctggcattcaagatttggtactct

ctcacaacaacacacgatccagttttttgacagctaactgtgaaggacttgcttcactagcaggttacctagctatttattttgccggaatgtatattgg

tagaattatcagagatacagggagtcttttctgtgactatgtgcacttaacattaaaacttggacttggaattgtgattatgtgggaaagtacaatttta

gcagagtactattttggggtatcacgcagacttgcgaatactggctatgtcatttgggttctgtgtgtatcactgatattcttcgaaatgtttttgtttt

gctctcttgcagttcattctataaaaatatatacaactaaaatgacgagacagcttgaaattaattatgtaccaatgctgtatgatgctgtgaattttaa

tggcctggcatgtttcctgttcgcaaatattcttactggtgtagtaaatttatgcttctacacactaaccttttcaaaattttctagttttatcatggtc

atattgtacatgggcactgtatgtttaagtattgcaacactgtactggaagaaaataaagcttaaattttggtgaaaattgttttggtttgaaacatgaa

tcatgtgaaatgtagatgatatgatgg

>45716|AGAP009281-PA

cttcacattattttattttcctactaattttttgctcatatgttttataattacattattactctccatcgtagtaaaaatgaaaagactcgtcagacgt

ttaattaatgtcatatacagcaactttcacattattttcttcaaatgtcatttaattaatatcacatacaacaactttcacattcggtgatagtctcatt

atatttatctggtgttacaaagcatcagatacaatgattatgtcactgattaaatgtacttcatcattctaggaagtttcatcaccgataatcagttttc

acaggtttggttggtttttcgcagtctctcagtgtccagaccattcgttgtcataacagagaacatattttggatgagttcgtcggtggggtttttttct

cttgtgagcagccacgagaactgcatgttagccgcgcctccaatatcgatacacgaccagaccacggagtagctcccgtagtccgtgtcgagaatccagt

atggcgcctcaaatgaaccggtctgtgggaagataacgaccaggcgaccttccgttgtgttcccatccacactggcttgtccattgacgatttctggatc

gcctgtttctgggtcaatcatggcgttttcgactcccacggtgccgtccccattgtcagtgtatgtggcggtcacacactctccgttcccctcgaagatg

gcgaagtagcggtactgctcgtaccatttccccaggtaagcagctgcgtcgaagttggagacaagtgtgacgttgggacagggtccggtactgccgatct

gggccagtgacggtgtcgctaccagggccagaacggttacgaaagaaactgctgaagccattggttcctgtgaaacagatcagcggagaagtggggccac

t

>45769|AGAP009314-PA

tctttgtctcatcttttctttcaaaaatggtttagaaattgcaaacacagtagcaaaaagtgatggtgcatttacaatatggacagccttcagccttaaa

ggaaagccatcctggaacattttaatgaaaagtgataagtttgatggtgttgcaactaggaaatgttgaagtccataccctgtcatatccacaataaata

ctgcaccagagatttgaacgtgaggatcttcacaacaaaatgaaagcactgttgctattgtctggaagcattcttctactgcaatctttctgtaatccag

tttttcaaatcgcatgatcactacaatcctcccatcattatctttttggtctagaactgtcattatgtccatgtcataaacatatcctttttttgatgga

aggcaacattcaagatgttttggataagtcttcttcactttgtaaaagttcttgagcagtctgaaggcttcattcacattgtgtttcctcgctcttaaaa

aagctaagaggaatgtgtcatcagttcttgaatagagccctttttcgccaaacaaaagcgctggcaggtcgatagacacacaaacaaacacaaacataca

cacaaaattc

>45770|AGAP009314-PA

actgatcagtacactattttggcagtacattaaataattgagtttttgtaacaagaaacataaatttactttttaattgcaaatctatattttttctgca

gtaattcatattatggctttttaaagaatatgaattaatctttattttcaagcattaaggtaaactgaattttgaagttctcacaaccaacagtttctta

aacattttcaaccaccgttgctgattcccccatgttatattttagcagtaatcaattaacttctttcttggactttccatagctggaaaacttctatgaa

atgaaggagtcttttccagaacaccttgaaccttaccttccatcagagaaaagtaacatttatgatatagaagcaatgactgttttagaacaaaaagatg

atgatgaaagagctgtagtcatcataagagtggaaaacacagatttcagaaaagttacagtagaagagtgttttcagctaataacaacagtgattttatt

ttgttgtgaagatcaatatgttcaaatttctggtataactgtcattgtggatctaaaagcatttggatttcaacacgcaattcatgcaacaccaaaaaca

ttgtctttatttattaaaatgtttcag

>45771|AGAP009314-PA

tggaatccgaccacatcagattgtgaaacatttcaataacattaggaacactgaaaaatccaaccaaaacaacaacgattccaacacaaactttaaagcc

gtgataattcacgtagggaccaactctattagaagcagcagacaagaagaaatcataaatgatattcgtaatgtaattcggtctgccaaagatttgtata

caaactccagaattgttatcaacggaattctacaaagaagatcggcgaaaaagggctctattcaagaactgatgacacattcctcttagcttttttaaga

gcgaggaaacacaatgtgaatgaagccttcagactgctcaagaacttttacaaagtgaagaagacttatccaaaacatcttgaatgttgccttccatcaa

aaaaaggatatgtttatgacatggacataatgacagttctagaccaaaaagataatgatgggaggattgtagtgatcatgcgatttgaaaaactggatta

cagaaagattgcagtagaagaatgcttccagacaatagcaacagtgctttcattttgttgtgaagatcctcacgttcaaatctctggtgcagtatttatt

gtggatatgacagggtatggacttcaacatttcctagttgcaacaccatcaaacttatcacttttcattaaaatgttccaggatggctttcctttaaggc

tgaaggctgtccatattgtaaatgcaccatcactttttgctactgtgtttgcaatttctaaaccatttttgaaagaaaagatgagacaaagaattcatgt

tcatggtagaaacatgtcttccttgcatgaatatgtgccttatgagatactaccaaaacagttaggaggaatggtacctgatttcaacttcaattcattc

agacacaaactacaacaaaatgaaagtatgatcaaagcatggaacaaatatggatatggtatggaaccccaagacaatttcgaaattggaatgtcaaaat

gaatgtcaagattaaaaccttactatgtctaggtagtacagtcaatgaaaactgaaacatttgtgccatatgtattaacaacatcattgaaacaggacag

aatttatatgttagaaacaaaccattttgaacattatctcatataaattcttagtcattataaattgcagtaagaattgaaatgtctcctatcaaagtgt

aaaatatattcattttgtctcaggaaatctgttggagttaatcttagtgacattacatcatatgtccatttattgtatatttttcagtattaaaatgctt

>45795|AGAP009324-PA

aagctggtattccattgagtggtttcttgcagtcgccacatttaaccttgcatccagctttagagattctttgatagactagcctattaccgggggtctt

gacaaccttctgcttattagaagctgtcttgtaagtgttacgtgttctataacaaagtctctg

>45873|AGAP009385-PA

gactgtaattgtactttcagcaaaatctgccctgtttcataacaagcatatgaacctttgacaaaactgttcacaggatgccatgccgctgagactgaag

gaggtgcatatcgtaaaacagccgtacattttcaacatggtg

>45917|AGAP009428-PA

gccttctcccaacgatagagtggactaaagaacggtgtcgaaatgagtaagtctccagatcagtcctacttccatacaagaggagcagaaagaagcacgc

gaggtggtcatgggacgccgcaatgtgtaaaggatgtacagggtacccttctaatgctgcgccgtattctagcagcaactttagtccagcactgaagcca

ttttgacaagcaatctggagaggggacgagagattctctctgctaccatcagggtcagctccagcttctaacagcagtctgcacacttctgtatgactat

tgtacactgctacaaacaggggtgtttgtgccttaatgtcaacaacatctacttctgctcctcgcctcagcagctcttgaacacatacagtacttcccag

aagaactgcttatacgttgaaaaatgtttgtgcaaacattaactaaataaattccaatatgatctccatctgataatcaaaacacttcaacgtcaattct

ttcgtgcaatatctgtgccttcacgactaaatgaaactatgggtgcagtaggcgtaacacctcttcgttgtgcagctgcag

>45964|AGAP009431-PA

gaagtgtccgtcatacgtcctgtagctggatccacatcagcaatattactctgtatagacgcatgatcctttgcgtgaatgatgccatccttttttgcaa

gccttgctatacaatcatcagactcccc

>45965|AGAP009431-PA

ctgtagctggatctacatcagcaatattaatctgtatagacgcatgatcctttgcgtgaatgatgcggttgctggcggaaagtgcactgccttgtggtat

tcacatcagattgcttctgataacgagcattattacgctatgtacacaaataaacacagtatgtagctacacttggttttacactggctcatagaatgta

aggattcgaacttttacaaagaatata

>45979|AGAP009432-PA

agctgtaaacggattagaataacataacagagagggtactgacttaaaatatgactatgtcaaagcataaagagcatttttgatttaacagaaacacaaa

actttcacagccagtctgagaacttttcaccataccattatgccaccttctgttagacagaatggtggtattttatttaattcagtacaagccctttata

taaatatatgactgttttcaagaacctatactttcaatatataaatcgagtctaataaaattttctgtacaaatcacaaataaaaaatcacacctgatta

caaagggcaaaaaataatatacgtgccctttctatctgcaccaaatgtatatatattaactgtatacatattaaaaacttacaactaaagagtaaataac

cttcatgtgcaaagcaagcactgttggtgttaaatatttggttggtattcaaatgtcatccccagtgggtaaaaacactggcttactgtcactctaagat

aatgacatcgttatcactgtcattatcaatctctggatgtgttgttaagtgcctttccacctgttttgattcatgtgaatgaaattttgatgtattattc

ttggactgttcacattcttgcagcataccattatgtctgtcctgcagtttagatggtctcatttcagaagaaaaatttgacaccctaacatcgattttat

gtggcataccatgtgcttcaactttgattgaagctgtggcaattaccactctctctttaggttctcttcttggcgattcctgtagcaaattacatttttc

attatttttgctcctttcacgaagtaactttgccttattgtcactattatgttctgattttcgttcatgaattaccacaatttcttcttcattttctgct

tgtttgctgttattatcttcacacacagtttcctctgtctgtaggtcttctccatccacgttgccgtcactctcctcatcggattcttgttcattttcac

tggtatcattgtcattgttatccatatctgccaaaatcttatcaacaaagtcatcttctgtttcgcttccatcaacagtctctttttcactaccatcctc

ttcttcatcctctgatttgcttgctatttcatcatcagcaacctcttctggtatcatgtcttttgtttgatcagcaaatttgtcaataacctcatttata

cgactcccaaactccttcttgttctctctaagtttctcatccagctcctggtcgtcttcagctggatccctgttatcacccaaataatatgagtgtgcat

tccacgtagagaattgtcgctgccgctgcagttgctctccaatggtctggaatgctgctttagcttggtttcttagtctctcaggagaaatattcatttt

ctcctcctcatatgcagacttgaaaagtttcaaaacatcttgataatcagggaactgtcctgttttattcacatacttttcaatagcacggttgacactt

ggaaatgcagttcctgaatatgttactgctcttctctcctgaagttttggtatggcaccacattcatactgttgcagctttttgtacactgcaactgcac

gctctttatacctttgcaactgtatgtatgtggagtcattttcttcatcccagtctacttctttttgttctagtttagtaattcttctctgaaggcaatg

cagcattttgctcaatttctttgcggctctctcttgaaaatgtggttcttctttaaccgtctttacttcttcagtcggcttttctaggttggtactatct

ggtgaatctgtttcaagttttatttttttccttggtggatctttcttacctctcagtttcagttcatcgacaacttctttgacatacacataaagattct

taggatccttgtctatcagttccattcttgtttgcagaagcttcttgaaggtttcagacgatgtataagcagcattacatgcatgataatatttcttgac

ttttgtgtcaattattttgcacatgtctgctgacttatcatattttaaacatttcatgatgaacatttctaaggacatctttgaattccaaaaactgact

tatacagaatacacgataacaaaacttatgacgttcac

>46023|AGAP009441-PA

aaaccgcacccccctcatgttttcttctgcaagcacacccgccttggtagcccacgggaacccagcaacaacagaatccttg

>46036|AGAP009441-PA

gagggtgtgcttgcagaagaaaacaggaggggggcgaggtttaacatctctgatgtcgcccttcacccagatgctatacacaga

>46035|AGAP009441-PA

tcaccagcctcctcaatgatacattgcgccatggggtccgatttggcaaggcgcttcaaaccttccccaagctttggtagatctgca

>46033|AGAP009441-PA

ggcttaattcgttcagcaattgcttgacgcagaacattctcagtctgtacacactcacctgacacacaatccacagcacttgtctcgcc

>46038|AGAP009441-PA

catagatgttaaacctcccccccctcctgttttcttctgcaaccacccccccctccgtagccccctggaacccagcaacaacagaatcctt

>46022|AGAP009441-PA

gacatcatagatgttaaacctcacccccctcatgttttctcctgcaagcacaccctcctttgcagcccactcgaacccagcaacaacagaatcctttatc

ccattcaggtattggacacc

>46020|AGAP009441-PA

catagatgttaaacctcacccccctcattttttcttctgcaagcaccccctcctttgtagccccctggaacccagcaacaacagacccaggcccgtcact

ccgggtggacccaagcccccgccaccccgcc

>46029|AGAP009441-PA

cccatggtgcaatgtatcattgaggagtctggtgaacacatctttgcaggagctggtgaactgcacttggaaatttgtctgaagtatcttgaggaggacc

atgcttgtattcccaataagaagtctggtgaacacatcattg

>46024|AGAP009441-PA

taccagtcattctgtgagaaattaaactgccagatggcggtaagaaagggcattaaagaattaaagaatggtgtgtgtcaggtgtgtgtgtacagactga

gacttttctgcgtcaagcaattgctgaacgaattaagcctgtc

>46028|AGAP009441-PA

catgttggattactatggatttccttcctcactaacgaacacggtcgtccaacaatggcggacttcacggtagatgagattcgtgtgatgatgtacaaaa

agcggaatatccgcaatatgtccgtcatcgcacacgtggatcacggaaaatcaacact

>46031|AGAP009441-PA

catcatagatgttaaacctcacccccctcatgttttcttctgcaagcaaaccctccttcgtagcccactggaacccagcaacaacagaatccttctcatc

tgcatactttgacgccgcctacgacaacgttcgcctcgtgatgcccgtagcaaaagcc

>46032|AGAP009441-PA

caaggagggcacggtccatcttgttcatgaacaggatggaccgtgccctccttgaactgcagttcaaggagggcacggtccatcttgttcatgaacagga

caggcttaattcgtccagcaattgcttgacgcagaacagtctcagtctgtacacaaaacacaaacactac

>46026|AGAP009441-PA

cttgaggaggaccatgcttgtattcccattaagaagtctgatcctgtagtgtcttatagggagactgtctctgaagagtcagatcagatgtgcctttcaa

agtcacccaataagcacaacaggcttttttatgaaggctgtcccaatgccagatggccttgcagaagacattgacagtggagaagtaaacccg

>46019|AGAP009441-PA

tgcagttcttaacagcaccagctacttcctcatcatggggaacttcatacaacatttccattctgtatttttgtgcaatcacaggagatggcagatgaat

tgctatcatctgaagcaaagcttcacctgctgggagccaggtcctcatcacatctgactcctcaatgatacattgcaccatggggtccgattttgctagg

cgcttcaaaccttccacaagctttggtagatccgcaggg

>46021|AGAP009441-PA

tattgcaagtgctaaagcttttgacaccagggagtccgccattgttggacgaccgtgttcgttagtgaggaaggaaatccatagtaatccaacatggtga

acttcacggtagatgagattcgtgttatgacggacatattgcggatattccgctttttgtccatcatcacacgaatctcatctaccgtgaagttcaccat

gttggatttctatggatttccttcctccctaacgaacacggtcgtccaacaatggcggactccctgcgaaaggactgtgccacggagtgcag

>46081|AGAP009450-PA

cgtgttggtcgtattatttctgaactctcaggtccatgtcttgagtgcatgtgtactgaagtaggggttcaatgcagaccacttgactgctgacataaga

tctttctttagtgtcagtgcaaagcagcataagacttatgacagcaggtgcacaaggactacacattataatgttcactcagatgggctcactattgtgt

gttactgtgcacatggttttgaatattttatctgtaaatttagtataagagtttcatgtagataaacaaatacttctgaggacagagactccag

>46127|AGAP009463-PA

cccagaacggcgtgtggcccatggcgatggtatccaggaagacactggggatgcggtagtggcagtagaaaacggagcagtccagcttgggccggtccat

gccgtagatggcagtcacgtagccctcgagcccatagcgcaggtagctgatgtatgagccccagtacaagtatgagggcagatctcgcaacgtcacgccg

aagcccgcgaacatcatcatgggcaccgacagcgtcggtcccaggaacgtgccattctgcaatagtaaagaggaacgttacatagtgagggtaatttttt

gtcagcagataacgatgataagcacacgtaggagaaataactcaaaacctcatttctgacagtactttattacagataaaagcgtcaactgtggcaaatc

tgaagttactacaccactggccattaaaattgctacaccacgaagatgacgtgctacagacgcga

>46147|AGAP009471-PA

tattattgttgttgttatgcttgggaagtcagtagtagtgctaatgtataatgtctacaatatgttaagtttatatgaaagcaaattattttttgagcca

gtagatttaatataaggtgcttcagttatatttaatagatatttatcttccctacagcttggaacatttatactggctgcagtaacaattccaatgttcc

ttttctctggtttctttatgaggctggatgatataccgtcatattttgtgcctctgacttatttaagtctcttccgatatggatttgaggcaattgtgtt

gtgcatttatggatttggacggaagaatcttgtgtgttcagaaatatattgctattacaaggatccctccaagtttcttgaaaacatggggatggcccat

agtgactactggatggatatatgtggccttgttacatggctagtgatactacaactgacactgtttgtgaccttgaaaattaggctacataaatctaggt

gaagaatactactgtactttgtttcttctttgtgaagagaatgaatgcaagaggcaactataaataatttttgggtgaatggggaatgccagaagcatca

gacactttatataatataaagtggacaatgtgcactgctattggtattctaggttgtttgtaagaacacttatgtacatatttgctgcaaggctacttgc

agcagaaaaaaattaagatctggaacagacggacattgtctctaatatttatttttgcaaaacatgttgtgtgacactagatttaatgcacttttttaaa

tgatgacctgagtgtaatatattattttggaataagctaacatgctcgctatattaggcaaaattataaacacaagaaatgaatcctcataaactattca

tttttgattctttttatacttacaagaattttgctatattagccctaacgtaatatagaatgtttggaactaacttttatagctgtacttcctcttaact

tgggaagagactggtggaaagatatgacaattttctattgaagatatttttccattttagcatctgtctaatcagtgtacagtaactgtatatggctttt

cctcagtattttttactagtactataattcatgtgataaaataagtcttcttccaaaactttgttttcataatgtacctgatttgtgtaataaaaagcag

gataaattaaatatctgtcctcatgaccttttattgttgtgatttttataagaacatggtgctgatgaccacactgtttagcactcaacaattctaaata

catccatcgatttgtaacccacctgctggtcaaatctgtggcatttccatcatattttgctataaacaagactgtttccaactttcctagacagtattac

attcatagtaaataaataaatatcatgtattatataaatcaactgaaatttcctttgtacaacttgtaattaaaaatgtg

>46174|AGAP009479-PA

ttactgaaagaacaaacagtacagatttttgtccatgtaaaaatgcctttcttgctgttcatggttttgggatgcattgtcattgccttttacaatggct

gtcatgtatattaagaccaaaacttgaaatatctcatgaggccttggaatgaaactacagatttatgtcttaaactgtatgagaagtctgtcgatgattg

acaaaaaatatttcttttggagaaacgttgatacctctttatgatgatggtatgatgtacaagattgtaggtggatgcttgttgtttattgtttagttct

tcttgggattacttgtggttgtttttaacttgctctgtttctttttttcctgctcctgaaatttgagagcgatgtattggtcaacaagaggtccatgaaa

atcctccggaaaactgggagtgactggaaggatgtatttgtataggcgctcatttagaacatacaactgagttgtggacgagcagcgcacgttgaaccac

caatcacatgtcagtgctacttgactaaaaattgttccattaggacagagaaatgatgcttgtccaccgttgagatcacagtagtgccaaacctggaaga

agaaaattataaagattgtaattttacaccaccagtggag

>46195|AGAP009482-PA

ggaaggacttcgagcacagaccgcacttgtgctgcttctccttgatgtgcgtcatgcggtggtagtagaggttggagctggcggtgaacctcttgccgca

gacctggcactgatgcggcttctccccgctgtgga

>46194|AGAP009482-PA

caaggcgttccacaggactacatccagcatctctacgatcctctccatgggagaatagcagcctgcattgctgcgaaaggtggatatacactgtactagt

gccgacattgtgcttgctctgttgcctgtgcctatgtgcctgtggttctgtcagtgtgatcatgtgatgtatctgaccccaggaatgtgtcaataatgtt

tccccttcctgggacaatgaattcacagtgttcttatttcaatttccaggagtgtatttgcgtaatctaacaaatacttgcattttctttctgatgtgaa

gaaatgcatgtgtcttgagagtatccaagtaagtgaaacttttgtcacagataccacacttgtgaggtccactaccagtgtgttcaaatgcatgtgtctt

gagatcacctgatgtagcaaaagattccccacagatcttacatttgtgaggttactttccactgtgaattaatgagtgtttcttgagattatgtagcaca

gtaaaagatttcccacaaatctcgcacatgtaaggtttctttccagtgtggattaatgtatgtaccttcagatagcatgacaacgcaaaagctttcccgc

acatctcacatttgtgaggtttctttccagtgtgaattaaaacatgatttttaagatagcctgacgcagcaaaagatttcccacaaatctcacatttgtg

>46757|AGAP009483-PA

gacttgaggtccccgggcgtggggaaggacttcgagcacagaccgcacttgtgcggcttctccttgatgtgcgtcatgtgggcccgcaggttacacgcca

g

>46756|AGAP009483-PA

ggtgccgatgaggttgacgtcttccgtgtactcgtggacggcgcgtaggaaacgtacccagttacagtggcggacacgccgcctgtctacttctgtgatc

tcgtcgtaacagccgaaccggtgtcggatctgcaaca

>46758|AGAP009483-PA

attaattcacactgggaagaaaccccacaaatgcgatatttgtgggaaatcttttgctgcggtggtcagtctcaagacacactcactaatccactctgga

aagaaacctcacagatgtgagatttgtgggaaatgtttcactgtgtccagcaatctcacgagacatgcattaattcacactgggaagaaaccccac

>46797|AGAP009495-PA

ggaccaagacgagttaatcatgcagctgttgttgttcttgataagatttactcttttggtggttactgcacgggggagaactaccagaggaagcggccaa

tggatgttcacattcttaacacggttgaccaccctcacaaccataagggatctaatattccacactcctgctcattgaataccagcttcacccagagatc

cgtttggatgttattttacctccagaatattttaatgatacaaaagagctatatgatacacatctcttaatttagtttttaatcaagtcatgaagtgata

ctaaggtatttgtagtgttggctatggatatttgttttgaagctatacgccg

>46798|AGAP009495-PA

tgtggagtttatgcgtaagggtacaaggaatatttcgtgaagttttaaactggcatagacaacattaaaaagcttacagaatgtattggacagtacactt

agaaggtggaccaagacgagttaatcatgcagctgttgttgttcttgataagatttactcttttggtggttactgcacgggggagaactaccagaggaag

cggccaatggatgttcacattcttaacacaggtaactacaggtggcacgctttacctacaccagaatacaatgatgtgacaaaaaccactgtcccatatc

agaggtatggacacacagctgttgaacatgagcagaagatttacatatggggtggacgtaatgatgaagctgcgtgcaacagattattctgttttaatcc

agataatttaacttggagcagtcccaaagtgagtgggatgattcctgctgcaagagatggccattcagcttgtgtaattaataatcagatgtttgtcttt

ggaggttttgaggaacagatggacagattttcacaagatgtctatgctttagacctgcgtacaatggtgtggagatacataataactcagggtgaccctc

catcatacagagatttccattcggcg

>46799|AGAP009495-PA

attatgaataacaaaagttttggtttatttttatttttaaataaaattgtaagcaaagtcacggcaggacagtgtaatgatccatcagtgtcaatttcaa

atcacacgagtagacctacaagataatgttttcgtgcagtcagactgtttagcaataggtgtgagcacatttatctcattttcttacttcatgaacaatc

tgttaatagcaaatgcatgatcatatgtacatgtacatttgaaaatgactcatgttgaatttggccaaaagcactgaatgaattatcttattcaattagt

atacaggctcactttgatgtatgagcttacaagcataaggaaaattgtaaatgttactgttcactttgatgatgctttcgaaacaaacaaaacacttttg

atccaaataatgacgatgtgactgcaaaagatgtcacaaccagacactgttcataaaatacatattcattaagtatgcaatggactgaaaatagtattac

aactaatattcttaagcaacagataaaccccaattgagtgctacactatcataattttcatactgagagctgtatttagcttggatcttttttttacact

gttttcgtgaaccacttctcaaacctcaatgttcaatgctcctgtactccatatttgaccttgaaactcactcaaaaggcagagtgggactatgggctta

aatacatactctaggatgtgttacttcacttttatcacattacagtatgtagttttctctgtaacaaacccatgggtttatattaattgctagctccagg

acatctgtttcaataagtgttccaaatgtccccaaaaactaattactgacaaccacaaaaagcaattgccattcagtctgcaaatgtcaacgtctgatga

tgatgatgatgacaatgatgataatgatgatgatgcttgagtgctcactgggcattgcacattctctgccatttagtgatacctcgatttatgactgcca

tgataaatgaatacaagatctgcctagccccgttcaagtgggcaaagcaaacagtatgtcttgttactgagagaccagtccaaatactagttattagtct

gtaaaaattccacagcttatatattcatactgaaataagtgaccccatcaatcaaatctgatgagaaaccactgaattgtggagtacatccattagtagc

agttcctccacttatggtaactaccaaaaattgaccggtaccaaagattcaactgtgcaaaggagtgtgaacaccagggtagtaacaactaatagtttca

cacaaaagggattttaaacatagtacactactaaataaacttaactttttggctatttagtggggaacaaaaccactgcagtgtgaaaggaataatgccc

attacatagcgcaaggaacaggacataaacatcaacatcgcagcattctgaaattctaatgcagtatttcatttctacatgacatgagatggacacatta

tgtgcatgcaatgaaactgaaggtacaggctgtaacagtgtcatgcatcaatggagggataataattttcttataaataggaactggtgtaatttgtctc

cctttttggagaaagaaggaagttagatatcatatatggtaaagacttagttgtacgaatgccgagatcagatgtgtaaaacaatgtttaatgatacttg

ggggtaagtacaaaaaaaactggacacagtgttgtaatgagcaacaatgagtcagaaatatcaagtcacatggagtatgaggtaacaatcatgcagagat

cttatcatccgcagccacagttgacagtcggcgttgtggaaaagtcgtccccgccctcatagtatatgctgcgcgccctccgctcgacctggtcttgctc

gaggcgtcgcctcaagagctcacgcagggagcgtggcagagtgtctagctctgatttgattcgaggatcgtccactagtgaaagtacaaagtctacacat

aacctgcgcaaagacttaggtgtgaagttcagaagtatcagctcttccgcatgatccttcccagcaaacatttcgtcggtgccaaagatatttttggcat

agccgcctatgacgacaatttcgccagttgggaccagtggggccttgtgccagagccgtggcttccttttgtggatcgagggtatgtccttcagctctga

ccatgcgatatccggcacctccagcacccagcaatcgttcagaatagtgttcatttggttcagacccccgtacaagatagccctttgtggagagatgaat

gttagactgtgccatgatcggccacaaggttttgcgcactggaggctattgttcagatctccactccaagtcatggtgtccaggtcaaggcagtggaggt

cagacattcgtctgcccctgtaccgcccaccaaacaggtacaagcgcgatcccgtgcgatcggcagcatgcgccgcacgtggttcggggcaacggccgtg

cgtctttggccacgaccatgaatctgtctctgggcagtagcagaccagttggttgctccaccctcgctcgtagagtgaactcgtcgacggatctatgacg

tgcttgaagggcgggtcgtagctgggtggggggccgaagccgccgaaaaagtacagcctgccttcgtactcccagccaacgagcttgtcacaggccgcag

gctctggtttcccactcggatgcagctgctcccaggtgagggatgtgaggtccagccggtacaggccgttcgtgttgttgttacggtcatcagtggtgtc

agttgtgtaccctgcaagcaatgcaaaacctccaaaaacatacaaatgttgcttcaatatcacaccgcaggcaccagagttccgaggtggatgttcacct

gttgttacttcccggtcccaaagttctgtattgctgtcataaatccatatttcatctgttggaaaatattcagtccgaatttgtgaagtcgatgttctct

gaacatgttccgtgtaacctccccatactacaacgtaatttttgtagactacggcgacgtgtccacttcttctatggattctatcgtaaggaacagtata

cacttcttctgctgacatattcaatttttttcgctttggctcacattcactatttcacataccagtcacttcttcttttgccaaacaccacaatcggcaa

acatgacgtgcactctttctgttgaaagtcagggcataaactacgcatcagaaatatgatatactacaagttaaagacactgttgcaccgttgtcgtccc

cttaactaatgcgccacatagccttgcttgcgatgacagtccattattaatggaattaatgctggacagcaagagcggcgacaaacgcacgtcggccgac

attttcgcaggatgccaccttccccacactcacccgctcacacacgcacgttcgcacgcacgccgctagaccccctgcgtcgcctagctggctgccggcg

acgacacccgcgccgggg

>46828|AGAP009498-PA

gcccagatagctggcgtcgacagcaagcttctccagggcacgggcagcttttgctttgcactcgattgtatagtggcctcgatgtatcgacgctcatctg

ggtcgatgttcttgtgctgtgctggcgaatcggaagccagcagccaccatggcagacaccatagtagtccgacgccg

>46827|AGAP009498-PA

gagaattacgaatattaaactttgaaatgtgccagtacagaaagtgcagtccacgtataatgaaacatatgtgataagtaatatgaattccacaagaatt

agtgatctgtatgaccacactactgaatacatctccaatataaaccattttacactgtcgctaagacctacggtatacatgtacatataacagctgactc

tgatgagtgtgttcattaactgaataacagctatttgtctcagctgttatcaaaatcacacttaaaggtatgaccagttgttgagtagtcacatgagatt

tgattgtgtttgttcaaaagtgaaaagttttccccctgttgtataagtggcctgtcagctgctcattgagtaagatttacttgtgtgaatcatgtttgac

tttaagaaagattcaaaattcaaataaatgtgtaacattttgctgaattcagtttataaactgcttctgcattagatacaataagcagctggagtcagaa

tttgaataggatgacatcatgaaaattaaatgtctttcaaatgaatgattaaaatattctaccttgcccctcgttctcactcatatgcagtctaaccaca

tggctcctgctacttccacactccattccaaggttgttggcttccttcactatgattcatgatctgtatctctatcaccctaggttaaaatccaagaggt

gccttggaaacaaatattcaataacgtaattaagtttgcaaaacttgacattcataatataaaatatggtggcacgtatatcatgcttctgagaacagaa

atatccaatttacgaaggataactttttcttgaaagggattaaacacacattaataaagcccattgttcatagtagatttgtctgtgtcaaatcctggat

gaacaaaatttgcaggattcacaattcacaacttcattgttttcagagcagtctgttggttggtatgtgaactgagtgagtatcttagctctttaatgtc

actaattgtaccaagcaggcaaaatacacaattctgagatactgtgattgtagtcttgttgcctttgggctacagatatggcagatttgttgtgcttaga

gctttcgcagtggtgttgcctcctcctcagcgtccccttcctcagcaaggtccttgccattagcggttgcagtactgcctggtgggttgttccagtgctg

gagctctccagatccaagcaccagataggccagtgctcctgctgtgtatgtgcccactaggatcccaaacaccttctgccactggccgaacgtttgattg

ccttgcgtcagagatcccaccatggctgatgacaagaaacccgtgaaagaagacagggtctgggccataccgaagatggttcctgagaagttgggagcga

tgtcgagcccgttgcccaggtatccagcggtgacggcgccgttgaatgtgagtgcgatggtgaagcaggcgacggcggccgcgcgctgctcacccagcag

cagctgcactgccattgctattgccggcatcaccagtgcgatggtggtgaagagcttgcgcgccaccgtggtgctgaggcggcccgattggcgcagatgg

tccgcgagcgcgctcatggcgacggcgaacaggtacttgcccaggtacggcaagctggacagcagcccgttctctttgatcttgaagtcgaggatgtact

tcatgtaggtgggcagctggttgatgacggtgaagtagccgaaaatgctgcaggcgtgcgcgatgatgatggcccaaacggggcctgacgtcagcacatg

ccgccacggcagcggcagcgccttgcccttcttggtggtggagccgatcgccttctcgatgtactcgcgctcctcgtacgagatgcgcgggtgctgggcg

ggcgagtcgtagatgaggaagaaccaggcgatgctccagacggtgcccaccaggccagtagagtagaagaccgactcccagccgaaggaggcgatcagca

gcccgcacaccggcatcgtgatggccgcgcccagcgccgacgccatggtgttggcgatgaacttgctgcgctccataggtggaatccacttggccaccat

ggggtgaatggacggccacgtcactcccagcgagagccccagcacgacgcgcagcaggatgacgac

>46826|AGAP009498-PA

ctggtgggcaccgtctggagcatcgcctggttcttcctcatctacgactcgcccgcccagcacccgcgcatctcgtacgaggagcgcgagtacatcgaga

aggcgatcggctccaccaccaagaagggcaaggcgctgccgctgccgtggcggcatgtgctgacgtcaggccccgtttgggccatcatcatcgcgcacgc

ctgcagcattttcggctacttcaccgtcatcaaccagctgcccacctacatgaagtacatcctcgacttcaagatcaaagagaacgggctgctgtccagc

ttgccgtacctgggcaagtacctgttcgccgtcgccatgagcgcgctcgcggaccatctgcgccaatcgggccgcctcagcaccacggtggcgcgcaagc

tcttcaccaccatcgcactggtgatgccggcaatagcgatggcagtgcagctgctgctgggtgagcagcgcgcggccgccgtcgcctgcttcaccatcgc

actcacattcaacggcgccgtcaccgctggatacctgggcaacgggctcgacatcgctcccaacttctcaggaaccatcttcggtatggcccagaccctg

tcttctttcacgggtttcttgtcatcagccatggtgggatctctgacgcaaggcaatcaaacgttcggccagtggcagaaggtgtttgggatcctagtgg

gcacatacacagcaggagcactggcctatctggtgcttggatctggagagctccagcactggaacaacccaccaggcagtactgcaaccgctaatggcaa

ggaccttgctgaggaaggggacgctgaggaggaggcaacaccactgcgaaagctctaagcacaacaaatctgccatatctgtagcccaaaggcaacaaga

ctacaatcacagtatctcagaattgtgtattttgcctgcttggtacaattagtgacattaaagagctaagatactcactcagttcacataccaaccaaca

gactgctctgaaaacaatgaagttgtgaattgtgaatcctgcaaattttgttcatccaggatttgacacagacaaatctactatgaacaatgggctttat

taatgtgtgtttaatccctttcaagaaaaagttatccttcgtaaattggatatttctgttctcagaagcatgatatacgtgccaccatattttatattat

gaatgtcaagttttgcaaacttaattacgttattgaatatttgtttccaaggcacctcttggattttaacctagggtgatagagatacagatcatgaatc

atagtgaaggaagccaacaaccttggaatggagtgtggaagtagcaggagccatgtggttagactgcatatgagtgagaacgaggggcaaggtagaatat

tttaatcattcatttgaaagacatttaattttcatgatgtcatcctattcaaattctgactccagctgcttattgtatctaatgcagaagcagtttataa

actgaattcagcaaaatgttacacatttatttgaattttgaatctttcttaaagtcaaacatgattcacacaagtaaatcttactcaatgagcagctgac

aggccacttatacaacagggggaaaacttttcacttttgaacaaacacaatcaaatctcatgtgactactcaacaactggtcatacctttaagtgtgatt

ttgataacagctgagacaaatagctgttattcagttaatgaacacactcatcagagtcagctgttatatgtacatgtataccgtaggtcttagcgacagt

gtaaaatggtttatattggagatgtattcagtagtgtggtcatacagatcactaattcttgtggaattcatattacttatcacatatgtttcattatacg

tggactgcactttctgtactggcacatttcaaagtttaatattcgtaattctccattgacacttatatcagttgtaattctgcacatttaacccatgtgc

aaacagtgcattccatttatttacaaatttctgtcagttattcctcttaatattccatttatttctgactaaagccatgagtttgtgatgactaaaatgc

aatatcttacatgatgaaatccagacaaatacgtatgaatactcactgccactttagcaaagattttttatttttagtccttccattgctctgaataata

gacagaatggaagtcacagtgtaaagttaaataaactgtagctactaatcagagagagaaatacattattgaaaaatgtttgttttaataactgccaaaa

tgagggcttatgagattcttttccctctttaggttgtgagcaagtatctatttcctatctacttacacaagttataattgtctttattataagtgaggat

cacatatctcctgtctgtattgtgatacttaataattccgtttgcttaatttctattgtggttttgtgaaacttaattttttttttaaattttgtttctt

cttactgtaactttttagcagcaagccatgtaaatgaactacagcaactcagtatgatgctataagcagtgttatgttacattaaggctcgtgatgtgag

ttcacgttaacaatgggagtcatattaatgttgtttccaactgttgttagtattgtatttctatacgtaatttgtgatatattgcttgtgaatgaagata

tttgagagagatatatgcaagttcatcttaggctttctcatactctgttacagaaagagatactgaatgagtaaaaacatttgccattgtgattctgttt

cacagctgcaccatccatgtctttactttttgtgtatactcagttttgtaaggtatgttattagttataaatttatgcatacattccacgatctgacatg

tgtaaataataaatacatggtgtgttatatatacagccaagacattacctctacatgaa

>46846|AGAP009503-PA

tcctcctccatcacctgttagttcaagctattcagagctgcgcagggcaaattaccaacctgactttgggacctatggaccttcatctcagagctctgcg

tacgaatctatatatgaacctattaatccgcggccaccaagccagatgtcatctcgttccaactattctttgtatgctccttacatgagtggctcacaaa

acaaatcagatgaagcaaatacagcaccttcgttaggaaaggaagcagaagttgatgctttgactaatttactagttcaatccatgcacgattcctcaga

tcccgatgacatatatggtgtgtgtacagcctgtggacaaaaggtggtgggagaaggatctggatgtacagccatggaccaagtgtaccacatttcatgc

ttcacatgtcaacactgtgcaatccagttacaaggaaaacctttttatgctcttgatgggaaaccatattgcgaagaagattatttgaacacactcgaaa

aatgttctgtttgcatgaaacctattttggaccgtatattaagagcaacaggaaagccataccatccacaatgcttcacttgcatagtatgtggaaaaag

tctagatggtatccctttcacagttgatgcaacaaaccagatacactgcattgaagattttcataggaaatttgcaccacgatgctgtgtctgtggtctg

cctatcatgcctgaacctggccaagatgaaactgttcgtgtggtggctttggatcgaagtttccatatagcttgttacaagtgtgaggactgcggtctca

tactgtcttcagaagctgagggccgtggatgttaccctctggatgaccatgtattatgcaaaagctgcaatgctaaaagagtgcaggctcttacatctca

catgactactgaattgtagaattaaatgctagactgctcagagaaactgaatttgagtgtcaggttttggttaagccatatgttttatttgtgaaagttt

tgtgtgtcggggagcaaatatctagttcattgtattaagtctgtgataaaaataaccaatgtaaattgcttacatactgggtgatccacatgtggcacaa

tttttgagagcgccttcacttattttgtagatacgagcaatcacattttaaactgttaactccgaaatgaatgaaacgctggatacgtaacaatttttat

ttatagcacttctgataagtcttcaatgttaagcaatgtgtgtggtgatatttgttgatctacatatagtacgtactgatcttgattcgtgatttccctc

cacttttcgcaaattgaacatctaattttcattactgtcattttaatgattaattatgtgtgttaaatattgtttaacacatttattaaattacgaaagt

ggcactttcatatggccacaatgtgcaacagactgttttaatggtgcattgtatttcacagttcattctgttgaggtcactgtttgtagaaagtattaac

tgacttgtgatatgttatgattgactgatagtgttgtaaagctcccagtttcattttaagttgttattgtaaacctgtgtgtcatttttatagcaaagta

tagatgttggtcaattgagaggctaacaagttgaaaaaactgaaaattttctctccaagctttcatttgattttgtttatggctaatgtacattactgat

tttgcaatcaatcttagtgttttgtaattaaagttgctctgtattttgagactttatgtgcacttgtaatttcgtaatgtggacccatagggatataagt

gtatttagatgaaatgaagtagaattgtactcaaattgctctacaggtcattaggttcaatagctaagtttataatcattatttgacacatctttgcata

aagcatttgcattctgttcgtgatttcagcaaaagttaatgtcccctaatttcaacaaaagcttgataagtgaatggtttttccactgtatttgtctcct

ttgtgttgtatagatgataaatttataattattggcacatgatttgatattagaatgaatggtgatctcttgcagtcatataaagaaagactgttagttt

gagtagtttaaacatgtttatgaaatttggtgtcatatgtgaatcaccctctagatagtactaatatattgatgagggactagaactgcaatatttatgg

acgtgttttgagaaacattgaaatcttgaggcaactgctgcttgccgttgtagcatagttatcatgtaaacatcttttatatgaattagtttaaaaaatc

tgttatcagtttattgtgttatgatcgtacatgacttacttgcaagatattatgtatttactgtcaccaaggcaaggcccaagttgtgttttacaggagc

ttattttccaagtgctgttttccaataccttcttcaaagtaagcttacctactgctcacagtaattatgtatataagttctagaccacttacttttcttt

atagtaactactctgtgttcatattgatatactgagcagtttctgaacagagagaaggctggatatactggcatacagattttcagcttcataataccga

tacagtttttaaaaattcactagagcttgtaacattttgagttgaaactgttacaagggacattaagccgaattttacctttaaaagatctttttgcatc

atgtatcagttttataatataccagaatattgttatgaatgtaaaactctcacatgtatacaccagaatttattttatttgtctcttgtcgtgtagaaga

tgtaaactgttcatgccctagaatcactgtgcaaattaaatttggtgcaaagtttttaaatttcagttgtttgaaatctacttttattgtggagacacat

gaccagaacatgtgtgcagtaataattgtgtgttgttttgtatgctggttgtgtcccatattgtgttagagatcaaaatatatagtatagtgagtaatat

tattgtctctgtgccagtggtattcgttctactttggggttagtctgtaaaggaataccaaagatgcaaacacacagtaaatatgactaacaattgtgaa

aagtgagcgattgcttcacattgtttaaatacagtatattgtactatgtgtgaatggaaattttagaatcattctctatgcccttcagtgaacgtgaatt

attagctcccattaaacaaatattgcaatgctcatgagaacataatttcaagttttaagattgaatgtctggattctaatgacaagattcacgaatcgga

attaaattataaa

>46863|AGAP009518-PA

tttttttctcacatatcacatatgaacacctggaggaatgttcacagctgaatacaaatgttcataaatatgccaactctggaaagaaagtgacaaagca

ccaggagatatgaggagatgtgcactaaatgcatacttcgtgcagaacttgatgaaagcagagtttttctggagactgttgttacagaatgctctttggt

gtagtcgctcagacctgcttcatttctggcagccaaccttacagtatactttgtatcctgcattagtccctctattttgtatggtggagcttccaattcc

acaacattagcattattccagtttgccaaagctccttgctgcactggaatgtactcaacacggtatgaaagtatcttcaaatccccagcatctggtgcag

taatatccaatacaatggtatcatcagtatgttccttaatactaaatgttggaacttctggcttatttccttgctttaggcgaattttttgttcccggct

accaaaattatttgtagcaatgcatttgtattcaccaaaggaacttgcattgtgtatcaccaactgcagtacacttgtgtcattatcactgattatagtt

atgttttcatcttcattgattgtgacatcatctttcatccatctgaacacagggggaggttcagctacaacctcacatgtcagattttttgttccattga

tgaaaccgtatgctgtatccttcttattcttttcttttggttcatgaagaactttgacaaaaattgtcttttgctgcatgtttgagatatgtggtgagag

ttggaaagcttgacactgataataaccacggtcatccagtgtaacattactaatgaacagtccatctcctccaacttcatattttggattaccatctact

gaagttccattaacttcccacttgatagttggttcaggatctccttgaacttcacattttaccaaggcatttgatttctctttcacctcttgaatctccg

gtgtgtcttcaaaatgtatgggtgtgtagacaaacagctcgaagtgtaaggaacgttcttgtccctgtatgcgagagacacatgtgtattttcctttgtc

cttcttctcaattctggagaacacaagggacagtgtctcctttccaccttcaacatgtacccttcccttctcgtagatgcggcccttgtcgggtcccagc

cagctcatctgctcgccgccgcggctgacacacgtaaccacgtatgactcgtcgacgaaccgcacaactggctcgctcttgttcggcgacagatggaagt

cggaagtgtcgccagctgccgtccctagaacgaagacgacggtggagagcagcagtatcctggccatcatgacgcaccggctcgtagcacacatcacctg

ctatagataccacgacgcactgccaaccgaacgcgacgcgcttaagtcctcggggcactggcggagccacggcccgtacgtagcgcgtag

>47024|AGAP009566-PA

cacggaactcatctgtcctaacaaaagtagggtaattactataaattctttctcacactgtttgatacttgtgtttggtatcttcgattttaaagacaga

atacagacaattcatacaaaagcttgttatatagtatttattatacaatttgtttttatacagccagtcacttaacataaaaattcatagcactaaccaa

aataatagaatcattttacataaagagaaattctcatataatcaatactgtaaataaaatgggaattccatttcaagttcaaattatgcacatacaaaaa

ggaattctaactttcatattttcaaacatataggtagttgataagatagcacaattcaaaatcacggcatgagtttacactttagccacaacgatcaatt

tctttaagtatcttaacatctccagaatacattaagtagaaaaacctaacacgttattttttaaactgatgtgcatctcttttactatcagacttacaat

ttaagtatacatatacggacacaccctgtttgctccagaatctactctgttttccaactaataaaactatatgaaacgtaaggtggagcagatatagcaa

aatcatgacgacatattaaacatgaccaacagtatttcaaacgccaaaagtagttggcaaaggtgctaacagaaatatgaaataattttcatcagctgta

atccacagtgcataatatattactgtcatcatacaatctctgaaccattaatgcagaaggaagtatgtccattaacggcattactgcctgaggaacatgg

cactgtgttctagaacctcagaacaaaagattactctaaacacacaataaacatgctctttgtgaactaaagtttatgaaaaaactgtttaacaatcttc

tctgtgaagtttcatatgatttacatatgagcctttccgtacatatttcttattacataaattacattcaaaattttcttgtttcacatgcattttcata

tgattctttaagtgagatgaacgaacaaatttcttagtacatatattacactcatatctcttttcaatatgaataaggacatgtttagcaagatctgctt

ttccaaaaaatgattttttgcacacatcacattcaaaacatctttctttggtatgcacagacctaacatgcaaattcagtcttgatggtacagtaaatct

tttattacataactggcactggtaattcccatcaccagcatgcccttgcttatgaaaaatcaatgcagagcggcaggaaaacgttcgtttacaaactacg

cattcatttggcttctctccttcatggagccgtaaatgatatttcaacaaataatttgatgaatatgactttgagcaaattgtgcaagcatagcgtttct

tgttatggacatttaaatgaacagttaaagcagattttgccgaaaatttcttgttacatacactacactgataagctttgatgccagtatgcagtattaa

gtgacgtaacatatcatttttctgggaaaatccagaaccacatacagaacaaaagaaagactttcctttagcatgtctcaaaacatgattcttatagtca

aaactgctagcaaactgttttccacagactgtacaaacatatggtttgtcatctgaatgtttcctagctacatgtagagttaaatgttctttgcgagcaa

aacctctagaacaggtttcgcactcatactttttaattcctttatggagtgcagtatgaacctcgaaatttcttttcaaagcatagtttttcccacaaac

attacaaacaaagggtttctcccctgtgtgagaacgtatatgccccttaagagctctattagtggtataatgcttggtgcataacgaacagctaaatttc

tttttattttgctttttaatatcgtcagcgctaaaataaactcctagtgaatcgtcattgctggtatcatacacaaaatcagtcaagaagatatcacttg

gctctaccttcaccgttgcgaaaaattctgcatcatccgtatataagacattctgattcggactctccttaacacgggcagcaatttctttttcattctc

tgttgtgagatcaggaaatacgttttcagttttcaaatctgtagctaacatagctacaatcaacgagcttactatattaatttaaaggcatagggatcag

cgagcagcctctccaagaattccagaacatgccgtgctatatgtcaaacgcatttatccgaaagaagagaagtatgaaaacatttttctgctcacagtta

ttaagacactataatatcaccaaagcagggaaataatttacaactgtaagtgtagttataccacataaacatcttcgaaatacaaaagcaactctaccgc

tcatttcaaagataac

>47025|AGAP009566-PA

gagagatattgcatgcacacatgcatgctcggtctagctcaatttcgtccggaaggaagttaccttgttctgtgtgcacctatgaacgactcaatgtttc

tgctagtgggtgtatgtctcctttactcctaagttatttatgttcaataagaaacttactaacaacttgtgctttgaatttattatgtttttatgaacaa

aaaatagcagaaatcataaactctgaatgtgtttatactatagattatttacaattaaatatatattcatttcacagttccaaaaccagacatttataca

tattatgaaacaaaaactttatcatcttataaagttcctcaaactcaaagtgacaaacaaccacgagctgttcaacaatatgtcactccagatccacgct

tacaattacatgcaacagccatagacatgaaacaaagtgaactgttacagaaatataacaaacttctgtttctcatttgcaaaattgaatttttctaagc

aataaagattgctacagtgctcatagcatacaatagtttaaaaaaatatcatgtgaaaaatgtaattatgtaagttgtcaaggaccacagtccttgtaaa

tagaatcacattgaaagctcattccgtactagggtcaagacactcgttccacaataacagaggaaggactgaacccacatcacattcttctcagagagag

ggggagactagcatttttaagcaccaacttttgtaattttctatgttttttttctttctttcttcctcttcccttccctcccctcccctccccctaaaac

ttcagtgttctctgatatcctttcattattatatctccctcacaagacaattgcagaggaaattgtatgagctaatcttgcagcttatcagcaacaataa

aaggaggtgatatatatatatataaaagagtctattaattatctatgccacttcccctacacacagagcaccaaaacattagcgagtcttcacaactttg

ctccacagtacatacattcatactgtgctagtatacgggacaactgtactatcaatttcgccgggatgtgccttcctgatatgcaccgtcaacctgtatt

tacgagcgtatgctttgtgacaatatggacaagctgctttctcccgatgacattcagcatgcactctcaggtaggctatcgacgtaaaatttctgtggca

caccaggcactcaaagggccgttcacccctgtggactcgttcgtgatcttccagtctcgctttgtccgcaaataccttcccacagtcagaacaccgatgc

ggagcgtccggatctggatggagtgcggccatatgtcttaagagatgggccttgtccgagaacctacggccacagtaagcacactcgtaaggcttctctc

ccgtgtgcgtcctcatgtgtatcgccatgtgtgacgtgttcgagaatcccttcccgcactcggggcacgagtgcggccgggctaccgacagcggtacact

gccagtgtcaacggcatgcgttcgcaggtggcggatgagacatggctgctgccggaagcggtgaccacatatgcggcactcgtagggccgctctcccgtg

tgtatcctccggtggacgttcagcctggcccggtcgtagaaacggcgcccgcagtcagcacactcgtgcttgagtgcattggcatcgtgtgtggcgacgt

ggcgttcgaagcccggacgtgtcgtcagctgcttgccgcactgcgggcacttgaacatgcaggaggcagcttcttctccgtcagaatgggcagttcgttg

gtgtcggaagagctcgactggccgtaagaaggcacgtccgcagtctgtgcatgagtgctttgccctggcactctggtgttcttgcgtacggtgcacattc

agatctttccggctcgggaaagccatttgacagtaatggcagatgaagattccctgagcatcgggctgcacggttttccgagatgtcccgtcagcacctg

cacctaaatctacgctgccatgtgcctttgcatagtgggtatccaactcgtgctggctggcaaaagtggtcatacacacgacacacacaattgtcctttc

caccattaccttgacagtgctgttcaactgagacacagagttacgtggccgcaacctccctaccacgcagagatgaatatcgaggtgttcctgcttcgtg

aaaactcgcttgcagagactgcacttaaatctttcctcctcgtgtcgatttgggcatcttcgggcagtggccaatacgtagttttccattaaaggcagat

cctttctttggagtgaaagtcctgtgagatcttgtgcatcttccctctgagagtgaatatgtatactaaacctcggatcagcctcctcaactttgctcat

cagaggtccactgttgacatcattccaatgcttctgtcctgtgcttgtcaccggtgcgggcatctccagattctgcaactgcgacgtgaccagcacggat

ccatcacctgaagtttggctcaaaagttgtcgttcatcatctttgtaatgatccttgcccagaactggagga

>47079|AGAP009579-PA

ggaaatcagagcacaagtttgcattttgtttttaaaaaagaattgatagcaatcactctttgttaaaaagtgtagaactttattccagtgtgaacatttc

atttttaatatgtgtatattgtttttacatcttcatctagtctggttctataacatttgtgcagcaatagcaaataaacacaagtgttgaacagtaatat

ggatcaccataccatgagattcttatcttggaatttctggaccatataattaaataaatgtaagtcaggtgaacaaaacctgacttgtgaacaggtattt

atataacttgtgactgttctgagtcgcccaaaacagtcacagccagcatcagagtatcttggaggtgacatataacataaccccacacagactcagagca

aactggctgacttttgtttctgcagctgccccacagtacttatccagttcctcccttggatcttcagtgaaacatgcagagaaccgactccgaggtagtt

tgttcagtggtagtttgtggcaggggtgttctgtatcattgtagtccactaattcagacttagcactcagatggctaaagcaagttggacgcatagaatc

caccacaaccagtagtagacttgtgtgtggaacacggcatggaatgaatggtctcgaacagtgacttgggatgaactggaaatcttcacattccaggttc

tctggatgaaacacatataagttgtacttctgatcgcatggtttggcaaccctcttcattttcatctttttctttggggcctcagtagtaggcccaaatg

ttgtctgttctggctgttcagtgacttcctcctccagatagtcatcatcgtagtactcaagaggtgtggctgttgcccagtttagagaccagagctgctg

tacatttattcggagcaaagccaacagtagctcactgataagccactgcacatatgcaataggtgtccagagtccaccagcagcactactgtctgggacc

atggtgaagcacagtgattgataatcatagattggaatttctctgaaaatgcctagctccaccatttctttcatcacagcaccctccacttcaccaaaaa

attggccagtgtgttccagcgtctctgagatcactatatagccattgctatcaagtaagtagcagtccagtccttcatgtgcacaagtttcattgtagtg

gtgtgtaccctcttctgtgatggacatgaatcgatcataaagagccctgtgctgtatctgaagtcctactacagatgctggtgcctccttaccagcatct

cgtggaaatatggcatgactcagagttatgggaacatcatcctttctacctgcatcaaaaggagcagaaatgacaactgactctggatcatgtttatgct

caatgactgcagctttgtaccatgcctcctccaccgaatgagggaaaaggtcaccaaactctgagatttcatccttgccttcttctgtatccattatata

ctgccatcttgttaatccactttgtgttgccataaatttaaggctagtgttgtaaagttttattaaatactcttcagtgttctcaaacgaccaactgtca

ccaaagaattcatgggtcagtcttccatcaaacactaacatttgcatcagttctttctcacagtaatattcatcttctgtaatttctggtctgtcacagt

tcacaggtgtagttgagcgtgtggagtaatcctcttcttgacctatgggatcaccagcaaacatttccttccactgaaagttcgtactgctcatactctc

caagaagtggtacacttctctttctggtgaagtaaagttgtggagagagccatggaagtatctgcaatacaaccagtttggatgcactttccagttattt

ccttggaagtaattggtaactggctttcctttccgagcatgatcttttattatgtcattcactcttaaataaaatttgccataaccttcaggcaaagaaa

tcccaagactaaatggagtgccttccaacccggagaaatagtagttccgttcttcgctcatcacacgccgcatgtcatcatagtgatatttcatggaaat

gtttaatatttctccatcttcatggtttaccatcctctccctcagctctagtattttttctggcaactctcgaggtggtgcagcaggtgacatgctgtca

agaagttccacttcagtcaggtctacactattatagttgtctttcaggtaaccatatgacactgggcgaaggtcaggatgaagtaaaacataaccattat

ttgtaactatgaaagcatagccattgacaccaattttgtaaggcattgtgagtttctcaatttcaggaataggaacatcaattcctgcaataccaagaag

agaaggagcttctgtcttgttatctgtgttatactccattcttctttcaaatgctggaacagacactgaggtaacaagatggtattcctggaatttgatt

tcatctggttcccatactttcttgactacagtctgcttataaacattgtcctcctcacgttggctgaaatacagttctttatttcggaaatgtttatcta

gcttttctttctgttctaaattttccatcacatcaaatagccattttgctagctttggatcctcaacatcagcatctgcatgggtccacacaacaggatg

tatctcgccttgcaagactaatggttgtgcaattattgggatgtactgaaacaccgtctctcgaacttcattgagactctgaatgtgtttgaatacacct

ctgtttagacacgccatccacttaatttccatgacttttgtaacttctctaccaactagaaatgtaaagactctgacaggaattgtgctaccattattta

aacggttatatatctcaaaaacctcagttatgttccctgttacactatctgtaatcagcatcacagcctgattacaggtcagtcttgtaccatttcctcc

acagtcacgctcgtttctagatttctccagaagtttgaaggcctggataaatgcctgggaaaaattggcaactccttctgggtcaattatttccaaggaa

tctttcagggtgtttacattttccaaagtagcctgtactagcatattcttgaaacacggcacagtctcctgggctctctctgagaagttgaagacattaa

caaagtcattattggaaagtgtatcaagaatagtgcgcactgtgtgtttggaaatcgtgtgcctcaggcctttcatcgatcccgtggtgtccatcaggac

gacaacatctttgctgcaagttgaagcttcaatataccaggagcgcgtgcggcaatcaaacaggtctattggttgtgcccacttcatggctggaaacatt

cgcataaatcctgtagcaccaccaaatagttgccaagatatggtaggatcactcttaagattctgaatgaatacttcatctagcccctctgaccacttaa

ttccatcatacacatcccctcctaaatcaaaaacgttggtgggcacgtggactgaactgtgactcctgtttacaggaatgctactaaactgtcgatcagg

ttccagatacattggagcatacggatattcatcatcacatgattctgagtctgatggctcatcattgacaggagaacattttgcactgtgatatgtgaag

ttcacatactcaagagggtcaaaactttcagccagctgctctgctttctgcattatacacttgacagaaacaatttttgacttgagcattcgttcaacat

tctcaatcatagctttaagtagttcagcaccacttctctccttgacacttgcattcaagtatttgtatctagctcgaacatcatgtgctcttgttactga

tgtagttagtctccatagttcatccccaagctggtctgcccattttaacaccaagtgcttcttgggcggttcggcgtagtcgagctgtgcggcg

>47105|AGAP009588-PA

tgtagacttaattaattaaaagtagcactgtcatgcagatttttaaattttgaagttctccaggaatctatgctagcaggtatttggtttgatgtccaca

gagtgagagttgtcatgcagaacattttctcttcctttctgtttctgtgccacgttcctaattccagcagtgattttattctctagtgaggcagttgaaa

gccgtgatacagtgctcacgggattaaaaatataacaccatattaccgccccacaaaacaatgttgtgaagcacttcagtgttgcatatttcaggccagt

aaatcatttcagaatcaccttaagataggcccatttccgtttttctgtgtataaattttctctcttcttggagggaaaacgtaagaacttctgtgcccat

gatcatgattcctgtgacattatcctatggtcccaaaaattgtaggcagcattcaaaatttcacttcccttctcttcatgttgcccacctttaagatatg

gtggagtgaactcccgataagcaatacacacccttctttctgaaatgtcttcacgtaaaacacagtgttcccaaccataccgtgctggaccataaatcac

caa

>47128|AGAP009600-PA

aaaatgttctaccacttcttcgaagagggattggtattcaccatggaggattattgccaattttgaaggaaactatagaaattctatttgctgaaggttt

aataaaagctttgtttgcaactgaaactttcgcaatgggtcttaatatgcctgccagaacggtgttatttactggccctcgaaaatttgatggaaaggat

tttcgttatcttacatcaggagaatatattcagatgtctggaagagcaggtcgacgaggtggaatgaaaggcgaaacaaacatctataaaattgtaaaaa

tgatcatggagagacaatttgcaccagtcattattttcagtttcagcaaaaaggactgtgaactttatgctatgcagatggcaagattggattttaatac

tgctgaagaaaagaaattagtggatgaagtattcaataatgcaatggatgttttatccaaagaggatcgtcagcttcctcaggtg

>47147|AGAP009602-PA

ctagagatcaaaactaacaaactccattttattttacaatagcataatattactgtacagtgaagttttctacattgaagatactgattatatcggtgag

tttttgaagcccctggaagcatagtctgcaggcaattgtctatcagacagcttaggatattctgggtctccgggaacgacgcgcttgcgactgacaagta

ctctccagcctcctttccttgtccagtcgtttgcattgtatttgaagtagtacgtcgttgcatatatggcgaacaacgatataaagacttttcctgtgac

gtaccgtgcaatctgagctcggcgctctccaatagtaggcttaagagcattaaatataacgtccaatggctttctgtacgcccttcgaatagggttcatt

aactctcgttccaattcgggtacacgcctgggttcgttaggagacagtatttgatcttgcaaccattgttttctccatgctctttcctcatttgtcatac

ccactagtctctctctttcgcggaccatacggcccgcaatgggcattggctttacgccacccgtatctgaggccatttttcttaataaccttttaaagtt

aataagctgccccagccagccgacttcttgagttcttcgtgtaggacaggttagtgaggttacctattatgtcgatagttacaccag

>47165|AGAP009604-PA

cttttatttctgctttctacagattcatccgtcccacggcgtgtgctacatcccatatggtggcagagagataggcaagagtgagtacgaggtgctgatc

acaaattagtctgcgcgggaataacaagatgtgtgctctcatttataccacttagtttctgtaaataaactgccag

>47190|AGAP009609-PA

gccaatccacgggactttctcacacctgttgcatggtttgaagaccgttcagttgaagaaaatctgtaccgagttattagtaaattccaaggacatctgt

ttgttgcatggtttgaagaccgttcagttgaagaaaatcagtaccgagttattagtaaattccaa

>47191|AGAP009609-PA

cttgtttactcgaggggatacaccgcccggccttgggtccctggcaaccgctccgttgctgcgctgttgacaaccggcagctgccgcagacgaacgcgcc

atggacttcgagggcctcaagtacttaactggctttggcagtgaattcacctctgaagatccaaggtgtccaggagccctgccacatgggcaaaacagtc

ctcagaagtgtccttatggactgtatgcagaacagttatctggctctgcatttacagctccaagagaaggaaataagagatcctggttatatcgtatccg

gccatcagtcatacatgaaccctttcaaccatttgcaaaaaaacactttgggaataattggaatgaaaatccaccaaacccaaaccagatgagatggcta

ccatttgatttgccaagtgatgatgaacaagttgattttgttgaaggtcttcatacagtctgtggagctggtgatcctcgaacaagacatggaatagcta

tacatgtatatctatgtaacaaatcaatgggaaacaaatgcttttataattcagatggagattttcttctggtgccacagcaaggtgttctgtatattac

aacagaatttgggaaaatgactgtttctccaaatgaaatctgtgtcatccagcaaggtatgaggtttaatgtgcatgttaccaaaccatcacgaggctat

atactggaagtttttgataatcacttccagctcccatacctaggacctattggtgcaaatggtcttgccaatccacgggactttctcacacctgttgcat

ggtttgaagaccgttcagttgaagaaaatcagtaccgagttattagtaaattccaaggacatctgtttgttgctcagcaagggcacagcccttttgatgt

cgttgcatggcatgggaattatgtgccatataaatatgacttgtctctcttcatggttatcaatgcagtgcagtttgatcattgtgatccatcaatcttt

actgtacttacctgcccttcaaacaaaccaggaacagctattgctgattttgtcatattcccacctagatggtcagtgcaagaacatacatttcgtcctc

cgtattatcacagaaactgtatgagtgaatttatgggtcttatcatgggcaactatgaagcaaaggaagaaggcttcctgcctggtggggcttctcttca

ctcaatgatgacaccccatggacctgatacacagtgttttgaggcagcatcaaatgcaaaacttgtcccttgtagagtggcagatggaacacaggccttc

atgtttgagagctcacttagtctggcagtaacaaaatggggagaagagacatgtcaaaaattagatgcttcatattataagtgctggcagactcttaaaa

aacactttaatccaaaagaaaaagaaccacagtagatgttttacagctgtggtatttcattcagaagcagaagctgtaaaccaatgtttctgtaaaattg

tgtttctataattatgatgtcaccttcattgttggtgctggtccctacctacactataaaatatattgtactaatcactcaaacaatatactcacctatg

gtgtaattgaactgcttgccataactcatgaatgcagctgcaaacggcaccaatgtactgcaaatgatgaattaatatttcttgtggaatttgacaccat

atttcactctatatgtaccagaaagtagctctgaaatatgcaat

>47241|AGAP009613-PA

gttttgtctgtgccatcaagacccttgaaatgcagcaaagagtttagctgtgacgcgtagagtgatgtctggatggtgaagtttgttttcgatttgaacg

agtgtatattttcgtatgaatactacgaaggttggagattaaacagacaaatatgctattgtgtaaagtgttcgaaacgtatgctggaacaataaatgtc

attaacaacagtgtcagatgctgtaatgttgtgaaacaagcatcgtttagttcatacgcaccatattttattgaccctgtatttaagcagaatgaacaga

atagattaatagaagaaggagaagcccagaagctctcacatttacctgtgaaagccgccctaaatgatcagacttcgtctgtttttcatgatgatattgt

caggaaatttacaaatcatgttatgaaatcgggacggaaagctttagcccgagagctggtacaaaaggcatttgaaaacataaaaaggatacagcttgaa

cgatataacaaagcaaatgagcttgaaaagtcaaaaattgaaactaatccacaaacaatttttcataaagctattgctaattcaagacctgtgctgcatg

ttgcaccaataaaacgaggtggtgcaacttatcaagttcctgtgcctatcactgaaaagcgctcatacttcatggctatgaaatggttaattgaagcagc

taaagacaaagaacgcacagttcattttccagaaaagcttgcgtgggaattagtggatgctgcaaataatcagggaaaagttgttagaaggaagcaagaa

ctacataaactctgtgaagctaacagagcatacgcacattaccgatggggatagatggaagagagagaggggggggggggaaattgtactttttggtaat

tgctttccgtgctgcaacatcaattgaaatttacattgacactataagtctgtaaatacatttgtagctaataaaacattactttatttgaccaaaga

>47254|AGAP009614-PA

agaacatctttgacattttatgggactgcgatcgcaaagtgcaaataatcatgttaacagtgaagtgcgtgttggttggaggcatgagagattccgaagt

agtttgttgttacgtgctcgaagctataatctaatgaacatgctcagcatttcggacgttagtaggatagtagtacgttacgggagaacaaaggtgcatt

ttagggggctgaagactatgcttctaaatacgaagtgtagttgtgacatgtttcgatgttttggaaatactataaacaacgttgacaatagtgcttcaag

cataaatacaaaaggcgcttctgatgtatatacaccgttttataaatttcagtatgttcttgctgcaagaaatctatccagactgaagtggtatcaaaca

ttttgtaccgcgctgagtattccaacagcgctgtgtttggaagtagctggtttggtgccaactgatacagtgaaagcggtttttattttaggtgcaatgg

ggacacttgctctacacacaattggtgtacttggcactaatgtagtaggagcattgtacctcaattcatctgagagccttgtaaagatttcttatctgga

tttctatggtcgccgtattgataccatctgtggtaaagaagatataatgttgttgtctgagttagcagatgaaccaagtgacgtgtatattcacctcagg

cgttactccacttcacagaaacttaagctgagtttaagatttggaaaaattttacacatggataaatttgtatcagtctttggtcaaataaagtaatgtt

ttattagcagatgaaccaagtgacgtgtatattcacctcag

>47277|AGAP009620-PA

ttacttttaaggaattcttcaatgaatatctagtcaatgacaagaaaatgcttaacttaaaactacatggtttcatcacatatttcaagactcaatagtg

gatttagtcatttcaaaagaaaaattaattatatgcatagtctctgtcacatatgatatgcatacacgtcactttcaataaacacaatagcaccaaacag

tgaaatcatctttttacaattagcagtatgtctagcagaaatcaactgcaagctgcaaggacaaaacagcatcttttcgggagcttccccaacctaagca

aatgaagaatacaacaattagcagaactctcatttgcataatctaagggtaaatcaacctgaaagaaagtgtatcggcaatgatgacacatggaatctct

tacgcaactgttttcagcctgtaatcataaaccacaaatgccacatcacgatcaggtatcttcaggccggctcatgttccacagttgttggcgcttgtac

agtgacaaaccatcgtaactttgaatctttcctattggttttacaatcacctttggtatacaacagtattctggcattggctgtggtttgaaaaagtctg

acagttgtggttctttgtctttaggaaactttttccatacccgagatgaaggacggtctccaatggcaggaacaatgtcttcttcacgcacgttaagggc

tatcaagtcccggtacagatgttcctcgacaggaaaattcaatttcttggcaatcgtttcttccgctacttgacattttacaggtggtaaatccttggtg

cttttgatcgtcgcctttggtactgtttgagctgttttacggatttcatttgccaagcataaggttgtgttcaattctgggacagcatatttatgcggag

gttcgcctaggtcactagttttactgcttgttgttgaatacaaactatggacatcgacacgttgggatagtcccttatgtcggctgactgatactggttg

ctcgacatttctaactaacgattttttcgcctgagatttcttgggcgattttttaattgctccagttcttgaactacagctatgccgtgatacggagttt

gatctaacgggtatccgagacggaactgacacttgttgtgtattaagttttatgtcatctttgaaagtcac

>47299|AGAP009623-PA

tcatcatacgtcgctggcttcgcaagcttgcaagtgaggtcgacaactgacacatctacggtaggaacacggaaagccataccagtg

>47298|AGAP009623-PA

tcttataagctgcatgattcacgcccatcacaaacatgggtacattgtcctttggaggagctgatattatgaccttcttggcacctccagcaatatgttt

cttagcaagttcttctgtgcagaaaattcct

>47300|AGAP009623-PA

tggcgcctccagcaatgtgcttcttggctgcctcttcggtgcagaagatgccagttgattcacagataatctgggcattcacagaaccccaggggatggc

agacggctccttttctctgaagacagcgatttcactaccgttcacaacgagtttatcgcctttgatttcg

>47317|AGAP009641-PA

cctgttgctcagcatggtatcgaaaaaatttgcagggtctctcttcattcggtgtaaggtctttttgttctggatcatcatatcttacagtcaaaatttt

atcaatacatttatccattatagaacgaagctcagtcttgaacataactgaatcaatttttactgagataagcttttccattggtactgcatccaaacaa

atcatttttggaatcaaaggaatgtgtcgtgcacggttctctagatcaacttttatccaatgaataaatatctcaagtagcctactttcatcatcaatgt

ttatgtaatctgaaaaaagaatatctgacaacagttcaatagttacatccaagaactcaggctgttcacacactgatgagaaattacgaagtatgcagcg

caaagcttgatgggcaagatcattgtaaccactacctatagcagtctggtacactgataggcagttgtctacacagagacttgtctgtagatactggcag

caggggtggcttaaagctggaatctgtaggaaatctgccatactgatcacagaataaatattttcctcagaaatctttatttgacctgtgtacataaaat

gtacgatttccttaaaggtaggtagttcaatgtcatggagaactatcttttcttggtggctttccattaaacttccacaaaacatagtcctaaaatatag

agatgaagctgctaaaacaattttatgagctcttatctcctcattctcaacagtgagtgtgatgtcacataactcctttgaatgttgaaatagggccaca

ttctggagcactatgttagcatgcaatttgtcatgatggtacagtacatggcccataactgctggcagttccgcgagacgcctcgaacaaaacactaact

tagccggatatgtcagtgtcagtcggacgattctcgcgcaatagcgtccacata

>47349|AGAP009649-PA

gcttcgggcgcacagacagttcgctggggacagccatgcaggacgctaaggacgccgagttgcagcgcgagcggctcatgaagcagcccgtcgctcagat

ggaggtgcatgagcacaagtacccgatcggagggaacctctttgggtgcagttttttcaaggcccggtacctgttcgccgtgctagcagcggtgtcgttc

gccatcatctatgggctgaaggtgaacctgagcgtgtgtatcgtggcgatggtcaaccagacgtacataatgcaggagctgcagcaccctaacggctccg

tcacctccggccacgagtgcccggatgaccccgacgttcacacctcatcagagggacagatagacgaggatgggccattcaactggtctgagacgcagca

aggtctcctgctgggtgcctatttctgggggtacatggtgagcgagctgcctggcggtcggatggctgaggtatacagcgcgcgctgggtcatgttcagt

gccgtcgccatcaatgtgctgggcactgtactcacgccacccgcggcctacttccatttctactgcctggttgtgctgcgtgtgctcgagggccttggag

gggg

>47378|AGAP009652-PA

aggagatcgagatgagaagtggacgatagttcaagtagtatgcatacaatccagcagcggttattacaaagtatcctagtatcatagtcgaaccgctaaa

atatgtcttggcaggtatccttgcgaactttattgactaaggcgagaccttcactttcattacgcctttaggtttcgtacagcccaatgactcgc

>47380|AGAP009652-PA

tcggccaacaacattggaaaaactgcaagtcgtccacttctcatctcgatctcctcacgacgaataagacgatagttcaagtagtatgcatacaatccag

cagcggttattccaaagtatcctagtatcatagtcgaaccgctaaaatatgtcttggcaggtatccttgcgaactttattgactgatagcctcctggggg

aggcatatcttgtacttttgctgttgacattttgattcacaataaatactgttttttctatgcaatagcgattacctacaccacaaaacctatcacctcc

acctccccccagcaaacggctcaacaaacttaaagataga

>47453|AGAP009675-PA

ctgttatgttacactgtggttttggtgacatattgacttgtagcctagtccactttttgtacattcgtgccataatttaatcggtcgatttcaaccgaac

gccagttggattgtactcagtgtagttgcacgtgaacctggccgtaaataggtatatctttcttgtgacttaacccacctttgacagctagagagccttc

agtctttcaatcagtgtgttcggtaggaaggagtggagatgtggaagttgcgtgtgtatgtgatttgtaactggaaacttcgacagaatcatgtattgta

aacatttttgtttaaaacggaccgaagacgatgaaaacatcgtgtatttaaggagtatacttcacaatgttcattgtcgctcttaatctggaagaaatcc

ggtgtgcaacaagtaaaataatttttcctttctttacacttaatgtttgagaagagtaagagcttacactgaaacatacagtatggatggtattgtggaa

tatctaaaaagtccaagcttggtagcaaaaattcagttgtattttggcgtgtttccgaagaaagtactccatgacaaatgcgaattaaacggactgacga

agtcacttagcaccttgcaatcaaaaagtgcatgtaatattcataatagagtgggaaaccaatgttctggtggtgttagtaacggccgctctgagtcaga

aggcctaataggtacagaaagtaaacaagactgtccggaaacagacgtggatgatgaaacagtttcttcggaatctgaaaccgaagctagtgattatgtt

gttactaagttgttctggcattatttgttcattgttgggacggcattaggtgacgaaatattttatgcttcctttattcccttttggttttggaacattg

atggtgctgtcgggagacgagttgttttagtgtgggcaattataatgcatattggccaaagtattaaggatgttgttcgctggcctcgtccatcataccc

ggccatcagactgcaaaaaaagtgggcacaagaatatggaatgccatcgactcacgcaatggttggtgtttccataccattttctgttatcctgtacaca

atgaaccggtatcagtattcagtcactctaggactcatagttgctgtcgtctggtgtac

>47501|AGAP009683-PA

aacacctcgggatccgaccagtgcgtcgcctggcccagcggcttgccgcgcacgtcgaagctgtacagagggatgccggcgtcgtcgcggaaccagaaga

ccatgtagagcttgtcatgacccggcggcgtgacgttgcaaggtagctccacgctgcggccttccacggcctgcacgtccaccgtcaccactgtctgcgg

cgtgccgtggttgccggcgtctgtggagtcggccgctgcagcgcggatgacggcgaagacgcagctggcccacaggactcggaagaccgttgcgctacgg

ccggacatcgcgaagaatgcggcgcacctcagtcgactctcgtggcttccactcgcagatctcggcacatcgtccttcacggggaacccgacgagagagt

gcacgtatcagagtgcaaagtgtggaagtcacttgttctgttccctgtgcaagctatcttcgatactttctagatgacgcctttggacacgggagtcgca

cttgttccagtcagctcgcttgatagttgtcgcatttgaaggtcttgaaattattcgtttctgtcagaaaccactcagaaaaacatgtagctaactatga

taaatcacaagctaccagaacgtcaaaaatcacattgctcctctaggaaggaatatgtaaatgtttgtgtgtgagttacggaaacagttcgaagaacccc

agccagtaggtataaaatttttacgatagaggtggcagccgaaattcggagctgcacaattaatagcagcaatgcagtcccctgtggtcgtatggagaac

tggttaattcattgttgtgtgggggaacatgagtcggctatcgctgcaaaggtggggcgccacacgggcacgcccccacgcggcgaagtgtgcacttttc

gtacggccccggccgccgtgcgtcgcgcgctgaccaccgcgaaatagcgtgggcagcgtctggctgctcgccggtccgcgtccgat

>47573|AGAP009688-PA

atatttattgagtttcactcaccatctgggatattgcacaaattaggaaacaatggctcaatattaataaggcagattagagaaactgtgtgcaaattta

agtgtactcactatgactccagcgatgatttaaaaaaaaaagcatgcatgtgtagacctactgcttctcctagcatacaatgcttattgcagcggcaggt

gatggtacttgatgtatgtggtggtgttatgtttgattatgtatataccgtgatctggagggtttactttaactttcattttatgctataaagtattgat

tcgtaatgtacatactcagagataaagtacctgtttttgttgtattgtttttcgttcggcgattgtatatttgcaatgtaggaattgagtgaaaatggtg

gcgaacacctgagttgttattgtgttccctcagtggaatgagtattgtaaaatatggcaaacttaatactattgtttacttgccatattatgttcactgt

gatacagttggaagcttctaataatgatccttgttcatcttcatgttcctgcttgggtcggtttattgactgtagtaggaaaaagttgcaacacatacca

gctgatttaccaacctggacagaagaactgaatttgaataaaaactacttaactgaaataccagctgtggatgctcagttgaatctcacaacgttgagcc

tggctcacaatcaaattagaaaaattgatgcaaatgcactccacaatcttcccacacttaagtcattggatgtaagttataataatataagagaagttac

attggaaagctttccggacaactcatcaattgaaattataaatcttagtagcaacaaaatatctgatattgaggaagggacatttgagaagctctcagcc

ttgcacgaattgaagatcaacaggaaccatttgacaggatcagttaaggagctattcaaaaacttggtcaacttaaaagtattggaattaaacagaaaca

acataactgaaattgaaggactttcgtttgaaggatcaaataaattaagtgtgctaaaactcagaagaaatttcatcagcatgctgggggatggcgcgtt

tttcaaattggaaaatttgcatacactgtatctggatttcaacaatataagtgccattagaaaaggatggttatatggtctggtgttactaaatcatttg

aacctaagtaacaatcaaatttcagccattgaagctgatggatggcagttctgtcaacagttgacagttatggatctttctcagaaccgccttcaagctg

ttacaaagggaatgttcgaacagttaacaagattacaagttcttcgtttgaatggcaatataattgaatatattgctgacggcgcttttgatggcctgat

atcactcgaagttct

>47627|AGAP009693-PA

ggggccggcgacgcgtcgtccggcgccaaggcgggcgagaaacgcaaggcggacgacagcgagcccagcaaggacctcaaaaaggccaaactagagacga

aggctttgaaagcgaagaggccaggttttacggacgaccgctacaacgagacgtcgtactacattgaaaatggtcttcgcaaagtctatccgtactactt

cactttcaccacattcactaaaggaagatgggttggtgagaagatcctcgatgtctttgcccgggaatttcgtgcgcatcccgcggaagaatatgaacgt

tgcatacgtgccggcacattgacagtgaactatgagaaagtggatgtagactacaggctgaaacacaatgatctcctggccaatgttgtccacaggcatg

aagtccctgttacctccgaccctatcaccatagttcacatggatgaggatgt

>47785|AGAP009725-PA

atctaacggcttcctcagaactatccatcctgattcttgttgaatctcaaaatattccaagtctgacgggttatatggagcactcagagaataaacaata

gcaccattattgtcagcatcttcatcagaagctgagacacgcaaaatgttggtaccaatgcttgcatcctgcttgacattttcaatgtatttctgtctgt

caaacaaaggtggattgtcattaacatcagtaatctccacagtaaatgagcatacaccttccagggaaggttctccttggtcagtggctttcaccgttac

tgacacaaattttccatcatcaccttcacgatcgaacaccttatttgttgagacttcacctgtttcttcatcaacagtgaatttagttcccttttgatta

ggttgttggacaatggaatatttcacctgtccattaactcccttatcttcatctgttgcgtgtaccttaatgacagggcttccatttggtgcaccttctt

caactttaggtgagtaagttccacagtccttaaaaactggtttgttgtcattcacatcagttatgaaaacaacaacaacagctgtgcttgtgtgaatagt

gctatccccgttaacacaacatgaaccatcatccattgctgttacattcagttcatatttatcacga

>47812|AGAP009726-PA

acggtgctcaccgtcaccgccaaggaccacgacgagtcgtcccgtattcgatatgagatcacgagcggtaacataggcgga

>47809|AGAP009726-PA

gagataccggaatgataacaatgcgtcatggaacacaagatgggaggtataacttgcgattcaaagtatatgacagaaaacacatctgtttgag

>47816|AGAP009726-PA

aaaggacaaagcatacttgacatagctctgtgtcttgaacatagtgggcacagtaggcagagtacagctgggacctgcccatcactattttgactcacac

>47807|AGAP009726-PA

cagcaaagacatccacagcaactttgttagtcattgttgaagatgttaatgataatccaccacatctcttggagaattataaccctgttttatatgagca

tacaccacctacagaaattatagaaatacttggagcagatgctgatgattattcaagaaataatgggc

>47815|AGAP009726-PA

cgatcagctttgctcctctgaagactttgtgtcctgtagttccacactctaatgaaatcttcgtctgtaataccagctattcttatagagccagaattaa

taacagcatcgtgtggaatctctctgacaatgactgttgcgtttgcttgatacagaaagggaaaatgttgatgtttttcgtaactgtacactaa

>47814|AGAP009726-PA

cgcaggtatagttggaacagtacaactaggtcctgtccatcccggttcacatttgcagcgggcttgtgaaaagctacccacacaagaaccatgttcccag

caacgagcagtcgcatcttgtgcttcacatagtgcttctgtttgaggacagccagcgacactgttccgaaagagtcctgggtgcgctagatcatacagct

tactgttgtgaaacacatttttgatacatccatcgaaacctttgcctgatggtaatgttgtccattcatatagttctgcttcaaaattttgcacatacca

accaccaatctgtaaaggagcattgacatttaggtactcattgaattgtggcactgttccatgtgcttggcagctagaatcatcaaattctggtggtgtc

ccatcctccagctctactacatcagctgatttgcagtaatccaccaccaaacgtacattctctgtatcccagaaaatatcaatattatgccattctccat

catctaatgtcttttttgttttaactctcaactccaatgttcctgaaccaaaattaatgaacagctttggcattccaccttcaagctccagtgaaataaa

at

>47813|AGAP009726-PA

ccagacacagaaattggacgtgtttatgtttatgatctggatgattgggatttgccagacaaaaagttttactgggaaggaatagatcatcccagattta

aactgaatgaagattcaggcatgataatcatgagacatggcacacaggatggcaggtataatttaaggttcaaggtgtatgaccgtaaacacactcagac

tgatgtacaagcaaacgtaacagtcattgtcagagagattccacacgatgctgttattaattctggctctataagaatagctggtattacagacgaagat

ttcattagagtgtggaactacaggacacaaagtcttcagaggagcaaagctgatcgattcagagacaaactagccgaattgcttgatacagaaagggaaa

atgttgatgtttttagtgtacagttacgaaaaaagcatccgcctgtcacggatattagatttgcagcccatggctccccatactacaaacctgtgagact

gaatggactcgtgcttatgcacagagaggagattgaaaaagatgttggtataaatataactatggtaggaattgatgagtgcctttatgaaaatgtgatg

tgtgaaggaagttgtacaaatatactagacattagtaatttaccatacatggttaatgctaacaagaccgcactggtaggagtgcgtgttgatgtcatag

cagaatgtacttgtggtgcacgtaatttcagtaaaggagagagctgtagattgagtccatgttataatggaggacgttgtatggagggcagatttggact

gagttgttcatgtccaactggttatagtggtccaaggtgtcagcagacaggtcgtagtttccgaggaaatggatgggcgtggtatcctcctctggaaatg

tgtgatagttctcatctcagccttgagtttataaccaaaaagaaagatggaatgctgctttataatggacctattgtgcctccagagtctaatgaaatta

ttgtgtcagattttatttccttagagcttgaaagtggaatgcctaagcttttaattgattttggatcaggcactctggaactacgtgtgaaaacaaaaaa

gagtttagatgatggagagtggcacagagttgatatattttgggatactgagaatgttcgcatggtaatagattactgcaaatctgctgatgtagttgaa

ctggaagatggaactccaccagattttgatgattcaagctgtcaggcacatggtaccattccaccatttaatgaatacctgaatgtgaatgcgccattgc

aacttggaggttggtttgtggaagctattaacccagaattgtaccactggcatgcagcaccatctggaaaaggttttgatggttgtattaaaaatgtctt

ccacaacagcaagctttatgaccttgctcatcctggccatcatcgcaacagtgtagctggttgccctcagacagaggaaatatgcaacacacaggactct

actattcgctgttgggaacatggtacttgtgtaggtagtttctcagaggggcgttgtgagtgcaaacctggatggacaggtcccagctgtactctgccta

ctgtgcccactatgttcaa

>47806|AGAP009726-PA

attccacagatcaacgcactcaaatggatcaggacagatgacattagcacatggtttattagaagggcagttacgctctaagtttctagccattgttgcc

tgtccccattgagtcccgttcattgcaggaggcagaggcagatgtttcccttccagtctgatgtcatccaggcagcctttctggtaatcagcataaactt

caaatgttcgaactcctgtatattctgcttttcctccagcataaacaccttcctgcttatctaccagtaaaagctggtgtccctcaaaggtgaaagtttc

gttatatctacggccttcgccaccatcgagttccaaagttgcagctgaaccatgtcgtgaaaccttagctgtatgccattgtccatcatccactgcaact

gcacttaaccagagatctttctcctcagtacgaagactattaagattatagcggaaatgtaatctgctatctttaatctcaagaattccatattcccggt

tgtgctggtcactgacacggaatagttcaccatgttgctctcttgtgcgaaacctaagctgcatctgagtagagaagcgatcgggctcaaaggacaaagc

atacttgacatagctctgtgtcttgaacatagtgggcacagtaggcagagtacagctgggacctgtccatccaggtttgcactcacaacgcccctctgag

aaactacctacacaagtaccatgttcccaacagcgaatagtagagtcctgtgtgttgcatatttcctctgtctgagggcaaccagctacactgttgcgat

gatggccaggatgagcaaggtcataaagcttgctgttgtggaagacatttttaatacaaccatcaaaaccttttccagatggtgctgcatgccagtggta

caattctgggttaatagcttccacaaaccaacctccaagttgcaatggcgcattcacattcaggtattcattaaatggtggaatggtaccatgtgcctga

cagcttgaatcatcaaaatctggtggagttccatcttccagttcaactacatcagcagatttgcagtaatctattaccatgcgaacattctcagtatccc

aaaatatatcaactctgtgccactctccatcatctaaactcttttttgttttcacacgtagttccagagtgcctgatccaaaatcaattaaaagcttagg

cattccactttcaagctctaaggaaataaaatctgacacaataatttcattagactctggaggcacaataggtccattataaagcagcattccatctttc

tttttggttataaactcaaggctgagatgagaactatcacacatttccagaggaggataccacgcccatccatttcctcggaaactacgacctgtctgct

gacaccttggaccactataaccagttggacatgaacaactcagtccaaatctgccctccatacaacgtcctccattataacatggactcaatctacagct

ctctcctttactgaaattacgtgcaccacaagtacattctgctatgacatcaacacgcactcctaccagtgcggtcttgttagcattaaccatgtatggt

aaattactaatgtctagtatatttgtacaacttccttcacacatcacattttcataaaggcactcatcaattcctaccatagttatatttataccaacat

ctttttcaatctcctctctgtgcataagcacgagtccattcagtctcacaggtttgtagtatggggagccatgggctgcaaatctaatatccgtgacagg

cggatgcttttttcgtaactgtacactaaaaacatcaacattttccctttctgtatcaagcaattcggctagtttgtctctgaatcgatcagctttgctc

ctctgaagactttgtgtcctgtagttccacactctaatgaaatcttcgtctgtaataccagctattcttatagagccagaattaataacagcatcgtgtg

gaatctctctgacaatgactgttgcgtttgcttgatacagaaagggaaaatgttgatgtttttagtgtacagttacgaaaaaagcatccgcctgtcacgg

atattagatttgcagcccatggctccccatactacaaacctgtgagactgaatggactcgtgcttatgcacagagaggagattgaaaaagatgttggtat

aaatataactatggtaggaattgatgagtgcctttatgaaaatgtgatgtgtgaaggaagttgtacaaatatactagacattagtaatttaccatacatg

gttaatgctaacaagaccgcactggtaggagtgcgtgttgatgtcatagcagaatgtacttgtggtgcacgtaatttcagtaaaggagagagctgtagat

tgagtccatgttataatggaggacgttgtatggagggcagatttggactgagttgttcatgtccaactggttatagtggtccaaggtgtcagcagacagg

tcgtagtttccgaggaaatggatgggcgtggtatcctcctctggaaatgtgtgatagttctcatctcagccttgagtttataaccaaaaagaaagatgga

atgctgctttataatggacctattgtgcctccagagtctaatgaaattattgtgtcagattttatttccttagagcttgaaagtggaatgcctaagcttt

taattgattttggatcaggcactctggaactacgtgtgaaaacaaaaaagagtttagatgatggagagtggcacagagttgatatattttgggatactga

gaatgttcgcatggtaatagattactgcaaatctgctgatgtagttgaactggaagatggaactccaccagattttgatgattcaagctgtcaggcacat

ggtaccattccaccatttaatgaatacctgaatgtgaatgcgccattgcaacttggaggttggtttgtggaagctattaacccagaattgtaccactggc

atgcagcaccatctggaaaaggttttgatggttgtattaaaaatgtcttccacaacagcaagctttatgaccttgctcatcctggccatcatcgcaacag

tgtagctggttgccctcagacagaggaaatatgcaacacacaggactctactattcgctgttgggaacatggtacttgtgtaggtagtttctcagagggg

cgttgtgagtgcaaacctggatggacaggtcccagctgtactctgcctactgtgcccactatgttcaagacacagagctatgtcaagtatgctttgtcct

ttgagcccgatcgcttctctactcagatgcagcttaggtttcgcacaagagagcaacatggtgaactattccgtgtcagtgaccagcacaaccgggaata

tggaattcttgagattaaagatagcagattacatttccgctataatcttaatagtcttcgtactgaggagaaagatctctggttaagtgcagttgcagtg

gatgatggacaatggcatacagctaaggtttcacgacatggttcagctgcaactttggaactcgatggtggcgaaggccgtagatataacgaaactttca

cctttgagggacaccagcttttactggtagataagcaggaaggtgtttatgctggaggaaaagcagaatatacaggagttcgaacatttgaagtttatgc

tgattaccagaaaggctgcctggatgacatcagactggaagggaaacatctgcctctgcctcctgcaatgaacgggactcaatggggacaggcaacaatg

gctagaaacttagagcgtaactgcccttctaataaaccatgtgctaatgtcatctgtcctgatccatttgagtgcgttgatctgtggaat

>47861|AGAP009745-PA

cacatgtgctcctccaaagaaattcagcatgtcaaaataatactgtatggtaacaaacatgactatatgagcaaatgacataagtatgccatgtgcaaga

cctcttatctgggttggaaaaagttccgctgtcatagtccatggaattccaagtagtcctatcatacttgcacacatatatcccaaaatacatgccacag

ggatccatgtcccaccacctccattttttatatgcatcgttgacagtcctgaagctgtcatacaagctgccatactaaggctactagttataactagttt

ccgtcgaccaaaatgtttcatcaaaattgtgcttactatactcattccaaatctcattagtcccaaaaatactgttgcaagattaggatcaagttcagtt

ccaacctcctcaaaaaaatttattgcataaaataaagtcatgtaaattccagaaaactgctgaaagatgaaaagcactgacataattgctattggcttat

atgctgtgggtttcagaaatactttcaatccctgaaagaaatttggagctgttagtgctctcatatcctgtgatctgaccatagctgccagttgtttctt

gtggcttgttttattgtttcctcctaaccatttcagtgctttttctgcctcatcaattcttccttttgtaacaagccacactgggctctcaggtatccag

atagccatcactattattggcgcaaatgtgtagattaaattgagccaggcacagaggcgccaatctaatattgcaccctctccataaataattagcatcc

ccagtgatgcaagtgatggccctgatgctgtaagtgctctcatatcctgtgatctgaccatagctgccagttgtttcttgtggcttgttttattgtttcc

tcctaaccatttcagtgctttttctgcctcatcaattcttccttttgtaacaagccacactgggctctcaggtatccagatagccatcactattattggc

gcaaatgtgtagattaaattgagccaggcacagaggcgccaatctaatattgcaccctctccataaataattagcatccccagtgatgcaagtgatggcc

ctgatgctgtaagtgctctcatatcctgtgatctgaccatagc

>47860|AGAP009745-PA

ggcaaacactggacacaggacggctcgcagctcgccatggacgttagcagcctcagtctacacgatccacgacaatatgttcctgatttggtcaggctgg

aggatgaaacccttgaaaatgaacatcaagaactcaaaacactaaataatgtttattcctcgacaacaagtgagatagacaagacatcggcagtacctca

gattctggccaccgctgctgttacaggcttccacgttgtatgtggagtgtcactgggtttctctgcgatatttatagccagtgtgatgaaggaaggaagc

gatctcaaactttcgagtacacaatcagcatggacagcaagtgtcttagttcttgtcgttccgattggagcattacttggaggtatcctcatggaagcat

atggacgactgaacgccataaaaatggctgctatcccatgtgcagctggatggatattagttgctgttggacaaagtttcctacctatatttattggacg

tgtactgactggaatatcaggaggtatgggaacgagtccagctgttgtgtacacaactgaaatcggaagaccagaaatcagaggagcacttacagcatca

gggccatcacttgcatcactggggatgctaattatttatggagagggtgcaatattagattggcgcctctgtgcctggctcaatttaatctacacatttg

cgccaataatagtgatggctatctggatacctgagagcccagtgtggcttgttacaaaaggaagaattgatgaggcagaaaaagcactgaaatggttagg

aggaaacaataaaacaagccacaagaaacaactggcagctatggtcagatcacaggatatgagagcactaacagctccaaatttctttcagggattgaaa

gtatttctgaaacccacagcatataagccaatagcaattatgtcagtgcttttcatctttcagcagttttctggaatttacatgactttattttatgcaa

taaatttttttgaggaggttggaactgaacttgatcctaatcttgcaacagtatttttgggactaatgagatttggaatgagtatagtaagcacaatttt

gatgaaacattttggtcgacggaaactagttataactagtagccttagtatggcagcttgtatgacagcttcaggactgtcaacgatgcatataaaaaat

ggaggtggtgggacatggatccctgtggcatgtattttgggatatgtgtgtgcaagtatgataggactacttggaattccatggactatgacagcggaac

tttttccaacccagataagaggtcttgcacatggcatacttatgtcatttgctcatatagtcatgtttgttaccatacagtattattttgacatgctgaa

tttctttggaggagcacatgtgttgcaatggttttttgcagtggtctgtataatggctgctgtacatgtatggattttcctgccagaaacacatggtgtt

acgctatcagatattgaaaagtatttccgtgaaaacacattttattttatgtattctaggaaacaggcaagggaaacaaaagtcacaaattcttaaaata

aaacaactaaaaaaaataaaataaccaggtagccattatttgacatttcaattattacattgtcctcaattgcgcaaattcagtgaatgtgtattaaact

tagtctagctttgtatgatacacctagcaagacctaaataggccacaaaccaataacaaggctttttttgctgattaaatcatttttatgagctaggacc

catttgctcactttggataagttgtatattttgtatttgtaaatagtgttacttttttgtcagtgttacaggtgtacaataaggtcagtggagatttcaa

atcccacaaaattatttattgatatcaattagtaaacaaattatgttaaattaaatattacatcatacgttgtatcatcagatgttaatgaggataattg

gaaagatctaggaagaattgttgttttaaatgatcaatttaattactgtgtggattaccaatgctctgtataacacataaagcttcataggtgttattgt

taatgtttgggtatcatacaagatctgcttcttcttccccctccccccccccc

>47899|AGAP009747-PA

tggattttgagtccttcacactcagtttggccgtggtcgcatatgaaagtgagaggattcgtgctattgaccacagtgttgccaaatggccccagtataa

cggtgatgcgatctggagcatttctatacgccggaagcagctggtttatgaagaattctctgcagcctgggcttagcgttt

>47901|AGAP009747-PA

tttctgccgttcttgcacctcttccgatacgtcgacccctcccgcctctacttgttgttgttgtttacttgcagggcgtgagctcctccccggtgctggc

gaccgtctactacgaggccaagtgccccgactcgcgcgacttcgtggtccagcagctgctgccggcggttctccgggacccggagctggtgcagccggtg

ctgattcctttcgggaaggcgaccgtaaccactacctcaagcggctacgagtttagttgtcagcacgggccaacggaatgtctgggcaacaagctacacg

cgtgtggcatcgcactggcagagagcaggacgctgcaggtttcgtacgccgcgtgtatgatgagcaactacaccgacgttgagtcagcttctgaaaagtg

tgctggcgagttgggcctgtcgtttccaaccatttcagactgcgcaaacggccaggccggcagtgaactcctgagagcgtacgggaacgttaccagcgac

ctcagtcctgcgctcacctttgttccgacagtcaccctgaacgggctcagaggcaatcagacggccgtgttgggtgacttctgggaacaagttgttgaaa

cgtccttagaaacaaagtcggagactaggcgctccgccggccactagagtcctggcagccgccgcctgcgtggcccctgg

>47900|AGAP009747-PA

agttgtacatttattgtttaatacagagcacatttgtgtccgtcctcttaagctgaagatcgcagaattttgttcctgcagacgtgcggcccgcggcgtt

ggcagctcctcgcgggtgtcggtgtgagcctgctggagaggcggcaggcagagccaggcgggatggcagccagagcggctccaggggccacgcaggcggc

ggctgccaggactctagtggccggcggagcgcctagtctccgactttgtttctaaggacgtttcaacaacttgttcccagaagtcacccaacacggccgt

ctgattgcctctgagcccgttcagggtgactgtcggaacaaaggtgagcgcaggactgaggtcgctggtaacgttcccgtacgctctcaggagttcactg

ccggcctggccgtttgcgcagtctgaaatggttggaaacgacaggcccaactcgccagcacacttttcagaagctgactcaacgtcggtgtagttgctca

tcatacacgcggcgtacgaaacctgcagcgtcctgctctctgccagtgcgatgccacacgcgtgtagcttgttgcccagacattccgttggcccgtgctg

acaactaaactcgtagccgcttgaggtagtggttacggtcgccttcccgaaaggaatcagcaccggccgcaccagctccgggtcccggagaaccgccggc

agcagctgctggaccacgaagtcgcgcgagtcggggcacttggcctcgtagtagacggtcgccagcaccggggaggagctcacgccctgactgataagag

cggtggcaacgaggcagaaggccgcgacgctacgcggcaggctcatcctacagggagacagcgtccagtggagggatgcgggacggatggagctggaact

cacagcagaggcgcgaccggcgggttccagcagaaacgtcaggggccgtctcacgggctcgtagctcccaccaggactgcgtttcggcctcggtttcag

>47979|AGAP009763-PA

tgcacttcccgttctgacaaccattgtcacacactgggtcacaaaccattttattatttgcatttttctggtgcccaacattacaggaacagatgtctgg

tccaacacacaacacataaacacaccaccagagagcaccaccgatcacatcaaaaggac

>47994|AGAP009772-PA

tttacaatggaacaatacattgttaacaacactaggtatcttaaaagaaagagttgttaagaggctacaaatgcattctcattctcatattctctcttgg

taatgttaaaagagacaagagtaattgaatgtttctgtttgtaagcatcaaatcagtctgcaaaagtctcttgagtctgacactacgtgtgaggaacatt

caacatgtttaaactcacagtaatattctatatcagcatatacaattattttcaaaattatcatttattttctgataaattaggtaggatttttgccatt

cccctgtttaagacagtggctaatctttcaagtgttgtactatccaactgcagtttttctttcaatttagcacccatattttcagcttctgaataaccgg

catcagcacagtagcggatgcagacaactgctttttcactattgttgactgcaatgcaagcatccagaagttggtttaaaactgtaacttctggcaggcc

aacaattttgtgatgttccttgtcaagcttgtccaatactgcaaatgccttttcaatctcttgaccattcagcaacagaataatgatgtttccaagattt

tgcccagtccaactgagtgcagatttaacatcaactttttcttgaaatttgttaaaaatttcccaagctacatcagcaaactgtagagcagttggtgaat

ccactggtggtaaatttctggccattgactctaaaagattggatatcaatttttccctatcagtgtgatcaaaagtgaccatatctgaccacactcttgg

aagatgatcaacctggccacttaagtcaatggctttaatcacctcctccataacacttggttctggaatgtatatatgtggaattagtttattgtaatat

tccataaggacatctagaggctcacttacgcacaggagagcaaaaaaatgccgatagtagattgactctttatacgagtctccaatcaaatcataattat

ttcctctgtgcagaaatttatccaacctgtatgccagttctttatcctgaagatgatttctgcaaacatccattgcagtaacaaagaaaaatgtgtcttt

gggatgtgtaactttatattctttattttctatgtagttcattatgtctaaaagaatggagctcacaggacctctttccttgcagaatatcataagtaaa

taataataagatgctaaacttggttcaatgtttatccttctgaactctgatagtatttgcagagcatatgtcttactgagtttc

>48023|AGAP009780-PA

gtaggcaatggtcaaatgtcatagtaaaagcagtaccaatatacacaaaccattactagatgcacttacaaagatcaaaccttttaaatgaatattacaa

tatcagcaaacaaaacatcatgttcatatttagttgcaatcacagaacaggttatttttatgtgttacaacaatacaaacaatgtcaccacagctgatgc

atctgtaatgacaaaatacagaatccatggaagggatgtagttaattcaaaatatctgtcatgcactgacctgtagtcagttccaggaacaaagggcttg

tactcgtaactaagtgagaaaagaggttcatctgatggcccagctactgctgttgctgcacgtgtattaagtagcaatctctggataccagatgcaggcg

ttcgtccacctggtcctggtcgaggaagggctgatgcagaatcaggacgattctgaggtgcaattaacacaggaaacacggagtcttgtgtcagctgatc

acgtaagtatatggcaccaagttcaatatcgaaacgatgtgatccagttcgtggccgtgattcaaaatcaagttttacattctcaaactgcagctcaatg

aatggtgtcttcttcctatcttcagtacttgtgcacaagttaaaagttccccgcttcaatgtgaaattgcactgtccaaatacaacatcacggcgtagca

aagtgttattttctagtgtgtctgccaatgcatcaagtaactgatcttctatgggattctgttctgtctgctgctgggacgatgctgaagtagaagctga

tgtagacgggctggtgtaccaccactggggaaaccactgcatcagcacactacggcccgaacctgtttgcggtgcagcagctggaagttgagggggacgc

actcgtttcattgctatctcacgaagaatccgtagttcatccaagcttcgttcagtttctatgcgggtgcgcagctgcttgtctaatggggcagcacccg

ggttggccagactatgggaatacagttttacatactgcacattatctttggcacgtgaaagtgctcgctgccaacctgcactacctgtcaagggtgtatc

tggcagaagcaag

>48410|AGAP009824-PA

gaaatgaaaatgatggattgtttggaggcttatggtgttgagaggggaaaggagaagtgcaagacattaattgaagatttcaaagagtgtgcattgctag

ctaaacagaaactccgtattcgagaaatgagattagagcgccaccgacagtcctttgctggggaacgtccacgagaagaactttatgcaaaaccaccaac

agatgatagtttttaaagag

>48425|AGAP009835-PA

aggtcaagctgcaggcctcagtcatgaggttactgaaggtggtgagaatctcagtgttgggcaacgtcagctggtttgcttggccagagccct

>48424|AGAP009835-PA

aaggataacattttgtttggtaaatccctagaccagtcctcatacctacaaataatagatgcatgtgcccttaaaccagatctgcagatgcttccaggtg

gtgatcagacagaaattggagaaaag

>48426|AGAP009835-PA

aatcccacgataccagcatttatggactcatggcccactactgcaaacagtgatgaaaatagaattattaaatttccaatcatctctaatctgactgcaa

gccatctggaatgacacagaaaatcttttactcaggattaaaaccatatatagggaagcaagagaaaaatcaaacagc

>48428|AGAP009835-PA

cccctgtaaccaacataaatgtgggtggtgtgaaacaattccaagacatattcagtgccatgagaaatcaaaaagagaaagaaagattgaaacaaagaaa

aagcaaagagaagcataatggtggggaaggcctacaactaagattggttcaggctcacctgtacgacaaacactattaacataggaagcatggtcagggg

catacggatcatgttgaacagggttaggcatacaaatgccttctcaggatccagaacatttttctcatcccacataatgtatgtggcaaaggttgttagg

gataccaagaaaggagcacaggaccatataaatgatgttattgcattgagat

>48427|AGAP009835-PA

aatttgcaaatgtaagtcatcagcaataaaactccaaagaatatttcacagtttaaggacatgtttgtgtgttgtggttattggttcaggtttactataa

attacacaagtattatgtgaataatgttcattaaatgtttttatgacatgcattttaggtatccctaacaacctttgccacatacattatgtgggatgag

aaaaatgttctggatcctgagaaggcatttgtatgcctaaccctgttcaacatgatccgtatgcccctgaccatgcttcctatgttaatagtgtttgtcg

tacaggtgagcctgaaccaatcttagttgtaggccttccccaccattatgcttctctttgctttttctttgtttcaatctttctttctctttttgatttc

tcatggcactgaatatgtcttggaattgtttcacaccacccacatttatgttggttacaggggggtgttcatggttatgtgaaggttgtagtaatgtgta

acacttgcttgtgcttaaggatttgattggtaaagtgggaaagatttgtaacttctttagttggttttctgatagagtactgtactataattctttacag

ggattggttcgttaaatgtgggtaatggttggaatattcacaacattagttttgtaccttagtaatatcagaagagtccaacaacgtattactctgtata

aaactgttttattaggttaaatgcttgtattcatttaaa

>48454|AGAP009850-PA

ttctttgatgaaccaacaagtggtctggacagttcatcatgcttccggtgtatatcccttctaaaataactagctcgtggtggacggaccatagtatgca

caatccatcagctagcgattttagaagggatatacactggaagcatgatgaactgtccagac

>48453|AGAP009850-PA

tcattaagatcttattactttgcaatcacaatatcggacataccattccaggccattttctgcgtaatacactattgacacataaattacgcagaaaatg

gcgtggaatggtatgtccgatattgtgattgcaaagtaataagatcttaatgaataccatctgttgaaatt

>48475|AGAP009864-PA

attacatattttatagtgataatgattttgttgataatacgattaatgcagtatttgaaatgtttaaacctagattcaaatacactaattacactcatgc

ttgtataaatcaaactgaatgcaactttgatttatccttttggtcaaatgaagtggtaatactggaaatgcctcattacagtgccattgagactgaacga

aaattgtatttgatttccagctgttaccctcgaatggcagtgtatatagtatttccaatttctgtcttgttcttgatattaggttgcgcttttctgtgaa

tggatatgtggcctatgttgtacattataataatgtccttggacaacagactagaaattggtctggagtgctgtcattatgcaactcactttcatataac

atagtatggatttaataagagcttaagttgtgattcttaggtgttgcgatctgcttataagtttttcattattttactgcatacttggatatctaagtta

acagaaattagctcaggtgaaagtagttgcagcagattttcacattacggacatagtctcaaaacagcaattagagcgtagaaaggcgaaccagctataa

attatggacatccaggtataaaagggaaacaaaagtaaagaataacagttgagcatcggctttcccaatccccttt

>48474|AGAP009864-PA

accctccatgcaacagtctagtatcataactacaacgccacattgctactcctgttatttcattacagtttcactcagaagattattgtcacaaaggata

tttcaggcctggattaagcatttaaatgagtgatttacaacagatacttcattaagtggcccaagtgacaactcaactggtttcaaacagaagtactagc

tttgacagtatgttaactagaactagaaaggtacaaagaggattggcatggccaattgggtagatgtactttcaaggacaatggtgcagtatggtaagaa

attgatagcttctataacgtaggatagaagattgcagacactggatttcacacgctagtcaaataagacttaagtcacattatagtgaaccttcaaccaa

gtaaatcgtggctgattttcagtaatgtggaagtgactaaatatgagagaaatgtttttcaaaactccatccctccatctctcaggtatgttctgttgaa

ctgcaccagtggtagaacttttgccttttattagaatcttcagaactgaatttcagttaatcaaaatctcctatagcaataaaggcgtgttcttatcata

ctgttgtatggttattcacaaatctgtggaaacacatcgtagcccttacaacaaaaaggtcattccctcatcattcagtttctgcatttgatgaaaagtt

acagagtgctgaactaacagctgtgtgagcctggcagaacacagtagatattccatctactgtttcaaggaccagaatttcatcctcagcaacttcaaac

attctgtctgagaaaacactgtgacgcaggtacaggggtataacaaggaggacagttggaaggactgctgtcaacaagcagaaagttgcaactcgtcgtg

ctccatgcatcgctcccttctcttgtttaccacaaaattcatgaataaagttacaaagtcttgaaaataaaacttagttgtttttcactataagcatcag

ctgtcgatgtttacattctat

>48488|AGAP009865-PA

gctacacctcctggagacctcgcaaattttttcggttggtaatgctgtttcatgcataactctctttcaccttcctctagcagcactctggcttctctca

tatctttctctctctgtctttctccacttcggcttgctttaaaatcgattgata

>48490|AGAP009865-PA

aatctaaaacccagtcaggtggttccacttctcttgcatacgctacacctcctggagacctcgcaactgtcagcaacaacagatttttggatttaactca

cattttttcggttggtaatgctgtttcatgaataactctctttcaccttcctctagcagcactctgg

>48511|AGAP009875-PA

ctttctccattttaacaaatgaaggaatatacatttccatttcatttacatcagtccttccaggtctgcagagcacaagagacactctcagcttctccac

caccaccaccactaccaccgccatgttcttcaagctggtaaccgtactgtgcctggccgccgctgccatggccctgccccagaggcctgtcgctggagga

cgaggcaaggacgccgtcattgtctctgccaccaacgatgtcaactttgatggctcctaccgttacagcttcgagacctccgacggccagcgcgcctcgc

aggagggcgctctgaagcaggtgtctgcccctggccccgacggtgacggcctgggtgaagcagtgcgcggcgacttctcctacaccgacgacgccggcaa

ccagttcgccatccagtacaccgccgacgagaacggctacgtgcctcagggcgcgcacctgcccacccctcccccaatcccagaggccatccagaaggcg

ctcgcctacatcgccagccagccgcaggcccggtcttaagcgtcgccacgccacctcatggacaccgctcctctcgctcttcttctcctctgtcttttgt

acaaacgcgcaactcgtgaccaataaagtttcg

>48538|AGAP009896-PA

gagcacgacgacatcatcttacggcaccaaccagcacaccgtcgtcaacgtgagcgacctcaaaggcgccgcctgcaatgttaacgccgccaagacgccg

cagcagcaacaggcaccgcctcccgcttacaacagtatcagcagcaaggaagcagaagctgcaggatatggagctagtacaggcatcttcactgtggggg

atgctttatcacggaaggcgagtgcagcgcagcaagagtacaaaccacatttagagcgtgatgttgaaaagcctatcaccaacatggagacattgctgca

tctactgaagggcagcctaggaacaggcatcttggcaatgccaaatgccttcatgcatgcaggatatgccgtgggtatagttggaacatttattattgga

gtgctgtgcacctactgtatccatctcctagtgaaggcacaatatgagctctgcaggcggcgccgtatacccagcctaggctaccaggaaactgcaatgg

ctgctcttgaggagggacctcgcttccttagacctctagctccatacactgggcacattgtgaatatcttcttgctgatctaccagctgggaacttgttg

tgtgtatgttgtatttgtcagcagtaatataaaatcagtggttgatgaatactacagtgggctggatttaaggatgtacatgctgattttgctgctgcca

ttgataatgataaacatggttcgcaacctgaaacttctggcaccattcacaacactagccaatttactcactctgattggcttcggcatcacactgtatt

acctggtcaacagtgctcctacacttgcagacaaggagccttttgggactcttaagggtttacccctcttctttggaactgtgctttttgcattagaagc

aataggtgttattctgcctttggagaacagcatgaagacaccaaagagttttggtggatcactgggtgtacttaatagagccatggttgtcatcataact

ttgtatattggaattggattttttggttacatgaaatatggcagttgtgtgaaagggagcataacacttaacctccctaatgaagagatacttgcacagt

gtgtgaagataatgttggctatctcaatttttataactcacagcttacagaactatgtggccatagatataacgtggaatggctacttgagtgaaaagct

tgagaaacatcctcataaagttatttttgaataccttgttcgtgtttccctggtcattgtcacattcctgttggctgttgcaattccaaatctggagctg

ttcatatcactgtttggtgccctctgtctgtcagcacttggaattgctttccctgcaataatacaaagctgtacattttggaacaaagaaactactcaac

tcaagttcatcttaatggttatgaagaacagtttcctagtcctgtttgcagcactgggtcttattgttggaacatatatatcaatttcagatataattaa

ggagttcagctaagatagtattgtaatgagtcctacaaacttgttgggacaccagttttaggcttaagaagtttggagggatgagcaccattgttcataa

ctttcctgtaaacctgattcctccagaaattctgttggccctaaacatggttgttacattacaatctcatgtagagctgtggtatgttttatctgagcac

atttagatgcaataccagttctcaaataagaatgaagaaaaaaggcagcagttgaaggatgtaaaataatggaaagctctgttcagatgtgttgcaaaac

actgcaggagctctagtctcaagtattcatcagttagggcagtattactggaccacgatattattgcacctagtctgccatggcaagaaacatgtgatga

cattacaataaagtctagtggtgactaaggaaagtgaatcacactgagcgatttaaagacctttgtgcatatgatacaaagtggttcagcatgtcctcat

gaaatatcatgagaatccacaataagccaaatgaagtgccctcacttccatgtgcaataatattgtggtatactaataaaactggttggcattttagctg

cctgaagtgtggctacatgaaaatgattgaaaactgttcactaccacaattgcaagagttaggaaagtttttactaaaattaatgtgaaaaatttgtgaa

acaatgttgaagaatactaattagttacctgtcattcttcagcaacagttactactgtaactgcaacagatttatgcagtaaaaatactcaacaaagagg

gctagggaggagaggaggggaatgagtggggaaaggtagagagaagaaaagctttgatataaaatatttatcatcatcagccttcaaaatttaaatctca

ctgagtacatagttcaaacttcgttttactcctgactagatcataaatagtgatgtgccacttgtgtacaatagaatgtttggttataaatgcaagcagt

ttactgtgaaataatttttaccagaaatgatgaaattaattgtaagtgctgtggaagtgatttctgttcatgttgttaaaaataatgttcactcaaatga

>48539|AGAP009896-PA

cagcgcagcaagagtacaaaccacatttagagcgtgatgttgaagcctatcaccaacatggagacattgctgcacctactgaagggcagcctaggaacag

gcatcttggcaatgccaaatgccttcatgcatgcaggatatgccgtgggtatagttggaacatttattattggagtgctgtgcacctactgtatccatct

cctagtgaaggcacaatatgagctctgcaggcggcgccgtatacccagcctaggctaccaggaaactgcaatggctgctcttgaggagggacctcgcttc

cttagacctctagctccatacactgggcacattgtgaatatcttcttgctgatctaccagctgggaacttgttgtgtgtatgttgtatttgtcagcagta

atataaaatcagtggttgatgaatactacagtgggctggatttaaggatgtacatgctgattttgctgctgccattgataatgataaacatggttcgcaa

cctgaaacttctggcaccattcacaacactagccaatttactcactctgattggcttcggcatcacactgtattacctggtcaacagtgctcctacactt

gcagacaaggagccttttgggactcttaagggtttacccctcttctttggaactgtgctttttgcattagaagcaataggtgttattctgcctttggaga

acagcatgaagacaccaaagagttttggtggatcactgggtgtacttaatagagccatggttgtcatcataactttgtatattggaattggattttttgg

ttacatgaaatatggcagttgtgtgaaagggagcataacacttaacctccctaatgaagagatacttgcacagtgtgtgaagataatgttggctatctca

atttttataactcacagcttacagaactatgtggccatagatataacatggaatggctacttgagtgaaaagcttgagaaacatcctcataaagttattt

ttgaataccttgttcgtgtttccctggtcattgtcacattcctgttggctgttgcaattccaaatctggagctgttcatatcactgtttggtgccctctg

tctgtcagcacttggaattgctttccctgcaataatacaaagctgtacattttggaacaaagaaactactcaactcaagttcatcttaatggttatgaag

aacagtttcctagtcctgtttgcagcactgggtcttattgttggaacatatatatcaatttcagatataattaaggagttcagctaagatagtattgtaa

tgagtcctacaaacttttgggacaccagttttaggcttaagaaatttggagggatgagcaccattgttcataactttcctgtaaacctgattcctccaga

aattctgttggccctaaacatggttgttacattacaatctcatgtagagctgtggtatgttttatctgagcacatttagatgcaataccagttctcaaat

aagaatgaagaaaaaaggcagcagttgaaggatgtaaaataatggaaagctctgttcagatgtgttgcaaaacactgcaggagctctagtctcaagtatt

catcagttagggcagtattactggaccacgatattattgcacctagtctgccatggcaagaaacatgtgatgacattacaataaagtctagtggtgacta

aggaaagtgaatcacactgagcgatttaaagacctttgtgcatatgatacaaagtggttcagcatgtcctcatgaaatatcatgagaatccacaataagc

caaatgaagtgccctcacttccatgtgcaataatattgtggtatactaataaaactggttggcattttagctgcctgaagcgtggctacatgaaaatgat

tgaaaactgttcactaccacaattgcaagagttaggaaagtttttactaaaattaatgtgaaaaatttgtgaaacaatgttgaagaatactaattagtta

cctgtcattattcagcaacagttactactgtaactgcaacagatttatgcagtaaaaatactcaacaaagagggctagggaggagaggaggggaatgagt

ggggaaaggtagagagaagaaaagctttgatataaaatatttatcatcatcagccttcaaaatttaaatctcactgagtacatagttcaaactttgtttt

actcctgactagatcataaatagtgatgtgccacttgtgtacaatagaatgtttggttataaatgcaagcagtttactgtgaaataatttttaccagaaa

tgatgaaattaattgtaagtgccgtggaagtgatttctgttcatgttgttaaaaataatgttcactcaaatgaaaggaattagtgttcattattacagtg

gaaaagagctttccattgttaacaaaaaagggaagctgtgataggaataaatgttagttgatgacattaccacaacaaagtagcacaataaaaagtacat

ctggtgcagaatgcaaaaataacatatgtaaaatgtccataagatttgataaaatggaataaattgttcttgtataagagttctagatacagtgcagcca

cttttaagaattacctattttttcctaagataaatgctctatgaatgccttaaagcatttttagagctgaaagaaaatgtttaccaggtcataaattcaa

gtcccaaaataaattttcagtacagcaagagttaaggtagtgaatttactttgaattttcctaaaatttaacagtgtgggtacttttgggcacatccagt

tgcaaataacttagtgataatttgcgtagttttccaggtaaaaatcattggtcgcaaatagttcaaactaaggagacacaaccattttccagaaatggaa

atcaatctgttttcaaatcacaataataatggtgctcagttgtagatgtgaaaatgttgtatattagtgtatatcttgtaaataatgttttgttatttat

taatgtgctgtgctactttctaagtaaccttatttaaaataagggatttttaaattgtataatgtgttgtgaaagactgaaagtgtttttctctattcca

ctgtgcctccttctcaaaactgttgaactcagctgtgaaagcactacctaatgcaagaagcatcagttcacccaagatgactgattaatctaaacacagt

tctgacccttcaaagtgacttatttggaatttttagcatgttaacagttggtcttacaatggcagccatattttttaagaaataatgttaactacctatt

ttttcctgtggagttagtattacagcctaaattgttcaaacatcatgaatctgaactgtttttctgaggcaaagatgactcactgtggtaaacattaaaa

ttttatatgcgattgcaggctttgtcagtgtacttcagctatgacatcttcacgggttatcagctgagtggcgttgtcttctagtcgcaacatttcaatg

gattgcatatcagtcatcttcaggtgaagattaccttcaccagaagatgatggataagcaatccattgaaatgttgggactagaagaaaccactcggctg

ataactcatgaatatttcataattaaaattttagcatgcctccataattcactgcaactatttagcaactgaaactaaatgtgatattttatttctgtgt

ggttaatgtgatgagaaatgaatctgtccaattatctttaataagttgtgttaagacatgtcatcatatcggaactgttcattg

>48564|AGAP009897-PA

ggccatctcgatggccgctggacacagcaggcccagcgtggagaagcacacggcccccaccagcgacaccacgggccccagctccggtacagcagccgcc

acgcccactgtgagcaggacgacacagacgcggagagtgtactcgaagcggttccagcgcttctgcgacacgcggtcgcccaccatcttcagcacgatct

ccgtggccacgtggaactgcagcgggtacgtgaacatgatggccagcccgatcaggatcttcaccgcctggcccagtggctcttgggggaggttgagggt

gacgctgcctaaggtaaattcaccgtaggcgaggtagccgaagaagccgatgaaggcgtacagcaagatgacgatggtcatggcgatggtgaggacgcct

cggcagcccaggaagtgctgtgggtgtttcatcgtgttctccaccggcatcaccactcctattccttccatggcgaagacgacggtactgaagaagagtg

ggaaacgggcgatgtcttcgttcagcggcagccgcccgggatccttcatgtcgcggaacatgtagtagagcgtgcagccgaagccaaccacgatgaacag

gttggcggccgtcgagaagggcaccaggaacttgaggttgcgcaccaggcagctgggcaccagcggcagcagcatcatggcaatgtagacgcgcacgtcc

aggatagcccacgggaatagcacgtcgcacacctgcttaatactggtagaaacgaagatgacgtagacgcagcaggcgctgtagtaggtgataaccagcg

ccaagttgacgaacgtc

>48563|AGAP009897-PA

tagaggcgaaagcggtcacgtgaccccgctacccggcgaacacctgacgggggacatcgcacgcgccgccgccgacgcagccagtgggaccgtgacgccg

ccgacggaaggacaaacagcagcgcagcgccttcgccgccctgccgactcgcaccatgggagcctcagccgctaagcagcaggagaaagcagccagctat

gcagtggaaaccagcaagcagcctacaaaaagcaatgggtccgtgttcgacatgacagcattctcgtccactgcgaaactcgcagatggagaccccgaga

aaccaggcctaccagcgtacgatcccttccaggagagagatgtgtcacacccaaacagcgacgtgggcgcgctggcgcacctgctcaagagcagcctggg

cagcggcatcctggccatgccgctggccttccgcaacgcggggctcgccttcgggatggtcgctacgctcgtcgtcggctacatctgcacctactccgtg

cacatgctggtgaactgttcgcaagtgctgagtcagcgagtgcggcagcccagcctgactttcgccgggacggcgggccgcgccttcgaaaccggaccgc

ccgccctgaggcggtactcccgagctgcacataatgcggtggacgtgctactgctggcgacctactacagcatggtctgcgtctacatcgtcttcatcgc

cacgtcgttgcgacaggtgctggtcttctacttcccggaggacgccaacttcgacgtgcggctgtatatcgtggcgacggcgccgctgctgctgccgctg

tgcatgatccggcagctcaagttcctggcgccgttctcggccgtcgccaacctgctcatctgtgtcagcttcgccatcacgctgtactacatcttcacag

accttccgccgctctccagccgcgactccttctctagcatcggccagctgccgctcttcttcagcaccgtcatcttcgctgtagagggcatcggcgtggt

actgccagtggagaacagcatgaggaacccgacgcatttcctgggctgtccaggagttctcaacatcgccatgggcatcgtcatcggcctctacgccgtc

atcggcttctttggatacctgaaatacggagacaagactgagggcagcattacactaaatttgccatcaaatgaaccgctggcgcaagccgtcaaagtgc

tgatcgccgtggccaccatcttctcgtacgggctgcagttctacgtgccaaccgagatcgttatgaggcggatgaagccgcgtctgccgcacgcctgggc

caatcccgccgagttcggctaccgcttcctcacggtcatgctcacagtggccgtggcggcggcggtgcccgacctgggccccatcatcacgctggtgggc

gccgtcttcgcgtcgacgttggggctgctgtgcccggcggtgatcgacagcgccaccttctgggagacggcgcgctggtggcgccacgcgcgcaacgcct

tcgtctgcgtcttctcgctgttcgcgctcgtcactggcgtctacgccagcctgctcgacatcatcgccacctactgaggcgccgctgctcaacacttatc

ctaccgcatcgcccggcgcgtcgacattcgtgtcacgccgaaagcaccgtccacgttcgcaattacggacaataaaaagctgttcacttcctcgtgaagg

agtcggctgaagttttacctgttgccagtgaaagcgactgaggctccaagtgtcgtctacagcgaaaaaatctgatgtattgcgtcacttcgcagttaac

tgtgctgtccaccttgcacaggagaacatgaggaatcggtaaaagtgtgaatgaaaactttaagagagcgccgtaagtacctctcctcttggcagagtct

gcactactggctgcacttcaaacgttgtgaaggtaactctaaatgataatccaacgaaagttagctgcagacagtctagttgtgtgaaagctttgcagag

cacatatgtgcaggttctattacgcgctactgattccactgcaagcgagctggagctgtcttttgagaaagtacagaacggaaatggcagcattactatg

caccagctttgggtatttaccaataagagtaggaattattcttacgtctgcaatgagaaaatagaattgttacgttgtcaagagatagcagaaactgtga

gtgtaaaactgtttactgaatgtaattctttggtagccttcacctcctcctccagaggggctaacagcatgaagtgcctgccgagcaagagaaaggcgga

aacactgatgcagcagagcgtcagggaaagtcagatgaaaagccttcccggtgcaacgcctctttgccccatctgaccacactcttcgttcgcacatatt

gtaacatacgttacagtccagaagcgtctgtcacaattgagatagtcagcgaaatgatttcgtgcgaacttgcaaaaatgtaattttattcacaaatttt

gttatttggcaataggtaaattttttttaatgtgcagaaagtcagggcgacaccaagaaccgataacgtaagagacattcttgtaacgctggaatctgtc

tagctgctgccagagcagagtaacagattagtacctgatatcgagttttacacatcaataatttgaatgcgtcgaccgtgatatctttaacgctgtgtgt

aaaacaatgaaaaatatgggactaccacgaaactgtttctggacgtttgaaaaacggatcataactaactgactcgtcggtaatgccgaaagagctttgt

acagtattaactataaaactatttttgtacctgtgataatattttatagttaaattcacaatcctaataaaattttgttgtttatgcaa

>48581|AGAP009903-PA

gccaagattatgccaatctgtctttttttatgcatattgtgaaactgcttttttttttatacatgaaatcagtcatcttacaaatagaacaccatccatt

acatgcaaccaaacacacactatttaccaagaatttgtctcagttaaccagaggaacatacctttctcagtattacacttttattgatgaaaacattaca

ttaaattttgtgatcttgtcaacctcccttcctctacaaggactactactcactacagtcatattttcattgtgattcccctgctgccctttgtcatatc

attatcttgaagcagcaggagtagtagtagtagtagtagtagtagtagtagtagtttgaaggcatttgcaactaaccatattacattttgtaaaattact

ggtgattttaaaattttaagcagttacttctgctcactaccttgcatttttgttgccacattcttatgaaaagaaccttaaggtactctgtcaaaccatc

tttttaatacttcttacagacaaatttttatcataatatgtggcaggacagcgattttttaaaaatacatttatttttcatgtaacatgtaatttttctg

tttataaataagacaaaagcaattaacaatgtcttccttgaagagttcattaaataaaatttttatttgctgcccatgaaatttcctcagataactaatt

tcatgcacaatggcttaatttacaacacagtataatatgttacttgaacacacaaagttaaatattatgatttaatacagaaagaaaataacattcttgt

aaagtggattgtattgtaagattccttactttacatctgttccataccacatgcacagaaacatcacacacattatgttcaggcaaaatttaaatttctt

tataatgtcatctacctgtagctgactggtgtgtgacaacactacacatacctgttaattgattttactacctttcagttatcactttttttattttatt

ttattttattttatgtatacacatgcagccaccgtgtgtgtgtgtgtgtgtgtgtgtgtcagcatctaattttgttttcttacgtttatgtaaggaatgg

aaattccttgttggtgtatgctcagtattaatagcatttatttttaggcatttacatatgttggtagagtattagtaacataagacctcttgctgagttc

ataaaataaccctgcttacttaaaatatgttgctgttattcaagaacaaggatgctctgctttcttccaaactaatcagtccaaataaatttatgcatca

caagcagcaccttcatcactgtaatgtaatctcagttgaatgtgacctaaaggaattatttgactctcagcagttttaacttgtaaatggaaatctatca

tgaagttgagagtatttttaaccagcaggcttagatcttatacataacaccctgtaccattaagatattgatgactcaggatattgtaatcataacagtc

acagtactacaacatcacaatgtcgatttatggcattcattgccaatgaacaaaatgtgttactgaatgtttttcctaaaataaaataaaaataaaaaac

caaccttaaatggagaatgaactaaggtgcagaagtcatcgtgtaccattccacagcttagtggcttagccaatgtaacaaaaatgtatatgaaatgtat

atttgtaaaaaatatttaaacttttcaattttttttttaacaagctgttttaatttggagactctgaaggggtttaaatactgtaagaatttgctcatac

tgaattcactggggacctgataatttatactgatgtacttaatatatacatacattatttatatttttgcacacattccaaatttgtttttacgctggag

attattatataatatatcaatgttgatactattaaattgtggtttttaatgaattttcgtactgtttaagttgtttgataaacccatcatttggtctaat

tgcttcccttctctccttcaagtacgtaaatgcttctgtaaatgtcatatcacattctttcatgaggtaagctattacaacagtagcagatctagatata

ccagcattgcagtgaataaacacacatccattactgtctttcactttttttaaaaactcaaagcttggctctaaaaatgatacaatgtctgtgtcaggta

gatccagagcttcaacaaatttatactcaaaaccatcaaactgttcaactctcacacctaaacttaatatgtgtgttatttcataattatgaagaagatt

aacatcttgtacaacatcctgggagccacagaaaagacctggtaatacgagagcaaccttcagatcaggatttgtatcaacaacatagccaggtgctaat

gaatctgtctgtgtcaagtgcacgcatttaccatctttaataatctctctgtgaactctgccatctggagaagttactgtggtttgacaaggccgaagat

gttgctttttttgtattaaactacttaagaagctcattgcaagatttataaacatttaacacatctctacaaattgttgttctctgtccactgattatca

gtttatttcatataaaagatcttgtggatcatattctacattaacaaggaacaacccataggggggtactgtaacagccttaggattccaactgtcttta

ctaggtacatctaacatgtactggacgtcttccacagttagtcttccttgagccactgcaatcagagctgctacagttcttcgtacctgtcgatacaaaa

atgactgccctttacacgttacttcccaaaaatcataatgcttacaacttggcatataatcatcgctcagaaattgctggccaggtttgatacagagttc

tttcatacttcttacagtcctgatgtcccgatcatctcgcgaagcacccataaatgacctgaaatcttttattcctgggaaaagtgcggcggcttgtttc

attgcatcaacattgaattctggattctgtataaaataacatctcctccattcagaaaatggtatttcagccaagtatttgaagcccatcttttgtagtg

ttggtgcattttgtttcctcacagctagacggtacaagtaggttctgcactttgcactatgtcttgaatgaaatgtatctgggactagctgacacttttg

cactctaagtccaactttggccttctcaaaatacctgttgaaacctacagtcagacactctgggtcatacccaccacctgtatggcgctctagatcaaca

tgggcagatgaaagtaacgcatggacacctttgtcagttctgctagaaacatacacttttggttcatttgccggcttaaatcttctacatgctatttcca

gaattccttgcacagactccggatctttgacttcaggttctgtccggaaagtttgcttctgcactgccctg

>49278|AGAP009904-PA

ttgatttaataaatacttaatttgtaagaaaccaaagtaacaataccttcaactagttcttacatgttttaagtaagcaatctccttaactcgcattgta

cattgctaaaaaagacctaaaaaccgctatttattagtgccttttccactctgatgctttctttcttttctaggaacccatgctcagtgctccaaggaaa

atatgatgaaaatcggtgactatgtagtcatacagaggaagaattattcaaaaattcacaaaatttccaacaaattgtcagtgatgattggaaacaacca

attggaactggataacatcattgggcgaccattttccacaacattcagaatggtgccaaaggaaaagaaaagaaatgttttcatgttggaggagtgtagc

aaggctgaaagcctcgttgaaaagcttataaaggatgttgccagtggaacagacaatagaaatatagttagtgataacagttctcaaaaattatccaagg

aggagattgtcagcttgcgtgatagtggaatgtctggttgtgacatagtaagtcagcttttagagaacagtaaaacatttcaggagaaaaccgagtattc

tcaaaagaaatatttaagtaagaaagaaaagaagtattttgattatgtgttaattaggaaacctactctacgattggtgtgtgacataatttataaacaa

gaccctatgaaaataataggtttgcgttgtgacacactctctcaactgattactggtgtaggtgtcagttcaggtgggacatacatagtttatgaaagct

cctgtgagggattaccgacagcagcacttctcaattatttaggtgacgatgggtatttagtacatactttcccaggaatgacacctcaaagacaggcaat

ttttgctatgaattatagtgagtcccagaaaaagcgactaatcagtgttagtatcatgagtctcatgg

>49313|AGAP009910-PA

atggaaaagatcctcctctccacaattctttgaacacatcgtttgttttctagtgtatggtagtgttgttattatgccgcgcgcagcgaaagtggatgca

gttataattgtaatagatgttggacactttgactccaagcatgttatacagaaacgcagtgattttttacagaaggcaaaactatgtgttcaaagaattg

tggagaggaggatatttaccaatggaaaagatgaaattgggttgattgtacttggttctgacaagacgcagaatccacttgattatgcaaatgtgagtgt

ggtatttcctcttgcactgcctacatggcagatgatatcctttgtggagaaatctttacatgaaagtgaaataaaaacagactggatagatggtgttgtt

gttggcatgcaagtcttgaaagatgaactagagggtaaacagttcacgacaactacattggtgttgattagtgactgtgctggtgatgttgataaccaac

atgttgatggaataataaatgagttgaattccatgaatgtttcatttgttgccataggtccagatctgataccaagtgatgaagttagtaacggtgatga

aagcaagaatctgaaaattggtcagtgtacatcaggagctgttctggccaaaataattgatgaggttaatgattctatggtgtgcaattttaatgatgca

atagagcatctgttgcacttgcaaaagaagagtgtcaaaccaaacccatggaatataatcttggagatagggcctgacatccgtataccattgactggat

accgcaaaatttctgaggacatgtttcccacttgggacaaagacagcaatagtgcgatgattgtgcgagaaacgagttacttcttgaatcccgatgccga

tgatgaagacaacatggctacaggagaagcagtcgatgaaaaagatgtgtgttctgcctactctctaagagcaaccattgtgcccacaagtgaggtggtc

gaacaggcaatgtcatacagttctccagcaaagtcactgactgtgttgtgtttcacagcagcagaaaatgtgcctcagtacctgttcatgggcaatggtg

tcctgtacttcactctccaaaatggcagaacagaggcaggctctgcattctctgctgttatccaagccatgcagaatttagatatggtagctgttgttcg

aagagtttatcagagtaactatgacccctgtgtgggtgtgcttttcccaaagattgaagacggattcgagtatcttgtatacacagagctaccatttgca

gaggacgttcgtgatgacttgattcttgaaccattggatcgagagataaatgctctgacacctgagcaactgaatgttgtagacgaactgattgattcca

tggacttaatgggtgctttaccagatggaactgaggcatttaaatcggatgagataactaatataactcatcacaaattgttcagttgcatttcctaccg

tgccttacacccaggagaaaaaataccacctatggtggaagaaataagagagtttgttactagcccttttgtcgattcagggcatgtcaatgacataatc

ctgaaaatgaaaaagacattcgacttgaaggaagtagtgcggcggcggcgtgaaaaggactctgctgacgtaaaacaagcagttacaaatggcacaaaat

ctgagaaaccgaaggattttgaccagcctggaacatctgctatgagcagttcacaagcagcgaagccaagtgggtttttagctcacggtagagtgaaggt

agttggaaccacaaacccggagagcgatttccaggaattactaagtcaaggagaaccattctcccaagcattcaacagtatgaagagtgcaatagagaag

cttgtggaacgttcattctcgccccagcagtatcagaaggccctctctgctgtcaaaactctcaggagtaaatctgtggaaatagaaccactcttatata

atgagtggatcactcagcataagaaaatgctcaaaatgttgatgaaggaagaattctggaacatgattgttgcagcaaaacttggtctcatatcatcctc

cgagtccgtgagaagttctgtgagtgaaaaagaagcgaatgactttttcagtgcagatgttagagagtctataaagagtgaagatgatggctttgaagat

gaagatgatttactcatggcattgtgaagagtgcctagagcaccatagccaagataagaagaaagacagagaattttttcttcgtgatgaaaataagata

attcttatgtctgcgttaccagttgttcctaatcaccaaatgcagctgtatattttgaaggtgtaaaactgtgcaaat

>49360|AGAP009913-PA

cactcaactgatgttattgagaactgggcacggtaaaaatgtacttcatgaagctggtcgcctccaataacgtacagaagaccatctgctgcagctactg

ctggtgtagcacgccccaccctcattggtggtacaacagtccatttgttctcttggaaagaatatttttccaccgactgcagcacttcattatgtctatt

agtgccccctacaatataaagatggtcattcaggactgcaacacccatctgacttcggggaaccgacataggtcgcaaatttgcccattccttagttaca

ggattatagctgtagagctctcttccatggcggctacgatgtgtacaaccaccaaccatgtagatcaaaccttcatatgatacaacacccatgctgaaac

gagcttcaggcaggtttccttccagttgccactcattttgcatgggatcatatcgctcaatggagtcaccaatgtcttcaccaacccagccacctgcctt

tcctgcaggttcaccatctgtttctgtgcacatagtcaaatcaccagctccagctgcctcacccctggtaaccccctctccccattctgtgtcaccctgc

atcactgatgacgaattgatggacgaagcattggtcggaatgggtaaatgaggtacggcacctgcccagtcaagctcctcctcgggtttgtcgtgggcat

cagctggtgatatgtgaactttcatgtcattgttactgtgctacataccacaa

>49413|AGAP009920-PA

tggtgcctcacttaggaagatggttaagaagatggaaattacgcagcattccaaatgcacttgcaccttttgtggaaaggatgcaatgaagaggagttgt

gttggaatgctgcgtaatttccatcttcttaaccatcttcctaagtgaggcaccata

>49465|AGAP009937-PA

tggagaggggacgagagattctctctgctaccatcagggtcagctccagcttctaacagcagtctgcacacttctgtatgactattgtacactgctacaa

acaggggtgtttgtgccttaatgtcaacaacatctacttctgctcctcgcctcagcagctcttgaacacatacagtacttcccactgcagctgcacaacg

aagaggtgttacgcctactgcacccatagtttcatttagtcttgaaggcttttcatttaacagaaataccattttctcgaagtcacttttcaagactgct

tcatgcagagaaatgtcttcatctggatcaatcatgaagttaaatacttcagccatttccctgattccagtacatgaatcagtgcgaaggactgcttata

cgttgaaaaatgtttgtgcaaacattaactaaataaattccaatatgatctccatctgataatcaaaacacttcaacgtcaattctttcgtgcaatatct

gtgccttcacgactaaatgaaactatgggtgcagtaggcgtat

>49467|AGAP009937-PA

tgtgtgtgtgtgtgtgtgtgtgtgtgtgtgtgtgtgtgtgtggttttttttctgataaaaagcagatttttcatgcattgtaatactggtgaaacatatt

gtacattgcatggtgtgttagtaacaaaaagacaaaatattcttggtgaacattgttttgtaatagtaagcttgctttcaaggtgagaataactgtttat

atacatcttgttttagtttgcataataagtgaattatgtcagaatgttttataaaaagattcctgttgaaaatattttgtaaaatattcactttgtcttt

aattctgcttatttgttagaaacagagtagttacattgattgcttaatctttcaaatgttggtgaagtttcttttcacttagcttccgaccaagtgggca

cgagaatgtcaatagtctgtgttcaatttaaaaaacattgtcatatttatttgtgtttaataaaagtattttgaagatactttagaagactgtatagtac

aattcagttattttgtaatgtttaatgttagttctctcacagtagcctgtgcctttatttgtaatttggcatgtacctgtaacaggcactgattcatgta

ctggaatcagggaaatggctgaagtatttaacttcatgattgatccagatgaagacatttctctgcatgaagcagtcttgaaaagtgacttcgagaaaat

ggtatttctgttaaatgaaaagccttcaagactaaatgaaactatgggtgcagtaggcgtaacacctcttcgttgtgcagctgcagtgggaagtactgta

tgtgttcaagagctgctgaggcgaggagcagaagtagatgttgttgacattaaggcacaaacacccctgtttgtagcagtgtacaatagtcatacagaag

tgtgcagactgctgttagaagctggagctgaccctgatggtagcagagagaatctctcgtcccctctcca

>49466|AGAP009937-PA

ctccgtacgccgcttttgtgttgcttgtcaaactgcgcagcggaggaaattccgcaaaacgcatgtattgtgagtgtgcgtgcgggatactgctcaaata

actaagctgtatctgtgaagataatttctctcgcacgcgagagttcaaataagtgaagttaaatcattcataagaacgaaatatatattcgtacttttgc

ctgtagcgacttgtaagtaatcggcaaaggctcggtgtggaaaatcaacggacaaaggaacctttgtgctaatatgactctcacactgctgcgcagcttc

gacaaagatgaaattcttggctggtcacctgtccgtttcgcagaggaaacggagcattgggagtctgtggaaatgattcttattgaaggagcttctatga

gtgacttagctgtgacgaagaggaagctacaggactcggaacgtgcggcactgatctttggcgagctctgcaggcgtaggctggtgagactgctcacctt

cgcccttcaacagagccctggtctggtgcaagccgagctgccactcaaccgttcaccactacacgaagcggcccgctctggctgcccagcaactgtccgc

tgtctcatcgcagcagacgctgcggtcaatgctgctgatgatcacggtatgactccactacatgaagcagcagcccattccaacgacttcaccgtgcgca

tacttctagatgctggcgctgttcctgacaccacagacaatggagggagaacggctctgcactatgcatccagaagtggttccgctgcctgcatccagtt

tctgctggcgaagggttcgcctgtggaccatgtaagtgctgatggctgtactgctctccacgaggccgccaccagccccagtgcccacgctgtccgcttg

ctgctggacgctggtgctgacaaacatgcaaagtctgcttctggttggacagctctacactgggctgccagcaggggtcgactgtacgctgtcatgatct

tactggaagctggcattagtgtggatgtgaagacagtggatgaggaaacgcctcttcaccttgcagcaaccctcacatcgtgtgatatttgggaggctct

cgtcagggctggagcagatgaaggagctttggataagaatggtcggacgccaaaggaacgagctgcagataagaccgtgcctcatgttgttgggtatgac

gttggtggaatgggcaccagcaatatatcaccaaaccagtctcatattcctctgggagtggcaaatgatgcaccattgacttccaatggtaacgtgtgtg

attcagagaatctctctgctgaagattcagttgttccattaagcagtgcatcaccaaattctacaacacaacaagcagtaacagcaactgctgaagaaga

taacggaacaggtgaagagggtgcaccctcttcacctgttccgttatcttcttcagcagttgctgttactgcttgttgtgttgtagaatttggtgatgca

ctgcttaatggaacaactgaatcttcagcagagagattctctgaatcacacacgttaccattggaagtcaatggtgcatcatttgccactcccagaggaa

tatgagactggtttggtgatatattgctggtgcccattccaccaacgtcatacccaacaacatgaggcacggtcttatctgcagctcgttcctttggcgt

ccgaccattcttatccaaagctccttcatctgctccagccctgacgagagcctcccaaatatcacacgatgtgagggttgctgcaaggtgaagaggcgtt

tcctcatccactgtcttcacatccacactaatgccagcttccagtaagatcatgacagcgtacagtcgacccctgctggcagcccagtgtagagctgtcc

aaccagaagcagactttgcatgtttgtcagcaccagcgtccagcagcaagcggacagcgtgggcactggggctggtggcggcctcgtggagagcagtaca

gccatcagcacttacatggtccacaggcgaacccttcgccagcagaaactggatgcaggcagcggaaccacttctggatgcatagtgcagagccgttctc

cctccattgtctgtggtgtcaggaacagcgccagcatctagaagtatgcgcacggtgaagtcgttggaatgggctgctgcttcatgtagtggagtcatac

cgtgatcatcagcagcattgaccgcagcgtctgctgcgatgagacagcggacagttgctgggcagccagagcgggccgcttcgtgtagtggtgaacggtt

gagtggcagctcggcttgcaccagaccagggctctgttgaagggcgaaggtgagcagtctcaccagcctacgcctgcagagctcgccaaagatcagtgcc

gcacgttccgagtcctgtagcttcctcttcgtcacagctaagtcactcatagaagctccttcaataagaatcatttccacagactcccaatgctccgttt

cctctgcgaaacggacaggtgaccagccaagaatttcatctttgtcgaagctgcgcagcagtgtgagagtcatattagcacaaaggttcctttgtccgtt

gattttccacaccgagcctttgccgattacttacaagtcgctacaggcaaaagtacgaatatatatttcgttcttatgaatgatttaacttcacttattt

gaactctcgcgtgcgagagaaattatcttcacagatacagcttagttatttgagcagtatcccgcacgcacactcacaatacatgcgttttgcggaattt

cctccgctgcgcagtttgacaagcaacacaaaagcggcgtacggag

>49464|AGAP009937-PA

taaagatttatgtcatttataaattacctaaattaattgtacagaactctaagacacacgaaattaagccaacagaatgagaattaaaaaactattacac

attacacaaaattgagtaaaccaatgaaaattaaattccataaaataaacatagcaaaataaatggtcactgatacctaaaaaatgcagtaagatattta

aatagcacattaaacacactaaacatactaatatatcatggaaggttgaaagatacaaaaatcaatgtatagacaaggcactgaatggatgagacacata

caaatgctgaaattgttgttaggtgttcggagaatgttcctcttcagagctgcagagccagtacaatgtagaatgtagtgtttatgtgcctatcaactgc

tcaatgcctcaactactgagttgttacctttactcccctcaggacattctacttatcttatttacacaaatatttcaacaacacataactaatcattcag

tacaccaattaaatccagtcttgagagtacatagggaattccagattctgaaaactgtgttacagctgataaaagtttcaaaagcctctcatgaacaaaa

aagaaagatatagagttccactgtgatctttagattccacaatcatgtttacttaacatcagaataacatcatcctaaattacagcagtacaaaacatga

actgctccttgctagcacattacataaaactattctgcagtataatccagaggtaaaaatttactctcatttatagtttttcatataggagcatgtttcc

tacatactcatgcatgtggtgctagttaggtaccagtaaatgtacacttgtctaagatgggtcacttctgtgatgccaagatttcagtcatgtccccttt

ctgaaacacaaatgtttcatgtgaaaacagctacagtctgagcaaatattcagcacatatgtcacagcaggcagtcattgtatactctcacttacataca

tacacactgtgaatgcacaaaagtgttttaaagcttttttatagaaagtttcttttttagttttatttagaaaaaatacgtgtttgaaaagtaaaaacga

cagtaaccataaatcgcaaagcttgcttgaacccaatagtgctcatataactttgccctcttcacagctgtgagacagcttacagaacctgttaagattt

taacagggaatccatagtattgtaccacatgacatgcctcatgtacaggaatgacctccacaggaaacagcagtttccacattaaaataaattggatgtt

ccatttatccatcttaatgtaattatatcagcactaacacacacattcacagttgggtctggtgacattcttacattgttctttacacccagaggagtgt

tcccagtagagtattcaactctgagatctgcagcatcagtcctcccctgtaacacatttgtgcatgaagcacaaattctgcaacaataaaaactacagct

taccactgttcatcgctcttatgtttcctactaaacattctctatattctttcttccaatgtaggtgtaaatgttttattgtgtacagatattaaaataa

aaatccattataccacagctgcctactgtattgacagttgcagcaactgtacacttgtttgctgtagattcagatttgtattcctctttttattctcagc

ttatgtttatgatatagttggcagcaaaccatctactcagtaacttatcaactcttacctcctcgacttctgccatggctcttaccaccacggcccgcac

cattccacttaccagcccgactttccccttgtcgattagcataatgaccattactatggtgaccaccagactgattagcgtaatgagcttcaccatgtcg

attacccctatggccttcaccatgtcgattaccgttatgagtttccccatattgattactgctacgactttcactatatttattactatgatgccctttg

ccatgtcgattgccataatgacttccaccatgttgattaccatggtgaccttcactgttttgattaccagaaccttcaccacgctgactgccatggtgac

tttcaccatgtctctttttgtgatgacctacaccatgctgggaatcaggtcgttttttcttcttgtgttctgcgttagaatctccttgctgttttgatac

agattttgggtgttttatctttttgtttttcttgtcttctggcgtatgaggctctctgttcttatagtcagctctcacactaggttgttgtttactatgt

ggctcgattgatccacctccaaacagaactgtatttggttggccacaatttccatcacattgccttcttgcatgttgatatacagaaccattaaaatttc

ctcttctgattcctttctcattgctcctaccatgctgtctacttccttgtgcatgtggagattcattgacattttcttgtcttgatgttgataccactga

tctggtgttatatgaagctgcattcccacgagtacctctaggagctccgcgtcctcttcctctaaattttccttgaaattttgttgcttgttggttgtat

tttgtggaagtgccaacagttcccaaagtatggaacaatggaggtccctcataatacattattgtattagttgtcacaataggggagaaaagttccttaa

ctgaccctctttctgagactttatctatttcttcatttgctgagctgatgtaggaagaagcatcttcttcatctgcatcactgtctgtttcatcatcact

gcccgtttcttcatcactcgccatttcatcaccactgtctgtttcttcatcactctccagtagctcaccactatacgtttcatcagcactccctgtttca

tcactcagttcactgtattcctcacacgatgaactatgcatgctgtctgttctttgactagaaattgctgacatacactctacacttgctccttcatctt

cttggaagtctgttacggtttgttgtgttgtagaatttagtgtcacactgcttaaaggcacagtaaaattttcaacagttagttcctcagactcacagat

actattgcttgatgtctcatacaataagctacccttattgtctgctgtttggccagaaactgatggtgcaccctcttcacctgttccgttatcttcttca

gcagttgctgttactgcttgttgtgttgtagaatttggtgatgcactgcttaatggaacaactgaatcttcagcagagagattctctgaatcacacacgt

taccattggaagtcaatggtgcatcatttgccactcccagaggaatatgagactggtttggtgatatattgctggtgcccattccaccaacgtcataccc

aacaacatgaggcacggtcttatctgcagctcgttcctttggcgtccgaccattcttatccaaagctccttcatctgctccagccctgacgagagcctcc

caaatatcacacgatgtgagggttgctgcaaggtgaagaggcgtttcctcatccactgtcttcacatccacactaatgccagcttccagtaagatcatga

cagcgtacagtcgacccctgctggcagcccagtgtagagctgtccaaccagaagcagactttgcatgtttgtcagcaccagcgtccagcagcaagcggac

agcgtgggcactggggctggtggcggcctcgtggagagcagtacagccatcagcacttacatggtccacaggcgaacccttcgccagcagaaactggatg

caggcagcggaaccacttctggatgcatagtgcagagccgttctccctccattgtctgtggtgtcaggaacagcgccagcatctagaagtatgcgcacgg

tgaagtcgttggaatgggctgctgcttcatgtagtggagtcataccgtgatcatcagcagcattgaccgcagcgtctgctgcgatgagacagcggacagt

tgctgggcagccagagcgggccgcttcgtgtagtggtgaacggttgagtggcagctcggcttgcaccagaccagggctctgttgaagggcgaaggtgagc

agtctcaccagcctacgcctgcagagctcgccaaagatcagtgccgcacgttccgagtcctgtagcttcctcttcgtcacagctaagtcactcatagaag

ctccttcaataagaatcatttccacagactcccaatgctccgtttcctctgcgaaacggacaggtgaccagccaagaatttcatctttgtcgaagctgcg

cagcagtgtgagagtcatattagcacaaaggttcctttgtccgttgattttccacaccgagcctttgccgattacttacaagtcgctacaggcaaaagta

cgaatatatatttcgttcttatgaatgatttaacttcacttatttgaactctcgcgtgcgagagaaattatcttcacagatacagcttagttatttgagc

agtatcccgcacgcacactcacaatacatgcgttttgcggaatttcctccgctgcgcagtttgacaagcaacacaaaagcggcgtacggag

>49488|AGAP009942-PA

cagacttatatcactgatatctgtaccactggaacttctatgtctcacatggagttagaggtggtcacacttaatttcaaattaaagccatcatacaaca

gcacaaagatttcataatactatgtacagtatgaaaaggtggcaccagttacatgatgttttagattcggacacaaaactacctgtctgcaaataaacac

tttttcgttggaaaccttcaaattgaaaaatttctgcatgacagggatacatcagaatttacaacatttagtacataggtacaacaacatttagtatagc

attaaaagttgaacagaaagatgaaataaacacaagaacacaatgataaataaaaatatttaaacatcaagatttataattgtttaccacatcctttcaa

aaaggatagaagtaggagatatccttgacaaagacaaactgtctattacatgtaatgatacacacattaaccttcggatatcttacttttgcacagttta

taggaacaaataatacccttccactcttcagtgagaagaagggtgttttaactattttgtacataaataaataaggaaataaatgtacatggtatatctt

tcttaaaacccaaagaacaccttagaaacacacctgaaggtatcttatattaaataaatgagttgtaatgaataaaatacattttttccttagttgttat

aaacagattaaaaaggaaagtgaacatctaagatattcactatcctacctgccagtattattcagtactatgttacatgtttattaacaaagagtttctg

tagcagcatatctcaacactggtgattacagcattattgctcataaaataaaaacaaaacatcagaaataaaaggcaaattgcagattatttaagtgtac

catctgttaagatacaagaattcctggcataacactacagtagtgatgttcacagctttgccttcattaactgagatttcacactatatgtacatcccac

caagcaagcacaggaatgtagcaccaatgctgccttgaaaaatatctttcttacaatatgaaatatttcagttcctccacagcatcttttgtacttggac

cacagggaactacagaattctgcagccttttactacgaatggcgttcatcaacaataaaaagtttccataaaaaggctctatgtacaaaattatgttcag

tagtctctcactcatttcaacaacttagcaacaggcagttgctactttgtctcagattttaaaataactttttgggctatttcttcttgaaatcagagac

ccaaaaaactgcaaagcatgaatgtgagaacaagtgttattaccacgagcaggaccgccagccataacggtattgtacttctaaatggaacatgatgtat

gcagacacggttatccacagaaatactgataacagctgtttcatatcctcctaagcagtcacttgggaggaattctaggccagtaacgaacatattgtgg

acaccttgcacattcagcacttgctgcaaactgaatgcaatatgtatgtgcacgctaccagagaacatagttccaactgctacaaatttgccatcatctg

acacactaagtgcagaaagactttcatcaaattcaaatgactttttgagtgatgcctctgctggatcccacttttgaagatatgactttagctttccagc

ttttccaaaagggttgcttagagtaaacaaacagctattttttgagtttccttcagttgttccaaacctgcatctcttgtatagatatttgacattttct

ggtgcattccattttagttcagacagcttctttcctgtctgtgtactccaaattacagcaagtccatctttcgatacacttaataccattgtttcatctg

gactaaagtcaatgtcatcaatttctttcttgtgggcagcaatatcgaggtgttttcttaatgaaggaaactgccaaagacgaatatggccatcagtgcc

tcctgtagccataagtttgccagaaagacttattcgaacaactcgctgtaatggctcatcatcaccaaaatcagtctgtatgctgtcatttggcttaata

tcaaatatgagtcgatggaaggaattgctgttcctgtcagtttgctcttttgttggttttatttcttggatcttctcatgtggatcagtacttcgtttcc

tacttcttactgtttcatttttgtggtctttcagcactgtttcgtcgttttcatattcaaccttcatgtttaccgtgtacagttggcaatggctttcctg

accagctaccaaaaaggtccgtttaccgttattataagacgcacagttcattactacacttggacctgtttcatgacggataacttcttcagcaatgtac

ttttctccatcatgagataactcaaatatttcaaaaccattggcgacgcctgttttcgaagagccacctcctcctgcgacaagtatatgatgatttgtta

tcattttaactacgtacaacggaaaattaactcttgcgagtaaaccttctttatttcttcgatttggtgacatactgaatttatatgatacgcgaatgtg

gacacagaagaatttcacatgtaatttaattattcaaacacgataaacatgctgacggcttgtcctccacgtactttttt

>49521|AGAP009944-PA

tcaccttctgcaacatcagcaatcgtttcacctgtagaagggtttatgacagggaacttctttccagatgctgagtcaacaaattcattattgatgaata

gcttactgtaatgacgaacaatgtttgtcttcttcttcctgtgggaggt

>49523|AGAP009944-PA

tttcaccacaagcaccatttggtggctacaagcagtctggaattggcagggaactgggagaagacacactgcagaaatatctgcaggtaaagactgtgtc

tgtgaaacttccaagcaaatcgtaagatctttcagccatcaaataagaattttgttctgttgaacaaatcattattgtgaagctttaacaaacttttgtg

ctgatcatattgtaaaaccagtaatatggaaataaatttacatatatttttttatcagagtatttactttcttcgtaatttgcaccttttgctcattttg

gcaataaaactataactatcatacg

>49520|AGAP009944-PA

aaacaattcctgctgatggtccattctttacatatacacgaaaggaaccagttggtgtagtggggcaaattgtcccatggaactttccactgacactgac

agcactgaaatggggccctgcacttgccactggatgtacaattgtattaaagcctgcagagcagacccctctctgcatactgtatttggctgctcttacg

aaagaggctggatttcctaatggtgttattaatgtcgtacctggttatggacccactgctggagcagctgtttcttctcatccagacattgctaaagttg

ctttcacaggttctacacaggttgggaaaataattatggaaactgccgcaaaatctaatttgaaaaaagtctcgcttgagcttggagggaagagcccact

tatcgttcttgatgacgctgatgttg

>49597|AGAP009958-PA

cttttatttacaaaataacaaatacagtgatgagtttggaaggtactttcagtttctatatctgtaagttatcgagtctttttcttttcttatctgatat

gtaaatttgttgcaattgtctgtttcgttgtatgttgttcgtttgctgattgatttacaatgttaccctgatttgtaagtcagagggacaataagatttt

cattctaggagacaatggacaatgataaatatgcttgaatgaatattatgtatcaattatgagagaaagagagagagagagagagagagagagagagaga

gagaggtgtcaagtgtgaatctgcccaattctgttaatttttttatcatgttacaattatttcaatatggtatcctttagatacattcccataaaatgaa

aaaaaaaatcagtaatagttatagcaaaaatcagtatttgctacagaagataatctcaagagctgaaaaatttatgaattttcatggaaatagtaaatga

aaacacacagtggaactgaaaggcattactagtttggttatactgaataacaatctgaaagctacacaatatcttgttactgtacagacattaccctttc

aagattacaaaagactataccgcaatgctgtaaaaaaatctaaaattgtcatatgcatatcagacaagaactgtacaaaatttccaggcagcaacttttg

tcattttgtgtctttcaggggcttgcattcatgccctttatcatagatgaatagtttccactcccgattcaacatcactaatgccacagcaaatatattt

tcttttaaatttttacagcttgatgtttaaattatatcgccgggtaaaacagactgaaaaacttcaagtacagggtaaaataagaatatctcattattac

ctgaaaactgaacatttaagtcctgagtcaactcctgttctcattaagcacccacatggagtgaaaaatgaaatcctcgatcatgcaaacacacatttct

tttgaaatatgattacacacacgcaaacacgatggacttgttggacctgttcacgatacagctggcttcaatgtaaaggacaacttgggtcgtgaattgt

tctttcccaaaattttcttctgctcttccttgattcgtttttcttgctcttttctcagcctttggcgttcctcttccatctttctctgttcctcaaccat

agccaagcgctcttctgccagtttcttctgggcttcctcaattttacgattattttctgccataatctgttctaactcttcacgcttccttctttcttca

gcctacaaagtaatttttcatgtcaatattggatggttatatatgagcaaacatgcaaactgataactaaaagaaagagtagttacattaatactatgtg

atgaaatactgtggcactgcgaatctttgagcaaatgctaccaagcatcatatcaatcaggtaaatgattctgtactatacacacgattaaatcatttgc

tgcctgcacactgcatatttttgcaaagatttaatcaagaatattcactgaattgattatacaaaaagattaaaaacctctatgatatcatcttttacct

atgtacattaccccatcccaccccacaatttactaattataac

>49598|AGAP009958-PA

agagagagagagagagagagagagaggtgtcaagtgtgaatctgcccaattctgttaatttttttatcatgttacaattatttcaatatggtatccttta

gatacattcccataaaatgaaaaaaaaaatcagtaatagttatagcaaaaatcagtatttgctacagaagataatctcaagagctgaaaaatttatgaat

tttcatggaaatagtaaatgaaaacacacagtggaactgaaaggcattactagtttggttatactgaataacaatctgaaagctacacaatatcttgtta

ctgtacagacattaccctttcaagattacaaaagactataccgcaatgctgtaaaaaaatctaaaattgtcatatgcatatcagacaagaactgtacaaa

atttccaggcagcaacttttgtcattttgtgtctttcaggggcttgcattcatgccctttatcatagatgaatagtttccactcccgattcaacatcact

aatgccacagcaaatatattttcttttaaatttttacagcttgatgtttaaattatatcgccgggtaaaacagactgaaaaacttcaagtacagggtaaa

ataagaatatctcattattacctgaaaactgaacatttaagtcctgagtcaactcctgttctcattaagcacccacatggagtgaaaaatgaaatcctcg

atcatgcaaacacacatttcttttgaaatatgattacacacacgcaaacacgatggacttgttggacctgttcacgatacagctggcttcaatgtaaagg

acaacttgggtcgtgaattgttctttcccaaaattttcttctgctcttccttgattcgtttttcttgctcttttctcagcctttggcgttcctcttccat

ctttctctgttcctcaaccatagccaagcgctcttctgccagtttcttctgggcttcctcaattttacgattattttctgccataatctgttctaactct

tcacgcttccttctttcttcagcctctctcctcctttcctcttccaactgtagttctcgccgtctttccatttcttccatcatttgcttttccattattt

tcttagcttcctcaactctcctgagtacttctgcctcaatttcctcttttcgtttttccaattcctcctcaaccctcttcttcacttgctcttcaattcg

ttttgcagtttcttcctccaccatcttttgttctagctccctttgtctcctctgtctttccatctccgccagtctttcaacttcatccattttccgatca

tggtaacttcttttcctagagtgtccaccagttcttggtacgtcgtctgaagcatcagaactacagcttgatgatccagaatgtgaccttttccttgatt

tgttgctcctttcccttgtcttttcactgtgtttgctgtgagagcttctgtcacgagacttcgatcttttccgagagtgtttacttttgtgccgcctttt

cggtgatttgcttcgtgttctcgatcttcccatgtcgtaaaaatatcaccaaggagaataaaacgaactggccctagtgtcaggaacacaacagaatcta

acgacaacacatatccgacattactccaacgcttcactccccatgttcagtatatgcagtgttgccaagctgattaattgcgccaaggaatactatcaac

gaaaaattaaaacacatgcagactcttaaatctttcatttccagctaaccaacaagaaaacatgcattaatgacaagtaataggaatttataaacaac

>49600|AGAP009958-PA

caatatttcttcacaggatacgtcattaagaacttacgttcaaatttgttacatgctggttcattctcaaagcgttgttacctgttggcagtgcaaagtt

cagaagagactgaaacacctgtcttctctcctactgaattagccgatttgattctggatatctaggctatatggagaacagttttatcatgtttttgaaa

ctcttgtgacttgattaagattttttgtatggaatgccaagccacacgcagtaagaatctccatattttctccttaaaaatgtaactcattttttgagaa

atgtctgtagtattcttactgatatgagatagtttcccctatgattgctgtttgcatttggagaggcatggtatgaatactggcatatttcttacttatg

ttagctgttttatataagaaatgtagataagtttcacttaagacaatgattgatttagtaaatctttttaatttacaatgtgatgaagtgttaagattaa

ctactagttttgaaattatttcttgcattcatagacttttaccatatttaatttcctccatggtacatcttgatatgttgggtggttgtctatttagttt

gcagttagctgctcgagttagaaagtaaaatgtgtgttggttttactactataaatggttgctactgtgttgcacccacatatcactcacaacaaattgc

caagaaaaacttgccactcaaagttttgaattttgttgtgtatctcaggggaaaaggtttacagaacctgtaggttgagcataaactcttatatacagca

ctaaatttgaacacaagtactgtgattcgcttatacaaaatatttaaaatactgatgttgccaattcatccttaaacatcacatagtgtctgtgtgatac

cattactactgtaaaattaaatagcctgaagaggggaacagcaaaaaacttgaagctttgtatattttagggtaagataactttcattcatattaggttt

tgattttggtaacttagtctcactatggctgatcagtacttattggaagttttttcatttggatctaaaatatagttatttgattggttgctttgccaca

taccatattggatttttggtttaccaggagaatgttatttttggttccccagtttacaattttctgataaaatcttctgtacaacagtaggtgccatgcc

acaacttttaagaaggagctaatacaattgaaaatgcctggagatacctttcttccatttaatttgctggtgtttcatccagcactgtgtaagcaaaaaa

taatttaatcgtgtgaatattaaagaatcatttacctaaatgatatgaacattacatttaatgggatatgaacatcacatttaatgggttataattagta

aattgtggggtgggatggggtaatgtacataggtaaaagatgatatcatagaggtttttaatctttttgtataatcaattcagtgaatattcttgattaa

atctttgcaaaaatatgcagtgtgcaggcagcaaatgatttaatcgtgtgtatagtacagaatcatttacctgattgatatgatgcttggtagcatttgc

tcaaagattcgcagtgccacagtatttcatcacatagtattaatgtaactactctttcttttagttatcagtttgcatgtttgctcatatataaccatcc

aatattgacatgaaaaattactttgtaggctgaagaaagaaggaagcgtgaagagttagaacagattatggcagaaaataatcgtaaagaaggaagcgtg

aagagttagaacagattatggcagaaaataatcgtaaaattgaggaagcccagaagaaactggcagaagagcgcttggctatggttgaggaacagagaaa

gatggaagaggaacgccaaaggctgagaaaagagcaagaaaaacgaatcaaggaagagcagaagaaaattttgggaaagaacaattcacgacccaagttg

tcctttacattgaagccagctgtatcgtgaacaggtccaacaagtccatcgtgtttgcgtgtgtgtaatcatatttcaaaagaaatg

>49599|AGAP009958-PA

cttttatttacaaaataacaaatacagtgatgagtttggaaggtactttcagtttctatatctgtaagttatcgagtctttttcttttcttatctgatat

gtaaatttgttgcaattgtctgtttcgttgtatgttgttcgtttgctgattgatttacaatgttaccctgatttgtaagtcagagggacaataagatttt

cattctaggagacaatggacaatgataaatatgcttgaatgaatattatgtatcaattatgagagaaagagagagagagagagagagagagagagagaga

gagaggtgtcaagtgtgaatctgcccaattctgttaatttttttatcatgttacaattatttcaatatggtatcctttagatacattcccataaaatgaa

aaaaaaaatcagtaatagttatagcaaaaatcagtatttgctacagaagataatctcaagagctgaaaaatttatgaattttcatggaaatagtaaatga

aaacacacagtggaactgaaaggcattactagtttggttatactgaataacaatctgaaagctacacaatatcttgttactgtacagacattaccctttc

aagattacaaaagactataccgcaatgctgtaaaaaaatctaaaattgtcatatgcatatcagacaagaactgtacaaaatttccaggcagcaacttttg

tcattttgtgtctttcaggggcttgcattcatgccctttatcatagatgaatagtttccactcccgattcaacatcactaatgccacagcaaatatattt

tcttttaaatttttacagcttgatgtttaaattatatcgccgggtaaaacagactgaaaaacttcaagtacagggtaaaataagaatatctcattattac

ctgaaaactgaacatttaagtcctgagtcaactcctgttctcattaagcacccacatggagtgaaaaatgaaatcctcgatcatgcaaacacacatttct

tttgaaatatgattacacacacgcaaacacgatggacttgttggacctgttcacgatacagctggcttcaatgtaaaggacaacttgggtcgtgaattgt

tctttcccaaaattttcttctgctcttccttgattcgtttttcttgctcttttctcagcctttggcgttcctcttccatctttctctgttcctcaaccat

agccaagcgctcttctgccagtttcttctgggcttcctcaattttacgattattttctgccataatctgttctaactcttcacgcttccttctttcttca

gccttgtagcggcgcagcccaagccttcttcctctgcaaactgtaaactcagcctcaaattcattctttagtactaatgatgcagtcttctttttacaga

tgatgaaccttacttagggactgacaatcagtctctggacccccggaaaaatgtttgttagattccccagtaaattacatgtatagtgacaagactatgg

gaaaaatattgaaagaaatctatagtttacaaatgggtttaaagataaggaagttagaagatttttttcaacggatataaaatcatacaggacctatttt

ttttacagaggtaaaaataatggttcatttacgttaaacaaaaaagatgagaacttacaaggataagaacttaaattctgtccaaattagtaacaattct

catataaaaacaaactatttctacatgcaactgatcaattactgctacgctgaagcttttaacaagtaatggaaattgacaatacaatgaggcctgttgt

ctcaaaataaaagcacactatatgtttttaatccatttcatatttaattcagtgaagttcaaatatccaaatataagcaaactacaaaaagaaaaacaca

aaaagtgtaaatactttttcagtctctttctttttttttttaattgcatgtctgtggcactggaactttgtccatattatactgagaaacaagagcagaa

aaatcttgtcatcgtaccacttcttcaatcacaaactggaacagtgaagcagtttaaataaaagaaatattttcaactgtacttctgagcagattaatgg

gactgcagagatattatatgcaaacaagtgcatcactcagctctttcaaatgttcccattaatctgtcc

>49623|AGAP009960-PA

aaacattcctgtgtcagctctcttatcctgtcttctgtccacaacaactttaattacatcttccaaacattgtgaacacattctagcagtaacacttgtt

gtaggtgccatgcatgggcacgaatttatctctattaaccatgggcagaaatcttctgtt

>49679|AGAP009973-PA

agagtctatttcatcaataaaaataacccatggggcccgatctttttctgccttgaagagatcacgaactcttct

>49723|AGAP009978-PA

agtctcacaagccgtccatctatcactgtcctaatctgttgctgtagttaagcaagcatcaggaaggaaatgggtcttccatttcctggcagctgctctg

tttattcactgctatgtatgtgcttgggagtgcattaacagtcttcacatgttgagatcacagtctttcaatacatcatatggatagataaactacacca

tggactctttcaaatacatatgcttgatgtggcatttgatttttttggtggtaaatgtatgtagtcacacaataaatatatgttttccacgaaattcact

ggaattgtcaacagttgccatgttgtatacattgccattcatagcagggtctgttggacaagaaagaagattaaaaccaccaggagaggttttggctaat

cgaaatgatcataatgcaagaggataccttgcacagtggcttgttagtggatgttggggtgaagactgtcatgaaggaggaaagcctaactcaaacgatg

agactgaatatgaaggttatgatggattttataacaacatggctaaacctgaccttggagccatagatacaccactcttacgaagggttccagcagtgta

tgcagatggtgtttaccagccagcaacaaagaacaggcccaatcctttggttctcagcgaacagctcatgagtggaaacattggaaacagatcgaatact

ggaaaatcagctctgcttgtatactttggtcaacaagttgtggaagaaattctagatgcacaaaggccatcatgtccaccagaatatttcaatattgata

tacccatgggccacaaatacagagaggagagtggtctgactattatgccagtcctcagaacaagatttgatatgaacactggcttatctcctaataaccc

tcggcaacagctgaatgaaataacaccttggatagatggaggtttagtatatggaacttcaaaaacatggtcagatgtactgcggactgatgaagatggg

aatcttgtacctcatggagagcttgcttcatctgcaggaggactcttcccagcactgaacaagcaacgtctacccatggccaatccaccaccaccttttt

atcacagtacccacaccaagcgacattctacagatccggtggaaagatttttcaaacttggtaacccaagaggaaatgagaatccattcctactaacatt

tggaattctatggtttcgatggcataataaactggcacgtgaactacatgagctgaatccggaatggactgatgaaaaagtgtacaatgaagcacgcaag

tgggttatagcaactcaacaacacataactgtcaacacatggttgccacaattgctgggggaaccattgccagactacaatggatatgatccatctatca

atccacagattgatcagttttttcagtcagctgcatttcgatttggacacactcttgtgacaccaggtgtttacatcagagactatgcacgaaatggatg

taaagtgggtgtccttggtgacaaattcaaaacagtgcggacctgccaacattactggagaccacatgaatccatcacaaggaaatataataatgaaaca

tcagaatttgttgacattgatcgtctgctaatgggaatggcaatgcagatgtgtgaaagagaagataatgtgattgttgaagatctcagaggtagagtat

tcggtcccttggaattttcacgtcgtgacctcatggctctgaatattcaacgtggccgagaccacgggctgcccgatttcaacacagctcgacgggcata

caaactgaagcctattgaagacttaacatatttcaaagataaagtacctgataaaatttgggaaaatctcaattcactttacaatggttccgtggataat

ttggacgtctgggttggaggcctgcttgaaacgacaaagaggggacctggtgaactcttctcagctattatcacagaccagttccagagaatacgcgatg

gtgatcgcttttggtatgccaacataaacaatgggcatttcactggagaagaaattcaacgtatagaaaacctgacagtttatgatattattatgtctgt

gacagacatggaccatgatgatattcaaaaaaatccatttgttgtgcctttacatggagacaatataaattccaaatgtaaattagaaaaaagtacatgc

ctgagtatgacaacagatgaaaatttaacatgctaccatctaccacttcttaattcatctacagatgttgaagcctgctccactttacagacatatgact

acttcagaaacagtgaaaaagcatttattgtaacataccttgcttgtggagttctcataattgttttcggagtatcactgtgggtgttgggagccaacaa

gcagaggaaagtgatggagatgaagcaaacaactgcaagtcaaatgagagaatccttccggaaggaatgtcagcagcagataactccatactccgttctt

gaatgggtggatcacaactgcgcagcacgtgaggtactacttgtgctagagaaatcccgcaagcagattcagattttaaaccaccgtgggcaactgctac

gtgccatggaccttacacatgctggagttctccaagtatttattatagctgatggccaccatgttatgctgcgtgtcacaaatgattatgatctcgtcat

aaaatttgaagcagatctgatgcgtacaaacttccttcggaatctggaggagtttctgaaacaattgaatgtaaggcaagagaacttcacagctccactt

cagcctgctcttcggcaagctatcactcgaaaggcacgccaaatgaggcttgagaagtttttcagagttgtcttctcacaggctttcaacatttcccaat

ctttggctgtaaaagagccactcagtgctggagccgcaaaggaagtaatttatacagaattaacaataaatgagtttgctgaagcactgagtatgagggc

cgactcagaatttgtaagaaagatgttcgctttagtcgacaaagatcacaatggctttatttccttcagagaatttcttgacatgcttgtaatattttca

aaaggaacagccgaagataaagtgaagttgatgtttgatatgtatgacataaatggtcatggtaaactgacaagagaagacttcaaagacatgatcaagt

cactgattgaaactgctaactctgatgttgaccaaactcatctggatgacctcatagtgtcaatgttgcgtaatgctggtttggcagacaagaatgtcat

aacgcttgaagactttcaaagcctccttattgattataaggacaagctgggatatacgcaactaaatttcccaggaattcaaggatatcaaaatgttctt

cccaacagaagtacacagttcgagaaggctagatatacagtattcagcatgtacggtggttctaaatattctggaacagagagagagccactgaacgtta

caagcagtatcatgccggaccaagatattacattacatacaaagagcctagatgattcgcaaaacacaacatacttcacactggtatcaactgttgcaaa

ttatagccgtgaaatcttctgggtcactttgtacacacttgttgtgattgcaatatttgctgagcgagcttactactattcagtggaaagagagcacact

ggtttacggcgcatagcaggctatggagttactataacacgtggtgcagcctcggccatgatgttcacctactctaccttgttagtcactatgtgccgta

acactataactacactacgtaacactgcactgcatctttatgtgccttttgatcgcgctgtacaaatgcataaatatattgctgtctgggctgttgtttt

cactgtaatccatatagttggacatgctttcaacttctacagcatcagcacacagccatctgatgatctctcctgtttgttccgtaattttcagcattca

acacatgagattcctaaattccaatactggtgttggcagacaatcactggtgtaactggaatcctcctgacactgctccttgctctggtctgcatcttct

ccacaagatatgcacggcagcatctgtataatgcattttggttgatacattcactgtatccaattttctacatacttatgttccttcatggaatgggtcg

gcttgtacaggaaccatttatggtttacttcttcattggtccgtgtcttctattcacaattgaccgtttcattacggtagccactaagaaagtggaactt

ccagttgtgagagcggaattgctaccatcagatgtgacactccttgaattcaaaaagccaactgattgtgaatacaagtctggacagtggttaagactgg

cttgccctgcacttaatacaagcgagtatcatccattcaccatatcttctgcacctcacgagacaaatctgactgtgcacattcgagctgttggcccttg

gactcaaaatatacgttctcttttcgatgactgcctttctcacgaaaagccgctgcctaaggtgcatatcgagggaccatttggcgaaagtcatcaagac

tggtacagatatgagacagctgtcctcattggaggtggaattggagttacaccatttgcctccattcttaaagatattgtcttcagagctaactctaacc

gtttttcaagctgtaaaaaggtgtactttctgtggataacaaagacacaaaaacagtttgaatggctggtagacatcattagagaggtggagaatgctga

catcagtaatgttgtgtctgtacacattttcataacacaattctaccacaaatttgatctcaggactattcttctgtacatctgcgaaaggcacttccag

cgaatatgcaaccactcactgttcactggtctacaagcagtcacccacttcggcagaccagattttccagttttcttcaggtcactgcaggccttgaacc

aaaaggtgcgcaaaataggagtttttagctgcggaccacaaccaatgacacaaagcatcgacaaagtatgtgccaaaatcaataaagagacgatacatgg

gcccatcttccaacattattttaagaatttctagaagttaa

>49724|AGAP009978-PA

ctgctatgtatgtgcttgggagtgcattaacagtcttcacatgttgagatcacagtctttcaatacatcatatggatagataaactacaccatggactct

ttcaaatacatatgcttgatgtggcatttgatttttttggtggtaaatgtatgtagtcacacaataaatatatgttttccacgaaattcactggaattgt

caacagttgccatgttgtatacattgccattcatagcagggtctgttggacaagaaagaagattaaaaccaccaggagaggttttggctaatcgaaatga

tcataatgcaagaggataccttgcacagtggcttgttagtggatgttggggtgaagactgtcatgaaggaggaaagcctaactcaaacgatgagactgaa

tatgaaggttatgatggattttataacaacatggctaaacctgaccttggagccatagatacaccactcttacgaagggttccagcagtgtatgcagatg

gtgtttaccagccagcaacaaagaacaggcccaatcctttggttctcagcgaacagctcatgagtggaaacattggaaacagatcgaatactggaaaatc

agctctgcttgtatactttggtcaacaagttgtggaagaaattctagatgcacaaaggccatcatgtccaccagaatatttcaatattgatatacccatg

ggccacaaatacagagaggagagtggtctgactattatgccagtcctcagaacaagatttgatatgaacactggcttatctcctaataaccctcggcaac

agctgaatgaaataacaccttggatagatggaggtttagtatatggaacttcaaaaacatggtcagatgtactgcggactgatgaagatgggaatcttgt

acctcatggagagcttgcttcatctgcaggaggactcttcccagcactgaacaagcaacgtctacccatggccaatccaccaccacctttttatcacagt

acccacaccaagcgacattctacagatccggtggaaagatttttcaaacttggtaacccaagaggaaatgagaatccattcctactaacatttggaattc

tatggtttcgatggcataataaactggcacgtgaactacatgagctgaatccggaatggactgatgaaaaagtgtacaatgaagcacgcaagtgggttat

agcaactcaacaacacataactgtcaacacatggttgccacaattgctgggggaaccattgccagactacaatggatatgatccatctatcaatccacag

attgatcagttttttcagtcagctgcatttcgatttggacacactcttgtgacaccaggtgtttacatcagagactatgcacgaaatggatgtaaagtgg

gtgtccttggtgacaaattcaaaacagtgcggacctgccaacattactggagaccacatgaatccatcacaaggaaatataataatgaaacatcagaatt

tgttgacattgatcgtctgctaatgggaatggcaatgcagatgtgtgaaagagaagataatgtgattgttgaagatctcagaggtagagtattcggtccc

ttggaattttcacgtcgtgacctcatggctctgaatattcaacgtggccgagaccacgggctgcccgatttcaacacagctcgacgggcatacaaactga

agcctattgaagacttaacatatttcaaagataaagtacctgataaaatttgggaaaatctcaattcactttacaatggttccgtggataatttggacgt

ctgggttggaggcctgcttgaaacgacaaagaggggacctggtgaactcttctcagctattatcacagaccagttccagagaatacgcgatggtgatcgc

ttttggtatgccaacataaacaatgggcatttcactggagaagaaattcaacgtatagaaaacctgacagtttatgatattattatgtctgtgacagaca

tggaccatgatgatattcaaaaaaatccatttgttgtgcctttacatggagacaatataaattccaaatgtaaattagaaaaaagtacatgcctgagtat

gacaacagatgaaaatttaacatgctaccatctaccacttcttaattcatctacagatgttgaagcctgctccactttacagacatatgactacttcaga

aacagtgaaaaagcatttattgtaacataccttgcttgtggagttctcataattgttttcggagtatcactgtgggtgttgggagccaacaagcagagga

aagtgatggagatgaagcaaacaactgcaagtcaaatgagagaatccttccggaaggaatgtcagcagcagataactccatactccgttcttgaatgggt

ggatcacaactgcgcagcacgtgaggtactacttgtgctagagaaatcccgcaagcagattcagattttaaaccaccgtgggcaactgctacgtgccatg

gaccttacacatgctggagttctccaagtatttattatagctgatggccaccatgttatgctgcgtgtcacaaatgattatgatctcgtcataaaatttg

aagcagatctgatgcgtacaaacttccttcggaatctggaggagtttctgaaacaattgaatgtaaggcaagagaacttcacagctccacttcagcctgc

tcttcggcaagctatcactcgaaaggcacgccaaatgaggcttgagaagtttttcagagttgtcttctcacaggctttcaacatttcccaatctttggct

gtaaaagagccactcagtgctggagccgcaaaggaagtaatttatacagaattaacaataaatgagtttgctgaagcactgagtatgagggccgactcag

aatttgtaagaaagatgttcgctttagtcgacaaagatcacaatggctttatttccttcagagaatttcttgacatgcttgtaatattttcaaaaggaac

agccgaagataaagtgaagttgatgtttgatatgtatgacataaatggtcatggtaaactgacaagagaagacttcaaagacatgatcaagtcactgatt

gaaactgctaactctgatgttgaccaaactcatctggatgacctcatagtgtcaatgttgcgtaatgctggtttggcagacaagaatgtcataacgcttg

aagactttcaaagcctccttattgattataaggacaagctgggatatacgcaactaaatttcccaggaattcaaggatatcaaaatgttcttcccaacag

aagtacacagttcgagaaggctagatatacagtattcagcatgtacggtggttctaaatattctggaacagagagagagccactgaacgttacaagcagt

atcatgccggaccaagatattacattacatacaaagagcctagatgattcgcaaaacacaacatacttcacactggtatcaactgttgcaaattatagcc

gtgaaatcttctgggtcactttgtacacacttgttgtgattgcaatatttgctgagcgagcttactactattcagtggaaagagagcacactggtttacg

gcgcatagcaggctatggagttactataacacgtggtgcagcctcggccatgatgttcacctactctaccttgttagtcactatgtgccgtaacactata

actacactacgtaacactgcactgcatctttatgtgccttttgatcgcgctgtacaaatgcataaatatattgctgtctgggctgttgttttcactgtaa

tccatatagttggacatgctttcaacttctacagcatcagcacacagccatctgatgatctctcctgtttgttccgtaattttcagcattcaacacatga

gattcctaaattccaatactggtgttggcagacaatcactggtgtaactggaatcctcctgacactgctccttgctctggtctgcatcttctccacaaga

tatgcacggcagcatctgtataatgcattttggttgatacattcactgtatccaattttctacatacttatgttccttcatggaatgggtcggcttgtac

aggaaccatttatggtttacttcttcattggtccgtgtcttctattcacaattgaccgtttcattacggtagccactaagaaagtggaacttccagttgt

gagagcggaattgctaccatcagatgtgacactccttgaattcaaaaagccaactgattgtgaatacaagtctggacagtggttaagactggcttgccct

gcacttaatacaagcgagtatcatccattcaccatatcttctgcacctcacgagacaaatctgactgtgcacattcgagctgttggcccttggactcaaa

atatacgttctcttttcgatgactgcctttctcacgaaaagccgctgcctaaggtgcatatcgagggaccatttggcgaaagtcatcaagactggtacag

atatgagacagctgtcctcattggaggtggaattggagttacaccatttgcctccattcttaaagatattgtcttcagagctaactctaaccgtttttca

agctgtaaaaaggtgtactttctgtggataacaaagacacaaaaacagtttgaatggctggtagacatcattagagaggtggagaatgctgacatcagta

atgttgtgtctgtacacattttcataacacaattctaccacaaatttgatctcaggactattcttctgtacatctgcgaaaggcacttccagcgaatatg

caaccactcactgttcactggtctacaagcagtcacccacttcggcagaccagattttccagttttcttcaggtcactgcaggccttgaaccaaaaggtg

cgcaaaataggagtttttagctgcggaccacaaccaatgacacaaagcatcgacaaagtatgtgccaaaatcaataaagagacgatacatgggcccatct

tccaacattattttaagaatttctagaagttaaaaggaacagtttctctgtgtatcaaaacattgtgcggtacagcagcaagcctaaattattaattttt

ttgaaatataattgtcactgtgatttaaagtggtcctttttttaattgtatctgacaacaatcaccacaacttaagaagccttgtcacacagaggtgcac

gagttgtcaaagcgcttacaagtatgttgtacctctgtgttacagatgaaaacagtcatcataaaataaaactagcaactgccatcaggtataataacag

aaggaaaaggtacaataaaagaaggaaaaaagtataatgtagaaagaacctatgattgtgttacattgtaattatagatatgatctacccaaattgatag

aaaataaagctaaatgcatattattatggaaaaggtacaataaaag

>49744|AGAP009983-PA

ttcgtgactagaggtggggggcgggggcggccggcgtcagtcgtccttcttgcggcgcaggcgaccctcggtctggatggtgcgagacacctggtccacg

cactcggccaggccgggccggctgccgtcgcggctgtagtcgtagccggcgaggcgcgccagcgtcgcgatgtaggcggacgcgatgcgcagcaccgaca

gcttgctcagcttctgcgcgtgcgcgtacgacggcacggcgcgccgcagcgtgtcgaacgccgcgctgatcgtgtgcacgcgcgtgcgctcgcgggcgtt

cgcctcgatgcgccgctcgcgcgtcatgttcttgtagttgcgcggtgcctgctgctgctgctgctgctgctgcgagggctg

>49776|AGAP009990-PA

cagtacatcacttgcattctttaaattgtatattgtatcttacagatatgtgtagaatgtgttatttaaaatctgattgtaatttttcattttcaggctg

catatgtggtgcatatctttccgtcctgtgagtttgctaatgaaaacctagctagaatagc

>49775|AGAP009990-PA

cgcccctgcttctccaagtgttcatagaaagcttcctggcattccctggatgcaaaatcaacttgcaacttgtgacctcgcaatccgatgcctcgcattt

ccctaacagcttcctgagcagaggcgagctgctcataatagatgagagcctggcctcgtacacgatccaatgtaatatgcagaattgttccataagggtt

gaactgcatattcaggtacttctcagaaacatgttctgtgatgccatccacccaaacacatgttgtaggcatagattttccaaaccccaatttaatgcgg

ttgttgcccatatgttcgccatccatagcacgtatagccttcacaacactactgatgtcagtgaattgacagaaggcatatggtgatgcaacgccagact

ttttaatatcaatttcaattatttctccaaattgttcaaaatgttttcgaagctcagatgaagtgacgtctttctccaggtttccaatgaataaagttcg

ggttgctttaggatgaaattcatcttgttcagcttcatacagtctcttgtattcgtggaagagtccatccttaagactcgtatcactggagcgggtagga

agatttcgaacacagatggcgagagggcgg

>49892|AGAP009997-PA

gtcagagatgataacttatactttgtttattaacattgggttgtttatttaggaaaaagcatgtcgtttaagaaagatttgattttacttttaaaacctt

actatagcataaacattttgttaagtttgtcttatatactatgcaagaaagttccttttgtgtgtgggattttgtttccaggcagctatccacaatgtga

attagataccagggaatctgagatcctattcttcctcctaatagtagtaatgatgaaaaccaagaaatctggaagcgtcactatgataaactatctgact

tccagcttcatttacaataaagtagcaaacatgatattatggttttatgctgacgtacgcatgggaataatatttagcgtaatttgtatattctttgcaa

tggtgtttcctgagccaacatacagcggcccagataaagttgtctacttcagaactgctgcaggcttggaagaacaacttgagagagacaggagagttac

gtggctggttgtgttctatactgcatggaatcctgcctgtgttacttttgcaccaatatttgctgaattgtctgctgaatatggtcttgaaaatttgaaa

tttgggaagattgatgttggccgttatcccgatgcagccaaaaagtataatgttaatgactcatctatgagtcgccagttgcctacactgatacttttta

aggatgggaaggaagttacaagacgccctggatttgataacaaggggaaagttatcaagttctttttctctggtgataacgtcaaagcatcatttgatct

gaataacttgtataatgagtgtaaatcaaacccgttgaaaaaaaggaaacctctcactgaaaggactgaaatgacagaaactgtgaataatcatgtcaaa

tctgaatagtgctgaatgctgtattaaggctagcatctgttagtattttttctctttttttgcaattggctgtgctttaagtgttttattgtcaccctag

tgattcatacaagagaacaagagccacaaccataaaaggtttatgctatgagcaagcgcacacagtggtccacggactttttttaagccaaaaatgaaac

tgtttgcttagtttgctgtacacaggtattatatagaatgtagctctaaaccaaagattcttacaaaatatcggtagagtatgaagttatgagtgcatac

atgtgaaagaacagtgcatttttctagagtaattgtttcattctccaggaagttctcttcactgactgaaatattgtcagtttcagtaccataaatatat

taaaactggatgtgttatgtcataataagctgatgctgctgaacatgtaacatgtgaaccattgcagtatcataagaagagaattatgacttctattttg

tggatta

>49912|AGAP010001-PA

gcagacatgcgccgccgcgtgctcaggttgctgatggcggccagtgtgctgatggtctcctatggcaagactgccagcaagtatccgctgcaggtgcacg

ggccgtcgcagcagcggctgggcgcgcccggccactgggctccatggtcagcgtggtcgccctgctccaggacctgcggcggcggcgtcgccgtgcagac

cagggactgccggcctatggagacccggcgccgtcgccggttgacggagccgccgcctcgcagccactgcgtgggcgtctataagcgcgtccacgtctgc

aacgaccaggaatgcccaccagggtcaaaggacttccgtagcgcgcagtgcgaagctttcaacggaaaacccttcatggggcgcctctacctctgggagc

ccttcctgcaggcgcccaacccgtgcgagctgaactgccgcgcggcgggcttccgcttctacgcaacgctcaacaagactgtggtggacgggacgccttg

ccgcagg

>49998|AGAP010005-PA

gaaacattagcggtgtgacaaaatttatgtttgacgtgtgtgagtgaagcttgtatacctatacaacaaatgtaaacataacgtcgtcgtcaggaagaca

tttggcaagggcgcagtgaaagccatttggaatgcaacagtcaactggaaaataaccataatgaacccatttgactggttccgaaagctgtggtcattgg

ctcatgtgaatttgacataccgccgatatgccattgtgtttatattgattttcttatttcttcggtattttgggccaagcatgactcagtggatatctgg

ttcaacgcctagtaatgaaggtgaagacgaacagtgcttatcaaatagtcttgctcagttctctgcagattcagaagaattcagtgctaatatcagacat

tatccagccaaagatgacgagaaaccttacttagcttttgtgggaaatggtgtttttggcttgactgtagatccagcaagtcccgtctatatcaagagtg

ggcgtacattatctttacctgcatactggtttcctatagtgtcagttacatcacaagcagaagcagcatatgaagctactgttgttcactataccacagg

cattgttcacagaattcagtgtttgccccgaggaactagtgtgtctcatcagtattatgcacataggacattgccgaacgtcttcgttcaagaaataaca

ataaacaatccaacgaatgacatacagtcattcagtctgcgccaggggacataccttaattggcctactgcagtgtcacatgttgaaccattttcaaatg

gaaaagaaagcttcagatttcaagttgtaactggaatggttgaagtatcttccaaaactgctaatgtaatgggtgtttcaattgtatcaaagtcaatccc

acagactgttgagatgaaagcacgaagcacgaagaccattcagtgtctcacagtaataaagtactctgaggctataacaaaagtggaatatgcaacaagg

aaagatattcttgagaaggaagcattaaatataatggaaagagctgtggtaaagccagatttgaaagatgatcatgttaatgtgtggaaacagttatgga

gcactggtgtcacaataagtcagtcaaagcacacacaagcaattaatggagacaaaattaatgcaacattctattatgttctatctcatgttgcatcacc

atatcatgaagatggaacttcaaaggaaaagaaagcggaactgatgaacagtttgttttatgcagaaggatgtttcggcggtcatcacacattgtaatta

tgatttgtgtattctttctgtttggagctgacctttttgtatttttatgtactcagttccttggaactttttttttcctgtgctaaaaatcgccctggac

tctttttagattactgtttgatgaaactaattctctttcattgtatggaagcacaaaaaataagtactaacaagtacaggtttact

>49997|AGAP010005-PA

gtttctgctactttctgtaccattccggggtaaaattgtaatcaaatttttaatgccattcaaattttacatcattccaagttcttttttacagcatttt

gactggaactttaattataatcaccttgtacaactgtcagtaaacctgtacttgttagtacttattttttgtgcttccatacaatgaaagagaattagtt

tcatcaaacagtaatctaaaaagagtccagggcgatttttagcacaggaaaaaaaaaagttccaaggaactgagtacataaaaatacaaaaaggtcagct

ccaaacagaaagaatacacaaatcataattacaatgtgtgatgaccgccgaaacatccttctgcataaaacaaactgttcatcagttccgctttcttttc

ctttgaagttccatcttcatgatatggtgatgcaacatgagatagaacataatagaatgttgcattaattttgtctccattaattgcttgtgtgtgcttt

gactgacttattgtgacaccagtgctccataactgtttccacacattaacatgatcatctttcaaatctggttttaccacagctctttccattatattta

atgcttccttctcaagaatatctttccttgttgcatattccacttttgttatagcctcagagtactttattactgtgagacactgaatggtcttcgtgct

tcgtgctttcatctcaacagtctgtgggattgactttgatacaattgaaacacccattacattagcagttttggaagatacttcaaccattccagttaca

acttgaaatctgaagctttcttttccatttgaaaatggttcaacatgtgacactgcagtaggccaattaaggtatgtcccctggcgcagactgaatgact

gtatgtcattcgttggattgtttattgttatttcttgaacgaagacgttcggcaatgtcctatgtgcataatactgatgagacacactagttcctcgggg

caaacactgaattctgtgaacaatgcctgtggtatagtgaacaacagtagcttcatatgctgcttctgcttgtgatgtaactgacactataggaaaccag

tatgcaggtaaagataatgtacgcccactcttgatatagacgggacttgctggatctacagtcaagccaaaaacaccatttcccacaaaagctaagtaag

gtttctcgtcatctttggctggataatgtctgatattagcactgaattcttctgaatctgcagagaactgagcaagactatttgataagcactgttcgtc

ttcaccttcattactaggcgttgaaccagatatccactgagtcatgcttggcccaaaataccgaagaaataagaaaatcaatataaacacaatggcatat

cggcggtatgtcaaattcacatgagccaatgaccacagctttcggaaccagtcaaatgggttcattatggttattttccagttgactgttgcattccaaa

tggctttcactgcgcccttgccaaatgtcttcctgacgacgacgttatgtttacatttgttgtataggtatacaagcttcactcacacacgtcaaacata

aaatttgtcacaccgctaatgtttcaaaacaaacttacaaacccaaag

>49996|AGAP010005-PA

catctgcacaggaatctgcgaagctacatagctgcgtggaaactgccgcctgttttgcgttgttgtgtcagagatgtcttacaagtacttagattctagt

tggaatatctttgggtttgtaagtttgttttgaaacattagcggtgtgacaaaatttatgtttgacgtgtgtgagtgaagcttgtatacctatacaacaa

atgtaaacataacgtcgtcgtcaggaagacatttggcaagggcgcagtgaaagccatttggaatgcaacagtcaactggaaaataaccataatgaaccca

tttgactggttccgaaagctgtggtcattggctcatgtgaatttgacataccgccgatatgccattgtgtttatattgattttcttatttcttcggtatt

ttgggccaagcatgactcagtggatatctggttcaacgcctagtaatgaaggtgaagacgaacagtgcttatcaaatagtcttgctcagttctctgcaga

ttcagaagaattcagtgctaatatcagacattatccagccaaagatgacgagaaaccttacttagcttttgtgggaaatggtgtttttggcttgactgta

gatccagcaagtcccgtctatatcaagagtgggcgtacattatctttacctgcatactggtttcctatagtgtcagttacatcacaagcagaagcagcat

atgaagctactgttgttcactataccacaggcattgttcacagaattcagtgtttgccccgaggaactagtgtgtctcatcagtattatgcacataggac

attgccgaacgtcttcgttcaagaaataacaataaacaatccaacgaatgacatacagtcattcagtctgcgccaggggacataccttaattggcctact

gcagtgtcacatgttgaaccattttcaaatggaaaagaaagcttcagatttcaagttgtaactggaatggttgaagtatcttccaaaactgctaatgtaa

tgggtgtttcaattgtatcaaagtcaatcccacagactgttgagatgaaagcacgaagcacgaagaccattcagtgtctcacagtaataaagtactctga

ggctataacaaaagtggaatatgcaacaaggaaagatattcttgagaaggaagcattaaatataatggaaagagctgtggtaaagccagatttgaaagat

gatcatgttaatgtgtggaaacagttatggagcactggtgtcacaataagtcagtcaaagcacacacaagcaattaatggagacaaaattaatgcaacat

tctattatgttctatctcatgttgcatcaccatatcatgaagatggaacttcaaaggaaaagaaagcggaactgatgaacagtttgttttatgcagaagg

atgtttcggcggtcatcacacattgcaagcgaacaacctgtggcaaggcctttcatctatagaagaagtgaacacagcagtatcttattggttacttaca

cttgaaaaacagggctgccataacttactcaaagcaggagctcctggtgtaatgcaagcaatggttttgagtattggaggccttcgctttagcaaccagc

atttagaattgaacatgcatccaagtgacattcatagagaatatgtcttcaggcggatcagctatggaaacctaactcatgtgaacataagtgtggaaat

tcaggacaacaaacctgtgctgtatgtagcacttgatcgaagtgataaatattattatggatgtgatgccggttgcctggaccctccagttttgctaaga

tcagagaagacaaagttccaggtcaagctgactgagccattaacagcaatactgtatattacatatgacaagcaacacatggaagatctcaggcatgcaa

tacatgtgaaaaacgtagaggaagctcctccactgcatagccatataatttcactgcataagcatggacatcatcttggattctcaacaatgttttgggt

gttcatagcattaggaattacaacatttcatatttttgtttttagattaatttacaatgaacttttccaccgaaatgacaaattaccgcaaaggactcga

tatacaggagatctaaacaatatgcaccggctataatgttgtatttgtttaagctacttgtggaacagttatattctgataacaaatatttgaacatcct

gcatttatagtattgtggctgtttcattagtatttaagcaaaagatattcatctgatcaagaatgtcatgaactggcatggtgctctgaatttttcagtt

aaatttggtttactttgtttcaaacttttgagatgatggtatagaagtgcatttagtagagttcagcaaagttcagtgatctagtgaaataggctgttat

gaaaagtcatgtgatgcagacagacaatatcaaatatagaataattcttgaaagggaaaaatagaagaaaagaaacagataatatgagttacttgtaaat

agtatttaaattgtagctttatcttttggagtgggtggtggtgagtaatatcctgtgttgtgcttgaaagagaatctgttgctgttgtattaatcattct

catctgagaatgtgtttggtttcctcagtggcaagaattttaaaattgagtatattcgagatgaagaggaagatatagggtcggtcagatgaagtaaaaa

gcaatgtgataatacttgtgtgaatagtaaatgaacagaaacattcataaaacaaaggagtgcattaaagaaacaaacaaacaaaatcaaatttaaacca

tagaaagagtgactgaatgttatgtaacaaaaagtagtttcagaacagtacaagatgatgtgtcatcctctaattgtgtagggaaaaaatttggaatgga

aagtaagaaagaaatattaacagtaaaagacacttcaaaatttgtataacacacacacaacttataattttggctaactcatcaacagaagggtacacta

aattataatgttgttagagaaaactcaccccacagagattttatggggtgagaatacacccatactatttcgtgtagcgtacttaattatatcagcactg

ttagctattttttagtcctctccaaagctactatttcattctccacccaacttctgttaacaaaatgtagttatgcgtgttttacttccaggctgctttt

atgcgaagtttctttaattttaaagtatttgtaattcatgaagcttagtgattggtattgttttaaatttcatttgcttacacataacagtgagaaggtt

ctgttctctttacaaacagactgccatggttttgtcatctacagtttgcagtttcattgcttttctgcagtattttcaatgtaatgtaagctccactgac

taggtggatgcaaagtgcatttaagtactaagttattttgtttgaaatttcgtatctaccagattttcacttttagtttcacttctgttttgtctgtcac

tttttaaattttcatgtcatgaagtaaagttgtagtttctatttttatgttgcttgtacatcagttttccgttgtcttctttgttatctttggtgctttt

gtttgttctcttttttatttactcctcattgccccaattttattctgttctagcttcctcacttttctcctttcataattctactcttttaatttttttg

actactttttttttgtttaatcttacttctgtttggtttctttttatttttaattgaatgaccttttgcccactttgtagcaacctataaatatgtatca

ccagtctagtccattgctatttaccatacagacaagaaactcacatgtacttacttcattgccttcattatcaactttttctcctgatttttttgtttta

aaattcttgccaccattgggcattcctggagaggttaacttctttactttctttctttttaattatagaagcatactgcacttcaaaagtagagttattc

tctgttgttatttgttacacattttatcaatgtacagtatttctttatcctgagataggcattgaaatatagatcttgcagtgcaatgctaatatggagg

ttagaaagatgtaaataaatgaagtttggatgccttatttttttggaaaattttgtacgtgatgacaaaatatacatgtatgtttcaaatatttcaagac

tgcgcccacttggggtttgtacaaagcataaactagcagaagtaatactgattaaattataacaataatgttaataaaaactttacagttatctggtagt

cacacttattgatgtaaataaactttattactgacgacacaacgtatggttatggttgtacttagcacaagtttggaaatttgtccttatctgtttaggt

ataatggaattgaaaaaaaaaaaatagaaagaacttaaaccatatgtaactaacaggtgccttacagagttactacttttttaatatacagcctttaaaa

cttcgtctgtttgtacaaagtatgtgttaaatattgcatcagcagcctcctctccatgtgacaattgatagctagggcctttcgtacttactatgtgtat

aattaaacatccagaaatgttttgttgtgcagtgtttaactttttgttggaaagtgcataattgcaatacccatattatacttatattgttgttgtgaag

tccagtattttatgggattgtttacatgtttggagaaaattgtacatgtttcttgtatttattcaaaattacagatcaactctgaaccacagattgaatt

agactttacttccag

>50543|AGAP010021-PA

ctaatatcttcatttgtagctttacgatagtaaggaaatctttcatctaaataaaactgcagaatccaaaacaataccaaacgtattgtccaaaataaga

gatttacagtgataagtacgcccaggcgtttctgccagcaacagacctggcgttcgccgtgcctggctgacgtgacgtcgccaacgggcgccgagtgcat

caccatcgcgggcggcgtcagctactgcgcctgccccaagggcttccgcacacgccacgacggctcctgcgaagacgtggacg

>50770|AGAP010024-PA

ggccggcatgtgcacgatggcaggtgattgacaacctggcagtctgcgttcgggccacaagtgccagggcacgggtcttggcacttgttacg

>50771|AGAP010024-PA

tgcatgcgccgtgctttctggatttctgcacttctgattgttgcatgcttcattctgactgcaatctgagctcacaacacactctggcctgcatgatgga

ggacttccaagg

>50772|AGAP010024-PA

ggcggaggtggaataggtctgcattgaataaatgcattgccagacatgccctctggacaactgcagattggaatatgattgacaacatcacagcgtgcat

cctgcccacaagtccctggacaaggatctgcacacttgtttcttatgcaagcctttgt

>50773|AGAP010024-PA

ataaatgtgtggatccatgtccaggaacttgtggtcaagatgcccaatgtgatgttgttaatcacattccaatatgcagttgcccagagggcacatctgg

caatgcttttattcagtgtagacctattccacagccaccggtgacacaaccgtgcaacccatctccatgcggacc

>50768|AGAP010024-PA

gaacaggatgcccgctgtgatgttgtcaatcacatcccaatatgcagttgtcctgaaggaatgtctggcaatgcatttatccagtgcaggcctattcctc

ctcctgtggtgacgcagccatgcaacccatctccgtgtggaccaaacagccagtgtcgggagattaatggccaagcagtgtgttcttgtgtgccaggata

ccttggaagtcctccatcatgcaggccagagtgtgttgtgagctcagattgcagtcagaatgaagcatgcaacaatcagaagtgcagaaatccatgccca

ggcacatgtggtgtttcagctcgctgtgaagtagttaaccacaatccaatttgcagttgtcctacacgatatactggtgatccattcgtcagatgccagc

caatctccgatactcctccagcacaattaaatccatgtgctccatcgccttgtggtccaaacgcacaatgtcaggttattggagacactccatcatgcac

atgccttccagaatacatgggatcaccacccaattgta

>50767|AGAP010024-PA

cggcatccttcatacgggttgcccacgtactctggcagacaagtgcaagacccggcgccgttctgttctctgcattcagcatttgagccgcagggcgaag

gcaggcatggcgtcggtctctcttgcaccggtggctgtgaagaaacaagacacttagagaatgggtccccagtgaatccaactgcacacacacatgtagg

cgtgtggctgaccactctgcactctgcgtttgacccacaagctcctggacaggggtctttgcacttctgcccgatacaggccagatggctggcacactcg

ctgttgctgatgcactcgggccggcagtttggaggcgagcccttgaattctggcaggcatgaacatgatgggctgtctccagctaccttgcactgagcat

ttggaccacatggtgaaggctggcaagggttcactggctcgctgaccacagggttctcaggtatgggcagacatctggagaacggatcgccggtgtaacg

aggtggacagctacaaattgggttgtgattgaccacctcacaacgcgctccaattccacacgttcctgggcagggattcctacatttctggttactacat

gcttcgttctggctgcaatcggagctcacgacgcactctggtctgcacgccggaggacttcccaagtatccaggtacacaagagcacactgcctggccat

taatctcccgacattggctatttggtccacatggagatggccgacatggtagtgtaactgcaggaggtggtactggtcggcactggatgaaagcgtttcc

agaggtgccttccggacaggtacagatggggatgtgattaactacatcgcaccgagcatcctgtccacaagtccctgg

>50769|AGAP010024-PA

ggacattcagcatttatactacactcaggtctgcagtttggtggcattccaatgaagtttgctagacaggcacatgaagctgtaccacctgcatttatgc

actgagcattgggaccacagggtgatggagagcatgggttaactggtcgaagcacatctgctggaggaggagctggcacagcaaaacagcgtgtgaaggg

atcaccagtgtacccagcttggcagctgcaaattggactatgattaatgacttcacagcgagtgttgagaccacaggtaccagggcatgggtcagcacat

ttctgtctaatgcaagccctattttgctgacattctgcactcactgtgcactcaggtctgcaattaggaggtgtgcccatgtaatccggaagacaagaac

atactgcttgaccatttacctcacgacatgcactgttaggcccacaaggagatggcacacaagggttaccaatttcaactggaacatgttctattatagg

actgcagtacttgaatggatctccagagtacccttgaatgcaagtgcatgaaggcaggtgattgaccacttggcaatcagcatttaggccacaagttcct

gggcatggatcctgacatttgttacgtatgcatgctctattggatggacagtcagtattcaaaatacattctggtcggcagacctcataaggattaccaa

cataatctgggagacatgtacaagagccagctccattctgctctttacaaactgcatttgagccacatggtgatggtatacatggtgttggtctttcttc

tggcacaggtggttcctgtaataaacactggatgaatggatctccagaaaatcctggaagacatgcacagttaggtgtgtggctaataactctgcattct

gcattagatccacatgaacctggacaaggatctttacagtgttgcccaatacatgcaaggtggcttgcacactctgtgttgctaatacactctggtcggc

aattaggaggtgatcctttaaaatcgggcaaacatgaacaagatggactgtcaccagaaacttggcaaactgcatttggtccacagggagacggctggca

agggtttgttggttcccgtaatggagtttcagggactggatagcatcttgcaaatggatcgcctgtatagtgtgagggacaactgcatataggattgtgg

ttaacaacctcgcaacgagctccaataccacatgttcctgggcaagggttccgacatttctggttgttacaggcttcattttgtgcacaatctgagctca

caatgcattctggtctacatgctggtggacttcctaagaagcctggcacacatgaacacactgcctggccattaatctctcgacattggctgttagggcc

acaaggag

>50825|AGAP010026-PA

tttttgttataattttgcagataatgataccactctcactttatgtgacaatagaaatggcaaaacttatacaagtgtaccatattcataatgatgtcaa

tttgtatgacccagaaactaacaaaaggatagagtgtcgtgctttgaacataccagaagagctgggccaagtgagtatcaccacataaatgattgtaaat

aaaagcaccgcc

>50943|AGAP010039-PA

gcttcccctgctttccttccagtgattcaataagtgcagtttcttctacaaatggtcggagcaacagccacagtctcatcatttgcaacagttgtagggc

agccaaacactccaacatcggctgg

>50944|AGAP010039-PA

acctgctgccacctaactattgctccagccatagtccctccccaaataaagagcaaatacggacccctttctgatgaagatcgcatcttcacaatcttgt

atggtcgacatgactggaggctgaagggtgcattgaaacgtgg

>50958|AGAP010044-PA

cgactctctctctgacaccccactacaccccacacgacaagccggctcccccgccgtgctggtgcgacgcgagggcgcggtgtgtctcgccacatcggca

gttcgtccctcgatgagcgtcaatccaaacatgcagacctggcttaccggcctcggtttgatactttgtgtccattaactttccttctcgaattttaacg

tctcttaagcacacatccaaacgccccccctccaccaagtctctctctctctctctctctctctctctctctctctctctctaagaaactttgccgtttc

catgtaactattctgttttccgcaaatgtatagaatgccagtgtagattacgcgttggtgtatgtgtgtgacttagtgttggagctaaaagctggtattg

ttgtaacccgtagattgcgagcagccgcgtcggaaggaaggctgggtttcggtagcgaacagcgggtagcggtttgagtgtcgcgtagcgtagtgagagc

ccgttgacggcaccgtggctgtgttgcgaaggttgcagcagcagcagacgtcgccgccgctggagctggagcagtcggtgtcgtcgccggcggggcagcc

tccagccggcgccgcccacggccgccgccccctcaaggcgggggcggctctgcactcgacggtggcgcacgtgctgtgcagtgcctccggtaccagcgca

gttgccatcgataacaagattgaacaagcaatggatcttgtgaaaagccacctcatgttcgcagtgagggaggaagtcgaagtactgaaggaacgtatcg

cagagctcatggaccgaatctgtcagctcgagacggaaaacacgatcctgaaagcgcacgcctctcaggaagccctggcacagttgggcctaaataatca

gggccagcaacagcaacaaactccgtctcagcagcagcagctacaacagcagcagcagcagcagcagcagcagc

>50993|AGAP010064-PA

ctgaactgctgatcaacatcacagagcatgaacttgtacctgagcatgtcgtaatgacaccagaagaaaagcaagagttgcttgcaagatacaaattgaa

ggagaaccaacttcctcggattcagacaggcgatcctatttgtcgatactttggtcttcgtcggggacaggtggtgaaaattatcaggccttccgaaaca

gctggtcggtacatttcataccggctggtgtgttaactgcaactgatgatagaatgctaatttcttgttaatcattctgtcttaagttgaaaatgtaaca

taaggtttttgtaaataaattatgcctattttatgtagcatttctgtaagagttttaagtatggacaactctctgtaaactccctttttttgcattaagc

aagacatgaatttcaatttctcaacaggtttaaaatctaattatgtattttgtaccaacaggaagcaaccatactctttaatatggctagtaatttgaag

atagtactaacatgcatgggtttgtttctgatcacttattatttaattaatttaaataaatgattatcag

>51058|AGAP010078-PA

agtacatcggcgcgtccgccgacctacgcggcggcttcgactggaacctggtcttcaagtgggagtacctcagcgccgaggagcgggctgcgcgcaagaa

ggaccccacagcccccatacggactcctatgattgccggaggtctgttcgtcatcgacaaagcctactttgagaagctgggcaagtacgacatgatgatg

gacgtgtggggcggcgaaaacctcgagatctcgttccgcgtgtggcagtgcggcggcagcctggagatcatcccgtgcagccgcgtgggccacgtcttcc

gcaagcgcca

>51057|AGAP010078-PA

ccaatctttgctgattaaaaacaatccaccagcaatggttggactcgtgaaaggttcagatggatcttttttactttctttctcttcatttgagagaggc

aaccatttgaaatgaagactccaatcaaaacctcctttcagttgagatgaagaagagtggtattcaaatgtgtcttggtttatcacatcaatgacaggac

tagcaactagcagaggattctgtgaaagcctgttgagaagtggctccagccatccagtgttaacctcacagtggctgtccagaaaaactaaatagttgcc

tcttgcctccctggctccaatgttgcgtgacctgattaggccttctctgacagtattccttatgacccgaacttttggaatggcttcaagaagcgtaccg

tcctcctcgttgtcactgaagtcgtctacaagcagtatttcctccagcagctcggcgggtgtgcggttgattatgctgacgacagtccttaggagggcgg

agcgtgcctcgttgtggaaggaaatcactacgctcgttgtcttgtcgctgtctatggtatatatcatctctctgcaactgtggtgcctggtgtcgggcaa

gtggcggtcgcagcggaggcgggacctctccttcgagtcgggttgcttgagagtgcagctccaggggggtggtaagctccatctgagactaaatatggcc

acgaggccgatagcgaac

>51079|AGAP010089-PA

ctacctgaccgacgatgaggacaccaaggtgttcgttcgactacctgaccgacgatgaggacgccaaggtgttcgtgggctgcatcttcgcgtggtcct

>51082|AGAP010089-PA

tgggatcattgtcgacagaggcgtaagggcagcccttttgccgaaggcgccgatcatggccaccacggcgtacggtgtccaggagcagaggagtcagaag

aaagc

>51081|AGAP010089-PA

ccacttcatccgcttctgcacctccgctctgaacctagggtgttcgtgggctgcatcttcgcgtggtcctatgcattcccactgtgcctaatctgctgct

tctactaccgcctcatcggagccgtccgggag

>51083|AGAP010089-PA

ggtcgcgcagcatcttctcgtgctcccggacggctccgatgaggcggtagcagaagcagcagatgaggcactgtgggaatgcataggaccacgcgaagat

gcagcccacgaacaccttggttaacttcgtcggtctaggtactcggtagaggctgaagtacttattg

>51080|AGAP010089-PA

taacggtgcctcccgcgttgacggtggtcggcgtcacagttatggtgtagggcgacggctttgtgctgggtctggacgctgcccttcacgctgatgccgc

ttctccgcatctggagcaggttcaccgcagagggtttcctgacgacgtgttcgttcgactacctgaccgacg

>51137|AGAP010133-PA

cctggagggcaaacactgtgcgccggcggggctcatgaacgcgtccgcctgtcgcttcaacgcgcccgtcttcgtctcgaggcctcacttcctgctctca

gacccgtcgctgggcgctgccgtccaggggctgcagcccgacccggacaagcacaacttcacgctcggcattgagccgttcacgggaatccccctgtacg

tgaacgcgcgtctcatgctgaacgtcatgctggagcctgtggagctagtttcgttctacgaaaagctgcccggaagaatttattttccggctttctggtt

taatcaaaaggcagtcctaacagaagatttagctatgctggcaaagtttattaagaatgcgcctgccctcggcatgggtcttttctttggcatggcaggc

atcggcttgataatggtgttcgtcggcggaatcttgacgctgtgccatggatggaaagattcgtaactggaccatttccctcagaataaactacacctgg

agtcgccggtatgacattttgtgtcggatatgacaagtgcactggacgaggactgtctttctaagtacgcctcacagcgtgcattagatcgagccacggt

cgtcgctcccgcggcatcagattcagcagcatctcacataagaaagacaagaatagacaaatacttttctgaacgaattatcagtcctgatacaacgtaa

catagagtctgtggtgtacatcacctgcctgaagcttcttttgtgatttttaggggttctcaaatgactgcttgaagttatgcactcgggagagacagat

ttaatgtctgttatgtcacacagtctgaaccagccgtcgcttcattacagcaaacactcgactgcctgttgaaaatttgatcctgggacggaatttgacg

ctggtgtagatgcttaatgctaattaaaaagtaaatatacaaaactgacaaatatttaaatttcttcaatgttatacgtttgttacagattctttgtgat

acttttatactgccaattatcttgtctaccacgagcactttttcttttcctgtcaacagcgtccagctcagccggacgagtcggaaaaattcagcaacta

ctagtcactgtagttagacaacttttaacgctgtaacagtcaatgtgtaagacgtaactttttgtgtacggttttctatatcataaaaaatacaccaaac

>51167|AGAP010141-PA

atgtttcaggcataggacggatgctttagccttcttcgaaaacattaaatataacgatgcaaaattccagttttgcttcgtttgcttgtcatctatgcaa

tgctgcactccgaaatttccactcgtcaaggtatttacagaaaacccattacgaagttctgaaaatagcacgaaatagctcaaataaggacatcaaggag

gcatttattaaattatcgaagaagtttcatccagacatcaacaaaaataacccagacacccacaatgagtttgtaaaactgaatgaagcgtacagcatct

taagtcgtccagacaagaggcgggagtatgacctcagcttgcaggttgctcacccatcaccacaccccactggtggccccaatgtatacgagaatgacat

ccagcaggacccatggaaggacccatcattcttcaggaaccgtgatcgcagccgggacgatgagaaccgcagcaagccatactacggcatcggcaagctg

ggccggctgcccaactcgtggctggttgccttcctgtttgctgtaactgctgtcggtgttgctctgcaagctgtagctatcaggcactcgatgacactgc

gccgaagcgctctagatgaggcgagcgcacgccacagcgtgcttcacacaaagatgcaggagcaggcgcacgacgccggcaaccacaataacctgcgcat

gatgcactcgcgtctcggtggcaccagtgcggggtttgcactcctcatgaaagatggcaatcgggagccggcagaaaagcaaccggtggaaaagccgcca

tcacccatcaaagttgtggatgatcttccagcagtggatgtgaaagcagcttcaatcggctgaagatccggatgtggtctaatgcctgacattcacagtg

ccctgctgatgattccttttatttatgcttgaagcacagaaattttaaatgaagatttatttttctaagtcctttatataaagatgttgaaatattagtt

gttaattcttttgctgttatgtgtcactgcaagatacgtgaaactgaaacctagtacaatgaactgtgataattttgtaagtgaaaagtaggtaaaattg

agtttgctcaaaatattgatatcttactctgatgtaaacaagaaattacttagtgcagctacatataacaataaggatacttaaacacaaaagattatta

cagttgtacagtggtactctctctgttagtaacgctcagacatcttgtctctttactactgctaagtagtgaagatggctgtgataccatatatactcat

cccattttccctctctttgatctctgtatggctgtctaggttttaagatttccatagtttccgtaaacagcatgacaatcaccaggatggtgtctttgga

aagtactggctagtgcccttcctcaatccaaacttgtgttacatcaacatacacactccgtaagccaccatatggtgtgtatggtggaggtatcctgtat

gaccactagtcatttcctttcctgtttcacttgcaaatatagtgagggcgaaaccctctgtaagtctcccatcattctcggcctcaaaaagcagttctct

aaatttgtctttcctccagtgattcccatctgagttcccaaatcatctccaaaatacttgcatgttgttcgaacctactggtaacaaatcttgctcttga

ccattttgcccatggaacattgttgagctatccagatttacattccctaaattgtgttaggtaaatgccagactggtaccttccgaaaagacattgcaaa

tgttattcctttctaatccaatcttctgctctgtctattaataatcctgttgttaatgggaaattaagatctaatctcacttcccttttatgattcctgc

gaaagcattttttgttgtaaagcatgatgtttaacactgttgcagatatttgtaagccatcttgttggtttgatgtcataaatgcagaagactatctatg

atcatgtttaaatatcagtactacataatttttaggtgaaaagaaagaaggaatatccatggaatattagtcttcaaaattgtacacttattgtcggtac

ataagtagttcttagttagatcgattccaagttttgtttgggaaagctattacactacaaggagtatttacctccatgaggtaaaattaaaagtttcacc

aacccaaaggttggtttgagcatcatgaagctttagtgggtgtaatcctactggagattgaggagaagctgttgcctggtttgtgcgagcacccagccat

tggctaaacgatgcttactatttaaagtggactctgacctgtaatggcactcagcattttttacaccaggagtgaaaggaatgataagttttgtaggact

gaccatggacctgacgccttcagattgcgatgcccgacccagttctctccttatacggatcattttttgtctataaatgactgggggtggcaattggact

tgtaattatagtgggcagacactcaaatgcagtaagacctcagtatttatttcactaaatgctctaagcattaattttgaacagctactgtgatggtgtt

gtttgtaatttttgtaagaaacatctttacacaggattgaccactgactgaaaatgagtttttacactgaggttaccatgtaatttttaatgtgaagaac

ttcttaaactgatagtacttcagtgcaatgtaacaataatttactgcacttttgttgaaacataaatttactttgtcacacatcatcaatcagtaaaatc

atatgttctgaacaccacagtaataatctttcttttagtaatgaaaatgtgcacaataccttttgttgtcttctacatctgcttttattatgcttttcat

tttaataaagtagctggtattttttacacagtgatgctttgtttaatatggcaaaaagaatattaagcactgtatacatagcta

>51200|AGAP010147-PA

gaagaagacccagggcgtcatcaacgagaagaccgctgaggggttgcgggctgctgaagacaaggtgacccacttgaacaaggtgaaggccaagctc

>51195|AGAP010147-PA

ttcgcttggccttctcaacgtcgccacgaagtttcttctcacgctccagagagtcctctagttcgtccagcgtctgctcgagcttggccttcaccccggg

ctgtgctggatggtctgctcgagttccttcttgttacgctcgaggtcggccacggcttcctg

>51196|AGAP010147-PA

tttcgcttggccttctcaacgtcgccacgaagtttcttctcacgctccagagagacctccagttcgtccagcgtctgctcgagcttggccttcaccttgt

tcaagtgggtcaccaaggaccaccagatccgcaacctcaacgacgagatcgcccaccaggacga

>51198|AGAP010147-PA

tcttcagcagcctgcaactcctcagcggtcttctggttgatgtcgccctggttcttcttctccttgttcaagtggttcaccttgtcttcagcagcctgca

actcctcagcgggcttctggttgatgtcgccctggttcttcttctccttgttcagcttgttgatcagctcgtcctggtgggcg

>51194|AGAP010147-PA

ccaccagatccgcaacctcaacgacgagatcgcccaccaggacgagctgatcaacaagctgaacaaggagaataagaaccagggcgacatcaaccagaag

accgctgaggagttgcaggctgctgaagacgagctgatcaacaagctgaacaaggagaagaagaaccagggcgacatcaaccagaagaccgctgaggag

>51205|AGAP010159-PA

gaattctttgtggcgaacgcgccaagaattgtttacgcgcgaaagctggaggtggaagtcacagaggaccgagatccttgtgcataaggactttaatctt

gttgttcacggtacgttacaatttgccagatgcctattgccactgcgacactccggtttgtgaagctaacggaaaatgcatttgctccttcgaaagaatc

ttcaacagcagcaggatatgacctgaaaagtgcatatgatatagttgtccctccacgtggaaaagaacttgtaaaaactgatattaagatacaactaccc

aaaggttgctatgggagaatagccccacggtcaggtctagctttaaaacatcacatagacgtcggagccggtgttgttgatgaggactacagaggaaatg

ttggcgttgttctttttaatcattcggataagcccttcaacgttcagcgtggcgacagaatagcacagctaatatgtgaacagatattctatccagatct

ggaggaagttcaggaactagaagataccgatcgcggctccggtggatttggttcaacaggcacaaagtgaagttccatttctacaatcagtgcattatcc

ttacattaagtggtaatttttctgatctgtttttt

>51204|AGAP010159-PA

ctccataccacttgaggcgctgctgtcggtctgatttttcccactttcaagacaggagttgcgtttctcttttttcttgttctgtgtttatttttccgct

tctggcagcactagtgaaatcgtaaagttatggtatccaaatagtatgggttcgttgtcgtaacagaataatagatgttgggctgtgacgtaagaagtcg

gagatgtgtgcattaaatgcgataggaagaaagctttaaaaagttgattacgcatgaatattcttgctgttacattcacgtaacataagccccttgttct

caatggatttatagccgaacacacacctactgttaatcgttaactgctgacagttgtttgtatataaaatgacaggacggagaaatgcaatattgaaatt

tgtgaaactgacagagaatgcattttcgccgataaaactctcaccaaaatctgcgggttatgatctcaggagcgcttacgatctttgtgtgccaccgcac

gggaaggaactagtgaaaatagacattaaggtacagcttcctgagggatgttatggtagaattgcaccgagatcaggccttgcactaaactatcacattg

atatcggagcaggtgttattgatgaagattatcgtggtaatattggtgtggtactgttcaaccattctgacaaggaatttgttgtgaaacgaggtgatcg

catagcacagttaatttgtgaacagatactacatccagaattggaggaagtacaggagctggatgacacacagcgaggatctatgggtttgggctcaaca

ggtataaagtaattgttatgaataaaaattatttcctattgtcgttttcagtattattgcattagagcagtgtcagtacacagtacagg

>51203|AGAP010159-PA

cgctgctgtcggtctgatttttcccactttcaagacaggagttgcgtttctcttttttcttgttctgtgtttatttttccgcttctggcagcactagtga

aatcgtaaagttatggtatccaaatagtatgggttcgttgtcgtaacagaataatagatgttgggctgtgacgtaagaagtcggagatgtgtgcattaaa

tgcgataggaagaaagctttaaaaagttgattacgcatgaatattcttgctgttacattcacgtaacataagccccttgttctcaatggatttatagccg

aacacacacctactgttaatcgttaactgctgacagttgtttgtatataaaatgacaggacggagaaatgcaatattgaaatttgtgaaactgacagaga

atgcattttcgccgataaaactctcaccaaaatctgcgggttatgatctcaggaggtgcatctctcacttgcttgctatcaattaaaatcctacactgca

cattagcgcttacgatctttgtgtgccaccgcacgggaaggaactagtgaaaatagacattaaggtacagcttcctgagggatgttatggtagaattgca

ccgagatcaggccttgcactaaactatcacattgatatcggagcaggtgttattgatgaagattatcgtggtaatattggtgtggtactgttcaaccatt

ctgacaaggaatttgttgtgaaacgaggtgatcgcatagcacagttaatttgtgaacagatactacatccagaattggaggaagtacaggagctggatga

cacacagcgaggatctatgggtttgggctcaacaggtataaagtaattgttatgaataaaaattatttcctattgtcgttttcagtattattgcattaga

gcagtgtcagtacacagtacaggatgacagtctcttgtatattgttcatcattctgtaattaaagaaactgat

>51202|AGAP010159-PA

ggaccgagatccttgtgcataaggactttaatcttgttgttcacggtacgttacaatttgccagatgcctattgccactgcgacactccggtttgtgaag

ctaacggaaaatgcatttgctccttcgaaagaatcttcaacagcagcaggatatgacctgaaaagtgcatatgatatagttgtccctccacgtggaaaag

aacttgtaaaaactgatattaagatacaactacccaaaggttgctatgggagaatagccccacggtcaggtctagctttaaaacatcacatagacgtcgg

agccggtgttgttgatgaggactacagaggaaatgttggcgttgttctttttaatcattcggataagcccttcaacgttcagcgtggcgacagaatagca

cagctaatatgtgaacagatattctatccagatctggaggaagttcaggaactagaagataccgatcgcggctccggtggatttggttcaacaggcacaa

agtgaagttccatttctacagtcagtgcattatccttacattaagtggtaatttttctgatctgttttttctacgttggatttgtttgttcattgcagta

tttatagttttaaatgcatttattgtttgtgatgttcaaaagtagtgaaaatagggattacgtagatattttttttcgtctttccttgaagttttaaatt

aaagatgtgtttgtaaaatttttattaatgcatctgggataaacaagtacaaaaaattcaatctttccgttttaacagtatatcattcattttatagaac

tggaaaaaactttgaactcgattttggacaatgtaacacctatggttgttaagtagactatatttcagggttaagtggacttctgttaaataaattatag

cacactaaatataaaaacatgctgcaacattagcaaatgagtgatgtgtgaatggggagaaatcatggcagtaacaaaagtagtgcccatggtcttcaac

aacagatatcatatcgagcccactcatgccctgtgttggattggatagtttgacaattatttcacatggagaaacaatggtggtgtcttcctccgcagct

tcagattttgtaatagtaactggtactgtatcttctaaaaatttaattttacagcagccatcacattttaatgacttcaacataattgtgactacttggg

gcagttttttcccgtacttcaattacagtgaagtc

>51207|AGAP010167-PA

tgtggctccagtctgtgtttgtgtgtttataagtatgaagcacgcgtgacgtcggccgtcaaatggggcagggtgcctcgtcgcatcagccgctggagag

gggccgtgggacggccaacgctgccaatggcgtccccacaaggagcaagtccttccgccgcagcaagcggcaggcggggtcggcgccgcgcggcatggac

cggctgcggaggtcgctacgggactctctgcgccggcgcaaggagcacgtgcccgagagctccaagccgcaccaatggcaggccgacgaggcgggcgtgc

gcgccggcacctgcagctttcacgtcaagtatttgggatgtgtggaagtattcgagtcaagaggaatgcaagtctgcgaagaggcactgaaattattacg

ggtaagtaaaaacaagaatgggtagctgtcatagataaagtttccacatactgtatttgttcgtaatgttaagatgacatttaacatttgtttgctgaaa

ggatgaccgaaatatttgtacatagcatcaaaatgaaaataaagatcattttgtgcacaagagtgaggtacaaatgaaagaaacactcagtaacactgga

aatttgtggatggaataatatgtaaaataaagggaaggcaaccacacagcaatagccgtctgatgttcggtgcatagagacatgcaacagaaaacagctt

ttgattcttgctgtg

>51214|AGAP010198-PA

gaaatacaccgtggcgggtagttgtgtacgttaggcgtataagcggcaaacgtaggtttttctgagatggttgttgaaggctctggtaaaatgtttgttt

tgaagagagacggaaggaaagaggaagtccattttgacaagatcacatccagaatacagaaactgtgctatggattgaacatgggttttgtggatccacc

agcgatcactctgaaagttatcagtggtctatactctggtgtgacaacagtggaactggacaatcttgcagcagaaactgccgccacaatgacaaccaga

catcctgattatgctgtactggcagcaagaattgcagtttcaaacctacacaaagaaactaagaaggctttcagtgatgtaatggatgatttgtataata

tggtcaatcctgtgacaaaaaggaaaagtccacttatatcagaattccaccacaatgtcattatgaaaaatgcagacaggcttaattctgcaataattta

tgatagagatttcagttacaactactttggattcaagacactagaacgttcatatttgctacgcattgatggaaaagttgttgaacggccacaacatatg

ttgatgcgtgtatctcttggcatacatggtgaggacatagatgctgccatacaaacatataatctgttatctgaaagatacttcacacacgcttcaccta

cactcttcggagctggtactcctagaccccagttatcaagctgttttctgctgtgcatgaaggaggacagcattgaaggtatttatgatactttgaagca

gtgtgcaatgatttccaaatcggcgggaggtattggtcttagtgtacattgcatacgagctgctggaagctacattgctggaaccaatggcacgtccaat

ggcttggttcctatgctgcgtgtttataacaatacatccagatatgtggaccagggcggtaataagagacctggagcatttgcaatctacctggagccat

ggcatgcagacgtctttgaattcctggacctgaaaaagaacactggtaaagaggaagttcgtgctcgtgacttgttttatgctttgtggatcccagatat

atttatgcgtcgtgtggaggccaatgaagaatggagtttaatgtgtccccacaactgtcctggtctcgaagattgttggggtgaagaatttgaaaaactc

tatacaaaatatgaggctgaaggaagatatgtacgcaaagtgagggctcagcaattgtggtatgctattatagagtcacaagtggaaacagggacacctt

acatgttgtacaaggatgcctgcaaccgaaagtctaatcaacaaaatctgggtacaattaaatccagcaacctgtgtacggagattattgagtacacatc

accaacagagattgctgtatgtaaccttgcatccatagctgtcaatatgttcgtgaaacctgataagacgtatgattttgacaagctgaaagaagtctca

aaaattgtgaccaaaaatttaaataaaataattgaagtcaatttttatcctgtaccagaggcaaggacgtcaaacatgcggcatcgtcctattggtattg

gtgttcagggccttgcagatgcatttattctaatgagatatccttttgagagtgaagaggctcagaagttaaatatccagatatttgaaactatctacta

tggagctctagaagcaagctgtgaactggcagagaccttgggtgcttatgagacttacgaaggaagtcctgtcagcaaagggattttgcagtatgatatg

tgggatgttaaacctacatcactttggaattgggctgctctcaaggaaaaaattgcagcccatggtgtccgcaactctctcttgttggcacctatgccaa

ctgcatcaactgcccaaattcttggaaacaatgagtcgtttgagccatataccagcaatatctatacacgtcgtgttttgtctggagaatttcaggttgt

aaatcaacacctcttgagggacttgactgactgtggtctgtggaatgatgatatgaagaatgaaataatagcaagtggtggatcaatacagaacattgct

ggcattcctgatgacctgaaggcactgtacaaaactgtgtgggagatacctcaaaaaactattatcaagatggcagctgatagaggagctttcattgatc

agagccagtctctcaacatacatattgctgaaccaaattatggaaagctcacatcaatgcatttctacgcatggcgaatggggctgaagacgggaatgta

ctatcttcgtactaagcctgcggctcaagcaatccagttcacagtcgataagagtaaattatcacgcaatgttagcacctcaacaaatggccattccacc

ccaaaggtgcagaacaacagcgatgaagcgtacaatatgagtgcaatggtttgttcattagccaacaaggatgaatgtttaatgtgtggttcatagagta

tttacatacaatgcttaaaagtgtatcaatgtgtataacatacaacattgcaattgttctgagccatattggctaaggagaaacatatttttgtgctgtt

ttgtttaatcagaactagtcacatagatgtttattacatcctgtatatctttttgaatgctgcttacatccattaactattacatatttttgataaatct

gaagcttttcttttttatggacatgtatgttttgtgggaggcattttagtccttcattttatatttttcttgtagtgcatgtgaaacaggattccatcct

tgtggctaactatggttaaatgaaatatgccaggaaaacagttatgtcagatatttttcaagaaatgtaatacatcacatgaacacatcttaaggttctg

ttacaaacacgaatacttcgggtttgattttgaagtgtgcaatatttgatgttgcagataactgaaactatcagatcatctttttcatgtacagtgcaat

ccatagtgtaatatgtctatccttaccagttctcccccccccccccaaaaggattctgtaatttcagttaatcagacaaatgactggtagcatgaaagtt

cactatattgtttggagtagggaagaagtgcagcagttccctgtaaatctctgtctgtttacagtttatttctgtatttaagtttttggttaccttgtac

ttaactggtttaatagaaa

>51218|AGAP010215-PA

tccactgaatatcttacgttaataactgtaacaagcatcttaattaagaatttctcactgtttctcatatagtgaaacatcaaaatcttcataaaaacgt

gggtcacttaatcgtaacactgttctcttgtagaagtaatagtaaaatacagccaaaattcgctgacacacataaagcgccagtatgcagttggggaact

gaagactccacatatgctgccagcgaaacctgacgactaaaatatggtatgataccgtaagtgtaacgtaagtagcgcatataaaaactgtagcacgaaa

ctggcggaataaggatcccagaaagccagcctggaagacaaatgtggaaaacagactcaagaagaaggtaatagcgccaaatactagaagaacgtcctgg

attatgaacaatatcaacaaggttactgcatcattgtacgcaaagtctgaaaaggtattgatacaaagatctatcgctagaaggaagagttgtataaaca

ccataaaaccatagcgacttaaagttatcatctcagtaatgaatttctatttgcaataactgtagcatattaatagttctgaaaataaaaatcaatagag

aacattgcaattctcttccaatttcatgcatttcgctgccgctgcagttcatggcagccacgacgcgttgccatgacaacacttgtgtaagcgaatatgc

aaagacaaaatgaaacgttcacagtcgagatcacctcaaagtattgaagacacaagtggacaacttttcag

>51217|AGAP010215-PA

ttcacagtaacattcaaaatttattatcattgaagacttacaacaactccactgaatatcttacgttaataactgtaacaagcatcttaattaagaattt

ctcactgtttctcatatagtgaaacatcaaaatcttcataaaaacgtgggtcacttaatcgtaacactgttctcttgtagaagtaatagtaaaatacagc

caaaattcgctgacacacataaagcgccagtatgcagttggggaactgaagactccacatatgctgccagcgaaacctgacgactaaaatatggtatgat

accgtaagtgtaacgtaagtagcgcatataaaaactgtagcacgaaactggcggaataaggatcccagaaagccagcctggaagacaaatgtggaaaaca

gactcaagaagaaggtaatagcgccaaatactagaagaacgtcctggattctgcatcatacacaaaaaccttaagtgtcaaacactttatagaaaatata

aaatagtatctaattacacatgtcttacatgaacaatatcaacaaggttactgcatcattgtacgcaaagtctgaaaaggtattgatacaaagatctatc

gctagaaggaagagttgtataaacaccataaaaccatagcgacttaaagttatcatctcagtaatgaatttctatttgcaataactgtagcatattaata

gttctgaaaataaaaatcaatagagaacattgcaattctcttccaatttcatgcatttcgctgccgctgcagttcatggcagccacgacgcgttgccatg

acaacacttgtgtaagcgaatatgcaaagacaaaatgaaacgttcacagtcgagatcacctcaaagtattgaagacacaagtggacaacttttcag

>51220|AGAP010216-PA

cgtcgcacccagaaatttcacagagctattgttggtgcatctcttactgacatcatggaaaaaaggaatatgaagcctgaaattaggaagtgcagcctgc

ttctttgctgctttgac

>51224|AGAP010217-PA

cggaaccgcgccgccgcccggacgaactccggcttcgccctcttgcagtggccgcaccagggtgcgtagaacatgacgaggacgtgcttcttcttgcgca

ggaagggcttgaag

>51225|AGAP010217-PA

ggactacggcggcgagcggacggaggagtacttcgtgcggttcatgcgcgaccccctgggcgccgccgcagactctccggcggacacggcggaggcggag

gggtgggccgcgctgccgggcggcgaccacgtccggctgctgactgaggcggacttggacgctgccgtcgcctcgcctgcacccacgctcgtgctgttct

acgccccttggtgcgggcactgtcgcagcatgaaggcg

>51226|AGAP010220-PA

ttttcagtgaaagggcgtctcgtggtacaggtctttgtgtaggaagccgggcaggtttagggcttttgttagcaagtgttccacattgcagtcaaaatgt

tcgaagcaagacttgtgcagagttcaatcctgaagaaagttttggaggcaatcaaagaattgctaaatgaagcaacatttgattgctccgattccggaat

acagttacaagccatggataactctcacgtcagcttggtttcactcaatatgagaagtgacggattcgacaaatatcgatgcgatagaaatctgtcgatg

ggaatgaatttggcaagtttaacaaagatttttaaatgtgcgtcaaacgatgatatagttaccatcaaagcccaagataatgccgatacagtaacttttg

tcttcgaatctccaaatcaggagaaagtctcagactatgaaatgaaactgatgaacttggatcaggagcatttgggaattccggagactgattactcttg

cacaataaggcttccatcagcagaatttactcgcatttgccgagacttgagccagtttggcgaatcgtgtgtaatttcatgttcgaaagagggagtaaag

ttttctgcagcaggagacattggttcagctaatataaaattggcacagacagctaatgttgacaaagaggaagaagcggtgaccattgaaatgcaagaac

cagtcactttgacatttgcatgcagatacctatcatcttttacaaaggcctcacctctgtcacctgttgttcagctttcgatgtctgctgatgttccttt

agttgtggagtataaaataacagatatgggctacatccgttactatctggcacctaagattgaggatgacgagaataattaatacgtacttgaaggccat

gttaagtttttgtgtctgttttaaagtaactcaggaaaagtcttctatgaagtttttatgatcatgtttgcctgtgtattatgtgtggaaggaaaagtga

atagcagttttagtacagctggatttaataatattcaaagataattattttaaagaatttttcaactgtgaatctcgtggtaatgtactggtattacata

tttcaccctttgtgaagtaataaactttgtaatttatgtagctggcttttttacagtcaaataaatttttatacaaaacattttctagtgtctattttga

tttcagatttttttttaaatagttgcatactttgtgatttgtgaagtaccctctattgcttaacttagaacaccagtgagtaaataataaagcaagttca

ttccttcaagatttcacaatattgtttatattatatttgaatgtagatataaaatgtatttaaaatatcaaatcaactatttaagtttatgacatactgt

ctgtaagtgtcgaca

>51227|AGAP010239-PA

aagctttccgaaaattagcgctccattatcacccggacaagaacaaggatccagcagcaggtgaaaagtttcaggaaatttcacacgcttatgaagtttt

gtccgatccgcagaagagacagcattatgatgtttttggcaaggaaagtaatcagtttgaccgaacaattttcgctgattttgaaggaatgttcagagat

ttcgcaagaaatatgtttcattttcagcacgaagcccataacgctaagaaagactttaagcgcgagtcttttggattttacgattttcttgatgaaccaa

atgtgcacttccatcaccctaatgatcactaccaaaggtacggtcaccggagtcattttcaccagcatcaccatccacatggatctccattctcttgggg

cccatatcaccattctccacatttccaagggcaccatggctgccaaacaatagtgcaacaagttggaaaccatatgagggtaatgacaaggtgtccacat

aaccaaaatgaatgagag

>51229|AGAP010243-PA

tggagacgatgccgtactgtgtggacccgacaaccagcggtccgccgctgtccccattgcacgcacctctgccatattcaccagcgcagatcatgttctc

cgtgatgccgcccaagtagtagatattgcagtcacgacgtgcgacgatgacggtggtgacagcttgaagctgctca

>51230|AGAP010260-PA

tttgctaaaaatttagattctttattcataagacttaaatgtcacaatgaacaaaactgacaattatatgtaatttaaatattgtggaaaatgttttgta

ttatttattctcatttataacatccctttcaaatcttaaatgtccttatttaatcgttactaagaattctgaatattcgtgttacacataatgtgaaatt

tacaacagacatcatcttgcaaacacttttaaacacactggaggaaaactggaattaatgttgttcccaattctgtttataattagaaatataaatgagt

tcagaataattttagcatattacacgatttattttcaaaaaaaatatattttatatgttggttttattttaaattaaatctaaattggtatttctccatg

cacccaagtaccttcgaagtagaattctgctaacagcatttccattcataaacgccatatgtgaatgatacgaaaacgtcactgagatgtgtttggtatg

tgctgggaaggggaagacagagtaaggagctagaaaattaattagtgccttgttgttcatcaataaaattctaaatcagaagagggatagtacacagatt

acgcaatgtactagatgttggattgtgtcaggtcgttatctagatttgaattaaaaacttcaaaaatatacatacgtaaaatcatgttcattccatcttc

tgattaattaaaacagtgcttctgacatatttacttctgccagtcttgattcaatcctgaaaggaagttatttccttacggtgatgatttatggattcaa

cacagcacaagcaggctagttaataaatagattatcttttaaagaatgatatacacatttcctacgaaagtttcagttaaggttcgtttgaaatatagtc

attagtcatgttataattagaatacatttatctacatacattggcttataattgttagtgccagtacactcaataaattacaagtggaaaagaaataaca

tcgaagaacaagcacacatatatacagtcactttataattaattggagatacagtacagtctatcgtaagagtgaccttcaaataccaaacaaaaataac

attattataagtatatggctactgccagttgaggttagtagtaagcagctcttctttggttatacatttcataaaggacatacgctgggtatgggtcaag

tttggttatccaaaagactggaaactgtctttctttccttcaaggtaaaatgggtcgtatattcactctaatctttcactcgtgtgggagatcaaggctg

gttaactatgtagttaacagcacacaggatcaatggataacagctaatgctagaagatatcttggattagctcctaatgcgatttgaatgttattacgaa

catgagccaatacaatttctgttaatagaggtaatgtgcgggattgtatcgtagtggttagattttcacaattaatcatgtaacatgtaacgtaaatcgc

caagttttcattaaaattcttagacacagtgtagagctgatgaagcaaagaaaatgtgaagtgtctagtgtctacgtgacatctactgctttaaactttc

tgtgttgcggaactacagcaaagtaaaaaataaagagtgtgatacatccatttttcagtttgaaatatcatatcaacttcccagttttttctgacagaac

acgtattgcccttacagaaaaattcttaaaatcaaaagatttgatgaacatgacattcctaaacaaaagtaactctacacgaatttacagttaagataca

tgttctacatggttagcagtcaatgttcctctcttatgttttgagaatattctccaaattgtacataacgtaaaatatgctactttatggtagcgatttc

agttcttcatgttctacaaatcacagccagaacacaataaaatgaacatgcttgtttcacgttcaagctttgtcattctacgcaaagggaacattaccat

ggctagagatattactctagagagaaacattccagtgatctatttcatgtgtctttactcacacagaaggattcatggacgtttgtactacacatccttt

acagttatgttcaaatgacatgaagaattaaggctataactgtacagttagaaggtaccacatgcatgcacttaatacatatttctgatacgataagcgt

gacatctagatacacattagttactaagaacgactacacacataatggccacagcaaagatattcataataattgtgaacttgagtagacttccagacaa

tggatgattaggatgatacatgatacattatattgatagacgttactagacgataagttattttgaatagatcttcatggagacaaggaaactaatacat

ttccattatgccatatttacaaagcgtgacaactttggcagaggtcactccttaagtgtatcaaatcattaggacacccttttccagtgtagaagaaaac

gactgatacaattataagagaaatatcattgacaatcttagtaatacgcaccagagaggaaatcagttacgtgacctgtcagtagagtatctcagtgcag

aaggaccacgcagttatataattttagatgtggtgttcagcatgttaatcacattactcattcctacgtgtataaaactacaacaaatagaatatgtagt

gctggctggtggatgttttccaaaatcatcaccgaactatggtaacatttacaataaaaagaggtaaaagcaatttatcctgttctggcgatactttcat

atggtaaggatgtgtttatctgtgtgaaacaaacatctcatgacggaatgtgtttctgatatggattttttatagttcaccttcttgacctctatgatgt

actatttctggttacattgggtaatcgaaatgcattattgcagtaatgtgtgcaacatggtgcttacttcaaattctggcataaaacaccacgtcagtgt

ttctcaacccgagatacaatagggacattttcacaataaaattgcaatgtagaaatatattctaagactggaccgcatacacctttattataaagtaaat

aacaaggtgaaatgaccttagtcccacacagttttgttttccactacatgttacattaattatgaaaaggtaaaagcatagaaactgttataatcatgca

tggtgatttattacaacctctatttataaaataattttcaacgacttttataactctatgtattacacattgtaacaattttctgaagaggagaatatta

gacaactaatttaaagatatgtttcatgagttacagcaggctattaatacgtgaagcataggagtctaagtatgtttcgaaacagtatatagaggaaaat

ttagcttgtggtgaggttgagaaacgccaaacactggtggggagccactgttgtgacacggtaggtcacttcagtccaagaatccggtattgacgagctt

ttccaggaagaattttacgtcgcccagctctgagtcctttattccaagactcttgagcattcccagatcgccgttctccacagccatgaagactgagcgc

aggtgttccaagtccgatctcatcagatgtagcgccgccccgaagtcctcgatggtctgtgattctgaaagagcaatagcgaccttctcgactcctccga

gtggtgccagggcgtctctggctttgtttccaaagagtttctcaaggtagtttggtccgtggctcgccagaagtgactgtagaaaggatgctgtctcttc

aactggtggtcgttttgctgcggtgcagagccgtgtgtcctcgtcgtatccgtcagggcagtctggagcgccatcgcacagatactggatcgagatgcag

atgccctttccttcaccaccaggacatttgaatggctcgtatggatgacaggcctcgttcctt

>51231|AGAP010280-PA

cgccgagggcgagtcagcgcgcatcacgtccgcctaccgcgtcagggctgacgcgtgcgaccggctgtgggtgatggacgccggcaccgccgacctggga

cagggcgctctggtgcgcctcaccccgccacagttactcgtctacgacctgcgcaccgacaccctcctactcaggtactacctgcggagagacgacttcg

acgcgacgtcgttcttcgccggtatcgcggtggacgttgaggcgggccactgcggcgaggcgttcgcctacctgccggg

>51252|AGAP010324-PA

catccgtgctgagctgcgatatttcagctgatgacaagtatattgtgacaggctctggtgacaagaaagcaactgtgtatgaagttatctactgaccaat

ttatccattcttctgaaatactgcagatgtgatactgtgaatgtgtttccggcatgaagaactaagtgtaattttgacaatacacgccaataagaaagtg

cttggatcctgagcatactgctgcacagttaaatatgacatattgtgagtataaaaaaggaggaagtgtttggaaactctgaaagaactcaggtgcatta

tgttgaggaacaaactgttattaatggacaacatggagttaaagataaaacaatgggcctattgtatagagaaaatatatggcaccattcattttctaat

tgttgactacacacatgtaataaattttattgatgtcgtacacctttaaaatagttttatctgaagttcttgtgctcttaatgtaaatgtgtatgtagtt

tcttataaaatagatttttagttttgctcacaaaaaagactgctgcataatatttatgatattggcatgtatgtgtgggaaagattttgagcttcataca

cttgtcactggatctgaagaaatatattttccataaatttaggattatcacatgttggtaactaaagcttagagatgtaaactctgtttcttttgatgtc

ttattaccattactcattttatggtaactttctttaatacttcagatgtccctaagttattttaattgtttttgcttatcttgtttctacagaattagtc

aagatttgtaaaatgtgagtcaaattgcttagtttttgaggtattttctcattatgtcatattatgaaagcctttgtcattgttgaataaccaatattaa

gtagaccacatatgatcactgtttgaaatgattattgtgtttgacagttgtggagaatattgtctgaagctcaaagttgttcactcaaatatcttgaatg

atactacaatggtaaaacctttgtccgtagttttgtttgactgtattgaaagttatatcagcctggatgaatcatccttttttgttatttggtatctttg

tagggtgctgaacaccataacgcaaaagctgatgttttttgacctaaaaataatttattattcacaaacactgttttattgtgaatgcttttgttaatta

catcttcatactttgataaatattgttactctactcaactgtacaatgtgagaataatagatttctgtcattattgacttttttattttttcattcataa

ttataaatatgatgaaaataaaataaatttgacatttttgtttgtaagcagtgttccatgtggtctctaaattataatgcattgatatttatatagttct

acctaagtgagcagctcactctataatcactttgttcaaatttctttattatgtttctattgatttaatcaattgaactgtacagctttattacctttct

atgtagtaattcagtaactaaatcacaaagtggttataggtatgagaaaagcttatgggtggttctcctagctaggtttatacttgcatggtattgggta

ctgcttgtatttatcagcaggacctgccacctaaatttatcttcatcctatggtcagacataatagtacatttttccaatgtacaatacagtttgtgatg

ggactagagatgatcatttttcgtgcttcaatgaacattgttaaagtaaaggtgttgctaggtgttatcatacagcaaacatgaaaagcacaaaactttt

aatgtgaagacaaaaactggcttcttcattgactaaatgtgcatgcattttgtgactaccttgtgccaataagtcacagtttcaagcactgctgattaca

tgtaccaattatattaagaaaagttaatgcattgcttaaatatttgttggcagaatgattacaactgttgtttgttaggttgagaatgtaatttgccaca

tggactgtattgtttcaaattatttaaagaacagaaccttatattaaatattcaaacatgttacacatttttcaagtgtgatacccatttgaaaatgcaa

acataaaacacacacacacatcagttttgatctagacaactaccagtgtggtatactgtatcacctgaatgttacatttgatggagataggtggaatttt

tatttttatgtcttttctgtgggacaggcttgtggtggtgtgttttctatcactaaattactctctttattactaacagatttgctatgtttctgttctg

gtttttaaaattt

>51254|AGAP010325-PA

gttcaggtagagtagattcgtgctgcagtaaatgctacaaagagtaggtgatcaaattaatcacactagaagtctcaatggaaatcatccatgatccatg

tcaataaaacactcgagctcaaacagtattccttcttcgaaacttcgtggtagattcagactgtgtgtcggaccaggtctcgattcattgaaagaattct

caagaagtgtagcctacaagaaaggaggcagcttacgaacccctaggtcgactgatacttgaatattgcttctcagtctgttaactcttggcaaatagga

gaatcgatggaggaaacagagaaaactcaaaggtgagcagcgacttaccgttaaacgtacatttaggaatggtgacgctcatccaactgagcgacaaacg

ttacaagagaggcagtgtacagcaagatttggtttactgttaaaattcagacagcatacgtacctaaagcagtcaatgaacatattgcttccacctgcct

ttatctcgcgaaatgatcgtgaaggcaaaattagagaaatccgaggacacacggaggcttaccaacaaccgattttcccgcatatcattcgtgactgtaa

cagaaaagaggacattgacagtgatgcacaaagtactcttcgtaacacagcgtactatggtttgcggagtataggtgtagaagtagatgtagagcccgga

aacttgcctttagttggcaataccttcaccgactaagttacgcagatatgactcacgagatgcagcagtggctgtacagttcagtcagtaacagcattgc

tcaaaggagattaggtttcggtttagagtctcactctagtacacagtttaaatctgccaggaaatttcaaaacggtgcacactcaactgcggagtgataa

ttctttctggacatttcttaacgtagtacgtcgttggaaaacaaaagcaaataagttcaaacaatttgattaagtttaaagtcctgaaaacataagccaa

cccttaatcgtctaatacttgcaagcattttatctcgtatctcacattaacggcacagtggatatcggtctcgagccttgaagctcttaatactaacaat

tcaagcatgatttgaaacgcataggcaataaaaacatttcaatgatttcgtgtattcttagagctctgttgagcttagagagcagttattggcacattga

gagggcagggctgatcactgagaggaaacagtgcctgagtgatccattgccattatgcaacgacggatcgaaagggtactgccatgaagtatccatttgc

gacgagtttcttttgtctttcacaaggattttgttatttcaggtacgatgatctcgctttcctcgaggttacgttcttggcttttcagtcctcatcgact

cctgcacagtgaggatagaacagactaagggcttgcaggttctgtacagttcgtgatcgctgtgcagccaacacagaggctcccaggtctggtctgagaa

cagtgaagaggcgcgcccgctgctagaacctgtggctctcagtcaccacagagtcgtccgcgtctgtgctacctccggcgcagcagccgacgctgttgtc

gctgttcttagaggccacctcagatggccgctcctcgaacaccagctggtacaggagggtcgccaccgctgaacccgacaggggacccacccagtagatc

cagtggtccgtccagacgttcttccagagcgctggagccagtgtcctcactgggttcatgctggctcctgtgtaagggcccgcagcgagtgagatgatgg

tgatgtagaagccgaacttgagcggcgaggagtcggtgttgcggctgttgcggccgtcccagaggcccatgcagagcagcaccagcacggccgtggcgac

ggtctccaccagcagcgcctgcagcggcgtcagcggcgccgcggggaccgtcgtgcactcgcatgccacgctggccggcgtcgccaccagcagcaggccg

aagcctagcacggcgcccacgccctcagccaccaggtacaccagggcggctg

>51256|AGAP010337-PA

gagcatttgctggaattttaagtggtgggctgcccaacctttttaacaggatacgagataccatcttcagagttgcaccacccttcatcattggttatct

ggtatcctgttaaaaaggttgggcagcccaccacttaaaattccagcaaatgctcgctgtt

>51258|AGAP010353-PA

gtgcatttaataaaacctcgtaaagttcatataaattctgtaaaaaactttaacaatcatattgtcttactacattttataaaaagtaaattgaagcaac

acctgctttaaacttctgtaaaattataaaatttattgcacagagagataagaggacagtttcttctgagtttttgaagaaactggttgagacagccacg

aagaaaagtatcgctgcaaaatagcatgttttccaacattcactgtcggaacaatttgttctggctgcagaaactgtacaaactcacgtaattctgaata

actggagtgttcgctataggggacaccataaatagtgacattatcatcagctgttcgcctgaaggttagtgaggatcctgagtcttgcaattcccatcct

gatggccgaaaggcaagaacattggaatatattccctgtaagctgttgagatggtccttcaagtcccgggtgttgagctctttcatagccagcacatgca

cctgagcttcatgtggttttgttacaagtcttgtttcaatgtttggatcacacaaacattttagcacacctctcttatcagggcgtgcccacagttttgc

tcctattgctttactgatacctagaaaaactttttcctttccaatgagatatgttccacacactaccaaagttttaggacatctttgcaatgttgctttt

gttatttctatggcctttgcaataacttcttcctgtgatgggaaatcataccttggttcacaatatgtggtgtcgaggaatagcttgtttatagagcagt

gttgaatggctggaaatgacttcatgattccgtgataacgaaaatcgcctacatgtaaaaaatttctaccatcaggtagccgaaacagcagcataattga

accaggacaatgatttgcatctaatgctgtaacccgaaccattccaataactctttcatcattaagttctaggacagtcaagtacttctcatccacacca

atttttttcttgacaagggcagctgtaactgcactgcagtagattggtttactgaagcctttacgaagacctgtatagtggtccaaatgaaaatgtgtca

ggaagtatgaagtaatgcctggaatgatcccatactggaatgcatcaacaacaaatgttgtatctggaatcttcttataaaaaggacatttcttctcttt

tgaagtatcagacacattgtttcttccagaaatgtttcttggagcactgtttcttggagcaatactttcgctacttgatgacacactttctgatgaaaca

acctgcatatcttcatacccttttgccttcatacgatccataagttttttccactcatcttttgctgacttttttctcttttgaagtatcagacacattg

tttcttccagaaatgtttcttggagcactgtttcttggagcaatactttcgctacttgatgacacactttctgatgaaacaacctgcatatcttcatacc

cttttgccttcatacgatccataagttttttccactcatcttttgctgacttttttctctctttatcatcagctacaactggtttttgtgtatatgaaac

atttgtagagtaggtgcctcctgaactaacacatgacacaacagatacttctttcataggctgtacaagtgcttgtttcccagaaaatcccagcccatca

agagatgattcaaatcttggctccaagcacctcctctttggtgatggtgtttttactgtacttctttgatttgccactgtacattctacatgcatagcac

tcctgcagttgcacatttctacatttacctcatcaagtaaagttccagggacagtatcaatgccgagaccttgccacccagttttgttgtaagtaagatg

aactttgacaggacttactagggacttctttggtgaattaatctccatttctacagaaggtacttcagaagtgatcccatggacacaattgcatggaata

gtcttagacttaactggacttttaacattggtggtatcagcactgtcatatttgcctttaaatttaatgctacctttaaactgaagacttttctgaacaa

aaatttctgttttatcagttattgcagaactaccactgtcttcatcacagctcaggcccactgtatttgatttcagactattggaactgtcgcaagttga

aatttctttttcagtatccaccaatgcagcttccactgactgatgatcttccagattacctgaaaactgattgtccttaccaccactcaagtttctcaag

atgttactaggctttttaactttgaatgggtttttggaaagttgttcaacaggtgaagaaactttgagaattgaaacagatttggacgattcatcagaac

ccgaaaaaacatctgttctgctagttattcggtcattatggtgtataaatcgttctgagggcactttagtagaactatttccttcaaaatgcttggacga

gatactttttgtatgatttgttttgttaaaatctgctactgattcactagtatcgacattgccattatcaacatcagcttcaaaatatgttgactgtgat

gaagaagaaactgatacttctttcctcccatctgaagcagcatttacacaggcagctcgataagttgtttgctcaatacagcctccttcatctccctcat

ccgaaaataaatctaaagaactctcagtactggtctctgatattatcctcttactggccgacaactgacttctgcccacatcgcacaaatcatctttcga

taggcgacgattgtagtcaatgactgttgctcgaatttctgccaataatttgtgggaaaagtctttaaagtgaaaaatgcttgtttcactacactccttt

ccttggggacattctctgagatcatcccaaaccactgagcatgtactaacatgaacttcaggcaaaatatttagtgacgtcaaaggcatctggcacagtg

gacaaaactgctgctcgtcatccggagtctctctcttttgtgcacggttctttttacagcccttctgcaagtttttgtctactttcttagcacaatcgga

aaacttcgcatttgcagtgtcaagactactctgagaagctttcagtcgcctgcgaactttctgctgtgaaaaagaaagcttggtccttgttacatcctta

cgaattgggctcgattgcaactgagacgtcctcgcaaatgaaacaaaatcttcagataaatcgtcactcattgatgtaaacagaatcttcatttgggaag

tcacaaattatgaaacgacttcgtcgcataagtatatacaccccacaatataaatattttcagtaccacactagtgccgcaacccgcttcatgttttgtt

ttgaacacagcac

>51261|AGAP010370-PA

cattgttcccaagtttttatttctataataaagttgaattacaacattcaaaatcatgtattatatcttgagtatatacctgcgtgctttctgaccccac

tggaaaaattttaaattgttacgttacaaatcgtgatacaaccataaatattttgtctatttataataaaatgtaaagagacgataagacaagaagaagt

gctgtgtgctcttctgagaacagtagaggccatctaaattggtggcgatgttcatgatttcctttgtgaattactgagaactgattttaaactgttgtaa

ttatattgtgtactgctcgaaatctagtggcgaactgtaataggatgtatggtgaactgctgaaaatgtattatgaggtttcatgtcttttccatgtgtc

cgcagaatgccataccgatggcggagagatgattgtatttcacacagtactgggtcgtttttggtcctgtcatgaacctgctctaatttttgtgtctatg

gtccaactaactaataacagtttccagggacagtaattagtaaactaaattgtagtgtgatagtaataccaacaaagtgtacttcaaaaactttattgct

acagtagcatctttgtactggcattgcgtccagcgaagagtgtgcagtgtttggtgcaggtatcaagagggtgtcgcttcctcctttttcttgaggttcc

acggctgcgtctcagcagagccaaagaagataaacacaagaccgctcacgaggtacagcccagacccgatgaagaagatgatttgccactgctgtaaggt

actacctccttctaagatgtacccgaccatggccggcatgatgaaacccgtgcatgtgcctactgcgttcattataccatacagagatcctgcgtagttg

ggggcgaggtcgtggtggttggcaagactggtgacggtggcagctccgttcagacccagcgacagactcaggatcgccacggcgagtgcctcgttcttcc

ccacgtagcccatccca

>51260|AGAP010370-PA

cgataagacaagaagaagtgctgtgtgctcttctgagaacagtagaggccatctaaattggtggcgatgttcatgatttcctttgtgaattactgagaac

tgattttaaactgttgtaattatattgtgtactgctcgaaatctagtggcgaactgtaataggatgtatggtgaactgctgaaaatgtatcatgaggttt

catatcttttccatgtgtccgcagaatgctataccgatggcggagagatgattgtacttcacgcagtactgggacgtttttggtgctgtcatgaacctgc

tctaatttttgtgtctatggtccaactaactaataatagtttccagtgacagtaattagtaaactaaattgtagtgtgatagtaataccaacaaagtgta

cttcaaaaactttattgctacagtagcatctttgtactggcattgcgtccagcgaagagtgtgcagtgtttggtgcaggtatcaagagggtgtcgcttcc

tcctttttcttgaggttccacggctgcgtctcagcagagccaaagaagataaacacaagaccgctcacgaggtacagcccagacccgatgaagaagatga

tttgccactgctgtaaggtactacctccttctaagatgtacccgaccatggccggcatgatgaaacccgtgcatgtgcctactgcgttcattataccata

cagagatcctgcgtagttgggggcgaggtcgtggtggttggcaagactggtgacggtggcagctccgttcagacccagcgacagactcaggatcgccacg

gcgagtgcctcgttcttccccacgtagcccatcccagccatcatcacggccggtaagaagtgcgatgggatgacgaacagctttcggaccacggtcttgt

ttctgtacgggtcggagagaatccagtcgcccacagaactgaaaactactccaaacagcagcctcatcaggtatggcagacttgcgaaacctcccgactg

tttgatgtcgaagccgagaacttcagacatgtacttgggtccttcagtcatcaggaagaagagtcctcccagattgccgaagtgcagtgccaccagcgcc

aggaacggacaggaagttatcaccgacaagaacggcagcaccctcttctccgtggagacggtgtccgcgatgctggtggtgatgtaggtcaactcgtcgg

gcgagatgcgcgggtgtctggcgggagagtcgtacaccgtcacgaaccacatgatggtccagacgcccacgatgccgccgaggatgtagaagctcgcctt

ccaccccagggcctcgataatggccgagcagatgggccacgtcaccaccgtgccgaaggtccctccgagcatactccagacgaacttgcccttctcctgt

ggcggtccccactgcgccaccagagcgtgcagtgatgggtacaaagggcccccacagacgccaatgaacaggcgggcggcaaagagccagccgaccccag

catcagctgcgagcgggcagagaatggtgaggacagcaccagccagcgacgtgacacccaccacaagccgtgggcccagcagctcggccaacatcccacc

agggatgtgcgccaccaccgagccgtagaagtaggctgacagcacgtggccctgtgcagactgcgaccagctgtacctaggtccatagtcaggcaggggg

gcctccgagtaggtcgtggtgaggttgtccgcggaggccggcagcaggatgatgtcgcggggctccacggcctcctcggtcctggaggggtacaccatgg

cgagcagcaccaggctcaggttgacacgcagcatgtaggtgccccaggtgccgaggaagcactggatggcgacgatcagccgcgccggcaccgcctcatt

tattctacgtaaccaggaccctgatttatcggctggaggctccattttgtcagccgctaacgggacagcgcgctcgcgagtgtcgctccacggactccgg

actcggctgcagaggtgcgtactcctctctgccggctgctagactagcaggtgttttcgccttgtggctctc

>51276|AGAP010390-PA

gctgcgccagggcgtcgtctccgggataaaagtgatcaccgaaagccggcgaccaatcagcgcctaccttgggataccatacgccatccctccggtcgga

gagcttcgatttgcgccaccgacaccgcacgccggctggaacggcacgctgttggctggccgctacggtgccccatgtccgcagctgccggcgccgggac

ctcc

>51277|AGAP010393-PA

aatctatcaatgcaaccagatcgaaaaatgtggaagtttctcttcaggccagaaaatgcatgttttccattgccagcaacagagctgttgctaacagaaa

gattgaaatgactcagatcagtctgatcaggtattccaaagttgaatctgagaaatgcattaagaactacactgcagtcatcacacaaaggtgctgcgca

gcaaacaaagaaccaaaaaacctcatgtttcaaacgaatcaactgttcatttttggtgttgctaataactagtggctgtagtaaaagtttcaggtgcttc

ttaatatcttgaccatttgaagcacacaacttttcaattaggtacttccaacatttgaaagctggagctctatcactgcgt

>51278|AGAP010394-PA

cgtcgatgccgattcctagcatgaggaagggcgcggccaggttgatgccgatgaactcgacgcccaagtacatgacgagcccgaaggcagctccggtgcc

c

>51280|AGAP010394-PA

gccggggtacttcatcgtggtgccgatcctgctgtcggggctgttcgccacgggctaccagcgcatgcgctacgagatcgaccccgagtacctgttcagc

cccgtcaacggcgagggcaaggccgaacgcgccatcgtcgagcagtacttcaagcccaactacaccagccgcttcaacgtgggacgcatcacgaggccag

gccggttcgggcgcgtgatcgtgacgccgcgcgaggggcacgacatgctgcggcaggagatctggg

>51279|AGAP010394-PA

ctgattttgagacgggcagcacgcgcatgcaggtgaaagagtgtaggttgcgctgttcggcgtatcccgagagggccatgcagcccgcgaagaacgttat

gtgccacaggaaagtgaagcacacggcgaagcctgtgtagatgcagaagatgcgcacagaggggaaggggctgatgatgccgatccagaagctgatcatg

tcggtgaaggaggtgatggtgatggagacggccgcctcgctcagcgtccgcgccatcctctcgggcacggtgagcgtcaccggcgtgcgccgccacgccg

ccagcatcacgaatgtgtcgtcgatgccgattcctagcatgaggaagg

>51282|AGAP010403-PA

agaataattacagaaagatatgaattaatcaaaactaaaaacttgagtttctgccattaagtgaaagcgaaagacaaatactattacccaatatattgac

agagacaacaagaaaaatatgtccgacaaaaagatgaaactgactgataagagaaatgcaccactagataaatacctagatcttctgcaggaacaacttt

gctcacaagacctgctcttaaagcttcttcagctgaaagaggaagacctgtaaacaacatgtgagctgctactttttttggcactgcacgaccaacagca

attccaggtgtagagcagaatattccaaagtttgctcctggagttgagaaggagctgttatcagagcaaattgcaatgtcacagctagcaacaagttggc

aaccagcagcagcagccagtccttgaacaacagctatgacaggcacaggacattgtaagatgcctagcatcagacgagaacaggttgcaaaaacctcata

gtggaattcagacccatctttgctcgtcagttctttcaaattgtggccagctgagaacacccgtccttctccatacagtaaaatacacctgagggaaggg

tcattctgattgcaggtgatgcctgcaatcaagtcttccatcattttcatggacagtgcattccttgttttttgacttgacatgattattttacgaacac

catcatattgtacagtctttgtcaatggttcatttgcaataatacatgaagtactaagtgacatattatgttttggtttccctcggattcttgccacatg

ctttcccattgccaaggacatacgtgtggtaacagacattattagtacgaacgattcgtaaatattatattttaaaatatacttcacaaccaacaactct

tctatgtacttggtttcacgcttagaaggcctcaactgtttatacgcgtgacaaccgttgccatagcagcagttgccacctacgaaaggtgaccaatcac

ctgctagctttcagttgctcggagcaacgaaaaatggcagatgcagtagaagcagaacctccagcacaagtatttttgttgccatgtgcagtatgtggac

gaacattccgtccagaatcactacaaaa

>51283|AGAP010425-PA

gagaaactttttttctatttctacttgaccgcctggtcctgcaagcatccccagctgatctgtgtttacattccgtggaacgtaataagctatgtttt

>51284|AGAP010425-PA

ctctattaatcactgacaactgtaacaggtctgctaccaaatatctgttaagtcacttaggactagcaggctgacactacactcaatactttgaagtaca

atctgcatttattataaattttgtcaatatattcagagatcatgcacaaaacttttgggtaatacacaaacatagctcgcctttctttaacagttccttc

actgagtacatgagttacattttcataaataacacagtcatcaaatgatattaaaaaatgtttattggtacattaattgcctaactgttactcatgtata

agttctccataatatgctgtgactgccacaagtttcttgtcgagaaaattttgttctatttctacttgaccacctggacctgcaagcatcaccagctgaa

atataaaataaccatatcattgtgtgttgcccaagttgcaaaggttagtaagtgagcatcatagtgatatgccataattattatgaaacaattacatcaa

ttaccagacttttttaaaatataatatgtaccaaggtttttgttttgtaagagaccaaactcttacctgatttaaaacaatactacaactccagaaatcg

cttacactgttgttacaaccaatgagtttgtaaaacagaagggtcattaagctctttttcaaacaggtgcaccaacagtacactgttatgcatgatgatc

cttttctacacaccagcagtatttatcacagttgatccacttcctcagatttcaaatataaaatagatccttgaaagttgaagtaagcagacaagattaa

agttttagaaaacacaaactaccctcacagtttgggcagcaagctttccagtttcgtttgaaagatgcacaaacttgactatacaatcactgatcaatta

agagcaccactaattaaagtgtcagctttagctgattaacacaagcaaattttattttttctctccaatgagtatttgcctaaagccaaatatataacat

cgttcgtgtgaaggatcatttcacaggcacggtaattatttttcttctaagaggttcactctcaaacagagagaccacaggaatctaactctttgggtca

cctaaatacacaatttttaccatttaaaattatggcaagtttcaaattagaaaatattcctccattgtagttgatgtagaatgcagccttaataaagatg

aagtacattttcaatatatgtcaaatccatgagcaaacagctgtactttccatttcaaacactatcgcccacgtagctgtgacatttgcaggacacctac

ctgtttaaatgataatttcctataggaatatgtggaaattctcatcagtgacttcactatttgctattacctgagtagcctggtacaaaacaaaatttct

cccccacagaggcgcgcgagcttcgccccccccctcccctcctgagatcaccactctcaacttcatagatacatacttcactatacgaactaatggatct

caccttacactgatggctgcacccttctttcattgtcataaatacttttatactacatttagatacttattattcgatgttacatttttccttactgttt

ctcaattacattgccaggaaaaaaaaaattagtacaccctgttagaatttccaattcactcaagatttattgttgcaacagtgcatatggtgtacatgaa

atgacttgattacatttacagatcactgactctggcatccagtcagttgtaaagatgagcgaatagtgtcctgtgacacattatgccacacctgctcgac

tctgtcacatagctctgaaagaattgttggttggagggtcatttattgtcccgtcttatccaacacttgaccgactggagacatgtccatataccatgcc

ggccagagaagttgctgcacatcttgcatggcacactgag

>51286|AGAP010433-PA

cgtcaaggtaactcagtgttcagtcatcggtgttgttgactgccttttttaggtgaattaggtacatatctttgccgatagtgctgttgcgctaaacgag

aagaatacaaatgggaatgctagctacttgtgtaaatttcatggacgctcggagcgttgaaaggtagatattttccattgacagataggcatgagcgctt

catcgaaaaatgttgtcgttgtggtttgacaactttgttgtatattatgacaatttatccatgtactacgttgtaagtgtaattatgtcattcatggcac

tttttctggcgtggtggtacagttactacaataatggaacagaactaacttctgaccaagctctgatgagcgaaaatatccagtgtgaaggcgccgtatc

taagagcggactgctaaagaaaataaaattggctcgtcaggaggcaattgaacaagaattggcagcaggactcacagaccagcagctccaggaagagaga

gaaacagaacaggctcagttggctgctatttttaaattactgcaggagcaagaggaaaaatttcaagtatcttcaatggaagaattgcaggagcagctga

aactttatcgccgataaaaggagctagctaattctgctgctgaagaatgtataatttataattaacaggtccatttgttgtatgtaacatgaagacagtt

actttcttaaaatggaacacagttggtgctaaaaaatgttaaaatgcactgatatgcataaactatttttatatgtcctttacaagtgtgaacaaatgat

ttacataatatttataaaagatttaataaaagttatttgttgtatgagcctctcccttctttattaaagtcattgaacctgcaagaacaacacacatttt

acactaggtaaacatctactttattattgg

>51288|AGAP010435-PA

cgtaacaaaaagtcaagttctgctgggtctatctcattatccattaaaagaatctgaatagccatctgagccataaaaattaatttgtcacattca

>51287|AGAP010435-PA

attctcacagtcttagtttgaatagggtaactacgaactcctcacgtctgtaaaccctattttcggcttgcaactattatgagacagtctaataggtggc

acagcatttgtatatgtgaaatttcagctatgggagttactttcttaaatcgcg

>51304|AGAP010444-PA

tttgttcaggcaagaagcaagcctctctattgccctgcagttacgccaacatgaggagaaaacagcacacctggtacagagacagactacacttctgcag

cagcaagaacagcaattacagttcctcattgctcaacaaattgagaggcagaagaaactagacgatgcccttgttgaccaacaaaaacgcataacattat

acttggaggctctttcagctcaaccactgattcctgaaagtctgcctcaaacttccttcatccctccaagagaagagaaaaaatatccagagactaagga

agaagtactaattcagccagatactttgaggtgggagcaagaaaagaagagtttggaagaaatcattgaatctctcaaaagccgacatgctcaagaactg

cagcttttggaagattcctacaagagacagttatctactctgcagaaaagcttagaaacacaag

>51305|AGAP010450-PA

gattatccaatgttatgcatggaaaaactccaattggaagcgcaacaagaaatcgagaactaggaaaccgaccacgatctggactatggcaaaggaaaga

tggacgccatggtcgcaagataaggcgtctgcctccagtccgagtaattggagaagctgccccgaaacctccaactccgattcatctgacactgaatatt

gcttagtttatgcagtgtacttttgtagatttttaagtgtctaatgagcaacataaatgtacaggttgtgaaatattaaaaactgaaatatttatatatt

agtaaatgacagatagtagtttttttattgtagaaatatgccaacatttgaaactgactggaaccagaatgcctcagatctattgctgtgctgctggttg

taactgaaaaattatgtgcctgatactgtacaaaattcatggtgtacgagttgcaacctcttggtggcatcatgcccttcataatctacatgctgttatg

tgtaacatccttagatctagatcagttcacctgcacagcaaaaggggcagcagtgaccaaagagaaaataattatgtttacttttcaaatttgcctgaac

tctctaaaagacatcagcagatacttaatgacagcagattagattagattattttttcgttccaaacacatttactttgttaacatgaattcatctgatt

atgtgagctgtatgctcttacaaaggaaattgtttattttgataccactgtttgtacctttaataaataatttagcaagtcagttccaggcatgtagctg

gtaggttaccctgcagatttctttattttactgtacatgcaaggtgattgttagccattacatcattttttgttgtgttctcttcataaagtagtctcat

cttcatcgtagactatgcatattattttgaatgtttataaaaagcactatctcagcattaaatagtgcagatgcatactagctcatacccatttcaaatt

gcttgctgctgactagctaactcttcatctgcttttcactgcagatgagtgatttcgtacaaaaatggataaacattgggactgagttccttgtcccaaa

acatggggaaaagaacatttgtagtgatgttatcgatacaagccaacctgcaatgaattgtgtatatccag

>51309|AGAP010462-PA

gtaattttttcattaattgaatgagcaatgctgtaatatgtctgttcttcagtggtgttcttatattcatcatcttcagctttagctttctgaattactg

cctgcatcatctcctcatctgataaattctctccagttctcatgtataaaatctttagcta

>51308|AGAP010462-PA

aggatacgatccagaagttcaaatttcccagaagctcggcaaagatctggtcctactataacacttccttgataaccaatatgatcgcagtaggcctttt

caatgtgttgaaacaggaaaggatgattacataattttctaagttgcataatggtgttcataagtgcttttgtgccacctttaccttgctttc

>51306|AGAP010462-PA

gtaattttttcattaattgaatgagcaatgctgtaatatgtctgttcttcagtggtgttcttatattcatcatcttcagctttagctttctgaattactg

cctgcatcatctcctcatctgataaattctctccagttctcatgtgttggttttcgttcttgtgtaaatctagaacatgtttacagatatcaaag

>51307|AGAP010462-PA

tagccttcttcgtcttctgccattagtcgcctccatctctctcttgctctcctctctagctccttcttttgttccctctctgcattagcgtggtgagtca

tgacagcccgattaagacgttgtatccgcgcataattgttccggaagtattccttcacatctttaccatgctgaagaacagcatttaggaactcctggtg

tttctggcgtctttttcgttctgcctcaggcctctgttgtttctccaatttctccgtagccctcgcttcacgtaaactaaatttttttggtctctttttt

gcaagtgaagcagtgtccaacatggtgtccttttgcatgcatgcaataacctctgctcttagtgacctctggaagtttaataaccttaaagctctcagtt

caatctcagccttaatcctcaaatcttctggcattgctgctgggagactagttaattcttgaagtctcagtgctattcgagcattcaacctgcgttcacg

ctcagctgttaaaagttcaggtggtaattttgacggccttgaaacaggagtaatacgattttgtatttgtggttgctgtggaatggatgatgcagcacca

gtattagtgttagtgctggtaacatcagcacactgaacatggctgggcccagcaactggtccaaaactacttgaagcttgtgagcttaaaggtgtagacg

tagtacctgtgataatcaaccaacataatgcttaacagtaac

>51311|AGAP010483-PA

accttagcaaattacacattttcttgaaaatcatacttagtattaactggttacgtcttaagttatttcttggcttggaatcatgtgatatttacaactc

aaagaaaaattttactaccccaagcgacttggtaacaaacaatatggcattaaatattttatttttatgcacatcagaattaaatgacacaaaacagctc

tatggatccatatatccgctattttaaaaatccgtatggttcatacactcctccttcatttaccttttctgcttctctaagtaaggcattcttcagtgtc

ttgttaagattgtagcctatgtctctgccttcaattagagcatcccgaaatgtatgggggctcaggcttccttccccagtcttgaaaactgaggtcacta

aaccggattcagttactgctatgaccacacttgcaacgctacacgcttcctcttcggctgtcggatcaactacacagttatcgcctattttacaaagtgt

aattagaacaggagtatttgaaacatccaaatgggtacagtcgttataattctgcgataacacaagcatttcctcgtcaccatccttgccttcattttcg

acttttggcactctggtattgtgcaaggctgctttaactgccaatgagactgcatcaaaaaggttacctccacattctaagataagaatgtcaacatata

gcttccagcaatgaacaccaggtgtaattatcaaaggcgaaaggttgaaaacttgtgaagacatgtacgctcttgccaaacttgcactaatttctgtggc

taagtcttcacctcctctaccttcgaatgcaggcgtcgcatttgcagagcagtcaacgaagaactctatcttcccctcgtttggtctctcggggaaagga

gtgtcaatttctgtttttacgcccactaaaatatcggtgtttgctaagcgcaagcgagccgaaccatttgcatgaattaccacttctgtttctaattcga

tgggtctgtacgttcgtctgttcctcccgtccgttctaaaatcctcgtcaacaccgtgcaaaatgtaagtcttttcggctgtactcaggagtacttccgc

catatggacgaggctttgaacagcagcagatggtgaaatctttttacaaaacctaaataacacgataatacaccaataactacacgaacatgcaccacaa

gtttaaatacttccccgcgaacattacatataaacaaaacatatacacgtataatcaaagatgttgcccctgctgggagcgttgagtgcagagatgaatg

ttttgtgtggatattcacagccgaccttattaatacgttcgttcaaaatacagttgcaacaagtgcaatcacgcggatgcgttcgacgcaggcattctcc

cagttctcgtggtttctatccgaattgcagtcaatggactactgcatacaatttcgactgatcaaaggaagttttctg

>51312|AGAP010483-PA

caacgctcccagcaggggcaacatctttgattatacgtgtatatgttttgtttatatgtaatgttcgcggggaagtatttaaacttgtggtgcatgttcg

tgtagttattggtgtattatcgtgttatttaggttttgtaaaaagatttcaccatctgctgctgttcaaagcctcgtccatatggcggaagtactcctga

gtacagccgaaaagacttacattttgcacggtgttgacgaggattttagaacggacgggaggaacagacgaacgtacagacccatcgaattagaaacaga

agtggtaattcatgcaaatggttcggctcgcttgcgcttagcaaacaccgatattttagtgggcgtaaaaacagaaattgacactcctttccccgagaga

ccaaacgaggggaagatagagttcttcgttgactgctctgcaaatgcgacgcctgcattcgaaggtagaggaggtgaagacttagccacagaaattagtg

caagtttggcaagagcgtacatgtcttcacaagttttcaacctttcgcctttgataattacacctggtgttcattgctggaagctatatgttgacattct

tatcttagaatgtggaggtaacctttttgatgcagtctcattggcagttaaagcagccttgcacaataccagagtgccaaaagtcgaaaatgaaggcaag

gatggtgacgaggaaatgcttgtgttatcgcagaattataacgactgtacccatttggatgtttcaaatactcctgttctaattacactttgtaaaatag

gcgataactgtgtagttgatccgacagccgaagaggaagcgtgtagcgttgcaagtgtggtcatagcagtaactgaatccggtttagtgacctcagtttt

caagactggggaaggaagcctgagcccccatacatttcgggatgctctaattgaaggcagagacataggctacaatcttaacaagacactgaagaatgcc

ttacttagagaagcagaaaaggtaaatgaaggaggagtgtatgaaccatacggatttttaaaatagcggatatatggatccatagagctgttttgtgtca

tttaattctgatgtgcataaaaataaaatatttaatgccatattgtttgttaccaagtcgcttggggtagtaaaatttttctttgagttgtaaatatcac

atgattccaagccaagaaataacttaagacgtaaccagttaatactaagtatgattttcaagaaaatgtgtaatttgctaaggtgtcatttttgtcaggc

ttgaaagtgtaggctaatttcaaattgatttgatttattattttgtatcttagtttttgtacaataaaatagctgcatattttccaaagacatcctaata

ctgcttctttaattgcctgtaagattaaaattatcatgttgaaagttaagtaatgcacttcacaggtgcaattatgatgaagtgacatttttatcagccg

caccattagtacctcagtgaatgatgatattccctgccatgggcagaattttgatgcagtatggattggattgagaatttctggatgaaattttgctaaa

cagaaaaaaaggaaaagaaaaatatgttcctctgtttaagaatatcagtaataaaatttttaaacaca

>51314|AGAP010490-PA

ctcagtcagcatcaacacagaatcatagtaatccctctacaccacaacctacacttagcagccagacaggacgaggagcctcagtgataatgaagcacaa

agtggaaggggtgagaagactgtgctctcacccaatgcttgggatgtacctcacaggctctcaagatggatcagtgcagatgtgggaatggtgtcagcag

cattctgttgccactcccagaccatctggaacatttgcaaaggtgactcgagtgcgtttcagtgaacatggaaacaaatttggggttgctgatggagatg

gaaatgtcagcctttggcaggttggcttgtcaagcaatggcagccgtcctttctttacgtaccagtgccacaataaaataacaagtgattttgtattcct

tgggtcatgtagcatgctggctacagctggccacagctcagaaagtaaaagcgttgcattgtgggattcattactacctcagaagaaggcgttgataaca

gcattttccattcatgatcaaggagcctcaagtcttgtctatgcaccgcaacaccagttaattatttctgctggaaagaagggtgatgtagccattctgg

atgttcgacagcgacaagtgagacatcgctttgtagcccatgaatcgcccataaagtgccttgcaattgatcccagtgaagagttttttgtgactggttc

agcagatggagacattaaggtgtgggcgttatcagtccataatctgctctattcatttcctggtgaacatgcacggtcaagtttcttcaagaatatagga

caaggtgttacacagctacatgttgatggagcaagtcgtttgttttcatgtggtgcagatggctcaatgaaagtgcgccagctgccagagagagaagctg

ctgtacatactttgtactagtttgcttttcattagtgggtgacttaaatttcggctgtgcttcaaattgtataaaatgcctcttacatttgtactcttta

tttgaaaacttagcagtgtgacatgtgcatataaaatgttaatctgctgtgtgtttcattgttgctcctgtatcagtgttcaagaatgaggctggcatgt

aagattagtgctaaacgtgggcaaaatggatgtgtttgtccgtattaagtggactagcatacatattattcatgttcttatgataattcgtgtgctgtaa

tggactttgagtgactatcttatttcagaattatttataaaacaacatgatattattgtgttgtggacaagactgttttactttgaatggcttaagcctt

aacattaatgggactgcattcctttttgtaagttgtacttgatgttagactgtttgagccaagacatgtatttctgtactaaagttttctaaacagggtc

accacgtgtgaatttatttaatttcggtattcattgagatctgtgaacaatagaatatatatttttagcaaccgtattttgcccccatacataaactgta

gtgatattataagaaagtgggttcatgttgtgcctcccaggcagcacggaagtatcccaaaatatttgcacagtatgaatatatcctctagtgacatgaa

caataaaatcaagtgttcacattttgtgggtacaactgttctatttacatatgatgacaaaagaggtaatcatctcttttgtttaaacattgattcttat

aatattgctacttagtttgattctattattgcaagtgagaggaatgtggaaataggctaattgagctttatgcaagtgcatcaataaagtgctgtgacag

gaaattctcagtatattattgcaagacgtagttcagttggtagccttaaattcaataaccagaaaatgtgattaaaggtacaataatttttactccaaag

gtctagtcctgtatgatactttactagagaaacactaattgttcatcatgtgctggatatgaacaattcctgagattgtgtagataaatactttatctag

tagtatgtgtccatgcccagttattgtatatacacattgtaaaatgcgctgtatcaaagacataatcagtgtaaatattcctcttccattttgacatctg

atattttattctgtagatattagtaactgtaaatctcccataatccttctggtttgttgatcagtattccaatttagtaatacagttttagtactttaag

ttgtttgaaatgtgcagattttattttgtgaatttcattagtattttgttacctaagtgtacatccaattatattatatagacaaggaagcagattgaca

acaagagagaatatttttttagtatgtttaaaatagatttttcccactttgggaaacaggattttggttctatactgattctttctgcctttatattgtt

tttgaaaagagaatattcttcttagaataattcccttctataagccaggatcataatgccatgatctatacat

>51322|AGAP010539-PA

ccagaaaataaaattgtaactagagctttatttttaaaaaataattattttataattaaaaacaatattacagctttttatataacattccatatacaaa

tatacatatctatttataaaaacaatattgatgaaataaacacacatgtaattcctacagtgcaaaacaggattctgaaacttaatacatacaataatac

atgtgccctgaaaatattattgtccaatacagtgattatcatttacagagcattttttgtctttccagttttctactggtaacagcacaaaggaaagaaa

taaaaatgtcacaacatgaacacagccagccaaaagaggaacaatgatgtgtcagaacattttcacatagaatcaataacaagccacttttcttgcactt

gttcaacttgctttgtaacaattggatttcgtattctgattttcacttctagcttccc

>51325|AGAP010552-PA

cgaggacgaagaacagcaccaccagtggaagaggaaattatgcgcagtgttgctattatggaaatgaaaaagaaaaatgctgcagaacttgcaagaaaag

aaggaaagaaagatcctggtgttttagataaagaggttaaaggaatggaatcatttccaaagtatgatgaatatgaagtaatgccaggtaaaagtaagga

agcagataaataacacacattgagacaatttgttgtttatatagactgtaccagtcaaaaaaagttgattgtgttgaactatagcgtgtttttttttaaa

tatgttattcataaaatgcagggactagttaccgtataatgattctcactgatttctcataagaactttgtttatgcatgtaaaacaataaagtgcagtg

aattattttttgtgaacttttctcatgcattccattacagaactggtttacaaaagaaacatcagagtaattatatttcaattaaagcaaaacatagttg

ctgttgtgataaccttttctgctatttaaatgagac

>51328|AGAP010552-PA

ttcactgcactttattgttttacatgcataaacaaagttcttatgagaaatcagtgagaatcattatacggtaactagtccctgcattttatgaataaca

tatttaaaaaaaacacgctatagttcaacacaatcaacttttttgactggtacagtctatataaacaacaaattgtctcaatgtgtgttatttatctgct

tccttacttttacctggcattacttcatattcatcatactttggaaatgattccattcctttaacctctttatctaaaacaccaggatctttctttcctt

cttttcttgcaagttctgcagcatttttctttttcatttccataatagcaacactgcgcataatttcctcttccactggtggtgctgttcttcgtcctcg

gagccatgcttcccattctgctggaatttcctgctcaaagttatctttcacagctggttcaaaccagcgtgaagcttttctcttgccaacgcttggattg

gccggtatctcgaaatatttatttcctaagtaatctgttcctactggagttcctcttatctgtcgtggctttaaggactggatgaaattcttgaatatca

tgccaataatgcttctaccttctttagccatcccgtacctctccgctgactaaaacggtctaaagacgcagtggtaatttctccagatctgttgagttat

caatttactgagtggctgaaatgtagttcgggtaccaatgacaggtttgttgagacgcaattttctgtgtgggcagatttaactttttcatacggtagta

agtcgcagaagtcacagaaatcgcggaagtagcaatggcggaggg

>51326|AGAP010552-PA

ttcactgcactttattgttttacatgcataaacaaagttcttatgagaaatcagtgagaatcattatacggtaactagtccctgcattttatgaataaca

tatttaaaaaaaacacgctatagttcaacacaatcaacttttttgactggtacagtctatataaacaacaaattgtctcaatgtgtgttatttatctgct

tccttacttttacctggcattacttcatattcatcatactttggaaatgattccattcctttaacctctttatctaaaacaccaggatctttctttcctt

cttttcttgcaagttctgcagcatttttctttttcatttccataatagcaacactgcgcataatttcctcttccactggtggtgctgttcttcgtcctcg

gagccatgcttcccattctgctggaatttcctgctcaaagttatctttcacagctggttcaaaccagcgtgaagcttttctcttgccaacgcttggattg

gccggtatctcgaaatatttatttcctaagtaatctgttcctactggagttcctcttatctgtcgtggctttaaggactggatgaaattcttgaatatca

tgccaataatgcttctaccttctttagccatcccgtacctactgattacagcagtgacaagaaggtgctgagtcaatacatagaaaaacaggtgcagtga

tgaagtaaaagttacaatgactgtcactcatgaaataccacttacacgcaatgccttcagccttctaagccacgtttttatccaccaatggtgcaagaat

tatttgtgcacagcacactacatgtcgtctgctttctgcttatcagaaagaaatagtgggcaggaaatcgcgtatgttagaaatcacagtgattg

>51330|AGAP010566-PA

cgacgtttgatcgtaataattttttttccaaatgttccagatgacgaaataagtaagattgctgtcgcagttatcgttttgcagacacgcatgttcgaaa

aagtgttttgttgtagactgacattctcgattattgtgttatttcatagcacttttcgattgacacttctcgtttagaaatctgtacttttcatttagaa

aatcttccccataatttgcagaaatgctagttggcggccacggcgaatctaatccgaacacaaactggctggacagtcgtggtttatggattagttacgc

tgtggggatgcttgtgcttcatctgattcttctaagtataccagttttcagtgtagcatttgcttggactcttacgaatatcatccataatttgtgtcac

ttttttttccttcacttcgtgaaaggctcaccatgggttcctcaagatcaaggggacagtagggaacttacccactgggagcaaattgattatggagagc

agtttactgttactcggaaattcttgacggttgtgcccattgtactgtttttcctggctagctactacacaaggtatgcaactggacatttcgtagtgaa

ttttgtatcgatgatgttggtcttgctgccgaagctaccacagttccacagagtgagactttttggtataaataagtactaagtatttttctgatgactt

actgtgtgtaaaattatacaatgttacagacgtcactgcatcttttgcagatgcactgtacttacagtataaactgccattatgtttgagattgcagtta

agtgtgtgtgttttttaaacactctcaagctttgagaagtgtatggcaggtattgttacttcattttctgtgtaaaactttccatataaacacgatattg

ttcagactttctttgcattgattttacctctgttgttggacaaatttcttttggttaggatatttgtgtaaaaagaaagaaatcgaatattgtttaaata

aactcactgaaatggtgacattctgcattatcatcaccctctgcatttgtgtgaagcagctcttacatctttcacatatttaaactagattcaggtttgt

aactgctgtttaacacaattaat

>51331|AGAP010568-PA

aaaaaactctcctgccttgtatgaactgcgaaccagtggaccactcgcagtgtacaagaaccccatctcatttcccaccctctcccagtgtttaaatttt

tcaggatgaacatactccactacttttaggtgacgttttgttggctgcatatattgtccaagagtgacacagtcaactcccattgcccgcaaatctggaa

acaaacagctggcattaaaaacttgcatttctggaaagcatttaagaactgctgattgttcattcattcaggattggggaataaataaaattacatgtta

gcaggcaaaagcaaccactgttggctaattctgttaagtataccagcaacagaaaataagcattagtgctaatttaaaaaaatgtaataactataggaac

tacatgtagattactttgtgtgtctgagggggtagtactaaaagttttaattatcacaatgtaatcaattttttgtttgtttgcagccctggtacaaatt

aaatgcttgcaccaatgacactgtaacgcattcctagag

>51333|AGAP010572-PA

caatttcagatgcaaaaacggatgatggaaggtaaatttttgtgacttgtgagagactttcttccaaattttcgttctgtttttgactggatgggtgttt

ctcttccttagtttttttcttttcactaacaggctcccagacacattcagcactgctgacttccttgagatgctgaaggtagtcgtagtcatcatcaaag

aaaattccatattttctctgttcttctttccgtttttcaacttcttcctgcttaggcctcttctcagcagctggaacgagaacatgctgtggggcagttt

catctgctataagcggatcttgctgacttctgtgtacaagatggaaggtcacagcattcttgcgatcaatgaacttcttctttgcttttggcatggttga

ctacaatcgttacccttcctggcttgctactctcacatcactcaataataacaatattagtataatttcatatactatgcaaacaagacaccatgtgttt

cagatttgtaaacacaataccacagacgtatacacatagcggaataccactagtactagagtctcgactgtggcgaaagtctctgatgtgcatagtatat

gaaattatactaatattgttattattgagtgatg

>51337|AGAP010573-PA

cccaaatcaaagcatatctttttactcagagaaagtggtttatttacacagacataacaactacatatttacaaataaaatgcattaggcacaacagcat

tcactttttctcagcttgctccagagctttcagcatttctacgtctctcttgcgttgcagctgttctgcaaactcttggatttgttttctatgctgtgca

ctctcagggggatacaattctcttcttgtcttaataacccactcttcaaagtattctggctgattaaaaaaatgaaacatagcaacaggaaatgaaagat

atattgccatttttgcagcttcaagtttccagcctcccattatctgctccttttttataacgaccttgtgaatagagaagagttcactagtcccacttgg

acaccctttcagcacaagcatggatttcttacggatgcactctcttgcctgggcactcggtatacctcatcgattctactcctcctccgcacaaaagcca

ctagcagtttgctcacctataaaattcataacataaacattttcatccagatttgtttagtggaaagaaataacacactttactcgtgatatgtccgttt

ccgattgtcgattcactataggacgctagttgggaaa

>51334|AGAP010573-PA

ttcccaaatcaaagcatatctttttactcagagaaagtggtttatttacacagacataacaactacatatttacaaataaaatgcattaggcacaacagc

attcactttttctcagcttgctccagagctttcagcatttctacgtctctcttgcgttgcagctgttctgcaaactcttggatttgttttctatgctgtg

cactctcagggggatacaattctcttcttgtcttaataacccactcttcaaagtattctggctgattaaaaaaatgaaacatagcaacaggaaatgaaag

atatattgccatttttgcagcttcaagtttccagcctcccattatctgctccttttttataacgacctgaagtaataaatagactgtagagtttgttctg

tcacattaatatcttctatgataataaaggaaatgttccccatagaaacaatataccttgtgaatagagaagagttcactagtcccacttggacaccctt

tcagcacaagcatggatttcttacggatgcactctcttgcctgggcactcggtatacctcatcgattctactcctcctccgcacaaaagccactagcagt

ttgctcacctataaaattcataacataaacattttcatccagatttgtttagtggaaagaaataacacactttactcgtgatatgtccgtttccgattgt

cgattcactataggacgctagttgggaaa

>51338|AGAP010577-PA

gtcaacactgtttgacgcgtgttgaatgataggcgccaggttttaatggtttgggacgtagggttacgttgatcaaatggaacagcatacaatgtaagtg

atgtgtttctctcaagtaaagcatcaaacaatacggatctttgtgcattcatccggacagagctgtaaaatgtgtcgtgagaagtaagagaaacgacttc

aaaaattatgtttacaataaacattgctcaggtggtcgtcagaacattttgtggttccagcaagtaatcacgttattggacatgtcttcatcaagtcctg

cactaaaacgcccatacgatagtgggagtgccagttcaagcccagcgccacaaaagaagtcacggacatgtacttttggtgaaagttgctacagaagaaa

tccaaatcatttctcagagtatgagcatcctcacttggataagctactagagcaacaagaagatgcagaacctaaactgcctccagattataagccaaag

actacaccatccattgttttggcacagttgatgatactacaggaccttaatgccaaaaatggaataaaaggaaaagggaatgagcctaaaaataaacctg

cagtgcaggaagctgctagttccagcacatttatgagggatgtctacaagcaatctcaagctcctaagaaacaacctcatacaacagtaaatagcccaag

cacatccgttggatctagaagttccagttcagattccagagaatctaagaggaatgtccaacagaagacatccacagaaaaattgaaagaacgtgtttcg

gaagcatcctcttcaagtaattattcaaataaccaagcaacacatgataaaggtgctgagagactgagtaaggaatcccgcttggagatgaaaaggaaga

tcagggaacagtggaacaacatctcgtcagtggtacctgtcagtgacaatgtactggagaagctagcagctggtgcgccgtacaattttttccttacaac

agttctcgattctaggccaactcattcagagatgacatcgattacatttgctgatttactacatccatcgcttggtgaattgaaagcatcattgcaagtg

aattttatggttgaactggaatggctgttggagcagtacgactcccatggctacaagaaaaaaccgttactcattctttatgaagatgatggtgatgatt

tcaaaaagtgcaagaacatcccagataatgtgaagggttatcgagtaaagccaggacatgcatttggacatcatcacacgaaaatgtccatcttctgtta

tgatgatgacagtgtgagagttgttataagtacagctaacctcattgcatctgacttcgagaacagaactcaagggttatgggtaagcccaagatgtcca

aggatcccactgaatgcagacactaaagcgggtgactctataacagaattccgcgctgacttaatgcgatatctatccgcctaccagatgccagttctgc

aggaatggattacaagaatacgttgtgccgatttctccaaagtcagagtttgtcttgtgggttccgtgccagggacacatcgtggagcagatgtagatag

atggggtcaccgtcgaatggcagtccaccttaagcgctttgctgaggttccaagtacatcgtggcctctattcttccagtgttcctctattggcagctta

ggcgcaactccagattcttggatgtgtggagaatttcgagctagtcttgctcagtccgtcaacaagcagcccctctcacagcagccaccagtgaaagtga

tctatccctgttttcgtaatgtggcccaaagtatacatggccttttgggtggtgactgtctaccatattccataagaactcatgggaagcaggcttggtt

tacaggatacctacatgagtggaagagtgacagccgctatcgttcagaggctatgccacatatcaagacatatggccgagcatcaccagactggaccagc

ctagcctggtttcatttgagcagtgccaacttgtcaaaagcagcttggggtatggccagcaaaggaaagacatctggttctgggcactacattatgtcct

atgaactgggtgtacttttcctgccacaattactgattgggaagtcagagtttcgtgttggtaatacactggggaatagttcagcaccttcatttcctct

accatttgatgttccacctacaccatatggcgaaagagataaaccatgggttactgaatatatgactgttaaattttaatatgtaatattttgtgtcaag

cactaatgtattgtcacattttgtattagtgcctttatgtgaagcacagttgcaaagaacattaaaacattaatttcataaaagaaatgtccaatat

>51372|AGAP010591-PA

ggtcctgttcgaatgccccctaagatattgaggattacaactcgtaacacgccctgtggtgaagggtcaaagccttg

>51381|AGAP010591-PA

agaagcagaagctacgcgtgaacggccctgttcgaatgccctctaagatattgaggattcctacccgtaagaccccctgtgg

>51388|AGAP010591-PA

aagggtcctcttcgaatgcccactaagatattgaggattaatcctcgtaatatcccctgtggtgaagggtcaaagacttggg

>51363|AGAP010591-PA

cgtgtgaagggtcctgttcgaatgccccctaagatattgagggttactgctcgtaacatgccctgtggtgaagcgtcaaagacttg

>51371|AGAP010591-PA

tacgtgtgaagagtcctgttcgaatgcccactaagatattgagttttactactcgtaagacgcccggtggtgaagggtcaaaggaatggg

>51382|AGAP010591-PA

tacgtgtgaagggtcctgttcggatgcccactaagatattgaggtttcccgctcgtaagtccccctgtggtgaagggtcaaagacttggg

>51362|AGAP010591-PA

agaagcagaagctacgtgtgaagggtcctgttcgaatgccaaataagttattgaggattactactcgtaaggccccctgtggtgaagggtcaaa

>51355|AGAP010591-PA

gcccactaagatattgaggattacttctcgtagggccccctgtggtgaagggtcgaagacttgggtcagatttcaaatgagtattcacaagaga

>51383|AGAP010591-PA

tcctgttcgaatgcccactaagatattgaggattactaatcgtaagcccaactgtggtgaagggtcaaagacttgggacagatttcaaatgagg

>51365|AGAP010591-PA

gaagcagaagctacgtgtgaagggtcctgttcgaatgcccactacgatcttgaggattactcctcgtaagaccccctgtggtcaagggtccaagacttg

>51389|AGAP010591-PA

gaagcagaagctgcgtgtgaagggtcctgttcgaatgcccaccaagatagtgaggagtactaatcgtaagtccccctgtggtgaagagtcaaagacttgg

>51366|AGAP010591-PA

aagcagaagctacgtgtgaagggtcctgttcgaatgccccctaagaaactgagtattactacaagtaagaccccctgtggtgaagggtcaaagacttggg

>51356|AGAP010591-PA

gaagcagaagctacgtgtgaagggtcctgttcgaatgcccattaggatatttaggattactcctcgtaagcccccctgtggtgaagggtcaaagacttgg

>51386|AGAP010591-PA

gcttctgcttcttagccccaaatattagggcactgcaaactttctctaatcttcttacatcccgcgacgtgagagttattctgatacgatgaattgttgg

g

>51370|AGAP010591-PA

tagcttctgcttcttagccccaaatattaggtcactgcaagctttccctaaactcctgacagtccttgacgctagagttattctgatacgatgaattgtg

g

>51376|AGAP010591-PA

taagaagcagaagctacgtgtgaagggtcctgttcgaatgtcctctaagatattgcggattcctactcgtacgcccccctgtggtgaagggtcaaagact

g

>51377|AGAP010591-PA

agaagcagaagctacgtgtgaagggtcctgttcgaattcacactaaggtattgagaattactccaagtaagcccccctgtggtgaagggtcaaagacttg

g

>51391|AGAP010591-PA

gaagcagaagctacgtgtgaattgtcctgttcgaattcccactaatatatttaggattactactcttaataccccctgtggtgaagggtcaaagacttgg

g

>51375|AGAP010591-PA

gaagcagaagctacgtgcgaagggtcctgttcgaatgcacactaggatattgaggattactgctcgtaacaccccctgtggtgaagggtcaaagacttgg

g

>51374|AGAP010591-PA

agaagcagaagctacgtgtgaagggtcctgttctaatgcccactaagctattgaggattactcctcgtaaggccccctgtggtgaagggtcaaaggcttg

g

>51378|AGAP010591-PA

gaagcagaagctacgtgtgaagggtcctgttcgacttcccactaagatattgaggattactacccgtaagtccccctgtggtgaagggtcaaaaactggg

g

>51354|AGAP010591-PA

agaagcagaagctacgtgtcaagggtcccgttggaatgcccactaagatattgaggattgctacttgtaagccctcctgtggggaagggtcaaagacttg

gg

>51373|AGAP010591-PA

agaagcagaagctacgtgtgaagggtcctggtcgaatgcccactaagacattgaggattactccccgaaagaccccctgtggtgaagggtcaaagacttg

ggc

>51349|AGAP010591-PA

agcagcagctacgtgtgaagggtcctgttcgagtgcccagtaagatattgaggattactcctcgtaagaccccctgtggggaagggtcaaagtcttggga

cagatttcaaat

>51352|AGAP010591-PA

tgaattgttggggctaagaagcatcagctacgtgtgaagggtcttgttcgaatgccctctaagatattgaggattacgtctcgtaagaccccctgtggcg

gagggtcaaagacttg

>51351|AGAP010591-PA

cccaagtctttgacccttaaccacagggggccttacgagaagaaatcctcaatatcttagtgggcattcgaacaggagccttcacacgtagcttctgatt

cttagccccaaatattaggacactgcaaactttctctaaa

>51350|AGAP010591-PA

agggtcctgttcgaatgcccactaagatattgagaaagtttgcagtgacctaatatttggggccaagaagcagaagctacgtgtgaagggtcctgttcga

atgccctttaagaaattggggaccactgctagtaacaccccctctcgtgaaggcgcacagagtgg

>51353|AGAP010591-PA

ccaagtctttgacccttcaccactgggggacttacgagtagtaatcctcaatatcttagtgggcattcgaacaggacccttcacacgtagcttctgcttc

ttagccccaacaatttatcgtatcagaataactctcacgtcaaggaatgtaagaagtttagagaaagttattctgatacgacgaattgattatctcgacc

aggtgatctattaactgcagaaatggctttcaataagg

>51393|AGAP010599-PA

tgctgcaagagttgggacgtgctgagctccgcaaagtggctcgagaaaggcttcttaaacttctacgcaaggatgcaaataacgaaagagtgcacttcaa

tctggggatgttggcaatggatgaccgggatattgctggagcagagcactggttcagacaagcagtccagttaaaagatgacttccgaagtgccctcttt

aatctcgcacttctccttgctgatgatcatcgtccactggaggcagcaccttttctaaatcaacttgtaaaatttcatccagatcatataaaaggactca

tcctgctgggagacatttatattaacaacataaaagatctggatgctgcagaagcttgttataaaagaattctgcaactagacccagaaaatgttcaagg

gctgcacaatctttgtgttgtgtatgttgagcgtggaaatctcctgaaggcagaaacatgcctgcttcgtgcccatcgacttgcacctgaagaggattat

gttttacgccatcttggtattgtacagtcacgtattaataagctacaactccggcaagatccagatgcactggcagcagcagcagccagtgatgatggct

tttcatttgaagatccagtctctacagaaggcaggttgcccattgaaaacagaagcattgactcagcatcagcagaaggcagtgagacaaaattagcaga

taatgctgcaagtcaaagggtacacgagacaggtcagaagaatgtacatcatggaaggaatagtattccagagtccttcagtccagcatttgtcgagtac

cccaagaaagatacacgcaggaagacagtaatgagccgtaacacagaaatgaataggttgtcatcatcttagcacataggaatgatccttagttattgca

gacacaaaaataatactgtatagatttttgtttctatgtgaaggactatttttgtgcatctaacaatgtaagactggaagggtggtgttggcacatccag

tagtagcatcaaatgattttgtttcagttcagacagagtatgtatgtacacatattttgtcatctatccaatagtcacatccttctgcctgtccctgtcc

ctttatttatatctgcatccattaacctctttttttcttggatggatggatggatgacagtagagaaattggcaaagcattctatcaaaccaaattgtca

tgttccacaatgaatttttaacatacaataaatagtaagtagagtctgctcaggatatagctgtgacgtgcaatgaccagttttcatcaaaagtactgga

cgagttcattcatttgttcagaagtggagtgtacacctgcttttgcccagtcaccaacatgctgtgaagatagaatcaaggcacatgccgaacagtattt

ctcaaacaattttacagaaactatttggtaaaaaaaattagttattgcattacttgtaggtttttatgtcggcttcatgataatgtggcaccatttcatt

aacggtcatagttattgttatatttccatttaagtaagatactgaacaaaattcaaaaaatgaaaaacaggggtcgctacaattttgtgtttcgtacata

ttacataatatgttgatatgaaattcagctaagatattgaatttttcagtagattgaaggtccctgtcatcaatctcacgaaaactgatctgatgtagct

cattgatttgcagtcatatgtgttagcaataaagccataaagaaatagccacaacattctttgtatttcataaattgtgtgagatatcgaacagagattt

ggaatatgatagcaagcaaagatgagagtattttgccacatggttattatgcaaaactctattatctgccatgttattccattaactacagacctttcta

gggaaagaatataattttaagggtagtcaatagctggtgttacaaaaaagttgctggatttggaacaacataacagaagacatggcacaaattattttta

taccgaggatatgttttttcataagatattaacctcagttttactcatttcctaacttaataaaccatgttttcagaattctcttgcagttacatctgca

caagacgttagcgaggtgatatgtaaactccactgcgttttggggaaaaatgcaaacattaatgtggtaacaagagaaatgtaggttttacagcaaattt

gccacttatgatacttctctgaaaattacacatggtgaaaaagccaggcaaaaataaatcaaggcttttgttgcattatcagtggaattctttctagata

ccaaccaaatcaggagtgacagtagatatttttgaattgtcaccatgcaagtgtgggcatggagggactccaattaatatttacagaaaatgtgaataca

gacttcagtttagaacagtacttcccctccagtgctcagcttttcccatacatcacctgcccgcaacccctaccactaccagtctcaccatgcttgccct

gcctgttgtgcatctaccagccacattattctccatggattctaatgaagagttccatggcttatttctaattggggtgctgtgaaagcatttttatcct

ctgtagcttcctttagtgagggctcacattttattgaaaattacttcttgttaaagtttcattccatgtttttggaggataatttgtattgttttattgg

aactcaatcagaaacggagaattcatagatggaagcccaccagttgatgaaaaactgtctgctcatctgataggttgttatgtgcatcattaccagttgt

gggatgatatatttgattgctatgctagtttgtattgactgaaaagaaattcagtcttacagcagaatatcatatcatgctatatcaactaaagggccta

ggacgaagcatctgattgtaataagaactatttaaatacttctgtctttcttatatcaattttagaagaggatatttgcattttatacaaaagaacaaca

aacaatataaacattaccttattaaaatttgtacacaactggcaggctgacagtcatcaaagtaggttacctgatggaaaaattaacagaatgtgagagg

ctgtcagacaatactctgttaataggtagatagggctgcacagatgtcaactaactcttatattcctgaacatacaaaggttttagccccatgttgacac

aaaaatatgaaagtaaggactacttacataataattgcttgtacatgcaaagaaaagattttaagcagtggccatgtcctacaggtgcatttctggatta

ttaaaaaaaatatacattgtaacacagtgctgttaaggacaaattgttttgtaggaacattattttagtttatccacattttttgtggatttacctataa

actgacactttccattagaaaatgtcaaggcaatcagatcttgcatactctgtctgaaatttttaatactgatgtg

>51396|AGAP010613-PA

caagtattgttcttgatgtgaacccatgggatgatgagactgatatgagagaaaagaaaaacaaagttagaagtattacaatggatggtcttgtatgg

>51394|AGAP010613-PA

ccatccattgtaatacttctaactttgttttccatttctttcatatcagtctcaacatcccatggtttcaaaacaagaacaatacttgattttgcaataa

gtgctcgctattttgatttgttgaactaatgaccatattttttcctttagtcac

>51398|AGAP010672-PA

gtggattatttattatcctaagcaatacctcccattacataatcatatactatggtttcttgaattcacaaatattttaaaataatattgaagttagaac

ctgcaaagcagttatgtacacaaaagtgttatgtacacagattctcctcataaataaatactgttgcatactctttaagcacactacaccaagcactact

tccagtgtgattgttatgagaaaagactttttatattacagtgacagtagccatgctgtcacaattgttgctgcagttaacatagcataccctgtgtaat

acacttcctttattgtcagtgcctttcctaaatcccaaaccaagtgacggacaccattagatatatgatatgccagaggaaacgccatcattgctttcat

ggctgtgattactgctggattcaaatgcagttcttgcatccaagtgatgaaatcagtgaaccggtaaggtgatacaagagtacctagacccaaagttgtc

atgtagaaactcaaagctagtcctgtcattcggtgagtgattgaaagcattgatgttagttgcattttgtaaatggtaaggtgaggtgactgtggccgac

ccagcctagcatttctctcatcgtgggtttcattgacaggagattcaatctttgttacttttagtgtaatgcaccgacacggctgcgagtatacattgcg

taccaggctccgacacaattgcctgcctcccagtaatctgtaacacaatgccatagccctattgtgtttttgtcttgagcgctatcaaagatgtgacaca

cgtttggcgtcttttttgttttgttattttttttgttactaatgatcttaggcgtgtttaaccaattgtacgccaaaattcaagttattcgaatattccc

aacaaccgtgactgagcttgacgacgtctctttcaca

>51399|AGAP010687-PA

cttgagtagtattcagaatctgaaattgtattagttctagcataactaaaaactgaagaaatgaagcaaaaaatattaatgccctgttaaaaatacagtc

ataaaatgataaagttgtatcaaaaattgtacctgtgctcattatcatcccagcggccatagcagagatcgatgcccccgacaaaggcatatgtctgatc

tattacaaccagcttttcatgatgtgcccagaaaagcacaccagcttttgcatgatctggatgacgcagaaccttcagagtggtagaggaaagagtcaaa

tcgcactgttagatgttaatacaacagaaatgtgtaaaaagttcctagctttatattatgactggcaataaatatagaaaacatttggcacgtacttgtt

tcctcattcattatgcagaaatcagtttctgaccagtaaaat

>51400|AGAP010694-PA

ctctttccctacacgacgctcttccgatctccagccgctgcagatcgaggagactgacggatacgttcccgagagggaagcgaacttctcgcggctggtg

ctgtacttccggttcgagcggcagatcgggcaccacctcatccagacgttcgcgcccag

>51401|AGAP010694-PA

tgatgctgtcgtggttcagcttctggatcggcctggacgccatcccgggccgtgtcacgctgctcgtcacctccatgctgactctcgtcaccatgttcac

cggcctcaagtccgacatcccgcccgtcgcctacgtcaaggcgctggacctctggatggccggctgcatggtgttcgttttcgcggccctgggcgagttc

gtcgtggtgaaggtgttggaggtccgctaccagcgagagaagtcccaccgcggcgtcaacctgccacgcctgctgcccatgcgcatccaggcgatggaga

aatcgccgtgcatgtctgcttgggacgtggagtcgggttcgggcggccaacagtccgggcagcaggcacagacgtcttccgctgcagccagccgacgaag

ctcagtcgggtccgtactgcgcatgcgcggccgaccgctgctgcagacgtcttggcgggatcacaccacgggccgggagaaggtgctgtggagagagatt

gataggcattcacgtacaatctttccagcattcttcctgctctttgttgttttctactggcccatattgctgctgaagaagtcttaacactcagtgatga

caagattctttcactatttcagagtttattgaaaaagtcaaatggagaaaaggcttgtagccctttctgcacagacatgaatgtctaaagagcacactgc

aatgtgtcactggctgtgatactgaaagatgttttatcagagtgataagccagattatgaggattaaaataatgtgattagtagtgaatattgtgccttt

aatcactgtaaataattatagctctaaattactgatctttcaagcctatgttcaaattatggttgttctacagtggtgtgaataaagaaatctctttagt

tactgttcagttaatgtttgagatgagaatatagctaaaaagtgttgcactctatataaaatcaaagtgttcctgtgatatgcatttgtaaagattgtga

gtgagatttgtcttgcagtctcttctagtatatag

>51402|AGAP010700-PA

tgtttccagatttgacccttcgtaatataaacggtagcagtaactggtgcactttattgataatcgatcctttatcctcctccatctcagcatttccttg

aaagtcagaaaccttgaggtaatcctcaagtagattttcttcaggaaatattttgggcaacacaaaccttagcaatgcccacaattcattaatatcattc

tgtaaaggtgttccagtcaataaaagtctactgcggcttctgtggaattttagggtgttgtaaagcttggacttcgcattcttcaacctatgaccttcat

caacaataatgaacctccagtcgaattttgcaagtattagctctctcaaagctatatcataacttgtcagaactactggagtcatatcagtagtcagaac

tgtcgtc

>51414|AGAP010701-PA

ccggcacagccgctgcgagcagtccaccagaatgtggacgcagtgtgtgcagatgtatcccaccagggcggtgcccacgaggcccacggccagacctgcg

ctccggaaggccataggcatcgccaggattcccgtgccgaggctgcacttgagcaggtgcatcagcgcacccacgtccgatacgggcctctccacctggc

ggtgggcgaacggg

>51416|AGAP010701-PA

gcggaatatttccgatggcgatggcggcaaggcggcggcaccacggcggcgcaccaggtgtggtactacagagatgggagcggaccacgaaaaaggcgcg

gacagcgggccgccaatccccatggagaacttctcctcgacggcccagttgacggaggacaccgtttccaagcagcatgagagcgctccggaatacgacc

ccttctctgaaaggagcatacagcaccccaactcggacctgggggcgctgatgcacctgctcaagtgcagcctgggttcggggatcctggcgatgccgat

ggccttcaggaacgcgggcaccgccgtcggcttcgtcggcacggtgctcatcggcatgctctgcggccattgcgtctacatgctggtgcggtcgtcccac

atcgtgtgccgaagggctcgtgtcg

>51417|AGAP010710-PA

caccccttgaaacagaaaattaagcgaaacaatgtaaagtctacagcagttttacttacctatggaaaaaaaggcatcttcattgtgtcttcttatctga

taatgaattatttgtccttgatactgaactgacagcacataatcccctgcagctgtgttgctttcacggaccaaaaagtagccatcttccacttcatctt

cttggagcagaagctgttcagctgcttccctagatatctttccatggtaccaacagatttcatcatccctgttcattgtcacatctgagttgcacttcag

ttcctcgagcggtacaaataaacttcgacttcgatagatgcatgtaagcaaagctgtagttgaaccatacagatcacatatcaacaggtgtttacaaacc

agagtcgtaattcagat

>51418|AGAP010710-PA

tacataccttcttttgtggctcgatgaagaagattcatccttccatgtcgccttgaatcatgaggtggtggatccttcttacacacttttgtaagcttac

acagtaatccatgtgaagcttcttgataatgctctatgagagtttcaagtccatggattatggtgtgttcgtctatggaaaaaaaggcatcttcattgtg

tcttcttatctgataatgaattatttgtccttgatactgaactgacagcacataatcccctgcagctgtgttgctttcacggaccaaaaagtagccatct

tccacttcatcttcttggagcagaagctgttcagctgcttccctagatatctttccatggtaccaacagatttcatcatccctgttcattgtcacatctg

agttgcacttcagttcctcgagcggtacaaataaacttcgacttcgatagatgcatgtaagcaaagctgtagttgaaccatacagatcacatatcaacag

gtgtttacaaaccagagtcgtaattcagatcgtacatttcatttcctcacatccggatgtaagtaaacactgtgacgaagagtctaattacccgtcataa

agaaaagcagattttccgatagtggataaatgttgtgaatatggtttttaagagaacgcgatttaactaatacgtcccgcccggatgttataaataataa

taaaaaaaaaacatcgcgaagtacaccgtacagtagtccacttaccttaagttgtaggaaccttgacactagctgtttcaacattgttgacgtacagtag

caatttgaatgtccatatcacatttgagtctttgtgtgtattctgtatacctgtcgaccttgtttttgcaccagtaacccacacaacagggcctcagaaa

tattcgcgggagcaagaggcggttggttgaatcaacaccgctgtgcatgaagtggaaagatgaatcaactccattcgtcattaataaatatccagcgtcc

accttggtaggatagcatcagaataacaattggcctagagaagtttaccttcactttatgagtcatacctgtcctggtaatttacctgtgttctccagct

actggaactaactcataggattaagttgattgattcatctttaagtatgttccatgtaattgatgtcttgttacatacatttatataattgcaaaataag

tcatacattttagatattaaaaaaatgtaattatggtttatactagacacatataataattat

>51420|AGAP010716-PA

gcaatagtgcaactgacagcctgtctttgtgctaatatactttgcatctaaatagttttgtatactatcagagaaaagcaagtgcatacagagtctctct

ctctctctctctctctctctctctctctctctctctctctctctctttcactctcactcactcaccacacacaatcaggtcttgtcatttcatttcacaa

ctcgagtatctcctcttctcacaaattattgcaacagttcacataaagcaggaactatacttaatatcagatacaattcatacagcagaaataaatatgc

aaaatatttgcagcatctaggaaagaaaaatattcgcaacactcatggacaacattttctcccaataatcactgaattaccaataatcactgaattacca

cctcactgaggtgagctgaaaatgttgtcagagctgcaaatactaaaacaattcagctgtcacacatatgaaacaaagtttcattctgatgatttctttc

ggtcaggactgaggtagtctttgtacatctgatatccaagaccaagagtcatgcaaccaacaaccatactttgcgcagcaactcgaagctgcatcaggaa

cactgatgtcgacatctgtccacgatggcggtacttgtaagcaccaaaagcacatgccgcaacaagaccagctagaccaataggtacgaacggtgcttcc

ttaatttttcttgacagtttatccgtttgcgattcgtcacgatacaggattggcggagacgatgccatttcttctgattcagcaaccactttccttgcag

aaatttagcgcagccgacagttgtgcgccacactgacccccgcacaaagattactgaaggacaccaaagactactaccaactcgagcagtgacgtaggtc

ggtgaatcgtttcgtcagcccagctgtcacgtgaaattacaaaa

>51419|AGAP010716-PA

ttttccataaaataaatattttttttattccacaacagcatcattatctagaaataaaatatccattccagagatgaagtatacatatgtaatacataca

tgatgatgtaatttaaatgtattgatgtttgacaaataggagtagcagtttgtacctcaacatgtctatcacaatgtaatacagatttttttcacagagt

actctattttctcacacttgcttcccacaatatatgtaatcagtgattacgtattaatgtttaaagactttttttccaagtttctggcacatgcagctta

aaaattaattctcaaacacctataacatgatttaaaaactggcaacatcattacagttaccatacacaacatcacacagttgtaacaaagactgtaacta

ttatgtaatactgaacatttatcatgtgaacagctaaccaggataaaaaagttaaaacttacatcaaagctaatatctggcaatttatattttgcgaaca

tctgaaaatactaacacctgacactttgtgaatctgaacatttatgacttgtagatcacatgtttctctagtgcactgaagcaatgatctgcttaaagga

tagagtgtaatgtaaaaccctgcactatttctgatcacttggcctgtacacgtagcacagattttaattaattgaaaatggcatgaattacaatttcaaa

taattgcaatagtgcaactgacagcctgtctttgtgctaatatactttgcatctaaatagttttgtatactatcagagaaaagcaagtgcatacagagtc

tctctctctctctctctctctctctctctctctctctctctctctctctctctctctctttcactctcactcactcaccacacacaatcaggtcttgtca

tttcatttcacaactcgagtatctcctcttctcacaaattattgcaacagttcacataaagcaggaactatacttaatatcagatacaattcatacagca

gaaataaatatgcaaaatatttgcagcatctaggaaagaaaaatattcgcaacactcatggacaacattttctcccaataatcactgaattaccaataat

cactgaattaccacctcactgaggtgagctgaaaatgttgtcagagctgcaaatactaaaacaattcagctgtcacacatatgaaacaaagtttcattct

gatgatttctttcggtcaggactgaggtagtctttgtacatctgatatccaagaccaagagtcatgcaaccaacaaccatactttgcgcagcaactcgaa

gctgcatcaggaacactgatgtcgacatctgtccacgatggcggtacttgtaagcaccaaaagcacatgccgcaacaagaccagctagaccaataggtac

gaacggtgcttccttaatttttcttgacagtttatccgtttgcgattcgtcacgatacaggattggcggagacgatgccatttcttctgattcagcaacc

tatgcgaacaaagtgattattgcataacacgcaaagtaaaattatttacttatgtaatctattggaagtaagtgttgctcaagaattgcaaacttctgaa

atcgaaacctcgcaaagtagaacgcttaattgggggaatttacaaaagatccagtgcaagaagctacagtactgtcattaaaacagtaggataaaagtag

taatcgccggccataaatacagtttgacataaaagcacatagcaaggtaggtgcaaggcagcattataatttcttgaaaaagttttctattgcattattt

tttttatttaacgcgtgtgcaaaagaattaggcctacatagaaagcagttacttagctcgtggcctacaatctccaagcaataaatggcctaggttctaa

atgttaccgataatgaatatcatataccttgttaggcattccaagttccgcttacagacctattaaaaaggcgaaagaaaaattatcagctgataccgaa

aatgaatttgcggacattacaaatttttcaatttattacatcacgagcttaacactttcactgctgaagattatcagttatcacaatagttgtgttttcc

ttgttctagttatgttatcactcacaacttaccactttccttgcagaaatttagcgcagccgacagttgtgcgccacactgacccccgcacaaagattac

tgaaggacaccaaagactactaccaactcgagcagtgacgtaggtcggtgaatcgtttcgtcagcccagctgtcacgtgaaattacaaaatctcgctcag

atgaccaataactgaaagtatcactcttgtcgcgatgtttcctcgattccgcactcactggcttccacacagatcaactgaggtgtccttcagtaatctt

tgtgcgggggtcagtgtggcgcacaactgtcggctgcgctaaatttctgcaaggaaagtggt

>51421|AGAP010723-PA

cttcaaggcttcaatcctacaaatgttataacattacgcactataaaagcagatacaatatgtggtttgacacctggacaggcagaagacacagcagcaa

aaaatggtgattggctgataacaggaaagtatgcagttattcagttctgtggtaaagcatgaactctaatctgttttattacttgttaactatggtcttg

tctgtgatatggctctgagaatttttatattctttaagagctttaattttgtatctttttgggtactggctgaatttatttagtatagtgttcttgaata

aagtatattgattcatgtgagagtgaatactttcttctgttaatataagaaccctattatagcggatatacagtcagcaaatgtaggggcatgctttact

tacatcaggtgttacataattattaattccacatcatgaccaatattaaaaattacaggacctagagaaagaaaaaaatttaatgttgtatgttaattta

cataatcctttttatcaccctgtgctgtttcattttatacagattttatttttcttcaccactttatggtgtcatatcttgttctgttaatatggcatcc

ttttaacatagatgattttataaggtatgcaagttatcccttaaataagttcttggattgtaaatatacctggaaattccacagttcatctgtataatag

tttcagcttctgttacactgcttggttttactaggtcactatgtttagcactattaaggtaagatttcagtttgaatgctagtaatacctcctatgctgt

tgggaatatctgtgcactgcagataaaccctcctccctttttcttcccaactactgattgtctgtcctcattttttaagattttaactgaacgttgagtc

acttctgaagcatacaagagacacttaaaacattcataaattacaaatgtaattgaaaactgtctcttttattgcattcctattaaccctagggaaaatt

accctctcggagttaaaaccttctactatcattacgtgcactagttacttccatcagttttgta

>51422|AGAP010729-PA

ccatgatggattgcatgccacaacgtaagagatcactgatgtatattaaatcatcactggcatcattaaatccatatgatctccacaagaagctcatcaa

tgaatatttgttaagccgtaagggcagcacatcactgttaagaagagacaggtctcgagataaatgtgatagtgatgttatccgggagaatcatcggttt

ctgtgggatgatgatgatattccggattcctgggaagcccaattagctaagaaatattatgacaaattattcaaagaatattgtatttgtgaccttagca

gatacaaggaaaacaagcactcgactggatttttcacctgtttgaactgactgtaaatttgattgaaggtggctatgcgatggcgtacagaacaagaagt

tgttgtagggaaaggccagtttagctgtggagaaaaacactgctcatctaaggaggacttacatacatgggaagtgaactttggttacattgaaaatggt

gaaaagaagaatgcactagttaaagcaagactttgtaccatgtgttcagaaaaattgaattacaaacataaaaggcgtgaaattacacgtaagaaaaaaa

gaagactaaaaggtcttcctgaaatcgaaccgaataaaaaattgcgaaaggatgaggatgatgatagggacgtgaaccaggaattaaatccagagataaa

agaagaggatgaaagtgcattgtggagggaggctcatcagggaacagaagagaaaactagagatcaagagttcgaagaatatttagaggatttattcatg

taatataagcatgtgtgtggcacagccctcacagtgttttgtatgtgatatattatgtatgaatgttgcaatgttttcttgttatgcgaataaaatatgc

taaatgtcaattcac

>51426|AGAP010765-PA

aaggcttgcatgtcctgtgctattttctgcataacttctccacctggaaataattttactattaatgacacagtttccttatgccgcttgactttatgct

ctagttccacagtcatacgatagatttcactcatgaagttttctcctagagctgatgctcgttgtacatctacggagaggaccttgaagtcttgataatt

gttcgaatccttcagagatttttcagtaaactccttgtccagccacaagggcagacaatctgtggtgttagtcgtcatcacttctatagcggacgtaata

ctttttgtgagaccggtcgaagcgtcaacgttcagccttggcgactgtgctcttcgagga

>51428|AGAP010786-PA

ctgcaattcacatttgttgttgttgttgttgttgttgtctgaatcgttggagttttgcgtcgcggtgttcgctggcgtgcgtgaacttgtcaatttttca

aggctaggtgaactgttattgttcacgactcataatggatgaggagggagaggttagaaattcgaaaaagcaaacattgctagagaaatacgatattcct

gttgagcatctaaacttcgagtatgtggagaagtgtaatgatgagaaagaactagaaaaaatagtgaagatattaaggtcgggtgaagaaggcttctatc

cccagctcatcgcatctgccgaagcgaggctgcgtaaagtgaagcccgacagtcgtgtacttcgtacagaggaacctgctctcaggaaggagaccacgag

ttcagaacagtgggaggaggtcacctcggaggtggagaaatggagtgacgagatgcgtgcaaaagagaaagagttgaagctggctgcagcagaaccgatg

gatgtgtcggaattgccagcagtgcggtctagagctaaagtgtctgcctcaacaaaacctgcaagtgccacgacacagtctaaaactggacagcaagtac

ctccagcgaagcgcagtccggtgccgcgagactactcggcctgggagaagtacgatgcagacaaggagttgacgttgatggagatagaggagcagcagca

gcaagaggagagggagaggaagcggcgggaggagcagctgcaggtgcagcgcctgcaggacgaaatgaagaaagcagggaagggaaatgctgagatggtg

ataccagcaaacttgacaccgacagagcgggagctgttagccactagagagaaggataaaggcaatgatttcttcaaagtggccgattacagtaacgcca

tcaagcactactctgcctcaattcagattctcccgacacctgtcgcctacaacaatagggccatagcaatgattaaactgcatcgctatgacgcggcatt

gaaagactgcaatgaagttcttaaagtggaacctgacaatgtgaaagctctcatgagaagaggaatttctcatcaaaacaggaacagttaccagaaggcc

tatgaggacttcaaccgggtagtggcactgcagccagagaatggtcttgccaaatctttggccaaccagatgaagaacaagtgccttgccaagaggaaag

gagtcaggatgaagatcgaggaagagggaagtccttcccagatcagcgttgtgtcgaaaaggagggccaccaaggtcacagagcagaagctggagaatct

gccatcgtcagagaggacgtgggagggccgacaggagccatggcagccgccgccgctgcccgacaagatcttggaggctccgtggggcggcaggatgaac

cccccactcaaagagggctgcaacacctcctggccttacgctccacctgtaaaggggcctatgacggattacgggctgccgaagaacttctgcaattgta

atggagagccaccgttcgtgcgccacccgccccagatcatctgcctcatgtgccacgagcgcaaggtactcggcctgccgccactgccgggtggcgactg

caactgtcccaagcagccgccacaaaaaaagccagttgtgaacggtggtgccagtggcgaggggactgcacctgccaaagacgaatctaattttgaccga

gagaaacggggcagtgagacgaatgtggaagagcccaaaccgaccacttccgacgacacgggggacagactcgctgcggccgagcctacgggagaagccc

cacagcctgctggtagcagcacgacaggcgaatcgggcagaagaagacaaaggaacattccacatccatctgcatcattcctgccacaaatcccgggact

gggcagccctgtaaagatgaaggttgcacccaagtacatttgtgcaacgaaacgagaggacaagtcagccgaaagttcgtctgaagacagtgggacgacc

acagtgagggggaccagagagaacacaccagaggccggaccttcggcggcccagcgggacagggatggggacgggttgcagaaggtagcggccctcggcc

ggctgtcgctgcagccgggcgccaggatcacgtcgccctacgagttcacacgcgcctggggcggctcccggcccgactccagcctcgaggggtacgccac

ggtgctcagggccgttgccgtcgaggacctcgccaccgtggttggtacaaagttagacggtcagatgctcagtttgattctgcgcacactggagaagcac

tttatagatgaaccagaatatttggtgcagatgttgcaccatgtcgctctcctgccccgcttctcaattgcgtgggccttcctcgacagcattgacaaag

aagctgtggataaaattgtttctacgttgaagaagagcgggcacgagctgagccctgtcgctgaaaaggcgttcgagctgtgaggctggtgctgctcact

cgtgaggagagctgcggcagtgagctgcagctgctattctccattagtcgttgcagaggtcctttctctgtgtgtccgtcgtattgtttcctaataaatg

tgctaggtgttaatcagcagaaattaaatgtgttgacctcattgccgtattatggtataattttaggagttaatgaagaaatagggtcttcgagagagtc

ataagcaaacatctatgtgtgtgctgttgattaaattatggcgtgatgaaaagtagaaaaatctgtgactgtcattttgaggaagaagtaagctatctga

ggtgagctgagcttccagtaaaaagaaatttcagtagctgttgatctgtaactcagaaaaaaattattgttttgtgtgattgtattctccctcgatttac

tggccagctcaagtctgtttctcttgcgtgactgttaatcgtattgttggtttaaatactttttttattgtgctatgctacttttgtttccagtaaaatt

aggcaaaaatgtcacatttttgtctttaatgtgaaactttttggattgaacattaatggtgaagaattctatgtaacattttctacctacttcagccata

atcccccttcccccccataccctatctgtttgctttagtgcagtaaggaaccccagctaggatgtttacattgtatttcgtccttttctgatttgctcat

ttgacaacttactgcaataaatgttgagagattgtgtttattttttgtctttcattgtctgctgtgtgctcgtactgtaactttagacttggtgtgggtg

ctgctgggaatggaggaatgtttacacttcctttacattcactgcaagaagcatgcatgtcaagtgtcgagttactttgcacatacagcattcaacctaa

tgtaagaagtaccataacgaagtcaaaacattagagttaaaatcaattagtcaatttgcagtcaatcgtattttctgctcacttatttaatgtgtataca

tgataacggattgtaaatttccaatgatagctttcgtaagagccaagagttttgcagaagtgttacttgttatattttatgttaggtaagcattgtaaat

aaacctcagtgtc

>51440|AGAP010851-PA

cccccgcagccgtccaggtcttccgtctctgtgacgggcgaagctgagcacgcgctcttcttccacgggaagcgctgccggaactcgcgcttgaccctct

cgttgtaaattccgtagatgaacgggttatagcaactgttgctcatggccagccagtcacaacagaagaagatgatgt

>51443|AGAP010876-PA

ccttctgcgttttcgattacttttactttatcgccttcaagtacagcaacacatgagttggtagtacctaagtcaataccaataatttttgccatttggt

gtgttcctcgaaaagtctt

>51442|AGAP010876-PA

aaagaaagaccgtgtggaagcagttaatcaagcagaaagtatcactcatgatattgagcccaagatggaggagtttaaggaccaattaccaaaagaagag

tgatactttctgcttgattaactgcttccacacggtctttctttaccttatcctg

>51446|AGAP010884-PA

caatgacggaagctttcttctgagtgccgctcactgctgcgcgtcgactattctcccaagaacctgaaacatttttgatctgtataatgaaagtgtgtat

cgggctgtttccatcataaggcatctcccagctaatattggctgttctgctcgaaaaattaatcaaacgaacgttaaat

>51445|AGAP010884-PA

aagacaggtgcaagtcttcatgaagtacagctttcacctgttgtgtgaaagatgctatttgtgcaggtacatgacttgctggagcctgcacgatatgtct

ggacccagcactttctccaactgctgtaaatgctgacacccagaattcataacggtggtgcaactctaaatgagtgatagtgtaaaaggtctgctcagca

ggaatgtcaaagctgtccttgtccaaacctattccatccagtgatctcacatacacttt

>51444|AGAP010884-PA

ggatggaataggtttggacaaggacagctttgacattcctgctgagcagaccttttacactatcactcatttagagttgcaccaccgttatgaattctgg

gtgtcagcatttacagcagttggagaaagtgctgggtccagacatatcgtgcaggctccagcaagtcatgtacctgcacaaatagcatctttcacacaac

aggtgaaagctgtacttcatgaagacttgcacctatcttgccactctgttggagaacctgcaccacttcgtctctggagaaagaatgaaaaaccagtaca

ag

>51447|AGAP010895-PA

agatgaacacatctgagaagccaagggatgtgagtggtgtggagctcctaatgaacaaccaccagagcctcaaagcagaaatagatgcgagggaagacaa

ctttacctcgtgcatatctcttggcaaagagctgttggcacgtaaccactatgcgagtgcagaaatcaaagaaaagctgttgtcgctgaccaatcagcac

aagtctctgctccacagatgggaggaacggtggcttaacttgcagctcatcttggaagtgtaccagtttgccagagatgcggccgtggcggaagcttggc

tgatcgcacaggaaccatacctcatgagtcaggaattggggcatacaattgatgaggttgaaaatctaataaagaagcatgaagcattcgaaaaatctgc

ggcagcccaagaagaacgcttcagtgcacttgaaagacttacgacgtttgaattgaaagaactaaaacgccgccaagaagaggaagagcgcaaacgtcaa

gaggaactggctgccaagcagacacctccacctagctctcctgatgaacaaccatctgaaaaggtggatggagaaatgtcaccagaggctgagcaagtgc

aagtgaatggtgaggatacagaggccagagaagtgcatgaagtggacacgggaatgggagcgggagcgggctactcggagtcgggccggccgcccacccc

cgtgccagacccggcagtgcccacagctccggtcctcgtgccctcgcctccgccagcgtctccggtgcgaaccgcacccccggcaactacccctcggccc

cgtccgacaaaggagccggtggccgtcacgtcgccgccggtgacgccggccggcccgggcgccacggccagcctggacaggaagaaagaccgctcgcgtt

ccaagtcacctttccgcagtctgcgctcgtggcgcaggtctccgaaggctggcacgtctggcgcacacagtgacgacgagggcacgttggagcgcgctgc

agctgagcggcccagcccaggcgcagaggaggatcacatggagggcaaccttgtgaggaaacacgagtgggaaaataccacaaagaaagcatcaaacagg

tcatgggacaaggtgtatgcagtcctcagaggtaacctgctaatgttctacaaggaccagaagacgtacaagacatcacccgacaccttcttcaagggag

aaagcccagttgatttgcgtggaggtgcttgccaagttgcctcagattacacaaaaagaaagcacatatttagggtgaagctgtcaaatggagcagaata

cctcttccaggcacgtgacgatgatgagatgaacacttgggtgactcgcctgtcccagcaatgcgaacgtga

>51448|AGAP010896-PA

gcactgcgaccgtaagaaagactgcccggatggagttgacgaactgcactgtgacaacataaattgtgaggctccattgtactggaagtgtgaagatgga

agatgtatacagaccagattcaaatgtgatgggaacaatgattgtgaagatggaagatgtatacagaccagattcaaatgtga

>51449|AGAP010924-PA

catccacccaacatctttggagtttgtaaatgcacgtggatattgtcaacaacagctgatcgaagctgaaacatagtaaatattcccagtaacaggtaca

aagcctgctgacaataaagactgacgagaaaaaaatgatccatggacaatcttttatcgtcaaacacaattcgtcgaggaactgtttccttcacttttca

gcttactggttacgattattaaatgccagtaaggtagcggttggaggattaaaaatggaatatggtaatgagcaggcagcttttgtttcttggagtcctt

atgcagacagttctattgaacaaaatgaagtttctgttagctctgtctttgcacagaaactagggttaaaaaatgaggatacagtgcttatttcttattt

tccaaatgttgctgcagtgagcaaagtgtgcgtttcaccactgtcatctgaagactgggatcttttagtaagcgaataaattgttacatattgtctatat

taactgcattgggctcagacactcaaaaacctttgtttatattca

>51450|AGAP010928-PA

tgttataaagaacagtgtttattttgataatcaaatatacagtatgaccttttccctgaattaaccattaaccattttcattagcaattttttccttttt

tgccattcctcagaacaccttgaaagatactgttaatatcattatttagtaatttccataattatcttcagttggtgttgcgtgcttagtatcccttctc

ccacatgtttaatgtaacagagtcagaagttgaaaaagtattgccagtttccgtttatccatatgacacaactgagtgttttatgcttcctgtgcctgta

attttttcataacatcatgaagcatctgattgcagagtgctgcatatggattcatttccactagctgatgctttgcaaatttgcttcccaagtttgctgc

agctttgaaatcttcctttgctaggtcatcattgccctccttacgatgaataatacctctttgacaaagtgcttggcaccccgatttaccacggccgttg

ctcaactgtatcgccaagtttaagtctgcaacagcttctgaaatttttccaccaagcctgtatgcttgcgctcgattgttgtatgcagaagatctagaag

gtgctgtagctatggccttagtaaacaggttaatggcagcctcaatatttccagattctgcaagggtgactgcatccatttccagttgctttgcagcttt

gacagtgggatcactttcatcttcttcatctttaacagattcgtcagttgcctcgtctggcaatttgtctgcgaggggccctaaaggattaaatatggta

tcaagtatttccctatcgtgttgcgttgacatttttacctttttttggttttttgtttgtctttctatatttaatgatgatgttttgtt

>51452|AGAP010935-PA

cccgattaccagatccagctttgataagatactccttaatagcaccaacacggccatccatcatgtcagatggtgcaattatatgtgcaccagccttcgc

gtatgatactgcaacttctgca

>51460|AGAP010954-PA

gttactgatcttagtggcagagggaaaggataattccacattctccctcaaacgaaacttatccaacctctgtatttctgggatggttctttaggattca

gataatgtcgtcctctccctctgcgcccctcccctctattgtccaccagccagaagctccgacatggcagcaacccttctgaagactggcaggcgtgtct

ccggcgacggacggaagcacgccggaagagtctgcagtgaaggcgccgcagtggagggcggctgcgtgaataccatgttgaaagtgaaagcatcggtcgg

ggactgcggcacagcaggagaggtggcagctggcggcgaggacggcttgctgcccggtgggggtgaagtagaggtggacgacgcagaggcgacgcccaat

gcagcaaccagggggcttccgggggccagtgggctgccaggagctagtgggcttccaggagctagaggactacctggtgctagtgggctacccggagcca

ctggcgtgacaggtgacactggagaaaccagcagaggtgactgcggaggagaagctatggtcttggggcgggcctcgtcagcattatgaatgaagtgaca

gcgtggcccatacgggcagaaaccaatcgtgtggaaagtgcgacagagctccgtcttgtacttggggtgtctagcaagcgagcgcagctctcgggcgcca

tgcgcgaactggcacttgtccccgtacttgcaggagccattttcctcgaaggggcggcagagctctgtcttgtagcgagaagtgttgacggctgcagagg

cagaggctgcagatgcaggctccgactggctacgatccagcttgcggtgcggttggggctgcggcgtttgggggccactcgctggcgcgtgcgagtcccc

actcagagggttggagctgcggcggtagacgccgcccttgccgcacagtaccacagggcagcccaagttggtagggctgctacacgtgctgccagaagag

ctggacgctgaggacgggggcggggacgacccgccagccttgctgctgctgctgctgccggagctcgtggtgctcgctgcactagacgcggctgaacttc

cgctgctcatcccgtggctcagtatgagaggcgacggcacaccagccaggcgcaactggcagatgtccgtgcaggggcactgcgagagacacatggcggc

tgcccacggtccacgttccagctgacacgaccacacttgacggcacactgagacgagactgacaaacaccgccttatgcaaggcgcgcgccacgacacac

tataggccgttatctgtccaccgccttctctgctccactgcgccggcgcgagaacttccag

>51459|AGAP010954-PA

tggtacattaaattacacttcatgaccagtttactacatcaatagcaaaagaaaacaactggagaaacaaaccactgaattgagaatacatcatgtgcac

ggtaaacaaagtacacttatttacatttaatgaacctcaagataaataaaaaacttaattaaatgcccaagaatcatcacagcaaatggtaaaaggcacc

taatttctgccattaagcatgtcaaaagaaagggaattaatgtgagagtgttactgatcttagtggcagagggaaaggataattccacattctccctcaa

acgaaacttatccaacctctgtatttctgggatggttctttaggattcagataatgtcgtcctctccctctgcgcccctcccctctattgtccaccagcc

agaagctccgacatggcagcaacccttctgaagactggcaggcgtgtctccggcgacggacggaagcacgccggaagagtctgcagtgaaggcgccgcag

tggagggcggctgcgtgaataccatgttgaaagtgaaagcatcggtcggggactgcggcacagcaggagaggtggcagctggcggcgaggacggcttgct

gcccggtgggggtgaagtagaggtggacgacgcagaggcgacgcccaatgcagcaaccagggggcttccgggggccagtgggctgccaggagctagtggg

cttccaggagctagaggactacctggtgctagtgggctacccggagccactggcgtgacaggtgacactggagaaaccagcagaggtgactgcggaggag

aagctatggtcttggggcgggcctcgtcagcattatgaatgaagtgacagcgtggcccatacgggcagaaaccaatcgtgtggaaagtgcgacagagctc

cgtcttgtacttggggtgtctagcaagcgagcgcagctctcgggcgccatgcgcgaactggcacttgtccccgtacttgcaggagccattttcctcgaag

gggcggcagagctctgtcttgtagcgagaagtgttgacggctgcagaggcagaggctgcagatgcaggctccgactggctacgatccagcttgcggtgcg

gttggggctgcggcgtttgggggccactcgctggcgcgtgcgagtccccactcagagggttggagctgcggcggtagacgccgcccttgccgcacagtac

cacagggcagcccaagttggtagggctgctacacgtgctgccagaagagctggacgctgaggacgggggcggggacgacccgccagccttgctgctgctg

ctgctgccggagctcgtggtgctcgctgcactagacgcggctgaacttccgctgctcatcccgtggctcagtatgagaggcgacggcacaccagccaggc

gcaactggcagatgtccgtgcaggggcactgcgagagacacatggcggctgcccacggtccacgttccagctgacacgaccacacttgacggcacactga

gac

>51458|AGAP010954-PA

ttttcactcatttttgtaattatctgtgcacaatgcaggtttaggtacttcataaggataaagagtgtggtgtccaagaggaaaggaaattgttaaataa

aaacaataatttaatcaaaatgctataaaattatattcatccactttataaatatttcattcattgcatacacactgagcttgtgcacatattaaacgtc

tataaataatgacattttacatgagttgtatattgcagatataataacacacaatatacaattatgtcagactaagtaacacacaatctgatgacatcca

aactccttaaatgagccacaaaaacgaaacaaaaaaaaaaaactttcccaatgcaaaattgtaatatagtgccaagcaaacataaatacaattgtggttt

cacaggttcagctttttaattaaatattttaatgagataataatctgataaatatattttattcatataaataagtcacataaatataacattgtgtaaa

cacaagaacaaaaaataccccccatcccattacttaaaaaattataaaaactaagttttcattttgtggaaaggagtgtgttgttgctacaacagcatca

ctgaaagttaaatgactgcattaacgcattcccaaactgaattgaagaatagtaacatactaacattctgcaagcaacatattcgaacttcactcccttt

aaagataataaataaatagaattcagtacatttaaatttgtacagattttatttcttcagcctttcacatcattttgcatgcaaaactatccactttatc

attaataaataagaaacttacatttgaatcaatataattaaaaaacaaagttcatgatgtcattatgaaataaatatgtttaaataatgctcagatattt

taaaaatggaatgaaaatgttgtctgtacataaaataaataaaacaatacaagaacccaaatgacttcacacaaatatttaaatgagcttttcgaaacat

gaaaaggagaaatttgtcaatattgctgtttggacttccctcaaatattagaagcggaacaaaaattttgaggcaattttgtattaattaaataaagtct

tgccagtcaattctataccttccaaataccataacagatcaacacacaatataatttctttccacaaggcacatttcaattaatcagttcagtagcataa

tcacttccaaaactagtgccacaacagcgtcacttaacagcattttgttaaccagaatttttagatactcatatcaagaataagattactataaataacg

aaagtccaaagcatcatagaaagagaaagaaaataatgttatgcatcatgtaacagaatcaaaatataatttaatgtgtataaattaaataatgtggcac

cccactttcacatatatatgtatacataatatattatatttataataaaacattctcctccaatttagtacggtatcagaaagacagcaggtttatgtac

aatgtggtatacaaagttttgtacaactgaatcttattatcagtctgttcaaggagaggaaacaaaaattaaaactcaaagcaatactgtaaccataaat

aaaatgaatgtacagaagtttcctgtttggaatgttgctgatttctcatttgaaaactaaccagatttcagcaacgtttggtcctcacccatgctagaga

cactataataccggataattacaatctatcagtctatagttcaatagtgtgtaaaaaatctgatacactgagtaatttggaaaatttcgtgtgcttctga

ttcaagcacttttatgtgcagatatacacattcgtgaaaaattattattgcaggcatttatgatatgcacactgccaaatttctttgatatgaaggttag

ttgcagctgaaacatcaataaaataataagtctaccactaaatatttttcttcctcttatggcttaattatcacaccagaaatatattatatgccttggc

tattgcctctcttacttctacattggctaatttattgtaactgtttttccatgcaatcaaatatacacatgtttatatatataaacacacaaaaatattt

tactgtacgagaatgcatgtatagcgtcaagtgcatgaattttattcccagggatttcagacctggatctcaaaagagaatcttaatcatattactggta

cagtattagtcaaaaaggcacttagatcttaaaaataaatggtgtttattgctgcctatagggaattatttgtttcattgctgctgagtgtctgaaacaa

ctgcacatatatgagagagaatgtggaatgttcaagaaatgtgcatggaagacattgttacttgccaaactttgaaactttgatgtactagtttcaagaa

actctaccaccattttcaatctggctcagttgttaagtttcacgatattggtacattaaattacacttcatgaccagtttactacatcaatagcaaaaga

aaacaactggagaaacaaaccactgaattgagaatacatcatgtgcacggtaaacaaagtacacttatttacatttaatgaacctcaagataaataaaaa

acttaattaaatgcccaagaatcatcacagcaaatggtaaaaggcacctaatttctgccattaagcatgtcaaaagaaagggaattaatgtgagagtgtt

actgatcttagtggcagagggaaaggataattccacattctccctcaaacgaaacttatccaacctctgtatttctgggatggttctttaggattcagat

aatgtcgtcctctccctctgcgcccctcccctctattgtccaccagccagaagctccgacatggcagcaacccttctgaagactggcaggcgtgtctccg

gcgacggacggaagcacgccggaagagtctgcagtgaaggcgccgcagtggagggcggctgcgtgaataccatgttgaaagtgaaagcatcggtcgggga

ctgcggcacagcaggagaggtggcagctggcggcgaggacggcttgctgcccggtgggggtgaagtagaggtggacgacgcagaggcgacgcccaatgca

gcaaccagggggcttccgggggccagtgggctgccaggagctagtgggcttccaggagctagaggactccctggtgctagtgggctacccggagccactg

gcgtgacaggtgacactggagaaaccagcagaggtgactgcggaggagaagctatggtcttggggcgggcctcgtcagcattatgaatgaagtgacagcg

tggcccatacgggcagaaaccaatcgtgtggaaagtgcgacagagctccgtcttgtacttggggtgtctagcaagcgagcgcagctctcgggcgccatgc

gcgaactggcacttgtccccgtacttgcaggagccattttcctcgaaggggcggcagagctctgtcttgtagcgagaagtgttgacggctgcagaggcag

aggctgcagatgcaggctccgactggctacgatccagcttgcggtgcggttggggctgcggcgtttgggggccactcgctggcgcgtgcgagtccccact

cagagggttggagctgcggcggtagacgccgcccttgccgcacagtaccacagggcagcccaagttggtagggctgctacacgtgctgccagaagagctg

gacgctgaggacgggggcggggacgacccgccagccttgctgctgctgctgctgccggagctcgtggtgctcgctgcactagacgcggctgaacttccgc

tgctcatcccgtggctcttataatggaaatcaccaaagtcgaagacgcttgctccggcattcatcactgcggcggacattgcggagcgaaataattcctt

cggaacggacgactatcgtagaaaaataaaaaaaaaaacagctgcactcacaaaactgaggaatagggggagggagggtgaagtgggaaacaaaaaaaaa

aatatggaaacaaaaacacccctgttctatgggaggatctgtcggagcaccgggtaaaaaaaatataaacacttgcgcgtaccgcggggacgcagcacgc

tacgacgccggtatcgacacttgttgcgagagggtgagcaggtgaggggggagtccgccacggaacagcactggagtctcactcggcgcgcctctagcga

ctttctgtgttgccttcgccacttgcgaggatgggcaggaccggacttcgcgcgctagcactcggcgactctcttaccgcccaactgacgccgcgcggga

gcgcgcgcccgcacgtcatgagcggcgcacggcgcatgcgcgcg

>51462|AGAP010956-PA

ttcaagtgagttatggagacgtgtgttcagaggcaatctacattgcctgagactgacagtgtctatcaattgattactcaactttgtaaatatgaactga

gagagaaggggtcttcttatgttctgaagaaagcacgctctaatgcatttgggattctacttaagaagacacctgctgcctttggtagagaaaatgtgaa

ccatccaccatggtttgacccattgtttgcattggcaacactgcaactagatcttccagatggcaaggcttctcagcttgcacattgtttgcagcatctt

gaatttttgaaaaatgtgtactcaacagataacttgaagaaaattgtgcattttattttggctttatccaactcaagaccaccacaaaaggaagattttg

tcagtttcaaaccaggtcaattagatgctgaattttcaaaacggtatccaaatgcatatccacagtatggccctcaactgtttatcctgccacctgttga

gaggtttgtttcaactgtgtatggaactgaatgtccttatttaccaccaggaggatggtaccctttttctgagccactaggtacaggactcagcaagttc

ttccagattaatcatcaaagtggaactgcacaaggtgaaggtgttttaccatatgatattccagacttcccagaggtaaaaagcccaaaagaactgtgta

ttccaaaagcaagtaaagaacgagataggactccagatgaagggtatgggacgccagcagcagaacatgccaatgcctctcctgagtgtgattttcatga

ccatcatgcaccaagtgatcaagagatcttagctcgggtaaaccatagaacttgggaagcatatgggtcacgtctgcctccacttgaaaaaccattctta

agtgaaaaaccagaagcaattcacaggttgtgggtgctgcatatgagacagcaggctgtaagaaggcgtactgttgttgtacctggtgcatcccacttga

aagtactgcaaaaggatcagttgctggctgatattcggtatcttctggatggagttgctagtgatacgttcccttataataagggcttggatgcattcac

agcagcacaaaatatctgtgtagaaggaatttctcctgacacattacagaattttctcacaaattttttggcagttggatcactgatatatcagttggaa

atattggtgcagtcacctccagaaggttgttgtctggcttatcaggccatttgtgagtgtgtacgtcattatctttgcctctatcggggtgaactactca

gacaaaagttcagcggaagtcttctctcattagctaactccatcaggcgattcataatgcctcaactgttttgcatttcacatgtcctgtcacagcatac

caccaccactgcttacttgttgtcacaacttcatgatgctgctatgcaggcagtgtgcccaatcacagctcccctgcttactgttatgctgcatgcaggg

acttccatttatctcaagtatgtggagctgtggctctttgaaggtaaactagatgacagccaagaagagttctttatttgtgttcgaccaactatgtctc

tgtcacgaacccgcgattattggaattgggccttttatgtgaataagaaagcagttccaaattttctattgggccttgaacatgatatactggactgtgg

cagagccattaacctactcagaatctgctgcccaaagaatccacttctccaagagctggaaaaaggaatcccacatttgagactttgtggaagtacagaa

cagctcaagcagttagaagttgagtgcttaagatttgaagaaagagtttcagaagcatgtggtttgacaggccttgcagccagagacttccttctcaaaa

gtcaagctgcaaaatcagatctgatagataaggcttccgatgccaggcaacaacacgagaaaaaaattctagctgaaatagaggagaaacgtgttcaaaa

gattcatgataaaaggcaacaaatggaacagttgcaggcacaaattgtggaagctaaagaacaccgcttagcagaacgcagaagacaactagctgaggat

gaggcatttagacgactaaatgaatatcaacaacaagaggaaaagcgcatgaaacaactggagaaggagaagataattatgttctacgaagagcagtcaa

aagcagcagacaggaaaaagttccgagcagagtggtgcatgagaagaatgaaactaaatgagaagcggaagaaatttttatcagatgagtctttgtaccc

agcattaacacaggaagaggacactaagcagcacatgtgcactgatcttaatcccgatggagtaaaaaaccatttagagactccagatcaaacaaataat

aatgtaaaaaacatagacactgagaagttagtgccagcttattccacaaccattaccagcaatgtggaaagaattgagaatggtgacataggtaactcag

aaaagcccatatgtgtagataatgtttctttagaaagcaatgcagacaaacaagaaatgtcagatgcccagaagaatcgtctcaaagttctacagagtga

atttggcattttgctctctcttcctagtaatgttacaaatcagcaaaaagattgcaaagacttagtgactgatgcagaacgtaacagattcaaaatgatg

tggagtgaatatgatattattacaggcaataacaatataacatcactgtataacaagactccagaaatgtggaatatgactggagattcagaccatgtca

ttgcttatagtgaatcatgtgccatcactgataaaataaatcataattttatagacacaaatgtagagtttagtggtgatactacattggtgacttctgg

agatgtaataagatatgatggtgtgaacaatattttggcaccagaatatgaaaccaaattatcacccacaaaagaaatgagtcttttagctgtttcatcc

ccaattggtttacaaacctccagtcacattatggtggaaaaaagtaatgaaaatagtaatttgacatcacaagaaaatgggatagcagtagcagcggaac

tgccagccagttcagatcagttaacaaataactctgaacaagaaaaccagaatgttgtaaagtgctttcaagaggaggcatcagtttcccaggcttcatt

caccggtgttttgaagaatgatgataacaccaacgcatcatcttttctcagcgaaagttctccagagtttatgaagttgaatgaaatgggggaaccagtc

agtgacaagccagtatactatggcttaagtgtttttaatgctgtgcatccttcatggacatttgaaatgccatctgaagcaccatttcccataagggata

ttacagatcgcttctttcatggttgtttaaatcaagaaagtgtacattacaaaccagagactgagacactcgaaactgaagctcttcagcgctcagtatt

attaccactgtacatacacagcaagttggttaatggtgctctccttcagttctttttacaagatgaatctttgctgagccatctagaaagtctaagaaac

tattttctcctgcaagatggtgaattctctcaacttctctgtactcagttattccaaggagcacatgaatcagttcattcatcccgactcctaaatgtat

caacgttgaacagagtcctggcacgtgctgtgagtggttcaccacaggcagagcgtgatgtgaattcgtcacgtctctgcttctcattgtcaagtacagc

aacaggtcatttgtcccatacctctccaaatatgtttgcttcattagtgctggggtactcagtggagtggccattgtgcctcattcttacggagagtgcc

ctggccaactacaatagcatttttggatttctgctgaaagttcggcgcattgcatgggttcttgaacaaaactttaaggaccttaagctgcctgcaaatt

acactagaaatgaacatcatcggatacatctgtgtcgtcatgaaatgatgcagtttgtccatggcctccaaatttatataatgtccactgtgttgcaagt

tagctgggcagaatttgaagtattacttggaaatgctgtttctcttgatgacctttatcgcttccacgcaagctatctcaagacaatattatacaagtgc

ctgctgtttgaaaaaacaaaacgaatgatggatcatctaatgcgggtgtttacactgattctaaagttctgtgaaatggtgcgcagtggccttaagatgg

atgatttactaaaaacatttaatgactatcagaaggctgtcaattattggttgcattttgttagccgtatggcggacagtggtttccaaccacactttgc

aaatcttgtgttaagcctcaatgtaagtggctattataagaatgtataatgccaattagtgaaatatttaataagacagttgttgcacccaactttgtag

tcctgtttttattcatgactgtgtatatatgcttttttgtaacaatttctcgtttatgcttgtaaatagttattacaattaaactaaatttgaaaataac

ctataactaaatgtcaaagctgtg

>51461|AGAP010956-PA

ctgccaactcaatttgaatttttattcatgatccaccacaaatcattaacaatctgcagactgtcttcacatgtgttggcagagtgtctgagcacagctt

tgacatttagttataggttattttcaaatttagtttaattgtaataactatttacaagcataaacgagaaattgttacaaaaaagcatatatacacagtc

atgaataaaaacaggactacaaagttgggtgcaacaactgtcttattaaatatttcactaattggcattatacattcttataatagccacttacattgag

gcttaacacaagatttgcaaagtgtggttggaaaccactgtccgccatacggctaacaaaatgcaaccaataattgacagccttctgatagtcattaaat

gtttttagtaaatcatccatcttaaggccactgcgcaccatttcacagaactttagaatcagtgtaaacacccgcattagatgatccatcattcgttttg

ttttttcaaacagcaggcacttgtataatattgtcttgagatagcttgcgtggaagcgataaaggtcatcaagagaaacagcatttccaagtaatacttc

aaattctgcccagctaacttgcaacacagtggacattatataaatttggaggccatggacaaactgcatcatttcatgacgacacagatgtatccgatga

tgttcatttctagtgtaatttgcaggcagcttaaggtccttaaagttttgttcaagaacccatgcaatgcgccgaactttcagcagaaatccaaaaatgc

tattgtagttggccagggcactctccgtaagaatgaggcacaatggccactccactgagtaccccagcactaatgaagcaaacatatttggagaggtatg

ggacaaatgacctgttgctgtacttgacaatgagaagcagagacgtgacgaattcacatcacgctctgcctgtggtgaaccactcacagcacgtgccagg

actctgttcaacgttgatacatttaggagtcgggatgaatgaactgattcatgtgctccttggaataactgagtacagagaagttgagagaattcaccat

cttgcaggagaaaatagtttcttagactttctagatggctcagcaaagattcatcttgtaaaaagaactgaaggagagcaccattaaccaacttgctgtg

tatgtacagtggtaataatactgagcgctgaagagcttcagtttcgagtgtctcagtctctggtttgtaatgtacactttcttgatttaaacaaccatga

aagaagcgatctgtaatatcccttatgggaaatggtgcttcagatggcatttcaaatgtccatgaaggatgcacagcattaaaaacacttaagccatagt

atactggcttgtcactgactggttcccccatttcattcaacttcataaactctggagaactttcgctgagaaaagatgatgcgttggtgttatcatcatt

cttcaaaacaccggtgaatgaagcctgggaaactgatgcctcctcttgaaagcactttacaacattctggttttcttgttcagagttatttgttaactga

tctgaactggctggcagttccgctgctactgctatcccattttcttgtgatgtcaaattactattttcattacttttttccaccataatgtgactggagg

tttgtaaaccaattggggatgaaacagctaaaagactcatttcttttgtgggtgataatttggtttcatattctggtgccaaaatattgttcacaccatc

atatcttattacatctccagaagtcaccaatgtagtatcaccactaaactctacatttgtgtctataaaattatgatttattttatcagtgatggcacat

gattcactataagcaatgacatggtctgaatctccagtcatattccacatttctggagtcttgttatacagtgatgttatattgttattgcctgtaataa

tatcatattcactccacatcattttgaatctgttacgttctgcatcagtcactaagtctttgcaatctttttgctgatttgtaacattactaggaagaga

gagcaaaatgccaaattcactctgtagaactttgagacgattcttctgggcatctgacatttcttgtttgtctgcattgctttctaaagaaacattatct

acacatatgggcttttctgagttacctatgtcaccattctcaattctttccacattgctggtaatggttgtggaataagctggcactaacttctcagtgt

ctatgttttttacattattatttgtttgatctggagtctctaaatggttttttactccatcgggattaagatcagtgcacatgtgctgcttagtgtcctc

ttcctgtgttaatgctgggtacaaagactcatctgataaaaatttcttccgcttctcatttagtttcattcttctcatgcaccactctgctcggaacttt

ttcctgtctgctgcttttgactgctcttcgtagaacataattatcttctccttctccagttgtttcatgcgcttttcctcttgttgttgatattcattta

gtcgtctaaatgcctcatcctcagctagttgtcttctgcgttctgctaagcggtgttctttagcttccacaatttgtgcctgcaactgttccatttgttg

ccttttatcatgaatcttttgaacacgtttctcctctatttcagctagaatttttttctcgtgttgttgcctggcatcggaagccttatctatcagatct

gattttgcagcttgacttttgagaaggaagtctctggctgcaaggcctgtcaaaccacatgcttctgaaactctttcttcaaatcttaagcactcaactt

ctaactgcttgagctgttctgtacttccacaaagtctcaaatgtgggattcctttttccagctcttggagaagtggattctttgggcagcagattctgag

taggttaatggctctgccacagtccagtatatcatgttcaaggcccaatagaaaatttggaactgctttcttattcacataaaaggcccaattccaataa

tcgcgggttcgtgacagagacatagttggtcgaacacaaataaagaactcttcttggctgtcatctagtttaccttcaaagagccacagctccacatact

tgagataaatggaagtccctgcatgcagcataacagtaagcaggggagctgtgattgggcacactgcctgcatagcagcatcatgaagttgtgacaacaa

gtaagcagtggtggtggtatgctgtgacaggacatgtgaaatgcaaaacagttgaggcattatgaatcgcctgatggagttagctaatgagagaagactt

ccgctgaacttttgtctgagtagttcaccccgatagaggcaaagataatgacgtacacactcacaaatggcctgataagccagacaacaaccttctggag

gtgactgcaccaatatttccaactgatatatcagtgatccaactgccaaaaaatttgtgagaaaattctgtaatgtgtcaggagaaattccttctacaca

gatattttgtgctgctgtgaatgcatccaagcccttattataagggaacgtatcactagcaactccatccagaagataccgaatatcagccagcaactga

tccttttgcagtactttcaagtgggatgcaccaggtacaacaacagtacgccttcttacagcctgctgtctcatatgcagcacccacaacctgtgaattg

cttctggtttttcacttaagaatggtttttcaagtggaggcagacgtgacccatatgcttcccaagttctatggtttacccgagctaagatctcttgatc

acttggtgcatgatggtcatgaaaatcacactcaggagaggcattggcatgttctgctgctggcgtcccatacccttcatctggagtcctatctcgttct

ttacttgcttttggaatacacagttcttttgggctttttacctctgggaagtctggaatatcatatggtaaaacaccttcaccttgtgcagttccacttt

gatgattaatctggaagaacttgctgagtcctgtacctagtggctcagaaaaagggtaccatcctcctggtggtaaataaggacattcagttccatacac

agttgaaacaaacctctcaacaggtggcaggataaacagttgagggccatactgtggatatgcatttggataccgttttgaaaattcagcatctaattga

cctggtttgaaactgacaaaatcttccttttgtggtggtcttgagttggataaagccaaaataaaatgcacaattttcttcaagttatctgttgagtaca

catttttcaaaaattcaagatgctgcaaacaatgtgcaagctgagaagccttgccatctggaagatctagttgcagtgttgccaatgcaaacaatgggtc

aaaccatggtggatggttcacattttctctaccaaaggcagcaggtgtcttcttaagtagaatcccaaatgcattagagcgtgctttcttcagaacataa

gaagaccccttctctctcagttcatatttacaaagttgagtaatcaattgatagacactgtcagtctcaggcaatgtagattgcctctgaacacacgtct

ccataactcacttgaa

>51466|AGAP010977-PA

aagacggcgctgcccacgatcgtgccgaccccgatgtcgccctccgtgatgaacgtgccgatcacgttcacgaacagctccggcgccgacgtcgccgccg

ccatgaacgtggcccccgccacgtcgttcgacatgttcagtgctcggctgggctgcggtgacggcgatggaggcgccaaacggcacgctgccggggtatg

gcgcggcgcaggggctgcccaactgcacgccgcccgccatcgacgacttcccgcgcgacctcttctcggagcagcagcgccaggagggcgccgtcgtgct

gcacgtgctcgcctcgctctacctgttcgtcgcgctcgccgtcgtctgcgacaggtacttcgtgcccgccgtcgagcgcatctgccaagcactgaacatg

tcgaacgacgtggcgggggccacgttcatggcggcggcgacgtcggcgccggagctgttcgtgaacgtgatcggcacgttcatcacggagggcgacatcg

gggtcggcacgatcgtgggcagcgccgtctt

>51467|AGAP010977-PA

ggtcttttttttttttatctcacgtcgttcgctgttgtacgaggcggagtgttacaggacacccgaataaaaagcggaaagcaaaaactgtgacgtgtga

gcggcaaaaccaccggcaaaaaatccaccctttggcgacttccaagttcggggaaatcctggacgttgctgttgttgaccctgcgtgatggaaggtgtgg

aacaggacgaaggcgcggcgcaggggctgcccaactgcacgccgcccgccatcgacgacttcccgcgcgacctcttctcggagcagcagcgccaggaggg

cgccgtcgtgctgcacgtgctcgcctcgctctacctgttcgtcgcgctcgccgtcgtctgcgacaggtacttcgtgcccgccgtcgagcgcatctgccaa

gcactgaacatgtcgaacgacgtggcgggggccacgttcatggcggcggcgacgtcggcgccggagctgttcgtgaacgtgatcggcacgttcatcacgg

agggcgacatcggggtcggcacgatcgtgggcagcgccgtcttcaacatcctcgccgtggccgcctgctgcgggctgggcgccggcatcgtggtgcccct

ggactggtggcccctgacgagg

>51472|AGAP011047-PA

acaagaccgtacaccatctgaaatatagatatcccaaagtcaatacgaagtgctgctatctgaaagggttaggtaagcagagagtgcacaacgatgtttg

gcgcatcgggcacggttgccttgaaatctgcgtactggcagctgtacgagtatcccttgctcgtgttgctggcgtcccaggacagccagctgatgttgcg

gcacacgtctgggcagactatgtagtcaggggttagcttgtgaacgtatgggttatttacgcctaggagcaccacagcttctttagtgatcgcattgtac

acgatcttccagaaaattttcggcactggaatatacctcctgccgtgagcataaaggtacatctgcgtctcgacattattcgcattgggcagcgtcgtga

tgccgtgagtgcccgtgtagatttccaggtcgaccaacctgttcttagcgtaggaacgtacgttgttctccatggtgttccagttggcactgttgaacga

attccactgcggcgcggcgttcacgtagaagtacggagcacgaaatcggacgtagcggcgaggtgtcccctggtaaggaagttagatttagaaatgtacc

tgctcgccctgggacccagtagtgtggtgaacgtattgacctgcgtattgaaactgtactgcatatgcatgtcgatgttcccgaagaatccgccgcccaa

ccacgtggactggaacgtaccgttttgcttgccccctatgttggcgaccattgtgaaactgtcatagactgtattgtaggagcgatcatcgaagcagaca

tcaatgagtgtgtagaagtctgagccgacctggtagccaagtcggatcatctggtactgacgtttgctgccacactttctgcccgtcacctgggccgatt

gctctggcggggacaggcagtcgaggtcagcaaaggcgtatcgccgtcttttgactttgaaagtggaggccgaaacgcaggaggctgtctcttgctgtcc

attgatgagccggagcctgctgccaggacacgccagggtgattttctggttataactgagcgagattattcccgaagagtctggcttcacaaagccgtgc

aagccttttctgctactgggcattagcagtagtggctgtgggttgggcatcacggttctgttattcacgtcca

>51486|AGAP011050-PA

aaaatctgtgacaaaatcgcggattgcagataatttcgaaatctttgactttgagccctcagctgatcatatggcattcatggataaatttaactgcgag

>51487|AGAP011050-PA

tgcgtccaatgatcaatcagatcgaaattagtccttggatcttacctgacccggaacttgaacaatattgcaatgatcatggaattattcttgaatctta

ttcacccatgggatctggcagtcgagtcaaggagtgtatggaagatcctatcatacaagaattagcagcaaagtataacaagtcgccagctcatatcata

ttgaggtctatgcttgacagaggctatctaatcatacccaagtccagcaacccaaagcgtgtattatctaattatgaggtctttgactttgctttgacta

aagaagacctggattacatggctgaaaagctcaacaagcagatacgtacttgccctgatccaaatgaagttgcataggctagtttgaagctgtgattcgt

gtacagttgttccctatctttgtgattcatgtctgcttttaattattcttttatgtgagtattgaagaaacaaaacaaaataaaacagtgtacaagatc

>51488|AGAP011053-PA

ctttggatttgcgtgactcaatatcctcaattaatggttgcaagtgattgatcatgaaattactcacaccaatagctcgaacacgtccgcttgcatattg

tctttccatctcatgccatgcgtccaagcggcggtcagcattcttgggatcacgcatctcatactcttgtccaccaaaatgagttgtagctcccggccaa

tgaagaaccagcatatcaagctgagtcctgttgcaatccttcatgaaacgatcaacagtctctttgactttctgggcgctcaatgttggccacactttgg

tagtgacgaatattgtgtgttgactcttctttccatgaggatgactaaagttatcaaggaactctttttcagagacaaccttgcagcccttaggaatctt

gatattctctgttgctggacaattagtttcacccttgaactcagcctcaaagcgatctagcgcagacctcacagccggataattctgatagaaatcagaa

gtatcaacggctttgtaaccatgcttgaatgcagacaagatcacatcactagcatcgtcttttgattcataggagccaagcccaacagctggcatgagaa

taccattaggtagctcgcgtacaaggaccttcatagccggtagtcaagtcaagaagtgaatgtgagtgtttgtg

>51489|AGAP011061-PA

gatggtttcagaaacctctcattctcatgtgcgtaggtgcactgcatactagtaggaaaagttatcacaaacaaggtcattgaggcaataggctgtcacg

tggggttttgataagacaatatagaaacgaacctgtaaaaattggccagacgatgacgtttcaaatccaaacagttcgaggaccagttctacctaacaaa

cttggaataacattgacccatgagcacttttctctaacatttcaaaaattttatgtggagccaccacaacagttgcaaaattatttgcatggtgaaatta

ctcttgaaaatgctggccttattagacaatatccttacagcaataagtataatctcacatttgacgacaaggaaacacatgctgctgttctcacagaaat

gcaaatgtttaatgaatttgggggaggaaccattgtagagaatacatcacatggtcttaacagaaacatacctctcatgattgaagtttcacagaaaact

ggcatccatgttgtagcaggcactggttattacgtggctggacttcagccgcagagcactctccagatgtctattgaagatctggccaatgttatgcgta

gtgagctcacagaaggttgttttgaagacaaaagtgttagatgtggtttcattggggaagttggatgttcttggcctctgcatgaatttgagaaaaaggt

tattaaagctacagctgaggttcagagcagtctagactgccctgtcagctttcatcctggccgtcatccaaaagcaccatttgaaataatgcgactgttt

gcagaagctggtggtaaagctgaaaaaacaataatgtcacacttggacaggacaattctagaaacagaaactttgctggaatatgcttcacttggatcat

atctacagcaagatctttttgggactgaatgctcttggtatcagcttgaaccaaacactgatatgttgtctgatgctcaaagaatacaaaggattgttga

acttatttcaaatgggttaacagatcgcatacttatggcacatgatattcacacaaaacatcgactggttaaatttggaggtcatggattttcacacatc

ataaataatgttatacccaagatggaaataaaaggcattgaaagagaaaaaataaatcagatacttatgaaaaacccccaggcatggctgtcgtcaaaag

tgataagctaaagaataactggttgaaagttggtgccgtatttctggatacataatgcaattgttatgcatataaaatgtagtttattgcaaaactgtgt

attcatgtgtacacaaaataattcagcttcaaataaatggatgccattttttcatgtcaca

>51490|AGAP011077-PA

tccactagttaacgcagttttcagaacttcttgaagagcagtgtttatgtccattcctccggcacccgttgccggaggaatggacataaacaccgctctt

caagaagttctgaaaactgcgttaactagtggaacgctagcgcatggtcttcacgaag

>51497|AGAP011104-PA

gctgctgctgctcctcctggcggcgatactggccgctgccggcgctacggcgcacgcgctccaggaccccgccgataccactaagtctacgtactgggga

tccgacaatcggcttatccaaggctatacggacagcaaggggcgccattacccacggctgccctactatccaattgacgacgacgacaatatggacaacc

gcatatcaccgccgtctgcgtcgctgtacgagaggctgccgtcgccgcccccgcagcgccgcccacagcagccacgcagacggatgaggccggccggccc

tcgcgagggcgtcttccgcacctcgggccacggcagcgtgcgcatcccgtacccgggggcccacgtccacgtcaacaacgacgccccttcgcgcaagctg

tactggggcgccatcaatgcgtctgtgacgccaccggagcaggcacctctacgcaggtacccgcccgagaagcgcagtggatcaggtgcgcggccgtgcg

tgaggtgcccgggcagccgggcagtcgtggccagccgcgggaagcggagggtgctcgtcgagcagccggaactgtcgccctgccccaacagcccagtgtt

cccactgacagagctcatgctggactgggttcatggaccaaagcctggaactctggctgaagagggtgatcacaccgttgttgtacgcatgttgtatcgt

cgaagggtgctgcagaaatgccgttatcgctacagtgttgtagttagaagatgtcctgtgctctctctgcccagcaccttgatagcaaactgctcaatgg

ataatgtctggggaagcacatgtgacttgagatgtcctcatggctactttttgaaggggcattcaagaattgaatgtggtgattcactaatgtggtcaca

tccaattcctcattgcgaagagatgtcagcatgtccaatgcctatgagtcctgagcatggtcgcctcagctgtaagactcttggaagcagcccacctggc

acactacctgaacatagtgtttgtcgttacgaatgtgatgaccattatactataccagatgcacagcacagcttgatggttattgaatgcaaaggagggg

aatggaacagtacagctgatccaagttgccttgagatggctgagaagaggcacagcccacaagttcctgcaaaaatacgaagacatcgtagaaatcgccg

caggcatcgagcacatagaagcagaattccatttccaagagcaactgcacaggggttgactgctgatccgtgcaggcccaacccctgtcagggagggggc

acttgcttgcagggtcctccttacgcaccagttatctgcctgtgcccacctcacagacaaggagatctatgtgaaagggcacaatgtcataatgatacat

gccagaatggaggaaaatgtatggttatcgccaatgaaactgcttgctactgtcctcctgggtattctggatcccagtgccagttaaaggatgccaggta

gctgatatgatgtattaacaagtcttgcctgaagtacaaccattggcatatggaaacgtatcaactgtctcctacagtatcatctacagaaccagtagtg

ctatcaaagtaaagaaaagattgtgagagtggaaataaaagattctgaaatctgtaggagccaggaactcaccaatctagtctacttatatataagaatt

ttagccttaaggagaaatcagattttagaataaatgttaaatagtgagatcctcatgaaacctaacaccttctgaatatgtagagcgaagcttttagtac

ttagagcaccataaataattatacaaatacagcaaattttttttgtccagtacataataaacctatttgtgaactaggatataaatagcgcaagcattct

ttctgtgatttagctttgaattacatacattttaataattccatacattataagttatttttattgtttcttgttagtagagaagaaaatacagctgcac

tgttatcatgcagtctcttcagaaacagtagtcttaatgagtattccatttaatactcatcatttacttattataaatcctccaaaacaataatttgaag

tcaaactcaaggcaaagcatcttttgaccactcattgacactgaaacaatttcaatagggctgataaaaacatagcgc

>51496|AGAP011104-PA

gttatagttgatcagaacagtgtaccctgcattgttatctctcttttagttctacaaatcttttccagagcagttatgtacatcctgatgtacacaatag

gtgtattatgatatgagaaatgttgttgcatttgattgtgttgttttttgttggcactttgtatatacaaattttcatgacactacatcattgtcatctg

tcacaacatttctcatatcataatacacctattgtgtacatcaggatgtacataactgctctggaaaagatttgtagaactaaaagagagataacaatgc

agggtacactgttctgatcaactataaccctggtcttgttctaagtgctttgtagaggtattttatgcccattatataagtgcaagaatggggtgtataa

gattaaagaagtagcttgattgtgaactctaatcagagacagattgaaggtaacatgtttatacattgatgccaaatttcttggttttaattacatcaag

ttattcaaaaatgacatccttctgatagacctttgaggcgtcagtgtatctaagtatagcactctctttgtatgccaatagtaatgcaaaaactaccaaa

tacagaatgaaattaaacctggcataaaaatcaattttggattgacaactggcactacagctgaccaagcaacgaaagagatatgatatgcattcaggca

gtgtgaaaagttccagtggcttgacagctgttcatcaccatgcatgcacaatacagccagtttgctaacattcaagtaacaatgacaatcagtcatgatc

gtgtgaatgtgttcagttgaaactttcttgcagttccagtgtgtcatgactatctgaaagctgtctgagtaccagccaatggatggtcatgacaagtctt

gactggccacaggaatttcatctgacatttccaaagcatttaataatgttattccaaagaaaaagacaacacatctgcataacatttttttatttggcct

gttggtattcttgttgaagaagtcaactggtaggttcctattggtattttaaagggacagtacatgcccccttgtgaaaatttaatacaccacagtgcac

ttctattgccttaagaggtttttttaagcagaaatatactgtgtatatggatcctagggtatggaaaacgaataatttttccctcttctataaaatggtg

agatcgcaaaagctgtcagttgtggctcacatactacaattattgctataattaaatgtgcacagtaacttggttcacttaagctgcataccagatttgc

aaccactgttggcaatgagcacacaaagcatcatctgctcaaaatggatggatgtacaaacatgcatatttaatactcatttttaaatcaacagaagtgc

acacaaatatgaaatacttgcttaatgttttgctcagaattataataatattacactgccactgcaaaagacctgtcattgtaatgataaatttttgaaa

gacaagtatgtgacaatgtacttcttagtagcatactgatgtatggtcttgaccattaaaactgatgttacattagttgtttctagctttctaaatacaa

gtaactttttcaaatgtaattaattcattgatactagctgggaacaccaaaggtcattaatataagtaatgacaataacactaaccatacgtttgtacat

tggatacacagcagccattgtctaccaactcaaccagtgacagtttttcttgaaattagataatttccagcaactctaatgtttggtcatttactacctt

gtagtttatcaacaactacatcagtcagaagataattaataccaattaccatcaaaattatgcattactaaaactgatgagacaatcttatcaaatttgt

cagaatatgcatataatcaaaatatgtaaaacataatgtgatgttaatgaggaaagaggtaagtgacaggattaattcagaactacttcaattaacattt

tgtatcaattctgctcattgaattatatgactgcttcaagaagtgacaaccacaaattgtgagtagaataatttgagcaatgataaatagaataaaagtg

ccaactagtgctgccagtggtcaaacagcaaaacatactgaatatgagatttgataatgtaatggtattatgatgatcaagacgatcttgaagctgcgca

tgtgaagtagagacagtttgcaaacagtgatgattgatcatgatacctttgctcgaatgaacccaagtttctgcatatttctgctgtgaaaacattatgt

ctgaacatgattgttttaaattaatgacagtggtggtgctgtgtacatttggccagaaggtacattagaaagtactcaaatattaatggctaagtgaccc

atatgcaatgtaaaagaaaaaatgcatatttaaagaactgaaaatttaatgtagcaagaagagggaactccaaaaagtagtctgaggagttatttgtaac

atttactgttcttttgttgtaagcattagttaaaatttttaatggatgttatttcatagttctgctattctaaacagattataatgaagggtgggaaata

aataaaaagtgaatgtcattctttggtaattaactaaacaggacttggccagatcagttaagaatgatttgtctttctcaggttccttgggatcatatca

accaaaactattcagtaacggaatgagtcattatttgagtcacagaatactggtaaaataaaactaaatgaaataaataactgagaagttacttcttatg

atattacttaaattaattacagtgaaaagatccaaactactgtagcacctgtttgtattaaaacgtttcagcttcgcttcaagtctattgcagatgaaac

cacagaacagttccctcactcctacagaactgttaaggaagagtttttcaagtgcagatcaagtgttcattgaatgaatgttacagtccattttgaagga

atccttattactaaccacttatttcatgtgggtctaaacatggaagttaggtagagttaaaatagcaacaatgatacacatgcagctcatgaactgacag

cacagattggcctgactgctattttatgagatgataaagtaacaaaaatacaaatgaaatcataaaaaattaaatttgtaataactgcctgaacataaag

gagaaaattcttgcagtcagctcaacagaagaaatgtcccagacagccagtagaggtttgtaaaaactcccaaaaagttgattacacgtacagaaaagta

tcaaaatgtttttaaagcttttttcattgtactctgtcacactgtaacataccaaatattttgatgtgtcttctgcattgtaatctgattatatgtaaac

atatgttatgagacgaatacatcactattcactattaataggtcatctttgcaaaaagacaggacaatgtttttgctgcagttttctgaaaaatggagtc

tagagaacatattaataattaataatcagatcttctggctgacatcctctcgaaaattacataatctgtcaactacaattttcattccagaacgatcttt

tccaatatatatgtaatttagcagctcatcaaatatgaaacacttcatttggataacaaaatctttttgagtgatatcctctgagacctgcataaaacat

gactatgagaaacaaacatttatgatttgctgtcataaatatgataaatgtcattaattgtacaactggaaactacttcatctaattttcaaccaagaaa

atgcactgatatcagtaactatatgtatgtctagcagctttatatttattcctttattcgtgaagtgttcatctcaagcttaaatattgcctccaagagc

tcacctaattaatggaatattctgtcaagcataggatgtcatttctgattttggtgacaaaacctccaaaacacaaaacctatgttctctatttttactc

tgcaatataatttgaaataatattctaaagcaatactacattttgatacaatgtttatcttacacaacaatatgtaataagagtgacataaacaacacac

tctccttaattctgtgataatttgtattcataaatttacgggctggttaatatcatagctgactatttttctgctacataccaaggcaacttggatcagc

tgtactgttccattcccctcctttgcattcaataaccatcaagctgtgctgtgcatctggtatagtataatggtcatcacattcgtaacgacaaacacta

tgttcaggtagtgtgccaggtgggctgcttccaagagtcttacagctgaggcgaccatgctcaggactcataggcattggacatgctgacatctcttcgc

aatgaggaattggatgtgaccacattagtgaatcaccacattcaattcttgaatgccccttcaaaaagtagccatgaggacatctcaagtcacatgtgct

tccccagacattatccattgagcagtttgctatcaaggtgctgggcagagagagcacaggacatcttctaactacaacactgtagcgataacggcatttc

tgcagcacccttcgacgatacaacatgcgtacaacaacggtgtgatcaccctcttcagccagagttccaggctttggtccatgaacccagtccagcatga

gctctgtcagtgggaacactgggctgttggggcagggcgacagttccggctgctcgacgagcaccctccgcttcccgcggctggccacgactgcccggct

gcccgggcacctcacgcacggccgcgcacctgatccactgcgcttctcgggcgggtacctgcgtagaggtgcctgctccggtggcgtcacagacgcattg

atggcgccccagtacagcttgcgcgaaggggcgtcgttgttgacgtggacgtgggcccccgggtacgggatgcgcacgctgccgtggcccgaggtgcgga

agacgccctcgcgagggccggccggcctcatccgtctgcgtggctgctgtgggcggcgctgcgggggcggcgacggcagcctctcgtacagcgacgcaga

cggcggtgatatgcggttgtccatattgtcgtcgtcgtcaattggatagtagggcagccgtgggtaatggcgccccttgctgtccgtatagccttggata

agccgattgtcggatccccagtacgtagacttagtggtatcggcggggtcctggagcgcgtgcgccgtagcgccggcagcggccagtatcgccgccagga

ggagcagcagcagcagcggcggcggccggaccgcgccggtggcgtgcgaagcgcgtgcctgccgcatcg

>51503|AGAP011130-PA

ggtggtggaggacatagagcgcgtggacatggaccagtcggacaagcacgaccccaagttccacgactggaaggagctggccgccgagtgggagtggaca

gaggcacgcactctctacaccaaccagaaggacgacctcatgtgtttcttcaacgtgccccgtttcgactgccccgaactcaacgtcgacccctacgatg

ccaagaagtatgaggagctctgggacaatgtgagtaatcctcccggctgctgctatttgtagtagtgaatcaaagtggaggtacagctgaaaaaaattcc

agggaaagcttcaaagattcagtagttaatgacactcagtagtataatgatggctgagattgtaaagggtttgttcggataatcgaactgtacatgttta

tttgccaaaaaaaagtgctgtg

>51504|AGAP011152-PA

ccatacttcacaatctttgagtttatggattttcttttctcgaagcaaaatgaaatttgggatgaacgatatgataaagtctaccaagacatgacgaaac

cgttgtcacactattggattgcctcatcacacaatacatatttaacaggtgaccaattctcaagtgaatcatccactgaagcttatgttcgctgtttacg

aatgggttgccgatgcatagaactagactgctgggatggtcctgatggtatgccattcatctaccatgggcatactttaacgacaaggataaaatttctg

gatgttgttaaaactatcaaggaacatgcatttgttacatcagatgctcatgccataacagcagatataagtcttgatgagactattaaagcagcagaat

ttttcatgtctgatgg

>51505|AGAP011160-PA

aaaaaattgaacgcatttcaacaactgtgattgggtctacagagaatgtgaaggaaggaaatgaacaactacatcaggtcatgcaaaagagtgcaggtct

tcgtgcatgggcactgttctttttcattgtcatgtccttctccctgttgtttctgcactggtaccatgactgactgttgttgctattctatgagtttatt

gcggtttggcacctggacaggaagaaggatggaagatgtgtggtatatttcaccctttattcaacgaagattgcatactatctacagactttcatgatac

gaaatgttaataattcatgaaaccaatactgatgtttacaaatatatgcatttgaatacctcatgtttatattctgtatgtcaagaagctgttacttatt

tctcagtgcatatatattttgtggtaacaatgctcaagtttgtggttcttgtattttgaaatgacacttagtgtttctcttggttttatacttttttttt

ttaaagaaatgtacatttttggagcctaacttttgctgttttttttctaaatacttattcagtattctgtgctttaggtaattttctgtttgtcttttta

cactgtattacaccagttacaaggacaacctcttaaaagaaaagtgttaactcatgagatgttataggccacaagagtatcaatgaattaataataataa

taataataataataataagggaagaaactgaagcatatttatgttccagatgttaatctttctgatcagaaaatgtaattcatttaagcctctggttctc

tgtaattcaatgcctttctcttattgtgcggaacatgtacctgacagcttaagagtattgtttatgtctgaaagaatcatcagcggtggtttcttagtct

tataacaaacataatctacatcatttgattcaggtacaggagacatgtcaaaactgtggtaaaatttcactataaacatagaatacctgatgttaacaaa

cagcatcataataaagttttaaacatggctgtgtgaccagcgactgtttacaaattaatggtaaagcaattacagtcctcagtcacaaaaattaagtatt

ttgacctggtttcagcaccactgtgagtgcctcatcagaaattaaacattcaaattggcctacaaaagaagtacaaaattatgggaaaatttttattata

taaaggtgtaagtactgactgacagtacaagaaagaaacaacacttatatctcacatataaaataaatatcaagcgacaaagctttagtcacaaaatgtt

ttaaagtagaatgcgtggaaagcaagccactatgggctgcttgtatatgtcgagctacaggtagcatcagactgaggcgcccgagttaacaactgtgtgc

aggtgaacgttgcaccgggagtgccatctagcagccacaagtacaaactggctacttaacattcaaatgaaaagactaatattatgatgaacattattca

gtatatgaaataaaagacaatattttgtttgacatctagcagccacaggtacaaactggctacttaacattcaaatgaaa

>51507|AGAP011163-PA

tgagaaaagaagactcaagcgctcaaggacagatcgtcaccgaaatattacagcatttacatgcgtgtaataattcagtgaaggtgttttatgggag

>51517|AGAP011173-PA

ttcttgaattccaaaaccaaagttaccagtttcagagaggttgtcttttcgtaattcatattctcgatccttcaacc

>51515|AGAP011173-PA

gatcatattttattcccaattcaatgtgttcttgaattccaaatccaaagtttccagtgtcagagaagttgtcttttcgtaattca

>51518|AGAP011173-PA

attttattcccaagtcaatgtgttcttaaattccaaatccaaagttaccagtgtaagataagttgtcttttcgtaattcatattctcgaaccttcaac

>51513|AGAP011173-PA

tattgaattaaaaaaaaaaagttacaagtgtaagagaagttgtcttttcgtaattcatattctcgaaaattcaaccctcgttcaaggatttcttctgcct

tg

>51512|AGAP011173-PA

caagtcaatgtgttcttgaattccaaaaccaaagttaccattttcagagaagttgtcttttcgtaattcatattctcgaacctccaaccctcgttcaagg

atttcttctgccttggcacctctcgctgtacaatgtacagcaatcttctcattacgtctaataccaaacgaccttacagtgtacctcgccttagaaaaaa

c

>51519|AGAP011174-PA

caatccgagcaagaagaaaagtggcaactgagaaagatttgctagaggcgagaaataaagggataaaatcatatgcaaaaggcgcagcaactccaag

>51521|AGAP011174-PA

cttctttctcggattgcaaacagtccagcttctggtcacacagatcgtatttccgcacctgtgctgtttggacagagtcgagcaagtagttcaaaacg

>51520|AGAP011174-PA

acttggaaggacggacacacatattcaagatacatgctcgttctatgagtgttgagagagatattcgttttgaactacttgctcgactctgtccaaacag

cacaggtgctgaaatacgatctgtgtgcacagaagctggaatgtttgcaatccgagcaagaagaaaagttgcaactgagaaagattttctagaggctgta

aataaagttataaaatcatatgcaaaattctcagcaactccaagatatatgacatacaattaaatttattttcagacttaatttaaaaatagtgattgtc

actgtgacagatatgtacatgatgatcagtaattctgaagttgtactgagtatgtttgtaaaaatacaaatttatacttcagaaatttgcattaattttt

ttgatttgatgtacaggttcaggaattcctgtgtacatttctctctacttggctgaaagtaagttctataatttttaagtaaattagttgattgacataa

caaacactaaaaaatcaaaactggaatgaaacaagatgtggaag

>51523|AGAP011207-PA

cttctggccaccactgatgagaaaatttcatatacaccttgtttactgttccaatggatagccccttaatagcattcatcttgtaagcaggtaatggggg

actgaacattgcctttgcttgtgccttaagcacaccaagagaaacagtcacaataacgtggtctgcaatatactgtgatccatcaagacaactgacaata

actttccctctgatttcattaggcaatgaatcccattcaatttttgttacttccttactaagttgtaaacgttccttcaaaggaagttcttggtcaggat

ttggatactttttcatcaaaacatccaaaatgtttctgtagcctccgtgtttccaggcccaaagcggatttccttcacattcccagtactctgtgagacc

ctttccagatgtttggaaccagctatcagaaccatcaatcgattgttcaaattttgaataccactcgaggaaagaatatgcaagaggtagatcttctgca

gaaattaatcctggttgtgtaatttgtctctgaaatctcgtttcttaggtagttgctgcactcgcctctcctgatagcagctactggaaaaattgcagaa

aagcagtgttgaagaaactgcaatttaatagccgctcaccggaggccgcgatacatgtttgctgaaatacaatctatgagcaa

>51524|AGAP011207-PA

ctttctgtttatggaattgtatatttattaaatttgaaggtctcaaacagagttacataacattattaagcaaatatttggaaaactgtgtatcacagca

cttggatgccaatgaagttacttgggttgtctcagacaggttcatctggcaaattcagagagatgatttcttaaataatctggcctcattaagaattcgg

tcagcctccctccagcctgtctctattgccccatgaacagtagagtagtagtgatcatgtgtggcctctccagcaaacagtaaaacctgattgccatcag

tgttggatattggttttgccagactagcagcatttgtaccatatctttctgtatccaaacttcggaaactgtaggacccacgaaaatggggatttgctgt

ccaacttgacctcaatattttttcaggatatggcactttgaaatgctttccaaggaaacgcttaagcaaactcattaaactgtcacagacttctgtatca

gacataagttccatttttcgagcctcaggaccaacaatccagccacacaaacaaagaggatgattatcaacagtattaaatgtgaacacctccttcagcc

aattctgctcatcacctccagatgtttctgatccagctttatcctctgcattccacaaaaatccaaaaccactacaatcttctggccaccactgatgaga

aaatttcatatacaccttgtttactgttccaatggatagccccttaatagcattcatcttgtaagcaggtaatgggggactgaacattgcctttgcttgt

gccttaagcacaccaagagaaacagtcacaataacgtggtctgcaatatactgtgatccatcaagacaactgacaataactttccctctgatttcattag

gcaatgaatcccattcaatttttgttacttccttactaagttgtaaacgttccttcaaaggaagttcttggtcaggatttggatactttttcatcaaaac

atccaaaatgtttctgtagcctccgtgtttccaggcccaaagcggatttccttcacattcccagtactctgtgagaccctttccagatgtttggaaccag

ctatcagaaccatcaatcgattgttcaaattttgaataccactcgaggaaagaatatgcaagaggtagatcttctgcagaaattaatcctggttgtgtaa

tatgtctctgaaattcccttgtgaaatagtccccaacagatccccagaaatttttcaagccacttttactgttttcttcaatttctgataggataccata

tatttttgctagtttttcaggtgctactggctttccagatgaatccacaaacataacattatcttgcgtaatacttgtaactgacgtctgaagaatgttt

agatctttaagtaatgaaaaaactacatttcctttttcaccatgcacccagtgtgccccaagatcaactatgttgccacagaaatctacactgtgtaccc

gtccaccaatgcggtcctctgcttccagaaccacaaaatcattcattcctcgctctaggagacgacaagctgcagcgattcctgaggctcctgcaccaat

gattactgcctttgtgggtatcatcttgacagaatgtttgaccagcagttgacaggaagtaaagaatacagagaaaccacttctcctgttgtagaacctg

ttatctgacttaattcttattaagacctcttatgtacctgtgtacagataagagtaccgaagaggaagtcatgtgaacgtttgtgtaataattttcctcg

tatgcatgatccggttccgctgaacttaaaagttataactatatgtatccattccgcttgctttattaacagtttaacgcgttgtgtgagcgaaagaaca

tgtattgccgtagaaaaaatagtgtcatttgtaaagccttgagattcaatcgcctgtaactcgttttataatccatatacaatatgttaccgagctgttc

attagcgctagtattttctgtacgatgaataaaaaaggatgaaaatc

>51527|AGAP011208-PA

actcgcaaataaacctcacattactgcagtcttggtctgttgcatgtttaaggccaagttcttccagcacttgccggcaatttgtgtaacttccccactc

ttaacctcttctttcattctgttctgcccaagcccccataacttgattttaatgctattccca

>51528|AGAP011218-PA

agggtatattttttggaggtctggaaccagaaatgagaagcacagtgtggccattcctgcttcactgttattcttatcagagcacacatgaagaaaggga

tcagattattgccatccgcaggcaggaataccatgagataactcgtagaagaaatgaaatggacccagtaagccaagagaatttttggcggaatatacag

tgtattgtggagaaagatgttgtccgcactgatcggggaaatccgtactttgcaggagaagacaatccaaacattgaagtaatgaaaaacattctgctaa

attatgctgtttacaatccaggatttggctacacccaaggcatgtcagatctcttagctccagtattagctgagatcagaaatgagtctgatgcattctg

gtgttttgttgggctaatgcaaaaagccatatttgtctgcacgcctacagataatgacatggacaaaaatctgaactacctcagggaactcattcgtttg

atggttccccaattctataatcatctgttgcgacacaatgatgctatggagctcctgttttgtcaccgctggatacttttgtgcttcaaaagggagtttc

cagaacccattgccttgaaaatgtgggaagcatgctgggcaaactatcttacagactacttccacctgttcctatgcttggctataatcagcatttattc

ggatgatgtcgtggcccaggacttgcgcacagatgaaatgctgctacacttcagctcgctggccatgtttatgaacggggagcttatactcaggaaggca

agaggtctactgtatcagtttagggagatgcagcgaattccatgcacggtcgctggcctttgtcagctgtgtggacctggcatgtgggactcgacacatg

cacctgttgttgaatgcacaagaaaccatcttcctgggcagtcgtgtcctcatcgtacttagagctggaaggacaataagatgtagtatgtggccctgcg

gttatttgaaaactcttaaccagtcattttaactacacaaaaaaagattcacttcttctattcacatgcacatcacaatcaaatatttggaagtttggag

aggcatttcacttataaattcatactctcctgcaccaggaaaaaaaagcagctaaataatctcaatttatacattatccacaaagtattttttataacca

gttttattggtgaaacactttcaagtatgattttagttatggaaggaagtgtttatctcttgtttacggaaagatttcaggtatgtaccctttgttatta

cagtgagaatattgtttttattagagaaactgtacattcccatcttcttgtttactatttttcagtattattcaacacatttataaagacagcaacaaga

agttaagtgatatatataagccactactgaattccatattatgtatattaaaggggacctgcaatgttatgcacctttaaaatctactacaaatgtaatg

gtactaagtatctcattaattatgggggcctaagtacaagaggtgctgtcaacatatcagagtaacatgattaaatgtcattttgtctattgcagtagat

tgtgctactgaagttcaagtataaagtgttgtacaatatataaagatttgttgcatgcttttaaaaataagaagtgaaatgggtgaaagcaaccactcat

gaatttatttttatgattaaattatgttatgcaatattttacagcaaaaaaatgtttttttaacagctaattcactgtgttcaaaaaaggacagtagaga

attttaaatgtgtgttggcctttgtaagtatcc

>51531|AGAP011284-PA

aacggaaagaaatacgaagagttgtgcccgtcaacacacaacatggaagtcccaaacgtagtgaggaccgattgccagctca

>51529|AGAP011284-PA

tctgtccattttcaagttgcttcttccaatcttccaattgtttatcgtccctccccagaggccactgcaaatcctgacgaagtgtaccgtcactagccaa

aagatggaggtgattgtcttcccagtgaataataccatattgagtccgggtaactataggttcctccatattatgtgaagtaggacaattttcatcatac

ttcttcccagtaaaaatatcaatgccggtgattgaagccttagcatgtccgtgcttcccagtcttagacgtagaatactcgacaactttgcatgggcgac

ccttcaacatgcaaaaagatcctttcttcacagcacccgcttgaatgggacgagtagcggacgatccagcattcgtagtttcgaactcatcagccatgat

gatgatatcttgtgtctagagtccacaaactacagaaaacttt

>51534|AGAP011298-PA

tgcctttctgttgctgttggtcatgtagccatgtcagaagatgagttagtacaaaatgagcacttggcaataaacttccttgtgtcattgttgaagaagc

actcattaaacagattccaagattgcttggaccaggcttaaataaggcaggca

>51535|AGAP011298-PA

cttatttaagcctggtccaagcaatcttggaatctgtttaatgagtgcttcacttgctaactcatcttctgacatggctacatgaccaacagcaacagaa

aggcacagcccctttttcatttgaaacttaattgttgctttcacttcatcaatttttgccatcattgctt

>51533|AGAP011298-PA

gaagcaatgatggcaaaaattgatgaagtgaaagcaacaattaagtttcaaatgaaaacggtgctgtgcctttctgttgctgttggtcatgtagccatgt

cagaagaataagaagctggttaaaaaattggcaaagaagtatgatgcatttttagcaagtgaggcactcattaagcagattccaagattgcttggaccag

gcctaaagaaggt

>51540|AGAP011319-PA

caaggaaggtattccgaagagcagatgctgttcaccggcaaactacttatcccaacctctgtcacagtagatgagggtgcagaatgcacctaagccatct

tctgcacacaaacacacgttgcattgctccatgtaggttgtgttggggacacagcatttcccttctggggcagccggcggtttcttctgggccaactgcg

gcgtcttggctcctcttttgttgcgacaagccatacgtgtacatgcccagacacgtgccggaagacacgtacatacgttgcagtcttccatcttcttctg

gccgggtatgcacccccaagcggcctggactgatacgatgaagagcaccactccgcatagtgcaagcgccaacttcatggtgggtgctgcgtcagcgttg

gtgatt

>51545|AGAP011319-PA

ctgctcactacattttatgaaggtgtttttcaaataaggttgatcaaaattgagtatatcaaattttatttgtagccattcgttaggtctacatcaaaat

tttaacgctattgaagtagtagatcactgctcagtaaggctccatgggcagctgacctaggcatcacatagaggtcacaccaatatagtacccccaccgc

cacccctggagggaggacagtatcggagcgtgcacgtccaggctccgccactggaacacctgcaccagttgcatccgtcgtatttccgggtgcccggcgt

gcaggcgctggagggcggacaatacttgagcgtgcagatccaggcgccgccactggaacacgtgcaccagttgcagccgtcgtacttgcgggagcccggc

gtgcaggctaaagcctgttcgcagaccagggcagcaatc

>51543|AGAP011319-PA

cggccgtcgagcatacactggcacaggttgcagcccctcttgaaaaccgaccccggagtgcacgcgatcgctctgcggctgcgcttcgggcagcccttga

gggtgcagagagctccggtgccagcgctgttacaagtgcacgtgttgcagtccagcttgaacgtcgtgttggggacgcagttctgcgcgttccgcttcac

agccaccttcacgccctcatcttcaggctgtggacaagccatacgagtacatccccatatgccagtaccactacatctgcaggtgttgcagtctttcatc

ttcacctcaccgggagtgcacggttcgtccaagcccgcagcgacgcccacgcagaggatgaccacgagggcggcggcagttagcgcggctcgagtcctca

ttgtctgccggtagctttgctgctgttgcggccgctgtcgcgagcg

>51541|AGAP011319-PA

cttcgcctgcaccttcaccgccagatagaacgctcgactgcaactgtaatttcgaccctatgacgccgttacaagactgagactggcatacagctgatat

accatcctctgagcagtagcagcggttacagccattcatgaatgagctgcctggaacacacttctgagtcgacattccaacagtagcagtcctcagagat

tgtggcgtgcagctctcatattgtattctggatcggcatacgtagcgagtacacgcccattgcctagcaaggctgcagatgcatctgttacactcaactg

ttgacacactccctggaagacaaccgtcacgcagttgtagttgattgcagttctttttggaacagagtgcactcgttccatcctgcctacacatacattt

gttacagtccatcatgaaggtcgatcctggcacacactgagtccgaggcaaaagcggagttccaccttctcgtgtgagagag

>51542|AGAP011319-PA

gatggattacaatcattgtgtttatttttcatgcttgaagacggtaagaaaattacaaatgacaaattggaagtagttgaaaataatacgcaacacagat

tgtaggcggaggttcgcatcacagaaatggcgttgttttcttttaactgctgagaacttcccggacaatctgtcaccttcatgaaggaacgctggttaat

acgagccagggggacacgctttcaaggtgcaggcggcgctcctgccgttggaactgcagcggcaggtgttacacttgtccttgaaggtcgttccgggtgt

gcagtttacctgtctacggccgcggctgagacatcccagctgagtgcagatggcggacgtcccactgctgttacacgtgcacgtgttacagtccttcttg

aaggtcgagttgggtgtacagctctgggcttcccgcttcacgatcggcgactcctctgcctctctcttcgtaacacatcctttgcgcgtgcagacccaga

cacctgttggagtgcacctacaagtgttgcagtcttcctttttcgtggttcctggagtacactctgatgcggcgtccacattggttgccatggcgacgaa

caaggccgccgctgctatcgctagtgtgaccttcatatctgctggtgctgcgtctgcctgctgccgctaatgctgtgtgaat

>51544|AGAP011319-PA

ttctcattgtttcctttgtgtttaataaagtagaagtacgactcttgagagagtagggaaacagtcagtgaatggcgaagatacacaaccctgacatttt

attaacagaaaatatattggtttttgtttgaaacacaaacagaaggtgcgaaagaggaggacagagttgatgagaaacgagtatcacaggctgttgctcc

cacgcccaggaaagtttccagcgtttcaccacatggctgttgagtcagggcagctccaaaatccttcgttgattggtaaagcagtactaattctctcgtg

ggcagtctatgtgagtgcacactgtggaacggccgtcgagcatacactggcacaggttgcagcccctcttgaaaaccgaccccggagtgcacgcgatcgc

tctgcggctgcgcttcgggcagcccttgagggtgcagagagctccggtgccagcgctgttacaagtgcacgtgttgcagtccagcttgaacgtcgtgttg

gggacgcagttctgcgcgttccgcttcacagccaccttcacgccctcatcttcaggctgtggacaagccatacgagtacatccccatatgccagtaccac

tacatctgcaggtgttgcagtctttcatcttcacctcaccgggagtgcacggttcgtccaagcccgcagcgacgcccacgcagaggatgaccacgagggc

ggcggcagttagcgcggctcgagtcctcattgtctgccggtagctttgctgctgttgc

>51547|AGAP011345-PA

gctgaattctttagtataattttcagtatggggatgagagtttttctatagacatagttaaaggagcacaacagcacaaggcaccactttaatctctaca

attttccagatagaacagttcctgttacacactacatatttttctactattacactattttcagtcttttgactctatagatgcttttgccactgcttca

gttaacttcgccattgcttttgcttctttggctttgcgttttgcttctttcgccaatttcttttcttctctttcctttttctccaacatttccttaaaac

gatcatcacggggatccactttaaagccaaaatgcctacggacttcttccattagtcgatcctttctcgcctttgcagcaagcatagcttcttctttctt

taaaactctagctttaagttcttccttccacttttccaaagattccatcttcttagaaatagcctcctctctttccctgatcgttgattccctcaacttt

ttttcagcgatacttctctcaatcatctcttcaagagtatgtgggtatgcgacagattcatattcacgaatgtcatctaaatcattcttagttggccaca

ttacgcctgggttgacagcactaatactaccataacgcccataaagcctacgtttatacttaatcgtatcatgaaaccagctcttactgctggaatatgg

tactttattgtgtaatatattgtgatgttgtggtaacagacgtgatttatttctcttcatttcgatgtattcctcataatttgtgctgtcatcagtgtcg

tcaataatacttgcttgtcgaattacagaatcttctacagcttcggtactaaattgccgcacggctttaaacaaacaacgttcagaacctttcttgacca

cactatgaaaagtatttgcaaggtacatttcaatatcaacactttcttagcaatcacaatacaaaatactcaacaccactctcacgtatgtatttcaaaa

caaacaaaacattcggaactcttctatgcaaacatcataagcaaaagatgttctacatgagcg

>51546|AGAP011345-PA

agaagagttccgaatgttttgtttgttttgaaatacatacgtgagagtggtgttgagtattttgtattgtgattgctaagaaagtgttgatattgaaatg

taccttgcaaatacttttcatagtgtggtcaagaaaggttctgaacgttgtttgtttaaagccgtgcggcaatttagtaccgaagctgtagaagattctg

taattcgacaagcaagtattattgacgacactgatgacagcacaaattatgaggaatacatcgaaatgaagagaaataaatcacgtctgttaccacaaca

tcacaatatattacacaataaagtaccatattccagcagtaagagctggtttcatgatacgattaagtataaacgtaggctttatgggcgttatggtagt

attagtgctgtcaacccaggcgtaatgtggccaactaagaatgatttagatgacattcgtgaatatgaatctgtcgcatacccacatactcttgaagaga

tgattgagagaagtatcgctgaaaaaaagttgagggaatcaacgatcagggaaagagaggaggctatttctaagaagatggaatctttggaaaagtggaa

ggaagaacttaaagctagagttttaaagaaagaagaagctatgcttgctgcaaaggcgagaaaggatcgactaatggaagaagtccgtaggcattttggc

tttaaagtggatccccgtgatgatcgttttaaggaaatgttggagaaaaaggaaagagaagaaaagaaattggcgaaagaagcaaaacgcaaagccaaag

aagcaaaagcaatggcgaagttaactgaagcagtggcaaaagcatctatagagtcaaaagactgaaaatagtgtaatagtagaaaaatatgtagtgtgta

acaggaactgttctatctggaaaattgtagagattaaagtggtgccttgtgctgttgtgctcctttaactatgtctatagaaaaactctcatccccatac

tgaaaattatactaaagaattcagctacatctttttaaatagcctgtctctttatttgtttacatcttgaaacttcctggcagattaaaactgtgtgccg

gaccgagactcgaactcaggacctttgcctttcgtgggcaagtgctctaccaactgagctacccaagcacgactcacgcccctcctcacagctttacttc

tgccagtatctcgtctcctgccttccaaactttacagaagctctcctgtgaaccttgcagaactaacacgcctgaaagaaaggatattgtggagaatcag

tcatattgtttattagctgttgaacatgactgagattttaatcttcagtacctcacacataacatgtctccattgtggcttaatttacatggaaggtaat

gggaatacttttccccacatacttattcgaagtcatatgtctttcagcagcacaactagagattgttattgcagtacacattactttgacaaatgcaact

tttgggcagttaatatgatattcattgctgcatgtaaagcatctttgcatcgctaaaatgtggatatcatttaatccatttctgtggattcatttatgat

atagcagctttcatgtgatacactacctgttactgcagctgtacttgcatatcagtgattttgttacattgagtgaaggtgagtaagcaagctattgtac

atgcaataacatcagctgcactcacatttctgcctgtttgagtaagtttagcttgcgatatgtggccttaatgcacatcgcagccaagcagtgcatgtgg

ggagcatcatgggcaggagcacatgtcgatgcatttttccagtggtatatgttcttcctcagggttaaagttatcgccatatcaaattttattgaaattg

attcagacgtttagtcatcaaaatgtaacaggcagaatttcttttgcacttatattattatcataggagagaagtaaagattgaacatgtttaggatcat

ataagcttgtatcatcttagaacatatcaacagtaacagtattttcaaaaatgtgataaaattcaaaagtcaaagagttaataggtttttccaaagagta

aatatttttccctaattcacataatagtgttcaaatcaataaatatcttgtcgtacacactttctggtagcaatctatgttctttctcggtgttcaaagc

tatctccacatcagatttcgtcaaaatcagttcagtggtttactcgtgataaggtaacagacaaacagacttttgcatttataatattagaatggattct

gtaaatgtatctgtatttcttggcgcatctacctttgcttatggcagataataaatgtgctgctcggggactgaaatgataatttaaccctagcaccact

aaggggatcaaaaattgc

>51550|AGAP011363-PA

tttttttttttttcaagctgaatttaggactgttgtaatgaaagcaaaggaaaacaaaaagtgaacaacatgaaaacaggacacttctatgtacacattc

tgaaacacatgatgtccattacaaattagacaaaattgagctaactttaaaaactgaattttacagaattgtatatgcattagaacacagtacaattctt

gcctcacaataacaaaattaaaacaagttatccagtgactactcagaactacttattttatgggatattaacagtttgtgttcagtttctgtttttatgt

taacattgtgctctttatgtaaatatcttatacgtaaagcataaatgtaagtaacatgtgaatccgaagtcctagatccaagttgatgaatgaaagtttc

agtaacagcatttttaaaaaggtaagtatgattatgaaatacaatgaagaccttatcaactgacggtgttcaataaaaaaatttaggcatcgtacagcaa

ctcaatatcctacattacttcgtttgaaataagcaagaattaacctcagtctttgcaacaatcacaatgacaacgagcttgttcccagatttgttgcaaa

tataactgattttcttccatttttcattcactttcaagttttaacacatacagccaccttcagaatcctgttggtcattttgctggggtgtctgtgtctg

aagatcaacagtgtcttctggtggtttgttttcagtagagtccatgcctggaagggctgcagcgacacgtcggaaaagctgcttaacattgtagccagct

tttgcactagtttctataaacatgacatttagttccttggctttccgttctccttcttcagtggagacctgtcgtttatctgagagatcagtcttgttgc

caacaagcataattattacgtcactaccacgttctgtccgaacatcgtcaatccatttagatgtctgatgaaaggaattcgcatttgtgatatcgtaaac

taccacagcaactgttgagtcacgtatatatgaaggaatcagactccggaacctttcctgtcctgcggtatcccaaagttgcaggcgtaccgttctgtct

tctaaatacatagtcttcgacaggaaatcaattccaattgtagcctgatatgtgttgtcgaagctgtcatacataaaccttgtgatgagagacgttttcc

caacgctttgctctcctaagaaaactaatttaaacttgcgtaatgggttgccgaaatcgcccgaggatgacatttttcggctcgtttatctaaattagta

cgtatattaaatgtaacttttatgcaaaccgtttcacagaaatcattaaaccacgaaaattgacagcccctcctattaatcactaatccgccacgttacg

acgttatagcccgtgacgtcactacaatcccacttctgtcata

>51556|AGAP011369-PA

ctcccacatagttgggtcccagtcagggaacaatgcagtgaagctaacaggttcttctccttgtttgactgtgatgataacactccgatcacggcgccta

tcactgggatccgtcttcaagtaggtctcagccattttgagactcttttccttttcttcttgtgttgatctgctaccaacccacacataaatttcatctc

cagagtccagcaccatgatatcatcttcatccaagtcctcctgcttataatgagggatctcttcaacccagacttttccattgcaggattacaattactg

gtagattcagagttgaagagatccctcattataagcag

>51555|AGAP011369-PA

ctaccataaagtcccaactgccacagatatttctaaagcactatgcaaataatgctatgtaaaaaaagaacgatatgtaatacatgcctgtaaattacag

catttataaaattagggtagtcaaataaacatacggagcgaaatgttattatatgaagaacagctgtttctgttacacctgacgtacctcccacatagtt

gggtcccagtcagggaacaatgcagtgaagctaacaggttcttctccttgtttgactgtgatgataacactccgatcacggcgcctatcactgggatccg

tcttcaagtaggtctcagccattttgagactcttttccttttcttcttgtgttgatctgctaccaacccacacataaatttcatctccagagtccagcac

catgatatcatcttcatccaagtcctcctgcttataatgagggatctcttcaactctgaatctaccagtaattgtaatcctgcaatggaaaagtctgggt

ccaagaagaggaattccttgagcatcaaattctgatagataatctcccttgcctcccagagctccccagaattcatctggctcatctccttcatttatag

tagttacagtaacatctggtgaaacaagggcacttgcatgtgctccaaattctttttcctcatcatttgaaagctttccaagccacacaaaggtttttcc

aggtatttccagcacaaacacatcatctgactgtagagaagacgctacttcaggaacttgtacagcccttacatcatctggacaggtaccacgaacctgg

aagagtcttgttccatcagtatcgtaagtgtcatgatcacgcaaatttttgaaaccactagcttttccacccatgaagatgatcattttacctttaaata

ttcttaagaaatgccttggttcattgccctgtgtaactctcacttgaacagcttttccacc

>51557|AGAP011369-PA

ctgcgaagccggcgagacaggtcatagctgcggccgctgccatggtgccaccagcattcgaggacgccggcaaacaggccggcatcgccgtatggagaat

cgagaattttgaaccagttgcctatcctaagaaagactatggaaagtttcacactggggactcctacatagtcctgaatacagtggagagaaagggtaca

tacaactgggatatccatttctggcttggaaaagaaacttcacaggatgaggctggcgcagctgctatcctatcagtagaactagatgatcatcttggag

ggtctcctgtccaacaccgtgaagtgcaggatcatgaatctcagcttttcttgtcctacttcagagctggtgtgaggtatctccctggaggggtggcaag

tggcttccaccatgtggatcctgatgctgtggagaagaggctcttccaagttaaaggcaaacgaaacatcagagtgagacaggttgatgtcaacatttct

tcaatgaacaaaggtgactgttttatactggatgcagggaaaaccatatatgtttatatgggtgccactagcaaaagaactgaaaaactgaaggcaatta

gtgcagcaaaccaaatcagggatcaagatcatgctggaagggctaaagttattgttattgatgagtacagtaatgacgttgaagtgataaaattttttga

agaattgggttctggatctccaactgaagtaccagatgaagatgaaggtggggatgatgtaaactttgagaaaacccaggaggcagttgtgactctatat

agggttagtgacgcagggggtgagatggtggttgaaaaagttggagagaaaccacttcatcaagacatgctgcaatcaaatgactgctttattttggata

cagtgacatcaggaatatatgtctggattggccgacgttgtacaaaggatgaaaaagtggaagcaatgaaaagagctcagtcatttttgtcaacaaataa

ctatcctgtgtggacaaaggttcaacgaatagtagaaggtggagaaccatcaacattcaaacaatatttcagtggctggagagaggcaggtgatcaaatt

ggtctaggaaggatctacacagtggagcaaattgcagcaagcatgcctgagccagactttgatcccagctctctacatgccgaaaaaataagacttctag

ctaagaatgctggcagagcctttggttttatgcctgatgatggatcaggaaaagtagaagtatatcgaattgaagattttgaattagtgccaattgatcc

tgctacatatggaatgttctttggtggcgactcttacgtgataaagtatacatatgagaaagacagacgagaaagctacataatctacttctggcagggc

aaagatagctctcaagatgaaagagcagcttctgcaattcatgctgtaagacttgatgatgaagttggtggaaaagctgttcaagtgagagttacacagg

gcaatgaaccaaggcatttcttaagaatatttaaaggtaaaatgatcatcttcatgggtggaaaagctagtggtttcaaaaatttgcgtgatcatgacac

ttacgatactgatggaacaagactcttccaggttcgtggtacctgtccagatgatgtaagggctgtacaagttcctgaagtagcgtcttctctacagtca

gatgatgtgtttgtgctggaaatacctggaaaaacctttgtgtggcttggaaagctttcaaatgatgaggaaaaagaatttggagcacatgcaagtgccc

ttgtttcaccagatgttactgtaactactataaatgaaggagatgagccagatgaattctggggagctctgggaggcaagggagattatctatcagaatt

tgatgctcaaggaattcctcttcttggacccagacttttccattgcaggattacaattactggtagattcagagttgaagagatccctcattataagcag

gaggacttggatgaagatgatatcatggtgctggactctggagatgaaatttatgtgtgggttggtagcagatcaacacaagaagaaaaggaaaagagtc

tcaaaatggctgagacctacttgaagacggatcccagtgataggcgccgtgatcggagtgttatcatcacagtcaaacaaggagaagaacctgttagctt

cactgcattgttccctgactgggacccaactatgtgggaggaagataaggaaacagccatttaaatcagcataaatgtccttggtcagctatgaggatgt

caagaatcaactggcagaatttaattcaacaattgaggattgaactagttcttaatatttctgtctcaagatatgttgcttattaaatgtaaagaaatat

ttatcattagattttttcattgaaggcttatgttttaaatgttagttacttgctgacatgtgtacgcaaaagtaggttgatatgcataattgtagaactt

tttattaaatctgtgatacctcacatggtttcatgtttattcaaagtgaatgaaatggagtacagtgtcatcgttaaatttttgccatcctgtagtgcag

aattggtttcagatgtggaaactcttctaattaaaattgttataactgctagcttcctaaaatatgaagttcctttctcacataagaaccaaaaatatat

agataaattatactgacaaactacaatatttatcaacaaaatataaaatatagtaggcatataatcactttatttttgcttaagataatagttttcattt

aatgaacataaatgtctgtagttcaaacaggtgtctctctaagactatggggccagattttttttttttttttttttttttttttggcgctcatctttgt

gtgactgtctttttcacagtgtaaagatgtaattccttttttatgaaaactgaaagaggctacttgtcaggcatatattagttgtagtaggacatg

>51559|AGAP011371-PA

acctcaggatgttaggacctccatcccagtgagatatttgattgtagtgtaggtccaacgtcaaatggctgctgctacttccaaactggctgcagtgtgg

tatagtgctgaggcgattgtgagcaaaactcagcgccgcctgggtctggagctgcagctccggatattcggtcagctggttgtctgaggcgtcgaggact

tgcagttggtggagtccacgcagtgccgctcccagtgtggtcagctggttctgcgagatatcaaggaacacgagattcgggaccccctggaaggcgtcct

gctcttgatgcaggcagcaaaggaacgtgttgcggagcgtcagttccttcagctgcggaagatgcggcagctggcccgcgagccgctgcacgcccgacct

ggacaggttcagcactttcagcttcttcattcccgcaatggccgccggtaccgtcaacagcgtgttgtctgctaggtccagttcttccagcgccggcatc

tggatgtgcgcatc

>51561|AGAP011396-PA

gcaaggctggactggaaggaagacttggtgacttgaaacgggaaccagtggcagatgaaattcacacattagaggcacttgttgctgatcaccgacagtt

catggaaaatgcaagtaaacgtcaaacagaagttgacagtgtctgcaaatcacggcagg

>51565|AGAP011426-PA

ctcctcccaaaacaagtacctggcatgaaagccctggtagcataggcacggtcagcgttgacaaaacgtccgtgccaaagaccctaactagtctcccgcc

gaaagaagccagttctacagttcctacgacagggctccagatgactaaagccgacaatacgcaagcacaacagctgacagggactggattgacaagttca

acatcggtgccgatgctgcctgtcgcaatggccctctctgcggaggaggatggccgctacgactgggatgagtaccagcaaggcctggtgctgggagctt

tcttctggctgcactggtccacgcagatcccgggcggcatgctggcgcagcggatcgggcccaagctggtgttcggcgtgagcaacctggtggcgtgcct

gctggccgtcctgctgccgctcgccgccagcatcgactaccgcgccctcatcgccgtcagggtcctccaggggttcatcgccggagctgcctggccttcg

atgcactccatgacggctaactggatccctcccaacgacaggagcaagttcgtcacagcgtatctgggcagcagcgtgggcgcggcgctgacgttcccgc

tgtgcggcttcctgatggaatggttcggctggccgaccgtcttctactccacggctctgctgggctccgcctggttcctcgcctggtggttcctcgtctt

cgaccacccgcacgtcca

>51571|AGAP011479-PA

gtacgacccgggctggttccccgcgctgctcaaggacagcagcggcaagctgcagccgcgcaacggaggcgcgccgcaggccgggaacctctccgcgcac

ctcgccgccttctcagaggagctcgaccagctcgttccggaccaagagtttgccggtattggagttatagatatggaatcttggcgtccagtgtggaaac

agttgtttggtgcttatgcaccatatccggaattttctagaaaattggtgaggtcccaacatccattctggagcaaagcaagcgtagagaaagcgcaggc

aagacatgattttgaaaagggagctcgcgaatttatggagacaacactagaactggcgaggcagcagcgaccattggctcagtgggggtatt

>51574|AGAP011493-PA

ccacaattacaaccaaagttttaaagcagttttgcttcattctaagtcaaacaccagaactggaaataccctcccacatattaacagtacagtgctccca

gctgctgatgtctggctgcagagagcagatctctaaaaacatctcacatttagtaaattctgcatgtaaaacttaatatttacaaatgttaaaggtacga

aattatgactgtggtccctgaaatttgtcaaaaatttaatacatagttttcttaacagttgacacccttctaagaaatccagtacaagccgaattgtcca

aatgactactttaaaattatgagcaaaattgctggtcaggaggcatgtccgtaatactaacctagctttgagaattaatagttccttccttgaacaggtg

atgtagactgtggaacgttgaagaagaagggcaggttagttactcagaccacgggatgagaaactgacatactggccagcaactctgttccacaatttta

tagttctccttttcttttcatccaatagtatacagtttgtcagttactgtcagctgttaaagcatttcatcatcactactaaagtcattcacaacaaatg

ccaaaatttaaaatcatcattttctctcatctggaacctgtatctacgcagctttaccacttattattattattattaatctttcttcttcgaaactggt

gtcaaggcacctctgattcttggtaccacttcagtgaatatgattgtacctgaaaagactagtatagttccaatccagtgaatgagggtgaagtcatttt

tgaaatagataattgaaaataacagacttataaactttcgtaaagttacaacaagggtgacagtaagagatgagcattctgtagtcaggacatagactga

gctgatgcacatgtatctggtcaaacgctaaggatttacttaacacaataatgtgaaacacttaagttgtaatgcataagaaactgaattatattttgga

ttctctaaatactaattcatgctccctagcagcatttacactccacttgcatttgtttataagttcagcaatcagaaatcacaaacatgtgaatgattac

atatagacaactgagactatcacattgctgtggttgataaaatattattaagcatttaaaaaaaaaaaaattgatgccctgtggatgtgcaaagggagta

aaaccattacacaggtgagatgtgtagatgatatgacatgtccagtctgcaaagtagttataaaattttgttgtaatcaaatagaaaacttacaagaaca

tcatttttcaacatagtctccttgcatttcaacgcacttggtccattgttgtacaagcttcctaatgccctcataaaagaaggttatctgttgagctgca

agccaggaatgcaccccttctctttcactgcttcgtccgaggcaaattgatggccccttaatacctttttgagtggacctaacaggtgatagtcagaggg

caagatcgggacaatatggaggatcataaagtacttaaaatttgagtttatggagcatttcagcagtgtgggcagcagtgtgtggacgggcattgttgtg

caacaacacagcatcttttaacagcaatcctcggtatttgctttgaattgcacattttagcctggcaataagcatctcactgttacatacactctattgc

tgtg

>51577|AGAP011493-PA

gtgtgacatagcatttattataaatgaatttcagcatagcatattgtagtgatatgaaaattttattttaatgattatatataaatatattttttgctta

acgtcaaatattacaagtttgaaaatattatattgctttatttttctgattcttctggcctcctgtaaggaaataacttgaacaaactcactttgtcaca

cacacagcagaaaataaaataaaattctcaagactcagttgcattctactttcatcacagacatattaacataagttctttatattaaagcatatacaac

agttctgcgagtacttatgcattttaaagtgctctacaaaactgaccattaacatcagtgtaacaaaagaaccctaacacacacacacacacacacacac

acacacacacacacagccagccagcatctgaaatgggaggcgacacactttagaaaccagagtaccacttaaatgcttttcaaaacttggagatcatcta

attcacaatgaccactagttagttgcacactctactaggtaatataaatcagcaaactatgaactctggaataaatttaaaaaaagacaaaatgataagt

gttcctgaagccgcacctcactttcctaaaaatattttctttggaaagaaacaccatgtacataatactgcacagagttcttaacttaaataatctgtat

aaaattaggcatctaagacattatctttcaagtctcaacttacatgaaacatttgcaggtacacttcagtacatgccaataaaactgacaactctgccat

acagagatgtaagcatttccataatttaaactggtacagcaataacccagttacaaataaaaatgaattcttttccacaattacaaccaaagttttaaag

cagttttgcttcattctaagtcaaacaccagaactggaaataccctcccacatattaacagtacagtgctcccagctgctgatgtctggctgcagagagc

agatctctaaaaacatctcacatttagtaaattctgcatgtaaaacttaatatttacaaatgttaaaggtacgaaattatgactgtggtccctgaaattt

gtcaaaaatttaatacatagttttcttaacagttgacacccttctaagaaatccagtacaagccgaattgtccaaatgactactttaaaattatgagcaa

aattgctggtcaggaggcatgtccgtaatactaacctagctttgagaattaatagttccttccttgaacaggtgatgtagactgtggaacgttgaagaag

aagggcaggttagttactcagaccacgggatgagaaactgacatactggccagcaactctgttccacaattttatagttctccttttcttttcatccaat

agtatacagtttgtcagttactgtcagctgttaaagcatttcatcatcactactaaagtcattcacaacaaatgccaaaatttaaaatcatcattttctc

tcatctggaacctgtatctacgcagctttaccacttattattattattattaatctttcttcttcgaaactggtgtcaaggcacctctgattcttggtac

cacttcagtgaatatgattgtacctgaaaagactagtatagttccaatccagtgaatgagggtgaagtcatttttgaaatagataattgaaaataacaga

cttataaactttcgtaaagttacaacaagggtgacagtaagagatgagcattctgtagtcaggacatagactgagctgatgcacatgtat

>51575|AGAP011493-PA

tattaaataggataaatgattttgctagtcctgatactagtactattgctttaattgctcctgttaatggtcaaggtctatagtctactaagtggaatgg

gtggtctgctgtgtggagttttggtgtatagatgtgacgtatggtccttaacgtgtgcgtgttttggcactaagattgaagatgaaagccgcactcgcaa

ttttgtttgtatttattggttgctgtagcaacgtcgtgtttttggagcttcttattaaggaggatccaagttctgggaatataatcacattttctcagtt

cttatttattgctctagaaggttttatatttacatcaagatgtggtacaaagaagccaagtattggtttaaaagattacagtattctagttgcaatgttt

ttcattgccaacctatgcaacaactatgcgtttaatttcaacattcccatgcctttgcacatgatatttagagctggctctctgatagcaaacatgatta

tgggaattataattttgaagaaaaaatacacattttcaaaatatctgtccgtcttcatgattacgtttggaatagttatgtgcacagtggtttctggtaa

ggaagtgaaagcaacatatgctgtaggtaaagagccagaagtaccagcatcacatttagaggtgttcttctggtggacagtaggtattactctcctgaca

gtagccctttttgtgtctgcaaggatgggaatataccaagaaactctatatgctagatatgggaagcacccaaaggaagctttgttctatactcatttgt

tacctctgccaggattcctaatagttggctacgatatttatcagcatataatatctgtttttgaaagtgctcctatggttcttccaatagtgggtgctat

accaaagatggttgtttatcttgctggaaatgttctgacacaatacatgtgcatcagctcagtctatgtcctgactacagaatgctcatctcttactgtc

acccttgttgtaactttacgaaagtttataagtctgttattttcaattatctatttcaaaaatgacttcaccctcattcactggattggaactatactag

tcttttcaggtacaatcatattcactgaagtggtaccaagaatcagaggtgccttgacaccagtttcgaagaagaaagattaataataataataataagt

ggtaaagctgcgtagatacaggttccagatgagagaaaatgatgattttaaattttggcatttgttgtgaatgactttagtagtgatgatgaaatgcttt

aacagctgacagtaactgacaaactgtatactattggatgaaaagaaaaggagaactataaaattgtggaacagagttgctggccagtatgtcagtttct

catcccgtggtctgagtaactaacctgcccttcttcttcaacgttccacagtctacatcacctgttcaaggaaggaactattaattctcaaagctaggtt

agtattacggacatgcctcctgaccagcaattttgctcataattttaaagtagtcatttggacaattcggcttgtactggatttcttagaagggtgtcaa

ctgttaagaaaactatgtattaaatttttgacaaatttcagggaccacagtcataatttcgtacctttaacatttgtaaatattaagttttacatgcaga

atttactaaatgtgagatgtttttagagatctgctctctgcagccagacatcagcagctgggagcactgtactgttaatatgtgggagggtatttccagt

tctggtgtttgacttagaatgaagcaaaactgctttaaaactttggttgtaattgtggaaaagaattcatttttatttgtaactgggttattgctgtacc

agtttaaattatggaaatgcttacatctctgtatggcagagttgtcagttttattggcatgtactgaagtgtacctgcaaatgtttcatgtaagttgaga

cttgaaagataatgtcttagatgcctaattttatacagattatttaagttaagaactctgtgcagtattatgtacatggtgtttctttccaaagaaaata

tttttaggaaagtgaggtgcggcttcaggaacacttatcattttgtctttttttaaatttattccagagttcatagtttgctgatttatattacctagta

gagtgtgcaactaactagtggtcattgtgaattagatgatctccaagttttgaaaagcatttaagtggtactctggtttctaaagtgtgtcgcctcccat

ttcagatgctggctggctgtgtgtgtgtgtgtgtgtgtgtgtgtgtgtgtgtgtgtgtgtgtgtgtgtgtgtgtgtgcgcacacgcacacacacgcgttt

ttttgtatgttggtctgttaactccgatgaaggacattgtatgatagcttaactatgagtttagacccg

>51576|AGAP011493-PA

atacatgtgcatcagctcagtctatgtcctgactacagaatgctcatctcttactgtcacccttgttgtaactttacgaaagtttataagtctgttattt

tcaattatctatttcaaaaatgacttcaccctcattcactggattggaactatactagtcttttcaggtacaatcatattcactgaagtggtaccaagaa

tcagaggtgccttgacaccagtttcgaagaagaaagattaataataataataataagtggtaaagctgcgtagatacaggttccagatgagagaaaatga

tgattttaaattttggcatttgttgtgaatgactttagtagtgatgatgaaatgctttaacagctgacagtaactgacaaactgtatactattggatgaa

aagaaaaggagaactataaaattgtggaacagagttgctggccagtatgtcagtttctcatcccgtggtctgagtaactaacctgcccttcttcttcaac

gttccacagtctacatcacctgttcaaggaaggaactattaattctcaaagctaggttagtattacggacatgcctcctgaccagcaattttgctcataa

ttttaaagtagtcatttggacaattcggcttgtactggatttcttagaagggtgtcaactgttaagaaaactatgtattaaatttttgacaaatttcagg

gaccacagtcataatttcgtacctttaacatttgtaaatattaagttttacatgcagaatttactaaatgtgagatgtttttagagatctgctctctgca

gccagacatcagcagctgggagcactgtactgttaatatgtgggagggtatttccagttctggtgtttgacttagaatgaagcaaaactgctttaaaact

ttggttgtaattgtggaaaagaattcatttttatttgtaactgggttattgctgtaccagtttaaattatggaaatgcttacatctctgtatggcagagt

tgtcagttttattggcatgtactgaagtgtacctgcaaatgtttcatgtaagttgagacttgaaagataatgtcttagatgcctaattttatacagatta

tttaagttaagaactctgtgcagtattatgtacatggtgtttctttccaaagaaaatatttttaggaaagtgaggtgcggcttcaggaacacttatcatt

ttgtctttttttaaatttattccagagttcatagtttgctgatttatattacctagtagagtgtgcaactaactagtggtcattgtgaattagatgatct

ccaagttttgaaaagcatttaagtggtactctggtttctaaagtgtgtcgcctcccatttcagatgctggctggctgtgtgtgtgtgtgtgtgtgtgtgt

gtgtgtgtgcattttttctcacgttaagctgtgctatgttaatgccattgttaaaaaccgaacaaattgtgcaaggaagttgatgtgacaccttgttatt

ttaacagatgcagcaatgaaatgggtgatttggcagctctgtaaatagcatgttacattaggaatcaggaaacaggaatggtgccacctagtcaacaaac

cttttgctgttgaccctgattactcaagatagcattgtgaaccagtgaacgacacactgctgccttgatggcattttacaaaaaccacaacagctatgtg

acagcacagtgtatattttatgctcattacaatcttgtgctccagtcttgtctgcactgctatcaaagtgtgggtatagaactttgagaaaaatgggaga

tagcgtgtgcaatactgcccttacaaacttcagataatgcaaaaactgaatgccagtgacctcccaaagagactacacttctgcagagaattgttggaac

gaccatcccaatccattcctctcggcatccattaacacattcgacaatatctttccaggtcatctcgtctcaaagaatggggacaattcttggcctccaa

gatcccctaacatttgagcatgtaattttggggttgtttgaaatcacagatctcccattgtgacccactgcacacaattgaggaactgaagtatcaagtt

tgggaagaatttaatcaaattccagtgccagtcctctaaagtgtgatgatgtcttaagttccgtaagactttgtgtgtgaaggaaaaattaagccatgta

aatgccattttcaggtgacggacattttaaattcaacggtggtttcaaaatcacccatttcttgccactcctgttgctcacagtttgaaagttcttagga

aattcctaggacatcactttgtaggaatgagtctccttttgtgtggcctgttgttgatgatacagaaacagtcaaattttcaaaatagaaaaaaaaatat

tactgaactcaacagttggaataatattccaatcaaattgttttgtactgaatggagtgtctgagttttgccaacagaatttcttgacgtaacattgtaa

ataatgcagaaatggtaaaaaatcttatttttgttgctttattataactcagcaggttttttctcccgtgaactgctgagactggtgctagtttaaaatt

gctgattttggctgtctcaacaaaaatctctttcttaccctatacaaaactctgtattttttatggtcttcaatagtgtaggcacctctttagtaactct

gttgcaccttcttagtattctgccaataaaatgctgtcattggtttaccttgctcacaacattatgtatgcaattttgcaatttttaagtagtttgtgat

tgtaaatcttagatatttaattgaattgatagccttttgatctgttgtgatttatcgtgtaactgaaattgcacagaatccttttatcgctaatgcggat

cacattacactgctgatcgagtgagttgtgcagtggttagcacactggactctcatttgggaggacggcagttcaatctcgcgtccggccaacctgattt

aggttttccgtgatgtccctaaatcgctccaggcaattgccaggatggttcctttgaaagggcagggccgacttccctaatccaatgagatcgatgacct

cgtagttttgtctcttcctccaaacaacccccaacattacactgctcattatttagggtctgttgccactttttgcaccttacaaatacctcgtctaaat

tgttttctagtgtttctgatgacttctcttgatggtaaatgacatcataatctgtggacagtctaagaggaccacccaaattgtctcctaaatcgccact

tgttttacttgattactttccatcacttaccacaaactgtgacctttctgacaggaaaccatgaatccagttgcacaggtgaaatgatactccataggca

cacaatttgatgagtagtctcttgtaagtatgtaagcctgtcgaaaataaagtgtttttcttctagttatctttggtgctttttctttttttaatatttg

ttactgcaaaaaggatgaaatcattgtatgctgtattcaacttaagaggaggaataattcactgaattatgacctttaatactaaataagagtgttataa

tggaaaaactttgatacaactgaaaaacactaactttcggctagctcttgcctctcagtggtttgtcagagtaaaaacctaaatattgaaatttgttgga

aatttaatgtactttcacacttgatagtttgagttacgagcaaaagaccataaaaaatgaatcttttcttccaggtgtcccccccccccaatgttgtcac

aaaattctgtaggcaaacctcagattcagcatcccaaaaacacatataggatagaataaatcagtactacgtaacttcccactaggggcccatttgagca

caaattgatcttttagaaagaggatatttctgttaaagttgacatgttggcattcagtgtatatagagtatttcttgtgtgtgtgtgtgtgtgtgtgtgt

gtgtgt

>51610|AGAP011514-PA

gcagcccgttgtagaaggtgttgtgccagatcttctccctgtcgtcccagttggtgctgatgccgtgctcgatgg

>51603|AGAP011514-PA

ccgactacctgatgaagatcctgacacagcgcggtttcagctccacgacctccgctgagcggtaaatcgtccgtgacatcaag

>51608|AGAP011514-PA

tggcataaagagaaagaacagcctgaatagccacatacatagcaggggcattgaaagtctcaaacatgatttgagtcatcttctc

>51587|AGAP011514-PA

gaaaaaatttggccccacaccctctacaacgagcctagggtggctcccgaggaacatcccgttcttctccctgtttgccttggggtt

>51588|AGAP011514-PA

acccttaaggaccccatcgagcacggcatcatcgccgactgggacgacatggagaagagctggcgccacaccttctacaacgagctgcgag

>51584|AGAP011514-PA

ccccatcgagcacggcgtcatcacaagctgggatgacatggagaagatctggtatcactccttatacaacgagctgcgagtcgctcctgag

>51596|AGAP011514-PA

atggagaagatctggcatcccacctcctacaacgagctgcgagtcgctccggaggagcgccccatcctgccgacggaggctcccctcaaccccaa

>51585|AGAP011514-PA

ctgaagtaccccatcgagcacggaatcatcgccaactgggatgacatggagaggatctggcctctcgccttctccaacgagctgcgagtcgctcct

>51592|AGAP011514-PA

acagctacgtgggtgatgaggcccagtgcaagagaggtatcttgcccctgaagtcccccatcgagcgcgggatcatcaccaactgggatgacatgga

>51609|AGAP011514-PA

aagagaggtatcttgaccctgaagtgccccatcgagcacggcattaccaccaactgggatgacattgagaagttctgtcatcaccccttctacaacgagc

>51590|AGAP011514-PA

tcttgaccctgaagtgccccatcgagcacggcatcacctccaactgggatgacatggagaagagctggcatcaccccttctacaacgagctgcgagtcgc

>51599|AGAP011514-PA

cgtacagcgaccgcacggcctggatggcgacgtacatgccgggcgcgttgaaggtctccaacatgatctgcgtcatcttatcgcggttagccttggggtt

g

>51583|AGAP011514-PA

ccctcaagtcccccatcgagcacggcatcatcaccacctgggacgccatggagaagatctggccccaccccttctacaacgagctgcgagtcgccccaga

ggaactccccatcctgctcaccg

>51602|AGAP011514-PA

ccgttgttctgcctgatgcgtacagagagagaacggcttggataccaacgtaagtgcatgggcaagaaaattgctcaaacatcatctgaaccattttctc

gcgattgctcttgggattgaggggagcttcagttaagagcactggatgctcctcgggatttatacgtaactcgttgaaaaacgtatgatgccagaccttc

tccatatcgtcccagttagtgatgatgccgtgcgcaatgggatatgtcaattgtagaattccacgctttgcttgtgcctcgtctccgacgtaggcgtcct

tcttctccataccaaccatgataccagcatgcttggggcgtccaataatagatggaaaaacgttagacggggc

>51640|AGAP011545-PA

aaaatcatatacagagcaattactcgagaggtggatacaagcgccaagccctgagaaagttatgcgggaaaagttggaaggggtggaagatagttgttca

gcagatcttatcacaactatttcatcaaaagttagtcataaagagtggatacaagcgccaagccct

>51638|AGAP011545-PA

ggcgaaactgccagtctctcccttcatctgacatgataaccgtttgatgtgcaggtgtctggcaatgagcacgcagtgcagctcgatcagggaaattgag

acagcaataactacaagccgttttctgatcattgctgttttctttactgactgccgagggcactaagacataaaaaacaacaaaaaccttaatttttat

>51641|AGAP011545-PA

tcttaattaactaatcaaatatacaaaatttctgaaggtgtgttgcattaaatatttgattatcacttacatagaaaatgttactactgtttcaggtgga

tacaagcgccaagccctgagaaagttatgcgggaaaagttggaaggggtggaagatagttgttcagcagatcttatcacaactatttcatcaaaagttag

tcataaagagtggatacaagcgccaagccct

>51639|AGAP011545-PA

ggtcagcagtggaaagcaaccaagacatgaaaagttttatccacttactttttttacaactcgaggagaagatattcaagatcagaattcaaccgctttc

tataacaactctgaggtgtatgaagttgttgaacgtgtttgtgaactgcgcaaaaagtggcctcctgcttggggaaaaatggacgatcagagtattggaa

taatgacaccttatgctgatcaagtttttagaattcggtcggaactacgaaaaaggagaatgggtggtatttcagtggaacgtgttctcaatgtccaagg

caaacaatttagagccattttcctgagtacagtgcggacacgccgtacttgtttgtcaggaacaaaaggttcaacgagtgaaatggactatggattttta

tccaattcaaagctacttaacactgcgataacacgggctcagagcctcgtagcagttgttggagatcctgttgctctttgctccattggaagatgcaggt

tagtttccttagacggacaaatgtagcacctggcacaccttaaaatgaaatgggggtccaacatgac

>51643|AGAP011562-PA

gtcaatcttcatcatgatcttgataagtgttagggtaaactgtttcatacttttatttttgtgtgatataaattacaaaattaatgtgtcaaattcaaat

acccaagcacaatcgttatattgtaagatatatcatctttaatgactggtcaatcttcatcatgatcttgataagtgttagggtaaactgtttcccagac

tgattccagctttaaaactttcagcaaaccatcttctcccccacaagcgatgtagccttgctctttattccaagccagacattttaatttagtattgttg

ggaatcgcaat

>51642|AGAP011562-PA

ggttttataaagccaaaagcacatgtcaaatctcagatgctacattactgcttcatacttttatttttgtgtgatataaattacaaaattaatgtgtcaa

attcaaatacccaagcacaatcgttatattgtaagatatatcatctttaatgactggtcaatcttcatcatgatcttgataagtgttagggtaaactgtt

tcccagactgattccagctttaaaactttcagcaaaccatcttctcccccacaagcgatgtagccttgctctttattccaagccagacattttaatttag

tattgttgggaatcgcaataaaggtacgaaatcatgggtgagatattaaaagattgcagcaggggaatgacctttttgcttaaataaataaacattttta

ttattattttctttcagatgacaaacagattcaaaataaacaaagtaactgacgccacgcaaagtacaaacgaggaag

>51644|AGAP011583-PA

cttttgtatctgacaagatcatttttctaaaatagagcacaccttgtcagcatcctgtggtcatgcttgctggagtgacagcacacgatctggaagctct

gctagaattcgtgtatcggggtgaggttagtgtggatccgaaccagttgccgtctttgcttcaggcagctcattgtctgaatattcagggcctggcacca

gggaccttaaatgcagaggtgagtgtgcatacaaacattttataaatatataaaaatatttatgaagttccaacataatgtaggagagaggccaagctac

ttggattatcaagtttattttttttcccatttgtcacagttgcattcaaaaatttatatttttagtcactaatttcagactacagagtccaaactcaaac

catag

>51645|AGAP011583-PA

ctcctgtttcatccgctgattgattactgctttcaccgtcgtcagtcttacgcgtatccggattagagaggacatcagactctggtatgtgagatgaatg

cgatgaattctgatggtgattagctggctgattttctgaatctgcagagagactatcatgacgggcccacttcgagccactagaccctgtgcttgaggca

tttcctgtagattctcttcgttttgtttttctcttcctcctaatcggtaaaaaggagttaatgacatctctcgacagagcttcatgagatgcaggagaag

gatgcatatcatcaagtttatgcaagctgtacatagtctctgcatttaaggtccctggtgccaggccctgaatattcagacaatgagctgcctgaagcaa

agacggcaactggttcggatccacactaacctcaccccgatacacgaattctagcagagcttccagatcgtgtgctgtcactccagcaagcatgaccaca

ggatgctgacaaggtgtgctctattttagaaaaatgatcttgtcagatacaaaag

>51646|AGAP011584-PA

ctcaccaatgaagcagatgtcatagacacaagactggaactctttctcttacataaaatgcaggctggccaatattgcaaccactactgccatctgcctc

ccagcagtcttcacagcactagatggagtttgaatggtcttcgcagcagaagatgagaactgagaggtcttcatagcagcagatgggaattgagaggtct

tctcagaaggagatggggactgagtggtcttctcagtaggagatggggactgagtggtcttctcagtaggagatggggactgagtgctcttctcagcagg

agattgggactgagtgctcttctcagcaagagatggggactgagtggccttctcagcaggagatggggaataagtggtcttcacagaagtagatggagac

tcagtggtcttcacaagaggggatggggattgagtgatcttcataagaggagatggggattgttcatcaaagcactcagtgaagaaggcatcaatagtgt

gatacaagtgagtcctcaatccgtttatccagtgatattcatctgggtaagtctgctgcctgaagagaacacccgcctcctcgagtgcgcgcgccagcat

catggagttctggtagaggacgttgtcatccgccgtcccgtggatcagcaggaacatcttgttggcgaagctgtcagccagtcgcgtgacgtcactcgca

ctgtaacctgccttgttgtcctctgcagtgggcagccccatgtatttctccgtatatttcgcatcgtagtacaaccagtcggcgacgggggccacagagg

caccgcacctgaagacgccctcgtggtcgcgcgccagacccatggccgtcacgtagccgccatagctccagccccagatggcgacgcgcgagctgtcgat

gaagcggtacttctccgttaaccgctttgtgacgtctatctggtcctggacctccacgccgcccagcctgcggtagacggaaaagagtacccggtcgcct

ttacaggcagacccccggccgtcgatgagcacgtacacatagcgccgcgtcgtcgcgaagtagctgtgcaggcccacctcgaaagagtcgt

>51647|AGAP011586-PA

gtgaacgttaaggcttcatttattgcctcaatattatccattccttttgaaagtctgacagcttcttctaatttctctgatgctgcttggtaaaaagtaa

ctcgttcgcccattttctgctgttcttcagcctgctgaccatggtacagtagagagatacatgtgtggtacaccattttgaatctcgcataacgtttcca

attctgtagcaaattaaataataaaaaaat

>51650|AGAP011634-PA

aataattagaggttaaacaggaacaaggaacaattttcatgtaaatttgtgatttttctctgtacagtgtcataaccttgcgccctggagatatcatttt

aaccggcactccagctggggttgggatgcacagaaagccaccagagtatttgaaggttggtgatgtt

>51649|AGAP011634-PA

ccttcaaatactctggtggctttctgtgcatcccaacaccagctggagtgccggttaaaatgatatctccagggcgcaaggttatgacactgtacagaga

aaaatcacaaatttacatgaaaattgttccttgttcctgtttaacctctaattattactattagggcatcacctaatattagtgcaagaattttggccat

aagaaagtattcataacttaatggataagagaagcagcaaaatgaaaccagtaagtaaaaatcacaggctgtacaaaatttacaagatattaaaagcttt

ggttcttgaaacaaacagaaatgtagtttttagacctgctagttattctcagtactataatacaagtgcaaaaagagcacgtctgtctgacatttgacga

ctttatagtccacatattgctactttccgacaatgatcctgttattcatacacaactaccatttaaaaaaatgtttgacagactccaaaatacttatgac

cttatcgtaactatccttgaacatatttaatgctagttacagtgtctaagcatacaacaaaatgacaaacaataacattcacatttgtcttataacaata

gaactgttt

>51651|AGAP011635-PA

atctatctaagaaaaaacttggaagagcaaagttttgtctccaaaatacatatattatttagactaatcattagagataatttaatacactttaggtttt

cttcttgtgtacaaaaattcttcccttgtgtggtacggtgcataaggatcatcaacatagtacttaggccgcttccagtattttgttaccatcttatcta

acagggtgctctgtgtggcattacagaagacatgttgcctaagtcgccgtcgcctgtttgatgattttgtccacattcgcttatgtcgtccagcttttgt

tcggatccagatgccccattgcaatctgaagaaacgtttcaaagctgctttaacagatttccttttcccctttttaagggaaaacttagttactgtcctt

gtcagcggctgttgaagaaaggtgccctgtcttgcgcaaatagccaaccgcgaaacagcagggctgagggcgttctcacatgacttaactaaactgcctg

agcattgtattggtaccgtaagtggtctccttgcaataactgtagcgatataagatccacgaacggctgtactgcactcagcaaggcgccgaagaccagt

tactgcagtacgtaacatattcgtaatgttgtttttgtttacttatggcagcctcaaatcaactacatttccattcttatagcttcaaagttc

>51652|AGAP011666-PA

cgaccacgtcgccgcatgcacgccaactctctctcccgctctccactgcacgccgctgccatgcgagctgtcttcgctatcgcctgctcagtgctgatgg

taactgctgctgccgccccagattgcggccaggatgaactgctgggttgcctcaagccaataaaccttctcaagtacgtcacggacagaagggaactgga

tgaagtgtgtcgggatctgatgaattctttgaattgtgtcgacgtgtacactcaacagtgcatgactaagctgcagcgtgatcacttcatgcacctgtac

tctggaacagtaacagtcactaaggaaatatgtcaggatggaaattataaagaagagtacctggtgcacgcaccgtgcatgcgcgaggtgaacctgcgga

acgaggactgcgcctcggactaccaggaggcgatccgccagaacgtgcccagcgccgagccgcggtcccagcaggagtcggacgccaagctgcagcaagt

ctgctgctcgttccagcagtacctaacttgctccacgacggcggcgctcagcggttgcggcgtagccactgccaacttcaccaagcacttcctcgaccgc

atggccaggccgcttgtcgagggccactgcgagaaattcgccaaggtatgcggagcagacggtgggagtgcgtctgtgggtccaacggcgctgctggtgg

agctgtccctggcgctggtggcagtgctgcacccggcattggctctgctgtaccggacgtgagctggtggcgcccacctggacctgtagcctacccaaca

accttagctgtcctagcggctgtacgggcttcacacgcccttttctcagtcctcctccgtagagactgaattcatgtatgcggcgaacttttaaatggtc

ccacttcggtttcatactgtccgtcttcgatgatttttattttctttttgcaaaaatgtttcctatcgttttggattgctcaagactaagagatttatcg

aatactatgtgtggctatttctgcataatcgatcgtcctaaaataacttacactctttcgtaccaaagtactcttagtgtttaaagactcgtaggttagg

tatatcagaaaagtattacatttcacctgcatcgtccttaactgtgataaatactggtggttcgactgatcgttgactgaactaaactgtgaagacaaca

aaatactcgataggcgtttgctgtaagctacagggaatggtgtgtgtgtgtgtgtgtgtgtgtgtgtgtgtgtgtcacagtgttatcagttcgcttgatg

tacacgttaagtagctatcgttcctcgtctgttatggttagactgtttttttcttaaaagtagcactgtcatccattgcacggtatagcttccgaaagtg

cacgcaaccacatttgtagataggaaaactggctgcaatggtggcggcttttaaatagcattacggaatgcagtaaaggattgtcacaaacaattccttt

tcctcacattcaagaaaagattttatgttttagaaaatcaatttgtacatatgtgttaagttattgttttcaactgtatagttgacggctagtaataagt

agagtgcacctaattactgttagtggtaaatacctctttttattgttaactgttgttcttgtggatgtatttactgtcgtagcgaaaatatatatgattg

gaaagtattaaagaatattgtgctgaaa

>51661|AGAP011673-PA

ggaggctaattttttctacgtggacaacattaaccctttcacactataataatgagcgacagtcagtttaactactggtttaatgtaactttcacgatat

cacacacaatgttctttctggttctttgaaagcttttggatcattgagttctttcgctttatgtgctaagtacgtgtctttgtactcattctgcatccat

tctggaagaggtttattccagtcttctggaggtgcatcccttttctcccaaatgtcgttgtcgatatgtgccttcaagcgttcatatcggcgcttcttct

cgctttttattagctcatcgcatgctttaaagtttttgctgtctctccagcggacacagttttcgtaatcgtgtttccactgagagcagtcaacgaattt

tccgaaaacgaaatattgatgaaatcttgctttaaagctcgcacagtcgctatattcatcatcataaatttcacacggacgtatctgaaaagcaaagtgt

tctggaaattacacttgattttgaaacactcatattaacgatataggttatggcaatagtaccatccatgcatctttgggactgtctgcttcatcagcct

tctccattcgcacacgtttttcg

>51660|AGAP011673-PA

ggataggttttaataatttatttgttcgctaaaattatttagtggaggctaattttttctacgtggacaacattaaccctttcacactataataatgagc

gacagtcagtttaactactggtttaatgtaactttcacgatatcacacacaatgttctttctggttctttgaaagcttttggatcattgagttctttcgc

tttatgtgctaagtacgtgtctttgtactcattctgcatccattctggaagaggtttattccagtcttctggaggtgcatcccttttctcccaaatgtcg

ttgtcgatatgtgccttcaagcgttcatatcggcgcttcttctcgctttttattagctcatcgcatgctttaaagtttttgctgtctctccagcggacac

agttttcgtaatcgtgtttccactgagagcagtcaacgaattttccgaaaacgaaatattgatgaaatcttgctttaaagctcgcacagtcgctatattc

atcatcataaatttcacacggacgtatcatccatgcatctttgggactgtctgcttcatcagccttctccattcgcacacgtttttcgtactgcctgtgt

ttagctttgggacgaactatttatttaatagggcattattccgtattcgaaaatttcgcgaatatcagagattaacttacattcagaattgaag

>51663|AGAP011680-PA

cagcacgctggacgaccacgtggcgaccatcatcgtgggcgcggtgcagctgctggccaacgtggcggcgctgttcgtggtggaccgcgccggccgccgc

ccgctgctgctcgcctccggggcgctcatggccgtctccatggcggcgctcggcgcctacttcaacctgcaggagcagaaccgcgaggagggcctggagc

tgctgccgctggccagcctggttgtgttcatgctgggctactcgctgggttactgcacgctgcccttcctactgatgggcgagctgctgccggaacgcca

gcgcggaccgctcagctcgcttgccggctccttcaaccttgccttcatgttcgtcgtcatcaagaccttccacaacttccagcaactgctcggcaacgac

ggcaccttctggctgtacggcgccatgtgcctcgccagctgcgccttcgtgtggctgctggtgcccgagacgcgcgccaaatcgctcgccgagatcgagc

agttcttcgaggacagcaaccaggcgcgcaaggagaagcgcgccgctgccgccagggcggtcgtagtcgccgccgccgcagctggcggcgctgacgtcgc

cgacggtgcgctggagaagactgcccagccggcgggtagcagctaacgccgggacatcgctcccgggcgatcgtgattaatgttcctgtacaagcgtctc

tccgttacagtgaaggactggtcattttatcgtggtcgctgtctttcggcgacttcattatgtagaactgaaggtttccaagtcaccgccgattaacttg

cctagagcacatcaggtcaaaaagtggatcgctctgtagctgtaccacacatccacctccacctactacgagcagccaatgaaatattgtctcccgctgg

gcacctagctaaacatcaatgtggacagcgatatcttctctcctccagactacactgtggcaataatttattgctgtccacagcatcactttatgaagta

>51662|AGAP011680-PA

aaaaaaagcagtaacaacaaagagagaatagcatggtgtggtgggagtttcagattgaaaatattcagtttatcgtaacttgacataagcggaatttgat

gatttttttccccgtatcgtgtgacaggtgcatacttcttgagcaacattgacacttgctttgggccacctgcgaaaggcaggttggacacctctggtat

agggaaggtcgaggtgaacaatttgattttgggcacttgtggactgtcaagtcgggaaactaccaaaagatggtctacagaaactaagtaagcaacagca

cccgcacatcactggaaattttcaaaattccctcgttctcattgtggaaactatgaccctagagaaaagactgaatagaaccttatttaattactttaat

tctctacaggattacattttcactataagccgcagatttcaagttgttcaaggaaagcgtaccacagtgacctccaaatgcacccccaccacaacactct

gttcttactagtcaggatttttagtatgttattcatgccgctacctcctacactgtgcaaaattcggaaattacaggaattattatccagattcgacatg

ttttgctcttgatattcatgtagggtctccagctggaacatatcaccatcttgacacaatatttacgcagttcacccagccgccatcttcaggtgaataa

gccatcacaccaactcaccgagttgacttggctgtttcgccattggcttaattatgcacttataagttgtagaattgatgtttgtgtatattttgctgac

agttttctgaattttaatactataaaattaacaattaagtcattttatttgtctggccttcttactacctaattagtgaggcataaatttaagtcgaact

gcaatcgtaattagcttttgcataactttcacaaaaatcttttttctttcattactctcacagttgttaatgaaatagctgacttatctggcggcgtagt

agacaactttgagttatttcccggcattacagactgcgagtgtctgtctcaaactgttcgtctcgattttgtctgcaccactgtgttatgttgctaacga

ttatggctcacgccatgtctgaaggcttaccacacaaagagagtcaaaaatagtacagcatggaggggaatacgttgcgtatgcacgttgacagagaggg

gaagtgaccttctctgtcgccatacttcataaagtgatgctgtggacagcaataaattattgccacagtgtagtctggaggagagaagatatcgctgtcc

acattgatgtttagctaggtgcccagcggaagacaatatttcattggctgctcgtagtaggtggaggtggatgtgtggtacagctacagagcgatccact

ttttgacctgatgtgctctaggcaagttaatcggcggtgacttggaaaccttcagttctacataatgaagtcgccgaaagacagcgaccacgataaaatg

accagtccttcactgtaacggagagacgcttgtacaggaacattaatcacgatcgcccgggagcgatgtcccggcgttagctgctacccgccggctgggc

agtcttctccagcgcaccgtcggcgacgtcagcgccgccagctgcggcggcggcgactacgaccgccctggcggcagcggcgcgcttctccttgcgcgcc

tggttgctgtcctcgaagaactgctcgatctcggcgagcgatttggcgcgcgtctcgggcaccagcagccacacgaaggcgcagctggcgaggcacatgg

cgccgtacagccagaaggtgccgtcgttgccgagcagttgctggaagttgtggaaggtcttgatgacgacgaacatgaaggcaaggttgaaggagccggc

aagcgagctgagcggtccgcgctggcgttccggcagcagctcgcccatcagtaggaagggcagcgtgcagtaacccagcgagtagcccagcatgaacaca

accaggctggccagcggcagcagctccaggccctcctcgcggttctgctcctgcaggttgaagtaggcgccgagcgccgccatggagacggccatgagcg

ccccggaggcgagcagcagcgggcggcggccggcgcggtccaccacgaacagctccatggcaacgggactgacgag

>51665|AGAP011686-PA

tccatgcccccgaaaacatacttgcaaagtatggactttgagaaagatatattttgtgcagtttccactgtttgtccaatgcattcacggtcacatcact

cccctttccctcagtgaacaaggtcttgtgaatgtatttgacaaacagtggaaactgca

>51664|AGAP011686-PA

cgacattttagcagataatgtagaggcgtcagcaaagagcgtcgcggccgttgaaagttacgcacagtgagatggtagcatcgcttgaaataatttttat

aatggaattaattatttgttaatgacatcacgacatcgtcacgcattgaaagtgaacagctatattaccgtgagggcgcactatttatttccgaaacaga

catgggctcaacgtttgcaaaattgttgagagacaattgcccccccgacattttagcagataatgtacggcagcttgttgggaagaaaagaaaacgaagc

gaagaacccgaacctgaattgaacgaccgaatgcgggcgcccaaaaagaaaaagctgatgtgtaccccaaaatacattcacaagaccttgttcactgagg

gaaaggggagtgatgtgaccgtgaatgcattggacaaacagtggaaactgcacaaaatatatctttctcaaagtccatactttgcaagtatgttttcggg

ggcatggagagaagctgatgaagatactgtgaacataaaaattgttgatccaaaaataacagtcagtgctttacagacggtatttggatctttgtatatg

gatgaaactactttggagccttctgacattgtttccgtagtagcggcagcaacacttttccagttagagggcattattgagaagtgttgtgaagttatgc

tggaaaccatgtgtgctcagacggctgttgtttactatgaggcttcttgtgagtatggcctcaatgtagtgaaggagaatgcatttaaattcatcctaat

tagcctcctgtcacgtctcaatgagttccctgatcagataaaggcaatcagtgaagaactgatgacaaaacttgtaagaagtgtagatctttttgtgata

tcatcagaactttctctgtatctcttcctgaggaagtggttgtacctgcggttgaacattccacctgatccaaatgtgccagaaggagtatctcagcctt

gccattattttcaaaatataagaaaaggtttgttaccct

>51671|AGAP011722-PA

ttttttttaaaaaaaaagtcatttttatgcaccaccaattggattcagacctatggcaggtcttggtggtagacacacacaatttcctttccctaattat

gatcttcatcatacgacctttacaaaatatattggtgcaattttttctcatctacttctgttcaagaagcatagcagctgtttgaaggggaatagattac

cgacagtgcaatgtctctcattaggaacgtaactgctccagtttcccacattctttacactgtgtaagtaacacgaagcaaaaatatctgagactaaata

ttatattaagtggaagtagcaagtcttacctgtgcattatagaccatatggaccccttgaaagacaatacttttggcatgttctccaagaaattccagta

tatgttcaacctgagcttcatatggaagccctcgcagtcttatgcagtctttccgagtcccggaagtaataatgtgctgagggagcaaaggcacttgtgg

aatttgtgctattagtggctgttgctgctcatacgttttaggatccatagatcgattcaacac

>51673|AGAP011728-PA

cgaagtcgccacagagctaccccagccaagttggttccggctggctcagggctggaagacatcaaacttgctatccagcagctgacaatgagatcacaca

>51672|AGAP011728-PA

tccccctggagggcagcatatgtaagggcagtccagtgttgggtctctgcatgcactgctggacagttagtggaaccatgaggaggtgtctcaatgtcag

gatctgctcctgcatccagcagattacgtacacatgattcatcatgggatataactgctatcatgagag

>51685|AGAP011737-PA

tctggaatggagtagatttacgatacaaattactgaagggaccaaagatacggatcagcattgctggaattattatatctaggggccgtgatgcaacacc

atacctggagagaaaccgtgtgggtcgtgatgccattgactctgcagctgctctcacagatatgggcaagtacattccttaaattcttttataggtaaat

ttttttctctctctattgcagttttccggtttctgttggtttcacatttttcactttcttggtgttcctccctccactgcttagtattcatgtatttttc

ctcaactgtgtcctttggctcatctgcccttgtatttccagtctttgctttactgtaacttctaatttctcaggtttgcaaataaatctcttaaatttct

tctgtggccccataacttccattttttctgtcagtttcatttaatatatgggatgtattcttgcactgccttgataaatcacaatatgcatttacatagg

aaagaacactgccctttctcatggaagatctcttcatttcttctctttcacactccaaagtccatgtgattgcactgcagacatgatgggttacaccatt

actgctgacttatatgtcc

>51687|AGAP011754-PA

ctattggagaggtgccttctgatgtggacagtaaaatgacagaggaggaagaacttggaaggacatccgtcatgtgtgccattaatggtattttagtggc

aatggtgagtgtcgcagatgaagtcaaacctgaggcgcacttagcagtatttaccctgaagaagatggggcttgaggtgatcctcctaacaggtgacaac

cgccagacagctgtttctattgcaagacaggtgggaatcggaagagtctttgcagaagtactgccatcacacaaagtggcaaagatccagcgcttgcaag

agagtggcgcccgtgttgccatggtgggtgatggtgtcaatgactccccagcactcgctcagtcagatgtcggcattgccattgcctcagggacagatgt

agctgttgcagcagcagatgttgttttgatgaggaacgacttgctcgatgtgattgggtgcctcgatctgtcacgaagaacagttcgcaggatacgactc

aactttgtattcgccagtatgtacaatctcttggggattcctatagctgctggtgtgttcagcccatttggcttcagcctgcagccatggatggcctctg

ctgcgatggcactaagctcagtctctgtcgtcgcttcctcgctgctactaaagcggtaccggaagccgacgcgcgcatccctgcagacggcggagtacag

cagcgcgatgcgggcgcgcgccgccgccctgtccgagctcgactccatctcggtgcatcgcggcctcgacgacatcgagcgtcccgtcttctccaggtcc

accagcagcaccatcagcaggaaagaggacaaaacccctgtgggatgaaggtgtgaacagttttttgcacgaccaaagaatgaggcagagggtttgcttc

tcggctcagatgacccagatgatattgatgtagacttcagtggctttgataaaaagaagcaagatgttatacaaatgacaccagtttgaagctttatgtg

tctcaaatgtgtttcttgccataaagataggtcattgcagactgcaggagacctgcatgttgtcttgatccatgattcgg

>51694|AGAP011816-PA

agtgaacttgtctcatactagtagaaggtctgtgttgtgatgcgcaagaatagtcgttggagggagaataaaagctgtcgttttggtttggttgaagatg

gatttttatttgaaataaatgttctctgttcatagagtggttgcataattctttgatacttcccttactgtacataatttccaactatgcctccaaagat

cccggcaacaatgaatcttaaacagtttatgcttcgtcagaaagttttgaaactgtatcgagacatattacgggaaatacgcagagtaccggatgaagga

agtaaagcagaacttcgcctttgggctagaagtgactttagaaagtaccaaaatgagaaagatgagtacgctataaagatgctgatcttcaatggtgaga

gatccttaaaggaactgaagcaatccattgagttgagcagtgcaaagaataattaaaagtagcatgtgatgttaatggcacatgtgtgctgtgactgaac

aatagaaaacaatgtgtaagtatttacgttaggtaccatctttaagatgaaaatacttctttatgtggctgctgatttgtagtttccatccattgtttga

ccaaaaaacgtaaatttgtcatcaacctaattgttccatttgaagtcttctcttttattccaccattgtgggagctggcacaagtgtcaacttatctgga

gacattgtttaacatgtaattcaaaccactgtgcatgcaggcagtttccacaaatgcagaacatgaacagaagtgagagttgtgtgcaagtgtcagggaa

aagccgtataacaacccatgtatcaaacacatgagttttgtgttgtgccattgatgaagcccgtgagctgtgaatcatttgagtgttgtgaaaataaaaa

gagtgaagttgagattttaactgtggtgttctcaattctacgtctggaagtttaatgcaattatgcatgcatgcatgatgcatttgtatgaaatgatatg

attttctgtaaatgtgtagacttattattgagaaggcaacatctagaatactggcg

>51693|AGAP011816-PA

tgttaccttcacagtttattttgctggtgatgattcaactatcatagatgtattatatggttacactggacacacacaaggactggcatagctttgatca

aaaatcccccaaggcaataaactgtaagtgagatgtataattcatgtcaatttaaatcagccagtattctagatgttgccttctcaataataagtctaca

catttacagaaaatcatatcatttcatacaaatgcatcatgcatgcatgcataattgcattaaacttccagacgtagaattgagaacaccacagttaaaa

tctcaacttcactctttttattttcacaacactcaaatgattcacagctcacgggcttcatcaatggcacaacacaaaactcatgtgtttgatacatggg

ttgttatacggcttttccctgacacttgcacacaactctcacttctgttcatgttctgcatttgtggaaactgcctgcatgcacagtggtttgaattaca

tgttaaacaatgtctccagataagttgacacttgtgccagctcccacaatggtggaataaaagagaagacttcaaatggaacaattaggttgatgacaaa

tttacgtttttttggtcaaacaatggatggaaactacaaatcagcagccacataaagaagtattttcatcttaaagatggtacctaacgtaaatacttac

acattgttttctattgttcagtcacagcacacatgtgccattaacatcacatgctacttttaattattctttgcactgctcaactcaatggattgcttca

gttcctttaaggatctctcaccattgaagatcagcatctttatagcgtactcatctttctcattttggtactttctaaagtcacttctagcccaaaggcg

aagttctgctttacttccttcatccggtactctgcgtatttcccgtaatatgtctcgatacagtttcaaaactttctgacgaagcataaaatattagcct

atcaaaggataaaattgacaagtcaacctcccatgtagtgacgtctttgtgagttgtcctgctataggc

>51695|AGAP011816-PA

cctggcaagtattactgcataatgcataatcaaaaaatttgtctctatgtttggaaaaaaagactgttccaattctctgacatgtggagcagtgacatgt

accttttatatttcctttaaaatctcttacatactacaacagattttatttctcacttcacttatacaaatacatgcaacaatcaaatgtctgaacaatt

tttcaatctcatgctccaaaaaattgtttttactatacttgtgactacactcaactactgactactgagatcttgcagtgagtctctacatcagacctaa

gtaatctaaaacatttgagtcctggagttttgtattactgtgatgacaattcactgctactcacactcacaaatgggagctactgatattagcactcctg

atattgcaaaacaactgagactacctaaagtgaacaagataccaagtgacagagtttcagctaaatgtaacacagaatacactgctgaattatccccact

actggctcacatttatcaagaatttttcatgcactgaatagtcccacattactggaaatgagtataggtcactctcattttaaaaaatggtaaaggattg

gatgcacataattacagactaatatcgctgatctctatctgttgcaggatcctagaccacattctatgctcaaatatcatgacggtcctggacgaaaaca

aagttctctcaaagaatcagcattaattcatgagaaatcatttgtgagaaacacagcttgctttattcatacacaatacctttccatgcaggtgaacaga

taggttctgccttcttgaatttctgaaaggtattctgacacagtaccacactgtcaattactatccaagatacaaaaatacagaatatctttgcaagtat

gtgattgtctcaatgaatatttgacaactgtaaggcagtatgtaataatacttggagatgaatgtgttacagtaattaaagtcatattgggtgtagccca

agaaaatgtaataatgactataatattgtcaatatacataaaaggatttttcaggtagaatcagtaacattaagaggctctccttaaatgtggatatgtg

taaaacaaggcctataacaaagagaaagaacctgattacaagattagctgtgaacatcttgagtgtatcgcattttccatctacaaatcagcatggattc

tgagagcatgacacatctgaaacttagcttcctcttttcttgcacacagcacttcacactgcagactgttcacaaaatctgagcatacagaataggttct

cagacatgtgagtagctcaaggattttttaagtaatggaaaccagtatggtgtcctggatagcaagtggtcatcaaaagtatcaccgggagtggtccagg

gctgtgtgatgggactgctcttgttctctacttgactagcagtgatataagttacttccccaatgcaccatatggtgaggtatatgtgtagatagatgta

gacattaagtatttagaggtaatactaaaaagtgatactaaatgggataaacacatacaattagtattagggaaggtgaacgaaagacttggatttgttg

gataggttctgtgaaaaagtgtactacattcgtaaaggaaattgtatataacatgctagggcagccaattctaaagtattgttccatagtttggtgtcat

taagtaggcatgacaatagacagcaaacaaattcagacaagttgctagggttgtagcaggttggtattgccctcacaaaagtgtaacagaattgctcaga

gaacttcaattggaatcattggagggcagatggtatagttcttgtgaagccctttgggaatatttacagaacttgtattcaaagaagactgtacaaccat

tctgggatcatgagaataagataaagcgagattaaggcatgtacacaggtatatagagcatctttttctctcgtttaatatggtgcagagtactttcagc

cattcactgtttaatggcttgcagagtacatacatagatctagaaagaacggccaaaatttgtaaaagcaccttgtgctcatctatatcaacaaggcaca

gggagtacacacacaaaccagttatcaatcacattaattaggtttctgccccccactttatatcacttttgctcacacaagcaaaatatgtgacaataca

tgaccaatggaaaggaacagtagcattcctcgtgtaaaggatataaaaaataattagtttccatcattatgtcaaagtattctggcttcaggctgtttgt

taacagcttcatttataaccaaatcaaataacagctgcaataattaaacaaattggtcccagctataattattataagttacaacatctatcttatttat

ttcaccatctctctgttgtcaggatgatgtcaccataaatattacattgttttctattgttcagtcacagcacacatgtgccattaacatcacatgctac

ttttaattattctttgcactgctcaactcaatggattgcttcagttcctttaaggatctctcaccattgaagatcagcatctttatagcgtactcatctt

tctcattttggtactttctaaagtcacttctagcccaaaggcgaagttctgctttacttccttcatccggtactctgcgtatttcccgtaatatgtctcg

atacagtttcaaaactttctgacgaagcataaactgtttaagattcattgttgccgggatctttggaggcatagttggaaattatgtacagtaagggaag

tatcaaagaattatgcaaccactctatgaacagagaacatttatttcaaataaaaatccatcttcaaccaaaccaaaacgacagcttttattctccctcc

aacgactattcttgcgcatcacaacacagaccttctactagtatgagacaagttcac

>51699|AGAP011824-PA

ggcggggggggccaccaccttcagccggtccgtcagctgcagggagccgatcacgcgcagcagctcatcgacattgcggccgcaggacgctgg

>51697|AGAP011824-PA

tacggcactcacctcgcccaggcccggcaccaaggtgatgatcctgccccccgtgcccgaccaagacctgcccaagctcttccccggcggcgtcgagcgc

gtctccatgccctccggaaacaactacgtgcgcaccactaccgactactgagatgttcaatatttcgtcaccaatatcatcatacagtttttgtatttca

ttgtaatatcataataaaaattctcgttttctc

>51696|AGAP011824-PA

ccccgccaactggacgcccggcaccaaggtgatgatcctgccccacgtgcccgaccaagacctgcccaagctcttccccggcggcgtcgagcgcgtctcc

atgccctccggcaacaactacgtgcgcaccaccaccgactactgagaaaacccagttttacaagtcgtaatattaatgttgtttatgtaccaaatgtatt

tgttttaaaaatacatctttaataaaagaactgcaaat

>51698|AGAP011824-PA

cggcccagctctgtggtacacaccggcgtgaagtcagccgggtgcgaaaacaaaacacaccacgagtttcccttccacttgtagaagtcgagaggaccct

gcgtggagggcgcctggaagttgggcacggtgctctcgagcttcatggtgacgtttggctggcttgtgagttgtgctctgctaccgtggtgacagcggac

tcggagagaaagtgccagttaccgcttcgcaacgccgaaa

>51700|AGAP011824-PA

tggacggcgatgcggcccagctctgtggtacacaccggcgtgaagtctgccgggtgagagaacagaacgcaccacgagtctcctttccacttgtagaagt

ccagtggcccctgcgtggagggcgccttgaagtttggtacgatgctctcgagtttcatgttgctgcctggaagctcgtatgccaatcgccagctgcgtgc

agattgactctggacctcgctgcagacgcttatatctgctgcggaacgagcttcg

>51720|AGAP011825-PA

atataaggaaaatcaaaaaaataaacgcaagggcataaaacaagaatagatacaacctggaaatatgaaggtttggtcagaaccctttagca

>51723|AGAP011825-PA

tttcattccttctttctgctaaagggatctgaccaaaccttcatatttccaggttgtatcctattattatatatcccatgcaacacgtacaggaatataa

ggaatttcaaaaaattaatcgcaagggcataaaacaagaagagattcaacctggaaatatg

>51721|AGAP011825-PA

tttccaaagtgtgcaaacttgcatgttttttatttccaaactctcctataacggtaccagtattataaatatatagaatttttcatatcatattaattcg

ctaggccaataaattatcaaaagatgatagctgttaactacaaatttctgacaagacagcggatatccgaatattttcggcgtcatcattctcgaaataa

aaatgtacaatgttgcaagcaccggtactcctacggcacacacaaatgaactgcacacataaaagtggaaaactttctttcattccttctttctgctaaa

gggatctgaccaaaccttcatatttccaggttgaatctcttcttgttttatgcccttgcgattaattttttgaatttcctgctatcagactaatttttca

ttcatatagcttactatattcctgtacgtgttgcatgggatatataataataggatacaaggcaccaccaatgaaaccaactactgtgcctacaaatg

>51724|AGAP011825-PA

taacggtaccagtattataaatatatagaatttttcatatcatattaattcgctaggccaataaattatcaaaagatgatagctgttaactacaaatttc

tgacaagacagcggatatccgaatattttcggcgtcatcattctcgaaataaaaatgtacaatgttgcaagcaccggtactcctacggcacacacaaatg

aactgcacacataaaagtggaaaactttctttcattccttctttctgctaaagggatctgaccaaaccttcatatttccaggttgaatctcttcttgttt

tatgcccttgcgattaattttttgaatttccttatattcctgtacgtgttgcatgggatatataataataggatacaaggcaccaccaatgaaaccaact

actgtgcctacaaatgcgtaatatttccatcctttcagtatcattttaaactcctttttattaacatcgtcaagatactttattgactattcacttagct

taaccgtttacagacatttctccgcataaatttcacattacaaagttgtgtacaaatatcacaggtaatgttttgcaacttgcactcaacgcggaaggca

gagatcatcaagtgaat

>51725|AGAP011827-PA

ttttgggtttggggaaaattacaagggtgtgcgggtttcagccgctgaacaactgtctaaactgaagcctgcttttgtgaagccacatgggactgtaact

gcagcacatgcatctttcttgactgatggtgcttcagcatgcctgatcatgcctg

>51732|AGAP011844-PA

caacttctcgtaatttttcaattaatgcattgtatgcagctgtctttttgttcctgtcactgtactctttacttttaatcttccacaaacatgggtcatt

tcta

>51728|AGAP011844-PA

gggggggggggggcggtcgcataatttaatgacctatagtagtaaatcttctatttcgtaagttgaaaataatactgcataacatagtggagatgacctt

ccctgatgtgagtggacagatgtagcgggaaatctgatatttggatgaatgcagctctctgtcactattaagattgatcgtttaacttcatcttcatttc

aaagaggagattgtcgattctattcattgcaatatctctactcctttcgtccatcttttttaactttgttgcaatcagttggccatacaggtcgcattcg

tctgcctcaccaacgtacctttcatactgtgtggtatatgcagcatcgtttctctctttgcattcctctgtttcatattttcttttcttcgcttgtggta

catagtcagaagtgctaactcgtgatgtgtaccaatgatctctgttctcatttcctataaacacagtgtttccttcctcaatgacatcatcttcgctaac

ggtttcttggacactttcaacactttgctcagttataaattcctgtgacactgattcctcgtaatttgatactgtcattccatgctgcgtttcttcgcca

tctaggaattgaagtagatggtagtaccaaagcctgggaatgtgcacgtcatcctcgcttgcgccagacatcatcgactcgtttgttttcctcttttcct

tgcgataagaactccggagtgagttgattttctttattactgaatccctgtctgcgctaggatccatctccttcaatttggcaaccattttacgatatgc

caagtttttcttatttcggtcgttgtattctttcgattttaccttccaaaggcaaggtagagatttgtacagttcaataaattctgttataaatgcctta

ggataacaacgcacgtccgtcatttttaaacatacgcaaaaaagtcttcggtaactgcaatggtaatggaaccaacttcgctaacgtttatcgccttcac

actcgagagcttcgccgtgcaaactgagtttgtgcagttaaggccttcgccaggccgacggttccacaagcgacacttatcgacctgcgccagctgggct

gttctcgtccgtgcgcgcgcagcttttccgttacaaaccggtttgcacaggctagcacgcggcgcagtactcgtgtc

>51737|AGAP011886-PA

gatctaccctctttccctacacgacgctcttccgatctcgtcaaaacgcattaagcctcagaagcgtctttcgcagcgtcatcgccttttgcttgagcgt

tcgcttgagattgagcgtattctttaccagcaagaattgcatcagccatagctgaagcatataaagttactgcacggatcgcatcgtcgttaccaggaat

aacgtaatctacgttgtctgggttagagtttgtatcaacgataccgataacaggaatacctaagtttttcgcttctttgattgcaatcgcttcg

>51749|AGAP011899-PA

tgactctaactctacactgtgtaagagatcatgtcgatccaatacccgcaaaattctgtcttcacatgccaaaactgccaacagcttattgttacccttt

tctgcccccagagcaatgacatcaattatgcgatctccacaatggaaagaattaacatctttacagtcaaaatattgattatatgcatgatcaccacata

ctaaaagattgcttccac

>51748|AGAP011899-PA

gtcatacttgcgtaggatttccagtaactgaggaatctttccttttacattctgtcctttaaatttatgcctgtcaatataaaggtccgagaccaagcct

ggaataggaattgtgtaagtactgtatagatatccacaacaaaacatgcttgagtacaatgttgtgcctcaaaacactgttttagaaggcactgtgcagt

aaataatatatttgagaagtcaaatgaat

>51755|AGAP011952-PA

gttggacgccagggaccgcgagtacaacgtcagcgaactgtggcagccgttcacagccaggctgtgccccactctcgctggcaaacccaagctcttcttc

atacaggcgtgtaagggaacagatactgatgccggcgtgatggtgagcgacctcactacagacgccccagacagtggctatgccgaggagctcacttacc

cagtgccttcttacgccgacatcatcgtttcatactcgacattcgaagaaatcgaggagcctgcaaacgagaggaaggagattcgagtgccac

>51754|AGAP011952-PA

cactttccactacagccgcagcacgcagccggccgctgcctgatcagccagaaggagacaggcaagtagaggacgcggattcaatctcggcgtgaatgtc

agcaatatcgtcagcgacacctcggacgaggggcgtgattcctacgccgacatcctagtctcgtattctgcattcgaaggagtcacacccacccgtgaca

cacaaaatgcctcgcggtttgtgcagactctgcacgacgaactggtagcccataggggagtcaaggacctggtgtcgtcgctcacagacgtgaaccgcac

cgtggctcagcgagtacgcttacgcaccgacagggagcatctgttacatggcgggctgcttcagccgtgcttctcctcaaccttgacgcgccgtgtctac

ttccgctagatcagtctactgc

>51753|AGAP011952-PA

cttacgttttacagaatagcagcaactgaatgtgataataaattgttattggaacaggcatatttcaaaataaactaaccgataaaccttgatagatata

caatgaaaatatatatttatgagcattagagatgctcttaataaagtataaacagatatttaaaatgtgtaaagatacaggaataaatttacattttata

agagaataaaataagaatgttttcatactaaaacaattgcatcaaaattgtgattcttgacggagaacaagattaagttggacaaaggtgagaaaagaat

acagcagtgacacctaaagaaaatcatttataattttccttaaacactctaccaaaatctcagaaaatttaaatctagatggggctttgaaccctgcacc

ttttgagtgctaatctagtgccacagcccctgcattattttatacatttggaacgaagggcaagtatttgttagggggaaaggaaagatggaattaagaa

agtctcagaggtattgtctatctgtgttccaggcatcacatggagtcggttaatgtttttttctcagtgaatctgtttttcaaaatttaacattcagtca

taactgattctcagattacagtttactaacttaatttctgaagttgtaaagtattgtaaatataagcacaagagtaaagcatactaccagtactgctgat

atgcgaagattaaatgtattcgaggttgcaagaaactcatattacatatagcgtaagaaatctccagtggaacagaatggaatattaataatcaaaaatg

agacacttgcaggagacacagggacatcataatatctgtgttataacctacactaagcctacagggctctgttggtctgtgttgcaatgaatatcagaaa

tcaccgtactgcattactctgaataagtccacacagcatgtgttaaaagaaacaatgtgcatattattatttataaagagttgtgggaacattaaaggaa

aaagaggctgtttcatgtctcacagaatttatacaaggttgcatcattgtagtttacagtgttttttaaaagaaacgtttgtagaatgtataattcaaat

ccataatatggaagtttccttcatttcgatgatgtgttggtgattttgtttctactcccctaccgagagcaatagacaagaggaagtcaatgggtttcca

acattccacttcagtatcaacaatttttgtagtgtgcttcctttgatatctctgttatacatctgtctcgagtgagcatgcatccatatctaatttataa

gtatacacaaattagtaatacagcattaagatacatcttataaatgagttactgtaaaccagttgaactgtgatgatcaaaacgagtatgtgtctcattg

cagcaacataggctggaggaagacttcagccgtttgactggttgcagaagtcgaagtttctggggctgcagtgggtgcgctgttgccggcgcgcggcagt

gacaaggaaagcgtcactgccggtggcctggtgagtgcagtagactgatctagcggaagtagacacggcgcgtcaaggttgaggagaagcacggctgaag

cagcccgccatgtaacagatgctccctgtcggtgcgtaagcgtactcgctgagccacggtgcggttcacgtctgtgagcgacgacaccaggtccttgact

cccctatgggctaccagttcgtcgtgcagagtctgcacaaaccgcgaggcattttgtgtgtcacgggtgggtgtgactccttcgaatgcagaatacgaga

ctaggatgtcggcgtaggaatcacgcccctcgtccgaggtgtcgctgacgatattgctgacattcacgccgagattgaatccgcgtcctctacttgcctg

tatgaagaagagcttgggtttcccgccgaggctcgggcagcgcctgggtgtgaacggctgccacagttcgctgacgtggtagtcgcggtctctggcgctc

agctgcccgtccctcccgcagtgagacagcactgccactgcaacgcagtcgcgactgtcgtggtcctgctccgccacatgcctgagttcgctgaatattt

cctcatgacacggatcgtcgaaacacagaacttcaaagccgagcgttgtgaatgtgttgcggagtgcgtctctgtcgccttccgtgccgtctctcacggg

aggctggtagtctccgtccacgtgtcgccgaacatcgaagttcttgtggctgaagacgagtgccacaccgcgctctcggtgtcccatgaagtactccaca

tcgttagaacttagcaggtcatagagagtagatgtcggaaacgatgcagcctccggaataaggggtctgtctccttctggctgatcaggcagcggccggc

tgcgtgctgcggctgtagtggaaagtgtgtcagtgtcggcagtagccatgacaactgccacgaaaatgtaggtgaactctctagcgcgtctccagaacat

aatgcaaggacaaaggtgtcaggccaaatgaagaacttttccctcaaaatatcttcttcagatttgcacactgtcaacaacgggaggcgtcgcgccgggt

cccgcgccgcggcggctgcgccggcagtggacgtcggcgccggctgcctcctctttatgccgacactcgccccggcggcggcgccgcaggtccgacgata

acctctccgcgtgtgcgcagcgttcccagacctccggcgctgcg

>51757|AGAP011961-PA

cgccactattgtgatttgtgaacatattcgaatcagctgggccgtgaactgtatgtatttgttttggagtgaaagttgtaatcgtgataagtaaatttta

atatgaaggcttaatcctgcattagactcagtgcaagattactgaaccagtagtttgtttctggaatctggcaaataatctcgtgtcgccagcttctgcg

gtatgactgccaatgtcggagtgccgttgagttgaaataatgtttgtgggtgatataaaaaaggacttttacggtgaattaattggaaagagaattttgt

tgctcgtcaaccatgaggtggatgccatatgtgcatgtaggattttgcattgcttgttaagggctgacagtgttgtttatacgccgtttccaaatctttt

attccttcaattggaacaagcgtataaacaacactgtcagcccttaacaagcaatgcaaaatcctacatgcacatatggcatccacctcatggttgacga

gcaacaaaattctctttccaattaattcaccgtaaaagtccttttttatatcacccacaaacattatttcaactcaacggcactccgacattggcagtca

taccgcagaagctggcgacacgagattatttgccagattccagaaacaaactactggttcagtaatcttgcactgagtctaatgcaggattaagccttca

tattaaaatttacttatcacgattacaactttcactccaaaacaaatacatacagttcacggcccagctgattcgaatatgttcacaaatcacaatagtg

gcg

>51758|AGAP011965-PA

ggccgccgcgtaggagtttgtgtgttgacagttcagagtaagcatggctgttgcgttccatagccggtgcggcgatagagtgacgctgataaatagtaat

agaacagcagtcagaaacttttcagagtttaataacgggttggtgctgagcagtcagcctcttgcagacaatcagctttttcaagttcgcattgataaga

agataaactcctggagcggaagtatcgaaattggtgtaacatgcttgaacccagagacaatggaattcccgtctactgcgtcggagcttcgaggaggatc

ttggattctatcagggacttcggtgctcaaagacggccagtctatcgtggaggtgtacggcgaagacttggagaagctgtcggaaggtgactgcgtcggg

gtcatgcgtacctcagagggtgagctggtgttctacgtgaatgaggagtcgcagggagtggccacgagggacgttccgagcggcgtctacggcttcgtcg

acatgtacgggaagtgtgcgcaggtgacagtcatcgatgacgacgacgagacccgtggagattccgatgacagatccgacttcgacgtcgagctgagtgc

gagctgcggaacgtacgagccggccgacgggcg

>51759|AGAP011972-PA

cgacgcgccgcgtgagtcagctgccactgcgccgggcggcaataacagccggcactagggcccgttgcgccgacttcctcagtcgccgcgatggacttcg

tcacgttgctgaccagggtagccatcttgctcaccgcctgtgtgtacggcgcgctcttcctggcgcgcaatgtctggcgctggctgaagaaccccaccaa

cttcttctggagggtggccaagcggccgacgccgcctgctgtactcagcgaccccgccctcggggagcatcgctatgctcaactcaaggacttgaaaata

cattacgttgagaaaggagataaatcgcacccgctgatgttattcgtccacggctacccagagttttggtactcatggagacatcagatcaaggaattct

cgagagactactggacagttgcaatggatatgagagggtttggagactccgacaagccagagggtaaacagaactataagatcgacctgcttgtcgagga

tataaggaacctcgtgcaagctctagggcgcgaaaagttcaccctcgtcgcacacgactggggaggcgtggttggctgggcttttgtgtttaaatactac

cacatgctggacaactacatcatcctcgacgctcctttcccagatgcttggtcgaatatggtattttcggacaaggatcagttcttaaaatcttggtatg

tgtttttctatcaactgccgtttcttcctgaactcgctctacgcctggatgacttcggcatctttgatacgatttttcgcatgtatgccaaggttccaat

aacaaaagaagaaattgaagcatataaatatacttttggaaagccaggagcgctgacgccgcccgtcaactactaccgggcccttatggaggcgccgtgg

caccagccgtcaccgccgagaggcaagccgctgccccccggactcttcctgttcggcgagaaggagttcgcgctctccttcgacttcctgtctcacgcca

gggaccagataggagtccccaccgccatcgtcaaggaggcgggacacttcgtgcagcaggaaaacccagaggaagtcaacaggctcatcagggacttcct

gtccagccaggaagctcgctgacgcctggtacctgcacctacaaaaggtttcccttttcagtgccattaaagcagggaagacctgtgcctcagaac

>51768|AGAP012010-PA

cactggtgttagcttaatgattgtatgtagaggaacttgcaggtacggtagctcatctttgcatccatatgccactggtgttagcttaatgattgtatgt

cgaggaacttgcaggtacggtagctcatctgctgacttgtcaaggaaataagcaagcaa

>51770|AGAP012028-PA

tgccatatcgacagtaaactcatgaaccttcagcggcatcctggtgtgttggttccaatatattccacagtctttccagttttcttaattgtttccagca

atttgtctgatgttagtgttgaagtcactctctccttcttgtcctccagtgaaatggtaacatcttcaac

>51773|AGAP012047-PA

acgccgcagcaggaggtaatgcgtccgacctgccggacggcctggacggggtgaacatgtggccgcggctggtctctggagagcccagccagcgcacgga

gatactcctcaactacgacgaggtggctgagaccggcgccgtccgcgtcggcgactggaagctggtcaggggtgtgcagaaccac

>51774|AGAP012048-PA

tatgaaacatttcattcacatacgtttcaggagcttggagaggtaatttgtttccacagacatggacaatacatgtattaagatacagatctccacactg

tcttgaaatcacataccctgaacactctccaatagttcataacaaatttataacaacacaattcatgttaagtatgatgtacactacattttaatttgtg

attaaacaaatcatgttgccccactaacacccttccctttaaagtaagctttgttggagacaataccacactacccttttacaggacacagcagagcaat

ggaatttctagttgaggcagccatcacataactatttgtgtgcacaaaagctgcctccaatggtcgtttcaaattttgaggcagatccttctcttataga

aacaagatctgcaaaagatgtccaacatgtatgctaccgaaactgggttcgacctacatcactaataaggtaatggtgaaatataggctcagcgtctgaa

gatgggatgaaatagtgctatgaggtactgagaagtccgaggttgacaattgtcacaactataactaccgcatcgcctattaagtacgtgggctgttcct

tcagaggaactgtctgctcctggcagtttggaaacttattactatttgttcctgtaatgtccaccgcaacacaaacacagtggataattgaagacggcat

tgtgaaaatttacaactctgtagtgcttcgacgcaattacaaataaaccgtctaaactgcttttttacttaataattcctgcactgctgctgcaaacaaa

gcgtggaacctcgtgtcaattaccgacgtgtactacagttcgaagatagctaagccgagcagcttcctagatgtgcacatccagaaagcctacattattg

tctgctacttcctttccatctaaggcagggaatttacacctccactacacgacgctacggcaatctctctactactgttggtacattccacgactgtgac

tgcatttctgtgcttcccctcccacttgcaaatatcacgagtgcaattttgttcattcccacagacgtacaatcattcacacagccactaacattttcat

taaacactcggtagaaacaagacaacactcacacttattaactatcaaccgtcagcactcacactacacacttccctctcattctcatttttgtttcgcc

taaaagatttagcagggccgacgggcaggacacgatctccgacgccaccccactcgacagacgcgcgcttcgcgagcgtctatgcggccttcgccgaacc

cttcaccatcttcatgaggccgtcggtcgacatcgacttgggcctctcgatgggcaggccgaagatgcggtcccagacgagtgacgccatcacgcccagg

gcacgcgacactccgaacaggaccgtgtagtagttcatctccttaagtccgtaatactgcaggaggatgccagagtgggcatccacatttggccacgggt

tcttcaccttgcctgtctccatcaggatggggggaaccaccttgtacacctgggccaccaacttgaacatcgggtcatcgggcaagtgcttcatggcgaa

ctccctctggcaggtgtagcgcggatccgtcttgcgcagcaccgcgtggccgtagccgggcaccacctggcccgacttcagtgtcttccagatgaactcc

ttcagcttgtcgtccgacgggttgtcacccagcgctgcctgcaccttcttcaggaagattagcacctcctggttggcaagcccgtgaagaggtcctgcga

ggccatttattccagcggcgaaggagaggtaaggatcggacagcgccgagcccactaagtggaccgtgtgtgcactcacgttgccaccctcgtggtcact

gtggattgtgaggtacagcctcatgagctcagtgaactccgggttgtcgtagccaagcatctggcagaagttggcgctccagtccttcttggggtctata

gctccgatgcccttcgactccttgtaagtgttgcggtaaatcatggcagccacgacgggcagtttcgcaatgagatccatcgcatcttcgtatacgtact

cccagtatttggccttcgggacaccctcgctgtatgcctggaggaacttgctctccgtgttgagggcggtgatggcactgctgaactgtgacatggggtg

caggttggtggggaagttgttcagcatcgtcaccacgtgtgaaggcaggccagcccggttagcccactccttcgacaggtacttcacctgagcctcgttg

ggtatgtcaccagtgatgagcagccagaaaaggccctcggggaggggttcgttgccacccggagccttcggcagcagcttctggcactccgggatcgagt

agccacggaagcggatgccttcttctggatccagcactgaagtttcacacaccagacccttgatgccacgcataccaccatacatcatgtcgacggtgac

ctcgccgaccttggtgctgccgtactgcttgcggaaggccttgactcgctccagctcgcgcgccgccttctcggtgagcaccgccttcaggtccgtcgag

tcgctggccacgccgcgcgacagcaccgacagcacaggcagcgccaccttctggtgtgcttcgcataatctcgatgcggatatgcggaatagtgccatta

tatctgcagttcgcttcctcctctcgttccggtttctgttacttctgaccgctcgtccaacccggcttgcctacctaatcaagcgctgcagacagtcgta

tccgtgagataccaaagtaggccaaaccctacacctaacctgcttcaagtaatactgttggttcacgccaacttttctg

>51781|AGAP012114-PA

aagagaagctgcatgctggccatcatgtttacatggacaatttttacaacagctttgttttagctaaaaccctgttgaatataaaaacacattgaactgg

aatacgaagggtaaataggggaaataccactaaagacgtggtatgtgcaaaactggagaaaggacg

>51776|AGAP012114-PA

gccacttacctatcataactgcttctgcaaatctgccaacagcttcaccttttcgtaattttgccttgggcgtattcttcttattcatcctcagagttcc

tgtgcagtgagtcttcttatccaagagcaactttgccaatgcaaagctattatactaattgtccatgtacaagtggtgaccagcattcaacttttcaccc

agtaaatgaagaacaatttcttgcgcatggcctcttcc

>51778|AGAP012114-PA

gcattttgtactgcaacattcatcagccttctaaacattttcatacaccactttacacctttcttcctttccgtgagaaaaggttgtagtttctgatctt

tcaaatctacaccgcccatgaaattgttgtactcttttttgcgtgaggctctcttacctcattgccccttttggttgccgtaacaactgcatcattatga

aatgtaaaaataaaggcaacctgcattttatccatccatttcggtgccattacgccgtgtgaatgatatgctatgagttcacctttcttcagttttgcat

gtctcaaggcatcaggtacattgttcgtattcagtcgatgtgtgccagctacattcatacctctttccttcaaaaagtggcacaaaaggatactgttata

ataattgtctaaatagagcatatgccctaagttgaaaagagg

>51782|AGAP012114-PA

gaagtggtaacaagcggtaacatagctgatacaagtgttgcatgggaaagtaagccagttggaatggtaaattccaactggtaaattttataaaaaatga

gggactgctaattcaaccaacaggaaacactcccttagattattttcatcttttgctgatgaacgaatttctattgaaagttgtagaaggaacaaattga

aacacaattgaattattcctttttgtgggaaccaaagaacaatgacgcataaattgttggaaagatgtgactattggtgaagtgttagtgttcttgggag

ttttcctgcacatgggaaatgtaaagatgaggaagttgcaggactattggaaaaagaaccctttgtttcacatgaaaggaattgccgatggtatttcacg

aaatcgttttcttctaatactacgtgcattgcatttttctgaaaatccaaacaggacaaactaaaaccatccgacaggttgtttttcagtgaaaggatgt

gccaaatgtactaccatggatggtaactg

>51783|AGAP012114-PA

cgaataaacatgttataatattctacttcttcaacacagcattgtgaatgccactaaaacagtcgttgcacataaaactgtcacttttattgcatttaga

gcacaccctgttcactttcttcgcagcgtctttcctttcttctgttcttcgcagccgcttgtaacagccctaacatcatctccgactgctcttctttggt

ccctctactgtcacaaggcgacggctgttagcttcgtggaacgtttgtagtgttgctcgtggttgtggatctttggcattcaactgcagtagagtcaagc

gctctctgaaaggaattatttgtatccgagacttggatcatttgtttgccaagttgtccagcccttgagtatttactacagatgttccccaacaggattt

caaacccatatttctgtaccatttaaccgtacgccaaggtgtcgctgagtatgagttcacctgatcggatacatcagtaaatgatgtggtggcattgtag

tccttaactgcttggggtttcaaaacttccacgccgttttta

>51785|AGAP012114-PA

gtgcatcacattccttgttttatttctgttactgtcgggcatagcattttctgtgaacaaccatttggtaatgattttcctgaaattttcttatgcctgg

ttttgtatttgtggctctctggtacacggagcaagcatttactgtatttgttcctaaaagcatatgcagtgctaatttctgataccacttgctaaccttc

cttatcactgtggcatatgacaatctggtcacctctgccagtgctaggttttctattagtgtatgctagtatcaccaaaggctgtttcggaggacgacct

cttgtgtgacattgtaataggtgcatgttttgttgataacatactaaaattgtcaacatctctcccttttagcaccacaataccattatcatattttgct

gtcatttcatctctcttcaatgggtatgacattacaaaacgtagcataaaccttttgttgtactgcacagttccagtgatttgaattttagacattggct

actaattcatacctcttgtactagttaccaacaaaattcttccttcatttagcaattcacctatgggttcactgcaagcattttcagctgtgccatcttg

ctttcttccagtggtgtctaatcaagaatatattttcacacactgtgtatacctttcatggggcactgtttgaaaagtttttcagcatacttacgcaatt

ttgttggtatgtactgcctgaacttcagtcatttgctccaaggaaccatggtctcgtcaactacagtatcttcaactggtgaaaagactttctccgtcaa

catgtttaggaatggtaaaacttatctgatactctcatttttagtttcattatttgtgaaatgcaagtttgctaacagtaattcaaaacgattaccggac

atgctgctgcaaggaaatggaaagttgtaaatagggtccattgaccaacatcttatgaacggtagctgtactagatccatccaaataatcagttgtatga

aatttctaatttcctcagtctgttcagttgaacatgtttagtcatttcagtttgtgccctgcttatttattagtctctctgactaaaaggcttatcactt

tgtcatttaagaatagcaaaagcacttcacctaagctaatgtcacatagcatatccaatcagaagcccaccttttctagtgaagggaaatgactattgcc

tgttagtgaatttgttccatagagttcagtccctcttcttcatctgcagcaagttcttccaacatgtcattgagatctttactagcatataattgctcca

aataagaatctgaactcgatgaaattttgttcatcactttttatctctaatgcactactatgtcatcactactttcttcactttgactcagtcttttatg

ctgtcaaagatacaaaggccactggtgtgtgtctcagagtatgcttcttgacttgatgacaaatctgtgataattgcagtgagatcacaggcagtgctct

ggccaaagcgtttattgcttcccacattttggaactgagaacttcgttctttgtaatgagtcaagggctgtggggcattaagtcaccggcaacttactgc

cctatttgagttacatcgttgctattcccacttatcgctttgtttactttccagcattccaacagttggctttgggtcctgcatgaaactcaactgaatt

gcgccaggtgacgaatgtggtaaactggagtttatgtaggtgtaatatgcagccagtcactttttgaatttggtttgtcgtcttgtaccattaataacaa

taatggaaacttctggatggaaaaatacataaaattggggaaaggcaaccatttacctatgaggattgtgcgccatacatgagaaataatgtaatagaaa

acattgaacactagctttcaagctcttgctctttttctagtaggaatacacacaccaacttttaatgtgtgtactcatactagataagagcaagtgtgtg

aaagctagtgttaagcattttctgttgcacatgtgtgtgccacataccaatcctctgtacacacacacacacaaacacacacacacaatacgtaaactca

aagtcatgttattgttgagttttatttaattgattttcgataaagaataattctctgtagtttaacggaatagtaaattacgctaagaaagctgagggtg

caaacaacaaatgagaattgccaataggcaatcagtgacctgaatacaaatgatgggtggccctctgaaacacttaattgttcatgttataataatatgg

ctctgaatggtaaaagctctggtgatctagtgggtagggcacttgtgtctatctctttcaggtcctaggttcaatcctgagtaggacaatattttggagc

agttagtctcgtgatcatcaaatgtcatgctgtaaattattggaccttattgtaggattcagcacctttttcctatttgtatataaatgaatatgccttg

ctgtagttcatatttaatttattgtttagacccaaaaaactttaccacagagaaattatccatgtttttgtcaatttttttacataactttattattctg

tataccactgttcagcatattgtgtgggattctgagtataagtatttttcttcattaattcatggatgctttgcagaaaaaaggttacttacatgattat

tcactgacatgtctagctttgaaattctgattcttatgcagcacgactgcagtacaaatagcatatacaagaggaactattgtgttgtgcacattaaaag

acagtaagaattgggaagacaaacaaaagaaataaactaactggtaacaaaataagatgtagtattttaacatttttagagtttattggtaatttcagtc

accgtcttcacaaaaatctcagagcctttcacataggacatgagagactctgtgctccagatcttttttgtgacgtatcggatttagtattttataaatt

tactgtgtaatctgtagcaacagtgcttttccaagctcccagctgcgaataacagttactgtagacgatatacttcaaagtcgcgagtagagttgatgct

tcaatttttttccacagtatttcagtgactgagtctgccttcactctctgattctgcaagcatatgtcttttgtattaactcatctaacacaattattaa

taggtcctaataaaatgaatgtcactcattaaaatattatgcaaaaaaatggagatgtcaactctgatgcacaatgagaaagtttacagcgacatgaaga

aaactgcagaaatcagaaaaactttcaccattatataatgacttcgcactacgcaatgaaagttattatgttttgccatttatgatgtcctttctgtaca

tttcacttttttgatcctcaaaaatgtgtagcatttcaaatttactctccattatattaatgccagtcttgttgttgactgatttctgctcattctttaa

ccactgcagatatttcaggcatcataaatctagacaaaagtaagaaacaaaatattcaagctaaaattcatatgcactatagaaataagtttttgtttta

ttttattggtgctttcttgatgtagtgttattacctttggcactttctgtgaattttcagtaactgttttctctgagcgttaatgatgttcctgattaat

attcctggtcatggtaaccgaggaaaaatgcagcggctttactggatctgtaatagtaccatgtttatctatgtatagaaaccatgagtgttcacaatta

tttctccatactaattatacactaacagatgtatacataagtaatgttttcacttttttgctcacctacaaacccaaaaaaaactacttttttcaaagag

gaatgaatatttaagtatttcctgaaactctttgtacaaatttt

>51777|AGAP012114-PA

tgattttatttttatatactgagacatctctgtacataacagcactatatttacaatgtacattaattagatacagttttggcacttcaacaaagatggc

acacaacagtacactagcatccttggcatgggtaaaatttgtacaaagagtttcaggaaatacttaaatattcattcctctttgaaaaaagtagtttttt

ttgggtttgtaggtgagcaaaaaagtgaaaacattacttatgtatacatctgttagtgtataattagtatggagaaataattgtgaacactcatggtttc

tatacatagataaacatggtactattacagatccagtaaagccgctgcatttttcctcggttaccatgaccaggaatattaatcaggaacatcattaacg

ctcagagaaaacagttactgaaaattcacagaaagtgccaaaggtaataacactacatcaagaaagcaccaataaaataaaacaaaaacttatttctata

gtgcatatgaattttagcttgaatattttgtttcttacttttgtctagatttatgatgcctgaaatatctgcagtggttaaagaatgagcagaaatcagt

caacaacaagactggcattaatataatggagagtaaatttgaaatgctacacatttttgaggatcaaaaaagtgaaatgtacagaaaggacatcataaat

ggcaaaacataataactttcattgcgtagtgcgaagtcattatataatggtgaaagtttttctgatttctgcagttttcttcatgtcgctgtaaactttc

tcattgtgcatcagagttgacatctccattttttttgcataatattttaatgagtgacattcattttattaggacctattaataattgtgttagatgagt

taatacaaaagacatatgcttgcagaatcagagagtgaaggcagactcagtcactgaaatactgtggaaaaaaattgaagcatcaactctactcgcgact

ttgaagtatatcgtctacagtaactgttattcgcagctgggagcttggaaaagcactgttgctacagattacacagtaaatttataaaatactaaatccg

atacgtcacaaaaaagatctggagcacagagtctctcatgtcctatgtgaaaggctctgagatttttgtgaagacggtgactgaaattaccaataaactc

taaaaatgttaaaatactacatcttattttgttaccagttagtttatttcttttgtttgtcttcccaattcttactgtcttttaatgtgcacaacacaat

agttcctcttgtatatgctatttgtactgcagtcgtgctgcataagaatcagaatttcaaagctagacatgtcagtgaataatcatgtaagtaacctttt

ttctgcaaagcatccatgaattaatgaagaaaaatacttatactcagaatcccacacaatatgctgaacagtggtatacagaataataaagttatgtaaa

aaaattgacaaaaacatggataatttctctgtggtaaagttttttgggtctaaacaataaattaaatatgaactacagcaaggcatattcatttatatac

aaataggaaaaaggtgctgaatcctacaataaggtccaataatttacagcatgacatttgatgatcacgagactaactgctccaaaatattgtcctactc

aggattgaacctaggacctgaaagagatagacacaagtgccctacccactagatcaccagagcttttaccattcagagccatattattataacatgaaca

attaagtgtttcagagggccacccatcatttgtattcaggtcactgattgcctattggcaattctcatttgttgtttgcaccctcagctttcttagcgta
[truncated: 54,553,248 more chars]
